# Comprehensive genome based analysis of *Vibrio parahaemolyticus* for identifying novel drug and vaccine molecules: Subtractive proteomics and vaccinomics approach

**DOI:** 10.1371/journal.pone.0237181

**Published:** 2020-08-19

**Authors:** Mahmudul Hasan, Kazi Faizul Azim, Md. Abdus Shukur Imran, Ishtiak Malique Chowdhury, Shah Rucksana Akhter Urme, Md. Sorwer Alam Parvez, Md. Bashir Uddin, Syed Sayeem Uddin Ahmed

**Affiliations:** 1 Department of Pharmaceuticals and Industrial Biotechnology, Sylhet Agricultural University, Sylhet, Bangladesh; 2 Department of Microbial Biotechnology, Sylhet Agricultural University, Sylhet, Bangladesh; 3 Department of Molecular Biology and Genetic Engineering, Sylhet Agricultural University, Sylhet, Bangladesh; 4 Department of Biochemistry and Chemistry, Sylhet Agricultural University, Sylhet, Bangladesh; 5 Department of Genetic Engineering and Biotechnology, Shahjalal University of Science and Technology, Sylhet, Bangladesh; 6 Department of Medicine, Sylhet Agricultural University, Sylhet, Bangladesh; 7 Department of Epidemiology and Public Health, Sylhet Agricultural University, Sylhet, Bangladesh; Management & Science University, MALAYSIA

## Abstract

Multidrug-resistant *Vibrio parahaemolyticus* has become a significant public health concern. The development of effective drugs and vaccines against *Vibrio parahaemolyticus* is the current research priority. Thus, we aimed to find out effective drug and vaccine targets using a comprehensive genome-based analysis. A total of 4822 proteins were screened from *V*. *parahaemolyticus* proteome. Among 16 novel cytoplasmic proteins, ‘VIBPA Type II secretion system protein L’ and ‘VIBPA Putative fimbrial protein Z’ were subjected to molecular docking with 350 human metabolites, which revealed that Eliglustat, Simvastatin and Hydroxocobalamin were the top drug molecules considering free binding energy. On the contrary, ‘Sensor histidine protein kinase UhpB’ and ‘Flagellar hook-associated protein of 25 novel membrane proteins were subjected to T-cell and B-cell epitope prediction, antigenicity testing, transmembrane topology screening, allergenicity and toxicity assessment, population coverage analysis and molecular docking analysis to generate the most immunogenic epitopes. Three subunit vaccines were constructed by the combination of highly antigenic epitopes along with suitable adjuvant, PADRE sequence and linkers. The designed vaccine constructs (V1, V2, V3) were analyzed by their physiochemical properties and molecular docking with MHC molecules- results suggested that the V1 is superior. Besides, the binding affinity of human TLR-1/2 heterodimer and construct V1 could be biologically significant in the development of the vaccine repertoire. The vaccine-receptor complex exhibited deformability at a minimum level that also strengthened our prediction. The optimized codons of the designed construct was cloned into pET28a(+) vector of *E*. *coli* strain K12. However, the predicted drug molecules and vaccine constructs could be further studied using model animals to combat *V*. *parahaemolyticus* associated infections.

## Introduction

*Vibrio parahaemolyticus*, a highly reported pathogenic bacteria of aquatic environment, has emerged as the leading cause of seafood-associated gastroenteritis and a significanthazardfor global aquaculture [1–3]. The overgrowing population, with increased purchasing power worldwide has enhanced the demand for and export potential of seafood, resulting in the steady expansion of the aquaculture industry [4]. However, the sector has continuously been challenged by aquatic animal health problems, which is a significant constraint to the development of this sector [5]. Besides, Multiple drug resistance (MDR) has been recognized as an essential global threat issue to food safety [6]. The continuous and inappropriate use of antibiotics in the aquaculture industry favors the development of a variety of resistant isolates and the dissemination of resistance genes within the bacterial population [7]. *V*. *parahaemolyticus* has been reported to show multidrug resistance during aquaculture production [8], which raised the concern about public health and economic threat of this bacterium [9].

Though *V*. *parahaemolyticus* was first isolated in 1952, reports demonstrated the recent outbreaks of *V*. *parahaemolyticus* are more severe [10,11]. On the recent outbreak in the city of Osaka (Japan), acute gastroenteritis was reported in 272 individuals, 20 of whom died [12]. To date, *V*. *parahaemolyticus* has been responsible for 20–30% of food poisoning cases in Japan and sea foodborne diseases in many Asian countries [13]. A total 802 outbreaks of food-borne diseases have been reported in 13 of the coastal provinces of eastern China, causing more than 17,000 individuals to become ill [14], where *V*. *parahaemolyticus* attributed the most significant number (40.1%) of these cases [15,16]. The leading cause of human gastroenteritis associated with seafood consumption in the United States is *V*. *parahaemolyticus* [17]. Centers for Disease Control and Prevention (CDC) declared it as a significant foodborne bacterium compared to other *Vibrio* species, which was responsible for approximately 34,664 foodborne cases annually in the USA [18].

The food poisoning caused by *V*. *parahaemolyticus* usually occurs in summer and is predominantly associated with different kinds of seafood, including crab, shrimp, shellfish, lobster, fish and oysters [19,20]. *V*. *parahaemolyticus* is usually found in a free-swimming state, with its motility conferred by a single polar flagellum affixed to inert and animate surfaces including zooplankton, fish, shellfish or any suspended matter underwater [21]. Among the whole range of seafood, shellfish is regarded as a high-risk food because it is infested with large populations of bacteria, including *V*. *parahaemolyticus* [22]. Illness is inevitable, once consumers eat undercooked contaminated seafood [23]. The symptoms of the disease include diarrhea, vomiting, abdominal pain, nausea and low-grade fever. In most cases, the disease is self-resolving. However, *V*. *parahaemolyticus* may cause a more debilitating and dysenteric form of gastroenteritis [24]. Uncommonly, in immunocompromised patients, it may progress into a life-threatening fulminant necrotizing fasciitis characterized by rapid necrosis of subcutaneous tissue [25]. In rare cases, *V*. *parahaemolyticus* causes septicemia, which is also associated with a high mortality rate [26]. Also, *V*. *parahaemolyticus* is one of the major pathogens of cultured mud crabs and cause acute hepatopancreaticnecrosis disease (AHPND) in shrimp [27]. Usually, 99% of clinical *V*. *parahaemolyticus* isolates are known to be pathogenic, whereas the majority of the environmental isolates are non-pathogenic [28]. Nonetheless, around 0–6% of the environmental isolates are identified as pathogenic carrying virulence genes [3]. During infection, *V*. *parahaemolyticus* uses the adhesion factors to bind to the fibronectin and phosphatidic acid on the host cell, thus releasing different effectors and toxins into the cytoplasm, causing cytotoxicity and serious diseases [21]. Thermostable direct hemolysin (TDH) and TDH-related hemolysin (TRH) are considered two major virulence factors of this pathogen due to the invasiveness and roles in disease pathogenesis [3]. TDH, which is prevalent in 95% of clinical *V*. *parahaemolyticus* isolates, can lyse red blood cells when secreted [29]. Whole-genome sequencing of *V*. *parahaemolyticus* confirmed that the pathogen possesses two sets of type III secretion system (i.e. T3SS1 and T3SS2) genes [30], where T3SS1 is involved in cytotoxity and T3SS2 is responsible for enterotoxicity [31].

Many antibiotics are no longer effective in hospitals to treat *V*. *Parahaemolyticus* infections [32,33]. First-generation antibiotics, including ampicillin are extensively used in aquaculture resulting in reduced susceptibility andlow efficacy of ampicillin for *Vibrio* sp. treatment [34]. Literature also reported higher resistance to third-generation antibiotics such as cephalosporin, cefotaxime, carbapenemsand ceftazidime by *V*. *parahaemolyticus* isolates [20,35] which enhanced the necessity of searching safe and more effective drugs for combating infections caused by *V*. *parahaemolyticus* in the future. However, the development of new antibiotics is difficult and time-consuming. Recent progress in the field of computational biology and bioinformatics has generated various in silico analysis and drug designing approaches. Thus eliminating the time and cost involved in the early trial phase before going into the drug development phase [36]. Subtractive proteomics is one such *in silico* strategy that helps to facilitate the selection, processing, and development of strain-specific drugs against various pathogens [37]. It can be utilized to identify drug targets based on the determination of essential and nonhomologous proteins within the pathogenic organism [38,39]. The term ‘Druggability’ is used to describe a biological target (e.g. protein) with the potential to bind with high affinity to a drug [40]. The concept is often utilized in drug discovery which reflects the ability of a druggable target to be modulated by small drug-like molecules. Various novel drug targets have already been successfully identified for *S*. *typhi meningitides* sero group B using the mentioned approach [39].

Moreover, *in silico* docking studies between the identified drug targets and existing drugs with slight modification may lead to the discovery of novel drugs for the treatment of infections [41,42]. As a result, a wide range of drug targets and lead compounds can be identified before laboratory experimentation to save time and money. The study was designed to employ a comprehensive genome-based analysis of *Vibrio parahaemolyticus* for identifying novel therapeutic targets as well as suitable drugs and vaccine molecules through subtractive proteomics and vaccinomics approaches.

## Materials and methods

The whole proteome of *V*. *parahemolyticus* was analyzed according to subtractive proteomics approach to recognize novel drug targets as well as vaccine candidates. The overall workflow for subtractive proteomic analysis and vaccinomics approach has been illustrated in results section.

### Retrieval of complete proteome and identification of essential proteins

The whole proteome of *V*. *parahemolyticus*strain was retrieved from NCBI Genome database. Paralogous sequences were excluded from the proteome of *V*. *parahemolyticus* by using CD-HIT [43]. With a cutoff score of 0.6, proteins with more than 60% identity were excluded. Remaining proteins were subjected to BLASTp against *Homo sapiens* human Refseq proteome in ‘Ensemble Genome Database 92’ using threshold expectation value (E- value) 10^−3^ as the parameter. Proteins were assumed as homologous were excluded if any significant hit above the threshold value 10^−4^ was found. The remaining non-homologous proteins were subjected to the Database of Essential Genes (DEG) [44]. Proteins hit with expectation value ≤10–100, identity ≥ 25% were listed for metabolic pathway analysis considering as essential non-homologous proteins of *V*. *parahemolyticus*.

### Analysis of metabolic pathways

Kyoto Encyclopedia of Genes and Genomes (KEGG) which contains complete metabolic pathways present in living organisms [45]. Metabolic pathways of *V*. *parahemolyticus* were analyzed against the human metabolic pathways through the KEGG server. All metabolic pathways present in the pathogen (*V*. *parahemolyticus*) and host (*H*. *sapiens*) were collected from the KEGG PATHWAY database using three letters KEGG organism code ‘vpa’ and ‘has’ respectively. A comparison was made in order to recognize the unique metabolic pathways only present in the *V*. *parahemolyticus*, while the remaining pathways of the pathogen were grouped as a common one. Identified host non-homologous, essential proteins of *V*. *parahemolyticus* were subjected to BLASTp through the KAAS: An Automatic Genome Annotation and Pathway Reconstruction Server at KEEG. Proteins present only in the unique metabolic pathways of the pathogen were listed for further analysis.

### Druggability analysis and identification of novel drug targets

A ‘druggable’ target needs to have the potentiality to bind to the drugs and drug-like molecules with high affinity. Shortlisted unique proteins were screened through the database of DrugBank 5.1.0 [46] using default parameters to identify both druggable proteins and novel therapeutic targets.

### ‘Anti-target’ analysis and prediction of subcellular localization

This analysis was performed to avoid any kind of cross-reactivity, and toxic effects due to docking between the drugs administered for the pathogen and the host ‘anti-targets’.‘Anti-targets’ are gene products that show cross-reactivity with administered therapeutics. Novel drug targets were subjected to BLASTp analysis in the NCBI blast program against these human ‘anti-targets’ setting an E-value <0.005, query length >30%, identity <25% as parameters. Proteins were showing a <25% identity that was listed for subcellular localization analysis. Besides, proteins functioning in cytoplasm can be used as putative drug targets, while surface membrane proteins can be considered both as drug targets and vaccine candidates. PSORTb v3.0.2 CELLO v.2.5), ngLOC servers were used to predict subcellular localization of shortlisted pathogen-specific essential proteins.

### Human microbiome non-homology analysis

Both membrane and cytoplasmic proteins were subjected to BLASTp through NCBI protein blast server against the dataset present in the Human Microbiome Project server (https://www.hmpdacc.org/hmp/) (BioProject-43021) [47] with a cutoff score 0.005. Membrane proteins showing <45% similarity were selected for vaccine candidacy, whereas cytoplasmic proteins showing <45% similarity were selected for protein-protein interaction analysis.

### Analysis of Virulence Factors (VF’s) and Protein-Protein Interactions studies (PPIs)

Virulence factors are responsible for modulating or degrading host defense mechanisms by bacteria. Novel cytoplasmic proteins with the least similarity with the human microbiome were subjected to BLASTp search against the database of protein sequences from the VFDB core dataset [48] with default hit with cut-off bit score >100, and E-value was 0.0001. The protein-protein interactions studies (PPIs) of selected shortlisted proteins were predicted using STRING v10.5 [49]. PPIs with a high confidence score (≥90%) were considered to avoid false-positive results. Only characterized proteins were subjected to BLASTp.

### Screening of drug molecules against novel cytoplasmic proteins

All the pharmaco-metabolites reported in the Human Metabolites Database (www.hmdb.ca) were used for the screening of suitable drugs. Molecular docking was performed against predicted drug targets (novel cytoplasmic proteins) by using AutoDock Vina tools [50]. The size of the grid box was set to 54 A° x 74 A° x 126 A° (x, y and z) and 65A° x 85 A° x 65 A° (x, y and z) with 1 A° spacing between the grid points for two cytoplasmic therapeutic target proteins (Q87TC9 and Q87165 respectively).

### Screening of novel outer membrane proteins for vaccine construction

The VaxiJen v2.0 (http://www.ddg-pharmfac.net/vaxijen/) was used for the investigation of protein immunogenicity to find the most potent antigenic outer membrane proteins [51]. Proteins were prioritized based on their antigenic score (threshold value 0.4) and sequence similarity with human microbiota.

### T-cell epitope prediction, transmembrane topology screening and antigenicity analysis

MHC-I (http://tools.iedb.org/mhci/) and MHC-II prediction tool (http://tools.iedb.org/mhcii/) prediction tool of the Immune Epitope Database were used to predict the MHC-I binding and MHC-II binding peptides, respectively. To predict the transmembrane helices in proteins [52] and to determine epitope antigenicity [51], TMHMM (http://www.cbs.dtu.dk/services/TMHMM/) and VaxiJen v2.0 server (http://www.ddg-pharmfac.net/vaxijen/) were utilized.

### Population coverage, allergenicity, toxicity and conservancy analysis

Population coverage for each epitope was analyzed by the IEDB population coverage calculation tool (http://tools.iedb.org/population/) [53]. The most potent antigenic epitopes were selected and allowed for determining the allergenicity patternvia four servers named AllergenFP [54], AllerTOP (http://www.ddg-pharmfac.net/AllerTop/) [55], Allermatch (http://www.allermatch.org/allermatch.py/form) [56] and Allergen Online [57]. Moreover, the ToxinPred server predicted the toxicity level of the proposed epitopes (http://crdd.osdd.net/raghava/toxinpred/) [58]. The conservancy level determines the efficacy of epitope candidates to confer broad-spectrum immunity. For revealing the conservancy pattern, homologous sequence sets of the selected antigenic proteins were retrieved from the NCBI database by using the BLASTp tool. Further, the epitope conservancy analysis tool (http://tools.iedb.org/conservancy/) at IEDB was selected for the analysis of the conservancy pattern.

### Prediction of 3D structures for superior epitopes and analysis of molecular docking

Top-ranked epitopes were subjected to the PEP-FOLD server to predict peptide structures [59]. Depending on the available structures deposited in Protein Data Bank (PDB) database, HLA-A*11:01 and HLA-DRB1*04:01 were selected for docking analysis with MHC class I and class II binding epitopes respectively. MGLTools were used to visualize and analyze the molecular structures of biological compounds [60]. The grid box was set to 28 A°, 18 A°, 20 A° (x, y and z) with a default value of 1.0 A° spacing by Autodock Vina at 1.00- °A spacing. The exhaustiveness parameter was kept at 8.00, and the number of outputs was set at 10 [60]. Output PDBQT files were converted in PDB format using Open Babel. The docking interaction was visualized with the PyMOL molecular graphics system (https://www.pymol.org/).

### Identification of B-Cell epitope

B-cell epitopes were predicted for both proteins to find the potential antigens that would interact with B lymphocytes and initiate the immune response. Several tools from IEDB i.e. Kolaskar and Tongaonkar antigenicity scale [61], Karplus and Schulz flexibility prediction [62], Bepipred linear epitope prediction analysis [63], Emini surface accessibility prediction [64], Parker hydrophilicity prediction [65] and Chou and Fasman beta-turn prediction [66] were used to identify the B-cell epitopes antigenicity depending on six different algorithms.

### Epitope cluster analysis and vaccine construction

Epitope cluster analysis tool from IEDB was used to identify the epitope clusters with overlapping peptides for both proteins using the top CTL, HTL and BCL epitopes as input. The identified clusters and singletons were further utilized to design construct. Vaccine sequences started with an adjuvant followed by the top CTL epitopes, top HTL epitopes and BCL epitopes respectively for both proteins. Three vaccine constructs i.e. V1, V2 and V3, each associated with different adjuvants including beta-defensin (a 45 mer peptide), L7/L12 ribosomal protein and HABA protein (*Mycobacterium tuberculosis*, accession number: AGV15514.1) [67] were constructed. PADRE sequence and different linkers, for instance, EAAAK, GGGS, GPGPG and KK linkers, were also used to construct effective vaccine molecules.

### Allergenicity, antigenicity and solubility prediction of different vaccine constructs

The AlgPred v.2.0 [68] and AllerTOP v.2.0 [55] servers were utilized to predict the non-allergic behavior of the vaccine constructs. For proposing the superior vaccine candidate, the VaxiJen v2.0 server [51] was utilized. The probable antigenicity of the constructs was determined through an alignment-independent algorithm. Protein-sol software [69] predicted the solubility score of the proposed vaccines.

### Physicochemical characterization and secondary structure analysis

The ProtParam, a tool from Expasy's server (http://expasy.org/cgi-bin/protpraram) [70,71] was used to characterize the vaccine proteins functionally–including molecular weight, isoelectric pH, aliphatic index, hydropathicity, instability index, GRAVY values and estimated half-lifeand other physicochemical properties were investigated. The PSIPRED v3.3 [72] were used to predict the alphahelix, betasheet and coil structure of the vaccine protein. The polar, nonpolar and aromatic regions were also determined.

### Tertiary structure prediction, refinement, validation and disulfide engineering of vaccine construct

The I-TASER server [73] was employed for determining the 3D structure of designed vaccine constructs based on the degree of similarity between the target protein and available template structure from PDB. Refinement was performed using ModRefiner [73]. The refined protein structure was validated through the Ramachandran plot assessment by the MolProbity software [74]. Residues in the highly mobile region of the protein exhibit the potential to be mutated with cysteine. Pairs of residues with propergeometry and the ability to form a disulfide bond were detected by the DbD2 server to perform disulfide engineering [75]. The value of chi3 considered for the residue screening was between −87 to +97 while the energy considered was <2.5.

### Protein-protein docking and molecular dynamics simulation

The binding affinity of the vaccine constructs with different HLA alleles and human immune receptors, ClusPro 2.0 [76], hdoc [77] and PatchDock server [78] were applied. Desirable complexes were identified according to better electrostatic interaction and free binding energy following refinement via the FireDock server [79]. The iMODS server was used to explain the collective motion of proteins via analysis of normal modes (NMA) in internal coordinates [80]. Essential dynamics is a powerful tool and alternative to the costly atomistic simulation that can be compared to the normal modes of proteins to determine their stability [81]. The server predicted the direction and magnitude of the immanent motions of the complex in terms of deformability, eigenvalues, B-factors and covariance. The structural dynamics of the protein-protein complex was investigated [82].

### Codon adaptation, *in silico* cloning and similarity analysis with human proteins

A codon adaptation tool (JCAT) was used to adapt the codon usage to the well-characterized prokaryotic organisms for accelerating the expression rate in it. Rho-independent transcription termination, prokaryote ribosome binding site and cleavage sites of restriction enzyme ApaI and BglI were avoided while using the server [83]. The optimized sequence of vaccine protein V1 was reversed, followed by conjugation with ApaI and BglI restriction site at the N-terminal and C-terminal sites, respectively. SnapGene [84] restriction cloning module was used to insert the adapted sequence into the pET28a(+) vector between ApaI (1334) and BglI (2452). At last, human sequence similarity analysis of the proposed vaccine with human proteins was done by using NCBI protein-protein Blast (https://blast.ncbi.nlm.nih.gov/Blast.cgi), and here blast was done against *Homo sapiens* (taxid: 9606) dataset.

## Results

**Fig 1:**
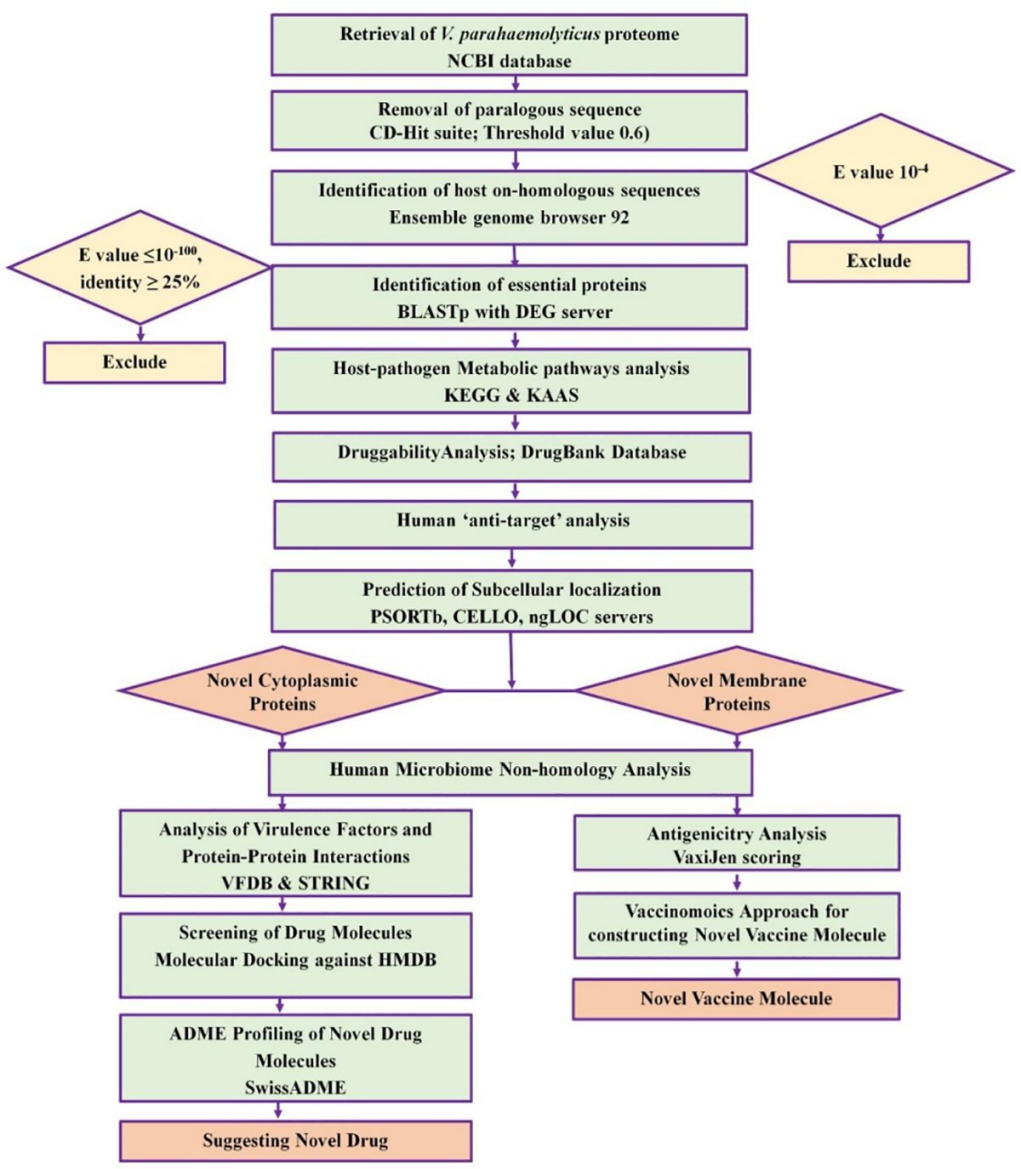
Proteome exploration of *Vibrio parahaemolyticus* to identify novel drug targets.

**Fig 2:**
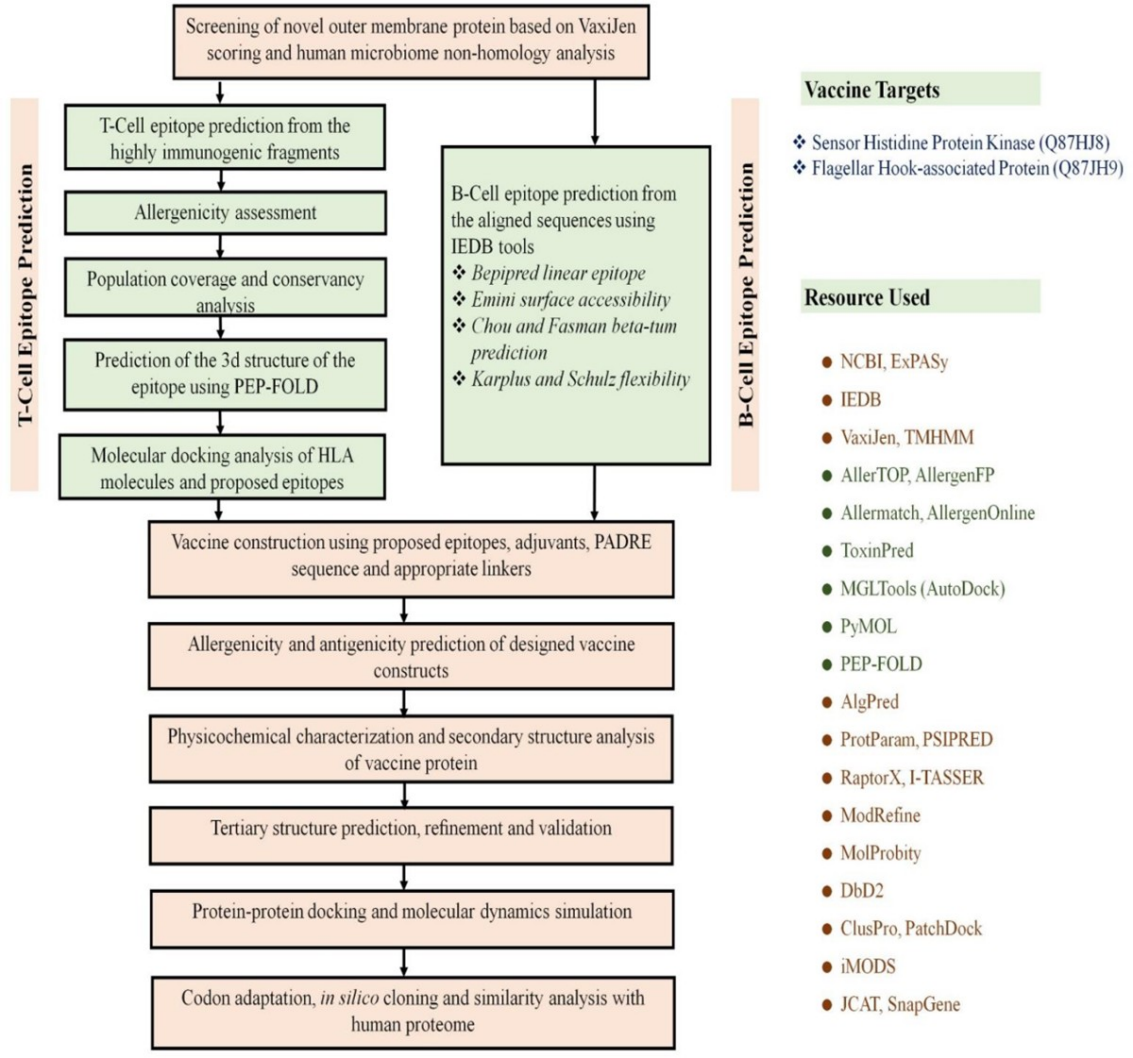
Flow chart summarizing the protocols over multi-epitope subunit vaccine development against *V*. *parahaemolyticus* through reverse vaccinology approach.

Various bioinformatics tools and databases were used to analyze the entire proteome of *V*. *parahemolyticus* through subtractive proteomics and vaccinomics approach. The step by step results (or workflow in Figs 1 and 2) from the complete computational analysis was presented in Table 1.

**Table 1:** Subtractive proteomics analysis scheme.

Sl. No.	Steps	No. of proteins	Protein sets
1	Total number of proteins	4822	Set 0 (S1 File)
2	Non-paralogous (>60% identical) in CD-Hit	4729	Set 1 (S2 File)
3	proteins with >100 amino acids	4123	Set 2 (S3 File)
4	Number of proteins nonhomologous to *H*. *sapiens* using BLASTp (E value 10^−3^)	3164	Set 3 (S4 File)
5	Essential proteins in DEG 15.2 server (E value ≤10^−100^, Bit score >100)	1107	Set 4 (S5 File)
6	Essential Proteins involved only in unique metabolic pathways (KAAS at KEGG)	96	Set 5 (S9 File)
7	Essential proteins found to be novel in DrugBank 5.1.0 (using default parameters)	41	Set 6 (S10 File)
8	Novel drug target proteins non-homologous to ‘anti-targets’ using BLASTp (E value <0.005, Identity <25%, Query length >30%)	41	Set 7 (S11 File)
9	Essential cytoplasmic proteins using PSORTb, CELLO, ngLOC, PSLpred	16	Set 8 (S12 File)
10	Proteins showing <45% similarity with human microflora proteins	9	Set 9 (S13 File)
11	Identified virulence associated novel proteins by VFDB analysis	2	Set 10 (Table 4)
12	Essential membrane proteins using PSORTb, CELLO, ngLOC, PSLpred	25	Set 11 (S14 File)
13	Identified vaccine targets having less similarities with human microflora proteins and antigenicity	2	Set 12 (Table 7)

### Retrieval of complete proteome and identification of essential proteins

The whole proteome of *V*. *parahemolyticus* strain O3:K6 was extracted from NCBI database (S1 File) containing 4822 proteins (Set 0). Paralogous protein sequences of the pathogen. A total of 93 paralogous sequence above >60% similarity were identified through the CD-hit server and removed, leaving 4729 non-paralogous protein sequences in Set 1 (S2 File). Among these proteins, proteins with >100 residues (4123 proteins) (Set 2) were only considered (S3 File) for further analysis. Again, proteins showing significant similarity with human RefSeq proteins (1143 proteins) were excluded from the list designated as Set 3 (S4 File). Analysis of remaining proteins throughthe DEG server revealed only proteins that are essential (Set 4) for the survival of the pathogen (S5 File).

### Analysis of metabolic pathways

The KEGG server contains 131 metabolic pathways for *V*. *parahaemolyticus* (S6 File) and 325 pathways for humans (S7 File). Through manual comparison, 40 metabolic pathways were found to be pathogen-specific and are provided in S8 File. Proteins involved in these unique pathways can be selected as drug targets. Non-homologous essential proteins subjected to BLASTp in the KAAS server at KEGG revealed that 96 proteins among 1107 assigned both KO (KEGG Orthology) and metabolic pathways that further deputed as Set 5 (S9 File).

### Druggability analysis and identification of novel drug targets

Only 56 proteins showed similarity with the available drug targets, while the remaining 41 showed no hit. These 41 proteins (Set 6) were considered as novel drug targets which include both cytoplasmic and membrane proteins (S10 File). Besides, the results of druggable proteins are provided in S1 Table. Furthermore, the other 41 proteins were considered as novel therapeutic targets and subjected to human ‘anti-targets’ analysis.

### ‘Anti-target’ analysis and prediction of subcellular localization

A total of 210 ‘anti-targets’ reported in the literature were fetched from NCBI (S11 File). All novel drug target proteins were successfully screened through BLASTp analysis, and no evidence of similarity was seen. Hence, all these novel drug target proteins were listed for human microbiome analysis considering non-homologous to host ‘anti-targets’ (S10 File). Moreover, The results of subcellular localization analysis by four servers are provided in S11 File. The result revealed that among 41 specific proteins involved in pathogen-specific pathways, 16 were cytoplasmic proteins assigned as Set 8 (S12 File, Table 2), while the remaining 25 sequences were membrane proteins.

**Table 2:** Pathogen specific essential cytoplasmic proteins as novel therapeutic targets.

Sl. No	Protein Id	KO assignment	Description	Pathways
1	Q87L83	K09823	Fur family transcriptional regulator, zinc uptake regulator	Quorum sensing
2	Q87GW9	K07674	Two-component system, narl family, nitrate/nitrite sensor histidine kinase narq	Two-component system
3	Q79YW1	K02410	Flagellar motor switch protein flig	Bacterial chemotaxis
4	Q87HD9	K11904	Type VI secretion system secreted protein vgrg	Bacterial secretion system
5	Q87TC9	K02461	General secretion pathway protein L	Bacterial secretion system
6	Q87NV3	K07780	Arac family transcriptional regulator required for anaerobic and stationary phase induction of genes	Two-component system
7	Q87K78	K07718	Two-component system, sensor histidine kinase yesm	Two-component system
8	Q87I65	K07688	Two-component system, narl family, response regulator, fimbrial Z protein, fimz	Two-component system
9	Q87TD5	K02455	General secretion pathway protein F	Biofilm formation
10	Q87MI1	K03567	Glycine cleavage system transcriptional repressor	Biofilm formation
11	Q87HC5	K11892	Type VI secretion system protein impk	Bacterial secretion system
12	Q87Q12	K02053	Putative spermidine/putrescine transport system permease protein	Quorum sensing
13	Q87LE2	K03092	RNA polymerase sigma-54 factor	Biofilm formation
14	Q87HC6	K11891	Type VI secretion system protein impl	Biofilm formation
15	Q79YX4	K03408	Purine-binding chemotaxis protein chew	Bacterial chemotaxis
16	Q87NG0	K11617	Two-component system, narl family, sensor histidine kinase lias	Two-component system

### Human microbiome non-homology analysis

Cytoplasmic proteins showing <45% similarity with reported human microbiome proteins were selected for protein-protein interaction analysis, whereas membrane proteins were selected for further vaccine candidacy. However, microbiome analysis revealed that a total of 9 proteins (Set 9) of the pathogen showed <45% similarity with human microflora (S2 Table, S13 File).

### Analysis of virulence factors (VF’s) and protein-protein interactions studies (PPIs)

**Fig 3:**
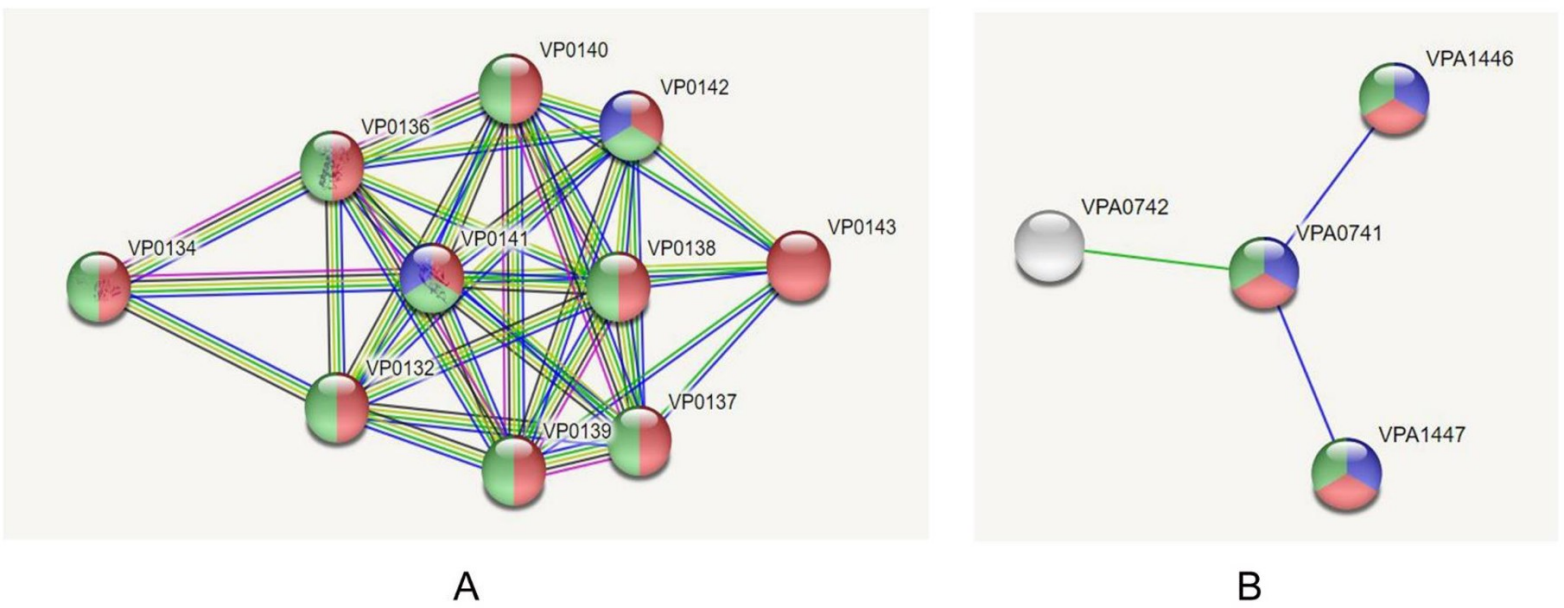
Investigation of PPIs through STRING v10.5 server; (A) UDP-N-acetylmuramoyl-L-alanyl-D-glutamate—2,6-diaminopimelate ligase (murE), (B) Trigger factor (tig).

From 9 cytoplasmic novel proteins, five uncharacterized proteins were removed and the remaining four proteins were considered for VFDV analysis. The VFDB result showed that two proteins (Set 10) i.e., VIBPA Type II secretion system protein L (Q87TC9) and VIBPA Putative fimbrial protein Z (Q87I65) were associated with virulence of *V*. *parahaemolyticus* (Table 3). These proteins were subjected to protein-protein interaction study. STRING v10.5 revealed that Type II secretion system protein L confers interactions with nine proteins (Fig 3A), while putative fimbrial protein Z exhibits interactions with three other proteins (Fig 3B). These proteins are mainly responsible for protein transport, involved in biofilm formation and bacterial secretion system, or act as regulatory proteins (e.g., transcription regulator, signal transduction response regulator).

**Table 3:** Predicted therapeutic targets (novel cytoplasmic proteins) showing virulent properties.

Novel drug targets	Accession ID	VFDB analysis	Interacted proteins
VIBPA Type II secretion system protein L	Q87TC9	5 hits	9
VIBPA Putative fimbrial protein Z	Q87I65	4 hits	3

### Screening of drug molecules against novel cytoplasmic proteins

A total of 335 unique metabolites were retrieved from Human Metabolites Database for docking analysis against predicted therapeutic drug targets (novel cytoplasmic proteins). Docking scores were analyzed to screen the top drug candidates with the lowest binding energy (S3 Table). Among top 10 metabolites (Table 4), Eliglustat (DB09039) was found superior in terms of free binding energy for both protein targets, followed by Simvastatin (DB09039) and Hydroxocobalamin (DB00200) for VIBPA Type II secretion system protein L (Q87TC9) and VIBPA Putative fimbrial protein Z (Q87I65) respectively. ADME analysis was performed to get an insight into how the predicted pharmaceuticals will interact with the body as a whole (Table 5).

**Table 4:** Top 10 metabolites predicted as suitable drug candidates against VIBPA Type II secretion system protein L and VIBPA Putative fimbrial protein Z.

Novel Targets	HMDB ID	Binding Energy	Name	Drug Bank ID	Drug Name	Drug Group
***VIBPA Type II secretion system protein L (Q87TC9)***	HMDB0004971	-10.6	Glucosylceramide (d18:1/16:0)	DB09039	Eliglustat	Approved
	HMDB0004972	-10.5	Glucosylceramide (d18:1/18:0)	DB09039	Eliglustat	Approved
	HMDB0004970	-10.3	Glucosylceramide (d18:1/9Z-18:1)	DB09039	Eliglustat	Approved
	HMDB0004976	-10.3	Glucosylceramide (d18:1/26:1(17Z))	DB09039	Eliglustat	Approved
	HMDB0008646	-10.3	PC(22:4(7Z,10Z,13Z,16Z)/22:4(7Z,10Z,13Z,16Z))	DB00641	Simvastatin	Approved
	HMDB0011300	-10.1	PC(P-18:1(11Z)/P-18:1(11Z)	DB00334	Olanzipine	Approved, Investigational
	HMDB0008443	-9.7	PC(20:4(5Z,8Z,11Z,14Z)/20:4(5Z,8Z,11Z,14Z))	DB00641	Simvastatin	Approved
	HMDB0008138	-9.6	PC(18:2(9Z,12Z)/18:2(9Z,12Z))	DB00641	Simvastatin	Approved
	HMDB0004973	-9.5	Glucosylceramide (d18:1/20:0)	DB09039	Eliglustat	Approved
	HMDB0010348	-9.5	Dehydroepiandrosterone 3-glucuronide	DB01708	Prasterone	Approved, Investigationa, Nutraceutical
***VIBPA Putative fimbrial protein Z (Q87I65)***	HMDB0004972	-9.6	Glucosylceramide (d18:1/18:0)	DB09039	Eliglustat	Approved
	HMDB0002308	-9.5	Hydroxocobalamin	DB00200	Hydroxocobalamin	Approved
	HMDB0004970	-9.3	Glucosylceramide (d18:1/9Z-18:1)	DB09039	Eliglustat	Approved
	HMDB0004971	-9.3	Glucosylceramide (d18:1/16:0)	DB09039	Eliglustat	Approved
	HMDB0002174	-9.1	Cobalamin	DB14098	Cobalamin	Experimental
	HMDB0008646	-8.9	PC(22:4(7Z,10Z,13Z,16Z)/22:4(7Z,10Z,13Z,16Z))	DB00641	Simvastatin	Approved
	HMDB0060546	-8.9	Norbuprenorphine	DB01026	Ketoconazole	Approved, Investigational
	HMDB0041936	-8.7	Morphine-3-glucuronide	DB00295	Morphine	Approved, Investigational
	HMDB0004974	-8.6	Glucosylceramide (d18:1/22:0)	DB09039	Eliglustat	Approved
	HMDB0008443	-8.5	PC(20:4(5Z,8Z,11Z,14Z)/20:4(5Z,8Z,11Z,14Z))	DB00641	Simvastatin	Approved

**Table 5:** ADME profiling of top drug candidates.

ADME analysis		Top drug candidates		
		*Eliglustat*	*Simvastatin*	*Hydroxocobalamin*
***Physicochemical parameters***	Formula	C23H36N2O4	C25H38O5	C62H89CoN13O15P
	Molecular weight	404.54 g/mol	418.57 g/mol	1346.36 g/mol
	Molar Refractivity	120.69	118.47	352.74
	TPSA	74.52 Å^2^	72.83 Å^2^	468.89 Å^2^
***Lipophilicity***	Log *P*_o/w_ (iLOGP)	4.07	3.74	0.00
	Log *P*_o/w_ (XLOGP3)	4.02	4.68	-3.48
	Log *P*_o/w_ (WLOGP)	3.57	4.59	-0.99
	Log *P*_o/w_ (MLOGP)	2.27	3.77	-3.84
	Log *P*_o/w_ (SILICOS-IT)	4.88	3.77	-4.08
	Consensus Log *P*_o/w_	3.76	4.11	-2.48
	Log *P*_o/w_ (iLOGP)	4.07	3.74	0.00
***Pharmacokinetics***	GI absorption	High	High	Low
	BBB permeant	Yes	No	No
	P-gp substrate	No	No	Yes
	CYP1A2 inhibitor	No	No	No
	CYP2C19 inhibitor	No	No	No
	CYP2C9 inhibitor	No	Yes	No
	CYP2D6 inhibitor	Yes	No	No
	CYP3A4 inhibitor	No	Yes	No
	Log *K*_p_ (skin permeation)	-5.91 cm/s	-5.53 cm/s	-16.98 cm/s
***Water Solubility***	Log *S* (SILICOS-IT)	-5.05	-3.56	-7.11
	Solubility	3.57e-03 mg/ml; 8.83e-06 mol/l	1.15e-01 mg/ml; 2.74e-04 mol/l	1.04e-04 mg/ml; 7.73e-08 mol/l
	Class	Moderately soluble	Soluble	Poorly soluble
***Druglikeness***	Bioavailability Score	0.55	0.55	0.17
	Lipinski	Yes; 0 violation	Yes; 0 violation	No; 3 violations: MW>500, NorO>10, NHorOH>5
	Ghose	Yes	Yes	No; 4 violations: MW>480, WLOGP<-0.4, MR>130,
	Veber	No; 1 violation: Rotors>10	Yes	No; 2 violations: Rotors>10, TPSA>140
***Medicinal Chemistry***	Synthetic accessibility http://www.swissadme.ch/index.php	4.76	5.80	10.00
	PAINS	0 alert	0 alert	0 alert
	Brenk	2 alerts: imine_1, imine_2	1 alert: more_than_2_esters	1 alert: phosphor
	Leadlikeness	No; 3 violations: MW>350, Rotors>7, XLOGP3>3.5	No; 2 violations: MW>350, XLOGP3>3.5	No; 2 violations: MW>350, Rotors>7

### Screening of novel outer membrane proteins for vaccine construction

From the 25 novel outer membrane proteins (S14 File) designated as Set 11, two were selected based on the highest antigenicity score and human microbiome analysis to develop a novel chimeric peptide vaccine against *V*. *parahemolyticus* (Table 6). The schematic diagram summarizing the protocol over *in silico* vaccinomics strategy has been elucidated in Fig 1. Sensor histidine protein kinase (Q87HJ8) and flagellar hook-associated protein (Q87JH9) possessed better antigenicity (0.64 and 0.53 respectively) while showed less percentage similarity (<45% and <41%, respectively) when compared with gut microbiome data (S4 Table).

**Table 6:** Novel vaccine targets proteins showing higher antigenicity.

Accession No.	Protein name	VaxiJen score	Similarity with human microbiome (%)
Q87HJ8	Sensor histidine protein kinase UhpB	0.65	<45
Q87JH9	Flagellar hook-associated protein	0.53	<41

### T-cell epitope prediction, transmembrane topology screening and antigenicity analysis

A plethora of CTL and HTL epitopes were identified for both proteins that can bind to the different large number of HLA-A and HLA-B alleles using MHC class-I and MHC class II binding predictions of IEDB (S15 File). Top epitopes (MHC-I and MHC-II binding peptides) for both proteins having the capacity to elicit strong T-cell responses were selected as putative T-cell epitope candidates according to their topology screening by TMHMM and antigenic scoring (AS) by Vaxijen server (S16 File).

### Population coverage, allergenicity, toxicity and conservancy analysis

**Fig 4:**
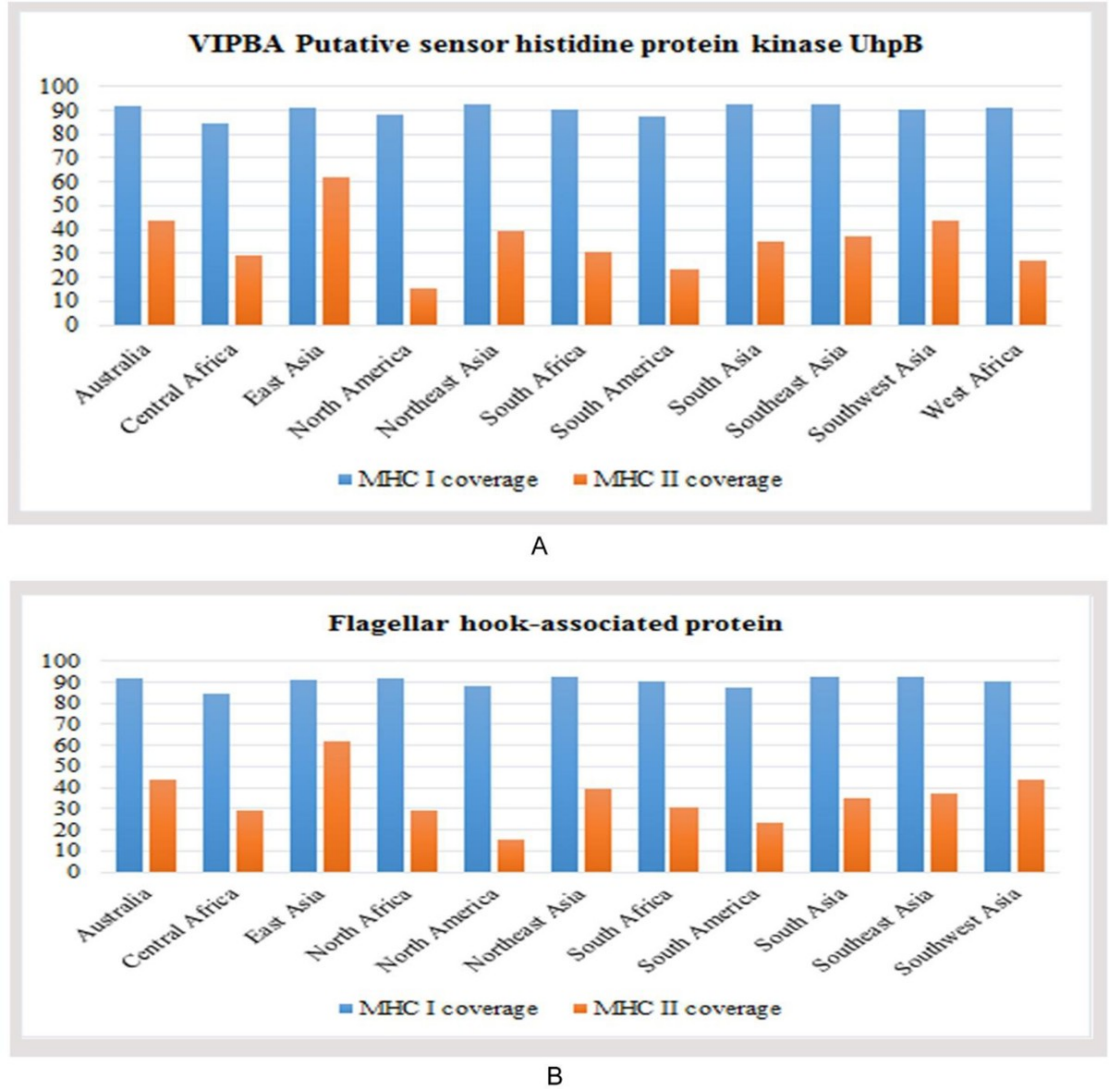
Population coverage analysis of (A) VIPBA putative sensor histidine protein kinase UhpB, and (B) Flagellar hook-associated protein.

Two different population coverages were calculated from CTL and HTL populations for MHC class I and MHC class IIrestricted peptides, respectively (Fig 4). Epitopes, found to be non-allergen for humans, were identified according to the allergenicity assessment via four servers. However, epitopes predicted as a toxin was removed from the proposed list of epitopes. Several epitope candidates from both proteins were found to be highly conserved within different strains of *V*. *parahemolyticus* with maximum conservancy level of 99% for histidine protein kinase and 100% for flagellar hook-associated protein respectively (S5 Table). Top 3 epitopes (CTL and HTL) for each protein were considered based on the above mentioned parameters to design the final vaccine construct (Table 7).

**Table 7:** Predicted final CTL and HTL epitopes of histidine protein kinase and flagellar hook-associated protein.

Protein	MHC	Epitope	Start	End	Vaxijen	Conservancy
Sensor histidine protein kinase UhpB	MHC-I	AILLFPFAL	36	44	2.993	99.00%
		ILLFPFALR	37	45	2.9721	99.00%
		HDDGVGFKV	448	456	2.363	99.00%
	MHC-II	ILLFPFALRLGIALH	37	51	2.1484	87.00%
		AILLFPFALRLGIAL	36	50	1.9451	87.00%
		LLFPFALRLGIALHT	38	52	1.8268	87.00%
Flagellar hook-associated protein	MHC-I	FNAQDEEGH	125	133	1.8425	98.00%
		GGRHNNLDL	234	242	1.7855	99.00%
		KPSPNFQAEV	203	212	1.4978	45.00%
	MHC-II	DSIESSFNAQDEEGH	199	133	1.304	88.00%
		IGGRHNNLDLMDGAH	233	247	0.801	59.00%
		KLSDDPMASIKLLNL	38	52	0.887	88.00%

### Prediction of 3D structures for superior epitopes and analysis of molecular docking

The epitopes, showing conservancy pattern at a biologically significant level, were only allowed for further docking analysis. 3D structures were predicted for top epitopes (6 from Flagellar hook-associated protein and six from sensor histidine protein kinase) to analyze their interactions with different HLA alleles. The PEP-FOLD3 server modeled five 3D structures for each epitope, and the best one was identified for docking study. The result showed that ‘AILLFPFAL’ epitope of Flagellar hook-associated protein was superior in terms of free binding energy while interacted with HLA-A* 11:01 (−8.3 kcal/mol). Demonstrated energy was −9.1 kcal/mol for epitope ‘GGRHNNLDL’ of Sensor histidine protein kinase (Table 8).

**Table 8:** Binding energy of predicted epitopes with selected MHC class I and MHC class II molecules generated from molecular docking by AutoDock.

Protein	Epitope	MHC Class	Binding Energy
Sensor histidine protein kinase	FNAQDEEGH	HLA-A*11:01	-8.6
	GGRHNNLDL		-9.1
	KPSPNFQAEV		-8.2
	DSIESSFNAQDEEGH	HLA-DRB1*04:01	-7.3
	IGGRHNNLDLMDGAH		-6.5
	KLSDDPMASIKLLNL		-6.3
Flagellar hook-associated protein	AILLFPFAL	HLA-A*11:01	-8.3
	ILLFPFALR		-8.1
	HDDGVGFKV		-8.1
	ILLFPFALRLGIALH	HLA-DRB1*04:01	-7.0
	AILLFPFALRLGIAL		-6.4
	LLFPFALRLGIALHT		-7.2

### Identification of B-Cell epitope

B-cell epitopes of both proteins were generated using six different algorithms from IEDB (S1 and S2 Figs). The epitopes were further investigated to reveal their non-allergenicity pattern, and the top one epitope from each prediction was selected for vaccine construction (S6 Table).

### Epitope cluster analysis and vaccine construction

The construction of vaccine protein was based on identifying larger cassettes containing multiple epitopes. Epitope cluster analysis tool from IEDB predicted 21 epitope clusters among the top epitopes (6 CTL, 6 HTL epitopes, and 12 BCL epitopes) proposed in Table 8 and S2 Table. Each vaccine construct was occupied by a protein adjuvant, PADRE peptide sequence, T-cell and B-cell epitopes with their respective linkers (S7 Table) Constructs V1, V2 and V3 were 370, 455 and 484 residues long, respectively. PADRE sequence was used to enhance the potency and efficacy of the peptide vaccine.

### Allergenicity, antigenicity and solubility prediction of different vaccine constructs

**Fig 5:**
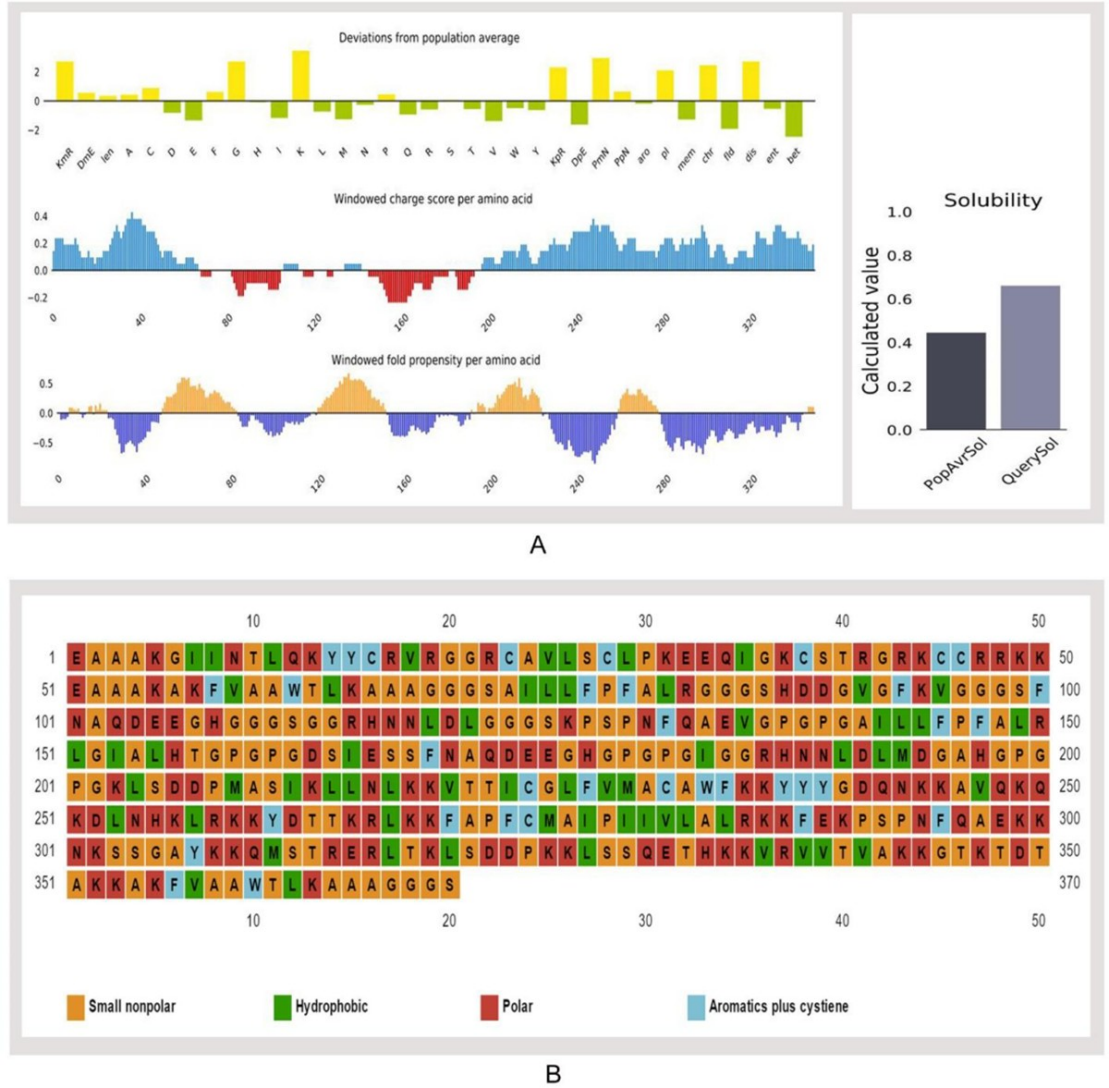
Solubility prediction of vaccine constructs. (A) Solubility prediction of designed vaccine construct V1 using via Protein-sol server, and (B) prediction of polar, nonpolar, hydrophobic and aromatic regions.

Results revealed V1 as the most potent vaccine candidate with better antigenic nature (1.18) and non-allergic behavior that can elicit a strong immune response (S7 Table). All three constructs showed solubility above the threshold value (0.45). Again, construct V1 was superior in terms of solubility potential. The surface distribution of charge, hydrophobicity and stability were calculated at 91 different combinations of pH and ionic strength (Fig 5).

### Physicochemical characterization and secondary structure analysis

The molecular weight of the designed construct V1 was 39.45 kDa. The theoretical pI 9.95 implied that the protein would have a net negative charge above this pI and vice versa. At 0.1% absorption, the extinction coefficient was 26,930, while assuming all cysteine residues reduced. The estimated half-life was predicted to be >10 h in *E*. *coli in vivo*, whereas 1 h within mammalian reticulocytes in vitro. Hydrophilic behavior and thermostability of the protein were represented by the GRAVY value and aliphatic index that were −0.510 and 67.62, respectively. Instability index (37.49) and various physicochemical features classified the protein as a stable onewith the capacity to induce a robust immunogenic reaction in the body. The predicted secondary structure confirmed to have 35.6% alpha helix, 11.89% sheet and 52.43% coil region (S3 Fig). Around 34.59% polar, 16.21% hydrophobic and 9.46% aromatic regions were identified in the structure (Fig 5).

### Tertiary structure prediction, refinement, validation and disulfide engineering of vaccine construct

**Fig 6:**
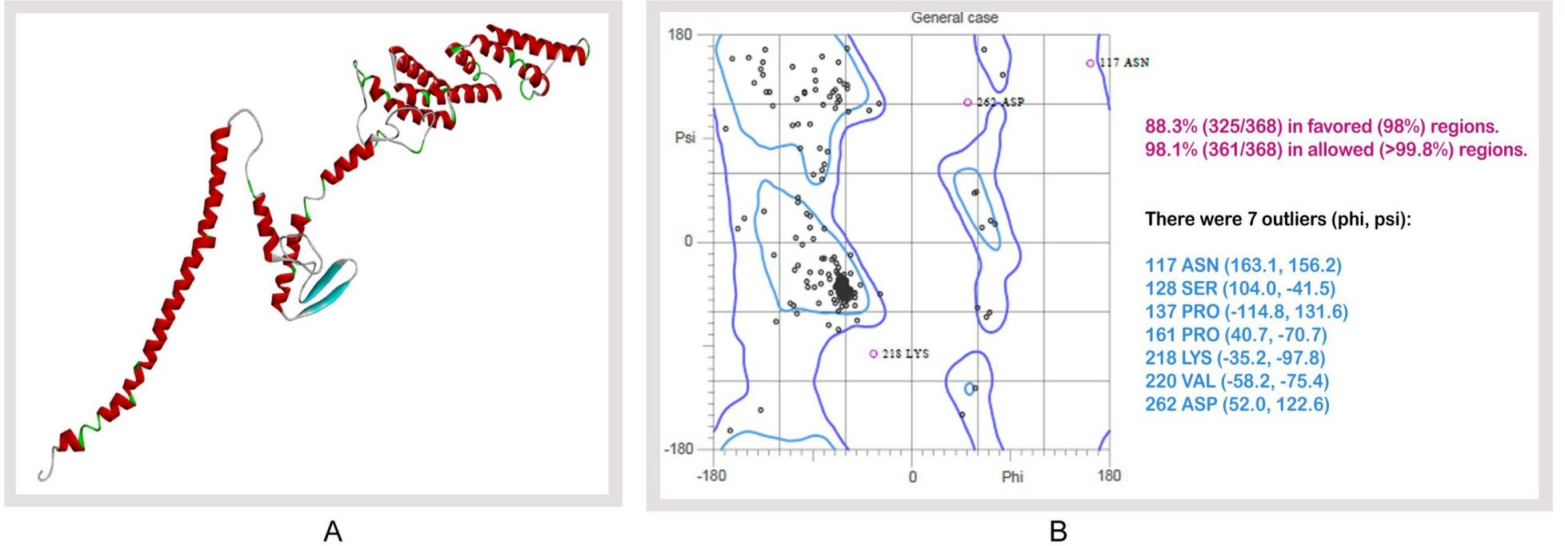
Tertiary structure prediction and validation of vaccine protein V1. (A) Tertiary structure of modeled construct V1, (B) Ramachandran plot analysis of vaccine protein V1.

I-TASSER predicted five models for each proposed vaccine candidates, which were ranked based on cluster size. Ten best templates (with the highest Z-score) selected from the LOMETS threading programswere used to predict the tertiary structures. Homology modeling was performed by using 1kj6 from RCSB Protein Data Bank) as a best suited template for Vaccine protein V1. Results showed that model 1 had the highest C-Score of -2.11 while the estimated TM-score and RMSD were 0.46±0.15 and 11.6±4.5Å (Fig 6). After refinement, 88.3% and 98.1% residues were in the favored and allowed region revealed by Ramachandran plot analysis (Fig 6). The modeled tertiary structure of designed construct V2 and V3 have been shown in S4 Fig. A total of 22 pairs of amino acid residues were identified with the potential to form disulfide bonds by DbD2 server. However, only two pairs (i.e., ARG 82-Gly 85, Lys 347-Thr 350) were compatible with disulfide bond formation considering the energy, chi3 and B-factor parameter (S5 Fig). All these residues were replaced with a cysteine residue.

### Protein-protein docking and molecular dynamics simulation

**Fig 7:**
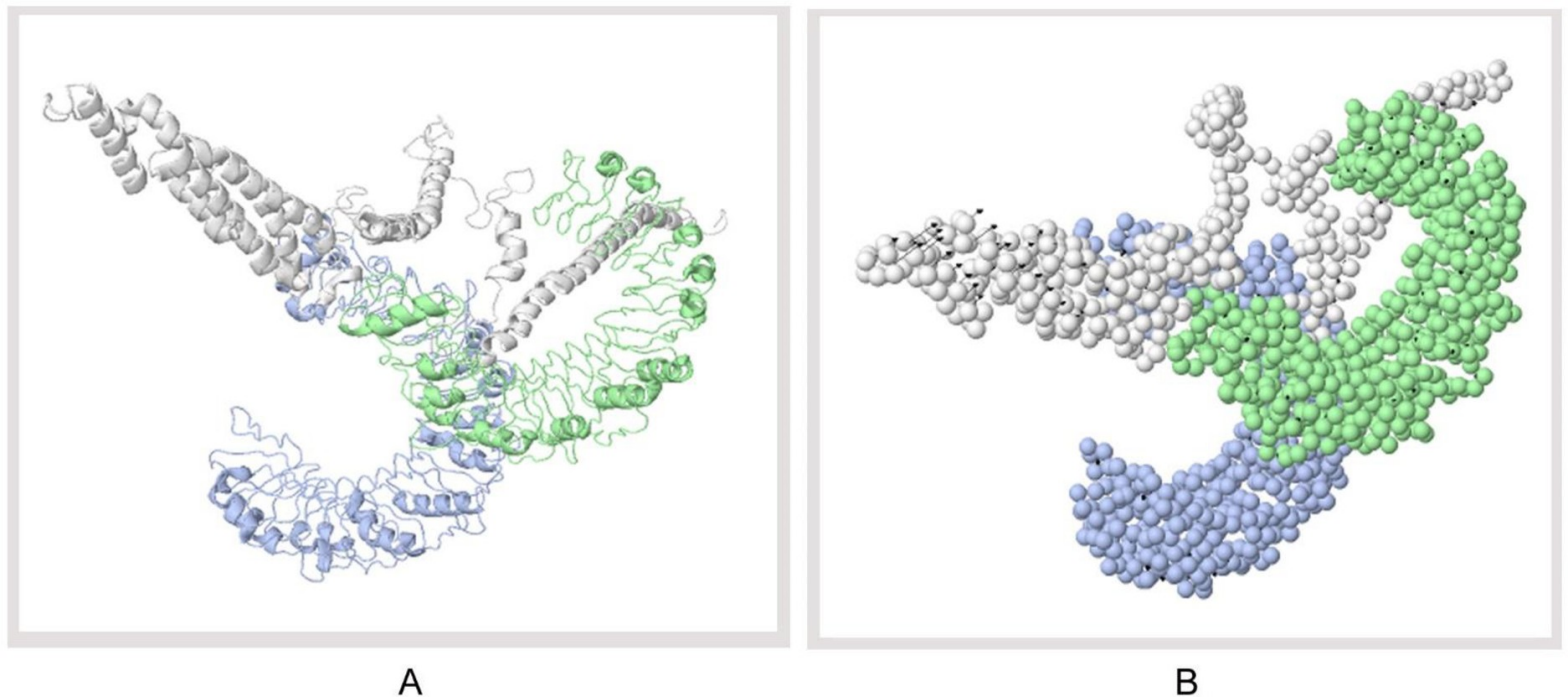
Docked complex of vaccine construct V1 with human TLR 1/2 heterodimer. (A) Cartoon format, and (B) Ball structure.

**Fig 8:**
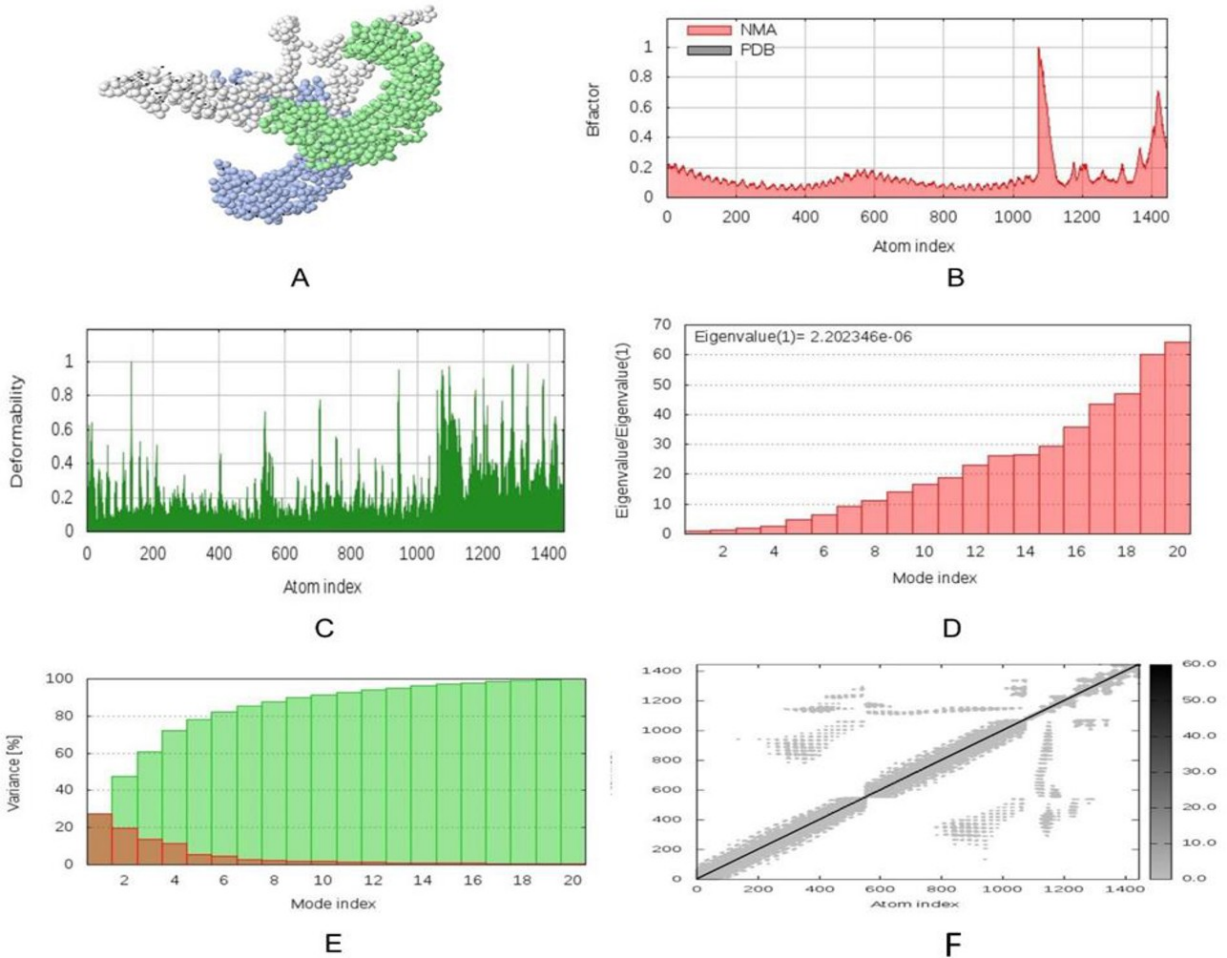
Molecular dynamics simulation of vaccine protein V1-TLR8 complex. Stability of the protein-protein complex was investigated through (A) mobility, (B) B-factor, (C) deformability, (D) eigenvalue, (E) covariance and (F) elastic network analysis.

Docking study was conducted between three vaccine constructs (i.e., V1, V2, V3) and different HLA alleles. Construct V1 showed biologically significant results and found to be superior in terms of free binding energy (S8 Table). Besides, the binding affinity of the predicted vaccine and TLR-1/2 heterodimer complex was also analyzed. The 3D structure of human TLR-1/2 heterodimer was retrieved from the RCSB protein data bank. ClusPro generated thirty protein-ligand complexes (clusters) as output along with respective free binding energy. The lowest energy was −1257.9 for cluster 1 (Fig 7). FireDock output refinement of the PatchDock server showed the lowest global energy of −7.08 for solution 5. Normal mode analysis allowed the demonstration of large scale mobility and the stability of proteins. The analysis was performed based on the internal coordinates of the protein-protein complex. In the 3D model, the direction of each residue was given by arrows, and the length of the line represented the extent of mobility (Fig 8A). The eigenvalue found for the complex was 2.4784e−05 (Fig 8B). The vaccine protein V1 and TLR1-2 heterodimers were oriented towards each other. The B-factor values deduced from normal mode analysis was analogous to RMS (Fig 8C). Hinges in the chain indicated the probable deformability of the complex measured by the contortion of each residue (Fig 8D). The variance associated with each normal mode was inversely linked to the eigenvalue. Covariance matrix explained the coupling between pairs of residues was correlated, uncorrelated, or anti-correlated motions were represented via red, white and blue colors, respectively (Fig 8E). The result also generated an elastic network model (Fig 8F) that identified the pairs of atoms connected via springs. Each dot in the diagram was indicated one spring between the corresponding pair of atoms and colored based on the degree of stiffness.

### Codon adaptation, *in silico* cloning and similarity analysis with human proteins

**Fig 9:**
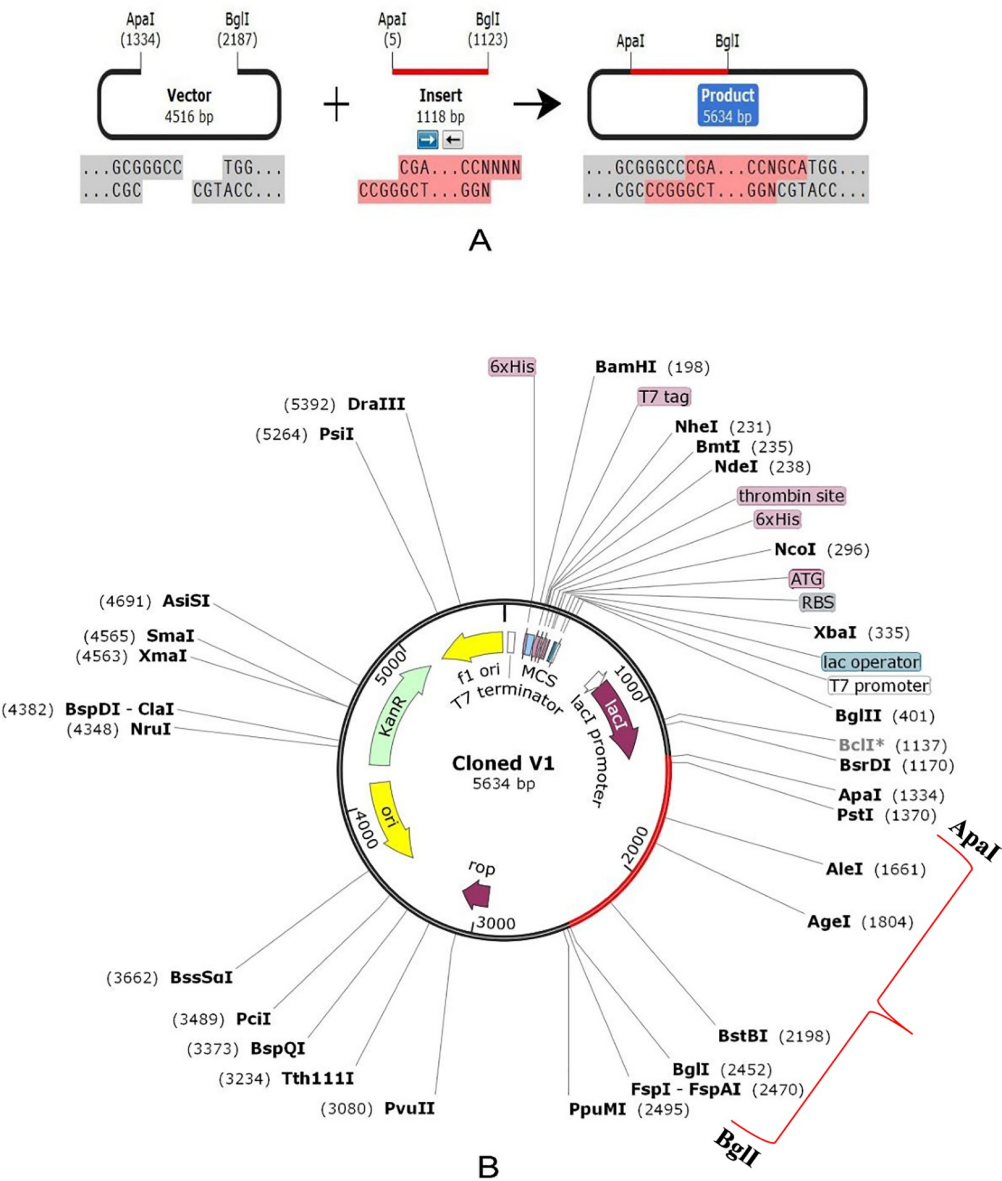
*In silico* restriction cloning of the gene sequence of construct V1 into pET28a(+) expression vector; (A) Restriction digestion of the vector pET28a(+) and construct V1 with BglI and ApaI (B) Inserted desired fragment (V1 Construct) between ApaI (1334) and BglI (2452) indicated in red color.

*E*. *coli* strain K12 was selected as the host for the cloning purpose of the vaccine construct V1. Vaccine protein V1 was transcribed reversely, where the Codon Adaptation Index (CAI) was found 0.994, and the GC content of the optimized codons (50.55%) was also significant. The construct did not hold restriction sites for ApaI and BglI, which ensured its safety for cloning purposes. The optimized codons were incorporated into the pET28a(+) vector along with ApaI and BglI restriction sites. A clone of 5634 base pair was obtained, including the 1118 bp desired sequence and the rest belonging to the vector. The desired region was shown in red color in between the pET28a(+) vector sequence (Fig 9). Sequence similarity analysis of the proposed vaccine with human proteins revealed that there was no similarity between predicted vaccine constructs and human proteins.

## Discussion

Emergence of rapid antibiotic resistance [35,85], severe effects on human health [14,86], and economic importance for substantially impairing the aquaculture production [4] have made it necessary to identify effective drug targets and vaccine candidates against *V*. *parahaemolyticus*. Different computational approaches are now being widely practiced to identify proteins those are essential for the survival of the pathogen and not involved in the metabolic pathways of the host, thereby choosing the proteins associated only in the metabolic pathways of the pathogen is important [38,87]. Essential proteins are most promising for new antibacterial drug targets since most antibiotics are designed to bind essential gene products. Here, subtractive genome approaches (removal of paralogous proteins, identification of non-homologous proteins against the host, identification of essential proteins and metabolic pathways analysis of the pathogen), and vaccinomics strategy were employed for identifying novel drug and vaccine molecules through the comprehensive proteome exploration of *V*. *parahaemolyticus* genome.

The complete proteome of *V*. *parahaemolyticus* (4822 proteins) was retrieved from the NCBI database, and the homologous proteins were removed based on their identity with human proteins. Proteins encoded by essential genes and unique to an organism can be considered as species-specific drug targets, as they play vital roles in its metabolism. The study revealed 96 unique, essential proteins (Set 5) of *V*. *parahaemolyticus*, which can be considered as suitable drug targets for combating *V*. *parahaemolyticus* infections. Among the unique proteins, 55 proteins were druggable and can be targeted using existing drugs (92) that are already approved and available in the market (S1 Table). In the case of a broad-spectrum drug, for avoiding mutational changes as well as the emergence of resistant bacteria, the DrugBank databases were screened, which contains entries for 2556 approved small molecule drugs, 962 approved biotech drugs, 112 nutraceuticals and over 5125 experimental drugs. A total of 41 proteins of *V*. *parahaemolyticus* showed no similarity after passing through the DrugBank database and listed for the prediction of novel drug targets and vaccine candidates (Set 6). To avoid severe cross-reaction and toxic effects in human, identification of nonhomologous proteins to essential human proteins (referred to as ‘anti-targets’) was a crucial step considered in this study. However, the identified novel drug targets (41) showed no evidence of similarity with the ‘anti-targets’. Although both cytoplasmic and membrane proteins serve the purpose as therapeutic targets [86], membrane proteins are best suited for vaccine candidates [88,89]. Hence, in this study, membrane proteins (25) were used for vaccine construction, whereas cytoplasmic proteins (16) were proposed as suitable drug targets. Targeting human microbiome non-homology proteins is suitable since drugs or vaccines designed and administered for these targets will be less harmful to other commensal microbial strains. Among the novel cytoplasmic proteins (16), only the proteins (9) conferring <45% similarity with human microbiota were retained. The VFDB (virulence factor database) analysis studies confirmed that ‘VIBPA Type II secretion system protein L (Q87TC9)’ and ‘VIBPA Putative fimbrial protein Z (Q87I65)’ were associated with virulence in the host (Set 10). The protein-protein interaction studies also strengthened the superiority of these two proteins as suitable drug targets (Table 4).

Pharmacologically active metabolites can be conveniently used as leads during the lead optimization phase of drug discovery [90–92]. Many drugs are converted to metabolites that can retain the intrinsic affinity of the parent drug for the pharmacological target. The contribution of active drug metabolites efficacy is relative to the contribution of the parent drug, target affinity, functional activity and plasma protein binding [93]. In this study, molecular docking of 350 human metabolites against ‘VIBPA Type II secretion system protein L’ and ‘VIBPA Putative fimbrial protein Z’ was conducted to screen superior drug molecules (S2 Table). The study revealed that ‘Eliglustat (DB09039)’ was the top drug candidate for both protein targets in terms of free binding energy (Table 4). Therefore, it can be suggested as a suitable drug to treat infections caused by *V*. *parahaemolyticus*. Eliglustat was first approved in August 2014 by FDA for the treatment of Gaucher's disease [94]. The compound belongs to the class of ‘enzyme inhibitors’ which is believed to work by inhibition of glucosylceramide synthase [95,96]. Eliglustat prevents the formation certain fatty substance in the body which may causes liver, spleen, bone, and blood problems. Mistry et al. reported that oral substrate reduction therapy by Eliglustat resulted in significant improvements in platelet count, liver volume, hemoglobin level and spleen volume, when compared with untreated adults with Gaucher disease [97]. Hydroxocobalamin (vitamin B12), on the contrary, is often used as dietary supplement to treat pernicious anemia, cyanide poisoning, toxic amblyopia and Leber's optic atrophy [98,99]. However, antibacterial activity of vitamin B12 is not new. There is also experimental evidence of synergistic antimicrobial effects of vitamin B12 with other antibiotics [100]. In the present study Hydroxocobalamin showed significantly higher binding affinity to VIBPA Putative fimbrial protein Z (Table 4). Therefore, it can be used as supplement with other drugs to combat *V*. *Parahemolyticus* associated infections. Simvastatin (belongs to the group of medicines called statins) is another approved drug which is often used to treat patients with high blood cholesterol. It is also prescribed against rheumatoid arthritis, type-1 or type-2 diabetes [101,102], and to prevent strokes and heart attacks [103,104].

Recently, the possibility of Simvastatin as a therapeutic option for COVID-19 was reported due to its ability to block the key factors required for infectivity [105,106]. Usually, it is considered to be a very safe medicine and unusual to have side effects [107]. However, cautions should be taken for the repurposed use of these drugs to minimize the risk of potential toxicity.

Moreover, drug profiling (Physicochemical parameters, Lipophilicity, Pharmacokinetics, Water Solubility, Druglikeness, Medicinal Chemistry) of ‘Eliglustat (DB09039)’ along with other top candidates, i.e., ‘Simvastatin (DB09039)’ and ‘Hydroxocobalamin (DB00200)’ were also performed through ADME analysis (Table 5), which showed no undesirable effects that could reduce their drug-likeness properties.

Several advantages help the researchers to select membrane proteins both as drug and vaccine candidates as their functions can be easily studied through computer-based approaches than wet-lab process [89,108]. In this study, two vaccine targets, ‘Sensor histidine protein kinase UhpB (Q87HJ8)’ and ‘Flagellar hook-associated protein (Q87JH9)’ were selected after screening the novel outer-membrane proteins (25) based on their antigenicity score and human microbiome non-homology analysis (Table 6). Both the proteins further analyzed to design a potent, highly immunogenic vaccine candidate against *V*. *parahaemolyticus*. Numerous antigenic epitopes were generated which were investigated extensively for antigenicity, allergenicity, toxicity, conservancy and other physiochemical properties using a number of bioinformatics tools and software. The final vaccine constructs were designed with the help of different adjuvants and amino acid linkers [84]. It has been reported that the PADRE sequence reduces the polymorphism of HLA molecules in the population [108,109]. Linkers in vaccines also enhanced the immunogenicity of the vaccines in previous studies [110,111]. Therefore, all the important that could induce the immunogenicity of the designed vaccine constructs were taken. Also, disulfide engineering was employed to enhance the stability of the designed vaccine. The purpose of the molecular docking analysis was to show the proposed epitopes could interact with at least one MHC molecule at minimum binding energy. Therefore, it was done to explore the binding affinity of promiscuous epitopes with different HLA alleles including HLA-DRB1*03:01 (1A6A), (HLA-DRB5*01:01 (1H15), HLA-DRB3*01:01 (2Q6W), HLA-DRB1*04:01 (2SEB), HLA-DRB1*01:01 (2FSE), and HLA-DRB3*02:02 (3C5J). The OmpU, one of the major outer membrane porins of *V*. *parahaemolyticus*, is recognized by the Toll-like receptor 1/2 (TLR-1/2) heterodimer in THP-1 monocytes [112]. So, a docking study was also performed to analyze the affinity between the designed construct and human TLR-1/2 heterodimer. The vaccine receptor complex showed deformability at a minimum level, which also strengthened our prediction. Finally, the optimized codons of the designed construct been cloned into the pET28a(+) vector of *E*. *coli* strain K12.

The idea of subtractive genomic analysis using various bioinformatics tools has brought a revolution in the drug discovery process. The present study will help to develop novel therapeutics and preventive measures against *V*. *parahaemolyticus*, thereby help to reduce the mortality and morbidity caused by it. However, further *in vivo* trials using model organisms are highly recommended to validate our prediction.

## Supporting information

S1 Fig B-cell epitope prediction of Sensor histidine protein kinase UhpB (A: Linear, B: Beta-turn, C: Flexibility, D: Surface Accessibility, E: Antigenicity, F: Hydrophilicity). For each graph, X-axis and Y-axis represent the position and score. Residues that fall above the threshold value are shown in yellow color while the highest peak in yellow color identifies most favored position.
(PPTX)

S2 Fig B-cell epitope prediction of Flagellar hook-associated protein (A: Linear, B: Beta-turn, C: Flexibility, D: Surface Accessibility, E: Antigenicity, F: Hydrophilicity). For each graph, X-axis and Y-axis represent the position and score. Residues that fall above the threshold value are shown in yellow color while the highest peak in yellow color identifies most favored position.
(PPTX)

S3 Fig Secondary structure prediction of constructed vaccine protein V1 using PESIPRED server. (PPTX)

S4 Fig 3D modeled structure of vaccine protein. (A) V2 and (B) V3 generated via RaptorX server.
(PPTX)

S5 Fig Disulfide engineering of vaccine protein V1. (A) Initial form, (B) Mutant form.
(PPTX)

S1 Table Pathway dependent metabolic proteins with druggable properties. (DOCX)

S2 Table Predicted binding energy (docking score) of novel cytoplasmic proteins with human metabolites. (DOCX)

S3 Table Antigenicity of novel membrane proteins (Vaccine targets) and similarity analysis with human microbiome. (DOCX)

S4 Table Antigenicity and similiarity analysis of novel outer membrane proteins with human microbiome (%). (DOCX)

S5 Table Allergenicity pattern and toxicity analysis of top epitopes for Sensor histidine protein kinase and Flagellar hook-associated protein. (DOCX)

S6 Table Allergenicity assessment of the predicted B-cell epitopes generated from histidine protein kinase and flagellar hook-associated protein. (DOCX)

S7 Table Allergenicity, antigenicity and solubility prediction of the constructed vaccines. (DOCX)

S8 Table Docking score of vaccine construct V1, V2 and V3 with different HLA alleles. (DOCX)

S1 File All protein sequences. (DOCX)

S2 File Non-paralogous (>60% identical) in CD-Hit. (DOCX)

S3 File Protein sequences with >100 amino acids. (DOCX)

S4 File Number of proteins nonhomologous to *H. sapiens* using BLASTp (E value 10−3). (DOCX)

S5 File Essential proteins in DEG 15.2 server (E value ≤10−100, Bit score >100). (DOCX)

S6 File Metabolic pathways for *V. parahaemolyticus* in KEGG server. (DOCX)

S7 File Metabolic pathways for humansin KEGG server. (DOCX)

S8 File Pathogen-specific (40) metabolic pathways. (DOC)

S9 File Essential proteins involved only in unique metabolic pathways (KAAS at KEGG). (DOCX)

S10 File Essential proteins found to be novel in DrugBank 5.1.0 (using default parameters). (DOCX)

S11 File Novel drug target proteins non-homologous to ‘anti-targets’ using BLASTp. (DOCX)

S12 File Essential cytoplasmic proteins using PSORTb, CELLO, ngLOC, PSLpred. (DOCX)

S13 File Proteins showing <45% similarity with human microflora proteins. (DOCX)

S14 File Essential membrane proteins using PSORTb, CELLO, ngLOC, PSLpred. (DOCX)

S15 File Predicted MHC-I epitopes for VIBPA Putative sensor histidine protein kinase UhpB. (DOCX)

S16 File Top MHC-I epitopes of VIBPA Putative sensor histidine protein kinase UhpB. (DOCX)
