# Supplementary material for: Comprehensive genome based analysis of Vibrio parahaemolyticus for identifying novel drug and vaccine molecules: Subtractive proteomics and vaccinomics approach
Source: PLoS One. 2020 Aug 19;15(8):e0237181. doi: 10.1371/journal.pone.0237181 (PMC7444560; doi:10.1371/journal.pone.0237181)
Supplement: S1 Fig — B-cell epitope prediction of Sensor histidine protein kinase UhpB (A: Linear, B: Beta-turn, C: Flexibility, D: Surface Accessibility, E: Antigenicity, F: Hydrophilicity). For each graph, X-axis and Y-axis represent the position and score. Residues that fall above the threshold value are shown in yellow color while the highest peak in yellow color identifies most favored position. (PPTX) [file pone.0237181.s001.pptx]

## Slide 1
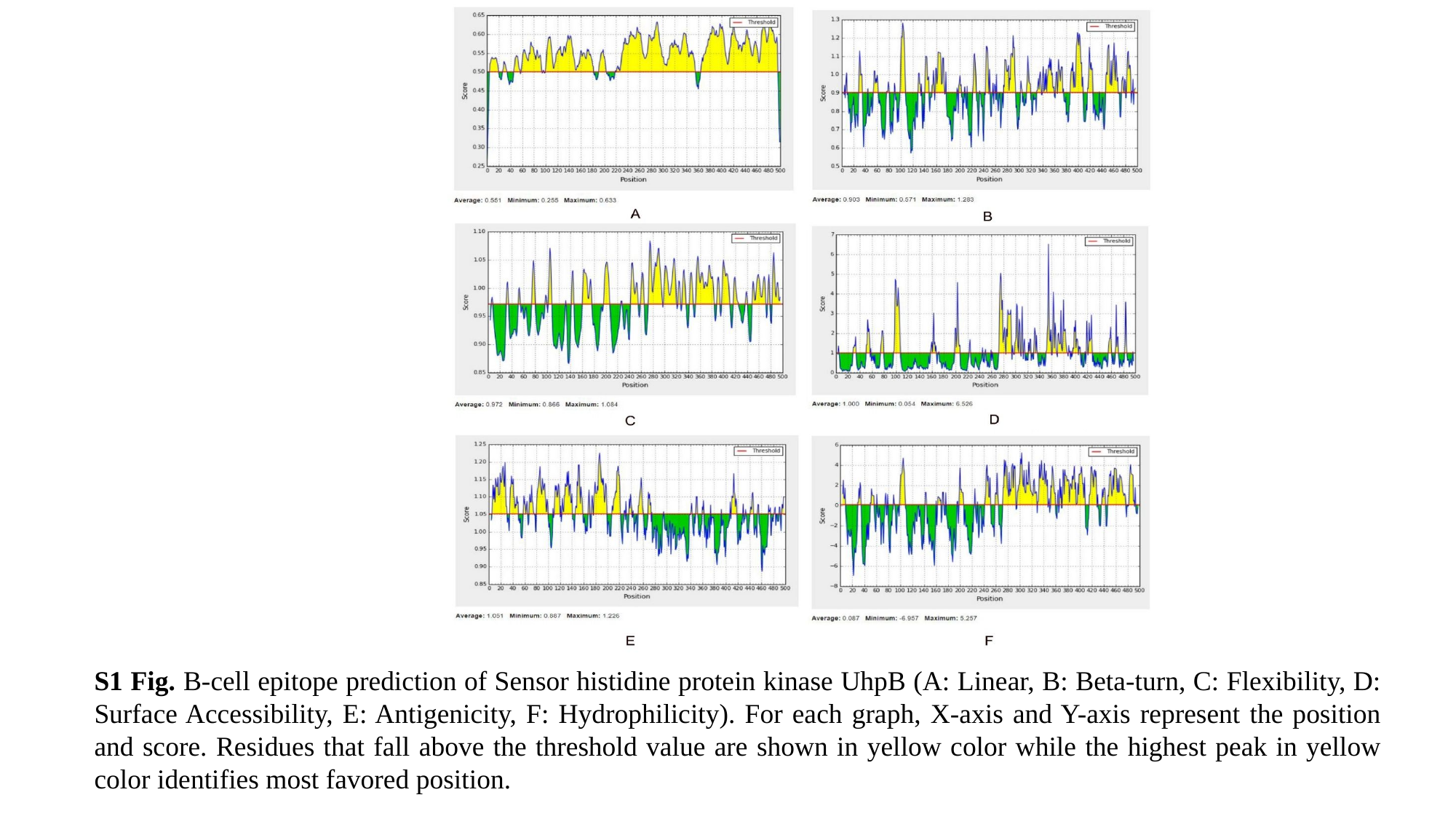

S1 Fig. B-cell epitope prediction of Sensor histidine protein kinase UhpB (A: Linear, B: Beta-turn, C: Flexibility, D: Surface Accessibility, E: Antigenicity, F: Hydrophilicity). For each graph, X-axis and Y-axis represent the position and score. Residues that fall above the threshold value are shown in yellow color while the highest peak in yellow color identifies most favored position.
