# Supplementary material for: Comprehensive genome based analysis of Vibrio parahaemolyticus for identifying novel drug and vaccine molecules: Subtractive proteomics and vaccinomics approach
Source: PLoS One. 2020 Aug 19;15(8):e0237181. doi: 10.1371/journal.pone.0237181 (PMC7444560; doi:10.1371/journal.pone.0237181)
Supplement: S3 Fig — (PPTX) [file pone.0237181.s003.pptx]

## Slide 1
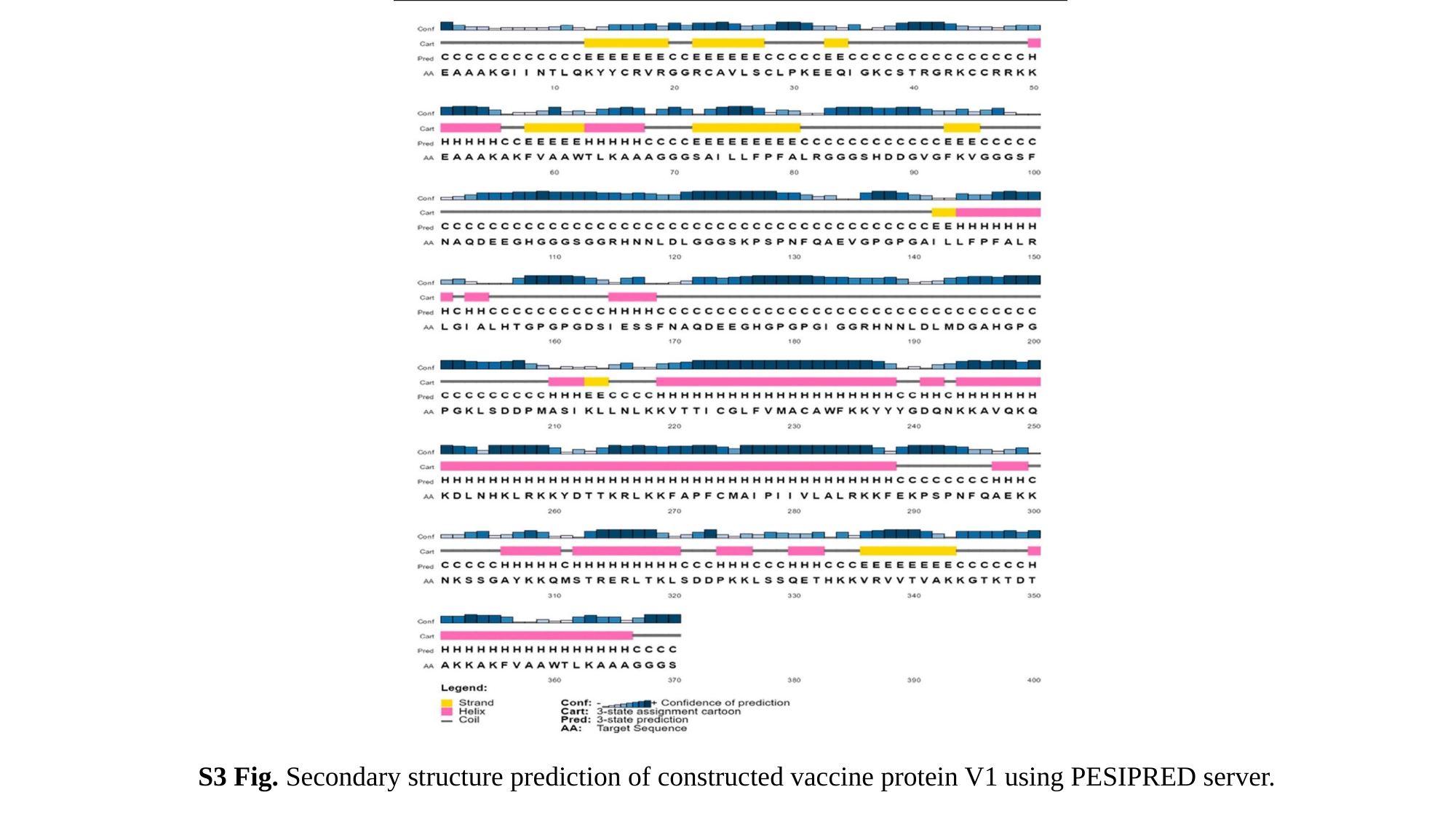

S3 Fig. Secondary structure prediction of constructed vaccine protein V1 using PESIPRED server.
