# Supplementary figures and images for: Comprehensive genome based analysis of Vibrio parahaemolyticus for identifying novel drug and vaccine molecules: Subtractive proteomics and vaccinomics approach
Source: PLoS One. 2020 Aug 19;15(8):e0237181. doi: 10.1371/journal.pone.0237181 (PMC7444560; doi:10.1371/journal.pone.0237181)

## Slide 1
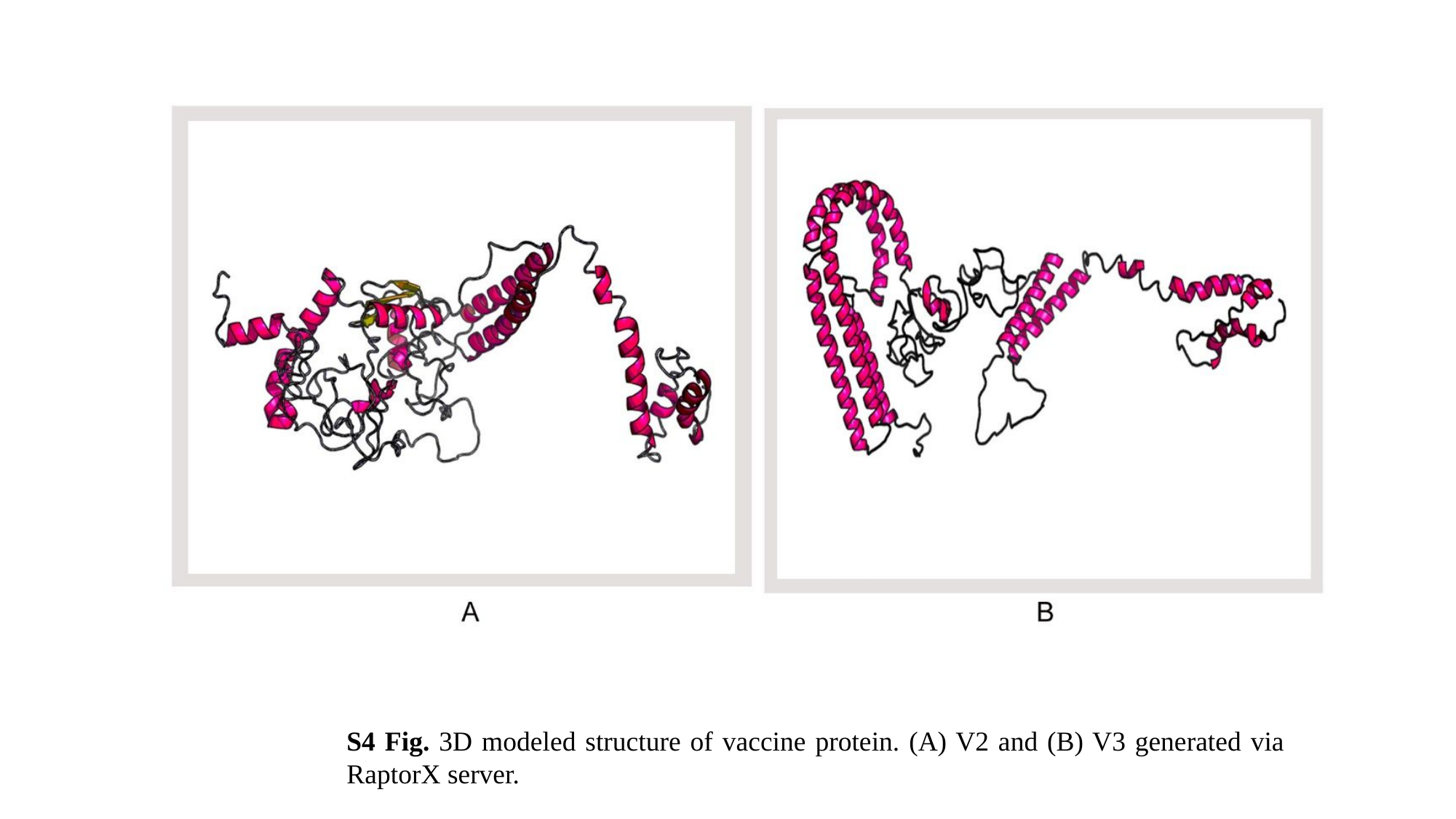

S4 Fig. 3D modeled structure of vaccine protein. (A) V2 and (B) V3 generated via RaptorX server.

Supplement: S4 Fig — (A) V2 and (B) V3 generated via RaptorX server. (PPTX) [file pone.0237181.s004.pptx]

## Slide 1
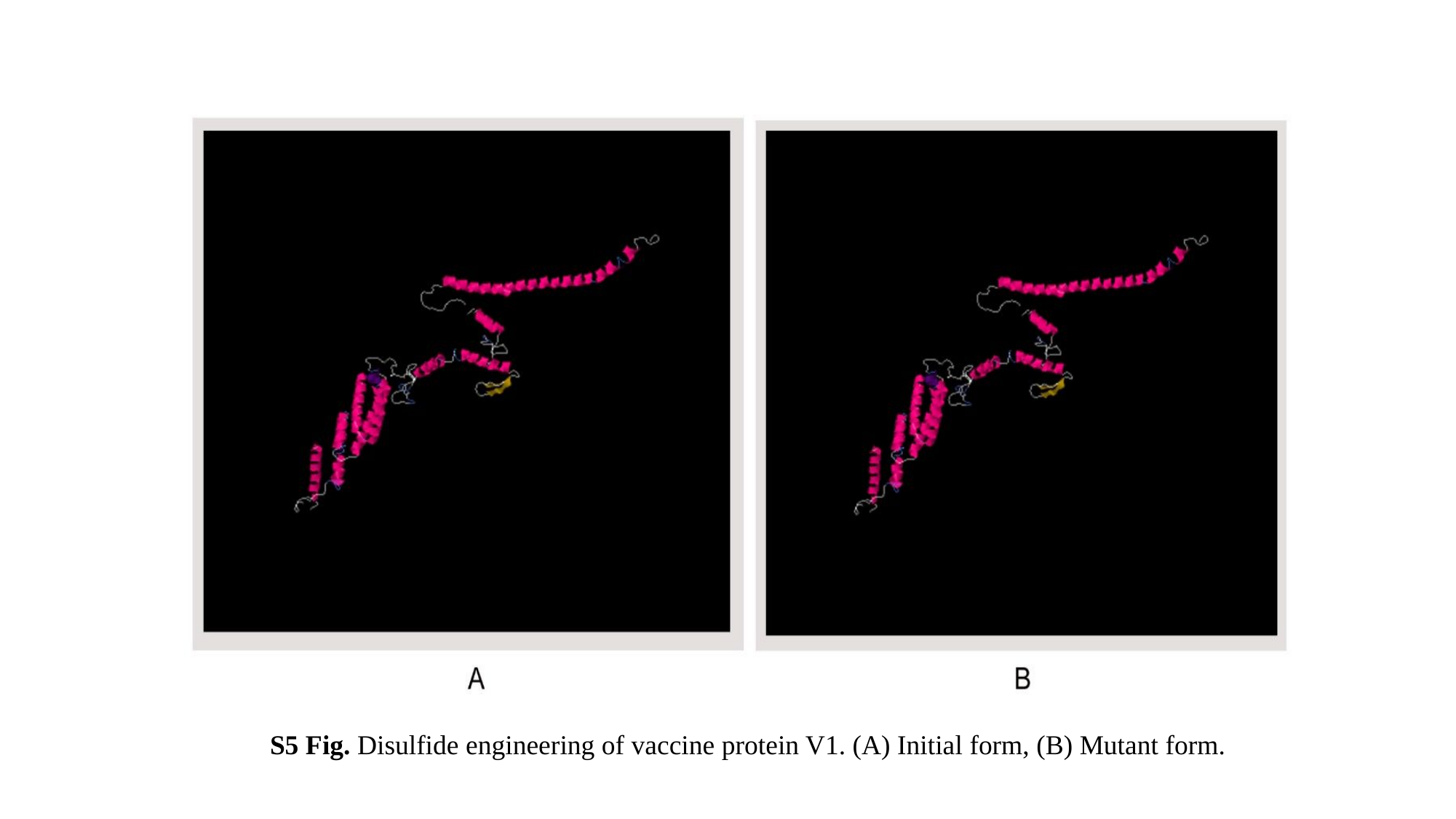

S5 Fig. Disulfide engineering of vaccine protein V1. (A) Initial form, (B) Mutant form.

Supplement: S5 Fig — (A) Initial form, (B) Mutant form. (PPTX) [file pone.0237181.s005.pptx]
