# Supplementary material for: Comprehensive genome based analysis of Vibrio parahaemolyticus for identifying novel drug and vaccine molecules: Subtractive proteomics and vaccinomics approach
Source: PLoS One. 2020 Aug 19;15(8):e0237181. doi: 10.1371/journal.pone.0237181 (PMC7444560; doi:10.1371/journal.pone.0237181)
Supplement: S1 Table — (DOCX) [file pone.0237181.s006.docx]

**Supplementary Tables**

**S1 Table.** Pathway dependent metabolic proteins with druggable properties.

| **Accession**  **Id** | **Pathway involved** | **Drug bank id** | **Drug name** | **E value** |
| --- | --- | --- | --- | --- |
| Q87T41 | Biosynthesis of antibiotics | DB03161 | Thymidine-5'-Diphospho-Beta-D-Xylose | 6.48766e-14 |
|  |  | DB03751 | 2'deoxy-Thymidine-5'-Diphospho-Alpha-D-Glucose |  |
| Q87LD8 | Phosphotransferase system | DB01899 | Nd1-Phosphonohistidine | 6.74952e-08 |
| Q87GN8 | Two-component system | DB04395 | Phosphoaminophosphonic Acid-Adenylate Ester | 1.72082e-18 |
| Q79YV7 | Two-component system | DB02671 | 1-Methylimidazole | 2.24743e-12 |
|  |  | DB03366 | Imidazole |  |
| Q87R58 | Two-component system | DB09462 | Glycerin | 6.34798e-11 |
| Q87GX4 | beta-Lactam resistance | DB09462 | Glycerin | 4.25465e-42 |
| Q87RK0 | Phosphotransferase system | DB08357 | 1-Ethoxy-2-(2-Ethoxyethoxy)Ethane | 3.64017e-78 |
| Q87MK4 | Two-component system | DB02524 | 2',3'-O-{4-[Hydroxy(oxido)-λ5-azanylidene]-2,6-dinitro-2,5-cyclohexadiene-1,1-diyl}adenosine 5'-(tetrahydrogen triphosphate) | 1.75895e-105 |
|  |  | DB03909 | Adenosine-5'-[Beta, Gamma-Methylene]Triphosphate |  |
|  |  | DB04395 | Phosphoaminophosphonic Acid-Adenylate Ester |  |
| Q79YV8 | Two-component system | DB01857 | Phosphoaspartate | 7.12121e-92 |
| Q87I33 | Microbial metabolism in diverse environments | DB01676 | Trinitrotoluene | 3.17226e-103 |
|  |  | DB02060 | Cyclohexanone |  |
|  |  | DB03247 | Flavin mononucleotide |  |
|  |  | DB03651 | Picric acid |  |
|  |  | DB04528 | 2,4-Dinitrophenol |  |
|  |  | DB07373 | Androsta-1,4- Diene-3,17-Dione |  |
|  |  | DB02508 | Isopentyl Pyrophosphate |  |
|  |  | DB11090 | Potassium nitrate |  |
| Q87GN9 | Two-component system | DB09462 | Glycerin | 3.29994e-07 |
| Q87RQ5 | Beta-Lactam resistance | DB01326 DB00923 | Cefamandole Ceforanide | 3.70867e-68 |
| Q87QE7 | Two-component system | DB02355 | Adenosine-5'-Rp-Alpha-Thio-Triphosphate | 3.22519e-09 |
|  |  | DB02596 | Alpha,Beta-Methyleneadenosine-5'- |  |
|  |  | DB07706 | Triphosphate 2-Hydroxyestradiol |  |
| Q87FT7 | Microbial metabolism in diverse environments | DB03247 | Flavin mononucleotide | 2.93774e-98 |
|  |  | DB03793 | Benzoic Acid |  |
| Q79YX1 | Two-component system | DB09462 | Glycerin | 9.53705e-09 |
| Q87SD5 | Two-component system | DB01857 | Phosphoaspartate | 2.98609e-09 |
| Q87NJ3 | Folate biosynthesis | DB01942 | Formic Acid | 3.87202e-178 |
| Q87H50 | Biofilm formation | DB01972 | Guanosine-5'-Monophosphate | 3.60354e-26 |
| Q87SQ6 | Phosphotransferase system | DB08357 | 1-Ethoxy-2-(2-Ethoxyethoxy)Ethane | 5.27414e-78 |
| Q87FY2 | Two-component system | DB01857 | Phosphoaspartate | 2.44477e-78 |
| Q87QT0 | Two-component system | DB02355 | Adenosine-5'-Rp-Alpha-Thio-Triphosphate | 9.6571e-23 |
|  |  | DB02596 | Alpha,Beta-Methyleneadenosine-5'-Triphosphate |  |
|  |  | DB07706 | 2-Hydroxyestradiol |  |
| Q87K89 | Two-component system | DB02355 | Adenosine-5'-Rp-Alpha-Thio-Triphosphate | 7.00351e-35 |
|  |  | DB02596 | Alpha,Beta-Methyleneadenosine-5'-Triphosphate |  |
|  |  | DB07706 | 2-Hydroxyestradiol |  |
| Q87LQ8 | Biofilm formation | DB08874 | Fidaxomicin | 6.79493e-67 |
| Q79YZ2 | Bacterial chemotaxis | DB02461 | S-Methyl Phosphocysteine 3-Aminosuccinimide | 2.39514e-07 |
|  |  | DB03487 | Aspartate Beryllium |  |
|  |  | DB04156 | Trifluoride |  |
| Q87TF1 | Two-component system | DB01857 | Phosphoaspartate | 0.0 |
| Q87PF5 | Two-component system | DB02355 | Adenosine-5'-Rp-Alpha-Thio-Triphosphate | 8.10461e-33 |
|  |  | DB02596 | Alpha,Beta-Methyleneadenosine-5'-Triphosphate |  |
|  |  | DB07706 | 2-Hydroxyestradiol |  |
| Q87P07 | Two-component system | DB01857 | Phosphoaspartate | 1.93619e-07 |
| Q87TN0 | Beta-Lactam resistance | DB09462 | Glycerin | 9.07529e-20 |
| Q87N38 | Two-component system | DB02355 | Adenosine-5'-Rp-Alpha-Thio-Triphosphate | 8.70626e-06 |
|  |  | DB02596 | Alpha,Beta-Methyleneadenosine-5'-Triphosphate |  |
|  |  | DB07706 | 2-Hydroxyestradiol |  |
| Q87H65 | Two-component system | DB04395 | Phosphoaminophosphonic Acid-Adenylate Ester | 1.61933e-07 |
| Q87L60 | Biofilm formation | DB03793 | Benzoic Acid | 7.34907e-131 |
| Q87KT8 | Biofilm formation | DB01972 | Guanosine-5'-Monophosphate | 3.51963e-28 |
| Q87H96 | Biofilm formation | DB01972 | Guanosine-5'-Monophosphate | 5.15207e-21 |
| Q87G70 | Biofilm formation | DB03793 | Benzoic Acid | 5.53374e-08 |
| Q87P19 | Biofilm formation | DB03142 | Alpha-L-Arabinose | 7.45968e-10 |
|  |  | DB04062 | Beta-D-Fucose |  |
| Q87GX0 | Two-component system | DB02355 | Adenosine-5'-Rp-Alpha-Thio-Triphosphate | 5.03724e-06 |
|  |  | DB02596 | Alpha,Beta-Methyleneadenosine-5'-Triphosphate |  |
|  |  | DB07706 | 2-Hydroxyestradiol |  |
| Q87SA3 | Phosphotransferase system | DB08357 | 1-Ethoxy-2-(2-Ethoxyethoxy)Ethane | 0.0 |
| Q87LW1 | Peptidoglycan biosynthesis | DB01329 | Cefoperazone | 0.0 |
|  |  | DB00430 | Cefpiramide |  |
|  |  | DB00438 | Ceftazidime |  |
|  |  | DB09050 | Ceftolozane |  |
| Q87LZ0 | Peptidoglycan biosynthesis | DB00760 | Meropenem | 4.1457e-104 |
|  |  | DB01329 | Cefoperazone |  |
|  |  | DB01331 | Cefoxitin |  |
|  |  | DB01328 | Cefonicid |  |
|  |  | DB00303 | Ertapenem |  |
|  |  | DB04570 | Latamoxef |  |
|  |  | DB00417 | Phenoxymethylpenicillin |  |
| Q87TB5 | Two-component system | DB04395 | Phosphoaminophosphonic Acid-Adenylate Ester | 1.89214e-141 |
| Q87R82 | Two-component system | DB02671 | 1-Methylimidazole | 3.37532e-25 |
|  |  | DB03366 | Imidazole |  |
| Q87TM1 | Quorum sensing | DB03374 | 3,5-Diiodotyrosine | 1.06301e-15 |
| Q87SH0 | Beta-Lactam resistance | DB01598 | Imipenem | 2.19002e-36 |
|  |  | DB01329 | Cefoperazone |  |
|  |  | DB01327 | Cefazolin |  |
|  |  | DB01163 | Amdinocillin |  |
|  |  | DB01328 | Cefonicid |  |
|  |  | DB01413 | Cefepime |  |
|  |  | DB01415 | Ceftibuten |  |
|  |  | DB00948 | Mezlocillin |  |
|  |  | DB00438 | Ceftazidime |  |
|  |  | DB00303 | Ertapenem |  |
|  |  | DB06211 | Doripenem |  |
| Q87IT0 | Beta-Lactam resistance | DB04233 | (Hydroxyethyloxy)Tri(Ethyloxy)Octane | 1.48083e-11 |
|  |  | DB07084 | N-(6,7,9,10,17,18,20,21-octahydrodibenzo[b,k][1,4,7,10,13,16] hexaoxacyclooctadecin-2-yl)acetamide |  |
|  |  | DB13092 | Meclocycline |  |
| Q87G68 | Two-component system | DB02365 | 1,10-Phenanthroline | 3.05491e-08 |
| Q87HG5 | Bacterial chemotaxis | DB02365 | 1,10-Phenanthroline | 7.39316e-13 |
| Q87G84 | Two-component system | DB02671 | 1-Methylimidazole | 5.05136e-06 |
|  | Two-component system | DB03366 | Imidazole |  |
| Q87NX1 | Quorum sensing | DB02451 | B-nonylglucoside | 9.12499e-09 |
| Q87QR1 | Bacterial chemotaxis | DB02365 | 1,10-Phenanthroline | 1.83281e-14 |
| Q87KZ7 | Bacterial chemotaxis | DB02365 | 1,10-Phenanthroline | 2.0555e-14 |
| Q87G25 | Bacterial chemotaxis | DB02365 | 1,10-Phenanthroline | 5.82633e-15 |
| Q87TN1 | Beta-Lactam resistance | DB03825 | Rhodamine 6G | 1.21402e-137 |
|  |  | DB04209 | Dequalinium |  |
|  |  | DB03619 | Deoxycholic Acid |  |
| Q87IW5 | Two-component system | DB02365 | 1,10-Phenanthroline | 3.83631e-13 |
| Q87QG4 | Bacterial chemotaxis | DB02365 | 1,10-Phenanthroline | 2.14253e-08 |
| Q87K74 | Bacterial chemotaxis | DB02365 | 1,10-Phenanthroline | 7.72671e-13 |
