# Supplementary material for: Comprehensive genome based analysis of Vibrio parahaemolyticus for identifying novel drug and vaccine molecules: Subtractive proteomics and vaccinomics approach
Source: PLoS One. 2020 Aug 19;15(8):e0237181. doi: 10.1371/journal.pone.0237181 (PMC7444560; doi:10.1371/journal.pone.0237181)
Supplement: S2 Table — (DOCX) [file pone.0237181.s007.docx]

**S2 Table.** Predicted binding energy (docking score) of novel cytoplasmic proteins with human metabolites

| **Protein** | **HMDB ID** | **Energy** | **Protein** | **HMDB ID** | **Energy** |
| --- | --- | --- | --- | --- | --- |
| ***VIBPA Type II secretion system protein (Q87TC9)*** | HMDB0004971 | -10.6 | ***VIBPA Putative fimbrial protein Z (Q87I65)*** | HMDB0004972 | -9.6 |
|  | HMDB0004972 | -10.5 |  | HMDB0002308 | -9.5 |
|  | HMDB0004970 | -10.3 |  | HMDB0004970 | -9.3 |
|  | HMDB0004976 | -10.3 |  | HMDB0004971 | -9.3 |
|  | HMDB0008646 | -10.3 |  | HMDB0002174 | -9.1 |
|  | HMDB0011300 | -10.1 |  | HMDB0008646 | -8.9 |
|  | HMDB0008443 | -9.7 |  | HMDB0060546 | -8.9 |
|  | HMDB0008138 | -9.6 |  | HMDB0041936 | -8.7 |
|  | HMDB0004973 | -9.5 |  | HMDB0004974 | -8.6 |
|  | HMDB0010348 | -9.5 |  | HMDB0008443 | -8.5 |
|  | HMDB0002308 | -9.4 |  | HMDB0010348 | -8.5 |
|  | HMDB0060824 | -9.4 |  | HMDB0004973 | -8.4 |
|  | HMDB0001993 | -9.3 |  | HMDB0008376 | -8.4 |
|  | HMDB0002961 | -9.3 |  | HMDB0008138 | -8.3 |
|  | HMDB0004974 | -9.3 |  | HMDB0010321 | -8.3 |
|  | HMDB0042005 | -9.3 |  | HMDB0010339 | -8.3 |
|  | HMDB0000077 | -9.2 |  | HMDB0041917 | -8.3 |
|  | HMDB0004246 | -9.2 |  | HMDB0000063 | -8.2 |
|  | HMDB0007934 | -9.2 |  | HMDB0041937 | -8.1 |
|  | HMDB0000015 | -9.1 |  | HMDB0003033 | -8 |
|  | HMDB0000234 | -9.1 |  | HMDB0004969 | -8 |
|  | HMDB0008376 | -9.1 |  | HMDB0008036 | -8 |
|  | HMDB0001926 | -9 |  | HMDB0008172 | -8 |
|  | HMDB0004969 | -9 |  | HMDB0060947 | -8 |
|  | HMDB0000063 | -8.9 |  | HMDB0000015 | -7.9 |
|  | HMDB0000374 | -8.9 |  | HMDB0000374 | -7.9 |
|  | HMDB0000626 | -8.9 |  | HMDB0000626 | -7.9 |
|  | HMDB0010339 | -8.9 |  | HMDB0004976 | -7.9 |
|  | HMDB0041936 | -8.9 |  | HMDB0011300 | -7.9 |
|  | HMDB0004975 | -8.8 |  | HMDB0004975 | -7.8 |
|  | HMDB0011334 | -8.8 |  | HMDB0008070 | -7.8 |
|  | HMDB0000253 | -8.7 |  | HMDB0012458 | -7.8 |
|  | HMDB0001032 | -8.7 |  | HMDB0000518 | -7.7 |
|  | HMDB0002103 | -8.7 |  | HMDB0001375 | -7.7 |
|  | HMDB0004979 | -8.7 |  | HMDB0011334 | -7.7 |
|  | HMDB0008172 | -8.7 |  | HMDB0000016 | -7.6 |
|  | HMDB0014440 | -8.7 |  | HMDB0000637 | -7.6 |
|  | HMDB0000016 | -8.6 |  | HMDB0002103 | -7.6 |
|  | HMDB0000518 | -8.6 |  | HMDB0000067 | -7.5 |
|  | HMDB0000637 | -8.6 |  | HMDB0009637 | -7.5 |
|  | HMDB0002174 | -8.6 |  | HMDB0000037 | -7.4 |
|  | HMDB0008070 | -8.6 |  | HMDB0000053 | -7.4 |
|  | HMDB0060546 | -8.6 |  | HMDB0000077 | -7.4 |
|  | HMDB0001170 | -8.5 |  | HMDB0001032 | -7.4 |
|  | HMDB0008036 | -8.5 |  | HMDB0001926 | -7.4 |
|  | HMDB0010321 | -8.5 |  | HMDB0001993 | -7.4 |
|  | HMDB0041937 | -8.5 |  | HMDB0002961 | -7.4 |
|  | HMDB0003550 | -8.4 |  | HMDB0014440 | -7.4 |
|  | HMDB0000561 | -8.3 |  | HMDB0042005 | -7.4 |
|  | HMDB0003033 | -8.3 |  | HMDB0000153 | -7.3 |
|  | HMDB0004977 | -8.3 |  | HMDB0000234 | -7.3 |
|  | HMDB0006736 | -8.3 |  | HMDB0000253 | -7.3 |
|  | HMDB0009637 | -8.3 |  | HMDB0001170 | -7.3 |
|  | HMDB0012458 | -8.3 |  | HMDB0001452 | -7.3 |
|  | HMDB0000067 | -8.2 |  | HMDB0001830 | -7.3 |
|  | HMDB0001903 | -8.2 |  | HMDB0001903 | -7.3 |
|  | HMDB0000053 | -8.1 |  | HMDB0004977 | -7.3 |
|  | HMDB0000908 | -8.1 |  | HMDB0004979 | -7.3 |
|  | HMDB0001452 | -8.1 |  | HMDB0006278 | -7.3 |
|  | HMDB0004659 | -8.1 |  | HMDB0007934 | -7.3 |
|  | HMDB0010369 | -8.1 |  | HMDB0060824 | -7.3 |
|  | HMDB0001830 | -8 |  | HMDB0000319 | -7.2 |
|  | HMDB0001425 | -7.9 |  | HMDB0000145 | -7.1 |
|  | HMDB0041917 | -7.9 |  | HMDB0001980 | -7.1 |
|  | HMDB0060676 | -7.9 |  | HMDB0004659 | -7.1 |
|  | HMDB0000319 | -7.8 |  | HMDB0001314 | -7 |
|  | HMDB0000852 | -7.8 |  | HMDB0001547 | -7 |
|  | HMDB0001547 | -7.8 |  | HMDB0060676 | -7 |
|  | HMDB0006278 | -7.8 |  | HMDB0000058 | -6.9 |
|  | HMDB0010404 | -7.8 |  | HMDB0000151 | -6.9 |
|  | HMDB0000054 | -7.7 |  | HMDB0010404 | -6.9 |
|  | HMDB0001980 | -7.7 |  | HMDB0000054 | -6.8 |
|  | HMDB0002869 | -7.7 |  | HMDB0001425 | -6.8 |
|  | HMDB0000037 | -7.6 |  | HMDB0003424 | -6.8 |
|  | HMDB0000153 | -7.6 |  | HMDB0000908 | -6.7 |
|  | HMDB0000706 | -7.6 |  | HMDB0041867 | -6.7 |
|  | HMDB0006335 | -7.6 |  | HMDB0004246 | -6.6 |
|  | HMDB0000145 | -7.5 |  | HMDB0041818 | -6.6 |
|  | HMDB0000151 | -7.5 |  | HMDB0000706 | -6.5 |
|  | HMDB0001375 | -7.5 |  | HMDB0029898 | -6.5 |
|  | HMDB0041867 | -7.5 |  | HMDB0000195 | -6.4 |
|  | HMDB0000305 | -7.4 |  | HMDB0014967 | -6.4 |
|  | HMDB0000918 | -7.4 |  | HMDB0000248 | -6.3 |
|  | HMDB0000265 | -7.3 |  | HMDB0000265 | -6.3 |
|  | HMDB0001893 | -7.3 |  | HMDB0001220 | -6.3 |
|  | HMDB0010370 | -7.3 |  | HMDB0015372 | -6.3 |
|  | HMDB0000430 | -7.2 |  | HMDB0000095 | -6.2 |
|  | HMDB0014967 | -7.1 |  | HMDB0000852 | -6.2 |
|  | HMDB0060538 | -7.1 |  | HMDB0006335 | -6.2 |
|  | HMDB0001314 | -7 |  | HMDB0060538 | -6.2 |
|  | HMDB0006725 | -7 |  | HMDB0000133 | -6.1 |
|  | HMDB0009331 | -7 |  | HMDB0000258 | -6.1 |
|  | HMDB0013302 | -7 |  | HMDB0001438 | -6.1 |
|  | HMDB0041818 | -7 |  | HMDB0002869 | -6.1 |
|  | HMDB0000058 | -6.9 |  | HMDB0003337 | -6.1 |
|  | HMDB0001220 | -6.8 |  | HMDB0001893 | -6 |
|  | HMDB0010368 | -6.8 |  | HMDB0008714 | -6 |
|  | HMDB0015532 | -6.8 |  | HMDB0000045 | -5.9 |
|  | HMDB0015372 | -6.8 |  | HMDB0000299 | -5.9 |
|  | HMDB0014488 | -6.7 |  | HMDB0000939 | -5.9 |
|  | HMDB0001238 | -6.6 |  | HMDB0002028 | -5.9 |
|  | HMDB0005453 | -6.6 |  | HMDB0003333 | -5.9 |
|  | HMDB0061040 | -6.6 |  | HMDB0000430 | -5.8 |
|  | HMDB0000248 | -6.5 |  | HMDB0002886 | -5.8 |
|  | HMDB0000763 | -6.5 |  | HMDB0014703 | -5.8 |
|  | HMDB0000929 | -6.5 |  | HMDB0006725 | -5.7 |
|  | HMDB0008714 | -6.5 |  | HMDB0013302 | -5.7 |
|  | HMDB0013609 | -6.5 |  | HMDB0010369 | -5.6 |
|  | HMDB0000939 | -6.4 |  | HMDB0012110 | -5.6 |
|  | HMDB0001438 | -6.4 |  | HMDB0000296 | -5.5 |
|  | HMDB0006461 | -6.4 |  | HMDB0000767 | -5.5 |
|  | HMDB0029898 | -6.4 |  | HMDB0013609 | -5.5 |
|  | HMDB0000197 | -6.3 |  | HMDB0000378 | -5.4 |
|  | HMDB0002028 | -6.3 |  | HMDB0000561 | -5.4 |
|  | HMDB0000299 | -6.2 |  | HMDB0000929 | -5.4 |
|  | HMDB0001285 | -6.2 |  | HMDB0001285 | -5.4 |
|  | HMDB0009467 | -6.2 |  | HMDB0006736 | -5.4 |
|  | HMDB0012110 | -6.2 |  | HMDB0010370 | -5.4 |
|  | HMDB0014703 | -6.2 |  | HMDB0060994 | -5.4 |
|  | HMDB0060947 | -6.2 |  | HMDB0000305 | -5.3 |
|  | HMDB0000840 | -6.1 |  | HMDB0000840 | -5.3 |
|  | HMDB0003337 | -6.1 |  | HMDB0000885 | -5.3 |
|  | HMDB0007578 | -6.1 |  | HMDB0001238 | -5.3 |
|  | HMDB0013339 | -6.1 |  | HMDB0001347 | -5.3 |
|  | HMDB0000195 | -6 |  | HMDB0001434 | -5.3 |
|  | HMDB0000714 | -6 |  | HMDB0003072 | -5.3 |
|  | HMDB0003252 | -6 |  | HMDB0004148 | -5.3 |
|  | HMDB0005464 | -6 |  | HMDB0014488 | -5.3 |
|  | HMDB0005474 | -6 |  | HMDB0029865 | -5.3 |
|  | HMDB0009093 | -6 |  | HMDB0000068 | -5.2 |
|  | HMDB0029865 | -6 |  | HMDB0000639 | -5.2 |
|  | HMDB0000095 | -5.9 |  | HMDB0000640 | -5.2 |
|  | HMDB0000159 | -5.9 |  | HMDB0000763 | -5.2 |
|  | HMDB0002043 | -5.9 |  | HMDB0000918 | -5.2 |
|  | HMDB0004148 | -5.9 |  | HMDB0012109 | -5.2 |
|  | HMDB0000122 | -5.8 |  | HMDB0013339 | -5.2 |
|  | HMDB0000181 | -5.8 |  | HMDB0000125 | -5.1 |
|  | HMDB0000258 | -5.8 |  | HMDB0000181 | -5.1 |
|  | HMDB0000296 | -5.8 |  | HMDB0000318 | -5.1 |
|  | HMDB0000684 | -5.8 |  | HMDB0000472 | -5.1 |
|  | HMDB0000944 | -5.8 |  | HMDB0000684 | -5.1 |
|  | HMDB0001336 | -5.8 |  | HMDB0000714 | -5.1 |
|  | HMDB0001347 | -5.8 |  | HMDB0000824 | -5.1 |
|  | HMDB0003070 | -5.8 |  | HMDB0001336 | -5.1 |
|  | HMDB0004095 | -5.8 |  | HMDB0001490 | -5.1 |
|  | HMDB0005066 | -5.8 |  | HMDB0006461 | -5.1 |
|  | HMDB0005784 | -5.8 |  | HMDB0010368 | -5.1 |
|  | HMDB0013338 | -5.8 |  | HMDB061695 | -5.1 |
|  | HMDB0000045 | -5.7 |  | HMDB0000122 | -5 |
|  | HMDB0000068 | -5.7 |  | HMDB0000197 | -5 |
|  | HMDB0000209 | -5.7 |  | HMDB0000216 | -5 |
|  | HMDB0001123 | -5.7 |  | HMDB0000289 | -5 |
|  | HMDB0001999 | -5.7 |  | HMDB0000736 | -5 |
|  | HMDB0005393 | -5.7 |  | HMDB0001847 | -5 |
|  | HMDB0006275 | -5.7 |  | HMDB0002013 | -5 |
|  | HMDB0006709 | -5.7 |  | HMDB0002030 | -5 |
|  | HMDB0000259 | -5.6 |  | HMDB0003070 | -5 |
|  | HMDB0000318 | -5.6 |  | HMDB0006351 | -5 |
|  | HMDB0000640 | -5.6 |  | HMDB0013338 | -5 |
|  | HMDB0001434 | -5.6 |  | HMDB0013856 | -5 |
|  | HMDB0002030 | -5.6 |  | HMDB0000292 | -4.9 |
|  | HMDB0003072 | -5.6 |  | HMDB0000660 | -4.9 |
|  | HMDB0003080 | -5.6 |  | HMDB0001123 | -4.9 |
|  | HMDB0004685 | -5.6 |  | HMDB0002012 | -4.9 |
|  | HMDB0008957 | -5.6 |  | HMDB0004685 | -4.9 |
|  | HMDB0012109 | -5.6 |  | HMDB0006275 | -4.9 |
|  | HMDB0000125 | -5.5 |  | HMDB0009467 | -4.9 |
|  | HMDB0000378 | -5.5 |  | HMDB0000020 | -4.8 |
|  | HMDB0000660 | -5.5 |  | HMDB0000044 | -4.8 |
|  | HMDB0000824 | -5.5 |  | HMDB0000159 | -4.8 |
|  | HMDB0001490 | -5.5 |  | HMDB0000291 | -4.8 |
|  | HMDB0006351 | -5.5 |  | HMDB0006709 | -4.8 |
|  | HMDB0010383 | -5.5 |  | HMDB0009093 | -4.8 |
|  | HMDB0013856 | -5.5 |  | HMDB0011635 | -4.8 |
|  | HMDB0062769 | -5.5 |  | HMDB0041870 | -4.8 |
|  | HMDB0000020 | -5.4 |  | HMDB0000118 | -4.7 |
|  | HMDB0000118 | -5.4 |  | HMDB0000132 | -4.7 |
|  | HMDB0000472 | -5.4 |  | HMDB0003252 | -4.7 |
|  | HMDB0000767 | -5.4 |  | HMDB0003933 | -4.7 |
|  | HMDB0000848 | -5.4 |  | HMDB0004369 | -4.7 |
|  | HMDB0001043 | -5.4 |  | HMDB0010383 | -4.7 |
|  | HMDB0002012 | -5.4 |  | HMDB0062555 | -4.7 |
|  | HMDB0002183 | -5.4 |  | HMDB0036062 | -4.7 |
|  | HMDB0003333 | -5.4 |  | HMDB0000209 | -4.6 |
|  | HMDB0005454 | -5.4 |  | HMDB0000259 | -4.6 |
|  | HMDB0061864 | -5.4 |  | HMDB0003414 | -4.6 |
|  | HMDB0000073 | -5.3 |  | HMDB0004095 | -4.6 |
|  | HMDB0000132 | -5.3 |  | HMDB0005065 | -4.6 |
|  | HMDB0000222 | -5.3 |  | HMDB0005784 | -4.6 |
|  | HMDB0002013 | -5.3 |  | HMDB0009331 | -4.6 |
|  | HMDB0002712 | -5.3 |  | HMDB0000098 | -4.5 |
|  | HMDB0004667 | -5.3 |  | HMDB0001403 | -4.5 |
|  | HMDB0000133 | -5.2 |  | HMDB0002043 | -4.5 |
|  | HMDB0000216 | -5.2 |  | HMDB0003080 | -4.5 |
|  | HMDB0000736 | -5.2 |  | HMDB0004812 | -4.5 |
|  | HMDB0001388 | -5.2 |  | HMDB0008957 | -4.5 |
|  | HMDB0003208 | -5.2 |  | HMDB0000158 | -4.4 |
|  | HMDB0004978 | -5.2 |  | HMDB0000679 | -4.4 |
|  | HMDB0041870 | -5.2 |  | HMDB0002712 | -4.4 |
|  | HMDB0000292 | -5.1 |  | HMDB0003208 | -4.4 |
|  | HMDB0000529 | -5.1 |  | HMDB0061040 | -4.4 |
|  | HMDB0001085 | -5.1 |  | HMDB0000072 | -4.3 |
|  | HMDB0062558 | -5.1 |  | HMDB0000073 | -4.3 |
|  | HMDB0000044 | -5 |  | HMDB0000157 | -4.3 |
|  | HMDB0000207 | -5 |  | HMDB0000207 | -4.3 |
|  | HMDB0001403 | -5 |  | HMDB0000283 | -4.3 |
|  | HMDB0001847 | -5 |  | HMDB0000568 | -4.3 |
|  | HMDB0003933 | -5 |  | HMDB0000807 | -4.3 |
|  | HMDB0005065 | -5 |  | HMDB0000848 | -4.3 |
|  | HMDB0007008 | -5 |  | HMDB0000904 | -4.3 |
|  | HMDB0007158 | -5 |  | HMDB0002183 | -4.3 |
|  | HMDB0011635 | -5 |  | HMDB0005066 | -4.3 |
|  | HMDB0000306 | -4.9 |  | HMDB0005454 | -4.3 |
|  | HMDB0002068 | -4.9 |  | HMDB0000208 | -4.2 |
|  | HMDB0002259 | -4.9 |  | HMDB0000867 | -4.2 |
|  | HMDB0004369 | -4.9 |  | HMDB0000943 | -4.2 |
|  | HMDB0006710 | -4.9 |  | HMDB0002917 | -4.2 |
|  | HMDB0000157 | -4.8 |  | HMDB0005457 | -4.2 |
|  | HMDB0000283 | -4.8 |  | HMDB0005474 | -4.2 |
|  | HMDB0000289 | -4.8 |  | HMDB0006710 | -4.2 |
|  | HMDB0000291 | -4.8 |  | HMDB0062558 | -4.2 |
|  | HMDB0002231 | -4.8 |  | HMDB0056381 | -4.2 |
|  | HMDB0003418 | -4.8 |  | HMDB0000222 | -4.1 |
|  | HMDB0000158 | -4.7 |  | HMDB0000510 | -4.1 |
|  | HMDB0000162 | -4.7 |  | HMDB0000695 | -4.1 |
|  | HMDB0000573 | -4.7 |  | HMDB0000958 | -4.1 |
|  | HMDB0000885 | -4.7 |  | HMDB0001085 | -4.1 |
|  | HMDB0003231 | -4.7 |  | HMDB0003231 | -4.1 |
|  | HMDB0005457 | -4.7 |  | HMDB0007008 | -4.1 |
|  | HMDB0000673 | -4.6 |  | HMDB0007098 | -4.1 |
|  | HMDB0013622 | -4.6 |  | HMDB0061864 | -4.1 |
|  | HMDB0029581 | -4.6 |  | HMDB0000162 | -4 |
|  | HMDB0060994 | -4.6 |  | HMDB0000172 | -4 |
|  | HMDB0000208 | -4.5 |  | HMDB0000247 | -4 |
|  | HMDB0000679 | -4.5 |  | HMDB0000306 | -4 |
|  | HMDB0000695 | -4.5 |  | HMDB0000517 | -4 |
|  | HMDB0000826 | -4.5 |  | HMDB0000744 | -4 |
|  | HMDB0033923 | -4.5 |  | HMDB0000784 | -4 |
|  | HMDB0000172 | -4.4 |  | HMDB0001539 | -4 |
|  | HMDB0000562 | -4.4 |  | HMDB0002005 | -4 |
|  | HMDB0000639 | -4.4 |  | HMDB0004667 | -4 |
|  | HMDB0000847 | -4.4 |  | HMDB0005453 | -4 |
|  | HMDB0001863 | -4.4 |  | HMDB0031067 | -4 |
|  | HMDB0002886 | -4.4 |  | HMDB0031125 | -4 |
|  | HMDB0004812 | -4.4 |  | HMDB0033923 | -4 |
|  | HMDB0035159 | -4.4 |  | HMDB0062556 | -4 |
|  | HMDB0040598 | -4.4 |  | HMDB0000078 | -3.9 |
|  | HMDB0062436 | -4.4 |  | HMDB0000529 | -3.9 |
|  | HMDB0000098 | -4.3 |  | HMDB0000562 | -3.9 |
|  | HMDB0007098 | -4.3 |  | HMDB0000883 | -3.9 |
|  | HMDB0062555 | -4.3 |  | HMDB0003229 | -3.9 |
|  | HMDB0000177 | -4.2 |  | HMDB0003334 | -3.9 |
|  | HMDB0000182 | -4.2 |  | HMDB0005393 | -3.9 |
|  | HMDB0000807 | -4.2 |  | HMDB0005464 | -3.9 |
|  | HMDB0000958 | -4.2 |  | HMDB0006483 | -3.9 |
|  | HMDB0010378 | -4.2 |  | HMDB0007158 | -3.9 |
|  | HMDB0000134 | -4.1 |  | HMDB0007368 | -3.9 |
|  | HMDB0000254 | -4.1 |  | HMDB0062769 | -3.9 |
|  | HMDB0000744 | -4.1 |  | HMDB0000167 | -3.8 |
|  | HMDB0000904 | -4.1 |  | HMDB0000575 | -3.8 |
|  | HMDB0002005 | -4.1 |  | HMDB0000687 | -3.8 |
|  | HMDB0002368 | -4.1 |  | HMDB0000806 | -3.8 |
|  | HMDB0003229 | -4.1 |  | HMDB0000847 | -3.8 |
|  | HMDB0003339 | -4.1 |  | HMDB0001565 | -3.8 |
|  | HMDB0006483 | -4.1 |  | HMDB0001999 | -3.8 |
|  | HMDB0031125 | -4.1 |  | HMDB0002000 | -3.8 |
|  | HMDB0000060 | -4 |  | HMDB0002231 | -3.8 |
|  | HMDB0000072 | -4 |  | HMDB0002259 | -3.8 |
|  | HMDB0000078 | -4 |  | HMDB0003339 | -3.8 |
|  | HMDB0000112 | -4 |  | HMDB0004136 | -3.8 |
|  | HMDB0000192 | -4 |  | HMDB0004978 | -3.8 |
|  | HMDB0000247 | -4 |  | HMDB0007578 | -3.8 |
|  | HMDB0000357 | -4 |  | HMDB0062436 | -3.8 |
|  | HMDB0000510 | -4 |  | HMDB0000134 | -3.7 |
|  | HMDB0000784 | -4 |  | HMDB0000168 | -3.7 |
|  | HMDB0000827 | -4 |  | HMDB0000177 | -3.7 |
|  | HMDB0000883 | -4 |  | HMDB0000182 | -3.7 |
|  | HMDB0007368 | -4 |  | HMDB0000220 | -3.7 |
|  | HMDB0000039 | -3.9 |  | HMDB0000573 | -3.7 |
|  | HMDB0000517 | -3.9 |  | HMDB0000827 | -3.7 |
|  | HMDB0000696 | -3.9 |  | HMDB0000944 | -3.7 |
|  | HMDB0000806 | -3.9 |  | HMDB0001388 | -3.7 |
|  | HMDB0000943 | -3.9 |  | HMDB0029581 | -3.7 |
|  | HMDB0001539 | -3.9 |  | HMDB0035215 | -3.7 |
|  | HMDB0003424 | -3.9 |  | HMDB0000254 | -3.6 |
|  | HMDB0031067 | -3.9 |  | HMDB0000826 | -3.6 |
|  | HMDB0000161 | -3.8 |  | HMDB0001863 | -3.6 |
|  | HMDB0000190 | -3.8 |  | HMDB0002068 | -3.6 |
|  | HMDB0000220 | -3.8 |  | HMDB0013622 | -3.6 |
|  | HMDB0000243 | -3.8 |  | HMDB0015576 | -3.6 |
|  | HMDB0000568 | -3.8 |  | HMDB0040598 | -3.6 |
|  | HMDB0000687 | -3.8 |  | HMDB0000060 | -3.5 |
|  | HMDB0000870 | -3.8 |  | HMDB0000187 | -3.5 |
|  | HMDB0002917 | -3.8 |  | HMDB0000192 | -3.5 |
|  | HMDB0003334 | -3.8 |  | HMDB0000357 | -3.5 |
|  | HMDB0000043 | -3.7 |  | HMDB0000696 | -3.5 |
|  | HMDB0000187 | -3.7 |  | HMDB0003418 | -3.5 |
|  | HMDB0000214 | -3.7 |  | HMDB0010378 | -3.5 |
|  | HMDB0001310 | -3.7 |  | HMDB0000043 | -3.4 |
|  | HMDB0001565 | -3.7 |  | HMDB0000214 | -3.4 |
|  | HMDB0002000 | -3.7 |  | HMDB0000870 | -3.4 |
|  | HMDB0015576 | -3.7 |  | HMDB0002994 | -3.4 |
|  | HMDB0062556 | -3.7 |  | HMDB0035159 | -3.4 |
|  | HMDB0000056 | -3.6 |  | HMDB0000112 | -3.3 |
|  | HMDB0000131 | -3.6 |  | HMDB0001043 | -3.3 |
|  | HMDB0000167 | -3.6 |  | HMDB0002368 | -3.3 |
|  | HMDB0000168 | -3.6 |  | HMDB0000131 | -3.2 |
|  | HMDB0000251 | -3.5 |  | HMDB0000190 | -3.2 |
|  | HMDB0000867 | -3.5 |  | HMDB0000243 | -3.2 |
|  | HMDB0000575 | -3.4 |  | HMDB0000251 | -3.2 |
|  | HMDB0001881 | -3.4 |  | HMDB0002329 | -3.2 |
|  | HMDB0002329 | -3.4 |  | HMDB0000039 | -3.1 |
|  | HMDB0002994 | -3.4 |  | HMDB0000056 | -3.1 |
|  | HMDB0004136 | -3.4 |  | HMDB0000097 | -3.1 |
|  | HMDB0000115 | -3.2 |  | HMDB0000161 | -3.1 |
|  | HMDB0000097 | -3.1 |  | HMDB0000673 | -3.1 |
|  | HMDB0006112 | -3.1 |  | HMDB0001310 | -3.1 |
|  | HMDB0000257 | -3 |  | HMDB0000257 | -3 |
|  | HMDB0000925 | -3 |  | HMDB0000115 | -2.9 |
|  | HMDB0001429 | -3 |  | HMDB0001429 | -2.9 |
|  | HMDB0001448 | -3 |  | HMDB0001448 | -2.9 |
|  | HMDB0000595 | -2.8 |  | HMDB0001881 | -2.9 |
|  | HMDB0002878 | -2.8 |  | HMDB0002786 | -2.8 |
|  | HMDB0000294 | -2.6 |  | HMDB0002878 | -2.8 |
|  | HMDB0000149 | -2.4 |  | HMDB0006112 | -2.8 |
|  | HMDB0002786 | -2.2 |  | HMDB0000595 | -2.7 |
|  | HMDB0000464 | -1.9 |  | HMDB0000925 | -2.7 |
|  | HMDB0000547 | -1.9 |  | HMDB0000294 | -2.3 |
|  | HMDB0000586 | -1.9 |  | HMDB0000149 | -2.2 |
|  | HMDB0015531 | -1.9 |  | HMDB0003125 | -2 |
|  | HMDB0001875 | -1.6 |  | HMDB0001875 | -1.6 |
|  | HMDB0003338 | -1.6 |  | HMDB0003338 | -1.6 |
|  | HMDB0003378 | -1.6 |  | HMDB0003378 | -1.6 |
|  | HMDB0003125 | -1.5 |  | HMDB0002500 | -1.4 |
|  | HMDB0002500 | -1.3 |  | HMDB0000464 | -1.2 |
