# Supplementary material for: Comprehensive genome based analysis of Vibrio parahaemolyticus for identifying novel drug and vaccine molecules: Subtractive proteomics and vaccinomics approach
Source: PLoS One. 2020 Aug 19;15(8):e0237181. doi: 10.1371/journal.pone.0237181 (PMC7444560; doi:10.1371/journal.pone.0237181)
Supplement: S3 Table — (DOCX) [file pone.0237181.s008.docx]

**S3 Table.** Antigenicity of novel membrane proteins (Vaccine targets) and similarity analysis with human microbiome

| **Protein ID** | **Vaxijen Score** | **Similarity** | **Prediction** |
| --- | --- | --- | --- |
| Q87P28 | 0.4458 | 68 | ANTIGEN |
| Q87HY1 | 0.3452 | 42 | NON-ANTIGEN |
| Q87TD7 | 0.5575 | 56 | ANTIGEN |
| Q87GB4 | 0.4450 | 52 | ANTIGEN |
| Q87P22 | 0.5721 | 49 | ANTIGEN |
| Q87JA2 | 0.7064 | 72 | ANTIGEN |
| Q87Q13 | 0.5984 | 50 | ANTIGEN |
| Q87R85 | 0.4509 | 62 | ANTIGEN |
| Q79YZ4 | 0.3205 | 68 | NON-ANTIGEN |
| Q87P56 | 0.4949 | 76 | ANTIGEN |
| Q79YT9 | 0.5180 | 74 | ANTIGEN |
| Q87FY4 | 0.6195 | 45 | ANTIGEN |
| Q87P44 | 0.4034 | 67 | ANTIGEN |
| Q79YY3 | 0.6429 | 60 | ANTIGEN |
| Q87FM8 | 0.4523 | 51 | ANTIGEN |
| Q87J60 | 0.6522 | 60 | ANTIGEN |
| Q87LX8 | 0.4736 | 63 | ANTIGEN |
| Q87LX7 | 0.5144 | 59 | ANTIGEN |
| Q87HJ8 | 0.6461 | 45 | ANTIGEN |
| Q87GB2 | 0.5797 | 48 | ANTIGEN |
| Q87JH9 | 0.5311 | 41 | ANTIGEN |
| Q87GY3 | 0.5303 | 45 | ANTIGEN |
| Q87Q18 | 0.3866 | 42 | NON-ANTIGEN |
| Q87FX8 | 0.7299 | 71 | ANTIGEN |
| Q87IQ2 | 0.3443 | 47 | NON-ANTIGEN |
