# Supplementary material for: Comprehensive genome based analysis of Vibrio parahaemolyticus for identifying novel drug and vaccine molecules: Subtractive proteomics and vaccinomics approach
Source: PLoS One. 2020 Aug 19;15(8):e0237181. doi: 10.1371/journal.pone.0237181 (PMC7444560; doi:10.1371/journal.pone.0237181)
Supplement: S4 Table — (DOCX) [file pone.0237181.s009.docx]

| **Accession No.** | **Vaxijen Score** | **Similiarity analysis with human microbiome (%)** |
| --- | --- | --- |
| Q87P28 | 0.4458 | <68 |
| Q87HY1 | 0.3452 | <42 |
| Q87TD7 | 0.5575 | <56 |
| Q87GB4 | 0.4450 | <52 |
| Q87P22 | 0.5721 | <49 |
| Q87JA2 | 0.7064 | <72 |
| Q87Q13 | 0.5984 | <50 |
| Q87R85 | 0.4509 | <62 |
| Q79YZ4 | 0.3205 | <68 |
| Q87P56 | 0.4949 | <76 |
| Q79YT9 | 0.5180 | <74 |
| Q87FY4 | 0.6195 | <45 |
| Q87P44 | 0.4034 | <67 |
| Q79YY3 | 0.6429 | <60 |
| Q87FM8 | 0.4523 | <51 |
| Q87J60 | 0.6522 | <60 |
| Q87LX8 | 0.4736 | <63 |
| Q87LX7 | 0.5144 | <59 |
| Q87HJ8 | 0.6461 | <45 |
| Q87GB2 | 0.5797 | <48 |
| Q87JH9 | 0.5311 | <41 |
| Q87GY3 | 0.5303 | <45 |
| Q87Q18 | 0.3866 | <42 |
| Q87FX8 | 0.7299 | <71 |
| Q87IQ2 | 0.3443 | <47 |

**S4 Table.** Antigenicity and similiarity analysis of novel outer membrane proteins with human microbiome (%)
