# Supplementary material for: Comprehensive genome based analysis of Vibrio parahaemolyticus for identifying novel drug and vaccine molecules: Subtractive proteomics and vaccinomics approach
Source: PLoS One. 2020 Aug 19;15(8):e0237181. doi: 10.1371/journal.pone.0237181 (PMC7444560; doi:10.1371/journal.pone.0237181)
Supplement: S5 Table — (DOCX) [file pone.0237181.s010.docx]

**S5 Table.** Allergenicity pattern and toxicity analysis of top epitopes for Sensor histidine protein kinase and Flagellar hook-associated protein

| Protein | Epitope | Vaxijen | Allertop | Allergen FP | Allermatch | Allergen Online | Toxin Pred |
| --- | --- | --- | --- | --- | --- | --- | --- |
| **Sensor histidine protein kinase** | DPELAILLFPFALRL | 2.2869 | Allergen | Non-Allergen | Allergen | Non-Allergen | Non-Toxin |
|  | LMLVPMCYLLWNYLF | 2.1611 | Allergen | Non-Allergen | Non-Allergen | Non-Allergen | Non-Toxin |
|  | ILLFPFALRLGIALH | 2.1484 | Non-Allergen | Non-Allergen | Non-Allergen | Non-Allergen | Non-Toxin |
|  | LAILLFPFALRLGIA | 2.1118 | Non-Allergen | Non-Allergen | Allergen | Non-Allergen | Non-Toxin |
|  | PELAILLFPFALRLG | 2.0796 | Allergen | Non-Allergen | Allergen | Non-Allergen | Non-Toxin |
|  | VNDPELAILLFPFAL | 2.0618 | Allergen | Non-Allergen | Allergen | Non-Allergen | Non-Toxin |
|  | ELAILLFPFALRLGI | 2.0569 | Non-Allergen | Non-Allergen | Allergen | Non-Allergen | Non-Toxin |
|  | ACAWFCLWVIAYYFV | 1.9635 | Allergen | Non-Allergen | Non-Allergen | Non-Allergen | Toxin |
|  | AILLFPFALRLGIAL | 1.9451 | Non-Allergen | Non-Allergen | Non-Allergen | Non-Allergen | Non-Toxin |
|  | WFCLWVIAYYFVNDP | 1.9417 | Allergen | Non-Allergen | Non-Allergen | Non-Allergen | Non-Toxin |
|  | NDPELAILLFPFALR | 1.8981 | Allergen | Non-Allergen | Allergen | Non-Allergen | Non-Toxin |
|  | MLVPMCYLLWNYLFQ | 1.844 | Allergen | Non-Allergen | Non-Allergen | Non-Allergen | Non-Toxin |
|  | LLFPFALRLGIALHT | 1.8268 | Non-Allergen | Allergen | Non-Allergen | Non-Allergen | Non-Toxin |
|  | CAWFCLWVIAYYFVN | 1.8066 | Allergen | Allergen | Non-Allergen | Non-Allergen | Non-Toxin |
|  | VMACAWFCLWVIAYY | 1.7285 | Allergen | Allergen | Non-Allergen | Non-Allergen | Toxin |
|  | AILLFPFAL | 2.993 | Non-Allergen | Allergen | Non-Allergen | Non-Allergen | Non-Toxin |
|  | ILLFPFALR | 2.9721 | Non-Allergen | Allergen | Non-Allergen | Non-Allergen | Non-Toxin |
|  | FCLWVIAYYF | 2.8883 | Allergen | Non-Allergen | Non-Allergen | Non-Allergen | Non-Toxin |
|  | CYLLWNYLF | 2.8253 | Allergen | Allergen | Non-Allergen | Non-Allergen | Non-Toxin |
|  | ELAILLFPF | 2.7265 | Non-Allergen | Allergen | Allergen | Non-Allergen | Non-Toxin |
|  | LLFPFALRL | 2.7071 | Allergen | Non-Allergen | Non-Allergen | Non-Allergen | Non-Toxin |
|  | LAILLFPFA | 2.6785 | Non-Allergen | Allergen | Allergen | Non-Allergen | Non-Toxin |
|  | WFCLWVIAY | 2.5591 | Allergen | Allergen | Non-Allergen | Non-Allergen | Non-Toxin |
|  | LWVIAYYFV | 2.4774 | Non-Allergen | Allergen | Non-Allergen | Non-Allergen | Non-Toxin |
|  | HDDGVGFKV | 2.363 | Non-Allergen | Non-Allergen | Non-Allergen | Non-Allergen | Non-Toxin |
|  | YLLWNYLFQ | 2.3399 | Allergen | Allergen | Non-Allergen | Non-Allergen | Non-Toxin |
|  | DDGVGFKVQ | 2.325 | Allergen | Non-Allergen | Non-Allergen | Non-Allergen | Non-Toxin |
|  | DGVGFKVQD | 2.1287 | Allergen | Non-Allergen | Non-Allergen | Non-Allergen | Non-Toxin |
| **Flagellar hook-associated protein** | DSIESSFNAQDEEGH | 1.304 | Non-Allergen | Non-Allergen | Non-Allergen | Non-Allergen | Non-Toxin |
|  | PNFQAEVDASLNAID | 1.146 | Allergen | Non-Allergen | Non-Allergen | Non-Allergen | Non-Toxin |
|  | SGAYVVEGNSDVRVV | 1.06 | Allergen | Non-Allergen | Non-Allergen | Non-Allergen | Non-Toxin |
|  | AEFEKPSPNFQAEVD | 1.056 | Allergen | Non-Allergen | Non-Allergen | Non-Allergen | Non-Toxin |
|  | GGRHNNLDLMDGAHS | 0.964 | Non-Allergen | Non-Allergen | Non-Allergen | Non-Allergen | Non-Toxin |
|  | GAYVVEGNSDVRVVT | 0.92 | Allergen | Allergen | Non-Allergen | Non-Allergen | Non-Toxin |
|  | KLSDDPMASIKLLNL | 0.887 | Non-Allergen | Allergen | Non-Allergen | Non-Allergen | Non-Toxin |
|  | NFQAEVDASLNAIDD | 0.8431 | Allergen | Allergen | Non-Allergen | Non-Allergen | Non-Toxin |
|  | EVDASLNAIDDTMAN | 0.832 | Allergen | Non-Allergen | Non-Allergen | Non-Allergen | Non-Toxin |
|  | MMLQSLQSNSAGLGK | 0.819 | Non-Allergen | Non-Allergen | Non-Allergen | Non-Allergen | Non-Toxin |
|  | IGGRHNNLDLMDGAH | 0.801 | Non-Allergen | Non-Allergen | Non-Allergen | Non-Allergen | Non-Toxin |
|  | KVSGDLSALDYGEAS | 0.751 | Non-Allergen | Non-Allergen | Non-Allergen | Non-Allergen | Non-Toxin |
|  | LNKSSGAYVVEGNSD | 0.701 | Non-Allergen | Non-Allergen | Non-Allergen | Non-Allergen | Non-Toxin |
|  | GGGKNVLNQIDALIA | 0.639 | Allergen | Non-Allergen | Non-Allergen | Non-Allergen | Non-Toxin |
|  | VDASLNAIDDTMANV | 0.625 | Non-Allergen | Non-Allergen | Non-Allergen | Non-Allergen | Non-Toxin |
|  | LDIGGGKNV | 1.9185 | Non-Allergen | Allergen | Non-Allergen | Non-Allergen | Non-Toxin |
|  | GANGSLTDQ | 1.8499 | Allergen | Allergen | Non-Allergen | Non-Allergen | Non-Toxin |
|  | FNAQDEEGH | 1.8425 | Non-Allergen | Allergen | Non-Allergen | Non-Allergen | Non-Toxin |
|  | SFNAQDEEG | 1.8224 | Allergen | Allergen | Non-Allergen | Non-Allergen | Non-Toxin |
|  | SPNFQAEVD | 1.7972 | Allergen | Allergen | Non-Allergen | Non-Allergen | Non-Toxin |
|  | GGRHNNLDL | 1.7855 | Allergen | Non-Allergen | Non-Allergen | Non-Allergen | Non-Toxin |
|  | PNFQAEVDA | 1.7403 | Allergen | Non-Allergen | Non-Allergen | Non-Allergen | Non-Toxin |
|  | KPSPNFQAE | 1.7093 | Allergen | Non-Allergen | Non-Allergen | Non-Allergen | Non-Toxin |
|  | DIGGGKNVL | 1.662 | Non-Allergen | Allergen | Non-Allergen | Non-Allergen | Non-Toxin |
|  | NFQAEVDAS | 1.5455 | Allergen | Allergen | Non-Allergen | Non-Allergen | Non-Toxin |
|  | SSFNAQDEEG | 1.5143 | Allergen | Allergen | Non-Allergen | Non-Allergen | Non-Toxin |
|  | KPSPNFQAEV | 1.4978 | Non-Allergen | Non-Allergen | Non-Allergen | Non-Allergen | Non-Toxin |
|  | LQSNSAGLG | 1.4717 | Non-Allergen | Allergen | Non-Allergen | Non-Allergen | Non-Toxin |
