# Supplementary material for: Comprehensive genome based analysis of Vibrio parahaemolyticus for identifying novel drug and vaccine molecules: Subtractive proteomics and vaccinomics approach
Source: PLoS One. 2020 Aug 19;15(8):e0237181. doi: 10.1371/journal.pone.0237181 (PMC7444560; doi:10.1371/journal.pone.0237181)
Supplement: S6 Table — (DOCX) [file pone.0237181.s011.docx]

**S6 Table.** Allergenicity assessment of the predicted B-cell epitopes generated from histidine protein kinase and flagellar hook-associated protein

| **Protein** | **Start** | **End** | **Peptide** | **Length** | **Allergenicity** | **Algorithm** |
| --- | --- | --- | --- | --- | --- | --- |
| Histidine protein kinase UhpB | 6 | 20 | VTTICGLFVMACAWF | 15 | Non Allergen | Linear epitope |
|  | 100 | 106 | YYYGDQN | 7 | Non Allergen | Beta turn |
|  | 272 | 283 | VQKQKDLNHKLR | 12 | Non Allergen | Surface accessibility |
|  | 353 | 359 | YDTTKRL | 7 | Non Allergen | Flexibility |
|  | 207 | 222 | FAPFCMAIPIIVLALR | 16 | Non Allergen | Antigeneicity |
|  | 271 | 277 | AVQKQKD | 7 | Non Allergen | hydrophilicity |
| Flagellar hook-associated protein | 201 | 211 | FEKPSPNFQAE | 11 | Non Allergen | Linear epitope |
|  | 147 | 153 | NKSSGAY | 7 | Non Allergen | Beta turn |
|  | 29 | 43 | QMSTRERLTKLSDDP | 15 | Non Allergen | Surface accessibility |
|  | 73 | 79 | LSSQETH | 6 | Non Allergen | Flexibility |
|  | 161 | 167 | VRVVTVA | 7 | Non Allergen | Antigeneicity |
|  | 138 | 144 | GTKTDTA | 7 | Non Allergen | Hydrophilicity |
