# Supplementary material for: Comprehensive genome based analysis of Vibrio parahaemolyticus for identifying novel drug and vaccine molecules: Subtractive proteomics and vaccinomics approach
Source: PLoS One. 2020 Aug 19;15(8):e0237181. doi: 10.1371/journal.pone.0237181 (PMC7444560; doi:10.1371/journal.pone.0237181)
Supplement: S7 Table — (DOCX) [file pone.0237181.s012.docx]

**S7 Table.** Allergenicity, antigenicity and solubility prediction of the constructed vaccines

| **Constructs** | **Composition** | **Complete Sequence of Vaccine Constructs** | **Antigenicity**  (Threshold 0.4) | **Allergenicity** | **Solubility**  (Threshold 0.45) |
| --- | --- | --- | --- | --- | --- |
| V1 | Predicted CTL, HTL & BCL epitopes  with β defensin adjuvant & PADRE  sequence | **EAAAK**GIINTLQKYYCRVRGGRCAVLSCLPKEEQIGKCSTRGRKCCRRKK**EAAAK**AKFVAAWTLKAAA**GGGS**AILLFPFALR**GGGS**HDDGVGFKV**GGGS**FNAQDEEGH**GGGS**GGRHNNLDL**GGGS**KPSPNFQAEV**GPGPG**AILLFPFALRLGIALHT**GPGPG**DSIESSFNAQDEEGH**GPGPG**IGGRHNNLDLMDGAH**GPGPG**KLSDDPMASIKLLNL**KK**VTTICGLFVMACAWF**KK**YYYGDQN**KK**AVQKQKDLNHKLR**KK**YDTTKRL**KK**FAPFCMAIPIIVLALR**KK**FEKPSPNFQAE**KK**NKSSGAY**KK**QMSTRERLTKLSDDP**KK**LSSQETH**KK**VRVVTVA**KK**GTKTDTA**KK**AKFVAAWTLKAAA**GGGS** | 1.18 | Non Allergen | 0.661 |
| V2 | Predicted CTL, HTL & BCL epitopes  with L7/L12 ribosomal protein  adjuvant & PADRE sequence | **EAAAK**MAKLSTDELLDAFKEMTLLELSDFVKKFEETFEVTAAAPVAVAAAGAAPAGAAVEAAEEQSEFDVILEAAGDKKIGVIKVVREIVSGLGLKEAKDLVDGAPKPLLEKVAKEAADEAKAKLEAAGATVTVK**EAAAK**AKFVAAWTLKAAA**GGGS**AILLFPFALR**GGGS**HDDGVGFKV**GGGS**FNAQDEEGH**GGGS**GGRHNNLDL**GGGS**KPSPNFQAEV**GPGPG**AILLFPFALRLGIALHT**GPGPG**DSIESSFNAQDEEGH**GPGPG**IGGRHNNLDLMDGAH**GPGPG**KLSDDPMASIKLLNL**KK**VTTICGLFVMACAWF**KK**YYYGDQN**KK**AVQKQKDLNHKLR**KK**YDTTKRL**KK**FAPFCMAIPIIVLALR**KK**FEKPSPNFQAE**KK**NKSSGAY**KK**QMSTRERLTKLSDDP**KK**LSSQETH**KK**VRVVTVA**KK**GTKTDTA**KK**AKFVAAWTLKAAA**GGGS** | 1.00 | Non Allergen | 0.603 |
| V3 | Predicted CTL, HTL & BCL epitopes  with HABA adjuvant & PADRE  sequence | **EAAAK**MAENPNIDDLPAPLLAALGAADLALATVNDLIANLRERAEETRAETRTRVEERRARLTKFQEDLPEQFIELRDKFTTEELRKAAEGYLEAATNRYNELVERGEAALQRLRSQTAFEDASARAEGYVDQAVELTQEALGTVASQTRAVGERAAKLVGIEL**EAAAK**AKFVAAWTLKAAA**GGGS**AILLFPFALR**GGGS**HDDGVGFKV**GGGS**FNAQDEEGH**GGGS**GGRHNNLDL**GGGS**KPSPNFQAEV**GPGPG**AILLFPFALRLGIALHT**GPGPG**DSIESSFNAQDEEGH**GPGPG**IGGRHNNLDLMDGAH**GPGPG**KLSDDPMASIKLLNL**KK**VTTICGLFVMACAWF**KK**YYYGDQN**KK**AVQKQKDLNHKLR**KK**YDTTKRL**KK**FAPFCMAIPIIVLALR**KK**FEKPSPNFQAE**KK**NKSSGAY**KK**QMSTRERLTKLSDDP**KK**LSSQETH**KK**VRVVTVA**KK**GTKTDTA**KK**AKFVAAWTLKAAA**GGGS** | 1.03 | Non Allergen | 0.625 |
