# Supplementary material for: Comprehensive genome based analysis of Vibrio parahaemolyticus for identifying novel drug and vaccine molecules: Subtractive proteomics and vaccinomics approach
Source: PLoS One. 2020 Aug 19;15(8):e0237181. doi: 10.1371/journal.pone.0237181 (PMC7444560; doi:10.1371/journal.pone.0237181)
Supplement: S8 Table — (DOCX) [file pone.0237181.s013.docx]

**S8 Table**. Docking score of vaccine construct V1, V2 and V3 with different HLA alleles.

| **Vaccine**  **Construct** | **HLA alleles PDB ID’s** | **Global Energy** | **Hydrogen Bond Energy** | **ACE** | **Score** | **Area** |
| --- | --- | --- | --- | --- | --- | --- |
| V1 | 1A6A | -5.77 | 0.00 | -2.93 | 15800 | 2614.80 |
|  | 1H15 | -32.57 | -1.48 | -3.79 | 16644 | 2074.50 |
|  | 2SEB | -26.95 | -2.39 | 2.40 | 17546 | 2082.60 |
|  | 2Q6W | -16.57 | -1.50 | 3.50 | 15770 | 2538.40 |
|  | 2FSE | -19.27 | -5.69 | 8.65 | 16988 | 2105.90 |
|  | 3C5J | 11.34 | -1.87 | 2.10 | 16094 | 2066.20 |
| V2 | 1A6A | -22.98 | -2.20 | -3.85 | 15152 | 2094.40 |
|  | 1H15 | -18.21 | -2.99 | 11.23 | 16964 | 2574.80 |
|  | 2Q6W | -1.13 | 0.00 | 4.11 | 18528 | 3097.60 |
|  | 2SEB | 4.86 | 0.00 | 4.62 | 17698 | 2379.80 |
|  | 2FSE | -43.58 | -5.18 | 2.59 | 16638 | 2311.70 |
|  | 3C5J | -4.64 | -1.04 | 1.32 | 18134 | 2927.80 |
| V3 | 1A6A | -0.58 | -1.24 | 4.86 | 17262 | 2533.10 |
|  | 1H15 | 11.12 | -1.51 | 8.43 | 18698 | 2572.80 |
|  | 2SEB | 17.22 | 0.00 | 3.91 | 21978 | 3151.80 |
|  | 2Q6W | -16.11 | -4.54 | 13.23 | 16068 | 2515.50 |
|  | 2FSE | -11.82 | -6.92 | 18.45 | 16308 | 2064.50 |
|  | 3C5J | -5.73 | -0.98 | 6.61 | 17746 | 2873.70 |
