# Supplementary material for: Comprehensive genome based analysis of Vibrio parahaemolyticus for identifying novel drug and vaccine molecules: Subtractive proteomics and vaccinomics approach
Source: PLoS One. 2020 Aug 19;15(8):e0237181. doi: 10.1371/journal.pone.0237181 (PMC7444560; doi:10.1371/journal.pone.0237181)
Supplement: S2 File — (DOCX) [file pone.0237181.s015.docx]

**S2 File.** Non-paralogous (>60% identical) in CD-Hit

>sp|Q87P32|VOPS_VIBPA Adenosine monophosphate-protein transferase VopS OS=Vibrio parahaemolyticus serotype O3:K6 (strain RIMD 2210633) OX=223926 GN=vopS PE=1 SV=1

MISFGNVSALQAAMPQARNEILNEGKLSIGGKEYTINAATQEFTRANPTSGAVARFFEAT

GKLFREGSTQSVAKAITKAVFDNEQGQAQRLQTSSSVEHGQMLFKDANLKTPSDVLNAFA

KLDSKMVKSHAAELSQLAERAMTEVMLETDSGKNLKALIGDDAVKSLAVRVVKDYGGGVA

AAQKNPEVRINQMQAVFDMEVMHLKAAQRHIEGLASTDLNQGVYAEGLPEDAFNKAGVTN

NVERAAAWIINASNSKGNDAENITSLLKEYATNGKDLLNMDNLKELHARLVPNVERDYRG

PNISGGTLPSSIGGEGMLKQHIEGFLKENPVADKDLGKHLFAGVIGYHGFTDGNGRMGRM

LYAIAELRNDSFNPLAMNAENSLHGIK

>sp|Q87TN7|TRKH_VIBPA Trk system potassium uptake protein TrkH OS=Vibrio parahaemolyticus serotype O3:K6 (strain RIMD 2210633) OX=223926 GN=trkH PE=1 SV=1

MQFRSIIRIVGLLLALFSVTMLAPALVALLYRDGAGVPFVTTFFVLLFCGAMCWFPNRRH

KHELKSRDGFLIVVLFWTVLGSAGSLPFLIADNPNISVTDAFFESFSALTTTGATVIVGL

DELPKAILFYRQFLQWFGGMGIIVLAVAILPVLGIGGMQLYRAEIPGPVKDTKMTPRIAE

TAKALWYIYLSLTIACAVAFWLAGMTPFDAISHSFSTIAIGGFSTHDASMGYFDSYAINL

ITVVFLLISACNFTLHFAAFASGGVHPKYYWKDPEFRAFIFIQVLLFLVCFLLLLKHHSY

TSPYDAFDQALFQTVSISTTAGFTTTGFADWPLFLPVLLLFSSFIGGCAGSTGGGMKVIR

ILLLTLQGARELKRLVHPRAVYTIKVGGSALPQRVVDAVWGFFSAYALVFVVCMLGLIAT

GMDELSAFSAVAATLNNLGPGLGEVALHFGDVNDKAKWVLIVSMLFGRLEIFTLLILLTP

TFWRS

>sp|Q87KB0|GLMU_VIBPA Bifunctional protein GlmU OS=Vibrio parahaemolyticus serotype O3:K6 (strain RIMD 2210633) OX=223926 GN=glmU PE=3 SV=1

MKFSAVILAAGKGTRMHSNMPKVLHTLAGKPMVKHVIDTCTGLGAQNIHLVFGHGGDQMQ

TTLADETVNWILQADQLGTGHAVDQASPRFEDDEKILVLYGDVPLISPETIENLLDAQPT

GGIALLTVMLDNPTGYGRIIRKNGPVVAIVEQKDASEEQKQIKEINTGVMVATGGDLKRW

LSGLNNNNAQGEYYLTDVIAAAHDEGRAVEAVHPVNAIEVEGVNDRAQLARLERAFQSMQ

AQKLLEQGVMLRDPARFDLRGELQCGMDCEIDANVIIEGNVSLGDNVIIGTGCVLKDCEI

DDNTIVRPYSVIEGATVGEECTVGPFTRLRPGAELRNDAHVGNFVEVKNARIGEGSKANH

LTYLGDAEIGQRTNIGAGTITCNYDGANKFKTIIGNDVFVGSDSQLVAPVTIADGATIGA

GTTLTKDVEEGELVITRVKERKITGWQRPVKQK

>sp|Q87TN9|FADB_VIBPA Fatty acid oxidation complex subunit alpha OS=Vibrio parahaemolyticus serotype O3:K6 (strain RIMD 2210633) OX=223926 GN=fadB PE=3 SV=1

MIYQADTLQVKEIQDGIAELSFCSPKSVNKLDLATLESLDKALDALTSHQGLKGLMLTSD

KDAFIVGADITEFLGLFAKTDAELDQWLQFANSIFNKLEDLPVPTISVLKGHTLGGGCEC

VLATDMRIGDKTTSIGLPETKLGIMPGFGGCVRLPRVIGADSAMEIITQGKACRADEALK

IGLLDAVVETDALYESALQTLTSAINEKIDWQARRKQKTSPLTLSKLESMMSFTMAKGLV

AQVAGPHYPAPMTAVITIEEGARFARNEALDIERKYFVKLAKSEEAKALVGLFLNDQYIK

GIAKKAAKSASKDTERAAVLGAGIMGGGIAYQSALKGVPVLMKDIAQPSLDLGMTEASKL

LNKRLAQGRIDGFKMAGILASITPSLHYAGIENSDVIVEAVVENPKVKATVLSEVESHVG

EDTVITSNTSTIPINLLAQSLKRPENFCGMHFFNPVHRMPLVEIIRGEKTSDETINRVVA

YAAKMGKSPIVVNDCPGFFVNRVLFPYFGGFSMLLRDGADFTKVDKVMERKFGWPMGPAY

LLDVVGIDTAHHAQAVMAEGFPERMGKQGRDAIDALFEANKYGQKNGNGFYSYTIDKKGK

PKKTFTEDILPVLADVCADKQEFDEQTIIQRMMIPMINEVVLCLQEGIIATPQEADMALV

YGLGFPPFRGGVFRYLDSVGIAEFVEMAKQHADLGAMYHVPQMLIDMAAKGESFYGAQQQ

GSI

>sp|Q87K41|PPNP_VIBPA Pyrimidine/purine nucleoside phosphorylase OS=Vibrio parahaemolyticus serotype O3:K6 (strain RIMD 2210633) OX=223926 GN=ppnP PE=1 SV=1

MSIKENSYFAGGVKSLGFNQHGQDVSVGVMLPGEYTFGTQAPERMTVVKGALVVKRVGEA

DWTTYSSGESFDVEGNSSFELQVKDATAYLCEYL

>sp|P40609|HMP_VIBPA Flavohemoprotein OS=Vibrio parahaemolyticus serotype O3:K6 (strain RIMD 2210633) OX=223926 GN=hmp PE=3 SV=2

MLSNQTIEIVKATAPLIAETGPKLTAHFYDRMFTHNPELKDIFNMSNQRNGDQREALFNA

ICAYAANIENLPALLGAVEKIAHKHTSFLITKDQYQIVGKHLIATIDELFNPGQEVLDAW

AEAYGVLANVFIQREEQIYQANASQEGGWRGLREFELVGKQLESEHICSFVFKPTDGSKV

TKYKPGQYLGIYINSDKFENQEIRQYSLSSSVQENTYRISVKREQGGKVSNYLHDELNIG

DKVKLAAPAGDFFMDVDTNTPVVLISAGVGLTPTLSMLESLTEHHAPVTWVHATENSKHH

AFKEHVNQLVTAKENMNALIWYNQPTAEDKIGEDFHFTGFVNLHEIEAALKQDNVQVYFC

GPVGFMQHVAKQLQELGVPQEQFHYECFGPHKVV

>sp|Q87SJ9|HLDE_VIBPA Bifunctional protein HldE OS=Vibrio parahaemolyticus serotype O3:K6 (strain RIMD 2210633) OX=223926 GN=hldE PE=3 SV=1

MKPILPDYNSAGVLIIGDVMLDRYWYGPTGRISPEAPVPVVKVENNEERPGGAANVAMNI

ASLGGHAHIVGLTGEDEPAKVLTETLSALNVKCDFVALPDYPTITKLRVMSRGQQLIRLD

FEDKFENTDATPVLSRMDAALPNVKAVIMSDYAKGSLEHVQAYIQKARAANIPVFIDPKG

ADFERYRGATLLTPNMKEFEDVVGKVKSDQELVEKALALVKEFDFEALLVTRSEHGMTLI

RRGQEPFHLPTQAKEVYDVTGAGDTVISVLAASVAAGKSFEEACALANAAAGVVVGKLGT

STLSEIELAEAVHGSQDTDFGVISEKALIEAVKKARARGEKVVMTNGCFDILHAGHVSYL

NHAAELGDRLIVAVNTDESVKRLKGPGRPVNPTDRRMAVLAGLGAVDWVVPFSEDTPQRL

ISEVLPSMLVKGGDYKPEEIAGGKEVIAAGGEVRVLNFEDGCSTSEIINAIKGGKG

>tr|Q87H05|Q87H05_VIBPA Bifunctional NAD(P)H-hydrate repair enzyme OS=Vibrio parahaemolyticus serotype O3:K6 (strain RIMD 2210633) OX=223926 GN=nnrD PE=3 SV=1

MDFNLALKLYTAEQVKNGEVVAAHMAGVSMYSLMQRAGMAVYERFLHLYPRAKNVLVVCG

KGNNGGDGYVFASLAKQAQLNVQVFQIGDVTQLQGDALRAYQDWQTVDGKNSSWDDWNTA

LLEAEVIIDAMLGTGLKGEVRTEYRRYIEQINQIQCPVIAVDIPSGLCANTGSVLGDAIQ

ADHTVTFIGVKQGLCTAQARDRIGELHFCGLGVNVEFDAIEEESALGIDHKVIPRLLPKP

KATAHKGDNGKLLCVGGNQGMSGAIRLCASAAVRSGAGLTASITHPDSFIPLQVSCPEVM

SQSITTDLLRDTENALTKRIRWADVLVFGPGFGDDEWAYQAYQYLSQEQKPKVVDADGLN

ILAMLSQRCDGTLVCDNQRVITPHPGEAARLLNVTTKQIESDRYSAARQLQERYGGVVVL

KGAGTLVFDGVRMYVCLAGNPGMATGGMGDVLSGVIGALLAKGLPISIAARLGVMIHSHA

ADLNAEKHGEVGLLATDVVETLRAATHIQRAKN

>sp|Q87RT6|THII_VIBPA tRNA sulfurtransferase OS=Vibrio parahaemolyticus serotype O3:K6 (strain RIMD 2210633) OX=223926 GN=thiI PE=3 SV=1

MKFIVKPHPEIFVKSESVRKRFTKILECNIRNIVKSRTESVAVFNRRDHIEVTSESNEYH

AEVLEILTHTPGIHHVLEVKQSEFKDLHDIYEQVLELSRPLIENKTFVVRAKRRGKHDFT

SIELERYVGGGLNQAVESARVKLHNPDVTVKVEVSGDKLNQVLARHKGLGGFPLGTQEDV

LSLISGGFDSGVSSYLHIKRGSKVHYCFFNLGGPAHEIGVKQVSHYLWNKYGSSAKVRFI

SVDFEPVVAEILEKVDDGQMGVILKRMFMRAAGMIAEKFKIEALVTGEALGQVSSQTLTN

LRHIDNVTDTLILRPLINWDKEDIINLAREIGTEDFAKTMPEYCGVISKKPTVKAVKEKL

EAEEAKFDFSILEKVVYEARQMDIRDIAKESEQAAPEVEQVQAVEEHAVVLDIRSPDEED

DNPLEIAGVDVKHIPFYKLGTQFGDLDQSKTYLLYCDRGVMSRLQALYLQEQGFNNVKVY

RP

>sp|Q87RN8|KPRS_VIBPA Ribose-phosphate pyrophosphokinase OS=Vibrio parahaemolyticus serotype O3:K6 (strain RIMD 2210633) OX=223926 GN=prs PE=3 SV=1

MPDMKLFAGNATPELAQRIADRLYISLGDASVSRFSDGEVAVQINENVRGSDVFIIQSTC

APTNDNLMELVVMIDAMRRASAGRITAVIPYFGYARQDRRVRSARVPITAKVVADFLSNV

GVDRVLTIDLHAEQIQGFFDVPVDNIFGTPVLLEDMQSRGLDNPVVVSPDLGGVVRARAT

AKALGDIDIAIVDKRRPRANVSEVMNLIGDVEGRDCVIVDDMIDTGGTLCKAAEALKERG

AKRVFAYATHAVFSGNAADNIKNSVLDQVIVTDSISLSKEMAATGKVTTLSLSRMLAEAI

RRISNEESISAMFN

>sp|Q87T89|COABC_VIBPA Coenzyme A biosynthesis bifunctional protein CoaBC OS=Vibrio parahaemolyticus serotype O3:K6 (strain RIMD 2210633) OX=223926 GN=coaBC PE=3 SV=1

MQTLAGKKILLGISGGIAAYKCAELTRRLIERGAQVQVVMTKAAKEFITPLTMQAVSGRP

VSDSLLDPAAEASMGHIELAKWADLVLLAPATADLIARMSAGMGNDLLTTLVLATDSPVA

VSPAMNQQMYRNIATQENIATLARRGMNIWGPAAGEQACGDVGPGRMLEPMQLVHLCEQF

FQPKVLEGKSILISAGPTREAIDPVRYITNHSSGKMGYALANAAAQLGAKVTLVSGPVNL

STPMGVERINVSSAQEMYEAVMAQAISHDAFISCAAVADYRPEAIASQKLKKTADNDQMT

IKMVKNPDIVASVAALTDKRPFTVGFAAETNDVETYARGKLAKKNLNMICANDVSVEGQG

FNSNDNAITLFWPDGELALALESKEALSFKILEKMRELM

>sp|Q87KN2|LEXA_VIBPA LexA repressor OS=Vibrio parahaemolyticus serotype O3:K6 (strain RIMD 2210633) OX=223926 GN=lexA PE=3 SV=1

MKPLTPRQQQVFDLIKSKIDDTGMPPTRAEIARELGFRSANAAEEHLKALARKQAIEIIP

GASRGIRILLEDAANDEQGLPLIGQVAAGEPILAQEHVEAHYQVDPAMFKPQADFLLRVN

GESMKDIGIMDGDLLAVHKTQDVRDGQVVVARVDDDVTVKRLERKGSTVLLHAENEEFAP

IQVDLTSQHLTIEGLAVGIIRNTDWM

>sp|Q87SG9|MURE_VIBPA UDP-N-acetylmuramoyl-L-alanyl-D-glutamate--2,6-diaminopimelate ligase OS=Vibrio parahaemolyticus serotype O3:K6 (strain RIMD 2210633) OX=223926 GN=murE PE=3 SV=1

MTKAISMDALLSPWVDCPSLASVLVSELELDSRKVQPGTTFVALVGHVVDGRKFIASAIE

KGANAVIAQACDVKAHGTIDIIDDIPVVYLDALDKCLSEIAGQLYTYPDMKLIGVTGTNG

KTTITQLIAQWIGLVGSKAAVMGTTGNGFLDDLKEAANTTGNAVEIQHTLASLAEQQAQY

TALEVSSHGLIQGRVKSLSFAAGVFTNLSRDHLDYHGTMEEYANAKLTLFTQHQCAQAII

NVDDEVGAAWAKQLTNAIAVSLAPTTEFEHALWASQVAYAESGITIRFDGQFGEGTLHAP

LIGEFNAANLMLAFATLLSLGFDKSDLLATAAQLQPVLGRMELFQAEHRAKVVVDYAHTP

DALEKALQALRVHCDGQLWAIFGCGGDRDAGKRPMMAEIAERLGDKVVLTDDNPRSEDPV

LIVKDMLAGLSKPAEAIVQHDRFKALFYALENAAPQDIILLAGKGHEDYQIRNGETIHYS

DRESAMQLLGLSS

>sp|P19250|HLY2_VIBPA Thermostable direct hemolysin 2 OS=Vibrio parahaemolyticus serotype O3:K6 (strain RIMD 2210633) OX=223926 GN=tdh2 PE=1 SV=2

MKYRYFAKKSFLFISMLAAFKTFAFELPSVPFPAPGSDEILFVVRDTTFNTNAPVNVEVS

DFWTNRNVKRKPYKDVYGQSVFTTSGTKWLTSYMTVNINDKDYTMAAVSGYKHGHSAVFV

KSDQVQLQHSYDSVANFVGEDEDSIPSKMYLDETPEYFVNVEAYESGSGNILVMCISNKE

SFFECKHQQ

>sp|Q87LP9|PYRG_VIBPA CTP synthase OS=Vibrio parahaemolyticus serotype O3:K6 (strain RIMD 2210633) OX=223926 GN=pyrG PE=3 SV=1

MTTNYIFVTGGVVSSLGKGIAAASLAAILEARGLKVTMMKLDPYINVDPGTMSPTQHGEV

FVTEDGAETDLDLGHYERFIRTKMTKRNNFTAGRVYADVLRKERRGDYLGATIQVIPHIT

NAIKDRVIAGSEGHDIAIVEVGGTVGDIESLPFMEAIRQLAVELGRERAMFMHLTLVPYL

AAAGEVKTKPTQHSVKELLSIGIQPDILVCRSDRMIPANERKKIALFCNVPEKAVISMKD

VDSIYKIPQLVKSQGLDDLVCTRFGIDAPEADLSEWEQVIYEEANPTGEVTIGMVGKYIE

LPDAYKSVNEALKHAGLKNRLNVTIKYVDSQDIETKGVELLEGLDAILVPGGFGDRGVEG

KIRAAQYARENKVPYLGICLGMQVALIEYARNVAGMEGAHSTEFNKDTKYPVVGLITEWV

DETGNVEERTESSDLGGTMRLGSQLCHLEKGTKARELYGSATIHERHRHRYEVNNVLRPQ

IEKAGLKVSGLSADKKLVEMIENPAHPWFVAAQFHPEFTSTPRDGHPLFAGFVKAAGQYS

RGEFEK

>sp|Q87S19|RLMN_VIBPA Dual-specificity RNA methyltransferase RlmN OS=Vibrio parahaemolyticus serotype O3:K6 (strain RIMD 2210633) OX=223926 GN=rlmN PE=3 SV=1

MTTEKINLLDFDRKGMRQFFAEELGEKAFRADQVMKWIYHFGVDDFDNMTNINKKLREKL

QHKCEIKAPTVAEAQHSSDGTIKWAMKVGDQDVETVYIPEEDRATLCVSSQVGCALECKF

CSTAQQGFNRNLKVSEIIGQVWRAAREIGLQKETGRRPITNVVMMGMGEPLLNMKNLIPA

LEIMLDDLGFGLSKRRVTVSTSGVVSGLDQMTGKIDVALAISLHAPNDKLRSEIMPINDR

WDIQDFLASVRRYIASSNANRGKVTVEYVLLDHVNDDMGHARELAELMKDTPCKINLIPF

NPYPGSPYKKPSNSRIDRFQKTLMQYEHTVTVRKTRGDDIDAACGQLVGDVIDRTKRTAA

LKAARGAETIDVKAV

>sp|P22848|5NTD_VIBPA 5'-nucleotidase OS=Vibrio parahaemolyticus serotype O3:K6 (strain RIMD 2210633) OX=223926 GN=nutA PE=3 SV=2

MNQRLIIKTALSAAILASLAGCASQPAHEWNADTTYKLTVLHTNDHHGRFWQNKHGEYGM

AARKTLIDDLRDEIQAEGGSVLLLSGGDINTGVPESDLQDAEPDFKGMSKIGYDAMALGN

HEFDNPLDVLFKQQDWANFPMLSANIYDKKTGKRLFQPYAMFNKQGIKIAVIGLTTEDTA

KLGNPEFIGQVDFRDPKVEAKELIAELKKTENPDLIFAVTHMGHYENGNRGINAPGDVAL

ARYLNEGDLDMIVGGHSQEPVCMEGPNVIKKNFKPGDECQPDQQNGTYIVQAHEWGKYVG

RADYEFRNGELSMVSYDLIPVNLKKKINVDGQSQRVFVQDEITQDKAMLDFLRPFQEKGQ

SQLNVKIAESNGKLEGDRDVVRFQQTNLGRLIATAHMERAKADFAVMNSGGVRDSIEAGD

ITYKDVLTVQPFGNMVSYVDMSGQEVLDYLNIVATKPVDSGAYAQFAGISMRIENDKVTN

VFIGNKQLRLDGRYRFTVPSYNASGGDGYPKIDTHPGYVNTGFTDAEVLKDYLESHSPID

VNEYAPSGEVMYQTNNVVNQ

>sp|Q99289|HLT_VIBPA Thermolabile hemolysin OS=Vibrio parahaemolyticus serotype O3:K6 (strain RIMD 2210633) OX=223926 GN=VPA0226 PE=1 SV=2

MMKKTITLLTALLPLASAVAEEPTLSPEMVSASEVISTQENQTYTYVRCWYRTSYSKDDP

ATDWEWAKNEDGSYFTIDGYWWSSVSFKNMFYTNTSQNVIRQRCEATLDLANENADITFF

AADNRFSYNHTIWSNDAAMQPDQINKVVALGDSLSDTGNIFNASQWRFPNPNSWFLGHFS

NGFVWTEYIAKAKNLPLYNWAVGGAAGENQYIALTGVGEQVSSYLTYAKLAKNYKPANTL

FTLEFGLNDFMNYNRGVPEVKADYAEALIRLTDAGAKNFMLMTLPDATKAPQFKYSTQEE

IDKIRAKVLEMNEFIKAQAMYYKAQGYNITLFDTHALFETLTSAPEEHGFVNASDPCLDI

NRSSSVDYMYTHALRSECAASGAEKFVFWDVTHPTTATHRYVAEKMLESSNNLAEYRF

>sp|Q9LCJ0|NQRF_VIBPA Na(+)-translocating NADH-quinone reductase subunit F OS=Vibrio parahaemolyticus serotype O3:K6 (strain RIMD 2210633) OX=223926 GN=nqrF PE=3 SV=2

MDIILGVVMFTLIVLALVLVILFAKSKLVPTGDITISVNGDADKAIVTQPGGKLLSALAG

AGVFVSSACGGGGSCGQCRVKVKSGGGDILPTELDHITKGEAREGERLACQVAVKTDMDI

ELPEEIFGVKKWECTVISNDNKATFIKELKLQIPDGESVPFRAGGYIQIEAPAHHVKYAD

YDIPQEYREDWEKFNLFRYESKVNEETIRAYSMANYPEEHGIIMLNVRIATPPPNNPDVP

PGIMSSYIWSLKEGDKCTISGPFGEFFAKDTDAEMVFIGGGAGMAPMRSHIFDQLKRLHS

KRKMSFWYGARSKREMFYVEDFDGLAAENDNFVWHCALSDPLPEDNWDGYTGFIHNVLYE

NYLRDHEAPEDCEYYMCGPPMMNAAVIGMLKDLGVEDENILLDDFGG

>sp|Q87TN4|ILVC_VIBPA Ketol-acid reductoisomerase (NADP(+)) OS=Vibrio parahaemolyticus serotype O3:K6 (strain RIMD 2210633) OX=223926 GN=ilvC PE=3 SV=1

MANYFNTLNLREQLDQLGRCRFMDREEFVSEADYLKGKKVVIVGCGAQGLNQGLNMRDSG

LDVSYALRQAAIDEQRQSFKNAKDNGFEVGSYETLIPQADLVVNLTPDKQHTNVVETVMP

LMKEGAALGYSHGFNVVEEGMQIRKDLTVVMVAPKCPGTEVREEYKRGFGVPTLIAVHPE

NDPKGEGWDIAKAWAAATGGHRAGCLESSFVAEVKSDLMGEQTILCGMLQAGSIVCYEKM

IAEGIDPGYAGKLLQYGWETITEALKFGGITHMMDRLSNPAKIKAFELSEELKDLMRPLY

NKHMDDIISGHFSSTMMADWANDDKNLLGWRAETGETAFENYPETDVEISEQEYFDNGIL

MVAMVRAGVELAFEAMTASGIIDESAYYESLHELPLIANTIARKRLYEMNVVISDTAEYG

NYLFANVATPLLREKFMPSVGTDVIGKGLGETSNQVDNATLIAVNETIRNHPVEYIGEEL

RGYMTDMKRIAVGG

>sp|Q87LQ0|ENO_VIBPA Enolase OS=Vibrio parahaemolyticus serotype O3:K6 (strain RIMD 2210633) OX=223926 GN=eno PE=3 SV=1

MSKIVKVLGREIIDSRGNPTVEAEVHLEGGFVGMAAAPSGASTGSREALELRDGDKARFL

GKGVLKAIEAVNGPIAEALVGKDAKDQAAIDAVMIELDGTENKSKFGANAILAVSLANAK

AAAAAKGMPLYEHIAELNGTAGQFSMPLPMMNIINGGEHADNNVDIQEFMIQPVGAATLK

EAVRMGAEVFHNLAKVLKSKGYNTAVGDEGGFAPNLKSNAEALEVIAEAVAAAGYELGKD

VTLAMDCAASEFFDKEAGIYNMKGEGKTFTSEEFNHYLAGLVEQFPIVSIEDGLDESDWD

GFAHQTQLLGDKIQLVGDDLFVTNTKILAEGIEKGIANSILIKFNQIGSLTETLAAIKMA

KDAGYTAVISHRSGETEDATIADLAVGTAAGQIKTGSMSRSDRVAKYNQLIRIEEALGER

APFNGLKEVKGQA

>sp|Q87KV8|NHAP2_VIBPA K(+)/H(+) antiporter NhaP2 OS=Vibrio parahaemolyticus serotype O3:K6 (strain RIMD 2210633) OX=223926 GN=nhaP2 PE=1 SV=1

MDADTINSFFLIGALLIALSVLLSPVSSKLGIPILLVFLAVGMLAGEDGLGGILFDNYSI

AYLVSNLALAIILLDGGMRTRVASFRVALWPSVSLATIGVAITTLLTGLMATWLFDLDLL

QGILVGAIVGSTDAAAVFSLLKGRSLNERVGSTLEIESGTNDPMAVFLTVTLIAILSSTG

TGLSAGFLALSFVKQFGIGALLGFAGGWVLWKVINRNQLPDGLYSILTVSGGLIIFALSN

SLGGSGILSIYLVGLLLGNRPTRSRHSILHVLDGMTWLAQIGMFLVLGLLVTPSNLLSIA

VPGLALAFGMILFARPISVWIGLLPFKSFTPREKWFVSWVGLRGAVPIILAVFPMMAGLP

DAQLYFNLAFFVVMVSLIVQGGTLTKAMSLAKVELPPKPEPISRTGVEIYPTSEWELFIY

RLKADKWCIGEPLRSLSMPEGTRIAAVFRNQELLHPSGSTRLEEDDTLCVLAQEKDLAAL

SLLFSEAPEKASLTRFFGDFFLDIEVKLADVAMMYGLNLGYELQDKTLSNIVEEQLGSTP

VLGDQFEWQGLQWVIADVVDHQVTKVGLRLPNEEEEGEEED

>sp|Q87SK9|CCA_VIBPA Multifunctional CCA protein OS=Vibrio parahaemolyticus serotype O3:K6 (strain RIMD 2210633) OX=223926 GN=cca PE=3 SV=1

MQVYLVGGAVRDQLLGIDSYDNDWVVVGATPEMMLAQGYTAVGKDFPVFLHPKNKEEHAL

ARTERKSGSGYTGFDCFFDPSVTLEEDLIRRDLTINAMAMDDSGQLYDPYGGQADLNNRI

LRHVSDAFVEDPLRVLRVARFSAKLASLGFTVAKETMQLMRDIADSGELNTLTPERVWQE

WHKSLSTPRPDVFLSVLRDCGALAVVLPEIEALFGVPQPEKWHPEIDTGIHTLMVAEQAA

KLSTSLPVRFAAQVHDLGKGVTPESEWPSHKMHCHTGLKLIKKLCERVRVPNEFKELALM

VCEQHSNIHRAAELKPQTIIKILNKFDVWRKSERLKDILICCQADHAGRKGLEDLPYPQA

GIFMLAYQAAASVDVQAIIQDGFKGPAIRDEQEKRRIEAVKVALNK

>sp|Q87LJ1|IXTPA_VIBPA dITP/XTP pyrophosphatase OS=Vibrio parahaemolyticus serotype O3:K6 (strain RIMD 2210633) OX=223926 GN=VP2621 PE=3 SV=1

MKKIVLATGNQGKVREMADLLSDFGFEVLAQSEFNVSEVAETGTTFIENAIIKARHAAQE

TGLPAIADDSGLEVDFLKGAPGIYSARYAGEKASDQENLEKLLAAMEGVPEAERTARFHC

VLVLMRHENDPTPIVCHGKWEGRILTEAHGENGFGYDPIFFVPEDNCASAELEPARKKQL

SHRGKALKSLFAQLSAQTAQ

>tr|Q87MV7|Q87MV7_VIBPA Aspartate-semialdehyde dehydrogenase OS=Vibrio parahaemolyticus serotype O3:K6 (strain RIMD 2210633) OX=223926 GN=asd PE=3 SV=1

MRVGLVGWRGMVGSVLMQRMVEEKDFDLIEPVFYSTSQVGIPAPNLGKDAGMLQDAFDIE

SLKQLDAIITCQGGSYTEKVYPALRQAGWKGYWIDAASTLRMAEDSIITLDPVNLKQIQH

GIHGGTNTFVGGNCTVSLMLMGLGGLFEKGLVEWTSAMTYQAASGAGAQNMRELISQMGV

INDAVSSELANPASSILDIDKKVADTMRSASFPTDKFGVPLAGSLIPWIDVKRDNGQSKE

EWKAGVEANKILGSQGAPVPIDGTCVRIGAMRCHSQALTIKLKQNVPMDEIEEIIATHND

WVKVIPNDRDITAQELTPAKVTGTMSVPVGRLRKMAMGDDFLNAFTVGDQLLWGAAEPLR

RTLRIILSEKA

>sp|P74956|LON_VIBPA Lon protease OS=Vibrio parahaemolyticus serotype O3:K6 (strain RIMD 2210633) OX=223926 GN=lon PE=3 SV=2

MNLERSERIEIPVLPLRDVVVYPHMVIPLFVGREKSISCLETAMETNKQVLLVAQKQADT

DEPTVDDLFEVGTVATILQLLKLPDGTVKVLVEGQQRAKINHFKESDFFLAEAEFIVTPE

LDEREQEVIVRSAINQFEGFIKLNKKIPPEVLTSLNGIDEAARLADTIAAHMPLKLVDKQ

QVLEIIDVTERLEFLMGQMESEIDLLQVEKRIRGRVKKQMEKSQREYYLNEQMKAIQKEL

GEMEDAPDEFETLQKKIDESKMPQEAREKTEQELQKLKMMSPMSAEATVVRSYIDWMVSV

PWTKRSKVKKNLAKAEEILNEDHYGLERVKERILEYLAVQNRINKLKGPILCLVGPPGVG

KTSLGRSIASATGRKYVRMALGGVRDEAEIRGHRRTYIGSLPGKLIQKMSKVGVKNPLFL

LDEIDKMSSDMRGDPASALLEVLDPEQNNSFNDHYLEVDYDLSDVMFVATSNSMNIPGPL

LDRMEVIRLSGYTEDEKLNIAKRHLVEKQVQRNGLKPNEIVIEDSAIIGIIRYYTREAGV

RGLEREISKICRKAVKNILLDKDIKSVTVTMDNLKEYLGVQRFDYGKADESNRIGQVTGL

AWTEVGGDLLTIETQSMPGKGKLTQTGSLGDVMQESIQAAMTVVRSRADKLGINSDFYEK

KDIHVHVPEGATPKDGPSAGTAMCTALVSALTGNPVKAEVAMTGEITLRGEVLPIGGLKE

KLLAAHRGGIKTVLIPKDNERDLEEIPENVIADLQVIPVQWIDEVLKVALERDPTGVEFE

AKK

>sp|Q87KS8|PUR2_VIBPA Phosphoribosylamine--glycine ligase OS=Vibrio parahaemolyticus serotype O3:K6 (strain RIMD 2210633) OX=223926 GN=purD PE=3 SV=1

MRVLIIGSGGREHALGWKAAQNPNVETIFIAPGNAGTALEPKLENVNIDVEDIAGLVAFA

KEKAIELTIVGPEAPLVIGVVDAFREAGLPIFGPTQAAAQLEGSKAFTKDFLARHQIPTG

AYANFTEIEPALAYVREQGAPIVVKADGLAAGKGVIVAMTLEEAEDAIKDMLAGNAFGDA

GSRVVIEEFLDGEEASFIVMVDGENVLPMATSQDHKRVGDKDTGPNTGGMGAYSPAPVVT

PEIHNRIMEEVIYPTVRGMASEGNPYTGFLYAGLMIDKDGTPKVIEYNCRFGDPETQPIM

MRMESDLVDLCLAAIDEKLDQVESKWDPRASIGIVLAAGGYPAAYNKGDVISGLPQVEIE

GEKVFHAGTDNQDGDIVTNGGRVLCATALGNSVSEAQQRAYELAKQISWDGMFHRNDIGY

RAIAREQEK

>sp|Q87KE0|PURK_VIBPA N5-carboxyaminoimidazole ribonucleotide synthase OS=Vibrio parahaemolyticus serotype O3:K6 (strain RIMD 2210633) OX=223926 GN=purK PE=3 SV=1

MHVLVLGSGQLARMMSLAGAPLNIQISAYDVGSGNVVHPLTQQVLGHGLENAIEQVDAIT

AEFEHIPHDVLDICELSGKFLPSTAAIKAGGDRRVEKALLDNAGVRNAKHYVIETREDFE

RAIEHVGIPMVLKSALGGYDGKGQWRLKEAAQIETIWAEMAECIAATPTQAIVAEEFVPF

NREVSLVGARGKDGSVEVYPLAENVHTNGVLSLSTAIDAPELQAQAKQMFTAVADSLNYV

GVLALEFFDVEGTLLVNEIAPRVHNSGHWTQQGAETCQFENHLRAVCGLPLGSTKLIRET

SMVNILGEDTLPEALLAMDGCHIHWYGKEKREGRKMGHINVCGDYPGELHRRLCALAEVL

DPMIFPAVHEFAKQAQR

>sp|Q87HS9|PXPA_VIBPA 5-oxoprolinase subunit A OS=Vibrio parahaemolyticus serotype O3:K6 (strain RIMD 2210633) OX=223926 GN=pxpA PE=3 SV=1

MTLNKQQLTLNCDMGESFGSWKMGADDSVMPHVDMANIACGFHASDPNVMHDTITLANLH

DVDIGAHPGYPDLQGFGRRSLSMSSDEITNMVIYQVGALQALCRAQYTDIGYIKPHGALY

NDMMKSDAVFRAVVKAAALFKVPLMILASQENEKYLEIADDYDVPLLFEAFADRLYQDDG

MLTPRRHPNAVLKDELAILEQVRTLADSGRVKTASGSYILLEADTICVHGDNEESIALIQ

KIRQSLYSGGN

>sp|Q87N49|PYRF_VIBPA Orotidine 5'-phosphate decarboxylase OS=Vibrio parahaemolyticus serotype O3:K6 (strain RIMD 2210633) OX=223926 GN=pyrF PE=3 SV=1

MIDQKVIVALDYDNQADALAFVDRIDPASCRLKVGKEMFTLFGPDFVRELHKRGFSVFLD

LKFHDIPNTCSKAVRAAAELGVWMVNVHASGGERMMTASREILEPYGKDRPLLIGVTVLT

SMEQSDLAGIGLNVEPQEQVIRLATLTKNSGLDGVVCSAQESSLLKNELGKEFKLITPGI

RPAGSDQGDQRRIMTPVDAIQAGSDYLVIGRPITQATDPAAVLKSINDSLASK

>sp|Q87KE1|PURE_VIBPA N5-carboxyaminoimidazole ribonucleotide mutase OS=Vibrio parahaemolyticus serotype O3:K6 (strain RIMD 2210633) OX=223926 GN=purE PE=3 SV=1

MKVGIIMGSKSDWPTMKLAADMLDQFGVSYETKVVSAHRTPQLLADYASSAKERGIKVII

AGAGGAAHLPGMAAAFTSLPVLGVPVQSRALKGMDSLLSIVQMPKGIAVGTLAIGEAGAA

NAGILAAQILGTHDESIMAKVEAFRNEQTETVLANPNPAED

>sp|Q87LF8|PYRB_VIBPA Aspartate carbamoyltransferase OS=Vibrio parahaemolyticus serotype O3:K6 (strain RIMD 2210633) OX=223926 GN=pyrB PE=3 SV=1

MANSLYQKHIISIPELSREELELIVETAGNLKKEPRPELIKNKVVASCFFEPSTRTRLSF

ETAIQRIGGDVIGFDNDGNTSLAKKGETLSDSVQVISSYVDAFVMRHPQEGAARLASEFS

NGVPVINAGDGANQHPTQTLLDLYTIAETQGRLDNLNVAFVGDLKYGRTVHSLTQALAKF

NNIRFYFVAPDALAMPEYICEELDEAGIEYSLHTEMEAVIPELDILYMTRVQKERFDESE

YAHIKSAYILTAALLKGARENLKVLHPLPRVDEITTDVDKTPHAYYFQQAQNGVYARQAL

LALVLNETL

>sp|Q87LF7|PYRI_VIBPA Aspartate carbamoyltransferase regulatory chain OS=Vibrio parahaemolyticus serotype O3:K6 (strain RIMD 2210633) OX=223926 GN=pyrI PE=3 SV=1

MAKETQLQVEAIKNGTVIDHIPAQIGIKVLKLFDMHNSSQRVTIGLNLPSSALGHKDLLK

IENVFINEEQASKLALYAPHATVNQIENYEVVKKLALELPEKINNVFECPNSNCISHNEP

VESSFKVFEKKEEIRLKCKYCEKVFSREIVTER

>sp|Q87S37|QUEA_VIBPA S-adenosylmethionine:tRNA ribosyltransferase-isomerase OS=Vibrio parahaemolyticus serotype O3:K6 (strain RIMD 2210633) OX=223926 GN=queA PE=3 SV=1

MQVSDFHFDLPDELIARYPQPERTASRLLQMDGNTGELIDGTFTDVLNQVQAGDLVVFNN

TRVIPARMFGRKESGGKLEVLVERMLDEKSILAHVRCSKSPKPGTTIIVGENDEYSAEMV

ARHDALFELKFNSDKTVLDILEEIGHMPLPPYIDRPDEDADKERYQTVYNQKPGAVAAPT

AGLHFDDVLLDKIKAKGAEFAYVTLHVGAGTFQPVKVDNINDHHMHAEYVEVPQEVVDAI

NATKARGGRIIAVGTTSVRSLESAAQDALKKGTELVPFFGDTEIFIYPGYEYQLVDCLIT

NFHLPESTLIMLVSAFAGYENTMNAYKHAVENKYRFFSYGDSMFIKKKTI

>sp|Q87T92|PYRE_VIBPA Orotate phosphoribosyltransferase OS=Vibrio parahaemolyticus serotype O3:K6 (strain RIMD 2210633) OX=223926 GN=pyrE PE=3 SV=1

MKAYQREFIEFALEKEVLKFGEFTLKSGRKSPYFFNAGLFNTGRDLARLGRFYAAALADS

GIEFDVLFGPAYKGIPIATTTAVALADHHDIDTPYCFNRKEAKNHGEGGNLVGSALEGRI

MLVDDVITAGTAIRESMEIIKANGADLAGVLVAIDRQEKGKGELSAIQEVERDFGCAVIS

IVSLGDLITYLEEKGNATEHLEAVKAYRAEYGI

>sp|Q87Q87|PUR7_VIBPA Phosphoribosylaminoimidazole-succinocarboxamide synthase OS=Vibrio parahaemolyticus serotype O3:K6 (strain RIMD 2210633) OX=223926 GN=purC PE=3 SV=1

MSLANQVLAVNDDLPIRTHKPVHSGKVRSVYWLTEEDSARLIKEKGYDVAPDAPLAIMVI

SDRISAFDCIWHGEGGLKGVPGKGAALNAISNHWFKLFKDNGLADSHILDIPHPFVWIVQ

KAKPVKIEAICRKYITGSMWRAYANGEREFCGIQLPEGLEKDKALPNLLMTPSTKGILKG

IPGVPEADDVNITRQNIVDNYEAFNFSSAEDIAQYEKLLKEGFNVISQALEGIDQIFVDT

KFEFGYVHDAAGNEKLIYMDEVGTPDSSRIWDAKEYQAGNIVENSKEGFRQFLLNHFPDP

DILLNKERMPEREALARDNDLPVESLMDISRTYIGIAEKITGKPITLSENPKAEIIEILS

KEYGLID

>sp|Q87Q53|PURT_VIBPA Formate-dependent phosphoribosylglycinamide formyltransferase OS=Vibrio parahaemolyticus serotype O3:K6 (strain RIMD 2210633) OX=223926 GN=purT PE=3 SV=1

MFGTATSANATRVLLLGSGELGKEVAIECQRLGLEVIACDRYADAPAMQVAHRSYVLDML

DGKALEEIINKEQPAYVVPEIEAIATDKLVDLEEKGLNVVPTAKATKLTMNREGIRRLAA

EELDLSTSPYRFADNYDDFKAAVEHVGIPCVVKPVMSSSGKGQSVIKTEDDIEKAWDYAQ

EGGRTGAGRVIVEGFIDFDYEITLLTVRAVDGVHFCAPIGHRQEDGDYRESWQPQAMSEN

AIKAAEYTAEKVVNALSGYGIFGVELFVKGDKVIFNEVSPRPHDTGMVTMISQEMSEFAL

HVRAFTGMPINKIVQYGPSASAVILGQGTSTDIRFDNLAKALAQPQTQVRLFGKPEIDGR

RRLGVVLTRRKTIEESVQDAVENAKKVKIVY

>sp|Q87ME0|PYRH_VIBPA Uridylate kinase OS=Vibrio parahaemolyticus serotype O3:K6 (strain RIMD 2210633) OX=223926 GN=pyrH PE=3 SV=1

MTTNPKPAYQRILLKLSGEALQGEDGFGIDPAILDRMAQEVKELVELGVQVGVVIGGGNL

FRGAGLAEAGMNRVVGDHMGMLATVMNGLAMRDALHRAYVNARVMSAIPLKGVCDDYNWA

DAIRELRQGRVVIFSAGTGNPFFTTDSAACLRGIEIEADVVLKATKVDGVFTADPVANPD

AELYDKLSYAEVLDKELKVMDLAAFTLARDHKMPIRVFNMNKPGALRRVVMGEAEGTLIN

SDA

>sp|Q87RN9|PTH_VIBPA Peptidyl-tRNA hydrolase OS=Vibrio parahaemolyticus serotype O3:K6 (strain RIMD 2210633) OX=223926 GN=pth PE=3 SV=1

MTQPIKLLVGLANPGPEYAKTRHNAGAWVVEELARVHNVTLKNEPKFFGLTGRIMVNGQD

LRLLIPTTFMNLSGKAIAALAKFYQIKPEEIMVAHDELDLPPGVAKFKKGGGHGGHNGLR

DTISKLGNNKDFYRLRIGIGHPGHKDKVAGFVLGKAPAKEQELLDAAADEAVRSLDILIK

DGLSKAQNRLHTFKAE

>sp|Q87QW9|PURR_VIBPA HTH-type transcriptional repressor PurR OS=Vibrio parahaemolyticus serotype O3:K6 (strain RIMD 2210633) OX=223926 GN=purR PE=3 SV=1

MATIKDVARLAGVSTTTVSHVINKTRFVAEATQEKVMKAVDELNYAPSAVARSLKCNSTR

TIGMLVTQSTNLFFSEVIDGVESYCYRQGYTLILCNTGGIYEKQRDYIRMLAEKRVDGIL

VMCSDLTEELKEMLDRHSDIPKVVMDWGPESSRADKIIDNSEEGGYLATKYLIDNGHTDI

ACLSGHFEKLACQERIAGFRRAMAEAKLPINEDWILEGNFECDTAVLVADKITAMEKRPT

AVFCFNDTMALGLMSRLQQNGIKVPDDVSVIGYDNIELAEYFSPPLTTIHQPKRRVGKNA

FEILLERIKDKEHEKRVFEMQPEIVIRNTVKKLN

>sp|Q87J46|PYRC_VIBPA Dihydroorotase OS=Vibrio parahaemolyticus serotype O3:K6 (strain RIMD 2210633) OX=223926 GN=pyrC PE=3 SV=1

MTTLTITRPDDWHVHLRDGEVLKDTVRDISRYNGRALIMPNTVPPVTNTEMALAYRDRIL

KEQHGEQFEPLMALYLTDNTTPEEIRAAKATGKIVAAKLYPAGATTNSDSGVTDAKNIYH

VLEAMEEVGMLLLVHGEVTHHHVDIFDREKEFLDTVLAPIVNDFPNLKIVLEHITTADAA

QFVNNASDNVAATITAHHLLFNRNHMLVGGIKPHFYCLPILKRNTHQQALIEAATSGSKK

FFLGTDSAPHAKGAKESACGCAGSYTAHAALELYAEVFEKEGKLENLEAFASFNGPDFYG

IARNADTVTLEKSAWDVPESMPFGNDIVVPIRANEQIEWKVK

>sp|Q87PB7|PYRD_VIBPA Dihydroorotate dehydrogenase (quinone) OS=Vibrio parahaemolyticus serotype O3:K6 (strain RIMD 2210633) OX=223926 GN=pyrD PE=3 SV=1

MLYRLARTGFFQLDAEKAHDLAIKNFQRFNGTPLDLFYRQQLPNRPVECMGLTFRNPVGL

AAGLDKNGECIEAFDAMGFGFVEVGTVTPRPQPGNDKPRLFRLVEAEGIINRMGFNNLGV

DHLVENVKKAKFNCVLGINIGKNKDTPIENGAEDYLICMEKVYEYAGYIAVNISSPNTPG

LRSLQYGEALDELLSELKAKQSELAEKHGKYVPLALKIAPDLSDDEITQICESLLKNNID

GVIATNTTLDRTVVEGMKHANEAGGLSGRPVQSRSTEVVRKLHEALGDKLPIIGVGGIDS

YVAAKEKMMAGAQLVQVYTGFIYHGPGLVRDIVKNL

>sp|Q87MH0|PUR5_VIBPA Phosphoribosylformylglycinamidine cyclo-ligase OS=Vibrio parahaemolyticus serotype O3:K6 (strain RIMD 2210633) OX=223926 GN=purM PE=3 SV=1

MSGNNSSLSYKDAGVDIDAGNALVDRIKGAVKRTRRPEVMGGIGGFGALCELPTKYKQPV

LVSGTDGVGTKLRLALDMNKHDTIGIDLVAMCVNDLIVQGAEPLFFLDYYATGKLDVDTA

ADVVSGIADGCVQAGCALIGGETAEMPGMYEGEDYDVAGFCVGVVEKEDVIDGTKVAAGD

ALIAVGSSGPHSNGYSLIRKILEVSGADKNEELAGRTIGEHLLEPTKIYIKSALKMIEKH

DIHAISHITGGGFWENIPRVLPEGTKAVIDGNSWEWPIIFKWLQEKGNVETHEMYRTFNC

GVGLVVALPKDQADAAVALLKEEGENAWVIGEIAQAEANEEQVEIN

>sp|Q87KT0|PUR9_VIBPA Bifunctional purine biosynthesis protein PurH OS=Vibrio parahaemolyticus serotype O3:K6 (strain RIMD 2210633) OX=223926 GN=purH PE=3 SV=1

MNNARPIRRALISVSDKTGIVEFAQALAERGVDILSTGGTARLLAEQGIAVTEVSDYTGF

PEMMDGRVKTLHPKVHGGVLGRRGQDDDVMAKHGINPIDMVVVNLYPFAETVAKDGCTLA

DAVENIDIGGPTMVRSAAKNHKDVTIVVNASDYDRVIAEMDANDKSLTLETRFDLAIAAF

EHTAAYDGMIANYFGTMVPSYGENKEGDEESKFPRTFNQQFEKKQDMRYGENSHQAAAFY

VEANPQEASVSTARQIQGKALSYNNIADTDAALECVKEFNEPACVIVKHANPCGVALGKD

ILEAYNRAYQTDPTSAFGGIIAFNQELDAETASAIVERQFVEVIIAPSVSAEAIEVVAAK

KNVRLLECGEWTTKTTGFDVKRVNGGLLVQDRDQGMVSLDDLKVVSKRQPTEEELKDALF

CWKVAKYVKSNAIVYAKGDMTIGVGAGQMSRVYSAKIAGIKAADEGLEVAGSVMASDAFF

PFRDGIDAAAEAGIKCVIQPGGSMRDDEVIAAADEHGMAMIFTGMRHFRH

>sp|Q87RW0|PUR4_VIBPA Phosphoribosylformylglycinamidine synthase OS=Vibrio parahaemolyticus serotype O3:K6 (strain RIMD 2210633) OX=223926 GN=purL PE=3 SV=1

MRILRGSPALSEFRVNKLLELCREQDLPVTGIYAEFMHFADLKSDLDDQELEKLEKLLTY

GPTIEEHEPEGLLLLVTPRPGTISPWSSKSTDIAINCGLDTVKRLERGTAYYVESSVVLS

EAQVDAVKALIHDRMMETVFTELEAASALFTVAEPKPVAHVDILAGGRLALEEANVSLGL

ALAEDEIDYLVENFTKLGRNPNDIELMMFAQANSEHCRHKIFNADWTIDGVDQEKSLFKM

IKNTFETTPDHVLSAYKDNAAVMTGSKVGRFFPDPKSRQYTYHHEDAHILMKVETHNHPT

AISPWPGASTGSGGEIRDEGATGIGGKPKAGLVGFTTSNLRIPGFEQPWETDFGKPGRIV

NALDIMLEGPLGGAAFNNEFGRPNLLGYFRTYEEKVTSHAGEEVRGYHKPIMIAGGMGNI

RDEHVQKKEIPVGASLIVLGGPAMNIGLGGGAASSMASGQSAEDLDFASVQRENPEMERR

CQEVIDRCWQLGEENPIAFIHDVGAGGISNALPELCDDGERGGKFQLRDVPNDELSMSPL

EIWCNESQERYVLAVAPENMEAFDAICKRERAPYAVVGVATEERHLTLEDSHFDNTPIDM

PMDILLGKTPKMHREATTLKVDSPAIARDGIEIDEAADRVLRLPTVAEKTFLITIGDRSV

TGLVARDQMVGPWQVPVANCAVTAASYDTYHGEAMSMGERTPVALLDFGASARLAVGESL

TNIAATDIGDIKRIKLSANWMSPAGHPGEDAGLYEAVKAVGEELCPALGLTIPVGKDSMS

MKTKWEENGESKEVTSPLSLVITAFGRVEDVRKTVTPQLRTSDTLEGLGDTSLVLVDLGN

GKNRLGATALAQVYKQLGDKPADVDNAEQLKGFFDAMQNLVRNDKLLAYHDKGDGGLFVT

LAEMAFAGHCGVKADIAELGEDALAVLFNEELGAVVQVKNDDLDSVLSTLAANGLEACSH

VIGSVEASDDFVFTSGDDVVLKRSRTELRVIWAETTHKMQALRDNPACADQEFEAKKDNT

DPGLNVSLSFDVNEDIAAPYIAKGAKPKMAILREQGVNSHVEMAAAFDRAGFEATDIHMS

DILTGQAVLDEYQGLVACGGFSYGDVLGAGEGWAKSILFNAQAREQFQAFFNREETFSLG

VCNGCQMLSNLKELIPGADLWPRFVRNESERFEARFSLVEVQKSDSVFFDGMAGSRMPIA

VSHGEGRVEVRDGEHLNAIEASGTVALRYVDNNGNPTQQYPNNPNGSPNAITGLTTADGR

VTIMMPHPERVFRTVANSWAPEGWGENGAWMRMFQNARKNIG

>sp|Q87J80|PSRP_VIBPA Putative phosphoenolpyruvate synthase regulatory protein OS=Vibrio parahaemolyticus serotype O3:K6 (strain RIMD 2210633) OX=223926 GN=VPA0373 PE=3 SV=1

MQINNQSRDVFYVSDGTAITCETLGHVVLGQFPFIPNEKTFPFVESQDKVADVVKEIETS

YQRNGVKPLVFFSIVVPGVREMLLEAPAYSYDVLESIVQKVQDDIQMAPAPKLQRSRSVG

KDSDTYFDRIAAIEYTLAHDDGITLKGLEQADIILLGVSRSGKTPTSLYMAMQFGLRVVN

YPFIAEDVKMMRLLPEFEVHRHKLFGLTITPERLNEIRENRLSGSDYASEEQCKLELDTV

EALFRREAIPYINTSSLSVEEISTRILERAGMKRRLFG

>sp|Q87FD5|PTYBC_VIBPA PTS system N-acetylmuramic acid-specific EIIBC component OS=Vibrio parahaemolyticus serotype O3:K6 (strain RIMD 2210633) OX=223926 GN=murP PE=3 SV=1

MAKITSNTVSQLLSAVGGSSNVSKCGNCMTRLRLSLANNGLADQSVIKKIPGVMGVVESD

EQFQIILGPGKAQQAAEMMNQLIDSLTSGDSEEPDMPQQDLSAVAAEQKKQMKSKQTSAV

QRFLSKFATIFTPLIPGFIAAGLLLGFATLLEQMFVLDQTPSQFMLDLIAYMKVFGKGLF

AFLSILIGYNAQQAFGGSGVNGAILASLFVLGYNPEATSGIYSGMNEFFGFAIDPRGNII

GVLLAAIIGAQVERKVRQYMPDDLDMILTSVITLLIMGAVTFLIIMPIGGELFKGMSWLF

LNLNDNPLGAAILAGLFLISVVFGIHQGFVPVYFALMEAQGFNSLFPILAMAGGGQVGAS

MALYFKAKKDALLRTQVKGAIIPGLLGIGEPLIYGVTLPRVKPFVTACIGGAAGGFFIGL

VSYLGLPVGLNTVFGPSGIVAIPLMTSENGIFPGMMVFVAGLLISYIVGFLATYFFGCKD

VDLS

>sp|P40607|PURA_VIBPA Adenylosuccinate synthetase OS=Vibrio parahaemolyticus serotype O3:K6 (strain RIMD 2210633) OX=223926 GN=purA PE=3 SV=3

MGNNVVVLGTQWGDEGKGKIVDLLTEDAKYVVRYQGGHNAGHTLVIDGEKTVLHLIPSGI

LRDNVKCVIGNGVVLSPEALLKEMKPLEERGIPVRERLFISEACPLILPYHVAMDQAREI

ARGKKAIGTTGRGIGPAYEDKVARRGLRVGDLFDMEAFAEKLKEVMEYHNFQLVNFYKAE

PVSYEAVLEEAKGYAELLTSMVIDVTDELDAARKRGDKIMFEGAQGTLLDIDHGTYPYVT

SSNTTAGGVAAGSGFGPRHIGYILGIAKAYCTRVGAGPFPTELYDGLEKQDPVGKHLGTV

GHEFGATTGRLRRTGWFDAVAMRRAIQINSVSGFCLTKLDVLDGLEELKICTGYKMEDGS

VLEVSPMAAEAFEKATPIYETMPGWSENTFGAKSLDALPQAALNYIKRIEELTGVPVDII

STGPDRNETIIKVHPFEA

>sp|Q87RV0|PROB_VIBPA Glutamate 5-kinase OS=Vibrio parahaemolyticus serotype O3:K6 (strain RIMD 2210633) OX=223926 GN=proB PE=3 SV=1

MTTNQQNAVVSQPQTVVVKLGTSVLTGGTLALDRAHMVELARQCAELKKQGHSVVMVSSG

AIAAGREHLGYPALPNEMASKQLLAAVGQSRLIQTWESLFGIYGIKIGQMLLTRADLDDR

ERFLNARDTINALVANDIIPIVNENDAVATSEIKVGDNDNLSALVGILCGADKLLLLTDQ

KGLFTADPRKDPNAELIKEVKTIDDTLRKIAGGSGTTLGTGGMATKLQAADIARRAGIEV

IIAAGSAPNVIFDSLSTEPQGTRFLPCSEALENRKRWILAGPAASGDIIIDDGAVNAVVG

KGSSLLAKGVIKVSGDFARGEVARVTNSHGKLVARGISAYSSEDLAKITGKHSKDIISIL

GHDYGSEVIHRDDLVVIQE

>sp|Q87RL6|SYE_VIBPA Glutamate--tRNA ligase OS=Vibrio parahaemolyticus serotype O3:K6 (strain RIMD 2210633) OX=223926 GN=gltX PE=3 SV=1

MTVKTRFAPSPTGYLHVGGARTALYSWLYAKNQGGEFVLRIEDTDLERNSQEAVDAILEG

MEWLGLEWDEGPYYQTQRFDRYNEMVDKLLAEDKAYKCYASKELLDEIRAEQEANKEMPR

YDANHPKIKAANEAAKDGDPCVIRFRNPKEGSVVFEDQIRGRIEIRNDQMDDLIIRRTDG

SPTYNFCVVVDDWDMGITHVVRGEDHINNTPRQINIYEALGAPVPTFAHCAMILGDDGAK

LSKRHGAVSVMQYRDMGYLPAALNNYLVRLGWSHGDQEIFSQEEMINLFSLNAVSKSASA

FNTDKLQWLNNHYIKNSDPAYVAEHLQWHLDQQKLDVTNGPAITDVIKLVGERCHTLVEL

AEQIRYFYEDFSEFEAGAAKKHLRGVAKEPLEVALAKVEAITEWTTENLHQMIADVCAEL

EIGMGKIGMPLRVAVTGGGQSPSVDAVMALIGKERCVARIKMALEFIAEREANA

>sp|Q87TP8|SYGB_VIBPA Glycine--tRNA ligase beta subunit OS=Vibrio parahaemolyticus serotype O3:K6 (strain RIMD 2210633) OX=223926 GN=glyS PE=3 SV=1

MAKEFLIELGTEELPPTQLRTLAEAFAANFEAELKGAELAHEGVKWFAAPRRLALKVAAL

AESQSDKVVEKRGPAVSAAFDAEGNPTKAAQGWARGCGITVDQADRMVTDKGEWLLFKQE

VKGQPTSEIVVELAAKALANLPIAKPMRWGNKTTQFIRPVKTLTMLMGSDLIEGEILGVA

SSRTIRGHRFMGEKEFTIDSAEQYPAILEERGKVMADYEARKAIILADAQKAAAAVGGIA

DLEDDLVEEVTSLVEWPVVLTAKFEEEFLKVPSEALVYTMKGDQKYFPVYDENKKLLPNF

IFVSNIESKEPRYVIEGNEKVVRPRLADAEFFFNTDRKRPLIDRLPELEQAIFQQQLGTI

KDKTDRITELAGYIAEQIGADVEKSKRAGLLAKCDLMTSMVFEFTDTQGVMGMHYARHDG

EAEEVAVALNEQYMPRFAGDELPSNGVSTAVAMADKLDTIVGIFGIGQAPKGSDPFALRR

ASLGVLRIIVEYGYNLDLVDLVAKAKSLFGDRLTNDNVEQDVIEFMLGRFRAWYQDEGFS

VDIIQAVLARRPTKPADFDQRVKAVSHFRELEAAESLAAANKRVGNILAKFDGELAEEID

LALLQEDAEKALAESVEVMTEALEPAFATGNYQEALSKLADLREPVDAFFDNVMVMADDE

ALKKNRLTLLNNLRNLFLQIADISLLQK

>sp|Q87S90|SYI_VIBPA Isoleucine--tRNA ligase OS=Vibrio parahaemolyticus serotype O3:K6 (strain RIMD 2210633) OX=223926 GN=ileS PE=3 SV=1

MSEYKDTLNLPETGFPMRGNLANREPEMLERWYKEDLYGEIRKAKKGKKSFVLHDGPPYA

NGDIHIGHALNKILKDIIIKSKTLSGFDAPYIPGWDCHGLPIELMVEKKVGKPGQKVTAA

EFREKCREYAAGQVEGQKESFKRLGIMGEWDKPYRTMDFATEANIIRALGKIASNGHLLK

GFKPVHWCTDCGSALAEAEVEYKDKVSPSIDVRFKTADEAALLSKFELTEGHEGKGDVSI

VIWTTTPWTLPANRAVCLRDDLEYVLIQVEGDNPERIIVAAELAKDVMDRAGIEHFHNLG

FAKGADLELSQFQHPFYDFTVPAILGDHVTTDSGTGVVHTAPGHGQEDFAVGQKYNLEVA

NPVGSNGVYLPDTELFAGQHVFKANDAVVETLKEKGALLHHHAYEHSYPHCWRHKTPIIF

RATPQWFVSMDQAGLRAKALESIKGVQWMPEWGQSRIEGMIEGRPEWCISRQRTWGVPIA

LFVHKETAELHPNTLELIEKVAKLVEEKGIQAWWDVDAAELLGDEAEQYEKVLDTLDVWF

DSGVTHFSVVDAREEYNGNSADLYLEGSDQHRGWFQSSLISSIAMKGVAPYKQVLTHGFV

VDGHGRKMSKSIGNVVAPKDVTNKLGADILRLWVASTDYTGEVAVSDEILKRSADAYRRI

RNTARFFLANLNGFNPATDIVPAEEMVALDRWAVGRALAAQEEIIKAYDEYNIHAVTQRL

MQFCSIEMGSFYLDVIKDRQYTAKQGGHAQRSCQTALYYIVEALVRWMAPIMSFTADEIW

NEMPGEREKFVFTGEWFDGLFGLAEGEELNNEFWTEIQKVRGAVNKLLEAARAEKTIGGA

LQAELTLFADDALAAKINKLEDELRFVLLTSAAAVKPLSEKSDAAQATDIEGLFVEVKAT

EAEKCDRCWHHTPDVGTIAGHEKICGRCVSNVDGKGEVRKFA

>sp|Q87RQ0|SYL_VIBPA Leucine--tRNA ligase OS=Vibrio parahaemolyticus serotype O3:K6 (strain RIMD 2210633) OX=223926 GN=leuS PE=3 SV=1

MQEQYNPQDIEQKVQKHWDDNKTFVVSEDPNKEKFYCLSMFPYPSGRLHMGHVRNYTIGD

VVSRFQRLQGKNVMQPIGWDAFGLPAENAAVKNNTAPAPWTYENIEYMKNQLKLLGFGYD

WNREFATCTPEYYRWEQEFFTKLYEKGLVYKKTSSVNWCPNDQTVLANEQVEDGCCWRCD

TPVEQKEIPQWFIKITEYAQELLDDLDKLEGWPEMVKTMQRNWIGRSEGVELKFEVKGQQ

DLEVYTTRPDTLMGVTYVGIAAGHPLATLAAENNPELAAFIEECKNTKVAEAELATMEKK

GMATGLTAIHPLNGREVPVYVANFVLMDYGTGAVMAVPAHDQRDFEFATKYGLDIIPVIK

PADGSELDISEAAYTEKGVLFDSGEFDGLEFQAAFDAIAAKLEAEGKGTKTVNFRLRDWG

VSRQRYWGAPIPMVTTEDGEVHPVPADQLPVILPEDVVMDGVTSPIKADKEWAKTTFNGE

PALRETDTFDTFMESSWYYARYCSPQADDILDPEKANYWLPVDQYIGGIEHACMHLLYSR

FFHKLLRDAGYVTSDEPFKQLLCQGMVLADAFYFENEKGGKEWVAPTDVAVERDGKGRII

SAKDNEGRDVTHSGMIKMSKSKNNGIDPQEMVDKYGADTVRLFMMFASPADMTLEWQESG

VEGANRFLKRVWKLVKEHAEKGAAEAVDTAALSGEQKALRRDVHKTIAKVTDDIARRQTF

NTAIAAIMELMNKLAKAPQESAQDRAILDEALKAVVTMLYPITPHISYELWTALGESDID

NAAWPTFDEKALVEDEKTIVVQVNGKLRAKLTVAADATKEQVEELGLNDENVTKFTDGLT

IRKVIYVPGKLLNIVAN

>sp|Q87MC3|SYP_VIBPA Proline--tRNA ligase OS=Vibrio parahaemolyticus serotype O3:K6 (strain RIMD 2210633) OX=223926 GN=proS PE=3 SV=1

MRTSNYLLSTLKETPNDAEVVSHQLMLRAGMIRKLASGLYTWLPTGLRVLRKVENIVRQE

IDNAGAVETLMPVVQPFELWEETGRSEKMGPELLRFTDRHVRPFVLSPTAEEVITSLVRN

EVSSYKQLPLNLYQIQTKFRDERRPRFGVMRAREFCMMDAYSFDIDKAGLEKSYQAMHDA

YCKAFDRMGLEYRPVLADSGAIGGSGSQEFHVLADSGEDLIAFSTESDYAANIEKAEALA

PAVERAEPTQEMTLVDTPNAKTIAELVEQHGLPIEKTVKTLFVKASDEIDAPIIALIVRG

DHELNEVKAENLPQVASPLEMASEEEIRELIGAGPGSLGPVGLELPFIVDRSVAVMSDFG

AGANIDGKHYFGINWGRDVELGQVEDLRNVVEGDPSPCGKGTLMLKRGIEVGHIFQLGNV

YSEAMNCSVLGPDGKNVILEMGCYGIGVSRVVASAIEQNHDKYGIIWPDAIAPFQVAIVP

MNMHKSERVKEAAEKLYAELTAMGIEVLFDDRKERPGVMFSDIELIGIPHTIVIGDRSMD

EGNFEYKNRRTGEKTPVAMADIVEHVKSQLK

>sp|Q87RE8|SUCC_VIBPA Succinate--CoA ligase [ADP-forming] subunit beta OS=Vibrio parahaemolyticus serotype O3:K6 (strain RIMD 2210633) OX=223926 GN=sucC PE=3 SV=1

MNLHEYQAKQLFAEFGLPVPEGYACDTPQEAFEAAGRISTAKKVVKCQVHAGGRGKAGGV

ELHDTKEGVKEFAQKWLGKNLVTYQTDANGQPVTKILVEEASNIANELYLGAVVDRASRK

IVFMASTEGGVEIEKVAEETPELIHKAAIDPLVGPQAYQGRELAFKLGLEGDQIKQFVKI

FMGLGTMFSQYDLALLEINPLVITAEGNLLCLDGKINIDSNALYRQPKLREMHDPSQEDE

REAHAAQWELNYVALDGNVGCMVNGAGLAMGTMDIVNLHGGKPANFLDVGGGATKERVAE

AFKIILSDDNVKAVLVNIFGGIVRCDMIAEGIIGAVKEVGVSVPVVVRLEGTNADLGREV

LANSDVDIIAAESLTDAAQKVVAAAEAK

>sp|Q87ST4|SURA_VIBPA Chaperone SurA OS=Vibrio parahaemolyticus serotype O3:K6 (strain RIMD 2210633) OX=223926 GN=surA PE=3 SV=1

MKIWKSILFTTLLSCGAVAAPVELDKVAVIVNDGVILQSDINTAMKTLQANARQSGKSLP

SASVLKEQVVEKLIIDTLQGQEADRIGVRIDDNRLNQAIAEIARNNNQSVEELAASVQAE

GLSYPEFREQIRKEIAASEARNALVRRRINILPAEVDSLADQLAKETNATVQYKIGHIQL

RFTDGQDKSEVEAQAKALVKKLNDGADFTEMAYTYSKGPKALQGGDWGWMRKEEMPTIFA

DQIKMQNKGSIIGPFRSGVGFHILKIEDVKGLETVAVTEVNARHILIKPTVILSDEGAKK

QLNEFVRRIKAGEATFAQLASQYSQDPGSAAQDGELGYQTPDLYVPEFKHQVETLPVGSI

SEPFKTVHGWHIVEVLDRRQVDRTDSAMKNKAYRILFNRKFNEEAGAWMQELRASAFVEI

VDDNNDN

>sp|Q87LQ5|SURE_VIBPA 5'-nucleotidase SurE OS=Vibrio parahaemolyticus serotype O3:K6 (strain RIMD 2210633) OX=223926 GN=surE PE=3 SV=1

MELDSLNTKPLRILISNDDGVHAQGIHALADELRSIAEVIIVAPDRNRSGASNSLTLEQP

LRVSEIAPNTYSVQGTPTDCVHFALNELMKDDLPDLVLSGINHGANLGDDVLYSGTVAAA

MEGHFLGVQAIAFSLVGKRHFESAAKIARQLVEQHLAAPIPTNRLLNVNVPDLPLESLGE

IEVTRLGARHHAENMIKQKDPRGHDIYWLGPPGKEQDAGEGTDFYAIEHGRVSITPLQVD

LTAHESLRAMDSWLKEEK

>sp|Q87RS5|SYDP_VIBPA Protein Syd OS=Vibrio parahaemolyticus serotype O3:K6 (strain RIMD 2210633) OX=223926 GN=syd PE=3 SV=1

MTQTVTQALQDFSLRYQQAWQNKHNELPRNEELADLVSPCVEEKCDGAVLWKAFPREEMA

DFTNVENAIELTLHEDIKAFYGSQYSADMDATWQTNPLTLLQVWSDDDFVRLQENILGHL

VTQRRLKLKPTVFIAATDAELDVISICNLTGNVILERLGTDKREVLAENVVEFLAKIEAA

V

>sp|Q87QV2|SYD_VIBPA Aspartate--tRNA ligase OS=Vibrio parahaemolyticus serotype O3:K6 (strain RIMD 2210633) OX=223926 GN=aspS PE=3 SV=1

MRTHYCGHLNKSLAGQTVELCGWVNRRRDLGGLIFIDMRDREGIVQVVVDPDMADAYEVA

NTLRNEFCIKLTGEVRVRPESQVNKDMATGEVEILAKGLEIINRSDVLPLDFNQKNSEEQ

RLKYRYLDLRRPEMSDRIKLRAKASSFVRRFLDDNGFLDIETPVLTKATPEGARDYLVPS

RVHKGSFYALPQSPQLFKQLLMMSGFDRYYQIVKCFRDEDLRADRQPEFTQIDIETSFMT

ADQVREVTEKMVREMWQELLNVDLGEFPVMPFSEAIRRFGSDKPDLRNPLELVDVADLVK

DVEFKVFSGPANDEKGRVAVIRVPGGAELTRKQIDGYAEFVGIYGAKGLAWMKVNDRAAG

VEGIQSPVAKFLSEDVINGILDRTQAESGDIILFGADKANIVAEALGALRLKLGKDLGLT

KEGTWAPLWVVDFPMFEEDDEGNLHAMHHPFTSPLGVTAEELKANPAVANSNAYDMVLNG

YEVGGGSVRIHNAEMQAAVFDILGIDAEEQQLKFGFLLDALKFGTPPHAGLAFGLDRLVM

LLCGTENIRDVIAFPKTTAAACLLTDAPSIANPAALEELAIAVTAAKAKDAE

>sp|Q87QP1|SYS_VIBPA Serine--tRNA ligase OS=Vibrio parahaemolyticus serotype O3:K6 (strain RIMD 2210633) OX=223926 GN=serS PE=3 SV=1

MLDSKLLRTELDETAAKLARRGFKLDVETIRKLEEQRKSIQVEVENLQSTRNSISKQIGQ

KMAAGDKEGAEEIKKQIGTLGSDLDAKKVELEQVMAQLDEFTLSVPNIPADEVPDGKDEN

DNVEISRWGEPKTYDFDLKDHVDLGEMGGGLDFASAVKITGARFIVMKGQFARLHRAIAQ

FMLDLHTEEHGYTEMYVPYLVNSDSLFGTGQLPKFGKDLFHTEPLAEKVNDEEPRKLSLI

PTAEVPVTNLVRDTITDEADLPIKMTAHTPCFRSEAGSYGRDTRGLIRMHQFDKVELVQI

TKPEDSMTALEELTGHAEKVLQLLELPYRKVVLCTGDMGFGARKTYDLEVWVPAQETYRE

ISSCSNMWDFQARRMQARFRRKGEKKPELVHTLNGSGLAVGRTMVAILENNQEADGRIAI

PAVLQKYMGGATHIG

>sp|Q87Q70|SYT_VIBPA Threonine--tRNA ligase OS=Vibrio parahaemolyticus serotype O3:K6 (strain RIMD 2210633) OX=223926 GN=thrS PE=3 SV=1

MPIITLPDGSQRQFDNPVSTMEVAQSIGPGLAKATIAGRVNGNRVDACDLIEEDASLEII

TVKDEVDGLEIVRHSCAHLLGHALKQLYPQAKMAIGPTIDNGFYYDIDLDESLTQEDLEK

IEKRMKELAKTKYEVVKKKVSWQEARDTFESRGEPYKVEILDENVSRDDRPGLYHHEEYI

DMCRGPHVPNMGFCQHFTLLNVAGAYWRGNSDNKMLQRIYGTAFHDKKALKAHLTRLEEA

AKRDHRKIGKQLDLFHMQQEAPGMVFWHHNGWSIFRDLEVFVRDKLNEYDYQEVKGPLMM

DRVLWERSGHWDKYADAMFTTSSENREYAIKPMNCPGHVQIFNQGLKSYRDLPLRMAEFG

SCHRNEPSGALHGIMRVRGFTQDDAHIFCTESQIQEEVTSCIKMVYDTYQTFGFDNIVVK

LSTRPEKRVGSDEIWDQSEEALKQSLESMEIPYEIQEGEGAFYGPKIEFTLYDCLDRAWQ

CGTVQLDFNLPGRLGATYVGENNERLVPVMIHRAILGSLERFIGILIEEYAGFFPTWLAP

EQAVLMNITDKQSGYVQEIVQKLQKSGIRAKADLRNEKIGFKIREHTLKRVPYMLVVGDQ

EMEAGEIAVRTRKGKDLGKFKVDDFIAYIQDEISSRKLNLEE

>sp|Q87LR3|SYA_VIBPA Alanine--tRNA ligase OS=Vibrio parahaemolyticus serotype O3:K6 (strain RIMD 2210633) OX=223926 GN=alaS PE=3 SV=1

MYMSTDEVRNAFLKFFESKGHQIVESSSLVPHNDPTLLFTNAGMNQFKDCFLGLEKRAYT

RATTAQRCVRAGGKHNDLENVGFTARHHTFFEMLGNFSFGDYFKEDAISFAWEFLTDVLK

LPADRLLVTVYETDDEAFDIWNKKVGVPADRIIRIGDKKGGKPYESDNFWQMGDTGPCGP

CTEIFYDHGEHIWGGRPGTPEEDGDRFIEIWNNVFMQFNRHADGTMEPLPKPSVDTGMGI

ERISAIMQGVHSNYEIDVFQALIKAAAEVIGYEDLSNQSLRVIADHIRSCSFLIVDGVMP

SNEGRGYVLRRIIRRAVRHGNKLGAQGAFFHKLVGVLADIMGTAGEELKRQQAVVEKVLR

IEEENFGRTLERGMAILNEALDNLDGKVLDGETVFKLYDTYGFPADLTNDVAREREFAID

EEGFEKAMEEQRQRAREAGQFGTDYNAAIKVDTQTEFCGYVGTKGSSSVAAMFVEGNEVD

SLSAGDKAIIVLGETPFYAESGGQCGDAGEIRTEAGVFRVEDTQKLGNAIAHHGVMAEGV

LAKGDEVATIVDAERRAAISLNHSATHLLHAALRQVLGEHVTQKGSLVKAENLRFDFSHL

EAVTAAELKEVERLVNAQIRRNHVIETNVMDIESAKKKGAMALFGEKYDDEVRVLSMGDF

STELCGGIHASNTGDIGLFKITSESGIAAGIRRIEAVTGEAALDAIEAQAAKYEEKLAES

AQKAKTLEKELQKLKDKMAAAESANIMGKAVEVNGTKVLVAALEGADSKNLRTMVDDIKN

QMGSGVVLLANVTDDKVGLIAGVTKDLVGKVKAGDLVKMVAEQVGGKGGGRPDMAQAGGT

DVSALPEAIKTVQPWLEERL

>sp|Q87Q60|SYFA_VIBPA Phenylalanine--tRNA ligase alpha subunit OS=Vibrio parahaemolyticus serotype O3:K6 (strain RIMD 2210633) OX=223926 GN=pheS PE=3 SV=1

MQHLEEIIASASTAIEAAESLVALDEVRVQYLGKKGELTAQLQSLGKLPPEERREAGQEI

NKAKGVVQQAIAARKDALQSAELEAKLAAETIDVTLPGRRIENGGLHPVTRTVERIEKFF

GELGFNTEAGPEIEDAFHNFDALNIAADHPARTDHDTFFFNPDLMLRTHTSGVQIRTMEN

GKPPFRFIAPGRVYRNDYDQTHTPMFHQVEGMLVDENVNFAQLKGILHDFLCNFFEEEVE

VRFRPSYFPFTEPSAEVDVKGKNGKWLEVLGCGMVHPNVLRSVGIDPEKYSGFAFGMGVE

RLTMLRYGVNDLRAFFENDLRFLKQFK

>sp|Q87Q59|SYFB_VIBPA Phenylalanine--tRNA ligase beta subunit OS=Vibrio parahaemolyticus serotype O3:K6 (strain RIMD 2210633) OX=223926 GN=pheT PE=3 SV=1

MKFSESWLREWVNPAVTTDELTHQITMAGLEVDDVLPVAGSFTGVKVGHVVECGQHPDAD

KLRVTKVDVGEEELLDIVCGAHNCRQGLKVAVATVGAVLPGDFKIKKAKLRGQPSHGMLC

SFTELGIDVESDGIMELAEDAVIGTDFREFLGLDDVTVDVDLTANRADCFSIRGLAREVG

VLNRADVTEPSVEAVAPSIEDKVSIEVKAPAACPRYLGRVVKNVNVQAETPLWMQEKLRR

CGIRSIDPVVDITNYVLLEQGQPMHAFDLAKIEGGIVVRMAEQGEKLTLLDGSEAELNAD

TLVVADHNKALAIAGIFGGEESGVTTETKDVLLECAFFAPDHIRGRARSYGLHTDSSMRF

ERGVDYALQVSAMERATQLLVEICGGEVAPVVAVESEADLPKPNKVALRRTKLDNLLGHH

IADADVVEILERLGLTVEASEEGWVAVAPTWRFDIAIEQDLIEEVGRIYGYDNIPNQNPA

AALKMHNHVEADLPLKRVRDLLVDRGYHEAITYSFVEPEQQKLVVPGVEPLVLPNPISAD

MSAMRLGLIQGLLNTVVHNQKRQQPRVRLFEYGLRFIPCESAENGMRQEPMLAGVIAGTR

GEEHWDIETNTVDFFDLKGDLEAVLELSANEKAYSFAALSPESKKANPALHPGQSAAIIV

DGKEVGVIGTVHPELERKFGLNGRTIVFEIEWSAINSKVIPEAVALSKFPSNRRDIAVVV

DEAVASGDIVNACLEQGGEFLKDAKLFDVYVGKGVEEGKKSLAIALTLQSLERTLEDADI

AGAVDAIVAHVSEKFGAALRD

>sp|Q87LA3|SSB_VIBPA Single-stranded DNA-binding protein OS=Vibrio parahaemolyticus serotype O3:K6 (strain RIMD 2210633) OX=223926 GN=ssb PE=3 SV=1

MASRGINKVILVGNLGNDPEIRYMPNGGAVANITIATSESWRDKATGEQREKTEWHRVVL

FGKLAEVAGEYLRKGSQVYVEGQLQTRKWQDQSGQDRYSTEVVVQGFNGVMQMLGGRAQG

GAPAMGGQQQQQGGWGQPQQPAQQQYNAPQQQQQAPQQPQQQYNEPPMDFDDDIPF

>sp|Q87FP8|SSTT_VIBPA Serine/threonine transporter SstT OS=Vibrio parahaemolyticus serotype O3:K6 (strain RIMD 2210633) OX=223926 GN=sstT PE=3 SV=1

MQHNSLVARYARGNLVLQILVGIVFGILLATVSPSHAESVGLIGSLFVGALKAIAPILVF

ILVAASIANQKKNQHTYMRPIVVLYLMGTFFAALAAVVLSFLFPTHLTLVTGAEGATPPQ

GIAEILRTLLFKLVDNPVNALVEANYIGILAWGVGLGLALHHASATTKAVFEDLSHSVSH

IVRFIIRLAPFGIFGLVASTFATTGFSALASYAHLLAVLLGAMMIIALVVNPAIVFFKTK

QNPYPLVFQCLRESGVTAFFTRSSAANIPVNMALCEKLDLDEDTYSVSIPLGATINMAGA

AITITTLTLAAVHTMGIEVDLMTALLLSVVAAVSACGASGVAGGSLLLIPLACGLFGISN

DVAMQVVAVGFIIGVIQDSAETALNSSTDVIFTAAVCRSEQQKSQ

>sp|Q87KN5|STHA_VIBPA Soluble pyridine nucleotide transhydrogenase OS=Vibrio parahaemolyticus serotype O3:K6 (strain RIMD 2210633) OX=223926 GN=sthA PE=3 SV=1

MAHVNHYDVIVIGSGPGGEGAAMGLTKAGLNVAIVEKESSVGGGCTHWGTIPSKALRHAV

SRIIEFNSNPLFCRNNTSLHATFSDILGHAKTVIDKQTRLRQGFYDRNDCTLLFGTARFI

DTHSIAVMQNDGTEETYSADKFVIATGSRPYRPSDVDFLHERIYDSDSILSLKHDPRHII

IYGAGVIGCEYASIFRGLGVKTDLINTRDRLLEFLDNEVSDALSYHFWNSGVVIRNDETY

EKIEGTEDGVIIHLQSGKKMRADCLLYANGRTGNTDKLSLDVVGLESDSRGQLKVNRNYQ

TAVEHIYAVGDVIGYPSLASAAYDQGRFVAQAITKGQAENYLIEDIPTGIYTIPEISSVG

KTEQELTAAKVPYEVGRSSFKHLARAQIAGKDVGSLKILFHRETKEILGIHCFGERAAEI

IHIGQAIMEQKGQANTIEYFVNTTFNYPTMAEAYRVAALNGLNRLF

>sp|Q87QJ9|SYC_VIBPA Cysteine--tRNA ligase OS=Vibrio parahaemolyticus serotype O3:K6 (strain RIMD 2210633) OX=223926 GN=cysS PE=3 SV=1

MLKIYNTLTRQKEEFKPITAGKVGMYVCGVTIYDLCHIGHGRTFVSFDVVSRYLRYLGYD

LTFVRNITDIDDKIIKRAAENGESCESLTERLIGDMHADFDALNMKRPDVEPRATQFIAE

IIELVEKLIERGFAYVADNGDVMFEVGKFDEYGKLSKQDLDQLQAGARVDIETAKRSPLD

FVLWKMSKPGEPTWESPWGPGRPGWHIECSAMNSTILGDHFDIHGGGSDLQFPHHENEIA

QSCCAHDTKYVNTWMHSGMVMVDREKMSKSLGNFFTIRDVLGHYDAETVRYFLMSGHYRS

QLNYSEDNLNQARASLERLYTSLRGLDLNAAPAGGEEYVSRFTAAMNDDFNTPEAYSVLF

DMAREVNRLKTESVEKASELGALMRELADVIGILYQDPEAFLKGNAGNDDEVAEIEALIK

LRNDSRASKDWANADMARDKLTEMGIVLEDGPEGTTWRRK

>sp|Q87S93|RS20_VIBPA 30S ribosomal protein S20 OS=Vibrio parahaemolyticus serotype O3:K6 (strain RIMD 2210633) OX=223926 GN=rpsT PE=3 SV=1

MANSKSAKKRAIQAEKRRQHNASRRSMMRTYMKKTVAAIEAGDKEAATAAFAVVTPILDR

MATKGLIHKNKAARHKSRFAAQIKAL

>sp|Q87L72|RS6_VIBPA 30S ribosomal protein S6 OS=Vibrio parahaemolyticus serotype O3:K6 (strain RIMD 2210633) OX=223926 GN=rpsF PE=3 SV=1

MRHYEIVFMVHPDQSEQVAGMIERYTGSITEAGGKIHRLEDWGRRQLAYPINKLHKAHYV

LMNVEADQAVIDELETAFRFNDAVLRNMIMRTKAAITEQSIMLKQKEERAPRREERSEAK

PEAKSEAAE

>sp|Q87QU8|RUVA_VIBPA Holliday junction ATP-dependent DNA helicase RuvA OS=Vibrio parahaemolyticus serotype O3:K6 (strain RIMD 2210633) OX=223926 GN=ruvA PE=3 SV=1

MIGRLRGILLEKQPPEVLIEVNGIGYEVQMPMSCFYELPNIGEEAIIYTHFVVREDAQLL

YGFNTVKERALFREVIKANGVGPKLGLGILSGMTASQFVSCVEREDVSTLVKLPGVGKKT

AERLVVEMKDRLKGWGAGDLFTPFTDAAPTDSAAASSNSAEEEAVSALLALGYKPTQASK

VVSQIAKPDMSSEQLIREALKSMV

>sp|Q87TP7|SYGA_VIBPA Glycine--tRNA ligase alpha subunit OS=Vibrio parahaemolyticus serotype O3:K6 (strain RIMD 2210633) OX=223926 GN=glyQ PE=3 SV=1

MQKYDIKTFQGMILALQDYWAQNGCTIVQPLDMEVGAGTSHPMTCLRALGPEPMSTAYVQ

PSRRPTDGRYGENPNRLQHYYQFQVALKPSPDNIQELYLGSLEVLGIDPLVHDIRFVEDN

WENPTLGAWGLGWEVWLNGMEVTQFTYFQQVGGLECKPVTGEITYGIERLAMYIQEVDSV

YDLTWNIAPDGSKVTYGDIFHQNEVEQSTYNFEHADVDFLFSFFDQCEKESKELLELEKP

LPLPAYERILKAAHAFNLLDARKAISVTERQRYILRIRNLTKAVAEAYYASREALGFPMC

KKEQA

>sp|Q87NH5|SYN_VIBPA Asparagine--tRNA ligase OS=Vibrio parahaemolyticus serotype O3:K6 (strain RIMD 2210633) OX=223926 GN=asnS PE=3 SV=1

MTYAPVSDVLSGKLAVDSEVTVRGWIRSRRDSKAGISFLAIYDGSCFDPIQAVVPNNLNN

YDNEVLKLTTGCSVEVTGKIVESPAQGQDFELAATDVKVVGWVEDAETYPMAKTRHSIEY

LREVAHLRPRTNVIGAVARVRNCLSQAIHRFYHEQGYFWVSAPLITASDAEGAGEMFRVS

TLDMENLPRTDAGKVDYNEDFFGKETFLTVSGQLNAEAYACALSKVYTFGPTFRAENSNT

SRHLAEFWMVEPEVAFAELDDVAKLAEDMLKYVFKAVLEERRDDLEFFAQRIDKQAITRL

EQFVSSDFAQVDYTDAIQILLDSGREFEFPVEWGIDMSSEHERFLAEEHFKAPVIVKNYP

KDIKAFYMRMNDDGKTVAAMDVLAPGIGEIIGGSQREERLDVLDARMREMGIDPEHMSWY

RDLRRYGTVPHAGFGLGFERLVSYVTGMGNVRDVIPFPRTPRSANF

>sp|Q87L13|SYW_VIBPA Tryptophan--tRNA ligase OS=Vibrio parahaemolyticus serotype O3:K6 (strain RIMD 2210633) OX=223926 GN=trpS PE=3 SV=1

MSKPIVLSGVQPSGELSIGNYLGALRQWQQMQDDYDCQYCVVDLHAVTVRQDPKALHEAT

LDALAICLAVGVDPKKSTLFVQSHVPEHAQLGWLLNCYTQMGELSRMTQFKDKSARYAND

VNVGLFDYPVLMAADILLYGAHQVPVGSDQKQHLELARDIATRFNNIYSPESPIFTVPEP

YIPTVNARVMSLQDATKKMSKSDDNRKNVITLLEEPKSIIKKINKAQTDTETPPSIRHDV

ENKAGIANLMGLYSAATGMSFEEIEAKYKGVEMYGPFKKDVGEAVVAMLEPIQEEYRRIR

ADRAFMDEVMKQGAEKASARAAETLKKAYEAVGFVARP

>sp|Q8GRF5|RRF_VIBPA Ribosome-recycling factor OS=Vibrio parahaemolyticus serotype O3:K6 (strain RIMD 2210633) OX=223926 GN=frr PE=1 SV=1

MINEIKKDAQERMDKSVEALKNNLSKVRTGRAHPSLLSGISVEYYGAATPLNQVANVVAE

DARTLAITVFDKELTQKVEKAIMMSDLGLNPMSAGTIIRVPLPPLTEERRKDLVKIVRGE

AEGGRVAVRNIRRDANNDLKALLKDKEISEDEDRKAQEEIQKLTDVAVKKIDEVLAAKEK

ELMEV

>sp|Q87M05|RS15_VIBPA 30S ribosomal protein S15 OS=Vibrio parahaemolyticus serotype O3:K6 (strain RIMD 2210633) OX=223926 GN=rpsO PE=3 SV=1

MSLNAETKAAIVAEYAQSEGDTGSPEVQVALLTASINHLQGHFKAHKGDHHSRRGLLRMV

SRRRKLLDYLKGKDLARYQDLIKRLGLRR

>sp|Q87RQ4|RLMH_VIBPA Ribosomal RNA large subunit methyltransferase H OS=Vibrio parahaemolyticus serotype O3:K6 (strain RIMD 2210633) OX=223926 GN=rlmH PE=3 SV=1

MKIQLIAVGTKMPKWVEEGFQEYRRRFPHDMPLELVEISAGKRGKNADIARILQKEGEAM

LAAVPKGNRIVTLDIPGKKWDTPQLAEQLEAWKLDGRDVSILIGGPEGLAPACKAAADQS

WSLSALTLPHPLVRIVMAESLYRAWSITANHPYHRE

>sp|Q87LN9|RNC_VIBPA Ribonuclease 3 OS=Vibrio parahaemolyticus serotype O3:K6 (strain RIMD 2210633) OX=223926 GN=rnc PE=3 SV=1

MNSPIDKLERKLGYQFKDAGLINLALTHRSANSKHNERLEFLGDSILSFVIADDLYHRFP

KVNEGDMSRMRATLVRGHTLAELGREFDLGDYLKLGPGELKSGGFRRDSILADAVEAIIG

AIYLDSDIEKVRSIVLSWYNSRLEAIKPGVSQKDPKTRLQEFLQGRRKPLPVYTVTNIKG

EAHNQEFTVECEVAGVDKPVIGKGTSRRKAEQAAAETALEQLTNG

>sp|P46232|RNT_VIBPA Ribonuclease T OS=Vibrio parahaemolyticus serotype O3:K6 (strain RIMD 2210633) OX=223926 GN=rnt PE=3 SV=2

MTIENEALTLKKRFRGYFPVVVDVETAGFNAQTDALLEICAVTLRMDEEGVLHPASTIHF

HIEPFEGANLEKEALEFNGIRDPFSPLRGAVSEQEALKEIYKLIRKEQKASDCSRAIMVA

HNAAFDLSFVNAANERCKLKRVPFHPFATFDTATLSGLAYGQTVLAKACKTAGMEFDNRE

AHSALYDTQKTAELFCGIVNKWKALGGWPLVNEE

>sp|Q87SD2|RRAAH_VIBPA Putative 4-hydroxy-4-methyl-2-oxoglutarate aldolase OS=Vibrio parahaemolyticus serotype O3:K6 (strain RIMD 2210633) OX=223926 GN=VP0492 PE=3 SV=1

MRDITPDICDQFEDQVTLLNLPLQNFGQRTAFHGEIVTVRCYHDNSKVREVLEQDGTGKV

LIVDGHGSCQKALLGDQLAILGIENGWEGIIVYGAVRDVAQMSQMDIGVQALGTCPFKTE

KRGVGEVNVTLTMLNQIVQPKHHVYADWNGVLISKEALDF

>sp|Q87KQ4|RPOB_VIBPA DNA-directed RNA polymerase subunit beta OS=Vibrio parahaemolyticus serotype O3:K6 (strain RIMD 2210633) OX=223926 GN=rpoB PE=3 SV=1

MVYSYTEKKRIRKDFGTRPQVLDIPYLLSIQLDSFDKFIEQDPEGQYGLEAAFRSVFPIQ

SYNGNSELQYVSYRLGEPVFDVKECQIRGVTYSKPLRVKLRLVIFDKDAPAGTVKDIKEQ

EVYMGEIPLMTDNGTFVINGTERVIVSQLHRSPGVFFDSDKGKTHSSGKVLYNARVIPYR

GSWLDFEFDPKDNLYVRIDRRRKLPASIILRALGKSTEEILDIFFEKVNFEVKDQTLLME

LVPDRLRGETASFDIESNGKVYVEQGRRVTARHIRQLEKDGVDHIEVPVEYIVGKVASKD

YINEATGEIIVNANQEISLEALANLSQAGHKALEVLFTNDLDHGPFMSETLRIDSTVDRI

SALVEIYRMMRPGEPPTKEAAEALFESLFFSEERYDLSTVGRMKFNSSIGREDAQEQGTL

DETDIIEVMKKLIAIRNGKGEVDDIDHLGNRRIRSVGEMAENQFRVGLVRVERAVKERLS

LGDLDAVMPQDLINAKPISAAVKEFFGSSQLSQFMDQNNPLSEVTHKRRISALGPGGLTR

ERAGFEVRDVHVTHYGRLCPIETPEGPNIGLINSLSAFARCNEYGFLETPYRRVVDGVVT

DEVDYLSAIEEGQFVIAQANAKLNEDGTFADELITARQKGESGLHPREHAQYMDVATNQV

VSIAASLIPFLEHDDANRALMGANMQRQAVPTLKADKPLVGTGIERNVAVDSGVTAVAKR

GGVIQSVDASRIVVKVNEEELVPGEAGIDIYNLTKYTRSNQNTCINQRPCVMPGEPVARG

DVLADGPSTDLGELALGQNMRIAFMPWNGYNFEDSILVSERVVQEDRFTTIHIQELTCVA

RDTKLGSEEITADIPNVGESALSKLDESGIVYIGAEVKGGDILVGKVTPKGETQLTPEEK

LLRAIFGEKASDVKDTSLRVPNSVSGTIIDVQVFTRDGVEKDKRALEIEQMQLKEAKKDL

TEEFQILEGGLLNRVKAVLIEGGYSEAKLDATDRKKWLELTLEDDALQTQLEQLAEQWDE

LKADFDKKFETKRRKITQGDDLAPGVLKIVKVYLAVKRRIQPGDKMAGRHGNKGVISKIN

PVEDMPYDEKGQPVDIVLNPLGVPSRMNIGQILEVHLGLAAKGIGDKINQMVKEQQELAK

FREFLQKVYDLGDTRQKVDIASLSDDEVRTLIKNLRGGLPIATPVFDGAPEASIKALLEL

ADLPTSGQLTLFDGRTGDAFERPVTVGYMYMLKLNHLVDDKMHARSTGSYSLVTQQPLGG

KAQFGGQRFGEMEVWALEAYGAAYTLQEMLTVKSDDVNGRTKMYKNIVDGNHSMEPGMPE

SFNVLLKEIRSLGINIELEDEE

>sp|Q87IJ9|RNB_VIBPA Exoribonuclease 2 OS=Vibrio parahaemolyticus serotype O3:K6 (strain RIMD 2210633) OX=223926 GN=rnb PE=3 SV=1

MFQDNPLLAQLKQQIQENLPKKEGSIKATDKGFGFLEVDSKTSFFIPPAYMKKCIHGDKV

VAIIRTENEREVAEPQELIEQSLTRFIGRVKMFKGKLNVVPDHPQLKKLSLKAKLKKGLK

PDNFAEGDWVVAHLVRHPLKGDNTFFVEISEKITDADDKIAPWWVTLAQNDLPNSEPAGI

ENWELKDDADLERIEMTHVPFVTIDGESTKDMDDALYAKKTESGDFELTIAIADPTAYIT

PEDEMDKVARERGYTIYLPGRNIPMLPRDLADNLCSLIEGEIRPAICCTVTVSKDGVIGD

DIKFFAANIKSHARLAYDHVSDWLENGNSDAWQPSEEIATIVRDLYEFSLARAEWREKNA

VVFPDRPDYRFELSEDNDVIAIHADMRRSANRLVEESMITANICAGRTLREKFETGVFNT

HAGLKPEKIEEVVQLVNPEGTLEFTAESIATLEGFAALRRWLAVQETSYLDNRIRKFQAY

SEVGNQPLPHYAMGLDIYATWTSPIRKYGDMINHRMLKAVILDKEPVQKPDDQVGEELAL

HRKHHKIAERNVSDWLYARTLADEPSKQTCFTGEIFDINRAGARVRLLENGAAAFIPGAL

ILDNKERIECNGDNGTISIDKEVVYKLGDTLEIVLADVNQENRSLVAKPTQVFADQPAPQ

TEQTVSEE

>sp|Q87MX0|RNFG_VIBPA Ion-translocating oxidoreductase complex subunit G OS=Vibrio parahaemolyticus serotype O3:K6 (strain RIMD 2210633) OX=223926 GN=rnfG PE=3 SV=1

MLTAIRKNGLTLAIFACATTGLVALTQYLTEDQIKLQEQKQLLSVLNQVIPETMHDNALT

QSCTLVTSPELGTMHAMPTYIATKNGEPTAIAIESIAPDGYNGEIKVITGIDNQGKILGT

RVLSHQETPGLGDKIDLRVTSWILGFTGKQVTEDNWNSWKVRKDGGDFDQFTGATITPRA

VIKAVRNTVNYVNQSRDEILSQPLNCAGDNQ

>sp|Q87SZ0|RPOA_VIBPA DNA-directed RNA polymerase subunit alpha OS=Vibrio parahaemolyticus serotype O3:K6 (strain RIMD 2210633) OX=223926 GN=rpoA PE=3 SV=1

MQGSVTEFLKPRLVDIEQISSTHAKVTLEPLERGFGHTLGNALRRILLSSMPGCAVTEVE

IEGVLHEYSTKEGVQEDILEILLNLKGLAVRVAEGKDEVFITLNKSGSGPVVAGDITHDG

DVEIANPEHVICHLTDDNAEIAMRIKVERGRGYVPASARIHNEEDERPIGRLLVDATYSP

VDKIAYAVEAARVEQRTDLDKLVIDMETNGTLEPEEAIRRAATILAEQLDAFVDLRDVRV

PEEKEEKPEFDPILLRPVDDLELTVRSANCLKAEAIHYIGDLVQRTEVELLKTPNLGKKS

LTEIKDVLASRGLSLGMRLENWPPASIAED

>sp|Q87SZ2|RS13_VIBPA 30S ribosomal protein S13 OS=Vibrio parahaemolyticus serotype O3:K6 (strain RIMD 2210633) OX=223926 GN=rpsM PE=3 SV=1

MARIAGINIPDQKHAVIALTAIYGIGKTRSQAILAEVGIAEDVKISELTEEQIDQLRDGV

AKYTVEGDLRREVSMNIKRLMDLGCYRGLRHRRSLPLRGQRTKTNARTRKGPRKPIKK

>sp|Q87T23|RRAA_VIBPA Regulator of ribonuclease activity A OS=Vibrio parahaemolyticus serotype O3:K6 (strain RIMD 2210633) OX=223926 GN=rraA PE=3 SV=1

MEYNTSALCDIYLDQVDVVEPMFSNFGGRASFAGQITTIKCFEDNSLIRETLEQDGLGRV

LLIDGGGSLRKALIDAEIAAIAEENEWEGIVVYGCVREVDELEDMNLGIQALASIPVGAA

NQGIGELDVPVNFGGVSFLPEDYIYADNTGIILSPEPLNIDLELDDTVEE

>sp|Q87L43|RS12_VIBPA 30S ribosomal protein S12 OS=Vibrio parahaemolyticus serotype O3:K6 (strain RIMD 2210633) OX=223926 GN=rpsL PE=3 SV=1

MATINQLVRKPRAKQVVKSNVPALEACPQKRGVCTRVYTTTPKKPNSALRKVCRVRLTNG

FEVTSYIGGEGHNLQEHSVVLIRGGRVKDLPGVRYHTVRGALDCAGVNDRKQGRSKYGVK

RPKS

>sp|Q87T00|RS14_VIBPA 30S ribosomal protein S14 OS=Vibrio parahaemolyticus serotype O3:K6 (strain RIMD 2210633) OX=223926 GN=rpsN PE=3 SV=1

MAKQSMKAREAKRAKLVAKFAEKRAALKAIISDVNASEEDRWNAVLTLQSLPRDSSASRQ

RNRCNQTGRPHGYLRKFGLSRIKVREACMKGEIPGLRKASW

>sp|P66346|RS10_VIBPA 30S ribosomal protein S10 OS=Vibrio parahaemolyticus serotype O3:K6 (strain RIMD 2210633) OX=223926 GN=rpsJ PE=3 SV=1

MQNQRIRIRLKAFDYKLIDASTAEIVETAKRTGAQVRGPIPLPTRKERFTVLISPHVNKK

ARDQYEIRTHKRLIDIVEPTDKTVDALMRLDLAAGVDVQISLG

>sp|Q87T09|RS19_VIBPA 30S ribosomal protein S19 OS=Vibrio parahaemolyticus serotype O3:K6 (strain RIMD 2210633) OX=223926 GN=rpsS PE=3 SV=1

MPRSLKKGPFIDLHLLKKVEKAVESGDKKPIKTWSRRSMIIPTMIGLTIAVHNGRQHVPV

FVTDEMIGHKLGEFAPTRTYRGHAADKKAKKR

>sp|P66478|RS18_VIBPA 30S ribosomal protein S18 OS=Vibrio parahaemolyticus serotype O3:K6 (strain RIMD 2210633) OX=223926 GN=rpsR PE=3 SV=1

MARFFRRRKFCRFTAEGVQEIDYKDVATLKNYITEAGKIVPSRITGTSAKYQRQLARAIK

RSRYLALLPYTDKHQ

>sp|Q87T07|RS3_VIBPA 30S ribosomal protein S3 OS=Vibrio parahaemolyticus serotype O3:K6 (strain RIMD 2210633) OX=223926 GN=rpsC PE=3 SV=1

MGQKVHPNGIRLGIVKPWNATWFANTKDFADNLDGDFKVRQFLTSELKKASLSRIVIERP

AKSIRVTIHTARPGVVIGKKGEDVEKLRAAVAKIAGVPAQINIAEVRKPELDAQLVGDSI

ASQLERRVMFRRAMKRAVQNAMRLGAKGIKVEVSGRLGGAEIARSEWYREGRVPLHTLRA

DIDYATSSAHTQYGVIGIKTWIFKGEILGGMPAANAVEPKGDKPKKQRKGRK

>sp|Q87SI4|RS9_VIBPA 30S ribosomal protein S9 OS=Vibrio parahaemolyticus serotype O3:K6 (strain RIMD 2210633) OX=223926 GN=rpsI PE=3 SV=1

MAENQYYGTGRRKSSAARVFIKPGSGNIVVNKRSLDEYFGRPTSRMVVKQPLELVELTEK

LDLYVTVKGGGISGQAGAIRHGITRALMEYDESLRPALRAAGYVTRDARCVERKKVGLRK

ARRRPQFSKR

>sp|Q87SZ4|RL15_VIBPA 50S ribosomal protein L15 OS=Vibrio parahaemolyticus serotype O3:K6 (strain RIMD 2210633) OX=223926 GN=rplO PE=3 SV=1

MRLNTLAPAAGSKHAPKRVGRGIGSGLGKTGGRGHKGQKSRSGGKVRPGFEGGQMPLKQR

LPKFGFTSRKSLVSAEVRLAELAKVSGDVVDLNSLKAANIITKNIEFVKVVLSGEINKAV

TVKGLRVTKGAKAAIEAAGGKIEE

>sp|Q87MQ4|RECR_VIBPA Recombination protein RecR OS=Vibrio parahaemolyticus serotype O3:K6 (strain RIMD 2210633) OX=223926 GN=recR PE=3 SV=1

MRTSHMLEQLMEALRCLPGVGPKSAQRMAFHLLQRDRKGGLQLADALSQSMTEIGHCAEC

RTFTEEEVCHICTNPKRQENGQICVVESPADIAAVEATGQYSGRYFVLMGHLSPLDGIGP

SDIGLDVLDYRLRRGDISEVILATNPTVEGEATAHYIAELCREHQVEASRIAHGVPVGGE

LELVDGTTLSHSLLGRHKI

>sp|Q87LS9|RIMM_VIBPA Ribosome maturation factor RimM OS=Vibrio parahaemolyticus serotype O3:K6 (strain RIMD 2210633) OX=223926 GN=rimM PE=3 SV=1

MSMKGKETMSNEKIVVGKFGATYGIRGWLKVFSYTDNAESIFDYSPWYINQKGKWVEYKV

ESWKRHNKGMVAKLEGMDVREDAHLMTNFEIAIDPAVLPELSEDEFYWRELFGMHVVTTK

GYDLGVVTDMLETGSNDVLVVKANLKDAFGQKERLIPFLEEQVIIKVDREAQRIEVDWDP

GF

>sp|Q87GR5|RIBB_VIBPA 3,4-dihydroxy-2-butanone 4-phosphate synthase OS=Vibrio parahaemolyticus serotype O3:K6 (strain RIMD 2210633) OX=223926 GN=ribB PE=3 SV=1

MNQSSLLAEFGDPITRVENALIALKEGRGVLLLDDEDRENEGDIIYSVEHLTNEQMALMI

RECSGIVCLCLTDAQADKLELPPMVVNNNSANQTAFTVSIEAKVGVTTGVSAADRVTTIK

TAANPHAKPEDLARPGHVFPLRARPGGVMTRRGHTEGTIDLMQMAGLQPAGVLCEVTNPD

GTMAKAPEIVAFGHLHNMPVLTIEDMVAYRNQFDLKLA

>sp|Q87KQ2|RL10_VIBPA 50S ribosomal protein L10 OS=Vibrio parahaemolyticus serotype O3:K6 (strain RIMD 2210633) OX=223926 GN=rplJ PE=3 SV=1

MALNLQDKKAIVAEVNEAASGALSAVVADSRGVEVGAMTSLRKQAREAGVYMKVVRNTLA

RRAVQGTDYECLTDTFTGPTLIAFSNEHPGAAARLFKDFAKENKDFEIKAAAFEGALTDA

EVLATLPTYDEAIARLMMCMKEASAGKLVRTIAAIRDQKEAA

>sp|Q87KQ1|RL1_VIBPA 50S ribosomal protein L1 OS=Vibrio parahaemolyticus serotype O3:K6 (strain RIMD 2210633) OX=223926 GN=rplA PE=3 SV=1

MAKLTKRMRVIREKVDVTKEYEINEAVALLQELATAKFVESVDVAVNLGIDARKSDQNVR

GATVLPHGTGRDIRVAVFTQGANAEAAKEAGADIVGMEDLAEQVKKGEMNFDVVVASPDA

MRVVGQLGTILGPRGLMPNPKVGTVTPNVAEAVKNAKAGQVRYRNDKNGIIHTTIGKANF

SAEQIKENLEALLVALKKAKPSSAKGTFLKKVSISTTMGAGVAVDQASLNTQA

>sp|Q87NE7|RIBA_VIBPA GTP cyclohydrolase-2 OS=Vibrio parahaemolyticus serotype O3:K6 (strain RIMD 2210633) OX=223926 GN=ribA PE=3 SV=1

MAEVRARVDFKVGAKSNIDAEILSFRGLKTDKEHVAVIFKQADQTQDTPLVRMHSECLTG

DVFHSSRCDCGEQLEETIQRMGESGGVILYLRQEGRGIGLYNKIDAYRLQSQGMNTYEAN

NHLGFDDDLRDFTEAAQMLEALGIKKIRLVTNNPKKIRELAEYGIEIVEVVNTSAHIKDG

NENYLRAKVSHGKHNLKV

>sp|Q87SI5|RL13_VIBPA 50S ribosomal protein L13 OS=Vibrio parahaemolyticus serotype O3:K6 (strain RIMD 2210633) OX=223926 GN=rplM PE=3 SV=1

MKTFVAKPETVKRDWYVVDAEGKTLGRLASEIASRLRGKHKAEYTPHVDAGDYIIVINAE

KVAVTGNKAKDKVYYRHSEFPGGLKSITFEKLIDRKPEMVLELAVKGMLPRGPLGRAMYR

KLKVYAGAEHNHVAQQPQVLDI

>sp|Q87SZ7|RL18_VIBPA 50S ribosomal protein L18 OS=Vibrio parahaemolyticus serotype O3:K6 (strain RIMD 2210633) OX=223926 GN=rplR PE=3 SV=1

MDKKASRIRRATRARRKIAELGATRLVVHRTPRHVYAQVIAANGSEVIAAASTVEKAIRE

QVKYTGNVDAAKAVGKAVAERALEKGVTAVAFDRSGFQYHGRVAALAESAREAGLKF

>sp|P0A481|RL20_VIBPA 50S ribosomal protein L20 OS=Vibrio parahaemolyticus serotype O3:K6 (strain RIMD 2210633) OX=223926 GN=rplT PE=3 SV=1

MPRVKRGVQARARHKKVLKQAKGYYGARSRVYRVAFQAVTKAGQYAYRDRRAKKRQFRQL

WIARINAASRQNGLSYSRFINGLKKASIEIDRKILADIAVFDKAAFAVLVEKAKAAL

>sp|Q87SU4|RL21_VIBPA 50S ribosomal protein L21 OS=Vibrio parahaemolyticus serotype O3:K6 (strain RIMD 2210633) OX=223926 GN=rplU PE=3 SV=1

MYAVFQSGGKQHRVSEGQTLRLEKLDVETGATVEFDKVLLVANGEDIKVGAPLVEGGKIV

AEVVQHGRGDKVKIVKFRRRKHSRKQQGHRQWFTEVKITGINA

>sp|Q87T08|RL22_VIBPA 50S ribosomal protein L22 OS=Vibrio parahaemolyticus serotype O3:K6 (strain RIMD 2210633) OX=223926 GN=rplV PE=3 SV=1

MEAIAKHNFARISPQKARLVADLIRGKSVDQALEILTFSNKKAAALVKKVLESAIANAEH

NEGADIDDLNVAKIFVDEGPTMKRIMPRAKGRADRILKRSSHITVVVADR

>sp|Q87QD9|RL25_VIBPA 50S ribosomal protein L25 OS=Vibrio parahaemolyticus serotype O3:K6 (strain RIMD 2210633) OX=223926 GN=rplY PE=3 SV=1

MKFEAVVRTELGKGASRRLRHAGKFPAVVYGGEEAAVAIVLNHDDIVNQMDKPEFYEGIV

LVIDGKEVKVKPQDVQRHAFKPKVEHMDFIRI

>sp|Q87T10|RL2_VIBPA 50S ribosomal protein L2 OS=Vibrio parahaemolyticus serotype O3:K6 (strain RIMD 2210633) OX=223926 GN=rplB PE=3 SV=1

MAIVKCKPTSPGRRHVVKVVNADLHKGKPYAPLLEKNSKNGGRNNNGRITVRHIGGGHKH

HYRVIDFKRTKDGIPATVERLEYDPNRSANIALVLYKDGERRYILAPKGVVAGDVIQSGV

DAPIKAGNTLPMRNIPVGSTVHNVELKPGKGGQLARSAGAYAQIVARDGAYVTIRLRSGE

MRKVLSEGRATIGEVGNSEHMLRELGKAGASRWRGVRPTVRGVVMNPVDHPHGGGEGRTS

GGRHPVSPWGMPTKGFKTRKNKRTDKYIVRRRNK

>sp|Q87KQ0|RL11_VIBPA 50S ribosomal protein L11 OS=Vibrio parahaemolyticus serotype O3:K6 (strain RIMD 2210633) OX=223926 GN=rplK PE=3 SV=1

MAKKVEAYIKLQVAAGAANPSPPVGPALGQHGVNIMEFCKAFNAKTESLEKGLPTPVVIT

VYSDRSFTFVTKTPPAAVLLKKAAGVKSGSGRPNTEKVGTVTDAQIQEIAETKAADMTGA

DIEAMKRSIAGTARSMGLVVEG

>sp|Q87T06|RL16_VIBPA 50S ribosomal protein L16 OS=Vibrio parahaemolyticus serotype O3:K6 (strain RIMD 2210633) OX=223926 GN=rplP PE=3 SV=1

MLQPKRTKFRKVQTGRNRGLAKGTDVSFGEFGLKAVGRGRLTARQIEAARRAMTRHVKRQ

GKIWIRVFPDKPITEKPLEVRQGKGKGNVEYWVAQIQPGKVMYEMGGVPEELAREAFRLA

ARKLPFKTTFVTKQVM

>sp|Q87LT1|RL19_VIBPA 50S ribosomal protein L19 OS=Vibrio parahaemolyticus serotype O3:K6 (strain RIMD 2210633) OX=223926 GN=rplS PE=3 SV=2

MSNIIKALEEEQMKSGLPKFAPGDTVVVQVKVKEGDRERLQAFEGVVIAIRNRGLHSAFT

VRKISNGEGVERTFQTHSPIVDSIEVKRRGAVRRAKLYYLRERSGKSARIKEKLAKK

>sp|Q87T11|RL23_VIBPA 50S ribosomal protein L23 OS=Vibrio parahaemolyticus serotype O3:K6 (strain RIMD 2210633) OX=223926 GN=rplW PE=3 SV=1

MITEERILKVLRAPHISEKATMAAEKANTIVFKVAKDATKKEIKAAVEKLFEVEVKSVNT

LITKGKTKRQGLRQGRRSDVKKAYVTLKEGQDLDFVGGAE

>sp|Q87T02|RL24_VIBPA 50S ribosomal protein L24 OS=Vibrio parahaemolyticus serotype O3:K6 (strain RIMD 2210633) OX=223926 GN=rplX PE=3 SV=1

MAAKIRRNDEVIVLAGKDKGKKGKVTKVLATGKVIVEGINLVKKHQKPVPALGIQGGIVE

QEAAIDVSNVAIFNAATGKADRIGFRFEDGKKVRFFKSNGETVSN

>sp|Q87JS5|RIMK_VIBPA Probable alpha-L-glutamate ligase OS=Vibrio parahaemolyticus serotype O3:K6 (strain RIMD 2210633) OX=223926 GN=rimK PE=3 SV=1

MRIAILSRNENLYSTMRLKQAGEERGHQIDVIDTLHCYMDITSNNPMIRYKGEELPQYDA

VIPRIGASITFYGTAVVRQFEMMGTFCVNESVAISRSRDKLRSLQLLSRKGIGLPRTGFA

HHPDNIQDVIKNVGGAPLVIKLLEGTQGIGVVLAETNKAAESVIEAFMGLKANIMVQEFI

EEAKGADIRCFVVGNKVIAAMKRQAKEGEFRSNLHRGGSAQLVRLSKEERATAVNAAKVM

GLNLCGVDILQSKNGPVVMEVNSSPGLEGIELATGKDVAGMIFDFIEKNAKPNSNRTRGK

G

>sp|Q87M00|RIMP_VIBPA Ribosome maturation factor RimP OS=Vibrio parahaemolyticus serotype O3:K6 (strain RIMD 2210633) OX=223926 GN=rimP PE=3 SV=1

MTGLERQLTEMLEAPVEASGYELVGLEFIRAGAHSTLRIYIDHENGINVDDCAEVSHQVS

AVLDVEDPISVAYSLEVSSPGLERPLFKAAHYEQFIGHEVSIVLKMAVANRRKWKGIIHG

VDGETVTVTVEGQQEEFALSNISKANLIPKF

>sp|Q87SZ5|RL30_VIBPA 50S ribosomal protein L30 OS=Vibrio parahaemolyticus serotype O3:K6 (strain RIMD 2210633) OX=223926 GN=rpmD PE=3 SV=1

MATIKVTQTKSSIGRLPKHKATLRGLGLRKINHTVELEDTPCVRGMINKVYYMVKVEE

>sp|Q87MC5|RL31B_VIBPA 50S ribosomal protein L31 type B OS=Vibrio parahaemolyticus serotype O3:K6 (strain RIMD 2210633) OX=223926 GN=rpmE2 PE=3 SV=1

MKPGIHPDYRPVVFHDTSVDEYFVVGSTLKTDRTIEWKDGKTYPYFTLDVSSSSHPFYTG

KQRVVQAEGRIANFNRRFGQFTSEKE

>sp|Q87Q69|RL35_VIBPA 50S ribosomal protein L35 OS=Vibrio parahaemolyticus serotype O3:K6 (strain RIMD 2210633) OX=223926 GN=rpmI PE=3 SV=1

MPKMKTNKGAAKRFKKTAGGIKYKHATKRHILTKRTTKNKRQLRPNAILPRCEVAAVIRM

LPYA

>sp|Q87MA4|RLMG_VIBPA Ribosomal RNA large subunit methyltransferase G OS=Vibrio parahaemolyticus serotype O3:K6 (strain RIMD 2210633) OX=223926 GN=rlmG PE=3 SV=1

MKTELNLHGRSLTLHRFPKRSNETLQAWDAGDEYLINHVEEMALPDHQNIVVINDNFGAL

ACWFSEKHHVTFMSDSFVSHKGAQKNLEDNQCNKVAFLTTMDSIPANTDLVLVQLPKSNR

HLVWILSQLRKILPTACPVIAVNKAKEIHTSTLKLFEKYLGETKTSLAWKKHRLVFSQAN

AEPRIEVDPITAWDVEGEHIQLKNLPNVYSGESLDLGARFMLQHIPQDASINHIIDLGCG

NGVLSVKAGQLNPNVRLTCVDESFMALESAKQNLLDNLGEGRDIQCVANNCLDGFKPDSC

DLIMCNPPFHQQHAITDHIAWQMFCDAKQILNQNGKLLVIGNRHLGYDAKLKRLFGDKNV

KLIASNNKFVILQATKNPAKLSAKQ

>sp|Q87RT2|RLMM_VIBPA Ribosomal RNA large subunit methyltransferase M OS=Vibrio parahaemolyticus serotype O3:K6 (strain RIMD 2210633) OX=223926 GN=rlmM PE=3 SV=2

MLYCRSGFEKECAGEIQDKATQLEVYGFPRVKKNSGYVVFECYQDGDAEKLVKGLDFSSL

IFARQMFAVAAEFEALPSEDRISPILAELSEFESFPRCGDLRIETPDTNEAKELLKFCRK

FTVPMRQALRGKGLMWNKDNAKKPVLHICFVAPGHCYVGYSLPGNNSQFFMGIPRLKFPA

DAPSRSTLKLEEAFHVFIPRDEWDERLAPGMWGVDLGACPGGWTYQLVKRSMFVHCVDNG

MMADSLMETGQIKHHMVDGFKFEPDRKNVTWIVCDMVEKPARVAHLMGQWLLKGWAKEAI

FNLKLPMKGRYDEVLQDLENLKMFLIENKVKFKLQAKHLYHDREEITIHIQCLSNISPH

>sp|Q87SZ8|RL6_VIBPA 50S ribosomal protein L6 OS=Vibrio parahaemolyticus serotype O3:K6 (strain RIMD 2210633) OX=223926 GN=rplF PE=3 SV=1

MSRVAKAPVAIPAGVEVKLNGQEITVKGAKGELTRVLNDAVVIAQEENNLTFGPKEGVAN

AWAQAGTARALVNNMVVGVTEGFTKKLTLKGVGYRAAIKGNAVGLTLGFSHPVEHELPAG

IKAECPSQTEIVITGCDKQLVGQVAADIRSYRQPEPYKGKGVRYADENVRTKEAKKK

>sp|Q87L75|RL9_VIBPA 50S ribosomal protein L9 OS=Vibrio parahaemolyticus serotype O3:K6 (strain RIMD 2210633) OX=223926 GN=rplI PE=3 SV=1

MQVILLDKIGNLGGLGDTVNVKSGYARNFLIPQGKAVMATKGNVEMFEARRAELEAKVAE

QLAAAEARAEKVNALEAVVIASKAGDEGKLFGSIGTRDIAEAITAAGVEVAKSEVRLPEG

ALRTTGEFEISVQLHSEVFATAKVQVVAAE

>sp|Q87LP5|RLMD_VIBPA 23S rRNA (uracil(1939)-C(5))-methyltransferase RlmD OS=Vibrio parahaemolyticus serotype O3:K6 (strain RIMD 2210633) OX=223926 GN=rlmD PE=3 SV=1

MARIFQPKKKTQLNTRHQAVQVERLDHHGAGIAYLKKKPLFIDGALPGEEVVTQLVEEKS

KFARGKLIKILKPSDARVEPFCPHYHECGGCDLQHLNYDQQLTHKQQTLRQLMRKFAGSD

IDLDAPVLGESLGYRRRARVSLFVDKKTRQLHFGFRKKQSKQIAQVTDCPVLAPELNVLL

PEIYSVLKAFKKPDQLGHVELVLGDNGPCITLRHLSNLADDEVSALVELATRHQASLYLM

PETDQLNLVAGEVPFYQEAGVKIPFDPNNFIQVNQAVNQKMVEQAIKWLDPQSDERVLDL

FCGLGNFSLPIAKRAKHVVGVEGVAEMVEKASNNASLNQINNAQFYHANLEQDFDGQAWA

AEKFDKVLLDPARAGASGIIDQVSALGAQRVVYVSCNPATLARDSQSLLDQGYQLTKLGM

LDMFPHTSHLESMALFEKS

>sp|Q87LZ4|RLME_VIBPA Ribosomal RNA large subunit methyltransferase E OS=Vibrio parahaemolyticus serotype O3:K6 (strain RIMD 2210633) OX=223926 GN=rlmE PE=3 SV=1

MSKQKHSASSGRWLKEHFDDKYANEARKKGYRSRAYFKIDEIQTKDKLLKPGMTVVDLGA

APGGWSQYAAKIVGDSGQIIACDLLPMDPIAGVSFLQGDFRDDAVLEALLDRIQPLMVDV

VMSDMAPNIAGNNSVDQPRAMYLVELALDMCRQVLAPNGSFVVKVFQGEGFDQYVKEVRD

MFKVVKIRKPDSSRARSREVFVVATGYKG

>sp|Q87Q26|RLMF_VIBPA Ribosomal RNA large subunit methyltransferase F OS=Vibrio parahaemolyticus serotype O3:K6 (strain RIMD 2210633) OX=223926 GN=rlmF PE=3 SV=1

MKNNSHNAKQAPSKAAKPKHDNDVNKAKPKRVKKKAAVKAKLSAEKSDVDFIKIAKSGLH

ESNAHRGRYDFKKLIASEPALASFVIKNPKGEDSINFSDPNAVKMLNKALLAAYYNIDFW

DIPEHYLCPPIPGRADYIHRVAELLDGEVKGKYRHQNVRALDVGVGANCIYPIVGVTQYG

WHYTGSDVDPKSIDSAANIVERNVALNGKIELVQQMSESHIYRGVIKPNDRFDVTTCNPP

FHRSAEDAAMGSQRKLDNLKANQRKKGVKQQNSPVKQGKPTLNFGGQNAELWCEGGEAAF

IRRMANESQAFSSQVLWFTTLISKKDNVRPMRKQLEKLGVKAIRVVEMSQGQKISRFMAW

SFMDKQQRKTWIELK

>sp|Q87PC0|RLMKL_VIBPA Ribosomal RNA large subunit methyltransferase K/L OS=Vibrio parahaemolyticus serotype O3:K6 (strain RIMD 2210633) OX=223926 GN=rlmL PE=3 SV=1

MNQYLAVTSNGMENLLVEELTKLGIENAKPVQAGVKFKATNEQIYRCCLWSRLASRFVRV

LSEFTCNDDMDLYLSTSSINWVNQFHSSKRFVVDFNGTNREIRNSQYGAMKVKDGIVDCF

EKKGLPRPNISKERPDIRVHVRLHKDKAILGVDMVGSGLHQRGYRPESGRAPLRETLAAA

IIMRCGWDGHQPLLDPMCGSGTLLIEAAMMAANMAPGVKRKQWCFESLEDFEPDTWAEIK

SEANVQARRGVKKVDAKFFGFDNDPKVLKVAQENARRAGVEELIEFAQGDVATITRLSGF

ENGVIVSNPPYGERLGTEPGLIALYTAFGGQLKAEFGGCKASIFSSSDELLSCLRMRADK

QFKLNNGALPCHQKNYSIAERSADEVKGADTNTQIAPDFSNRLKKNIGKIGKWARKEKLD

CYRIYDADLPEYNVAIDVYGDQIVIQEYAAPKNIPEEKAKRRLTDIIRATIQVTGVEANK

VVLKVREKQKGRSQYQKLGQVSETLEVNEYGVKLIVNLHDYLDTGLFLDHKITRRRLGEM

AQGKDFLNLFAYTGSATVHAAVGGARSTTTVDMSNTYLNWAKDNMQLNGCIGRQHRFEQA

DCLQWLENAKGEYDLIFIDPPTFSNSKRMETSFDVQRDHIKLMTNLKRLLRAGGTIVFSN

NKRHFKMDEEGLAELGLKAQNISSQTLPLDFSRNKHIHNCWLVTHAE

>sp|Q87RD6|SYR_VIBPA Arginine--tRNA ligase OS=Vibrio parahaemolyticus serotype O3:K6 (strain RIMD 2210633) OX=223926 GN=argS PE=3 SV=1

MNIQALINDKVSQALEAAGAPAGSPAAVRQSAKPQFGDYQANGVMGVAKKLGTNPREFAQ

KVLDVLDLDGIASKTEIAGPGFINIFLSEEFLAKQADAALADSRLGVAAEEAQTIVADYS

APNVAKEMHVGHLRSTIIGDAVVRTLEFLGHKVIRANHIGDWGTQFGMLIANLERVQQES

GEVSMELADLEGFYRESKKLYDEDEEFAVKARNYVVKLQSGDEFCAEMWKKLVDVTMIQN

QRNYDRLNVSLTRDDVMGESMYNDMLPKIVADLKAQGLAVEDDGAQVVFLEEFKNKDGEA

MGVIVQKRDGGFLYTTTDIACAKYRYEELGADRVLYFIDSRQHQHLMQAWTIVRKAGYVP

ESVSLEHHAFGMMLGKDGKPFKTRAGGTVRLADLLDEAEVRAAQLIESKNPELDAEEKEK

ISKTVAMAAVKYSDLSKHRTTDYVFDWDNMLAFEGNTAPYMQYAYTRVASIFAKAGVAMD

ELQGDIQITDEKEKALIAKLLQFEEAVQSVAREGQPHIMCSYLFELAGQFSSFYEACPIL

VAEDEAVKQSRLKLAALTAKTIKQGLSLLGIETLERM

>sp|Q87SB1|SYK_VIBPA Lysine--tRNA ligase OS=Vibrio parahaemolyticus serotype O3:K6 (strain RIMD 2210633) OX=223926 GN=lysS PE=3 SV=1

MTDAVQNETVQEENKLIAERRAKLDEIRKSCKANGHPNDFRRDALAGDLQKEFGEKTKEE

LEELNHVVAIAGRIMAKRGPFLVIQETSGRIQAYADKEVQKELKEKYQGLDIGDIIGVKG

ALHKSGKGDLYVNMEEYELLTKALRPLPEKFHGLTDQEMRYRQRYVDLIVNEDSRNAFVV

RSKVMSAIRNFMISKQFMEVETPMMHVIPGGASARPFITHHNALDMPMYLRIAPELYLKR

LVVGGFDRVFEINRNFRNEGLSPRHNPEFTMMEFYMAYADYKDLMDFTEELLSSVALEVL

GSTSMPYGEDTVEFGGKYARMSMFEAIKHYNPDHAQIQALTEEDLQNRELMVSIAKSVHV

EVEPFWTCGQLLEEIFGETAEPKLMQPTFITGYPADISPLARRSDDNPFFTDRFEFFIGG

REVANGFSELNDAEDQDARFKAQVEAKESGDDEAMFYDADYITALEHGLPPTAGQGIGID

RLVMLLTNTHTIRDVILFPAMRPQA

>sp|Q87RG4|SYQ_VIBPA Glutamine--tRNA ligase OS=Vibrio parahaemolyticus serotype O3:K6 (strain RIMD 2210633) OX=223926 GN=glnS PE=3 SV=1

MSEADARPSNFIRQIIDKDLADGKHTSVHTRFPPEPNGYLHIGHAKSICLNFGIAQDYQG

QCNLRFDDTNPEKEDIEYVESIKKDVNWLGFEWDGEVCYSSNYFDKLYEYAIELINKGLA

YVDELSPEQIREYRGTLKEPGKPSPYRDRSVEENLALFEKMRAGEFEEGKACLRAKIDMG

SSFMVMRDPVLYRVRFATHHQTGDKWCIYPMYDFTHCISDALEGITHSICTLEFMDNRRL

YDWVLDNITIDCRPHQYEFSRLNLEYTVMSKRKLNQLVTEKLVNGWDDPRMPTVSGLRRR

GFTPASIREFCKRIGVTKQENMIEFSSLESCIRDDLNENAPRAMAVLDPVKVVIENFEVG

AVENLTLANHPNKPEMGEREVPFTREVWIEREDFREEANKKYKRLVLGKEVRLRGAYVIK

AERVEKDAEGNITTIYCTYDPETLGKNPADGRKVKGVIHWVSADKALPAEIRLYDRLFTV

PNPAAAEDFASTINTDSLVVINGFVEPSLASAEAEQGYQFERMGYFCADSKDSTADNLVF

NRTVGLRDTWAKIENQ

>sp|Q87TH0|TATB_VIBPA Sec-independent protein translocase protein TatB OS=Vibrio parahaemolyticus serotype O3:K6 (strain RIMD 2210633) OX=223926 GN=tatB PE=3 SV=1

MFDIGFWELVLISVVGLVVLGPERLPHAIRSVSRFIGAAKNMANSVKDELSHELKVQELQ

ENLRKAEQMGMEDLSPELKSSVEELKKAAQSVNRPYADKAQSETETAKAEPVTESAEKVE

EIKVSAADKKAE

>sp|Q87TH1|TATA_VIBPA Sec-independent protein translocase protein TatA OS=Vibrio parahaemolyticus serotype O3:K6 (strain RIMD 2210633) OX=223926 GN=tatA PE=3 SV=1

MGGISVWQLLIIAVIVVLLFGTKKLRGIGGDLGSAVKGFKKAMSDEDSAKNEKDADFEPK

SLEKQQQKEAAPETKKDKEQA

>sp|Q87GY5|TAL_VIBPA Transaldolase OS=Vibrio parahaemolyticus serotype O3:K6 (strain RIMD 2210633) OX=223926 GN=tal PE=3 SV=1

MSNKLEQLRKLTTVVADTGEIDAIKKYQPEDATTNPSLILKAAQIAEYAPLIDASIEYAK

AQSNDKAQQVQDTCDMLAVNIGKEILKTIPGRISTEVDARLSYDMEGSVAKARQLVKMYN

DAGITNDRILIKLASTWEGIRAAEILEKEGINCNLTLLFSFAQARACAEAGVFLISPFVG

RIMDWYKAKEGRDFEASEDPGVLSVTKIYNYYKEYGYKTVVMGASFRNIGEILELAGCDR

LTIAPALLAELEAAEGEVVEKLVDSKGAAERPAPMTHAEFLWEHNQDPMAVEKLAEGIRN

FAVDQGKLEAMIEAKL

>sp|P59410|TDH_VIBPA L-threonine 3-dehydrogenase OS=Vibrio parahaemolyticus serotype O3:K6 (strain RIMD 2210633) OX=223926 GN=tdh PE=3 SV=1

MKIKALSKLKPEQGIWMTEVDKPEVGHNDILIKIKKTAICGTDVHIYNWDEWSQKTIPVP

MVVGHEYVGEVVAIGQEVRGFEIGDRVSGEGHITCGHCRNCRGGRTHLCRNTIGVGVNRE

GAFAEYLVIPAFNAFKIPDEISDDLASIFDPFGNAVHTALSFDLVGEDVLITGAGPIGIM

AAAVAKHVGARHVVITDVNEYRLELARKMGVTRAVNVAEEKLEDVMAELGMTEGFDVGLE

MSGNPAAFNSMLTTMNHGGRIALLGIPPSDMGIDWNQVIFKGLVIKGIYGREMFETWYKM

ASLIQSGLDLSPIITHHYKIDDFQEGFDVMRSGMSGKVILDWE

>sp|Q87KF0|THIC_VIBPA Phosphomethylpyrimidine synthase OS=Vibrio parahaemolyticus serotype O3:K6 (strain RIMD 2210633) OX=223926 GN=thiC PE=3 SV=1

MSSRKQARLEAKQFIDTLSVQPYPNSTKVYIEGSRPDIRVPMREISLADSLIGGTKEAPI

FEPNEPVRVYDTSGVYTDPDYAIDLYSGLPKLREGWIEERNDTEILEDVSSVYAKERLDD

ETLDDLRYGNLPRIRRAKAGKCVTQLHYARKGIVTPEMEYIALRENMGRAQYRDDVLTQQ

HPGQSFGANLPKDITAEFVRKEVAEGRAIIPSNINHPESEPMIIGRNFLVKVNANIGNSS

VTSSIEEEVEKLVWATRWGGDTVMDLSTGRNIHETREWILRNSPVPIGTVPMYQALEKVN

GIAENLNWEVMRDTLIEQAEQGVDYFTIHAGLLLRYVPMTAKRVTGIVSRGGSIIAKWCL

AHHQESFLYTHFREICEICAKYDVALSLGDGLRPGSVADANDEAQFAELRTLGELTKIAW

EYDVQVIIEGPGHIPMHMIKENMDQQLEHCHEAPFYTLGPLTTDIAPGYDHITSGIGAAM

IGWYGCAMLCYVTPKEHLGLPNKEDVKTGMITYKLAAHAADLAKGHPGAQVRDNALSKAR

FEFRWEDQFNLALDPDTARAFHDETLPQESGKVAHFCSMCGPKFCSMKISQEVREYAKDT

EQVAADQAISIKMLDDPLEGMRKKSEEFRATGSELYHPAVHAEADE

>sp|Q87JW8|THIE_VIBPA Thiamine-phosphate synthase OS=Vibrio parahaemolyticus serotype O3:K6 (strain RIMD 2210633) OX=223926 GN=thiE PE=3 SV=1

MNAYRLYLVTDDQQDLPTLKHVVRKAVEGGVTMVQVREKHGDVREFIERAQAVKTILEGT

GVPLIINDRVDVALAVDADGVHLGQSDMPAEIARQLIGPNKILGLSIETEDQLAEADSLP

IDYIGLSAIFATPTKTNTKKHWGIGGLKMALNTTSLPIVAIGGINETNIPALSATGVHGL

ALVSAICHAENPTKAAEYLLSLMD

>sp|Q87J85|SYY1_VIBPA Tyrosine--tRNA ligase 1 OS=Vibrio parahaemolyticus serotype O3:K6 (strain RIMD 2210633) OX=223926 GN=tyrS1 PE=3 SV=1

MTTPLLQDLQDRGLIAQASDLEEIQTLLSQPQTVYCGFDPTAGSLHIGHLVPLIMLKRFQ

DAGHQAVALIGGATGMIGDPSFKATERSLNSAEIVSGWVNDLSNQIQQLMNHQLSKPMIM

VNNADWMRAINVIDFFRDVGKHFSINTMINRESVKQRLQRPDQGISFTEFSYALLQSYDF

AELNRQYGCRLQIGGNDQWGNIVSGIDLTRRQNGEQVFGLTLPLITKSDGTKFGKTEGGA

VWLDPSKTSPYAFYQFWLGAEDADVYHFLRYYTFLSCEEIASIEAQDQASQGKPQAQRIL

AEEMTRFVHGEEGLASAERITQALFSGNVQQLSLGELKQLELDGLPSIESAQQDLVELLI

ESGLASSKRVAREHISNNAISVNGEKVSADNPSLSFPLFDQYWLLQRGKKHFCLVKRAA

>sp|Q87LY8|SYY2_VIBPA Tyrosine--tRNA ligase 2 OS=Vibrio parahaemolyticus serotype O3:K6 (strain RIMD 2210633) OX=223926 GN=tyrS2 PE=3 SV=1

MASIEAALAEIKRGVEELIPEEELIAKLKEGRPLRIKLGADPTAPDIHLGHTVIFNKLRL

FQELGHEVTFLIGDFTAMVGDPTGKNTTRPPLSREDVLRNAETYKEQVFKILDPAKTKIQ

FNSEWLSELGAEGMIRLAANQTVARMLERDDFKKRYAGGQPIAIHEFMYPLLQGWDSVAM

ETDVELGGTDQKFNLLMGRELQKSHGQKPQVVLMMPLLVGLDGEKKMSKSAGNYIGISEA

PSEMFGKIMSISDDLMWSYYELLSFRPLEEIEQFKADVQAGKNPRDIKVLLAKEIIARFH

SEADADAAEQEFVNRFAKNQIPDEMPEFDFDAGTPVANLLKDAGLCASTSEAMRMVKQGA

AKVEGEKVADAKFAPEAGTYVFQVGKRKFARITIK

>sp|Q87SU3|RL27_VIBPA 50S ribosomal protein L27 OS=Vibrio parahaemolyticus serotype O3:K6 (strain RIMD 2210633) OX=223926 GN=rpmA PE=3 SV=1

MAHKKAGGSTRNGRDSESKRLGVKRFGGESVLAGNIIVRQRGTKFHAGNNVGIGKDHTLF

ALTEGKVKFEVKGPKNRKFVSIEAE

>sp|Q87N18|RL32_VIBPA 50S ribosomal protein L32 OS=Vibrio parahaemolyticus serotype O3:K6 (strain RIMD 2210633) OX=223926 GN=rpmF PE=3 SV=2

MAVQKNRKTRSKRGMRRSHDALTTAALSVDATSGETHLRHNVTAEGYYRGQKVINK

>sp|Q87T01|RL5_VIBPA 50S ribosomal protein L5 OS=Vibrio parahaemolyticus serotype O3:K6 (strain RIMD 2210633) OX=223926 GN=rplE PE=3 SV=1

MAKLHDYYKSSVVAELTKQFSYTSVMQVPRIEKITLNMGVGEAINDKKLLENAASDMATI

SGQKPLITKARKSVAGFKIREGYPIGCKVTLRGERMWDFLERLINIALPRVRDFRGVSAK

SFDGRGNYSMGVREQIIFPEIDFDKVDRVRGLDITITTSAGTDEEGRALLAAFNFPFRK

>sp|Q87LY1|RSMC_VIBPA Ribosomal RNA small subunit methyltransferase C OS=Vibrio parahaemolyticus serotype O3:K6 (strain RIMD 2210633) OX=223926 GN=rsmC PE=3 SV=1

MSAYIAPSQIAQRQLEYFNGKHVLVAGEVEDMFPLELTAHCESVEVFTSNYSYFRQIRHS

DKIKSHFGSEFDVETQADMLLLYWPKAKAEAEYLLAMLMAKLGVNTEIVVVGENRSGVKS

IEKMFKEYGPVNKYDSARRCSFYWGNCLNEPKPFNQEEWFKSYTVTLGEQSLTVKSLPGV

FSHGEFDLGSRLLLETLPNLSGKVLDFGCGAGVLGAFMAKANPEIAIEMCDINAYAITSS

QATLEANGLSGRVFASDIYSDTANDYRFIISNPPFHSGLDTNYNAAETLLGHAPQHLSNH

GEMIIVANSFLKYPPIIENAFNNCETLNKTNKFSIYYAKKS

>sp|Q9AJH1|RSMH_VIBPA Ribosomal RNA small subunit methyltransferase H OS=Vibrio parahaemolyticus serotype O3:K6 (strain RIMD 2210633) OX=223926 GN=rsmH PE=3 SV=1

MTETFQHISVLLNESIDGLAIKPDGIYIDGTFGRGGHSRTILSKLGPNGRLYSIDRDPQA

IAEAGKIDDPRFTIIHGPFSGMAQYAEEYDLVGKVDGVLLDLGVSSPQLDDAERGFSFMK

DGPLDMRMDPTSGIPVSQWLMEADLDDITWVIREFGEDKHARRIARAIVEYRENEENEPM

VRTGQLAKLISEAAPKSFKEKKHPATRAFQAFRIYINSELEEIDTALKGAARILAPEGRL

SVISFHSLEDRMVKRFIRKESKGPEVPHGIPLTEAQIKELGSANMKTVGKAIKPSKQEID

MNPRSRSSVLRIAEKL

>sp|Q87MX3|RNFB_VIBPA Ion-translocating oxidoreductase complex subunit B OS=Vibrio parahaemolyticus serotype O3:K6 (strain RIMD 2210633) OX=223926 GN=rnfB PE=3 SV=1

MSTILIAIIALAVLAAIFGAILGFASIRFKVEADPIVDQIDAILPQTQCGQCGYPGCRPY

AEAIANGDNINKCPPGGQATIEKLADLMGVEPEESAHDLDSKVPTVAFIHEDMCIGCTKC

IQACPVDAIVGGTKAVHTVIKDECTGCDLCVAPCPTDCIEMIPVETTTDSWKWQLNAIPV

VNITDAASDAANVKDSIK

>sp|Q87MW9|RNFE_VIBPA Ion-translocating oxidoreductase complex subunit E OS=Vibrio parahaemolyticus serotype O3:K6 (strain RIMD 2210633) OX=223926 GN=rnfE PE=3 SV=1

MSENKQLMKNGMWSNNPALVQLLGLCPLLAVSSTITNALGLGIATLLVLVGSNVTVSLIR

NYVPKEIRIPVFVMIIASLVTCVQLLMNAYAYGLYLSLGIFIPLIVTNCIIIGRAEAYAS

KNDVLPAALDGLWMGLGMTSVLVVLGSMRELIGNGTLFDGADLLLGDWAAALRIQVFQFD

SSFLLALLPPGAFIGVGLLIALKNVIDSSIQARQPKEEKPAIERARVTNA

>sp|Q87LL9|RPIA_VIBPA Ribose-5-phosphate isomerase A OS=Vibrio parahaemolyticus serotype O3:K6 (strain RIMD 2210633) OX=223926 GN=rpiA PE=3 SV=1

MTQDEMKKAAGWAALKYVEKGSIVGVGTGSTVNHFIDALGTIKDDIKGAVSSSVASTERL

KELGIEVFECNDVIKLDVYVDGADEINHAREMIKGGGAALTREKIVAAISEKFVCIVDDT

KAVDVLGQFPLPVEVIPMARSYVARELVKLGGDPAYREGVVTDNGNIILDVHNMQITNPK

EMEDKINGIAGVVTVGLFAHRGADVVITGTPEGAKIEE

>sp|Q87TB0|RPOZ_VIBPA DNA-directed RNA polymerase subunit omega OS=Vibrio parahaemolyticus serotype O3:K6 (strain RIMD 2210633) OX=223926 GN=rpoZ PE=3 SV=1

MARVTVQDAVEKVGNRFDLVLIAARRARQMQTGGKDALVPEENDKPTVIALREIEEGLIT

KEVLDARERQEQQEQEAAELAAVSSIAHNR

>sp|Q87SA4|RPPH_VIBPA RNA pyrophosphohydrolase OS=Vibrio parahaemolyticus serotype O3:K6 (strain RIMD 2210633) OX=223926 GN=rppH PE=3 SV=1

MIDGDGYRLNVGIVICNNHGQVFWAKRYGQHSWQFPQGGIDEGETPEQAMFRELYEEVGL

TKKDVKIIATSRHWLRYKLPKRLVRWDSKPVCIGQKQKWFLLRLECDESRINMQRGKSPE

FDGWRWVSYWYPVRQVVSFKRDVYRRAMKEFASLAMPFRERKTKGKRKKQQRRG

>sp|Q87T05|RL29_VIBPA 50S ribosomal protein L29 OS=Vibrio parahaemolyticus serotype O3:K6 (strain RIMD 2210633) OX=223926 GN=rpmC PE=3 SV=1

MKAQDLREKSVEELNAELMNLLREQFNLRMQAATGQLQQTHTLKAVRRDIARVKTVLTEK

AGA

>sp|Q87T15|RL31_VIBPA 50S ribosomal protein L31 OS=Vibrio parahaemolyticus serotype O3:K6 (strain RIMD 2210633) OX=223926 GN=rpmE PE=3 SV=1

MKAGIHPEYKAVNATCSCGNSFEFNSTLGKESIHLDVCDKCHPFYTGKQRIVDTGGRVDR

FNKRFGALSSGKK

>sp|Q87T12|RL4_VIBPA 50S ribosomal protein L4 OS=Vibrio parahaemolyticus serotype O3:K6 (strain RIMD 2210633) OX=223926 GN=rplD PE=3 SV=1

MELMVKGADALTVSETTFGREFNEALVHQVVVAYAAGARQGTRAQKTRSEVSGGGAKPWR

QKGTGRARAGTIRSPIWRTGGVTFAAKPQDHSQKVNKKMYRGAMKSILSELVRQERLIVV

DNFSVEAPKTKELVAKLKELELTDALIVTSEVDENLFLAARNLYKVDARDVAGIDPVSLI

AFDKVVMTAEAVKQVEEMLA

>sp|Q87P89|RLMI_VIBPA Ribosomal RNA large subunit methyltransferase I OS=Vibrio parahaemolyticus serotype O3:K6 (strain RIMD 2210633) OX=223926 GN=rlmI PE=3 SV=2

MTAAIYLVKGREKSVKRKHPWIFSRGIGKVEGEPALGETVDVFTHDGKWLAKAAYSPESQ

IRARIWSFEKEEINKAFFVKRFQNAQLLREDVIERDGLTGYRLIAAESDGLPGVTIDRYQ

NFFVCQLLSAGAEYNKQAIVDALVECFPDCNVYERSDVAVRKKEGLKETTGVLHGEEPPK

SVVIEENGVKISVDIVGGHKTGFYLDQRDSRQQAMKYVKDKEVLNCFSYTGGFGLYALKG

GAKRVINADVSQPALDTAKYNAELNEFDISKKRAVFLNADVFKLLREYRDQGTQFDVVIM

DPPKFAESKAQLNGACRGYKDINMLALQILKPGGTLLTYSCSGLMDQVLFQKIIADAAVD

ANRQVKFVERFEQAADHPTDTAYPEGFYLKGFACKVL

>sp|Q87MF1|RNH2_VIBPA Ribonuclease HII OS=Vibrio parahaemolyticus serotype O3:K6 (strain RIMD 2210633) OX=223926 GN=rnhB PE=3 SV=1

MVAKAKTTKAKVELPPFEYPQGYQLIAGVDEVGRGPLVGDVVTAAVILDPNNPIEGLNDS

KKLSEKKRLALLPEIKEKALAWAVGRCSPEEIDELNILQATMVAMQRAITGLKVQPDLAL

IDGNRCPELPMDSQAVVKGDLRVAEISAASIIAKVVRDQEMEELDKQYPQFGFAKHKGYP

TKAHFEAIEQHGVISEHRKSFKPVKKALGLD

>sp|Q87TR4|RNPA_VIBPA Ribonuclease P protein component OS=Vibrio parahaemolyticus serotype O3:K6 (strain RIMD 2210633) OX=223926 GN=rnpA PE=3 SV=1

MNTYAFNRELRLLTPEHYQNVFQQAHRAGSPHFTIIARNNKLSHPRLGLAVPKKQIKTAV

GRNRFKRLARESFRNSQHQLPNKDFVVIAKKSAQDLSNEEIFKLFDKLWQRLSRPSRG

>sp|Q87T93|RNPH_VIBPA Ribonuclease PH OS=Vibrio parahaemolyticus serotype O3:K6 (strain RIMD 2210633) OX=223926 GN=rph PE=3 SV=1

MRPNDRKADQVRPIKITRNYTAYAEGSVLVEFGNTKVLCNATVEESVPRWLKGQGRGWVT

AEYGMLPRATHSRTRREAANGKQGGRTMEIQRLIARSLRAVVDLQAMGEFMITVDCDVIQ

ADGGTRTASISGASVAMADAFQHLVDSGKLKANPMKGHVAAVSVGLLGDEVLCDLEYVED

SAADTDMNVVMTEEGKMIEIQGTAEGEPFSHEQLMALLESAKVGITEIVAAQKAALAN

>sp|P40611|RNR_VIBPA Ribonuclease R OS=Vibrio parahaemolyticus serotype O3:K6 (strain RIMD 2210633) OX=223926 GN=rnr PE=3 SV=2

MSDNIPNDPFADRESQNYENPIPSREFILEFLEQAGVPMNRNDLFEALKLAGEEQYEGLR

RRLRAMERDGQLVFTRRQCYALPEKLEMVKGYVIGHKDGHGWVRPEGSVGKDDDILLPHH

QMKNIIHGDFVLVQPTDNSKRGRREGRLVRVLEERNSQIVGRFFLEYGYSYVVPDDSRIS

QDILIPNEHKAGARMGNVVVIEITDRGSRSRGMMGKVVEVLGENMAPGMETQIAIRTHQI

PYEWPEAVEKQIVNLGEEVPEEAKVGRVDLRELPLVTIDGEDARDFDDAVFCEKKKDGGW

RLWVAIADVSYYVRPDSALDKEAINRGNSVYFPSQVVPMLPEVLSNGLCSLNPQVDRLCM

VCEMTISESGKLSSYKHYEAVMNSHARLTYSKVSAILEGDEELRERYQPLVSHLEELHAM

YKVLKEARDQRGAIEFETVETKFIFNAERKIESIEPVIRNDAHKIIEECMILANIASASL

VEKAKEPALYRIHESPGELRLQGFRDFLSELGLELKGGLEPSPTDYADLARQIAGRQDQE

LIQTMLLRSMKQAVYNADNAGHFGLALKRYAHFTSPIRRYPDLLLHRAIKYLIAKEEGRN

QDRWTPTGGYHYSFDDMDFYGEQCSMTERRADDATREVADWLKCEYMQDHVGDELEGVIA

NVTSFGFFVRLTDLHIDGLVHISTLANDYYQFDPIGQRLIGESFGNIYRLGDAVKVKVLA

VNLDDKQIDFELVETSRKLRGEGKTAKKRAAEAKRKAKEKKRAATRSSSKESATARAVPA

IEPTKRPEQTDSGRKRKGPKRGDDDSAKKPKVKKAHKKKPHSKPKKTKRTKQDAQ

>sp|Q87KQ5|RPOC_VIBPA DNA-directed RNA polymerase subunit beta' OS=Vibrio parahaemolyticus serotype O3:K6 (strain RIMD 2210633) OX=223926 GN=rpoC PE=3 SV=1

MKDLLNFLKAQHKTEEFDAIKIGLSSPDMIRSWSFGEVKKPETINYRTFKPERDGLFCAR

IFGPVKDYECLCGKYKRLKHRGVICEKCGVEVTQTKVRRDRMGHIELASPVAHIWFLKSL

PSRIGLLMDIPLRDIERVLYFEMYVVTEPGMTDLEKGQMLTEEEYLDRLEEWGDEFTAKM

GAEAIKDLLGSMDMHAEAEQMREELETTNSETKRKKVTKRLKLVEAFIASGNNPEWMILT

VLPVLPPDLRPLVPLDGGRFATSDLNDLYRRVINRNNRLKRLLELAAPDIIVRNEKRMLQ

ESVDALLDNGRRGRAITGSNKRPLKSLADMIKGKQGRFRQNLLGKRVDYSGRSVITVGPY

LRLHQCGLPKKMALELFKPFIYSKLETRGLATTIKAAKKMVEREEAVVWDILDEVIREHP

VLLNRAPTLHRLGIQAFEPVLIEGKAIQLHPLVCAAYNADFDGDQMAVHVPLTLEAQLEA

RTLMMSTNNILSPASGDPIIVPSQDVVLGLYYMTREKINVKGEGMYLSGPAEAEKAYRTK

QAELHARVKVRITETVVDEDGNSTTETKMVDTTVGRAMLWQIVPAGLPYSIVNQKLGKKQ

ISNLLNEAYRKLGLKDTVIFADQIMYTGFAYAALSGVSVGIDDMVVPPAKYTEIAEAEEE

VREIQEQYQSGLVTAGERYNKVIDIWASTNDRVAKAMMENLSSETVVNREGEEEQQESFN

SIYMMADSGARGSAAQIRQLAGMRGLMARPDGSIIETPITANFKEGLNVLQYFISTHGAR

KGLADTALKTANSGYLTRRLVDVAQDVVVTEHDCGTHEGVDMMPHIEGGDVKVALSELAL

GRVVAEDVLKPGTEDVLIPRNTLIDEKWCQIMEENSVDSMKVRSVVTCDSDFGCCAQCYG

RDLARGHLVNQGEAVGVIAAQSIGEPGTQLTMRTFHIGGAASTAAAENSIQAKNNGSVKL

HNAKFVTNKDGKLVITSRASELTIIDEFGRTKEKHKLPYGSLLSKGDNDAVEAGETVANW

EAHTLPIITEVAGRIQFVDMIDGVTVSRQTDDLTGLSSSEVTDAAARPAAGKDMRPAIKL

VDEQGNDVMIPGTEMPAHYFLPGKAIVNIEDGAEVGVGDTLARIPQKSGGNKDITGGLPR

VADLFEARKPKEPAILAEHTGTVSFGKETKGKRRLVITRDSGEVYEEMIPKHRQLNVFEG

ERVERGDVIADGPESPHDILRLRGVHAVTQYIANEVQEVYRLQGVKINDKHIETIVRQML

RKCTITHAGDSEFLPGEQVEYSQVKIANRNLEAEGKEPARFERELLGITKASLATESFIS

AASFQETTRVLTEAAVSGKRDDLRGLKENVIVGRLIPAGTGFAYHQERQAKRAEAQEGPS

AEQATDNLAALLNAGFSSDE

>sp|Q87LD3|RLUA_VIBPA Ribosomal large subunit pseudouridine synthase A OS=Vibrio parahaemolyticus serotype O3:K6 (strain RIMD 2210633) OX=223926 GN=rluA PE=3 SV=1

MAMLEYNPPTDPWTDIVFEDDHILAVNKPSGLLSVPGRLAEHHDSMWSRLQEEYPDIQVV

HRLDMSTSGLMVLAKNKRAESALKKQFQFRLTHKIYYARVWGHVEQEEGEIDLPLICDWP

NRPLQKVCFEDGKPSKTLFQVAKREEQEDGTKTTIVRLLPITGRSHQLRVHMQALGHPIV

GDEFYATEEAKVFSERLELHASELSFYHPKSHWLRSIFVPCDFYPEAEEMIFDYFDPERK

LPDYKTLPRP

>sp|Q87MX4|RNFA_VIBPA Ion-translocating oxidoreductase complex subunit A OS=Vibrio parahaemolyticus serotype O3:K6 (strain RIMD 2210633) OX=223926 GN=rnfA PE=3 SV=2

MTEYVLLLVGTVLVNNFVLVKFLGLCPFMGVSKKLETAIGMGLATTFVLTLASVCAYLVE

SYILRPLGIEYLRTMSFILVIAVVVQFTEMVVHKTSPTLYRLLGIFLPLITTNCAVLGVA

LLNINENHNFIESIIYGFGAAVGFSLVLILFASMRERIAAADVPVPFKGASIAMITAGLM

SLAFMGFTGLVK

>sp|Q87MG2|RNH_VIBPA Ribonuclease HI OS=Vibrio parahaemolyticus serotype O3:K6 (strain RIMD 2210633) OX=223926 GN=rnhA PE=3 SV=1

MTKHVEIFTDGSCLGNPGPGGYGIVLRYKDVEKTLSKGYTLTTNNRMEMLAAVVALQTLK

EPCRVTLTTDSQYVRQGITQWIHNWKKRGWKTADKKPVKNADLWQALDKETARHQVDWHW

VKGHAGHRENEICDELARTAAENPTEEDTGYQPS

>sp|Q87TR3|RL34_VIBPA 50S ribosomal protein L34 OS=Vibrio parahaemolyticus serotype O3:K6 (strain RIMD 2210633) OX=223926 GN=rpmH PE=3 SV=1

MKRTFQPTVLKRKRTHGFRARMATKNGRKVINARRAKGRARLSK

>sp|Q87NB7|RLUB_VIBPA Ribosomal large subunit pseudouridine synthase B OS=Vibrio parahaemolyticus serotype O3:K6 (strain RIMD 2210633) OX=223926 GN=rluB PE=3 SV=1

MSEKLQKVLARAGHGSRREIESLIKSGRVSVNGVVAKLGERLEDESSVVRIDGHIVSAKV

QEEVICRVLAYYKPEGELCTRHDPEGRRTVFDRLPKIRGSRWISVGRLDANTSGLLLFTT

DGELANRLMHPSRQVEREYLVRVFGEVTEQKVRNLVKGVELEDGLARFEDVVYAGGEGMN

HTFYVVINEGRNREVRRLWESQECTVSRLKRVRYGDIFLDKKLPRGGWMELDLKEVNYLR

ELVELRPEKETMLDLSKDNTSRKRERARSQKIRRAVKRHEERVSTSKGRSNNPARRKPKK

NAGEQGARNKHR

>sp|Q87N15|RLUC_VIBPA Ribosomal large subunit pseudouridine synthase C OS=Vibrio parahaemolyticus serotype O3:K6 (strain RIMD 2210633) OX=223926 GN=rluC PE=3 SV=1

MSEIRTQVQFVDIDEDMAGQRIDNFLRNQLKDIPKSMIYRIVRKGEVRVNKKRIKAEYKL

KAGDLVRIPPVTVEKKEEDVAPSTKLNKVAELEHMIIYEDDHMLILNKPSGTAVHGGSGL

KFGAIEALRALRPQARFLELVHRIDRDTSGILLVAKKRSALRHLQAQFREKTVKKFYFAL

VMGQWKSSCKVVNAPLLKNEVNSIVRVNPNGKPSETRFKILEKFEQATLIQASPITGRTH

QIRVHTQYTGHPIAWDDRYGDRRFDAYTGQLGLDRLFLHAANIKFQHPSNDEWMEINAPM

ESKLEKVLVGLRKAN

>sp|Q87S65|RLUD_VIBPA Ribosomal large subunit pseudouridine synthase D OS=Vibrio parahaemolyticus serotype O3:K6 (strain RIMD 2210633) OX=223926 GN=rluD PE=3 SV=1

MAQQIVLTNTVKDSQLGQRLDQAIAELFADFSRSRLKEWLLDGKVQVNGEVVTKPRTKVM

GGEEITLQAELEDEERWEAQDIPLDIVYEDDDIIVINKPRDFVVHPGAGTPDGTVLNALL

HHYPDIAEVPRAGIVHRLDKDTTGLMVVAKTVPAQTRLVRALQKRNITREYEAIAIGRMT

AGGKVDQPIGRHSTKRTLMAVAPLGKPAVTHYRVAEHFREHTRIRLRLETGRTHQIRVHM

SYLQHPLLGDTAYGGRARIPTGASQELTDMIRGFDRQALHAVMLRFEHPITGEELEFHAP

VPDDMVAMTEALRKDTEEYGLPDEF

>sp|Q87QY8|RLUE_VIBPA Ribosomal large subunit pseudouridine synthase E OS=Vibrio parahaemolyticus serotype O3:K6 (strain RIMD 2210633) OX=223926 GN=rluE PE=3 SV=1

MSSRTGHEKGRRTSQSKTGHFKRSGKPNRVEDQTTRRHPTRTAKAKAKAKANKPRVDLQN

RKVIIFNKPYDTLSQFTDGDGRKTLADYIPVKDVYAAGRLDRDSEGLMVLTNDGILQAKL

TQPKSKSPKTYWVQVDGAPQEQDLEKLRKGVELKDGMTLPAKVEVIDAPTIWERNPPVRF

RANIPTTWLAITIIEGRNRQVRRMTAHIGFPTLRLVRYSMGDITLGDLQPGQWKEIQL

>sp|Q87MX1|RNFD_VIBPA Ion-translocating oxidoreductase complex subunit D OS=Vibrio parahaemolyticus serotype O3:K6 (strain RIMD 2210633) OX=223926 GN=rnfD PE=3 SV=1

MSFFIASSPHAHSRRSTPDLMKWVALCAIPGLAAQTYYFGWGTLIQLIFAIAVAVSLEAL

VMICRKRSPMRALRDNSAIVTAWLLAVAIPPWSPWWIIVIGLIFAIVIAKHLYGGIGQNL

FNPAMVAYVVLLISFPVQMTSWSAPTLLIPDHVNFADTLSLIFTGFDYDGLSLQQVRAGV

DGVTMATPLDAFKTGIHTGATPSEVLSQPIFGGLAGIGWQWVNIAYLIGGLVMIKKRIIQ

WYIPAGFLASLTLFSLIFSVITPGETASPIFHLLSGATMLGAFFIATDPVSASTTVKGRL

IFGALIGALVFIIRSWGGFPDGVAFAVLLANMCVPLIDYYTKPRTYGH

>sp|Q87RU4|RISB_VIBPA 6,7-dimethyl-8-ribityllumazine synthase OS=Vibrio parahaemolyticus serotype O3:K6 (strain RIMD 2210633) OX=223926 GN=ribH PE=3 SV=1

MKVIEGGFPAPNAKIAIVISRFNSFINESLLSGAIDTLKRHGQVSEDNITVVRCPGAVEL

PLVAQRVAKTGKYDAIVSLGTVIRGGTPHFDYVCSECNKGLAQVSLEYSLPVAFGVLTVD

TIDQAIERAGTKAGNKGAEAALSALEMINVLSEIDS

>sp|Q87LR1|RECA_VIBPA Protein RecA OS=Vibrio parahaemolyticus serotype O3:K6 (strain RIMD 2210633) OX=223926 GN=recA PE=3 SV=1

MDENKQKALAAALGQIEKQFGKGSIMRLGDNRAMDVETISTGSLSLDIALGAGGLPMGRI

VEIYGPESSGKTTLTLELIAAAQREGKTCAFIDAEHALDPVYAKKLGVDIDALLVSQPDT

GEQALEICDALARSGAIDVMVVDSVAALTPKAEIEGEMGDSHMGLQARMLSQAMRKLTGN

LKQSNCMCIFINQIRMKIGVMFGNPETTTGGNALKFYASVRLDIRRTGAIKEGDEVVGNE

TRIKVVKNKIAAPFKEANTQIMYGQGFNREGELIDLGVKHKLVEKAGAWYSYNGDKIGQG

KANACNYLREHPEIAKTIDTKLREMLLSPAQPEAPAAGEKPEQEEEF

>sp|Q87LR2|RECX_VIBPA Regulatory protein RecX OS=Vibrio parahaemolyticus serotype O3:K6 (strain RIMD 2210633) OX=223926 GN=recX PE=3 SV=1

MYQKRQAPTLSSKEAAIQLLSRRDHGLYELHQKLAMKGYEEADIEAAINFCLEHNYLDDL

RYAKSQVRQHVYKGHGERRIRQELNQKRVAESVIEQAMAEEPQDWFELAKLAAEKKFKGI

KAKDQKEYAKQVRFLQYRGYSFDQISYALSFEDED

>sp|Q87T85|RL28_VIBPA 50S ribosomal protein L28 OS=Vibrio parahaemolyticus serotype O3:K6 (strain RIMD 2210633) OX=223926 GN=rpmB PE=3 SV=1

MSRVCQVTGKRPVTGNNRSHARNATKRRFLPNLQTHRFWVESEKRFVKLRLTAKGMRIID

KKGIDTVLADIRARGENV

>sp|Q87T84|RL33_VIBPA 50S ribosomal protein L33 OS=Vibrio parahaemolyticus serotype O3:K6 (strain RIMD 2210633) OX=223926 GN=rpmG PE=3 SV=1

MAKKGVREKIRLVSSAGTGHFYTTDKNKRNMPGKFEIKKFDPVVRQHVMYKEAKIK

>sp|P0A498|RL36_VIBPA 50S ribosomal protein L36 OS=Vibrio parahaemolyticus serotype O3:K6 (strain RIMD 2210633) OX=223926 GN=rpmJ PE=3 SV=1

MKVRASVKKICRNCKVIKRNGVVRVICSEPKHKQRQG

>sp|Q87KQ3|RL7_VIBPA 50S ribosomal protein L7/L12 OS=Vibrio parahaemolyticus serotype O3:K6 (strain RIMD 2210633) OX=223926 GN=rplL PE=3 SV=1

MSITNEQILDAVAEMSVMQVVELIEAMEEKFGVSAAAAVVAGGAAAGAAVEEQTEFDVIL

ESAGGNKVAVIKAVRGATGLGLKEAKALVDGAPAPLKEGVDKAEADALKAQLEEAGATVA

VK

>sp|Q87L11|RLMB_VIBPA 23S rRNA (guanosine-2'-O-)-methyltransferase RlmB OS=Vibrio parahaemolyticus serotype O3:K6 (strain RIMD 2210633) OX=223926 GN=rlmB PE=3 SV=1

MSNEFIYGIHAVKAVLEREPERFIEAYVLKGRQDDRLMPILNDLQVCGVSIQQMTRKTLD

DKAHGANHQGIIARVKVAKQLNENDIDDILAQHETPLLLVLDGVTDPHNLGACLRNADAA

GVAAIIVPKDRSAPMNATVSKVACGAAEVVPLIRVTNLARTMRTLQEQGIWFVGTAGEAT

HDIYQAKLTGPLAIVMGAEGDGMRRLTRETCDDLIKIPMAGSVSSLNVSVASGICLFEAV

RQRLAAK

>sp|Q87M18|RF3_VIBPA Peptide chain release factor 3 OS=Vibrio parahaemolyticus serotype O3:K6 (strain RIMD 2210633) OX=223926 GN=prfC PE=3 SV=1

MSNTPFLSEVSKRRTFAIISHPDAGKTTITEKVLLFGNAIQKAGTVKGRGNAQHAKSDWM

EMEKERGISVTTSVMQFPYNDCLVNLLDTPGHEDFSEDTYRTLTAVDSCLMVIDAAKGVE

DRTRKLMEVTRLRDTPIVTFMNKLDRDVRDPMEVLDEVENELGMMCAPITWPIGCGKEFK

GVYHIHRDETILYESGHGHEIQEVRIIKGLDNPELDEKVGESLAASVREELELVMGACPE

FDKELFLTGELTPVYFGTALGNFGVDHMLDGLTEWAPAPKTRQAVERDVEATEDKFSGFV

FKIQANMDPKHRDRIAFMRIVSGTYTQGMKMNHVRLGKQVSISDAVTFMAGDRSRAEHAY

AGDIIGLHNHGTIQIGDTFTQGESLKFSGIPNFAPELFRRIRLKDPLKQKQLLKGLVQLS

EEGAVQVFRPLQNNDLIVGAVGVLQFDVVVARLKSEYNVEAIYEGVNVATARWVECGDAK

KLDEFQRKNQANLALDGGDNLTYIAPTMVNLNLAKERFPDVEFRATREH

>sp|Q87SZ9|RS8_VIBPA 30S ribosomal protein S8 OS=Vibrio parahaemolyticus serotype O3:K6 (strain RIMD 2210633) OX=223926 GN=rpsH PE=3 SV=1

MSMQDPISDMLTRVRNGQAANKVAVKMPSSKLKVAIAALLKAEGYIVDFAVEGEAKPELE

VTLKYFQAKPVIEQLKRVSRPGLRVYKKKDELPSVMGGLGIAIVSTSKGLMSDRAARKAG

LGGEIICYVA

>sp|Q87TJ6|RSMJ_VIBPA Ribosomal RNA small subunit methyltransferase J OS=Vibrio parahaemolyticus serotype O3:K6 (strain RIMD 2210633) OX=223926 GN=rsmJ PE=3 SV=1

MQLQLICEDPSQQSHLDELAARWQLSHTDESDFALVLTAERLELRKVDEPKLGAIFVDLI

GGAVGHRRKFGGGKGQAIAKAAGLNKGATPTVLDGTAGLGRDAFVLASLGCKVQMVERHP

VVAALLDDGLARAKQDPEIGTWVSERMSLIHASSHDALDQLAQDREFVKPDVVYLDPMYP

HPENKKKSALVKKEMRVFQSLVGADLDADGLLEPALALATKRVVVKRPDYANWLNEQKPS

MAIETKKNRFDVYVKASMA

>sp|Q87SZ1|RS4_VIBPA 30S ribosomal protein S4 OS=Vibrio parahaemolyticus serotype O3:K6 (strain RIMD 2210633) OX=223926 GN=rpsD PE=3 SV=1

MARYLGPKLKLSRREGTDLFLKSGVRAIDTKCKIDNAPGVHGARRGRLSEYGVQLREKQK

VRRMYGVLEKQFRNYYKEAARLKGNTGENLLQLLEGRLDNVVYRMGFGATRAEARQLVSH

KAILVNGKVVNVPSFKVAANDVVSIREKAKQQARIKAALEVAEQREKPTWIEVDGGKMEG

TFKRMPERSDLSADINEQLIVELYSK

>sp|Q87L44|RS7_VIBPA 30S ribosomal protein S7 OS=Vibrio parahaemolyticus serotype O3:K6 (strain RIMD 2210633) OX=223926 GN=rpsG PE=3 SV=1

MPRRRVIGQRKILPDPKFKSELLAKFVNILMVDGKKSTAEKIVYTALDSMAEKSGKDHLA

IFEEALENVRPAVEVKSRRVGGSTYQVPVEVRPVRRNALAMRWLVEAARKRGEKSMAQRL

AAEMLDASENKGTAVKKREDVHRMAEANKAFAHYRW

>sp|Q87FP9|RSGA2_VIBPA Small ribosomal subunit biogenesis GTPase RsgA 2 OS=Vibrio parahaemolyticus serotype O3:K6 (strain RIMD 2210633) OX=223926 GN=rsgA2 PE=3 SV=1

MNQTHTFCDTLISTSNPLKQLGWKPFFQQQLTLDDYDNTIFARVIAHHRSGYLLATEAGQ

VHLNVHHSLPNMTVGDWVILNEDQQFVRLLDRLSLFSRKAAGSKVAEQLIAANVDTVFIV

CSLNHDFNLSRIERYLALVHEADVEPVIVLSKADLCDDVDELKSQVQKLDPLLVIETVNG

LDAESTSKLMSWCNEGQTVAFIGSSGVGKSTLVNALLGQQEQSTGHIREDDSKGRHTTTS

RSIHLLPAGGILIDTPGMREIQLVDCEAGVSEAFADVEALADHCRFGDCKHQTEPGCAVQ

AAIENGSLEVRRFNNYQKLLREQAFNGATLAEQRAQSRQFGKLTRNVMSDKRKRQQSY

>sp|Q87PA5|RSMF_VIBPA Ribosomal RNA small subunit methyltransferase F OS=Vibrio parahaemolyticus serotype O3:K6 (strain RIMD 2210633) OX=223926 GN=rsmF PE=3 SV=1

MHPNVYIPDAFLEKIQTILPANLNMEDFISACQRPLRKSIRVNTLKMSVEDFVKRAEDKG

WTLSPVPWCDNGFWIEADESVVPLGNTAEHMSGLFYIQEASSMMPVSALFMNDESYDAVL

DTAAAPGSKTTQIAALMKNEGVLVANEYAASRVKVLHANIERCGVRNAALSNFDGRVFGG

WLPEQFDAVLLDAPCSGEGTVRKDEDAMKNWTQASVLEIADTQKDLIESAFHALKPGGVL

VYSTCTLSTEENQQVCHHLKETFGDAVEFESLDGLFENANAALTEEGFLHIFPQVYDCEG

FFVARIRKHHSVEAPQVKKRMGKFPFVKASKKESEEISKQLHNALDIELPSESTVWLRDK

DVWLFPDALEPMIGELRFSRMGIKIAEAHKNGYRWQHQVATALATGAESNAIELTIEEAR

EWYMGRDVRPQIIPEGLKTGKGEVLVKYQGAIIGLGKWVSNRIKNGLPRELVRDKNLF

>sp|Q87QD4|RSUA_VIBPA Ribosomal small subunit pseudouridine synthase A OS=Vibrio parahaemolyticus serotype O3:K6 (strain RIMD 2210633) OX=223926 GN=rsuA PE=3 SV=1

MRLDKYLCDALGATRKQATKIIKSGEVLVDGEVQKSGSFKVPEGGVVEWDGREVGAPGPR

YIMLYKPADFVCSHEDGYNPTAFVLLDEPKVENLHFAGRLDVDTTGLVLITDDGQWSHRI

TSPKHKCKKTYRVWLADAIQPDYVEKFAQGIELRNEREATLPAHLEIVNEAENEVLLTIV

EGKYHQVKRMFAALGNKVELLHRERIGDIVLDDTLEPGDYRYLTQEEVDSVWN

>sp|Q87LM9|SDHE_VIBPA FAD assembly factor SdhE OS=Vibrio parahaemolyticus serotype O3:K6 (strain RIMD 2210633) OX=223926 GN=sdhE PE=3 SV=1

MYTPEEKARIKWACRRGMLELDVVIMPFFEECFDALNEQEQRDFVSLLECDDPDLFTWVM

GHGRSENLGHAAMVDKIVAHNLSKVR

>sp|Q87L36|SLYX_VIBPA Protein SlyX homolog OS=Vibrio parahaemolyticus serotype O3:K6 (strain RIMD 2210633) OX=223926 GN=slyX PE=3 SV=1

MTEKLIEQLEARINDLECQVAFQEQTIEDLNSALSQQQLQITKMQDQMKYVVGKVKNMDS

SNMEDPANEPPPPHY

>sp|Q87QV1|RUVC_VIBPA Crossover junction endodeoxyribonuclease RuvC OS=Vibrio parahaemolyticus serotype O3:K6 (strain RIMD 2210633) OX=223926 GN=ruvC PE=3 SV=1

MSIILGIDPGSRITGYGVIRQQGRHLQYLGSGCIRTSEKELPGRLKQIYAGVTEIITQFQ

PDVFAIEQVFMAKNADSALKLGQARGSAIVAAVNADLPVYEYAARLIKQAVVGTGGADKV

QVQHMVQHMLKLPAKPQADAADALGVAICHANTNKTLVALAGKASSARKGRYR

>sp|Q87QA3|SERC_VIBPA Phosphoserine aminotransferase OS=Vibrio parahaemolyticus serotype O3:K6 (strain RIMD 2210633) OX=223926 GN=serC PE=3 SV=1

MEQNTDNVFNFSAGPAALPKAVMQQAQQELIDWQGLGTSVMEISHRSKEFIKVAQEAEQD

LRDLLNIPDNYKVLFCQGGARAQFAAVPLNLLGDAETATYIDGGYWAESAVEEAKKYCEP

DVFDAKTEIDGKVAVLPASEWKIAPEAAYVHFCPNETIDGIEINDLPITDKPIVADMSST

ILSREIDVSKYGVIYAGAQKNIGPSGIAIAIVRDDLLGMAKQVLPSILDYKVLAEKESMF

NTPPTFAWYLSGLVFKWLKAQGGVKSIETVNREKAKLLYDYIDQSDFYRNGVHPSNRSLM

NVPFQLAKPELDATFLELADAKGLKALKGHRVVGGMRASIYNAMPLEGVQALVDFMKEFE

QNYA

>sp|Q87T90|SLMA_VIBPA Nucleoid occlusion factor SlmA OS=Vibrio parahaemolyticus serotype O3:K6 (strain RIMD 2210633) OX=223926 GN=slmA PE=3 SV=1

MAGSKKSNRREEILQALAQMLESAEGASRITTAKLAKQVGVSEAALYRHFPSKARMFEGL

IEFIEEALMTRINRILDDEKDTLERIRMVMHLILAFSERNPGLTRILSGHALMFENERLR

ERINQLFERIETQLRQILRERKIREGKSFPVEERILAAQILGQVEGSLNRFVRSDFKYQP

TANFDEYWALLSAQIK

>sp|Q87QA2|SDHD_VIBPA Probable D-serine dehydratase OS=Vibrio parahaemolyticus serotype O3:K6 (strain RIMD 2210633) OX=223926 GN=dsdA PE=3 SV=1

MNKLNVEVLKKQFPLVEQLINLDEVCWFNPNVTSLEEGLPHVGLNAEDIHVASARLKRFA

PYLMKAFPETKAASGLIESPVVDIPKMKAALETQYNVPIFGRLMLKLDSHLPISGSIKAR

GGIYEVLVHAEKLAIATGLLSESDDYSKLLSGEFRQFFQQYSIAVGSTGNLGMSIGMMSA

KLGFSVSVHMSADAREWKKNKLRSHGVNVVEYEQDYGVAVEQGRKQAESDPNCFFIDDEN

SQTLFLGYSVAGERLKQQFDDLGIVVDERHPLFVYLPCGVGGGPGGVAFGLKVAFGDHVH

CIFAEPTHSPCMLLGVHTGLHDEIAVQDIGIDNLTAADGLAVGRPSGFVGRAMERLIDGY

YTVTDERMYQLLGELSEREGINLEPSALAGMMGAVHVSGSLHYQTRLQLTDERLKNATHL

VWATGGGMVPEAEMSADLAKSGR

>sp|Q87KZ3|SECB_VIBPA Protein-export protein SecB OS=Vibrio parahaemolyticus serotype O3:K6 (strain RIMD 2210633) OX=223926 GN=secB PE=3 SV=1

MAEAAPQEAQQNFAIQRIFLKDVSFEAPNSPVIFQKEWNPDVKLDLDTQSRELGEGVYEV

VLRLTVTVKNEEETAFLCEVQQGGIFTAEQMEAGQLAHCLGAFCPNILFPYARETISSLV

VKGTFPQLNLAPVNFDALFMNYLQQQAQQGEAQA

>sp|Q87SF7|SECA_VIBPA Protein translocase subunit SecA OS=Vibrio parahaemolyticus serotype O3:K6 (strain RIMD 2210633) OX=223926 GN=secA PE=3 SV=1

MITKLLTKVIGSRNDRTLRRLRKIVKEINNYEPTFEALSDEELKAKTVEFRERLEQGETL

DKLLPEAFATVREASKRVYGMRHFDVQLIGGMVLNAGQIAEMRTGEGKTLTATLPAYLNA

LPGKGVHVVTVNDYLAKRDAETNRPLFEFLGMTVGVNVPNMPPQAKKEAYQADILYGTNN

EFGFDYLRDNMAFRNEDRVQRERFFAVVDEVDSILIDEARTPLIISGPAEDSSELYTRIN

LLIPHLQKQDKEDSEEYRGDGHYTVDEKSKQVHLTETGQEYVEELLVKNGLMEEGDTLYS

PANISLLHHVNAALRAHVLFERNVDYIVNDDGEVVIVDEHTGRTMPGRRWSEGLHQAVEA

KEGVKIQNENQTLASITFQNYFRLYEKLSGMTGTADTEAFEFQSIYGLETVVIPTNKPMI

RNDMPDVVYRTEAEKFAAIIEDIKERVAKGQPTLVGTVSIEKSELLSNALKKAKIKHNVL

NAKFHEKEAEIVAEAGMPGAVTIATNMAGRGTDIVLGGSWQAKVESLQDPTKEQIDAIKA

EWKKVHDQVLDAGGLHIIGTERHESRRIDNQLRGRSGRQGDAGSSRFYLSMEDSLLRIFT

SDRMASLIQSGMEEGEAIESKMLSRSIEKAQRKVEGRNFDIRKQLLEYDDVANDQRKVVY

ELRDELMNVDDISDMIEQNREDVLTAIIDEYIPPQSLEDMWDVEGLQERLKADFDLDAPI

KQWLEEDDKLYEEALREKIISLAVEVYKAKEEVVGAQVLRNFEKSVMLQTLDTLWKEHLA

AMDHLRQGIHLRGYAQKNPKQEYKRESFELFEGLLEALKTDVITVLSRVRVQQQEEVERM

EEQRRAQAEEAARRAQAQHAAAENQLADGEESEGSNQPVVRDERKVGRNEPCPCGSGKKY

KQCHGQIN

>sp|Q87LV9|SFSA_VIBPA Sugar fermentation stimulation protein homolog OS=Vibrio parahaemolyticus serotype O3:K6 (strain RIMD 2210633) OX=223926 GN=sfsA PE=3 SV=1

MQFKPALESATLLKRYKRFLADIEFDNGEVRTIHCANTGAMTGCATPGNKVWFSTSDNPK

RKYPNSWELSETEQGHRICINTARANQLAVEAIENNVISELCGYDTLQTEVKYGNENSRI

DILLSASDKPKCYIEVKSVTLLDETGSAGQGYFPDAVTTRGQKHLRELTEMAQNGSRAIL

LFTVLHSGIEKVSAAHHIDAKYSLLLKQAQDAGVEVLCYKAELSNTEMKLISAIDFIN

>sp|Q87LK4|SPRT_VIBPA Protein SprT OS=Vibrio parahaemolyticus serotype O3:K6 (strain RIMD 2210633) OX=223926 GN=sprT PE=3 SV=1

MDIELSYKAKQVMVDCISLAQQAFKRSFPIPSITFNVRGKAAGKAYLQLNQIRLNPILFR

ENPQAFLEEVIPHEVAHLITYQVYGRVRPHGKEWRGVMESVFGISANTTHRFEVTSVQGK

TFEYRCGCMTYPLSIRRHNKVLRKEATYSCQKCHQPLNFTGVQLS

>sp|Q87JS8|SPEA_VIBPA Biosynthetic arginine decarboxylase OS=Vibrio parahaemolyticus serotype O3:K6 (strain RIMD 2210633) OX=223926 GN=speA PE=3 SV=1

MRIELEKATKLDRIRADYNVHYWSQGFYGIDDQGEVYVSPRSDRAHQIPFSAIVNELEAQ

QLNLPVLVRFPQIVHQRVHGICHAFNQAIEEYQYPNKYLLVYPIKVNQQREVVDEILASQ

AQLETKQLGLEAGSKPELLAVLALAQQGSSVIVCNGYKDREYVRLALIGEKLGHKVFIVL

EKLSELDLVLEEAKSLGVKPRLGLRIRLASQGAGKWQASGGEKSKFGLSASQVLSVIERL

KREGSLDAMQLVHFHLGSQMANIRDVRNGVNESARFYCELRALGANIEYFDVGGGLAVDY

DGTRSQSSNSMNYGLAEYARNIVNTVGDVCQQYEQPMPVIISESGRSLTAHHAVLISNVI

GTETYQPEEVHELGVDAPLLLQNMWRNWENLQDGTDARALIEIYNDTQSDLAEVHSQFAT

GVLNLEQRAWAEQLSLRIYFELSRKMSTKNRFHRPILDELSERLADKFFVNFSLFQSLPD

AWGIDQVFPVLPLSGLGDAEERRAVMLDITCDSDGAIDHYVDGQGIESTLPVPAWSKDKP

YLMGFFLVGAYQEILGDMHNLFGDTHSAVVNVDEHGQFEISYINEGDSVEDMMRYVHIDV

DAIRDNYKQLVSQRVEANEQAQILAELEQGLAGYTYLEDF

>sp|Q87RY2|SSRP_VIBPA SsrA-binding protein OS=Vibrio parahaemolyticus serotype O3:K6 (strain RIMD 2210633) OX=223926 GN=smpB PE=3 SV=1

MAKKKSKQKAGSNTIALNKKARHEYFIEDEIEAGLELQGWEVKALRQGKANISESYVFMR

DGEAFVSGMTITPLNQASTHVVANPTRVRKLLMSRRELDNLLGRINREGMTLTALSLYWS

RSWVKIKIGVAKGKKLHDKRTDLKEKDWAREKARVMKSALR

>sp|Q87KD8|SMG_VIBPA Protein Smg homolog OS=Vibrio parahaemolyticus serotype O3:K6 (strain RIMD 2210633) OX=223926 GN=smg PE=3 SV=1

MMMDILMYLFETYIHSDAELQVEQDELEEELLRAGFQQKDIYKALVWLEELAALQQSDTS

SAISACIASSSTRIYTAKEMQRLDLECRGFLLFLEQINVLTTETREMVIDRVMGLETSEF

ELEDLKWIILMVLFNVPGNENAYTLMEELLYTKEQGILH

>sp|Q87S36|TGT_VIBPA Queuine tRNA-ribosyltransferase OS=Vibrio parahaemolyticus serotype O3:K6 (strain RIMD 2210633) OX=223926 GN=tgt PE=3 SV=1

MKLKFDLKKKNGNARRGQLTFERGTVQTPAFMPVGTYGTVKGMTPEEVKGTGAEILLGNT

FHLWLRPGQEVMKMHGDLHDFMNWHGPILTDSGGFQVFSLGKMRTITEKGVHFRNPVNGD

KIFMDAEKSMEIQKDLGSDIVMIFDECTPYPATHNEAKKSMEMSLRWAQRSRDHFDKLEN

PNNLFGIVQGGVYEDLRDVSVKGLTEIGFDGYAVGGLAVGEPKEDMHRILEHTCPQLPED

KPRYLMGVGKPEDLVEGVRRGIDMFDCVMPTRNARNGHLFVTGGVIKIRNAKHKTDTTPL

DPHCDCYTCQNYSKSYLHHLERCNEILGARLNTIHNLRYYQRLMESIRKAIDEDRFDEFV

QEFYARRDREVPPLSKA

>sp|Q87KF4|THIG_VIBPA Thiazole synthase OS=Vibrio parahaemolyticus serotype O3:K6 (strain RIMD 2210633) OX=223926 GN=thiG PE=3 SV=1

MLKIGDKEFKSRLFTGTGKFSNSHLMAEAIQVSGSQLATMALKRVDVHDQQDDILQPLIH

AGVNLLPNTSGAKNAKDAVFAAQLAREALGTNWVKLEIHPDPKYLMPDPIETLAAAEQLV

RDGFIVLPYCHADPVLCKRLEEVGCAAVMPLGAPIGSNKGIASHDFLEIIIDQANVPVVV

DAGIGAPSHAARAMEMGADAVLVNTAIAAASNPVAMAKAFKMAVESGRMAYKAGLAGKVS

HAVASSPLTAFLDEL

>sp|Q05939|TOXS_VIBPA Transmembrane regulatory protein ToxS OS=Vibrio parahaemolyticus serotype O3:K6 (strain RIMD 2210633) OX=223926 GN=toxS PE=3 SV=1

MKIKVASAVLAVSILFSGWLYWGSDLKVEQVLTSNEWQSTMVTVITDNLPDDTVGPLRRV

NVESNVKYLPNGDYIRVANIKLFAQGSTAESTINISEKGRWEVSDNYLLVSPSEFKDISS

SQSKDFSEAQLRLITQIFKLDAEQSRRIDVVNEKTLLLTSLNHGSTVLFRN

>sp|Q05938|TOXR_VIBPA Cholera toxin homolog transcriptional activator OS=Vibrio parahaemolyticus serotype O3:K6 (strain RIMD 2210633) OX=223926 GN=toxR PE=4 SV=2

MTNIGTKFLLAQRFTFDPNSNSLADQQSGNEVVRLGSNESRILLMLAERPNEVLTRNELH

EFVWREQGFEVDDSSLTQAISTLRKMLKDSTKSPEFVKTVPKRGYQLICTVERLSPLSSD

SSSIEVEEPASDNNDASANEVETIVEPSLATTSDAIVEPEAPVVPEKAPVASAVNPWIPR

VILFLALLLPICVLLFTNPAESQFRQIGEYQNVPVMTPVNHPQINNWLPSIEQCIERYVK

HHAEDSLPVEVIATGGQNNQLILNYIHDSNHSYENVTLRIFAGQNDPTDICK

>sp|Q87R81|TIG_VIBPA Trigger factor OS=Vibrio parahaemolyticus serotype O3:K6 (strain RIMD 2210633) OX=223926 GN=tig PE=3 SV=1

MQVTVETLEGLERRLNITVPAANIEDAVTAELRNIAKNRRFDGFRKGKVPLKMVAKMYGK

AVRQDVLGEVMQRHFIEAIVKEKINPAGAPTFAPVENKEGADLVFTATFEVYPEVELKGL

ENITVEKPLTEVKEADVEEMIETLRKQQATWVEVEEAAEAGKRVSIDFVGSIDGEEFEGG

KAENFPLEMGAGRMIPGFEDGIAGKTAGMEFDIDVTFPEDYHAENLKGKAAKFAIKVNKV

EARELPELNDEFVAKFGVAEGGVDALKAEVRKNMERELKQAVKTRIKEQAIEGLVKENEI

DVPAALIEQEIHVLRQQAAQRFGGNPEAAAQLPRELFEEQAKRRVVVGLLLGEVIKSEEL

KADDEKVKALIEEMATAYEDPSEVIAYYEQNEQMMNNMRNVALEEQAIDAIIAKAQVTEK

EVGFNELLNQQPAA

>sp|Q87MF4|TILS_VIBPA tRNA(Ile)-lysidine synthase OS=Vibrio parahaemolyticus serotype O3:K6 (strain RIMD 2210633) OX=223926 GN=tilS PE=3 SV=1

MESLYQQFSHVLNTYYQPQTKVVVAFSGGVDSRLLLELLRRYREENSLSCHAVYVHHGLS

ENADIWADKCQVWAKQAGISCSVERVNLDTNSGESIELLAREARYEALARHINRGDLLLT

GQHADDQVETFLLALKRGSGPKGLSSMAESMPFAGGTLVRPLLNTKREQIEATAKNIGLE

WVEDESNQDTRYDRNFLRHRIVPELSERWPSIHQAVQRSASLCAQQEALLDELLGSVFAR

ALQADLSLSIDELAIHSELAQARLIRMWLSKLNANMPSQTQLRLIWQEVALAQQDANPKL

KLKQGEIRRFQNKLYWVTHRADVTSWQGHIQIDEPLILPESLGTLTLSSGSHQPNISLPS

HPELLRVTFNPEGLSAHPTTRSRSRKLKKLFQEYNVPSWLRRQIPILMYKDQVVAVADLF

VDQTFSGQDCELIWRKPL

>sp|Q87QI7|TORA_VIBPA Trimethylamine-N-oxide reductase OS=Vibrio parahaemolyticus serotype O3:K6 (strain RIMD 2210633) OX=223926 GN=torA PE=3 SV=1

MAITRRSFLKGVATTSAASVIGPSLLASASANAAESTGTWKVTGSHWGAFRAHIYAGKVQ

EIKPLELDKNPTEMLNGIKGIIYSPSRVRYPMVRLDWLKKHKYSADTRGNNRFIRVTWDE

ALDLFYRELERVQKEYGPWALHAGQTGWNQTGSFNNCTAHMQRAVGMHGNFITKVGDYST

GAGQTIMPYVLGSTEVYAQGTSWSEILENSDNIILWANDPVKNLQVGWNCETHESFKYLA

ELKEKVAKGEINVLSVDPVKNKTQRYLENDHLYINPMTDVAFMLAVAHVLYNENLYDKKF

IDTYCLGFEEFIQYVQGKTKDKVEKTPEWAAAICGVKADKIREFARMLVSGRTQILMGWC

IQRQEHGEQPYWAAAVVAAMVGQIGLPGGGISYGHHYSSIGVPSTGFAGPGGFPRNLDQG

MKPKWDNNDFNGYSRTIPVARWIDCLLEPGKEINYNGGKVKLPDFKMMVISGCNPWHHHQ

DRNRMKKAFRKLQTVVTIEFAWTATCRFSDIVLPACTQWERNDIDVYGSYSNKGLIAMHR

LVDPLFQSKPDFQIMSELTQRFGRREEYTRGMSEMEWIESLYNDCKKANEGKFEMPEFNE

FWEKSVLDFGEGKPWVRHADFRKDPELNPLGTPSGFIEITSRKIGRYGYEHCQEHPMWFE

KSERSHGGPGSDKYPFWLQSCHPDKRLHSQMCESEEFRATYAVQGREPVYINPIDAKAKG

IKDGDLVRVFNGRGQLLAGAVLTDSYPRGVIRIEEGAWYGPLNEKEGAICTYGDPNTLTQ

DIGSSELAQATSANTCIVDFEKFTGKVPPVTSFGGPIEVA

>sp|Q87JV8|TRMYL_VIBPA Putative pseudouridine methyltransferase OS=Vibrio parahaemolyticus serotype O3:K6 (strain RIMD 2210633) OX=223926 GN=VPA0140 PE=3 SV=1

MRSFVLRARAAPTTSKALLEGVGNEAHTEILAHTMMNTMFVAQSHREDVVVHLVLESTKD

YSRTITIRSNDITNIGGFHESTLIAAVARALDASVGMGKEQLREVEPGITVRTVSFERLV

QELAEDHQLYMLDKKGEFVRDAEIGENPCFLLTDHIPMPKKSFNSLKRLGTEKISLGPKM

LFASQCVVLIHNELDIREF

>sp|Q87QT9|TOLB_VIBPA Tol-Pal system protein TolB OS=Vibrio parahaemolyticus serotype O3:K6 (strain RIMD 2210633) OX=223926 GN=tolB PE=3 SV=1

MIKRLLLGMFVLLGSLTNVAHAALELVITDGIDSARPIAVVPFKWEGSQPLPTDISAVIA

SDLQRSGKFSPVPTNKMPQTPFNESEVNFDSWTSLGVDALLTGSIKQNEQGDYVVNYQLV

DVVRGQLTGGQSKALGSDGELVLSKDHVLFNKVATVKGPRMREYAHRISDLVYEQLTGER

GAFMTRIAYVVVNDKDRFPYQLRVADYDGYNERLVLRSKQPLMSPAWSPDGQKLAYVSFQ

NGQAEIFIMNIYTGEREKITSYPRHNGAPRFSPDGNKLALVLSKTGTLQVYTFDLKTRKL

TQITRSRSNNTEPFWHPDGKSLIFTSDRGGKPQIYQVNLGSGSIDRLTWQGSQNLGGQIT

PDGRFLVMVNRSDSGFNLAKQDLETGALQVLTKTLLDESPSIAPNGGMVVYSSIYNKKNV

LSMVSIDGRFKARLPATNGRVRAPAWSPFL

>sp|Q87QW8|TORD_VIBPA Chaperone protein TorD OS=Vibrio parahaemolyticus serotype O3:K6 (strain RIMD 2210633) OX=223926 GN=torD PE=3 SV=1

MQEVKAFNEKRAEIYWWFSSLFAKELSEKELETYHSVEIRSFLAGLGENESLKPAVDSLV

DALNRLQDRNDAQLELAADFCELFLKTDKYGALPYASMYIGESGLLNDKPAEEMEKLMAD

FGVQVDENLKEPADHLAVELDFLGNMIIRSNELEQEKHMEEAFVKQNDFIQNQLMSWLPK

FAEKCKQFDEFGFYLSVAQLLIAFCKLDSAYLLGE

>sp|Q87LK8|TKT1_VIBPA Transketolase 1 OS=Vibrio parahaemolyticus serotype O3:K6 (strain RIMD 2210633) OX=223926 GN=tkt1 PE=3 SV=1

MSSRKHLANAIRALSMDGVQQANSGHPGAPMGMADIAEVLWRSHLNHNPANPEWADRDRF

VLSNGHGSMLIYSLLHLSGYELSIDDLKNFRQLHSKTPGHPEYGYAPGIETTTGPLGQGI

TNAVGMALAEKALAAQFNKEGHDIIDHFTYVFMGDGCLMEGISHEACSLAGTLGLGKLIA

FWDDNGISIDGHVEGWFSDDTPKRFEAYGWHVIPAVDGHDSDAINAAIEAAKADPRPTLI

CTKTIIGFGSPNKSGSHDCHGAPLGAEEIAAAREFLGWEHPAFEIPADVYAEWDAKAAGA

EKEAAWNAKFEAYAAAYPTEAAELKRRLNGELPAEWEEKANQIIADLQANPANIASRKAS

QNALEAFGQMLPEFMGGSADLAPSNLTMWSGSKSLEANDFSGNYIHYGVREFGMTAIMNG

IALHGGFVPYGATFLMFMEYARNAMRMAALMKIQNIQVYTHDSIGLGEDGPTHQPVEQMA

SLRLTPNMNTWRPCDQVESAVAWKLAIERKDAPTALIFSRQNLAQQERTAEQVTDIAKGG

YILKDSDGKPELILIATGSEVELAVKAAEQLTAEGKKVRVVSMPSTDAFDKQDAAYREAV

LPSDVTARIAIEAGIADFWYKYVGFDGRIIGMTTFGESAPADQLFEMFGFTVENVVNTAK

ELLA

>sp|Q87JW7|THIM_VIBPA Hydroxyethylthiazole kinase OS=Vibrio parahaemolyticus serotype O3:K6 (strain RIMD 2210633) OX=223926 GN=thiM PE=3 SV=1

MLIEQITQALTAVRQQKPLVVNITNYVVMNNTANALLAIGASPIMAHSKQEMAEMMSFAG

ALVINIGTLDSVWTPRMSYAVEQANSNGKIVVLDPVGCGASSLRTETSREIARLANKLII

RGNASEIIALAGEQAQSKGVDALDSSDAALGAAHFLVKEYGASVVISGETDYIVTKEQTV

QLNNGHEMMPYVTGMGCTLTALTGAFAAIGDETGLAAAAVLGVVGEIAAEQARGPGSLQM

HLLDELYQLDEETLATRLKLQVR

>sp|Q87SV4|THIQ_VIBPA Thiamine import ATP-binding protein ThiQ OS=Vibrio parahaemolyticus serotype O3:K6 (strain RIMD 2210633) OX=223926 GN=thiQ PE=3 SV=1

MLVLDDVQYTYQRELFRFELSIERGQIVSLMGPSGAGKSTLLALVAGFIHPDQGDIRVDG

ESIVRKEPYQRPFSMLFQEHNLFSHLSVRDNIGLGLHPGLKLTVDQKRQVEQAAQQVGVA

EYLDRLPEHLSGGQRQRVALARCFVQPHPMWLLDEPFSALDPVLREEMLSLVKKLAAERG

ITVLMVTHHLSDAKAIASHFAFVANGKVEAVGEIDALTAEHPSKTLQAFVRAAG

>sp|Q87JQ6|TNAA_VIBPA Tryptophanase OS=Vibrio parahaemolyticus serotype O3:K6 (strain RIMD 2210633) OX=223926 GN=tnaA PE=3 SV=1

MKRIPEPFRIKMVEPIKMTTLADREIALKEAGYNPFLLRSDDVYIDLLTDSGTGAMSDNQ

WAGIMLGDESYAGSRNYYNLCSAVEHFFGYKLTVPAHQGRGAEQILFPALIERMRQVRGG

ETPVFISNYHFDTTAGHIELNGGKAINVVTEEAFDTTTPYDWKGNFDLNKLVETIEEHGS

KNICAIITTVTCNSSGGQPVSMENMRSVYEIATKYDIPVVIDSARYCENAYFIKQREEGY

ADKSILEIIREMYQYGDMLTMSAKKDPMVNIGGMCCIRDHQELFQAVQTRCVPMEGFVTY

GGMAGRDMEALARGLYEGADEDFLHYRISQVQYLGERLREGGIPIQYPTGGHAVFVDAAK

ILPHIPADQFPAQALCNALYLEAGIRAVEIGSLLLGRDPETGEQKASELELMRLTIPRRV

YTNDHMDYIADALIELKNRAHELKGLMFEYEPPVLRHFTARFREID

>sp|Q87KH5|RHLB_VIBPA ATP-dependent RNA helicase RhlB OS=Vibrio parahaemolyticus serotype O3:K6 (strain RIMD 2210633) OX=223926 GN=rhlB PE=3 SV=1

MKKTHITEQKFADLGLQPQVTEGLEKKGFEYCTPIQALALPVLLTGQDIAGQAQTGTGKT

LAFLTATFNHLLTTPEHEGRQPTQPRAIIMAPTRELAIQIFNDAEPLIASTGLKAALAYG

GESYDKQLAKLQDGVDILIGTTGRIIDFYKQRVFNLNNIQAVVLDEADRMFDLGFIKDIR

FLFRRMPEPKERLNMLFSATLSYRVQELAFEHMHNPEHVVVEPAQKTGHRIQEELFYPSN

EDKMALLQTLIEEEWPDRAIVFANTKHKCESVWGHLAADGHRVGLLTGDVPQKKREKILE

QFTKGDVDILVATDVAARGLHIPQVTHVFNYDLPDDCEDYVHRIGRTGRAGASGHSISFA

CEEYAINLPAIEEYIEHTIPVSEYDASALIQDLPAPIRMRAPRVQQRRTNTGGTRSGNRK

PQGRRPRQPRQSAPKQS

>sp|Q87T03|RL14_VIBPA 50S ribosomal protein L14 OS=Vibrio parahaemolyticus serotype O3:K6 (strain RIMD 2210633) OX=223926 GN=rplN PE=3 SV=1

MIQMQSMLDAADNSGARSVMCIKVLGGSHRRYAHIGDVIKVTVKEAIPRGKVKKGDVMKA

VVVRTRKGVRRPDGSVIRFDRNACVLLNNTTEQPIGTRIFGPVTRELRGDKFMKIVSLAP

EVL

>sp|P66368|RS11_VIBPA 30S ribosomal protein S11 OS=Vibrio parahaemolyticus serotype O3:K6 (strain RIMD 2210633) OX=223926 GN=rpsK PE=3 SV=1

MAKQPTRARKRVRKQVADGVAHIHASFNNTIVTITDRQGNALAWATAGGSGFRGSRKSTP

FAAQVAAERCAEMAKEYGLKNLEVMVKGPGPGRESTVRALNAAGFRITNIVDATPIPHNG

CRPPKKRRV

>sp|Q87LS8|RS16_VIBPA 30S ribosomal protein S16 OS=Vibrio parahaemolyticus serotype O3:K6 (strain RIMD 2210633) OX=223926 GN=rpsP PE=3 SV=1

MVTIRLARHGAKKRPFYQIVVADSRNAATGRFIEKVGFFNPTAKGQEEGLRLDLDRVNHW

VGQGASVSDRVAKLVKDAQKAA

>sp|Q87T04|RS17_VIBPA 30S ribosomal protein S17 OS=Vibrio parahaemolyticus serotype O3:K6 (strain RIMD 2210633) OX=223926 GN=rpsQ PE=3 SV=1

MSEVKRTQQGRVVSDKMDKSITVAIERFVKHPIYGKFVKRTTKVHAHDENNECGIGDTVE

IAECRPLSKTKSWTLVKVVEKAKM

>sp|Q87ST6|RSMA_VIBPA Ribosomal RNA small subunit methyltransferase A OS=Vibrio parahaemolyticus serotype O3:K6 (strain RIMD 2210633) OX=223926 GN=rsmA PE=3 SV=1

MRNDVHLGHKARKRFGQNFLNDPYIIDGIVSAINPKPGQNLVEIGPGLGAITEPVGREVD

KFTVIELDRDLAERLRNHPDLADKLTIHEGDAMRFDFTQLVKPNNKLRIFGNLPYNISTP

LMFHLFEFHKDIQDMHFMLQKEVVNRLAAGPGSKAYGRLTVMAQYYCKVVPVLEVPPTAF

VPPPKVDSAVVRLVPYEELPCPAKDLRLLDRVCREGFNQRRKTVRNCYKSLLSAEVLEEL

GVNPSMRPENLTLQQFVAMANWLADNPQH

>sp|Q87KD3|RSMB_VIBPA Ribosomal RNA small subunit methyltransferase B OS=Vibrio parahaemolyticus serotype O3:K6 (strain RIMD 2210633) OX=223926 GN=rsmB PE=3 SV=1

MNVRAAAANVLYLVVDKGHSLSSALPAAQQTVRPRDHALLQEICYGALRYLPRLEMIANQ

LMDKPLKGKQRVFHHLILVGIYQLSFMRIPAHAAVGETVEGTKELKGPRLRGLINAVLRN

YQRNQEELDQMAVSNNAGKYGHPSWLLKLLQDAYPQQWESIVDANNQKAPMWLRVNHQHH

TRDEYLALLKNENIDSTPHTEAMDAIKLAAPCDVMKLPGFDKGWVSVQDAAAQLSINYLK

PQDGELILDCCAAPGGKTAHILERTSGSEVVAIDCDDTRLKRVHENLKRLNLQAKVVCGD

ARNPQEWWQGEQFDRILLDAPCSATGVIRRHPDIKWLRRADDIAALAELQSEIFDAMWTQ

LKPGGTMVYATCSITPQENVEQVKAFLARTADAQLLDSDPDQPGRQILPGEEDMDGFYYA

VLTKTRA

>sp|Q87QU7|RUVB_VIBPA Holliday junction ATP-dependent DNA helicase RuvB OS=Vibrio parahaemolyticus serotype O3:K6 (strain RIMD 2210633) OX=223926 GN=ruvB PE=3 SV=1

MIEADRLIAPENPAFRDEDVIDRAIRPKKLADYQGQDHVRDQMEIFIKAAQLRSEALDHL

LIFGPPGLGKTTLANIVANEMEVNIRTTSGPVLEKAGDLAALLTNLEENDVLFIDEIHRL

SPMVEEVLYPAMEDYQLDIMIGEGPAARSIKIDLPPFTLIGATTRAGSLTSPLRDRFGIT

QRLEYYKVQDLQNIVQRSADCLGLSMEPEGALEVARRARGTPRIANRLLRRVRDYAEVKG

NGHICADVADKALNMLDVDAQGFDYMDRKLLLAIMEKFGGGPVGLDNMAAAIGEEKDTIE

DVLEPYLIQQGYLQRTPRGRIATDRAYLHFGIEK

>sp|Q87MD8|RS2_VIBPA 30S ribosomal protein S2 OS=Vibrio parahaemolyticus serotype O3:K6 (strain RIMD 2210633) OX=223926 GN=rpsB PE=3 SV=1

MATVSMRDMLKAGVHFGHQTRYWNPKMKPFIFGARNRVHIINLEKTVPMFNEALAELAKV

GEKKGKVLFVGTKRAASEAVKEAAIASNQFYVNNRWLGGMLTNYKTVRQSIKRLKELEIQ

SQDGTFDKLTKKEALMRTREMEKLEKSLGGIKDMGGLPDALFVIDADHEHIAIKEANNLG

IPVYAVVDTNSNPDGVDYIIPGNDDAIRAVQLYLNAAASAVTEGRNKDVAVVAEKDGFVE

AE

>sp|Q87SZ6|RS5_VIBPA 30S ribosomal protein S5 OS=Vibrio parahaemolyticus serotype O3:K6 (strain RIMD 2210633) OX=223926 GN=rpsE PE=3 SV=1

MAKEQQVQANDLQEKLIAVNRVSKTVKGGRIMSFTALTVVGDGNGRVGFGYGKAREVPAA

IQKAMEKARRNMTTIALNEGTLHHPVKGRHSGSKVYMQPAAEGTGVIAGGAMRAVLEVAG

VHNVLSKAYGSTNPINIVRATIDALGSMKSPEMVAAKRGLTVEAISE

>sp|Q87L00|RSGA1_VIBPA Small ribosomal subunit biogenesis GTPase RsgA 1 OS=Vibrio parahaemolyticus serotype O3:K6 (strain RIMD 2210633) OX=223926 GN=rsgA1 PE=3 SV=1

MAKKKKLTKGQVRRVRSNQQKRLKKQEESIQWDENMLGASKQGLVITRFGQHADIEDLET

GEVQRCNLRRGIESLVSGDRVLWREGLESMAGISGVVEAVEPRTSMLTRPDYYDGLKPVA

ANIDQMVIVSSVLPELSLNIIDRYLVAAETLNIAPLLVLNKVDLLEVDDRAMYEEWLKEY

ERIGYKVLFVSKNSGEGISDLEVQLRDRINIFVGQSGVGKSSLVNALMPELEQEVEEGAI

SENSGLGQHTTTAARLYHIPTGGDLIDSPGVREFGLWHLEAEEVTKAFVEFRPYLGGCKF

RDCKHNDDPGCILREAVEKGEVSEVRFENYHRILESMMENKANRQYSRNKKADL

>sp|Q87K99|RSMG_VIBPA Ribosomal RNA small subunit methyltransferase G OS=Vibrio parahaemolyticus serotype O3:K6 (strain RIMD 2210633) OX=223926 GN=rsmG PE=3 SV=1

MSALRTKLDSLIAQTDLDVSEKQREQLVGYVELLDKWNKAYNLTSVRDPLEMLVKHILDS

IVVGTHLQGERFIDVGTGPGLPGIPLAIMHPEKTFFLLDSLGKRIRFIKQVVHTLGLKNV

TPIQSRVEEFQPEEKFDGVLSRAFASMTDMVEWCHHLPKEQDGVFLALKGQHPKDEMDLL

PEWCSVTEIISLAVPELEGDRHLVILSRKEN

>sp|Q87SY9|RL17_VIBPA 50S ribosomal protein L17 OS=Vibrio parahaemolyticus serotype O3:K6 (strain RIMD 2210633) OX=223926 GN=rplQ PE=3 SV=1

MRHRKSGRQLNRNSSHRKAMFSNMASSLVRHEVIKTTLPKAKELRRVVEPLITLAKTDSV

ANRRLAFARTRDNEVVAKLFNELGPRFAARQGGYTRILKAGFRAGDKAPMAYIELVDRPA

AEEAAE

>sp|P66533|RS21_VIBPA 30S ribosomal protein S21 OS=Vibrio parahaemolyticus serotype O3:K6 (strain RIMD 2210633) OX=223926 GN=rpsU PE=3 SV=1

MPVVKVRENEPFDVALRRFKRSCEKAGILSEVRRREHYEKPTTVRKRAKAAAQKRHAKKL

ARENARRVRLY

>sp|Q87T13|RL3_VIBPA 50S ribosomal protein L3 OS=Vibrio parahaemolyticus serotype O3:K6 (strain RIMD 2210633) OX=223926 GN=rplC PE=3 SV=1

MIGLIGRKVGMTRVFTEEGVSIPVTVVEVEANRVSQVKTLETDGYAAIQVTAGSKKANRV

NKAEAGHFAKAGVEAGRGLWEFRLENGEEFEVGAELTVELFNETKKVDVTGTSKGKGFQG

AVKRWNFRTQDMTHGNSLSHRAPGSIGQCQTPGRVFKGKKMAGHMGAERVTTQNLEIVRV

DAERNLLLIKGAVPGATGGNVIVKPAVKA

>sp|Q877T5|EFTU_VIBPA Elongation factor Tu OS=Vibrio parahaemolyticus serotype O3:K6 (strain RIMD 2210633) OX=223926 GN=tufA PE=3 SV=1

MSKEKFERTKPHVNVGTIGHVDHGKTTLTAAICTTLAKVYGGEAKDFASIDNAPEERERG

ITIATSHVEYDTPSRHYAHVDCPGHADYVKNMITGAAQMDGGILVVAATDGPMPQTREHI

LLGRQVGIPYIIVFMNKCDMVDDEELLELVEMEVRELLSEYDFPGDDLPVIQGSALGALN

GEEQWEAKIVELAEALDTYIPEPERAVDQPFLMPIEDVFSIQGRGTVVTGRIERGILTVG

DEVAIVGIKDTTTTTCTGVEMFRKLLDEGRAGENVGALLRGTKRDEVERGQVLAKPGSIT

PHTKFESEVYVLSKEEGGRHTPFFKGYRPQFYFRTTDVTGDISLPEGVEMVMPGDNIQMV

VELIAPIAMDEGLRFAIREGGRTVGAGVVAKIFE

>sp|Q87L45|EFG1_VIBPA Elongation factor G 1 OS=Vibrio parahaemolyticus serotype O3:K6 (strain RIMD 2210633) OX=223926 GN=fusA1 PE=3 SV=1

MARKTPIEHYRNIGICAHVDAGKTTTTERILFYTGLSHKIGEVHDGAATMDWMEQEQERG

ITITSAATTTFWRGMEAQFPEHRVNIIDTPGHVDFTIEVERSLRVLDGAVVVFCGSSGVE

PQSETVWRQADKYHVPRMVFVNKMDRAGADFLRVVDQIKNRLGANPVPIQLNVGAEEDFR

GVIDLIKMKMINWNDADQGMSFTYEDIPADMIDLAEEWRNHMIESAAEASEELMDKYLEE

GELTEAEIKQALRTRTLNNEIVLATCGSAFKNKGVQAVLDAVIEFLPSPSDVPAIKGVDE

KDNEIERHADDNEPFSALAFKIATDPFVGTLTFMRVYSGVVNSGDAVYNSVKEKKERFGR

IVQMHANKREEIKEVRAGDIAAAIGLKDVTTGDTLCDQSHKVILERMEFPEPVIQIAVEP

RSKADQEKMGIALGKLAAEDPSFRVETDDETGQTLISGMGELHLDIIVDRMKREFSVDCN

VGKPQVAYRETIRGKAEVEGKFVRQSGGRGQYGHVWVKLEPSEPGEGFVFVDEIVGGVIP

KEYISSVAKGIEEQMNSGVLAGYPVLDIKATLFDGSYHDVDSSEMAFKIAGSMAFKKGAL

EAQPVILEPMMNVEVTTPEDWMGDVVGDLNRRRGMIEGMDEGVAGLKIIRAQVPLSEMFG

YATDLRSATQGRASYSMEFNEYAEVPKNFADKIIAERGY

>sp|Q87M30|EFG2_VIBPA Elongation factor G 2 OS=Vibrio parahaemolyticus serotype O3:K6 (strain RIMD 2210633) OX=223926 GN=fusA2 PE=3 SV=1

MTDLSKYRNIGIFAHVDAGKTTSTERILKLTGKIHKIGDTHDGSTTTDFMEQEAERGITI

QSAATTCFWNDHRLNIIDTPGHVDFTIEVYRSLKVLDGGIGVFCGSGGVEPQSETNWRYA

DESHVSRLIFVNKLDRMGADFYKVVDQVQNVLGATPLVMTLPIGIEEDFVGVVDVLSQQA

YVWDESGQPENYEVQEIPADMVDKAAEYREMLIETALEQDEDLMMAYLEEGEEPSVEDIK

RCIRKGTRDLAFFPTYCGSAYKNKGMQLILDAVVDYLPSPTEVDPQPLTDPDTGEATGEV

ATVSADEPLKALAFKIMDDRFGALTFIRIYSGKMKKGDTVLNSATGKTERIGRMVEMHAD

ERNEIDSAQAGDIIAVVGMKNVQTGHTLCDPKHECTLEPMIFPEPVISIAVKPKDKGGSE

KMGIAIGKMVAEDPSFQVETDEESGETILKGMGELHLDIKVDILKRTYGVELEVGAPQVA

YRETITQAIEDSYTHKKQSGGSGQFAKIDYRIKPGEVGSGFTFKSTVVGGNVPKEFWPAV

EKGFAGMMETGVLAGFPTLDVEVELYDGGFHAVDSSAIAYEIAAKGAFRQSMPKAGAQLL

EPIMKVDVFTPEDHVGDVIGDLNRRRGMIKDQQAGTTGVRIKGDVPLSEMFGYIGTLRTM

TSGRGQFSMEFSHYSPCPNNVAEQVIADVKERNAKK

>sp|Q87SC9|KHSE_VIBPA Homoserine kinase OS=Vibrio parahaemolyticus serotype O3:K6 (strain RIMD 2210633) OX=223926 GN=thrB PE=3 SV=1

MGVVVYAPASIGNVSVGFDVLGAAVSPVDGTLLGDRVQVKAGTEPFSLNTAGHFVSKLPT

DPKENIVYDCWVVFARELDKKGIELKPLEMTLEKNMPIGSGLGSSACSIVAALDALNRFH

DQPLNETELLALMGEMEGKISGGIHYDNVAPCYLGGVQLMLEELGIISQEVPCFDEWYWV

MAYPGIKVSTAEAREILPSQYRRQDIIAHGRHLAGFIHACHSGQPELAAKMIKDVIAEPY

REKLLPGFANARQYAASAGALATGISGSGPTLFSICKQKDVAERVARWLEQNYVQNEEGF

VHVCRLDKQGSKVTGSEL

>sp|Q87N44|KCY_VIBPA Cytidylate kinase OS=Vibrio parahaemolyticus serotype O3:K6 (strain RIMD 2210633) OX=223926 GN=cmk PE=3 SV=1

MSSQTPVVTVDGPSGAGKGTLCMLLAKKLGFQLLDSGAIYRVLALAAIHHGVDTESEDAL

VPLATHLDVQFIAEGDLVKVILEGEDVSGELRKEETGMAASKVAALPRVREALLRRQRAF

EAAPGLVADGRDMGTVVFPSAQAKIFLDASAEERANRRLKQLQDKGLDVRFADLLSEIQE

RDDRDRNRPVAPLRPAEDALVLDSTSMTIDEVVEKALQYIESKLAE

>sp|Q87T74|KDKA_VIBPA 3-deoxy-D-manno-octulosonic acid kinase OS=Vibrio parahaemolyticus serotype O3:K6 (strain RIMD 2210633) OX=223926 GN=kdkA PE=3 SV=1

MIQQYRDSNQVIWFDEELIEDPSQPIFDAEYWQSTNKVTGSASGRGTTWFVQLDTMQAAL

RHYRRGGLFGKLVKDNYLFSGWEQTRCAQEFQLLLTLINAGVHVPRPIAARAVKSGLTYQ

ADLLSERIPNARDLVSILQEKPLPEGMYQKIGQEIAKMHNAGVNHTDLNIHNILIDDKDK

VWIIDFDKCRKQEHGDWKKQNLERLLRSFKKELLKRQIHWKERDFAVLTEALSCLDIK

>sp|Q87RN0|KDSA_VIBPA 2-dehydro-3-deoxyphosphooctonate aldolase OS=Vibrio parahaemolyticus serotype O3:K6 (strain RIMD 2210633) OX=223926 GN=kdsA PE=3 SV=1

MEQKIVNIGDIQVANDKPFTLFAGMNVLESRDLAMQICEHYVKVTDKLGIPYVFKASFDK

ANRSSVHSYRGPGLEEGMKIFQELKDTFGVKIITDVHTEAQAQPVADVVDVIQLPAFLAR

QTDLVEAMAKTGAVINVKKPQFMSPGQVGNIVEKFAECGNDKIILCERGSCHGYDNLVVD

MLGFGVMKNASKGSPIIFDVTHSLQMRDPSGAASGGRREQTVELAKAGLATGIAGLFIEA

HPNPDQARCDGPSALPLDKLEPFLAQMKSLDDLIKSFENIDIK

>sp|Q87N26|KTHY_VIBPA Thymidylate kinase OS=Vibrio parahaemolyticus serotype O3:K6 (strain RIMD 2210633) OX=223926 GN=tmk PE=3 SV=1

MMKANFIVVEGLEGAGKSTAIKTVLDTLKQAGIENIVNTREPGGTPLAEKMRALVKEEHE

GEELKDMTELLLLYAARVQLVENVIKPALANGQWVVGDRHDLSSQAYQGGGRQIDASLMK

NLRDTTLGDFKPAFTLYMDIDPRIGLERARGRGELDRIEKMDISFFERTRERYLEIANAD

PSIVVINAEQSIEEVSRDIQDALNEWLSRQ

>sp|Q87R14|KDSB_VIBPA 3-deoxy-manno-octulosonate cytidylyltransferase OS=Vibrio parahaemolyticus serotype O3:K6 (strain RIMD 2210633) OX=223926 GN=kdsB PE=3 SV=1

MSFTVVIPARYSSSRLPGKPLADIGGKPMVQWVYEQAMQAGADDVIIATDDERVSAAVEQ

FGGKVCMTSPNHESGTERLAEVVEKMAIPADHIIVNVQGDEPLVPPVIIRQVADNLAASD

APMATLAVEIESEDEVFNPNAVKVVADERGYAMYFSRATIPWDRDNFAKQDKAIVNPLMR

HIGIYAYRAGFINTYVNWAPSALEQIECLEQLRVLWYGEKIHVAVAKEAPAAGVDTPEDL

EAVRAIVAKKA

>sp|Q87RH4|KAD_VIBPA Adenylate kinase OS=Vibrio parahaemolyticus serotype O3:K6 (strain RIMD 2210633) OX=223926 GN=adk PE=3 SV=1

MRIILLGAPGAGKGTQANFIMDKYGIPQISTGDMLRAAIKAGTELGKQAKAVIDAGQLVS

DEIILGLIKERIAQDDCEKGFLLDGFPRTIPQADGLKEMGVEVDYVIEFDVADDVIVERM

AGRRAHLPSGRTYHVVYNPPKVEGKDDVTGEDLVVRDDDKEETVRARLGVYHEQTAPLID

YYGKEAAAGKTKYLKFDGTKQVAEVSADIEKALA

>sp|Q87TA9|KGUA_VIBPA Guanylate kinase OS=Vibrio parahaemolyticus serotype O3:K6 (strain RIMD 2210633) OX=223926 GN=gmk PE=3 SV=1

MGKGTLYIVSAPSGAGKSSLISAMLERNPTYAMKVSVSHTTRNMRPGEEDGVHYHFVAKE

EFETLIAKGDFLEYAEVFGNYYGTSRVWIEETLEKGIDVFLDIDWQGARQIREQMPKAKS

IFILPPSNGELERRLNTRGQDSAEVIAKRMAEAKSEISHYSEYDYVIVNDDFDTALMDFK

AILRAERLKEEKQAAKYKGMLDALLAE

>sp|Q87LQ3|ISPF_VIBPA 2-C-methyl-D-erythritol 2,4-cyclodiphosphate synthase OS=Vibrio parahaemolyticus serotype O3:K6 (strain RIMD 2210633) OX=223926 GN=ispF PE=3 SV=1

MIRIGHGFDVHKFGGEGPVIIGGVAIPYEQGLIAHSDGDVALHALTDALLGAIAAGDIGR

HFPDTDDKWKGANSRELLKDVYRRVKEQGYRLGNADVTIMAQAPKMAPHIDAMCAAIAED

LETDISNINVKATTTERLGFTGRKEGIATEAVVLLFKQ

>sp|Q87S16|ISPG_VIBPA 4-hydroxy-3-methylbut-2-en-1-yl diphosphate synthase (flavodoxin) OS=Vibrio parahaemolyticus serotype O3:K6 (strain RIMD 2210633) OX=223926 GN=ispG PE=3 SV=1

MQHESPIIRRKSTRIYVGDVPIGDGAPIAVQSMTNTRTTDVEATVAQIRALEKVGADIVR

VSVPTMEAAEAFKLIKQQVSVPLVADIHFDYRIALKVAEYGVDCLRINPGNIGNEERIRS

VVDCARDKNIPIRIGVNGGSLEKDLQMKYGEPTPEALVESAMRHVDHLDRLNFDQFKVSV

KASDVFLAVDSYRLLAKKIDQPLHLGITEAGGARAGAVKSAVGLGMLLSEGIGDTLRISL

AADPVEEIKVGFDILKSLRIRSRGINFIACPSCSRQEFDVIGTVNALEQRLEDIITPMDV

SIIGCVVNGPGEAEVSHLGLAGSNKKSAFYEDGKRQKERFDNNDLVNQLEAKIRAKASMM

DSENRIEIKVQD

>sp|Q87I38|KATG2_VIBPA Catalase-peroxidase 2 OS=Vibrio parahaemolyticus serotype O3:K6 (strain RIMD 2210633) OX=223926 GN=katG2 PE=3 SV=1

MSNTNGGSVGKCPVMHGGQTSTDKSVMDWWPNALNLDILHQHDSKTNPFGPDFNYKEELK

KLDVDALKQDLKDLMTNSQVWWPADWGHYGGLMIRMAWHAAGSYRIADGRGGAATGNQRF

APLNSWPDNANLDKARRLLWPIKRKYGNKLSWADLIILAGNMAYESMGFKTFGFGFGRED

IWHPEKDTYWGSEQEWLAPSGAKNSRYSGERDLENPLAAVMMGLIYVNPEGVDGNPDPLK

TAQDMRVTFARMAMNDEETVALTAGGHTVGKCHGNGDAANLGPDPEGADVHEQGLGWMNH

KTRGIGRDTVTSGLEGAWTTHPTQWDNGYFYLLFKYDWELKKSPAGAWQWEPIDIEEQDK

PVDVEDGSKRYNPIMTDADMALKMDPEYRKISERFQQDPAYFEDMFARAWFKLTHRDMGP

KSCYLGPDVPSEDLIWQDPTPAGKTDYNVDLVKGKIEASGLSIADLVATAWDSARTYRGS

DRRGGANGARIRLAPQKDWQGNEPERLSRVLAVLESIAAEEGCSVADAIVLAGNVGIELA

ARAAGHDVSVPFAPGRGDASQDMTDVESFEVLEPVADGFRNWLKKDYVVKPEELLLDRAQ

LMGLTAPEMTVLIGGLRVLGSNYGGSKDGVFTDRVGTLSNDFFVNLTDMAYTWKPAGENQ

YEIRDRKTDQVKWTATRVDLVFGSNSILRSYAEVYAQDDNQEKFIHDFVAAWTKVMNADR

FDLQ

>sp|Q87N03|DSBB_VIBPA Disulfide bond formation protein B OS=Vibrio parahaemolyticus serotype O3:K6 (strain RIMD 2210633) OX=223926 GN=dsbB PE=3 SV=1

MTIFSSLNQFSKGHVSWLLLLLFIIFFEACALYFQHVMMLAPCVMCIYERVAMMGIGGAA

IIGLIAPNNALFRWLGLIGWGLSSYKGLMLAMQHVDYQFNPSPFATCDLFVTFPSWAPLN

QWVPWMFEAYGDCSKIVWQFFDLSMPQWLVVIFAGNLVALALIVIAQFFPVKRKNPIR

>sp|Q87SC6|END4_VIBPA Probable endonuclease 4 OS=Vibrio parahaemolyticus serotype O3:K6 (strain RIMD 2210633) OX=223926 GN=nfo PE=3 SV=1

MTNMKNKFGNKLIGAHVSAAGGVDQAPLRAREIGANAFALFTKNQRQWVAKPLEAKTISA

FKANCKMLGFGAEHILPHDSYLINLGAPEAEKLDKSRAAFIDEMERCNQLGLTLLNFHPG

SHLKKVSEQECLATIAESINLAHKTVPDVVAVIENTAGQGTNLGWKFEHLAEIIEQVEDK

DRVGVCIDTCHTFTAGYDLRTKEDCERTFAEFDRIVGMHYLRAMHLNDSKVEFASKVDRH

HSLGKGEIGWDCFEYIAKDSRFDGIPLILETIDPDIWQQEINTLRQFHLAAINNQ

>sp|Q87TG0|ENGB_VIBPA Probable GTP-binding protein EngB OS=Vibrio parahaemolyticus serotype O3:K6 (strain RIMD 2210633) OX=223926 GN=engB PE=3 SV=1

MSVKIHYQNTHFITSAPDIRHLPEDEGVEIAFAGRSNAGKSSALNRLTNQKSLAKTSKTP

GRTQLINLFKVEEGCHIVDLPGYGFAQVPVEMKNKWQKSLGEYLQKRECLKGLVVLMDIR

HPMKDLDQQMIFWAIDSRIPVQVLLTKADKLKSGARKQTLLKIRKQVETFGGDVSVDVFS

SLKGLGVDQLRAKLDTWFAPALAHLIEEDDLEMPESNEE

>sp|Q87PC5|FABA_VIBPA 3-hydroxydecanoyl-[acyl-carrier-protein] dehydratase OS=Vibrio parahaemolyticus serotype O3:K6 (strain RIMD 2210633) OX=223926 GN=fabA PE=3 SV=1

MQNKRDSYNRDDLLASSQGELFGPGYPQLPAPNMLMMDRVTKMSETEGDFGKGLILAELD

ITPDLWFFDCHFPGDPVMPGCLGLDAMWQLVGFFLGWVGGKGKGRALGVGEVKFTGQILP

TAKKVTYEIHMKRVVNRKLVMGLADGRVCVDGKEIYVAKDLKVGLFQDTSSF

>sp|Q87N20|FABH1_VIBPA 3-oxoacyl-[acyl-carrier-protein] synthase 3 protein 1 OS=Vibrio parahaemolyticus serotype O3:K6 (strain RIMD 2210633) OX=223926 GN=fabH1 PE=3 SV=1

MYSKILGTGSYLPSQVRTNADLEKMVDTSDEWIVARTGIKERRIAAEDETVADMAFYAAE

NAIDMAGIDKNDIDLIIVATTSSSHTFPSSACQVQAKLGIKGCPAFDLAAACSGFVYALS

VADQHIKSGMCKNVLVIGADALSKTCDPTDRSTIILFGDGAGAVVVGASQEPGIISTHIY

ADGQFGDLLSLPVPERGKDVDKWLHMAGNEVFKVAVTQLSKLVKDTLEANDMHKSELDWL

VPHQANYRIISATAKKLSMSLDQVVVTLDRHGNTSAATVPTALDEAVRDGRIKRGQTLLL

EAFGGGFTWGSALVKF

>sp|Q87ME8|FABZ_VIBPA 3-hydroxyacyl-[acyl-carrier-protein] dehydratase FabZ OS=Vibrio parahaemolyticus serotype O3:K6 (strain RIMD 2210633) OX=223926 GN=fabZ PE=3 SV=1

MTTEKKTMNISEIQELLPHRYPFLLIDRVIDFQEAKYLHAIKNVSVNEPQFTGHFPQLPV

FPGVLILEAMAQATGLLAFKSFGAPTENELYYFASVDGAKFRKPVVPGDQMVIEVEFLKE

RRGIAAFSGVAKVDGEVVCSAELKCARREF

>sp|Q87KY6|EPMA_VIBPA Elongation factor P--(R)-beta-lysine ligase OS=Vibrio parahaemolyticus serotype O3:K6 (strain RIMD 2210633) OX=223926 GN=epmA PE=3 SV=1

MQTNWQPTASIEQLRQRATLIAAIRQFFAERQVMEVDTPAMSHATVTDIHLHTFQTEFVG

PGYADGSKLFFMTSPEFHMKRLLAAGSGCIYQINKAFRNEENGRYHNPEFTMLEWYRVGF

DHHKLMDEMDDLLQLVLKCGAAQRMTYQQAFIDVLGVCPLEGSMTELKAAASKLGLSDIA

EPEEDRDTLLQLLFSVGVENKIGQDVPAFVYDFPASQAALAKINPQDHRVADRFEVYFKG

IELANGFHELDNPKEQLARFEQDNAKRIEMGLKPQPIDYHLISALEAGLPDCAGVALGID

RLIMLALGCDHIDQVTAFPFPIA

>sp|Q87RX2|DNAJ_VIBPA Chaperone protein DnaJ OS=Vibrio parahaemolyticus serotype O3:K6 (strain RIMD 2210633) OX=223926 GN=dnaJ PE=3 SV=1

MSKRDFYEVLGVSRDASERDIKKAYKRLAMKFHPDRNQGDESAADKFKEVKEAYEVLTDS

QKKAAYDQYGHAAFEQGGGGFGGGFGGGGADFGDIFGDVFGDIFGGGRRGGGGHRAQRGA

DLRYNMELTLEEAVRGVTKEIEVPTLVHCDSCDGSGAKKGSSAETCGTCHGHGQVQMRQG

FFAVQQTCPTCHGKGKIIKDPCNECHGQGRKQKTKTLNVKIPAGVDTGDRIRLSGEGEAG

EMGAPSGDLYVQVHVKEHHIFERDGNNLYCEVPVSFAMAALGGEVEVPTLDGRVSLKVPS

ETQTGRMFRMRGKGVKGVRGGGIGDLIVKLVVETPVNLSSRQKELLKEFEESCGGEAATK

HKPKSEGFFNGVKKFFDDLTS

>sp|Q87KW0|DSBD_VIBPA Thiol:disulfide interchange protein DsbD OS=Vibrio parahaemolyticus serotype O3:K6 (strain RIMD 2210633) OX=223926 GN=dsbD PE=3 SV=1

MRALLSVLLLGLITFSTPSLALFGKDQFNNSQNNSFGSSNDSFVPVDQAFPFNFYQQDDK

LMLDWQVRDGYYLYQERLSVSGENISLGELQMENGTPHKDEFFGDVHIYTTPLFVNVPLD

EWQEGARVIVQYQGCAKAGFCYPPETRIIPIDAFTASIADSAVATKPTQSTANNNQTNAT

SPTASTSVSSNNTPAPVTEQDSLAANLADNWWTPLLFLALGVGLAFTPCVLPMYPILTSI

VLGSGKLSQRRALGLSLVYVQGMALTYTLLGLVVASAGMQFQAAMQHPYVLIGLSILFVT

LALSMFGVYTLQLPSSVQTWLNNLSNKQQGGSSAGVFAMGAISGLVCSPCTTAPLSGALL

YVAQSGDLLTGGIALYALAMGMGIPLILVAVFGNKLLPKAGGWMDRVKTLFGFILLAAPI

FLLERILPEMWSTALWSALGIAAFGWLYHIKNSLEFGGWKQSAIGIIAVLGLFASAQPAL

NYWFGNHETQAQQTTVSFTRIANVAELEEQLALAKAAGKPVMLDFYADWCVACKEFEKYT

FHDPKVEAKLQDFVLLQADVTKNQVQDIELLKHMNVLGLPTIEFWDAKGEHVSNARLTGF

MQAEPFLEHINRF

>sp|Q87LP0|ERA_VIBPA GTPase Era OS=Vibrio parahaemolyticus serotype O3:K6 (strain RIMD 2210633) OX=223926 GN=era PE=3 SV=1

MADNEFDIDAFFASHGEVSSPENQHCGFIAIVGRPNVGKSTLLNKILGQKISITSRKPQT

TRHRIMGVDTDGDYQAIYVDTPGLHIEEKRAINRLMNRAANSSLSDVNLVFFLVDGTHWT

KDDEMVLTKLQKSNFPVVLCVNKVDNVQDRNEVMLHMAEMSKKMDFVDVVPISAKQGKNI

DVLRKHVRNHLPKATHHFPEEYVTDRSQRFMASEIVREKLMRFTGDELPYSVTVEIERFD

YNPETDGFHINALILVERNGQKKMVIGKGGEKIKTIGREARLDMEELFGRKVYLETWVKV

KSGWADDERALRSLGYIDDL

>sp|Q87LY4|ERPA_VIBPA Iron-sulfur cluster insertion protein ErpA OS=Vibrio parahaemolyticus serotype O3:K6 (strain RIMD 2210633) OX=223926 GN=erpA PE=3 SV=1

MSEVNVPLSFSDAAASRVKALIAEEENPALKLRVYITGGGCSGFQYGFTFDENVNDGDTT

IENSGVTLVVDPMSLQYLIGGIVDYTEGLEGARFFVNNPNATTTCGCGASFSV

>sp|Q87QB9|FABV1_VIBPA Enoyl-[acyl-carrier-protein] reductase [NADH] 1 OS=Vibrio parahaemolyticus serotype O3:K6 (strain RIMD 2210633) OX=223926 GN=fabV1 PE=3 SV=1

MIIKPRIRGFICTTTHPVGCEANVKEQIAYTKAQGPIKNAPKRVLVVGASSGYGLSSRIA

AAFGGGASTIGVFFEKEGTEKKPGTAGFYNAAAFEKLAREEGLYAKSLNGDAFSNEAKQK

TIDLIKEDLGQVDMVVYSLASPVRKMPETGELIRSALKPIGETYTSTAVDTNKDVIIEAS

VEPATEEEIKDTVTVMGGEDWELWINALSDAGVLAEGCKTVAYSYIGTELTWPIYWDGAL

GKAKMDLDRAAKALNEKLGATGGSANVAVLKSVVTQASSAIPVMPLYIAMVFKKMREEGV

HEGCMEQIYRMFSQRLYKEDGSAAEVDDMNRLRLDDWELREDIQQHCRELWPQITTENLK

ELTDYVEYKEEFLKLFGFGVEGVDYEADVNPAVETDFIQI

>sp|Q87HT6|FABV2_VIBPA Enoyl-[acyl-carrier-protein] reductase [NADH] 2 OS=Vibrio parahaemolyticus serotype O3:K6 (strain RIMD 2210633) OX=223926 GN=fabV2 PE=3 SV=1

MRIEPLIQGVVARSAHPYGCHASIKEQIEYVKKAPKIKSGPKRVLIIGASSGFGLAARIA

LTFGGAEADTIGVSFERGPSEKGVGSAGWYNNIFFKQEATHAGRTAINIVGDAFSDSVRN

EVIEAIETYFEGEVDLVIYSLAAGVRPKPHSDTFWRSVIKPIGESVTGASILLENDQWVE

TTLEPATEEEAEATIKVMGGEDWESWIDTLINTESVAQGCKTIAFSYMGPEVTHPIYLDG

TLGRAKIDLHQTSHALNLKLANFDGGAYATVCKALVTKASVFIPALSPYLLALYRVMKEK

GTHERCIEQMQRLFTTKLYDQPKVPVDGERLIRIDDLELDPQTQAEVSHLLEQMNTENFK

ECGDYQGFKDEFMKLNGFNFDDVDYSQDISLETLASLKP

>sp|Q87TP0|FADA_VIBPA 3-ketoacyl-CoA thiolase OS=Vibrio parahaemolyticus serotype O3:K6 (strain RIMD 2210633) OX=223926 GN=fadA PE=3 SV=1

MTNQTRNVVVVDCLRTPMGRSKGGAFRHTRAEDLSAHLMKGILARNPQVNPSEIEDIYWG

CVQQTLEQGFNIARNAALLAGLPIEIGAVTVNRLCGSSMQALHDGTRAIMTGDAEICLIG

GVEHMGHVPMNHGVDFHPGMSKNVAKAAGMMGLTAEMLGKLHGISREQQDEFAARSHARA

HAATLEGRFKNEILPTEGHAADGTLFQLDYDEVIRPETTVEGLSQLRPVFDPANGTVTAG

TSSALSDGASAMLIMSEEKANELGLKIRARIKGMAIAGCDPSIMGYGPVPATQKALKRAG

LAIEDMDVVELNEAFAAQSLPCAKDLGLLEVMDEKVNLNGGAIALGHPLGCSGARISTTL

INLMEAKDAKYGLATMCIGLGQGIATVFERP

>sp|Q87S09|EX7L_VIBPA Exodeoxyribonuclease 7 large subunit OS=Vibrio parahaemolyticus serotype O3:K6 (strain RIMD 2210633) OX=223926 GN=xseA PE=3 SV=1

MLSKTNQNIFTVSRLNAEVRLLLENEMGIVWLVGEISNFSAPVSGHWYLTLKDSRAQVKC

AMFRGNNRRVTFKPANGNQVLVKARLSLYEPRGDYQLIIESMQPEGDGRLQQEFEELKMK

LAAEGLFAQTNKLPLPEHPKRVGIITSKTGAALYDILDVLKRRDPSLPVVIYPTMVQGDD

AAIQIAQAIGRANSRNECDVLIVGRGGGSLEDLWCFNNEILARTIAASQIPIISAVGHEV

DMTIADFVADVRAPTPSAAAELVSRDNSHKDQSLVAKQHKLASAMRYYLSQQKQQSAQLL

HRLERQHPSYQLQRQSQQLDELDMRLRRAMQRFIDTRQQAVERKHHRLQLNSPVKHLAQQ

KSRLERVEHKLLDTMDRKLLTMRHQLAIAAEKLDTVSPLATLKRGYSITQTEQGKVVTSA

DDVKTGDLLVTRLANGEIHSTVS

>sp|Q87RT8|EX7S_VIBPA Exodeoxyribonuclease 7 small subunit OS=Vibrio parahaemolyticus serotype O3:K6 (strain RIMD 2210633) OX=223926 GN=xseB PE=3 SV=1

MAVKKPENMTFEATIEELDSLVDQLENGDLALDDALRKFERGIALARAGQTKLSDAEQRV

SILLSEDDEAPLNDFKPDSE

>sp|Q87SW0|F16PA_VIBPA Fructose-1,6-bisphosphatase class 1 OS=Vibrio parahaemolyticus serotype O3:K6 (strain RIMD 2210633) OX=223926 GN=fbp PE=3 SV=1

MSGMRTLGEFIVEKQADFPHASGDLSSLLASIRLAAKIVNREINAAGLGDITGAVGTENV

QGEAQQKLDVYANDKFKAALEARDQVCGVASEEEDEAVAFNKELNQNAKYVVLMDPLDGS

SNIDVNVSVGTIFSIYRRVSPIGTPATEEDFLQPGHKQVAAGYVIYGSSTMLVYTTGNGV

NGFTYDPSIGSFCLSHENMMIPEDGKIYSINEGNYIRFPQGVKKYIKYCQENVPEDGRPY

TSRYIGSLVADFHRNLLKGGIYLYPSTQSHPQGKLRLLYECNPMAFLIEQAGGIASDGVN

RIMDIKPTELHQRVPFFVGSKNMVRKVEEFLELHRDEE

>sp|Q87HJ2|FABH2_VIBPA 3-oxoacyl-[acyl-carrier-protein] synthase 3 protein 2 OS=Vibrio parahaemolyticus serotype O3:K6 (strain RIMD 2210633) OX=223926 GN=fabH2 PE=3 SV=1

MTNYYAEITGWGKCVPPTVLSNDDLSTFLDTSDEWIRTRTGIENRRISHVNTSDMATVAA

QHALARAGVEASEIDLIIVATCSPDSLIPNIASKVQQNLGIRSAAAFDLNAACTGFVYGL

ETGTRLIQSGNYRHAIIIGAERLSFYIDWAMRDTAVLFGDGAGAVILSRTEKETGLLQSQ

IGCDAKGRDILSVPKFGTCMDRFAEDNGYWDFNFVGQEIFKRAVKGMGLAAKTVLKASQL

TTEQIDVVIPHQANIRIIQTLCDLSGIPQDKAFVNIQNYGNTSAATVPIALCEAVEQGRV

NPGDNMLLAAFGAGLTWGAAVLKWGDRVTPIGESDAALPECEQSALELLERAIKLCNERR

RDEV

>sp|Q87MM2|FADI_VIBPA 3-ketoacyl-CoA thiolase OS=Vibrio parahaemolyticus serotype O3:K6 (strain RIMD 2210633) OX=223926 GN=fadI PE=3 SV=1

MGKQEVKTRNGERVAIVAGLRTPFARQSTEFSQVPAVDLGKMVVSEMLARTDIDPKLIEQ

VVFGQVVQMPEAPNIAREIVLGTGMNIHTDAYSVTRACATSFQAAVNVAESIMAGTIEIG

IAGGADSSSVLPIGVSKKLAANLLALSKTKTLGQKLNILKSLSFKDLMPVPPAVAEYSTG

LSMGQTAEQMAKTHGISRAEQDALAHRSHTLASQAWKEGKIQGEVMTAFPEPYKKWISED

NNVRHDSTLEGYAKLRPAFDRQYGSVTAANSTPLTDGGAAVMLMREGKAKELGMEILGYI

RGYAFSAIGVEKDMLMGPTYATAKVLENTGLELSDLTLIDMHEAFAAQALANVKMFASDK

FAQENLGRSKAIGEIDMDKFNVLGGSIAYGHPFAATGARMMTQTLRELKRRGGGIALNTA

CAAGGLGAAMILEVE

>sp|Q87QX5|GLGA_VIBPA Glycogen synthase OS=Vibrio parahaemolyticus serotype O3:K6 (strain RIMD 2210633) OX=223926 GN=glgA PE=3 SV=1

MATNNLSILFVASEVEGLIKSGGLADVAKALPEALQNLQQDVRITIPAYTSIERLADAEV

VLETNLTSWPHTKYRVLLLTLGNNPVYLIDCDPYFNRPSMYAENNQAYTDNGERFAFFSA

ACLDMLPKLAFQPDIIHANDWHTGLVPFLLKHRYGNDPFFAHTKSVISIHNAVFKGVFSY

DDVQCLPEFHCRNVPDAAVSATHITMLKAGVMNADKINAVSPTYAEELKTELGSHGMAWE

FQQRAGDLVGILNGCDYSAWNPETDIYLPMNYSADKQSMVLGKNTCKRALQQRLNLAEKD

VAMFGMVCRLTQQKGVHYLLPALADFLKHDVQVVVVGTGDPVLAAQLEEVAAQFSDKFVF

VEAYDNELAHLVEAGSDFFLMPSEFEPCGLNQIYSMAYGTLPIVRGVGGLKDSVNDYDVD

PCDATGFVFYEPTSQALLLTMLRALLLYAQNLTEVQRVQLHAMQKDFCWRKAAESYLQLY

RSALN

>sp|Q87LV6|GLUQ_VIBPA Glutamyl-Q tRNA(Asp) synthetase OS=Vibrio parahaemolyticus serotype O3:K6 (strain RIMD 2210633) OX=223926 GN=gluQ PE=3 SV=1

MARYVGRFAPSPSGPLHFGSLIAALGSYFQAKANNGIWLVRIEDLDPPREMPGASQLILE

ALKAYQLHWDGEVVYQSERHGLYQAQIDAWLDNGDAYYCQCTRKQIKEHGGFYPGTCRDK

NLKEGAIRLKMTKPVARFLDQKHGMIEIPEQLVNEDFIIKRRDGLFAYNLAVVLDDIDQG

VTEVVRGADLIEPTGRQISLYQILGQPEVSYLHLPLAMDDNGNKLSKQNHATAIDIENPK

PALLHAMTFLGFDVPEEIKAASMNEILSWGCENWRLEQLPSEIEITPRFSNGTV

>sp|Q87KZ2|GPDA_VIBPA Glycerol-3-phosphate dehydrogenase [NAD(P)+] OS=Vibrio parahaemolyticus serotype O3:K6 (strain RIMD 2210633) OX=223926 GN=gpsA PE=3 SV=1

MTQANTNNAYGKDIAMTVIGAGSYGTSLAISLARNGANVVLWGHEPEHMARLEADRANHA

FLPGVDFPESLIIESDLEKAVQASRDLLVVVPSHVFGIVLNSCKPFLREDSRICWATKGL

EPETGRLLKDVAYDIIGENYSLAVLSGPTFAKELAMGLPTAISVASPDAEFVADLQEKIH

CSKTFRVYANNDFIGMQLGGAVKNVIAIGAGMSDGIGFGANARTALITRGLAEMSRLGAA

LGAKPETFMGMAGLGDLVLTCTDNQSRNRRFGLALGQGQDVDTAQEEIGQVVEGYRNTKE

VWMLSQRMGVEMPIVDQIYQVLYQGKDARLAAQDLLARDKKAEGK

>sp|Q87L12|GPH_VIBPA Phosphoglycolate phosphatase OS=Vibrio parahaemolyticus serotype O3:K6 (strain RIMD 2210633) OX=223926 GN=gph PE=3 SV=1

MTQQDIKLIAFDLDGTLLDSVPDLAVAADQAVQALGYPAVSEEQVRDYVGNGADVLIGRA

LSQSMTISSDLSEDLRAKGRELFDDFYAQSGHQLSHLYPTVKETLEELHQAGFTMALVTN

KPSKFVPEILEQHGIAKYFVDVLGGDAFPEKKPNPVALNWLMEKHQVKASEMLMVGDSKN

DILAAKNAGCASFGLTYGYNHGEPISASNPDFVADSLSELLEVVAVSA

>sp|P65129|IF1_VIBPA Translation initiation factor IF-1 OS=Vibrio parahaemolyticus serotype O3:K6 (strain RIMD 2210633) OX=223926 GN=infA PE=3 SV=1

MAKEDVIEMQGTVLDTLPNTMFRVELENGHVVTAHISGKMRKNYIRILTGDKVTVEMTPY

DLSKGRIVFRAR

>sp|Q87N46|IHFB_VIBPA Integration host factor subunit beta OS=Vibrio parahaemolyticus serotype O3:K6 (strain RIMD 2210633) OX=223926 GN=ihfB PE=3 SV=1

MTKSELIERLCAEQTHLSAKEVEDAVKDILEHMASTLESGDRIEIRGFGSFSLHYREPRV

GRNPKTGDKVELEGKYVPHFKPGKELRERVNLG

>sp|Q87KB6|ILVD_VIBPA Dihydroxy-acid dehydratase OS=Vibrio parahaemolyticus serotype O3:K6 (strain RIMD 2210633) OX=223926 GN=ilvD PE=3 SV=1

MPKYRSATTTHGRNMAGARALWRATGVKDEDFGKPIIAVVNSFTQFVPGHVHLKDLGQLV

AQEIEAAGGIAKEFNTIAVDDGIAMGHGGMLYSLPSRELIADSVEYMVNAHCADAMVCIS

NCDKITPGMLMASMRLNIPVIFVSGGPMEAGKTKLSDQIIKLDLVDAMIQGADPKVSDEQ

SEQIERSACPTCGSCSGMFTANSMNCLTEALGLSQPGNGSLLATHADRKELFINAGKRIV

ELTKRYYEQDDETALPRNIATKAAFENAMALDIAMGGSTNTVLHLLAAAQEGEVDFDMTD

IDRMSRQVPHLCKVAPSTQKYHMEDVHRAGGVVGILGELNRAGLLHNQSKTVLGLTWEEQ

LAKYDIMLTDSEEVKSFYRAGPAGIRTTQAFSQDCRWDTLDDDRAEGCIRTKENAFSQDG

GLAVLKGNIALDGCIVKTAGVDESILKFTGPAVVFESQEDAVDGILGGKVKAGDVVVIRY

EGPKGGPGMQEMLYPTTYLKSMGLGKECALLTDGRFSGGTSGLSIGHASPEAANGGAIGL

VQDGDLIAIDIPNRSISLEISEQELAERRVKQDELGWKPANRQREVSFALKAYASMATSA

DKGAVRDKSKLEG

>sp|Q87S29|ISCR_VIBPA HTH-type transcriptional regulator IscR OS=Vibrio parahaemolyticus serotype O3:K6 (strain RIMD 2210633) OX=223926 GN=iscR PE=3 SV=1

MKLTSKGRYAVTAMLDVALHSQQNPVPLADISERQGISLSYLEQLFSKLRKAGLVASVRG

PGGGYRLGADAHSIAIGTVIAAVDESVDATKCQGKGDCQGGTRCLTHTLWRDLSSRISDF

LNNITLGELMTDNEVLEISDRQDIGLAVTHGLSNKNTTAAPIGVNVRS

>sp|Q87S87|ISPH_VIBPA 4-hydroxy-3-methylbut-2-enyl diphosphate reductase OS=Vibrio parahaemolyticus serotype O3:K6 (strain RIMD 2210633) OX=223926 GN=ispH PE=3 SV=2

MKILLANPRGFCAGVDRAISIVERALELYQPPIYVRHEVVHNRFVVEGLKQRGAIFVEEL

HEVPDNNIVIFSAHGVSQAVRQEAKQRDLTVFDATCPLVTKVHMEVARASRRNMEVVLIG

HAGHPEVEGTMGQYSSETGGMYLVETPADVEKLKAIVKDPSDLHYVSQTTLSVDETADVI

EELRRVFPDIQGPRKDDICYATQNRQDAVRELAGDVDVMVVVGSKNSSNSTRLKELAEKL

GTPGYLTDCPEDIKPEWFEGKTKVGVTAGASAPEELVNQILERIKELVGARSVDEVLGRE

ENMFFEVPKELQIKQVD

>sp|Q87Q56|IHFA_VIBPA Integration host factor subunit alpha OS=Vibrio parahaemolyticus serotype O3:K6 (strain RIMD 2210633) OX=223926 GN=ihfA PE=3 SV=1

MALTKAELAENLFDKLGFSKRDAKETVEVFFEEIRKALESGEQVKLSGFGNFDLRDKNER

PGRNPKTGEDIPITARRVVTFRPGQKLKARVENLKKEQ

>sp|Q87RN7|ISPE_VIBPA 4-diphosphocytidyl-2-C-methyl-D-erythritol kinase OS=Vibrio parahaemolyticus serotype O3:K6 (strain RIMD 2210633) OX=223926 GN=ispE PE=3 SV=1

MEHHPMIETSTRWPSPAKLNLFLYINGRTENGYHELQTLFQFVDHGDELTIQANHSGDVT

ISPEIEGVPLQDNLIWKAATALQNYAHCSFGAHIELHKVLPMGGGIGGGSSNAATTLVAL

NYLWQLNLTDDELAEIGLKLGADVPVFVRGFSAFAEGVGEKLSPANPEEKWYLVVRPNVS

IATADIFRHPDLTRNTPKRDLETLLNAPSVNDCEKIVRMLYPEVDKQLSWLLQYAPSRLT

GTGSCVFAEFSSKSEAETILAQLSDKVSAFVAQGRNISPLKETLAEYQSASHRPI

>sp|Q87S28|ISCS_VIBPA Cysteine desulfurase IscS OS=Vibrio parahaemolyticus serotype O3:K6 (strain RIMD 2210633) OX=223926 GN=iscS PE=3 SV=1

MKLPIYLDYSATCPVDPRVAEKMVQYMTMDGTFGNPASRSHRYGWQAEEAVDTAREQIAD

LLNADPREIVFTSGATESDNLAIKGAAHFYSKKGKHVITCKTEHKAVLDPCRQLEREGFE

VTYLEPESNGLIDLEKLKAAMRDDTVLVSIMHVNNEIGVIQDIAAIGELCRERKIVFHVD

AAQSAGKLPIDVQEMKVDLISLSAHKVYGPKGIGALYVRRKPRIRLEAQMHGGGHERGFR

SGTLATHQIVGMGEAFRIAKEEMQKDYDHALALRNRLLNGVKDLEAVTVNGDLEQRVPQN

LNISFAFVEGESLLMSLKDLAVSSGSACTSASLEPSYVLRALGMDDELAHSSVRFSFGRF

TTEEEIDYAIEQIRVAVTKLRDMSPLWDMYKEGVDLSTVEWAHH

>sp|Q87SW1|IPYR_VIBPA Inorganic pyrophosphatase OS=Vibrio parahaemolyticus serotype O3:K6 (strain RIMD 2210633) OX=223926 GN=ppa PE=3 SV=1

MSLNHVPAGKSLPEDIYVVIEIPANADPIKYEVDKDSGAVFVDRFMSAPMFYPCNYGYVN

NTLSLDGDPVDVLVPTPYPLMPGSVIRCRPVGVLKMTDESGEDAKVVAVPHSKISKEYEH

IQDVGDIPELLKAQITHFFERYKELESGKWVKVDGWADVEAAKAEILQSYERAQNK

>sp|Q87LQ2|ISPD_VIBPA 2-C-methyl-D-erythritol 4-phosphate cytidylyltransferase OS=Vibrio parahaemolyticus serotype O3:K6 (strain RIMD 2210633) OX=223926 GN=ispD PE=3 SV=1

MSENVSSHIAIVPAAGVGSRMKADRPKQYLLIDGKTVLEHTVEKLLAHPQIAKVVVAVTE

GDPYYPELSIALHPDVIRVAGGKERADSVLSGLNYVSAQLPCEWVLVHDAARPCVTLNDI

DRLIDVCCAHPTGGILASPVRDTMKRANKENNIDHTVDREALWHALTPQMFKTQQLTRAL

ADALQQGVAITDEASALEWLGETPALVQGSANNIKITQPEDLALAEFYLSRERG

>sp|Q87KY1|FRDD_VIBPA Fumarate reductase subunit D OS=Vibrio parahaemolyticus serotype O3:K6 (strain RIMD 2210633) OX=223926 GN=frdD PE=3 SV=1

MKPNYSVNTSPKRSDEPIWWGLFGAGGTWFAMLTPITVLVLGILVPLGVIDAEAMSYERV

SAFATSIIGALFIIGTLALPMWHAMHRVHHGMHDLKFHTGVIGKVACYAFAGLITALAVI

FIFMI

>sp|Q87N05|FADR_VIBPA Fatty acid metabolism regulator protein OS=Vibrio parahaemolyticus serotype O3:K6 (strain RIMD 2210633) OX=223926 GN=fadR PE=3 SV=1

MVIKAKSPAGFAEKYIIESIWNGRFPPGSILPAERELSELIGVTRTTLREVLQRLARDGW

LTIQHGKPTKVNQFMETSGLHILDTLMTLDVDNATNIVEDLLAARTNISPIFMRYAFKAN

KENSERTIKNVIESCEALVNASSWDDFIASSPYADKVLQSVKEDNEKDEAKRQEILIAKT

FNFYDYMLFQRLAFHSGNQIYGLIFNGLKKLYDRVGSYYFSNPASRDLALRFYRQLLETC

ETGQREQLPVVIRHYGMESAQIWNEMKKQLPTNFTEDDS

>sp|Q56712|FLAC_VIBPA Polar flagellin C OS=Vibrio parahaemolyticus serotype O3:K6 (strain RIMD 2210633) OX=223926 GN=flaC PE=3 SV=2

MAVTVSTNVSAMTAQRYLNKATNELNTSMERLSSGHKINSAKDDAAGLQISNRLTAQSRG

LDVAMRNANDGISIAQTAEGAMNEATSVMQRMRDLAIQSSNGTNSPAERQAINEESMALV

DELNRIAETTSFGGRRLLNGSFGEAAFQIGASSGEAMIMGLTSIRADDTRMGGVTFFSEV

GKGKDWGVDPTKADLKITLPGMGEDEDGNVDDLEININAKAGDDIEELATYINGQSDMIN

ASVSEDGKLQIFVAHPNVQGDISISGGLASELGLSDEPVRTSVQDIDMTTVQGSQNAISV

LDSALKYVDSQRADLGAKQNRLSHSINNLANIQENVDASNSRIKDTDFAKETTQMTKAQI

LQQAGTSILAQAKQLPNSAMSLLQ

>sp|Q9X9J5|FLGH1_VIBPA Flagellar L-ring protein 1 OS=Vibrio parahaemolyticus serotype O3:K6 (strain RIMD 2210633) OX=223926 GN=flgH1 PE=3 SV=1

MKRICLLALITTMSGCAMLEPIETDEVTQATTVVDAVEGDKSKDESSGIVDTLRGRNDPV

AGDPAWAPIHPKQKPEHYAAATGSLFSPEHITDLYDDSKPRGIGDIITVTLDETTSATKS

ANADLSKTNEAQMDPLQVGGEELKVGGKYNFSYDLNNTNTFAGDSSAKQSNSISGYITVE

VIEVLANGNLVIRGEKWMTLNTGDEYIRLSGTIRPDDINFDNTIASNRVSNARIQYSGTG

LSQDMQEPGFLARFFNVAL

>sp|Q87JI2|FLGI1_VIBPA Flagellar P-ring protein 1 OS=Vibrio parahaemolyticus serotype O3:K6 (strain RIMD 2210633) OX=223926 GN=flgI1 PE=3 SV=2

MKPINTFFSSFLLALTLGLPATSQAEVEIPIMDLVDVRGIRENQLVGYGLVVGLAGQGDR

NQVKFTSQSITNMLRQFGVQIDDSMDPKLRNVASVSVTASVDPMAGPGQTLDVVVSSIGD

AKSLRGGTLLLTPLRGIDGEVYAIAQGSVVVGGLSAEGKSGSKVEVNTPTAGRVPNGATL

EREIKTDFNQRDEITLNLRKPSFTTAKNIAREINNTFGPNVAVAINKARVDMRAPKDTQQ

RVIMMSMLEEMSVVEGRKPARIVFNSRTGTVVIGKNVKVGEAAVSHGNLTVRISESEKVS

QPNAFADGETKVVNQTDIDVNEELAQMVIWPPGTELNTIVDAVNSLGATPTDLMSILQAL

NEAGALNAELVVI

>sp|Q9X9J4|FLGI2_VIBPA Flagellar P-ring protein 2 OS=Vibrio parahaemolyticus serotype O3:K6 (strain RIMD 2210633) OX=223926 GN=flgI2 PE=3 SV=2

MKRIVLLLMSVALFSTAAQAARIKDVAQVAGVRSNQLVGYGLVSGLPGTGEANPFTEQSF

AAMLQNFGIQLPPGTKPKIKNVAAVMVTAELPPFSKPGQQVDVTVSSIGSAKSLRGGTLL

QTFLKGLDGQVYAVAQGNLVVSGFSAEGADGSKIVGNNPTVGLISSGATVEREIPNPFGR

GDYITFNLLESDFTTAQRMADAVNNFLGPQMASAVDATSVRVRAPRDVSQRVAFLSAIEN

LEFDPADGAAKIIVNSRTGTIVVGKHVRLKPAAVTHGGMTVAIKENLNVSQPNSFSGGQT

VVVPDSDIEVTEEKGKMFKFEPGLTLDDLVRAVNEVGAAPSDLMAILQALKQAGAIEGQL

III

>sp|Q87M60|GAL1_VIBPA Galactokinase OS=Vibrio parahaemolyticus serotype O3:K6 (strain RIMD 2210633) OX=223926 GN=galK PE=3 SV=1

MSELIQNVKASFEQVLGYAPSHIIQAPGRVNLIGEHTDYNDGFVLPCAINYQTVVAAAKR

EDNIVRVVSVDYGNAVDEFDITQAITFQQDKMWANYIRGVVKCLLARGYQFTGADISVSG

NVPQGAGLSSSAALEVVIGQTFKVLFNLEISQAEIALNGQQAENEFVGCNCGIMDQMISA

EGRENHAMLLDCRSLETEAVSMPEDMAVVIINSNKKRGLVDSEYNTRRQQCEEAARIFGV

KALRDVTIEQFNEKVAELDEMVAKRARHVITENDRTVEAAQALRAHDMKRMGELMAESHA

SMRDDFEITVKEIDTLVEIVKEVIGDQGGVRMTGGGFGGCIVALVPPALVDDVKAEVEAK

YQAATGLKESIYVCQAQNGAGLVEVL

>sp|Q87MM3|FADJ_VIBPA Fatty acid oxidation complex subunit alpha OS=Vibrio parahaemolyticus serotype O3:K6 (strain RIMD 2210633) OX=223926 GN=fadJ PE=3 SV=1

MSEQKAFSLNVDEQNIAWLAIDVPNEKMNTLQAAFADEMKEIFAQLKDSSGIKGMIIHSL

KPDNFVAGADVRMLEACTTANEAQALAKQGQELFQQLSDLPYPVVAAIHGPCLGGGLELA

LACDYRVCTDFDKTRLGLPEVQLGLLPGSGGTQRLPRLIGLLPSLDLILTGKQLRAKKAK

KLGVVDACVPDTILLDVAKQFIDKGKNKGKKKQSTKEKLMSGSGLGRKLVFEQAAKKTNQ

KTRGNYPATVAILEVIQHGLEKGFAQGQELEAKRFGELVMSSESKALRSIFFATTEMKKE

HGTDAQPAAVKKVGVLGGGLMGAGISHVTVAKAKVPVRIKDVSNDGVLNALNYNYKLFEK

QRKRRILSKADLQAKMLQLSGGVDFTSYNHIDVVIEAVFEDLDLKQQMVADIEANAKSET

IFATNTSSLPIHKIAEKAERPENIVGLHYFSPVEKMPLVEVIPHETTSDETISTVVALAK

KQGKTPIVVKDKAGFYVNRILAPYMNEAAHILLANEPIEKLDGALLDFGFPVGPITLLDE

VGVDIGAKIMPILVNELGERFKGPDVFDILLNDGRKGRKSGKGFYTYKGKKKEVDKSIYK

LLKLTPESKLSDNDIALRCVLPMLNEAVRCLDDGIIRSPRDGDIGAIFGIGFPPFLGGPF

RYMDQFGLKELVEKMNEFASKYGDRYAPCDGLLTRAGEGRTFY

>sp|P64128|FIS_VIBPA DNA-binding protein Fis OS=Vibrio parahaemolyticus serotype O3:K6 (strain RIMD 2210633) OX=223926 GN=fis PE=3 SV=1

MFEQNLTSEALTVTTVTSQDQITQKPLRDSVKASLKNYLAQLNGQEVTELYELVLAEVEQ

PLLDTIMQYTRGNQTRAATMMGINRGTLRKKLKKYGMN

>sp|Q56705|FLIDP_VIBPA Polar flagellar hook-associated protein 2 OS=Vibrio parahaemolyticus serotype O3:K6 (strain RIMD 2210633) OX=223926 GN=fliDP PE=3 SV=2

MSLGPVGMSGGMDINSMVSKIVDAERVPKQQRIDNDRTTINASISAYGRLRESLDTMKNL

MANFRQEKAFAVRTVETTDDNIVSATATTDAIAGKYAIDVLQLAQSHKVASDVLPEDAKF

GPGKLQISLGDDRFNIEVRSRSKLIDVVRGINGAKDNPGVRASVINDVEGPRLILASNLS

GKDHQIKVSVEAERGNPLKYFEYQTLEDRVNALEEARAAAEEVLGPLQAPQQPDQPEILD

ENGNPLPPEAQKAADNAQDDAQDDASQEPISAAGAEAAKAGQEAIDKANQRSSLRPEERI

PGWTETASGTLLDSYEEPELELDEKAIEKAPDVPGWNNAASGTLTDSYVTTKEAKQLLEQ

EKAEIEQKIADEKQELDAKVERGELSEEQAKQIHRAKLDPQERERLEKIDEAEAKIAKAQ

SSFEEYLGMTEVQAGQDSEVLLDGVAKLSSHNNVIEDAIEGVDLTLKGKSEPNKPPAEIG

VEYDRQSVRSDIENFVSAYNSFYQTSQALSSVDPTTGQKGPLAGDSTVRSADSRLKAVFS

SRIDQAPENLKSLTEFGITTTRQGTLEINYDMLDRQLNNNFNELEKFFGGNTGFAKRIED

AIHGITGITGSIRTREKSLTEQNYRLNDDQAALDRRMEGLEKRTHAKFTAMQDATGKMQG

QLGALMSALG

>sp|Q87JI3|FLGH2_VIBPA Flagellar L-ring protein 2 OS=Vibrio parahaemolyticus serotype O3:K6 (strain RIMD 2210633) OX=223926 GN=flgH2 PE=3 SV=1

MKWLSKSWAVAVVLLVGCAGRQEFIPPQPNAEEYAPPKLDYTLPDAQSGSLYRHQYTMTL

FQDRRAYRVGDVLTVVLSEETSSSKKAGTKFGKSSAVNFAAPTIGTKKFDELGVSIDGSR

NFDGSASSSQGNKLQGAITVTVHDVLPNGVLRISGEKWLRLNQGDEFIRLTGIVRVDDIT

RNNQVSSQRIADARITYAGRGALADSNAAGWLTQFFNSPWVPF

>sp|Q9X9J3|FLGJ_VIBPA Peptidoglycan hydrolase FlgJ OS=Vibrio parahaemolyticus serotype O3:K6 (strain RIMD 2210633) OX=223926 GN=flgJ PE=3 SV=1

MMKNPNDIGFIHDISSLDSLRQKAVKEGKDGEQEALHAAARQFESIFTSMMLKSMREANE

GFESNIMNSQNEKFYRQMLDEQMASELSANGSMGLADMIVAQLTAGQGNDKSETAMRDAA

NSAVEYRRVDPKKAREIEKRLIESGELSRTSHTPAKFDSPESFVNSMKPYAEKAAKALGV

EPSLLLAQAALETGWGQKVVQNARGSSNNLFNIKADRSWQGDKVTTQTLEFHDNTPVKET

AAFRSYSNYQDSFNDYVRFLNDNPRYETALQQRGDSESFIRGIHRAGYATDPTYADKVLQ

VKQKIESM

>sp|Q87I04|GCSH_VIBPA Glycine cleavage system H protein OS=Vibrio parahaemolyticus serotype O3:K6 (strain RIMD 2210633) OX=223926 GN=gcvH PE=3 SV=1

MDKTLKFTDSHEWVRDNGDGTVTIGISEHAQEMLGDVVFVDLPDVEDEVEAGESFSLVES

VKAASDIYSPVTGEVVEINEELEDSPELINEEPYEGGWIVKVKLSDPSELDDLKDAEEYL

SSIEEE

>sp|Q87QM0|HFLD_VIBPA High frequency lysogenization protein HflD homolog OS=Vibrio parahaemolyticus serotype O3:K6 (strain RIMD 2210633) OX=223926 GN=hflD PE=3 SV=1

MANTLYDRTIAFAGICQAVALVQQVARNGHCDQDAFETSMNAILNTNPANTIGVFGREAD

LKLGLECLVKGIDSTPSGSEITRYIISLMALERKLTARTDAMSQLGDRIQMAKRQTEHFE

LLEDQMISNLASIYLDVVSPIGPRIQVTGTPSVLQQTANQHKVRALLLSGIRSAVLWRQV

GGKRRHLIFGRKKMVEQAQILLARM

>sp|Q87L07|HFQ_VIBPA RNA-binding protein Hfq OS=Vibrio parahaemolyticus serotype O3:K6 (strain RIMD 2210633) OX=223926 GN=hfq PE=3 SV=1

MAKGQSLQDPFLNALRRERIPVSIYLVNGIKLQGQIESFDQFVILLKNTVNQMVYKHAIS

TVVPARPVSHHSGDRPQGDRPQEKSED

>sp|Q87QK6|HIS6_VIBPA Imidazole glycerol phosphate synthase subunit HisF OS=Vibrio parahaemolyticus serotype O3:K6 (strain RIMD 2210633) OX=223926 GN=hisF PE=3 SV=1

MLAKRIIPCLDVRDGQVVKGVQFRNHEIIGDIVPLAKRYAEEGADELVFYDITASSDGRV

VDKSWVARVAEVIDIPFCVAGGIKSAEDAARILEFGADKVSINSPALANPQLITDLADKF

GVQCIVVGIDSYYDKDTGKYQVYQFTGDEERTKATKWETRDWVQEVQKRGAGEIVLNMMN

QDGVRSGYDIEQLNMVREVCKVPLIASGGAGAMEHFAEAYQKANVDGALAASVFHKQVIN

IGELKQYLKQQGIEVRL

>sp|Q87QK9|HIS7_VIBPA Histidine biosynthesis bifunctional protein HisB OS=Vibrio parahaemolyticus serotype O3:K6 (strain RIMD 2210633) OX=223926 GN=hisB PE=3 SV=1

MSKQQKILFIDRDGTLIVEPPIDFQVDRLDKLKLEPFVIPSLLSLQDAGYRLVMVTNQDG

LGTDSYPQEDFDAPHNMMMEIFESQGVKFDDVLICPHFEEDNCSCRKPKLGMVKEYLQGG

KVDFQNSVVIGDRQTDLQLAENMAIRGIQYNPETMGWKQILKDLTVKARVAEVIRTTKET

DIKVAVNLDEQGGNDISTGLGFFDHMLDQIATHGGFQMVCKVDGDLHIDDHHTIEDTALA

LGQALKEALGDKRGIGRFGFSLPMDECLAQCALDLSGRPYLKFDAKFSRDQVGDLSTEMV

VHFFRSLTDTLACTLHLSSAGDNDHHIIESLFKAFGRTLRQAIKVEGTELPSSKGVL

>sp|Q87QL0|HIS8_VIBPA Histidinol-phosphate aminotransferase OS=Vibrio parahaemolyticus serotype O3:K6 (strain RIMD 2210633) OX=223926 GN=hisC PE=3 SV=1

MEKLARKQVQELTPYLSARRIGGTGDVWLNANESPFNNEYKTDFARLNRYSECQPKALIS

AYAAYAGVKPEQTLTSRGADEGIELLVRAFCEPGQDAILYCPPTYGMYAISAETIGVERK

TVPLTSDWQLDLAGIESNLDNVKLVFVCSPNNPTGNLVKREDIVSLLEMTKDRAIVVMDE

AYIDFCPEASTVDLLAQYPNLAILRTLSKAFALAGLRCGFTLANEELINVLLKVIAPYPV

PVPVAEIAVQALSEAGLARAKFQVLDLNANRAYLQVGLSMIPGLQVFEGWGNYLLVKFPD

GDALFKAAWDTGIILRNSPIENCVRISVGNRDECEKTLGFIRNYYS

>sp|Q87TE0|HSLO_VIBPA 33 kDa chaperonin OS=Vibrio parahaemolyticus serotype O3:K6 (strain RIMD 2210633) OX=223926 GN=hslO PE=3 SV=1

MANNVLNRYLFEDLSVRGELVQLDEAYQRIISSKEYPAAVQKLLGELLVSTTLLTATLKF

EGSITIQLQGDGPVSLAVINGDHNQQVRGVARWEGDIADDASLHEMMGKGYLVITIEPKK

GERYQGVVGLEGENLTEVLEGYFANSEQLKTRLWIRTGEFEGKPHAAGMLIQVIPDGTGS

PDDFEHLEQLTNTVKDEELFGLEANDLLYRLYNQDKVRVYEPQPVAFHCGCSRERSGAAI

ITVEKAEIYDILAEVGSVSLHCDYCGTTYTFDETEVTELYTQASGGNKTLH

>sp|Q87T21|HSLU_VIBPA ATP-dependent protease ATPase subunit HslU OS=Vibrio parahaemolyticus serotype O3:K6 (strain RIMD 2210633) OX=223926 GN=hslU PE=3 SV=1

MSEMTPREIVHELNRHIIGQDKAKRSVAIALRNRWRRMQLEESLRVEVTPKNILMIGPTG

VGKTEIARRLAKLANAPFIKVEATKFTEVGYVGKEVETIIRDLTDVAVKMTHQQAMEKVK

FRAEEQAEERILDALLPPARDSWGQVEQKEDTSNTRQIFRKKLREGQLDDKEIEIDVAAP

QMGVEIMAPPGMEEMTNQLQGMFQNLAGDTKKKRKLKIKDAMKALAEEEAAKLVNQEELK

EAAIFNVENNGIVFIDEIDKICKRGESSGPDVSREGVQRDLLPLIEGSTVSTKHGMVKTD

HILFVASGAFQVAKPSDLIPELQGRLPIRVELEALSSNDFKRILTEPKASLTEQYIALMK

TEDVDIEFTEDGITRIAEAAWTVNETTENIGARRLHTVMERLMDEISYDAAEQSGAKFVI

DAAYVQARLGDTIEDEDLSRFIL

>sp|Q87T20|HSLV_VIBPA ATP-dependent protease subunit HslV OS=Vibrio parahaemolyticus serotype O3:K6 (strain RIMD 2210633) OX=223926 GN=hslV PE=3 SV=2

MTTIVSVRRNNKVVIAGDGQVSLGNTVMKGNARKVRRLYNNKVLAGFAGGTADAFTLFER

FESKLQMHQGHLTKAAVELAKDWRSDRALRKLEALLAVADETASLIITGNGDVVQPENDL

IAIGSGGAYAQAAATALLENTDLDAREIAEKALNIAGDICVFTNHHHTVEELDSTTEPET

PAA

>sp|Q87RH5|HTPG_VIBPA Chaperone protein HtpG OS=Vibrio parahaemolyticus serotype O3:K6 (strain RIMD 2210633) OX=223926 GN=htpG PE=3 SV=1

MSETVSQNKETRGFQSEVKQLLHLMIHSLYSNKEIFLRELISNASDASDKLRFQALSNPD

LYEGNADLGVKLSFDESANTLTISDNGIGMSRNDVIEHLGTIAKSGTAEFFSKLSEEQSK

DSQLIGQFGVGFYSAFIVADAVTVRTRAAGLPADEAVQWHSAGEGEYTIENITKESRGTD

IILHMRDEGKEFLNEWRLRDVISKYSDHIGIPVSIQTVVRDEDGKETDEKKWEQINKAQA

LWTRNKADISDEEYQEFYKHVSHDFADPLVWSHNRVEGKNDYTSLLYIPSKAPWDMMNRD

HKSGLKLYVQRVFIMDDAEQFMPSYLRFVRGLIDSNDLPLNVSREILQDNKVTQSLRNAC

TKRVLTMLERMAKNDEEKYQSFWKEFGLVLKEGPAEDFANKEKIAGLLRFASTEVDSAEQ

TVGLASYVERMKEGQDKIYYLTADSYAAAKNSPHLEQFKAKGIEVILMFDRIDEWLMNYL

TEFDGKQFQSITKAGLDLSKFEDEADKEKQKETEEEFKSVVERTKSYLGDRVKDVRTTFK

LASTPAVVVTDDYEMGTQMAKLLAAAGQAVPEVKYIFEINPEHELVKRMADEADEEAFGR

WVEVLLGQAMLAERGSMEDPTQFLGAINKLLTKV

>sp|Q87QN1|HTPX_VIBPA Protease HtpX OS=Vibrio parahaemolyticus serotype O3:K6 (strain RIMD 2210633) OX=223926 GN=htpX PE=1 SV=1

MKRIMLFLATNLAVVLVLSVVLNIVYATTGMQPGSLSGLLVMAAVFGFGGALISLMMSKG

MALRSVGGMVIESPRNETEHWLLETVGRQAQQAGIGMPTVAIYDSADINAFATGAKRDDS

LVAVSTGLLHNMTRDEAEAVLAHEVSHIANGDMVTMTLMQGVVNTFVIFLSRFIANIVAS

NDDEEGQGTNMMVYFGVSMVLELVFGFLASFITMWYSRHREFHADAGAARLVGKEKMIAA

LERLKMSQESKLDGTMMAFGINGKQSLTELLMSHPPLDKRIAALRNQ

>sp|Q87Q75|HUTG_VIBPA Formimidoylglutamase OS=Vibrio parahaemolyticus serotype O3:K6 (strain RIMD 2210633) OX=223926 GN=hutG PE=3 SV=1

MSQSDLHQQKFHWQGRHDAEDGKLGQRIHHVVKHQKTSELEKASQGVSILGFATDAGVAR

NKGRIGAKKAPDLIRRALANLAWHKDAPLYDLGTVVCDDDLLEESQSRCANLISEALPHT

PTIVLGGGHEIAWASFSGLAEYFKTHHPEKKPKIGIINFDAHFDLRAFESSLADVKPSSG

TPFNQIHHFCQRNDWAFHYACLGVSRSSNTQALFQKADELNVWYVEDSQLNYLNHSYHLT

QLQHFIDDCDYLYLTIDLDVFPAATAPGVSAPAARGVSYDTIAPFLERILHYKNKLMLAD

IAEYNPNYDVDSQTARLAARLCWDIANAMADKEHKRQPIK

>sp|Q87Q74|HUTI_VIBPA Imidazolonepropionase OS=Vibrio parahaemolyticus serotype O3:K6 (strain RIMD 2210633) OX=223926 GN=hutI PE=3 SV=1

MDLLIENARLVSMERGEAGYLPTPPARVGIQAGKIAAISAHPVGRDTPQIEALLSPQHYS

QTIDLQGQLLTPGLIDCHTHLIYAGNRANEFEMRLNGVPYQEIAKQGGGILSSVKATRAA

TEEQLIELALPRLDGLLASGVTSVEVKSGYGLTLKDELKMLRAAKALEQERNVKITTTLL

AAHALPPEFEGRADDYIEHVCQEIIPIVAEENLATSVDVFCESIGFNLEQTEKVFATAKL

YGLHVKGHTEQLSNLGGTELTARYKGLSADHIEYLDEDGVVALSKSDTVATLLPGAFYFL

RETQLPPIELLRKYHVPMAIATDVNPGTSPFSDLTLMMNMACTLFRLTPQEALRGVTQHA

ATALGYTNSRGVIKTGFDADLAIWDIEHPADLSYQVGAKRLVGRIVNGEYVSHGGF

>sp|Q87J32|HMUV_VIBPA Hemin import ATP-binding protein HmuV OS=Vibrio parahaemolyticus serotype O3:K6 (strain RIMD 2210633) OX=223926 GN=hmuV PE=3 SV=2

MNHVVLSGRNISMKYGHRLVLDDISIDIRAGEVTALLGPNGAGKSTLLKLLCGEVPSHNE

IDYFGEPKEAWKPEEIAKHLAMLPQHSTLTFPFLAREVVELGAIPLSLSNKETTELALHY

MQKTDVLHLAESLYPALSGGEKQRLHLARVLTQLHQSGDKKILMLDEPTSALDLAHQHNT

LKIAREAAKAQNAAVVVVLHDLNLASQYADRLVLLHNGKLVCDDNPWQALTPERIEQVYG

YRSIVTKHPTLDFPQVHAAA

>sp|Q87M02|IF2_VIBPA Translation initiation factor IF-2 OS=Vibrio parahaemolyticus serotype O3:K6 (strain RIMD 2210633) OX=223926 GN=infB PE=3 SV=1

MTQLTVKALSDEIGTPVDRLIEQLADAGMKKASSDNVTDEEKQKLLSHLKKEHGDKSGDA

EPTRLTLQRKTRSTLSVNAGGGKSKNVQVEVRKKRTYVKRSTIEDEAKREAEEVAKREAE

EAAKRVAEETAKREAEEAAKREAEDAVKREAEEKAKREAEEKAKRDADTNAQRNAEEKAK

RDAEEKIKQEAARKEADELKRRQEEEAKRKAEEESQRKLEEARELAEKNKERWSAAEEKK

GDMEDTDYHVTTSQYAREAEDEADRKEEAGRRNKKKKKPSTKDEQARNSGRSQRGGKGGR

KGKLAKPTSMQHGFDKSATVAKQDVVIGETIVLSELANKMSVKATEVIKVMMKMGAMATI

NQVIDQETAQLVAEEMGHKVVLRKENELEEAVLSDRDTNAESVPRAPVVTIMGHVDHGKT

STLDYIRRTHVASGEAGGITQHIGAYHVETENGMITFLDTPGHAAFTAMRARGAQATDIV

VLVVAADDGVMPQTVEAIQHAKAAGVPLIVAVNKIDKEDANPDNVKNELAQYDVIPEEWG

GENMFVHISAKQGTNIDGLLEAILLQSEVLELTAVKEGMASGVVVESRLDKGRGPVATVL

VQSGTLHKGDIVLCGQEYGRVRAMRDELGQEITEAGPSIPVEILGLSGVPASGDEATVVR

DERKAREVANYRAGKFREVKLARQQKSKLENMFSNMTAGEVAELNVVLKADVQGSVEAIA

DSLLKLSTDEVKVNIVGSGVGGITETDVVLAEASNAIILGFNVRADASARRAVEAAAVDL

RYYSIIYQLIDEVKQAMGGMLAPEFKQEIIGLAEVRDVFKSPKLGAIAGCMVTEGLIKRN

NPIRVLRDNVVIYEGELESLRRFKDDVQEVKNGYECGIGVKNYNDVRVGDQIEVFEIVEI

QRTLD

>sp|Q87JH5|IDI2_VIBPA Isopentenyl-diphosphate delta-isomerase OS=Vibrio parahaemolyticus serotype O3:K6 (strain RIMD 2210633) OX=223926 GN=fni PE=3 SV=1

MAPQTNRKDLHLDAVLHHDMSMKKKTAGFESVEFEHCALPECDFNTIDLSTEFLGHRLAL

PFLISSMTGGARDAETINCRLAEAASELGIAMGVGSQRISLEESQHSGLGKTIRELAKGV

PLYSNLGAAQLRDKGKLDNAQRAVEAIQADALFVHVNPMQEAFQKNGDHNWIGVLHAIEQ

LKPRVNVPIIIKEVGFGISGDVAQRLVDAGVDAIDVAGAGGTSWSAVEGYCQDNPHMQRA

AELFRDWGIPTATCLAQIRAQHPKLPLIASGGIHNGLEAAKAIHLGANLVGQAGAVLKAA

TISTQLVVDHFEQMALELRLACFGTGSAKVNALTKARRL

>sp|Q87S24|HSCA_VIBPA Chaperone protein HscA homolog OS=Vibrio parahaemolyticus serotype O3:K6 (strain RIMD 2210633) OX=223926 GN=hscA PE=3 SV=1

MALLQIAEPGQSSAPHEHKLAAGIDLGTTNSLVASVRSGDATTLNDEQGRSILPSVVNYS

AESTVVGYDAKAKAEFEPENTIISVKRLIGRSLKDIQSRYPSLPYRFKESDNGLPVLQTA

QGDKNPIEVSADILKALGKRAEETLGGDLAGVVITVPAYFDDAQRAGTKDAAKLAGLHVL

RLLNEPTAAAIAYGLDSGQEGVIAVYDLGGGTFDISILRLSKGVFEVLATGGDSALGGDD

FDHLLADYLMEQAGLEAPLSAEKNRALLNIATATKIAFSEQDSVEVDVFGWKGTVTREQF

EDLIRPLVKKTLMSCRRALKDADVEAEEVLEVVMVGGSTRTLLVREMVGEFFGRTPLTSI

NPDEVVAIGAGIQADILAGNKPDSEMLLLDVIPLSLGIETMGGLVEKIIPRNTTIPVARA

QEFTTFKDGQTAMSVHVVQGEREMVDDCRSLARFSLKGIPPMAAGAAHIRVTYQVDADGL

LSVTAMEKSTGVQSEIQVKPSYGLSDNEVANMLRDSMTHAKEDMQARALAEQRVEADRVI

EGLIAAMQADGDELLSEQEKQDLLKAIEALIELRNGDDANAIEQGIKDTDKASQDFASRR

MDKSIRAALSGQSVDDI

>sp|Q87S25|HSCB_VIBPA Co-chaperone protein HscB homolog OS=Vibrio parahaemolyticus serotype O3:K6 (strain RIMD 2210633) OX=223926 GN=hscB PE=3 SV=1

MNHFELFGLPSQFQLDGSLLSSKFRELQKRFHPDNFATASERDRLMSVQKAAQINDAYQV

LKHPISRAEYILAEQGMEIRGEQQTMQDPMFLMEQMELREELEDIADCSDPESALFDFDS

KVSKMYKQHLASVEQELNDGLWAEAADRVRKLKFIAKLKNEIELVEDKLLG

>sp|Q87Q77|HUTH_VIBPA Histidine ammonia-lyase OS=Vibrio parahaemolyticus serotype O3:K6 (strain RIMD 2210633) OX=223926 GN=hutH PE=3 SV=1

MLNLTLKPGHISLNELRQVSRSPVNLTLDPEAIPGIEESTQVVDRVIAEDRTVYGINTGF

GLLANTRIAPEDLETLQRSIVLSHAAGIGKFMSDETVRLMMVLKINSLARGFSGIRLKVI

NMLIDLVNAQVYPCVPQKGSVGASGDLAPLAHMSTVLLGEGQARHNGQIVSGYEALKIAG

LEPITLAPKEGLALLNGTQASTAFALEGLFIAEDLFASATVCGAMSVEAALGSRRPFDPR

IHRVRGHRSTMDAAMAYRHLLDTSSEIGESHTNCEKVQDPYSLRCQPQVMGACLQQIRNS

AEVLQVEANSVSDNPLVFAEDNDIISGGNFHAEPVAMAADNLALAIAEIGSLSERRMALL

IDSALSKLPPFLVDNGGVNSGFMIAQVTSAALASENKTLAHPASVDSLPTSANQEDHVSM

ATFAARRLKEMGENTRGILAVEYLSAAQGLDFRAPNKSSERIEIAKQMLREKVSFYDKDR

YFAPDIEQANTLLKLALHNALMPENLLPSVH

>sp|Q87Q76|HUTU_VIBPA Urocanate hydratase OS=Vibrio parahaemolyticus serotype O3:K6 (strain RIMD 2210633) OX=223926 GN=hutU PE=3 SV=1

MTQRQGQDPRLDTSRTIRAPHGNKLRAKSWLTEAPLRMLMNNLDPDVAEHPHSLVVYGGI

GRAARNWECYDKIVEVLERLEDDQTLLVQSGKPVGVFPTHKNAPRVLIANSNLVPHWANW

EHFNELDKQGLMMYGQMTAGSWIYIGSQGIVQGTYETFVSVAKKHFNGDAKGRWVLTGGL

GGMGGAQPLAGTMAGFSMIAVECDESRIDYRLRTGYVDQKATSLDEALAIITESDTPVSV

GLLGNAADVFSELVERNITPDVVTDQTSAHDPLNGYLPQGWSMSHAAEMRLQDEAAVVKA

AKKSMAVQVQAMLDLQSRGAATLDYGNNIRQMALEEGVENAFDFPGFVPAYIRPLFCEGI

GPFRWAALSGDPEDIYKTDQKVKELIPDNPHLHNWLDMARERIQFQGLPARICWVGLKDR

ERLGQAFNEMVKNGELKAPIVIGRDHLDSGSVASPNRETEGMMDGSDAVSDWPLLNALLN

TAGGATWVSLHHGGGVGMGFSQHSGMVICCDGSDDASQRIARVLHNDPATGVMRHADAGY

DIAKQCAAEQGLDLPMLNEELKKLK

>sp|Q87KH4|GPPA_VIBPA Guanosine-5'-triphosphate,3'-diphosphate pyrophosphatase OS=Vibrio parahaemolyticus serotype O3:K6 (strain RIMD 2210633) OX=223926 GN=gppA PE=3 SV=1

MSQAGSSPLYAAIDLGSNSFHMLVVRHIDGSVQTMAKIKRKVRLAAGLDEHNSLSMEAMQ

RGWDCLSLFAERLQDIPTQNIRIVGTATLRTATNVDVFLEKANQILGQPIEVISGEEEAA

TIYKGVAHTSGGSGRRLVVDIGGASTELIIGEGFEAKALTSLKMGCVTWLENFFKDRQLN

ARNFEAAIEGAKQTIKPILEQYTDLGWDVCVGASGTVQALQEIMLAQGMDEVITHSKLKR

LQKQAMLADHLEELDIEGLTLERALVFPSGLSILIAIFELLEIDAMTLAGGALREGLVYE

MVDELRQNDIRARTICSVQSRYQLDCQYGEQVATLAGKLLEQAGGDEWIAEPQGKVLLET

TAKLHEIGLTIDFKKGGEHSAYLLQNLDLPGYTRAQKFFIGEIARRYREQLSSLPEQHAI

SGTSAKRVLRLLRLAVLLTHRRNPSLEPQVELLAEGDKLTLSIDAKWLEANPLTAAELEI

ESNRQTDIGWPLTITAC

>sp|Q87SC7|GRCA_VIBPA Autonomous glycyl radical cofactor OS=Vibrio parahaemolyticus serotype O3:K6 (strain RIMD 2210633) OX=223926 GN=grcA PE=3 SV=1

MIQGIQITKAANDELLNSIWLLDSEKNEARCVAAATGYEADQVIAISDLGEYESREVAIE

TAPRIEGGQHLNVNVLKRETLEDAVAHPEKYPQLTIRVSGYAVRFNSLTPEQQRDVIART

FTETL

>sp|Q87S07|GUAA_VIBPA GMP synthase [glutamine-hydrolyzing] OS=Vibrio parahaemolyticus serotype O3:K6 (strain RIMD 2210633) OX=223926 GN=guaA PE=3 SV=1

MTKNIHDQRILILDFGSQYTQLVARRVREIGVYCELWSWDVEEADIREFNPDGIILSGGP

ESVTEENSPRAPQYVFDSGVPVLGVCYGMQTMAEQLGGKVAGSNEREFGYAQVKVSGESA

IFKDLEATQDVWMSHGDKVVEIPADFVKVGETDTCPYAAMANEEKKYYGVQFHPEVTHTK

GGLQMLENFVLGVCGCERLWTSESIIEDAVARIKEQVGDDEVILGLSGGVDSSVVAMLVH

RAIGDKLTCVFVDNGLLRLNEGQQVMDMFGDKFGLNIIKVDAEERFLEALKGKSDPEEKR

KTIGHVFVDVFDEESKKLKNAKWLAQGTIYPDVIESAASKTGKAHVIKSHHNVGGLPDDM

EMGLVEPLRELFKDEVRKIGLELGLPYNMLYRHPFPGPGLGVRVLGEIKKEYCDLLRRAD

AIFIEELHAADLYDKVSQAFTVFLPVRSVGVMGDGRKYDWVVSLRAVETIDFMTAHWAHL

PYDFLGKVSNRIINEVEGISRVVYDISGKPPATIEWE

>sp|Q87H06|GUAC_VIBPA GMP reductase OS=Vibrio parahaemolyticus serotype O3:K6 (strain RIMD 2210633) OX=223926 GN=guaC PE=3 SV=1

MRIEQELKLGFKDVLFRPKRSTLKSRSQVNLTRDFTFKHSGRQWSGVPVIAANMDSVGSF

EMAKALAEHGVMTAVHKHYTVNDWADFVKSADNATLKNVMVSTGTSDADFQKTKDIMALS

DELIFICVDIANGYSEHLVEYVERVRAEFPDKVISAGNVVTGDMCEELILAGADIVKVGI

GPGSVCTTRVKTGVGYPQLSAIIECGDAAHGLGGMIIGDGGCSCAGDVSKAFGGGADFVM

LGGMLAGHEESGGEIIEKDGETFMKFYGMSSQSAMDKHSGGVAKYRAAEGKTVLLPFRGS

VHGTISDILGGVRSTCTYVGAAKLKELTKRTTFIRVQEQENNVFGKEK

>sp|Q87LZ2|GREA_VIBPA Transcription elongation factor GreA OS=Vibrio parahaemolyticus serotype O3:K6 (strain RIMD 2210633) OX=223926 GN=greA PE=3 SV=1

MEKVPMTLRGEQMLRTELERLLKLRPQISEAIAEARELGDLKENAEYHAAREEQGICEAQ

IRDIEYKLSVAQVIDVTKMENTGKVIFGSTVTLIDVDTDEEKTYQIVGDDEADIKAGRIS

VSSPIARGLIGKMEGDEVAIQTPGGAKDFEIDRVEYI

>sp|Q87LS2|GSH1_VIBPA Glutamate--cysteine ligase OS=Vibrio parahaemolyticus serotype O3:K6 (strain RIMD 2210633) OX=223926 GN=gshA PE=3 SV=1

MTDFAARLKKVASNPEVFKQFGRGVERETLRYRQDGHLATTPHPEGLGSAFTNKWITTDF

SESLLEFITPVSHDIPELMAQLKDIHHFTQTKMGEEKMWPLSMPCYVGSEDDIQLAQYGS

SNSAKMKTLYREGLKRRYGSLMQIISGVHFNFSFPESFWDALYGEQDEQARQDTKSAAYF

ALIRNYYRFGWMIPYFFGASPALCGSFIQGRETKLPFESIGGTLYLPKATSLRLSDLGYT

NSAQSVLKIGFNSIDQYLEGLGDAIRRPSEEFAKIGVKVDGEYRQLNTNILQIENELYAP

IRPKRVAKSGEKPSDALSRAGVEYIEVRSLDVNPFSAVGVSEEQVRFLDLFLTWAALSDS

DPMDNCELECWRDNWNKVIISGREKGLMLQIGCQGERLSLQDWAKRVFVELRQIAVEMDS

AAGGDAYQAVCDKLEAWIDNPELTISGQLLELTKELGGLGKVGCALGMKFREENLAHGYQ

HYSQDIMETEVASSVEKQRKAEESDTLSFDEFLENYFAYLKQ

>sp|Q87QG0|HCP_VIBPA Hydroxylamine reductase OS=Vibrio parahaemolyticus serotype O3:K6 (strain RIMD 2210633) OX=223926 GN=hcp PE=3 SV=1

MFCIQCEQTIQTPAVKGCSFAQGMCGKTSEVSDLQDVLVYTLQGVSFWASKALEFNIIND

EINQWAPKAFFSTLTNVNFDPERILELTSQAANYKALLKEQVMSAATLSNTNLADIPAVA

NFELPNSAEAILAFAPQVAVNRGKDQVHEDVIGLRLLCLYGLKGAAAYMEHARVLEQTNN

DIYAEYHEIMAWLGTDPEDLGELLDCSMRIGLMNYKVMEMLDQGETTTFGHPEPTTVNVK

PVKGKCILVSGHDLHDLEKILQQTEGKGINVYTNGEMLPAHGYPELKKYPHLVGNYGSAW

QNQQKEFANFPGAIVMTSNCLLNPNVGQYADRLFTRSIVGWPGVAHIEGDDFSQVIECAL

AQDGFQHDEIEHHITVGFSRNALMNAAPAVIDQVKQGNIKHFFLVGGCDGDKAERSYYTD

FTAEAPEDTLILTLACGKFRFNKNTFGDINGIPRLLDVGQCNDAYSAIQLALALAKEFDC

DINELPLTLVLSWFEQKAIVILLTLFALGVKGIYTGPTAPAFLTPNLIAIIQEKFDMRSI

GNVQDDLKAILAA

>sp|Q87LY3|GSA_VIBPA Glutamate-1-semialdehyde 2,1-aminomutase OS=Vibrio parahaemolyticus serotype O3:K6 (strain RIMD 2210633) OX=223926 GN=hemL PE=3 SV=1

MTKSSELYQKAQQTIPGGVNSPVRAFNGVGGSPLFIERADGALIFDADGRAYIDYVGSWG

PMILGHNHAVIREAVIDAAQRGLSFGAPTEMEIAMAELVSELVPSMEQIRMVSSGTEATM

SAIRLARGFTGRDKIMKFEGCYHGHADSLLVKAGSGALTLGQPSSPGVPADFAKHTLTAT

FNDLDSVRELFAANKGEIACIIVEPVAGNMNCIPPVEGFHEGLREICDQEGALLIFDEVM

TGFRVALGGAQAHYNIKPDLTTLGKVIGGGMPVGAFGGRKEVMQYVAPTGPVYQAGTLSG

NPVAMAAGFACLNLLKEEGNEKRLASKTKQLADGFKSLAEKHGIPLVVNQVGGMFGFFFT

DQETVTCYEDVTKCDIERFKRFFHLMLDHGVYLAPSAFEASFTSLAHGSKEIDATLEAAD

RCFAIIAAEAK

>sp|Q87KZ5|GPMI_VIBPA 2,3-bisphosphoglycerate-independent phosphoglycerate mutase OS=Vibrio parahaemolyticus serotype O3:K6 (strain RIMD 2210633) OX=223926 GN=gpmI PE=3 SV=1

MSAKKPLALVILDGYGYREDTASNAIANAKTPVMDALIANNPHTLISASGMDVGLPDGQM

GNSEVGHTNIGAGRVVYQDLTRITKSIADGEFEQTPALVEAIDAAVKAEKAVHIMGLMSP

GGVHSHEDHIYAAVEMAAARGAEKIYLHCFLDGRDTPPRSAENSLQRFQDLFAKLGKGRV

ASLVGRYYAMDRDNNWERVQVAYDLLTQAKAEFTAETAVAGLEAAYARDENDEFVKATAI

KAEGQEDAIMQDGDAVIFMNYRADRARQITRAFVPGFDGFERAVFPAINFVMLTQYAADI

PLATAFPPASLENTYGEWLSKQGQTQLRISETEKYAHVTFFFNGGVENEFEGEERQLVAS

PKVATYDLQPEMSSPELTEKLVAAIKSGKYDTIICNYPNADMVGHTGVYEAAEKAIEALD

ESVGKVVEAIKEVGGQLLITADHGNAEMMIDPETGGVHTAHTNLPVPLIYVGDKAVEFKE

GGKLSDLAPTMLSLAGLEIPAEMSGDVLVK

>sp|Q87TB8|GREB_VIBPA Transcription elongation factor GreB OS=Vibrio parahaemolyticus serotype O3:K6 (strain RIMD 2210633) OX=223926 GN=greB PE=3 SV=1

MKTKLITREGYNKLKQEHDYLWNEKRPEITKIVTWAASLGDRSENADYTFNKRLLRQIDR

RVRFLRKFLPEVTIVDYSPQQEGKVFFGAWVEIENEAGDVKKFRIVGPEEIYGDAKDYIS

IDSPMARAMLKKQVDEEFTVRTPEGDKEWFINSIEYNKQSAES

>sp|Q87RX5|GRPE_VIBPA Protein GrpE OS=Vibrio parahaemolyticus serotype O3:K6 (strain RIMD 2210633) OX=223926 GN=grpE PE=3 SV=1

MSNEENKVTEEELDQIIEEAEKVEAAAQEAEAELEEIGDEKDAKIAQLEAALLSSETKVK

DQQDAVLRAKAEVENMRRRTEQEIDKARKYALNKFAEELLPVIDNLERAIQAADTENEVI

KPILEGVELTHKTFVDVVAKFGLKEINPEGETFNPEFHQAMSIQESPDHESNTVMFVMQK

GYELNGRVIRPAMVMVAK

>sp|Q87LK1|GSHB_VIBPA Glutathione synthetase OS=Vibrio parahaemolyticus serotype O3:K6 (strain RIMD 2210633) OX=223926 GN=gshB PE=3 SV=1

MIKLGIVMDPISSINIKKDSSFAMMLEAQRRGYEIHYMEMNDLHLDQGKAIADTKVVELK

EDPNGWYEFKSEQMIELSELDAVLMRKDPPFDTEYIYATYILERAEEQGALIVNKPQSLR

DCNEKLFTAWFPELTPTTIVTRKAEKIKAFREEHGDVILKPLDGMGGASIFRVKENDPNV

SVIIETLTNHGQNYAMAQTFVPDISNGDKRILVVDGEPMPYCLARIPAKGETRGNLAAGG

TGEARPLSETDMKIAQAVAPTLKEKGLIFVGLDVIGDKLTEINVTSPTCIREIEAAFDIS

ITGKLMDAIERRVKGE

>sp|O51859|GYRB_VIBPA DNA gyrase subunit B OS=Vibrio parahaemolyticus serotype O3:K6 (strain RIMD 2210633) OX=223926 GN=gyrB PE=3 SV=2

MSENYDSSSIKVLKGLDAVRKRPGMYIGDTDDGTGLHHMVFEVVDNSIDEALAGHCKDIV

VTIHEDNSVSVSDDGRGIPTEMHPEEKVSAAEVIMTVLHAGGKFDDNSYKVSGGLHGVGV

SVVNALSEKVVLTIHRGGHIHTQTYRHGEPEAPLAVVGDTDKTGTQIRFWPSAETFSNTE

FHYDILAKRLRELSFLNSGVSIKLIDEREADKQDHFMYEGGIQAFVQHLNTNKTPIIEKI

FHFDLEREDGISVEVAMQWNDGFQENIFCFTNNIPQRDGGTHLAGFRAALTRTLNSFMDK

EGFSKKAKTATSGDDAREGLTAVVSVKVPDPKFSSQTKDKLVSSEVKSAVESAMGEKLSE

FLVENPSEAKMVCSKIIDAARAREAARKAREMTRRKGALDLAGLPGKLADCQEKDPALSE

LYIVEGDSAGGSAKQGRNRKNQAILPLKGKILNVEKARFDKMLSSQEVATLITALGCGIG

RDEYNPDKLRYHNIIIMTDADVDGSHIRTLLLTFFYRQMPELIERGYVYIAQPPLYKVKK

GKQEQYIKDEEAMNQYQVSLALDNASLHVNAEAPALAGEALEKLVQQYNAGIKLADRMSR

RYPRALVHELIYTSRLTAEQCHDAAAVEAWTKQLVEQLNAKEVGASQYSYEVELHAELGL

SLPKIIVRTHGVTHEHALSVDFLNSKEYGKLADLSEVLDGLLEEGAYIKRGERTLPVSSF

AEALEWLVKESMRGLSRQRYKGLGEMNPDQLWETTMDPETRRMMQVTIEDAVGADQLFTT

LMGDQVEPRRHFIEENALKVANLDV

>sp|Q87RN5|HEM1_VIBPA Glutamyl-tRNA reductase OS=Vibrio parahaemolyticus serotype O3:K6 (strain RIMD 2210633) OX=223926 GN=hemA PE=3 SV=1

MSLLAIGINHNTASVDLREKVAFGPDKLGPALEQLREHEAVNGSVIVSTCNRTELYCDVK

QGARNKLIDWLAQFHQVSREDLMPSLYVHEEQAAIKHLMRVSCGLDSLVLGEPQILGQVK

QAFSDSRDHQAVDSSIDKLFQKTFSVAKRVRTETDIGGNAVSVAYAACTLAKHIFESLSD

STVLLVGAGETIELVAKHLASNGCTKMIVANRTKERAQGLAEQFGAEVISLNEIPDYLAR

ADIVISSTASPLPIIGKGMVETALKQRRHQPILLVDIAVPRDVEAQVGELNDAYLYSVDD

LQSIIDSNIEQRKVEAIQAEAIVSEESASFMTWLRSLQAVDSIRDYRKSANEIREELLSK

SLQSLAAGADPEKVLRELSNKLTNKLIHAPTRALQSAAEQGEPAKLTIIRQTLGLDDL

>sp|Q87SK0|GLNE_VIBPA Bifunctional glutamine synthetase adenylyltransferase/adenylyl-removing enzyme OS=Vibrio parahaemolyticus serotype O3:K6 (strain RIMD 2210633) OX=223926 GN=glnE PE=3 SV=1

MQLPSSLVSVAESAVQNAQEAGYLQSWPNEVVEQFHYVSALSQFITETIHRDEALAQQLP

TMLSELSRHQAYRTRLAALLAECPDEMSGHRVLRQFRNREMVYIAWKDFLHAWTLEESLR

HLSQLAEAMIFETYQWQYKICCAEWGTPTNAEGEAQPMLIIGMGKLGGGELNFSSDIDLI

FTYPENGETQGARRSIANAQFFTRLGQRIIKALDQQTFDGFCYRVDMRLRPFGESGPLVM

SYAALEDYYQEQGRDWERYAMIKARVMGCEMYPQYQELRKMLRPFVFRRYIDFSAIQSLR

RMKSMISSEVRRRGLTNNIKLGAGGIREIEFIAQVFQLIRGGREPSLRNRGLLETLSGIE

ELALLTPQEVSNLEAAYKYLRQLENLLQAMADKQTQTLPDCDIERLKLATAMQLESWDLL

IEQTQQHMNKVHQVFETLIGDDEEDEGSTIARHFHELWDMANKQDVLELILEQDIQVEEP

AIFSKAIINFKADLAKKTLGPRGREVLNRLMPKVFDAVFAHPDAQFGLPRVLHLLHNICT

RTTYLELLDEHPAALVQLVRLCTASPMISEQLSRYPILLDELIDPQQLYNPIPLDSYRTE

LRDFLARIPEDDMEQQMEALRQFKQICILRIAAADIAGVLPVMKVSDHLTYLAEAIVEAV

VSQAWLQVSEKYGEPTHVKDREGKGFAVIGYGKVGGWELGYNSDLDIVFMHDCPVNVYTD

GKKEIDGRQFYLRLAQRIIHIFSTRTASGILYEVDTRLRPSGASGLLVSPTDAFDDYQHQ

DAWTWEHQALVRARMIYGDEPLAIAFHNTRHDVLCKPRDEQTLKKEVVEMREKMRDHLGG

KKSGRFMIKQDVGGITDIEFLAQYLVLNYSHEKPKLTRWCDNVRIYETLIAQGVMEEDQA

MQLIRAYTAMRNEIHHRNLLNLDADVVEDKFVAEREWVKQAWNQWFA

>sp|Q87NZ6|ECTA_VIBPA L-2,4-diaminobutyric acid acetyltransferase OS=Vibrio parahaemolyticus serotype O3:K6 (strain RIMD 2210633) OX=223926 GN=ectA PE=3 SV=1

MYFKMITSAPWVLYPEIGEDPSKKWIFREPKISDGDGIYSLIADCPPLDMNSSYCNFLQS

THFSKTSILVEHKGDIAGFISGYQKPDEQDVLFIWQVAVSPRFRGNGLAFRMLKELLERE

ALSEVKSVETTITEDNQASWALFKKLDAMNGNHGQVSTFLDEKAHFKGKHDTEFLYRIPL

K

>sp|Q87L81|G6PI_VIBPA Glucose-6-phosphate isomerase OS=Vibrio parahaemolyticus serotype O3:K6 (strain RIMD 2210633) OX=223926 GN=pgi PE=3 SV=1

MLKNINPTQTQAWKALTAHFESAQDMDLKELFAQDAARFDKYSARFGSDILVDYSKNLIN

EETLKHLFALANETELKSAIEAMFSGEAINQTEGRAVLHTALRNRTNTPVMVDGEDVMPA

VNAVLEKMKSFTDRVIGGEWKGYTGKAITDIVNIGIGGSDLGPYMVTEALAPYKNHLNLH

FVSNVDGTHIVETLKKVNPETTLFLIASKTFTTQETMTNAHTARDWFLESAGDQAHVAKH

FAALSTNATAVSEFGIDTANMFEFWDWVGGRYSLWSAIGLSIALAVGYDNFVELLDGAHE

MDKHFVSTDLESNIPVILALIGIWYNNFHGAESEAILPYDQYMHRFAAYFQQGNMESNGK

YVDREGNAVTYQTGPIIWGEPGTNGQHAFYQLIHQGTKLIPCDFIAPAISHNPAGDHHQK

LMSNFFAQTEALAFGKSEETVKEELVKAGKNAEEVAAIAPFKVFEGNRPTNSILVKQITP

RTLGNLIAMYEHKIFVQGVIWNIFSFDQWGVELGKQLANQILPELADASQINSHDSSTNG

LINAFKAFKA

>sp|Q87KD4|FMT_VIBPA Methionyl-tRNA formyltransferase OS=Vibrio parahaemolyticus serotype O3:K6 (strain RIMD 2210633) OX=223926 GN=fmt PE=3 SV=1

MSQSLRIVFAGTPDFAARHLAALLSSEHEVIAVYTNPDRPAGRGKKLAAPPVKQLALEHN

IPVYQPESFKSDEAKQELADLNADLMVVVAYGMLLPQAVLDTPKLGCINVHGSILPRWRG

AAPIQRSIWAGDAETGVTIMQMDIGLDTGDMLKIATLPIEATDTSASMYEKLAELGPEAL

IDCLADIAAGKAVPVKQDDELANYAKKLNKEEARINWNDDAAHIERCVRAFNPWPMSHFE

AAENSIKVWQSRVAEQTSDKPAGTIVQADKTGIYVATGNGVLVLEQLQVPGKKAMSVQDI

LNSRAAWFEVGTLLV

>sp|Q87RB8|FOLD_VIBPA Bifunctional protein FolD OS=Vibrio parahaemolyticus serotype O3:K6 (strain RIMD 2210633) OX=223926 GN=folD PE=3 SV=1

MTAQNIDGTLISQTVRSEVAARVKARVAAGLRAPGLAVVLVGEDPASQVYVGSKRRACEE

VGFVSKSFDLPASTSEEELLALIDELNNDNEIDGILVQLPLPAGIDTTHVLERIHPEKDV

DGFHPYNVGRLAQRIPKLRSCTPKGIITLLDRYNIELRGKHAVVVGASNIVGRPMTLELL

LAGCTTTTCHRFTKDLESHVRQADVVVVAVGKPNFIPGEWIKKGAVVVDVGINRLDSGKL

VGDVEYDKARESASFITPVPGGVGPMTVASLIENTMLACEQFHTEQ

>sp|O24755|FUR_VIBPA Ferric uptake regulation protein OS=Vibrio parahaemolyticus serotype O3:K6 (strain RIMD 2210633) OX=223926 GN=fur PE=3 SV=1

MSDNNQALKDAGLKVTLPRLKILEVLQQPDCQHISAEDLYKKLIDLGEEIGLATVYRVLN

QFDDAGIVTRHHFEGGKSVFELSTQHHHDHLVCLDCGEVIEFSDDIIEERQREIAAKYNV

TLTNHSLYLYGKCSDGGCKENPDAHKPAK

>sp|Q87KM5|GLPE_VIBPA Thiosulfate sulfurtransferase GlpE OS=Vibrio parahaemolyticus serotype O3:K6 (strain RIMD 2210633) OX=223926 GN=glpE PE=3 SV=1

MDQFQHIDVQGAQALLEQGEAKLVDIRDPQSFAVAHAESAYHLTNDTIVAFMEDVEFEQP

ILVMCYHGISSQGAAQYLVNQGFEQVYSVDGGFEAWQRAQLPIVRS

>sp|Q87GZ6|GCH1_VIBPA GTP cyclohydrolase 1 OS=Vibrio parahaemolyticus serotype O3:K6 (strain RIMD 2210633) OX=223926 GN=folE PE=3 SV=1

MSGLSESAKLVKEALEQRGLETPMRPNAVSREEKKEKIEHHMREILTLLQLDLTDDSLEE

TPHRIAKMYVDEIFSGLDYSNFPKITVIENKMNVSEMVRVKDITVTSTCEHHLVTIDGKA

AVAYIPRGKIIGLSKINRIVRFFAQRPQVQERMTQQILVALQTLLESDDVAVTIDATHYC

VKSRGVMDATSETTTTALGGIFKSNPATRAEFLHGLR

>sp|Q87QX6|GLGC1_VIBPA Glucose-1-phosphate adenylyltransferase 1 OS=Vibrio parahaemolyticus serotype O3:K6 (strain RIMD 2210633) OX=223926 GN=glgC1 PE=3 SV=1

MAGVLGMILAGGEGSRLRPLTESRSKPSVPFGGSYRLIDFALNNFVNADLMRIYVLTQFK

SQSLFHHLKKGWNINGITDRFIDPIPAQMRTGKRWYEGTADAIYQNLRFMELEEPDQVCI

FGSDHIYKMDIKQMLNFHTEKKASLTVSALRMPLKEASQFGVIEVDAEGRMIGFEEKPAN

PKSIPGEPDFALVSMGNYVFEAQVLFSELVEDADNEASSHDFGKDIIPKMFPRGDVFVYD

FSTNRISGEKEEVYWRDVGTIDAYWQAHMDLLEKDAPFSLYNRKWPLHTYYPPLPPATFT

DSDNGRVQIIDSLVCNGSYVRGSRIEKSVLGFRSNIASACDISECILLGDVKIGEGCVLR

RVIVDKDADIAPGTQIGVNLQEDKKHFHVSEEGIVVIPKGARVGY

>sp|Q87MD6|GLND_VIBPA Bifunctional uridylyltransferase/uridylyl-removing enzyme OS=Vibrio parahaemolyticus serotype O3:K6 (strain RIMD 2210633) OX=223926 GN=glnD PE=3 SV=1

MPLQSPLTFSDEQINIGELKQELEKFSSTQKQEFLNHHPVTSLVLARAEYMDLLLTRLWQ

YFGFNDIYNISLVAVGGYGRGELHPLSDIDILVLSNNKLPTALEAKISEFITLLWDLKLE

VGHAVRTVNECAQIGRDDLTVATNLQEARLLCGSEDTFQALKKVVLSDSFWPSETFYRAK

IQEQRERHARYHDTTYNLEPDIKSTPGGLRDIHTLSWVARRHFGATSLLEMSRYGFLTDA

EYRELVECQDFLWRVRFALHIELRRYDNRLTFAHQAQVAENLGYVGEGNRGVEMMMKEFY

RTLRRVAELNKMLLKLFDQAIINGGATENAEILDADFQRRGSLIEARKPALFQARPETIL

DMFLHIANDSTIEGVSPPTLRQLRTARRRLNKFLHTIPAAREKFLALCRHPNALHKAFSL

MHRLGVMAAYLPQWSQIVGQMQFDLFHAYTVDEHSIRLLKHINTFNNPDNHAKHPICCDI

YPRMQKKELLIIAAIFHDIGKGRGGDHSVIGEGEAYDFCIEHGLSKPEAKLVGWLVRHHL

LMSVTAQRRDIYDPDVITEFAKQVRDEESLEYLVCLTVADICATNPELWNAWKRTLLAEL

FYSTQRALRRGLENPVDVRERIRHNQQMASALLRKEGFSARQIEVLWQRFKADYFLRHTH

TQIAWHCAHLLRMDDPNKPLVLISKKATRGGTEVFVYTKDQPALFATVVAELDRRNFNVH

DAQIMTSKDGHVIDTFMVLDQHGEAIDESRHAAVIKHLTHVLEAGRPTKIKTRRTPNKLQ

HFNVKTKVDFLPTKGKKHTLMEFVALDTPGLLAKVGRTFADLNINLHGAKITTIGERAED

LFILTSEAGGRLSEEQQNELRDKLIEKLSDAVTA

>sp|Q87RR2|GLYA1_VIBPA Serine hydroxymethyltransferase 1 OS=Vibrio parahaemolyticus serotype O3:K6 (strain RIMD 2210633) OX=223926 GN=glyA1 PE=3 SV=1

MLKRDMNIADYDAELFAAIQEETLRQEEHIELIASENYTSPRVMEAQGSQLTNKYAEGYP

GKRYYGGCEYVDKAEQLAIDRACKLFGCEYANVQPHSGSQANSAVYMALLNPGDTVLGMS

LAHGGHLTHGSPVNFSGKHYNVIPYGIDEAGQINYDEMEQLALEHKPKMIIGGFSAYSQI

VDWKRMREIADKVDAYLFVDMAHVAGLIAAGEYPTPVPHAHVVTTTTHKTLAGPRGGLIL

SNAGEDMYKKLNSAVFPGGQGGPLMHVIAGKAVAFKEAMEPEFKAYQARVVKNAKAMVGQ

FQERGYKIVSNGTENHLFLVDLIDKDITGKDADAALGAANITVNKNSVPNDPRSPFVTSG

IRVGTPAITRRGFTEEDAKDLANWMCDVLDNIGNEEVIEATKQKVLEICKRLPVYA

>sp|Q87I05|GCSP_VIBPA Glycine dehydrogenase (decarboxylating) OS=Vibrio parahaemolyticus serotype O3:K6 (strain RIMD 2210633) OX=223926 GN=gcvP PE=3 SV=1

MTELLQSLSTQNEFVGRHNGPKLSDQQKMLEAINAVSLDALISETVPANIRLEQPMTLAE

AKSEADMLATMKQFAKQNQVKRTFIGQGYYNTFTPNVILRNVLENPGWYTAYTPYQPEIS

QGRLESLLNFQQMVIDLTGMEIANASLLDEATAAAEAMTLCKRAGKSKSNVFFVADDVHP

QTIEVVKTRAKFIGFEVLVGSLESLPEQDVFGALVQYPSTTGEVRDLTDIIAKAQANKTL

VTVATDLLACTLLKPAGEMGADVAIGSAQRFGVPMGYGGPHAAFMATRDKHKRTMPGRVI

GVSIDAKGNQALRMAMQTREQHIRREKATSNICTAQALLANMASFYAVYHGAEGLRTIAR

RTHHMTAILAAGLTKGGFELAHNSFFDTITINTGEKTQDLYTKALAADINLRVLPGKLGI

SLDETTTVADVEALFAIFGVKEDVTALSTEVAGNEFAAIPEALRRTSEYLTHPVFNTYHS

ETQMMRYLKQLENKDFSLTHGMIPLGSCTMKLNAAAEMIPITWPEFGSIHPFAPAEQAAG

YAALAKDLKEKLCEITGYDAFSLQPNSGASGEYAGLIAIQRYHESRGEGHRNVCLIPSSA

HGTNPATASMVSMKVVVVKCDDEGNIDIDDLAAKIEKHKDNLSSIMITYPSTHGVYEEKV

KEVCEMVHAAGGQVYLDGANMNAQVGLTSPGFIGSDVSHLNLHKTFCIPHGGGGPGMGPI

GVKSHLAPFLPGHIENGVEGEDFAVSAADFGSASILPISWAYIAMMGEAGLSNATKVAIL

NANYVMERLRPHYPVLYRGKNGRVAHECIIDIRPLKEETGISEEDIAKRLMDYGFHAPTM

SFPVAGTLMVEPTESEDLAELNRFCDAMISIREEMTKVKNGEWPLENNPLVNAPHTQVDL

SAEEWDRPYSRELGCFPSKATKSWKYWPTVNRVDNVYGDRNLICSCPSIDNYED

>sp|Q87LZ7|GLMM_VIBPA Phosphoglucosamine mutase OS=Vibrio parahaemolyticus serotype O3:K6 (strain RIMD 2210633) OX=223926 GN=glmM PE=3 SV=1

MSNKRRYFGTDGVRGKVGQYPITPDFVLKLGWAAGRVLAKQGTKKVIIGKDTRISGYMLE

SALEAGLAAAGLKATFTGPMPTPAVAYLTQTFRAEAGIVISASHNPYYDNGIKFFSSEGT

KLPDDIELAIEAELDKDIECVESSELGKATRLNDAAGRYIEFCKSTFPSTLSLSNLKIVV

DCAHGATYHIAPNVFKELGADVIAMGVEPNGININEEVGATDVRALQKRVVEEQAHLGLA

FDGDGDRIIMVDHLGNKVDGDQIAYIIARDALRRGELKGGVVGTLMTNLGMENGLKQLGI

PFVRAAVGDRYVMEQLLAKGWKIGAENSGHVILLDKVTTGDAIVAALQVLASVVGSDMSL

HDLSQGMTLYPQVLENVRFAGENNPLEAQAVLAAVEEVEAELGDKGRVLLRKSGTEPLIR

VMVEGEDAELVQSSALKIADAVKASC

>sp|Q87MG0|GLO2_VIBPA Hydroxyacylglutathione hydrolase OS=Vibrio parahaemolyticus serotype O3:K6 (strain RIMD 2210633) OX=223926 GN=gloB PE=3 SV=1

MLEIKSIPAFNDNYIWLIQNSDKRCAVVDPGDAQPVLDYLQANELTLEAILVTHHHNDHI

GGVPDLVRAFPHVTVVGPKAEPIPTLTTPMEEGDKLELFGEIFLVLGLPGHTLGHIGYVG

DSKLFCGDVLFSAGCGRIFEGTPEQMFESLSKIAALPEETQVFCAHEYTASNVAFALAVE

PDNEQLRQYRDDVNRLRALNIPTLPTTLRKEKWINPFLRTTNPDVVKSVANRIKNSDPCS

VFTALREWKNEF

>sp|Q87FT0|GBPA_VIBPA GlcNAc-binding protein A OS=Vibrio parahaemolyticus serotype O3:K6 (strain RIMD 2210633) OX=223926 GN=gbpA PE=3 SV=1

MKSFPNKSLVALAIASMSSGVLAHGYVSESNDGVAASRAALCKYPTSDTNERNTNCGAIQ

YEPQSVEGPDGFPETGPRDGKIASAETALAAALDEQTADRWVKRPIKSGTQTFEWTFTAN

HVTRDWKYYITKPNWNPNASLSRDSFDLNPFCVVDGNMVQPPKQMSHQCNVPEREGYHVI

LAVWDVGDTAASFYNVIDVKFDGDDPVIPEWTQGGQIIPTMNLKVGDSVYTRVFDQSGEN

VAYRTELAISNDVLTQAKNWSYALASKINQEQTKLQAGQYSEDKFTPVYGTNPIYLQSNS

GLERVEIGYNIETPVPDYSLTVDGLASEYIIGTEPTALDLTLTAEGDLTAELTVYNHHRE

PLASWTGSIQDGASEQVELTLSKSEPGHHMLVTRIKDTDGNLVDQQTLDFHLKSEEVTPP

PSGEYDFVFPEGLSSYTAGTKVLASDGAIYQCKPFPYSGYCVQWSESATQFEPATGSHWE

MAWDKLN

>sp|Q87SR3|GLMS_VIBPA Glutamine--fructose-6-phosphate aminotransferase [isomerizing] OS=Vibrio parahaemolyticus serotype O3:K6 (strain RIMD 2210633) OX=223926 GN=glmS PE=3 SV=2

MCGIVGAVAQRDVAEILVEGLRRLEYRGYDSAGVAIVDAEANLTRIRRLGKVQELADAVD

EAKVVGGTGIAHTRWATHGEPSEINAHPHMSGDITVVHNGIIENHEELRELLQSRGYVFE

SQTDTEVIAHMVEWELRTAESLLEAVQKTAKQLEGAYGTVAMDRKDPSRIVVARSGSPIV

IGFGVGENFLASDQLALLNVTRRFMYLEEGDVAEITRRDVTVFDVTGERVEREITESNAE

HDAGDKGQYRHFMQKEIYEQPKALINTMEGRITADSVVTDAIGVHAADILSKVEHVQIVA

CGTSYNAGMTARYWFEDIAGVSCDVEIASEFRYRKFVTRPNSLLITLSQSGETADTLAAL

RLAKEKGYMAAMTICNVAGSSLVRESDFAFMTRAGVEIGVASTKAFTTQLSALLMLVTAL

GKEQGRISKEKEKEIVEALHALPKQINAALSFEKEIEALATDFADKHHTLFLGRGEFYPI

AMEASLKLKEISYIHAEAYAAGELKHGPLALIDADMPVVVVAPSNDLLEKLKSNVEEVRA

RGGLLYVFADADAGFEGDETMKIITMPHVSEITAAIYYTIPMQLLSYYVALIKGTDVDQP

RNLAKAVTVE

>sp|Q87LI9|GLSA_VIBPA Glutaminase OS=Vibrio parahaemolyticus serotype O3:K6 (strain RIMD 2210633) OX=223926 GN=glsA PE=3 SV=1

MKPTAEILTDILAEVRPLIGQGKVADYIPALAKVPNNKLAIAVYTNEGEVIKAGDADESF

SIQSISKALSLTLAMCLYKQEEIWARVGKEPSGQAFNSLIQLEMEQGIPRNPFINAGAIV

VADLLQSRLSAPRQRLLEFVRQLSGDTHIVYDKVVAASEMMHGDRNAAIAYLMRSFGNFE

NEVIPVLQNYFHACALKMSCVDLAKTFSYLANKGTSVQTGKPVVSPTQTKQLNALLATCG

LYDGAGEFAYRVGMPGKSGVGGGIIAVVPGEMTIAVWSPELDASGNSLAGTKALELLSER

IGRSIF

>sp|Q87FR0|GLGB_VIBPA 1,4-alpha-glucan branching enzyme GlgB OS=Vibrio parahaemolyticus serotype O3:K6 (strain RIMD 2210633) OX=223926 GN=glgB PE=3 SV=1

MSSQRWLWRNQHSVQTNIGSREKDLKITKTKKIKKKHERAYELLAEAAYSDPFAALGPFI

DDGEGSLRVWMPGANKVELLVNGEPRVALERDGDSGFILKEQRDLHLTHYRLAVDWNGVE

QIIDDPYQYHNIYQEYEHLHTPKDMYHYMGAHFVTLERGGENISGVRFLVYAPHASAVSL

VGCFNQWDGRRHPMQRLDYGIWGLFIPGLEEGVQYKFELKGPNGEGLPHKQDPWGFYSEQ

YPSFASITYDHKRYQWQDAKWQNRAVTQKRDEALSFYELHAGSWKRDGKGDFLNYRELAE

QLVPYLVDMGYTHVELMPVSEHPFYGSWGYQPVGLFAPTSRYGSPDDFKFFVDACHQAGI

GVVLDWVPAHFPSDDHGLANFDGTPLFHDPDPRRGWHQDWNSYIYDLGREHVRRFLVSNA

LYWFEQFHIDGIRVDAVASMLYLDYSRSHDQWVPNVDGGNENYDAIATLKWMNEEVYKHF

PNAMTIAEESTAFPGVSAPTFMGGLGFGFKWNMGWMHDSLSYVKEDPVHRKYHHNTITFP

LVYAHSENYVLSLSHDEVVYGKGSIHNKMPGDEWQQTANLRAYYGYMYGQPGKKLNFMGA

EIGQTAEWNHDDQLQWFLLEYERHQGVQKLMRDLNHLYRNEAAMHDQDCVPAGFEWRLQD

EADASILAHERISKEGERILIITNFTPVPHERFRLGVPNVGQYELLLNTDDSKYGGSDFK

VLTSVKTEKVESESLPQSLELRLPPLSTVFYKLHK

>sp|Q87M72|GLPK_VIBPA Glycerol kinase OS=Vibrio parahaemolyticus serotype O3:K6 (strain RIMD 2210633) OX=223926 GN=glpK PE=3 SV=1

MTEQKYIVALDQGTTSSRAVILDHDANIVSVAQREFTQIYPQAGWVEHDPMEIWATQSST

LVEALAKSGIRSDQLAAIGITNQRETTIVWNKETGKPVYNAIVWQCRRTADICEDLKSRG

LEDYVRDNTGLVLDPYFSGTKVKWILDNVEGAREDAEAGKLLFGTVDTWLVWKMTQGRVH

VTDYTNASRTMLFNINDLCWDQKLLDEMGIPASMMPEVKRSSEIYGKTNIGGKGGTRIPI

AGIAGDQQAALYGQMCVEAGQAKNTYGTGCFLLMNTGQEKVTSKNGLLTTLACGPKGEPA

YALEGAVFMGGASIQWLRDELKILNGAEDSEYFATKVDTSNGVYVVPAFTGLGAPYWDAY

ARGTIVGLTRGVNSNHIIRATLEGIAYQTRDVLDAMQADSGIKLANLRVDGGAVANNFLM

QFQSDVLNTEVHRPQVTEVTALGAAYLAGLAVGYWNSIDELQDKAVLDRTFEPHDDEEKR

NRRYKGWKRAVKCAQTWSELHDEDD

>sp|Q87I03|GLYA2_VIBPA Serine hydroxymethyltransferase 2 OS=Vibrio parahaemolyticus serotype O3:K6 (strain RIMD 2210633) OX=223926 GN=glyA2 PE=3 SV=1

MNKSYPNHSLENFFSTNLSATDDAVFAGIQAEFTRQNEQIELIASENIVSKAVMQAQGTC

LTNKYAEGYPGRRYYGGCEHVDTVEAIAIERAKKLFNCEYANVQPHSGAQANGAVKLALL

QPGDTILGMSLDAGGHLTHGARPALSGKWFNAVQYGVDRETLEINYDDVRALALEHKPKM

IIAGGSAIPRTIDFAKFREIADEVNAILMVDMAHIAGLIATGAHPSPLPHAHVVTTTTHK

TLRGPRGGMILTNHEDIIKKINSAVFPGLQGGPLMHVIAAKAVAFGEALGPEFKTYIDSV

INNAKVLAEVLQTRGCDIVTGGTDTHLMLVDLRPKGLKGNKAEEALERAGITCNKNGIPF

DTEKPMITSGIRLGTPAGTSRGFGAEEFKLIGNWIGDVLDGLVNNPEGDAIVEKRVRKEV

KELCSRFPLYQ

>sp|Q87MG7|GMHA_VIBPA Phosphoheptose isomerase OS=Vibrio parahaemolyticus serotype O3:K6 (strain RIMD 2210633) OX=223926 GN=gmhA PE=3 SV=1

MYQDLIRSELNEAAEVLNKFLSDDHNIAQIEAAAKMIADSFKQDGKVLSCGNGGSHCDAM

HFAEELTGRYRDNRPGYAGIAISDPSHLSCVSNDFGYDFVFSRYVEAVGRKGDVLFGLST

SGNSGNILKAIEAAKAKGMKTVALTGKDGGKMAGLADVEIRVPHFGYADRIQEVHIKIIH

IIIQLIEKEME

>sp|Q87HX3|GLGC2_VIBPA Glucose-1-phosphate adenylyltransferase 2 OS=Vibrio parahaemolyticus serotype O3:K6 (strain RIMD 2210633) OX=223926 GN=glgC2 PE=3 SV=1

MQDALAVILAGGMGSRLSPLTDDRAKPAVPFGGKYRIIDFTLTNCLNSGLRKILVLTQYK

SHSLQKHLRDGWSIFNPELGEYITAVPPQMRKGGAWYEGTADAIYHNLWLLSRNDAKYVV

VLSGDHIYRMDYAAMLEEHKEKGAKLTVACMDVPVKDASAFGVMGIAENGLVKSFVEKPE

NPPTLPDDNAKSLASMGIYIFDMDVLKEALTEDAKLETSSHDFGNDIIPKLIDTESVYAY

KFCGSKGRVDKDCYWRDVGTIDSFYEANMDLLEPVPPMNLYQSNWAIRTYEPQFPPARTV

SSATGNEGIFINSIIATGVINSGGSVQHSIISSNVRIQDSATVVDSIIFDDVEVGEGSQL

VNCIVDKHVRIPPNTQIGINKVEDAKRFKISEKGIVVIPESYQF

>sp|Q87RP8|LNT_VIBPA Apolipoprotein N-acyltransferase OS=Vibrio parahaemolyticus serotype O3:K6 (strain RIMD 2210633) OX=223926 GN=lnt PE=3 SV=1

MMNLLFHRLKRPLAAAFVGASTTLAFAPYQLWPIAILSPAILLILLANQTPKRALWIGYA

WGLGQFATGVSWVYVSISGFGGMPLIANLFLMGMLIAYLAVYSGLFAWLNNKFFPQFSLS

KALLAAPALWLITDWLRGWVMTGFPWLWLGYSQIDAPLASFAPIGGVELLTLFVLISAGA

LAYAWIHKQWLMIIIPVVLMSAGFGIRQYDWVTPRPEDTTKVALIQGNVDQNLKWLPSQR

WPTIMKYADLTRENWDADIIVWPEAAIPAFEVEVPSFLSNIDSAAKMNNSAIITGIVNQS

EDRQFYNSILSLGVTPYGDYSFDMSERYHKHHLLPFGEFVPFEDILRPLAPFFNLPMSSF

SRGAFVQPNIVANGMHMAPALCYEIIFNEQVRQNVTDETDFILTLSNDAWFGHSIGPLQH

MEIARMRALELGKPLIRSTNNGLTAVTDYKGKIVEQVPQFETAVLRAELTPTDGTTPYRT

FGTWPLYFWVALSLMLAWWLPRKKD

>sp|Q87GB8|MALG_VIBPA Maltose/maltodextrin transport system permease protein MalG OS=Vibrio parahaemolyticus serotype O3:K6 (strain RIMD 2210633) OX=223926 GN=malG PE=3 SV=1

MAMVQGKSLKYRVWATHIAMWAFLALIIFPLLMIIAISFREGNFATGSLIPDNPTLDHWK

LALGFSITNADGTVTPPPFPVMTWLWNSVKVGGISAILIVALSTTSAYAFARMKFKGKNT

ILKAMMIFQMFPAVLALVALYALFDKLGQYIPFLGLNTHGGLIFAYLGGIALHVWTIKGY

FESIDSSLEEAAALDGATPWQAFRLVLLPLSVPILAVVFILSFIMVIGEVPVASLLLSDV

DSYTLAVGMQQYLYPQNYLWGDFAAAAVLSAVPITAVFLLAQRWLVGGLTAGGVKG

>sp|Q87Q92|MAO1_VIBPA NAD-dependent malic enzyme OS=Vibrio parahaemolyticus serotype O3:K6 (strain RIMD 2210633) OX=223926 GN=maeA PE=3 SV=1

MNNDKRPLYIPYAGPALMATPLLNKGSAFSAEERSSFNLEGLLPETTETIQEQVERAYQQ

YKSFESDMDKHIYLRNIQDTNETLFYRLVQNHISEMMPIIYTPTVGAACENFSNIYRRGR

GLFISYPNRDRIDDLLNNAANHNVKVIVVTDGERILGLGDQGIGGMGIPIGKLSLYTACG

GISPAYTLPIVLDVGTNNPQRLADPMYMGWRHPRITGPDYDAFVEEFIQAVQRRWPDALI

QFEDFAQKNAMPLLERYKDRICCFNDDIQGTAAVTVGSLLAACKAAGTQLSKQRITFLGA

GSAGCGIAEAIIAQMVSEGISDEKARSQVYMVDRWGLLQEGMPNLLDFQQRLVQKHSNTK

EWENEGNGFSLLDVMRNAKPTVLIGVSGAPGLFSQEVIEEMHKHCKRPIVFPLSNPTSRV

EATPNDIIRWTNGEALVATGSPFDPVVHEGRTYPIAQCNNSYIFPGIGLGVLAVNAKRVT

DEMLMESSRALATCSPLAINGRGALLPPLEEIHLVSKKIAFAVAKKAIEQGVALEITDEA

LNDAIDQAFWQPVYRRYKRTAF

>sp|P22100|LPW_VIBPA trp operon leader peptide OS=Vibrio parahaemolyticus serotype O3:K6 (strain RIMD 2210633) OX=223926 GN=trpL PE=4 SV=2

MLQEFNQNQKAKVAVCLNKTNSTDLAWWRTWTSSWWANVYF

>sp|Q87SF9|LPXC_VIBPA UDP-3-O-acyl-N-acetylglucosamine deacetylase OS=Vibrio parahaemolyticus serotype O3:K6 (strain RIMD 2210633) OX=223926 GN=lpxC PE=3 SV=1

MIRQRTLKEIVKTTGVGLHSGRKVTLTLRPAAANTGIIYRRTDVNPPVDFPADPASVRDT

MLCTALVNDEGVRISTVEHLNAALAGMGIDNIIVEVDAPEIPIMDGSASPFVYLLQQAGI

EMQNVPKRFIRIKKPVRFEDGDKWAEFVPFNGFRMDFEIDFNHPAIESDEQRLLFDFSSQ

GFVREISRARTFGFMRDIEYLQSQNLVLGGSFDNAIVLDDYRILNEEGLRFENEFVTHKV

LDAIGDLYMCGHPIIGEFRAYKSGHGLNNQLLRAVLADQEAWEWTTFEEEVGSPVAFAEP

NMVLA

>sp|Q87GB5|MALK_VIBPA Maltose/maltodextrin import ATP-binding protein MalK OS=Vibrio parahaemolyticus serotype O3:K6 (strain RIMD 2210633) OX=223926 GN=malK PE=3 SV=1

MASVTLKNVCKAYGDVLISKNVDLQIDEGEFVVFVGPSGCGKSTLLRCIAGLEDITSGDL

YIGDQRMNDVEPSKRGVGMVFQSYALYPHLNLYDNMSFGLKLAKADKAEIDKRVEHAAEI

LQLGHLLERQPKALSGGQRQRVAIGRTLVSQPNVFLLDEPLSNLDAALRVNMRAQITKLQ

RQLGCTMIYVTHDQVEAMTMADKIVVLDGGYVSQVGKPLELYHYPQNRFVAGFIGSPKMN

FMSVFIDEVESERVKVQLSNGVSFWIPVDGTTVNRGDRMSLGIRPEHLLSATEADATIHG

EVMIVEKLGNETQVYLNLEGADADVIYRQPDTLAVDTGDKLEIGIPAHRCHLFHSDGRAC

KRLFKENGVDFE

>sp|Q87SU7|MDH_VIBPA Malate dehydrogenase OS=Vibrio parahaemolyticus serotype O3:K6 (strain RIMD 2210633) OX=223926 GN=mdh PE=3 SV=1

MKVAVIGAAGGIGQALALLLKNRLPAGSDLALYDIAPVTPGVAADLSHIPTPVSIKGYAG

EDPTPALEGADVVLISAGVARKPGMDRADLFNVNAGIVKSLAEKIAVVCPKACVGIITNP

VNTTVPIAAEVLKKAGVYDKRRLFGITTLDVIRSETFVAELKGKDPSDIRVPVIGGHSGV

TILPLLSQVEGVEFTAEEVEALTKRIQNAGTEVVEAKAGGGSATLSMGQAACRFGLALVR

ALQGEEGVVECAYVEGDSEHAPYFAQPVKLGKEGVEEVLSYGELSDFEKAALDGMLETLN

GDINIGVEFAK

>sp|Q87FQ5|MALT_VIBPA HTH-type transcriptional regulator MalT OS=Vibrio parahaemolyticus serotype O3:K6 (strain RIMD 2210633) OX=223926 GN=malT PE=3 SV=1

MWIPSKLTRPGRLHNAIVRPRVLDLLQQAPYYKLVLFRSPAGYGKTTMAAQWLSDKPNVG

WYSIDDSDNDGFRFVNYLLQALNKATNFSCSNAQKLAEKRQISSLRSLFSEVFAEMADFH

QECYVVLDDYHLITNDEIHESMRFFLKHMPDNLTVVVTSRAAPPLGTANLRVRDLMIEIG

NEMLAFDTEETTRFFNQRIADGIDEDMANSLRTYVEGWPSAMQLIALQAQHQNRTLAQTV

ESVSQFNHAHLWDYLVEEVFDLLDHETRHFLMQVSVLDHFNDELVFALTQREDALGLIES

LNRYGLFIYPLEGEHNWFRFHNLFGEFLSHERQARIPQQEKDLHRNAAVAWLQQKSPHQA

IHHAQKSNDKDLVVEILNEFGWKMFNQGELSTLEHAINKLDAELLFSHPKLTMLRAWLAQ

SQHRYNQVGQLLEEAEEEHKKRNIELDIHYQGQANALLAQVAINSNQPEKALELAELALS

QLDNTIYRSRIVATSVVGEVNHVLGKLDRALPMMQQTEKLARQYQVYHQALWAILQQSEI

LIAQGYVQAAFELQDSGFRLIEDQQLQHVPLHEFLLRIRAQVLWCWNRLDEAEECAYRGL

QILENHSPSKHLHSYSMLARIAIGRGELDKAGKFIEHIQHLMKQSTYHVDWTANASLSLI

LFWQARGNTEAMQEWLNTAVRPESACNHFLQLQWRNIVRAHINLGQYEEARQALNFLQSE

ARRTNLITDTNRNLVVEAVLAARQKDEEQAKALLKEALVMTNQTGMVGNFLIDGATIGGL

LEKLSLRHELGDLERHRAQQLMKDISSNQRSRSIHFDEDFIEKLVNHPNVPELVRTSPLT

QREWQVLGLIYSGFSNEQIAQELDVAGTTIKTHIRNLYQKLNIANRKEAIVTAENLLQLM

GY

>sp|Q87NW7|METAS_VIBPA Homoserine O-succinyltransferase OS=Vibrio parahaemolyticus serotype O3:K6 (strain RIMD 2210633) OX=223926 GN=metAS PE=3 SV=1

MPIRIPDQLPASDVLRTENIFVMSETRAASQEIRPLRVLILNLMPKKIETETQFLRLLSN

SPLQVNVELLRIDNRPSKNTPTEHLDTFYRQFEMVKGKNFDGLIITGAPLGLVQFEDVIY

WDHLKTIMEWAKDHVTSTLYVCWAAQAGLKLLYDLPKKTRKEKLSGVYHHQIHNPFHPIL

RGFDDTFLAPHSRYADFSPHFLEEHTDLDILATSDVAGVYLATTKDKRNVFVTGHPEYDS

HTLHNEYIRDLGEGMEPAIPVNYYPNNNPDNPPIASWRSHGHLLFLNWLNYCVYQQTPYD

LDHFSEDAFTKDD

>sp|Q87RN6|LOLB_VIBPA Outer-membrane lipoprotein LolB OS=Vibrio parahaemolyticus serotype O3:K6 (strain RIMD 2210633) OX=223926 GN=lolB PE=3 SV=2

MTLRSFLIFFLSSLILAGCSSVPESVTSVEWQAHEQRLETIHDFQATGKLGYIGPDQRQS

LNFFWKHSTALSQLRLTTVLGQTALKLTITPQGATVETYDDQVLSARNANQLIYRLTGLM

MPVDHMPDWLLGLPTDADTFQLSPANTLQTLDKQIGLNDWKIAYERYGDVEWHEQTLPLP

NKLKLTTSDVKINLVITKWNITQ

>sp|Q87R20|LOLD_VIBPA Lipoprotein-releasing system ATP-binding protein LolD OS=Vibrio parahaemolyticus serotype O3:K6 (strain RIMD 2210633) OX=223926 GN=lolD PE=3 SV=1

MNKLLECRDIRKVYREGSLDTEVLKGVSFDIDKGELVSIVGSSGSGKSTLLHILGALDDA

TQGEVDFLGQNLSALSSNKQAALRNKHLGFVYQFHHLLADFTALENVAMPLLIGGIKVTE

AKQAAKALLEKVGLSHRMDHRPSELSGGERQRVAIARALVNKPDLVLADEPTGNLDHNTA

LAIYDLMRELNKESNIAFLVVTHDNELAAKMDRQMHMQDGLLVDRLMTESASVEG

>sp|Q87GU4|LUXP_VIBPA Autoinducer 2-binding periplasmic protein LuxP OS=Vibrio parahaemolyticus serotype O3:K6 (strain RIMD 2210633) OX=223926 GN=luxP PE=3 SV=1

MNKALVLSLISIAGMSPASQASQVLNGYWAYQEFLNEFPEQKHLTDALAAAVRDDPVPIA

PEKRHPLKISVVYPGQQISDYWIRNIDAFEKRLDKLNIDYQINQVFTRPNADIKQQSLSL

MEALKSNSDYLIFTLDTTRHRKFVEHVLDSTKTKLILQNITTPVREWETRQPFMYVGFDH

AEGSRELAVEFGKQFPKNTHYSVLYFSEGYISDIRGNTFIHQVNQDSQFELQSAYYTKAT

KQSGYEAAKASLKKYPDVEFIYACSTDVALGAVEALSELGREDVMINGWGGGSAELDAIL

KGELDITVMRMNDDTGIAMAEAIKWDLEGKPVPTVYSGDFEVVTKSDSPERIEALRKRAF

RYSDN

>sp|Q87GB7|MALF_VIBPA Maltose/maltodextrin transport system permease protein MalF OS=Vibrio parahaemolyticus serotype O3:K6 (strain RIMD 2210633) OX=223926 GN=malF PE=3 SV=1

MQSVQGTNAMTAPEASLPSSKKVFIKWSLLGTVGILNGYATILMYSRGEIAFALLTIILT

ALALFIFGSKKTYAHRYIYPGIAGMILFILFPLAYTIGLAFTNYSAKNQLSFDRAQSVLL

DRTYQSGDSYPFTLYNTDQGHQIVVEKDGELLATPVFQLQGFSETDLDLAPITEAAGDKE

PIKTIVKNRTALSSVDLHLPNGDDIRMSGLRKFAAVVPLYTLQEDGETLYNNRTQETLRP

NMEVGYYQPVDENGQFVGSTVSPGFVVNIGTHNFERVWKDDGIKEPFISIFIWTIVFSAL

TVVCTLVIGLVLASVVQWEALKGRSIYRLLLILPYAVPAFISILIFKGLFNQSFGEINML

LEGLFGISPAWFSDPFMAKTMILIVNTWLGFPYMMILCMGLLKAIPDDLYEASAIDGANF

ITNFTRITMPMMLKPLTPLLIASFAFNFNNFVLIQLLTGGGPNMIGTSEPAGYTDLLVSY

TYRIAFEGAGGQDFGLASAVATLIFLLVGALALINLRVTKVAQD

>sp|Q87S89|LSPA_VIBPA Lipoprotein signal peptidase OS=Vibrio parahaemolyticus serotype O3:K6 (strain RIMD 2210633) OX=223926 GN=lspA PE=3 SV=1

MSEKALTLKQSGVRWLWLAIVIFLADIGIKYVVMNNMGYGWANRIEILPFFNLLYVHNYG

AAFSFLSDQAGWQRWLFTGIAFVVTGLLTYWMSKLPAKEKWNNIAYAMIIGGAVGNVFDR

VIHGFVVDYLDFYWGNYHWPAFNLADMAICLGAAMIILDGFRKKDTAKA

>sp|Q87MX7|LUXO_VIBPA Regulatory protein LuxO OS=Vibrio parahaemolyticus serotype O3:K6 (strain RIMD 2210633) OX=223926 GN=luxO PE=3 SV=1

MVEDTASVAALYRSYLTPLGIDINIVGTGRDAIESLNHRIPDLILLDLRLPDMTGMDVLH

AVKKSHPDVPIIFMTAHGSIDTAVEAMRHGSQDFLIKPCEADRLRVTVNNAIRKATKLKN

EADNPGNQNYQGFIGSSQTMQQVYRTIDSAASSKASIFITGESGTGKEVCAEAIHAASKR

GDKPFIAINCAAIPKDLIESELFGHVKGAFTGAANDRQGAAELADGGTLFLDELCEMDLD

LQTKLLRFIQTGTFQKVGSSKMKSVDVRFVCATNRDPWKEVQEGRFREDLYYRLYVIPLH

LPPLRERGEDVIEIAYSLLGYMSHEEGKNFVRFSQEVIDRFNSYEWPGNVRQLQNVLRNI

VVLNNGKEITLDMLPPPLNQPLDRPSVSKLIEPKAMTVSEIMPLWMTEKMAIEQAIEACD

GNIPRAAGYLDVSPSTIYRKLQAWNGKEERQKV

>sp|Q87GU5|LUXQ_VIBPA Autoinducer 2 sensor kinase/phosphatase LuxQ OS=Vibrio parahaemolyticus serotype O3:K6 (strain RIMD 2210633) OX=223926 GN=luxQ PE=3 SV=1

MTITSKLKKRRSLSTLITKIIILVLAPIILGIFVQSYYFSKQIIWQEVDRTKQQTSALIL

NIFESHFAAIQIHHDSNSKSDVILDFYSERNEEALNYFFLSIDQSDPSHTPEFRFLTDHQ

GIIWDDGNAHFYGINDSMLDGLTSKVTFSNNWYYVTSITSMGARHLLLRRVPVLEPKTGE

VMGYSYNAVVLDNNFALMEKLKNEGNVDNVVLVANDIPVASSLAGDESYKIFDVLKRKET

QKKLDQLLIIQTPIEVNAAITNLKLLTVQDNQSVVTLQIQHFLAMLASVIGMIMIALMTK

EWIENRVVEELGSLMSYTRSAREEKGFERFGGSDIEEFDHIGSTLESTFEELEAQKRSFR

DLFNFALSPIMVWSEAGVLIQINPAARKELVIENDIETMHPVFKGFKDKLVPHLRMAAQG

ATLTGVNVPIGDKVFRWNLSPIRVDGDISGIIVQGQDITTLIEAEKQSNLARREAEKSAQ

ARADFLAKMSHEIRTPINGILGVAQLLKDSVEAEEQKNQIDVLRHSGEHLLAVLNDILDF

SKIEQGKFNIQKHPFSFADTMRTLENIYRPICENKGVELVIENQLDGNVEIFTDQVRLNQ

ILFNLVSNAVKFTPSGCVRLHAELEQFYGADNSVLVVEISDTGIGIESDKLDEMFEPFVQ

EEATTTREYGGSGLGLTIVKNLVDMLDGDVQVRSQKGQGTTFVVTLPVKDRERVLAPLDS

SQRVKPAELFDESLKVLLVEDNHTNAFILKAFCTKYKMQVDWAKDGLEAMEFLKDHSYDL

ILMDNQLPHLGGIETTKEIRQNLKLGTPIYACTADTAQETSDAFMEAGANYVLLKPIKEN

ALHEAFVDFKQRFLIERT

>sp|Q87MX8|LUXU_VIBPA Phosphorelay protein LuxU OS=Vibrio parahaemolyticus serotype O3:K6 (strain RIMD 2210633) OX=223926 GN=luxU PE=3 SV=1

MTILNQQKIDELSIEIGSDNVPVLLDIFLGEMDTYIDNLSQLEGSERLMYLKEISHALKS

SAASFGADSLCELAMSIDKKAKSGELVEQGSEVNTMLDRLNETRDAYRSWTQ

>sp|Q87JM4|MACB_VIBPA Macrolide export ATP-binding/permease protein MacB OS=Vibrio parahaemolyticus serotype O3:K6 (strain RIMD 2210633) OX=223926 GN=macB PE=3 SV=1

MSDVLLKVEDLTRRFVSGDESLTVLNHINLEIKRGEMVAIVGASGSGKSTLMNVLGCLDK

PSSGRYFINGQDVSTLESDQLAELRREYFGFIFQRYHLLGDLTAVANVEVPAVYAGVPHR

QRTERAQSLLARLGLEDRLTHKPSQLSGGQQQRVSVARALMNGGEVILADEPTGALDSHS

GQEMMALLKELHQLGHTIILVTHDMNVANFADRIIEIKDGEIIADTLNAQVVINEQAAKT

PSASFHRPAQAVSKWWKWDSFIDALKMALLAMSSHRMRTFLTMLGIIIGIASVVSVVALG

NGSQQQILSNISSMGTNTIDVRPGKGFGDRRSGRVKTLTADDAKSLESLPFVDSVTPSLS

NSLTVRYANQDATASVEGVGEDYFRVRGYEIAKGQFWDEESVNSLAQEAVIDDNTRKEMF

ADRNPIGEVIFLGSLPVRIVGVTQKKEDAFGNSDALKIWVPYTTMSGRMMGQRYLNGITV

RIDENAPSAAVEQSIINLLKMRHGTEDFFTINTDTIRQSIEKTTATMTLLISAIAVISLI

VGGIGVMNIMLVSVTERTKEIGVRMAVGARQADILRQFLIEAVLVCLCGGIAGIGLAFLI

GFAFSTSGSSFQMIYSMNSIIWAFICSTLIGIAFGFLPARNAAKLDPIEALARD

>sp|Q87ME9|LPXA_VIBPA Acyl-[acyl-carrier-protein]--UDP-N-acetylglucosamine O-acyltransferase OS=Vibrio parahaemolyticus serotype O3:K6 (strain RIMD 2210633) OX=223926 GN=lpxA PE=3 SV=1

MIHETAKIHPAAVVEEGAKIGANVTVGPFTYITSTVEIGEGTEVMSHVVIKGHTKIGKDN

RIFPHAVIGEENQDKKYGGEDTTVVIGDRNVIREAVQVHRGTVQDKATTVIGDDNLLCVN

AHIAHDVVVGNHTHIGNNAILGGHVTVEDHAGVMALSAIHPFCTVGAYAYVGGCSAVVQD

VPAYVLAQGNHATPFGLNLVGLKRNGFEKPEIRALQKAYKEIYRSGKTLEEVKPILAEMA

QEWPAVKRFSDILETTERGIIR

>sp|Q87MF0|LPXB_VIBPA Lipid-A-disaccharide synthase OS=Vibrio parahaemolyticus serotype O3:K6 (strain RIMD 2210633) OX=223926 GN=lpxB PE=3 SV=1

MEKPLRIGIIAGELSGDTLGEGFIKAVKERYPNAEFVGIGGPKMIAQGCESLFDMEELAV

MGLVEVLGRLPRLLKVKAELVKYFTQNPPDVFVGIDAPDFNLRLELDLKQAGIKTVHYVS

PSVWAWRQKRIFKIEAATNLVLAFLPFEKAFYDKFNVPCEFIGHTLADAIPLQSEQAPAR

DLLGLEQDKKWLAVLPGSRGSELKMLSQPFIETCKLLHQKYPGLGFVVALVNQKRREQFE

QAWKEHAPELDFKLVDDTARNVITASDAVMLASGTVALECMLLKRPMVVGYRVNTFTAFL

AKRLLKTKYVSLPNILADDELVKEYLQDDCTPDNLFNEVSRLLESDNKPMLDKFTEMHHW

IRKDADQQAANAVLKLIEK

>sp|Q87QK1|LPXH_VIBPA UDP-2,3-diacylglucosamine hydrolase OS=Vibrio parahaemolyticus serotype O3:K6 (strain RIMD 2210633) OX=223926 GN=lpxH PE=3 SV=1

MTTLFISDLHLTPSRPDITECFITFMRTEAKNAEALYVLGDLFEFWVGDDDKTPFANQIR

TEFKALTDQGVPVFFIQGNRDFLLGERFCKETGITLLDDVCTIDLYGTKAVILHGDTLCI

DDVEYQKFRKTVHQPWLQWIFKRIPWYLKKKIVSKVQSDIRDDKQMKSLDIMDVNQSEVE

KVMSQNCVNLMIHGHTHRPNTHFFDANGAKNTRIVLGDWYTQGSVLQVNSDGFELQNRPF

NT

>sp|Q87R15|LPXK_VIBPA Tetraacyldisaccharide 4'-kinase OS=Vibrio parahaemolyticus serotype O3:K6 (strain RIMD 2210633) OX=223926 GN=lpxK PE=3 SV=1

MVEKIWFENHPLKYLLWPLLWPLSVLFGAISRSKRQQFQTGRKQAYQAPVPVVVVGNITA

GGNGKTPVVVWLVEQLQHLGYKPGVVSRGYGAKAPQYPLVLNDDTPTQHCGDEPKLIHRR

TGAPVAVDPVRANAVKALVELDVDIIITDDGLQHYALERDVELVIVDGNRRFGNECLIPL

GPLREGVERLQEVDFIITNGGLAHQGEISMSLAPSKAINLKTKQQVDVSELKALVAFAGI

GHPPRFFNTLESMHADVKVTKGFADHQDFDQKELEALALQGANVIMTEKDAVKCSDYAQD

NWWYLPVSAQLEPKDAERILNRIKEVKATYGSPSA

>sp|Q87LS4|LUXS_VIBPA S-ribosylhomocysteine lyase OS=Vibrio parahaemolyticus serotype O3:K6 (strain RIMD 2210633) OX=223926 GN=luxS PE=3 SV=1

MPLLDSFTVDHTRMNAPAVRVAKTMQTPKGDTITVFDLRFTAPNKDILSEKGIHTLEHLY

AGFMRNHLNGDSVEIIDISPMGCRTGFYMSLIGTPSEQQVADAWLASMEDVLKVESQNKI

PELNEYQCGTAAMHSLEEAQQIAKNILAAGVSVNKNDELALPESMLKELRVD

>sp|Q87ME7|LPXD_VIBPA UDP-3-O-acylglucosamine N-acyltransferase OS=Vibrio parahaemolyticus serotype O3:K6 (strain RIMD 2210633) OX=223926 GN=lpxD PE=3 SV=1

MKKLTLAELATITGGELFGDESLVVGRVAPMDKAQEGDVTFLSNPKYAKHLSECKATVVM

VKAEHKDQCAGNALVVADPYVAFARVVQAMDTTPKPAEDIAPSAVIASDVKMGENVAIGA

NAVIETGVELGDNVVIGAGCFIGKNAKLGNNTKLWANVTIYHEVSLGDDCLVQSGTVIGS

DGFGYANDRGEWIKIPQLGSVRIGNRVEIGACTTIDRGALEDTIIEDNVILDNQLQIAHN

VQIGYGTVMPGGTIVAGSTKIGKYCQIGGASVLNGHITIADGVAITGMGMVMRSIEEKGL

YSSGIPLQTNREWRKTATRVHRIDEMNKRLKAVEKQLEQKEES

>sp|Q03476|LAFL_VIBPA Flagellar protein LafL OS=Vibrio parahaemolyticus serotype O3:K6 (strain RIMD 2210633) OX=223926 GN=lafL PE=2 SV=2

MTKQQMIAMFIAMIITSALVSAATIMGGIWYLNKQAQDSGETSSLLENSPLSFLVTEQPT

SKGPSFHPLDKVVLSIKGKKQTHFVMLELAIETRRPERIKDIDNYMPMVQNSLLKLFSDK

TFDELQQTGAIDILQNEVKQTLLVAFAKTDIVRDIDDVLLTKYVVQ

>sp|Q87SS9|LEUC_VIBPA 3-isopropylmalate dehydratase large subunit OS=Vibrio parahaemolyticus serotype O3:K6 (strain RIMD 2210633) OX=223926 GN=leuC PE=3 SV=1

MGKTLYEKVYDAHVAVAAEGETPILYIDRHLVHEVTSPQAFDGLREKGRKVRQVSKTFAT

MDHNVSTTTKDINASGEMARIQMETLSKNCEEFGVTLYDLNHKYQGIVHVMGPELGITLP

GMTIVCGDSHTATHGAFGSLAFGIGTSEVEHVLATQTLKQARAKTMKIEVKGKVAPGITA

KDIVLAIIGKTTAAGGTGYVVEFCGEAITDLSMEGRMTVCNMAIELGAKAGLIAPDETTF

EYIKGRKFSPQGADFDAAVEYWKTLKTDADAEFDAVVTLNAADIKPQVTWGTNPGQVIAV

DQPIPAPESFTDPIEKASAEKALAYMGLEAGKSLSDYQVNKVFVGSCTNSRIEDMRAAAV

VAKGRKVASHVQALIVPGSEQVKAQAEAEGLDVIFKEAGFEWRLPGCSMCLAMNNDRLGP

HERCASTSNRNFEGRQGRDGRTHLVSPAMAAAAAIAGHFVDIRELTFDKQD

>sp|Q87RR1|LIPA_VIBPA Lipoyl synthase OS=Vibrio parahaemolyticus serotype O3:K6 (strain RIMD 2210633) OX=223926 GN=lipA PE=3 SV=1

MSKPIQMEKGVKYRDADKMALIPVKNMPTEQKEVLRKPDWMKIKLPADSQRIQDIKAAMR

KNKLHSVCEEASCPNLAECFNHGTATFMILGAICTRRCPFCDVAHGRPLPPEAEEPQKLA

RTIADMKLKYVVITSVDRDDLRDGGAQHFADCNREIRELNPDIKIETLVPDFRGRMDVAL

DLMKDNPPDVFNHNLETAPRLYRKARPGANYKWSLELLKKFKEQHPDVPTKSGLMMGLGE

TKEEIIEVLKDLRAHGVTMLTLGQYLAPSRHHLPVERYVPPSEFDELKEVALELGFTHAA

CGPFVRSSYHADMQAQGLEIK

>sp|Q87LN7|LEPA_VIBPA Elongation factor 4 OS=Vibrio parahaemolyticus serotype O3:K6 (strain RIMD 2210633) OX=223926 GN=lepA PE=3 SV=1

MKHIRNFSIIAHIDHGKSTLSDRLIQVCGGLSDREMAAQVLDSMDLERERGITIKSQSVT

LNYTAKDGETYQLNFIDTPGHVDFAYEVSRSLAACEGALLVVDAGQGVEAQTLANCYTAI

EMDLEVVPILNKIDLPAADPERVAEEIEEIVGIDAMDATRCSAKTGLGVEDVLENIVSAI

PAPEGDPDAPLQALIIDSWFDNYLGVVSLVRIKNGSLKKNDKIKVMSTGQAWGVDRLGIF

TPKQVDTDVLNTGEVGWVVCGIKDILGAPVGDTLTLAKNGSDKPLPGFKKVKPQVYAGLF

PVSSDDYENFRDALGKLSLNDASLFYEPENSAALGFGFRCGFLGMLHMEIIQERLEREYD

LDLITTAPTVVYEVEKTDGELLYVDSPAKLPAINDIEEIREPIARCNILVPSDYLGNVIT

LCVEKRGLQVDMVYHGNQVAVTYDIPMAEVVLDFFDRLKSTSRGYASLDYNFQRFEASNM

VRVDVLLNGDKVDALALITHKDQSQTRGRQLVEKMKEFIPRQMFDIAIQAAIGNHIIARS

TVKQLRKNVIAKCYGGDVSRKKKLLKKQKEGKKRMKQIGNVELPQEAFLAILHVGKD

>sp|Q87QJ8|KITH_VIBPA Thymidine kinase OS=Vibrio parahaemolyticus serotype O3:K6 (strain RIMD 2210633) OX=223926 GN=tdk PE=3 SV=1

MAQMYFYYSAMNAGKSTTLLQSSFNYQERGMTPVIFTAALDDRYGVGKVSSRIGLQSDAH

LFRPDTNLYQEIAALHEVEKRHCILIDECQFLSKEQVYQLTEVVDKLHIPVLCYGLRTDF

LGELFEGSKYLLSWADKLVELKTICHCGRKANMVIRTDEHGVAIKEGDQVAIGGNDRYVS

VCRQHYKEALGK

>sp|Q87G18|LLDD_VIBPA L-lactate dehydrogenase OS=Vibrio parahaemolyticus serotype O3:K6 (strain RIMD 2210633) OX=223926 GN=lldD PE=3 SV=1

MIISASTDYRAAAKAKLPPFLFHYIDGGSYDERTLKRNTDDLGDVALRQRVLRDMTDLSL

ETEIFGEKLAMPIALAPVGLTGMYARRGEVQAAKAAEKKGIPFTMSTVSVCPIEEVAPAI

ERPMWFQLYVLKDRGFMKNVLERAKAAGVTTLVFTVDMPVPGARYRDMHSGMSGPNAAMR

RVFQAMRHPSWALDVGLLGKPHDLGNISTYRGEPTKLEDYIGWLGANFDPSISWKDLEWI

RDFWDGPMVIKGILDEEDAKDAVRFGADGIVVSNHGGRQLDGVLSTAKALPSIADAVKGD

LKIFVDSGIRTGLDVVRMLALGADCTLLGRSFVYALAAQGGAGVENLLDLYDKEMRVAMT

LTGAKTIADLSRDSLVKIP

>sp|P46235|LGUL_VIBPA Probable lactoylglutathione lyase OS=Vibrio parahaemolyticus serotype O3:K6 (strain RIMD 2210633) OX=223926 GN=gloA PE=3 SV=2

MSNGRILHTMLRVGDLDKSIKFYTEVMGMQLLRTNENKEYEYTLAFVGYGDESQGAVIEL

TYNWGKTEYDLGTAFGHIAIGVDDIYATCDAIKAAGGNVTREAGPVKGGTTHIAFVKDPD

GYMIELIQNKQASAGLEG

>sp|Q87QY1|LFTR_VIBPA Leucyl/phenylalanyl-tRNA--protein transferase OS=Vibrio parahaemolyticus serotype O3:K6 (strain RIMD 2210633) OX=223926 GN=aat PE=3 SV=1

MAIYLTELDSTFNFPSPYEALSDPNGLLAFGGDLDPHRILSGYYQGIFPWYGPGEPILWW

SPSPRAVFDPLTFKPSKSLKKFQRKHQYKVTINQATEHVIQLCSSTRPADETWLNEEMQA

AYIQLSNLGHCHSVEVWHDNTLVGGLYGISVGQLFCGESMFSLKDNASKIALWYLCTHLA

SKHGQLIDCQVMNPHLASLGAFELDRDEFIQKLLSLREKQTASDTFTPQVLQDSES

>sp|Q87SA1|LGT_VIBPA Prolipoprotein diacylglyceryl transferase OS=Vibrio parahaemolyticus serotype O3:K6 (strain RIMD 2210633) OX=223926 GN=lgt PE=3 SV=1

MSQGYLQFPNIDPVLVSIGPVSIRWYGLMYLVGFMFALWLANRRADKPGSGWTREQVSDL

LFAGFLGVVIGGRVGYVIFYNFELFLDDPLYLFKVWTGGMSFHGGLLGVITAMFWYAHKN

GRTFFGVADFVAPLVPFGLGMGRMGNFMNSELWGRVTDVPWAIVFPNGGPLPRHPSQLYE

MLLEGVVLFFILNWFIKKPRPLGSVSGLFLAGYGTFRFLVEFVREPDAQLGLFGGYISMG

QILSMPMIVLGILMMVWAYKRGLYQDKAQVKTK

>sp|Q87QP3|LOLA_VIBPA Outer-membrane lipoprotein carrier protein OS=Vibrio parahaemolyticus serotype O3:K6 (strain RIMD 2210633) OX=223926 GN=lolA PE=3 SV=1

MKKRFSAKLFSALVLSISFFSAANAASPKDELNKRLAMNEGFSADFSQQVISPEGETVME

GEGTVEIARPSLFRWSTTFPDENLLVSDGKTLWYYSPFIEQVSIYWQEQATEQTPFVLLT

RNRASDWDNYKISQKGNEFTLIPTAVDSTQGQFQINIDAKGVVKGFNVIEQDGQKGLFTF

SNVKLGKPKADRFTFTVPKGVEVDDQRN

>sp|Q03474|LAFS_VIBPA RNA polymerase sigma factor for flagellar operon OS=Vibrio parahaemolyticus serotype O3:K6 (strain RIMD 2210633) OX=223926 GN=lafS PE=2 SV=1

MLDMNPQETYTAPEEVNTPSRPIDENALLQRHQVMVKRVVNQLRVHATSHCSIEDMQQIG

LIALVEAGRRYGDIDDTHFPAFAVCRVRGAILDELRRLDWRSRKTRQQAHELNDVTRDLT

RSLGRMPTDSEIIKALGTDEQDYYNRQNAALAGEMQSLDQLMENSTDSHFGGQYDGMEHE

HIRRSLDSALGRLSKRDQLLLTLFYQHELNLHEIALVLDLTPPRICQLHKQALKQLNQLM

SS

>sp|P59494|LAMB_VIBPA Maltoporin OS=Vibrio parahaemolyticus serotype O3:K6 (strain RIMD 2210633) OX=223926 GN=lamB PE=3 SV=1

MKKVSVIAAAVAATLAAGSAFAVDFHGYMRAGVGVNADGGQQLTFEKNKVGRLGNESDIY

GEIQLGKEVYNNNGKTFYVDSMLAMTSNGSNDWEGTAANCGLDGTKVKCVDDAQFALRQF

NVQAKGVLNFAPEATLWAGKRYYQRHDIHISDFYYWNISGAGAGVEGIEAGPGKLSFAWV

RNDRNDNFNSGMGSGDTPDVGNGGSVNVNTLDVRYAGIPVWENGSLEVGLDYALVNETED

ASKAAKDAKDGVMFTAELTQGLDSGFNKTVFQYGTEGYSKAFAFYGDGSWYGAEARDGAS

GYRFINWGVIGLGDNWELGHQLVYGVGEDMWAADHKWEAMSAVVRPVFKWDDNHKTIFEA

GYAIDDNDGDENKYGKLTVAQAWSAGSSFWARPEIRLYASYLTADKADNSNTFDSGRSDD

TFQFGVQAEAWW

>sp|Q03477|LAFT_VIBPA Chemotaxis protein LafT OS=Vibrio parahaemolyticus serotype O3:K6 (strain RIMD 2210633) OX=223926 GN=lafT PE=2 SV=1

MQKFLGVLTILVCVFGGYMWAGGKLGAIWQPAEFLIIIGAAAGSLIIGNPPHVLKEMRQQ

VPATIKGPTEEYEYYMELMALLNNLLETARSRGFKFLDSHIEAPEQSSIFLMYPLVSEDH

RLISFITDNLRLMAMGQMSPHELEGLLEQEIEAIQNELLLPSRSLQRTAEALPGFGILAA

VGGIIITMQAIDGSIALIGYHVAAALVGTFIGIFGCYCGLDPLSNAMAQRVKRNMTAFEC

VRATLVAYVAKKPTLLAIDAGRKHIQLDIKPTFNQMEKWLAEQEG

>sp|Q03478|LAFU_VIBPA Chemotaxis protein LafU OS=Vibrio parahaemolyticus serotype O3:K6 (strain RIMD 2210633) OX=223926 GN=lafU PE=2 SV=2

MQKQEHVVFKRAKAHGHDEPHGGAWKVAFADFMIALMALFLVLWVMQVVDKEERKAIVAH

LHSSSVFDKSYGNPFDTSQSISPIDLAQDSSVPSKHNSNHVVSSYFQGDGDGPEINSLVP

GTFDTQEQLAALAKVIEEMTAQINAQGNVNVTVTPQGLRIVLQDDYKQHMFSRGGAELTP

FFEDLLLALAPLFEQVTNPLIISGHTDAIPFKKRFGRQSNWALSASRADVARKTLVEGGM

PDDRVMQVTGMSDRALLNPDEPDSSENRRIELFILTTPAAKVLETLFGNQDDSELQKAKQ

KAEFNQPVIRQEVIRYSADAEKQEAKIQAL

>sp|Q87RR0|LIPB_VIBPA Octanoyltransferase OS=Vibrio parahaemolyticus serotype O3:K6 (strain RIMD 2210633) OX=223926 GN=lipB PE=3 SV=1

MQHQLVVKRLGRQDYEPVWKAMHEFTDQRTEETPDEVWLVEHNPVFTQGQAGKAEHLINT

GDIPVVQSDRGGQVTYHGPGQLVAYFLINLRRKKLGVRDLVTTIENLVINTLKAYNIDSA

ARPDAPGVYVDGKKICSLGLRIRKGCSFHGLALNVNMDLTPFLRINPCGYAGMEMVQVSQ

FNGPSDVETVEKQLIEELVTLLDYERVEFSTEAPSQGNKA

>sp|Q87SS7|LEU1_VIBPA 2-isopropylmalate synthase OS=Vibrio parahaemolyticus serotype O3:K6 (strain RIMD 2210633) OX=223926 GN=leuA PE=3 SV=1

MNDQVIIFDTTLRDGEQALSASLTVKEKLQIAYALERLGVDVIEAGFPISSPGDFESVQT

IAKHIKNSRVCALSRAVAKDIDAAAEALKVADQFRIHTFLATSTIHVQDKLRRSYDDVLE

MAVNAVKHARNYTDDVEFSCEDAGRTPIDNLCRMVEAAIDAGASTINIPDTVGYTVPSEF

GGIIQTLFNRVPNIDKAIISVHCHDDLGMSVANSIAAVQAGARQIEGTINGIGERAGNCS

LEEIAMIIKTRQELLGVSTGIKHEEIHRTSKLVSQLCNMPIQSNKAVVGANAFSHSSGIH

QDGMLKNKNTYEIMTPESIGLKNQALNLTSRSGRAAVKSHMDSMGYKEDEYNLDALYADF

LKLADRKGQVFDYDLEALMHFSNLREEDDFYKLNYLSVQSGSVMATTSIKMQCGDAEMCE

AAVGNGPVDALYQCIYRVTGYEIVLDKFDLTAKGEGEDGLGQADIIANYKGRKYHGTGVS

TDIVEASGQALLHVINSIHRADTIAEMKQKKIATV

>sp|Q87SS8|LEU3_VIBPA 3-isopropylmalate dehydrogenase OS=Vibrio parahaemolyticus serotype O3:K6 (strain RIMD 2210633) OX=223926 GN=leuB PE=3 SV=1

MTDKSYKIAVLPGDGIGPEVMAQAHKVLDAIEQKHGISFEREEHDVGGIAIDNHGCPLPE

STVTACEESDAVLFGSVGGPKWEHLPPNDQPERGALLPLRKHFQLFCNLRPAQIHAGLEA

FSPLRADISGRGFDIVVVRELTGGIYFGQPKGREGEGANEKAFDTEVYHRYEIERIAKIA

FESARLRRKKVCSIDKANVLQSSILWREVVEEIAKDYPDVELSHMYIDNATMQLIKDPAQ

FDVMLCSNIFGDIISDECAMITGSMGMLPSASLNESKFGLYEPAGGSAPDIAGKNIANPV

AQILSAALMLRYSLGEEAAAQDIESAVSKALSAGELTADLASDKPALTTSEMGDKIAEYI

LNS

>sp|Q87ST0|LEUD_VIBPA 3-isopropylmalate dehydratase small subunit OS=Vibrio parahaemolyticus serotype O3:K6 (strain RIMD 2210633) OX=223926 GN=leuD PE=3 SV=1

MSGFKQHTGLVVPLDAANVDTDAIIPKQFLQKVSRLGFGKHLFHDWRFLDDAGERPNPEF

VMNAPRYQGASILLARENFGCGSSREHAPWALADYGIKAMIAPSFADIFYGNSINNQMVP

VRLTEQEVDELFQFVEANEGAQIEVDLEALKVRANGKEYDFEIDEFRRHCLLNGLDNIGL

TLQHEDKIAEYEANIPSFLR

>sp|Q87ST3|LPTD_VIBPA LPS-assembly protein LptD OS=Vibrio parahaemolyticus serotype O3:K6 (strain RIMD 2210633) OX=223926 GN=lptD PE=3 SV=1

MQHFSRTFIAASISTALFVPTTQAEANINDSVQEMPATDQCLVETSGEEDALNTPVVVEA

DNLQAINGDKAQYSGNVQVTQGPKKITADSVTLHQQDNVVVAEGNVTFNDGQVKARSDRV

TNDINQDTFSLENTEYQFLCQQGRGTAAYIARTGQSVYELEDGSITSCPEGDNSWRLVAS

GIDVDQDEETATLHHPRFEIMDVPVFYVPYMTMPIGNTRKTGFLFPSLSYGSSDGMEVEV

PFYWNIAPQYDMTLTALYMQQRGTKLDTDFRYLTDGWGEGKLKGEYLNSDKKYQDDSRWG

YQVKHDGIINKQWIVKVDYSQVSDIDYFLDLDSDIGNREDGQLVQEGHVQYRSDFWDASL

TVRDFQILLKEENRPYRLLPQLDLNYYTPLWGDHLNFDVKSQVSRFDTSDTARPNATRVH

IEPGLTMPLSNSWATWTTEARVLSTYYSQDLTGLTDVDLKNQLDEQVSRVIPEFRTHAQI

YLERDTSWVKGYTQTLEPQLQYLYVPEEDQTNIYNYDTTLLQTDYYGLFRSRKYSGIDKI

ASANQLSYGASTRFFDDDYKERLNVSFGQIYYFDKKTKISNSPNIPDETTNYSSWAVEAD

FNYNDYLFYHGGVQYDIDLSSMQLANSTLEYQFNGGFIQGNYRYVTREYIEDTIILENLD

TITRKGISQAGIVAAYEFNRNWSASGQYYYDLNENTDMEWLASLRYQSDCWYIGLTYSNQ

LLGWENQAIGSTGSSPEYENNFSVNFGIQGFATNQSTGTAVKELDGSDNAIKYGRPFYLN

N

>sp|Q87QK7|HIS4_VIBPA 1-(5-phosphoribosyl)-5-[(5-phosphoribosylamino)methylideneamino] imidazole-4-carboxamide isomerase OS=Vibrio parahaemolyticus serotype O3:K6 (strain RIMD 2210633) OX=223926 GN=hisA PE=3 SV=1

MIIPALDLIEGQVVRLYQGDYGQVTEYKVDPAEQFNLYHQAGADWLHLVDLTGAKDTSAR

QLDLIAKLLASTPANIQIGGGVRTEQDVVDLLEAGAQRVVVGSTAVKQPELVKGWMEKYG

AEKIVLALDINIDQDGTRKVAISGWQEDSGVTIEALINDYLTVGLQHVLCTDISRDGTLE

GSNVELYVDLCKQYPQVQFQSSGGIGSLADIEALKGSGVAGVIVGRALLDGKFTAEEAFA

CWQSE

>sp|Q87QK5|HIS2_VIBPA Histidine biosynthesis bifunctional protein HisIE OS=Vibrio parahaemolyticus serotype O3:K6 (strain RIMD 2210633) OX=223926 GN=hisI PE=3 SV=1

MSFKAAEVSSLSERINWEKVDGLVPAIVQDFQSSQVLMMGYMNQDALAKTGETGQVTFFS

RTKERLWTKGETSGNVLQLVNISLDCDNDTLLVRVNPIGPTCHTGTTTCWDGDAQEESQM

VWLHQLEQLLAARKSADPDSSYTASLYARGTKRISQKVGEEGVEVALAATSGDKAELVCE

SADLIYHLLVLLQDQGLSMNDVVNKLKERHK

>sp|Q87QK8|HIS5_VIBPA Imidazole glycerol phosphate synthase subunit HisH OS=Vibrio parahaemolyticus serotype O3:K6 (strain RIMD 2210633) OX=223926 GN=hisH PE=3 SV=1

MTEQKVVIIDTGCANVSSVKFAIERLGYDVTISKDPQVVLSADKLFLPGVGTASEAMKNL

EERDLINLVKQVEKPLLGICLGMQLLGKFSQEKGQKADELVECLGLCDGEVKLLQTGDLP

LPHMGWNTVSAKAGNPLFKDIEEGEYFYFVHSFAMPVGDYTIAECDYGNPFTAAVQSGNY

YGVQFHPERSSKAGAKLIQNFLEL

>sp|P40605|HFLK_VIBPA Protein HflK OS=Vibrio parahaemolyticus serotype O3:K6 (strain RIMD 2210633) OX=223926 GN=hflK PE=3 SV=1

MAWNEPGNNNGNNGRDNDPWGNNNRGGQRPGGRDQGPPDLDEVFNKLSQKLGGKFGKKGG

GGSSIGGGGGAIGFGVIAIIAIAVWIFAGFYTIGEAERGVVLRLGKYDRIVDPGLNWRPR

FIDEYEAVNVQAIRSLRASGLMLTKDENVVTVAMDVQYRVADPYKYLYRVTNADDSLRQA

TDSALRAVIGDSLMDSILTSGRQQIRQSTQETLNQIIDSYDMGLVIVDVNFQSARPPEQV

KDAFDDAIAAREDEERFIREAEAYKNEILPKATGRAERLKKEAQGYNERVTNEALGQVAQ

FEKLLPEYQAAPGVTRDRLYIDAMEEVYTNTSKVLIDSESSGNLLYLPIDKLAGQEGQTD

TKRKSKSSSTYDHIQLESERTQEETSNTQSRSTGTRQGRY

>sp|Q87QL2|HIS1_VIBPA ATP phosphoribosyltransferase OS=Vibrio parahaemolyticus serotype O3:K6 (strain RIMD 2210633) OX=223926 GN=hisG PE=3 SV=1

MQTQRLRIAIQKKGRLSKESQALLKQCGVKFNVMGERLVVHSENMPIDLLLVRDDDIPGL

IMDGVVDLGFIGENELEEVRLDRKALGEPCEFVQLRRLDFGGCRLSIAIDKDEEYNGPQD

LAGKRIATTYPQLLKAYMDEAGVPFSTCMLTGSVEVAPRAGLADAIADLVSTGATLEANG

LKEAEVIFRSKATLIQRIGEFDADKAELINKLLTRMQGVQQAKESKYIMLHAPAGKLEQI

KALLPGAEDPTVLPLSADKQKVAVHLVSTENLFWETMEQLKELGASSILVLPIEKMME

>sp|Q87T56|HLDD_VIBPA ADP-L-glycero-D-manno-heptose-6-epimerase OS=Vibrio parahaemolyticus serotype O3:K6 (strain RIMD 2210633) OX=223926 GN=hldD PE=3 SV=1

MIIVTGGAGMIGSNIVKALNEAGINDILVVDNLKNGKKFKNLVDLDITDYMDRDDFLTQI

MAGDDFGPIEAIFHEGACSATTEWDGKYMMLNNYEYSKELLHYCLDREIPFLYASSAATY

GETETFVEEREYEGALNVYGYSKQQFDNYVRRLWKDAEEHGEQLSQITGFRYFNVYGPRE

DHKGSMASVAFHLNNQINAGENPKLFEGSGHFKRDFVYVGDVCKVNLWFLENGVSGIFNC

GTGRAESFEEVAKAVVKHHNKGEIQTIPFPDHLKGAYQEFTQADLTKLRAAGCDVEFKTV

AEGVAEYLTIQNS

>sp|P40606|HFLC_VIBPA Protein HflC OS=Vibrio parahaemolyticus serotype O3:K6 (strain RIMD 2210633) OX=223926 GN=hflC PE=3 SV=1

MRKLMIPVLVIALALMLMSLFVIPEGERGIVVRFGRVLKDNNDITRIYEPGLHFKMPLFD

RVKQLDARIQTMDGRADRFVTSEKKDVIIDSYVKWRIEDFGRYYLATGGGNSLTAEALLE

RKVTDVLRSEIGAREIKQIVSGPRNDDVLPEDASSDEVNTEAAREALEIDGERDLIMSDV

LRDTRESAMKDLGVRVVDFRMKKINLPDEISESIYRRMRAERESVARKHRSQGREKAEII

RAQAELEVATILAEADKTARVTRGEADAEAAKIYANAYNKDPEFFSFLRSLRAYEKSFSS

KNDILVLDPKSDFFQYMNNAKGAKAE

>sp|Q87KI9|HEM3_VIBPA Porphobilinogen deaminase OS=Vibrio parahaemolyticus serotype O3:K6 (strain RIMD 2210633) OX=223926 GN=hemC PE=3 SV=1

MTQSTPIRIATRKSPLALWQAHFVKDALQAAHPGLEVELVTMVTKGDVILDTPLAKVGGK

GLFVKELEVAMLEGRADLAVHSMKDVPVDFPEGLGLVTICEREDPRDAFVSNTYSNINEL

PQGAVVGTCSLRRQCQLKEYRPDIIIKELRGNVGTRLGKLDAGEYDAIILAAAGLKRLKL

EDRIRSFIEPEQSLPAVGQGAVGIECRVDDERLLKLLEPLNHKDTADRVLCERAMNLTLE

GGCQVPIGSYALLEGDEIWLRALVGEPDGSEIVRGEVRGHRKDGEALGIQLANELLDSGA

RDILTKLYADHD

>sp|Q87RH3|HEMH_VIBPA Ferrochelatase OS=Vibrio parahaemolyticus serotype O3:K6 (strain RIMD 2210633) OX=223926 GN=hemH PE=3 SV=1

MQTKNKQGVLLVNLGTPDEPTAPAVKRFLSQFLHDHRVVDMTRWLWCPILHGVILPIRSP

KVAKLYESVWMEEGSPLMVYSKRQAKKLAQHLDMPVELGMTYGNPSLQSGFEALIAQGVE

EVIVLPLYPQYSGTTTAAVSDGITKAFKQLPVMPAFSFIRDYHDHPMYIEALAHSVRQYW

EEHGKGDYLLCSYHGIPKRYADNGDIYPQHCEATTRLLGEALGLSSDQIGMAYQSRFGRE

EWLQPYTDKTLETITSKGVKKIDIMTPAFSSDCLETLEEIAGENKEIFMEAGGEQFHYIP

CLNDDDMHIDMMAELVRSKL

>sp|Q87QL1|HISX_VIBPA Histidinol dehydrogenase OS=Vibrio parahaemolyticus serotype O3:K6 (strain RIMD 2210633) OX=223926 GN=hisD PE=3 SV=1

MRTVVWQSLSEEQQDAILERPAIAEGANITAAVADVIAKVRTQGDAALLELTEKFDRVKP

ESIRVPSKEINAASERLSAEMKQALEQAYSNIAKFHKAQKPQPIKVETQPGVMCEQVTRP

IQKVGLYIPGGSAPLPSTVLMLGVPAKIAGCRKVVLCSPPPIADEILYVAKLCGIDEVYN

VGGGQAVAAMAYGTKSVSKVDKIFGPGNAYVTEAKRQVSNDFRGAAIDMPAGPSEVLVIA

DETADPDFIAADLLSQAEHGPDSQVVLVTPSPIVADQVTDAVQRQLKALSRADIAQKALA

SSLIIISESITQAVSISNYYGPEHLIVQTKNPRELLPLLDNAGSIFLGDWSPESAGDYAS

GTNHVLPTYGYTRTYSSLGLADFSKRMTVQELSAEGLQNLAPTVVTMAEAEGLDAHKRAV

TIRVEKLTQNR

>sp|Q9ZBA2|FLAE_VIBPA Polar flagellin E OS=Vibrio parahaemolyticus serotype O3:K6 (strain RIMD 2210633) OX=223926 GN=flaE PE=3 SV=2

MVSLNTNVAAMMTQRHLSQAADQNVESQRNLSSGYRINSASDDAAGLQISNTLHVQTRGI

DVALRNAHDAYSVAQTAEGALHESSDILQRLRSLGLQAANGSHEQDDRKSLQQEVIALQD

ELDRVAITTTFADKNLFNGSYGSQSFHIGANANSISLALRNMRTHIPEMGGQHYLGDSLD

KDWRVTRDNQQFAFEYQDNEGQAQSKVLTLKVGDNLEEVATYINAQQSVVDASVTQDHQL

QFFTSTLNAPEGITWKGNFADEMDIGSGELVTVDDLDMSTVGGAQLAIGVVDAAIKYVDS

HRSEIGGFQNRVSGTIDNLNTINRSVSESKGRIRDTDFARESTVMVRSQVLQDATTALLA

QAKQRPSSALGLLS

>sp|Q03475|FLIDL_VIBPA Lateral flagellar hook-associated protein 2 OS=Vibrio parahaemolyticus serotype O3:K6 (strain RIMD 2210633) OX=223926 GN=fliDL PE=3 SV=2

MSSIDPATFAAQFAQIEIQPFKQRYQLQTNTYQSQLSALGKVESAMREFRTALNEMNSST

NSIIKNSTSISQEGYFTANADAKALSGSYQIFVEQVATSHQVSTGMPADLDATTEIPKTG

NLEFTINGKTMTIDLSTVDTDGDGVTTVSDLTKAINNNSDNPGVNATLVRSNGQTHFMLS

STETGVANQINVSATGTGQAWFEDAFTNLSQISAPQDAVIWLGAEKTGLKLTNSSNTFEG

VIDGVDITVTKAQTSGETAIGLGIGADDEATKEQLNKFVDAYNTLISTIDEHTQIGSEDK

KRGVLASDPTMRSIESQLSSLVRGEHGGMRLSEIGVTLDRHGKLKVDQEKFAEAQKNNSA

GLEAMFNGDGALLDSMDAMAEPFLKFSSGAFKSRKEALQANLDRLSDKQTTLERKYDMSY

KRYLKQFTQMNTLMTQMNQTMSMFG

>sp|Q87RU0|DXS_VIBPA 1-deoxy-D-xylulose-5-phosphate synthase OS=Vibrio parahaemolyticus serotype O3:K6 (strain RIMD 2210633) OX=223926 GN=dxs PE=3 SV=1

MTLDISKYPTLALANTPEELRLLPKETLPTLCDELRTYLLNSVSQSSGHLASGLGTVELT

VALHYVYNTPVDKLIWDVGHQAYPHKILTGRRDQMPTIRQKDGLHPFPWREESEYDTLSV

GHSSTSISAGLGLAISAQKEGKGRKVISVIGDGAITAGMAFEAMNHAGDVHPDMLVILND

NEMSISENVGALNNHLAKVLSGSLYTSIREGGKKVLSGVPPIKELVRRTEEHLKGMVVPG

TLFEEFGFNYIGPIDGHDVNELVKTLKNMRELKGPQFLHIMTKKGKGYEPAEKDPIGYHG

VPKFDPSHNCLPKSSGGKPTFSKIFGDFLCDMAAQDPKLMAITPAMREGSGMVRFSKEFP

DQYFDVAIAEQHAVTLATGMAIAGDHPIVAIYSTFLQRGYDQLIHDIAIMDLPVMFAIDR

AGLVGADGQTHQGAFDLSFMRCIPNMVIMAPSDENECRQMLYTGHKHTGPSAVRYPRGSG

MGTEIEKEFTALEIGKGRVVRKGEKVAILSFGTFLPNALEAAKNLNATVADMRFVKPLDE

ALIRQLADEHDVLVTLEENAIAGGAGAGVIEFMMKEKIIKPVLNLGLPDKFIHQGTQEEL

HEELGLDAKGIEKSIAEYLAK

>sp|Q87KE3|HEM6_VIBPA Oxygen-dependent coproporphyrinogen-III oxidase OS=Vibrio parahaemolyticus serotype O3:K6 (strain RIMD 2210633) OX=223926 GN=hemF PE=3 SV=1

MSAIDKYAVKQFLMSLQDSICQQLEQEDGKAVFVEDAWHREPGERLGGGGRSRVLRDGLV

FEQGGVNFSHVEGKEMPASATAHRPELAGRRFEAMGVSLVIHPKNPYVPTSHANVRFFIA

EKEGEEPIWWFGGGFDLTPFYPFEEDCQSWHDTAKAICAPFGEDVYAEHKAWCDKYFFLP

HRNETRGVGGLFFDDLNHWEFDKCFDYIKAVGEGYCQAYLPIVSRRKDIEYGEREREFQL

YRRGRYVEFNLVYDRGTLFGLQSGGRTESILMSMPPLARWEYRYEPEAGTPEAELYERYL

KPREW

>sp|Q87SG6|MURD_VIBPA UDP-N-acetylmuramoylalanine--D-glutamate ligase OS=Vibrio parahaemolyticus serotype O3:K6 (strain RIMD 2210633) OX=223926 GN=murD PE=3 SV=1

MERWQNIHNVVVVGLGITGLSVVKHLRKTQPQLTVKVIDTRDNPPGAERLPEQVELHRGG

WNTQWLAEADLVVTNPGIALATPEIQTVLAKGTPVVGDIELFAWAVNKPVVAITGSNGKS

TVTDLTGVMAKAAGLTVGVGGNIGVPALELLEQDADLYVLELSSFQLETTSSLKLKAAAF

LNLSEDHMDRYEGMADYRAAKLRIFDHAELAVVNRDDQETYPEVEMPVVTFGSDEQAYGL

EVDGSRTWLLDHGQRVIASDELKLVGKHNLANALVVLALLKAAGVDYHNALNALKNYTGL

THRCQVVADNRGVKWVNDSKATNIASTMAALSGLESTGKLYLLVGGVGKGADFTPLKPIF

ATLNLQLCCFGLDGDDFMPLHESAIRFNTMEDVIQQISSQLKSGDMVMLSPACASFDQFD

NFMARGDAFAVLAQKYA

>sp|Q87SG4|MURG_VIBPA UDP-N-acetylglucosamine--N-acetylmuramyl-(pentapeptide) pyrophosphoryl-undecaprenol N-acetylglucosamine transferase OS=Vibrio parahaemolyticus serotype O3:K6 (strain RIMD 2210633) OX=223926 GN=murG PE=3 SV=1

MKQNKRLMVMAGGTGGHVFPGLAVAKQLQEQGWEIRWLGTADRMEADLVPKHGIEIDFIK

VKGLRGQGVKRLLAAPFQIINAIMQARAHMKRWQPDAVLGMGGYVSGPGGIAAWLSGIPV

VLHEQNAVAGLTNQWLSKIAKKVFQAFPGAFPSAAVVGNPVREDVTQLDEPAQRMQEREG

PIRILVMGGSQGARILNQTLPAVMANLGQDYCIRHQAGKGAAQEVQAAYQANNVANAEVT

EFIDDVAQAYAWADLLVCRSGALTVSEVSAAGVGAIFIPFMHKDRQQALNADHLVECGAA

KMIEQPDLTVESLTQQIQQLDRQALLSMAQKARSAAKLDADKVVAQAIVALTEKR

>sp|Q87S81|MURQ1_VIBPA N-acetylmuramic acid 6-phosphate etherase 1 OS=Vibrio parahaemolyticus serotype O3:K6 (strain RIMD 2210633) OX=223926 GN=murQ1 PE=3 SV=1

MTNDALIAALSHLVSEGRNPDTMDIDLLPSLDIVQRINQQDKLVPLAVEKVLPEIAQAVD

KITDAFKVGGRLIYMGAGTSGRLGVLDASECPPTFGVSDKMVIGLIAGGPEAILKAKEGA

EDSPQLGEQDLKAIDFSSNDVLVGIAASGRTPYVIGGLEYANTIGATTVALSCNPDSPIA

DIADIAISPVVGPEALTGSTRLKSGTAQKLVLNMLTTASMIRLGKSYQNLMVDVKATNNK

LVARAARIVMQATECTKEEATEVLKQTNYEVKLAILMILTDLDIESARQHLHHQDGFLRK

AVESHKSN

>sp|Q87QT6|NADA_VIBPA Quinolinate synthase A OS=Vibrio parahaemolyticus serotype O3:K6 (strain RIMD 2210633) OX=223926 GN=nadA PE=3 SV=1

MSHILDKIDTVYPFPPKPIPLSEEEKSSYIASIKELLKQKDAVLIAHYYTDPEIQALAEE

TGGFVGDSLEMAKFGNRHPASTLIIAGVRFMGESAKILTPEKRILMPTLEAECSLDLGCP

ADKFSEFCDAHPDHTVVVYANTSAAVKARADWVVTSSIALEIVEHLDAEDKPIIWGPDRH

LGSYIANKTGADMLLWQGECVVHDEFSADALRKMKSVYPDAAILVHPESPASVVELADAV

GSTSQLIKAAKELPYQQMIVATDKGIFFKMQQLVPEKELIEAPTAGAGATCRSCAHCPWM

AMNGLKAIEKALSEGGEEHEIFVDEALRVKSLIPLNRMLDFAEQLNMQVKGNA

>sp|Q87L49|METJ_VIBPA Met repressor OS=Vibrio parahaemolyticus serotype O3:K6 (strain RIMD 2210633) OX=223926 GN=metJ PE=3 SV=1

MADWNGEYISPYAEHGKKSEQVKKITVSIPLKVLKVLTDERTRRQINNLRHATNSELLCE

AFLHAYTGQPLPTDEDLRKDRPDDIPTEAKALMTAMGIEFEAFDEE

>sp|Q87RC3|MINC_VIBPA Probable septum site-determining protein MinC OS=Vibrio parahaemolyticus serotype O3:K6 (strain RIMD 2210633) OX=223926 GN=minC PE=3 SV=1

MTHSPDLKGSSFTLSVLHLSDNEIANTVEFLQEKVSQAPSFFASAPLVINIAKVQGDIDF

PALKQGIADAGFIPVGITGSKDKRVQNLASEAGFAIMSASKSPSQAPAKMAPTKVVRTPV

RSGQQIYAKDGDLVVLAHVSAGAEVIADGSIHIHGTLRGRAIAGASGQQEARIICHDLQA

ELVSIAGDYWLSDQIESEYWQKKVMISKAEESLHLEVLAI

>sp|Q87RW1|MLTF_VIBPA Membrane-bound lytic murein transglycosylase F OS=Vibrio parahaemolyticus serotype O3:K6 (strain RIMD 2210633) OX=223926 GN=mltF PE=3 SV=2

MQIRHFNRLKRSVLLFASVLLLSACQIESQPKSEFEKIQERGVLRVGTLNNQLSYYIGPD

GPAGLDYELARKFAEELGVKLEIKPAFRQADLFPALKKGDIDIIATGLNQTSQAVKRFRP

GPAYYYVSQQVVYKKGQLRPRDIEQLIEYQASKDSQSEEDVNAGAQTLKIVEQSQYVPTL

TALKKQYPELQFEIVGDADTRDLLKHVSTGELRFTVTDSVELSLAQRLYPDLALAFELTE

DQPVSWFTRRSEDESLYAMLIEFFGNIKQSGELASLEEKYIGHIEAFDYVDTRAFIRALD

DKLPRWAPLFQKYSEEFDWRLIAALAYQESHWKPKAKSPTGVRGMMMLTLPTAKSVGVTD

RLNPEQSVRGGVEYLRRIVARVPDTINEHEKIWFALASYNIGYGHMMDARRLTKAQGGDP

NAWADVKDRLPLLRQKRYYSQTRYGYARGDEARNYVENIRRYYQSIIGHVSQKPSIDEDT

DDLQVIPPLNPELLISGAVETIAEEVSGASDITNEVDEDLDQEEE

>sp|Q87KU2|PRMA_VIBPA Ribosomal protein L11 methyltransferase OS=Vibrio parahaemolyticus serotype O3:K6 (strain RIMD 2210633) OX=223926 GN=prmA PE=3 SV=1

MPWIQIKLNATNENAEQIGDMLMEETGALSVTFLDAQDTPVFEPLPGETRLWGDTDILAL

YDAEADTNFIIDQIKASNMLAENFAYKVEQLEDKDWEREWMENFHPMKFGERLWICPSWR

EVPEPDAVNVMLDPGLAFGTGTHPTTALCLEWLESMDLSGKTVIDFGCGSGILAIAAIKL

GAEKVIGIDIDPQALLASKDNAERNGVADKLEVYLPQNQPEGLIADVVVANILAGPLREL

APIIKGLVKPNGALAMSGVLDTQAEDVASYYRDELHIDPIVEQSEWCRISGRKQG

>sp|Q87RU9|PROA_VIBPA Gamma-glutamyl phosphate reductase OS=Vibrio parahaemolyticus serotype O3:K6 (strain RIMD 2210633) OX=223926 GN=proA PE=3 SV=1

MDLTNMGKAAKGAAFELATASTAQKNQALAIIADELEANSAAILAANAKDIELGREAGLT

DALLDRLLLNEERLTGIANDVRNVISLNDPVGSEIDSKVLENGMSLSRRRVPLGVVGVIY

EARPNVTIDIAALCLKTGNASILRGGKETFFSNMELVKVIQSALAKANLPAASVQYIEKP

DRELVSQLLKLDDYVDMIIPRGGAGLHKMCKENSTIPVIIGGFGISHIFVDESADLEKSL

NVVENSKVQRPSACNSLDTLLVHEKVAAKFLPMIVERMSDKVTFVAEPKAKALMAQATQI

RDAVEGDFDTEWLSYTLGVKVVADVKEAIDHMRVHNASHSDAIMTNSLINSELFINSVGS

AAVYVNAATRFTDGAQFGLGAEVAVSTQKLHARGPMGLEELTSYKWVGKANYLARS

>sp|Q87PB0|PROQ_VIBPA RNA chaperone ProQ OS=Vibrio parahaemolyticus serotype O3:K6 (strain RIMD 2210633) OX=223926 GN=proQ PE=3 SV=1

MTEKLKNSKEVIAYIAECFPKCFTLEGEAKPLKIGIFQDLAERLNEDEKVSKTQLRAALR

QYTSSWRYLHGVKPGAVRVDLDGNPCGELEEEHVEHAKATLAESKAKVQARRKEQAQKAR

EEGKAKAKPAANKKPQQPRRTNKPKVQKPTKPVETRALNADEITVGNAVNVNMGKGNMAA

TIVEINKDDVRVQLANGLQMVVKAEHLRA

>sp|Q87PH3|POTA_VIBPA Spermidine/putrescine import ATP-binding protein PotA OS=Vibrio parahaemolyticus serotype O3:K6 (strain RIMD 2210633) OX=223926 GN=potA PE=3 SV=1

MGEIQTLNAKQQAGKPVIRLSGISKSFDGKEIIGNLNLDVNHGEFLTILGPSGCGKTTVL

RMIAGFETADNGQIVLDDQDVTQVPAEHRHVNTVFQSYALFPHMTVFDNVAFGLRMQKTP

AAEIEPRVMEALRMVRLEKMAQRKPHQLSGGQQQRIAIARAVVNKPKVLLLDESLSALDY

KLRKQMQIELKQLQRQLGITFIFVTHDQEEALSMSDRIIVMRDGVIEQDGSPREIYEEPK

NLFVARFIGEINVFNATMLERIDEKRIRAEIEGVESVVYYDKEAQAGDKLQVLLRPEDLR

IEEIKESEEKGIVGHVTERTYKGMTLDSVVQLDSGMRVMVSEFFNEDDPDVDHSLGQKVA

ITWVESWEVVLNDKQED

>sp|Q87S51|PPK1_VIBPA Polyphosphate kinase OS=Vibrio parahaemolyticus serotype O3:K6 (strain RIMD 2210633) OX=223926 GN=ppk PE=3 SV=1

MSAEKLYIEKELSWLSFNERVLQEAADKTVPLIERIRFLGIFSNNLDEFYKVRFADVKRR

ILINQERGGSDNSKRLLSKMQAKALKLNEQFDELYSELIREMARRRIFLVNEHQLDEAQE

KWITKYFRKEVMPHITPLLMKDEIDVLQFLKDEYAYIAVELRKEDHSQYALIEIPTDHLP

RFVMVPEQKGKRRKTIILLDNIIRYCLDELFKGFFDYDELAGYAMKMTRDAEYDLRNEIE

YSLLEQMSAGVNQRLTAMPVRFVYEREMPQEMLDFLCSKLRISNYDNLIPGGRYHNFKDF

IAFPNVGREYLENKPMPPMKCADFEGYANSFEAIKAKDILLYYPYHTFDHIGELVRQASF

DPKVLSIKINIYRVAKDSRLMNSLIDAVHNGKNVTVVVELQARFDEEANIEWSKVLTEAG

VHVIFGAPGLKIHSKLLMISRREGDDIIRYAHIGTGNFHEKTARIYTDFSLLTADQEITN

EVRNVFGYIENPYRPVKFNHLMVSPRNSRTQIYRLIDNEIANAKAGKKAGLTIKVNNLVD

KGIVTRLYAASNAGVKINMIIRGMCALVPGIEGVSENIRIISIVDRFLEHPRVVITHNDG

DPQVYISSADWMTRNIDHRIEVAAPVRDPRLKQRIIDITNIHFTDTVKARLIDKEMSNSY

VPRGNRKKVRSQVAIYDYLKNIEKQTRRQKSDVSDT

>sp|Q87L73|PRIB_VIBPA Primosomal replication protein N OS=Vibrio parahaemolyticus serotype O3:K6 (strain RIMD 2210633) OX=223926 GN=priB PE=3 SV=1

MTNRMELSGTIAKPPIRSKSPGGIEHCRFWLEHRSTVIEADLPRQVYCRMPVVVSGLRSQ

AITQNLVQGSNIKVSGFVAYQTGRNGVGKLVLHADNITQI

>sp|Q87KZ9|PSD_VIBPA Phosphatidylserine decarboxylase proenzyme OS=Vibrio parahaemolyticus serotype O3:K6 (strain RIMD 2210633) OX=223926 GN=psd PE=3 SV=1

MDKIKVGLQYWIPQHGLTRLVGKLASAKAGSLTTAVIRWFIKQYNVNMDEAKHSDPKHFK

TFNEFFVRELKEGARPITEGDEIITHPADACVSQFGPIEDGQLIQAKGHNYSAQELLGGD

EKLAEEFKDGSFATLYLSPRDYHRVHMPCDGTLRQMIYVPGDLFSVNPLTAENVPNLFAR

NERVVCIFDTEFGPMAQVLVGATIVGSIEQVWAGTITPPRGNTVYKWDYPAEGDKAVILK

KGEEMGRFKLGSTVINLFAKDAIEFDVSMENGQPTVMGTPYALKK

>sp|Q87S48|PSTB1_VIBPA Phosphate import ATP-binding protein PstB 1 OS=Vibrio parahaemolyticus serotype O3:K6 (strain RIMD 2210633) OX=223926 GN=pstB1 PE=3 SV=1

MFNFDNTLGYEPPLDVHNLTDEQTAISIENLNLFYGQAQALHDISMRIPKGRVTAFIGPS

GCGKSTLLRCINRMNDLVEGCKVTGKVRLHGKNVYHPNVDVATLRRRVGMVFQRPNPFPK

SIYENVVYGLRLQGVKNSRTLDDAVERSLRSAALWDEVKDRLHENAFGLSGGQQQRLVIA

RAVAIEPEVLLLDEPTSALDPISTLTIEELINELKTQYTVVIVTHNMQQAARVSDHTAFI

HMGKLIEYSDADSIFTSPMKKQTEDYITGRYG

>sp|Q87QW4|MUKF_VIBPA Chromosome partition protein MukF OS=Vibrio parahaemolyticus serotype O3:K6 (strain RIMD 2210633) OX=223926 GN=mukF PE=3 SV=1

MSEMTLNAAEQPIDELVGWVKQHDFSLNLTTERLAFLIAIAVLSNERFDEELGEGELHDA

FAIVTRLFDETGEASAFRANNAINEMVKQRLISRFVSEITDGASIYRLSPLAIGITDYYV

RHREFSRLRLSIQLSMVADEMAKAIEAAQKGGTPGHWKKNVYGVLKYSVGEIFDQIDLNQ

RVMDEQQQSVKQQIADLLNKDWREAINNCEALLSETSSTLRELQDTLQAASDELQTQILD

IQEIVYGDPELEFIEEALFGLQMKLDRITSWGQQAIDLWIGYDRHVHKFIRTAIDMDKNR

AFSSRLRQSIKDYFDMPWYLTFADAERLSDLRDEALVLRDDEVTGQVPMEVEYEEFQQVN

DELSERIGDMLKAHKEQGTPIDLSVVLRDYLAQHPYTHHFDLARIIVDQAVRLGYSESDY

QAIQPDWKAINEFGAKVQANVIDRY

>sp|Q87KP5|MURB_VIBPA UDP-N-acetylenolpyruvoylglucosamine reductase OS=Vibrio parahaemolyticus serotype O3:K6 (strain RIMD 2210633) OX=223926 GN=murB PE=3 SV=1

MQIKEHASLKAFHTFGIEQTCSYLAIVDSIDDVISLYQNPAFQSLPKLFLGKGSNVLFTE

HFDGLVIVNRLLGKSVSETHEDYLLHVQGGEDWPSLVAWCVAQGMGGIENLALIPGCAGS

APIQNIGAYGVELKDLCSYVDVLDLTTLKTRRMSAEDCEFGYRDSVFKHDLYEKCFVTAI

GLKLPKRWTPKNQYGPLQNIPENELSPNAIFERVCQVRMEKLPDPAKVGNAGSFFKNPVI

SQDHYDQLVRKHSDMVAYPANEGMKVAAGWLIDQCGLKGISVNGAQVNPLQALVLTNVDN

CSADDVVALASLVKRAVWDKYQIELEHEVRFMNRQGETNLAKIEAAQ

>sp|Q87RX6|NADK_VIBPA NAD kinase OS=Vibrio parahaemolyticus serotype O3:K6 (strain RIMD 2210633) OX=223926 GN=nadK PE=3 SV=1

MKNPCNVIAIIGKPRDQQAIQTHKELYEWLTSEGYKVFIDDRLAAILDEIPQNHFASLVE

LGKNADLAIVVGGDGNMLGAARILSRFDVPVIGVNRGNLGFLTDLNPDDFQAALKAVLAG

EYIEEERFLLEAEVHRHGQIKSHNAALNEAVLHPGQIAHMIEFEVYIDESFAFSLRADGL

IVSTPTGSTAYSLSGGGPILSPSLNAISLVPMFPHTLSSRPLVVDGKRRIKLIVSPENRG

TQEVSCDGQVSLPVSPGDEIHIYQSPNVLKLIHPKDYSYYHVLRNKLGWSSKLF

>sp|Q87L95|METH_VIBPA Methionine synthase OS=Vibrio parahaemolyticus serotype O3:K6 (strain RIMD 2210633) OX=223926 GN=metH PE=3 SV=1

MGSKVRQQIEAQLKQRILLIDGGMGTMIQGYKLEEQDYRGERFANWHCDLKGNNDLLVLS

QPQLIKEIHSAYLEAGADILETNTFNATTIAMADYEMESLSEEINFAAAKLAREVADEWT

AKTPDKPRYVAGVLGPTNRTCSISPDVNDPGYRNVSFDELVEAYSESTRALIRGGADLIL

IETIFDTLNAKACAFAVDSVFEELGVALPVMISGTITDASGRTLSGQTTEAFYNSLRHVR

PLSFGLNCALGPDELRPYVEELSRISESFVSAHPNAGLPNAFGEYDLSPEDMAEHVKEWA

SSGFLNLIGGCCGTTPEHIRQMAQAVEGVTPRALPDLPVACRLSGLEPLTIEKETLFINV

GERTNVTGSARFKRLIKEEQYDEALEVARQQVENGAQIIDINMDEGMLDAQACMVRFLNL

CASEPEISKVPIMVDSSKWEVIEAGLKCIQGKGIVNSISLKEGKEKFVEQAKLIRRYGAA

VIVMAFDEVGQAETRTRKLEICTNAYRILVDEVGFPPEDIIFDPNIFAVATGIDEHNNYA

VDFIEAVADIKRDLPHAMISGGVSNVSFSFRGNNYVREAIHAVFLYHCFKNGMDMGIVNA

GQLEIYDNVPEKLREAVEDVVLNRRDDATERLLDIAAEYADKGVGKEEDASALEWRTWPV

AKRLEHALVKGITEFIVADTEEARVNAVKPLEVIEGPLMDGMNVVGDLFGEGKMFLPQVV

KSARVMKQAVAHLEPFINAEKQSGSSNGKILLATVKGDVHDIGKNIVGVVLQCNNYEIID

LGVMVPCEKILKVAIEENVDIIGLSGLITPSLDEMVHVAKEMERLNFDLPLLIGGATTSK

AHTAVKIEQNYKNPVVYVNNASRAVGVCSSLLSDERRPAFIEKLDADYERVRDQHNRKKP

RTKPVTLEQARANKVAIDWDAYTPPVPAKPGLHIFDDFDVATLRKYIDWTPFFMTWSLVG

KYPTIFKHEEVGEEAQRLFHDANELLDRVEREGLLKARGICGLFPAASVGDDIEVYTDES

RTEVAKVLRNLRQQTEKPKGFNYCLSDYIAPKESGKQDWVGAFAVTGGIGERELADEYKA

QGDDYNAIMIQAVADRLAEAFAEYLHERVRKEIWGYAADENLSNDELIREKYQGIRPAPG

YPACPEHTEKGPLWELLNVEENIGMSLTTSYAMYPGASVSGWYFSHPDSRYFAIAQIQDD

QLESYADRKGWDRIEAEKWLGPNING

>sp|Q87LK6|METK_VIBPA S-adenosylmethionine synthase OS=Vibrio parahaemolyticus serotype O3:K6 (strain RIMD 2210633) OX=223926 GN=metK PE=3 SV=1

MAKHLFTSESVSEGHPDKIADQISDAVLDAILEQDPKARVACETYVKTGMVMVGGEITTS

AWVDIEELTRETVREIGYVHSDMGFDANSCAVLNTIGKQSPDINQGVDKADPKEQGAGDQ

GIMFGYACNETEVLMPAPITYSHRLVEKQAEVRKNGTLPWLRPDAKSQVTFQYDQGKIVG

IDAVVLSTQHCDSISTPDLREAVMEEIIKPVLPAEWISKDTNFFINPTGRFVIGGPMGDC

GLTGRKIIVDTYGGAARHGGGAFSGKDPSKVDRSAAYAARYVAKNIVAAGMADRCEIQLS

YAIGVADPTSIMVETFGTEKVSHDIIIEAVRQFFDLRPYGLQEMLNLLQPIYKKTAAYGH

FGREEFPWEATDKAELLRDFAGIK

>sp|Q87MA7|NQRB_VIBPA Na(+)-translocating NADH-quinone reductase subunit B OS=Vibrio parahaemolyticus serotype O3:K6 (strain RIMD 2210633) OX=223926 GN=nqrB PE=3 SV=1

MALKKFLEDIEHHFEPGGKHEKWFALYEAVATVFYTPGLVTKKSSHVRDSVDLKRIMIMV

WFAVFPAMFWGMYNAGGQAIAALNHMYAGDQLATVIAGNWHYWLTEMLGGSISADAGVGS

KMLLGATYFLPIYATVFLVGGFWEVLFCMVRKHEVNEGFFVTSILFALIVPPTLPLWQAA

LGITFGVVVAKEIFGGTGRNFLNPALAGRAFLFFAYPAQISGDVVWTAADGFSGATALSQ

WAHGGSGALINNITGAPITWMDAFIGNIPGSIGEVSTLALMIGAAMIVYMRIASWRIIAG

VMIGMIAVSTLFNVVGSDTNPMFNMPWHWHLVLGGFAFGMFFMATDPVSASFTNKGKWWY

GILIGAMCVMIRVVNPAYPEGMMLAILFANLFAPLFDHVVIEKNIKRRLARYGK

>sp|Q87MA9|NQRD_VIBPA Na(+)-translocating NADH-quinone reductase subunit D OS=Vibrio parahaemolyticus serotype O3:K6 (strain RIMD 2210633) OX=223926 GN=nqrD PE=3 SV=1

MSSAQNIKKSIMAPVLDNNPIALQVLGVCSALAVTTKLETAFVMTLAVTFVTALSNFSVS

LIRNHIPNSVRIIVQMAIIASLVIVVDQVLKAYLYDISKQLSVFVGLIITNCIVMGRAEA

FAMKSAPVPSLIDGIGNGLGYGFVLITVGFFRELFGSGKLFGMEVLPLVSNGGWYQPNGL

MLLAPSAFFLIGFLIWVIRVFKPEQVEAKE

>sp|Q87LR6|OADG_VIBPA Probable oxaloacetate decarboxylase gamma chain OS=Vibrio parahaemolyticus serotype O3:K6 (strain RIMD 2210633) OX=223926 GN=oadG PE=3 SV=1

MTNIGSLLVDAATLMVTGMAVVFIFLTILVYLVRLLSKLVPEEVPEPIAAPKTNTRVQST

SSAVSPQVVAAISAAIHQHRASIAK

>sp|Q87QL9|MNMA_VIBPA tRNA-specific 2-thiouridylase MnmA OS=Vibrio parahaemolyticus serotype O3:K6 (strain RIMD 2210633) OX=223926 GN=mnmA PE=3 SV=2

MSDNSQKKVIVGMSGGVDSSVSAYLLKQQGYQVEGLFMKNWEEDDNEEYCTAAEDLADAQ

AVCDKLGIHLHTINFAAEYWDNVFEYFLAEYKAGRTPNPDILCNKEIKFKAFLEFADEVL

EADYIAMGHYVRRSFPENGEKPQMLRGLDSNKDQSYFLYTLSHEQVARSLFPVGDLEKPE

VRRIAEEQGLITAKKKDSTGICFIGERKFTEFLGRYLPAQPGNIETPEGEVIGQHQGLMY

HTLGQRKGLHIGGRKGGGGNEDPWFVGEKDLDRNVLIAVQGKDHPMLKSEGLLASQLHWV

DREPIRDVMKCTVKTRYRQQDIPCTIIPIDDENIKVIFDEPQIAVTPGQSAVFYKDDVCL

GGGIIEQRIKYSQA

>sp|Q87SW6|MSRA_VIBPA Peptide methionine sulfoxide reductase MsrA OS=Vibrio parahaemolyticus serotype O3:K6 (strain RIMD 2210633) OX=223926 GN=msrA PE=3 SV=1

MLNKQTLISIEDALPGREQPMQIEDCHFVNQSSLTAPLAHHQQQILLGMGCFWGAERLFW

QLDGVVSTSVGYAGGFTPNPTYEEVCTGKTGHTEVVRVVFDERVISLAQLLAVFWEKHDP

TQGMRQGNDLGTQYRSAIYTYSQDQQEIADKSKLQYQQALEAELRSTITTEIVPAGPYYF

AETYHQQYLAKNPDGYCGIGGTGVCFPPSLQG

>sp|P59570|OMPK_VIBPA Outer membrane protein OmpK OS=Vibrio parahaemolyticus serotype O3:K6 (strain RIMD 2210633) OX=223926 GN=ompK PE=3 SV=1

MRKSLLALSLLAATSAPVLAADYSDGDIHKNDYKWMQFNLMGAFDELPGESSHDYLEMEF

GGRSGIFDLYGYVDVFNLASDKGSDKVGDPKIFMKFAPRMSIDGLTGKDLSFGPVQELYV

ATLFEWDGTDYKTNPFSVNNQKVGIGSDVMVPWFGKVGVNLYGTYQGNQKDWNGFQISTN

WFKPFYFFENGSFISYQGYIDYQFGMKEKYSSASNGGAMFNGIYWHSDRFAVGYGLKGYK

DVYGIKDSDALKSTGFGHYVAVTYKF

>sp|Q87RU3|NUSB_VIBPA Transcription antitermination protein NusB OS=Vibrio parahaemolyticus serotype O3:K6 (strain RIMD 2210633) OX=223926 GN=nusB PE=3 SV=1

MGASVKPAARRNARQFALQAIYSWQITKENVATIEEQFLSGDKYDEEELRASEPALAAPE

TDVAYFRELLSGVVLSHAELDSKIRPYVSRPMQDLDMMELALLRLAMYEMTRREDVPYKV

VINEAIELAKVFAAEDSHKFVNGVLDKAAPHVRKK

>sp|P40610|NSRR_VIBPA HTH-type transcriptional repressor NsrR OS=Vibrio parahaemolyticus serotype O3:K6 (strain RIMD 2210633) OX=223926 GN=nsrR PE=3 SV=1

MQLTSFTDYALRTLIYLASLPKDELTNITEVTDLFGVSRNHMVKVINRLGQLGYVHTVRG

KNGGIRLMKPASEITVGGVVRDLEPLDLVNCGVEFCHITPACRLKDKLAKAKSAFLAELD

ECTIESLLSDNSELLILLARP

>sp|Q87K03|QNR_VIBPA Pentapeptide repeat protein VPA0095 OS=Vibrio parahaemolyticus serotype O3:K6 (strain RIMD 2210633) OX=223926 GN=VPA0095 PE=1 SV=1

MLKTDLIFERENFSHHDFQNATFKNCHFYMCSFDHADLRDAKFIDCRFIESKALEGCSFR

FANLKDASFTNCMLAMSLFNGANCMGLELRKCDLKGANFQGANFANRVSNTMFFCSAFIT

GCNLTYCNFERVLLEKCDLFENRWNGANLAGATLKGSDLSRCEFSPEQWGTFNVEQCDLT

HVELDGLDIRRVSLFGVKICDWQQEQLLAPFGLIIL

>sp|Q87RS6|QUEF_VIBPA NADPH-dependent 7-cyano-7-deazaguanine reductase OS=Vibrio parahaemolyticus serotype O3:K6 (strain RIMD 2210633) OX=223926 GN=queF PE=3 SV=1

MSKYSDAKELAGLTLGKKTEYANQYDASLLQPVPRSLNRDDLELGDTLPFLGHDIWTLYE

LSWLNSKGLPQVAVGEVYIPATSANLIESKSFKLYLNSYNQTRFASWEEVAERLTQDLSA

CAGEKVLVEVNPVGHYTNQPIVTMEGECIDDQDIEINSYDFDADLLAGAAGEDQVEEVLH

SHLLKSNCLITNQPDWGSVEIRYQGAKIDREKLLRYLVSFREHNEFHEQCVERIFTDLMK

YCQPNKLTVFARYTRRGGLDINPYRSTEQDKPAHNHRMARQ

>sp|Q87H78|RBSD_VIBPA D-ribose pyranase OS=Vibrio parahaemolyticus serotype O3:K6 (strain RIMD 2210633) OX=223926 GN=rbsD PE=3 SV=1

MKKSTLINSGISYLVATLGHTDEITICDAGLPIPDHVQRIDLALTHGVPSFLDTVRVILS

ESQIEGVIIAEEFSDVSPVLHEALLKELSKESEETGKSIEIKYVSHEAFKARTEQSRAVV

RTGECTPYANVIFQAGVVF

>sp|Q87S56|RDGC_VIBPA Recombination-associated protein RdgC OS=Vibrio parahaemolyticus serotype O3:K6 (strain RIMD 2210633) OX=223926 GN=rdgC PE=3 SV=1

MWFKNCLVYRVNREVNFNADQLETQLAEFRFTPCGSQDKQKFGWVSAMGRHGDMMTHVSE

NRILICAKKEEKMLPASVIKESLNAKVDAMEAQEGRPLKKKEKDNLKDDIVMDLLPRAFS

RSSHTHVLIMPKEGFILVDASSYKKAEDVLALLRKTMGSLPVVPAIPEVAIETTLTEWVK

TGNTPQGITMMDEAELKSVLEEGGVIRCKKQELTTDEIRNHIAADKVVTKLALNWQDRIE

FIMTEDSGIKRLKFSDELKDQNDDIPREDQAARFDADFSLMCGEFSAFLPNLYEALGGLP

NPNA

>sp|Q87LP1|RECO_VIBPA DNA repair protein RecO OS=Vibrio parahaemolyticus serotype O3:K6 (strain RIMD 2210633) OX=223926 GN=recO PE=3 SV=1

MSNLSAEGFQRCFVLHRRPYSESSLILDVFSEEYGRITLMAKGARSKRSNLKGALQPFTP

LLLKWSGKGSMKTLRQAEPISLGLPLFGINLYSAMYVNELVGRVLMAEVPMPALFHDYLH

ALTELAQCENPEPALRRFELALLSSMGYGVDFLHCAGTGEPVDPEMTYRYREQKGFIASV

RRDNLTFLGNELIAISERRFVTKEQLKAAKRFTRIALKPYLGGKPLKSRELFIQTPRARS

NGK

>sp|Q87P80|QUEC_VIBPA 7-cyano-7-deazaguanine synthase OS=Vibrio parahaemolyticus serotype O3:K6 (strain RIMD 2210633) OX=223926 GN=queC PE=3 SV=1

MKKAVVVFSGGQDSTTCLVQALKEFDEVHAITFDYGQRHKLEIEVAQKIAKDLGVKAHKV

MDVGLLNELAISSLTRDDIPVSHELQENGLPNSFVPGRNILFLTLAGIYAYQIGADTVIT

GVCETDFSGYPDCRDDFVKSMNTALVKGMDRALTIKTPLMWLNKAETWALADQYNALQLV

RENTLTCYNGIIGDGCGDCPSCDLRKAGLDEYLNNKDAVMKSLVQKQESEGL

>sp|Q87H79|RBSA_VIBPA Ribose import ATP-binding protein RbsA OS=Vibrio parahaemolyticus serotype O3:K6 (strain RIMD 2210633) OX=223926 GN=rbsA PE=3 SV=1

MTQAILQLSEIEKAFPGVKALDKASLNVYPGRVMALMGENGAGKSTLMKVLTGIYHMDAG

SIQYQGQPAAFKGPRDSQEAGISIIHQELNLIPELTIAENIFLGREFTGSMGRIQWSKMY

AEADRLLQRLNVKHSSKTLLGDLSLGEQQMVEIAKALSFESKVIIMDEPTDALTDTETES

LFKVINELREQGCGIVYISHRLKEIFEICDDITVLRDGKFIGECRVADTDEDGLIEMMVG

RKLEEQYPRIDVKHGETCLEVVGLTGSGVHDVSFTLKRGEILGISGLMGAGRTELMKVIY

GALPSEHGVINLDNKTINPVSPQDGLANGIAYISEDRKGDGLVLGLSVKENMSLCALDKL

TKGVQIQHGEEVIAVEDFIKLFNIKTPTRDQIIGNLSGGNQQKVAIAKGLMTKPKVLILD

EPTRGVDVGAKKEIYQLINKFKADGMSIILVSSEMPEVLGMSDRILVMHEGRITGEFDAK

DADQEKLLACAVGKKINEEAA

>sp|Q87TQ5|RECF_VIBPA DNA replication and repair protein RecF OS=Vibrio parahaemolyticus serotype O3:K6 (strain RIMD 2210633) OX=223926 GN=recF PE=3 SV=1

MPLSRLIIQQFRNIKACDIQLSAGFNFLIGPNGSGKTSVLEAIYLLGHGRSFKSSLTGRV

IQNECDELFVHGRFLNSDQFELPIGINKQRDGSTEVKIGGQSGQKLAQLAQVLPLQLIHP

EGFDLLTDGPKHRRAFIDWGVFHTEPAFYDAWGRFKRLNKQRNALLKTASSYRELSYWDQ

EMARLAENISQWRSLYIEQMKTVAETICQTFLPEFEIQLKYYRGWDKDTPYQEILEKNFE

RDQSLGYTFSGPNKADLRIKVNGTPVEDVLSRGQLKLMVCALRVAQGQHLTAMTGKQCIY

LIDDFASELDSQRRKRLADCLKETGAQVFVSSITENQIADMLDDNGKLFHVEHGRIESN

>sp|Q87M03|RBFA_VIBPA Ribosome-binding factor A OS=Vibrio parahaemolyticus serotype O3:K6 (strain RIMD 2210633) OX=223926 GN=rbfA PE=3 SV=1

MSKEFSRTQRVAQQLQKELAMILQREVRDSRLGMVTISDVEVSRDLAYAKVFVTFLCVGE

QTPESCLAALREHEVHIRMMLGKRIRLRLTPEIRFYYDNTLVEGMRMSNLVTEVVNSDKQ

KQKNSGREDEE

>sp|Q87RN4|RF1_VIBPA Peptide chain release factor 1 OS=Vibrio parahaemolyticus serotype O3:K6 (strain RIMD 2210633) OX=223926 GN=prfA PE=3 SV=1

MKASILTKLETLVERYEEVQHLLGDPDVIGDQDKFRALSKEYSQLEEVTKCFQAYQQAQD

DLAAAEEMAKEDDEEMREMAQEEIKDAKEAIERLADELQILLLPKDPNDDRNCFLEIRAG

AGGDEAGIFAGDLFRMYSKYAEKRGWRIEVMSSNEAEHGGYKEMIAKVSGDGAYGVLKFE

SGGHRVQRVPATESQGRVHTSACTVAVMAEIPEADLPEIKAADLKIDTFRASGAGGQHVN

TTDSAIRITHLPTGTVVECQDERSQHKNKAKAMAVLAARIVQAEQERRAAEVSDTRRNLL

GSGDRSDRIRTYNYPQGRVSDHRINLTIYRLNEVMEGDLQSLIDPVVQEHQADQLAALAE

NA

>sp|Q87LD0|RAPA_VIBPA RNA polymerase-associated protein RapA OS=Vibrio parahaemolyticus serotype O3:K6 (strain RIMD 2210633) OX=223926 GN=rapA PE=3 SV=1

MTFALGQRWISDTESDLGLGTVVAMDARTVTLMFAASEENRVYARNDAPVTRVTFNVGDV

IDCQEGWSLKVEEVLEDEGLYTYFGTREDTQETAVVLREIFLSNQIRFNKPQDKLYAGQI

DRMDNFVLRYRALTNQFEQHKSPMRGLCGMRAGLIPHQLYIAHEVGRRHAPRVLLADEVG

LGKTIEAGMIIHQQVLSGRAERILIVVPETLQHQWLVEMMRRFNLHFSIFDEERCIEAFA

DAENPFDTQQYVLCSLDFLRKSRKRFEQALEGEWDLLVVDEAHHLEWSQDKPSREYQVVE

GLAERTPGVLLLTATPEQLGRESHFARLRLLDPDRFYDYEAFVEEEEQYAPVADAITSLF

SGEKLPDEAKNQITELLSEQDVEPLFRIIESNSDEEAKASARQELIDNLMDRHGTGRVLF

RNTRAAIKGFPTRNVHLMPMDIPQQYTTSMRVAGMIGGKMSSEARAMKNLYPEEIFQEFE

GDDASWWQFDSRVNWLLEKVKEKRGEKILVIASRASTALQLEQALREREGIRATVFHEGM

SILERDKAAAYFAQEEGGAQVLICSEIGSEGRNFQFANQLVMFDLPFNPDLLEQRIGRLD

RIGQNRDIDIHVPYLKGTSQAILARWFDEGLNAFAETCPTGRAVYDKYSDALIEILASGD

TSTLDEIIEESAKLNKELKSQLEQGRDRLLEMHSNGGEKAQQIVEKIESTDGDTNLVTFA

LSLFDTIGLNQDDKGENALVVTPSEHMMVPSYPGLPYEGATITFDRDTALSREDMHFISW

EHPMIQGGIDLLMSEGVGTSAVSLLKNKALPVGTILLELIYAVDAQAPKRSGITRFLPKT

PIRLMMDSRGNDLSAQVEFEGFNRQLSPVNRHLGSKLVTSVQKDVHRLIEAGDVLVEEKV

EAVRKQAQQDMQQSLNTELERLQALKAVNPNIRDEEIEAIEAQIKELTGYINQAQVQLDS

LRLIVVSHN

>sp|Q87ST5|PDXA_VIBPA 4-hydroxythreonine-4-phosphate dehydrogenase OS=Vibrio parahaemolyticus serotype O3:K6 (strain RIMD 2210633) OX=223926 GN=pdxA PE=3 SV=1

MTTNSIRRIVVTAGEPAGIGPDLVLALSKEDWAHQIVVCADKNMLLERAKMLGIDVQLFD

YNPEEAPKAQKAGTLIVDHVEIAENAIAGQLNEANGHYVLKTLERAALGCMNDEFDAIVT

GPVHKGVINRAGVAFSGHTEFFAEKSNTPLVVMMLATEGLRVALVTTHIPLAYVSKAVTE

ERLEKIIDILHKDLVEKFAIAEPNIYVCGLNPHAGEDGCLGREEIETITPTLEKIQKEKG

IKLIGPLPADTIFNEKYLNDADAVLGMYHDQVLPVLKYKGFGRSVNITLGLPFIRTSVDH

GTALELAGTGQADTGSFRTALTHAIELVEKKQ

>sp|Q87LP2|PDXJ_VIBPA Pyridoxine 5'-phosphate synthase OS=Vibrio parahaemolyticus serotype O3:K6 (strain RIMD 2210633) OX=223926 GN=pdxJ PE=3 SV=1

MSSIYLGVNIDHIATLRNARGTKYPDPVHAAEIAERAGADGITIHLREDRRHILDRDVRI

LRETIQTRMNLEMAVTEEMVEIALKTKPEFVCLVPEKREELTTEGGLDVVGQLDKVKAAT

QKLTEAGIKVSLFIDADRQQIEAAKQCGAPFIELHTGHYADAETEEEQQAELKKIAAGAS

YADDLGIIVNAGHGLTYHNVAPIAALPEIYELNIGHSIIGRAVFDGLEKSVAEMKALMIA

ARK

>sp|Q87MN8|PDXB_VIBPA Erythronate-4-phosphate dehydrogenase OS=Vibrio parahaemolyticus serotype O3:K6 (strain RIMD 2210633) OX=223926 GN=pdxB PE=3 SV=1

MKIIVDENMPYAEELFSQLGEVILKPGRTLTADDLIDIDALMIRSVTKVNAELISKANKL

KFVGTATAGMDHVDQALLKEKGIFFTAAPGCNKVGVAEYAFSVMMVLAQQQGFSVFDKTV

GIIGAGQVGSYLEKCLKGMGINVLINDPFKQEAGDPRSFTPLAELIEQSDIITLHTPITK

DGLHPTHHLIDEKVLNGLRGDQILINAARGPVVDNQALKQRLMKQDGFTAALDVFEFEPE

VDMELLPLLAFATPHVAGYGLEGKARGTTMIFNSYCEFLNNELRAHASDLLPTAPVPTMM

LDRAWDEATLHNITQLIYDVRKDDALFRREISKPGAFDLMRKNYWDRREYSAVTLVGNET

CNLAPLAELGFQIEVSQ

>sp|Q87FE9|PDXH_VIBPA Pyridoxine/pyridoxamine 5'-phosphate oxidase OS=Vibrio parahaemolyticus serotype O3:K6 (strain RIMD 2210633) OX=223926 GN=pdxH PE=3 SV=1

MELADIRREYTKGGLRRKDLKADPIDQFNLWLEQAIKAGLTDPTAMTVATVDENGMPFQR

IVLLKNVDKDGFVFYTNLGSRKAQHLEHNSNISLHFPWHPLERQVHITGVAEKLTAMENM

KYFTSRPKDSQLAAIASKQSSRISARGVLEGKFLELKQKFAKGEIPMPTFWGGFRVKPQS

IEFWQGGEHRLHDRFLFSQHDGEWDIDRLAP

>sp|Q87IL9|PCP_VIBPA Pyrrolidone-carboxylate peptidase OS=Vibrio parahaemolyticus serotype O3:K6 (strain RIMD 2210633) OX=223926 GN=pcp PE=3 SV=1

MKKVLITGFEPFGGDAINPALEAVKRLEETSLDGGIIVTCQVPVTRFESISAVIDAIEAY

QPDCVITVGQAAGRAAITPERVAINVDDFRIPDNGGNQPIDEPIIEQGPDAYFSSLPIKR

IAQTLHESGIPCQVSNSAGTFVCNHLFYGVQHYLRDKSIRHGFVHIPLLPEQATDGNHPS

MSLDMIVAGLKLVAQVVIDHESDVVVSGGQIC

>sp|Q87FP6|PDXY_VIBPA Pyridoxal kinase PdxY OS=Vibrio parahaemolyticus serotype O3:K6 (strain RIMD 2210633) OX=223926 GN=pdxY PE=3 SV=1

MQGILSIQSHVSFGHAGNSSAVFPMQRMGFEVWPIHTVQFSNHTQYQEGWTGRAFAAEDI

SELVRGLGNIGALEKCQAVLTGYQGSAEQCLAVEDTVAKVKQANPNALYVCDPVMGAPDK

GCIVAPGIAENLLTRLMPMADVIVPNQFELSQFAEMEIHSLDDAITACQRALAKGPKVVL

VKHLYCLENGSFNMLLATQEGIYLAKRPQFEFAKQPVGVGDLISAIFTSGLLKGWSPKQA

FQHCHDACYGVLSATYHAGEWELQTIAAQQEFVEPSKHFPIEEVELEMA

>sp|Q87GX2|PEPT_VIBPA Peptidase T OS=Vibrio parahaemolyticus serotype O3:K6 (strain RIMD 2210633) OX=223926 GN=pepT PE=3 SV=1

MKHLVERFLRYVTFDTQSNPHVAQCPSSPGQLVFAELLKQEMLDFGLSDVTLDEHGYLMA

KLPSNVDYDVPPIGFIAHMDTAPDASGKNVNPQFVEDYQGGDIALGLGDEVLSPVQYPDL

HNLHGHNLITTDGTTLLGADNKAGIAEILSAIAMLIENPDIPHGDICIGFTPDEEIGRGA

DLFDVEKFGAKWAYTIDGGPQGELEYENFNAASADVIFHGVSVHPGTAKGKMVNAMNLAA

QFQVKMPADQTPETTEGYEGFFHLKSGELGIARSELGYIIRDFDREGLEERKALMQKLVD

EMNAGLKHGSVELNITDSYYNMREMVEPYPHIIELAKQAMEACDVEPLIKPIRGGTDGAR

LSFMGLPCPNIFTGGFNFHGIHEFISVEMMEKSVLVIVKIAELTAKKHG

>sp|Q87S21|PEPB_VIBPA Peptidase B OS=Vibrio parahaemolyticus serotype O3:K6 (strain RIMD 2210633) OX=223926 GN=pepB PE=3 SV=1

MSTQMSVFLSQDSAAPHWGEKALLSFSETGATIHLGEGHDLGAIQRAARQLDGQGIHSVL

LSGEHWDLESIWAFHQGYRNPKKHGLLEWTALSEEDQTELQARIKATDFTRDIINKTAEE

VAPRQLATMAAEFIKSVAPEGTVTARIVKDKDLLAEGWEGIYAVGRGSDRTSAMLQLDYN

PTGDENAPVFACLVGKGITFDSGGYSLKPSNFMSAMKADMGGSGTITGGLGLAILRGLNK

RVKLILCCAENMVSGRALKLGDIITYKNGKTVEIMNTDAEGRLVLADGLIYASEHNPELI

IDCATLTGAAKNALGNDYHALMSFDDELSHQALTAANKEKEGLWPLPLADFHRGMLPSNF

ADLSNISSGDYSPGASTAAAFLSYFVEDYKKGWLHFDCAGTYRKSASDKWAAGATGMGVR

TLARLLNEQAEK

>sp|Q87KX0|PFKA_VIBPA ATP-dependent 6-phosphofructokinase OS=Vibrio parahaemolyticus serotype O3:K6 (strain RIMD 2210633) OX=223926 GN=pfkA PE=3 SV=1

MIKKIGVLTSGGDAPGMNAAIRGVVRTALSEGLEVFGVYDGYLGLYEGRIEKLDRSSVSD

VINKGGTFLGSARFPEFKQVEVREKAIENLKKHGIDALVVIGGDGSYMGAKKLTEMGYPC

IGLPGTIDNDIAGTDYTVGYLSALNTVIDAIDRLRDTSSSHQRISIVEIMGRHCGDLTLM

SAIAGGCEYIITPETGLDKDKLISNIQDGIAKGKKHAIIALTELMMDANELARDIEAATG

RETRATVLGHIQRGGRPTAFDRVLASRMGNYAVHLLLEGHGGRCVGIVKEQLVHHDIIDA

IENMKRPVRNDLYKVAEELF

>sp|Q87LL1|PGK_VIBPA Phosphoglycerate kinase OS=Vibrio parahaemolyticus serotype O3:K6 (strain RIMD 2210633) OX=223926 GN=pgk PE=3 SV=1

MSVIKMTDLDLAGKRVFIRADLNVPVKDGKVTSDARIIASLPTIKHCLEAGAKVMVTSHL

GRPTEGEYAEEFSLQPVVNYLNDALDCEVKLAKDYLDGLELNAGELVVLENVRFNKGEKK

NEEELSKKYAALCDVFVMDAFGTAHRAQASTHGVGMHAPVACAGPLLANELEALGKAMDK

PARPMVAIVGGSKVSTKLTVLESLSKIADQLVVGGGIANTFIAAAGHNVGKSLYEADLVD

TAKKLMDECAIPVATDVACAKAFDENAEAEIKHVSEVQDDDMIFDLGPESTAALAEILKN

AKTILWNGPVGVFEFKNFEAGTKGISEAIAASEGFSVAGGGDTLAAIDKFGIKADVSYIS

TGGGAFLEFVEGKVLPAVEMLEARAK

>sp|Q87JL6|PHNX_VIBPA Phosphonoacetaldehyde hydrolase OS=Vibrio parahaemolyticus serotype O3:K6 (strain RIMD 2210633) OX=223926 GN=phnX PE=3 SV=1

MSNSPIQAVIFDWAGTIVDFGSFAPTSIFVEAFKQGFDFDIDLEEAREPMGLGKWDHIQA

VGRIPAVDKRWNEKFGRSMTNEDIDAIYAAFMPLQKAKVADHAEPILNAVEVVNGLKDKG

IKIGSCSGYPREVMDVLIPVAADYGYQPDYVVATDDLPQGGRPAPFMALKNVIELDVTDV

KACVKVDDSAPGIFEGHNAGMWTVGLLLSGNEAGLTFEEYQAADEATLEKAREKARAKFI

KSAPHYLIDTISDLPEVIVDIEQRLAAGERP

>sp|Q87LF9|OTC_VIBPA Ornithine carbamoyltransferase OS=Vibrio parahaemolyticus serotype O3:K6 (strain RIMD 2210633) OX=223926 GN=argF PE=3 SV=1

MAFNLRNRNFLKLLDFSTKEIQFLLELSAELKKAKYAGTEQKTLQGKNIALIFEKSSTRT

RCAFEVAAFDQGAQVTYIGPSGSQIGHKESMKDTARVLGRMYDGIEYRGFGQSIVEELGT

HAGVPVWNGLTDEFHPTQILADFLTMQEHSRGKQLHEMTFAYLGDARNNMGNSLMVGAAK

MGMDIRLVAPKAFWPEEHLVATCQDIAKQTGAKITLTENVEEGVKGCDFLYTDVWVSMGE

AAEAWDERVALMTPYQINMDVIKQTGNPHVKFMHCLPAFHNDETTVGKEIAEKYGMKGLE

VTEDVFESEYSIVFDEAENRMHTIKAIMVATLGS

>sp|Q87PC8|MATP_VIBPA Macrodomain Ter protein OS=Vibrio parahaemolyticus serotype O3:K6 (strain RIMD 2210633) OX=223926 GN=matP PE=3 SV=1

MKYQQLENLECGWKWKYLIKKWKDGEAITRHIDTSEADAAIAELRRIEHEPTLVLAWIEK

HMSEELENKLKQAIRAKRKRHFNAEQVHTKKKSIDLDYRVWEKLSNRANELGCTLSDAIE

YLLSEASRSEKASQKVTSIKEDLSKLLSS

>sp|Q87RS1|METN_VIBPA Methionine import ATP-binding protein MetN OS=Vibrio parahaemolyticus serotype O3:K6 (strain RIMD 2210633) OX=223926 GN=metN PE=1 SV=1

MIEIKNVNKVFYQGSKEILALKDINLHIAKGTIFGVIGSSGAGKSTLIRCVNMLEAPSSG

SIIVDGVDLTTLSKKQLVETRRNIGMIFQHFNLLSSRTVFDNVALPLELAGKDKSQITTK

VTELLKLVGLADKHESYPSNLSGGQKQRVAIARALASDPSVLLCDEATSALDPATTQSIL

ELLKEINRKLNITILLITHEMEVVKSICHEVAIIGGGELVEKGTVGDIFAHPKTELAHEF

IRSTLDLSIPEDYQARLQPNRVEGSYPLVRMEFTGATVDAPLMSQISRKYNIDVSILSSD

LDYAGGVKFGMMVAELFGNEQDDSAAIEYLREHNVKVEVLGYVL

>sp|Q87L06|MIAA_VIBPA tRNA dimethylallyltransferase OS=Vibrio parahaemolyticus serotype O3:K6 (strain RIMD 2210633) OX=223926 GN=miaA PE=3 SV=1

MTDKLPLALFLMGPTASGKTELAIRLRQKFPVEIISVDSALIYKGMDIGTAKPDERELSL

APHRLIDILDPSESYSAADFRRDALQAMDDIVAEGKIPLLVGGTMLYYKALLEGLSPLPA

ANPEIRQQIEQEALTKGWSVLHDELKEIDPVSAARIHPNDPQRLSRALEVYRISGKTLTE

LTQTKGESLPYRVKQFAIAPKDRAELHRRIELRFDKMMEAGFEEEMKALYARKDLHPDLP

SIRCVGYRQMWEYLDGECTRDEAVFRGVCATRQLAKRQITWLRSWDDLTWLDSDNIEQAL

ETMSEAIASD

>sp|Q87FR1|MTGA_VIBPA Biosynthetic peptidoglycan transglycosylase OS=Vibrio parahaemolyticus serotype O3:K6 (strain RIMD 2210633) OX=223926 GN=mtgA PE=3 SV=1

MLKRLKSFLFKMVLILLIAPIVLVGVVKYVDPPIWGWKLSRIVAPPKNYPDSSQHEWVSL

TRISKNMQLAVIATEDQKFPHHYGVDFESLFDVISEAGDHGPSRGASTITQQAAKNVFLF

PSHSYVRKAYELYFALLMELMWSKERILEVYLNVVEFGPGIYGAEAAAQNYFGVSAKQLS

KWQAARLAVVLPNPYRIKVYPQSDYTARRTRWAMNQMSNLGSVQL

>sp|Q87SG3|MURC_VIBPA UDP-N-acetylmuramate--L-alanine ligase OS=Vibrio parahaemolyticus serotype O3:K6 (strain RIMD 2210633) OX=223926 GN=murC PE=3 SV=1

MTIQHTQDLAQIRAMVPEMRRVKCIHFIGIGGAGMSGIAEVLLNEGYEITGSDLSENPVT

ERLVSKGATVFIGHQASNVEKASVVVVSTAINEENPEVMAARELRIPIVRRAEMLAELMR

FRHGIAVAGTHGKTTTTALVTQIYSEAGLDPTFVNGGLVKSAGTNARLGSSRILIAEADE

SDASFLHLQPMVSIVTNIEADHMDTYGGDFETLKQTFIDFLHNLPFYGQAIVCIDDPVIR

ELIPRISRQVITYGFSDDADVRIENYHQEGQQGKFTVVRKGRANLDITLNIPGRHNALNA

SAAIAVATEDDIEDDAILKAMAGTQGTGRRFDHLGEFDTGNGHAMLVDDYGHHPTEVDVT

IKAARSGWQDKRLVMIFQPHRYSRTRDLYDDFANVLEQVDVLIMLDVYAAGEKPIAGADG

RSLCRTIRSRGKVDPIFVPEIEQLPSVLANVIQDGDLILTQGAGDVGKVAKQLANLELNI

NKMLG

>sp|Q87KP1|MURI_VIBPA Glutamate racemase OS=Vibrio parahaemolyticus serotype O3:K6 (strain RIMD 2210633) OX=223926 GN=murI PE=3 SV=1

MRASSKKKVLVFDSGVGGLSVFQEIHQLLPHLDYFYLFDNEAYPYGELDQNVLISRVNQL

VSALVAEHHIDIVVIACNTASTIVLPSLRDNLSVPVVGVVPAIKPASLLATQGVGLIATP

ATVTRQYTHELIRDFAQGKPVELLGSTRLVDMAEEKLRGESVPLDELKSILSPLCNKVDV

AVLGCTHFPLIKNEIQQVLGSNVVLIDSGEAIARRVKALLSCGELEEKEEGIKRIFASAP

PWQEDALNICLAKLGFNPVQIYRHLGVSDR

>sp|Q87SA6|MUTH_VIBPA DNA mismatch repair protein MutH OS=Vibrio parahaemolyticus serotype O3:K6 (strain RIMD 2210633) OX=223926 GN=mutH PE=3 SV=1

MKPEPQSEAELMERAHDIAGLSFAELADEAGMTVPENLKRDKGWVGQLLEWHLGAPAGSK

PQQDFSKLGIELKSIPIGYNGRPLETTFVCVAPLTGVQGLTWETSHVRNKLSRVLWVPVE

GEREIPLAERRVGTPLIWSPDKEEEQILRNDWEELMEMIVFGKFDQISARHGEALHLRPK

AANAKALTEAYSSNGKPMKTLPRGFYLRTQFTEQILLKHYINVQSE

>sp|Q87LQ9|MUTS_VIBPA DNA mismatch repair protein MutS OS=Vibrio parahaemolyticus serotype O3:K6 (strain RIMD 2210633) OX=223926 GN=mutS PE=3 SV=1

MKAEQKHTPMMQQYLKLKAENPEILLFYRMGDFYELFYDDAKRASQLLDISLTKRGSSAG

EPIPMAGVPFHAVEGYLAKLVQLGESVAICEQIGDPATSKGPVERKVVRIVTPGTVTDEA

LLSERLDNLIAAIYHHNGKFGYATLDVTSGRFQLTEPETEEAMMAELQRTAPRELLFPED

FEPVHLMSNRNGNRRRPVWEFELDTAKQQLNQQFGTRDLVGFGVEHASLGLCAAGCLIQY

VKDTQRTALPHIRSLTFDRQDHSVILDAATRRNLEITQNLAGGTDNTLAAVLDHCSTPMG

SRMLKRWLHQPMRCIDTLNNRLDAIGEIKDQGLFTDLQPTLKQIGDIERILARLALRSAR

PRDMARLRHAMQQLPELESLTASLTHPYLVKLAQYAAPIDEVCELLERAIKENPPVVIRD

GGVIAEGYNEELDEWRKLADGATEYLEKLEADERERHGIDTLKVGYNAVHGFFIQVSRGQ

SHLVPPHYVRRQTLKNAERYIIPELKEHEDKVLNSKSKALALEKKLWEELFDLLMPHLEQ

MQNLASAVSQMDVLQNLAERADSLDYCRPTLVKDAGIHIQAGRHPVVEQVTSDPFIANPI

ELSPSRKMLIITGPNMGGKSTYMRQTALIALMAHIGSYVPAESAQIGSLDRIFTRIGASD

DLASGRSTFMVEMTETANILHNATKNSLVLMDEIGRGTSTYDGLSLAWASAEWLATQIGA

MTLFATHYFELTELPNLLPNLANVHLDAVEHGDSIAFMHAVQEGAASKSYGLAVAGLAGV

PKTVIKNARTKLSQLEQLGQASDSPRPSTVDVANQLSLIPEPSEIEQALSNIDPDDLTPR

QALEELYRLKKML

>sp|Q87K60|NAGB_VIBPA Glucosamine-6-phosphate deaminase OS=Vibrio parahaemolyticus serotype O3:K6 (strain RIMD 2210633) OX=223926 GN=nagB PE=3 SV=1

MRLIPLTRAAQVGKWAAAHIAKRINDFKPTAERPFVLGLPTGGTPLATYKALIELYQAGE

VSFKHVVTFNMDEYIGIPADHPESYRSFMYNNFFNHIDIQEENINLLNGNTDNHEAECKR

YEDKIKSYGKINLFMGGVGNDGHIAFNEPASSLSSRTRIKTLTEDTRIANSRFFDGDINQ

VPKYALTIGVGTLLDAEEVMILVTGHNKALALEAAVEGCVNHLWTVSALQLHPKAVIVCD

EPSQQELKVKTVKYFSELEAENIKGF

>sp|Q87PK8|NAGK_VIBPA N-acetyl-D-glucosamine kinase OS=Vibrio parahaemolyticus serotype O3:K6 (strain RIMD 2210633) OX=223926 GN=nagK PE=3 SV=1

MYYGFDVGGTKIEFGAFNEKLERVATERVPTPTDNYELLVDTIAELVNKYDAEFGCEGTI

GLGLPGMEDADDATVLTVNVPAAKGKPLRADLEAKIGRSVKIENDANCFALSEAWDEELQ

DEPSVLGLILGTGFGGGFIYDGKVFSGRNHVAGEVGHTRLPIDAWFHLGENAPLLGCGCD

KKGCLDSYLSGRGFELLYAHYYGEEKKAIDIIKAHAEGEAKAVEHVERFMELLAICFANI

FTATDPHVVVLGGGLSNFELIYEEMPKRIPKYLLSVAKCPKIIKAKHGDSGGVRGAAFLH

IK

>sp|Q87S20|NDK_VIBPA Nucleoside diphosphate kinase OS=Vibrio parahaemolyticus serotype O3:K6 (strain RIMD 2210633) OX=223926 GN=ndk PE=3 SV=1

MALERTFSIVKPDAVERNLIGEIYNRIEKAGLRIIAAKMVHLTEEQASGFYAEHEGKEFF

QPLKEFMTSGPIMVQVLEGENAIARYRELMGKTNPEEAAAGTLRADYALSMRHNSVHGSD

SPESAAREIEFFFPESEICPR

>sp|Q87RP4|MIAB_VIBPA tRNA-2-methylthio-N(6)-dimethylallyladenosine synthase OS=Vibrio parahaemolyticus serotype O3:K6 (strain RIMD 2210633) OX=223926 GN=miaB PE=3 SV=1

MSKKLLIKTWGCQMNEYDSSKMADLLNAANGYELTEEPEEADVLLLNTCSIREKAQEKVF

HQLGRWKTLKDKKPGVVIGVGGCVATQEGDHIRERAPYVDVIFGPQTLHRLPEMIKQSQT

DDAPVMDISFPEIEKFDRLPEPRAEGATAFVSIMEGCSKYCTYCVVPYTRGEEVSRPMDD

VLFEIAQLAEQGVREVNLLGQNVNAYRGPMHDGEICSFAELLRLVASIDGIDRIRFTTSH

PLEFTDDIIAVYEDTPELVSFLHLPVQSGSDRILTMMKRPHTAIEYKSIIRKLRKARPDI

QISSDFIVGFPGETDKDFQDTMKLIKDVDFDMSFSFIFSPRPGTPAADYPCDIPEQVKKE

RLYELQQTINAQAMRYSRLMLATEQRVLVEGPSKKNLMELRARTENNRVVNFEGSADLIG

QFVDVKITDVFANSLRGELVRTEKDMDLRSVISPTQMMAKTRREDELGVATFTP

>sp|Q87MN6|MNMC_VIBPA tRNA 5-methylaminomethyl-2-thiouridine biosynthesis bifunctional protein MnmC OS=Vibrio parahaemolyticus serotype O3:K6 (strain RIMD 2210633) OX=223926 GN=mnmC PE=3 SV=1

MTSIKNAELGWNEAGTPVSDQFDDVYFSNVNGLEETRYVFLKQNHLPERWQEFDQRRFVI

GETGFGTGLNFLAVWQWFNEFRRNHPDAALKELHFISFEKYPLSLADLKKAHEAWPELAE

YAEKLQKHYPAAVPECHRIVLEDGAITLDLWFGDIKDCMPQVPYNEQGLIDAWFLDGFAP

SKNPEMWNQNLFNNMAKLAKQDCTVATFTAAGFVRRGLNEAGFAMKKVKGFGTKREMIAG

SMEQREKQSNHLAWFNRTASSNLDSIAIIGGGIASAALAKTLVRRGQNVTLYCKDTQPAE

GASGNRQGAVYPLLNGPHTGVSRVFAPAFLFARQFVEQAAQEIDFDHDWCGVTQLMWDDK

STDKLEKMLEGNFSPELIRKLSAEETAETIGLSIDMASVHYPMGGWLCPAELTRGLITQL

EKTELFEAKFEHQVESLTWDEARELWTLNANGQNFEHSTVVVANGNEFQTLSQTEALPMG

QVKGQVSHAPATKTLSKLKSVLCYDGYMTPVNPNNQHLCIGASYDRSHLDYEFDENAQRD

NVEKLVKCIPNQEWTKEVDTSGNLSRQGIRCVSRDHLPFVGNVGDFETIKMQYADLQNQA

EEEVEAIHQFPNLFCFLGLGSRGLSSAPLLAEVLASQICGDPLPLPVDVLTELHPSRMWV

RKLRKGKAITEL

>sp|Q87RC5|MINE_VIBPA Cell division topological specificity factor OS=Vibrio parahaemolyticus serotype O3:K6 (strain RIMD 2210633) OX=223926 GN=minE PE=3 SV=1

MSLLEFFRPQKKTSASLAKERLQIIVAERRSQNDPAPSYLPQLKEDILKVISKYVDIDPN

MVDLTFEHKDDDISVLELNVKLPDDEK

>sp|P40608|MOTX_VIBPA Sodium-type polar flagellar protein MotX OS=Vibrio parahaemolyticus serotype O3:K6 (strain RIMD 2210633) OX=223926 GN=motX PE=4 SV=2

MKLRTVAASLLLMLSATTVRASAADVGAPVPIYTEAELIKLIEQNKHLQRVRADNCQLVE

DIVARATRINLPAYEFLYGDMLAWGVCVEQDVELGLYYMENAAQQGLPAALEQIGRYYSR

GTLVQQDKERAIPYLREAASMGNLNARIHLAELLLRDYGSPLDYEDAYRWLYNSVTADQR

QHKRIAVLRRGLEQRMPQNIVARAKRRDMFW

>sp|P46233|MOTY_VIBPA Sodium-type flagellar protein MotY OS=Vibrio parahaemolyticus serotype O3:K6 (strain RIMD 2210633) OX=223926 GN=motY PE=3 SV=1

MNKWLITSGVMLSLLSANSYAVMGKRYVATPQQSQWEMVVNTPLECQLVHPIPSFGDAVF

SSRASKKINLDFELKMRRPMGETRNVSLISMPPPWRPGEHADRITNLKFFKQFDGYVGGQ

TAWGILSELEKGRYPTFSYQDWQSRDQRIEVALSSVLFQSKYNAFSDCIANLLKYSFEDI

AFTILHYERQGDQLTKASKKRLAQIADYVRHNQDIDLVLVATYTDSTDGKSESQSLSERR

AESLRTYFESLGLPEDRIQVQGYGKRRPIADNGTPIGKDKNRRVVISLGRTQV

>sp|Q87ID3|MGSA_VIBPA Methylglyoxal synthase OS=Vibrio parahaemolyticus serotype O3:K6 (strain RIMD 2210633) OX=223926 GN=mgsA PE=3 SV=1

MQKTTRTMPAHKHVALVAHDNCKPELLRWVKENKEKLQRHFLYATGTTGHMLSKETGLAI

KSMISGPMGGDQQLGALISEGKIDVLVFFWDPLNAVPHDPDVKALLRIASVWNIPVATNR

ATAKFLFDSPLLEQEVDIEVPDYEAYLAERM

>sp|Q87TR6|MNME_VIBPA tRNA modification GTPase MnmE OS=Vibrio parahaemolyticus serotype O3:K6 (strain RIMD 2210633) OX=223926 GN=mnmE PE=3 SV=1

MTTDTIVAQATAPGRGGVGIIRVSGPKANQVALEVTGKTLKPRYAEYLPFQAEDGTVLDQ

GIALYFPNPHSFTGEDVLELQGHGGPVVMDMLIKRILGIAGVRAARPGEFSERAFLNDKM

DLTQAEAIADLIDASSEEAAKSALQSLQGQFSQRIQTLVESLIHLRIYVEAAIDFPEEEI

DFLADGKVAGDLQAIIDNLDAVRKEANQGAIMREGMKVVIAGRPNAGKSSLLNALSGKES

AIVTDIAGTTRDVLREHIHIDGMPLHIIDTAGLRDASDEVEKIGIERAWDEIAQADRVLF

MVDGTTTDATDPKEIWPDFVDRLPESIGMTVIRNKADQTGEDMGICHVNDPTLIRLSAKT

GAGVDALRNHLKECMGFSGNTEGGFMARRRHLDALERAAQHLQIGQEQLEGYMAGEILAE

ELRITQQHLNEITGEFSSDDLLGRIFSSFCIGK

>sp|Q87GW6|NAPA_VIBPA Periplasmic nitrate reductase OS=Vibrio parahaemolyticus serotype O3:K6 (strain RIMD 2210633) OX=223926 GN=napA PE=3 SV=1

MKMTRRAFVKANAAASAAAVAGITLPASAANLIASSDQTKITWDKAPCRFCGTGCSVLVG

TQNGKVVATQGDPEAPVNKGLNCIKGYFLSKIMYGQDRLTQPLLRMKDGKYHKDGEFTPV

SWDVAFDTMAEKWKASLEKKGPTSVGMFGSGQWTVMEGYAAAKMMKAGFRSNNIDPNARH

CMASAVVGFMRAFGIDEPMGCYDDFENADAFVLWGSNMAEMHPVLWTRITDRRLSHPHVR

VNVLSTYYHRSFELADHGYIFNPQSDLAIANFIANYIIENDAVNWDFVNKHTNFTQADTD

IGYGLRDDDPLQKAAKNPNSGKLTSISFEEYKKSVAPYTVEKASEISGVEKEKLIELAKQ

YADPNTKVMSLWTMGMNQHTRGVWMNNLVYNIHLLTGKIATPGNSPFSLTGQPSACGTAR

EVGTFAHRLPADMVVANPKHRQIAEKIWKLPEGTIPPKPGFHAVLQDRMLNDGVLNCYWV

QCNNNMQAGPNINTERLPGYRNPENFIVVSDPYPTATAQAADLILPTAMWIEKEGAYGNA

ERRTQAWYQQVGTVGDAKSDLWQVMEFSKRFKMEEVWPEELLAKAPQYRGKTMYDMLFKN

GQVDKFPLEEARELNDDSHHFGFYVQKGLFEEYATFGRGHGHDLAPYDVYHTVRGLRWPV

VDGKETQWRFKEGSDPYAKAGSGWDFYGNADGKAKIISAPYEAPPEVPDSEFDLWLCTGR

VLEHWHTGTMTRRVPELYKAVPDAVCYMHPEDAKARNVRRGEEVVIANKRGEVRVRVETR

GRNRPPKGLVFVPFFDARILINKLILDATDPLSKQTDFKKCPVKITKVA

>sp|Q87MY2|MOAC_VIBPA Cyclic pyranopterin monophosphate synthase OS=Vibrio parahaemolyticus serotype O3:K6 (strain RIMD 2210633) OX=223926 GN=moaC PE=3 SV=1

MTQFTHINASGEANMVDVSAKAETVREARAEAFVHMAPETLQLIVSGQHHKGDVFATARI

AGIQAAKKTWDLIPLCHPLLLSKVEVQLEAIEAENKVRIESVCKLAGKTGVEMEALTAAS

VAALTIYDMCKAVQKDMVIGQVRLLEKTGGKSGHFKAES

>sp|Q87HN4|MODC_VIBPA Molybdenum import ATP-binding protein ModC OS=Vibrio parahaemolyticus serotype O3:K6 (strain RIMD 2210633) OX=223926 GN=modC PE=3 SV=1

MTIKIQFKQTLGETNFDIDLSLPRNEISALFGRSGAGKTTLINVISGLVTPQQGRIAIGD

HVLFDSEQGINLPTHKRKIGYVFQDSRLFPHYSVQGNLLYGVKEKDDAYFDAVTDLLSIK

PLLKRFPISLSGGEKQRVAIARALLSKPDLLLMDEPLASLDMPRKREVMPFLEELSDKVN

IPIIYVTHSLQEILRLAQHLAIIDKGQVTTSGKLEEVWASHAMRPWQSFSDQSSLFEGKI

EAHHSQYALTRVKLAPNASLWVQKIDGEPDTPIRLQVRANDVSIALEQPKATSIRNVLPA

EVHSIEAFNAGDDKQSINVSLQLDDGCYLWATITPWALDDLNLKVGDKVYAQVKGVSVTQ

RDVALAPH

>sp|Q87SG7|MRAY_VIBPA Phospho-N-acetylmuramoyl-pentapeptide-transferase OS=Vibrio parahaemolyticus serotype O3:K6 (strain RIMD 2210633) OX=223926 GN=mraY PE=3 SV=1

MIIWLAELLQPYLSFFRLFEYLSFRAILSVLTALGLSLWMGPIMIKRLQMLQIGQVVRNE

GPESHFSKRGTPTMGGIMILAAISITILLWTDLSNPYVWAVLTVLLGYGAVGFVDDYRKV

VRKNTDGLIARWKYFWQSLIAFVVAFALYAYGKDTAATQLVVPFFKDVMPQLGLMYIILT

YFVIVGTSNAVNLTDGLDGLAIMPTVLVAAGFAVIAWATGNVNFSEYLHIPYLPHASELV

VVCTAIVGAGLGFLWFNTYPAQVFMGDVGSLALGGALGTIAVLVRQELVLVIMGGVFVME

TLSVILQVGSYKLRGQRIFRMAPIHHHYELKGWPEPRVIVRFWIISMVLVLIGLATLKVR

>sp|Q87SQ3|MTLD_VIBPA Mannitol-1-phosphate 5-dehydrogenase OS=Vibrio parahaemolyticus serotype O3:K6 (strain RIMD 2210633) OX=223926 GN=mtlD PE=3 SV=1

MKNAVHFGAGNIGRGFIGKLLADAEVEVTFADVDVPLVDQLSHKQEYKVKVVGTECKIDT

VTHVTAVNSASEDVIDRIVKTDLVTTAVGPNVLDIIAKTIAKGIAKRFEAGNDAPLNIIA

CENMVRGTTHLKGEVYKHLDKSLHAKADELVGFVDSAVDRIVPPAEAANDDPLEVTVESF

SEWIVDEQQFKGDIPNIAGMEKTNNLMAFVERKLFTLNTGHCITAYLGCLKGHRTIREAI

EDPNIHAEVKQAMQESGEVLIRRYGFDHDMHNAYIEKILGRFANPYLVDEVDRVGRQPIR

KLGANDRLVKPLLGTIEYGTENQTLLKGIAAALKYTNDTDPQAVELQTSLKEVGVTKTLA

KYTGLAEDSDEVAQIETLYNQL

>sp|Q87FD6|MURQ2_VIBPA N-acetylmuramic acid 6-phosphate etherase 2 OS=Vibrio parahaemolyticus serotype O3:K6 (strain RIMD 2210633) OX=223926 GN=murQ2 PE=3 SV=1

MKIDLSRLVTESRNPASTEIDTLSTIEMLQVINEEDQKVALAVKAVLPQIAKTVNAITAA

FANGGRLVYMGAGTSGRLGILDASECPPTYGTHPDMVIGLIAGGHQAILKAVENAEDDVK

MGQDDLKALHLTKHDVVVGIAASGRTPYVLGGLEYAKSIGATTASIACNPECAMAKAADI

AILPIVGAEVVTGSSRMKAGTAQKLVLNMLTTGAMIRSGKVFGNLMVDVEATNAKLIQRQ

TNIVVEATGASKEEAERALNACDRHCKTAILMILADLDAEQAKSRLAAHNGFIRAALNNN

>sp|Q87L05|MUTL_VIBPA DNA mismatch repair protein MutL OS=Vibrio parahaemolyticus serotype O3:K6 (strain RIMD 2210633) OX=223926 GN=mutL PE=3 SV=1

MTIKILPARLANQIAAGEVVERPASVIKELVENSLDSGATRIDIDIEKGGAKLIRVRDNG

KGIAKDELGLALSRHATSKIHTLDDLEAIMSLGFRGEALASISSVSRLTLTSRPAAQEEA

WSAYSEGRDMQVKLQPAAHPIGTTVEVLDLFFNTPARRKFLRTEKTEFAHIDELLKRIAL

SRFDVSINVRHNGKVIRQYRAAKNQLQTEKRIAAVCGNAFVRNMLRIELEHQGLKLHGWI

TTPDGARQQSDLQYCYVNGRMMRDKLINHAIRQSYEMSLKPDQFAAYVLFIELDPHQVDV

NVHPAKHEVRFHQARLVHDFIYQALADALAQSSVIDKPQVNESAFHRAEPEERESQPETT

PQYSPQSVSTTVPERVYQAIDKTPTYPGRTDYEIKPRDRAPSDSSVREARIADSFKRTDW

IESKLAPKPNVGKERHAEPAPSKREVHAYHELLKTPDFESQQAEQPTSIESVREVSLPQV

TALGKALVVVDEQFVLMSSDSGVALVSLPRSEFYRTKGQLTPSEGALKAQPLLVPLSMKL

DTDLVRLAQDYQQDFAQLGIQLKARNDKALMVMGVPAPLRQQNLQNLVPDLLSYAQTWMK

GEKASTQMLPALIDWLAVQVTTVKSHYTLSEAIQIIAELEQLRHGQLPLDDKTFVSAVDF

SATIAKLKP

>sp|Q87LF4|MURA_VIBPA UDP-N-acetylglucosamine 1-carboxyvinyltransferase OS=Vibrio parahaemolyticus serotype O3:K6 (strain RIMD 2210633) OX=223926 GN=murA PE=3 SV=1

MEKFRVIGSDKPLVGEVTISGAKNAALPILFASILAEEPVEVANVPHLRDIDTTMELLKR

LGAKVSRNGSVHVDPSSINEYCAPYDLVKTMRASIWALGPLVARFGQGQVSLPGGCAIGA

RPVDLHITGLEQLGATITLEDGYVKAEVDGRLKGAHIVMDKVSVGATITIMCAAALAEGT

TTLDNAAREPEIVDTADFLNKLGAKISGAGTDTITIEGVERLGGGKHSVVADRIETGTFL

VAAAVSGGKVVCRNTNGHLLEAVLAKLEEAGALVETGEDWISVDMTDRELKAVSIRTAPH

PGFPTDMQAQFTLLNMMAKGGGVITETIFENRFMHVPELMRMGAKAEIEGNTVICGDVES

LSGAQVMATDLRASASLVIAGCIAKGETIVDRIYHIDRGYDKIENKLAALGANIERVS

>sp|Q87K98|MNMG_VIBPA tRNA uridine 5-carboxymethylaminomethyl modification enzyme MnmG OS=Vibrio parahaemolyticus serotype O3:K6 (strain RIMD 2210633) OX=223926 GN=mnmG PE=3 SV=1

MLYHENFDVIVVGGGHAGTEAALASARTGQKTLLLTHNIDTLGQMSCNPAIGGIGKGHLV

KEVDAMGGLMAEAIDHAGIQFRTLNASKGPAVRATRAQADRALYKAYVRNALENAPNLTL

FQQSVDDLIVEQDRVVGVVTQMGLKFHAKAVVLTVGTFLGGKIHIGMESSSGGRAGDPPS

IALADRLRELPFRVDRLKTGTPPRIDARTVDFSVLEAQHGDNPTPVFSFMGKREHHPRQI

PCFITHTNEQTHEVIRNNLDRSPMYAGVIEGIGPRYCPSIEDKVMRFADKNSHQIFIEPE

GLTTHELYPNGISTSLPFDVQVQIVRSMKGFENAHIVRPGYAIEYDFFDPRDLKQTYETK

FISGLFFAGQINGTTGYEEAAAQGLMAGLNASLYSQGKEGWSPRRDQAYMGVLIDDLSTM

GTKEPYRMFTSRAEYRLLLREDNADLRLTEKARELGLIDDVRWARFNEKIENMETERQRL

KSTWVNPNSAGIDELNKLLKTPMAREASGEDLLRRPEISYSQLTQLDAFAPALEDQQAAE

QVEIQVKYDGYIKRQQEEIEKSLRHEHTKLPADLDYKDVKGLSNEVVAKLSEAKPESIGI

ASRISGITPAAISILLVHLKKHGLLKKGEEE

>sp|Q87MY0|MOAA_VIBPA GTP 3',8-cyclase OS=Vibrio parahaemolyticus serotype O3:K6 (strain RIMD 2210633) OX=223926 GN=moaA PE=3 SV=1

MERCSVAQQFEDKFHRKFYYLRLSVTDVCNFKCTYCLPDGYKPSGNKNSSFLSLPEIKRV

VKAFADCGTSKVRITGGEPSLRKDFTDIIHSVATTPGIKKVATTTNGYRMAKQVADWREA

GLTNINVSVDSLDPRMFHQITGENKFTEVMNGIERAFEVGYEQVKVNVVLMKDLNHHELP

AFLNWIKDRPIQLRFIELMQTGEMDDLFSKHHVSGVAIRNQLIANGWLLKVRSHHDGPAQ

VFVHPDYKGEIGLIMPYEKDFCESCNRLRVSALGKLHLCLFGEHGVELRDLLERDDQENE

LIERIQSQLQTKSVSHFLHDGNTGMTPHLASIGG

>sp|Q87R16|MSBA_VIBPA Lipid A export ATP-binding/permease protein MsbA OS=Vibrio parahaemolyticus serotype O3:K6 (strain RIMD 2210633) OX=223926 GN=msbA PE=3 SV=1

MSINTDETTWQTFKRLWQFIRLYKSGLIVAVIALVINAISDTYMISLLKPLLDEGFGNAD

SDFLRTLPLIIFVMMFIRGTSGFVSTYCLSWVSGNVVMLVRRMVFNHFMHMPVSYFDKEK

TGNLLSRITYDSEQVSAATSQALVSIVREGASIIGLLVLMFYNSWQLSLVLFAVAPVVAW

GIGVVSKRFRKISKNMQTMMGNVTASAEQMLKGHKVVLSYGGQDIERQRFDKVSNQMRQQ

SMKLVTAQAAANPIIQMIASFAIVAVLYLASIDSIKEQLTPGTFTVVFSAMFGLMRPLKA

LTNVTSQFQRGMAASQTLFALIDLEPEKNEGKYTVERAKGDVSVKDVSFTYVGSEKPALE

HVSFDIPRGKTVALVGRSGSGKSTIANLFNRFYDVDSGSITLDGRDIRDYELKNLREQFA

LVSQNVHLFNDTIANNIAYATEDKYERSDIEHAAKLAHAMEFINKMENGLDTMIGENGAS

LSGGQRQRVAIARALLRDAPVLILDEATSALDTESERAIQAALDELQKDKTVLVIAHRLS

TIEKADEILVVDDGAIIERGNHADLIAKNGAYAQLHRIQFGE

>sp|Q87QW2|MUKB_VIBPA Chromosome partition protein MukB OS=Vibrio parahaemolyticus serotype O3:K6 (strain RIMD 2210633) OX=223926 GN=mukB PE=3 SV=1

MIERGKYQSLTMVNWNGFFARTFDIDGLVTTLSGGNGAGKSTTMAAFITALIPDQTLLHF

RNTTEAGSSQSSRDKGLYGKLQPGACYAALDVVNSRNQRLLFAVKLQQVAGRDKKVDIKP

FVIQGLPSHVKPTDILVESVSATQARVRQINEVKDAIAEFEGVQFKAFSSIVDYHAQMFE

FGVIPKKLRNSSDRSKFYRLIEASLYGGISSAITRSLRDYLLPQNGGVKKAFQDMESALR

ENRMTLEAIKTTQADRDLFKHLITESTNYVAADYMRHANDRRNKLEQTLSLRSELFGSRE

TLIEQNNLLNRVQEELELLIESESALEQDYQAASDHLQLVQNALRQQEKIERYQEDLEEL

SERLEEQMMVVEEAQERVMMVEEQATVAEEEVDSLKTQLADYQQALDVQQTRALQYQQAV

QALEKAKQLLGDDCLTAESAQALVSELKNKESESTNALLSVKHKLDMSSAAAEQFETALK

LVQSIVGQVERKDAAEQAKIVITKARESQQIAQNEQQWRAQHRDLERSLNQQRQARELVK

EYQKQFHVELTDEITFEQERERHAMQIETLEMTQEELREQRSEQRRLEQDAAAEINKLEA

IAPTWIAANDALEKLREQSGVDLEDRHAVMSHMQVVLEQEKELSLAKDKLAERRSQLESE

IERLASPGGSNDPRLKGLADTLGGVLLSEIYDDITIDDAPYFSAMYGPARHAIVVSDLSG

IEEKLVELDDCPEDLYIIEGDIDAFDDSSFDAEELEGAVCVRMNDRQMRYSRFPEIPLFG

RAAREQRLELLRNEREEVVEKHAKAAFDSQKMQRLYQAFNQFVANHIQVAFEADPEQALA

NVREKRGQIARVLADLEAKEQQHRSQLQTSKQALSSLDKLAPNMALIEDDTLQARFDELE

EKIAQLSEAKAFLNNHAKAVAELEKIASALDADPEQFDALEAEYKAADEQLQELKKQIFA

LSDLVERRHYFAYSDSVDLLNQSSELSEQLKAKLVQAEQMRTRSREELKQAQGQMNQYNQ

VLASLKSSHQAKLETVQEFKQELQEFGVNADEGAEERAIRRRDELHERLHTSRSRKSEYE

RTITSTELEMKGLAKRLKKVQKEYAELRTFVVAAKAGWCSVLRLARENDVERRLHKRELA

YMSADELRSMSDKSLGALRLAVANNDDLRDALRLSEDNARPERKVLFYIAVYQHLRERIR

QDIIRTDDPVEAIEEMEVELARLTEELTQRENRLAISSESVASIIKKTIQREQNRIRMLN

QGLSNISFGQVKGVRLNVKIRESHEVLLHGLSSQQEQHKDLFESPRFTFSEAMAKLFQRV

NPHIDMGQRSPQVLGEELLDYRNYLELSVEVNRGSDGWLQAESGALSTGEAIGTGQSILL

MVVQSWEEESRRLRSKDIIPCRLLFLDEAARLDAKSISTLFELCDRLDMQLLIAAPENIS

PEKGTTYKLVRKVFKDHEHVHVVGLRGFGQTDKPKSEVQEMIEEFES

>sp|Q87QW3|MUKE_VIBPA Chromosome partition protein MukE OS=Vibrio parahaemolyticus serotype O3:K6 (strain RIMD 2210633) OX=223926 GN=mukE PE=3 SV=3

MSDNLAKAISNPLFPALDSMLRAGRHISTEDLDNHALLSDFELELSSFYQRYNTELVKAP

EGFFYLRPRSTSLIGRSVLSELDMLVGKVLCFLYLSPERLAHEGIFTNQELYDELLALAD

EKKLMKLVTNRATGSDLDKEKLFEKVRTSLRRLRRLGMIINIGETGKFSISEAVFRFGAD

VRVGDDIREAQLRLIRDGEAVVHTKEPSQGSLLSEEDQEEQAQEEMTEEGEA

>sp|Q87SE5|MTNN_VIBPA 5'-methylthioadenosine/S-adenosylhomocysteine nucleosidase OS=Vibrio parahaemolyticus serotype O3:K6 (strain RIMD 2210633) OX=223926 GN=mtnN PE=3 SV=1

MKVGIIGAMEQEVTILKEAMTNCQTVNKAGCTFFSGQINDVDVVLLQSGIGKVAAAVGTT

ILLDEYQPDVVINTGSAGGFDSSLNLGDVVISTEVRHHDADVTAFGYEIGQMAGQPAAFK

ADEKLMDLAEKALEQMANTHAVRGLICTGDAFVCTAERQAFIRENFPSVIAVEMEASAIA

QTCHQFNTPFVVVRAISDVADKESPMSFEEFLPLAAKSSSEMVFKMLELVK

>sp|Q87PK5|MOBA_VIBPA Molybdenum cofactor guanylyltransferase OS=Vibrio parahaemolyticus serotype O3:K6 (strain RIMD 2210633) OX=223926 GN=mobA PE=3 SV=1

MLQPTQTSWVILAGGQASRMGGKDKGLIELNQKPLIEHVIERLSPQTPRILINANRNQDA

YSKFGFVFSDQFKDFPGPMGGIHAGLMHAETDWVGFVPCDSPQINTDLVERFCQAVKEDS

DILVAHDGDHQQPVFTLYHKRVLPKLTAFLERGDRKIILLYKECNTSYVDFSDSPNCFVN

LNTPEELAQFGQLES

>sp|Q87MS5|MSRB_VIBPA Peptide methionine sulfoxide reductase MsrB OS=Vibrio parahaemolyticus serotype O3:K6 (strain RIMD 2210633) OX=223926 GN=msrB PE=3 SV=1

MPKIVKKEPKFVEQSGKKVTKSDEQWREQLSDEEFRVCREQGTEPPFSGKLLHNKETGVY

ACTCCNAPLFISDNKYDSGCGWPSFDAPLNNEAIRYLEDLSHGMVRTEIRCASCDSHLGH

VFEDGPKTTGERYCVNSVSLIFNKSDE

>sp|Q87NA1|METE_VIBPA 5-methyltetrahydropteroyltriglutamate--homocysteine methyltransferase OS=Vibrio parahaemolyticus serotype O3:K6 (strain RIMD 2210633) OX=223926 GN=metE PE=3 SV=1

MATTTHILGYPRIGEKRELKFAQEKYWRGDIDQTELKKVGADLRAKNWQTQTEAGLSFTT

AGDFAWYDHVLTTTLLLGHVPKRHAGGFPNLDTLFKVGRGQSQAGCGCAGAAASDMTKWF

NTNYHYIVPEFSKDDTFEVSWPQLFEEINEAVQAGHKVKPVLLGPVSYLYLGKEVEEGFD

RLTLLPRLLTAYQAILAKLASQGVEWVQIDEPILSLELEKQWADAFKLAYQLIRSDVKVL

LTTYFDSVTDTLDKIVELPVDGLHVDLSAAPQQLDDVVAKLPEGWVLSAGVVNGRNVWRS

DLSAQLERLQPVKEKLGDKLWVASSCSLLHSPVDLELETELSEEVKSWFAFAKQKVTEVA

LLGRALDGDQNAILACDTYSQPIKARKTATHVNKPQVQVRLNNITASLAERSAPYAERAA

HQAEVLGLPLLPTTTIGSFPQTGEIRVQRSAYRTGQLSESDYIQALKGHIADAVKRQEAL

DLDVLVHGEAERNDMVEYFAENLAGFQTTKFGWVQSYGSRCVKPAIVVADIEREKPITVE

WSTYAQSLTSKQMKGMLTGPVTILCWTFPREDITRQEIAQQLALALRDEVSDLQDAGINI

IQIDEPAIREGLPLKKRDHKAYLEWAVNAFKISAASAKPETQIHTHMCYSEFNEIIDSVA

ALDADVITIETSRSNMELLKAFEEFNYPNEIGPGVYDIHSPNIPTEEWIEGLIKKAAEKI

PVQRLWVNPDCGLKTRNWAETEAALANLVSAAKKLRAELA

>sp|Q87R69|MEND_VIBPA 2-succinyl-5-enolpyruvyl-6-hydroxy-3-cyclohexene-1-carboxylate synthase OS=Vibrio parahaemolyticus serotype O3:K6 (strain RIMD 2210633) OX=223926 GN=menD PE=3 SV=1

MSYDQAVLNRIWSETILTELHRFGVKHVCIAPGSRSTPLTLEAAEQPNFSIHTHFDERGL

GFMALGLAKASQEPVAVIVTSGTAVANLLPAVAEAKLTGEKLVLLTADRPVELVGCGANQ

AINQLGIFSQHVSANLNLPSPSLNTPLNWLLTSVDEVMFNQQLHGSAVHINCAFPEPLYS

DGEKSAYQSYLSSVEAWRKGGQTYTQRFVSPSFRDIPFCADRKGVVVIGSLSAEHAQEAK

AFAQQMGWPVLADPQSGVSSDWSHYDLWLQQPKLASQLDECDLVLQFGSRIISKRLNQWI

NKQVSQSQQGRDVQYWFISPSLSRDNQTHLPQLHWVASPKSWVERVDVKSSSTQGWADGL

LTDIAHVRAHISDEFLFSSASTLNEIALAADIEERTQSVDVFLGNSLFVRLVDMFGRLNT

EVFTNRGASGIDGLFATASGVQRSRGKPLLMYIGDTSALYDLNSLALFSRNDLPSVLVVT

NNDGGAIFDMLPVPQEHRTAYYQMPHGYQFEHAAKQFGLKYEKPTTLQMYQAMVADHLSS

GQGTMLVEVQTPPSQAAELIKAFNKSLHASL

>sp|Q87M06|PNP_VIBPA Polyribonucleotide nucleotidyltransferase OS=Vibrio parahaemolyticus serotype O3:K6 (strain RIMD 2210633) OX=223926 GN=pnp PE=3 SV=1

MFEKPVVKTFQYGNHTVTLETGVIARQATAAVMVTMDDTAVFVSVVGKKEAVAGQDFFPL

TVNYQERTYAAGKIPGGFFKREGRPSEGETLTARLIDRPIRPLFPDAFKNEVQVIATVVS

VNPDVQPDIPTMIGTSAALAISGIPFNGPIGAARVGHIDGQLVLNPSQTELNASRLDLVV

AGTESAVLMVESEADNLTEEEMLAAVVFGHDQQQVVINAINEFKAEVATPAWDWVAPEEN

TALKTKIAELAEAKLVEAYQITEKMARYDRIHEIAAEVNEALLAQDPEADTKEIHTIFHD

LEKTVVRRSIIAGNPRIDGREKDMVRALDVRTGVLPRTHGSALFTRGETQALVTATLGTQ

RDAQIIDELTGERKDHFLLHYNFPPYCVGETGFVGSPKRREIGHGKLAKRGIAAVMPSVD

EFPYTVRVVSEITESNGSSSMASVCGTSLALMDAGVPIKASVAGIAMGLVKEGDDFVVLS

DILGDEDHLGDMDFKVAGTSTGVTALQMDIKIEGITKEIMQIALNQAQGARKHILSVMDE

ALAGARDDISEFAPRIHTMKISAEKIKDVIGKGGAVIRALTEETGTTIEIEDDGTIKIAA

TEGAAAKEAIRRIEEITAEVEVGRIYTGKVARLADFGAFVTVLPGKDGLVHISQIAEKRV

EKVSDYLTEGQEVQVKVLEIDRQGRVRLSMKEAVEKPAEEAAAEAPAAKEE

>sp|Q87JL4|PHNW_VIBPA 2-aminoethylphosphonate--pyruvate transaminase OS=Vibrio parahaemolyticus serotype O3:K6 (strain RIMD 2210633) OX=223926 GN=phnW PE=3 SV=1

MKNEYLLLTPGPLSTSETVREAMLKDWCTWDDEYNKDIVEVIRTKLVKLATKHSGYTSVL

MQGCGTASVEATIGSAIGKEGKLLVVDNGAYGARIAQIADYLNIPCHVVSPGETSQPHLN

EVETALASDPAITHVAIVHCETTTGMLNPIEAFASAAKAHGKVVILDAMSSFGGIPMDIA

DLGIDFMISSANKCIQGVPGFGFVIAKQTELEKCQDQARSLSLDLYDQWHCMEVNHGKWR

FTSPTHTVRAFYQALLELEQEGGIEARHNRYQTNQKTLVAGMRSLGFEPLLSDDLHSPII

TSFYSPTHSDYQFKAFYTRLKEQGFVIYPGKVSNADCFRIGNIGEVYPADIERLIGAIEK

AMYWQVA

>sp|Q87LQ6|PIMT_VIBPA Protein-L-isoaspartate O-methyltransferase OS=Vibrio parahaemolyticus serotype O3:K6 (strain RIMD 2210633) OX=223926 GN=pcm PE=3 SV=1

MSNPHADRLIAFLISSGIKDQRVLDAMHCLPRESFVSQAMMHQAYDNNALPIGQGQTISQ

PYIVARMTELLELQRASNVLEIGTGSGYQTAVLAQIVDHVYSVERIKSLQWEAKRRLKQL

DIYNVSTKHGDGWLGWETKGPFDAIIVTAAAEVIPQALLSQLKDGGKMVIPVGDAEQQLL

RIERKGDEYLSTVVEMVRFVPLVAGDLA

>sp|Q87JE4|PNCB_VIBPA Nicotinate phosphoribosyltransferase OS=Vibrio parahaemolyticus serotype O3:K6 (strain RIMD 2210633) OX=223926 GN=pncB PE=3 SV=1

MTTTLFSSRIIQSALDFDVYKVNMMSAVAALYPDAMVSYKFIVRSEEDLSELLPEVKAEV

LKLQDVRFTEDEIAYMKRVAPYLKPEFVEALRHFRFNPQSDVSFHNKTMSDGSSQLRITI

NGLWKETILYETIIMSIVSEVRSRQRWSDIPFEQFQTVLEDKVRYLKAELERRNITNFKF

ADMSTRRRFSFQAQRTMLEYLSKELPQCLTGTSNYHLARELDLTPIGTVAHEWFMGHQAL

VNVRDSQKIALQRWQKMFNGALGIALTDTIGIDAFLKDFDEELSHAYVGVRHDSGCPFTW

GEKMIAHYESLGIDPMTKTLVFTDGLNFEQALDICEHFQGRVQVSFGIGTSLANDMGNYV

NDQGEAYQPLSIVIKMVTCNGSPVAKISDEPEKAMCEDIFFLMNLKRRFEQPLDLNECRK

LIDRLESEGQNYLIDA

>sp|Q87KN0|PLSB_VIBPA Glycerol-3-phosphate acyltransferase OS=Vibrio parahaemolyticus serotype O3:K6 (strain RIMD 2210633) OX=223926 GN=plsB PE=3 SV=1

MSSGQSFSRSLLKLPLSVMVKGTTIPSNPIDDLNIDLTKPIVYALPFRSNVDLLTLQKQA

MSLGLPDPLSPLEINGKTLNRFVFIASRPTVMGNDNDIPTDSVSLFTELLELHKLDSELD

VQMIPATVLWGRKPGKEESHRPYLQPMNGPQKAKAVMAAGRDCLVRFSPVVSLRYMADSH

GTDSAIAHKLARVARIHFSRQKLAASGPNLPQRQVLFARLLKSPAIEQAIEDEAKSKDIS

IEKARKEAHDIMDEIAADFSYGLVKNGDRILSWLWTKLYQGLHINNASTVRRLAQDGHEI

VYVPCHRSHMDYLLLSYVLYHEGMVPPHIAAGINLNFFPAGPIFRRGGAFFIRRSFKGNK

LYSTIFREYLAELFAKGYSVEYFSEGGRSRTGRLLQAKTGMLAMTIQAMLRGLNRPVTLV

PVYIGYEHVMEVGTYAKELRGKRKEKENAGLVLRTLRKLRNFGLGYVNFGEPIQLNQYLN

EHAPEWTKDIDSMGGSKPQWMNPVVNELANKMMTHINDAAAANALTLCATALLASRQRAL

SRDSLINQIECYLKLLKNNPYSSTSTIPTESAEELVDHAISLDKFVIETDSMGDIISLDR

SQSILMTYYRNNIIHLFALPSLIAQMIIRQRNLTVEKIQENVAQIYPFLKKELFLSYQEE

DLNDLVVKTLNEFAEQKMICLDGNKLEINQSNNQPLVLLGRTITETLQRYSIAMNLLVAY

PELGKSDLEQKSQDIAQRLGRLHGINAPEFFDKGVFTAMFNTLKQQEYLDSDGNCDKKKT

QKFAKLLFTLLYPEVKLTIEESIHQLQA

>sp|Q87SL3|PLSY_VIBPA Glycerol-3-phosphate acyltransferase OS=Vibrio parahaemolyticus serotype O3:K6 (strain RIMD 2210633) OX=223926 GN=plsY PE=3 SV=1

MDALALIMTMAAYLLGSVSSAVLICRLLKLPDPRNVGSNNPGATNVLRIGGKGAAVSVLL

CDMLKGTIPVWGGYFLGIDPIILGVIAIAACLGHMYPIFFHFKGGKGVATALGAIAPIGL

DLTGLVMLTWLSVAVLFRYSSLAALVTVLVTPFYTWMFKPQYTLPVAMLCCLIVFKHHQN

IRRLLSGEEPKIGEKKLTEKNSA

>sp|Q87IM9|PHS_VIBPA Putative pterin-4-alpha-carbinolamine dehydratase OS=Vibrio parahaemolyticus serotype O3:K6 (strain RIMD 2210633) OX=223926 GN=VPA0577 PE=3 SV=1

MLNEQKCEACSFDAIALTKEEQQSLLLQLSDWHLIERDDIPQLEKVYKFKNFKQAWAFSN

KIAELAEEEFHHPSILLEWGKVTVTWWSHSIKGLHKNDFICASKCDALVLSE

>sp|Q87N19|PLSX_VIBPA Phosphate acyltransferase OS=Vibrio parahaemolyticus serotype O3:K6 (strain RIMD 2210633) OX=223926 GN=plsX PE=3 SV=1

MQSITVALDAMGGDFGPRVTVPAAVQALSHFPELKVILIGDQSLITSQLSQLGTSTSSRL

TILHSEKVISNSEKPSLALRNSQNSSMRMAIDLVSDQEADACVSGGNTGALMALSRFILK

LLPGIERPALVSALPTISGKRTWMLDLGANVSCDADSLFQFAVMGSALAEEHLCRPPRVA

VLNIGAEEIKGNDLVKRCAEMLSQTDAINFVGYIEGNQILHDVADVIVCDGFVGNVCLKA

SEGTAQLFIEKLKTSMMASTIKGWIARKLFSRLFNELKTLNPDQYNGASLLGLRGIVIKS

HGSADVSAIVNALGEAVHEVKRQVPSRISDRLEAVLLERHY

>sp|Q87SU2|OBG_VIBPA GTPase Obg OS=Vibrio parahaemolyticus serotype O3:K6 (strain RIMD 2210633) OX=223926 GN=obg PE=3 SV=1

MKFVDEAVVKVQAGDGGSGVVSFWREKFITKGGPDGGDGGDGGDVYIQADENLNTLIDYR

FQRFYEAERGENGRGGNCTGKRGKDIVLRVPVGTRAVDIHTNEIVAEVAEHGKKVMVAKG

GWHGLGNTRFKSSVNRAPRQRTLGTKGEIREIRLELLLLADVGMLGLPNAGKSTFIRAVS

AAKPKVADYPFTTLIPSLGVVSVVPEKSFVVADIPGLIEGAADGAGLGIRFLKHLERCRV

LLHMIDIMPIDQSDPVQNALTIIDELEQYSEKLASKPRWLVFNKVDLMPEEEANEKIQEI

LDALGWEDEYFKISAINRSGTKELCYKLADFMENLPREEEEVAEEDKVNFMWDDYHKDAM

AGKDVVTEDDDDWDDWDDEEDDGHVVYVRD

>sp|Q87LZ1|OMPU_VIBPA Outer membrane protein U OS=Vibrio parahaemolyticus serotype O3:K6 (strain RIMD 2210633) OX=223926 GN=ompU PE=3 SV=1

MKKTLIALSVSAAAMATGVNAAELYNQDGTSLEMGGRAEARLSMKDGDAQDNSRIRLNFL

GTQAINDNLYGVGFWEGEFTTNEQGGVDGDVNKDSSNLDTRYAYAGLGGAWGEFTYGKNE

GALGVITDFTDIMAYHGNSAADKLAVADRSDNMMSYKGQFENLSVKASYRFADRKLNDAG

TEYTDNGQDGYSLSAIYAVADTGLELGAGYADQDEANEYMLAASYTMGDLYFAGIFTDGE

KAKTEGDYTGYELAGAYTLGQTVFTTTYNNAETNNETSANNFAVDASYYFKPNFRGYVSY

NFNLIDSGDKLGKVGGNTTASKADAEDELALGLRYDF

>sp|Q87TC4|NFUA_VIBPA Fe/S biogenesis protein NfuA OS=Vibrio parahaemolyticus serotype O3:K6 (strain RIMD 2210633) OX=223926 GN=nfuA PE=3 SV=1

MSNITITETAQTHFANLLGQQPEGTNIRIFVVNPGTQNAECGVSYCPPEAVEATDTEIPY

AGFSAYVDELSLPFLEDAEIDFVTDKMGSQLTLKAPNAKMRKVSDDAPLVERVEYVIQTQ

VNPQLAGHGGHVNLVEITEAGVAIVAFGGGCNGCSMVDVTLKEGIEKELLNQFVGELTAV

RDATEHDRGDHSYY

>sp|Q56725|NHAA_VIBPA Na(+)/H(+) antiporter NhaA OS=Vibrio parahaemolyticus serotype O3:K6 (strain RIMD 2210633) OX=223926 GN=nhaA PE=1 SV=1

MNDVIRDFFKMESAGGILLVIAAAIAMTIANSPLGETYQSLLHTYVFGMSVSHWINDGLM

AVFFLLIGLEVKRELLEGALKSKETAIFPAIAAVGGMLAPALIYVAFNANDPEAISGWAI

PAATDIAFALGIMALLGKRVPVSLKVFLLALAIIDDLGVVVIIALFYTGDLSSMALLVGF

VMTGVLFMLNAKEVTKLTPYMIVGAILWFAVLKSGVHATLAGVVIGFAIPLKGKQGEHSP

LKHMEHALHPYVAFGILPLFAFANAGISLEGVSMSGLTSMLPLGIALGLLIGKPLGIFSF

SWAAVKLGVAKLPEGINFKHIFAVSVLCGIGFTMSIFISSLAFGNVSPEFDTYARLGILM

GSTTAAVLGYALLHFSLPKKAQD

>sp|Q87N04|NHAB_VIBPA Na(+)/H(+) antiporter NhaB OS=Vibrio parahaemolyticus serotype O3:K6 (strain RIMD 2210633) OX=223926 GN=nhaB PE=1 SV=1

MPISLGNAFIKNFLGKAPDWYKVAIIAFLIINPIVFFLINPFVAGWLLVAEFIFTLAMAL

KCYPLQPGGLLAIEAIAIGMTSPAQVKHELVANIEVLLLLVFMVAGIYFMKQLLLFIFTK

ILLGIRSKTLLSLAFCFAAAFLSAFLDALTVIAVVISVAVGFYSIYHKVASGNPIGDHDH

TQDDTITELTRDDLENYRAFLRSLLMHAGVGTALGGVTTMVGEPQNLIIADQAGWLFGEF

LIRMSPVTLPVFICGLITCALVEKLKVFGYGAKLPDNVRQILVDFDREERKTRTNQDVAK

LWVQGIIAVWLIVALALHLAAVGLIGLSVIILATSFTGVIEEHSMGKAFEEALPFTALLA

VFFSIVAVIIDQELFKPVIDAVLAVEDKGTQLALFYVANGLLSMVSDNVFVGTVYINEVK

SALMEGLITREQFDLLAVAINTGTNLPSVATPNGQAAFLFLLTSALAPLIRLSYGRMVVM

ALPYTVVLAIVGLMGIMFFLEPATASFYDAGWIAPHTGDLTPVVSGGH

>sp|Q87ND9|NRFA_VIBPA Cytochrome c-552 OS=Vibrio parahaemolyticus serotype O3:K6 (strain RIMD 2210633) OX=223926 GN=nrfA PE=3 SV=1

MSIKHWMSAPIAVATLFASQLLLAGSVLAAENNDRLDPRNDAFEQKHPDQYHSWKATSES

KHIEDALSEDPNMVILWAGYGFAKDYNKARGHFYALDDVRQTLRTGAPADENSGPMPMAC

WSCKSPDVARVIEERGEDGYFSGKWARLGSEIVNPIGCSDCHDTRSEKFNQGEPELALTR

PYVERAFDVIGKNFDDQSRLDKQASVCAQCHVEYYFTGPTKAVKFPWDMGTTVGDMEKYY

DALDFKDWTHAVSKAPMLKAQHPGFETWREGIHGKNKVVCVDCHMPKVTKADGTVYTDHK

VGNPFDRFEDTCAQCHTQTKEQLRNIVSSRKALVLNMKLTAEKQIVAAHFEAGEAWKAGA

TEEEMKPILQDIRHAQWRWDYAIASHGVHMHAPEVALEVLGTAVDRAADARTKLVRLLAT

KGITEPVQIPDISTKAKAQEALGMDMEKMNADKKHFLDTVVPDWDKAAAEREATY

>sp|Q87MB0|NQRE_VIBPA Na(+)-translocating NADH-quinone reductase subunit E OS=Vibrio parahaemolyticus serotype O3:K6 (strain RIMD 2210633) OX=223926 GN=nqrE PE=3 SV=1

MEHYISLLVKSIFIENMALSFFLGMCTFLAVSKKVKTSFGLGVAVVVVLTIAVPVNNLVY

NLVLKENALVEGVDLSFLNFITFIGVIAALVQILEMVLDRFFPPLYNALGIFLPLITVNC

AIFGGVSFMVQRDYNFAESVVYGFGSGVGWMLAIVALAGIREKMKYSDVPPGLRGLGITF

ITVGLMALGFMSFSGVQL

>sp|Q87RU8|NRDR_VIBPA Transcriptional repressor NrdR OS=Vibrio parahaemolyticus serotype O3:K6 (strain RIMD 2210633) OX=223926 GN=nrdR PE=3 SV=1

MHCPFCSENDTKVIDSRLVADGHQVRRRRQCLACSERFTTFETAELVMPKVIKSNGNREP

FDEDKMVGGIQRALEKRPVSADSIELAISMIKSQLRATGEREVPSQMIGNLVMDQLKELD

KVAYIRFASVYRSFEDIREFGEEIARLED

>sp|Q87PH8|NPD_VIBPA NAD-dependent protein deacylase OS=Vibrio parahaemolyticus serotype O3:K6 (strain RIMD 2210633) OX=223926 GN=cobB PE=3 SV=1

MNFPYRNIVVLTGAGISAESGIQTFRAQDGLWENHRIEDVATPEGFARDPDLVQSFYNQR

RQKLQDETIKPNAAHLALGRLEAELEGKVTVITQNIDNLHERGGSQNVIHMHGELLKARC

SESNQVIEHTEDIKTGELCHCCQIPSQMRPHIVWFGEMRLRMGEIYAALEEADLFVSIGT

SGVVYPAAGFVHDAKMHGAHTIEINLEPSAVESEFEEKRYGKASVEVPKLVDEILALDTK

A

>sp|Q87MV3|NDPA_VIBPA Nucleoid-associated protein VP2128 OS=Vibrio parahaemolyticus serotype O3:K6 (strain RIMD 2210633) OX=223926 GN=VP2128 PE=3 SV=1

MSLHLSNVILHQLCKNDQDELVVKLRPASLENDASTENLVAELHRVFHSKAGKGFGSFQS

DSEFQFWLQEMRKGERDFYDFSQISANRLKEELIKYPFADEGILVFAEYQSLATDYLFIG

ILPMNQSLKVTEGLDISATDYLDITKMDIAARIDLSSYETDKESNRYLQYIKGRVGRKVA

DFFLDFLQADIGLDTKQQNLVLMQAVDDFCADSKLEKQEVNEYKKQVYNYCNEQIKSGEE

VQISELSGELPPSQDGTSFMDFTKEQGYELEESFPGDRSTVRKLTKYVGAGGGLNISFDS

LLLGERIFYDPETDTLTIKGTPPNLKDQLSRN

>sp|O82855|NORM_VIBPA Multidrug resistance protein NorM OS=Vibrio parahaemolyticus serotype O3:K6 (strain RIMD 2210633) OX=223926 GN=norM PE=1 SV=2

MHRYKEEASSLIKLATPVLIASVAQTGMGFVDTVMAGGVSATDMAAVSVASSIWLPSILF

GIGLLMALVPVVAQLNGSARREKIPFEIQQGVVLALLISIPIIGVLLQTQFILQLMDVEA

VMAGKTVGYIHAVIFAVPAFLLFQTLRSFTDGMSLTKPAMVIGFIGLLLNIPLNWIFVYG

KFGAPELGGVGCGVATTIVYWVMFALLLAYVMTSSRLKSINVFGEYHKPQWKAQVRLFKL

GFPVAAALFFEVTLFAVVALLVSPLGPIIVAAHQVAINFSSLVFMLPMSVGAAVSIRVGH

RLGEENVDGARVASRVGIMVGLALATITAIITVLSRELIAELYTNNPEVISLAMQLLLFA

AVYQCTDAVQVIAAGALRGYKDMRAIFNRTFIAYWILGLPTGYILGRTDWIVEPMGAQGF

WLGFIIGLTAAALMLGVRLRWMHRQEPDVQLNFSLQ

>sp|Q87FV4|MDTL_VIBPA Multidrug resistance protein MdtL OS=Vibrio parahaemolyticus serotype O3:K6 (strain RIMD 2210633) OX=223926 GN=mdtL PE=3 SV=1

MSRFLLCSFALVLLYPTAIDLYLVGLPQIASDLNASESQLHIAFSVYLAGMATTMLFAGK

IADSVGRKPIAVVGAMIFVLASFLGGMAEQPNTFLIARFCQGIGAGSCYVVAFAILRDTL

DDERRAKVLSMLNGITCIIPVIAPVIGHLIMLKFPWPSLFTTMAGMGILVSVLAIFVLKE

SLPSQQGEEQTTPESHQETFFERFFISRLIITALGVTTILTFVNASPIVVMSMLGFDRGG

YSSIMAGTAMISMLISFSAPLALGIFKQRTLMMTSQVLLACAAIVLSAAHFHDGQSHYYV

FGLGLICAGFACGFGVAMSQALSPFSQQAGVASSLLGIAQVCSSAFYIWFMGFIGVSALN

MLVFILVLGSVISLALILLIPKPVHDTHYEEIPSAT

>sp|Q87J41|NADE_VIBPA NH(3)-dependent NAD(+) synthetase OS=Vibrio parahaemolyticus serotype O3:K6 (strain RIMD 2210633) OX=223926 GN=nadE PE=3 SV=1

MEQSIRDEMRVLPSIDPHFEIERRIAFIKRKLQEAGCKSLVLGISGGVDSTTLGRLAQLA

VDQLNEETGSNDYQFIAVRLPYGEQKDEDEAQLALSFIKPTHSISVNIKQGVDGMHAASN

IALEGTGLMPEDAAKVDFVKGNVKARARMIAQYEIAGYVGGLVLGTDHSAENITGFYTKF

GDGACDLAPLFGLNKRQVREVAATLGAPEVLVKKVPTADLEELAPQKADEDALNLTYEQI

DDFLEGKPVSQQVVDRLVSIYKATQHKRQPIPTIYD

>sp|Q87S70|NCPP_VIBPA Non-canonical purine NTP phosphatase OS=Vibrio parahaemolyticus serotype O3:K6 (strain RIMD 2210633) OX=223926 GN=VP0554 PE=3 SV=1

MSKKIMATQKVVIASLNPAKINAVKSAFQSAFPQQVFEFVGISVPSEVADQPMTNEETHR

GALNRVKNAKLEMPTADFYVGLEAGIEGNVTFAWMVIESDTHRGESRSASLMLPPEVLAQ

LADANELGDVMDKVFGTENIKQKGGAISLLTQNQLTRSSVYHQALILALIPFTNPDHFPA

NL

>sp|Q87KX9|EFP_VIBPA Elongation factor P OS=Vibrio parahaemolyticus serotype O3:K6 (strain RIMD 2210633) OX=223926 GN=efp PE=3 SV=1

MATVSTNEFKGGLKLMLDNEPCVILENEYVKPGKGQAFNRVKIRKLLSGKVLEKTFKSGD

TCEVADVMDIDLDYLYSDGEFYHFMNNETFEQIAADAKAVGENAKWLVENNTCMITLWNG

NPITVTPPNFVELEVTDTDPGLKGDTQGTGGKPATLATGAVVRVPLFIAIGEVIKVDTRT

GEYVGRVK

>sp|Q87MD9|EFTS_VIBPA Elongation factor Ts OS=Vibrio parahaemolyticus serotype O3:K6 (strain RIMD 2210633) OX=223926 GN=tsf PE=3 SV=1

MATVTAALVKELRERTGAGMMECKKALVEANADIELAIENMRKSGAAKAAKKAGNVAAEG

AIIIKEENGSAVLLEVNCQTDFVAKDGNFTAFAQEVAAAALASKATVEELQAQFEEARVA

LVAKIGENINIRRVQYVEGTAIASYRHGEKIGVVVAGEGDAETLKHVAMHVAASKPEYVN

PEDVPADVVAKEKEVQVEIAMNEGKPAEIAEKMVVGRMKKFTGEISLTGQAFIMEPKKTV

GEMLKEKGASVATFVRLEVGEGIEKAEGLSFAEEVALAQKG

>sp|Q56704|FLAG_VIBPA Protein FlaG OS=Vibrio parahaemolyticus serotype O3:K6 (strain RIMD 2210633) OX=223926 GN=flaG PE=4 SV=2

MEISFYASNIQPYGTPNGTNVANKNGNGIGTPSTASSTGDVSPQKAKGTEHDFSVQAAIE

MAESRQELNREEREKMVEQMNEFVSSINKGVAFRVDEESGRDVVTIYEANTGDVIRQFPD

EELLVVLRRLAEHTANSGLLVEKV

>sp|Q87LI5|FETP_VIBPA Probable Fe(2+)-trafficking protein OS=Vibrio parahaemolyticus serotype O3:K6 (strain RIMD 2210633) OX=223926 GN=VP2627 PE=3 SV=1

MSRTVFCARLKKEGEGLDFQLYPGELGKRIFDNISKEAWAQWQHKQTMLINEKKLNMMDP

EHRKLLETEMVNFLFEGKDVHIEGYTPPSE

>sp|Q03473|FLAL_VIBPA Lateral flagellin OS=Vibrio parahaemolyticus serotype O3:K6 (strain RIMD 2210633) OX=223926 GN=lafA PE=3 SV=2

MALSMHTNYASLVTQNTLNSTSGLLNTAMERLSTGFRVNSASDDAAGLQIANRLEAQTRG

MSVAMRNAQDGISMMQTAEGAMEEMTNITYRMNDLATQSLNGSNSDKDRAAMDAEFKQLS

AELNNIMGNTSFGGQKLLAAGGGFEAGAVTFQIGASSAETLDVDASASIKKVAATLADAA

ITDGIGDATKAKAALDKISDAGGLIEDIGATRAQFGANINRLEHTMTNLGNMVENTSAAK

GRIMDADFAVESSNMTKNQMLMQAGTTVLSKTNQLPSMAMSLLR

>sp|Q87T81|FPG_VIBPA Formamidopyrimidine-DNA glycosylase OS=Vibrio parahaemolyticus serotype O3:K6 (strain RIMD 2210633) OX=223926 GN=mutM PE=3 SV=3

MPELPEVEVSRMGISPHMVGQTIKAFVFRTPKLRWDIPQELKLLEGQVIRNIRRRAKYLL

IDTDQGTAIVHLGMSGSLRVLDADFPAAKHDHVDLKLTNGKVLRYNDPRRFGAWLWCAPG

ESHAVLEHMGPEPLTDAFNSEYIADKAQGKRVAVKQFIMDNKVVVGVGNIYANESLFKSR

ILPTRQAGQVTPQEWVLLVENIKATLKIAINQGGTTLKDFAQADGKPGYFAQELLVYGKA

GEPCPECGEPLQELKIGQRNTFFCNECQQ

>sp|Q87KY2|FRDC_VIBPA Fumarate reductase subunit C OS=Vibrio parahaemolyticus serotype O3:K6 (strain RIMD 2210633) OX=223926 GN=frdC PE=3 SV=1

MSNRKPYVREVKRTWWKNHPFYRFYMLREATVLPLILFTLFLTFGLGCLVKGPEAWQGWL

AFMANPIVVAINIVALLGSLFHAQTFFSMMPQVMPIRLKGKPVDKKIIVLTQWAAVAFIS

LIVLIVV

>sp|Q87HX2|FTHS_VIBPA Formate--tetrahydrofolate ligase OS=Vibrio parahaemolyticus serotype O3:K6 (strain RIMD 2210633) OX=223926 GN=fhs PE=3 SV=1

MQSDIEICRNTPLSSIDVIAEKAGLLPEEFDTHGKYKAKVHPKCLARLNDNQNGKLVLVT

AITPTPLGEGKTVTTIGLAQGLAKLKQSVMACIRQPSMGPVFGIKGGAAGGGYSQVAPME

ELNLHLTGDIHAVTAAHNLASAALDARLFHEQREGYDAFEARTGLKALKIDVESITWKRV

MDHNDRALRMVKIGLNEHGKTINGFERNEGFDISAASELMAIIALAKNLKDLRQRIGKIV

VAYDLDGQPITTEDLQVAGAMAVTLKEAIAPTLMQTLEGVPTLIHAGPFANIAHGNSSII

ADEIALKLSRYTVTEAGFGSDMGFEKACNIKAAAANKAPDCVVIVATLRGLKANSGHYDL

RPGMAIPDSIFSPDQAALVAGFENLKWHIKNVHKYGIPAVVAINQFPQDCEQELTALQDL

IHAFDPNVKVAISTAFAQGGEGTRDLAQYVVDACEKTTNFRPLYQKHQSLQEKLMSVCEA

GYGATNVEMSELATKQLAHFEKLGFNELAVCIAKTPLSVTTDSSVKGAPVGFTVPIRELR

LCAGAGFVYALSGSVMTMPGLPDKPAFMNLDLDEDGNIIGLS

>sp|Q87LQ1|FTSB_VIBPA Cell division protein FtsB OS=Vibrio parahaemolyticus serotype O3:K6 (strain RIMD 2210633) OX=223926 GN=ftsB PE=3 SV=1

MRIFVIALTLLFGWLQYTLWFGKNGVSDYYTVKDEIEVQQQVNSKLQARNNEMFAEIDDL

KQGLDAIEERARHELGLVKEGETFYRIVGEENP

>sp|Q87QP4|FTSK_VIBPA DNA translocase FtsK OS=Vibrio parahaemolyticus serotype O3:K6 (strain RIMD 2210633) OX=223926 GN=ftsK PE=3 SV=1

MFKENAKKVETIIKTSEEPQSSRLNGFQRLKECCFIVGVLSSVLLAVALFTFSPADPSWS

QTAWGGEIDNAGGLFGAWLADTLFFTFGSLAYPIPFLLAAAAWVICRKRGEDEPIDFMLW

GTRLLGLTVLIMTSCGLADINFDDIWYFSSGGVVGDVLSSLALPTLNVLGTTLVLLFLWG

AGFTLFTGISWLNIVEWLGDRSLAVLAAIANKFRGSEQETLEPQLDEFVEDKVSTKHVED

DQQDDETLPHLTAYEVEEPKEKAAVHEYPIYMPQAKSETSAVKPTPEPQPQRVAAVNATP

TYVEPEPQLKAVSTDNVDPMVERTKQLNVTIEELEAAAQQADDWASEEQTSQSYADTNAV

YQEQVQAKHEEVVEHDTPQLESSYAEYAQFAAQQEQQLHVEPTPHEEPVIDTRALDDITD

HAEPSEHIEPTISDFDVVDEEETYVAPQPQSRSPEPQPMVQPQSVSQIQPEQAPEPSVAF

EPAPQEVEVEEVQDGDQDVAAFQSMVSSAQAKVAATQNPFLMKQEQNLPVPEEPLPTLEL

LYHPEKRENFIDREALEQVARLVESKLADYKIKADVVGIYPGPVITRFELDLAPGVKVSR

ISGLSMDLARALSAMAVRVVEVIPGKPYVGLELPNMSRQTVYLSDVISSPQFEQAKSPTT

VVLGQDIAGEAVIADIAKMPHVLVAGTTGSGKSVGVNVMILSMLYKASPEDLRFIMIDPK

MLELSIYEGIPHLLAEVVTDMKDASNALRWCVGEMERRYKLMSALGVRNVKGFNEKLKMA

AEAGHPIHDPFWQEGDSMDTEPPLLEKLPYIVVVVDEFADLMMVVGKKVEELIARLAQKA

RAAGIHLILATQRPSVDVITGLIKANIPTRVAFTVSTKTDSRTILDQGGAESLLGMGDML

YLPPGSSHTIRVHGAFASDDDVHAVVNNWKARGKPNYIDEIISGDQGPESLLPGEQMESD

EEMDPLFDQVVEHVVQSRRGSVSGVQRRFKIGYNRAARIVEQLEAQGIVSAPGHNGNREV

LAPAPPKD

>sp|Q87NC0|EFPL_VIBPA Elongation factor P-like protein OS=Vibrio parahaemolyticus serotype O3:K6 (strain RIMD 2210633) OX=223926 GN=VP1948 PE=3 SV=1

MPKASEIKKGFAIESNGKTLLVKDIEVTTPGGRGGAKIYKMRCTDLTTGARVDERYKSDD

VVETVEMNKRAVVYSYADGDEHIFMDNEDYSQYTFKHNEVEDDMLFINEDTQGIHIILVD

GSAVGLELPSSVELVIEETDPSIKGASASARTKPARFASGLVVQVPEYIATGDRVVINTA

ERKYMSRA

>sp|Q87N01|DUSC_VIBPA tRNA-dihydrouridine(16) synthase OS=Vibrio parahaemolyticus serotype O3:K6 (strain RIMD 2210633) OX=223926 GN=dusC PE=3 SV=1

MRVVLGPMEGVLDHLMRQILTEINDYDLCVTEFVRVIDQVLPDHVFHRLCPELMQGSQTT

SGVPVHVQLLGQEPHWMAENAIRAAELGARGIDLNFGCPAKMVNKSKGGAALLQHPELIH

SVVKACRDAVPANIPVSAKIRLGWENPEDCFEIVDAIQSAGANELTVHARTKQGGYKASE

IKWEYINKIRERFSIPLIANGEIWNFEDGQRCIETTGVDSLMVCRGAFNIPNLGNMVKHN

HTPMVWSDVVDLLIYYSKFEMKGDKGLYYPNRVKQWFAYLRQSYPEANELFREIRTFNKA

APIVEHIQRYRDELQSRQSQVA

>sp|Q87ME3|DXR_VIBPA 1-deoxy-D-xylulose 5-phosphate reductoisomerase OS=Vibrio parahaemolyticus serotype O3:K6 (strain RIMD 2210633) OX=223926 GN=dxr PE=3 SV=1

MQKLTILGATGSIGASTLKVVEQNPELFSVVALAAGTNVEKMVALCRQWQPKFAVMADKA

AAVALQSEIHTISPNTEVLGGVDALCHVASLEEVDSVMAAIVGAAGLLPTMAAVKAGKRV

LLANKEALVMSGQLFIDAVEQYGAELLPVDSEHNAIFQCLPQQVQTNLGRCNLDEHGISS

ILLTGSGGPFRYADIADLDSVTPAQAIAHPNWSMGPKISVDSATMMNKGLEYIEAKWLFN

AARDQLKVIIHPQSVIHSMVQYRDGSVLAQMGEPDMATPIALTMSYPSRVDAGVKPLDFT

QVGELTFLQPDFARYPCLKLAIDACYEGQHATTALNAANEVAVDAFLNNRLGFTDIARIN

ELVLHKITASCKPENANSLESLLELDRMSRTIALEIIRERS

>sp|Q87LL0|E4PD_VIBPA D-erythrose-4-phosphate dehydrogenase OS=Vibrio parahaemolyticus serotype O3:K6 (strain RIMD 2210633) OX=223926 GN=epd PE=3 SV=1

MLKVAINGFGRIGRNVLRAVYESGKHQQIKVVAVNELAQPEAMAHLLQYDTSHGRFGKKI

SHDQEHLNVHHESGEYDAIRILHLSEIELLPWRDLEVDIVLDCTGVYGSKADGLAHIEAG

AKKVLFSHPGANDLDNTIIYGVNHETLKDEHRVVSNGSCTTNCIVPIIKVLDEAFGIESG

TITTIHSSMNDQQVIDAYHNDLRRTRAASQSIIPVDTKLHKGIERIFPKFSNKFEAISVR

VPTVNVTAMDLSVTINTNVKVNDVNQTIVNASQCTLRNIVDYTESPLVSIDFNHDPHSAI

VDGTQTRVSNGQLVKMLVWCDNEWGFANRMLDTALAMKASSQVEL

>sp|Q87NZ7|ECTB_VIBPA Diaminobutyrate--2-oxoglutarate transaminase OS=Vibrio parahaemolyticus serotype O3:K6 (strain RIMD 2210633) OX=223926 GN=ectB PE=3 SV=1

MDIFKKQESNVRSYSNNFPVVFRKAKGCWLETEQGERYLDFLAGAGSLNYGHNNPVLKQA

LLEYIEMDGITHGLDMHSEAKAGFLAALDNYILKPRKLDYKVQFTGPTGTNAVEAALKLA

KKVKGRSSVVAFTNGFHGCTAGALAATGNQHHRQGNGSSLTNVTRIPFEGYAGVDGLALF

ETMLNDNSAGMDKPAAVLLETVQGEGGLNAASNEWLQRLSKICKANDILLIVDDIQAGCG

RTGTFFSFEPSGIEPDIVTLSKSIGGYGLPMAVVLLKPELDQWKPGEHNGTFRGNNHAFI

TAAKALEIYWSNDDFETHIKQCSQNVSEVIDRCVRRFPQMFVQKKGRGMMIGIECIHGDL

AAEIAKACFDDGMVIETAGPDDEVVKFFCPLTISESELNQGLSIFERAVETIAAKHFKQA

S

>sp|Q87NZ8|ECTC_VIBPA L-ectoine synthase OS=Vibrio parahaemolyticus serotype O3:K6 (strain RIMD 2210633) OX=223926 GN=ectC PE=3 SV=1

MIVRTLDECRNSERRVVADNWESVRMLLKDDNMGFSFHITTIYEGTETHIHYQNHLESVF

CMSGEGEIEVVGGETYPIKPGTLYILDKHDEHYLRAYKNKEMVMACVFNPPITGAEVHDE

NGVYPLVD

>sp|Q87LV1|PANC_VIBPA Pantothenate synthetase OS=Vibrio parahaemolyticus serotype O3:K6 (strain RIMD 2210633) OX=223926 GN=panC PE=3 SV=1

MQTFAEISAVRGHLKTFKREGRKIAFVPTMGNLHEGHLTLVRKAREYADIVVVSIFVNPM

QFDRADDLNNYPRTLEEDLSKLTAEGVDVVFTPTPEIIYPEGLDKQTFVDVPGLSTILEG

ASRPGHFRGVTTIVNKLFNIVQPDVACFGEKDFQQLAVIRKMVDDLAMDIEIIGVPTVRE

MDGLAMSSRNGLLTLDERQRAPVLARTMRWISSAIRGGRDDYASIIEDANDQLRAAGLHP

DEIFIRDARTLQVITPETTQAVILMSAFLGQARLIDNQTVDMVVESKDEAESNDGTAANA

E

>sp|Q87LV2|PANB_VIBPA 3-methyl-2-oxobutanoate hydroxymethyltransferase OS=Vibrio parahaemolyticus serotype O3:K6 (strain RIMD 2210633) OX=223926 GN=panB PE=3 SV=1

MKKMTINDLIKWKQEGRKFATSTAYDASFAQLFESQEMPVLLVGDSLGMVLQGENDTLPV

TVDDIVYHTRCVRAGSPNCLLMADMPFMSYATPEQACENAAKLMRAGANMVKIEGGDWLV

DTVKMLTERAVPVCAHLGLTPQSVNIFGGYKIQGRDQEKADRMVKDALALQEAGAQIVLL

ECVPAELAERITKVLDVPVIGIGAGNVTDGQILVMHDMFGISANYMPKFSKNFLAETGDM

RKAVAKYIEDVANGVFPDDAHTIA

>sp|Q87TE1|PCKA_VIBPA Phosphoenolpyruvate carboxykinase (ATP) OS=Vibrio parahaemolyticus serotype O3:K6 (strain RIMD 2210633) OX=223926 GN=pckA PE=3 SV=1

MTVMEHTKAATLDLTKHGLHNVKEVVRNPSYELLFEEETRADLTGYERGVVTELGAVAVD

TGIFTGRSPKDKYIVKDATTEEHMWWTSDTVKNDNKPITQEVWNDLKELVTNQLSGKRLF

VVDGYCGANPDTRLSIRVITEVAWQAHFVKNMFIRPTEEELATFEPDFVVMNGAKCTNPK

WEEQGLNSENFTVFNLTERTQLIGGTWYGGEMKKGMFAMMNYFLPLKDIASMHCSANMGK

DGDVAVFFGLSGTGKTTLSTDPKRALIGDDEHGWDDDGVFNFEGGCYAKTIKLSKEAEPD

IYNAIRRDALLENVTVRSDGSIDFDDGSKTENTRVSYPIYHIENIVKPVSKGGHANKVIF

LSADAFGVLPPVSKLTPEQTKYHFLSGFTAKLAGTERGITEPTPTFSACFGAAFLTLHPT

KYAEVLVKRMEAAGAEAYLVNTGWNGTGKRISIQDTRGIIDAILDGSIEDAPTKHIPIFN

LEVPTSLPGVDPSILDPRDTYVDPLQWESKAKDLAERFINNFDKYTDNAEGKALVAAGPQ

LD

>sp|Q87KQ7|NUDC_VIBPA NADH pyrophosphatase OS=Vibrio parahaemolyticus serotype O3:K6 (strain RIMD 2210633) OX=223926 GN=nudC PE=3 SV=1

MLRKGDVNRVANAYWCVVAGSEIWLVDGAVPFGSAEQFSLPEENARQIGDYLGSPVMWIN

FADLEQDLPLVSLRDCLHFPEPLFMLLSKAIQYGHMTQSLRFCPQCGGRNFLNNNQFAMQ

CGECRTLHYPRIFPCIIVAVRKENQILLAQHPRHRNGMYTVIAGFLEAGETLEDCVAREV

HEETGIHVKNIRYFGSQPWAFPSSMMMAFLADYDSGELNPDYTELSDAQWFGVKEMPPVA

PTGTIARALIEQTISDILSD

>sp|Q87KP9|NUSG_VIBPA Transcription termination/antitermination protein NusG OS=Vibrio parahaemolyticus serotype O3:K6 (strain RIMD 2210633) OX=223926 GN=nusG PE=3 SV=2

MSEAPKKRWYVVQAFSGFEGRVAQSLREHIKMHGMEELFGEVLVPTEEVVEMRAGQRRKS

ERKFFPGYVLVQMIMNDESWHLVRSVPRVMGFIGGTSDRPAPITDKEADAILNRLEKASE

APRPRTMYEAGEVVRVNEGPFADFNGTVEEVDYEKSRLKVSVSIFGRATPVELEFGQVEK

LD

>sp|Q87MA6|NQRA_VIBPA Na(+)-translocating NADH-quinone reductase subunit A OS=Vibrio parahaemolyticus serotype O3:K6 (strain RIMD 2210633) OX=223926 GN=nqrA PE=3 SV=1

MITIKKGLDLPIAGTPSQVINDGKTIKKVALLGEEYVGMRPTMHVRVGDEVKKAQILFED

KKNPGVKFTAPAAGKVIEVNRGAKRVLQSVVIEVAGEEQVTFDKFEAAQLSGLDREVIKT

QLVESGLWTALRTRPFSKVPAIESATKAIFVTAMDTNPLAAKPELIINEQQEAFIAGLDI

LSALTEGKVYVCKSGTSLPRSSQSNVEEHVFDGPHPAGLAGTHMHFLYPVNAENVAWSIN

YQDVIAFGKLFLTGELYTDRVISLAGPVVNNPRLVRTVMGASLDDLTDSELMPGEVRVIS

GSVLTGTHATGPHAYLGRYHQQVSVLREGREKELLGWAMPGKNKFSVTRSFLGHLFKGQL

FNMTTSTNGSDRSMVPIGSYERVMPLDMEPTLLLRDLCAGDSDSAQALGALELDEEDLAL

CTFVCPGKYEYGQLLRECLDKIEKEG

>sp|Q87MA8|NQRC_VIBPA Na(+)-translocating NADH-quinone reductase subunit C OS=Vibrio parahaemolyticus serotype O3:K6 (strain RIMD 2210633) OX=223926 GN=nqrC PE=3 SV=1

MASNNDSIKKTLGVVIGLSLVCSIIVSTAAVGLRDKQKANAVLDKQSKIVEVAGIEANGK

KVPELYAEYIEPRLVDFATGEFVEEAADGSKAANYDQRKAAKDNATSIKLTAEQDKAKII

RRANTGIVYLVKNGDDVSKVIIPVHGNGLWSMMYAFVAVETDGNTVSGITYYEQGETPGL

GGEVENPSWRAQWVGKKLFDENHKPAIKVVKGGAPAGSEHGVDGLSGATLTGNGVQGTFD

FWLGDMGFGPFLAKVRDGGLN

>sp|Q87L01|ORN_VIBPA Oligoribonuclease OS=Vibrio parahaemolyticus serotype O3:K6 (strain RIMD 2210633) OX=223926 GN=orn PE=3 SV=1

MSFSDQNLIWVDLEMTGLDPETHKIIEIASIVTDSELNILAEGPVLAVHQPEEELAKMDD

WCTNTHTASGLVERVRNSKISEQDAVAQTIEFLEKWVPKGVSPICGNSIGQDRRFLYKHM

PELEEYFHYRYLDVSTLKELTRRWKPEVLDGFSKQGTHLALDDIRESIAELKYYRETIFK

I

>sp|Q87NA8|OPAM_VIBPA Acyl-homoserine-lactone synthase OpaM OS=Vibrio parahaemolyticus serotype O3:K6 (strain RIMD 2210633) OX=223926 GN=opaM PE=3 SV=1

MSLKLSLVSLSNTDLPIETKQQALIDIVLRFLTPQERASLFESITHQRETNLLARYPEYQ

SKSLSVLFELMDYRDLVRLDPNNLHDDVYLLELTVAECFPHWLDFWCACEIEAIKQKYSL

ENREPATELSFEDASYSAMLIDDISKSSMRVQLPSYPVAMTLSDAVALSNLELFVQGEKW

YEILPLLSLSQKGKHFILLQTQTTPVLVASALIQDWNQRNTWLSYAPQFNSEKWRFCLPL

HGYQELNRLDILASDLVTDYGSLTVFDQAFQTHITKTEMVCEVLRLTVSGSVQHKLYFLY

LAQKELMNVLFQSGYKVGFTIIEQAFMLNFYQSIDSKAYFHSGYCDINGDGINTYRGFWN

FESMVDTFKRTDFRDYKRRIRVIRQNTQVNEHA

>sp|Q87KN8|TRMA_VIBPA tRNA/tmRNA (uracil-C(5))-methyltransferase OS=Vibrio parahaemolyticus serotype O3:K6 (strain RIMD 2210633) OX=223926 GN=trmA PE=3 SV=1

MATLDVNPQRYQEQLAEKVERLTDMFAPYNVPELEVFESPEQHYRMRAEFRVWHEGEDLY

YIMFNQETREKYRVDQFPAASRLINDLMPLLVEAMKDNESLRRKLFQVDFLSTLSGEILV

SLLYHRQLDEEWIENAKALKQRLNDEGFNLNIIGRARKMKIILDRDYVIEKLDVNGQSYI

YQQVENSFTQPNGKVAEKMLEWAVDCTQESTGDLLELYCGNGNFSLALAQNFDRVLATEL

AKPSVESAQYNIAANKIDNVQIIRMSAEEFTEAMEGKREFRRLKDNGVDLKSYNCNTIFV

DPPRAGMDVDTCKMVQGYERIMYISCNPETLKENLDILSETHNVTRFALFDQFPYTHHME

AGVLLERKA

>sp|Q87KE2|TSAC_VIBPA Threonylcarbamoyl-AMP synthase OS=Vibrio parahaemolyticus serotype O3:K6 (strain RIMD 2210633) OX=223926 GN=tsaC PE=3 SV=1

MDNFEQVLNALQQGEVIAYPTEGVFGVGCDPDNPDAIQKLLDLKQRPVEKGLILIAASYE

QLLPYIDESQLTPEQLATVHATWPGPYTWIMPASDKVSNWVSGQFDSIAVRVTDHPLVQK

MCNAFGKPLTSTSANLSGLPPCMTTEEVEQQLGDKLVAILRGETSGRDKPSEIRDAKTSQ

ILRQG

>sp|Q87SL5|TSAD_VIBPA tRNA N6-adenosine threonylcarbamoyltransferase OS=Vibrio parahaemolyticus serotype O3:K6 (strain RIMD 2210633) OX=223926 GN=tsaD PE=3 SV=1

MRIIGIETSCDETGIAIYDDEKGLLAHKLYSQVKLHADYGGVVPELASRDHVKKTIPLIK

EALKEANLTSQDIDGVAYTAGPGLVGALLVGATIGRSIAYAWGVPAVPVHHMEGHLLAPM

LEDNPPPFPFVAVLVSGGHSMMVEVKGIGEYKILGESIDDAAGEAFDKTAKLMGLDYPGG

PLLSKLAEKGTPGRFKFPRPMTNVPGLDMSFSGLKTFTANTIAANGDDEQTRADIAYAFE

EAVCATLAIKCKRALEQTGMKRIVIAGGVSANRRLRAELEKLAKKIGGEVYYPRTEFCTD

NGAMIAYAGMQRLKNGEVADMSVEARPRWPIDQLTPIA

>sp|Q87PG9|TTCA_VIBPA tRNA-cytidine(32) 2-sulfurtransferase OS=Vibrio parahaemolyticus serotype O3:K6 (strain RIMD 2210633) OX=223926 GN=ttcA PE=3 SV=1

MNQKDTRKETLEFNKLQKRLRRNVGNAITDYNMIEEGDVVMACISGGKDSFAMLDILLNL

QKAAPIKFEVVAVNLDQKQPGFPEHILPEYFETLNIPYYIVDKDTYSVVKEKVPEGKTTC

GLCSRLRRGTLYSFAEKIGATKLALGHHMDDIVETMFLNMFHGSRLKAMPPKLRSDDGRN

VVIRPLTYCREKDLIKYAEHKDFPIIPCNLCGSQENLQRQAIKAMLIDWDKKTPGRVEAI

FKSIQNVSPSQLADRELFDFVNLPLDRDGSREEYEFSEAVVSSTNIDESLFIDVTNI

>sp|Q87S71|TRPR_VIBPA Trp operon repressor homolog OS=Vibrio parahaemolyticus serotype O3:K6 (strain RIMD 2210633) OX=223926 GN=trpR PE=3 SV=1

MSHEPEYRDWQQIVELIRSSVDSQQHEMLLTMLMTPDERESLTARVNILNELLKGELSQR

QISQMLGVGIATITRGSNELKSKSEAEKDKLKLLLEQVAQVAK

>sp|Q87RD1|TSAB_VIBPA tRNA threonylcarbamoyladenosine biosynthesis protein TsaB OS=Vibrio parahaemolyticus serotype O3:K6 (strain RIMD 2210633) OX=223926 GN=tsaB PE=1 SV=1

MSAKILAIDTATENCSVALLVNDQVISRSEVAPRDHTKKVLPMVDEVLKEAGLTLQDLDA

LAFGRGPGSFTGVRIGIGIAQGLAFGAELPMIGVSTLAAMAQASYRLHGATDVAVAIDAR

MSEVYWARYSRQENGEWIGVDEECVIPPARLAEEAQADSKTWTTAGTGWSAYQEELAGLP

FNTADSEVLYPDSQDIVILAKQELEKGNTVPVEESSPVYLRDNVTWKKLPGRE

>sp|Q87N09|URK_VIBPA Uridine kinase OS=Vibrio parahaemolyticus serotype O3:K6 (strain RIMD 2210633) OX=223926 GN=udk PE=3 SV=1

MSDNNQCVIVGIAGASASGKSLIASTIYNELRAKVGDHQIGVITEDCYYNDQSHLSMEER

VKTNYDHPSALDHDLLCEHLEKLVRGEAVEVPEYSYTEHTRTSNTTTMTPKKVIILEGIL

LLTDPRLRDLMHATVFMDTPLDICLLRRVKRDVEERGRTMESVLKQYQQTVRPMFMQFIE

PSKQYADIIVPRGGKNRIAIDVLKAHIAKLLKA

>sp|Q87MX6|UVRB_VIBPA UvrABC system protein B OS=Vibrio parahaemolyticus serotype O3:K6 (strain RIMD 2210633) OX=223926 GN=uvrB PE=3 SV=1

MSKVYELVSEYQPSGDQPTAIKQLLEGLDAGLAHQTLLGVTGSGKTFTLANVIAQAQRPA

ILLAPNKTLAAQLYGEMKSFFPNNAVEYFVSYYDYYQPEAYVPTTDTFIEKDASVNAHIE

QMRLSATKALLERKDAIIVASVSAIYGLGDPESYLQMMLHLRRGDVIDQRDMLRRLAELQ

YSRNDVAFERGQFRVRGEVIDIFPAESDQDAVRVEMFDDEVDCISVFDPLTGVVKQRDLP

RYTIYPKTHYVTPRDRILEAIESIKVELEVRKKQLLENNKLIEEQRISQRTQFDIEMMNE

LGFCSGIENYSRYLSGRSEGEPPPTLFDYLPHDGLLIIDESHVTVPQIGAMYKGDRSRKE

TLVEFGFRLPSALDNRPLKFEEFESLAPQTIFVSATPGNYELEKSAGEIADQVVRPTGLL

DPILEVRPVATQVDDLLSEIRIRAAKEERVLVTTLTKRMAEDLTEYLHEHDVRVRYLHSD

IDTVERVEIIRDLRLGEFDVLVGINLLREGLDMPEVSLVAILDADKEGFLRSERSLIQTI

GRAARNIEGKAILYADNITKSMKKAMDETNRRREKQQAYNEKMGITPQALKRNIKDIMEL

GDITKSKRQRNTKQVPLSKVAEPSQTYEVMSPQQLEKEISRLEAAMYQHAQDLEFELAAE

KRDEIEKLRAQFIANS

>sp|Q87RT0|XNI_VIBPA Flap endonuclease Xni OS=Vibrio parahaemolyticus serotype O3:K6 (strain RIMD 2210633) OX=223926 GN=xni PE=3 SV=1

MSIHLVIIDALNLIRRVHSAQPDPTDIARTITTTQRTLTRILSEAKPTHIIAVFDHHEQD

RGWRAEILPDYKQNRKPMPEPLMQGLDAIQQAWWEQGIDSLLSEGDEADDLVATLATKVA

SHGEKVTIVSTDKGYCQLLSPTLQIRDYFQHRWLDEPFIEKEFGVKPSQLADYWGLTGIS

SSQVPGVPGIGPKAAKEILTQFEDIEAAYASDELAPKYRKKLDEHIESARLCKRVAALKC

DIELGFNLQDIRFTGPNKAE

>sp|Q87Q20|Y1330_VIBPA Uncharacterized protein VP1330 OS=Vibrio parahaemolyticus serotype O3:K6 (strain RIMD 2210633) OX=223926 GN=VP1330 PE=1 SV=1

MRQGTFFCIDAHTCGNPVRLVAGGVPPLEGNTMSEKRQYFLEHYDWIRQALMFEPRGHSM

MSGSVVLPPCSDNADASILFIETSGCLPMCGHGTIGTVTTAIENRLITPKEEGRLILDVP

AGQIEVHYQTKGDKVTSVKIFNVPAYLAHQDVTVEIEGLGEITVDVAYGGNYYVIVDPQE

NYAGLEHYSPDEILMLSPKVRTAVSKAVECIHPNDPTVCGVSHVLWTGKPTQEGATARNA

VFYGDKALDRSPCGTGTSARMAQWHAKGKLKSGEDFVHESIIGSLFNGRIEGITEVNGQT

AILPSIEGWAQVYGHNTIWVDDEDPYAYGFEVK

>sp|Q87TH2|UBIB_VIBPA Probable protein kinase UbiB OS=Vibrio parahaemolyticus serotype O3:K6 (strain RIMD 2210633) OX=223926 GN=ubiB PE=3 SV=1

MTPAELKRLYHIIKVQLEYGLDELLPDHQLTKAPLLMRKSLFWIKNQHPEKPLGERLRLA

LQELGPVWIKFGQMMSTRRDLFPPHIADPLALLQDQVAPFDGELAKQQMEKALGGPLENW

FTEFDIKPLASASIAQVHTARLKDTNQEVVLKVIRPDIRPVIDSDLKLMHRMARIVAGAM

PEARRLKPVEVVREYEKTLLDELDLRREAANAIQLRRNFEGSEELYVPEVFPDFSNETVM

VSERIYGIQVSDIEGLEANGTNMKLLAERGVSVFFTQVFRDSFFHADMHPGNVFVKPEHP

ENPMWIGLDCGIVGTLNSEDKRYLAENFLAFFNRDYRRVAELHVDSGWVPADTNVDEFEF

AIRIVCEPIFAKPLCEISFGHVLLNLFNTARRFNMEVQPQLVLLQKTLLYVEGLGRQLYP

QLDLWETAKPFLEEWMMNQVGPQALVNSIKDRAPFWAEKLPELPELLYDSLKQGKAMNQR

MDLLYQGYRQSKRQQATGKFLFGVGATLVVCSAILVDNAYEQLSMASGIAGVTFWLLSWR

AYRQ

>sp|Q87TH4|UBIE_VIBPA Ubiquinone/menaquinone biosynthesis C-methyltransferase UbiE OS=Vibrio parahaemolyticus serotype O3:K6 (strain RIMD 2210633) OX=223926 GN=ubiE PE=3 SV=1

MTDTSLQSNTALENETTHFGFTTVAKEEKVTKVAEVFHSVAAKYDIMNDLMSGGIHRLWK

RFTIDCSGARPGQRILDLGGGTGDLTAKFSRIVGDQGHVVLADINNSMLNVGRDKLRDNG

IVGNVHYVQANAEELPFPDDYFDVITISFCLRNVTDKDKALRSMFRVLKPGGRLLVLEFS

KPVLEPLSKVYDAYSFHLLPKMGELVANDAESYRYLAESIRMHPDQETLEGMMQDAGFEN

TKYYNLTGGIVALHRGYKF

>sp|Q87M04|TRUB_VIBPA tRNA pseudouridine synthase B OS=Vibrio parahaemolyticus serotype O3:K6 (strain RIMD 2210633) OX=223926 GN=truB PE=3 SV=1

MARRRKGRPINGVILLDKPTGISSNDALQKVKRIYFAEKAGHTGALDPLATGMLPICLGE

ATKFSQFLLDSDKRYRVIAKLGERTNTSDSDGEVVETRPVDVTLEKLEACIEKFRGESDQ

VPSMFSALKYQGKPLYEYARKGIEVPRESRKITVYEIILHRFEGDEVEMEVHCSKGTYIR

TIVDDLGEMLGCGAHVTMLRRTAVAKYPYEKMVTLEQLNELLEQAHREEIAPRELLDPLL

MPMDTAVEDLPEVNLIPELADMVQHGQPVQVLGAPEQGFLRLTMGEEHLFIGVGEMNDDG

KIAPKRLVVFRDEE

>sp|Q87LQ4|TRUD_VIBPA tRNA pseudouridine synthase D OS=Vibrio parahaemolyticus serotype O3:K6 (strain RIMD 2210633) OX=223926 GN=truD PE=3 SV=1

MSDILSSLAYLTGKPVASAKIKAQPEHFQVREDLGFAFTGEGEHLMVRIRKTGENTSFVA

NELAKACGVKSKDVSWAGLKDRHAVTEQWLSVHLPKGETPDFSTFLAQYPSIEILATDRH

NKKLRPGDLIGNEFVVTLSEVTDMADVEQRLEKVKQVGVPNYFGSQRFGNDGNNLDEARR

WGRENVRTRNQNKRSMYLSAARSWIFNRIVSARLENGVFDKFIDGDIAQTSQGLLAVDAS

NLADMQNKLALSEVEITAALAGDNALPTQADALALEQPFIDEEPDLMALIRGNRMRHDRR

EIALKPKDLAWNVEGNNITLTFSLDAGSFATSIVRELVNEVKVEREY

>sp|Q87M23|TYPH_VIBPA Thymidine phosphorylase OS=Vibrio parahaemolyticus serotype O3:K6 (strain RIMD 2210633) OX=223926 GN=deoA PE=3 SV=1

MYLPQEIIRKKRDGEVLTADEINFFIQGVANNTVSEGQIAAFAMTIFFNEMTMDERIALT

CAMRDSGMVIDWSHMNFGGPIVDKHSTGGVGDVTSLMLGPMVAACGGFVPMISGRGLGHT

GGTLDKLEAIPGYNITPTNEVFGQVTKDAGVAIIGQTGDLAPADKRVYATRDITATVDNI

SLITASILSKKLAAGLESLVMDVKVGSGAFMPTYEASEELAKSIVAVANGAGTKTTAILT

DMNQVLASSAGNAVEVREAVRFLTGEYRNPRLLEVTMASCAEMLVLAKLAENTDDARAKL

MEVLDNGKAAACFGKMVAGLGGPADFVENYDNYLEKAEIIKPVYATETGIVSAMDTRAIG

MAVVSMGGGRRVATDEIDYAVGFDNFIRLGEVADSDKPLAVIHARSEGQWEEAAKALRSA

IKVGGEYTPTPEVYRQIRAEDI

>sp|Q87TP4|TUSA_VIBPA Sulfur carrier protein TusA OS=Vibrio parahaemolyticus serotype O3:K6 (strain RIMD 2210633) OX=223926 GN=tusA PE=3 SV=1

MSFNPELATKTLEAEGLRCPEPVMMVRKTIRNMQDGEVLLVKADDPSTTRDIPSFCRFMD

HQLIAAQTEQLPYQYLIKKGLE

>sp|Q87LI7|TRMB_VIBPA tRNA (guanine-N(7)-)-methyltransferase OS=Vibrio parahaemolyticus serotype O3:K6 (strain RIMD 2210633) OX=223926 GN=trmB PE=3 SV=1

MSEVTTNEYNEDGKLIRKIRSFVRREGRLTKGQENAMNECWPTMGIDYKAELLDWKEVFG

NDNPVVLEIGFGMGASLVEMAKNAPEKNFFGIEVHSPGVGACLSDAREAGITNLRVMCHD

AVEVFEHMIPNDSLATLQLFFPDPWHKKRHHKRRIVQLEFAEMVRQKLIPNEGIFHMATD

WENYAEHMIEIMNQAPGFENIAQDGDFVPRPEDRPLTKFEARGHRLGHGVWDIKYKRIA

>sp|P22099|TRPE_VIBPA Anthranilate synthase component 1 OS=Vibrio parahaemolyticus serotype O3:K6 (strain RIMD 2210633) OX=223926 GN=trpE PE=3 SV=2

MRLCTAGRLKQLQGGLVNKAIEIKKLGQLEVLKASVPYTQDPTRLFHTICENKTDSLLLE

SAEIDSKQNLKSLLIVDSAVRIVCYGHTVSFHALTENGKNLLTHVNQNVRGEVASQFDGE

TLTLEFIQPCDTIDEDSRLREASSFDALRLVQHSFDLSSQDKHAIFLGGLFAYDLVANFE

PLGDAVATNQCPDYVFYVAETLLVVDHQTESCQLQATLFVDGSQKAALESRIEDIRAQCT

SPKRLPDATQVANITAQPSVPDQDFCQIVRDLKEFVVKGDIFQVVPSRRFTLPCPSPLAA

YKELKQSNPSPYMFYMQDELFTLFGASPESALKYETDTNQIEIYPIAGTRRRGKRPNGEI

DFDLDSRIELELRSDKKENAEHMMLVDLARNDVARISQAGTRHVADLLKVDRYSHVMHLV

SRVVGQLRDDLDALHAYQACMNMGTLTGAPKIRAMQLIRDVEGARRGSYGGAVGYLTGEG

TLDTCIVIRSAYVENGIAQVQAGAGVVFDSDPQAEADETRGKAQAVISAIQAAHSQPANK

E

>sp|Q87MP1|TRUA_VIBPA tRNA pseudouridine synthase A OS=Vibrio parahaemolyticus serotype O3:K6 (strain RIMD 2210633) OX=223926 GN=truA PE=3 SV=1

MRIALGIEYNGTNYFGWQRQREVKSVQEELEKALSIVANHPVEVQCAGRTDAGVHGTGQV

VHFDTNVNRKMVAWTMGANANMPSDIAVRWAKEVPDDFHARFSATARRYRYIIFNHALRP

GILNSGVSHYHGELDEKKMHEAGQYLLGENDFSSFRAAHCQSLSPCRNLMHLNVTRHGDY

VVIDIKANAFVHHMVRNITGSLIKVGRGEEKPEWIKWLLEAKDRKLAGATAKAEGLYLVD

VDYPEEFELPCVPIGPLFLPDNLN

>sp|Q87KM8|UBIC_VIBPA Probable chorismate pyruvate-lyase OS=Vibrio parahaemolyticus serotype O3:K6 (strain RIMD 2210633) OX=223926 GN=ubiC PE=3 SV=2

MNQPTSLYLASLLEVEWQKPEEFEFPNEFAKHWLEEQGSLSRRLKQHCQELTVELLQNHI

VTAKTLKSDESQLLSEQDCLLREVVLCGDDCPWVVGRTLIPRTTLVDQQYDLAQQGDIPL

GLTVFSADNVERDSLQMGWVHLPQGRFLARRSRLWMNHKPMLVAELFLSNSPIYAKERV

>sp|Q87LT0|TRMD_VIBPA tRNA (guanine-N(1)-)-methyltransferase OS=Vibrio parahaemolyticus serotype O3:K6 (strain RIMD 2210633) OX=223926 GN=trmD PE=3 SV=1

MWVGVISLFPEMFRSVTDFGVTGQAVKKGLLSIETWNPRDFTHDKHRTVDDRPYGGGPGM

LMMVQPLRDAIHTAKKASPGKTKVIYLSPQGRKLDQKGVEELATNENLLLICGRYEGVDE

RIIQSEVDEEWSIGDFVMTGGEIPAMTLIDSVSRFIPGVLGDFASAEEDSFANGLLDCPH

YTRPEVLDDKEVPSVLMSGNHKDIRQWRLKQSLGRTWLRRPELLENLALTDEQEQLLAEF

ISEHNAK

>sp|P22095|TRPA_VIBPA Tryptophan synthase alpha chain OS=Vibrio parahaemolyticus serotype O3:K6 (strain RIMD 2210633) OX=223926 GN=trpA PE=3 SV=2

MSRYEKMFARLNEKNQGAFVPFVTVCDPNAEQSYKIMETLVESGADALELGIPFSDPLAD

GPTIQGANIRALDSGATPDICFEQIGKIRAKYPDLPIGLLMYANLVYSRGIESFYERCAK

AGIDSVLIADVPTNESAEFVAAAEKFGIHPIFIAPPTASDETLKQVSELGGGYTYLLSRA

GVTGAETKANMPVDHMLEKLNQFNAPPALLGFGISEPAQVKQAIEAGAAGAISGSAVVKI

IEAHVEQPQIMLDKLGEFVSAMKAATQK

>sp|Q87IM1|TRPB2_VIBPA Tryptophan synthase beta chain 2 OS=Vibrio parahaemolyticus serotype O3:K6 (strain RIMD 2210633) OX=223926 GN=trpB2 PE=3 SV=1

MKNSFDHNMPNNEGYFGEYGGSFVPPELEQIMRDINAAYEECCQDPEFKDELARLYKHFV

GRPSPIFHAANLSKKYGADIYLKREDLNHTGAHKINHCLGEAILAKKMGKKKLIAETGAG

QHGVALATAAALVGLECDIYMGEVDIAKEHPNVVRMRILGANVIPATHGRKTLKEAVDAA

FEAYLKDPETQLYAIGSVVGPHPFPKMVRDFQSIIGNEARVQFKEMTGKLPNNLVACVGG

GSNAMGLFSAFLEDENVAIHGVEPAGRSLDKVGEHAATLTLGEPGIMHGFKSYMLKDEQG

EPQEVHSVASGLDYPSVGPQHSYLKDIGRVNYGSINDDEAIDAFFELSREEGIIPAIESS

HAVAYAIKLAQQGESGSILVNLSGRGDKDIDFVVENYGAKYGIESLI

>sp|P22098|TRPC_VIBPA Tryptophan biosynthesis protein TrpCF OS=Vibrio parahaemolyticus serotype O3:K6 (strain RIMD 2210633) OX=223926 GN=trpC PE=3 SV=2

MKMTDFNTQQANNLSEHVSKKEAEMAEVLAKIVRDKYQWVAERKASQHLSTFQSDLLPSD

RSFYDALSGDKTVFITECKKASPSKGLIRNDFDLDYIASVYNNYADAISVLTDEKYFQGS

FDFLPQVRRQVKQPVLCKDFMVDTYQVYLARHYGADAVLLMLSVLNDEEYKALEEAAHSL

NMGILTEVSNEEELHRAVQLGARVIGINNRNLRDLTTDLNRTKALAPTIRKLAPNATVIS

ESGIYTHQQVRDLAEYADGFLIGSSLMAEDNLELAVRKVTLGENKVCGLTHPDDAAKAYQ

AGAVFGGLIFVEKSKRAVDFESARLTMSGAPLNYVGVFQNHDVDYVASIVTSLGLKAVQL

HGLEDQEYVNQLKTELPVGVEIWKAYGVADTKPSLLADNIDRHLLDAQVGTQTGGTGHVF

DWSLIGDPSQIMLAGGLSPENAQQAAKLGCLGLDLNSGVESAPGKKDSQKLQAAFHAIRN

Y

>sp|Q87SC4|UNG_VIBPA Uracil-DNA glycosylase OS=Vibrio parahaemolyticus serotype O3:K6 (strain RIMD 2210633) OX=223926 GN=ung PE=3 SV=1

MNQSPTWHDVIGEEKKQSYFVDTLNFVEAERAAGKAIYPPAKDVFNAFRFTEFNDVKVVI

LGQDPYHGPNQAHGLCFSVLPGIKTPPSLVNMYKELAQDIEGFQIPQHGFLQSWAEQGVL

LLNTVLTVEQGKAHSHSKTGWETFTDRVIEAINQHQHGVVFLLWGSHAQKKGRFIDRSKH

HVLAAPHPSPLSAHRGFLGCKHFSQANQLLASQGKEPINWHLPLTV

>sp|Q87TJ1|USPB_VIBPA Universal stress protein B homolog OS=Vibrio parahaemolyticus serotype O3:K6 (strain RIMD 2210633) OX=223926 GN=uspB PE=3 SV=1

MISGDTILFALMVVTGVNMIRYLTALRSLIYIMREAHPLLYQQVDGNGFFTTHGNVTKQV

RLFHYIKSKEYHHHHDEIFTGKCERVRELFVLSTALLGVTLLAAFIL

>sp|Q87SL0|UPPP_VIBPA Undecaprenyl-diphosphatase OS=Vibrio parahaemolyticus serotype O3:K6 (strain RIMD 2210633) OX=223926 GN=uppP PE=3 SV=1

MSYFEAFILALIQGLTEFLPISSSAHLILPSAIFGWADQGLAFDVAVHVGTLMAVVIYFR

HEVITLFRALFASIFKGDRSKEAKLAWMIVIATIPACVFGLLMKDVIEVYLRSAYVIATT

TIVFGLLLWWVDKNAKLVADEYQTGWKKAVFIGIAQALAMIPGTSRSGATITAALYLGFT

REAAARFSFLMSIPIITLAGSYLGMKLVTSGEPVHVGFLLTGILTSFISAYICIHFFLKM

ISRMGMTPFVIYRLILGVGLFAFLLSA

>sp|Q87ME1|UPPS_VIBPA Ditrans,polycis-undecaprenyl-diphosphate synthase ((2E,6E)-farnesyl-diphosphate specific) OS=Vibrio parahaemolyticus serotype O3:K6 (strain RIMD 2210633) OX=223926 GN=uppS PE=3 SV=1

MQNSQAFSDSLPKHIAIIMDGNGRWAKSKGKPRVFGHKKGVNAVRKTVAAASKLGIKAMT

LFAFSSENWRRPEEEVGLLMELFITVLSSEVKKLHKNNLQLRVIGDTSRFSERLQKKIVE

AENLTASNTGMVINIAANYGGKWDITEAAKALALKARNGEIRVEDINEQLITEHLTMADL

PEVDLLIRTSGECRISNFMLWQMAYAEMYFTPEFWPEFDEDSLVEAVTWFINRERRFGCT

GEQVKALMTAQ

>sp|Q87NC4|UVRC_VIBPA UvrABC system protein C OS=Vibrio parahaemolyticus serotype O3:K6 (strain RIMD 2210633) OX=223926 GN=uvrC PE=3 SV=1

MNPPFDSASFLKTVTHQPGVYRMYNADAVVIYVGKAKDLQKRLSSYFRKKVDSEKTRALV

SNIAKIDVTVTHTETEALILEHNYIKQYLPKYNVLLRDDKSYPYILISGHKHPRLSMHRG

AKKRKGEYFGPYPDSGAVRETLHLLQKIFPVRQCEDTVYSNRTRPCLMYQIGRCAGPCVS

SIISDEEYAELVGFVRLFLQGKDQQVLKQLIEKMEVASQQLRFEDAAKFRDQIQAIRRVQ

EQQYVSEDSMDDMDVLGFAQENGIACIHILMIRQGKVLGSRSHFPKIPQNTSQQEVFDSF

LTQYYLSHNEARTIPSRIILNQELADDLEPIQKALSEVAGRKVHFHTSPTGARGRYLKLS

NTNALTAITTKINHKMTINQRFKALRETLGMESIMRMECFDISHTMGESTIASCVVFNNE

GPVKQEYRRYNITGITGGDDYAAMGQALERRYSKQLDVEKIPDIIFIDGGKGQLNRAHEI

IAQYWGDWPKRPIMIGIAKGVTRKPGLETLITVDGEEFNLPSDAPALHLIQHIRDESHNH

AIAGHRAKRGKTRRTSALEGIEGVGPKRRQALLKYMGGLQELKRASVEEIAKVPGISHSL

AEIIFQALKQ

>sp|Q87FH3|UXAC_VIBPA Uronate isomerase OS=Vibrio parahaemolyticus serotype O3:K6 (strain RIMD 2210633) OX=223926 GN=uxaC PE=3 SV=1

MKNFLCEDFLLSNETARRLYHEHAFHQPIYDYHCHLNPAEVTQNRQFDNLGQIWLEGDHY

KWRGMRSAGIEERLITGDASDYDKYMAWAKTVPQTLGNPLYHWTHLELRRPFGITNTLFS

PDTADQIWHQCNELLATPEFTARGIMQQMNVVMAGTTDDPIDSLEHHKAIAEDDTFNVKV

LPSWRPDKAFKIELDLFADYMHKLGEVADIDIRRFDDLLSALDKRLAHFDSHGCRAADHG

IEIVRYAPIPSEADLDALLARRLSGEVLSELECAQFSTAVQVWLGKRYAQLGWVMQLHIG

AQRNNSTRMFQLLGADAGFDSIGDRPFAFELAHLLDEMDQTNELPRTILYCLNPRDNEMM

ATMIGNFQGGGIAGKVQFGSGWWFNDQKDGMQRQMEQLSQLGLLSQFVGMLTDSRSFLSY

TRHEYFRRILCDMVGRWAENGEVPNDLSLLGPMVEDICFGNAKRYFEERA

>sp|Q87FH9|UXUA_VIBPA Mannonate dehydratase OS=Vibrio parahaemolyticus serotype O3:K6 (strain RIMD 2210633) OX=223926 GN=uxuA PE=3 SV=1

MEQTWRWYGPNDPVSLDDIRQAGATGIVNALHHIPNGEVWSKEEILKRKAIIEEKGLTWS

VVESVPVHEEIKTLTGNFQQWIDNYKQTLRNLAECGVDTVCYNFMPVLDWTRTDLEFEMP

DGSKALRFDQIAFAAFELHILKRPGAEADYTEAEQAQALEYFNNMTADQIQQLTSNIIAG

LPGAEEGYTLEEFQAQLDRYAGISKDKLREHMAYFLSQLMPVCEAHGLKLAVHPDDPPRP

ILGLPRIVSTIEDIDWLTEKVPSKMNGLTMCTGSYGVRGDNDLVKMIKKHGERIYFTHLR

STKREESNPMTFHEAAHLDGDVDMYNVVMAILDEEQRRAEVGDHRLIPMRPDHGHQMLDD

LKKKTNPGYSAIGRLKGLAEVRGLEMALKRAFYTK

>sp|Q87T31|TPIS_VIBPA Triosephosphate isomerase OS=Vibrio parahaemolyticus serotype O3:K6 (strain RIMD 2210633) OX=223926 GN=tpiA PE=3 SV=1

MRRPVVMGNWKLNGSKAMVTELLTGLNAELEGVEGVDVAVAPPALYIDLAERLIAEGGNK

IILGAQNTDLNNSGAFTGDMSPEMLKDFGATHIIIGHSERREYHNESDEFIAKKFNFLKE

NGLTPVFCIGESEAQNEAGETEAVCARQINAVIDTYGVEALNGAIIAYEPIWAIGTGKAA

TAEDAQRIHASIRALIAAKDEAVAAQVIIQYGGSVKPENAEAYFSQPDIDGALVGGASLD

AKSFAAIAKAAAAAKA

>sp|Q87SB8|TRMN6_VIBPA tRNA1(Val) (adenine(37)-N6)-methyltransferase OS=Vibrio parahaemolyticus serotype O3:K6 (strain RIMD 2210633) OX=223926 GN=VP0506 PE=3 SV=1

MKSGKFQTKGFKFKQFCIEGGESGMPVSTDGVMLGAWMESPSPAHILDIGTGTGLLALMC

AQRFPDAKITAVDIETTAVEAASHNFSHSPWHDRLSVQHTDVLIFSPPQRFQRIVCNPPY

FNTGEQAKQSQRATARHTDSLRHDALLKCCYQLLDAEGKASFVLPITEGELFIELALTQG

WSLSRLCRVQPSEKKPVHRLLFELAKQPCDTQESHLIIHSSDGYSDDFVRLTHEFYLKM

>sp|P22096|TRPD_VIBPA Anthranilate phosphoribosyltransferase OS=Vibrio parahaemolyticus serotype O3:K6 (strain RIMD 2210633) OX=223926 GN=trpD PE=3 SV=1

MEAIINKLYEQQSLTQEESQQLFDIIIRGELDPILMASALTALKIKGETPDEIAGAAKAL

LANANPFPRPDYDFADIVGTGGDGHNTINISTTAAFVAAACGLKVAKHGNRSVSSKSGSS

DLLDSFGINLAMSAEDTRKAVDDIGVAFLFAPQYHGGVRHAMPVRQTMKTRTIFNILGPL

INPARPNIELMGVYSEELVRPIAETMLQMGMKRAAVVHGSGLDEVAIHGTTTVAEIKDGK

ITEYTLTPEDFGLESHPLEAIKGGDPEENKAIITNILTGKGTDAQLGAVAVNVALLMRLF

GHEDLKANTQQAIEAMNSGKAYQLVQQLAAHA

>sp|Q87MD4|TRUC_VIBPA tRNA pseudouridine synthase C OS=Vibrio parahaemolyticus serotype O3:K6 (strain RIMD 2210633) OX=223926 GN=truC PE=3 SV=1

MAPVELEIVYQDEYFVAVNKPAGMLVHRSWLDKHETQFVMQTLRDQIGQHVFPLHRLDRP

TSGVLVFALSSEVASQVMPMFAEHKMEKTYHAIVRGWIEEEGVLDYALKVELDKIADKFA

SQEKEAQEAVTAYKPLAKVEVPYSTGKFPTTRYCLMEMKPKTGRKHQLRRHMAHLRHPIV

GDTSHGDGKHNKLFRNEFDSHRLLLHASELRFVHPFTNEELVMKASIDDTWQQLFTRFEW

DEELVK

>sp|Q87L41|TUSC_VIBPA Protein TusC homolog OS=Vibrio parahaemolyticus serotype O3:K6 (strain RIMD 2210633) OX=223926 GN=tusC PE=3 SV=1

MSQLTYLFRSAPHGSAAGREGVDALLAASAYCEDITVIFIGDGVYQLLLGQEPSGILSKD

YAPMLKLFDLYDIEQVFVCSESLAQRGLAQADLVIDAQALSLEQVKEKLQQAGKLLSF

>sp|Q87L40|TUSD_VIBPA Sulfurtransferase TusD homolog OS=Vibrio parahaemolyticus serotype O3:K6 (strain RIMD 2210633) OX=223926 GN=tusD PE=3 SV=1

MGGLTYTLVVNGSVYGSQSARSAYQFAQAVIEQGHTLVSVFFYQDGVTNGTALSVPANDE

FDLTKAWQGLAKEHDVRLETCVAAALRRGIISEDEATQHGLTQNNLAEGFVQAGLGSLAE

AMLTQDRVVQF

>sp|Q87KJ6|XERC_VIBPA Tyrosine recombinase XerC OS=Vibrio parahaemolyticus serotype O3:K6 (strain RIMD 2210633) OX=223926 GN=xerC PE=3 SV=1

MTTTPNTPLPNSLQKPLERFYEFLRSEKGLSLHTQRNYKQQLETMAQHLAEMGLKDWSQV

DAGWVRQLAGKGMREGMKASSLATRLSSLRSFFDFLILRGEMSANPAKGVSAPRKKRPLP

KNLDVDEVNQLLEVNEDDPLAIRDRAMMELMYGAGLRLAELVSVDVRDVQLRSGELRVIG

KGDKERKVPFSGMATEWVGKWLRVRGDLAAPGEPALFVSKLGTRISHRSVQKRMAEWGQK

QSVASHISPHKLRHSFATHMLESSNNLRAVQELLGHENISTTQIYTHLDFQHLAQAYDQA

HPRARKKNGE

>sp|Q87TI7|Y082_VIBPA UPF0391 membrane protein VP0082 OS=Vibrio parahaemolyticus serotype O3:K6 (strain RIMD 2210633) OX=223926 GN=VP0082 PE=3 SV=1

MVRWMFIFLALALVSAVLGFSGIAGAAAAVAQVIFYLFLLSLIVSIVFVILGKKNVNR

>sp|Q87QY9|Y1009_VIBPA UPF0319 protein VP1009 OS=Vibrio parahaemolyticus serotype O3:K6 (strain RIMD 2210633) OX=223926 GN=VP1009 PE=3 SV=1

MRLKTWIVAFFLGLFGTTVNADTTHKTDVSVTLSTAQGVQVLFVNGVSTDELSTPYTLID

GSNQVVIKVNKAIGRGDKRTQVYSAPYILGFSSGAGELYIDAPSFRDKRQADKLFEKDTM

DWKVSINDKSIDYSQYKMPGKKGAFPYSNLDEQLAEYNELNGVYFSNGKRVELSELQATG

TAKETHRVNSPLTKAKIAYLEMTDEERQLFMKWVSQQ

>sp|Q87NB9|Y1949_VIBPA UPF0263 protein VP1949 OS=Vibrio parahaemolyticus serotype O3:K6 (strain RIMD 2210633) OX=223926 GN=VP1949 PE=3 SV=1

MSDLISYDDVIDAAYDIFLEMAPDNLEPADVILFTAQFEDRGAAELVETGEDWVEHVGFE

VDKEVYAEVRIGLVNEENDVLDDVFARMLISRDPEHKFCHMLWKRD

>sp|Q87QK4|Y1145_VIBPA UPF0225 protein VP1145 OS=Vibrio parahaemolyticus serotype O3:K6 (strain RIMD 2210633) OX=223926 GN=VP1145 PE=3 SV=1

MASSCPCGANRTYQQCCEIAHNNHADVKTPEQLMRSRYSAHVLGLVDYVVKTYHPSCNAE

EQREGIAQSIDSDWCKLEVVKAEAGSHENEGFVEFNAYFNEDGQRYCLSERSRFVKENGL

WYYIDGTFPEEESEQDPRLNQSISSLKVGRNDPCICGSGKKFKKCCG

>sp|Q87T86|Y184_VIBPA UPF0758 protein VP0184 OS=Vibrio parahaemolyticus serotype O3:K6 (strain RIMD 2210633) OX=223926 GN=VP0184 PE=3 SV=1

MILKALPNESMPREKLLQRGPQALTDAELLAIFLRTGTQGMNVIELADFLIQDFGSLRQL

FSASEQEFCQHKGLGQAKYVQLQAVLEMTQRYLAETLKRGDALTSPEQTKLYLSSILRDR

QREAFYILFLDNQHRVIKDEILFEGTLDAASVYPREVVKRALHHNAAALILAHNHPSGVA

EPSQADRRITRRLIDALALVDIRILDHFVIGDGESVSFAERGWI

>sp|P46231|Y2115_VIBPA Uncharacterized membrane protein VP2115 OS=Vibrio parahaemolyticus serotype O3:K6 (strain RIMD 2210633) OX=223926 GN=VP2115 PE=4 SV=2

MNPVVISVCVMLVLALMRVNVVVALTFSAIVGGLVAGMSLGDTVAAFESGLGGGATIALS

YAMLGTFAVAISKSGITDLLAKSVIKRLNGKESAASTTGLKYAVLVALVLVTMSSQNVIP

VHIAFIPILIPPLLGVFAKLKLDRRLIACVLTFGLITPYMVLPVGFGGIFLNNILLKNLH

DNGLENVVASQVPTAMLLPGAGMIFGLLLAIFVSYRKPREYKETELTVVHETDHSINKQH

ILVAALGIIAALGVQLYTGSMIIGALAGFMVFTFGGVIAWKETHDVFTKGVHMMAMIGFI

MIAAAGFAAVMKQTGGVETLVQSLSTSIGDNKPLAALLMLVVGLLVTMGIGSSFSTIPIL

ATIYVPLSLAFGFSPMATIALVGTAAALGDAGSPASDSTLGPTSGLNADGQHEHIWETVV

PTFIHYNIPLIIFGWIAAMVL

>sp|Q87MM7|Y2204_VIBPA UPF0115 protein VP2204 OS=Vibrio parahaemolyticus serotype O3:K6 (strain RIMD 2210633) OX=223926 GN=VP2204 PE=3 SV=1

MSKKDTEHDDDFALFKEAVQGVKKLRQDTIIQQPKKNTKQKEIKRSNREASDSEFYFSDE

FVPRLNEEGPTRYARDDVSTYEVKRLRRGVYVPDVFLDMHGMTQQEAKRELGAMIAYCVK

NEIHCACVQHGIGKHILKQKAPLWLAQHPDVMAFHQAPLEFGGDGALLVLLSIPEK

>sp|Q87MD5|Y2321_VIBPA UPF0325 protein VP2321 OS=Vibrio parahaemolyticus serotype O3:K6 (strain RIMD 2210633) OX=223926 GN=VP2321 PE=3 SV=1

MYPHLTGLGIHDPKQIERYSLRQEAHKDVLKIYFHKQKGEFFAKSVKFKYPRQVKNVLVD

SGSHKYKEVTEINRNLTLVIDELNKITKPAKVSELDVKQKILSDLRHLEKVVSSKIAEIE

ADLEKLK

>sp|Q87TR5|YIDC_VIBPA Membrane protein insertase YidC OS=Vibrio parahaemolyticus serotype O3:K6 (strain RIMD 2210633) OX=223926 GN=yidC PE=3 SV=1

MDSQRNILLIALALVSFLLFQQWQVAKNPAPQAVEQAQSSSSLPAPSFADELDPVPGQQQ

ASAKTITVTTDVLTLSIDTVGGDVVHADLNQYSAELDSSDPFVLLKDTQGHQFIAQSGLV

GPQGIDLSSSNRPHYNVSADSFTLADGQDELRVPMTFTANGIEYTKTYVLKRGSYALNVE

YDVANNSGNNATFGMYAHLRQNLMDAGGSITMPTYRGGAYSTEDTRYKKYSFEDMQDRNL

SINLADGQGWAAMIQHYFAAAWIPRNEPGTNLYTRVIGNLGDIGVRMPNKTIATGDQAKF

EATLWVGPKLQQEMAAVAPNLDLVVDYGWLWFIAKPLHSLLAFIQSFVGNWGVAIICLTF

IVRGAMYPLTKAQYTSMAKMRMLQPKLQAMRERIGDDRQRMSQEMMELYKKEKVNPLGGC

LPLVLQMPIFIALYWALMESVELRHSPFFGWIHDLSAQDPYYILPLLMGASMFLIQKMSP

TTVTDPMQQKIMTFMPVMFTFFFLFFPSGLVLYWLVSNIVTLIQQTLIYKALEKKGLHTK

>sp|Q87RV3|XGPT_VIBPA Xanthine phosphoribosyltransferase OS=Vibrio parahaemolyticus serotype O3:K6 (strain RIMD 2210633) OX=223926 GN=gpt PE=3 SV=1

MSKKFIITWDAMQTYCRELAEKQMPAEQWKGIWAVSRGGLVPGAILARELGIRYVDTICI

SSYDHDHQRDMTVLKAPEGDGEGYLIVEDLVDSGDTARKLREMYPKAKMIAVCAKPSGKE

LLDDYVVDIAQDTWIEQPWDMSIQYAEPVNRKQK

>sp|Q87TE5|Y125_VIBPA UPF0761 membrane protein VP0125 OS=Vibrio parahaemolyticus serotype O3:K6 (strain RIMD 2210633) OX=223926 GN=VP0125 PE=3 SV=1

MNQLSESYKARLSNLLPSCVAFFKYLLKRLTHDRVNVNAGYLAYITLLSIVPMLTVLLSI

LSKFPVFANVGEVLQGYIIENFVPASGEAVHTALQEFVANTGKMSAVGGGFLFIAALMLI

SNIDKNLNYIWRVKDKRRLVFSFSMYWMVLTLGPILVGASIAATSYVTSLQILENETLSG

AFNLFLRWLPLLLSFFAFLGLYILVPNKKVHLAHGAVGAAVAAILFELSKKGFALYITQF

PSYQLIYGALAAIPILFVWVYLCWMIVLLGAEVTAALGEQEHWSEDLDMIHSSAESQLAN

EGSESSDSANSTSQ

>sp|Q87N16|Y2060_VIBPA Maf-like protein VP2060 OS=Vibrio parahaemolyticus serotype O3:K6 (strain RIMD 2210633) OX=223926 GN=VP2060 PE=3 SV=1

MKNYQLVLASTSPFRQQLLEKLSVPFICLSPDCDETPYESEAPLDLVQRLAVNKATSCSI

KKPSLVIGSDQVCVIDGKIVGKPLNRENAINQLLAQSGKAITFYTGLAVYNSVTNLTEVG

YDTFEVHFRNLNREQIERYVDREEPFYCAGSFKSEGMGICLFEKLVGKDPNTLVGLPLID

LIDMLQKQGFEIL

>sp|P46234|Y2110_VIBPA Uncharacterized protein VP2110 OS=Vibrio parahaemolyticus serotype O3:K6 (strain RIMD 2210633) OX=223926 GN=VP2110 PE=4 SV=2

MISEWEKHTLLADTALQLDDPVRSILHYQQALSLSEDISECVEIEADERLLISVISCHNL

AQFWRWAGDTEYELKYLQLASEKVLTLIPQCPNQNCASFIDSIGCCTKALIDFMKRHPNP

AIAKQVEKIDTATNCEVIAKFRLN

>sp|Q87K87|Y4011_VIBPA Probable transcriptional regulatory protein VPA0011 OS=Vibrio parahaemolyticus serotype O3:K6 (strain RIMD 2210633) OX=223926 GN=VPA0011 PE=3 SV=1

MGRSFEVRKASMAKTAGAKIKVYSKYGKEIYMCAKNGGSDPDMNLSLKHLIAKAKKDQVP

AHVIEKALDKANGGGGEDYVPARYEGFGPGGTSVIVDCLTDNGNRTFQDVRQCFVKVGAK

IGVEGSVSHMFDHQAVFQFKGEDDEVILETLMMEDVDVTDVELEDGVITVFAPHTEFFKT

KTALNAAFPDLTIDVEEITFVPQTQTPVAGEDAEKFQKFLDLLDDCDDVQQVYHNAEL

>sp|Q87K77|Y4021_VIBPA Uncharacterized response regulatory protein VPA0021 OS=Vibrio parahaemolyticus serotype O3:K6 (strain RIMD 2210633) OX=223926 GN=VPA0021 PE=3 SV=1

MNTGFTAIIADDEPLLRHHLDKSLAEVWPELDIVAKVADGEQALLAIEQSQPDIAFLDIR

MPVLDGMSLAQKLNRLANPPLIVFVTAYDDYAIKAFEQNAADYLLKPISDARLQTTCERV

KARLSQRGSDNSHVQMNSLLEQLQQLSAPQTPQYLQWIKATQGDDIHLIATSDVLYFKAE

EKYVSVYAQQGKGEVQEYLIRTSLKELIGQLNPEQFWQVHRSSVVQVSKISKVNKDFAGR

MFVYVGETKLPVSRASQSLFKGM

>sp|Q87HV6|Y4850_VIBPA UPF0312 protein VPA0850 OS=Vibrio parahaemolyticus serotype O3:K6 (strain RIMD 2210633) OX=223926 GN=VPA0850 PE=3 SV=1

MKKSLFATGLAIAMALPLGAQAADYVIDTKGAHASINFKVSHLGYSFIKGRFNTFSGDFS

FDEKNIADSKVNVVVDTTSLDSNHAERDKHIRSGDFIDAGKYSEATFKSTKVVDKGNGKL

DVTGDLTLHGVTKPITIEAEFVGAGNDPWGGERAGFVGTTRLELADFDIPVMGSSSYVDM

ELHIEGVKK

>sp|Q87RV2|Y674_VIBPA UPF0255 protein VP0674 OS=Vibrio parahaemolyticus serotype O3:K6 (strain RIMD 2210633) OX=223926 GN=VP0674 PE=3 SV=1

MSEDVSKNLSETLFVKHKQAKETSALTQYMPTSKKILDDREQQEDRAWYRHLRRLQWAWQ

GLSPIEMEGVLSRIASSTHSRTHDDWLDTVMGYHSGNWTFEWIKLGMEHQRRANDLKGED

AADELFTASLCFSIAGYPHLKNDNLALQAQVLANKAYSEGAEKTQYTIKQIEVPYQKRKI

IANLHLPRTDKQLPVVMVSAGLDSLQTDMWRLFRNHFAPKDIAMLTVDMPSVGHSSHWPL

TEDSSCLHQAVLNELYSIPYVDHHKVGLVGFRFGGNAMVRLSFLEQEKIKACVALGAPVH

DLFTSPKKLQKMPKMYLDMLASRLGKSAVDINSMAGQMMAWSLKVQGFLSSRKTKVPILA

LSLEGDPVSPYSDNQLVALFSHYGQAKKISSKTITKGYEQSLDLAIKWLEDELLR

>sp|Q87MZ5|Y2081_VIBPA UPF0208 membrane protein VP2081 OS=Vibrio parahaemolyticus serotype O3:K6 (strain RIMD 2210633) OX=223926 GN=VP2081 PE=3 SV=1

MSNKVGLIHSLKDGQSYMEIWPVRKELGAIFPEQRIIKATRFGIKVMPAVAAISVLTQMA

FNNYNALPQSIVVALFAISLPLQGIWWLGARSNTKLPPSLASWYRELHQKIVETGFALEP

VKARPRYKELAIILNRAFRQLDKSSLERWF

>sp|Q87MR2|Y2169_VIBPA UPF0260 protein VP2169 OS=Vibrio parahaemolyticus serotype O3:K6 (strain RIMD 2210633) OX=223926 GN=VP2169 PE=3 SV=1

MSTPFWQSKSLEHMSEEEWESLCDGCGKCCLHKLMDEDTDEIYYTNVACSWLNSKTCSCK

DYPNRFTSGEECTKLTREDIDDFTWLPHTCAYRLLAENQPLPEWHPLITGSKSAMHAAGE

SVRNKVVYEIDVVDWEDHILNHPNRP

>sp|Q87MC8|Y2328_VIBPA UPF0178 protein VP2328 OS=Vibrio parahaemolyticus serotype O3:K6 (strain RIMD 2210633) OX=223926 GN=VP2328 PE=3 SV=1

MKLWVDADACPKVIRETIVRAAERTGVECTFVANHVVPVPKRANIHSLQVPAGFDIADNE

IVRRVEPNDLVITSDIPLADEVITKGALALSSRGELYTKDTIKARLNIRDFMETMRSSGI

QTGGPAALSQTERREFANHLDRILAKR

>sp|Q87SC0|Y504_VIBPA UPF0246 protein VP0504 OS=Vibrio parahaemolyticus serotype O3:K6 (strain RIMD 2210633) OX=223926 GN=VP0504 PE=3 SV=1

MLIVVSPAKTLDYESPLATEKFTQPELIEYSKELIDVCRKLTPADVASLMKVSDKIADLN

VGRFQEWSETFTTENSRQAILAFKGDVYTGLEAETLSDADFEYAQKHLRMLSGLYGLLKP

LDLMQPYRLEMGTKLANDKGSNLYQFWGNVITDKLNEAIAEQGDNVLINLASNEYFKAVK

PKNLDAQVITPIFKDCKNGQYKVISFYAKKARGMMARYIIENRIESVADLTKFDTAGYYF

VEEESTPTDLVFKREEQN

>sp|Q87GT9|Y5226_VIBPA UPF0265 protein VPA1226 OS=Vibrio parahaemolyticus serotype O3:K6 (strain RIMD 2210633) OX=223926 GN=VPA1226 PE=3 SV=1

MSSVFEIVNQARRKNKLKRELLDNEKKVRDNRKRVDLLENLLDYIKPEMSHDEIVAIIKN

MKADYEDRVDDHIIKSAEISKARRDISRRIRELTEEDKQTSGKK

>sp|Q87FZ3|Y5527_VIBPA Probable phosphatase VPA1527 OS=Vibrio parahaemolyticus serotype O3:K6 (strain RIMD 2210633) OX=223926 GN=VPA1527 PE=3 SV=2

MEFKVDTHTHTYASGHAYSTLIENAKSAKENGLTMFCSTDHAESMPGAPHYWFFSNQKIL

PRFLEGVAIVRGVESNILNIEGEIDIPPSVDRNLDWVIASFHEPVFPPANKEAHTEALIN

VIKSGRVDALGHLGNPNFDFDFEQVLKCAKEHNVAIEINNTTLKGNSRVGSVDRCHEIAQ

IGKALDVYFTTGSDAHFCHDVGNLELVSELMDNLDIDSNKVITHSAKQFLSFLELRGRLP

IAEYDEIRNA

>sp|Q87RY0|Y646_VIBPA UPF0125 protein VP0646 OS=Vibrio parahaemolyticus serotype O3:K6 (strain RIMD 2210633) OX=223926 GN=VP0646 PE=3 SV=1

MSIESDMIHVEVVYALPHEQRVFNLVVNNHATVEEIIRQSGVLELYPEIDLAKNKVGVFS

RNVKLDATVRDKDRIEIYRPLLADPKEIRRKRAEQAKAAGNADPVTGGKPNALRK

>sp|Q87NA5|YCIB_VIBPA Probable intracellular septation protein A OS=Vibrio parahaemolyticus serotype O3:K6 (strain RIMD 2210633) OX=223926 GN=VP1970 PE=3 SV=1

MKQILDFIPLIIFFALYKMYDIYVATGALIVATAVQLIVTYALYKKVEKMQLITFVIVTI

FGSMTIFFHDDNFIKWKVTIIYVVLAVGLTASHLMGKSVVKGMLGKEITLPDAIWAKINW

AWVGFFSFFAGLNIYIAYELPLDVWVNFKVFGMLIATFAYMIATGVYIYKHMPKEEKNNS

SDVSVDD

>sp|Q87T24|ZAPB_VIBPA Cell division protein ZapB OS=Vibrio parahaemolyticus serotype O3:K6 (strain RIMD 2210633) OX=223926 GN=zapB PE=3 SV=1

MSFEVLEQLESKIQTAVDTITLLQMEVEELKEDKVKLEAEANELRSQREDLEQKAQQAQQ

EHAQWQERIRALLGKMDEVE

>sp|Q87R62|Y936_VIBPA UPF0324 membrane protein VP0936 OS=Vibrio parahaemolyticus serotype O3:K6 (strain RIMD 2210633) OX=223926 GN=VP0936 PE=3 SV=1

MNRKYIPFGLALLFCLTPFVSSPIALVIGFLLASFGLVPTELPIASFTKKLLSYSIIGLG

FGINFEQALSVTSDGIGLIIATIVGTLVIGSLIAKVIKLETTTAYLISSGTAICGGSAIA

AVAPAIRAKDEQIGLALATVFVLNSLALFIFPVIGHALNLDQHTFGTWAAIAIHDTSSVV

GAASAYGEEALTTATTLKLARALWIIPVALISAVIFSRGNKENGSKKLVIPYFIFWYCAA

IAFSDFFPQLEVVYHGIFTIAKQALVVCLFLIGCSISISKLKSSGPKPLLFGVTLWVLIS

TTSLSWLVLR

>sp|Q87R12|Y986_VIBPA UPF0229 protein VP0986 OS=Vibrio parahaemolyticus serotype O3:K6 (strain RIMD 2210633) OX=223926 GN=VP0986 PE=3 SV=1

MAQFIDRRLNGKNKSAVNRQRFLKRHKEQIKESVADAVNRRSITNTETGEDVSIPHKDIN

EPIFHQGKGGVRERVHPGNDQFITGDKIERPKGGGQGSGSGEGNASPDGEGQDEFVFQIS

KDEYLDILFEDLELPNLEKNQIAKITEWKTHRAGFQTAGIPSNISVIRSLQQSLARRTAM

TAGKKRLLKELEDELTRIKNIEPAQQLEENRLKKEIEELRKKIENVPFIDTFDLRFKNYE

KRPVPSSQAVMFCLMDVSGSMDQATKDIAKRFYVLLYLFLTRTYENVDVVFIRHHTQAKE

VDEHEFFYSQETGGTIVSSALKLMDEIVKERYPVGQWNIYAAQASDGDNWADDSPRCRDL

LVNKLLPNCQYYSYIEITRRSHQTLWHEYEKLTDEFPNFAMKNIRSVEDIFPVFRELFQK

ETA

>sp|Q87TF6|YIHI_VIBPA Der GTPase-activating protein YihI OS=Vibrio parahaemolyticus serotype O3:K6 (strain RIMD 2210633) OX=223926 GN=yihI PE=3 SV=1

MSRKKKSRKPGAAGAPEFVVTRNRTESDVEGRLRKRAKKRKGLKTGSRNSEVNEQKKQSS

EQNRDPRLGSKKKIPLIVEPVKKMTKQERRLSAEQELEMLENDAQLNVLLDRIEAGENLG

TGLQKYVDEKLDRIEKLMDQLGLLEPEEEEDFTASSAKGSRNDDDLLADFDDINFDDYKG

>sp|Q87RQ9|Y718_VIBPA UPF0250 protein VP0718 OS=Vibrio parahaemolyticus serotype O3:K6 (strain RIMD 2210633) OX=223926 GN=VP0718 PE=3 SV=1

MLTINSDAKLKDLLEFPCSFTYKVMGHAKPELPELVLEVIQRHAPGDYSPKVKPSAKGNY

HSVSINITATSIEQVETLYKELGEIDIVRMVL

>sp|Q87RL7|Y761_VIBPA UPF0319 protein VP0761 OS=Vibrio parahaemolyticus serotype O3:K6 (strain RIMD 2210633) OX=223926 GN=VP0761 PE=3 SV=1

MKKTTTLLGICAILSAPAFAAQLTLQKELVPRVINGEFVYPEWISDNNSIELKDGENQLA

VTVGQIVFEDGKRRKFDSQPLLLEFDAEKDAELSLTYKTFRTIEEAKAFELDPKVVLKDK

NGKEVDFSMVQLRKGGLQGFRDYEREVADYNNAVNKQATKSSIAQSPAVTKTLKESFNEL

SREEQQEFMQWAMRNLK

>sp|Q87RJ5|ZIPA_VIBPA Cell division protein ZipA OS=Vibrio parahaemolyticus serotype O3:K6 (strain RIMD 2210633) OX=223926 GN=zipA PE=3 SV=1

MQELRFVLIVVGALAIAALLFHGLWSSKKEGKAKFGNKPLGKLDVDQGDKDSVEQERSFA

PATEDDFEIIRKDRKEPDFGMENTFDSKFEADPLLGGVAEEKHSVKEEAEEIPSFVAMKN

DVEDVAIQPSEVEEPMQEVVEEEIMPSAFDAPKQEMEMVEEVAPAVVEQPEEPKPEPEMQ

VIVLNVHCAGEEPFIGTELFDSMQQNGLIYGEMHIFHRHVDLSGNGKVLFSVANMMHPGT

LEHGDPAEFSTKGISFFMTLPCYGEAEQNFNLMLRTAQQIADDMGGNVLDDKRNLMTPDR

LAAYRRQIVEFNAANA

>sp|Q87RE5|ZNUC_VIBPA Zinc import ATP-binding protein ZnuC OS=Vibrio parahaemolyticus serotype O3:K6 (strain RIMD 2210633) OX=223926 GN=znuC PE=3 SV=1

MSSLVSLEQLCVEFDDRRVLDNISMELEKGKITTLIGPNGAGKSTLVKVILGLQKPTSGK

LVKAKKLKIGYVPQKLKLNDSLPLNVIRFLNLAGKYSKQECLDALRLVGAEHLIKSNMHR

LSGGENQRVLLARALLQRPDLLVLDEPAQGVDVQGQIDLYDLIESIRHRFDCAVFMVSHD

LHLVMAKTDDVICLHHHVCCSGSPATITQHPSYIALFGNAARESLAFYHHDHEHHHHDLS

GSPVSGDATSCSNHNHGHHHHD

>sp|Q87LJ9|YQGF_VIBPA Putative pre-16S rRNA nuclease OS=Vibrio parahaemolyticus serotype O3:K6 (strain RIMD 2210633) OX=223926 GN=VP2613 PE=3 SV=1

MSRTIMAFDFGTKSIGSAIGQEITGTASPLKAFKANDGIPNWDDIEKQIKEWQPDLLVVG

LPTDLHGKDLETITPRAKKFAKRLQGRYGLPVELHDERLSTSEARAELFSMGGYKALSKG

NIDCQSAVVILESWFEALWGE

>sp|Q87LT3|ZAPD_VIBPA Cell division protein ZapD OS=Vibrio parahaemolyticus serotype O3:K6 (strain RIMD 2210633) OX=223926 GN=zapD PE=1 SV=1

MTTHKFEHPLNEKTRIYLRVESLLRQAHLASGFADNHQYQLFFRALFDMVEIFEQIQLKS

ELAKDLEKQRLSYRHWLNVEGVDQEALNSLLNEIDVVHSQLMGAERFGQALKEDRFLSSI

RQRFNLPGGSCCFDLPALHYWLHLPIERKKHDANQWQKSLKPLSDALTLWLKLARETGHF

KAQIARAGFFQSDADEANILRLHIPMKYGVYPMISGHKNRFAIKFMAFENGQACSQDVEF

ELAVCS

>sp|Q87RP6|YBEY_VIBPA Endoribonuclease YbeY OS=Vibrio parahaemolyticus serotype O3:K6 (strain RIMD 2210633) OX=223926 GN=ybeY PE=3 SV=1

MAIELDLQLAVENEEGLPSQQDFQLWLDKTIPLFQPQAEVTIRIVDEKESHALNHEYRGK

DKPTNVLSFPFEAPPGMEIDLLGDLIICRQVVEKEAIEQNKPLLAHWAHMVVHGSLHLLG

YDHIEDDEAEEMESLETEIMQGMGYEDPYIAEKE

>sp|Q87IB3|YEGS_VIBPA Probable lipid kinase YegS-like OS=Vibrio parahaemolyticus serotype O3:K6 (strain RIMD 2210633) OX=223926 GN=VPA0693 PE=3 SV=1

MKTIRAILNGKKAGNPELRDAIMKMRDRGVDVQVRVTWESQDMPRLVKEAVTDGIERIVV

AGGDGTVNEAASALIHIDHESRPELAIIPLGTANDFATANHIPDSIADALTLAVEGQALS

VDCVKANDRCFINVAAAGFGAEVTAETPVELKNFLGGGAYTLTGVVKALGFKPYDGSITI

EGGRYDGEMLLGAFCNSRLAGGGQQLAPNAMIDDGLMDLTLVRPFLPHELPKVIEEINNP

SEKGEFVKHTRASWLEIDFPNPLPLNLDGEPYHSRKIRFEVQPKSLKLVLPKDCPCVTH

>sp|Q87Q54|TPMT_VIBPA Thiopurine S-methyltransferase OS=Vibrio parahaemolyticus serotype O3:K6 (strain RIMD 2210633) OX=223926 GN=tpm PE=3 SV=1

MRDQEFWHSKWASNQIGFHLEDVNPLLPAYWHHANPKREDKVLVPLCGKSEDLVWLATKH

DSVEGVELSQIAVRSFFAEHFYTPTVTPISGMHELYQFDELSIYTGDFFTAPVSQADIVY

DRAALVALPQDMREEYVARLKQLLNPGGRILLVTLNYPQEEMAGPPFSVPLEEIQQLFAG

YKVTCLNVDQADEHHPKIAKKGLSRFSEEVYLIEAQ

>sp|P22097|TRPB1_VIBPA Tryptophan synthase beta chain 1 OS=Vibrio parahaemolyticus serotype O3:K6 (strain RIMD 2210633) OX=223926 GN=trpB1 PE=3 SV=2

MAKLNAYFGEYGGQYVPQILVPALKQLEQAFIDAQEDPEFRSEFMTLLQEYAGRPTALTL

TRNLTKGTKTKLYLKREDLLHGGAHKTNQVLGQALLAKRMGKHEIIAETGAGQHGVATAL

ACALLGLKCRVYMGAKDVERQSPNVFRMKLMGAEVIPVHSGSATLKDACNEALRDWSGSY

EDAHYLLGTAAGPHPFPTIVREFQRMIGEETKNQILAREGRLPDAVIACVGGGSNAIGMF

ADFIEEESVRLIGVEPAGKGIDTDQHGAPLKHGKTGIFFGMKAPLMQDENGQVEESYSVS

AGLDFPSVGPQHAHLNAIGRAEYDNVTDDEALEAFQELARSEGIIPALESSHALAHALRM

ARENPEKEQLLVVNLSGRGDKDIFTVHAILEEKGVI

>sp|P22101|TRPG_VIBPA Anthranilate synthase component 2 OS=Vibrio parahaemolyticus serotype O3:K6 (strain RIMD 2210633) OX=223926 GN=trpG PE=3 SV=1

MANIVFIDNFDSFTYNLVDQFRSLGHSVKIYRNHIPAETIEQAINELENPVVLLSPGPGA

PSEAGSMPELIQRMKGKVPMIGICLGHQAIVEAYGGTVAGAGEIIHGKVSMMEHQDHAIY

QNLPSPLAIARYHSLVATKVPDSLTITAEVDNLVMSVVHEQDKVCGFQFHPESIMTTYGA

TLLGNAIEWALEKNNA

>sp|Q87MQ3|Y2178_VIBPA Nucleoid-associated protein VP2178 OS=Vibrio parahaemolyticus serotype O3:K6 (strain RIMD 2210633) OX=223926 GN=VP2178 PE=3 SV=1

MFGKGGMGNLMKQAQQMQERMQKLQEEIANMEVTGESGAGLVKVTITGSHSVRRVDIDES

LMEDDKEMLEDLIAAAFNDAARRVEETQKEKMASVTGGMQLPPGMKMPF

>sp|Q87LD9|Y2673_VIBPA Nucleotide-binding protein VP2673 OS=Vibrio parahaemolyticus serotype O3:K6 (strain RIMD 2210633) OX=223926 GN=VP2673 PE=3 SV=1

MRLIVVSGHSGAGKSIALRVLEDLGYYCVDNLPVNLLDAFVHSIADSKQNVAVSIDIRNI

PKKLKELTGTLEQLKTELDVTVLFLDANKETLLKRYSETRRIHPLSLGGQSLSLDQAIER

EKEILTPLKAHADLILNSSGQSLHELSETVRMRVEGRDRKGLVMVFESFGFKYGLPSDAD

YVFDVRFLPNPHWEPALRPLTGLDAPIAAFLEQHQSVLSLKYQIESFIETWLPLLEKNNR

SYLTVAIGCTGGKHRSVYLTQQIGEYFADKGHQVQIRHTSLEKNAKE

>sp|Q87LD5|Y2677_VIBPA UPF0307 protein VP2677 OS=Vibrio parahaemolyticus serotype O3:K6 (strain RIMD 2210633) OX=223926 GN=VP2677 PE=3 SV=1

MARKNQKAPWEPEEEIIWVSKSEMKRDMEELQKLGEELVGLKPAVLEKFPLSEDLREAIA

DAQRFKNEARRRQLQRIGKLMRYEDPEPIQAALDKVRNKHSQATAALHKLEMLRDRVVEE

GDKAIDDVMELYPEADRQRLRQLARQAAKEKKAGKPAKSYREIFQILKALNEEEI

>sp|Q87MH1|UPP_VIBPA Uracil phosphoribosyltransferase OS=Vibrio parahaemolyticus serotype O3:K6 (strain RIMD 2210633) OX=223926 GN=upp PE=3 SV=1

MKVVEVKHPLVKHKIGLMREGDISTKRFRELATEVGSLLTYEATADFETEKVTIEGWNGP

VEVDQIKGKKVTVVPILRAGLGMMDGVLEHMPSARISVVGIYRDEETLEPVPYFNKLASN

IDERIALVVDPMLATGGSMIATIDLLKEKGCQSIKVLVLVAAPEGIEALEKAHPDVELYT

AAIDEKLNDKGYIVPGLGDAGDKIFGTK

>sp|Q87MV2|Y2129_VIBPA UPF0352 protein VP2129 OS=Vibrio parahaemolyticus serotype O3:K6 (strain RIMD 2210633) OX=223926 GN=VP2129 PE=1 SV=1

MPITSKYTDEQVEKILAEVALVLEKHAASPELTLMIAGNIATNVLNQRVAASQRKLIAEK

FAQALMSSLETPKTH

>sp|Q87LJ3|Y2619_VIBPA UPF0235 protein VP2619 OS=Vibrio parahaemolyticus serotype O3:K6 (strain RIMD 2210633) OX=223926 GN=VP2619 PE=3 SV=1

MSAAVWQDGEDIVLKLYIQPKASRDKIVGLHGEELKIAITAPPVDGKANAHLTKFLAKQF

KIAKGLVHIEKGELGRHKQIRIESPVQIPAEVKAIL

>sp|Q87LC4|Y2688_VIBPA Maf-like protein VP2688 OS=Vibrio parahaemolyticus serotype O3:K6 (strain RIMD 2210633) OX=223926 GN=VP2688 PE=3 SV=1

MKKSLSLVLASGSPRRKELLAQLGYDFDIVLPDIEEAKQADEQAQDYVLRLSLEKAQAGL

ALAKPDSVVLGSDTVVVCDDRVLEKPKSFEDSKRMLTDLSGRRHQVMTAVSVVSSEQQHS

VVVTTDVWFKPLTHEEIEQYWQSGEPCDKAGSYGIQGLGGRFVTRIEGSYHAVVGLPLFE

TDQLIQEFL

>sp|Q87HP7|Y4916_VIBPA UPF0181 protein VPA0916 OS=Vibrio parahaemolyticus serotype O3:K6 (strain RIMD 2210633) OX=223926 GN=VPA0916 PE=3 SV=1

MFDDLPPISHKEQQEAVERIQELMAKGTSTAEAIKIVADQIRAEYAAKQQGS

>sp|Q87LA0|UVRA_VIBPA UvrABC system protein A OS=Vibrio parahaemolyticus serotype O3:K6 (strain RIMD 2210633) OX=223926 GN=uvrA PE=3 SV=1

MDKIEVRGARTHNLKDINLTIPRDKLTVITGLSGSGKSSLAFDTLYAEGQRRYVESLSAY

ARQFLSLMEKPDVDHIEGLSPAISIEQKSTSHNPRSTVGTITEVYDYLRLLYARVGEPRC

PTHHAPLAAQTVSQMVDKVLELPEGSKMMLLAPIVKERKGEHVKTLENLAAQGFIRARID

GETCDLSDPPTLELHKKHTIEVVVDRFKVRPDLQQRLAESFETTLELSGGIAVVAPMDGD

GEEIIFSANFACPQCGYSMQELEPRLFSFNNPAGACGTCDGLGVQQYFDPSRVIQDDSLS

LAQGAIRGWDQKNYYYFQMLTSLADHYGFDLHAPFNSLPKKTQDVILKGSGRTEIEFKYI

NDRGDIRVKRHPFEGILNTLERRYRDTESNSVREELAKYISTKSCSSCGGTRLRLEARNV

FIADTTLPEIVELSIADALTFFQTLKLEGQRAQIAEKVMKEINDRLQFLVNVGLNYLNLS

RSAETLSGGEAQRIRLASQIGAGLVGVMYVLDEPSIGLHQRDNERLLKTLTHLRDLGNTV

LVVEHDEDAIRCADHVIDIGPGAGVHGGNVVAEGTMDEIIANPNSLTGQYLSGAKEIAVP

KERTPRDPKKTVELLGATGNNLKNVDLSIPVGLFSCITGVSGSGKSTLINDTFFKIAHTQ

LNGATTAHPSPYKSIKGLEHFDKVIDIDQSPIGRTPRSNPATYTGIFTPIRELFAGTQES

RSRGYKPGRFSFNVRGGRCEACQGDGVIKVEMHFLPDVYVPCDVCKGKRYNRETLEVRYK

GKTIDEVLEMTVEDARTFFDPVPAIARKLQTLMDVGLSYIRLGQAATTLSGGEAQRVKLA

RELSKRDTGKTLYILDEPTTGLHFHDIQQLLTVLHRLRDHGNTVVVIEHNLDVIKTADWI

IDLGPEGGQGGGEIIAQGTPEDVSQIEGSHTARFLKPMLK

>sp|Q87SA0|TYSY_VIBPA Thymidylate synthase OS=Vibrio parahaemolyticus serotype O3:K6 (strain RIMD 2210633) OX=223926 GN=thyA PE=3 SV=1

MKQYLDLCQRIVDQGVWVENERTGKRCLTVINADLTYDVANNQFPLVTTRKSFWKAAVAE

LLGYIRGYDNAEDFRKLGTKTWDANANLNDAWLNNPYRKGEDDMGRVYGVQGRAWAKPDG

GHIDQLRKIVDDLTRGVDDRGEILNFYNPGEFHMGCLRPCMYSHHFSLLDDTLYLNSTQR

SCDVPLGLNFNMVQVYVFLAIMAQITGKKPGQAFHKIVNAHIYEDQLELMRDVQLKREPL

QAPTFHINPEIKSLEDLETWVTLDDFWVEGYEHHDPIRYPFSV

>sp|Q87KM9|UBIA_VIBPA 4-hydroxybenzoate octaprenyltransferase OS=Vibrio parahaemolyticus serotype O3:K6 (strain RIMD 2210633) OX=223926 GN=ubiA PE=3 SV=1

MSAEKAKAYWQLMRMDRPIGSLLLLWPTVWALVIAAQGIPSWDVLIVFVLGVFLMRSAGC

VINDFADRKVDGHVKRTKQRPLPSGKVTAKEAIGLFLVLAVSSFLLVLTMNPLTIQLSFA

GLVLAFIYPFMKRYTHIPQLFLGLAFSWAIPMAWAAQTGELPVMVWFVFVINALWTIAYD

TQYAMVDRDDDLKIGIKSTAILFGRHDKLIIGVLQLVTLAMLVGLGQFYQLGQSYYWTVL

IAASLFVYQQHLIRHRERDLCFRAFLNNNYVGMVIAIGLLVAFW

>sp|Q87KI0|UBID_VIBPA 3-octaprenyl-4-hydroxybenzoate carboxy-lyase OS=Vibrio parahaemolyticus serotype O3:K6 (strain RIMD 2210633) OX=223926 GN=ubiD PE=3 SV=1

MSFKDLREFIDHLEQKGRLKRITHPVDPAYEMTEISDRTLRAGGPALLFENPIGYNVPVL

TNLFGTPERVAIGMGREDVKELREVGKLLAYLKEPEPPKGFKDALEKLPVFKQVLNMPAK

RLRKAPCQDIVWQGDEVDLDKIPVMSCWAEDVAPLLTWGLTVTKGPNKKRQNLGIYRQQK

IAKNKIIMRWLAHRGGALDLRDWMETNPGKPFPVSVAFGADPATILGAVTPVPDTLSEYA

FAGLLRGSKTEVVKSISNDLEVPASAEIVMEGYIDPNEFADEGPYGDHTGYYNEKEKHHV

FTITHITMRKDPIYHSTYTGRPPDEPAVLGVALNEVFVPILQKQFPEIEDFYLPPEGCSY

RMAVVTMKKQYPGHAKRVMMGVWSFLRQFMYTKFVIVCDESVNARDWNDVVKAMTEHMDP

VRDTLMIDNTPIDSLDFASPVVGLGSKMGLDATIKWDAELATRPQISKQDSKVITEADLE

SLKQQRPEIIDIYLPPTTNNRFAVVTMKKDQAGQSQALMEYLWDFFAQYTDNKFVILCDE

DVNARDWNDIIWAVTTRMDPDRDTTRVSGKAESSSKLGLDATNKFESEVTREWGTPIKKD

PKLVAKVDEIWDQLGIL

>sp|Q87QB8|Y1232_VIBPA Putative transport protein VP1232 OS=Vibrio parahaemolyticus serotype O3:K6 (strain RIMD 2210633) OX=223926 GN=VP1232 PE=3 SV=1

MNIDVVQLLDQNPILLIFVVLAIGLAIGKIRFGNLQLGNSIGVLITSLIMGHLGFSFNAE

ALTIGFMLFIYCVGIEAGPNFFGIFFRDGKHYFILSMTVLVAAVSLTYGLSHYFGLDFGL

SAGMMAGALTATPVLVGAQDALNSGLATIPRNMDFSLVLENLSVGYAMAYLIGLISMIMF

AKLLPKLQKQNLSDSAQQIAQERGLGNSSQRKVYLPIIRAYRVGPELIDWTDGKNLRELG

IYRQTGCYIERIRRNGILAHPDGDAILQEGDEIALVGFPDSHARLDPSFRNGKEVFDRNL

LDLRIVEEEIVVKSDAIAGKRLSDLNLSEFGCFLNRVVRAQIEMPMDLDIVLAKGDVLQV

SGEKSRVHGLAEKIGFISIHSQMADLLAFCSFFILGIMFGLVTMTFGQVSFSLGNAVGLL

LSGITLGFLRANHPTFGYVPQGALNMVKDLGLMFFMVGIGLSAGGKMFEHLTQVGPQVIG

LAFIVSVVPVVIAYLVGAYILKMNRALLFGAIIGARTCAPAMDVVNEYAKSTIPALGYAG

TYAIANILMTLAGTILIILS

>sp|Q87Q67|Y1283_VIBPA UPF0145 protein VP1283 OS=Vibrio parahaemolyticus serotype O3:K6 (strain RIMD 2210633) OX=223926 GN=VP1283 PE=3 SV=1

MIYTTTDTIPGKEIAEVRGVVTGNVVQSKHIGRDLMAGLKSIVGGEIRGYTEMMTEARDI

AIQRMVEQANQKGADAIVGIRFTTSSIVDGSSEILAFGTAVKLVE

>sp|Q87Q50|Y1300_VIBPA UPF0299 membrane protein VP1300 OS=Vibrio parahaemolyticus serotype O3:K6 (strain RIMD 2210633) OX=223926 GN=VP1300 PE=3 SV=1

MIKDRFLQLIQLLISLFLIMGALGIGITIQKFTGVSVPGSVIGMLVLFFSMTLGLVKVDW

VKPGATLFIRYMILLFVPISVGLMQHFDMLLANALPIIASAVGGSLIVLVSLAWLLDYLL

KEKH

>sp|P59562|Y1617_VIBPA UPF0234 protein VP1617 OS=Vibrio parahaemolyticus serotype O3:K6 (strain RIMD 2210633) OX=223926 GN=VP1617 PE=3 SV=1

MPSFDIVSEIDTVELRNAVDNANRELSTRFDFRNVNASFELVEENVKVSAEGDFQLKQMR

DILRGHLAKRNVDANAMDAQNPEVTGKNWHQNILFRQGIDTPTAKKLVKLIKDAKLKVQA

SIQGDKVRVTGKKRDDLQATIAAIREAELGQPFQFNNFRD

>sp|Q87MC1|Y2335_VIBPA UPF0253 protein VP2335 OS=Vibrio parahaemolyticus serotype O3:K6 (strain RIMD 2210633) OX=223926 GN=VP2335 PE=3 SV=1

MQVYGCCELVRELYAQIGSGDQAYIPQAISCAVKALNDVAADESLPKEVREKAAFAAANL

LISDFEDK

>sp|Q87LW4|Y2494_VIBPA UPF0231 protein VP2494 OS=Vibrio parahaemolyticus serotype O3:K6 (strain RIMD 2210633) OX=223926 GN=VP2494 PE=3 SV=1

MEFEFIRNTLMGEYYVKCSMGHEIVGRWLQEEIGKDPTMIAQVEALIDKAFSLPSQEHTL

TGTEISLMIQGDEVLVQENALSHDYDVEMESEFDFYDAESTASCGIEDFVALIEQWKDFL

NI

>sp|Q87LM3|Y2588_VIBPA UPF0149 protein VP2588 OS=Vibrio parahaemolyticus serotype O3:K6 (strain RIMD 2210633) OX=223926 GN=VP2588 PE=3 SV=1

MSEITLPEYQSIAAELQSASLAVTPAELHGLLVGMLSGGLAINDQTWQPILFDYTNDGMG

WPTTALAFAQTVFKVTANELTGSSMELSLLLPDESGEEGLFALADSLSDFVNHFISGLGL

AGIALNNASDDAKEALADLEEIAKLGIDEDDDFGEQAQLLEQVIEHVKACVLTIHAEFGA

RPESSESKPTIH

>sp|Q87JZ5|Y4103_VIBPA Macro domain-containing protein VPA0103 OS=Vibrio parahaemolyticus serotype O3:K6 (strain RIMD 2210633) OX=223926 GN=VPA0103 PE=4 SV=1

MNAISLVQGDITTAHVDAIVNAANPRMLGGGGVDGAIHRAAGPALINACYAVDDVDGIRC

PFGDARITEAGNLNARYVIHAVGPIYDKFADPKTVLESAYQRSLDLALANHCQSVALPAI

SCGVYGYPPQEAAEVAMAVCQRPEYAALDMRFYLFSEEMLSIWQHALTQH

>sp|Q87SH5|Y448_VIBPA UPF0102 protein VP0448 OS=Vibrio parahaemolyticus serotype O3:K6 (strain RIMD 2210633) OX=223926 GN=VP0448 PE=3 SV=1

MGLFSKRQIGNQYETLAKQYLQRQGLRFLDQNYLTKFGEIDLIFQQDETIVFVEVKYRKN

DHFGSAAEMVTNAKMRKLIKTAQVWLSQQRTMNTIDYRFDVIAIHDSGRDINWIQNAISE

G

>sp|Q87S15|SYH_VIBPA Histidine--tRNA ligase OS=Vibrio parahaemolyticus serotype O3:K6 (strain RIMD 2210633) OX=223926 GN=hisS PE=3 SV=1

MAKTIQAIRGMNDCLPTQSPLWQKLENTVKNVISAYGYNEVRMPIVEETNLFSRAVGEET

DVVSKEMYTFDDRNGDSLTLRPEGTAGCVRSCIQNSLINRDEQRLWYMGPMFRHERPQKG

RYRQFHQCGVEVFGLNGPDVDAELIMMTARLWRELGIDKHVRLELNSIGSQEDRADYRTA

LVAFLEQHIDVLDEDCKRRMHTNPLRVLDTKNPDIQAILGDAPRLSEYLGEESKAHFAGL

CELLDAAGIEYTVNERLVRGLDYYNRTVFEWITESLGAQGTVCGGGRYDGLVEQLGGKPT

PAVGFAMGLERLVLMLETLELTDVRRSVDVYVVTAGEGTMMAGMKLAEQLREAISGVRVM

NHFGGGNFKKQFKRADKVGAVVALVLGENEVADNTVVLKDLVGGEQETYNQAEVAEKIAA

LI

>sp|Q87N07|SYM_VIBPA Methionine--tRNA ligase OS=Vibrio parahaemolyticus serotype O3:K6 (strain RIMD 2210633) OX=223926 GN=metG PE=3 SV=2

MANDPRHLPSRKLLVTCALPYANGSIHLGHMLEHIQADIWVRYQRLRGNTVNFICADDAH

GTPIMLKAQQMGITPEEMIAAVSEEHQKDFAGFDISFDNYHSTHSEENRELASHIYLELK

KNGFISSRTISQLFDPEKEMFLPDRFVKGTCPKCKSEDQYGDNCDNCGETYSPTELINPK

SAVSGATPVMKDSEHFFFDLPQFESMLKEWTRSGSLQSETANKMQEWFESGLQQWDISRD

APYFGFEIPGEKDKFFYVWLDAPIGYMGSFKNLCNKRDDLDFDEYWNKDSKTELYHFIGK

DIVYFHSLFWPAMLEGSGFRKPNNVFVHGYVTVNGAKMSKSKGTFVKASTYLDHLDPECL

RYYYAAKLNSRIDDLDLNLEDFTQRVNADVVNKIVNLASRNAGFIAKRFEGKLSDNFAEP

ELYNEFVAAADRIAELFEAREFGRAIREITALADKANQYVDEKAPWVVAKEEGKDQELQE

ICSVGINLFRVLMTYLKPVMPALAARTEAFLNQELTWEGIAQPLTGHEITKFKALFNRID

PKNIEAMIEASKEDAAAEMAAKEKAEAAKTETELSKDPIADEIEFDTFAQVDLRIARIIS

CEEVPKANKLLKFQLDIGGETRQVFSGIKSAYKPEELEGKLTVMVANLKPRKMKFGMSEG

MILAAGPGGSDLWILEPHEGAQPGMRVM

>sp|Q87LG6|SYV_VIBPA Valine--tRNA ligase OS=Vibrio parahaemolyticus serotype O3:K6 (strain RIMD 2210633) OX=223926 GN=valS PE=3 SV=1

MEKTYNPTSIEQALYQTWEEKGYFKPHGDTTKESYSIMIPPPNVTGSLHMGHAFQDTIMD

TLIRCERMKGKNTLWQVGTDHAGIATQMVVERKIAAEEGKTKHDYGRDAFIDKIWEWKGE

SGGTITKQLRRLGASVDWDRERFTMDDGLSNAVQEVFVRLYEDDLIYRGKRLVNWDPKLH

TAISDLEVENKDTKGHMWHFRYPLADGVKTADGKDYIVVATTRPETMLGDTGVAVNPEDP

RYKDLIGKEIILPIVDRRIPIVGDEHADMEKGTGCVKITPAHDFNDYEVGKRHQLPMINI

LTFDANIRGAAEVFNTNGEPSDAYSTELPAKYHGMERFAARKAIVAEFDELGLLEEVKDH

DLQVPYGDRGGVVIEPMLTDQWYVRTAPLAKTAVEAVENGDIQFVPKQYENMYFSWMRDV

QDWCISRQLWWGHRIPAWYDNQGNVYVGRTEEEVRKNNNLESVIELHQDEDVLDTWFSSA

LWTFGTQGWPEQTDDLKVFHPSDVLVTGFDIIFFWVARMIMMTMHFVKDENGKPQVPFKT

VYVTGLIRDENGDKMSKSKGNVLDPIDMIDGIDLESLVEKRTGNMMQPQLAKKIEKNTRK

TFENGIEAYGTDALRFTLAAMASTGRDINWDMKRLEGYRNFCNKLWNASRYVMMNTEEQD

CGFNGGEIEYSLADKWIESQFELAAKAFNNHIDNFRLDMASNTLYEFIWNQFCDWYLELT

KPVLWKGTEAQQRGTRRTLITVLEKTLRLAHPVIPYITETIWQSIKPLVEGVEGETIMLQ

ALPQFDEANFNQEALDDIEWVKAFITSIRNLRAEYDINPGKPLDVMLKAANAEDAARLEA

NKQVLMSLAKLESVRVLAADEETPACATALVAKSELMIPMAGLIDKDAELARLDGEIKKT

HGEIKRIEGKLGNEGFVAKAPEAVVAKEREKLEGYKETLAKLEEQKKTIAAL

>sp|Q87Q30|TARI_VIBPA Ribitol-5-phosphate cytidylyltransferase OS=Vibrio parahaemolyticus serotype O3:K6 (strain RIMD 2210633) OX=223926 GN=VP1320 PE=3 SV=1

MNVALIFAGGVGTRMQNSTKPKQFLELYNKPVIIYTLEKFEENKNIDAIVVVCVEPWIDY

LRKLLFKFDIQKVKFVIPGGETGQESIFNGLCKIEEEFDHNSVVLIHDGVRPLINDEIIN

RNIEAVKSHGSAITTCPPVETFVLVDNEDVVKDVHDRSLSRLAKAPQSFYLRDILKVHRQ

AREDCYTEAIDSCSLMTKYGYDVRLVSGISENIKITSPIDFFIFKAIIDTQENLQVFG

>sp|Q87MF7|Y2298_VIBPA UPF0294 protein VP2298 OS=Vibrio parahaemolyticus serotype O3:K6 (strain RIMD 2210633) OX=223926 GN=VP2298 PE=3 SV=1

MFKRFVLSLAFLIMAAVVSFQVIFTVPEHPQIITQTGSEITEDIRCIEFSGAKALDDNGE

LNLLVWNIYKQNRANWQSELELFSADKQLLLLQEASMTDSFKQWLVDGSWVSNQVSAFKA

LGSGAGVISIAQKQPIKACAYTSKEPWLRLPKSALYSQYRLSNGESLAVINVHAINFTVG

TEEYTSQLSALETLLKQHSGPIVFAGDFNSWSEYRITAMKQALREANLREVQFSPDHRTQ

FITGLPLDHVFYRGLTLKNAKAPQSDASDHNPLLVSFTLND

>sp|Q87G36|Y5481_VIBPA UPF0397 protein VPA1481 OS=Vibrio parahaemolyticus serotype O3:K6 (strain RIMD 2210633) OX=223926 GN=VPA1481 PE=3 SV=1

MNLSAKTVVVIAIGAALYGIGGLPMFGIPVFANTTLKPAMAVLALFSVLFGPLVGFLVGF

IGHWVTDLFAGWGVWLTWVLGSGIVGLIIGLFPSLTRYRLEKGDFNMKDFALFVVLALLG

NVFGYGCSAFLDTILYAEPFTKVFTQLSIIAIGNTILIAIVGFFILKSVAKRNKQSRNLT

EA

>sp|Q87FT1|Y5597_VIBPA UPF0176 protein VPA1597 OS=Vibrio parahaemolyticus serotype O3:K6 (strain RIMD 2210633) OX=223926 GN=VPA1597 PE=3 SV=1

MSQYVVCALYKFVELNNYQELREPLLALMEKHHIRGTLLLAGEGINGTVASDRAGIDTLL

EWLNTEPRLTGTVYKESYSETQPFNRTKVKLKKEIVTLGVEGIDPRHVVGTYVKPQDWND

LIADPEVFVVDTRNDYEIEIGTFKGAVNPNTETFREFPDYVKENMDPAKHKKVAMFCTGG

IRCEKSTAYMKEQGFEEVYHLEGGILKYLEEVPQEESMWEGDCYVFDGRVAVNHQLEKAD

YDLCNACRLPITDEDKQSELFEQGVSCPKCHGKHSEEQVERFREREKQVSLANQRGEQHV

GGESAKQRAQRREAKLAKKAAQRKQA

>sp|Q87R88|Y909_VIBPA UPF0061 protein VP0909 OS=Vibrio parahaemolyticus serotype O3:K6 (strain RIMD 2210633) OX=223926 GN=VP0909 PE=3 SV=1

MSIWQRVSFTHRFSQLPSAFYTLVEPQPLDNTRWVAWNGEFAQQFGLPAAQNDELLAVFS

GQSEFEPFRPLAMKYAGHQFGVYNPDLGDGRGLLLAEIEHQNGTWFDIHLKGAGLTPYSR

MGDGRAVLRSTIREYLCSEAMAGLGIPTTRALGMVVSDTPVYREKTEFGAMLIRMAETHV

RFGHFEHLFYTNQLAEQKLLADKVIEWHFADCASAEKPYAAMFGEIVQKTADMIAYWQAY

GFAHGVMNTDNMSILGQTFDYGPFGFLDDYEPGYICNHSDYQGRYAFEQQPRIALWNLSA

LAHALSPLVEREDLEQALSQFEGRLSQQFSRLMRSKLGLKTKIAEDGRLFESMFELLNQN

HTDYTRFFRALSNLDKQPAQEVIDLFIDREAAQAWLDLYLARCELEVDEIGEPISAEQRS

EQMRQTNPKYILRNYLAQLAIDKAEEGDFSEVHRLAEILRHPYDSQPEFEAYAKLPPEWG

KKMEISCSS

>sp|Q87R08|Y990_VIBPA UPF0304 protein VP0990 OS=Vibrio parahaemolyticus serotype O3:K6 (strain RIMD 2210633) OX=223926 GN=VP0990 PE=3 SV=1

MEMSNAQRLILSNQYNLMSQLDPNNAAKYKRLQTIVERGYELQMRELNKDFGCLSEAECR

EIIDIMEMYHAMQESNNMLDAEERSKVDQRRLQFLGFDIATEAQQVHYVRFLVDSEGLYP

QFDKADHHFNSQMPMLDKYRRMLQTWRNCPRQYHLCENELAQIFSA

>sp|Q87L25|Y2791_VIBPA UPF0270 protein VP2791 OS=Vibrio parahaemolyticus serotype O3:K6 (strain RIMD 2210633) OX=223926 GN=VP2791 PE=3 SV=1

MIIPWQDIAPETLENLIREFVLREGTDYGAIEISLQNKIDQVKTQLEKGEAVIVFSELHE

TVDIQLKAKF

>sp|Q87GU2|Y5223_VIBPA UPF0502 protein VPA1223 OS=Vibrio parahaemolyticus serotype O3:K6 (strain RIMD 2210633) OX=223926 GN=VPA1223 PE=3 SV=1

MKVELTAIEARVIGCLIEKEVTTPDQYPLSLNALTNASNQKSNREPVMALSESDVLDAVD

ALIERRLVSDESAFNSRVSKYQHRFCNTEFGDLKLSEQEKGIICCMLLRGAQTPGEIRTR

TNRLATFHDVKEVEAVLEHLASEEKGPLVVKLPREAGKRESRYMHLFCGEVDVSAMAAAV

PATSSSSVRIAQLEQEVAELREELDALKAQVESLLS

>sp|Q87RC2|Y875_VIBPA YcgL domain-containing protein VP0875 OS=Vibrio parahaemolyticus serotype O3:K6 (strain RIMD 2210633) OX=223926 GN=VP0875 PE=3 SV=1

MLCSIYKSSKKEGTYLYIPKKDDFSQVPDTLMQMFGKPIPVMTIKLDGRKLAQVDIEKVK

ASLQNDGFFLQVPPPPENLLEKYKEQKAQQKNEQ

>sp|Q87LK0|Y2612_VIBPA UPF0301 protein VP2612 OS=Vibrio parahaemolyticus serotype O3:K6 (strain RIMD 2210633) OX=223926 GN=VP2612 PE=3 SV=2

MNLTNHFLVAMPGMKDPYFQNSVIYVCEHNEEGAMGLMINAPVDITVGNMLKQVDVQPVH

PRLFEASLDRPVYNGGPISEDRGFILHKPKDYYESSIQMTDDLAVTTSRDILSVLGTEAE

PSDYLVALGYSGWSAGQLENELVENSWLTIEATPEIIFDTPITERWKKAVEKLGIDPSQL

SADAGHA

>sp|Q87JD1|Y4321_VIBPA UPF0251 protein VPA0321 OS=Vibrio parahaemolyticus serotype O3:K6 (strain RIMD 2210633) OX=223926 GN=VPA0321 PE=3 SV=1

MARPKIERRICGRAAHHCFKPNGVPFHQLEQVAILPEELEALRLADLEGLSQQQAADQMG

ISRQTFGNTVKSARFKVAKSLVEGHALVFPNEESNL

>sp|Q87ND5|UBIG_VIBPA Ubiquinone biosynthesis O-methyltransferase OS=Vibrio parahaemolyticus serotype O3:K6 (strain RIMD 2210633) OX=223926 GN=ubiG PE=3 SV=1

MTKAQNVDPSEIKKFEEMASRWWDLEGEFKPLHQINPLRLNYVLEKADGLFGKKVLDVGC

GGGILAESMAKEGATVTGLDMGKEPLEVARLHALETGTKLTYIQSTIEDHAAENAGTYDV

VTCMEMLEHVPDPLSVIRSCAALVKPGGHVFFSTLNRNIKSYLFAIVGAEKLLKIVPEGT

HDHEKFIKPAEMMKMIDQTDLTEMGITGLHYNPLNDSYKLGRNVDVNYIVHTKKY

>sp|Q87PM1|Y1481_VIBPA Putative kinase VP1481 OS=Vibrio parahaemolyticus serotype O3:K6 (strain RIMD 2210633) OX=223926 GN=VP1481 PE=3 SV=1

MWQAISQQLSDTLLFEYQITEKVRLSGGDISESYMINDGEQRYFVKINDREFLHKFEVEA

ESLHLLRETSTIFVPEVVLVGKTKNNAFIILNYLPTKPLDDPENSFKFGQQLAQLHQWGE

QKEFGFDTDNYLGSTLQPNQWHKKWCMFFAEQRIGWQLQLLKEKGVTLVDIDDFIDVVKQ

LLANHTPEPSLLHGDLWNGNVALTAFGPICFDPACYWGDRECDIAMTELFGGFQPEFYQG

YESVMPLLPGYHERKDIYNLYHILNHCNLFGGHYLEQAQLTINKIISY

>sp|Q87NJ8|Y1870_VIBPA UPF0283 membrane protein VP1870 OS=Vibrio parahaemolyticus serotype O3:K6 (strain RIMD 2210633) OX=223926 GN=VP1870 PE=3 SV=1

MSELKQKQIFSEKALEKEQQSDSPELTAQKTFSEKETFVPVKIEEDRIETEQELQLEHVI

RPRPGRKWLATSVFATFAGLVGWQAVDSVVTAVQTADWLALGWVGFITAVASLGLGAIGK

ELWKLRKLRNHFSIQEEAELLVHSDSVGKGKVFCEKVAEESGVLAENPGFDRWQNSINPA

HSDAEILDMYDSMVVSQQDKLATKVVSQHATESAALVAVSPLAAADMLLVAWRNFKMIDN

LSKVYGVELGYASRIKLLRAVFVNMAAAGASELAIDAGMDLMSMDLAGKVSARAGQGLGV

GILTARLGLKAMALLRPLPWYPDRQVKLGTIRKAVVAKVASITMKP

>sp|Q87N95|Y1980_VIBPA UPF0313 protein VP1980 OS=Vibrio parahaemolyticus serotype O3:K6 (strain RIMD 2210633) OX=223926 GN=VP1980 PE=3 SV=1

MTNNITPIHEYKKYWAECFGTAPFLPTSRKEMDALGWDSCDIIIVTGDAYVDHPSFGMAI

IGRLLEAQGFRVGIIAQPEWQNKNAFMQLGKPNLFFGITAGNMDSMINRYTADKKLRHDD

AYTPNNEGGKRPDRATLVYSQRCREAYKEVPIVLGGIEASLRRVAHYDYWSDKVRRSVLF

DAKADILLFGNAERALVEVAHRIANGEDISTMTNIRGTAVNLPAAPEGYTVIDSSRIEKP

RKEAFVPKNPYEVETQCETKKDEPVAQPITIRPSRHDAATTAVRLPSFEKLRNDRILYAH

ASRVLHLETNPYSGRALLQSHGDRELWVNQAPIPLTTEEMDFVFGLPYARVPHPMYGKAK

IPAYDMIKTSVNIMRGCFGGCSFCSITEHEGRIIQNRSKESIINEIEEIRDKVPGFTGTI

SDLGGPTANMYRLGCKDPKAEANCRRPSCVFPGICNKLNTDHKHTIDLYREARKVEGVKK

VMVASGVRYDLAIESPEYVKELVTHHVGGYLKIAPEHTEKGPLDLMMKPGMGTYDRFKEM

FEKYSAEAGKKQYLIPYFISAHPGTEDEDMLNLALWLKKNNFECDQVQNFYPSPMCNATS

MYYSETNPLKRVKYKQREDIPVAKGERQRRLHKALLRYHDPANWPLIREALINMGKKHLI

GDKPTCLVPAEDIDAQTPAQRRKSGRHGANRFATKHTKNQPGFGGHLNKRAEGGSKDGKP

SGNRNGSGKVQGGQRPASNGQRPSGNGANRPAGSKPQGQGRPQGQGKPAGQRKPKRR

>sp|Q87MQ8|Y2173_VIBPA UPF0597 protein VP2173 OS=Vibrio parahaemolyticus serotype O3:K6 (strain RIMD 2210633) OX=223926 GN=VP2173 PE=3 SV=1

MNQQWTQYIQIIKQVVKPALGCTEPIAAAYAAAVARKELGTSDIDAIEVRVSDNLFKNSM

GVFVPGTGKIGLKIAASVGALAGDPTAELEVLARINEQDVAAAQQLIDEERVTVARMDTQ

EFIYCSVTLTSGDDVVSVTISGGHTNIIQIMRNGDVIFDAPPQQRVATASVCEGVDISIK

QIYEFATQAPFEEIKFILQAAELNTLLAQEGIDRGYGLEIGRTLKGNIEQGLLGNDLMSR

IQMMTSAASDARMGGATLPAMSNFGSGNQGIAATMPVVVAAEVFQNDEEQLARALIMSHL

GAIYIKSYYPPLSAFCGNTVTSAAASMALVYLAGGTFEQSCYAIQNVISDSSGMVCDGAK

SSCAMKVCTSSTTAVRSYLMAMGNHSVKNQGIVGEEVEQTIRNVGSMVRFGMPYTDKSII

DIMSA

>sp|Q87KG2|Y3015_VIBPA UPF0276 protein VP3015 OS=Vibrio parahaemolyticus serotype O3:K6 (strain RIMD 2210633) OX=223926 GN=VP3015 PE=3 SV=1

MKHYDFHPLVGVGLRTPHLDFFQQQRPELSWLEIHSENYFQPNAAERKYLHTLREQYQIS

CHGIGLSLGSVERVSQVHLAQLKALIDAIDPMFVSDHLSWSENGGHYFNDLLPLPYTEEA

LNVFTRNVLEVQDYLQREILIENPSSYVKFQHSTISEWEFLAEVQQRTSCRLLLDLNNVH

VSAFNHGFDCNTYLSAIPADKVDEIHLAGFTIKQLDKGEIWIDTHSRPVSTEVWKLYQHW

IEQYGPRHTLIEWDLDIPAPEVLLAEAEKASLLLSQITTPLSATRKAS

>sp|Q87GU3|Y5222_VIBPA UPF0213 protein VPA1222 OS=Vibrio parahaemolyticus serotype O3:K6 (strain RIMD 2210633) OX=223926 GN=VPA1222 PE=3 SV=1

MSESVEQHWSVYLIRNNRNALYCGVTNDIERRFKQHQLGKGAKALKGKGPLVLEWSTSFD

SKVTAMRAEYFIKQLTKSKKEQLVELKALIAVDDNQQVMFESCSQS

>sp|Q87S86|Y538_VIBPA Uncharacterized response regulatory protein VP0538 OS=Vibrio parahaemolyticus serotype O3:K6 (strain RIMD 2210633) OX=223926 GN=VP0538 PE=3 SV=1

MLTALVIDDEQFAREELAELLDETGQVEVVGDASNAILGLKKINELKPDVVFLDIQMPQV

TGIELLGMLDPETMPYVVFVTAYDQYAIQAFEDNAFDYLLKPVDPCRLNKTVKRLNKVIS

QSALTQQLSAITPDTLDQIPCIGHNRIVIMATETVECAYSDISGVHVRSTSQTASTQLTL

KTLEEKTPLVRCHRQYLVSIKAISEIKLLENGLAEIITKTGFEIPVSRRYLKVLKEMLGI

SH

>sp|Q87GA3|Y5414_VIBPA UPF0267 protein VPA1414 OS=Vibrio parahaemolyticus serotype O3:K6 (strain RIMD 2210633) OX=223926 GN=VPA1414 PE=3 SV=1

MSSHPTKITFFEFLTPLITSGQKTITIRDESESHYVPNTEVEVFTLETDRKVCDIKILSV

EPLNFDEINEFHAEQEAIELPKLKQLIREIYPNIDKLFVIEYELIKK

>sp|Q87FU4|Y5584_VIBPA UPF0319 protein VPA1584 OS=Vibrio parahaemolyticus serotype O3:K6 (strain RIMD 2210633) OX=223926 GN=VPA1584 PE=3 SV=1

MKLIKPLTCALALAMSGMAFADVTVSVPDDVSVLAANGEKAKLSGGFFASEKALTLPDGV

NQVVFRYAPYFNQGNDRLSVESDVIVARFDTANAELTIEVPKYRNMRDAEENIKDLDWKL

VDGSGKAVAVDQDKLIKPGMQIGRDYVREIEDYNRAGGTAAVAFAGAATMQPVTLPAKIP

EDMKQARATAVKADSTAEEMLHFWYQKADAETKARFKAYINQQ

>sp|Q87LH9|YDJC_VIBPA Carbohydrate deacetylase OS=Vibrio parahaemolyticus serotype O3:K6 (strain RIMD 2210633) OX=223926 GN=VP2633 PE=3 SV=1

MKVIFNADDFGLTQGVNNGIVKSHQDGVVKSTTMMVGMDAEQNAIELAHQNPDLKIGVHL

RFTAGAPLTEHPNLTNGRTHFVKYSELWNKQDFEAQAVYDEAKAQIDHFLSLGLTLSHLD

SHHHAHTHPQILPIVQKLAKEHRVPLRGSGICHQPMTTSYFFTDEFYDQKVSLDGLMQHL

LSLKENYDVVEVMCHPAYADQPLIMKSGYALQRELELQVLTSPILKEQLAQHGIAVTDYS

ALVSTSQVVGV

>sp|Q87G35|Y5482_VIBPA Putative ABC transporter ATP-binding protein VPA1482 OS=Vibrio parahaemolyticus serotype O3:K6 (strain RIMD 2210633) OX=223926 GN=VPA1482 PE=3 SV=1

MTIEFSNFSFRYESLDKPTLKNINLRIEKGEKIVIIGPSGSGKSTLGQCLNGLIPHAIKG

ETSGTLTIYGQDTAPFDMHQYTEQVGTVLQDTDSQFVGLSIGEDIAFALENQLTSNIDMY

PLVKATAKMVDLEQMLDRSPHDLSGGQKQRVSLAGILVDDVDILLFDEPLAALDPKTGKK

TIEIIDDLHRETGKTIVIIEHRLEDVLHRSVDRIILMESGEIIADTTPDEILASPLLEDY

GIREPLYISALKEAGCAIEGDAKPSSLTTLPLEQYKPTVQAWFDGSTAQPPKTPAETLLE

VRGLTYSYDGEKNALEDVSFDIKRGEFVSVLGKNGSGKSTITKLIMGVIEADSGSMSMNG

QDLNELTIFERSQKVGVVMQNPNHMISHHMIFDEVAFGLRNRGIKEKQIEAKVLEVLELC

GLSKYRHWPIEALSYGQKKRVTIASILVLEPELLILDEPTAGQDYRNYTSMLSFIEKLNR

ELGVTVMIISHDMHLVLEYTTRSIVIADSKLVADAPMTQVFSSPELLDQANLTTTSLFDL

ATKVGIEDTNGFMQHFINVEKAHRQQKSGEVNQPEKAVA

>sp|Q87R29|Y969_VIBPA UPF0227 protein VP0969 OS=Vibrio parahaemolyticus serotype O3:K6 (strain RIMD 2210633) OX=223926 GN=VP0969 PE=3 SV=1

MIIYLHGFDSTSPGNHEKVLQLQFIDDDVRFINYSTLHPKHDMQHLLKEVSKVIDQSDDP

NPLICGVGLGGYWSERIGFLCGIKQVMFNPNLHPENTMAGRIDRPEEYEDIATKCVDQFR

AKNQGRCLVILSKEDEIHDNTKTASELEKHYDIIWDESQSHKFKKISQHLQAMKEFKNT

>sp|Q87LT2|YACG_VIBPA DNA gyrase inhibitor YacG OS=Vibrio parahaemolyticus serotype O3:K6 (strain RIMD 2210633) OX=223926 GN=yacG PE=3 SV=1

MSKITIVQCPQCGTDVEWGEQSPHRPFCSKKCQMIDFGEWADEENAIAGAPDMSDSDGWS

EDQY

>sp|Q87LM8|YGFZ_VIBPA tRNA-modifying protein YgfZ OS=Vibrio parahaemolyticus serotype O3:K6 (strain RIMD 2210633) OX=223926 GN=VP2583 PE=3 SV=1

MEWQTRFSPLNLSTQDALPELSISRLDHLGMITMVGDDKKSYLHGQVTCDVVSLEKDQST

LGAHCDAKGKVWSVFRLFHHGDGYGMIQPKSAIEIELKEIKKYAVFSKVTIEESNDVILG

VAGVNADAFVSALNEDAGDVRIINGGTAVKVEANRWLLVVTEEAAQALIENSDATLTTRE

LWTRFDIESALPFVSATAQNEHIPQALNIQALGGISFTKGCYTGQETVARAKYRGTNKRA

MYIVKGVTSTALNDDAIELERSVGDNWRSVGTLLTHYQFSDNQAMGLIVLPNNLDDDTRL

RLTSQPDCEWTIAELPYSLDDE

>sp|Q87SF4|CARA_VIBPA Carbamoyl-phosphate synthase small chain OS=Vibrio parahaemolyticus serotype O3:K6 (strain RIMD 2210633) OX=223926 GN=carA PE=3 SV=1

MSKLALLVLEDGTVFRGVSIGADGVSVGEVVFNTSMTGYQEILTDPSYSQQIVTLTYPHI

GNTGTNSEDEESSSIHAQGLVIRDLPLIASNFRNEQSLSDYLKSQNIVGIADIDTRKLTR

ILREKGAQNGCIVAGNNLDEALALAKAKEFPGLKGMDLAKEVTTKEAYQWKQGSWTLESG

LPEAKDDSELPYHVVAYDFGAKRNILRMLVDRGCRLTVVPAETSAEEVLALNPDGVFLSN

GPGDPEPCTYAIEATKVFLEKGLPIFGICLGHQILALASGAQTVKMKFGHHGANHPVKDL

ERNVVMITSQNHGFAADEATLPENLRATHVSLFDGSLQGIHRTDKPAFSFQGHPEASPGP

HDAAPLFDHFIELIKKHSA

>sp|Q87JE8|CATA_VIBPA Catalase OS=Vibrio parahaemolyticus serotype O3:K6 (strain RIMD 2210633) OX=223926 GN=VPA0305 PE=3 SV=2

MQMSKSFLLITVGLASTSLQAQTLTRDNGAPVGDNQNSITAGENGSVLLQDVHLIQKLQR

FARERIPERVVHARGTGAHGEFVASGDFSDLTVSAPFTEKGKVTPVFVRFSTVIHSKGSP

ETLRDPRGFATKFYTEQGNWDLVGNNLPVFFIRDSIKFPDMVHSLKPSPVTNVQDPNRFF

DFFSHEPSATHMLTWVYSNLGTPASYRTMDGFGVHAYKWINQQGDVNYVKFQWKSQQGIK

SLRPNKVTEMQGKDFNHLTNDLYAAIGRGNYPKWDLYVKVLSPEALSKLDYNGLDATKVW

LNVPDRKVGTMTLNRLPENFFLETEQSAFAPSNLIPGIEPSEDRLLQGRLFAYADTQLYR

LGANLFQLPVNRPLTSVNNHNQNGLSNNAQLSNGDVNYEPSRKLNLAEDNQFKAVETKLV

GTVQQKAISKPRDFYQAGVLYRSMNEQDRSDLIANLAGDLNKVIDKDIKATMVSYFYRAD

KEYGSRLAEATDTNLSQVKNKAMM

>sp|Q87KA9|ATPE_VIBPA ATP synthase epsilon chain OS=Vibrio parahaemolyticus serotype O3:K6 (strain RIMD 2210633) OX=223926 GN=atpC PE=3 SV=1

MAPITFHLDVVSAEKRIFSGRVETFQVTGSEGELGIFHGHTPLLSAIKPGMVRIVKQHGH

EEFIYVSGGMVEVQPGTATVLADTAIRGEDLDAAKAEEAKRRAEEKIQNQHGDMDFAQAA

SELAKAIAQLRVIELTKKRR

>sp|Q87NZ5|BCCT2_VIBPA Glycine betaine/proline/choline transporter VP1723 OS=Vibrio parahaemolyticus serotype O3:K6 (strain RIMD 2210633) OX=223926 GN=VP1723 PE=1 SV=1

MSTDNNGGIKRPDGKVNAIDTDYQIGQDNVALKVGPFGLDIHNRVFAISGMAIVLFVVAT

LTFRQQVEPFFAGLRAWLVSNLDWFFLASGNVFVIVCLVLIVTPLGRVRIGGTEATPDYS

YAGWLAMLFAAGMGIGLVFFGVSEPMSHFSSALGGVNIENGVRTDWAPLGGAVGDTDAAS

ALGMAATIYHWALHPWSIYALLALGLAIFSFNKGLPLTMRSIFYPLFGERVWGWVGHIID

ILAVVATVFGLATSLGYGASQAATGLNFLFGVPMTDTTQVVLIVVITALALISVVAGLDS

GVKRLSEINMILAAMLLFFVIIVGPTMAILTGFFDNIASYITNIPALSMPFEREDVNYSQ

GWTAFYWAWWISWSPFVGMFIARVSRGRSVREFIICVILIPSTVCVLWMTAFGGTAISQY

VNDGYEAVFNAELPLKLFAMLDVMPFAEITSVVGIILVVVFFITSSDSGSLVIDTIAAGG

KVDAPTPQRVFWCTFEGLVAIALMLGGGLAAAQAMAVTTGLPFTIVLLVATVSLIKGLMD

EPRLSTKAVKKDK

>sp|Q87H52|BETB_VIBPA NAD/NADP-dependent betaine aldehyde dehydrogenase OS=Vibrio parahaemolyticus serotype O3:K6 (strain RIMD 2210633) OX=223926 GN=betB PE=3 SV=1

MEMKTHYIDGAMYIGCSEEHFTTYNPANGEPLANIKQANQSDMEAAIESAKRGFEVWSAM

TAIERSRILNKAVAILRERNDELAALEVADTGKPIQEAIAVDITTGADVIEYYAGLAPSL

QGEQQPLNENQFFYTRREPLGICAGIGAWNYPIQIAMWKSAPALAAGNAMIFKPSEETPL

TALKLAEIYSEAGLPDGVFNVVQGDYRVGQMLTAHPDIAKVSFTGESGTGKVVMGDSAKT

LKQVTMELGGKSPLIVFDDAKLDDAVSAAMVANFYTQGEVCTNGTRVFVHESIYDDFVAQ

LKTRTEKLVVGDPLDENTQIGALISKEHESKVLSAIESAKASGATLLTGGYKVTDNGLQN

GNFVAPTVFIDCDDSMSHVQQEIFGPVMSVLKFSEEAEVIERANDTDYGLAAGVFTQNLS

RAHRVIHKIQAGICWVNAWGDSPAEMPVGGYKQSGIGRENGVETLKHYTQTKSVLVQLSD

FESPYA

>sp|Q9L7P5|CH601_VIBPA 60 kDa chaperonin 1 OS=Vibrio parahaemolyticus serotype O3:K6 (strain RIMD 2210633) OX=223926 GN=groL1 PE=3 SV=2

MAAKDVKFGNDARVKMLEGVNVLADAVKVTLGPKGRNVVLDKSFGAPTITKDGVSVAREI

ELEDKFQNMGAQMVKEVASKANDAAGDGTTTATVLAQAIVNEGLKAVAAGMNPMDLKRGI

DKAVAAAVEQLKELSVECNDTKAIAQVGTISANSDASVGNIIAEAMERVGRDGVITVEEG

QALQDELDVVEGMQFDRGYLSPYFINNQEAGSVELENPFILLVDKKISNIRELLPTLEAV

AKASRPLLIIAEDVEGEALATLVVNNMRGIVKVAAVKAPGFGDRRKAMLQDIAILTGGTV

ISEEIGLELEKVTLEDLGQAKRVSITKENSTIIDGAGEEAMIQGRVAQIRQQIEDATSDY

DKEKLQERVAKLAGGVAVIKVGAATEVEMKEKKDRVEDALHATRAAVEEGVVAGGGVALI

RAASKIVDLEGDNEEQNVGIRVALRAMEAPIRQITKNAGDEDSVVANNVKAGEGSYGYNA

ATGEYGDMLEMGILDPTKVTRSALQFAASVAGLMITTEAMVTDLPQKESAGMPDMGGMGG

MGGMGMM

>sp|Q87MK5|CHEB1_VIBPA Chemotaxis response regulator protein-glutamate methylesterase of group 1 operon OS=Vibrio parahaemolyticus serotype O3:K6 (strain RIMD 2210633) OX=223926 GN=cheB PE=3 SV=1

MAIKVLVVDDSSFFRRRVSEIINSESRLEVIDVAVNGREAVEKAKALKPDVITMDIEMPV

MDGITAVREIMAASPTPILMFSSLTHDGAKATLDALDAGALDFLPKKFEDIARNRDEAVS

LLQQRVIQIASKRAFMRRPVARPAAATSSARPLASRTAAPAASAPARPATTKFRASGKKY

QLTAIGTSTGGPVALQKILTRLPMNYPHPIVLIQHMPATFTAAFASRLNTLCKIQVKEAQ

DGDVLQAGVAYLAPGGKQMMIDGRAGAARLRIIDGGDRMNYKPCVDVTFGSAAKVYGDKV

LSMVLTGMGADGREGARMLKSAGSTIWAQDEESCVVYGMPQAVAKAGISSEDLPLDRIAE

RMLVEVGLA

>sp|Q87JG7|CH102_VIBPA 10 kDa chaperonin 2 OS=Vibrio parahaemolyticus serotype O3:K6 (strain RIMD 2210633) OX=223926 GN=groS2 PE=3 SV=1

MKIRPLNDKLIVERQEVENKSEGGIVLTSQSVKKSNRGKVIAVGLGKRFENGERAAMEVK

VGDQIIFNDGYGVKTEKIDGAEYLILSESDVLAIVE

>sp|Q9LB13|CHEZ_VIBPA Protein phosphatase CheZ OS=Vibrio parahaemolyticus serotype O3:K6 (strain RIMD 2210633) OX=223926 GN=cheZ PE=3 SV=1

MISLEQAKSLVQMLENGEQDQANMLVASLYEGTENPVLQEIGTLTRDLHDSLKQFNLDQR

MTEIAKDEIPNARDRLHYVIEKTELAANKTMDAVDCCLPIADNLHDCLQQVRPQWNELMY

GRIELSEFKALCHRIDKLLVQVEGDSTELRGQLTEILMAQDFQDLTGQIIRRVITLVNEV

EGRLVEILTVFSGQKPAEQVQVLSEPADKKIKQSSEAEGPILHPELREDAVSSQDEVDDL

LSSLGF

>sp|Q87Q52|CDD_VIBPA Cytidine deaminase OS=Vibrio parahaemolyticus serotype O3:K6 (strain RIMD 2210633) OX=223926 GN=cdd PE=3 SV=1

MKSRIEQALASAPEALSKQLAPIVLADDFDATLSAQQFEQLLSATSLSDKELRVALLPFA

AAYSYAPISEFYVGAIVRGLSGRLYFGANMEFFGVQLGQTVHAEQSAISHAWMKGEHGVK

DITINFSPCGHCRQFMNELSTAKELKVQLPERDEKSLHEYLPEAFGPADLGIESGLMAEV

KHQFVCDDKDALIQQAVEAMNMSHAPYTNNLSGLALELANGRVFKGAYAENAAFNPSLPP

LQVALIQVLLAGETFDSIKAAALVENSEGKISHLADTQSTLEALNPDIPVSFVNV

>sp|Q87KD5|DEF1_VIBPA Peptide deformylase 1 OS=Vibrio parahaemolyticus serotype O3:K6 (strain RIMD 2210633) OX=223926 GN=def1 PE=3 SV=1

MSVLQVLTFPDDRLRTVAKPVDAVTPEIQKIVDDMIETMYDEEGIGLAATQVDIHKRIVV

IDISETRDEPMVLINPEILEKRGEDGIEEGCLSVPGARALVPRAAEVTVKALDRDGKEFT

FEADDLLAICVQHELDHLQGKLFVDYLSPLKRKRIQDKLAKIKRFNEKQQNA

>sp|Q87M25|DEOD1_VIBPA Purine nucleoside phosphorylase DeoD-type 1 OS=Vibrio parahaemolyticus serotype O3:K6 (strain RIMD 2210633) OX=223926 GN=deoD1 PE=3 SV=1

MATPHINAEMGAFADVVLMPGDPLRAKYIAETFLEDVVQVCDVRNMFGYTGTYKGRKISV

MGHGMGIPSCSIYATELIKDFGVKKIIRVGSCGAVNEDIKVRDVVIGMGACTDSKVNRIR

FKGHDFAAIADYKMVRAAEDAAKARGIDVKVGNLFSAELFYTPDPEMFDVMDKYGIVGVE

MEAAGIYGVAAEYGAKALTICTVSDHIKTGEQTTSDERQTTFNDMMLIALDSVLLGDAE

>sp|Q87S12|DER_VIBPA GTPase Der OS=Vibrio parahaemolyticus serotype O3:K6 (strain RIMD 2210633) OX=223926 GN=der PE=3 SV=1

MVPVVALVGRPNVGKSTLFNRLTRTRDALVADFPGLTRDRKYGQARLDEEHEFIVIDTGG

IDGTEEGVETKMAEQSLAAIDEADVVLFLVDGRAGLTPADEAIAAHLRKIEKPAMLVVNK

IDGIDADAACADFWQLGVDDMYQIAAAHGRGVTALLERALAPFFDDLLTSESEEGEIEDL

TEFEDAEIAPDEYTEEEAEAEFQRLQEQPIKLAIIGRPNVGKSTLTNRILGEERVVVYDM

PGTTRDSIYIPMERDGREYVIIDTAGVRRRGRINETVEKFSVVKTLKAVEDANVVLLVID

ARENISDQDLSLLGFALNAGRSIVLAVNKWDGLDNEVKENVKKELDRRLGFVDFARIHFI

SALHGTGVGHLFESIQEAYKSATTRVGTSVLTRIMKMATDDHQPPMVRGRRIKLKYAHAG

GYNPPIVVIHGNMVRELPDSYKRYLMNYFRKSLEIMGTPIRINFQNSENPFENRANKLTL

SQERKRKRMMSVVKNRKK

>sp|Q87HS0|DDL_VIBPA D-alanine--D-alanine ligase OS=Vibrio parahaemolyticus serotype O3:K6 (strain RIMD 2210633) OX=223926 GN=ddl PE=3 SV=1

MIKNILLLCGGGSSEHEISLLSANFVEQQLNLIQNVKVTRVEIKNEGWVTDQGELVYLDL

NTKQLCSNESNQTIDFIVPCIHGFPGETGDIQSLFEIAGIPYLGCGPEASSNSFNKITSK

LWYDALDIPNTPYLFLTRNDEHAHRQAEQAFEKWGKVFVKAARQGSSVGCYSVAEKQAIA

KAVNDAFGYSDQVLVEKAVKPRELEVAAYEMNGELHITKPGEVIAPDGAFYSYDEKYSSS

SHSLTEVEAKNLTQEQIDKIRHASETVFKQMNLRHLSRIDFFLTEDNEIYLNEVNTFPGM

TPISMFPKMLQNNGHKFHEFLEDCINSAK

>sp|Q87KR0|DCUP_VIBPA Uroporphyrinogen decarboxylase OS=Vibrio parahaemolyticus serotype O3:K6 (strain RIMD 2210633) OX=223926 GN=hemE PE=3 SV=1

MTELKNDRYLRALLKEPVDYTPVWMMRQAGRYLPEYKATRAQAGDFMSLCKNAELASEVT

LQPLRRFPLDAAILFSDILTIPDAMGLGLRFAAGEGPVFDNPITCKADVEKIGLPDPEGE

LQYVMNAVRQIRKDLNGDVPLIGFSGSPWTLATYMVEGGSSKAFTKIKKMMYAEPQTLHL

LLDKLADSVIDYLNAQIKAGAQSVMVFDTWGGVLTPRDYNLFSLQYMHKIVDGLIRENDG

RRVPVTLFTKNGGMWLEQIAATGCDAVGLDWTINIADAKARIGDKVALQGNMDPSMLYAS

PERIREEVAGILEGFGDAGTGHVFNLGHGIHLDVPPENAGVFVEAVHELSKPYHK

>sp|Q87M24|DEOB_VIBPA Phosphopentomutase OS=Vibrio parahaemolyticus serotype O3:K6 (strain RIMD 2210633) OX=223926 GN=deoB PE=3 SV=1

MKRAFILVLDSFGIGATADAKEFGDVGSDTLGHIADQCEKGLADNDKRQGALRLPNLSKL

GLAMAHKESTGRFAPGLDADAEIIGAYGHAAELSSGKDTPSGHWEIAGVPVLFDWGYFTD

KANSFPKELTDRILERAGLDGFLGNCHASGTQVLDDLGEEHMKTGQPIFYTSADSVFQIA

CHEETFGLDRLLELCQIAREELEDYNIGRVIARPFIGPGKGQFERTGNRRDLSVEPPSAT

VLQKLVEEKQGNVVSIGKIADIYANCGITKKVKATGIPALFEATLEQIKEAGDNTIVFTN

FVDFDSAYGHRRDVAGYAAALEYFDGRINEVLELMGEDDVLILTADHGCDPTWPGTDHTR

EHIPVLVYGQKVPAGSLGRRETFADIGQTLASYFGTSPMDYGKNFL

>sp|Q87IR2|CYOE1_VIBPA Protoheme IX farnesyltransferase 1 OS=Vibrio parahaemolyticus serotype O3:K6 (strain RIMD 2210633) OX=223926 GN=cyoE1 PE=3 SV=1

MSKEIALTLDSRKRLGSTYLKLTKPKVVALMLVTAIVGMSLAPVTDFPWIQASIGLIGIG

LMAGSAAAFNHLIDRRIDARMARTHTRPLPSGDTNPLSVAIFAVAIGVVGFVLLYAWVNE

LTAWMTFLSLLGYAVVYTMYLKRATPQNIVIAGIAGAMPPLLGWTAVTGELHGNAWLLVM

IIFIWTPPHFWALAIHRVEDYRKVDIPMLPVTHGIEYTKTSILLYTVLLTLVCVMPVLVG

MVGFIYLFSALLLNAGFIYHAWKLKFAPEPNSAIETFKFSIYHLLALFVALLADHYIGMV

LQ

>sp|Q87SX6|CYSC_VIBPA Adenylyl-sulfate kinase OS=Vibrio parahaemolyticus serotype O3:K6 (strain RIMD 2210633) OX=223926 GN=cysC PE=3 SV=1

MTAETPVKDENIVWHQHTVDKQFRAELKKQKPAVLWFTGLSGAGKSTVAGALENRLAELG

YHTYLLDGDNVRHGLCSDLGFSEQDRRENIRRIGELAKLMADAGLIVLSAFISPHRAERQ

LVRDLLPEGEFIEVFVNASLEVCEGRDPKGLYKKARAGEIPNFTGIDSEYQAPINPEIDL

PAGEKSVEELVELCLNELKQRRVIS

>sp|Q87L92|CYSH_VIBPA Phosphoadenosine phosphosulfate reductase OS=Vibrio parahaemolyticus serotype O3:K6 (strain RIMD 2210633) OX=223926 GN=cysH PE=3 SV=1

MLDSVASKPELAELLTLTKTEQILRLAQINVELEPLSAQERVKWALENLDGEFAVSSSFG

IQAAVMLHLVTQEKPDIPIILTDTGYLFAETYRFIDELTEKLNLNLKVYRAEQSAQWQEA

RYGKLWEQGVEGIEKYNKINKVEPMRRALKELNVGTWFSGLRREQSKSRAGLPILSIQNG

VFKFLPVIDWTNKDVHYYLEQHGLTYHPLWEEGYLSVGDTHTTRKWEPGMSEEETRFFGL

KRECGLHEDDGNEQDGSGI

>sp|Q87L91|CYSI_VIBPA Sulfite reductase [NADPH] hemoprotein beta-component OS=Vibrio parahaemolyticus serotype O3:K6 (strain RIMD 2210633) OX=223926 GN=cysI PE=3 SV=1

MTFSTENNKQIVLGEELGPLSDNERLKKQSNLLRGTIAEDLQDRITGGFTADNFQLIRFH

GMYQQDDRDIRNERTKQKLEPLHNVMLRARMPGGIITPTQWLAIDKFATEHSLYGSIRLT

TRQTFQFHGVLKPNIKLMHQTLNNIGIDSIATAGDVNRNVLCTTNPVESELHQEAYEWAK

KISEHLLPKTRAYAEIWLDGEKVESTEEDEPILGKTYLPRKFKTTVVIPPQNDVDVHAND

LNFVAIADNGKLVGFNVLVGGGLAMTHGDTSTYPRRADDFGFIPLEKTLDVAAAVVTTQR

DWGNRSNRKNAKTKYTLDRVGTDVFKAEVEKRAGIQFEASRPYEFTERGDRIGWVEGIDG

KFHLALFIENGRLLDYPGKPLKTGVAEIAKIHKGDFRMTANQNLIVAGVPKSEKAKIEKI

AREHGLMDDNVSEQRKNSMACVAFPTCPLAMAEAERFLPQFVTDVEGILEKHGLPENDNI

ILRVTGCPNGCGRAMLAEIGLVGKAPGRYNLHLGGNRAGTRVPKMYKENITDKQILEEID

LLVARWSKEREEGEAFGDFTIRAGIIQEVFVSKRDFYA

>sp|Q87S45|CUTC_VIBPA Copper homeostasis protein CutC OS=Vibrio parahaemolyticus serotype O3:K6 (strain RIMD 2210633) OX=223926 GN=cutC PE=3 SV=1

MVTHLEVCIDNIESLHYAIAGGATRIELCSSLALGGLTPSYGFMQQAAKQSSVPVYAMIR

PRQGDFFYNEEELDMMRWDIEAAHQSGLDGVVLGVLTQEGDIHIPFATALCEFAQALGLG

ITFHRAFDQCRNAEQALEDIIHLGCERILTSGLAPSAPAGESVLKSLVEQAQGRIAIMAG

AGVNADNARDLVKNTNVQEVHLSGKTTRPSQMTFIAGQSKMGASDVDDFAIPITSTQAIA

NVAAALK

>sp|Q87L90|CYSJ_VIBPA Sulfite reductase [NADPH] flavoprotein alpha-component OS=Vibrio parahaemolyticus serotype O3:K6 (strain RIMD 2210633) OX=223926 GN=cysJ PE=3 SV=1

MSFQKNEYSHKNVSEDNNGQGGNPPIASPLNDQQFNSLQQTVSELSSQQLAWVSGYFWGL

AQHQPSAAATPIAQAAAAVSAKPAGKLTIIFASQTGNAKGVAEALEQEAKAEGIAVELFD

ASDYKGKNLAKETHVIIVASTNGEGEAPDNAIELHEFLQSKKAPKLSNLQYGVIALGDSS

YEFFCQTGKDFDTYLAKLGATSFIERIDCDVDYEAAAEEWRKNALGKVKETLSSGNEAEI

VQLPVGQAAASHSQYNKQNPYTATLLTSQKITGRDSGKDVRHIEIDLDGSGLTYQPGDAL

GVWYENSSELASDILGKVGLSGVETVDVDGESLSIHSALVSKFEITTSNPQLVAKFAELS

GSKKLQKLAEDKDKLREYSANTQIVDVFAEKKTKLTADELVGLLRRLTPRLYSIASSQAE

VDEEVHLTVGLVEYDHNDEKRYGGASSFLAQRLEEGGDVKVFVEHNNNFKLPEDDTTPII

MVGPGTGIAPFRSFIQERENRDAEGKNWLFFGDRTFTQDFLYQVEWQKYLKSGVLSRLDV

AFSRDQVEKVYVQHRILENAAQVWQWIQDGAYIYVCGDATRMAKDVHDALVIVAEQEGKM

PRDDAEQFINDLRKAKRYQRDVY

>sp|Q87KJ1|CYAY_VIBPA Iron-sulfur cluster assembly protein CyaY OS=Vibrio parahaemolyticus serotype O3:K6 (strain RIMD 2210633) OX=223926 GN=cyaY PE=3 SV=1

MNDTEFHQLVDAQMQIIEESIDDSGADIDYEVSGNVMTLEFEDRSQIIINRQEPMHEIWL

ASKSGGFHFKLVEDKWTCSKTGMELFEMVKQECEKHAGEEIDWA

>sp|Q87IH5|CYOE2_VIBPA Protoheme IX farnesyltransferase 2 OS=Vibrio parahaemolyticus serotype O3:K6 (strain RIMD 2210633) OX=223926 GN=cyoE2 PE=3 SV=1

MLKSYLSITKPGIIFGNLISVAAGFFLAAKSEPASLMLFLTTLAGVGLVIASGCVVNNIF

DRDIDQKMARTQNRETVKGNINIDVAFVYALAMLLLGTALLFQLVNPLSAVVVLLGYVYY

VFFYTMWYKRNSVYGTLVGSISGAVPPLVGYLAVTNFISLEAILLFTMFCLWQMPHSYAI

AMFRMQDYREAGIPVLPVKDGIHKAHRHMKAYVVAFGAVSLGLFLLGEAGYEYLAVAAVV

CLMWTKVTFRSIDESNYVVWSKSVFKVSLLVVMGISGVLGVELIPLAL

>sp|Q87SY0|CYSD_VIBPA Sulfate adenylyltransferase subunit 2 OS=Vibrio parahaemolyticus serotype O3:K6 (strain RIMD 2210633) OX=223926 GN=cysD PE=3 SV=1

MDQQRLTHLKQLEAESIHIIREVAAEFDNPVMMYSIGKDSSVMLHLARKAFYPGKIPFPL

LHVDTDWKFREMIEFRDRTAEKYGFELLVHKNPEGIAMGCSPFVHGSSKHTDIMKTQGLK

QALNKYGFDAAFGGARRDEEKSRAKERVYSFRDKNHTWDPKNQRPELWKTYNGQVNKGES

IRVFPLSNWTELDIWQYIYLENIEIVPLYLADKRPVVERDGMLIMVDDDRMELQPGEVIE

EKSVRFRTLGCYPLTGAIESEANTLTGIIEEMLVATSSERQGRAIDHDQSGSMELKKRQG

YF

>sp|Q87SF5|DAPB_VIBPA 4-hydroxy-tetrahydrodipicolinate reductase OS=Vibrio parahaemolyticus serotype O3:K6 (strain RIMD 2210633) OX=223926 GN=dapB PE=3 SV=1

MVRIAIAGAAGRMGRNLVKASHINPDASVTAGSERPESSLVGVDIGELCGEGKFDVFLTD

DLEKEVDNFDVVIDFTVPVSTLANLELCKQHGKSIVIGTTGFSEEERALIDAVAKHVPVV

MAPNYSVGVNLVFKLLEKAAKVMGDYCDVEIVEAHHRHKVDAPSGTAIGMGEAIAGAMGN

KLSDVAVYAREGITGERTKDEIGFATIRAGDIVGEHTAMFADIGERVEITHKATDRMTFA

NGAVKAAVWLHSKPAGFYTMTDVLGLNEL

>sp|Q87MI6|DAPE_VIBPA Succinyl-diaminopimelate desuccinylase OS=Vibrio parahaemolyticus serotype O3:K6 (strain RIMD 2210633) OX=223926 GN=dapE PE=3 SV=1

MTDSPVLALAKDLISRQSVTPEDAGCQDLMIERLKALGFEIEVMVFEDTTNFWARRGNEA

PLFAFAGHTDVVPAGKLEQWDTPPFEPTIIDGYLHGRGAADMKGSLAAMVVAVERFIAEH

PDHKGSIGFLITSDEEGPFINGTVRVVEALMERGENIDMCIVGEPSSTEIVGDVVKNGRR

GSITGDLTVKGTQGHVAYPHLANNPVHASLLAIHELATTEWDKGNDYFPPTSFQIPNVSA

GTGASNVIPGEFNVQFNLRFSTELNNDTIVQRVTETLDKHDLNYDLHWTFNGDPFLTDTG

ALLDAVVAAVAEVNNTKPALLTTGGTSDGRFIARMGGQVVELGPVNATIHKVNECVKVDD

LEKLTDMYENTLKHLLAK

>sp|Q87KJ4|DAPF_VIBPA Diaminopimelate epimerase OS=Vibrio parahaemolyticus serotype O3:K6 (strain RIMD 2210633) OX=223926 GN=dapF PE=3 SV=1

MHFHFSKMHGLGNDFMVVDCITQNVFFSQDLIRRLADRHTGVGFDQLLVVEAPYDPETDF

HYRIFNADGSEVEQCGNGARCFARFVRLKGLTNKYSISVSTKKGKMILDVEDDGEVTVNM

GVPEFEPNKIPFKAKQKEKTYIMRAGDKTLFCGAVSMGNPHVVTVVDDVDTADVDTLGPL

LESHERFPERVNAGFMQVVSRDHIRLRVYERGAGETQACGSGACGAVAVGILQGLLDESV

KVSLPGGELHISWQGPGKPLFMTGPATHVFDGQLSC

>sp|Q87GZ9|CLCA_VIBPA H(+)/Cl(-) exchange transporter ClcA OS=Vibrio parahaemolyticus serotype O3:K6 (strain RIMD 2210633) OX=223926 GN=clcA PE=3 SV=1

MTKRERIVKSVLAHVPKDAINQFVSRGSTPFSVLIMAAIVGTLAGFVGTYFELAVHFVSE

TRTEWLRSEIGSVLPLWLAAVLISALLAFIGYFLVHRFAPEAAGSGIPEIEGAMDNIRPV

RWWRVLPVKFFGGMGALGSGMVLGREGPTVQMGGAVGRMVTDIFRVKDDDTRHSLLASGA

AGGLAAAFNAPLAGIMFVVEEMRPQFRYSLISIRAVIISAIMANIVFRAINGQDAVITMP

QYQSPALQTLWLFLLLGALFGVFGVIFNKLITVAQDSFVAIHKNDRKRYLITGSILGGVF

GLLLLYVPQLTGGGIALIPDVTTGNYSISILVLLFIGRVVTTLLCFGSGAPGGIFAPMLA

LGTLFGYAFGASADVLLPTLDIEPGVFAIAGMGALFAATVRAPITGILLVIEMTNNYYLI

LPLIITCLGAVIVAQLLGGQPIYSQLLHRTLKNDKLRQQDLPENQAS

>sp|Q87QY5|CLPS_VIBPA ATP-dependent Clp protease adapter protein ClpS OS=Vibrio parahaemolyticus serotype O3:K6 (strain RIMD 2210633) OX=223926 GN=clpS PE=3 SV=1

MSKNFEWVTPDSDLLERESTKVQPPKLYNVVLNNDDYTPMDFVIEVLERFFSHDIDKATQ

IMLKVHYEGKAVCGTYSAEIAETKVAQVTMYARENEHPLLCTMEQA

>sp|Q87Q46|COBT_VIBPA Nicotinate-nucleotide--dimethylbenzimidazole phosphoribosyltransferase OS=Vibrio parahaemolyticus serotype O3:K6 (strain RIMD 2210633) OX=223926 GN=cobT PE=3 SV=1

MDCSFSADIQTRIDNKTKPLGALGVLEKVALQLALIQSQDQAQAVEEIVIRKPTMLVFAG

DHGVAKEGISIAPSEVTQQMVANFLAGGAAINCFCDVNQIEFKVIDCGMLAPIEVMVPEF

KSHPNLIEQRLGNGTANFSKQAAMSSEQVALGLEYGARVAQSTIYSGSNLLMFGEMGIGN

TSSASALLAALSPLEVNHCVGLGTGINSEQLSRKLKLVAQGVSRCRGLDAKAVLSQVGGF

EIVQMVGAFLEAKRLKTPVLVDGFIVSVAAYVATLLDEETRDYMLFAHRSEENGHKFVLE

SLKAEPLLDLGLRLGEGTGAALALPLLKAAAQFYNKMASFESAGVTV

>sp|Q87SX9|CYSN_VIBPA Sulfate adenylyltransferase subunit 1 OS=Vibrio parahaemolyticus serotype O3:K6 (strain RIMD 2210633) OX=223926 GN=cysN PE=3 SV=1

MNSAVEAQLAELGIEGYLKQHQYKSLLRFLTCGSVDDGKSTLIGRLLHDSKQIYEDQLAA

VHSDSQRVGTTGEKPDLALLVDGLQAEREQGITIDVAYRYFSTQKRKFIIADTPGHEQYT

RNMATGASTCDLAVILVDARKGILDQTRRHSFISNLLGLKHFVVAINKMDLVDYSQARFE

EIRDEYLKFSENLTGDIDIQIIPISALEGDNVVDKGQNLNWFEGPSLLELLETVDVDYEK

GAGEFRFPVQYVNRPNLDFRGFAGTVSSGSVKVGDAIKALPSGKTSTVARIVTFDGDIEE

AQAGLAVTLTLNDEIDISRGDLIVLENAQVQTTNHLLADVVWMTEQPLQPGRDYDIKIAG

KKTVGHVESIRHQYDINNLSTHGAAELPLNGIGLCEWSLNESVALDNYQDCADTGGFIII

DRLTNVTVGAGMVKESLTELERGLADVSAFELELNALVRKHFPHWEAKDLSQLLKK

>sp|Q87RJ6|CYSZ_VIBPA Sulfate transporter CysZ OS=Vibrio parahaemolyticus serotype O3:K6 (strain RIMD 2210633) OX=223926 GN=cysZ PE=3 SV=1

MKINNQQRTGFGYFLYGIQLALSPEIRRFVVLPLLANIILVGGAIFYLFSHLNMWIEGWI

GQLPEFLSWLTYILWPLLALTILATFSYFFSTLANFIAAPFNGLLAEKVEETLTGKKIND

DGFTAVLKDVPRVLAREWRKLLYILPKAIGLFLLLLIPALGQTVGPVLWFIFTAWMLAIQ

YCDYPFDNHKIPFNDMRYKLKQKQGKAYGFGVLVSVFTTIPILNLIVMPVAICGATAMWV

AEFKHQR

>sp|Q87RV1|CRL_VIBPA Sigma factor-binding protein Crl OS=Vibrio parahaemolyticus serotype O3:K6 (strain RIMD 2210633) OX=223926 GN=crl PE=3 SV=1

MSETTQGPTHFRLMSKLKAIGPYLREPQSQEGRYYFDCLSVCVDDKKSPEKREFWGWWMD

LESIEGGFTAKYHIGKYNKEGKWVSEALPQKVVEEVYFTQSTFHQKLIDTLAEHFKLEVE

YHTESFDFA

>sp|Q87LR5|CSRA_VIBPA Translational regulator CsrA OS=Vibrio parahaemolyticus serotype O3:K6 (strain RIMD 2210633) OX=223926 GN=csrA PE=3 SV=1

MLILTRRVGETLMIGDEVTVTVLGVKGNQVRIGVNAPKEVSVHREEIYMRIQAEKGNGNV

ASGNY

>sp|Q87FK7|ARAG_VIBPA Arabinose import ATP-binding protein AraG OS=Vibrio parahaemolyticus serotype O3:K6 (strain RIMD 2210633) OX=223926 GN=araG PE=3 SV=1

MIMINSPSYLEFCNISKHFPGVKALSNISFRANKGSIHALMGENGAGKSTLLKTLSGLHQ

PTEGELVVDGKALVFNSATDALEQGIAIIYQELNLVPELSVAENIYLGQLPTKGGSVDVE

TLNARAREQLKRLGEDFDPSRPLKEFSIGQWQMVEIAKALSRNAQIIAFDEPTSSLSQRE

IQNLFKVIRELRDDGKIILYVSHRMEEIFDLCDAITIFKDGTHVQTFDDMTDLTHEKLVE

LMVGREINDIYNYRSRSLGESGLRIENLEGKGLTQPVSLDIRQGEILGLFGLVGAGRTEL

TRLIFGAEKAQAGQIYIHGQPISVRSPQDAIRAGITLCPEDRKADAIVPILSVEENTNIS

ARPWNLKLGGLIDFKWERDNAEQQRKALNVKTASLQQAIGQLSGGNQQKVILGRWLSTDM

SVILLDEPTRGIDVGAKSEIYELIFNLAERGVTVLVVSSDLPEVLGISDRVMVMKEGAVT

GELQRHEFKEQTALSLAMLGNNQAAA

>sp|Q87ST7|APAG_VIBPA Protein ApaG OS=Vibrio parahaemolyticus serotype O3:K6 (strain RIMD 2210633) OX=223926 GN=apaG PE=3 SV=1

MDVIQPCIKIQVHTKYIEEQSNPELQRFVFAYVITIKNLSQQTVQLISRRWLITDSNGKQ

MTVEGEGVVGQQPFIPSNDEYTYSSGTALETPVGVMQGHYKMLDEKGQEFITEIEPFRLA

IPNVLN

>sp|Q87LL6|ARGP_VIBPA HTH-type transcriptional regulator ArgP OS=Vibrio parahaemolyticus serotype O3:K6 (strain RIMD 2210633) OX=223926 GN=argP PE=3 SV=1

MRGLDYKWIEALDAVVYQGSFERAAEHLFVSQSAISQRIKQLEKFLAQPVLIREQPPKPT

PIGKKLLGLYRRVRLLEHEILPEIKNDTTTRPVQLSLATNADSLATWLLPALQDVMKTRQ

VELKLTIYGESRSIEKLKSGEVAGAISLESQAIPNCRADYLGRIDYVCVANPEFYQRYFS

EGVNNQTLAKAPAVSYDQYDDLHKKFLTEHFNVRPDSVIHHNISSSEAFLKMALAGVAYC

LIPRLQITDELEQGSLIDITPGFLMSYRIYWHHWQLETGVLQEISQAIVNYAQRHLPQ

>sp|Q87ST8|APAH_VIBPA Bis(5'-nucleosyl)-tetraphosphatase, symmetrical OS=Vibrio parahaemolyticus serotype O3:K6 (strain RIMD 2210633) OX=223926 GN=apaH PE=3 SV=1

MATYIVGDIQGCFDELQQLLKRVNFSTQHDQLWLAGDLVARGPKSLETLRFVKSLGDSAK

VVLGNHDLHLLAVSYGLKKRKDKDKTTPIFLAKDREELLSWLAKQPLLAEHDEFVMCHAG

ISPQWDLETARQCAREVERIIQGEELPWLLKNMYSNLPDLWDDSLEGLDRYRYIINAFTR

MRFCFSDGRLDMDCKLPPQEVTGDQLVPWFELPHRIPLEKTVLFGHWAALQGYIDEKVIG

LDTGCVWGGSLTMIRWEDKQLFTQDALD

>sp|Q87LG8|AMPA_VIBPA Probable cytosol aminopeptidase OS=Vibrio parahaemolyticus serotype O3:K6 (strain RIMD 2210633) OX=223926 GN=pepA PE=3 SV=1

MEFSVKSGSPEKQRSACIVVGVFEPRRLSPVAEQLDKISDGYISSLLRRGDLEGKPGQML

LLHQVPGVLSERVLLVGCGKERELGERQYKEIIQKTISTLNETGSMEAVCFLTELHVKGR

DTYWKVRQAVEATKDGLYTFNQFKSVKPETRRPLRKLVFNVPTRRELSLGEKAITHGLAI

ASGVKASKDLGNMPPNVANPAYLASQARRLADDYESVTTKIIGEQEMEKLGMTSYLAVGR

GSKNESMMSIIEYKGNPDSDAKPIVLVGKGLTFDSGGISLKPGEGMDEMKYDMCGAASVF

GTMKALAKLNLPINVIGVLAGCENMPGSNAYRPGDILTTMSGQTVEVLNTDAEGRLVLCD

ALTYVERFEPDCVVDVATLTGACVIALGHHISGVLSNHNPLAHELVNASEQSSDRAWRLP

MADEYHEQLKSPFADMANIGGRPGGTITAGCFLSKFAKKYNWAHIDIAGTAWKSGAAKGS

TGRPVSMLVQFLLNRSGQETEE

>sp|Q87L55|ARGC_VIBPA N-acetyl-gamma-glutamyl-phosphate reductase OS=Vibrio parahaemolyticus serotype O3:K6 (strain RIMD 2210633) OX=223926 GN=argC PE=3 SV=1

MLKTTIIGASGYTGAELAFMVNKHPQLTLSGLYVSANSVDAGKTIAQLHGKLANVVDMVV

NALTDPKQVAQDSDVVFLATAHEVSHDLAPIFLEAGCQVFDLSGAFRVKSDGFYDTFYGF

EHQFNNWLDKAAYGLAEWNQEEIKNAPLVAVAGCYPTASQLAIKPLLVDGLLDTQQWPVI

NATSGVSGAGRKASMTNSFCEVSLQPYGVFNHRHQPEIAQHLGCDVIFTPHLGNFKRGIL

ATVTMKLAQGVTEQQVAQAFEQAYQGKPAVRLKGDGIPRIQDVENTPFCDIGWKVQGEHI

IVISAIDNLLKGASSQAMQCLNIHYGYPELTALL

>sp|Q87L20|ARGD_VIBPA Acetylornithine aminotransferase OS=Vibrio parahaemolyticus serotype O3:K6 (strain RIMD 2210633) OX=223926 GN=argD PE=3 SV=1

MTTEIKVERGLFDEVMVPCYNPMEMIPVRGKGSRIWDQDDNEYIDFAGGIAVSCLGHCHP

VMVDALTEQGNKLWHLSNVMTNEPALRLAKKLTEVSFAERVFFANSGAEANEAALKLARR

YAADVHGPEKSEIIAFKQGFHGRTFFTVTVGGQAAYSDGFGPKPGDVTHLPYNDIEALQA

HMSDRTCAVMMEPLQGEGGIVPPTPEFAQAVRELCDKHNALLIFDEVQTGNGRTGHFYAY

QGLGITPDILSTAKSLGGGFPIGAMLTTAKLAEHLKVGTHGSTYGGNPLACAVAEAVVNE

VTKPEVLAGVLEREALFRAGLEKINAKYNLFSEVRGKGLLLGAALNEEWQGRARDVLVAA

GKQGLLVLVAGANVVRFTPSLVITQQEIEEGLAKLDKAIATLV

>sp|P59601|ARGE_VIBPA Acetylornithine deacetylase OS=Vibrio parahaemolyticus serotype O3:K6 (strain RIMD 2210633) OX=223926 GN=argE PE=3 SV=1

MQLPTFLEVYEGLISTSSISSTDPSWDQGNAKVIEKLATWFKDLGFHVEVIEVESGKHNM

IARMGEGEGGLLLAGHSDTVPFDEGRWSFDPHKLTEKDNRFYGLGTADMKGFFAFIYEAV

KKVDWSKQNKPLYVLATCDEETTMLGARHFTTNAPFKPDYCIIGEPTSLVPIRGHKGHVA

NAIRVTGKSGHSSDPALGVNAIEIMHEVLFAMMQLRDKLIKEYHHPGFAIPSPTLNLGHI

HGGDSANRICGCCELHYDVRPLPGISLDGLENMLRSALQEVEAKWPGRIDIVPLHEPIPG

YECQHDHPFIGGVEEICQTSSQTVNYCTEAPFLQQLCPTLVLGPGSIEQAHQPDEFLSFD

FIDPTIDVLSKAMVKYCC

>sp|Q87S80|ANMK_VIBPA Anhydro-N-acetylmuramic acid kinase OS=Vibrio parahaemolyticus serotype O3:K6 (strain RIMD 2210633) OX=223926 GN=anmK PE=3 SV=1

MKFNELYIGVMSGTSMDGVDTALVEITDNHVRLIAHGDYPMPAAMKEMLLSVCTGQATNL

KAIGELDHQLGHLFADAVLQLLNKSGYVAEQIRAIGNHGQTVFHQPTGDLPFTTQLGDAN

IIAVKTGIDTVADFRRKDMALGGQGAPLVPAFHKSIFAMQDSTTVVLNIGGIANISVLHP

QQPVHGYDTGPGNMLMDAWCERHTGHGFDKDAQLALRGSVNEALLAHLLKEPYLAMSAPK

STGRELFNMDWLHHQLANYDVSVEDVQRTLCEYTAITIAHDVTKFTYGETPQLLVCGGGA

RNPLLMQRLAELLPQWHVTTTTDKGVDGDYMEAMAFAWLAQRHIHDLPSNLPEVTGASRL

ASLGVLYSKN

>sp|Q87GM3|ADE_VIBPA Adenine deaminase OS=Vibrio parahaemolyticus serotype O3:K6 (strain RIMD 2210633) OX=223926 GN=VPA1292 PE=3 SV=1

MNAFIQGLPKVELHLHIEGSLEPELMFKLAKRNGIDIPYSSPSELREAYQFEDLQSFLDL

YYQGANVLRTEQDFYDLTWEYLEHCKADNVIHTEIFFDPQTHTERGIDFDTVLNGISRAL

TDGREKLGITSQIIACFLRHLSEESAMETLQSVLKHRDKIIGVGLDSSEKGHPPAKFLRV

FQQAKEAGLLTVAHAGEEGPAQNITDAIEMLEVSRVDHGVRCVEDEALVGSLIETKMPLT

VCPLSNIKLCVFDEMGQHNIVELLRKGVAVTINSDDPVYFGGYMTDNFLAVNQAHPMIKE

ELAKFTLNAIDASFIDNELKAQYRHKVEQYVAQHSSM

>sp|Q87NU5|AGUA_VIBPA Putative agmatine deiminase OS=Vibrio parahaemolyticus serotype O3:K6 (strain RIMD 2210633) OX=223926 GN=aguA PE=3 SV=1

MKLSTTPAQDGFYFPAEFQPVSEVWLAWPERKDNWRDDALPAQETFARIANLIAEVTKVC

VAVCSHNFDRARQMLHSDVRLVEIPFNDAWMRDIGPTVLVNQAGERRGISWQFNAWGGEY

NGLYDNWQQDDLVAGSVCDIIGIDYYRAPFVLEGGAIHTDGEGTLYTTEECLLSPGRNPQ

LSKAQIEEQLKVYLGIEKIIWLPNGLFNDETDGHVDNLMHVIAPGKVVLSWTDDPSDPQY

ALSRQAEQVLKSQHDAKGREIEIVRLPLPGPLHYSEREANGIDASSGMSRQAGERLSASY

ANFLIVNGHVFLPMLDEDTDAIAIDILQNAMPEYQIIAIPSREVLLGGGNIHCITQQIPA

>sp|Q87HG4|ALR2_VIBPA Alanine racemase 2 OS=Vibrio parahaemolyticus serotype O3:K6 (strain RIMD 2210633) OX=223926 GN=alr2 PE=3 SV=1

MRLKKTLLSIAIAAATFTPAMHSIAAPLQLQATLDQESQIQSSNTWLEIDLGQFKQNIEQ

FKSHMNDQTKICAVMKADAYGNGIAGLMPTIIEQQIPCVAIASNAEAQVVRDSGFKGQLM

RVRSAEIGEIEGALDLNVEELIGTLDQAKAIAALSKKANKTVKVHLALNDGGMGRNGIDM

TTENGKKEALAIAKQSGVEIVGIMTHFPNYNAEEVRAKLGSFKESSAWLIKEANLKREDI

LLHVANSYTALNVPEAQLDMVRPGGVLYGDLPTNLEYPSIVSFKTRVASLHHLPKNSTVG

YDSSFTTTKESVMANLPVGYSDGYPRKMGNTADVLINGQRAKVVGVTSMNTTMIDVSDIK

GVKPGSEVVLFGNQKSQTINAAEIEKNADVIFPELYTIWGTSNPRVYVK

>sp|Q87L78|ALR1_VIBPA Alanine racemase 1 OS=Vibrio parahaemolyticus serotype O3:K6 (strain RIMD 2210633) OX=223926 GN=alr1 PE=3 SV=1

MKAAKACIDLSALQHNLQRVKAQAPESKVMAVVKANGYGHGLRHVAKHANHADAFGVARI

EEALQLRACGVVKPILLLEGFYSPGDLPVLVTNNIQTVVHCEEQLIALEQADLETPVVVW

LKIDSGMHRLGVRPEQYDEFISRLKTCPNVAKPLRYMSHFGCADELDSSITPQQIELFMS

LTSGCQGERSLAASAGLLAWPQSQLEWVRPGIIMYGVSPFSDKTAQDLGYQPVMTLKSHL

IAVREVKQGESVGYGGIWTSERDTKVGVIAVGYGDGYPRSAPNGTPVWVNGRTVPIAGRV

SMDMLTVDLGPDATDKVSDEAILWGKELPVEEVANHIGTIAYELVTKLTPRVEMEYTK

>sp|Q87L54|CAPP_VIBPA Phosphoenolpyruvate carboxylase OS=Vibrio parahaemolyticus serotype O3:K6 (strain RIMD 2210633) OX=223926 GN=ppc PE=3 SV=2

MNEKYAALKSNVRMLGHLLGNTIRDAHGEEIFEKVETIRKLSKSAQAGNQADRESLIEEI

KHLPDEQLTPVTRAFNQFLNLTNIAEQYHTISRHCEEHICEPDAINSLFSKLVQNDVSKL

DTAQAVRDLNIELVLTAHPTEITRRTMINKLVKINECLSKLELSDLSSKERKKTERRLEQ

LIAQSWHSDVIRQQRPTPLDEAKWGFAVVENSLWEAVPDFLREMNDRLKSYLGEGLPIDA

RPVHFSSWMGGDRDGNPFVTHSVTREVLLLSRWKAADLYLNDINELISELSMTVSNDQVR

ELAGEDQHEPYRAILKQLRALLNETKDILDAKIHGQKLAVKAPLQKVEQLWDPLYACYQS

LHECGMGVIADGSLLDTLRRVKAFGVHLVRLDIRQESTRHADVLSELTRYLGIGDYEQWS

EQDKIAFLTNELASKRPLLPRDWEPSEPVKEVLDTCKIIALQPREAFGAYVISMARTASD

VLAVHLLLQEAGCPYRMDVCPLFETLDDLNNAESVIKQLMSIDLYRGFIQNHQMVMIGYS

DSAKDAGVMSAGWAQYHAMESLVKVAEDEGVELTLFHGRGGTVGRGGAPAHAALLSQPPK

SLKGGLRVTEQGEMIRFKLGLPDVAVNSFNLYASAILEANLLPPPEPKQEWRDLMEVLSE

VSCEAYRGVVRGEPDFVPYFRQATPELELGKLPLGSRPAKRNPNGGVESLRAIPWIFSWS

QNRLVLPAWLGAGEAIQYSVDKGHQALLEEMCREWPFFSTRLGMLEMVYTKCNMEISRYY

DQRLVEPQLQPLGDRLREQLQRDIKSVLNVENNENLMQSDPWGQESIRLRNIYVEPLNML

QAELLYRTRQTEEASANLEEALMVTIAGIAAGMRNTG

>sp|Q87SF3|CARB_VIBPA Carbamoyl-phosphate synthase large chain OS=Vibrio parahaemolyticus serotype O3:K6 (strain RIMD 2210633) OX=223926 GN=carB PE=3 SV=1

MPKRTDIQSILILGAGPIVIGQACEFDYSGAQACKALREEGYRVILVNSNPATIMTDPEM

ADATYIEPIQWEVVRKIIEKERPDAVLPTMGGQTALNCALDLEKHGVLAEFGVEMIGATA

DAIDKAEDRSRFDKAMKSIGLECPRADTAKTMEEAYKVLDMVGFPCIIRPSFTMGGTGGG

IAYNKEEFEEICRRGLDLSPTNELLIDESLIGWKEYEMEVVRDKADNCIIVCSIENFDPM

GIHTGDSITVAPAQTLTDKEYQLMRNASLAVLREIGVETGGSNVQFGINPKDGRMVIIEM

NPRVSRSSALASKATGFPIAKIAAKLAVGFTLDELQNDITGGATPASFEPTIDYVVTKIP

RFNFEKFAGANDRLTTQMKSVGEVMAIGRNQQESLHKALRGLEVGATGFDEMVDLDAPDA

LTKIRHELKEAGAERIWYIADAFRAGMSVDGVFNLTNIDRWFLVQIEELVKLEEQVKAGG

FAGLTEEVLRQMKRKGFSDARLSKLLGVAESEIRRLRDQFDIHPVYKRVDTCAAEFSSDT

AYMYSSYDEECEANPTDKDKIMVLGGGPNRIGQGIEFDYCCVHASLALREDGYETIMVNC

NPETVSTDYDTSDRLYFEPVTLEDVLSIARVEKPKGVIVQYGGQTPLKLARALEAAGVPI

IGTSPDAIDRAEDRERFQAAVERLGLLQPQNATVTAMEQAVEKSREIGFPLVVRPSYVLG

GRAMEIVYDEQDLRRYFNEAVSVSNESPVLLDRFLDDATEVDIDAICDGERVVIGGIMEH

IEQAGVHSGDSACSLPAYTLSQEIQDKMREQVEKLAFELGVRGLMNTQFAVKDNEVYLIE

VNPRAARTVPFVSKATGAPLAKIAARVMAGQSLESQGFTKEIIPPYYSVKEVVLPFNKFP

GVDPLLGPEMRSTGEVMGVGATFAEAYAKAELGCGSVYPEGGRALLSVREGDKQRVVDLA

SKLVKLGYQLDATHGTAVILGEAGINPRLVNKVHEGRPHILDRIKNNEYTYIVNTAAGRQ

AIEDSKVLRRGALAEKVNYTTTLNAAFATCMSHTADAKASVTSVQELHAKVKASLEA

>sp|Q87MK8|CCMA_VIBPA Cytochrome c biogenesis ATP-binding export protein CcmA OS=Vibrio parahaemolyticus serotype O3:K6 (strain RIMD 2210633) OX=223926 GN=ccmA PE=3 SV=1

MLEVSNLTAIRDERVLFENLQFEIKPGELVQIEGRNGTGKTTLLRIITGLGDREEGMIKW

KGEEVEKSRDVFHQDLLFLGHQTGVKRELTAFENLRFYQSIQNNSTSDEEIFTALTQVGL

AGREDVPVAQLSAGQQRRVALARLWLSKQILWILDEPLTAIDKQGVKVLEALFAQHADNG

GIVMLTTHQDMFADSPKLRKIKLGD

>sp|Q87ML2|CCME_VIBPA Cytochrome c-type biogenesis protein CcmE OS=Vibrio parahaemolyticus serotype O3:K6 (strain RIMD 2210633) OX=223926 GN=ccmE PE=3 SV=1

MNPRRKKRLGIVLAIFIGISATIGLMLYALNQNMDLFYTPTELVNGKPDGTKPEVGQRLR

IGGMVVVGSVRRDPNSLKVSFDLHDVGPKVTITYEGILPDLFREGQGIVAQGVLKDATTV

EAFEVLAKHDEEYMPPEIAEAMKKTHEPLQYSSEQKQGSGE

>sp|Q87Q38|BTUD_VIBPA Vitamin B12 import ATP-binding protein BtuD OS=Vibrio parahaemolyticus serotype O3:K6 (strain RIMD 2210633) OX=223926 GN=btuD PE=3 SV=1

MMRVKHIAVGSRLLPLSFECKDGEVVHVVGPNGSGKSTLLAAISGTLTGRDGASGEVHVD

DKNLLTLSLSEQAHVRGYLCQQSRPAFNVDVFQYLALSLPSGTAITDGKVRDAVNMVVEL

VQLQDKLHRSIQTLSGGEWQRVRLAGVCLQVWRTINPYSQLLILDEPAAPLDIAQEGLLY

QLINAIAAQGIGVLVANHDLNRTLKHADKVLLLSNGVLHSSGRADDVLSEAGLAEVFKTQ

ARKVMIDERPYLIFD

>sp|Q87R72|5DNU_VIBPA 5'-deoxynucleotidase VP0926 OS=Vibrio parahaemolyticus serotype O3:K6 (strain RIMD 2210633) OX=223926 GN=VP0926 PE=3 SV=2

MKESHFFAHLARMKLIQRWPLMRSVSPENVSEHSLQVAFVAHALALIKNKKFGGTLNPER

IALLAMYHDSSEVLTGDLPTPVKYYNPEIAKEYKKIEAAAEHKLLSMLPEEFQEDFAPFL

LSHSSHEEDSQIVKQADSICAYLKCLEELSAGNHEFALAKKRLDVTLQERKTPEMEYFLN

TFAPSFELSLDEIS

>sp|Q87L68|AROB_VIBPA 3-dehydroquinate synthase OS=Vibrio parahaemolyticus serotype O3:K6 (strain RIMD 2210633) OX=223926 GN=aroB PE=3 SV=1

MERITVNLAERSYPISIGAGLFEDPAYLSQVLSNKNTNQKVVVISNVTVAPLYADKILHQ

LKQLGCDASLLELPDGEQYKNLDVFNQVMNFLLEGSYARDVVIIALGGGVIGDLVGFASA

CYQRGVDFIQIPTTLLSQVDSSVGGKTAVNHPLGKNMIGAFYQPKAVIIDTNCLSTLPER

EFAAGIAEVIKYGIIYDGAFFDWLEENLDRLYTLDEDALTYAIARCCQIKAEVVAQDEKE

SGIRALLNLGHTFGHAIEAELGYGNWLHGEAVSSGTVMAAKTSLLRGLISEEQFERIVAL

LRRAKLPVHTPDSMSFDDFIKHMMRDKKVLSGQLRLVLPTGIGSAEVIADTSQEVIQQAI

DFGRNI

>sp|Q87KA7|ATPG_VIBPA ATP synthase gamma chain OS=Vibrio parahaemolyticus serotype O3:K6 (strain RIMD 2210633) OX=223926 GN=atpG PE=3 SV=1

MAGAKEIRNKIGSVKSTQKITKAMEMVAASKMRRSQDAMEASRPYAETMRKVIGHVANAN

LEYRHPYLEEREAKRVGYIIVSTDRGLCGGLNINVFKKAVTDIQTWKEKGAEIELAVIGS

KATAFFKHGGAKVAAQVSGLGDSPSLEDLIGSVGVMLKKYDEGELDRLYVVFNKFVNTMV

QQPTIDQLLPLPKSDSKEMQREHSWDYIYEPEPKPLLDTLLVRYVESQVYQGVVENLACE

QAARMIAMKAATDNATNLIEDLELVYNKARQAAITQELSEIVGGASAV

>sp|Q87PP9|AZOR_VIBPA FMN-dependent NADH-azoreductase OS=Vibrio parahaemolyticus serotype O3:K6 (strain RIMD 2210633) OX=223926 GN=azoR PE=3 SV=1

MSRVLALKSSILGDYSQSNKLVEDFIKNVDQDKLTVRDLAANPLPVLDFAVATALRATED

LSQEQQAVVDLSDTLIEEVKAADTLVIAAPMYNFTIPTQLKNWIDLIARAGVTFKYTENG

VQGLIEGKKAIVVTTRGGIHKDSPTDNVTPYLRTVLGFVGITDVEFIYAEALNMGEDAAS

KGISDAQSQLATMA

>sp|Q87J97|BCCT4_VIBPA Glycine betaine transporter 2 OS=Vibrio parahaemolyticus serotype O3:K6 (strain RIMD 2210633) OX=223926 GN=VPA0356 PE=1 SV=1

MHGFRALKSEVIMSNMTNAAPHTPIQEADYSAIHPPSLLKRLELTNPVFWLSGSFLSLFV

LLALTNTESLTAMVNAGFGFATKYFGAYWQVLLLLNFLIGLALAFGRTGYVRLGGLAKPD

IDTFKWLSIVLCTLLAGGGVFWAAAEPIAHFVTAPPLYGEASPKTSAINALSQSFMHWGF

LAWAILGCLSSIVLMHLHYDKGLPLKPRTLLYPIFGDKAIHGWIGNLADACSIIAVAAGT

IGPIGFLGLQISYALNSLFGFPDNFITQSMVIVAAIVMYTLSALSGVSKGIQLVSRYNII

LSVLLIGYILFFGPTSFIIDGYVQGVGRMVDNFFPMALYRDDTGWLSWWTVFFWGWFIGY

GPMMAIFIARISRGRTIRQLILSISIAAPLITCFWFSIVGGSGLAFELANPGLISSAFEG

FNLPAVLLAITGELPFPMIISVLFLILTTTFIVTTGDSMTYTISVVMTGSAEPNAVIRSF

WGLMMGVVAIALISMGSGGITALQSFIVITAVPVSFILLPSILKAPGIANQMAKDQGLV

>sp|Q87QN6|BIOB_VIBPA Biotin synthase OS=Vibrio parahaemolyticus serotype O3:K6 (strain RIMD 2210633) OX=223926 GN=bioB PE=3 SV=1

MEVRHNWTHAEVRDLMEKPFMDLLFEAQLVHRQYQQTNHVQVSTLLSIKTGACPEDCKYC

PQSARYTTDIEKERLMEVERVLDAAQKAKNAGSTRFCMGAAWKNPKERDMPHLTDMIKGV

KDMGLETCMTLGMLTPEQAKQLANAGLDYYNHNLDTSPEFYGNIITTRTYQDRLDTLSHV

RDAGMKICSGGIIGMGESANDRAGLLVELANLPTHPESVPINMLVKVKGTPLETVDDVDP

FDFIRLIAIARIMMPQSAVRLSAGRENMNEQMQALCFMAGANSVFYGCKLLTTPNPSEDK

DMMLFKKLGINSQEVSQKPDEIEENELLDRVVERVAARPTKDDLFYDASV

>sp|Q87PP5|BCCT1_VIBPA Glycine betaine/proline/choline/ectoine transporter VP1456 OS=Vibrio parahaemolyticus serotype O3:K6 (strain RIMD 2210633) OX=223926 GN=VP1456 PE=1 SV=1

MIKFASFLKFRVQINGGRYWSSSPLRSVSNYVKFVFMDNAFKKYSIDTTDYQVGQDNVQK

WGFDIHNPVFGISAGLVVFCLISLLLVEPVTARDALNGIKNGIIEQFDAFFMWSTNFFLL

FAVGLLFSPLGKIRLGGKEATPDHSTVSWLSMLFAAGMGIGLLFWSVAEPTAYFTDWWGT

PLNAEAYSADAKSLAMGATMFHWGVHGWSIYALVALALAFFAFNKGLPLSLRAAFYPIFG

DRAWGWLGHVIDILAVLSTLFGLATSLGLGAQQATSGINHVFGLNGGIGTQMVVIAFVTF

IAVLSVVRGIDGGVKLLSNVNMIVAFALLIFITFITFDTAMGSLVDTTMAYIQNIIPLSN

PHGREDETWMHGWTVFYWAWWVSWSPFVGMFIARVSKGRTVREFLFAVIVIPTLVTLVWM

SVFGGIALDQVVNKVGELGANGLTDISLTLFHVYDVLPYSSVISILSIVLILVFFITSSD

SGSLVIDSITAGGKIDAPVPQRIFWACIEGSIAAVMLWVGGKEALQALQSGVVATGLPFT

FVLLLMCVSLVKGLRTELSAYR

>sp|Q87QN3|BIOD_VIBPA ATP-dependent dethiobiotin synthetase BioD OS=Vibrio parahaemolyticus serotype O3:K6 (strain RIMD 2210633) OX=223926 GN=bioD PE=3 SV=1

MIDAFFIAGTDTDVGKTVASKAVLQALAAKGLNTIGYKPVAAGSEKTEQGWRNSDALHLQ

KAATLEVAYEDVNPYALELPASPHIAAKHEQVEIEYDLLSEKLAQHKEQADVVLVEGAGG

WRVPVSDTDSLSTWVQQEQLPVVLVVGIKLGCLSHALLTAEIIKADGLNLVGWIANRVNP

GTEHYADIIEMLESRIDAPKLGEIPYIPSAKRKELGKYINVEPLLNID

>sp|Q87SE7|BTUF_VIBPA Vitamin B12-binding protein OS=Vibrio parahaemolyticus serotype O3:K6 (strain RIMD 2210633) OX=223926 GN=btuF PE=3 SV=1

MMNKICLYLPLFFSSLTMANEPVERVISLAPHATEIAYAAGLGDKLIAVSEMSDYPKEAG

ELEKVSNYQGIKLERIIALQPDLVIAWPAGNPAKELEKLKQFGVPIYYSTTGTLEDIANN

IEQLSQYSDDPSKGQKAARDFREELTALKAKYNTTEKVRYFYQLSEKPIITVAGKNWPSE

VFNFCGGENVFANTAAPYPQVSIEQVITRQPEVLFTSRHAMSDDGMWAQWKNELPALRNN

HVWSLNSDWINRPTPRTLNAIIEVCEHFESVKRKR

>sp|Q87QN5|BIOF_VIBPA 8-amino-7-oxononanoate synthase OS=Vibrio parahaemolyticus serotype O3:K6 (strain RIMD 2210633) OX=223926 GN=bioF PE=3 SV=2

MPAFKSRIESALAARKAQGLNRSMNVVFAGNQSILEHEGRRYINFSSNDYLGLANDQALV

RAWQQGLSVYGSGSGASPMVTGFSAAHSNLEAALTEWLGFERAILFGSGFSANQALLFTL

LEKSDVLIQDRLNHASLMEAGALSTAKMKRFKHNDIKHLETLFTNEGNHLVVTEGVFSMD

GDCAPLKDIAEVARLRNAWLAVDDAHGIGVLGEAGGGSCELANVKPEILVVTFGKAFGMS

GAAILCDQATGDFLTQFARHHVYSTAMPPAQAYALTHAVSMIQEQSWRREKLVELNEVYQ

TNLSDLDGFVETDTPIKPFVIGESELALQVANACRQNGIWVTAIRPPTVPKGTSRLRITL

TANHSTEQVKTLSIALKQALGAL

>sp|Q87QY2|BPT_VIBPA Aspartate/glutamate leucyltransferase OS=Vibrio parahaemolyticus serotype O3:K6 (strain RIMD 2210633) OX=223926 GN=bpt PE=3 SV=1

MSTDLQHIRIGLTNNHPCSYLPERQERVAVALDAELHTEQNYQLLMANGFRRSGDTIYKP

HCERCHACQPIRISIPDFVLSRSQKRLLSKAKSLRWEMKTEMDAEWFELYSRYICKRHKN

GTMYPPKRDEFSRFAQTTWLTTLFLHIYDESNQLLGVAVTDVMAQCSSAFYTFFEPDYPL

SLGTLAVLYQVNYCQQNNEQWLYLGYQIDECPAMNYKTRFQRHQRLVNQRWQG

>sp|Q87KN9|BTUB_VIBPA Vitamin B12 transporter BtuB OS=Vibrio parahaemolyticus serotype O3:K6 (strain RIMD 2210633) OX=223926 GN=btuB PE=3 SV=1

MKKSALAITLASLLSPVSYLQAQEISVDETIVVTANRFKQIDGAVLAQTVTVTKEDIRRQ

QADSLFDVFRTLPSIEVAQYGGRGQSASIFVRGGSATQVLVLVDGVRMPRAIMGGIDFNQ

FPINSIERIDYIRGARASIYGSEAISGVINIITRASIDDDASRVSAGYGSNNHKKGTFAV

SKPVGEGKHIKGVLGYEKTDGFNVKPLPGLNDGDEHGFETLNLKLGYQQNFSDNFSGYVG

FSTYSNEYDYDNSSYGNPGWGTVDKHEKKTGEVEYVGADLSLEYSKDVYTSELKLAYGQQ

DNYDLKSGQSKSTGDHVAIEQFNAIWLNSYSINDELSIGGGLDYRNEKLAKGYLAPSDWG

PAKDYNPEKNPRTNIGISAIAQYALNAWTFEASVRNDENNQFGNNTTWQTAAGWKVYEGY

ELTLSHGTAFRAPSFVDLYYPGYEMPNLKPEESKNTELSLSGVASIVDWTVTGYYNQIEN

MLIWKGAGMQNIGEAEIKGIELEVKLDTDIVSHEFYLDYKDPVDKSGAEDTQLAYRSKRG

AKWNAYATFDQWTLGSQYLYQGERFNGSTRLPSYSLWNFTASYAVNSSWDINAKLSNAFD

KNYEMYSGYATPGRQYFVSADYRF

>sp|Q87Q39|BTUC_VIBPA Vitamin B12 import system permease protein BtuC OS=Vibrio parahaemolyticus serotype O3:K6 (strain RIMD 2210633) OX=223926 GN=btuC PE=3 SV=2

MDFQQLILNKERRWQRNLVIMSVVLVLLSTIHLMVGEVFLSPFQSLSVFEQKLLLDLRLP

RLVAAAMIGAALAVSGATLQVLLGNVLAEPGVLGISGGASLAMVLVMFALPVLPTPMIFM

LAAIAGSMLFTLILVGIARAMHLTTARLLLVGVALGILSSAIVTWAFYFSDDLSLRQLMY

WLMGSIGGASWYQHTVTLVMLPVLVWLCCQGKPLDKLMLGEIHATQLGVDVHQMRWKLIL

AISVLVGCSVALGGIISFVGLVVPHLLRLAFGTENRYLLPLSAIFGAALLVFADIGARLL

LDSAELPLGVMTTSIGAPIFIWMLVKSHDAR

>sp|Q87H51|BETI_VIBPA HTH-type transcriptional regulator BetI OS=Vibrio parahaemolyticus serotype O3:K6 (strain RIMD 2210633) OX=223926 GN=betI PE=3 SV=1

MPKVGMPEIRKPQLVKATMSVIDRVGLHAASISLISKEAGVSTGIINHYFGGKHGLLEET

MREILRQLSSTITGKLRALPADAHHQRINAIIDGNFVGYQAENKVAKTWLAFWSYSMHDE

QLKRLQRVNERRLLSHLRRELKALLSAEQAELVAHGIASLIDGIWLRGTLNPQGIEADKA

RIIINDYLDKQLTFYSHKI

>sp|Q87TC2|BIOH_VIBPA Pimeloyl-[acyl-carrier protein] methyl ester esterase OS=Vibrio parahaemolyticus serotype O3:K6 (strain RIMD 2210633) OX=223926 GN=bioH PE=3 SV=2

MSTNLHWQSFGQGPDLVLLHGWGMNGAVWQQTVESLQADFCVHVVDLPGYGFSAEHHGED

LAQIAAMVLKDAPEKAVWLGWSLGGLVATHIALNAPQRVSKLITVASSPKFAAEKPWRGI

QPNVLSAFTSQLLEDFSLTIERFMALQAMGSPSARKDVKQLKQAVLSRPQPNPESLLVGL

NILADVDLRDALISLTMPMLRLYGRLDGLVPIKVATDLSQQLPHTQQFVFSQSSHAPFMT

EHDEFCAQVRDFAQD

>sp|Q87Q45|COBS_VIBPA Adenosylcobinamide-GDP ribazoletransferase OS=Vibrio parahaemolyticus serotype O3:K6 (strain RIMD 2210633) OX=223926 GN=cobS PE=3 SV=1

MSESKSSRQATLRYQMELFLLAVSFFSRLPVPSDLPYSEERMNQAGRYFALVGVILGVLC

ALVFYFTQLIFPDSVAIVLTMAFSLLLTGAFHEDGLTDMADGIGGGMTVERRLSIMKDSR

IGTYGAATLVMALLAKFVLWSELVHLPDFWLVIVVAYTTSRALAATLIYDMPYVSDSDTS

KSKPLASKQSSSEVAILLFTAGVASLFLGVIQTSFIVIVLFAFRFAFKRWLTKRIGGFTG

DCLGAAQQLSELLVYLTLIAFYQNI

>sp|Q56696|COLA_VIBPA Microbial collagenase OS=Vibrio parahaemolyticus serotype O3:K6 (strain RIMD 2210633) OX=223926 GN=prt PE=1 SV=2

MSHIRFFPRHRLALACMLASVSSFSFAQNQCAVADLQQSRDLAAAVSGAEYDCYHAWFSA

PSATLNDIYSEASLSRIQVALDQEIARYRGEAEQARVLENLGEFVRAAYYVRYNAGTGTP

EFSEALSQRFAQSTNLFLNNPHALDQGREQVGAMKSLTLMVDNVKQLPLTMDSMMAALMH

FNRDTAKDTQWVDGLNNLFRSMAGHAANDAFYRYMANNTHHIDTLARFASDNAWALDTDA

NFIVFNALRETGRLLASPDQETKRKALAVMQQVMQRYPLGSEHDKLWLAAVEMMSYYAPE

GLNGLNLEQAKQDLAARVMPNRFECQGPAIIRSEDLTDAQAAKACEVLAAKEADFHQVAN

TGNQPVADDLNDRVEVAVFASNDSYVDYSSFLFGNTTDNGGQYLEGTPSRADNTARFVAY

RYANGEDLSILNLEHEYTHYLDARFNQYGSFSDNLAHGHIVWWLEGFAEYMHYKQGYKAA

IDLIPSGKLSLSTVFDTTYSHDSNRIYRWGYLAVRFMLENHPQDVESLLALSRSGQFAQW

AQQVTVLGQQYDAEFERWLDTLEVVVEPEQPGTDPEEPSEPTDPEVQVTELAANQSLQLS

GEAYSEKLFYVDVPANTVRFNVSIEGAGDADLYMSYNKVAHYYDFEMSQYADGSNEEIQF

APEQNGYVKAGRYYISLTGRDSYDSVNLVAALEVEAQTPPTQVQDDLAPVVLESGEAKVL

TVHQQRYAAVYVPEGVKEVRVWMSSQSNANDPYGAGNVDLYASRKHWPTAEQHEYASNYA

GSNEYLAIPVTEAGYVHFSLQAPQQGDDVEMLVYFF

>sp|Q87T80|COAD_VIBPA Phosphopantetheine adenylyltransferase OS=Vibrio parahaemolyticus serotype O3:K6 (strain RIMD 2210633) OX=223926 GN=coaD PE=3 SV=1

MNRSVHPMKVIYPGTFDPVTNGHLNLIERTHEMFDEVVIGVAASPSKNTMFTLEERVALM

EEVVAHLPGVTVKGFSGLLVDFARQEQAKVLIRGLRTTVDFEYEFGLTNMYRKLLPGIES

VFLTPEEEFAFLSSTIVREVAIHGGSIEQFVPAAVANAIEKKVNERQ

>sp|Q87LT4|COAE_VIBPA Dephospho-CoA kinase OS=Vibrio parahaemolyticus serotype O3:K6 (strain RIMD 2210633) OX=223926 GN=coaE PE=3 SV=1

MALVIGLTGGIASGKTTVANLFKQQFKIDIVDADIVAREVVEPGTPGLNAIIQHFGQDIT

HDDNTLDRAKLREKIFSNPEEKAWLNALLHPIIREKMIEDLQQVTSDYALLVVPLLVENN

LDSLCDRVLVVDVEPETQISRTVKRDNVSEEQAHAILASQASRQQRLDIADDVVKNNPND

PDLLLQITDLHEKYLAMCKKNLRK

>sp|Q87HN1|COBQ_VIBPA Cobyric acid synthase OS=Vibrio parahaemolyticus serotype O3:K6 (strain RIMD 2210633) OX=223926 GN=cobQ PE=3 SV=1

MKSAIPSLMVQGTTSDAGKSVLVAGLCRVLARKGINVAPFKPQNMALNSAVTKDGGEIGR

AQAVQAQACNIEPTVHMNPVLIKPNSDTGAQIILQGKALSNMDAASFHDYKKVAMNTVLD

SFSKLTKEFDSIMIEGAGSPAEINLREGDIANMGFAEAADVPVIIVADIDRGGVFAHLYG

TLALLSESEQTRVKGFVINRFRGDIRLLQSGLDWLEEKTGKPVLGVLPYLHGLNLEAEDA

ITAQQELNSEVKLNVVVPVLTRISNHTDFDVLRLNPDINLSYVGKGEKIDKADLIILPGT

KSVRDDLAYLKSQGWDKDILRHIRLGGKVMGICGGYQMLGKTIDDPDGVEGEPGSSEGLG

LLNVHTVLTGSKQLTKTEAVLNLNNQKAKVKGYEIHVGRSQVLDEQPLELDNGECDGAIS

ECGQIMGTYLHGFFDEAEALNLITEWVNGTQVKQQDFEVLKEQGINRIADAIEQHMNLDF

LFK

>sp|Q87S63|CLPB_VIBPA Chaperone protein ClpB OS=Vibrio parahaemolyticus serotype O3:K6 (strain RIMD 2210633) OX=223926 GN=clpB PE=3 SV=1

MRLDRFTSKFQIAISDAQSLALGRDHQYIEPVHLMVALLDQNGSPIRPLLTMLDVDVTHL

RSKLGEMLDRLPKVSGIGGDVQLSSSMGTLFNLCDKVAQKRQDSYISSEVFLLAALEDRG

PLGQLLKEVGLTEQKVSQAIEKIRGGQKVNDPNAEELRQALEKFTIDLTERAEQGKLDPV

IGRDDEIRRTIQVLQRRTKNNPVIIGEPGVGKTAIVEGLAQRIVNNEVPEGLRGRRVLSL

DMGALVAGAKYRGEFEERLKSVLNELAKEEGNVILFIDELHTMVGAGKGEGSMDAGNMLK

PALARGELHCVGATTLDEYRQYIEKDAALERRFQKVLVDEPTVEDTVAILRGLKERYELH

HHVEITDPAIVAAASLSHRYISDRQLPDKAIDLIDEAASSIRLQIDSKPESLDKLERKII

QLKIEQQALSNEHDEASEKRLQALNDELNEKEREYAELEEVWNTEKAALSGTQHIKSELE

QARMDMEFARRAGDLNRMSELQYGRIPELEKQLDLATQAEMQEMTLLRNKVTDNEIAEVL

SKQTGIPVSKMLEAEKEKLLRMEDVLHNRVVGQSEAVAVVSNAIRRSRAGLSDPNRPIGS

FLFLGPTGVGKTELCKTLASFMFDSEDAMVRIDMSEFMEKHSVARLVGAPPGYVGYEEGG

YLTEAVRRKPYSVILLDEVEKAHPDVFNILLQVLDDGRLTDGQGRTVDFRNTVVIMTSNL

GSSRIQENFATLDYQGIKSEVMDVVSKHFRPEFLNRVDEIVVFHPLGQEHIKSIASIQLE

RLAKRLEEKGYQLEVSDKALDLIAQVGFDPVYGARPLKRAIQQNVENPLAKSILAGEIVP

DKKVQLIVTNDQILAHQ

>sp|Q87R80|CLPP_VIBPA ATP-dependent Clp protease proteolytic subunit OS=Vibrio parahaemolyticus serotype O3:K6 (strain RIMD 2210633) OX=223926 GN=clpP PE=3 SV=1

MSPIMDALVPMVVEQTSRGERSYDIYSRLLKERVIFLTGQVEDHMANLVVAQLLFLESEN

PDKDIFLYINSPGGSVTAGMSIYDTMQFIKPNVSTVCMGQACSMGAFLLAGGAAGKRYVL

PNSRVMIHQPLGGFQGQASDIQIHAQEILTIKQKLNNLLAEHTGQPLEVIERDTDRDNFM

SAEQAVEYGIVDAVLSHRGA

>sp|Q87QV4|CMOA_VIBPA Carboxy-S-adenosyl-L-methionine synthase OS=Vibrio parahaemolyticus serotype O3:K6 (strain RIMD 2210633) OX=223926 GN=cmoA PE=3 SV=1

MNPKSNPDTIFSAPIDKIGDFTFDERVAEVFPDMIQRSVPGYSNIISAIGMLAERFVKPH

SNIYDLGCSLGAATLSMRRHIKQEGCKIIAVDNSPAMVERCKLHVNAYRSDTPVTVVEAD

IRNIEIENASVVVLNFTLQFLSPEDRYALLEKIYAGLRPGGILILSEKFVFEDEVSNELL

IDLHHDFKRANGYSELEISQKRSAIENVMRPDSKKDHKERFAKIGFSSYDVWFQCFNFGS

MFAIK

>sp|Q9X9K2|CHER_VIBPA Chemotaxis protein methyltransferase OS=Vibrio parahaemolyticus serotype O3:K6 (strain RIMD 2210633) OX=223926 GN=cheR PE=3 SV=1

MTAITISDQEYRDFSRFLESQCGIVLGDSKQYLVRSRLSPLVTKFKLASLSDLLRDVVAG

RNRELRIAAVDAMTTNETLWFRDTYPFTVLADKLLPEVAANKRPIKIWSAASSSGQEPYS

IAMTILETQQRKPGLLPSVSITATDISASMLEMCRAGVYDNLALGRGLSPERRRTFFEDA

GDGRMKVKDNVKRLVNFRPQNLMDSYALMGKFDIIFCRNVLIYFSPEMKSKVLNQMASSL

NPGGYLLLGASESLTGLTDKFEMVRCNPGIIYKLK

>sp|Q87R79|CLPX_VIBPA ATP-dependent Clp protease ATP-binding subunit ClpX OS=Vibrio parahaemolyticus serotype O3:K6 (strain RIMD 2210633) OX=223926 GN=clpX PE=3 SV=1

MTDKSKESGSGKLLYCSFCGKSQHEVRKLIAGPSVYICDECVDLCNDIIREEIKDVLPKK

ESEALPTPKQIREHLDDYVIGQDYAKKVLAVAVYNHYKRLRNGDTTSEGVELGKSNILLI

GPTGSGKTLLAETLARFLDVPFTMADATTLTEAGYVGEDVENIIQKLLQKCDYDVAKAER

GIVYIDEIDKISRKAENPSITRDVSGEGVQQALLKLIEGTVASVPPQGGRKHPQQEFLQV

DTSKILFICGGAFAGLDKVIEQRVATGTGIGFGAEVRSKNETKTVGELFTQVEPEDLVKY

GLIPEFIGRLPVTTTLTELDEEALIQILCEPKNALTKQYAALFELENAELEFREDALRAI

AKKAMERKTGARGLRSILESVLLETMYELPSATDVSKVVIDESVINGESEPLLIYSNADN

QAAGAE

>sp|Q87QV5|CMOB_VIBPA tRNA U34 carboxymethyltransferase OS=Vibrio parahaemolyticus serotype O3:K6 (strain RIMD 2210633) OX=223926 GN=cmoB PE=3 SV=1

MFNFANFYQLIAQDTRLQPWLNVLPQQLTDWQNAEHGDFGRWLKALNKIPEGSPDQVDIK

NSVTISNDTPFHEGELKKLENLLRTFHPWRKGPYTVHGIHIDTEWRSDWKWDRVLPHISP

LKNRSVLDVGCGNGYHMWRMLGEGARLCVGIDPSHLFLIQFEAIRKLMGGDQRAHLLPLG

IEQLPKLEAFDTVFSMGVLYHRRSPLDHLIQLKDQLVSGGELVLETLVIEGDENAVLVPT

SRYAQMRNVYFFPSAKALKVWLELVGFEDVHIVDENVTSVDEQRTTDWMTHNSLPDYLDP

NDPSKTVEGYPAPRRAVLVARKP

>sp|Q87JP5|CRYD_VIBPA Cryptochrome DASH OS=Vibrio parahaemolyticus serotype O3:K6 (strain RIMD 2210633) OX=223926 GN=cry PE=3 SV=1

MKKKIGLYWFTFDLRLHDNSLLVDASSFLDELVCLYCRPSVTPFLHHFAQEVTLGRARQK

FIDASLCELNDALGQLGQRLWTLDLPPYQALKYAIQYLSVTHLYSDAMAGSDEQSILHKL

QDEYPHLVIVQHSVRSLFDESKLPFTLPDLPETFTQFRKCVEGIDIAHPIDAPSRLPPMP

KGAQLPTLSSFYFDESALFSGGEWSGLAHCRRYFFSGLASSYKETRNGLDGMAYSTKFSP

WLALGCVSPRMIHAMLKQYEQTQGANDSTYWIYFELLWREYFYWYARCYQQRLFRFGGIR

NQPPLTSFYAHRFQQWKNGTTPYPIVNACMHQLNHTGYMSNRGRQLVASCLVHELGLDWR

YGAAYFETQLIDYDVGSNWGNWQYLAGVGADPRGSRQFNLEKQTQMYDPNHEFIERWQGR

DSRAQQDVVDMVGWPITTQQENGDK

>sp|Q87MF3|ACCA_VIBPA Acetyl-coenzyme A carboxylase carboxyl transferase subunit alpha OS=Vibrio parahaemolyticus serotype O3:K6 (strain RIMD 2210633) OX=223926 GN=accA PE=3 SV=1

MSLNFLEFEKPIAELEAKIEALRDVSRHGGDSAIDLDKEIEQLEKKSLELKKKIFSDLGA

WETAQLARHPLRPYTLDYVQHVFEEFDELAGDRAFADDKAIVGGIARLEGRPVMIIGHQK

GRETKEKVRRNFGMPKPEGYRKALRLMEMAERFNMPIITFIDTAGAYPGVGAEERGQSEA

IAKNLKVMSGLKVPVICNVVGEGGSGGALAIGVGDYVNMLQYSTYSVISPEGCASILWRD

SDKAPQAAEAMGLTAPRLKELELIDEIIEEPLGGAHRDHVKMAENMKATLLRQLEDLEQL

DEESLRERRYQRLMNYGYC

>sp|P0A2W2|ACP_VIBPA Acyl carrier protein OS=Vibrio parahaemolyticus serotype O3:K6 (strain RIMD 2210633) OX=223926 GN=acpP PE=3 SV=2

MSNIEERVKKIIVEQLGVDEAEVKNEASFVDDLGADSLDTVELVMALEEEFDTEIPDEEA

EKITTVQAAIDYVNSAQ

>sp|Q87MP2|ACCD1_VIBPA Acetyl-coenzyme A carboxylase carboxyl transferase subunit beta 1 OS=Vibrio parahaemolyticus serotype O3:K6 (strain RIMD 2210633) OX=223926 GN=accD1 PE=3 SV=1

MSWLEKILEKSNLVSSRKASIPEGVWTKCTSCEQVLYHAELERNLEVCPKCNHHMRMKAR

RRLETFLDEGNRVELGTELEPQDKLKFKDSKRYKERISAAQKSSGEKDALIVMQGELLGM

PLVACAFEFSFMGGSMGSVVGARFVKAVEAAIENNCALVCFSASGGARMQEALMSLMQMA

KTSAALERLSEKGLPFISVLTDPTMGGVSASLAMLGDINIGEPKALIGFAGRRVIEQTVR

EDLPEGFQRSEFLLEHGAIDMIVDRREMRQRVGGLIAKMTNHKSPLVVSVNESPNEEPYS

VPEVDEKG

>sp|Q87IJ5|ACKA2_VIBPA Acetate kinase 2 OS=Vibrio parahaemolyticus serotype O3:K6 (strain RIMD 2210633) OX=223926 GN=ackA2 PE=3 SV=1

MSNSFVLVINSGSSSLKFAVIDSVSGDAVLSGLGECFGLSDARMSWKFNGEKKEISIEGD

DSHHKIAIGKLVGLTEELGLAQDIVAVGHRIVHGGEKFTKTVRITEEVTQEIEKLADLAP

LHNPAGAIGIRAAVEAFPSLPQFAVFDTAFHQTMPQRAFTGAIAKELYTDFGIRRYGFHG

TSHYFVSREAAKMINKPIEESSFISVHLGNGASVCAINNGESVDTSMGFTPLSGLMMGTR

CGDLDPGIIEYLLKKGWSQEKVFNSLNKASGFLGVSGLTSDARGILEAMEEGHEGAALAF

QVFTYRVSKYIASYLAALDSFDGIIFTGGIGENSMPIRREILKNLKLLGFVEDVKGNEDA

RFGNAGVIATSELLGAKALVIPTNEEWVIAQQSVELL

>sp|Q87P91|ACYP_VIBPA Acylphosphatase OS=Vibrio parahaemolyticus serotype O3:K6 (strain RIMD 2210633) OX=223926 GN=acyP PE=3 SV=1

MNVKCERFIVKGHVQGVGFRYHTSHQGLKLGLTGYAKNLNNGDVEVMACGPKEKIDQFCE

WLQEGPRTATVESVTRESVSYKPFRGFKIL

>sp|Q87LP3|ACPS_VIBPA Holo-[acyl-carrier-protein] synthase OS=Vibrio parahaemolyticus serotype O3:K6 (strain RIMD 2210633) OX=223926 GN=acpS PE=3 SV=1

MAILGLGTDIAEIERIEKALGRSGEPFAQRILSEDEMAKFSQLKQQGRYLAKRFAAKEAA

SKALGTGIAQGVTFHDFTVSNDELGKPVLMLSGVAQKMAQTMGVNHVHLSISDERHYAVA

TVILES

>sp|Q87KU7|ACSA_VIBPA Acetyl-coenzyme A synthetase OS=Vibrio parahaemolyticus serotype O3:K6 (strain RIMD 2210633) OX=223926 GN=acsA PE=3 SV=1

MSEAHVYPVKENIKTHTHADNDTYLAMYQQSVTDPEGFWNEHGKIVDWIKPFTKVKSTSF

DTGHVDIRWFEDGTLNVSANCIDRHLAEHGDDVAIIWEGDDPADDKTLTFNELHKEVCKF

SNALKDQGVRKGDVVCLYMPMVPEAAIAMLACTRIGAVHTVVFGGFSPEALSGRIIDSDA

KVVITADEGVRGGRAVPLKKNVDEALTNPEVKTISKVVVLKRTGGNVDWHGHRDVWWHEA

TAKVSDVCPPEEMKAEDPLFILYTSGSTGKPKGVLHTTGGYLVYAAMTFKYVFDYQPGET

FWCTADVGWITGHTYLIYGPLANGAKTILFEGVPNYPNTSRMSEVVDKHQVNILYTAPTA

IRALMAKGNEAVAGTSRSSLRIMGSVGEPINPEAWEWYYKTIGNENSPIVDTWWQTETGG

ILIAPLPGATDLKPGSATRPFFGVQPALVDNMGNILEDTVAEGNLVILDSWPGQMRTVYG

DHERFEQTYFSTFKGMYFTSDGARRDEDGYYWITGRVDDVLNVSGHRMGTAEIESALVAH

HKIAEAAIVGIPHDIKGQAIYAYITLNDGEFPSAELHKEVKDWVRKEIGPIATPDVLHWT

DSLPKTRSGKIMRRILRKIATGDTSNLGDTSTLADPSVVDKLIAEKAELV

>sp|Q87MZ4|ACKA1_VIBPA Acetate kinase 1 OS=Vibrio parahaemolyticus serotype O3:K6 (strain RIMD 2210633) OX=223926 GN=ackA1 PE=3 SV=1

MSKLVLVLNCGSSSLKFAVVDAENGEEHLSGLAECLHLPEARIKWKLDGKHEAQLGNGAA

HEEALAFMVETILASKPELSENLAAIGHRVVHGGEQFTQSALITDDVLKGIEDCATLAPL

HNPAHIIGIKAAQKSFPALKNVAVFDTAFHQTMPEESYLYALPYNLYKEHGIRRYGMHGT

SHLFITREVAGLLNKPVEEVNIINCHLGNGASVCAVKNGQSVDTSMGLTPLEGLVMGTRC

GDIDPAIIFHLHDTLGYSVEKINTMLTKESGLQGLTEVTSDCRFVEDNYGEKEEATRAMD

VFCHRLAKYVAGYTATLDGRLDAITFTGGIGENSAPIREMVLNRLGIFGIEVDSEANLKA

RFGGEGVITTENSRIPAMVISTNEELVIAEDTARLAGL

>sp|Q87TF3|ADD_VIBPA Adenosine deaminase OS=Vibrio parahaemolyticus serotype O3:K6 (strain RIMD 2210633) OX=223926 GN=add PE=3 SV=1

MITKNLPLTDLHRHLDGNIRTKTILELGQKFGIALPAYDIESLTPHVQIVEAEPSLVAFL

SKLDWGVAVLGDLDACRRVAYENVEDALNAQIDYAELRFSPYYMAMKHKLPVAGVVEAVV

DGVQAGMRDFGIKANLIGIMSRTFGTDACQQELDAILSQKDHIVAVDLAGDELGQPGERF

VSHFKQVRDAGLNVTVHAGEAAGAESMWQAIQELGATRIGHGVKAIHDPKLMDYLAENRI

GIESCLTSNFQTSTVDSLANHPLKQFLDHGVLACLNTDDPAVEGIELPYEYEVAAPAAGL

SQEQIRQAQINGLELAFISDAEKAELKEKVKDRV

>sp|Q87M22|DEOC_VIBPA Deoxyribose-phosphate aldolase OS=Vibrio parahaemolyticus serotype O3:K6 (strain RIMD 2210633) OX=223926 GN=deoC PE=3 SV=1

MSDLKAAALRALKLMDLTTLNDDDTDAKVISLCHDAKTAVGNTAAICIYPRFIPIAKKTL

REQGTPEVRIATVTNFPHGNDDIDIAVAETKAAVAYGADEVDVVFPYRALMAGDEKVGFE

LVKQCKEACGDILLKVIIETGELKEEALIKKASQICIEAGADFIKTSTGKVPVNATPEYA

RMMLEVIRDMGVAETVGFKPAGGVRTAEDAAAYLAMADEILGDNWVDARHYRFGASSLLT

NLLNTLEVSDDVADPTAY

>sp|O50286|DLDH_VIBPA Dihydrolipoyl dehydrogenase OS=Vibrio parahaemolyticus serotype O3:K6 (strain RIMD 2210633) OX=223926 GN=lpd PE=3 SV=2

MSKEIKAQVVVLGSGPAGYSAAFRCADLGLETVLVERYSTLGGVCLNVGCIPSKALLHVS

KVIEEAKAMADHGVVFGEPQTDINKIRIWKEKVVNQLTGGLSGMAKMRNVTVVNGYGKFT

GPNSILVEGEGESTVVNFDNAIVAAGSRPIKLPFIPHEDPRIWDSTDALELKEVPEKLLI

MGGGIIGLEMGTVYHSLGSKVEVVEMFDQVIPAADKDIVKVYTKRIKDKFKLMLETKVTA

VEAKEDGIYVSMEGKKAPAEAERYDAVLVAIGRVPNGKLIDGEKAGLEIDERGFINVDKQ

MRTNVPHIFAIGDIVGQPMLAHKGVHEGHVAAEVISGKKHYFDPKVIPSIAYTEPEVAWV

GKTEKEAKAEGIKYEVATFPWAASGRAIASDCSDGMTKLIFDKETHRVIGGAIVGTNGGE

LLGEIGLAIEMGCDAEDIALTIHAHPTLHESVGLAAEVFEGSITDLPNKKAVKKK

>sp|Q87TQ7|DNAA_VIBPA Chromosomal replication initiator protein DnaA OS=Vibrio parahaemolyticus serotype O3:K6 (strain RIMD 2210633) OX=223926 GN=dnaA PE=3 SV=1

MSSSLWLQCLQQLQEELPATEFSMWVRPLQAELNDNTLTLFAPNRFVLDWVRDKYLNSIT

RLLQEYCGNDIPNLRFEVGSRPVSAPKPAPTRTPADVAAESSAPAQLQARKPVHKTWDDD

PQAIAAINHRSNMNPKHKFDNFVEGKSNQLGLAAARQVSDNPGAAYNPLFLYGGTGLGKT

HLLHAVGNAIVDNNPNAKVVYMHSERFVQDMVKALQNNAIEEFKRYYRSVDALLIDDIQF

FANKERSQEEFFHTFNALLEGNQQIILTSDRYPKEISGVEDRLKSRFGWGLTVAIEPPEL

ETRVAILMKKAEDHQIHLADEVAFFIAKRLRSNVRELEGALNRVIANANFTGRPITIDFV

REALRDLLALQEKLVTIDNIQKTVAEYYKIKVADLLSKRRSRSVARPRQLAMALAKELTN

HSLPEIGDAFGGRDHTTVLHACRKIEQLREESHDIKEDYSNLIRTLSS

>sp|Q87N39|DNAE2_VIBPA Error-prone DNA polymerase OS=Vibrio parahaemolyticus serotype O3:K6 (strain RIMD 2210633) OX=223926 GN=dnaE2 PE=3 SV=1

MSYAELFCQSNFSFLTGASHAEELVLQAAFYRYHAIAITDECSVAGVVKAHATIEQHKLD

IKQIVGSMFWLNEECQIVLLCPCRKAYAEMCRIITNARRRSEKGSYQLSEWDLMSIRHCL

VLWLPTHQASDHYWGRWLNQHHNNRLWVAIQRHLGGDDDAYTNHCEKLAHELQQPITACG

GVLMHTAERLPLQHILTAIKHGCSVDQLGFERLSNAERALRPLNKLVRIYKPEWLEESKY

IADLCEFKLSDLKYEYPTELIPNGYTPNSYLRMLVEQGKERRFPEGVPEDINQTIENELR

LIEDLKYHYYFLTIHDIVMFAKQQGILYQGRGSAANSVVCYCLEITAVDPRQISVLFERF

ISKERKEPPDIDVDFEHERREEVIQYIYKKYGRERAALAATVISYRFKSAVREVGKALGI

EETQLDFFIKNVNRRDRSQGWQAQIIELGLQPESLKGQQFIQLVNEIIGFPRHLSQHVGG

FVISSGPLYELVPVENAAMEDRTIIQWDKDDLESLELLKVDVLALGMLNAIRKCFQLIEK

HHQRSLSIAEITRRQDDPHVYRMLQKADTVGVFQIESRAQMSMLPRLKPACYYDLVIQIA

IVRPGPIQGDMVHPFLKRRNGEEPVSYPSEAVKSVLERTMGVPIFQEQVIKLAMVAAGFS

GGEADQLRRAMASWKKNGDLAKFKPKLLNGMQERGYDLAFAERIFEQICGFGEYGFPESH

SASFAVLAYCSAWLKYYYPAEFYTALLNSQPMGFYSPSQLVQDARRHGVEVLPICVNHSY

YQHHLIQRPNGRLGVQLGFRLVKGFNEEGATRLVERRPKTGYHSIQEVKQILRSRRDIEL

LASANAFQILSGNRYNARWAAMDSLSDLPLFHHIEEPSVGYQVQPSEYESLIEDYASTGL

SLNRHPITLLEEAGILPRFTRMKQLVDKEHKSLVTVAGVVTGRQSPGTAAGVTFFTLEDD

TGNINVVVWSATARAQKQAYLTSKILMVKGILEREGEVIHVIAGKLIDCTHYLSNLQSKS

RDFH

>sp|Q87RJ4|DNLJ_VIBPA DNA ligase OS=Vibrio parahaemolyticus serotype O3:K6 (strain RIMD 2210633) OX=223926 GN=ligA PE=3 SV=1

MSESVHQRLEELKESLHYHAVRYYVEDNPEIPDAEYDRLMRELLEIEAQHPDLVTVDSPS

QRVGGKPLSEFSQVTHEVPMLSLDNAFDDSELDSFHKRAQDRIGGESIKQYCCEPKLDGL

AVSLLYENGILVQAATRGDGTTGENITENVRTINAIPLKLRGDDWPARLEVRGEVFMPKA

GFEKLNELARQKGEKVFVNPRNAAAGSLRQLDSRITASRPLSFYAYSVGVVQGADLAASH

YERFLQIKSWGLPMCPETKRVDSLADVKTYYQHILQRRDALPYEIDGVVIKIDDIAVQER

LGFVARAPRWAIAYKFPAQEEITTLNEVEFQVGRTGAITPVAKLEPVFVGGVTVSNATLH

NADEIERLQVKIGDQVVIRRAGDVIPQVVSVIKERRPETARDIIFPTQCPVCGSHVERIE

GEAVTRCTGGLVCQAQRKQALKHFVSRKALDVDGLGDKVIEQLVDREMVETPADLFKLSA

GVLTVLERMGPKSAQNIVNALEKSKLTTLPRFLYSLGIREVGEATAANLAQHFKSLEAIQ

AATEEQLIAVQDIGVVVAKHITTFFEEEQNQAVVQDLLVQGIHWPEVSAPEQGAELPLEG

KTVVLTGTLSQLGRTEAKEALQSLGAKVTGSVSKKTDILFAGENAGSKLAKAQELGIEIK

TEQDLLELIN

>sp|Q87ST2|DJLA_VIBPA Co-chaperone protein DjlA OS=Vibrio parahaemolyticus serotype O3:K6 (strain RIMD 2210633) OX=223926 GN=djlA PE=3 SV=1

MHIFGKILGAFFGLLLGGPFGLLFGLFIGHQFDKARRLSQAGFSTGGFGKGPSQAQRQEE

FFKAAFAVMGHVAKAKGQVTKEEIQLATAMMDRMNLHGEQRRAAQDAFREGKESDFPLED

VLVRVKISTAGRFDLLQFFLELQISAAFADGAIHPSERNVLHKIARGLGFSSEQLERRLQ

MQEAAFRFQHQGGFHGQQQGQYQSSGWQQASQADQLADAYKILDVSPEADGKTVKRAYRK

LMNEHHPDKLMAKGLPPEMMNVAKEKSQEIQNAYDLIKKVKGFK

>sp|Q87L85|DUSA_VIBPA tRNA-dihydrouridine(20/20a) synthase OS=Vibrio parahaemolyticus serotype O3:K6 (strain RIMD 2210633) OX=223926 GN=dusA PE=3 SV=1

MTHSCRLSVAPMLDWTDRHCRYFHRLMTKETLLYTEMVTTGAIIHGKGDFLAYNEEEHPL

ALQLGGSNPEDLAKCAKLAQERGYDEINLNVGCPSDRVQNGRFGACLMAEPQLVADCVAA

MKEVVDVPVTVKTRIGIDDQDSYEFLTDFVSIVSEKGGCEQFTIHARKAWLSGLSPKENR

EIPPLDYPRAYQLKQDFSHLTIAINGGVKSLEEAKVHLQHLDGVMIGREAYQSPYLLASV

DQELFGSNAPVKKRSEIVEEMYPYIEAQLAKGAYLGHITRHMLGLFQNMPGARQWRRHIS

ENAHKPGSGLEVLQDALAKIPKELNV

>sp|Q87I22|DEF2_VIBPA Peptide deformylase 2 OS=Vibrio parahaemolyticus serotype O3:K6 (strain RIMD 2210633) OX=223926 GN=def2 PE=3 SV=1

MAVLEILSIPDPRLKVKAEKVTDVSTIQTLIDDMLETLYATGNGIGLASTQVGRKEAVVV

IDISDERNDPLILVNPEVVSGENKALGQEGCLSVPEYYADVERYTSVVVSALDRDGNPIT

IESDEFLAIVMQHEIDHLSGNLFIDYLSPLKQKMAMKKVKKYVKAQAK

>sp|Q87R73|DGTL1_VIBPA Deoxyguanosinetriphosphate triphosphohydrolase-like protein OS=Vibrio parahaemolyticus serotype O3:K6 (strain RIMD 2210633) OX=223926 GN=VP0925 PE=3 SV=1

MSFELHSLWQERHDDEHKIRRDDHRSPYQRDRARILHSAAFRRLQAKTQVHGNSLEDFHR

SRLTHSLEAAQLGTGIVAQLKKKQSEFKELLPSDSLIDSLCLAHDIGHPPYGHGGEVALN

YMMRDHGGFEGNAQTFRIVTKLEPYTEHFGMNLSRRTLLGLIKYPALISQTRSVKLPNPA

EHQRRLKAKEWSPAKGIYDCDKDLFDWVIAPLSENDKSLLSQMRYRPDSDLEHSKTRFKS

LDCSIMELADDIAYGVHDLEDAIVLGMVTRQQWQEGAASQLADCGDPWFEEHIGSIGQML

FSGKHHQRKDAIGGMVNALLTSISIKVVDEPFQNPLLAWNACLEPHMAKALDVLKHFVSQ

YVIQVPQVQIVEYKGQQIIMDIFEALSADPERLLPIHTKELWQSATSDSGKMRVIADYIS

AMTDGHAQKLHRQLFSSIVL

>sp|Q87TE4|DTD_VIBPA D-aminoacyl-tRNA deacylase OS=Vibrio parahaemolyticus serotype O3:K6 (strain RIMD 2210633) OX=223926 GN=dtd PE=3 SV=1

MIALIQRVSEAAVRVDGEVVGEIDTGLLVLLGVEKDDDEAKAKRLMERVTTYRVFEDDEG

KMNLNVKQVNGKVLVVSQFTLPADTKKGTRAGFSRGAHPADAERLYDYFSDLCEQELPTE

RGRFAADMKVSLINDGPVTFWLQV

>sp|Q87KU1|DUSB_VIBPA tRNA-dihydrouridine synthase B OS=Vibrio parahaemolyticus serotype O3:K6 (strain RIMD 2210633) OX=223926 GN=dusB PE=3 SV=2

MKIGNYQLKNNLIVAPMAGVTDRPFRELCLRYGAGMAVSEMMSANPKLWKTSKSKQRMVH

EGESGIRSVQIAGSDPQLMADAAQFSVENGAQIIDINMGCPAKKVNKKLAGSALLQYPTI

IEEILKAVVNAVDVPVTLKTRTGWDTDNKNCVQIAKLAEDCGIQALALHGRTKACMYKGE

AEYDSIKAVKEAISIPVIANGDIDSPEKAKFVLEYTGADALMIGRPAQGRPWIFQEIHHY

LENGTTMDELPTQEVKAIMLGHVNALHEFYGEYLGPRIARKHVGWYLKEHEQASEFRRTF

NAIDAAPLQIEALEGYFDNVAS

>sp|Q87RX3|DNAK_VIBPA Chaperone protein DnaK OS=Vibrio parahaemolyticus serotype O3:K6 (strain RIMD 2210633) OX=223926 GN=dnaK PE=3 SV=1

MGKIIGIDLGTTNSCVAVLDGDKPRVIENAEGERTTASVIAYTDGETLVGQPAKRQAVTN

PTNTLFAIKRLIGRRFEDEEVQRDIEIMPYKIVKADNGDAWVEAKGQKMAAPQVSAEVLK

KMKKTAEDFLGEEVTGAVITVPAYFNDAQRQATKDAGRIAGLEVKRIINEPTAAALAYGL

DKKGGDRTIAVYDLGGGTFDISIIEIDEVEGEKTFEVLATNGDTHLGGEDFDNRLINYLV

DEFKKEQGIDLKNDPLAMQRVKEAAEKAKIELSSTSQTDVNLPYVTADATGPKHMNIKVT

RAKLESLVEDLVQRSLEPLKVALADADLSVNDITDVILVGGQTRMPMVQAKVAEFFGKEA

RRDVNPDEAVAMGAAVQGGVLAGEVKDVLLLDVTPLSLGIETMGGVMTKLVEKNTTIPTK

ANQVFSTAEDNQSAVTIHVLQGERKQAMYNKSLGQFNLEGIQPAPRGMPQIEVTFDLDAD

GILHVSAKDKQTGKEQKITIQASGGLSDDEIEKMVQEAEANKEADKKFEELATARNQADQ

MIHGTRKQMEEAGDALPAEEKEKIETAISELEEARKGEDKEAIDAKVQALMTAAQKLMEI

AQQQAQAQQAQGADAGAQSKDDDVVDAEFEEVKDDKK

>sp|Q87MB4|DPO4_VIBPA DNA polymerase IV OS=Vibrio parahaemolyticus serotype O3:K6 (strain RIMD 2210633) OX=223926 GN=dinB PE=3 SV=1

MSERIRKIIHVDMDCFYAAVEMRDNPNYRDIALAVGGHEKQRGVISTCNYEARKFGVRSA

MPTARALQLCPHLLVVPGRMHIYKQVSLQIRAIFERYTSLIEPLSLDEAYLDVTDATACR

GSATLIAESIRNDIRNELGLTASAGIAPIKFLAKVASDMNKPNGQFVIPPEKVQEVVDKL

PLEKIPGVGKVSLEKLHQAGFYLCEDIKNSDYRELLRQFGRQGASLWKRSHGIDDREVVV

ERERKSVGVERTFSQNISTYDECWQVIEEKLYPELEKRLERASPDKSIIKQGIKVKFADF

QLTTIEHIHPQLELEDFKLLLKDILKRQNGREIRLLGLSVMLKPEEQARQLSFF

>sp|Q87KE9|CRCB_VIBPA Putative fluoride ion transporter CrcB OS=Vibrio parahaemolyticus serotype O3:K6 (strain RIMD 2210633) OX=223926 GN=crcB PE=3 SV=1

MGQFSILGFIALGGAIGACSRYLVSEFCVLLFGRGFPYGTLTVNVVGSFIMGLLIAAFEN

EILATEPWRQVIGLGFLGALTTFSTFSMDNVLLMQQGAFFKMGLNILLNVVLSISAAWIG

FQLLMRS

>sp|P0A308|ATPL_VIBPA ATP synthase subunit c OS=Vibrio parahaemolyticus serotype O3:K6 (strain RIMD 2210633) OX=223926 GN=atpE PE=3 SV=1

METLLSFSAIAVGIIVGLASLGTAIGFALLGGKFLEGAARQPEMAPMLQVKMFIIAGLLD

AVPMIGIVIALLFTFANPFVGQLG

>sp|Q87L22|ASTD_VIBPA N-succinylglutamate 5-semialdehyde dehydrogenase OS=Vibrio parahaemolyticus serotype O3:K6 (strain RIMD 2210633) OX=223926 GN=astD PE=3 SV=1

MTHWIAGEWVQGQGEEFVSLSPYNQEVIWRGNGATAEQVDQAVAAARAAFVEWKKRPFAE

REAIVLAFAEKVKENSEKIAEVIAKETGKPIWETRTEAAAMAGKIAISIRAYHDRTGEAT

REAAGNQIVLRHRPLGVMAVFGPYNFPGHLPNGHIVPALLAGNTVVFKPSEQTPWTGELA

MKLWEEAGLPKGVINLVQGAKETGIALADAKGIDGILFTGSANTGHILHRQFAGQPGKML

ALEMGGNNPMVISDNYGDLDATVYTIIQSAFISAGQRCTCARRLYVPFGEKGDALITKLV

EATKNIRMDQPFAEPAPFMGPQISVAAAKFILDAQANLQSLGGESLIEAKAGEAAFVSPG

IIDVTNIAELPDEEYFGPLLQVVRYEGLDKAVELANDTRFGLSAGLVSTDDQEWEYFVDH

IRAGIVNRNRQLTGASGDAPFGGPGASGNLRPSAYYAADYCAYPMASMEGQETELPATLS

PGVTL

>sp|Q87Q40|ASTE_VIBPA Succinylglutamate desuccinylase OS=Vibrio parahaemolyticus serotype O3:K6 (strain RIMD 2210633) OX=223926 GN=astE PE=1 SV=1

MTKSLFRQSFLTDTLDVHIDVAPAEQVLSNGVQLKLYQRGVLEVIPENPTQETKNIIISC

GIHGDETAPMELVDSIIKDIESGFQKVDARCLFIIAHPESTLAHTRFLEENLNRLFDEKE

HEPTKELAIADTLKLLVRDFYQDTEPKTRWHLDLHCAIRGSKHYTFAVSPKTRHPVRSKA

LVDFLDSAHIEAVLLSNSPSSTFSWYSAENYSAQALTMELGRVARIGENALDRLTAFDLA

LRNLIAEAQPEHLSKPCIKYRVSRTIVRLHDDFDFMFDDNVENFTSFVHGEVFGHDGDKP

LMAKNDNEAIVFPNRHVAIGQRAALMVCEVKTRFEEGELVYD

>sp|Q87KA4|ATPF_VIBPA ATP synthase subunit b OS=Vibrio parahaemolyticus serotype O3:K6 (strain RIMD 2210633) OX=223926 GN=atpF PE=3 SV=1

MNINATLLGQAISFALFVWFCMKYVWPPLMQAIEERQKKIADGLQAAERAAKDLDLAQAN

ASDQLKEAKRTATEIIEQANKRKSQIIDEAREEAQAERQKILAQAEAELEAERNRARDEL

RKQVATLAVAGAEKILERTIDKDAQKDILDNITAKL

>sp|Q87KA6|ATPA_VIBPA ATP synthase subunit alpha OS=Vibrio parahaemolyticus serotype O3:K6 (strain RIMD 2210633) OX=223926 GN=atpA PE=3 SV=1

MHCSLNGDWSMQLNSTEISDLIKQRIESFEVVSEARNEGTIVSVSDGIIRIHGLADVMQG

EMIELPGGRYALALNLERDSVGAVVMGPYADLKEGMKVTGTGRILEVPVGPELLGRVVNT

LGEPIDGKGPIEAKLTSPVEVIAPGVIDRKSVDQPVQTGYKSVDSMIPIGRGQRELVIGD

RQTGKTAMAIDAIINQKNSGIFSIYVAIGQKASTIANVVRKLEEHGALANTIVVVASASE

SAALQYLAPYAGCAMGEYFRDRGEDALIVYDDLSKQAVAYRQISLLLKRPPGREAFPGDV

FYLHSRLLERAARVNEEYVERFTNGEVKGKTGSLTALPIIETQAGDVSAFVPTNVISITD

GQIFLQTELFNAGVRPAVDPGISVSRVGGSAQTKIIKKLSGGIRTALAAYRELAAFAQFS

SDLDEATKKQLDHGQKVTELMKQKQYAPMSVFDQALVIFAAERGYLDDVELNKVLDFEAA

LLSYARGQYAELAAEIDKSGAYNDEIEAQLKKLTDDFKATQTW

>sp|Q87KA5|ATPD_VIBPA ATP synthase subunit delta OS=Vibrio parahaemolyticus serotype O3:K6 (strain RIMD 2210633) OX=223926 GN=atpH PE=3 SV=1

MSDLTTIARPYAKAAFDFAVDKGQLDQWGQMLSFAAEVAKNEQMNELLTSSFSAEKMAEI

FVAVCGEQVDAHGQNLLKVMAENGRLAALPDVCEQFFILKKEHEKEIDVEVISASELSDE

QLANIGSKLEARLERKVKLNCSVDETLLGGVIIRAGDLVIDDSARGRLNRLSDALQS

>sp|Q87KA3|ATP6_VIBPA ATP synthase subunit a OS=Vibrio parahaemolyticus serotype O3:K6 (strain RIMD 2210633) OX=223926 GN=atpB PE=3 SV=1

MAAPGEALTSSGYIAHHLSNLSLYKLGLVGSETSFWNVHIDSLFFSWFTGLIFLGIFYKV

AKRTTAGVPGKLQCAVEMIVEFVADNVKDTFHGRNPLIAPLALTIFCWVFLMNVMDLVPI

DFLPYPAEHWLGIPYLKVVPSADVNITMAMALGVFALMIYYSIKVKGLGGFAKELALHPF

NHPLMIPFNLLIEVVSLLAKPLSLGMRLFGNMFAGEVVFILCAAMLPWYLQWMGSLPWAI

FHILVITIQAFVFMMLTIVYLSMAHEDSDH

>sp|Q87KA8|ATPB_VIBPA ATP synthase subunit beta OS=Vibrio parahaemolyticus serotype O3:K6 (strain RIMD 2210633) OX=223926 GN=atpD PE=3 SV=1

MATGKIVQIIGAVVDVEFPQSEVPSVYDALNVTDSKERLVLEVQQQLGGGVVRCIVMGSS

DGLRRGVEVVNTGAPISVPVGTKTLGRIMNVLGDAIDERGEIGAEEVYSIHREAPSYEEQ

SNETALLETGVKVIDLVCPFAKGGKIGLFGGAGVGKTVNMMELINNIALQHSGLSVFAGV

GERTREGNDFYFEMQEAGVVNVENPEESKVAMVYGQMNEPPGNRLRVALTGLTMAERFRD

EGRDVLLFVDNIYRYTLAGTEVSALLGRMPSAVGYQPTLAEEMGVLQERITSTKQGSITS

VQAVYVPADDLTDPSPATTFAHLDATVVLNRNIAAMGLYPAIDPLDSTSRQLDPLVVGQD

HYDIARGVQQTLQRYKELKDIIAILGMDELSESDKQVVSRARKIERFLTQPYHVAEVFTG

DPGVYVPLKETLRGFKGLLAGEYDDIPEQAFMYCGTIDDAIENAKKL

>sp|Q87MQ1|APT_VIBPA Adenine phosphoribosyltransferase OS=Vibrio parahaemolyticus serotype O3:K6 (strain RIMD 2210633) OX=223926 GN=apt PE=3 SV=1

MTTETISLIKSSIKSIQDYPKPGILFRDVTSLLEDAKAYQATIGLLVERYKDMGFTKVVG

TEARGFLFGAPLALELGVGFVPVRKPGKLPRPTIAQSYELEYGIDTLEIHTDAIVEGDKV

LVVDDLLATGGTIEATTKLIRQLGGEVEHAAFVINLPEIGGDKRLEALGLNVFSICDFEG

H

>sp|Q87LG0|ARCA_VIBPA Arginine deiminase OS=Vibrio parahaemolyticus serotype O3:K6 (strain RIMD 2210633) OX=223926 GN=arcA PE=3 SV=1

MSKLYVGSEVGQLRRVLLNRPERALTHLTPSNCHELLFDDVLAVEAAGEEHDAFARTLRE

QDVEVLLLHDLLVETLAVPEAKQWLLNTQISDFRYGPTFARDLRQYLLEMDDEHLATILL

GGLAYSELPIQSSSMLPKMKRPLDFVIEPLPNHLFTRDTSCWVYGGVSLNPMMMPARQRE

TNHLRAIYRWHPIFAGQDFIKYFGDDDLHYDNANVEGGDVLVIGKGAVLIGMSERTTPQG

VENLAASLFKAGQASEVIAIDLPKHRSCMHLDTVMTHMDVDTFSVYPEIMRKDLDTWRLT

PKGTDGEMHVEASHNYLHAIESALGLDQLKIITTGGDSYEAEREQWNDANNVLTVKPGVV

IGYERNVYTNEKYDKAGIQVLTVPGNELGRGRGGARCMSCPIERDDI

>sp|Q87SU8|ARGR_VIBPA Arginine repressor OS=Vibrio parahaemolyticus serotype O3:K6 (strain RIMD 2210633) OX=223926 GN=argR PE=3 SV=1

MRNSEKQDNLVRAFKALLKEESFGSQGEIVDALKQQGFESINQSKVSRMLTKFGAVRTRN

AKMEMVYCLPAELGVPTVSSSLRELVLDIDHNAALVVIHTGPGAAQLIARLLDSLGKSEG

ILGVVAGDDTIFITPTMPVSTEQLFKSVCELFEYTG

>sp|Q87FK3|ARAA_VIBPA L-arabinose isomerase OS=Vibrio parahaemolyticus serotype O3:K6 (strain RIMD 2210633) OX=223926 GN=araA PE=3 SV=2

MKIFNDKQVWFVTGSQHLYGPQVLESVAQNSEEIIAGLNSSDDISVSIANKGTVKTPDEI

LAVCRAANNDPDCIGLMLWMHTFSPAKMWIAGLTQLNKPFLHLHTQFNAALPWDEIDMDF

MNLNQSAHGCREFGFIGTRLNIERKVVVGHWQEPQVHRDIDDWCRAAIGVNAGQHLKVAR

FGDNMRQVAVTEGNKVSAQIQFGYEVNAYGLGELSDVVNSISDADVNHQLDKYACMYEMS

PDLFNDSDLKKLMAQEARLELGMESFLKSVGAGAFTNTFENLTGLTNLPGLATQRLMAKG

FGYGGEGDWKTAAMTHIMKVMGQGKPGGTSFMEDYTYNFGEKGQVLGAHMLEVCPTIAAA

KPRLEVHRHTIGCRCDIPRLIFSGQSGEALNVSIIDLGDRFRMIVNVIDTVTPPQSLPHL

PVAHALWEPQPNLNIAAAAWIHAGGAHHAVYSQAVTLPMLADYAEILGIEMVVIDNSTNL

RQFKQELRNNGVYYRLG

>sp|Q87M87|ARGA_VIBPA Amino-acid acetyltransferase OS=Vibrio parahaemolyticus serotype O3:K6 (strain RIMD 2210633) OX=223926 GN=argA PE=1 SV=1

MKIRSTALVKGFRQSTPYVNAHRGKTMVIMLGGEAVAHNNFGNIINDIALMHSLGIKVVV

VYGARPQINQLLEKQDLTTPYHKNIRITDEAALSVVMQAAGQLQLAITARLSMSLNNTPM

AGTQLNVVSGNFVIAQPLGVDDGVDYCHSGRIRRIDTDAINRTLDQGSIVLLGPIASSVT

GECFNLLSEEVATQLAIKLGADKLIGFCSEQGVIDDNGNAVAELLPIEAEHVIKTLSENH

ASDSDYNTGTLRFLKGSIAACRAGVPRSHLISYKVDGALIQELFSFDGIGTQVVMASAEQ

VRQAGIDDIGGILELIHPLEEQGILVRRSREQLEQEIGKFTIIEKDGLIIGCAALYPYSE

ERKAEMACVAIHPDYRDGNRGLLLLNYMKHRSKSENINQIFVLTTHSLHWFREQGFYEVG

VDYLPGAKQGLYNFQRKSKILALDL

>sp|Q87KU6|AROQ_VIBPA 3-dehydroquinate dehydratase OS=Vibrio parahaemolyticus serotype O3:K6 (strain RIMD 2210633) OX=223926 GN=aroQ PE=3 SV=1

MSAKSRILVLNGPNLNLLGLREPTHYGNNTLAQIVDALTEQAHNAGVELEHLQSNREYEL

IEAIHAAYGKIDFIIINPAAFTHTSVALRDALLGVAIPFIEVHLSNVHAREPFRHHSYLS

DKAEGVICGLGAQGYEFALSAAINKLQAK

>sp|Q87L56|ARGB_VIBPA Acetylglutamate kinase OS=Vibrio parahaemolyticus serotype O3:K6 (strain RIMD 2210633) OX=223926 GN=argB PE=3 SV=1

MTQTNQAPLVIKLGGAALSCTQTLSQLFGAIAAYQKSAQRQIAIVHGGGYLVDELMAKLQ

LKTVKKHGLRVTPYDQIPVIAGALAGTANKLLQGQAIADGLNAVGLSLADGGLCHVEELD

PELGAVGKATPGDSSLLQAILNTGALPIISSIGLTAEGQMMNVNADQAAVAVAGALDAEL

VLLSDVSGVLDGKGHLLKSLSKKEANALIEGQVITDGMIVKVKAALEAANDLGRPIEVAT

WRYPEKLTQLFAGESIGTQFLPQ

>sp|Q87QX9|AROA_VIBPA 3-phosphoshikimate 1-carboxyvinyltransferase OS=Vibrio parahaemolyticus serotype O3:K6 (strain RIMD 2210633) OX=223926 GN=aroA PE=3 SV=1

MESLTLQPINKIQGEVNLPGSKSVSNRALLLSALAKGTTRLTNLLDSDDIRHMLNALTKL

GVKYTLSADKTECVVEGLGRPFSVSEPVELFLGNAGTAMRPLAAALCVGQGEYVLTGEPR

MKERPIGHLVTALQKAGADIEYLENTNYPPLKIVGTGLKAGTVSIDGSISSQFLTAFLMS

APLAEGEVRIKIEGDLVSKPYIDITLHIMKQFGVEVINNDYQEFVIPAGQHYVAPGDFLV

EGDASSASYFLAAAAIKGGEVKVTGIGKNSIQGDIQFADALEKMGAEIEWGDDYVISRVG

KLKGIDMDYNHIPDAAMTIATTALFAEGTTAIRNVYNWRVKETDRLSAMATELRKVGAEV

EEGEDYIIVKPVPHLKHAAIDTYDDHRMAMCFSLLALSDTPVTINDPKCTSKTFPDYFDK

LKALSC

>sp|Q87MM9|AROC_VIBPA Chorismate synthase OS=Vibrio parahaemolyticus serotype O3:K6 (strain RIMD 2210633) OX=223926 GN=aroC PE=3 SV=1

MAGNSIGQHFRVTTFGESHGIALGCIVDGCPPGLEITEADLQTDLDRRRPGTSRYTTQRR

EPDEVKILSGVFEGKTTGTSIGLLIENTDQRSKDYSDIKDKFRPGHADYTYHQKYGIRDY

RGGGRSSARETAMRVAAGAIAKKYLKDEFGVEIRAYLSQMGDVSIDKVDWDEIENNAFFC

PDADKVEAFDQLIRDLKKEGDSIGAKIQVVATNVPVGLGEPVFDRLDADIAHALMSINAV

KGVEIGDGFDVVNQKGSQHRDPLSPQGFGSNHAGGILGGISTGQDIVANIALKPTSSITV

PGDTITKEGEPTQLITKGRHDPCVGIRAVPIAEAMLAIVVMDHLLRHRGQNHGVTTETPK

I

>sp|Q87L67|AROK_VIBPA Shikimate kinase OS=Vibrio parahaemolyticus serotype O3:K6 (strain RIMD 2210633) OX=223926 GN=aroK PE=3 SV=1

MAEKRNIFLVGPMGAGKSTIGRHLAQQLHMEFVDSDTVIEERTGADISWVFDVEGEEGFR

KREEAVLEDLTQEQGIVLATGGGSVKSKENRNRLSARGVVVYLETTIEKQLARTNRDKKR

PLLQTDNPREVLEQLAEERNPLYEEVADYTVRTDDQSAKVVANQIVKMLEER

>sp|P59605|ASSY_VIBPA Argininosuccinate synthase OS=Vibrio parahaemolyticus serotype O3:K6 (strain RIMD 2210633) OX=223926 GN=argG PE=3 SV=1

MSKVNVNKVVVAYSGGLDTSVIIPWLKENYDCEVVAFVADVGQGAEELEGIEAKAKASGA

SECYIADLKEEMVADYIYPTLKTGAYYEGKYLLGTSMARPIIAKAQVEVARKVGADALCH

GCTGKGNDQVRFEGAFAALAPDLHVIAPWREWDLVSREQCLDYLAERNIPCSASLTKIYS

RDANAWHISTEGGVLENTWNAPNEDCWVWTVDPEQAPNEAEYVTLKVEKGEVVAVDGEAM

TPYNALVYLNEKGAKHGVGRIDIVENRLVGMKSRGCYETPGGTIMMEALRAVEQLVLDKS

SFEFREELGLKASHLVYDGRWFTPLCKSILAASEELAQDVNGEVVVKLYKGQATVTQKRS

DNSLYSEEFATFGEDEVYDQSHAGGFIRLYSLSSRIRALNSQKK

>sp|Q87MQ5|AQPZ_VIBPA Aquaporin Z OS=Vibrio parahaemolyticus serotype O3:K6 (strain RIMD 2210633) OX=223926 GN=aqpZ PE=3 SV=1

MNKYLAEAFGTFWLVLGGCGSAVLAAGFPDVGIGLLGVALAFGLTVLTMAFAIGHISGCH

LNPAVTVGLWAGGRFDTKDVAPYIIAQVIGGLIAGGILYVIATGQAGFDVVGSGFAANGY

GEHSPGQYSMLAALVSEIVMTMMFLIVIMGATDKRAPQGFAPIAIGLCLTLIHLISIPVT

NTSVNPARSTAVAMYVGDWAVSQLWLFWVAPIVGGVLGAVIYKNLLGKESND

>sp|Q87FK5|ARAB_VIBPA Ribulokinase OS=Vibrio parahaemolyticus serotype O3:K6 (strain RIMD 2210633) OX=223926 GN=araB PE=3 SV=1

MDTINTHQQHVIGLDFGSDSVRALIVNAETGQEVSSSVVYYSRWMKGLYCQPAQSQFRHH

PQDYLDAMTSAIQEVLATVPQTLADSVVGIGVDTTGSTPAPIDENGTVLALLPEFEHNPN

AMFVLWKDHTSVAKADRINELAHSGKFTDYTRYVGGVYSSEWFWAKAAWVSEQDEQVAKR

AFSWVELCDWIPAILADTQHPQKLRRGICAAGHKAMWHESWGGLPEQAFLSAISPTLDGI

RDRMFTEVFTSDQAAGYLSKAWAIKLGLPEGIAIAIGEFDCHMGAVGAGAGANDLVKVIG

TSTCDILMVESQNVGDRTIHGICGQVEGSAMPELLALEAGQSAFGDMYAWFKNVLMWPLQ

AYAEHNPDFALTAEEIASELLPMLSQAAEQQGIDQYTPVAMDWLNGRRTPYANQRLKGAI

CDLNLGSSSPAIFSALVESTAHGAKAIVDCFIEQDVTVERVIAIGGIAQKSPYVMQMCAD

VIGRDIIVVESDQCCALGAAIFAAVAAGVYPTTKSAQAVMASPVRQTYSPTPKVQTLRAQ

RYATYRELGQHMEQIAEFHQSQEREDV

>sp|P59620|ARLY_VIBPA Bifunctional protein ArgH OS=Vibrio parahaemolyticus serotype O3:K6 (strain RIMD 2210633) OX=223926 GN=argH PE=3 SV=1

MALWGGRFTQAADTRFKEFNDSLRFDYRLAEQDIVGSIAWSKALLSVGVLSAEEQQKLEL

ALNELKLEVMEDPHQILRSDAEDIHSWVEQQLISKVGDLGKKLHTGRSRNDQVATDLKLW

CRQQGQQLLIALDRLQSQMVQVAKQHQGTVLPGYTHLQRAQPVTFAHWCLAYVEMFERDY

SRLSDALQRLDTCPLGSGALAGTAYPIDREQLAHNLGFHRATRNSLDSVSDRDHVMELMS

VASISMLHLSRLAEDMIFYNSGESNFIELADTVTSGSSLMPQKKNPDALELIRGKTGRVY

GALAGMMMTVKALPLAYNKDMQEDKEGLFDALDTWNDCMEMAALCFDGIKVNGERTLEAA

KQGYANATELADYLVAKGIPFREAHHIVGVAVVGAIAKGCALEELSLQELQEFSDVIDND

VYDILTIESCLEKRSALGGVSPKQVAYAVDQADKRLAQRDSSAVKVRPARLTDIETLEGM

VAYWANMGENLPRSRNELVRDIGSFAVAEHHGEVTGCASLYVYDSGLAEIRSLGIEAGWQ

GQGQGSAIVNYLVDKARQMAIKKVFVLTRTPEFFMKQSFLPTSKSLLPEKVLKDCDQCPR

QHACDEVALEINLVEQIIQRSHVA

>sp|Q87KE4|AROE_VIBPA Shikimate dehydrogenase (NADP(+)) OS=Vibrio parahaemolyticus serotype O3:K6 (strain RIMD 2210633) OX=223926 GN=aroE PE=3 SV=1

MTPQIDRYAVFGNPIGHSKSPFIHTLFARQTNQSLTYTAECAPVGGFIEAAKAFFADGGK

GCNVTLPFKEDAYQFASRLTERAQLAGAVNTLKKLDDGEIIGDNTDGAGLVQDLLQHQVV

LEGARILIIGAGGAARGVIKPLLDQKPTSLTITNRTFSKAEELAELFSVYGPVTAKEMNI

VAEEFDIIINSTSASLSGELPAISSSVFAANSTSYDMMYGKGDTTFNQWAKQHGAAHAYD

GLGMLVGQAAESFMLWRGLRPGAKQILRELRKNLEGQ

>tr|Q877M0|Q877M0_VIBPA Uncharacterized protein OS=Vibrio parahaemolyticus serotype O3:K6 (strain RIMD 2210633) OX=223926 GN=VP0639 PE=4 SV=1

MNDVLTIKASNHLDTQTLLTTQDSQLHQEVWGHVKALVELGVFEDATNGIENVLAPYMAE

VNAELI

>tr|Q877N6|Q877N6_VIBPA Uncharacterized protein OS=Vibrio parahaemolyticus serotype O3:K6 (strain RIMD 2210633) OX=223926 GN=VP1581 PE=4 SV=1

MLRFTSASISWVILSMVLIGLSFYLVAEIVVASNIPNGLNQS

>tr|Q877K3|Q877K3_VIBPA Uncharacterized protein OS=Vibrio parahaemolyticus serotype O3:K6 (strain RIMD 2210633) OX=223926 GN=VP1584 PE=4 SV=1

MNLSIWKSRRNQRQLVASQDNGTHIYFDSFELEAVEASLWLYQGMTLVACIKAQNDTLAD

ITTTANRMATLGAQNNGQPLHEIRKQDEAPQVGADSSL

>tr|Q877T2|Q877T2_VIBPA Uncharacterized protein OS=Vibrio parahaemolyticus serotype O3:K6 (strain RIMD 2210633) OX=223926 GN=VP1570 PE=4 SV=1

MVGHGGVNGRVVHTRNGNAEVVTTNGILTANVSGVTGITRLGFRTAHLPHPQI

>tr|Q877U7|Q877U7_VIBPA Uncharacterized protein OS=Vibrio parahaemolyticus serotype O3:K6 (strain RIMD 2210633) OX=223926 GN=VP0640 PE=4 SV=1

MQRLPLSVLVGSNHRGINALIPCLYALFESQRNANRITTAYRAQPDTVSLTGC

>tr|Q877N5|Q877N5_VIBPA Putative phage structural protein (Alteromonas PM2) OS=Vibrio parahaemolyticus serotype O3:K6 (strain RIMD 2210633) OX=223926 GN=VP1566 PE=4 SV=1

MPLFIFVIILLGIGAFTMTTSTTSSVRGVRIHNPLNIRIAGNAWKGKVTPSRDKAFETFK

APEWGFRAGAILLRNYQQRHELHTLTEIIHRFAPPNENHTANYARFVAGRVGVGMDERID

LVNNKPLLVEVLHAMSIMEVGRHYSKHTVLKGVNLV

>tr|Q877L0|Q877L0_VIBPA Putative phage-related protein OS=Vibrio parahaemolyticus serotype O3:K6 (strain RIMD 2210633) OX=223926 GN=VP1569 PE=4 SV=1

MLNSNERISLEAMDTTHDIAETAIDEVVDFAGNSLATYASTNSENLDMLAGLAGSQAAQN

SKNLEAMMDLAKFKQDGGQVETSKMMVVLAIVLVLVLGYVMVKKR

>tr|Q877R3|Q877R3_VIBPA Uncharacterized protein OS=Vibrio parahaemolyticus serotype O3:K6 (strain RIMD 2210633) OX=223926 GN=VP1582 PE=4 SV=1

MEAEVKRSKGGVFWRLLLVWMKEVNKQIQKKPNTDDELAEA

>tr|Q877Q6|Q877Q6_VIBPA Uncharacterized protein OS=Vibrio parahaemolyticus serotype O3:K6 (strain RIMD 2210633) OX=223926 GN=VP1574 PE=4 SV=1

MAQEQSMWGSIWDGVLETGGELLTDVTDLGKDWLGVKIENEAQRVESSNPDEQRKHNNDY

QQPTGEPVYTSAFAGVTTTHLMMGAMLFVLLLLAVFYVAKGKK

>tr|Q87P17|Q87P17_VIBPA Putative exoenzyme S synthesis protein C OS=Vibrio parahaemolyticus serotype O3:K6 (strain RIMD 2210633) OX=223926 GN=VP1701 PE=4 SV=1

MSARQTIDEVLQKFAHQIGLPELHLTDNELSLAFDDHLKVHFIFHPETNTLQLEAEIVGL

QIVNSDLYRSFLAFNYHWPEHQLFFSLDNHRHVLCLNKLIGIEQLDYEYFENALAELLTQ

SESWESLLSAHVVAEETPAMPQSLDLRV

>tr|Q87S17|Q87S17_VIBPA Uncharacterized protein OS=Vibrio parahaemolyticus serotype O3:K6 (strain RIMD 2210633) OX=223926 GN=VP0607 PE=4 SV=1

MTEHENTNEVPLSIEAGTLLKNKRESLGMTQKQVADRLRLRVSVIEDIENNRFESLQVAT

FTRGYLRSYAKFVGLDEKVVLVALEQTADVKPKEQEIEMQSFSRKTKHEKHNSRIMLLTW

VIAIVIIGISAAWWWQNQQENSLAQVVAEANVETTQPSADEIADIDLMTEEELIASTPAE

LAASNNTASESSINAAQTDEVVPAETEESTTEATQEPVALIEAAEEVQEASPVVPEGMTL

LTMKFKADCWIQVKDTNGKTLVSGTQKPGQDVELTGKAPFKVILGAPEGVTMTFASEPVD

LSGYTSGKVARFTLPL

>tr|Q87HU6|Q87HU6_VIBPA Long-chain fatty acid transport protein OS=Vibrio parahaemolyticus serotype O3:K6 (strain RIMD 2210633) OX=223926 GN=VPA0860 PE=4 SV=1

MNKKHCSLLTISILFACNAHSAGFQVAEHSASGLGRAFSGEGAVADNASVLARNPAAMTL

FKEAQFSGALSIVDPEVNVYDTLHKEQSDDVAPLQVVPAGYYISPINDNWAWGIGMFTTY

GVATDYPDDISAGDMAGDTSLLSVNINPNIAYRINEKFSVGGGINLVYAEAELTRHKGAL

APLFGSGSSKSDNLIGMEGETFAWGWNVGALFELNENNRFGFGYRSKVDLDFDDGEFSSY

DSGIATAAKVDGRLKISLPSIIELSAFHQLNDQWAIHYGWQQTDWSTFKELKATSNECKG

GVCFFKPEKYEDNNRYSVGATYTLNTEWTFRAGLAYDEQAGEATLSIPDSDRMWYSAGLT

YAMNENLSFDAGFALVQSESGSFTETNAAGQEIKFDSEGTAYISAVQMNYTFK

>tr|Q87S43|Q87S43_VIBPA Transcriptional regulator, LysR family OS=Vibrio parahaemolyticus serotype O3:K6 (strain RIMD 2210633) OX=223926 GN=VP0581 PE=4 SV=1

MNKWPSLKQLHYLVTLHETRHFSEAAERCFVSQSTLSKGIQNLEELIGCPLYEKKDKKSP

LVFTQTGEMVVKQGRELLAKGQDLVELGRLCQGDDMKGQLKVGCIPTIAPFLLCDLVQEV

NYRFPQLNLLLREDTTTNLLQALRHGELDVLILALPVEIDGMESRVVGKDPFRMVISANQ

VDSIPVPIRYDDLPDESVFLLEKEHCLTEHAVSACKLTDKEKINPFTATSLHTLVQMVAN

GLGTTFIPQMAIEHGLLHNQNLVVIDPPGQQAYREIGLVWRPSSSRTNTFNQLADVVSEL

L

>tr|Q87JZ2|Q87JZ2_VIBPA Uncharacterized protein OS=Vibrio parahaemolyticus serotype O3:K6 (strain RIMD 2210633) OX=223926 GN=VPA0106 PE=1 SV=1

MKQKLLVTLLGLSALGASPVFAANTNVDLAEDAYIYGYSIDEAYKFFYHTAVENNYPLNR

FQNIRALADDSYTAHPTINNDTLHLMGWLDVAAEPVIVSVPDMDEGRYWILHTMDMGHYT

NAAFSSRTRGTKGGQFMFAAQDWQGEVPASVDEVVRVDSNLVKLMGRIMAVNDEDAKVAL

NYMDQWNIRTLSEYLGKNGPKPVQRTYPDPKKSTWLERVNFVLCDGSMGNADKQWLDKYQ

SIGVEPCKTDFTPEQLKLAKVGEKKGMEHLVELAPKMTDARTLLGTRDTLGDAPRDIFAE

GTYLGQWGLPPIEASYRKSDFDSIGQKLDGSKHDYVMRFKAPNVSEFWSVTIYGNDNRLM

AKNDLNRHSRGDRTMKADKDGYYTIYMSANEKGRADDPNFLPVPEKPFYAIMRFYGADDA

IQSGEYQMPEIKVVK

>tr|Q87J23|Q87J23_VIBPA Uncharacterized protein OS=Vibrio parahaemolyticus serotype O3:K6 (strain RIMD 2210633) OX=223926 GN=VPA0430 PE=4 SV=1

MRPVSKELSEICYPFIYEDSPVCDFEITADELCSRIGLVLSMELDDFSRDILTRLQPNVY

HLNGSVRGKLAITESEVEELKADYEAIKARIDGGFKGFVLPGGHATSSQLHLCRSQAKKT

VRALVAIEHAGKKQPAPILFRYANLMANVIYSLASYINHVYQVEETEFVSRSYTMPKK

>tr|Q87LJ6|Q87LJ6_VIBPA Pyridoxal phosphate homeostasis protein OS=Vibrio parahaemolyticus serotype O3:K6 (strain RIMD 2210633) OX=223926 GN=VP2616 PE=3 SV=1

MSSIQQNIEHITSQIRRDEQKCGRSPESVQLLAVSKTKPVEAILEAYQAGQTAFGENYVQ

EGVSKVQHFAEHYPDNRIEWHFIGPIQSNKSRLVAEHFDWVHTIDRAKIAQRLNEQRPQE

LKPLQVLIQVNTSGEDSKSGVSDAEIFELAELISRLPNLTLRGLMSIPANVSDYDAQLRE

FEKLATLKQTLEQQYPEIDTLSMGMSGDMTAAIEAGSTMVRIGTAIFGARDYSTKSE

>tr|Q87GP6|Q87GP6_VIBPA Uncharacterized protein OS=Vibrio parahaemolyticus serotype O3:K6 (strain RIMD 2210633) OX=223926 GN=VPA1269 PE=4 SV=1

MGQKGKTQDGRTSDLTFSWMLTSLGAEWQQWQELAAEWMMAQNGDIDTKLVALSRFFESY

VLEHASYAIDVSLFFKGYKGHLCSSEELEKVTRKKVNSPYKVQKYVNHASNFIDFVIEKV

FSEEDDKGNLIPLVQNPLRKIKVQRNATETVRNPLPYRYIQDLRQILCPLPDKTELTVLE

QNLKQGEPLLPAYHYRHFKHWTWAQQQTGQGNNGGDWFEVEPELIDKSDPDCVWRTKEVT

RYIKGEGTKKITVHQIWSPVKAMVIFIKLHLPLRTYQVRMLDSGEADTWRYEQGQWILNT

KHDFALGSEKRPFGKGIFRRIHDTMTGLHSTGLYINTNKTADQNKDELERGYIIPWQNEE

VLYWLEKLRNWQEKYNPIAKPTDCTTLLRKHTGKKKSDKQLQSMGEIAFLFRDASAKGED

KFKPIQYTAIDSFWYHLLFELENNLAEQGNTLDNGERLKLVVDYPEGTPELHKLATLFPL

HSLRVSLITAYTMDTQLPLPVISKILAGHTRLLMTIYYNKITPSVMAEKMDEAHGELDAK

SKQSVRNFLKDASMEQIQCKMVYHSDDSIQAALVNRNPIGWEERSCGVCLVGGNTVKSDE

VSTLGGCWNGGELIRDAKTAANRVYASVPHGPENCPRCRWFITEARYLPALNAQFNQLSY

KAHQAANLSVEIEGELETLKDEQFFCEEQGTPFIKHDELQALQRRYEKQKVEADEYTKDW

IACFELINKIIRVEETRNKDDTKDKLIAVGSEQDVSHALKFIETDSELLHLSLICDDAEF

YPDLQDELRKTPVIQKRSMQLSRVLMKKGFEPIFLEMDEKQQLIAANAMLRQMAKIADPD

DKLEGYRKVANYIEAGEYLNDNKLLSQGIHALTDKAIHLDGIALPNLLED

>tr|Q87I16|Q87I16_VIBPA Putative YhfP protein OS=Vibrio parahaemolyticus serotype O3:K6 (strain RIMD 2210633) OX=223926 GN=VPA0790 PE=4 SV=1

MDPLTQGLLGAALPQSASNKRHIVAAGVLGVAAGMAPDLDVLIRSSSDPLLFLEYHRHFT

HSLLFIPFGSFLCALVLYPLFAKRRGLNFQQSWFYCALGYSTHALLDACTSYGTQLFWPI

NNTRYAWNTISVIDPAFSLPILILLLFAALKRNRSYARVAFLWSLAYLTLGVIQRDRAEA

AGWKIVQERQHSAVQLKAKPTFGNLLVWKVIYETEDNYYVDAVRVGRSIKIYPGESTPKL

SVKTQFPWLDPQSQQAKDIERFRWFSNGFVVQDPDDEMRIVDVRYSIVPNQINALWGINL

SPKASADEHVEYTAHRGSTAEDRQTFLNMLTDFD

>tr|Q87QL3|Q87QL3_VIBPA Putative transcription regulator TxR OS=Vibrio parahaemolyticus serotype O3:K6 (strain RIMD 2210633) OX=223926 GN=VP1136 PE=4 SV=1

MMKLRKGSTKQTVQFSEAELYPLIGNSACIHELRNAIKKYAQCDAEVLIQGETGVGKGLC

ARLIHQLSSRRSSPFVEVNCGAIPSGLIASELFGHEKGAFTGAISDRIGFIQKAHKGTLF

LDEIGDMPSDLQIHLLHFLESKQIHKVGADKFIDVDCRVIAASHVDLKSEVVQGEFREDL

FYRLNILPLTIPPLRKRGKDILMLSEHFLTDLSHGQVHNMSEEVKAKLLKHRWPGNVREL

RNVIQRAIVMCEDNTLLVADLGLENSERQLLPSVDQIDLDYLLQAIEENKHNMSAAARHL

GISRTTLYRLIKKYNINI

>tr|Q87JK3|Q87JK3_VIBPA Uncharacterized protein OS=Vibrio parahaemolyticus serotype O3:K6 (strain RIMD 2210633) OX=223926 GN=VPA0246 PE=4 SV=1

MKVVGADFRLVLASLFQAQIQKVSNRMKIFSNFDSGSIHVVKADDKNDIQLKIPNDNMSE

FYQWFHFRLETEAEQSHTIKLLDLAKSAYPEGWQGYDVVASYDREEWFRIPAEFDGDTLT

FTVIPERSSIYFAYFAPYTYDRHLDLLHMAQSAHHCKLDTLGHTLDGNDMSLLTFGEPEE

GKKKIWMIARQHPGETMAEWFMEGMIQRLLDENDTVARALLEKAVLYVVPNMNPDGGIRG

HLRTNAVGVNLNREWQTPSMEKSPEVFLVRERMLETGVDMFLDVHGDEAIPYNFVAGSEG

IPSYDENLAALENAFKQALLTITPEFQDEIGYDKDEPGKANLTVGSNWVAEQFKCLSYTI

EMPFKDNNNHPDPLYGWSPERSVMFGQDVLAATLAVTDKI

>tr|Q87MQ7|Q87MQ7_VIBPA Putative regulatory protein OS=Vibrio parahaemolyticus serotype O3:K6 (strain RIMD 2210633) OX=223926 GN=VP2174 PE=4 SV=1

MKELISSEHAPAAIGPYSHGTSYGDLIFTSGQLPVDKATGKVVEGGISAQSHQSLTNLKH

VLEAGGGSVDTVLKTTCYLSNINDFAEFNKVYAEFFQQDCPARSCFAVKDLPLGVLIEVE

AIAHKK

>tr|Q87SN8|Q87SN8_VIBPA Uncharacterized protein OS=Vibrio parahaemolyticus serotype O3:K6 (strain RIMD 2210633) OX=223926 GN=VP0384 PE=4 SV=1

MSKVLVYSKRNREMSYLSVREERETKLNNFKVIYNEISDEEALRRANSINLDEKNNVPKV

RLKRKDIKEKKECRVPGLYGCNRYKSRKNEALNVF

>tr|Q87PT4|Q87PT4_VIBPA Uncharacterized protein OS=Vibrio parahaemolyticus serotype O3:K6 (strain RIMD 2210633) OX=223926 GN=VP1417 PE=4 SV=1

MGHWFWNENLMDKSTMHDYEVFHRSKYYQGNNRLMQANKERMMEIQPDLVVDFCQCPRLF

FAEKYADILSDMEPLGIERIKNPYRPHLDAEVQIDIGDEEEHEIIDDVDYVFFHVFNRYD

VLDNEATVWNCETPSIGAVVTPVLDESKLHAIPLQQRLIFKLDQDPSFMFVHDSVVDAML

NANMQDIWVRWAD

>tr|Q87QY7|Q87QY7_VIBPA Isocitrate dehydrogenase OS=Vibrio parahaemolyticus serotype O3:K6 (strain RIMD 2210633) OX=223926 GN=VP1011 PE=4 SV=1

MPTEKPTIIYTITDEAPALATYSLLPIIQSFTASSGINVDTRDISLAGRIIANFPEHLKE

EQRIGDALTELGELAQTPEANIIKLPNISASIPQLKAAIKELQAKGYDLPNYPEEPSTYE

EKAIKSAYDKIKGSAVNPVLREGNSDRRAPLSVKNYAKKNPHSMGAWSSDSKSHVSSMAG

DDFFGSEKSTTISGATEVKIEFVGADGSVKELKSAFPLLDKEVIDSSVLKKKALVEFFEK

EIADAKEQDVLLSLHMKATMMKVSDPVIFGHAVKVYYKDVFAKYGKLFEELGVDVNNGLG

DVYSKIESLPAAQKEEIEAAIQAVYQTQPELAMVDSDRGITNLHVPSDIIVDASMPAMLR

SSGQMWGPDGKQKDTKAMIPDRCYAGIYQAVIDFCKEHGAFDPTTMGSVPNVGLMAQKAE

EYGSHDKTFILDAAGTVRVVDASGAALLEQAVEEGDIFRMCQVKDAPIQDWVKLAVTRAR

ATGVPAVFWLDENRAHDAELIKKVNAYLPDHDTTGLEIKILAPVEACKYSLERMKAGLDT

ISVTGNVLRDYLTDLFPILELGTSAKMLSIVPLMNGGGLFETGAGGSAPKHVQQVEKENH

LRWDSLGEFLALAASLEHLSSVTGNAKAQALADTLDKATGEFLDKNKSPSRKVGELDNRG

SHFYLALYWAKALAEQTVDADLATEFAPIAKSLAEQEEKIVAELNSAQGVKGELGGYYLL

DDALVSKLMRPSETFNAIIDK

>tr|Q87J16|Q87J16_VIBPA Uncharacterized protein OS=Vibrio parahaemolyticus serotype O3:K6 (strain RIMD 2210633) OX=223926 GN=VPA0439 PE=4 SV=1

MIVFKGLDMTYVNNSKLKAKLACALEYSDLSRRQMAVFDNACRLSGVGRGLSRSRIFRLL

QTLDTLTYDSLERRLNVSLKALSRESYSKRTIALYLSVCRKVASQLAQMYGIPRHETTIP

ESEFQYRPSVSDKRVFTVAERMLPKGAKYLIVSMSTLEEALRLKPRTIRESLKRLDHHGY

IVCLGGDEGANYSITFKRSIHATSALSLQAA

>tr|Q87MT7|Q87MT7_VIBPA Putative transcription regulator protein OS=Vibrio parahaemolyticus serotype O3:K6 (strain RIMD 2210633) OX=223926 GN=VP2144 PE=4 SV=1

MLITSPKQLGIYLRDVRRNQRRNQTKVGDIVGLKQTTVSKLERDPASSSIESLMRLLSSL

DLELHIQDKAVSREQAKLRDPDDW

>tr|Q87NM5|Q87NM5_VIBPA Uncharacterized protein OS=Vibrio parahaemolyticus serotype O3:K6 (strain RIMD 2210633) OX=223926 GN=VP1843 PE=4 SV=1

MILWEEESLNDREEIFEFLYDFNPDAAEKTDNLIEAKVENLLKQPLMGVQRDGIRGRLLI

IPEISMIVSYWVEGDIIRVMRVQHQKQKFPTD

>tr|Q87N70|Q87N70_VIBPA Putative thioredoxin OS=Vibrio parahaemolyticus serotype O3:K6 (strain RIMD 2210633) OX=223926 GN=VP2005 PE=4 SV=1

MSSTKQHRTFRYWLIQIVQMVLIVTAISIAMDWYRTKDIPKQETPPLSSVLSDGEYFDAI

AQSHEQPVVVYFWATWCPACKFVSPSVSWLSDDYAVVGVSGSSGPNERVAQFMQAKEYRF

ENINDPSSDIMREWKISVTPTIYILRNGEVTSITTGITTPIGILARICLAR

>tr|Q87H72|Q87H72_VIBPA Uncharacterized protein OS=Vibrio parahaemolyticus serotype O3:K6 (strain RIMD 2210633) OX=223926 GN=VPA1093 PE=4 SV=1

MSHQAWMSSNLSDLIRGLFMKKFTAAALTALLFTAVPTFANTDTNADKMTNETLLECANA

SNARNAIPEHRAVFRHYLMSERGYSFSQLSSSETEFKAQDNNDSEIEQFYQTQCKDVGEQ

LYRVGY

>tr|Q87NL9|Q87NL9_VIBPA Uncharacterized protein OS=Vibrio parahaemolyticus serotype O3:K6 (strain RIMD 2210633) OX=223926 GN=VP1849 PE=4 SV=1

MVEFSVSETDLFVESLLGDSVCLKELFGVVGSFSTALRVPSGFSDRRFETAPDLSLPKHV

KQVLAKRQSASNQSFRFGN

>tr|Q87S13|Q87S13_VIBPA Outer membrane protein assembly factor BamB OS=Vibrio parahaemolyticus serotype O3:K6 (strain RIMD 2210633) OX=223926 GN=bamB PE=3 SV=1

MKRVFKKALLGALTVGILAGCAGEEDTIVMAPLPQVDSQFTTKSEWSTSIGDGVGHYFSK

LSPVYANDMLFVASRDGLVKALDPENGKQLWQTDLEEDVIARLSGGITAAYEHLYIGSEN

GEVIALSQETGEIIWRIKVGGEVLAKPVADSGLIIVNTSRGMLIALDESSGEQRWALSTE

VPNLTLRGDSTPTAIGGGVFWGTANGRLAAAIVERGQLIWQQPVGTPKGATEIDRLVDVD

SSPIVLGGTLFTIGYNGQLIAIDLRSANPIWKRNYSSANDIATDGSRIFVITEKDHVVAV

DARSGTELWENSLLEHRQLTAPVIVGNYLVLGDTLGYLHWLDRSTGEFVAQQFVDDSGFA

VGPTLLSDGYVITTRNGDVKKLTINE

>tr|Q87PU5|Q87PU5_VIBPA Uncharacterized protein OS=Vibrio parahaemolyticus serotype O3:K6 (strain RIMD 2210633) OX=223926 GN=VP1406 PE=4 SV=1

MEKLNRAADDSMINTLSEKSNEFSFYQAVLLLEKHYQFEPNSNFVAVGENKYFHQERIEF

SVSPNLCFPKSDMEFIVHMERNGQQYSRVETNFLGLHGSSSPLPVSYTEKLTGREPDDNP

VKDFFDFFHNRYTSLLYRVWKKYRYHVQYQSGATDAFSGRMLHLAGLSGVMQDCGVAELD

RAKVLSYVNQLSTRTRSPKLISGIVSHYFSLPSVRIEEWVYRRVEIADSQRNKLNRANCI

LGQSFHLGQSIADLNGKFNLCIDDIDFETFKQFSYEGELHKTLVGLMRFILRDPMSWDLK

LTVNLDSIPNNQLGNGEGNNLGQTFWLGNPTDEDVKIRVIGSI

>tr|Q87N86|Q87N86_VIBPA Putative secretion protein OS=Vibrio parahaemolyticus serotype O3:K6 (strain RIMD 2210633) OX=223926 GN=VP1989 PE=4 SV=1

MTADQKFKHWMRTLIVLFIVLFLYIIIADRHAPLTTEGRVQGYVVQVAPEVSGKVTDVLI

ENNQSVQKGDVLFTIDDRKYKIALEQAELSLQSAYEKEATLYSQREAALANIARAQATFD

NAHREYTRLLTLSKQKVISQSTLDNAFAQNQVSRAALKAERQNLKVIEAQLGDQKGQSTA

VRIAKNGIEKAQLDLANTAVLAPSDGVVTNLQLEVGTMANTNMPLLTFVPTGSLWVAADF

REKSVANVDKTFHALVTFDANPGSVYDFDLASRDYGVAAAQQTPNGALTKVEVNNRWVRD

AQRTRVNLTSSEELPPALFVGSRATIVLYPDNSIFWQLMAQAQIHLASWFHFIY

>tr|Q87RZ1|Q87RZ1_VIBPA Putative integral membrane protein OS=Vibrio parahaemolyticus serotype O3:K6 (strain RIMD 2210633) OX=223926 GN=VP0633 PE=4 SV=1

MEHINALLISMKPLLEQYGYLALIVSIFFEGIGIPMPGQSLMIAASILASEQVMNLSMVM

AISWLSCFLGNTCGYLLGYYFEGWLDKKGYISGPKMQKLQSTIQKYGPACLVVSRFIEGM

KQFMPLACGIAKMPRREFLLGNALAATIWVLVFSLLTHFAFEHLSALSHLYSHYKYAIWA

SAAVVFALMLLGLLKRKKAKSSL

>tr|Q87N08|Q87N08_VIBPA Iron-sulfur cluster carrier protein OS=Vibrio parahaemolyticus serotype O3:K6 (strain RIMD 2210633) OX=223926 GN=VP2068 PE=3 SV=1

MHQFTSKQDFCHWFNQFQHPQLVENWADIHGVVTITAQGEVKITLPFASNELQRTLQEWI

QQQQAFKAVAAFPYHIELGVKALETQVSNAVKGVKNIIAVSSAKGGVGKSTTAVNLALAI

AQSGAKVGLLDADIYGPSVPMMLGQEDAKPEVRDGKWMEPIFAHGIYTHSIGYLVNKSEA

AIWRGPMASKALAQLLNETDWPELDYLVIDMPPGTGDIQLTLSQQIPVTGTVLVTTPQDL

ALADARKGAAMFNKVNVPVVGVVENMSYHICSQCGATEHIFGMGGAEKMSQEFGLALLGQ

IPLHISMREDIDAGVPTVVRRPDSEHSGYYKQLADRVCSTMFWQGKAKPDAISFTMVN

>tr|Q87SK4|Q87SK4_VIBPA Uncharacterized protein OS=Vibrio parahaemolyticus serotype O3:K6 (strain RIMD 2210633) OX=223926 GN=VP0419 PE=4 SV=1

MTMPVNTIMGLFAKSPIKPLQRHVVCVNECCSHLVKFFEVSSKGDWEKASEIRAQISHLE

KEADVLKREIRLKLPRGLFMPVDRTDMLELLTQQDKLANLAKDIAGRVYGRQLVIPEALQ

PNFLAYVQRCLDAANQAQKVINELDELLETGFKGREVTLVAEMIHQLDVIEDDTDAMQIE

LRQQLMAIESEMNPIDVMFLYKILEWVGGIADQAQRVGARLEVMLSRS

>tr|Q87P55|Q87P55_VIBPA Putative YscY OS=Vibrio parahaemolyticus serotype O3:K6 (strain RIMD 2210633) OX=223926 GN=VP1663 PE=4 SV=1

MLSTKDIELLLVHAALQVQYQQPDQAIAILDAVLELEPEREDALHTLAVACLQTGRYTRA

VAVCEGLLKSQSQSVQAAGIWFCLSQARWKQNDVEGARQAHRRYLQSLHSESHE

>tr|Q87QV6|Q87QV6_VIBPA Uncharacterized protein OS=Vibrio parahaemolyticus serotype O3:K6 (strain RIMD 2210633) OX=223926 GN=VP1042 PE=4 SV=1

MHPDNKAMATTGVNRINIENLGIGLSCLFFKSLAYIDKGSQVRIFLYLLLSEE

>tr|Q87PG5|Q87PG5_VIBPA Uncharacterized protein OS=Vibrio parahaemolyticus serotype O3:K6 (strain RIMD 2210633) OX=223926 GN=VP1537 PE=4 SV=1

MNPDFLGALLIGLVGAGHCMGMCGGIASLLSLGSSSSKSSPTIPLFYNLGRLSSYALIGA

LVGGAISGLSELSGLAQSLAWLRFVAAIFMILVALYIAKWWQGLLIVEKMGQHLWKFISP

AGKSLLPLKKPIHALPFGFVWGWLPCGLVYSALTWSAVSGSAVNGGLIMLAFGLGTLPSM

LAVGYGASHFQKLQKSLIFRNISALILIVYGAYTAAGAMQMLGFI

>tr|Q87G06|Q87G06_VIBPA Superoxide dismutase [Cu-Zn] OS=Vibrio parahaemolyticus serotype O3:K6 (strain RIMD 2210633) OX=223926 GN=VPA1514 PE=3 SV=1

MLKGLLCISAAAAFVMTPNALAQSITMTDLGTNQAVGTVDLSESDYGIVFTPNLSGIPAG

LHGFHVHANPSCDSAEKDGKTVVGGGAGGHYDPDNTGQHGFPWTDGNHLGDLPALYADME

GNANQPVLAPRLKMSDLKGRALMIHAGGDNHSDQPAKLGGGGARIVCGVID

>tr|Q87JR9|Q87JR9_VIBPA Uncharacterized protein OS=Vibrio parahaemolyticus serotype O3:K6 (strain RIMD 2210633) OX=223926 GN=VPA0179 PE=4 SV=1

MSKETFDATRYNQSDNKPFEEVLEASLSRRSILKGGLGISAMTAFGAFGLAGCNSSSSGT

SASNGSGVSKAVLNFDSIPGSLTDAVSIPQGYTAQVLVPWGTPLNAQGSAWKNDGSNTSS

DQLNALGMHHDGMHFFPLNDSTTDGLLCINHEYIDTSALHPNGPTVANGVRTIVDEVRKE

INAHGVSVVRIQLEDNMWKLADTDPLNRRYTGATVMDLSGPVAHTALTVTRFSPDGSQAR

GTLNNCGNGYTPWGTYLTCEENWPGYFVNAGTRTEEQDRIGVDDKSTRYLWETLAGNSEE

RLDEFTRFNVAPTGTSSADDYRNEANGHGYIVEIDPYTQNSRAKKRTALGRFRHEGCAFG

KLEAGKPVVFYSGHDSRFEYLYKFESAAAWDPADANPANRLATGDKYMDEGTLYVARFNE

DSTGTWLPLTLDSVTTSGGTLADHFNSLAEIIINTAGAADLVGATPMDRPEWCSVDPFTG

SVYLTLTNNTRRTDETNPANPRLNNKFGHVIRWDEGTSATDFTWDIFVFGSPANGDADTN

RSGLNELNQFASPDGLAFDGRGILWIQTDNGADEVTSYTNDQMLAVVPSKLTNENGAQAV

IGADNQAELKRFFVGPNGCEVTGFTISPDYKSLFVNIQHPGNWPYSDDAAQETPAGTTVR

PRAATVVIRREDGGEIAV

>tr|Q87K93|Q87K93_VIBPA Uncharacterized protein OS=Vibrio parahaemolyticus serotype O3:K6 (strain RIMD 2210633) OX=223926 GN=VPA0005 PE=4 SV=1

MSINSIDHEEMTNIAHKWELPEEEETVVKPEKTMKSAEARRRIEALREIRESGLSIEEAR

ELGLIH

>tr|Q87PE6|Q87PE6_VIBPA Uncharacterized protein OS=Vibrio parahaemolyticus serotype O3:K6 (strain RIMD 2210633) OX=223926 GN=VP1564 PE=4 SV=1

MFIPINFGDRVAENEAENLNSYFVETHAWKELYEGKKDVVFGSKGAGKSALYTLLLQKES

ELLSSRNTFLLSAEKPQGQTVFSDISSTPPTSEKEFVYLWKIYFCQLIVDYLQKNNQCKG

KANEVRDRLVEAGLIQETNNLKRLLNGAASFAKKLANIESLEGGGSLVEASVSGRITFRT

PSFEESQKGLLSVDELLVHLDEHLRSISMSCWLLCDRLDVAFDESLELEKNALRALFKVY

RDVEEYQQIALKVFLRDDIWARITKEGFREASHITRTTTISWDSKNLLNLIIRRALCDTN

LVKHFNVNTSEIMNDHNQQEKFYYEIFPKQVDLGEKKSETFEWIKSRIRDGLMNTPPREL

IHYYNEIVTQEQREQEIGNDKVEEPNIVSRAAIKNATIEVSKVRTEQTLFAEFPELKEKI

LCFENKKAEHNLSTLQEIWGEDEEVSRSIANKLADIGFFENRPARNERIFKIPFIYRPYL

NITQGKAY

>tr|Q87MG3|Q87MG3_VIBPA DNA polymerase III subunit epsilon OS=Vibrio parahaemolyticus serotype O3:K6 (strain RIMD 2210633) OX=223926 GN=dnaQ PE=4 SV=1

MNTSSNSEHNRIVVLDTETTGMNQEGGPHYLGHRIIEIGAVEIINRKLTGRHFHVYIKPD

REIQPEAIQVHGITDEFLVDKPEYASIHQEFLDFIKGAELVAHNAPFDTGFMDYEFEKLD

PTIGKTDDYCKVTDTLAMAKKIFPGKRNNLDVLCERYGIDNSHRTLHGALLDAEILADVY

LLMTGGQTSLEFNANKQEGGVETIRRIEGRKALKVLRATADELEAHQKRLELVNDCIWHQ
[truncated: 1,827,432 more chars]
