# Supplementary material for: Comprehensive genome based analysis of Vibrio parahaemolyticus for identifying novel drug and vaccine molecules: Subtractive proteomics and vaccinomics approach
Source: PLoS One. 2020 Aug 19;15(8):e0237181. doi: 10.1371/journal.pone.0237181 (PMC7444560; doi:10.1371/journal.pone.0237181)
Supplement: S3 File — (DOCX) [file pone.0237181.s016.docx]

**S3 File.** Protein sequences with >100 amino acids.

>tr|Q87IB9|Q87IB9_VIBPA Uncharacterized protein OS=Vibrio parahaemolyticus serotype O3:K6 (strain RIMD 2210633) OX=223926 GN=VPA0687 PE=4 SV=1

MSGLPTPPSTYILRELHDVAVPPSVSWYPQTIGWKILAAVALIALVYVAYRLAQQWWCNR

YRQEALLTISQIKPSDKGMPKALFSVLKIVLIHVDSRNAKLFDTAFLRKLDALYPQTGDS

QANSQVVFNDELSKRWLQSIVDPSVMLTNEERVTLIARAKNWVSEHRCDAQKNAAKKSPL

LKRKANQGGQHE

>tr|Q87LH1|Q87LH1_VIBPA Uncharacterized protein OS=Vibrio parahaemolyticus serotype O3:K6 (strain RIMD 2210633) OX=223926 GN=VP2641 PE=4 SV=1

MTTSTTLPPAGFFRRLAALFYDTLIVLAIEMMAAGVVIAVVFALNAAGLLSYGEYVDAAD

MLGKHPVLSPIFTLYLAVVWIYFFVFFWTRAGQTLGMRAWKMQIRNAKDGAPITATQALI

RLATSGFGLANLTVPIDPQKRGFHDIWAKTEVVVLPKAK

>tr|Q87TL7|Q87TL7_VIBPA Uncharacterized protein OS=Vibrio parahaemolyticus serotype O3:K6 (strain RIMD 2210633) OX=223926 GN=VP0051 PE=4 SV=1

MEMKKAHLSSRIVQSGLSLGGLLLCAQFSAPVLADDFLCQATQASDKELPMLEKSCPIGQ

GVWGKKVPQRGNDFYWIQCGLLPKPMPLAKAKPIYSKITTDVWMKPETKGYRCLIGPYTE

FSKASADLRGVKTLSNYREAFIRVVGKGSDNTVKQTQPSSKPAPVAKPNVAPKPVAPVVA

SRPDTEAFKASATKTTAQPVTAKEPKPSTTTKPVAKAKPTPSVKGNGEVEVRLRASLHGK

TYVVPYLLDNQFYMEYGKPWNRLNYESSQQICQQLGMSLATATEFKALRDSGVMEKNKWP

LQLPYWGKDKKGLFADREPNQLTGTSLLNVMCVK

>tr|Q87MZ9|Q87MZ9_VIBPA Putative maltodextrin glucosidase OS=Vibrio parahaemolyticus serotype O3:K6 (strain RIMD 2210633) OX=223926 GN=VP2077 PE=3 SV=1

MSLPFLFHSQTVDGLAFDGKRVKVCLKTEALTFDAVYVRIEPDNEEYLVAMVQCGRAGEL

LLWEVSFEPNRDRDITHYVFKVVRNGQQYWLDARGVQKRIPPKEFHFKLNVNHQPPKWVQ

EQVFYQIFPDRFAASQSESAIREAYYEYDPDAEIKMWGEPVGNHQNSGAREFFGGDLKGV

EQKLGYLEQLGVTALYFNPIFSSPSNHKYDTTDYFTIDPMLGTNEHFASLCEKIRSKNMK

IVLDAVFNHTSVHHPWFDIQQKGKGAYGNPKSDFRDYYFFDGESNHYIGWKGIDNLPVLN

FENHEVREYIYQGEASVIKHWLKPPYSVDGWRFDVIHMLGEGEGAKNNAHYVKAFRQATK

SVNPNAYVLGEHFFEATQWLQGEQEDGAMNYYGFAHPVRAFIAHQDITYDPIDIDGFEFK

AWLDEARAKVPFANQLSQLNQLDSHDTARFLTLVNGDEKKMKIALALLMTYVGAPCIYYG

SEVGLEGSFDPDNRRCFPWHLVQGSPWLHLYQQWIEIRKQFQALQSGSMQWLYCDERAFA

YARQLGEETIIVAVNIGLQESTIDLPLWQLGLNAKHLHQLLDKCEIIDYEGHVSVNMAAL

SINLWQVK

>tr|Q87GW7|Q87GW7_VIBPA Chaperone NapD OS=Vibrio parahaemolyticus serotype O3:K6 (strain RIMD 2210633) OX=223926 GN=napD PE=3 SV=1

MSLNEVHISSLVVHVLPEHLDEIKTQIETYENAEIYGDSPEGKIVVVLETENQGFITDTI

DAINNLPNVLSTVLVYHQIETALEETDEEDTGTQHSQIEGEV

>tr|Q87G15|Q87G15_VIBPA Carbonic anhydrase OS=Vibrio parahaemolyticus serotype O3:K6 (strain RIMD 2210633) OX=223926 GN=VPA1502 PE=4 SV=1

MKKSLTAIGLSLVFVGSANAANWGYEGSHGPEHWGEFASECAQGKNQSPIDIHSATQAEL

AKLQLDYQGKVVALTNNGHTLQTSIEGENVLTIDSKAFTLKQFHFHTPSENHVDGKSYPL

EAHYVHADEQGNLAVVAVFFEQGKANPALANLLENVPERDQNVAIRAPFDANALIPSDKD

YYRFNGSLTTPPCSEGVRWLVIKDPQSISAEQIAQFEHVMGENNRPVQPLNARMVLTK

>tr|Q87PX6|Q87PX6_VIBPA Putative transporter OS=Vibrio parahaemolyticus serotype O3:K6 (strain RIMD 2210633) OX=223926 GN=VP1374 PE=4 SV=1

MWLEMPNVMANQLRKSSSTMRPYLKYIIPTVIPLLIWLMPLSAFPFDGITIVQQRVIAIF

LLAALCWVFEPIPIYATSVVIIVLELLLISNKGIVLFRTQEGQPHFGELLQYHEIMATFA

SPIIMLFLGGFFLAMAATKYRLDVNLARVLLKPFGQNPKFVMLGLMLITAIFSMFMSNTA

TTAMMLSILTPVIAVFGPKDPGRIAFALCIPVAANIGGIGTPIGTPPNAIALKYLVGDNL

ITFGEWMVFGVPFVVIMMALAWLLIGYLYKAEQTKIDLNIKSKFLKTPKAMIVYVTFAGT

IILWLMGSAHGMNSYTVALIPVAVFSLTGIINKEDLKKISWDVLWLVSGGIALGLALDKT

GLAKLVVHSIPFDEFSPYVVLFGAAFLCLLMANFMSHTATANLLMPIMAALGTSMASLTP

LGGEVTLILVVTFAASLGMSLPISTPPNALAHATGHVESSQMARVGAVLGIVGVLLSFAM

VWILHTVGHIG

>tr|Q87MS4|Q87MS4_VIBPA Glyceraldehyde-3-phosphate dehydrogenase OS=Vibrio parahaemolyticus serotype O3:K6 (strain RIMD 2210633) OX=223926 GN=VP2157 PE=3 SV=1

MTIKVGINGFGRIGRFVFRAAQERADIEVVGINDLIDVEYMAYMLKYDSTHGRFNGTVEV

EGGNLIVNGKTVRVTAERNPADLKWDEIGVDVVAEATGLFLTDETARKHIEAGAKKVVLT

GPSKDATPMFVMGVNHATYAGQDIVSNASCTTNCLAPIAKVLNDKFGIESGLMTTVHATT

ATQKTVDGPSAKDWRGGRGASQNIIPSSTGAAKAVGVVLPELNGKLTGMAFRVPTANVSV

VDLTVNLKEGASYEAICAAMKEASEGELKGVLGYTEDQVVSQDFIGEVQTSVFDAKAGIA

LTDNFVKVVSWYDNEIGYSNKVLDLIAHISK

>tr|Q87IL0|Q87IL0_VIBPA Putative methyl-accepting chemotaxis protein OS=Vibrio parahaemolyticus serotype O3:K6 (strain RIMD 2210633) OX=223926 GN=VPA0596 PE=4 SV=1

MYMSEKEYDYPASYSLVSITDPSSYIKYASAHFNEVAGFEEGELIGKPHNVVRHPDMPKQ

AFKDLWSHLKAGKHWMGMVKNRRKNGGYYWVDAFASPIKYNGEIVEYQSVRFKPERIYVK

RAEKAYAKLRNDKKPLQLYLPRTRLWMRAAFFLFISNFLGLLLLGMNQSPMVAFSAAALL

SALSIYMLTRPIEKLAKQARQDFNNPLMEYIYCGKVNDLAEIELAMKMRKQFANALLGRV

GVSVKDSCEETKTNADSAADNSERVTENLSAQKIEIDSVATAVNEMQASSSEISHNAQAT

ADATLKAQNTMTDSRNVIDNVNHSVLDLVGELGEISKVVMRLNQQTDQIGTVIEVINGIA

EQTNLLALNAAIEAARAGEQGRGFAVVADEVRTLAKRTQESTTEIKSAIESIQHGSKSAV

SALEKGNEMSTGTVTLLGTALESIQQLESLIQDVVERNQQIAVAIEEQVYVTDEINENIQ

SLNLKYSESYDLMTETNQMNSRVQNCTNELSDMIKKFA

>tr|Q87NW4|Q87NW4_VIBPA Uncharacterized protein OS=Vibrio parahaemolyticus serotype O3:K6 (strain RIMD 2210633) OX=223926 GN=VP1754 PE=4 SV=1

MLNKLSLETKYIFATISVVVLVVVVNLVLYMHGYDTYSNNLAQQITQNSQDKTESYVKQK

GLVYAELLSKQLFDPLYNDNISQVYGQITATLSQPDVSKIHVVDNDGLVFHDGTPQLAMF

AQPHYRSDFILKAIEQERVQVALSREQLEIAAPIHQSDVTLGAVYLELNLAHLLEEKEQN

IAQVAAITSKDKNNMFILLLAISIISIFMGSLFAFAVGRSLVKPIKQLSEQFSEFDTQAL

PVTDIQRKDEIGELITAYNKMSNKVNTYTTRVEFMAYHDILTSLSNREKLLIDLQEQISA

KRAPTLAVLFVDLDDFKHINDNYGHNVGDKLLVHLSALLRKELPIYIEPDYRPWILASRV

GADEFVVVFPCNNSLEAREIANQIHRQVLSPLRLDSHNIRLTTSLGVAVYPEFGCNADSL

LQLSSLAAQESKRRGKDIMSVYDPAFDATVKQRLYIERELSRSIHDLSQFELWYQPKFDL

KTYQLVGVEALVRWNHPEKGYIGPELFIPIAEQNDLILDLGEHLIETAIKQRAEWAEAFD

HDFHIALNLSPRQIYRQDLSLIFEHFLSQYDVSAKDIHVEVTESLLMDDIEKAHFVLRKL

QATGIEVWLDDFGTGYSALSYLQQVNFDGLKIDRSFIAQTQQDNKDDSLVRAIISMAHNL

GMKVVAEGIETQKQLALIERLNCDVAQGFFLGKPVPAAQLLELENTCQAMKHNASSIVG

>tr|Q87MV4|Q87MV4_VIBPA Uncharacterized protein OS=Vibrio parahaemolyticus serotype O3:K6 (strain RIMD 2210633) OX=223926 GN=VP2127 PE=4 SV=1

MHQLRAGIKRIIWFLFILWCVFSATLWFQAKQTMQTLSTLNELGSKVEEVRNFFNFELPY

RVKHVDQVSLKLQLVYAVRLQLESEVSDANGPDVTQLLYSTDRFLESARAFIGSDGELVS

LAEQLHTSRGAENNSQQIENMYYRLGALVLESIFSDSNTNTDTYRELDLLFIESDSLSTG

ERSAFQRRLAQTSSVLAANAQGSYLANQLLKPDFPNQLVSMKATLEQKLVSFIFWLIVVS

GCLLALVSWAVFSKNAVANSSSSEPAVSQTENASSSLEHENKARELASDAQANKTQELAP

DSRQESLAPQEPFIDINRMLDSLSGDEGAVRMLLEVFIQDHAEDGSKLHKLLNEDIDNAQ

RAAHSLKGVSGSLGAMPLHYISGEIELLIKQGKEVPDNKLCQLRDVLQQTTLFAEKVLNS

EKIREVLTD

>tr|Q87FM5|Q87FM5_VIBPA Putative ferrichrome ABC transporter (Permease) OS=Vibrio parahaemolyticus serotype O3:K6 (strain RIMD 2210633) OX=223926 GN=VPA1654 PE=3 SV=1

MSASIISESPSSVSKSHWKASLFVCAMALCTLSGYATSMIGWSNFSLSVNDLTSYWFAFD

EGNMLHQILATLRAPRAYAGVLIGASLAVSGVLMQGLTRNPLASPSILGINAGAACFMAL

ASIGVPFFSQLNPIINAVFGALLSGGAVMLLGGFFSARSHPLRLVLAGIAISALLIGLTR

ASVILADDMAYSVLHWLTGSLSAVDSQQWLQLWPLATLGLVLAMGLAGNLNLLALGDEVA

VGLGGNIRLTRLISGLAVVLLAGTSVAIAGPIGFVGLLVPHLVRPIVGHNYHILIPVSAL

CGAALVTWSDALSRAIAFPAETPVGVITALLGTPCFIVIAMRKSS

>tr|Q87MM1|Q87MM1_VIBPA RNA polymerase sigma factor OS=Vibrio parahaemolyticus serotype O3:K6 (strain RIMD 2210633) OX=223926 GN=VP2210 PE=3 SV=1

MEAPVAILKMFGKKNSNSPVNSDMDKQRKYEALVRGYHRDLYRYAYWLCKDKAIAEDLVQ

ETCLRAWKSLDSLQDEKAAKSWLITILRRENARRFERKQFDLVDIDDYGNDAKVSDDPHH

QQEWLQAQIMRLDVEYREPLFLQVIGGFSGEEIGDILDLNKNTVMTRLFRARNQLKEMLD

SQDTKRGQKNG

>tr|Q87IP4|Q87IP4_VIBPA Putative chemotaxis transducer OS=Vibrio parahaemolyticus serotype O3:K6 (strain RIMD 2210633) OX=223926 GN=VPA0562 PE=4 SV=1

MSLYLRLIVVRSLWMNLTISGKLQLSFVLLAVVFIASAIFTYRNINIVEQHASSLLSSDL

PTVDTSRGIQQSVQASLSSIRAYMLLGSDEAESLRLSQEVQSIMASTDDALPTLQALISE

SDFQSIHQQWEAMKVSLSQLIELSHSAENLPAHNLFINEAAPIAEVALDQIQSLINEESG

NEMGGERKRLFKVYADSYTSLANALSALRDFLLYGQQTHLEKYQDLIKFHNQSVAEIDAK

LDMLSDNDQSLWSLFKEMQQMYFPLADQVITLRQSDEWNKANLLMANELLPAANALNQSL

ETIVLAQQAKANQSGIGISQSIKNVLTSMLVAALLVVIAAIAISKYMGNTIGRRVQRLSK

RAKIIASGDVSQPPLNVVGKDELADLTSSINRMNESLAGIVAGVNGKANQVDTSMGALLE

SAQVTSNNVEQQQSNISEMGRQLEEIAFAASNTLDQANQSVQKLSDSKDEIEQGRDALEQ

NKATMESLHSSIESASTQVTQLSKESEAIGRVTEVIEGLAEQTNLLALNAAIEAARAGEQ

GRGFAVVADEVRMLATRTTQSTTEINGIINAIQRSTHTVVQEIEHSQSLAEQGAEHIDNA

VTRLVSTTEQIGSLNEQMAELAAAAEQQSHATQSINGLMAGITQSVEMVASNSQHASETS

LQVKQEVTELNQQMAMFKTA

>tr|Q87P64|Q87P64_VIBPA Probable membrane transporter protein OS=Vibrio parahaemolyticus serotype O3:K6 (strain RIMD 2210633) OX=223926 GN=VP1654 PE=3 SV=1

MFMLLESALLFAAGVVGGIMNSIAGGGSFITFPALMAVGVPPIMANATNTYAACAGYISG

AVGFREEILKNKQELLFTVSFSLVGGAVGAYLLLNTPESLFLEAIPWLLLFATLLFLTGS

RLTMLIKSITREHKHAGVLGLIALSLLLVGVSAYGGFFNAGLGVIVLSYLVVAGHQDINL

MNGLKLLVSTCVSLIAIVIFVANGSIDWSKGSMVMVGTLVGGYLAARVSRQLNPSHVKGF

VALSSIIITIYFFIDVYA

>tr|Q87G72|Q87G72_VIBPA Putative secreted calcium-binding protein OS=Vibrio parahaemolyticus serotype O3:K6 (strain RIMD 2210633) OX=223926 GN=VPA1445 PE=4 SV=1

MSTSDNDTYLGDAGANNNIADFDGGDDLFIGYGGNDSFVGGGGDDYIIGGAGNDRLTGGA

GDDVLRAGTGNDYLGGGTGNDLLLGLLGDNKLNGGVGEDILVAGSGDNVLVGGSDSDKFV

FTDKFGGHGEAKVVDFTIGEDSLHIISDTVTEFGDLTFSYDAAGNAVFTDGADLTVKLIG

VTETDINTYGADLFVI

>tr|Q87FI9|Q87FI9_VIBPA RNA polymerase sigma factor OS=Vibrio parahaemolyticus serotype O3:K6 (strain RIMD 2210633) OX=223926 GN=VPA1690 PE=3 SV=1

MEKQKRYEALTKERYQDIYRLAYWLCKDQHIAEDLAQETFLKAWRFFDNLNDPNAEKSWL

ITILRRENARRFDRKQYDFVDIDECAIQTKTYPGSDHDMAQDWLQKQMMILKPKYREPLL

LSLVAGLNHDEIAQALRLNHNTVATRLFRARNQLIAQQGNH

>tr|Q87MK3|Q87MK3_VIBPA RNA polymerase sigma factor FliA OS=Vibrio parahaemolyticus serotype O3:K6 (strain RIMD 2210633) OX=223926 GN=fliA PE=3 SV=1

MNKAITYDQHGKFNSQQAFIERYSVLVKRIAHHLLGRLPPSVQVEDLIQAGMIGLIEAQQ

NYDGSKGASFETYAGIRIRGAMLDDMRKGDWVPRSVHKNNREINQAVAELEGVLNRDPTD

SEVAAHLGMSLDQYHSALTDINCSRLVGIEDLGVSDDVISPADETDDSNPFKGVADESFR

KALVESIKQLPEREALVLSLYYDEELNLKEIGDVLGVSESRVSQILSQAMQRLRTKLSAW

TQND

>tr|Q87IU5|Q87IU5_VIBPA Methyl-accepting chemotaxis protein OS=Vibrio parahaemolyticus serotype O3:K6 (strain RIMD 2210633) OX=223926 GN=VPA0511 PE=4 SV=1

MKFRHKVVTASSILLLITVSLLSTQQVMTIRSQTQEHINSSVKEILTSVSNTVQSEMNAK

KDLARSITEIIELSPNDRTYVKDILEKPTPKSSFLAIGFGYESNGYVIENDDGWDAGPDY

DPRQRPWFIAAKNKGDLVVTDPYVDASSKNVIISVGTPVKQNGQFLAGMFYDLELTTLSD

LVNQVNLFDAGYLFLVTDDGTTIAHPQSKYNGEKLNSYLPQVDLNKATQHIEVDNNPYMV

SLTHIPSENWYVGAIIDETAAYSVVGELRNSAIIYSIIAVLASVIALTLLIRTLMRPLDT

LNTAIKDVASGKGDLTQRLETDTDQEFSELAKNFNTFMENLQQQIIESKSISDQILTGTQ

ITAEGARDSAGAIQTQLQELEQLATAMHEMSVTATEVANNAQGAASAAKEADQATIEGSS

VVSESTQTINMLSDSIDLAVEEVQVLESATANIETILKVINDIADQTNLLALNAAIEAAR

AGESGRGFAVVADEVRTLAQRTQESTTEIRSMIEQLQSGASSVASAMHQSKGSAVEAVEK

ADLANDALQRIRDAIQRISDMNLQIASAAEEQSLVAEEINNNTVNIKDLSTQVADSANRT

NEAMQSQHDNVRKQDEILNRFTV

>tr|Q87GW8|Q87GW8_VIBPA Ferredoxin-type protein NapF OS=Vibrio parahaemolyticus serotype O3:K6 (strain RIMD 2210633) OX=223926 GN=napF PE=3 SV=1

MVDLSRRRLFARKTHTDDAVRLPWLARPEQFTDGCTRCGKCIDACETKIITKSDGGFPSV

DFSIDECTFCYQCADVCPEPLFLAETEQPWQAKAVINDSCLAKQNVECRSCGDMCDPMAI

QFKLELGKVAQPNLNLDECNGCGACVSVCPTSSINVSNITA

>tr|Q87KD9|Q87KD9_VIBPA DNA topoisomerase I-related protein OS=Vibrio parahaemolyticus serotype O3:K6 (strain RIMD 2210633) OX=223926 GN=VP3038 PE=4 SV=1

MSSKIDNTLFSAHEHALEHEPCPKCGSELTLKHGKHGPFLGCSHYPSCDYIKALHNNDGH

IVKELGVPCPDCGNELVLRQGRYGMFVGCSNYPECHHIESINQPEPEKQSQEHHACPECG

KGHLVERKTRFGKTFYACDNYPKCKFAVNLPPVKGRCEECGFTLLVEKKLASGVKLQCAN

RKCQHTQQG

>tr|Q87MK6|Q87MK6_VIBPA Uncharacterized protein OS=Vibrio parahaemolyticus serotype O3:K6 (strain RIMD 2210633) OX=223926 GN=VP2226 PE=4 SV=1

MSSNSVLSSEQALDDYFSALLGEEVEVTDLEHELDLLAQELSSVEPEPEPEPEPEPEPEP

EPEPEPEPEPEPEPEPEPELQMQYAESDSYQFRENYAPQLEAPNLEDVQRLLSQLESTNV

VDELDIDDILEQNTIDIAIHAKSIQSDVEVAVESPIEVVEEIQEWVIEEPVSEVIQTEVA

QVEAVEVVPEIEQDIAIEQQTETQLETEASPAIPNTWEGTQRSEDFQVLYFDVNEVTFAV

PLDELGGIHRITELNHLIGRPAWYLGLQTNREQQFDVVDTAKWVMADKLRGDEYKDNYQY

VVMLGESMWGLASNQLLGTETLNGDNVRWREQAGKRPWLAGMVKEKMCALIHVQALIDML

NAGLDVKSLN

>tr|Q87ID1|Q87ID1_VIBPA Histidine kinase OS=Vibrio parahaemolyticus serotype O3:K6 (strain RIMD 2210633) OX=223926 GN=VPA0675 PE=1 SV=1

MLLATASIGRKLLLSFIAMAMLVMLSALIGVSGFSLVAKTERNVVDAAIPAMIEARQVSE

LSTRIISSVQMLSNAQNEQERKEAGRVLFEQLESLLTHIKELGGESFDSKLLDALESNVQ

NVINNLAELGVTVERKLWLAKEIDTRVEEMRLLSEELEQLTRTQVQNTSTIAVANVTHIY

DLLEANKKDQVYQALDALVEVDLDLTERLHELHLLAFKMLNQIEEARTLTNVDRIQQIQT

AFENNLKIMKRRVLAVEDPTRSKQMSQLLTELGKRQVVFTILLQQYENNEQSQQLMQKTL

ELFSELNSTVNKLVDDSNKTTTFAVDQLTNTLKFAQWSLTVISIVGLIVVVLILWRVVYV

SVVKRLAEYSAALLSVAQGNLAVELEVKGKDELAHMGQAIITARNTAQALKVVAEGEAKA

KRELEEHKEHLEELIEQRTSQLRQANLRLNEEVVNHAQARNEAEQASRAKSAFLATMSHE

IRTPMNGVLGTARLLMDSGLNPIQKRYAEIINRSGKTLLAILNDVLDYSKIEAGHLEIRR

LGFDLHQMVEDTFQLMNSRAQEKQLLFSYHIESDVSRYWKGDVTRISQVLNNLVGNAIKF

TEDGEIDIYVSLNPEDESQVLFEVSDTGIGISKKDQKTLFDAFTQAEGGLNQIGGTGLGL

AISKRIVEAMGGVLEVDSEEGEGSRFWFSIPLEESEPVEIGVVASARCKVKAKVLLVEDN

EVNRVVAEGFLQSMGHQVVMAEDGLQAERIIDKQDFDIALVDINLPDCDGTDLIQRLKRI

ERNKPGDKALSPTPMIAVSAHVFAEEVERYLAAGFDGYLPKPVEKEALATLIQDVLDGKQ

LLLPQSGECLPLSETSDTNLTIENQVEQDHQQREEPEMVIINPSVIQSDMKILGREKMLH

IIDLFRNTSADVLGQLVESAEKNDSLAVKNLAHKLKGSAGSLGLTALMNTCQSIEIAAEP

LDTYNAQQGLLDEQVATSVNALDELMAE

>tr|Q87MF6|Q87MF6_VIBPA Nitrogen regulatory protein P-II OS=Vibrio parahaemolyticus serotype O3:K6 (strain RIMD 2210633) OX=223926 GN=VP2299 PE=3 SV=1

MNMKKIEAIIKPFKLDDVREALAEVGITGMTVSEVKGFGRQKGHTELYRGAEYMVDFLPK

VKLEIVVTEDVADKCVETIIETAQTGKIGDGKIFVTDVERVVRIRTGEEDEDAI

>tr|Q87FE4|Q87FE4_VIBPA Uncharacterized protein OS=Vibrio parahaemolyticus serotype O3:K6 (strain RIMD 2210633) OX=223926 GN=VPA1735 PE=4 SV=1

MTLYKKLVVGMVTVFILLMASVFVIEFNTTRTSLEQQQRSEVNNTINTVGLALAPYLKDK

DKVAVESVINALFDGSTYSVVRLTALDSDYQIVRSYPVKPSTVPQWFIDMNLFKAIHDKR

VVTSGWMQLAEVEIISHPGAAYEQLWQGFIRLLSAFSVIFLAGLIAISYILRRSLKPLAA

IVKKMHDIANNQFGEPLTRPKTKDLIAVVDGINMMSAQIELSFKEQAKEAQRLRAQAYLD

PISKLGNRSFYINQVDQWLAEQTQGGIALLHAEYIGDIYETKDYQRADAHVKELSQRLKN

TIDVPGATIARLSSDEFGLLFPHMDESELRILADSIVNCVNGLNTDPTGLAKPKASLGVA

HSKDQKTRSEVLSMVDNALSKAKAQPDKPYGFIGSDTPSNLMGKQQWKALVEEAIHSDWV

KFRFQAAKNTWGKVFHNEVFSSIEKDGETYRANQYLFALEQLDATNIFDQYVIESMIKML

ESGELNDPVAINIAQSSLSQPSFIRWTTNMLEKHSKVASKLHFEIPEECFIDHPHHTALL

CNAIRQSGAEFGVDNYGRNFQSLEYIQEFRPNYVKLDYLFTHNLHDEKQKFTLTSISRTA

HNLGITTIASRIETQIQLDFLTEHFVEVFQGFIVDKE

>tr|Q87I60|Q87I60_VIBPA Chemotaxis protein CheV OS=Vibrio parahaemolyticus serotype O3:K6 (strain RIMD 2210633) OX=223926 GN=VPA0746 PE=4 SV=1

MSNVTSTILTESGTNELEIIEFHLEKQLPDGSTKTCYYGINVAKVREVIRVPETTDYPNA

QPHMIGVFSSRDVLTPLVDLAGWLGVPTRQDLERKFVIVTDFNKMTNGFLIDSISRIHRI

SWNDVESPSQFLEAGEQDCVVAVVRKDGNLIMILDFEKIIADINPELSMEKYDVKVDKSV

DLNQRMVGKRNAKTIMVVDDSAFIRSLIQDTLASSGYNVITCKDGGEAHEKLMSLIEVAK

EEQLPVRELLDAVVTDVEMPRMDGMHLVKRLRETEAFRQMPIIMFSSLMSEDNRAKALSL

GANDTITKPEIGRMVNMMDKYVFA

>tr|Q87I70|Q87I70_VIBPA Putative sensory box sensor histidine kinase/response regulator VieS OS=Vibrio parahaemolyticus serotype O3:K6 (strain RIMD 2210633) OX=223926 GN=VPA0736 PE=4 SV=1

MFSILRKIIILTATVTSLAVSFNALAKENTKDSIVVGVITQNDSSNSVVDSVGPFYGINL

DYLSNIAKVLGLNLELRAYQHIPPLLNDVENGVIDGAVGFSKTPEREKRFLFSKPFFSST

IAAWYRDASYKDRDIRDIKWVCVEGSVYCDNLTSQGIDNIIYVKTRLEAFDDVRRGKANA

LIYTYVGITEYLDANDIVKGIVDIPNWLQEEEVSFIASLDNQKLIDNIDKILEWEQSGKN

IRSVASKNPYHINDKLLVAYRRNHKSNLTITYSSSDEAYPFLYRDSHTGKLDGFFPDFID

LIQSRTGLAFSYVKPTSSLSNGLTAFDADIVPVSYVGPVPKSDWLITKPFMHSNYVSIQA

EESERDKSSPEKAGILLSLKKQGLVHLGVWQQDRFDRYDDLKQLLTDLKSGVLDVAYIPD

DVVHSMIAQDQVDGLVINEQDVLTFSNAFAVAKHNTQLQHMLNSIIETIDSNEIEKLLRS

HRSFNLVYGYDDEQMAKFFLAGAVAFSLMFAAAYFILAHLRLKVKLAELNANNEEAEKQW

LMGIIQEINSLVFIHGEDNQLEMSNCANYKSKQCQECKLKSCSTNAPLVNNSDELATVIG

GQRISEEVASAGCELGIKHVYRERKTIASPSGKKKFVLTVIQDITQQKEREQALIDAQKE

AQTAVRARENFLATMSHELRTPLSAAHGILDLLNRQVTADSNRELIAQAMRSLNHLNVLV

DEVLDYSKLEAGQLTVAPAKTDLLKTLCDVFRSFEPRALAKGLDYKVTIKPFSDAFIEID

ALRLVQITTNLLSNAVKFTAEGEIGISVILHEKQLILKVADTGIGMTDGQLEGILNPFVQ

ADDSITRKYGGTGLGLSIVDRLIDCMGGVLCIDSQFGLGTTITVKLPIERCDSEPEVRLN

WTYSEALPLSIRQWCDAWKMQPATLARSMANLYAAQDNEAGFDGVMLRDDREILGSLTLR

ELQYPDALFNLLSQTQQETKLDELKSEEVSWILGTVLVAEDNPINQSVISMQLRELGIEP

VIVNNGREAWEYINQDENVVLLLTDFHMPEMDGYELIKHVRASGFKSLPIIGVTAEDARL

ASERTQDIGIDDVLYKPYDLNKLKTVLVPFIGKKEKTRLPDWIKRFKTQDAKEIARVFSQ

SMAMDIENLQAAPTERDKKRVIHGIKGAVGAIGISMLTELCIEAERVTAAEFDRRAAELI

LRIEQEIDNIDYWAEMNEYTA

>tr|Q87T87|Q87T87_VIBPA Methyl-accepting chemotaxis protein OS=Vibrio parahaemolyticus serotype O3:K6 (strain RIMD 2210633) OX=223926 GN=VP0183 PE=1 SV=1

MVLKSRNKLLLITLIPLLLITTLISVVYYINSSKSLEAELVRDRQELIDARKKELKAYMM

MGVTAIKPLYDSDVNGSNKQAAKEILKAMRFESDGYFFAYDSQGINTLHAIKPSLEGKNL

YDLKDENGVAVIAGLIDASQKGDGFLYFSWHKPTINAQAPKLGYAEYLQKWDWVLGTGIY

IDDIDQQVAMQRELRTQELNQHTLSAVTISVIGLIITSILTSIAVSKGIKPLQHVADSLK

DVAAGGGDLTARLKVESKDEVGEVAAAFNEFMDKLHPLMQDIHQSASAVQTVSEELNDQT

RTASGQMQSHCLETDKVVTAVTEMSMTAKEVASNTNATAQAIDDANDQVTEAQREVEQAI

QGITELVTEINSTSDAISELSQQTEQITKVLDVIGEIAEQTNLLALNAAIEAARAGEQGR

GFAVVADEVRSLASRTQNSTHEIGDMLKQLQSGVSRAVTTMSASQERGVKTAEESALIQQ

SLSGVHHSIGTIRDMGIQTASAAEEQSAVAEDINQNLVAIQQIVNNINDTLQHAETISTQ

LSQSSTEIHDLVGNFKL

>tr|Q87HE3|Q87HE3_VIBPA Putative exonuclease SbcC OS=Vibrio parahaemolyticus serotype O3:K6 (strain RIMD 2210633) OX=223926 GN=VPA1022 PE=4 SV=1

MKPIKLTMQAFGPFAKTETIDFEKLGSNALFLINGPTGSGKTSILDAICFALYGETTSNE

RQGIQMRCDLASPQLLTEVILDFSLHGKVYRVTRAPEQEAPKARGEGLTVRKHTASLYEL

GETEKLITSKTTQVKTEITNIIGLNETQFRQVMVLPQGKFRELLLATSKEREEIFGQLFE

TDIYKKIEYALKDKASSISKAKDEFDNQIRGALQVAGVTSEAELTEQRETLSVQFASAQK

REQESLAQLNAVKTEIQKAETLSSEFKKREQAEAALHLHLQQTETISARQLQLDNAKKAS

RIELPYITLQNAAQQVQDLNQKVAVLSQDLKAADDVLKTKEQAFQQAKEQANRVPTLTEQ

QYQLESMKSKLVEKVELDSAIAAGLQQKSEFEETLKKYIVFREKLTQDALQAQKVLEQAR

IDVASIGGVDAEIKQQQRLMSDLQKLGNLNQELSKLDALTSSKQASVTQAKSYYENLQRE

ADALELSWHNAQAAVLALRLQTGEKCPVCGSTEHPQPAQFVGDEVTKEQVQQARHQEREA

QITLNQLSNQLEQHNIAIAQYTQQIEQMVSELGQNANLELQALQASIHQLNERLQHLNAI

NIAQLEQSVNELNQRCVVGEGKINDLQNQMAANESTIKANQQQLAKLSASLDDKYTSLEQ

LENDLTQIHKQIVELNNGLESAQAQLQQAVLAKSNVDSQLTTHQQWLLEANSKLENAKTE

WESALKASAFADEAHFLQSKVSEQQTQTWQQEIDNFKQTKIQLEQTLADLNQALKAVEKP

NLEALNGSLSTAQHSYLEIRNQLDSTRSLFEGLEKVRNEISNLHDKNRKLEEEYKVFGTL

YDVASGKTGSRISLHRFVLGVLLDDVLIQASQRLSLMSKGRYILARKTEGFKGAAGRGLD

LVVEDGYTGKMRDVATLSGGESFMAALALALGLSDVVQSYSGGIRLDTLFIDEGFGSLDP

ESLDLAIQTLVDLQQTGRMIGVISHVSELKEQMALRIDVEPSRFGSTVSVKSQMLISK

>tr|Q87QU2|Q87QU2_VIBPA Tol-Pal system protein TolQ OS=Vibrio parahaemolyticus serotype O3:K6 (strain RIMD 2210633) OX=223926 GN=tolQ PE=3 SV=1

MTADISILDLFLQASLLVKLVMLTLLGMSIASWAMIIKRSKVLSQASKNAEQFEDKFWSG

TDLSQLYQKVKGRKDEIAGTEEIFFAGFTEFARLRKSNANSPAYIMEGTGRAMRVAVARE

VDELETSLPFLATVGSISPYIGLFGTVWGIMHAFIALGEVKQATLSMVAPGIAEALIATA

MGLFAAIPAVMAYNRLSNKVSKLEHTYATFSEEFHSILHRQAMAGRDSADKE

>tr|Q87GW4|Q87GW4_VIBPA Cytochrome c-type protein OS=Vibrio parahaemolyticus serotype O3:K6 (strain RIMD 2210633) OX=223926 GN=VPA1201 PE=4 SV=1

MKILKAFWKRLKNPSKAAAGVVLFLGFAGGLLFWGAFNTGMEATNTEEFCSGCHAPIVKE

IQETIHYSNRSGVRAICSDCHVPHEWTDKIVRKVQASKELFAHYVLGTIDTPEKFQARRG

HLAEREWARMKKNDSLECRNCHEFDYMDYSQQGSRAAAQHSTALASGDKTCVDCHKGIAH

KLPDMSGVEGWQ

>tr|Q87JM7|Q87JM7_VIBPA Carbonic anhydrase OS=Vibrio parahaemolyticus serotype O3:K6 (strain RIMD 2210633) OX=223926 GN=VPA0221 PE=4 SV=1

MNKTLLAFSLSLLTLSAAQASEWGYGNDKHGPEHWGEIAKDCATTKNQSPINIENPADAK

LEALNPSYTGQVIGLTNNGHTLQAQVNGRNSFTIDGETFELQQFHFHTPSENQIKGRQYP

LEAHFVHANEDGELAVISVMFDAGDQNAALSKLINAIPQENQTTFFKDTFEINDLLPKTA

NYYRFNGSLTTPPCSEGVRWFVLKDTQTLSKDQAAKLMEVMGQNNRPLQPLNARVVLSN

>tr|Q87J31|Q87J31_VIBPA Putative hemin ABC transporter, permease protein OS=Vibrio parahaemolyticus serotype O3:K6 (strain RIMD 2210633) OX=223926 GN=VPA0422 PE=3 SV=1

MLLRRIPLSTTLITLSGFLAFIAIASITVGPMNISFTDSLRSLVGAHSELAPHIQLVINE

IRLPRTILCMFIGAILAICGVVMQGLFRNPLAEPGIIGVSAGAALGGAFAIVVFAEFSQN

HPQLMNLAALPLFAFLGGALTTVLVYWLGTNKFGTSVTIMLLAGVAISALSGAAIGFLNF

SADDQMLRDLTLWSMGSLAGANWAGIGLSSVTLVVLLFWFHKKAMSLNALLLGESEARHL

GVPVQKLKRQLILLSAVGVGVTVSICGAIGFIGLVIPHLGRMLAGPDHRTLLPISALLGA

LLLTCADMIARVLLTPAELPVGIVTALIGAPFFIYLLFQQRGKIL

>tr|Q87SB2|Q87SB2_VIBPA Peptide chain release factor 2 OS=Vibrio parahaemolyticus serotype O3:K6 (strain RIMD 2210633) OX=223926 GN=VP0512 PE=3 SV=1

MEEKLAQLEFRRMFSGDHDSSDCYIDLQAGSGGTEAQDWTNMMLRMYLRWAEAKGFKAEV

IEVSEGEVAGLKSATVRISGDYAYGWLRTETGVHRLVRKSPFDSGGRRHTSFASAFIYPE

IDENIAIDINPADLRIDVYRASGAGGQHVNTTESAVRITHVPTNTVVQCQNDRSQHKNKD

QAMKQLRAKLFELELQKQNAEKQANEDAKSDIGWGSQIRSYVLDDSRIKDLRTGVENRNT

QAVLDGDLDKFIEASLKSGL

>tr|Q87GX6|Q87GX6_VIBPA Methyl-accepting chemotaxis protein OS=Vibrio parahaemolyticus serotype O3:K6 (strain RIMD 2210633) OX=223926 GN=VPA1189 PE=4 SV=1

MTIRQKLYFLGVIAILGIVTLLGTSSHFANQSNELNHAVKLVGDLEIRLLNLRRNEKDFL

LRSNVKYLDKFDSNVDKFLSTEKELSQILNRYELPSSQRFKQDLLAYQKGFQALVSASQK

FGLDKESGILARYENLLLEAKKSADHQQILSLIQFDNAVKMGEFDSSKLSDLYVPELLES

AKQLAAQKQVIGVAYNKGLLGETRALSHAVEEQFAAFSSSIDSAATQRDEKMASIKQAIT

AFILVVIFALIWQISRSINVRVGSLLATIKNISESNNMGLRSDLAGKDELFDISHHLNDL

LEKLERLIHNTQEKSMQLTASTDNMHRELEGVMEQFHAQTDHTASMATAVQQMVATIGEI

SESTSVAVEGVHQAATNAEQGRSVVEMTVTNVGQLTGILSNSQQSIGSLNQHVDKIGGAV

NIIQDIAEQTNLLALNAAIEAARAGEQGRGFAVVADEVRALASRTHQSTEEITRVVMDIQ

AQMSMVVSDIDQCNEQGQQTLSASEQLDASLQQIITDMHAIQGNSERIASAIEEQGIVMN

QVSDSITELNVISENNMHSAQECLHEVNSVSAQAHDMDEAVAEFKTRLK

>tr|Q87ID8|Q87ID8_VIBPA Uncharacterized protein OS=Vibrio parahaemolyticus serotype O3:K6 (strain RIMD 2210633) OX=223926 GN=VPA0668 PE=4 SV=1

MSTQTVPQNAIVNAVKGEVLVLDLTGKIRVVSAGDALNTGDVIVTENNASLDVLINNELY

LVDQNCVACLPEPSSEQPETLVQTPVDGQIAFDPTAIDSANFDANDVAAIQQAILEGADP

TAILEATAAGGDASGSANAGYVTVEYNNPEMLASTFFETSATRTGVQDREETDDINVTIF

ADGGQSLESEVTEGSISLTTYPQSIATSVSVEAGDLPLDPSSFVPSAASLESLLAELNTD

IQSGGKAVEFSYDEAQNAIIGVQDGNEVLRIEIEATSLGRDIELEVTTTISQGIDHIASV

DDGQVSIVGDRISIEFEMTGTDIGGNSIRTPIDFVTTIIDGDNPAPQDISFENEESSSTR

ITGTFVEIGSDQLASVSFNQESLSQFDGLLTDNQVTVATLSEDGSNITLTVAGTGEVVLS

ITLNTDGTYQFEQFKPLEQTNDSDTIALSLPTTIVDFDQDTVSNTFVITIADGDNPVINN

VTSLSLDESGVEQGSLQGIAITSGTGSISAAAGSDIIDHFEVEPTEFNVSGELQSQGQNV

LLELTSDVNGVRTYEGYIELDGVRITVFDISIDSPYQGEYQFNLYEQLDHTGANDESLTF

TIPVYAVDADGDRSSLTLGSNDAKAAEIVIEVKDDAPSIDGVEALTVDEDDLASIGSDQN

DSVSVDGKFTTTEGSDRVVSYQLDASTNPIDGLTSHGEAVELVETANADGSFTYTATANG

NSVFTLVVNTDGSYNFTLEGPIDHASGSDALTLNFPITATDFDGDTSTMVLPVTIQDDVP

TIENVVPLTVDENDLITSGGQSNALLVEGQFTTTQGSDGVVQYQLETGSDPLNGLTSNGQ

VITLAEVSNADGSFTYTATANGNPVFTLQVNSDGSYSFELQGAVDHAPNSDTLTLDFSII

ATDFDGDTSKITLPVTIVDSLPVVKDFETISVDEDDLAGIGSDQNDPVSIDGQFTTTEGA

DRVVSYQLDSNATPVDGLTSQGVAATMTETANSDGSFTYTATAGTNPVFTLTVNPDGSYN

FTLEGAIDHAANSDSLTLNFPITVTDFDGDTTSAVIPVTITDDQPTITNVEAISVDEDDL

ANIGSDQNDSLTIDGQFTTTQGSDRVVSYQLDTSVDVVAGLTSQGNPVTLIETANPDGSF

SYVAAADGNPVFTLVVKTDGSYNFTLEGPIDHAINSDELTLNFPIIATDFDGDTTSATIP

VTIVDDKPVITNVDAIQVDEDDLTGIGSDQSDALSINGQFAITQGSDGVVSYQLDSSADP

VAGLTSHGEAVDLVETANVDGSFTYTATANGNPVFTLVVNVDGSYNFTLEGPIDHASGSD

ELTLNFPIIATDFDGDTSSAVIPVTIVDDQPTITNVDAIMVDEDDLSGVGSAQDGVVSID

GKFTTTEGSDRVVSYQLDSSTDPVAGLTSHGEAVVLVETANADGSFTYSATADGNPVFTL

IVNADGSYNFTLEGPIDHAINSDELTLNFPIIATDFDGDTSSAVIPVTIVDDQPTITNVD

AITVDEDDLTSIGSAQDGVVSTDGKFTTTEGSDRVVSYQLDGSTNPVAGLTSHGEVVDLV

ETENADGSFTYTATADGNPVFTLVVNTDGSYNFTLEGPIDHVTGSDELTLNFPIIATDFD

GDTSSATIPVTIVDDQPTINDVQAITVDEDDLEQIGSAQDGSVSIDGHFTTNQGSDGVVS

YQLDASATPVDGLTSQGVAVTLSETANPEGSFTYTATAGTNPVFTLTVNPDGSYNFTLEG

PIDHASNSDELTLSFPIIATDFDGDSSVMVLPVTIQDDVPTIENVVPLTVDENNLITNGG

QSNALLVEGQFITTQGSDGVVQYQLETGSDPLNGLTSNGQVITLVEVSNANGSFTYTATA

NGNSVFTLQVNNDGSYSFELQGAVDHAPNSDTLTLDFSIIATDFDGDTSQVTLPVTIVDS

LPVISAVDAINVDEDDLVNVGSDQNDPVSIDGKFTTTEGADRVVSYQLDSNAKPVDGLTS

QGNSVTLIETANPDGSFSYVATADGNPVFTLVVKTDGSYNFTLEGPIDHAINSDELTLNF

PIIATDFDGDTTSATIPVTIVDDKPIITSVDAIQVDEDDLTGIGSDQNDALSINGQFATT

QGSDGVVSYQLDASADPVAGLTSHGEAVDLVETANADGSFTYTATANGNPVFTLVVNVDG

SYNFTLEGPIDHASGSDELTLNFPIIATDFDGDTSSAVIPVTIVDDQPTITNVDSITVDE

DDLSGVGSAQDGVVSIDGKFTTTEGSDRVVSYQLDSSTDLVAGLTSHGEAVVLVETANAD

GSFTYSATADGNPVFTLVVNVDGSYNFTLEGPIDHAINSDELTLNFPIIATDFDGDTSSA

VLPVTIVDDQPTITNVDAITVDEGDLTIIGSAQDGVVSIDGKFTTTEGSDRVVSYQLDGS

MNPVAGLTSHGEIVDLVETANADGSFTYTATANGNPVFTLVVNTDGSYNFTLEGPIDHVT

GSDELTLNFPIIATDFDGDTSSATIPVTIVDDQPTINNVQAITVDEDDLAQIGSAQDGSV

SIDGHFTTNQGSDGVVSYQLDASATPVDGLTSQGVAVTLSETANPDGSFTYTATAGTNPV

FTLIVNPDGSYNFTLEGPIDHAINSDELTLNFPIIATDFDGDTTSATIPVSIVDDQPVIT

NVAPITVDEDDLINIGSDQKDALSIDGKFTTTQGSDSVVSYQLDASAMPVDGLTSQGVAV

TMTETANADGSFTYTATAGANAVFTLVVKPDGSYNFTLEGTLDHPIGADELTLNFPIIAT

DFDGDTTSATIPVTIVDDRPTLDGIDANSVLTVDEDDLPTVGSDGNEPTSIIGKFVATEG

ADHIVEYHIVDLNTPVQGLTSGGQSLILVEVSNSGGVSVYEAIIDGTTTPAFRVTLDVSD

GSYKFELLEPLDHPTSNGQNDIVINLPIAATDFDGDVSNTLTLPITVVDDVPTIDGLAQG

SEQTVDEDDLPQGTDRAQDTIIGGTFDVTEGADQVTSIQLSDLTTPVSTLMSGGEAITLV

LTSSTGGVNVYQGITVNSQEVVFELTLNASNNSYEFDLRKALDHPDGNQQNNIIIELPIT

VTDGDGDVSPVFTLPITVVDDVPVVTNIDRLQVHEDDLPLGSDETKEPLTVSGQFEVTSA

DGIDSFVLDLNSNLLPALTSGGESIAITQDATASTSDALVYVGKTAGGDTIFTLTLHQDG

RYDFELSGALDHATNSDDLTINLPIVITDGDNDSVNATLPVTILDDKPTIEAIRPGSHLS

IDEDDIPNKGSDGQGDHIIGGHFDVVDGADSIVSFQLEDLVLPVAGLTSAGQPLELVEYS

NANGVIEYRAYVQGTTDIVFKLTLNAGEDRYQFELFAQLDHPNGNGENELVIDFPVNATD

FDGDVSNTISLPITVVDDVPSITGVDNSSQLTIDEDDLPAGSDTSGLRVLDGHFNVVAGA

DEIVSYHVSDLAGAVAGLQSNGQDVELRLVSEADGVSTYEAVIVGTNTQIFTLTLDAKDN

SYQFELVGPVDHPAGLGENSLTLDIPISVTDFDGDTSASVNLPITIVDDVPEIKTATPLF

LDEDDLPSGSELSKDSLAASGSFDSVEGADTIVSYQLDLSGNPISGVTSGGQAVMLVQTG

VNNNNYTYQGQTPDGKSVFTLVLNADGTYKFTLEGVLDHGVQGEDLLTLNLPVFATDVDG

DTAGINLPVTITDDIPTIYDSSITRVEGQGTRTVQLFQDPVEGDLNYGADGSELTSFSAD

DSGIYFKQNGIDMTTVDLNGSNQTVFVHKTLNGVDTEIGRLIVRTDGSISFRPNDDLDHT

DAASIDFTVHVTATDGDGDTSTADLDISVTDRNAQIDTSSVLSFEDKGRDGSILGTDNAN

TQDNLSGLDTTPAKVDLVINLHDLDRNESLGDITIRDASTHNGTFYYRNVNGEYIELTPV

NGSVVLDGSNVIQSFNGEFVTLENLYFVPDRHFATGDSGIDPRIRVEILNNGVSDHIING

RLNIQVESVADIATWTANSTFNYTVDEDGNNVSLNISAQTQDSSNPESIVYELVFTQGEG

NATLVYSDGSAIAQTGGVYLVDASRIGDVQVDPIDNFSGEIKIDVTAITTESVNPLTGKE

TARSETETIVIDVSPVADAGSFTVNRINIFEDNARTQDTVDPVTDHDPLQLSEVISMKPS

ADMDGSEELFVRISNFSIDGVTLVWLDGANPSQIVEVTDGNGNVLYYEIPESQLTNVEVL

PPLHSNDDFTFNVEGIVKDNASLSTGSAQDVLSLGNKTVIVDVKGVADIPIVELNDKSGI

WQEFDDGNVRGVQTFVDENDQVDISFSILSGEFKDNPNDHSETVTVLLSNIPDGVEIFDN

DGNSVDLTFVGYDGNNQPIYEANITQANINSGIVIKPEASSTENIHITATTIVTENDGHT

RTSTGEIRVIVGPVIDARNNYTVVSEGDEDTRFNIDWKPTTSQSPDADEFFSEVTISGFP

PSCTVFVDGVAQTLVAGTLTLTPQANESEQDFSARVTQSGYVQVELEQDSSTDFDLSTTL

TVKEIDHEYVDAANPGQGIAEAVITGSVHVQVNPVVEPEDTSGAIGDQTRLLVTESNGTA

IDVVKSDAQGAIDFTINTSAGGQVGANIIKYQEFDASSDEVVTELVVQLHTTDPAILNQL

VIIGALNEGDGRWTIIDEENFCIKAPSGLDLTPNDDTDNGDNGGLSQIGLTIYARVNDLG

EDSVEKDATEIRQTDVVLEFPTVLTPQTSVAAEIDVTDDVQIEGSEDNFVDLGAQLTSKI

DVINPDGVEDVLTIIIDPSSPGIPPGLIITGTDVDFINGKYVFQADIDSSGNIVGLEDLT

MRVAEDYAGDFVLPVRFVTKDTGSADEKSSTELIPVQILPVADVPSSAGDQPLDRNITPD

VTVDITGTLGLDANKQPVDDLNNDVPTADGVGYEDGLIQLNLNVDFADGFNNTLGGRETL

TNIKLTLNDTTQGEFVDSNGNSLGTSIEFNEADILAGALDNVLFKPSENYPVGGGQNTVK

INIEGEITDVAVFDQSTLINPGDNVDVRTFTDDVTFEVTPVVDDIVITGTDPSQPIVVTG

DEDTLISLNQSGTGVTISLTDDDGSESFVSLKLTGIPNDFVVESNSSDYIVKNAGNGEWS

IQVKDLTQTSIDLSDIQIKPPKHFSGEVEIGISVFIQEELLKVPTERNSNFTLVVNPIGD

DVDVNPDTQVSGNEGEYITINVNALVVDNKESIGDGANYQENDPETLRVEISNVPDGASL

SLPDGTTFVDQGSGVFVLEINAQDLDQIVFNSGDRNDNSWAGSLHFKVQAVDTGLDGSQS

LGNAEEFDVSVDVTAVNDRPDFVNVVDVETPEDNALLLNSFGISDVDAVLDNPNAEYVLN

IAVDSGYLALNANVISKYGLTVQGDGTGAVELKGSVADLNAAIAEGLIEFNPDLNFFGDV

TVNITVDDQGNEGIVISGVDDTLSTNSSSFVIDVTAVNDAPETSPVTLTSIGEDSGVFAI

SASDLLVNATDVENDNLTVSNVQLVDPSSGSLAFNSATGNWEFTPAPGYNGPVELTYDIT

DDGTTNGVSDPKTVSGSASFEVFDVNDAPQTSEVTLSSIEEDSGAVSITASELLANATDP

ENDNLAVSNVTLADPSAGTITQISATEWRFEPSANFNGDVSFVYDITDDGTTNGAPDPIT

ISGSAVMNVQAVNDAPEIDGSLVTSTIFESAGQKISGITIADVDFTGIHENEIMTISLST

SEGDVSVIAPSGSGVTQGVGLAGETVLMGTLSQLNAVLTSTAPNVGVFVDASDVNSSAIS

LTVTADDNGIYYENSTGTSLQTTETFDINVTPVADKPTLAFDANFNYIQRITASQSASFQ

GVALVGIIAALTDLDEVLALEVTGVPRGATLTSDATTSSISFDSATSTWTVPADEIDTLH

IDNVRQGDHDITLTAVSTESNGDQAFSDPIDISINVTSNNRDIDVSSETEDNLLLGSDRG

ISLIGGAGDDRIIGGDGDDILIGGLGSDILTGGGGNDIFKWTQDTVDEGAVDTITDFTLN

EDTIDLKDVIADLNDPMAGIDELLAHIQADYDATTDNVSLSITTDANVHQTIVVENLGQA

IDFNGLSSNEIVESLLNRGVIDNG

>tr|Q87LP4|Q87LP4_VIBPA Histidine kinase OS=Vibrio parahaemolyticus serotype O3:K6 (strain RIMD 2210633) OX=223926 GN=VP2567 PE=4 SV=1

MTRYGLRARVITLTLAPTLIIGLLLSGFFSFNRYQDLEKQVITTGNSIIEPLAIASESHL

LAESREAVRRLISYAHRKNSKLVRSIAVFDEHHELFVTSNFHPNFEALMFPKDKPIPKLG

NSEIYENSLILRVPILSEGHYLSELSNENQGTKAIGYIAVEMDLSSLRLQQYQEVFSAFL

VLILGLGLASVFASRLMHDVTQPITHMKNVVDRIRRGHLDVRIEGKMHGELDQLKNGINA

MAVSLSEYHVEMQHSIDQATSDLRETLEQLEIQNVELDIAKKRAQEAARVKSEFLANMSH

ELRTPLNGVIGFTRQMLKTQLSNSQTDYLQTIEKSANNLLNIINDILDFSKLEAGKLALE

NIPFDFRESLEEVINLQATSAHEKGLEITLKVDPKIPPGLVGDPLRIQQILTNLVGNSIK

FTERGNIDVSVEMRSQAGDSVELQFMVRDTGIGISERQQAQLFQAFSQADASISRRYGGT

GLGLVITQKLVSQMGGEISLTSRLHQGSTFWFTLRLNSTEMPMSDLIEVELLTGKQLLLV

EPNMQAASVTQQILSQEGILVTYRSSLPENEEHYDYVLLNLAANQTYDEQSVAAWIEQAK

RMAPSVIMGTPSTELALADQIMTEHQIQCLTKPLSRRKLLQSLINEHVEAPQAITAPVET

EESERLPLTVLAVDDNPANLKLISALLKERVETVTACSNGQQAVNLATEKKYDLIFMDIQ

MPLMDGVTACQNIKELELNKDTPVIAVTAHAMAGERDRLLAAGMDDYLTKPIEEHVLQQV

LVHWNPNTCTESIGKVAPSYVDEPESEAPATAENTQPDVIIDWQAALKQSANKEDLAKDM

LRMLIDFIPEVEEVVDHALEQDDYSREELIHVIHKLHGSSSYCGVPRLKSLCATLEHALR

SGASIEEIEPEMFELQDEMIKVTTTAKLYLEQ

>tr|Q87TC6|Q87TC6_VIBPA 3'(2'),5'-bisphosphate nucleotidase CysQ OS=Vibrio parahaemolyticus serotype O3:K6 (strain RIMD 2210633) OX=223926 GN=cysQ PE=3 SV=1

MPMTKDLSHLLPQVIEIARSAGQMILDIYEKKQYEAYTKSDETPVTSADIAAHKLITERL

SELTPDIPVLSEEAADISLEQRAQWQRYWLVDPLDGTQEFIARSGDFATIIALIDNNKPA

MGVVYGPVSGVTYYAYSGKGAWKIPDMSESVKIHTHKHEQAGQNIAIAISRRQDINRITS

RMSSAWNYDLIPLGSAALKACLVAEGAVDCYLRLGPTGEWDTAATQCIVEEAGGRILSTH

LEPLSYNERETLENPNFIVLGDTNLPWDDILQRKD

>tr|Q87TH3|Q87TH3_VIBPA Uncharacterized protein OS=Vibrio parahaemolyticus serotype O3:K6 (strain RIMD 2210633) OX=223926 GN=VP0096 PE=4 SV=1

MPFEPLITAVIETSLNTLIKDDPALGRRLARLKGQVIQVHLKEIDKTLTFVFSQQIDVLG

NYEGQPDCYLSLNLSVLPELRDQANITRLIKQDKLELEGDIQLAQKFSQLMTDCKPDIEE

WLSRVTGDVVAHTLVQGTKNVGGFFVSQAEKHQRHLAQVVTEEWKLAPAPLEVAYFCDQV

DEVRSQASRVEARLNALLSKVGSDKPALENP

>tr|Q87QR6|Q87QR6_VIBPA Putative ATP-dependent DNA helicase OS=Vibrio parahaemolyticus serotype O3:K6 (strain RIMD 2210633) OX=223926 GN=VP1083 PE=3 SV=1

MDSIESARQIASDLHLKAIGRGVSPWNSLKFAIAEADRRDYDVEAVAAGATVLNGSKATL

IADEYLILHENSGTEFQKAFLIAHEIGHIELGDTKDNVSVEEIDPARTIEASPIGIDRVV

DYSAQQRREVQMDLFAREFLLPRTWVKKLHVDDGLTATEIAEKLDAPYEVVAQQLLDALL

LPAVNPGTKEGKSEVILNEQQKHAADHRGKPYLLEAGPGTGKTQTLVKRVEGLLDEGVDP

RRILILTFSNKAAGEMAERLASKNKEAVASMWIGTFHAFGLDLVRRFYSELGFEKEPRLL

ERTEAVEALEDEFPRLGLEHFRNLYDPTEHITNLLSAISRAKDEVVDHRDYFRLAQEMLD

SADTEESKTAALKAKEVATVYEAYERLKRAKGCIDFGDLVLLPVKFLENNVELFKHFQSL

YDHVLVDEYQDVNRSSIRLLMLLCGEGENLWAVGDARQSIYRFRGASSVSMSRYCTQDFP

TALQGRLKINYRSSEEIIRAYSKFAFDMSANIGSPELIADRGETGCLPEFKVVEKADDQA

VVLAETIKDMQESGYSYRDQAVLCSGNERLSQLAEQLELLGVPVLYLGSLFEREEVKDMF

SFLSLLTDSWGSGLVRIGRWPEFELSLEDLDVILNHLRTERCEDKGWLLDSQDITGISSS

GKEALSKLGWTFEGIDKTDHPWKVLATLIFDKTRIAARLSSSEVVSDKNRSIAIWQLMNF

VRTAISAETASGLPVTRLLERVRRLLRLGDDKDLRQLPASAQSIDAVRLMTMHGAKGLEF

KVVHLPGMSQGTLPRTYQKPACEPPFGMITGEDDTSVTMLKAEHEKEQECLFYVSMSRAQ

DKLFMYAPAKKSNNSQWKHSPFISRIQDKVRTSNVVETYNLPRIDLDTDMDIAVDRDFIF

ESNHLGLYQQCPRRYLYTHLLKTGGKRTKTSYVKLHDAVRFVVQRHMEEGFDESELKSVV

ESALAINGLSEHGYIKEFTDLACSMSNYFVSSRKSMEKATISPITLELGGEKVIVRPDDI

LSEPSGRKVVRRIRTGHARKKDDSVGTAAFILASEQAYPSAKVETLYLADELTSESSLTA

KVLSNRKKTLIDIITQIRLGQFPAKRSSRSCPSCPAFFICGMVPSGSIKKKLKKITGST

>tr|Q87L27|Q87L27_VIBPA Uncharacterized protein OS=Vibrio parahaemolyticus serotype O3:K6 (strain RIMD 2210633) OX=223926 GN=VP2789 PE=4 SV=1

MTNKHAEYTLTLEHLWQFSLQFYGVREVKEACLSLQNNYHGNVNLLLLLRWLDEQQLIFQ

EKDWHLVQSCLGRSETLLHSFRDLRRHLKSQVNDALYRESLQFELQLEKQQQADLVDCIN

SLTLIKNDGDPLTLRYCRQLGGEHLQQAFAIPVPNIHPPKHS

>tr|Q87R25|Q87R25_VIBPA Putative ribosomal-protein-serine acetyltransferase OS=Vibrio parahaemolyticus serotype O3:K6 (strain RIMD 2210633) OX=223926 GN=VP0973 PE=4 SV=1

MSPDFEIITPRLALRLIPADDAHSLQRLLSQSPSLHTWLDWCDAEVSLKDAQDFLLATRL

NWVKTEAFGFGIYDRDSNTLLGMAAVNELYHTFNMASIGYWVADRYQRQGYAQEAIKALA

EFCFAKLSLTRIEIVCDPDNNASQALIESVGAKKEAIARNRFIFHGKPKDGVVYSLLPTD

LL

>tr|Q87I53|Q87I53_VIBPA Uncharacterized protein OS=Vibrio parahaemolyticus serotype O3:K6 (strain RIMD 2210633) OX=223926 GN=VPA0753 PE=4 SV=1

MVVLMKKTIRVVGVASLLCCQSVNAMSEKDWDVLTDIGTYGLVATAAAVPAYKGDWEGFW

QAGLSIGTASGVGLIGKKTIDEERPDKSDNDSFPSNHTANAFASATNLYLRYGWEAGLPA

YSMAALVGVGRVEAKKHYWRDVLAGAAIGTLSAYIFTDAYDENVQLVPWVTSEDAGISIT

YRW

>tr|Q87T18|Q87T18_VIBPA Transcriptional repressor, LacI family OS=Vibrio parahaemolyticus serotype O3:K6 (strain RIMD 2210633) OX=223926 GN=VP0252 PE=4 SV=1

MATMKDVAQLAGVSTATVSRALMNPEKVSSSTRKRVEDAVLEAGYSPNSLARNLRRNESK

TIVTIVPDICDPYFSEIIRGIEDAAMEHGYLVLLGDSGQQKKRESSFVNLVFTKQADGML

LLGTDLPFDVSKPEQKNLPPMVMACEFAPELELPTVHIDNLTSAFEAVNYLTQLGHKRIA

QISGPDTAVLCQFRQQGYQQALRRAGISKDPQYSVITEFSFDGGAKAVRKLLELPEPPTA

IFCHCDTMAIGAIQEAKRLGLRVPQDLSVVGFDDINFAQYCDPPLTTISQPRYEIGRQAM

LMMLELLKGHDVHSGSRLLETKLVVRGSAAPPQRA

>tr|Q87LU8|Q87LU8_VIBPA Transport permease protein OS=Vibrio parahaemolyticus serotype O3:K6 (strain RIMD 2210633) OX=223926 GN=VP2510 PE=3 SV=1

MYQLYWTAFRSLLGKEVTRFSRIWVQTLVPPAITMTLYFIIFGNLIGSRIGEMSGFSYME

YIVPGLIMMSVITNSYSNVASSFFSAKLQKNIEELLVAPVPNYVIIAGYVMGGVTRGLLV

GAIVTCVSLLFVDLQVEHWGIIVATVFMTSVVFALGGLINAVYAKTFDDISIIPTFVLTP

LTYLGGVFYSISLLPEFWQGVSKINPIVYMVNAFRYGFLGVSDVGIVTSFSVLGVFVVVL

YAIAHYLVTKGIGLRS

>tr|Q87SM7|Q87SM7_VIBPA Type I restriction enzyme R protein OS=Vibrio parahaemolyticus serotype O3:K6 (strain RIMD 2210633) OX=223926 GN=VP0395 PE=4 SV=1

MESTINKSKLSETDIITKFILPAIKGAGWDDMSQIRQEVKLRDGKVIVRGQAAARKKVKS

ADIVLYHKPSMPLAVVEAKANKHEIGKGMQQGLDYASLLEVPFIFASNGDGFIFHDKTNP

AQLETEIRLEDFPTPQQLWDKYCVWKGYKTEHLPVITQDYHDDGSGKSPRYYQLQAINKT

VEAVAAGQNRVLLVMATGTGKTYTAFQIIWRLWKSRAKKRILFLADRNILVDQTKTNDFQ

PFGTAMTKVTGRTVDPAYEIHLALYQALTGPEDHQKAYKQVDPDFFDLIVVDECHRGSAA

EDSAWREILEYFGSATQIGLTATPKETDIVSNTEYFGDAIYTYSLKQGIEDGFLAPYKVV

RVDIDVDLQGWRPTKGQVDKHGEVIEDRIYNQKDFDRTMVIDERTELVAQTITSYLKRTD

PMAKTIVFCNDIDHAERMRRALINCNPEQVAKNEKYVMKITGDDEIGKAQLDNFINPKKA

YPVIATTSELMTTGVDAKTCKLVVLDQGIQSMTKFKQIIGRGTRIDDRYGKLWFTILDFK

KATELFADERFDGVPERVKETKPEDFETDEGLDEIIDGEDELEQDDPFEGEDIDPESIQE

PEASYDSSSSPSGASNAEDDWQDEHRVRKFHINGVTVKAIAERVQYYDADGKLVTESFKD

YTRKTMAKQFTSMDDFVKRWQDADKKQAIIDELAEEGIIWEALELEVGKDLDPFDMICHV

VYDQPALTRKERADNVKKRNYFTKYGETAQQVLNNLLDKYADEGVQEIENIHVLKVKPFD

EMGRPAEIVKKCFGGKEQYLEAISELEAEIYQSA

>tr|Q87KL0|Q87KL0_VIBPA Uncharacterized protein OS=Vibrio parahaemolyticus serotype O3:K6 (strain RIMD 2210633) OX=223926 GN=VP2967 PE=4 SV=1

MKKTLLASLVCVVALAGCNQKEETEAPAQSQQEANPICNLEKLPGGWSQSDMTDEAQPLL

DVVLAQMNTSAKLKQILSVRTQVVSGMNYAIEFEMDNGEIWNTIVYRSLDDEIKMTQPAK

LGRFCS

>tr|Q87HK1|Q87HK1_VIBPA Sulfur carrier protein FdhD OS=Vibrio parahaemolyticus serotype O3:K6 (strain RIMD 2210633) OX=223926 GN=VPA0962 PE=3 SV=1

MRCTTQSYFELSDFNQAPPQPSAFNYRELIDGRPVQQMSLASESALAISYNGISQAVMMV

TPGHLEDFVKGFSLSTGIVHDFKEIKDLEIGGDHESHYAEVEISNRAFWALKTQRRNLAG

TTGCGICGVEALEQALPDLAPLTPSLPPRSDVFEGLRDKVALHQEAARESGALHAALFAD

LQGNIKLCREDIGRHNALDKLIGALANNHIEPQSGFAIMTSRCSLELIHKAVRARIPTLV

CLSAPTALTVEWARRNHLNLIHLPKHGSPRLYSPAP

>tr|Q87RH0|Q87RH0_VIBPA Asparagine synthetase B, glutamine-hydrolyzing OS=Vibrio parahaemolyticus serotype O3:K6 (strain RIMD 2210633) OX=223926 GN=VP0826 PE=4 SV=1

MCSVFGILDIKSDAAALRPIALEMSKKLRHRGPDWSGIYSSERAILAHERLAIVGLNSGA

QPLYSPDKKLILAVNGEIYNHKEIRARYEGKYEFQTDSDCEVILALYQDMGADLLEELNG

IFAFVLYDEEKDEYLVGRDHIGIIPLYQGYDEHGNYYVASEMKALVPVCKTVSEFPPGSH

YSSKDAEPQRYYIRDWNEYAAVQGNSTSKEELTEALEAAVKRQLMTDVPYGVLLSGGLDS

SITSAVAKRYAAMRIEDDEKSEAWWPQLHSFAVGLEGAPDLKAAREVAEKIGTVHHEMTY

TIQEGLDAIRDVIYHIETYDVTTIRASTPMFLMGRKIKAMGIKMVLSGEGADEIFGGYLY

FHKAPNAKEFHEETVRKLLALNMFDCARANKSLAAWGVEGRVPFLDKEFIDVAMRLNPAD

KMCGNGKMEKHILRECFEHYLPDSIAWRQKEQFSDGVGYSWIDTLKAVAEEKVTDQQMET

AQYRFPYNTPTTKEGYVYREIFEELFPLPSAAECVPGGPSVACSSAKAIEWDESFKNCVD

PSGRAVQTVHNDAY

>tr|Q87IX6|Q87IX6_VIBPA Putative cation efflux system transmembrane protein OS=Vibrio parahaemolyticus serotype O3:K6 (strain RIMD 2210633) OX=223926 GN=VPA0480 PE=3 SV=1

MIGAIIRWSLNNRFMILIATLMLVLAGLYSVKNTPVDAIPDLSDVQVIIKTSYPGQAPQV

VEDQVTYPLTTAMLAVPGAETVRGYSFFGDSYVYIIFNDDTDMYWARSRVLEYLSQVAPK

LPPNAKPTLGPDATGVGWVYSYVLQDKTGQHDLAELRSLQDWFLKYELQTVDGVSEVATV

GGMVKQYQVQIDPAKLRAYNLTLQQVNMAIQNGNQETGASVVEVAEAEHMVRTTGYLTSI

EDIKSLPLKVTDKGTPLLLGDIADINLGPQMRRGISELNGEGEAVGGVIVMRYGENASEV

ISKVKDKLEDLQRSLPDGVEIVTTYDRSTLINAAVDNLWKKLAEEFIVVAIVCALFLFHI

RSSLVIALSLPVGILAAFIVMHSQGINANIMSLGGIAIAIGAMVDGAIVMIENVHKHIER

TPLTDKNRWQVIGKAAEEVGAPLFFSLLIITLSFVPVFALEGQEGKMFSPLAFTKTYAMA

SAAALAITLVPVLMGYFIRGKILPEHKNPVNRGLVALYRPLLNLSLKYPKAMIVLALGLL

ASAYYPTSQLGSEFIPPLDEGDLMYMPTTYPGISIGKARELVQQTNKLIKTVPEVKTVWG

KIGRAETATDPAPLTMIETVIQLKPHDQWREGVTTESLRKEFDNLVQFPGLTNAWVMPIK

TRIDMLATGIKTPIGIKIAGPDLKEIEKIGAQLEPILNNVRGTSSVYAERVAGGRYVTID

IKRRAAARYGLSIKDVQQVISTAVGGMNVGETIEGLERYPINVRYPQDYRDSVVKLQNLP

LVTPNGARIALADVADIRYEDGPPMIKTENARPNGWVFVDIDGRDLGSYVVEAQQAVADQ

LVLPAGYSLAWSGQYEYMERAKDRLSVVVPITLVIIMMLLYFSFRRVGEVLIIMLTLPLA

MVGGLWLMHYLNYNFSIAVGVGFIALAGVAVEIGVIMLVYLNQAWHHKKLHAEENQQALQ

IGDLTDAIREGAGLRVRPVMMTVLTVIIGLIPIMYGSGTGSQVMQRIAAPMIGGMASALL

LTLLVLPAIFKLWKQREVNKTQNPEK

>tr|Q87MZ6|Q87MZ6_VIBPA ABC transporter substrate-binding protein OS=Vibrio parahaemolyticus serotype O3:K6 (strain RIMD 2210633) OX=223926 GN=VP2080 PE=4 SV=1

MKTKRLLATAVLSATAILSSTHVFANTATVAVSQIVEHPALDATRQGLLDGLKAKGYEEG

KNLEFDYKTAQGNPAIAVQIARQYVGESPDVLVGIATPSAQALVSATRTIPVVFTAVTDP

VGAKLVKSMKQPGKNVTGLSDLSPVAQHVELIQEILPQVKSIGVVFNPGEANAVTLVNLL

KESAEAKGIKVVEATALKSADVQSATQAIAAKSDILYAPTDNTVASAIEGMIVAANQAKK

PVFGGATSYVEKGAIAGLGFDYYQVGVQTADYVAAILDGQEPGKLDVKVATGSDLVINES

AASKLGIQLPSSVVQRATSMQ

>tr|Q87LT6|Q87LT6_VIBPA Type IV pilin biogenesis protein PilC OS=Vibrio parahaemolyticus serotype O3:K6 (strain RIMD 2210633) OX=223926 GN=VP2525 PE=3 SV=1

MKTTTPQLKNFRWKGVNSSGKKTSGQTLAMSEIEVRERLDAQHIKIKKLKKSSISFLTKL

SHRVKGKDITVFTRQISTMLVTGVPLVQALKLVSDNHKKAEMKSILMSVTRAVEAGTPMS

KAMRTASNHFDPLYTDLIATGEQSGNLAEVFERLATYREKNEQLRAKVIKALIYPAMVIL

VALGVSFIMLTKVIPEFEKMFVGFGAELPWFTRQVLDLSAWTQNWSPFIALGSISVFISA

RVLSKRSDSFRLMLNRSVLKFPVLGAVLSKAAIAKFSRTLATSFTAGIPILTSLKTTSKT

SGNMHYQLAIEEVYRDTAAGMPMYVAMRNCNVFPELVLQMVMIGEESGRLDDMLNKVATI

YEFEVDNTVDNLSKILEPLIIVFLGIVVGGLVTAMYLPIFNLMSVLG

>tr|Q87PL4|Q87PL4_VIBPA Uncharacterized protein OS=Vibrio parahaemolyticus serotype O3:K6 (strain RIMD 2210633) OX=223926 GN=VP1489 PE=4 SV=1

MAQQLKGKVIDFVSTPLQSFLQGSRQNKVRSQTVFLSLKRTIESHLRTRLCHHQIVVGRR

HSIEHLFYSGALTSSSLLNLQLATSLTPPLGLSVFELFPVRAEKQYAPVRKYRSLYRAKK

TQNHFRHRLSYETSMNPLGLHTHTKQNAMLDFESHCPMNINTERR

>tr|Q87Q64|Q87Q64_VIBPA Putative integral membrane protein OS=Vibrio parahaemolyticus serotype O3:K6 (strain RIMD 2210633) OX=223926 GN=VP1286 PE=4 SV=1

MMPHHKAQQHQGIGLGKIPTKYSLLVLLATIFLIIVATIGGKNLYFRGDYDIFFDGTNKQ

LLAFDEIQTTFAKTDNLAIVIAPEDGDIFTPQTLSLIQKITVDAWQVPYSSRVDSIANYQ

HTEAFDDDLLVEDLLYSEYELTPERISKVKSIALSEPVLKSALVSEKGDVTVVNITVQLP

EMDKTAEVEEVVSSINAMIDRYQRAYPDVTFHKAGIIAMNHAFMTAAQDDSSTLVPTMLV

VILVFLTIMLRSILSVIATLIVIIGSVMATMGISGWAGMFLSTATVNVPTLIMTLAVADC

VHVIATKRQSMKNGFTKVQSIERSIALNFVPILITSVTTAIGFFMMNMSDSPVLRDFGNL

SALGVMVACFLSVTLLPALLKLLPIHVKMETSQDQKHVMDRLGDFVVSQRRALLPLSVVV

IVVCASLIPLNKVNDESVEYFGQRNEFRQASDFMEERISGMTNISIAIKTNESQGIAAPD

FLNTIGEFSSWLRDQPETDHVATLADVYKRLNKNMHGDDEAYYLLPQERELAAQYLLLYE

MSLPYGLDLNNQINVDKSSIKMVLTVANLGSVELVDLENRIYQWFAEHAPQYQVVASSPS

LMFAHIGETNMASMLSTLPITLVLISALLIFALRSVRLGLISLMPNIAPAVIGFGLWALI

SGEINLGLSVVVTLTLGIVVDDAVHFLSKYQRARREGQTAEQAVRYAFHTVGRALWITTV

VLVAGFSVLAMSSFRLNADMGQLSAIVIFIALVVDFLFLPTLLMLFDKKAYLQKSPSDNA

RLKASSTITATQS

>tr|Q87GG0|Q87GG0_VIBPA Uncharacterized protein OS=Vibrio parahaemolyticus serotype O3:K6 (strain RIMD 2210633) OX=223926 GN=VPA1356 PE=4 SV=1

MVRCTLYIELDASQKAIPKVGFVRPRHSNLIKRSELATYQNSKVLIMALEEKVKEYQNLL

EEQVLMMLEEKEQLLNQVIVDEYQKVSEAWKEQQIEWFKVAEHELSRHLKEQEEAILDVK

RELKHQIALEVQARLTKLTQSEKLISHLVEVLHSEMDDACKVLQVETEQHEDGVTLSIED

DDRVISIDSKTIIEELKRGLDSI

>tr|Q87K94|Q87K94_VIBPA Peptide ABC transporter, ATP-binding protein OS=Vibrio parahaemolyticus serotype O3:K6 (strain RIMD 2210633) OX=223926 GN=VPA0004 PE=4 SV=1

MIIIIFCIGWVFLMSEINLSHAFHSEPVIQIRELCVDYITDNGDFNAVKSVSFDIGKGEV

FGLAGESGCGKSTIAFSINRLHKPPAFISGGQILFQGKDILRLSDKELSALRWSEIAMVF

QSAMNSLNPVLTIQEQFADVLRHHQGLSNEQAKDRAEKLLDLVNIPRDRLGEYPHQFSGG

MRQRLVIAIALSLNPKLIIMDEPTTALDVVVQREILQQIYQLREEFGFSVLFITHDLALM

SQLCDRIAIMRHGEIVEVNHAYEIRNHPQHPYTQKLWASFPNIHDVHKQPEQQGAPA

>tr|Q79YW5|Q79YW5_VIBPA Flagellar motor switch protein FliM OS=Vibrio parahaemolyticus serotype O3:K6 (strain RIMD 2210633) OX=223926 GN=VP2242 PE=3 SV=1

MTDLLSQDEIDALLHGVDDVDDIDEPLDNDTEGAVSFDFSSQDRIVRGRMPTLELINERF

ARHMRISLFNMLRKTAEVSINGVQMMKFGEYQNTLYVPTSLNMVRFRPLKGTALITMEAR

LVFILVENFFGGDGRFHAKIEGREFTPTERRIIQLLLKIVFEDYKEAWSPVMGVEFEYLD

SEVNPSMANIVSPTEVIVVSSFHIEVDGGGGDFHVVMPYSMVEPIRELLDAGVQSDKMET

DVRWSSALREEIMDCPVNFRVNLLEKDISLRDLMELQPGDIIPIEMPEHATMFIEDLPTY

RVKMGRSEDKLAVQVSQEIERPHVVKTDLAFLGKDIMSELENDDDRDD

>tr|Q87QR8|Q87QR8_VIBPA Uncharacterized protein OS=Vibrio parahaemolyticus serotype O3:K6 (strain RIMD 2210633) OX=223926 GN=VP1081 PE=4 SV=1

MIVVNPLRKCKLTGELYRRLPEIESKLSELLQLPEETLVERSLINDKTSVQFVPIECLVH

IVRNVGGRASKKSYERLYKILMGRVLKLIPKRATRGDLDSVTNTEVKSQILGQFAELIAN

DCLEYNDKLDFYEVRFLSAFSTLKTDAIRKVTKGFTKQESMEVEESGGIIKPEVEYAVDG

YNPFDVHISSVSDYQIYLDSAIDTLPDLQKRIMQLMKLGMPIDSKDPNTESISATLGKSE

KTIRTHRNKAFAALKKKLTGGDL

>tr|Q87HM0|Q87HM0_VIBPA Putative AcrA/AcrE family protein OS=Vibrio parahaemolyticus serotype O3:K6 (strain RIMD 2210633) OX=223926 GN=VPA0943 PE=3 SV=1

MKLDTKWLGVILFAILALLFLYMAGFFTEKLPTEHDVKTNQIDVNNAETHQLMLTSEPVV

REFPGVVVAEQHADIAARLTASVMEVLVKVGDRVKQGDVLARLESDDLDARVRQSEQALS

SAQAQLNAARKEFSRVRALLNKKLIPQSQFDQAESALQTAQANFNKAQAAVSEAETTFGY

SIITAPFDGLITQKPINKGDTATPGALLLSMYNPNSLEIEVNFAESVMPYVTYDKEVDVV

FPSYNLNAQAAVKEVTPSADANSRSYTVKLQFEPPTAIYPGTYAKVALTLTDDVILRVPK

EAVYQVGQLDYVKVVQDSGEVETRLIQLGELGRVRTGLKQGDIVLLNPRAL

>tr|Q87M64|Q87M64_VIBPA Sodium:galactoside symporter family protein OS=Vibrio parahaemolyticus serotype O3:K6 (strain RIMD 2210633) OX=223926 GN=VP2394 PE=4 SV=1

MTTLQNKLTNKELMSYFGYGVGQCFSFGLVGSFILYFYTDILGISPIAASTIFLIARVWD

AVNDPLIAGYMDTLDSRFGKFRPYMLFTPVLIVLVTVAAFYNIDADTSTKVMYAGITYIL

WGTLYTISDVPFWSMSSVMTDEPQERAKAATCAMLGVNAGIGATLVVFPKLSAYFADGRA

DQGYLPAVIVLMIAGLFFMLNGFYNTKERISTKSCEKVTLKQTFQAVRKNKPLFFILAAF

FMNVFFNLVNGLYIFFFTYNMGDAGLVSLIGTITLVSAIACLATPVLTKRFKKRDIFIAL

CVLEIIARIGFYFTGYDNPTVVMAWLAVITGIFMMTNPLISAMIADTVEYSYYHSGKRTA

AITFSGQTFTGKLSVAVAGGLTGLILTMIGYVPNAAQTETALNGMFFCIALLPALGALVR

IFIMSRYTFTEDQHAILREKLQRGEFAEGVDVKSVKPHEALS

>tr|Q87K11|Q87K11_VIBPA Putative transporter OS=Vibrio parahaemolyticus serotype O3:K6 (strain RIMD 2210633) OX=223926 GN=VPA0087 PE=3 SV=1

MEYDYLVIAGYFALMVAISLLFKKMASNSTSDYFRGGGKMLWWMVGATAFMTQFSAWTFT

GAAGKAFNDGFAVLAVFVGNMVAYVFAYFYFARRFRQMRVDTPTEGVKRRFGNTNEQFFT

WVIIPLSVINAGVWLNGLGVFASAVFNADIVTTIWVTGLAVLAISLLSGAWGVVASDFIQ

TLVVAVISIACAAVALYVVGGPGEIVENFPGGFIMGPDMNYPLLLVCTFIFFVVKQLQSI

NNMQESYRFLNAKDSKNASKAALMALVMMLFGAVIWFIPPWASAILYPDAATQYSSLGAK

ASDAVYLVFARETMPLGTVGLLMAGLFAATMSSMDSALNRNSGIFVRSFYSNIVRKGQAS

DKELLRAGQIACLVNGILVIMMAQFFNSLKHLSLFDLMMQVATLLQSPILVPLFLGIIIR

RTPKWAPWATVVVGMFVSWSVVKIFTPEFVGSWFGMDELTRREAGEMRTMITIAAHLVFT

AGFFCLSTLFYKEETDTHKETTAEFFKDVDTECVAEEGQDIVDRMQRAKLGTLVIYMAAG

LTLMVLIPNPLWGRLLFLACAASVFAVGYGLKKSAKLDTQLSKVAATTTQ

>tr|Q87KH7|Q87KH7_VIBPA Transcription termination factor Rho OS=Vibrio parahaemolyticus serotype O3:K6 (strain RIMD 2210633) OX=223926 GN=rho PE=3 SV=1

MNLTELKNRPVSDLVKLGESLGLENLARLRKQDIIFAILKAHAKGGEDIFGDGVLEILQD

GFGFLRSADSSYLAGPDDIYVSPSQIRRFNLRTGDSIAGKIRPPKDGERYFALLKVNTVN

DDKPDNARNKILFENLTPLHANERMVMERGNGSTEDITARVLDLASPIGKGQRGLIVAPP

KAGKTMLLQNIAQSIAYNHPECELMVLLIDERPEEVTEMQRLVKGEVVASTFDEPASRHV

QVAEMVIEKAKRLVEHKKDVVILLDSITRLARAYNTVVPSSGKVLTGGVDANALHRPKRF

FGAARNVEEGGSLTIIATALVDTGSKMDEVIYEEFKGTGNMELHLNRKIAEKRVFPAIDF

NRSGTRREELLTKTDELQKMWILRKIVHPMGEIDAMEFLIDKLAMTKTNDEFFDAMRRQ

>tr|Q87LG5|Q87LG5_VIBPA Uncharacterized protein OS=Vibrio parahaemolyticus serotype O3:K6 (strain RIMD 2210633) OX=223926 GN=VP2647 PE=4 SV=1

MKKTLTFATLHFTIAFSVAYVLTGDILIGSLIAMIEPSVNTVAFYFHEKTWASVPALKAR

QFMTKLKTASFATVHFSVAFTVVYLLTGDAFIGGVMAMLEPSLNTVAYYFHEKVWLRKSS

DQSSAPHFCFHKHA

>tr|Q87MH7|Q87MH7_VIBPA Putative beta-barrel assembly-enhancing protease OS=Vibrio parahaemolyticus serotype O3:K6 (strain RIMD 2210633) OX=223926 GN=VP2278 PE=3 SV=1

MLKRTRSLVCLCLAAAISSPTTYANSIDLPDIGTAAGGTLSIDQELIYGDAYMRMLRASK

PIVNDPVINEYIDSLGHRLVANANDVKTPFHFFMIRDRNINAFAFFGGYVALHSGLFLHA

RSESELASVVAHEIAHVTQRHLARSMEDQARRSPATLAALAGALLLSIASPEAGMAAITA

ATAGNMQSQINYTRSNEKEADRFGIATLAKAGFDVQAMPRFFGRLADEYRYASKPPPMLL

THPLPEDRVADSRQRAQAYPPMRIAPSIDYHLARARIVARYAGIDGQAALGWFERTQKKA

SADIMPSFDYGRALVHLDNKRLSQAEPILKKLLAQDPGNPFYLDAMTDLYIEKKQPQKAV

ELLNSALKRNPQNKVLTINYANALLEANQNDQAVRTLQRYTHDNPEDSNGWQLLSKGYHA

LGRSDEELAARAEILALRANWNKAIQYYSEASKMAELGSLEQARYDARIDQLMIQRDRFM

ALQ

>tr|Q87RW4|Q87RW4_VIBPA Uncharacterized protein OS=Vibrio parahaemolyticus serotype O3:K6 (strain RIMD 2210633) OX=223926 GN=VP0662 PE=4 SV=1

MSNQAVNKAPSSKPSGMDRFLNFIERAGNKIPDPAILFFWALIITWAASALLSNVTFDLP

NPRTGEALTITNLLTGEALASFLANMVTTFTGFAPLGIVLVAMLGVGVADSSGFITTGLK

KMLNFTPAKLLTPMLILVAIISHTAADAGYVLVIPLGGIIFHAAGRHPLAGIAAAFAGVS

GGFSANFIPSGIDPLLAGFTQTAAQVLDPEYVVNPLANIFFTGLSSVIIVAIGWYVTEKI

IEPRLAKMPIDEDAETAPNLGSFTELESKAFRYAGWAMMAGIVLLVAALLPENSALRSPE

GEITAFSAPIMKSIVPLIFILFIIPGYVYGKVSGTFKTSNDIIKAMADTMSTMGAYIVMS

FFCAQFLSAFAQSNIGTMLALYGAEGLKAMNLPGEATIIGMILLTAAVNLLIGSASAKWA

LIGPILVPMLMAVGISPELSQAAYRVGDSVSNIISPLMVFFPLVVVYCQRYVKSTGIGTL

ASLMMPFSIAMLIGWSIFLVLYWMVGIPLGIQAPYTYTM

>tr|Q87S79|Q87S79_VIBPA Beta-hexosaminidase OS=Vibrio parahaemolyticus serotype O3:K6 (strain RIMD 2210633) OX=223926 GN=nagZ PE=3 SV=1

MGPLWVDVAGYELTAEDKEILEHPTVGGLILFTRNYHDSEQLQALTQSIRKAVKRPFLIG

VDQEGGRVQRFREGFSLIPAADEYAKHHNGEELARMGGWLMAAELIAHDIDLSFAPVLDK

GHQCKAIGNRSFGEDADTILRYSTAYMQGMKSVGMATTGKHFPGHGGVIADSHLETPYDE

RSDIFEQDMAIFKAQIDAGILDAMMPAHVIFPNYDSQPASGSEYWLKHVLRQKLGFKGLI

FSDDLTMEGAAIMGSPAERGAQAMKAGCDMLLMCNKREAQVEVLDNLPVSTVPLADALLK

KQSFSLSDLKLSHEWKAASEAMKRLTS

>tr|Q87K97|Q87K97_VIBPA Uncharacterized protein OS=Vibrio parahaemolyticus serotype O3:K6 (strain RIMD 2210633) OX=223926 GN=VPA0001 PE=4 SV=1

MTADEKILIKAPRSHKDGHLFEVHESSADWVEQYQHFKGVTKSILELLNLISLRGFSSKD

GLVSTTEIVEATDGQLTRAALQQRLRAAVNIGLFTQTPVRFEEGLAGKTMLHKFVNPNQL

ISVLGATSLVTDKVRQNEKQKRSKALAQTQVNKRLLNEHGLNTPPMMKDEADQFVVSPTN

WAGIIDQALAPPRTRKSYQKSMVSISGTKAVIETRSSKNIMTVDDLMTLFALFTLTVQYH

DHHKDQYQMDAAHVPNKTPLYITDILSLRGKKDSGPARDSIRDSIDRIEFTDFQLHELTG

RWLSENMPEGFKSDRFRFLARTITASEEAPVEGSDGEIRIKPNLYILVWEPSFYEELLTR

DYFFLFPPEILKQHTLVFQLYSFFRSRMVRRHTDCMLLSELNQKLARNIEWRRFSMDLIR

ELKRLSDGKGTDDLFVVNLWGYHLTIETMIENDKVMDYQIDIKCDVEEVLRYSRARTTNA

GKRNMAPTLPNPLRNEMVTRQQLEALSGIIDGEFEPIQRKTPSPRGNLGRRVKQRKHLVE

INADEITITLSKYTSPEALERSITALSAMTGHSYASIKEECSEYIEKLDWLRVGDEPIPY

ETLSKTVELFNTQNEVKHLTIERLIAGLAVRRKVCRQVFDGHMDEMVFRALDEMAI

>tr|Q87HD5|Q87HD5_VIBPA Uncharacterized protein OS=Vibrio parahaemolyticus serotype O3:K6 (strain RIMD 2210633) OX=223926 GN=VPA1030 PE=4 SV=1

MSEEFLKYYNRELAYLRHKGQEFGEQYPKIAARLRISEEQVEDPHVERLLEGCAFLTARI

RQSLDNSYPQFTESLMGQLYPDFHAPIPSMSVIKLNCSESTTSSFAIPVGERVEVSAPGY

QDCQFRTGYPTTMYPLDIISGSFENAPFKPTGSKWEHNAHSVLRCQLSAHDSESSINSMG

IDCLRLYLNGQTQLTLKLYQMLFQSLIGLSIVLDGKEIMPLTKRHLQSVGFGDEEQVVPY

SKRSFSGSRLLVEYLHFPEKFMFFDLIELGLDKLEIQGQVEICFYFDTSDDWLPKQVDEE

SVMVGCVPIVNLFKSTMEPKRLLPTEYEYQLSPQYQDAESNEVVSISDVTLRNWNKTYEH

LPCYHSGEHTHYMSASEVYWLMRREDKNWAGGYDEPGRETYVSFVDKQYQLFSPESRDNW

LMYVEAECCNRNLPQKIPFGGGLPKVQLPNVDDNFKSIRCLTSLSETLRPEMDESTRWQL

TKLLTLNHFTEADGLATLKQTLNLYAFAGTAETKAVIDALVKLEFEHTTGRVSQKGKVGF

AHGVKLVLTVSDQILPKEQIFLLGSVLSVYFAQYAEINVFTQLEIKLKSTSSSFHVWPAL

TGDKVLL

>tr|Q87FM0|Q87FM0_VIBPA Uncharacterized protein OS=Vibrio parahaemolyticus serotype O3:K6 (strain RIMD 2210633) OX=223926 GN=VPA1659 PE=4 SV=1

MNHNALYLTQRLIDTCLREDMFGILSNAKFSSAAPKGVIVPQLEQVWLTFDNPDFTLYLP

VVPTYYMQHWCYGQANGEAPLGWWVEKNGQVEHQQHYQEWIALLTTLAHESSHELLAGYL

QELECAEKHKALCDQAFRHHADHISQPISELGSWHKKLLLADQIASYLDHPYYPTARAKF

GLSDEDLAKYAPEFAQSFALHWIAIEKSLVTLTSEQPDCWPTMEQVGFPADFALSHVLFP

AHPLTLRSLETLPEGMIEAPFTYLDVTPTLSVRTVVVNDAPHIHIKVPLIMRSLGTKNIR

LIKPSTLYDGHWFERLLSHLEQTDADLNSRLFHCNEKHGGHVGEDKTFAYIVREYPLSHC

EDKALVPVAALASPMPDDRLFLEHLAEQYYQQDTLAWFQDYVDLLCQVHLTLWLRYGIAL

ESNQQNAIVAFDQQGKMTLAMKDNDAARIWPERFNFATEHAAQKHGAGTTPVNCDELLDQ

RIKVDNELALGQMFTTITLQLDIAAIVEAMAAKGIASSASLYAIVAKSIAEQLNRLEGEG

LETKLARELLQEAPDLYAKYLLSSGSLLSKEASGASDINKFYGLSAPNFLLLSSEEAQRA

YLEAIKKSVR

>tr|Q87LY0|Q87LY0_VIBPA Histidine kinase OS=Vibrio parahaemolyticus serotype O3:K6 (strain RIMD 2210633) OX=223926 GN=VP2478 PE=4 SV=1

MFKFYKKQKFKRLQSTLMTAFLVLSITPLTITAIFFLQSHSKDLQEQSTSHLLSVRDTKQ

QQILDYFEAQETEVMGFVRSELAYASGGRFYGLVNAFSRLGNDIEEARENAQQRYIEGSG

DQIKTSILPESSNYVGSERYRLLHKRYHWAYLELLKRSDFNDILLVDINGNVTYSINKDD

NYGTNLLTGRYKDSALGKTFKRLADDVNERRKVNEDYTPVIISDFELEHGRQVAWLGAPI

VQQGYLHSYAMFRLPNNGITKLIADKNRESSINTLLVGSDQQPRTINTKQDSIQNSLEVI

DKALAGQTDVGTYTNGLGEEIIAAFAPIQTRGITWALVVQLPEKEAFSRIHQLEKLFVIA

MLIAIILVVIASHYLSNFITSPLLKLTWAAEKVSAGDLDETTFNTERKDEIGRLAISFER

MQRSIREKIQTIKQQNEELESNIKLIQKQNDELQLADKLKDEFLATTSHELRTPLHGMVG

IAETLVSGANGVIPASQKYQLDIIIKSGQRLANLVDDLLDYHKMRYGSMDIQKSAVSLAS

ATRLVLELSNHLLGNKTIRIINQVPADLKAVSADPQRLEQVLYNLIGNAIKYTSEGKIVI

SATIVDDHVRVQVVDTGQGIPAEHLEHIFEPLIQAGQDASRYRQGAGLGLSISRQLIELM

GGSLYVSSQPMVGTTFSFTLPLASEEEIQATQTLVARGHFQIPEINLENGDDLSLPENPD

GPLLYVADDEPVNLRVLESFLRLEGYRVRTVSDGPETLALVEQEKPELLLLDIMMPGMSG

YQVCSELRETYDHAELPIIMLTALSQTEDRVRGFEAGANDYLSKPFNKQELAARIQAHLT

ASKAEMRHMENKVLESELRQRAVVEASLLETQGRLLEQLESAPEAIICLREDQRIRFANE

AACKLFKRSLEQLKRSSADELIAPKYLTVKQPHYCGKIDIYIEDIRQNIEADILKLPEGS

GLDVMYIFNVGGGANAARIHNLETAVEVLSSYAFDGDRDQLQKLKELGGEFTRLADKALG

NKKDKQDLMREVLVDAMTHALDYWESVTGESKFAFAEQSGLWRVYLDRSTLQTRTLDKYM

RIETLPKTPRWRTVLSSIEFILEHCKEQSPERAYIEAQRDKLQRLLTS

>tr|Q87HP3|Q87HP3_VIBPA Sensor histidine kinase OS=Vibrio parahaemolyticus serotype O3:K6 (strain RIMD 2210633) OX=223926 GN=VPA0920 PE=4 SV=1

MIKQFLLGVASILPLFSAPALSDSLPERIDTFTELFNYEVALKSYDIRILQSNYPTKLLS

PDSLLPQTSDYPLKDIQQLYSLANTCRGKLPLSPLITEPLVFTRAICKGTQLTPRWFSRS

GLIHPGGGTYAARYVEKYPELRPKLAQYMHIKERDNEEGDELLESLQNMDDDAINALIAG

ASMFIEGKEMWLRRGDRYFVFSKDVWQENVANAGLSYTLASQSKSCFVKRGNICWDVEDH

SQILRISMIILVIANILLVIGWAVYRWNSKREELRSRMLVLQILTHELRTPIASLSLTVE

GFRREFEHLPESVYDEFRRLCEDTRRLRQLAEASKDYLQSDNQMLATDWVPSVAEWLEYK

IEEEFDNAVLFNINEDVAAKVNVYWLGTCIDNLLRNALKYGVPPVELNVQTSEEKIVFQV

RDAGDLTSKDWANLRKPFVSKSGLGLGLTIVESMVGRMGGKMSLMGPPTTFILEIPCETD

TASR

>tr|Q87HV1|Q87HV1_VIBPA Putative 5'-nucleotidase OS=Vibrio parahaemolyticus serotype O3:K6 (strain RIMD 2210633) OX=223926 GN=VPA0855 PE=3 SV=1

MKMNKNHKPVRIKLAHINDTHSYFEPTSLQLKLKINKDLILEPYVSAGGFSRIATRVEQL

RDDAQRQGQGMLFLHAGDCFQGTLYFSLFKGKANADLLNALNIDAMALGNHELDMGNEPV

ALFCQRTNFPLLAGNWDLSNESKNKAHRLSDCNNVLSYQPDSRSAQYLVKEFNGVKVAIF

GLSIDKMSDIANPDADTPFMTAIETAKATVAQIHNDGIKNIILLSHLGYEGDLELAEQVE

GIGIIVGGHSHRLQGDFTAIGLGEDDPYGIKVNGTYVVQAGFHALTMGHCVLEFDENGQA

TMLNGQNELLLGRRIFWDSTLNQQLDQGVFETACDFIHGQPNVVVCKKHPETQAILRDKY

IPRVRALQSQVIAHVESKKRHVRIPDELGGSELAGAVARSFLHSLNKRGHNVQFAIHNAG

GVRTSLNPGDITVADVAGRLLPFAVPIGFYDVKGAVIRRALEGAINNALNNGVEGTGSGS

YPYTYNLNFDYQADAPKGERIQRLEILDGYRWVKVDDDAWYRGTSSAYTMKGKEGYEALL

DMKGEGCVSNLSMADCFIELLTDEPNCLNESHKLYSQQNR

>tr|Q87MF9|Q87MF9_VIBPA Membrane-bound lytic murein transglycosylase D OS=Vibrio parahaemolyticus serotype O3:K6 (strain RIMD 2210633) OX=223926 GN=VP2296 PE=4 SV=1

MRLKYSWALVLLLSGCQLTQPNSSTTPTPETNKSEQTAKQNATKTKSKNKQSLAKDSAKD

TPKVLSPQEQEDVWQRIAMQLEMDIPNNKKVDYYRTWYLKHPNHLYTVSKRAAPFLYMIT

QRIEERGLPMELALLPVVESSFDAFAYSHGSAAGLWQFVPGTGKMMGLEQNYWYDGRRDV

AASTDAALDYLVQLNERFDGNWEHAIAAYNSGGGRVSSAIRKNKKLGKPVDFFSLDLPKE

TSSYVPKLLALADVVANQEKYGIDIPSIPNKPVLTLVDPKEQLDLAIAADYAGIGVKELQ

SYNPAYNQWSTAPEGPHQLLIPVEKKDAFLTQVESNRGKGMKVARYKVKPGDSLSMLAKK

YGTTSKVIRRANGLSNNNIRIGQYLLIPTSTKDDSKYALTAQNRLNKTQSQARGQLKLSH

VVQSGESLWSIARDNKVSHKSLAKWNGMGPKDTLRVGQKLVIWKKSDQGAVIRTVFYNVR

SGDTISGIASKFKVKSNDIVKWNSLHKQKYLKPGQKLKLYVDVTKVSV

>tr|Q87N11|Q87N11_VIBPA Cob(I)yrinic acid a,c-diamide adenosyltransferase OS=Vibrio parahaemolyticus serotype O3:K6 (strain RIMD 2210633) OX=223926 GN=VP2065 PE=3 SV=1

MSIEENKEARHKARQQKVKEQVDAKIAAAQEEKGLLLIITGNGKGKSTSGFGTIARAVGH

GLKCSVAQFIKGTWDNGERNLLEKLGVEFQVMATGFTWETQNKTADTEAAQLVWKECKRM

LQDDSIDVILFDELTYMVSYGYIDLDEVVEALNNRPKMQSVVITGRGAHRTLIEMADTVS

EVKNVKHAFESGVKALKGVDW

>tr|Q87GV3|Q87GV3_VIBPA Uncharacterized protein OS=Vibrio parahaemolyticus serotype O3:K6 (strain RIMD 2210633) OX=223926 GN=VPA1212 PE=4 SV=1

MINKHYWMLILILFPLLGFANVQCNPSSWDDNLTQFNRLESNYNQHVKVFNTLLSEHKQR

QLLSQTFSTDELSLLWRAKYNQNLFQNQLKASVQYKEELTQKANELIKLSTESQWAANGW

EKLAQSCRHNNETANQISAEWYRENAQQLAKDYTTLSSQFLGLAHLYDKEASALKYARGS

RH

>tr|Q87QH4|Q87QH4_VIBPA Phage shock protein C OS=Vibrio parahaemolyticus serotype O3:K6 (strain RIMD 2210633) OX=223926 GN=VP1175 PE=4 SV=1

MLSHQHGDVSMNNRELYRDPVNGKISGVCAGFANYFGAEVWLIRIVVISAALLGGTFLVV

LAYIAMAFMLEKQPVRYSENIREQQEHTLKSKPWQKGQSPDQLLSVLERDFDRLDGKIRN

MEAYVTSDTFRVNREFSKL

>tr|Q87JV3|Q87JV3_VIBPA Uncharacterized protein OS=Vibrio parahaemolyticus serotype O3:K6 (strain RIMD 2210633) OX=223926 GN=VPA0145 PE=4 SV=1

MGVLVMSIKWCALILCGLVTFSTHAAVDLVKVDKSKRRMYLIEDGIILKEYRIALGASPK

GHKQEEGDNRTPEGSYTLDYVIEDSAFYRSVHISYPDAIDTLEAHRRGVSPGGNIKIHGL

KNGETQDPSFIQSFDWTNGCIALTNEEMDEFIQLVTMGTPITIEW

>tr|Q87HP2|Q87HP2_VIBPA NAD(P) transhydrogenase subunit beta OS=Vibrio parahaemolyticus serotype O3:K6 (strain RIMD 2210633) OX=223926 GN=VPA0921 PE=3 SV=1

MSAGLVQAAYIVAALFFIMSLAGLSKQESARNGNYYGIAGMAIALIATIFSPDAQGFGWI

IIAMAIGGAIGIFYAKKVEMTEMPELVAILHSFVGLAAVLVGYNSYLDAPEAATHAEHVI

HLVEVFLGVFIGAVTFTGSIVAFGKLRGVISSSPLNLPHKHKMNLAAIVVSTLLMIYFVK

ADGSMFALIVMTLIAFAFGYHLVASIGGADMPVVVSMLNSYSGWAAAAAGFMLANDLLIV

TGALVGSSGAILSYIMCKAMNRSFISVIAGGFGQEIIISSDEEQGEHRETTAEEVAEMLK

NSKSVIITPGYGMAVAQAQYPVHEITDALRSQGIEVRFGIHPVAGRLPGHMNVLLAEAKV

PYDIVLEMDEINDDFSETDTVLVIGANDTVNPAALEDPNSPIAGMPVLEVWNAKNVIVFK

RSMNTGYAGVQNPLFFKENTSMLFGDAKESVEAIFKAL

>tr|Q87N35|Q87N35_VIBPA Uncharacterized protein OS=Vibrio parahaemolyticus serotype O3:K6 (strain RIMD 2210633) OX=223926 GN=VP2040 PE=4 SV=1

MIRNSGIITESHTELDIEVYEHFRHGKTALVTQGGGQRGIFTAGVLDALLLSNFDPFDEF

YGTSAGALNLCSYLCRQHGMGKAFITELTTSPEFFNLFRYIRKQQYLSLEWALERIQDFP

YKLDLDLGRKTLAGRGAFAAVTNVENLSDQYLPILGDDWFHTMLATCAIPKLYHGPVDVN

GQKFVDGGVSASIPVQEAWRQNARNIIVIRTEPFSEIEARAQSEIGDVPVEWYRESINSV

QQSWQQKVSQWKSDWTSFFQQKINTAHQSKLTGKMLLNGGRWLFGADNLYRLSHLIGENF

DSGLADMLMIHYQTFELTQAFLSNPPDDTFILQIAPKEGLKSSSLLSEPEALEHDYQLGL

QAGFNLVQLYDQLNMDFEGRESA

>tr|Q87PL9|Q87PL9_VIBPA Uncharacterized protein OS=Vibrio parahaemolyticus serotype O3:K6 (strain RIMD 2210633) OX=223926 GN=VP1483 PE=4 SV=1

MSDITLDIADFHWVTQILDTMDSGLIVLDPEYNVCVWNSFMQSYSGVLSQDILGQCLFDH

FDELPRTWLETKLKASADLETRSFSSWENRPYLFKFNNFSPVSNSCDFMFQDIVITPLRS

LSGEVSHIAIQVNDVSETARNRIHLRETNQHLSEISRKDGLTGLFNRAFWEQSLKDEFAH

LKVIDGPCSLVIFDIDHFKKVNDTYGHPTGDEVIRRTSALLRKTARSSDICGRFGGEEFT

VLLPHTNQEQACYFAERLRKRIEQEIVKVEDFLINYTISIGVCEYKPYFESHTQWLKSAD

AALYRAKENGRNQTCLHECD

>tr|Q87IY6|Q87IY6_VIBPA Putative multidrug efflux system transmembrane protein OS=Vibrio parahaemolyticus serotype O3:K6 (strain RIMD 2210633) OX=223926 GN=VPA0470 PE=3 SV=1

MKAKYKFALVSIAISSALLSGCKTGDIATKTAQAPSVETVTLTETKITPYHTFIGRTEAV

NDVDIMPRVGGELTAIHFKDGDMVEKGQLLFEIDDRPYKAALAYAKASLQKAKAQLVQTT

RDAERVKKLIKDKSISEQQYDDAIAAHAAAIASVEEAKATLVSARLDLEFSSVRAPFSGR

VGFSNFRVGDRISKIQLVPLVSITQIDPIRFGFDVDEKLYRRVRSAIDVAHRNDDKLDVD

LTLTLSDGSLYPLDGKIYAVGNKIDLETGSIRAEAQFDNPTYALMPGEYGNLTIKLRNKT

IDGLLIPSAAVQQDQAGDYVMVVGDDHVVSRRNVELGQTYGVKRAVLTGLAANEKVIVNG

LQKVRPGATVKDVELQSEQG

>tr|Q87LX6|Q87LX6_VIBPA Peptide ABC transporter, ATP-binding protein OS=Vibrio parahaemolyticus serotype O3:K6 (strain RIMD 2210633) OX=223926 GN=VP2482 PE=3 SV=1

MTAPLISIRNLCVDYITDAGDVRACNNVSFDIAPGEVFGLAGESGCGKSTVAFSLMRLHK

PPAFITGGEVIFNGEDILQYSDQRMQSFRWSEMSMVFQSAMNALNPVLTMEEQFCDVIMR

HTGLTRAQARVRAEGLLEIVDIHPSRLSDYPHQFSGGMRQRLVIAIALALNPKMIIMDEP

TTALDVVVQREILQKIYALKEEFGFSILFITHDLSLMVEFSDRIGIMYSGELIEVAPSKQ

ILESPYHPYTKGLGSSFPPLTGPKTKLTGIPGNPLNLLEIPQGCRFQARCDRVHEACTRV

PTQLRQIEPGRYSNCHLYGDTIAQAKV

>tr|Q87NY4|Q87NY4_VIBPA DNA-binding response regulator OS=Vibrio parahaemolyticus serotype O3:K6 (strain RIMD 2210633) OX=223926 GN=VP1734 PE=4 SV=1

MRLLLVEDDKLLGQSMVTSLSRHGYTVDWVEKGSGVTSALKTEAFTAVILDLTLPDIDGL

EVLRNIRKGGFKLPVMILTARDDIRDRVQGLDGGADDYLGKPFALEELLARLRVLIRRQS

GSAEEIIQVGQLSLSLSEQSIRYDDAPLKLTRNEFKILTSLMTNAGRVQSKEQLQQSLHG

WDEGSSDNAIEVHIHNLRKKAPNVAIKNIRGVGYILEK

>tr|Q87HF1|Q87HF1_VIBPA Putative S-transferase OS=Vibrio parahaemolyticus serotype O3:K6 (strain RIMD 2210633) OX=223926 GN=VPA1014 PE=4 SV=1

MSHTHAQPSESAATKQSWQMPDTLILIFFVGIFAAILTYLIPAGHFDNQQVSYVVDGAEK

TRTVIDPSSFSYATDENGELVYNKVGLFASGGGIGLMNFPFEGLVSGSKWGSAIGVIMFM

LVIGGAFGVVMRTGTIDNGILRLIDKTKGNEALFIPVLFLLFSLGGAVFGMGEEAVAFAI

IIAPLMVRLGYDGITTVMVTYVATQIGFATSWMNPFSVAIAQGIAGIPVLSGMTVRMALW

VGFTLIGIAFTMVYASRIKANPEYSYSRRTDKYFRQQELGSHDSRWNFGDTLVILTVIAT

TIWVVWGVVAKAWYIPEIASQFFTMGFVVAIIGTIFRLNGMTLNCAADAFKEGAAIMLAP

ALLVGCAKGVLLILGGGTTDEASVLNSILNSAGGVISGLPDVAAAWLMYVFQSIFNFFVT

SGSGQAALTMPLLSPLADIAGVTRQVAVLAFQLGDGFTNVIVPTSASLMATLGVCRIDWG

DWAKFCWRFMLLLFTLSSIVVVAAHVMGFA

>tr|Q87LR7|Q87LR7_VIBPA Oxaloacetate decarboxylase, alpha subunit OS=Vibrio parahaemolyticus serotype O3:K6 (strain RIMD 2210633) OX=223926 GN=VP2544 PE=4 SV=1

MSKPLAITDVVLRDAHQSLFATRMRIEDMLPIAAELDKIGYWSLETWGGATFDSCIRFLG

EDPWERLRELKKAMPNTPMQMLLRGQNLLGYRHYADDVVEKFVERAHANGMDVFRIFDAM

NDVRNFQKAVKSAVDVGAHAQGTLSYTTSPVHNTDTWVDLAKRLEDLGCHSLCIKDMSGL

LKPYEAQELITRIKASCDVPLALHCHATTGLSTATAIKAVEAGIDILDTAISSMSCTYGH

TPTETVVAMLQGTERDTNLKLDQLEPIAAYFREVRKKYAKWEGQLKGVDSRILIAQVPGG

MLTNMEGQLKEQGAADRIDEVLEEIPRVREDLGFIPLVTPTSQIVGTQAVINVLTGERYK

SITKETAGVLKGEYGAAPAAVNAELQAKVLEGKEPITCRPADLLESEMEALTVDLMEKAQ

SEGIKLASERVDDVLTYALFPQVGLKFLKNRGNPDAFEPAPTLESAKPVAAPAAPVASGS

VETYSVRVDGQVYEVEVGPKGQLTSVTPSSASVPVAAPVAPVTTNAESVPAPLAGNIFKV

NVQPGAEVAEGDVLLILEAMKMETEVRAARGGIVQELNVKEGDAVTVGAPLLSLA

>tr|Q87GI5|Q87GI5_VIBPA Putative OspC2 OS=Vibrio parahaemolyticus serotype O3:K6 (strain RIMD 2210633) OX=223926 GN=VPA1331 PE=4 SV=1

MLSDDQASLIITRTLRKNIAQKAYARMFSEGDYWKRKMADPNGLATMEKLSAGNISKLVK

SLGPLDDKEIRFFEEILNVKFAITHATDANVINENSTLTLFSRKKLDERDISYRSGLSTA

RDIQEFKNDDFVFLP

>tr|Q87IF7|Q87IF7_VIBPA Uncharacterized protein OS=Vibrio parahaemolyticus serotype O3:K6 (strain RIMD 2210633) OX=223926 GN=VPA0649 PE=4 SV=1

MLTILLIALALYIFWLLFRNGPAFAERLPDHKRNWVGPTANQEEWRGYCADVFQPFSSRT

TFYAIAYFANYIAVANAISWSIVYEGNYPVMMSRFGSYFIVSTHDAMLVAGFVSLAFFII

NWQLAMHCASLLSQYLVRILIINRTRFVAMSLVFSSYFAYLLTIGIFWLFHDTYSIWQGL

KLTPAFLLLALLLAVVLPAMKKETSESE

>tr|Q87ND3|Q87ND3_VIBPA Ribonucleoside-diphosphate reductase, beta subunit OS=Vibrio parahaemolyticus serotype O3:K6 (strain RIMD 2210633) OX=223926 GN=VP1935 PE=4 SV=1

MAYSTFNQNKNDQLKEPMFLGQSVNVARYDQQKFEIFEKLIEKQLSFFWRPEEVDVSSDR

IDYNKLPDHEKHIFISNLKYQTLLDSIQGRSPNVALLPLVSLPELETWIETWSFSETIHS

RSYTHIIRNIVNDPSVVFDDIVENEHILKRAKDIAHYYDDLIQTTNDYHRYGEGEHVLNG

ETVKVSLYDLKKKLYICLMSVNALEAIRFYVSFACSFAFAERELMEGNAKIIKLIARDEA

LHLNGTQHMINLLRNGQDDFSFMQIAEEAKQDCFDLFKEAAEQEKEWAEYLFKDGSMIGL

NKDILSQYVEYITNIRMQAVGLPAAYPEATTNPIPWINAWLSSDNVQVAPQEAEISSYLV

GQIDNEVRADDFEGFEL

>tr|Q87PV5|Q87PV5_VIBPA Uncharacterized protein OS=Vibrio parahaemolyticus serotype O3:K6 (strain RIMD 2210633) OX=223926 GN=VP1396 PE=4 SV=1

MRQRIGVEHLKAPITNQEVEGALAKAERAVKDLSQLPDTWLDFCNEKLAITSESLGFLIR

QRVQLHKRGYPSRELDYLKLIERQIEELKKVYLSFYRLAPGLIHQLRGREPEIYAWLTLQ

KELGSEFDNLLCGLSLLEELDAQTAMVIVTQSPVENMDSVMSELIEGKAKSSTFYFEYLR

VRQALSVALIKRWHKAEIIRSNIALPLLALQDVEEGIEWINDNANTEQYLFERLITKRDR

GTWFRQCFGIFHLPPTH

>tr|Q87G77|Q87G77_VIBPA Uncharacterized protein OS=Vibrio parahaemolyticus serotype O3:K6 (strain RIMD 2210633) OX=223926 GN=VPA1440 PE=3 SV=1

MKTTIRLLSGIMLGALLTGCATETYVSQENREKFSDVNVSKFLISECLAPQREIHIAVAE

HFDFDKFNIREADATSLDAFIRDIQGLSGRITIVGHTDYKGSNEYNDALSLRRAQSVAAY

LKQQLDPTFYDWEIKHFGETQPLKLDTSDQARAENRRAYVMFEEAQKYDEMPFCEPPKPE

RKVYMTMTPHFDFDQSELKAEDLTQLDDFIEQLQGLEGSILVAGHTDQVGLLSYNEKLAE

RRAQTVVEYLKTKLDASRLVWEVKAFGELQPVINQTTSEANALNRRAFIVFKESEHGQLT

E

>tr|Q87GI0|Q87GI0_VIBPA Uncharacterized protein OS=Vibrio parahaemolyticus serotype O3:K6 (strain RIMD 2210633) OX=223926 GN=VPA1336 PE=4 SV=1

MSNINNSVSLFIRDTVDGEFDKATSKQSNTDDDFSKILNQMSKVESRDIDLSFVLDQEEE

DEDEECDTELLQNTRSIESVKERGLLNFLFRHPTKNVYIRPTNKKRDIEQNEIVLTLQYQ

QSNYNFKWRKIEIEGVKVRLEKNTPGLRVFNSLHFDNNNFVSIIDEKIYSKNNEFAYLSS

DFKKYINVENYTRSIAIPLASTMSFDLSVNYFNQINTLNKYRVLYKKKYYIFEFENGKLV

NFMRGYNDGY

>tr|Q87JX6|Q87JX6_VIBPA Uncharacterized protein OS=Vibrio parahaemolyticus serotype O3:K6 (strain RIMD 2210633) OX=223926 GN=VPA0122 PE=4 SV=1

MQIRPIKVSDHERFTALWNRVYSEGEFLISPAPDNATLRSILQRVENESIPQFVAFDRQE

LIGSVEIFPAEMCGYEGGQLIRVGILGIHIDKSYRGKGLGRKLLSMAIARGWAFGYDQIV

LNVYKTNTPAIALYERFGFEHQGELGEVILPNGKVLMSQKMVLNKAQHRSNNPGL

>tr|Q87RS9|Q87RS9_VIBPA Uncharacterized protein OS=Vibrio parahaemolyticus serotype O3:K6 (strain RIMD 2210633) OX=223926 GN=VP0698 PE=4 SV=1

MITHISPAGSMDLLSQLEVERLKKTASSDLYQLYRNCTLAVLNSGSHTDNSKELLDKHQS

FDVNVVRRERGIKLELTDPPEHAFVDGEIIKGIQEHLFSVLRDIVYVNMHLADSQRLNLT

NPTHITNLVFGILRNAGALTPGIEPNLVVCWGGHSINGVEYQYTREVGNELGLRELNICT

GCGPGAMEGPMKGAAIGHAKQRYTEQRYLGLTEPSIIAAEPPNPIVNELIIMPDIEKRLE

AFVRMAHGIVIFPGGPGTAEELLYILGIMMHPDNADQPMPIVLTGPKESEEYFRSIDAFI

RDTLGEEGQKHYQIVIDDPAEVARIMKRSMEDVRLHRKEKGDAYSFNWSLKIEADFQLPF

DPTHTSMEGLNLNLDQEPQVLAANLRKAFSGIVAGNVKAEGIREIECKGPFSIHGDPVLM

KKLDKLLQDFVEQHRMKLPGGSDYEPCYRIAHP

>tr|Q87SE1|Q87SE1_VIBPA Glutamate synthase, small subunit OS=Vibrio parahaemolyticus serotype O3:K6 (strain RIMD 2210633) OX=223926 GN=VP0483 PE=4 SV=1

MGKPTGFLEHGRELPQKIDPAERIKNNKEFVLNEEFGSKINTQASRCMDCGVPFCHNGCP

IGNIIPEFNDAVYRDSWEEAWNILSSTNNFPEFTGRVCPAPCESACVLGINQDPITICNI

EKTIVETAYREGYAKPKTPRSRTGKTVAIIGSGPAGLAAAEQLNSAGHTVTVFERDEKVG

GLLRFGIPDFKLSMEVIDRKINLMAEAGVEFRVNQHVGVDVNAQQLRQEYDVVLLTGGST

VPRDLPVPGRDLKGVYFAMQFLGQNNRRANNMDLKGEEIHAAGKHVVVIGGGDTGSDCVG

TSNRHGAASITQVEIMPIPPEKRPANMPWPQYPMILRTSTSHEEGVDRHWNILTKEFIGN

DKGEVTGLRLADIVWEDAKPGERPSFKEVEGSERVIPCDMAFLAMGFLHPEPTGVLAQLD

IALDDRGNVATQGFATNQEGVFAAGDMRTGQSLVVRCINEGRECARAIDEYLMGGTNLEA

KANSLMLSA

>tr|Q87LY2|Q87LY2_VIBPA Putative permease OS=Vibrio parahaemolyticus serotype O3:K6 (strain RIMD 2210633) OX=223926 GN=VP2476 PE=4 SV=1

MPNNVKITSSHRVLMIALLAAAFACYLLVEPYINSIVMAFIISLLMFPVHEWFERKMPKH

KNSASLLTCVVLTFIIVIPLLFVFAAIVQQGSVFSQNTYKWVTNGGIQDIFQHPWVIKGL

SIVNEYLPFDKIEPKAIAEKIGQFATTFGSNLVAISAKILGDATNFLMDFFLMLFVLFFL

LRDHDKIISAIRHILPLSRSQEDRILTEIEQVSKSAVMGSFLTAIAQGLAGGIGMWLAGF

PGLFWGTMMGFASFIPVVGTALIWIPAAAYLFLTGDMTWAIFLTAWSVVIVGSIDNLLRP

LLMQGSAGMNTLMIFFSLLGGLHLFGLIGLIYGPLIFAVTMVLFNIYEEEFKDFLNQQDN

S

>tr|Q87KR6|Q87KR6_VIBPA Uncharacterized protein OS=Vibrio parahaemolyticus serotype O3:K6 (strain RIMD 2210633) OX=223926 GN=VP2910 PE=4 SV=1

MTFSNLGNVKLRLVEVDNELETLSAGYLLQRYENGFKPDTIKKDAQGIQHLYRFCINQGI

NLHQLVASKSPLSMGDIEEYASFCSVNYASYCSPSKDTYELVSVDYYKQRMRISWAFIKW

LWLFYQNRTKGKLDDLKAAQIQFAAMELGFKAYMKSPINTTISQKTGLSPELRNRFFDII

NPLPENTQNPWKSQRVRWRNYALLLTMVLGGNRKGESLLLKLNHFSLSGNRKYFEILKSS

DLDYPRSEAPSVKTLGREIELNSMMADIFEHYISVWRKEFKNAKKSMYMFLSSRDGLPLS

VQTPNAILKQLINMYPEYEGVLSPHRLRNTFHDVLNDALNLMNAAESPLSRKLKKAPIQE

YAGGWKRGSEMPNHYPKGSIQREVARMHFIIQDRILSTTEYAEAKRKEELDHSEAGFYEW

MDN

>tr|Q87JD6|Q87JD6_VIBPA Putative transporter binding protein OS=Vibrio parahaemolyticus serotype O3:K6 (strain RIMD 2210633) OX=223926 GN=VPA0317 PE=4 SV=1

MTQEVWSLSFLCYFWFLEKRSGSTVNDVNLRYLDFLLKHFEPHKHHLVTLSELESVICTS

RRNVSIVMKKLAAYGWVDWQPAIGRSNPSRLSITITLQQAMTEFLVEELARGRMNSITKL

IDWFGQTAVRSLTIASEKVNELNESKNAILISSYPWVSNIDPAETFRHTELHVVKSVYDG

LLTQNSNGGIEPALAHEWKMEENVMTLWLRPGIFRHDGELLSIDDVVWSLERLKQIKGPV

YELWQCIETVTAETPNCLKIVLKRANQFFPYMLATPHASILCRDTASFGSGYSYHIGTGP

FRISSWNKESITLQAHKEYFSARALLDQITLSHGDVQNLNMLSFNQVSDHVEIEKISALS

YLTYRERRDSQLTKQEWQQLANYINQQKRHHEPQSAVDSIQLSPLEPSCSAETAPTLQGK

VVLAEPIWTIPNLMRHSEWLHEVIRSTGLELEVIKVEDISFPESVSHLADLLFIEEVMEA

PFEYGVFEWLSVATGLRFSFEQNQMERHREQLQEAIAYEQPISHLLGIEEALRAQYVYLP

LFIGYEEVTKTQQVRGVQVKNTGYSDLHRLWLAHSS

>tr|Q87KV2|Q87KV2_VIBPA Fumarate hydratase class II OS=Vibrio parahaemolyticus serotype O3:K6 (strain RIMD 2210633) OX=223926 GN=fumC PE=3 SV=1

MTTEFRIETDSMGEVKVPANALYQAQTQRAIDNFAFSQHTMPSGFIQALAHIKQTAALTN

AQLGLLEGDIANAIADAAQAIIDGQHLDQFPIDLFQTGSGTSSNMNANEVIATLASELLG

GNVTPNDHVNMGQSSNDVVPTAIQVSSALAIEHKLLPALAHLSQSLETKKQALQGVVKTG

RTHLMDAMPITFAQELGGWQFQIEHAKQAIEQTLPMVKALAQGGTAVGTGINADPRFASL

FADNLTQSTRVTFTSSDNFFFNLSSQDAIVALSGQLKTAAVAIMKIANDLRWMNSGPLAG

LGEIELQGLQPGSSIMPGKVNPVIPEAAAMASAQVIGNDATITIAGQSGNFQLNVMLPVI

AHNILESIELVANSATALADKAIATFDVRQDNLDIALAKNPILVTALNPVIGYLKAAEVA

KKAYKQGRAIIDVAEEETDLNRETLERLLNPAKLTQGGVAE

>tr|Q87I74|Q87I74_VIBPA Uncharacterized protein OS=Vibrio parahaemolyticus serotype O3:K6 (strain RIMD 2210633) OX=223926 GN=VPA0732 PE=4 SV=1

MSQSMKCKHIFVSIFLFFGNAYHSYAEVEKPAMSAQLEEYVYNPRILKDVSDTLLGIDLN

KNNIRDDVDKVVNHLEVTEADKEFMLRYIQYATSILSYDFAKDRLENPLIASELYKELNY

INSCYKESGVDGKDLYDSINALNVLMFNTDARIIAYLHYEKYLDIRNTYELLSYDCKNKK

YHQV

>tr|Q87MA3|Q87MA3_VIBPA Putative lipoprotein OS=Vibrio parahaemolyticus serotype O3:K6 (strain RIMD 2210633) OX=223926 GN=VP2354 PE=4 SV=1

MKKLLLAASVALLAACSAPQQPQLNLMPESTLSTNPIVQGKTYSLTSKDVRAAQYVALVD

NGRSNILPLHAKQNLRIALEEALEKQFSSQGFHSDLNSNNAIELNVQEALVNVKHSVMEN

QMDAKVILEITAETPQGKLVKTYTGTAKRSGTLSASDSDIEETLNDVVTLTLKEIANDPE

LRQYMQERF

>tr|Q87T96|Q87T96_VIBPA Oligopeptide ABC transporter, ATP-binding protein OS=Vibrio parahaemolyticus serotype O3:K6 (strain RIMD 2210633) OX=223926 GN=VP0174 PE=4 SV=1

MSVSEGETLGLVGESGCGKSTLGRTILKLYEPTEGKIFFEGKDITKFSVKEMRSLRKDMQ

IVFQDPMESLNQRHTIGMILEEPYIIHKIGTPAERKQWVKELLVKVGLPETAVNRYPHEF

SGGQRQRIGIARAIALKPKLLICDESVSALDVSVQAQILNLLLQLQQEMNLAIIFISHDL

SVVKHVSDKVAVMYFGKVVEAGNAKEIYANPQNDYTKKLLSAIPITHPKYRKKKRVAEKK

QQVA

>tr|Q87IC4|Q87IC4_VIBPA Putaive transcriptional regulator, LysR family OS=Vibrio parahaemolyticus serotype O3:K6 (strain RIMD 2210633) OX=223926 GN=VPA0682 PE=4 SV=1

MSNVMDLNLIQTFLVVAEFQSYTKAAEQLGLTQPAVSAAIKRLEQVVDKQLFVKKGRGIT

LTSAAYQLLPQFEQAVSIIDNAIMEKKHFEVCCSEILLHIMSPIKNAIFYESPPEKYILF

ELLRQQKVDLVIDTVVTKDAAFVMEEAYEEKAVIICREQHPRIQGTLSKEQFYDETHCLF

SGKWNNMSGFEQLAQETILERKVDLVTSSLAGMALYVAQRDCLGLVSQSFANKWSKALKL

QVLPCPISICTIPYKFVYHKRELNNPAHIALRERIKTHLAAAHYEPISL

>tr|Q87PL8|Q87PL8_VIBPA Uncharacterized protein OS=Vibrio parahaemolyticus serotype O3:K6 (strain RIMD 2210633) OX=223926 GN=VP1484 PE=4 SV=1

MVVETDGYLALIEHLSLNLDIFTTEIGDTGSESIEDVVTDMVASNIMAIFEQNPELHSSV

RFKLLKEADAVVEDLGEVLAGVWSKKATNEQIAFLDEYIALVKNLFDTAVATYD

>tr|Q87S76|Q87S76_VIBPA Putative ToxR-activated protein TagE OS=Vibrio parahaemolyticus serotype O3:K6 (strain RIMD 2210633) OX=223926 GN=VP0548 PE=4 SV=1

MSKKIIMQIPTKNGDQQFYLGRGSVLTILTSIIALPAVVGGAWYLNQQQIHNQTTLAQTI

TKLESEKAEITALYEEQLDTNHSLSQALTDKNNQIQLLGKRVFDVESVLGLADEELSIDE

NNLALEERIDAAAIDSAVRATMFRLIPNDSPVTYQRISSSYGSRINPISGRRHVHTGIDL

TCKRGEEILAPADGVVETVRPGNKGYGNYLTLRHSFGFSSSFAHLNKFNVKSGQFVSKGD

VIAQCGNSGNSTGPHLHYEVRFLGRTLNPQSLMEWTPENFNYVFEKEKMVKWGPLVQLID

NVVRLQINLTNSPYRDTTINTVSSEDNNETIIN

>tr|Q87GK8|Q87GK8_VIBPA Putative adhesion protein OS=Vibrio parahaemolyticus serotype O3:K6 (strain RIMD 2210633) OX=223926 GN=VPA1307 PE=4 SV=1

MGRTVLMRSKSLTFISKGLGTMAAIAAIVSSPTWALDIFVCEPEWKALLQRHAPEANIYS

ATTDKQDPHYVQARPSLIAKMRQADLAMCSGAELEVGWLPMLQARSSNAAVQNGAKSMIY

AASFVRMLDTHQHVDRSMGDIHAHGNPHVQFAANDMITLSRVVTQCLQAVDPENAPTYQL

NGMKFRAHWRKKLNEWEAKAKPLNGKQVVGYHSTYRYLFDWLGMVQVADLEPKPGVSPTT

SHLQSLTKLDASSFDVIVYSSHQDQRPAMWLQQRTNKPVVQLPLTVSKEQNLDELYDEVI

AELLDVFVSPSAVKF

>tr|Q87N33|Q87N33_VIBPA Uncharacterized protein OS=Vibrio parahaemolyticus serotype O3:K6 (strain RIMD 2210633) OX=223926 GN=VP2042 PE=4 SV=1

MKNKLSWLVGGMLMSCALSAQANVIVTPFIGFTGGGQVEDENKNTYDIDPALSYALSIET

PFEMGKIGLFYSAQPTELKELSNSADIHYLQFQSSIYYPLAEGWQSYVGLGVGGSYTDVD

WADKKYGFSTSAFAGLEYAFSKNFALTAQVRWLGTVVDNDTSGACALPSDGSSCIIKFDT

DWMNQFQSNVGFSFRF

>tr|Q87G63|Q87G63_VIBPA Uncharacterized protein OS=Vibrio parahaemolyticus serotype O3:K6 (strain RIMD 2210633) OX=223926 GN=VPA1454 PE=4 SV=1

MTDLSQWLNIEFVWWWALFLLPLPLLIYKLLPEETRQAEIKLAYLPESKNSRKPKQWLQK

SLAVGVWTLLVVACARPVWFGEPVEFQPKYRDLMLVVDLSGSMQKEDMNLDGEYIDRLSA

VKKVLSDFVAKRKGDRLGVVLFGDHAYLQTPLTADRQTVIQQIKQTVIGLVGQRTAIGDG

IGLGTKTFVDSDAPQRVMILLSDGSNTAGVLDPIEAAEIAKKYNATIYTVGVGAGEMMVK

DFFMTRKVDTAADLDEQTLTKIAEMTGGQYFRARDAEQLEKIYDTINKLEPVSSDTQTWR

PQSEWFPYPLSAALVLSMLLFLLRRKHG

>tr|Q87Q24|Q87Q24_VIBPA Uncharacterized protein OS=Vibrio parahaemolyticus serotype O3:K6 (strain RIMD 2210633) OX=223926 GN=VP1326 PE=4 SV=1

MKFQYSIATAVTLALLQGCGGGGDGASPSINTSTNSAWTQGDYSKTSNEFANRCETPRTG

LDPYAGYQPYPDKAGSALQEKLFLRAFTNETYLWYDEVPDRNPSFYSSVIAYFHSLVTSE

TTATGKTKDRFHFAIPYDDEMKSSQSGLVAGFGFNWSFIQATEPRDVRISYTEDNSPAAL

NGIVRGDKLIKIGSIDVVNTSNNAEINTINTTLFSPEINKEYSFTFETVDGYRKSVILKA

ADVATSPVRNAKVIEHNGKKVGYLQYNSFEPSAQTPLIDAFNKLAEAQVDEIVIDLRYNG

GGLVLQSSQVGYMLSGADKNRVFSELIHNDKIMKTIPNGDNVYPFTNVAIDWAAGKFSDQ

MLPTLGKQQVYVLTSGGTASSSELLMNALRGIDVNVIQIGSTTMGKPYGFAPEQNCGTMY

YTIQFKSANEKQFGDFADGFVPTPKSQISSNLGLDNRVEGCWVDDDLTKPLGDTSERMLS

AALNYMDTGTCPQVSPASFNTMPLNSDSPALKDHRSPLRTEAIHVEIN

>tr|Q87I50|Q87I50_VIBPA Molecular chaperone SugE OS=Vibrio parahaemolyticus serotype O3:K6 (strain RIMD 2210633) OX=223926 GN=VPA0756 PE=3 SV=1

MAWGILFIAGLCEIAWAVGLKYSQGFTKFGASAFTIVFMLLSFWLLGIALRTLPLGIAYG

VWVGIGAIGTAIVGIYLFNEPATLIKALSLLMIVAGIAGLKISA

>tr|Q87PY6|Q87PY6_VIBPA Possible ABC transport system periplasmic substrate-binding protein OS=Vibrio parahaemolyticus serotype O3:K6 (strain RIMD 2210633) OX=223926 GN=VP1364 PE=4 SV=1

MDDSKSSYKLGLFVVSALVSLFVVLFILGGRSLFEPKIIVETYFDESVSGLEVGAPVRFR

GITAGEVVSIELSDALYEAAVPRENRKSYVVVRSEITGAKRTIEEWNRSIEISIERGLRA

TTQLAGITGQQYLSFDYTSMDEGLSFNWKPNYPYVPSTKSSAGKIVSGIQSLIARLDEAD

INTLVANINMLIETLNQSISALDADALNAQLLALLINSNQMVKSVDGVISDPEVKEIVSS

LAQISKSLNSSLKDKGDINKLIKHLDRAAVRLDVIMADNQSDINYVIKDLRVTVENLKSF

SETLKNQPSSIIFSSEPEKLKIN

>tr|Q87NB5|Q87NB5_VIBPA Putative translation factor OS=Vibrio parahaemolyticus serotype O3:K6 (strain RIMD 2210633) OX=223926 GN=VP1953 PE=3 SV=1

MSQFFYVHPENPQARLINQAVAIIRNGGVVVYPTDSGYALGCQLENKQALERICQIRRLD

DKHNFTLLCRDLSEISLYARVDNGAFRLLKNNTPGPYTFIFKGTKEVPRRLMNAKRKTIG

IRVPNNQIALDLLEALGEPLMSTSLILPGSDVTESDPEDIRDKLEHAVDVILNGGYLGEQ

PTTVIDFSEGEPEVVRIGSGDPSPFE

>tr|Q87S02|Q87S02_VIBPA Sodium/alanine symporter OS=Vibrio parahaemolyticus serotype O3:K6 (strain RIMD 2210633) OX=223926 GN=VP0622 PE=3 SV=1

MQSLVDFLNGIIWSPVLIYLCLGAGLFYSIMTRFVQIRHFFEMWRLLLSGKSSTKGISSF

QALAVSLSGRVGTGNIAGVAAAIGFGGPGAVFWMWVVAFFGAATAYAESTLAQIYKEEDE

GEFRGGPAYYIEKAMGQKWYAWIFAISTIFACGVLLPGVQSNSIGNAVEAAFGSGDMIET

AIGTFSFAKIFTGTVVCVILAFIIFGGVKRIANFTQIVVPFMALAYIITAFVIILLNIGE

IPRIIGMILGDAFTPMAGVGAAIGWGVKRGVYSNEAGQGTGPHAAAAASVDHPAQQGLVQ

SFSIYIDTLLVCSATAFMILITGAYNVHGAVEGAFLVQNLPADIGANGPVFTQMAIESAL

PGVGKPFIAVALFFFAFTTILAYYYIAETNIAYIRRTFKVNGLMFILKLVLISAVFYGTV

KTANLAWAMGDVGVGLMAWLNIVGILIIFFMSKPALKALTDYEEQQKQGVTEFTFNPVAL

GIKGADYWEEKYKRKTGQAPTTETTATDTVEQPST

>tr|Q87T17|Q87T17_VIBPA Primosomal protein N' OS=Vibrio parahaemolyticus serotype O3:K6 (strain RIMD 2210633) OX=223926 GN=priA PE=3 SV=1

MRPSIARVALPVPLDKQFDYAIPGHLFPIIGGRVSVPFGRQTLVGIVTAMVNHSDFPKDQ

LKPIKAVLDSQPVWPEKLYSLLIWCSQFYQYPLGDTLHNAMPAALRKGKPADFATLQEWQ

ITESGKDKLMQGLDRRAIKQQKVLQMLVNGALPHQEFVDQEITSTVLKSLEDKGWIERIE

KKPAITQWGQHVECDVEKPKLNHEQALAIASVNSQTGFACYLLEGVTGSGKTEVYLNLIK

PVLEKGKQALVLVPEIGLTPQTINRFKRRFNVPVDVIHSGLNETERLNAWLSARDKAAGI

IIGTRSALLAPFADLGIIIVDEEHDTSYKQQDSLRYHARDVAVMRAHKEQVPIVLGSATP

ALETLHNALSGKYHHLTLTQRAGSAVPTSNKVLDVKGQYLESGLSAPLIAEMRKHLKAGN

QVMLFLNRRGFSPALMCHECGWIAECKRCDAYYTFHQYSNEIRCHHCGSQQPVIHQCQGC

GSTQLVTVGVGTEQLEQQLAQLFPEYKAIRIDRDSTRRKGSLEDALDSIRKGEYQILIGT

QMLAKGHHFPNVTLVALLDVDGSLYSSDFRASERLAQLFIQVAGRAGRASKPGEVVLQTH

HPEHSLLQALLEKDYRHFAMTALEERKLAQLPPYSFLTLFKAEANQSEIVEDFLRQVRFT

LESHPLFDDTCMVLGPTPSPLAKRAGKYRWQLLIQTQHRSLMQKLLTSAKPAIELLPNAK

KVRWNLDIEPQDLS

>tr|Q87FR2|Q87FR2_VIBPA Alpha-amylase OS=Vibrio parahaemolyticus serotype O3:K6 (strain RIMD 2210633) OX=223926 GN=VPA1616 PE=4 SV=1

MKLNLITTTLLLASASAIAEPNITISTTTNSRDFPLSAEQPLVVPLTKDGYTLKITGVEG

DCVAPDGQKVKFNKPIALNCGKPTELPLKVRFTGDYSFAYDDAAKTLLFKREPKKAAKTE

FKRPIPQVQCEVYQGGEVTIAVGDSFPEGTKLRDAYSGQMVEVKEQKVSLTPSKDSGGLV

LLEPVQKTKEAVLFDYRNANIYFVMVDRFNNADASNDNSYGRKKDGKDEVGTFHGGDLKG

VIEKLDYIKSLGTDAIWLSPIVEQVHGFVGGGDSGSFPFYAYHGYWTRDFTKIDENFGNE

EDLKTLVEEAHKRGLKVLMDAVINHSGYSTLADLQFDGIDVLKPNAELPKKWGDWQPKAG

ENWHSYHQNIDYQSLNWAKWWGGDWVRTGLPGYPAPGSSDITMSLAGLPDFITELDKAVK

PAQWLLDNPGTRVEARDNYTVSDYLIEWQTDWVKRFGIDGYRVDTVKHVEGEVWKRLKEE

ASNSLEQWRKENGKSGQPFWMMGEVWGHTAYRSPYFDDGFDALINFDMQKKLDKGAACFS

QMADTYQSYADTIANEKDFNPVSYMSSHDTELFFSRFKDYGMQRNAANALLLSPGAVQVY

YGDEVGRNIGPYADDFHQGTRSDMVWKLSDDKQALLKHWQTLGQFRQAHPAIGAGQHRVI

EQEGAYVFSRSLGNDKVVVAFVGRDK

>tr|Q87Q28|Q87Q28_VIBPA Putative LicD1 protein OS=Vibrio parahaemolyticus serotype O3:K6 (strain RIMD 2210633) OX=223926 GN=VP1322 PE=4 SV=1

MKKLTFEQLRSVQMSILDRVHLFCERHDLEYSLAGGTLLGAIRHKGYIPWDDDIDIMMPR

EDYEYLLQNFAKEYPDFTFFNLDTKHNPYPFLFSKLSLNDSVISNGRIKGVGINIDIFPI

DSLGKEKGAAKLRFSLMKAINFFARIQRSRHDRANPIVNIAFRLFQKCLKLLPKKWFLSA

IKGLCSTCSVNDDGYAVSIGSSYLAKEFTESSLYHDYTSCPFEDRQYKCIARYDDYLSMI

YGDYMQLPKEQDRENHDNVGFIKEHLLPM

>tr|Q87GX5|Q87GX5_VIBPA Efflux pump membrane transporter OS=Vibrio parahaemolyticus serotype O3:K6 (strain RIMD 2210633) OX=223926 GN=VPA1190 PE=3 SV=1

MLSRFFIQRPKFALVISIILTLAGAISLAILPVAEYPQISPPSVSVTAYYTGASAEVVEQ

AIADPIETSVNGVENMIYMSSKSANDGSYSLNVTFDVGTDPDMAQVNVQNRVSQIESKLP

QEVRMVGVTVKKRSPDLLMVLNFYSPDGKYDDQFLINYINLNVKDQLARAKGISEVNVIG

GGEYAMRVWLDPEKMANLKLTTSDVYAALAEQNVQVAAGRVGAAPYNNPQEVQFNLVTKG

RLESVSEFENVVLRANPNGSTVYLKDVARVELGKKFYDGNGKFRGQDASIVALSLQSDAN

ALESGKAVMELLERLSENFPEGMTYETSYDTTVFVAESIKGVVKTLVEAILLVIAVTYLF

LGSARATLIPVVAIPVSLIGTFAIMQATGFTINTVTLFGLILAIGIVVDDAILVIENVDT

TMAKDPTISPRKATLLAMKEVTGPIITSTLVLLAVFLPVAMLPGITGIMYRQFALTICIS

VVISSINALTLSPALCSLVLKQGGGNTANWFQAFNRGLERVTTRYGQVAGFLVKKSLLLV

TFFIVAVAAVSFFAKTTSTAFVPQEDKGILLVNVQLPDSASLSRTEEVTSDLMKMIEEEP

GVDGVTVANGFSFMTGAAASNGASLFIKLHDWETRNSLGGEHSANAIAARINGRAAMELP

QAVVFAMGPPAVPGMGAASGFEFVLEDTLGRSRTDLSMVMGEMIQAANQAPEIAYTFSTF

RANVPHYYVDIDREKAQQLGISLSSIFQTLQGNLGSLYVNDFTMFGKNFRVTMQADSQHR

SGMQDLERFHVRSSMGEMVPLSTLVSYEQVFEPDVAWRYNMYRSAIIQGQPAPGYSSGDA

IAAMERVAAEVLPQGYTYEWTGMAYQEVLAGNQAIYAFALALIFIYLFMVAQYESWSIPL

AIILVVPVATLGSFLALNLTGTPLNLYAQIGLVLLIALAAKNAILIVEFAKQEREEKDVS

IDDAAVKGGTLRFRAVNMTSWSFILGIFPLIFAAGAGHVSQNSLGVSLIGGLLCVLLAGT

FLIPGFYALVQRQREKIHGGNTKLVPLDDE

>tr|Q87HY5|Q87HY5_VIBPA Amino acid ABC transporter, permease protein OS=Vibrio parahaemolyticus serotype O3:K6 (strain RIMD 2210633) OX=223926 GN=VPA0821 PE=3 SV=1

MIVRVIKPVLQALLQMMLLFIAIVWVLDSGADAMGYQWQWERVPDYIAFYEDGEWWPAEL

IDGLLVTLNISAISLFFTLVLGLVTALLRLSDSKVGNVIGTTYVEVIRNTPLLVQIYLLY

FVFGPVIGLDRFSTAVLALSLFQGAYTAEIFRAGLNSIPKGQFEAAQTLGLSPFYTYKDV

ILPQVLQRTLPPLTNEVVSLIKNSSIVSVMAIFDLTTQARNIVSETAMPFEIWFTVAAIY

LALTLSLSGLSAWLEHKLGASWRKL

>tr|Q87MY4|Q87MY4_VIBPA Molybdenum cofactor biosynthesis protein E OS=Vibrio parahaemolyticus serotype O3:K6 (strain RIMD 2210633) OX=223926 GN=VP2092 PE=4 SV=1

MDPRVSVQVEDFSVDQEYQRLSEGTASGAVVTFIGKVRDMNLGDNVIGLHLEHYPGMTEK

SLSDICDEAEARWPLLGVRVIHRVGDMDSGDQIVFVGVSSAHRGAAFDACEFIMDYLKTK

APFWKKERTTNEDRWIESRDTDHQAARRWEK

>tr|Q87Q96|Q87Q96_VIBPA Uncharacterized protein OS=Vibrio parahaemolyticus serotype O3:K6 (strain RIMD 2210633) OX=223926 GN=VP1254 PE=4 SV=1

MSLIATGTLNKMRASLDGAVSYRLPVGDEEVDLSPFLGKTLTLTHTGNIFCCSCGKKTKK

SYSQGHCFVCMKKLASCDMCIMKPETCHYDQGTCREPQWGEENCMVDHFVYLSNTSSLKV

GITRHTQIPTRWIDQGATQGLPIFKVKTRHISGLIEVELAKHIADKTNWRTLLKGDGEPI

ELQDRFAELLPLVQDKIAEIKQQFGEDAIEVLSETITDLSYPVQQHPTKITSHNFDKNPV

VTGILQGIKGQYLIFDTGVINVRKFTSYEVEVSA

>tr|Q87Q04|Q87Q04_VIBPA Oligopeptide ABC transporter, permease protein OS=Vibrio parahaemolyticus serotype O3:K6 (strain RIMD 2210633) OX=223926 GN=VP1346 PE=3 SV=1

MIWYILRRLAIAVPTLLFIALVSFWLMHIAPGGPFDMERPMPEIVRANIEAKFHLDEPFL

VQFWIYITNFVQGDLGPSFVYQDFSVTQLVAQSWPVSAMLGMLSFCISVPLGMLLGTIAA

LKRNSRLDYGLMTLSMTGVVVPAFVLAPVLVTIFAIQLGWLPAGGWEGGQLAFLVLPVLS

LAIGSVASIARVMRGAMIETLNQPYIRTAKAKGLSTAYILFHHALRPSLIPVVAMLGPAF

VSVVTGSVIIDIFFGTGGMGQHFVSGALNRDYGLVMGITLIVASLTIFFNLVVDLLYTVI

DPRIRV

>tr|Q87NQ5|Q87NQ5_VIBPA Uncharacterized protein OS=Vibrio parahaemolyticus serotype O3:K6 (strain RIMD 2210633) OX=223926 GN=VP1813 PE=4 SV=1

MKVGKLLVFLSFFSMTSQADTVLDEFKQIESEASQLRMVVVKCYVQMKLLKSEGWKSQAC

VDYKSIASVDGEKLKVDLKESSLKFKKNQKVGKYSYEETAERMELMYSIKTHFDGFKGIP

SKIKELRKT

>tr|Q87QP7|Q87QP7_VIBPA Uncharacterized protein OS=Vibrio parahaemolyticus serotype O3:K6 (strain RIMD 2210633) OX=223926 GN=VP1102 PE=4 SV=1

MQTQFQFINDCLIENQPLWRFEPFQSSIQPSLPWQETHPQLCQWLESLSPSQIETLKADP

DLALDEISVFLPDLPSLLRHTQLESMALDGLALERGLDSGIPGRKLEQITAMGEAAIQSH

QGEEWLEWCSGKGYLGRILTTQTNQPVTSFEYQQALCDSGQQAANEHHWKMTFIQGDAFD

PQTKAVFKPTQHAVALHACGDLHVRLMEYGSEAGIAAMTISPCCYHLIQSETYQALSQSV

RASELILSKHELRIPLQQTVTGGERVRRHRQQEMVFRLGFDLLTREILGVDEYQPVPSIR

KSQLSDGFEALCLWVAKEKGIEINQAIDFAEFEKRAIKRFWHMERMSLVQLVFQRPLEIW

LALDKVLYLEERGYRVRLAEFCPKSVTPRNILICAYKN

>tr|Q87TQ0|Q87TQ0_VIBPA Valine-pyruvate aminotransferase OS=Vibrio parahaemolyticus serotype O3:K6 (strain RIMD 2210633) OX=223926 GN=VP0019 PE=4 SV=1

MQFSKFGEKFNQYSGITQLMDDLNDGLRTPGAIMLGGGNPAAIPAMLDYFHQASEEMLAS

GELVAALTNYDGPQGKDVFVKALAQLFRETYGWDISEKNISLTNGSQSGFFYLFNLFAGQ

QPDGSHKKVLLPIAPEYIGYGDAGIDEDIFVSYHPEIELLDNGLFKYHVDFEKLTVDDSV

AAICASRPTNPTGNVLTDEEVRKLDKLARENNIPLIIDNAYGLPFPNIIFEDVEPFWNEN

TILCMSLSKLGLPGVRCGIVIASEEITQALTNMNGIISLAPGSVGPALANHIIAKGDLLK

LSSEVIKPFYKQKSQRAVELLQQAITDERFRIHKPEGAIFLWLWFDELPITTMELYQRLK

ARGVLIVPGEYFFIGQKDEWDHAHQCLRMNYVQDDEMMQKGIAIIAEEVEKAYQQGQ

>tr|Q87RQ8|Q87RQ8_VIBPA D-alanyl-D-alanine carboxypeptidase OS=Vibrio parahaemolyticus serotype O3:K6 (strain RIMD 2210633) OX=223926 GN=VP0719 PE=3 SV=1

MNKNKFVKSILVSSVALSATFAQSAFASPVVVPDAPQIAAKGYVLMDYHSGKVLAEKEMN

TKLSPASLTKMMTSYVIGQELARGNISEDDDVTISKNAWAKNFPDSSKMFIEVGTTVKVR

DLNRGIIIQSGNDACVAMAEHIAGSEDAFVDLMNAWANTLGMKNSHFANVHGLDNSELYS

TPYDMALLGKALIRDVPDEYRVYSEKKFTYNGITQYNRNGLLWDKSMNVDGIKTGHTSNA

GYSLVSSATEGQMRLVAVVMGTKDANARKSESKKLLSYGFRFFETVAPHKAGETFVEEKV

WMGNKDTVALGLDQDTYVTLPRGEAKNLKASFVLEKELEAPINKGDVVGKLYYQIDGEDI

AEYPLMALETVEQGSLFSRLWDYIVLLFKSFF

>tr|Q87SR0|Q87SR0_VIBPA Sensor protein OS=Vibrio parahaemolyticus serotype O3:K6 (strain RIMD 2210633) OX=223926 GN=VP0362 PE=4 SV=1

MMPKFSLPFSMKNKLVLMFVSMTFVTYFSLAFILQYAIERHFYSQDFSYISSKFNAIENE

LKSSLEDVFKQANNSTLYMWVFEGKLNVYQNSSLTIPQNRAYSISSPQVLNHERAIEWSE

DSLNIRAFAFQAGEYQVVLGMSINHHILFLDKLDWILFWALGLAFVISSALSGWMVKRGL

KPIVGLNQHIQQISPEQMGIRLDPDSLPIELRELANKHNAMLDRLQTGFRRLSEFSSDIA

HELKTPLTNITTQNQVILGACRTSEEYQDAIASTLEELNRITKTINDLLYIAKAENKLIH

RHDEVFAVQEEIDRLVAYFEILAEDAEVRIVSSGDGQLYMDKNMFERAVGNLLSNAIRHA

YADSTIVIDVQEVDEQVTVSVSNQGDTIAEQNLPYLFDRFYRADKSRQHVGSVGAGLGLS

ITQSIVQAYEGQIKVTSDKDKTRFVMMLPSAGVNGSDKSVEA

>tr|Q87JX3|Q87JX3_VIBPA Oxidoreductase, short-chain dehydrogenase/reductase family OS=Vibrio parahaemolyticus serotype O3:K6 (strain RIMD 2210633) OX=223926 GN=VPA0125 PE=4 SV=1

MNKVVIVTGASRGIGAATAKKLAEQGYRVCVNYRTQQEAADQVVAEIQANGGKAIAIAAD

VADEQQVLSLFNETEREFGAVTHLVNNVGVLFTQSPLADISLERFQTVMNTNVVSCFLCC

REAAKRFSAGGAIVNVSSGASRSGAPFEYVDYATSKGAMDTLTKGLSLELAERNIRVNGV

RPGFIYTDMHADGGEPNRVDRLSPQIPLKRGGTVEEVANAIAWLLSDEASYVTGSFIDIA

GGR

>tr|Q87NC3|Q87NC3_VIBPA Transcriptional regulator, LuxR family OS=Vibrio parahaemolyticus serotype O3:K6 (strain RIMD 2210633) OX=223926 GN=VP1945 PE=4 SV=1

MINVFLVDDHELVRTGIRRIIEDVRGMNVAGEADSGEDAVKWCRSNHADVVLMDMNMPGI

GGLEATKKILRVNPDVKIIVLTVHTENPFPTKVMQAGASGYLTKGAGPDEMVNAIRVVNS

GQRYISPEIAQQMALSQFSPASENPFKDLSERELQIMLMITKGQKVTDISEQLNLSPKTV

NSYRYRLFSKLDINGDVELTHLAIRHGMLDTETL

>tr|Q87RA7|Q87RA7_VIBPA Putative enzyme OS=Vibrio parahaemolyticus serotype O3:K6 (strain RIMD 2210633) OX=223926 GN=VP0890 PE=4 SV=1

MSHVSDYRPCFLIPCYNHGSTIADVVASLSSFDLPVLVVDDGSNEDTKQALSALSEQGLI

KLISLSENQGKGGAVMAGLHQAHQLGFTHTIQIDADGQHDLEALPKLLSASETKPDNLIS

GQPIYDESVPKARLYGRYATHIWVWIETLSLSIKDSMCGFRAYPVAKTVAVLNKYNIGKR

MDFDIEILVRMYWEGVDIDFVPTRVIYPEGGISHFDALWDNVKISWMHTRLFFGMLPRIP

SLLARKRRHTDDSNAHWSKRKEQGTILGIKTLLAVYRLFGRKAFDLILKLVMRYYHFTGK

SAREASEIYLQNLQRYAAQQNIALPKQLDSYHHLLSFGRTMLDKLAAWQGDFNVDNLTIH

GQHHFEEMAQKKQGVLILGSHLGNIELCRALGRRHSHVKINALVFTQHAEQFNAVMKAVN

PNSELNLIQVSSMGPDTAILLQQKIEQGEWVVIVGDRTSTSKENRVVWADFLGKPAPFPQ

GPFMLASVLKAPVYLLFGLRDDEANTPHFNVYFEHFSDQITLPRKERQQALEHVVQQYAH

RLEHHTLQAPLQWYNFFNFWTLSNQPHDKQQ

>tr|Q87MZ8|Q87MZ8_VIBPA ABC transporter, ATP-binding protein OS=Vibrio parahaemolyticus serotype O3:K6 (strain RIMD 2210633) OX=223926 GN=VP2078 PE=4 SV=1

MIELNNIQVTFNPGTILENRALKGVSLEVPEHQFLTVIGSNGAGKSTLLGAVTGETPMIG

GQVIIDGKDVTKQTVAQRATQCARVFQDPLAGTCGELTIEENMALAYMRGKKRGWGLALS

AKRRKLFQERISILGLGLEDRLGDSIGLLSGGQRQAVSLVMATLSESKLLLLDEHTAALD

PRMAAFVIDLTKRIVNEFDLTVMMVTHSMKDALACGDRTVMLHQGEIVLDVAGEQRANMQ

VPDLLDMFSKVRGEELSDDSLLLN

>tr|Q87ML7|Q87ML7_VIBPA VacJ lipoprotein OS=Vibrio parahaemolyticus serotype O3:K6 (strain RIMD 2210633) OX=223926 GN=VP2214 PE=4 SV=1

MYNKGKSYLMLLLALGLVGCSSAPEEAVTSEGETNQTTSDVYDPLEGFNRTMWEINYEYL

DPYLVRPVSIAYVEYTPVPIRSGIANFLSNLDEPSSMVNNLLMGNGSKAVDHFNRFWINS

TFGILGVFDIATAAGITKYDNKEFSSAVGHYGVGNGPYFMIPGYGPYTLREVTDTVDGMY

LPLSYLNIWAGLGKWALEGLEKRALLVPQEAQLDSSPDPYVLTRDVYIQRQNFKAEIDTV

EEVNPEEEALLDEYLDEF

>tr|Q87KU4|Q87KU4_VIBPA Acetyl-CoA carboxylase, biotin carboxylase OS=Vibrio parahaemolyticus serotype O3:K6 (strain RIMD 2210633) OX=223926 GN=VP2881 PE=4 SV=1

MLDKVVIANRGEIALRILRACKELGIKTVAVHSTADRDLKHVLLADETVCIGPARGIDSY

LNIPRIISAAEVTGAVAIHPGYGFLSENADFAEQVERSGFIFVGPKADTIRMMGDKVSAI

TAMKKAGVPCVPGSDGPLDNDEDKNKAHAKRIGYPVIIKASGGGGGRGMRVVRAEKDLVQ

AIAMTRAEAKAAFNNDIVYMEKFLENPRHVEVQVIADGQGGAIHLGERDCSMQRRHQKVV

EEAPAPGITEEMRKYIGERCTRACVEIGYRGAGTFEFLYENGEFYFIEMNTRIQVEHPVT

EMVTGIDLIKEQLRVAAGQPLSFTQDDIKIRGHAIECRINAEDPERFLPSPGKIQRFHAP

GGMGVRWESHIYSGYTVPPHYDSMIGKLITYGENRDVAIARMKNALGEMIIEGIKTNVPL

QVSIMNDENFQHGGANIHYLEKKLGLQ

>tr|Q79YX3|Q79YX3_VIBPA Soj-like protein OS=Vibrio parahaemolyticus serotype O3:K6 (strain RIMD 2210633) OX=223926 GN=VP2227 PE=4 SV=1

MIVWSVANQKGGVGKTTSTVTLAGLLSQKGHRVLMVDTDPHASLTTYLGYDSDTVSSSLF

DLFQLKTFTRDTVKPLILETELEGMDIIPAHMSLATLDRVMGNRSGMGLILKRALQAVSQ

DYDYVLIDCPPILGVMMVNALAASDRILIPVQTEFLAMKGLERMIRTLTIMQKSRPDGFK

VTIVPTMYDKRTRASLQTLTQLKKDYPNQVWTSAVPIDTKFRDASLKHLPASHFASGSRG

VFAYKQLLIYLERLAFDEQ

>tr|Q87IR3|Q87IR3_VIBPA Putative cytochrome c oxidase assembly protein OS=Vibrio parahaemolyticus serotype O3:K6 (strain RIMD 2210633) OX=223926 GN=VPA0543 PE=4 SV=1

MTLTKLVRLSLCLTLVVIMLGAYTRLSDAGLGCPDWPGCYGHFSVPHHEDDVLRANINFP

EREIEHEKAWLEMIHRYFAGTLGMVIFAITVIAIRTERVNPSIPILLSFLVVGQAMLGMW

TVTLKLMPVIVMLHLLGGFTLLALQAVFYCQLKARDNLYFSPSSRSVRLFSVFSFLVVFS

QVLLGGWTSSNYAALMCTTLPICEGDWMNYLDWKEAFSFWQTGHDNYEFGVLEYPARMTI

HVSHRIGAMVTAVVVLTYCVVLMKQDSHHSQKVGMWLGMALACQITLGISNVVFQLPIYI

AVAHNLGAAIMLSLICVSQFYLWQGKADWNYAVKGVRYE

>tr|Q87JY8|Q87JY8_VIBPA Putative phosphatase OS=Vibrio parahaemolyticus serotype O3:K6 (strain RIMD 2210633) OX=223926 GN=VPA0110 PE=4 SV=1

MKLDAILWDYDGTLVNSVPKNIEITKAILAIVAPHLTGDNLPKYLHSEANYHYANHAAKN

WQELYVDYYGMSHDEMLQAGGLWAEHQEKNQTPVTLFEGIDGVIKEFAQLPHGICSQNSQ

SNIRRVLSDNGISAPFKSIVGYDDVSNGHQKPDAYSGIKCVESIFGHAENRQLMYIGDHE

ADTQFARNIEQQLGGQSKVIAVAAAYSGAMPEHWSVQPDYVARTVDELFSIIAQHT

>tr|Q87PI6|Q87PI6_VIBPA Uncharacterized protein OS=Vibrio parahaemolyticus serotype O3:K6 (strain RIMD 2210633) OX=223926 GN=VP1516 PE=4 SV=1

MNKHHNGIWIAYILSCFTPFTFLISGVIAIIYAGYRLDKGEDSDVVISHYYGLIRTFFLY

LTFFVVLIVTVATSNGVLVGVSDYWVKSTLIEDIAYFIPWVGMVFAALAILVWFIRMFQG

MQQLRNNQPHTPSTGPNL

>tr|Q87KG9|Q87KG9_VIBPA RarD protein OS=Vibrio parahaemolyticus serotype O3:K6 (strain RIMD 2210633) OX=223926 GN=VP3008 PE=4 SV=1

MTPEEQQRARQGVLLAVGAYTMWGIAPMYFKSIAQVSPLEILSHRVIWSFFLLAALLHFG

RHWRSVYHIATDKKKIAYLLSSSVLIGGNWLIFIWAVNSNHMLDASLGYYINPLINVLLG

MVFLGERLRKLQWFAVVLAGCGVLVQLIVFGSVPIVAMALAMSFGFYGLLRKKVAVDAQT

GLFVETLILLPAAAVYLLFIASSPTANMIENPWQLNTLLIAAGVVTTLPLLCFTGAATRL

KLSTLGFFQYIGPSLMFLLAVLIYGETFTMDKAITFAFIWGALVVFSFDGLRNNRRSRRA

MQPQ

>tr|Q87TC0|Q87TC0_VIBPA Uncharacterized protein OS=Vibrio parahaemolyticus serotype O3:K6 (strain RIMD 2210633) OX=223926 GN=VP0150 PE=4 SV=1

MSQAICRMIAQELNVRPEQVNAAVTLIDDGNTVPFIARYRKEVTGGLDDTQLRTLDSRLS

YLRELDDRRQTILKSIQEQGKLTPELEQEITQADSKTRLEDLYLPYKPKRRTKGQIAIEA

GLEPLADTLWNQPQTEPESEATKYLDADKGIADTKAALDGARAIIMERIAEDANLLEKIR

AHLNRNAELVSRVVEGKEQEGEKFKDYFNHNEPLSKVPSHRALAMLRGRNEGFLTLAMNA

DPEQEEGARQSYCETIIADHYGVTLSSAPADAWRKQVISWAWRIKVSMHMETELMGAMKE

RAEIEAIEVFATNLKDLLMAAPAGPRATLGLDPGLRTGSKIAIVDPTGKVLATETIYPHP

PQKQYDKSAQIVDQLVRKYNVDLIAIGNGTASRETDSFVADVIKRGNLKVQKIIVSEAGA

SVYSASELAAKEFPNMDVSLRGAVSIARRLQDPLAELVKIDPKSIGVGQYQHDVSQSMLV

KRLDAIVEDCVNAVGVDVNTASAALLTRVAGLSSTIAQNIVDFRDENGRFEARTTLKKVP

RLGPKAFEQCAGFLRIMDGKNPLDASAVHPEAYPVVKAIAEKNRKDIKALIGDSTFLKGL

HAVDYTDEHFGVPTVTDIIKELDKPGRDPRPEFKTATFAEGVNSVADLEPGMILEGVVSN

VANFGAFVDIGVHQDGLVHISALTDRFVSDPREVVKAGDIVKVKVMEVDVQRKRIGLSMR

LNDEPGQDNRSQRSSAAPRRNDQSQRRQPRRDDSSSNNAMGGAFAAAFAKAKK

>tr|Q87HC0|Q87HC0_VIBPA Uncharacterized protein OS=Vibrio parahaemolyticus serotype O3:K6 (strain RIMD 2210633) OX=223926 GN=VPA1045 PE=4 SV=1

MAEGRVAVGDINILVVDDCSTSSLLVKHQLIALGAKASNITCVTNTQAALLAAKTRFYSF

LVIDYHLAEKYTGLDLVHLLSRAQLISDTTAVLMISSDATKETVLTALSSSGRVRHLLTK

PLQTKALYTKMLQALQEQQHIAAVTKRLLASQPLLLSDVILLHKTHASSICVESLIIDTL

VERRDYTLLEDYLPLCSQKEHASKVCATAFLLHHQGHISEAVKVLADYVTRNPLCLAAID

SLIGLYESLGQQRHALCLAKRAFSLTPSNGSRFLSASRITAKLGLFEDLYELGRTYAAHL

SQTDAQWLNVLSSYVDLVSDHFKTLTHIHTKRKVLVQLNELCLLTQKQLGKEQQVNLLAF

KQLMQCKLLLVESRAAEAHLKLLESLSYFYDIPTQMPIALLKQALPLLAFFGEFSIRRSL

LGVINQSCSSVRLQECNIVPHEYDYPFSVETKLNALAAPDQQYHSNSESVVNFLKQRALP

PNWSRWLSDYLSGSFSSQIPEPFSYHITDRE

>tr|Q87K66|Q87K66_VIBPA Putative membrane protein OS=Vibrio parahaemolyticus serotype O3:K6 (strain RIMD 2210633) OX=223926 GN=VPA0032 PE=4 SV=1

MMRSVVAMLRKEWLENPLVLRLPLFMLACGALLFISLMSNTTLQHNMFFEMSLGGDVSDI

HKELGDDVNMLITGGAGLLSMLLAIQYFPRTLRKERAEGSIMFWRSMPVSDLKTHLVKLT

FGLLVIPLVCSGLVLAADFMLWLLNVSTDQQLALLYRQTSLGYVLLHWVEFLARMALAGV

LMLPLALCAMAISQKVNSPLVILFIAIYALRWMPIAMFGYYGLDQFFSAVLYLPLHAIIA

SNPFSAIPEAGLANVGIYAFIGILAWTASLKFSRTIQ

>tr|Q87LB4|Q87LB4_VIBPA Putative V10 pilin OS=Vibrio parahaemolyticus serotype O3:K6 (strain RIMD 2210633) OX=223926 GN=VP2698 PE=4 SV=1

MKRQGGFTLIELVVVIVILGILAVTAAPRFLNLQSDARESALQGLKGAIDGASGIVFGKS

AIEGIETKAPASDVKVEDVLVGYGYPVAADNGLNNAVTGLDEDWALALEGTTPNKLVATF

ISGDLKKGLDAGNTAAKVKAGNCYVTYTEATSAAVSTTAVTDTGC

>tr|Q87RW3|Q87RW3_VIBPA tRNA-specific adenosine deaminase OS=Vibrio parahaemolyticus serotype O3:K6 (strain RIMD 2210633) OX=223926 GN=tadA PE=3 SV=1

MRRALALAEQAELEGEVPVGAVLVKDGEVIAEGWNRSICSHDATAHAEIQTLRNAGAVLE

NYRLLDTTLYVTLEPCPMCAGALLHSRVKRVVFGAPDLKAGAAGTVLNLFESQAAYHYAT

VEKGLLEDECREQLQAFFKRRRKEIKAKKQEKKVLEGKQED

>tr|Q87QZ9|Q87QZ9_VIBPA Amino acid ABC transporter, periplasmic amino acid-binding protein OS=Vibrio parahaemolyticus serotype O3:K6 (strain RIMD 2210633) OX=223926 GN=VP0999 PE=3 SV=1

MEMKKWLLAATLAATAVSGVAQAKEWKTVRFGIEGAYPPFSWTETDGSLKGFDVDMANAL

CEEMQVKCQIVAQDWDGIIPSLLARKYDAIIAAMSITEERKKKVDFTGKYALIPNKFIAK

KGANLDFANLDGQKIAVQRATTHDKYLTDNYGDKVEIVRYGSFDEAYLDLANGRVAAVLG

DASALEEGVLNKAGGDAYEFVGPSLTDAKWFGEGFGIAVRKQDKDLTKKLDAAILSLRDK

GVYQEIAGKYFNYDVYGE

>tr|Q87T95|Q87T95_VIBPA Uncharacterized protein OS=Vibrio parahaemolyticus serotype O3:K6 (strain RIMD 2210633) OX=223926 GN=VP0175 PE=3 SV=1

MAMPSNLPKSFSRPFLKMFHILEAVLLVAITLATIYAMAQEFIHVFVEKRVLLTDILLMF

IYLEVLAMVQQFVMNGKIPVRYPIYIAMMAIARYITLGMKELDATLVVWLSIAAFILAAA

TLVIRVGHHYWPYVDNRTLEKDE

>tr|Q87JQ0|Q87JQ0_VIBPA Putative GGDEF family protein OS=Vibrio parahaemolyticus serotype O3:K6 (strain RIMD 2210633) OX=223926 GN=VPA0198 PE=4 SV=1

MRYSPRKQCYFALFFFAFVALCVGVIELLHQNQQDLQQERVQVEAKEQLSILRSNLEAVL

MADIYKASTLATLITLLPNSEEEELSIAADRILNKSKHITVIGIAENDVISHIFPTQGNE

RVLGLDYRKVPAQWVQVQKAKDIQEIFIAGPVSLVQGGRGLIVRVPVFRDPPINKEYWGV

ISAVVDFDGLMRETGVFDFSYHYPLTIRGYDSGGEYGDMFFGDPNQTGKLYAKEHVHFPY

GGWSMAAYAGGQLEQQISWFELNIVRIIGYPILIALSIALIVIYRLYIIADDRALHDELT

HLPNRRYFMQSYKQQFEIAKRYKKRYSFALINIDLDRFKHINDTFGHDAGDKVLIATAER

IKSSLRRSDMVARMGGDEFLVVVHNLGMEEHVQKLLQKLRLALCSTPVIYDEELIYLRVS

IGYAIFDPQMTSPEQMMKIADERMYQQKHGG

>tr|Q87GV5|Q87GV5_VIBPA Uncharacterized protein OS=Vibrio parahaemolyticus serotype O3:K6 (strain RIMD 2210633) OX=223926 GN=VPA1210 PE=4 SV=1

MPWIYLRKLILLLFAMVLLPVHVSAAQIDHKAHVPHFSKLQPFVAASVSPNSSVDFSEVS

EESSQSPVSEGHASLDSVALFNSQRWTSYLREGLDDEHVDFVGDLTTPFYADAGYAYSLM

DINWRHNQSTFYHFTSDHRISGWKETNAMYVALNSQFSA

>tr|Q87IV9|Q87IV9_VIBPA Putative regulatory protein, ArsR family OS=Vibrio parahaemolyticus serotype O3:K6 (strain RIMD 2210633) OX=223926 GN=VPA0497 PE=4 SV=1

MSNKCNETCQIAFETTQQQLDKEQALAAQAKALSHPARLRILHILHTLDTMGGCLNSDLV

SELGLAQSTVSEHLRILKQAGFISAEPNPPKVCYRIQREKLNAFNAQFSGLFS

>tr|Q87JF9|Q87JF9_VIBPA Uncharacterized protein OS=Vibrio parahaemolyticus serotype O3:K6 (strain RIMD 2210633) OX=223926 GN=VPA0294 PE=4 SV=1

MTFRQEYNGCKSFVCPNCGVPDLSLYSRSNRLGYDAWHCPECGAYPPVLINEPILALAHQ

LQQQTFELKLLPHCECRLPAWQRYGRTAVGSPRVKCRCCQKTATLPNPIKESRTLQPLLD

ALLAEVSPKDLQYKLGLNHRRFSQSLERLASMLDTFSRLYERHLSFSNIQTRSFVQVARS

GFRHHGREQRAAHIWTLCSADAQTGYVLLLSDNAWFPQTEMSEHVIPQPLWEQSRYQLTQ

QEEMPNESDIFLQAQRTYDKILSRSQFDQLAYCDGSHAKSKEVLLTRPVFAAHAHMQNLA

KYFANKVPKRFVLEHESFLRGAAITAFTSAIKRGTTQLYYCHIGQSEENLSLNHAKQKMS

WWDETWRRGNIQNQYGSWQVGMGWLTQKSARPSESIDELIPAHPNWNSEFWNRYTQWLPP

SYAAHLSLQRIKQWQAIYRFLYNYSAMQSPPKVGVDEIDLSRVDSIAESINRSVNAPVHL

>tr|Q87T55|Q87T55_VIBPA Putative OtnG protein OS=Vibrio parahaemolyticus serotype O3:K6 (strain RIMD 2210633) OX=223926 GN=VP0215 PE=4 SV=1

MNLIRTSLSLSLIGPALVWAQDMPFEAPPLQPSQMDFGGVGLMQMPTGRMAPEGEFNFAI

NNSNEYTFYNVTLQVMPWLETTIRYTTAKDVLYSEDPNFSGNNEYTDKGIDFKVRLLQES

KYLPELSVGVRDFAGTGLFDGEFVAATKRYSNPNLGTFDFTLGIGWGYLGTRDNISNPAC

KLSDSFCERPSDFKGNGGSVDFERWFKGPAALFGGVEYQTLHAPLRFKLEYDSNDYSEDF

PVVRGGVDMTPHTPWNFGVLYRLGHMADLRLSYERGDTLVAGVSLYTNFNNMPSFWRDTP

TPEVEDKQPEQLSDVDWERVTEELDKIAGYQNTQLYVEDNTVSVVGEQKKYRDRNEAHEK

AAAVLYNEMPDNIDTFTINERSSGLIGDQTVISKEKYRDFAEVNYINPNIEDATSTSSDK

PQGKPAYDGFERFDWSFSPKLAQTLGNPEEFYLFSVGLSGSASYWLTDNLEIGGSLYWDW

YNNYDKFNYVTPPDGTSIPRVRTMFRAYQNEHAVTMSNLQLTWFQEYSDTMDQQFYAGYL

ESMFAGVGTEFLYRPKGANWAIGADVNLISQRDPQSYFGVYDEKWQYVPEYGRPFQVVDK

GFTGFVSGYYYPQWDFLQDLMIQVDVGQFLAGDVGTQVNVSKQFKSGVIAGAFASISDLS

ADEFGEGSFTKGFYISIPFDIMTVKPSNNRAFFSWQPLTRDGGQKLGRKYSLIELTDERN

PWYQRPNASNAK

>tr|Q87H36|Q87H36_VIBPA Putative hydroxymethylglutaryl-CoA lyase OS=Vibrio parahaemolyticus serotype O3:K6 (strain RIMD 2210633) OX=223926 GN=VPA1129 PE=4 SV=1

MSVKIRSTNMLPAKVNIVEVGARDGLQNETAVTLVDKIRLINLLSNCGLKHIEAGSFVSP

KWVPQMADSDQVFAGITQKPDVVYSALTPNLAGLERALESGVKQIAVFGSASEAFSQKNI

NCSIAESLSRFEPVIELAKQHNIPVRGYLSCTMVCPYEGEMKPEQTTSVANTLFDMGCYE

ISLGDTVGKATPNRVIAMLDSLLTQLPKDALAVHFHDTYGQALANIYQALLMGINTIDSA

VAGLGGCPYAKGASGNVATEDVLYLCEQLGIETGVDLNIINEAGWQICHALGKRPSSKVA

LALGDPHSL

>tr|Q87M83|Q87M83_VIBPA RecBCD enzyme subunit RecC OS=Vibrio parahaemolyticus serotype O3:K6 (strain RIMD 2210633) OX=223926 GN=recC PE=3 SV=1

MFTVYHSNQVDVLKSLLVELIRISPLENPFEKEQILVQSPGMSQWLKMELAKEFGVAANI

DFPLPATFIWEMFTQVLPDVPKRSAFNKEAMTWKLMHLLPGLLNQEVFSPLAQYLKDDSD

HSKLYQLAEKIADIFDGYLVYRPEWIASWEAGQSVPELEDEHPWQPILWQALYDHTVSLG

QSPCHRANLYEHFIDTLASFNGNFDHLPKRLFVFGISSLPPRYMDALKAIGEHIDVHLMF

TNPCRYYWGEVRDRKYLARLAAKHRQHVVWQDDHSEVQGETEQLKGSLEDNVIDELHTDV

VGNSLLASMGKLGRDNMYLLSQLESHEIEAFVDVERDSLLHQLQADILNLEEHQDDQQIE

NSHHKQVVSLGDKSLSLHACHSPMREVEVLHDQLLAMFDADPTLKPRDIIVMVADINAYS

PAIQAVFGNAPGERFIPYSISDRTADQESPILAAFMQLVNLPNTRCLASELLELLETPAI

LKRFELSEDDFLQAKQWVEESGIRWGLDANTGREFELPETRQNTWQFGIQRMLLGYAMPE

SAGLFETEQGSLSPYNEVQGMGAELAGKLAHFIQQISTYRSKLSHVQTIDGWRETLVSLL

DDFFSVELEGEMALKSIRDTLSQLKEQLADAAFDDALSPSIISQYLQNKLSGTRVSQRFL

AGQVNFCTLMPMRSIPFRTVCLLGMNDGVYPRSMPPEGFDLMTGRTKPGDRSRRDDDRYL

FLEAMLSAQQTLYISYVGRSIQDNTERVPSVLVSELMEYCHQNYCLSGDEALPVDESGDN

LLNALVNTHAMVPFSPSAFQGTHASYAKEWIPAALTQSSQSQGYVRRDFNRALDDYLFGA

TFPLELDLVELQRFWRLPVQYFFNRRLKVIFEPPLPVMEDDEPFVLGGLESYQMRDELLE

TLLETTLMQPEKKTEVLKHFMSQQRAQGKLPVGAFGDIEFETNRVQAEELVDKLAFLSGA

PQDDLEIDLTFDSLFERNEGEEKNVRLTGWLTQCYQSGLIRYRSGKIRSQDYLAAWIDHL

AMSASGYAKKTHLIGYDRKEGALHLIYPEIADAQYAKQLLTELVRLFFEGMTKPLPYFPK

TALACVEAGFSRGQWVDDEEKSLKKMADAFNDGFMSPGEGNNAYISRIWPAWNDELASEV

RLLTALVLQGARLAVQDADDVKA

>tr|Q87NU2|Q87NU2_VIBPA Aldehyde dehydrogenase OS=Vibrio parahaemolyticus serotype O3:K6 (strain RIMD 2210633) OX=223926 GN=VP1776 PE=4 SV=1

MNQKVQYVRCGGDVDGQGAFVSPTILDNVDNKMRVAQEEIFGPVLCVIPFKDEAQAIEIA

NDSKYGLGAALWSSNINRVHRVAKRLQAGSVWVNNYNEGDMTVPFGGFKMSGNGRDKSLH

AIEKFTETKTTWIRLHP

>tr|Q87JN1|Q87JN1_VIBPA Uncharacterized protein OS=Vibrio parahaemolyticus serotype O3:K6 (strain RIMD 2210633) OX=223926 GN=VPA0217 PE=4 SV=1

MKYDWILFDADETLFHFDAFKGLQLMFSRKGVDFTEQDFAHYQTVNKPLWVDYQDGKVTA

DELKHKRFTEWAEKLNTTTADLNSAFLEAMADICSLLPGAKELMEALQGKAKMGIITNGF

TELQAIRLERTGMTEYFDKVVISEQVGVAKPDLGIFEYAMQQMGNPCKTRVLMVGDNLHS

DILGGNNFGIETCWLNTTGASVDERIAPNYTVESLSELKNILVA

>tr|Q87Q13|Q87Q13_VIBPA Putative permease of ABC transporter OS=Vibrio parahaemolyticus serotype O3:K6 (strain RIMD 2210633) OX=223926 GN=VP1337 PE=3 SV=1

MSRELTSLDSVLEIERHVNYQQSELRAQLQKAERNKNLRSIMLTLPLVCFILLTFAFPIL

EMLYRSVDNRDIPQAMPKTIQALAHWDYQGLPDSEVVEAFSVELLALYETKALPKIANRM

NIEVSGMRSLMMKTGRKLSRLEVLPTSIKELSRLDKRWADAKHWVAFKNLSGAITVNHYL

AALDMQVNEMGEIEAQPEKRQIYVDLFFKTFWMSILITVICLLMAYPVAYLLANLPDKRA

NLLLIVVLLPFWTSLLVRTTSWIVLLQNQGVINDLLIWSGLTSERIQMIHNTFGTVVSMV

HILLPFMILPLYSVMKGISPTYFRAARSLGATPLVAFVKVYMPLTLPGIGAGALLTFILS

IGFYITPALVGGRSGQMISNMIAYHMQTSLNWGMAGALGGLLLFVVLALFYVFNRVVGIN

NIKVGG

>tr|Q87IC2|Q87IC2_VIBPA Putative arylsulfatase regulator OS=Vibrio parahaemolyticus serotype O3:K6 (strain RIMD 2210633) OX=223926 GN=VPA0684 PE=4 SV=1

MSKISPRHSTTMPIVPLKTASKAPVSGAYDRRFHVMAKPGGAKCNIDCQYCFYLHKENLL

HQEKQPKMDDATLEAFVKSYIESQDGEEIVFSWQGGEPTLLGLDYFRNVVALQKKHQPKG

VRIENDLQTNGILLNDEWCAFLKEHNFLVGLSIDGPRELHDKYRKTRSGKPTFDLVMKAV

DKLQAHGVKFNALVTVNRHNAKYPLEVYRFLTQELGVTYIQFAPVVEANDFQTTAPQFWN

EQMIPTKGSDLAKPGHLMSVVTDWSVDPEDWGRFLMATFEEWVNNDLGRVLVNLFETAVA

QVMGKPSQLCVTAEFCGKGLAIEHNGDVFSCDHYVYPEYKLANIHEHSLNDMAFSTRQYT

FGMAKRESLPTYCKQCPYLPYCWGECPKNRLIKTPNGEAGLNYLCSGIKMFFDYALPMLV

GLAQLLQSEEPQR

>tr|Q87J87|Q87J87_VIBPA Uncharacterized protein OS=Vibrio parahaemolyticus serotype O3:K6 (strain RIMD 2210633) OX=223926 GN=VPA0366 PE=4 SV=1

MWYTKLIINYRNINKDFNMLRFIQRRRIKKVIKLMSTRLIVGYGSREYFSVGQVKTSTSE

LSACQQKIALALYANPQDLDLENQPELQAIRSDVAHDFFAGVDYTAQDVLHLFGAGGWKG

GRMEDGMSHHFGMHSRY

>tr|Q87L51|Q87L51_VIBPA Bifunctional aspartokinase/homoserine dehydrogenase OS=Vibrio parahaemolyticus serotype O3:K6 (strain RIMD 2210633) OX=223926 GN=VP2764 PE=3 SV=1

MSVQRQLHKFGGSSLANPECYLRVADILKEYSAENDLVVVSAAGKTTNRLIEFLEGLDKD

GRIAHEALQGLRQFQSELIESLLEGEVQTQLLASLHDEFSTLAELTAPLTDAQKAAVLGH

GEVWSSRLLAALLSQQNVPAVAQDARAFLRAEAGTQPEVDRARSYPLIKEALAQHSHKRV

IITGFMAQNEAGETVLLGRNGSDYSATVIGALAEVTTVTIWSDVAGVYSADPRLVSDACL

LPLLRLDEASELARLAAPVLHSRTLQPVAQSTMDLSLKCSYQPESGSTRIERVLASGRGA

KIITSLDEVLLIQLSFRHGHDFNKTQSDVLKSLQRAQLEPLSYEVQADQQKLRLAYTAEI

ATGALKYLQDLAVEAEIKLKEGYSLVAAVGAGVTKNANHCFGFYQKLKHAPVEFVSETES

GLSLVAVLRRTDTEALVQLIHSQLFQAQKRVAVTLCGKGNIGSSWLNLFAIQKTELEKRH

GMSFDLVAVVDSQTYWFDEKGIDAAAVADKFDDESIENDGTWLSRLGDLQGYDEAVVLDV

TASKELAQRYVDIAQQGIHLISANKVAGSADSQYYHQVQDAFAKIGRYWLYNATVGAGLP

INHTVRDLRESGDEIVALSGIFSGTLSWLFQQFDGSVPFNELVDLAWQQGLTEPDPRADL

DGSDVMRKLVILARESGLDIEPDSVKVESLVPEELRSLSLDEFFDNGALLSEILQERLTK

AQRDEQVLRYVARLEKNGKATVGVEALPREHALANLLPCDNIFAIESKWYKDNPLVIRGP

GAGREVTAGAIQSDLNRLAGLF

>tr|Q87ID4|Q87ID4_VIBPA DNA helicase OS=Vibrio parahaemolyticus serotype O3:K6 (strain RIMD 2210633) OX=223926 GN=VPA0672 PE=3 SV=1

MQLTATSKAQFFIQNEYYHVEIKENSVLLSSIGSEEHIPFTVWNGKVNVKRGLFWSSLQF

FAHEQDGKQQSWLVQGLPWPQCRKFALEAVRQYQDWHNTQCRKLAEYLPKWEEELYQLKH

LPAYLSHSQVMAWVEKLNQELAEIKTSLDEAKMRMPNRMGEIEPWLVDTSHTLGERNHEW

LENERPNWEVLFNRIESSPLNLSQQYAVLLNDDHNLVLAGAGSGKTSVLTARVAYLLQSH

QAQAEELLMLAFGRDAAKEMKERLVDKVGLAAEGVRVNTFHQLGLYILNQVEQQPVEISP

LALDDNQRTAWCVDWLKKHWMTPTNFKRWQKHLDKWPIAYLKGDDELGSHSENPKLIAWL

DSQLSHLAAVGLTKKQVQEKLVDHQDYTRLNSELALCWPCFSAWQKMLKESNQVDFPTMI

SRATDYVNKGKFVSPWRFVMVDEYQDISPDRLALIEALCESTEKQPGATLFAVGDDWQAI

YQFAGADVDLITGFKERFAHSTVHHLDTTYRFNNQIGDVANTFVQQNPSQLPKTLNSHKQ

RKQKSVHTAPSNQVEKILDQLNQQAKQTKSVLLLGRNHYHKPDLYDDWLRRFPNLDIRFM

TCHASKGREADFVIILAVDEGQFPAKKKQIHIDGALTESKDKFPYAEERRLFYVAVTRAK

EKVWITHTGAGSAFVQELVSGDYPIVTSR

>tr|Q87GI3|Q87GI3_VIBPA Uncharacterized protein OS=Vibrio parahaemolyticus serotype O3:K6 (strain RIMD 2210633) OX=223926 GN=VPA1333 PE=1 SV=1

MKLNIKRLHLSLTLMSVVMLLVIIYNNFFQPVHFYETSYKYQAADSTYMHDVAINVSIKG

NHFTSDIIIRELVKSENKNYYNVIGHGDIIQKNTHQYYLNFDNIDVYTGTNKANMKPYKE

PTSISSLINKSNNIRVVYLSEEYVVVEFFFYDGQIITLHRY

>tr|Q87J94|Q87J94_VIBPA Uncharacterized protein OS=Vibrio parahaemolyticus serotype O3:K6 (strain RIMD 2210633) OX=223926 GN=VPA0359 PE=4 SV=1

MVSKLEFSHAVAAIRKERGLTQGQLADELARSYSAFESLNQPTLSQWESGKVTPSLLKRL

AFAHYIGKQYQYTSSEYKRVKASQSKSIYLSFKDIVYEYRVTDVKNCALSQISLTEYEQI

NAVHKQLITPGGIEDTLNQFNESPSKARLYYCEGMLVGHLIYQELRSEFRILSFWHLGRS

ILKYLVTDILQVMGNTIIHFPVHEPVIKQLLFDIFIRDFYQHRRVTFFRAPATQIFGNPM

VKYCFDGLLDLVLYRFHQVGNEFMQSRQEVH

>tr|Q87NR6|Q87NR6_VIBPA Uncharacterized protein OS=Vibrio parahaemolyticus serotype O3:K6 (strain RIMD 2210633) OX=223926 GN=VP1802 PE=4 SV=1

MDSRKNNSRLMNDSVTQVFNGTINTNRSSVLKEIHRHKSNVEYFINIEINCISIDFLTYD

SWDMANANRIFEYKYEGTNLEDEFLLGVFDQANSVMLEKGYVLSQYYLHVDLSALTVDIL

DIVKPNIFLDMLRCATEIFIYTYDKNRLNSNDTFSVLQGQVNCAVSCSSGFYHTSKMNFD

CKLPLGKQNVSRFNGEIMTRKLIIFGNGLGMALDAQHFSLKAALKDIWDKPDFLTLEQQQ

LIERCLGRAGAPEGEDELDLLHQAVTHCKSLNRIGDGDIHWLTEDGQSFPEITATYIHKV

ATKLHNFDGSLPHAFETALVDFIQQTKSHVATLNYDKLLYNSFIDNDLVDGYNGSLIDGM

LNRGFSADALERKYDNNFGYYLHLHGSPLFMDYEGSIVKLSRDSLNLQLNIPSRHIVLTH

VKHKPSVIAASHALSTYWDYLQFALSEAEEIILFGYSGFDTHLNMLLKPYLKVTPLRVVE

WSGAGEQVQRETYWANCLGRSVVVERMDNISDFVAW

>tr|Q87SF2|Q87SF2_VIBPA Uncharacterized protein OS=Vibrio parahaemolyticus serotype O3:K6 (strain RIMD 2210633) OX=223926 GN=VP0472 PE=4 SV=1

MKQEDDLDLTSTLLISLLFLLLGLSCIYFFYNDVINYVKYIYNKDEVIVISQGALYGLFG

SWIFISCFIFLITVFLNKRKLSHKKEALFVRVIISFSIITIVAPTALMFLTKEHLIRQGY

SFSIDEDKSWLQDRVYIFKK

>tr|Q87SX3|Q87SX3_VIBPA Uncharacterized protein OS=Vibrio parahaemolyticus serotype O3:K6 (strain RIMD 2210633) OX=223926 GN=VP0299 PE=4 SV=1

MWKKVLASCLLAIAFTTPVMASDEPLTLDWIDLVPEAERKLFDSVGMPASDHSGGAAQQS

KIGNVRPELNGSQVKIPGFVIPLEGDANTVTEFLLVPYFGACIHVPPPPPNQIIYVKFPK

GAPVQELWDVIYVVGTLKTETINHELAETAYVIEGSKIEAYDDM

>tr|Q87HD3|Q87HD3_VIBPA Uncharacterized protein OS=Vibrio parahaemolyticus serotype O3:K6 (strain RIMD 2210633) OX=223926 GN=VPA1032 PE=1 SV=1

MTQWKNALSEGQLQQALELLIEAIKASPKDASLRSSFIELLCIDGDFERADEQLMQSIKL

FPEYLPGASQLRHLVKAAQARKDFAQGAATAKVLGENEELTKSLVSFNLSMVSQDYEQVS

ELALQIEELRQEKGFLANDTSFSDVRDIDDRLGGYIELFSTAGNYFLVPIASINTLEIKS

ATSLLESVWRPVEFDIDGLGEGEGHMPMTYVDSESDAQKLGRETDWKQIADKEVYLGLGL

KCWLVGEMALPISDLQNLQVIKELA

>tr|Q87QS8|Q87QS8_VIBPA Uncharacterized protein OS=Vibrio parahaemolyticus serotype O3:K6 (strain RIMD 2210633) OX=223926 GN=VP1071 PE=4 SV=1

MLSNQRLSSRKSLYLITSRTGVFTFRWNLRVNGKHHQPTLSLKTRDYLQAVKLASEIAIR

IQQITLPTLEDIKSIYSDFKGSQSKKALLLQSIDISNHLTDLSIKSQTEYRNCWNSFVSA

LSVSTTLDSVRQSHIEQWKKTQTCSLTTMKKKLRLLSSCFGRVGHKVEQDWFKIAVEKTP

VRPKRAITKQELEKLLKATQCYKKSKDEWKYYLPRIAALTGCRLNEIAQLRVCDVHLGNE

PTLNINDDHDDKKLKNSSSKRVLPVTAPLLNLLSELIAGRGKNEHLFYLPYSKQNGYAGK

PSKYFSELLKSLDIKGVSFHSLRHYVVTELFNAGVKEELIGSLMGHSVGKLTTGKIYLSG

FSYSNKNNALNILGVGFF

>tr|Q87LS1|Q87LS1_VIBPA Protease, insulinase family OS=Vibrio parahaemolyticus serotype O3:K6 (strain RIMD 2210633) OX=223926 GN=VP2540 PE=4 SV=1

MRMFWVGACSLLVITGCASNSPVSSLPKGVTFVESSKAEDGKVKIPYQKYKLDNGLTVIL

APEGSDPLVHVDVTYHVGSAREEIGKSGFAHFFEHMMFQGSENVGDQEHFKIITEAGGTL

NGTTNRDRTNYFETVPANQLEKMLWLESDRMGFLLDAVSQRKFEIQRSTVKNERAQRYDN

RPYGLIWERMSEALYPEGHPYSWQTIGYVEDLERVDVNDLKAFFLRWYGPNNATITIGGD

LDVEQTLAWVNKYFGSIPRGPEVENAPKQPAKLQEDKYITLEDRIQQPMVMIAWPTTYSG

EESQASLDTLSEVLGGGTNSVLYQDLVKTQKAVDAGSFHDCAELACNFYVYAMGDSGDKG

DLSTLYGELMKSMSKFAEKGVTDDRLEQLKGKAEADAIFALESVKGKVTQLASNETFFGQ

PDLIEKQLEQIRAVTPQSVEKVYQNFIQGKSKVTLSVVPKGKTDLAVKSATFTTPERTLP

EYKKITDDQLAYRRATDNFDRSVQPPVGEPVKATMPELYNVHFDNGSELLGTVSNETPTV

MMQFSLPAGTRFVEKGKEGLAQLTAAMLQEGTTKRSVEQIQAELDKLGSMISVDATGYTT

NISVSSLEKNLEPTLKIVEEMLLSPAFKQEDFDRVKMQALEGLVYEHQNPSWMASQASRQ

VLYGDSVFARPKDGTQAGVSALTLDDVREFYAKHYTPQSAQVVVVGDIAKQDIEQKLAFW

AEWKDEAAPLYAPQTIPALGEQKIHLVDKPGAPQSVVMMVRQGMPYDATGDFYLSQLANF

NLAGNFNSRINQNLREDKGYTYGAYGYFSGNPETGSVVFTAQVRADSTVASIIEMENELN

EYAQSGMTDEEMKFMRQAVGQKDALKYETPTQKAELISDILKYNLDQDYLQQRNAIVEKV

DKQTLNALAQKWFDPNDYQIIVVGDAKSLRPQLEKLGKDVEELEIIR

>tr|Q87IK1|Q87IK1_VIBPA Uncharacterized protein OS=Vibrio parahaemolyticus serotype O3:K6 (strain RIMD 2210633) OX=223926 GN=VPA0605 PE=4 SV=1

MALGCILKTTYQENVMSVSQMATSRLYKLVQILCSARNANLPVEQGKCLYEPIENGVELH

HAHFLLDSQHSEYTSPIAKILFDSQTQLWRFYVPASRSEDIRWIPYKKLPHSHTLEDLLA

ELESDPQACFWE

>tr|Q87GX3|Q87GX3_VIBPA Putative deoxycytidylate deaminase OS=Vibrio parahaemolyticus serotype O3:K6 (strain RIMD 2210633) OX=223926 GN=VPA1192 PE=4 SV=1

MISKWAKRFYQMAELVASWSKDPSTQVGAVITNQNRIVSVGFNGYPHGVSDSVDTDEREL

KYLKTLHAEENAILFSKRDLDGCDIWVTHFPCPNCAAKIIQTGISRVHCPEQSEDFLSRW

GDKIQVSQDMFDQAGVEVDWLPLDDINFEDKDVR

>tr|Q79YX0|Q79YX0_VIBPA Flagellar biosynthetic protein FlhB OS=Vibrio parahaemolyticus serotype O3:K6 (strain RIMD 2210633) OX=223926 GN=flhB PE=3 SV=1

MAESDGQERTEEATPRRLQQAREKGQVARSKELASASVLIVGAIALMWFGESLARSLFSI

MSRLFDLKRDEIFDTTKLFDIALGAMTDLLFPLFLILITLFVAATIGAAGVGGISFSAEA

AMPKLSKMNPLSGLKRMVGMQSWVELIKSILKVVLVTGVAMYLIQASQADLIQLSMDVYP

QNIFHALDILLNFILLISCSLLIVVAIDIPFQIWQHADQLKMTKQEVKDEYKETEGKPEV

KGRIRMLQREAAQRRMMADVPQADVIVTNPEHYSVALRYKQKTDRAPVVIAKGTDHMAMK

IREVAREHDITIVPAPPLARALYHTTELEQEIPDGLFTAVAQVLAFVFQLKQYRKRGGQR

PKIQDYDLPIPPEHRH

>tr|Q87KJ2|Q87KJ2_VIBPA Putative lipoprotein L OS=Vibrio parahaemolyticus serotype O3:K6 (strain RIMD 2210633) OX=223926 GN=VP2985 PE=4 SV=1

MKKLVTVLFVMAAAMLAGCGQSGSLYIPDDAQQNEQSQ

>tr|Q87IW8|Q87IW8_VIBPA Transport permease protein OS=Vibrio parahaemolyticus serotype O3:K6 (strain RIMD 2210633) OX=223926 GN=VPA0488 PE=3 SV=1

MMKPIARMKAVMIKEIRQLSRDRITFGMVVMIPLIQLLLFGFAINTDIRNIPVGVVDQSG

STAGRIITQSVKVTQVVDIKETYATAQEAEQAIQDGIVRAVLILPSDLTQRMMQGRELGQ

WIVDGSDTMISSAILSLQTMPLTDFDFEIRPAPSKTFEVALYYNPSRRSAVNIVPGLLGV

ILTMTMILFTSAAIVRERERGNLELLITTPIHSIELMVAKIVPYIFVGLIQVFIILGLGH

WIFGVPINGAISQILFGTLLFIAASLTLGLVISTIATTQLQAMQMTVFILLPSILLSGFM

FPYEGMPVAAQWISEALPATHFMRMIRGIVLRGADLMDLWRDTLWLLGFTVFGLVVASLR

FKKSLD

>tr|Q87TA4|Q87TA4_VIBPA Putative TolR OS=Vibrio parahaemolyticus serotype O3:K6 (strain RIMD 2210633) OX=223926 GN=VP0166 PE=3 SV=1

MKGFKTLAAGLLASLFMVSGVQAETPQTLSELLKNVKSESIVESKENKEREAVFKRDRDQ

QAVLLQQAREELAAQQALGDQLKATFDENDKQLTELTETLRVRSGTLGEMFGVVRQYAGE

FKGLFAASQNAVQFPERDALLTKLAESKELPSTQELEAFWHTILQQVVVSGDTSTTQATV

VYGEGKEAVRDVTLVGEFNAIADGKYVIYVPQTGKFEELSRQPSKNITSQVAGFESAKGT

YEPLFLDPSRGVILSLLVQSPTVQERIDQGGIVGYVILAMGAVGVIIALLCFLRLQIIGG

KMRKQAKSDTVIPGNPLGEVIQAYQDHKGDNLEDLEAKLDEIILRNAPSIERFISSIKLF

ASVAPLLGLLGTVMGMIGTFQAITLFGTGDPKLMAGGISEALVTTMLGLVVAIPLLFLYT

IVHSKGRRLVQTLEEQSAGFIARYQEKLHKAES

>tr|Q87PK4|Q87PK4_VIBPA ABC transporter, ATP-binding protein OS=Vibrio parahaemolyticus serotype O3:K6 (strain RIMD 2210633) OX=223926 GN=VP1498 PE=4 SV=1

MTIKITAEQLSMRFKERVLFHIPELAIGPNDAIYLKGDNGVGKTTLLKILAGLLKPSTGD

VVAPKDTWLKRLTRRNGRVDVIYLHQSPYLFDGTVYENVVYGVKYQQDTPKDKRAQVIHA

LRMVGLETLADEHISVLSGGEKQRVAMARAWILKPSILLMDEPSASLDQESIERLVVMAK

DLLDRGSSIVVTSHQTNALTDLCKKQWWIKDKTLIESPLLYVIPKETSQENAYASTNTN

>tr|Q87GA9|Q87GA9_VIBPA Putative lipopolysaccharide biosynthesis protein OS=Vibrio parahaemolyticus serotype O3:K6 (strain RIMD 2210633) OX=223926 GN=VPA1408 PE=4 SV=1

MMKTKILHIINDLSQNGGAQKFLVDLVLEHNPEYEIKILVLCAENDYLEQLSARGIQCFN

WQTLSLSEKWSLLRWPDLVHGHLYPSIYLALLAIGKKRIQTEHCSYNRRRDYPLFKFIEY

VLYWGHHYTVSISEKVQEELVKFMPRYQHKYRVVHNGVDLNRFPMTAKQAHDISEKATIN

IGMVGRLHEHKDHETLIHAIAKLPECYHLHLAGDGDRKASLQRLAQSLNVSPRVHFHGVV

SDIPRFLSDMDIYVQSSHVEGFGLAAVEAMAAGLPVLSSDVPGLDEVTGSSEYLFEVGDS

NLLAQKVAELCEDAALYNSASHYSVNRAKLYTIDKFREGYYGIYQQLCTDK

>tr|Q87L30|Q87L30_VIBPA Glutathione-regulated potassium-efflux system ancillary protein KefG OS=Vibrio parahaemolyticus serotype O3:K6 (strain RIMD 2210633) OX=223926 GN=kefG PE=3 SV=1

MAVAASILPRVLVIYAHPEPHNSIANQVMIKRVQSLEHVTVHDLYGAYPDFFIDVNHEHD

LLMNHDVIVFHHPLYMYSCPALLKEWLDRVLGKGFAFGDGSALEGKYWRSVITTGGKEEA

FSARGYNKYPLEQILQPFELTAALCQMHWMEPLVLYWSRNVSDIERYEHAEQYRRWLSNP

LDEWEALHQGGQHGDNQ

>tr|Q87T35|Q87T35_VIBPA Putative epimerase/dehydratase OS=Vibrio parahaemolyticus serotype O3:K6 (strain RIMD 2210633) OX=223926 GN=VP0235 PE=4 SV=1

MQRLQFIWQLSRTNKRIVSVLIDIILIFMAFHLAILVRSGDSNYFADPAVWGIQVAVTIV

TITVFARLGLYRAVLRYLTFQALFVVAAGAVISATLVAALSFYMSDPFPRTVPIIYGAFL

ALFCGGPRVIVRSLIAQSYSTQSKEVLIYGAGSGGRQLAMALRSSGDYRVRAFIDGDSTL

CNTMILGLPVIAIEDAMPIINKYDVSQVLLAVPSAKRSRRKVILDELAKLPVEVLTVPDM

TDIVSGKAKIDELKDVAIEDLLGRDPVAPQQVLLEANIKDKVVMVTGAGGSIGSELCRQI

VEQSPKSIILFELSEFGLYQIDRELNQLKLEKGLTCDIIPLLGSVQRQHRLETTMSSFKV

QTVYHAAAYKHVPLVEFNVIEGVRNNIYGTYYTACAAIKAGVESFVLISTDKAVRPTNVM

GTTKRMAELGLQALADQENAKPNGTRFCMVRFGNVLGSSGSVIPVFKKQIASGGPVTVTH

PEITRFFMTIPEAAQLVIQAGAMGKGGDVFVLDMGESVKITDLACNLIQLSGLEVKSDAN

PHGDIEIQFSGLRPGEKLYEELLIGDNVKQTAHERIMTAHEVHLPLKEYELLLNDLDFAC

HSMDHENIRTLLLSAPTGFNPTDGIGDLVWNHNQSEIDYSDAIQFMSQS

>tr|Q87J05|Q87J05_VIBPA Uncharacterized protein OS=Vibrio parahaemolyticus serotype O3:K6 (strain RIMD 2210633) OX=223926 GN=VPA0450 PE=4 SV=1

MSTIQINSQHRGNLDLADIQNIKTNAKEGDTVKFGSVFGKEYSVTKSNDGEISLKQKENR

SFFNRFFSKTDSSKNSDLKLNLMNQQLHKKENNGNVKVLTLTYNQANQKMPAETKNYFQN

LIQKGDYDVVLFAEQESKLLANDLELDGMNLLSQNKMKVMTKGLGEGISYTSMSVFAKDG

VDINVKNESEYRHGIGGRNMEFFMGITGNKGGVKTALEINGQPLNVISAHLDSNKEVKRE

FEGNKLMEGINPNEEVLITGDLNEREKRVAEGSDVLYDPIAHDDTHLAKHGFKFKPLDSH

TYMQLDKHTGNIKQKEGRDRPDFGELDNTGLTNKTGNLQNHQTSVITDGFENVSDHKPVQ

STFEVRSFSQKLIENAFTQNANDFKNDAAYLKPGTNPTNATFDDVTSANQARLGLENLNP

NEQAFVKENFASFIIGKDAIFSQLTSGFMEEMSQLHASDLAKNPTHLQAQQIALSEKYEQ

LSDKVNAEFNKQFVANL

>tr|Q87JI0|Q87JI0_VIBPA Flagellar hook-associated protein 1 OS=Vibrio parahaemolyticus serotype O3:K6 (strain RIMD 2210633) OX=223926 GN=flgK PE=3 SV=1

MNLVNIALSGLNANRVALDVTAQNVANVNTPGYSRQQALMATVGDGKSNRLSSGMGVEVT

SIRRVTDQFLVKQTWSTNSLAAYAAGYTSNMSQLENTLGADGFSISAGLDSLNAALNDAT

VKPESMPYRQQIINESEALARRFNTLTQSLHNQHKDMNDQRTAALTHANSLMSNIAHVNK

QIVEMQGTGGNPAQLMDTREKLIGELSQVIEVKTTDQPDGSMQVTLVSGQPLVMGSDFGQ

LSAIPDPSDPYLADLHVNFANQSFAIGDSVGGKLGAINDYQTEVLKPNQVALDDMAKALA

DEYNAVLATGKDLKGNAGKPLFNYDPDNPAASLTITDLSAEELAFSSDGTPGNANVLKSL

IDLSNKPVAVTGYGSVSLNDAFTSMVGQTAIKARQADADYQAKLAMSKQAHTARDNVSAV

NSDEEAANLMTFANAHNANMKVISTANQLFDSVLQLF

>tr|Q87QE7|Q87QE7_VIBPA Putative two-component response regulator OS=Vibrio parahaemolyticus serotype O3:K6 (strain RIMD 2210633) OX=223926 GN=VP1202 PE=4 SV=1

MSRNLCPLYIVDDEVPVLESMAFMLESYGYSVDVYSNGQDFLQSVNLHQAGCVLLDSRMP

EMRGQELHLLMRNQCSPISVIYLTGHGDIPMAVEALKEGALDFFQKPVDGNALVAAIDNA

MECSLKNQEKQSAKLTLQSLTRREKEVLILVVKGMKNQDMANQLCVSLRTIEVHRSNVMK

KLEVESLAALIHKVGHVI

>tr|Q87QQ3|Q87QQ3_VIBPA Putative tryptophanyl-tRNA synthetase OS=Vibrio parahaemolyticus serotype O3:K6 (strain RIMD 2210633) OX=223926 GN=VP1096 PE=3 SV=1

MNIKETHQQREIILTGDRATGPLHLGHYVGSLQQRVALQSEHDQTILVADMQGLTDNAHN

PSKVSSNILNVVADYLAVGIDPIQTTICLQSQLPALAELTMYYSNLVSIARLERNPTVKN

EIQSKGFERSIPAGFLTYPISQAADITGFRATLVPVGDDQLPMLEQTNEIVRKINHLGGQ

EILKECRPLLSDAPRLPSTDGKNKMSKSMGNAINLGATEKEISVAVKSMYTDPSHLRIED

PGQVEGNIVFTYLDAFHSDKEHVEQLKAHYRRGGLGDGTTKKVLEECLQEMLRPIREKRA

LYLNDKAQLIEILKHGSQVSREKTEQVLSDVKSVFGLNLF

>tr|Q87NL4|Q87NL4_VIBPA Uncharacterized protein OS=Vibrio parahaemolyticus serotype O3:K6 (strain RIMD 2210633) OX=223926 GN=VP1854 PE=4 SV=1

MEHVDLFPLLHNRMNREEQSVPEYIGEFDGVASLPTGDYPAIYFWVDRENSNSVRLSFNT

PKLERWCMEEAVANGFNTDLIEQNVKQALVTLQQVSTPAFEAMIAEMLNSKPDSPNLENS

QVTDSSKTKKWWQFW

>tr|Q87SH0|Q87SH0_VIBPA Peptidoglycan D,D-transpeptidase FtsI OS=Vibrio parahaemolyticus serotype O3:K6 (strain RIMD 2210633) OX=223926 GN=ftsI PE=3 SV=1

MIRKKTNAKQGNKRKSSAKPKAAVNDTKTAPKAAKVEVESSFLIRWRFHLLLFFVFCAFA

LLVARVAYIQIIEPDNLIKQGDLRSIRVKSIPSARGIISDRNGEPLAVSVPVEAVWADPA

TIFKENALSQKQNWYALADVLGVDRQGLIDKIKRNEKRRFIYLQRQVSPAMANYIRELKL

PGIGLKSESRRYYPAGEVSAHLVGVTGIDGHGLEGVERSYDEWLTGEEGKKTIRKDRYGR

VVENIAWQDKQEGKSLQLTIDQRLQAIAYRAIKQAVADHRATSGSVVMLDVKTGAVLAMV

NAPSYNPNNRTDWQSYKMRNRVITDSMEPGSTIKPFVILAALENGVADKDTIVDTGNGVL

RLGGSRVRDVSWVGKASLSMILKKSSNIGVTKLAMQMPVEALLGLYSSVGFGELSGLNLV

GEVTGIFPTRTRWSPIERATIAFGYGLSITPIQLAHAYATLGNLGKYEPIHIIESNDRDM

SRQVVSKENARLVLDMLETVTQKGGSARRAAVPGYRVGAKTGTSRKASAGGYSDEYITYT

AGLAPVSDPRIALVVIVNEPQGDDYYGGSVASPVFSEIMKGALQILNVAPDENKFQQ

>tr|Q87PE3|Q87PE3_VIBPA Uncharacterized protein OS=Vibrio parahaemolyticus serotype O3:K6 (strain RIMD 2210633) OX=223926 GN=VP1568 PE=4 SV=1

MNTQLIAGQVIPLTPDGNWLYLKAAQAEIEIYRESSGERVTLGKSAVFNVGEGKHLGRLL

ISSRTDNEIEIQFGFGTFMPPVEGQSVVVQALPNVVIEQLPAVEIAPNQQLAVNQLPAVE

LAANQQLGVTTLPPVEFKAPQPVNVQSLPAVTLEATQVVKVDEQVSSNLITEAVSVFPHN

MAQNATRKAITIKASKANTASVFVDAFELEAGERITIESTAAMTLTGTAGDTVTIMEI

>tr|Q87KB2|Q87KB2_VIBPA Putative cyclohexadienyl dehydratase signal peptide protein OS=Vibrio parahaemolyticus serotype O3:K6 (strain RIMD 2210633) OX=223926 GN=VP3065 PE=3 SV=1

MKKILFALGICLLMVTQVVHAQSRLQEILDKGVLRVGTTGDWNPMTMKDPATNSYRGFDI

DVTTELAKDLGVKVEYVATDWKTLVNGITANKYDITGSASLNMSRAKVAGYSQPYFYLAF

VPVVQKKDLAKFSDWEDFNKPDVKVAATLGTVQEKMVKEFFPSAQHIVIEAPARDFQELL

ARRADVSVTSNVEAATLVDKFKQLAIVPVKAPRRPTPIAMLLPQDDQVWINYINHWVELK

KTQGFFKQTAQKWGLKSL

>tr|Q87KV9|Q87KV9_VIBPA Transcriptional regulator, LuxR family OS=Vibrio parahaemolyticus serotype O3:K6 (strain RIMD 2210633) OX=223926 GN=VP2866 PE=4 SV=1

MDSTYTIIIADDHPLFRNALFQSVHMAVSGANLLEADSLDALLALLAKEEEPDLVLLDLK

MPGANGMSGLIHLRAEYPDLPIVVVSASEEPTVVSQVKSHGAFGFIPKSSDMRELVNALN

QVLNGDPYFPEGLITNNAACNDLAEKIAALTPQQYKVLGMLSDGLLNKQIAYELNVSEAT

IKAHMTAIFRKLGVKNRTQAVIMLTEMND

>tr|Q87K14|Q87K14_VIBPA Putative transporter protein OS=Vibrio parahaemolyticus serotype O3:K6 (strain RIMD 2210633) OX=223926 GN=VPA0084 PE=3 SV=1

MTLLDSMIVMIYLLFIGIAAWLFRRFSTSSNDFLQGGGTMMWWMAGATAFMTQFSAWTFT

GAAAKAYEDGISVMFIFWGNALGFFVAAAFFARRYRRLRVETAMEVIRVRFGHTSEQLFT

WLSFPLTLIACAIWMNGLASFFSAVFAIEMSTTILAMGVIVTLIAVSGGAWTVSVTNVIQ

LVLLIAITLVIGYTALQHFDSPSEVFAYTTPIYGEQIRMPSLFVMWAIAMLLQQTMSNNH

TLSCYRFLTTANDKDATKAAFVAGVLFVFAPVLWFMPAWFVAAQNIDLLAIYPALEQSAN

NAAYLYYIQHYMPQGLLGLVMVALVAATIAPLSSAHNRNAGIVVKSLYQVWFNPNATSRQ

QLMVGKIATIVSGILATVTALLLASVESYSLFDMMIVFAAFIQMPINIPSLLALVSLKTP

NWSGWATVMVGCVVSLFMMFIFDVQWISSSPLTPRESKDLSVVATLAVHLLVTGGFFLST

RVFYAKESPSIQQSRTALSIQLATPISDEELSPINHKQGLYVGSITALAGMLLILAGVVL

AGSSQSIFVAIGCIVLLFSLFLLQPTLHSKRAWQKQKTK

>tr|Q87J07|Q87J07_VIBPA Uncharacterized protein OS=Vibrio parahaemolyticus serotype O3:K6 (strain RIMD 2210633) OX=223926 GN=VPA0448 PE=4 SV=1

MTPQLPKWVTDLAIVGLIFTVGGYATRIELAINGINEDIAEMEKKIVSFDTAISIHHGPN

WTERARSNDLKRVEDLEKTIGGISEKFEERVTTLGSGFTSTSKDMSDIQRKVEAINRWTQ

QGDVLIKKSRLDKFRALANYATYESPDPTQVSINKQHSRGKNFKIGDRLILENPANGSSV

EVGVKSMLDDPNSSNILVQLPQDLLLELGLTTQGGRYELYIQGKPEALRWKSLDQIYQEL

SAQQ

>tr|Q87T78|Q87T78_VIBPA Uncharacterized protein OS=Vibrio parahaemolyticus serotype O3:K6 (strain RIMD 2210633) OX=223926 GN=VP0192 PE=4 SV=1

MKTTLIITTYNWKEALKAVLESVKRQTVLPDEVIVADDGSREDTKATVDQMREGFPVPLI

HSWHEDNGFQLSMSRNRAIAKASGDYLIMVDGDMVLSQTFIESHKRVAKPNWFVQGGRVL

TDETCSRDIMENGLVPSVFSKGIRNRKNCITNSLLSNWFSYERNNDKATRGCNMAFWRQD

VIEVNGFNQDFVGWGREDSEFVHRMLNAGKSRLYLKFAGVGYHLYHVENSRASLSQNDEI

LENTIKNKLKRCENGVDQFLEA

>tr|Q87JB6|Q87JB6_VIBPA Uncharacterized protein OS=Vibrio parahaemolyticus serotype O3:K6 (strain RIMD 2210633) OX=223926 GN=VPA0337 PE=4 SV=1

MVAGSICTLHAKGRFMRLFIALFFSLILSLPTLASEEKYSESDLLDRPLMERYILDELKS

LRQDYQSLEKRMITEITDRELTVADKSLNYANVTVTYFFYIIAGVASLIAFVGWQSLREF

KHNTKEMADKRLEKIAQEYEKKFVALERDLKRKTRIISENNKEIEKINEIHNLWLRAQSS

QTPEQRIEIYDEILVIRPGDLEALTYKADAAMEINEYHWALSICNRVLEVDYTNGPALYQ

RACAYSRLGIEEQAIEDLERAIDYSPSIRDLLAEERDLELLHGNERFEKLLQLSNNNE

>tr|Q87SU0|Q87SU0_VIBPA Uncharacterized protein OS=Vibrio parahaemolyticus serotype O3:K6 (strain RIMD 2210633) OX=223926 GN=VP0332 PE=4 SV=1

MGNLMNIFDLTLGLLNDMFFAAIPAVGFALVFNVPQRALIYCAVGGAIGHGSRYLMMQFG

VPIEWATFFAATLVGMIGVHWSHRFLAHPKVFTVAALIPMVPGVFAYKAMIAMVEINHLG

YSPELIATCMENFLKAMFIIAGLAVGLAVPGLLFYRRRPIV

>tr|Q87NI5|Q87NI5_VIBPA 6-carboxy-5,6,7,8-tetrahydropterin synthase OS=Vibrio parahaemolyticus serotype O3:K6 (strain RIMD 2210633) OX=223926 GN=VP1883 PE=3 SV=1

MNAVKVYIKQRFNKLSSHLSEIYKEFMFEAAHHLPHVPEGHKCGRLHGHSFLVRLYVEGE

VDPHTGWVVDFAEIKAAFKPIYNRLDHYYLNDIEGLENPTSEVLAKWIWQQLKPNLPLLS

KVEIKETCTAGCIYKGE

>tr|Q87H33|Q87H33_VIBPA Uncharacterized protein OS=Vibrio parahaemolyticus serotype O3:K6 (strain RIMD 2210633) OX=223926 GN=VPA1132 PE=4 SV=1

MLITFRCRSYANVTMFGDIALEMIKMMGHSGTVPGSISAQDVPDALSKLTSALSAKNAAE

ENLPTDVDVDEKEEQVEPAVSLGRRAFPLIELLKSAIKEECEVMWDNQ

>tr|Q87NM7|Q87NM7_VIBPA Uncharacterized protein OS=Vibrio parahaemolyticus serotype O3:K6 (strain RIMD 2210633) OX=223926 GN=VP1841 PE=4 SV=1

MPRVEFGMKTESGEEFTINGELTDDELLTITNFVQSAEKLLECRLVQCGNFLPKKITWNQ

VSGFDICEVNEIDFEALFACLHLSRPFILQKEPWSYNSVSGILGRVFSHHSEVKRELKGL

RKIYSDGALSYYFDISIQEKSIFDESRFLSWLNSYQYHRDQDKSVLYDELKSSFSENGAH

HIMLIRLHGKLQAIEQLTHLCKVIIKSSAKQS

>tr|Q87QA0|Q87QA0_VIBPA Transport ATP-binding protein CydD OS=Vibrio parahaemolyticus serotype O3:K6 (strain RIMD 2210633) OX=223926 GN=VP1250 PE=4 SV=1

MDKKKQSSLNKWLKQQSKLAKRWLMIAIGLGVLSSVFLLAQAALLASILHQLIIEQVDKS

ELIWHFVGLVGTVIGRAACTWGREIAGYRCGEQIRVYIRQLILDKVHKLGPAYIKGKPAG

SWATLILEQVENMQDFFSRYLPQMSLSVLVPFIILVVVFPVNWAAGLIFLVTAPLVPIFM

ALVGIKAAEANRKNFKAMQRLSGHFYDRLQAMTTIRLFDRTKSETETLKGASEVFRTRTM

DVLKIAFLSSAVLEFFTSISIAITAVYFGFSYIGELNFGYYGAGVTLFAGLFILILAPEF

YQPLRDLGTFYHAKQQAVGAAESIVEFLELEVDQVKDGTQSLEQNTPIQIEAQDLVVFSP

EGKQLAGPLSFSLEANQSTALVGPSGAGKTSLVNTILGFMPYKGSLKINGCELSQLDLST

WREAVSWIGQNPMLLHGSIRDNVTLGKHDIPDAQVNTVLNDSYAAEFVEMHGLNYAISDR

SGGLSVGQAQRLALARAMLQNGRFWLLDEPTASLDARSEKLVMEGINKYTQSTTNLMITH

QLAPLQNVSQILVMQDGQIVQRGDYATLASQEGLFKDMLAANLAQRESDKGNLDA

>tr|Q87JP6|Q87JP6_VIBPA GGDEF family protein OS=Vibrio parahaemolyticus serotype O3:K6 (strain RIMD 2210633) OX=223926 GN=VPA0202 PE=4 SV=1

MTDEFKKSTANLKKAVPLMIKNHVAATPANYALWYTYVDKTIPELNFEMDEVLEHYGICP

PAANKQLYNNYVASRAETSLEDLKTNVEVLLHEVSSSMSDTLSDTSSFSAMVDKSFNKLE

KVEDNSLSIEEVMVVIRQLVAESHEIRHSTKFLNRQLTNASEEISRLKHQLAEVQKDALF

DGLSNLYNRRAFDTDLSMLINANQAMTLILLDIDHFKAFNDNYGHLFGDTVLKAIARRLQ

QSCRDGVSAYRFGGEEFALIVPNKSLRIARQYADTLRRMIEKLRVRDKRSGTQVHSITAS

FGVAEFEVGDTVDSLVNKADKQLYEAKQLGRNRVMPI

>tr|Q87PC2|Q87PC2_VIBPA ABC transporter, ATP-binding protein OS=Vibrio parahaemolyticus serotype O3:K6 (strain RIMD 2210633) OX=223926 GN=VP1595 PE=4 SV=1

MALLTIHNAQLAFGDHPLLDRAEFALQENERVCLVGRNGAGKSTLMKVLAGDILLDDGKI

QITQDVVVSRLEQDPPRNQEGTVYEYVSGGLAEIGEQLKIYHDLLDLVAQDPSEKNINRL

AKTQEQLDHSNAWRFDDRVKNVLSALKLSPDTLLRDLSGGWQRKAALARALVCDPDVLLL

DEPTNHLDVTTIEWLENFLKDFKGSIIFISHDRAFIKSMATRIVDLDRGQLSSFPGDYDN

YLLEKEEMLRVEEMQNAEFDKKLAQEEVWIRQGIKARRTRNEGRVRALKKLREERRDRRE

VQGKVNLNIDDASRSGKIVFEAENVSFAYDGKQIVDNFSFNIMRGDRIALIGPNGCGKST

VLKLLLGQLEAQSGRLHCGTKLEVAYFDQYREILDPEKTVIDNLADGKQEVMVGGRQRHA

LSYLQDFLFAPKRARTPVKALSGGEKNRLLLARILLKPNNLLILDEPTNDLDIETLELLE

EMLANYQGTLLLVSHDREFVDNTVTTSWIFEGDGVIEEFVGGYHDAKQQRDQALAVRFST

EKPAKKEKVVEETPKTTQPKNNSKKLSYKLQRELEALPAKLEQLESDIETLQEQVNDPEF

FAKPVEQTQPVLEQLAALEQELEIAFERWEELEAMQQDS

>tr|Q87HL6|Q87HL6_VIBPA Transcriptional regulator, ArsR family OS=Vibrio parahaemolyticus serotype O3:K6 (strain RIMD 2210633) OX=223926 GN=VPA0947 PE=4 SV=1

MSYTDMDVAAMKGNANEAADLLKVMAHPERLMVLCQLTQGEVGVGELQKNSFLSQSAFSQ

HLTVLRKHKLITARKASQQVFYSLSEPRVESLIKALHGVFCN

>tr|Q87PZ3|Q87PZ3_VIBPA Uncharacterized protein OS=Vibrio parahaemolyticus serotype O3:K6 (strain RIMD 2210633) OX=223926 GN=VP1357 PE=4 SV=1

MKLKIGLVFLTTLFTQLSFAQDSFFPIWGDEAEKRGYSLPKPYGLSLSYMDMSNPITVNS

IDLTGHPVLEALDIDANHADFKGSNITLRGDVWIFPFMNIYGIIGYTQGTSTAKINALSC

DTSSVQGIGNKVLCSLLDAAGDELPDSAAFELEMDGGTYGAGTTLAGGVGNWFALLDMNY

TYTSIAAIDGNIKTFVAAPRVGYRWQYDGGRELRVFVGAMYQNVQQELSGDLKSLNLPPQ

LLGLVENLSPDAGFNVSQSADENWNSVLGFQYAFNKDWEILMEAGFGERETLFFSLGRRF

>tr|Q87IM3|Q87IM3_VIBPA Putative ATP-dependent RNA helicase OS=Vibrio parahaemolyticus serotype O3:K6 (strain RIMD 2210633) OX=223926 GN=VPA0583 PE=3 SV=1

MTDKTQQATFADLGLIPTLVERLEALEYNQPTPIQSHAIPHVLDGRDMIGGANTGSGKTA

AFSLPILQKILQQGESNDRRGNFVSHLILVPTRELASQVAYNVKSYSYHLRDKIKTVAVF

GGVSVNPQMLALRGGCDIIVATPGRLLDLVSSNAIKLDQVKTLVLDEADRMLSLGFTEEL

NKILALLPEKKQTLLFSATFPEKVTALAQHLLNDPVEVQLQSAEASTLVQRVFSVNKGEK

TAVLAHLIKQHQWRQTLIFVNAKNACNHLAQKLSKRGITAEVFHGDKGQGARTRVLDGFK

SGEIQVLIATDIAARGLDIEKLPVVINFDLPRSPADYMHRIGRSGRAGEVGLGLSLIDYD

DYHHFKVIEKKNKFQLEREQVEGFEVEDDQSEAYFLPMKPRAKPAGTGKKKKKRNQ

>tr|Q87MN9|Q87MN9_VIBPA Aspartate-semialdehyde dehydrogenase OS=Vibrio parahaemolyticus serotype O3:K6 (strain RIMD 2210633) OX=223926 GN=asd PE=3 SV=1

MSQQYNVAILGATGAVGETILEVLQERKFPVGELFLLASERSEGKTYRFNGKTVRVQNVE

EFDWSQAHIALFSAGGDLSAKWAPIAADEGVIVIDNTSHFRYEYDIPLVVPEVNPEAIAE

FRNRNIIANPNCSTIQMLVALKPIHDAVGIERINVSTYQSVSGAGKAGIDELAGQTAKLL

NGLPADKKQFSQQIAFNCIPQIDQMMENGYTKEEMKMVWETQKIFNDPSITVNPTCVRVP

VFYGHAEAVHVETRSPIDAQEVINLLEQTEGVEVFHGDDFPTQVRDAGGKDHVMVGRIRN

DISHHSGVNLWVVADNVRKGAATNAVQIAEVLIRDYY

>tr|Q87KC0|Q87KC0_VIBPA ComM-related protein OS=Vibrio parahaemolyticus serotype O3:K6 (strain RIMD 2210633) OX=223926 GN=VP3057 PE=4 SV=1

MGLAIIHSRASVGVQAPSVSVEVHISNGMPGFTLVGLPETTVKESKDRVRSAIINSNFQF

PAKRITVNLAPADLPKEGGRFDLPIALGILAASEQIATDRLKNYEFVGELALSGGLRPVK

GVLPAALAASKVGRHLVVPHVNGDQAALVGKEQHKSAQSLLEVCAELCGQHRLNLYQTPK

RKTVEKHGRDLQDIIGQQQGKRALEIAAAGNHNLLFLGPPGTGKTMLASRLCDLLPEMSD

EEAMETASVASLTQSEINEHNWKSRPFRAPHHSSSMAALVGGGSVPRPGEISLAHNGLLF

LDEMPEFDRKVLDSLREPLESGEIIISRAQGKTRFPARFQLVGALNPSPTGYYEGNQART

NPQAILRYLGRLSGPLLDRFDMSLEIPSLPKGTLAEGGDRGEPTELVKERVSLARESMLN

RNGKVNALLGSREIEAFCPLQKSDAEFLETALHRLGLSIRAYHRIIKVARTIADLEGAEQ

IERSHLAEALGYRAMDRLLKQLTAQAV

>tr|Q87RC7|Q87RC7_VIBPA Long-chain-fatty-acid-CoA ligase OS=Vibrio parahaemolyticus serotype O3:K6 (strain RIMD 2210633) OX=223926 GN=VP0870 PE=4 SV=1

MDKPWLSRYPSDVPETINPEQYESLVEMFEQSVQKYADQPAFMNMGSVMTFRKLEERSRA

FAAYLQNELKLQKGDRVALMMPNLLQYPVALFGILRAGCIAVNVNPLYTPRELEHQLNDS

GATTIVIVSNFANTLEQIVDNTPVKHVVLTSLGQMLPRAKGTIVDFVVKYVKGMVPKYDL

PGAISMRKALRKGRRLQYVKPFMSGDDIAFLQYTGGTTGVAKGAILTHRNMIANVLQAKG

AYGPVLSPGRELVVTALPLYHVFALTVNCLLFIEMGGRNLLITNPRDIPGFVKELQKYQF

TAITGVNTLFNALVNNEDFHELDFSNLRLAVGGGMAVQRAVAEKWLKTTGCYLLEGYGLT

ECSPLVAAYPHDLVEYNGSIGLPVPSTEVRMVDEEGNVLPNTGTGELQVRGPQVMQGYWQ

RPEATKDTINEDGWLSTGDIVKFDDEGFLHIVDRKKDMILVSGFNVYPNEIEDVVALHGK

VLEVAAIGQPHEVSGELVKIYVVKRDPSLTKEEVIAHCRQHLTGYKVPKLVEFREDLPKT

NVGKILRRVLREENDAELAKKSA

>tr|Q87IP9|Q87IP9_VIBPA Uncharacterized protein OS=Vibrio parahaemolyticus serotype O3:K6 (strain RIMD 2210633) OX=223926 GN=VPA0557 PE=4 SV=1

MPKAVAVLFFLLFTSLSYAESAFDIVQKSDQAMRGKSSYSEATMEIVRPDWTRSMTMKSW

TKGTELSLVLVTAPAKDKGSASLKRHREMWNWVPSIERVIKIAPSMLSQSWMGSDFTNDD

LINQSSIVVDYQHALLGNDSFEGDKVWVIEATAKPDAPVVWNKVTLWISQSTYLQRKVEF

YDEFDERVNVLTTYDVKELGGRKIATRMEMKPVDKPGNKTIFTTHQAQFDFDINDDFFSQ

QQMKSLRD

>tr|Q87KG1|Q87KG1_VIBPA Uncharacterized protein OS=Vibrio parahaemolyticus serotype O3:K6 (strain RIMD 2210633) OX=223926 GN=VP3016 PE=4 SV=1

MKLATLQNQFAKALHYQALGDDCDIASDQFTADERMQIYRNNFIISLSEVLSATYPMVEA

LLGETCFAQIARQHVLTHPLQEGSVIHYGKDFHHTVMLFGQVMAQAPYSPEVALFEWNID

LARQARCEHQADTAVQPLTELPHVPESQHPHLVFHLRPGCKSFDAHYAVFDLFAAVRTQQ

FDQLNINQLQQGVIRIEPNGEAQCFALAAEAYQLLASLEQKQCLSDIPEPLLAHLNHIMT

LDLIEGFTLQTA

>tr|Q87MV1|Q87MV1_VIBPA Uncharacterized protein OS=Vibrio parahaemolyticus serotype O3:K6 (strain RIMD 2210633) OX=223926 GN=VP2130 PE=4 SV=1

MVDSGNSYGERVSRLVGWGHWFAFFNIVASMLIGTRYIVQSPWPETLLGQFYLAVSWVGH

FGFLVFALYLLVLFPLTFVLPSRKLFRLVAVIFATVGQTILLIDTQAYQSINLHLTPVVW

ELLFSDDKSALSSDLQHLFVVMPLIFLVQLALSEWVWRKQRKLSHKHVGRPLAAVFFLSF

MTSHLVYIWADAYFYNPITSQRSNFPLSYPMTAKSFMEKHGLLDREEYLKRLAENENNVE

LVNYPLEKLEFNRRVNKLNVLMISVNNLRADALNQEEMPNLYEFAQQNQNFRKHYSSSND

TYGAFGLFYGLPTSYASSIKAQGASPVLLDVLKDQGYTFGLFSGSGFEDDLYSEIVFRGV

NLAEKLDGTQAHTDKQSIADWNMWLTEKANQPWFSYIEVTTVDNFESIPSNSKEDMSASE

RFKNAYEFAVKSADDKVGGIIKTLEDAQLLANTVVIVTSNHGSEFNETNTNSWGANSNYS

RYQLQVPMVIHWPGMMAGEFNHSTSHLDLSVTLLQDMLGVSSNPYDYSSGRNLFDESRRR

WILAGDTRELALITNNQTTVIDKFGNYKLYDENYKRMRDETPKLPVLMQGLTELQRFYSK

SE

>tr|Q87NK2|Q87NK2_VIBPA Uncharacterized protein OS=Vibrio parahaemolyticus serotype O3:K6 (strain RIMD 2210633) OX=223926 GN=VP1866 PE=4 SV=1

MSYLPLDEYQRKWIFTHQSMPVPEEDLAFIKPMSQARSAQFWKENISAQSPDMDRLSSQD

WPMSESNWLESVDWMAAWESDEPEMPQEVDAHIDWQDDVTVYFCYEKYNVIETKWSIFKK

HWKNFLFYDDGPILIGRRRSQALWFDSKGMVKLGERH

>tr|Q87FZ5|Q87FZ5_VIBPA Uncharacterized protein OS=Vibrio parahaemolyticus serotype O3:K6 (strain RIMD 2210633) OX=223926 GN=VPA1525 PE=4 SV=1

MRRLIPFYRSMLFASALSWVLVTLMPVINAHGNSMGVWATLCTLNGFELVQIEEGDSSVT

HKGKPCPFSHFSTFHQDSIPTTPILSRLSSVISERYTFLALSVRFETPVPRAPPVASFS

>tr|Q87RF3|Q87RF3_VIBPA Succinate dehydrogenase hydrophobic membrane anchor subunit OS=Vibrio parahaemolyticus serotype O3:K6 (strain RIMD 2210633) OX=223926 GN=VP0844 PE=4 SV=1

MVKHVSSFGRNGVHDFLLIRATAIIMTLYTIYLVSFCAFSDISYASWTQFFGGTFTKVFT

MLALASVLIHAWIGMWQVFTDYIKCAKLRGALQLGVIAVLFGYVFSGLFILWGA

>tr|Q87G01|Q87G01_VIBPA Uncharacterized protein OS=Vibrio parahaemolyticus serotype O3:K6 (strain RIMD 2210633) OX=223926 GN=VPA1519 PE=4 SV=1

MKKRTQLSLVMTSVALAVGAASAQAAELEITVTNATKGIYFTPLIVAAHDSDLHMFKVGE

SATAELEAMAEGGDISGLSTVIGNAGGAVVENPAGGILNPGAATTFTLDSGDHGYLSLSA

MLLPTNDGFVGLDSWKIPTEAGTYKATLNSYDAGTEANDELASSIPNPPFITFGSGGTGV

ETAVSNDKVHIHPGNLGDSDPAGGKSDLDSSHHRWLNPVATITIVVK

>tr|Q87QI2|Q87QI2_VIBPA Peptide ABC transporter, ATP-binding protein OS=Vibrio parahaemolyticus serotype O3:K6 (strain RIMD 2210633) OX=223926 GN=VP1167 PE=4 SV=1

MSALLEVDNLSKQFVTRSRLFRRQVNEAVKPVSFTLEPGQTIGFIGQNGSGKSTLARMLA

GVVEPSSGEIRVNGERLEHKDYATRCKLIRMIFQDPNTSLNPRIQIGRILEGPLKRNTNM

PPDARMKRVKETLLRVGLLPEHAYFYPQMLAAGQKQRVCLARALILQPSIIVADEALNGL

DMAMRSQIINLFLELQEEMGLSFVYVSQHIGIVKHITDKVMVMHEGEVVEFGETNQVLTN

PEHAITQRLVESHFYKAPSH

>tr|Q87SS5|Q87SS5_VIBPA Uncharacterized protein OS=Vibrio parahaemolyticus serotype O3:K6 (strain RIMD 2210633) OX=223926 GN=VP0347 PE=4 SV=1

MVTAKQLGRSALALTLISLSGCQLFQSNTNAEVASPVIVNDHAQSIMVISDQMDMYLSSE

GRESFLNQMLVALESDSRDKFKGKDPDTYSWWKIRVQPNEWQQAEIVRPVEEGVDTSNKL

EFMDVSYRSKTGLNLRSKPSVESTKLGQLEKGEVFNALARVEGEPWILVEQKGVIKGYVH

QDYVRSNVVNRDILSTQPNPLFDAQQATHSVSSARHELLGSYTCRALSYELTKDGDVTTG

SLRACRKKRKVWYIDTPSASPANPS

>tr|Q87H18|Q87H18_VIBPA Putative phenylacetate-CoA ligase OS=Vibrio parahaemolyticus serotype O3:K6 (strain RIMD 2210633) OX=223926 GN=VPA1147 PE=4 SV=1

MNELFDAKESLSPAEREDSLFQRLPTLIENAKANSEHYGNIFSGIDASIASNREGLAQFP

ITRKFNVPSQQQLKPPFGGLNSVAIGQMARVFQSPGPLYEAQTDESDFWRMGRAFHAAGF

RRGDLVHNTLSYHFSPGGFIMDGGARACGCAVFPAGVGNTEAQIEAIKQLQPTGYTGTPS

YLLTLLQKYQEQYGELPSFTKALVSGEAVTADMQQAFTDKGIAVFQAYASAELGLIAYQV

AGEQGLVIGEDIIVEIVDPDGMPVPPGEVGEVVVTSLDEKFPLIRYATGDLSAYIENDAN

SPYTNMRIKGWMGRADSAVKVKGLFVYPHQIQEVCRRHEITGSLKIERDGFNDTITFCCH

DVHVSAEDIQATLQSVTKLKGNVQFVANDADQPLINDLRS

>tr|Q87M38|Q87M38_VIBPA Putative pilus assembly protein OS=Vibrio parahaemolyticus serotype O3:K6 (strain RIMD 2210633) OX=223926 GN=VP2420 PE=4 SV=1

MSRTQVIMLLLLSIVFGLAAVLIAKQWMDGRNQPTVELEEVERHPVVVATMELEPGTILD

EKHLTTKLMEVDWIDDETLKVPEQALGRIVANTIYAGELVNPNRLAQPGEGATLAALIPE

NKRAVTIRVNDVIGVAGFLLPGNKVDVLSTVSYGKGKQATTLTVLKNINVLAVDQTAKTN

DNKPVIVRAVTLEVTPKEAEKLLSANSKGEIQLALRNPHEVDKPVVAKKYTPRPSVTIIK

GSQASSVRVSN

>tr|Q87NC8|Q87NC8_VIBPA Carboxynorspermidine/carboxyspermidine decarboxylase OS=Vibrio parahaemolyticus serotype O3:K6 (strain RIMD 2210633) OX=223926 GN=VP1940 PE=3 SV=1

MQHNELKTPYFMINEDKLIANLEKAKQLKEISGVKLVLALKCFSTWGVFDIIKPYLDGTT

SSGPFEVKLGYETFGGETHAYSVGYSEDDVREVADICDKMIFNSQSQLAAYRHIVEGKAS

IGLRLNPGVSYAGQDLANPARKFSRLGVQADHIKPEVFEGIDGVMFHMNCENKDVDAFIG

LLDAISEQFGEYLNKLDWVSMGGGVFFTWPGYDIEKLGLALKAFSEKHGVQMYLEPGEAI

ITKTTDLVVTVVDIVENVKKTAIVDSATEAHRLDTLIYNEPASILEASDNGEHEYVIGSC

SCLAGDQFCVANFDQPLEIGQRLHILDSAGYTMVKLNWFNGLRMPSVYCERSSGEIQKLN

EFDYSDFKRSLSQWSVK

>tr|Q87NK5|Q87NK5_VIBPA Uncharacterized protein OS=Vibrio parahaemolyticus serotype O3:K6 (strain RIMD 2210633) OX=223926 GN=VP1864 PE=4 SV=1

MSKTVQQQIGNIALVVENYDDAIEFYTQKLQFTLVEDTDLGGGKRWVQVSPPNSSGTNLL

LAQASTPEQMQAVGNQTGGRVFLFLQTNDFWRDYELMKTNGIVFNEEPRVEEYGTVVVFQ

DLYGNKWDLLQLNRATK

>tr|Q87SD8|Q87SD8_VIBPA Sensory box/GGDEF family protein OS=Vibrio parahaemolyticus serotype O3:K6 (strain RIMD 2210633) OX=223926 GN=VP0486 PE=4 SV=1

MDQLLNWLWNGQESYGYIVLLSIVSAIAIALIYFRFQNALKQLITDSPYPVLVLDASHGQ

ILLSNQAAMQLLGIRSLGTGFLYPALFELHKLSSLLDSFSSRYFRHQVFDWRLSENESIK

VELSGRKSLLRGHRVWIMYAQPYQVTLQEQQQELAQLEMARSALDSLSELICLQDCQGNL

LTTNRAFDQFWEGRREEAITATASRVATRKSERKWTTDTQGRSCLLEVNLSSLVSPSGEL

LGTLSISHDVTEWHKIQQNLRDEMERRKDTEVALAQRDTILQTILDASPDSIGIFNENMV

YQACNKPFVNALGISEVSDLIGKRLQDVIPVEMYTRLETSDLHALRQAEPVRYIDKVVSS

DGHITWFDVVKSPFKDKASGTNGVLIMARDISERYLAEQKLEQANLELEKLSFMDSLTQV

ANRRRFDERLHVLWYHHIREKLPLTIMLCDIDFFKDYNDCYGHQQGDEALIRVAAVFKQV

VNRSSDCVARYGGEEFGFILPNTTTEGAEQVAQRIHQQIRQLDMEHGSSEASEHLSVSIG

FVSYVPQHGDEPEMGIAMADSALYQAKADGRNRTCIHPSSF

>tr|Q87NF5|Q87NF5_VIBPA Uncharacterized protein OS=Vibrio parahaemolyticus serotype O3:K6 (strain RIMD 2210633) OX=223926 GN=VP1913 PE=4 SV=1

METLHLTNSHFKSDSPINRLILLLVALITLFLVVISPGWKQAMLSVILMAIIVGFAVLMI

KKSQVTFTLTASHFQQHLFKGGWVVRWRNIESIGICSYQQDGWHQPLPWIGIRLKHYSPY

LDAICPRIATEILLSQRALLYLGARQNHCEEKFEDMVLDPQPYTSKAGKQYDGLQAMLAN

RMKYQREFYGYDVFISASDLDREADEFVGLTRRYLAAAEPE

>tr|Q87Q48|Q87Q48_VIBPA Sodium/dicarboxylate symporter OS=Vibrio parahaemolyticus serotype O3:K6 (strain RIMD 2210633) OX=223926 GN=VP1302 PE=3 SV=1

MDFAVIASLAVFIGILFFLFTQQQKQNTLSRLVLIGLVTGSLFGLALQLVHGEGSDVISK

TLEWVGIVGSGYVGLLKMVIMPLVLVSMISAVVKLEKGGSLGKISGLTISVLLVTTAISA

LIGILVTSAFGLSAEGLTEGARETARIAVLESRAGTVSDLTIPQMLVSFIPTNPFADLTG

ARSTSIIAVVIFGVLTGIAARKVMMEKEELESPIRTFVEAAQSIVMRLVKMIMALTPYGI

AALMAKVVATSNASDILNLLGFIVASYVAIGLMFVVHGILVSFVGVSPVEYFKKIWPVLT

FAFTSRSSAATIPLNIEAQVTKLNVPPAIANLSASFGATIGQNGCAGIYPAMLAVMVAPT

MGINPLDVNFILSLIAIITISSFGIAGVGGGATFAALIVLPAMGLPVTIAALLISIEPLI

DMARTALNVSGSMTAGTIASRVLKSSEAETALEETKA

>tr|Q87QX4|Q87QX4_VIBPA Putative beta-ketoadipate enol-lactone hydrolase OS=Vibrio parahaemolyticus serotype O3:K6 (strain RIMD 2210633) OX=223926 GN=VP1025 PE=4 SV=1

MTQPLLFHKTYLHPTSNEWVVFVHGAGGSSSIWFKQIKAYKQHFNLLLIDLRGHGKSNQL

LKELITSHYTFTEVTQDILKVLDHLKIQSAHFVGMSLGTIIVRNLAELSSERVRSMVLGG

AVTRLNTRSQILVKLGNFCKHILPYMWLYKLFAYIVMPQRSQRESRHLFIREAKKLCQKE

FKRWFILAADVNPLMKYFKDRELPIPTLYLMGDRDYMFIQPVKEMVAAHKLSILREIPNC

GHVCNVERPEDFNQHSIEFIKQQSIVA

>tr|Q87M55|Q87M55_VIBPA Beta-galactosidase OS=Vibrio parahaemolyticus serotype O3:K6 (strain RIMD 2210633) OX=223926 GN=VP2403 PE=3 SV=1

MNNWENFQHLHENRMAPRAYFFSYDSVQSAQTFQRELSRRFMLLSGQWTFRYFTNPMLVP

DEFYSQTMNDWGHITVPNMWQMEGHGDLQYTDEGFPFPIDVPFVPTDNPTGAYQRTFTLG

PQWDNQQTIIKFDGVETYFEVYVNGEYVGFSKGSRLTAEFDISKFVQQGENLLSVRVMQW

ADSTYIEDQDMWWTAGIFRDVYLIGKENIHVQDLTIRTDFADDYQSATLSAQIELENLST

AIASGYTLEYALHDKGTVVASGQCDSLTIQNHHSTSFAIDMVSPTHWTAENPYLYHLFIT

LKDHQGNVVEVIPQRVGFRDIKVRDGLFYINNQYVMLHGVNRHDNDHLKGRAVGMDRVEK

DLILMKQHNINSVRTAHYPNDPRFYELCDIYGLFVMAETDVETHGFANVGDLSRITNDPA

WEAVFVDRAVRHVHAQKNHPSIIMWSLGNESGYGCNIRAMYTATKAIDDTRLVHYEEDRD

AEVVDVISTMYSRAQLMNYFGEHPHEKPRIICEYAHAMGNGPGGLTEYQNVFYAHDHIQG

HYVWEWCDHGILARDEHGQEFYKYGGDYGDYPNNYNFCMDGLIYPDQTPAPGLKEYKQVI

APVKIRAVEGCHDRFIVENKLWFTNLDDYTITADVRAEGETLRSVQFKVEALVANSEREV

TIDLPELDEREAFVNFTVRKDSRTLYSEANHEIAVYQFQLKDNTATLPALVNNNVQPLVL

EESRLEHVITGHNFALTFSKVNGKLTSWRVNGEEIIQSEPRLNFFKPMIDNHKQEYEGLW

HPAHLQIMQEHFRTLAVEATDDSVLITTTSIIAPPVFDFGMRCTYRYQINAQGHLNVELS

GERYGDYPHVIPVIGLDLGINGSFDQVSYYGRGPEENYQDSRQANLIDVYHSTVEDMFEN

YPFPQNNGNRQHVRWASLTNRHGTGLLVKPQQEINFSAWFYTNQNLHEAQHTIELEKSGY

ITLNLDHQVMGLGSNSWGSEVLDSYRVYMDEFRYGLTLMPLQAGNCNAHVMANHDFDNAF

FTQTNTQPVNEA

>tr|Q87I49|Q87I49_VIBPA Uncharacterized protein OS=Vibrio parahaemolyticus serotype O3:K6 (strain RIMD 2210633) OX=223926 GN=VPA0757 PE=4 SV=1

MVPIGVRFMVLSAFGFALMSATVKHVSLHGIPVFEIVAARALVSLVISYLDVKRKRISVW

GNNKPLLLARGAVGTFALMCVYYSVTTLPLAEATIFQYIHPVFTALLAVIFLKERIQPTT

FICIALCLLGVYVMVRPETGVESAQTLPMFSVMIAILGAFGSSIAYVIVRKLSQTEDSSV

IIFYFPLVALPTSILFMGDNFVMPDLYLTCMLVLVGVFTQIGQLGLTKAMQTQQAGKASA

YSYVQIVFSVLLGIVFFGELPSLWTYIGGALIVAGALINVFGHHLRLPFIKRSA

>tr|Q87QN4|Q87QN4_VIBPA Malonyl-[acyl-carrier protein] O-methyltransferase OS=Vibrio parahaemolyticus serotype O3:K6 (strain RIMD 2210633) OX=223926 GN=bioC PE=3 SV=1

MDNAENMVLDNVHQDKEAIASSFGKAADTYDKHAAFQRDVGHRLLEKLPSDLTNKRVLDL

GCGTGYFSQLLLERGASVVCADLSQGMLDKARERCGDHNVRYVVADAESLPFEDASFDYV

FSSLALQWCVDLSYPLREIRRILAANGKGCFSTLVDGSLCELRYSWAKIDTYQHVNNFIT

LNQVKIALAQSRCHNHHLDSTTITVWYDSAFSVMRDLKGIGANHVSGRSHGLTSRRTLLQ

VERAYQAFKNDQGLLPATYQVCFGVIHL

>tr|Q87IJ2|Q87IJ2_VIBPA Uncharacterized protein OS=Vibrio parahaemolyticus serotype O3:K6 (strain RIMD 2210633) OX=223926 GN=VPA0614 PE=4 SV=1

MANTEHQETLDFLDKLSVCLYRAGISVFAIALLGLAALESQWLDGMDGRYRAVFAVFAIS

GAMSASNIHVYSKSVRTIISWSGWIGVILMVCDPELNREWLALGFIFVTFSGIALKESFC

FKVLGLKLVPTLLVVTTFALWFEQSTIVAISALLAGLIMAYLSIAKWRMPLHFDIGIKAN

YEV

>tr|Q87GZ0|Q87GZ0_VIBPA Putative thiosulfate sulfurtransferase SseA OS=Vibrio parahaemolyticus serotype O3:K6 (strain RIMD 2210633) OX=223926 GN=VPA1175 PE=4 SV=1

MQALISAKELHALLDQQNVKLLDASISFQIPSESKKITDKWIPNTYRFDYDNDFCLPDTS

LPHMMPTEAGFNVSAQKLGLNNEDLIIVYDNSGTLASPRAWWMLKAMGHENVKVLNGGLP

AWIEAGLPVVDSLAKPEQLGNFAGKLNKNAFLDANAVLEYSNNCSAHIVDARSRARFFGE

VPEPREGLRSGHIPNSLCLPFQELLDNGYIKPNSELKQAFSELSLYNDNSIIFSCGSGVT

ACILLLAAHQLGLRNLSVYDGSWTEWGADCSLPITR

>tr|Q87HD9|Q87HD9_VIBPA Uncharacterized protein OS=Vibrio parahaemolyticus serotype O3:K6 (strain RIMD 2210633) OX=223926 GN=VPA1026 PE=4 SV=1

MKKASQDNNFIRISSPFGKDALILNSFEYREGISELFSLRAKAYFNDQKNELNEIVGKEV

TISVENSSRVSKSPRFFHGIVSAAKLEGQRVMNSHNGENYKNIEIIVEPKVKFAAYRNNC

KIFQKKNIKDIISEVLSEHGVAFKFELKNTYPQYSYKVQYEESDLAFVQRLLAEEGLSFC

FSHSKSSHVLDIFDDVSFYKPSPEFMVDFDTGSSESSHISAWNETQVLTTKSSQKSGFNM

LKPASQPKNVAAGDTALFTVPASEYFEYLGETESDDQYSLRNTHAIESLQQNVYLCSGEA

SCRTFSVGKCFKFKKHEDKSRVGKEYVLASVTIFASVFNQTGLGGTASQGVRVAFTCVDS

KTILRPAVTYPKPQIKGLQTAIVTGNKDGEVYVDKHGRIKVQFHWDRLGKYDVNSSCWIR

VAQSVAGNGWGAVFHPRVGQEVIVEFVNGDPDQPIVTGALYNGSQLPPYSLPEKSSQSGF

KSRSVQKGNANFNELRFEDKPGEEHIYLHAEKLFQMLVEDCVDIVVENNKVEKVTNDVTQ

DVGKNATLKVGENYTSDTGKVLSLNAGKSIEIKVGGASIQMSSSGEINIKGNKISINGSA

IALKAGQISLN

>tr|Q87PL5|Q87PL5_VIBPA Putative MutT/nudix family protein OS=Vibrio parahaemolyticus serotype O3:K6 (strain RIMD 2210633) OX=223926 GN=VP1487 PE=4 SV=1

MQLLQRASLILVNHQQELLLIQRFQNDRHYWVFPGGSVEVGELLVEAAKREALEETSLEL

NRVQKVFEIENQGRLETYFLSYVGNSKVKLGVGPEQTRQSDVNQYHLKWVRLDQLHTIPL

YPEQAKVFCLDNEEWFYDR

>tr|Q87SF1|Q87SF1_VIBPA Putative membrane protein OS=Vibrio parahaemolyticus serotype O3:K6 (strain RIMD 2210633) OX=223926 GN=VP0473 PE=4 SV=1

MEKNMYPVIQQDKTSNAKEIHILSVNTLDSLISINIKRKDIASKWTILKDKFDNTANYYA

TADDLVFLSRLANNLTGAVNPVHINYYSGQAHFVFTGTPALKIKLRVLGSRAPTPIIANF

AVGSKQAIQSIKSGTIITVVLVSSFRALEHFFSENNKTTLLSLFGTITSDIIKLGISSAI

AAIAVKGISGATTIFALTSGPLFLAIGVSLVVGNILNHIDNKYEITQKFKLALEKTLNES

LKKEEMRINDVIGRSYIKNGVFL

>tr|Q87N55|Q87N55_VIBPA Uncharacterized protein OS=Vibrio parahaemolyticus serotype O3:K6 (strain RIMD 2210633) OX=223926 GN=VP2020 PE=4 SV=1

MSLVTSKCRQVWQSDDYYQTLWFLLFLAFIIIFSGIGLRDPWPADEPRFVEVAREMVQSG

NWFFPTRGGELYPDKPPVFMWSMAAFYWLTGSLKATFMLPNAIVSLIALVCCYDISAKLW

NVKTARNVGFLLLLAPQFIIQAKAAQIDAMVAAWITISMYGLLRHFYIKTNWVWYCLAWV

FMGLGIITKGVGFLPALLLIPVLFLHFSGRHHFEDAVSWKLLLGPVFMLLTIACWFVPMV

SIVEISHNPDFAAYRDNILFKQTGERYVNSWGHIQPWYYFIVSVIPVMWFPLYLVFFNKK

CWQQLRLSPALLSLFAWVVLVIAFFSLSPGKRGVYILPALPMLAVIAGYALTNQAWPKWT

DKLLKAIVLILGVVLFSAAVVAGLEVKSVTKHLGEYDSIFPYVVFFLVAAAIWVGAFFKT

RTVQARYTFWVALALTWVWYSVAGYTLLNPVRTPAKEIMSEAQKAIGKDGELGLTLFKEQ

FLLFSPVSVTHFSYLSDHKEQERNAWLWLQEKPNRYILTQGGNEMECFDANKAKKLGEAH

RRDWILFDAESALESCQPPRSIKRYELHIDKPYS

>tr|Q87KH0|Q87KH0_VIBPA ATP-dependent DNA helicase RecQ OS=Vibrio parahaemolyticus serotype O3:K6 (strain RIMD 2210633) OX=223926 GN=VP3007 PE=4 SV=1

MTSTLLAEQSDSPVTPQRVLEEVFGYQTFRDGQQEVIESAVEGKDSLVIMPTGGGKSLCY

QIPALVRSGITLVISPLISLMKDQVDQLKANGVAAECVNSTMSREELLSVYNRMHSGQLK

LVYVSPERVLMRDFIERLENLPLAMIAVDEAHCISQWGHDFRPEYAALGQLKQHFSHVPF

MALTATADDATRRDILERLRLHEPHVHLGSFDRPNIRYNLVEKHKPISQIIRYLDTQKGN

CGIIYCGSRKKVEMVTEKLCNNHIRAAGYHAGMDADERAYVQEAFQRDDIQIVVATVAFG

MGINKPNVRFVVHFDIPRNIESYYQETGRAGRDGLPAEAMMLYDPADISWLRRMLDEKDD

GPQKQVESHKLNAMSAFAEAQTCRRQVLLNYFGEYREKPCGNCDICLDPPKHFDATEEAR

KALSCVYRVNQSFGMTYVVEVLRGMQNIRVREHGHDKISTYGIGRDHSHDYWVSIFRQLI

HKGLLFQNITRNSTLQLTEEARPLLRGDVSLELAVPRLDTAARAAKSDKLTSKNYDKKLF

AKLRKLRKSIADEDGLPPYVVFSDATLIDMAEILPTSYGEMLAVSGVGQRKLEKYADPFL

DLIQEHITHHG

>tr|Q87FT2|Q87FT2_VIBPA Putative acetyltransferase OS=Vibrio parahaemolyticus serotype O3:K6 (strain RIMD 2210633) OX=223926 GN=VPA1596 PE=4 SV=1

MQQFHIRRLREEDIPLLKGLLVELDTSHYEAEPNFYRSPEEMAQLREERGVFDKYFDDSM

IAFIACSHRQVVGFISGTVRDIDSMVSVAKRVGFVNELVVSESHRNLGIGLSLMDKIESD

LCNQGIEEIGLTVASFNHEGEDFYHKMGYRVMTKMMSKRVKD

>tr|Q87PG1|Q87PG1_VIBPA Cbb3-type cytochrome c oxidase subunit OS=Vibrio parahaemolyticus serotype O3:K6 (strain RIMD 2210633) OX=223926 GN=VP1541 PE=3 SV=1

MTTFWSLWIIVITIGTLVGCAILLTWCAKDKMGVEEGEDMGHEYDGIRELNNPLPKWWTY

LFVSTFVFAAVYLALFPGLGSFKGFLNWQSSAQTVRSLEESKEAIAQAQENKQLNQYAKE

LDDADAYFGEAFRKLAHNANGLRPVPEIAQDPEALKVGQRLFLQNCSQCHGSDARGQKGF

PNLTDDAWLYGGEPAAIVTTIMHGRIGQMPAWKDALGEQGVQEVVSYTLSLSGRKVNARE

AAAGKARFVVCAACHGTDGKGNPAVGAPDLTDQDWLFGDSRAAVTETVMNGRSGVMPAWK

DILGEDKVQLVASYVWSLSNSDNK

>tr|Q87HV2|Q87HV2_VIBPA Uncharacterized protein OS=Vibrio parahaemolyticus serotype O3:K6 (strain RIMD 2210633) OX=223926 GN=VPA0854 PE=4 SV=1

MRLERIEISGFRGIKRLSLSFDELTTLIGENTWGKSSLLDALSIALPANGSLYEFELKDF

HVDYSISHPQSQHIQIIVCFKAQDKHEANAGRYRRIKPAWCTTEDGEHVIYYRISASRVD

HDITTKYAFLDRDGKPKQIHHSEKLAVELMTLHPVIRLRDSRRFPRPSHINGDSKNARIE

KRINNTCRRLLAMPGHVNKGEIRSSLDAMKTLVEHYFAFRSIGKGNPRKPRDGLFYTSPS

SDKGLSQYLKETNDKQSRLLLMGLLSAYLQAKGPTDLRRCARPILIIEDPEGRLHPTHLA

RAWSLLQLLPMQKILTTNSGSLLGSVPLYSIRRLIRLSDKTIAKSLNTSKFGGDELRRIG

FHIRFHRAGALFARCWLLVEGETEVWLFNELARQCGYDLAAEGVQIVEFAQSGLRSLIKV

AKEFGIDWHVVTDGDPAGKKYAHTVRAQLGHDQERHRLTELPDRDIEHFLYNHGFELFFK

DMIKVPHDHPIPAKKVVNRVLKKHAKPDLALAIVSHCEEKGMECIPVLLRWTLKRIVTMA

SGNT

>tr|Q87T44|Q87T44_VIBPA Putative rhamnosyl transferase OS=Vibrio parahaemolyticus serotype O3:K6 (strain RIMD 2210633) OX=223926 GN=VP0226 PE=4 SV=1

MKLVFVTVNYNNIDETRKVLNCFSGLSHGENSVLYVVDNSDSKSECQSLDKENTNNKIKF

LYPKENLGYLTGASFGVDFHYNNHGFDFDYLIILNNDIIFNVNEVLKSLEIFSQNNDAMM

ISPLVIDRGVNVNPYLTVRKSKRYMMFWNFILSNFFTFKVFHKINKYRALLSSKNNFSST

VQPFQKIYGSHGSVFILKNEYFLRGGSLRRLPFLYGEEIYIAEQVRKLGGDCVYYPSIKF

EHKGSATLGNDTSRFKYLCIKQGHSFLYREFYS

>tr|Q87KM7|Q87KM7_VIBPA Flagellar protein FliL OS=Vibrio parahaemolyticus serotype O3:K6 (strain RIMD 2210633) OX=223926 GN=VP2950 PE=3 SV=1

MYKRYVAQIIFALTILISAPSWAETEETGPKLAYFTLEPDLTTNFYTKGKKLGYVQVRID

IMVMSQQDLAIVEHHQPLIRDAVIELLGKQTEDTIKSLSGREDLRKTLVKELNETLLPET

GKTVIADLLFTKYLYQ

>tr|Q87IH8|Q87IH8_VIBPA Cytochrome o ubiquinol oxidase, subunit I OS=Vibrio parahaemolyticus serotype O3:K6 (strain RIMD 2210633) OX=223926 GN=VPA0628 PE=3 SV=1

MFGRLTLDSIPYHEPIIIITLAIVALVGLAVVVAVTKAGKWQYLWNEWFTSVDHKKLGFM

YIAVAMLMLVRGFADAVMMRSQQLLSSAGEAGYLPPHHYDQIFTAHGVIMIFFVAMPLVI

GLMNIIVPLQIGARDVAFPYLNNLSFWLFVVGVILTNMSLGLGEFGRTGWLAYPPLSGIE

ASPGVGVDYWIWALQISGVGTTLTGVNFFATILRMRTPSMPMMKMPVFTWASLCANILII

ISFPILTVTIALLTLDRYLGMHFFTNDLGGNVMMYVNLIWAWGHPEVYILVLPIFGVFSE

VTATFSRKKLFGYTSLVWATIVITILAFVVWLHHFFTMGSGANVNAFFGIATMIISIPTG

VKIFNWLFTMYKGRIRFTTPMMWTVGFLITFTVGGMTGVLMAVPGADFVLHNSVFLIAHF

HNVIIGGVVFGCFAAITYWFPKATGFTMNETWGKRAFYLWIVGFLMAFLPLYALGFMGMT

RRLSQDINPEYFPLLAVAAAGTVVIALGVLSQFIQIYVSIRDREQNRDLTGDPWGGRTLE

WATSSPPPFYNFAHLPKGDVLDAFWYQKQSGEFDPMKEVEYERIHMPKNTATGIYVSAWA

LVFGFAMIWYIWWLAAASLVGIVVTCIQHSYNDDVDYYVEVEEIKAIEAARRAQLEEAKK

GTVKDNENSDDLEVTYAN

>tr|Q87HH4|Q87HH4_VIBPA Uncharacterized protein OS=Vibrio parahaemolyticus serotype O3:K6 (strain RIMD 2210633) OX=223926 GN=VPA0991 PE=4 SV=1

MGRIIIIIISGEAVIDKLRMNKHALNKGIFFLLVLFLFLQNIQYAKAAGSSIGQQDTLVM

LLNFKENPNDQPITSEEAHALVFGEVNDFYQENSYGKTWLSGQVAGWYTLPVSDQVCDYP

SVQAEADKMARADGIVLEDYQRIIYIMTQSGCAGGGSATTGKTFPSRAYIDGTLSARVIA

HEFGHNLGLSHAKALDCGDASVSNNCSVIEYGDSYDVMGTPDMGYINTYYKERMGWLNDA

ESPNILTATQDGLYEIAEYETQDITQNIALKIPRGVNPNSGLKEWFYVEYRQAMGYDQFL

DDRSYMLFRGDVTDGVIVRLVEEGAEESYILHMKPNSDYSQVYGRKDWKDTALPVGDSFT

DPVSGLTINLASAANGFAGVNVSFGDTVTPSVCEMSAPSVSVKATSDTQVNAGDTVEYQL

TVTNTANDVCGTIRFDVSAQVESGWQASSQSISLASGESSVATIAVTSALSATSGDYPVT

FNVVNTKDEAYNVATQATYAVAEQQTGSGDVVAVDDNVQMSKVSTVTINVLGNDIIADGV

SVEISAMSAPSKGTVKLLSDGSIQYTPAKRFKNQDSFSYTITSGNVTSTAMVTVALQSSP

DSGGSSPGKGKNK

>tr|Q87HW2|Q87HW2_VIBPA Uncharacterized protein OS=Vibrio parahaemolyticus serotype O3:K6 (strain RIMD 2210633) OX=223926 GN=VPA0844 PE=4 SV=1

MNFLAHLHIADHCNSHFMGNLLGDFVKGDPSKLYDSDITNGIKLHRFVDRITDHHPIVEE

CKPHFKGVARRFAPIALDMFWDHCLAKHWNDFHQESLDDFVEFAFQKVDQDVHEPLPARF

MLLHQRMWRGGWLQSYQDLSNIEFALHRMSQRSPRMADLTTTFEVLDGEYAALESNFSEL

YQDVLRQSLEFHQQLSSS

>tr|Q87LL3|Q87LL3_VIBPA Uncharacterized protein OS=Vibrio parahaemolyticus serotype O3:K6 (strain RIMD 2210633) OX=223926 GN=VP2598 PE=4 SV=1

MAGESMGIDTTLTDGLTQAETWLTNNSDLLIQYGVNIISALIILFIGNIIVKAVAGSVSK

VLEKKNMDKAVVEFIHGLVRYLLFVIVLIAALGRLGVQTASVVAVIGAAGLAVGLALQGS

LSNFAAGVLIVAFRPFKSGDYVEIGGVAGSVEAIQIFQTVLKTPDNKMVVVPNSSVIGGA

ITNYSRHATRRVDLVIGVSYKADLKLTKKVIRETLEKDPRILKDPDMTIGVLTLADSSVN

FVVRPWVKTADYWGVYFDSMQGIKEALDENGIEIPFPQMDVHLNRVES

>tr|Q87N61|Q87N61_VIBPA Putative tetrathionate reductase, subunit A OS=Vibrio parahaemolyticus serotype O3:K6 (strain RIMD 2210633) OX=223926 GN=VP2014 PE=3 SV=1

MDNKRRQFLKSGLAVGGLGAFAAGYATTTKHMVEGALEGTAGQKTRDIHHGNSLEPEYAV

NDQGNLIPNPNQRVAPSMCFGCWSLCGLRVRIDNRNDEILRITGNPYHPLSHQQHIPFET

PVKDAYLSLAGEAGLDGRSTACARGNGMLEIRNSPYRITQPLKRVGKRGEGKWIPISYEQ

LIEEVVEGGDLFGEGHVDGLRDIRDLTTPVDPENPEYGPKANQLLMTNAGNEGRDDIFKR

FAFNSYGTRNFGHHGSYCGYAFRAGSGAFMNDLDKNAHLKPDFDNAEYIIFIGMSPAQAG

NPFKRQARQLANARTEGSLEYTIITPSLPAGSSSLAAGDNNRWIPIKPGTDSALVLGMIQ

WIIENQRYNKTFLAQPGANAMKRAGTAHWCNATHLVISDEQHPRNGTFLRASDLNLPFEG

EALSDSDPYVIVEEQSGQFGVHTQTEEATLFVDKTVSLASGKVRVKSSLQRLKEEAFKYS

LAFYSQQCEIPVEQIAELAKRFTSCGTKAVVDTHGGNMHTNGFYNSFTIMMLNALIGNIN

MKGGAMAKAGGYPTSAAGPRYDFTKFAGKVGPKGVFLSRSKFPYHKTSEYKRRVEAGKSP

YPTRAPWYPFAAPLLTEHLSAAIDGYPYRVKAWINHMANPMYGVPGLKTLLEDKLKDPKQ

LGLIVSVDAFINETTALSDYIVPDTVTYESWGMATPWHDVPVKTITARWPIVEARTEKTA

DGRSICLENFLIDVAKKMQLGGFGDNAIQDAQGNWHALHSAEDFYLRSAANLAYVKGGVP

EVSAEDIAWSGLERLMPAMQSTLTADEMKRVAFIFARGGRFENATEAYQGEQMKHKWTRP

VAIWNERVGSARNTMTGDFYTGCPTWHPQKLADGTPMEEQFPSSEWPFSLTNFKSNIHSA

VSNLSPRLNSIKGVNPVYIHPIDAERAGIKTGDEFVIETPSASTKALAMIVSGIRPGTLG

FEHGFGHTELGQRSHWIGDKQQPVKTRSQDGVNINDIGLIDPTREGKGVLLDWVVGAAAR

QSLPAKIRKA

>tr|Q87ST9|Q87ST9_VIBPA Dihydrofolate reductase OS=Vibrio parahaemolyticus serotype O3:K6 (strain RIMD 2210633) OX=223926 GN=VP0333 PE=3 SV=1

MIISMIAAMADNRVIGKDNQMPWHLPADFAWFKRCTMGKPVVMGRKTYDSIGRPLPGRLN

IVISRDESLSIEGVTTVTSVEKALEAAGDVEEVMIIGGGAIYASCLAMANKLYVTHIDAV

VEGDTQFPDWGNEFKETYSETYQADEKNAYNMRFTILEKQ

>tr|Q79YT7|Q79YT7_VIBPA CsuC OS=Vibrio parahaemolyticus serotype O3:K6 (strain RIMD 2210633) OX=223926 GN=VPA1505 PE=4 SV=1

MMFRRDEMEFILKKSLLIATFLFVFISNANANSLLIWPIYPKITAETNATYLWMVNKGNV

DSYLQVRIFKWGQNEGEDNYSNQNQLIATPPFVKIEPGEKQLIRILNQDEATAGKEYAYR

VIIDELPPTHDPLTDSMENELAVKVRMRYSIPLFIYGKGLNNTQQQNKDSELWNGLQAQL

IKKNNQRFIHIKNSGNHHVRLVNLQLDSPNKEPLMISEGLAGYILPEQYKVWPISYKNIN

NMSLSANIGGKRYKIPLTQ

>tr|Q87GE6|Q87GE6_VIBPA Uncharacterized protein OS=Vibrio parahaemolyticus serotype O3:K6 (strain RIMD 2210633) OX=223926 GN=VPA1371 PE=4 SV=1

MRGFAGWRQAGLLRQFNFKHKEDPFIDSELSLPRWLLVVFLLFVKQPKLSVVLIFLCWFG

CEKKRRKVVGFAFGLKGGGVTFNNFHEALFETVVKINHLR

>tr|Q87PA8|Q87PA8_VIBPA ABC transporter, ATP-binding protein OS=Vibrio parahaemolyticus serotype O3:K6 (strain RIMD 2210633) OX=223926 GN=VP1609 PE=4 SV=1

MIPKNDTISRSWLITQVKKHKSKLILANLIAIVATLISVPIPLLMPLMVDEVLLDKPASG

LSAMNAVLPEAWHTPTGYIFFTLFLVVLMRAASQALNIVQSRQFTLVSKTITFEMRSKMI

DKLGRISIRQYETKGSGGINAHLITDIETIDQFVGSTLAKFVISFLTVIGTAIVLLWLDW

RLGLFILLVNPIVIYFSRKLGSMVKHLKRKENHSFEIFQNRLVETLDGIYQLRAANKERE

FLQQLKESANQVRVDADKYAWQSEAAGRVSFLLFLIGFELFRAVAMLMVMFSDLTIGQIF

AVFGYLWFMLSPVQELLGIQFSWYSAKAALKRINDLLELEEEDRPVSKVNPFNETREVDV

KIEHVDFSYNNENKVLDDLSLHIPAGKKVALVGASGGGKSTLIQLLIGVYRQNAGSIRFN

DELTEDIGFEVIRDKIAVVLQQPILFNDTLRHNLTLGGHFDEMSLWRALEVAQLQDVISQ

LNHGLDTQIGRNGIRLSGGQRQRLAIARMVLSNPQFVILDEATSALDTATEAALHKALTE

FLRGRTTLIVAHRLSAVKQADLIYVLEDGKVTQTGTHGELVEQEGLYQTLYGGIQSQA

>tr|Q87IU9|Q87IU9_VIBPA Uncharacterized protein OS=Vibrio parahaemolyticus serotype O3:K6 (strain RIMD 2210633) OX=223926 GN=VPA0507 PE=4 SV=1

MHVDPIVEALIIRRKEHGLSREQMANIAGMSVKTYQRIERGESDLKLSQYRSILRSLNLT

DLDIALDIRGIEHVTAEDLVTVARLLSPEAQSLLVRLLSLVLEQRSDPNAS

>tr|Q87RR8|Q87RR8_VIBPA Trehalose operon repressor OS=Vibrio parahaemolyticus serotype O3:K6 (strain RIMD 2210633) OX=223926 GN=VP0709 PE=4 SV=1

MSKKLTILDIAKLAGVGKSTVSRVLTNDPKVKPQTREKVEQIIRESGYVPSKSAQSMRGG

SQKVIGVIISRLDSPSENRAVGTMLNALYSAGYDAVIMESQFDRDKTNEHLNVLKRRNVD

GVIVFGFTGCDEQALAEWGNRIVVIAMDTNNVSSINYDNQGVIDMALSHLEQQSLSRIAY

IGVDPEDKTTGLARLNAYKAWCQRKQLTPCFQTGKLSHESAYQLVDHVLKADTQAIVCAS

DTIALGVIKRLQELGREDVVVTGVGGNELLSFLFPKVFSVDPGYTLAGEKSAKMLINQLN

GDEEVVHFTQHPVCR

>tr|Q87IT0|Q87IT0_VIBPA Putative OmpU OS=Vibrio parahaemolyticus serotype O3:K6 (strain RIMD 2210633) OX=223926 GN=VPA0526 PE=4 SV=1

MKKAALTTAILTALVSAPSFAATVYKNDGTELKVGGRVEFRGDFIGSDGAEVEGSMEDQS

RARLNLKGKTDIGNGMSAFGVYEAEQKTGKSEFKNRYMYAGVNTDVGAFSVGRQDMAAVI

ISDMTDITEFSGVQQVIDSSSDKQDSVFAYRGEFDALQLQATYQANSGDSQDKYGISGMY

SLPMGLDLGLAYSGGDVDKSNSEDQILGGIAYSLDNLYLAGTYSQGSLTDSEDFTAYELV

ASYKVATKVTLAALYTAQENDPDNGSKYDSVEGIELVGYYKLNSNFRTYLSYYINQLDEV

KDATSGLVTEGEDTLRLGVRYDF

>tr|Q87JC6|Q87JC6_VIBPA Putative translation elongation factor G OS=Vibrio parahaemolyticus serotype O3:K6 (strain RIMD 2210633) OX=223926 GN=VPA0328 PE=4 SV=1

MLINKIRNIAFVGQTGTGKTTLIEKLLFTCDATHHLGSVEKGDTVTDFDPQSIQYQHSIE

ATPVALRWNKHRLNIIDTPGQNELLGRTISVFPAVETSALVIDPQTPLNQTSDRLFTFAK

EQQKCQMIIINKLDNHGNQLERLLENIVEHFGDHCLPINLPSADGESVVDCYFEPELGRE

TLLATVDEAHETLIDQVIEVDEELMELYLEQGSELTAEQLHDPFEEALRTGHVIPVCFVS

AQTGAGVELLLRTLAEIMPMPNEGNPPLLEKNGKKIKVNCETLEHTVAHVYKVSVDPYMG

KLAYLRVYQGEINAGSQLYIGESNKAFKVGHLYQLQGKDRTEIPRALAGDFCVLAKVDDL

EFDSIVHDSHEEDGVQLKTLDLPDSMYSLCLKPLKRGDEQKLGDVLNKIASEDPSLRIEH

RARTNETIISGQGEFHLKVALEKMANVYKLDVETCQPSVEYFETITKPAEGHYRHKKQSG

GAGQFGEVQLKVRPLERGAGFKFINKVVGGAIPTALIPAVEKGILQALEEGAISGNPIKD

VEVTVYDGKYHSVDSKEIAFVIAGKKAFLDAINNADPIVLEPIVQMELHIPTANVGDVSG

DLSGNRGLIEGTEPIDSSFTLLRAKSPVNELQDYARRLRSITGGEGSFSMTLSHYEPAPP

AVQKQVCQGSTQQG

>tr|Q87HR9|Q87HR9_VIBPA Uncharacterized protein OS=Vibrio parahaemolyticus serotype O3:K6 (strain RIMD 2210633) OX=223926 GN=VPA0887 PE=4 SV=1

MAISNYRGFNLQSGGPDGSIWKVKIKNHVLQGSLTAVKKSIDWWCDTASIIDPKEFASLG

QRAHPTGGVQSEVFNGYTIKNDTGEPNGWYCMFNGKLIKGGKAAIQRHIEAYLVAKQKAM

QAQAQQQKK

>tr|Q87KR2|Q87KR2_VIBPA D-isomer specific 2-hydroxyacid dehydrogenase family protein OS=Vibrio parahaemolyticus serotype O3:K6 (strain RIMD 2210633) OX=223926 GN=VP2914 PE=4 SV=1

MQHPNQIFLLSEHRDTYETLLAEKNLPDLNITDKPQEAHIVLADPPLLSQRLDEFSQLEW

VQSTYAGVNALITPEFRQDYTLTNVRGVFGPLIAEYVLGYCISHYRHFMHYHQQQQNKQW

QPHLYTSLQSKTMVILGTGSIGKCLAQTAQTMGINTIGINRTGIPTANDEFAQTFHINEL

ANALRKADIVVNTLPSTPNTRALLNRETLQHLNQALLFNVGRGDVLDEASLLLAIKNRWV

EHAFLDVFEQEPLPPAHPFWKLPQVTITPHIAALSFPEQVVDIFADNYRLWRDGFSLNNQ

VDFEKGY

>tr|Q87NF1|Q87NF1_VIBPA Uncharacterized protein OS=Vibrio parahaemolyticus serotype O3:K6 (strain RIMD 2210633) OX=223926 GN=VP1917 PE=4 SV=1

MQPMVTDQTRRTLLKAALFGAATPVLPFGCAATTKREPALIGCSIVRRDKFAAVVADEHG

MPISTLPIPERGHGVATNQHGHAVVFGRRPGTFFMVFDYRTEQMLKLELAKPERHFYGHG

VYSHDGLYLFATEGERGTSRGIIGVYDVQNQYRKIAELSGFGIGPHEVIMMPDGTLAIGV

GGVHTNGREPLNLDSMKPSLSYLSQNGELLDQVALPDHKLSIRHLAHDGSETVLCGQQYR

GEPDEYPALLAMHERGGEMQTLQAEPEEWARFNHYIASIAATNDWILATSPPGSCYGIWS

KETGKLVELNALPDASGVVIYGDEFRVSSGAGKVVEQKPSQFKNAYSSGVQWDNHWSRII

>tr|Q87QG7|Q87QG7_VIBPA Cystathionine beta-lyase OS=Vibrio parahaemolyticus serotype O3:K6 (strain RIMD 2210633) OX=223926 GN=VP1182 PE=3 SV=1

MSEGKKTKLITAGRDKKWTNGVVNPPVQRASTVVFNTVAEKNQAAINRANKTLFYGRRGT

NTHFAFQDAMVEIEGGAGCALYPCGTAAISNAILSFVEAGDHILMVDTCYEPTRDFCNVI

MKKMGVETTYYDPMIGENIRDLIQPNTKILFLESPGSITMEVQDVPTMARIAHEHDIIVM

LDNTWGAGVNFSPFEHGVDISIQAATKYIVGHSDVMLGTAVASEKYWDQLREQSYLMGQC

VSPDDAYLGLRGIRTLDVRLRQHAENSLKVAQWLANRPEVDHVRHPALETCPGHEFFERD

FTGGNGLFSFVLKTSYPKATTALLDGMKHFSMGYSWGGYESLILANEPRSFNSLRTVANP

NFEGTLIRIHVGLEDVEDLIADLEAGFVRYNALVLEKEVSLK

>tr|Q87G71|Q87G71_VIBPA Putative transcriptional regulator, LuxR family OS=Vibrio parahaemolyticus serotype O3:K6 (strain RIMD 2210633) OX=223926 GN=VPA1446 PE=4 SV=1

MRNIMEQYTEKPEILMLTQQSLQSENFKEMLSKNTETKITIIDAKNPSYHELIPDRYFLL

VDFSVDTPSDTLVYLKDSNKVLGTIMLNLGYDLDTEELASWPHVKGIFGPLDSMEKVCRG

LGAIVKGDNWLSRRLLDQLVNYYKGKESNNVSEPAIEVELTRREIQVLKMLKEGGSNMEI

ADSLFISEHTIKSHLYNIFRKLEVKNRTQATSWAKRNL

>tr|Q87LB0|Q87LB0_VIBPA MSHA biogenesis protein MshN OS=Vibrio parahaemolyticus serotype O3:K6 (strain RIMD 2210633) OX=223926 GN=VP2702 PE=4 SV=1

MESTLSAINKALSELAEKTSPSELTRAEIPNVSRSKPWLWLVAGFSLSLAVGGWAVSQGP

APSDEITSFQPSATQPLDTTNADRLASSPTNKMTPETSNVVYSKPVEKPQAVAALPTPQP

SEGVFGSRADADMSSDTPMLIAQVANAPSNTTTNLVEESSMRIEQVELSHRQLSLKAISR

AEKAIDANDMETALSAYTEALRYEPSNIETRQKLAALYFGKGDTRKSYEILQEGINLDKD

NQPLRLALSKLLVKANQPSAALSPLVHLPPMPSRDYLAMRAALAQKQKQNDIALESYQLL

TQREPDNARWWLGLAIQQERALTFTAAINSYNEALGKVGISNQSQAFIRDRLTILKQLES

AQ

>tr|Q87NG2|Q87NG2_VIBPA Transcriptional regulator, MarR family OS=Vibrio parahaemolyticus serotype O3:K6 (strain RIMD 2210633) OX=223926 GN=VP1906 PE=4 SV=1

MEKYEEVLVSIRQIIRAIDLHSKKLSKESGLTAPQLILMRAINELDNVTIKQLSNHTNMS

QATATTILDRLERNQLVERRRSVEDKRKVHAVLTEKGEEALRQAPTPLQEHFINRFQKLE

EWEQTLLLSSVQRISTMMNAEDIDVAPLLEIGSITKPE

>tr|Q87FQ3|Q87FQ3_VIBPA Uncharacterized protein OS=Vibrio parahaemolyticus serotype O3:K6 (strain RIMD 2210633) OX=223926 GN=VPA1625 PE=4 SV=1

MKVLKIAAIAVALVAGFYAPTLLKSFSSSSTVKPLDEYCLLSTTACVQQGVTMTLNVDNA

QPLIPAKLDVEWNKSDAEQLVLSLSGREMEMGKPKFLLKKIALGKYSGEVVLPVCTQESM

TWVGELTDGQSTIYPAIKMQR

>tr|Q87NN7|Q87NN7_VIBPA Uncharacterized protein OS=Vibrio parahaemolyticus serotype O3:K6 (strain RIMD 2210633) OX=223926 GN=VP1831 PE=4 SV=1

MKLSELQSHIKEFDYAPEQSGHYFLKLIEEVGELSESIRNGKSGQPTLDELKGSIAEELY

DVLYYVCALANIHGVNLEKTHELKEVLNKVKYNR

>tr|Q87GF8|Q87GF8_VIBPA Putative dimethyladenosine transferase OS=Vibrio parahaemolyticus serotype O3:K6 (strain RIMD 2210633) OX=223926 GN=VPA1358 PE=4 SV=1

MGKTLFSLEPAYSLNSDIFSKVNILAGALFDNGIISLSFLGGIVFSLSNETLIQCKLHSF

DLDMTFYVKQKEIERLTGIEFSHMDEKYLSYLISQHFLKYGISFGNVSDRDVNDDNKIFI

KSMLWVDNKKIEILVDLNEINLDEGCLIYQKNKLPGTLRLKTSLNILDTVLDTTEIASLT

TDDVVLVYPK

>tr|Q87RW8|Q87RW8_VIBPA Uncharacterized protein OS=Vibrio parahaemolyticus serotype O3:K6 (strain RIMD 2210633) OX=223926 GN=VP0658 PE=4 SV=1

MFKQKGASLVEFMIASALGLISLATVGSIYVSGQRVIMERSKELMLLQNSESVVQMLKSD

IQRAGFDGNDGHSIKISGSANTIYTLDDVDRGLIAYAYYIGVSGSAPLYKNVAYEQRVNT

PESLFVCEKKQITIWDVNDVVNLAGTGSCNTLFDKKVIHVNRFDLASELLESTDAKSALV

TITLGTELKDATDIRTQQSFTTMQRNWQ

>tr|Q87KF8|Q87KF8_VIBPA Putative multidrug resistance protein OS=Vibrio parahaemolyticus serotype O3:K6 (strain RIMD 2210633) OX=223926 GN=VP3019 PE=4 SV=1

MALLTLLVLFSPLAIDIYLPALPLIASTFQVDNALAQDTITWFLFAMGVGQLFAGPLADK

LGRRTVALGGITIYAMSALLAWSAQNIEWMLMSRLLQGLGACATSVAAFATVRDIFGPEK

SGKMISYLNGAICFIPALAPILGSWLTQQFDWRANFSFMAGFAVVSGTLMFLMMKETNPA

TEKQAVFKVSRYWSVLSTPSFIFHASLCLMAMAVILAYVTSAPSVLMTGMGLSMNEFTFW

FGINAVINIAGCMLAPKFMERFGTHNTLVVGISTLALAGVVMLVMNGQNTALSFMLPIFM

SSVGFALILGGAAGKALEPFGDKAGTAAALLGLFQMSGSGLLVGTLQRLPFDPQTLIAIH

MWVLLPALVILFSKAGKSWHARFANA

>tr|Q87NJ2|Q87NJ2_VIBPA Uncharacterized protein OS=Vibrio parahaemolyticus serotype O3:K6 (strain RIMD 2210633) OX=223926 GN=VP1876 PE=4 SV=1

MTGNSSLERKLKREIASRKAAELLLEQKSYELYESTQKLSVALKKLELRSESDLRKFEFE

EKIDATLIRFGRTFLSSTFDETMIASFLEQLTSNSVITASYLYLDPVQLTSLRRHHFGHL

DLKSDKPIARTPNWQNEALHLPIIIDERVVGELIFSVSLEQIEQAFISKQMELVSDLVHG

VISRHLSLEREVELRKRAEESEKATKEFVAMINHELRTPLNGVLGSADLLSKTQLGEDQR

QYLGNLIQSGDLLRVIINDLLDFSKMNAGMMEIIDKVFAWDDLEKAITGVFAAKAAEKRI

HFSIDKKLGIPEFLIGDLERITQILVNLIGNAIKFTHLGGVVLRVEWVNGTAFFEVEDTG

IGIPLAAQSSLFDPFVQVDRSAKRSFEGSGLGLAICKNLVDLMQGEISFESEERKGTTFK

VSIPLKEGQAQGAASGELAVVERSDLAGRSILVVDDIRMNQVIVTQMLKKLDITPDLKAN

GLEALEAVKNKDYELIFMDCRMPEMDGYEATAHLRQKGFDRPIIALTAGTTIEERQKCID

SGMDDILTKPYTATDIEQIMCKWLEQ

>tr|Q87MA2|Q87MA2_VIBPA Peptidyl-prolyl cis-trans isomerase OS=Vibrio parahaemolyticus serotype O3:K6 (strain RIMD 2210633) OX=223926 GN=VP2355 PE=3 SV=1

MLRKAILSLALLSCSVWAGPKVAFETTLGSFTVELNEEKAPITVANFLKYVEDGSYEGTI

FHRIIPGFMAQGGGFNQDMQMVKTYAPIKNEGNNGLNNDRATIAMARTNAPDSATRQFFV

NLVDNDFLNYGARPPGYAVFGEVTEGFDVIEKMAQQPTTTVGRMRDVPETQIVITKATLL

K

>tr|Q87QZ6|Q87QZ6_VIBPA Uncharacterized protein OS=Vibrio parahaemolyticus serotype O3:K6 (strain RIMD 2210633) OX=223926 GN=VP1002 PE=4 SV=1

MKKTIISLGLAAVAAGCGGGENKSQSNSQVTPTPVPVQQALETGNALLVSDPNDFIRESR

QVVEAHKKQSNAIKSAIAKNLSGLYWDPTHDAAIFAPTYGFNDTILMTNKAMASGYKDQA

LSIGIAGEQTNGQRYAVLGSNPFRTAQRFPDSSNAAMTQWLKNLVTWLSGGATSNVVIAQ

MDQSYYFPDEQATRSWLNNNISPDLTFNEANLCDGSKLLSCLKADKPNLLILSQHLLSGD

TNQQVLDALAYAEQAKIPVLYLHWDGGLTDLGRDILAKFHVDYVGDNYWRKLGLVDWAPS

SLMNFVPDSVITQQALLSRFETQNFNVDLSQCDDKSCPEAANMDSQFYDAANSIRQWLKS

LDEQKISLFEQDGYQYEKLMVLLADHYRQTASFPMDKQSTGTTEFLKSYFADYVQYNSRR

INPKQPNMGNFSRSEFGADVKRIDTTVNMESKHNFRSAGVYALPGETFTVTRKDNNDVTT

KIVINSLRSGATHEFSKDGYTRPKLLTSFAYEVKAGETITLTSPYGGPVQVHFDKNDIPV

ELRLNHVAQHPVWRSEKDNDTFIQQLEANLFDWAELITPGFEVHSKRDKMLESVNDEMWS

TPAEMALATEEYVHNYPHVLAGFQGPGIDEVPEIIQYAQNQGWEIANIDMVKHMNADQAT

CGYGCSGNPYDAYWAFSPLGHGDLHELGHGLEKGRFRFAGWEGHSTTNYYSYYSKSRFFQ

NTGKESTCQSLDFKGQFELLQTSRTQSDPNAYMAEQNQTGWSWGARVYIQMMMATQHEGV

LKNGWHLLARLHLIEREFNRLKADEALWNAKQSSIGFSMYTKDEASSISNNDWLLIALSY

VAQRDMTNYLDMWGFSFSEKAKQQVVALNLTPMPLTYFASSNTGYCLNEFAQMPVSIDGQ

TVWPLN

>tr|Q87QL6|Q87QL6_VIBPA DNA-binding protein OS=Vibrio parahaemolyticus serotype O3:K6 (strain RIMD 2210633) OX=223926 GN=VP1133 PE=3 SV=1

MSELTKTLLNIRSLRAFSRELTLEQLEEALDKLTTVVEERREAEEAEREALAQQEAKLAE

IAEKIAQDGIDVEALISALSGETKTKAKSKRAPRPAKYKYIDTNGQEKTWTGQGRTPSAI

QEQLDAGKSLDDFLI

>tr|Q87JA1|Q87JA1_VIBPA Putative permease of ABC transporter OS=Vibrio parahaemolyticus serotype O3:K6 (strain RIMD 2210633) OX=223926 GN=VPA0352 PE=4 SV=1

MSSSVLSPSASRAAKPTTSVWLKRIKPAIWLVPFALFFYLFQLAPMIWVLFNSFIYDGEF

ALDNYIEVLDSAFMLQAFGNSLWLSVWSSIFGLAIATLLVSSLRRVDSKLRDAVIAFTNM

SSNFAGVPLSFAFIIILGTNGAITLLLKQYGLLGDFDLYGKWGLLAIYIYFQIPLAVLLL

YPAFDALSDDWQAAAALLGARTAQYWAKVALPVLSPALFGTFIILIANAIGAYASVYALT

SGNYNVITIRIASLVSGDLFLEPNLAAAISVILMALLAFITVINQWLIAKSYAAKKSRK

>tr|Q87K50|Q87K50_VIBPA Uncharacterized protein OS=Vibrio parahaemolyticus serotype O3:K6 (strain RIMD 2210633) OX=223926 GN=VPA0047 PE=4 SV=1

MFFKMDFDAFMSECWQLHEEKPEQVYAQLETALNEVEALSQVERIVALMLHTAIGHLEHP

ERFLACLDRLDDRAITESLAFQRSIAVARFFVDDTEDVALLEEQDQRRVFSLIANELSAL

TQLEPASQWLAKAALGMTPHDAEEVLARSIAITANNLACQYEELSERTDEQKARMLESAR

LALDYWKIAGGWMQEERAEYRLAMSLLKADAPKEAKVHAERCEAICLQNGGDAFELFYAH

DLLMQVHFQLSQQCKKQLDAQVQQYCTTSELA

>tr|Q87M82|Q87M82_VIBPA Uncharacterized protein OS=Vibrio parahaemolyticus serotype O3:K6 (strain RIMD 2210633) OX=223926 GN=VP2376 PE=4 SV=1

MFRKLSSNSAWLIAFFACFAVYLSIACYPFVLSNNQNEPLTLLQGYYLVSTQYGWLGLIA

LPFMLLSLLLSFLPTRAFKGIVIAIAICLLIMFKVDILVFQQYKLHINALLIRMFFEGGG

DVFDISWMSWLIFISEVAVLVIGLACTVWVSNKLAQSRAKFVLISVWFAVLVSTQMIHAY

KNALYDDEVSQFSNNWPLYYPLTARKFIYKHDLVDENIASKNRVELTNIERSSLNYPLAP

IQVQSKEKQPNVLFILVDAWRYSDATPEVMPNVSKFAEKTVNFTQHMSGGNSTQAGIFSL

FYSLPATYWESFYASQRSPVFMDTLQAEGYRMGIFGSAPLTSPPLSRTVFKKVSDLTLKQ

TGETAVERDQQITDKFIEFQKQDSDKPYFGFLFYDAAHGTVFPEPEFAKFKPYWERVDHI

LLNNDFDASLYHNRYKNSLYYIDSLIDDVLKNVDLDNTIVVISSDHGEEFNDHKMNYWGH

TGNYSDVQVHVPLYVYMPNRQPEQIDYRTTHFDVVPTMMNTLFDVQGTTSSYSVGHNLFD

SSAPRDWYIAGSYYNYALVGKELMLVVNPGGHSQQLNRQLKVEKEQKVPVDIIRHSLDEM

SRFYKKG

>tr|Q87P48|Q87P48_VIBPA Putative translocation protein in type III secretion OS=Vibrio parahaemolyticus serotype O3:K6 (strain RIMD 2210633) OX=223926 GN=VP1670 PE=4 SV=1

MRIKPSTDTPSTQPHSPQSPMPIDDHAMLQSRFERALKENPDQTKPQPNETNNQAALEAK

KPFTENYSERSLASLHSTGSNQRKTAEKSAFAQNNDVVTENINNESDTSSPIALNTQDKM

PSDMDANMKPDIRIPTTGDKKLPMEPSNKREKNADEQDAFERLVEDHDDSKTELAAAKTA

ESHESTQSPKDTKHIPTAGTKPNTVETVSDALASTLASTTVASATSASLASHPVTSTVHK

TATKTEVNKHEGDSSLGFNSRNLKTPIPNDEHPKASQAERTLLDRAMLDSEKSNDKPMHQ

PVTTPITQGDVILKGISPPQPTQPTREVNQLIQQLVDKVYVALPTASNEKEVRLFLSEGQ

LKGGEIHIKLDSQGYSVTIRQEHALSIINQQARQDLAERLNRMGFEQPLRVSISEQTSQH

GHTQQDQQQSRQQRSVYEEWQPEADQ

>tr|Q87S84|Q87S84_VIBPA Putative carbon starvation protein A OS=Vibrio parahaemolyticus serotype O3:K6 (strain RIMD 2210633) OX=223926 GN=VP0540 PE=4 SV=1

MMWFLTCVAALIGGYFIYGAFVEKIFGINEKRQTPAHTKTDGVDFVPMSTKKVYLVQLLN

IAGVGPIFGPIMGALYGPAAMLWIVVGCIFAGAVHDYFSGMLSVRNGGASVPTITGRYLG

NGAKHFMNIFAIVLLLLVGVVFVSAPAGMITNLINDQTSLNVSMTTMVVVIFAYYIIATI

VPVDKIIGRFYPLFGALLIFMSVGLMTAVALSSEHTVMGDFQVTDMFSNLNPNDMPLWPA

LFITIACGAISGFHATQSPLMARCMENEKNGRFVFYGAMIGEGVIALIWCAIALSFFGSL

EALSEAVKNGGPGNVVYSASFGLLGVFGGIIAFLGVVILPITSGDTAFRSSRLILAEYFN

MEQKTLRNRLLMAVPLFVIGGVLTQVDFGIIWRYFGFANQTTAVMMLWTASAYLLRHNKL

HWITTIPAMFMTTVCVTFILNNSSLGFGLPMQVSTIAGVIFSLVVTGYVIKTSKGKGESE

LADEEKPQAVTETA

>tr|Q87R85|Q87R85_VIBPA C4-dicarboxylate transport protein OS=Vibrio parahaemolyticus serotype O3:K6 (strain RIMD 2210633) OX=223926 GN=VP0912 PE=4 SV=1

MDILLLFLMVIGFMLIGVPIAISLGLSSVLFLMLHSDASLASVAQTLFNAFAGHYTLLAI

PFFILASSFMSTGGVAKRIIRFAIAIVGWFRGGLAMASVVACMMFAALSGSSPATVVAIG

SIVIAGMIKNGYSKEFAAGVICNAGTLGILIPPSIVMVVYAAATDVSVGRMFLGGVIPGL

LAGVMLMIAIYIAARIKNLPKQPFVGWKETFDAAKDASWGLLLVVIILGGIYGGIFTPTE

AAAVAAVYSFLIANFIYKDMGPFADKQNTKPAIVKVIQTFVHEDTKHTLYEAGKLTIMLL

FIIANALILKHVLTEERIPQMITESMLSAGLGPITFLIVVNLLLLIGGQFMEPSGLLIIV

APLVFPIAIALGIDPIHLGIMMVVNMEIGMITPPVGLNLFVTAGVARMSMMNVVKAALPW

VGVMFLFLIIVTYVPWVSTWLPTTLMGPEIITK

>tr|Q87JC2|Q87JC2_VIBPA Putative transcription regulator protein OS=Vibrio parahaemolyticus serotype O3:K6 (strain RIMD 2210633) OX=223926 GN=VPA0331 PE=4 SV=1

MNLRQLEVFYAIMQAGTVSGAARLLHVSQPNVTRVLAHTEQQLGFALFERVKGRLVPTQE

AKALLPEAEKVYQQLGQFRSLTNKVKQGTQHLRVGAPPVLAAHLLAPTVALLSKEHGISF

ELLTANRDELCSGLLKHELDVAIAFGEETPPAIMGHVLLKENLALIAPKSAAIPAEKTVI

LKELISHDLPIIGLDSRDPLGLLLHQTLSARDEHYQHAITVRSYSAAAELVKHQAGFAIV

DPWTAKQYRQDDAVSVHALEPNMSFSVSILFAEHTPQSIATKQFITALKQQMV

>tr|Q87J12|Q87J12_VIBPA Uncharacterized protein OS=Vibrio parahaemolyticus serotype O3:K6 (strain RIMD 2210633) OX=223926 GN=VPA0443 PE=4 SV=1

MVRCLRSGYMSNVIRPDTFSLEEDGFSTSGFYQIRKDKGTSDSRYLEQVFLAKEQQAHLN

SASDDFVGVITHATLHNSVDDGDVVNINQFGQLRVILSRRANHNTLLVTERCNNLCLFCS

QPPREERDDWLLSYGALALAAFGFDGDVGISGGEPLLYGDAFIKFLEFVSDHAPNTRLHV

LTNGRAFSDSEFTGQLALMASRLDITFGIPLYSVRGRIHDHLVGRDGAFSQTVVGMINAG

NSGIKMEVRFIPTQHNASELPQVAEYVARVFSGVVQLSIMNLEATGWAKRNWSALKCAPE

DYEDALIKGLLTAEMAGLNPTLFNFPLCHLPSSIRSYAVKSISDWKNYYPSECSDCQLKD

SCGGYFSSSRGKYHASPRRII

>tr|Q87GD3|Q87GD3_VIBPA Uncharacterized protein OS=Vibrio parahaemolyticus serotype O3:K6 (strain RIMD 2210633) OX=223926 GN=VPA1384 PE=4 SV=1

MDDLSSAVDAVAVTIGVALSAEVKIGFMEQYPFKYALIGCLGLATYLYCLLKIQNVIEIR

TAHRFATANVSYFGITLCFLTLIIVPFVPSIVANVVWGAALFSQILIPVRTAFKVLTREA

KQALD

>tr|Q87H95|Q87H95_VIBPA Uncharacterized protein OS=Vibrio parahaemolyticus serotype O3:K6 (strain RIMD 2210633) OX=223926 GN=VPA1070 PE=4 SV=1

MKKKLIFALLSPVVLSACSNQQASQMGMRSSSVNMYAQQMSNTQLCETLYYNRSTNQTKV

AIGAEFNRRGLNKKWCDNELNKWYLEKFVDSVVPKKKPAPRKPMATIQPASTK

>tr|Q87KR8|Q87KR8_VIBPA Uncharacterized protein OS=Vibrio parahaemolyticus serotype O3:K6 (strain RIMD 2210633) OX=223926 GN=VP2908 PE=4 SV=1

MNELVNFIRLKRDSEGAQLAELEALSKVHFKSLDILCSGDWDDDEWSYLKVSDKLKFKSI

GGGTLPIELSTLSKIFIVEVLWNQRLRGEPYSHSYIADLLNPFKVWAEMGIQELSELNQD

SYDATIIYLRDRYTVSEPYGIKVNKVIKFLNDKNILNAHIDTHNIRKVLGNTDEYGKVLA

KKNKMPLPELVKAIIHLKWAVDDSFDGSTKSVNDKLCILTQVFQYGLGLRLGETLRLPID

PLIEVNGEMFCLVWTEKGHIPMARYVPSIWRHPLSEAVAEIRKITQPYRDVAIQIEQRNN

LCFVDERLDCLHQEKAALLAHKIDELKVLLELKKQDSVKLWQLKREVKPTERYELKDLNE

VLPIASNAKDSSSLITYYRQMGIDIVSESLGGRKHKHYVTGKDIVNVIDELVAKRASNVT

LKELLTIINPQRSDILNSSKDKLIKENIDKLYGAANYLTFTFGDEKYSVNGTCIGVVSFE

NAIKILESYVLGGYDNNRFVSISDFNDMFPELFNITSSSKNNIENILVNKKECFYVKHGG

ERGFQKVRGYLVDIDKLKNYLISEYIRINVGIESDLLDASIEEYLEDGVEITSKSFRIKQ

KPSDYLFIRAGMRGGNYFDYLPQILGYNAVNYFFKGNEIQEDAFTRYDISVELHVIESWQ

SHKGRHWQTTSLFRAGLAELVVNKWMGRTSGQGENYDHNTGRERAKVIGKAMLENTERFL

GYVPDKIRKWKEQEIPIETMPDHLNDNLKSVQYSPLGYCLRDLYLKPCEFNLRCLTGNEG

KGCKHYIYDLYDPSHRERVTAERDKSSLELSRLLEVYDRGIEAAAMHIEHHMTILRNTTS

ILEDAELILRDQQLEELQDFMPFKINGSYPDDCPFQCGDSE

>tr|Q87LA8|Q87LA8_VIBPA MSHA biogenesis protein MshL OS=Vibrio parahaemolyticus serotype O3:K6 (strain RIMD 2210633) OX=223926 GN=VP2704 PE=4 SV=1

MRKLVVGITIASLMGCSMGHRDPVEVKQALNESINQANSRALEEIPSSVEADLMPNLDSN

VSSDNGTSKRFRIQANAVEARSFFASLVKGTEYSVAIHPAVQGNITVNLSDVTLDEVLSV

VQNMYGYDVMKSGKVIQVYPAGMRTVTIPVDYLQFKRSGRSLTSIVTGSVTSAGTSNSGG

SSDSSDSSDSNNSNNGNDSSTTSTGGTRIETITESDFWPMLQQAVANLIGSGKGQSVVVT

PQAGVITVRAFPDDIREVREFLGVSQERMQRQVILEAKILEVTLSDGYQQGINWSNLSAS

IGNSGSIIVNRPASALPPLDAIGTLLGGQTNVTISDGNFEAVLNFMSTQGDLNVLSSPRI

TAANNQKSVIKVGTDQYFVTELSSNAGNGENSNAVPEVELTPFFSGISLDVTPQIDNKGN

VFLHVHPAVIEVTEEVKQLNLGGDFQNIQLPLAKSSIRESDSVIRAKDGDVVVIGGLMKQ

QNVEQVSKVPFLGDVPALGHLFRNTSNVTQKTELVILLKPTVVGVNSWQKELERSRDLLQ

EWFPDAQ

>tr|Q87FT7|Q87FT7_VIBPA Oxygen-insensitive NAD(P)H nitroreductase OS=Vibrio parahaemolyticus serotype O3:K6 (strain RIMD 2210633) OX=223926 GN=VPA1591 PE=4 SV=1

MDIVQAAKSRYSTKVFDPTRKLPQEKIDAVKELIRFSPSSVNSQPWHFILASTEEGKQRI

AKAAQENYAFNAAKILNASHVLVFCAKTGIDNDYIEALMAQEEKDGRFPTEEAKAAVRGG

RSYFVNMHRFDLKDANHWMEKQVYLNVGTLLLGASTLEIDAVPIEGFDPKVLDEEFGLRE

KGYTSVVIVPLGYHAEDDFNAKTPKSRWDAETVFTEI

>tr|Q87GJ5|Q87GJ5_VIBPA Cytotoxic necrotizing factor OS=Vibrio parahaemolyticus serotype O3:K6 (strain RIMD 2210633) OX=223926 GN=VPA1321 PE=4 SV=1

MPILNISKFSNTEYAFVNNRKLKEPCIKVKSVRTSREGGEILFINMPSKSKYKDLRAMVK

SSVVVSESNQTAASKFENNSRFNRNDINVKKADAKSITALKSGDLHILKGKGIIGMKGGD

NKLPFKCTIVNDDKNGAHLSQGTNLATNGIKSMAGDSVRAAQLIPGTPLGQFYNSAPLDD

SFNVVHLPNGQRGVNGLKIPLSEFYSEKKFLFSNGALSGCMTCTAIDKNNLYIFHVGKDG

NDTSPWKTNVDGSSLIQKNMKMLLGQNSDSLNNGIQGLIDYCSKNFDKAIIQYCGHGEQY

SGRKNIHLFDYNTPQKNNPLRVGNNLTLISHSDNGSLSISTLCDDMIINSKTCETNSVNS

KLVLLKNG

>tr|Q87HT4|Q87HT4_VIBPA ABC transporter, ATP-binding protein OS=Vibrio parahaemolyticus serotype O3:K6 (strain RIMD 2210633) OX=223926 GN=VPA0871 PE=4 SV=1

MQTPVLKAPIVQANLVSKQVSTNQEQLTILKDVNVDIRKGETIAIVGTSGAGKSTLMTLL

AGLDTPTSGEITLLGHELSKLDDEARAKIRADSLGFVFQSFLLIPSLSALRNVTLPCLLR

GEAEDEARATELLSAVGLEKRLDHLPSQLSGGEQQRVALARAFMTQPELLFADEPTGNLD

HQTADKVIELLFDLNSQHGTTLILVTHDVNLASRCDRIYRMEAGELKEEM

>tr|Q79YV6|Q79YV6_VIBPA Flagellar secretion chaperone FliS OS=Vibrio parahaemolyticus serotype O3:K6 (strain RIMD 2210633) OX=223926 GN=VP2254 PE=3 SV=1

MRGSLQAYKKVSVDSQLSAASPHKVIQMLMAGAIERLIQGKAAMQAGNIPAKGERLGKAL

DIIIALRSCLSMEDGGDIASNLDSLYEFMITQISAANHQNDPQPIDDVIDIIREIKSAWD

QIPTEFHNLTAAEVGI

>tr|Q87JS6|Q87JS6_VIBPA Uncharacterized protein OS=Vibrio parahaemolyticus serotype O3:K6 (strain RIMD 2210633) OX=223926 GN=VPA0172 PE=4 SV=1

MNQKMIIGNAEAICLPELGITHLEARIDTGAQTSSLHVDNIDCFEKDGQSYVAFDLHPDV

YHLEQVVRCTAKLKANRKVKSSNGTFEHRCVITTMLRLGDQEWPIDITLTNREKMTYMML

LGRQGMADKVLVDPSQSHLLAS

>tr|Q87SI9|Q87SI9_VIBPA Uncharacterized protein OS=Vibrio parahaemolyticus serotype O3:K6 (strain RIMD 2210633) OX=223926 GN=VP0434 PE=4 SV=1

MPWIYAIVGLLVGTIVGVVISRLTTPEYKKQKSVQKELEAAKFELEQQRQELVDHFAQTA

EMLDTLGKDYTKLYQHMAKTSSELLPNLPEQDNPFDKKTAMSSEQIKEDQPEVNEQPKDY

ANGATGLLKDQEKEIINAPDVVTAKAS

>tr|Q87JJ5|Q87JJ5_VIBPA L-serine dehydratase 1 OS=Vibrio parahaemolyticus serotype O3:K6 (strain RIMD 2210633) OX=223926 GN=VPA0254 PE=4 SV=1

MLSIFDIFKVGVGPSSSHTNGPMLAGFHFTQLLSDSMAKVRRVQVDLYGSLSLTGRGHHT

DRATVLGLMGNKPDTIKMTSAKSALQHTIENHTLSLAGSHQIEFSYDHDIVFHSDNLPLH

ENGMTITALDAAGAQVAFETYYSIGGGFIATAAELEHGGQQASAEVPFPFSSADEMLEKA

EKNGLSLGGMILQNELAFREQEEIDQRAEQIWKVMSLCMQRGFDTEGILEGGLNVTRRAP

NLLKKLEANAAVENDPMEIMDWINLFAFAVSEENAAGGQVVTSPTNGAAGVIPAVLMYYH

RFIKELDLKQLKDFLAVAGAIGILYKTNASISGAEVGCQGEVGVSSSMAAAGLTALRGGS

NEQICIAAEIAMEHSLGMTCDPIGGLVQVPCIERNAMGAVKAINASRMALKRTSKCLISL

DKVIETMYQTGKDMNKKYRETSLGGLAIIHMAPPCE

>tr|Q87J88|Q87J88_VIBPA Putative transcriptional regulator protein OS=Vibrio parahaemolyticus serotype O3:K6 (strain RIMD 2210633) OX=223926 GN=VPA0365 PE=4 SV=1

MKIDDLKLFTKVVQLGSFTAAANALDLPRANVSRRIGELERYLGTQLFHRTTRSLSLTNK

GDAYYQDLQKVLTLLDSANQQASNESIEVRGKIKLGLLPETYEHLQPILFEFQDQYPQVE

LDIRNINNGFIDMFQQGLDIALHGGMLFDSDIVARKILTLDRCVVASPNYLQTHGTPATL

EELSDHQCICFRWPNGNVDNLWHFQEQNVALKPKLISNNIGFIKSSTVLDRGISYIPKLL

VKNELESGALVQVLSQYAVTEETGWLLYPQPKTLNQASRLLIEHLCNKIPKLL

>tr|Q87PV2|Q87PV2_VIBPA Uncharacterized protein OS=Vibrio parahaemolyticus serotype O3:K6 (strain RIMD 2210633) OX=223926 GN=VP1399 PE=4 SV=1

MHNSTGRYVLGMEVMTPTGMNLDIATSVANADLARFDQVVGEDGIERFTFAKIEYTKESD

LFEKTCELTANLLDSLLTQLPRSLKPIPLLIAVPTTISLVKIQEWLGESDYSDFLSVVEA

VHDSGPSFVLQAMKSLDKYDAMMCISVDSMVNRIQELIDDTMVMSTNNPWGVIPSEGGAG

LIFCRRNTVETLKLKPLAQLGYIDTELNTSDRRGMYRLVQRASKKLTAFGEVYSDMTNLR

AHSEDYGFALGAKAERFINPEQPLLINELWGTMGNCSSLALGAFAVKYHHFNQPVTLLMF

DFGGDKALLQLLAC

>tr|Q87GI7|Q87GI7_VIBPA Putative traA protein OS=Vibrio parahaemolyticus serotype O3:K6 (strain RIMD 2210633) OX=223926 GN=VPA1329 PE=4 SV=1

MPINRLQSFCAPSMKRTSSTVKENSLLVNKVSHKAIDEAIFKTMKIAKNSPSSERKEKYG

ILKNHVSFVCNLEINRKLTENAASISNGKLLNLKRDIINAQVLSGFKMDYLMCSAPLQNY

LASQLDKNQEIKALSKTDREKVLQIAYELLDNKIDEKIIEKNLKTPTIAAAKELIEIVCK

EQLSSIEKDKDLRKRVSTLVSNELKFDFSLDKFAETTLVELDEVFHKYQ

>tr|Q87NZ1|Q87NZ1_VIBPA Glycine betaine/L-proline transport system permease OS=Vibrio parahaemolyticus serotype O3:K6 (strain RIMD 2210633) OX=223926 GN=VP1727 PE=3 SV=1

MSTEQTNNDPWSQAAPTQEDPWSQGSEAATNNDWLNSEAVQETPFDIMNPFQDAVLPLDN

WVESGLNWLVEHGRPVFQAIRVPIDFILSSFETALVSTPSPIMVLILFLLAWQFSNFKLG

LSTLVSLVFIGLIGAWSQAMTTLALVLTSVFFCLLIGLPMGIWLARSQTAAKFVRPILDA

MQTTPAFVYLVPIVMLFGIGNVPGVVVTIIFALPPVVRLTILGIQQVPEELIEAGHSFGA

NKKQMLYRIQLPLALPTIMAGVNQTLMLSLSMVVIASMIAVGGLGQMVLRGIGRLDMGLA

AVGGLGIVILAILLDRITQELGVNAGNTKLRWYHAGPISYLLKFKTNKNQNDLKLRRNTN

E

>tr|Q87QH8|Q87QH8_VIBPA Peptide ABC transporter, periplasmic peptide-binding protein OS=Vibrio parahaemolyticus serotype O3:K6 (strain RIMD 2210633) OX=223926 GN=VP1171 PE=4 SV=1

MNAFIRLSLSLIGISLLTACGEEIDHNDIRQAGFVFCGQGKPTTFNPQLIDSGITAEALS

PQLYDTLLTLDPKTHQPIASIAESWSVNKEGTEYTFKLKHGVQFQTTPWFSPTRPLNSKD

VVFSFKRIIDVDHPFHQVGGGFYPWFAGLDFQNLLQDVIAVDDYTVTFKLAQPNFSFLAN

IATSHAVILSAEYGQQLAAKDNKEQIDQLPIGSGPYQLKEYQVNDLIRLERHPNYWNSPA

KMEQVVFDISHRGTGTLAKLLRNECDVLSSPISSQIPIIQEDENLELTATPANNVSFIAI

NTETPALRDPRVRQALNLAINRQNILDSVYYGTGTLAYTLLPPNSWAYQKDSAKIRYDRN

YALALLREAGYEDGLKLSMWVPLEPRAFNPSPRKTAELIQANFADIGIKLTLLTDDRFER

VDLENINDIDLLLTGWIGDTGDPDNFFRPLLSCESDRVGLNVSMWCNDDFDFLLDLALET

QEQRYRLNLYRQAQNILNEEFPVIPLAHGVQFRVHDKSLTGFKSSPFNAQPFDRVERIH

>tr|Q87IF6|Q87IF6_VIBPA Heat shock protein 70 family protein OS=Vibrio parahaemolyticus serotype O3:K6 (strain RIMD 2210633) OX=223926 GN=VPA0650 PE=4 SV=1

MYIGFDYGTANCSVAKIESGKPVLLNLEGDSPFIPSTLAAPTRESVSEHLFRHRDIKPFD

QVGEQVLCRAINLNREESIELEPEDIAFGQAALNRYLEDPRDIYYVKSPKSFLGASGLRD

VQLSFFEDLVCAMMDNIKAQAENHAQQSITDAVIGRPINFHGRGGETANIQAENILLRAA

KRAGFKNIEFQFEPVAAGLEYEATLSEDKRILVVDIGGGTTDCSLIQMGPSWRGKADRTA

SLLAHSGQRVGGNDLDIALAFKQLMFPFGMDSKMESGIVMPTTQFWNPIAINNVEAQNDF

YSRENLAALKLLQKQAQEPEKLARLLEVYHESLGYNIVRRAEEAKVALSDQTAYCTGINL

LSELIEVDVQRDEMIEAIESPKNKMTALVTEAVEQAGVKPDAIFMTGGSARSPILRDAVQ

TVLPNIPVVSGNYFGSVTAGLARWADVCFK

>tr|Q87GM1|Q87GM1_VIBPA Putative DamX-related protein OS=Vibrio parahaemolyticus serotype O3:K6 (strain RIMD 2210633) OX=223926 GN=VPA1294 PE=4 SV=1

MHKILSLTLIFSLMGCAMPHQPIDNGQCVGVMSSQDAYGYLNPNHYTMQVLALKKEKDVK

EYIRYIDPKHPVWVNWKNSRGTRWYTVTAGDFKSKQEAYNALSSLPSHVKQSSPFVLTFA

EMQRKQQTSVVRMR

>tr|Q87G49|Q87G49_VIBPA ATP-dependent RNA helicase, DEAD box family OS=Vibrio parahaemolyticus serotype O3:K6 (strain RIMD 2210633) OX=223926 GN=VPA1468 PE=3 SV=1

MENTLQFKDLGLDNRLLKNLKHYDFKKPTDIQKQAIPVAIAGKDLLASSKTGSGKTLAFV

LPMLHKSLKTKSFSAKDPRAVILAPTRELAKQVYGELRSMLAGLSYDAALILGGENFNDQ

VKALRRYPKFIVATPGRLADHLEHRSLYLDGLETLILDEADRMLDLGFAPELRRIHNAAK

HRRRQTLMFSATLDHAEVNDIASEMLNAPKRIAVGVSNEEHKDITQKFYLCDHLDHKEAI

LERVLSEAEYRQVIIFTATRDDTERLTAKLNEKKLKAVALSGNLNQTQRNTIMSQFERAV

FKILVTTDVASRGLDIATVTHVINFDMPKHTEEYVHRVGRTGRAGNKGDAISLVGPKDWD

SFKRIETYLQQDLAFSVFEDLKGKFKGLKPPKKDFRNKKATTKKARPQAKKVAKQPAKRD

KSFYKNVSVGDDVFIPKKKPAAPSSEES

>tr|Q87LT7|Q87LT7_VIBPA Type IV pilin assembly protein PilB OS=Vibrio parahaemolyticus serotype O3:K6 (strain RIMD 2210633) OX=223926 GN=VP2524 PE=4 SV=1

MHSNLSTILRQKGLLTFSQEESLIEQVKASGISMPEALLSSGFFTSSELAEHLSSIFGLS

QPELSQYEYASLCQQLGLRELITRHNALPLHRTPSTLLLAVADPTNQQAEDDFRFATGLQ

VELVLADFRELSTAIRRLYGRSLSHEKSGLKEINQEELASLVDVGADEIDNIEDLSQDES

PVSRYINQILLDAVRKGASDIHFEPYEKMYRVRLRCDGILIETQQPPNHLSRRLSARIKI

LSKLDIAERRLPQDGRIKLKLNQDTAIDMRVSTLPTLFGEKIVLRLLDSSSASLDIDKLG

YSEQQKQLYLEALRRPQGMILMTGPTGSGKTVSLYTGLNILNKPEINISTAEDPVEINLS

GINQVQVQPKIGFGFAEALRSFLRQDPDVVMVGEIRDLDTAEIAIKASQTGHLVLSTLHT

NSAAETIIRLSNMGVESFNLASSLSLIIAQRLARKLCPYCKQPQEHTVQLQHLGIQTTDN

IFRANPDGCNECTHGYSGRTGIYEVMRFDESLSEALIKGASVHELEKLAIANGMSTLQMS

GIEKLKQGITSFSELQRVLYF

>tr|Q87FU7|Q87FU7_VIBPA Putative membrane protein OS=Vibrio parahaemolyticus serotype O3:K6 (strain RIMD 2210633) OX=223926 GN=VPA1581 PE=3 SV=1

MKKLFVIALNLVILGGAAWLGYQKFEEYFNNPWTRDGQVRANVIKVAPRVSGPIVNVSVQ

DNQEVKTGDLLFEIDPTTYEVALSQAEVALEKSIVSSRGKKIEYDRLKDIRAKDRGAVSH

KDLIRREIAYEESLLQIKSAEEQLKSARLNLSFTKVYATVDGFVSNLDIRNGTQAVANQP

LLALIDKNSFWVFGFFRENQLAQIEPGSRARVTLMSHPDTPIDATVDSIGWGIAPKDGTV

GYNLLPNVNPVFQWIRLAQRIPVRISIQELPEDVDLRFGLSASIMVMKDSSAENEQ

>tr|Q87RI0|Q87RI0_VIBPA Uncharacterized protein OS=Vibrio parahaemolyticus serotype O3:K6 (strain RIMD 2210633) OX=223926 GN=VP0814 PE=4 SV=1

MSNQTIVRVRDVMAATYVMVDGLMTVYEGITLAKKYQVKALVVKKRDDNDEYGIVLMNDI

AKKVLANNRAPQRTNIYEIMTKPALCVDPDMNVKYCARLFERFGISRAPVIEDGEVIGMV

SYNNIVINGMVREDG

>tr|Q87RX1|Q87RX1_VIBPA Uncharacterized protein OS=Vibrio parahaemolyticus serotype O3:K6 (strain RIMD 2210633) OX=223926 GN=VP0655 PE=4 SV=1

MLNHQVLAGTKKLQMQKGLRFSRPLFFELLVNLS

>tr|Q87LL2|Q87LL2_VIBPA Fructose-bisphosphate aldolase, class II OS=Vibrio parahaemolyticus serotype O3:K6 (strain RIMD 2210633) OX=223926 GN=VP2599 PE=3 SV=1

MSKIFDFVKPGVISGDDVQKVFEVAKENHFALPAVNCVGTDSVNAVLEAAAKVKAPVIVQ

FSNGGAAFFAGKGLKLEGQEAQILGAVAGAKYVHAVAEAYGVPVILHTDHAAKKLLPWID

GLLDAGEEYFAQTGKPLFSSHMIDLSEESLEENIEISAKYLERMAKMNMTLEIELGCTGG

EEDGVDNSDMDASELYTSPEDVAYAYEKLNAISHRFTIAASFGNVHGVYKPGNVVLTPTI

LRDSQAYVSEKFGLPANSLNFVFHGGSGSSLEEIRESIGYGVIKMNIDTDTQWATWDGIR

QYEAENHDYLQGQIGNPTGEDAPNKKYYDPRVWLRAGQASMVTRLEQAFADLNAIDVL

>tr|Q87RC0|Q87RC0_VIBPA Transcriptional regulator, LysR family OS=Vibrio parahaemolyticus serotype O3:K6 (strain RIMD 2210633) OX=223926 GN=VP0877 PE=4 SV=1

MLSERAAQMVIFSALIKHKNFTVAAKSLGVSVSHVSKQLSQLEESLGIKLVQRTTRSFTP

TEAGKTFYHHCEQVVQAVSSATLEMESQRDEVAGLVRLGLSQSFGTLHIIPAIQELRELY

PQLQVEVHLFDYKVDMLAEGLDLWVTNNEHLPEGYIAQRLTDCQFVVAASPDYLLKYDTP

TEPNDLSLHNCLIYRSWERDYTGWAFTKGQQELNVKVSGNYSVDLAEAVRDAAIAGWGIA

YLATYLLGDEFRTGKLIQLLPDWKASQSMPFYAVYPSRQHMPKKISAVINFIKQKIGNPC

HWDQRLAPYISIPK

>tr|Q87S03|Q87S03_VIBPA Putative nonspecific acid phosphatase OS=Vibrio parahaemolyticus serotype O3:K6 (strain RIMD 2210633) OX=223926 GN=VP0621 PE=4 SV=1

MKNYLLNVFTLAVAVISFPILAQNSDPLPSWNDGQTKQAIIQFVSDVTDANSPNFVAPQD

RIATFDNDGTLWSEKPMYFQILFMLDQIKEQAASHPDWKTTKPYSLVLSGQIDKLSLEDV

LTMVQKTHSGMSSDEFTSIVQAWISTAKHPATGRPYTDMVYQPMLELLDYLDSNGFKNFI

VSGGGNAFMRAWATDVYNIPSERIIGTRLSTEFVNVDGSYQVKRVPGIEVNNDKAEKPQQ

IYQHIGKRPIASFGNSDGDLQMLQWTTSGTGPRLAMYVHHTDDQREWKYDRTSSIGKLDK

GLDEAKAKGWLIADMKNDWKQVYPTK

>tr|Q87IE2|Q87IE2_VIBPA Putaive Fe-regulated protein B OS=Vibrio parahaemolyticus serotype O3:K6 (strain RIMD 2210633) OX=223926 GN=VPA0664 PE=3 SV=1

MESPACSSIKLSSLCLAVGMALANPVLAQESDSHFEEVVVWGTKVSSNTESMFADDMSLK

QADHMSDLLRDIPGVDVGGTHSVNQRITIRGLGETDLDIRLDGASQHASMFHHIGNLTLN

PDILKSADIQVGNNSVTQNGLGGSVYFETKDARDLLRYDETFGARVYGGFASNDNQQGSL

TLYGLLSENVDAMVYGHYVTRDDFKDGNGDKTFGSAGDVYNILAKLGYEFNDIHRFELSY

DMYRDSGDYSPRPDMAGSANNGLSSKLLIPTDYDRDTVTLSYELRGEKHQGNVTLYNTET

EIKRDETVMAPRWPSNRLSKNTAKNQNLGLNAKFQSDFALAQFDNRVTYGFDYMDKTSSS

YYGSSKFMDESAVSTALFVEDQFFFSDAFFITAGLRFDDYKRKAETGTSNFDDVTWALAA

EWAVTQDWTLFASSRSLFKGPELMETFIAYQDVAYLDEGMKAETGQNTQGGLRFNKTLDK

HFFGANVTVFQTNIDDYIADKYQDATQTYLIYNIGDVEIKGFEASASYGYDKFNSKLSYS

QSDTKNKNTGGPVAGGNGRSIDMGDSIALTLDYQSDSLETIFGWTSMFVLEEDNVFDGQP

AKDSYDVHNLYAQWVPSNVDGLSVTFGIDNIFDEVYTSHASRSGTVRGFTLDDYEPGRNF

KLSAAYQF

>tr|Q87QA1|Q87QA1_VIBPA Transport ATP-binding protein CydC OS=Vibrio parahaemolyticus serotype O3:K6 (strain RIMD 2210633) OX=223926 GN=VP1249 PE=4 SV=1

MRDLLPYLKLYKKHWFGLSLGMLLAFLTLAASVGLLTLSGWFISAAAVAGLTVARETFNY

MLPGAGVRGAAMARTAGRWGERVVSHNATFKLLTDLRIFFFKKLAPLIPGRVSTMRDADL

LNRLVADVDAMDHVYLRLISPVIIGIFGILSLTAVLAFFDWHLALLLGSILTIMLLVWPV

LFYKLGKNNGEHLTQNKAQLRVATLDWLQGYSELSIFGAEERYRNLILEKQKQLLSNQFV

NANLTGMASGLLMLVNGLTLVVMLWFAADGVGGRAPDPWIALFAFATMASFEILMPIAGA

FQYLGQTLTSARRLNEVILAEPDVVFPEQSKREEKPLDIEFINIDFAYPDGQQKVLKDLS

LSLPLGSKTAIVGQTGSGKSTVIQLLCRYWDVDQGEVRIGGASLKDWCESDLRSAITVVS

QRVDVLNGTLRDNLLMASPNATDEQLADILTKVELGALLEEPGLDAWLGDGGRQLSGGEK

RRIGIARALLRDAPILLLDEPTEGLDKRTEQKVMALFNEYFANKTVVFITHRLIELENMD

NICLMEQGEIIEQGDHQTLLAQHGRYYQLLQSL

>tr|Q87S61|Q87S61_VIBPA Uncharacterized protein OS=Vibrio parahaemolyticus serotype O3:K6 (strain RIMD 2210633) OX=223926 GN=VP0563 PE=4 SV=1

MPHLRFRAVEPQAVQALSKPLTDELQPLMNSPREDFTFEYIYTTFFSEGEVSPAYPFVEV

LWFDRGQETQDKVAKVITQQVRGVVGEDIDVAVIFSALSPNAYYDNGEHY

>tr|Q87QG1|Q87QG1_VIBPA Putative ferredoxin oxidoreductase protein OS=Vibrio parahaemolyticus serotype O3:K6 (strain RIMD 2210633) OX=223926 GN=VP1188 PE=4 SV=1

MFFEWQGNQPVTLRCIDKYFETHDTVSIKLAELTESLLFQFKPGQFINLGVEIDGKMEFR

AYSISSINEDDHLQLTIKRVSGGKVSNYIVDSLLLGDTVQALPPAGEFNCIDHPPVLRDG

ETRALLISAGCGVTPVFSMAKHWLSNQDENDNDVDIAFLHIARSPEETIYYDQLETFDAV

YPNFHLKLLLKNREGTSHPQGRLNADWLKELVPDFKQRTVYLCGPSQFMQDVHGYLNDLG

FDMTNFYQESFTPATGAETSVSDEVVHVSVPDFAQTIDAQKGQVLADVLEGAGLPLIVAC

RSGICGSCKCKVRQGSVSSTSLETLTPEEIEQGYVLACSSTIEADLEVQIG

>tr|Q87NF3|Q87NF3_VIBPA Putative exported protein OS=Vibrio parahaemolyticus serotype O3:K6 (strain RIMD 2210633) OX=223926 GN=VP1915 PE=4 SV=1

MFLFMVSRNFSRRDFLKMTAGGVVLASAMPTLSWASLPDEPRVLAMNNLNTGELLETCYF

DGNRYVGKELQRLNEFCRDHRRNEVHPMDKRLFDQISQIQKLIGTESEVIVISGYRSPVT

NASLRSGSTGVAKKSLHMEGKAIDFRLDGVKLSTVRDAAISLKAGGVGYYPGSNFVHIDT

GAVRSW

>tr|Q87Q43|Q87Q43_VIBPA Putative alpha-ribazole-5'-phosphate phosphatase CobC OS=Vibrio parahaemolyticus serotype O3:K6 (strain RIMD 2210633) OX=223926 GN=VP1307 PE=1 SV=1

MKTLNIYLMRHGKVDAAPGLHGQTDLKVKEAEQQQIAMAWKTKGYDVAGIISSPLSRCHD

LAQILAEQQLLPMTTEDDLQEMDFGDFDGMPFDLLTEHWKKLDAFWQSPAHHSLPNAESL

STFSQRVSRAWSQIINDINDNLLIVTHGGVIRIILAHVLGVDWRNPQWYSTLAIGNASVT

HITITIDDQIYASVRSIGVPLVED

>tr|Q87QM8|Q87QM8_VIBPA Uncharacterized protein OS=Vibrio parahaemolyticus serotype O3:K6 (strain RIMD 2210633) OX=223926 GN=VP1121 PE=4 SV=1

MKIAIVGTGISGLTCGYYLHQQHDITLFEANDYIGGHTATVDVRVGIEDYAIDTGFIVYN

DRTYPNFIKMMNEIGVEGIPTQMSFSVRNDGNGLEYNGHTLTTLFAQKRNWLNPKFYRFV

LEILRFNKLAKASATEQISSSQSLGAFLDEHQFSDFFSDNYILPMGAAIWSSSLADMRAF

PLMFFLRFFLNHGLLDVTNRPQWYVIKGGSRAYIPPLTQGFADKIRLNSPVEKVVRSEQG

VAIYANGQCEWFDEVIFACHSDQALAMLSDASSCENDILSNMAYQANDVVLHTDTSVLPK

RKSAWASWNYLLEGGEEEQQRLPSLTYNMNILQHIDSSHTFCVTLNRTEDIDENKILRQF

TYHHPVFTTESIAAQQRKEEIQGAQHTWFCGAYWYNGFHEDGVRSALDVVKGIAAKHNEK

NDTLYEQGAA

>tr|Q87G24|Q87G24_VIBPA Endopeptidase La OS=Vibrio parahaemolyticus serotype O3:K6 (strain RIMD 2210633) OX=223926 GN=VPA1493 PE=3 SV=1

MAILRLEPQQLYSVADLENMPCKSTKELPPIDEIVGQERAQKAVEFAMSIKEKGYNIYAI

GQNGLGKRTMILRYLNRHQHDADALFDWCYVANFEDTRTPKVLKLPCGIGNKLRVDIEAL

MGKLLNALPLAFDNEMYFSRADRLKNQLANKQQNELDRISREAKEHGISLTITSQGDYQF

VAMNGDELHTEESFDALSKKEQEQFSDSIDALEVSLRSMVRELTEWEDTYSDKIKKLNDE

VTLDVITHFIKKLKVDYSEYSEIKTYLTELQKDIVENADIFLDQSGEQGEIAAASLDKKL

PRRYKVNVLVSRNNSDFPIVVEENPNYHSLFGSIETATFKGTVFTDFSLIRAGSLHKANG

GVLLMDAQKVLEQPYVWDGLKRALRARQLSFTSLEKEVTLTGAVSLDPEPIPLDIKIILF

GDYRTYQLLQHYDPEFSELFRVTADFEDEMKRTPESELHYARFISSVVHDNNMLHCDRKA

IARIIEYSSRLSGDQSKLSLHSANIANLLRESNYVARQANSNMIRIGHVEEAMKNQEMRV

SRLRDSVMETFLNGTTLILTEGSAIGQVNALSVLSTSDYMFGAPNRITATTCYGDGDVID

IERSVDLGGSIHSKGVMILSAYLSSVFGKTARVPLTTTITFEQSYGGVDGDSASMAEFCA

VVSAFSKQPNRQDIAITGSMNQFGEAQPIGGVNEKIEGFYDVCVIKGRHSGQGVIIPRSN

VHNLMLRADIVKAVEKGEFHIWAIDHVTEAIELFTGKVAGEPTDEGSYPIDTIFGLAQAK

LNSLRK

>tr|Q87JH7|Q87JH7_VIBPA Putative integral membrane protein OS=Vibrio parahaemolyticus serotype O3:K6 (strain RIMD 2210633) OX=223926 GN=VPA0276 PE=4 SV=1

MFTTLALITAFFAILIGLKAASRISLLEKELSKLQKEVASLRVSTRRDSKLEASQPEPDT

SESSLGAASSMRSDSGDSALSAPSNIEPEFEPSYLATQQHHSEQEQTELNQSQQTQVQLA

TFTEQEDPLIQKHAQKLIANFQENWLVWVGALAMLIGGGYLVQVIGSHIEFSPLMRVSFA

FSLSLLMVLAGEWFHRKEQNNPDRAARAQGFTYVPAAVTGTGLTGIYCTVIFAFVVYQML

TPSVSLIVLAAAAFCSLALSLRQGPLMAVLGLIGGYTAPLWIGGNEPNYFLLAGYICAIS

FAATLLMQKVHRAWIAPSIAVPHVIWMLLLIESIPTELLFSWLAIYLSITLYLIFAVPRM

GWMLNPRFRHYQNKWTNQPTATSLAVALLTFSAITRMPELNTLQMAYCYTLLVAIIWLPA

LRTGWSLRVFLPSILVSATALIVLTIAFDALYMLEREASILFALAVSIALIGFRTYCQSL

SDRSQISNVLLLVLAPTMGLVTLFYADEFMHGYALYWTLFCALIAAHYAFLSQRLRPLAL

ECSAVVHALISGVAFVWLSDTWLTTTISIQVAVMALQIQAGFFRPASWAVKIVMGILVVR

LTLLPFIPQWQPVESGHWAWVILSYVPSLAILAYARSVLAKQDSDLTNWFEGAFLHVFLM

ALFTQTNYWLTGQYGYLGHIDFTSAVVFANQALVMGLVYSYRSQFAQQLTLVYQAYSYLL

WGAFALLTVWLNSVESPLISNNVSAQAMPVFNMLSLGWLLPACILIAAVVRRWNTLRVHR

LAVACLGFALAAIWLGMSIRQFWQTTSMTLFQPTSMAELFSYSVAGLMVGGALTWRGVTH

KAMMMQRIGLVILAFVALKVFLWDVSSLDGFWRAISFLGLGASLIALGWLFQKLHRSVTQ

TSEP

>tr|Q87I86|Q87I86_VIBPA Uncharacterized protein OS=Vibrio parahaemolyticus serotype O3:K6 (strain RIMD 2210633) OX=223926 GN=VPA0720 PE=4 SV=1

MSRALIFVFVAITMFLAFFYVSDFFDQPEPVAVVENEVFVQVAFVNRTVNKADPVNIDAI

DIREFSESEAKQHSYTPYTSIKIESGALWAKDVTEGTQLTSNLVSNPGEKDYMYLSLKKD

QVPYYYLSQGSSIVEALPIQPGDRVSFVATTSSQSNLLESGYSDIDSLTSNVIIDGALVL

QAMNVSEDDSSNEDYGLIIALTVKQVLKLEMAQKIGAITLVPAKMVHKYLSVKSSDLIEH

RFGVRQLRGN

>tr|Q87M49|Q87M49_VIBPA Uncharacterized protein OS=Vibrio parahaemolyticus serotype O3:K6 (strain RIMD 2210633) OX=223926 GN=VP2409 PE=4 SV=1

MLWIKKYVIISLLVLTACATSEQASDFDSSLYSGKPVESLTNDEPPKTEEEAISRGDIAL

TNKNVDLALYEYIRSLSFPNAVHKDKTLYTVGRIHLARENYELADKAFRASLAENPDNIG

SLQELGTLYTKRGEKDVGKSYYIRALNADQIRMKGNPNITNDNITAESVATYRYDDKSPV

AAYSGLGVLYDVRGDHGVAQALIRKGLDIDPRNVNALVSLGYSYYMEKEYSKAAHFTQAA

LSIKPSDERAINNLGLIALAKDQPRQALSIFSRHMTEPEALNNVGYFLILQGKPDEAIPY

LQQAIDKNPAYYELANKNLERALAIVRERQDTSKVFLQ

>tr|Q87QT2|Q87QT2_VIBPA Uncharacterized protein OS=Vibrio parahaemolyticus serotype O3:K6 (strain RIMD 2210633) OX=223926 GN=VP1067 PE=4 SV=1

MFRREIQERENWRELARQFGFGFHSMYGQPYWDESAYYQFTLEQIEHDLESPTEELHQMC

LSIVDEVVRCEQLLTKCAIPELMWEQVASSWQRKEPSLYSRLDFAYNGTSPAKLLENNAD

TPTSLFETGFWQWVWLEDVVNKGRIHQAADQFNILQDFLIERFAEIAKYQPGQTLHFSCC

KYTEEDKATVQYLEDCAREVGLATAFVYVEDIGVTEDGKFVDVDRRAIRWMFKLYPWEFM

FEEEYAKYLATANVNWLEPMWKSILSNKALLPLLWERFPNHPNLLPAYFANDPNASCLND

YVIKPLFSREGANIEIVKDGKRLVKTPGPYESKFNIVQKYHPLPKFGQNHTLIGSWLVND

RAAGISIREDSSLVTQDMARYIPHVILT

>tr|Q87FZ7|Q87FZ7_VIBPA Glyoxylase I family protein OS=Vibrio parahaemolyticus serotype O3:K6 (strain RIMD 2210633) OX=223926 GN=VPA1523 PE=4 SV=1

MFNAIHHVAIICSDYPTSKRFYTEVLGLRIIAENYREMRDSYKLDLALPDGSQIELFSFP

GSPERPSFPEAQGLRHLAFQVDNVEEVKAYLESKHIAVEPIRIDEFTGKAFTFFQDPDGL

PLELYQK

>tr|Q87PL3|Q87PL3_VIBPA Uncharacterized protein OS=Vibrio parahaemolyticus serotype O3:K6 (strain RIMD 2210633) OX=223926 GN=VP1488 PE=4 SV=1

MLRHTSVYKKLTWRRESTLLWCLPRFRMTKLILVVCIYGISTTDSGYEALDVRTIVSFRR

RKNLFLYLRSRRSVECDISLSSRAQQPFSPNLEGKLADLSEVPKQTLCFYQKRIDKEEQS

LLFFRTPRVRFKGTVDLKNGYEITNV

>tr|Q87QZ8|Q87QZ8_VIBPA Amino acid ABC transporter, permease protein OS=Vibrio parahaemolyticus serotype O3:K6 (strain RIMD 2210633) OX=223926 GN=VP1000 PE=3 SV=1

MLDLQGYEASIFKGALVTIEVAVLSLLLAVILGMLGALAKLAPYKWARAIATLYTTIIRG

IPDLVLMMLIFFGGQILLNNSLYSINETLNEWFASSDPNHEWTSYLPDYIDVSPFIAGVL

TIGFIFGAYMAETFRGAIMAVDKGELEAAKAYGMSPVLSFRRILLPQMIRHALPGFGNNW

LVLLKTTALVSIIGLEDMVRMSSLAAGTTKMPFTFYMAVALIFLFFTSISTGLLKLVERK

FSIHAR

>tr|Q87L65|Q87L65_VIBPA Putative fimbrial assembly protein PilP OS=Vibrio parahaemolyticus serotype O3:K6 (strain RIMD 2210633) OX=223926 GN=VP2747 PE=4 SV=1

MRNKSLLTVLVSALLVGCQANDESLTDFIRGVESQARRDVEKLKPADKYIAVAYAPEVMR

APFELPKEATIATQPVARKDCWQPPNRARSGKLEKFPLSQLRLKGVMGMGNTVSGLVQAP

NGTVYKVKPGQYLGRNNGKVTHVTHSYLLINETLPDGLGCWQKRKVKLALR

>tr|Q87NZ2|Q87NZ2_VIBPA ABC superfamily (Glycine/betaine/proline transport protein) OS=Vibrio parahaemolyticus serotype O3:K6 (strain RIMD 2210633) OX=223926 GN=VP1726 PE=4 SV=1

MDPILEVKGLYKVFGEAPERAFSLIEKGVDKDDIFEQTGLTVGVNDVSLTINEGEIFVIM

GLSGSGKSTLVRLLNRLIEPTKGSVYLKGIDIAHISEEELREVRRNNISMVFQNFALMPH

MSVIENAAFGLELAGVDVTARHESALSALQRVGLDTYAESFPDELSGGMKQRVGLARALA

CDPDILLMDEAFSALDPLIRSEMQDELIRLQNDDKRTIVFISHDLDEAMRIGDRIAIMQN

GEVVQVGTPDEILHNPANDYVEAFFRGVNVASVLTVKDIARKKPAAVFKKSEHDGPGSAM

QILMDHDRDYGIVVDKSSRYSGIVSLDSLRLAHKENRSLASAQLEDDVTLQPDQSVNDIL

GVVASVPYAVPVVDEQGTYFGVVTKSRLLQTLDKD

>tr|Q87I51|Q87I51_VIBPA Putative metalloproteinase OS=Vibrio parahaemolyticus serotype O3:K6 (strain RIMD 2210633) OX=223926 GN=VPA0755 PE=4 SV=1

MDLGSKYQRKLVVGFVLVSPLLVSPSLSAAQLERLEPNHGRVVSLEKQNLAATQNVAKAL

GLKPGNNVQKLKANQDKNGDVHVRYQQTYKGIPIWGKQIVLHRDKQGKIKRFGGTLVHDI

GQDISNTTPQLSLERIRSKVQKPYLDVGYHIEDQQQGLRIYIDDKDVAHLAYEIQFFADS

EQALNPTRPTYLVDAKSGEVLLQYEGLAHAEADGPGGNQKIGFYEYGKEYDPLLVQQSGS

TCIMDTPNIVQTINLDGRTSGSVHTFNCPTNTVKEINGAYSPLNDAHYFGKVVYDMYKDW

LNTAPLTFQLQMRVHYRKRYENAFWNGSSMTFGDGASYFYPLVSLDVSAHEVSHGFTEQN

SNLIYSGQSGGINEAFSDMAGEAAEFYSRGSNDWKVGFDIRKSPTGALRYMDNPPLDGRS

IDHASQYVSGMDVHYSSGVFNKAFYLLAVDYDWGTENTFKAFAHANQNYWTPSATFDSAA

AGVLAAAQDLSLPASDVTAAFAQVGVSTDGGVVEPPSSACDAVSLSNGTSSNIESASTGQ

WHCFTIDVPANGSDLTITTAGSNGDADLYVKLGSAPSLSNYDCRSISSNSNEVCSFATPS

EGTWHIGVYAYSGISNVSVTASYTEQEAPPPSGGVTTQSINNGKTWTAIVTGSGLHDGVW

NNNPSDSCGNDSECSKSGIDKKTGSVSFTLSDGQTFVILKP

>tr|Q87LH8|Q87LH8_VIBPA 6-phospho-beta-glucosidase OS=Vibrio parahaemolyticus serotype O3:K6 (strain RIMD 2210633) OX=223926 GN=VP2634 PE=3 SV=1

MSREAIKLAIIGGGSSYTPELVEGVIKRLDYLPVKQIHFVDIESGAEKLEIIKGLAQRMV

DKAGADIEIKADFDRRAAIKGADFVMTQFRVGGLAARANDERIPIKYNVIGQETTGPGGF

AKALRTIPVILDICKDIEELAPNAWMLNFTNPAGLVSEAVSKHTKVKSIGLCNVPVSMEM

MIAEMMDCEPKELQLEFAGLNHLVWVHKAWLKGEDITQTVLEKVGDGANFSMKNIWEEPW

DPAFLKALGAIPCPYHRYFYQTDAMLAEEKQSAGEKGTRAEQVMETENALFKLYQDPNLD

HKPKELEERGGAYYSDASLNLVDSIYNNRNSIHVVNVLNNGAINGLPDDAVIECSAVIGS

WGAKPLAVGELSNNIKGLLHQVKAYEQLTIEAAVEGNYDKALMALTNNPLVPDIGRAKAI

LDELLAVNAPYLPQFKLTAL

>tr|Q87T63|Q87T63_VIBPA Putative oxidoreductase (Flagellin modification) OS=Vibrio parahaemolyticus serotype O3:K6 (strain RIMD 2210633) OX=223926 GN=VP0207 PE=4 SV=1

MLKDKKIVIAGAGGLLGASVVKSILEAGGSVVATDVSLEHLKARLSSVGVNLADTHLTMH

ELDITRSDALTRFWQEAEGVTGAVNCTYPRTKSYGAKFFDVTLDSFNENVSIHLGSAFLF

SQQCAAYFVNKEQPFSLVNISSIYGVIAPKFSVYDNTPMTMPVEYAAIKSAIVHLNKYVV

SYINDSRFRVNSVSPGGILDGQPEAFLDAYRKNTHGAGMLNVEEMTGSIVYLLSDQSKYV

TGQNIIVDDGFSL

>tr|Q87R71|Q87R71_VIBPA Putative aspartate aminotransferase OS=Vibrio parahaemolyticus serotype O3:K6 (strain RIMD 2210633) OX=223926 GN=VP0927 PE=4 SV=1

MQNIGMSSKLDNVCYDIRGPVLKHAKRMEEEGHKILKLNIGNPAPFGFDAPDEILVDVIR

NLPTSQGYCDSKGIYSARKAVVQHYQRKGIRSLDVEDVYVGNGVSELIVMAMQALLNNGD

EMLIPAPDYPLWTASVALSGGNPVHYLCDEQSDWYPDLDDIKKKITPKTRGIVLINPNNP

TGAVYSRDFLLEVIEIARQHKLIIFADEIYDKVLYDGATHTSVATLTEDVLVMTFNGLSK

AYRVCGFRGGWMFLTGPKHLAQGYVNGLELLSSMRLCANVPMQHAIQTALGGYQSINELI

LPGGRLLEQRNRAFELINQIPGVSCVKPKGAMYLFPKIDTKMYNIKNDQQMVLDFLKQEK

VLLVQGSGFNWPKPDHFRIVTLPHVEDLETAIGRFERFLSTYSQ

>tr|Q87HC1|Q87HC1_VIBPA Uncharacterized protein OS=Vibrio parahaemolyticus serotype O3:K6 (strain RIMD 2210633) OX=223926 GN=VPA1044 PE=4 SV=1

MMEEKNKDKSINSSDSGDKTKIVKTQKRENAIEPAKNVNKQDSAENINETDTKLSQKENT

IPPINKSDNKNTKNKSGINSNLIGQTVKNRYEIESILGHGGLCDVYLAKDKILESSGSES

PYVALKVLQKEFASQPETARMLIREAQQTQRLSHPNVIRVFDFGVDGDIYFLVMEYIDGE

TLESLIQRSRPNGLKYHAMLSILNQILDALSYAHSLGVVHADLKPANVILTSEGHVKLLD

FGVSKTHQIKHDQYAAKRKTEDLETLGYTPNYASLNLLSGKEPNISDDMFALCCITYELL

SCKHPYSRTPVNEALKKNIKVSKPANMPIAKWPIFSKIFSTGTIPADFNAKTVKRRLNKQ

YWPLAAGVAASLLFMAGLGLVYNQQQVAISDLEAKLSQQNEVIQNAELLLSTSPEHSRSL

IDGDASLHPLIKAGLLRLHKPYLLGQFESEIDDVLNSDATSYPNYDEIEAILQKAKGYYP

DSHKIEVLALDIQSSKHSTLLSIARQINSHLEKSRYDKEEDTKSIYELKEELNQIHQDYP

FVPSSLSSDVFGKHLSDALKDRDAAALVTLIKVGNTFFTNSDEHKANLEISNAMKDAVLE

MKLYETAIDSSNPLPFPIDAARILYQDEFDGLYYRLKQARTTVHLDKLVKDVDKFSENFP

VGFQDINDLRFQTADKYLQFSDILLNKRKTTSARRAMKKANDLMKQIEQDSEQS

>tr|Q87RN2|Q87RN2_VIBPA Uncharacterized protein OS=Vibrio parahaemolyticus serotype O3:K6 (strain RIMD 2210633) OX=223926 GN=VP0745 PE=4 SV=1

MYVALKHIHLVTIALSATLLSIRFALMMMNSPKRNHRFLKVFPHIVDTALLLSGVALIFV

TGFIPFTDSAPWLTNKITCVLAYIALGFFALKLAKNKLLQIFGFFGALGWLVMAANIAVS

KSPTLFG

>tr|Q87LE5|Q87LE5_VIBPA Lipopolysaccharide export system protein LptC OS=Vibrio parahaemolyticus serotype O3:K6 (strain RIMD 2210633) OX=223926 GN=lptC PE=3 SV=1

MSFTRLIYLLLVFVVAWCGYYLLTPKGNDAIQVAPDTELPLFSGTGLENITYGEDGVRSY

IIRSNNLDYYAKSGETIFNSPTLMVYREGSVVEWKVTATRAVLDEDQVLTLYDKVLMQNL

LPGASFDTMATDKLVINLTNRDFKADQQVMLVGPQFETTGGAMQGNLKQHTATLTNEVQG

RYETVTP

>tr|Q87P37|Q87P37_VIBPA Uncharacterized protein OS=Vibrio parahaemolyticus serotype O3:K6 (strain RIMD 2210633) OX=223926 GN=VP1682 PE=4 SV=1

MNTIQPLLDEFCRLNELPPLILEDGNRCQLLVDDRFVLYFTATEDDALMLSVAFGGLEKS

GELRVRGLELLARANYQRVGSGNLALSLAPNGRQLVLAGRQPTEHLNSANLTVWFHEIIE

QTELWQARFAMLDQDLSATSNHEQSHVQPLRV

>tr|Q87LI1|Q87LI1_VIBPA Putative HD-GYP hydrolase domain containing protein OS=Vibrio parahaemolyticus serotype O3:K6 (strain RIMD 2210633) OX=223926 GN=VP2631 PE=4 SV=1

MAAIKLTVDRIQPGLHIRLPLKWNDHPFLLNSFKIKDQEQIEMIRHLGVKFVYFNPEQSD

AAPLPVNQIQTDSTTSNESLDLETQKLWQEKQKRIEKLSAYRRRVIQCEKEFERSLARMR

SVMTKIRNRPTAAVDEAQLLIEDIVEKLMCDDNVTLHLMNGKNEFEDIYFHSLNVAVIAM

MIGRAKGYSAKQLKELSFAALFHDMGKIKIPTAILRKQVPLTEPETNYLKLHTKYGLDLA

NQIEGFPEPAKTVIAQHHELRDGSGYPEGLKGDEIDELAQIVIVANAFDNLCHTPIASEQ

KIPYTALSHLYKNCKHLYKEENLNILIKFMGVFPPGTVVQLSNNMVGLVISVNASNLLFP

NVLVYDPSVPRTQAPIIELASKDLRIVNAIHPSKLPDKIKEYLNPRSRISYFFDSDE

>tr|Q87J61|Q87J61_VIBPA Uncharacterized protein OS=Vibrio parahaemolyticus serotype O3:K6 (strain RIMD 2210633) OX=223926 GN=VPA0392 PE=4 SV=1

MVVRTNAMSLKNKSFYWVGEPPTSNFCEGVILVNQIEEVPVGLGGMVCISYQDTQQINHA

LKAFFKEKGRWSWAVYVTVETPYSRCIADGVFEEVESKKVWRSIQSKIDSIDEPDVLDPL

IGWLGVNRQRRVSALKSLESTSIYSFPITDLLFPDIYSTYRYVLSEQGRGVLEPEALIDR

IRVCSHCNSGHLNYVEVCPDCSSIDIDSQSSLHCFTCGHVGEQHSFQRRGKLECPKCLTQ

LRHIGVDYDRPLENHVCHSCSSLFVEAATISQCLSCDSKIKVEELVVRKIYQYRLGEVGE

YIFQHGKSIQAPELSIKGKVEVSFFQNLLAWLNKVALRHKDQHLLLGLHLPTIDEYGKQY

GDAKLFSLMDQLTCRLSGLFRDTDICCQYKQDVLLVLMPNTTNASLSVLQQKLSDLGDLV

EDEEFELDVFAWDLPDPVIEGGVSVWIESLMGEIYAAR

>tr|Q87Q84|Q87Q84_VIBPA Uncharacterized protein OS=Vibrio parahaemolyticus serotype O3:K6 (strain RIMD 2210633) OX=223926 GN=VP1266 PE=4 SV=1

MSFKTIALSFTLSSCLSVSAFASQAPTSPAEQFELAQQLALSPKSDSPSDVRYWLEQSAS

QGYLPAQKQLAEDYSRGLTGAVNYPQATYWFTSVALNDPRDRGFLLADFVQHHQKDITTS

DLVEAWYQLASDTNPQAEEAYNHYLEERFNQLRAKQVSEIVELDKEATKEEQPVAPQSTV

TSDNGTIMWLAIGGLVIALAGFGGCVVLRKQQRALAAQSSQEANQTQQLNAKVKELEFTN

KQLKRQLEKVFKEFKKTRSQSDNHKVAVACAMFGYTPQTIPDNQAVKLRYRQLSKLYHPD

TRGSEEEMKRLNQAFKIISQNVTK

>tr|Q87I94|Q87I94_VIBPA Ribosome biogenesis GTPase A OS=Vibrio parahaemolyticus serotype O3:K6 (strain RIMD 2210633) OX=223926 GN=VPA0712 PE=3 SV=1

MVNNSIQWFPGHMHKARKEIEEAIPQVDVIIEVLDARIPFSSENPMISKIRGEKPVVKVL

NKRDLADPELTQLWIDHLEKEQNVKAMAITTSEPQEVHKIMELCRKLAPHREEVGKNIRT

MIMGIPNVGKSTIINTLAGRTIAVTGNQPAVTRRQQRINLQNGIVLSDTPGILWPKVENP

HSGFRLAATGAVKDTAMEYDEVAFYTVEYLAAHYPERLKERYQIEELPESDIEIMEEIGR

RRGALRAGGRVDLHKVSEILLHELRQGTLGQITLELPEMITQELIEVEIETARKEEEKAK

RKEERRKRYLRNKR

>tr|Q87SJ3|Q87SJ3_VIBPA DNA topoisomerase 4 subunit B OS=Vibrio parahaemolyticus serotype O3:K6 (strain RIMD 2210633) OX=223926 GN=parE PE=3 SV=1

MTEQYNAGAIEVLNGLEPVRRRPGMYTDTARPNHLGQEVIDNSVDEALAGHASKVQVILH

ADQSLEVIDDGRGMPVDIHPEEKVSGVELILCKLHAGGKFSNKNYQFSGGLHGVGISVVN

ALSKRVEVTVRRDGQVYEIAFEHGDKVSDLTVTGTCGRRNRGTSVHFWPDAKYFDSANFS

VTRLVNNLRAKAVLCPGLEITFTDKVNNKDYQWYYEDGLKDYLAEGVKGYPVLPEEPFTG

EFSADTEAANWAVIWQPEGGEMITESYVNLIPTAQGGTHVNGLRQGLLDAMREFCEFRNL

LPRGVKLTGDDVFDRCSYVLSVKIQDPQFAGQTKERLSSRQTAAFVSGVVKDAFSLWLNE

KPQLAEQLAEVCIANAHRRMRASKKVVRKKVASGPALPGKLTDCSVQDLNRTEIFFVEGD

SAGGSAKQARDREFQAVMPLRGKILNTWEVSADQVLASQEVHDISVALGIDPDSDNLDSL

RYGKICILADADSDGLHIATLLCALFTRHFRALVEAGHIYVAMPPLYRIDCGKEVFYALD

DDEKDGILERLSKKKAKINVQRFKGLGEMNPIQLRETTMDPNTRRLVQLTIDDSEATMEM

MDMLLGKKRADDRRSWLQNNGDLAEV

>tr|Q87FP2|Q87FP2_VIBPA Uncharacterized protein OS=Vibrio parahaemolyticus serotype O3:K6 (strain RIMD 2210633) OX=223926 GN=VPA1636 PE=4 SV=1

MRIKTFGSNFFITCFNRKWSIFHLLCFISIRDLCMLRIISADKFISSRFILEYSKAEFAS

LLGVSVAKITNIESGKSSIPPVYDYAINHLLSEHPFKESAVEALCSHETLNFARRIDVNS

KVQRLSCKRCDSRRLHVMAGMSGLYFVECMDCKHTMYSSGIAVKRYQQWSNNGVKLDSHL

FN

>tr|Q87TC9|Q87TC9_VIBPA Type II secretion system protein L OS=Vibrio parahaemolyticus serotype O3:K6 (strain RIMD 2210633) OX=223926 GN=VP0141 PE=1 SV=1

MEGSVSEFLTVRLSSEQQSTIPWVVWSTEQQEVIASGELAGWEHLDELVSYAGQRQVIAL

LASNDVVLTQVDIPPGATRQFDSMLPYLIEDEVAQDVDSLHFTVLGKQADKAQVCAVERA

WVQTVLQRFASQGLTIKRILPDVLALPVSDDNSSAALIGEQWLIRHSETEGAVVDSAWLD

LYLSSYLQNHEGWQLDCYSSVPESTVESVWVPKPEEMTMALLAKGVASSKTNLLTGEFKP

KSSWGKYWKVWQKAAIAAGVLLVVVVAQQLLVVHKYEAQAQAYREESERIFRQVFPNKNR

IPTVSYLKHQMTDEERRLSGGSTDVAMLSWLAALPATLGQVKDLEITSFKYDGQRGEVRI

HARSSDFQPFEQARVKLAEKFNVEQGQLNRSDNVVMGSFVLKRQ

>tr|Q87KT2|Q87KT2_VIBPA Transcriptional regulator, MerR family OS=Vibrio parahaemolyticus serotype O3:K6 (strain RIMD 2210633) OX=223926 GN=VP2894 PE=4 SV=1

MFQIGELAKRCGVTSDTLRFYEKNALIKPAGRSESGYRLYNEENQKQVRFILKAKELGLS

LDEIKDLLEIKLEATEHSCAEVKAITSAKLELIDEKINELTKIRRALKKINDACCGHTND

DASHCSILAALE

>tr|Q79YZ5|Q79YZ5_VIBPA BipA protein OS=Vibrio parahaemolyticus serotype O3:K6 (strain RIMD 2210633) OX=223926 GN=VP0122 PE=4 SV=1

MATPQIDKLRNIAIIAHVDHGKTTLVDKLLQQSGTLESRGEAEERVMDSNDIEKERGITI

LAKNTAINWNDYRINIVDTPGHADFGGEVERIMSMVDSVLLIVDAVDGPMPQTRFVTQKA

FAHGLKPIVVINKIDRPGARPDWVMDQVFDLFDNLGATDDQLDFKVVYASALNGWASLEE

GETGENMEPLFQTIVDVVDAPNVDLDGPLQMQVSQLDYSSYVGVIGVARVTRGSVKANQQ

VTIIGADGKTRNGKVGTVMGYLGLERHEVEQANAGDIIAITGLGELKISDTICAQNAVEA

LPALSVDEPTVTMTFQVNTSPFAGKEGKFVTSRNILERLEKELVHNVALRVEQTDDPDKF

RVSGRGELHLSILIENMRREGFELAVSRPEVIIKEEDGQLMEPFETVTIDVMEEHQGGIM

ENIGLRKGELKDMAPDGKGRVRMDFIMPSRGLIGFQTEFMTLTSGSGLLYHTFDHYGPHK

GGNIGQRVNGVLIANAAGKALTNALFNLQERGRLFIGHGVEVYEGMVIGIHSRDNDLTVN

ALKGKQLTNVRASGTDDAQVLTPPIVMTLEQALEFIDDDELVEVTPESIRIRKKFLTESD

RKRASRSAK

>tr|Q87RG9|Q87RG9_VIBPA Putative glutathione-regulated potassium-efflux system protein KefB OS=Vibrio parahaemolyticus serotype O3:K6 (strain RIMD 2210633) OX=223926 GN=VP0827 PE=1 SV=1

MEIILITTAFLAGFIALKCNLPPLVGFLLAGFGLHAFGYQSNDVIVTLADLGVTLLLFTI

GLKLDVKTLLSKEIWGGATAHNILSTVFFALALSGLKLLGLTSLASMESGQILLLAFALS

FSSTVFAVKTLQEKGELNATYATLAIGILVMQDIFAVVFLTVSTGKVPEWYAIGLFALPL

LRPLFYKLLDKVGHGEMLVLFGIFFALVVGAGLFELVGMKPDLGALILGMMLAGHRKASE

LSKSLFNMKELFLVCFFLNIGLSASLSLTGIALALLFIVLLPIKGLLYFLTINHFKFRVR

TSLLASLSLFNYSEFGLIVGGLAYKMGWMPSDMLAAIAVAVSLSFIISAPLNRLGHKIYQ

HSGKWLQETAAEKLNQRDQLINPGHAQVLILGMGRIGTGAYDELRARYGKISLGIEIREE

AAQQHRSEGRNVISGDATDPDFWERILDTGHVKLVLLAMPHHQGNQTALEQLQRRNYKGQ

IAAIAEYPDQLEGLLESGVDAAFNIYSEAGSGFARHVCKQLEPQFTSIK

>tr|Q87RF6|Q87RF6_VIBPA Uncharacterized protein OS=Vibrio parahaemolyticus serotype O3:K6 (strain RIMD 2210633) OX=223926 GN=VP0841 PE=4 SV=1

MNNLQLEKILNSKLSPEFIKDYAPNGLQVEGCPQIKRVVTGVTASQALIDKAIELRADAL

LVHHGYFWKGEPEPIRGMKGKRIRSLIKSDINLYGYHLPLDIHPELGNNAELARLLDIEI

DGGLEGHPQSVALFGRLKKPMTGSQFASKINQVLNREPLHIAPDNAEKMIETVGWCTGGG

QDFIELAVQHGLDAFISGEISERTTYTAREMDIHYFAAGHHATERYGIKALGKWLADEYG

LDVEFVDIDNPV

>tr|Q87FI2|Q87FI2_VIBPA Putative transcriptional regulator OS=Vibrio parahaemolyticus serotype O3:K6 (strain RIMD 2210633) OX=223926 GN=VPA1697 PE=4 SV=1

MKRQSTMLTRQIDSKSGVITSNEMMAANPHSPVLVKTIDMPKGYIDALHQHTWHQIIFPI

KGLLQTQTEHYQHLVPHTSALFVPAGVQHESIALSNTIFVGIYLNPEFGATYEPQVRTIA

LTPFLNELLQEIRRQCEGETSHEEVLHLLAVLHDQILKSNVQTFQLLLPQDRRLKLIFEQ

LTDEPALSFSLKEWGEKIGASERTLSRLFAKEFNTSFLLWRQQIRLIYSLSLLDESLPIQ

AIADLVGYQNDSSYIKAFKAYFDMTPQQFRVNGASRR

>tr|Q87GI4|Q87GI4_VIBPA Putative transcriptional regulator ToxR OS=Vibrio parahaemolyticus serotype O3:K6 (strain RIMD 2210633) OX=223926 GN=VPA1332 PE=1 SV=1

MTSKKYRIDQKILSSDSPFLISLGSQDRVKLGTHEHLVLLALCEQPGTLLDKETLIEKGW

PGKFVTDSSLTQAIRNIRAHLNDNGKSQKHIKTIAKKGYLIEKDYVQSLEVIDDKNINET

ESIRKLVTLTKRNILLISIILQLAFIIYVAYSYTSIFVSSTAKDDYPSLSFQQDYVYIFS

SDFQLSEELGVALINALSAKEIVPERLYVMLNDKTISFSFISKNKKSKNRVLSTEKKLNY

KHISEYIVNEIEY

>tr|Q87HE7|Q87HE7_VIBPA Outer membrane lipoprotein Blc OS=Vibrio parahaemolyticus serotype O3:K6 (strain RIMD 2210633) OX=223926 GN=VPA1018 PE=3 SV=1

MKSIKAWMLLCLIAMFGCTSKPDDVEPVNNFALQPYLGKWYEIARLDHSFERGLSNVTAE

YELREDGGVTVINRGYSQEDKKWTQAEGKAYFVGDENIGHLKVSFFGPFYSSYIVFELGE

NYEYAFVSGFNHDYLWLLSRKPNVDKATVERFKRVAKEKGFALDELSLVDQKQ

>tr|Q87LC1|Q87LC1_VIBPA Rod shape-determining protein MreB OS=Vibrio parahaemolyticus serotype O3:K6 (strain RIMD 2210633) OX=223926 GN=VP2691 PE=4 SV=1

MFKKLRGMFSNDLSIDLGTANTLIYVKGQGIVLDEPSVVAIRQDRVGSAKSVAAVGHAAK

QMLGRTPGNISAIRPMKDGVIADFYVTEKMLQHFIKQVHDNSILKPSPRVLVCVPCGSTQ

VERRAIRESALGAGAREVYLIDEPMAAAIGAGLRVSEPTGSMVVDIGGGTTEVAVISLNG

VVYSSSVRIGGDRFDEAVINYVRRNYGSLIGEATAEKIKHEIGSAYPGDEVQEIEVRGRN

LAEGVPRSFSLNSNEILEALQEPLSGIVSAVMVALEQCPPELASDISENGMVLTGGGALL

KDLDRLLMEETGIPVVIAEDPLTCVARGGGKALEMIDMHGGDLFSEE

>tr|Q87RQ6|Q87RQ6_VIBPA Peptidoglycan glycosyltransferase MrdB OS=Vibrio parahaemolyticus serotype O3:K6 (strain RIMD 2210633) OX=223926 GN=mrdB PE=3 SV=1

MKMDPSTGKNRALFERFHIDLPLLLGIFALMGFGLVIMYSASGQSLEMMDRQAMRMVLSL

VVMVVLAQLSPRTYESLAPLMFVAGVVLLFGVLFFGEASKGAQRWLNLGFVRFQPSELLK

LAVPLMVARYIGRQPLPPTFRTLIVALIMVCLPTILIAKQPDLGTSILIAASGIFVIFLA

GISWKIIAAAAIALGGFIPILWFFLMREYQKVRVRTLFNPESDPLGAGYHIIQSKIAIGS

GGISGKGWLHGTQSQLEFLPERHTDFIFAVIAEEWGMIGFLCLLAIYLFIIGRGLYLASQ

AQTAFGRMMAGSIVLSFFVYIFVNIGMVSGILPVVGVPLPLISYGGTSMVTLMAGFGILM

SIHTHRKAFSKAT

>tr|Q87MN7|Q87MN7_VIBPA 3-oxoacyl-[acyl-carrier-protein] synthase I OS=Vibrio parahaemolyticus serotype O3:K6 (strain RIMD 2210633) OX=223926 GN=VP2194 PE=3 SV=1

MKRVVITGMGIVSSIGNNVEEVLASLKAGKSGITASEQFKEHGLRSQVWGDLKINPAEHI

DRKQMRFMGDAAAYAYLSMQQAIEDAGLTEEQVSNDRTGIVAGSGGASALNQTVATDIMR

EKGVKRVGPYMVPRTMSSTVSACLATPFKIRGVNYSMSSACATSAHCIGHAMELIQLGKQ

DIVFAGGGEELDWSQTMMFDAMGALSTKYNDTPEKASRTYDADRDGFVISGGGGMLVIEE

LEHALARGAKIYGEIVGYGATSDGYDMVAPSGEGAVRCMKMAMQDVDSIDYVNTHGTSTP

VGDVKELGAIQELFGDNSPAISATKAMTGHALGAAGVHEAIYSTLMLDNNFIAPSINIEN

LDEAAKGLDIVTEARDAELTTVMSNSFGFGGTNATLVIKKYQA

>tr|Q87I90|Q87I90_VIBPA Putative transcriptional regulator protein OS=Vibrio parahaemolyticus serotype O3:K6 (strain RIMD 2210633) OX=223926 GN=VPA0716 PE=4 SV=1

MKETNKKNLRVVALAPTGRYFASIISSLEILETAAEFAEFQGFMTHVVTPNNRPLIGRGG

ISVQPTAQWQSFDFTNILIIGSIGDPLESLDNIDPALFDWIRELHLKGSKIVAIDTGIFV

VAKAGLLQQNKAVMHSYFAHLFGELFPEIMLMTEQKALIDGNVYLSSGPYSHSSVMLEIV

EEYFGKHTRNLGNQFLSTIESSGNSHSYCDVFRYMQHRDELILKIQKWILTTDLDIVSIS

DLANEACLSERQLKRRFKEATSISPLKFIQLGRLSFAKELLRSTKLSIDEVASRSGYVDT

QFFRQIFKRENDCSPLEYRKRNQVKAE

>tr|Q87S01|Q87S01_VIBPA D-amino acid dehydrogenase, small subunit OS=Vibrio parahaemolyticus serotype O3:K6 (strain RIMD 2210633) OX=223926 GN=VP0623 PE=4 SV=1

MEVIVLGSGVIGLTSAWYLSQAGYQVTVIDRQPSSAMETSFANAGQISYGYSSPWAAPGI

PLKAIKWLMEEHAPLKIKPSLSTDMISWASKMVANCTLPRYQINKARMLSIANHSRACLE

QLRNEHAIEYQGRQFGTLQVFRTTQQLTAIEKDLKLLEQSGTRFELMDVEQCLRQEPGLA

LVKDKIVGGLYLPDDETGDCFQFCQQLTELAKAHGVTFKFNTEVSNWVTVGKKIIGVQTN

HGLFKADQFVVASGSFSTALLKQLDIDIPVYPVKGYSLTLPIENEEYAPRSTVMDETYKV

AMTRFDDRIRVAGTAELAGFDPSLPQKRKNTIEMVVRDLFPRGGDFSQAEFWTGFRPMTP

DGTPIIGATPYDNLFTNTGHGTLGWTMACGSGHLLANIMTGEKAKIMENSELNLLRYA

>tr|Q87PV4|Q87PV4_VIBPA Uncharacterized protein OS=Vibrio parahaemolyticus serotype O3:K6 (strain RIMD 2210633) OX=223926 GN=VP1397 PE=4 SV=1

MVFFIFHPLIEPSGLPSAQVLTFAKLLEIKEFEAFDISSSLAPVDFALSGDWKLVPEIIE

HLETLEEDEGEIWIQALYVVYGKLLPLTPQGVGVEYEWEEIVDLLNEWVEDEKYIQNLPS

RLGYALSFESTLAAMQDANVDVLFRDWLWRQICIQSRAYVPWDMAMPVYQQDWNFNNLKA

APSASERFNLRNSNAVMGY

>tr|Q87JJ3|Q87JJ3_VIBPA Uncharacterized protein OS=Vibrio parahaemolyticus serotype O3:K6 (strain RIMD 2210633) OX=223926 GN=VPA0256 PE=4 SV=1

MPNITQIHISSTFFWKQVVRMKTQIVKQAETAQNNVITDESKRIIYKSCLSALMIVAISI

LVNLSIRLDYVVLGSSLGEVSITETLQVIMLAIASWSFFQLSKQEEQVKHAAILISGFFA

VLIIRELDYWMDMIRHGSWVFPALTVTALACAKAYQGGKGTVNEMARILQVPHMKLLIGA

VVLLLVFSRLYGMGSFWQQVMNESYIRDVKNISEEGMELLCYCLIALSAVRMRREITE

>tr|Q87SL1|Q87SL1_VIBPA Putative 2-amino-4-hydroxy-6-hydroxymethyldihydropteridine pyrophosphokinase OS=Vibrio parahaemolyticus serotype O3:K6 (strain RIMD 2210633) OX=223926 GN=VP0412 PE=4 SV=1

MITTYIGVGTNIDREQHAKVAYLELQKLGEDLLVSPIYECEPIGFSSQNFYNFVIAMRTK

LSLEELSHHLREIEYKWGREENAQKYQDRTLDLDIVLFGECISAQKPELPRSDIYKYPFV

TKPLYDLEPHLVIPGDGRTVADIWHAMQPVDSLKPVSFSL

>tr|Q87RV7|Q87RV7_VIBPA Uncharacterized protein OS=Vibrio parahaemolyticus serotype O3:K6 (strain RIMD 2210633) OX=223926 GN=VP0669 PE=4 SV=1

MARSKTSKVFELLGHKIQPGQRLEVEFEAAQLYTHSPLSIPVEIIHGKQEGPVLMVNAAI

HGDELNGVEIVRQMINQLNPLKLKGTVIAVPIVNVFGFIHKSRYLPDRRDLNRCFPGSEK

GALASRMAYGFFNNIAKRCDYILDLHTGAIHRTNLPQIRANLSNPETLRIAKAFATPVIV

DSALRDGSLRSEAEKCDIPVLTYEAGEALRFDPLSISAGVLGVQRVMQAIGMLRASRKKL

PEPVIAKSTSWVRAPGNGILRTVVNLGDKVEKGETLAYISSPLGHDEIELKAPKSGLVIG

QQTLPLVNEGDAIFHLAYFSQSDDEVEQAVESYIEGLTEFDVEQVTTGQIPLDTQS

>tr|Q87R32|Q87R32_VIBPA Uncharacterized protein OS=Vibrio parahaemolyticus serotype O3:K6 (strain RIMD 2210633) OX=223926 GN=VP0966 PE=4 SV=1

MRAWLIGLAVIIGLAGCADNTAGIRVDGQTQNVFFNDNVLGSRLLVDNITTTYVDDRPRG

VVQLSSNYKGDQHILYRFYWYDNNGLEVNTKPGPWRKMIVRGFESVTLSEVTVNPNGTHF

RVQIREAQDD

>tr|Q87L38|Q87L38_VIBPA Peptidyl-prolyl cis-trans isomerase OS=Vibrio parahaemolyticus serotype O3:K6 (strain RIMD 2210633) OX=223926 GN=VP2778 PE=3 SV=1

MKSVLKVSLLAATVMLAVGCQKEETKPEAAPQAEQVQAETGKAVHFKTDDDKAAYAIGVS

FANYLSTSIEKPSEIGINLNKDLVLKGIEDVFKGNAELNEEETRAALESLDKRVAEKMQA

QAAEKAAAAKKAGDDFRAEFEKQEGVVKTDTGLLYQVITPAEGEKPKDTDTVQVHYKGTL

IDGTQFDSSYDRGEPATFPLNRVIPGWTEGVQLMPVGSKFKFVIPPELAYGDQDTPSIPA

NSTLVFEVELLKVENDKAEQ

>tr|Q87QG9|Q87QG9_VIBPA Putative lipase activator protein OS=Vibrio parahaemolyticus serotype O3:K6 (strain RIMD 2210633) OX=223926 GN=VP1180 PE=4 SV=1

MKKVALIAVPILSLLGVGAASLYVSDSPQPKMAQSQHDTTIDLSSQKDFFEYSLSGLGEH

SLEEIQENIEESISQQNSLGIDVELFQTYLAYKRALSKLEPLEDTTLSLSQLQRLNEAIL

NLQLEYFNDQQISQLFDEENRLRQLAIEKLVIKTYEQDSDSQQLLLNQALSEQPEYIQKS

ERNNALTRQLDQTELLSSQDKYLARVELVGEEGAQRLQKLDEQRATFEIELTNYLEKRAD

ILNDEFLDSEQKQLEIANLRKQSFETTQWRRIEALERIHDSQN

>tr|Q87HI8|Q87HI8_VIBPA Uncharacterized protein OS=Vibrio parahaemolyticus serotype O3:K6 (strain RIMD 2210633) OX=223926 GN=VPA0975 PE=4 SV=1

MTNNTDIKNQTNPMSGDLLRQIIRLGFPVAIQSALVAILALADVLMVSDFGKEATAAVGI

ASKWHFVAIMIMAGLASANGTLVAQYWGRNDAVSAKTVTGIAIRFGLKVLIPVTLIITLG

SELIMRLQTSDTRVIELGATYLWYAFPVLLLTHIVIVLEASMRSSGDTVTPLLMGAMTIV

LNIGLNFWLIKGGFGIPAMGVAGAAFATTISRLFQVLAMIAYMRWRKHWLLEVKEGSERP

SLWLSYRRLALPMTLGALLWAIGTMVYQMIFGHMGTTELAVFSMLGPFESLCYSIFFGIS

VACSVMIGQSLGRDAFEQAEHMAKFFIKAVFVFGISIGALLLLNRELIIAALNLDNPELY

PLAAPAVMILCCAIWLRMLNMIIINGIIRAGGDNVFCLRMDFIAMWMTGLPLCAIAAFVL

GWEFKYVYALMIAEEVVKLALCYRRYTQKYWIKNLTVVTTS

>tr|Q87TH7|Q87TH7_VIBPA Uncharacterized protein OS=Vibrio parahaemolyticus serotype O3:K6 (strain RIMD 2210633) OX=223926 GN=VP0092 PE=4 SV=1

MFRFAIPSPLMTDWQHALQQCCDATQAQGQLYTLSYTKQWLSSEVSTSPQNHRAIVAMIE

QNCAQFSAPHFDVVCSQFDDENLSLCPVWLPNEELFAVVTLTHSTQQKQTVSAWLSALAG

RISFDVSQHYHQELKRHHSHANKPLPTLQEFIDCLDDHIWIKTTDGLYAMTNRSVEQAWK

KSNDDIVGKNDFDLFSAERAEKFIDADRLVVATGTQNIVEECRQIDENNNPTWLETIKSP

VRNQAGDLIGILGMTRNITRRKMVETQLSLASKIFNNSQEGMVITDSNANIIDVNNAFSQ

ITGFSAEEVIGKNPNILRSGHHDDAFYQQLWQQLETKGQWKGEFINRKRDGSIYPQLATI

SAVMDDKNHLINYICVFEDISVRKAHEEKLQRMAFYDPLTNLPNRTHLISLLEQHIEMEQ

PFATLFLDIDHFKHINDSMGHFCGDQLLSKLAVRLQDILHLNAHVARIGGDEFVIVLPDI

DGDSPLLETLSSILGVFRRPFDLANHDSLRISTSIGIALYPNDGQDSETLLKNADTAMYL

AKKNGRNGYAFYSPDLTDKSVSHVRIQSALHEAIEKEQLHLVYQPQYNLAQNAIIGVEAL

VRWEHPEFGLVPPADFIPIAEKTGLIQNIGEWVLKDACLQGTDWLARGVQFGKIAVNVSA

LQLQQSRFINKLKTILRETGFPAHRLELEITESFLLIDPKLAIASLNQLCELGIEISLDD

FGTGYSSLSYLKGLPINKLKIDRSFVSDVPNDNDSNAIVNAIIAMGTTLSLKVVAEGIET

PEQVAYLVDKGCIYGQGYLFSPPVAAEKLFN

>tr|Q87PD5|Q87PD5_VIBPA Uncharacterized protein OS=Vibrio parahaemolyticus serotype O3:K6 (strain RIMD 2210633) OX=223926 GN=VP1579 PE=4 SV=1

MSRPPPMSKHPNPIRGHVSCPVCHTASTVHRVGEGKLIAEGEPTKNGRNLGLLYYKCPNC

GNSPMSKSINACVESNMVDSVEQLEVSDAVTIDVELPTVEPVPEIVASTDMHSIAVAEPV

EAPSVETEPPTEPEPVKKPPFPVKKVLAGIAFVALLIWAIRQLMPTKQPTEQGEPINAG

>tr|Q87J89|Q87J89_VIBPA Putative efflux protein OS=Vibrio parahaemolyticus serotype O3:K6 (strain RIMD 2210633) OX=223926 GN=VPA0364 PE=3 SV=1

MNYKMAPKLWSHLMNHQIKTAALSTIALSLLGCNQAQPQVEEPSASRPVQVIEVNERNEE

LNKSFSGIVKAKETASLSFRVPGTVESVFVNKGSVVEKGQVIATLDKHDYQVSLEELQAR

MLEAQSAHKLAKAELKRVKQATSDDAIASVNLDRAISSYERSLSAVKVVEKNIQRAKDAL

RYTTLRAPFTGIVADVSVDPHEQTLPGGSVVSIQQEDSWEVDIDVPENMIAQFALGQPAS

LTWYDSEQSYRATVAEIAPKKHLLKQTYTVTLDIDSISSALFNGKAVTVNTALNTSHSSH

CFPYSAILGEKQTLHVNVVRDAVIHSEPVNLDSIDAYQACVTGSFEQGDYVVISGSHYLS

EGDAAPNLTIKTL

>tr|Q87QR4|Q87QR4_VIBPA Uncharacterized protein OS=Vibrio parahaemolyticus serotype O3:K6 (strain RIMD 2210633) OX=223926 GN=VP1085 PE=4 SV=1

MSLQLINLNSDLKRLRDEGYFIQVKNGFLIMRDVPYVNSNRHVCRGTIISSLSLAGDRTR

IPDTHVVHFDGDMPCNAEGEALNAVVLQSSIFDLGRGITAKHMFSSKPKSGYTDYYHKMT

TYASILSGHAEVLNSGISPKVFSTPEDEEDSVFNYTETASGRVGIGALSDLLTEESVAII

GLGGTGSYILDLVAKTPVREILLFDSDEFLQHNAFRAPGAPTLEALRDAEKKVEYFKSIY

SNMHKRISTSSTYIDEENLELLNGVTFAFICIDAGTSKKSIVQKLEELDIPFVDVGMGVE

LTDGSLGGILRVTASTSGKRQHVHEGRVSFGGGEGNDVYSSNIQVADLNALNAALAVIKW

KKIRGFYRDLEQEHHSTYTTDGNLLLNGESCA

>tr|Q87RI4|Q87RI4_VIBPA Uncharacterized protein OS=Vibrio parahaemolyticus serotype O3:K6 (strain RIMD 2210633) OX=223926 GN=VP0811 PE=4 SV=1

MRSTPITIAIPTEKATVIRRPTPYSVMDCSELGIIPPSAATSEASSATAVNAGSAIVVPN

PKQNAKINNQNTLPFRAKACAIFSPMGNKPSSSPKIKNVRPKMTNNKPKRIETKLGKGSC

RTTNWKKVITITIGRRSRALLNRARNKAVSASMQILPLTNKLLALKARPVR

>tr|Q87NT7|Q87NT7_VIBPA Putative glutamine synthetase OS=Vibrio parahaemolyticus serotype O3:K6 (strain RIMD 2210633) OX=223926 GN=VP1781 PE=3 SV=1

MVISNFYSSGYLEKKMESYLQEVQNFKQKWPDIEFIDLIFTDINATPRGKRIPIDALEKL

DKGVALPLSTITLDTKGNVVETAGLGEDLGEPDHLCYPISGTLMPTAKEQVGQLLLSMMD

ETGQHPNPLFIRNILASMLQTLHAKDQYPCVALELEFYLIDKTRGENGEPLTAINPTKKT

REKDTEVYDLDGLDDYADFLTDLNRIALEQGLNTSGALSESAPGQFEINFNHSKDVLRAC

DEIIIAKRLIRQVAHQHGFDATFMAKPFGEQAGNGMHIHLSLVDKDGNNHFSQTDGQPSP

FFYQTMAAMLSQTSDAMGLICPNVNSFRRFLPGAYVPTKADWGENHRGVALRVPISDSKN

RRIEHRIAGADVNPYILAAVVLSAVLASDNYTKDQCPPTLSDDAVELPLRMSEALERLEH

SELAQYISQDFIDLYLACKRSELAEFERAITPLEIDWMLHSA

>tr|Q87MS0|Q87MS0_VIBPA Uncharacterized protein OS=Vibrio parahaemolyticus serotype O3:K6 (strain RIMD 2210633) OX=223926 GN=VP2161 PE=4 SV=1

MRFSILGLAFASVLSGCVSTTPTTSALSSTDVTTFYDYQLHSPSGNALALQNLPDELKNA

DVILVGEWHTHAGIHRFQTDLLRQLSSGERRIALSMEQVTRDKQSVLDAYLKGEIGEQYF

MSQSNAWPNYESDYRPLVEFAKQANLPVIAANTPKYIVRCIGRQGVDYLNKLDDEERSFV

AENVNTANSPYKEKFMASMHHGKPEQTEKQYAAQVTWDETMAESIVKYLAQNPNAQVVHV

AGKFHTEAGLGTAASILQRNPELKVVVVNPTSEISTNSPDYQLEVLEPPVRFVQDANRMA

AYKHLSTRNDDLQCK

>tr|Q87LL7|Q87LL7_VIBPA Uncharacterized protein OS=Vibrio parahaemolyticus serotype O3:K6 (strain RIMD 2210633) OX=223926 GN=VP2594 PE=4 SV=1

MKLYSCLLASALSLSSVSVLADVPEFAHVTTTGYGEVVATPDMATFSVKVVDTTMTAEQA

KQSVDKTVEDFLQYLSDAGLSKDNITSSNLYLAPQYHYPKSGKAELVGYRASRSINVTVT

DLADLNQYLDMALKAGINQVDNIQLKVSNQAEYQQKARMAAIKDAREKAASLASGFDKKL

GGIWQINYNQMHVQPVLMRSMAMDSKQGSNSYQDSTLVIRDQVDVVYKLK

>tr|Q87GJ7|Q87GJ7_VIBPA Uncharacterized protein OS=Vibrio parahaemolyticus serotype O3:K6 (strain RIMD 2210633) OX=223926 GN=VPA1320 PE=4 SV=1

MLSECRNHIPFVLSEVCVFHVFYFLAGANFMIITSHCIYVQRCRTSYLNSRSHTAHTDEF

ATTSENDWGDCSEGADKADIDFLVERRDVFDSSRIEFEEDEQDRELVKQRENSPEIGVDI

DEL

>tr|Q87H28|Q87H28_VIBPA Cytochrome BD2, subunit I OS=Vibrio parahaemolyticus serotype O3:K6 (strain RIMD 2210633) OX=223926 GN=VPA1137 PE=4 SV=1

MFGLDAFMLARIQFAFTVSFHIIFPAITIGLATYLAVLEGLWLKTRNPDYQKLYHFWSKI

FAVNFGMGVVSGLVMAYQFGTNWSGFSDFAGSITGPLLTYEVLTAFFLEAGFLGVMLFGW

RRVGEKLHFFATSMVALGTIISTFWILASNSWMQTPQGYEIVDGRVVPTDWFAIVFNPSF

PYRLAHMSVAAFVSSALFVGASAAWHLLRGNQSTAVKTMFSMSLGILVFLAPLQAVIGDV

HGLNTLEHQPAKIAAIEGHWDNSDGKPTPLILFGMPNMEQERTDYALEIPVLGSLILRHS

LTEPIPALKDFPKEERPNSPIVFWSFRIMVGLGLLMIFQSFYSMWLRKKNTLYTSRWFLK

FSLFMGPSGLIAILAGWFTTEVGRQPWVVYGVQKTRDAVSAHGDLQMSISLLCFLVVYSL

VFGFGYYYMIHQIKKGPDAIEHDEHDMTTVSGRI

>tr|Q87SF6|Q87SF6_VIBPA Mutator MutT protein OS=Vibrio parahaemolyticus serotype O3:K6 (strain RIMD 2210633) OX=223926 GN=VP0468 PE=3 SV=1

MKRIHIVAAIIFNQDKSQIFITKRPDDKHKGGFWEFPGGKVELGETVEQAMVRELEEEVG

IRVIEQAPFEHLEYDYPDKSLKFDFITVSQFENEPYGREGQEGRWVAVGELGDYTFPEAN

VPILQRVVKEFA

>tr|Q87KL8|Q87KL8_VIBPA UPF0056 inner membrane protein OS=Vibrio parahaemolyticus serotype O3:K6 (strain RIMD 2210633) OX=223926 GN=VP2959 PE=3 SV=1

MDILSAATMLFLIMDPLGNLPIVLSILKHIDPKRRRKVLIRELIFALLILMLFLFAGQSI

LSFLHVQPETLSISGGIILFIIAIKMIFPSAGSITGLAAGEEPFIVPMAIPMIAGPSVIA

ALLLLSSQHPDKLVELSASVVIAWGATFFILMFYGFFNRLLGERGLKAVERLMGLLLVMI

STQMFLDGVKSYLG

>tr|Q87J08|Q87J08_VIBPA Uncharacterized protein OS=Vibrio parahaemolyticus serotype O3:K6 (strain RIMD 2210633) OX=223926 GN=VPA0447 PE=4 SV=1

MSVVKECIAMSRFYVDYWSREWVDENDFPEAELVSFKEDYANRDLGVAFSGGGTRSAACT

VGQLKALDELGLLPRVKYISAVSGGGWAATPFTYTKNTEQYFGEIRDPENITCSNSKSVH

PKSMQSAITNSPLISNLITGGLKLKGDESFAYSLGEVFLKPYGLHDSSRYFTFNEETEAL

TRQGFPPNTKTDFYLVRKGAPYLILGATLLNEDGLASDKKYHVEYTPFYSGVRVGHIDKD

LWSSNDYFGGGYVTSCGYDCIGPYNKRTLEGREQLLVKQAPVLSFDITNEKAFSLNDIIA

STGAAPQEITNNIRLGALGFPEFNHIPLNTLEGDNSVSEEYPHSDGGHLENLGIMPLLAR

KMTKIVVFVNTKKPFTPNKKKPLDSGFNKSVKALFVPIDNLFKKGDFATNVVFDNGEEEL

TKLIGQFSQLVKKESSSDEYIAKTALFAKTKLVTTSNEHYGINAGHEVEITWVYNCRSSA

WEKKLKDKDLGKLIANKKRLIGDQKGLEDFPHYGTFFENFRGVVELTKVQTNLLTNLSYW

VAKKALNGV

>tr|Q87LJ0|Q87LJ0_VIBPA Oxygen-independent coproporphyrinogen-III oxidase-like protein OS=Vibrio parahaemolyticus serotype O3:K6 (strain RIMD 2210633) OX=223926 GN=VP2622 PE=3 SV=1

MLTPPALSLYVHIPWCVQKCPYCDFNSHALKAEIPEEEYISALLEDLDTDIDKYRLNDAP

RPLHSIFIGGGTPSLISAEGIERLLKGIEARIPFKPDIEITMEANPGTIEAERFVGYRKA

GVTRISIGVQSFEQEKLERLGRIHGQDEAVNAAKLAHQIGLNSFNLDLMHGLPDQSIEQA

LADLDKAIELAPPHLSWYQLTIEPNTMFYYKPPTLPDDDDLWDIFEQGHEKLAAAGYVQY

EISGYSKPGYQCQHNLNYWRFGDYLGIGCGSHGKLSFADGRIIRTTKVKHPRGYLAAYQN

MVKPYLHTEQLVADEDRPFEFFMNRFRLMEACPKQDYVDTTGLPLSTIQDTIDWALEMGY

LSETETHWQITEKGKLFLNDLLEAFMAEEDEE

>tr|Q87QF3|Q87QF3_VIBPA Putative ABC transporter, periplasmic substrate-binding protein OS=Vibrio parahaemolyticus serotype O3:K6 (strain RIMD 2210633) OX=223926 GN=VP1196 PE=4 SV=1

MKKLLSTIGILATTFATSSFADSSAASEWPKIEQQADGQTVYFHAWGGSQEINRYIQWAG

KELKSRYNVTLNHVKVTDISETTTRLIAEKAAGKNSGGSVDMVWINGENFKSMKDNQLLF

GPFVEGLPSWQYVDKSLPIDVDFSEPTEGLEAPWGVGQLVFIHDEHTLHNPPRSFAEMLS

YAKAFPNRLTYPRPPEFHGTSFIKALLIELTNNDPALQKPVTGETFEQITQPLWAYLDEF

HKVAWRGGKQFPGGTAETLQLLDDGQIDLAITFNPNAVFSAQSSGNLAETTKAYAMDAGA

LSNIHFLAIPWNANASAGAQVAINFLLSPEAQSRKGNLNIWGDPSVLSSQYLTGSAKNTQ

QFKSIAEPHPSWQSALEKEWLKRYGN

>tr|Q87HR1|Q87HR1_VIBPA Uncharacterized protein OS=Vibrio parahaemolyticus serotype O3:K6 (strain RIMD 2210633) OX=223926 GN=VPA0898 PE=4 SV=1

MEKEVIEVVTQSKRALGELKPAFRACPQAYTELCKAVNEGRVSLYRLKSADCSLVIAGER

DGDNYFLWGVAGRGLRSGIKQLVNVVKAAGMQSLSADTAFQGVARLVRSIGVTAKQDGDF

IRLDLGV

>tr|Q87R43|Q87R43_VIBPA Ferredoxin OS=Vibrio parahaemolyticus serotype O3:K6 (strain RIMD 2210633) OX=223926 GN=VP0955 PE=4 SV=1

MAFVVTDNCIQCKYTDCVAVCPADAFHEGPNFMVINPIECIDCGLCVDECAAAAIFQEDE

LPEDQTIYKELNAELAEIWPVQTEVKPAMDEAEKWNGVPNKLDMLEK

>tr|Q87KA0|Q87KA0_VIBPA ParA family protein OS=Vibrio parahaemolyticus serotype O3:K6 (strain RIMD 2210633) OX=223926 GN=VP3078 PE=4 SV=1

MGKIVAIANQKGGVGKTTTCINLAASMAATKRKVLVIDLDPQGNATMASGVDKYMVDATA

YDLLVEETPFDQVVCTQTTGKYDLIAANGDVTAAEIKLMEVFAREVRLKNALSTVRDNYD

FIFIDCPPSLNLLTINAMAAADSVLVPMQCEYFALEGLTALMDTISKLAAVVNENLKIEG

LLRTMYDPRNRLSNEVSDQLKKHFGNKVYRTVIPRNVRLAEAPSHGKPAMYYDKYSAGAK

AYLALAGEMLRREEIPV

>tr|Q87R89|Q87R89_VIBPA Putative sugar transport system (Permease) OS=Vibrio parahaemolyticus serotype O3:K6 (strain RIMD 2210633) OX=223926 GN=VP0908 PE=4 SV=1

MSTEDFSVSAICREAAVMVRRNLLAIVIACIPLIIVHSIFVFMYPEMTGQDQASVQEGNI

AGFYFVTIVLYPLVAAMAAVRVHRIYLVGDYMHSALDVFRLSAREFRFIGWWILFGLMMM

LVIGIPIFVLSFIYIAESGEPDFVAMAFITTVASIPGYWVMARWSFVLPATAMDHQPRSL

RRSWNQSRPYNKQVFILMGLIPLGAGLLSQLIFSHFTNFFMLSFVGVAYGIFGAYQLALL

SLSYKTVVDIERARFDTPSEPPKTGEEFSA

>tr|Q87JB4|Q87JB4_VIBPA Uncharacterized protein OS=Vibrio parahaemolyticus serotype O3:K6 (strain RIMD 2210633) OX=223926 GN=VPA0339 PE=4 SV=1

MDAKAKNYLNRYLDALSLEQKSAIPSVSADYYCADEYNANVCAELVRIGQKTASCSMELW

YSEHGEPMPEVGHLQVVTNWDGEPVCVIQMTDVSTCRYDEVTAEFAYAEGEGDRTLNWWR

DAHAAFFKAECDELNVDWHEQRLLVLERFKVVYPFE

>tr|Q87TD2|Q87TD2_VIBPA General secretion pathway protein I OS=Vibrio parahaemolyticus serotype O3:K6 (strain RIMD 2210633) OX=223926 GN=VP0138 PE=4 SV=1

MLEVLVALAIFATAAISVIRSVSQHINTVSYLEEKMFAAMVVDNQMANVMLDTGALKAKN

GTEELAGRTWYWKVTPVATTQPLLKAFDVSVATAKNASPVVTVRSYVAQ

>tr|Q87NF4|Q87NF4_VIBPA Glyoxylase II family protein OS=Vibrio parahaemolyticus serotype O3:K6 (strain RIMD 2210633) OX=223926 GN=VP1914 PE=4 SV=1

MALKYQVVPVTSFSQNCSIVWCDETMKGIVVDPGGDEKQLAMLIKELGVDVVKLVLTHGH

LDHVGGTEPLSALLGGTEIVGPHKADNFWLQGLEGQSKMFGFPLTEAFEPNQWLNEGDVV

TFGNQKLNVIHTPGHTPGHVVLYSEEARLAFVGDVLFNGSIGRTDFPQGDFNTLISSIKE

KLWPLGNDVTFVPGHGPQSTFGHERKTNPFVADEMPLY

>tr|Q87JF5|Q87JF5_VIBPA PTS system, fructose-specific IIA component OS=Vibrio parahaemolyticus serotype O3:K6 (strain RIMD 2210633) OX=223926 GN=VPA0298 PE=4 SV=1

MITELTNVNLIKGNLQANNKKEVFEELAKMLFENNRISSKEAFLTDIEARETLSVTSMDG

IAYPHAKSKAVTEPAIAVGVKREGIEYGDEEGVKPTVFFMIASPDNGADHHIYVLQELFG

KFSEEFIEDIHNAKDENQILNILINS

>tr|Q87PF0|Q87PF0_VIBPA Bacteriophage f237 ORF5 OS=Vibrio parahaemolyticus serotype O3:K6 (strain RIMD 2210633) OX=223926 GN=VP1556 PE=4 SV=1

MNKSLFLLLFSCLFLSLNASAAQPTYKVSDVSAYPDCKLLLGLRVNPASYVSCYENKFVN

YKDFSTKSCYLRHGKYVVDIMCHTTSPSWPLYRAAGFLQNSAQCPPDYEKVEDEYVVSCE

PIVPACEYGENPDGSCMDACQFKQSIGDTVKLYWHPAIYGELVTGACYGDYGATRCEVTK

NESTIICTGVPDGQYTPDSQCSLRFAYTGRQCDGGTLFWGVNGPDEPIIPPDTPEDPTHD

PDDPTDEIEDPTVLPDDSTNTVNPGVVDDKPDVEDPDTDESTDTAVLSAIKGLNVDVNKG

IHDLNVDINQSHADITNAVIDVKGSLVDNTQAIQEQQINDNKIYNNTKALIQQANGDITT

AVNNNTNATIGIRDDLKGLGDSVGELDSSLNAIEGLLTGSEFGTPTGTAITGEIFTAEDF

ANLQTTIDEKAESIQGYVDDIKGLITIGTNFNNGTLSDKSFNIKGATVESGLQRFDAVSG

YVRPVVLFICALIALWVLFGNRSK

>tr|Q87GL1|Q87GL1_VIBPA Putative 3-oxoacyl-(Acyl carrier protein) reductase OS=Vibrio parahaemolyticus serotype O3:K6 (strain RIMD 2210633) OX=223926 GN=VPA1304 PE=4 SV=1

MKSEKVAVVTGAARGIGQAICEQLLEDGFHVVGLDISPVEWSHSAQLSSYQVNLCDAAAV

SEVVDSIVEQHGRIDALVNNAGITRDALLPDMLEQDWDSVIDVNLKAVFLLTQRVAPVML

EQGTGSIVSISSIVGTDGNIGQSNYGASKGGVIAMSKGWAKELSRKGAQIRVNCVAPGFI

TTDMTKNLPEKVIQMMESKTPLGRMGSVQDIANGVAFLVSDKSAFITGQVLKIDGGLVL

>tr|Q87Q49|Q87Q49_VIBPA Exodeoxyribonuclease I OS=Vibrio parahaemolyticus serotype O3:K6 (strain RIMD 2210633) OX=223926 GN=VP1301 PE=4 SV=1

MHQDNQPTFFFFDYETWGTNPAKDRPSQFAGVRTDENFNIIGEPLVMYCQLPADYLPSPE

AALITGITPQKAMQEGLSEPEFIAKIHAELSKPKTTSLGYNSIRFDDEVTRYTCYRNFID

PYAWSWQNGNSRWDLLDVLRACHALRPEGVEWPENEDGFTSFKLEHLSVKNGIEHSNAHD

AMADVIATIEMAKKVKAAQPKLFDYFFSMRHKRKLNELVDIVNMTPLMHVSGMLGRECQY

TSWIVPVAWHPTNNNAVITIDLAKDPQPILELSTEELHERLYTKREDLGDLLPVPVKLVH

LNKCPILAPAKTLTAENAENIGIDRQKCLDNLALLRQHPEIREKLIGLFSIERQFEKSDD

VDTQLYDGFFSPADRAAMDIIRETDPNNLAALDIEFDDKRIKPLLFRYRARNFPGTLDEQ

EQRRWALHCREVFESQIEEYMLNLENLVHEHESDEKKIAILKSVYRYVESLAS

>tr|Q87N24|Q87N24_VIBPA 4-amino-4-deoxychorismate lyase OS=Vibrio parahaemolyticus serotype O3:K6 (strain RIMD 2210633) OX=223926 GN=VP2051 PE=3 SV=1

MFWVNGVPQTQVSLGDRSFQYGDGCFTTIQTKNGELEYWQAHVERLEACLKTLHIPLPDW

SIVSEWAHLATLKDEHAGIKIHISRGIGGRGYGTKGIEGPMVTISNFAFPVHYVDWQQNG

VNLGVCETRLGIQPLLAGHKHNNRLEQVLAKAEIEHSNFADAVTLNVQNHVIETTMANLF

WVKDNKVYTPSLNLSGVAGVMRRKVLEFFEANNTEVEVGDFSLVELLSSDEVWMCNSLLG

VAPVTSITASNNHKTVFPIGKLTQRLQGNLNT

>tr|Q87P34|Q87P34_VIBPA Uncharacterized protein OS=Vibrio parahaemolyticus serotype O3:K6 (strain RIMD 2210633) OX=223926 GN=VP1684 PE=4 SV=1

MLCAYFNKPSSNLTGHNMDTGKINILLAELGDSLNLERVTEFEHQMWSLCFSEDNAIDVQ

FVQEKNEVLVSKSLVVEKDILTADKLQVLLEYNFLYRETGGVQFCINPTTKDILLQIAIT

ADTEISQLANVISQLNELSDTWVQLFQNNTVVDFSQQTGSHPFIQV

>tr|Q87LN1|Q87LN1_VIBPA L-aspartate oxidase OS=Vibrio parahaemolyticus serotype O3:K6 (strain RIMD 2210633) OX=223926 GN=VP2580 PE=3 SV=1

MNTNREHECDVLVVGSGAAGLSLALRVANHCKVMVLSKGPRSEGATYYAQGGIAAVFDES

DTIESHVEDTQIAGDGICDEETVRFIAEHSKECVQWLIDGGVPFDREDDDSDDEPRYHLT

REGGHSRRRILHAADATGMAMQTSLQDNAHNHPNIHVLERHNALDLITEDKIGGDKNKVI

GAYIWNRNEEHVETVRAKFVVLATGGASKVYQYTSNPDVSSGDGIAIAWRAGCRVANLEF

NQFHPTCLYHPEARNFLLTEALRGEGAYLRRPDGTRFMPDFDERKELAPRDVVARAIDFE

MKRLGADCMYLDISHKPADFITKHFPTIYSRLMDLGIDMTKEPIPIVPAAHYTCGGVMVD

QNGHTDLKNLYAIGEVSYTGLHGANRMASNSLLECVVYAWSAAKDILKHHTSVELPSRVP

CWDESQVTNSDEEVIIQHNWHELRLFMWDYMGIVRTDKRLERALRRIQLLQQETHEYYSN

FRVSNNLLELRNLLQVAELMVRCAMQRKESRGLHYTLDYPNQLENSGPTILVPAKQLTDN

>tr|Q87KS3|Q87KS3_VIBPA Putative phage protein OS=Vibrio parahaemolyticus serotype O3:K6 (strain RIMD 2210633) OX=223926 GN=VP2903 PE=4 SV=1

MTQDTQLTFEARDEFNRKSIAEKVITLLRSDITVSPLVIDGSWGLGKTEFCQKLLSLMST

EETHHLIYIDAFKADHADEPLLTVLAKVLEVLPSQEEQQGLIQKAIPALRYGLKTGGKAL

VAHILRQDTDSIINGFDEEVKQVADKAIDASVESLLKDHVEAESSLQALQQALKSIAEQK

PIVLFIDELDRCRPNFSVLMLETIKHTFDVEGVQFVLITNTNQLKASINHCYGPTIDAQR

YLDKFIRFSFTLPHTTNENRHDVTMASVTHYKNLVAKSERLQGLSLDRDSDFWLVAQVIN

TNNISLREVETLVRHIEIYQALFDDQPLSNHTQWAYRLMALLGVLLISFKPNLAKSALIN

NLDAKDLGNFLGVHSLPKLENNQHPTHCDTLVFMLASECKYNSDFYVPDNPQDKERWEGE

ANEYFRGRYNLTRNGRMSLVTGAIKGLGLSVN

>tr|Q87LV8|Q87LV8_VIBPA RNA polymerase-binding transcription factor DksA OS=Vibrio parahaemolyticus serotype O3:K6 (strain RIMD 2210633) OX=223926 GN=dksA PE=3 SV=1

MPEAKKKTIGILAIAGVEPYQEKPGEEYMSPAQVEHFTKILTAWRDQLRAEVDRTVHHMQ

DEAANFPDPVDRASQEEEFSLELRNRDRERRLIKKIEKTLDKIKEDDFGFCESCGVEIGV

RRLEARPTADLCIDCKTLAEIKEKQMQG

>tr|Q87NS4|Q87NS4_VIBPA Uncharacterized protein OS=Vibrio parahaemolyticus serotype O3:K6 (strain RIMD 2210633) OX=223926 GN=VP1794 PE=4 SV=1

MEVVEAEKSDLDSFFAYLENQLSENGNGEVPLFQPMSREQSEIPEATKDKFIKGFSHHFG

DIGWRKLWVVKDTSGSIKGHIDLRHHGDESRSHRVLLGMGVDVSCRKQGIGQRLIDAVIN

FCQEKAEIDWLDLCVLSENFPAKNLYLKAGFDVVGEFKDQYRIDGLSVSETAMTKYVKNH

A

>tr|Q87NM6|Q87NM6_VIBPA RelB protein OS=Vibrio parahaemolyticus serotype O3:K6 (strain RIMD 2210633) OX=223926 GN=VP1842 PE=4 SV=1

MFVSCPSQSLVLTFVHTPTKEYIMDTRIQFRVDEETKRLAQQMAESQGRTLSDACRELTE

QLAEQQRKTLSHDAWLTEQVNLAFEKFDSGKSVFLEHQTAKSRMEERKARIRNRGKQ

>tr|Q87FU9|Q87FU9_VIBPA Putative outer membrane protein OS=Vibrio parahaemolyticus serotype O3:K6 (strain RIMD 2210633) OX=223926 GN=VPA1579 PE=4 SV=1

MMLPKLTKPSLAILSLLGLSACTTVGPDYVHPPQTPLPSDWSVQKAAKDTQQSEQQLQQW

WQQFNDPTLNRLVELANEQNLDIEAAGLRIVQARSLLGISTGLQYPQVQTVSGNLARAYV

NDQGVNNAALSFDAAWEMDIWGKYARGIESAEAGYYASIASYHDIMVTITAEVARNYINY

RTFQERILLSRRNIEIQERVVHITQVQFDSGNVTELDVQQAKNQLYTTKAAQPSLEVAMK

QSRTALALLLGVLPEEVEKLLQSDGLTQQIADYEAQFKSTGRKSALSGTDEHSIVPRPPL

LDTQVDANLVMRRPDLQVSEMQARAQSAQIGVAETALYPSFSLFGSIGIDSTVPDGSSFS

FSDSLTMVVGPTFSWNIFQYGRVKNNIRFEDARFQETLTNYNKKVLQAVNEVTNAIEAYD

LYLQQKDLRLQSVNSSIRAFNISMTQYENGQISFERLLNSVEKMTRAEDSYAQIKGNVAN

QVIALYKALGGGWQAQTGKPFLEESIAKQMQDRTDWDGYLDEENRTLPPLPIETPTENGD

KNKTEDL

>tr|Q87K58|Q87K58_VIBPA Uncharacterized protein OS=Vibrio parahaemolyticus serotype O3:K6 (strain RIMD 2210633) OX=223926 GN=VPA0040 PE=4 SV=1

MSDTEFRHGKKRFYDNVKFPRGFAKSGDFTLSEEEILTIYGDTMLGLESGELTPENSEEK

HFVKVLENPGKAKTKIERTWLKYTQLARGRKRFHTLNGRNKPDAADDYAEESLAEDD

>tr|Q87M16|Q87M16_VIBPA DNA polymerase III subunit psi OS=Vibrio parahaemolyticus serotype O3:K6 (strain RIMD 2210633) OX=223926 GN=VP2442 PE=4 SV=1

MSINEKQYLHEMGITSWELIHPERLAGYQPPTIDLPSSCKLLLVSPICPTNETAILFEKI

LKSMKLTLEQAMHIEPERLAMLGEHQLEWVWFAGCESNSMENAKQLTSPLLQDIDGNNEQ

KRALWQQICSYS

>tr|Q87HZ2|Q87HZ2_VIBPA Fructose repressor OS=Vibrio parahaemolyticus serotype O3:K6 (strain RIMD 2210633) OX=223926 GN=VPA0814 PE=4 SV=1

MTLDEIAKLAGVSKTTASYVINGKAQKYRISEKTQKKVMAVVDEYNYKPDHAASSLRAGN

SRSFGLIIPDLENTSYARLAKLIEQNSRKAGYQILIGCSDDDPETEKKVAEALISRRIDA

LFVASGMPSANEYYLKLQNSGTPVIALDRPMDDEHFCCVISEDFDAAFELTESVLSPEIK

TIGLIGALQDLQVSKERELGFRSACQKDDTAMTVGYGLHFSREEGKKVLDKWVQADSLPD

AIVTTSYTLLEGVLDVMLEKPELMSQVRLATFGDNRLLDFLPVKINSLPQQFELIADSAM

ELALNASAKRYKPGVELIPRKIVKRQ

>tr|Q87IT2|Q87IT2_VIBPA Glutaredoxin-related protein OS=Vibrio parahaemolyticus serotype O3:K6 (strain RIMD 2210633) OX=223926 GN=VPA0524 PE=4 SV=1

MTQPIKITLYRWAGNWGPFKVNIPCGECTLTKDILVDTFANELADVPVELEVKDWLSHWW

EPLKVGAWHAPILLVEGKVVSQGEALNRGVLVQSVISEWVKRDELKGNIVYGKATCPYCV

KTKQLLDDAGIKYEYHDVVKDSAALYRMIPEVKAIIGEKTPVTVPQIWLEGKYVGGADNL

EKWLAAKGLDKVPNNVVEIPSQSA

>tr|Q87RA0|Q87RA0_VIBPA Uncharacterized protein OS=Vibrio parahaemolyticus serotype O3:K6 (strain RIMD 2210633) OX=223926 GN=VP0897 PE=4 SV=1

MRRRMSPLSKLAVQTAIELLKQHQADYLVFSSRHGELHRSVALVADIISGEEASPMAFSQ

SVHNTAAGLATIATKQPIPLTSIAASENTFQSAILEAWLFLTDNPDKKVLLVDFDEPLPD

SYTEFEQQQYPGFGLGLVLSNGDDFTLRSSHSVETQGHLLPQGLAFLQHYLSDKTQWTIH

APQQSWEWRKQ

>tr|Q87TM2|Q87TM2_VIBPA Peptide ABC transporter, ATP-binding protein OS=Vibrio parahaemolyticus serotype O3:K6 (strain RIMD 2210633) OX=223926 GN=VP0047 PE=3 SV=1

MSLLEVKNLRIEYPSRHGVHAAVKSLSFNIERGEIVGVVGESGAGKSTVGNAVIDLLSPP

GRVASGDVFLDGEKISGLTPEAMRKVRGSKIGFIFQDPMTSLNPLFTVEQQLKETIHANM

KVSDDEAYQRALSLMQQVGIPQPENRLKQYPHQFSGGMRQRVVIAIALAGEPDLIIADEP

TTALDVSIQDQILNLIRELCIQNNVGCMLVTHDMGVVSNVTDRVAVMYRGDLVEFGPTAK

VLGNPDHPYTRSLISAVPRSDRKLDRFPLVSYIEEATEMKSLDIKNHWLGQSQDQREYTG

PLLDVKNVNLRFVTKDSLFESRREYVQASNNVSFEVHEGETFGLVGESGSGKSTIARVIA

GLYEPNSGQVKFEGIDLTAMKSEKERRPMRRQMQMVFQNPYTSMNPRMKVADIIAEPIRF

HRLTKNEAETRQIVNDLLDHVGLGRMAGVKYPHEFSGGQRQRISIARALATRPRLLICDE

PTSALDVSVQAQILNLLKDLQSELNLTMLFISHDLPVIRQMCDRVGVMQMGTLLEVAPTE

QLFTAPQHEYSKKLISLMPEFTGLREEIKTA

>tr|Q87T59|Q87T59_VIBPA KDO transferase OS=Vibrio parahaemolyticus serotype O3:K6 (strain RIMD 2210633) OX=223926 GN=VP0211 PE=4 SV=1

MLIRIIYTALLALASPFLLLGLYKSKPNKPKFGGRWKEHFGITPQLKTHQRPIWIHAVSV

GESIAATPLIKELKQQYPEQPIVVTTTTSTGAEQIAKLGDLVEHRYMPIDFGFAVKSFLK

AIQPKKMLIIETELWPNTLNVVKQANIPITVVNARLSEKSCQNYAKVQWLFNQLHPCLTQ

VLCQTDSDAERFERLGVNKEKLSVTGSIKFDIQISDHVKQQGKALRAQLGKDRPVWIAAS

THKGEDEQVLEAHKQILESHPHALLILVPRHPERFDDVFALCKKQGFETVRRTEKQPAEN

TTQIYLGDTMGEMLVLIGAADICFMGGSLIGDKVGGHNVLEPAALGVPVITGPSYYNFQD

LVDTMQREDCITLIYNARDLATSITLLMQGDYQHRRFRKYITKFVENNTGALKKTLRALK

ENS

>tr|Q87L37|Q87L37_VIBPA Uncharacterized protein OS=Vibrio parahaemolyticus serotype O3:K6 (strain RIMD 2210633) OX=223926 GN=VP2779 PE=4 SV=1

MQRISHLFLYLIVITTLNGCFFTEHEVQRWPLEPQGSTSFGLSRDGRFALLYSEEHHLVL

WDLKQNKKLATLGKLDADGNAVSHIRISDNGRFAVTAGQMNFAVWDLGWTQAQGLWSISD

ALIRDIDITSNGEQVLLGLSNGKAIYVNLVTGRRLEFLAHREKVNSVAISPNGRFALTGG

NDYKAYLWDTESGQVLRTFEHEQRVVRVALQRDGKLAMTSDGGNQAIIWNLETGQKVSQL

SSWSRQLIFSTARFSDDGSQLVTGTPSGRVSIWDTQSGKRIDGFEVEPKKDTRPPRAVVY

DAAFDSKQRVITATSAGIAQAWQLENGS

>tr|Q87JB2|Q87JB2_VIBPA Uncharacterized protein OS=Vibrio parahaemolyticus serotype O3:K6 (strain RIMD 2210633) OX=223926 GN=VPA0341 PE=4 SV=1

MLHTLSKFIFASMFSVLSACSSIESNQPSEKSTTFHFGYQQDSVLAHYFEAYGQDPKTIT

GFYPLNQGHDALLARTSLIESAQKSLDLQYYIYRGDETSQLITWRLYEAAKRGVRIRLLL

DDMQKRNDNVMAALNAHPNIEIRLFNPHQYRSARIFALTSDFERLNRRMHNKSLIADSVS

AIVGGRNIGNEYFSFESEVEFGDFDLLLYGEAVQQTADQFDLYWNSVHAVPMEWISPQSQ

SVSDAAIQKQVTKLNLQEKFSSGRYDFTALDMYQDLKQGKLNLYWGDGQVWFDLPDKVTT

HDSQLVGNLTELLKSVEHSFVLISPYFIPTEAGTKALTNAAKRGVDITIVTNSLASNDVF

AVHGWYAKYREDLLESGIKLWEVKSSAKLKSKWSLTGSSRASLHAKAMTIDDKTLFVGSM

NWDPRSAALNTEMAVVIEQPEYVQAFLAKLPSQLKDNAYRLTLRDGDIVWTNTKTGEEYD

SEPEAGVFRRLGAWFSGILPIEDQL

>tr|Q87TK0|Q87TK0_VIBPA Ribosomal RNA large subunit methyltransferase J OS=Vibrio parahaemolyticus serotype O3:K6 (strain RIMD 2210633) OX=223926 GN=rlmJ PE=3 SV=1

MLSYRHSFHAGNHADVVKHIVQSLILDALKQKDKPFVYHDTHSGVGRYDLTHEWSEKTGE

YKQGIARIWDNPNIPEDIKSYIDAIKALNNGDKLRYYPGSPRVARAQIRPQDRMVLTELH

PADHPLLEQEFHRDRQVSIYKEDGFKRLKGSLPPQERRGLVLIDPPYELAKEYRDVVQAI

YQSHKRWATGIYAIWYPVVNRHDIEDMIEGLESLGIRKILQIELGVAPDTNERGMTASGM

IVINPPWKLESQMKEILPFLQDAIAPATGHWKVDWIVPE

>tr|Q87SB9|Q87SB9_VIBPA ATP-dependent RNA helicase SrmB OS=Vibrio parahaemolyticus serotype O3:K6 (strain RIMD 2210633) OX=223926 GN=srmB PE=3 SV=1

MIRTFAELDLDQNLLEAIEEMGYERPTKIQAEAIPQALDGRDILASAPTGTGKTAAFVLP

ALQYLQDFPRRKPGPARILILTPTRELAMQVADQARALAKNTKLNIFTITGGVQYQEHAD

ILATTQDIVVATPGRLREYIDAERFDCRAIEWLVLDEADRMLDMGFAPTVDRLSNECRWR

KQTLLFSATLEGKGVEGFTADLLNEPAEIDAKSPLRERKKIAQWYHRADSAEHKLALLKH

IITEQAERTIVFLKTRERLAELRAQLESAQIPCSWIQGEMPQDRRNNAIARFRDGTVNVL

LATDVAARGIDLPDVSHVINYDMPRTADVYLHRIGRTARAGKKGNAISIVEAHDQPMMDR

VARYVKEDIKERFIKEMRPKHKKPVFKKKKKKEDKKKTAKKKTAKKK

>tr|Q87LC0|Q87LC0_VIBPA Uncharacterized protein OS=Vibrio parahaemolyticus serotype O3:K6 (strain RIMD 2210633) OX=223926 GN=VP2692 PE=4 SV=1

MKTLNALLALMLMLPSFVYASQCSVKGQDNFTVSFDVEGDDEYQAVKLKQGSHGYVLWYT

GYKRNNTNNYDYLFNEQQLINDVDYNIQITHEAGSNTLKYYRKFEGAANYKLIETQTVNL

DNGQYWVVDVGDDVDNIQCSNTVDPGNPGGPINRSPDFEFGTVDNSTCSMTGGKYTCTIH

FENTYDASHPKPLVFVMPTIDKTLSSKNPRKTEYPSSISVVHTTHNSATIVQEFPPHQKA

DRNVTFLDKNSSQVQKELAKVDYFVIEPGVLELNNGAKIVAGTIKTNVAASQYKNNNKGI

NEQNNGITIDFDDYGLTGFDGKPGVLVQPQTKNNDGINNWFTGMARDANTTSFKLALEKS

EVYKKNNQGHETFNILSDNETVAFVAGEGFGYINGQRFWLGQGRTKYTLDQQDPVIDPIY

EGCKVYTPFPNTAGFVSPPVLVANKNSRRGNNGGWLRRCDIKKDSVAFIVEEDMQKDRER

GHLDEDVGWFMFEKANPNPICDAFNAPVQTWRRELVNDAVDGTLVLSNTSKILGAPVLTV

GGDRKRVVGFMPRTVSGENKSDACDGYECHGDEGLLIGKEGLENFPITTSWNNQIIGAND

RVTFSEGTNVKHLNVDGVLTLEPGKYWFDSVKINTGGKLLIKEGTEVIINTKALALANYS

YMGMDVNVENTPVFSGNMRVNVYGLTPVAGSTIHDRVDIANHSKVVGLIYSEDKVYLSDH

SVIYGAVTAKDIDMNNNAEVHAATSCLPPLDDYELTVSPKAQYALMCGVEKPTFTIETRN

QGELESAWVSVEVLPANSANNFTISVANDIGSGTYPRFRTSMNEGSKGELEISVSVKNTV

KVDLDQTYSLKVTLEEDGNQSQTATFKYVPFKFHVDEQRVIAGQTKPVTAQVLACSDGEQ

TVVKSYIGTPDVSFKLETPNSGRSDASLLSYEPNFVKNDNGSSTEDFKLIESGEYTVTLT

DPNFVCDPQYADDCPIAPEEGDESQQETVILNGSFNVKSRPWKIAICDVKSEEGDVSNPS

TQGAGNGFIPSATSFNVTYKPVVHSQSRGNATEICDYPLTQNYFSSDNTNAPLEVKFSVT

YPAGGDLANLSEDNGFIGSSTFTKAEASSGKEGEYVWNEVGSLSLTTNATYLASDFKLDE

DSRVIGRFYPKYFQVIASDWNYPGSQSFAYMNQPFDAVEFSVEALNANKAAIKNYAGFTT

KAEFNLDDIDRYSGRFDAPSFGAGSWSNESDKSIGEFSISNSGQCIGSACWNKDLGGNYP

DGPFNSVIGTAKSEIGLIYTNNADPVEYISNEGSNSRLVKQPDIRFGRIDLDDVGGNQGL

TLHVPLRVEYWNGSRFIANPNDNQTDVKGVTAAERHIWPTGADADPKAVTLGAGGEVSSG

SSRSVTATQAEPYRQQTRVWLDLDDSTNGLPWLKYNWDNKNAGEENPSSVVTFGIHRGND

RVIYRGEPGLTGQ

>tr|Q79YU9|Q79YU9_VIBPA Basal-body rod modification protein FlgD OS=Vibrio parahaemolyticus serotype O3:K6 (strain RIMD 2210633) OX=223926 GN=VPA0266 PE=3 SV=1

MSLAQYTALSSDAPVKAAAKENSGIAPNPTDKNSASSLQNEFISLMVAQIQNQDPLNPLD

GTEYVGQLAQFSQVQSTENMSKVMQNSMVLLDNMQVLSTAGLVGQTVYVNSKEFELGDGA

QNGKIELSHGSNQVTLVVKDQFGQKTNVPLGAHGAGDVEFSINPEELGLKPGKYTVSVEV

QEGQASPNVLLAGTVEQVRIPSSGGAALVNVNGVGNVPFYQITQFGA

>tr|Q87PR2|Q87PR2_VIBPA DnaK-related protein OS=Vibrio parahaemolyticus serotype O3:K6 (strain RIMD 2210633) OX=223926 GN=VP1439 PE=3 SV=1

MNQENLSQETAQEQQSPKFSVGIDLGTTHCVMSYVDTQDEDARVQVMPIPQLTAPGTVET

RSQLGSFLYQPHEHEMNPQSRVLPWSSEPKALVGAIARNLGSKTPIRLVASAKSWLCHAG

VNRRDAFLPTGSPEEVEKVSPLRATELYLEHLKDAWNHANPNHKLADQDVTITVPASFDP

AARDLTAEAARNVGFVHLTLLEEPQAALYNWIDNSNDKWRDEVEVGDIVLVVDIGGGTTD

LSLVEVTEDEGNLTLNRIAVGEHILLGGDNMDLALAYRLKMKLAQEGKELQPWQVQAMTH

ACRDAKEALLNDSALQSVPIVVPSRGSKLLGATLKTELTQEEVQQTLVDGFFPQVAITDH

PVQRNRGALTQMGLPYAQDAGITRHIAAFLSKQAHAQSGGESAQAQDFNPFANMPGMPGS

DAAQSADFIKPTAILFNGGVLKSKLLATRLEDTINKWLIEADAEMAKRLTGVDLDLAVAS

GAAYYGSVRRGQGVRIRGGIASAYYVGIESAMPAIPGMAPPMEALCVAPFGMEEGSSVDV

PSQEFGLIIGQPVNFQFFGSTVRRDDLAGTHLDYWAPEELEELPEIQVTLPVSEGRREGE

VVPVTLASRVTELGTLYLEAIAADNGQKWHVEFDVREDAKSDSNEEQ

>tr|Q87FW1|Q87FW1_VIBPA Putative pyruvate formate lyase OS=Vibrio parahaemolyticus serotype O3:K6 (strain RIMD 2210633) OX=223926 GN=VPA1567 PE=4 SV=1

MDLHTLPERIKAHKSALVNIVTPPICTERAEAYTRAYQANEDKPVIVQRALALQEHLRTR

TIWIKHDELIVGNQASKVRAAPIFPEYTVRWIEAEIDELADRPGAGFSVSEEDKQSIHAI

TPYWRGKTVQDRCYGLFTDEQQEILASTIIKAEGNMTSGDAHLAVDNEKILKIGMNGLLN

EVRQHRANNDVSTYEGLKKEQFYKAVEIVLLAIQEHMVSYADLALEMAQNETRPERKAEL

ETIAENCRHVAFHAPTNFWQALQLSYFVQLMLQIESNGHSVSFGRMDQFLNDYYVRGLES

GAMNKAFALELLQSCWLKLLEVNKIRSGAHSKASAGSPLYQNVCIGGQKLNENGEPEDAV

NPLSWAILESCGQLRSTQPNLSVRYHEGLNQEFLMGCIEVIKCGFGMPAFNNDEIVIPEF

IKLGVEKADAYNYASIGCIETAVPGKWGYRCTGMSFINFARILLAALNEGVDATTGKAFL

SHDKSLAKGNFESFEQVTASWAEQIRYYTRKSIEIDTVVDSVLEQQAQDIFCSSLVDDCL

ARGKTVKEGGAKYDWVSGLQVGIANLGNSLAAIKHLVFEEAQISQTELAKALEEDFDGIE

NEQLRQRLINFAPKYGNDDDYVDQLLADAYQVYIDELAQFVNTRHGRGPIGGGYYAGTSS

ISANVPFGASTMATPDGRKAKTPLAEGASPASGSDRLGPTAVYNSVGKIQANKILGGVLL

NQKLSPAAVASEGDKLKLSMLIRTFFNHHKGWHVQYNIVSRETLLAAKKNPEQYRDLVVR

VAGYSAFFTALSPDAQDDIIARTEHEL

>tr|Q87JU6|Q87JU6_VIBPA Biopolymer transport protein ExbB-related protein OS=Vibrio parahaemolyticus serotype O3:K6 (strain RIMD 2210633) OX=223926 GN=VPA0152 PE=3 SV=1

MIKKWLSVALISTAAMMPHTTFASDTLLQKAQQENRQQQTHNASRESGFKKTEQELQAIK

NKLVAERAALQAEADTLSVTFSENEAELAQLEEKLRLETGSLGELFGVVRQNAKELESEL

KHAVTGVDANSYQKDIEAIVAAKSLPTLKQLQAMWRSMEEQVTASGELAKVSFTLLDGEG

KEQTVNGVRLGAMALLDDNGYVKWNGQRGDAVNYLRQPENGPTASAFTNGELDSLVIDPS

RGILLEQLANSPTLKDRLNAGGVVGQIILVLLAIGLIIALVRGVSLFIARQKIAKQLKNP

TKAEDNPLGRVLSVYQNDKHRSVEALELRLLEAVVDEQTHLEKGLSMLKLLAALAPMLGL

LGTVTGMIETFQVITQFGNGDPKVMAGGISMALVTTVLGLVAAMPLLLAHNVLSSQAESI

RNILEKQGIGLVAEQAERDMSNNKGNRNTIAENAA

>tr|Q87LA2|Q87LA2_VIBPA Transcriptional regulator, LuxR family OS=Vibrio parahaemolyticus serotype O3:K6 (strain RIMD 2210633) OX=223926 GN=VP2710 PE=4 SV=1

MRKSAYARKLFLISMEDDAAQKVASLEKYIDMSIPVISTDALMEAKPEHRNKILLIDFSE

HKSLVQSIKNLPLVWKNFETVVFNVPKRLTTDELLAFGQLKGLFYSEDSLEQVGEGLKGI

VNGQNWLPRNVTSQLLHYYRNVINTHTAPATVDLTIRELQVLRCLQAGASNSQMAEELFV

SEFTIKSHLYQIFKKLSVKNRVQAIAWADQNLMS

>tr|Q87S88|Q87S88_VIBPA Peptidyl-prolyl cis-trans isomerase OS=Vibrio parahaemolyticus serotype O3:K6 (strain RIMD 2210633) OX=223926 GN=VP0536 PE=3 SV=1

MTTITNDSVVTLHFTIKMKDGSVADSTHNMGKPAKFVMGDGSLSENFEQCLVGLQSGENK

AIELKAQDAFGMPNPDHIHHMDRTKFVGEAEVEVGTIMAFSGPDGMEIPGIITEIAGDSV

TVDFNHPLAGQDVTFEVEILSVE

>tr|Q87FS5|Q87FS5_VIBPA Putative phosphatase OS=Vibrio parahaemolyticus serotype O3:K6 (strain RIMD 2210633) OX=223926 GN=VPA1603 PE=3 SV=1

MFSNILVVCVGNICRSPTGERLLQQLLPDKQISSAGIAVEKSRLTGKPADETARLVASER

GIYLLDHKAQQLTAQLCAKQDLILVMEQGHIDALTELVPEARGKAMLFGHWIGSVDIPDP

YRQSREAFEHALSLIENAAHAWAKKI

>tr|Q87FF4|Q87FF4_VIBPA Uncharacterized protein OS=Vibrio parahaemolyticus serotype O3:K6 (strain RIMD 2210633) OX=223926 GN=VPA1725 PE=4 SV=1

MVHQITYFKDAFSAWEQWNLTDFDYKCEHVLALKSALEGQNAVVAKVVSYHLQQASALLA

EPHQLVGPTGETNELYAAGRGVALVICENNIENTENALYSTFAMITCALIAGNSVVICSD

NAELNRLVEQAVSSTNFPSNLVQVVAYDASAPLLESDVRSVGYVGHSQVEHALNLQLAKR

DGAIVGLVSETDLESPKLAHDPHLSLRFITERTRTINITAVGGNATLLELGNETH

>tr|Q87JA4|Q87JA4_VIBPA Uncharacterized protein OS=Vibrio parahaemolyticus serotype O3:K6 (strain RIMD 2210633) OX=223926 GN=VPA0349 PE=4 SV=1

MSKPLYVFDMDETLINADCAMIWNAFLVEKGIATQPNFIEEDQRLMALYAEGKMDMEDYL

EFCMAPLADMPIAHVLMLVEECVEHHILPKQFEQSKTLIAQLDRDDIDMVIISASVTFLV

EAVGRRLGIPVALGIDLVEKHGSYSAEIAGVPSYREGKVIRLKAWLDAQPESYSEVHFYT

DSINDLPLCEYADYAYLVNPCPQLKARADKANWTLLRWN

>tr|Q87ME6|Q87ME6_VIBPA Chaperone protein skp OS=Vibrio parahaemolyticus serotype O3:K6 (strain RIMD 2210633) OX=223926 GN=VP2309 PE=3 SV=1

MIKAAGIGLLVLSSSMFANAAEAAQKIAYVNTAQVFQALPQREVVLQKMQKDFKSKADEL

KSIQAQAKTKIEKLQRDGELLGQEEIEKLRIDIAKLDSEYKVKAQALEKASARREAEEKA

KLFKTIQDAVKKVADKKGYDLVIDISALQYGKPEYNISEDVIKALK

>tr|Q87KY9|Q87KY9_VIBPA Uncharacterized protein OS=Vibrio parahaemolyticus serotype O3:K6 (strain RIMD 2210633) OX=223926 GN=VP2835 PE=4 SV=1

MNYDIFNGDADGIIALLQLRLADPIDSQLITGVKRDIKLVEKVDVQAGDELTVLDISMEK

NMAGLEQALAQGAHVFYADHHKAGDIPQHGNLDAHIDLDANMCTALIVDKLLEGRFHTWA

ITAAYGDNLIAKADVLADQAGLNNEQKAQLKELGTLINYNGYGSKVDDLHFHPADLYRAL

VQYISPFEVIEDKASTYYQLQSAYQQDMDAAQAVPATHESDTLKLFELPNTAASRRISGV

YGNWLANQNPDSAHAVLTENADGTYTVSLRAPLNNKQGAVAVCGQFPTGGGREAAAGINA

LRKEDVNAFIDAVETYYA

>tr|Q87L62|Q87L62_VIBPA Putative fimbrial assembly protein PilM OS=Vibrio parahaemolyticus serotype O3:K6 (strain RIMD 2210633) OX=223926 GN=VP2750 PE=4 SV=1

MDKLIVTGIDIGHNSLKAVVLKPIGDQYALLGYKEILLKEGIVAENNTINHQEIVKTLKQ

MKKELPFGAKRVAISVPDNSVISKKLQIEQSLDESEIEFAVVQAFSHQSPFPVEELSLDF

VRLRAEEGMRGTDSYQVFATRKDVVESRVEALQQSGLKPVLVDVHSQSLGHIWKLAAERF

PEKNKYCLLDIGSLASSFTMFTEQGELFHKEFACGTRISMGTSQEDLLSDDAQAKTEQFN

RQVVERVKRQMQLYTSINGSQNIKGIWLSGEGASTPMLAEELSHQLALECELLNPLGLFE

MKVSKRKRRAADWQHFSTAAGLAVRGIHWLGGVRALSH

>tr|Q87K56|Q87K56_VIBPA Uncharacterized protein OS=Vibrio parahaemolyticus serotype O3:K6 (strain RIMD 2210633) OX=223926 GN=VPA0042 PE=3 SV=1

MITVRHAEDRGRANFGWLDSKHTFSFGSYYDPKHMGFSELRVINDDLVQPNAGFDTHGHR

DMEIISYVLEGVIAHKDSEGNVQTLPAGEFQLMSAGKGIYHSEYNASETDPLRFLQIWIQ

PNTFGNKPGYQQKDFGRNAGLTTIATPTGESGTLHIKQDAYLYQLILEPNRELAFDIHSG

RNVYVHQVSGELHVDNTLLTGGDGAKIEAQSKVTFRNLTNQSVTALIFDLP

>tr|Q87N14|Q87N14_VIBPA Ribonuclease E OS=Vibrio parahaemolyticus serotype O3:K6 (strain RIMD 2210633) OX=223926 GN=rne PE=3 SV=1

MKRMLINATQKEELRVALVDGQRLFDLDIESPGHESKKANIYKGRITRIEPSLEAAFVDY

GAERHGFLPLKEIAREYFPEGYTYQGRPSIKEVLTEGQEVIVQVEKEERGSKGAALTTFI

SLAGSYLVLMPNNPRAGGISRRIEGDERTQLKAALSTLELPQGMGLIVRTAGVGKSAEEL

EWDLNVLLNHWSAIKGAADSNPAPFLIHQESNVIVRAIRDYLRRDIGEILIDSNTIYERA

KEHIQLVRPDFINRVKKYEGEVPLFSHYQIESQIESAFQREVRLPSGGSIVIDPTEALTS

IDINSARATKGGDIEETALNTNLEAADEIARQLRLRDLGGLVVIDFIDMTPVRHQREVEN

RLREAVRLDRARVQIGRISRFGLLEMSRQRLSPSLAEASHHICPRCSGTGVVRDNESLAL

SVLRLIEEEALKDNTAQVLAVVPVPIASYLLNEKRRSVNHIERIQEVKITVVPNSDMETP

HFEVIRVREGEEFDLISYLLPKKLEAMKEAEGKEPSETDIKPKRIEEPVLKGFAAPAQTA

PAPVSKPAPVAKKKEVEQEAKPGLFSRLFKALGSFLFGGSEEVKEEPKQEEKKSNRDNKR

NKRDRNDRRRSNQRDNREGRENRDNRRRRKPREEQANEQNEAQQPANKQQPNRSQKRKPK

PQQAEKAQEPKANAKVAEKGLQLAAEARSEVKPEENKAKANAKTEKVKERRQRRKLNKSV

RVKDQLADEQVEETAVKAIEPVESQVDQAVDTAADAQQDNKQGESKQRRNRRSPRHLRAS

GQRRRRGRDRRPNPFRLRKGGVASPEMAMGKVMPRYIPKPAPKKEERVQEETVVAIATVP

AMQGGFACPEMAMGKVIIRREQPAVAEAPVVEPKVQETVEAKAESPIVEAVAPEKAEPVA

VDQAPVEPIVEVAEAPVAEATTETVVEEAPVQEPVVAETVEEAATEVAVETPVAAPEMKA

EVINVVAKPRASAPMTKAPGPQELREIEVAAAPFKAERYQAKGAGSQVATNRASAAMTKP

NFN

>tr|Q87FW5|Q87FW5_VIBPA Putative transcriptional regulator OS=Vibrio parahaemolyticus serotype O3:K6 (strain RIMD 2210633) OX=223926 GN=VPA1563 PE=4 SV=1

MKIGELAQKTGLAPSRIRFYERIGLLKTVTRKANGYREYPQEALMILDMITTGQQAGFSL

DELSALLPSDLSNWDHHQLLTTLQQKVQELDELERKIAQSKSKLKEVLKEIEAKPDDMDC

AMNAKRVMSQLGQTILTKENPSNKNKAVTKAKQR

>tr|Q87GK3|Q87GK3_VIBPA Uncharacterized protein OS=Vibrio parahaemolyticus serotype O3:K6 (strain RIMD 2210633) OX=223926 GN=VPA1312 PE=4 SV=1

MYFIGYHGTSEKSAINILNTGIRRDCLPKTGQIGHGFYVAKVKGALPEWGAEQATSAGRH

NLSICQRALNKMLGERNNLFLPNEAKRTILKIYSTKYISHCNWNTMNPVDLSCLNEILKE

TSQSRGDALNNLIKERSEWLQMVIAPEDFKYIFARRDDSSKEKNSNWFSKEAPF

>tr|Q87P65|Q87P65_VIBPA Putative tricarboxylic transport TctA OS=Vibrio parahaemolyticus serotype O3:K6 (strain RIMD 2210633) OX=223926 GN=VP1653 PE=4 SV=1

MLDGILQGLSTAVMPMNIMMVIVGCFVGTFIGMLPGLGPISAIALMIPITYGLEPSSGLI

LMAGVYYGAVFGGSTSSILINAPGCSSTVVTAFDGYPMAQKGQAGKALALAAYSSFTGGT

LSAIMLLVAAPALANVSLSFQSSDYFALMLLGLSAVAAFAGKGQVIKAWMMTILGLMLST

VGIDKGVGVERFTFGLTDLMDGFSFLLLAMATFALGETLMGILKPSNDTRDEEQDKLSNI

GSMKVTKEEIKEVAPVSIRSSILGFFTGVLPGAGATIAAFLSYGLERNLAPKEKKEEFGK

GSIRGLVAPESANNAASSGSFVPLLTLGIPGSGTTAIMLGALIAYGIQPGPRLFVEHPDV

FWSVIISMYFGNIVLVILNLPLIPYISKLLAVPRTVLLPMILFFSITGVYLVSFNTMDVF

VMLLVAMAAIALRLANFPLAPLLLGFILGGLMEENLRRALMISDGELSFLWERPITLTFT

VLAVLVLGSPLFVKLFQKLKAQPVKVEQ

>tr|Q87LD4|Q87LD4_VIBPA 2-hydroxyacid dehydrogenase family protein OS=Vibrio parahaemolyticus serotype O3:K6 (strain RIMD 2210633) OX=223926 GN=VP2678 PE=3 SV=1

MSLPKVVFLDRATIPNHIQVPRPEFPHHWMEYALTPPEFVVERLADADIVISNKVVLDQS

VLSQLPQLKMIAVAATGFNNVDVNYCAEHGIAVANVRGYATRSVPEHVIAMLFALRRNLF

GYHQDIAAGEWQRNKQFCFFTHSIGDVGGSTLGVVGSGALGQATANLAQALGMTVLFAER

KGAAECRPGYVPFEEVLKHSDALTLHCPLNEHTQNLIGKAELQQMKPNAILINTGRGGLV

DEQALVDALKQGEIAAAGFDVFTQEPADESNPLIANIHLPNLLLTPHVAWGSDSSIQRLA

DILIENINAFQRGDLLNRLA

>tr|Q87Q91|Q87Q91_VIBPA Putative ATP-dependent helicase OS=Vibrio parahaemolyticus serotype O3:K6 (strain RIMD 2210633) OX=223926 GN=VP1259 PE=4 SV=1

MFQLPIDSLQAEFDQHVKHHHLVVEAETGSGKSTRLPLWAAKHGRVLVIEPRRIACTSLA

EFLAEQSGEPIGNKIGYAIKLHAYFDENTEVVFVTPGVALRWFAEDKLASFDIVIVDEFH

ERRWDIDLLTAILKQENQHRLIVTSATLEGEKLAHYLNAHRLCSEGRCFPVSVTHRVIDS

RHLPNKKGCENDVVKTVKEALEDEEGDILVFLPGRKEITQCAQMLQHLDDVLVVKLHASV

SDEERHRALTVQAQRKVVLATNVAETSLTIPNIRIVIDSGLERRTVQRNGRTALTLTNIS

KASAVQRMGRAGRVAEGTCIRLFGEHAPLELVTPPELHREELVEPMLAAACCGYRLSELH

FLDAVPEKSLNAALLCLQAMGALDDNGDVTEHGKKVYPLPIDALFADLVTRIPTKAEKEA

MIDLAAALSVPAQLYQLQRGEAAEALEREEPLGCDASLMIRLVRGEQLPAVIVDTSVLEE

AQGLAKQMRDVFELPQLEVASRYRRDQLTQAIAALHPELMFVRRERRRDALGNGSMEMVV

GRNSRFPDKSEAALVLDSHSVPGRGVKQTLNLASVMMPISLELIKTLELGEWHQGDTQYE

DDTPLATMHLVYAGRTIFTEHQALQGEVAIQSIADMIEQEMLLPGFSPLRKQQIQHWKIY

NSLGLNTELVDKKVLDELCFSAWLTEQLATLGVESVDDIELFEADDLPFEGIPEWEYNDF

AEQYPLKLVLAELKLDVEYFVSRKLVHVIYTEGNRKGDPKRWELPRWAGWKVQYKKASRV

VDVK

>tr|Q87H64|Q87H64_VIBPA Putative cytochrome c551 peroxidase OS=Vibrio parahaemolyticus serotype O3:K6 (strain RIMD 2210633) OX=223926 GN=VPA1101 PE=4 SV=1

MRLTTAIKWGAAVLFVCTASSVSVAKAEVELVLPIEGDPVVDVKLARIGWHLFRDPNLSS

NGKVSCESCHNLQTNGAQNTAVAAGVKGVGIRNAITVFNASLNYRLLWDGSSNTLVSQMD

GPIHDPLEMDSNWPKIESYVQNNEQYQALFKRAGNLPISIDNIKASIVEFVKGLETPGAP

FDAYLLGYTHVLNEKAKRGWKTFQKAGCVQCHQGKNVGGAMIQRFAYFEHKPVQKDTGRH

LLTTEGDEGYYFRVASLRNVALTGPYFHNGQVTTLAEAIQIMAQTQLGITMSDSNIEDIE

AFLTSLSAPRPVILEVLENE

>tr|Q87SE0|Q87SE0_VIBPA Glutamate synthase, large subunit OS=Vibrio parahaemolyticus serotype O3:K6 (strain RIMD 2210633) OX=223926 GN=VP0484 PE=4 SV=1

MRINKGISPRIVFLAILFLRDTERNQDQSTSTGGDNKYKGQDASCVTRQLRSASYLCVAL

NWKDVSMVDREQNSQGLYTPELEHDACGIGFVAHLKNRKSHEVVTQALDMLARMEHRGGQ

GCDPCSGDGAGILLQKPHEFLLEEAVKLGIKLPSFEKYGVGVVLFPKDEYKREQCRDILE

RNAKRLDLEVIGYRVLPTDNSMIGADPLSTEPQFEHVFISGGPGITPEELERKLYVLRNY

TVRVCLESVSNIGDDFYINSMSYKTLVYKGQLTTEQVPQYFLDLQNPTMVTALALVHSRF

STNTFPKWRLAQPFRYIAHNGEINTVRGNLNWMKAREAILESDLFTQAEIDMLLPICQEG

SSDSSNFDMALELLVLSGRSLPHALMMMIPEAWQENKNMDPKRRAFYQYHANIMEPWDGP

ASVCFTDGVQVGATLDRNGLRPSRYTVTKDNFLVMASESGVVDIAPENVEFRGRLQPGRI

FVADLEQGRIISDEEVKDTIATAQPYEKWVEENLLSLKKLPDASNQFSQPKPEHLLHRQQ

AFGVSTEEVNEIIVPMANEGKEPLSAMGADWPLAVLSHQSQHLSNYFKQLFAQVTNPPID

PIRERMVMSLNTYLGKDQNLLTETPLHCQKVELESPVLANSELEKLRAIDNEHLQAKTLD

IVFQASEDQGKLERALKRICQYAEDAVIDGYSIILLTDRAVNSNHAAIPAMLAVGAVHHH

LIRKGLRAKCDIVVETGDARETHHFATLLGYGANAVNPYLVIETIIELQRTKKLDPEANP

RDLFNNYRKAINGGLLKIFSKMGISTLQSYHGAQIFEALGIHKSVVDKYFTGTVSRIQGL

TLDDIAKEVLIRHRIGYPQREIPIQMLDVGGVYQWKQRGEKHLFNPETISLLQQSTRNKD

YEQFKQYASAVDKQGDNAVTLRSQLEFIKNPAGSISIDEVEPIESIVKRFATGAMSFGSI

SYEAHSTLAVAMNRLGAKSNSGEGGEDPMRFERKENGDWERSAIKQVASGRFGVTSYYLT

NAEELQIKMAQGAKPGEGGQLPGDKVDDWIGATRHSTPGVGLISPPPHHDIYSIEDLAQL

IYDLKNANRAGRVNVKLVSEAGVGTIASGVAKAKADVVLIAGFDGGTGASPMSSIRHTGL

PWELGLAETHQTLLKNGLRNRIVVQADGQMKTPRDLAVATLLGAEEWGVATAALVVEGCI

MMRKCHKNTCPVGIATQNKTLRERFDGRVEDVVTFFQYMAQGLREIMAELGFRTIDEMVG

QGQKLKVRQDVSHWKYKNLDLSPVLHVEQPREADGVFNQAQQNHNLEAVLDRKLIQAAIP

ALEKGEAVNADFPIVNTDRSVGTMLSNEISKVYKDQGLPQPMNVKFKGSAGQSFGAFLAK

GVKFEVEGDANDYWGKGLSGGTLVLYPDAKSSIVAEDNIVVGNVCFYGATSGESFIRGMA

GERFCVRNSGAKVVVEGVGDHGCEYMTGGAAIILGSTGRNFAAGMSGGVAYVWDKSCDFE

SKLNPELVDLDPIEQEDKDLLLDMLTKHVQFTGSEVAQSFLDNFEASLASMVKVMPRDYK

AVLQKRKAEETQSQGNETQAEAV

>tr|Q87IQ6|Q87IQ6_VIBPA Uncharacterized protein OS=Vibrio parahaemolyticus serotype O3:K6 (strain RIMD 2210633) OX=223926 GN=VPA0550 PE=4 SV=1

MTPLSELIKQMGIKKIPFVDEHKAAKKRWLKEQAPLFARVCENKPATNPVLHLLGLLTKS

HIEASALYEQHAHSTQQMQKVLADTLGDEQADKFTNQSAEDLVLITHLWLFTQGYLNMDF

SLAHDHAEQTQNTLQHELVIKRIDVDAFRTELMQSFYLGKEANPTASNGFFGWLKRLFSS

>tr|Q87S46|Q87S46_VIBPA Uncharacterized protein OS=Vibrio parahaemolyticus serotype O3:K6 (strain RIMD 2210633) OX=223926 GN=VP0578 PE=4 SV=1

MSKLKKKEYEQELKKLQVELVKLQEWVKHKGLKVVVLFEGRDAAGKGGVIKRITEKLNHR

VCRVVALPAPTEKEKTQWYFQRYVAHLPSAGEIVLFDRSWYNRAGVEKVMGFCTPDEYEE

FLRSCPEFERMLQRSGIILLKYWFSVSDEEQEKRFLGRINTPIKRWKFSPMDLESRHRWA

EYSEAKDKMFAYTDTKNCPWWVVPSDSKQKARLNCISHLLSQIEYKALEYPPIELPEINK

EGYVRAPIDDQTFVPDKY

>tr|Q87JD7|Q87JD7_VIBPA Putative outer membrane protein OS=Vibrio parahaemolyticus serotype O3:K6 (strain RIMD 2210633) OX=223926 GN=VPA0316 PE=4 SV=1

MLKKTWLTAALLATCSMANAAPYIGASIGKATFDDISGSIKSNTYNASEKLEVKDNESLS

GKVFGGYTFNEYISLEGAIGGYDALDGSVVTVGDMKFLAIQPKLTLPIGDRFNLFAKAGL

SYFNAEFKVSNSVLTGDEGHTTFSETTVTGLYGLGAEFAITKNLALQVEWEYMKPELDVA

KLGSEKVTVEAEISAFSVGMSYRF

>tr|Q87NS2|Q87NS2_VIBPA Uncharacterized protein OS=Vibrio parahaemolyticus serotype O3:K6 (strain RIMD 2210633) OX=223926 GN=VP1796 PE=4 SV=1

MQIGIYMNLPKKELVVYNKARNFLLANTPDEVTDEILDSYLSLPEPSYKCLSINELYKRL

LFSAQNANMKTGVIGGSIGGVEKLSSVLFDFDPEQTFLRYQGNAEKLLDEIVLNLKPKGK

IRRTPRSLWPKYCETIISASEFLRQFSDSQEFFEWAESFHRDKKSLAALPMLIDAEVQGV

GFPLACDFLKELGFTNYGKPDVHIIEIFEASGLVAKGATNYQILKAISRIADSTGFSSYN

VDKLFWLIGSGYFYNHPGIGNKGRVGKMKEAFITSLESENA

>tr|Q87IL5|Q87IL5_VIBPA Uncharacterized protein OS=Vibrio parahaemolyticus serotype O3:K6 (strain RIMD 2210633) OX=223926 GN=VPA0591 PE=4 SV=1

MMRLYCVIATAMLMPLTAQATVYDVAKPSELNQICSTHSSFDIQRTDTTFFCHGKIVLPA

GDSIISSSPENEVILEAHQGIVLEGNNRIGEPNKRISLRSLSVELKVNNAQASSIEDYQN

KHTVIYGDLLSAYPTKLHNVLIDGDIELTGSTLHIDGKYNTIIGKILTHSTTDIFNTNVC

GTIESKGHQLHLKTNNPFQQHFVVGDIVAHSSLLIDDMAVYGTTESKGASAALNGSFYAK

DTAIKYFQTLNFNQGVGSQVCGEIQQTPGSSHPHSVTGKYSQFCGVGQSRCDYSSSICPS

TATPPPECSMLPPSNDDLGLTVTPSDDMALMCGDDLPQFTAITTNNDEVVSAPVMAMLSD

PDLFTLEVVKGKPTSTENQFQSNDNGELVVRVVPNDIDKIALDANYYLTFTMVGDSAKEQ

TVNFMFTPFMFEAYSNERRTLNEIRVIAGKPENVHTRLLACASTGEPVVASNYNGKPKVV

HPLIKPLGGSEGDFSYSAEFKDGLSEHGLITNESGLFEVTLSDQFECKGFSECPDDGTVE

VTGKFNVYSRPWTLAICENQNTLPSGTSEQGDKFIAAGEHFSLTVKPVIWQKGGSISDPI

NSSAYCDALVTTNFMHDGAPAASVVLSSEQHSPLNTANQTSTLLKSNYALTQGHQSAKNH

RFVFNGLYWAEVGSLKVKANLDSTYFGMKVNEGYRHIGRFYPKYFKVQSQDWTYPKSQGF

VYMNQPFEKVTYDVVALNANKEDVKNYAHFAPSLQQHFYLGELGGYQDRFVPPAPQKAEW

KRIGDASIGQFVIEKASTNATCHNSPCWEKDKTKGQYPDGPFNRGANSKSSKIGLVTTHV

VDEVNFFDGGEILTKQPDIRFGRLNFKDVGGNQGMKIKVPLDVEVWQNGRFVTNFDDNST

TANGAYYTSTPIWSNAPVNNAQLSGVAKMSVGRTNDIIASQIDAAREQIQFSLNLDHSGN

QLPWLKYDWETSTPEEENPPTIVTFGIHRGNDRIIYRGEPNMLGLN

>tr|Q87SX1|Q87SX1_VIBPA ABC transporter, ATP-binding protein OS=Vibrio parahaemolyticus serotype O3:K6 (strain RIMD 2210633) OX=223926 GN=VP0301 PE=4 SV=1

MTDSASNVVELKQAQFVWPGSETAVINIPDLQIATGEHVFIKGPSGCGKSTLLALLTGIN

TLSSGSLSVLNTNLATLSSSRRDRFRADHIGYIFQQFNLLPYLNVIDNVLLPCQFSKVRR

DRVTPNASKAELLVQATTLLERLHLPTNLHSRPVSELSIGQQQRVAAARALIGHPALVIA

DEPTSALDHDNRMAFIELLMEQANQANATLIFVSHDPTLESLFDRTINLPEINQSSNMEV

LA

>tr|Q87ML4|Q87ML4_VIBPA Thiol:disulfide interchange protein DsbE OS=Vibrio parahaemolyticus serotype O3:K6 (strain RIMD 2210633) OX=223926 GN=VP2217 PE=4 SV=1

MNKKILFIPLVAFLVLAGIFATQLVRNQDGDDPTKLESVLVGKPVPKFHLEDLAEPGKQY

DQSIFKGEPLLLNVWATWCPTCYAEHKYLNELAGKGVKIIGMNYKDDRNKAIGWLNDLGN

PYLISLFDGSGMLGLDLGVYGAPETFIIDANGVIRYRHVGDVNPRNWADTLAPMYNQLVE

EAKQ

>tr|Q87SB6|Q87SB6_VIBPA Flavodoxin OS=Vibrio parahaemolyticus serotype O3:K6 (strain RIMD 2210633) OX=223926 GN=VP0508 PE=3 SV=1

MKIGLFYGSTTCYTEMAAEKIRNIIGADLVDIHNVKETPLSLMAEYDLLLLGISTWDFGE

IQEDWSAIWQDIASTPLKGKVVALFGLGDQEGYGEWYLDAMGLLHDELKATGAQFIGYWP

NEGYEFEASKALTEDGSQFVGLALDEDSQYDLSDERIEKWCEQVLVEFHDTL

>tr|Q87PV6|Q87PV6_VIBPA Uncharacterized protein OS=Vibrio parahaemolyticus serotype O3:K6 (strain RIMD 2210633) OX=223926 GN=VP1395 PE=4 SV=1

MMEKQTLIKETITKQQADTPACFSRFGTIVAINESTGSVRVDFDGNPFNQPLTARLGRGF

RRSELQMAIDNRLNCRIEFLNDDLSLPLVTDIFFSILDDSDEFVLRAKKMVIETEQELIV

KSGETETRYSGRDGRITTKAKYVTSQAEKAQKIQGGTIAIN

>tr|Q87LU5|Q87LU5_VIBPA Sulfate permease family protein OS=Vibrio parahaemolyticus serotype O3:K6 (strain RIMD 2210633) OX=223926 GN=VP2513 PE=3 SV=1

MFGSRFKDINFKGDIFGGVTTAIISLPLALAFGVASGAGAEAGLWGAIMVGLFASLFGGS

NTLISEPTGPMTVIMTAVLTSMMAKYPETGMAMTFTVVMMAGAFQILLGTLKMGKYVTLM

PYSVISGFMSGIGVILIILQLSPLLGHAAPAGGVLGTLSALPETISNLKFNELFLGLLTL

GILFFFPKKYRKYVPAQLVALVAVTLLSVMLFDTEDIRRIGEIPAGLPSLVAPHIDPDMF

VEMVIDALVLGTLGCIDTLLTAVIGDSLTRKEHDSDKELRGQGLANMISGLFGALPGAGA

TMGTVTNIQVGARSPLSGVVRALVLALVVLVAGGLTEPIPMAVLAGIAVYVGFNILDWSF

IQRAHKVSFSGMAIMYGVMLLTVFVDLIVAVGLGVFVSNIMIIERLSREQARQVKAISDA

DEDDVPLTDSERGLLDRANGRVLFFYLSGPMIFSVSKAISRQHTSISDYDVMILDLTDVP

MLDVTVGLALENAIKDALDARCEVYLLCPNQRTREQLEKFHVIDLVPDNNMYQFRYEALN

AAVAHVESDQYQRMTA

>tr|Q87JI8|Q87JI8_VIBPA LfgN OS=Vibrio parahaemolyticus serotype O3:K6 (strain RIMD 2210633) OX=223926 GN=VPA0261 PE=4 SV=1

MASAASQRIQYFVRSISEDIKLYQQLLTLLQQQKALYLKFDGEALNTNVQQQTPILNKLS

RSSNERSQCIRELGLPCNDSSVTRIFNALPAKIGAQARKQWTLLETLIKQCQQYNQSNGQ

SSAAFHELVSQLKQPVQHTYEDKSF

>tr|Q87KZ4|Q87KZ4_VIBPA Uncharacterized protein OS=Vibrio parahaemolyticus serotype O3:K6 (strain RIMD 2210633) OX=223926 GN=VP2830 PE=4 SV=1

MQEYIEFFQQNMILSLVWVGLLVAFIMNIVKSATAAYKEINVNQLTHLMNRENGVVVDIR

TKDEFKKGHITDSLHILPSDIKAGNLGSLENHKSDPIIVVCKTGQTAQESANLLVKAGFE

KVSLLKNGLIAWNEANLPLVRGKK

>tr|Q87H25|Q87H25_VIBPA Uncharacterized protein OS=Vibrio parahaemolyticus serotype O3:K6 (strain RIMD 2210633) OX=223926 GN=VPA1140 PE=4 SV=1

MIKAQIYDVFCDELAQGNPCGVVELNHWLSDSELLQITHQVAQPVTSFVVKSGSDYHIRW

FTLAGEINLCGHGSLGAGAALISKYHQNEVILQSSHGYITVSELDGMYRIVMPSWQAKPH

AIPLGISGFSNRPIDAFTTRDLVVVLESEDDVVCYQPNFDELIKIDEFHAVIVTAQSSDS

GYVLRYFAPKIGISEDLATGSAQCSLAPYWFEKLGKEKLTARQLSRSGGYFEVARGSEST

IVLSAQVKSRT

>tr|Q87KK5|Q87KK5_VIBPA Uncharacterized protein OS=Vibrio parahaemolyticus serotype O3:K6 (strain RIMD 2210633) OX=223926 GN=VP2972 PE=4 SV=1

MNTTYVARQPIFNRKRQTLGYELLFRDGESNAYPAHIESNRATYRLIVENFLSLGTNPVI

ASSRCFINFPHQSLVRRLPRSLPKNKIVVEVLETCQPTDDLLDAIRELYREGYLIALDDF

TLTPEWRRFLPYVHIVKLDIMAMGLEKACELVKTHLAKRVKYHFLAERVETAEEFEQAKA

AGFKFFQGYFFSKPLVSQTKYVSPEQVLALQLFREVCAPEPDFDRIESIITQDVALSYKL

LRFVNTQITTLEVEISSFRQALIYLGQDKLKQFVSLVVASYISSNKPRELYNLSLQRAQF

CELMSRYQPFSHLNEQGFMVGLLSILDAMLDLSIEALVKQLPLSDSVQQALLHRKGEYGV

LISLEECYERADWHGVEKLSGQLGLSFDEVKASLSEAVRWSQSVVTV

>tr|Q87LW2|Q87LW2_VIBPA Uncharacterized protein OS=Vibrio parahaemolyticus serotype O3:K6 (strain RIMD 2210633) OX=223926 GN=VP2496 PE=4 SV=1

MFKTLSSSPMSRYFALWVSLFLIATPSVAQVKNEDTPTRPKVAVVLAGGGAKGAAHIGVL

KALEEMHIPVDIITGTSMGAYVGGLYATGMSADEIESFIYSVDWNSGYRDRVDRSQRRVR

DKEYEDRYQITTDLGLRFGEVRAPTGVVQGQNMLRVLRETTGNLGRFDSFDELAIPYRSV

ATDILELDEVVIGNGYLVDAMMASMSVPGALPPYKLNGHMLVDGGVVNNMPVDVARAMGA

DVVIAVDISTDYKTEDDFTGLFTVADQLSNYLVRRSTQQQVETLQEHDVYIRPNVGQMET

VEFDKMPWAFQSGYDITREMESKLAGLRLSNAEYQKYIDHKQEVRKKLVYGDDRVVDEIV

IVNNTHYSDVLLTNRLELETGRKIETAEIEKAVENLYALDRFELITYHFEEVDGSNLLVF

DVNEKSWGPNYLNFRFFLEDDFDTDSQYGIGMSTNFTNLNSHGAEMALNVEMGTDKLIEA

ELYSPVLSSQEFFVAGKVAYSSEGRNLPVSDDDSSLSSVNDFLPVSYTEFVSEIAIGIQP

TLWQELRLGGRYSSGSIELSTLASVGNLDFERRGLFANYRLDTLDDFAFPTRGLLVDLEY

LVSHDTSPEEIGQSKPKDIVEDTVYEIDARFKGAMSYQRHTLVGQAEYSFVQSKNSSITL

DPRELGGFLHLSGIPRNSLIGQNLFFSSLVYRYKWFDNDFGLFEAPVYVGASLEHGGTWS

DNDLKLNEAPLYNAASIFFGVDSPIGPIMLAYGRTEQDMEAVYLIVGTSFK

>tr|Q87LE6|Q87LE6_VIBPA Arabinose 5-phosphate isomerase OS=Vibrio parahaemolyticus serotype O3:K6 (strain RIMD 2210633) OX=223926 GN=VP2666 PE=3 SV=1

MSNPFDFRTAAKQVLDIEVAALQELDKYFDEQFEQACELILSNSGKVVVMGMGKSGHIGN

KIAATLASTGTSAFFVHPGEAAHGDLGMISAGDIVIAISNSGESHEILSLFPVLKRLNIK

IISMTGKPESNMAKLADLHLQITVPQEACPLGLAPTSSTTATLVMGDALAVALLQARGFS

AEDFALSHPGGALGRKLLLKLSDIMHFGNALPKVSPDALIRDALLEISEKGLGMTAIVDE

HDAMLGIFTDGDLRRTLDKRIDIHTTAIGEVMTKNPTTAHPEMLAVEGLNLMQNKNINAL

ILCKEDKIVGALNMHDLLKAGVM

>tr|Q87TR2|Q87TR2_VIBPA Amino acid ABC transporter, ATP-binding protein OS=Vibrio parahaemolyticus serotype O3:K6 (strain RIMD 2210633) OX=223926 GN=VP0006 PE=4 SV=1

MIKLENIHKRFGDTEVLKGIDLDIKQGEIIVIIGSSGTGKSTLLRTVNFLEQADEGRITI

DDISVDTQKHTKAEVLALRRRTGFVFQNYALFAHMTARQNIAEGLITVRGWKKQEALTRA

QQILDDIGLGEKGDSYPAALSGGQQQRVGIGRAMALQPELLLFDEPTSALDPEWVGEVLS

LMKKLANQHQTMLVVTHEMQFAKEVADRVIFMAEGNIVEQGSPQDIFDNPQDPRLKKFLN

QVGIE

>tr|Q79YT8|Q79YT8_VIBPA Uncharacterized protein OS=Vibrio parahaemolyticus serotype O3:K6 (strain RIMD 2210633) OX=223926 GN=VPA0982 PE=4 SV=1

MQSDHKEKIAILVDVQNVYYTCREAYRSNFDYNQFWYVATQEKEVVSAKAYAIASNDPKQ

RQFHHILRGVGFEVMLKPYIQRRDGSAKGDWDVGITLDAIEIAPDVDRVILVSGDGDFSL

LVERIQQRYNKKVTVYGVPRLTSQTLIDCADNFVAIDDDFLL

>tr|Q87S53|Q87S53_VIBPA Putative phosphate ABC transporter, periplasmic phosphate-binding protein OS=Vibrio parahaemolyticus serotype O3:K6 (strain RIMD 2210633) OX=223926 GN=VP0571 PE=4 SV=1

MGRRVSRAIACIALTCSSMAVAQDKPLSNYHKTTGIVGNLLSVGSDTLAGMTTLWVEEFK

SIYPNINAQVQASGSSTAPPALTEQTAQFGPMSRPMRLKEIEAFERQHGYKPTALRVAID

AIGIFVHQDNPIQGLNFNQLDAMFSATLRCGESQFITNWQQLGLKAEWAKRNLQLFGRNS

VSGTYGYFKSNALCGGDFKTRVNEQPGSASVVQSVASTISGVGYSGVGYRVAGVRLVPIA

KRGVNYVSPTRTNIVSGKYPLSRYLYVYVNKHPDYPLSPIEAEFIRFMFSAQGQALVEKD

GYVPITADFAAEELKKVGL

>tr|Q87M51|Q87M51_VIBPA Chromate resistance protein-related protein OS=Vibrio parahaemolyticus serotype O3:K6 (strain RIMD 2210633) OX=223926 GN=VP2407 PE=4 SV=1

MFTIFKTFFWLGWFSFGGPAAHIGYFRQTFVEKLKWLDDSEYAQIVALSQFLPGPGSSQV

GFALGYKRGGLGGACAAFVGFTLPSVIIMLALAMVSSQITDTAVFQNIVHGLKLLAVVVV

ADATWGMYKNFCQSKLTAGLCVATAIALLVAPSIMTQMFVLLVAGFVGTRYLKKDSAPSA

EPFKPSIAPLALFAVLLLGLPLVAHSLPLLGLFSDFFQAGSLVFGGGHVVLPLLQNIVGD

QLSQDAFLTGYAAAQAVPGPMFTFATFIGYELSDTPILGALIATLGVFLPGFLLLLGVLK

NWQALAGKPLVSGAINGVNASVVGLLLAALYQPVFSSAVVAPIDMVLVVAGFYLHKKLNL

SVLWMIVFFVAAGLVTGMM

>tr|Q87SK5|Q87SK5_VIBPA Phosphate transporter OS=Vibrio parahaemolyticus serotype O3:K6 (strain RIMD 2210633) OX=223926 GN=VP0418 PE=3 SV=1

MDILANYGTVLIIVAAIFGFMMAIGIGANDVANAMGTSVGSKALTVKQAIIIAMIFEFAG

AYLAGGEVTDTIRKGVIETSLFASQPDILVFGMMSALLAAGTWLLLASYMGWPVSTTHSI

IGAIIGFACVSVGTEAVDWSSVQGIVGSWIITPVISGFFAYVIFVSAQRLIFDTENPLFN

AKRFVPVYMFITTMVIALVTIKKGLKHVGLHLTNGEAWMWAVAVSALVMAGGYFYIQKKF

ANREEDRSFAGVEGIFSVLMVITACAMAFAHGSNDVANAIGPLSAVVSTVEHMGEVTAKS

TIAWWILPLGGIGIVVGLATMGHKVMATVGTGITELTPSRGFAAQLATACTVVLASGTGL

PISTTQTLVGAVLGVGFARGIAALNLGVVRNIVASWIVTLPAGALLAVVFFYGIQAMFS

>tr|Q87GW0|Q87GW0_VIBPA Acetoacetyl-CoA reductase OS=Vibrio parahaemolyticus serotype O3:K6 (strain RIMD 2210633) OX=223926 GN=VPA1205 PE=4 SV=1

MKKVALITGSKGGIGSAISSQLVNDGYRVIATYFTGNYECALEWFNSKGFTKDQVRLFEL

DVTNTAECAEKLAQLLEEEGTIDVVVNNAGITRDGVFKKMTAQAWNDVINTNLNSLFNVT

QPLFAAMCEKGGGRVINISSVNGLKGQFGQANYSAAKAGMIGFSKALAYEGARSGVTVNV

IAPGYTGTPMVEQMKPEVLESITNQIPMKRLATPEEIAASVSFLVSDAGAYITGETLSVN

GGLYMH

>tr|Q87KP0|Q87KP0_VIBPA Uncharacterized protein OS=Vibrio parahaemolyticus serotype O3:K6 (strain RIMD 2210633) OX=223926 GN=VP2937 PE=4 SV=1

MTKPKRVVISWSSGKDSTLTLERLNENPNYQVVGLYTTYVGKEVPFQATPLHVVQMQAEL

LGFPLITIELPEVFPSNDLYQSAIVNALQSSGLNVEAVAFGDMFCNGIADYRRSYIEPAG

WECVFPLLGENSLSLAMEVIERGIQAMLITIGGSVLSPEWCGRWYDQVLIESLPRHIDPC

GENGEFHTLVTSTPSFQGHIELTKLEVEIGERFSHQRYRAKALPKQI

>tr|Q87JA7|Q87JA7_VIBPA Uncharacterized protein OS=Vibrio parahaemolyticus serotype O3:K6 (strain RIMD 2210633) OX=223926 GN=VPA0346 PE=4 SV=1

MITTMLTLAPLVALVVSLSPSLLSVLYGRSTTSKMNRHPIWCPFYARRGNSLRAFFFLSG

GHLLGGKYKKASLKGWQVEQQGQRLLLTSVELVLDTSFLLIVHNRRL

>tr|Q87M54|Q87M54_VIBPA Evolved beta-D-galactosidase, beta subunit cryptic protein OS=Vibrio parahaemolyticus serotype O3:K6 (strain RIMD 2210633) OX=223926 GN=VP2404 PE=4 SV=1

MIVLDSLEQFKQVYRNGRKWNRCVEAIGNIGSIKDGVMHSIGDSLVYMVEDGIAKQTETF

IGNRRYFDVHYYLEGQETVEFADKTALTLEQTYRDETDREFFSGQGEIRELCEGQVAIFE

NSEAYRFHGDNRVRKVVLKVTIEDGYFLNK

>tr|Q87FG2|Q87FG2_VIBPA Transcriptional regulator, AsnC family OS=Vibrio parahaemolyticus serotype O3:K6 (strain RIMD 2210633) OX=223926 GN=VPA1717 PE=4 SV=1

MSYQLDRIDLHILRVLHSRGRIPVVELAKQINLTTSPCSDRVKRLEKEGYINGYHAELNA

EKLGLDVQVFIHIRLDQTSFSIFEKFAKAVELMPEIEECYSLSGDFDTMIKVRVKNMKAY

QEFMSSKLGTLPGVIQTRSEVVIEEHKTGFGVNPELLG

>tr|Q87MP0|Q87MP0_VIBPA Uncharacterized protein OS=Vibrio parahaemolyticus serotype O3:K6 (strain RIMD 2210633) OX=223926 GN=VP2191 PE=4 SV=1

MRQNYKRLLAALVLMSATQTSSVVQAEGIRLVGPSGEVQSSPSYAEEIERALPTPPENTQ

PSRFFGPTGENQTLWSIASELRPSRNVSVQQTLLAIYRINPQAFDNQNIHELIPGSRLRV

PSLEQVRSATTEQAVAIMKAHELRLKQPKPEKPAPVKQIEVKPKPVAEPTAPKTPVVTPP

KPEPVKPVETTANVPPVSTSPQGEKQVSDLKDKLQGSQSELESLEEKNHRLRLMLSQVQS

EVETLKSELNDEERIRSEVEKLLAEERQRVAEQQRMQPSTMDKILSNGWLVGLAALIPGA

LLALLVVMLLGRRSKAKEEEAAQQQTQDIDPLAAPIGLAAADQLDDELSLDDDLFGDDDS

EPLLEQDEKAELEEDVFANLDDEELDFNLEGDDGEDPFAGIGDDGDLDVGFDEFDSSTSG

IQVNGDEKALGLEEMERALDEVTPELEVLDEDETGFDLSDDDNAISDDEFAKLLADDEPS

EDLSSGSVDQAMLDDLFAELGDDDLDLDVEDTKPEPQVSDEDFSAGEASDDDIDKLLAQY

DQPAEETSTELDPLDELESLAGLTDDTDIDENSTELLDELIDFDEDETEEEFDPLNELEA

LSDFEAEEVIEELDENSVDLLDELLQDDDDEPVLEDETTSELDPFDDLIREDEVVTADES

QLDDDLVADFGFEEIEPQTEQNKALEPEEEKPLTAQVEEDASELEFESGLDIDALLSEAQ

QQPVAPEPTESLQEESVPFNSNDFLGDLEEMSPEHDPLMTELDELFESDNTVLEETEQES

ADFAAELDALLEGKDGFAEELENSSVKENLMPSAEELPSENGEYQLREEQTEPEITSKEE

EPAFTPTPNTVENEFGVPQEEDWLLDDVEPEAENKLEQEPVAATSTDVDEDEFNFDELEL

PEFDEEDALASMADEPKLPESEREAAAPAAEAEEEFNFDELELPEFDEEDALASMAGEPE

LPEAEVQTAEPSADAEEEFNFDELELPEFDEEDALASMTAEPELPEAEVQTAEPTVDAEE

DFNFDELELPEFDEEDALASMADEPELPEAEVQAAAPTVDAEEEFNFDELELPEFDEEDA

LASMADEPELPESEREAAAPAAEAEEEFNFDDTELPEFSEEDAIAAMAEASSSSESEVAP

KPILQADNDHDALFEVFAQNSFDQEVEQPTAIESPSELDDFDESTMANLLADEPSAEAFD

GKLDSDTIASAGMDFETMLDVGDDWDGFKPASDMPEPSVTEEVPEDQQEVWSSSEALTQP

EIAQENWAEQEDLDDFDPKKNQFMTIDELMAQVDKEGGEFEEQELMLDVGLNEFPDVIGD

IGDVDVDTNAEAAGKLDLAKIYLEMNDPQGAIKLLEEAIVYGEDDIRREAKKLIDTINGR

>tr|Q87SM2|Q87SM2_VIBPA Putative transmembrane protein OS=Vibrio parahaemolyticus serotype O3:K6 (strain RIMD 2210633) OX=223926 GN=VP0400 PE=4 SV=1

MARKNDGIIWHLMDAPWWVSILFSAGIYVGLSFLLPGLAARSDNFIFNAIGPNLPQMAPY

FAFLFLIPAPIAFFKQYQRKHNYLATTSQIRAHKNTTPLNSLSWIEFESYIGEYFKQQGY

DVKQSFSKQADGGVDIWLTKDGELSLVQCKHWKARKVGVQVLREMYGVMIDNRASKMIIV

TSGDFTSEAIAFAQEKRMWLVNGSELVHMIEDGRSFMNKPVIGEPAPEPHSKVCPNCQSN

LVIRVAKKGRNAGKSFYGCSAYPKCRYTCDC

>tr|Q87J72|Q87J72_VIBPA Putative transcription regulator OS=Vibrio parahaemolyticus serotype O3:K6 (strain RIMD 2210633) OX=223926 GN=VPA0381 PE=4 SV=1

MHIVRSGAADRFDSLVSELGQNPVEIMAAVGLSAAQFRDPDTYLAYPRLAELLEEAAARC

QQPLFGALLAERQNLQSLGDLPMLVSRAETVGEALVRVNDFLYLHSSGVTLNMTPQDDWV

RLSLSIDVHSERGIAQLMQLSVSHLAMFIASLLDIEASHFSLHLTQHASFEAEQSAFAQQ

NKLRFGDKFDGILLKASLLNAKNHQDEDALERHFQQHLKELQTRYPNNLSDQAANMIGRL

LSTGECSVERVARALDLHPRMLQSKLKQQGTSYRQLLQQVRQDFAEQRLSENSQSITDIA

LQLGYAETAVFSRHFRRWTGKSPRQWRLDKMAAR

>tr|Q79YZ4|Q79YZ4_VIBPA Sodium-driven polar flagellar protein MotA OS=Vibrio parahaemolyticus serotype O3:K6 (strain RIMD 2210633) OX=223926 GN=VP0689 PE=4 SV=1

MDLATLIGLIGGFAFVIMAMVLGGSIGMFVDVTSILIVVGGSAFVVLMKFTLGQFFGAAK

IAGKAFMFKADEPEDLIAKIVEMADAARKGGFLALEEMEINNSFMQKGIDLLVDGHDADV

VRAALQKDIALTDERHTQGTGVFRAFGDVAPAMGMIGTLVGLVAMLSNMDDPKAIGPAMA

VALLTTLYGAVLSNMLFFPIADKLSLRRDQETLNRRLIMDGVLAIQDGQNPRVIDSYLKN

YLNEGKRALEIDE

>tr|Q87TG7|Q87TG7_VIBPA Uncharacterized protein OS=Vibrio parahaemolyticus serotype O3:K6 (strain RIMD 2210633) OX=223926 GN=VP0103 PE=4 SV=1

MIDTHAHIYASEFDNDRDEVVERALTQGITKILLPNIDLDSIEPMLQTEAAYPEVCHSMM

GLHPCYVDSNVDQTLEIIRGWFEKHNFIAVGEIGIDLYWDKTYRAEQEMAFVTQLNWAKE

MDLPVVIHTRDSIEETLTLLRQEQDGRLRGVFHCFGGSLEEARAINELGFHLGLGGVSTF

KNGGMDQVIPHLDMQWVILETDCPYLAPVPHRGKRNEPAYTSLVAQRVADLRGEEVATID

AITTENAKSLFKI

>tr|Q87SC1|Q87SC1_VIBPA Sodium/alanine symporter OS=Vibrio parahaemolyticus serotype O3:K6 (strain RIMD 2210633) OX=223926 GN=VP0503 PE=3 SV=1

MTDLINLMNDLLWGSILVYLLVGVGIYFTVRLGFIQFRHFGHMFSVLKNSRKADKAGISS

FQALCTSLAARVGTGNMAGVAVALTAGGPGAIFWMWLIAMLGMATSFAESTLAQLYKTKD

DDGNYRGGPAYYMEKGLGMRWMGVLFSVFLIIAFGLVFNAVQANSIANAMSNAFGWNDLY

VGIAVVALSAVVIFGGIKRIAKVAELIVPIMALLYLVLALFVVFSNLEKLPDVLMLIFKS

AFGLQEAAAGGLGYAIAQAMINGIKRGLFSNEAGMGSAPNAAASATPYPPHPASQGYVQM

LGVFMDTIVICSATVAIILMSGEYVGQATEVTGIELTQRALSSQVGDWGGIFVAVAIFFF

AFTSIIANYSYAETNLIFLEHNHKAGLSIFRVVVLGMVMFGALASLPVVWSLADVSMGLM

AIVNLVAILLLSGIVIKLAKDYNRQLGEGKVPTFDANDFPELKSQLEDGIWDNTKKD

>tr|Q87GK0|Q87GK0_VIBPA Transposase OS=Vibrio parahaemolyticus serotype O3:K6 (strain RIMD 2210633) OX=223926 GN=VPA1316 PE=4 SV=1

MPKPRYKTTNWKQYNKALINRGSLTFWIDEEAIRQWKQSKQDKRGRPRQFSDLAITTALM

VKRVFSMPLRALQGFIDSVFSLANVPIVCPHYSCISRRAKQVEVSFKPKTRGAIQHLAID

ATGLKVYGEVEWKVKKHGIDGKRRVWRKLHLAVDTSTHEIVAAELSLLNVTDAEVLPNLL

KQTRRRIIEISGDGAYDTRDCHDAIRFKRAVPLIPPREGAAFWENGHPRNLAVGCKRLYG

SNNKWQKRYGYHKRSLSETAMFRVKQLLGGRLSLRNYNAQRH

>tr|Q87IL6|Q87IL6_VIBPA Putative ATP-dependent RNA helicase OS=Vibrio parahaemolyticus serotype O3:K6 (strain RIMD 2210633) OX=223926 GN=VPA0590 PE=3 SV=1

MSFSSQGFAPEVVKALAECGYEKLTPIQQKAIPMARKGHDIFATAQTGTGKTAAFSLPMI

QQLLDSGRTASRKTARALILAPTRELAEQIADNIKAYTKYTDLSVVAVFGGRKMSSQVSA

LENGVDILVATPGRLEEHIEQGNVSVANLEFLVFDEADRILDMGFIHAVRKIMLDVDTDP

QIMMFSATTSSQLNLLAKDILRKPKRIEVERANTTAHTVAHVLYPVDQERKTELLSELIG

RKNWQQVLVFVNYKETANDVVKELKLDGIKAVVCHGDRAQSARRRALEEFKTGKVRVMVA

TDVAARGLDIEDLPHVVNYDMPFLAEDYVHRIGRTGRAGKQGHAVSFVNRDEELTVVQVE

NLIQQRIRRIELAGYEPKQRESYIEKLNSKPAFKNRQGRRNNPNQSATDQGSAERRLAMV

KRLKARRGTN

>tr|Q87HD6|Q87HD6_VIBPA Uncharacterized protein OS=Vibrio parahaemolyticus serotype O3:K6 (strain RIMD 2210633) OX=223926 GN=VPA1029 PE=4 SV=1

MSSEDFYRELKQRSLFEAIHLIEQELLKVESQIGTDALPKNEKLNLKVNASLGFENAQLV

STKPLGKDKLALETNLIGLTGEQGVLPQHYSELALHRLKEGDHAMVDFYDIFNHRLLSLY

YRSWQLSQLTIQARAHAKNQRSPLTDCMSSLTGGGNDLALHYGGMYASPTRSKGALKSIL

ECLSGCQIRIHEFQGQWMRLSKSEQTRLVSKSMPEGQFAQLGAGASLGKKAWNINASTTI

EFLPKESKQVTNLLPKTNTLKTIKQVAGDFIGKHKHVKWQLTTKHSLLPQVQISKQQGQL

GVGSVLKKHERTEDRNITITV

>tr|Q87GT8|Q87GT8_VIBPA Uncharacterized protein OS=Vibrio parahaemolyticus serotype O3:K6 (strain RIMD 2210633) OX=223926 GN=VPA1227 PE=4 SV=1

MYNKTILESQHTLIPMPSTSTNAQPTLAKQLFTMTWPMLFGVLSLMSFQLVDSAFIGQLG

VLPLAAQGFTLPLQMVIIGIQVGLGIATTAVISKALGANDARYAKQLGGLVLMIGSIGVA

VFGLLIWLLRYPILSLLSAPETVMPIIDSYWPWWLLSSWAGAVLYFYYSICRANGNTMLP

GTMMMVTSGVNLVLDPLFIFTLDLGINGAALATLVAFGFGILVVAPRVKRNHWATTQWQD

LNVMKSVTSIGNIMGPAMVSQLLPPLSSMLATKLLASFGTAAVAAWALGSRYEFFAIVSV

LALTMSMPPMVGRLLGAKNYRDIQSLVGIAVKFILGFQLLIAAITFLLANPLALLMTSDT

QVEQVLHMHLMIVPISLGSLGVCMLMVSICNALGKSYTALTISALRLFVFFLPCLWLGAQ

LDGIRGLLFGACVGNIMAGISAYLTYRKTIHKLAIKQATA

>tr|Q87FZ6|Q87FZ6_VIBPA Uncharacterized protein OS=Vibrio parahaemolyticus serotype O3:K6 (strain RIMD 2210633) OX=223926 GN=VPA1524 PE=4 SV=1

MLRNSTKPQLKEASRAKSIYFMTWRWHFYAGLFVIPFMLMLSVTGLVMLFDDEIELARYE

TTLKVVQQEHKVPVSVQLESVKQAYPDFSVTQFVPAKTAHLANRFSIKAEDGRSLVAAVN

PYTGEVQGTIDRSDSVYELMNNIHGTLLIGEFGDRLIEISASLGILLLVSGLYLWLPRDN

ASRAGFLKIRIAQGSRILLRDVHANLGGVLSLVLLFFLISGLSWAGIWGAKMVQAWNTFP

TYYTWGEKPESILTHKDLNHGSSEEMPWNLELAAVPESKDKPAHDHANMEKGASYTASHR

ALSIDDIILKAEMMGFTSYKIFLPRSDNGVYTVAANSMGGDISDPRQDRTSHFDQYSGRL

LVDVTWQDYSWFAKLMAAGVSLHQGDVSIINKALNVLFCLAFILIAISGVVMWWLRRPSR

SASLGAPPQFQHDGVWKLGLATLVAICAAFPMGGLAIVSVLLLDWVVFNRVEKLKAALN

>tr|Q87RF4|Q87RF4_VIBPA Succinate dehydrogenase, cytochrome b556 subunit OS=Vibrio parahaemolyticus serotype O3:K6 (strain RIMD 2210633) OX=223926 GN=VP0843 PE=4 SV=1

MSKPVKERKSRPVNLDLQTIRFPITAIASILHRVSGVITFVAVGILLWLLSISLSSHMGF

MEAADIVDGFFVKFILWGILTALAYHIAGGIRHLLMDLGHFEELDSGAMSAKVAFGATAV

LSLLAGILVW

>tr|Q87PP2|Q87PP2_VIBPA Putative intercellular adhesion protein A (Biofilm formation) OS=Vibrio parahaemolyticus serotype O3:K6 (strain RIMD 2210633) OX=223926 GN=VP1459 PE=4 SV=1

MTWLVATHVWLRSRLMNDLIWIAGFGLSAFLIIYHHVGYPLLLKWLPLKPKANDEGEHFA

ERHYKASASDNKLPSVTIIIPAYNEEQWIAEKIRNLASLDYPRDKLKVVIACDGCTDKTA

EIAQDTIQEAICSDTLFIVNDHSINRGKVALINEEMKHVTSDITALSDTSALVSCDSLLL

ASQHYQNEKVGVVNATYQIMRTSNQGEAAYWQYQSRVKHQESLLGSTIGSHGAFYTFRTK

LFEPLEAGTINDDFILPMRIVLRGFTSVYEPKMLALELEQSSDDADFKRRLRISAGNMQQ

LMQLKKLLLPRYRGTAFAFLSGKVLRLATPYLMIVCLVCSLLLAQHPVFLLLLLAQVGIY

GIALMTYLIPALNTVKPFKLISYIVVGHIANFVGGMKYLLGMENGRWKRVNQ

>tr|Q87PY5|Q87PY5_VIBPA Putative M20/M25/M40 family peptidase OS=Vibrio parahaemolyticus serotype O3:K6 (strain RIMD 2210633) OX=223926 GN=VP1365 PE=4 SV=1

MDMTKHSKIFAGVASAIALTVGVAAWAAGDQDFSSMQMKGVEQIEVKVDLDGAAKRLSKA

VQFPTISNQDLSDFDEQAFNDYHNFIEQSYPLVHKTLKREVVGDPRPFSLIYTWEGKNPA

LPPAVFMAHQDVVPVAEESRSQWKEDPFSGAIKDGYIWGRGVLDDKNQIHAILEAAEMKI

KEGFQPERTILFVFGHDEEVGGPEGAKHAADIIEQRYDKIAFVIDESAPLVPGIFPGIRE

NTALIGIAQKGFVSLEIAINAVGGHSSQPPAESNIVALAKAVTKVEEAQFPYKIHDAIRY

QYRYMGPELPEEQQPMYKAVAYGNNDSITELEQKFLDVMSKNQVTRAMLHTTTAVTMFNA

GIKDNVLPPAATAVVNFRPMPGDTPEVIIEHVKKAINDDRITVRDISASTPATNVADPSS

DGYKILEKSIRQTWGNDLIVSPFFVIGGSDSKHFQARDFAPDVYTITAIQLENTTEFEGF

HGVNERIRVDEYGRSIGFFYQLMDNLDYL

>tr|Q87GQ3|Q87GQ3_VIBPA Uncharacterized protein OS=Vibrio parahaemolyticus serotype O3:K6 (strain RIMD 2210633) OX=223926 GN=VPA1262 PE=4 SV=1

MINYLNTLFADFRLYSLFGDDDNFTVTQLWVLEIERESSSELRFLYARTLPSTYQSDTWQ

GSVSTKTQLYNNCSVKTHTLTLHTSTKKLKVFLEHFINGAPLQKASQLAEVNISDKLANT

VGVNTFGESPLIRSVMHLPTRDYYQFQTSRLSPTSYCSVDSGAISPEDKPKIFSVPEGCD

MMIAEAACQALDADTGLDFSKTDSWRISDFEFICAPGLNAAERCKYDISLKGKQSSLTLF

ESLTREPSDLLVIIKAYSEGSIQSSYITNLSKSSSYPLHHQFELKVFQNQSSTAYTMEIY

ALGPNGEHSSLLLQTGNHFMRQMNYNLQLVEPIRAYEQFSWLDKKVPKREKVKLEAAKQV

GRAIRPSRSQMSDYTADPWVPLNRLIQDRVRQLCPNKSDGRFFPTLNNSNGMSRLELKDW

LKSIFEQHHDAKIAWIDPYMEDVGIELLNRLGTASADYLIITTEKTSNDDSTKESGQPTR

VDNLLARCSGWNNGYFGSVHLKVLAVPESKLHDRMILIRSANGQPLSGYHLSNSVQRASE

NHPLLVTPIPLDVVPHVFEYVDQIIQNTLHRDDKTPPPARIIFNSTDIKPRDEEKPKGLS

HNSSFAEPQCAGSVIAWWLDDEQLSDFSGSELMEKMSTKGYVKDGQLDPEHFDALPAKFW

KEGLPMADFHSAWDALGCVIANSPANRYTGSLYNKEQSVLSEQVKLALLEHISPSRANAL

QPRLTNKQLDIEHYRSQGLIELLLSKCDPFSTFTYSPVDTSYSDYFSIQLLWSEAPKQFV

SWLNTILSKPIKQPRSHALVVEALKHICLVVSFDKHQEQIDALLQSNVSVITWIGLHALA

KNINGGDWGIEALSKIEHITPSAVRRTIQCWLINEANYFNSDIKPQLIASLTQSLEAPLK

DNELKDILLPVRNHNGRLHHFKPWILESMLVPMLEQRIIDITQVAHQWLTELINQWQTAL

KNNRLDFMLETDGAFTDELAVLTKYLSPDVREKIFSKLQKVFNTLARTIRRPMSAQISWN

SYSNAHRVNLWLYAFANRMAELLPDKPSPLNELLLESKEIIERISPSTWDIFSINKLSIY

AMDDPKHIRSHNLHQIIKNTLTTH

>tr|Q87IB1|Q87IB1_VIBPA Accessory colonization factor AcfA OS=Vibrio parahaemolyticus serotype O3:K6 (strain RIMD 2210633) OX=223926 GN=VPA0695 PE=4 SV=1

MNKTLLALLVTCSVSVSAAPYVGLEYGLGTTDHDLEPHFSADNVTLNPELEDGIFSGFVG

YAFNDNWALELGYSQFDLDDSRSKNLGIKPIDGKDYHHEMDWDASIKAKQVSLAPVFSYA

LNDKWTTKFKAGLTYTQYDAKVSKHQEYELVANDDVEMTNTLFHSAEKNNEFGAMFSVGA

EYQVFPKLTIGANAKYQLDSYANTASFNVGTTYYF

>tr|Q87JD2|Q87JD2_VIBPA Uncharacterized protein OS=Vibrio parahaemolyticus serotype O3:K6 (strain RIMD 2210633) OX=223926 GN=VPA0322 PE=4 SV=1

MLQLMEWHAIGLETMVRCTAAYSSFYFWSCHYCPSQTLCYVTPKLWHMPITFLAYANNLS

LQKSYLIRCWLFSHHNASSIATCLKPAQYSVLQFKHLHAFDDSDYFVSKSWRDAWITPKT

PVLLTQIDHSIALL

>tr|Q87QQ1|Q87QQ1_VIBPA Putative phospholipase C OS=Vibrio parahaemolyticus serotype O3:K6 (strain RIMD 2210633) OX=223926 GN=VP1098 PE=4 SV=1

MKFSQWSLLLSTLVVLPSLADTDVYLTNNSDQPLTIQVKHDGSDLLQYGEEWQQHVEVLG

PWETKPVLSFNRWEGVKSDQNYRFETVVSNPQGESITLHQVMEGHWYNSTIEYGLSAADV

NLALKDDRTIHRSNTEAFGSRSAELAFKSDSTARYDDLYYTVTPSKVAETVDNNEQNLKL

MTYNIWALPAIASHIGDRYELIPDYVKGYDVLAFQEVFASGRDAFLRELAKEYPYQTKML

DKDGFNVYDGGVIIVSRYPIVNEAQYVFPDCSGTDCFADKGVNYAEIIKGGQAYHVFATH

TASFDTDTAREYRQRQFQQMRTMAQSLNIPNNETVVYSGDFNVNKLKFPGDYQQMIANLS

AMEPQYSGYTASTFDPRINNFAGEALSGGENVEYLDYVMVSNEYGVKSFNDNRVDVPRST

AEGLWKHYNLSDHFPVSAVIKP

>tr|Q87MJ6|Q87MJ6_VIBPA Polar flagellar protein FlaK OS=Vibrio parahaemolyticus serotype O3:K6 (strain RIMD 2210633) OX=223926 GN=VP2253 PE=4 SV=1

MQGLAKLLVIDDDASSRLNLSNILEFVGESCEAVGSEQLGDVDWSSVWSGCIVGNISAGR

AATAVMARLNDAYHIPLLVLGSFPLPVDDLPNFVGELEQPLNYPQLSEALRHCKDFLGRK

GVNVVASARKNTLFRSLVGQSRGIQEVRHLIEQVSGTEANVLILGESGTGKEVVARNIHY

HSSYRNGAFVPINCGAIPPELLESELFGHEKGAFTGALTARKGRFELADGGTIFLDEIGD

MPMSMQVKLLRVLQERCFERVGGNSTIKVNVRVVAATHRNLESMIEEGTFREDLFYRLNV

FPIEMPALKERKQDIPLLLQELMTRLEAEGGQPICFTPRAINSLMEHHWPGNVRELANLV

ERMIILYPNSLVDVNHLPTKYRYSDIPEFQPEGNPFTSIEEQERDVFQDIFSEDFSFDEQ

SDLDHNMNAPQALPPEGVNLKELLADLEVNMISQALEAQGGVVARAADMLGMRRTTLVEK

MRKYNLQR

>tr|Q87NA2|Q87NA2_VIBPA Uncharacterized protein OS=Vibrio parahaemolyticus serotype O3:K6 (strain RIMD 2210633) OX=223926 GN=VP1973 PE=4 SV=1

MKKLLCLFLAFGALAGCSSSNDNQRQLELMASNRAGVLSAGLPLEYGPLQIMRVSSNKNV

VEMMMIYNDDALGAKPLNQVLNTSIYTYCNTPSVREQIDMGLMYRLKVRNSRGQLIADEL

VSEQTCSKIK

>tr|Q87K85|Q87K85_VIBPA Uncharacterized protein OS=Vibrio parahaemolyticus serotype O3:K6 (strain RIMD 2210633) OX=223926 GN=VPA0013 PE=4 SV=1

MLAFLDLLKVKAQQACSQMRRKGVRERRCSIICCGTSTGNVVKRINTPALIFSPLFLSYL

VLNPAIIDNSLGCYCVFTQNHLSPLTPIYSLKIPLVASYS

>tr|Q87QI3|Q87QI3_VIBPA Uncharacterized protein OS=Vibrio parahaemolyticus serotype O3:K6 (strain RIMD 2210633) OX=223926 GN=VP1166 PE=4 SV=1

MSEQLTQEQIDTINRYNEEQRLKYCVKEIVANRKVWILKDEHGCVMLNTEDDDCVPVWPN

EEFAKQWATGDWEECEPEAISLNKWHSRWTTGLEDDELSVVVFPNQNEEGVVLFPDEFDF

ELRKQASRR

>tr|Q87ST1|Q87ST1_VIBPA Uncharacterized protein OS=Vibrio parahaemolyticus serotype O3:K6 (strain RIMD 2210633) OX=223926 GN=VP0341 PE=4 SV=1

MKRFLLLLCALLSFSALAAPKADLWPYWQQSNGVNQTSISHLEWQQLLDSYLVTRGDNTL

FRYNQVSFADKTKLKQYIQRLASLNPLQYRQAEQYAYWVNLYNALTVHLILDNYPITSIT

KLGGLFSFGPWDQGVIIINGKSLTLNDIEHRILRPIWQDPRTHYAVNCASLGCPNLQTQA

FTAENTQTLLESAAKTFINSKKGVSIEGDTAKISSIYEWFAVDFGGEKEVFNHIRKYAPQ

YNHFSGRVKYDYDWSLNQAD

>tr|Q87RV5|Q87RV5_VIBPA Aminoacyl-histidine dipeptidase OS=Vibrio parahaemolyticus serotype O3:K6 (strain RIMD 2210633) OX=223926 GN=VP0671 PE=4 SV=1

MSEFHSEISTLSPAPLWQFFDKICSIPHPSKHEEALAQYIINWATEQGFDVRRDPTGNVF

IKKPATPGMENKKGVVLQAHIDMVPQKNEDTDHDFTKDPIQPYIDGEWVTAKGTTLGADN

GIGMASCLAVLASKDIKHGPIEVLLTIDEEAGMTGAFGLEAGWLEGDILLNTDSEQEGEV

YMGCAGGIDGAMTFDIKRDAIPTGFVTRQLTLKGLKGGHSGCDIHTGRGNANKLLGRFLA

GHAQELDLRLVEFRGGSLRNAIPREAFVTVAVPAENQEKLAELFNYYTELLKAELGKVET

GIVTFNEEIVTESQAFTAADQQRFIAALNACPNGVMRMSDEIEGVVETSLNVGVITTEEN

KVTVLCLIRSLIDSGRSQVESMLRSVAELAGAQVEFSGAYPGWKPDADSEIMAIFRDMYE

GIYGHKPNIMVIHAGLECGLFKEPYPNMDMVSFGPTIKFPHSPDEKVKIDTVQLFWDQMV

ALLEAIPEKA

>tr|Q87Q36|Q87Q36_VIBPA Uncharacterized protein OS=Vibrio parahaemolyticus serotype O3:K6 (strain RIMD 2210633) OX=223926 GN=VP1314 PE=4 SV=1

MPTCSTQETPNTDINAITQQVDQWLNDVVIGLNLCPFAAKPQRNKQIKIFVSEATQEEAL

LEDILLQLIELSTTEPEKLETTLVVVPNMLQDFWDYNFCIDWVEGLIKQQDWEGIFQVAT

FHPDYCFGGAAPEDDENLTNRSPYPIFHLIREESMEKVLKHYPDPESIPDTNIARVSALS

EEERKKLFPYLFR

>tr|Q87NX9|Q87NX9_VIBPA Uncharacterized protein OS=Vibrio parahaemolyticus serotype O3:K6 (strain RIMD 2210633) OX=223926 GN=VP1739 PE=4 SV=1

MTSQKNIEEQDEVVVIEERDKRTHIYIAIAAVLGLAFGGLAGSVLTANKWESTYQVLEDK

YQALAQDKTALVSQVKTREESLDKEIQEKVDALLAEKDAAHQKALKKLQTQLTEVEKVNL

SLESQVKQQNDKLSSSKTENEKLTRQADMQATMFERSREVFQKELKISQDLEALEKEREK

LLPKIDKLKKECDVFLEGKSWDVKSDACDKHDEANSRLSQIDQLIEVYKMDLKQIKEITS

DMGL

>tr|Q87QI0|Q87QI0_VIBPA Peptide ABC transporter, permease protein OS=Vibrio parahaemolyticus serotype O3:K6 (strain RIMD 2210633) OX=223926 GN=VP1169 PE=3 SV=1

MLTNNVYQEERIPTQFERFWRSYRANGLAMFGLWCLGLIVLITILAPWLAPFDPQAQSGE

LLVPPSWAPAGTVDYFLGTDDLGRDILSRLIMGSQLTFGAAVGITAIATIVGCFIGVLAG

MTKGLLSSTLNHLLDTVMSIPSLLLAIIFVAFLGFGEFNILLAICLALIPRFIRSVYIAV

HTEVEKDYIMAARLDGANDFYLLWSSILPNILTVIAAEITLALSAAILDITALGFLGLGA

QAPSTEWGAILGDSVELIYIAPWTVTLPGLTIMFTVIILNLVGEGVRQALNAGIE

>tr|Q87J04|Q87J04_VIBPA Uncharacterized protein OS=Vibrio parahaemolyticus serotype O3:K6 (strain RIMD 2210633) OX=223926 GN=VPA0451 PE=4 SV=1

MDIYQESISFLGNDANFDANGLFQCELSSNTNDHDEALFLTIFKNETTINLHMSLTSPVE

LPTPLPEAIAIAIGEHALEPFRGGFGVGLMPDSRRLTVYKVISLANKPQSYVQNTFQQLL

EKIEQWHLFIEQTDNEEAIPEQLPAGIFI

>tr|Q87TI0|Q87TI0_VIBPA Uncharacterized protein OS=Vibrio parahaemolyticus serotype O3:K6 (strain RIMD 2210633) OX=223926 GN=VP0089 PE=4 SV=1

MNDSEHTINERQGSATADSSRALNGAKNQEVEQAINGLRAILSQIDDLSTSLRTWSGSTL

DLFLLEMKVNVAAARQIVMFSVIFTLLSVLFIFSVCLAAGVVTYHFTAQLLLSVGVFIVS

LGLALVGLAWWQKRLTHFLGFKNTTDQLQEGWHAFSNQTRPSHADKTHRG

>tr|Q79YX5|Q79YX5_VIBPA Bacteriophage f237 ORF9 OS=Vibrio parahaemolyticus serotype O3:K6 (strain RIMD 2210633) OX=223926 GN=VP1562 PE=4 SV=1

MKYHEMTKNYIFREFECGLTVEEAAKLCLKNVRTVKEWDKGKSIPPECKRLMRMNKGREL

SSCEEWENFVMRHDRLELPTGQLVTAQQVLIGVALLELGASNDIKVAHQILKYARALKRF

I

>tr|Q87P71|Q87P71_VIBPA Citrate synthase OS=Vibrio parahaemolyticus serotype O3:K6 (strain RIMD 2210633) OX=223926 GN=VP1647 PE=3 SV=1

MPQTVAKKAELGGAGLRGQSAGSTALCTVGKTGTGLTYRGYDITDLANNAQFEEVAHLLL

RGHLPSQQELDAYKTHLLSLRGLPTELKQALELIPASAHPMDVMRTGCSVLGNLEQEMSF

DEQLQATERMLALFPAIICYWYRFSHDGVRIDTEDQTEDCIGGYFLRLLTDKEPSVLHKQ

VMHCSLILYAEHEFNASTFAARVCASTLSDIHSCITAAIGTLRGPLHGGANEAAMEMIED

WQTPDEAEANIMQMLANKDKIMGFGHAIYRESDPRNALIKRWSEELSKHVGDTHLYAVSE

RVESVMKREKGLFCNADFFHASAYHFMDIPTKLFTPIFVMSRLTGWAAHVYEQRANNRII

RPSADYTGPDHQEWVPIENR

>tr|Q87NV3|Q87NV3_VIBPA Uncharacterized protein OS=Vibrio parahaemolyticus serotype O3:K6 (strain RIMD 2210633) OX=223926 GN=VP1765 PE=4 SV=1

MKALIEVLDGQVKHCPINQKFDISSTDFYIVLNHNCALELCDSKGIKQLIDPPCLVAIGA

DFKGQIAINSFVENANISGFRLAACFIEKLNQQLNFRDLCDGVFSGNVAVSFTLRPGIVD

IYSALKAMVKKGCGNRSNDELLDINSMLLYLLMHFDQSASQAETRSYSSLSSRIRALISK

DLTKPWTLKEIAKLVYMSESTVKRKLNKEGTTFTDVLQAARLDTAQKMVCNSDASVSAIA

ELCGFKHASYFGACFRKEYGVTPLAYRKQAQLRAN

>tr|Q87NE4|Q87NE4_VIBPA Thiol:disulfide interchange protein DsbE OS=Vibrio parahaemolyticus serotype O3:K6 (strain RIMD 2210633) OX=223926 GN=VP1924 PE=4 SV=1

MSPKTMPKLFGLFIVVAVFSVVVVAALSDRPHVEQAKESTLMVPNFSAVSVISGKEVTQV

LFQDNGYKLLNVWASWCGICKKEHETLLQIADAGVPIIGLNYRDNNSAAQNYLSSKNNPY

QDVISDPNGELAVELGVIGTPETYLIDQNGKVLIKYRGAITIDKWNSIFSRYFNGAI

>tr|Q87K23|Q87K23_VIBPA Uncharacterized protein OS=Vibrio parahaemolyticus serotype O3:K6 (strain RIMD 2210633) OX=223926 GN=VPA0075 PE=4 SV=1

MGTSVTPNIFDFLIKIDPFDKLPEPVVESIANSVKVKYLVRGETIGFSALCDQRYLYIIR

TGAIEQRTPNGELRARLGQDDQFGFTFLDPLENAEDGYQAEAIEDSLLYLIPHSELKLVS

EEHPEFADYFAAQANVRLSSAVKYVCRKEEKGLFFRTVGEIASENIAIVDVNDSIRSVAQ

TMCGKQRSSCAVVMKEGEIIGLVTDRDMTKSVVAQDMDTNQPIADVMTPNPVLIEDDAKV

IQAISLMLQYNIRCLPVVHGKQVKGLLTTTHLVHNHRTQSLFLIEKIKYASSLNALAALK

EERQTIFQALVESGVSAEIQGRVMSMIMDAFTRRIIQLNEELLGPPPCDYAWLVAGSHAR

NEVHMLSDQDSAIVLSDDATEEHKLYFTHLAMRVCNGLAACGYPLCDGKYMAASPKWCQP

IRRWKEYYQKWVASPEYNKLLSISVFLEVRAIHGNREFVDQIQQHLHDCIQKSPSFIPAL

VRDAIDTQPPLGIFNNLVLEKGGENSNTLNIKKYALNLIIDLARIFSLAAGGSLTGTEER

FRYAAQHGTLSKDSVENIIGAYRFITQVRFRHQLNAILAGTTPDNHIAPDDFSSFERKHL

KDAFKIISELQDVAKLKFVKGA

>tr|Q87PW1|Q87PW1_VIBPA Uncharacterized protein OS=Vibrio parahaemolyticus serotype O3:K6 (strain RIMD 2210633) OX=223926 GN=VP1389 PE=4 SV=1

MNLRHTLCLITLAVLSGCNQESAKEPEQHAPKPQTVQDRIAEKNAQQWEDGRVYITENNY

VIYDKETGKPKHFIFKAKEWPREDLTNEGSYDLPRVVFRWDNARNEAKDHYDQRDKVWSI

KTDGTDLRLVADEFVGKVRLMRVSPNNRYLALAYSASEGMFKVIKDLKTGEYIELGRSRG

YPEFLWAEDSSYLYYVDKFKDWKYTLATGEKEETEVNFNEYSVIYNGKRIVVVAYGVAVF

DEQTNELLYSVAPKKRGGDLDAKEFRKKAISPTGRYVWSETRTHRYLIDVKNRTFQSEEI

VPRAVGKLRYFEILGKDVNYTRDGAARVILSKTTENNEFGPYLKWEQIGTGHSAGNSSLY

NAFANNGDFVKEGDL

>tr|Q87H46|Q87H46_VIBPA Putative enoyl-CoA hydratase/isomerase OS=Vibrio parahaemolyticus serotype O3:K6 (strain RIMD 2210633) OX=223926 GN=VPA1119 PE=4 SV=1

MTGTVNVSKLECADGSVIGVLELDNPAALNALSYVMIEQLYKQLNEWQMDDSIVAVFLHA

KGDKAFCAGGDIQAIYRALENKEEDFDSAFSAMETFFDLEYRCDHLIHTYPKPIIAWGHG

YLMGGGVGLFMGASHRVAETTTRFAMPEIKIGLYPDVGATYFLNQLPKHLALFLSLTACQ

INATDMKALGLSQWITKSESKQALLDLFASVSWKGEANINEKIATTLRSVQVDEPSEGML

MPHAGLIEQLCSGDLDSIVRAISSADVDNKWFVNAQKALNYGSPLSINITYQQLTQYQNL

SLKACFDMEFNLTLRCGLEGDFREGVRALIIDKTNQPTWQYRTVSDVTPQALERFFAPID

SAEYPLTASSVATAVL

>tr|Q87PI8|Q87PI8_VIBPA Formate dehydrogenase, iron-sulfur subunit OS=Vibrio parahaemolyticus serotype O3:K6 (strain RIMD 2210633) OX=223926 GN=VP1514 PE=4 SV=1

MARMKFLCDTKRCIECNGCVTACKNENDDALEWGIQRRRVVTLNDGEPGENSISVACMHC

TDAPCMAVCPADCFEHTEDGIVLHNKDLCIGCGYCLFACPFGAPQFPKQEAFGERGKMDK

CTFCAGGPNTEPGSEEERQKYGANRIAEGKLPMCASLCSTKALLAGDAEKVSDIFRQRVV

ERGAKNAGWTDGNDLSYDATKS

>tr|Q87T50|Q87T50_VIBPA OtnA protein OS=Vibrio parahaemolyticus serotype O3:K6 (strain RIMD 2210633) OX=223926 GN=VP0220 PE=4 SV=1

MLKGYFSIAACVLASVFAPFSHAQTPTSEQMQMFQSLPADQQQALASKYGISLPSGSSSQ

PSNYQNPQVIDPRPEVSNANQTDKMDLAKDEELKRFGLDLFAGSPTTFAPVSDVPVPTDY

TVGAGDEIVIQLFGKENTTHRLRVNRAGIINFPSLGPVQVAGMTFSDVRDSLNQRVKEQM

IGVRSDISLGEMRTMQVFVMGDAYKPGAYTVSALTTISQAIYYSGGFSESGALRNVQLKR

NGQVIRKLDMYDLLLKGDARNDIRLLPGDVVFIGALGNTISIDGEVNRPAIYEIKPGETY

KQAIQMAGGFTANAYSDQIEVKRYAEKGARDALTLNFSQSHDQQTKVKDGDAVNVLKKNE

ELTRYVQIEGDVRHPGFIEWKSGLRIADLFQSVDTSFNSTADVSYAVVVREINPQRDIEV

YQVNLANAILSPTSKDNLKLNSRDRVLVFNRFNNEDLDTLADQETVTKAKTLEQAQLQAQ

QEQLKEQEVMSSSVAVSSAMPLEKDSKQPKIVFRGKEITKDDFEALKQNTRRTLLAPVLL

QLQQQSRLGLAPQIAEVFGEVKHPGRYPITPRMTISTLIEAAGGLTYNAFTINAELARTV

INSKDERASIDVERIDLRQAIQGSTADDAIIVGRDRLNILEKPNVKLQSTVTLQGEVRFP

GTYTVRQGETLGELLERAGGLTEFAHPQGAIFTREALRLQEQKLLNQYAADMRAETAKKT

FRADSNMGSVISDPDKTLKFVEEASRSKALGRMVVQLNRILKDERSADFMLEDGDFLFVP

TFRNTVSIMGEVQVPITYLLDNKLDVDDYLNKAGGAKKQADEDRIFVVRADGSVYKPTSG

YWFGNNHEELKAGDTIVVPIDTDYRDALSTWTAATQILYQTGVAINALK

>tr|Q87LU1|Q87LU1_VIBPA Pyruvate dehydrogenase complex repressor OS=Vibrio parahaemolyticus serotype O3:K6 (strain RIMD 2210633) OX=223926 GN=VP2520 PE=4 SV=1

MAYQRIRQPKLSDVIEQELERLIVEGTLSPGQQLPPERELAKQFDVSRPSVREAIQRLEA

KRLLTRRQGGGTFVSENIWKSFSDPLLNLLSSHSETQLDLLESRHAMEGISAYFAALRGT

EEDFARIQGCLERISKEQANNDIEAESAEVMQFLIAITEAAHNVVLLHIVRSLAPLLEQN

ILQNFKLLRRRPEAVEKVSKHRANIVDAIVSGQPEKAREMSHSHLAYIEETLLDLTREES

RRERSLRRIQQGNNS

>tr|Q87HF3|Q87HF3_VIBPA Cytochrome c554 OS=Vibrio parahaemolyticus serotype O3:K6 (strain RIMD 2210633) OX=223926 GN=VPA1012 PE=4 SV=1

MSFPLGEALFYGQLSLNRESIMLRTLLLTSLIFTSTVYANPFGDVEKGKVKAPSCVYCHG

TNGLSSNDAYPNLAGQSPKYLYDSMKAYQDGLRTGPLAEMMKAQLRMLNDEDLRDVAAFF

AEQSGD

>tr|Q87JI7|Q87JI7_VIBPA Flagella basal body P-ring formation protein FlgA OS=Vibrio parahaemolyticus serotype O3:K6 (strain RIMD 2210633) OX=223926 GN=VPA0263 PE=3 SV=1

MLLGILSTGQTFASPDQTTFSSEELKAVVAKHFEQEVDRVAKSNQWGEYQLEYDLWVPGS

ANHLPKCDAKLVITGRDNQPLPVGNLKRSVSCEDLTAPWRINVTIKSSVTLPVVVAATTV

GRSEVVTANHLKLETRTISRSDDFYTAPKHAIGLETTRRLRAGQVVDPTSLSAPALIEKG

NEIIIIASKDGFSASTKGVALEDGAQGQQIEVENLSSGRVIRAVVTGRNQVHTQF

>tr|Q87KQ9|Q87KQ9_VIBPA Uncharacterized protein OS=Vibrio parahaemolyticus serotype O3:K6 (strain RIMD 2210633) OX=223926 GN=VP2917 PE=4 SV=1

MKKTLLSLFMVSVSFAVVGEEARYTMDDLKALNGSKNWNELLAHAEDIRPSQRNSEWESL

VQNAALGAFEHYVASGAKDDAIGLGQQLILSYPFLSQSKSFTQQFSKELVPAAQPCIQYA

IEGCVENYGQLLNTLAPSAEVSYEEGTKVFQNVSKSLSVPFFAAAVQQAENYCADENVAN

ALLYTLDRPNNTNFALAKEVATQRCANTALTNFENYIIESQTVREALCPTYLSKGHVKGL

MKKVCQS

>tr|Q87MT0|Q87MT0_VIBPA 2,4-dienoyl-CoA reductase OS=Vibrio parahaemolyticus serotype O3:K6 (strain RIMD 2210633) OX=223926 GN=VP2151 PE=4 SV=1

MYPNLLKPLDLGFTQLRNRVLMGSMHTGLEEHKEGLQKLAAFYEERAKGGVGLIVTGGFS

PNLRGRLHPLSAEFSKPKHAKAHRVVTDAVHKHGGKIALQILHAGRYAMHPFAQSASGIK

APIAKFAPSEMSERQIKKTIDAFANSAELAQLAGYDGVELMGSEGYLINQFLCKRTNMRY

DDWGGSYKKRMRFPLEIVKAVRKAVGEKFIIIFRLSMLDLVEQGSTFEDVIELAKGLEDA

GVTIINTGIGWHEARIPTIATQVPRGAFTWVTEKVKPHVSVPIVTCNRINTPDEAERILA

SGQADMVSMARPFLADPYFVAKAEQEKAQFINTCIGCNQACLDNVFKGKRASCLVNPLAC

YETEIVVEPTFKKKNIAVVGAGPAGLACATTLAERGHKVDLFEKNDRIGGQFRLAMQIPG

KEEFRETIRYFANRIDETGVNLHLSTEANYDLLTEYDEVVMASGVEPRKVNIEGIDNAEK

VVDYQTLIRDKTPVGEKVAIVGAGGIGVDVATMITEPAGQTLDDWLHEWGIDKEMSQPGG

LYPYPDAVSDKEVWVLQRRKGRVGKGPGKTTGWIHKRTLEKRGVHLLGGVSYDKIDDNGL

HIHVDGKEQLLEADKVVICAGQESVRPFEDKWSELGEKLHIIGGADHAGELDAVRAIRQG

VKLAVKL

>tr|Q87KK0|Q87KK0_VIBPA Uncharacterized protein OS=Vibrio parahaemolyticus serotype O3:K6 (strain RIMD 2210633) OX=223926 GN=VP2977 PE=4 SV=1

MKALKNPVALATLLVLQACSSQPPQSGAETPTSPPASLDKPETIQPQTFMLRGKVIIGHE

SQYIMPCGSDKQYWLQLSPQQIQRAIKLGNEPYQTMYGEVIGHLNPPGIDGFSADFDANF

VVEQVNFLTTENPNRCSQPQKPTRVFGNEPSWAASFEANALKFQQMGKTTEILSIQSSQL

QPRQRTYRLDDGELRMTENLCSDTMSDSLYGWKATLKHDGNTYQGCGMAANVDATLSWAN

TYVATSTQSQGFEVQMTLNPDHSATTKYSYSNGQDPLVERGFWQQLSPSQVQVVMTHHQQ

QRLMSERLFTREGNQLKATKEKVGSMVYPIADGGLVLYPATVRDAGVQQPAAKRADQPIG

SADVPSSADFDSKVDAAVRNYFFIHQTDPSNNQYRWLTYDLNGDGNEELLVQLDWCGSGG

CTLLVFENHEKEWRFNSRITLVRSPIMLGQQTSHGWRDLIFDVSGGGATPAKHVMQYTGV

SYPLNPSMAPTATPEQISGVRLFSDGISPVREGVRL

>tr|Q87M66|Q87M66_VIBPA Methionyl-tRNA synthetase-related protein OS=Vibrio parahaemolyticus serotype O3:K6 (strain RIMD 2210633) OX=223926 GN=VP2392 PE=4 SV=1

METIAYSDFAKLEIRTGKIIEVARHHNADKLYIVQIDIGEKTLQTVTSLVPYYTEEELMG

KQVVVLCNLAKAKMRGETSECMLLCAETDDESESVLLTPERAMPTGVRIV

>tr|Q87GR3|Q87GR3_VIBPA Uncharacterized protein OS=Vibrio parahaemolyticus serotype O3:K6 (strain RIMD 2210633) OX=223926 GN=VPA1252 PE=4 SV=1

MVTTRLTMLRLGYLGLVPFLFSLLLIVSDTTLFNLSGQQFFIAYSAVILSFLSGVLWGNG

IDHYYHRLSRNILVLSNLFVLLAWGALLQGNTHYIAAILLLATGYAAVWYAEKLIRNVEL

EVDPKGYQGMRNKLTCGVILMHGLVLIA

>tr|Q87H27|Q87H27_VIBPA Cytochrome BD2, subunit II OS=Vibrio parahaemolyticus serotype O3:K6 (strain RIMD 2210633) OX=223926 GN=VPA1138 PE=4 SV=1

MHFDLSVIWFAIIVFATLMYIIMDGFDLGIGILMPFIKDEKQKDVMVNSVAPVWDGNETW

IVLGGASLFGAFPMAYAVIIEALTIPLTLMLIALIFRGVAFEFRFKALENHLKFWDRSFI

VGSILTTFFQGIVVGAVIQGFTVENRVFVGSQLDWLTPFSIFCGLGLVATYALLGSTWLI

MKTEGALQNTMYRFTNKTLLAMISALIIVSAWTPIAYPAIAERWFSLPNLFYLLPVPVIT

GLVCLKIADSVKKRKERSPFVMALVIVILGFAGLGISIWPNIIPPSISIWEAAAPASSQR

FMLVGAVIIIPIILAYTFWSYYVFSGKVKEDEAYH

>tr|Q87JQ1|Q87JQ1_VIBPA Uncharacterized protein OS=Vibrio parahaemolyticus serotype O3:K6 (strain RIMD 2210633) OX=223926 GN=VPA0197 PE=4 SV=1

MQGQGDLFESTDWQEVRQGKLFYQPNFLSQQEADTYFSTLMSTLPWQQERITIFGKSVLQ

PRLQTWHGDAPYTYSGLTMAPHPWTSELSSLKARCESLANTPFNSVLANLYRDGQDSMGW

HQDNEPELGRNPVIASLNLGDTRRFVLRNLHCKTQIEYELGHGALLIMAGELQHHWRHCV

PKTAKPKGERINLTFRHILNY

>tr|Q87T82|Q87T82_VIBPA Uncharacterized protein OS=Vibrio parahaemolyticus serotype O3:K6 (strain RIMD 2210633) OX=223926 GN=VP0188 PE=4 SV=1

MSKKVYRRRRWNNILILGIIVFMVILNAPTWIKTYLIDEQADPYPNVLRSDQDVSAIYYA

GWELDKRNGQWQSNIKANIPIQEIVTRWQSLEGTELNSQQYEQLKPSLSSPESIEVWYQG

LEEPQRITFYRLDDFWLFKNWQDKWIAISVDSSYIMPK

>tr|Q87L94|Q87L94_VIBPA Putative Na+/H+ antiporter OS=Vibrio parahaemolyticus serotype O3:K6 (strain RIMD 2210633) OX=223926 GN=VP2718 PE=4 SV=1

MSVYHTLCFLSAAAMLIAFVNSKIGKMQTTIAITAGSMVLSLLILIAGQNNWFHLTEIAT

ETVTRINFEDFLLKGILGFLLFAGGLGIKLPNLKDQKWEITVLALGATLFSTFFIGFVLY

GLCMLIGIQFDLVYCLLFGALISPTDPIAVLAIVKKLDAPKRISTQIEGESLFNDGFGLV

IFVTLFTIAFGSEAPTVGSVTLLFIQEAIGGIVYGFLLGLVFHYLISATDDHSMELLLTI

GVPTAGYAFAEYIHVSGPLAMVVSGIMIGNWTRFIGFSKESEDHLDHFWELVDEFLNGVL

FLLIGMSMLLFKFHEEDWIMMAIAVPLVLASRYLSVFISYIGFKRYRKYNPWSVKILTWG

GLRGGLALAMALSIPSGIWVVQDKLIDVKEIILVMTYSVVVFSILIQGSTITPMIEKAKA

EEKKMEDEKLAQSSLAEQREQTEA

>tr|Q87G12|Q87G12_VIBPA CsuB OS=Vibrio parahaemolyticus serotype O3:K6 (strain RIMD 2210633) OX=223926 GN=VPA1506 PE=4 SV=1

MTLVVRGFSLVVMSWCSSTVLADVTETFQVSATVDTGCLINGAVQEESATQAGQIGTLDF

GEHSSVYAAEVQGSVTYSSSLTLSCTPGIAMNVSLNGGLNSSDGVRKLKHTEEVTTVDYF

LFQDLDYTQVLDIDTRYSVDTTQDPDNIQFPIWAKAIINGNEIAGIYMDTLTLTLEW

>tr|Q87K78|Q87K78_VIBPA Uncharacterized protein OS=Vibrio parahaemolyticus serotype O3:K6 (strain RIMD 2210633) OX=223926 GN=VPA0020 PE=4 SV=1

MCSAKSMVASGWQRSVIVTTLFCLFISGMTLSVWGGPYYVHVLVSFGFGYSALFFSWLID

KLFPTIPRMLEIALSLTACLLFGVINAQFWLGEYFGISGMLPVLLMGLLFSGMCYFYFHS

REKEAIAQRELESIKRENAEQERALLLSQLKQMQSQIEPHFLFNTLANISALMSQDVDKA

KQMLDQLTALLRATLKNSREEHTTVENEITLIDAYLGIQKIRLGERLSYTIEVQEGLGNT

ELPPMMLQPLVENAIIHGIEPKREGGEVQLLIKQEKQLLQIEVKDTGVGLSHVSNHSGSG

IGLSNLKQRVDALFAGKGQVSISESSEGGVSVRLSWPMISKEQ

>tr|Q87IP1|Q87IP1_VIBPA Putative glucose dehydrogenase-B OS=Vibrio parahaemolyticus serotype O3:K6 (strain RIMD 2210633) OX=223926 GN=VPA0565 PE=4 SV=1

MLAFKASTHFNMEGPMRIVTIALLACLSFSLHAAQYHADEVANGFQVPWGIEFIDSQRVV

VNEKNGTISLLNVMTGQRQKIHTVQNVNTSGQAGLLDVALAPNTDKKNPQLYFTYSKRTQ

KGNTVALATATLNGEQLTGWKDLFIADAITDTGRHFGSRIAFIDGKIYFTIGDRGERDNG

QNTQTHAGSILRLNLDGTVPTDNPFTSSKAKPEIWSFGHRNPQGLFYDKETDQLWSIEHG

PRGGDEINLIKKGANYGWARVSQGKEYWGPLDVGEAKSLPGMEDPKLVYIPSIAPSNMVL

YRGDKYSELDGKILVGALKLTHINVVGINDGKLTESQRLLEKLGERIRDITISPDGYIYF

STDSGKIFRLTEQ

>tr|Q87GS2|Q87GS2_VIBPA Putative cytosine deaminase OS=Vibrio parahaemolyticus serotype O3:K6 (strain RIMD 2210633) OX=223926 GN=VPA1243 PE=4 SV=1

MTTLLIKNVTLKGQEGLQQILIEDGQFKRIESNDVELNHNGDTIDAEGGMAVAPFCEPHI

HLDTTQTAGEPSWNISGTLFEGIERWAERKEMLSIEDVKARAKQTLKWQIANGVQHVRTH

VDVSDPTLIALKAMLEVREEMKKWVDIQIVAFPQEGILSYPNGKELLEEAVKLGADVIGA

IPHFEFTREYGIESLHYAFELAQKYDRLIDVHCDEIDDEQSRFVETLAALAHKFDMGNKV

TASHTTAMGSYNSAYASRLFRLLRMSGINFVANPLVNIHLQGRFDDYPKRRGVTRVKEML

NANINVCFGHDDVFDPWYPLGTANMLQVLHMGLHVCQVMGYEQINHSLDLISTNSARTLN

IQDKYGIEEGKPGSLLILPAENGFDAVRRQVPVRYSVRHGKVIAETQPATTRIHLEQPEQ

VTFKR

>tr|Q87P56|Q87P56_VIBPA Low calcium response protein OS=Vibrio parahaemolyticus serotype O3:K6 (strain RIMD 2210633) OX=223926 GN=VP1662 PE=4 SV=1

MNLMNKLIDILNKVGQRKDIMLAVMLLAIVFMMILPLPTALVDVLIGANMSIAVVLLMLA

IYITTPLEFSAFPAVLLITTLFRLSLSITTTRLILLQGDAGQIVYTFGNFVVGGNLVVGI

VVFLIITIVQFMVITKGSERVAEVSARFSLDAMPGKQMSIDGDMRAGVIDVHEARHRRSL

IEKESQMYGSMDGAMKFVKGDSIAGLVIIIVNILGGVTIGVTQKGMSASEALELFAILTV

GDGLVSQIPALFIAITAGIIVTRVSHEDSADLGSDIGGQVTAQPRALLIGGVLLVLFALI

PGFPKITFLVLALVVGGGGFYLFYQQKKQTESESSDLPSFVAQGAGSPAAKPNKPTPSRG

SKGKLGEQEEFAMTVPLLIDLDSSLQESLEAVALNDELARVRRALYLDLGVPFPGIHLRF

NDGMKNGEYLIQLQEVPVARGRIEKDKLLVTEGSDQIELLGVPFEQDDDFLPGVSSLWVA

QSYQEKLTASHVGFLTPDRILTFHLSHVLKEYAQDFIGIQETRYLLEQMEGSYSELVKEA

QRIVPLQKMTEILQRLVSEDISIRNLRVILEAMVEWGQKEKDVVQLTEYIRSSLKRYICY

KYASGQNMLPAYLLDQSLEDTIRSGIRQTSAGSYLALDPSVTQQFVSDVKQTVGDLSRMP

NKPVLVVSMDVRRYVRKLIESEYYDLPVLSFQELTQQINIQPLGRVGM

>tr|Q87FS1|Q87FS1_VIBPA Putative transcriptional regulator OS=Vibrio parahaemolyticus serotype O3:K6 (strain RIMD 2210633) OX=223926 GN=VPA1607 PE=4 SV=1

MRLKTTLEQWQTLQAIDQEGSIQSAALLLNKSHTTLIYSVRKLEDQLGIQLIEVRGRKAG

LTEHGKTILRRAQSMLEQARELEVISEQLKSGVESQITVAVDHLCDPCWLYAPLSQFLED

NNTTSVQVVETSLSKTTEMVVNELADIAIINLPITNYPAEAFGVTTMLPVIAAHHPLAKK

PHVSIDDFATTSQIVIRDLGDSDKTKRDVGWLRARQRITVDNFDHAFHAVEQGAGFCRLP

AHLVAARKSDKIKVLEIEHSHQYQVALHLTLPKGAKSGPATQALYRTLLKSVETRA

>tr|Q87TF8|Q87TF8_VIBPA Uncharacterized protein OS=Vibrio parahaemolyticus serotype O3:K6 (strain RIMD 2210633) OX=223926 GN=VP0111 PE=4 SV=1

MPRKNGIKKATVKLSLSKCHQVAKLCCTHLVHQESFCFSCFPCQKVLFFIFLMSFGYPFT

SCLLSNFLCLILSTVLTIRRDDHPYRAGSLPT

>tr|Q87GX8|Q87GX8_VIBPA CbbY family protein OS=Vibrio parahaemolyticus serotype O3:K6 (strain RIMD 2210633) OX=223926 GN=VPA1187 PE=4 SV=1

MSTIDFRPYGGFIFDMDGTLIDTMPAHLAAWQATADRFDFPFCQEWLHSLGGMPSFKIVA

ELNRHYGLSLDPQTVSKFKMETFAAMELQGDVIQCTGIVLEEYLGQKKIAVGTGSQRESA

MRLLSHAGLIDRLDAVVTASDVENHKPCPDTFLLAADRLGIDAQNCLVFEDTELGKRAAH

SAGMDCVMVEGNNLVFYPKR

>tr|Q87Q05|Q87Q05_VIBPA Oligopeptide ABC transporter, permease protein OS=Vibrio parahaemolyticus serotype O3:K6 (strain RIMD 2210633) OX=223926 GN=VP1345 PE=3 SV=1

MFSIKKLDTQSLQDQVVDNIEAVEGRSLWQDAWARFRKNRAAMTSVYVLAFITLCITFGP

SIAAFGHDEIDWDVLADPYELGKPSLSSGHYFGTDDLGQDLFARTMQGGRLSILVGFVGA

LVAVVIGTIWGSVSGYLGGVVDSVMMRIIEVLDSVPFMFMVILFVTLFGNNIYLIFVVIG

MVSWLGIARVVRGVTFGIKKREFIEAAHSIGVSKFTIVRRHVMPNVLGIVMVYSSLMVPG

FIMFESFLSFLGLGVQPPDTSWGILISEGAKTIDVALWMLLFPSLFLVTTLFCFNFIGDG

LRDALDPKDR

>tr|Q79YT9|Q79YT9_VIBPA Anaerobic C4-dicarboxylate transporter OS=Vibrio parahaemolyticus serotype O3:K6 (strain RIMD 2210633) OX=223926 GN=VPA0981 PE=3 SV=1

MLYLEFLFLLVMLYIGSRYGGIGLGVVSGIGLVIEVFIFKMPPTSPPVTVMLIILAVVTC

ASILEAAGGLKYMLQVAERVLRKNPKRVTLIAPFVTYFMTFLLGTGHAVYSIMPIIGDVA

LKNGIRPERPMAAASVASQIAITASPISAAVVYYLAQLSDIQHEITLLSILLVTVPATLF

GTLLMSLYSIKRGKELEDDEEYQERLKDPVWREKILNTTATSLDEVLPTSARNSVLLFIA

SILVIVVIAMWPDIRTIVDGAKPISMAVVIQMMMLCFGGIILLATKTDPRDVPNGVVFKS

GMVAAIAIFGIAWMSDTYFQYAMPQFKSGIVEMVTNYPWTFALALFIVSVVVNSQAATAR

MMLPVGLGLGLDPALLIGLMPAVYGYFFIPNYPSDIATVNFDTSGTTKIGKWYFNHSFMS

VGLIGVIGACCLGYVLGQIIIPS

>tr|Q87M92|Q87M92_VIBPA Putative GGDEF family protein OS=Vibrio parahaemolyticus serotype O3:K6 (strain RIMD 2210633) OX=223926 GN=VP2366 PE=4 SV=1

MQFTLEHAQDVLSGMPDPTFILSEDGIYIDVFGGTDKKAYHDGSNLIGKTLHSVLEKEQA

DWFIEQINRSLNQDSIVTIEYKMCADSIQGIDANSGPKGDLYYEGKICPLTINYQGRRVV

LILTRNISNRHRVETLLRHQSQIDALTNIYNRRVFFEKLTEEVIKSQTPDYQSALLLFDL

DFFKVINDKRGHDAGDYVLETLADLVSHLLVKDEIFARVGGEEFAIILPETSLSAAKEVG

EEVRSHIQNCQFEFEKIEIPVTVSVGVVKIGRNETTKRLYAHADKALYAAKRNGRNCVAT

LDRQME

>tr|Q87SB3|Q87SB3_VIBPA Single-stranded-DNA-specific exonuclease RecJ OS=Vibrio parahaemolyticus serotype O3:K6 (strain RIMD 2210633) OX=223926 GN=VP0511 PE=4 SV=1

MIEIQRRPEPDLSLLPDSIPPILKRIYINRGITDIAQLETSARGLHSYQKLGGIEQAVEL

LFQAIQEQKRIIVVGDFDADGATSSALSVLALRMLGSNNVDYLVPNRFEDGYGLSPEVVD

QALELGAEMIMTVDNGVSSIEGVRYAKENGITVLVTDHHLPGQVLPEVDAMVNPNLDSCT

FPSKALAGVGVAFYLMMALCVHMRKHNWFAQQGMQEPKLMELIDLVALGTVADVVPLDEN

NRILVHQGLQRIRAGKARPGIQALIEVAKRDARRLVASDFGFALGPRINAAGRLDDMSFG

VELLMCNNIHAARRMASELDGLNQTRKEIEEGMKQEAMAFCERLQFGENSELPYGLSLFQ

RDWHQGVIGILASRIKEKFHRPVIAFADGGEGTIKGSCRSIPGLHMRDALDFIDTQNPGL

IIKFGGHAMAAGLTIKEQDFERFSRLFDEVVKKELDEAALKGVILTDGELKPEEFSMHIA

EQLRAGGPFGQAFPEPIFDGEFKVLHQKLVGEKHLKLMLEPLYKGHPTNVMIDGIAFNVD

LRRWPDASVKTVRLAYKLDVNEFRGNQSLQLMIDHIEAK

>tr|Q87PN8|Q87PN8_VIBPA Putative glycosyltransferase OS=Vibrio parahaemolyticus serotype O3:K6 (strain RIMD 2210633) OX=223926 GN=VP1463 PE=4 SV=1

MTDIVVFGEDFGGLPSSTQHIVQRLATNHRILWVNSIGLRQPKPTGKDIQRLVSKISRVI

GNTGSDKPTMHCDAVDNIFTVNLLTIPAPHSAFSRKVAAKMMQHQLEKHLKALNFDNPLF

WTSLPTAADVCNAMNKRGLIYYCGDDFGALAGVDHQTVMEHERTLVDSADFILAASDKLA

ARFPDNKTTTLPHGVDFSLFSTPAEKASDLPNNGRKILGFYGSLSDWLDYRLIDQVAQHA

PDWDLVFIGPNEFAHNPLPQRDNVHYLGPRAHHLLPSYSQHWDASWLPFVDNAQIKACNP

LKLLEYLATGTPVISTPFPALMPYKHMLHIVADVEDVCASLSHLLPPPNGSTSYVQQQSW

EARADQVEKLVRAL

>tr|Q87I29|Q87I29_VIBPA Uncharacterized protein OS=Vibrio parahaemolyticus serotype O3:K6 (strain RIMD 2210633) OX=223926 GN=VPA0777 PE=4 SV=1

MAPVFFLTQQAALPSRKVLRKVIQFATALAQPIVVLVEDHRRSEDHAFAHFLSFNSDHIE

KRQAEFEAWTSKFIDFVIELCGEEEVETPTLSYEHFKGSHWIAQVAKRLVDHTDLLLVGH

FNKHNIQTLLTELAKNDCDLLLLSERSWPSHANMLCAIDPLHRGDAKSVVDKAIVSRGSK

LEKTLSGKMTLVYCRYVAPYLSRYRAEILSNQKAGITDFITAQKLTRVPLLLPEGNPETA

LPKAVKQSQAAILIMGACKRSMSSQFWSGSTVDTLLDQPPCDLLLVNSTKD

>tr|Q87LV4|Q87LV4_VIBPA Poly(A) polymerase I OS=Vibrio parahaemolyticus serotype O3:K6 (strain RIMD 2210633) OX=223926 GN=pcnB PE=3 SV=1

MNTNDYTPSEPATVPELALNVITREEHNISRKQISDNALKVLYRLNGAGFDAFLVGGGVR

DLLLGQKPKDFDIATNATPEQIKQLFRNCRLIGRRFRLAHIMFGRDIIEVATFRGHHQEQ

KNKNISQQSKEGMLLRDNVYGTIDEDAERRDFTINSMYYNIADYSIHDYARGIEDLEDRL

IRLIGDPETRYREDPVRMLRAVRFAVKLDFDIEEDTAAPIEEMAPLLREIPSARLFEESL

KLLQSGHGLETYHLLREYNLFQQLFPAISEHFTEDYSSHTEQMLDLVLDSTDMRIEDGKR

INPAFMFAAMLWYPLCALADKLMDQHNLCHYDAIMEASNIILDDQVRTIAIPRRHTATIR

EIWQLQLRLPRRNGKRAFRLMELNKFRAGFDFLEMRGEIEGGDTLKLAKWWETFQNAGRE

MRQAMAADLDGQAPKSGQRRRKTFRKKKSKPKS

>tr|Q87GV6|Q87GV6_VIBPA Protein translocase subunit SecD OS=Vibrio parahaemolyticus serotype O3:K6 (strain RIMD 2210633) OX=223926 GN=secD PE=3 SV=1

MKKTPKQQKNRKLMNHYSAWKYVVLITTVIILTLSAIPTWFGEQPSIQLTFADQNNSEMS

VVHLNQLLKTNHISADKIIEKDGKTTIVFDGEDAQSKARALLNKELNKDDSITFSYVSVA

PEWLNEMGFSPIKLGLDLRGGVQFLLNVDVNKAFEEQRDALIDEINASRDQRVRGVQVRA

ESGNRITVNSDSEQALSEVNKFLRQNYPGWISKSSSRGFVLEPSEQNIQEFQSSTLQQNL

KIMRGRIEELGITEALVQRQGKESIRIELPGVQDPAQAKNVIGATASLAFYEVKDASQAH

SSQDLVLQDNDGRTVILAKRPVLTGEHIVNARAGVDKMGMSEVNISLDHAGGKKMSDFSA

THIGKPMATVYREYKTNDRGVTERSERVISVATIQSQLGSQFRITGAGSMDDAQQLALLL

RAGSLTAPVTIVEERTIGASLGAENIENGFAALALGMGLTLTFMALWYRRLGWVANVALC

ANMLCLLGLIALLPGAVLTLPGIAGLVLTVGMAVDTNVIIFERIKDKMREGRSFAQAIDL

GFDSAFSTILDANITTMITAVILYAIGNGPIQGFALTLGLGLLTSMFTGVFASRAMINLI

WGRDARRDVRV

>tr|Q87PX1|Q87PX1_VIBPA Putative homoserine/homoserine lactone efflux protein OS=Vibrio parahaemolyticus serotype O3:K6 (strain RIMD 2210633) OX=223926 GN=VP1379 PE=4 SV=1

MASNHDWKGAPMQLDTWIYYTLAILVLTASPGPSSLLCLSKGVSSGFRLALTTALGSLSA

ITIILTLSFTGLGVVIASSEFVFNIIKWCGAAYLIWLGIQAFRSKQNDFAKSDSAQVSTS

HVSAYTSGFIVGSSNPKAIIFFTALFPQFIDPTASLLTQYAIFAGTFVVFELSWLTFYAL

LGVKTSNWLFEAGRAKLFNRLTGGVFISAGVMLSTANRS

>tr|Q87HW1|Q87HW1_VIBPA ATP-dependent RNA helicase, DEAD box family OS=Vibrio parahaemolyticus serotype O3:K6 (strain RIMD 2210633) OX=223926 GN=VPA0845 PE=3 SV=1

MRFIMSVNFADLGIEQQLVETLNNMNIVTPTPVQEKSIPHVLEGKDLLAAAQTGTGKTAA

FGLPIIQAVQQKKRNGTPHALILVPTRELAQQVFDNLTQYAEHTDLRIVCVYGGTSIGVQ

KNKLEEGADILIATPGRLLDHLFNGNVNISKTGVLVLDEADRMLDMGFWPDLQRILRRLP

NDKQIMLFSATFEKRIKTIAYKLMDSPVEVEVSPANTTAETVKQMVYPVDKKRKRELLAY

LIGSRNWQQVLVFTKTKQGSDELAKELKLDGIKAVSINGDKSQGARQRALDEFKQGKVRA

LIATDVAARGLDIQELEQVVNFDMPFKAEDYVHRIGRTGRAGKSGLAVSLMSRDEEYLLR

AIETLLDQRLPQEWLEGFEPSLIEEVEPERDGGGRRKSRSSEKRKLKAKLAIHKNRGKHR

K

>tr|Q87P14|Q87P14_VIBPA Sigma-54 dependent transcriptional regulator OS=Vibrio parahaemolyticus serotype O3:K6 (strain RIMD 2210633) OX=223926 GN=VP1704 PE=4 SV=1

MELQHISDNNWLSTSWIRSEQAGLKQRRRPEDIRVTPATLQDRRHQLNFLLETVTQFALP

LFNQLFAHSDSRLILTDADGVIIGSWGQPKFREKLTEIALSSGACWQEKVKGTNAIGTAI

IEAKPVSVIGDQHFIQHHRFISCSANPIFDHLGHLIGVLDITSEQKKHDFSTQVLVQNMV

QQVENQLLNLIPQGHIRVDLACEKGLLNSGWQGIIIANEDGQILAHNQVASQLLAQQNVI

GQSLDDILSIQSADHPFVFKTKPLTDKKVKSRSVTASNDLHYGDSTVEHCWQQANRVIDK

DISLLILGETGVGKNEFVKALHKNSQRKTGPLVSVNCGALPKDLVESELFGYVAGAFTGA

NSKGYQGKIRQAHKGILFLDEIADLPLEAQSRLLHVLQDKTVLPVGSNQSVQVDTQIIAA

THKDLDSLVSEGLFRQDLYYRLNGLIIELPRFEERDDKQQLIENIHRRHAESEQQLCPHL

LSLLLSYSWPGNLRELDSLIKVSALMAQGEETLELAHVPTHLSKKLSQAQDVTTAAEPTL

KLRATVEDKLLKTYQANQGNISKTSRMLGVSRNTIYRKLKGLGILG

>tr|Q87LC5|Q87LC5_VIBPA Cytoplasmic axial filament protein OS=Vibrio parahaemolyticus serotype O3:K6 (strain RIMD 2210633) OX=223926 GN=VP2687 PE=4 SV=1

MSAELLLNVTPSETRVAMIEGGVLQEIHIERESRRGIVGNIYKGKVSRVLPGMQAAFVDI

GLDKAAFLHASDIVPHTECVAENEKQQFQVRDISELVRQGQDIVVQVVKDPLGTKGARLT

TDITLPSRYLVFMPGASHVGVSQRIESESERERLKKVVNHYCDEHGGFIIRTAAEGADEK

ELSQDAAFLKRLWLKVMERRSKYKTRSTLYGELGLAQRILRDFVGTELDKILVDSRLEYE

NLKEFTSEYVPELTDKLELYEGDKPIFDMYDTENEIQRSLERKVELKSGGYLIIDQTEAM

TTIDINTGAFVGRRNLEETIFNTNIEATQAIARQLRLRNLGGIIIIDFIDMASEEHRQRV

LTSLEAALSKDRVKTNINGFTQLGLVEMTRKRTRESIEHILCSGCPTCEGRGTVKTVETV

CYEILREITRVNRAYDADNFVVYASPFVAEALLGDESHALAELEVFIGKQVRIQAEPLYI

QEQFDVVMM

>tr|Q87MY1|Q87MY1_VIBPA Molybdenum cofactor biosynthesis protein B OS=Vibrio parahaemolyticus serotype O3:K6 (strain RIMD 2210633) OX=223926 GN=VP2095 PE=3 SV=1

MGHAESKFQPANIAVLTVSDTRTEENDTSGRYLVEHAQEAGHNVVDKQIVIDDMYKIRAI

VSQWIADENIQAIMITGGTGFTSRDSTPEALKPLFDKEVEGFGELFRMVSYEEIGTSTIQ

SRAIAGFANHTVIFAMPGSTGACRTGWTKIIKQQLDASHRPCNFMPHLSV

>tr|Q87NP2|Q87NP2_VIBPA Uncharacterized protein OS=Vibrio parahaemolyticus serotype O3:K6 (strain RIMD 2210633) OX=223926 GN=VP1826 PE=4 SV=1

MKLSDFSIEALKGFVTGDDSDAPYMSGPDLVKFFNLFGVRDVYSFQNGGLPESASRKDYA

LKTVKSLNGTNQMKLMVEGLVDSRRSENYAELAVGINEIIKHDGYSLEQNSLGVFIVSGC

NIEDSTVDVEAYFQEIRQRILESILKAKFSIWVAMAWFTDKEIGNALLNKHRDGLNIQVI

VNDDSTTSKYGLDFSSKGIEYYKIAPSSPWGKKIMHNKFCVIDFKKVVHGSYNWTKNAQY

NNESITITDSRELAEDFAEQFIALKQAHKSAHA

>tr|Q87P38|Q87P38_VIBPA Uncharacterized protein OS=Vibrio parahaemolyticus serotype O3:K6 (strain RIMD 2210633) OX=223926 GN=VP1680 PE=4 SV=1

MVNTTQKISQSPVPDLEQFRAIAAQKDDRVISKRGEVKEPSTFHKGHKFASVSEGVLRKK

YTKFFQENIKTHLDLKQALLKEEKPETALLAYSLVSPSGYRGEPLTERKILEVVSLLDEV

KVEGDTYQQLKNTFDSISKDPRMQVSLENQYPGKMDGFGAQLLEMGKEKLKGSGVNAAIN

LALPGVGLLVATGRELHKASVNGDAEAYHHQLEQISQLPGRDQRLSMPMQQTLAIGHAML

SAEGAVGATLGMATGGLGTFGVSSVATAGVTPIAKEAIGTALATGIISGGGFVAGQAGAY

GLNNEVQDQLKQGPMSGVLPRLEISNVKGDFTFSMQEPAAVRALMAYLGPKEDTSMSSPQ

APKEAQEMEAARLTLKQMLGSSPNEHLVPDVDSLLKLSDEDMPSQTESTANGAFKKLLSE

DWDWLMPAVRAMDKGEANKINEKLTYKLPLDAANGRVYLDKSPNLSGAQLDALDKLGSPS

QLRLMYLAEGWI

>tr|Q87P43|Q87P43_VIBPA Translocation protein in type III secretion OS=Vibrio parahaemolyticus serotype O3:K6 (strain RIMD 2210633) OX=223926 GN=VP1675 PE=4 SV=1

MSGEKTEQPTAKKLRDARKKGQVAKSQEIVSSALILALIAVLFAFADYYMSHISALLLLP

SELAYQGFQDALIDVAIAIAKEIAYLLAPIILVAALIAIFSNMGQFGFLFSGESIKPDIK

KINPVEGAKRIFSLKSVIEFIKSILKVSLLSCIIWVTLRGNINTLMQIPTCGLECVPAVT

GVMIKQLMIISSVGFVVIAAADFAYQKFDHTKKLKMSKDEVKREYKEMEGSPEIKSKRRQ

LHQELQASNQRENVKRSNVLVTNPTHIAVGLYYKKGETPLPVITLMETDAMAKRMIAIAR

EEGVPVMQKVPLARALYADGNVDQYIPSELIEATAEVLRWLASLESDGTQR

>tr|Q87IS9|Q87IS9_VIBPA Outer membrane protein N, non-specific porin OS=Vibrio parahaemolyticus serotype O3:K6 (strain RIMD 2210633) OX=223926 GN=VPA0527 PE=4 SV=1

MNKKFLAAAIAAATFGTQAVAVELYNNDGTTFSIGGHVSVNLNGSEQGDTDVGTNSPRIN

FTATQDLGNGFTADARGEWALNYLDGGENSFKTRLGYLGLTHDTYGRAVGGTQWAPYYDV

AGVADMPIAFANDFIYDDHGNLGTGRAEKMISYRNAIELGEAGAINFGLGWQGSKDEFAR

DENNQIVGGADYDDRLQVALSYSIMGAKIGYTYNGGDINQGLRSESAESHLVSAAYGSYG

QGLYLAAVYGSNENMNFDGKTRLAESDAYEALIAYALPNSLNLSINYEMVEGKELKGAKT

ETAREEMALQAEYNFTPKFVGYAGYQFDLNDANDRKTDDKWALGARYYL

>tr|Q87HU7|Q87HU7_VIBPA Putative lipase OS=Vibrio parahaemolyticus serotype O3:K6 (strain RIMD 2210633) OX=223926 GN=VPA0859 PE=4 SV=1

MKHTFKLSLLCSAILLAGCGDNTSSSGTTDKVTFESEVQALLDRGTSISFTIQGANADVP

APSYLLMDTSDGTLGLPTNGDDALTNPRASMNTMDGWSTSMPIVLNFNGDGFAPGMLASG

VKVIKINQRLTEWDGKTNPIAKVLVQNQDYVVQANGKSLYVQFADSLDESSEYIFAVTQD

VTDVNGEPIGTSSSYAVAKSKQVKYESGDLASVQAVTEAVEGIFGLAQVNPADIVYSSWF

STQSVGDTLAAVKGVTATGFATGANTLNTIYKNDTNENAVDLATAYTMVLGETQDFMTAL

DSDSDFNKYVSDLDAAKTAIKGLYVQSGASVNVTQGQVRLPHYLEKGANWNMQPMVSAMP

SLAKLNAALTDPAEQANMVQQLSSGVFASSPVDVSKLATDPTEQLKLVGSKLYLNDGTQL

DSERVITRYSPVPEVKSLENVEFLLFTPQNGVAKDVVIYQHGITSAKENAYAFAFNMVQT

GVAVIAIDLPIHGTRSLDEQRSANANVLAYLNLSNLAVARDNLRQSVLDVLGLRASLVVS

AQGGLLAGGPLQGFNPMTGSQVKMLGHSLGGIVGTSAVAAANNTLGSPTADALYTFSAAS

IQNSGGQIGNLLLGSSDFGPQIKHNLAYAASTDYKSYADAQCAQLDDKACYEVFEGLATP

EQLAALSAGFSQFIYAAQTTLDTVDPFTNAADLVASGTLTTPFLMTEVEGDKTVPNNVAN

APFAGTEPLAKKLGLTEVNSLNTAVAATSSFVQFNAIASHSTFASPSGTLADVNHHAEMQ

KENADFLMDNALSDVSDTTVLK

>tr|Q87KF7|Q87KF7_VIBPA Transcriptional regulator, LysR family OS=Vibrio parahaemolyticus serotype O3:K6 (strain RIMD 2210633) OX=223926 GN=VP3020 PE=4 SV=1

MNIEKLARLDLNLLVCFKVLIEELNVTRAAHRLCLSQSAVSKSLAKLRIQFDDPLFTRNS

HGLTPTPRALFLKPKLDLLINQLEVLTQPEEFSPQSSEYRFQIAAVESVYPLILPHFLPA

IFQQAPGVTISTHPWSDNTFKMLQRGELDLGLTGKDIDINDAKLTLLPPDDICELEIYRD

HQMCIIRKNHPALKQKWNLEAYLALRHVQVRCDGNDRWLLDYRLADIGAERDIAVTVPDF

NSAASLCSYTDFIFTAPSHFVQLAAKQLDLVVLPLPLEFPPMAYTLFWHRDRENDPALNW

LRTMITQKTDHLR

>tr|Q87HC8|Q87HC8_VIBPA Probable phosphoprotein phosphatase OS=Vibrio parahaemolyticus serotype O3:K6 (strain RIMD 2210633) OX=223926 GN=VPA1037 PE=4 SV=1

MNWNYFTFAKTHAGKVRPYNEDALLDMTEQHIWVVADGMGGHSAGDIASQMLVDRIARHM

QDVPNTNIDDIRAAVQQANFEIYQYAQMNLDGKTMGTTLVLLFIQDGYYHCLWVGDSRIY

QLRNGTFVQKTRDHSQVMELVEQGLLAFEDAESHPLANVITRAVGVDEMVTIDQVSGQLA

DGDQFLLCSDGLSKELSCDEMHKTLCADSVNQAGLALMHSALVRGASDNVTCALIKVRQE

PSEFDSARRDDATIPVFLRGRV

>tr|Q87NE6|Q87NE6_VIBPA Uncharacterized protein OS=Vibrio parahaemolyticus serotype O3:K6 (strain RIMD 2210633) OX=223926 GN=VP1922 PE=4 SV=1

MDSVILQLKKDFYSQIRAFQTPKLPQVTSTLAVLTDEEIQELEAVWIELAVWKRSQTH

>tr|Q87J38|Q87J38_VIBPA Uncharacterized protein OS=Vibrio parahaemolyticus serotype O3:K6 (strain RIMD 2210633) OX=223926 GN=VPA0415 PE=4 SV=1

MNYAIEFHDANYPFLNVAARKKSLKHSLLSVVSGLAIIKLGKQEYAIEPGQYFWIPQGCL

SSLTFLPNTHVHRCDFSVRLNDAFAQQAGFIKPSILLKALIEKLAKTETRSDIQLDLLAV

VKHEVLAISPNLSNSALSQAINQWKPGCEARISRELCLVLTLREARKMKLSGKKESQIVE

HLFAGNTEEYEQLCFLVFGEAL

>tr|Q87QH3|Q87QH3_VIBPA Putative multidrug resistance protein OS=Vibrio parahaemolyticus serotype O3:K6 (strain RIMD 2210633) OX=223926 GN=VP1176 PE=3 SV=1

MRKLLIPIAVTAALSGCGKELPPVPEPESRPAKLFTVSVGNAQFERHFPATTEAGDRAVL

AFRVPGLLQSIEVNEGQVVKKGDVLAVLNPDEYSLLEKQARANFALADVQYKRYKKLRKD

QVVSEQDFDEAKANHNSAKAQWDQAKANLRYTQLVAPYDGTISYLPAENHEYVAAKEGVM

NIQTNQVMKVIFQLPDYLLNRYTQGVNVSAKMIFDAFPERSFDLTFQEIDTEADPKTGSY

KVTMVMERPPELGILPGMSGNAYLVSQNSGATKIPDSALFEENGKTYVWRVDDQGVVAKA

DITMNDNGQILQGLADGDQIIISGINVIEPGIKVRAWVKERGL

>tr|Q87RB5|Q87RB5_VIBPA Putative beta-ketoacyl-ACP reductase OS=Vibrio parahaemolyticus serotype O3:K6 (strain RIMD 2210633) OX=223926 GN=VP0882 PE=4 SV=1

MTRQVLVTGASKGIGKAIAVQLAKDGFQIIVHYMGDQQGAQDTLDTIEQHGGSGRLIQFD

ISDRADCREKLEADIAEHGAYYGVVNNAGITKDTAFPAMTEEEWDGVIHTNLDSFYNVLH

PCVMPMVQKRKGGRIVTLASVSGLMGNRGQTNYSAAKAGVIGATKSLALELAKRKITVNC

VAPGLIDTGMVDEHVKEHAMPQIPLRRMGEPEEVAGLVSYLMSDIAGYVTRQVISVNGGL

V

>tr|Q87SN5|Q87SN5_VIBPA Putative HsdS polypeptide, part of CfrA family OS=Vibrio parahaemolyticus serotype O3:K6 (strain RIMD 2210633) OX=223926 GN=VP0387 PE=4 SV=1

MNKLITEHIDIWTSAVKTKSISGRGSSKKLELYGIKKLRELIIELAIQGKLTASLDFKGS

AKKSVEDAIVAKFNLSKERNIRGDKETYSSDRAKGFPLHWETICVGQVAHVLGGKRVPKG

YKLSEQPTDFVYLRVTDMKNQSIDESDLRYISEEVFKQISRYTINTGDVYVTIAGTIGAV

GTIPPHLDGMSLTENAAKLVFSGLSKKYLVTVLQSSFVTRQFNDAVNQMAQPKLSLNSIK

HTCIPIPPLEEQEYIADKVDELMALCDQLEQQTEASIEAHQVLVTTLLDTLTNSADADEL

MQNWARISEHFDTLFTTEESIDQLKQTILQLAVMGKLVPQDPSDEPAAELLKRIAEEKAQ

LVKEKKIKKQKALPPIAEDEKPFELPSGWEWCRLDDICFGITSGSTPPKVNFNESEGIPY

LKVYNIREQKIDFEYKPQFVDNDCHKTKLARSVLYPGDVVMNIVGPPLGKIAIIPDTYPE

WNCNQAITFFRPIVPQLNKYIYTYLTAGSFLDSIELIGTAGQDNISVTKSRSILLPTPPL

REQKRIVNKVHELFLLCNSLKMRLRKRQELKLCITDTIVEQAV

>tr|Q87TG2|Q87TG2_VIBPA DNA polymerase I OS=Vibrio parahaemolyticus serotype O3:K6 (strain RIMD 2210633) OX=223926 GN=polA PE=3 SV=1

MASIPENPLILIDGSSYLYRAFHAYPGTMSNGEIPTNAVYGVVNMLRSMMRQFASERIAV

VFDAKGKTFRDEMYSEYKANRPPMPDDLRCQIEPLHNVIRAMGLPLICVPGVEADDVIGT

LAYQASQQGMPVLISTGDKDMAQLVDDNITLINTMTNVVMDREGVVEKFGIPPELIIDYL

ALMGDKVDNIPGVPGVGDKTATALLQGIGGLTKLYENLDDIAALGFRGSKTMAKKLVDNK

DNAMLSYELATIKLDVELEETPESLLKAEPNKDELIKLYGQLTFKSWLNELLEGGSGTVE

AVELAGSAQASSSSSSHAEMETSAVTIDRSQYETILDEASFNAWLEKLKAAELFAFDTET

DSLDYMVANLVGLSFAIDEGIAAYVPVAHDYLDAPEQLDRDWVLEQLKPILEDDAQAKVG

QNLKYDASVLARYGIEMKGIKYDTMLASYVYNSVGGKHDMDSLALRFLQHSCISFEQIAG

KGKNQLTFNQIELEQASPYAAEDADVTLRLHNRLFANIEQDEKLKSVYEEIEMPLVPVLS

RIERTGVLIDDMKLSAQSVEIAARLEELEQKAYEIAEQEFNMNSPKQLQAILFEKMGLPV

VKKTPSGTPSTNEEVLQELALDYPLPKLILEYRGLAKLKSTYTDKLPKMINPSTGRVHTS

YHQAVTATGRLSSTDPNLQNIPIRNEEGRRIRQAFVAPAGYKVLAVDYSQIELRIMAHLS

GDQALLDAFRDGKDIHAATAAEIMGVSIDQVSSEQRRRAKAVNFGLIYGMSAFGLAKQLG

IPRGEAQAYMDKYFERYPGVMQYMEDTRSAAADKGYVETIFGRRLHLPEIKSRNGMRRKA

AERAAINAPMQGTAADIIKKAMLLVDQWIQEEGNGRVKLLMQVHDELVFEVEESSLSEIE

SKVQKLMESAAELKVPLVAEAGHGDNWDQAH

>tr|Q87NZ0|Q87NZ0_VIBPA ABC superfamily (Glycine/betaine/proline transport protein) OS=Vibrio parahaemolyticus serotype O3:K6 (strain RIMD 2210633) OX=223926 GN=VP1728 PE=4 SV=1

MNNSWKKALSVGALSAIAFSTYGIAGELPGAGVAVQPVQSTVAEETFQTLIVNRALEALG

YDVQPTKEVDYNVGYTSIAKGDATFLAVGWFPLHADKYTMSGGDDKFFREGQYVSGAAQG

YLIDKKTAEKYGITNIGQLKDPKLAKLFDANGDGKADLTGCNPGWGCEMVVEHQLDAFKL

RDTVTHNQGNYAAIIAYTISRYKKGDPILYYTWTPYWVSGVLVPGKDVVWLEVPFSSLPG

ERKDIDTTLKNGKNYGFEMNSMRIVANKEFTKKNPSAAKLFEIIKLNINDVSAQNMMMSQ

GKNSAADIEAHVNGWIKANQTTFDGWIEEAKKAAM

>tr|Q87JT5|Q87JT5_VIBPA Putative ATP-binding component of ABC transporter OS=Vibrio parahaemolyticus serotype O3:K6 (strain RIMD 2210633) OX=223926 GN=VPA0163 PE=4 SV=1

MLGPSISIDNLSLQYGDNVILQNVTTTFEPGKCHVIMGPNGGGKTSLLRSVLGLTPFTGD

ISVHWPNKTHNKLGGNVGYVPQKAMFEASLPLTVMDFVLLNQTRIPLFWRRKTKQTQRAL

VQLDRVGMAARSDRRMGQLSGGEQQRVLFAQALLDDPSLLVLDEPTTGMDEQGVRYLEGL

IHEVVAQGKTVLAVHHDVTAVRRLDAEVHVVNRQIVASGHHSDVLSPDRIETLFKHYTTK

VEAA

>tr|Q87LA6|Q87LA6_VIBPA MSHA biogenesis protein MshJ OS=Vibrio parahaemolyticus serotype O3:K6 (strain RIMD 2210633) OX=223926 GN=VP2706 PE=4 SV=1

MNEFWSSLEERFGDMSAREKMLVALCGFVTVVMLLFTLVLEPKLNQISNNERQLSNLKQS

NQKTEIDILRVKAQLKKDPNADIDLEISNLLTESQHLSMQLSQIIEHLVTPSQMAGVLES

VLEQQSGIHLVSLQTLPSEPITEDKEASPYSGYYVHPVRMELTGDYFSIANYLNKLESLP

ASYFWRSFSYKVEEYPKAKLVLEVYTLGSREEFIGG

>tr|Q87KE6|Q87KE6_VIBPA Carbonic anhydrase, family 3 OS=Vibrio parahaemolyticus serotype O3:K6 (strain RIMD 2210633) OX=223926 GN=VP3031 PE=4 SV=1

MSSIRSYKGIKPQLGERVYVDSTSVLVGDIRIGDDSSIWPLVAARGDVNHIHIGDRTNIQ

DGSVLHVTHKNSENPNGYPLIIGNDVTIGHKVMLHGCEIHDRVLVGMGAIVLDAVVIESE

VMIGAGSLVPPGKRLESGYLYVGSPVKQARPLSETERAFLQKSANNYVQNKNDYLHEVED

LN

>tr|Q87GA4|Q87GA4_VIBPA Uncharacterized protein OS=Vibrio parahaemolyticus serotype O3:K6 (strain RIMD 2210633) OX=223926 GN=VPA1413 PE=4 SV=1

MTSTIARLGFISISLLTIGLSLWKSSELNHAVYLNMENYVGGSSTLHFTFSMLIGFLAVF

TFPRFTHATKMDAFGIRLLFCLLLIISAEEFSQLFIESRSFSFDDLSTNWIGMILGYFGA

KAITLFRTSRSRA

>tr|Q87Q94|Q87Q94_VIBPA Transporter, NadC family OS=Vibrio parahaemolyticus serotype O3:K6 (strain RIMD 2210633) OX=223926 GN=VP1256 PE=4 SV=1

MTALAVTLKNWFFTRNSMILNANILLFIILFNTLPFEPQVVTGISILVFVAILWLTEAIH

VSITALLIPMLAVFLGVFNTQAALNNFSNSIIFLFLGGFALAAALHKQKLDQALADKVLL

IARGRMSVAVFMLFGVSAGLSMWISNTATAAMMLPLVLGVMTKLDAKKNHNTFLFVLLGI

AYSASIGGIATLVGSPPNAIAAAEVGLNFTEWMKLGLPISLILMPIAILVLYTMTKPDLS

HKFELDHKPVEWTNGKMVTLAIFLLTVTLWIFSKPINTMLGGFAKFDTLVAIGAILLLGA

SRAVEWKDIEKTTDWGVLILFGGGICLSNVLKATGTSVFLAHSLTGFLEQAGVLLTILSV

VAFVVFLTEFASNTASAALLVPVFATIAEALGLSPVILSALIAVAASCAFMLPVATPPNA

IVFGTGHIKQKEMMRIGFVLNIACIGALTLFAWLFW

>tr|Q87SR9|Q87SR9_VIBPA Acetolactate synthase III, small subunit OS=Vibrio parahaemolyticus serotype O3:K6 (strain RIMD 2210633) OX=223926 GN=VP0353 PE=4 SV=1

MRHIISLLLENQPGALSRVVGLFSQRGYNIESLTVSPTDDETLSRLNITTTSDEMQLEQI

QKQLHKLIDVLKVQEVTEFEHIERELMMVKVKASGFARAEVKRTADIFRGQIVDVTASQY

TVQLAGTSEKLDAFIQAISEVTEVVEVARSGVVGIARGERALKP

>tr|Q87SS4|Q87SS4_VIBPA Uncharacterized protein OS=Vibrio parahaemolyticus serotype O3:K6 (strain RIMD 2210633) OX=223926 GN=VP0348 PE=4 SV=1

MRTIETITQPIATIAKFDLAFSSVCLQHRFNQQVANISKGVSHSGDGHLYVVLGLLAWYL

DKEHGSWFMLAGLLAFAVELPIYWTLKNSFKRRRPEELSALLPAFITPSDRYSLPSGHTA

AAFVMATLINHYYPELGYAAFFWASLIGTARILLGVHFFTDVIIGALLGSACGALAIAIV

GG

>tr|Q87HT0|Q87HT0_VIBPA Uncharacterized protein OS=Vibrio parahaemolyticus serotype O3:K6 (strain RIMD 2210633) OX=223926 GN=VPA0876 PE=4 SV=1

MHQTKFSIDLVSECSILIRFDETISENSIGQLARAMNQHLSHIVMNIVPSYRTVLIDYLP

FRISEFQLVDILHNILVAFDPNKADKAEPNEISIPVYYSEETALDLERFEEQGIDLGALI

DAHTKPIYTVSAIGFAPGFAFLSDVEESIAMPRLKTPRTFVPSGSVGIADSKTAVYPSDS

PGGWNIIGRSPLSLFSDTPPFIPFEVGDKVTFYTVSKQEFLELGGSL

>tr|Q87GW1|Q87GW1_VIBPA Acetyl-CoA acetyltransferase OS=Vibrio parahaemolyticus serotype O3:K6 (strain RIMD 2210633) OX=223926 GN=VPA1204 PE=3 SV=1

MEKVFIVAAKRTPIGAFGGSLKNTSAGDLAAVAIKGALEAAKLAGDKVDEVIVGNVVGAG

QGMGVGRQAALFAGIPESVPAYGVNMVCGSGMKTVMDAVSHIRSGDAEIVVAAGVEVMSQ

IPFAAPSSIRDGNKMGNLELKDLLVADGLTDVYNQYHMGVTAENVAKEIGLTRQQQDEYA

LSSQQKAVAAIEAGKFKDEIVPVEVQQRRETVLFDTDEYPKANATMEALSKLRPAFDREG

TVTAGNASGINDGASAIIVASESAVKAHGLTPLAEIVSYAQSGLDPKIMGLGPVESVNKA

LAKAELTVNDIDVYELNEAFAAQALGVIHQLAETNQVPVNAIQDKANFNGGAIALGHPLG

ASGNRILVTLVHELHKQNGQYGVASLCVGGGMGTAVVVKAVK

>tr|Q87KW8|Q87KW8_VIBPA Putative periplasmic protein CpxP OS=Vibrio parahaemolyticus serotype O3:K6 (strain RIMD 2210633) OX=223926 GN=VP2857 PE=4 SV=1

MKSAKKLVLAAVVLPLTLGTASAFAFGGKDHHKGPRDECGMGMDRGIMRDLNLTDAQKDQ

LQSFRDANRAEMKGKYSQNREARMAERQAHHAKMQSLLLADTFDEAQATALAKEMVERQT

EHRVKMLERKHQMLSVLTPEQKAEFVKLQNERMQECGDQMQQRMGKHRNN

>tr|Q87HU5|Q87HU5_VIBPA Uncharacterized protein OS=Vibrio parahaemolyticus serotype O3:K6 (strain RIMD 2210633) OX=223926 GN=VPA0861 PE=3 SV=1

MKVWIKASALLTLAGLTACSASPTGRNQILMFSDQEMSSLGAKSFDQMKKDIPISKDKKT

NAYVQCVAKQITNAIPPQAGFKDWEVVVFDSDQVNAFALPGGKIGVYTGLLKVAKNQDQL

ATVIGHEVAHVLAEHSNERLSQSQLANAGLSLANVAIGASEYKQYQQLTMAALGVGVQYG

VILPYGRTQESEADIVGLEYMAKAGFNPNQSVDLWKNMSAASGGAQPPEFFSTHPSHSTR

IKDLQATINKLPKYNVKAPKCG

>tr|Q87RZ2|Q87RZ2_VIBPA Putative Na+/H+ antiporter OS=Vibrio parahaemolyticus serotype O3:K6 (strain RIMD 2210633) OX=223926 GN=VP0632 PE=4 SV=1

MTIKVGEISLLTSLIIIFVVYMSGSQQTTKTVAPSAVALIPLIVFLALFIGVGTYLSLQG

VEFAFYQLPAPIAVLPAIIIAFLLSKEKLNRSIEHFMRGVGHQDIIAMCMIYLLAGAFAA

VAKASGGVDATVNLGLSAIPTSMILPGIFMISAFIATAMGTSMGTIAAVAPVALGIAQSA

GMSLPLTAGVVLSGAMFGDNLSIISDTTIAATRSQGCEMKDKFKENIRIALPAALVAMGI

FAFNSTATQVPETGPIEWLKVLPYVTILILAVSGLNVFVVLTIGILLAGGVSLVSIDDYG

LTNLAQDVYSGFGNMQEIFLLSMLIGGLSELMRRQGGLAFLTNLVSSMIHAFGSSHSKHA

NSRASELGIAGLVSMVNGCTANNTVAIIVSGSVARQLAEENNVSPRRSASLLDIFSCVVQ

GILPYGAQVLLLGSVFNLSPLEVVANSYYCFALAIAAIVAVFIKHPARQPAVQTEN

>tr|Q87K34|Q87K34_VIBPA Uncharacterized protein OS=Vibrio parahaemolyticus serotype O3:K6 (strain RIMD 2210633) OX=223926 GN=VPA0064 PE=4 SV=1

MKKFTLIAASVLMASPVLAANHSPLTLEVYNADGNSFHVNSTLVYGETDAMVIDTGFTKA

DAMRIAAKVLDSGKTLKTIFISQADPDYYFGAEALHQQFPDAQIIATPAVQKIIKEKLAG

KLAYWGPKLGANAPVKPVIPVAYDKASLELEGHKIEIRGNHGTSAHRPYLWIPDNKAILG

NVAVYSNVHLWMADAADQTAINAWEQQLSEMLALKPQVVIPGHMKAGTKLNADTIRYSQQ

YLQDFQQAKKHSNNSAQLIDTMSAKYPEAQLPIALEIGAKVHTGEMSW

>tr|Q87TG4|Q87TG4_VIBPA Uncharacterized protein OS=Vibrio parahaemolyticus serotype O3:K6 (strain RIMD 2210633) OX=223926 GN=VP0105 PE=4 SV=1

MLIREGTLEEALQVVTRVKEFANGETLDSMSQRLGDKNSLILVAEKNGAIVGFKIGYELD

EDTFYSWFGGVAPQARNEGVAQMLLEAQEEWVAEQGYKTLKVKSRNQFPAMLRLLLRNGY

LIEKFEEKINLRESRVHFIKAM

>tr|Q87MT5|Q87MT5_VIBPA Putative oxidoreductase protein OS=Vibrio parahaemolyticus serotype O3:K6 (strain RIMD 2210633) OX=223926 GN=VP2146 PE=3 SV=1

MAHSVFITGANRGIGLSLTELYLERGWNVHATSRNVTDSDELQALRARYSSLSLHELDVT

DYAKIALLAQSLPPIDLLINNAGYYGPKGYGFGNTDVDEWRKVLEINTIAPLKLVETLYP

QLQQGQLKKIACISSKVGSMTENTSGGGYIYRSSKAALNSVVKSLSNDLTPDGFTVLALH

PGWVRTTMGGPNALIDAETSAQGLAQVIDQSTVEHSGQFINYDGTSLPW

>tr|Q87ML8|Q87ML8_VIBPA Long-chain fatty acid transport protein OS=Vibrio parahaemolyticus serotype O3:K6 (strain RIMD 2210633) OX=223926 GN=VP2213 PE=4 SV=1

MKTNKTFLSTAVALSLLGTSGMANAAGFQLAEYSATGLGRAYAGEAAMADNASSQWRNPA

MLTYLEGTQVSVGAIYVNPNIDINGQTTLKHPVNQSVINSSSTSSDDFAHDAIIPNFYLS

HQYNEQFAIGLAFGTNYGMETDLGKGFAASHFGNEASVTTMEANLNAAYKINDAVSIGGG

IRYIIGEGSFGATTPKVNVLQKPQGTTLKYMEGDDTAWGWQLGTAWQINENNRIGFTYKS

EVELKLEGHAEGLGFGFGTPKLPQLRDNGYMYLNLPATAELASFHQVTDQLALHASFNWT

DWSSFEKLEAHLETAGTHMVKVENWEDNYRFAVGATYQLQPKVALRTGIAYDTSAVSDKN

RTITIPETDRTWLSIGATYDWTKDFSLDAGFTYIIAKDAPIVESRGYKSDDSAEVVGGQF

VGETTGNVWLIGVQANYRF

>tr|Q87IE9|Q87IE9_VIBPA Iron(III) ABC transporter, periplasmic iron-compound-binding protein OS=Vibrio parahaemolyticus serotype O3:K6 (strain RIMD 2210633) OX=223926 GN=VPA0657 PE=4 SV=1

MKKLVSAAVFSLLTISPSFAANVVIDHYMGKTELEQSPKRVVVIGFGPLDALDNFGIDPV

AVSNASHLPSYLSKYSKENYTSAGSLFEPDFEAIYMQKPDLILVGPRGSAKYEELSEIAP

TVVFAAKEGEGYWEGTQAQWRNIGKIFNIEDKVEQKIETLDAQFKAIRDYNQTNNNDALT

ILSIGDNISAFGAKSRFGAIYNDFGFIETVKNIKTGTHGDVISYEFIREADPKNILVIDR

NSLHAKPSQDLRKSMDNDLVKATTAYKNQKITFLDVDAWYLAMSGVTATEKMVNEIKNTV

DL

>tr|Q87FJ4|Q87FJ4_VIBPA Putative lactoylglutathione lyase OS=Vibrio parahaemolyticus serotype O3:K6 (strain RIMD 2210633) OX=223926 GN=VPA1685 PE=4 SV=1

MIQLEHINLVVKNIPEMLKFYQTAFPHWYVRDEGEGTWSGKHRNWLHFGDEYQYIAMSDH

GEGENRELGGHQVGLAHFAYVTNNVDAIIKRLTDAGYPIAQPGADEPYRKNVYFVDPAGF

EIEFVEYLADDPKLRNLTS

>tr|Q87QN9|Q87QN9_VIBPA Uncharacterized protein OS=Vibrio parahaemolyticus serotype O3:K6 (strain RIMD 2210633) OX=223926 GN=VP1110 PE=4 SV=1

MKSLFLLLGLSSSSLFTNPISIEPFYSPYNNSDRFFVEKVTFLTQDNVRAAYGAEWYFEF

PLKSPSEEVSVAKSCSELEQKSKKKFSAKMDSEYSALMATKVLCESWKVMGALKPSNTSY

ISDSIISERLPDISPSELSFIISDEQLRKSKEAHRWADMDRITSFERLNDFQAVFYDNSD

GYQKVTLLATGDYNDDGLEDVILLKENSVLSGSYGSTHGYVLSRLQENGSYELIKEW

>tr|Q87N37|Q87N37_VIBPA Transcriptional regulator, ROK family OS=Vibrio parahaemolyticus serotype O3:K6 (strain RIMD 2210633) OX=223926 GN=VP2038 PE=4 SV=1

MYMAQPGHIDHIKQVNAGRVYKLIDLKGPISRIDLSKQSELAPASITKITRELIEAHLIH

ETTVQEATSRGRPAVGLQVNNEGWQFLSMRLGRGYLTIALHELGGDVLIDTKIEIHERDQ

DDVLERLLYEIDDFFQTYADQLDRVTSIAITLPGLVNSEQGVVLQMPHYNVENLALGPEI

YKATGLPVFIANDTRAWALAEKLFGHSQENDNSVLISIHHGLGAGIILDGRVLQGRHGNI

GELGHIQIDPNGKRCHCGNIGCLETVASSQAIREEVVRRISEGEGSILADQEEMSIESIC

EAAANGDPLAVDVIEKLGRYLGSAIAIVINLFNPEKILIGGVINQAKEVLYPAVRRCIEE

QSLPVYHQDLELVESRFYKQATMPGAALIKQALYDGQLLMKVVEG

>tr|Q87QR0|Q87QR0_VIBPA Uncharacterized protein OS=Vibrio parahaemolyticus serotype O3:K6 (strain RIMD 2210633) OX=223926 GN=VP1090 PE=4 SV=1

MLEITLFCVQVDGSKSEQFDDKNGECLYRIWGSSPDLID

>tr|Q87GA1|Q87GA1_VIBPA Putative oxidoreductase OS=Vibrio parahaemolyticus serotype O3:K6 (strain RIMD 2210633) OX=223926 GN=VPA1416 PE=4 SV=1

MTNATNRRIVLASRPHGAPTTENFRLEEVAKPVPQAGEMLLRTVYLSLDPYMRGRMSDAE

SYAEPVALDDVMIGGTVCQVEASNHPDYEAGEWVLAYTVGWQDYAISTGEMVIKLGKEPQ

NPSYALGVAGMPGFTAYMGLLDIGQPKPGETIVVAAATGPVGATVGQIGKIKDCRVVGIA

GGEEKCRYAKEVLGFDECIDHKADDFEQQLKDACYNGIDVYFENVGGKVFEAVMPLLNTS

ARIPLCGLISQYNATELPEGTDHLPLLMGKLLTKRIKVQGFIIFDDYGHRYGEFAQDINQ

WLAEGKIQYREHLVEGLDNAPEAFIGLLEGKNFGKLVVKINDPL

>tr|Q87GH7|Q87GH7_VIBPA Putative type III secretion system EscC protein OS=Vibrio parahaemolyticus serotype O3:K6 (strain RIMD 2210633) OX=223926 GN=VPA1339 PE=3 SV=1

MRFSQLLLALCCLTFGAQADVFIKQEDMDLKQALEAIAKDMQLKLVSDIDSKTSSQPITQ

TLSGSGPMLLSKLSSVFDFDWYTFGGTLTIQSGQQYANYTYKPKNISPSQLLSEMKGAFK

TNSTTKIKLVERGHSLLFSGTRKFVNDAMGYASMVDRNQFLEKGNNLEVARIEFNYVSVM

DRNIATYDGQVNFPGAQSLIAAAITNIGQFTNLNDEELTSRAYRVKLSQGEKQALEEDEK

STKVQALPGTNALLIRGTQEEVKLAKRIAALIDVKRRQLLFSLKVYDISAERKENLGIDS

GWLNGNRGIYDIVVPPFTETKDFVKNFQALYSNGIARSVYETNLVALENQQSQFGKKQTA

TLTLISDKQVKTEKIEADNSLYVTGRLLPSGQVQARIQYVEESLDEDDNEDSNRPPRVSS

QSLNSEVYIQPNQTVILGGFDNTVTQQQESGVPIISSIPLLGELFKYTSESKHKYKRYVS

ISFQVIE

>tr|Q87KK4|Q87KK4_VIBPA Uncharacterized protein OS=Vibrio parahaemolyticus serotype O3:K6 (strain RIMD 2210633) OX=223926 GN=VP2973 PE=4 SV=1

MDTHVWLAYVVTAIVFSLAPGSGTVNSISNGLSYGTRKSLGAIVGLQIGLAIHIALVGAG

IGALVAQSATAFTIIKWVGAAYLVWLGIQKWRDTSGLIANESHKEMSGSALLYKAVLINL

TNPKSIVFLVALFPQFIDPAKDQLTQLLALGVTTVVIDACVMLGYTTLAAQMGRFIRSDK

IMGKINKVFGSMFMGCGALLAAAKS

>tr|Q87IY8|Q87IY8_VIBPA Prolyl endopeptidase OS=Vibrio parahaemolyticus serotype O3:K6 (strain RIMD 2210633) OX=223926 GN=VPA0468 PE=4 SV=1

MAFTTRLMKLDSFCRYIVEFRCIAQKDNRKIKRNFMKKNTLAFATALALGSVLSVAHANE

AAQSSVEQVTPKAITYPTTQKVDVVDDYFGTKMSDPYRWLEDDLSPETAEWVKAQNAVTF

DYLSKIPYREQIEERITKLMDYEKQSQPFKEGRFTYFYKNDGLQNQDVLYRQLGDGDAEI

FLDPNTFSEDGTTSLAGVSFSRDGSLVAYSISEGGSDWRKVIVLDTETKKPVGETLVDIK

FSGISWLGNQGFYYSSYDKPKGSELSAKTDQHKLYFHKLGTKQSEDQLIFGGFEEEKYRY

VGGYTSDDERYLFISASVSTSGNKLFFKDLSKPNSPLKTILDNTSSDTWVIDNQGTKLYL

VTNLNAPNKRVVTVDASQPQPKNWKDLIPETKNVLRVSTAGGNLFASYIVDAISMVKQYD

MNGKLIREIKLPDIGSAYGFSGKKDETEVYYSFTNYKMPSTTYRLNIKDGDSEVYYKSKA

PFDPALYDSRQVFYTSKDGTKVPMIITYKKGTPMDGCAPTILYGYGGFNISLTPSFNPTR

AAWLELGGVYAVANIRGGGEYGKEWHNAGIQMQKQNVFDDFIAAAEYLIDEKVTSSNKLA

INGGSNGGLLVGAVMTQRPELFKVALPAVGVLDMLRYHTFTAGAGWAYDYGTAEQSKEMF

QYLKGYSPVHNVKAGVEYPATLITTGDHDDRVVPAHSYKFAAELQSKQAGSNPTLIRIET

NAGHGAGTPTRKIIETNADIYSFALFNMGIEKLQ

>tr|Q87NV8|Q87NV8_VIBPA Putative adenylate cyclase OS=Vibrio parahaemolyticus serotype O3:K6 (strain RIMD 2210633) OX=223926 GN=VP1760 PE=1 SV=1

MVSDAHFQGQFEVELKYRVKNHDAFLNMVKQIEHEVMFENNQESDWFYDTPQRTLTQQGK

SLVLREIQPAGIKLWIVKGPEADRCEATNITKLDSAQSMLENMGYEVIQCSKKIRSIFFV

GEFHITLDFLDGFGHFAEFAIMTDDETALARYRERLVALAQQFHLSEADREHRSYKEILS

A

>tr|Q87QP5|Q87QP5_VIBPA Leucine-responsive regulatory protein OS=Vibrio parahaemolyticus serotype O3:K6 (strain RIMD 2210633) OX=223926 GN=VP1104 PE=4 SV=1

MADNYKKPSKELDRIDRNILNELQKDGRISNVELSKRVGLSPTPCLERVRRLERQGYITG

YTALLNPQFLDASLLVFVEITLNRGAPDVFEQFNSAVQKLDDIQECHLVSGDFDYLLKTR

VSDMGAYRRLLGDTLLRLPGVNDTRTYVVMEEVKQSNQLVIKTR

>tr|Q87MF2|Q87MF2_VIBPA DNA-directed DNA polymerase OS=Vibrio parahaemolyticus serotype O3:K6 (strain RIMD 2210633) OX=223926 GN=VP2303 PE=4 SV=1

MSDPKFVHLRIHSDFSMVDGINKVPPIVKKVAELGMPAMALTDFTNLCGLVKFYGTAHGS

GVKPIIGADFKMQSDEFGEELTQLTVLAADNAGYKNLTLLISKAYLRGHVQHQPVIDRAW

LAEMSEGLIVLSGAKNGDIGKALLKGNRKLVESCVEFYKQHFQDRFYLELIRTGRPDEET

YLHFAIDLAEQHDLPVVATNEVVFLSADLFDAHEIRVAIHDGYTLEDPRRPKNYSPQQYL

RTEEEMCELFADIPEALENSVEIAKRCNVTVRLGEYFLPAFPTEGMEETEFLVMKSREGL

EERLEFLFPNEEERKKRRPEYDERLQIELDVINQMGFPGYFLIVMEFIQWSKDNAIPVGP

GRGSGAGSLVAYALKITDLDPLEYDLLFERFLNPERVSMPDFDVDFCMDKRDQVIDHVAE

MYGRDAVSQIITFGTMAAKAVIRDVGRVLGHPFGFVDRISKLVPPDPGMTLEKAFKAEPA

LPELYEADEEVKELIDMCRLLEGCTRNAGKHAGGVVISPTTITDFAPIYADAEGHFPVTQ

FDKNDVETAGLVKFDFLGLRTLTIIDWALGLINPRLEKEGKPPVRIESIPLEDPASFRLL

QNSETTAVFQLESRGMKELIKRLQPDCFEDIIALVALFRPGPLQSGMVDNFIDRKHGREA

ISYPDEKWQHESLKEILDPTYGIILYQEQVMQIAQVLSGYTLGGADMLRRAMGKKKPEEM

AKQRATFEEGAVKNGVDGELAMKIFDLVEKFAGYGFNKSHSAAYALVSYQTLWLKTHYPA

EFMAAVMTADMDNTEKVVGLVDECFRMKLTVLPPDINSGLYRFNVDENGAIVYGIGAIKG

VGEGPIDAILEARNKGGHFKDLFDFCARIDLKRVNKRVIEKLIYAGALDRLGPHRAAMMA

SLNDAVKAASQHHQAEAFGQGDMFGVLTDAPEEVENKYTQVPPWPEKVWLEGERETLGLY

LTGHPINAYLKELNKYTSCRLKDATPTRRDQSVTVAGLVIAARVMTTKRGTRIGIMTLDD

RSGRMEVMLFSDALDRYAELLEKDRILVVSGQVSFDDFNGGLKMSAREVMDLGSAREKYA

RGLSVSIDANQINDQFFEQFSRILEPHKAGTVPVNVYYQRADARARLTLGTEWRVTPSDT

LIDDLKQLLGKSQVELEFN

>tr|Q87FF2|Q87FF2_VIBPA Transcriptional regulator, AraC/XylS family OS=Vibrio parahaemolyticus serotype O3:K6 (strain RIMD 2210633) OX=223926 GN=VPA1727 PE=4 SV=1

MSDSDNPKAPPYKESMLPKPAELITLPSYMDCHDHAYTQIVIGLKGQAEFEVRGQGNLVG

PGQGCVVTACSDHAFGGVIHQSDILVLNMPKPTNDDPEMLQKINNLERSHLYFQLDGQIQ

KLIQMLVQEMRNHPDDLLLSRACNDTVIALMQRHISAFETSRKDSRFDLEAIDRYIEQHL

SHKISVAQLAGRVFLGESQFHSLFKDQMGITPHQYVLGKRIDMARELIEQGHLSLGQIAE

FTGFSSQSTFTHTFTRLQGLSPSQYKKQVG

>tr|Q87M62|Q87M62_VIBPA LacI-family regulatory protein OS=Vibrio parahaemolyticus serotype O3:K6 (strain RIMD 2210633) OX=223926 GN=VP2396 PE=4 SV=1

MAKKITMSDIAAAAGVSQSTVSLVLNGSTSVKIAEATKRKVLETAESLGYKNKKVAHALG

RPKKIALVINGLTSYDPFIDAINAAREEAWSNDYILVTFNYSHDEVLASKIESEINGGDY

AGLVYASSMTRELEQQRVTTSLPTVLLNCTNEEHTETTSILPADMIGAYKAVSHLTTQGY

RRIAMLSGESWMLASKQRADGYRQALIDADIIPNSEYLVDANWSLKEAHQQTLALLNMEQ

PPEAIFCGSDYMAMGCYQAIAELGLKIPHDVAVVGYDNQQIASESFPALTSVELPYSDMG

KLAIESLMALIEGQPLLSNKRKVEGELIVRTSSTKTKKVGM

>tr|Q87SQ5|Q87SQ5_VIBPA Putative glycerol metabolism operon regulatory protein OS=Vibrio parahaemolyticus serotype O3:K6 (strain RIMD 2210633) OX=223926 GN=VP0367 PE=4 SV=1

MLSIEDTKTKWEFFQKHHWVSPEFSRTPVLESWERCIKQCSPYTWSKPHIASGFTFSSLM

KRNEHLIECATTVLEDTYDMLGDEDLLLMITDGNGCVLSVVGHHSMQQEMQALGIKQGCF

LSEGKIGTNAVNLCISTHIPSEVFAAEHFNRHLHSYASVAAPVFDQFGKLRGTTCLLKKA

ETYKKENLVIIASSAKEVSLQIHIQSEQENINRLTCAHNATLECMDDGLLAWDEESRITM

VNYQSERLLNIEASQVLDKEVFSVLRFPPNVLNSLESGASINRKLTTVEVRGEFVEAIIT

LRPLNDGTHLLFLHPIDKIRELAQQQIGGSARYTFSTLPVISRKMKHVITVAKRAIKSKS

PILITGEEGVGKATLAMAIHNESTYKEGPFITLNCRSINTEQLIIETLGYDEGQGMPSKF

ELAHGGTLFLEKVEYLSPDLQAVLLKLLKTGLVSRSDSLRLIPVDFQLITSTASEISEYV

TQRSFGRQLYYEISSNELHIPPLRKRKEDIEFLVQQLISHYERRHNVTISIEPVALEALM

NFRWSGNNSELRNRTERILLNRSSNLIKLNDIPEDIKLNSRHTAENTPVITLEEAERRAI

VQAWNQCDGKMHDMAKALQIGRTTLWRKINKFGLQEQVKLY

>tr|Q87RM7|Q87RM7_VIBPA Uncharacterized protein OS=Vibrio parahaemolyticus serotype O3:K6 (strain RIMD 2210633) OX=223926 GN=VP0751 PE=4 SV=1

MSHDDDFELFQQMMGDVKPINQDTAEHKKVHQVTDAHIAKREAAIWLTEDDPEYLSLDHA

EMLKPDDFVEFKRDGVQDGVFRKLRLGKYPIQARLDLHRKTLKEARDEVVKFLKQCISMD

IRTVVIVHGRGERSNPPALMKSFVSSWLQQIKEVQCVHSAQRFHGGSGAVYVLLRKSADK

KLENRERHQKRLG

>tr|Q87G91|Q87G91_VIBPA Uncharacterized protein OS=Vibrio parahaemolyticus serotype O3:K6 (strain RIMD 2210633) OX=223926 GN=VPA1426 PE=4 SV=1

MKSEVSGLYWSLRMKFKIGLISAAILLSGCATQKMAFPTQNKLTITGNTLVYDGMITGDA

VLEAIRMVNDSDQKVTTLRITSSGGDIGVGIEFGHFIKNNDMDVIVSQLCFSACANYILP

AAKSVVIGKDALIGWHGGANQSDEMWDQSVPSQYEKQFYRYLTRLRIKEAEFYNKVGVDP

NITVYGHTRTNTCQRSTLANGWYYTESDMRHMGIKNLTVQGELLNTLNYNNSEINSCLMP

QIFN

>tr|Q87J65|Q87J65_VIBPA Putative regulatory protein OS=Vibrio parahaemolyticus serotype O3:K6 (strain RIMD 2210633) OX=223926 GN=VPA0388 PE=4 SV=1

MDKSLQQFLLVARCQSISAAARMAGLSQPTVTSNLKKLEENLGVTLFERHPNGMVLTEYG

RILYRHSNAMQHEYNQMMQSIDERKQYQVGKIKMGTGDAWWPLFVKQALNEHLTKQPSAS

THVEFGNHLGLMDSLINEQIEFFIGHEIVGLSSKCDVTFIPLFQTVDAYFVSSNHPLLGK

TVTEEMLHEYPALSVTYDAKKFAHVIDNPIPKQNERERQQLDDRPTYEVDSLLASIDVLK

ESVAVMSYTRHLQSYLESFGLRCLTMENEPNPGTVGIYRHRRETSESHLDLIAGITQRAK

LLN

>tr|Q87LW9|Q87LW9_VIBPA Iron(III) ABC transporter, ATP-binding protein OS=Vibrio parahaemolyticus serotype O3:K6 (strain RIMD 2210633) OX=223926 GN=VP2489 PE=3 SV=1

MSCALSIKNLTCQYDAQTVLESLSLEVEQGQIVCLLGASGCGKTTLLKAIAGLLPLTSGQ

MSLNCQTIDDGENWLPPEERNIGMIFQDYALFPHLTVAQNIAFGLKHQSAQEKRAKVDEM

LELVHLQGYADRYPHQLSGGQQQRVAIARSLAYSPDLLLLDEPFSNIDTQVRHELISEIR

KIFKAQGVTAIFVTHSREEAFAFADKMAVMNHGVIEQYGTASELYYQPSSKFVADFLGGG

SYLAATRISDTEYETHIGVVEASAQQEIQMQSACSLLLRPQHVQIQSDEDSAVTVLEQHF

MGDHCRYVIDANGDRLLAIASQPLHIGERVAVKIETQGVLAFA

>tr|Q87Q51|Q87Q51_VIBPA Uncharacterized protein OS=Vibrio parahaemolyticus serotype O3:K6 (strain RIMD 2210633) OX=223926 GN=VP1299 PE=4 SV=1

MWLIVTIVVFLIARQIAIKINSPLANPLLISIAVLIPLLTYLNVPFETYYADNEGISFLL

QPAVVALAYPLYEQLPQIKANWRIITFACTLGSIMSMATTTLIAVAFKADISLIASLVGK

SVTTPIAMEISSHLGGETAVAAILVLIVGLLGAILAYPIYNLIGIKHPIARGLTMGTVSH

ALGTATCAEKQPGDAAFSSLALVLCGIITSILAPSFFGLAVWLYQ

>tr|Q87PN7|Q87PN7_VIBPA Putative membrane protein of ExoQ family, involved in exopolysaccharide production OS=Vibrio parahaemolyticus serotype O3:K6 (strain RIMD 2210633) OX=223926 GN=VP1464 PE=4 SV=1

MLWTVMPHPILIVAVCFAPLALLFVLNQTFWLVTLFVIFSFFRIHEAFPAIYSFKIPLLL

SLGALAALAFHLLLSRQLTAFWHPSLKWLCIFWGLVIIGIVFASSRDIAITVFKNTYWKI

IVMTLAITWLITKPQQLAQMSKAIIVAGMLIGLVALNNAANGIDLVEGTRVSIGRSIGSV

LGDPNDLALVLMFPLAFTVSQLMEKRSPTLLRLFALITCFILFFAVIQTQSRGGLLGSLS

VFAYFAFKHIKSKTLVLVLGAVAALALYAFAGISGRESGGAAEDGIDASAMGRLYAWEAA

FKMALHHPFTGVGLDNFYFNYYFYSPHWDGINHAVHSTWFGVLAETGFVGLAVFITLIVS

LIRTSLKSISLLTPDSDYALTVGANAVLSGLIGTIVSGTFLTQGFTWPIYILAALTVVVS

RIVQSDCQNEKS

>tr|Q87HM8|Q87HM8_VIBPA Uncharacterized protein OS=Vibrio parahaemolyticus serotype O3:K6 (strain RIMD 2210633) OX=223926 GN=VPA0935 PE=4 SV=1

MNNWKLILPLMMSGSLIACTSTQTTTEPVEPETPKVEQPVTVEPEVKPEVEPKPEVKPKP

ETKPEKKPEPKPEKPVKVQKTPDGMLILGSEEWVYVPAIDQTFKARVDTGATTSSISATE

IVPFERDGKDWVQFKIDINGKTSKEFKVPVERWAKVKQSSSDEVDKRAVVVAYIQVGDYK

EKTEFTLADRDHMKFPILLGRSFFRDIAVVDVSKKYIQDKPTKSTKK

>tr|Q87II6|Q87II6_VIBPA Putative acyl-CoA thiolase OS=Vibrio parahaemolyticus serotype O3:K6 (strain RIMD 2210633) OX=223926 GN=VPA0620 PE=3 SV=1

MGNDIWIVSAKRTPIGCFQGLLQPYSAPQLGAFAIQAAMKSLGENVLSETVIDEVLMGCV

LPAGCGQAPARQAALGAGLPPSTGCTTINKVCGSGMKSIMLAHDLIKAGTIRTAVAGGME

SMTNAPYLLKEARNGMRLGHHSTYDHMFLDGLQDAYEGHLMGVYAQQIANRLKFSREKMD

EWATMSAKRALEAQERGLFDDEIAPIDIRTKTATTHLDYDEHPRSIDLEKIPQLKPAFDK

NGSVTAANSSAISDGAAALILMDEETARHQNLTPLAIIKSHATHARKPDEFTLAPVYAIE

QLLSQLDWSIDEIDLWEINEAFAVVTQIAVAELGLDSSKVNIKGGACALGHPIGASGARI

LVTLIHSLRQLQALGIDGDASNKKVMRGVASLCIGGGEATAIGIEIPL

>tr|Q87KJ0|Q87KJ0_VIBPA Adenylate cyclase OS=Vibrio parahaemolyticus serotype O3:K6 (strain RIMD 2210633) OX=223926 GN=VP2987 PE=4 SV=1

MQAYTYTIIQRLDNLNQQRIDRALALMDSQSQQVFHLIPALLNYNHPVIPGYYDADVPFG

VHGLELNPIQQQFIDDIQLAIGQPLKTAEKPAILGLYTMGSTSSIGQSTSSDLDIWVCIS

PEMDCDERELLTNKCLLITDWAQSQGVEANFFLMDEERFRSNHSEEMTGDNCGSSQHLLL

LDEFYRSAVRIAGQRLLWQIVPPEMEECYDEYVSQLCSDGYIDCSEWIDFGKLNRIPAEE

YFGSNLWQLYKSIDSPYKSVLKAILLEAYSWEYPHTQLLSIDTKRRFFAHEPDLYGMDAY

YLMLEKVTRYLERIQDDTRLDLVRRCFYLKTHEKLSREPDVGSVAWRREALSDMIAKWNW

DDSVVAELDDRRNWKVEQVKVVHHALLDALMQSYRNLIQFARRNDITSAISPQDISILAR

KLYAAFEVLPGKVTLLNPQISPDLHEADLSFIEVKEGGVNKSGWYLYKQPLIAHRILGQP

CLEHHEYLSKLVSWAFFNGLITESTRLHAVVREAQLDIDKFYQMVSDLRNTFALRKRRPT

MQALASPCEISQLAMFINFENDPTSELSGRSLKVDVKNTDIFSFGPEHKNLVGSVDLVYR

NSWHEVRTLHFKGETAMLDALKTILGKMHQDALPPESVDVFCYAKNMRGVMRNMVYQLLA

ECIDLRLKPVEQEKRRRFKAMRLGNQTYGLFFERRGVSVQKLENSIDFYRSISTNKLKGS

PLLMLDREQEYQLPEAVDGFASEGLVQFFFEDNEDGFNIYVLDESNQVEVYHQFSGSKDE

MIASVNSFYTSVKDDSRVASKFINFNLPQYYQIIHPEEGNAYIIPYRNDGCSPHRPTKAV

NA

>tr|Q87FY4|Q87FY4_VIBPA Flagellar M-ring protein OS=Vibrio parahaemolyticus serotype O3:K6 (strain RIMD 2210633) OX=223926 GN=VPA1536 PE=3 SV=1

MSELTPQVAGNTAMTTSTTQAFSPAGNMDDVTNKLKQLWSSSQRNLVLSAVLAAIVAAII

VVALWSSSQSFRPLYSQQERFDIGEIVSVLESEGVSYRMQEQNGQVLVPEGEVARIRMLL

ASKGVKAKLPTGLDSLKEDSSLGTSQFMETARYRHGLEGELVRTIMSLNSVANARVHLAI

PRQTLFVRQNGENPSASVMLELKPGEDLKPEQVEAIINLIVGSVTAMKPEFVSVIDQYGR

LLSADVASAEAGKVNAKYLEYQKNVEKQIIQRAADMLTPIVGPSNFRVQVAADMDFSQVE

ETREILDNAPVVRNEHTIQNNSIDQIALGVPGSLSNQPPVTGEAATNDSQNTNARSEVNR

QYAVGSSVRRTQYQQGQIEKLSVSVLLNSKASPDGVAWSDADKAQISTMITDAVGISAAR

GDSLSLMSFNFTPIDIDAPTALPWWQDPTVQQPLRYVIGGMLGLAMIFFVLRPLIMHLTG

ADKPVPELNFAEPPQEEPDYDNLQTREEREHEEVLNRRLSEKGISASTGLDVNSDMLPPA

GSPLEIQLKHLQLIANEEPERVAEILKQWVNINEHSSVDVKTNA

>tr|Q87TK6|Q87TK6_VIBPA Gluconokinase OS=Vibrio parahaemolyticus serotype O3:K6 (strain RIMD 2210633) OX=223926 GN=VP0063 PE=3 SV=1

MAGSSVIVMGVCASGKSTIGEHLAKKLGRKFIDGDDLHPRANIQKMASGQPLNDDDRKPW

LERIRDAAYSLESKNEHGIIVCSALKKIYRDQIREGNDNVTFLFLDGDMELILNRMRMRK

GHFMKENMVKSQFETLERPDNEPQTLIISIDGDISDVVERSAAELIKMQAIEVA

>tr|Q87T73|Q87T73_VIBPA Putative capsular polysaccharide biosynthesis protein D OS=Vibrio parahaemolyticus serotype O3:K6 (strain RIMD 2210633) OX=223926 GN=VP0197 PE=4 SV=1

MNTILSLIGRTDALFTQDIAHHEAELFRIVSESRFLVLGGAGSIGQAVTKEIFKRNPKKL

HVVDISENNMVELVRDIRSSFGYIDGDFQTFALDIGSLEYDAFIKADGKFDYVLNLSALK

HVRSEKDPYTLMRMIDVNVFNTDKTIQQSIDAGAKKYFCVSTDKAANPVNMMGASKRIME

MFLMRKSEQIAISTARFANVAFSDGSLLHGFNQRIQKRQPIVAPNDIKRYFVTPQESGEL

CLMSCIFGENRDIFFPKLSEALHLISFADIAVKYLEQLGYEPHLCDSEEEARQLAHMLPE

QGKWPCLFTASDTTGEKDFEEFFTDKEELDMSRFENLGIIKNEPLYEQELLTLFENSISE

MKGEQAWSKEEIVKLFFTMIPDFGHKETGKYLDSKM

>tr|Q87GG4|Q87GG4_VIBPA Uncharacterized protein OS=Vibrio parahaemolyticus serotype O3:K6 (strain RIMD 2210633) OX=223926 GN=VPA1352 PE=4 SV=1

MKLGSKRMENRKFSLLINEFAQMTKQGRLTLETEQGLICHYKNRDIRIENGQRIGLKGQI

VIVAPLKQKVTSRVQGKINGTFSLSKDGYLTEIKSLGCCLVRHISMPEHPRMLLEEVNQS

LVILNQILKEIESNVAT

>tr|Q87NH8|Q87NH8_VIBPA Ribonuclease R OS=Vibrio parahaemolyticus serotype O3:K6 (strain RIMD 2210633) OX=223926 GN=rnr PE=3 SV=1

MKLNISAQSLNYTKYDNVIPSRDAILGLFDKVKSYLSYEQISHSSGLNDEPEKDALKKRL

RAMERDGQLIFTRRKGYKRVDQSSLVTGKISIHVDGFGFLTYDDNEKDLFLPKHQLSHVF

DGDIILVLKGHTQHQGRSNHRFIKIVERKTTHIVGLLKRKGSKLYLLPENSKLTQTIYVT

PNELIAKNVGKLVHCKINTYPDYRQPTTVEVNEVLGRPGEAGIEVKLALRRHGINDKWDK

DVLDAASAFGSQVEEKDKTSRVDYRDLPFVTIDGDDAKDFDDAVYGYQMDNGQWKLFVAI

ADVSHYVKPNDHLDLEAQSRATSVYFPGCVVPMLPESLSNGLCSLNPNEDRLVMVCEMTF

DDEGNMLDSEFSEGIIHSHARLTYDDANRIIMQSSAKTTYQSNNPKNNIAQYLVNLHRLY

LNLSGQRKVRGAIDFDTQELAFKLNNKKKIASIVPVVRNDAHRMIEEFMLCANVATAQFL

ELNKIPSLYRVHSGPQMKKLTSLRMLLAEKGLTLAGGDKPTSHDYNALLDQVRDLDECDI

IRTLLLRSQSQAEYSPKNLGHFGLAYDAYAHFTSPIRRYPDLLIHRAIRAKLREKTTGKL

RSLLMKLKPIRQLAGISNSYPYDTKEIEQLSVHCSHQSRQADEVSREVESALKCHYMKPF

IGRNFMGSVSGVTHFGVFVELEENRIEGLIPLSSFQNGDFEFDAIKQKISSQTTTFTLGT

QVNIIVKEIDSKQRKIIFNFA

>tr|Q87SY5|Q87SY5_VIBPA ElaA protein OS=Vibrio parahaemolyticus serotype O3:K6 (strain RIMD 2210633) OX=223926 GN=VP0287 PE=4 SV=1

MTTWITLPFAQLTTLQLYEMLRLRVDVFVVEQTCPYPDLDGKDIIDGVHHLLGYHNDELV

ACARLLPAGTTYDNVSIGRVVTKQTARGGGLGHRLITQAIQACSTLWPEKTIDIGAQEHL

IDFYAHHGFEVMSDSYLEDGIPHVDMRRKHSTQ

>tr|Q87IH7|Q87IH7_VIBPA Cytochrome o ubiquinol oxidase, subunit III OS=Vibrio parahaemolyticus serotype O3:K6 (strain RIMD 2210633) OX=223926 GN=VPA0629 PE=3 SV=1

MQTNVAAHHNHDHHHDTNGNKLFGFWVYLMSDCVLFATLFATYAVLSSNSIAGPTGKEIF

ELPFVFVETMLLLFSSITFGFGIIAMKRNDVAGLKRWMLVTFALGLGFICMEVYEFHHLI

QEGYGPQTSAFLSAFFTLVGTHGLHVTFGLIWLAVAYHQLSTKGLNDNMAMRFNCLSLFW

HFLDIVWICVFTIVYLMGVM

>tr|Q87GZ8|Q87GZ8_VIBPA Uncharacterized protein OS=Vibrio parahaemolyticus serotype O3:K6 (strain RIMD 2210633) OX=223926 GN=VPA1167 PE=4 SV=1

MNWKRLVQFYSVPPSQAALALGMIGLGQAWALYVPDIGTPIRPFLASFGALLLAPVLIKY

ATNPRLFMADIKHPLSGSLMAPMSMALLILTDYLASVAPLIAYPLWCLAVLLHFTMMVLF

FGFQLMNFKMSNIVPSWFLYPVGLISSSLAGSQFGHNLFSETLAIMCIGIYFFMLPLVLY

RLVFFGSLPRRARPTLAIMAAPVNLSLAAYLVNFPQPDPILTGALAGIAITMTLLIYLCY

FRLLRLKFQPSIAAVTFPSVISAIAMHRLTSFFAQSHPQWHWLHDFGFLELSIATVLVVW

VSAGYVKMYWPEIVRTPTKQA

>tr|Q87M97|Q87M97_VIBPA Uncharacterized protein OS=Vibrio parahaemolyticus serotype O3:K6 (strain RIMD 2210633) OX=223926 GN=VP2360 PE=4 SV=1

MKEVMLFPLTSVVLPEGKMNLRIFEPRYKRMVKECSLQNVGFGVCLVGSEGDPKDVGNVS

SIGTLVRIVDFETLSDGLLGITVAGEKRFVIKRVRADSDGLRHAEVEWLDNWQTPSQQLD

FGYLSQQLAQVYEQFPQLGTLYQHRFYDDPIWVTQRWLELLPLDSHLFESLVGAQDCRPA

LRFLNQAIEAPSNKEARI

>tr|Q87MQ2|Q87MQ2_VIBPA DNA polymerase III subunit gamma/tau OS=Vibrio parahaemolyticus serotype O3:K6 (strain RIMD 2210633) OX=223926 GN=dnaX PE=3 SV=1

MSYLALARKWRPNKFDQVVGQKHVLTALENALAQNRLHHAYLFSGTRGVGKTSIGRLFAK

GLNCETGITATPCGECATCREIDEGRFVDLLEIDAASRTKVEDTRELLDNVQYKPARGRF

KVYLIDEVHMLSRHSFNALLKTLEEPPEYVKFLLATTDPQKLPVTILSRCLQFHLKPISV

DDIHQQLDYILGQEQVSAESKALGMISHAADGSMRDALSLTDQAIALGNGAVQTDIVSHM

LGTIDTDQAIHLLESISSKQPQQAMDKIHQLAANGVEWDGLLQQIATQLHRIAMYQALPA

SLDRAQPDAEKIELLSKALSPQDVQLYYQIALKGREDLTLSPNGRVGMEMIVLRMLAFRP

SANNGANIVTAGSQPQLSQMPNASLTNSAPAQQAPVAQQQSRPNPVQAQQSVSQPVRPSV

NQPASMNEEPQYQPDYQTGGYDMSPPQYDMAPPMSENPAPEAQPQQESAPAPSRMGAAGL

RHQLRSQRKQGLQQNTTQNGGTKKPKAASAKPESVIDRVAQLHGNSAQVSPNSTPKPTEP

EVEEAYRWRPSKPVETKVNTKLTPTQLKQALEHEKTPEMAQKLIDESLEQNEWAKMISLM

NLPKLVEQLALNSHYSKEGSTVSLQLRSSQAHLNTDRAQSELLNALNTVLGEECHLSIEV

GESGETPLELRDRLYQEKLQQALNSLENDPNVDFIERRFAAELDKDSVRPI

>tr|Q87FJ2|Q87FJ2_VIBPA Putative transcriptional regulator OS=Vibrio parahaemolyticus serotype O3:K6 (strain RIMD 2210633) OX=223926 GN=VPA1687 PE=4 SV=1

MDKIRSLRFFIATLEGGSFAAAAKAYGTDPSTVSKAIHRLESDLGIQLFQRSTRQIRLTE

AVRRYANTARFVLDELAACEDSLKSHNDALSGLLKINVPVSYGRLYIRPLLKEFCRRYPS

ITIDIHYDDAYVDIIEQGIDVSIRSGVVQDSQLIVRQLSPIDFIICGSQDYLTRHGVPSG

PDAFNDHSWVRFRFKQTGKLLPIRMPEPDGTSEYNPERNFIVNDGESMAELCAEGLGLTQ

IPHFIARDWLKVGRLVPIFPSMRQAGNGVYLLYANREYVPARVRVFIDFITQAIQDMDET

PFHTWAETLPIYQPNQGD

>tr|Q87I79|Q87I79_VIBPA Putative nonspecific tight adherence protein TadD OS=Vibrio parahaemolyticus serotype O3:K6 (strain RIMD 2210633) OX=223926 GN=VPA0727 PE=4 SV=1

MNIKNYFSLLVLLFLSGCSTINQELVTKEKLLTNSGQTEQLIEFYKANLVEVPSYKIKLI

NLYLDNKDQKSAELYINTLEKKDLKDPDIIYILAKLEYLKSNYDASERYLEEYLDNGGPE

GDYYLLKGKILARKKDYDGAISHFNDSKKNGASDREANNNIAVVMMLQGRPEPAMELLYG

LFAANPNDEKVRSNLLLASTRSNRPDVALEVLKHDSSEQEARVKLKKLMKSVKPLSKKID

TKVPPKQVAKSKEKKNMEMDTKSVDLKPKYVARSVLDPQNLRPKAPSIYRIQVLATYKVI

PSDYLNYLKGNYGKVYSYTHGLWKRYCVGEFSDIDDAKKFLENMNIKGAFVVDYTKKRYV

EL

>tr|Q87P44|Q87P44_VIBPA Translocation protein in type III secretion OS=Vibrio parahaemolyticus serotype O3:K6 (strain RIMD 2210633) OX=223926 GN=VP1674 PE=3 SV=1

MSYDDLHQALFLYSLTLPRLMACFIFLPILSKQMLGGAMIRNGVLCSLALFIFPVVNEQA

LPAETDGLWLIVILGKEVLLGMLIGFVAAIPFWAIEATGFLVDNQRGAAMASMFNPTLGS

QSTPTAVLLTQTLITLFFSGGGFVAFIYALFKSYTTWPILGFFPMVTDAWVSFFYDQFQQ

LMWLGVLMSAPLVLAMFLAEFGLALISRFAPQLNVFFLAMPIKSAIASVLLIVYLGLMMD

HFEALFYGITRFGDQLNTIWK

>tr|Q87GS6|Q87GS6_VIBPA Putative ABC transporter OS=Vibrio parahaemolyticus serotype O3:K6 (strain RIMD 2210633) OX=223926 GN=VPA1239 PE=4 SV=1

MSGKERNLPTHRISRFSKFASLATRVAGNVIAEGTKQIAKGNRPKAKDLLLTPQNIARLT

DQLAHLRGAAMKLGQMLSMDAGDILEPELAEILARLRSNADPMPSKQLNSVMVNALGEQW

KSAFLAFNFKPIASASIGQVHQAYSDAGDNLAVKVQYPGIRKSIDSDVDNVGTLLKIVGL

IPESVDYKGLLEEAKKQLYDEANYEREAQFACRYHEALQGHPHFVVPKIYPEISSQSVLA

MSFIQGTPIEKIANYDQETRDFVMHNLLELLFKELFEFKMVQTDPNFANYLYLEESKQIG

LLDFGATREYSERFSSGYRQAFSSVVNDDEQGLNDALEQIGFFAQSIKPEQRMAILDLVK

MACEPMLIDEAYDFKASGLAQKLREAGTILSMEQDYWHTPPADAIFLHRKIGGMYLLAAR

IGAKVNIRRLVQPYLKINSG

>tr|Q87JU3|Q87JU3_VIBPA Protein TonB OS=Vibrio parahaemolyticus serotype O3:K6 (strain RIMD 2210633) OX=223926 GN=VPA0155 PE=3 SV=1

MGRLLIALPASLLIAVSLFSFMAWMVDNGNQRAPKPSEAVRFDMVMVENDADVQRRQRSV

PEQPEPPQAPEPMELSQANTQVEPMSQVTPISALGLNTALDGIAINAPNLKGTMGNQQAL

PLYKVEPRYPSKALKRKVEGYVIMRFTIDTTGRPKDIEVIDAEPKRMFEKEAISALKKWK

YQPKVENGVSIEQFGQTAKVEFKLGK

>tr|Q87TE6|Q87TE6_VIBPA Uncharacterized protein OS=Vibrio parahaemolyticus serotype O3:K6 (strain RIMD 2210633) OX=223926 GN=VP0124 PE=4 SV=1

MPYLLAIVLSIFTLTGCQSAYYSAMEQVGYHKRDIMVDRVEDAKESQQEAQEEFTSALEA

LSALTNFDGGELESVYNNINDKYEDSEKAAQDVRDRIAAIEDVSDALFEEWQGELDLYTS

AKLRRSSEQKLRETKASYKTMLSAMKRAEKKMTPVLNTLRDNTLYLKHNLNASAIGSLQG

EFSSLEKDIQFAIKQMNEAIAESDKFLQKLNQK

>tr|Q87FG7|Q87FG7_VIBPA Uncharacterized protein OS=Vibrio parahaemolyticus serotype O3:K6 (strain RIMD 2210633) OX=223926 GN=VPA1712 PE=4 SV=1

MVNHTANTQIPQSLKAGVFNGRGIFDFGAKNEAYADYFTGTSYLALLNQPGLIVANVTFE

PGCRNFWHIHHEGGQILLVTGGRGWYQESGQPAQALNVGDVVHIAPGTKHWHGAAKDSWF

SHVAIEIPAEGASNEWCEPVTDEEYAELG

>tr|Q87HG1|Q87HG1_VIBPA Putative transmembrane protein OS=Vibrio parahaemolyticus serotype O3:K6 (strain RIMD 2210633) OX=223926 GN=VPA1004 PE=4 SV=1

MNIETSLGGTLFIIIAMGLVTLATRWGGVYVMSFIPISERVQRFITAMSGSVLIALLAPL

AVEGDNGARAALFSTAVVMFIVKKPLPAIAAGIIAAAAVRAF

>tr|Q87IN9|Q87IN9_VIBPA Glyoxalase OS=Vibrio parahaemolyticus serotype O3:K6 (strain RIMD 2210633) OX=223926 GN=VPA0567 PE=3 SV=1

MKKVAVILSGSGVYDGSELHEAVLALHAIEKAGATWHCFAPNIDQLHVINHLTGEEMDEP

RNVLVEAARIARGNIEDVARLNVEDFDALLLPGGFGAAKNLTDFAVSGAECSINTHVAQA

CRAFANANKPAGYLCIAPVIIPMIYEHGVKGTIGNDDATAAAFHQMGGEHVECNVDEYVF

DEKHNVLSTPAYMLAQNISQAASGIDKLVSKLVEIA

>tr|Q87I65|Q87I65_VIBPA Putative fimbrial protein Z, transcriptional regulator (LuxR/UhpA family) OS=Vibrio parahaemolyticus serotype O3:K6 (strain RIMD 2210633) OX=223926 GN=VPA0741 PE=4 SV=1

MRFTLNNVLIIDDQPLYSEALASLVENAINTAEVIQTTDSAEVMELVRSQRIDLIILDVV

LGDRDGMRLAKNILATGYRGRLLFVSSRDYSSLSKAAYEMGANGFLNKNEARETIADAIV

SVSRGYSMFKSTHTPSSGDVTLSNREAMVFHYLAQGYSNKKISEQLSLSAKTISTYKTRI

LKKYHADSLIELLHTIPQSENIQFCR

>tr|Q87KK9|Q87KK9_VIBPA Putative transporter OS=Vibrio parahaemolyticus serotype O3:K6 (strain RIMD 2210633) OX=223926 GN=VP2968 PE=4 SV=1

MISQLSKSIRQYMLVTFNYWNFTVTDGALRMLVVLYFHDLGYSTLAIASLFLFYEFFGVV

TNLIGGWLGARLGLNKTMNIGLAMQVFALLMLAVPNAWLTIPWVMAAQALSGIAKDLNKM

SAKSAIKTLVPDEQQGALYKWVAILTGSKNALKGAGFFVGGLLLSWVGFQNSMFIMASVL

AVVFIFSMIWLEADMGKAKNKPKFSHIFSKSESVNILSAARMFLFGARDVWFVVALPVYL

GSVFGWDHLWVGGFLASWVIAYGFVQGFAPRITGKAQGRVPDGSAALVWAGILALITGGI

AYGVQIGWQPEIVIVVGLMIFGAVFAINSSLHSYLIVSYAKGDGVSLDVGFYYMANAMGR

LIGTVLSGWIYQEAGLAACLWVSFAFLALTTLISIKLPKANIATA

>tr|Q87JF4|Q87JF4_VIBPA Putative transcription regulator protein OS=Vibrio parahaemolyticus serotype O3:K6 (strain RIMD 2210633) OX=223926 GN=VPA0299 PE=4 SV=1

MSPSRKLTKGLLHQLDLNLLKVFIVLAEEQKTVLAAKRLNMTQPAVSRALSRLRQHFNDE

LFVRTRHGLKPTRKGQLLADNLPRIIDELSIVLEGLDDFDAGSHAAHIRIAINNFFGVSL

PAKFYLKINKLAPNMVFSIENWGPTTLNRLVNGDIDLGINYSLNNVPKEISRRYLANDHF

QIFAREGHPLTEKIVTVQDIASFPIVSAIVPDWNEKKPRIIQLDQSQTLGFNVAFRSESI

ASLLEITATSDVLFPTSMYLLQNHHPRLSKLTLAESIVETLVSESQEIELYMHYRNRSNP

MFKWLLSIINELFEYDFPQD

>tr|Q87J66|Q87J66_VIBPA Transcriptional regulator, LysR family OS=Vibrio parahaemolyticus serotype O3:K6 (strain RIMD 2210633) OX=223926 GN=VPA0387 PE=4 SV=1

MDKFSDMTLFVSIVKHQGLAAAGRELGLSPATVTARLQAIEERYGVKLLNRSTRHVSLTD

AGAMYHQACLNIIDSVKETENLLQTGISEVRGTLKISAPRDIGKQIISPMVSAFSEQYPD

VTPYLYLNDNLSNLAESGLDLVIRYGELADSNLISRRLASSQRVLCASPDYLSKQGVPNC

PQDLAAHRCLAMVRSNEELKTWHFKDEESHQSITVTPKRFSDDGEVIRQWALDGAGIALK

SILDIQQDLKQQRLVTVLDGYMKNFSAFSQGAEADLHVIYQSRQYQPKRVRLFLDFLVEQ

FSALSDTSNQI

>tr|Q87PT9|Q87PT9_VIBPA Uncharacterized protein OS=Vibrio parahaemolyticus serotype O3:K6 (strain RIMD 2210633) OX=223926 GN=VP1412 PE=4 SV=1

MRSIAILLGCALLSGCAMWDQFKESTGITPETSSIELVIEASSVLNVREGGQSSPVILRV

HELTSPVLFRSLDFFALFENDKASLGDEYIKRYEYQMQPGEKIHEFLELDPATRAVGFSV

AFRDIDGSSWRKVEVIEEKSEYYIKLKLEGSELISDNTRGIEQVYF

>tr|Q87P09|Q87P09_VIBPA DevB protein OS=Vibrio parahaemolyticus serotype O3:K6 (strain RIMD 2210633) OX=223926 GN=VP1709 PE=4 SV=1

MINHKIYQTAEQVVESLANDMKAFSEMGRPVHISLSGGSTPKMLFKLLASEAYATSIQWQ

NLHFWWGDERCVAPDDAESNYGEANTLLFSQVNIPAENIHRIRGEDEPKAEAERFAKEMA

EVIPTENGTPVFDWILLGVGADGHTASLFPGQTNYDDANLSLVAAHPESGQLRVSKTARV

LEAAKRISYLVLGAGKADIVEEINSSPANVLPYPAAKIHAKSGLTEWYLDLDAAAKIA

>tr|Q87GU8|Q87GU8_VIBPA Uncharacterized protein OS=Vibrio parahaemolyticus serotype O3:K6 (strain RIMD 2210633) OX=223926 GN=VPA1217 PE=4 SV=1

MKLWDHPMSENDFRQCLRIATGATIGFTLCKLFGWNYGVFFTVTPMLLLGMVPVVNLHAS

RQLIFSAVVCGLEVGILGGLFGSHPVLMTLIAFGLFLYKFACMSKGSLFLFGANSVLSLS

IMLHFASYPTTDLNDLIFSNLQANVLSIIVAYLVTFLIPDAEPRQPLPKPSQAKQSHRMR

HEALMGASIATMSFLVFQIFDLNDSMSAQATTLLLLFPMHWNGALGYARKRAMGTLLGVT

FGIVGQLILYDWSNTLLFIVPLLWIGAMIFSYMHVKESSGSGAGFGGLTTLGILFGQYLT

PDGDLIFSALYRVSSILFAIVVTLLVTYAVHRLLNSFAATRFSQS

>tr|Q87NZ4|Q87NZ4_VIBPA Putative DNA polymerase III, epsilon subunit OS=Vibrio parahaemolyticus serotype O3:K6 (strain RIMD 2210633) OX=223926 GN=VP1724 PE=4 SV=1

MMWLEKSKASTPLLDTSQTPEWSVLFEQLAEQAQDQRLKRYYSTPMVNGDTPLKEVPFVS

VDFETTGLNAEDDAILTIGLVPFTIDRVQCSGSAHWIVNPNRELNEESVVIHGITDSEVK

NAPQLTQILGEILDALAGKVVLVHYKNIERQFFYNALLNTIGEGIQFPVVDTLDIEYALQ

RRECSGIWNKLKGKKPGSVRLGHARERYGLPAYQPHHALTDALATAELFQAQLQYHFNRD

MPISAIWQ

>tr|Q87KM6|Q87KM6_VIBPA GlpG protein OS=Vibrio parahaemolyticus serotype O3:K6 (strain RIMD 2210633) OX=223926 GN=VP2951 PE=4 SV=1

MKRLVTLNNPRMAQAFIDYMASRQIDIEMMPEGEGQFALWLTDSQHEVEAEAELKQFLAN

PSASKYSAASWDVADTRKSQFHYASPSIIGMIKAKAGPVTLLIMTVCAVIYGLQMLGFGN

GVFALLHFPAFEGQQWQLWRWVSHALLHFSVTHIIFNLLWWWQLGGDIERRLGSGKLLQI

FVVSAALSGAGQFYVEGANFGGLSGVVYALLGYLWVLGYRLPHLGLTLPKSIIGFMLVWL

VLGFVQPFMAIANTAHLAGLLAGMAIALFDSGKQKYQQQA

>tr|Q87J49|Q87J49_VIBPA Transcriptional regulator, AraC/XylS family OS=Vibrio parahaemolyticus serotype O3:K6 (strain RIMD 2210633) OX=223926 GN=VPA0404 PE=4 SV=1

MKNSARNLHPSLSIDRAPSDVFMNFEAFLSNTETRIHSHPWGQVQLISGGILEMDAEDTR

FLAPPHLAIWVPAGIRHTSYNRKPIEYCSLNIAPELTAHFPTKTSLIKVTPIVSAIIEDF

RQRDINVAQSDEDKRLVRVLLDQLAKQEVEHHFLPTTDNKYLQPILKAVEESPTDEISLA

EWAAKVHTTERTLARHCQSELGMSFTEWRLRVRYLHSMELLRKGQTVKEVALTLGYNQAS

PFIAMFKKYSGMTPEQYKNRLL

>tr|Q87GH4|Q87GH4_VIBPA Putative Type III secretion protein Spa24 OS=Vibrio parahaemolyticus serotype O3:K6 (strain RIMD 2210633) OX=223926 GN=VPA1342 PE=4 SV=1

MYINAGACSGVYFFAIGGAMVELLSPTNAYAGVNLVSLLVLVLLSAVFFICFTGFVKYSI

VLNIIKNAVGTQQIPPAIVVNLLAALMALNAVWPQIQPGIERVKPYFSQQEKQELNQDVD

NDMPKNVTMYSLVSEWSTFFPELLEHAKQKTDKLSEVIELPFNFDKEHDTFKYFVGSLIY

DLYKGFELGLKLYIVFVSIDFLIAVILSGVGMTMLSPTVISTPVKLAVFYFSDSWTILFK

ALG

>tr|Q87LZ6|Q87LZ6_VIBPA Dihydropteroate synthase OS=Vibrio parahaemolyticus serotype O3:K6 (strain RIMD 2210633) OX=223926 GN=VP2462 PE=3 SV=1

MIITANNKSLDLSRPHVMAILNVTPDSFSDGGKFNSLELALAQVEKMITAGVSIIDVGGE

STRPGAPDVSLEEELQRVVPVVKAIREKYDVWISVDTSKAEVMRQAIAVGADLINDIRAL

QEPGALQVAAASKLPVCLMHMKGQPRTMQESPQYENLMDDVAQFLEERIAACEAVGINKS

QLILDPGFGFGKTIEHNYHMLAHLEKFHEFGLPILAGMSRKSMIFKLLDKPAADCTNASV

VCATIAAMKGAQIIRVHDFEETIEAMKIVEMTQNNI

>tr|Q87HR8|Q87HR8_VIBPA Sui1 family protein OS=Vibrio parahaemolyticus serotype O3:K6 (strain RIMD 2210633) OX=223926 GN=VPA0888 PE=4 SV=1

MTLVYSTETGRIKPEEEKVARPKGDGIVRIQRQTKGRKGKGVCIVSGLDLDDAPLKLLAA

ELKKVCGCGGSVKDGTIEIQGDARDKIKTHLEKKGMTVKLAGG

>tr|Q87FZ2|Q87FZ2_VIBPA Uncharacterized protein OS=Vibrio parahaemolyticus serotype O3:K6 (strain RIMD 2210633) OX=223926 GN=VPA1528 PE=4 SV=1

MLTLNYYVEITATPQRVWRVLTDAELYKRWAQAFSPQSQFEGEWEEGSDITFFDPDMGGT

RAVIDSVQPLHRLEFHHVAIFNPDNRQQLDADIAAKWIGSREIYQIDTEDERLLLNITIH

THSDFVSMFNNGWEKALPLIKSICEETDSQ

>tr|Q87LS3|Q87LS3_VIBPA Uncharacterized protein OS=Vibrio parahaemolyticus serotype O3:K6 (strain RIMD 2210633) OX=223926 GN=VP2538 PE=4 SV=1

MGLNIKPLVVSVLGSVLLAGCATAPPKQQDNLCEIFREKSGWYDDAKYMEKEWGTPIHVA

MAIIKQESSFRHDAKPPKDYVLGFIPWGRVSSAYGYAQAQDPAWDDFQDSTGQGGSRSNF

DDSIMFVGWYTHETRRQLGISLWDPYNQYLAYHEGRGGYKRGTYKRKPSLMKVARRVEQT

AKTYGWQLKQCRQELEDNSSWFF

>tr|Q87QL5|Q87QL5_VIBPA Uncharacterized protein OS=Vibrio parahaemolyticus serotype O3:K6 (strain RIMD 2210633) OX=223926 GN=VP1134 PE=4 SV=1

MNLIDFSHSPVSLLPPIVALTLAILTRRVLVSLGVGIALGAVLLNSWSIGGTASYVGTQV

SSVFIEDGGINTWNMSIVGFLILLGMTTALLTLSGGTRAFAEWAQSRVKSKRGSKLLAAF

LGVFIFVDDYFNSLAVGAISRPVTDRFYVSRAKLAYILDSTAAPMCVIMPASSWGAYIIT

IIGGILVSHGITEYSALGAYVRLIPMNFYAVFALLMVFAVAWFGLDIGKMREHEIAASQG

RGFDKDKENDTQEAHELNEELDIRESEKGKVSDLILPIVTLIIATIASMMYTGGQALAAD

GKEFALLGAFENTDVGTSLIYGSLLGLAVALFTVLKQGLPLTEITRTLWIGAKSMFGAIL

ILVFAWTIGSVIGDMKTGSYLSTMAQGNINPHWLPVILFLLSGLMAFSTGTSWGTFGIML

PIAGDMAGATDIALMLPMLSAVLAGAVFGDHCSPISDTTILSSTGARCNHIDHVSTQLPY

ALSVALVSCVGFIALGMTTSIAFSFIAASITFVIVCAVLSWLSKSKIESCQSA

>tr|Q87P26|Q87P26_VIBPA Putative type III export protein OS=Vibrio parahaemolyticus serotype O3:K6 (strain RIMD 2210633) OX=223926 GN=VP1692 PE=4 SV=1

MRVDPSFVGQTPAHSTDVRHYERDDAKRMSELMTRETTAEVSRSAPKDTLTKVEEKLNAI

KDWYASIKEAETVSKQSVLSSLKDVISDPQTQKEALWYAFHQAKSAKGTDDAVPELLSVL

KQELLGDFAGQLMAEPPTDRAALKAMLAQSFPLGAQKEQALWHCWAELKSLPEMTSTVDL

VREELSFVIQKNAMVKNIMTHSHKLDLS

>tr|Q87IU7|Q87IU7_VIBPA Putative tyrosine-specific transport protein OS=Vibrio parahaemolyticus serotype O3:K6 (strain RIMD 2210633) OX=223926 GN=VPA0509 PE=3 SV=1

MNLKLVGSSLIVAGTALGAGMLAIPMVLAQFGLLWGTLLMLFIWAGTTYAALLLLEASCK

VGGGVSMNAIARETLGKGGQLVTNGLLYALLVCLLMAYIIGAGDLVQKITASVGLSVSTV

SSQVGFTILVGLIVSAGTGVVDKLNRGLFIGMIVALVLTLFALAPSVSFEGLNEVVSSDK

MALIKTSSVLFTSFGFMVVIPSLVTYNKEASKTQLRNMIVVGSTIPLVCYLLWLFAVVGN

LPPHELVQYSNVTELISVLGQQYNGLEFILSMFTGLALLTSFLGVAMALYDQNADLLKTS

KPVVFVTTFILPLLGAVFAPEHFLAILSYAGIILVFLAVFVPLSMTMKVRRVPVEDNSVY

EAGGGVMGMSMIFLFGCFLLFAQAV

>tr|Q87QY0|Q87QY0_VIBPA Putative outer membrane protein OS=Vibrio parahaemolyticus serotype O3:K6 (strain RIMD 2210633) OX=223926 GN=VP1019 PE=4 SV=1

MMTRVRKFLKVCPWIILFLPLFANAAYQRNVAKPVNEVVYGKIDSVRYITQQEVVQSKSN

GWKTLLGATIGGLVGNQFGGGTGKEVATAVGALAGAVVAQNQSNYQYTVEYKLVELLIKV

KGDKLINVIQDVDKNMLFSRGDEVRILYFDDGVRVDLAY

>tr|Q87QJ4|Q87QJ4_VIBPA Putative membrane protein OS=Vibrio parahaemolyticus serotype O3:K6 (strain RIMD 2210633) OX=223926 GN=VP1155 PE=4 SV=1

MTRNERIFHAVLFELMALAIIVPAAALITGKGSSDLALVGIGLSLYTVVWNYIYNLYFDK

WFGSNRADRSLAMRLGHTVGFEGGLIFISIPVIAWFLEITFLRALMLEAGFLVFFLFYAT

GFNWLYDKVQPFGKMRKLLV

>tr|Q87QE9|Q87QE9_VIBPA Sulfurtransferase OS=Vibrio parahaemolyticus serotype O3:K6 (strain RIMD 2210633) OX=223926 GN=VP1200 PE=4 SV=1

MKKSTIALGVLAAVAAVGYSAYNTMFSAEAVIVDAAAQEQKFTQYAHPEHFISAQQLKSL

MDGDKDVVVIGALNPIKPDSPISGSYTMWRNDYSATEDAYDFGGMSNSTEEMETILGSFG

ATTDSTIVVYAAGSHHDAARLYWQIHNLGHQDVRYLDGGLNAWMGAGYPTGSANQSVAAV

EYNAPNTQQENNALATLDMVIAAQNNPDWVILDTRGNDEFNGEVAVSGAYGPGTIPSSVH

INWTKALNEDTTLKSAEELQALYGDIIKGKKVIAYCQSGVRSAHTTMILTEVLGAEGVYN

YDGSWIEYSHAHYEQKNPEVNVINGKS

>tr|Q87HV4|Q87HV4_VIBPA Transcriptional regulator, LysR family OS=Vibrio parahaemolyticus serotype O3:K6 (strain RIMD 2210633) OX=223926 GN=VPA0852 PE=4 SV=1

MRYSLKQLAVFDAVADTGSVSQAADKLALTQSATSMSLAQLEKMLGRPLFERQGKQMALT

HWGMWLRPKAKRLLQDALQIEMGFYEQHLLSGEIRLGASQTPAEHLVPDLISIIDNDFPE

MRISLGVQSTKGVIDGVLDYKYDLGIIEGRCDDNRLHQEIWCRDHLTVVAASHHPFARNP

SVSLAQLEQAKWVLREHGSGTRKTFDSSIHHLIEDLDVWREYEHVPVLRSLVANGQYLTC

LPYLDVERYIEAGRLVALNVPDLKMERTLSFIWRADMAENPLVDCIKREGLRMMKGKPSV

L

>tr|Q87T69|Q87T69_VIBPA Putative acetyltransferase OS=Vibrio parahaemolyticus serotype O3:K6 (strain RIMD 2210633) OX=223926 GN=VP0201 PE=4 SV=1

MLVDLLRNQNRTILAVMSPDDISTRQAFDGIMQLSNDKDISRYSPDEVRLVNGIGMMPKS

LLRRKVNQYFLDLGYQFETVISDQALVSKFAHLQDGAQILKGAIVQCGAVIGEHSIINTG

AVIEHDTVVGEHNHIAPRAVLCGGIVTQSDVYVGANATVIQNLKLAQNVVVGAGAIVTCH

LDAHQVCYSGRATIKNSK

>tr|Q87K05|Q87K05_VIBPA Uncharacterized protein OS=Vibrio parahaemolyticus serotype O3:K6 (strain RIMD 2210633) OX=223926 GN=VPA0093 PE=4 SV=1

MLDTTKPSLALFDFDGTITREDMFSLFLHYSAYGLRKRVGKLAIMPFYALYKLGVLPARV

MRPLSSFIAFSGKETQHIEAIGATFAHEVIPLYLRPEAMERLAWHQHRGDTIVVVSASLN

AYLKPWCEANGYHLLCSELISEQPKLSGFYQQGDCSLERKVSRVKAAFSLDEFASVYAYG

DTHEDIPMLKLADYAMLNWSEWRASE

>tr|Q87KB1|Q87KB1_VIBPA Putative amino acid ABC transporter, permease protein OS=Vibrio parahaemolyticus serotype O3:K6 (strain RIMD 2210633) OX=223926 GN=VP3066 PE=3 SV=1

MTFRTLWLISLLLLTGCSDYQWGWYVLDPSTEQGITNLKFLVAGFNDTIQVSLLSMCFAM

TLGLLIALPALSRSPTLKWMNRIYVEVIRSIPVLVLLLWVYYGMPTLLDVSLNHFWAGVI

ALTIAESAFMAEVFRGGIQAINRGQHEAAESLGLNYWQKMRLVILPQAFRQILPPLGNQF

VYVLKMSSLVSVIGLSDLTRRANELVVNEYLPLEIYTFLVLEYLLLILFVSQAVRWLEKR

IAIPSY

>tr|Q79YY3|Q79YY3_VIBPA BfdA OS=Vibrio parahaemolyticus serotype O3:K6 (strain RIMD 2210633) OX=223926 GN=VP1393 PE=4 SV=1

MPTPAYMSINGETQGHITKDTYSADSVGNTWQEAHVDEFLVQELDHVLTVPRDPQSGQPT

GQRVHRPLVVTKVQDRSSPLLFNALVSGEKLPECLIRFYRTSVQGKQEHYYSIKLIDALL

VDIQTRMNHCQDAATADRVTEEVLKFTYRAIEVTHENCGTAGNDDWRAPREA

>tr|Q87J54|Q87J54_VIBPA Putative aminotransferase OS=Vibrio parahaemolyticus serotype O3:K6 (strain RIMD 2210633) OX=223926 GN=VPA0399 PE=4 SV=1

MEIAQSLQQTKSSYIREILAAASDKNVISLAGGLPDEKTFPIDLMKPTLENLANMPEVFQ

YGATAGYAPLLNFLTDYYQLPESHLAMITTGSQQGLDLIARAYVNPGDKVVMEAPSYLGA

MQVFGLVQANIVTVSQTECGPNLEELEQCFATQQPKMFYAVPDFHNPTGVCWALETRQKV

AELCIQYDVAFIEDAPYRELRFSGEALPLVSGFCPQHSIVLRSFSKIASPGLRIGVVTGK

RSYLEPLIKIKQGADLHSSIPMQALLHGLLQHDNFETHISTICALYKSRYDVMFAELQKQ

LPESCLLKPVDGGMFVWVEIPECDTFELAKTLLANWVAVVPSPVFYPEGSKVKAALRLNF

TNATPEELTTAVTRLADVLNAL

>tr|Q87JZ1|Q87JZ1_VIBPA Putative transcriptional regulator OS=Vibrio parahaemolyticus serotype O3:K6 (strain RIMD 2210633) OX=223926 GN=VPA0107 PE=4 SV=1

MRKAKEITLMNFDYNLLKVLAVILETRNTTTAAERLCTSQPAVSRSLRKIRDLFNDDILV

RKGTNMELTPKAEEIKAQLSGIINDIDKLVNVSHSFDPATESSVLRVAINSSIAQWFSAA

FTQLLAKEAPFMNLVIEDWTETTPDKIDAGEIAFGINYFPMELPKHLVQKKGGRDDFALA

CRATHPHGGKRMYLDDISEYAYAVHIIQHWNEKEDHISRLLQPFSVVPRIQLRTTHINTI

LNLVADSDVLFPCSRHLINQLDKRFSFIEFDDALPKLEGNFGYVYSVKRRNDPLILWVNN

TVESLMKSLGIES

>tr|Q87SV8|Q87SV8_VIBPA UDP-N-acetylmuramate--L-alanyl-gamma-D-glutamyl-meso-2,6-diaminoheptandioate ligase OS=Vibrio parahaemolyticus serotype O3:K6 (strain RIMD 2210633) OX=223926 GN=mpl PE=3 SV=1

MHIHILGICGTFMGGAAILARQLGHKVTGSDANVYPPMSTLLESQGIEIIEGFDPSQLDP

QPDLVVIGNAMSRGNPCVEHVLNSNMRYTSGPQWLNEFLLHDRWVLAVSGTHGKTTTSSM

LAWILEDCGYQPGFLVGGVLGNFGVSARLGESMFFVVEADEYDSAFFDKRSKFVHYHPRT

LIMNNLEFDHADIFDDLEAIKRQFHHLVRTVPGNGLILAPKQDQALTDVLERGCWTEKQF

SGEDGDWQAHKLVLDGSKFEVALQGEKVGTVEWDLVGDHNVDNALMAIAAARHVGVTPEL

ACQALGRFINTKRRLELKGEEQGITVYDDFAHHPTAIELTLGGLRNKVGEKRILAVLEPR

SATMKRGVHKNTLADSLHSADEVFLFQPDNIEWSVQDIADQCKQPAFVDADMDNFVAKIV

ERAQPGDQILVMSNGGFGGIHGKLLEQLKLKA

>tr|Q87I85|Q87I85_VIBPA Rough colony protein RcpA OS=Vibrio parahaemolyticus serotype O3:K6 (strain RIMD 2210633) OX=223926 GN=VPA0721 PE=3 SV=1

MFNKNILLVTLGVALLLPASVSAGELLSLDKGAAKTINVKRNIDTVFVADTQIADYKVIA

NGKLVIYGIGRGATSIIAYDRAGNEIYNAEVVVNKSLRLLKQTIIARYPDEDIKLTNIGE

QIVIDGVVSSEEIKDKVYRHVGEMLKKSKQRNTFELSGANGESVDPLDYTATYVFEDIIN

NLKVLTTDQINVKLTVAEVSSSFLTELGVSYAETNGKSIGGAGQFVNKILDFTAEDIVAV

ISASGNDSIGQVLAEPNLSVISGESASFLVGGEIPITVRDNDGISVTYKEYGVKLSMVAK

VTDSENIRLSLLPEVSSIDKTNGVNSGLVSVPSLRTRKAQTTVQLKDGQSFVLAGLLTSE

EQESLAKIPYLGDIPILGALFSKTNTERRKTELIIVATVNLVDPVKETDIKLPKFERTSD

LERLLKLDLSKVDDEELENTIKAGGFN

>tr|Q87MH3|Q87MH3_VIBPA Uncharacterized protein OS=Vibrio parahaemolyticus serotype O3:K6 (strain RIMD 2210633) OX=223926 GN=VP2282 PE=4 SV=1

MRYLALLLIGWLSLPVYALTQVDIYRAEVVIDSEQNDGESAAREQGMKDVIVRATGSQSS

LSNPVIQKALSSSSRYISQLGKSQVDGKASLKMLFNSGQIQSLLTQAQLPSWSPNRANIL

VWLVEEQDYDRAIAWEHSDSANVAALKKATETRGLPITIPVGDFDDVTGVNTSDLWGGFI

EPISQASQRYPADAVLIIRAQGEQIRWTLYDQAPAKIIDAQTSPRSGSATGADAISAMVD

GIADYYASKNAVVVSGKSSKAVNVKVLNVTSAADFFRLENALTKLNSVAGTEIKRVQGSE

LTLTIHLLASQQAFEQEASSISQLMEFEDPLGDVEEVTPSEEQSVETLPSEAQAIEGQPK

TVVQAVEETVPAVQVQQPVVIPQVQDQYDLIYEWNSTSQS

>tr|Q87KG7|Q87KG7_VIBPA Uncharacterized protein OS=Vibrio parahaemolyticus serotype O3:K6 (strain RIMD 2210633) OX=223926 GN=VP3010 PE=4 SV=1

MNESTILLTLASIHFIALMSPGPDFALVVQNATRHGRQTGLYIALGLSVGILLHSLFSLT

GVSYLVHQHPLLYSVLQLLGGSYLLYLGVGALRGVIATIKNPQTDQQNKTNSFVISNKRQ

AFAKGFATNILNPKALVFFISLMSSLVPAGMSVSGKSIALVILFSLSLVWFSSLAWMLST

QRLQRRLQQAGIYIDGICGVVFTLVGGSILVQTISTLIG

>tr|Q87HH5|Q87HH5_VIBPA Uncharacterized protein OS=Vibrio parahaemolyticus serotype O3:K6 (strain RIMD 2210633) OX=223926 GN=VPA0990 PE=4 SV=1

MGFWFFQDDVQALDDGSFSGQHVVGDVLVLIDYPQGANDEPYAALVTWDPTCSKADSNDP

APGDCAAKNLRLQAETSGANPADCSAVNDGDFGCATTNKGETPIPSPWPYTAKNGTMNAF

PYESFYEGGVNLTQLLGGIDGTASCFSSFMAETRSSASFTAALKDFVLGQFQLCGMELVK

TCPTGALSPSGDSIIYNYEIKVTNTGFGALYDLYVEDITAGDSFTADVLAAGETATFTGS

FVSLINGVENEATASAAIKDGGEPVLTKSDTDSCPPLTPVGALSITKDCTTYVEENASGA

YGLRVNYEGKVCNDSKVKLNDVKITEMHDGMTVVKDVGTLNPESCMNYDGTYIPAPGSDV

SGGPVVAHDVRTFKDTVVAEGVNAITGVTVDTGMPVEASCPLCPAP

>tr|Q87FU1|Q87FU1_VIBPA Uncharacterized protein OS=Vibrio parahaemolyticus serotype O3:K6 (strain RIMD 2210633) OX=223926 GN=VPA1587 PE=4 SV=1

MDTLLKDFPVITEIKVAWGEMDALQHVNNVVYFRYFETARLDYFNKINLLVDLQTSQIGP

VLSETQCRYKLPVTYPDTLLVGSRVIDMQEDRITMEYQVLSKKWGKITTVSTATGVMFDF

KNNEKAAIPDHVRQSILELESTVGSTHHLE

>tr|Q79YX1|Q79YX1_VIBPA Chemotaxis protein CheY OS=Vibrio parahaemolyticus serotype O3:K6 (strain RIMD 2210633) OX=223926 GN=VP2231 PE=4 SV=1

MNKNMKILIVDDFSTMRRIVKNLLRDLGFNNTQEADDGLTALPMLKKGDFDFVVTDWNMP

GMQGIDLLKHIRADAELKHLPVLMITAEAKREQIIEAAQAGVNGYIVKPFTAATLKEKLE

KIFERL

>tr|Q87HS2|Q87HS2_VIBPA Putative acetyltransferase OS=Vibrio parahaemolyticus serotype O3:K6 (strain RIMD 2210633) OX=223926 GN=VPA0884 PE=4 SV=1

MNKLHKLFKPSSVAVIGASQKDLRAGQVVMRNLLQSGFDGAIMPVTPRYKAVSGVIAYRD

VASLPYTPDIAILCTNASRNEQLLKELDERGTPFAIVISDDAQTLDLSSLNIRVLGPNSL

GIILPWHNFNCTFSPVAAKPGKIAFISQSAAVCTTVLDWANDKNIGFSSFISIGRGQDID

FADLLDYLSMDGNTEAILLYVDSIQDARRFMSAARAASRNRRILVLKAGRSKEMNTFEQQ

DGDTLDVIYDSAIRRTGMLRVSNTHELFAAVETLTHSVPLRGERLAIITNGGGPAVMAVD

TLVERGGNLATLDEVTTDQLRAILPSNWRGVNPIDLSGDATKKRYVDAINAVMNNDCADA

ILIMHSPSAVSDSYETALAVIEAIKNHPRHKHFNVLTNWSGEQTSRDARLAFTQAGIPTY

RTPESAVVAYMHLVEYRRNQKQLMETPTTAEPLHSGSVNSAKEWVDERLLDKNTVTLDTH

QTSPLFKLFGFNVLPTWIASDEIEAVHMAENIGYPVAVKLRSPDIPHKSDVHGVALNLRN

SREVSNAAQSILDTVKFSYPSANVHGLLVQGMAKLGSAEELRISIAVDKVFGPVILLGQG

GSEWNIAQDAVAALPPLNMTLARYLVVVALKSGKIRLQKHKDALDITELSKFLVRISQMA

VELPEIQRLDIHPVLVSGDDLTILDADVTLCKYEGDAQKRLAIRPFPAEFVETVTLRDGQ

PILLRPILPEDEPLHAQFINSVSKEDLYKRFFSEVGEFNHEALANFTQIDYDREMAFVAV

AFDKSGPSIIGVARALITPDNSDAEFAILVRSDLKGKGLGKILMEKIISYCKIKGTKQMS

GMTMPTNRGMLMLAQRLGFEVDVQFADGTADMVLPLN

>tr|Q87LH4|Q87LH4_VIBPA Deacetylase DA1 OS=Vibrio parahaemolyticus serotype O3:K6 (strain RIMD 2210633) OX=223926 GN=VP2638 PE=4 SV=1

MKLNKLAIATLVSAALSQYAFAQTDTKGTIYLTFDDGPINASIDVINVLNQEEVKATFYF

NAWHLDGIGDENEDRALEALKLALDSGHIVANHSYDHMVHNCVEEFGPNSAAECNATGDH

QINSYQDPAYDASMFAENLSVLEKYLPNITSYPNYKANEFARLPYTNGWRVTKDFKADGL

CATSDDLKPWEPGYACDTANPSNSVKAAIAVQNILANNGYQTHGWDVDWAPENWGIAMPA

NSLTEAEPFLGYVDSALNTCAPTTINPINSKAQEFPCGTPLHADKVIVLTHEFLFEDGKR

GMGATQNLPKLAKFIQLAKQAGYVFDTMDNYTPNWQVGNNYSAGDYVLHLGTVYQAVTSH

TAQQDWAPSPTSSLWTNADPATNWTQNVSYKQGDVVTYQGLRYLVNVPHVSQADWTPNSQ

NTLFTAL

>tr|Q87SQ2|Q87SQ2_VIBPA PTS system, mannitol-specific IIABC component OS=Vibrio parahaemolyticus serotype O3:K6 (strain RIMD 2210633) OX=223926 GN=VP0370 PE=4 SV=1

MISPDAKIKIQNFGRFLSNMVMPNIGAFIAWGFITALFIPTGWLPNETLASMVGPMITYL

LPLLIGYTGGKLVGGDRGAVVGAITTMGVIVGTDIPMFMGAMMVGPMGGWAIKKFDNYID

GKVKSGFEMLVNNFSAGIIGMLCAILAFFLIGPFVKVLSGALAAGVNFLVSAHLLPLTSI

FVEPAKILFLNNAINHGIFSPLGIQQASETGQSIFFLIEANPGPGLGILLAYMVFGKGTA

RQTAGGASIIHFFGGIHEIYFPYILMNPRLILAAIAGGMTGVFVLTMFNAGIVSPASPGS

IFAILLMTQKGSIVGVLASIAAATGVSFAVASLLMKTQTSTEEDGDEAALEKATSQMKDM

KSSSKNGAVVNNESKGDVDLATVQSIIVACDAGMGSSAMGASMLRKKVQDAGLNVHVTNL

AINSLPESADIVITHKDLTGRARKHAPNAHHISLTNFLDSEMYNQLVTKLLAAQKQSAAN

DDQMVKVSVLAANDDSFEPQQPSVFQIQRENIHLGLKAANKEEAIRFAGNKLVELGYAEP

EYVDAMFEREALVPTYLGESIAVPHGTVEAKDRVKKTGIVICQYPSGIQFTEDDDDVAKL

VIGIAAKNDEHIQVITTITNALDEPEAIEKLTSTNDVEEILNILGGQQAA

>tr|Q87LA7|Q87LA7_VIBPA MSHA biogenesis protein MshK OS=Vibrio parahaemolyticus serotype O3:K6 (strain RIMD 2210633) OX=223926 GN=VP2705 PE=4 SV=1

MVKKLIISIAAIALSGSVLANQDPTAPLGWQKPAEVSAKPKAKQYRLPTLNSIVCKPNTE

CVAIMNNRLVEQGELFNGYRVASINSEFVTLKRGSRQWNLELFGLNVKK

>tr|Q87L70|Q87L70_VIBPA Site-specific DNA-methyltransferase (adenine-specific) OS=Vibrio parahaemolyticus serotype O3:K6 (strain RIMD 2210633) OX=223926 GN=VP2742 PE=3 SV=1

MKKQRAFLKWAGGKYGLVEDIQRHLPPARKLVEPFVGAGSVFLNTDYDHYLLADINPDLI

NLYNLLKERPEEYISEAKRWFVAENNRKEAYLSIRAEFNKTDDVMYRSLAFLYMNRFGFN

GLCRYNKKGGFNVPFGSYKKPYFPEAELEFFAEKAKKATFVCEGYPETFRRARKGSVVYC

DPPYAPLSNTANFTSYAGNGFTLDDQAALADMAERTATERGIPVLISNHDTTLTRRLYHG

ADLSVVKVKRTISRNGSGRNKVDELLALFKAPESDSAAS

>tr|Q87MN5|Q87MN5_VIBPA Uncharacterized protein OS=Vibrio parahaemolyticus serotype O3:K6 (strain RIMD 2210633) OX=223926 GN=VP2197 PE=4 SV=1

MKIVAFGASTSSTSINKTLATYAAEQVKGAQVNVLDLNYYNVPMFSEDKEKEIGQAEGAI

AFLRELEQADAIVVSFAEHNGSYAAAYKNLFDWATRIERNVFQNKPVVYLATSPGPGGAQ

SVLAAATGSAPFFGADVKASVSVPSFYENFDLESGEIVNQEIAEQVKQAVALLK

>tr|Q87NU1|Q87NU1_VIBPA Putative aldehyde dehydrogenase OS=Vibrio parahaemolyticus serotype O3:K6 (strain RIMD 2210633) OX=223926 GN=VP1777 PE=3 SV=1

MKTQKQWIELKNNLNIENRAYINGEYSAALTGQTIPVVNPATDEIFTEIARCQSEDVDLA

VSCARQAFQSGQWSESSPAHRKAVLKQFADLIDQHQEELALLETLDTGKPISHSFSTDIP

GAANSLRWYAEAIDKVYGEVAPTEKDVHAFVSHQPIGVVAAVVPWNFPLWLACWKLGPAL

AAGNSVLLKPSEKSSLTAIFLGQLASQVGLPTGVFQVVTGFGHEAGDALAKHQDVDCIAF

TGSTRIAGQLMVSSGESNLKRVFAEAGGKNANIVFEDCDDLDRAAAETAAGCFYNQGEVC

VAATRLLVHESIKDQFIEKVIQASKAFTPKDPMNPSSSMGALIDQDHKSKVLEYISLGES

EGAIRALWR

>tr|Q87SA2|Q87SA2_VIBPA Probable membrane transporter protein OS=Vibrio parahaemolyticus serotype O3:K6 (strain RIMD 2210633) OX=223926 GN=VP0522 PE=3 SV=1

MTVSLFLLLLALGAFVGVMAGLLGIGGGLIVVPALLFLLPWAGISPEMSMHMALATSLAS

IIVTSGSSALNHLKLGNVDMFVVKWLMPGVVIGGFVGANIAEWIPTHYLPKVFGVIVLCL

AVQMFRSIKVKSEKPMPSSPVTMMYGTGIGVVSSLAGIGGGSLSVPFLNKHGVEMRKAVG

SSSVCGCIIAISGMIGFILHGYKVEGLPAYSIGYVYLPALLAIAMTSMLTTKVGAKLATN

LPTAVLKKIFAIFLMFVAATMLL

>tr|Q87QH1|Q87QH1_VIBPA Transporter, AcrB/D/F family OS=Vibrio parahaemolyticus serotype O3:K6 (strain RIMD 2210633) OX=223926 GN=VP1178 PE=3 SV=1

MSEQNKEPQSDDDVTGIAAYFIRNRVISWMVSLIFLIGGIAAFFGLGRLEDPAFTIKDAM

VVTSYPGATPQQVEEEVTYPLEKAIQQLTYVDEVNSISNRGLSQITVTMKNNYGPDDLPQ

IWDELRRKVNDLKVTLPPGVNEPQVIDDFGDVYGILLAVTGDGYSYKELLDYVDYLRREL

ELVDGVSKVSVSGQQQEQVFIEVSMKKLSSIGLSPNTVFNLLSTQNIVSDAGAIRIGDEY

IRIQPTGEFQSVDELGDLLITESGAQGLIFLKDVAEIKRGYVEVPSNIINFNGSLALNVG

VSFAQGVNVVEVGKAFDRRLAELKYQQPVGVEISEIYSQPKEVDKSVSGFVISLAQAVGI

VIIVLLFFMGLRSGLLIGLILLLTVLGTFIFMKYLAIDLQRISLGALVIALGMLVDNAIV

VVEGILIGTQKGRTRLQAATDIVTQTKWPLLGATVIAVTAFAPIGLSEDSTGEYCGTLFT

VLLISLMLSWFTAISLTPFFADIFFKGQKIKQGEGEENDPYNGIIFVAYKKFLEFCMRRA

WLTVVVLIVGLGASVYGFTLVKQSFFPSSTTPIFQLDVWLPEGTDIRATNDKLKELESWL

AEQEHVDHITTTAGKGLQRFMLTYAPEKSYAAYGEITTRVDNYEALAPLMARFRDHLKAN

YPEINYKLKQIELGPGGGAKIEARIIGSDPTVLRTIAAQVMDIMYADPSATNIRHDWRER

TQVLEPQFNESQARRYGITKSDVDDFLSMSFSGMTIGLYRDGTTLMPIVARLPEDERIDI

RNIEGMKIWSPAQSEFIPLQQVTMGYDMRWEDPIIVRKNRKRMLTVMADPDILGEETAST

LQKRLQPQIEAIQMPPGYSLEWGGEYESSGDAQESLFTTMPMGYLFMFLITVFLFNSIKE

PLIVWLTVPLALIGVTTGLLALNTPFGFMALLGFLSLSGMVLKNGIVLLDQIEIEMKSGK

EAYDAVVDAAVSRVRPVCMAAITTILGMIPLLPDIFFKPMAVTIMFGLGFATILTLIVVP

VLYRLFHKVSVPK

>tr|Q87IF8|Q87IF8_VIBPA Putative DOPA-dioxygenase-related protein OS=Vibrio parahaemolyticus serotype O3:K6 (strain RIMD 2210633) OX=223926 GN=VPA0648 PE=4 SV=1

MYHVHVYFPLQQLEKAQALNEIIRQERQDVLRVYPLVDRLVGPHKMPMFEMHLESISEEF

LAWLDTIRGDFSVLIHPVSERELRDHTESAIWLGRELGVFEEKLEN

>tr|Q87I20|Q87I20_VIBPA Uncharacterized protein OS=Vibrio parahaemolyticus serotype O3:K6 (strain RIMD 2210633) OX=223926 GN=VPA0786 PE=4 SV=1

MLFLIGLQAALIGIGATLIMDLWAWLQRRVFGIPSLNYALVARWVLCMPKGKLVHAPIMS

TDPMPGEKALGWFLHYAIGVVFALAHIAVFGHHWLVEPSLTPGLITGAVTLVFPFLVIQP

CLGFGFAASKTPTPWKARFLSTLAHLAYGLGLFITAFAIKGVSQYFM

>tr|Q87MM5|Q87MM5_VIBPA Peptidase, insulinase family OS=Vibrio parahaemolyticus serotype O3:K6 (strain RIMD 2210633) OX=223926 GN=VP2206 PE=3 SV=1

MHLSPNDSNQYRYLTLSNGLRVLLIHSDTAQQSAAALAVNVGHFDDPVDRQGLAHYLEHM

LFLGTEKYPKVGEFQSYISQHGGTNNAWTGTEHTCFFFDVTPSAFENALDRFSQFFTAPL

FNEEALDKERQAVDSEYKLKLNDDSRRLYQVNKEVINPEHPFSKFSVGNLDTLGDRDGQS

IRDEIVEFHHSQYSADLMTLTLFGPQSLDEQQAWVEAMFADIPNHQLSGKSIDVPIGTED

STGILVQIEPIKEFRKLILTFPMPGMDKHYGVKPLSYFAHLLGYEGEGSLMLQLKSKGWI

TSLSAGGGASGSNYRDFTVSCTLTPEGLDHVDDIIQAVFQYLTMIKQDGMNEWRYLEKQA

VLESAFRFQEPSRPLDLVSHLVINMQHYQPHDIIYGDYKMSGYDEDLQRSLLQYLSVDNV

RVTLIAKGLEYNRTAEWYFTPYSVTTFSSEQKRFFQQIDPRWQFVLPEKNPYICYDLDPM

PFENGGSLPELIEDLEGFRLWHLQDDEFRVPKGVVYVAIDSSHAVASPKNIVKTRLCVEM

FLDSLAKETYQAEIAGMGYNMYAHQGGVTLTLSGFSQKLPQLLEMILRRFAAREFNPTRF

ETIKQQLLRNWRNSSQDRPISQLFNALTGLLQPNNPPFATLAEALEEIEVDELSTFVESI

LAELHVEMFVYGDWQRQQAHDMATTLKDALRVKEQRYEEALRPLIMLGQNGSFQREVHCN

QQDSAVVIYHQCEDIEPRSIALYSLANHLMSATFFHEIRTKQQLGYMVGTGNMPLNRHPG

IVLYVQSPNAAPAELVTSIDEFLNAFYMVLLELNDYQWHSSKRGLWNQIATPDTTLRGRA

QRLWVAIGNKDTEFNQREKVLAELKKLTRADMIRFVVNELKPRTANRLVMHSQGQAHVDA

PRIHLGQEIGSIEEFQLRPKDCGLG

>tr|Q87HN0|Q87HN0_VIBPA Uncharacterized protein OS=Vibrio parahaemolyticus serotype O3:K6 (strain RIMD 2210633) OX=223926 GN=VPA0933 PE=4 SV=1

MKKTIIATIAALTIAPSIALAKDHSQTPSNVQFGGPVTIEKLDTLLKDSNMFTEKDVVVE

GNLLRQVRADTFIFSDGTGEVMVELDDDIRLNSPIDQTTKVRLFGEFEGGNKPEIEVEQL

VIM

>tr|Q87JI6|Q87JI6_VIBPA Flagellar hook protein FlgE OS=Vibrio parahaemolyticus serotype O3:K6 (strain RIMD 2210633) OX=223926 GN=VPA0267 PE=3 SV=1

MSFNIALSGLDATNTELNTISHNIANASTYGFKGARTEFAAVYNGMQPGGVEVASISQNF

DKNGSITGTGRSMDLAINGSGFFVTKDHMGQTLYTRSGVFGTDKSNFVTANNGAKLQGYS

VDSNNNLMTGSVGNIQVSTSSLNAKATDKLDFVANFDASAKAIDKAVTPFDPADPTSFNS

SYTTQVYDSLGNSHTVTQYFTKTADNAWEVNVQVDGGKTPVSTIPVTFNKDGTLAAPTGS

FNVAFPAAGANAMSVDINLKGSTQFGAAFGVSTNSPNGYTSGELAGVRVEDNGMVYATYT

NGQSQLQGQVVLADFANTQGLAKVSGTAWTQSFSSGAPIMGVPGSGTLGNLTPGALEGSN

VDLTSELVALMTAQRNYQANAKTISTSDKLTQALFNAV

>tr|Q87QS2|Q87QS2_VIBPA Integrase, phage family OS=Vibrio parahaemolyticus serotype O3:K6 (strain RIMD 2210633) OX=223926 GN=VP1077 PE=3 SV=1

MSHLIHRLKDKEIEQAQGRDRIYRLPDGGGLYLLVKPNNLKYWEFRYTKPGTKNRTFLGL

GSLDMLNVDGARDKAFEMRKLLKEGIDPKLDRIEKRTQIQQEQACTFKSVADEWVKTKTK

LKPKTVQGNWRKLELYAFPKFGEIPVKSLTPIIVQEAFRPIARRGKLETVKRTIQLVNEI

MRFAINSGLIQHNVLSGVGETFASPDVKHMAVLKPDELTELLQTVATANMQLATKCLIEW

QLHTMTRPSEAAGARWDEIDIENRLWIIPDTRMKMKREHRIPLTDQTIAILERIHPISGH

RVHVFPSMRFPKRSIDSETINKALGRIGFKDRTTAHGMRSLASTTLNERGFDPDIIEAAL

AHQDRNAIRAAYNRTDYLERRRKMMEWWSSYIDDAAVGSLSVTGAVHLRAIG

>tr|Q87I54|Q87I54_VIBPA Glutathione S-transferase-related protein OS=Vibrio parahaemolyticus serotype O3:K6 (strain RIMD 2210633) OX=223926 GN=VPA0752 PE=4 SV=1

MITALYASILALLLVWLAFQVIKQRRLNKVAYADGGVEALQIARSAQSNASEYIPITLIL

MALLEFNGAAPIWIHLTGIIFVIGRIIHARGILQESFKGRVKGMQLTFLVIVSLVVLNMF

YFPYGKLW

>tr|Q87FX3|Q87FX3_VIBPA Uncharacterized protein OS=Vibrio parahaemolyticus serotype O3:K6 (strain RIMD 2210633) OX=223926 GN=VPA1547 PE=4 SV=1

MVNLISTHAIYEYLCSLAVQHIVTDDDQLVFEALKHNDIQHACQAIRETQSGEVVYQHLT

LRFGLRGEQSVYQFELSEQMQYCLDLFSLYLALRQTQFQLEAFHPDLISNVVVPIRVDAL

LWDPGVYFLEQMVQFHSAAFQHVIPSLQADETLCLHERMETLVEKLKENAFALWFEVATS

QTHFERIAEFDPDMIKLSVTIENKQDQHSFLPIARFLRKHKYQWVAGRVASQVELNRYRL

LGASYYFGYFSDIPTSLSFRSFDEE

>tr|Q87GA6|Q87GA6_VIBPA Putative glycosyltransferase OS=Vibrio parahaemolyticus serotype O3:K6 (strain RIMD 2210633) OX=223926 GN=VPA1411 PE=4 SV=1

MKKVLHITEAFGGGVQTALYSYVHSSRHEPYEHFLLARARHNDLTKDSNHDVFAQTKWVE

GGLLQFVRDANQTINDIKPDIVHLHSSKAGFLGRFLKLNRARLVYTPHCYAFEREDISGF

ARQLYKALEKVMLNKIDVVAGCSQRECDLAISIGAKRAELLNNYVSYQSATRVGRDHQAA

LNIVVLGRVAPQKDPQFLLNTLRCLNRFALNRQLDITWIGGGDRELEQALRAEDVQVTGM

LPRDEVVKKLQASDLYLHTAAWEGMPLTILEAAKLHLPMVIRSIGATKDLNYPFLAQTPE

DMAKQMTHCINHYDDIDFRQYTTELNETFSEEKQRLALNNIYS

>tr|Q87NV4|Q87NV4_VIBPA Putative transmembrane protein OS=Vibrio parahaemolyticus serotype O3:K6 (strain RIMD 2210633) OX=223926 GN=VP1764 PE=4 SV=1

MNILLAMIPAFFWGTTYAVTQFTLPDWPPVLLGALRALPAGLLLLAIKPSLPKKHEWKVL

LVLGTINIAFFFGLIFVMALTLPSAISGVGMISVPVFAMLFGWAVYKRQPSAIQGISGAV

LIALAWFLFDPSSISLNPIGLGAMLAAIMCIVIGSSVTKSLGTKMHWWTVLTWQLILGGV

LLSIAAAVLATINPQPYVHAVQNIDMTNTLGLLWVIVLNTALGYGMYVWLLQRMSVVDFT

FGGIANPIAGIVSGLLLLGENFTPLQYSLMVGMIVMSLLPQILTSLRARKEAITV

>tr|Q87SM4|Q87SM4_VIBPA Uncharacterized protein OS=Vibrio parahaemolyticus serotype O3:K6 (strain RIMD 2210633) OX=223926 GN=VP0398 PE=4 SV=1

MDLDQKRNALRVQLETAINDLKKQAEYRGCKIAQRRRSGYLYAVDAARNIVLEPWFTKLL

RQHGTILKKYSRSNAIHLAEIIESYGGLNKTIKLIETVLALRGFSLHTQRRVNYADQVLL

GLKDLRSLTPQHQEEEVRWNSVLPYCALCWRLRSRSHYYCEKHHPIKSTKLYKQQKYAVI

TALKHLPNQNNTAYEKYLAQPNKQKKLGRQLYDLVSGYAPHPRVFLRHCKDSAKAGDWLS

LSKNIVQTCKVNYPASYKKINQIKSDDFGQWSSWCIAIVRCLDPKEPNAWSDKECLTLFN

ELNTWTTLIGILHRFECVERINSIETKRGPDVGYGANLEQHQLIKELLKQQLATNSKTNL

SDIARTLGLSRQRVHQIIKKHQLLS

>tr|Q87LU9|Q87LU9_VIBPA Lipid A biosynthesis lauroyltransferase OS=Vibrio parahaemolyticus serotype O3:K6 (strain RIMD 2210633) OX=223926 GN=lpxL PE=3 SV=1

MNIVPPPLFSYQLLKPKYWSVWLAFGALAIIVNVLPYVVLRILGRSIGCVAMQLMKRRYK

IALRNLQLCFPDYTDWQCKDVVRKNFQYTGMALIETGIAWFWPDWRINRITSIVGKDRLL

TEEKNGRGVLVVCSHHLNLEITARIFSQFAKGYGVYRPNSNPAYEFIQHRGRTRFGHQMI

DRKDVKSMLKVLKNGHRLWYLPDHDYGASHSVFAPFFAVEQAASTVGSSVLIDATKCAVI

SGVTVSRNHHYTLYIGKDLSEYFEKRNAMKAASILNQELEKMIRRDIPAWMWLHKRFKTR

PEGFDCVYT

>tr|Q87SX2|Q87SX2_VIBPA Uncharacterized protein OS=Vibrio parahaemolyticus serotype O3:K6 (strain RIMD 2210633) OX=223926 GN=VP0300 PE=4 SV=1

MSAVIKLAWKSLMNRKATAVLTIMTVAISVILLLGVERIRTQAKDSFANTISGTDLIVGG

RSGQVNLLLYSVFRIGNATNNIDWKSYQKFSQHRAVDWAIPISLGDSHKGFRVMGTNHSY

FEHYKYGSKQPLTFSKGKEFNGLFETVLGSDVAKQLGYQIGSEIIIAHGISDVGFSRHDK

LPFKVVGILAPTGTPVDKTVHVSLEAIEAIHVGWESGARLGPTPDAKVLQERDFQPKQIT

AMLVGLKSRIQTFALQRQINNYPKEPLSAIMPGVALHELWGMMSVAEQALMAVSGFVVIA

GLLGMLSSLLTSLQERRREMAILRAMGARPRHVFSLLISEASLLTAAGIVTGVLGLYAIL

ALLQPLIQQHYGINLTLSTLSAYEWMLLSFVQCAGIVIGFIPAFRAYRQSLSDGMTIRI

>tr|Q87GG8|Q87GG8_VIBPA Putative transcriptional activator ToxR OS=Vibrio parahaemolyticus serotype O3:K6 (strain RIMD 2210633) OX=223926 GN=VPA1348 PE=4 SV=1

MLRNYLLGNQVIFDTLKREVLTTDKIISLGGREAAILKLLCENANTVIAKEEINDKVWGK

VFVSETSLTKAISNLRKSLQLIEGVMCEIKTIPKEGYMLILEGENLGLMVAEDEPPLEVK

RIESKDLALLKAPVGNNRFLSTLAKSDNKMNEGHIKPSWMLLAVLSSAFLSSVTSTAMIL

LLK

>tr|Q87GM9|Q87GM9_VIBPA Putative transcriptional regulator OS=Vibrio parahaemolyticus serotype O3:K6 (strain RIMD 2210633) OX=223926 GN=VPA1286 PE=4 SV=1

MSELEKLNQMLTEFYDKMSSWEQSVVKETGYSLAQVHTIEVLGGHGALRMKELAEKLGIT

TGTLTVQIEKLVNANLIERCPHPEDRRAIVVRLTPEGEKIHRHHNQLHLDLVRDLTRHIE

PEQQSTFLSCLEKMNREF

>tr|Q87IK0|Q87IK0_VIBPA Putative AraC-type regulatory protein OS=Vibrio parahaemolyticus serotype O3:K6 (strain RIMD 2210633) OX=223926 GN=VPA0606 PE=4 SV=1

MPNIEIIRFNHFTARKSERYTATHNGLYVVEEGALVVHQPNGEQFELQAGDFTLYNSSDL

RSAEAIPGENGFKAVALVFDISLFCEFKKAHPGLHSEAEHRRFYPFSPESNSEITQLKNT

LLALASRNAPDYTQSHIAMALLSLMVEVQPDILSIIDDASSLTASQKAIKYIEKNIEKDI

TLEGLAEHMSMSIATLKRRLAAENLSFSQILKVKRINYAATQLRVSQKSITEIAFESGFK

SAAHFSTAFKSIYNITPKDFRNQVVRG

>tr|Q87SQ7|Q87SQ7_VIBPA Putative dihydroxyacetone kinase OS=Vibrio parahaemolyticus serotype O3:K6 (strain RIMD 2210633) OX=223926 GN=VP0365 PE=4 SV=1

MQIEKQHIVHWLELCAKTYQENQDFLTDLDRDIGDADHGLNMNRGFKKVAEKLPQFADQN

IGSILKNTGMTLLSSVGGASGPLYGTLFIRTAAAVGTREQLTFEELVDALKSGVDGVVSR

GKAELGDKTMCDVWLPVLVSVKASLENGMSADHLLNEMVELAETNAVATIEMLAKKGRAS

YLGERSIGHQDPGATSSLLMIKALQQAIAGN

>tr|Q87MD0|Q87MD0_VIBPA Acetyltransferase-related protein OS=Vibrio parahaemolyticus serotype O3:K6 (strain RIMD 2210633) OX=223926 GN=VP2326 PE=4 SV=1

MAIVTRRMLLLPYNESLQLEFVMLNCCAKNRAEMNGPHTVASAKQLFEKILDDENIYSMA

VLESTSRDYMGHVFISHLDSEPELGFIFDKAYWGKGLATEALKAFFPKACRELELHKVKA

NVNSNHQASMAVLEKLGFVKTRESKDLFGPYFEMEFTSDVAVGESSAA

>tr|Q87TC7|Q87TC7_VIBPA General secretion pathway protein N OS=Vibrio parahaemolyticus serotype O3:K6 (strain RIMD 2210633) OX=223926 GN=VP0143 PE=4 SV=1

MKRAVLYVVIFIVCFSVSLIMGLPVSWVLQQAPTVKGLDIQGAHGSVWQGQASSVRWQRQ

NLGQVNWDFQWSSLFTGKAEFSVRFGRGSDMNIRGRGLVGYSLSDGLYAENLVASIPASK

AVEQARLPVPIGVDGQLELNIRHATYAAPWCKTGEGTLVWSASGIQSPVGSLELGPVIAD

LKCQDSVLTASGEQTSTQVSSAFSAEMMPNQRYSTKAWFKPGADFPSSMGEQLKWLGQPN

AQGQYEFDYKGRF

>tr|Q87TR0|Q87TR0_VIBPA Amino acid ABC transporter, periplasmic amino acid-binding portion OS=Vibrio parahaemolyticus serotype O3:K6 (strain RIMD 2210633) OX=223926 GN=VP0008 PE=3 SV=1

MKNWVKVAVAAIALSAATVQAATEVKVGMSGRYFPFTFVKQDKLQGFEVDMWDEIGKRND

YKIEYVTSNFSGLFGLLETGRIDTISNQITMTDERKAKYLFADPYVIDGAQITVRKGNDS

IKGVDDLAGKTVAVNLGSNFEQLLRQYDKDGKINIKTYDTGIEHDVALGRADAFVMDRLS

ALELIKKTGLPLELAGEPFETIQNAWPFVNNEKGQKLQAEVNKALAEMRADGTVEKISVT

WFGADITK

>tr|Q87G97|Q87G97_VIBPA PTS system, fructose-specific IIABC component OS=Vibrio parahaemolyticus serotype O3:K6 (strain RIMD 2210633) OX=223926 GN=VPA1420 PE=4 SV=1

MDITNLIELETICLDLKAQTKDEALKELVEMLEAAGKLNSQSQFLADIWKREEIGNTGFD

DGIAIPHAKSDAVAKPAVAVGISRNGIDYGAEDGELSDVFFMLASPDGDDHHHIEVLAQI

STKIIEDGFVEKLKQAQSREEALEMLTDIQTQSNEFLPTSLEFASEPLSPWAQKLGRIKE

HLLFGTSHMIPFIVAGGVLLSLSVMISGHGGVPQEGILADIAQMGIAGLTLFTAVLGGYI

AYSIADKPGLAPGMIGSWIAVSHYNTGFLGAIVVGFFAGLVVWLLKKIQLPDSMSSLGSI

FIYPLVGTFVTCGAVMWVIGAPIASAMTTMNEVLTGMAGSGKVMLGTVLGAMTAFDMGGP

INKVATLFAQTQVNTQPWLMGGVGIAICTPPLGMALATFLAPSKFKRDEREAGKAAGIMG

MIGISEGAIPFAAGDPARVLPAIVAGGIVGNVIGFMFHVMNHAPWGGWIVLPVVDGKIGY

IIGTIAGSVTTALIVIALKKTVTEDESYTGHSQVYGSVQGEGEADVLAVTSCPSGVAHTF

LAAKSLEKAACALGIKIKVETQGANGVINRITEKDIEKAKFVIFAHDVAIKEPERFRKIK

VLDVTTKDAMLNATALLQARRVS

>tr|Q87KJ3|Q87KJ3_VIBPA Diaminopimelate decarboxylase OS=Vibrio parahaemolyticus serotype O3:K6 (strain RIMD 2210633) OX=223926 GN=lysA PE=3 SV=1

MDYFNYQDDGQLWAEDVPLQALAEQYGTPLYVYSRATLERHWKAFDSAVGQHPHLVCYAV

KANSNLGVLNALARLGSGFDIVSGGELERVIAAGGDAKKVVFSGVGKTPAEMKRALELGI

KCFNVESEPELERLNKVAGELGVIAPISLRINPDVDAKTHPYISTGLRDNKFGIAFDRAP

EVYQFAQSLPNLNVQGIDCHIGSQLTSIDPFIDATDRLLALIDDLKAQGINIRHLDVGGG

LGVVYRDELPPQPSDYAKALLGRLENHQDLELIFEPGRAIAANAGILLTRVEFLKHTEHK

NFAIIDAAMNDLMRPALYQAWQDIVPVSPRNGEPQTYDLVGPICETGDFLGKDRALVLQE

GDLLAVRSAGAYGFVMSSNYNTRTRAAEVMVDGNQSHLVRQREELTSLWQLEQILPE

>tr|Q87H12|Q87H12_VIBPA Putative high-affinity branched-chain amino acid transport ATP-binding protein OS=Vibrio parahaemolyticus serotype O3:K6 (strain RIMD 2210633) OX=223926 GN=VPA1153 PE=4 SV=1

MKGRSTMVSETPLLQVNDISLAFGGVKALTDVSFHVNEKEIFSIIGPNGAGKTSMLNCIS

GRYTPNKGAVIFAGNDVTKRTPSQRAELGLGRTFQNLALFSHMSVLDNIMVGRHHLLKNN

FVTGPLYWFSNAQKEEMAHRKYVEDVIDFLEIQHIRKATAGTLSYGLRKRVELARAIALR

PKLLLLDEPMAGMNLEEKEDMARYIMDLNEELDITIIMIEHDMGVVMDISNRVLVLDFGK

HIAMGEPEEVMANPHVKQAYLGEELPTLEESA

>tr|Q87HH8|Q87HH8_VIBPA Nitrite reductase (NAD(P)H), large subunit OS=Vibrio parahaemolyticus serotype O3:K6 (strain RIMD 2210633) OX=223926 GN=VPA0987 PE=3 SV=1

MSKLKLVVIGNGMVGHRYIEDLVEKADVSQMDITVFCEEPRVAYDRVHLSSYFSHHTADE

LSLVKEGFYEKHGINMLIGERAININRENRIVYSSTGREVQYDKLILATGSFPFVPPIKG

HESKDCFVYRTIEDLKAIEACAKKSKSGVVIGGGLLGLEAAGALKALGVETHVVEFAPKL

MAEQLDLAGGNQLRQKIERMGVNVHTSKNTLEIAAEGKNARNVMRFADGTELETDFIVFS

AGIRPQDKLARQMELELGPRGGVAINDHCQTSDENIYAIGECASWNEMFYGLVAPGYKMA

TVAVDHLLGNTESKFEGADMSAKLKLLGVKVGSIGDANGRTEGCKSYVYQNEEQEVYKRL

IVSEDNKKLLGAVLVGDTSDYGDLLQLMLNEIDLPEHPDALILPAHAGAEKPALGADALP

ESAVICSCFDVTKGKIAQAVADGHHTLGDIKAVTGAGTGCGGCIPLVTSVLNAELAKSGI

EVKNDVCEHFAYSRQELFHLIRIEEIKTFDELLEKYGKGYGCEVCKPLAGSILASCWGEH

ILKPQLVKLHDTNDNFLGNIQKDGTYSVIPRMAGGEVTPQALGALANVAAEYNLYTKVTG

AQRIGLFGAQKDDLPEIWRKLIDAGFETGQAYAKALRMAKTCVGSTWCRYGVQDSVGLGS

YIENRYKGIRTPHKMKFGVSGCTRECAEAQGKDLGIIATDAGWNMYVCGNGGMKPRHADL

LASDLDKETLIKYIDRFMMFYIRTAAPLQRTSVWMENMEGGVDYLRAVIVEDKLGINAQL

EADVAKLVDEYECEWTATINDASQLTRFAHFINSDKRDDNVVFVQEREQHRPATFTEKHP

EAKGDILHVAITEA

>tr|Q87JJ1|Q87JJ1_VIBPA MutT/nudix family protein OS=Vibrio parahaemolyticus serotype O3:K6 (strain RIMD 2210633) OX=223926 GN=VPA0258 PE=4 SV=1

MRHLQSTIHPELDHLDDKIIFQRNAARAIVLDGEDVLLLYTERYHDYTLPGGGIDEGEDV

IAGLVRELEEETGAQNIHSIKPFGIFEEFRPWYKDDADVMHMISYCYTCKIDRELGETAY

EDYEVKNGMRPVWMNVHEAIAHNEKTMAESPKKGMSIERETFLLHLIAKELL

>tr|Q87MU3|Q87MU3_VIBPA Uncharacterized protein OS=Vibrio parahaemolyticus serotype O3:K6 (strain RIMD 2210633) OX=223926 GN=VP2138 PE=4 SV=1

MVNSMARPKTYTDEDLIQVANELISRGKKPSGWRIREYLQRGKSSSIQADLERLIADGRI

PEAQNDIPSATTQVRTSYELPAEIQELLDRKEYDISTSLRDIVIAMNDSIHTHFESITYT

RIREAEAISQHAIKQKEQSEGEALDIEQTLQRATDANDQLEEKIEALQSEVVESQIEKSE

LLKNISQLTSSLNQTNDRLENQQDTICGLQASLSKVEKMNSALTVQLEHALNDAKKLESQ

LTELSERYESTCIKLTESSSKLQSMTEALDNSEHKLSTVQDEYTDLSVNYRILESKLHES

QSNISELKLANHELEKQLTYFLEETDSICDAVDDSSSGH

>tr|Q87R92|Q87R92_VIBPA KtrB OS=Vibrio parahaemolyticus serotype O3:K6 (strain RIMD 2210633) OX=223926 GN=VP0905 PE=4 SV=1

MTQFHQRGVFYVPDGKRDKAKGGEPRIILLSFLGVLLPSAVLLTLPVFSVSGLSITDALF

TATSAISVTGLGVVDTGQHFTLAGKILLMCLMQIGGLGQMTLSAVLLYMFGVRLSLRQQA

LAKEALGQERQVNLRRLVKKIVTFALVAEAIGFVFLSYRWVPEMGWQTGMFYALFHSISA

FNNAGFALFSDSMMSFVNDPLVSFTLAGLFIFGGLGFTVIGDVWRHWRKGFHFLHIHTKI

MLIATPLLLLVGTVLFWLLERHNPNTMGALTTGGQWLAAFFQSASARTAGFNSVDLTQFT

QPALLIMIVLMLIGAGSTSTGGGIKVSTFAVAFMATWTFLRQKKHVVMFKRTVNWPTVTK

SLAIIVVSGAILTTAMFLLMLTEKASFDKVMFETISAFATVGLTAGLTAELSEPGKYIMI

VVMIIGRIGPLTLAYMLARPEPTLIKYPEDTVLTG

>tr|Q87JL0|Q87JL0_VIBPA ABC transporter, permease protein OS=Vibrio parahaemolyticus serotype O3:K6 (strain RIMD 2210633) OX=223926 GN=VPA0239 PE=3 SV=1

MDAKTMTMNSLTTQDKAKSMMGSISRDNIVLFGLLAGLSTMMILFILMPLWAMLAKSVQN

SDGEFVGLANFATYFSSSSLWVSVGNTFSLGLVVTTVVGILAFGYAYALTRSCMPFKGLF

HILGTAPILAPSLLPAISLIFLFGNQGVAKELLGGHSVYGVIGISMGLIFWTFPHALMIL

TTSLRTSDARLYEAARALKTSPMKTFFMVTLPAAKYGLISTLIVVFTLVITDFGVPKVIG

GSYNVLATDIFKQVVGQQNFAMGAVTSIMLLFPAVMAFGADRWVQKKQKSLFDTRSVPYQ

PEPNKTRDGLCFVYCSLISVAVLAVLGMAVYGSLVTFWPWNKALTLNNYNFAEMSTYGWS

PFFNSLTLAGWTALIGTAVIFVGAYCIEKGRAFGPVRQAMQMLSVVPMAVPGMVLGLGYI

FYFNDVNNPLNVLYGTMAFLVINTVVHYYTVGHMTALTALKQLPSEIEATAASVRLPQYK

LFFKVTLPVCMPAVLDIATYLFVNALTTTSAVVFLYSTDTIPASVSILNMDDAGQTGAAA

AMAVMIMVAAAIAKIVQMTLGKWLESRTQAWRKR

>tr|Q87SA7|Q87SA7_VIBPA Uncharacterized protein OS=Vibrio parahaemolyticus serotype O3:K6 (strain RIMD 2210633) OX=223926 GN=VP0517 PE=4 SV=1

MQKHQLDMWLHGQHKDTYTLPKVFVIGCSDVSDYLLAVEYKHQLEPIKDGEEPLHFGSLD

LVKEELLRLGIDKAYLRLHNAYDECGSEGTASYCDIELSLVTH

>tr|Q87HE8|Q87HE8_VIBPA Transcriptional regulator, AraC/XylS family OS=Vibrio parahaemolyticus serotype O3:K6 (strain RIMD 2210633) OX=223926 GN=VPA1017 PE=4 SV=1

MALIEKNTQFDADKLTANVVGIAADVGKHDSGMHQHHKGQLLYAPQGCMTFALNDSICIL

PPTKAVWIPPHTPHRAVMTNVVAYRSVYFDCDKFSCPKSIVMIEVNDLLKALINKMALWE

WDIEQTKTNATTILFWEEFYQAKQFELTLPLPSDRRLASFRNAMTKDGFIAPDLNHLSKT

IGASGKTITRLFKSETGMSYQDWRQQWRLLKAIELLCEERQVSDVAHCLEFSSDSAFIAF

FKKQTGQTPLSFLKHRTLL

>tr|Q87PK2|Q87PK2_VIBPA Uncharacterized protein OS=Vibrio parahaemolyticus serotype O3:K6 (strain RIMD 2210633) OX=223926 GN=VP1501 PE=1 SV=1

MKKIALTLAATSITLVSYSAFSAQDAEHVRLATTTSTYHSGLLDYLLPQFEKDTGYKVDV

IAAGTGKALKMGENGDVDLVMTHAPKAEGTFVEKGYGVLPRKLMYNDFVIVGPKADPAKI

KDDESVLDVFKEIANKNATFISRGDDSGTHKKEMGFWAQTKIEPNFGGYRSVGQGMGPTL

NMASEMQGYTMSDRGTWLAYQNKLDLEILFQGDEKLFNPYQVILVNPERYPTINYQGAKA

FSDWLVNPRGQELINGFRLNGKQLFVANAESK

>tr|Q87MD3|Q87MD3_VIBPA Uncharacterized protein OS=Vibrio parahaemolyticus serotype O3:K6 (strain RIMD 2210633) OX=223926 GN=VP2323 PE=4 SV=1

MTTSTKLVELLYQLEAQLQKHELWQQTMPSPEALQSVEPFAIDTLDPHEWLQWIFIARMH

ALVESSQPLPRGFSIEPYFAEVWKQEPQYAELLNTIRTIDELCK

>tr|Q87FK8|Q87FK8_VIBPA ABC-type arabinose transport system, permease component OS=Vibrio parahaemolyticus serotype O3:K6 (strain RIMD 2210633) OX=223926 GN=VPA1671 PE=3 SV=1

MSVTSSTKILEQQDAKRSWNFAGIWDRFGMLMVFAGLFLLCAFFVPYFATFINMKGLGLA

ISMSGMVACAMLFCLACGDLDLSVASIIACSGVVTAVAINATSSVVLGVGAGLLSGVAFG

LLNGFVIAKLQINALITTLATMQIARGLGYIISDGKAVGITEESFFALGNSSFIGIPTPI

WLTIITFGVFAFLLNRTVYGRNTLAIGGNEEAARLAGVNVVKTKMIIFTVSGFISALAGV

ILAARMTSGQPMTSVGFELVVISACVLGGVSLKGGIGKVSYVIAGVLILGTVENAMNLLN

MSPFAQYVVRGAILLAAVIFDRYKQTSRA

>tr|Q87LU2|Q87LU2_VIBPA Pyruvate dehydrogenase E1 component OS=Vibrio parahaemolyticus serotype O3:K6 (strain RIMD 2210633) OX=223926 GN=VP2519 PE=4 SV=1

MSDMKHDVDALETQEWLQALESVVREEGVERAQYLLEQVLDKARLDGVDMPTGITTNYIN

TIPADQEPAYPGDTTLERRIRSIIRWNAIMIVLRASKKDLELGGHMASFQSSAAFYETCF

NHFFRAPNEKDGGDLVYYQGHISPGIYARAFVEGRLTEEQLDNFRQEVDGKGIPSYPHPK

LMPEFWQFPTVSMGLGPISAIYQARFLKYLEGRGMKDTSEQRVYAFLGDGEMDEPESRGA

ISFAAREKLDNLCFLINCNLQRLDGPVMGNGKIIQELEGLFKGAGWNVVKVIWGNNWDSL

LAKDTTGKLLQLMNETIDGDYQTFKAKDGAYVREHFFGKYPETAALVADMTDDEIFALKR

GGHESSKLYAAYKNAAETKGRPTVILAKTVKGYGMGEAAEGKNIAHQVKKMDMTHVLHLR

DRLGLQDILTDEAVKELPYLKLEEGSKEYEYLHARRKALHGYTPQRLPNFTQELIVPELE

EFKPLLEEQKRDISSTMAFVRALNVLLKNKNIGKNIVPIIADEARTFGMEGLFRQIGIYN

PHGQNYTPQDRDIVSYYKEATSGQVLQEGINELGAMSSWVAAATSYSTNDLPMIPFYIYY

SMFGFQRVGDMAWMAGDQQARGFLLGATAGRTTLNGEGLQHEDGHSHIMAGTVPNCISYD

PTFAYEVAVIMQDGIRRMYGPDQENVFYYLTLMNENYAMPAMPEGAEEGIRKGIYKLETY

TGDKAKVQLMSSGTIMNEVRKAAQILSEEYGVASDVYSVTSFNELTRDGQDAERFNMLHP

EAEAKVPYIQTVMGTEPAIAATDYMKNYAEQVRAFIPAESFKVLGTDGFGRSDSRENLRR

HFEVNAGYVVVAALTELAKRGEVEKSVIAEAIKKFDIDTEKTNPLYA

>tr|Q87L08|Q87L08_VIBPA GTPase HflX OS=Vibrio parahaemolyticus serotype O3:K6 (strain RIMD 2210633) OX=223926 GN=hflX PE=3 SV=1

MFDRYESGERAVLVHINFTQEGEWEDLAEFEMLVSSAGVETLQVVTGSRQSPHPKYYVGE

GKAQEIATTVHLTGAEIVIFNHSLSPAQERNLEALCKCRVLDRTGLILDIFAQRARTHEG

KLQVELAQLRHISTRLIRGWTHLERQKGGIGLRGPGETQLETDRRLLRERIKAILRRLEK

VAKQREQGRRARNRAEIPTISLVGYTNAGKSTLFNRITEAGVYAADQLFATLDPTLRKME

LADVGPAILADTVGFIRHLPHDLVAAFKATLQETQEADILLHVVDASDERFRENIQAVHE

VLEEIDAHEVPTLVVMNKIDNLESQTPRIERDEEGVPRAVWVSAMEGLGIELLFDALTER

LASQMVEHQLRIPPQYQGRFRSTFFQMKCIQREEYDQDGNLLIDIRMQQVDWSRLEKREG

AVLTDFIVT

>tr|Q87N68|Q87N68_VIBPA Putative membrane protein, suppressor for copper-sensitivity A OS=Vibrio parahaemolyticus serotype O3:K6 (strain RIMD 2210633) OX=223926 GN=VP2007 PE=4 SV=1

MIGWVIVVCLMQNSGLVSACSMSDDQTLVSALSSDDAHVVKADKVKEPSKCELSEKLIQF

TQHQLETFIVVLFIGIVLAAVWRCSAFVNARQWTEPIYDKHRIHLTFCVFRE

>tr|Q87RA1|Q87RA1_VIBPA Putative phospholipid biosynthesis acyltransferase OS=Vibrio parahaemolyticus serotype O3:K6 (strain RIMD 2210633) OX=223926 GN=VP0896 PE=4 SV=1

MAQTMNRLNQFWRVLATGFCFSVFGLGGLALSFIVIPTIRIFVSDQTEREYKVQGAIQYS

FNTFCKLMKFSGAIDYEIIGADLLQQDRNCLIVANHPSLIDYVLIASQLNRCDCLVKSAI

WSNPFMKHIVKAAGYIPNETPDDLLALCEERFNKGNVLLIFPEGTRTTPGVKPKLQRGAA

QIAVRTERDLRVVHITVTPSFLTKEKKWYQVPTTKPFFKIEVKDKVEIEPFIKQTTSPTI

AARRLQQHLADNIFPENFY

>tr|Q87FT5|Q87FT5_VIBPA Putative cyclodextrin glucanotransferase OS=Vibrio parahaemolyticus serotype O3:K6 (strain RIMD 2210633) OX=223926 GN=VPA1593 PE=4 SV=1

MNKIKILPLAIALALSGCGSDETTPIDTGSHVASPDWQDQIIYFLMIDRFNDGDSALNDQ

GQNEFDPTSDKKFSGGDLVGVSDKLSYIEDLGATSIWITPPVANQWWDAEQNYGGYHGYW

ARDFQKVDEHFGDMESYQALAREVHARNMFLIQDIVTNHVGNFFTYDDPYSYDPSLPCQG

FRLIKNALPAGQELPYPLNMNECKTDGTGSYHWTPTITDHNDPVQEKTWQLSDLDDLNTS

DPEVRAYLKESYRKWIREVGVDGFRIDTVKFVEHDFWSDFLHADDGVMTQAVDTGRDNFL

TFGEVFETSTPYHTEGEEKMLTYIGESGSPKQLTSVLNFPLQATMTRVFASGQPTDYLRF

RLEKMMEMFPNPYIMPNFIDNHDMPRFLSQGSVNDMKQALITMMSVPGIPVIYQGTEQAM

SAARDAMFAGGYREDGNYVDSFDQNSEMYLFIQELAKLRTENKVMTRGEIKVIGSDKAGA

GVFAFTRTLDNDEVLVVMNTSNSPMLMNQLDVEQAGGTVFKQQIMSNWADAPEQLVADEN

GHVTLELPAKSAVIYSKTSSNQSVNTPDLNIQLAQDWEGKTITGDIVIAGSANANEKLNL

VVDGNLETAQTITVGADGQFSVTLSTRHFAIGEQQHRFAIYSTEKKAGIEDVNFVSNLSW

SNTPDDTIDDAGDAQDGVGGPNGNYSLPTDPTFDKDNSQLAINKAEVFTVGSNVRLTFTM

DKITDTWLPPNGFDHVGFTIFIDLPEEAATNLSELPKINASMPNGTWSRNAVVFGWQSSI

YNTKGANATTWGEAVTPAPTVTVDKANNTISMDFASDALGRPESLDGIRFYVTTWDLDGL

SATYRPLEQDKGPWNFSGGASDESKIWDDLPIITLSE

>tr|Q87FQ2|Q87FQ2_VIBPA Sco1-related protein OS=Vibrio parahaemolyticus serotype O3:K6 (strain RIMD 2210633) OX=223926 GN=VPA1626 PE=4 SV=1

MSRNWSLFLVVAFVLGFGTKTYLDSLDKPADVSETTSSEQSVLFGENNQPVNVFDVNDPR

IRIVYFGFTRCPDVCPTSLAMLAGALNQISDEQKAQLRPMFISLDPERDEADASAKYAHY

FHPMIEGLSAPLDITTPLAHRYGVIFRKTELEGSELKYTLDHSSYFYFLKPDGTLITKVP

HTLTPAPIVEAISKLTTKEGSNEG

>tr|Q87FS8|Q87FS8_VIBPA UPF0056 inner membrane protein OS=Vibrio parahaemolyticus serotype O3:K6 (strain RIMD 2210633) OX=223926 GN=VPA1600 PE=3 SV=1

MKELILHTVTVFMGFFAIMNPIANIPIFLSLTADEDKETVRSIALRSVFIAFVIVAIFAI

AGKVIFDLFGITLYALRITGGILVFMIGFNMLQGDSTHQKTKEKAYSPAQQQAALSIAVS

PLAMPILAGPGTIATAMNFATTGGFDQTIITIVSFAVLCIITYILFLFGDKLVKAVGPSA

LNVVTKMMGLILAVIGTQMFIDGAGEAYKTVFA

>tr|Q87N59|Q87N59_VIBPA Uncharacterized protein OS=Vibrio parahaemolyticus serotype O3:K6 (strain RIMD 2210633) OX=223926 GN=VP2016 PE=4 SV=1

MKRFYSLFALPLITAATWANEPLPDRQTELPAVKKENHGEYLQPRDLSKIPNTAFGDQVK

RGYSLFVNSQQMRGKYVGNEQNCVNCHMEAGRKANAAPLWAAYMAYPAYRKKNDRVNSYA

DRIQGCFEYSMNGKAPAYDSPEIVALSAYAYWLAMGGLLDSYGMNDEAVPELDIKALQVG

GKAQDFPLPDAIAQALPVKERGNLAGRGYPKIAAPKQEPSPERGALVYEKNCETCHRADG

SGIKGTDGHSYIPPLWGEFAYNWGAGMHRINTAAYFIYENMPLGKSVQLTEQEAWDVAAY

INSHERPQDPRFKGNVTANAEKYHQHQGYYGKTLNGKQIGKNAFASGTVE

>tr|Q87H22|Q87H22_VIBPA Putative molybdenum containing oxidoreductase OS=Vibrio parahaemolyticus serotype O3:K6 (strain RIMD 2210633) OX=223926 GN=VPA1143 PE=4 SV=1

MSENEKNKPQGIYKYYENNPKSADEKVFGRVSYPDRRGFLKGAGLATMAAALGGFIPFHR

NMPAGIIPAAFAEGLDDVMIEGKDGLTVLNDRPMNAETPPHLLNDDVTPTRRHFIRNNGI

PPTDVNPETWTLTIDGLVDKPMSLSIADLKKNFDVVEQQLVVECGGNGRAFFDPKASGNQ

WTYGAVACSSWTGVRLADVLKAAGVKDGAIYTAHYGADKHLSGKEGKLPISRGVPIAKAM

GSENLIAFAQNGEALHPMNGAPLRLVVPGWPGSCSQKWLTRIQIRDQIHDGPKMTGTSYR

VPNRPVAPGENVAKEDFEIIERMPVKSLITSPQTNTEVNGNEIAIRGHAWSGDRKVTKVQ

ISIDFGATWMDADLAAPANDGAWQTFNAKVKFPQAGYYEVWAKATDDQGVSQPFAIAWNP

KGYLNNTFHRIALIVRS

>tr|Q87IU8|Q87IU8_VIBPA Uncharacterized protein OS=Vibrio parahaemolyticus serotype O3:K6 (strain RIMD 2210633) OX=223926 GN=VPA0508 PE=4 SV=1

MQLFQSVLIKMEKHQSYKSITAKVSIRKMQRILDQLLNEIDEKHRASKENVVTLTRQSQH

RLMSYKELYLHREAIAESELLLAYESMSDTEKQIADMGLSELTYAIEALDRAC

>tr|Q87PY3|Q87PY3_VIBPA Putative transcriptional regulator, LysR family OS=Vibrio parahaemolyticus serotype O3:K6 (strain RIMD 2210633) OX=223926 GN=VP1367 PE=4 SV=1

MTFNFEQLLAFVTVYEELSFSKAAVKLNKHRTTTGQVISNLEDQLAITLFDRVGRSVEPT

EDGHLLYHYAKKTVEQARIFENIALSLSFGGLERVVIAYSSFMPPSALFMIRKQLAKDFP

TMRVELLVRDRAEIKRGIVDGSIHFGLVNVHESSAMHSMDSVFLGHVEFVPYVQAGGRLA

KLDQSSVVDAMVTERQFILKSLVDEGLKEKFIFSANNEQVDQLSVVINMVREGLGWSWLP

KALTKPQFKAEGIEPVSIDLMLEGMKLPLSMWCPHAKYLKDIKASIMHAVHEHVESYNP

>tr|Q87PD0|Q87PD0_VIBPA Uncharacterized protein OS=Vibrio parahaemolyticus serotype O3:K6 (strain RIMD 2210633) OX=223926 GN=VP1587 PE=4 SV=1

MTHKKLQSVHLSKMDLRMRYVVTLFLLLLPTASTLADDSETNPVAKKIKSTLQKKVDKQF

DQYDGYCDLMIEMEHKGKVAIVKRVTGSGDTKVCRFARSNLKTGKRYRYKYPEKYIRIHI

TTGS

>tr|Q87LM4|Q87LM4_VIBPA UbiH protein OS=Vibrio parahaemolyticus serotype O3:K6 (strain RIMD 2210633) OX=223926 GN=VP2587 PE=4 SV=1

MKQYDVVIAGGAMAGATLALAIEHLSQGALRVAVVEPFKAQSDQHPGFDSRSIALSYGTV

NLLRHLELWSAIEPFSTPIEHIHVSDRSHAGMTDITKQDVGVEALGYVVELADVGRVYQE

LLTHSTAIDLYCPNSAKHITRTQENVTIELASGELLNAKLLVAADGAVSQCCQQIGLELS

EHDFDQVAVIANIVTQEPHQGRAFERFTENGPVALLPMSDNRMSLVWCLRPDEAQIVMEL

SESEFLERLQQDFGWRLGAMQKVGLRASYPLLLRHRKQNISHRFAIVGNAAQTLHPIAGQ

GFNLGIRDVVTLAEELVKQGEDVGRYQGLIRFSQRREADRNETIWLTSSLVHVFSNDLLA

MRIGRNTALAAMDNLSIFKQQLLRHTLGLVKR

>tr|Q87N29|Q87N29_VIBPA PTS system, glucose-specific IIBC component OS=Vibrio parahaemolyticus serotype O3:K6 (strain RIMD 2210633) OX=223926 GN=VP2046 PE=4 SV=1

MFKNLFANLQKVGKALMLPVSVLPVAGILLGVGAAHLSFIPEIVSNLMEQAGGSVFGQMA

LLFAVGVALGFTNNDGVAGLAAIVGYGIMTATLGVMAGVMGVEKIDTGVLGGILVGGVAA

WAFNRFFKIQLPEYLGFFAGKRAVPIITGFAAIILGVILSVIWPPIGGAISAFSDWAAHQ

NPQLAFGIYGVVERSLIPFGLHHVWNVPFFFEAGTCVNAAGETQHGVLTCYLVADEASRA

AGNGFGQLAGGYMFKMFGLPAAAIAIAHCAKPENRAKVMGIMASAALTSFLTGITEPIEF

SFLFVAPVLYGIHALLAGSAYIVANTLGFVHGTSFSHGLIDFLVLSGNAQKMGLMIAVGL

VYAVIYYVVFRAVITALDLKTPGREDETEEAAATSSSDMAGELVAAFGGKANITGLDACI

TRLRVAVADTAAVDQDKLKQLGAAGVVVVAGGVQAIFGTKSDNLKTEMDEWIRNHG

>tr|Q87SH3|Q87SH3_VIBPA Ribosomal RNA small subunit methyltransferase I OS=Vibrio parahaemolyticus serotype O3:K6 (strain RIMD 2210633) OX=223926 GN=rsmI PE=3 SV=1

MTDKNKLPNEGPTLYIVPTPIGNLADITQRAIEVLSNVDIIAAEDTRHTGKLLSHFNIQT

KTFALHDHNEQQKAQVLVEKLLSGQSIALVSDAGTPLISDPGYHLVTKCRQAGVRVVPLP

GACAVITALSASGLPSDRFSFEGFLPPKSKGRKDKFLEIASVERTCIFYESPHRILDSLQ

DMLDVLGPEREVVLARELTKTFETIQGMPLGELIEWVKSDDNQQRGEMVLLLHGHRETSD

EALPDEALRTLGILTKELPLKKAAALVAEIHNLKKNALYKWGLENLD

>tr|Q87SI3|Q87SI3_VIBPA Uncharacterized protein OS=Vibrio parahaemolyticus serotype O3:K6 (strain RIMD 2210633) OX=223926 GN=VP0440 PE=4 SV=1

MLSVTVSLARLWNTMSLYVLLYVQLVTLLVTLVALNVRKLVYVKHVVVHSSLSVNFLLPE

IMLSRIILGLCTKSSAICRAFCFLLSDKTSVLSGYLRTLPLNIHATFYEGFVAIFTLSLC

YKSLNLRVSDFCHLLKTYSV

>tr|Q87SJ6|Q87SJ6_VIBPA Uncharacterized protein OS=Vibrio parahaemolyticus serotype O3:K6 (strain RIMD 2210633) OX=223926 GN=VP0427 PE=4 SV=1

MAQVAEKQRYHVDLAGLMRTYETNYAKLNALLPVSAEVGDVRCYQAANMVYQLTVNEITK

YTTVVEICQSDETPVFPLPTMSVRLYHDARVAEVCSSGEFSRIKAKYDYPNDQLMQRDEK

HQLNTFLGEWLTFCLRSGISRTPLAFN

>tr|Q87QC9|Q87QC9_VIBPA Putative 83 kDa decaheme outer membrane cytochrome c OS=Vibrio parahaemolyticus serotype O3:K6 (strain RIMD 2210633) OX=223926 GN=VP1220 PE=4 SV=1

MNGLKSIATLIFFILLLTGCGPDSKNDQNTSPPALGDFEISVSEPSLVTQETGETKLVVD

FTVKDGSGRSHELDETKDFRIALLKAMPSRVDTQNSSDPAFAFNGRHGNTYWKSFHHSSN

TTNNRASMESVWDGTLVKTDEGYRYTFAIPDVLKVSDPYTADSSSNNGFIAWDADKLHRI

VMAYGEQGNGFTYVFEWVPQESSDAAVSRNVIETGTCENCHMGEPLHHGPGYRSIDNNIA

VCTACHNDSNPGAAPARRPLAAVVHQYHGNVFKLGSDRNNTDTYKQPVDENDVLVTDING

LVIEGNPFPQDARNCTTCHSTDVAKASDANNWFEHPSQVACETCHLYRDRGAHDNQIGTA

WVRNGEPQNSCSGCHRPYDRDDNGDPIIGQDASRSAKTVHVVRLENLAKARDSLEISVES

ARFIDDQFEVELRVSKAGSGIGSINELTPFINEHGHLNLLLNWDNGQGPMVANNSLNVAD

DGALGDGCEAQGEGLFLCHKDFTDAATKPTSNSTLTVNIADMPLCANRRDGELAECVTFE

GIDLIKSPFVIAANNASGSFDVSGINKQRKLPVGADISSCNDCHKELTIHKLGEHPHAAT

DFQQCKNCHNSERSAFYPGMAADLKYHVHSFHAFGSAHSGEASFPGAVNNCEACHTNTQY

NLPSQQNTRPSLASGKYFSPALVACGACHLESSLANADPDTVAGDAPLNHMLNHGAVFGA

DTAAQAMGSEQCATCHAIGQSQGIDKVHKVYDYR

>tr|Q87P73|Q87P73_VIBPA Uncharacterized protein OS=Vibrio parahaemolyticus serotype O3:K6 (strain RIMD 2210633) OX=223926 GN=VP1645 PE=4 SV=1

MENNTPNQMSQIKVPATYMRGGTSKGVFFNLEDLPSEAQVAGEARDKLLLRVIGSPDPYG

KQIDGMGGATSSTSKTVIVSRSSRDDHDVDYLFGQVSIDKPFVDWSGNCGNLSAAVGSFA

IHAGLIPQERILENGIVTVRVWQVNISKTILVHVPIVNGFVQETGEFELDGVTFPAAEIQ

VDFVDPADGEGSMFPTGNLVDDLVVPDVGTFNATFINAGIPTIFIDAESIGYQGTELQDQ

INNDDAALAMFESIRAHGALKMGLISDLEEAQTRQHTPKVAFVSKPKSYQSSSGKAVNES

EIDVLVRALSMGKLHHAMMGTAAVAIASAACVPGTLVNLAAGGGEKESVTFGHPSGTLKV

GAQAKQTEQGWVVQKAIMSRSARILMEGFVRVPSDVFE

>tr|Q87I72|Q87I72_VIBPA Putative transcriptional regulator, LysR family OS=Vibrio parahaemolyticus serotype O3:K6 (strain RIMD 2210633) OX=223926 GN=VPA0734 PE=4 SV=1

MQTRVRLTPPERNRMKNKDKNKKPLHNLDLNLLKIFRVVSEEKKTVAAAKRLNITQPAVS

RAMARLREHFNDPLFVRTRYGLKPTDKGQLLSDSLPKIMDDLSNLILELDEFSPESSSAN

IKIAINGFFGVSFPAKLHLKLLDIAPNITIETESWSSNTISKLINGELDLGINYPLNNVP

KELQTKHLATDEFQILARDAHPLSDQAIDCNDLSLYPIVTAIVPDWNENRTLIECWAERE

NIETETAFRSSSILSLLEVISETDALFPTSAFLSRKHLSGLKSLSLSPQLKASFKGESQD

IYLYMHYRHRNSPIYKWLIPIIESTLSSINDN

>tr|Q87IE6|Q87IE6_VIBPA Iron(III) ABC transporter, ATP-binding protein OS=Vibrio parahaemolyticus serotype O3:K6 (strain RIMD 2210633) OX=223926 GN=VPA0660 PE=4 SV=1

MIVIEKLTKAFGSSKVVDSADTQFEKGKVTSIIGPNGAGKSTLLSMASRLTERDGGSVII

DCKEIAEWDTKELAKRLAVLRQANSITMRFTVKELVSFGRFPYSKGNLTKDDHAVIAQAI

DYLDLQDIQDKYLDELSGGQRQLAFIAMVIAQDTDYVFLDEPLNNLDIKHSLQIMKVIQR

LAHDMNKAMVVVIHDINFAACYSDVILALKQGKVVASGAVEEVIQATTLEDIYETPFNII

ELNGKRMCTYH

>tr|Q87MK2|Q87MK2_VIBPA Site-determining protein OS=Vibrio parahaemolyticus serotype O3:K6 (strain RIMD 2210633) OX=223926 GN=VP2233 PE=3 SV=1

MTENMIHDQASGLRRLTQPSLTKVIAVTGGKGGVGKSNVTLGLAICMARQGKKVMVLDAD

LGLANVDVMLGIRSKRNLGHVLAGECELKDAIVEGPYGIKIIPATSGTQSMTELSHAQHA

GLIRAFGSLEDEMDILLIDTAAGISDMVISFSRAAQDVVVVVCDEPTSITDAYALIKLLS

KEHQVQRFKVVANMVRSYREGRELFAKLTLVTERFLNVSLELVACIPLDDKVRQAVKRQK

IVVDAFPRSPAALAISSLANKALMWPLPKTPSGHLEFFVERLLNRTEFAEDPFGE

>tr|Q87LW8|Q87LW8_VIBPA Iron(III) ABC transporter, permease protein OS=Vibrio parahaemolyticus serotype O3:K6 (strain RIMD 2210633) OX=223926 GN=VP2490 PE=3 SV=1

MKDRHSLWKTSSGAITLLLVLPILAIFYTAIGETDNLFTHLMSTVMPTYIYNTVVLTIGV

MGLSLIFGIPSAWLMAMCKLPTEKWLQWALVLPLAMPGYIIGYIFTDWFDFAGPIQIFLR

DVTGWGPGEYWFPDIRTLPGATFVLSLVLYPYVYLLCRAAFMEQNVSLLQSARLLKCSPW

ESFWRISMPLVRPSIAVGLSLVAMETIGDFGTVSYFAVNTLTTAVYDTWLGYSNLNAAAK

ISAIMLLIVVLLLSTERYSRRKQKLFQSQFNSHEDFRYELSGWKKWAALVWCWGLVAVAF

ILPLLQLIDYSITYFEQSWTPEFREYAWNSLVVSVIAAIIGVAVALIVNFTHRVNGKRES

LAFMRLSSMGYAVPGTVLAIGVMVAVLFMDYRVNDIAKAMEWGRPGLIFSGSMFALIFAM

VVRFSAVAIGSIESNLNKISPSLDMASRTMGCTPNTMLWRVHFPLVKRGALIAALLVFIE

SMKELNASLLLRPFNFETLATYVYNFASDEHLELAALPAVLLVLVGLIPLVVVNRSLEQN

H

>tr|Q87QW5|Q87QW5_VIBPA tRNA 5-carboxymethoxyuridine methyltransferase OS=Vibrio parahaemolyticus serotype O3:K6 (strain RIMD 2210633) OX=223926 GN=cmoM PE=3 SV=1

MTEDRNFDDIAHKFAKNIYGSDKGEIRQIIVWEDFLQILSELDASQQPLEVLDAGGGLAQ

MSQKLAKLGHRVSLCDLSSEMLQLAKQDIEKNGLLEQYRLIHSPVQSIAEHMEEQVDLVM

FHAVMEWLVDPKTALETVLEQVKPGGIASVMFYNHHGLVYKNVVCGNIPHILDGMPHRKR

FKLQPQKGLKPEDVYQWIEEAGFSVCGKSGIRSFSDYIGNMQYMGDYEFEDVLALEKQLC

RQEPYLSLGRYIHVWAKKNDKQE

>tr|Q87I75|Q87I75_VIBPA Uncharacterized protein OS=Vibrio parahaemolyticus serotype O3:K6 (strain RIMD 2210633) OX=223926 GN=VPA0731 PE=3 SV=1

MKKLIPFAIIFTSNVALANCINQSENYVTSKVLKSSDTKITQVDGKLASVINEDLSIVQD

EKIEKVEATNATESCTFDKNSMSLVLHYDTNEHYLTNVHKQAISKYLELVNEDKRILVEG

HTDDVGSQAYNKSLSLRRASTASKYLKKDLGLGNRIVEKAFGESAPICKVNENKLSGCNR

RVVLTIE

>tr|Q87IC6|Q87IC6_VIBPA Arylsulfatase OS=Vibrio parahaemolyticus serotype O3:K6 (strain RIMD 2210633) OX=223926 GN=VPA0680 PE=4 SV=1

MTAKYGVKRRLAILAAALIGATSSSIAAEKPNILVIWGDDIGQSNLSAYTFGLMGYKTPN

IDSIAKEGMMFTDYYGEQSCTAGRSTFITGQTVLRTGLSKVGLPGADLGLKAEDATIAEM

LKPMGYMTGQFGKNHLGDKDEHLPTNHGFDEFFGNLYHLNAEEEPENVDYPKDPEFRKKF

GPRGVIRSYADGKIEDTGPLTRKRMETVDEETLDAALDFMDRAVKAKKPFFVWWNATRMH

FRTHVKPDNQGKTGISTYADGMVEHDNHVGQLLKKVDDLGIKDNTIVFYSTDNGPHMNSW

PDAGTTPFRGEKNTNWEGAYRVPAMVRWPGKIKAGSVSNDIMHHMDWMPTFVAAAGDDNI

KEKLLKGYSAGDKKFKVHLDGYNFLPYLTGKEEKAPREEIFYFSDDGDLTALRYNKWKLV

FMEQRAKGTLRIWAEPFTKLRVPKIFNLRMDPYEVADVTSNTYYDWMLDRAYMLVPAQTY

VGRFLETFKEFPPRQKAASFSLDQVMEKLQENPNK

>tr|Q79YY1|Q79YY1_VIBPA Bacteriophage f237 ORF2 OS=Vibrio parahaemolyticus serotype O3:K6 (strain RIMD 2210633) OX=223926 GN=VP1552 PE=4 SV=1

MAKSVFVLGMDITWNSARGDSAQLNVSRPLREINSEKFKRRTIGESGDVNPQWDQPLMID

HEYALLLERTGALVPRREYQLRLEINPEDPLAGAIVTELIPVDQEIKKHFEASMKPVQG

>tr|Q87LX0|Q87LX0_VIBPA Putative phosphoglucomutase/phosphomannomutase OS=Vibrio parahaemolyticus serotype O3:K6 (strain RIMD 2210633) OX=223926 GN=VP2488 PE=4 SV=1

MIKFGTGGWRAFIGEEFTKDNVRLVAQAVANITNRESVADRGFVIGYDRRFLSDKAGRWF

AEVLAANGIVVSFIDKFVPTPIVMFKAKEMGCAYSACITASHNPADYNGIKVFIEGGRDA

DEIITEKIETQISTLTAQDVKSVDFDQAVEDKLIEIINPMNEFVDSIIDFIDIEAIKKAN

LRVLIDPMFGVAKNALQTVLINGRCEVDVINDGKNPDFGGLMPSPSAATLYRLMHLVEQE

GYDIGIGTDGDADRLGIIDEKGNFIHPNEVLILLYYYLLKYKGWKGSVVRNIATTHLLDK

IAEDHGEKCFEVPVGFKHISSQMEADDSLIGGESSGGLTIRGHIKGKDGVFASSLLVEMI

SVTGKKLSELLDEIYGKYGYAYMAEGDCKFKPAQKAVLFNKIYVEKQLPEFEFDIEKVSY

EDGAKVYFKNGGWIIARFSGTEPLLRIFAEMQDKDTAERVLQQFKDFLSL

>tr|Q87T32|Q87T32_VIBPA Uncharacterized protein OS=Vibrio parahaemolyticus serotype O3:K6 (strain RIMD 2210633) OX=223926 GN=VP0238 PE=4 SV=1

MTTPISDIASVIHHGGKHTVTGSCHELKLPHGSILIDCGLFQGKDIHFGNRRASLDIEFP

VKHIKALVLTHAHIDHIGRLPWLLAAGFKGPIYCTKATAELVPLMLEDGLKLQLGLNYHQ

RQQVLNVIKKQLKPHDYQQWLPLGKQCYLRFQHGGHILGSAYVEFKLPNHEIIVFSGDLG

PSNTPLLPDPKPPKRADYLFIESTYGNKEHEDIATRPERLNAIIDHALQDGGVILIPAFS

VGRTQELLFDIEQLIHQRDLSSSLPIILDSPLAKRVTKTYRRFKKLWGKEAKQKLNNHRH

PLAFEQCITVENHREHQALVNRLASTDEPAIVVAASGMCEGGRIVNYLKALLPDGRNDVL

FAGYQAQGTLGREIQSGSHTVDIDNQPIEANAQIHTISGYSAHADQSDLLKFVTGVPKQP

KAVHLIHGEKEAKKELGEKLEAEGIEVTY

>tr|Q79YT5|Q79YT5_VIBPA Flagellar secretion chaperone FliS OS=Vibrio parahaemolyticus serotype O3:K6 (strain RIMD 2210633) OX=223926 GN=VPA1551 PE=3 SV=1

MLMDSGYDSYQQVDLDAQAAAANPHQLVIMLIDGLLDEIERIRGHLAAKRLAEKGAGINK

CMNILIGLTSALDDENGGEIAENLRQLYDFCQVELYYASVQNDADRLMNVERVMGNIREG

WMNFGQQA

>tr|Q87HL9|Q87HL9_VIBPA Putative cation efflux system (AcrB/AcrD/AcrF family) OS=Vibrio parahaemolyticus serotype O3:K6 (strain RIMD 2210633) OX=223926 GN=VPA0944 PE=3 SV=1

MPDKLKRSFVQSVMNSFFPPIMILLALVVGAAALWLTPKEEDPQIVVPMADVLVSAPGLS

ASQVENQITEPLEKLVSQIDGVEYVYSSSMEGAAQVIVRFYVGENREDALVKLYNKLYSN

QDKVPPSVTNWLVKPVEIDDVPIVVAAIYSTDPDILDRHQLRRIADQATLGIKSLDATNK

VEVIGGEPRKIQIELDSVAMANFKVTIDDLEQAIQLSNSKTQGKNVRVNGQNFTLESGRF

LTNAEEVGDLVIAVLNGKPVYLKDVARIYDGEGEATSDTWYRDKNHDEAYPAVFISVAKQ

KGSNAVNVAQSVRDKLAALQSEQFPPQVQVAVIRDYGETANAKVNNLVSSLGISILTVVV

FVGLFLNWRSALVVGIAIPISYGAALGMDLAFGYSINRVTLFALILALGLIVDDPIASID

NIERYLKRKNLTRTNAIVLAMAEIRSALLMSTVAIVIVFTPMFFITGMMGPYMAPLAFNV

PISVIFSTVVAFMITPWLAKKLLKGAEENGHYDIQSSPMYRLYRGVLIPLLESRKKAWLF

LGLVALLFVLAALLPALRLVPLKLLPYDNKNEFQLVLNMPESSSFVDTSNALSSFTDYLM

SVPEVTSVSGFAGTASPMDFNGMVRHYFMRSEPYQGELRVVLAEKNRRAMQSHELVTRLR

ADLEQIADKFDADVQLVEVPPGPPVIATITAEVYGDEATSYEDLMIQAEKVADRLRKEQL

VSEVDTSIQGDLETWQFIVDQEKAALSGVSVADINNTLITAANAKVLGYIADPREVDPLP

IEVQLRREDRDNLNQLEQLYVRGRPGIAKVESNGAVVDAPQPIVQLSEVGHFVKRAADKP

IFHKNLKPVVYVYAEVVGRVPGEVIADVMADQDTTHAEDVHRHWQDRTYLSNGAGVTWSV

PDNIEVVWSGEGEWKITVDVFRDLGIAYGAALLGVFVVMLIQTGLPAVSGIIMLAIPLTV

IGIMPGFWILNVLSSDIGNYPNPALFTATAMIGMIALAGIVVRNSLVLIEFVQQSLAEGR

SLHDALIESGVVRMRPILLTAGTTLLGNVVITLDPIFNGLAWAIIFGITASTIFTLLVVP

VVYNLAYQNTKGHGLPQMEEEQ

>tr|Q87GA8|Q87GA8_VIBPA Uncharacterized protein OS=Vibrio parahaemolyticus serotype O3:K6 (strain RIMD 2210633) OX=223926 GN=VPA1409 PE=4 SV=1

MESTNSSVPISNTVPLLAAITLALFFVPVKFAVGGVALAPSDIASLLSVGLIALIILEGR

AKQLLHPCIGFLVLFTGYVFINGLLNRVPLAPLLIETVQWLAILCLLSLLYAYGAFDDER

VMVYFTYLLFIICGLVAAWHFAQGYQSGFKLLGVSKYGFGVLCSLLYLYRDKIRLFPFLM

LVAFALLVLSQERKALLGYCLLFFLDQIFIKNWMRKTISETYTWMLLLALAFVVIGTVST

TLYVGFDALADKLEITQEDILFANQSEARWVSNLHRKLLLANGMDILTQHPILGVGAKML

PNFMIDYFNYDELAIYTHNFVLDTAIEYGLLGIAFLFGGYFLFIKFCFRSIDDNRKSLLL

AVYALIMVFFVAVNTTIILILLLPVMISRKRTPEPQTVIHSLPVNKSHEF

>tr|Q87JY7|Q87JY7_VIBPA Uncharacterized protein OS=Vibrio parahaemolyticus serotype O3:K6 (strain RIMD 2210633) OX=223926 GN=VPA0111 PE=4 SV=1

MHQQIIKFWFEELTPQNWFENNPELDKHIASRFASVLEQAARCELFNWRDSAQGRLAEII

VLDQFSRNVYRNTPKAFAQDPLALALAQEAIRLGHDQELAPEQLSFLYMPFMHSESRLIH

VEAEKLFRASGLENNYDFELKHKAIIDQFGRYPHRNAILGRESTPEELAFLQQPGSSF

>tr|Q87T58|Q87T58_VIBPA ADP-heptose-LPS heptosyltransferase II OS=Vibrio parahaemolyticus serotype O3:K6 (strain RIMD 2210633) OX=223926 GN=VP0212 PE=4 SV=1

MKKILIIGPAWVGDMVMSQSLYITLKQLHPESQIDVIAPGWCKPILERMPEIHQAIEMPI

GHGEFNLLGRREIGKSLREKQYDHAYILPKSAKSALIPWFANIPLRTGWKGEMRYGLLND

LRPNMKSFQYMVERYVALAYSKSEMVDSSSLGGLDTLPRPSLSLNKEEQQTTINKFNLDQ

KRPAVGLCPGAEFGPAKKWPETHYAEVAAQMCKTGHQVWLFGSQKDLETCNNIRALIPTQ

FHEHIHVLAGQTSLIEAVDLLAACKTVVANDSGLMHVAAAVGCNVVAVYGSTSPKYTPPL

AEKVEMVHTDIDCRPCFKRECQYQHLKCLTELSPKQVLDSIQKLEAIATSSC

>tr|Q87SD5|Q87SD5_VIBPA Aerobic respiration control protein FexA OS=Vibrio parahaemolyticus serotype O3:K6 (strain RIMD 2210633) OX=223926 GN=VP0489 PE=4 SV=1

MQTPQILIVEDEQVTRNTLKSIFEAEGYAVFEASDGEEMHQVLSDNSINLVIMDINLPGK

NGLLLARELREQANIALMFLTGRDNEVDKILGLEIGADDYITKPFNPRELTIRARNLLSR

SMSTNAVQEEKRSVEKYEFNGWVLDINSRSLVSPAGDSYKLPRSEFRALLHFCENPGKIQ

TRADLLKKMTGGELKPHDRTVDVTIRRIRKHFESVSGTPEIIATIHGEGYRFCGDLED

>tr|Q87T39|Q87T39_VIBPA Putative UDP-galactose phosphate transferase OS=Vibrio parahaemolyticus serotype O3:K6 (strain RIMD 2210633) OX=223926 GN=VP0231 PE=4 SV=1

MMKRLFDFLVSLIALILLSPIIVLVAWKIRKKLGSPVLFRQTRPGLNGKPFEMVKFRTMK

DAVDEQGNLLPDSDRMTPFGEKLRNSSLDELPGLWSVLKGDMSLVGPRPLLMRYLPLYNE

EQARRHDARPGVTGWAQINGRNAISWEEKFALDVWYVDNQTFWLDIKILLLTVKKVFVKE

GISADDHVTMPEFEGSKDDK

>tr|Q87GR0|Q87GR0_VIBPA Uncharacterized protein OS=Vibrio parahaemolyticus serotype O3:K6 (strain RIMD 2210633) OX=223926 GN=VPA1255 PE=4 SV=1

MINGISKIKSLRSFGIYENHINDGCDEFAKFNLIYGWNGSGKSTLSRLFRCIENKSLDGT

NYTESAFEIEYSLDGQAQSVLTQQNLSQNQLNIRTFNNDFVRENIDWSGTVKSILLVDQQ

KIEERKQLDEQNKTLKLKESASEKETKIAGDLDTEINKFLSRTAKSIKTSLKVIGTEDSK

YLNYNKTSLENLIRTSSAEVVDPASVLTDEQIILHTKSASPVEKDKISFPASTIRKDYFE

SSFNALTKLLGKSAVNEVIEFLRDNPDVQTWVSSGMAVHDKHKSEECHFCGNKIDESRLT

SLNNHFSNEFKLLKQEITEATKYCCDLPELTLPSVESFFDEFQTEYKKVVAPLEGVVTEI

NSIVEQWRECIDKKSNDPFDISLKISSVTVDLIDQYNNVILDIAACVKKHNDKSGNFQAV

TTAHKKALELHYAADEVIDFDYSKKVEDRKTANGNVLKLSDEISAIKKEIQRLEAELSNE

SIAVDAFNAELAKFIGRTELTLKFDAKQKGYRISRNGSDRHAQNLSEGEKTAIAFVYFAT

KLEEHDNDIKNTIVVIDDPVSSFDSNHLFHSYSFLKKHCEQAKQLFVMTHNFSYFKLVRD

WMLKKNKMRKQPPVIKARAYTIETKSDGVRQSKLVNAGGSLTDYNSEYHYLFSKLYSLKE

KQELNLDEVYLCANLSRKLLESFLCFKFPKKRSDFRQLVDDGVRHYPAIKPEDVEKAYRF

INKYSHNQEIELEDNADNLLGESPAILNIILDIVKTIDEAHYTEMETVVTA

>tr|Q87PK9|Q87PK9_VIBPA Uncharacterized protein OS=Vibrio parahaemolyticus serotype O3:K6 (strain RIMD 2210633) OX=223926 GN=VP1493 PE=4 SV=1

MRIHAFHRLYQYRQSISTKPFNARGCKVQRCPFCQVSEQHCLCELQPDIDSNVACMLIVS

ENEVFKPSNTGRLIADTVKETYVYQWNRTEPSQEMLDLLSNEDYLPVIVFPADYVDQPER

LLDGLHTEHLHRTDGNSKKWLLIFIDGSWREARKIFRRSEFLQSLPVLSIEPECLSEYIM

RRSENEQHLSTAEVATLVLKQAGENKASECLQLWFEAFRETYMLTKTRVKNDPNRPHLKR

FKEWLKTES

>tr|Q87IR8|Q87IR8_VIBPA Putative cytochrome c oxidase assembly transmembrane protein OS=Vibrio parahaemolyticus serotype O3:K6 (strain RIMD 2210633) OX=223926 GN=VPA0538 PE=4 SV=1

MADKSSHKKLTIKLVLATFAMFGFGFALVPLYDVMCDALGINGKTSDVAAIQPTGMQPDL

SRTIRVEFMAHVNPDMPWEFKPKVISMNVHPGEVVQTEYLAFNESGQRLVGQAVPSVSPG

NGAAYFNKIECFCFTQQPLDGKQHAQMPLIFYIEPDLPDSIHTLTLSYTLYKLPPPTGS

>tr|Q87NL7|Q87NL7_VIBPA Uncharacterized protein OS=Vibrio parahaemolyticus serotype O3:K6 (strain RIMD 2210633) OX=223926 GN=VP1851 PE=4 SV=1

MLINLRLQKVSQELTENKIMQALDWAYDKAINGVAGLDSAQELALSYMKESSDPISQANS

LIRWQNTKAGTSGFLTGLGGLITMPVTLPANITSVMYVQIRMIAAIAHMGGHDLKDDRVK

AMVYACLTGNAAKDILKDIGIVVGRKLTENAIKSISGKTITKINQAVGFRLLTKFGEKGA

INLGKAIPLVGGIVGATFDSVTTNTIGNMARDTFIALPEFA

>tr|Q87FH2|Q87FH2_VIBPA Putative 2-dehydro-3-deoxygluconokinase OS=Vibrio parahaemolyticus serotype O3:K6 (strain RIMD 2210633) OX=223926 GN=VPA1707 PE=4 SV=1

MKHIAIIGECMIELNGKPFGSMHQTFGGDTLNAAVYLSRGCEANSKPDKVKVSYVTALGN

DPISTGMLTRWEDEGISTDLVLRDEIRTPGLYLIQLDDQGERTFLYWRNQSAARYLLQHP

NFGIVKQALTQVDAVFLSGISLAILPEEDRISLLNLLVELKAQGVEIAFDSNFRPALWPQ

DNNQTVKNVYQAMYQLTDVALVTFDDEQLIWGDETPEQTIERLTSLGVGKCIVKLGADGC

LVQDPTNQGSSAALAPQAVPTQPVAQVLDTTSAGDSFNGGFLSAYLAGADLATSCQRGNA

LAGAVIQHRGAIIPKAFTQPALGVV

>tr|Q87KH1|Q87KH1_VIBPA Gene 3 protein-related protein OS=Vibrio parahaemolyticus serotype O3:K6 (strain RIMD 2210633) OX=223926 GN=VP3006 PE=4 SV=1

MQKEFGLTEYIAEEGRLLIAAPRFDLDTFPALGERLVGLLSATVIEKQWDADIHSWLIDF

EGCRLFMKAEHYSEAIWFEALNIEESREELDYLAGLFQRGF

>tr|Q87KS4|Q87KS4_VIBPA Uncharacterized protein OS=Vibrio parahaemolyticus serotype O3:K6 (strain RIMD 2210633) OX=223926 GN=VP2902 PE=4 SV=1

MHTFGKVQQLTANIKSVDLFNLFSLFDVPSELEESFQAIHSRRNKRRIELAKSALESGLD

NSIEVPQPSLTFVVGEVLSHKNLGRGLIEIEYDPLDTLIVDGVITLFAIMQLSGFSHPFE

KKRVSKELILKNDVARQELAHCPIQVNLLFSPTEPLSKKTCITLYKKYSQTEKNIHAPLI

ESVNAELPINTYVREVAKTIDLQAFGGMNTTSIRLSVKDPYVTTEATMIRLVLGAIGGAD

YQDKNKVDVFGSGPFSAKHTNVIKPYICIFMEAWLKSVKGQLFSHKSGFHYSTTLWQSLG

LVIHKLFLNEEPINEFAKAGAFLGQLDYSKSAKHWGDCDALELDASGKLYKNATGGGRAI

RIAMAHYLFNVYQNGKK

>tr|Q87FM1|Q87FM1_VIBPA Uncharacterized protein OS=Vibrio parahaemolyticus serotype O3:K6 (strain RIMD 2210633) OX=223926 GN=VPA1658 PE=4 SV=1

MKSTMIIVSHVVNDAVTHGFVPAAKAMDLHVVLITDHKLSHLELASRDSHFNPDTILECD

VFNPLEIIETITENELKPDAIFSNSDHLQTSTAICAQFFGLPAKDWSVTLKAKNKHLTRQ

VLNEKALPNTQSCLLHRHSNVALGMAFPVVAKPKEGVASLDVQRCDSQSELDAYCDAFWQ

KYPNAAILVEQFLQGPLVTLETLGDGERLIAVGGFDVSLSEPPYFIETAADWNGPNGIAH

REACLAQLKAFGVGFGVCHSEFIITENGPVLVEINYRSIGDGREFLLNNLAHFGWFSTIL

GLHLGRRLDGDYQIHGSAHVHYVVAEHSGVIDGDSTSFIQHDGDVITQQQVLKTAGELFQ

QSYSNKDYLARISIVSPSGRNLDQALQNALNNFNLAPTREVAA

>tr|Q87PK0|Q87PK0_VIBPA Sigma-54 dependent response regulator OS=Vibrio parahaemolyticus serotype O3:K6 (strain RIMD 2210633) OX=223926 GN=VP1502 PE=4 SV=1

MSTQSHISTNQQYNAFSVLVVDDELGMQAILKKALGKLFSHVDTAGSIEEAEQLRNSRHY

DLILLDINLPGRSGIEWEEAFEDDEKRADVIFMTGYADLEIAIRALQLGASDFILKPFNL

EQMLKAVSRCMDRRLNERMQYAMKRDYQRHNTSEIIGGSEKTRQLKQLITQFAPSRASVL

VEGESGTGKELVARGIHQASGRTGPFVPINCGAIAPELLESELFGHTSGAFTGAKKSREG

LFRVANGGTLFLDEIGEMPLSMQASLLRVLEQRTIRPVGSEREISIDVRIVAATNRNLQE

EVNQGNFRSDLYYRLNVLKIEVCPLRERKTDLFELVPFFSNMLTRELGMPAPKWAHEDME

AMSEYDWPGNIRELKNLIERCILLGKPPAHYWRELNGGHSLPNVSITVSHSAELPTFREG

KEFTGEGYPNNWTLKEVEKAHIKQVVNLHEGNKSAAARDLGVARKTLERKYKEWDSEDEG

YAD

>tr|Q87TL3|Q87TL3_VIBPA Uncharacterized protein OS=Vibrio parahaemolyticus serotype O3:K6 (strain RIMD 2210633) OX=223926 GN=VP0056 PE=4 SV=1

MYTRSVVFWLSALTATSTLAADASAATSHPPASTLKQGYFIDSPVTGLYYKTSSNITGVT

QQGAFHYQPGDVISFFLGNDDKGYLLTTLSSQEVLTPTMATTKPSRSINMTRLLLSLDST

PENREEIVLANRVLSDPRFQSQLKSLDLNYLDDAKRHLNLDWVSVDEAIDHLNESQQYIE

ENFASDEIIFEPRGITFKNIVIKKKDWQGRACAYDLRYKHHPKYRPPFGETRFTITKDSL

IEHPSVGDHFQGCFISPNHQITEDIVEPISKFADWFNLVGCSITGCTRNDLNGFSLEDFD

DEGDWKYRSVAMNFDPSTELFMEKVQGLGRNKHIQHSNRTEMLWFSYPNTSEHDIDYLGV

WQQTQYHQQSMTQSCLLMRHQQVMRLPRSAETCPTDASLYTQDVTREFADMWWVNNDEPK

ANLAQMNIMVRWSTTLAEINYTTWEYLPAGANWEQGILYRYQQNVSRNRDGSDHIETHTI

SEFVKVSEEV

>tr|Q87JF1|Q87JF1_VIBPA Uncharacterized protein OS=Vibrio parahaemolyticus serotype O3:K6 (strain RIMD 2210633) OX=223926 GN=VPA0302 PE=4 SV=1

MKIEHVAIWTERLEELKGFYEKYFNAVSNDKYHNPKKHFSSYFLSFESGARLELMSMEGV

TTCENSHSMQVTGLAHFAFALGSEQAVDQITKTLVEDGYQRIDGPRYTGDGYYESCVLDP

DGNRIELTV

>tr|Q87KL3|Q87KL3_VIBPA Uncharacterized protein OS=Vibrio parahaemolyticus serotype O3:K6 (strain RIMD 2210633) OX=223926 GN=VP2964 PE=4 SV=1

MSDIEKVVMRTRTIEKLLRTQYHAEGKGLHQLISSCEERLPHDVIAKLRFIATVRNKVVH

EDNYKLDDRKGFMAACDDCEKELTPRSSRFIWRTAILLMALITLAALGFYYMHWDILSEH

IQ

>tr|Q87KJ9|Q87KJ9_VIBPA Uncharacterized protein OS=Vibrio parahaemolyticus serotype O3:K6 (strain RIMD 2210633) OX=223926 GN=VP2978 PE=4 SV=1

MSQPCPHCGFQFNCICSLVPKLTSKHEILLLMHPNELTRDTNTGQLLQHCQLNVEQAIWD

RKQPPAELLTRLANPSLYPVILFPSEESITLEHVEMQSQQQAKMPLYIILDATWQEARKM

INKSRWLEGIPTMGLSALADSQYQLRRNQQQGNLCTFEVAAHLLGQLGEQDNQQQMHTFF

THYLALFQAEKSGHALKSSPRSLE

>tr|Q87K81|Q87K81_VIBPA Uncharacterized protein OS=Vibrio parahaemolyticus serotype O3:K6 (strain RIMD 2210633) OX=223926 GN=VPA0017 PE=4 SV=1

MGKLIPVSVRLAWLNLQRNRRRSLLSMLIIAIAVFALTSAGGFGLYTYDSLKESTARDTG

HLTLTTPGYFAKEEEMPLSNGLSHADEITKQLIGLSEVRGVQPRVEFSGLISNGSKSSIF

IGIGVNEREFDMKGPFLDVRDGQTLSNIHASRYDASEPEVMLGVDLASNLSVNVGDWITL

LATTTDGALNAYDFKVRGIYSTGVPELDKRQLYVHINSAQSLLGSDKVSALSVFLFDTCL

TNKVEQQVATILNKQKPSFGDQEVEITPWQERAFFYLKVKDLYDRIFGIMGAVMALVVFV

ALFNTMTMSVTERTREIGTLSALGSYPREIIAGFLREAGLLAVIGSLIGALFTALVTIFL

MMVDVQMPPPPGRTEGYPLTIYFSWELVAAAGLSVLLICLVAAFFSARKGVNKPITEALI

YV

>tr|Q87TK7|Q87TK7_VIBPA Phosphogluconate dehydratase OS=Vibrio parahaemolyticus serotype O3:K6 (strain RIMD 2210633) OX=223926 GN=VP0062 PE=3 SV=1

MTHSVVLEVTQRLTERSREARAAFLARTEVQAEAGKGRVGLSCGNLAHAVAASCSSEKKN

ILDFTHANVALISAYNDMLSAHQPYQDYPAQIKQVLADYGHTAQVAGCVPAMCDGVTQGQ

AGMDMSLFSRDLIAQSTALSLSHNVFDATLLLGICDKIAPGQLMGALSYAHLPTAFVPAG

LMATGISNEEKVDVRQKYAAGEVGKDALLDMECRAYHSAGTCTFYGTANTNQLVFEAMGL

MLPGSAFIHPHTQLRKALTDHAALKIASMTAGSAHFRPLAEVVTEKSLVNGIIALLASGG

STNHTIHMIAVARAAGILLTWQDISDLSDVVPLLARVYPNGPADMNAFQDAGGVPALLHR

LNESELLHRDVKPVFGKFEDQMTLPSLVDGQLTWTPCQGSQDGDVIAKPDATFQNTGGTR

VLTGNLGKAVVKVSAVKEEQRVIVAPAIVFQCQHEVEAAYKRGELNKDCIVVVTHNGPAA

NGMPELHKLMPILGNVQKAGFKVALVTDGRLSGASGKIPSAIHVSPEAIRGGAIGLVRNG

DLIRLDCQTGELNNLSDTTGRELIHFDTESTQQTWGRGLFSVIRQNVSSAEEGASFIV

>tr|Q87SI2|Q87SI2_VIBPA Ubiquinol-cytochrome c reductase iron-sulfur subunit OS=Vibrio parahaemolyticus serotype O3:K6 (strain RIMD 2210633) OX=223926 GN=VP0441 PE=4 SV=1

MSNAPLNNGRRRFLTATTAVVGGLGAVAVAVPFIKSWNPSAKAKAAGAPVEVDISKIEPG

QLVRVEWQGKPVWIVRRTQSVLDNLKTISDKLRDPQSEMEQQPEYAQNEFRSIKEEYFVA

VGFCTHLGCSPTYLPDSFSEQVQGVRSGFFCPCHGSKFDMAGRVFQGVPAPLNLVVPKHM

YLSDTKLIVGVDEGDA

>tr|Q87M84|Q87M84_VIBPA RecBCD enzyme subunit RecB OS=Vibrio parahaemolyticus serotype O3:K6 (strain RIMD 2210633) OX=223926 GN=recB PE=3 SV=1

MDQMSGAECPLHTLDRTEQPQPTPLEPMTFPLHGARLIEASAGTGKTFTIAGLYLRLLLG

HGSAETRHRVPLTVDQILVVTFTEAATAELRDRIRARIHDARIAFARGQSSDPVIQPLLN

EFDDHKQAAEILLQAERQMDEAAVYTIHGFCQRMLTQNAFESGSRFNNEFVTDESHLKAQ

VVADYWRRNFYPLPFTLAGEIRQLWSSPSALLSDISNYLTGAPLSLSVPAMKGSLADLHT

ENLKKIDELKAQWRESQDDFFTLISDSDINKRSYTKKSLPTWLEAVNAWAATETTGYDYP

DKLEKFAQNVLLEKTPKGSAPQHAVFEAIETFLANPISLKAPLLAHAIEHCRVMLANAKN

QKQWLSFDDLLTQLSASIDTDESELLAARIRTLYPVAMIDEFQDTDPLQYSIFSRIYLND

PECGLFMIGDPKQAIYGFRGADIFTYIKARNQVSAHYTLGTNWRSSADMVQAVNQVFALP

DSPFIYDSDIPFLPVKYSPNAEKRIWTMGGQKQPALTYWLQEADDKPLPKGEYLTRMAEA

TASQIQTILTQAQQGQACLVNGEKQKAVQAGDIAVLVRTGSEGRMVKQALADQGIASVYL

SNRDSVFTSSVAQDLQRLLQAVLTPENDRALRASLASELFALDAASLDALNNDEVVWENA

VNEFKEYRKLWVQRGVLPMLRAVISKRHIAERLLEEGASSQGENGERVLTDLMHIGELLQ

QASNELDSDHGLLRWLAQSISDAENGLGGSDDQIQRLESERNLVQIVTIHKSKGLEYDLV

FLPFVFSYREASEAKYYDAANDRTVLDITGNDASMKQADKERLAEDLRLIYVALTRAVYA

CFIGASPLRNGRSTKEPTGVHRSAIGYLIQNGQEGGINDLHQGLTKQQDELDCVVVADPP

QQLEDKYVAPQEEIHDLSAKELQNPIDRNWRITSYSGLVKQGSHHAEHDATIEITGFDID

SSEEQDEADLVEPERSIFTFPRGARPGTFLHSLFEEIEFTQPATTEENTQIILGLMESEQ

LDEEWLPILQQLIDTVLVTPLDGKSLLLNQKAPSQRLVEMEFLLPIEVLSAPALNRVIQR

HDPLSAKAGDLGFQTVQGMLKGFIDLVFEHQGKYYVLDWKSNHLGDDVTHYHGEALKSAM

ADHRYDLQYQIYALALHRFLRSRLANYQYEQHFGGVYYLFLRGMDGQSDHGIFAAKPTLD

FLREMDRLIDGQVLETRSTQAGQMELL

>tr|Q87Q66|Q87Q66_VIBPA Uncharacterized protein OS=Vibrio parahaemolyticus serotype O3:K6 (strain RIMD 2210633) OX=223926 GN=VP1284 PE=4 SV=1

MRDLTPEYLLAALRQAIKTKGLTYRELSEKMGMPLSTFKRHLTSTNLALDKLLEYCRAID

CTLDELQKLANQLQGEDEDYFSRTQDEVFFQFPHLYDFYRELRMLRGKDGYTILKKKYDL

SEQSMSDYLGALELLDLVYVDENKSITLHGPLYYSYAENSKLNDKYTEIIKEQTTTHDKC

VRVALARMKITEEQLLELEDQVAKTVIDFHSKNVVAENFNVSDFTNVVLLAGPHQPVTFS

DGIVETESRFIDDIRYAIAAAGDKPSLSI

>tr|Q87H15|Q87H15_VIBPA Putative high-affinity branched-chain amino acid transport permease protein OS=Vibrio parahaemolyticus serotype O3:K6 (strain RIMD 2210633) OX=223926 GN=VPA1150 PE=3 SV=1

MAQLSMRPCGDFRTTYKSDTPIFETKTIRSLAIAGVIAMLAAPLVLDIYFLNLFIQIAYL

GIAALGLNILVGFTGQISLGHGAFFGFGAFASAWLNNQFNIPVVFAIPLAGYLTMIVGML

FGLPAARIKGLYLAIATLAAQFILEDFFARAEWFSGGSYGASASPINLFGFEFSTDESFF

YVALFALIFMYLWASNLIRSRDGRAFVSVRDHYLSAEIMGINLTKYRLLSFGVCAFYAGI

GGALYGHYLGFVSAEGFTIMMSIQFLAMIIIGGLGSVKGTLMGTIFIVLLPEVLEFGVTG

LAAFSDNTSFIDGLAYFKEMAIGLVIMLFLIFEPQGLSHRWQQIRAYWKHYPFSY

>tr|Q87I95|Q87I95_VIBPA Aminotransferase, class II OS=Vibrio parahaemolyticus serotype O3:K6 (strain RIMD 2210633) OX=223926 GN=VPA0711 PE=4 SV=1

MCTKNETKPLPSFIEERLNFHIQDLIKSNENQKHLVLGKRPSENAVVMQSNDYLSLSHNE

LIQKAHRDAISERDDNVVMSAIFLQDDQSKPAFEHQLATFVGMESCLLSQSGWAANIGLL

QTICAPNLPVYIDFFAHMSLWEGARTAGAQIHPFMHNNMNHLRKQIQRHGAGIIVVDSVY

STIGTIAPLRAIYEMAKEFDCGLVVDESHSLGTHGPEGSGLLQKLGLTQMVDFVTVSLAK

TFAYRAGAILGPNKLAQSLPFVAYPAIFSSTVLPQEVVRLEKTLEVIKAADDKRECLFKR

AKELAIGLKRIGFNIRSESQIIALECGSERNTERVRDFLEERDVFGAVFCRPATGRNKNI

IRFSVNADMTAQQVDHVLSACQEAFDHPDLEFV

>tr|Q87TQ6|Q87TQ6_VIBPA Beta sliding clamp OS=Vibrio parahaemolyticus serotype O3:K6 (strain RIMD 2210633) OX=223926 GN=VP0012 PE=3 SV=1

MKFTIERSHLIKPLQQVSGALGGRPTLPILGNLLIKVEENVLSMTATDLEVELVSKVTLE

GDFEAGSITVPSRKFLDICRGLPDDAIITFVLEGDRVQVRSGRSRFSLATLPANDFPNIE

DWQSEVEVSLSQADLRTLIDKTQFSMANQDVRYYLNGMLFEIDGTTLRSVATDGHRMAVS

QTQLGADFAQKQIIVPRKGVQELVKLMDAPEQPVVLQIGSSNVRAEVNNFIFTSKLVDGR

FPDYRRVLPQHTNKTLIASCDELRQAFSRAAILSNEKFRGVRVNLAGSEMRITANNPEQE

EAEEMLDVTFEGDPIEIGFNVSYVLDVLNTLRCEKVQVSMSDANASALIENADDDSAMYV

VMPIRL

>tr|Q87SL7|Q87SL7_VIBPA DNA primase OS=Vibrio parahaemolyticus serotype O3:K6 (strain RIMD 2210633) OX=223926 GN=dnaG PE=3 SV=1

MAGHIPRSFIDDLLARLDIVDIIDARVKLKKKGKNYGACCPFHNEKTPSFSVSQEKQFYH

CFGCGAHGNAIDFMMEFERLEFVEAIEELASYLGLDVPREQRSGGSGQFKSGPQASSSEK

RSLYDLMGSIAQFYRNQLKQPSSKVAIEYLKDRGLSGEIVQKFGIGYVADEWDLVRKNFG

QNKDNQDMLVTGGMLIENDKGNRYDRFRGRIMFPIRDRRGRVIGFGGRVLGEGTPKYLNS

PETPIFHKGKELYGLYEVLQAHREPAQILVVEGYMDVVALAQYGVDYSVASLGTSTTGDH

IQMLFRQTNTVVCCYDGDRAGKEAAWRALENALQFLKTGNTLKFLFLPDGEDPDSYVRKY

GKAAFEQQIEQATPLSSYLFDNLIELHQINLGNNEGKSALRAYASALIDKIPDPYFQELL

EKLLDERTGFDNRLRQPRKKISETRPQPHKEIKRTPMREVIALLIQNPSYAQMVPDLSSV

RDLSIPGLSLFADVLDKCQAHPHINTGQLLEHWRNSQNEALLSRLASWDIPLDEDNQEEI

FLDSLDKIIAQCVEKQIENLQAKARSVGLSAEEKRELLALMLDLKA

>tr|Q87JV9|Q87JV9_VIBPA Putative PmbA-related protein OS=Vibrio parahaemolyticus serotype O3:K6 (strain RIMD 2210633) OX=223926 GN=VPA0139 PE=4 SV=1

MSQEQQLLNAVDYVLSEAKRQGAEADVIVNRNSSFSLKANQGKLDEYKVSSSQVLGVRVI

KDARVATSYSESLEQPSLDLMLTNALQSARFSKQDEHQTISCVNSKITTDIAEIAQDDTT

SVDEKIELSLALEQGVVALPHASSSPYNGYSDGETQLIIANTQGTLCQHFERSFTCYAYT

LFEKDGKQSMAGRMSLGRRFDELNPTYCIEGGYNLARDLLDGVPVATGNYPAIFHINALA

SLFGAFGSAFSGVSAMKGISPLGDKLGQSVASELLTFTDAAYMPNGMAIAGFDSEGFATQ

DNVLIANGQLNTLLHNSQTASYLGAVSTASASRSAKSSLDVSANHKVIATGNSSASEVKA

GEYLELVELQGVHSGADAVSGDFSFGASGFLCRDGQRVQPVRGITVAGNFYKMLQEVEAV

GDTQLINDSRTFFAPDVRFARLSIGGK

>tr|Q87N02|Q87N02_VIBPA Preprotein translocase SecA subunit-related protein OS=Vibrio parahaemolyticus serotype O3:K6 (strain RIMD 2210633) OX=223926 GN=VP2074 PE=4 SV=1

MQYTLIEKGEVQLDESSLFIEGAVLAANLTTKPLAPEAWLEPLFGEGFKSIQPAVEEQIH

KQHNRILRNEYSALELTEKDPEQLADFAEGFMSVWPLIEEQWQEVELNDGLQRMLQALLT

TMMLAIDEESTQKHMRDAGIETPPALTDLVDQLDLMLVEVALGADELMVGNKSQSLNPFK

DVGRNDPCPCESGKKFKQCCGK

>tr|Q87SK3|Q87SK3_VIBPA Uncharacterized protein OS=Vibrio parahaemolyticus serotype O3:K6 (strain RIMD 2210633) OX=223926 GN=VP0420 PE=4 SV=1

METEIELKFFVSPEFSETLRAKISETKVLQHSCRDLGNTYFDTADNWLRQHDIGLRIRRF

DDVFVQTVKTAGRVVAGLHQRPEYNAEHTNNEPDLSLHPSDIWPSGKDVATLQSELVPLF

STNFTREQWLIGMPDGSQVEVAFDQGMVVAQGEDGEERQEPICEVELELKSGQTDALFTL

ARSFCEQGGMRLGNLSKAAKGYRLATGYTGDEVKPLPLVDSNKTDTVEYCLINSLEHALS

HWHYHEQIYSERDSVEALREISNAIRFIRQTLTIFGGVVPRRASAILRQELKWLEEELVW

LDEHAHLEELLDDKGNVLRKLDARKFLVSELTQQLEELPSREEMLTLLSSARYTGLLLDL

SRWILARGWQPFLDEKAREKMASNIMPFSVTQLDRTWAELMEAFPAERDLSAQEYVDQRY

RLLRNLYTGIGFASLYNFDERNSFRLPWADLVHGIDDLLMLNHLLPLVDMLENEEKEQLE

RWLHRQERSILHAMDQTRAISVETQPYWREK

>tr|Q87K37|Q87K37_VIBPA Hypothetical membrane protein OS=Vibrio parahaemolyticus serotype O3:K6 (strain RIMD 2210633) OX=223926 GN=VPA0061 PE=4 SV=1

MPKRQNINQRMSIVALAWPILVEILLRTALGTSDVFMLSGYSDKAVSAVGVITQITFFLI

IVSTFVSSGTGILIAQYNGAGREQESVNVGVASIALSVIIGVLLSVIAVFGAIFLLPYYG

LEAQVEQYAREYLLISGAMTFNVTIGIVFTTILRSHGYSRSPMVVNLISGVFNIIGNYIA

LYQPFGLPVYGVQGVAIATVVSQVIGTLMLWFILARSSIELPMSTMKQVPAEIYKKILKI

GGMNAGEVLSYNVAQICIVYFVVQMGTASLAAFTYAQNIARFSFAFALAIGQAAQIQTGY

YIGKGWVSSILKRVQIYFLVGFVASTAATTLIYLFREEILRVFTDQPEILALAGSLVMGS

ILLEAGRVFNLIFIAGLKGAGDIKFPVQMGILSMWGLGVLFSYIFGIHLGYGVLGAWMAI

ALDEWVRGIIMARRWRSQVWTKFKVS

>tr|Q87MZ1|Q87MZ1_VIBPA Uncharacterized protein OS=Vibrio parahaemolyticus serotype O3:K6 (strain RIMD 2210633) OX=223926 GN=VP2085 PE=4 SV=1

MNNSINNSFLKFGLAACLFSFSAFSFSSPSIEGNDQDMSTGTVTAKMAHADENGWMVVHR

TDESMKPGPVIGYAPLKMGQNENVNAILMEPVESGDMLMLMVHGEKGGMKTGVFEYSLGA

KEDGPVKVDGKLVMDIVRAK

>tr|Q87IW0|Q87IW0_VIBPA Putative heavy metal membrane efflux protein OS=Vibrio parahaemolyticus serotype O3:K6 (strain RIMD 2210633) OX=223926 GN=VPA0496 PE=4 SV=1

MTTEVLNSASEKMSFLDRYLTVWIFVAMAIGVGIGAVYPQVAEWNEAMSVGSTNVPLAIG

LILMMYPPLAKVNYGLLGTVTRDKKAITLSLVMNWIVGPILMFVLALTFLGDHPGYMVGI

ILIGLARCIAMVLVWNDIGGGNKEYGAALVALNSAFQILTYSVMAWLFITVLPPFFGYEG

FVVDISMGDIAESVLIYLGIPFLAGFLSRKWLVAAKGEKWYNDVFIPQISPITLIALLAT

IVLMFSLKGEMILQLPMDVFRVAVPLAIYFVLMFFASFFIGKRMGIPYDKNASIAFTATG

NNFELAIAVSIAVFGLNSDQAFAGVIGPLIEVPVLIALVNVALRMKTKYQS

>tr|Q87H85|Q87H85_VIBPA Putative membrane transport protein OS=Vibrio parahaemolyticus serotype O3:K6 (strain RIMD 2210633) OX=223926 GN=VPA1080 PE=4 SV=1

MDLFALLDINNTLVNIPIGDGYAMSWIEAFGTVFGLLCIWFASQEKTINYVFGLLNVTLF

AVIFFQIQLYGLLLLQLFFFCANIYGWYAWTRPNAQGETLEVRWLSKQKLMATAVVCVVS

IALLTIYIDPFFFALANIAVDSLNIFGAGLSEPVLEPDAFPFWDATMTVLSVVAQILMTR

KYVENWILWVVINIISVGIYATQGVYAMSFQYAILMFIAANGTREWARSAKRNGDKTLAQ

ATA

>tr|Q87PG7|Q87PG7_VIBPA Putative stress protein OS=Vibrio parahaemolyticus serotype O3:K6 (strain RIMD 2210633) OX=223926 GN=VP1535 PE=4 SV=1

MSIYSKILVVADINNDEQPALARAVQLAQKSVSRSRITFFLSIYDFSYDMTSMLSVDERD

AMRRGVIHQREQWMRKIAQPYLNDSFDFDVCVVWHNRPYEAIIAEVYAGGHDLLIKGTRK

HDVLESVIFTPTDWHLLRKCPTPVLLIKNSDWPEQANILASVHVGSENPTHIDLNDAMVE

RLKEMSNRLDAEPYLVNAYPVTPANITIELPEFDPTTYTDAVRGHHLTAMKALRQKHGID

EEQTIVEQGLPEDVIPAAAERLNAAMVILGTTGRTGLSAVFIGNTAEHVIDKINCDVLAL

KPKGYISPLDPNTAT

>tr|Q87LS6|Q87LS6_VIBPA Uncharacterized protein OS=Vibrio parahaemolyticus serotype O3:K6 (strain RIMD 2210633) OX=223926 GN=VP2535 PE=4 SV=1

MDSLIAIAAAMLYVLAIATIIPGLSQQSGIKAKTVFASAACALVFHAWILSDLIFDGSGQ

NLSILNVASLISFIISLVMSIAMLKNRLWFLLPVVYSFAAINLTAATFLPSTFVKHLEND

PKLLIHISFALFSYATLTIGALYALQLAWLDHKLKAKKSLAINPNLPPLLMVERQLFKII

LIGNLLLTGTLITGFVFVQDMFAQGKAHKGILSFIAWIVYSILLWGHYHKGWRGRKVTWF

AVAGATLLTLAYFGSRFVKEIILN

>tr|Q87HY6|Q87HY6_VIBPA Amino acid ABC transporter, periplasmic amino acid-binding protein OS=Vibrio parahaemolyticus serotype O3:K6 (strain RIMD 2210633) OX=223926 GN=VPA0820 PE=4 SV=1

MKLFKATITALLGLAIAMPTLAKEEAATTPNIDQIKERGTLRVGMSTFVPWAMRNKQGEL

IGFEIDVAKRLAEDSGLKVEFVPTAWDGIIPALLAKKFDVIIGGMSVTPERSKSVLFTEP

YSHSGVQVAANKELASGFSEFSDFDSRRVKIAARRGAFTVQVARETFPKAKILQFDDDAQ

AFQEVLNGNAHAVIASSPKPEHETVKHSDKLFLPFSERLSKGNEAFAVRLGEEDKKAFFN

KWIEERTQDGWLKQRYEYWFSTLDWQDQVAQGQ

>tr|Q79YW0|Q79YW0_VIBPA Flagellar M-ring protein OS=Vibrio parahaemolyticus serotype O3:K6 (strain RIMD 2210633) OX=223926 GN=VP2249 PE=3 SV=1

MADKSTDLTVTDGGSDGALVSSSDMDVESQNPDLEERSASKFDMAVGDLDLLRQVVLVLS

ISICVALIVMLFFWVKEPEMRPLGAYDTEELIPVLDYLDQQKINYKLDGNTVSVESSEYN

SIKLGMVRSGVNQATEAGDDILLQDMGFGVSQRLEQERLKLSRERQLAQAIEEMKQVRKA

RVLLALPKHSVFVRHNQEASASVFLTLSTGANLKQQEVDSIVDMVASAVPGMKTSRITVT

DQHGRLLSSGSQDPASAARRKEQELERSQEQALREKIDSVLLPILGFGNYTAQVDIQMDF

SAVEQTRKRFDPNTPSTRSEYALEDYNNGNMVAGIPGALSNQPPADASIPQDVAQMKDGS

VMGQGSVRKESTRNFELDTTISHERKQTGTVARQTVSVAIKDRRQVNPDTGEVTYTPMSE

GEINAIRQVLIGTVGFDQSRGDLLNVLSVKFAEPETEQLVDQPIWEHPNFNDWVRWFASA

LVIIVVVLVLVRPAMKKLLNPAGDDDDEMYGPDGLPIGADGETSLIGSDIESSELFEFGS

SIDLPNLHKDEDVLKAVRALVANEPELAAQVVKNWMNENG

>tr|Q87GK6|Q87GK6_VIBPA Uncharacterized protein OS=Vibrio parahaemolyticus serotype O3:K6 (strain RIMD 2210633) OX=223926 GN=VPA1309 PE=4 SV=1

MWRKPLEREAKESGHVTSVVKYEAGSRFSPHLHPYGEEIFVLDGVFSDENGDYPAGSYLR

NPPGSLHAPFSEKGCVILVKLNQFDPNDLKTVRINTRETEWLPGIGGLQVMPLHDFKHEH

VALVKWPKGEKFQPHKHFGGEEIFLLSGEFRDELGIYPTHTWLRSPHMSEHFPFVEEETV

IWVKTGHLPLGS

>tr|Q87GT3|Q87GT3_VIBPA Uncharacterized protein OS=Vibrio parahaemolyticus serotype O3:K6 (strain RIMD 2210633) OX=223926 GN=VPA1232 PE=4 SV=1

MTLTVWLSLFTVCLLGAMSPGPSLAIVAKHALAGGRMNGLATAWAHAFGIGIYAFITLIG

LAVVLQQSPMLFKTISLAGAAYLAYLGFNALRSKGGVAAKLESGEETTVLQSAREGFLIS

ILSPKIALFFIALFSQFVALGNDLSNQMIIVATPFVVDGLWYTFITLVLSSSRVVDKIRS

KAVLIDRLSGVVLMLLALRVVVTV

>tr|Q87K15|Q87K15_VIBPA 4-hydroxy-2-oxoglutarate aldolase/2-deydro-3-deoxyphosphogluconate aldolase OS=Vibrio parahaemolyticus serotype O3:K6 (strain RIMD 2210633) OX=223926 GN=VPA0083 PE=4 SV=1

MKDLNQQLSEIKVVPVIAIKDAGKAVKLAQVLIENGLPCAEVTFRTEDAALAIKNMREAY

PEMLIGAGTVLTSAQVDEAIDAGVDFIVSPGFNPTTVKYCQQRNVTIVPGVNNPSLVEQA

MEMGLRTLKFFPAEPSGGVAMLKALSAVYPVKFMPTGGVSPSNVKDYLSISSVLACGGTW

MVPGDLIDNEQWDELAKLVREVAGIIE

>tr|Q87G85|Q87G85_VIBPA Putative two-component response regulator OS=Vibrio parahaemolyticus serotype O3:K6 (strain RIMD 2210633) OX=223926 GN=VPA1432 PE=4 SV=1

MSYKVLVVDDEPRIHTFIRISLSAEGFDYIGASTIAEAKACFEAYSPHVILLDLGLPDGD

GTEFLTTLRQTYKTPVLVLTARDQEEEKIRLLEAGANDYLSKPFGVKELIARIKVLVRDL

VDEQSIADELVAGRVKIIKSTHQFWLDQREIPLTKKEFSFIEQLILKPGKLIEQTHLLAV

IWGKSHVEDTHYLRVLVSQLRKKLNDSADEQRLLKTEPGLGYRLVLETSKHH

>tr|Q87IM6|Q87IM6_VIBPA Uncharacterized protein OS=Vibrio parahaemolyticus serotype O3:K6 (strain RIMD 2210633) OX=223926 GN=VPA0580 PE=1 SV=1

MALGFGMKMELQQFLDALASSPEKIEFETTMAVIEDNYDFTPAAFTNGNTQNDANENNGS

CKIFAFGLLNALDKEATLACFGRFYREDVLLHPENNDHQNIRNFMVTGWEGIQFETSALT

AK

>tr|Q87FS3|Q87FS3_VIBPA Uncharacterized protein OS=Vibrio parahaemolyticus serotype O3:K6 (strain RIMD 2210633) OX=223926 GN=VPA1605 PE=4 SV=1

MEDKEMKTNRTFLTVHGVIYTVFAFALFFVPTMIWPMYGVQINDQYALFLSQHTSIFLGG

IAAVSLMLRDVESGKTAKQLFKALLITNGLGAVITTYASITGVFVGFGWSDPIFFVSLSL

LTYKQLRVQ

>tr|Q87TI2|Q87TI2_VIBPA Uncharacterized protein OS=Vibrio parahaemolyticus serotype O3:K6 (strain RIMD 2210633) OX=223926 GN=VP0087 PE=4 SV=1

MRNENIASIIELIHSGNDIYKKAIDNIDNTSLNHVLQDLVHVREHAALELKPYAIANPQT

ADDTPASYTIKAREVYSDVMKEVSSDKESMYLEQLEGVESKVLKEIENVITAQPEQADNA

VLLKVRSDIKSCKEKLSNISLIS

>tr|Q87MA0|Q87MA0_VIBPA Putative transcriptional activator ChrR OS=Vibrio parahaemolyticus serotype O3:K6 (strain RIMD 2210633) OX=223926 GN=VP2357 PE=4 SV=1

MNKHPDNNLLEAYASGSIDAVSGLVVATHLETCSKCRAYVNQVEASQANTVSESPSEYSP

EFDDMLNDIINAEPVNDNVVIQDTAFVNVAGKSFELPKTLVRFSDLVGSWRSYGGKVFSA

QIDLGEDARVSLMYIGENVQIPQHTHRGLESTLVLHGGFSDEDGQYEEGDLMVRDASVKH

SPFTQEGEDCLCLTVLTEPMIFTQGVARIFNLFGKGLYP

>tr|Q87S77|Q87S77_VIBPA T-protein OS=Vibrio parahaemolyticus serotype O3:K6 (strain RIMD 2210633) OX=223926 GN=VP0547 PE=4 SV=1

MAVELNALRDQIDAVDKQMLELLAQRLELVEKVGEVKSEHGLPIYAPDREAAMLASRRAE

AEKMGVPPQLIEDILRRTMRESYASEKDSGFKCLNPELRSVVIIGGNGQLGGLFGRMFKL

SGYQVKVLGSKDWGRADEILKDAGLVVVTVPIHLTEGVIEKLGNLPQDCILCDLTSIKSK

PLQAMLNVHAGPVVGLHPMFGPDVPSLAKQVIVYCDGRGKEQYQWLLQQFGIWGASLCQI

DAQEHDHGMTLIQALRHFTSFAYGMHLSKENPNIEQLLKLSSPIYRLELAMVGRLFGQDP

NLYGDIILASQENIDMIKRFHQRFGEALAILDSKDKAKFVESFEQVSDWFGQYSQQFMNE

SQNLLKQANDNIHRG

>tr|Q87LW3|Q87LW3_VIBPA Aconitate hydratase B OS=Vibrio parahaemolyticus serotype O3:K6 (strain RIMD 2210633) OX=223926 GN=VP2495 PE=3 SV=1

MLEAYRKHVEERAAEGVVPKPLDAEQVAGLVELLKNPPQGEEEFILDLLENRIPPGVDEA

AYVKAGFLTAVAKGEVSSPLVSREKAAELLGTMQGGYNIAPLVELLDDEALAEIAVKALS

HTLLMFDAFYDVEEKAKAGNAHAQKVLQSWADAEWFLSKPKLEEKITLTVFKVTGETNTD

DLSPAPDAWSRPDIPVHALAMLKNEREGINPDQPGTIGPIKQIEELKSKGHQLVYVGDVV

GTGSSRKSATNSVLWFMGDDIPYVPNKRAGGYVLGGKIAPIFFNTMEDAGALPIEVDVTK

LNMGDVIDVYPYEGKVCNHETGEVLAEFSLKTDVLIDEVRAGGRIPLIIGRGLTDKARQA

LGLESSDVFRKPGEVADSGKGYTLAQKMVGKACGVAGVRPGTYCEPKMTTVGSQDTTGPM

TRDELKDLACLGFSADLVMQSFCHTSAYPKPVDVNTHHTLPDFIMNRGGVSLRPGDGIIH

SWLNRMLLPDTVGTGGDSHTRFPLGISFPAGSGLVAFAAATGVMPLDMPESILVRFKGKM

QPGITLRDLVHAIPYYAIQQGLLTVEKAGKVNEFSGRILEIEGLETLTVEQAFELSDASA

ERSAAGCTVKLSQESIEEYLNSNIVMLKWMISEGYGDRRTIERRITAMEEWLAKPELMSA

DSDAEYAHVIEIDMAEIHEPVLCAPNDPDDARLLSEVQGTAIDEVFIGSCMTNIGHFRAA

GKLLDKFNGQLATRLWVAPPTKMDKDQLTEEGYYGIFGRAGVRIETPGCSLCMGNQARVA

DKSTVMSTSTRNFPNRLGTGANVYLSSAELAAVGAILGKIPTKEEYLEYAKQIDATATDT

YRYLNFHKMEQYTKKADEVIFQEPA

>tr|Q87TL0|Q87TL0_VIBPA Putative transcriptional regulator, LysR family OS=Vibrio parahaemolyticus serotype O3:K6 (strain RIMD 2210633) OX=223926 GN=VP0059 PE=4 SV=1

MAHQLDALDLNLMRLLKAVVENRSIKLAAMQLGISQPSASRGVMKLKQVFDDPLFVRKAH

GVEPSPMAIRLAAEFDNMIAPLEKVVQEFEVFDPQQYQGQIAIVTDPYLMDEQGQRLLTG

CHRAFPKAHFAFSSWNSYSHDEMLEGEHDYCILDQETELSKDIYMRPLFVEKRVILARKN

HPTLSKVSNDWDTVSKLPLVSLPAPASYKPLCTVESEYRRMGYEPVVLLKSYNLRVACQM

LQETDAIMYASQSSALLMPELASYAMPLVNREFSQFVVSGGFLQTNRNHPLHRHLHKVVR

QTLNTPLIFTQ

>tr|Q87KB9|Q87KB9_VIBPA Acetolactate synthase OS=Vibrio parahaemolyticus serotype O3:K6 (strain RIMD 2210633) OX=223926 GN=VP3058 PE=3 SV=1

MTGAQLVVAALRQQGIKTVFGYPGGAIMPIYDALYDGGVEHILCRHEQGAAMAAIGMARS

TQDVAVCMATSGPGATNLVTGLADAFLDSVPLVAITGQVASSHIGTDAFQEMDVIGMSLA

CTKHSYLVTDIEDLAPTLAEAFEVAKTGRPGPVIVDIAKDVQLAQAPTELLPPYVAPEIE

DVSAEDIKRAQDVLAASTRPVLYVGGGVQLAKATDAVREFLRLNPMPAVSTLKGLGTIER

HDPHYLGMLGMHGTKAANLVVQEADLLIVVGARFDDRVTGKLDTFAPHAKVIHIDIDAAE

IHKLRHANAPLRGDINTILPQLELTQDISSWVHHSESLRSGFKWRYDHPGDLIYAPLLLK

QLSDMMPDSAIVSTDVGQHQMWAAQHIQPRDPQNFITSAGLGTMGFGLPAAMGASVARPD

DQSILITGDGSFMMNVQELGTLKRRQIPVKIVLLNNQRLGMVRQWQSLFFDGRHSETILD

DNPDFVMLAKAFDIPGKTITKKEEVEPALKEMLASETSYMLHVLIDEEENVWPLVPPGAS

NSDMLENT

>tr|Q87NR1|Q87NR1_VIBPA Uncharacterized protein OS=Vibrio parahaemolyticus serotype O3:K6 (strain RIMD 2210633) OX=223926 GN=VP1807 PE=4 SV=1

MSINIKWDGDCRFKVSTEGGFTFNVDATSETAPCPTEVLLSALGSCSATDVVLLLQDQGF

EVKGLKNKVTFALTESEPRLYKSANLHFTVNGSGFKESDILRAAQEAVEKHCHVCLMLSP

TIDITCSAEVGKNCT

>tr|Q87QA8|Q87QA8_VIBPA Uncharacterized protein OS=Vibrio parahaemolyticus serotype O3:K6 (strain RIMD 2210633) OX=223926 GN=VP1241 PE=4 SV=1

MDMTDQAFFKYVRNFNEYQKRSMFGGIGLFSDDAMFALVSNDCCYLRGGNGLDEEFTLLN

CEKYKHVKKQTTATVNYYDVTDLFESGFTGLDDLLRKSIDCSIKERKYQKSSASKRLRDL

PNMQLTLERMVKKAGIDDVETFLELGPVEVFNKVRVAYGNDVDVKLLWKFAGAIDGVHWK

LIQEPRKKQLLALCE

>tr|Q87SG0|Q87SG0_VIBPA Cell division protein FtsZ OS=Vibrio parahaemolyticus serotype O3:K6 (strain RIMD 2210633) OX=223926 GN=ftsZ PE=3 SV=1

MFEPMMEMSDDAVIKVVGVGGGGGNAVEHMVRESIEGVEFISVNTDAQALRKTSVGNVIQ

IGGDITKGLGAGANPQVGREAALEDRDRIKDSLTGADMVFIAAGMGGGTGTGAAPVIAEV

AKELGILTVAVVTKPFSFEGKKRLAFAEQGIDELSKHVDSLITIPNEKLLKVLGRGVTLL

EAFASANDVLKNAVQGIAELITRPGMINVDFADVRTVMSEMGHAMMGSGIAKGEDRAEEA

AEMAISSPLLEDIDLAGARGVLVNITAGLDMRLDEFETVGNTVKAFASDNATVVIGTSLD

PDMTDEIRVTVVATGIGNERKPDITLVAGGKAKVASAPQAQPQQVAATQAEEKPAQTLQN

QVQEKPQVTPQPTNTVSSSPAAGQSSAAPKQEKESGYLDIPAFLRRQAD

>tr|Q87JW5|Q87JW5_VIBPA Putative ABC transporter substrate-binding protein OS=Vibrio parahaemolyticus serotype O3:K6 (strain RIMD 2210633) OX=223926 GN=VPA0133 PE=4 SV=1

MNKNKLMSAAALLASMVSTNVLAAEKELTLMLDWFVNPNHGPIVIAQERGYFKQQGLKIN

IQEPADPSTPPKLVAAGKVDMAISYQPSLTIDVAAGLPLIRSATLIATPLNTLMVLDNGK

NDNLGDLKGKKIGIAIAGNEEATIGTMLAQENVKFSDVQIINVGWALSSSLASGKVDAIW

GGLRNFETNQLALEGYKAKAFFPEEHGVPAYDELVFVANAKTYDKAAIKAFNKALEQATT

YIVNHPKESWKEFVAYSPDTLNNELNQRAWNDTLTRFALRPSAVDLKRYDEYAEFMYSQK

IIETLPKAKDYVPSFD

>tr|Q87PD3|Q87PD3_VIBPA Putative phage replication initiation protein (Alteromonas phage PM2) OS=Vibrio parahaemolyticus serotype O3:K6 (strain RIMD 2210633) OX=223926 GN=VP1583 PE=4 SV=1

MDFDTKAIEIKMAGKTFANDAIHQSAFSRRFFPRLPAIVRNDVRRKVEARTQRQNATREN

VIKTAKDAVKFGLKCAHHIENRYSFVDSRKGAHSEPLTHNILMRDDALTKFAEKYADQCA

EILSSLNAEGYASFIEALAAVYSEQKALLKTIHIKPPYVNFNAKDVEVLEQMLTAAVLKM

QSEKWVERRLLRLRGDYIEYAQITMSRVGDKGHQSKYVSEISFSNWKRKQRESEKYMKSM

SVYNEETGEHFPLEEVAKRTIANPENRRIEMMVRSRGFEELADELEYTALFITWTLPSRY

HRNSPKWDGSSVKDGHAELMRQWSLARAKLAKLEIEYFGFRVAEPHKDATSHAHYFLFCS

HKDKANIIRILRGEAIAPDREELGDDITPRFDVKEADPSKGGATAYIAKYVSKNINGKHM

PDTEAEESAFKVRAWASVHRIRQFQQFGGEPVSLWRSLRRATAEQTQKDDQLEELRQAAD

SSKWALFCQLAKGAKLAYKENKNDYGEPIKKIIGFEWCGQVIETASECYSLVKTKDVKRL

LKSRGATSWSTENNCNSPLITELKKLTGWSFEGVKCLLEPLANGATVSIDQYCSIKLQNN

TLRLI

>tr|Q87SN6|Q87SN6_VIBPA Putative inner membrane protein OS=Vibrio parahaemolyticus serotype O3:K6 (strain RIMD 2210633) OX=223926 GN=VP0386 PE=4 SV=1

MELQALRYASMISTMTFDKACDYYAQYLKKEGLVVEAREAILEFVDLDENSLDDFGNDVR

IVLASADFGKELTTSVLWLRDKSIDISCVRLTPYRYREDVLINAEQIIPVPEVEEYQVKF

REKRAEQRTSVQKGEKDYSEYRYNGHTYKKRHLALALVTDWIEKHQPQSLNDVLNAFNEP

VRRRIAILADEIPQGRIRRFHNDEDALITLPNDEVIAITNQWSLSNITRLILFAEQSGMV

VEKAD

>tr|Q87LY5|Q87LY5_VIBPA Uncharacterized protein OS=Vibrio parahaemolyticus serotype O3:K6 (strain RIMD 2210633) OX=223926 GN=VP2473 PE=3 SV=1

MSGTSFGRRFVERPWLVSLILILLLVAWLAAGQLKAQGDHTLSPSLQSENTPLAKVMFDT

FTAKPTSKTIELYGRTAPNRQARLGAEVAGKIVSLSINKGQLVKQGQVIANIDKRDLDSQ

LKRAQAMLRVKEKEFNAAKSLKSRGLQGEVAFATAEAALVDARANLNNVQTALKNTEVKA

PFDGIVDHHFVEVGDFVGVGDPIATVIDLETLVIEADVSERHIQYLKEGLQADVRTINGQ

HHLGTLRYIGRVSSVSTNTFPIEIEIDNRNSLIPAGISAEVQLPLNEVLAIKITAAMLAL

DEEGNLGVKTLQDEHVKFVPIQLVKAEEDGVWLSGLGEQADIIVLGQGFVRDGETVIANK

VGDVAADAAEK

>tr|Q87KH6|Q87KH6_VIBPA Thioredoxin OS=Vibrio parahaemolyticus serotype O3:K6 (strain RIMD 2210633) OX=223926 GN=VP3001 PE=3 SV=1

MSDKILQLTDDGFENDVINAAGPVLVDFWAEWCGPCKMIAPILDEIAEEYEGKLTIGKLN

IDHNAGTPPKFGIRGIPTLLLFKDGNVAATKVGALSKTQLKEFLDANL

>tr|Q87S85|Q87S85_VIBPA Uncharacterized protein OS=Vibrio parahaemolyticus serotype O3:K6 (strain RIMD 2210633) OX=223926 GN=VP0539 PE=4 SV=1

MELVISLLQQMCVYLVLAYMLSKTPIILPLLSISSRLSHRLICYVLFSGFCILGTYFGLH

INDAIANTRAIGAVMGGLFGGPVVGFAVGLTGGIHRYSLGGFTDLACAISTTAEGVIGGL

LHVYLIKRNKGALLFNPSVVFSVTFVAEVVQMILLLAVAKPFDQAYELVSAIAAPMIIAN

SFGAALFMSILQDRKTIFEKYSATFSRRALTIADRSVGILSNGFNTENAEKIARIIYEET

KVGAVAITDQEKILAFVGIGDDHHRPNTPISSQSTLDSMEKNDIIYLDGTERPYQCSLAK

DCKLGSALIIPLRAGKAVIGTIKLYEPKRKLFSTANMSMAEGIAQLLSSQILYGDYQQQQ

ALLAQAEIKLLHAQVNPHFLFNALNTISAITRRDPDKARELIQNLSHFFRSNLKQNINTV

TLKEELAHVNSYLSIEKARFTDRLEVEIDIDPELLDIKLPSFTLQPLVENAIKHGISNML

EGGKVKIYSEMHPQGHLITVEDNAGSFQPPKDNHSGLGLEIVDKRLTNQFGRDSALKIAC

VTHQFTKMSFIIPPKS

>tr|Q87PA9|Q87PA9_VIBPA Uncharacterized protein OS=Vibrio parahaemolyticus serotype O3:K6 (strain RIMD 2210633) OX=223926 GN=VP1608 PE=4 SV=1

MNIEQYQRLTKQAVALIESEPDFIANLANLSSLLFMELEDLNWAGFYLTKGDELVLGPFQ

GKPACVRIPMGRGVCGTAAKTNTTQRVYDVHEFEGHIACDAASNSEIVIPFSIKGKVAGV

LDIDSPSIGRFNETDEEGLTHFMSEVEKLLNSHANDA

>tr|Q87GY9|Q87GY9_VIBPA Uncharacterized protein OS=Vibrio parahaemolyticus serotype O3:K6 (strain RIMD 2210633) OX=223926 GN=VPA1176 PE=4 SV=1

MVGLKKNIWTLYMMLLTVSIVTFSLFGYYHYQATLDKYKDKQLLQLELFASSVESLLKGQ

ESLLEVVGHQLVEQNNFTRTAAIQTRPMLDKLLNIHPAIIGFGLTNPNGDYISVSSNLIL

EKLHNLKQDPLTRETFLEALNSDRMVIGRTYFMEAQDSLVIPIRKAIPDKNGVVQAVMTA

GFNMNTSSVFRNDIHANEHNRVSLIRNDGYLTFSSSEDTTIKDYQKPADDLNKQALLDQI

QQDYGWDTDQVKQLTRAINVVVDTQRLNELITLKYLPDYGLWAASSTDLGFIKRGFYSQF

AFYCIVFLIVQAAFYALFRSIANNEHETKERLLYQACHDHLTRLPNREYLRSNIQRWMCG

SSNPFTLMFIDIDNFKSVNDTHGHEFGDEVLKQISTRLNHFSGEGRLIVREASDEFIFIV

NRTDEETIKDLASELIQTLSKPYNVNDNQFLLSCSIGIAFYPMHGDNLDALLLSADIAMY

QAKKQRNAYSLFNQEMQASHLHKMKVEQRLRLAIEKQTLFMAYQPQLNINGKIYGVEALV

RWEDEELGKVPPNEFVPVAESSGLMVRLGELIIEKSLEDMGLLTTHLATPIQMSINISVK

QFLHAKFIERLMAAMDKYHLDCNRITLEITENLFIEDLEKFSPTCERLHALGFKISLDDF

GTGYSSLSMLRTLPIDEVKIDKSFVDNIEHDKKALNMVKNIIAIGKNFEMKVLAEGVETQ

RQRDQLEACGCDLIQGYFYSKPLSFDQLVSFVKDNKEEKAIID

>tr|Q87MT1|Q87MT1_VIBPA Putative nitroreductase OS=Vibrio parahaemolyticus serotype O3:K6 (strain RIMD 2210633) OX=223926 GN=VP2150 PE=4 SV=1

MEALDLLLNRRSIAKLSAPAPEGKALENIIRAGLRAPDHAGLTPWRFVIAQGDGLKKLSD

ILVKAAIADHSDEAVIEKVKNAPFRAPIVITVIAKVTEHEKVPALEQYLSAGCAVQAMQM

AAVAQGFQGFWRSGKWMFHPEVHQAFGLEGEDEIVGFLYLGTPGCTPMKVPERDLSKFVE

FQ

>tr|Q87IE1|Q87IE1_VIBPA Uncharacterized protein OS=Vibrio parahaemolyticus serotype O3:K6 (strain RIMD 2210633) OX=223926 GN=VPA0665 PE=4 SV=1

MKGPQVIYLRAFLLSKIRFYRIQSSSSVSLRENISTCDSIMPFTCSNCFSESASHAIMRQ

SPFKLNSAFPAGNVTKPLCSSHVAGTLRAKCRHSVTKYLLACSLSIWVVSLIILSLCLFV

EKGPLSRAL

>tr|Q87GL9|Q87GL9_VIBPA D-alanyl-D-alanine carboxypeptidase OS=Vibrio parahaemolyticus serotype O3:K6 (strain RIMD 2210633) OX=223926 GN=VPA1296 PE=3 SV=1

MRKITLFNTTFLTGLVTFSHFAFAAPTVVPNAPELSSRGYVLMDYHTGKVLVERDADKRL

NPASLTKLMTAYVAGQEVNAGNISLDDQVVISRNAWAKNFPDSSKMFIEVNTSVPLSDLY

RGLVVQSGNDASVAIAEHVAGSEAGFVSLMNSWASQLGLTNSSFTNPHGLDSDGLYSTPH

DIAKLGQAIIRDLPDIYPMYSETSFTYNGITQYNRNGLLRDRSMNVDGMKTGYTSGAGYS

LATSATNGDMRLIAVVMGAKSQSVRESESKQLLSYGFRFYDTLMPTAAGTDIANARVWMG

QKDELKVGVNRDVYLTLPKGDVNKLKAEVEYNGDLLAPIAQDQVVGTLLYKVDGKVVKET

ELVALEPVEEGGIFKRIMDWFKRLVASWF

>tr|Q87KK2|Q87KK2_VIBPA Uncharacterized protein OS=Vibrio parahaemolyticus serotype O3:K6 (strain RIMD 2210633) OX=223926 GN=VP2975 PE=4 SV=1

MSTSLPCKEITKIVASDLDGTLLAPNHQLSAYSKETLKALHEKGYTFVFATGRHHVDVAS

IRRQVGIPAYMITSNGARVHDQNDQLMYSENVPADLVQGVIDTIKHDHEILIHMYQNDSW

LMNKDDETLRDFHDEFTYVLFDEDQAPTDGIAKIFFTHPAQDHERLVVFENKLREQFGDK

LNIAFSTPWCLEVMSAGVSKGHALQAVAETLGLTLENCIAFGDGMNDVEMLSMAGKGLVM

GTSHEKVMKALPNNEVIGSNADDAVAHYLQDHLL

>tr|Q87TC1|Q87TC1_VIBPA Uncharacterized protein OS=Vibrio parahaemolyticus serotype O3:K6 (strain RIMD 2210633) OX=223926 GN=VP0149 PE=4 SV=1

MIVSPTNVSVPLIAPSVNVQTEQAARDNRVREPVTPTVALAKTNAERKVKSDEKRRRQSS

WDPSEHPDYEVDHEVDARYHEEPEDTLERLFDLLALKSYSEDQGKGYAIRFRLPKRILDA

AINQGLMEKRRKVIKFHYGHSVVPHTPSEVIAVL

>tr|Q87TF0|Q87TF0_VIBPA Nitrogen regulation protein OS=Vibrio parahaemolyticus serotype O3:K6 (strain RIMD 2210633) OX=223926 GN=VP0119 PE=1 SV=1

MDTSLPSAILNNMVTATLILDDGLAIRYANPAAELLFSQSAKRIVEQSLSQLIQHASLDL

ALLTQPLQSGQSITDSDVTFVVDGRPLMLEVTVSPITWQRQLMLLVEMRKIDQQRRLTQE

LNQHAQQQAAKLLVRGLAHEIKNPLGGLRGAAQLLEKMLPDPSLTEYTHIIIEQADRLRA

LVDRLLGPQKPGKKTQENLHQILEKVRQLVELESQNSIIIERDYDPSLPEILMDADQIEQ

AMLNIVSNAAQILAHQEHGNITIRTRTVHQANIHGKRCKLAARIEITDNGPGIPPELQDT

LFYPMVSGREGGTGLGLSISQNLIDQHNGKIDVESWPGHTTFTIYLPI

>tr|Q87PV8|Q87PV8_VIBPA Putative ClpA/B-type protease OS=Vibrio parahaemolyticus serotype O3:K6 (strain RIMD 2210633) OX=223926 GN=VP1392 PE=3 SV=1

MININLSSLIQRLHPIAKVALEDAAALAVSEKANEVQIEHYLLSLLERPNSDFDVLLSHF

DCSENLLRQSVRSTLDTNAKGNGSKPVFSALLIEWLQESWLVSSLDLSETQIRSGALLLT

LVSNPLRYGQHGYASILEAVNPDSLKRNFAELTSHSIEAQVATSEKTQAREDGSALSKFT

TDFTGKARKGEIDPVFCRDQEIRQIVDILARRRKNNPIAVGEPGVGKTAVVEGLALKIVQ

GDVPDNLKGVELYGLDMGLLQAGASVKGEFEKRLNAVLDEVKNSPTPIILFIDEAHTLVG

GGNQAGGSDAANLLKPALARGEVKTIAATTWSEYKKYFEKDPALARRFQLVKLDEPSPEQ

AALIIRGLRPAYEKSHNVYVRDDAITAAAALSARYISGRQLPDKAIDVLDTACARVNISL

NAIPASVETLQQELAAQQRELEALERDALQQTGDKHSLANIPDLKLAMETTKEELAEQEV

QWHKEKEQIQEMIALRSRLHELVFGKVNEEELVVDEDSEQDVSPYTEMDEEAVRIAISAC

QEQLDAIRNGNPLVHFEVGPDEVSHVISDWTGIPMGKMLQDEAETTLKLKESLTQSIKGQ

EYAIDALSEGIQTAKAGLGNPDAPTGVFLLVGPSGVGKTETARAIADQMFGGERFMTTIN

MSEFQEKHTVSRLIGSPPGYVGYGEGGMLTEAVRQRPYSVVLLDEVEKADPEVLNLFYQV

FDKGTLNDGEGRTIDFKNTLIIMTSNLATHEIESLVHQSKEIDANIIAEAIRPTLNQHFK

PALLARMSVLPFVPLSDEAMTEIIHHKLNKVSQRLHSHHKLSLSYEESLVEFVLGNCRLA

ETGARNIDAVINRQLLPQLSTQLLVHDKDDSHTQITVSVDEQGTLTYAFS

>tr|Q87GP9|Q87GP9_VIBPA Putative ATP-dependent exoDNAse (Exonuclease V), alpha subunit OS=Vibrio parahaemolyticus serotype O3:K6 (strain RIMD 2210633) OX=223926 GN=VPA1266 PE=4 SV=1

MNTALHEDQMRVTSIPYRSTKMVIFSGVPLAKDSYKTNSGKYYVTIKADPDSIPVLPTLG

QHWSVKGARQIESVEMGDYVMQQHTYESPKHIECTLPETGEQLIRFIARESDFKGIGESK

ARALWQLLGKDFHTTLRNDTLESRKRLTSILSEDSVEALFKGYAKYKNLAHCNWMSEHNI

PASVQQRLLKHHGEASIEVIKDNPYALMGFGLSFSAIEDIIKVTDFKSDVAKDDPRRLSA

ALEMAIRKEIEKGHTYTTHANVRHYLSKLLKDKTLVTQAFQSGHDKAQYILNPDTGAYHP

TAQLLMESVVAKRLNTLIKRNDLFDENANAAYCAAVTELPYELTLKQIEAVTTCLDNSVS

CITGGAGTGKTTVLRTTLRAYHQLGFEIHAVALSGRAAMRLHESIGFVTSTIAKLLREDP

IEPSVEKTNHLLVIDEASMIDLPTMYRLVNHIHPSVRLIFTGDPDQLPPIGCGKVLADIV

EAKTVANTMLDIVKRQEGSTGIPEYSKLINQGVMPDQLSTGAIHFHETNKADIAKVCCEL

YQQCPENSRVMAPTKAIVTEINKLTQQAVNPNSDRLEFEINGDKFFLPLRMNDAVLFTQN

HYDKGIQNGSLGMLTNAKTSGDSYGEVTLDTGEKVEITQSVLDCMELGYAITLHKAQGSQ

FPRIIIALQKGRIVD

>tr|Q87NX5|Q87NX5_VIBPA Uncharacterized protein OS=Vibrio parahaemolyticus serotype O3:K6 (strain RIMD 2210633) OX=223926 GN=VP1743 PE=4 SV=1

MDILNFEAFLIAITILTLTPGLDTALVLRNTSRSGLKDGCTTSLGICFGLFVHAFFSAVG

ISAILAQSAELFQIVKMIGAAYLIWLGISSLKALMASGGGITVAEQIQQVYSGKRSFREG

FLSNVLNPKTAVFYLAFLPQFVNPEGSPLLQSMTMAAIHFVIAMVWQCGLAGALNSAKNL

LKNASFMKWMEGVTGAVLVALGIKLLIEEPL

>tr|Q87HP1|Q87HP1_VIBPA NAD(P) transhydrogenase subunit alpha OS=Vibrio parahaemolyticus serotype O3:K6 (strain RIMD 2210633) OX=223926 GN=VPA0922 PE=3 SV=1

MQIGVPREILAGESRVAASPKSVEQLIKLGFDVVIESQAGVLASFDDAAYEAAGAKVVSS

DEVWASGLILKVNAPIVDEEKGIDEIALLQDGATLVSFIWPAQNAELMEQLSSKNINVLA

MDSVPRISRAQALDALSSMANIAGYRAVVEAAHEFGRFFTGQITAAGKVPPAKVLVAGAG

VAGLAAIGAAGSLGAIVRAFDVRPEVKEQVESMGAEFLTVDFQEDSGSGDGYAKEMSDDF

NKKAAELYAEQAKDVDIIITTALIPGRPAPKLITKEMVDSMKAGSVIVDLAAANGGNCEY

TVKDQVIMTDNGVKIVGYTDMVGRLPTQSSQLYATNLVNLLKLLCKEKDGNINIDFEDVV

LRGVTVIKEGEITWPAPPIQVSAQPQAKQAQPVKKEPKVEEPTSPTKKLIGLVAAVGAFA

WIASVAPAAFLSHFTVFVLACVVGYYVVWNVTHALHTPLMSVTNAISGIIVVGALLQIGQ

GNGVVSFLSFIAVLIASINIFGGFTVTKRMLEMFRKDK

>tr|Q87N52|Q87N52_VIBPA Nucleotide sugar epimerase OS=Vibrio parahaemolyticus serotype O3:K6 (strain RIMD 2210633) OX=223926 GN=VP2023 PE=4 SV=1

MKYLVTGAAGFIGSATIRKLNSLGYEVIGIDNINDYYDVELKYARLNFIKNPLFRFFNMD

ISNKNKNEIERLFEKEKFDRVIHLAAQAGVRYSLVNPHCYAESNLSGFLNVLEACRKSHI

KHFIYASSSSVYGLNKKVPFSTSDNVDHPVSLYAATKKSNELMAHSYSHLYQLPTTGLRF

FTVYGSWGRPDMAPFIFTEKIINGQSIDINNNGDMWRDFTHINDIVEGIVRISDVIPRIN

QRWQFENSTPADSSAPYSIYNIGYGSPICLMDFIKAIENELGIEAKKNYREMQPGDVYQT

YADTTAFYQATGYRPSVSVEEGIAEFVAWYRNFYNK

>tr|Q87LC6|Q87LC6_VIBPA Uncharacterized protein OS=Vibrio parahaemolyticus serotype O3:K6 (strain RIMD 2210633) OX=223926 GN=VP2686 PE=4 SV=1

MNSGFNRFGRFCAWTLVTVLVFLAILVTTLRVTLPQLNHFQDEIKTWVKQGTGFDFSISS

VAGSWRNSHPSIALLGLEANLPNNQEARFAVDEIQIEFDLIQSLLQFKPVVADLTIHNLA
[truncated: 1,701,891 more chars]
