# Supplementary material for: Comprehensive genome based analysis of Vibrio parahaemolyticus for identifying novel drug and vaccine molecules: Subtractive proteomics and vaccinomics approach
Source: PLoS One. 2020 Aug 19;15(8):e0237181. doi: 10.1371/journal.pone.0237181 (PMC7444560; doi:10.1371/journal.pone.0237181)
Supplement: S4 File — (DOCX) [file pone.0237181.s017.docx]

**S4 File.** Number of proteins nonhomologous to *H. sapiens* using BLASTp (E value 10^-3^).

>tr|Q87HM8|Q87HM8_VIBPA Uncharacterized protein OS=Vibrio parahaemolyticus serotype O3:K6 (strain RIMD 2210633) OX=223926 GN=VPA0935 PE=4 SV=1

MNNWKLILPLMMSGSLIACTSTQTTTEPVEPETPKVEQPVTVEPEVKPEVEPKPEVKPKP

ETKPEKKPEPKPEKPVKVQKTPDGMLILGSEEWVYVPAIDQTFKARVDTGATTSSISATE

IVPFERDGKDWVQFKIDINGKTSKEFKVPVERWAKVKQSSSDEVDKRAVVVAYIQVGDYK

EKTEFTLADRDHMKFPILLGRSFFRDIAVVDVSKKYIQDKPTKSTKK

>tr|Q87KJ0|Q87KJ0_VIBPA Adenylate cyclase OS=Vibrio parahaemolyticus serotype O3:K6 (strain RIMD 2210633) OX=223926 GN=VP2987 PE=4 SV=1

MQAYTYTIIQRLDNLNQQRIDRALALMDSQSQQVFHLIPALLNYNHPVIPGYYDADVPFG

VHGLELNPIQQQFIDDIQLAIGQPLKTAEKPAILGLYTMGSTSSIGQSTSSDLDIWVCIS

PEMDCDERELLTNKCLLITDWAQSQGVEANFFLMDEERFRSNHSEEMTGDNCGSSQHLLL

LDEFYRSAVRIAGQRLLWQIVPPEMEECYDEYVSQLCSDGYIDCSEWIDFGKLNRIPAEE

YFGSNLWQLYKSIDSPYKSVLKAILLEAYSWEYPHTQLLSIDTKRRFFAHEPDLYGMDAY

YLMLEKVTRYLERIQDDTRLDLVRRCFYLKTHEKLSREPDVGSVAWRREALSDMIAKWNW

DDSVVAELDDRRNWKVEQVKVVHHALLDALMQSYRNLIQFARRNDITSAISPQDISILAR

KLYAAFEVLPGKVTLLNPQISPDLHEADLSFIEVKEGGVNKSGWYLYKQPLIAHRILGQP

CLEHHEYLSKLVSWAFFNGLITESTRLHAVVREAQLDIDKFYQMVSDLRNTFALRKRRPT

MQALASPCEISQLAMFINFENDPTSELSGRSLKVDVKNTDIFSFGPEHKNLVGSVDLVYR

NSWHEVRTLHFKGETAMLDALKTILGKMHQDALPPESVDVFCYAKNMRGVMRNMVYQLLA

ECIDLRLKPVEQEKRRRFKAMRLGNQTYGLFFERRGVSVQKLENSIDFYRSISTNKLKGS

PLLMLDREQEYQLPEAVDGFASEGLVQFFFEDNEDGFNIYVLDESNQVEVYHQFSGSKDE

MIASVNSFYTSVKDDSRVASKFINFNLPQYYQIIHPEEGNAYIIPYRNDGCSPHRPTKAV

NA

>tr|Q87FY4|Q87FY4_VIBPA Flagellar M-ring protein OS=Vibrio parahaemolyticus serotype O3:K6 (strain RIMD 2210633) OX=223926 GN=VPA1536 PE=3 SV=1

MSELTPQVAGNTAMTTSTTQAFSPAGNMDDVTNKLKQLWSSSQRNLVLSAVLAAIVAAII

VVALWSSSQSFRPLYSQQERFDIGEIVSVLESEGVSYRMQEQNGQVLVPEGEVARIRMLL

ASKGVKAKLPTGLDSLKEDSSLGTSQFMETARYRHGLEGELVRTIMSLNSVANARVHLAI

PRQTLFVRQNGENPSASVMLELKPGEDLKPEQVEAIINLIVGSVTAMKPEFVSVIDQYGR

LLSADVASAEAGKVNAKYLEYQKNVEKQIIQRAADMLTPIVGPSNFRVQVAADMDFSQVE

ETREILDNAPVVRNEHTIQNNSIDQIALGVPGSLSNQPPVTGEAATNDSQNTNARSEVNR

QYAVGSSVRRTQYQQGQIEKLSVSVLLNSKASPDGVAWSDADKAQISTMITDAVGISAAR

GDSLSLMSFNFTPIDIDAPTALPWWQDPTVQQPLRYVIGGMLGLAMIFFVLRPLIMHLTG

ADKPVPELNFAEPPQEEPDYDNLQTREEREHEEVLNRRLSEKGISASTGLDVNSDMLPPA

GSPLEIQLKHLQLIANEEPERVAEILKQWVNINEHSSVDVKTNA

>tr|Q87T73|Q87T73_VIBPA Putative capsular polysaccharide biosynthesis protein D OS=Vibrio parahaemolyticus serotype O3:K6 (strain RIMD 2210633) OX=223926 GN=VP0197 PE=4 SV=1

MNTILSLIGRTDALFTQDIAHHEAELFRIVSESRFLVLGGAGSIGQAVTKEIFKRNPKKL

HVVDISENNMVELVRDIRSSFGYIDGDFQTFALDIGSLEYDAFIKADGKFDYVLNLSALK

HVRSEKDPYTLMRMIDVNVFNTDKTIQQSIDAGAKKYFCVSTDKAANPVNMMGASKRIME

MFLMRKSEQIAISTARFANVAFSDGSLLHGFNQRIQKRQPIVAPNDIKRYFVTPQESGEL

CLMSCIFGENRDIFFPKLSEALHLISFADIAVKYLEQLGYEPHLCDSEEEARQLAHMLPE

QGKWPCLFTASDTTGEKDFEEFFTDKEELDMSRFENLGIIKNEPLYEQELLTLFENSISE

MKGEQAWSKEEIVKLFFTMIPDFGHKETGKYLDSKM

>tr|Q87GG4|Q87GG4_VIBPA Uncharacterized protein OS=Vibrio parahaemolyticus serotype O3:K6 (strain RIMD 2210633) OX=223926 GN=VPA1352 PE=4 SV=1

MKLGSKRMENRKFSLLINEFAQMTKQGRLTLETEQGLICHYKNRDIRIENGQRIGLKGQI

VIVAPLKQKVTSRVQGKINGTFSLSKDGYLTEIKSLGCCLVRHISMPEHPRMLLEEVNQS

LVILNQILKEIESNVAT

>tr|Q87SY5|Q87SY5_VIBPA ElaA protein OS=Vibrio parahaemolyticus serotype O3:K6 (strain RIMD 2210633) OX=223926 GN=VP0287 PE=4 SV=1

MTTWITLPFAQLTTLQLYEMLRLRVDVFVVEQTCPYPDLDGKDIIDGVHHLLGYHNDELV

ACARLLPAGTTYDNVSIGRVVTKQTARGGGLGHRLITQAIQACSTLWPEKTIDIGAQEHL

IDFYAHHGFEVMSDSYLEDGIPHVDMRRKHSTQ

>tr|Q87GZ8|Q87GZ8_VIBPA Uncharacterized protein OS=Vibrio parahaemolyticus serotype O3:K6 (strain RIMD 2210633) OX=223926 GN=VPA1167 PE=4 SV=1

MNWKRLVQFYSVPPSQAALALGMIGLGQAWALYVPDIGTPIRPFLASFGALLLAPVLIKY

ATNPRLFMADIKHPLSGSLMAPMSMALLILTDYLASVAPLIAYPLWCLAVLLHFTMMVLF

FGFQLMNFKMSNIVPSWFLYPVGLISSSLAGSQFGHNLFSETLAIMCIGIYFFMLPLVLY

RLVFFGSLPRRARPTLAIMAAPVNLSLAAYLVNFPQPDPILTGALAGIAITMTLLIYLCY

FRLLRLKFQPSIAAVTFPSVISAIAMHRLTSFFAQSHPQWHWLHDFGFLELSIATVLVVW

VSAGYVKMYWPEIVRTPTKQA

>tr|Q87FJ2|Q87FJ2_VIBPA Putative transcriptional regulator OS=Vibrio parahaemolyticus serotype O3:K6 (strain RIMD 2210633) OX=223926 GN=VPA1687 PE=4 SV=1

MDKIRSLRFFIATLEGGSFAAAAKAYGTDPSTVSKAIHRLESDLGIQLFQRSTRQIRLTE

AVRRYANTARFVLDELAACEDSLKSHNDALSGLLKINVPVSYGRLYIRPLLKEFCRRYPS

ITIDIHYDDAYVDIIEQGIDVSIRSGVVQDSQLIVRQLSPIDFIICGSQDYLTRHGVPSG

PDAFNDHSWVRFRFKQTGKLLPIRMPEPDGTSEYNPERNFIVNDGESMAELCAEGLGLTQ

IPHFIARDWLKVGRLVPIFPSMRQAGNGVYLLYANREYVPARVRVFIDFITQAIQDMDET

PFHTWAETLPIYQPNQGD

>tr|Q87I79|Q87I79_VIBPA Putative nonspecific tight adherence protein TadD OS=Vibrio parahaemolyticus serotype O3:K6 (strain RIMD 2210633) OX=223926 GN=VPA0727 PE=4 SV=1

MNIKNYFSLLVLLFLSGCSTINQELVTKEKLLTNSGQTEQLIEFYKANLVEVPSYKIKLI

NLYLDNKDQKSAELYINTLEKKDLKDPDIIYILAKLEYLKSNYDASERYLEEYLDNGGPE

GDYYLLKGKILARKKDYDGAISHFNDSKKNGASDREANNNIAVVMMLQGRPEPAMELLYG

LFAANPNDEKVRSNLLLASTRSNRPDVALEVLKHDSSEQEARVKLKKLMKSVKPLSKKID

TKVPPKQVAKSKEKKNMEMDTKSVDLKPKYVARSVLDPQNLRPKAPSIYRIQVLATYKVI

PSDYLNYLKGNYGKVYSYTHGLWKRYCVGEFSDIDDAKKFLENMNIKGAFVVDYTKKRYV

EL

>tr|Q87P44|Q87P44_VIBPA Translocation protein in type III secretion OS=Vibrio parahaemolyticus serotype O3:K6 (strain RIMD 2210633) OX=223926 GN=VP1674 PE=3 SV=1

MSYDDLHQALFLYSLTLPRLMACFIFLPILSKQMLGGAMIRNGVLCSLALFIFPVVNEQA

LPAETDGLWLIVILGKEVLLGMLIGFVAAIPFWAIEATGFLVDNQRGAAMASMFNPTLGS

QSTPTAVLLTQTLITLFFSGGGFVAFIYALFKSYTTWPILGFFPMVTDAWVSFFYDQFQQ

LMWLGVLMSAPLVLAMFLAEFGLALISRFAPQLNVFFLAMPIKSAIASVLLIVYLGLMMD

HFEALFYGITRFGDQLNTIWK

>tr|Q87JU3|Q87JU3_VIBPA Protein TonB OS=Vibrio parahaemolyticus serotype O3:K6 (strain RIMD 2210633) OX=223926 GN=VPA0155 PE=3 SV=1

MGRLLIALPASLLIAVSLFSFMAWMVDNGNQRAPKPSEAVRFDMVMVENDADVQRRQRSV

PEQPEPPQAPEPMELSQANTQVEPMSQVTPISALGLNTALDGIAINAPNLKGTMGNQQAL

PLYKVEPRYPSKALKRKVEGYVIMRFTIDTTGRPKDIEVIDAEPKRMFEKEAISALKKWK

YQPKVENGVSIEQFGQTAKVEFKLGK

>tr|Q87TE6|Q87TE6_VIBPA Uncharacterized protein OS=Vibrio parahaemolyticus serotype O3:K6 (strain RIMD 2210633) OX=223926 GN=VP0124 PE=4 SV=1

MPYLLAIVLSIFTLTGCQSAYYSAMEQVGYHKRDIMVDRVEDAKESQQEAQEEFTSALEA

LSALTNFDGGELESVYNNINDKYEDSEKAAQDVRDRIAAIEDVSDALFEEWQGELDLYTS

AKLRRSSEQKLRETKASYKTMLSAMKRAEKKMTPVLNTLRDNTLYLKHNLNASAIGSLQG

EFSSLEKDIQFAIKQMNEAIAESDKFLQKLNQK

>tr|Q87FG7|Q87FG7_VIBPA Uncharacterized protein OS=Vibrio parahaemolyticus serotype O3:K6 (strain RIMD 2210633) OX=223926 GN=VPA1712 PE=4 SV=1

MVNHTANTQIPQSLKAGVFNGRGIFDFGAKNEAYADYFTGTSYLALLNQPGLIVANVTFE

PGCRNFWHIHHEGGQILLVTGGRGWYQESGQPAQALNVGDVVHIAPGTKHWHGAAKDSWF

SHVAIEIPAEGASNEWCEPVTDEEYAELG

>tr|Q87HG1|Q87HG1_VIBPA Putative transmembrane protein OS=Vibrio parahaemolyticus serotype O3:K6 (strain RIMD 2210633) OX=223926 GN=VPA1004 PE=4 SV=1

MNIETSLGGTLFIIIAMGLVTLATRWGGVYVMSFIPISERVQRFITAMSGSVLIALLAPL

AVEGDNGARAALFSTAVVMFIVKKPLPAIAAGIIAAAAVRAF

>tr|Q87I65|Q87I65_VIBPA Putative fimbrial protein Z, transcriptional regulator (LuxR/UhpA family) OS=Vibrio parahaemolyticus serotype O3:K6 (strain RIMD 2210633) OX=223926 GN=VPA0741 PE=4 SV=1

MRFTLNNVLIIDDQPLYSEALASLVENAINTAEVIQTTDSAEVMELVRSQRIDLIILDVV

LGDRDGMRLAKNILATGYRGRLLFVSSRDYSSLSKAAYEMGANGFLNKNEARETIADAIV

SVSRGYSMFKSTHTPSSGDVTLSNREAMVFHYLAQGYSNKKISEQLSLSAKTISTYKTRI

LKKYHADSLIELLHTIPQSENIQFCR

>tr|Q87KK9|Q87KK9_VIBPA Putative transporter OS=Vibrio parahaemolyticus serotype O3:K6 (strain RIMD 2210633) OX=223926 GN=VP2968 PE=4 SV=1

MISQLSKSIRQYMLVTFNYWNFTVTDGALRMLVVLYFHDLGYSTLAIASLFLFYEFFGVV

TNLIGGWLGARLGLNKTMNIGLAMQVFALLMLAVPNAWLTIPWVMAAQALSGIAKDLNKM

SAKSAIKTLVPDEQQGALYKWVAILTGSKNALKGAGFFVGGLLLSWVGFQNSMFIMASVL

AVVFIFSMIWLEADMGKAKNKPKFSHIFSKSESVNILSAARMFLFGARDVWFVVALPVYL

GSVFGWDHLWVGGFLASWVIAYGFVQGFAPRITGKAQGRVPDGSAALVWAGILALITGGI

AYGVQIGWQPEIVIVVGLMIFGAVFAINSSLHSYLIVSYAKGDGVSLDVGFYYMANAMGR

LIGTVLSGWIYQEAGLAACLWVSFAFLALTTLISIKLPKANIATA

>tr|Q87JF4|Q87JF4_VIBPA Putative transcription regulator protein OS=Vibrio parahaemolyticus serotype O3:K6 (strain RIMD 2210633) OX=223926 GN=VPA0299 PE=4 SV=1

MSPSRKLTKGLLHQLDLNLLKVFIVLAEEQKTVLAAKRLNMTQPAVSRALSRLRQHFNDE

LFVRTRHGLKPTRKGQLLADNLPRIIDELSIVLEGLDDFDAGSHAAHIRIAINNFFGVSL

PAKFYLKINKLAPNMVFSIENWGPTTLNRLVNGDIDLGINYSLNNVPKEISRRYLANDHF

QIFAREGHPLTEKIVTVQDIASFPIVSAIVPDWNEKKPRIIQLDQSQTLGFNVAFRSESI

ASLLEITATSDVLFPTSMYLLQNHHPRLSKLTLAESIVETLVSESQEIELYMHYRNRSNP

MFKWLLSIINELFEYDFPQD

>tr|Q87QE5|Q87QE5_VIBPA Uncharacterized protein OS=Vibrio parahaemolyticus serotype O3:K6 (strain RIMD 2210633) OX=223926 GN=VP1204 PE=4 SV=1

MEQLEFFTVPSPCVGVCTSDEKGYCKGCMRKREERFNWLNLTPAQQLHVIKLCRQRYRRK

MLARKTKPEQLQDNSSPQQDLF

>tr|Q87J66|Q87J66_VIBPA Transcriptional regulator, LysR family OS=Vibrio parahaemolyticus serotype O3:K6 (strain RIMD 2210633) OX=223926 GN=VPA0387 PE=4 SV=1

MDKFSDMTLFVSIVKHQGLAAAGRELGLSPATVTARLQAIEERYGVKLLNRSTRHVSLTD

AGAMYHQACLNIIDSVKETENLLQTGISEVRGTLKISAPRDIGKQIISPMVSAFSEQYPD

VTPYLYLNDNLSNLAESGLDLVIRYGELADSNLISRRLASSQRVLCASPDYLSKQGVPNC

PQDLAAHRCLAMVRSNEELKTWHFKDEESHQSITVTPKRFSDDGEVIRQWALDGAGIALK

SILDIQQDLKQQRLVTVLDGYMKNFSAFSQGAEADLHVIYQSRQYQPKRVRLFLDFLVEQ

FSALSDTSNQI

>tr|Q87PT9|Q87PT9_VIBPA Uncharacterized protein OS=Vibrio parahaemolyticus serotype O3:K6 (strain RIMD 2210633) OX=223926 GN=VP1412 PE=4 SV=1

MRSIAILLGCALLSGCAMWDQFKESTGITPETSSIELVIEASSVLNVREGGQSSPVILRV

HELTSPVLFRSLDFFALFENDKASLGDEYIKRYEYQMQPGEKIHEFLELDPATRAVGFSV

AFRDIDGSSWRKVEVIEEKSEYYIKLKLEGSELISDNTRGIEQVYF

>tr|Q87GU8|Q87GU8_VIBPA Uncharacterized protein OS=Vibrio parahaemolyticus serotype O3:K6 (strain RIMD 2210633) OX=223926 GN=VPA1217 PE=4 SV=1

MKLWDHPMSENDFRQCLRIATGATIGFTLCKLFGWNYGVFFTVTPMLLLGMVPVVNLHAS

RQLIFSAVVCGLEVGILGGLFGSHPVLMTLIAFGLFLYKFACMSKGSLFLFGANSVLSLS

IMLHFASYPTTDLNDLIFSNLQANVLSIIVAYLVTFLIPDAEPRQPLPKPSQAKQSHRMR

HEALMGASIATMSFLVFQIFDLNDSMSAQATTLLLLFPMHWNGALGYARKRAMGTLLGVT

FGIVGQLILYDWSNTLLFIVPLLWIGAMIFSYMHVKESSGSGAGFGGLTTLGILFGQYLT

PDGDLIFSALYRVSSILFAIVVTLLVTYAVHRLLNSFAATRFSQS

>tr|Q87NZ4|Q87NZ4_VIBPA Putative DNA polymerase III, epsilon subunit OS=Vibrio parahaemolyticus serotype O3:K6 (strain RIMD 2210633) OX=223926 GN=VP1724 PE=4 SV=1

MMWLEKSKASTPLLDTSQTPEWSVLFEQLAEQAQDQRLKRYYSTPMVNGDTPLKEVPFVS

VDFETTGLNAEDDAILTIGLVPFTIDRVQCSGSAHWIVNPNRELNEESVVIHGITDSEVK

NAPQLTQILGEILDALAGKVVLVHYKNIERQFFYNALLNTIGEGIQFPVVDTLDIEYALQ

RRECSGIWNKLKGKKPGSVRLGHARERYGLPAYQPHHALTDALATAELFQAQLQYHFNRD

MPISAIWQ

>tr|Q87KM6|Q87KM6_VIBPA GlpG protein OS=Vibrio parahaemolyticus serotype O3:K6 (strain RIMD 2210633) OX=223926 GN=VP2951 PE=4 SV=1

MKRLVTLNNPRMAQAFIDYMASRQIDIEMMPEGEGQFALWLTDSQHEVEAEAELKQFLAN

PSASKYSAASWDVADTRKSQFHYASPSIIGMIKAKAGPVTLLIMTVCAVIYGLQMLGFGN

GVFALLHFPAFEGQQWQLWRWVSHALLHFSVTHIIFNLLWWWQLGGDIERRLGSGKLLQI

FVVSAALSGAGQFYVEGANFGGLSGVVYALLGYLWVLGYRLPHLGLTLPKSIIGFMLVWL

VLGFVQPFMAIANTAHLAGLLAGMAIALFDSGKQKYQQQA

>tr|Q87J49|Q87J49_VIBPA Transcriptional regulator, AraC/XylS family OS=Vibrio parahaemolyticus serotype O3:K6 (strain RIMD 2210633) OX=223926 GN=VPA0404 PE=4 SV=1

MKNSARNLHPSLSIDRAPSDVFMNFEAFLSNTETRIHSHPWGQVQLISGGILEMDAEDTR

FLAPPHLAIWVPAGIRHTSYNRKPIEYCSLNIAPELTAHFPTKTSLIKVTPIVSAIIEDF

RQRDINVAQSDEDKRLVRVLLDQLAKQEVEHHFLPTTDNKYLQPILKAVEESPTDEISLA

EWAAKVHTTERTLARHCQSELGMSFTEWRLRVRYLHSMELLRKGQTVKEVALTLGYNQAS

PFIAMFKKYSGMTPEQYKNRLL

>tr|Q87GH4|Q87GH4_VIBPA Putative Type III secretion protein Spa24 OS=Vibrio parahaemolyticus serotype O3:K6 (strain RIMD 2210633) OX=223926 GN=VPA1342 PE=4 SV=1

MYINAGACSGVYFFAIGGAMVELLSPTNAYAGVNLVSLLVLVLLSAVFFICFTGFVKYSI

VLNIIKNAVGTQQIPPAIVVNLLAALMALNAVWPQIQPGIERVKPYFSQQEKQELNQDVD

NDMPKNVTMYSLVSEWSTFFPELLEHAKQKTDKLSEVIELPFNFDKEHDTFKYFVGSLIY

DLYKGFELGLKLYIVFVSIDFLIAVILSGVGMTMLSPTVISTPVKLAVFYFSDSWTILFK

ALG

>tr|Q87LZ6|Q87LZ6_VIBPA Dihydropteroate synthase OS=Vibrio parahaemolyticus serotype O3:K6 (strain RIMD 2210633) OX=223926 GN=VP2462 PE=3 SV=1

MIITANNKSLDLSRPHVMAILNVTPDSFSDGGKFNSLELALAQVEKMITAGVSIIDVGGE

STRPGAPDVSLEEELQRVVPVVKAIREKYDVWISVDTSKAEVMRQAIAVGADLINDIRAL

QEPGALQVAAASKLPVCLMHMKGQPRTMQESPQYENLMDDVAQFLEERIAACEAVGINKS

QLILDPGFGFGKTIEHNYHMLAHLEKFHEFGLPILAGMSRKSMIFKLLDKPAADCTNASV

VCATIAAMKGAQIIRVHDFEETIEAMKIVEMTQNNI

>tr|Q87HR8|Q87HR8_VIBPA Sui1 family protein OS=Vibrio parahaemolyticus serotype O3:K6 (strain RIMD 2210633) OX=223926 GN=VPA0888 PE=4 SV=1

MTLVYSTETGRIKPEEEKVARPKGDGIVRIQRQTKGRKGKGVCIVSGLDLDDAPLKLLAA

ELKKVCGCGGSVKDGTIEIQGDARDKIKTHLEKKGMTVKLAGG

>tr|Q87FZ2|Q87FZ2_VIBPA Uncharacterized protein OS=Vibrio parahaemolyticus serotype O3:K6 (strain RIMD 2210633) OX=223926 GN=VPA1528 PE=4 SV=1

MLTLNYYVEITATPQRVWRVLTDAELYKRWAQAFSPQSQFEGEWEEGSDITFFDPDMGGT

RAVIDSVQPLHRLEFHHVAIFNPDNRQQLDADIAAKWIGSREIYQIDTEDERLLLNITIH

THSDFVSMFNNGWEKALPLIKSICEETDSQ

>tr|Q87LS3|Q87LS3_VIBPA Uncharacterized protein OS=Vibrio parahaemolyticus serotype O3:K6 (strain RIMD 2210633) OX=223926 GN=VP2538 PE=4 SV=1

MGLNIKPLVVSVLGSVLLAGCATAPPKQQDNLCEIFREKSGWYDDAKYMEKEWGTPIHVA

MAIIKQESSFRHDAKPPKDYVLGFIPWGRVSSAYGYAQAQDPAWDDFQDSTGQGGSRSNF

DDSIMFVGWYTHETRRQLGISLWDPYNQYLAYHEGRGGYKRGTYKRKPSLMKVARRVEQT

AKTYGWQLKQCRQELEDNSSWFF

>tr|Q87QL5|Q87QL5_VIBPA Uncharacterized protein OS=Vibrio parahaemolyticus serotype O3:K6 (strain RIMD 2210633) OX=223926 GN=VP1134 PE=4 SV=1

MNLIDFSHSPVSLLPPIVALTLAILTRRVLVSLGVGIALGAVLLNSWSIGGTASYVGTQV

SSVFIEDGGINTWNMSIVGFLILLGMTTALLTLSGGTRAFAEWAQSRVKSKRGSKLLAAF

LGVFIFVDDYFNSLAVGAISRPVTDRFYVSRAKLAYILDSTAAPMCVIMPASSWGAYIIT

IIGGILVSHGITEYSALGAYVRLIPMNFYAVFALLMVFAVAWFGLDIGKMREHEIAASQG

RGFDKDKENDTQEAHELNEELDIRESEKGKVSDLILPIVTLIIATIASMMYTGGQALAAD

GKEFALLGAFENTDVGTSLIYGSLLGLAVALFTVLKQGLPLTEITRTLWIGAKSMFGAIL

ILVFAWTIGSVIGDMKTGSYLSTMAQGNINPHWLPVILFLLSGLMAFSTGTSWGTFGIML

PIAGDMAGATDIALMLPMLSAVLAGAVFGDHCSPISDTTILSSTGARCNHIDHVSTQLPY

ALSVALVSCVGFIALGMTTSIAFSFIAASITFVIVCAVLSWLSKSKIESCQSA

>tr|Q87P26|Q87P26_VIBPA Putative type III export protein OS=Vibrio parahaemolyticus serotype O3:K6 (strain RIMD 2210633) OX=223926 GN=VP1692 PE=4 SV=1

MRVDPSFVGQTPAHSTDVRHYERDDAKRMSELMTRETTAEVSRSAPKDTLTKVEEKLNAI

KDWYASIKEAETVSKQSVLSSLKDVISDPQTQKEALWYAFHQAKSAKGTDDAVPELLSVL

KQELLGDFAGQLMAEPPTDRAALKAMLAQSFPLGAQKEQALWHCWAELKSLPEMTSTVDL

VREELSFVIQKNAMVKNIMTHSHKLDLS

>tr|Q87IU7|Q87IU7_VIBPA Putative tyrosine-specific transport protein OS=Vibrio parahaemolyticus serotype O3:K6 (strain RIMD 2210633) OX=223926 GN=VPA0509 PE=3 SV=1

MNLKLVGSSLIVAGTALGAGMLAIPMVLAQFGLLWGTLLMLFIWAGTTYAALLLLEASCK

VGGGVSMNAIARETLGKGGQLVTNGLLYALLVCLLMAYIIGAGDLVQKITASVGLSVSTV

SSQVGFTILVGLIVSAGTGVVDKLNRGLFIGMIVALVLTLFALAPSVSFEGLNEVVSSDK

MALIKTSSVLFTSFGFMVVIPSLVTYNKEASKTQLRNMIVVGSTIPLVCYLLWLFAVVGN

LPPHELVQYSNVTELISVLGQQYNGLEFILSMFTGLALLTSFLGVAMALYDQNADLLKTS

KPVVFVTTFILPLLGAVFAPEHFLAILSYAGIILVFLAVFVPLSMTMKVRRVPVEDNSVY

EAGGGVMGMSMIFLFGCFLLFAQAV

>tr|Q87QY0|Q87QY0_VIBPA Putative outer membrane protein OS=Vibrio parahaemolyticus serotype O3:K6 (strain RIMD 2210633) OX=223926 GN=VP1019 PE=4 SV=1

MMTRVRKFLKVCPWIILFLPLFANAAYQRNVAKPVNEVVYGKIDSVRYITQQEVVQSKSN

GWKTLLGATIGGLVGNQFGGGTGKEVATAVGALAGAVVAQNQSNYQYTVEYKLVELLIKV

KGDKLINVIQDVDKNMLFSRGDEVRILYFDDGVRVDLAY

>tr|Q87QJ4|Q87QJ4_VIBPA Putative membrane protein OS=Vibrio parahaemolyticus serotype O3:K6 (strain RIMD 2210633) OX=223926 GN=VP1155 PE=4 SV=1

MTRNERIFHAVLFELMALAIIVPAAALITGKGSSDLALVGIGLSLYTVVWNYIYNLYFDK

WFGSNRADRSLAMRLGHTVGFEGGLIFISIPVIAWFLEITFLRALMLEAGFLVFFLFYAT

GFNWLYDKVQPFGKMRKLLV

>tr|Q87HV4|Q87HV4_VIBPA Transcriptional regulator, LysR family OS=Vibrio parahaemolyticus serotype O3:K6 (strain RIMD 2210633) OX=223926 GN=VPA0852 PE=4 SV=1

MRYSLKQLAVFDAVADTGSVSQAADKLALTQSATSMSLAQLEKMLGRPLFERQGKQMALT

HWGMWLRPKAKRLLQDALQIEMGFYEQHLLSGEIRLGASQTPAEHLVPDLISIIDNDFPE

MRISLGVQSTKGVIDGVLDYKYDLGIIEGRCDDNRLHQEIWCRDHLTVVAASHHPFARNP

SVSLAQLEQAKWVLREHGSGTRKTFDSSIHHLIEDLDVWREYEHVPVLRSLVANGQYLTC

LPYLDVERYIEAGRLVALNVPDLKMERTLSFIWRADMAENPLVDCIKREGLRMMKGKPSV

L

>tr|Q87T69|Q87T69_VIBPA Putative acetyltransferase OS=Vibrio parahaemolyticus serotype O3:K6 (strain RIMD 2210633) OX=223926 GN=VP0201 PE=4 SV=1

MLVDLLRNQNRTILAVMSPDDISTRQAFDGIMQLSNDKDISRYSPDEVRLVNGIGMMPKS

LLRRKVNQYFLDLGYQFETVISDQALVSKFAHLQDGAQILKGAIVQCGAVIGEHSIINTG

AVIEHDTVVGEHNHIAPRAVLCGGIVTQSDVYVGANATVIQNLKLAQNVVVGAGAIVTCH

LDAHQVCYSGRATIKNSK

>tr|Q87K05|Q87K05_VIBPA Uncharacterized protein OS=Vibrio parahaemolyticus serotype O3:K6 (strain RIMD 2210633) OX=223926 GN=VPA0093 PE=4 SV=1

MLDTTKPSLALFDFDGTITREDMFSLFLHYSAYGLRKRVGKLAIMPFYALYKLGVLPARV

MRPLSSFIAFSGKETQHIEAIGATFAHEVIPLYLRPEAMERLAWHQHRGDTIVVVSASLN

AYLKPWCEANGYHLLCSELISEQPKLSGFYQQGDCSLERKVSRVKAAFSLDEFASVYAYG

DTHEDIPMLKLADYAMLNWSEWRASE

>tr|Q87KB1|Q87KB1_VIBPA Putative amino acid ABC transporter, permease protein OS=Vibrio parahaemolyticus serotype O3:K6 (strain RIMD 2210633) OX=223926 GN=VP3066 PE=3 SV=1

MTFRTLWLISLLLLTGCSDYQWGWYVLDPSTEQGITNLKFLVAGFNDTIQVSLLSMCFAM

TLGLLIALPALSRSPTLKWMNRIYVEVIRSIPVLVLLLWVYYGMPTLLDVSLNHFWAGVI

ALTIAESAFMAEVFRGGIQAINRGQHEAAESLGLNYWQKMRLVILPQAFRQILPPLGNQF

VYVLKMSSLVSVIGLSDLTRRANELVVNEYLPLEIYTFLVLEYLLLILFVSQAVRWLEKR

IAIPSY

>tr|Q79YY3|Q79YY3_VIBPA BfdA OS=Vibrio parahaemolyticus serotype O3:K6 (strain RIMD 2210633) OX=223926 GN=VP1393 PE=4 SV=1

MPTPAYMSINGETQGHITKDTYSADSVGNTWQEAHVDEFLVQELDHVLTVPRDPQSGQPT

GQRVHRPLVVTKVQDRSSPLLFNALVSGEKLPECLIRFYRTSVQGKQEHYYSIKLIDALL

VDIQTRMNHCQDAATADRVTEEVLKFTYRAIEVTHENCGTAGNDDWRAPREA

>tr|Q87JZ1|Q87JZ1_VIBPA Putative transcriptional regulator OS=Vibrio parahaemolyticus serotype O3:K6 (strain RIMD 2210633) OX=223926 GN=VPA0107 PE=4 SV=1

MRKAKEITLMNFDYNLLKVLAVILETRNTTTAAERLCTSQPAVSRSLRKIRDLFNDDILV

RKGTNMELTPKAEEIKAQLSGIINDIDKLVNVSHSFDPATESSVLRVAINSSIAQWFSAA

FTQLLAKEAPFMNLVIEDWTETTPDKIDAGEIAFGINYFPMELPKHLVQKKGGRDDFALA

CRATHPHGGKRMYLDDISEYAYAVHIIQHWNEKEDHISRLLQPFSVVPRIQLRTTHINTI

LNLVADSDVLFPCSRHLINQLDKRFSFIEFDDALPKLEGNFGYVYSVKRRNDPLILWVNN

TVESLMKSLGIES

>tr|Q87SV8|Q87SV8_VIBPA UDP-N-acetylmuramate--L-alanyl-gamma-D-glutamyl-meso-2,6-diaminoheptandioate ligase OS=Vibrio parahaemolyticus serotype O3:K6 (strain RIMD 2210633) OX=223926 GN=mpl PE=3 SV=1

MHIHILGICGTFMGGAAILARQLGHKVTGSDANVYPPMSTLLESQGIEIIEGFDPSQLDP

QPDLVVIGNAMSRGNPCVEHVLNSNMRYTSGPQWLNEFLLHDRWVLAVSGTHGKTTTSSM

LAWILEDCGYQPGFLVGGVLGNFGVSARLGESMFFVVEADEYDSAFFDKRSKFVHYHPRT

LIMNNLEFDHADIFDDLEAIKRQFHHLVRTVPGNGLILAPKQDQALTDVLERGCWTEKQF

SGEDGDWQAHKLVLDGSKFEVALQGEKVGTVEWDLVGDHNVDNALMAIAAARHVGVTPEL

ACQALGRFINTKRRLELKGEEQGITVYDDFAHHPTAIELTLGGLRNKVGEKRILAVLEPR

SATMKRGVHKNTLADSLHSADEVFLFQPDNIEWSVQDIADQCKQPAFVDADMDNFVAKIV

ERAQPGDQILVMSNGGFGGIHGKLLEQLKLKA

>tr|Q87I85|Q87I85_VIBPA Rough colony protein RcpA OS=Vibrio parahaemolyticus serotype O3:K6 (strain RIMD 2210633) OX=223926 GN=VPA0721 PE=3 SV=1

MFNKNILLVTLGVALLLPASVSAGELLSLDKGAAKTINVKRNIDTVFVADTQIADYKVIA

NGKLVIYGIGRGATSIIAYDRAGNEIYNAEVVVNKSLRLLKQTIIARYPDEDIKLTNIGE

QIVIDGVVSSEEIKDKVYRHVGEMLKKSKQRNTFELSGANGESVDPLDYTATYVFEDIIN

NLKVLTTDQINVKLTVAEVSSSFLTELGVSYAETNGKSIGGAGQFVNKILDFTAEDIVAV

ISASGNDSIGQVLAEPNLSVISGESASFLVGGEIPITVRDNDGISVTYKEYGVKLSMVAK

VTDSENIRLSLLPEVSSIDKTNGVNSGLVSVPSLRTRKAQTTVQLKDGQSFVLAGLLTSE

EQESLAKIPYLGDIPILGALFSKTNTERRKTELIIVATVNLVDPVKETDIKLPKFERTSD

LERLLKLDLSKVDDEELENTIKAGGFN

>tr|Q87MH3|Q87MH3_VIBPA Uncharacterized protein OS=Vibrio parahaemolyticus serotype O3:K6 (strain RIMD 2210633) OX=223926 GN=VP2282 PE=4 SV=1

MRYLALLLIGWLSLPVYALTQVDIYRAEVVIDSEQNDGESAAREQGMKDVIVRATGSQSS

LSNPVIQKALSSSSRYISQLGKSQVDGKASLKMLFNSGQIQSLLTQAQLPSWSPNRANIL

VWLVEEQDYDRAIAWEHSDSANVAALKKATETRGLPITIPVGDFDDVTGVNTSDLWGGFI

EPISQASQRYPADAVLIIRAQGEQIRWTLYDQAPAKIIDAQTSPRSGSATGADAISAMVD

GIADYYASKNAVVVSGKSSKAVNVKVLNVTSAADFFRLENALTKLNSVAGTEIKRVQGSE

LTLTIHLLASQQAFEQEASSISQLMEFEDPLGDVEEVTPSEEQSVETLPSEAQAIEGQPK

TVVQAVEETVPAVQVQQPVVIPQVQDQYDLIYEWNSTSQS

>tr|Q87KG7|Q87KG7_VIBPA Uncharacterized protein OS=Vibrio parahaemolyticus serotype O3:K6 (strain RIMD 2210633) OX=223926 GN=VP3010 PE=4 SV=1

MNESTILLTLASIHFIALMSPGPDFALVVQNATRHGRQTGLYIALGLSVGILLHSLFSLT

GVSYLVHQHPLLYSVLQLLGGSYLLYLGVGALRGVIATIKNPQTDQQNKTNSFVISNKRQ

AFAKGFATNILNPKALVFFISLMSSLVPAGMSVSGKSIALVILFSLSLVWFSSLAWMLST

QRLQRRLQQAGIYIDGICGVVFTLVGGSILVQTISTLIG

>tr|Q87HH5|Q87HH5_VIBPA Uncharacterized protein OS=Vibrio parahaemolyticus serotype O3:K6 (strain RIMD 2210633) OX=223926 GN=VPA0990 PE=4 SV=1

MGFWFFQDDVQALDDGSFSGQHVVGDVLVLIDYPQGANDEPYAALVTWDPTCSKADSNDP

APGDCAAKNLRLQAETSGANPADCSAVNDGDFGCATTNKGETPIPSPWPYTAKNGTMNAF

PYESFYEGGVNLTQLLGGIDGTASCFSSFMAETRSSASFTAALKDFVLGQFQLCGMELVK

TCPTGALSPSGDSIIYNYEIKVTNTGFGALYDLYVEDITAGDSFTADVLAAGETATFTGS

FVSLINGVENEATASAAIKDGGEPVLTKSDTDSCPPLTPVGALSITKDCTTYVEENASGA

YGLRVNYEGKVCNDSKVKLNDVKITEMHDGMTVVKDVGTLNPESCMNYDGTYIPAPGSDV

SGGPVVAHDVRTFKDTVVAEGVNAITGVTVDTGMPVEASCPLCPAP

>tr|Q87FU1|Q87FU1_VIBPA Uncharacterized protein OS=Vibrio parahaemolyticus serotype O3:K6 (strain RIMD 2210633) OX=223926 GN=VPA1587 PE=4 SV=1

MDTLLKDFPVITEIKVAWGEMDALQHVNNVVYFRYFETARLDYFNKINLLVDLQTSQIGP

VLSETQCRYKLPVTYPDTLLVGSRVIDMQEDRITMEYQVLSKKWGKITTVSTATGVMFDF

KNNEKAAIPDHVRQSILELESTVGSTHHLE

>tr|Q79YX1|Q79YX1_VIBPA Chemotaxis protein CheY OS=Vibrio parahaemolyticus serotype O3:K6 (strain RIMD 2210633) OX=223926 GN=VP2231 PE=4 SV=1

MNKNMKILIVDDFSTMRRIVKNLLRDLGFNNTQEADDGLTALPMLKKGDFDFVVTDWNMP

GMQGIDLLKHIRADAELKHLPVLMITAEAKREQIIEAAQAGVNGYIVKPFTAATLKEKLE

KIFERL

>tr|Q87HS2|Q87HS2_VIBPA Putative acetyltransferase OS=Vibrio parahaemolyticus serotype O3:K6 (strain RIMD 2210633) OX=223926 GN=VPA0884 PE=4 SV=1

MNKLHKLFKPSSVAVIGASQKDLRAGQVVMRNLLQSGFDGAIMPVTPRYKAVSGVIAYRD

VASLPYTPDIAILCTNASRNEQLLKELDERGTPFAIVISDDAQTLDLSSLNIRVLGPNSL

GIILPWHNFNCTFSPVAAKPGKIAFISQSAAVCTTVLDWANDKNIGFSSFISIGRGQDID

FADLLDYLSMDGNTEAILLYVDSIQDARRFMSAARAASRNRRILVLKAGRSKEMNTFEQQ

DGDTLDVIYDSAIRRTGMLRVSNTHELFAAVETLTHSVPLRGERLAIITNGGGPAVMAVD

TLVERGGNLATLDEVTTDQLRAILPSNWRGVNPIDLSGDATKKRYVDAINAVMNNDCADA

ILIMHSPSAVSDSYETALAVIEAIKNHPRHKHFNVLTNWSGEQTSRDARLAFTQAGIPTY

RTPESAVVAYMHLVEYRRNQKQLMETPTTAEPLHSGSVNSAKEWVDERLLDKNTVTLDTH

QTSPLFKLFGFNVLPTWIASDEIEAVHMAENIGYPVAVKLRSPDIPHKSDVHGVALNLRN

SREVSNAAQSILDTVKFSYPSANVHGLLVQGMAKLGSAEELRISIAVDKVFGPVILLGQG

GSEWNIAQDAVAALPPLNMTLARYLVVVALKSGKIRLQKHKDALDITELSKFLVRISQMA

VELPEIQRLDIHPVLVSGDDLTILDADVTLCKYEGDAQKRLAIRPFPAEFVETVTLRDGQ

PILLRPILPEDEPLHAQFINSVSKEDLYKRFFSEVGEFNHEALANFTQIDYDREMAFVAV

AFDKSGPSIIGVARALITPDNSDAEFAILVRSDLKGKGLGKILMEKIISYCKIKGTKQMS

GMTMPTNRGMLMLAQRLGFEVDVQFADGTADMVLPLN

>tr|Q87LH4|Q87LH4_VIBPA Deacetylase DA1 OS=Vibrio parahaemolyticus serotype O3:K6 (strain RIMD 2210633) OX=223926 GN=VP2638 PE=4 SV=1

MKLNKLAIATLVSAALSQYAFAQTDTKGTIYLTFDDGPINASIDVINVLNQEEVKATFYF

NAWHLDGIGDENEDRALEALKLALDSGHIVANHSYDHMVHNCVEEFGPNSAAECNATGDH

QINSYQDPAYDASMFAENLSVLEKYLPNITSYPNYKANEFARLPYTNGWRVTKDFKADGL

CATSDDLKPWEPGYACDTANPSNSVKAAIAVQNILANNGYQTHGWDVDWAPENWGIAMPA

NSLTEAEPFLGYVDSALNTCAPTTINPINSKAQEFPCGTPLHADKVIVLTHEFLFEDGKR

GMGATQNLPKLAKFIQLAKQAGYVFDTMDNYTPNWQVGNNYSAGDYVLHLGTVYQAVTSH

TAQQDWAPSPTSSLWTNADPATNWTQNVSYKQGDVVTYQGLRYLVNVPHVSQADWTPNSQ

NTLFTAL

>tr|Q87SQ2|Q87SQ2_VIBPA PTS system, mannitol-specific IIABC component OS=Vibrio parahaemolyticus serotype O3:K6 (strain RIMD 2210633) OX=223926 GN=VP0370 PE=4 SV=1

MISPDAKIKIQNFGRFLSNMVMPNIGAFIAWGFITALFIPTGWLPNETLASMVGPMITYL

LPLLIGYTGGKLVGGDRGAVVGAITTMGVIVGTDIPMFMGAMMVGPMGGWAIKKFDNYID

GKVKSGFEMLVNNFSAGIIGMLCAILAFFLIGPFVKVLSGALAAGVNFLVSAHLLPLTSI

FVEPAKILFLNNAINHGIFSPLGIQQASETGQSIFFLIEANPGPGLGILLAYMVFGKGTA

RQTAGGASIIHFFGGIHEIYFPYILMNPRLILAAIAGGMTGVFVLTMFNAGIVSPASPGS

IFAILLMTQKGSIVGVLASIAAATGVSFAVASLLMKTQTSTEEDGDEAALEKATSQMKDM

KSSSKNGAVVNNESKGDVDLATVQSIIVACDAGMGSSAMGASMLRKKVQDAGLNVHVTNL

AINSLPESADIVITHKDLTGRARKHAPNAHHISLTNFLDSEMYNQLVTKLLAAQKQSAAN

DDQMVKVSVLAANDDSFEPQQPSVFQIQRENIHLGLKAANKEEAIRFAGNKLVELGYAEP

EYVDAMFEREALVPTYLGESIAVPHGTVEAKDRVKKTGIVICQYPSGIQFTEDDDDVAKL

VIGIAAKNDEHIQVITTITNALDEPEAIEKLTSTNDVEEILNILGGQQAA

>tr|Q87LA7|Q87LA7_VIBPA MSHA biogenesis protein MshK OS=Vibrio parahaemolyticus serotype O3:K6 (strain RIMD 2210633) OX=223926 GN=VP2705 PE=4 SV=1

MVKKLIISIAAIALSGSVLANQDPTAPLGWQKPAEVSAKPKAKQYRLPTLNSIVCKPNTE

CVAIMNNRLVEQGELFNGYRVASINSEFVTLKRGSRQWNLELFGLNVKK

>tr|Q87L70|Q87L70_VIBPA Site-specific DNA-methyltransferase (adenine-specific) OS=Vibrio parahaemolyticus serotype O3:K6 (strain RIMD 2210633) OX=223926 GN=VP2742 PE=3 SV=1

MKKQRAFLKWAGGKYGLVEDIQRHLPPARKLVEPFVGAGSVFLNTDYDHYLLADINPDLI

NLYNLLKERPEEYISEAKRWFVAENNRKEAYLSIRAEFNKTDDVMYRSLAFLYMNRFGFN

GLCRYNKKGGFNVPFGSYKKPYFPEAELEFFAEKAKKATFVCEGYPETFRRARKGSVVYC

DPPYAPLSNTANFTSYAGNGFTLDDQAALADMAERTATERGIPVLISNHDTTLTRRLYHG

ADLSVVKVKRTISRNGSGRNKVDELLALFKAPESDSAAS

>tr|Q87MN5|Q87MN5_VIBPA Uncharacterized protein OS=Vibrio parahaemolyticus serotype O3:K6 (strain RIMD 2210633) OX=223926 GN=VP2197 PE=4 SV=1

MKIVAFGASTSSTSINKTLATYAAEQVKGAQVNVLDLNYYNVPMFSEDKEKEIGQAEGAI

AFLRELEQADAIVVSFAEHNGSYAAAYKNLFDWATRIERNVFQNKPVVYLATSPGPGGAQ

SVLAAATGSAPFFGADVKASVSVPSFYENFDLESGEIVNQEIAEQVKQAVALLK

>tr|Q87SA2|Q87SA2_VIBPA Probable membrane transporter protein OS=Vibrio parahaemolyticus serotype O3:K6 (strain RIMD 2210633) OX=223926 GN=VP0522 PE=3 SV=1

MTVSLFLLLLALGAFVGVMAGLLGIGGGLIVVPALLFLLPWAGISPEMSMHMALATSLAS

IIVTSGSSALNHLKLGNVDMFVVKWLMPGVVIGGFVGANIAEWIPTHYLPKVFGVIVLCL

AVQMFRSIKVKSEKPMPSSPVTMMYGTGIGVVSSLAGIGGGSLSVPFLNKHGVEMRKAVG

SSSVCGCIIAISGMIGFILHGYKVEGLPAYSIGYVYLPALLAIAMTSMLTTKVGAKLATN

LPTAVLKKIFAIFLMFVAATMLL

>tr|Q87QH1|Q87QH1_VIBPA Transporter, AcrB/D/F family OS=Vibrio parahaemolyticus serotype O3:K6 (strain RIMD 2210633) OX=223926 GN=VP1178 PE=3 SV=1

MSEQNKEPQSDDDVTGIAAYFIRNRVISWMVSLIFLIGGIAAFFGLGRLEDPAFTIKDAM

VVTSYPGATPQQVEEEVTYPLEKAIQQLTYVDEVNSISNRGLSQITVTMKNNYGPDDLPQ

IWDELRRKVNDLKVTLPPGVNEPQVIDDFGDVYGILLAVTGDGYSYKELLDYVDYLRREL

ELVDGVSKVSVSGQQQEQVFIEVSMKKLSSIGLSPNTVFNLLSTQNIVSDAGAIRIGDEY

IRIQPTGEFQSVDELGDLLITESGAQGLIFLKDVAEIKRGYVEVPSNIINFNGSLALNVG

VSFAQGVNVVEVGKAFDRRLAELKYQQPVGVEISEIYSQPKEVDKSVSGFVISLAQAVGI

VIIVLLFFMGLRSGLLIGLILLLTVLGTFIFMKYLAIDLQRISLGALVIALGMLVDNAIV

VVEGILIGTQKGRTRLQAATDIVTQTKWPLLGATVIAVTAFAPIGLSEDSTGEYCGTLFT

VLLISLMLSWFTAISLTPFFADIFFKGQKIKQGEGEENDPYNGIIFVAYKKFLEFCMRRA

WLTVVVLIVGLGASVYGFTLVKQSFFPSSTTPIFQLDVWLPEGTDIRATNDKLKELESWL

AEQEHVDHITTTAGKGLQRFMLTYAPEKSYAAYGEITTRVDNYEALAPLMARFRDHLKAN

YPEINYKLKQIELGPGGGAKIEARIIGSDPTVLRTIAAQVMDIMYADPSATNIRHDWRER

TQVLEPQFNESQARRYGITKSDVDDFLSMSFSGMTIGLYRDGTTLMPIVARLPEDERIDI

RNIEGMKIWSPAQSEFIPLQQVTMGYDMRWEDPIIVRKNRKRMLTVMADPDILGEETAST

LQKRLQPQIEAIQMPPGYSLEWGGEYESSGDAQESLFTTMPMGYLFMFLITVFLFNSIKE

PLIVWLTVPLALIGVTTGLLALNTPFGFMALLGFLSLSGMVLKNGIVLLDQIEIEMKSGK

EAYDAVVDAAVSRVRPVCMAAITTILGMIPLLPDIFFKPMAVTIMFGLGFATILTLIVVP

VLYRLFHKVSVPK

>tr|Q87IF8|Q87IF8_VIBPA Putative DOPA-dioxygenase-related protein OS=Vibrio parahaemolyticus serotype O3:K6 (strain RIMD 2210633) OX=223926 GN=VPA0648 PE=4 SV=1

MYHVHVYFPLQQLEKAQALNEIIRQERQDVLRVYPLVDRLVGPHKMPMFEMHLESISEEF

LAWLDTIRGDFSVLIHPVSERELRDHTESAIWLGRELGVFEEKLEN

>tr|Q87I20|Q87I20_VIBPA Uncharacterized protein OS=Vibrio parahaemolyticus serotype O3:K6 (strain RIMD 2210633) OX=223926 GN=VPA0786 PE=4 SV=1

MLFLIGLQAALIGIGATLIMDLWAWLQRRVFGIPSLNYALVARWVLCMPKGKLVHAPIMS

TDPMPGEKALGWFLHYAIGVVFALAHIAVFGHHWLVEPSLTPGLITGAVTLVFPFLVIQP

CLGFGFAASKTPTPWKARFLSTLAHLAYGLGLFITAFAIKGVSQYFM

>tr|Q87HN0|Q87HN0_VIBPA Uncharacterized protein OS=Vibrio parahaemolyticus serotype O3:K6 (strain RIMD 2210633) OX=223926 GN=VPA0933 PE=4 SV=1

MKKTIIATIAALTIAPSIALAKDHSQTPSNVQFGGPVTIEKLDTLLKDSNMFTEKDVVVE

GNLLRQVRADTFIFSDGTGEVMVELDDDIRLNSPIDQTTKVRLFGEFEGGNKPEIEVEQL

VIM

>tr|Q87JI6|Q87JI6_VIBPA Flagellar hook protein FlgE OS=Vibrio parahaemolyticus serotype O3:K6 (strain RIMD 2210633) OX=223926 GN=VPA0267 PE=3 SV=1

MSFNIALSGLDATNTELNTISHNIANASTYGFKGARTEFAAVYNGMQPGGVEVASISQNF

DKNGSITGTGRSMDLAINGSGFFVTKDHMGQTLYTRSGVFGTDKSNFVTANNGAKLQGYS

VDSNNNLMTGSVGNIQVSTSSLNAKATDKLDFVANFDASAKAIDKAVTPFDPADPTSFNS

SYTTQVYDSLGNSHTVTQYFTKTADNAWEVNVQVDGGKTPVSTIPVTFNKDGTLAAPTGS

FNVAFPAAGANAMSVDINLKGSTQFGAAFGVSTNSPNGYTSGELAGVRVEDNGMVYATYT

NGQSQLQGQVVLADFANTQGLAKVSGTAWTQSFSSGAPIMGVPGSGTLGNLTPGALEGSN

VDLTSELVALMTAQRNYQANAKTISTSDKLTQALFNAV

>tr|Q87QS2|Q87QS2_VIBPA Integrase, phage family OS=Vibrio parahaemolyticus serotype O3:K6 (strain RIMD 2210633) OX=223926 GN=VP1077 PE=3 SV=1

MSHLIHRLKDKEIEQAQGRDRIYRLPDGGGLYLLVKPNNLKYWEFRYTKPGTKNRTFLGL

GSLDMLNVDGARDKAFEMRKLLKEGIDPKLDRIEKRTQIQQEQACTFKSVADEWVKTKTK

LKPKTVQGNWRKLELYAFPKFGEIPVKSLTPIIVQEAFRPIARRGKLETVKRTIQLVNEI

MRFAINSGLIQHNVLSGVGETFASPDVKHMAVLKPDELTELLQTVATANMQLATKCLIEW

QLHTMTRPSEAAGARWDEIDIENRLWIIPDTRMKMKREHRIPLTDQTIAILERIHPISGH

RVHVFPSMRFPKRSIDSETINKALGRIGFKDRTTAHGMRSLASTTLNERGFDPDIIEAAL

AHQDRNAIRAAYNRTDYLERRRKMMEWWSSYIDDAAVGSLSVTGAVHLRAIG

>tr|Q87I54|Q87I54_VIBPA Glutathione S-transferase-related protein OS=Vibrio parahaemolyticus serotype O3:K6 (strain RIMD 2210633) OX=223926 GN=VPA0752 PE=4 SV=1

MITALYASILALLLVWLAFQVIKQRRLNKVAYADGGVEALQIARSAQSNASEYIPITLIL

MALLEFNGAAPIWIHLTGIIFVIGRIIHARGILQESFKGRVKGMQLTFLVIVSLVVLNMF

YFPYGKLW

>tr|Q87FX3|Q87FX3_VIBPA Uncharacterized protein OS=Vibrio parahaemolyticus serotype O3:K6 (strain RIMD 2210633) OX=223926 GN=VPA1547 PE=4 SV=1

MVNLISTHAIYEYLCSLAVQHIVTDDDQLVFEALKHNDIQHACQAIRETQSGEVVYQHLT

LRFGLRGEQSVYQFELSEQMQYCLDLFSLYLALRQTQFQLEAFHPDLISNVVVPIRVDAL

LWDPGVYFLEQMVQFHSAAFQHVIPSLQADETLCLHERMETLVEKLKENAFALWFEVATS

QTHFERIAEFDPDMIKLSVTIENKQDQHSFLPIARFLRKHKYQWVAGRVASQVELNRYRL

LGASYYFGYFSDIPTSLSFRSFDEE

>tr|Q87GA6|Q87GA6_VIBPA Putative glycosyltransferase OS=Vibrio parahaemolyticus serotype O3:K6 (strain RIMD 2210633) OX=223926 GN=VPA1411 PE=4 SV=1

MKKVLHITEAFGGGVQTALYSYVHSSRHEPYEHFLLARARHNDLTKDSNHDVFAQTKWVE

GGLLQFVRDANQTINDIKPDIVHLHSSKAGFLGRFLKLNRARLVYTPHCYAFEREDISGF

ARQLYKALEKVMLNKIDVVAGCSQRECDLAISIGAKRAELLNNYVSYQSATRVGRDHQAA

LNIVVLGRVAPQKDPQFLLNTLRCLNRFALNRQLDITWIGGGDRELEQALRAEDVQVTGM

LPRDEVVKKLQASDLYLHTAAWEGMPLTILEAAKLHLPMVIRSIGATKDLNYPFLAQTPE

DMAKQMTHCINHYDDIDFRQYTTELNETFSEEKQRLALNNIYS

>tr|Q87NV4|Q87NV4_VIBPA Putative transmembrane protein OS=Vibrio parahaemolyticus serotype O3:K6 (strain RIMD 2210633) OX=223926 GN=VP1764 PE=4 SV=1

MNILLAMIPAFFWGTTYAVTQFTLPDWPPVLLGALRALPAGLLLLAIKPSLPKKHEWKVL

LVLGTINIAFFFGLIFVMALTLPSAISGVGMISVPVFAMLFGWAVYKRQPSAIQGISGAV

LIALAWFLFDPSSISLNPIGLGAMLAAIMCIVIGSSVTKSLGTKMHWWTVLTWQLILGGV

LLSIAAAVLATINPQPYVHAVQNIDMTNTLGLLWVIVLNTALGYGMYVWLLQRMSVVDFT

FGGIANPIAGIVSGLLLLGENFTPLQYSLMVGMIVMSLLPQILTSLRARKEAITV

>tr|Q87SM4|Q87SM4_VIBPA Uncharacterized protein OS=Vibrio parahaemolyticus serotype O3:K6 (strain RIMD 2210633) OX=223926 GN=VP0398 PE=4 SV=1

MDLDQKRNALRVQLETAINDLKKQAEYRGCKIAQRRRSGYLYAVDAARNIVLEPWFTKLL

RQHGTILKKYSRSNAIHLAEIIESYGGLNKTIKLIETVLALRGFSLHTQRRVNYADQVLL

GLKDLRSLTPQHQEEEVRWNSVLPYCALCWRLRSRSHYYCEKHHPIKSTKLYKQQKYAVI

TALKHLPNQNNTAYEKYLAQPNKQKKLGRQLYDLVSGYAPHPRVFLRHCKDSAKAGDWLS

LSKNIVQTCKVNYPASYKKINQIKSDDFGQWSSWCIAIVRCLDPKEPNAWSDKECLTLFN

ELNTWTTLIGILHRFECVERINSIETKRGPDVGYGANLEQHQLIKELLKQQLATNSKTNL

SDIARTLGLSRQRVHQIIKKHQLLS

>tr|Q87LU9|Q87LU9_VIBPA Lipid A biosynthesis lauroyltransferase OS=Vibrio parahaemolyticus serotype O3:K6 (strain RIMD 2210633) OX=223926 GN=lpxL PE=3 SV=1

MNIVPPPLFSYQLLKPKYWSVWLAFGALAIIVNVLPYVVLRILGRSIGCVAMQLMKRRYK

IALRNLQLCFPDYTDWQCKDVVRKNFQYTGMALIETGIAWFWPDWRINRITSIVGKDRLL

TEEKNGRGVLVVCSHHLNLEITARIFSQFAKGYGVYRPNSNPAYEFIQHRGRTRFGHQMI

DRKDVKSMLKVLKNGHRLWYLPDHDYGASHSVFAPFFAVEQAASTVGSSVLIDATKCAVI

SGVTVSRNHHYTLYIGKDLSEYFEKRNAMKAASILNQELEKMIRRDIPAWMWLHKRFKTR

PEGFDCVYT

>tr|Q87SX2|Q87SX2_VIBPA Uncharacterized protein OS=Vibrio parahaemolyticus serotype O3:K6 (strain RIMD 2210633) OX=223926 GN=VP0300 PE=4 SV=1

MSAVIKLAWKSLMNRKATAVLTIMTVAISVILLLGVERIRTQAKDSFANTISGTDLIVGG

RSGQVNLLLYSVFRIGNATNNIDWKSYQKFSQHRAVDWAIPISLGDSHKGFRVMGTNHSY

FEHYKYGSKQPLTFSKGKEFNGLFETVLGSDVAKQLGYQIGSEIIIAHGISDVGFSRHDK

LPFKVVGILAPTGTPVDKTVHVSLEAIEAIHVGWESGARLGPTPDAKVLQERDFQPKQIT

AMLVGLKSRIQTFALQRQINNYPKEPLSAIMPGVALHELWGMMSVAEQALMAVSGFVVIA

GLLGMLSSLLTSLQERRREMAILRAMGARPRHVFSLLISEASLLTAAGIVTGVLGLYAIL

ALLQPLIQQHYGINLTLSTLSAYEWMLLSFVQCAGIVIGFIPAFRAYRQSLSDGMTIRI

>tr|Q87GG8|Q87GG8_VIBPA Putative transcriptional activator ToxR OS=Vibrio parahaemolyticus serotype O3:K6 (strain RIMD 2210633) OX=223926 GN=VPA1348 PE=4 SV=1

MLRNYLLGNQVIFDTLKREVLTTDKIISLGGREAAILKLLCENANTVIAKEEINDKVWGK

VFVSETSLTKAISNLRKSLQLIEGVMCEIKTIPKEGYMLILEGENLGLMVAEDEPPLEVK

RIESKDLALLKAPVGNNRFLSTLAKSDNKMNEGHIKPSWMLLAVLSSAFLSSVTSTAMIL

LLK

>tr|Q87GM9|Q87GM9_VIBPA Putative transcriptional regulator OS=Vibrio parahaemolyticus serotype O3:K6 (strain RIMD 2210633) OX=223926 GN=VPA1286 PE=4 SV=1

MSELEKLNQMLTEFYDKMSSWEQSVVKETGYSLAQVHTIEVLGGHGALRMKELAEKLGIT

TGTLTVQIEKLVNANLIERCPHPEDRRAIVVRLTPEGEKIHRHHNQLHLDLVRDLTRHIE

PEQQSTFLSCLEKMNREF

>tr|Q87IK0|Q87IK0_VIBPA Putative AraC-type regulatory protein OS=Vibrio parahaemolyticus serotype O3:K6 (strain RIMD 2210633) OX=223926 GN=VPA0606 PE=4 SV=1

MPNIEIIRFNHFTARKSERYTATHNGLYVVEEGALVVHQPNGEQFELQAGDFTLYNSSDL

RSAEAIPGENGFKAVALVFDISLFCEFKKAHPGLHSEAEHRRFYPFSPESNSEITQLKNT

LLALASRNAPDYTQSHIAMALLSLMVEVQPDILSIIDDASSLTASQKAIKYIEKNIEKDI

TLEGLAEHMSMSIATLKRRLAAENLSFSQILKVKRINYAATQLRVSQKSITEIAFESGFK

SAAHFSTAFKSIYNITPKDFRNQVVRG

>tr|Q87MD0|Q87MD0_VIBPA Acetyltransferase-related protein OS=Vibrio parahaemolyticus serotype O3:K6 (strain RIMD 2210633) OX=223926 GN=VP2326 PE=4 SV=1

MAIVTRRMLLLPYNESLQLEFVMLNCCAKNRAEMNGPHTVASAKQLFEKILDDENIYSMA

VLESTSRDYMGHVFISHLDSEPELGFIFDKAYWGKGLATEALKAFFPKACRELELHKVKA

NVNSNHQASMAVLEKLGFVKTRESKDLFGPYFEMEFTSDVAVGESSAA

>tr|Q87TC7|Q87TC7_VIBPA General secretion pathway protein N OS=Vibrio parahaemolyticus serotype O3:K6 (strain RIMD 2210633) OX=223926 GN=VP0143 PE=4 SV=1

MKRAVLYVVIFIVCFSVSLIMGLPVSWVLQQAPTVKGLDIQGAHGSVWQGQASSVRWQRQ

NLGQVNWDFQWSSLFTGKAEFSVRFGRGSDMNIRGRGLVGYSLSDGLYAENLVASIPASK

AVEQARLPVPIGVDGQLELNIRHATYAAPWCKTGEGTLVWSASGIQSPVGSLELGPVIAD

LKCQDSVLTASGEQTSTQVSSAFSAEMMPNQRYSTKAWFKPGADFPSSMGEQLKWLGQPN

AQGQYEFDYKGRF

>tr|Q87TR0|Q87TR0_VIBPA Amino acid ABC transporter, periplasmic amino acid-binding portion OS=Vibrio parahaemolyticus serotype O3:K6 (strain RIMD 2210633) OX=223926 GN=VP0008 PE=3 SV=1

MKNWVKVAVAAIALSAATVQAATEVKVGMSGRYFPFTFVKQDKLQGFEVDMWDEIGKRND

YKIEYVTSNFSGLFGLLETGRIDTISNQITMTDERKAKYLFADPYVIDGAQITVRKGNDS

IKGVDDLAGKTVAVNLGSNFEQLLRQYDKDGKINIKTYDTGIEHDVALGRADAFVMDRLS

ALELIKKTGLPLELAGEPFETIQNAWPFVNNEKGQKLQAEVNKALAEMRADGTVEKISVT

WFGADITK

>tr|Q87G97|Q87G97_VIBPA PTS system, fructose-specific IIABC component OS=Vibrio parahaemolyticus serotype O3:K6 (strain RIMD 2210633) OX=223926 GN=VPA1420 PE=4 SV=1

MDITNLIELETICLDLKAQTKDEALKELVEMLEAAGKLNSQSQFLADIWKREEIGNTGFD

DGIAIPHAKSDAVAKPAVAVGISRNGIDYGAEDGELSDVFFMLASPDGDDHHHIEVLAQI

STKIIEDGFVEKLKQAQSREEALEMLTDIQTQSNEFLPTSLEFASEPLSPWAQKLGRIKE

HLLFGTSHMIPFIVAGGVLLSLSVMISGHGGVPQEGILADIAQMGIAGLTLFTAVLGGYI

AYSIADKPGLAPGMIGSWIAVSHYNTGFLGAIVVGFFAGLVVWLLKKIQLPDSMSSLGSI

FIYPLVGTFVTCGAVMWVIGAPIASAMTTMNEVLTGMAGSGKVMLGTVLGAMTAFDMGGP

INKVATLFAQTQVNTQPWLMGGVGIAICTPPLGMALATFLAPSKFKRDEREAGKAAGIMG

MIGISEGAIPFAAGDPARVLPAIVAGGIVGNVIGFMFHVMNHAPWGGWIVLPVVDGKIGY

IIGTIAGSVTTALIVIALKKTVTEDESYTGHSQVYGSVQGEGEADVLAVTSCPSGVAHTF

LAAKSLEKAACALGIKIKVETQGANGVINRITEKDIEKAKFVIFAHDVAIKEPERFRKIK

VLDVTTKDAMLNATALLQARRVS

>tr|Q87JJ1|Q87JJ1_VIBPA MutT/nudix family protein OS=Vibrio parahaemolyticus serotype O3:K6 (strain RIMD 2210633) OX=223926 GN=VPA0258 PE=4 SV=1

MRHLQSTIHPELDHLDDKIIFQRNAARAIVLDGEDVLLLYTERYHDYTLPGGGIDEGEDV

IAGLVRELEEETGAQNIHSIKPFGIFEEFRPWYKDDADVMHMISYCYTCKIDRELGETAY

EDYEVKNGMRPVWMNVHEAIAHNEKTMAESPKKGMSIERETFLLHLIAKELL

>tr|Q87MU3|Q87MU3_VIBPA Uncharacterized protein OS=Vibrio parahaemolyticus serotype O3:K6 (strain RIMD 2210633) OX=223926 GN=VP2138 PE=4 SV=1

MVNSMARPKTYTDEDLIQVANELISRGKKPSGWRIREYLQRGKSSSIQADLERLIADGRI

PEAQNDIPSATTQVRTSYELPAEIQELLDRKEYDISTSLRDIVIAMNDSIHTHFESITYT

RIREAEAISQHAIKQKEQSEGEALDIEQTLQRATDANDQLEEKIEALQSEVVESQIEKSE

LLKNISQLTSSLNQTNDRLENQQDTICGLQASLSKVEKMNSALTVQLEHALNDAKKLESQ

LTELSERYESTCIKLTESSSKLQSMTEALDNSEHKLSTVQDEYTDLSVNYRILESKLHES

QSNISELKLANHELEKQLTYFLEETDSICDAVDDSSSGH

>tr|Q87R92|Q87R92_VIBPA KtrB OS=Vibrio parahaemolyticus serotype O3:K6 (strain RIMD 2210633) OX=223926 GN=VP0905 PE=4 SV=1

MTQFHQRGVFYVPDGKRDKAKGGEPRIILLSFLGVLLPSAVLLTLPVFSVSGLSITDALF

TATSAISVTGLGVVDTGQHFTLAGKILLMCLMQIGGLGQMTLSAVLLYMFGVRLSLRQQA

LAKEALGQERQVNLRRLVKKIVTFALVAEAIGFVFLSYRWVPEMGWQTGMFYALFHSISA

FNNAGFALFSDSMMSFVNDPLVSFTLAGLFIFGGLGFTVIGDVWRHWRKGFHFLHIHTKI

MLIATPLLLLVGTVLFWLLERHNPNTMGALTTGGQWLAAFFQSASARTAGFNSVDLTQFT

QPALLIMIVLMLIGAGSTSTGGGIKVSTFAVAFMATWTFLRQKKHVVMFKRTVNWPTVTK

SLAIIVVSGAILTTAMFLLMLTEKASFDKVMFETISAFATVGLTAGLTAELSEPGKYIMI

VVMIIGRIGPLTLAYMLARPEPTLIKYPEDTVLTG

>tr|Q87JL0|Q87JL0_VIBPA ABC transporter, permease protein OS=Vibrio parahaemolyticus serotype O3:K6 (strain RIMD 2210633) OX=223926 GN=VPA0239 PE=3 SV=1

MDAKTMTMNSLTTQDKAKSMMGSISRDNIVLFGLLAGLSTMMILFILMPLWAMLAKSVQN

SDGEFVGLANFATYFSSSSLWVSVGNTFSLGLVVTTVVGILAFGYAYALTRSCMPFKGLF

HILGTAPILAPSLLPAISLIFLFGNQGVAKELLGGHSVYGVIGISMGLIFWTFPHALMIL

TTSLRTSDARLYEAARALKTSPMKTFFMVTLPAAKYGLISTLIVVFTLVITDFGVPKVIG

GSYNVLATDIFKQVVGQQNFAMGAVTSIMLLFPAVMAFGADRWVQKKQKSLFDTRSVPYQ

PEPNKTRDGLCFVYCSLISVAVLAVLGMAVYGSLVTFWPWNKALTLNNYNFAEMSTYGWS

PFFNSLTLAGWTALIGTAVIFVGAYCIEKGRAFGPVRQAMQMLSVVPMAVPGMVLGLGYI

FYFNDVNNPLNVLYGTMAFLVINTVVHYYTVGHMTALTALKQLPSEIEATAASVRLPQYK

LFFKVTLPVCMPAVLDIATYLFVNALTTTSAVVFLYSTDTIPASVSILNMDDAGQTGAAA

AMAVMIMVAAAIAKIVQMTLGKWLESRTQAWRKR

>tr|Q87SA7|Q87SA7_VIBPA Uncharacterized protein OS=Vibrio parahaemolyticus serotype O3:K6 (strain RIMD 2210633) OX=223926 GN=VP0517 PE=4 SV=1

MQKHQLDMWLHGQHKDTYTLPKVFVIGCSDVSDYLLAVEYKHQLEPIKDGEEPLHFGSLD

LVKEELLRLGIDKAYLRLHNAYDECGSEGTASYCDIELSLVTH

>tr|Q87HE8|Q87HE8_VIBPA Transcriptional regulator, AraC/XylS family OS=Vibrio parahaemolyticus serotype O3:K6 (strain RIMD 2210633) OX=223926 GN=VPA1017 PE=4 SV=1

MALIEKNTQFDADKLTANVVGIAADVGKHDSGMHQHHKGQLLYAPQGCMTFALNDSICIL

PPTKAVWIPPHTPHRAVMTNVVAYRSVYFDCDKFSCPKSIVMIEVNDLLKALINKMALWE

WDIEQTKTNATTILFWEEFYQAKQFELTLPLPSDRRLASFRNAMTKDGFIAPDLNHLSKT

IGASGKTITRLFKSETGMSYQDWRQQWRLLKAIELLCEERQVSDVAHCLEFSSDSAFIAF

FKKQTGQTPLSFLKHRTLL

>tr|Q87PK2|Q87PK2_VIBPA Uncharacterized protein OS=Vibrio parahaemolyticus serotype O3:K6 (strain RIMD 2210633) OX=223926 GN=VP1501 PE=1 SV=1

MKKIALTLAATSITLVSYSAFSAQDAEHVRLATTTSTYHSGLLDYLLPQFEKDTGYKVDV

IAAGTGKALKMGENGDVDLVMTHAPKAEGTFVEKGYGVLPRKLMYNDFVIVGPKADPAKI

KDDESVLDVFKEIANKNATFISRGDDSGTHKKEMGFWAQTKIEPNFGGYRSVGQGMGPTL

NMASEMQGYTMSDRGTWLAYQNKLDLEILFQGDEKLFNPYQVILVNPERYPTINYQGAKA

FSDWLVNPRGQELINGFRLNGKQLFVANAESK

>tr|Q87MD3|Q87MD3_VIBPA Uncharacterized protein OS=Vibrio parahaemolyticus serotype O3:K6 (strain RIMD 2210633) OX=223926 GN=VP2323 PE=4 SV=1

MTTSTKLVELLYQLEAQLQKHELWQQTMPSPEALQSVEPFAIDTLDPHEWLQWIFIARMH

ALVESSQPLPRGFSIEPYFAEVWKQEPQYAELLNTIRTIDELCK

>tr|Q87FK8|Q87FK8_VIBPA ABC-type arabinose transport system, permease component OS=Vibrio parahaemolyticus serotype O3:K6 (strain RIMD 2210633) OX=223926 GN=VPA1671 PE=3 SV=1

MSVTSSTKILEQQDAKRSWNFAGIWDRFGMLMVFAGLFLLCAFFVPYFATFINMKGLGLA

ISMSGMVACAMLFCLACGDLDLSVASIIACSGVVTAVAINATSSVVLGVGAGLLSGVAFG

LLNGFVIAKLQINALITTLATMQIARGLGYIISDGKAVGITEESFFALGNSSFIGIPTPI

WLTIITFGVFAFLLNRTVYGRNTLAIGGNEEAARLAGVNVVKTKMIIFTVSGFISALAGV

ILAARMTSGQPMTSVGFELVVISACVLGGVSLKGGIGKVSYVIAGVLILGTVENAMNLLN

MSPFAQYVVRGAILLAAVIFDRYKQTSRA

>tr|Q87N68|Q87N68_VIBPA Putative membrane protein, suppressor for copper-sensitivity A OS=Vibrio parahaemolyticus serotype O3:K6 (strain RIMD 2210633) OX=223926 GN=VP2007 PE=4 SV=1

MIGWVIVVCLMQNSGLVSACSMSDDQTLVSALSSDDAHVVKADKVKEPSKCELSEKLIQF

TQHQLETFIVVLFIGIVLAAVWRCSAFVNARQWTEPIYDKHRIHLTFCVFRE

>tr|Q87FS8|Q87FS8_VIBPA UPF0056 inner membrane protein OS=Vibrio parahaemolyticus serotype O3:K6 (strain RIMD 2210633) OX=223926 GN=VPA1600 PE=3 SV=1

MKELILHTVTVFMGFFAIMNPIANIPIFLSLTADEDKETVRSIALRSVFIAFVIVAIFAI

AGKVIFDLFGITLYALRITGGILVFMIGFNMLQGDSTHQKTKEKAYSPAQQQAALSIAVS

PLAMPILAGPGTIATAMNFATTGGFDQTIITIVSFAVLCIITYILFLFGDKLVKAVGPSA

LNVVTKMMGLILAVIGTQMFIDGAGEAYKTVFA

>tr|Q87N59|Q87N59_VIBPA Uncharacterized protein OS=Vibrio parahaemolyticus serotype O3:K6 (strain RIMD 2210633) OX=223926 GN=VP2016 PE=4 SV=1

MKRFYSLFALPLITAATWANEPLPDRQTELPAVKKENHGEYLQPRDLSKIPNTAFGDQVK

RGYSLFVNSQQMRGKYVGNEQNCVNCHMEAGRKANAAPLWAAYMAYPAYRKKNDRVNSYA

DRIQGCFEYSMNGKAPAYDSPEIVALSAYAYWLAMGGLLDSYGMNDEAVPELDIKALQVG

GKAQDFPLPDAIAQALPVKERGNLAGRGYPKIAAPKQEPSPERGALVYEKNCETCHRADG

SGIKGTDGHSYIPPLWGEFAYNWGAGMHRINTAAYFIYENMPLGKSVQLTEQEAWDVAAY

INSHERPQDPRFKGNVTANAEKYHQHQGYYGKTLNGKQIGKNAFASGTVE

>tr|Q87IU8|Q87IU8_VIBPA Uncharacterized protein OS=Vibrio parahaemolyticus serotype O3:K6 (strain RIMD 2210633) OX=223926 GN=VPA0508 PE=4 SV=1

MQLFQSVLIKMEKHQSYKSITAKVSIRKMQRILDQLLNEIDEKHRASKENVVTLTRQSQH

RLMSYKELYLHREAIAESELLLAYESMSDTEKQIADMGLSELTYAIEALDRAC

>tr|Q87PY3|Q87PY3_VIBPA Putative transcriptional regulator, LysR family OS=Vibrio parahaemolyticus serotype O3:K6 (strain RIMD 2210633) OX=223926 GN=VP1367 PE=4 SV=1

MTFNFEQLLAFVTVYEELSFSKAAVKLNKHRTTTGQVISNLEDQLAITLFDRVGRSVEPT

EDGHLLYHYAKKTVEQARIFENIALSLSFGGLERVVIAYSSFMPPSALFMIRKQLAKDFP

TMRVELLVRDRAEIKRGIVDGSIHFGLVNVHESSAMHSMDSVFLGHVEFVPYVQAGGRLA

KLDQSSVVDAMVTERQFILKSLVDEGLKEKFIFSANNEQVDQLSVVINMVREGLGWSWLP

KALTKPQFKAEGIEPVSIDLMLEGMKLPLSMWCPHAKYLKDIKASIMHAVHEHVESYNP

>tr|Q87PD0|Q87PD0_VIBPA Uncharacterized protein OS=Vibrio parahaemolyticus serotype O3:K6 (strain RIMD 2210633) OX=223926 GN=VP1587 PE=4 SV=1

MTHKKLQSVHLSKMDLRMRYVVTLFLLLLPTASTLADDSETNPVAKKIKSTLQKKVDKQF

DQYDGYCDLMIEMEHKGKVAIVKRVTGSGDTKVCRFARSNLKTGKRYRYKYPEKYIRIHI

TTGS

>tr|Q87N29|Q87N29_VIBPA PTS system, glucose-specific IIBC component OS=Vibrio parahaemolyticus serotype O3:K6 (strain RIMD 2210633) OX=223926 GN=VP2046 PE=4 SV=1

MFKNLFANLQKVGKALMLPVSVLPVAGILLGVGAAHLSFIPEIVSNLMEQAGGSVFGQMA

LLFAVGVALGFTNNDGVAGLAAIVGYGIMTATLGVMAGVMGVEKIDTGVLGGILVGGVAA

WAFNRFFKIQLPEYLGFFAGKRAVPIITGFAAIILGVILSVIWPPIGGAISAFSDWAAHQ

NPQLAFGIYGVVERSLIPFGLHHVWNVPFFFEAGTCVNAAGETQHGVLTCYLVADEASRA

AGNGFGQLAGGYMFKMFGLPAAAIAIAHCAKPENRAKVMGIMASAALTSFLTGITEPIEF

SFLFVAPVLYGIHALLAGSAYIVANTLGFVHGTSFSHGLIDFLVLSGNAQKMGLMIAVGL

VYAVIYYVVFRAVITALDLKTPGREDETEEAAATSSSDMAGELVAAFGGKANITGLDACI

TRLRVAVADTAAVDQDKLKQLGAAGVVVVAGGVQAIFGTKSDNLKTEMDEWIRNHG

>tr|Q87SH3|Q87SH3_VIBPA Ribosomal RNA small subunit methyltransferase I OS=Vibrio parahaemolyticus serotype O3:K6 (strain RIMD 2210633) OX=223926 GN=rsmI PE=3 SV=1

MTDKNKLPNEGPTLYIVPTPIGNLADITQRAIEVLSNVDIIAAEDTRHTGKLLSHFNIQT

KTFALHDHNEQQKAQVLVEKLLSGQSIALVSDAGTPLISDPGYHLVTKCRQAGVRVVPLP

GACAVITALSASGLPSDRFSFEGFLPPKSKGRKDKFLEIASVERTCIFYESPHRILDSLQ

DMLDVLGPEREVVLARELTKTFETIQGMPLGELIEWVKSDDNQQRGEMVLLLHGHRETSD

EALPDEALRTLGILTKELPLKKAAALVAEIHNLKKNALYKWGLENLD

>tr|Q87SI3|Q87SI3_VIBPA Uncharacterized protein OS=Vibrio parahaemolyticus serotype O3:K6 (strain RIMD 2210633) OX=223926 GN=VP0440 PE=4 SV=1

MLSVTVSLARLWNTMSLYVLLYVQLVTLLVTLVALNVRKLVYVKHVVVHSSLSVNFLLPE

IMLSRIILGLCTKSSAICRAFCFLLSDKTSVLSGYLRTLPLNIHATFYEGFVAIFTLSLC

YKSLNLRVSDFCHLLKTYSV

>tr|Q87SJ6|Q87SJ6_VIBPA Uncharacterized protein OS=Vibrio parahaemolyticus serotype O3:K6 (strain RIMD 2210633) OX=223926 GN=VP0427 PE=4 SV=1

MAQVAEKQRYHVDLAGLMRTYETNYAKLNALLPVSAEVGDVRCYQAANMVYQLTVNEITK

YTTVVEICQSDETPVFPLPTMSVRLYHDARVAEVCSSGEFSRIKAKYDYPNDQLMQRDEK

HQLNTFLGEWLTFCLRSGISRTPLAFN

>tr|Q87QC9|Q87QC9_VIBPA Putative 83 kDa decaheme outer membrane cytochrome c OS=Vibrio parahaemolyticus serotype O3:K6 (strain RIMD 2210633) OX=223926 GN=VP1220 PE=4 SV=1

MNGLKSIATLIFFILLLTGCGPDSKNDQNTSPPALGDFEISVSEPSLVTQETGETKLVVD

FTVKDGSGRSHELDETKDFRIALLKAMPSRVDTQNSSDPAFAFNGRHGNTYWKSFHHSSN

TTNNRASMESVWDGTLVKTDEGYRYTFAIPDVLKVSDPYTADSSSNNGFIAWDADKLHRI

VMAYGEQGNGFTYVFEWVPQESSDAAVSRNVIETGTCENCHMGEPLHHGPGYRSIDNNIA

VCTACHNDSNPGAAPARRPLAAVVHQYHGNVFKLGSDRNNTDTYKQPVDENDVLVTDING

LVIEGNPFPQDARNCTTCHSTDVAKASDANNWFEHPSQVACETCHLYRDRGAHDNQIGTA

WVRNGEPQNSCSGCHRPYDRDDNGDPIIGQDASRSAKTVHVVRLENLAKARDSLEISVES

ARFIDDQFEVELRVSKAGSGIGSINELTPFINEHGHLNLLLNWDNGQGPMVANNSLNVAD

DGALGDGCEAQGEGLFLCHKDFTDAATKPTSNSTLTVNIADMPLCANRRDGELAECVTFE

GIDLIKSPFVIAANNASGSFDVSGINKQRKLPVGADISSCNDCHKELTIHKLGEHPHAAT

DFQQCKNCHNSERSAFYPGMAADLKYHVHSFHAFGSAHSGEASFPGAVNNCEACHTNTQY

NLPSQQNTRPSLASGKYFSPALVACGACHLESSLANADPDTVAGDAPLNHMLNHGAVFGA

DTAAQAMGSEQCATCHAIGQSQGIDKVHKVYDYR

>tr|Q87P73|Q87P73_VIBPA Uncharacterized protein OS=Vibrio parahaemolyticus serotype O3:K6 (strain RIMD 2210633) OX=223926 GN=VP1645 PE=4 SV=1

MENNTPNQMSQIKVPATYMRGGTSKGVFFNLEDLPSEAQVAGEARDKLLLRVIGSPDPYG

KQIDGMGGATSSTSKTVIVSRSSRDDHDVDYLFGQVSIDKPFVDWSGNCGNLSAAVGSFA

IHAGLIPQERILENGIVTVRVWQVNISKTILVHVPIVNGFVQETGEFELDGVTFPAAEIQ

VDFVDPADGEGSMFPTGNLVDDLVVPDVGTFNATFINAGIPTIFIDAESIGYQGTELQDQ

INNDDAALAMFESIRAHGALKMGLISDLEEAQTRQHTPKVAFVSKPKSYQSSSGKAVNES

EIDVLVRALSMGKLHHAMMGTAAVAIASAACVPGTLVNLAAGGGEKESVTFGHPSGTLKV

GAQAKQTEQGWVVQKAIMSRSARILMEGFVRVPSDVFE

>tr|Q87I72|Q87I72_VIBPA Putative transcriptional regulator, LysR family OS=Vibrio parahaemolyticus serotype O3:K6 (strain RIMD 2210633) OX=223926 GN=VPA0734 PE=4 SV=1

MQTRVRLTPPERNRMKNKDKNKKPLHNLDLNLLKIFRVVSEEKKTVAAAKRLNITQPAVS

RAMARLREHFNDPLFVRTRYGLKPTDKGQLLSDSLPKIMDDLSNLILELDEFSPESSSAN

IKIAINGFFGVSFPAKLHLKLLDIAPNITIETESWSSNTISKLINGELDLGINYPLNNVP

KELQTKHLATDEFQILARDAHPLSDQAIDCNDLSLYPIVTAIVPDWNENRTLIECWAERE

NIETETAFRSSSILSLLEVISETDALFPTSAFLSRKHLSGLKSLSLSPQLKASFKGESQD

IYLYMHYRHRNSPIYKWLIPIIESTLSSINDN

>tr|Q87LW8|Q87LW8_VIBPA Iron(III) ABC transporter, permease protein OS=Vibrio parahaemolyticus serotype O3:K6 (strain RIMD 2210633) OX=223926 GN=VP2490 PE=3 SV=1

MKDRHSLWKTSSGAITLLLVLPILAIFYTAIGETDNLFTHLMSTVMPTYIYNTVVLTIGV

MGLSLIFGIPSAWLMAMCKLPTEKWLQWALVLPLAMPGYIIGYIFTDWFDFAGPIQIFLR

DVTGWGPGEYWFPDIRTLPGATFVLSLVLYPYVYLLCRAAFMEQNVSLLQSARLLKCSPW

ESFWRISMPLVRPSIAVGLSLVAMETIGDFGTVSYFAVNTLTTAVYDTWLGYSNLNAAAK

ISAIMLLIVVLLLSTERYSRRKQKLFQSQFNSHEDFRYELSGWKKWAALVWCWGLVAVAF

ILPLLQLIDYSITYFEQSWTPEFREYAWNSLVVSVIAAIIGVAVALIVNFTHRVNGKRES

LAFMRLSSMGYAVPGTVLAIGVMVAVLFMDYRVNDIAKAMEWGRPGLIFSGSMFALIFAM

VVRFSAVAIGSIESNLNKISPSLDMASRTMGCTPNTMLWRVHFPLVKRGALIAALLVFIE

SMKELNASLLLRPFNFETLATYVYNFASDEHLELAALPAVLLVLVGLIPLVVVNRSLEQN

H

>tr|Q87QW5|Q87QW5_VIBPA tRNA 5-carboxymethoxyuridine methyltransferase OS=Vibrio parahaemolyticus serotype O3:K6 (strain RIMD 2210633) OX=223926 GN=cmoM PE=3 SV=1

MTEDRNFDDIAHKFAKNIYGSDKGEIRQIIVWEDFLQILSELDASQQPLEVLDAGGGLAQ

MSQKLAKLGHRVSLCDLSSEMLQLAKQDIEKNGLLEQYRLIHSPVQSIAEHMEEQVDLVM

FHAVMEWLVDPKTALETVLEQVKPGGIASVMFYNHHGLVYKNVVCGNIPHILDGMPHRKR

FKLQPQKGLKPEDVYQWIEEAGFSVCGKSGIRSFSDYIGNMQYMGDYEFEDVLALEKQLC

RQEPYLSLGRYIHVWAKKNDKQE

>tr|Q87I75|Q87I75_VIBPA Uncharacterized protein OS=Vibrio parahaemolyticus serotype O3:K6 (strain RIMD 2210633) OX=223926 GN=VPA0731 PE=3 SV=1

MKKLIPFAIIFTSNVALANCINQSENYVTSKVLKSSDTKITQVDGKLASVINEDLSIVQD

EKIEKVEATNATESCTFDKNSMSLVLHYDTNEHYLTNVHKQAISKYLELVNEDKRILVEG

HTDDVGSQAYNKSLSLRRASTASKYLKKDLGLGNRIVEKAFGESAPICKVNENKLSGCNR

RVVLTIE

>tr|Q79YY1|Q79YY1_VIBPA Bacteriophage f237 ORF2 OS=Vibrio parahaemolyticus serotype O3:K6 (strain RIMD 2210633) OX=223926 GN=VP1552 PE=4 SV=1

MAKSVFVLGMDITWNSARGDSAQLNVSRPLREINSEKFKRRTIGESGDVNPQWDQPLMID

HEYALLLERTGALVPRREYQLRLEINPEDPLAGAIVTELIPVDQEIKKHFEASMKPVQG

>tr|Q79YT5|Q79YT5_VIBPA Flagellar secretion chaperone FliS OS=Vibrio parahaemolyticus serotype O3:K6 (strain RIMD 2210633) OX=223926 GN=VPA1551 PE=3 SV=1

MLMDSGYDSYQQVDLDAQAAAANPHQLVIMLIDGLLDEIERIRGHLAAKRLAEKGAGINK

CMNILIGLTSALDDENGGEIAENLRQLYDFCQVELYYASVQNDADRLMNVERVMGNIREG

WMNFGQQA

>tr|Q87HL9|Q87HL9_VIBPA Putative cation efflux system (AcrB/AcrD/AcrF family) OS=Vibrio parahaemolyticus serotype O3:K6 (strain RIMD 2210633) OX=223926 GN=VPA0944 PE=3 SV=1

MPDKLKRSFVQSVMNSFFPPIMILLALVVGAAALWLTPKEEDPQIVVPMADVLVSAPGLS

ASQVENQITEPLEKLVSQIDGVEYVYSSSMEGAAQVIVRFYVGENREDALVKLYNKLYSN

QDKVPPSVTNWLVKPVEIDDVPIVVAAIYSTDPDILDRHQLRRIADQATLGIKSLDATNK

VEVIGGEPRKIQIELDSVAMANFKVTIDDLEQAIQLSNSKTQGKNVRVNGQNFTLESGRF

LTNAEEVGDLVIAVLNGKPVYLKDVARIYDGEGEATSDTWYRDKNHDEAYPAVFISVAKQ

KGSNAVNVAQSVRDKLAALQSEQFPPQVQVAVIRDYGETANAKVNNLVSSLGISILTVVV

FVGLFLNWRSALVVGIAIPISYGAALGMDLAFGYSINRVTLFALILALGLIVDDPIASID

NIERYLKRKNLTRTNAIVLAMAEIRSALLMSTVAIVIVFTPMFFITGMMGPYMAPLAFNV

PISVIFSTVVAFMITPWLAKKLLKGAEENGHYDIQSSPMYRLYRGVLIPLLESRKKAWLF

LGLVALLFVLAALLPALRLVPLKLLPYDNKNEFQLVLNMPESSSFVDTSNALSSFTDYLM

SVPEVTSVSGFAGTASPMDFNGMVRHYFMRSEPYQGELRVVLAEKNRRAMQSHELVTRLR

ADLEQIADKFDADVQLVEVPPGPPVIATITAEVYGDEATSYEDLMIQAEKVADRLRKEQL

VSEVDTSIQGDLETWQFIVDQEKAALSGVSVADINNTLITAANAKVLGYIADPREVDPLP

IEVQLRREDRDNLNQLEQLYVRGRPGIAKVESNGAVVDAPQPIVQLSEVGHFVKRAADKP

IFHKNLKPVVYVYAEVVGRVPGEVIADVMADQDTTHAEDVHRHWQDRTYLSNGAGVTWSV

PDNIEVVWSGEGEWKITVDVFRDLGIAYGAALLGVFVVMLIQTGLPAVSGIIMLAIPLTV

IGIMPGFWILNVLSSDIGNYPNPALFTATAMIGMIALAGIVVRNSLVLIEFVQQSLAEGR

SLHDALIESGVVRMRPILLTAGTTLLGNVVITLDPIFNGLAWAIIFGITASTIFTLLVVP

VVYNLAYQNTKGHGLPQMEEEQ

>tr|Q87GA8|Q87GA8_VIBPA Uncharacterized protein OS=Vibrio parahaemolyticus serotype O3:K6 (strain RIMD 2210633) OX=223926 GN=VPA1409 PE=4 SV=1

MESTNSSVPISNTVPLLAAITLALFFVPVKFAVGGVALAPSDIASLLSVGLIALIILEGR

AKQLLHPCIGFLVLFTGYVFINGLLNRVPLAPLLIETVQWLAILCLLSLLYAYGAFDDER

VMVYFTYLLFIICGLVAAWHFAQGYQSGFKLLGVSKYGFGVLCSLLYLYRDKIRLFPFLM

LVAFALLVLSQERKALLGYCLLFFLDQIFIKNWMRKTISETYTWMLLLALAFVVIGTVST

TLYVGFDALADKLEITQEDILFANQSEARWVSNLHRKLLLANGMDILTQHPILGVGAKML

PNFMIDYFNYDELAIYTHNFVLDTAIEYGLLGIAFLFGGYFLFIKFCFRSIDDNRKSLLL

AVYALIMVFFVAVNTTIILILLLPVMISRKRTPEPQTVIHSLPVNKSHEF

>tr|Q87JY7|Q87JY7_VIBPA Uncharacterized protein OS=Vibrio parahaemolyticus serotype O3:K6 (strain RIMD 2210633) OX=223926 GN=VPA0111 PE=4 SV=1

MHQQIIKFWFEELTPQNWFENNPELDKHIASRFASVLEQAARCELFNWRDSAQGRLAEII

VLDQFSRNVYRNTPKAFAQDPLALALAQEAIRLGHDQELAPEQLSFLYMPFMHSESRLIH

VEAEKLFRASGLENNYDFELKHKAIIDQFGRYPHRNAILGRESTPEELAFLQQPGSSF

>tr|Q87T58|Q87T58_VIBPA ADP-heptose-LPS heptosyltransferase II OS=Vibrio parahaemolyticus serotype O3:K6 (strain RIMD 2210633) OX=223926 GN=VP0212 PE=4 SV=1

MKKILIIGPAWVGDMVMSQSLYITLKQLHPESQIDVIAPGWCKPILERMPEIHQAIEMPI

GHGEFNLLGRREIGKSLREKQYDHAYILPKSAKSALIPWFANIPLRTGWKGEMRYGLLND

LRPNMKSFQYMVERYVALAYSKSEMVDSSSLGGLDTLPRPSLSLNKEEQQTTINKFNLDQ

KRPAVGLCPGAEFGPAKKWPETHYAEVAAQMCKTGHQVWLFGSQKDLETCNNIRALIPTQ

FHEHIHVLAGQTSLIEAVDLLAACKTVVANDSGLMHVAAAVGCNVVAVYGSTSPKYTPPL

AEKVEMVHTDIDCRPCFKRECQYQHLKCLTELSPKQVLDSIQKLEAIATSSC

>tr|Q87SD5|Q87SD5_VIBPA Aerobic respiration control protein FexA OS=Vibrio parahaemolyticus serotype O3:K6 (strain RIMD 2210633) OX=223926 GN=VP0489 PE=4 SV=1

MQTPQILIVEDEQVTRNTLKSIFEAEGYAVFEASDGEEMHQVLSDNSINLVIMDINLPGK

NGLLLARELREQANIALMFLTGRDNEVDKILGLEIGADDYITKPFNPRELTIRARNLLSR

SMSTNAVQEEKRSVEKYEFNGWVLDINSRSLVSPAGDSYKLPRSEFRALLHFCENPGKIQ

TRADLLKKMTGGELKPHDRTVDVTIRRIRKHFESVSGTPEIIATIHGEGYRFCGDLED

>tr|Q87T39|Q87T39_VIBPA Putative UDP-galactose phosphate transferase OS=Vibrio parahaemolyticus serotype O3:K6 (strain RIMD 2210633) OX=223926 GN=VP0231 PE=4 SV=1

MMKRLFDFLVSLIALILLSPIIVLVAWKIRKKLGSPVLFRQTRPGLNGKPFEMVKFRTMK

DAVDEQGNLLPDSDRMTPFGEKLRNSSLDELPGLWSVLKGDMSLVGPRPLLMRYLPLYNE

EQARRHDARPGVTGWAQINGRNAISWEEKFALDVWYVDNQTFWLDIKILLLTVKKVFVKE

GISADDHVTMPEFEGSKDDK

>tr|Q87GR0|Q87GR0_VIBPA Uncharacterized protein OS=Vibrio parahaemolyticus serotype O3:K6 (strain RIMD 2210633) OX=223926 GN=VPA1255 PE=4 SV=1

MINGISKIKSLRSFGIYENHINDGCDEFAKFNLIYGWNGSGKSTLSRLFRCIENKSLDGT

NYTESAFEIEYSLDGQAQSVLTQQNLSQNQLNIRTFNNDFVRENIDWSGTVKSILLVDQQ

KIEERKQLDEQNKTLKLKESASEKETKIAGDLDTEINKFLSRTAKSIKTSLKVIGTEDSK

YLNYNKTSLENLIRTSSAEVVDPASVLTDEQIILHTKSASPVEKDKISFPASTIRKDYFE

SSFNALTKLLGKSAVNEVIEFLRDNPDVQTWVSSGMAVHDKHKSEECHFCGNKIDESRLT

SLNNHFSNEFKLLKQEITEATKYCCDLPELTLPSVESFFDEFQTEYKKVVAPLEGVVTEI

NSIVEQWRECIDKKSNDPFDISLKISSVTVDLIDQYNNVILDIAACVKKHNDKSGNFQAV

TTAHKKALELHYAADEVIDFDYSKKVEDRKTANGNVLKLSDEISAIKKEIQRLEAELSNE

SIAVDAFNAELAKFIGRTELTLKFDAKQKGYRISRNGSDRHAQNLSEGEKTAIAFVYFAT

KLEEHDNDIKNTIVVIDDPVSSFDSNHLFHSYSFLKKHCEQAKQLFVMTHNFSYFKLVRD

WMLKKNKMRKQPPVIKARAYTIETKSDGVRQSKLVNAGGSLTDYNSEYHYLFSKLYSLKE

KQELNLDEVYLCANLSRKLLESFLCFKFPKKRSDFRQLVDDGVRHYPAIKPEDVEKAYRF

INKYSHNQEIELEDNADNLLGESPAILNIILDIVKTIDEAHYTEMETVVTA

>tr|Q87PK9|Q87PK9_VIBPA Uncharacterized protein OS=Vibrio parahaemolyticus serotype O3:K6 (strain RIMD 2210633) OX=223926 GN=VP1493 PE=4 SV=1

MRIHAFHRLYQYRQSISTKPFNARGCKVQRCPFCQVSEQHCLCELQPDIDSNVACMLIVS

ENEVFKPSNTGRLIADTVKETYVYQWNRTEPSQEMLDLLSNEDYLPVIVFPADYVDQPER

LLDGLHTEHLHRTDGNSKKWLLIFIDGSWREARKIFRRSEFLQSLPVLSIEPECLSEYIM

RRSENEQHLSTAEVATLVLKQAGENKASECLQLWFEAFRETYMLTKTRVKNDPNRPHLKR

FKEWLKTES

>tr|Q87PY0|Q87PY0_VIBPA Uncharacterized protein OS=Vibrio parahaemolyticus serotype O3:K6 (strain RIMD 2210633) OX=223926 GN=VP1370 PE=4 SV=1

MMPKVMLNGFILVPEEELETVKSALPIHMRLTKSEAGCLVFQVVENPDNPLRFDVYEEFT

NRDTFEHHQLRVKDSDWGRVTVNVERHYEILDVQE

>tr|Q87NL7|Q87NL7_VIBPA Uncharacterized protein OS=Vibrio parahaemolyticus serotype O3:K6 (strain RIMD 2210633) OX=223926 GN=VP1851 PE=4 SV=1

MLINLRLQKVSQELTENKIMQALDWAYDKAINGVAGLDSAQELALSYMKESSDPISQANS

LIRWQNTKAGTSGFLTGLGGLITMPVTLPANITSVMYVQIRMIAAIAHMGGHDLKDDRVK

AMVYACLTGNAAKDILKDIGIVVGRKLTENAIKSISGKTITKINQAVGFRLLTKFGEKGA

INLGKAIPLVGGIVGATFDSVTTNTIGNMARDTFIALPEFA

>tr|Q87KH1|Q87KH1_VIBPA Gene 3 protein-related protein OS=Vibrio parahaemolyticus serotype O3:K6 (strain RIMD 2210633) OX=223926 GN=VP3006 PE=4 SV=1

MQKEFGLTEYIAEEGRLLIAAPRFDLDTFPALGERLVGLLSATVIEKQWDADIHSWLIDF

EGCRLFMKAEHYSEAIWFEALNIEESREELDYLAGLFQRGF

>tr|Q87KS4|Q87KS4_VIBPA Uncharacterized protein OS=Vibrio parahaemolyticus serotype O3:K6 (strain RIMD 2210633) OX=223926 GN=VP2902 PE=4 SV=1

MHTFGKVQQLTANIKSVDLFNLFSLFDVPSELEESFQAIHSRRNKRRIELAKSALESGLD

NSIEVPQPSLTFVVGEVLSHKNLGRGLIEIEYDPLDTLIVDGVITLFAIMQLSGFSHPFE

KKRVSKELILKNDVARQELAHCPIQVNLLFSPTEPLSKKTCITLYKKYSQTEKNIHAPLI

ESVNAELPINTYVREVAKTIDLQAFGGMNTTSIRLSVKDPYVTTEATMIRLVLGAIGGAD

YQDKNKVDVFGSGPFSAKHTNVIKPYICIFMEAWLKSVKGQLFSHKSGFHYSTTLWQSLG

LVIHKLFLNEEPINEFAKAGAFLGQLDYSKSAKHWGDCDALELDASGKLYKNATGGGRAI

RIAMAHYLFNVYQNGKK

>tr|Q87FM1|Q87FM1_VIBPA Uncharacterized protein OS=Vibrio parahaemolyticus serotype O3:K6 (strain RIMD 2210633) OX=223926 GN=VPA1658 PE=4 SV=1

MKSTMIIVSHVVNDAVTHGFVPAAKAMDLHVVLITDHKLSHLELASRDSHFNPDTILECD

VFNPLEIIETITENELKPDAIFSNSDHLQTSTAICAQFFGLPAKDWSVTLKAKNKHLTRQ

VLNEKALPNTQSCLLHRHSNVALGMAFPVVAKPKEGVASLDVQRCDSQSELDAYCDAFWQ

KYPNAAILVEQFLQGPLVTLETLGDGERLIAVGGFDVSLSEPPYFIETAADWNGPNGIAH

REACLAQLKAFGVGFGVCHSEFIITENGPVLVEINYRSIGDGREFLLNNLAHFGWFSTIL

GLHLGRRLDGDYQIHGSAHVHYVVAEHSGVIDGDSTSFIQHDGDVITQQQVLKTAGELFQ

QSYSNKDYLARISIVSPSGRNLDQALQNALNNFNLAPTREVAA

>tr|Q87PK0|Q87PK0_VIBPA Sigma-54 dependent response regulator OS=Vibrio parahaemolyticus serotype O3:K6 (strain RIMD 2210633) OX=223926 GN=VP1502 PE=4 SV=1

MSTQSHISTNQQYNAFSVLVVDDELGMQAILKKALGKLFSHVDTAGSIEEAEQLRNSRHY

DLILLDINLPGRSGIEWEEAFEDDEKRADVIFMTGYADLEIAIRALQLGASDFILKPFNL

EQMLKAVSRCMDRRLNERMQYAMKRDYQRHNTSEIIGGSEKTRQLKQLITQFAPSRASVL

VEGESGTGKELVARGIHQASGRTGPFVPINCGAIAPELLESELFGHTSGAFTGAKKSREG

LFRVANGGTLFLDEIGEMPLSMQASLLRVLEQRTIRPVGSEREISIDVRIVAATNRNLQE

EVNQGNFRSDLYYRLNVLKIEVCPLRERKTDLFELVPFFSNMLTRELGMPAPKWAHEDME

AMSEYDWPGNIRELKNLIERCILLGKPPAHYWRELNGGHSLPNVSITVSHSAELPTFREG

KEFTGEGYPNNWTLKEVEKAHIKQVVNLHEGNKSAAARDLGVARKTLERKYKEWDSEDEG

YAD

>tr|Q87TL3|Q87TL3_VIBPA Uncharacterized protein OS=Vibrio parahaemolyticus serotype O3:K6 (strain RIMD 2210633) OX=223926 GN=VP0056 PE=4 SV=1

MYTRSVVFWLSALTATSTLAADASAATSHPPASTLKQGYFIDSPVTGLYYKTSSNITGVT

QQGAFHYQPGDVISFFLGNDDKGYLLTTLSSQEVLTPTMATTKPSRSINMTRLLLSLDST

PENREEIVLANRVLSDPRFQSQLKSLDLNYLDDAKRHLNLDWVSVDEAIDHLNESQQYIE

ENFASDEIIFEPRGITFKNIVIKKKDWQGRACAYDLRYKHHPKYRPPFGETRFTITKDSL

IEHPSVGDHFQGCFISPNHQITEDIVEPISKFADWFNLVGCSITGCTRNDLNGFSLEDFD

DEGDWKYRSVAMNFDPSTELFMEKVQGLGRNKHIQHSNRTEMLWFSYPNTSEHDIDYLGV

WQQTQYHQQSMTQSCLLMRHQQVMRLPRSAETCPTDASLYTQDVTREFADMWWVNNDEPK

ANLAQMNIMVRWSTTLAEINYTTWEYLPAGANWEQGILYRYQQNVSRNRDGSDHIETHTI

SEFVKVSEEV

>tr|Q87JF1|Q87JF1_VIBPA Uncharacterized protein OS=Vibrio parahaemolyticus serotype O3:K6 (strain RIMD 2210633) OX=223926 GN=VPA0302 PE=4 SV=1

MKIEHVAIWTERLEELKGFYEKYFNAVSNDKYHNPKKHFSSYFLSFESGARLELMSMEGV

TTCENSHSMQVTGLAHFAFALGSEQAVDQITKTLVEDGYQRIDGPRYTGDGYYESCVLDP

DGNRIELTV>tr|Q87KL3|Q87KL3_VIBPA Uncharacterized protein OS=Vibrio parahaemolyticus serotype O3:K6 (strain RIMD 2210633) OX=223926 GN=VP2964 PE=4 SV=1

MSDIEKVVMRTRTIEKLLRTQYHAEGKGLHQLISSCEERLPHDVIAKLRFIATVRNKVVH

EDNYKLDDRKGFMAACDDCEKELTPRSSRFIWRTAILLMALITLAALGFYYMHWDILSEH

IQ

>tr|Q87K81|Q87K81_VIBPA Uncharacterized protein OS=Vibrio parahaemolyticus serotype O3:K6 (strain RIMD 2210633) OX=223926 GN=VPA0017 PE=4 SV=1

MGKLIPVSVRLAWLNLQRNRRRSLLSMLIIAIAVFALTSAGGFGLYTYDSLKESTARDTG

HLTLTTPGYFAKEEEMPLSNGLSHADEITKQLIGLSEVRGVQPRVEFSGLISNGSKSSIF

IGIGVNEREFDMKGPFLDVRDGQTLSNIHASRYDASEPEVMLGVDLASNLSVNVGDWITL

LATTTDGALNAYDFKVRGIYSTGVPELDKRQLYVHINSAQSLLGSDKVSALSVFLFDTCL

TNKVEQQVATILNKQKPSFGDQEVEITPWQERAFFYLKVKDLYDRIFGIMGAVMALVVFV

ALFNTMTMSVTERTREIGTLSALGSYPREIIAGFLREAGLLAVIGSLIGALFTALVTIFL

MMVDVQMPPPPGRTEGYPLTIYFSWELVAAAGLSVLLICLVAAFFSARKGVNKPITEALI

YV

>tr|Q87TK7|Q87TK7_VIBPA Phosphogluconate dehydratase OS=Vibrio parahaemolyticus serotype O3:K6 (strain RIMD 2210633) OX=223926 GN=VP0062 PE=3 SV=1

MTHSVVLEVTQRLTERSREARAAFLARTEVQAEAGKGRVGLSCGNLAHAVAASCSSEKKN

ILDFTHANVALISAYNDMLSAHQPYQDYPAQIKQVLADYGHTAQVAGCVPAMCDGVTQGQ

AGMDMSLFSRDLIAQSTALSLSHNVFDATLLLGICDKIAPGQLMGALSYAHLPTAFVPAG

LMATGISNEEKVDVRQKYAAGEVGKDALLDMECRAYHSAGTCTFYGTANTNQLVFEAMGL

MLPGSAFIHPHTQLRKALTDHAALKIASMTAGSAHFRPLAEVVTEKSLVNGIIALLASGG

STNHTIHMIAVARAAGILLTWQDISDLSDVVPLLARVYPNGPADMNAFQDAGGVPALLHR

LNESELLHRDVKPVFGKFEDQMTLPSLVDGQLTWTPCQGSQDGDVIAKPDATFQNTGGTR

VLTGNLGKAVVKVSAVKEEQRVIVAPAIVFQCQHEVEAAYKRGELNKDCIVVVTHNGPAA

NGMPELHKLMPILGNVQKAGFKVALVTDGRLSGASGKIPSAIHVSPEAIRGGAIGLVRNG

DLIRLDCQTGELNNLSDTTGRELIHFDTESTQQTWGRGLFSVIRQNVSSAEEGASFIV

>tr|Q87M84|Q87M84_VIBPA RecBCD enzyme subunit RecB OS=Vibrio parahaemolyticus serotype O3:K6 (strain RIMD 2210633) OX=223926 GN=recB PE=3 SV=1

MDQMSGAECPLHTLDRTEQPQPTPLEPMTFPLHGARLIEASAGTGKTFTIAGLYLRLLLG

HGSAETRHRVPLTVDQILVVTFTEAATAELRDRIRARIHDARIAFARGQSSDPVIQPLLN

EFDDHKQAAEILLQAERQMDEAAVYTIHGFCQRMLTQNAFESGSRFNNEFVTDESHLKAQ

VVADYWRRNFYPLPFTLAGEIRQLWSSPSALLSDISNYLTGAPLSLSVPAMKGSLADLHT

ENLKKIDELKAQWRESQDDFFTLISDSDINKRSYTKKSLPTWLEAVNAWAATETTGYDYP

DKLEKFAQNVLLEKTPKGSAPQHAVFEAIETFLANPISLKAPLLAHAIEHCRVMLANAKN

QKQWLSFDDLLTQLSASIDTDESELLAARIRTLYPVAMIDEFQDTDPLQYSIFSRIYLND

PECGLFMIGDPKQAIYGFRGADIFTYIKARNQVSAHYTLGTNWRSSADMVQAVNQVFALP

DSPFIYDSDIPFLPVKYSPNAEKRIWTMGGQKQPALTYWLQEADDKPLPKGEYLTRMAEA

TASQIQTILTQAQQGQACLVNGEKQKAVQAGDIAVLVRTGSEGRMVKQALADQGIASVYL

SNRDSVFTSSVAQDLQRLLQAVLTPENDRALRASLASELFALDAASLDALNNDEVVWENA

VNEFKEYRKLWVQRGVLPMLRAVISKRHIAERLLEEGASSQGENGERVLTDLMHIGELLQ

QASNELDSDHGLLRWLAQSISDAENGLGGSDDQIQRLESERNLVQIVTIHKSKGLEYDLV

FLPFVFSYREASEAKYYDAANDRTVLDITGNDASMKQADKERLAEDLRLIYVALTRAVYA

CFIGASPLRNGRSTKEPTGVHRSAIGYLIQNGQEGGINDLHQGLTKQQDELDCVVVADPP

QQLEDKYVAPQEEIHDLSAKELQNPIDRNWRITSYSGLVKQGSHHAEHDATIEITGFDID

SSEEQDEADLVEPERSIFTFPRGARPGTFLHSLFEEIEFTQPATTEENTQIILGLMESEQ

LDEEWLPILQQLIDTVLVTPLDGKSLLLNQKAPSQRLVEMEFLLPIEVLSAPALNRVIQR

HDPLSAKAGDLGFQTVQGMLKGFIDLVFEHQGKYYVLDWKSNHLGDDVTHYHGEALKSAM

ADHRYDLQYQIYALALHRFLRSRLANYQYEQHFGGVYYLFLRGMDGQSDHGIFAAKPTLD

FLREMDRLIDGQVLETRSTQAGQMELL

>tr|Q87Q66|Q87Q66_VIBPA Uncharacterized protein OS=Vibrio parahaemolyticus serotype O3:K6 (strain RIMD 2210633) OX=223926 GN=VP1284 PE=4 SV=1

MRDLTPEYLLAALRQAIKTKGLTYRELSEKMGMPLSTFKRHLTSTNLALDKLLEYCRAID

CTLDELQKLANQLQGEDEDYFSRTQDEVFFQFPHLYDFYRELRMLRGKDGYTILKKKYDL

SEQSMSDYLGALELLDLVYVDENKSITLHGPLYYSYAENSKLNDKYTEIIKEQTTTHDKC

VRVALARMKITEEQLLELEDQVAKTVIDFHSKNVVAENFNVSDFTNVVLLAGPHQPVTFS

DGIVETESRFIDDIRYAIAAAGDKPSLSI

>tr|Q87H15|Q87H15_VIBPA Putative high-affinity branched-chain amino acid transport permease protein OS=Vibrio parahaemolyticus serotype O3:K6 (strain RIMD 2210633) OX=223926 GN=VPA1150 PE=3 SV=1

MAQLSMRPCGDFRTTYKSDTPIFETKTIRSLAIAGVIAMLAAPLVLDIYFLNLFIQIAYL

GIAALGLNILVGFTGQISLGHGAFFGFGAFASAWLNNQFNIPVVFAIPLAGYLTMIVGML

FGLPAARIKGLYLAIATLAAQFILEDFFARAEWFSGGSYGASASPINLFGFEFSTDESFF

YVALFALIFMYLWASNLIRSRDGRAFVSVRDHYLSAEIMGINLTKYRLLSFGVCAFYAGI

GGALYGHYLGFVSAEGFTIMMSIQFLAMIIIGGLGSVKGTLMGTIFIVLLPEVLEFGVTG

LAAFSDNTSFIDGLAYFKEMAIGLVIMLFLIFEPQGLSHRWQQIRAYWKHYPFSY

>tr|Q87TQ6|Q87TQ6_VIBPA Beta sliding clamp OS=Vibrio parahaemolyticus serotype O3:K6 (strain RIMD 2210633) OX=223926 GN=VP0012 PE=3 SV=1

MKFTIERSHLIKPLQQVSGALGGRPTLPILGNLLIKVEENVLSMTATDLEVELVSKVTLE

GDFEAGSITVPSRKFLDICRGLPDDAIITFVLEGDRVQVRSGRSRFSLATLPANDFPNIE

DWQSEVEVSLSQADLRTLIDKTQFSMANQDVRYYLNGMLFEIDGTTLRSVATDGHRMAVS

QTQLGADFAQKQIIVPRKGVQELVKLMDAPEQPVVLQIGSSNVRAEVNNFIFTSKLVDGR

FPDYRRVLPQHTNKTLIASCDELRQAFSRAAILSNEKFRGVRVNLAGSEMRITANNPEQE

EAEEMLDVTFEGDPIEIGFNVSYVLDVLNTLRCEKVQVSMSDANASALIENADDDSAMYV

VMPIRL

>tr|Q87SL7|Q87SL7_VIBPA DNA primase OS=Vibrio parahaemolyticus serotype O3:K6 (strain RIMD 2210633) OX=223926 GN=dnaG PE=3 SV=1

MAGHIPRSFIDDLLARLDIVDIIDARVKLKKKGKNYGACCPFHNEKTPSFSVSQEKQFYH

CFGCGAHGNAIDFMMEFERLEFVEAIEELASYLGLDVPREQRSGGSGQFKSGPQASSSEK

RSLYDLMGSIAQFYRNQLKQPSSKVAIEYLKDRGLSGEIVQKFGIGYVADEWDLVRKNFG

QNKDNQDMLVTGGMLIENDKGNRYDRFRGRIMFPIRDRRGRVIGFGGRVLGEGTPKYLNS

PETPIFHKGKELYGLYEVLQAHREPAQILVVEGYMDVVALAQYGVDYSVASLGTSTTGDH

IQMLFRQTNTVVCCYDGDRAGKEAAWRALENALQFLKTGNTLKFLFLPDGEDPDSYVRKY

GKAAFEQQIEQATPLSSYLFDNLIELHQINLGNNEGKSALRAYASALIDKIPDPYFQELL

EKLLDERTGFDNRLRQPRKKISETRPQPHKEIKRTPMREVIALLIQNPSYAQMVPDLSSV

RDLSIPGLSLFADVLDKCQAHPHINTGQLLEHWRNSQNEALLSRLASWDIPLDEDNQEEI

FLDSLDKIIAQCVEKQIENLQAKARSVGLSAEEKRELLALMLDLKA

>tr|Q87JV9|Q87JV9_VIBPA Putative PmbA-related protein OS=Vibrio parahaemolyticus serotype O3:K6 (strain RIMD 2210633) OX=223926 GN=VPA0139 PE=4 SV=1

MSQEQQLLNAVDYVLSEAKRQGAEADVIVNRNSSFSLKANQGKLDEYKVSSSQVLGVRVI

KDARVATSYSESLEQPSLDLMLTNALQSARFSKQDEHQTISCVNSKITTDIAEIAQDDTT

SVDEKIELSLALEQGVVALPHASSSPYNGYSDGETQLIIANTQGTLCQHFERSFTCYAYT

LFEKDGKQSMAGRMSLGRRFDELNPTYCIEGGYNLARDLLDGVPVATGNYPAIFHINALA

SLFGAFGSAFSGVSAMKGISPLGDKLGQSVASELLTFTDAAYMPNGMAIAGFDSEGFATQ

DNVLIANGQLNTLLHNSQTASYLGAVSTASASRSAKSSLDVSANHKVIATGNSSASEVKA

GEYLELVELQGVHSGADAVSGDFSFGASGFLCRDGQRVQPVRGITVAGNFYKMLQEVEAV

GDTQLINDSRTFFAPDVRFARLSIGGK

>tr|Q87N02|Q87N02_VIBPA Preprotein translocase SecA subunit-related protein OS=Vibrio parahaemolyticus serotype O3:K6 (strain RIMD 2210633) OX=223926 GN=VP2074 PE=4 SV=1

MQYTLIEKGEVQLDESSLFIEGAVLAANLTTKPLAPEAWLEPLFGEGFKSIQPAVEEQIH

KQHNRILRNEYSALELTEKDPEQLADFAEGFMSVWPLIEEQWQEVELNDGLQRMLQALLT

TMMLAIDEESTQKHMRDAGIETPPALTDLVDQLDLMLVEVALGADELMVGNKSQSLNPFK

DVGRNDPCPCESGKKFKQCCGK

>tr|Q87SK3|Q87SK3_VIBPA Uncharacterized protein OS=Vibrio parahaemolyticus serotype O3:K6 (strain RIMD 2210633) OX=223926 GN=VP0420 PE=4 SV=1

METEIELKFFVSPEFSETLRAKISETKVLQHSCRDLGNTYFDTADNWLRQHDIGLRIRRF

DDVFVQTVKTAGRVVAGLHQRPEYNAEHTNNEPDLSLHPSDIWPSGKDVATLQSELVPLF

STNFTREQWLIGMPDGSQVEVAFDQGMVVAQGEDGEERQEPICEVELELKSGQTDALFTL

ARSFCEQGGMRLGNLSKAAKGYRLATGYTGDEVKPLPLVDSNKTDTVEYCLINSLEHALS

HWHYHEQIYSERDSVEALREISNAIRFIRQTLTIFGGVVPRRASAILRQELKWLEEELVW

LDEHAHLEELLDDKGNVLRKLDARKFLVSELTQQLEELPSREEMLTLLSSARYTGLLLDL

SRWILARGWQPFLDEKAREKMASNIMPFSVTQLDRTWAELMEAFPAERDLSAQEYVDQRY

RLLRNLYTGIGFASLYNFDERNSFRLPWADLVHGIDDLLMLNHLLPLVDMLENEEKEQLE

RWLHRQERSILHAMDQTRAISVETQPYWREK

>tr|Q87K37|Q87K37_VIBPA Hypothetical membrane protein OS=Vibrio parahaemolyticus serotype O3:K6 (strain RIMD 2210633) OX=223926 GN=VPA0061 PE=4 SV=1

MPKRQNINQRMSIVALAWPILVEILLRTALGTSDVFMLSGYSDKAVSAVGVITQITFFLI

IVSTFVSSGTGILIAQYNGAGREQESVNVGVASIALSVIIGVLLSVIAVFGAIFLLPYYG

LEAQVEQYAREYLLISGAMTFNVTIGIVFTTILRSHGYSRSPMVVNLISGVFNIIGNYIA

LYQPFGLPVYGVQGVAIATVVSQVIGTLMLWFILARSSIELPMSTMKQVPAEIYKKILKI

GGMNAGEVLSYNVAQICIVYFVVQMGTASLAAFTYAQNIARFSFAFALAIGQAAQIQTGY

YIGKGWVSSILKRVQIYFLVGFVASTAATTLIYLFREEILRVFTDQPEILALAGSLVMGS

ILLEAGRVFNLIFIAGLKGAGDIKFPVQMGILSMWGLGVLFSYIFGIHLGYGVLGAWMAI

ALDEWVRGIIMARRWRSQVWTKFKVS

>tr|Q87MZ1|Q87MZ1_VIBPA Uncharacterized protein OS=Vibrio parahaemolyticus serotype O3:K6 (strain RIMD 2210633) OX=223926 GN=VP2085 PE=4 SV=1

MNNSINNSFLKFGLAACLFSFSAFSFSSPSIEGNDQDMSTGTVTAKMAHADENGWMVVHR

TDESMKPGPVIGYAPLKMGQNENVNAILMEPVESGDMLMLMVHGEKGGMKTGVFEYSLGA

KEDGPVKVDGKLVMDIVRAK

>tr|Q87IW0|Q87IW0_VIBPA Putative heavy metal membrane efflux protein OS=Vibrio parahaemolyticus serotype O3:K6 (strain RIMD 2210633) OX=223926 GN=VPA0496 PE=4 SV=1

MTTEVLNSASEKMSFLDRYLTVWIFVAMAIGVGIGAVYPQVAEWNEAMSVGSTNVPLAIG

LILMMYPPLAKVNYGLLGTVTRDKKAITLSLVMNWIVGPILMFVLALTFLGDHPGYMVGI

ILIGLARCIAMVLVWNDIGGGNKEYGAALVALNSAFQILTYSVMAWLFITVLPPFFGYEG

FVVDISMGDIAESVLIYLGIPFLAGFLSRKWLVAAKGEKWYNDVFIPQISPITLIALLAT

IVLMFSLKGEMILQLPMDVFRVAVPLAIYFVLMFFASFFIGKRMGIPYDKNASIAFTATG

NNFELAIAVSIAVFGLNSDQAFAGVIGPLIEVPVLIALVNVALRMKTKYQS

>tr|Q87H85|Q87H85_VIBPA Putative membrane transport protein OS=Vibrio parahaemolyticus serotype O3:K6 (strain RIMD 2210633) OX=223926 GN=VPA1080 PE=4 SV=1

MDLFALLDINNTLVNIPIGDGYAMSWIEAFGTVFGLLCIWFASQEKTINYVFGLLNVTLF

AVIFFQIQLYGLLLLQLFFFCANIYGWYAWTRPNAQGETLEVRWLSKQKLMATAVVCVVS

IALLTIYIDPFFFALANIAVDSLNIFGAGLSEPVLEPDAFPFWDATMTVLSVVAQILMTR

KYVENWILWVVINIISVGIYATQGVYAMSFQYAILMFIAANGTREWARSAKRNGDKTLAQ

ATA

>tr|Q87PG7|Q87PG7_VIBPA Putative stress protein OS=Vibrio parahaemolyticus serotype O3:K6 (strain RIMD 2210633) OX=223926 GN=VP1535 PE=4 SV=1

MSIYSKILVVADINNDEQPALARAVQLAQKSVSRSRITFFLSIYDFSYDMTSMLSVDERD

AMRRGVIHQREQWMRKIAQPYLNDSFDFDVCVVWHNRPYEAIIAEVYAGGHDLLIKGTRK

HDVLESVIFTPTDWHLLRKCPTPVLLIKNSDWPEQANILASVHVGSENPTHIDLNDAMVE

RLKEMSNRLDAEPYLVNAYPVTPANITIELPEFDPTTYTDAVRGHHLTAMKALRQKHGID

EEQTIVEQGLPEDVIPAAAERLNAAMVILGTTGRTGLSAVFIGNTAEHVIDKINCDVLAL

KPKGYISPLDPNTAT

>tr|Q87LS6|Q87LS6_VIBPA Uncharacterized protein OS=Vibrio parahaemolyticus serotype O3:K6 (strain RIMD 2210633) OX=223926 GN=VP2535 PE=4 SV=1

MDSLIAIAAAMLYVLAIATIIPGLSQQSGIKAKTVFASAACALVFHAWILSDLIFDGSGQ

NLSILNVASLISFIISLVMSIAMLKNRLWFLLPVVYSFAAINLTAATFLPSTFVKHLEND

PKLLIHISFALFSYATLTIGALYALQLAWLDHKLKAKKSLAINPNLPPLLMVERQLFKII

LIGNLLLTGTLITGFVFVQDMFAQGKAHKGILSFIAWIVYSILLWGHYHKGWRGRKVTWF

AVAGATLLTLAYFGSRFVKEIILN

>tr|Q79YW0|Q79YW0_VIBPA Flagellar M-ring protein OS=Vibrio parahaemolyticus serotype O3:K6 (strain RIMD 2210633) OX=223926 GN=VP2249 PE=3 SV=1

MADKSTDLTVTDGGSDGALVSSSDMDVESQNPDLEERSASKFDMAVGDLDLLRQVVLVLS

ISICVALIVMLFFWVKEPEMRPLGAYDTEELIPVLDYLDQQKINYKLDGNTVSVESSEYN

SIKLGMVRSGVNQATEAGDDILLQDMGFGVSQRLEQERLKLSRERQLAQAIEEMKQVRKA

RVLLALPKHSVFVRHNQEASASVFLTLSTGANLKQQEVDSIVDMVASAVPGMKTSRITVT

DQHGRLLSSGSQDPASAARRKEQELERSQEQALREKIDSVLLPILGFGNYTAQVDIQMDF

SAVEQTRKRFDPNTPSTRSEYALEDYNNGNMVAGIPGALSNQPPADASIPQDVAQMKDGS

VMGQGSVRKESTRNFELDTTISHERKQTGTVARQTVSVAIKDRRQVNPDTGEVTYTPMSE

GEINAIRQVLIGTVGFDQSRGDLLNVLSVKFAEPETEQLVDQPIWEHPNFNDWVRWFASA

LVIIVVVLVLVRPAMKKLLNPAGDDDDEMYGPDGLPIGADGETSLIGSDIESSELFEFGS

SIDLPNLHKDEDVLKAVRALVANEPELAAQVVKNWMNENG

>tr|Q87GK6|Q87GK6_VIBPA Uncharacterized protein OS=Vibrio parahaemolyticus serotype O3:K6 (strain RIMD 2210633) OX=223926 GN=VPA1309 PE=4 SV=1

MWRKPLEREAKESGHVTSVVKYEAGSRFSPHLHPYGEEIFVLDGVFSDENGDYPAGSYLR

NPPGSLHAPFSEKGCVILVKLNQFDPNDLKTVRINTRETEWLPGIGGLQVMPLHDFKHEH

VALVKWPKGEKFQPHKHFGGEEIFLLSGEFRDELGIYPTHTWLRSPHMSEHFPFVEEETV

IWVKTGHLPLGS

>tr|Q87GT3|Q87GT3_VIBPA Uncharacterized protein OS=Vibrio parahaemolyticus serotype O3:K6 (strain RIMD 2210633) OX=223926 GN=VPA1232 PE=4 SV=1

MTLTVWLSLFTVCLLGAMSPGPSLAIVAKHALAGGRMNGLATAWAHAFGIGIYAFITLIG

LAVVLQQSPMLFKTISLAGAAYLAYLGFNALRSKGGVAAKLESGEETTVLQSAREGFLIS

ILSPKIALFFIALFSQFVALGNDLSNQMIIVATPFVVDGLWYTFITLVLSSSRVVDKIRS

KAVLIDRLSGVVLMLLALRVVVTV

>tr|Q87K15|Q87K15_VIBPA 4-hydroxy-2-oxoglutarate aldolase/2-deydro-3-deoxyphosphogluconate aldolase OS=Vibrio parahaemolyticus serotype O3:K6 (strain RIMD 2210633) OX=223926 GN=VPA0083 PE=4 SV=1

MKDLNQQLSEIKVVPVIAIKDAGKAVKLAQVLIENGLPCAEVTFRTEDAALAIKNMREAY

PEMLIGAGTVLTSAQVDEAIDAGVDFIVSPGFNPTTVKYCQQRNVTIVPGVNNPSLVEQA

MEMGLRTLKFFPAEPSGGVAMLKALSAVYPVKFMPTGGVSPSNVKDYLSISSVLACGGTW

MVPGDLIDNEQWDELAKLVREVAGIIE

>tr|Q87G85|Q87G85_VIBPA Putative two-component response regulator OS=Vibrio parahaemolyticus serotype O3:K6 (strain RIMD 2210633) OX=223926 GN=VPA1432 PE=4 SV=1

MSYKVLVVDDEPRIHTFIRISLSAEGFDYIGASTIAEAKACFEAYSPHVILLDLGLPDGD

GTEFLTTLRQTYKTPVLVLTARDQEEEKIRLLEAGANDYLSKPFGVKELIARIKVLVRDL

VDEQSIADELVAGRVKIIKSTHQFWLDQREIPLTKKEFSFIEQLILKPGKLIEQTHLLAV

IWGKSHVEDTHYLRVLVSQLRKKLNDSADEQRLLKTEPGLGYRLVLETSKHH

>tr|Q87IM6|Q87IM6_VIBPA Uncharacterized protein OS=Vibrio parahaemolyticus serotype O3:K6 (strain RIMD 2210633) OX=223926 GN=VPA0580 PE=1 SV=1

MALGFGMKMELQQFLDALASSPEKIEFETTMAVIEDNYDFTPAAFTNGNTQNDANENNGS

CKIFAFGLLNALDKEATLACFGRFYREDVLLHPENNDHQNIRNFMVTGWEGIQFETSALT

AK

>tr|Q87FS3|Q87FS3_VIBPA Uncharacterized protein OS=Vibrio parahaemolyticus serotype O3:K6 (strain RIMD 2210633) OX=223926 GN=VPA1605 PE=4 SV=1

MEDKEMKTNRTFLTVHGVIYTVFAFALFFVPTMIWPMYGVQINDQYALFLSQHTSIFLGG

IAAVSLMLRDVESGKTAKQLFKALLITNGLGAVITTYASITGVFVGFGWSDPIFFVSLSL

LTYKQLRVQ

>tr|Q87TI2|Q87TI2_VIBPA Uncharacterized protein OS=Vibrio parahaemolyticus serotype O3:K6 (strain RIMD 2210633) OX=223926 GN=VP0087 PE=4 SV=1

MRNENIASIIELIHSGNDIYKKAIDNIDNTSLNHVLQDLVHVREHAALELKPYAIANPQT

ADDTPASYTIKAREVYSDVMKEVSSDKESMYLEQLEGVESKVLKEIENVITAQPEQADNA

VLLKVRSDIKSCKEKLSNISLIS

>tr|Q87MA0|Q87MA0_VIBPA Putative transcriptional activator ChrR OS=Vibrio parahaemolyticus serotype O3:K6 (strain RIMD 2210633) OX=223926 GN=VP2357 PE=4 SV=1

MNKHPDNNLLEAYASGSIDAVSGLVVATHLETCSKCRAYVNQVEASQANTVSESPSEYSP

EFDDMLNDIINAEPVNDNVVIQDTAFVNVAGKSFELPKTLVRFSDLVGSWRSYGGKVFSA

QIDLGEDARVSLMYIGENVQIPQHTHRGLESTLVLHGGFSDEDGQYEEGDLMVRDASVKH

SPFTQEGEDCLCLTVLTEPMIFTQGVARIFNLFGKGLYP

>tr|Q87S77|Q87S77_VIBPA T-protein OS=Vibrio parahaemolyticus serotype O3:K6 (strain RIMD 2210633) OX=223926 GN=VP0547 PE=4 SV=1

MAVELNALRDQIDAVDKQMLELLAQRLELVEKVGEVKSEHGLPIYAPDREAAMLASRRAE

AEKMGVPPQLIEDILRRTMRESYASEKDSGFKCLNPELRSVVIIGGNGQLGGLFGRMFKL

SGYQVKVLGSKDWGRADEILKDAGLVVVTVPIHLTEGVIEKLGNLPQDCILCDLTSIKSK

PLQAMLNVHAGPVVGLHPMFGPDVPSLAKQVIVYCDGRGKEQYQWLLQQFGIWGASLCQI

DAQEHDHGMTLIQALRHFTSFAYGMHLSKENPNIEQLLKLSSPIYRLELAMVGRLFGQDP

NLYGDIILASQENIDMIKRFHQRFGEALAILDSKDKAKFVESFEQVSDWFGQYSQQFMNE

SQNLLKQANDNIHRG

>tr|Q87LW3|Q87LW3_VIBPA Aconitate hydratase B OS=Vibrio parahaemolyticus serotype O3:K6 (strain RIMD 2210633) OX=223926 GN=VP2495 PE=3 SV=1

MLEAYRKHVEERAAEGVVPKPLDAEQVAGLVELLKNPPQGEEEFILDLLENRIPPGVDEA

AYVKAGFLTAVAKGEVSSPLVSREKAAELLGTMQGGYNIAPLVELLDDEALAEIAVKALS

HTLLMFDAFYDVEEKAKAGNAHAQKVLQSWADAEWFLSKPKLEEKITLTVFKVTGETNTD

DLSPAPDAWSRPDIPVHALAMLKNEREGINPDQPGTIGPIKQIEELKSKGHQLVYVGDVV

GTGSSRKSATNSVLWFMGDDIPYVPNKRAGGYVLGGKIAPIFFNTMEDAGALPIEVDVTK

LNMGDVIDVYPYEGKVCNHETGEVLAEFSLKTDVLIDEVRAGGRIPLIIGRGLTDKARQA

LGLESSDVFRKPGEVADSGKGYTLAQKMVGKACGVAGVRPGTYCEPKMTTVGSQDTTGPM

TRDELKDLACLGFSADLVMQSFCHTSAYPKPVDVNTHHTLPDFIMNRGGVSLRPGDGIIH

SWLNRMLLPDTVGTGGDSHTRFPLGISFPAGSGLVAFAAATGVMPLDMPESILVRFKGKM

QPGITLRDLVHAIPYYAIQQGLLTVEKAGKVNEFSGRILEIEGLETLTVEQAFELSDASA

ERSAAGCTVKLSQESIEEYLNSNIVMLKWMISEGYGDRRTIERRITAMEEWLAKPELMSA

DSDAEYAHVIEIDMAEIHEPVLCAPNDPDDARLLSEVQGTAIDEVFIGSCMTNIGHFRAA

GKLLDKFNGQLATRLWVAPPTKMDKDQLTEEGYYGIFGRAGVRIETPGCSLCMGNQARVA

DKSTVMSTSTRNFPNRLGTGANVYLSSAELAAVGAILGKIPTKEEYLEYAKQIDATATDT

YRYLNFHKMEQYTKKADEVIFQEPA

>tr|Q87TL0|Q87TL0_VIBPA Putative transcriptional regulator, LysR family OS=Vibrio parahaemolyticus serotype O3:K6 (strain RIMD 2210633) OX=223926 GN=VP0059 PE=4 SV=1

MAHQLDALDLNLMRLLKAVVENRSIKLAAMQLGISQPSASRGVMKLKQVFDDPLFVRKAH

GVEPSPMAIRLAAEFDNMIAPLEKVVQEFEVFDPQQYQGQIAIVTDPYLMDEQGQRLLTG

CHRAFPKAHFAFSSWNSYSHDEMLEGEHDYCILDQETELSKDIYMRPLFVEKRVILARKN

HPTLSKVSNDWDTVSKLPLVSLPAPASYKPLCTVESEYRRMGYEPVVLLKSYNLRVACQM

LQETDAIMYASQSSALLMPELASYAMPLVNREFSQFVVSGGFLQTNRNHPLHRHLHKVVR

QTLNTPLIFTQ

>tr|Q87NR1|Q87NR1_VIBPA Uncharacterized protein OS=Vibrio parahaemolyticus serotype O3:K6 (strain RIMD 2210633) OX=223926 GN=VP1807 PE=4 SV=1

MSINIKWDGDCRFKVSTEGGFTFNVDATSETAPCPTEVLLSALGSCSATDVVLLLQDQGF

EVKGLKNKVTFALTESEPRLYKSANLHFTVNGSGFKESDILRAAQEAVEKHCHVCLMLSP

TIDITCSAEVGKNCT

>tr|Q87QA8|Q87QA8_VIBPA Uncharacterized protein OS=Vibrio parahaemolyticus serotype O3:K6 (strain RIMD 2210633) OX=223926 GN=VP1241 PE=4 SV=1

MDMTDQAFFKYVRNFNEYQKRSMFGGIGLFSDDAMFALVSNDCCYLRGGNGLDEEFTLLN

CEKYKHVKKQTTATVNYYDVTDLFESGFTGLDDLLRKSIDCSIKERKYQKSSASKRLRDL

PNMQLTLERMVKKAGIDDVETFLELGPVEVFNKVRVAYGNDVDVKLLWKFAGAIDGVHWK

LIQEPRKKQLLALCE

>tr|Q87SG0|Q87SG0_VIBPA Cell division protein FtsZ OS=Vibrio parahaemolyticus serotype O3:K6 (strain RIMD 2210633) OX=223926 GN=ftsZ PE=3 SV=1

MFEPMMEMSDDAVIKVVGVGGGGGNAVEHMVRESIEGVEFISVNTDAQALRKTSVGNVIQ

IGGDITKGLGAGANPQVGREAALEDRDRIKDSLTGADMVFIAAGMGGGTGTGAAPVIAEV

AKELGILTVAVVTKPFSFEGKKRLAFAEQGIDELSKHVDSLITIPNEKLLKVLGRGVTLL

EAFASANDVLKNAVQGIAELITRPGMINVDFADVRTVMSEMGHAMMGSGIAKGEDRAEEA

AEMAISSPLLEDIDLAGARGVLVNITAGLDMRLDEFETVGNTVKAFASDNATVVIGTSLD

PDMTDEIRVTVVATGIGNERKPDITLVAGGKAKVASAPQAQPQQVAATQAEEKPAQTLQN

QVQEKPQVTPQPTNTVSSSPAAGQSSAAPKQEKESGYLDIPAFLRRQAD

>tr|Q87JW5|Q87JW5_VIBPA Putative ABC transporter substrate-binding protein OS=Vibrio parahaemolyticus serotype O3:K6 (strain RIMD 2210633) OX=223926 GN=VPA0133 PE=4 SV=1

MNKNKLMSAAALLASMVSTNVLAAEKELTLMLDWFVNPNHGPIVIAQERGYFKQQGLKIN

IQEPADPSTPPKLVAAGKVDMAISYQPSLTIDVAAGLPLIRSATLIATPLNTLMVLDNGK

NDNLGDLKGKKIGIAIAGNEEATIGTMLAQENVKFSDVQIINVGWALSSSLASGKVDAIW

GGLRNFETNQLALEGYKAKAFFPEEHGVPAYDELVFVANAKTYDKAAIKAFNKALEQATT

YIVNHPKESWKEFVAYSPDTLNNELNQRAWNDTLTRFALRPSAVDLKRYDEYAEFMYSQK

IIETLPKAKDYVPSFD

>tr|Q87PD3|Q87PD3_VIBPA Putative phage replication initiation protein (Alteromonas phage PM2) OS=Vibrio parahaemolyticus serotype O3:K6 (strain RIMD 2210633) OX=223926 GN=VP1583 PE=4 SV=1

MDFDTKAIEIKMAGKTFANDAIHQSAFSRRFFPRLPAIVRNDVRRKVEARTQRQNATREN

VIKTAKDAVKFGLKCAHHIENRYSFVDSRKGAHSEPLTHNILMRDDALTKFAEKYADQCA

EILSSLNAEGYASFIEALAAVYSEQKALLKTIHIKPPYVNFNAKDVEVLEQMLTAAVLKM

QSEKWVERRLLRLRGDYIEYAQITMSRVGDKGHQSKYVSEISFSNWKRKQRESEKYMKSM

SVYNEETGEHFPLEEVAKRTIANPENRRIEMMVRSRGFEELADELEYTALFITWTLPSRY

HRNSPKWDGSSVKDGHAELMRQWSLARAKLAKLEIEYFGFRVAEPHKDATSHAHYFLFCS

HKDKANIIRILRGEAIAPDREELGDDITPRFDVKEADPSKGGATAYIAKYVSKNINGKHM

PDTEAEESAFKVRAWASVHRIRQFQQFGGEPVSLWRSLRRATAEQTQKDDQLEELRQAAD

SSKWALFCQLAKGAKLAYKENKNDYGEPIKKIIGFEWCGQVIETASECYSLVKTKDVKRL

LKSRGATSWSTENNCNSPLITELKKLTGWSFEGVKCLLEPLANGATVSIDQYCSIKLQNN

TLRLI

>tr|Q87SN6|Q87SN6_VIBPA Putative inner membrane protein OS=Vibrio parahaemolyticus serotype O3:K6 (strain RIMD 2210633) OX=223926 GN=VP0386 PE=4 SV=1

MELQALRYASMISTMTFDKACDYYAQYLKKEGLVVEAREAILEFVDLDENSLDDFGNDVR

IVLASADFGKELTTSVLWLRDKSIDISCVRLTPYRYREDVLINAEQIIPVPEVEEYQVKF

REKRAEQRTSVQKGEKDYSEYRYNGHTYKKRHLALALVTDWIEKHQPQSLNDVLNAFNEP

VRRRIAILADEIPQGRIRRFHNDEDALITLPNDEVIAITNQWSLSNITRLILFAEQSGMV

VEKAD

>tr|Q87LY5|Q87LY5_VIBPA Uncharacterized protein OS=Vibrio parahaemolyticus serotype O3:K6 (strain RIMD 2210633) OX=223926 GN=VP2473 PE=3 SV=1

MSGTSFGRRFVERPWLVSLILILLLVAWLAAGQLKAQGDHTLSPSLQSENTPLAKVMFDT

FTAKPTSKTIELYGRTAPNRQARLGAEVAGKIVSLSINKGQLVKQGQVIANIDKRDLDSQ

LKRAQAMLRVKEKEFNAAKSLKSRGLQGEVAFATAEAALVDARANLNNVQTALKNTEVKA

PFDGIVDHHFVEVGDFVGVGDPIATVIDLETLVIEADVSERHIQYLKEGLQADVRTINGQ

HHLGTLRYIGRVSSVSTNTFPIEIEIDNRNSLIPAGISAEVQLPLNEVLAIKITAAMLAL

DEEGNLGVKTLQDEHVKFVPIQLVKAEEDGVWLSGLGEQADIIVLGQGFVRDGETVIANK

VGDVAADAAEK

>tr|Q87S85|Q87S85_VIBPA Uncharacterized protein OS=Vibrio parahaemolyticus serotype O3:K6 (strain RIMD 2210633) OX=223926 GN=VP0539 PE=4 SV=1

MELVISLLQQMCVYLVLAYMLSKTPIILPLLSISSRLSHRLICYVLFSGFCILGTYFGLH

INDAIANTRAIGAVMGGLFGGPVVGFAVGLTGGIHRYSLGGFTDLACAISTTAEGVIGGL

LHVYLIKRNKGALLFNPSVVFSVTFVAEVVQMILLLAVAKPFDQAYELVSAIAAPMIIAN

SFGAALFMSILQDRKTIFEKYSATFSRRALTIADRSVGILSNGFNTENAEKIARIIYEET

KVGAVAITDQEKILAFVGIGDDHHRPNTPISSQSTLDSMEKNDIIYLDGTERPYQCSLAK

DCKLGSALIIPLRAGKAVIGTIKLYEPKRKLFSTANMSMAEGIAQLLSSQILYGDYQQQQ

ALLAQAEIKLLHAQVNPHFLFNALNTISAITRRDPDKARELIQNLSHFFRSNLKQNINTV

TLKEELAHVNSYLSIEKARFTDRLEVEIDIDPELLDIKLPSFTLQPLVENAIKHGISNML

EGGKVKIYSEMHPQGHLITVEDNAGSFQPPKDNHSGLGLEIVDKRLTNQFGRDSALKIAC

VTHQFTKMSFIIPPKS

>tr|Q87PA9|Q87PA9_VIBPA Uncharacterized protein OS=Vibrio parahaemolyticus serotype O3:K6 (strain RIMD 2210633) OX=223926 GN=VP1608 PE=4 SV=1

MNIEQYQRLTKQAVALIESEPDFIANLANLSSLLFMELEDLNWAGFYLTKGDELVLGPFQ

GKPACVRIPMGRGVCGTAAKTNTTQRVYDVHEFEGHIACDAASNSEIVIPFSIKGKVAGV

LDIDSPSIGRFNETDEEGLTHFMSEVEKLLNSHANDA

>tr|Q87GY9|Q87GY9_VIBPA Uncharacterized protein OS=Vibrio parahaemolyticus serotype O3:K6 (strain RIMD 2210633) OX=223926 GN=VPA1176 PE=4 SV=1

MVGLKKNIWTLYMMLLTVSIVTFSLFGYYHYQATLDKYKDKQLLQLELFASSVESLLKGQ

ESLLEVVGHQLVEQNNFTRTAAIQTRPMLDKLLNIHPAIIGFGLTNPNGDYISVSSNLIL

EKLHNLKQDPLTRETFLEALNSDRMVIGRTYFMEAQDSLVIPIRKAIPDKNGVVQAVMTA

GFNMNTSSVFRNDIHANEHNRVSLIRNDGYLTFSSSEDTTIKDYQKPADDLNKQALLDQI

QQDYGWDTDQVKQLTRAINVVVDTQRLNELITLKYLPDYGLWAASSTDLGFIKRGFYSQF

AFYCIVFLIVQAAFYALFRSIANNEHETKERLLYQACHDHLTRLPNREYLRSNIQRWMCG

SSNPFTLMFIDIDNFKSVNDTHGHEFGDEVLKQISTRLNHFSGEGRLIVREASDEFIFIV

NRTDEETIKDLASELIQTLSKPYNVNDNQFLLSCSIGIAFYPMHGDNLDALLLSADIAMY

QAKKQRNAYSLFNQEMQASHLHKMKVEQRLRLAIEKQTLFMAYQPQLNINGKIYGVEALV

RWEDEELGKVPPNEFVPVAESSGLMVRLGELIIEKSLEDMGLLTTHLATPIQMSINISVK

QFLHAKFIERLMAAMDKYHLDCNRITLEITENLFIEDLEKFSPTCERLHALGFKISLDDF

GTGYSSLSMLRTLPIDEVKIDKSFVDNIEHDKKALNMVKNIIAIGKNFEMKVLAEGVETQ

RQRDQLEACGCDLIQGYFYSKPLSFDQLVSFVKDNKEEKAIID

>tr|Q87MT1|Q87MT1_VIBPA Putative nitroreductase OS=Vibrio parahaemolyticus serotype O3:K6 (strain RIMD 2210633) OX=223926 GN=VP2150 PE=4 SV=1

MEALDLLLNRRSIAKLSAPAPEGKALENIIRAGLRAPDHAGLTPWRFVIAQGDGLKKLSD

ILVKAAIADHSDEAVIEKVKNAPFRAPIVITVIAKVTEHEKVPALEQYLSAGCAVQAMQM

AAVAQGFQGFWRSGKWMFHPEVHQAFGLEGEDEIVGFLYLGTPGCTPMKVPERDLSKFVE

FQ

>tr|Q87IE1|Q87IE1_VIBPA Uncharacterized protein OS=Vibrio parahaemolyticus serotype O3:K6 (strain RIMD 2210633) OX=223926 GN=VPA0665 PE=4 SV=1

MKGPQVIYLRAFLLSKIRFYRIQSSSSVSLRENISTCDSIMPFTCSNCFSESASHAIMRQ

SPFKLNSAFPAGNVTKPLCSSHVAGTLRAKCRHSVTKYLLACSLSIWVVSLIILSLCLFV

EKGPLSRAL

>tr|Q87GL9|Q87GL9_VIBPA D-alanyl-D-alanine carboxypeptidase OS=Vibrio parahaemolyticus serotype O3:K6 (strain RIMD 2210633) OX=223926 GN=VPA1296 PE=3 SV=1

MRKITLFNTTFLTGLVTFSHFAFAAPTVVPNAPELSSRGYVLMDYHTGKVLVERDADKRL

NPASLTKLMTAYVAGQEVNAGNISLDDQVVISRNAWAKNFPDSSKMFIEVNTSVPLSDLY

RGLVVQSGNDASVAIAEHVAGSEAGFVSLMNSWASQLGLTNSSFTNPHGLDSDGLYSTPH

DIAKLGQAIIRDLPDIYPMYSETSFTYNGITQYNRNGLLRDRSMNVDGMKTGYTSGAGYS

LATSATNGDMRLIAVVMGAKSQSVRESESKQLLSYGFRFYDTLMPTAAGTDIANARVWMG

QKDELKVGVNRDVYLTLPKGDVNKLKAEVEYNGDLLAPIAQDQVVGTLLYKVDGKVVKET

ELVALEPVEEGGIFKRIMDWFKRLVASWF

>tr|Q87KK2|Q87KK2_VIBPA Uncharacterized protein OS=Vibrio parahaemolyticus serotype O3:K6 (strain RIMD 2210633) OX=223926 GN=VP2975 PE=4 SV=1

MSTSLPCKEITKIVASDLDGTLLAPNHQLSAYSKETLKALHEKGYTFVFATGRHHVDVAS

IRRQVGIPAYMITSNGARVHDQNDQLMYSENVPADLVQGVIDTIKHDHEILIHMYQNDSW

LMNKDDETLRDFHDEFTYVLFDEDQAPTDGIAKIFFTHPAQDHERLVVFENKLREQFGDK

LNIAFSTPWCLEVMSAGVSKGHALQAVAETLGLTLENCIAFGDGMNDVEMLSMAGKGLVM

GTSHEKVMKALPNNEVIGSNADDAVAHYLQDHLL

>tr|Q87TC1|Q87TC1_VIBPA Uncharacterized protein OS=Vibrio parahaemolyticus serotype O3:K6 (strain RIMD 2210633) OX=223926 GN=VP0149 PE=4 SV=1

MIVSPTNVSVPLIAPSVNVQTEQAARDNRVREPVTPTVALAKTNAERKVKSDEKRRRQSS

WDPSEHPDYEVDHEVDARYHEEPEDTLERLFDLLALKSYSEDQGKGYAIRFRLPKRILDA

AINQGLMEKRRKVIKFHYGHSVVPHTPSEVIAVL

>tr|Q87TF0|Q87TF0_VIBPA Nitrogen regulation protein OS=Vibrio parahaemolyticus serotype O3:K6 (strain RIMD 2210633) OX=223926 GN=VP0119 PE=1 SV=1

MDTSLPSAILNNMVTATLILDDGLAIRYANPAAELLFSQSAKRIVEQSLSQLIQHASLDL

ALLTQPLQSGQSITDSDVTFVVDGRPLMLEVTVSPITWQRQLMLLVEMRKIDQQRRLTQE

LNQHAQQQAAKLLVRGLAHEIKNPLGGLRGAAQLLEKMLPDPSLTEYTHIIIEQADRLRA

LVDRLLGPQKPGKKTQENLHQILEKVRQLVELESQNSIIIERDYDPSLPEILMDADQIEQ

AMLNIVSNAAQILAHQEHGNITIRTRTVHQANIHGKRCKLAARIEITDNGPGIPPELQDT

LFYPMVSGREGGTGLGLSISQNLIDQHNGKIDVESWPGHTTFTIYLPI

>tr|Q87GP9|Q87GP9_VIBPA Putative ATP-dependent exoDNAse (Exonuclease V), alpha subunit OS=Vibrio parahaemolyticus serotype O3:K6 (strain RIMD 2210633) OX=223926 GN=VPA1266 PE=4 SV=1

MNTALHEDQMRVTSIPYRSTKMVIFSGVPLAKDSYKTNSGKYYVTIKADPDSIPVLPTLG

QHWSVKGARQIESVEMGDYVMQQHTYESPKHIECTLPETGEQLIRFIARESDFKGIGESK

ARALWQLLGKDFHTTLRNDTLESRKRLTSILSEDSVEALFKGYAKYKNLAHCNWMSEHNI

PASVQQRLLKHHGEASIEVIKDNPYALMGFGLSFSAIEDIIKVTDFKSDVAKDDPRRLSA

ALEMAIRKEIEKGHTYTTHANVRHYLSKLLKDKTLVTQAFQSGHDKAQYILNPDTGAYHP

TAQLLMESVVAKRLNTLIKRNDLFDENANAAYCAAVTELPYELTLKQIEAVTTCLDNSVS

CITGGAGTGKTTVLRTTLRAYHQLGFEIHAVALSGRAAMRLHESIGFVTSTIAKLLREDP

IEPSVEKTNHLLVIDEASMIDLPTMYRLVNHIHPSVRLIFTGDPDQLPPIGCGKVLADIV

EAKTVANTMLDIVKRQEGSTGIPEYSKLINQGVMPDQLSTGAIHFHETNKADIAKVCCEL

YQQCPENSRVMAPTKAIVTEINKLTQQAVNPNSDRLEFEINGDKFFLPLRMNDAVLFTQN

HYDKGIQNGSLGMLTNAKTSGDSYGEVTLDTGEKVEITQSVLDCMELGYAITLHKAQGSQ

FPRIIIALQKGRIVD

>tr|Q87NX5|Q87NX5_VIBPA Uncharacterized protein OS=Vibrio parahaemolyticus serotype O3:K6 (strain RIMD 2210633) OX=223926 GN=VP1743 PE=4 SV=1

MDILNFEAFLIAITILTLTPGLDTALVLRNTSRSGLKDGCTTSLGICFGLFVHAFFSAVG

ISAILAQSAELFQIVKMIGAAYLIWLGISSLKALMASGGGITVAEQIQQVYSGKRSFREG

FLSNVLNPKTAVFYLAFLPQFVNPEGSPLLQSMTMAAIHFVIAMVWQCGLAGALNSAKNL

LKNASFMKWMEGVTGAVLVALGIKLLIEEPL

>tr|Q87LC6|Q87LC6_VIBPA Uncharacterized protein OS=Vibrio parahaemolyticus serotype O3:K6 (strain RIMD 2210633) OX=223926 GN=VP2686 PE=4 SV=1

MNSGFNRFGRFCAWTLVTVLVFLAILVTTLRVTLPQLNHFQDEIKTWVKQGTGFDFSISS

VAGSWRNSHPSIALLGLEANLPNNQEARFAVDEIQIEFDLIQSLLQFKPVVADLTIHNLA

LDIRTVDLLPTSSNQEPTTDVDQNEVKLIDQLDSLLLRQFEDFTITNSRVWYKSVSGETR

RLDIEQLRWSNQGKRHLAEGTVSIADASLNSLLVNANFKDHGSLRDVSGEFYVSAQDVSV

TPWLTTYMQEESGAESGKVSLNTWLTLKHSQPNYAYVELQPSELVWNENGRHELFIESGV

FKLLPDKQGWKVNGHSLQIRTDDKSWPELDAAFDWQPDGWKLNLSQLDIEALIPLVKLAP

ESESTSDLLNKLAPKGRVEDIRLAMNGGLDTLRYSADLDELAMTQWELLPGFQHVQGSVA

GDLKQAKAKVTVIDDVFPYGDVFQAPLNIKQGEVDIIWQQDETGWRLWSDKVTAATPDLQ

VLGAFRLDFPKEQSPFLSFYAEADLYNAGETWRYLPTLALGQDLTDYLSTAIQGGKVNTA

KLLWYGELGDFPYKEHNGMFQAWVGLKDAKFSFDTAWPTITDLQLDLLFENDAMYLDSKS

ATLNGVHAKRITGRIPELAEDGHIEIEAKATAPGNEVRDYMMATPLVDSVGAALTALQVS

GPVYSEFQLNIPFDMEKDARAWGYADLKDNRVDIDAPPMMLEKATGRIQFDNDVVTTSGL

SAELLSQPISLDFHGESADQGYNVTINTLGDWDVEPLKPYLGERWLSLVSGHAPWQMDID

LQLNDVGFTYQVDVLAQLGRLASEYPYPLTKKVGEAGQAKLQASGNQESISARLQIPNAK

YQTEIDISGDVPVLTATNLVLGKGGFKISPVVGHDASIRLDEVNLDKWATLLDTPESKAQ

SVLANMKTPTIPLPTRIEVETPNLLLGGIEFHDVALNASKKNLSWQLDVSSQEVKGKATY

LKPYDLSVSLDHLHLYLPDFEEMTKERSSIFASEDQNAPLITNFDRKFHAEMPNLTLNID

DFWLQGYKVGKVNVDLQRQDDRLEWKNITFSSGKNRIDMNGWWELTKDRSHSNLTLKMKG

DNNTDLMERFGITSGIQQAPFEINANMNWDGAPWSMKTPTLQGNVSTEFGKGVVSEVSGA

ARLLGLFSLDSIIRKMQLDFTDVFDKGMAFNSITGTGKIQDGVFITNDIVMDALAGEMQI

RGIANMTSRMVDAEVKFTPDITSGLPMLTAFAVAPQTALYVLAISTVISPVVEVFTQVNY

EVKGPLDSPTVKEISRSKGEYKLPEKLREQAK

>tr|Q87RU1|Q87RU1_VIBPA Phosphatidylglycerophosphatase A OS=Vibrio parahaemolyticus serotype O3:K6 (strain RIMD 2210633) OX=223926 GN=VP0685 PE=4 SV=1

MTNPLSLISLKNPWHLLATGFGSGLSPVVPGTMGTLAAVPFFLLLAQLPFPAYVVVVLLS

CVIGIKICQVTSDDMKVHDHGSIVWDEFAGFWITMSIVPALNIPITEWKWLLTGFILFRF

FDMVKPWPIGWLDKRIHGGLGIMLDDIVAGIMAGIALFLVAKYAGWMS

>tr|Q87NF0|Q87NF0_VIBPA Uncharacterized protein OS=Vibrio parahaemolyticus serotype O3:K6 (strain RIMD 2210633) OX=223926 GN=VP1918 PE=4 SV=1

MKKTTLMSAVVATLSLVGCQSTTGSSDAQPEQTSHISQAVYEVEFHAAQSFLSQASQLEQ

SFADFCLAPKNDVEPVQQQWHSTMLAWMALQGQERGPATALEQSWNVQFWPDKKNTTGRK

MSALTKADKVWTVEEISTQSVTVQGLGALEWLLYDDASTLNTNSNVCASGVAIAENLHDK

AQIIANSWAENPWKSLQKTEWESEYISLLSNQLEYSMKKLSRPLAKIGHPRPYFSESWRS

ETSLSNLKANLESLHQLYFANGKGLDALLRAQGKTQLADRVAYQFDMALETWPEDKSLFS

ALQSVDGYRLVLAQYNKLEQLKYLIHEEVAIELGVVIGFNATDGD

>tr|Q87FE5|Q87FE5_VIBPA Uncharacterized protein OS=Vibrio parahaemolyticus serotype O3:K6 (strain RIMD 2210633) OX=223926 GN=VPA1734 PE=4 SV=1

MRSNLKRKVTLLISLSCFSFQIGALSTKEQRWVDAVTAVYGERAGLRVNTWRQNIDLFKQ

LETHEQLVGVNDFFNQLYFVDDIKLWGKTDYWATPLEFLGSNGGDCEDFTIAKYFSLLEL

GIPDSKMRLIYVKAIELNQFHMVLAYYPTPSSEPLILDNINPEIVRASKRSDLLPIYSFN

GTNLWLMKSSAGTGELAGKASRLSLWNDLRSRERKLQLNKPKINYDE

>tr|Q87HM1|Q87HM1_VIBPA Uncharacterized protein OS=Vibrio parahaemolyticus serotype O3:K6 (strain RIMD 2210633) OX=223926 GN=VPA0942 PE=4 SV=1

MKQANMRSLFVTALLIFSSTSLAQNSINLTSWNIEWLSINGGKVSRTSDDFVKLNQYVDK

TQADIIAFQEVDSKAAVQKAVGDGYAIYLSDRAQSNNKHLQFSDTNQYTGFAVRKDIEVS

DPADFSITRGNSKLRFASYIVVNPSQKDELHLLSVHLKAGCSGAYKNSRDCQTLSQQGEA

LAKWMSEREKKKEQYAVMGDFNHNLSYQRDWLWAIMTLGNDAQLVTRDTQADCKVRSNKN

PSKTHQFRSLIDHIIVSPQIKAKNAHQTLFSSQDVLDYKLSDHCPVNATVTLN

>tr|Q87SB0|Q87SB0_VIBPA Sigma-54 dependent transcriptional regulator OS=Vibrio parahaemolyticus serotype O3:K6 (strain RIMD 2210633) OX=223926 GN=VP0514 PE=4 SV=1

MAGQFKMDSIPGSLVVVGGTYEPWLSVLEQVGWKCHQVGDLRKANTLLEDIGPCIGIVDL

SHDEFSLNGLANLVSSHKHVRWLAFIRESQLGTDTICQFIVNFCIDFFTAPIPDAQLLST

IGHQLGMLKLEKKVWPSFGNSLDMGLIGESIPMKRLRDQVKRIGPTDVSILISGESGTGK

EAVARAIHKVSSRSHKPFMSINCRALNEQRFQAEVFGIAADAEMGPSLLEQADGGTVLFN

DILTISKDQQMNLLRFLQEGTIETREGVKNVNVRILAANSSDVEKALIDGDFNEELYHYI

NVLRINVPSLKERASDIALLARFYLQEFSKEYNSQAKSFSEDALKALTRYFWPGNVRELM

NQVKRAVLMSDSVMIEEHHLDLPQRNDSKRSLKSIREKSERDALLVVLESHSGQVSNAAK

ELGVSRATMYRLLNKHNLISDQAM

>tr|Q87NL5|Q87NL5_VIBPA Uncharacterized protein OS=Vibrio parahaemolyticus serotype O3:K6 (strain RIMD 2210633) OX=223926 GN=VP1853 PE=4 SV=1

MAAIKNLDFSIIESICKILGNTETGFTGTEIGKLLYESGIEDIDSANTKWKRLNSALANK

QSIDGCSNNILAFLQNAINPARHYDNLEWFNDTRYHLNQVLSFAGFNIGENGKIGTVTKT

NTISEAAARAYKLKDQLVSRNVHPDVLKYCKEELVVDNYFHAVFEATKSVADKIRNKTGL

TSDGSVLVDEAFSFKHIPHLALNSLVTESEKSEQKGFMNLLKGLFGTFRNTTAHAPKITW

SIEEIDALDILSMVSLVHRRLDKATQAKMIYESKI

>tr|Q87TJ7|Q87TJ7_VIBPA Sensory box/GGDEF family protein OS=Vibrio parahaemolyticus serotype O3:K6 (strain RIMD 2210633) OX=223926 GN=VP0071 PE=4 SV=1

MQRFLILVAFFWSACAWSELTLAQEPKVLVVHSYHQGFFWTDSIQRGIDQQLDDREMDMR

VLYLDSKRNQSEQFFTQLESLYRTKLSDERFDAILVTDNNALELMQHLAPLIKDTPVIFC

GINNYRPSFHQSLNATGVIENVDLEANLALIERLHPDNKQIYIISDHSVTGAILRDEIDD

FIQQHDRYRNKITQLVPDNVEALKKKIAQLNKENVVLFWTYYRDKDGVVGSERDWIQINK

ASNAPLFMVHDVGLGHGAVGGVIQSGYRQGVEAARLLEQVLDHPQEPLPPVVNGDSEIKL

DYQAVVRWGLGAEQESSAVFFNKPMEFSERFAKEIRLFGSLFVLMLVAILLLSYYLQRIR

RSETAARESQAILESIFDQSMQYMGILDKHGLLKSGNDRLQSLLYHQELRLDKPLWLHQN

WSEQARHAIADYFDAPQQQVSTFEAEIWSKEHGSMVLEISLKPFSQQVGKQKQYLFEARD

ITSRKLMEDKLYQRESSLRNYYEQQPVMMVTLDSHNRIQEVNRFAQLLLGYEPIEMLGHR

LREFYLDDKALFPRQVLLQPSHQVHDVWRREIEYRHADGHSVWVRENIRPLNDSDQLLIV

GEDISETKLLAEKLEYQARYDLLTGTYNRNHFELELKTALREVDSHRRVHAMLYLDLDQL

KVLNDTVGHDAGDAAIQFCASMLEEVLPYNSVLARMGGDEFAVLLKDCDEYGAINIAKVI

ISTLGEHLFIWEDTRLFISCSVGIRIIDHTAETPQMVHAQADSACHAAKEQGRNRYNLFS

LDNEELQRRQMEMQSVNLVHEALSNQRLELFAQRVMNLQSPESLMYFEILVRIRDAQGNY

VSPAIFIPASERYNIAHWIDKQVINQALEWFEQRPDVVEKLGRCSINLSGQSMGNQEFID

FLFERLKNSTMPCEKICLEITETAAMGNLDQAIDFFTRAKSLGCMIALDDFGSGLSSFGY

LKKLPVDIVKIDGLFVRDIDVNEMDRVMVRSINDLAKQLGKVTVAEFVENEQVMQHLIDL

GVDYGQGYGIGHPKPLAELVENLSSL

>tr|Q87GU7|Q87GU7_VIBPA Putative secretion protein OS=Vibrio parahaemolyticus serotype O3:K6 (strain RIMD 2210633) OX=223926 GN=VPA1218 PE=4 SV=1

MTPDQKFARWIKYSCVAFVLVFAYFLVADLAMPLTPQAMATRVVTKVAPRVNGQITHLYV

ANNQEIQKGDLLFQIDPQPYKLAVEKAQLNLQQVIQNNEQLDASITAAKADVEASKIVAE

QKIREANRLNTLFSRNGTSQQQLDDAQSSATAAKANLLAAKARLKELEVSRGELGEANVN

VRVAQNQLKQAELNLSYTQVSAEHDSVITNLQLETGAYAAAGTPLIALVSEQVDIIADFR

EKSLRHFNRDSRALVAFDSLPGEVFEARITSLDAGVSSGQFDADGRLATPTDSNRWVRDA

QRLRLHLSINEQPQAFPAGARATVQLLPDSTISGWLARLQIRFLSTLHYIY

>tr|Q87QA6|Q87QA6_VIBPA Response regulator OS=Vibrio parahaemolyticus serotype O3:K6 (strain RIMD 2210633) OX=223926 GN=VP1244 PE=4 SV=1

MTKYLILCVDDEREVLDSVLQDLAPFEEHFIVEGAESVTEAKQVIEEMAEEEVQLALILC

DHIMPEQTGISFLIELSQNGDTKAARKVLLTGQAGLEDTVEAVNHASLDYYIAKPWKGEE

LRQAITSQLTAFVIENDDNLLSWTTILDSEAILNAMADRRASFGE

>tr|Q87SD0|Q87SD0_VIBPA Bifunctional aspartokinase/homoserine dehydrogenase OS=Vibrio parahaemolyticus serotype O3:K6 (strain RIMD 2210633) OX=223926 GN=VP0494 PE=3 SV=1

MRVLKFGGSSLADADRFLRAADIIANNAQQEEVAVVLSAPGKTTNKLVAVIEGALRNGEA

ELQINELEESFKTLFADIQAVLPNLEGAAFDNQVKTSLSQLRQFVHGINLLGMCPNNVNA

RIISKGERVSIQLMKAVLEAKGQKANLIDPVEYLYAKGDHLEAMVDVDVSTQNFRQNPLP

KDHVNIMPGFTAGNEKGELVTLGRNGSDYSAAVLAACLRADCCEIWTDVDGVYNCDPRLV

EDARLLKSLSYQEAMELSYFGASVLHPKTIAPIAQFHIPCLIKNSFNPQGAGTLIGQDTG

EDNLAIKGITTLNDLTMVNVSGPGMKGMVGMASRVFGAMSSAGVSIVLITQSSSEYSISF

CIEADDKLKAQQVLADAFELELKDGLLEPVDFIDDVSIVTLVGDGMRTSRGVASRFFSSL

AEVNVNIVAIAQGSSERAISAVIPEDKISEAIKACHENLFNSKYFLDVFVVGVGGVGGEL

VDQIQRQQSKLAEKGIVIRVCGLANSKGLLLDSEGLPLEHWRDRMSAATEEFSLARLIAL

VQRNHIINPVLVDCTSSEDIANQYADFLAAGFHVVTPNKKANTASMAYYHQLRDVARSSR

RKLMYETTVGAGLPVIENLQNLIAAGDELERFSGILSGSLSYIFGKLDEGMSLSEATNIA

KENGFTEPDPRDDLSGMDVARKLLILAREAGMSLELEDVVVDQALPPGFDDSGSVDEFMA

RLPEADAYFKELSAKAAEEGKVLRYVGEINDGKCTVSMAAVDENDPMFKIKDGENALAFY

SRYYQPIPLVLRGYGAGTEVTAAGVFSDVMRTLGWKLGV

>tr|Q87IK5|Q87IK5_VIBPA Putative arylsulfatase regulator OS=Vibrio parahaemolyticus serotype O3:K6 (strain RIMD 2210633) OX=223926 GN=VPA0601 PE=4 SV=1

MVGVEESLMAGLHTTVKVVGSKCNYDCQYCFYLEKDLLLKSKKSMNIETLETYIKNYISS

QDTPIVEFAWHGGEPTLIGIDFFEKAVEFQKQYANEKLIKNTIQTNGSKLNSDWCRFFRE

NNFLVGLSLDGPEWLQEKYRTKQGKSAFKESFSALKLLQKMNVEYNVLACVTKEYCKHAE

AIYSFFRKNKVKHIQFSPLVESSPLETEQARGQHFGSNLIFSTDKHHTDPQKYEPIAWSV

DAKEYGRFLTDVFHLWVSQDVGKVFISNFEQALTQYLGNPSPNCIHAKKCGSSYAVEANG

DVYFCDHVAYPESKLGNVFETSLHEMSLNHELQFNKESRLSMRCKQCQFLSLCNGGCPKH

RYLSDTGEYENVLCDGYFYFFSSVQKYLQAMTTLLAHGYPASYVMQALDGPLILTPSNKR

>tr|Q87HJ0|Q87HJ0_VIBPA Putative MFS family transport protein OS=Vibrio parahaemolyticus serotype O3:K6 (strain RIMD 2210633) OX=223926 GN=VPA0973 PE=4 SV=1

MENTVVSAASPRVTVPVVALSLYAVASGYLMSLIPLMLGEYNISADYASWLASVFYGGLL

IGAMFIERIVRNVGHRKAFIGCLAAFSLTIVALPAFPNGLVWLVARFIAGVAVAGVFVIV

ESWLMSGDEASRAKRLSLYMLSLYGGSALGQFGIGVLGVSGGVPFVAITTLILMAMLVLM

FIDCEQPNSHESTSLSFKQIAKLNHAAIIGCVVSGLTLGAIYGLMPVELANRKITHQDIG

TLMALVILGGMLVQPMVTTLNKYMSRTVLMAFFCILGIFSIGLTFISTSTAVLAASLFLL

GMATFALYPVAINLGCEGLDERFIVSATQVMLFSYSVGSVAGPVVADKFMGQVHGLLGYL

FAALVATCVYMLLAASKTKQQMAAGL

>tr|Q87M45|Q87M45_VIBPA Putative pilus assembly protein OS=Vibrio parahaemolyticus serotype O3:K6 (strain RIMD 2210633) OX=223926 GN=VP2413 PE=4 SV=1

MLKPVKISDDTYKSLKTNLIVWVVYSTSNFKLHLSDELSGCVNVNVDWIELDLFNIDLVK

SKSIPDLIYIETGENWAQKVAHVYSGDGNLQHNHTALIVFGDESDTASLKMALRLGATDY

FSRSVDLGELYPLLKSTAEEKISNKKMGDLTLFINTKGGSGATTLAINTAIELSSYAKSK

VLLIDLDMQFSDAADYLNCKPKYNINDVIDSVNDLDELSLEGLVYQHPSGLNYLCFNQND

PRNNHKHAIHVSKLLPILRQFYSHIIVDLSHGVDHVYQQIVSPATHIFLIMQQNVTSVKH

AVSYIRSLELDYGLSNHQVELIVNRFEKKSTISLKDIENAVGGHAIHLVPNNFAIAIESA

NLGNPIVQSKKNSALKASLAEISHLLESPTQEHQSWIKKFFS

>tr|Q87SX4|Q87SX4_VIBPA Uncharacterized protein OS=Vibrio parahaemolyticus serotype O3:K6 (strain RIMD 2210633) OX=223926 GN=VP0298 PE=4 SV=1

MQQSVEAMRLSDVFNSARTVFDVTRFLVVVTVFLRIRLVFNHYRLFRWFCHVFSYQELAF

DACLNLHLNPRGDQRERLRF

>tr|Q87M41|Q87M41_VIBPA Uncharacterized protein OS=Vibrio parahaemolyticus serotype O3:K6 (strain RIMD 2210633) OX=223926 GN=VP2417 PE=4 SV=1

MIRSLIGGAMRRKMQTATRRTQKGITLVLISMVLLILLGVAAFGIDLNHQVLNKTRLQNA

VDTAALAGAVVADKTEDVDQAEAAVIATLSSIASESGNTELSFTDGNTSVTFSHDMQTFV

NAASFTPPTGEYDIYVRVAVTDMGISQYLSAVFGIVKNVSASAVAGRSAAIAYTCNLTPI

AMCGDPNGTVEDAWGYRPPGYDPNVDMDPSLVHELKVGDQNNTDMGPGNFQLLDFGQATG

NSGAALVRDALSGAYNGCAAVGNTVTTKPGNSAGPVAQGLNVRLNDFSGPIKNDGTVLPD

KYVREPDILAETDAEYAGDLYYANYRQELSSCESGGSCDYDSYTGDGGSNGRRILRIPIV

NCTESTGKSDFDVLGFGCFFLLQKVSNSGQASVFGQFLYDCLINNGSTGTDPTDKGFYRI

QLYKDPFSGAS

>tr|Q87RZ6|Q87RZ6_VIBPA Uncharacterized protein OS=Vibrio parahaemolyticus serotype O3:K6 (strain RIMD 2210633) OX=223926 GN=VP0628 PE=4 SV=1

MNKNWIPIALSGLALTGCFSEEAKEVEAVAEPLKVTAADKIYYGGDILTMAGDKPEYAEA

VATLGEKIIYVGSKDGAMEHKFGKTQLVDLKGKTMLPGFVDPHSHVYGVGLQAMVANVLP

SPDGEADTVAKIIETLKNAENNNTQRLFVEKTGWILGFGYDDAQLDYYPTKADLDKVSTD

KPVLIIHTSGHLSVANSKALELAGITSESEDPKGGIIRRMENSQEPNGVLEENAHFAMLF

NLNKLIDSELQDRMLEASQGLYAKYGYTTAQEGRATSEGYEAMKRASKNDKLMIDLVAYA

DMVSSSDFMDSEYNTPEYTNHFRIGGVKLNFDGSPQGKTAWLSQPYFHPPHGQDKDYAGY

PTFEDQQAYDYVETAFKNEWQVLTHANGDAAIEQFINAVTKANEKLGKQDRRPVLIHGQT

MRQDQVDRLAAQGIFPSLFPMHTFYWGDWHVDSVLGHPRADFISPTQAVRKAGLKFSTHH

DAPVALPSSFRVLDATVNRTTRTDKVLGADQRVDTYTALQAMTIWPAYQHFEESYKGSIE

VGKNADLIILDNNPMKIEPKALKDLNVEETISRGQSVYQRQ

>tr|Q87SG1|Q87SG1_VIBPA Cell division protein FtsA OS=Vibrio parahaemolyticus serotype O3:K6 (strain RIMD 2210633) OX=223926 GN=ftsA PE=3 SV=1

MTKAADDNIIVGLDIGTATVSALVGEILPDGQINIIGAGSSPSRGMDKGGVNDLESVVKS

VQRAIDQAELMAECQISRVFISLSGKHIASRIEKGMGTISDEEVSQEDMDRAIHTAKSIK

IGDEQRILHVIPQEFTIDYQEGIKNPLGLSGVRMEVSVHLISCHNDMARNIIKAVERCGL

KVEQLVFSGLAASNAVITEDERELGVCVVDIGAGTMDVAIWTGGALRHTEVFSYAGNAVT

SDIAFAFGTPVSDAEEIKVKYGCALSELVSKDDTVNVPSVGGRPSRSLQRQTLSEVIEPR

YSELMGLVNQTIDSVQAKLREEGIKHHLAAGVVLTGGAAQIDGVVECAERVFRNQVRVGK

PLEVSGLTDYVKEPYHSTAVGLLHYARDMQSSDDSDYNEPKRSSVTGFFDKLRNWIQKEF

>tr|Q87PH7|Q87PH7_VIBPA Putrescine-binding periplasmic protein OS=Vibrio parahaemolyticus serotype O3:K6 (strain RIMD 2210633) OX=223926 GN=VP1525 PE=3 SV=1

MKSKFYASALCAATLIATPAMAADQELYFYNWSEYIPNEVLEDFTKETGIKVIYSTYESN

ESMYAKLKTQGSGYDLVVPSTYFVSKMRKEGMLQEIDKKKLSHFSDLDTNFLDKPFDPNN

NYSIPYIWGATGIGINADMLDKSSVSKWDDFWDSKWEGQLMLMDDSREVFHIALTKLGYS

PNTTNPDEIKAAYEELKKLMPNVLVFNSDFPANPYLAGEVSLGMLWNGSAYMARQEGANI

DIIWPEKGTIFWMDSLAIPAGAKNVDAAHKMIDFLLRPENAAKIALEIGYPTPVKTAHEL

LPKEFANDPSIFPPQEVMDSGTWQDEVGEASVIYDEYFQKLKVNN

>tr|Q87GS7|Q87GS7_VIBPA Uncharacterized protein OS=Vibrio parahaemolyticus serotype O3:K6 (strain RIMD 2210633) OX=223926 GN=VPA1238 PE=4 SV=1

MTISHYCELVRETTMLSNTKRTEIKKGAHKAVTEVEKLHTIIDESLIAHIAITNESGPVV

IPMLAWRVDDFVYIHGANNSRLLRSLKQGQSTCLTFTLFDGWVLARSAFHHSAHYRSAVV

FGQFETVEDNQEKDRLLNHFLEQIAPGRTAHVRLSNERELSATMLLRISLEEASVKISRF

GVNDDAQDMDIPVWAGVLPYRTVVGPLEPCSDLYEGIEEPDYSSAYPNRWYASEKLS

>tr|Q87GQ5|Q87GQ5_VIBPA Uncharacterized protein OS=Vibrio parahaemolyticus serotype O3:K6 (strain RIMD 2210633) OX=223926 GN=VPA1260 PE=4 SV=1

MIKPYELSEEESQIVKSVQCDDLSPQQKWDDCRVTTMKSNIKKHYIAEQDKTCAYCQVNM

HTTHGMVWDTEHIIDKDSSPQWMFKPLNLCVSCKDCNGAKGTRAVTKSKSYKKFPSKSAN

YRIIHPHFDNYDEHIEVAVPGATYRYITEKGRYTIEVCGLLRYHQTVGRKKVDLGLQAVL

LAAANNQSPEMLQYAMEEIARRQAAVSQSTGSST

>tr|Q87K69|Q87K69_VIBPA Uncharacterized protein OS=Vibrio parahaemolyticus serotype O3:K6 (strain RIMD 2210633) OX=223926 GN=VPA0029 PE=4 SV=1

MCVSLASIRLNHHESELKNYDRNKQTQLFERDLGVLTVTAGRNFVR

>tr|Q87SJ4|Q87SJ4_VIBPA Uncharacterized protein OS=Vibrio parahaemolyticus serotype O3:K6 (strain RIMD 2210633) OX=223926 GN=VP0429 PE=4 SV=1

MSKPSLLLYIHGFNSSPLSMKANLMREYCAQHRPDIKVIVPQLPCFPEQAAQLLLDIVEQ

YKDDYNIGLVGSSLGGYMSTWLNAKYGFKAVVVNPAVKPYELLVDYLGEQTNPYTRETYT

LEACHIDELKALDVQSIASPHSFWLLQQTEDEVLDYRQAVDKFAKAKQTVEQGGDHSFVG

FERYPAKIIEFLEL

>tr|Q87GN7|Q87GN7_VIBPA Uncharacterized protein OS=Vibrio parahaemolyticus serotype O3:K6 (strain RIMD 2210633) OX=223926 GN=VPA1278 PE=4 SV=1

MYKPLLLVVLSSSLAACAYNQKPVVDMTNVDQAQYEQDFAYCQGYAEKVDKTEAAKSDAT

NGAMTGALIGAAAGALEDGIGGAAVGAVAGSAVGAGAGALGGANDSTKTQALVLRKCLQN

KGYTVYDLD

>tr|Q87J27|Q87J27_VIBPA TonB-like protein OS=Vibrio parahaemolyticus serotype O3:K6 (strain RIMD 2210633) OX=223926 GN=VPA0426 PE=4 SV=1

MNVQRYVIAGGASLVIHAALLFVSQENKVFAMPAGNPASSVSLNLVSAPPPTLEKPTPEN

VEPPKEPQPQKQSEPEKKIIKKQVVKKEAVKKKVVEKKPEPIKQPKKVEPTKTVAKQETQ

PKAEPKKKIEPVKKDTVTQQTASASQSKGATSQPILVDKPTFVSQPTQPRYPRSAQRRGI

EGAALYEVWLDENGNQIKHVLLESSGTESLDASALRAIKQWQFTPHILGGLKVAHRVQIP

VRFKLEG

>tr|Q87TI3|Q87TI3_VIBPA Uncharacterized protein OS=Vibrio parahaemolyticus serotype O3:K6 (strain RIMD 2210633) OX=223926 GN=VP0086 PE=4 SV=1

MTLNNMRVMELLIKMAHHRQTCLPLVDPHSHMNIARSAYRFVKIEKVMIKKMVDLFFDQN

GDDFIAEHANKTGIATLGNYKEMHFMNAQLLSELKQLLRELDDANLTALISYWVAALQVE

NDELEKHLPQGE

>tr|Q87IB4|Q87IB4_VIBPA Putative LysR-type regulator OS=Vibrio parahaemolyticus serotype O3:K6 (strain RIMD 2210633) OX=223926 GN=VPA0692 PE=4 SV=1

MIFEKFNSMKPTDLNLIPIFIAIYEERNLSRAAARLDITQPAVSKALARLRDIYDEPLFH

RSSSGVEPTSFAVDIYPAMVAAIKNFTSTLSASVEFDPKVSNRIFSIACVSILSYELLPQ

LMKQIHELAPNIALEVHPLFTEDYESDLRLQRYDLIIDLAPRGRTVLKVEPVITERLMVV

CNKDHPRIAESISQEQFFEEEHVAVSQWQSRKSMLSAEDIVDLDKRKIMYRAAGALEMLP

VICGSEYIGLMPESMIKLFSNTYHVKALPFPFDVTTYDLCTIWHPSRTNESAHQWLRAQI

KHAGKVIKK

>tr|Q87I66|Q87I66_VIBPA Putative transcription regulator protein OS=Vibrio parahaemolyticus serotype O3:K6 (strain RIMD 2210633) OX=223926 GN=VPA0740 PE=4 SV=1

MINLTKQKEWSFILEQPANNGCSNYFLTLHLVSRVNKDLDLNLLKILVLLDKHRQLKPVA

KAMGKSEASISKYLARLRLQLNDELFIRHPHHMEPTDYLKRQLPKIADALEKLETCLISG

EFEPENYEKDITVCLPQTAQYSFGHLLAMDLMELFPKAHISVATITEYTVEEIILGKVDA

QLHYFNDDLPKSVHQKFIGYAPAVVVVPEELGITTLEEAAQLPFIMLEIIGWKEKEQLAK

RAMESHGMVINRIAALDNVSSVLKLIKKKSAATVLLEYQAPIEGYNFIRVPESYYPNGWP

KIVVQMKQSHRHDAMHQLLTGAIAKYMSA

>tr|Q87IM2|Q87IM2_VIBPA Uncharacterized protein OS=Vibrio parahaemolyticus serotype O3:K6 (strain RIMD 2210633) OX=223926 GN=VPA0584 PE=4 SV=1

MTDEERIELQQNNPLHGLKLETMLQELVDFYGWDILDTAMRFNCFNTKPSIASSVKYLKK

TEWAREKLENFYLYRFKRMPRASEEEYNLPPRARTFPHGLEPKEPMPLTVDSILNSQAKA

ASSFKKRSSRGRPTRR

>tr|Q87JU4|Q87JU4_VIBPA TonB system transport protein ExbD2 OS=Vibrio parahaemolyticus serotype O3:K6 (strain RIMD 2210633) OX=223926 GN=VPA0154 PE=3 SV=1

MRLGRRPNQQDEAQVDLTSMLDIVFIMLIFFIVTSSFVRESGVEVNRPQASNVVSQKDAG

IFIAITAANDVYIDKRLVDLERVEASIEHLLLDQPDASLVIQADEHAYNGTVVKVMDAAK

GAGVKNIALAAEKP

>tr|Q87HN7|Q87HN7_VIBPA Uncharacterized protein OS=Vibrio parahaemolyticus serotype O3:K6 (strain RIMD 2210633) OX=223926 GN=VPA0926 PE=4 SV=1

MKQAVINSYAIATLSLLSAASQAETLNDLIVKEFEYNFALSVVLTDTDVFTFGFHDFDPN

QYFNLSNEDIGTADSLDLRKEIKEFTLPYSFDLAENETEKLTYRFNGRFYLLGIDQDVYF

NEEPIPDRSKEQFIGGYNEFEVEKQLTEHFSLSGALGAHLIHYKTDYTYRSDAITALQPI

LDGALINTSAWALLGQLNAKLKYLEQESWGKWYLWTSPHYFYGTGWGEANNGNVGKPEGW

YWVNGVKVFYDVAHIGTTVQSLYTSFSRIDIGGDTSDPLNTTNYYEGSIGWLMTPPFESD

WIDNIGIGLTINYGSDLKGGSLVLFFNQD

>tr|Q87G33|Q87G33_VIBPA Uncharacterized protein OS=Vibrio parahaemolyticus serotype O3:K6 (strain RIMD 2210633) OX=223926 GN=VPA1484 PE=4 SV=1

MNTSSHGWRTPQNFLLLISIIVPIAFSTWMALLNNFVIEKANFDGADIGLLQSVREIPGF

LAFTAVFVLLFIREQRFMLVSLAMLTLGTALTGYFPTLYGLLFTTLLMSTGFHYFETLKQ

SLSLQWLSKEEAPEMLGKFISVGALASLFTYGAIWILLEQLKFDFKTVYLLAGGVGFVLI

IVMALAFPQFKTAVPQNKKLVLRKRYWLYYALTFMSGARRQIFTVFAGFLMVEKFGYSAA

DITLLFLINYLFNFLFAKRIGRFIGVVGERKALTFEYVGLIFVFVGYGLVQTAEWAAALY

VVDHLFFALALAIKTYFQKIADPADMASTAGVAFTINHIAAVVIPVTFGMIWLVSPSSVF

YIGAGMAAVSLLLSLNIPAKPEEGNETRLLRWG

>tr|Q87S67|Q87S67_VIBPA Peptidoglycan lytic exotransglycosylase OS=Vibrio parahaemolyticus serotype O3:K6 (strain RIMD 2210633) OX=223926 GN=VP0557 PE=3 SV=1

MNVTISKFWAMLFFLLSASVYALEISPLKQPPYTGDLDVIKEKNVLRVLVSADLGFYYIE

GGTPKGIGAELLAHFEKDLRKIKPKLNVQIIPLARDQLLPSLENGSGDLVVANLTITEAR

KQKVDFSTPILSGIEEWVITNKNTPTITKLEQLSGKEIWVRASSSHFESIQAVNKTLNKK

GLPPIIVHFIEETLQDYELIGMLNNGYIKAIVLDSHKAKLWLNAMDDIKAHKKVPLRKNG

NIAWVMRQNSPQLKAVVNKFIQKSRSGTLLGNVIYSKYIDNTNWLNKALNPKKIAQLEKL

SALFSRYGEKYDLDYLLIAAVAYKESGFNNNLVGTRGAVGIMQVLPSTARDPNINIKNVR

QLENNIHAGAKYLAFLRRQYFGDKAITAENKIYFTLAAYNAGPGNVEKMRKRAAQQGYDP

NVWFNNVEVVMRKSLSSTVSYVTSVNRYYVIYKQLESLDLAKTLENVLLIPDDPVFMTPI

RKITQTPDTKMPLVN

>tr|Q87Q79|Q87Q79_VIBPA Uncharacterized protein OS=Vibrio parahaemolyticus serotype O3:K6 (strain RIMD 2210633) OX=223926 GN=VP1271 PE=4 SV=1

MRKLELKVPPVAVFMLVILLMYGLKVLTPSMNIRVPFVEFVVGALTLLSGYMGIAGVYEF

RKVKTTVNPVKPDAASSVVRTGVFAFSRNPMYMALLLLIIAVGLWWQHLSVVLCGVLFVS

YMNRFQIKPEEHVLERLFGEEYLDYKNQVRRWI

>tr|Q87PW4|Q87PW4_VIBPA Uncharacterized protein OS=Vibrio parahaemolyticus serotype O3:K6 (strain RIMD 2210633) OX=223926 GN=VP1386 PE=4 SV=1

MHVAEGTVAIIRHLRRNTGTISGNVFDAPVNGAKIEVFEFKDGKLGRKLASTSSNAFGDY

KVEFESSSMPLYVVAQQGSYTDPLTKEIVSSSVGKTLRLEGVVNFSEGSNQKLMLTPLTN

IVSGFAKYKIAGGVSGPDAVSQALDSINGMYGFDVNETTPIDITKGGQSSFATPGHQYGA

LLTAYSSYSYDMIQRYGQSEDNVYTSMHLADVQFRDVIADGLLDGLEISEATGAVTPLTF

GQQKITSDVYTQELSQQVLIVVNDPTLNISGTNADDYVAFSQQINSLGTAGETDGVIPPR

DDTDIDTIPPTATRTDNDVLAGKDIVDIKINDDIGVESVSAFVEYKLNGAWQGEFQCNDQ

LTSGSKFCSVDAQGFEVGLRETNIKVIIDTKAIDAVYVDQETGLSKVTAARLVLYTADVL

GNKLLPGGVKVIMSISLGITTRQLSP

>tr|Q87RW7|Q87RW7_VIBPA Uncharacterized protein OS=Vibrio parahaemolyticus serotype O3:K6 (strain RIMD 2210633) OX=223926 GN=VP0659 PE=4 SV=1

MKKQQGAAALLVVSVLLVAALMMSLGSYKSLFYQIKRANNQIEARQEHWRAEGGLECVYS

KTKINKTLPTNVNDCITAMGLDALTYSSGSSSLVTSTIGNREIKKTIKLPTSSGAGAIKS

KSDLVINGSYTSSPDPSKSLGNNLWECTSVLYYNFFYATSVSTFHPYQLSDKPYSSFPDS

PAGQEQKCASTHYSWGTSVAASESDYRQSSDMDPFKDVFDVPRSEWFDVMSDNSLFGYVP

LSLNSMKLNSKADLPSPVFNETCAEGIVSNIENGKDLIWVYGGCEINTADFTSIKNAIDN

SLGGSGIILVVQDGIFSTIGTQDFKGMLYHFISPLYTEPTTGATFDFTDWSGSANDAALD

GVIAALKTTVPMEKTKVGYFQHGAFNPLGGFVMDAPGTFALFNAALSFQYNRDVIEEPQE

KLKKAHWKKGSWNDL

>tr|Q87JX5|Q87JX5_VIBPA Putative hydrolase OS=Vibrio parahaemolyticus serotype O3:K6 (strain RIMD 2210633) OX=223926 GN=VPA0123 PE=4 SV=1

MLGYKGDSNMIYLFDWGNTLMVDFPHAQGKMCDWEHVETVPQATDTLAVLSQNNPIYIAT

SASDSNMEEVQRAFVRVDLDQFLNGYFCFANLGIAKNQPEFYLAVAKQLGVNVSEITMVG

DLPHKDIYPAMQAGVNTIWFNPTGADAPADPIPNQIRCLSELIP

>tr|Q87RX8|Q87RX8_VIBPA DNA repair protein RecN OS=Vibrio parahaemolyticus serotype O3:K6 (strain RIMD 2210633) OX=223926 GN=VP0648 PE=3 SV=1

MLAHLSVNNFAIVKSLQLELSKGMTTITGETGAGKSIAIDALGLCLGGRADAGMVRQGEE

KTEVSAAFLLDNNLHATRWLEDNDLLDGTECILRRIITKEGRSRAFINGSPVPLSQLKSL

GQLLINIHGQHAHHQLMKSEYQMAMLDQYAGHLNLLKSTRSAYQHWRQADNNLKQLKENS

QQNQAQKQLLEYQIKELNELSLGEEEFAELEQEHKRLSNSGELAATCQQALELIYEGEEV

NALGILQSANHSLIQLAELDEKLAELPNMLADAMIQLEETKNELRSYLDGIDVDPGRMAY

VEERFSKVMSMARKHHVMPDELYQHHQDLLAQIEALDCSDERLDELAQEVEQKYQSFLTQ

AEKLHKSRSRYAKELNKLITQSMHELSMEKAVFSIEVNNENTHPSPLGMDSVCFLVSTNP

GQPLQPIAKVASGGELSRISLAIQVITAQKVDTPSLIFDEVDVGISGPTAAVVGKMLRKL

GESTQVMCVTHLPQVAGCGHQQMFVAKHTKGGQTETQMRALDEEQRVAELARLLGGSQIT

DSTLANAKELLIAA

>tr|Q87FK2|Q87FK2_VIBPA Uncharacterized protein OS=Vibrio parahaemolyticus serotype O3:K6 (strain RIMD 2210633) OX=223926 GN=VPA1677 PE=4 SV=1

MNDIDVETVPTFQLEKTRWTLNLSTEQPASFELQRDGKTHFIGTMEVLNNGQLIAVHDAD

WRFRHKPEKLEQWCQLHHSETLSINMNYYFADDALVIEYLARNTVPTRLDIRHTIKQVVD

SPVEDNEQARSAKWQHQRDGLPSDFLSKTAKTDFFREAFSAKQWIVLENCEE

>tr|Q87KD6|Q87KD6_VIBPA Uncharacterized protein OS=Vibrio parahaemolyticus serotype O3:K6 (strain RIMD 2210633) OX=223926 GN=VP3041 PE=4 SV=1

MNRIFRNVASVTLPLLFSFNAVANVDAKPLTVKDDAPQTYVVVKGDTLWDISAMYLDSPW

LWPRLWQVNPDIDNPHLIYPGDKLSLVWINGQPVLSLKPVKKLSPKARITEKKAVPTVAE

GLVMPYLKSDRLLDQDALNNAAKVMGSSKGSKYLTAEDVLYIDGKQTATGWAIYRPVETF

SRDDVSKEITALRLIAKGELVEASSDYSGLKITSQLQEILRNDIALPNQSVENTDFTTTF

YPMPSPQGSSAKLLGNIEGIRFSSTNQVVVIDKGTSDSLKQGSVFDLKEDAHPVYKDGDE

FKKEFGVLDKKITLPQSKVGELLVIRPYEYFSLALITTSTKPINKEVSVVSPLTQESTTN

ETTQ

>tr|Q87KI8|Q87KI8_VIBPA Uroporphyrinogen-III synthase OS=Vibrio parahaemolyticus serotype O3:K6 (strain RIMD 2210633) OX=223926 GN=VP2989 PE=4 SV=1

MAVLVTRPGEQGSALCSLLERHGISAHHHPLIDIVADLTDTHLTTHLHQAQIVIAVSRHA

VQCAQQILTSNGASWPKQAVYLAVGQKTAHYLSKCTQQKVHYPEVSDSEHLLRLPALQNV

EQQQVLILRGNGGRELIKDALVRRGAKVHYSETYKREFILFDPVSCVSLWKTLQINQIVV

TSGEQLDYLCSQLTSEQLAWLNQQELYIPSQRIADIAIQRGFTRVRCTGSASNQELLAAL

QP

>tr|Q87J59|Q87J59_VIBPA Uncharacterized protein OS=Vibrio parahaemolyticus serotype O3:K6 (strain RIMD 2210633) OX=223926 GN=VPA0394 PE=4 SV=1

MKVNFHLDKKNKPQSESGMKVVYGQAKRGGYRLRWYLILAIVVSPLLFMAYYLFRTQVLV

TAPAIITSYPLTVTATRSAIVGPIPVNVGTEVAQDQALLLLKDNALNKEIDFIKEELIKL

SKNQVQSTDELYKNAIASSEEGLKKVQEIQKKYDVYRKKGQVSEVDYAAIVSVNNSLSNQ

LSSQKIAYVDAMRDLEELELAGPVTQEYRSLMRELVVKRAQQENLTFRSPMKGRVLDVHV

HEGQNVSENTPLLTIARNVTPEITAFLNPKYLQYSKMDTKAKVVFPDGRKFSATVSKPVE

VVNKLPSELQSPFEGQPAYLKVTLSFDEALEKSRWIEGVEVEVRF

>tr|Q87PR1|Q87PR1_VIBPA Uncharacterized protein OS=Vibrio parahaemolyticus serotype O3:K6 (strain RIMD 2210633) OX=223926 GN=VP1440 PE=4 SV=1

MNIDLNLIPQTFDMLHAGLAASSVLLLLIAVSRKSKVIEKVVEKPVEKIVEVEKPVEKIV

EVEKVVEVEKVVEKVVEVESKLATASTDSAMQLLSIMQQEARLIDFLKEDLTSFSDEEVG

AAARVIHTGGQKVLNDYVTLAHVRNEEEETRITVEEGFNPQEIRLTGNVTGSAPFHGTLV

HKGWKATSMNLPKLAENYDASVIAPAEVEL

>tr|Q87JQ2|Q87JQ2_VIBPA Uncharacterized protein OS=Vibrio parahaemolyticus serotype O3:K6 (strain RIMD 2210633) OX=223926 GN=VPA0196 PE=4 SV=1

MRIKPISLAVLLSCSLILNVQASQEEVPALNYSQSAQLIRDTYERELFTLPPFKEGHFGL

RMFRQTLDEKYYATIWTDMAQVASRLNRFANDVVKPEDIILYSSERLTRYQEKEDERSQR

RYTVTKHHPEYLYLGVDLLGAMARADEYGLKHQQDKTLREIIRRYDFTRYATDKEMIEAW

SAQLANQVYWLRQLGEQDVVNAFIEAFRATYPDDNDKKLSAQQYGNKIYGMTHIIFADSQ

YYQKRVNEADHQWIYDYFRDNIDTILLRAKEDIIAEVGISFLLAGLDHDPVVEKTRQAIR

DAISTEQGMIPSVDGDFDLKYGEHRNVLAIMLLDWKGVHKAPTYQAHPKAFKSIPYGLVP

KSPK

>tr|Q87G05|Q87G05_VIBPA Histidine kinase OS=Vibrio parahaemolyticus serotype O3:K6 (strain RIMD 2210633) OX=223926 GN=VPA1515 PE=4 SV=1

MSFKSRLVVFTTVWFCLAMAAIALTYNWQKETIELRTKQSLHQDLASHMRDDNPLMIGTD

YNPKALKSIFHTLMLIGPDFEIYFLDSQGNITTHAAPEGTELMGAVNLAPIRQFLSGEPF

PILGDDPRNRDEHKVFSVAAIEELGSTIGYLYVVIGSSRHTAIANAQVDSPYLALAGLVL

ISILGFAFGSYFLVKRSLLNPIERVTDQLQKQAEHDFRLQPDFAHQVPELVPIARSYQLM

AKHIQQQFLQLEYQSSHRRQSLLQLSHDLKTPLSSVLGYLETWRLQHPDPDPLIEVAFRN

SEKLSQQLHALLDVAKQEAPLPSYEYLPIDISQLMAECAETMQSQFQRKGVTLNITVDEP

IQVIGDKGLLERLILNLLENALRHSPSDATVSCDVKRGDNTSQVRFTFSNHIELNAQAGA

LGIGTKIVQSILMLHHSHLETDATSHQFTQRFTLPAA

>tr|Q87P96|Q87P96_VIBPA Amino acid ABC transporter, permease protein OS=Vibrio parahaemolyticus serotype O3:K6 (strain RIMD 2210633) OX=223926 GN=VP1622 PE=3 SV=1

MSTHQFQPDLPPPSNTVGVVGWLRKNLFNGPVNSIVTLILAYIVFNALWHIVDWAFINAD

WIGSTRDDCSREGACWVFISVRWEQFMYGFYPEAELWRPRLFYITLAIFTVLLAYEKTPK

RLWIWLFFVNIYPFIVAALLYGGVFGLEVVETHKWGGLLVTLIIALVGIVVSLPIGVALA

LGRRSDMPIIRSICTIYIEVWRGVPLITVLFMASVMLPLFLAEGSETDKLIRALRALIGV

VMFSAAYMAEVVRGGLQAIPKGQYEAADALGLSYWKKTGLIILPQALKITIPSIVNTFIG

LFKDTSLVLIIGMFDVLGIGQAANTDPEWLGFATESYVFVALVFWVFCFGMSRYSIWLEN

KLHTGHKR

>tr|Q87QH7|Q87QH7_VIBPA Psp operon transcriptional activator OS=Vibrio parahaemolyticus serotype O3:K6 (strain RIMD 2210633) OX=223926 GN=VP1172 PE=4 SV=1

MKQNLIGESPAFLAVLDKVSQLAPIERPVLIIGERGTGKELIAQRLHYLSKRWDKPLLSL

NCATLSEGLIDSELFGHESGSFTGSKGKHKGRFERAEGGTLFLDELATAPLLVQEKLLRV

IEYGEYERVGGHTALNADVRLVCATNADLPRLAEQGDFRADLLDRLAFDVIMLPPLRERK

EDILSLAEHYAMKMCRELQLEYFVGFTHQAQQALLDYSWPGNVRELKNVIERAIYQHGLN

AEPIDELIFNPFATGWNNALGHTAANEEPQEEASSQTTSIHFPLDYKQWQEEQDINLLNR

ALEEAKFNQRQAAELLGLSYHQLRGMVRKYGLVGQS

>tr|Q87HA2|Q87HA2_VIBPA Uncharacterized protein OS=Vibrio parahaemolyticus serotype O3:K6 (strain RIMD 2210633) OX=223926 GN=VPA1063 PE=4 SV=1

MSSILECIRTVGRGERGRKPLNFDQAFRVMDEYLNGECGDDQMAMLLMLIRVQNETQQEI

AGFVKAFQSRMPAIGADIDWPCYAGKREAAGQPWHLLAAKILADNGHKVLMHGYHDRQPG

RLHAEDYLDKFAIKKAESAEDAKRVLETQNIVYLPLSAFAPQAETMIGWKNRYGLRTPIN

TVVRALNPGQATVGIRGSFHPGFQQLHAEVEYEIGQTAHAVVSFKGQSGESEYNPKVSQT

VWLSQPSGVTSHYWTEQMLSEVPMPTVCPFGTEETDMNQMANTVLTTIAVVLFAEMHDRE

QAFERAFSYWASYCAAN

>tr|Q87G17|Q87G17_VIBPA Uncharacterized protein OS=Vibrio parahaemolyticus serotype O3:K6 (strain RIMD 2210633) OX=223926 GN=VPA1500 PE=4 SV=1

MEFTELDRNALYDIWMSQKAKMHITQMEMAKRLGLSLHEFSGLLRGNSPLTLGFIKQLCE

QLHVRPGQVIPSLTERELSAAGSVHLQNRVTVDGDIRNVFIEGNQVVIEYVHQVS

>tr|Q87KZ6|Q87KZ6_VIBPA Uncharacterized protein OS=Vibrio parahaemolyticus serotype O3:K6 (strain RIMD 2210633) OX=223926 GN=VP2828 PE=4 SV=1

MGYEWLALCAAFLWAVSSLISVIPAQHLGAFSYSRWRMGCTAVILSTMAWITGGWLSVSW

EHITPMMASGLIGIFIGDTALFACLNRMGPRQAGLLFSCHAVFSTILGYFLFSESMTAIE

LLGSALVFSGVVMAIFFGRRGQTNNVLEEIKGNIWVGVSLGLTAAICQALGGIIAKPVMQ

TSVDPIAASAIRMISAFVAHCLLLIVGVKVARSTQRITWRVFGITALNGFLAMAVGMTLI

LYALREGNVGMVALLSSTTPIMLLPLLWVYTKRRPNRFAWLGAALAVIGAGILVQ

>tr|Q87NJ5|Q87NJ5_VIBPA Fumarate hydratase class I OS=Vibrio parahaemolyticus serotype O3:K6 (strain RIMD 2210633) OX=223926 GN=VP1873 PE=3 SV=1

MTVIRKQDVISSVADALQYISYYHPLDFVQALEKAYHREESQAAKDAIAQILINSRMSAE

GHRPICQDTGIVTCFVNIGMGVQWDSTDMTVQQMVDEGVRQAYTNPDNPLRASVLMDPAG

KRINTKDNTPAVVHINMVPGDKVEIQIAAKGGGSENKTKMVMLNPSDDIAEWVEKTLPTM

GAGWCPPGMLGIGIGGTAEKAAVLAKESLMEHIDIQELIDRGPQNAEEELRLDIFNRVNK

LGIGAQGLGGLTTVVDVKIKTAPTHAASKPVCLIPNCAATRHVHFTLDGSGPAELTPPKL

EDWPDITWEAGENTRRVNLDTITKEEVQEWKTGETVLLSGKILTGRDAAHKRIQGMLDNG

EGLPEGVDLNGKFIYYVGPVDAVGDEVVGPAGPTTSTRMDKFTDMMLEKTGIMGMIGKAE

RGPATVESIKNHKAVYLMAVGGAAYLVAKAIKKARVVAFEDLGMEAIYEFEVEDMPVTVA

VDSNGVNAHQIGPDTWKVKIQEMEA

>tr|Q87PV7|Q87PV7_VIBPA Putative VgrG protein OS=Vibrio parahaemolyticus serotype O3:K6 (strain RIMD 2210633) OX=223926 GN=VP1394 PE=4 SV=1

MVNDVEFKFEVPGCGHEFRVESFQVNEELSKPFHISLSLLSLDPDISFDSLIRKAGTLTL

YGQGLSAARIFNGVVNEVRYLGTGRRFSRYQLILVPQAWFLSQRQDCRIFQQKSAKDIIT

EVLDDGSVTDYRFELSGIYPPKEYALQYRESDLHFVQRMMAEHGMWYYFDHTDSNHTMII

VDSNDAIAPLVSSPLNASYIGPIVYHADSGGVADREHISDLELVNRVRTGQVTYTDYNYE

QPKIPQEMTHAGDLDQDLKQFDYPGRYVDPVMGQVRTTEWMFEHIVDNQQVEASSDVMRL

ASGYSFNISDHPRSEINRDYIMLSVMHTGQDPQVHEDEASGMPTTYYNQFTCIPRDVVFK

APKLAAPVVDGPQTAVVVGPAGEEIYTDKLGRIKVQFHWDRYGNNDEHASCWIRVSQSMA

APTWGAVYLPRIGHEVVVTFLEGDPDRPLVTGAVYNGLHFPPYSLPENKTRTTFRTQTHK

GTGYNELSFEDEANQEEVYIHAQKDMSTKVLNNRYRDIGQDEFLKVARHQTNEVHGDHKE

TIDGHKTTQVNSTFTETVEQDVTVTYNANETQYVKNNSDLEIGDNRTTKIGKNDDLDVGE

NSNLTVGASKSSDIGADDNQTVGGNLTVSVKGNTSYKADGATQIISGDKIVLKTGGSSLV

MNSDGSIKLSGSSITIEGSDKVVVKGGNVAIN

>tr|Q87I46|Q87I46_VIBPA Putative ribosomal-protein-serine N-acetyltransferase OS=Vibrio parahaemolyticus serotype O3:K6 (strain RIMD 2210633) OX=223926 GN=VPA0760 PE=4 SV=1

MYPHDYQGNGSQARFSVSERVSIEPLTTKHATVLLEVVNTYRNSLSGYLPWTDFVTNRRE

AVNYISQRVNSKALDAHWCAIYFDEQFTGVIGIKGVDSHTHVTEIGYWLANNGRGHRVID

QVLAVLIPFLKQKGHAQFIQFHCMEDNIASINIAERAGATLKEYVDHEFETLDRSQRLGI

YELRLT

>tr|Q87L18|Q87L18_VIBPA Extracellular nuclease-related protein OS=Vibrio parahaemolyticus serotype O3:K6 (strain RIMD 2210633) OX=223926 GN=VP2799 PE=4 SV=1

MDTLIKPSILALTIASALSANANAEIIISQYVEGGSYNKAVEIANTGDAAVTLTGYELAK

SSNGGGTWGSKIDLSQVTLQAKQVFVLAHGDASDAIKAVADQTDKNVANFNGDDPLALLK

DGEVHDMVGVMGGVAFGKDATLVRNGDALTPSATFQSSQWTTLAKDNIDGLGELNAAEPP

AAFVCEVDGHAPTFTSIQDIQGEGASSPFIDGYPYITTEEHFVTGVVSAVTSGLTKGFYL

QAIENDNNDKTSEGLFIHTNAADTELKPGDVVCVKGKVQEYYNNTQLSSDATSYVKTGTS

DIPLVTPLVIKEGETLRDALERHEGMQVTLNSASDLFVTRNFSYDYDSKRNNMMLSHQAP

LFKPTQLHAADSAEAVALAKQNAENRVFLESDAKAPNGKIPYYPEFGQDLDQDGSSEQHI

RLGSRVEGLQGVVNYSYNEYRLIATNDVNNTNFVAAGDGFDVERKAAPAIAESDLRVASF

NVLNYFTSVADSGNNNPTGQNRGATNHDEFLIQQAKIVSAMNKMDADIIGLMEVENNGFG

DGSAVQNLVDALNAEIDDADDHYTYVEIADKDKYQEEYFGSDAIMVAILYRAKVVTPKEA

AKVIFTPEQHIAENTITRKDGAESNPAYDKYQRHSLLQTFTVQESGEDLSIVVNHFKSKG

SECIEEWIAGVEDSEPADLQGNCNNFRVSAAHAVGEALKDVKGDVLLMGDLNAYGMEDPL

LTLTDYSKEKYGRDIYTAGYTTINGGELQVEQTQIKQGYGLNNLNTLLHGTDTFSYTYSG

ELGNLDHALASNSLAQKVVAIEDWHINSLESNLFEYGSKYTGDMPKYKDAFSASDHDPVI

IAIDLPDTDIDLPKAGENLAVDVRLPPNAVAGDIVTVSLTVATQAAAVSSDAKAYSASKT

LDQTDIKARSVNVEFEHAPEQGNYQLEEKVTDSTGNVVKFSSARAVSMTQPETGTTDSKD

SDGGSFGFGALLAMLGLGLFRRRR

>tr|Q87HM2|Q87HM2_VIBPA Anaerobic ribonucleoside-triphosphate reductase OS=Vibrio parahaemolyticus serotype O3:K6 (strain RIMD 2210633) OX=223926 GN=VPA0941 PE=4 SV=1

MKPIVIKRDGSRAPFNRDRIQAAVEAAAENKDKDVAIYALNVALAVELQLKDHDEVHIHE

IQDLVENELMQGPYKALARSYIEYRHDRDIAREKQSALTREIEGLIEESNLDLINENANK

DGKVIPTQRDLLAGIVAKHYAKTHILPRDVVQAHEVGDIHYHDLDYAPFFPMFNCMLIDL

KGMLTHGFKMGNAEIDTPKSISTATAVTAQIIAQVASHIYGGTTINRIDEVLAPYVMTSY

EKHLEIAKEWNIAEPEEFAKARTEKECYDAFQSLEYEVNTLHTANGQTPFVTFGFGLGTS

WASRLIQQSILKNRIAGLGKNRKTAVFPKLVFGIKDGLNHKAEDPNYDIKKLALECASKR

MYPDILNYDKVVEVTGSFKTPMGCRSFLGTYEEDGELIHEGRNNLGVVSLNLPRIALRAK

GSEAKFYELLDDRLRLARKALETRISRLENVKARVAPILYMEGACGVRLKADDSIAEIFK

NGRASISLGYIGVHETINALFGIEAHVYDDAVLREKAVAIIKHLKDAVNQWADETGYGFS

LYATPSENLCSRFCRIDTKEFGLIEGVTDKGYYTNSFHLDVEKKVNPYDKIDFEMPYPEI

SSGGFICYGEFPNMQRNVEALENVWDYSYTRVPYYGTNTPIDECYECGYTGEFECTSKGF

TCPSCGNHDSTKVSVTRRVCGYLGSPDARPFNFGKQEEVKRRVKHL

>tr|Q87FX1|Q87FX1_VIBPA LafE OS=Vibrio parahaemolyticus serotype O3:K6 (strain RIMD 2210633) OX=223926 GN=VPA1553 PE=4 SV=1

MISSSLPSSNAASSSEGRITQAKDVQGFSQRSASGQTPLNQSAALSEQAVPSFRLAAQTF

SSEERKEATTATTEENAAVDTTGMNVLLSATTPTETPDLAAQAQTLTQGMLNMAEKSVAS

FNKVNPLEIKGEAKTTSPAQANALLNTSPQQQDIQTSQTTSANARVNTSAVDFQALLNQP

AQGQATTAPLNVQTSAPSAQAVSAATVAHAHTQGSEWAAVKVDTSAGKWGEQMMQVLHDR

VTLQAQQSVQEAKIRLDPPDLGKLDLLVRVEGDRLSVQINANTAATREALMQVSERLRTE

LQEQNFVHVDVNVGADQGQERHQQQMNDEDTTIFAARESSAFQSNTTTNYSEHWLNTQA

>tr|Q87SK7|Q87SK7_VIBPA Putative general secretion pathway protein B OS=Vibrio parahaemolyticus serotype O3:K6 (strain RIMD 2210633) OX=223926 GN=VP0416 PE=4 SV=1

MSVIKHVGLFLVPISVSAIAVAWHLDLLTPETQAVTPEVVVAEVVQPFEVLDYPESTDLA

QLPREWPSAELSRDIGTFLQPSEYANEGGAPDPTTATPVTSTASTHRRDDLGLSLDDLDL

SSLSPDLAKKVENALSQNDAPTSAPSQVNDLERNAQQWQGRLPALNLQTHMYASDANRRW

VKINNVEYHQGDVVDGQVTLKEIQPQAVIVEFQGEQIRIPALYEWDG

>tr|Q87HP6|Q87HP6_VIBPA Uncharacterized protein OS=Vibrio parahaemolyticus serotype O3:K6 (strain RIMD 2210633) OX=223926 GN=VPA0917 PE=4 SV=1

MKTQFKILATTITASLFANVASAATYAIEARSDAMGGVGTVSASYLTAPFFNPALTAIYR

RNDDAGMIIPSFGISYDDQYDLIDEIERISKLTDVDEIKKSMDAIDGKKMDFDLGGVVAV

GIPNKFLSATVYGKLYTESFVQPNVDTSGADPLTDSYVQGVSIGVAEAGVSLAKYTNFMN

QHLSFGITPKLQRIYTYSYATSFKNFDTKDLREHETAETMFNLDAGALWFFGPYRLGISA

KNLIGRDVKTKSFQYNTDTIQSYEYSMRPVYTVGAGIVADYFTMSVDYDLNEEKRFSSFD

DNTQMIRAGFEIDILRQMALRGGYMKNLARSNDEGTITAGIGLSPLNLIELDISARYTNE

NAMGASINFLATY

>tr|Q79YW9|Q79YW9_VIBPA Flagellar biosynthetic protein FliR OS=Vibrio parahaemolyticus serotype O3:K6 (strain RIMD 2210633) OX=223926 GN=VP2237 PE=3 SV=1

MEYPTQIILDWIANYFWPYVRISSMLMVMTVTGARFVSPRIRLYLGLAITFAVMPAIPAV

PQDIQLLSFRGFMTIAEQMIIGVAMGMVTQFMIQTFVLLGQILGMQSSLGFASMVDPANG

QNTPLLGQLFMFLTTMFFLATDGHLKMLQLVVFSFKTLPIGSGTLTAVDFRDMAGWLGIM

FKTALSMSLSGIIALLTINLSFGVMTRAAPQLNIFSLGFAFALMVGLLICWYILAGLYSH

YELYWALGEEQICSLIRLDC

>tr|Q87NL6|Q87NL6_VIBPA Uncharacterized protein OS=Vibrio parahaemolyticus serotype O3:K6 (strain RIMD 2210633) OX=223926 GN=VP1852 PE=4 SV=1

MRWFWWLRCYAEALVLRASHLNKALYIRSQYLETNDLIALIFSGIGAVFICIYYMDKKQS

VCCECNEVISHRKQNRYTLEKDGATLALCKKCFNKINKQASLKAQNCSCCKKPFTTRMKI

SEWKGEFQSYFLCVQCEKKVSKRVENTFLLNQLLSPDFIKKHSNFSDLESMVESSGVELQ

TQDDLNSDAWNTFIATNTSFSCWHEMKVGAEVLMLQRQNDIIVQSLRKQNV

>tr|Q87FM3|Q87FM3_VIBPA Ferric vibrioferrin receptor OS=Vibrio parahaemolyticus serotype O3:K6 (strain RIMD 2210633) OX=223926 GN=VPA1656 PE=3 SV=1

MSYQNLDGKVRVRAGLSTTYSVLAIAIASATSMSAVAAPAAKNETTVMETVVVTGSVIGN

SDIEDVKEYPGARTVITRDQIEKTAAGSIDNALQRVPGIKVQDESGTGVLPNISVRGLKA

SRSGHAQFLMDGVPLTLAPYGHTGQSIFPATLSMLDRIDIVRGGAAVQYGPNNVGGVINL

VTKPIPHTWQTEISNRLTVFDGGDAPLNDFYLRTGGWLSDTFALQLEGNFLKGESFREHS

DTDVKNFQAKAQWLLSDTQEIQAFLQRYDAETQMPGALSPQDYEQDRHQSKRPYDDYEGK

STRWSVKYIHDLPFADSAELEVLTFGHKSERLFKWGFNSAGGHWADPALPATDVRTSPRE

FTVYGIEPKMAMYFGEGKSVTQNWIVGTRYVNEDIDYKLTQTPIVGGATKVPRDWHLDTD

AFAGYVSNEIGLFNDTLKVTPGLRVESVRMTFTDRGKKQTADNKVTEWLPGLTVAYNVTD

QWVTYANAQKSLRAPQIAYIRGLGEEGSELAWNYEVGARYTQDATSFNAALYRIDFEDQL

QWQSSTQTFDNIGKTLHQGLELSARYVPEVLPALSLGASYNYLDATLEEEGANKGNQLPY

TSKHQLGWDATYAFYGMDTTLSGFYFSDSYTDNANTSAEDATGATGKVPSYMVWNFNLGT

DLYKDDKGKLRMNVAVNNLFDEEYYFRGIDTSPVGRYPAPGRSYTLDLNYQF

>tr|Q87FX4|Q87FX4_VIBPA Flagellar biosynthesis protein FlhA OS=Vibrio parahaemolyticus serotype O3:K6 (strain RIMD 2210633) OX=223926 GN=flhA PE=3 SV=1

MLTRLKQLQTSTKGYIGIPIVLLMILAMVILPLPPLLLDALFTFNIVLAILVLLVSTTAK

RPLDFSVFPTILLVATLLRLTLNVASTRIVLLEGHNGGDAAGKVIQAFGEVVIGGNYVVG

MVVFIILMIINFVVITKGGERISEVSARFTLDALPGKQMAIDADLNAGLIDQETARLRRK

EVANEADFHGSMDGASKFVRGDAVAGLLILFINIIGGISIGVFEHGLPASEAFKTYALLT

IGDGLVAQIPSLLLATAAAIIVTRINDSDNGMSETMQKQLLATPATLFTVAGIMAVIGMV

PGMPHLAFFAFAGALGFAGWRQSKKPVQDTQIEQVEALSQAMQEEDTPLTWDDIPHVHTL

SLALGYRLVHLVNKDQGAPLSQRIRGVRRNLSEQVGFLLPEVRIRDNLSLKPNQYTISLN

GEVIEQGFIEPERLMAIAVGDTYGEIDGILGSDPAYQLPAVWIEHQDKAKALNMGYQVVD

DGTVIATHISKIMKTNLAELFTHDDVEAMTQRLTQQAPKLAEALAAALNPAQQLKVYRQL

LLDQVPLKDIRTIANTMLESSENTKDPILLAADVRCALKRTLVNLIAGQKPELNVYALSD

ELEQMLLTSLQQAQASGTVVLDSFPIEPNILGQFQQNLPLIRQQLKQQGLPPILLVMPQL

RPLLARYARTFTQGLAVLSYNEIPENKQINVVGNLG

>tr|Q87NV6|Q87NV6_VIBPA Uncharacterized protein OS=Vibrio parahaemolyticus serotype O3:K6 (strain RIMD 2210633) OX=223926 GN=VP1762 PE=4 SV=1

MSMNIKELESYLEAFMCAESDFPFGPDALVFKVKGKMFAIIAERGGREYVSVKVKPEDGE

VLTSQFTDITPGYHLNKRHWVTVYFNGDVEDGLIQDLCERSYDLVVAKLPKAQRTLLEH

>tr|Q87JG4|Q87JG4_VIBPA Uncharacterized protein OS=Vibrio parahaemolyticus serotype O3:K6 (strain RIMD 2210633) OX=223926 GN=VPA0289 PE=4 SV=1

MEFVTLALLGVLIVISPGADFVLVLRNSLNQGREAGVYSAIGISMAISIHIAYSMLGISY

LISQNEWLFNLVRYLGAAYLVYLGIKGIFSSQPASNSETIQQSEYSMWRFFMQGFLCNLL

NPKTMLFFLSIFSQVISPDSSQQHIALFYGIYMIALHGIWFSIVAVLFTSLQLQAFLLKI

KHRLNQACGAGLVIFGAMLGLKA

>tr|Q87HD2|Q87HD2_VIBPA Uncharacterized protein OS=Vibrio parahaemolyticus serotype O3:K6 (strain RIMD 2210633) OX=223926 GN=VPA1033 PE=4 SV=1

MVVMTKIDALNFLDPEELTHEFTPMGLAEFDSHWLTQFVECSDSILAARIWLEGTQSKDF

ASFRHSIVSTINTIDTLLQKQLNEIIHHPDFQKLECSWVGVRYLCEQVDKPSSDSVKIKV

LSATWNEVSKDALKAIEFDQSALFKLLYQNEYGMAGGEPFGVVVGDYQLRYDPRTNYFDR

DISVLSKISQSAAAAFSPFVMSASPEIFGVNTFAELSSTRDVAAQFEQIDYVKWRQLRDN

DDTKFIGLTAPNVLFRQPYKSDGSRNDQFEFQENIDDSNNDLLWGSGAFCFAAVSIRAFQ

EHGWFTHMRGIKQGDYSQGIIVAPTRNKTRINGKNDRDRCPLNLKVSERKEQELSDCGFI

PISPVPETDMVGMTSNVSLYKPKLYEEKHVATNAKLTSMLQYTMCVSRIAHYVKVMGRDK

IGGYQDAASLEREFQTWLHQYTTASDEASDELRAKYPLNEAKIKVREKRDTPGHFYSVIH

LRPHFQLDQMVSSIKLITELSPEHLV

>tr|Q87QF4|Q87QF4_VIBPA Putative ABC transporter, permease protein OS=Vibrio parahaemolyticus serotype O3:K6 (strain RIMD 2210633) OX=223926 GN=VP1195 PE=4 SV=1

MLRALYLIIIAVCILPTIPGLLGVVVSALGYVPPIGLHHFSLDGFNAVFDWEGVWTSIGL

TFYSAIFSSYLACLITFAILQASWGCKFWRKIEVSLSPLLAMPHVAFAIGFAFLFAPTGM

GVRVLNQLFGYDPNAQTVNDLALLIKDPHALGLVIMLAIKEVPFLLLMSIPILQQLKVDK

IEKVSHSMGYSSAQAWWKCVFPQWIAKLRFPMLAVLAYSLSVVDVALIIGPTNPPTFAVL

VWQWFNDPDLSLLPRAAAGAVVLFAIASLLIGFARLMEWCITKGFKRWQYSGRNGFRLPG

KTLFSLIALLALLMIPLMVVWSFAQRWRFPDLLPSRYSLRFWEYEWHSILGTLEQSMLIA

VISATVALALALIAHEYRLRYRWQVPGYIIAIPMLIPQLSVLFGMQVVTLYLNSNAYFSW

VCWAHVFFAFPFIYLSLDGPWRSFDNGLTRVALSLGKSPMQAWLKVKLPILLPAIAFAWA

VGISVSLAQYLPTLVLGAGRISTITTEAVALSSGFDRRVTAIYAIWQALLPFLFFSFAIL

LSRLHVKYRRLSIKGLLLNESLSRKPRHP

>tr|Q87HI0|Q87HI0_VIBPA Putative formate dehydrogenase OS=Vibrio parahaemolyticus serotype O3:K6 (strain RIMD 2210633) OX=223926 GN=VPA0985 PE=4 SV=1

MSTDFKPAEFVQTMINVGEAKTNTSTRDLLLRGTMAGIILSLAVVVAITAMVQTGIGLVG

ALVFPVGFVILSVMGYDLVTGVFGLAPLAKFANRPGITWGRIFRCWGLVGLGNLIGSLIV

AYLVAISLTGNFSLELNAVAKKFVAVSTARSLGFENMGMDGWITCFVRGIFCNLMVCLGV

IGNMTARSVSGRVAMMWFPIFIFFALVFEHTVVNMFLFPLGMILGADFGIATWLNFNLIP

TILGNIIGGLVMTCLPLYLTHAKTAPSLSVEQDVIAEPAIAK

>tr|Q87J64|Q87J64_VIBPA Uncharacterized protein OS=Vibrio parahaemolyticus serotype O3:K6 (strain RIMD 2210633) OX=223926 GN=VPA0389 PE=4 SV=1

MKPSFIALAVASCIGLAGCGSDSSDAPVPVDKPIISADLYLRGLNGDWGTAEHAKLNYLG

NNEYETVLRVARGSNQFKIADPGWQIQYTYFEEPAAFDKAQEYLPKPDTNEACMNGDCNS

EITFEDKGYYKFSVSFADESRATMTVTKATQEEAEKYYSDAILDPAMVHEGHQNKEVKLF

ANYDDSSDTVIFSVKDPKAELREFGISTTTELRDALDQGLVINEQTGPRVVSGDVAFDAL

FALTMKELDQLAVSEIKDGNYNYNNPIKAEVFETGAKWHYVWTRDLAYAADLSLALMNPT

RVQNGLNFKLSAFRDTTDQQYDGEQIVQDTGTGGSWPISTDRTTWTLGAERLLSALDGEE

YNQFAERAYKAISNTLEADRLAAFDAKSGLYTGEQSFLDWREQTYSTWTPNDVNAIGSSK

ALSTNVVHYRAIQLAAKLAEKYDSTNAVKYTEWAAQLKTAINEQFWNAERGMYVSYLFDN

GKDIAVDKYDMLGEALAIISGVASDAQAKQIMTNYPHSEFGVPVYFPQQPDVPVYHNRAI

WPFVTAYSLRAAHQTQNVAAANNAIQSLVRGTATNLSNMENLEWLSGKSFIIHSDHGEDP

SLDGPVINSQRQLWSVGGYLNMVVETLFGIHTETGKLEVKPFITSWVRNKLLAQSEQITL

ENFAFKGKNYSVTIDLPEVSKDETGYYPVENVTMDEEGHFHVTLGAFVNVDSSITLVSGV

KPYVSEDSRVYSPQEPTLVVKSVDNSVSIDVTSKYNVNLYRNGELIQKNVIAQSYSDTPT

GFACYLAESINDKGFRSNPSQPVCVGEEIRIKLDGEYQPLKDNVSVVTVSKDSGIVKGAE

SFTAPVTGTYQFDAWYNNNLGQLNTGITNTVKLLEVLDDAGKVVGQGYLQMGHIGENQGM

RYSTPIEVALEQGKTYQFALKDHFNMSYLTSNETYIYAGGIEGEKNEAQIADIRITPLM

>tr|Q87PA7|Q87PA7_VIBPA PqiA family protein OS=Vibrio parahaemolyticus serotype O3:K6 (strain RIMD 2210633) OX=223926 GN=VP1610 PE=4 SV=1

MISLSKHAMQPNSVRLCQGCELPVDIVDLPNGKNAHCPRCGTQLYRGGSPSLSGNLAVAI

TCILLFIPSHFFNFISIRLFGVMIPATLPSGMITLMEEGFVLLSLLILFCSSLAPLTVCV

SVVTAHISLHQQWFKGLRVSLWLIQHLKHWVMLDVFLVSIAISCFKLQDYSDIFVGPGLI

GLVLLQIFTVLLVSRISVRRYWEAWKPENTYSFEHKDVHCHECHLSQPEGERCQRCHHEL

YHRKPNSIQKTWAYLVAATIAIFPANLIPISILITNGQRLEDTIFSGVASLVRSGMYGIA

IIIFVASIVVPVVKILGLAYIMLCIKFKRSVFRRQRMMVYFAVKWIGKWSVMDLFVISIM

MTLVDRGQILDFTPGYGAVAFGLVVVLTMLAAESIDPRLIWDEAPKQSEKESINE

>tr|Q87P63|Q87P63_VIBPA Immunogenic protein OS=Vibrio parahaemolyticus serotype O3:K6 (strain RIMD 2210633) OX=223926 GN=VP1655 PE=4 SV=1

MFKFKHIALAASLAALTAGYSVAAEETFVTIGTGGVTGVYYPTGGAICRLVNKSRAEHGI

RCSVESTGGSIYNINTIRAGELDLGIAQSDWQYHAYNGTSQFAENGPFKELRAVFSVHPE

PFTVVARQDSNIKTFDDLKGKRVNIGNPGSGQRGTMEVLMKEYGWSNDDFKLVSELKAAE

QSKALCDNKIDAMVYTVGHPSGAIKEATTSCDSNIVTVAGPKVDKLIADNSFYRVATVPG

GMYRGSDNDVMTFGVGATFVSSTAVPEEVVYNVVKAVFENFDDFRRLHPAFANLKKEEMV

KDGLSAPLHPGALKYYKEVGLIK

>tr|Q87N63|Q87N63_VIBPA Tetrathionate reductase, subunit B OS=Vibrio parahaemolyticus serotype O3:K6 (strain RIMD 2210633) OX=223926 GN=VP2011 PE=4 SV=1

MDSTKRRFLSAITAGAALVPIAGIGTATAGNVIRNNQSADRKGQVGKRYAMVIDLRKCVG

CQACTVGCSIENQAPIGQFRTTVKQYEVTLDDGSTTTQEAKAFMLPRLCNHCDNPPCVAV

CPVQATFQREDGIVMVDNSRCVACAYCVQACPYDARFINEDTLTADKCTFCAHRLEQGLL

PACVETCVGGARVIGDLNDPSSEVRRLITKHQDNIKVLKPDEKTKPHVFYIGMDERFTSH

IDGQPAIYAPQGDRA

>tr|Q87GU6|Q87GU6_VIBPA Putative transcription regulator OS=Vibrio parahaemolyticus serotype O3:K6 (strain RIMD 2210633) OX=223926 GN=VPA1219 PE=4 SV=1

MHLMIQDINILRDLSLAEKLSRVARLWKMVADRELEPLNLTYPRWTALWKLYRMGDNISQ

KQLAEALEIELASLMRTLKLLEEQSLVTRRCCEHDKRARIVSLTDEGKELLIQMEERILQ

VRRKLLSEINDQELKKLGLILEQIAHNALDSLGE

>tr|Q87KN6|Q87KN6_VIBPA Transcriptional regulator, TetR family OS=Vibrio parahaemolyticus serotype O3:K6 (strain RIMD 2210633) OX=223926 GN=VP2941 PE=4 SV=1

MKSMGIRAQQKEKTRRSLIDAAFSQLSADRSFSSLSLREVAREAGIAPTSFYRHFKDMDE

LGLTMVDEGGLLLRQLMRQARQRIVKEGSVIRTSVETFMEFIESSPNVFRLLLRERSGTS

SEFRTAVAREIQHFAAELTEYLMSTGMTREEAYTQAEASVTLVFSSGAEALDLDRRERDE

LAERLIMQLRMIAKGAYWYRKERERNRLKGGIE

>tr|Q87R30|Q87R30_VIBPA Uncharacterized protein OS=Vibrio parahaemolyticus serotype O3:K6 (strain RIMD 2210633) OX=223926 GN=VP0968 PE=4 SV=1

MAWFSWQQAKQLDPSLNALDGFFSEPPVKVQTVTGGLTNRCWRLESSQGLAYVWRPTSNV

CKAFSISRHNEYQVLHAIEPLNLGPKPIFVHEQGLLVEWIDGITLTKDGIELEELLPIAA

TIHQYHSSSIPVVPFSYISRIDHYWLELEGKYVGSEFETLYKQWRSEPSVEQIPLALCHF

DLGCYNLVRGDQGVKVIDWEYASLADPRLDLTLILQITDSPIEESVERYCQIRNIENTSV

WLEGVKAWQPRTLVMAMLWYLLAYKLWGDEQYLNSAQEFKDLLCLEDHCFENS

>tr|Q87LG7|Q87LG7_VIBPA DNA polymerase III, chi subunit OS=Vibrio parahaemolyticus serotype O3:K6 (strain RIMD 2210633) OX=223926 GN=VP2645 PE=4 SV=1

MQTATFYIINEESPQATTAGFEEYIVFLVQHFARQGAKVYLNCQDKPHAEQLAEAFWQID

ADQFMAHNLVGEGPKYATNIEIGHDGVKPSWNRQLVINLAENETTFANKFAQVVDFVPCE

EKAKQLARERYKIYRQAGYQLQTIEIQYP

>tr|Q87KL1|Q87KL1_VIBPA Putative inner membrane protein OS=Vibrio parahaemolyticus serotype O3:K6 (strain RIMD 2210633) OX=223926 GN=VP2966 PE=4 SV=1

MRERVLFFDLLRCVAAVAVIAIHVLAPYRNELGVIPMDQWLTAVGVNSVTRWAVPVFILI

TGALMLSDTRPFDGKYYVKRRLGKVLVPFLIWSTFYAYLSGWTAQGFDFETVKEVLSNSP

FHATYYHLGFFYYFIPLYFVIPLFQWMARNVDDNVLYTYLAFWMFTSTLFLFKIDGPWSN

QMWLYMGYLPLGYVLFQKVPLNRSMVTLFTGFGLVALAVTFTMVVTNSLEAEKYTVGRWL

SYKTLNVILAASMIFMLCRYFGEGLPKNVQKVVSFISQHSLGIYLLHPIFLWPMKEFGWY

TGHPGWVIPVWIVLSGAGALAMSYLFSKSAKTRWLLP

>tr|Q87TF7|Q87TF7_VIBPA Methyltransferase-related protein OS=Vibrio parahaemolyticus serotype O3:K6 (strain RIMD 2210633) OX=223926 GN=VP0112 PE=4 SV=1

MVCFASLFEEVSMHTCPLCHNQHTRHYFEDKHREYLQCEQCYLVFVNPDQRLDAETEKAH

YDLHENNPEDIGYRRFLSRIADPITERISPQSNGIDFGCGPGPTLSLMLEEAGHNMALYD

IYYHPDTAVLEKQYDFMTATEVIEHLYHPDVVWKQWLNLVKPGGWIGLMTKMVIDVEAFK

SWHYKNDPTHVVFFSRDTFQFLAERDKLELEFIGNDVILLRKTQ

>tr|Q87H88|Q87H88_VIBPA Uncharacterized protein OS=Vibrio parahaemolyticus serotype O3:K6 (strain RIMD 2210633) OX=223926 GN=VPA1077 PE=4 SV=1

MKKLALISVVGLALSGCGGSGSDNNDSSSTLPIAKPSAAIGSVESVDAKESTLTVNGYTY

RVSKVVYGTTPLALAVVQPNMMVKVDENIQKSEDGHAVVSLEPTITGRVTAIDYAKKAFT

VNGVELQFDGLSNEIGLGDWVMVSSLPTADAGYKVLSVVEIDVDHYPSIGDMYEIEGRIA

SIDANQSTFKLGANITVSYHNVDQLKVGQWVEAEGKMEGDIFVASDVEIEGYDNLENDSD

VEGIVTWVANDYSEFSLNYRGAFFVDNATRFEDGSKMSLKQGQEVDVSSVLRNGKRIATV

VEFEAPDFDDNHQWQGKGFECEGYVSNYDANARTFEIERCENDADQRLNNKLVVIDAQTQ

FQGIEELNLNGARVEVDGVIINNQNVAREIEREGYDD

>tr|Q87LF2|Q87LF2_VIBPA Putative anti-sigma B factor antagonist OS=Vibrio parahaemolyticus serotype O3:K6 (strain RIMD 2210633) OX=223926 GN=VP2660 PE=4 SV=1

MAQSHAQWQLQQDTLTLTGALDRDSVPSLWAFAQQWKPSQKELECSLEEIERVDSAGMVM

LIHLLEHAKKQNCHIMLSFVPAQLRTLFQLSNVESLVAKHIKNYQG

>tr|Q87G74|Q87G74_VIBPA Uncharacterized protein OS=Vibrio parahaemolyticus serotype O3:K6 (strain RIMD 2210633) OX=223926 GN=VPA1443 PE=4 SV=1

MSNQQPVVNELPAEKVYRFSSDANLSFKKPILLGVATLSFFLGGLGYWAATAKLESAAIA

YGDLSVLTKRQEIQHLEGGIIEKLYVQEGDLVKKGQPLVQLSQRQPMAKLDAVSGQYIHT

LAKENRLSAELDELADISWSGDLEAIPRVNIVKEAQLVQNKIFTARKRFFESKLSIIEQS

ISGASLELENLKQTKVIERERLNFIEEEIASNQALVQKGFSGKSTLLQLKRLAAEVRSTL

SQLDRQSLTVGKRLDENQAKIEELKLERLNEIVEELRNTKKEVVAIREEYRSAQDVVART

SINAPISGRVVNMQVFTEKGVIGSGQTLLELVPQDDKLLVEARVNPQDIDLINPGQQAHV

RLTALNARTLAPLDGTVLTVSADKLSQENQDDYYLARISISQDDVAKYRLTSGMNAEVLI

LSEPRTPLSYLIKPLTESMNRAFREE

>tr|Q87FI5|Q87FI5_VIBPA Putative D-serine dehydratase (Deaminase) transcriptional activator OS=Vibrio parahaemolyticus serotype O3:K6 (strain RIMD 2210633) OX=223926 GN=VPA1694 PE=4 SV=1

MDRKQQMLSNMYTFAVAGKCLSFTKAAEELFITQGAVSQRIKSLEEQLGFSLFVRMTRRL

ELTKEGERLLHALNQSFEVIFSELEDIKFNELRGELYIGVAPTFAQSWLLPRMVEFQRLY

PSLNIKLRVKASRLDFLHEPVDIAIYYSDSEHPGFHHQRLFDEELVPVCSPEYYQTHFSD

GVSSASQFEAVTFIHCTESLEANEPNHEWQSWLASQSNSELKSLNVMEKTYLFNHADMAM

IAAKNSMGIAMARASLVQTSLEKRELVAPFERVNAGRGYDLICLNGQQHRPKNAAFIEWL

ETQLPPLVSQ

>tr|Q87JA6|Q87JA6_VIBPA Uncharacterized protein OS=Vibrio parahaemolyticus serotype O3:K6 (strain RIMD 2210633) OX=223926 GN=VPA0347 PE=4 SV=1

MVLLVTTKSLTLRCKLQDCLFIFCVTVAIISYMLLPLSSKIINLLFRFEYILINVGLILS

LEPNMTWEILNQKPTESNARDVVVLNRFSNFLGQFDIDPRILDKREVKITRQAILVIQAM

TDFDRKNVIKDIYKIIDNPKRASLTKHRRNPFWRLARNIDPFRTYHFLILYRISDSGSVV

IEEIMLDSNLHGSKLESKKQRTMLYDVRKETKSRYSEDKSTNEDIQSALNSWDGKGANII

PKINCDHVTINGMNNDYDTASKLMGVHTDVAYRKDNIVRYNLFHNPTDGGVFDLAECIFD

QTRIFKSRNAKQLASLIIKTQSERRRVKWTVHSQGAIIFRSAIEEVKNLRPELRLDGHEL

AVHSPGGNYGILSMKAKHLGMKVQPLRANPADFVPNVIGLNGVTSITKMNLSLSPVFIWA

CFLSKTIGGSPHSLPYLGLNTYEKQLRESGARQKARVIGKYAKIGKH

>tr|Q87G08|Q87G08_VIBPA ScrC (Sensory box/GGDEF family protein) (Involved in swarmer cell regulation) OS=Vibrio parahaemolyticus serotype O3:K6 (strain RIMD 2210633) OX=223926 GN=VPA1511 PE=4 SV=1

MKKAVKKISSKKIITISAIIVSIYLAILIVVTSLGQNKLKDSQYRELDLKVKSYASTLDN

LFTIASEDVDNLSSDKTVQTFFANLASGMSMEYGLGASLINLKRRFDEKIDGKLINSSNI

YNKLTLVGSDGTTIVTTGPKDLKPMDIHDLMTQHGHPEEVCATRDGDNIRIQVIKQVKFS

GKPVAFLIADINKDIVIEQLTNQEHEDSYSRMVLKIKDVEMVVWDSITKNSTINADENHF

LSKILDGKIYFEVPVEQHHFKLMAWFEPLNERDIFTSRLFIVGLSILAVMIVIALCYAFV

TNNNKLELKIKLEEEKKRKDILSQQNHKLTKEIERRKASEKELAFQAKYDTLTELPNRSY

GSERLALELIRASRTGSKVLVMFIDLDHFKQINDSMGHFVGDEILKLSAQRLQNVARKTD

LLARIGGDEFLLVIPDLPDNDTAKRVASSVLSAFSEPFVWNNHEFFLTGSVGMSVFPDDG

DNAEQLLACADMAMYRVKQDGRDAFCFYNHNMNQDLQRYLDLESRLRNAISNQLLEMYYQ

PIIELKSGKIVGAEALMRWNDEKFGFVNPEEFISIAEKNGLIHQLGEFAIQQACHQASQW

QSISPLFVSVNFSSVQFRYCDRLLAFIRQSLEESGLPAEQFDVEVTESLLFNHDDELVDM

LDNLRALGTKLTIDDFGTGYSALSYLQKFPFDRLKIDRSFMQNVFENDSDRELVNVIIAM

AKALRLKIVAEGIEEQRHVDYLNELNCEFGQGFHYSRPVPAKEFEQLLNQPTWS

>tr|Q87S57|Q87S57_VIBPA Putative protease OS=Vibrio parahaemolyticus serotype O3:K6 (strain RIMD 2210633) OX=223926 GN=VP0567 PE=4 SV=1

MTTEKAFVPELLSPAGSLKNMRYAFAYGADAVYAGQPRYSLRVRNNEFNHENLKIGIDEA

HALGKKLYVVCNIQPHNSKLKTFIRDLKPVVEMGPDALIMSDPGLIMMVREAFPEMPIHL

SVQANAVNWATVKFWAANGVERVIVSRELSLEEIEEIREHCPETELEVFVHGALCMAYSG

RCLLSGYINKRDPNQGTCTNACRWEYKVEEGKENDAGDIVEKFDPTEAQSVEVQDERPET

TIGRGKPTDEVVLLSEAHRPEEKMAAYEDEHGTYIMNSKDLRAVQHVERLTKMGVHSLKI

EGRTKSFYYCARTAQVYRKAIDDAVAGKPFDESLMGTLESLAHRGYTEGFLRRHTHDTYQ

NYDYGYSVSDAQQFVGEFTGKRRGDMAEVEVKNKFVVGDSLELMTPKGNVVFTLEAMENR

KSESIEDAKGNGHFVFIPVPEDMDLEYGLLMRNLNAGQDTRNPTGK

>tr|Q87IX5|Q87IX5_VIBPA Putative cation efflux system transmembrane protein OS=Vibrio parahaemolyticus serotype O3:K6 (strain RIMD 2210633) OX=223926 GN=VPA0481 PE=3 SV=1

MNSNSIKIATIALLVGGALGFGVNQYLSGANHDMSGTGGSAASSSNEPLYWVAPMDPNYK

RDKPGKSPMGMDLIPVYAEDLAGEQDKPGTVKIDPSVENNLGVKTAQVSLEQLSPRIETV

GYIAFDESHLWQTNVRVAGWVEKLYINAVGEKVNKGDVLFTLYSPELVKAQEELLNAYRT

GRSGLVKGATERLVTLGVDRSQIRAITQRGKASQTIEIKAPEDGVIASLNVREGGYLSPA

QAVISAGPLENVWVDAEVFERQAHWMKAGSDATMTLDAIPGKEWQGSVDYVYPILDPKTR

TLRVRLKFSNPNGELKPNMFANIALQPVSDNAVLTIPKSSVIRSGGMTRVVLSEGDGKYR

STRIEVGREADDKIEVLQGLTQDDRIVTSAHFMLDSESSQTADLSRISSPTEATAQTAWT

KGEITDVMKGHRMLTINHQPVPEWDWPGMVMNFTLADGVDMGSLQKGQAIEFEIQKAASG

QYEIVDYKADDTVLATDVWVTGDISMLMADFGMITLNHLPVSEWNWDAGEMNFSVGDEVD

LSGFKEGQKVRFLVEKQGSDYVLKQIEAIEG

>tr|Q87M63|Q87M63_VIBPA Uncharacterized protein OS=Vibrio parahaemolyticus serotype O3:K6 (strain RIMD 2210633) OX=223926 GN=VP2395 PE=3 SV=1

MKRWSEEKAWQWQKEHGWLRGFNYLPRTAVNWNEMWQAESFAPEVMEQELAWAHDVGYNT

LRTNLPFIVWQADRDGLHARIERFLDICERNQIKVMLTPMDDCGFSGDHPYLGEQKAPVP

DLHNSQAAASPGRNVVMDKSMWGEVEAYIRDIISTYKNDPRIVIWDLYNEPTNRMIFTTT

GEIAYDEEMETFSHELMEQAFEWAREENPTQPLTVGAWHAPSILDRSLPIYEHPTDRKAM

ELSDIITFHAYLPLDLFHKAVEIVESYNRPMMCTEWLARHAQSYMHEQLPVFKQKNIGCY

QWGLVKGKTQTHLPWPEIKRSDANYASQWFHDLLDEQGQPYDASEVQLIKTLAEVD

>tr|Q87FK6|Q87FK6_VIBPA L-arabinose-binding periplasmic protein OS=Vibrio parahaemolyticus serotype O3:K6 (strain RIMD 2210633) OX=223926 GN=VPA1673 PE=3 SV=1

MKLKKLLTIAALSGMTMLSASANAFFGSNDDNVRLGYLVKQPEEPWFQTEWSFAEKAAKQ

YDFELVKMAVPDGEKTLNAIDTLAASGAKGFVICTPDPKLGPAIMAKAKSYDLKVITVDD

QFLNAKGEPMKEVPLVMMAASEIGYRQGSELFKEMTNRGWDAATTGVMAITADELDTARR

RVDGSVKALEDAGFPKSQIYRVPTKTNDIPGALDAANSLLVQYPKVKQWLIVGMNDNTVL

GGVRATEGQGFAAENVIGIGINGVDAVNELAKSNATGFFGSLLPSPDVHGFKSIESLYKW

VKEDVQPEKFVEVTDVVLITRDNFREELQKKGL

>tr|Q87LT5|Q87LT5_VIBPA Type 4 prepilin-like proteins leader peptide-processing enzyme OS=Vibrio parahaemolyticus serotype O3:K6 (strain RIMD 2210633) OX=223926 GN=VP2526 PE=3 SV=1

MEVFQYYPWLFVVFASIFGLIVGSFLNVVIYRLPKIMELEWRRECAESFPEYKIKPPQEV

LTLSVPRSSCQNCATPIRIRDNIPVISWLLLKGKCHHCHTAISPRYPLIELLTAACAGFV

AYHFGFSYFTVALIFFTFFLIAATFIDLDTMLLPDQLTLPLTWAGIALALTEISPVSLQD

AVIGAIAGYLCLWSVYWGFKLLTGKEGMGYGDFKLLAALGAWLGWQSLPMIILLSSVVGV

IFGLVQLRLQKQGIERAFPFGPYLAIAGWVSLIWGDQILSWYFTSILGV

>tr|Q87S33|Q87S33_VIBPA Protein-export membrane protein SecF OS=Vibrio parahaemolyticus serotype O3:K6 (strain RIMD 2210633) OX=223926 GN=secF PE=3 SV=1

MFQILKAEKSIGFMRWSKVAFVFSIFMIAASIFTLSTKWLNWGLDFTGGTLIEVGFEKPA

NLEEIRTALDAKGFGDATVQNFGSARDVMVRLRPRDDVSGETLGNQIIGAIKEGTGESVE

MRRIEFVGPNVGDELTEAGGLAILVSLICILIYVSMRFEWRLAAGAVMALAHDIIITLGV

FSFLQIEVDLTIVAALLTVVGYSLNDTIVVFDRIRENFRKMRKGDPADIMDASITQTLSR

TLITSGTTLFVVIALFVQGGAMIHGFATALLLGITVGTYSSIYVASALALKLGIQKEHLM

PPQVEKEGAEFDEMP

>tr|Q87J83|Q87J83_VIBPA Uncharacterized protein OS=Vibrio parahaemolyticus serotype O3:K6 (strain RIMD 2210633) OX=223926 GN=VPA0371 PE=4 SV=1

MKKPIVILTTLMLLTSPAHADWDEISNKMSELGEAVSDTAQDVWDDTKAFSKQAWQDFTN

WSEEAINTAGEWTDASIEKSKEWIDAADKKIDELMEGDTPEEARQAIDLMADSTLLKLFN

EKPEAKAIYDKAYGYAVFDSRKLSLLFHTNSGSGVAVDKTTEKRTYMNMFGMGVALGLGG

KFYQQVVLFESKQDFDTFVNEGWEATSEASAVAGEDAEEFSAQFNSGVAVYQLNSKGLLI

DANVSGSKYWVNEELTNAQ

>tr|Q87GK9|Q87GK9_VIBPA Uncharacterized protein OS=Vibrio parahaemolyticus serotype O3:K6 (strain RIMD 2210633) OX=223926 GN=VPA1306 PE=3 SV=1

MIMTEYTWLAPAILCGVIALIGNVVLGQQVLKRQIIFIDLAVAQVAALGAALSHYWLNSS

TLYVRIPWLSESWLGAMLGPWIMSLLLCGLIAALEKRNHPHLEPMIGSLFVVSASLAVLL

VSKDPHGADFIQGILNGQLLWSTWQDVWPLTVITGAMLLLVRTKPEFMRGSGFYLIFAIL

MPITVKLTGIYLEFALLVIPALCAASLKGRRFLTASLGIGTIGILLGIAASAKYDLPSGA

TIVITLFMMGLVFNLFSPLRKIVLLQMKS

>tr|Q87SU1|Q87SU1_VIBPA Uncharacterized protein OS=Vibrio parahaemolyticus serotype O3:K6 (strain RIMD 2210633) OX=223926 GN=VP0331 PE=4 SV=1

MNLETIPSGELTVSNQRSISRLVAQAGQMLLAHGAESTLVCDIMRRIGLACGVHEVAVAL

SANALVVTTVMDGHCITTTRSCADRGINMRVITQIQRICIMMERGLLDAAMAQKKLNTIS

PERYNRWLVVLMIGLSCAAFSHLAGGDWGVFIMTFLASAGGMIVRQEIGHRHFNPLLNFA

ATAFVTTLISAQAEIYQIGNLPTVAMASSVLMLVPGFPLINSVADMLKGHINMGIARFVM

ASLLTLATCLGIVAAMSVTGIWGWAI

>tr|Q87JK7|Q87JK7_VIBPA Uncharacterized protein OS=Vibrio parahaemolyticus serotype O3:K6 (strain RIMD 2210633) OX=223926 GN=VPA0242 PE=4 SV=1

MSYKYAVACLLSALSVTAHAEPTKHIVGANLGYGDVSYDVNNGHDDGDMFLGDVYYRYMF

DQNFGLEAGYKGAFNGIGSVLMSPISEITDVSYGGPRISGYVSYPLGAGFELYGKGGITY

YTLSYTLKNNGQNKEYDESSLGGEAAAGVAWGYKHFGLNLEYNYAKNSDFDSGGVMFGAQ

MRF

>tr|Q87PW5|Q87PW5_VIBPA Uncharacterized protein OS=Vibrio parahaemolyticus serotype O3:K6 (strain RIMD 2210633) OX=223926 GN=VP1385 PE=4 SV=1

MTSLILSSRNHNLALPLITIALTIIAGVSFSHYLLTPEEQSEVISHSYQGVLFDKEANNS

IEKTYDSHFQRTKITTGVINYAFVTSLINAGLSQQEIKSLIKLIESEFDIIGSVRKGDKF

SLKTRTNSYNEKYISSFYYSGSKKDFFIINDGKNNAYDEYGDRLTRKPYYSFPLAKEYKI

SSGFSLKRKHPITGLNTPHLGTDYAVPVGTPIYSIADGVIVKSRYNRFAGNYINIRHTNG

SISRYLHLSRSSVRKGDNVVKGQEIGRSGNTGRTTGPHLHLELFVDGAPVDYARYIKSNQ

APSLNIQMMLAAKTERAELIKELL

>tr|Q87H99|Q87H99_VIBPA Putative acetyltransferase OS=Vibrio parahaemolyticus serotype O3:K6 (strain RIMD 2210633) OX=223926 GN=VPA1066 PE=4 SV=1

MDILSARKEDLAAVYALENKLFGEHSYPQFFIRQAYDCWGESLLVAKEGEAVAGYVLLTT

SNVAKHYWIMSLAVNSEFRGRRIARSLVERVMAPLAEGSSVKLTVDPLNQPALSLYQSMG

FTVLEEEANYFGDEESRLVMEFVK

>tr|Q79YX6|Q79YX6_VIBPA Bacteriophage f237 ORF8 OS=Vibrio parahaemolyticus serotype O3:K6 (strain RIMD 2210633) OX=223926 GN=VP1561 PE=4 SV=1

MKGLDMKYVFRGKSYTTLTSCYRDNTDQVTVGIGTVRTRLKNGWSLNKALLHPKQKTIKT

KLGSHTVEGKVYENLPSIAEEYGMTLNTIYKRYSRGCRGDDLVPLKKRKSYVAPDDEANF

RFYANGVGYKSAADACRKLNVKYGTYRLHMSNGFTVEQALGIEKVQDGRKVRGTKFNVDG

KEYTINELSILHNVPEATIRDRLSRGATIIQSIGLDEIPKGTLKKQRDVAKNKRKPIRLT

VNGKLYTSYKALADAYGLPQYTVRQRIVVYGYSPEDAVTLDGKSKPLTVEGVDFSSKAAA

AEAYGLTPAVLLARLAGGSTIEQALGIEDKETSRTITYEGEMYNSLKDLADKKGISVGTL

RSRVQSGLSLEEAIKAGNRIINSGRYNLTILQRDNALANKPAWLYFVRIHIENKERFKVG

ITTQTVDKRLKQEAYEFQTIKVVDGTLLDCFLLEQEIIDLLFDKRDLEVTSDMLDGYSEI

FILNESDIEIVNEILDI

>tr|Q87HH9|Q87HH9_VIBPA Nitrite reductase (NAD(P)H), small subunit OS=Vibrio parahaemolyticus serotype O3:K6 (strain RIMD 2210633) OX=223926 GN=VPA0986 PE=4 SV=1

MNMSFEKICQISDIVPGTGVCALVNGQQVAVFRPTEAEEVLAISNTDPFFQSNVLSRGLI

CEHQGELWVASPLKKQRFNLTTGACMEDERFNVKAYKARVTNGEVEIQA

>tr|Q79YV1|Q79YV1_VIBPA Flagellar basal body rod protein FlgB OS=Vibrio parahaemolyticus serotype O3:K6 (strain RIMD 2210633) OX=223926 GN=VPA0264 PE=3 SV=1

MAITFENALGVHPEALNFRVQRTKVLASNLANVDTPGYLAKDLSFTTVMNQMSTNGVSNS

VPKLQVQAQYSVPYQNSKDGNTVELGVEQAKFTQNNMDFQTSLTFLNMKFNGLAKAIEGR

>tr|Q87LB2|Q87LB2_VIBPA MSHA biogenesis protein MshG OS=Vibrio parahaemolyticus serotype O3:K6 (strain RIMD 2210633) OX=223926 GN=VP2700 PE=4 SV=1

MPTFRYQGRTLDGSSTSGKVDAVNSEAAAEALMNKGIIPLNLRLEKEGVKNHVSLSKLLV

PAIPLEVIILFSRQLFSLTKAGVPLLRSMRGLLQNCENKQLKEALEDVVSELSNGRGLSS

AMQPHNKVFSPLFVSMINVGENTGRLDEALLQLANYYEQELETRKRIKAAMRYPTFVIVF

ITIAMFILNILVIPEFASMFTRFGVELPLPTRILIATSNFFVHYWGLLIAAMVGAFFVFK

AWVATAGGREKFDKFRLRLPIVGDIVNRAQLSRFARTFSLMLKSGVPLNQSLALAGEALG

NRFLENRILEMKAAIEAGSTISVTAINSNIFTPLVIQMIAVGEETGRIDELLLEVSDFYD

REVDYDLKTLTARIEPLLLVIVAGMVMVLALGIFLPMWGMLDIIKGG

>tr|Q87T27|Q87T27_VIBPA Uncharacterized protein OS=Vibrio parahaemolyticus serotype O3:K6 (strain RIMD 2210633) OX=223926 GN=VP0243 PE=4 SV=1

MKTVDRILQIVKRDGSVTAKQLSSELGMTTMGARQHLQGLEDEGILSIHDVKVKVGRPTR

HWSLTQKGHEQFADRHGELTIQFIEAVEHIFGKDGLDKVTSEREKLTLQNYRQHLDQCES

LESKLETLVFLREKEGYMAELEQDEHGFILIENHCPICKAATRCPSLCKSELSVFQSLLG

DDTTVERTEHIISGQRRCVYRIRA

>tr|Q87HC3|Q87HC3_VIBPA Uncharacterized protein OS=Vibrio parahaemolyticus serotype O3:K6 (strain RIMD 2210633) OX=223926 GN=VPA1042 PE=4 SV=1

MKQIYKSFLLFIFLVLMGCSAANLVVDPYADLEITANHNINPDSNGRPSPVVVYVFELTS

NTLFESQDFFSIYEEHEKVLGPDLVNKYEISLTPGQKEIYQASMSPKTEYLGIVAAFRDI

ENSNWRQVIKVDKTGYNTYQISLEDLSLVVQ

>tr|Q87IK2|Q87IK2_VIBPA ABC transporter, periplasmic substrate-binding protein OS=Vibrio parahaemolyticus serotype O3:K6 (strain RIMD 2210633) OX=223926 GN=VPA0604 PE=4 SV=1

MLRFTVATLLAMAVSLPSAMAKEVNISGSTSVARVMDVLAEEYNKTHPDNYIAVQGIGSS

AGITMVNKGVVKLGMSSRYLTESEKGEDLNVFPIAYDGLAVVVNRTNSVTNLSAQQLFDI

YKGEIKNWKEVGGADQPIAVVTREASSGSRYSFESLLGLTKIINDRLVSDISPNNLVVNS

NSMVKTIVNHNTRAIGFISVGSVDLSVKAIQFNGIEPTAANIANHKYKLARPFLVLYKVD

SLDQAGKDFVAFLKSEAGQKTIADYGYIPVKNFNQ

>tr|Q87G89|Q87G89_VIBPA Azurin OS=Vibrio parahaemolyticus serotype O3:K6 (strain RIMD 2210633) OX=223926 GN=VPA1428 PE=4 SV=1

MSLRILAATLALAGLSFGAQASAECEVSIDANDMMQFSTKTLSVPATCKEVTLTLNHTGK

MPAQSMGHNVVIADTANIQAVGTDGMSAGADNSYVKPDDERVYAHTKVVGGGESTSITFS

TEKMTAGGDYSFFCSFPGHWAIMQGKFEFK

>tr|Q87R61|Q87R61_VIBPA Uncharacterized protein OS=Vibrio parahaemolyticus serotype O3:K6 (strain RIMD 2210633) OX=223926 GN=VP0937 PE=4 SV=1

MSSDRQPSLLTQRKFLPYFVTQFFGAFNDNIFKNVLLLFVAFAGSSALPISSNLFINLAA

GLFILPFFLFSASAGVLADKYEKSWFIRKVKLFEIAIMLLGAVGFITESYGVLLLLLFLM

GTQSAFFGPVKYALLPQELNAKELVPGNALVEAGTFIAILIGTLGAGIIASADNAKYLAA

FCVVLFALLGYLSSRSIPFAAASAPELKFRWRPYQQTKHTLAIAKSDRIVFQCIMAISWF

WFLGAAYLTQFPNFTKVYLNGTESAVSFLLALFSVGIALGSLACNWISNHRIEVGIVPIG

ALGITIFGYLMATSVPTELPEFANFIEFVRFEPFWPLFFYLLMIGASGGLFIVPLYALMQ

HRAKETERAQVIAGLNIFNSLFMVGSAILGIICLSVVEMSIPQLFALLAVLNFLVAAYIF

LQIPIFVVRFAMWMLTHTIYRVKHKNLHNLPEHGGALIVCNHVSYMDALLLSAVCPRLIR

FVMEEDYANLPPLRRFLRRAGVIPISASNRSSIRRAFNDVEEALNEGHIVCIFPEGRLTS

DGEMNDFMRGIDIILRRSPVPVIPMALKGLWGSYFSRAKGRACRGFPTRFWSKLEIEAGE

PVDPSSATAQTMFEKVHALRGDWR

>tr|Q87R54|Q87R54_VIBPA Putative outer membrane protein OS=Vibrio parahaemolyticus serotype O3:K6 (strain RIMD 2210633) OX=223926 GN=VP0944 PE=4 SV=1

MKNAILLLSALTISVPTFAAQEQTHQSYFYLGTGNVGFDTPYFGSDRDNEWSTPHITVGW

GYYVNQYLAFEGVLRYSQNELKNDTLKTDLDLHYYQAGMSAVLTSDNLGDTPLSLFGRVT

ALGTQAEMYVPNAQKVTDDSGALFNVGAGVHWDMSKDIWLRAEYIYNVADMGFEDFYDSY

EGLQISLGKRF

>tr|Q87K24|Q87K24_VIBPA Putative DNA polymerase III, epsilon subunit OS=Vibrio parahaemolyticus serotype O3:K6 (strain RIMD 2210633) OX=223926 GN=VPA0074 PE=4 SV=1

MIKRWFKRWETPLSPEQKRQAIHVVDDWPMVLKDYLQRPLVDDSTTLKDLSFVALDFETT

GVDAQGDKILSIGVVDLTLDGIDIASSKEWYICHGQFIKPETAKINGLTPQDLAKGLPLD

EGMNRLLKRITGKVVLAHGCCIEKAFIHAYFSSRYQVEDFPAYFIDTLQIEKQFSYAGKS

GAHSSYQLDDLRRYYHLPGYLSHSAASDALACAELFLVQSKKLTALPDVTLRQLTR

>tr|Q87IR0|Q87IR0_VIBPA Putative disulfide oxidoreductase OS=Vibrio parahaemolyticus serotype O3:K6 (strain RIMD 2210633) OX=223926 GN=VPA0546 PE=4 SV=1

MFKTISKGVALFAAIAALSACDSGNSQPQQGKQYEVLPVSLQEYNLAPLTEAFALTCGHC

RSMEEFVPQIESLTEQKVEKMHVTFNESAQISAIIFYTAVMQLDATPDKAFMADLFAAVQ

MGADATAEERQQAVEKAFESRNLISPYHLDEAQQEKLFEYITKAESITTRGQINSVPAFI

VNGKYQVITGGHDSVEAMAETINYLLKQPK

>tr|Q87FX5|Q87FX5_VIBPA Flagellar biosynthetic protein FlhB OS=Vibrio parahaemolyticus serotype O3:K6 (strain RIMD 2210633) OX=223926 GN=flhB PE=3 SV=1

MSDQSSQDKTEKASPQKVKKARQEGQIPRAKEFTTAVIFMAVALYFYSQINSIWESMFGI

FRYNMSLTKADLENPQQMVEQVGQSLGMIIEMLLPLFTVIIIVTFGSSMVLGGWMFRPAN

MLPKLSKLNPLSGIKRIFSTRSLVELVKSTIKVTVIFGILYGYLDNHLQPLLGIQNLPLN

QGFSMVMSILFEGLLLMGFALLLFGVIDIPYQRWEHLKELRMTKQELKEEFKNNEGRPEV

KQRIRQIQQQFARRKIDKMVPTADVVITNPTHYAVALKYEPSLSDAPFVVAKGVDETAMH

IQRIARENQVEIINSPPLTRSIYHTTAIEQAIPSQLYIAVAHILTYVLQLKAFRKGDGKK

PTPLPHFSIPKHLQH

>tr|Q87L61|Q87L61_VIBPA Penicillin-binding protein 1A OS=Vibrio parahaemolyticus serotype O3:K6 (strain RIMD 2210633) OX=223926 GN=VP2751 PE=4 SV=1

MKFIKRLLFLTLICIILGVTTIFGFYQYVKPELPDVATLKDVELQTPMQVFSQDGKLIAQ

FGEKRRIPVTYDEIPQDLIHALIATEDSRFYEHPGIDPIGITRAAVVVALSGSAKQGAST

ITQQLARNFFLSNEKKLMRKIKEIFIAIHIEQLLSKQEIMELYVNKIFLGYRSYGFGAAA

QVYFGKSLNELSLSEIATLAGMPKAPSTMNPIYSLERATKRRNVVLMRMLDEKYITQEQF

DEARNEPIIARYHSAEIEVSAPYVAELARAWAVKEYGEEKAYTSGLNIYMTVDSKLQDAA

NKAAVNNLMAYDERHGYRGAEKVLWKEGEAAWDAEQIEKHLKGQPTYGDLYPAVVTKVEG

KTAQVVVKNNDAQTIPWTGINWARKFLTDNRQGSTPKSAQEVLAAGEQIWVRELTTTDED

GNTQSSWKLSQVPSANTAFVAMNPENGGILSLVGGFNFVHSKFNRATMSVRQVGSSIKPF

IYSAAIDKGLTLATLINDAPINKWDAGSGSAWRPKNSPPTYGGPTRLRIGLAQSKNVMAV

RTLREVGLDETRQYLTRFGFDINEVPRSETIALGAGSLTPMKVAQGYSVFANGGYYVEPF

YVERVEDAFGEVLFKANPKSVCHQDCPQMSPQPEMDRFASEFGEQDVAVDGQAPENALEN

DEPKYAPQVISEQNAFLMREMMYSNIWGGGNWREGTGWNGTGWRAQKLERRDIGGKTGTT

NDSKDAWYNGYGPGVVAIAWVGFDDHSRALGRTTVNSNLGQGQVSGAESGAKTAEPAWID

FMQVALEGKPEQGKNIPDDIVRVRIDRNSGLLTHKVDSTSMFEYFEKGTEPTEYVGNSLE

DSIYSSGSGGTTEELF

>tr|Q87SZ3|Q87SZ3_VIBPA Protein translocase subunit SecY OS=Vibrio parahaemolyticus serotype O3:K6 (strain RIMD 2210633) OX=223926 GN=secY PE=3 SV=1

MAKKPGQDFRSAQSGLSELKSRLLFVIGALLVFRAGSFVPIPGIDAAVLADLFEQQKGTI

VEMFNMFSGGALERASILALGIMPYISASIVVQLLTVVHPALAELKKEGEAGRRKISQYT

RYGTLVLATFQAIGIATGLPNMVDNLVVINQTMFTLIATVSLVTGTMFLMWLGEQITERG

IGNGISLLIFAGIVAGLPSAIGQTIEQARQGELHVLLLLLIAVLAFAVIYFVVFMERGQR

RIVVNYAKRQQGRKVFAAQSSHLPLKINMAGVIPAIFASSIILFPGTLAQWFGQNGESSA

FGWLTDVSLALSPGQPLYVMLYAAAIIFFCFFYTALVFNPRETADNLKKSGAFVPGIRPG

EQTAKYIDKVMTRLTLAGALYITFICLIPEFMMVAWNVRFYFGGTSLLIVVVVIMDFMAQ

VQTHLMSQQYDSVLKKANLKGYGR

>tr|Q87JM6|Q87JM6_VIBPA Putative long-chain fatty acid transport protein OS=Vibrio parahaemolyticus serotype O3:K6 (strain RIMD 2210633) OX=223926 GN=VPA0222 PE=4 SV=1

MRKNMQRQFTLSPIYLLIGLASSTAYAGGFQINEHSATGLGRAFAGDAVIGDNASVVSRN

AAAMTLFKQNALSFGVTYVKPDVTVKNAQYHRANINADVDVSMGGGLIPNLQPPSVSVTP

SETVADIDDVDGVGQPAVVPNFYFIHPINDDWYLGLSTYSNFGTDMEFKPNYGAPVFGGV

TSVASVNLGASLAYKVNDRFSIGGGIDVIYGSGELYRDMDVGVCVGGNILGNELEQRCGA

VKGNALDVEAGGIGLGANIGMMYEFNERHRLGLSYKHSPNIDAKGDIHFAGESYDSLAMP

LPDIAEFSGYHRVLPKFALHYSVQWIGWSAFDSLKADDQLLKDFQWQDSAHYSIGATWYA

NERWTLRTGYMFDKTPVDELTSLSIPDSNRHWLSAGASYQWSSDTTVDIGMTYLIGEDVN

VEEYAYEGVPAPMVTGVTHSNAFLIGAQLSHRF

>tr|Q87QT4|Q87QT4_VIBPA Putative membrane protein OS=Vibrio parahaemolyticus serotype O3:K6 (strain RIMD 2210633) OX=223926 GN=VP1065 PE=4 SV=1

MHMIAGLLAGFPNFLLYFSVSVIFVLAFKFIYVKLTPYDEWHLIKEKQNTAAAVALSGAF

LGYCIAISGAAKNSVNIVDFMVWGVVAMLAQIIAFAIVRFILLPRVTERIEKDELPAGIV

LAAVSISVGMLNAACMTY

>tr|Q87IX0|Q87IX0_VIBPA Uncharacterized protein OS=Vibrio parahaemolyticus serotype O3:K6 (strain RIMD 2210633) OX=223926 GN=VPA0486 PE=4 SV=1

MRVSSVFKWIILLLPALIWLAVRQIEHIWWAENLTSIPALIVFTYVILASAFFIARRWLQ

SAFSAFVAILFVSLLVPPQRSHVASCSHSLSVIQFNLFYENSDINQFINYLLRHPADLVV

MQELTPRVGSLLHMLDDVYPHYHGGQRDVGYPSNLMILSRHPITEVSVYRAPNGQEVIRA

VWLPKGQAPITLFAAHPPSPRTSELWHQRNALVRTIETLTALYPEPEVLVVGDFNLSSVS

PRFSTLFPSFQTAPVTSWPTNIKGINVPEPMMIGIDHLWLKSEQGQRIICSRESSALPQG

SDHRMVTTQIGVHIP

>tr|Q87HI2|Q87HI2_VIBPA Uncharacterized protein OS=Vibrio parahaemolyticus serotype O3:K6 (strain RIMD 2210633) OX=223926 GN=VPA0983 PE=4 SV=1

MGGKLLVILLLCVFASTALGYPAHTQYWPHRSVLYFAPENDDYVKQFLLEALMHECELVD

RDVVTLVITEDGYTVPSWLKEEFDLKILAEIYSVKKGEHTAILLGKDGLEKYRWGAETDW

QYINDLIDQMPMRKQEMQRQRSPCEI

>tr|Q87JY5|Q87JY5_VIBPA Uncharacterized protein OS=Vibrio parahaemolyticus serotype O3:K6 (strain RIMD 2210633) OX=223926 GN=VPA0113 PE=4 SV=1

MGYLLWRTLRLGADGIQFIKFLVAVVQSGNEMINKAKITLLTLGVVLAGCSSVTAPKKDA

IESIQQKCAVILSSDVSDDHRWQVYNELMQEYAVHAIKTQAQLDRFEAFVQRVQSDDSGQ

LITELIEVTDWGCSNGNYLEEMDMFIQEVQK

>tr|Q87TM0|Q87TM0_VIBPA Peptide ABC transporter, permease protein OS=Vibrio parahaemolyticus serotype O3:K6 (strain RIMD 2210633) OX=223926 GN=VP0049 PE=3 SV=1

MNFLSKFRDLWIVKGQGMFSFLVKRLFQALIVMFVISLVAFAIQDNLGDPLRELVGQSVS

ESERQALRDELGLNDPFITKYTRFVGNALQGDLGTSYFFKRPAVEVILDKLVATLELVFG

ATLIIIVLSIPLGVYSAIHPKSIFTKFVMAMSSVGISIPVFLTAIMLMYVFSIELGWLPS

YGRGETVNVLGWESGFFTIDGIKHLILPCIALASIMLPLFIRLVRSEMLEVLSSEYIKFA

KAKGLNLQKIYYQHALKNTMLPVLTVGGVQIGTMVAYTILTETVFQWPGTGFLFLEAINR

VDTPLITAYVIFVGLIFVVTNTIVDLLYGIINPTVNLTGKGA

>tr|Q87PS4|Q87PS4_VIBPA Uncharacterized protein OS=Vibrio parahaemolyticus serotype O3:K6 (strain RIMD 2210633) OX=223926 GN=VP1427 PE=4 SV=1

MAHRSFYPFAKPLNEQLQTLKNRMQAHLPCIDRVSFAKYHPHQDMLKSFAESEFDTWCLA

HHEAPFRKLHNLTISAENAIPRIVDDLHDINHCSRIQTLLKEGYQSSVAIPCYDNKAFTG

FVFLNSVQPQAFKLEALNGLKPYFEMVQFAVESEDHVVHAIETLADRMQGMMPGYSPEYY

AHTKRMKHYSQLIATQLADHHHFNDETVEHIGTFAQFHALSDIQLPIEIACSRTRYTREQ

EAILLQHIEQCIESADDIVARIGNPVHPSVTLFLQMITYQYENLNGSGYPYGLKKEAIPI

AAQIVAVANTFDVLTTHHPYRQAWSIPYALLELEKWVYQGLLSRECVNALREHQNYLKQI

IHKYPEHYTGLGLM

>tr|Q87L79|Q87L79_VIBPA Uncharacterized protein OS=Vibrio parahaemolyticus serotype O3:K6 (strain RIMD 2210633) OX=223926 GN=VP2733 PE=4 SV=1

MLALSASIFLSSPALSSEKDSAFVPFYFSTDTLGSTVGVAGVAKGVGQPQAALFGMGLYS

EKDSYIGFLSAFNYALSPNVLFSTQMYQARFNDSPYYLGEQGDNNSSFESQTITNSDEQK

YKFEFKYLLPWGAVKDHGLKGAFLPSRDVRNASPLASGVSSILLTPFYSSRALDVYNDKK

EQAYGFTLGFDWDNRDSARNTTRGSHTSLNLTTGAESGQSEDLWLKWEFQNSQYYSLGPL

GDWFDQQVLALDFYTADTPTWNQCDGVVCSRPPEQEQVRLGGLYRLRGYTAGRYHGRSAI

HYSAEYRVLPDWQPLDDIPIINYYDMPWWQWVAFVEVGRVADEYDLKTLHEDMKWSVGGA

VRFQVEGVVVRAEVAKGADEGTFRVMINQPF

>tr|Q87M80|Q87M80_VIBPA Transcriptional regulator, LysR family OS=Vibrio parahaemolyticus serotype O3:K6 (strain RIMD 2210633) OX=223926 GN=VP2378 PE=4 SV=1

MASHISLKQLKVFTTITQHKTLTAASDSLFLSKAAVSMALSELEKQIGHHLFDRVNNRLI

LNQEGQKLLPLADELLNRAKDIESIFDGDQALSGQLRIGASDTIGNQVAPYLLSEFRQQT

NHRSQSLFISNSAQICDMLTDYELDIALIEGKTLHPELQSTQFSEDEMCVICAPDYPAAH

EGPVNLTELENSEWILREAGSGSREFFMRVVAPRLEHWHEAFQLNTTEALINSVSAGLGL

GCLSRLSAEPAIRDGRVKLLDMPLDMKRRFWLLVHKEKYQSPLLKTFIEFCQDWERPTNL

PLGNKSAK

>tr|Q87G52|Q87G52_VIBPA Uncharacterized protein OS=Vibrio parahaemolyticus serotype O3:K6 (strain RIMD 2210633) OX=223926 GN=VPA1465 PE=4 SV=1

MKLKLLALSLALAGVQVTNAAQWDYPSAAITVTNQAEALRYLQANYASAGELKFRYQTRS

KLGDHYNFDVWVDGQYQPQRTIVLSTNQDRQIERIFKSLEDTVIRNGEAMPAAELELPVR

LEVTEPPALSSGELVTVPVSVFDPDLRTMQQQAAPDSAWNSLEDYPQPLQYVEKQIQVLQ

SDGKFYLANERVKQVDALALLPSPAVGAEPVRDTSSILPPEGVQSFADVKAMQSTQLGDN

AFLQLMAFYHLDNSLQYLSSLSYDLFEEPLRFDGRGLALDNSSYYTGSRALMLGVGGVSP

DAADADVILHELGHGIHYQIVPDWAYGHTGAIGEGVGDYWAGSASYRTQYLDAARRGQEF

EIDTVFNWDGMFGVRRATRSLWNQRARYFEGAEYRAHESVGGELGDELWSTPLFQALKAS

VARYGDGSDRVFRDFDSIVLEGMYGVGRGVKMHDLAESTVFAATMLFPEKEYAQILKDSF

AQHNLLKVPFNARYQSRYINAGETVELTLSHTGRSASVKGNWSLNNDTSKAVDVQLANSA

SFSANLPTGLTCGAPFESKVSLDYQFAEGLKAQSWQNVTALVNGIPLLLNKPKTIEDALT

DASINSRGQEVQGNKIFVQTLSDKTQKIDDSFAVYLDIEHANLADLHITLTSPQGQSVVL

FNHQPSQSGGFTGYFTVQHDPQLQALVGEPSWGTWRLEVSDRVVGNSGALKAWGVSHFNQ

YQCGADTTSKSNGNGSGGSGSPWALFGLLVLSMLRVVRSRNNDSSKRR

>tr|Q87KA1|Q87KA1_VIBPA ParB family protein OS=Vibrio parahaemolyticus serotype O3:K6 (strain RIMD 2210633) OX=223926 GN=VP3077 PE=3 SV=1

MSKRGLGKGLDALLSTSSFAREKQQIASQSQALSADGELTELAIGQLQPGIYQPRKDMAP

EALEELAASIQSQGIIQPIVVRQVVGGQFEIIAGERRWRAAKQAGLKRVPCLVKKVEDRA

AIAMALIENIQREDLNVIEEAQALERLQDEFSLTHQQVADVIGKSRTTVSNLLRLNQLDA

DVKRLVAEKQLEMGHARALLALEGEQQVEVAQMVAKKQMTVRQTEQLVKKCLAPQNEQKA

QQEDTEAEQMSHKLSQLLDAKVSLSRSANGKAKLTISIDEPHKLDQLIAKLEA

>tr|Q87IB8|Q87IB8_VIBPA Uncharacterized protein OS=Vibrio parahaemolyticus serotype O3:K6 (strain RIMD 2210633) OX=223926 GN=VPA0688 PE=4 SV=1

MNDLLASLTGLSGFEFAHPMWFLILPLPLVVYYLVPAYRTKQMAIKVPFFSQLVEAIGET

PSEGASQLTPSWWQRATLILSWLLVVCAMAKPTVLGEPQVRESLGRDVMVVVDLSGSMAE

PDFTSRTGEKISRLDAAKEVLTEFVQSRKGDRLGLVLFGDAAFVQTPFTADQKVWLELLN

QTDVAMAGQSTHLGDAIGLAIKVFEQSDKSRGALEQDQNREKVAIVLTDGNDTGSFVEPI

DAAKVAKAKGVRVHVIAMGDPETIGETALDMDTIHRIAKESGGEAFEALNRDELSAAYDE

IGKLEPQLYESTTYRPKQSLHHYLMALVVIMHLLAFSVATLKRRAATRSSLGESDV

>tr|Q87TA2|Q87TA2_VIBPA Uncharacterized protein OS=Vibrio parahaemolyticus serotype O3:K6 (strain RIMD 2210633) OX=223926 GN=VP0168 PE=3 SV=1

MYKNTTALSVAISLALGTAAAVAPLTAQAEEQQVEKLQKMKVTGSRLTRASMEGSTPVAV

IGRAEIERAGDVSIADVLRKSSFNSFGSHNERSGSSAQSQATISLRGLGSERTLVLINGK

RLPGSASMGGGAANINVIPSAIVERVEVMADGGSAVYGSDAVAGVVNIILKEEFDGINVT

LGSGIPSREGADEENVSIVLGTSGEKGNIMFSFEHDSKDEIYQRDRDYLSSTNTGSANYF

DMSGVSIYGRNVYHDGQLKALKGYDTDASCDPSKGFVGLTNYPGLGDICGYDYTSEAAQT

ASLERNTVFMNGNIFLSDDTTFNAQVLLNRNESFGRFAPAAGYFEVDPTTTGGAAFFAEN

GLDATKGPAAVYYRFNNVGTRDNSVTDFQADLKAGLDGTLYTDSFGEVIWETGYHLNFSN

SNETGTGYVFRSPAEQLANSGQFVNGEFSADATSQLSAGTGRETQMEMHQVYGGLQFDLA

EVGNIVIPLYVGAEYTTYDYFDQYDPQSEAGNIIGSSGNSAGGDRETYAIYAESLIAFTD

ELEMNIAARYDHYSDFGDEISPKVSFRYQPLDNLMFRTGAGLGFRAPTLSDLYGADSFSS

DYAKDYVFCSQNGISASECPETQYDVTRTSNEDLDAEKSVSFNFGISYSPIDDLDLTLDY

YNISIDDVITLNTLQSMIDQERSSGVSNPNIVRENGRIKEATAGLQNLGTLDTSGLDLKI

GYRYDFNVATIRYDFTGSYVLDYSSPEYVGGPVNNQVGRNGLPEYRFNTGVGVNFLEEHD

IYLSADHIADQAQDVDDNYNKTGHISSQTTWNIAYNYLAPWDAKFTAGVRNLTDEDPAFE

SDGVTYDKDLYSIQGRVYFLKYSQNF

>tr|Q87KG0|Q87KG0_VIBPA Putative transmembrane protein OS=Vibrio parahaemolyticus serotype O3:K6 (strain RIMD 2210633) OX=223926 GN=VP3017 PE=4 SV=1

MTDSVKSLISRYDEYTNSLQTHAMPLLLLFCRLWVAWVFFNSGLIKIASWDSTLYLFEFE

YQVPLLPWEFAAYSGTFAELVLPVFLALGLFTRPMAAMLFVFNIIAVLSYPLLWEKGFHD

HQLWGLMILIVTLWGAGPFSLDRLLKDKFVR

>tr|Q87Q57|Q87Q57_VIBPA Uncharacterized protein OS=Vibrio parahaemolyticus serotype O3:K6 (strain RIMD 2210633) OX=223926 GN=VP1293 PE=4 SV=1

MINQIINILFPVFALVGVGYLVGRHIKPDFRPINRINIDVFTPALVFSSLVSMPLDSEQG

PLLLAAIIAVLIPGLLMIPICKITGLTFKAWAPPHMFRNSGNLAIPLFTYTFGEVALSSA

VLLFVVSACLHISVGVMLLSNGNPLKQIMKMPIFLSASLALCLNLSGIGAWTPLYEATAL

LGQAAVPVMLLSLGSQMCHLRLDGLKVGVLCTVQSLTTGAIAFAIIYWLIPLPTMQLQMM

VLFTMLPPAVMNYLFAERLNIEPMTVASMVLFGNFFSVLTLPLLLSYTLSLT

>tr|Q87R31|Q87R31_VIBPA Uncharacterized protein OS=Vibrio parahaemolyticus serotype O3:K6 (strain RIMD 2210633) OX=223926 GN=VP0967 PE=4 SV=1

MKKSIIALLGLAVILGGCSNKVSYGDSQATETTTIDFGSTDLQKIAGEMVDSMMMSGSVA

AITRDQRPIVFVERIKNKTSEHIDTESITDTISTKMLNSGKFRFVDMDRVESVREQLNFQ

NTDELVNQSTAIQFGKMVGAQYMLYGNLSSIVKNAGSDKDVYYKMTMRLMDLETGLIEWA

DETEIRKEQSKSLFGL

>tr|Q87KT4|Q87KT4_VIBPA Uncharacterized protein OS=Vibrio parahaemolyticus serotype O3:K6 (strain RIMD 2210633) OX=223926 GN=VP2892 PE=4 SV=1

MTHINIHLACKKASESFSPHNSARHSIQSIEIEIEIEIEIEIEIEIEIEIEIEIEIEIEI

EIEIEIEIEIEIEIEIEIEIEIEIEKQTGYVFPLQACVVSSFVRHCPSFFTFRQRQKSPS

PK

>tr|Q87PV0|Q87PV0_VIBPA Uncharacterized protein OS=Vibrio parahaemolyticus serotype O3:K6 (strain RIMD 2210633) OX=223926 GN=VP1401 PE=4 SV=1

MSVIEIERLLAPISDVNPVGEDARYEFCYEMMESEVKKFGSLFGETVDWSVVKTHATEVL

EHHSKDLKAICYLVRALTEEFGLQGFEQGLKLLSEALNRFGVELYPSRKRGRDGAVEWLN

HQFKLVSSRFAESAQSWDLVSGCISTIEEVQRQYDDIYQDSEADFFEIRSQLNALSQQAA

VDEQSDGAMATIEQTAPLTASQPVAVETPAQSEVQTVVAKAVPSAPKPAPVKKTVTKEVD

VDTDFSSPTASKRTLKKVAEVMIHANPSEPLAYRIYRHLTWDDIDGLPDHQSNETPLSLA

VSSDQQAEYRDKASQESDIDTVKRLERTLTDAPFWLTGHYFVYSMLNNLGFNDAAFAVKQ

EVKRFVDSLEGIELLTFKNSIPFADEATLSWLSTQAATSSSAQSVVQTIVITDEDSLPME

DITLANLGEYAAELAHKLELDFSGRGQFMLHLQLIAAYQSVGLYPLCLPYLEKAWEVQQA

FNLASWEPHLSSQLEDLIRKTLHQLFRNKDLLPEQYEKWKAIYD

>tr|Q87HB3|Q87HB3_VIBPA Putative TetR-family OS=Vibrio parahaemolyticus serotype O3:K6 (strain RIMD 2210633) OX=223926 GN=VPA1052 PE=4 SV=1

MARKANFDREEKLLVAMDVFWRKGFANTSISDLTDELNINRFSLYNTYGDKQQLYYEALD

AYLKKVSLPSLTDLEKDGASLPELESFLKSFAELQRKRSCGCFIQNALVEHAGEDDDVLR

KGHFLFDHLIDIIADALTNAQKETLVPKTLKPEELARFVLNNMQGMRVLGKAKRYADLDT

ALSCLLVLIRK

>tr|Q87LC2|Q87LC2_VIBPA Cell shape-determining protein MreC OS=Vibrio parahaemolyticus serotype O3:K6 (strain RIMD 2210633) OX=223926 GN=VP2690 PE=3 SV=1

MKPIFGRGPSLQLRLFFAVIVSASLMLADSRLDTFSNVRYLLNSMVAPIQYAANMPRSMF

DGVFERFNTRQALVEGNRNLKREVLRLKSELILLDQYKEENQRLRKLLGSSFVRDEKKVV

TEVMAVDTSPYRHQVVIDKGQIDGVYVGQPVINEKGIVGQVTFVAAHNARVLLLTDAKNA

IPVQVIRNDIRVIASGNGEMDEIQLEHIPTSTDIQVGDLLVTSGLGGIYPEGYPVATVTN

VDHDTRQEFASIKAEPVVEFDRLRYLLLIWPNEDRQHKVLQSNANDGLEQEQEVTNGQ

>tr|Q87MQ0|Q87MQ0_VIBPA Inner membrane protein OS=Vibrio parahaemolyticus serotype O3:K6 (strain RIMD 2210633) OX=223926 GN=VP2181 PE=4 SV=1

MEFTIRIKRYFFNIVGGLCIALGIAGIALPLLPTTPFILLASACFMRGSPAFHHWLHNHK

TFGPILDNWHRHRAVSPKVKQRGAVFITLSFTVSIIVAPIIWVKIALLVMLIVLLSWFMR

LPVIELVADREENH

>tr|Q87R42|Q87R42_VIBPA Uncharacterized protein OS=Vibrio parahaemolyticus serotype O3:K6 (strain RIMD 2210633) OX=223926 GN=VP0956 PE=1 SV=1

MIRINEIKLPLDHEEGALLDAITKKLGIPAEKVISFNVFRRGYDARKKTNIHLIYTLDII

VEGDETALLAKFANDPHVRQTPDMEYKFVAKAPENLTERPIVIGFGPCGLFAGLVLAQMG

FNPIIVERGKEVRERTKDTFGFWRKRTLNPESNVQFGEGGAGTFSDGKLYSQVKDPNFYG

RKVITEFVEAGAPEEILYVSKPHIGTFKLVTMIEKMRATIIELGGEIRFSTRVDGLHMED

GQITGVTLSNGEEIKSRHVVLAVGHSARDTFEMLHERGVYMEAKPFSVGFRIEHKQSMID

EARFGPNAGHPILGAADYKLVHHCKNGRTVYSFCMCPGGTVVAATSEEGRVVTNGMSQYS

RAERNANSAIVVGISPEVDYPGDPLAGIRFQRELESNAYKLGGENYDAPAQKIGDFLKGR

DPSQLGDVEPSFTPGIKLTDLSKALPPFAVEAIREAIPAFDRKIKGFASEDGLLTGVETR

TSSPVCIKRGKDFQSVNLKGFYPAGEGAGYAGGILSAGIDGIKVAEAVARDIVAAMENA

>tr|Q87GI2|Q87GI2_VIBPA Uncharacterized protein OS=Vibrio parahaemolyticus serotype O3:K6 (strain RIMD 2210633) OX=223926 GN=VPA1334 PE=4 SV=1

MRTLREIVYKTLVDNLNVSPSLLSRVENNEPVSIELANGEEIFIHLNEHVLQSFIEIPLK

DSRNLRYKAPKIIEVLQEDSDIFMNIQKDKVILVTEIDKNTHSIEKELSAKLTLFNKMAS

EVKL

>tr|Q87RD0|Q87RD0_VIBPA Uncharacterized protein OS=Vibrio parahaemolyticus serotype O3:K6 (strain RIMD 2210633) OX=223926 GN=VP0867 PE=4 SV=1

MASINGLPPSLIPGTNRTNKVGKKGQVKKAQNKQSVGQPSKVANAVAHSIKQVDESQIHR

AQVQYDLPEGNARKAMEQYMDVMNRAKKEELAQLLGVDIYV

>tr|Q87SS3|Q87SS3_VIBPA Uncharacterized protein OS=Vibrio parahaemolyticus serotype O3:K6 (strain RIMD 2210633) OX=223926 GN=VP0349 PE=4 SV=1

MKILYGVQGTGNGHIARARAMSKAFESHDVQVDFLFSGRAPEKYFSMEAFGDYQTRRGFT

FVTEKGSVNYAKTALNNNLIHFFKEVNQLNLAPYDLIINDFEPVTAWAARQQNKPCIGIS

HQNAFRYPVPLKGASWLDKSVLEYFAPSQHQLGLHWYHFDQPLLPPIVHTLDNQADNDDF

VLVYLPFESIEDICDLLFHFNQQTFICYHPEVIEVEKVENIELRPLCHTNFQHHLHRCCG

VIANGGFELPSEALSLGKKLLMKPLAGQFEQQSNVATLEDLGLATAMDSLDISTVRQWLK

EQQAESVKYPDVAQAIVNWVLQGAWDSQEELSKQLWEQVDFPSYVSNI

>tr|Q87FM4|Q87FM4_VIBPA Putative FecB OS=Vibrio parahaemolyticus serotype O3:K6 (strain RIMD 2210633) OX=223926 GN=VPA1655 PE=4 SV=1

MKNKKQFTIKHFSTVLLMLLSSALMSFSAFSQARSVQDEQGTFELEAIPQRIVVLEFSFV

DALAAVDVSPVGVADDNDAIRVIPAVRAKIEPWQSVGMRSQPSLEAIAVLKPDLIIADAE

RHCAIYQDLQRIAPTLLLKSRGETYQENLESAQKIGVAIGKQAQMTQRIELHKQTMAEFK

QHFATQETIQFGVVSDKGMWLHSPVSYAGGVLSTLGIQSPLAPSERNAYIPTSFELLLKT

NPDWLLVGLYSQPNIVDEWRRNPLFKLLTASKKQQLVEVSPELWSLNRGMLAAEEIARNL

EALLGRS

>tr|Q87PN3|Q87PN3_VIBPA Putative hexosyltransferase OS=Vibrio parahaemolyticus serotype O3:K6 (strain RIMD 2210633) OX=223926 GN=VP1468 PE=4 SV=1

MKLISNNPDLPIVGEVWIFVDSRIFGGIESHILELAKGILSFGHAVRIVLPTEYIPQAQL

VEKAQAANIPTSYLPQISGVPLNTIAIKHLTSAVAHHQPSVLHTHGYKASILARTAKLLT

RTFPRLVSTYHAGESSSGKLWLYDGLDRCSGYLSDHCFAVSKSIQDKVLCPSQLLNNFVA

LPSMNNSYQEISFVGRLSHEKGADQFIELAKACPDYRFSIYGDGPERQNLETTAPANVIF

HGHQTDMNAVWENISVLIISSRFEGLPMAALEAMARGIVVISLKVGRLPDIIQSGNNGFI

ADDVPALALNLQYWMTMEKSQQERIRKNARQTIVEQFSTESVIPVLLTQYQIEMDN

>tr|Q87MQ9|Q87MQ9_VIBPA Uncharacterized protein OS=Vibrio parahaemolyticus serotype O3:K6 (strain RIMD 2210633) OX=223926 GN=VP2172 PE=4 SV=1

MLAQWFVSVCRILTNLLSILCRPNLQFLLREVTLLLKKLTKSDLDILNSMKNVVDGIARM

YGEHTEVVLHSLDAEAPEIIKIANGHVTERSEGAPITNLARMKLREGKDVSDSYLTKTSN

GKTLHSITTIVRNPKNKPIGLLCINVDMDAPMQAFLKAMLPQQHTECCVGGARSPETFAR

NIDETIISTIETVQTEVWENEAIAPSKRNRELVTRLHGLGIFKYKDAVLMVANHLGISRD

TIYLYLRELGND

>tr|Q87LJ2|Q87LJ2_VIBPA Uncharacterized protein OS=Vibrio parahaemolyticus serotype O3:K6 (strain RIMD 2210633) OX=223926 GN=VP2620 PE=4 SV=1

MSKWITALLISLLSLPTFAEQFKNMKDIEVHYVAFNSTFLTPKVARSYDIKRNGYVAVVN

ISVLDSASLGKPAVEAKVGGHAKNLIGQTQKLTFREIKEGEAIYYLAELPITHEETFTFD

IDVKAGNKGSGKLKFTQKFFVEE

>tr|Q87PI7|Q87PI7_VIBPA Formate dehydrogenase, cytochrome b556 subunit OS=Vibrio parahaemolyticus serotype O3:K6 (strain RIMD 2210633) OX=223926 GN=VP1515 PE=4 SV=1

MLNTLKRASLAMLSMMTALLLTFAMPASAEEQSSKVVQQEITQLAGADYWREVREGQEGY

TTSQSPEHGVLISKPGETWYILKEKWMSPAGAVAIFGSIAMVIMAYVFVGPLMLSKPRTG

KKMKRWSRLDRALHWSMAFTFLTLAFSGLMLVYGKHFLKPYVPTEFWGFIVMLAKQYHNY

MGPLFFILLMFVLLKWWRKSIPNMTDIRWFMKMGGMVGKHKGTHPSAGFSNGGEKAIYWL

LIFFGAIVAVSGLVLDFPIFGQTRRDMELSNLVHAISALILICGFIFHIYIGLFGMEGAL

EGMVTGEVDETWAKEHHDLWYEEVKSKEAMGETESKPTTEKGAKANEQTS

>tr|Q87PM6|Q87PM6_VIBPA Uncharacterized protein OS=Vibrio parahaemolyticus serotype O3:K6 (strain RIMD 2210633) OX=223926 GN=VP1475 PE=3 SV=1

MVNLIRRSVVSGLVVGMFGCSSFDYPDHGQGGLAESYQDISIENYQFSPVMPDEPLGPEH

GLRFDWQLTKLHLDALIQEGARWCFPAAVVQALEKQNRIARELEGGLLLDAANDLVIQRR

RLNQLEQQLDYVLTQTTCTPPDDIDALRNDLNIVADIYALLNVDNQFAIDSAEINPKYMG

HLAEAAYILRDHPALSLVVTGHADVTGTPEHNKKLAQERAEQVKRYLSVFGVTSDRVQTK

SVGDTLPLYEGQTPAVRLTNRRVSIEVLSANSDSNAPSNHNLPNKGNMPTSASGSIGGAL

>tr|Q87IZ4|Q87IZ4_VIBPA Uncharacterized protein OS=Vibrio parahaemolyticus serotype O3:K6 (strain RIMD 2210633) OX=223926 GN=VPA0462 PE=4 SV=1

MICLIGGDNFCHHSMSRYETRLKSHRVKPEKLAFKASFPIIETHLTLVSLEVSMELEAVA

KALKELGHPTRLCIYKEVVKAGYKGIAVGGVQEKLGIPASTLSHHISSLASAGLITQRRE

GRILFCVAEYECLDDVIDFLRAECCVNECQ

>tr|Q87QM6|Q87QM6_VIBPA Cyclopropane-fatty-acyl-phospholipid synthase OS=Vibrio parahaemolyticus serotype O3:K6 (strain RIMD 2210633) OX=223926 GN=VP1123 PE=4 SV=1

MLNTTSITLPRKLTTTQKAARGVIFQCLQKMEMGCLTIIESFHTETKERSERFTAPHGEY

SGQPVVATIEVKHPGFYSRILQGGSIAAGEAYMDGWWDSPDLTALMKLMALNIRALDKLE

EQGSWLTKLLYKFSHWTNRNSQENSRKNIHAHYDLGNDLYEAFLDTNMLYSSALYHTSDD

SLEQAQINKMERLCQQLDLQSTDHVIEIGTGWGAMAIYMAEQYGCRVTTTTISEEQHEYA

RQQIVQRGLADRITLLKEDYRNLTGTYDKLVSIEMIEAVGKQYLASYIKKCESLLKPSGL

MAIQAITIADQRYDYYSNNVDFIQKYIFPGGFLPSVTSLTQATTKHSDLVVRDLFDIGLD

YAKTLNEWHRRFNRAEDAVRGFGYDERFVRMWRYYLSYCEGGFLARTISAVHMTFQRP

>tr|Q87PJ4|Q87PJ4_VIBPA Uncharacterized protein OS=Vibrio parahaemolyticus serotype O3:K6 (strain RIMD 2210633) OX=223926 GN=VP1508 PE=4 SV=1

MSDKKDFENNYEKTEDAWPIGVELVEHEISTGIWVTTQWQLTGFALNPTEETPDVCLLQL

HKDERTDYRFNLSSQQPKLFLVMDNVESGEKPAIQLLTASQSVAAQYMDGDNLVLSNDMP

LAVQAWMEAFIGRHGELLEVRRKKRKGAGRANGN

>tr|Q87K72|Q87K72_VIBPA Uncharacterized protein OS=Vibrio parahaemolyticus serotype O3:K6 (strain RIMD 2210633) OX=223926 GN=VPA0026 PE=4 SV=1

MKIQSLLPVLLLCAASANAADNIRIEYMPAETTHDKLAEQSIQSSDVNPIFVRLSQDYFP

FRKPLTLIYGGEDGPMYDPDTHTIHIPYTFYLESLNYFSNNQYEDRYGKSPKTGALDTLL

HTLLHEAGHAYIEDQSIPVLGKEEDAVDNFATILLIDYLDDGADMAISAADMFAFESDDR

PDYYDFGEYIDEHSFDLQRYFSTLCLVYGSDPEQYKSLLDEVEKDYLRDRKDFCQYNYEN

IRTNWQHYLQHNEPKEASTRKNSEKPSSSPNPMTN

>tr|Q87J24|Q87J24_VIBPA Uncharacterized protein OS=Vibrio parahaemolyticus serotype O3:K6 (strain RIMD 2210633) OX=223926 GN=VPA0429 PE=4 SV=1

MDQQVKQERLQGRLGPEIKEFRQERRTLQLATVDSEGRPNVSYAPYVQNQEGYFVLISQI

ARHARNLLENPNVSLMMIEDEDSSKQLFARKRLTFDAVATVVERDTEMWQLVVGQMKARF

GDIIDGLSQLEDFVLFNLKAEKGLFVKGFGQAYQISGDDLVDFVHLQEGHKKIENA

>tr|Q87L97|Q87L97_VIBPA Uncharacterized protein OS=Vibrio parahaemolyticus serotype O3:K6 (strain RIMD 2210633) OX=223926 GN=VP2716 PE=4 SV=1

MASSTKMLVTPEQALLTTRRVFGLFSMMVAQRLIASKLATLVPPNLATLNALTAFLPRLP

KKYTHPMFCEERNRAKREVVRLSFENVPSEALHQTVLMTVDGTQPKSTDKPNPAILTYLG

TTPPE

>tr|Q87LB8|Q87LB8_VIBPA Putative type IV prepilin, MshO OS=Vibrio parahaemolyticus serotype O3:K6 (strain RIMD 2210633) OX=223926 GN=VP2694 PE=4 SV=1

MKTRGFTLMEMIVTIVIGSFIMLGIAGYVQLGMKGYADTIDRQRMQTQAQFVLEKMSREI

RHAVPNSFHVPTGSTNCLEFMPIEYSGFYTLTNNNLEFLLGNDSTLNPIPADRWMVINPS

RYEDLQSASPQSLAVGNLAKTGDVFVVSGAASTIGGTSISSRHYIYQNNSEVRYCFDNSQ

ITRNGITVADSVDTSVSDMHYLEPTLQRGGIVHINLEFTQNGERSVYQQDVQVLNVP

>tr|Q87HN3|Q87HN3_VIBPA Molybdenum ABC transporter, permease protein OS=Vibrio parahaemolyticus serotype O3:K6 (strain RIMD 2210633) OX=223926 GN=VPA0930 PE=3 SV=1

MTELEYQALMLSLKVGAYAVLWLIPLGVFLAWLLSRKEFFGKSILDSLIHLPLVLPPVVI

GYLLLVSLGRQGFLGRLLYEHLGLVFSFNWKGAVVACIVVALPLMVRSVRLSLESVDPKL

EHAASTLGASPLKVFLTITLPLTIPGIITGTMLSFARSLGEFGATISFVSNIPGETQTIP

LAMYNFIETPGAEMEAARLCIISIALALSTLMISEWLNRKAASRLGAKR

>tr|Q87J36|Q87J36_VIBPA Uncharacterized protein OS=Vibrio parahaemolyticus serotype O3:K6 (strain RIMD 2210633) OX=223926 GN=VPA0417 PE=4 SV=1

MRFIPLALALIAFSSSPSAYELDYYSKFGHIDNYGNLDLRNKPYTELPSGLVIKGNVDIS

QTPITQLPKGLDVGGSLDAKNSALKTIKSGVKIKGYANLLGSKIESWPKGVTLGGYLNLT

DTPLKTLPAKLRVKGDLSVIRTPLKALPEGLVVDGNLYIGGSALTEFPDTMTVKGNIFLG

GNKIIKWPKNLTLGGAVAR

>tr|Q87QJ5|Q87QJ5_VIBPA Putative transcriptional regulator OS=Vibrio parahaemolyticus serotype O3:K6 (strain RIMD 2210633) OX=223926 GN=VP1154 PE=4 SV=1

MFTIEQLQAFVATFESGSFSAAARKLGRAQSVISQHIMNMELDCGVDLFDRSGRYPKLTE

NGHALMPYAQAAISQLDRLNNKATQLFSAQASELVLAIDEGIPLTRLPDVLKNLEQQFPQ

LQVECLTASSPDIIELVKSERATTGIILSDLQMPRHIDFTNLGNIAFDVYVSSDHPLAAQ

RITHIDQLKQYRQLVIRSKSAEPGSLNQALSPDIWYADNYYILLELANKGFGWCFLPEHL

VADSPNTLKKVGDDFTKLAWQVNVDLIQHQKWHSDPLHQQAKAELITLFEQTVK

>tr|Q87RA2|Q87RA2_VIBPA Putative acyl carrier protein OS=Vibrio parahaemolyticus serotype O3:K6 (strain RIMD 2210633) OX=223926 GN=VP0895 PE=4 SV=1

MEQLHNDIKQLIINALNLEDLTVDDIETDAPLFGDGLGLDSIDALELGLAIKKQYNIVID

ADDSNTREHFASVANLAKYVSSQTNE

>tr|Q87SI0|Q87SI0_VIBPA Ubiquinol-cytochrome c reductase, cytochrome c1 OS=Vibrio parahaemolyticus serotype O3:K6 (strain RIMD 2210633) OX=223926 GN=VP0443 PE=4 SV=1

MKKWIVILFAMLPSLAMAAGANVPLDKANVDLTDKASLQNGAKLFMNYCFACHSTQYQRY

ERVATDLDIPADLMKENLIFNPEAKIGDLMVNAMPPKQAANWFGAPPPDLTLVARVRGAD

WLYTYLRSFYVDPSRPFGVNNIVFPSVGMPHVLEELQGIPTPVYGTKMVDGEEVKVVVGT

ETNGTGELSEGEYDKAVGDLVNFLVYAGDPVQLERHALGWWVMGFLVLLTIVVVMLKKEY

WRDVH

>tr|Q87HW0|Q87HW0_VIBPA Uncharacterized protein OS=Vibrio parahaemolyticus serotype O3:K6 (strain RIMD 2210633) OX=223926 GN=VPA0846 PE=4 SV=1

MMSISQLLGCISKQVDGQYIAQYEELTLRSVFQPIYKKDLSIIGLEALVRISTADGSMIR

PDLFFQSPSISEHVQLNVERLSRLIHIKNFGQSRYRNKKLFLNVLPRAAEMLAKDLSYSH

LLKQVICEADLCREQIVMELVELSAGDESFLYKATKELSDGGFKLAIDDYGINASTIERV

KCVRPDIIKMDRSLLLKYEDGDFSALIEALALAKELSSKTVIEGIETEHQLNLMKKLGFD

MYQGYLLAMPQTLEMYEEAKTA

>tr|Q87I63|Q87I63_VIBPA Putative response regulator VieB OS=Vibrio parahaemolyticus serotype O3:K6 (strain RIMD 2210633) OX=223926 GN=VPA0743 PE=4 SV=1

MSFDRNLCKVLVADDSRLVTSSVTAILRQLDLSDIYYAYKPFEVINLCKQMHFDLVICDY

NFQTQLNGFQLLEELKHAQILPAHTTFVFLTGENDHKIVRSIVDCDPDDYLLKPFNHTFF

RNRLLSAMKRRSVLLPIYEKLREMDFEGVIEATDTLLPFHPEYSKLIRRYRAHAMVQNKQ

FSSARSEYEKLLKEDNFDWIKTALANTLIETDNLEKAQEVLESLSSKKENPYYHDEMSNM

AVLSEDIPKAIKHLKQSTMLLDAGSERELVITNLSLASESYDDAVTYIKRYYEKNENTFR

GGIFTKLNFVRCHLYRSLNAPSNQCFENLLFGLNPLIGEIEKNSQFKAQFALIDAHIALI

RGDLKAAISQVRAVLASNQLSHFYDLYHLCVLLERCSFLSEMKSMLPRARRAIAEAQHPS

IFRSQVHMLKSLEMRLQESQTKIEQIRRQITAQKSISTSAITPHFDRYFQLHDLLPHSKK

ICLAIVRLASLRPFEYRGEYAIYRKLEHCDRVIRNLCDNRELQSIKYNNMYQYAKQNITV

SNG

>tr|Q87LI4|Q87LI4_VIBPA Membrane-bound lytic murein transglycosylase C OS=Vibrio parahaemolyticus serotype O3:K6 (strain RIMD 2210633) OX=223926 GN=VP2628 PE=3 SV=1

MKKIAFIATALLLAGCSREFVEKIYDVNYEPTNRFANNLAELPGQFEKDTAALDALINSF

SGNIKKRWGQREVKFAGKSNYVKYIDNYLSRAEVDFQKGLITIETVSPTEPQKHLKNAII

TTLLTPDDPANVDLFSSKEIKLEGQPFLYKQVLDQDKKPIQWSWRANRFADYLIANNIKT

KDVDFKKAYYVEIPMVEDHFSQRSYQYADIVRRASKKYDIPEDLIYAIIKTESSFNPYAV

SWANAYGLMQVVPKTAGRDVFKLVKNKSGQPSPEYLFNPENNIDTGTAYFYILKNRYLRE

VQHPTSLEYSMISAYNGGTGGVLNTFNRSDRKRAMRDLNSLQPNQVYWALTKKHPNAEAR

RYLEKVTNFKKEFNSEHTL

>tr|Q87FN1|Q87FN1_VIBPA Uncharacterized protein OS=Vibrio parahaemolyticus serotype O3:K6 (strain RIMD 2210633) OX=223926 GN=VPA1648 PE=4 SV=1

MKKLLLATAVVAAAGAAGYLYQNNLPQSGSSSLLAQIPADSLVVTFQTEPFKHYEYVNAF

GSANQMTMSDMFVGEQLSPKEQFAIDLFDGYLKSAASPETLKAYLGTGDEINPVLYTLGM

VPVYKLPIENPEALWKTLDHEEQMSGVTHEKVKLGTVEYRRYEMTDAVDPQDGIGLVVAV

IDNVLTVTVDIAELGDLNPLKMALGLESPTQSLADSGRAEAIQTQYGKNNNSFGYIDHRE

IIKGLTTVDGNMFARQLTRLNVDPNITEMRTPVCHNEFTQIAENWPQSVAFAEYKLNGEQ

ASIKGGFVVESKNKVIVDALKSIRGVLTESNSDKSFFSLALGIDINTLAPSIGQIWTEIT

QPQYQCALLSQMQKDMGGQNPAAAVSMGSGMLNGLKGLSMQLFDLNINPDAQFGEVFSQM

DGMFTLSAKDPNMLIQTAQMLVPELAQLQLQPDNQPVNVSDLLEMHTGMKIDVFARLNGS

HLTLYSGEYAAEASKDVMAQELKPNGLLSFSMDSERILEVLEKASMATGQPLPEDVLVSF

QNELTGGMKMDVTDKGIGFEFDYRSSAKKVSVAQQ

>tr|Q87HD0|Q87HD0_VIBPA Uncharacterized protein OS=Vibrio parahaemolyticus serotype O3:K6 (strain RIMD 2210633) OX=223926 GN=VPA1035 PE=4 SV=1

MTSIHSKLSRVRKPRVHITYDVETEGTTLKKELPFVVGVMGDFAGQNTEALKPLKDRRFI

QIDRDNFDDVLKKMSPSVKFKVANKLANDDSEFAVELSFKSMQDFEPAAIVNQVEPLRQL

METRNKLRDLMTKVDRSEELENILEEVLNNTDNLAKLAGELDLGKKAPSESTEGAE

>tr|Q87P78|Q87P78_VIBPA Uncharacterized protein OS=Vibrio parahaemolyticus serotype O3:K6 (strain RIMD 2210633) OX=223926 GN=VP1640 PE=4 SV=1

MYKLIALDMDGTLLNSDKAISEENKHAIAKAREAGVTVVLASGRPLEGMQAKLDELNIHS

EKDFVLFYNGSMVKNVGTNEIIHQQIIDGKAAKLVARKAKELGAYVHAFSQVHGLITNEN

NPYTDIEANINGLDVTEMNFESLEDDHPIIKAMMVAEPSKLTEVIAALPSEMREEFTVVQ

SAPFFLEFLNPASNKGIGVAAIAEYLGIQPEEVICMGDAENDHHMLKYAGLGIAMANAME

ETKKIADYITESNDDHGVAKAIEKFVLNA

>tr|Q87TB7|Q87TB7_VIBPA Putative exported protein OS=Vibrio parahaemolyticus serotype O3:K6 (strain RIMD 2210633) OX=223926 GN=VP0153 PE=4 SV=1

MKAVICGAVLTMAISLPTVAQEELKGCDAKAFALEQQIEYATVQGNQKRIDGLKRALAAI

EDECSEEDLREKLQAEVEQKAQKVKARELELAEAQTSGSSDKIEKKRRKLDEAQQEWLDA

KRELEWNYGQ

>tr|Q87GH1|Q87GH1_VIBPA Uncharacterized protein OS=Vibrio parahaemolyticus serotype O3:K6 (strain RIMD 2210633) OX=223926 GN=VPA1345 PE=4 SV=1

MLNVNLNATNQSLVTPPSTTAAQESTLPEPVLTLLNDSTTLEAPYSPETLQTQLQQQKAA

ARSTADDPRNKESDISDSLLDAINQSAEDLSVLQGWTDGGSGMFNAANKVMFEALKEAVQ

SNDSTKKGFALEDLFQLAVIDFMANSASKTDQAMKDKLAHYLESTGSGSHGVHENWNGQK

FADELQSVWDHIKTHAPENSLSKNILNYLETQAGGIDQLKIQYSTNFDARNGYAFRNNYD

KENFGLSPMLRLAVMAKFLTKKPDIVQADLEKLMIGTFKEMEDIITKHFGSETVENLLTD

GNSNGWQIINASGTTDKIVDWNGTGLDLQYFKDLYTKFPSRVLGDEDIKEINRIGDNVKM

IQQTLKYWYHILRDERLSIARNI

>tr|Q87I44|Q87I44_VIBPA Putative 2-oxoglutarate/malate translocator OS=Vibrio parahaemolyticus serotype O3:K6 (strain RIMD 2210633) OX=223926 GN=VPA0762 PE=4 SV=1

MNSSQMKMILLVAIGCILWFIPTPEGLTDQAWQMMAIFVTTVLSLILAPLPLGAMALMGL

TLATLLGVLPIKTALTGFAHPTIWMIAAAFFISRGFITTGFGRRVGYWFISKLGHNSLGL

AYGLVLTDLLFAPATPSTTARCGGIISPLFRSVASAYDSDPEKGTENRIGAFLVQCIFQC

NAITCAMFLTSMAGNPLAANFAKEQGVEITWAGWATAAIVPGLLCLFLIPLVMYMVFPPE

LKKTPEMREIARQKLAEMGKMTRDELMVCITFIGMVSLWVLGPTLGIHATVTALLGLVFL

LLTHTITWDAVLGEKEAWHTITWFAVLVMMAAQLNKLGFISWFSESMAESLSGFGWVTTI

VVLLLVYYYSHYLMASAMAHISAMYSAFLAIAISAGAPPMLAAIVLGIFSNLYMSTTHYS

SGPAPILFGAGFHTLQNWWKIGFIFSLIVIPIFVFVGGAWWKLLGLW

>tr|Q87HL7|Q87HL7_VIBPA Uncharacterized protein OS=Vibrio parahaemolyticus serotype O3:K6 (strain RIMD 2210633) OX=223926 GN=VPA0946 PE=4 SV=1

MLNVIPWESLFGGILLGISATILLLVNGKIAGISGIMNGIMSPKKGDYSWRLLFAVGMIA

GGLISVLMLGVAAPSTANLSLGMVIAAGLLVGIGTRLGNGCTSGHGICGMGRLSKRSIVA

TCVFMAVAGLTVFVRLHLV

>tr|Q87KH3|Q87KH3_VIBPA Probable queuosine precursor transporter OS=Vibrio parahaemolyticus serotype O3:K6 (strain RIMD 2210633) OX=223926 GN=VP3004 PE=3 SV=1

MSNFTPAQQRNALIYLVLFHLVIIASSNYLVQLPFTIFGLHTTWGAFTFPFIFLATDLTV

RIFGAQLARKIIFLVMLPALAVSYFLSVVFFEGQFQGFGHLGEFNLFVARIAIASFMAYL

LGQIMDVHVFNRLRQMKQWWVAPTCSTLFGNALDTIAFFAIAFYQSPDPFMAEHWTEIAL

VDYGFKLVISLGLFVPMYGVLLNYLIKKLTAVNPDFKVSTAA

>tr|Q87K76|Q87K76_VIBPA Uncharacterized protein OS=Vibrio parahaemolyticus serotype O3:K6 (strain RIMD 2210633) OX=223926 GN=VPA0022 PE=4 SV=1

MSYRVTLLNATGALSSVADYLYEQLNSLSLQLADHFELNNIDVTISPFGHGDVPQSGIGG

YCLSPYRVEVLLDTQRTDIKTVIENELAAVLAHELHHLFRMRAGENGLTLGEVLIMEGLA

CHFERQVNGGIIPSLFESIKDRDWRPFYTEMKDKLTSLDYNFDTYFLGSDESRWPKYMGY

WVGYNLVAEYLANFHGSELDLVGAKAEIFYQ

>tr|Q87HP4|Q87HP4_VIBPA Transcriptional regulator OS=Vibrio parahaemolyticus serotype O3:K6 (strain RIMD 2210633) OX=223926 GN=VPA0919 PE=4 SV=1

MKQTLLLVEDDKNLADGLLVSLEQAGYECLHVERIADVEPQWKKADLVILDRQLPDGDSV

QHLPEWKKIKDVPVILLTALVTVKDKVAGLDSGANDYLTKPFAEAELFARIRAQLRAPDS

ADQANADKVMTKDLEIDRATREVIFKGDLITLTRTEFDLLLFLASNLGRVFTRDELLDHV

WGYNHFPTTRTVDTHVLQLRQKLPGLEIETLRGVGYKMKA

>tr|Q87NQ3|Q87NQ3_VIBPA Uncharacterized protein OS=Vibrio parahaemolyticus serotype O3:K6 (strain RIMD 2210633) OX=223926 GN=VP1815 PE=4 SV=1

MSSLLVARHRATILSRTKLCKQQTLCALMHLNQAHSQSDYSMKPLGTNNAVENGLTTPKN

AFGKPISQCELSTKSLKPRGTYNTRENVISASKSAFVPTSSPSGVATKNRLSIVLLCQDQ

RTKQPLGHFQNLGLKIAKTEFW

>tr|Q87IE5|Q87IE5_VIBPA Transcriptional regulator, MerR family OS=Vibrio parahaemolyticus serotype O3:K6 (strain RIMD 2210633) OX=223926 GN=VPA0662 PE=4 SV=1

MNIGAVAKLTGLSSKSIRLYEDKGIISPPARSDSGYREYSDNHIQELNLVSRAKNAGFSL

QECKEFVQLAHNPNRKSSEVKERTMDKLREVEEKIAHLMEIKKQLEGWVSACPGDAKSRC

PIIDELTK

>tr|Q87T66|Q87T66_VIBPA Putative 3-chlorobenzoate-3,4-dioxygenase dyhydrogenase related protein OS=Vibrio parahaemolyticus serotype O3:K6 (strain RIMD 2210633) OX=223926 GN=VP0204 PE=4 SV=1

MELKMKTRALVIGLGSMGKRRVRNLLALGNIDVFGFDVRADRNEEASSKYQITTFDDVQK

AFEDVKPEVVVISTPPQLHMTYANMCFDARVPCFIEASVTDIEEIRELGKKALEASLVIA

PSCTMRYFPGPKKVKELLEQNVIGSPLSVNYQTGQYLPDWHPWEDIQDFYVSKRESGGAR

EIVPFELTWLNDLFSHDVKALASAVAKQTDMNADIDDIYHCLLQYDSGVLLNLTVEVISR

PLATREMLILGSKGKIKFSADSNSVSYCNLETDGWVESTWEEGTVESQYINPEEPYINEM

KDFVTAALAGDQSLFPNNLQDDVKVLEVLEALEEVDQLKGNKA

>tr|Q87G45|Q87G45_VIBPA Transcriptional regulator, MerR family OS=Vibrio parahaemolyticus serotype O3:K6 (strain RIMD 2210633) OX=223926 GN=VPA1472 PE=4 SV=1

MVCSSEEKLYAIRDVAEITGVKPVTLRAWQRRYNLIQPQRTEKGHRLYCQQDLDTIREIQ

GWLSKGIAIGKVKGLLGQGDVEISAENHSLEEVETVLSALSQLNRGKTESVLSSVLKEYP

LNLVVDQFINPVFEALDLVKGSLRSLQMGLFQTCLITRLALVIDSENKASTKGKCLLISF

EQSRDAESWIQAAKLCEQGYHITLIDKVDDVSGLVGHDVIERYQRVYVFSNKALPAKQVE

AFKALLARYPEQVQCSEVIEKLHLAQTQIQ

>tr|Q87SR7|Q87SR7_VIBPA Putative LuxZ OS=Vibrio parahaemolyticus serotype O3:K6 (strain RIMD 2210633) OX=223926 GN=VP0355 PE=4 SV=1

MYVSSPTLTDKVSKMICQDILTGELKPGQKLVVAELKEKYNVGASPIREALVQLSWSKYV

KLAPQKGCWVAPVCKHELNDLYESLRMTASFLLKKAIMTGDENWELDVLTSYHKLSRVNL

DQGFCWREWEERHYHFLEALLEGANSHNLFTFFRDILNQIKRYHYFAMQVLGTNNQGHCN

IDEQEMIMKLSLSKNSDDAVQFLDRYLLKSLQCVETAIDNPL

>tr|Q87RR5|Q87RR5_VIBPA Uncharacterized protein OS=Vibrio parahaemolyticus serotype O3:K6 (strain RIMD 2210633) OX=223926 GN=VP0712 PE=4 SV=1

MLNKMTLIAAATLALYGCGGGSSSGSANPNKVPQESFDIAIQNSIFAPETNTQLSYSFSL

DGQSEGDMKLSFQAMDSEQILAALAQIPNSEAIIQLVNDLVNYGAQQFYYSDEQFNNEEE

SNTLYFAGSDGALHEVTDVYSIGNQFIAMLMHSSPIFRLNGNDVKEKSPNIDIGEETMSL

QTTLTGNAVHHLLDSFEEHEWVQNLPMTASCQVVWQQQIRETGVRKTFSISGKTIEAAYL

TEENTYNLNCDDMDSMEFATSAERWFNPSLGLIEQIELLKVQQVQINEEKVQLTAIETK

>tr|Q87IH6|Q87IH6_VIBPA Uncharacterized protein OS=Vibrio parahaemolyticus serotype O3:K6 (strain RIMD 2210633) OX=223926 GN=VPA0630 PE=4 SV=1

MGQHVETSSSDYVKGFVASLILTVIPFYFVWAQTLPASATYVVMFTCALVQIFVHFKYFL

HMEAKTSDGRWNLVSLMFTAIVVLILIAGSIWIIYNMNVNMKL

>tr|Q87MF5|Q87MF5_VIBPA Cytochrome c554 OS=Vibrio parahaemolyticus serotype O3:K6 (strain RIMD 2210633) OX=223926 GN=VP2300 PE=1 SV=1

MKRVMIGAVAALTMLSGQALAGDAAAGQAKAAVCAACHGADGNATIPGYPNLKGQNEQYI

VSSIKAYKNKERSGGLAAVMQAQASLLSDDDIANLAAYYSSLK

>tr|Q87N58|Q87N58_VIBPA Paraquat-inducible protein A OS=Vibrio parahaemolyticus serotype O3:K6 (strain RIMD 2210633) OX=223926 GN=VP2017 PE=4 SV=1

MSIQPNSRQLISCEECGLVVRIPEIEQGQKAQCPRCSHSLTKINAKPYQSVIAVSSACLI

MLVLSISFPFMSFSVQGLSQEITLLHAAKMLAEFQNALLGALLLATVVVLPAIYVGLILF

LHLEALKARNHPPSKKQQRMAKVLCRILFRVEPWLMVDVFLIGVLVSLIKIASLADIGMG

SSFWAFCVYTILVVKCISMVDKSWLWGHFIPAIELPSVKEGDTHHNHNHIGCHTCHQLNP

IEDKKHQRCIRCYSRLHEYNPSENLQKAWALLFASVIFYIPANLYPMMYTVSLGHSEGST

IMEGVILLWHLGSYPIAMVIFFASVFIPMAKMLALAWLYYNAQKAQYLPPEESISRLKIY

RLTEFIGRWSMIDIFVVAILVALVQLQNLMAIYPGPAALSFAAVVIFTMLSAMIFDSRLL

WQLPQSEVQEPMTNNLTEKAKYE

>tr|Q87PI4|Q87PI4_VIBPA Uncharacterized protein OS=Vibrio parahaemolyticus serotype O3:K6 (strain RIMD 2210633) OX=223926 GN=VP1518 PE=4 SV=1

MISLSDIENLIQHIWEEPIFSDVTSKKVVVSLYGTLSKKIPDKFIIIEEVFPKDELEDIW

SNYEEYLDEYLIFPFLGTLGEAVICIGYGNDNKGKIFYFDFDFGACELDGDNLEAFLEKL

LES

>tr|Q87R47|Q87R47_VIBPA Methylated-DNA-protein-cysteine methyltransferase-related protein OS=Vibrio parahaemolyticus serotype O3:K6 (strain RIMD 2210633) OX=223926 GN=VP0951 PE=4 SV=1

MDQFLVQIFAVIHQIPKGKVSTYGEIAKMAGYPGYARHVGKALGNLPEGSKLPWFRVINS

QGKISLKGRDLDRQKQKLEAEGVEVSEIGKIALRKYKWQP

>tr|Q87FE6|Q87FE6_VIBPA Secretion protein, HlyD family OS=Vibrio parahaemolyticus serotype O3:K6 (strain RIMD 2210633) OX=223926 GN=VPA1733 PE=4 SV=1

MSRDNYNKLSNDELDFVDDKTAALLLNTPNSARLMLWVMVLFFVAAIGWASWAQIDQVTV

GQGKVIPSSQIQVVQNLEGGLVKEILVKEGQLVKKGQQLLLIDDTRFRSDYREREQQVAN

LTASVLQLSASINSVAVNRDFNIQDWEKSVVLDYGKLTFPPVLEETQPQLTQRQKAEYRE

DLDNLRNQLSVIDQQVEQKQQDLVEIEARVRNLRQSYQYAKKELDITQPLADEGVVPRIE

LLKLQRQVNDTRREMTSSELKIPVIKSAIKESMLNRIDVALKFRSEQQEKLNNAQDQLSA

LVESAVGLEDRVNRTVVVSPVTGKIKTLNVNTVGGVIQPGMDIVEIVPTEDTLLVEAKIA

PKDIAFLRPNLNAIVKFTAYDFTKYGGLVGELEHISADTTQDEEGNSFYIVRVRTEKTSF

GQDADLPIIPGMTASVDIITGKRTVLEYLLKPILSAKTNALKE

>tr|Q87TC8|Q87TC8_VIBPA Type II secretion system protein M OS=Vibrio parahaemolyticus serotype O3:K6 (strain RIMD 2210633) OX=223926 GN=VP0142 PE=3 SV=1

MMKNLISQAQAWWSGISQREQRLVLGCGAFAILGILYWGLLQPMSQRAELAQSRIQSEKQ

LLTWVQDKADDITALRKSGGVSFSNQPLNQLVSSSARRFKVELIRVQPRNDSVQVWIKPL

AFNQLVDWLRYLKEQQGIEVEFLDIDRTDQAGMIDVNRLQFKRG

>tr|Q87JD8|Q87JD8_VIBPA Putative transcriptional regulator, LysR family OS=Vibrio parahaemolyticus serotype O3:K6 (strain RIMD 2210633) OX=223926 GN=VPA0315 PE=4 SV=1

MKLPPLRAVHCFEVVARNLSFSLAAEELNVTQSAVSHQIRLLEDYLGESLFIRQGRKLSL

SDSGARYLEDISPAISSIAMASQKVREGDKGSIRLAIYSSLAVKWLIPRLADFKRLYPEI

DLTLNMVAGDPEQTDSVGDCFITVQRPKRNYMAVKLYAETLYPVCSHKIWKEIQDQSIPD

ALWQYPILSTDSIYRERGKDWAEWCKAGGYTLPNDVDVQHFSHMLLAIEAARYDQGIAFA

NDFMLNERDKAQDLVYIPSHGLETGDSFYFVHKKNRAKQAEIVKLTNWLKQQCL

>tr|Q87S39|Q87S39_VIBPA Putative acetoin utilization protein AcuB OS=Vibrio parahaemolyticus serotype O3:K6 (strain RIMD 2210633) OX=223926 GN=VP0585 PE=4 SV=1

MIKVEDMMTRNPHTLLRTHTLRDAKSMMDALDIRHIPVVDANHHLQGLVTQRDILAAQES

CLHPDDAEQSFTLDTPLYEMMHTNIMTAEPRAGLKESAIYMQKHKVGCLPIVHKGHLVGI

ITDTDFVTIAINLLELQEEVEPEEIEPDEEAL

>tr|Q87K86|Q87K86_VIBPA Peptidoglycan glycosyltransferase MrdB OS=Vibrio parahaemolyticus serotype O3:K6 (strain RIMD 2210633) OX=223926 GN=mrdB PE=3 SV=1

MNKRFFPRIDLPLLMAIIPIMLLSSLTLWSASGFDESMLFKHLARCALTLVCILVMSSIP

AASYQRSAPYLYFVAVSLLLAVALFGDSTNGSQRWLDIGFFRFQPSELIKLSIPIMIAWM

LHLEGGRPDFRKIALCLMITLVPAGLIALQPDLDGAIFTVIYALFVLFFAGMSWKIIGGF

VVSVLTLAPILWFFVMEAYQKSRVTQFLHPESDPLGSGYQIIQSLIAIGSGGMKGKGWMN

ATQGTLGFIPESHTDFIFSTYAEEWGFIGCVVLLALYLFITARVMLLACQSEHFFSRLVS

GTLAMSFFLYAFINTGMVSGLLPVMGSPLPFFSYGGTAMLTQGICFGVIMSLCYSKYRNT

>tr|Q87SQ9|Q87SQ9_VIBPA Glycerol dehydrogenase GldH OS=Vibrio parahaemolyticus serotype O3:K6 (strain RIMD 2210633) OX=223926 GN=VP0363 PE=4 SV=1

MDKIIISPSKYVQGEQVLTSIAHYVKTLGERPLVIADEFVTNLVGDDVKQSFADEKLPLT

MNIFGGECSRIEIERITDICATQKHDVIVGIGGGKTLDTAKAVAFYTKIPVVVVPTIAST

DAPTSALAVIYTPEGEFAEYLMIPKNPDMVIMDTSVIAKAPVRLLVSGMGDALSTYFEAR

ANMTSGKATMAGGLATRSAQALAKLCYETLLEDGLKAKAAVENGVSTKAVENIIEANTYL

SGIGFESSGLAGAHAIHNGLTKLEECHHLYHGEKVAFGTLVQLVLENAAMEEINTVLAFC

RSVGLPTNLFDMGVKELNHAKLREVAEASTAEGETIHNMPFPVTAENVYSAILTAHQLGQ

>tr|Q87FM8|Q87FM8_VIBPA Putative methyl-accepting chemotaxis protein OS=Vibrio parahaemolyticus serotype O3:K6 (strain RIMD 2210633) OX=223926 GN=VPA1651 PE=4 SV=1

MFKNLSLKNKLAISASAAIILGGVLVEGLSFRDSLQRLDAEVAQRLESTSASYNQYVSDW

LLSKERALTSLSAESEKRAIVTHLKQVRDSGAFDNVFLAYPDGSQDNANGVILPPGNNDP

RKWGWYTNAIANPSKVFMDNPTVAAATGANVVSLGKALQLHGQTTVLGADVEIGDILNSL

NQVILPGEGYMFIANDQGNIFTHNDSKLLNQPVSKLGLNNNDITNAARSGTERRVSISGT

DYVIYARPIEGTKLTTVTVLDHNSLVAPLYDAVWDQIIATAIVVIICVALFNLLCNILFR

PLYNVSNALSQIANGSGDLTQRIKVENRDEVGELAENFNQFVESLQQLIGHIRHQAEELS

QQSELSTTRANQSVSDLNHQQQEITMVATAVTEMASATQEIAAHAEQTAKAAQDSSASTQ

NGHELVINSKSSINNLSSEVNQASVVIGELNQHAQDISTVLSTIRDIAEQTNLLALNAAI

EAARAGEQGRGFAVVADEVRVLSQRTHTSTEEIRSTIETLQQTTQRAVTIMDKSSQLAQG

SVEDADRAALALDEINAAVALISDMATQIATAAEEQTHVTNEITQNVTSIKDVTDQLVVG

AEESMNQSAELKSQAEDLNSKVATFKLA

>tr|Q87PL7|Q87PL7_VIBPA Lipoprotein NlpC OS=Vibrio parahaemolyticus serotype O3:K6 (strain RIMD 2210633) OX=223926 GN=VP1485 PE=4 SV=1

MRRIYFPLIISALLSACSSGPEPSEQVEVTVPTNQLLSNNPDLFRFYNEWHGTPYRLGGT

QKSGIDCSAFVQRAFVEAYQLALPRTTKQQSTQGVELSWTDAKQGDLVFFKTRRSTYHVG

IYLGNKQFMHASTSKGVIISRIDNPYWASKFWQVRRVTL

>tr|Q87M99|Q87M99_VIBPA RNA polymerase sigma-70 factor, ECF subfamily OS=Vibrio parahaemolyticus serotype O3:K6 (strain RIMD 2210633) OX=223926 GN=VP2358 PE=3 SV=1

MMSDSPQKLGRNEWNAYMDKVKAKDREAFAFVFRFYAPKLKQFAYKHVGNEQVAMEMVQE

TMATVWQKAHLYDGKKSALSTWIYTIIRNLCFDLLRKQKGKELHIHSDDIWPSEYYPPDM

VDHYSPEQDMLKEQVVKFLDILPKNQRDVLQAVYLEELPHQQVAELFDIPLGTVKSRLRL

AVEKLRHSMHTEQL

>tr|Q87FM6|Q87FM6_VIBPA Putative ferrichrome ABC transporter (Permease) OS=Vibrio parahaemolyticus serotype O3:K6 (strain RIMD 2210633) OX=223926 GN=VPA1653 PE=3 SV=1

MMNQTKLMALLGLLLIASSLTLFVGAANLSAQQVFALLFSFSDSDFVIHQYRLPRMLLAI

GVGAGLGLSGVLVQGVIRNPLASPDLMGISAGAGLAATACLVLYPNAPVAMLPMVAMAGG

LLAAGFIAVLAYWSKPTPARLALIGVAVSAFLASGIDFLLIVHPIEINTAMVWLTGSLWG

RNWQQVPFIWSALMLLLPLAFWLAWRLDVMGLGEESATTLGMKPKQIQILALIAAVLLAS

ISVSVAGTISFVGLLAPHLARLLFGHNHKLLIPASATLGALLVICADGLARGLQPPIELP

AGVLTSVIGAPYFIFLLYRYRGW

>tr|Q87P88|Q87P88_VIBPA Putative calcium-binding outer membrane-like protein OS=Vibrio parahaemolyticus serotype O3:K6 (strain RIMD 2210633) OX=223926 GN=VP1630 PE=4 SV=1

MERVTFGSLMSNIVVIGLDGEIRTLEQGQLPKPGELIINEVSDTSELVIEKVTPQGSSIN

VTGDITALIEAIAAGQDPTQLGQEFETAAGEANGSSPQTTGNVARTGTELLASTNFETVG

TEGAGFSTTQILTLLDILPPTQAPAPTGIPIPVTITVQDISSPAVDEGQQVSFDVTLNQP

TEEETTIEFTLNDGSAVGGAASDVGADYVNTTVLITLSDGTTQEIQVNDDGSFTVSLPIG

EESFTVSLDTIDDNLAEGSETFSLSGSTPDQPTPVTGVATITDEAEQGPEDTVTVNMTGP

NSVSEGETTSEYTVTLSDPAPVGSIVTLAYSYTTASGDDITETTQAIIGADGVSATFTID

TVDDVYAEGDEVFRVSVSGIVDGDSNPIFEALDVSNAFVDTTINDETDPGPEDTVTVTMT

GPANVVEGDTTTEYTVTLSDPAPVGSIVTLAYSYTTASGDDITETAQAIIGADGVTATFT

IDTVDDVYAEGDEVFRVSVSGIVDSDSNPIFEALDVSNAFVDTTISDETDPGPEDTVTVT

MTGPANVVEGDTTTEYTVTLSDPAPVGSIVTLAYSYTTASGDDITETTQAVVGADGVTAT

FTIDTVDDVYAEGDEVFRVSVSGIVDSDSNPIFEALNLDNAFVDTTISDETDPGPEDTVT

VTMTGPANVVEGDTTTDYTVTLSDPAPVGSIVTLAYSYTTASGDDITETTQAVIGADGVT

ATFTIDTVDDVYAEGDEVFRVSVSGIVDSDSNPIFEALNLDNAFVDTTISDETDPGPEDT

VTVTMTGPANVVEGDTTTEYTVTLSDPAPVGSIVTLAYSYTTASGDDITETTQAVIGADG

VTATFTIDTVDDVYAEGDEVFRVSVSGIVDSDSNPIFEALNLDNAFVDTTISDETDPGPE

DTVTVTMTGPANVVEGDTTTDYTVTLSDPAPVGSIVTLAYSYTTASGDDITETTQAIIGA

DGVTATFTIDTVDDVYAEGDEVFRVSVSGIVDGDSNPIFEALDVSNAFVDTTISDETDPG

PEDTVTVTMTGPANVVEGDTTTDYTVTLSDPAPVGSIVTLAYSYTTASGDDITETTQAII

GADGVTATFTIDTVDDVYAEGDEVFRVSVSGIVDGDSNPIFEALDVSNAFVDTTISDETD

PGPEDTVTVTMTGPANVVEGDITTEYTVTLSDPAPVGSIVTLAYSYTTASGDDITETTQA

IIGVDGVTATFTIDTVDDVYAEGDEVFRVSVSGIVDGDSNPIFEALNLDNAFVDTTISDE

TDPGPEDTVTVTMTGPASVVEGDTTTEYTVTLSDPAPVGSIVTLAYSYTTASGDDITETT

QAIIGVDGVTATFTIDTVDDVYAEGDEVFRVSVSGIVDGDSNPIFEVLNLDNAFVDTTIS

DETDPGPEDTVTVTMTGPASVVEGDTTTEYTVTLSDPAPVGSIVTLAYSCTTASGDDITE

TTQAIIGADGVTATFTIDTVDDVYAEGDEVFRVSVSGIVDSDSNPIFEALNLDNAFVDTT

ISDETDPGPEDTVTVTMTGPASVVEGDTTTEYTVTLSDPAPVGSIVTLAYSYTTASGDDI

TETTQAIIGADGVTATFTIDTVDDVYAEGDEVFRVSVSGIVDSDSNPIFEALNLDNAFVD

TTISDETDPGPEDTVTVTMTGPANVVEGDITTEYTVTLSDPAPVGSIVTLAYSYTTASGD

DITETTQAVIGADGVTATFTIDTVDDSDDEPDEVYRVSVASIVDGDDNPIFEALDLTNAY

VDTTIIDNDEPNTPPTVRNFNILLDVEDGLFPIHDVDFDGTTSGGEQRVEDLEDDAVTAN

DDDIQIKVIDLPDYGELYYVDGNGQEVVIDGSSIFAENTDVKYRLTADFFEDQRFDSNDL

LEEFDKDFLADSVDVNGLSFYGGQITISGNDITFDSNAQVKVDFANQQVGLVVVSPGETG

NGDEISTHEYVAIKLDEGLEADKAKINLASLNDRFNNGGAWITAYIFLDGIVVDTQEIRA

EDISYSGNHEGTATIELANGSFDEIRLSPDTDSSSDKASFTLVGTEITSFTQVSDSFEYK

AIDSDGLESDSSATVAVDFENVTVDAVTALGYQTMALNSELNLIMGTDGDDILVGTDGND

MIIGGLGNDILTGGEGDDVFKWTEMQSATDTVTDFSDGDQLDFTDVFDDMTGTDISALLD

DLGSGDYRGRVDDITVEVTESGGNSTLTINKDGQQLEVNFDGASAADIANSLISNLEQLR

E

>tr|Q87G68|Q87G68_VIBPA Methyl-accepting chemotaxis protein OS=Vibrio parahaemolyticus serotype O3:K6 (strain RIMD 2210633) OX=223926 GN=VPA1449 PE=4 SV=1

MLKLHSLSIKQKVVLGITFAVLASTIIVGVMAQRHARDVLSHRLIDIELPAMLQQINTEI

DREVVQMQQAAKQLATNEFVVEALKNTDHPQFSETQLVQQLNNIKSQYGLNDASVANRKT

AYYWNQNGFLRQLNHSQDAWFFGFTSSGRETSVSVFQEANGEVKMFTNYQDLNGISMSGL

SKSMDDMVSLLNSFQIEDTGYVFLTNEKGDIQIHRQQGKNKTSIAQLFGSNANQLLNKNS

FNLINVEFEGKDNFIASLYVPSMNWFVIGVVPVDEVFADLNATGQKMMITTIVVALVFIL

MGVLLANSITKPIRLIADRFTDLGQGEGDLSQRIEIRGNDEIAQLSKGFNGFIEKIHATM

KEVSLTSGALSQAADSVSSKATSTYDNSQEQRDQTIQVVAAINQMGATISEIASNAATAA

DTANQASDNTQTGREVVMKAKEVISRLADDVETTNIVVTQLASTTKDIGSILGVIRDISE

QTNLLALNAAIEAARAGEQGRGFAVVADEVRNLASRTADSTEEIQRMINQLQSDAQDAVS

AMEAGKAVTFEGVASTDEAVEVLVNISERITDISDRNTQVATATEEQSTVVHTINQNIEE

INAINEMTTATAEELAGASRDLQELSSRLDKMVGSFKL

>tr|Q87MJ1|Q87MJ1_VIBPA Uncharacterized protein OS=Vibrio parahaemolyticus serotype O3:K6 (strain RIMD 2210633) OX=223926 GN=VP2264 PE=4 SV=1

MKKRTPATPVPSLETQTEAMKIAKATQKPGQTKEQTKLIAQGIEKGIAQYKKQQKERNRQ

ADKAKKKQQREKLRQHEEGVIESDNITDNTPTHTPSKLPWALLALSWIGFAAYIMLSK

>tr|Q87RR9|Q87RR9_VIBPA D,D-heptose 1,7-bisphosphate phosphatase OS=Vibrio parahaemolyticus serotype O3:K6 (strain RIMD 2210633) OX=223926 GN=VP0708 PE=3 SV=1

MAKPAVFIDRDGVINVDHGYVHDEHDFEYIDGVFEATKALKDKGYLLVLVTNQSGIARGK

FSEDRFLSLTQWMDWNFVDNGVEFDGFYYCPHHPEHGIGDYKQDCDCRKPKPGMFISARD

FLKIDMEKSVMIGDKAEDMMAAEAAGVGTKILVRTGKPITEQGEALATVVLDSIADVPAY

LQK

>tr|Q87PH2|Q87PH2_VIBPA Uncharacterized protein OS=Vibrio parahaemolyticus serotype O3:K6 (strain RIMD 2210633) OX=223926 GN=VP1530 PE=4 SV=1

MNNDFEKDFNLGGSVERALSGDYELKASAVFSEAWRATIQHFLSFSPAIILLLFVQLGIF

YIALQLQLGDPSVILDAIENPEAFTANIVSAIYIANFSYEVISAPIYAGISLMAMSHVAG

LKTKVRHVGKGLQFTVPVIIATLFSLTLQGVAGMILPPLSLYLAMAFSHSILLICEKRVP

PMQSLLLSLRAINKKIFVVAGLYMGVMLMFIVAAMFYGIGFIFVLPFFFHLKGILYREMF

GIKLKIVATQKNDNDHDGKSQVFNA

>tr|Q87JE3|Q87JE3_VIBPA Uncharacterized protein OS=Vibrio parahaemolyticus serotype O3:K6 (strain RIMD 2210633) OX=223926 GN=VPA0310 PE=4 SV=1

MNLVVHLQKKAIRKHGYEMATRQAWKCGIKANLVRRVIGLFKGQIVCVVEGCRAELSSVV

NNQLHDEEKQGRYVFVGGVCWEPNNIIAPGFPDFMFMHVRNLSHRHKYLTDDELFLSLA

>tr|Q87ID2|Q87ID2_VIBPA Periplasmic protein TorT OS=Vibrio parahaemolyticus serotype O3:K6 (strain RIMD 2210633) OX=223926 GN=VPA0674 PE=1 SV=1

MVINQRSIRTIFKHSVLAASLCASLPSVAAEKICAIYPHLKDSYWLSVNYGMVSEAEKQG

VNLRVLEAGGYPNKSRQEQQLALCTQWGANAIILGTVDPHAYEHNLKSWVGNTPVFATVN

QLDLDEEQSTLLKGEVGVDWYWMGYEAGKYLAERHPKGSGKTNIALLLGPRTRGGTKPVT

TGFYEAIKNSDIHIVDSFWADNDKELQRNLVQRVIDMGNIDYIVGSAVAIEAAISELRSA

DKTHDIGLVSVYLSHGVYRGLLRNKVLFAPTDKMVQQGRLSVMQAAHYLRHQPYEKQASP

IIKPLTPKTLHDDTIEESLSPSEYRPTFSIKAVD

>tr|Q87QC5|Q87QC5_VIBPA Adhesin OS=Vibrio parahaemolyticus serotype O3:K6 (strain RIMD 2210633) OX=223926 GN=VP1224 PE=4 SV=1

MTFLSQLSTHADKAFLRRLFAIALPITLQSIMFSSRSLVDVLMLGQLGEAEIAAVGVAAR

ATFVTTIMLVGVTTGGALLTAQYWGAGDKIGVRQSTSLTWMIAMVFAALAVCLFVFFPQP

IMGLTTDSQEVIELGSSYLVISSASMFAVACVASMAVGLRAMHQPGLSTFFSGIGILSNV

FLNWVLIFGHLGFPALGITGAAIATVISGAIEVGCLFGYLWLKKHIIAFGWGDIRASLVL

DKITRFLSLSLPTTFNFLAWAGGLFAYHAIMGQAGVQGLAALSVMTPVESIALAMMIGLS

NAAAVLVGNQLGAKNFEPVYYQAWATVILNVLIAFGVAVLLFFTNQLILDAFSALSAETR

HLAEQFMVILALGVILRSVPMMVIVGVLRAGGDVKFCLYQDVFAQWIIGIPLAAFAAIYL

GWEPQWVYLLFLTEEVVKWCICLPRMKSKKWMKNLIEK

>tr|Q87QY3|Q87QY3_VIBPA Uncharacterized protein OS=Vibrio parahaemolyticus serotype O3:K6 (strain RIMD 2210633) OX=223926 GN=VP1015 PE=4 SV=1

MKTFIIAWLLILLVGCDKHAVVVEASKPEFDPIIQRVEQGQAANLHSLLVWQDSQLTLEL

YRQGGGLDGNRQTPNVPVGQEVVHNVHSVTKSFVATLIFIAIDEGKIEGLDTSIFDFFPE

YTEPDRKAKLAITLRDVLNMSTGYALDELSVPYGQGNIFSRHYNAKDLKQQFLSTQLAFD

PGTKFAYSGLSTVGLSKVIEKVYGQSFTKVMKEKIFSPLGIEEYQWLPNIGSGEAGADWG

LRLTSRDMGKFGLMWLNKGQYNGTKIVGEEWLNELRKSKFYSYEMGYGLHFWQVSNVENG

VAAVGIGEQYIVAIPNKNAVIVTTAGNYETRSRPVLTLIQQLSSLL

>tr|Q87MC7|Q87MC7_VIBPA Efflux pump component MtrF OS=Vibrio parahaemolyticus serotype O3:K6 (strain RIMD 2210633) OX=223926 GN=VP2329 PE=4 SV=1

MSSSASIKQNSPKKPLFTRFLDTVEYLGNLLPHPITLFAIFCLAILVMSGIAGYFEVSVV

DPRPEGAKGRAADGMIQVVSLLNADGLELIVTNLVKNFVGFAPLGTVLVAMLGVAIAEYS

GLLSAAMRGLVMGASQRMVTVTVVFAGIISNTASELGYVVLIPLAAMLFHSLGRHPLAGL

AAAFAGVSGGYSANLLIGTVDPLLSGITETAAQMIDPTYTVGPEVNWYFMFVSTFFIAGM

GAFVTEKIVEPKLGKYNEEEAAEDLSNDKMGNLTDLEKKGLKVAGLAVLVVSALLAWTIV

PEDGILRSDAGTVAGSPFLKSIVAFIFVFFAIPGFVYGKVVGTMKTDRDVIDAMSKSMSS

MGMYIVLVFFAAQFVAFFKWTNFGQVFAVAGADFLQSIGLTGPMLFFAFILMCGFINLMI

GSASAQWAVTAPIFVPMLMLVGYAPETIQAAYRIGDSTTNIITPMMSYFGLILAVATRYM

KNLGIGTLIATMLPYSICFIVGWSLLFYLWVFVFGLPVGPGAATYYTP

>tr|Q87FL8|Q87FL8_VIBPA Putative AcsD OS=Vibrio parahaemolyticus serotype O3:K6 (strain RIMD 2210633) OX=223926 GN=VPA1661 PE=4 SV=1

MLQTQSSQTSELRAHDVQLNREKAQLNTIMGVLNCYLREFALPNQQVEWHYQSTSLPQTL

KRNYSAKQRVAVHLSQQNGVLVLPIHYASKLGKIKLAELPWAKMPGSGWAKLDATQTLTL

LLNYLKQVLAIPFNHELIEQLENSVLVTEQFLNVAPKSHHNEFIASEQSLIWGHTFHPTP

KSRSGVTMDDLLAFSPEVGAQVPLYWFEVDTSLLDVLSSDGEANPAQRMLQQLAPQHTTS

NGKTLYPCHPWESYTILSNPSVRRAIEQGKITPLGLGGEKMQPTSSVRTLYHPDMDWFAK

FSINVRLTNCVRKNAWYELDSAVQLTSILKPIRENEQLHNPVFKVMTEPYATTLNLESVA

ERNWDDNTKARESFGILYRENFSLSEVDILKPTLAGALFAYDKNGNSCIAQRLKNKARTT

QNRYSDIATLWFERYLHCLIPGVFNYYFKHGVAFEPHLQNTLIGFEQEMPCCVWIRDLEG

TKLLPEFWPAETLTDLSERARQSVYYSREQGWNRIGYCTFINNISEAIFFIAEGDEPLEQ

TLWNAVQSAIVRWQSVNGKQPELEALLNGGHFPSKNNFTTRLMQKADKESGYTQVAAPWQ

QHEKGAQHA

>tr|Q87J84|Q87J84_VIBPA Transcriptional regulator, LuxR family OS=Vibrio parahaemolyticus serotype O3:K6 (strain RIMD 2210633) OX=223926 GN=VPA0369 PE=4 SV=1

MPNQNFNAQLAEAISALNTPNFTPKLMSVIHSIFDFDCAIILGYREGKHPIYLYDSIENE

RELLFQRYLTNSFQNDPFFQNLNQHKQQGIFTLKDVAKKGIEYQTYRKQFYDQTGWKDEL

SMLVEIESGRWVILYFGCLREGKRFSAAQINNLRSHFSVLQSLCQQHWKQAEFNLSEPVV

RPDAYTGNMKVAIEQALSSFGIESLTRREQEVASLLAQGFDTKEISAHLHLVQGTVKNHR

KRIYSQLNVSSLSELFQLFLNHLIMHSK

>tr|Q87NJ3|Q87NJ3_VIBPA Para-aminobenzoate synthase, component I OS=Vibrio parahaemolyticus serotype O3:K6 (strain RIMD 2210633) OX=223926 GN=VP1875 PE=4 SV=1

MDNQFIDFKALEYAPEFALHLFSRIQHQPWTMLLRSASKTHIDSRFDVLVANPIATLETI

ADSTQVETPSNAYSIQDDPFTLLHQLQEQWLPHVELNKELDLPFVGGALGYFSYDLGRRV

ETMPEQAEKDLNTPDMAVGLYEWAVVVDHKLKKACLVGQNIEQAWQWLDKQEAEQSVDFA

LSGDWQSNMTKESYATRFDKVQEYLLSGDCYQINLAQRFNAPYLGSEWQAYLKLESANQA

PFSAFIRMPESSILSISPERFLELKDRVIETKPIKGTRPRSEDPKQDNANAHDLQTAEKD

QAENLMIVDLLRNDIGRVASPGSVHVPKLFDIESFPAVHHLVSTIRANLDEQYSPADLLR

ACFPGGSITGAPKVRAMQIIEELEPHRRSAYCGSIGYISRHGRMDTSITIRTLVAENHKL

YAWAGGGVVADSDCASEYQETLDKLSKILPALQS

>tr|Q87S05|Q87S05_VIBPA Putative chitinase OS=Vibrio parahaemolyticus serotype O3:K6 (strain RIMD 2210633) OX=223926 GN=VP0619 PE=4 SV=1

MFALKHLPIALSCVLAAQANASMNIQPDPQNPNGYVIARGDVQAMEQSKTSDPMYAIWSQ

ALETRPNSVVEAIVPGAATNPENVKRVERVFPESEWDFLTQMAAPEYTYTRFLRAIGKFP

AFCGEYTDGRDSDAICKKSIVTAFAHFAQETGGHISIDNVSDNPLGLEEWQQALVHVREM

GWSEGQEGYTTGCGQNDWQNKRWPCEPGQGYFGRGAKQLSYHFNYGAFSEVMFDGDASVL

LKNPGLVADSWLNLASAIWFFLTPQAPKPAMLHVIDRTWVPSQRELDAGIGYGFGTTINV

INGGIECGEQNKDKGQPVNRIRYWEGLAAHYQIPIEADEKNTCWQQTPYGSLNLNGATDV

LYTNWDGNWKYYSDRPGGHSFECELVGFQTAYSALVAGDYEKCVTNFYQSHAGWPEVRVV

DKLDPVDPVDPPVGGVAAWDSGKVYNTGDQVSYQGAVYEAKWWSQGNEPTKGDPWKLVSG

TPTEPPTTEPEPPVTEPEPPVTEPEPPVTEPEPPVTEPVPPVTGSFIQWEPGVTQVANGD

KVTYQNKCFVAKNGPGVWESPTQSNWFWDEISCQ

>tr|Q87H89|Q87H89_VIBPA Putative two-component response regulator OS=Vibrio parahaemolyticus serotype O3:K6 (strain RIMD 2210633) OX=223926 GN=VPA1076 PE=4 SV=1

MKILIVDDNHNVSETIADYLELEGMTIDCAYHGEAALALLEDNHYDVIIMDIMMPKLDGI

STVQKLRQEQFCGTPILFLTAKDTLDDKIAAFKAGGDDYLMKPFAMQELSLRIHALASRG

PRQDIGTLTFADICLDARTGKVTRDGKEIKLSRIQTKILKLLLKYAPASVSRTEVIESVW

GDEPPSSDALRSHIYGLRTALDKGFEESRLETIHGQGYRLKA

>tr|Q87HK6|Q87HK6_VIBPA Putative transporter binding protein OS=Vibrio parahaemolyticus serotype O3:K6 (strain RIMD 2210633) OX=223926 GN=VPA0957 PE=4 SV=1

MTETCLRRLSQLLKHYKHSQTYQVNLDDLELVFSTSRRNISNILRILDSYNWIHWEPGRG

RGKASTLKVTVTIHQALYFTIRNEINNGSFDVISRLLEHYRSTAVSALSQALAEVSEENK

DSNTLIVSQYPWVDELTPSLTYRFSELQVIRSLYDTLFTVDHYGQLKNHLACEYKNEGSC

IYIWLRPDIYCHDGLALQAEDVVHSLNKLITTDGPVQKLLQQVTHISFDNAKQAIRIDLK

QPNNLFIYCLATANASITTRRQKSFKGRSITIGTGPFVLRHWDTNKIVLKKHHNYFAKKA

LLEQITLSHQGTELDQYISYNQETDDTECYMIQAFSFLAHNRRAECSLSEQTWQRLFSFI

ESRRFEFAKANGLETMHILDDNSHNENVPQLEGTLVITHPKWTIDYLSKANQWIVDLIRQ

TGLHVEFVELTDASNPQLVKEQADLLFVEDVIEPPLTYGIYEWLLTGTGIRFALHSDEFE

QHVNHVHDAVSDLTPEKRLEGILSELRADTTILPLFWGQEKITRAKGVSGVQLRKSGYSD

FYKLRVRAGQD

>tr|Q87GY1|Q87GY1_VIBPA Putative drug transport transmembrane protein OS=Vibrio parahaemolyticus serotype O3:K6 (strain RIMD 2210633) OX=223926 GN=VPA1184 PE=4 SV=1

MDLNRNVWLLSLCQALLMTGNILLISVIALIGKTLAPSPSFITLPVALQFLGLMAATIPA

SLIMGKLGRRLGFSLGNLIGIVGASLATYALSQQTFYLFCFSTFLLGVGIGFGTLYRFAA

IEVCEESARHRAISISMAGGVLAAILGPNLAVYSQQWSDDGLYTSAFSVLIVLYITALVL

LQTIRFPPAHTQLSHVKADTISDIIRRPNFMIAVLAAMVAYAVMNILMTATPLAMIGCGF

DFDKAAGVIEWHVLGMFAPAFITGRLIEQFGAKKMILAGGVLFIACIGINIHGVSIWHFS

LALVLLGVGWNFMFIAATGLFSQSYEAKNRSKAQAFNEFFVFSCVTTTALLSGWLESTVG

WQNLNLYVLPFVLLVIVIFAMKTDNKTESQIA

>tr|Q87QU0|Q87QU0_VIBPA TolA protein OS=Vibrio parahaemolyticus serotype O3:K6 (strain RIMD 2210633) OX=223926 GN=VP1059 PE=4 SV=1

MKDKKKQSKEYTKPIVISVGLHALLVAALLWGTDFAMTKPEPTGQMVQAVVIDPKLVQQQ

AKEIRQQREKAAKKEQDRLDKLRREAEQLEKNRKAEEEQIRKLKEQQAKDAKAAREAEAA

RKQKEQERKAEEERVRQEKERTAKLEKERKAKEEAVRKAEQERLAKEAAIAKAEQERVAR

EKAAKEAEEKAKREREAAQKAEQERIAKEKAAKEAAEKARKEKERLKRLERERKEQEAAL

DDIFAGLESEASANQQAQGKFVADEVSRYSSIYIQLIQSRLLKDDYLLGKECRVNIKLIP

TGTDMIVSSVNVLSGDSRVCAAAKSAIAQVPSFPMSTDSTVNQRLKDINLTVALQQ

>tr|Q87NB4|Q87NB4_VIBPA Putative metal-dependent phosphoesterase YciV OS=Vibrio parahaemolyticus serotype O3:K6 (strain RIMD 2210633) OX=223926 GN=VP1954 PE=4 SV=1

MRIDLHSHTTASDGRLEPKDLVDRALGFDIEVLAITDHDTVDGLARAKQYVEENDLPIKI

INGIEISTVWQNKDIHIVGLNIDPENPALAALIEQQKQHRVARSELIASRLQKATREGVL

EEVQQLAGDAPITRAHFAKWLVDNGYAKTMQMVFKKYLTRNNPGYVPPNWCSMKEAVDAI

HAAGGKAVLAHPGRYQLTAKWIKRLLAAFVEANGDAMEVAQPQQAQQERRNLADYAIQYK

LLASQGSDFHYPSPWMELGRNLWLPAGVEPVWKDWGIDPSLDVSK

>tr|Q87L76|Q87L76_VIBPA Uncharacterized protein OS=Vibrio parahaemolyticus serotype O3:K6 (strain RIMD 2210633) OX=223926 GN=VP2736 PE=4 SV=1

MSRYWLLGLSLLCTTASYAEESVQPEQNIDVPNPLQTEVEFGYQAHTGNTDSRSLNARLS

AEYTSGRHRSNGEWKYYNLYKDGEEDKRSSTYSVQSDYKLGPKTYLYGSFKGVDSRYSAY

FKDYTLSGGLGYQFSYTENFILEAEIGPGFRYQEPNLDEIDDDDIVFPNIVREGIFRGNL

NTTWHALDNLSFAADITLVTGKSNTRVDSELSVTNDITEDIALKLAHSRQYHDKVPEGLS

KADSVFSVNLLFAF

>tr|Q87GL3|Q87GL3_VIBPA Uncharacterized protein OS=Vibrio parahaemolyticus serotype O3:K6 (strain RIMD 2210633) OX=223926 GN=VPA1302 PE=4 SV=1

MVQSVHCSLLSSNTVNKLTGALLIFTIFPAHVSANSCDIEGYTIGFFNGVATTLKGAKQG

QKKIESTLGIKQFNGEPVEYQLFYNDSYIESNGLNVLADFAETFDQRTQELGQKQFDRWE

AFWEIVSGRQDSSIIQKISATFSWFKGFVSDLLSQGMNTMIREFLQLLSTSIDSPDTEKT

QMQHKLINDSNTWKGKKLIYIAHSQGNLWVNQSYKYVVSQLGYDADNIHVVHIAPASPTL

TPDSEYILSTSDLVINGLQLTGIGSVPVSNTAIAPSTADIAGHGLIEIYLTHPDSINKIK

KSVGRAFDSLTKPDMEEHLFEVTYQYTPSFVESHAEPEIKFVDNPSGDWFEKAMYPHYDD

SYFEYDSAAYKYKLKPVAGLKYLSHQRDKKDRENQTFTVVQCSNIPNDDPFILGEKSEAL

IWYGYDEQPDNTKVIVTVRDRYGRKLQSGEQSVSELRRKGWRYQGIFLDLKAKNPYSQKE

KSFIEKQNLETSYELHSNSILLPTAS

>tr|Q87P47|Q87P47_VIBPA Putative translocation protein in type III secretion OS=Vibrio parahaemolyticus serotype O3:K6 (strain RIMD 2210633) OX=223926 GN=VP1671 PE=4 SV=1

MATRGGSMKLTIPTVDAESIALTNQLCAKQCHFQGRDEHSISITASQKPEFSGYRLTTLV

GGQTIQVDFCSAQLQQWLHSTLNATAFESLPNSLQLALLSTQIEPHADAFKRLFGQLPIL

SKLQPLEQSETQRNTLMLTLNKPSASLCLWVSEGQSVLVDALPPCSSQLTQHIALPVWLS

LGKTHLDLNQFHSLELGDVIFFDQCYIAQQQAIVQVSNKNLWRCQLEDNTLYIIEKETNM

NDVNTSETLTDHQQLPVELTFDIGHQTVTLEQLNQLQPGYVFELNQPVSKPVTLRANGKI

IGECELVNVNDHLGVRVLELFGGTQEPA

>tr|Q87PH1|Q87PH1_VIBPA Putative Bax protein OS=Vibrio parahaemolyticus serotype O3:K6 (strain RIMD 2210633) OX=223926 GN=VP1531 PE=4 SV=1

MPKSLKLMTLRVAAACIAATFISVGPYLHYDHQFKSANDPNVVQGPVTHPDLSHLPYIGD

IPDFNSIKDAKEKKAAFFDFLRPKIALENHRIEKERAFLSSLEIGEVSPDEEAYAERLAQ

LYGFPLHDGHVDEAWLTEMLKRVNVLPEALVLTQAANESAWGTSRFATQANNLFGQWCYK

QGCGIVPAQRAAGKTHEVQKFDSVQQSIHGYFMNVNRNPAYADLRDIRAMLADKHKNLFS

VATASELTHGLLSYSERGIAYVNDLRSMIRHNNAYWTQ

>tr|Q87Q78|Q87Q78_VIBPA Uncharacterized protein OS=Vibrio parahaemolyticus serotype O3:K6 (strain RIMD 2210633) OX=223926 GN=VP1272 PE=4 SV=1

MFLTPYFSNNNHQFQFTREQASHFAKRVAGDYNPIHDEDNKRFCVPGDLLFAVLLSKEGI

SQKMRFRFSGMVNDGIELHIENKCEKESAVVDEAGKEYLHMSREGETNHNPAFIEHVVTN

YVQFSGMNFPHIMVPLMEEKQMMINCQRPLVIYESMEVEFSRLDLTHPEVDFTGATFDVD

GKRGVVTLNFAFKEDGEVVGKGIKRMVASGLKPYDQEAVDDLVHRFNERKDAFLAKFVKA

A

>tr|Q87M26|Q87M26_VIBPA Putative smp protein OS=Vibrio parahaemolyticus serotype O3:K6 (strain RIMD 2210633) OX=223926 GN=VP2432 PE=4 SV=1

MSESLFSVRNALRILALILLCVMLFITIKNSVVISKGNEKIQAQQLETLTKVLISQASLS

ASEMILTKDQERLLKLTNQLAQDRLVFDATIYDSQGVRLASSDEALSVREVLGLDTPLAT

ASIGRQQLVEPVLADGAVIGFVRVTFETGRVTAISDHHYRKSDRYMYMMVLMSFVCGMLF

IMILRRQPIRRKKAENLLLTK

>tr|Q87FT6|Q87FT6_VIBPA Uncharacterized protein OS=Vibrio parahaemolyticus serotype O3:K6 (strain RIMD 2210633) OX=223926 GN=VPA1592 PE=4 SV=1

MLPQDNVITSFLCKKLGHNHLRIAGEYWHIEKLVALQCMMLKEAPNLQEGLLWWSKCVSL

FDRRLYVTLQHGDAQIHLQVECRTADKPSWAESVFSLLKMQLEMLELSESIQVQVHANNP

LAADIFLEETRDSKTSSSVFELVKHVYLLLSHQPIEQPELLSVLNALFIKNSNYALKLEQ

AARQLGVSKRTLQRRLQEKQMSYSQCVDFAKRKQALTLLADTQLTTQQIAYQLGYEEPSN

FHRTFRRWYPFSPMQYRQQCLDNRTQLNQQPIRLYYAKANTFDDNHIDQPVGKIWMEVDN

IAFEKVVSVECRDRDGAWRRYPAFFERFLSHGTELWATTELPVAHPLTFRLCYEVDGERY

IENNHQRDYVVSKGLLIGATEYIVPTRQLINIGAQYTLFVELACRLENVARIDGYVDDDP

TPHAMRLTQHSLHYACWVLQLSLTQTAKQCRFRLYDESGNELAKEHYPIQYPIVQPLN

>tr|Q87L24|Q87L24_VIBPA Phosphoribulokinase OS=Vibrio parahaemolyticus serotype O3:K6 (strain RIMD 2210633) OX=223926 GN=VP2792 PE=4 SV=1

MSAKHPIIAVTGSSGAGTTTTSEAFRKMFNMMNIRPAWVEGDSFHRFTRPEMDIEIRKAR

EQGRHISYFGPQANDFPGLEEFFRKFGHDGTGSVRRYLHTFDEAVPYNQMPGTFTPWQDL

PENSDVLFYEGLHGGVVDGDVNVAQHVDFLIGMVPIVNLEWIQKFVRDTRDRGHSREAVM

DSIVRSMDDYLNYITPQFSRTHINFQRVPTVDTSNPLNAKGIPSLDESFVVIRLRGIKNV

DFPYLLAMIDGSFMSRHNTIVVPGGKMSFAMELIVRPILQQLIETGKIG

>tr|Q87R59|Q87R59_VIBPA Uncharacterized protein OS=Vibrio parahaemolyticus serotype O3:K6 (strain RIMD 2210633) OX=223926 GN=VP0939 PE=4 SV=1

MKRLFSIVALLMFTVAVTPIAEAKKFGGGKSFGKSYKTAPAPKQQQQNTNTIGKEQTTKS

SSKKGLMGGLLGGLLAGGLLAAFFGGAFEGIQFMDILIIGLIAFIIFKLMRGMLGAKQGS

MNQQRQQPAFGGNASKFEQPNMQNFEQQPNTNAGGFTGFGAQTDVPHNYPPGFDQAAFIN

GSREHYRILQGAWNHNQLDTIEEYVSPSLFEDLKAERAKLEGEQHTDVMYVDAEIVRAEY

DANKAQLSLQFSGRYRDAAEGVEEDIEDIWHLERDLTVPNAPWLIVGIQG

>tr|Q87LU0|Q87LU0_VIBPA AmpD protein OS=Vibrio parahaemolyticus serotype O3:K6 (strain RIMD 2210633) OX=223926 GN=VP2521 PE=4 SV=1

MPPIIDNGWLTHAKHVPSPFFDARNSEHDISLLVVHNISLPPGQFGGSYIEDFFSGNLDP

NAHPFFEVIHKMGVSAHCLIKRDGEIVQFVSFLDRAWHAGQSSFAGRDRCNDYSIGIELE

GTEFVAYTEEQYQSLARLTQAIMHQYPQITLPRITGHQYIAPLRKSDPGLSFDWVKYRQL

VQR

>tr|Q87JR3|Q87JR3_VIBPA Branched-chain amino acid transport system carrier protein OS=Vibrio parahaemolyticus serotype O3:K6 (strain RIMD 2210633) OX=223926 GN=VPA0185 PE=3 SV=1

MLSARNIAALGFMTFAMYLGAGNLIFPPFLGYQAGENFLSGMSGFLLTGVGLPALALVMV

AIVNGSDKLTAALPKPLATSFWVMVFIVIGPAFVIPRAITVAYQFSFAPIFGEAALVPFT

IVFCVATIWFALYPGKLVDNLGKILTPALMAILIIMSVTALIYPAGELTQASGPYVSGAF

AEGLTQGYMTMDALGSIGFGWIIFRAIRSMGVDCPKATAKYTLIAALMYAVAMAFVYISL

SYIGSTSSYLGSEFSNGGDILTAFTFNHFGAFGSVLLGAVMVLACLTTAIGVTTAGSEFY

DNTFSKVNYKSCVVITMVLSGFIANIGLEQLLAITLPAVVALHPVAIALMMMAPVRNKMS

QFMLVLTAFTALAFGCVDALHILGYMPEAVDQWMSHNMPLYNEFASWIVPSVIMATIGLL

FTKKAEEIKEFELVNE

>tr|Q87J47|Q87J47_VIBPA Uncharacterized protein OS=Vibrio parahaemolyticus serotype O3:K6 (strain RIMD 2210633) OX=223926 GN=VPA0406 PE=4 SV=1

MAGASLLTLLDDIAAVLDDVALMSKMAAKKTAGVLGDDLALNAQQVSGVASEREIPVVWA

VSKGSFKNKLILVPSALLISAIIPWLIMPLLLIGGLFLCFEGAEKVLEKLFPHSHPHEEK

EELVDTGESLEDYEKRKVAGAIRTDFILSAEIIVIALGTVTGASLVTQILVVSLIAVIMT

IGVYGLVAGIVKLDDLGFYLEIRSKGKGWMAKVGSALVAFAPKLMKLLTIVGTAAMFLVG

GGIVVHNVPAIHHFVEPIIMNFSGHSVATAILPILLNGIIGFVAGLIVVAVWTVVEKLRG

K

>tr|Q87J60|Q87J60_VIBPA Uncharacterized protein OS=Vibrio parahaemolyticus serotype O3:K6 (strain RIMD 2210633) OX=223926 GN=VPA0393 PE=4 SV=1

MRSMLQDSLVLLDYLRGMLLHNEELWLLLFPVMIIIELPLYLLVLTGIFRWSYMREEPEL

KRFPSVSFVITCYGEGEAIGITIDTLVEQIYPGHIEILAVVDGAVQNQDTYKAALNGERR

HTGVRNRKVRVLPKWQRGGRVSTLNAGLSMASGEIVINVDGDTSFDNDMVFTMMKQFADK

NVIASGGALRVRNHNANLLTKMQSLEYMLSMQAGKTGMATWGVLNNISGAFGAFRKNLLK

QVGGWDTHTAEDLDLTMRLKQYKCRYPDNKLAFSTHSIGHTDVPDTLKGLVLQRLRWDGD

LLFLFLRKHNEGLSPRLLGWGNFVFTLAYGVIQNVLLPLLVVIFSVYMVIVYPLKFVLAL

MLMLYFVYLFLSALIFVVYIGLVSERKKEDLKSVKWLFLYPVYQFFMRLITAFSMVNEVV

RRSHEESSMAPWWVLKRGKKF

>tr|Q87NR7|Q87NR7_VIBPA Uncharacterized protein OS=Vibrio parahaemolyticus serotype O3:K6 (strain RIMD 2210633) OX=223926 GN=VP1801 PE=4 SV=1

MKDFTLQKISFAIITSVVGSYVFKKIEQVDSTSFIDNVVNAKVTVPVLGIVFTIILFAAV

LYIGVLRTKLSRTVSSTRTDARYDHAKYVGQVIYENGPKRSKELTDALIEKFDVDVQEAT

RIVSDMAHTKVLIKCLTGGFELCKNYTKRIDVHFKP

>tr|Q87RE6|Q87RE6_VIBPA Zinc ABC transporter, permease protein OS=Vibrio parahaemolyticus serotype O3:K6 (strain RIMD 2210633) OX=223926 GN=VP0851 PE=3 SV=1

MIEFLLPSILAGIGIAIIAGPLGSFVVWRKMAYFGDTLAHASLMGLALGFLLNVNLYLAL

LVCCLALAVLLVTLQRQQLVATDTLLGILAHSSLSIGLVSVSFLDNVRVDLMSYLFGDLL

AVSPEDLMFIYAGVIAVSACLYIFWRPLLSSTVSEELAAVEGVNTDLIRLVLMLMVGVVI

AVGMKFVGALIMTSLLIIPAATARKLSSTPEQMAFFASIIGAISVLMGLSLSWHFDTPAG

PSVVISATSLFMLSQLIQRKA

>tr|Q87LX8|Q87LX8_VIBPA Peptide ABC transporter, permease protein OS=Vibrio parahaemolyticus serotype O3:K6 (strain RIMD 2210633) OX=223926 GN=VP2480 PE=3 SV=1

MGYFLRRLSFYLVALLVAATLNFIIPRAMPGDPVTMMFANASVQVTPERIAAMKELLGFV

DGPIYIQYLSYIKNILSWELGTSIQFYPLSVNSLLGSAFGWSLFLAGTAVVLSFSIASVL

GIFAAWKRGSRYDAFVTPGTLIIQAIPQMVIAMLALFTFSIGLKWFPSGYAYTPGTVPDW

SSWAFIKDVGYHAVLPLFCATIVQIGGFLVNMRNNMINLLAEDYITMAKGKGLSENRVVF

NYAARNALLPSVTALSMSLGMAIGGQLIIEMIFNYPGLGTVLLNAIHARDYQVLQGQLII

MTMFMLCFNLMADMLYMILDPRLRKGGK

>tr|Q87R33|Q87R33_VIBPA Uncharacterized protein OS=Vibrio parahaemolyticus serotype O3:K6 (strain RIMD 2210633) OX=223926 GN=VP0965 PE=4 SV=1

MKLGVKLFGLISWVLMASGCANLSAGNLFSHYSDQNQEVYQAVVAGNYANAEQLQSSDVG

GEILGNFEKGRISFLAKDYPTSLKALEESDRAARVQQDRATISVSETATSVGSLAVNDNL

NEYQPADYELGFLHLYLGLNYLQKNDLEGALVEVRRANQVQEAARKAREKELKSAEKDLK

NQGMSANLGSVLASYPDAGKKLSAVQNGYLFYLSGLLYEASRDLNSAYVDYRRALAVMPD

NKQVIESTMYAAKKLGMREDLRLLEKRYGKAPTGLNKSQGRVIVIDEQGVVEALQGWRID

LPIFDSRGNGAIYSLALPYYPKRGAPRFSDVALNGKTLPSSTLADVNAMAQNDLNERLTT

IVIRQAIRVWAKDRIRKEAAKKGDDVGNILFNVWNTLTEQPDTRSWQTLPAQVKTASQIV

KPGTQQLNLGDQVYQFDVPAQQTTLVWVSRQGAHSTVWHKQLGRL

>tr|Q87P97|Q87P97_VIBPA Amino acid ABC transporter, permease protein OS=Vibrio parahaemolyticus serotype O3:K6 (strain RIMD 2210633) OX=223926 GN=VP1621 PE=3 SV=1

MKPTKDTSPSTMSKPSGSKSLIYNPAFRSAIFQIIAIAALVFFFYTIINNALNNLDARGI

ATGFGFLNQEAGFGIGLTLIEYNETYSYGRTFIVGLLNTALVSVLGIILATAIGFTMGVA

RLSTNWLVSRLAAVYIETFRNIPLLLQIFFWYFAVLQALPSARQSLSLGEAIFLNVRGLY

FPAPVFNEGSGVVIAAFAIGLIATISISIWARNKQRLTGQQTPMGRIGLGLLVGLPLLVY

FVSGMPISLEYPELKGFNFKGGISIIPELAALLLALSVYTAAFIAEIVRSGINAVSHGQT

EAAMSLGLPRAKTLKLVVIPQALRIIIPPLTSQYLNLTKNSSLAMAIGYPDLVSVFAGTT

LNQTGQAIEIIAMTMGVYLTLSLLTSALMNLYNRKVALVER

>tr|Q87TD4|Q87TD4_VIBPA General secretion pathway protein G OS=Vibrio parahaemolyticus serotype O3:K6 (strain RIMD 2210633) OX=223926 GN=VP0136 PE=4 SV=1

MKFKRSKQHGFTLLEVMVVVVILGILASFVVPNLLGNKEKADQQKAITDIVALENALDMY

KLDNSVYPTTDQGLDALVTKPSNPEPRNYRDGGYIKRLPNDPWGNAYQYLSPGDNGTIDI

FTLGADGQEGGEGPAADIGNWNMQDFQ

>tr|Q87FJ0|Q87FJ0_VIBPA Putative transcriptional regulator OS=Vibrio parahaemolyticus serotype O3:K6 (strain RIMD 2210633) OX=223926 GN=VPA1689 PE=4 SV=1

MGKNGLKNALKLLIATLVLFFGMLQTAHAADKRIGILVYDGVLTSDVTAPLEVFGVASRL

TWFSDYEVLTISVSDQKTITTEEGLEIGVDAWIGDQPELDVLVLTSSYDMDALIENKDLI

HFIQTTSKAADWMASNCSGAYLLAEAGLLNGKKATTWAGGENGLQKDYPAVDVQFNQNFV

VDDGVITSNGSLVSYQAALKLLHLMTSESKAQEVADALQYSRFSTQPF

>tr|Q87JU1|Q87JU1_VIBPA Uncharacterized protein OS=Vibrio parahaemolyticus serotype O3:K6 (strain RIMD 2210633) OX=223926 GN=VPA0157 PE=4 SV=1

MLLKSKGVQTWARRLHIYISMALLLVTLFFALTGITLNRPELFERKEPIIQQHTIIIPPQ

TLFLNGESFQPNRVALIDFLTQEVALRGTPSALDVYTEVEQGELVLGELSLDFKGPGYNA

TVFVDMTTGDADIETTNYGAIALLNDLHKGRNSGDVWKWFIDITALLMVFFVLTGVCLLL

PKKKTFRTSLQWMSFGSLLSLVIYFVAVP

>tr|Q87KU8|Q87KU8_VIBPA Putative DNA polymerase III, epsilon subunit OS=Vibrio parahaemolyticus serotype O3:K6 (strain RIMD 2210633) OX=223926 GN=VP2877 PE=4 SV=1

MNWLQRKYWHYKLKGSPYQSLFCAPHKTEFVSLDCETTSLDPNRAELVTIAATKIIDNRI

LTSQPFEVRLRAPQSLDSGSVKIHKIRHQDLADGISEKEALLKLLAFIGNRPLVGYHIRY

DKKILDLACQRQLGFPLPNPLIEVSQIYHDKLERHLPNAYFDLSLDAICKHLELPIQDKH

DALQDAISAALVFVRLTKGDLPNLTAPYT

>tr|Q87N97|Q87N97_VIBPA Uncharacterized protein OS=Vibrio parahaemolyticus serotype O3:K6 (strain RIMD 2210633) OX=223926 GN=VP1978 PE=4 SV=1

MIQQLLVTFPPMLFGAQALLTLLLIKGDICPGQRGRLHKMLPAIGVLWLAVASLRIEAFM

VVFAIFYFYSQVQTKKTREKGPLWALHLANGLAFAYVSIQVFEQPNWPASAAMAVMIFFL

GASFSQLLLTIARSRLQAFHRILPVTGIVSGMLLVLATLFSSYQLDEATLASATQPILIS

LALLISTIVVWCWHIFTHKAPEKVQLSIALLLALASMTSLQGLFALAV

>tr|Q87IT8|Q87IT8_VIBPA GGDEF family protein OS=Vibrio parahaemolyticus serotype O3:K6 (strain RIMD 2210633) OX=223926 GN=VPA0518 PE=4 SV=1

MDHVQISVQKLDEILKLNGSALLNRVTLDIHQQLKSHCTCVVEVAHLQHAAHTISFASGG

EISDNLSYHLSGTPCEKVAKDIGEHIFYQDQVYKRFPEDQMFQDDGVQAYLGLPLKTQSG

EVLGILLSTFTRSIHAKEAQDVLELHRFYANVIIHSLREKWVSERSDKLLNQLSYEVSHD

NLTGLLNRSCLADTLETLTQQATRPFNLAYLDIDNFKSINDINGNYIGDQIIKFTANAIQ

QSLSSPNNAFRVAGDEFAFITYDEDPVAVCQEVLDKIEAGYSDKSNRISFTVSIGIARAP

VHMLNSDELILNASLALKDCKKHRDTRIQHYDTHLSALYHRQTQLIEAMRIQLSTSITES

HELYVVLQPIVDVNNDRWDYFEILARWNSSVYGNISPAEFIEAAEQSGLIIELGERIIEL

ACIAKQELEAHLGYTVKLSINCSAHELVDSNRYINHLTTLLERYGHDASDFVIELTETVL

LSQSGREQMVLNSLRYLGFKVALDDFGTGYSSLNYIHNYPIDSIKIDATFVRNMLSNKTS

EQVVYLIAQLAQLLDVDLIAEGVEDDRALNKLIDMGCHYIQGYFYSRPHNVDELVHMIND

RQVKRA

>tr|Q87FU3|Q87FU3_VIBPA Uncharacterized protein OS=Vibrio parahaemolyticus serotype O3:K6 (strain RIMD 2210633) OX=223926 GN=VPA1585 PE=4 SV=1

MYSSIASKMQRLIGGFVDLFVVISVVVVIALGINFWLYDFRLSLQENFTLVYDTHAVYLL

TVPILLVINWYWLKEGQTIGMRFVGIKVVMKNGRAATKVTVSIRLGISYLLEMFIPGVPL

VNLVLLVFHPERRLLHDFLTKTKVVQAPDLLLSILHDQKKPH

>tr|Q87MI1|Q87MI1_VIBPA Putative glycine cleavage system transcriptional repressor OS=Vibrio parahaemolyticus serotype O3:K6 (strain RIMD 2210633) OX=223926 GN=VP2274 PE=4 SV=1

MKQHLVLTAVGTDRPGICNQVVKLVTQAGCNIVDSRIAIFGNEFTLIMLLTGNASHITRV

ETQLPLLGQEHDLITIMKRTSAHELLDNSYTMEVFIESEDRPGLTEKFTQFFADQQIGLD

SLSAQTISKSKLQLDADQFHIAITASVSADCNLMQLQEDFDELCKSLNVQGSLNFIKNTL

>tr|Q87QU3|Q87QU3_VIBPA Uncharacterized protein OS=Vibrio parahaemolyticus serotype O3:K6 (strain RIMD 2210633) OX=223926 GN=VP1056 PE=4 SV=1

MQGTSNPFRWPITVYYEDTDAGGVVYHSNYLKFFERARTEMLRAKGISQHVLLEQNIGFV

VRHMDIDFKQGARLDEQLTVLTRVSEIKRASLQFCQELVNDQGKILCKAFVKVACIDNKK

MKPIAIPTFINSELTNSDC

>tr|Q87FE1|Q87FE1_VIBPA Putative tryptophan repressor binding protein OS=Vibrio parahaemolyticus serotype O3:K6 (strain RIMD 2210633) OX=223926 GN=VPA1738 PE=4 SV=1

MSKIAIIYFSKTDVTGQLARAIAAGVEQQGIKQQGECEILSHRIEGSEIIEGRFVNPHLM

DELAECDAIIFGSPTYMGGVAAQFKAFADASSESWYHQKWANKVAAGFTSGGALNGDQSC

TLQYLQTFAYQHGMMWVGLDKISNSGEQNLNRYGVQGGIVAQGGEDGQLHSSDVATAEYL

GKRVAALVSKLSATSAT

>tr|Q87NT9|Q87NT9_VIBPA Putative glutamine amidotransferase OS=Vibrio parahaemolyticus serotype O3:K6 (strain RIMD 2210633) OX=223926 GN=VP1779 PE=4 SV=1

MSGTRKPIIGVVSCTKELGGYQIQAVNDFYLRAVKDFGGLPIMLAPEMSGDDVATILDMC

DGFLFPGSHSNVAPHRYNATHEESHKDEARDELSLTLIRHAVDQNIPCLGICRGFQEMNV

ALGGSLNPAVHDSGFNDHREATVEDFEQKYAPAHAVLVQKQSLFEQWLVQNHWENTTFFE

VNTLHNQGVDQLAPQLQVEAKAPDGLVEAFSLPQQKFFVGVQWHPEWKAKNNHFSQILFN

EFMMAASR

>tr|Q87H80|Q87H80_VIBPA Ribose ABC transporter, permease protein OS=Vibrio parahaemolyticus serotype O3:K6 (strain RIMD 2210633) OX=223926 GN=VPA1085 PE=3 SV=1

MSTNTMSKPTETSSKKLFTKEWLIEQKSLIALLFLIVVVSFLNPNFFTVDNILNILRQTS

VNAIIAVGMTLVILTAGIDLSVGSVLALCGAFAASLIAMEVPVLIAVPTALLAGAALGAI

SGIIIAKGKVQAFIATLVTMTLLRGVTMVYTDGRPISTGFTDTADAFAWFGTGYALGIPV

PVWLMVVVFAAAWYLLNHTRFGRYVYALGGNESATRLSGINVDRVKIGVYAICGMLAALA

GIIVTSRLSSAQPTAGMGYELDAIAAVVLGGTSLMGGKGRIMGTLIGALIIGFLNNALNL

LDVSSYYQMIAKAVVILLAVLVDNKNK

>tr|Q87LK9|Q87LK9_VIBPA Iron-regulated outer membrane virulence protein homolog OS=Vibrio parahaemolyticus serotype O3:K6 (strain RIMD 2210633) OX=223926 GN=VP2602 PE=3 SV=1

MSTLRLPVLTATGVLAFVSVPHVFANDSVSKMETVVVTASSYEQSQADAPASISVISREE

LDSRYYRDVTDALKSVPGVVVTGGGDTTDISIRGMGSKYTLILVDGKRQSTRETRPNSDG

PGIEQGWLPPLQAIERIEVIRGPMSTLYGSDAIGGVINVITRKDALEWTGNVQLGTVIQE

NSRSGGEQSANFFVNGPLAENLLLQVYGQYTAREEDDIDYGYEDKDMQSISSKLIYQIND

RHSVQLEGGTSAQSRRGNVGLSVPTTGCRRGCEDSLNEYRRNYVTLSHTGEWELLGNSDT

YLQREESENKSREMTIVNTTFKSSLVKGLGAHTLTTGVDATHAELEDFTSNKASSKTKAS

NTQWAVFIEDEWKIAEPFSLTLGGRLDHDENYGAHFSPRVYGVWRVAPAWTVKGGVATGF

RSPQLREITPGWAQVSGGGNIYGNPDLDPETSLNKEISVLYQGDSGLDVTLTAFHNEFKD

KITRVVCPDTICTDGPNQWGADPTYRINVDEAVTQGVEATLAKPLTETIYLSSSYTFTDS

EQKTGEYKGMPLQQLPKHLFNVDVTWQTTDNLESWTKVTYRGKEMDPVTGPSRNSIVEPA

YTFVDAGVTYQLTDNTKIKGAIYNLFDEDINYKEYGYVEDGRRYWLGLDVAF

>tr|Q87GT6|Q87GT6_VIBPA Uncharacterized protein OS=Vibrio parahaemolyticus serotype O3:K6 (strain RIMD 2210633) OX=223926 GN=VPA1229 PE=4 SV=1

MERWMLRLSIKTRLALLALLPAVVIVAFAIHQFYANSVRVDHLNQTVSNIQGFQLISQAS

HFIYSMEKDRRQYGQETPLSIEVDVQNNVVLSMHHKFSTNPHTSEYADDLKEAMLGMVSG

DLTNTDEVGDWAFQLLQEMSLSLLQNYQLYGNSDGHAMQNFIAYLAQLSYWTQKEAWLTY

RLVMDPDAKLNQSVFFQTIDRQQQNLDAFLHLGASYAHVDKLLGLFSSPRYQRNLESRAR

LMNGDMPRSDYAAYLDELDFRVQRLQMMIEGFTRQAEQSLLTQVNEQKRNVLLITCGVAV

VLLLLGWLGFATWYRVNSKIGAIIHALNALVNEEEKEKKVAVDGSDEFTLFAQQVNRVVE

EKQRQTHEILHAKESAVAANRAKSVFLANMSHEIRTPLNGIMGMTEILSQSELSPHQQEV

VDDIDTSSHTLLALLNDILDLSKIESGRLELSLVEADIREVVYQSVILFQSKATSKQLEL

DISLDENIPARVMVDDHRIKQIITNLVSNAVKFTEQGYILVDVSYEEALEQGRGSLTFLI

KDSGIGIERDKLATIFEPFTQEDEGVSRQFGGTGLGLAICRQLVSMMGGKLVATSTKGVG

TCFGFSIEVEALPLFGWHSDVVKRGLFICDNYAYAEQIVQECRLAQIELVGVNSLSEAKA

LDEDFDVIFLCNDGQMDIDSCLSELAEVYDVRRVVVCQHHLTSSYTNAENVHAVLTQPFL

GNRFKHAIEELAKLEKNTLRDNVTNIASRAESKISRTHRRILIAEDNLMNQKIASFFLDK

AGYDYLITSNGQEALDAITKGEQFDAILMDCMMPVMDGLTATKEIRRWEKKVGCKKTTII

ALTASVLEEDIHNCFAAGMDAYLPKPYKSNQLFELFNELKLA

>tr|Q87LU4|Q87LU4_VIBPA Carbonic anhydrase OS=Vibrio parahaemolyticus serotype O3:K6 (strain RIMD 2210633) OX=223926 GN=VP2514 PE=3 SV=1

MPEIKQLFENNSKWSEEIKSDRPEYFAKLAEGQKPDFLWIGCSDSRVPAERLTGLYSGEL

FVHRNVANQVIHTDLNCLSVVQYAVDVLKVKHIIVCGHYGCGGVNAAIDNPQLGLINNWL

LHIRDLYFKHRSYLDQMPVEDRADKLGEINVAEQVYNLGNSTIMQNAWERGQDVEIHGVV

YGIEDGRLEYLGIRSNSKETVEASYQKALSTILNPDNKLLCR

>tr|Q87FY8|Q87FY8_VIBPA Uncharacterized protein OS=Vibrio parahaemolyticus serotype O3:K6 (strain RIMD 2210633) OX=223926 GN=VPA1532 PE=4 SV=1

MKAKLKAVGKLQQMEEKQRDRVGQQLDAMRQRHNHLSTQLQQLSALKGHAGQSALSSPSL

NSAALMNFNRVDQMLQRMLRHHEQEQAVMQAECASVQKTLEHKHARVQGLEKVLERWRTK

QNYEKAKKEQKLIEDIINSRVKRKTL

>tr|Q87JN6|Q87JN6_VIBPA Uncharacterized protein OS=Vibrio parahaemolyticus serotype O3:K6 (strain RIMD 2210633) OX=223926 GN=VPA0212 PE=4 SV=1

MFKKLTLVSAITAALAGCGADDQAYDYVERDAKEVAVKDLKDGRWFYVPTTGAAPRFALN

QFPFLQGSPRYVELCFTKEGLEVRSYDKNYPDAHLSKDANNYCGEEKFVADDDGVNFAQV

LTIPGDFAAYRCQEDAHGDCTNKEETNEDASLNYKKKTHFTPRPEDLEIAEFNMEDLYGI

TEGIDEIGAPRLISWDFDPKNGLLNFELERTFRIDLDNISDYINFATKASLDEALVDGAF

KSRFYYSLVHESEVATEGYQPILYPVGDENDIGFFTTSTKKLNPVTNKYDRDVVYLNRFN

PDQGSIKYYLSDNFFEEKNKLFLDATLQSIDKMNQALNVFGADAGKPEIEIVNKTKAAGI

HPGDLRYNVINLIDEPLANGLLGYGPSVSNPMTGEIIKGHVNQYAGVARTGVPFYWDNLA

RFYNRNQLDLDGLEPLPTSSESVKTEVADRIEALSTMAAMASLKSDENIHAPLVSKEDIV

SGKAATKSPTQLMKNFNDDMEFEKVVEAEENRLSFWAENNVYPIEASWVSSTNKAMLDNL

KLDDERYFEIKLDDAGNEVSRQLKRWKYLPKELQVKAADAITVATYSNTLVHELGHNMGL

RHNFKGSNDKANYYTLEQAHQLGLNNIPAYSSTMDYAPSMLDETPTWGLYDIAAFKFGYG

RKVETIQDSSGSAPASVAKPADSASDEDKAAYARYLADQQAYQQSFAYKFGNNPDNTSLM

VCSEVKALTGNEKGKSLYNCDFSRFDTAALSDDPELNAKTRYGALYYLDKVNEIERKSYD

FCTDGNVSLNSDCNRFDEGTNLEEIVSYEWQNYLDSYDRRNLELYGTTGLFSSDYPGYLV

RRYMEMSAIRDKMEDLERIDNLYTNLGYTSSTDKPGDFLLRIASNPQYCSEGKADNSWFC

DYANGAKKSAAFFLDILRTPEHQCVIENAAGNQKVISFGQLLDNNSHQIPADYDLSTASC

FDDLAARFIEDSDEGYIAVAETANGRFLNSIGSFDPDYPWSNAVSVLGNWPDKALASHFL

ARRFSNRFTDEVSFASLLDIPGVQAEYEDIMGNIVANDALNTPVKLVGKDGKEYTNLKGV

TVNLHITEQMESLPPMPRGIARFLDIGAEGRGRVGDVILRMGVRQMKSYDYTVKTRGQSQ

FDTFTKQQDRYGIIAGDKVEFVIDGEKFVATKANKLAYDYANQLVTSKGYDLKTYLDSYE

PATLNKVAESIDSAWGPFQTAIVPAFHDPVLLLDAPMVNAMKRTIDADIAAGVLSFSQSF

INFTTANVTSGDLVIDATSGTLSYKGGKAHDELKAFEAYTDAMLLIYNDHTEDMMFAVDN

VYAGYMASSDADKELWLKDLNMIQAYLRGDIGAVAGDYYKMLDRLPTAVSFQ

>tr|Q87JY2|Q87JY2_VIBPA Permease OS=Vibrio parahaemolyticus serotype O3:K6 (strain RIMD 2210633) OX=223926 GN=VPA0116 PE=4 SV=1

MNLILILLGVIAFIVLTTTKFKLHPFLALIIAAFLAAFAYGLPADSIAKTIASGFGGILG

YIGLVIVLGTIIGVILEKSGAAITMADTVIKVLGERFPTLTMSIIGYIVSVPVFCDSGFV

ILNSLKESLAKRLKTSSVAMSVALATGLYATHTFVPPTPGPIAAAGNLGLESNLGLVIGV

GVFVAAVAALAGMLWANRFQHVEPDGIEAADEIQHDWQALKASYGKLPTASQAFAPIFVP

ILLICFGSIAKFPSLPLGEGFVFDVLTFLGQPLTALVIGLFLSVRLLKSDNKIEEFGERI

SQGITAAAPILLITGAGGAFGAVLKATPLGEYLGTTLSALGVGIFMPFIVAAALKSAQGS

STVALVTTSALVAPMLTQLGLDSEMGRVLTVMAIGAGAMTVSHANDSFFWVVSQFSRMSV

GLAYRAQTMATLVQGVTAMALVYILSLVLL

>tr|Q87I08|Q87I08_VIBPA Uncharacterized protein OS=Vibrio parahaemolyticus serotype O3:K6 (strain RIMD 2210633) OX=223926 GN=VPA0798 PE=4 SV=1

MSKDFEFTTEYTLDKPFFAECYDQTSQPVKFPQAYLKGILFLIFGVVLLEFELLPNGYVG

WFFIVLSVIEACSVYFKRTWWLWRQTISSSRGNKVVFEVNADSVRYKSGKIDRSLAWSEI

DQLEQTDLGFILHLGKQRQYISKSCLNEEVIAFMVEQHAGSKAS

>tr|Q87M36|Q87M36_VIBPA Uncharacterized protein OS=Vibrio parahaemolyticus serotype O3:K6 (strain RIMD 2210633) OX=223926 GN=VP2422 PE=4 SV=1

MLSELVIWSLLIAIGVSDAQKHRIPNKAVLLLLVAVTANVLYSPSVSLLDHAYGGLVAFA

VCFALYLVKAMAGGDVKLLAVIGAWLGLSNLWEASIGIILAGGIVGIFYLMLHIASTSVA

LTHQVKGYCIEKVTPGFRSNKPLVIPFAPVIVIGLAYFSYTH

>tr|Q87HC5|Q87HC5_VIBPA Uncharacterized protein OS=Vibrio parahaemolyticus serotype O3:K6 (strain RIMD 2210633) OX=223926 GN=VPA1040 PE=4 SV=1

MVSMEQTIVKPTPGGRAAVSKAQPQRSADSTVVISKNPELVNNDSVVAYGDNPLLAEANG

LLSIIGQIRATATHSDPLFLKETLAQKLRDYENRLRQHDVDLETIDTARYCLCCSLDEAV

LNTNWGSQSFWTHDSLLSSFYASSQGGEAFFKHLDSCLAHPESHLDLLELMYVCLSLGFI

GQYRLEKNGLEAHRRLRKQVVSVLKSHGRGVQQELSNKVEQHILAGAQVSERAPLWVVCS

VTSALLVCIFMYFSYELNKASNQTFAQLVNLIQPTPAVSNPMVESKSAPIAERISMYLAT

EIGKDLVTVEALQDRVRISLKAQDLFESGSASVVAYIQPVISKVARTLEATQGKIIITGH

TDDRPIFTSKYPSNWHLSLARATSLSEQLISNSALKGRVIPEGLGDARPLVENDSEKNRA

MNRRIEIDLIVGN

>tr|Q87H41|Q87H41_VIBPA Putative transcriptional regulator OS=Vibrio parahaemolyticus serotype O3:K6 (strain RIMD 2210633) OX=223926 GN=VPA1124 PE=4 SV=1

MNTFKISELAKEFDITTRSIRFYEDLGLLTPERKGNTRIYNGRDRIRLKLILRGKRLGFS

LADIKELFELYDTDQSTEQLNYMIRLIEEKKAALQQQANDIQAVMMELNAAQLRCQNTLR

SMKGEKVT

>tr|Q87N76|Q87N76_VIBPA Uncharacterized protein OS=Vibrio parahaemolyticus serotype O3:K6 (strain RIMD 2210633) OX=223926 GN=VP1999 PE=3 SV=1

MTKRWLVSAVSIALLGGGTYFYLQSAAQPELLPTLIVEKGTIEKQAVAVGKIVPAHSVSI

KSQIDGIVGEIYAKVGEKVKQGQPLIKVRPNPTPQALTDASAELMRSEADLESAKQKLSN

LESLVKQDIIPSNYDEYVSARSAVKSAQADVLQKRQNLELIRSGEASIGDARLTSTIYAP

IDGTVLNQKVEVGEPIISTQSSQAATEMMSLADMNSLIFKGSVSEHDAAQLSPGMPVMLT

VAPYPDVAISGVLTKVAIQSENLNSPEGNASAKSFDNGFEVEVGELKIPQDVLLRSGFSS

TAQIILKKSENVLTLPERALQFDGDAPNVLIPDSSEQGFHKQPVKLGLSDGINVEVLDGV

ELDEEVIDNSMMGAAHG

>tr|Q87QS9|Q87QS9_VIBPA L-alanine exporter AlaE OS=Vibrio parahaemolyticus serotype O3:K6 (strain RIMD 2210633) OX=223926 GN=alaE PE=3 SV=1

MKSRGPFCIRHAAADTFAMVVFCFITGMIIEILISGMTFEQSLASRTLSIPVNIAIAWPY

GMFRDWVLRQGNKLSPSSFMKNVSDLVSYVLFQSPVYAGILLAVGASYDQIVTAVASNAV

VSCGMGVLYGYFLDMCRRWFRVPGYHQGV

>tr|Q87GN4|Q87GN4_VIBPA Oxidoreductase, Gfo/Idh/MocA family OS=Vibrio parahaemolyticus serotype O3:K6 (strain RIMD 2210633) OX=223926 GN=VPA1281 PE=4 SV=1

MKIGIIGLGDIAQKVYLPVITQLPNVELVFCTRDAETLSSLAQQYRIMENCQDYRQLTAF

GVDAVMIHAATHVHFQIAEYFLKQSIPTFVDKPLADSAQQVEQLYEIAAIANQPLYVGFN

RRHIPLYNDYLPNVQKGDVANLKSLRWEKNRHNLPGELRTFIFDDFIHPLDSVNVVAKAD

LQDVYITHQFDGQQLARLDVQWQHGDTLLHASMNRHFGITNERVQACYANQAFEFDSFVE

GKLWQDNQERKLNLKDWTPMLTSKGFHAMLFDWFKVVESGKLATSTVQRNIASHQLAEQI

YQRIEQAVHCN

>tr|Q87IV3|Q87IV3_VIBPA Putative transcriptional regulator OS=Vibrio parahaemolyticus serotype O3:K6 (strain RIMD 2210633) OX=223926 GN=VPA0503 PE=4 SV=1

MSREDELLEKLDECENATAFLQVSNKIINLKLKALLPSVFVQDDLVKEYAVDPLLREDGP

LVTTDVVSKLMFAMGKISLQTYADIGLYDQVLEYVVTQPEKIAFTDDVIYDFIKNQAVLS

SQQDCFYLESINQLKFSSFESFSQMRYESLIKTVLKLSCEMLIERIEEEINQ

>tr|Q87LX7|Q87LX7_VIBPA Peptide ABC transporter, permease protein OS=Vibrio parahaemolyticus serotype O3:K6 (strain RIMD 2210633) OX=223926 GN=VP2481 PE=3 SV=1

MKNLFKLILGNSFARIGLAIITIFIFVAVAAPLITKHAPDKRTGNPHEYPSFVVKQAQSN

PDGWVAKNLADDRRTLIMSKKADHVLGTSRMGRDIWSQVAYGARVSLGVGFGAGIIVCFL

ATVIGISAGYFGGKVDDVLSAAMNIMLVIPQYPLLFVLAAFIGEAGPLTIALIIAGTSWA

WGARVVRSQTMALREKEFVKAAEVLGESPFRIIFVEILPNLIPIVGASFIGSVMLAINTE

AVISFLGLGDANTISWGIMLYNVQTSSAMLIGAWWEVLAPCIALTLLVTGLALLNFAVDE

IANPQLRSHKGMKRWKKLAAKDKKEREPELAPQNALWSGDK

>tr|Q87FF6|Q87FF6_VIBPA Putative ribosomal protein N-acetyltransferase OS=Vibrio parahaemolyticus serotype O3:K6 (strain RIMD 2210633) OX=223926 GN=VPA1723 PE=4 SV=1

MIFRSFFTHRIASMETKRLKLIPACLERAEEAHQAVVRSQKTLEVYLPWVPHVLTLEAMI

EGTEKAIANFENFEEELRYYIIEKESDRLLGAVGLMIRDPDVPSFEIGYWLDDAAVGNGY

VAEAVLEVERYAFDDLGAKRIAIHADSTNQKSRAVAERCGYEFEGELRNERLTTSGELSN

TVIYSKIRE

>tr|Q87M10|Q87M10_VIBPA Putative protease OS=Vibrio parahaemolyticus serotype O3:K6 (strain RIMD 2210633) OX=223926 GN=VP2448 PE=4 SV=1

MENSMKYALGPLLYFWPKQDIEAFYLQAKESSADIIYLGETVCSKRREMKPAHWFDIAKD

LSASGKQVVLSTMALLEAPSEVNIMKKYIDNGDFAIEANDVSAVQLASEHKVPFVVGPAI

NTYNAHTLNLFLKQGMTRWCMPVELSREWLSDTLTQCEDLGIRNKFEVEVFSHGYLPLAY

SARCFTARAENKAKDDCETCCIKYPTGIQVSSQEGQEVFNLNGIQTQSGYCYNLINDLPS

MQGLVDVVRLSPLGVSTFSELDRFRSNEQGSNPDKLSSRQCNGYWHQLAGLEVKNI

>tr|Q87M28|Q87M28_VIBPA Uncharacterized protein OS=Vibrio parahaemolyticus serotype O3:K6 (strain RIMD 2210633) OX=223926 GN=VP2430 PE=4 SV=1

MQQSEILSLAERLIPVYGSEDFDFVLGQLTEEEPPSAKILVKMELNRLMAPCKKSIDLRG

RVKGECREYVLDGISHWLDDVAFNAYHKNIKKFGTYTEGMWEALVNTRNNFRVMSKLATQ

EKSHSLTDPKSPFLAEPVHLGYDLKRQEKRLKVQSQVEIRRTKGQILHGLSIDLSSSGAK

FKVPSAFKYNLGEIIQVSFTEFAGKSQVAGIEKPLSYRVLAVDDCYENDAVRYLRTIRLT

ETNVVARVIDEALNSAAKRARHDNQDKIIRARTRGYEHIALKHTSNLPLFFDGNELKLAM

LTPNNQKLWQYWHDERNQQVFGSLFNEQRVASLIKQGVKGTSNVLYSFTHEHENKTYFYS

MLRPEATREQRQLFWHLGAKRESWRAFRISIFELSEQEKEDLALFSPELAETSQSLTHIG

ILQEIADEKSGQDYLLTEKPRLPANTLNAFRHPRKITGNPKGIYFDAQSRRKEPRYRFKT

PLELSSGELKAAGSTVDISKRGLGIRLDEPTMLRSGQEVQVNFRELQLYDKKLPLMAVPY

RVIRVSPDGCSVQLVIDENSKTIRVIAFFNSIIEHNQDKLIPQKEMLPSNALLERLHSVL

LSRTLSTPIFISKSTASALKTPVVGVNLPLPKHIELFARLGHDQKFSLEPIFKGRSSTLI

AEPIKRVDGAQARHQDIYISAAKIGNKITAIESKLHQEFNSVKERIQFIKKARMMGDFYV

FRMSTAPIFDPMTSLLRQDLSDLTQFGVHQASKLEKELTALVGYSEFEDITEEVVVRLEL

TE

>tr|Q87HT5|Q87HT5_VIBPA Arylesterase OS=Vibrio parahaemolyticus serotype O3:K6 (strain RIMD 2210633) OX=223926 GN=VPA0872 PE=4 SV=1

MIRLLSLVLFFCLSAASQASEKLLVLGDSLSAGYQMPIEKSWPSLLPDALLEHGQDVTVI

NGSISGDTTGNGLARLPQLLDQHTPDLVLIELGANDGLRGFPPKVITSNLSKMISMIKDS

GANVVMMQIRVPPNYGKRYSDMFYDIYPKLAEHQQVQLMPFFLEHVITKPEWMMDDGLHP

KPEAQPWIAEFVAQELVKHL

>tr|Q87RI1|Q87RI1_VIBPA Putative nitrogen regulatory protein P-II family protein OS=Vibrio parahaemolyticus serotype O3:K6 (strain RIMD 2210633) OX=223926 GN=VP0813 PE=3 SV=1

MRFKLILAFVEDSKTDKVLDAARDAGATGATVINNARGQGLNQKRTFFGLTLEVQKDVLL

FVVEEHLSRHILETINEVGEFDRESGQGIAIQIDIEDAVGVAHQVETLTKVVEDEL

>tr|Q87TL4|Q87TL4_VIBPA Putative RNA polymerase ECF-type sigma factor OS=Vibrio parahaemolyticus serotype O3:K6 (strain RIMD 2210633) OX=223926 GN=VP0055 PE=4 SV=1

MATTSALTHIIQQWQSGNKQAESELYQFAYLQLRKIAQQERERNAEKYGTDNMVLADSVN

STTALIHDAYLKMSNCDMSEIATKRDFFLMAAKVMRQILIDNARSHQAQKRQHITLMKDE

EDRFEQLIIMDKALDSFSTRYPRQSSALKLKYLMGMKNQEISELLECSASLIEKDLKFSR

SWLQSRMAHA

>tr|Q87TN5|Q87TN5_VIBPA Transcriptional activator IlvY OS=Vibrio parahaemolyticus serotype O3:K6 (strain RIMD 2210633) OX=223926 GN=VP0034 PE=4 SV=1

MNIKSLQLFIHLCESKSFAKTAAAMHISPSALSRQIQKLEEETNQQLFVRDNRSVELTTQ

GKKLMPVALKILSEWQQYQNHIKGTEGELKGEIRLFCSVTASYSHLPELISDFRLQHPFI

EFKLSTGDPAQAIDKILADEADIAISAKSEQMPNKIAFETISEIPLSVIAPVGVSSFAEE

LQKEQPDWSIIPFILPEAGTARDRANTWLKKMKIKPNIYAQTSGHEAIVSMVALGCGVGI

APDVVINNSPVREKINRLKVLPIKPFELGVCCTKNQLENPLVKAFWKVAESKYIAP

>tr|Q87NT9|Q87NT9_VIBPA Putative glutamine amidotransferase OS=Vibrio parahaemolyticus serotype O3:K6 (strain RIMD 2210633) OX=223926 GN=VP1779 PE=4 SV=1

MSGTRKPIIGVVSCTKELGGYQIQAVNDFYLRAVKDFGGLPIMLAPEMSGDDVATILDMC

DGFLFPGSHSNVAPHRYNATHEESHKDEARDELSLTLIRHAVDQNIPCLGICRGFQEMNV

ALGGSLNPAVHDSGFNDHREATVEDFEQKYAPAHAVLVQKQSLFEQWLVQNHWENTTFFE

VNTLHNQGVDQLAPQLQVEAKAPDGLVEAFSLPQQKFFVGVQWHPEWKAKNNHFSQILFN

EFMMAASR

>tr|Q87H80|Q87H80_VIBPA Ribose ABC transporter, permease protein OS=Vibrio parahaemolyticus serotype O3:K6 (strain RIMD 2210633) OX=223926 GN=VPA1085 PE=3 SV=1

MSTNTMSKPTETSSKKLFTKEWLIEQKSLIALLFLIVVVSFLNPNFFTVDNILNILRQTS

VNAIIAVGMTLVILTAGIDLSVGSVLALCGAFAASLIAMEVPVLIAVPTALLAGAALGAI

SGIIIAKGKVQAFIATLVTMTLLRGVTMVYTDGRPISTGFTDTADAFAWFGTGYALGIPV

PVWLMVVVFAAAWYLLNHTRFGRYVYALGGNESATRLSGINVDRVKIGVYAICGMLAALA

GIIVTSRLSSAQPTAGMGYELDAIAAVVLGGTSLMGGKGRIMGTLIGALIIGFLNNALNL

LDVSSYYQMIAKAVVILLAVLVDNKNK

>tr|Q87LK9|Q87LK9_VIBPA Iron-regulated outer membrane virulence protein homolog OS=Vibrio parahaemolyticus serotype O3:K6 (strain RIMD 2210633) OX=223926 GN=VP2602 PE=3 SV=1

MSTLRLPVLTATGVLAFVSVPHVFANDSVSKMETVVVTASSYEQSQADAPASISVISREE

LDSRYYRDVTDALKSVPGVVVTGGGDTTDISIRGMGSKYTLILVDGKRQSTRETRPNSDG

PGIEQGWLPPLQAIERIEVIRGPMSTLYGSDAIGGVINVITRKDALEWTGNVQLGTVIQE

NSRSGGEQSANFFVNGPLAENLLLQVYGQYTAREEDDIDYGYEDKDMQSISSKLIYQIND

RHSVQLEGGTSAQSRRGNVGLSVPTTGCRRGCEDSLNEYRRNYVTLSHTGEWELLGNSDT

YLQREESENKSREMTIVNTTFKSSLVKGLGAHTLTTGVDATHAELEDFTSNKASSKTKAS

NTQWAVFIEDEWKIAEPFSLTLGGRLDHDENYGAHFSPRVYGVWRVAPAWTVKGGVATGF

RSPQLREITPGWAQVSGGGNIYGNPDLDPETSLNKEISVLYQGDSGLDVTLTAFHNEFKD

KITRVVCPDTICTDGPNQWGADPTYRINVDEAVTQGVEATLAKPLTETIYLSSSYTFTDS

EQKTGEYKGMPLQQLPKHLFNVDVTWQTTDNLESWTKVTYRGKEMDPVTGPSRNSIVEPA

YTFVDAGVTYQLTDNTKIKGAIYNLFDEDINYKEYGYVEDGRRYWLGLDVAF

>tr|Q87GT6|Q87GT6_VIBPA Uncharacterized protein OS=Vibrio parahaemolyticus serotype O3:K6 (strain RIMD 2210633) OX=223926 GN=VPA1229 PE=4 SV=1

MERWMLRLSIKTRLALLALLPAVVIVAFAIHQFYANSVRVDHLNQTVSNIQGFQLISQAS

HFIYSMEKDRRQYGQETPLSIEVDVQNNVVLSMHHKFSTNPHTSEYADDLKEAMLGMVSG

DLTNTDEVGDWAFQLLQEMSLSLLQNYQLYGNSDGHAMQNFIAYLAQLSYWTQKEAWLTY

RLVMDPDAKLNQSVFFQTIDRQQQNLDAFLHLGASYAHVDKLLGLFSSPRYQRNLESRAR

LMNGDMPRSDYAAYLDELDFRVQRLQMMIEGFTRQAEQSLLTQVNEQKRNVLLITCGVAV

VLLLLGWLGFATWYRVNSKIGAIIHALNALVNEEEKEKKVAVDGSDEFTLFAQQVNRVVE

EKQRQTHEILHAKESAVAANRAKSVFLANMSHEIRTPLNGIMGMTEILSQSELSPHQQEV

VDDIDTSSHTLLALLNDILDLSKIESGRLELSLVEADIREVVYQSVILFQSKATSKQLEL

DISLDENIPARVMVDDHRIKQIITNLVSNAVKFTEQGYILVDVSYEEALEQGRGSLTFLI

KDSGIGIERDKLATIFEPFTQEDEGVSRQFGGTGLGLAICRQLVSMMGGKLVATSTKGVG

TCFGFSIEVEALPLFGWHSDVVKRGLFICDNYAYAEQIVQECRLAQIELVGVNSLSEAKA

LDEDFDVIFLCNDGQMDIDSCLSELAEVYDVRRVVVCQHHLTSSYTNAENVHAVLTQPFL

GNRFKHAIEELAKLEKNTLRDNVTNIASRAESKISRTHRRILIAEDNLMNQKIASFFLDK

AGYDYLITSNGQEALDAITKGEQFDAILMDCMMPVMDGLTATKEIRRWEKKVGCKKTTII

ALTASVLEEDIHNCFAAGMDAYLPKPYKSNQLFELFNELKLA

>tr|Q87LU4|Q87LU4_VIBPA Carbonic anhydrase OS=Vibrio parahaemolyticus serotype O3:K6 (strain RIMD 2210633) OX=223926 GN=VP2514 PE=3 SV=1

MPEIKQLFENNSKWSEEIKSDRPEYFAKLAEGQKPDFLWIGCSDSRVPAERLTGLYSGEL

FVHRNVANQVIHTDLNCLSVVQYAVDVLKVKHIIVCGHYGCGGVNAAIDNPQLGLINNWL

LHIRDLYFKHRSYLDQMPVEDRADKLGEINVAEQVYNLGNSTIMQNAWERGQDVEIHGVV

YGIEDGRLEYLGIRSNSKETVEASYQKALSTILNPDNKLLCR

>tr|Q87FY8|Q87FY8_VIBPA Uncharacterized protein OS=Vibrio parahaemolyticus serotype O3:K6 (strain RIMD 2210633) OX=223926 GN=VPA1532 PE=4 SV=1

MKAKLKAVGKLQQMEEKQRDRVGQQLDAMRQRHNHLSTQLQQLSALKGHAGQSALSSPSL

NSAALMNFNRVDQMLQRMLRHHEQEQAVMQAECASVQKTLEHKHARVQGLEKVLERWRTK

QNYEKAKKEQKLIEDIINSRVKRKTL

>tr|Q87JN6|Q87JN6_VIBPA Uncharacterized protein OS=Vibrio parahaemolyticus serotype O3:K6 (strain RIMD 2210633) OX=223926 GN=VPA0212 PE=4 SV=1

MFKKLTLVSAITAALAGCGADDQAYDYVERDAKEVAVKDLKDGRWFYVPTTGAAPRFALN

QFPFLQGSPRYVELCFTKEGLEVRSYDKNYPDAHLSKDANNYCGEEKFVADDDGVNFAQV

LTIPGDFAAYRCQEDAHGDCTNKEETNEDASLNYKKKTHFTPRPEDLEIAEFNMEDLYGI

TEGIDEIGAPRLISWDFDPKNGLLNFELERTFRIDLDNISDYINFATKASLDEALVDGAF

KSRFYYSLVHESEVATEGYQPILYPVGDENDIGFFTTSTKKLNPVTNKYDRDVVYLNRFN

PDQGSIKYYLSDNFFEEKNKLFLDATLQSIDKMNQALNVFGADAGKPEIEIVNKTKAAGI

HPGDLRYNVINLIDEPLANGLLGYGPSVSNPMTGEIIKGHVNQYAGVARTGVPFYWDNLA

RFYNRNQLDLDGLEPLPTSSESVKTEVADRIEALSTMAAMASLKSDENIHAPLVSKEDIV

SGKAATKSPTQLMKNFNDDMEFEKVVEAEENRLSFWAENNVYPIEASWVSSTNKAMLDNL

KLDDERYFEIKLDDAGNEVSRQLKRWKYLPKELQVKAADAITVATYSNTLVHELGHNMGL

RHNFKGSNDKANYYTLEQAHQLGLNNIPAYSSTMDYAPSMLDETPTWGLYDIAAFKFGYG

RKVETIQDSSGSAPASVAKPADSASDEDKAAYARYLADQQAYQQSFAYKFGNNPDNTSLM

VCSEVKALTGNEKGKSLYNCDFSRFDTAALSDDPELNAKTRYGALYYLDKVNEIERKSYD

FCTDGNVSLNSDCNRFDEGTNLEEIVSYEWQNYLDSYDRRNLELYGTTGLFSSDYPGYLV

RRYMEMSAIRDKMEDLERIDNLYTNLGYTSSTDKPGDFLLRIASNPQYCSEGKADNSWFC

DYANGAKKSAAFFLDILRTPEHQCVIENAAGNQKVISFGQLLDNNSHQIPADYDLSTASC

FDDLAARFIEDSDEGYIAVAETANGRFLNSIGSFDPDYPWSNAVSVLGNWPDKALASHFL

ARRFSNRFTDEVSFASLLDIPGVQAEYEDIMGNIVANDALNTPVKLVGKDGKEYTNLKGV

TVNLHITEQMESLPPMPRGIARFLDIGAEGRGRVGDVILRMGVRQMKSYDYTVKTRGQSQ

FDTFTKQQDRYGIIAGDKVEFVIDGEKFVATKANKLAYDYANQLVTSKGYDLKTYLDSYE

PATLNKVAESIDSAWGPFQTAIVPAFHDPVLLLDAPMVNAMKRTIDADIAAGVLSFSQSF

INFTTANVTSGDLVIDATSGTLSYKGGKAHDELKAFEAYTDAMLLIYNDHTEDMMFAVDN

VYAGYMASSDADKELWLKDLNMIQAYLRGDIGAVAGDYYKMLDRLPTAVSFQ

>tr|Q87JY2|Q87JY2_VIBPA Permease OS=Vibrio parahaemolyticus serotype O3:K6 (strain RIMD 2210633) OX=223926 GN=VPA0116 PE=4 SV=1

MNLILILLGVIAFIVLTTTKFKLHPFLALIIAAFLAAFAYGLPADSIAKTIASGFGGILG

YIGLVIVLGTIIGVILEKSGAAITMADTVIKVLGERFPTLTMSIIGYIVSVPVFCDSGFV

ILNSLKESLAKRLKTSSVAMSVALATGLYATHTFVPPTPGPIAAAGNLGLESNLGLVIGV

GVFVAAVAALAGMLWANRFQHVEPDGIEAADEIQHDWQALKASYGKLPTASQAFAPIFVP

ILLICFGSIAKFPSLPLGEGFVFDVLTFLGQPLTALVIGLFLSVRLLKSDNKIEEFGERI

SQGITAAAPILLITGAGGAFGAVLKATPLGEYLGTTLSALGVGIFMPFIVAAALKSAQGS

STVALVTTSALVAPMLTQLGLDSEMGRVLTVMAIGAGAMTVSHANDSFFWVVSQFSRMSV

GLAYRAQTMATLVQGVTAMALVYILSLVLL

>tr|Q87I08|Q87I08_VIBPA Uncharacterized protein OS=Vibrio parahaemolyticus serotype O3:K6 (strain RIMD 2210633) OX=223926 GN=VPA0798 PE=4 SV=1

MSKDFEFTTEYTLDKPFFAECYDQTSQPVKFPQAYLKGILFLIFGVVLLEFELLPNGYVG

WFFIVLSVIEACSVYFKRTWWLWRQTISSSRGNKVVFEVNADSVRYKSGKIDRSLAWSEI

DQLEQTDLGFILHLGKQRQYISKSCLNEEVIAFMVEQHAGSKAS

>tr|Q87M36|Q87M36_VIBPA Uncharacterized protein OS=Vibrio parahaemolyticus serotype O3:K6 (strain RIMD 2210633) OX=223926 GN=VP2422 PE=4 SV=1

MLSELVIWSLLIAIGVSDAQKHRIPNKAVLLLLVAVTANVLYSPSVSLLDHAYGGLVAFA

VCFALYLVKAMAGGDVKLLAVIGAWLGLSNLWEASIGIILAGGIVGIFYLMLHIASTSVA

LTHQVKGYCIEKVTPGFRSNKPLVIPFAPVIVIGLAYFSYTH

>tr|Q87HC5|Q87HC5_VIBPA Uncharacterized protein OS=Vibrio parahaemolyticus serotype O3:K6 (strain RIMD 2210633) OX=223926 GN=VPA1040 PE=4 SV=1

MVSMEQTIVKPTPGGRAAVSKAQPQRSADSTVVISKNPELVNNDSVVAYGDNPLLAEANG

LLSIIGQIRATATHSDPLFLKETLAQKLRDYENRLRQHDVDLETIDTARYCLCCSLDEAV

LNTNWGSQSFWTHDSLLSSFYASSQGGEAFFKHLDSCLAHPESHLDLLELMYVCLSLGFI

GQYRLEKNGLEAHRRLRKQVVSVLKSHGRGVQQELSNKVEQHILAGAQVSERAPLWVVCS

VTSALLVCIFMYFSYELNKASNQTFAQLVNLIQPTPAVSNPMVESKSAPIAERISMYLAT

EIGKDLVTVEALQDRVRISLKAQDLFESGSASVVAYIQPVISKVARTLEATQGKIIITGH

TDDRPIFTSKYPSNWHLSLARATSLSEQLISNSALKGRVIPEGLGDARPLVENDSEKNRA

MNRRIEIDLIVGN

>tr|Q87H41|Q87H41_VIBPA Putative transcriptional regulator OS=Vibrio parahaemolyticus serotype O3:K6 (strain RIMD 2210633) OX=223926 GN=VPA1124 PE=4 SV=1

MNTFKISELAKEFDITTRSIRFYEDLGLLTPERKGNTRIYNGRDRIRLKLILRGKRLGFS

LADIKELFELYDTDQSTEQLNYMIRLIEEKKAALQQQANDIQAVMMELNAAQLRCQNTLR

SMKGEKVT

>tr|Q87N76|Q87N76_VIBPA Uncharacterized protein OS=Vibrio parahaemolyticus serotype O3:K6 (strain RIMD 2210633) OX=223926 GN=VP1999 PE=3 SV=1

MTKRWLVSAVSIALLGGGTYFYLQSAAQPELLPTLIVEKGTIEKQAVAVGKIVPAHSVSI

KSQIDGIVGEIYAKVGEKVKQGQPLIKVRPNPTPQALTDASAELMRSEADLESAKQKLSN

LESLVKQDIIPSNYDEYVSARSAVKSAQADVLQKRQNLELIRSGEASIGDARLTSTIYAP

IDGTVLNQKVEVGEPIISTQSSQAATEMMSLADMNSLIFKGSVSEHDAAQLSPGMPVMLT

VAPYPDVAISGVLTKVAIQSENLNSPEGNASAKSFDNGFEVEVGELKIPQDVLLRSGFSS

TAQIILKKSENVLTLPERALQFDGDAPNVLIPDSSEQGFHKQPVKLGLSDGINVEVLDGV

ELDEEVIDNSMMGAAHG

>tr|Q87QS9|Q87QS9_VIBPA L-alanine exporter AlaE OS=Vibrio parahaemolyticus serotype O3:K6 (strain RIMD 2210633) OX=223926 GN=alaE PE=3 SV=1

MKSRGPFCIRHAAADTFAMVVFCFITGMIIEILISGMTFEQSLASRTLSIPVNIAIAWPY

GMFRDWVLRQGNKLSPSSFMKNVSDLVSYVLFQSPVYAGILLAVGASYDQIVTAVASNAV

VSCGMGVLYGYFLDMCRRWFRVPGYHQGV

>tr|Q87GN4|Q87GN4_VIBPA Oxidoreductase, Gfo/Idh/MocA family OS=Vibrio parahaemolyticus serotype O3:K6 (strain RIMD 2210633) OX=223926 GN=VPA1281 PE=4 SV=1

MKIGIIGLGDIAQKVYLPVITQLPNVELVFCTRDAETLSSLAQQYRIMENCQDYRQLTAF

GVDAVMIHAATHVHFQIAEYFLKQSIPTFVDKPLADSAQQVEQLYEIAAIANQPLYVGFN

RRHIPLYNDYLPNVQKGDVANLKSLRWEKNRHNLPGELRTFIFDDFIHPLDSVNVVAKAD

LQDVYITHQFDGQQLARLDVQWQHGDTLLHASMNRHFGITNERVQACYANQAFEFDSFVE

GKLWQDNQERKLNLKDWTPMLTSKGFHAMLFDWFKVVESGKLATSTVQRNIASHQLAEQI

YQRIEQAVHCN

>tr|Q87IV3|Q87IV3_VIBPA Putative transcriptional regulator OS=Vibrio parahaemolyticus serotype O3:K6 (strain RIMD 2210633) OX=223926 GN=VPA0503 PE=4 SV=1

MSREDELLEKLDECENATAFLQVSNKIINLKLKALLPSVFVQDDLVKEYAVDPLLREDGP

LVTTDVVSKLMFAMGKISLQTYADIGLYDQVLEYVVTQPEKIAFTDDVIYDFIKNQAVLS

SQQDCFYLESINQLKFSSFESFSQMRYESLIKTVLKLSCEMLIERIEEEINQ

>tr|Q87LX7|Q87LX7_VIBPA Peptide ABC transporter, permease protein OS=Vibrio parahaemolyticus serotype O3:K6 (strain RIMD 2210633) OX=223926 GN=VP2481 PE=3 SV=1

MKNLFKLILGNSFARIGLAIITIFIFVAVAAPLITKHAPDKRTGNPHEYPSFVVKQAQSN

PDGWVAKNLADDRRTLIMSKKADHVLGTSRMGRDIWSQVAYGARVSLGVGFGAGIIVCFL

ATVIGISAGYFGGKVDDVLSAAMNIMLVIPQYPLLFVLAAFIGEAGPLTIALIIAGTSWA

WGARVVRSQTMALREKEFVKAAEVLGESPFRIIFVEILPNLIPIVGASFIGSVMLAINTE

AVISFLGLGDANTISWGIMLYNVQTSSAMLIGAWWEVLAPCIALTLLVTGLALLNFAVDE

IANPQLRSHKGMKRWKKLAAKDKKEREPELAPQNALWSGDK

>tr|Q87FF6|Q87FF6_VIBPA Putative ribosomal protein N-acetyltransferase OS=Vibrio parahaemolyticus serotype O3:K6 (strain RIMD 2210633) OX=223926 GN=VPA1723 PE=4 SV=1

MIFRSFFTHRIASMETKRLKLIPACLERAEEAHQAVVRSQKTLEVYLPWVPHVLTLEAMI

EGTEKAIANFENFEEELRYYIIEKESDRLLGAVGLMIRDPDVPSFEIGYWLDDAAVGNGY

VAEAVLEVERYAFDDLGAKRIAIHADSTNQKSRAVAERCGYEFEGELRNERLTTSGELSN

TVIYSKIRE

>tr|Q87M10|Q87M10_VIBPA Putative protease OS=Vibrio parahaemolyticus serotype O3:K6 (strain RIMD 2210633) OX=223926 GN=VP2448 PE=4 SV=1

MENSMKYALGPLLYFWPKQDIEAFYLQAKESSADIIYLGETVCSKRREMKPAHWFDIAKD

LSASGKQVVLSTMALLEAPSEVNIMKKYIDNGDFAIEANDVSAVQLASEHKVPFVVGPAI

NTYNAHTLNLFLKQGMTRWCMPVELSREWLSDTLTQCEDLGIRNKFEVEVFSHGYLPLAY

SARCFTARAENKAKDDCETCCIKYPTGIQVSSQEGQEVFNLNGIQTQSGYCYNLINDLPS

MQGLVDVVRLSPLGVSTFSELDRFRSNEQGSNPDKLSSRQCNGYWHQLAGLEVKNI

>tr|Q87M28|Q87M28_VIBPA Uncharacterized protein OS=Vibrio parahaemolyticus serotype O3:K6 (strain RIMD 2210633) OX=223926 GN=VP2430 PE=4 SV=1

MQQSEILSLAERLIPVYGSEDFDFVLGQLTEEEPPSAKILVKMELNRLMAPCKKSIDLRG

RVKGECREYVLDGISHWLDDVAFNAYHKNIKKFGTYTEGMWEALVNTRNNFRVMSKLATQ

EKSHSLTDPKSPFLAEPVHLGYDLKRQEKRLKVQSQVEIRRTKGQILHGLSIDLSSSGAK

FKVPSAFKYNLGEIIQVSFTEFAGKSQVAGIEKPLSYRVLAVDDCYENDAVRYLRTIRLT

ETNVVARVIDEALNSAAKRARHDNQDKIIRARTRGYEHIALKHTSNLPLFFDGNELKLAM

LTPNNQKLWQYWHDERNQQVFGSLFNEQRVASLIKQGVKGTSNVLYSFTHEHENKTYFYS

MLRPEATREQRQLFWHLGAKRESWRAFRISIFELSEQEKEDLALFSPELAETSQSLTHIG

ILQEIADEKSGQDYLLTEKPRLPANTLNAFRHPRKITGNPKGIYFDAQSRRKEPRYRFKT

PLELSSGELKAAGSTVDISKRGLGIRLDEPTMLRSGQEVQVNFRELQLYDKKLPLMAVPY

RVIRVSPDGCSVQLVIDENSKTIRVIAFFNSIIEHNQDKLIPQKEMLPSNALLERLHSVL

LSRTLSTPIFISKSTASALKTPVVGVNLPLPKHIELFARLGHDQKFSLEPIFKGRSSTLI

AEPIKRVDGAQARHQDIYISAAKIGNKITAIESKLHQEFNSVKERIQFIKKARMMGDFYV

FRMSTAPIFDPMTSLLRQDLSDLTQFGVHQASKLEKELTALVGYSEFEDITEEVVVRLEL

TE

>tr|Q87HT5|Q87HT5_VIBPA Arylesterase OS=Vibrio parahaemolyticus serotype O3:K6 (strain RIMD 2210633) OX=223926 GN=VPA0872 PE=4 SV=1

MIRLLSLVLFFCLSAASQASEKLLVLGDSLSAGYQMPIEKSWPSLLPDALLEHGQDVTVI

NGSISGDTTGNGLARLPQLLDQHTPDLVLIELGANDGLRGFPPKVITSNLSKMISMIKDS

GANVVMMQIRVPPNYGKRYSDMFYDIYPKLAEHQQVQLMPFFLEHVITKPEWMMDDGLHP

KPEAQPWIAEFVAQELVKHL

>tr|Q87RI1|Q87RI1_VIBPA Putative nitrogen regulatory protein P-II family protein OS=Vibrio parahaemolyticus serotype O3:K6 (strain RIMD 2210633) OX=223926 GN=VP0813 PE=3 SV=1

MRFKLILAFVEDSKTDKVLDAARDAGATGATVINNARGQGLNQKRTFFGLTLEVQKDVLL

FVVEEHLSRHILETINEVGEFDRESGQGIAIQIDIEDAVGVAHQVETLTKVVEDEL

>tr|Q87TL4|Q87TL4_VIBPA Putative RNA polymerase ECF-type sigma factor OS=Vibrio parahaemolyticus serotype O3:K6 (strain RIMD 2210633) OX=223926 GN=VP0055 PE=4 SV=1

MATTSALTHIIQQWQSGNKQAESELYQFAYLQLRKIAQQERERNAEKYGTDNMVLADSVN

STTALIHDAYLKMSNCDMSEIATKRDFFLMAAKVMRQILIDNARSHQAQKRQHITLMKDE

EDRFEQLIIMDKALDSFSTRYPRQSSALKLKYLMGMKNQEISELLECSASLIEKDLKFSR

SWLQSRMAHA

>tr|Q87TN5|Q87TN5_VIBPA Transcriptional activator IlvY OS=Vibrio parahaemolyticus serotype O3:K6 (strain RIMD 2210633) OX=223926 GN=VP0034 PE=4 SV=1

MNIKSLQLFIHLCESKSFAKTAAAMHISPSALSRQIQKLEEETNQQLFVRDNRSVELTTQ

GKKLMPVALKILSEWQQYQNHIKGTEGELKGEIRLFCSVTASYSHLPELISDFRLQHPFI

EFKLSTGDPAQAIDKILADEADIAISAKSEQMPNKIAFETISEIPLSVIAPVGVSSFAEE

LQKEQPDWSIIPFILPEAGTARDRANTWLKKMKIKPNIYAQTSGHEAIVSMVALGCGVGI

APDVVINNSPVREKINRLKVLPIKPFELGVCCTKNQLENPLVKAFWKVAESKYIAP

>tr|Q87FF0|Q87FF0_VIBPA Transcriptional regulator, LuxR family OS=Vibrio parahaemolyticus serotype O3:K6 (strain RIMD 2210633) OX=223926 GN=VPA1729 PE=4 SV=1

MAWGQIAVVSPLVNIPVNTIEQSLLRQLPGCWGCKDKDSVFRYVNQEYAELLGHASPEEC

IGKTDFEMSSPTTECAQEFQRQDKHVIETGESLKILDIHPYPDGRWRAHIFTKTPWRDEQ

GNTLGTIFYGRELTDTAVIEVGYWVCRAIGTDMNHQSIFRFSNLNPKPEKLTCREQETLF

LLLYGKKPQFISQVMGISTKTVEGHVARLRNKFEANSKNELIDKAMEAGYGSVVPKTLLK

HQLSVVLNGER

>tr|Q87RD4|Q87RD4_VIBPA Uncharacterized protein OS=Vibrio parahaemolyticus serotype O3:K6 (strain RIMD 2210633) OX=223926 GN=VP0863 PE=4 SV=1

MTIQLVEKRLTPELMLQDLDVFMDKIEEFATLLKLDLSFAQADHIALRINDPETAKAAHE

AWNQYGKVISQAKINGRPIIVIEFDTALESRGWKIECLELPYPAEGKTYPTESWEHVEFV

IPSHAETADEFLADLKHTYPAFGARFDELEELGVKIKLSSPKGEGERLNNPTVAFKYQGI

CIKLHPHSLKRIVESEQAE

>tr|Q87QF2|Q87QF2_VIBPA Uncharacterized protein OS=Vibrio parahaemolyticus serotype O3:K6 (strain RIMD 2210633) OX=223926 GN=VP1197 PE=4 SV=1

MKFVKIALIVAVVAIALFAAKQTGILEIITDIKSLQDWIASFGVWGYAVFVAAFVFACVF

LLPGSAFTIVAGIVFGPIKGGILALFSATLGAVAAFIVARFLLRNTIMKKFGDNPIFKKI

DDGVAANGTSFLILTRLVPVFPFSLQNYAYGLTSLNLGTYALVSLLTMAPGAFIFAYMAG

DIATNGVSVMLLVKFAVAGLVLFGMSLIPKYIAKKKGINMEELAK

>tr|Q87LE4|Q87LE4_VIBPA Lipopolysaccharide export system protein LptA OS=Vibrio parahaemolyticus serotype O3:K6 (strain RIMD 2210633) OX=223926 GN=lptA PE=3 SV=1

MKPLHLSLLALVLAAPQAFALKSDTQQPVYINSDTQQVDMKSNQVIFKGDVSLKQGSINI

DADRIVVTRDPKTEAIKQIQAFGKPATFSQLMDDGKTLSGQATELDYRISTDELTMKGQA

QLAQDGNTIKGSSIRYQIGQQKLVADSSKNERVTTILQPNQIEN

>tr|Q87KY0|Q87KY0_VIBPA Uncharacterized protein OS=Vibrio parahaemolyticus serotype O3:K6 (strain RIMD 2210633) OX=223926 GN=VP2844 PE=4 SV=1

MESRSLGQGLKVEERRQLLHRGIKAALIGVPLLAVGLTINSSVLMTDAGYSYVHQNNITG

ELDVFTEPGIHFRMPFLSKITKYDQVITVSFGNSKGEDFYQRLDSVQVRFADTYIGQIPV

TFRFKLSNDPEALKKMHREFRNNNNLIDALLVKNARNVTVITATQYIGEEFFQGGLNQFK

SKLGDQLREGIYLTERRQVEVEELDLAPVGANQANANQLQRTNQLVWKTVPVVDKTGQPI

RQDNPLQQYGIQVTQVTIGDPQPEKQLDQLLADKKRLVADRIRAIQEQETSKAQAETEQL

RKEIQRTREVQDAQRQKELAIISQQKEVEVARQIAEREIVEVEKTKRLAEVEKEKELAIA

EANLAIQKANALSAEFEAKAILAKGRAESEVLKGKYAALGANREVYLAELNRDVANSLYN

NLQNFQVQMPQNYIGGSDETGLKTNLDVITGFGALGLMDQTKKVTQQ

>tr|Q87QT0|Q87QT0_VIBPA Sensor histidine kinase OS=Vibrio parahaemolyticus serotype O3:K6 (strain RIMD 2210633) OX=223926 GN=VP1069 PE=4 SV=1

MEIRSSLRKKSILALTLYLCFFIATIGSVVYLVVEPPVRDKLERNLDLRTQLLASQIKEP

LITSTGVLNSLVGLAQSSNQSDSLKSTIPQILRLSDEIIVSGGLWPKPELKEERWRFTSL

FFNKNSEGNIDQIHSYNNPESGGYDNEPWYRAAAEQSSGTVSWSAVYIDTFTQVQMITAS

APYYRNGEFAGVATVDLSLEALFQFIREHTNQYSLGVVIRDANSNVIIEHNFQITKQMYI

SKLDFGEFHWKLEVVNAKAKVADQVFEQVMSVEGGIIPFLLLCVLVGYYLLNRYIVEPIV

RIATKIDDSKTGGIIDIDYGSEDEIGHLITKFNEKTIYLEQERVKAQASTNAKTAFLATL

SHEIRTPMNGVLGTAQILLKTPLTDEQRKHLSTLYDSGDHMMTLLNEILDYSKIEQGHVE

FSNSPFPIESIIGSIKSVYHTLCAEKGLQFKVTSLVPAGRWYDNDKARLRQVLFNLLNNA

VKFTDRGIVEVTLSEQTHYDKTVLVIAIKDTGIGISKEAQKRIFRPFEQAESSTTRRFGG

TGLGLAIVKEIAEHMGGHVTVQSQENIGTTFTVEVEISPCEPGKVESGHRHKLNCNGLKA

LIVEDNRTNAIIMETFLRAKGFECSSVENGQLAVNKIAVEPFDLILMDNHMPVLDGVGAI

SAIRSMSSAAKSVLIFGCTADVFKETQERMLGVGADHIIAKPIVESELDDALYRHADLLY

QYQTKQNQQALEVLGTDSLLISFYVALDNGNLGDALDALLAIMDSLQPNTDEVLSEVITR

IKRDLLRQSPPDQEDIDTLTMLLATP

>tr|Q87IJ3|Q87IJ3_VIBPA Putative permease OS=Vibrio parahaemolyticus serotype O3:K6 (strain RIMD 2210633) OX=223926 GN=VPA0613 PE=4 SV=1

MFNSERKATVILVATTMIAALGWIFSKETIQGLPPFGFIGLRFTIASLCLLPLCFRPLKA

ARLKDVLAASGVGLLLGGAVMTWIYAISISDTLGEGAFIMSLSMLFVPIVAWVMFQQRPQ

RIFWISLPIAVIGLACLSLAGGWKQSASQLWFLGAALILALHFNVNSKYSQKLPVLLLTC

VQLFSAGCLGLLVSFLMESRPEQVDSSIWWWFALSTLLATSLRYVMQTMGQKFVQAGNAA

LIMILEPVWTVVLSILWYGEVLTANKLVGCLLILFSLVIYRTGGRFRFPKRA

>tr|Q87FH4|Q87FH4_VIBPA Mannonate oxidoreductase OS=Vibrio parahaemolyticus serotype O3:K6 (strain RIMD 2210633) OX=223926 GN=VPA1705 PE=3 SV=1

MKTIANSTLNAAVSLPDYDRSTLKSRIIHLGFGAFHRAHQALFTHEMLSKTSSDWGICEI

NLFGGEELIQSLRAQDHLYTVAEKGAQSTEVKVIGSVTESLHPHLDSIQAVLEKMAEPQV

AIVSMTITEKGYCADPATGTLDKNNPLVIADLANPTEPKSALGYIVQALKMRRERGLTPF

TVMSCDNVQENGHVAKAAVLEFAQLLDPELRDWIETNVTFPCTMVDRIVPAATEETLSEI

AELVGCEDPCGIACEPFRQWVIEDKFVAGRPDWNVAGAEFVADVVPYEEMKLRMLNGSHS

FLAYLGYLGGYAHISDTMTDEGYRKAAFDMMMQAQAPSLTMPEGTDLEGYAKLLIERFTN

PSLKHKTWQIAMDGSQKIPQRMGGSLRFHLAQGSNFSWLATAIAGWMRYVSGVDEQGNDI

DVRDPMAETLRQICDQHGLNVSVVPALLAVETIFPAELGQNPQVIDAVSSAYQSLIDNGA

RATVAALK

>tr|Q87K89|Q87K89_VIBPA Putative two-component sensor OS=Vibrio parahaemolyticus serotype O3:K6 (strain RIMD 2210633) OX=223926 GN=VPA0009 PE=4 SV=1

MDVKERTQRRFSIGNQLMLAVLTLSLIFTLVISAISLYRDFQEELSHLDTDLKQVESSYL

SSFSASLWVEDRELLLTQALGAMRLPSVDYLRIATKDEVIIELGTEITQDVVERRWPMQF

SVGEKTFELAELTVQSDLSAVYQDLWQQFFFLLTTEAIKILLLMVGVLWVAFRLLVNPLQ

LLSGAVSDFSGGNAPSTVTLPKRWCFDEVSLLAQKYNRSVQKVREHQAELEAERDKAEVA

NRKKSEFLATMSHEIRTPMNGIIGVASLLSDTKLDPQQKEFVEIIDNSSQSLMTIIDDIL

DFSKVEAGKVELASETYHFRQLLDDVISLHTVKAQQKNLQLLSDIDPKLPAEVQGDEGRL

KQVLNNLLSNAVKFTERGHVKLLVSLHEQNNDIAQVRFRVVDSGIGIAKEHQQAVFERFQ

QADGSTTRKYGGTGLGLAICAQLVHIMGGDIKLTSELGLGSCFDFTIPLTVVSGLPTYTD

PLNVLDFPRTEANEATNKNPDKPWVLIVEDTEVNQRVVRIMLEQLGLKVSVASHGEEAFQ

LCREHAFDLIFMDCQMPVMDGFIATEQIRDMNEWGAHVPIIALTANVVKEDQQRCFEAGM

NEFVAKPVTKARLQQIFEQYLPKALKNIATPK

>tr|Q87L39|Q87L39_VIBPA Uncharacterized protein OS=Vibrio parahaemolyticus serotype O3:K6 (strain RIMD 2210633) OX=223926 GN=VP2777 PE=4 SV=1

MTTTETINAEMLLEMESVNVMPFTEHDKIILRSYEAVVDGIASLIGPFCEIVLHSLEDLN

TSAIKIANGENTGRQVGSPITDLALKMLKDIEGSERNFSRSYFTRAKGGVLMKSITVAIR

NGENRVIGLLCINVNLDAPFSQVLQSFMPTQEAKEAASSVNFASDVEELVDQTVERTIEE

INADKSVSNNTKNRQIVMELYDKGIFDIKDAINRVADRLNISKHTVYLYIRQRKTEDE

>tr|Q87FF9|Q87FF9_VIBPA Putative acetyltransferase OS=Vibrio parahaemolyticus serotype O3:K6 (strain RIMD 2210633) OX=223926 GN=VPA1720 PE=4 SV=1

MTIQPTLTTERLVLRPFNIGDCQQVALLAGDKRIADMTANIPHPYELPMAQAWIDRHYPM

YQQHQGVAYAITLRNTGELLGAVSLPRIEEGYGTLGYWVGVPHWGKGYAFEASKVLLEFA

RMHFELNGITVMHLVDNQRSKSVIQKLKIPYVGDKTLRMQGKERKVCVYQLTFVASE

>tr|Q87HZ5|Q87HZ5_VIBPA PTS system, fructose-specific IIBC component OS=Vibrio parahaemolyticus serotype O3:K6 (strain RIMD 2210633) OX=223926 GN=VPA0811 PE=4 SV=1

MNIAIITACPSGVANSILAAGLLEQAVAKLGWNAKIECQSSVIAPTPLTDADIEQADAIV

IAANTTVDTSRFVGKKVYQAEISAVAKDATAFLTTAVESAATLEQATTVEAPVESASATK

KIVAITACPTGVAHTFMAAEALEEEGKRRGHQIKVETRGSVGAKNQLTDQEIADADLVII

AADIEVPLDRFNGKKMYRTKTGPALKKTAEEMDKAFEQATIYQHSGAASSASATDEKKGA

YKHLMTGVSHMLPVVVAGGLIIALSFVFGIEAFKEEGTLAAALMTIGGGSAFALMIPVLA

GYIAFSIADRPGLAPGLVGGMLASSTGAGFLGGIAAGFIAGYAAKLLADKVKLPQSMEAL

KPILIIPFVATLFTGLVMIYIVGGPVSGIMNGLTDFLNNMGSDSAVLLGIILGAMMCFDL

GGPVNKAAYAFGVGLLASQTYAPMAAIMAAGMVPALGMGLATFLAKNKFEPNEREAGKAS

FVLGLCFISEGAIPFAAKDPMRVIPSCMAGGALTGALSMLFGAKLMAPHGGLFVLLIPNA

ISPVLMYLVAIAAGTAVTGFTYAFLKNKADAKQEVAA

>tr|Q87LQ8|Q87LQ8_VIBPA RNA polymerase sigma factor RpoS OS=Vibrio parahaemolyticus serotype O3:K6 (strain RIMD 2210633) OX=223926 GN=rpoS PE=3 SV=1

MSISNTVSKVEEFEYDNASETTIDNELEKSSSTTEGKTAVREEFDASSKSLDATQLYLGE

IGFSPLLTAEEEVLYARRALRGDEAARKRMIESNLRLVVKISRRYSNRGLALLDLIEEGN

LGLIRAVEKFDPERGFRFSTYATWWIRQTIERALMNQTRTIRLPIHVVKELNIYLRTARE

LSQKLDHEPTAEEIAAQLDIPVEDVSKMLRLNERISSVDTPIGGDGEKALLDIIPDANNS

DPEVSTQDDDIKSSLIHWLEELNPKQKEVLARRFGLLGYEPSTLEEVGLRLKKWDERLVL

PVSVYVKFKWRVYVDFVKS

>tr|Q87HS8|Q87HS8_VIBPA Uncharacterized protein OS=Vibrio parahaemolyticus serotype O3:K6 (strain RIMD 2210633) OX=223926 GN=VPA0878 PE=4 SV=1

MSLRAKLIWPILVFITAIFVASKGYTSYTAYQSSKHELVEQTRKLINNVSYSIKDALTTK

NKRKAQTILADLIEQPNVSRVKLYDRANELFVLLEGSGESAPVPNQNERSKLDALGYALS

AKFLYVLEPIIHEGHVIGSIRVTLSYIPIINAQHSFLKDAGVLLLILAAGGIIFYITIDR

IILRPLLDLNGAIQDVTFGNASHVQIRHHSKDELGEVIHAFNRMMTKLRKREKQRQHSLA

TLEQKRAFSEEVIESIQYALVITDNLGTIIHSNAATQHIFQKTPEALENANIRDLIKTKM

PNELSQILSRCLECDDIHLQNIDNEQQLSLTTRRLSNHGYLLFAIQDITEIEEAMNRQRV

AGRVFEASQDGLIVLNHKGVITMVNPAVTKLVGLEIDQLVGQSFIQTIRWRKLQEMMPSI

IESIENYGVWQGEVIEQNHLGLLVPMFARVNRIVKCENNGFYDLVIILTDLSNAKEMERL

EYLAHHDALTGLANRSKFHLELEDLVQRSGYQRDEFAVLYLDLDGFKEINDTYGHDAGDE

VLKRVADRLTSATRHSDLIARLSGDEFVMLVNPANQKVVTRIAEQLLESICAPIEYKGNE

LKVGVSIGVKLVGVNERDATRILKSADTAMYQAKKAGKGQAILMGCELQETV

>tr|Q79YZ2|Q79YZ2_VIBPA Chemotaxis CheV OS=Vibrio parahaemolyticus serotype O3:K6 (strain RIMD 2210633) OX=223926 GN=VP0773 PE=4 SV=1

MTGILDSVNQRTQLVGQNRLELLTFRLMGRQRYGINVFKVKEVLQCPKLTSMPNLHPLVK

GIAHIRGHTVSVIDLSLAIGGRPTTDIDKCFVVIAEFNRTIQAFLVSSVERIINMHWEAI

LPPPDGAGKAHYLTAVTNIDNELVEILDVEKILAEIAPVDETMDSTIGEEIAQAEQEKPI

VRRILIADDSTVARKQVERAITSIGFEVVSVKDGKEAYNKLLEMAQEGSIYDQISLVISD

IEMPEMDGYTLTAEIRRNADLKDLYVILHSSLSGVFNQAMVERVGANTFIAKFNPDELGN

AVKSALTQ

>tr|Q87Q12|Q87Q12_VIBPA Probable permease of ABC transporter OS=Vibrio parahaemolyticus serotype O3:K6 (strain RIMD 2210633) OX=223926 GN=VP1338 PE=3 SV=1

MALPNYASKSERMAYAGYLVFCGLVLFFLIAPILTIIPLSFNATPYFTFTEGMLNLDADA

YSVRWYQEMFTNEQWLLALKNSTFIALMATLIATGLGTLAALGLANSNLPFRNAIMALLI

SPMIVPVIISAAAMYFFYTRLGLSQTYFGIILAHAALGTPFVVITVSATLSGFDQSLVKA

AASLGANPVYTFRHVTFPLIRPGMISGGLFAFGTSFDEVVVALFLTGAEQKTVPRQMWSG

IREQISPTILAVATLLIFMSVCLLVTLEILRRRNVRIRGIQE

>tr|Q87GF0|Q87GF0_VIBPA Uncharacterized protein OS=Vibrio parahaemolyticus serotype O3:K6 (strain RIMD 2210633) OX=223926 GN=VPA1366 PE=4 SV=1

MKRSEKWYFKLKHHCLGNEYLTVEQFEVLTGLTEAEIESFFKQSRRQIRQFLLHLGKVVR

YQKSKQVEISSDWSTLRHELGQRVCLYSDSIGIHKISQEGDDLEYGLALLANIDRRKAVT

WTIRLKKNLPDFAINVASIPRLTILLNQLNQD

>tr|Q87SQ0|Q87SQ0_VIBPA Putative hemolysin OS=Vibrio parahaemolyticus serotype O3:K6 (strain RIMD 2210633) OX=223926 GN=VP0372 PE=4 SV=1

MDSSTPFRLPRKTPFGIGENVAEWATGLSQLDKFYAQRPVNADTKTFLRFTLDILGIDYR

IAHGSLGSVPKQGATVIVANHPLGCVEGVILAELLLMVRDDIQILANQYLKTVPELDQLF

IGVDVFEGKDAVKSNMKALRAANKHLANGGLLLVFPAGEVSQLVDAKQQRLEDKEWSRSV

SALIRKNKAVTVPVFIRGQNSKRFYMAGKIHPLLRTLMLGRELLNKSAKTIELSFGQAIK

FKELNNLNDDQIVNYLRLNTYLLNRDVSATQQTVSDNDLLPIAAGLPIGQLLEELHSLPA

ETQLLQNGEFDVYCASAQQIPSLLHEIGRLREHNFRQVGEGTGQAIDIDHFDHDYLHLFV

WDRENQCMVGAYRLGLVDQLLAKYGVEGLYSRTLFNYDQRFLDQMGKSIEMGRSVIAEQY

QKSMSALLLLWKGIATFVHQHPEYTHLFGPVSISNDYSHTARQLLAQSMTLHHYDNDCAE

YVTPSNPLPETNLNWNTSMLTALGDLQLLSRVIARIDEGKGVPVLLRQYLSLNGKLVCFN

VDPAFNNALDGLIMVDLRDVPEKTLARYMGSENAREYLAMNN

>tr|Q87TF1|Q87TF1_VIBPA DNA-binding transcriptional regulator NtrC OS=Vibrio parahaemolyticus serotype O3:K6 (strain RIMD 2210633) OX=223926 GN=ntrC PE=4 SV=1

MSKGYVWVVDDDSSIRWVMEKTLSSANIKCETYADGESVLMALEREVPDVLVSDIRMPGI

DGLELLKQVQRDYPDLPVIIMTAHSDLDAAVNAYQKGAFEYLPKPFDIDETLTLVERAIA

HSHENKREQLSSEDAPADTPEIIGEAPAMQEVFRAIGRLSRSSISVLINGESGTGKELVA

HALHRHSPRANKPFIALNMAAIPKDLIESELFGHEKGAFTGANSVRQGRFEQANGGTLFL

DEIGDMPLDIQTRLLRVLADGQFYRVGGHSPIRVDVRIVAATHQNLEKLVHQGDFREDLF

HRLNVIRVQIPALRERKQDIEKLTQHFLVRAADELGVETKTLHPSTVEILNRLNWPGNVR

QLENICRWLTVMASGSEVLPNDLPSELLEEKKTVSDSTKGSWQEQLADWARQSLAAGDKE

LLSFALPEFERILLEAALEHTKGHKQDAAKVLGWGRNTLTRKLKELY

>tr|Q87HE2|Q87HE2_VIBPA Nuclease SbcCD subunit D OS=Vibrio parahaemolyticus serotype O3:K6 (strain RIMD 2210633) OX=223926 GN=sbcD PE=3 SV=1

MKFIHTSDWHLGRQFHNVSLLEDQQAVLEQLIQYIENNPVDAVIVAGDVYDRSVPPTIAI

ELLNRVVKRICGELNTPMILISGNHDGAERLGFGSEQMKRSGLHIISNFEDMLTPVVIET

KAAGHVAFYGMPYNDPEQVRYVYKEPVSTHDEAHKLLAEKITEQFQSEHRNILISHCFVD

GAIESESERPLSIGGSDRVSHEHFLNFDYVALGHLHQPQEKGEEYIRYSGSLMKYSFGEQ

NQKKGFTLVEIGKDGFIGAEHIELTAPHEMRIVEGELEQILEWGKTDPKNEDYLLVRLMD

KHAILNPMEKLRTVYPNVLHLEKPGMLIGVEQEMAQAKLARSEIDMFKDFFAEAQDSELS

NEQEQAISNIIKQLSQQ

>tr|Q87PF5|Q87PF5_VIBPA Sensor histidine kinase/response regulator OS=Vibrio parahaemolyticus serotype O3:K6 (strain RIMD 2210633) OX=223926 GN=VP1547 PE=4 SV=1

MKDKYLDTYQQEALQEALVELKQTKQREKLLADENKAILSAISAMSEAKNRNEIFSGLNS

VLKKYISFEDFIVITRDDNRYPFKTLISTNSVFDKVEWLHGNTMDRALNGECILLFEPAK

LLEFENLNSFVKTHVNSVILTGIRSEVTQSIILLIGAQKGHFSIENKETLRRFRPLIERA

VIDIETKEKLQRIVEVRTTQLARAREEAELANQSKSEFLAMMSHEIRTPLNSVLGMLDIL

RQSTLSDEQFDALNQMECSAELLLAIISDILDLSKIESGSFQLNEQWIHLNDTVTFVISQ

QKQVAITKNLSFNFDCQISSDKQYWIDSTRLSQILFNLIGNAIKFTDSGSVSVSVAEEND

EVVVSISDTGIGISRAKQAHLFTAFHQGDRSITRRFGGTGLGLAITKHLVEMMRGEISVK

SRENEGSDFTIRIPVLTRYNQSRPVKIEHNRPSKALNLLIVEDTQSNQLVIKLILNKLGH

NVHIASHGAEALTFLEENDTRIDMILMDVSMPVMDGITATRLIRKKGITIPIVALTAHAL

ESDKDKCLDAGMDSFVSKPVRRQDIYEAIQSLIETA

>tr|Q87J11|Q87J11_VIBPA Uncharacterized protein OS=Vibrio parahaemolyticus serotype O3:K6 (strain RIMD 2210633) OX=223926 GN=VPA0444 PE=4 SV=1

MKRNFNLAALLPGFFALNSGANAGTAAPEELDVKDELLLDKVVLAPLNEAIPLYIAAHRS

HSSHRSHSSHRSSAGSTYSAPVKKQKSQPLTQPSTPSYTAPATKRPVSTAEELEKRKELI

IRVQFALYTSGYYKGIIDGIMGKQTRKALNAYRVDNNIPVSQTLDTVTLNSLGIIAR

>tr|Q87I91|Q87I91_VIBPA Uncharacterized protein OS=Vibrio parahaemolyticus serotype O3:K6 (strain RIMD 2210633) OX=223926 GN=VPA0715 PE=4 SV=1

MIFILVDGYMKKIYFPIISTLMVSFAHANTLPAPTWHQFQLNDGSKKELKLYGDQESFWY

QDREGIIYVYDEETGWFYGNHEIVDGRFVVTSLGVEAKLEMTQAVRELLKERVTPTSSLL

PSEHVYPMNEPYLEQTPIRALNATNEPTHQPLLVVQVSFNNQDFVNDFTNVIFGDNQQSV

KDYFSKNSYGRYIVEPAKETEGTANDGVIDLTLDIAHPNCHSKNDATCDSKLNEAFKAAY

DKLDRYVDLSTYDLNNDDKITPDELSVMFVFAGYDKSAGSVNTPYIWPHRYSHNAIEIDG

KTIRDYCLFADFQGDHQSTMGVIAHELGHLMLGLPDLYSYKHSGSVGQWGLMGGGSWASK

QGDTYAGETPVNMLAWSKEAAGFIKPKVIEASGSSTIETRQGEGVVYLDPYLKQQGPRAY

FENRRKTEYDRALSGEGLLITAVSIDNRFNDTGPMQVQVFQADGLDELQTRGWSDQGDVF

PGSSNVVTISDDTTPSLKLITGGRETNISINDIASTDQSSTLRASIPSSTNKSAWITSLS

RTYVYEDSRNNTIGFGLENNDGYQSLVGFYLFAKPILPNSEMRYRMFKYELTTNRWGNAS

INVDTQVEVSSGVIPTFGGRVMFASPFDIDEGEQLLVIELENAKPEYSYSFLDAYLSNGE

KKKQFYGNASSYKTSGLSEGRGRNYPFAALLEKPQAKPSITVETDSVTVLSGESITIDVL

ANDHSSDANSTLKLDKITSKPSIGSAEISGQKIHYSVPVSDIPEQRAVMKYQVSDDFGTT

AEGEVVITINSPVITANSLQYKALEDTPLELNLHQQQGLEKGDTIVIKKPPMHGKIDGNT

YQSKENYFGNDQFEFSIMKENGRESNTAMAVITVEAVNDEPIFAVEASRKEGLSNEKVTL

SVLSLKDVDSTDHSFKWRQVSGTKAQFATTDTASVEVTLPKVSEKSEKLTFEVAVNDNDG

AIITKSVVTVVKQEVVVENDPGKSEEGGSMGLWVTLCMMLISLRTKVNNLYRFVIRNDIW

>tr|Q87SG2|Q87SG2_VIBPA Cell division protein FtsQ OS=Vibrio parahaemolyticus serotype O3:K6 (strain RIMD 2210633) OX=223926 GN=ftsQ PE=3 SV=1

MLNIALNEERLNTDNNRGRQDKILGALFFVVVVTLISSVLYSAISWMWDDQRLPLSKIVL

QGKLEYVKADDVQAAFSRIDHIGTFMSQDIDVLQQSVEALPWVAHAAIRKQWPDTVKVFL

TEHQPEAIWNGNELLDKNGLVFDGDVALLKDEKVKLYGPKDTGPEVLQTYRELSPKFQQL

GLAISSLVLNERRAWQIILDNGIRLELGKESLLERIERFFSLYNKLGSDTQRISYIDLRY

DTGAAVGWFPEEELEESTDD

>tr|Q87NE5|Q87NE5_VIBPA Formate-dependent nitrite reductase complex subunit OS=Vibrio parahaemolyticus serotype O3:K6 (strain RIMD 2210633) OX=223926 GN=VP1923 PE=3 SV=1

MALFKYIFITVFALALYSPASNGSEIKQNVDLFEFQSVEVQQRATSLAKTLRCPQCQNQN

LIESNAPAAKDLRLKVYTMVNEGSSDQQVKDYLVERYGNIVLYQPPFNYSTALLWIFPII

FLIFFALFSIRLIKRN

>tr|Q87PL6|Q87PL6_VIBPA Methyl-accepting chemotaxis protein OS=Vibrio parahaemolyticus serotype O3:K6 (strain RIMD 2210633) OX=223926 GN=VP1486 PE=4 SV=1

MQGRIDDSVGGFRYGLSSIGPEMNRISSFLSVDDPESSDAANRFIASASSMESTFLVMMM

HTDLLKAEKEYREMRNRIAGINLAYEDFKALHPEVSDYASLTAPYDMVKSGFDQEGILQL

ILAKLAQSEQQQREFHQASLLADETMRLLDSISQSASNLIDEREAMVNNTIGNVSMMVLV

AACVISLVILVSWFGLKTWTNRGLKNVLVRLSALTDHDFRAKADEIGPFELKEVARKLNQ

VIDSTHDSIQTVTRNCETLYQTAEISHDAAEQTNEGLTTQNEALASMVTTITQLEASIRE

IATVTNASSEDSLLATRHTEKGVQVVEQNRKRLESLESSLDVNEQSMLELDQRVKQIREM

VDMISGIAENTNLLALNAAIEAARAGEQGRGFAVVADEVRKLAKDTSQQTTNIREMMSEL

ITAAERSRQAVNDSREEMTHALHSSNEVKSTFSDINLAVKHIQERVEQISVATEEQERAT

ADVSQSINNISELGERTKLQLESMVESSEQVAEIAGHQQAMLHKYELHQSA

>tr|Q87LN3|Q87LN3_VIBPA RNA polymerase sigma factor OS=Vibrio parahaemolyticus serotype O3:K6 (strain RIMD 2210633) OX=223926 GN=VP2578 PE=3 SV=1

MNEQLTDQVLIERVQNGDKQAFNLLVTKYQNKVCNLISRYVSNPGDVPDVAQEAFIKAYR

AIPSFRGESAFYTWLYRIAVNTAKNHIVAQGRRPPATDVDAEEAEFYETGSALKEISNPE

NLTLSKELQRVVFSAIEALPEDLKTAMTLRELDGLSYEEIAEVMDCPVGTVRSRIFRARE

AVEKKIKPLLQR

>tr|Q87HG5|Q87HG5_VIBPA Methyl-accepting chemotaxis protein OS=Vibrio parahaemolyticus serotype O3:K6 (strain RIMD 2210633) OX=223926 GN=VPA1000 PE=4 SV=1

MSIRNLSIAKKISLSFLLIALINIVFGVFLSKELKEIKSELLNYTDDTLPAMERVDAIRD

DLSHWRRSQFATYTYKDADKIRNKIASNIREREKISKELEAYGSTIWPGEEQQTFQRLMR

QWKQYLVTMDQYNESMLAGNKTEALAVLSNSLNDFEAVDSDLNELIRLLKVAMDSNKNHI

LSSVNGLSSSSIASNVTILVIMIVMTLVLTRLICGPLQLVVEQANSIAKGDLSKDIDRKL

IGNDELGELADATTKMQNDLRQVIDNVIAAVTQLSSAVEEMNQISELSASGMKDQQLQIT

HVATAMTEMKAAVADVARNTEESASQANDANHRTQLGVRETQSMVDAIGEVANVIGAAGD

TVSELEQQSNQINVVVDVIRDIADQTNLLALNAAIEAARAGESGRGFAVVADEVRTLAGR

TQDSTSEITAIIEQLQSLAKDAKSATELSRTSIAECADQGIQSKQLMNDIEHAISDISDM

GSQIATACNQQDSVAEELSRSIENIHLASQEVAQGSEQTAQACRELSQLSVSLQDVMSRF

KLN

>tr|Q87LE2|Q87LE2_VIBPA RNA polymerase sigma-54 factor OS=Vibrio parahaemolyticus serotype O3:K6 (strain RIMD 2210633) OX=223926 GN=VP2670 PE=3 SV=1

MKPSLQLKLGQQLAMTPQLQQAIRLLQLSTLDLQQEIQEALDSNPLLEVEEGHEEPQANG

EDKSALETADNSANEPTEIEVPDSSDVIEKSEISSELEIDTTWDDVYSANTGSTGLALDD

DMPVYQGETTESLHDYLMWQLDLTPFSETDRTIAIAIIDAIDDYGYLTLSPEEIHESFDN

EDIELDEVEAVRKRIQQFDPLGVASRNLQECLLLQLATFPEDTPWLAEAKMILADHIDHL

GNRDYKLVIKETKLKEADLREVLKLIQQLDPRPGSRITPDDTEYVIPDVSVFKDHGKWTV

SINPDSIPKLKVNQQYAQLSKGNSADSQYIRSNLQEAKWLIKSLESRNETLLKVARCIVE

HQQDFFEYGEEAMKPMVLNDVALAVDMHESTISRVTTQKFMHTPRGIFELKYFFSSHVST

DNGGECSSTAIRALIKKLVAAENTAKPLSDSKIAALLADQGIQVARRTIAKYRESLGIAP

SSQRKRLL

>tr|Q87HX7|Q87HX7_VIBPA Iron-containing alcohol dehydrogenase OS=Vibrio parahaemolyticus serotype O3:K6 (strain RIMD 2210633) OX=223926 GN=VPA0829 PE=4 SV=1

MNFSYVNPTKIFFGQQQIAAIKDAIPADQKVLVIYGGGSIKKNGVYDQVAEALTGHEWSE

FSGVEPNPTKETLDKAVAIVKDQNIDFILAVGGGSVIDGSKYVAAASKYDGDGWDIMIGK

HQVTEATPLAAILTLPATGSESNMGAVITKAETQDKLPFMSPAVQPKFAVLDPDVMKTLP

ERQLINGIVDAWVHVCEQYITLPTDAMVQDGYAETLLKTLKMLGEQFAERDDDKWRANLM

WSANQALNGLIGSGVPHDWATHMIGHELTALWGVDHARSLAIIQPSLLRNQMQFKRAKLE

QMGRNVFGLESGDDLAERTIEAIEAFYHQLGVATQLDNYGESREQAIDAIIDQLNKHGMT

VLGENQAITLERSREILELAVS

>tr|Q87RQ7|Q87RQ7_VIBPA Endolytic peptidoglycan transglycosylase RlpA OS=Vibrio parahaemolyticus serotype O3:K6 (strain RIMD 2210633) OX=223926 GN=rlpA PE=3 SV=1

MQKRALYSLVFSALILAGCSSTSQKTQEGRYELESDVAPDTPLSVEHIEDAHPKYEPYSL

GGNTDYHLRGNDYKIVRDAKGFTEKGRASWYGKKFQGHLTSNGEIYDMYSMTAAHKTLPL

PSYVKVTNTDNGKTTVVRVNDRGPFHDGRIIDLSYAAAHKLDVIKTGTANVEIEVISVEK

PTDQKSLESHPKYVIQVASSKNEDRARTLGAELGQKLDTETFLENAKESYRLLLGPFTDY

SLTQATLDKVKLLGYSSAFIKKHNTAK

>tr|Q87FD8|Q87FD8_VIBPA Uncharacterized protein OS=Vibrio parahaemolyticus serotype O3:K6 (strain RIMD 2210633) OX=223926 GN=VPA1741 PE=4 SV=1

MSVINKIVARRTQLSQSGRLVGDWIVENAEKAAQLTSQELAAQVKVSQSSIVKFTQRIGF

KGYSEFKLALNEEIGRKHAMQSTPLHSDILADDPIAVISQKLVKAKTDAMFQTTNALSYE

ACHQAVKWLSEARRVQVVGIGGSALTAKDLSFKLLKLGITALSEQDSHVQIAVARTLSSE

DVQIAISYSGERKEILVAAEAAKEQGAKVIALSAPGRSRLRGIADITFDTIANETEHRSS

SIASRTAQNVITDLLFIILVQQRDESARQLISDISTDIKQILT

>tr|Q87M13|Q87M13_VIBPA Putative lipid carrier protein OS=Vibrio parahaemolyticus serotype O3:K6 (strain RIMD 2210633) OX=223926 GN=VP2445 PE=4 SV=1

MNVLNKIRSQLVKNAANILRSPVQLLPQTVQQKALLEGLKMVFKEALEDGDFEFLEDKWL

KVAIKDLNLAWYISYQDEKLVVAEKPVQEDVSFSGNLNDLVLIAGRKEDPDTLFFQRRLS

IEGDTELGLEVKNLMDSVDLEQLPKAMQVALNQLADFVQKGVQEPAQQPGVANAYSN

>tr|Q87QU6|Q87QU6_VIBPA Cytochrome d ubiquinol oxidase, subunit I OS=Vibrio parahaemolyticus serotype O3:K6 (strain RIMD 2210633) OX=223926 GN=VP1053 PE=4 SV=1

MSFSRHIGVTMIDVVDLSRLQFALTAMYHFLFVPLTLGMAFLLAIMESMYVMTNKQIYKD

MTKFWGKLFGINFALGVATGLTMEFQFGTNWSYYSHYVGDIFGAPLAIEALVAFFLESTF

VGLFFFGWDRLSKRQHLAVTWLVALGSNFSALWILIANGWMQNPVGADFNFETMRMEMVS

FSEVVLNPVAQVKFVHTVASGYTCGAMFILGISSYYLLKGRDIAFARRSFAIAASFGIAA

ILSTIVLGDESGYELGEVQKVKLAAIEAEWHTEPAPAAFTLFGVPNQETMHTDYAIKIPY

VMGIIATRSFDEQVTGLRDLRDEHVERIRGGMYAYELLEKLRAGDKSEENMTAFDEVKGD

LGYGLLLKRYTDNVVDATEEQIQAAADDSIPTVWPLFWSFRIMVACGFIMLFVFGAAFIQ

TCRQKIEQKQWILKAALLSIPLPWIAIEAGWFVAEYGRQPWAVGEILPVHVAASALTAGE

IWTSLFAILALYTVFLIAEVYLMLKFARKGPSSLKTGRYHFEQNADSVEDKVSRQVEA

>tr|Q87G93|Q87G93_VIBPA PTS system, fructose-specific IIABC component OS=Vibrio parahaemolyticus serotype O3:K6 (strain RIMD 2210633) OX=223926 GN=VPA1424 PE=4 SV=1

MITKLINEDLIKLDLKASSKEDVFKELVAVLHAQGRISDQTQFLADIKAREELGNTGFED

GVAIPHAKSAAVIEPAVVIGVSKSGIEYGAEDGLPSKLFFMIASPDGGDNHHIEVLAELS

SKLIEDGFIDAFLDAANSQDALALLLAKEEPQPVTDAPANQGFIIGVTGCPAGVAHTYLA

AEALEKGAAAMGYEIKVETNGSIGVKNSPTAEEIERADAIIVACDKQVDMNRFAGKRVVK

TNVKAPIRDAQGLINEALNAPTYQAESNGNTQASVADKASQARSDLYRYLMNGVSHMIPF

VVTGGLLIALALAVGGQPSEAGMAIPEGSMWNQILNVGVVAFTLMIPILAGYIAYAIADR

PALAPGLIGGWIANNGSFYGADAGTGFIGAIIAGLLVGYFVKWITSINYHKFIQPLVPIM

IAPITGSLFIAGLFIFVIGAPIASLMDGLTALLTSMSTGNVVLLGIVLGGMAGFDMGGPF

NKVAFLFSVGMIASGQTQFMGAMACAIPVAPLGMALATALGRKFDLFEESETEAGKAAGA

MGLVGISEGAIPFAAQDPMSVIPANVLGSMVAAVMAFSFGITNSVAHGGPVVALLGAMNH

PVLALICMTAGATVTAVTCVTLKKVRKAKMMQAAA

>tr|Q87TE3|Q87TE3_VIBPA Uncharacterized protein OS=Vibrio parahaemolyticus serotype O3:K6 (strain RIMD 2210633) OX=223926 GN=VP0127 PE=4 SV=1

MFKLITPTTENQLNKYYQFRWQMLREPWRMPVGSERDEYDVVSHHRMIVDSRGRPMAIGR

LYITPDNDGQIRYMAVKGNRRSKGMGSLVLVALESLARQEGAKRLVCNAREDAIAFYEKN

GFERRGELTDERGPVRHQQMVKPLDPMANVLRRPEWCTELQQRWEAQIPIADKMGIKINQ

YTGYQFECSAQLNPNLNPHNTMFAGSAFTLATLTGWGMTWLLLKERGLHGDIVLADSSIR

YRHPVEQNPVASTSLDGISGDLDRLASGRKARIVIHVVIYSGDTPAVDFVGTYMLLPNYS

QLLSC

>tr|Q87SL2|Q87SL2_VIBPA 7,8-dihydroneopterin aldolase OS=Vibrio parahaemolyticus serotype O3:K6 (strain RIMD 2210633) OX=223926 GN=VP0411 PE=3 SV=1

MALDKVFIEQLEVITTIGVYDWEQQIKQKLVLDIEMAHDNKPAGKSDDVQDALDYSQVSE

AVLNHIENGRFLLVERVAEEVAELIMQRFSVPWVKIRLAKPGAVPQARSVGVVIERGQA

>tr|Q87FC7|Q87FC7_VIBPA ParA family protein OS=Vibrio parahaemolyticus serotype O3:K6 (strain RIMD 2210633) OX=223926 GN=VPA1752 PE=4 SV=1

MKREKTIENLQELAELTQQVQADRIEIVLEERSDNYFPPMSKAMMETRSGLTRRKLDEAI

GKMEAAGHQFTKNNANHYSITLEEAHMLMDAAEVPKFYERKKNNGNKPWIINVQNQKGGT

GKSMTAVHLAACLALNLDKRYRICLIDLDPQGSLRLFLNPQISVAEHDNIYSAVDIMLGN

VPDGVEIDREFLHKNVLLPTQYPNLKSISAFPEDAMFNAEAWQTLSEDPSLDIVRLLKEQ

LIDKIANDFDIIMIDTGPHVDPLVWNAMYASNALLIPCAAKRLDWASTVNFFQHLPTVYE

MFPEDWNGLEFVRLMPTMFEDDNKKQVSVLTEMNYLLGDQVMMATIPRSRAFETCADTYS

TVFDLTVSDFEGGKKTLATAQDAVHKSALELERVLHSNWPSLNQG

>tr|Q87HS1|Q87HS1_VIBPA DamX-related protein OS=Vibrio parahaemolyticus serotype O3:K6 (strain RIMD 2210633) OX=223926 GN=VPA0885 PE=4 SV=1

MKKIAIIGLSVLLSACVSETYITDVTSESYREDYKSDAVSKPIMASESAVVEQDVKPEVV

KMTAKPEEKKVVKLTPKEQVKPVKIVPPSKKQAGIQRFGYTIQVVAVGSQAKVDQFANKL

PQNGQPIWENYKVVNGTKWFSVLYGDYATSAEAQQAISTLPTQFQQLKPFVKSIDAIKNS

SFPTLNKLN

>tr|Q87LH5|Q87LH5_VIBPA PTS system, cellobiose-specific IIB component OS=Vibrio parahaemolyticus serotype O3:K6 (strain RIMD 2210633) OX=223926 GN=VP2637 PE=4 SV=1

MKKILLCCSAGMSTSMLVKKMEQAAEIKGIECKIDAMSVNAFEEAIKEYDVCLLGPQVRF

QLEELRKTAQEYGKNIDAISPQAYGMMKGDEVLEQALELIN

>tr|Q87ND0|Q87ND0_VIBPA Uncharacterized protein OS=Vibrio parahaemolyticus serotype O3:K6 (strain RIMD 2210633) OX=223926 GN=VP1938 PE=4 SV=1

MGFLLRDNCEPDHQTINSETFVSKSSMFKKTLAGLLLTTSAVANASQYDMSRHSEFSYTP

DCMSGYCETTNLYTFESQNRSLAFEQNNDDSLNLDAGLQPRHLIVPQDKDWDYLMGQTYT

ILGLSVATVGLMTFLPESITKWDEEDRDMSKLGSKWKDNVSDGPVWDRDEHFLNYIMHPY

FGGVYYTAARHAGYNEFESFLYSATMSTFFWEYGVEAFAEVPSWQDIFITPFFGAVVGEM

MLTAEQDIVANGGEVLGSETVGDVSLFFLNPVGHIHHWVTDAWGGSAELKFNSSPWFGNQ

DVAKFAMDAGASYDSQFYGVELKVTF

>tr|Q87P07|Q87P07_VIBPA Transcriptional regulatory protein OS=Vibrio parahaemolyticus serotype O3:K6 (strain RIMD 2210633) OX=223926 GN=VP1711 PE=4 SV=1

MNMATRVMIIEDDIAIAELHHKYLSQLAGLDVVGIATTRLEAEMQLEVLKPDLLLMDVYL

PDGTGLEILNTLRSNNQTCDVILITAARDVDTLQQAMRGGVVDYLLKPVMFPRLETALKK

YITQRQQLDVAKSLDQGLVDRMLQSNTGTDSCPKRLPKGIDSVTLDKIRDLFVGEAALTA

DEAGEKIGASRTTARRYLEYLISSGELEADLNYGTVGRPERCYKKVIR

>tr|Q87Q27|Q87Q27_VIBPA Uncharacterized protein OS=Vibrio parahaemolyticus serotype O3:K6 (strain RIMD 2210633) OX=223926 GN=VP1323 PE=4 SV=1

MHPSLRYIQGYPQNIVEQVSSLVDNGKLIPWFEKRYPDRHQIKSEKALYEYAIAIKNRYM

KKASPISKVVYDKKIHAVNNALGLHSVISRNHGGKLKSKNEIRIANVFKDAPEALLRMLV

VHELAHTREKNHDKAFYQLCCYMEPEYHQLELDARLFMIYLDLKANSNEEQ

>tr|Q87QB4|Q87QB4_VIBPA Uncharacterized protein OS=Vibrio parahaemolyticus serotype O3:K6 (strain RIMD 2210633) OX=223926 GN=VP1236 PE=4 SV=1

MNTLEKIQKNLENFSKSERKVAEVIMASPQTAIHSSIATLAKMADVSEPTVNRFCRRLDT

KGFPDFKLHLAQSLANGTPYVNRNVEEDDGPDAYTHKIFESTMACLDVAKNSLDPMQVNR

AVDLLTQAKRISFFGLGASSSVARDAQNKFIRFNIPITCFEDIVMQRMSCINCSDNDVIV

LISHTGRTKSQVEIANLARENGATVIAITAKDSPLEKASSLAITLDIPEDTDVYMPMASR

VVQMTVIDVLATGFTLRRGTGFRENLKRVKDALKDSRYDKLSQY

>tr|Q87HJ8|Q87HJ8_VIBPA Putative sensor histidine protein kinase UhpB OS=Vibrio parahaemolyticus serotype O3:K6 (strain RIMD 2210633) OX=223926 GN=VPA0965 PE=4 SV=1

MRAYSVTTICGLFVMACAWFCLWVIAYYFVNDPELAILLFPFALRLGIALHTRTAYWPTI

YVSEWALTIALATLLEQPQWLTVLIASVASIPVTLIAKKYYYGDQNRHLAVMGIVIIITA

FINVMAVGFHVPSVYMVWLASISGGLMLVPMCYLLWNYLFQSRWSPLTSHLLNNTVVFSI

RHIVFYAVLLIGSILVQTSLPEELKRFAPFCMAIPIIVLALRYGWQGALLATMLNSIALI

AARSGVSNLEITDLLLSLSAQTITGIMLGLAVQKQKDLNHKLRGELSRNQNLSRQLIEAE

ESVRRDIARELHDEIGQNITAIRTQANIIKRIDNAEMSAHCADTIEGLSLNVYDTTKRLL

SKLRPKMLDDLDLKESVEQLTREMEFANHGTTVQLNWQGDYTSLSDTLKVTLFRLCQESL

NNAAKYAEAQLINIELTIGEAAVSLMIHDDGVGFKVQDSMKGMGVRGMQERVHALGGKMV

IYSTSDQVIGTQISITLPKV

>tr|Q87PR7|Q87PR7_VIBPA Putative V10 pilin OS=Vibrio parahaemolyticus serotype O3:K6 (strain RIMD 2210633) OX=223926 GN=VP1434 PE=4 SV=1

MRGSRGFTLIELIMCIIILGVVGVVALPKFLGIQKDARIAVLYGAREALMTANNLVYTKA

VIQSQENINSEDTRNIDLDNDGVNDLIGYFGLIKNVIPAKELAGFDPQLTINKWYGADSE

TEPYFLIGFANKPVSIHHLCYVEVYYPKTPGGQLRYGIQTEDC

>tr|Q87HH0|Q87HH0_VIBPA Uncharacterized protein OS=Vibrio parahaemolyticus serotype O3:K6 (strain RIMD 2210633) OX=223926 GN=VPA0995 PE=4 SV=1

MSAPPKSSSRLPLHLDSELCFVLPDFLAREFEYARSLEQGIMDNLHPEFTHQYRVTLRRM

RSLCVLLSEVIPCFELAILKPHLKTLMKQTNLLRDLDVFTLDTNQYLAMLPEQHSSLTRI

FADIDAMKNAEQVRVASWLASLAYQTHCAMVRNSLERTKQYDLLKNDIELVTFANQKIAD

QFRKVNNSQRKISPASKDDVIHTLRIKCKALRYLLECFSAIYPAQQHKENVQQLKLLQDK

LGDFNDTSTQIAFFTQLRKDKRYNKPDRQVLKSLIKEIKQAHEQSRQSTLLRLKSFDSFI

NDASTLEIYR

>tr|Q87FV9|Q87FV9_VIBPA Transaldolase OS=Vibrio parahaemolyticus serotype O3:K6 (strain RIMD 2210633) OX=223926 GN=VPA1569 PE=4 SV=1

MIELYLDTADVAEVKRFNQCLPLKGVTTNPSILAKSKQGLNQTLAGMQEALGGTPRFHAQ

VVSTTVEGMVEEARQIHELPYDMVIKVPATETGLSAIKLMKKEGIQVLATAIYSAQQGFL

AALCGADYLAPYVNRIDAMNGNGVEVVADLQLLLDQNQLPAKILAASFKNTQQAMEVMKL

GIEAITLPVDVAAQMFSHPAVQPAVDQFDKDWKSTFGNKLSFES

>tr|Q87Q99|Q87Q99_VIBPA Thioredoxin reductase OS=Vibrio parahaemolyticus serotype O3:K6 (strain RIMD 2210633) OX=223926 GN=VP1251 PE=3 SV=1

MSDVKHCKLLILGSGPAGYTAAVYAARANLNPVLVTGMQQGGQLTTTTEVENWPGDAEGL

TGPALMERMKEHAERFETEIVFDHINEVELSQRPFRLKGDSGEYTCDALIISTGASAKYL

GLESEETFKGRGVSACATCDGFFYRNQKVAVVGGGNTAVEEALYLSNIASEVHLIHRRDS

FRAEKILINRLMDKVENGNIILHTDRTLDEVLGDDMGVTGVRIKDVNTGTTEDLEVMGAF

IAIGHQPNTQIFEGQLEMKDGYIVVKSGLEGNATQTSIEGVFAAGDVMDHNYRQAITSAG

TGCMAALDAERFLDALSDK

>tr|Q87KT3|Q87KT3_VIBPA Putative transcriptional activator CadC OS=Vibrio parahaemolyticus serotype O3:K6 (strain RIMD 2210633) OX=223926 GN=VP2893 PE=4 SV=1

MVGVYFQINDWVLSVDENKLYRQDREVTVEPRLINLLHFLAEHANEVFNREELIQYVWAG

AIVTDQVVTQSIFELRKLLRDGREENTSYVITVPKRGYKLVANVTPMTHEEFVASRQSEE

EENAPLVEEPEEETLPIAPAVTFPAGPLTRAVCEMSKEKKAPQKKNKISPWRLGLMNFIW

ISVLVVVMGVFTYKQSEVRITQAIDTHLIEFKFQDNFTSEGLSYDLADGFAQKLMSDIAQ

VSDYRVMLKKASFTSGIVPGKSVVVRVRENDGGSFLEVEYRNNSSEKVLFSRQYALTSSH

LKTVLQQASLDLMQVLKVPDAKLKSTMLVAGMPMNPDALELFVQANHYLNVSDAKQFRQG

IDLMENILEIEPDNAYVQAELLIAYHVQKVLDSSLELKKGRVQQLSDALETNVKMMVGPI

QPRIYEALALHETIAGDMASAKQHLGQALRLRDSVLSYVIRGKHAELDGDLDLASESYSE

AFYIDTSVETYLLCENLVFPSNMKAIDYAMYRAVHPSVVRML

>tr|Q87R21|Q87R21_VIBPA Uncharacterized protein OS=Vibrio parahaemolyticus serotype O3:K6 (strain RIMD 2210633) OX=223926 GN=VP0977 PE=4 SV=1

MSCMYHPISLFIGLRYLRGRSGDRFSRFVSYMSTAGITIGVMALVTVLSVMNGFEAQLKE

RILGVLPHAVISQHDGRTPLTESAPQFIQAMSDVAEPEPVVRGEAVIQSSAQLTAGYLIG

IEPKKGDPIANHLIAGRLSSLQAGEYKVFLGHSLARSLKVSIGDKVRLMVTNATQFTPLG

RIPSQRNFTVAGIFNTGSDVDGQLMIVNMADAAKLMRLPKDTVSGWRVFFSDPFMVTDFA

DKPMPEGWQWSDWRAQRGELFQAVKMEKNMMGLMLGLIVGVAAFNIISALIMVVMEKQSE

VAILKTQGMTQSQVMTIFMVQGASSGVIGAIVGGAVGVALSLNLNVILESAGVALFSFGG

HLPIVIDSFQILLVVVLAIALSLAATVYPSYRASSVKPAEALRYE

>tr|Q87GB2|Q87GB2_VIBPA Putative polysaccharide export-related protein OS=Vibrio parahaemolyticus serotype O3:K6 (strain RIMD 2210633) OX=223926 GN=VPA1405 PE=4 SV=1

MNPLFKLIGLALLLFSTFVSANSNEQDYLLDTGDTISVQVYGEEDLSIKNILITSDGYFD

YPYLGRIKAINKTPKQLKYEIETGLKGDYLINPKVMVTINYFRLFYVNGEVRKPGGFEYR

PGLTIEKAIALAGGLTDRASRKSINLTKHKTGKTVEGVSMQRTVEPGDIVFIDQSFF

>tr|Q87K13|Q87K13_VIBPA Putative exported protein OS=Vibrio parahaemolyticus serotype O3:K6 (strain RIMD 2210633) OX=223926 GN=VPA0085 PE=4 SV=1

MKVRYLTSALLLAMTSGLAAAASVDIRHEYVPDRDGDEHRDRIYVSHRFDNQIGFSVEAK

WNYKDGHMGNAGHETGVSYRWKATDNFSLTPGINIDASPSGASVFKYNLTGAYKINDEWD

VAARIRHGYKNTDDSRYNQLNLYANRKFEWGKLGVEAEYKDIHGGEGGWKDKGHDQLIDF

KGEYTKLESGVIPFFAIAAITHKGDGSEFKDEYVPRFRVGLKYNF

>tr|Q87QS7|Q87QS7_VIBPA Putative helicase OS=Vibrio parahaemolyticus serotype O3:K6 (strain RIMD 2210633) OX=223926 GN=VP1072 PE=4 SV=1

MYPVPLVGESYMPYAFKTIQSEIEGNSNLREPQIECYRELKNFYQNQLKESEEREVGIVL

PVGCGKSGCITITPFAFQSKRALVIAPGVSIASQLLRDFEPLTSFYKKCSILKGNEFPEP

VEIRGKATNLSDLDESDVVVTNIQQLQGADNRWLDKLSDDFFDVIIFDEGHHSVADTWTN

LKSKFPKAVIVNFSATPERADGQIMSGKVIYTFPVQSAIQKGYVKELTAVRLNPKTLKYV

RNEDDKEIEVPLEEVIRLGEEDAKFRRSIVTSKETLDTIVNASIHQLIELRKATQNNKLK

IIASALNYEHCIQVVQAYRERGLAADYVHTREDSKKNNAVLTKLNNHELDVIVQVRKLNE

GFDHPFLSVAAVFSIFANLSPFVQFVGRVMRVIEQNSPRSPINQGIVVYHAGSNIAQRWS

DFKEFSTADREYFDQLLPEKEYTPTESSNFESLRPTIENDDKFVIKDQSEVSIDKSALID

DPKALEALRYLNSIGVTGDIDELLQQVDKIPVTKVRKRQAQRQALSDRVKNATGKILANL

EKKPMARGLDPSFRVNNYEYVCSRLNRHINEHVGRGAGERHDFSATELDSIDSAFPEIVL

FVEQGLK

>tr|Q87JX9|Q87JX9_VIBPA Uncharacterized protein OS=Vibrio parahaemolyticus serotype O3:K6 (strain RIMD 2210633) OX=223926 GN=VPA0119 PE=4 SV=1

MLIRAIPFVFVILWASGFVGARFGLQYAEPATLLSIRMAFNVLLFLVLVAVLRRRLPTGT

DFWHSCVVGALIHGFYLGGTYFAIALGMPAGLSSLLVGIQPILTAALLVAFVREEFKPSQ

WLGLALGFVGIAMVLMGKMEWQSEQHKVLAIGLCLLALVGITLGTLYQKKHCQQVDMVGG

ATVQYLAALIMFLPVAMQFETMQVQWELEFILTVLWLVVVLSCVAILLLLYMVRNGASSS

VASVFYLVPPTTAIQAWLAFGESFDWMGISGFVLAATAVYLVVKKPDLTIKKAIKTEYT

>tr|Q87RG5|Q87RG5_VIBPA PTS system, N-acetylglucosamine-specific IIABC component OS=Vibrio parahaemolyticus serotype O3:K6 (strain RIMD 2210633) OX=223926 GN=VP0831 PE=4 SV=1

MNILGYAQKLGKALMLPIATLPIAGLLLRLGQPDVFDIAFMAQAGNSIFSNLPLLFGLGI

AIGLSKDGQGAAGLAGAVAYFVLTATASTIDASVNMSFFGGIIAGIIAGHSYNAFHATRL

PEWLAFFSGKRLVPIMAGLFALVAGAVSGIVWPTIQGGLDALAHGISTSGAIGQFVYGTL

NRALIPVGLHHVLNSFFWFGMGSCQEVLVSGATAAGQALPALQQLCVDPALAKTLVAGQT

HTFEFANSVTPEITATVKEVTETVKSGDLHRFFGGDKSAGVFMNGFFPVMMFGLPGAALA

MYLAAPVEKRSQVGGALFSVAFCSFLTGITEPLEFMFVFLAPALYAIHAVFTGLSLVVAN

MLGTLHGFTFSAGLIDYLLNYGLATKPLLLGAVGLGFGALYFFTFSFAIRAFNLKSPGRE

DDDSQAAAPAGEAKSGDLARQYLKALGGHDNLTSIDACITRLRLTLKDRSVADEEVLKKL

GAKGVVKLGENNLQVILGPLAEIVAGEMKAIGANEDLSNVKLP

>tr|Q87FZ4|Q87FZ4_VIBPA Uncharacterized protein OS=Vibrio parahaemolyticus serotype O3:K6 (strain RIMD 2210633) OX=223926 GN=VPA1526 PE=4 SV=1

MLRPLVLGCAIAFGSTYAMAEAVDLKQNMKQMKMEFKQAAEASDVETMKSAIDSLQAIVE

QSKRGNYPPEKFDTYLEGFNKLSVTLDKIEADLDAGNLTEAKAQLREIDSLREEYHDKRN

PSIWSKLFG

>tr|Q87GQ2|Q87GQ2_VIBPA Uncharacterized protein OS=Vibrio parahaemolyticus serotype O3:K6 (strain RIMD 2210633) OX=223926 GN=VPA1263 PE=4 SV=1

MSYKVLPLSEVMSHEFGLIEGNFHNISEADIASAIPSHLTFEQLKQHLYEGNLALVSDTP

QTPALLSYNDPIGPKTWRLNSEVISELSDDAANNLLAITKTTRATGGYRSCIGETSTLER

VYTPQPIATESEEEITKEFEYSFEVGCSDATIKKMVHSDFALAKTEKENAVTRWEQTNTE

QGTRYTALCVFDEPKRLNIHIADDNLGLTPLEAVTLQKAGSCKTDEGFIPVVPAVRLGER

LGLPTEGYYYHFNDGELVQEYKILGEEKWAFYATQSTQDKLNDERGFTKDQSAILVYWKL

ADQIIENQYIIYLERQITREELDNLSEDWLCENGIKLDIPALFDAVKQPEEARSEGNNDE

TQAEQTAATHTVTVGEDWQSIAELYGMGAKALLLLNPVFEADPLSLAVGDEIIVAEKQQQ

QAPDKKNTFPPLRPQTYNNIRNSHYQHSDPLLGLTKYRAINTADCIEGDVVILNLKDATS

SIVFAKSCTRPEGCIEIGDQQESISNFGPWSFFFAQANANPAAVIPAIQATQAQMAMGSS

AAVAGSPEQMQQTQTAAMQLDKLAGTLKEKIVEGYRWQVEGIGALFAMQQSLFGDNTQYT

DQDLRQVTTAQSRVRVHITEPQGGEFYPHVQGYHVDDTRIPIKYVKQGNNGQLSVAIEEN

GPTIYWTPEENGEASWQTTPDHSDGFEKDDILVTPIHSDSDANVTVTPAPEEKDWRDAIL

VFPESSGIAPLYVVYKESPRDKPGVVTGKGEDIFGIWLADAGKDLGAPIPSQIADKLRGR

EFSSFDAFREGFWYAVGEDQTLINQFSRANQRLIKRGRSPFSLPSEQVGGRQRYELHHKE

EIQYGGEVFHVDNLCVLTPKRHISIHKDQ

>tr|Q87R27|Q87R27_VIBPA NADH dehydrogenase OS=Vibrio parahaemolyticus serotype O3:K6 (strain RIMD 2210633) OX=223926 GN=VP0971 PE=4 SV=1

MTRIIVVGGGAGGLELATKLGRTLGRKNRAQITLVDRKASHLWKPLLHEVATGSLDEGVD

ALSYRAHAKNHSFDFQMGSLQDIDRERKVIILSELKDEHGELLMPSRELEYDLLVMAIGS

TSNDFNTPGVRENCIFLDSPEQAHRFRTEMNNEFLKLHAKNGNGTVDIAIVGAGATGVEL

SAELHNAVKELRTYGFGDLDSSKLNVNLIEAGERILPALPPRISSAAHQELVKLGVNVRT

ATMVTKAEKDGLTTKDGEKIPAQIMVWAAGIKAPDFIKDIAGLETNRINQLVVKGTLQTT

RDDDIFVIGDLAQCTQPDGSFVPPRAQAAHQMASQAFSNIVAKLNGRELKNYVYKDHGSL

VSLSRFSTVGSLMGNLTKGSMMVEGRIARVVYISLYRMHLMALHGVFKTGLMMLVGRINR

VLRPNLKLH

>tr|Q87QA5|Q87QA5_VIBPA Response regulator OS=Vibrio parahaemolyticus serotype O3:K6 (strain RIMD 2210633) OX=223926 GN=VP1245 PE=4 SV=1

MNRYAVLCLDNNPISAEQFRLELSAFSSKFDIFSVESIEEAQSALEYLEEREQTVALVIA

SHHAHFNGVDFLIGLDKTPHTERARKILISCSSDIDAILTAVNEGRLDHCLTKPLPDNVL

FNTAQKELTQFILRNCKEDLLSYSQILDQHKLLRAHIENQMSNYQAGFLHDYHSMSDNEL

AEQVISALQQFFKENDETKACRTYSPEHLLTVEGEPNSFLWFITSGEVALYKRDEQGMQR

EVVRHTKGNIVGGMSFVTGECSFSTALTLTETEVIKLDRNVFRKVMQSDSNLLPLFTNLL

LRHFNRRLQRSINTKLQLQKTLESLESAHQQLIEREKMAMLGQLVAGVAHELNNPIAAIL

RGVENLTHTLEGLLTQLPTAEVQIKGVQLLTRAQTAKPASTAELRSRVKELNSTLPDRTI

AKKVVNLGLESDSELVSQLAKKKDNASSTLETLEQYHQAGASLRSINVCAARIADMVKSL

KGYARSDDERMHYADIHEGIEDTLVIFENKLKVHQVSTDYAPLPRMLCQPIALQQVWTNL

ISNAIDAFPDKGSLKIQTREVEKNQQRYAVISFEDNGCGIPDSQKKAIFELNFTTKKEGN

FGLGIGLSICQQIVAAHKGWIDVQSELNAFTCMTVWLPVIEEGDET

>tr|Q87I37|Q87I37_VIBPA Uncharacterized protein OS=Vibrio parahaemolyticus serotype O3:K6 (strain RIMD 2210633) OX=223926 GN=VPA0769 PE=4 SV=1

MATAALKSFKPKQPHKANFWFMLGIEDAETGRTDAVHKGFEPKVYRNIVERVKLSQNEFQ

NVTLIPVSTIKRRLKNDERFNTQESDAIYRLAMLLKLATELFDDEERALEWMKENVYGLG

GKRPLDMVSTTVDFEIVKDLIGRLEHGVFS

>tr|Q87NS9|Q87NS9_VIBPA Uncharacterized protein OS=Vibrio parahaemolyticus serotype O3:K6 (strain RIMD 2210633) OX=223926 GN=VP1789 PE=4 SV=1

MIYGKWGEIMKGIIVRWVDDRGFGFINSEELDGDIFVHVSKFKKGYRRPQIGDQVEFQLV

NNAPKLSASTAQLLGVEPNKVNPFSMLLSAVFIGIVGAGFYLFGIEPTLNPEYENMGFSC

QGKTYCSEMVSCNEAKFYLSNCPNVKIDGDNDGIPCESQFCGNW

>tr|Q87K42|Q87K42_VIBPA Uncharacterized protein OS=Vibrio parahaemolyticus serotype O3:K6 (strain RIMD 2210633) OX=223926 GN=VPA0056 PE=4 SV=1

MAIPARRKRKQDKEPQLSFWARHQLKMVKSAFVIFVCSLVAFFAYQLHVSYQDYIDPEHV

YGEWIEIGAPPYQTERLIFTSDGVYRNHRLITTEFAFDGKVITLNTGIGETAYQLSGSHL

SPQIRRIEPRIPDQRFIRKGFEHTVQGSEVGAASKRRAALSEHFSRD

>tr|Q87H16|Q87H16_VIBPA Uncharacterized protein OS=Vibrio parahaemolyticus serotype O3:K6 (strain RIMD 2210633) OX=223926 GN=VPA1149 PE=4 SV=1

MNKGHLATTLVLSSALFTTASWAKDSVFVGHLVDFSGPTAYVSKHYGSGVRDALLWINQN

GGINGTELEFETVDMAYKVPVAISNYKRWVARKNMVAMQGWGTADTEALISFVTKDKVPV

FSASYSGHLTDPTGKNPKTAKPAPYNFFYGASYSDACRALVKWAKEDWESKGNTTAPKFT

HIGDNHPFPNAPKDACAEYAKELGFEVQNPVVVSLKPGDFKAQCLSLKESGTNYGYIANL

GGSVISLVKSCNTVGTEFQYMANIWGGDKPVIEAAGDGLKDYVFPGMTAFWGDDSEGMKL

VQQISKLSESEPGEFRTHHYIRGVCSAFYMKEAMEWAKDNGGITGENIKKGMYVHKNWVP

KGLEGVCIPANWQPEDHRGTTTVNVFMGNNQGGAVDIKKVSQVTLSRRDDWLGY

>tr|Q87H50|Q87H50_VIBPA Uncharacterized protein OS=Vibrio parahaemolyticus serotype O3:K6 (strain RIMD 2210633) OX=223926 GN=VPA1115 PE=4 SV=1

MSQFYKLRALSLVFWFVFLTTKVYAQSPEPKDKFLQSLEGIESQIYALPQSSLAQIESLE

EDSLLQNQPKDLLIRYWLAKSTVLELLGRDKESLAVVDKGLSLTPEQSQEHLLFKLIQIR

AMMGNRDIDTALSSLDALLETSREKGDKKLESEVLLLKGRYYDEQGDYKKSYAALMSSME

AAESSGAQGLVERAALELGDVLVKIQGYDRSEVVLKQAYRYFKDRRMSFNELLSVLTIAK

LHKAQFQYDEAIKSYQAALKLAQIIGDGRFRFRVNLELAALYRETNNEKNMLRHLKLAEN

LQYRETSNAYLATFKLLQAEYMLERKQYQALLTMITPLLPEIIESRYIKQQQMELLKVAA

MAYAGDQNFELAYQTYGQYHEKFIQFSNQREVENLERQQTLFELERLEYENENLNWNNVL

QRLELENNRRTFYLLGEVLLIMIGILLLMALVFLYVNRSRLRMRRLAKTDMLTGLFNRRF

LEEWFAKPAEQKPKLIEKPIPETKKGKLVHKLNKQVMRVQYGYLALNHWVERKLDKQKMV

AKKPETGPITLVMMDVDHFKQVNDTYGHVFGDVVLTGVAKVLDSSVRESDIVARLGGEEF

VIVLPNTDLEEATALAERLRIALSQRGFVTENNQAVNVTCSFGVITSDDVDVAFEALCSQ

ADKLLYEAKSSGRNCVKALSFS

>tr|Q87TD5|Q87TD5_VIBPA General secretion pathway protein F OS=Vibrio parahaemolyticus serotype O3:K6 (strain RIMD 2210633) OX=223926 GN=VP0135 PE=3 SV=1

MAAFEYKALDAKGKQKKGTIEGDNARQVRQRLKEQGMIPVEVVEAKAKAAKSSGSVGFKR

GIKTAELALITRQLSTLVQSGMPLEECLRAVSEQAEKPRIRTMIAAVRSKVTEGYPLADS

LGDYPHVFDELFRSMVAAGEKSGHLDTVLERLAEYVENRQKMRSKLLQAMIYPVVLVVFA

VAIVSFLLATVVPKIIEPIIQMGQELPQSTQFLLAASEFVQEWGLIIFAVLVVCFYGLKL

ALQKPDFRLSWDRKIISLPLVGKISKGLNTARFARTLSICTSSAIPILEGMRVAVDVMSN

RYVKQQVLIAADNVREGASLRKALDQTRLFPPMMLHMIASGEQSGELESMLTRAADNQDQ

NFESTVNIALGVFTPALIALMAGLVLFIVMATLMPMLEMNNLMSG

>tr|Q87N54|Q87N54_VIBPA Uncharacterized protein OS=Vibrio parahaemolyticus serotype O3:K6 (strain RIMD 2210633) OX=223926 GN=VP2021 PE=4 SV=1

MKWLKLALLILAGVLLANQLDNPVLSHLTDRNWLVGYIQKNGALGHVVIFTFSVVFLSLS

GPKQAIALVFGYLYHVQLGVLITLFACVFGATFNYACARFLIGNWLYRRFPKKMTRFNSF

ASRKPFYKILLLRLFPIGNNVVTNVLSGSVRVPFFAFISASLLGYLPQIIIFALMGAGIH

SSSNTMIYLSIFFGIVSAVLTGFLYRDHIKSRVELLNMEEEV

>tr|Q87NY1|Q87NY1_VIBPA Uncharacterized protein OS=Vibrio parahaemolyticus serotype O3:K6 (strain RIMD 2210633) OX=223926 GN=VP1737 PE=4 SV=1

MLTINSFVLYVENIHVSQTFYSRLFDCEVTLLSPTFASMPLSHSVKLTLKQSDALTPISM

VKGGGAELSLSVADGHQLELVYNKWKEMGVDFIQPPMTSVYGLNFVATDPDKHRIRVFIS

E

>tr|Q87L21|Q87L21_VIBPA Arginine N-succinyltransferase OS=Vibrio parahaemolyticus serotype O3:K6 (strain RIMD 2210633) OX=223926 GN=VP2796 PE=4 SV=1

MLVVRPIAMSDYDALHTCAVESGHGFTSLPVNEELLTNRIKHSEYSFGKPDVTEPGDEGY

LMVGFDSESGEVAGTTGIEASIGWDVPFYSYHISKVVHSSPKLGVNNVVKLLTFGNNYTG

NSEICTLFLREKYRQGLNGRLMSKCRFLMMAEHPERFSKTIFAEMRGVSDANGNSPFWQW

LQEHFFSIDFTLADYLTGIGKKGFIADLMPKLPIYINLLSPEAQAVIGQVHDNTRPALKL

LEREGFTNRGYVDIFDGGPTVECDLRNIESVRHSFRAQVKISAHTSTQDFLMCNSSFENF

RAAAAKAAYDAETKSVVLAPELADALLVKEGDFVRLLPQ

>tr|Q87SQ6|Q87SQ6_VIBPA Phosphoenolpyruvate-protein phosphotransferase OS=Vibrio parahaemolyticus serotype O3:K6 (strain RIMD 2210633) OX=223926 GN=VP0366 PE=3 SV=1

MASNQIEGAVVGIRVNDGIAAAPVVLFTHEMPAVPERDFQSEQGEIERVKRAIGVVVQHL

QEQAKQPKGEIFSAHSMMLSDPELWASVESRIQTGMIAEQAWIESLQTLADEFRQAESQY

MREREADVHDIARQVMVEMTGVTPNAIDIQEPSILLARDLMPSDVAGLDKSKVLGICLSE

GGKTSHSAILARAMGIPAMVKAQGCLDAVRAGQVVTIDGFRGHLWFSPSDAIQQELEAQQ

IEWQSTRQSALASAQQAAATCDGVHIPVFANIGGPKDIDDALTSGAEGVGLFRTEFLFQN

SDELPTEEAQYQVYRDIAAALGDKPLTIRSLDVGGDKPLAAYPMPAEDNPFLGLRGVRLC

LQHESLFTAQLRAILRAFHEQPNIQLMIPMVAQVEEVRKVKALLAHQANQLGLDATHLPV

GIMIEVPAAVLNADALAQEVDFFSIGTNDLTQYVMAADRGNAAVAELVNYFEPSVLKAIE

LTCAAGDRAGIPVSMCGEMAGDPNATETLLRVGLQKFSASPSLLPGLKAQIRQLSVDV

>tr|Q87PM2|Q87PM2_VIBPA Riboflavin synthase, alpha chain OS=Vibrio parahaemolyticus serotype O3:K6 (strain RIMD 2210633) OX=223926 GN=VP1480 PE=4 SV=1

MFTGIVQGTAKVVQIDKKERFQTHVIELDGALIEGLEIGASVAHNGCCLTVTNIDGNRVS

FDLMQATLALTNLGLIEEGSAVNVERAAKFGDEIGGHSMSGHISLMANIVDVIDTPNNRT

IWFELPQESMKYVLAKGYIGIDGCSLTIGEVEANRFSVHLIPETLQRTLFGSRQVGDKVN

IEFDPQTQAIVDTVERVLAAKQL

>tr|Q87GC7|Q87GC7_VIBPA Uncharacterized protein OS=Vibrio parahaemolyticus serotype O3:K6 (strain RIMD 2210633) OX=223926 GN=VPA1390 PE=4 SV=1

MDWHYRTITFLPEYRNNEAIAAKCIKELHRFNYKYETRSIGVSFPLWGQETVGRKITFVS

TNKMELDFLISRRYFVQMTKLGYFSISTTQTVPDDCSYVLFKRAHSIDKGTFAGRARELK

RLERRALERGEIFDPIAYSKTTSHAFQSYHSLEEDSSSGNKFRLNIQMKERSGTVGTGKF

SSYGLGNTDNSLQVVPLI

>tr|Q87J01|Q87J01_VIBPA Putative DNA-binding stress protein OS=Vibrio parahaemolyticus serotype O3:K6 (strain RIMD 2210633) OX=223926 GN=VPA0454 PE=3 SV=1

MTHIDIGIDREHRLSTAEGLKQLLADSYTLYLQTHNFHWNVEGPNFRELHLMFEEHYTEL

ATAVDEIAERIRTLDVPAPGTFKEFAKLSVIEEVEGVPSATEMVDILTHNHEQVVKTARK

VLKLAQQADDESSVALVSDRMRIHEKTAWMLRSLQK

>tr|Q87J48|Q87J48_VIBPA Uncharacterized protein OS=Vibrio parahaemolyticus serotype O3:K6 (strain RIMD 2210633) OX=223926 GN=VPA0405 PE=4 SV=1

MKSGYSYPIGTPGQPWGEAERKAWVAQREVKRSYQEEVVSKIDALRDRFDVEQYGALSYD

QARFPLFCIKTRNWDSAKPIVLVTGGVHGYETSGVHGALKFVATEAEHYAEHFNIVVAPC

VSPWGYEVINRWNPNAIDPNRSFYADSPAEESANLIKLVSTLGDVLMHIDLHETTDSDET

EFRPALAARDGIEYIEGMIPDGFYTVGDTENPQPDFQKAVIESVAKVTHIAPADEHGEII

GSPVVQFGVINYPMVKLGLCGGVTNCVYGTTTEVYPDSPKVTDEECNDAQVAAVVGGLDY

VLAQL

>tr|Q87T91|Q87T91_VIBPA Lipid A biosynthesis lauroyltransferase OS=Vibrio parahaemolyticus serotype O3:K6 (strain RIMD 2210633) OX=223926 GN=lpxL PE=3 SV=1

MSKDKYTQPEFSLSLLHPRNWGVWLGFGLLAIIVNILPYRLLLSLGRSLGKLGMRYGKKR

VHIAKRNLELAFPEKTPEEVQHIVEENFKNTGMALIETGITWFWPTWRFKTLIVEKDIHA

LKEKGAEGKGVLLCCVHALNLEITARAFAVLGVAGYGAFRPHNNPAYNFIQYWGRTHNGN

KLIDRKDVKKMIRVLRSGERLFYLPDHDYGRNKSVFVPFFAIDDACTTTGTSILAYTSKC

TIIPGSGFRNDEGKYEIIADKCIEADYPQKDEVAAAAYMNKYVEEVILRAPEQWMWLHKR

YKTMQDENEPKGIRYK

>tr|Q87SE9|Q87SE9_VIBPA Transcriptional regulator, LysR family OS=Vibrio parahaemolyticus serotype O3:K6 (strain RIMD 2210633) OX=223926 GN=VP0475 PE=4 SV=1

MLLEGIETLLVLSKEKTMSRTGSQLYISQSAVSKRIANLEKKLGKKLIEPNGRHIKLTSD

ALALINTIEPTFNELRGQIYDQQELEDTSLIKMDCSETLVAGYLSMIMEEQLARDPYLRI

STNHTPRIVENVKSGRATLGFCAGYLPSHHGLMSVHLADEPFSIISKHPLTQLPKAILTN

DLNNPANTYQQAILSKYEIQPLMEMDSYTAAAQLALKGMAPALIPKSIIKTLNIASNFCF

EFEELEPLIRPIHICLRHNTYRNQRIKTVLEAIADVVAKEVLLPSA

>tr|Q87LS0|Q87LS0_VIBPA Uncharacterized protein OS=Vibrio parahaemolyticus serotype O3:K6 (strain RIMD 2210633) OX=223926 GN=VP2541 PE=4 SV=1

MLDAFNSAFEHLALWFSDSALWVLFLTGFLSATLLPGGSEAGLIATLSLNQYAVSSILLV

ATIGNTLGGLTNYWLGLWIPNKTQEEKHGHTALKWLAKYGYWGLFFSWLPIIGDPLCLAA

GWLRMKFLPCVILIFLGKAARYSLLAAIYLGLF

>tr|Q87JF0|Q87JF0_VIBPA Putative regulatory protein OS=Vibrio parahaemolyticus serotype O3:K6 (strain RIMD 2210633) OX=223926 GN=VPA0303 PE=4 SV=1

MNPRQNEILQLVNDRKRVQVTELSDIIGVSGVTIRQDLNFLEQQGYLKRVHGAATALQSD

DIDTRLEVRFDIKQTLANKAADLVAPNETVLIEGGSANALLARTLAERGDVTIITPSAYI

AHLIRNTSANIILLGGVYQHQGESLVGPLTKLCIENIHFSTAFLGIDGFHQDTGFTSRDM

MRADIAEAILAKKRRNIVLTDSSKFGQIYPSSIGKTNEISVLLTDKAAPKSDLEQLKKLG

VEVVLG

>tr|Q87FY2|Q87FY2_VIBPA Putative two-component response regulator OS=Vibrio parahaemolyticus serotype O3:K6 (strain RIMD 2210633) OX=223926 GN=VPA1538 PE=4 SV=1

MMTKTNILLVEPNEHLAQPVLDVLKNAGYTAKHTRTGRSALLEERASITLVSSTLPDMCV

REFVACHQKQRNAGVVIAIVDQEQGILAAETMKSGATDYLLRPFEANQLINLLKRVEALG

KPMANIVAESWRSKQVLQLAHRAACTNASVLITGESGTGKEVLARYVHEHSPRINGPFVA

VNCAAIPESMLEAVLFGHVKGAFTGATNSQSGKFEEANGGTILLDEIGEMSPAVQAKLLR

VLQEREVERVGSHKAIKLDIRVIAATNKDLREEVQKGTFREDLYYRLDVLPLHWPPLRER

KEDILPISQFFIEKYQDSSRCHLSQDAISALSQYHWPGNIRELENVIQRALVMRHGDYIT

AHDLMLPIELIAPVPSMEPKSSFGHVEAKKQAEYQFILDKLRQFGGNRTKTANALGVSTR

ALRYKLAAMREHGIDLQSALGSAA

>tr|Q87KX6|Q87KX6_VIBPA Alanine racemase OS=Vibrio parahaemolyticus serotype O3:K6 (strain RIMD 2210633) OX=223926 GN=VP2848 PE=3 SV=1

MGQTAAHAAPLLVDFDDSEREERVQASNAWLEIDTQAFSNNIQLLQKDLKGNTQICAIMK

ADAYGNGIAGLMPSIIANNVACVGITSNEEARVVREHGFEGKIMRVRAASRNEIENGVQY

EIEELIGTKMQADQIIEIAYNYNTVIPVHLALNTSGMGRNGLDLTTYEGQVEGVEIASDP

NLKIVGMMTHFPNEGLDEIRRKVDRFKVETKWLMDSAGLKRKDITLHVANSYITLNLPEA

HLDMVRPGGMLYGDYPATLPYERIVSFKTRIASLHFFPAGSTIGYGSTKVLERDSILANL

PIGYSDGFARSLSNKADVLINGQRARVMGKASMNTTMVDVTDIIGVQANDEVVVFGRQGF

EEITAEETEEKSGRILPEHYTIWGATNPRVYR

>tr|Q87PH6|Q87PH6_VIBPA Putrescine-binding periplasmic protein OS=Vibrio parahaemolyticus serotype O3:K6 (strain RIMD 2210633) OX=223926 GN=VP1526 PE=3 SV=1

MIWTTFGLELTSMKKWATLLAGSACALSLFSGSAAADDKELVFMNWGPYINSNILEQFTK

ETGIKVIYSTYESNETLYAKLKTHNQGYDLVVPSTYFVAKMRDEGMLQKIDKTKLKNFGN

LDKNYLDKPYDPNNDYSIPHVVAITGLAVNADMYDPNDFQSWADLWKPELEGQVMLMDDT

REVFHIALRKLGYSGNSTDPKQIDEAYAELQKLMPNVLVFNSDNPGAPYMSGEVGVGMLW

NGSAAAAQNEGLNLKLVFPKEGGIGWVDNFAISSGAKNVEAAHKMIDFLLRPEIAEQISR

DTGYLTAVTESNSKFKDVAPLFPSQEDLDRVEWQDSVGDMTVKYEDYFLKLKAGQ

>tr|Q87JL8|Q87JL8_VIBPA Phosphotransferase enzyme II, A component OS=Vibrio parahaemolyticus serotype O3:K6 (strain RIMD 2210633) OX=223926 GN=VPA0231 PE=4 SV=1

MLRELITSDVIRIHSDATDWKDAISKSCEALIENGAIEPSYVEAIYRSHEELGPYYVVGP

GMAMPHARPEDGVNRLSLAITVIQNGVNFNSEENDPVKMLVTLAATDSNSHVDAISKLAE

LFMNEEHVEAICNAQSKEDVLAIIDKY

>tr|Q87LK5|Q87LK5_VIBPA Putative cytochrome c oxidase, subunit I OS=Vibrio parahaemolyticus serotype O3:K6 (strain RIMD 2210633) OX=223926 GN=VP2607 PE=4 SV=1

MPRTVNPSDFQSKRKEVPDNEYARTIPCNTVNLSAPFHWLALGLHDFVRMPLISAFYGIC

FMAAAIGIVLLVQWQGTHLVVMPSLIVYMLIGPFLALGLYDASWERERGHKARLLHSMKA

IGRNSSSQWAFAVLLAVCMIFWMRIAALLHALYPSVQGAPLTEFLPFLVIGSLVGFVLAC

IVFSISAFSIPLMMERRVDMMTAVFTSFNAVKSNIPAMIVWAAIICGGILIGFATYGIGM

LFTMPILGYGTWHAYHATIKKKHTP

>tr|Q87QZ2|Q87QZ2_VIBPA Probable membrane transporter protein OS=Vibrio parahaemolyticus serotype O3:K6 (strain RIMD 2210633) OX=223926 GN=VP1006 PE=3 SV=1

MEMIEPTMLLVLALVAFVAGFIDAVAGGGGMLTVPALLSLGLPPHIALGTNKLAATFASS

TAAFTYYKKRLFKPQCWGRAFAATLVGATLGTLFVDAISTDWLEKVLPLIILAAALYTVF

HKTLHSPHQSPIPEPCPKLHKKQYLQGLSIGFYDGLAGPGTGAFWTVSSMALYRLNILLA

SGLAKAMNFTSNFTSLITFAILGHINWVLGLTMGVCLMAGAFVGAHSAIRFGSKFIRPVF

VTVVSVLAIKLAYDAWFVGLS

>tr|Q87SP6|Q87SP6_VIBPA Uncharacterized protein OS=Vibrio parahaemolyticus serotype O3:K6 (strain RIMD 2210633) OX=223926 GN=VP0376 PE=4 SV=1

MFTVEYSEGGRTTIRSERSCVTLEFLLQPIYLPKNGQTVYYEALSSVISDSGEHLNSEAF

FETINDEFIKTVLLLQMEHFSQSNSISQDILLNVNLSSLKDDDFIESIIKRNKSNKYHIE

VNEIDTPVRDVKILKNIKRLQKSGIYIILDDYYHENEVAHLSLGVIDWDYIKIDKSFLLY

NSGNDDCLKALIFVISPYCKNGLIIEGVETYFQNEFVKKYNLLAQGYYYSRPKNIFKENR

NHKKVLQMNL

>tr|Q87TF9|Q87TF9_VIBPA Cytochrome c4 OS=Vibrio parahaemolyticus serotype O3:K6 (strain RIMD 2210633) OX=223926 GN=VP0110 PE=4 SV=1

MKKLALILSLLASCSVWAQGNIEAGKAKSQTCVACHGADGNSAIAMYPKLAGQHAKYLEK

QLKDLKLGMTSGGKQGRYDPVMSGMAMPLSDEDIADLAAYYSSLPTSESSTPEDVVAKGK

VLYTAGDAERGLTACMACHGPRGNGTELSGFPKISGQHADYIKAQLEKFRDGNRGNDMNA

MMRDIAKKMTDEDIEVLSKYVGGLH

>tr|Q87QZ7|Q87QZ7_VIBPA Amino acid ABC transporter, permease protein OS=Vibrio parahaemolyticus serotype O3:K6 (strain RIMD 2210633) OX=223926 GN=VP1001 PE=3 SV=1

MDFSLIIESFPVYFQGLWTTVWLVGISLVIGLCVSVPLAIARNSTNYALSLPSWGFIYFF

RGTPLLVQLYLIYYGMDQFFPVKDTLWEHAWFCALVAFVLNTSAYTAEIIRGAINGLPKG

EVEAAKAYGMSKFMTYKRIILPSALRRALPAYSNEVIFMLHGSAVAGIVTIVDLTGAARL

VNSRYYAPFESFLTAGLFYMSLTFIILWCFKRAEKRFLAYLRPLS

>tr|Q87PT3|Q87PT3_VIBPA Uncharacterized protein OS=Vibrio parahaemolyticus serotype O3:K6 (strain RIMD 2210633) OX=223926 GN=VP1418 PE=4 SV=1

MPISEEYYVLSNDYSKVNFVVNMEDERGFSLLMDKAISTSMHVNFECISLENFSAIDAPF

DVSVNTEILFDLDFLEVLSELTLYKNSLVPAKIKDKEFVYLHCYNIIDIEDEVKGVNFDL

LSEIPIEERLVFKPSYGAIVVFFS

>tr|Q87TJ5|Q87TJ5_VIBPA Uncharacterized protein OS=Vibrio parahaemolyticus serotype O3:K6 (strain RIMD 2210633) OX=223926 GN=VP0074 PE=4 SV=1

MSKRLCKMNRKQIAANLGDIHRLVVAPKFVCRSCARSSASKDSLCKPAAIPPQKCQDKPL

NEQQACGLLAEALPAQSISVTPKHSAEKAAIVKRVVERVKEKKQAAKVVTPVSMAAPALF

DLADKKALKKAKKALKKHYKQQKKLLKVAKKQQKLLKRQRKLEARNAKLEVLLPTPPNAL

MGVSSQAKVH

>tr|Q87KH9|Q87KH9_VIBPA Uncharacterized protein OS=Vibrio parahaemolyticus serotype O3:K6 (strain RIMD 2210633) OX=223926 GN=VP2999 PE=4 SV=1

MQQGMLSSLLLSLSLLTLPVAVSAKEMQESVVEQWLQDTQIQTKVSELLEYVVRDEVDSL

KFSLDRLAFPQQEVVRFRLLEKLEQQNIILTPRMALFVESQVRLTPTYQMLERGDGYEFS

VPAFNYPAIASRLIKRWKQDQSTLDFVLQAERKELNLQQWLTGTSQQIQTRESLLIRELD

SLSPSALKALTTQLTQANVTSWLPSTAVVVRMAQVSQDKAMYDLLWRMRADYNSQQELKR

LADTGDAFSLQQLMNATINPSLKPHAIRLLTKSNPLSPEVKQFLIAKMALSEEATLVARQ

LAQQGHQTWLEELISSNRQVKARQIEQVLK

>tr|Q87HW7|Q87HW7_VIBPA Uncharacterized protein OS=Vibrio parahaemolyticus serotype O3:K6 (strain RIMD 2210633) OX=223926 GN=VPA0839 PE=4 SV=1

MVKRLARFLTLVLCIGTVAVQAEETNWELKRVLVLHSYDPSYQWTNDIQAGIEKGFKQTD

GEVKLSIEYLDAKRVHSEGYLQQMASYLRFKYRAYEFDGVIISDDAAMRFVRNYFQNPKR

NVPMVAIGINDKGSSLDTVSTNGSVIYEEDRVVENIALINRLRPRIKNLYYLADRSVTSE

LIYQRVKAEMNKFPRINLIEIRDASLQEAANQLSTVSANDAVLLTHYNTEGDSGKYHTYR

EVARAIGAKSRAPVFVMWELYLGEPGILGGFVNRSEQFGYEAAEIMASKMGMSLTSAAHA

LAITEAVLDYKALTKYEISHYDIPKNAEILNAPPPLFKVNLKTLLFTCGIIVLLSLVVVI

QFMTIRQRKEIDKKNRKIVLLQKRTLNVQKEMIHVLGEAIESRSGETGQHVKRVAKLSRR

LAQLCGLTHREVEMIEIISPMHDVGKISVPESILDKPGALTSSEREIMKQHTIKGYELLN

MKEGDITKLAAVVAHEHHEKWDGTGYPNNLKGEDIHLFARIVAIADVFDALLTERCYKRA

WTINEVVDWFKLEQGKHFDPILCQLLLDNLDDFIEVRYMYPDA

>tr|Q87SH8|Q87SH8_VIBPA Stringent starvation protein B OS=Vibrio parahaemolyticus serotype O3:K6 (strain RIMD 2210633) OX=223926 GN=VP0445 PE=4 SV=1

MDIAKMTARRPYMLRAFYDWLVDNDLTPHLVVDATMPGVRVPVEFVQDGQIILNIAPRAV

GNLELGNDAITFHARFSGRPHSVIVPVYAVQAIYARENGAGTMFEPEEAYTHIEEETIEE

EDLSPSFKAVTEETSEEIGAESQEEEAPRPKGRPSLRVIK

>tr|Q87R19|Q87R19_VIBPA Uncharacterized protein OS=Vibrio parahaemolyticus serotype O3:K6 (strain RIMD 2210633) OX=223926 GN=VP0979 PE=4 SV=1

MFSSLALMIGGRFSRAKKRNKMVSFISLSSTIGIAVGVAVIIIGLSAMNGFERELQSRVL

SVIPHGELEGVNGPLQNYTKTMNQALQHEHVVAAAPYVRFTGLAEKGSKLKAIEVRGVDP

AYEQAVSSMSDFIDPEAWQNFYSGQQQVILGRGVANELKVQVGDYVTLMIPQTGGTNKVQ

APKRVRVKVAGFLTLNGQIDHSLALVPLADAQQYVRLGDGVTGISLKTDDVLDAPSIVRE

VGNLVNVYVYLKSWQQQFGFLYRDIQLVRTIMYLVMVLVIGVACFNIVSTLMMAVKDRAA

EIAILRTMGAKDGLIKRIFVWQGVFSGVFGSLVGSLVGVLVALNLTPIIKGLEGLIGHQF

LSGDIYFVDFLPSQLHWPDVALVSTTAIVLSLLATWYPASRAAKLNPAAVLSAK

>tr|Q87TN8|Q87TN8_VIBPA Uncharacterized protein OS=Vibrio parahaemolyticus serotype O3:K6 (strain RIMD 2210633) OX=223926 GN=VP0031 PE=4 SV=1

MNDQPYLIPSAPALFEEEIKKSVFITYLAHTPSVDAAKAFVDQIKAKHSDARHNCWGFVA

GRPEDSMKWGFSDDGEPSGTAGKPILAQLSGSRVGEITAVVTRYSGGIKLGTGGLVKAYG

GGVQQALKLLQTIEKKITTKLRLTLDYGFMPIAQSIMPQFGAVEVAAEYSDQVILVVEIE

LREVSAFTQAIINKSGAKAIVTPLDGQ

>tr|Q87H83|Q87H83_VIBPA Ribose operon repressor OS=Vibrio parahaemolyticus serotype O3:K6 (strain RIMD 2210633) OX=223926 GN=VPA1082 PE=4 SV=1

MATMKDIAKLAGVSTSTVSHVINKTRFVSEEISERVNNAAKELNYYAPSALARSLKVNRT

KTIGMLVTTSTNPFFGEVVKGVERSCYHKGYSLILCNTEGDNERMRQSINTLLQKRVDGL

ILMCSSLEGERIDVFERYPDIPVVVMDWGPMLFTSDKIQDNSLRGGYLAAKYLIDCGHTE

IGCITGPLIKHQAQMRYEGYKRAMNEAGLEFNANWIIESDFECEGGYQAFKKMAERGTLP

SSIFVSNDMMAMGVINAANELGIKVPDDLSIIGYDDIHIAKFMSPSLTTIHQPKYRLGQA

AVETLVRRLDDKSNEAQVVQLEPTLVVRNSVTNFS

>tr|Q87RY8|Q87RY8_VIBPA Putative outer membrane protein A OS=Vibrio parahaemolyticus serotype O3:K6 (strain RIMD 2210633) OX=223926 GN=VP0636 PE=4 SV=1

MKSNVVTLVGLFSLTTFSTLSYAADSKDHGVYVGANYGYLKVDGQDDFDDDSDAMQALVG

YRFNRYLALEGGYIDFGSYGNNLANAETDGYTAALKVTAPITDRVDVYAKGGQMWYSTDY

NVAGFHGNKDDEGVFAGAGVGFKVTDNFLVNAEYTWYDVELNAENVFDGANTNTDFKQAS

LGVEYRF

>tr|Q87GE7|Q87GE7_VIBPA Uncharacterized protein OS=Vibrio parahaemolyticus serotype O3:K6 (strain RIMD 2210633) OX=223926 GN=VPA1369 PE=4 SV=1

MGLSTTVNITKPNLEIKAITHTSKRFNFEFLKLGKSYNVHHLEQRQREREYQMIEQACIY

DRRDISRQLPHINIRTTPAASSPTPNEIAILVLSQLGSRSAYCEEGVLSIKGSTLQAAIA

VNKDKQEIILVLNRNKKAAGLKSWLNKATKSQSDYLYASNISQELVKVRNTYFSNYAITI

TGEHKESKLINYVLTSAKKVNG

>tr|Q87KR3|Q87KR3_VIBPA Uncharacterized protein OS=Vibrio parahaemolyticus serotype O3:K6 (strain RIMD 2210633) OX=223926 GN=VP2913 PE=4 SV=1

MLKNPLQVRLEKLEPWQQITFMACLCERMYPNYAMFCENTEFAEPRAYRAILDSVWEILT

VKNAKVNFERQLEKLEELFPSADEYDFYGVYPAMDACQALSTLLHGLLDRDYLFDSMLKV

SQQSVQTVADLEQAQGSEPITNDNQKENEAVCEEWDVQWAIFRPLREATERDINLIKDLR

EELREEAVSNIGIAL

>tr|Q87PH5|Q87PH5_VIBPA Spermidine/putrescine ABC transporter, permease protein OS=Vibrio parahaemolyticus serotype O3:K6 (strain RIMD 2210633) OX=223926 GN=VP1527 PE=3 SV=1

MGRTVRFSFMALVYAFLYLPIIVLIVNSFNANKFGMKWGGFTTKWYETLVNNDSLMQAAW

HSLNVAVFSATAATIIGSLTAVALFRYSFKGKGAVNGMLFVVMMSPDIVMAISLLALFLV

LGAQLGFFTLLIAHITFCLPFVVVTVYSRLNGFDVKMLEAAKDLGASEWVILKQIILPLA

KPAVAAGWLLSFTLSLDDVIISSFVTGPTYEILPLKIYSMVKVGISPEVNALATVMLIVS

LVLVVISQLLAREKVK

>tr|Q87HN2|Q87HN2_VIBPA Molybdenum ABC transporter, periplasmic molybdenum-binding protein OS=Vibrio parahaemolyticus serotype O3:K6 (strain RIMD 2210633) OX=223926 GN=VPA0931 PE=4 SV=1

MKAWKTHACLAAILSISFSTNAATDLKVYAASSMTNAIDEIAQDFKEKYDVTVTPVYGGS

SSIARQIINGAPADVFISANTKWMDYLVDEGVIDSDNVTNLVRNSLVLIAPQSSSLAVFN

FADAKAWEAALNGSRLALGNPTSVPAGMYAKESLTTLGVWKEIQTKVAPAKNVRLALALV

ERGEAPLGVVYKTDAQLTSKVKIVGEFASDTHAAIVYPAAVVSDSTESRQFFQYLKSEDA

KRVFAHYGFQQ

>tr|Q87QS3|Q87QS3_VIBPA Uncharacterized protein OS=Vibrio parahaemolyticus serotype O3:K6 (strain RIMD 2210633) OX=223926 GN=VP1076 PE=4 SV=1

MSGYCSTVLLVSFKVHKELSFMSRNQFSITKQIIEANLKKSHGLTVAEQSVLVTLSSYFG

KRGNSNTYSCFPSQDRIAEQVGCTRPTVNNALQKLERLRFIRSSYRGTNTSKLYTWLGIP

TLEEEETEELSPTNERGDTVSSESQTGNDSDTENSDALASSTVNAVQLQREQHQATSDWW

NDFEEELEVSESPF

>tr|Q87T99|Q87T99_VIBPA Putative peptide ABC transporter, permease protein OS=Vibrio parahaemolyticus serotype O3:K6 (strain RIMD 2210633) OX=223926 GN=VP0171 PE=3 SV=1

MIKVSPLTQKKIRHFKEIKRGYWSFVILSIMLILSLFAELLINSKALIVKYDGSYYFPVV

SDVRLGSEFGQDSSSEADYRVLQQVFEEEGGDNFVILPIVPWNPYEQDFSGDFPPTAPSA

ENKHYLGTDVIGRDILARLVYGFRTAMGFALLTMAVSYAIGTAVGCAMGFWGGKFDLFVQ

RLIEVWSMVPFLYVIMILVSIVQPTFTLFVAINVLFGWMGMTWYMRTMTYKESAREYVMA

ARALGASTGRILFNHILPNTMVMIVTLAPFTIAANITALTALDYLGLGLMPPTPSWGELL

QQGKSNLDSPWIVASVVTSIVLVLVMVTFIGEAIRAAFDPKKFTRYV

>tr|Q87Q65|Q87Q65_VIBPA Putative transcriptional regulator OS=Vibrio parahaemolyticus serotype O3:K6 (strain RIMD 2210633) OX=223926 GN=VP1285 PE=4 SV=1

MVAFLFYIIVLQLSGVCGYTDQRIMFNFRCKKDGKRDGCRVSSGLTKKQQAIADREVELI

QLAKSLVQEQGFANLTMDKLTSASSYSKGTIYNHFCSKEDVILALCIHSLKSEAIFFERT

ANFDGNTREKIIAMHVGYRIYARMEPVLSTCAIVAKTPWVLEKASPERLNELNRLEEEVI

SGADALVNNAVECGDLKFSPAIGADAIVFANWSIAFGSNALAQNASNSRCIQRIQDPFSV

LHNANMLLDGLGWAPLSTEWDYRKTWRRVEQELFSEEIAYLETVNR

>tr|Q87QD5|Q87QD5_VIBPA Helicase-related protein OS=Vibrio parahaemolyticus serotype O3:K6 (strain RIMD 2210633) OX=223926 GN=VP1214 PE=4 SV=1

MYTLRPYQADSVKAVIHYFRKHSTPAVIVLPTGAGKSLVIAELARLAKGRVLVLAHVKEL

VEQNHAKYEGYGLKGAIFSAGLGRKETDQQVVFASVQSVVRNLDSFKNQFSLLVIDECHR

VPDDKNSSYQKVITHLRELNPGIKVLGLTATPYRLGMGWIYQYHTRGQVRTEESRFFRDC

IFELPIRYLLDENFLTPARMMDAPVLSYDFSQLKPANTGRYKEAEMDMVIDKAKRATPQI

VEQIIQYARERKGVMIFAATVRHAQEIHGLLPEGETAIVIGDTPTPERDAIIQAFKNREI

KYLVNVSVLTTGFDAPHVDLIAILRPTESVSLYQQIVGRGLRLSEGKNECLVLDYAGNSY

DLYQPEVGDPKPDSTSEIITIPCPACGFNNNFWGKLDSNGFLLEHFGRRCQGYFEDEDTG

EREHCGYRFRAKYCGECGADNDIAARICHECDATLVDPDKKLKEALNLKDALIFECTEMD

LSVFKSNDGKSQLKVTYSGEPYQGEGHALVHEFWSLNTKKQKQTFKDQFVRPHLADKHRP

FEEASPTRVVANQHRFRLPQFVIARKSGRFWKLRDKIFEDELK

>tr|Q87NU8|Q87NU8_VIBPA Putative exopolyphosphatase Ppx1 OS=Vibrio parahaemolyticus serotype O3:K6 (strain RIMD 2210633) OX=223926 GN=VP1770 PE=4 SV=1

MDLTTLSNNNTDNLVWVGHLSPDTDSAVSVILASHIYGGEAALTGEANPESKFVFEFCGM

DAPKVKADFSSHHIGLVDFNQSTQLAKSVDPTSIVAIIDHHAMGSSPISMPQIVTMDIRA

WGSAATILTANAEKLNVKLPKNIACAGLGAILSDTVVFQSSTTTEYDKQYAQKLADIAGI

KDIKGFGEQMLLAKSDLSHFSAETILTMDYKNFEFAGKKVGIGVAETLNAQQLIDRKQDF

NEAIQAYKKAQNLDYLFFSITDTKHKRANMLWADDADKKVLSKAFDVKIDNDMLVLDGVT

SRKRQIGPAIQQAIESL

>tr|Q87LH6|Q87LH6_VIBPA Permease IIC component OS=Vibrio parahaemolyticus serotype O3:K6 (strain RIMD 2210633) OX=223926 GN=VP2636 PE=4 SV=1

MDSMKLYDAIIGTVEKHIAPIAAKVGNQPHVRAMRDGFIVAMPFIIVGSFILIFAFPPFA

EDTTFTFGRLWLDFATTHFDTIMMPFNMSMGIMTIFVSLGVAYSLAKAYKMDGITSAVLS

LMCFLLVAAPAQDGSLAMKHMGGTGIFTAVMCAFFAVELYRFMKKHNITIRMPEQVPPAI

ARSFEVLLPVLAVFVTLYPLSIFVQTQYDMLIPDAVMAMFKPLISASNTLPAIIGALLMC

QLLWFAGIHGAAIVVGLLSPIFLTNISGNIDAFVAGQPVPNIFTQPFWDFYIFIGGSGAT

LALVLLMSFSRSVHLKSIGRMSAVPGFFQINEPVIFGSPIVMNPTLFIPFVFAPVINATI

AYFAVHLGFVGMGVATTPWTTPAIIGASWGSGWTFSPVLLVVGLLILDLLIYFPFFKMFE

KQMLEQELPTTKETKQAEQPAGEGVTA

>tr|Q87HF6|Q87HF6_VIBPA Uncharacterized protein OS=Vibrio parahaemolyticus serotype O3:K6 (strain RIMD 2210633) OX=223926 GN=VPA1009 PE=4 SV=1

MIQPSFASNAEKALLSERINKLAKALTDGVYEREHTIKLCLLAALAGESVFLLGPPGIAK

SLIAKRLIQAFDNSSYFEYLMTRFSTPEEVFGPLSIQELKDNGRYVRLTKGYLPTAQVVF

LDEIWKAGPAILNTLLTVVNEKTFKNGSDIERVPMRLLVSASNELPDEDSGLDALYDRML

VRVFVNRIQNKQNFKSMLTVGTSQEAQIPEGLAITDEEYHQWQQQLDKLTLTDNAFEKLF

QLKTMLETKVEAQFGDAAHTDMYVSDRRWKKAVKLLKASAFFNGRDEINPLDLLLLQDCL

WNSPESRDVVREVVQEFALKHAFDQQDAEQEIEYCRESLIDVQHELEDTYRMTLTSEAAT

GILRKETMRFDTSKAKTYKVGAAVNLVKLVILQSNMSVSESEKGDSRWVYVPKDELDRVI

KDGQGDVYGYVNQNTNMCRLRFDVDAANNLVIRDIANRGILVSLVTQQGLDIELYQKWLS

QSEKAVNQLSEAEHHMRKVKANFHDALPHSFVDPELPRLMESSLQQVSQRLESIQGESEK

IIQRFKNGHQFFS

>tr|Q87TL6|Q87TL6_VIBPA Uncharacterized protein OS=Vibrio parahaemolyticus serotype O3:K6 (strain RIMD 2210633) OX=223926 GN=VP0053 PE=4 SV=1

MNKVSLLAASVAIALTGCGGSDSGSSSASNGVVITGFDGYFKNAVVFEDTNNNGQWDTQE

SILGLTDEKGQLTLAAKPEKTLALQTLVPNGAKQKQLIALDAKKYAGTYTVDMDHPSQAM

AHEIVFRAPSSSNVISPITDLVAIEMAKDPSITEEDAKANVNKALGGSEEAPIDLYSDFV

EGATKNAELHKTAQILTESKAQNPTNYEKKATEFAQAANQEVDRLVASGENINDPSLRPV

ITDSTPNSDNLAPETLVNNKLTVNETVEDAAEDKLDKLPEIVKGAAFDGVELNIEGLFKD

KDQSLVSTKLTHNLAGTGIEVEQVGNLIVLHPTAIVEKSGDFEIVLTAQDKNSNGDVLST

VSTVFEIEIESANLPPMVVEAEQARLQSIVDGWYLQQGELFEQTLDISGLFQDKDGQITD

YSADYVGIEGLSAIEDGNAIVTIKGTPTKAGESGAALTISATDGHTAVQIALSMPEVKEG

VTPPPVVPTLGFTQAHFNGQEWKMGSFAQKDGEIGYAMLMQDSEGLLFCWGSNHDEMNQE

YKANISDVLENTWEHDAYAILADLDQQTGYAEYNNKDCEDVELKDGKLYNSYGEEYEMLY

QHKTGNNGTQIIVKISDDELFWMDSSNTPFAESLSVAEHIKHGTVEHDLMVESKGKDDDK

ILKYSSGTYKYDADTYNYSSFMPTGFQTPGNWEIKDDEGRELLILEETGSQSDYKTRHRY

INRDFGDFYIGIHWSKEQNGHESAPEYGLYSHNKEVMTSIAASLPILEPGDDD

>tr|Q87G62|Q87G62_VIBPA Uncharacterized protein OS=Vibrio parahaemolyticus serotype O3:K6 (strain RIMD 2210633) OX=223926 GN=VPA1455 PE=4 SV=1

MAEFTFLNPYWLLGLITIPFVLLINQKFRAKKSALIAPHLAKMLGQTTQSKPYFTIWGLA

WTIACVALAGPSWQSNTRPSFELSQNRMLVLDMSRSMYASDIKPNRLAQTRYKALDLLPK

WKEGATGLIVYAGDAYSLSPLTTDASTLAGIIENLSPELMPFQGSNLPAAIELSLSQFSQ

AGANQGDIVVLADDLDDSELARSLDLVKGKNIRVSVLAIGTANGAPIALPDGSLLKTTQG

STVVAKTHLKNLQTLANKTGGMFVPIQHNNRDVDSIAAYTNNTGNNLAAKQNQEVKTDSR

LNGGFWLLPLLLLPTVFLFRRGVIWVFVAALLPMTWTPDAQANPFLNADQKAANLYKQGE

YEQAKNLFTDPSWQGAASYQTGDYEAAIKAFSNDPSQTGRYNLANALAQNGQLEEADKQY

KKLLEENPDFEAAKKNLSVVEEKLKQQQQQQQQQQQQQQQQQQQQQQQQQQQQQQQQQQQ

QQQQQQQQQQQQQQQQQQQQQQQQQQQQQQQKSQNRTKQEQGQQSQNQQDAENKKEKQES

EHKAKQQKAETEVEKQGEQKKGEPMQAQAQPEKSHQDDPEFRRLEAVESARDPSFLIRAQ

MQLQAQQKQRPQQSQKEW

>tr|Q87RD2|Q87RD2_VIBPA ATP-dependent helicase, DinG family OS=Vibrio parahaemolyticus serotype O3:K6 (strain RIMD 2210633) OX=223926 GN=VP0865 PE=4 SV=1

MIAKTFSSDGALGKAIPGFQARQPQIDMAEAVSSAIKEQSQLVVEAGTGTGKTFAYLVPA

LLSGKKVIISTGSKNLQEQLYHRDLPLMVNALGFYGQVALLKGRSNYLCLDRLSRQMVES

HTNESDPTLLTQLVKVRSWSSETKTGDLGDCEDLPEDSMIIPTITSTNDNCLGKECPSYT

DCFVLKARKRAMDSDIVVVNHHLFLADLAIKETGFGELIPEADVFIFDEAHQLPDIASEY

FGQSVSSRQIHDLAKDIEIAYRTEAKDMRQLQKVGDKLLQSAMDMRIVLGEPGFRGNWRE

AMQSESIKRELVRLTDSLDLAIDVLKLALGRSQLLDTAFERANLIKGRIERVCDVDITGY

SYWYDTSPRHFTLHITPLSVADKFHEQIEIKQGAWIFTSATLAVSGDFKHFTDRLGLKPK

QQFSLPSPFDYEKQARLCVPRYLPEPNSPGLADKLVRMLAPVIEENDGRCFFLCTSHSMM

RELGEKFREVLDLPVLMQGEMSKQKTLAEFMELGNALLVATGAFWEGIDVRGDALSCVII

DKLPFTAPDDPLLKARIEDCRLRGGEPFAEVQIPDAVITLKQGVGRLIRDQKDHGALIIC

DNRLVTRDYGGTFLGSLPPIPRTRDLERIKAFLKAE

>tr|Q87QX3|Q87QX3_VIBPA Uncharacterized protein OS=Vibrio parahaemolyticus serotype O3:K6 (strain RIMD 2210633) OX=223926 GN=VP1026 PE=4 SV=1

MKIFIAKNPAEAHIVCELLKTEDICCEVRGEGLFGLKGELPFGDDTDPYVWLLDPEQQLK

AHSIIEAFRQQSQSNIYEDWQCPHCLEHNEGQFGACWQCGYQIGEP

>tr|Q87HF7|Q87HF7_VIBPA Uncharacterized protein OS=Vibrio parahaemolyticus serotype O3:K6 (strain RIMD 2210633) OX=223926 GN=VPA1008 PE=4 SV=1

MSGADGLNLALMVADSGIIDSAVNDLMARSQVMMMAENRGVKSSVKNHLLKWRGNVKKRI

TKVCETERFQQELALYQEVIHWDEDEFFERIEDVIKKLEWHSAFYMQARRMMEKNKGVNN

PMFPHYFCDQWYQSLADAIKQAQVSELEANKEKVLNDLYQRMETMRNMDKVTESGDAESV

GRLWDMASAKLSKTDLTVMKRHAEFLKKHKGLQEIAEQLGRMAGQVNDPDLNRAPAEELQ

MVEEKSDEATDDIVGIHESDDLNKLLPNETMFLAYPELEVVFYKHLVDKRLMNYRTQGKS

RTLRKVKAHRPDNKAVDIEKGPFIACVDASGSMSGFPEQCAKAMAYALMQIALAEQRDCY

VIIFSTEHITYELTKQDGLREAADFLTYSFHGGTDLEPTLIKSIDLMSGDKYKNADMVVI

SDFIAPKQQDELLAKVDALKAKKNRFHAISLSKYGNPALMSMFDHTWSYHPNVVGRLLKR

VSNG

>tr|Q87TH6|Q87TH6_VIBPA Uncharacterized protein OS=Vibrio parahaemolyticus serotype O3:K6 (strain RIMD 2210633) OX=223926 GN=VP0093 PE=4 SV=1

MNERRALGFGLSAVLLWSTVATAFKLTLAEFTPIQMLCIASMVSAIALTIVCGVQGKLNQ

LNATFLSNPWYYLLLGLINPLAYYLILFKAYDLLPASQAQAINYSWAITLTLMAAVFLGQ

KIRGKDWIACGFSYLGVIVIATQGNVLGMQFESPLGVGLALLSTLLWAGYWILNTKNKAD

PVVGVLLGFLVAIPFTIALSWYEGASWQSISIKGWAAVTYVGLFEMGVTFVLWLSALKLT

QNTARISNLIFASPFISLMLLATIIGEKIHPSTLIGLVLIISGLVIQQVTWGKKAKAANA

SQ

>tr|Q87RU5|Q87RU5_VIBPA 3,4-dihydroxy-2-butanone 4-phosphate synthase OS=Vibrio parahaemolyticus serotype O3:K6 (strain RIMD 2210633) OX=223926 GN=ribB PE=3 SV=1

MPISTPQEIIDDIRAGKMVILMDDEDRENEGDLIMAAEHITPEAINFMATYGRGLICLTM

TKARCENLGLPPMVQDNNAQYTTNFTVSIEAAEGVTTGISAADRARTVQAAVAPNAKAAD

LVQPGHIFPLAAQDGGVLTRAGHTEAGCDLARLAGLEPASVIVEILNDDGTMARRPDLEI

FAEKHGLKLGTIADLIEYRNNTETTIERVAECKLPTEHGEFTLVTYKDTIDSQVHYAMCK

GDLAGEAPLVRVHLQDVFTDVLRSDRNAERSWTLDKAMKRIGEEGGVLVVLGNEESTELL

IHRVKMFEAQDKGEAPTLAKKQGTSRRVGVGSQILADLGVHDMRLLSSTNKKYHALGGFG

LNVVEYVCE

>tr|Q87HE6|Q87HE6_VIBPA Uncharacterized protein OS=Vibrio parahaemolyticus serotype O3:K6 (strain RIMD 2210633) OX=223926 GN=VPA1019 PE=4 SV=1

MDIIFVRHGVPDFSLADERCMTQLEKDYAPLDRAYLSELHQKLTNAVFDDAQAIICSPYT

RALQTAEILNRRHGFELFVEHDLREWRADTAGGYISLAERDRRWHEYRELLKLRLPMSDE

RYEHVTALKARTMAVLERYRQYDKVIVVSHFNVFEALQGYQEQGLACGDSKIFRL

>tr|Q87IQ8|Q87IQ8_VIBPA Uncharacterized protein OS=Vibrio parahaemolyticus serotype O3:K6 (strain RIMD 2210633) OX=223926 GN=VPA0548 PE=3 SV=1

MKHWFFIVCGILSGCSSLNPDMVPGGNMLDTAPKTNTELRHPEWGYASASHVSTAPRVNQ

TARAAEKKSNNVTSLEMFLDRHNIPHETIGGGHLMIRLKEQVHFQTGSAQLSSNSQDWLR

KLGHYLAGRTDVDVVIDGHADSTGAASFNDTLSEKRAREVEKQMLASSIPRQRVFSRGFG

EYVPQCSNATSSGKACNRRVELMLIVDE

>tr|Q87RX0|Q87RX0_VIBPA Putative fimbrial assembly protein PilE OS=Vibrio parahaemolyticus serotype O3:K6 (strain RIMD 2210633) OX=223926 GN=VP0656 PE=4 SV=1

MIRISRCNSYKKRLRGMTLIELLIAVTIVGIIAAIAYPSYTNHVIKSHRTVALSDLSRIQ

LELETSYDGGYDWSHIISGGACTLCDSDTDRFSFDIASSASTAYTITATAKSDLGQDDDE

CLNGTTTITLTSTNVESPSACWN

>tr|Q87P20|Q87P20_VIBPA Uncharacterized protein OS=Vibrio parahaemolyticus serotype O3:K6 (strain RIMD 2210633) OX=223926 GN=VP1698 PE=4 SV=1

MRRRTQMKKQHWRRRSLFPDSIVTQRKVTVLQRGARYESASQPLQDLNVVHVNHRQLLSE

GVLNDDQLSLLQRLLDRSVVDSLCASQLVKTYLRLGTSIDRFAMRLFLEIGAQLSDSQRV

ATFEQRLEYINSRLGFRFNLATPKTLILCCYLALTEWIHRQTDQSALHASVKVEQLMNQL

DIQKEYWSKLSGEDTSAIFVEQQLALIESQQTQLKAQLNTLNEQQSQVIESHKALVDKWQ

PSLSNLKELADYTSTTDMFISDWKTWCSEARLQAPDLNEVWDACDVVYNDLNAVAKVWQW

FKDMQIVGDVDHYYFDIQSGQCGQACNHLSQI

>tr|Q87IT1|Q87IT1_VIBPA Putative transporter, DME family OS=Vibrio parahaemolyticus serotype O3:K6 (strain RIMD 2210633) OX=223926 GN=VPA0525 PE=4 SV=1

MNHHPVQGASWMLTAGLAFAIVNSFAQIASIHFGVSSTTFALFQYAIALFVILPYLRTLG

IRRSLRTQHFGWHAFRIFLSVIGIQLWLWALAYPVPIWQGIALLMTSPLFATVGSGLILK

EKVGTARWLATLTGFIGAMIILEPWSDSFTWATLLPVGAAFFWAAYSLMVKKMSVNDPPS

TMVVYLLLLITPFNIVLAIPSFTMPSDWTIWLILLFAGALTALAQWAIVKAYAVADASFV

QPFDHAKLPLNVLAGWMVFGWVPPGRLWLGAAIIVASIAFITHWEAKKTKLVERKI

>tr|Q87NF6|Q87NF6_VIBPA Uncharacterized protein OS=Vibrio parahaemolyticus serotype O3:K6 (strain RIMD 2210633) OX=223926 GN=VP1912 PE=4 SV=1

MRLAHKRKVQMKLQKRIKATVANLAAAPKTAKPVAEKAAVKPVVEKAVAEKKVAAAKTVD

VALTPKQQQVLDIVVANAEGINPKGIGLAAGQEDAKAASWATGALKKLLEENLVQKEQLA

GNKVIYKAV

>tr|Q87M12|Q87M12_VIBPA C-di-GMP phosphodiesterase A-related protein OS=Vibrio parahaemolyticus serotype O3:K6 (strain RIMD 2210633) OX=223926 GN=VP2446 PE=4 SV=1

MPSQQLQHWFATLTSNSPFFFAILDKKHNYRMVSDRYCDIAGLNHEEIIGLNDCQVLGEQ

FYKKLAPYYQRAFKGVHVEAEITLDETDLETSLHFSLSPVYEGNEVRFVVFHAVDTSEKQ

ILVRSLEEAENKFAKLTQLLPDGLLLIEDDTIISANPASARLLGLNSPHELLGEELSRLF

IDENTKKVFSHRLSTLISDKPFVCLTSARCGFERKVQLHADSTAILGSESQIILIQDADD

TPKHLSSASSEDSHIDSLTKLYNRFGFTKRLEQLIKSQTPLLVFYLDIDNFKNINDSLGH

HIGDKVIQEVSARLKRLLPNQAIIGHLGGDEFGIILPEPENSRMAEVLSDRIISLINQPF

DLHHFSKRLACSIGSVRYPEDGQDARILLQNADTAMYEAKDRGRNRLIKFNDQMNKEARM

RLWLEIELQKALQQNGLEVWYQPKVNARDFSINGAEALVRWKHPVEGYISPGSFIPVAER

AGLIEHLGRVVMRDVFNTVKRWKQQGILPGRVAINLSPEQFGNPQLIDFMEKLLRTTELD

PSCITFELTESAVMSDSEHTLQMLNAIKKLGFALSIDDFGTGYSSLSYLARFPIDELKID

RAFINDIDALPKQVTVIENIINLGKSLELTVVAEGVETHQQATLLSNLQCNSIQGFHFYR

PQPKHEIEELFVQNRRHKN

>tr|Q87ML6|Q87ML6_VIBPA Putative cytochrome c-type biogenesis protein OS=Vibrio parahaemolyticus serotype O3:K6 (strain RIMD 2210633) OX=223926 GN=VP2215 PE=4 SV=1

MTLFWISTVILTLIACALVALPLLKQKANNDDILRDELNKAFYKDRLSELQEETEEGLVE

SQEDLISDLKQSLLDDIPAQKMSQETKVSPMAVLIPSVILTVALSYGLYYKFGASQDVVK

WQEVTSNLPELSKKLMSSSSEPLSDDEMADLTLALRTRLHYQPEDSTGWLLLGRIALANR

DVTTAIDSMKKSYALEPKDPDVMLGYAQALMLSQDEMDQATARSLLGKLMQQDYVDLRVF

SLLAFDAFERKDYPAAIKYWGIMQQMIGPDDSRYESLSRSIDHARKQMGEVVSPDKSVAV

TINVAPEAQLDPNAVLIVSIHRADGSPMPVAAARYPLGSFPRTVVLDDGNAMMQGQKLSS

LEKLIVRVRADSDGNVATRDQDWHGESDVVEFGQPVAVTIDKQYQ

>tr|Q87GS3|Q87GS3_VIBPA Cytosine permease/transport OS=Vibrio parahaemolyticus serotype O3:K6 (strain RIMD 2210633) OX=223926 GN=VPA1242 PE=4 SV=1

MAGDNNYSLGPVPNTARKGVASLTMVMLGLTFFSASMWTGGSLGTGLSFNDFFLAVLIGN

LILGIYTSFLGYIGASTGLSTHLLARFSFGSKGSWLPSALLGGTQVGWFGVGVAMFAIPV

HKATGIDTNTLILVSGLLMTATVYFGISALMVLSAIAVPAIALLGGYSVVEAVNSVGGIR

ELQQVQPTEPLDFSMALAMVVGSFVSAGTLTADFVRFGKKPRSAVMITMVAFFIGNSLMF

IFGAAGASVTGQSDISEVMIAQGLLIPAIIVLGLNIWTTNDNALYASGLGFSNITGLPSK

YISMANGLVGTLCALWLYNNFVGWLTFLSLAIPPIGGVIIADFFTNRKRYANFEAAQFQS

VNWAGIIAVAIGVGAGHFLPGVVPINAVLGGAISFLILNPILNKKVLATQPA

>tr|Q87K09|Q87K09_VIBPA Putative siderophore utilization protein OS=Vibrio parahaemolyticus serotype O3:K6 (strain RIMD 2210633) OX=223926 GN=VPA0089 PE=4 SV=1

MKKKPQPKTLVVTDTETITPNMQRITLQGEALSHFPRDCEGSYIKLLFNDMGGADLSILS

EDDRPIMRTYTIRRFHPETSTIEVDFVRHVTQDLQCGFAARWAMAAQKGDTINIVGPGSI

SNLNTEADWFFMAADMTALPALSAKIRTLPEEAKGYAVISVLSPADIQPLHAPAGMELIW

LTEGQALADSVRELEWLDGNASIWCACEFDSMRALRQYFRNEKEVDRENIYISSYWKQGV

SEDGHKMIKREDAESNQ

>tr|Q87I00|Q87I00_VIBPA Transcriptional regulator, TetR family OS=Vibrio parahaemolyticus serotype O3:K6 (strain RIMD 2210633) OX=223926 GN=VPA0806 PE=4 SV=1

MNVRKQGRRSAEAAEQTKCLILKVAADMFCELGYERVSLRNISEKAGVSHSLIRHHFGSK

EKIWQAVSDAMDEFLQNYMAGLINEMPENTPSNRKIYLFMVRMLAFALVNPHPVQMIADS

IRQDDNALFDYFLKSKDEFAEIFTSIFAQYNSEFPEAQFDQWESKWQMLIFAHGAVSLAP

MMQETWPDIADNREQLLIKHWELFNTIMATQFRIAKEDMIHPSKLEDIVIDMCCTIDEAV

N

>tr|Q87GV4|Q87GV4_VIBPA Uncharacterized protein OS=Vibrio parahaemolyticus serotype O3:K6 (strain RIMD 2210633) OX=223926 GN=VPA1211 PE=4 SV=1

MKKVLIAVLILLLLGGAGAGYYFFFMQKDEPEKPVAAKTEKAPVDKGDASKPIMDLEAPA

PEITEYYVIERRVNVYNKPDDKSLIVDALYKGEKVSVLEKVEGWFRLSDYIVYEDGGEET

AEWVNSKGLSDAEPVIKEQERLEILDGYLQKSDDLKEHLDMFRNKTQQLLKDETCDPSDF

EELGGWVRSVTFKKRNVYFIYCGGLEQENKIYLDVDKGELFYR

>tr|Q87HI4|Q87HI4_VIBPA Ferric aerobactin receptor OS=Vibrio parahaemolyticus serotype O3:K6 (strain RIMD 2210633) OX=223926 GN=VPA0979 PE=3 SV=1

MTNRKFSLNLIVLAMSSGYAAHALAAEQAQQLASQNAADEQLVVVGSVMPKSISDIPGTV

WFVGQEEIAQQYRAGKTLGDILSATIPSLDVGTGGRTNYGQNLRGRAMLVMIDGVSLQSS

RPISRQLDAIDPFNIERIEVLSGATSIYGAGATGGVINIITKKAYSDELAFESFVGGTSG

FNSSDDFDYKVAQSVAGGNDIVKARGSVVYSETQGAFDGNGDIVTPDISQGSLQYNSTLD

VMGSAEIQISDASKLNLVAQYYDSQQDSPYGLYIVNSKFVDVRKGFDSDREHGTERVLLS

ANYAHDNVFGHQLIGELSYRSEDQTFTPYYQSSSQQETEVFAGRLALAKSWGAFSAVYGV

DAYLDRFDSNQALFDKTIADNSGNLINRTYAEVGRYPGVDVTSYAMFVQGDYHINQDWSV

QAGYRYQYMDNKIDDFVAYSVQKNIASGKGVSADAVPGGSTDYSVSLFNVGTIYQLNDES

QLWANFSQGFELADPAKYYGQGSYEAVDANGHYALKGSINVADSKMSGIKTDSYEIGYRL

DTDTVALQAAGYYSVSDNSIKYDKKTLLITNVDDEQRVYGAEANINYWVSSDILLGASGH

YVISELKTNGKWEDLSAGKASTSKANAWAAYYQDDYSVRLQSQTMFDYEDDANNKLDGYT

TFDLLGNVNLPVGQLGFGIQNLLDEDYTTAWGQRAQILYAAHYDAAAYDYKGRGRTFTLN

YSLEY

>tr|Q87IR6|Q87IR6_VIBPA Uncharacterized protein OS=Vibrio parahaemolyticus serotype O3:K6 (strain RIMD 2210633) OX=223926 GN=VPA0541 PE=4 SV=1

MTNRENTTTVKQTAIPNRFDSHNLCDNQDEETGGTMSTVFLFKLTLVLLLLFIIFNLAKA

LIEMVREDPDNPDKPTKPMSHYLGRRVMFSALVVILLLIALSSGWLAPNPRPY

>tr|Q87QQ5|Q87QQ5_VIBPA Uncharacterized protein OS=Vibrio parahaemolyticus serotype O3:K6 (strain RIMD 2210633) OX=223926 GN=VP1094 PE=4 SV=1

MTFFFFAADTYMDSVLHQLQFSLSITGPICLMLVLGVIFKRFGLINDNFIEIGSKLVFKV

TLPAMLFVSIVASEHDFSAASGFINYGVVASILFFIFTYMSVSVLFKSSPDQGVLIQGGF

RANTGIIGIAYVANAYGSQGVALAALYVAITTFIYNVQAVICLSPKGATSASQAAKMMGR

TLTKNPLIISIVLGMLFYLLSIPVPNVVIDAGNYLSKMTLPLALLCTGGSLDFSLMRKEK

GPSWFASSYKLIVAPVFITLGAYFVGFRGIELGILFFMNASPVAAASYVMARSMGGNAVL

AANIIALTTVLSTVTCTFGIVVLKSYGLI

>tr|Q87I88|Q87I88_VIBPA Putative membrane protein OS=Vibrio parahaemolyticus serotype O3:K6 (strain RIMD 2210633) OX=223926 GN=VPA0718 PE=4 SV=1

MNYINFALWLTCLVNVLLIFYGDIRYRIVKHRFLLIIFITSLLSLFFTPEPLTQLAVSTS

VFICFFILWLINIVGGGDVKLIGVLFLGVNDEYMLAAIVAIGLLGGIQILVMWLMSVYRK

KTPFENGIPYTIPIGISGLFFFYMSLI

>tr|Q87TL1|Q87TL1_VIBPA Gluconate utilization system Gnt-I transcriptional repressor OS=Vibrio parahaemolyticus serotype O3:K6 (strain RIMD 2210633) OX=223926 GN=VP0058 PE=4 SV=1

MILLWILCLAFGGLSASCVFVIMLHVTLLINHIFMAQQNKKTRTTLQDVADQVGVTKMTV

SRYMRNPESVAEKTRVKIAAAIEEMGYIENRAPAMLSKSSSKAIGILLPSLSNQIFASFV

QGIETVTKANGYETLLAHFSYDELEEERKIASLLSYQVDGLILTESHHTPRTLQMIKNAG

VPVVETMELPPQPIDMAVGLDHEDASYNAVKRMLDAGKRTIVYFGARLDTRTKLRMQGYD

RAMNEAGLEPKHVLTGVHSSFSLAHDLLERALKTYPTLDGVFCTNDDIAIGTMLSAQQRG

IQVPQQLAVVGYNALDIGQTISPKLTSVDTPRFQIGVKSAELLIARLKGEAQEEKVFDMG

YQITSGESV

>tr|Q87I56|Q87I56_VIBPA Putative chloramphenicol-sensitive protein RarD OS=Vibrio parahaemolyticus serotype O3:K6 (strain RIMD 2210633) OX=223926 GN=VPA0750 PE=4 SV=1

MNHRHYGNFMAALSFVIWGLLPVYYRFLHNAAMDELLAWRIIGSVPVGILIVYAVTRHWP

NWSQVWRDKKSLWYTFVASSLMCISWSAFTWALTHHRVIDASLGFFIGPLVSVALGVFVL

GDKLSKGQLIAILLATCGVLYQVFQYGQLPFVALTMGLFFALYGLYKKKINYDWSTTLFI

EALVLAPFALGYLLFKQWTTGELASSVDTTTLLLYFGSAPITILPLIFYSIAIRITNLST

VGLMQYIEPSLQFILAVVFFGELFDDVKAVTFAFIWVGLLLTIFEGVAKGIKRKKLANHS

V

>tr|Q87GF1|Q87GF1_VIBPA Putative two-component response regulator OS=Vibrio parahaemolyticus serotype O3:K6 (strain RIMD 2210633) OX=223926 GN=VPA1365 PE=4 SV=1

MSEFEIEVDKLSEDFFELLEGKVIDEHINKIKKEKKVTIEILKELLDLGDAHYQKYQLKE

AEIVYLSYTGLCPFDHRGPASLASIYLEQAQFQKALDMLNIVKTYPTADFDETLVNMALC

HYKLKQYIEAAAVFIIIKSEKLNEFNQKRYDFLRKQLNPYLI

>tr|Q87J58|Q87J58_VIBPA Uncharacterized protein OS=Vibrio parahaemolyticus serotype O3:K6 (strain RIMD 2210633) OX=223926 GN=VPA0395 PE=4 SV=1

MVAKNKLIRFTATEVADAFEKEDELIKQVQSNQLSQVLLLWQVKSPTLVLPAGKKWPVSN

ELEHALNTSGWKLFSRKTGGAPVPQVPGIINLSHIYHWPEGEPYDIKKAYLDLCAILTVF

FKQLGVKVDVHATPYSYCDGEYNLNIGGQKVVGTAQRVLLKKGGGQVVLSQACILINADV

EKIVKPVRLCNQLCGHDDDIRGDVHTPLFHHISDRPSVDSLFQQLTSAFLTHAKTE

>tr|Q87RP9|Q87RP9_VIBPA Uncharacterized protein OS=Vibrio parahaemolyticus serotype O3:K6 (strain RIMD 2210633) OX=223926 GN=VP0728 PE=4 SV=1

MPKRKTGYEEMLEDVIETLKQSPEEVNKALETSGKVVEAANDLTKDELALIAAYVKADLK

EFSESYEDSKSGPFYLTVADSIWQGLLEITDRTKVEWVELFDDLEHQGLYEAGEVIGLGT

LVCDECGHKTTYNHPIIIIPCIKCGHKGFSRQSLKP

>tr|Q87KD0|Q87KD0_VIBPA Uncharacterized protein OS=Vibrio parahaemolyticus serotype O3:K6 (strain RIMD 2210633) OX=223926 GN=VP3047 PE=4 SV=1

MYCSSLRFGAVKQTQIKGMTMKTWITLATLLATSSAYAAEVVTLADGREVKLNDDFTWEY

VMTSSAPKEAVAQTTTSAVETVAAPAIATIPVVTKTVGTTVVVNAKKPTMQLSDSGVDIL

IGSASYEGGELVLPTSITNQSSQSVIQVEVEVQVFDMSGKQLAKEKVTVWQSIKRMADTY

LRPQQAEQGKSIKLAVPQSQQYQFSAKVLEVKTR

>tr|Q87GL4|Q87GL4_VIBPA Uncharacterized protein OS=Vibrio parahaemolyticus serotype O3:K6 (strain RIMD 2210633) OX=223926 GN=VPA1301 PE=4 SV=1

MKSIASYQIKFKVLFTLTCSCIFATACNSDNTSTEIQSKLLVEKDFANNSRLRANPEQGT

VILFLEPPSATVAADDFNGESGSDVIPYRYSRSLYHTFCYEDDNSNSKHSTVLNDSSGAE

VLRISANEECVSAVISEGEYHLVMTHGQHVDSTDITFLVTTPDNGSQTEINSINYSITSR

VLRSIGSLPINSAYADAADNNVTTLISTNACKDCDLSGADLSSATLTFADLSGADLSDAI

LTNVDLFESTLTGTNFSGADLSNGDFRSSEMAYTDLSNANLSGAYFSNAHLSPSNLNHAT

VIDTNFDYANLVGATWIDGGICDITSVGFCNSTDGGDATPCDSLQQGTSDDGNIVYKCLL

PTVDKEVCTTELGGPDGGTLITSCEAKDASELVTSVDLVDIFDQASSSFSVTLDNDTPMA

ILAWGGEGGIGSSGGLWTSGGDGGRGGFASTVTTLTDFLDNYGQTYFIFYIGENGTLSNE

YGDGGSSTLVMTVENSPTSLEDDVVLIAGGGGGGESSSFFVDGTDGSMGGIAASSIMGQG

TIGVGQSFTDGASGGSSNEWGDGGNGADSGKDGIGGQGGQGFLGRNSEWVNGDPVVGSDG

RGGNADDSFSAGGGGGGGGGYGGGGAGDGGAGAGGGSWSIIPTITCNSAPTQDTMPSSPG

SSGDDYGSKNGAVEVWIFPNGC

>tr|Q87J68|Q87J68_VIBPA N-ethylmaleimide reductase OS=Vibrio parahaemolyticus serotype O3:K6 (strain RIMD 2210633) OX=223926 GN=VPA0385 PE=4 SV=1

MKQRPNKLFEPAQLKALSLQNRIVMAPMTRARTTQPGNIPNEMMATYYQQRASAGLIISE

ATQISDDSQGYSFTPGVYTDAQVAGWQGVTQAVKLQGAAMFCQLWHVGRVSHPTFQNGEQ

PIAPSALAPVETQVWIADEQGNGNMVDCVEPRAMTQADINRVVGDFANAAKRAIESGFDG

VEIHGGNGYLIDQFLRTNSNHRTDNYGGSRENRIRFLIEVVDAVIAAVGAHRVGVRLAPF

ITFKDMDCPDIVPTILEASKQLQERDIAYLHLSEADWADAPTIPETFRIELRKRFRNAII

VAGRYDPQRANEVLEKGYADLVAFGRPFVANPDLVSRLQHHHPLAELDGSTLFGGNERGY

TDYPALQQECAEQA

>tr|Q87MZ0|Q87MZ0_VIBPA Putative glutathione S-transferase OS=Vibrio parahaemolyticus serotype O3:K6 (strain RIMD 2210633) OX=223926 GN=VP2086 PE=4 SV=1

MGKLVEGVWHDVWYDTKANGGKFVREDAGFRDWIKNDSEAVFQPESGRYHLYVSLACPWA

HRTLIFRKLKGLEPHIDVTVVCPDMLSQGWQMGLPEPLFGHTRMHQIYTQAKPDYTGRVT

VPVLWDKKTNTIVSNESSEIIRMFNSAFNDLTGNHDDYYPEPLRGVIDEWNDYIYPNVNN

GVYRCGFATSQEAYEEAFESLFSALDKIDAHLATHRYLAGNKITEADWRLFTTLVRFDAV

YVGHFKCNKQRIADYVNIQGYLKELYQIDGIADTTDFYHVKRHYYFSHTGINPTQVVPKG

PDLDFSSPHQREMIG

>tr|Q87PT6|Q87PT6_VIBPA Uncharacterized protein OS=Vibrio parahaemolyticus serotype O3:K6 (strain RIMD 2210633) OX=223926 GN=VP1415 PE=4 SV=1

MRSLLLFNQQNSNGLVMGVTVGANGLSIVHKGSGGEANATLPDVCLTKVGKPIVPIPYGN

NAKSADLAGGTTTISMDGGNSVAIKGSTFSKSTGDAGGDKKGVASGTIEAEAKFISASPT

VKFEGKGVCRLSDQMTMNKANTMCLGGAQNPSVSVTEADDGTFTIDIDYRYPDGDPVYKA

DFKIIDSTGTEHVGTLSDKGKGTITGLPAGSISLFLGEDKRDIEPIQKPQPNKDYLELPG

PLDLVEFAGRGLVEYWDAVEMPSDYSQWAWGAIMGDFNQDRSAGQIAFDSAVTAIPFIDQ

IGDGRDISANLYAFYNADDIREDKEKYTDLVITLVGFIPTAGSLIKGLFKELKILGKAAD

LDMIAAFLRAGAKGDVVKWMQSLDMSVIKNDIYGQLDDITKQVKTLMDTLEQESRKRGYT

VVADAYKRCVEQLDDFFKKADGPISKVLSDFDDRLKMILPEAPLVTSGTSFSISAGTAQG

GGKAEVIKTRNKTKKKTDTCPLCNKKIGKGKKECEGAKVGSISKAVMDDGSSQKLWKEFE

IRNGWRDKTEHPWWHGAGAVQAHHLIPKNAFKLKNAKSKQTKEKMMFLRRIAQTCAYNID

FWKNGIGLPNKKETACFLRKPRHAGGHDRNEFNYTNECVKLVHDKLSSDIKKRTKEGSCS

KTTNSSLIMKFNESSKNIFDNIADFIWFITRDGKNYDPKVEPYVGCSSNCKKIHPIININ

TKRQIENYDLEIGK

>tr|Q87PX2|Q87PX2_VIBPA Putative HD-GYP domain containing protein OS=Vibrio parahaemolyticus serotype O3:K6 (strain RIMD 2210633) OX=223926 GN=VP1378 PE=4 SV=1

MQYNPEDSIKIAIADVAVGMFVTAIEHNKRVNLANAGRVSSAEGIKKLRASGVKFVWVDQ

SLSAQQCVFKPVEEPIDIEVGDKTLSVSSKVQRAYRSREVQHKRAKKLIAEAKGLAQKLL

NQTFEGKVIQVDEIESWADDMIESVFIDSDALQCVSALRQKDSYLLEHSVNVACLLVSFG

KHLGLDKDTLKQLAIGGIIHDVGKIKVDDKILHKPARLTPEEFEHMKLHQVFAGEIIVEV

KGLSDVSRDVCLMHHEKLDGTGYPRGLSGDEIPIHGRMSCIVDIYDALTADRCYKKGMSS

AEAFKILLSLTPFHLDPDLVYKFINCIGVYPVGSIVELSDGRVGIVWSSNPSQALKPEVK

CFYSRKYKRYIDVAMVDLKTSTHKIERAIAPSSLEIDPKPFYD

>tr|Q87SM8|Q87SM8_VIBPA Haemagglutinin associated protein OS=Vibrio parahaemolyticus serotype O3:K6 (strain RIMD 2210633) OX=223926 GN=VP0394 PE=4 SV=1

MMNLFQDDAVTWLSTLDAASVDLLITDPPYESLEKHRKIGTTTRLKVSKASSNQWFDIFP

NDRFEALLSEVYRVLKNHSHFYLFCDQETMFVIKPIAEKIGFKFWKPIVWDKVSIGMGYH

YRARHEYILFFEKGKRKLNDLSIPDILTHKRVYRGYPTEKPVSLLEVLVAQSSRDGELVV

DPFFGSGSTLVAAKNLNRRFKGNDISQSAHEHIHQRMDFGA

>tr|Q87KX4|Q87KX4_VIBPA Uncharacterized protein OS=Vibrio parahaemolyticus serotype O3:K6 (strain RIMD 2210633) OX=223926 GN=VP2850 PE=4 SV=1

MEIKMKKLMTSLAVAATIASPVALANSTPVMFSTIDHTNAPSNSAVGGVRLAVLHGQVNE

VKGVDFSVLGMSETDRTTGVNFGLFFGAAKVNQEMKGASLGLVNWNQGQTTGVNFGAVNI

TNNVKGLNWSAVNYSEGYTMADVGLASISKKSNFQLGFFNMTDQIDGVQIGLLNCADNGF

FKCFPIINFAK

>tr|Q87TN0|Q87TN0_VIBPA Uncharacterized protein OS=Vibrio parahaemolyticus serotype O3:K6 (strain RIMD 2210633) OX=223926 GN=VP0039 PE=3 SV=1

MKKWTFFMLLIAVLLFGSVIGFNLFKQQKIAEYMANRPEPEFPVTVTEVKAVDWVPVIEA

IGFIEPNQGVTVANETSGVIDKIAFESGTQVEAGQPLVLLDSEVEKANLKSSQAKLPAAE

AKYKRYQGLFKKGSISKEAYDEAEANYYSLKADIESLKATIDRREIKAPFAGVVGIRNVY

LGQYIQAGSDIVRLEDSSVMRLRFTVPQTDISRIKLDQEVDIFVDAYPDQPFKGSISAIE

PAVNVQSGLIQVQADIPNSDGKLRSGMFARANIIMPKLANQVTLPQTAITFTLYGDNVYI

VTEEEGEKRVKQHVVKVGERTKDIAHILAGVKPGDVVVTSGQVRLSNHAKVSIVESNAIT

PPAETPML

>tr|Q87S14|Q87S14_VIBPA Uncharacterized protein OS=Vibrio parahaemolyticus serotype O3:K6 (strain RIMD 2210633) OX=223926 GN=VP0610 PE=4 SV=1

MELYDTEEQQVEAIKDWWKENGKAVIIGAVVGLGGLFGWRYYQDTVIQASETASQSYTTA

MNTLQEKGVDAQSDVQAFIESNEVKEYSVLAALQLAKAQVEAKDFAAALEQLKWAQSNTK

DAALSPLISYRIARIETEMGNFDAANTELGKVTDTAWAGRIAELRGDIALRQGDKDAAYA

AYTEAQQAADASPTLQMKLDDLAK

>tr|Q87JG0|Q87JG0_VIBPA Transcriptional regulator OS=Vibrio parahaemolyticus serotype O3:K6 (strain RIMD 2210633) OX=223926 GN=VPA0293 PE=4 SV=1

MTKRQQTRDKILKAAWASFAQNGYDMTTTRQIAREAGVADGTVFSHFPTKLSILREGMLT

QLQQISQETLVSVEGKTAIEIGLALTEKYYRYYFANVELSRALLKEVIWDLDYYQSFNQA

LFQSASVSSVLEDKIPLIFDCYFMTLIAHLSRAEPDVEAALTDLHAKFRQIIPIESLG

>tr|Q87RS7|Q87RS7_VIBPA Uncharacterized protein OS=Vibrio parahaemolyticus serotype O3:K6 (strain RIMD 2210633) OX=223926 GN=VP0700 PE=4 SV=1

MFWDSSRLIKLIGFFCLLLAQPLHATILSSALLNEAQQLAEIEPAQAKQAAKNYLSQREL

TERKESGSPSAMSREETDRSIRTPASTIEAHKIIAQADYTMGNVRSAIDNLNEAEKLAKE

YQLPYMTLDVQLMRNQMIWMYDHNYSAAEDALNQIEKQLDEADSVLQRTDSVRYRLVMQR

ALLAAHRGDNVGAERLYAQAKSMLEDSRSDLALIDYYTSVGEFYLNSKKYNLALSELLYG

YWQSIESDSGTRLAKVNRLLARLFQERRVYDKAIEYLSQAADFYDSYPSSPILADVLEQM

GDIYFYQGKFNLALVHYFNVLDHESTSRNINRTIKIRLSLAATYLQLYNYALAEQYLERA

TELLEYADIPQLEAKAALLKSGLAYHQNDSKKVIENAKKALELVEKAPENKNFIKQQSYR

LLGLGYEQAGEYQLSLQAYKKYTSLVRLEQKQLNQISEDAFRQQKEFAEQTIHYVGQSER

LEQVEMEHRKFQKISFALFITVLVMFLFIMRRGVIMQRQAGEIEKLRNDLFTHSRSRLRN

LRMLNVKLSRSLKKSRDTFEQWQMGELIHEPLNDRLRFVMIDLPFLRSMYVQNGYKAGLE

LERAFGEYLAERIEKPARLYHFSDANLLYIEPNADRDASAEVMFEKFQAWVDEFATQHNV

NRIIRMGISDYPFLPRAYTAINDEELLDLLLLATHIAREVSLKDKQSHWVFLKAIDNAPA

ASFATGNIRTACQHAINQGLIKIHSSYKNEDDIKKILKNG

>tr|Q87IZ8|Q87IZ8_VIBPA Uncharacterized protein OS=Vibrio parahaemolyticus serotype O3:K6 (strain RIMD 2210633) OX=223926 GN=VPA0457 PE=4 SV=1

MPFQRVVMLEGRLYERYLPGYLESNSEEEKQRLWQQADPNTIYQLNSELQKLHGEGNDLI

SFSDPHRSHLPIAAWPDMLSADIAFWRKHQHWLLQSNDPVTPTNEDDLSVNYKIPLNYIN

QYNGEHLRLFYGSRLVYARLFSALHYILNHVKSVIEDWSHDRYPYQLPIELYIPTTSEPQ

SAVRSTYHHFNSANYQSLKNYFQQHYSSWIEEQYLNWMFELNTQLNHQTAATYIIEYEDE

FGIPRVDFICKNENTLRMIRPAHFVEDMRQMVSSTDYLDHQVQQVALRIERQCRAKGW

>tr|Q87SX0|Q87SX0_VIBPA Uncharacterized protein OS=Vibrio parahaemolyticus serotype O3:K6 (strain RIMD 2210633) OX=223926 GN=VP0302 PE=4 SV=1

MPSKQVLAIVIGLSLSTVATAEEYRQHSAHVHGHVEFNIAQDGSDLLLEITAPGADVVGF

EHAPENAEQEKTLQHAVATLEDSNALFAINPQAQCEIEEVHVEHSLGGQHEEHEHHDHEG

HDHDEHAHHDHDKHEHDGHEGHDHSEHSDHGEFTVQYRFHCAQVGELSHIQTDWFNQFPS

TESVNVNLFTDTTQSATSLTKSNTQIAIK

>tr|Q87G31|Q87G31_VIBPA Ferredoxin-type protein OS=Vibrio parahaemolyticus serotype O3:K6 (strain RIMD 2210633) OX=223926 GN=VPA1486 PE=4 SV=1

MKSSQSKTSQSRRRFLRDTVRTAAGVGAAACVLGLQSLQSQARETKGVPIRPPGALPEGD

FESACIRCGLCVQACPYDTLKLATLLSPVATGSPYFTARDIPCEMCEDIPCVVACPSGAL

DPTLTDIDDARMGTAVLIDHETCLNWQGLRCDVCYRVCPLIDEAITLEMVHNDRTGYHAK

LIPTVNSEVCTGCGKCEQACVLDVAAIKVVPTDLAKGKVGSHYNFGWKEIDKPLENILPS

ETPVPEGALESLKGGK

>tr|Q87G84|Q87G84_VIBPA Putative two-component sensor kinase OS=Vibrio parahaemolyticus serotype O3:K6 (strain RIMD 2210633) OX=223926 GN=VPA1433 PE=4 SV=1

MMYFMFAHWRDNTFLFSAIVIVGAVVASWLLDQLFDSTAAVLLILQLAVVVVAFQCNSRF

AYAAAVIEALSFNFFFTTPRYSLQMFRPEDIFNLVVFMVVAFITSTFADLYRRQQGELKQ

TKLQNSILLSVSHDLRTPLATIIGTLTTLNEYMPKLNDLERKELLDSATSESHRLHQYIE

NLLQATKLQHGTLKITKKDEPIANIVRDAVSRLPNYTEKVSMNMDDSVGYLSVSRSLIEQ

AIFNVLDNAMRFSPENESVEVSLSKQGLSCVIDVRDMGIGITAEDAEKIFSLFYSGANNK

SADSGTGMGLAVAKGIITAHQGEIQSMPVSEGTLIRIRLPLNQGAEQA

>tr|Q87QT3|Q87QT3_VIBPA Uncharacterized protein OS=Vibrio parahaemolyticus serotype O3:K6 (strain RIMD 2210633) OX=223926 GN=VP1066 PE=4 SV=1

MKRSSNVKRSSMDKSLKLGKIIPFAAFGGIFFLATQESKTEGYIFSDADECKSNSPEFSE

QCDIAYQEALARAERNAPRYNNEFECENDFYEDDCYYSSSSRAYVPHFGGFFYSRSVNDL

KGYNKSYYSEPMYRYKSKFYNGAGQFFGSYRNQSTKVATSNLNKRGGGTIGRAMSRGGFG

KAVSVSRGG

>tr|Q79YV9|Q79YV9_VIBPA Flagellar hook-basal body complex protein FliE OS=Vibrio parahaemolyticus serotype O3:K6 (strain RIMD 2210633) OX=223926 GN=fliE PE=3 SV=1

MKVDGIQAEMRAMMVEATNTTPTGTGAKVGADFNDLLTKAINNVNSLQKSSGDLQTRFDR

GDADVSLSDVMIARNKSSVAFEATVQIRNKLVEAYKDLMNMPV

>tr|Q79YY2|Q79YY2_VIBPA Bacteriophage f237 ORF1 OS=Vibrio parahaemolyticus serotype O3:K6 (strain RIMD 2210633) OX=223926 GN=VP1551 PE=4 SV=1

MSWCPSPTRPLNNVSTWGCLMTTATNILKSFDEQSVHIDYLCFTFAVKDLRHCHDAVRRL

HKHEEYKGFAKSGLLQRHCRAPKFPAPPVFNPTVAQTSDEIDAYNKAFDICYRNYLEDCL

RIFTNQVLGLSLSAPRGLGFQFYTESMKLTSPDGEDFCGFVGIGGNNDTVHFQINGTGCK

HVFARRPTWSLHDWLTNVLGVQTLARVDLAYDDYDGIFDCEYAYKAWRDDCFRTAERGRG

PVLHEDMTIASIGKDGKPIYTKEQYSIGSRTSRIYWRIYNKALEQKLANTGLVWYRSEVE

LKKWNVDVLLNPAGAYAALNDFAASISTAKKFNTKPVPTKRAALDLLASAHWMRRQYGKI

LNSLIEFHEGDIETVVGSLVRDGTKFTFPDTYGKLVTHILET

>tr|Q87NX7|Q87NX7_VIBPA Sodium/alanine symporter OS=Vibrio parahaemolyticus serotype O3:K6 (strain RIMD 2210633) OX=223926 GN=VP1741 PE=3 SV=1

MNDLQSLLQTIDNFVWGPPLLILLVGTGVYFTFSLGLIQFKHLPTALAMVFSKDKSSDKQ

GDVSSFAALCTALSATIGTGNIVGVATAIKLGGPGALFWMWLVALFGMATKYAECLLAVK

YRRVDDNGQMIGGPMYYLQYGVGSKALAIMFAVFSLGVACFGIGTFPQVNAILDASEISL

GVNRELAAFILTLLVAFVTLGGIKSIASVAGKVVPAMALFYVLACLSVIIMNADQLLNAV

ELVLVSAFTSTAATGGFLGASIMLAIQSGIARGVFSNESGLGSAPMAAAAAKTDSCVKQG

LISMTGTFFDTIIICTMTGLALILTGAWQSDLSGAAMTTHAFAVGLNAETFGPMLVSIGL

MFFAFTTILGWNYYGERCVVFLMGTKAVLPYKIIFLALVASGAFLHLDMIWILADIVNGL

MAIPNLIGLIALRHVVLAETKLFFNPSVQSDDLDAVKA

>tr|Q87TH9|Q87TH9_VIBPA Uncharacterized protein OS=Vibrio parahaemolyticus serotype O3:K6 (strain RIMD 2210633) OX=223926 GN=VP0090 PE=4 SV=1

MRSLTKLDRAMQIKRIEGKAALEQCLLDKQTLKFTSNRFVQENPWTALGLSAFAAIAVAK

AGRLSRVTSSFSSISLLLNGAMDLSGQLAKSSSSSAASSKEAGEEAAQPSDFPPHQ

>tr|Q87PA6|Q87PA6_VIBPA Uncharacterized protein OS=Vibrio parahaemolyticus serotype O3:K6 (strain RIMD 2210633) OX=223926 GN=VP1611 PE=4 SV=1

MSDQNNSQTSYVPDVKRSKGISPLWLLPILTMVLAGWLVVKSIHDAGQRVQIYFSDAAGL

VAGRTTIRYQGLEVGMVRDINLSEDLGSIYVDADIYPEATKLLNDKTRFWLVKPTASLTG

VSGLDALVSGNYISIQPGDGQEFETTFHALDSAPTDLRVSQGLNIKLKSRDLGGVSIGSQ

IVYKKIPIGAVYSYQLDEDAKSITIQANIQEQYRHIINDRSRFWNVSGIGASIGFEGVDV

RLESMSALLGGAIAVDSPDDGEPVEENTEFRLYKDLKTAGRGIAIKIALPDDNKVSSEGA

PIMYRGIEIGQVTDLSLSEGREVILASAAIQPAFSDMLTTGTRFVLEEAKVSLSGVENIA

NLVRGNFLTIVPGDGERSRRFTAIRKNVFNQQQEKSIAIRLISDNSFGLDSGANVLYKGI

VVGSIINVGLVDEKKQTKHEVFMDVLIDHEYKHLIKSNNRFYVTGSASAELTESGLSVTV

PPAKQLLTGSISFVSEGSESIQKEYQLFQNESLAELAQYNKTGSKTLMLFASELPPISKG

SPLLYRNLPVGNVSDFHLVDGGVLIKATIENRFAYLVTPQTVFWNRSGIEIDASLSGVSV

KAHPLKSLIEGGIAFDSVPGVENKVGERWKLYADQQKARKFGRVISLETDGTQEVLKGMP

IEYQGVKVGEVTLVVPNFRRNLVEVTARILPEYVENIAVEGTHFWLTEPEIGLGGVKNLG

ALVSKSISVEPGNGKAKFDFQLEKGFDRVEGVMFTLQSEQRGSVQVGTPVLYRQMEVGQV

TDVRLGEFADRVVSTIKIKPEYAYLVRQNSVFWNVSGVDVSIGITGANIKAGTIDSLVRG

GIAFSTPEQSQIPPAAKRGHSFYLYPRADESWVQWRTPIPKP

>tr|Q87NX1|Q87NX1_VIBPA Putative amino acid transporter OS=Vibrio parahaemolyticus serotype O3:K6 (strain RIMD 2210633) OX=223926 GN=VP1747 PE=4 SV=1

MSNIAKSAVKLSVFSVIMITVTSVDSIRNIPGAALFGSHAISFFLLAGLCFFVPTALVCA

ELSTTYPQQGGVYLWGKETIGPNFGFATVWYQYAENIVYYPPLISFIVATGAYPFFPELA

QNNIFMLIMINVIFWALTLVNIFGLRLSSMITNVFGTLGLIFPILLIIGLGGYWAYTNPG

ESHISLSHVSDWLPDFSQDGIGAGFTAVVLSLTGLEITTSYASEVENPQKAYPKALLAST

ALILVSLTACSLSISSVVSSDHASLSEGVILAFKTFFDDLNLSFMLPVIALAIVFGTLAS

LNNWIIAPTKSLHVAAKDQFMPLALSKENQNQAPVALLLLQGAIVSVLSLVFILVPNVNQ

GMWLLNILMTQLYMVMYVCIFISFLVSRRKHANIERPFRVPGGKVGMSVVAGLGLISCMI

TIVVSFDVPAGISAETGAYALVLGFIAFSLPAIAAVMYRNRKVRSQAQLIEALAS

>tr|Q87LD7|Q87LD7_VIBPA Magnesium transporter MgtE OS=Vibrio parahaemolyticus serotype O3:K6 (strain RIMD 2210633) OX=223926 GN=VP2675 PE=3 SV=1

MAEQIEFDQAHQALQEVTEALENGRFVHVRRQLQDMEPEDIAHLLEASPRRSREVLWQLT

DPEDYGEILDELNEDVKDSLVSKMAPEDLAEATEGMDTDDVAYVLRSLPDDLSREVLSQM

DSADRARVETALSYPEDTAGGLMNTDVITIRGDVDVDVVLRYLRMKGELPEATDALYVID

DESKLIGELPITTLITTQPDVKIIDVMEDADDAITVDTSDSDVASLFERRNWVSAPVVDE

NQHLVGRITIDDVVDVIREDAEHSMMSMAGMDDDEDTFAPVFKSARKRSVWLGANVLAAL

AAASVSNMFEATLDQMAAIAVLMTIVPSMGGVAGNQTVALVIRGLALGHIGDSNKRELLM

KEAAIGLLNGIMWALIIGGIVVVWKGNWMLGGIISAAMLTNLFVAGVAGVTIPVLLKKMN

IDPALAGGMALTTVTDVIGLSVFLGLATLLI

>tr|Q87R17|Q87R17_VIBPA Rec2-related protein OS=Vibrio parahaemolyticus serotype O3:K6 (strain RIMD 2210633) OX=223926 GN=VP0981 PE=4 SV=1

MTLLEKSWTLALFVASVISSAWWPTMPDWRWLLLGIITTGSIIKLRRGLISIGVIVGFMV

VIVHGNIMEYQRQALFQAGENSTIIGRVDSSFTQISHGYEGVVAIKQVNSHTLLPFLKPK

VRLITPFPLAVNSEFTTNVLIKPIIGLRNEAGFDAEKQSMGSGVVARAVVTKDSYWVIRT

SSSWREAIIQTVERDISRLEHFALIKALAFADRTGLTKEDWQSLRDSGLLHLVSISGLHI

GMALTFGLALGGLIRLAMPRYWFLPSVSGLAFAIVYAWLADFSLPTTRAVSVCIIYIALK

YWLVHWSPWRVLLLAVALQLFFQPFASFSLSFWLSYLSVGAVLFAVNTVQDSKEGRLGKL

RILLLTQLILSLLIVPISGYFFSGFSWSSLVYNLVFIPWFGFVVVPIMFAALIASLLFPM

LATVLWYLLDIFLVPLSWSVRYAIGTWQPISAEWTFVIAVVSAVLVSRHVMPRYVWMFVC

VIVVMTGLFPKQYNQTWRIDVLDVGHGLAVLVEKEGRVLLYDTGKAWQNGSIAEQVITPV

LHRRGYSSVDTMILSHADNDHAGGRKVIEQYFSPKHKLSSQSFLHYQPCIAAEKWKWQGL

NMEVLWPPKPVVRAYNPHSCVISLEDPSTGFKMLFTGDIEAISEWILLREPEKLRSDVML

VPHHGSKSSSNPKFINVVEPSLAIASTAKLNQWGMPAPEVVQAYTDSGVSWLDTGSDGQI

TILLDGNNWRFESKRRETIEPWYRQMLRNRVE

>tr|Q87IV5|Q87IV5_VIBPA Mannitol-specific PTS system enzyme II component OS=Vibrio parahaemolyticus serotype O3:K6 (strain RIMD 2210633) OX=223926 GN=VPA0501 PE=4 SV=1

MKANMRANVQAFGGHLTAMVLPNIGAFIAWGFITALFIPTGWMPNEAFGELVGPMITYLL

PLLIGYTGGQIVGEKRGAVAGAIGTMGVIVGADIPMFVGAMIMGPLSAWVVVQVDKRIQH

RIPSGFEMVVNNFSLGIVGMLLCLFAYEIVGPSVTAANLFVKSGIEALVATGFLPLLAII

NEPAKVLFLNNAIDQGIYYPLGLQAAAETGKSIFFMVASNPGPGLGMLLAYAKFGQGLSK

RSAPSAIIIHFFGGIHELYFPYVLMKPIMIVAMIAGAATGIATFNLFDAGLVAGPSPGSI

FSYLALTPKGSFIATIAGVTSATIVSFLVASAILKVSKSEEKESEFEKSVSDMKEMKAEG

AVKPVAAAATTQEPSKPISFVAFACDAGMGSSAMGASTFKRKLVQAGIDIEVKNFAIEKV

PSEADIVVTHESLESRAVNATGLPVVTIKNFLNDPALDELMDKIKQQTVATEQPA

>tr|Q87T79|Q87T79_VIBPA Putative lipopolysaccharide A protein OS=Vibrio parahaemolyticus serotype O3:K6 (strain RIMD 2210633) OX=223926 GN=VP0191 PE=4 SV=1

MKDSKFPYYFSNALLMLVPRFVYRAQAKRLLSTLTEAEKQYCLERVNYYNKCGQPFTVDS

REQSYAKIGEFKKTKGWTYFFDTRQVIRYFPSEFTFNYINGDVTHIPNVPSFIKSRPIHG

DNQNSVVLKLNQIRHFKFVDDEMSYHDKKDMVAWRGVGFQPHRCKVIQAFYDHPRCNIGQ

TRPQEGQPWEKGFMSIEEQLQYKFLLCIEGNDVATNLKWAMSSNSLVIMSKPKYETWFME

GKLEAGIHYVEVQDDYSDLPEKMDYYLANEQEALAIIENAHQWVEQFKDKRKERLISLMV

ADKYFTLSHQQ

>tr|Q87JB0|Q87JB0_VIBPA Putative membrane fusion protein OS=Vibrio parahaemolyticus serotype O3:K6 (strain RIMD 2210633) OX=223926 GN=VPA0343 PE=3 SV=1

MLAKPIVRWLLPFVVVGGSYAGYAAIAATAPEKESTKETITEPTVRASSLFPTDHKVVIT

SHGELVPFEKTHLSAQVSGEVISWHPNFVTGGIVKRGEVLFTIESDNYEAAVLQAEAGLA

SARASLIEEQAKAEVAKRQAKKLSDKQVTDLYLRKPQVLSAQAQVKSAQAALKRANRDLE

NCRVVAPYDALVVERDVGVGQFVTSGSRVATLNNIEVAEVHVPIAGFDSAFLPESIKELT

ATVTQQGILSTTREGKVVRDLGMIDSATRMINMVIQVEDPYGIDNQQPPIKFGSYVEVSF

TGKELKHIYRLPQELVKNRTVWVVNDENQLQPRTVTVLRAEGEFMLVGEGLEQSDQLVLT

LPEYPQKGMAVQIAKKNDDSETVVQ

>tr|Q87GZ3|Q87GZ3_VIBPA Uncharacterized protein OS=Vibrio parahaemolyticus serotype O3:K6 (strain RIMD 2210633) OX=223926 GN=VPA1172 PE=4 SV=1

MKRTSLILGSLFVSVYANAAPFDTCPSKAYLFQSTPVQVYGVNLVTGSTTLLADDVGLAS

GINAVGFDFTDRYIYGYDTTNLKIVRLGKDFQAETLNVAGLPSHTFFVGDVYNHVYYLYR

KGKGLFSIDLSPLDTDPNATLTLTQITATANVNLTDFAFHPGDGSLYGIDNNSGILYKFN

TANGQATMIGDTGETGTFGAGYFDVNGNYYVSRNQDGKIYRINLSADNASNINAGIVPAV

EFVSNGPSSSQNDGARCANAPVIDEDDPIDFGDAPDSYHTLLSSNGPRHEIDGVTWLGVI

APDGDPDGQIAPSGDNKVGTADEDGVGFVAALDPGLSSLVNVMASTSGYLSAWFDWNRDG

DFADEGEQVFTDQLLAAGNNTLPFIVSELATAGPSWSRFRFSQQTGLKYDGGSTSGEVED

HPITITENGVSVQHFPSAEGYATVAFEDNWPYTADYDMNDVVIQFRITETRMEGVVENIQ

ITGDLAAYGAGYKNGFAIRLPGIAKASIENGLTTLSFNGVQQASNGLETISEDAIFIISD

DLSQYTQSNCTYFRTQSGCDDNVAFSFTLTVYFKEGSDRSGLQAMPYDPFIFATPNTYHG

DGITFQPGRKWEVHLPDQAPTEQFDDTNLYGLGVDASDPVQGTYFKTSNNLPWALLIVED

WEWPLENVDLVDAYPQFATYAESAGSQNANWHASPKANKCYIP

>tr|Q87PN9|Q87PN9_VIBPA Putative polysaccharide export protein OS=Vibrio parahaemolyticus serotype O3:K6 (strain RIMD 2210633) OX=223926 GN=VP1462 PE=4 SV=1

MSDLRENISVLLHGAWRRRYMIVIPMLVLPILGFGVSKLVPTTYVAHTSMLIQETAKMNP

FLQDIAVSTMLKDRLSALSTLLKSRHVLYSVAKEQGLIDDDMGAKEQEFIIKDLASRLSV

QQLGKDFIQIQLRSGKAQGMESMLTSVSNRFVEQLLAPERSSIKDSSHFLTIHINKRREE

LDKAEHAFAEYKNTYSYATPEMQAQSLTRLASLKQTLAEKEAELAGVTRSLGSLDQQLSK

TNPVIGKIEEQIIEIRSELTLLRAKYTEAHSLVQGKLRELKRLEQERTVLLSSKQPELNS

NQLWDIASNATVSSLGEAQPLLVSQLHQLQIMRSRFESLTEETVSLQKMIQELESNAHRF

GSTATEINRLARDVAVKREMYDDLVERYEMAQLTGSLGVFEENKRVKIIDAPFTPTIPSN

LPSIVFIILGFIGGAGLGVGLAAILELADNSVRSRRALEKHLGVPVITTLPKVTFAS

>tr|Q87K32|Q87K32_VIBPA Uncharacterized protein OS=Vibrio parahaemolyticus serotype O3:K6 (strain RIMD 2210633) OX=223926 GN=VPA0066 PE=4 SV=1

MSQVRIKFIASDMDGTLLDQYGRLDPEFFDLFLQLEEQGILFSAASGRQYYSLRDTFAPI

KDRVLYVAENGTLVMYQDKELYSCTIPKAEVAEIVKAAREIDGANIVLCGKRSAYIETHD

QQSLEEFQKYYHRCETVTDLLEVEDEFIKVAICHFDGSEELLFPTMNAKFGATHKVVVSA

KIWLDVMNAEASKGAAIKHLQETMNFTPAETMTFGDYLNDLEMLQVSEHSYAVANAHEEI

KKIARYSAPSNQEAGVLKVIKEKVLAK

>tr|Q87L47|Q87L47_VIBPA Bacterioferritin OS=Vibrio parahaemolyticus serotype O3:K6 (strain RIMD 2210633) OX=223926 GN=VP2768 PE=3 SV=1

MKGDPIIIQHLNKVLGNELIAINQYFLHARMYKDWGLKHLADKEYHESIDEMKHADHLIE

RILFLEGLPNLQDLGKLMIGEDTKEMLECDLKLEMAAIPDLKDAIAYAEDIRDYVSRDLF

QDILEDEEEHVDWLETQLGLIEMSGIENYLQAQYVDEE

>tr|Q87QH9|Q87QH9_VIBPA Peptide ABC transporter, permease protein OS=Vibrio parahaemolyticus serotype O3:K6 (strain RIMD 2210633) OX=223926 GN=VP1170 PE=3 SV=1

MFWYTVRRFNLFFITLMILTLVGFSLLRLDPLSHWANVEFWSGWQSYLINLSQLDFGLSK

NGNAIYDELAVVFPATLELCFFAFLVSLLIGIPLGTIAGMKQGKWLDTGISFLSMSGYSA

PIFWVALLLIMVFSLEYHIFPVAGRYDLLYEIDHVTGFAIIDAFMAKGEYRAHALQSVIE

HMVLPCLVLALAPTTQVVGLMRASVAEVMSQNYIRAARIKGLSNREIVTQHVMRNAIPPI

IPKVGVQLSSMITLAIITESIFNWPGIGRWLLDALANQDYASIQAGVMVLATLVLTANIL

SDLIGAMINPLVRKEWYANK

>tr|Q87KF1|Q87KF1_VIBPA Thiamin-phosphate pyrophosphorylase OS=Vibrio parahaemolyticus serotype O3:K6 (strain RIMD 2210633) OX=223926 GN=VP3026 PE=4 SV=1

MAKILIPSSLIPLTGAVLQCLLLAKEQGFSIDEIELGVSPTQFIQLVLGQNTFRVGTDLI

DVCEEAETADFVLYYQSGLSVSECRQQPSSAIFIGIQDVESKLDDSVKTTSADVLDIWRH

PVNDEIRALSVASTSRTTTLQTDQHLAWTVTLLALDFPIEDALTLARPMTNVSRETLING

ETMVKQEWASQFADFPTPVLEDCRLGIKVGWSSHGQSVNFPHLSKQSLGLYPVVDDVSWI

ERLLPLGINTIQLRIKDPYQPDLEQQIARAIELGRQYDAQVFINDYWQLAIKHGAFGVHL

GQEDIEDSNLSQLSTAGICLGLSTHGYYELLRIVQINPSYIALGHIFSTTTKQMPSKPQG

LVRLALYQKLNDSIPYGESVGYPTVAIGGIDQSNAEQVWQCGVSSLAVVRAITLSESPKQ

VIEFFDQLMNTTSTSLVMEDYRAY

>tr|Q87H61|Q87H61_VIBPA Uncharacterized protein OS=Vibrio parahaemolyticus serotype O3:K6 (strain RIMD 2210633) OX=223926 GN=VPA1104 PE=4 SV=1

MQTPRLFNLNLRYKTALYFSLGLLGMIISFLFISRYFFLYSLDELENMEIDHSSQQAIAV

IDMMVTQQEGSSYDWAYWDETYDLFAHRDIAGYSERNLYVETLDALNLDLMSFITLDGQS

LVSLSRENTPESSSSLNNKVVSVPLLQQHILAMNSKLDVHRESLAGIFKINDNIWGLSLA

PVRNSEGDRPSNGWMLWGRNLSERFPGDFKAMMSASNTLVTKSFNPVDHAKTKSIDKTSE

SIIKWTPISDLAGEPIAWLKTQTKREHYSKGNTLFIYLFATVGVVASAIATSTFFIFKRK

VATRFSHFAEGINEIASQYQLEGLQSVKFDELELATKLVQKLSENTSMTQLQLKDSMEKF

GALYHSSSLGMLIVIEREIVDANHRALELLSYKKSDLLEQSLDTLCPTTADECRVDAMYR

ELQHGRNQFEAQMLDSHGETIDCLIEATLIQHNGHTALMLLLQDLSENKQQAEMIQKLTD

FDPVSGFCNRPVILGALEELVKHQPNHFSFIYITSKSLKQIAEVYGHLIFDEAIQYISTL

LRDHLGTHQIGRISEHEFIVILPNASGHSDALEAANRLLNQLSYKIELSGIMLSLDSKAV

MVDPKITHQSLEHLLLVARYSAQSMSGRHMHEVLVADGELSEQAETSMIIHRDLEVAIRQ

DNIIPYFQPIVNAKSGEIIGFEALARWPHPTLGIISPLIFIPLAEQGKLIVELGESILNQ

SCDFIGKLNTKRHSQGLPPLTVHVNLSAQHFYHSRLISFLQSMIEEHNISAGHLVLEITE

SMLMGGETESIHCINEIKQLGVQLALDDFGTGYASFSSVCNFPLDIVKLDKSYVDEIETN

DRAKTLVRNIANMSQELGLTIVAEGVETASQVRKLKVWNIDELQGFYFYKPMTREDAFAQ

FSGLPHC

>tr|Q87IE7|Q87IE7_VIBPA Iron(III) ABC transporter, permease protein OS=Vibrio parahaemolyticus serotype O3:K6 (strain RIMD 2210633) OX=223926 GN=VPA0659 PE=3 SV=1

MRDSTKIVLLGVIALVFAFLFIGIGLNADNYQYFLSRRVPKVLAIVLAGVAIAQSSMVFQ

TITHNRILTPSIMGFDALYVLTQVLIVLLFGGLSTLVLNIYVNFTIAACIMVAFSLLLFG

FYFSKGSRNLITLLLVGLIFGQLFSNVASFFSLLMDPNTFAFVQSKLYASFNNVKVNLVY

FSAPMLILACWLLFRMHRTLDVFWLDQDNAKSLGVDVPKVTRNVFILSAVLIAISTALVG

PIMFFGLLVTNLSREMFHSYQHKTLLIGCSLLAISSLLSGQWIIENVFNFETTLSVVINF

LGGIYFLYLLLKNKVV

>tr|Q87PQ0|Q87PQ0_VIBPA Putative ferredoxin-type protein NapF OS=Vibrio parahaemolyticus serotype O3:K6 (strain RIMD 2210633) OX=223926 GN=VP1451 PE=4 SV=1

MRQSESVDLSKRRLFSFRRAAVEQAQDPRVKARPPYAVEESMFTRLCDGCGKCASACPSQ

IIEMVDGVAALDISYSVCDLCGECKSACPTLALSNQTESTGLIATISNSCENLYGYCGSC

EDSCPYDALQWQDDAKPKIDAAKCKGCGQCAQSCYTSMISFDLKR

>tr|Q87QR1|Q87QR1_VIBPA Putative chemotaxis transducer OS=Vibrio parahaemolyticus serotype O3:K6 (strain RIMD 2210633) OX=223926 GN=VP1088 PE=4 SV=1

MFQNFTIKQKIVIPLSLIIGLFTVSSVLNVMTTSKQSELSDTLNEQIVPNLFTIEDAYRD

LYQATSAVQGIALAETQADIDHHIHEYKDNAYKALPRMEKVIELSRAGVMPASHGADVQK

LVSLGQKWLQSYEVMLSKPQSQWLSYYNEHKNTFEEQFVDVRAQLNVVKSAIEDKQGELK

SDISAATARAESILEMGIIVVILAALGMVFLLLRTVLKPLNDIKDAMAQIASGDGDLSQR

IQINTQDEIGQLAKAFNEFVSKIQATVSQVIDSSNTLRQEMANLSSLTETIADSTVSQQR

DSEAVAAAVHEMQVTSRNVSESANEAAVASQTANDELSNTNVILEQTVGSIRDLAGEIES

ASHVINTLDNDVSDIASVLDVIRGIAEQTNLLALNAAIEAARAGEQGRGFAVVADEVRSL

ASRTQQSTGEIQAMIEKLQSGAGQAVEVMRGSQNSSEETIQSAGRASESLAEILNAISRM

NEMNTHIATAASQQSTVSDEVNTNVQGIADSSTSIVDIVTQAQQSLAMLSQQTKRLDQQV

SQFRV

>tr|Q87KZ7|Q87KZ7_VIBPA Methyl-accepting chemotaxis protein OS=Vibrio parahaemolyticus serotype O3:K6 (strain RIMD 2210633) OX=223926 GN=VP2827 PE=4 SV=1

MRNTIKLKIQIAIAVIIAIVSGVQAWVSVNQLHEETTSTLNREIQNISESTNRYISDWLS

IRSDMMLANEQIIAGSDDADRELLLTKRAGKFLSVYAGFSDGAIAYGDKSESWPSDYDPR

TRPWYQDAMAQSGLIITEPYQDFDGSIVVSFAKAFNQNKQGVLAADLAVTDIINEVLNIQ

LDNNGFAFLVDGNNNLVAYKDEKLSQKPLTTLNPELTRDKMANLAQHAKLETITWPKQGD

QLIYVAQVPNTDWSLGVVQDKQMAFASVSEQVTFTAIASIVMYLIIAAISTYVITRLLRP

LQTLSDALSELSQGEGDLTQRIEIERMDEIGELATHVNQFLAQMQSMLKNIVENSQQLSE

QAQQANELSAMAAGRVEHQQNDVNQIATAIHEMSATAAEVASHAELTASASQNSASACVE

GQSVIQKNREAIVSLAEQVSDAANVISELEANTQSINQILSTIQGIAEQTNLLALNAAIE

AARAGEQGRGFAVVADEVRVLSQRTHGSTEEIRTMIETLQSNTKLAVNSMQASTSLADTS

VDYAQQAHDSLTSITNSITEINDMAMQIASAAEEQRAVSEDISRNTQGIKDDADVIAEQS

LKSSEGARRMFNTANTMRENISRFKV

>tr|Q87FI6|Q87FI6_VIBPA Uncharacterized protein OS=Vibrio parahaemolyticus serotype O3:K6 (strain RIMD 2210633) OX=223926 GN=VPA1693 PE=4 SV=1

MFGSKKLKMENEALKQELASLKDKYQTDVETLERQLKEAKQLLNTAQQRYESSDELMSSS

LKGGDMLQTIRTAMVESAQSMAHENEELKLLDDMFKQTHQALARLDDRAVKISSQATQSI

ESVQILDNTATSISHLVSTIQEISDQTNLLALNAAIEAARAGEAGRGFAVVADEVRNLAG

KASEASEQIDSLVNQVLTQVSSIKSAIDENQICAEEVSASSAQIGSIVNEVVVKSEHMKR

VIHFASTRSFLDTVKLDHAIWKNNIYRLLQSGSFGETVNSHSECRLGQWYYRGDGKAYSQ

LRSYAQLEAPHKGVHDSGRDAMNHAKSGNMAGMVTSINSMEDTSEQVVIHIDRLMDEIIA

N

>tr|Q87N38|Q87N38_VIBPA Chemotaxis protein CheV OS=Vibrio parahaemolyticus serotype O3:K6 (strain RIMD 2210633) OX=223926 GN=VP2037 PE=4 SV=1

MSGVLNTVDQRTNLVGENRLELLLFTLNSRQLFAINVFKVKEVIKLPPLTKLPGSHYNIR

GVASLRGEAVPVIDLRCSIGFPPLRGEAEEENLIVTEYNRSVQGFLVGPVRNIINTAWTE

IQPPPSTSGRSNYLTAITQVKDGDTSQIVEIIDVEKVLAEIVHYDVTISEDILDHDLSQS

MVGRNVLIVDDSSTARNQVRDTLSQLGMNIIECRDGLEALTVLKRWCDEGRDVEKELLMM

ITDAEMPEMDGYKLTHEVRSDPRMSKLFITLNTSLSGSFNEAMVQKVGCDRFISKFQPDL

LVEVAQDRLRQVLSANA

>tr|Q87IZ7|Q87IZ7_VIBPA Uncharacterized protein OS=Vibrio parahaemolyticus serotype O3:K6 (strain RIMD 2210633) OX=223926 GN=VPA0458 PE=4 SV=1

MSLWGFLGAGLAYLLMTFAFVFGGIFWLCAEGNTLRETKRQSSIMSGIIVCTMGTWVIAF

SIYIYGYFWDNSSHYYSYLLAPWPLAIVGITLRNHWVSQYSSVKQEKNEKWQRHWREILG

EDTEDLPPYRYDYGLYSGIWQANETLREQCFAALTHGNSVYERVKAFQKMTTHEHNTDDQ

ILLSKLAQLENEIIQALEQHSQKNVSIETGSGTLCKESKHNVYRHENGPTEEQLYNSINL

QHDLDRELRNIIYDRLGDDGLDEYFFLRAPLEELTENETAINWMLWGLVSDHFDVDPYQT

ALELNLMNAEPRWGQDERFVVVTTAA

>tr|Q87R95|Q87R95_VIBPA Putative hemolysin OS=Vibrio parahaemolyticus serotype O3:K6 (strain RIMD 2210633) OX=223926 GN=VP0902 PE=4 SV=1

MLLTCALLEGAMDILLLVGLIALNGIFAMSEIALVAAKSGRLKMMAEDNAPAALALELKN

NPTQFLSTIQIGITAIGLLSGIFGEATLSIPFEHWLVAQGLEREVATILATTSVVILITY

FAIIIGELVPKRFAQNNAESIAIVVAYPIHWLAKLARPFVFLLTVSTDALLKLLRQNENQ

GEIVTEEDIFAVVNEGSESGAIEPQEQLMIRKLLHLNDRLALSLMTPRCDIHFLDTNLPL

DAILKHLRQTQHSVWPVCKGGLDNIIGTISSKVLLDEYDHLSVSRLGKLLKHPRFVPESM

KGLPLLNYMQQTSAEMVFIVDEYGDVQGLVTLYDLLKSIAGELGMAPEQIWAKQQKDGSW

LMDALIPLNELKYKLGLTTIEGEESEGFQTLNGFLTWWLGRLPHAGELVDYEGWQFEVLN

VKHHRIVQVKVSRETESETTTDTNDGDANP

>tr|Q87JP9|Q87JP9_VIBPA Putative hemolysin secretion protein HylB OS=Vibrio parahaemolyticus serotype O3:K6 (strain RIMD 2210633) OX=223926 GN=VPA0199 PE=4 SV=1

MFAFVRQFPLYIMVSVVAGVPLLLAIFLAVQHTINLNTQSHQALKDQQLVTLITHYDNLA

HNLAVERGLTAGVLGSQGKTEIVQKLTQQRKKVDDAVNSLVQLNTPLISSSDSYDLLQDV

QVQLNRLNQVRQGVDRLSPQIAPFGYYSNLNQLIIDNIDILIAQTQSRELGTLGDALISV

IVMKERAGQARGALNGVFAKGSATSVLFSNIEGYIQSGDYASRKAQIAFPEQYRQSLQSH

QSNPAWKKVEQVQQSFLNQSANLDNIQGPQATEWFPMATERIGLMNQLRNQMVDQMLSAA

EHSAQQATLNRNLLMAATTVISLLMIMMVWGLVASLRSRVGRLKQTLRSMSEHHDMTVEL

DSRGKDEIASISNSINALISNFRKLLFDVTKTNNESSNRLQNIVESAQDLDSSSRSTIAK

CDNIATAMTELAQSSVEIAQSAERAMGDTTTMNNKVVDCQTQSELSYRSVQSLLEQINAT

EQCMAELANDTQSIGQIVETINSVSEQTNLLALNAAIEAARAGEHGRGFAVVSSEVRDLA

QRSQEATENISKLLDQIGEKTRFSVESMAKSKQASDDTFESVQQVNESVSLLESSIEHVN

NHISTITHSTIEQSKACEAIDQDIDVLASIAHKTGQHADDLNQIVSGYQAEAKELKHQLS

AFKLA

>tr|Q87SV7|Q87SV7_VIBPA Flavin prenyltransferase UbiX OS=Vibrio parahaemolyticus serotype O3:K6 (strain RIMD 2210633) OX=223926 GN=ubiX PE=3 SV=1

MHNKIQPSQKKAITLALTGASGAPYGLRLLECLVAADYHVYVLISSAARVVMATEHNLKL

PSGPEAAQQALVEHLNCNPDNITVCGKDDWFSPVASGSAAPKQMVVCPCSAGSVAAIAHG

MSDNLIERAADVVMKERGQLLLVVRETPFSTLHLENMHKLSQMGVTIMPAAPGFYHQPKS

IEDLVDFMVARILDHLGIEQGLVPRWGYDQRS

>tr|Q87G79|Q87G79_VIBPA Iron(III) ABC transporter, permease protein OS=Vibrio parahaemolyticus serotype O3:K6 (strain RIMD 2210633) OX=223926 GN=VPA1438 PE=3 SV=1

MRTKPYIGYVALIALLSLLSLQIDTSLSLSEQWQLFTQPESASEFRDVFFMQSQLPRLSI

TLLVGAMLGLTGSLMQQLTQNNLTSPLTLGTSSGAWLALVIVNIWFVDWVADYSAFAAMA

GALVAFGLIISIAGVRNMTGLPLVVSGMVINILLGSIATALIILNAQFAQNIFMWGAGDL

SQYSWDWFEWLLPRSTIAIVILIVAPRILTLMKLGQEGAAARGLAVLPAFGALMVMGIWL

VSASITAVGIISFIGLLTPNIARAMGARTPRDELISSMLLGAALLLITDSAAIYLSLLLE

ETIPSGVAAAAIGAPALIWFTRKKLTATDQLNLSMSQGKMALSNAAVWGIAVMGIIGILT

YSFVTHGISGIEFATPGEFQWQLRWPRMISAISVGVALSVAGIILQRIVYNPLASPDILG

VSSGATFAIIITGVMVGSVLAAFNWGVAFLGSLTVLMLLLIIGKRSHFNPSNFVLSGIAL

SALLQALVQFALAQGSGESYKILLWLTGSTYRVTSTSALMLLIAVLVLLAIVFALSRWLT

LISIGRVFSNARGLNPSSANTILLVIVALLCAFSTATVGPVSFVGLVAPHMAMMLGARKV

KEQLFVGSLIGATLMVWADWLGQIAIYPSQIAAGTLVAIIGSTYFLFLMLKSKFR

>tr|Q87IE8|Q87IE8_VIBPA Iron(III) ABC transporter, permease protein OS=Vibrio parahaemolyticus serotype O3:K6 (strain RIMD 2210633) OX=223926 GN=VPA0658 PE=3 SV=1

MKKLLLTLVVLSTVSLFVGVADMTPQQLFSGDAKALELFFTSRIPRLFAILLAGAGLSIA

GLVMQQISQNRFAAPSTTGTIECAMLGYVMSVVFFGDGDHLWLVFGISVLGTLTFVHFIQ

RIQFKSVVFVPLVGIIFGNVIESMTTFIAYKYDALQSLSAWSVANFANILRGDFELLYIA

VPMVILSYLFAARISAVGIGKDFAVNLGLNYQQVVTIGVLLVSIMSASVVMIVGQLPFLG

LIVPNLVSHFYGDNLKKNIPLTAMYGAILVLGCDLVSRLIIFPHEMPISIVISILGGVVF

IAMLLRGKQHA

>tr|Q87NH6|Q87NH6_VIBPA Methyl-accepting chemotaxis protein OS=Vibrio parahaemolyticus serotype O3:K6 (strain RIMD 2210633) OX=223926 GN=VP1892 PE=4 SV=1

MRSTITFKLLLALIVVFSCVLAASTAYQHYQQKTLINDVLSEQLHDKASNYFDSLNMMML

TGTMSQKETLRQKALAQEGIEQVRVLRADAVTKLYGAGQSNQQPIDEIDQRALAGELVIE

PITADWGKGIVVALPMKSSQNYRGTNCVSCHVAPEGEVLGAIRLEYNMNHVSSMINKQAM

YAMGIMSAIALVGFLITMGLIRKIIVRPIQKTSHFMSNVSASKDLSQRLVHKQNDEVGQL

SQSINSFMDTVSESLERVQDTSHSLAGSAGRLTDVAQSTDEAANNQQLETNEVQNNIIDM

LQQQVVVEEATINATTLVNHTVDVATNSASQAHNVSEDIKSLVSDIEQVREKITSLNQRT

EEVSSILGVIKGIAEQTNLLALNAAIEAARAGEQGRGFAVVADEVRNLASRTAEATSNIE

SIISQFQQGSEESLSSVDHVCQFAHQRSLDVEALSETMHNVVDEMHQVLKHAENIQLQTQ

TTSDVSKHIQSKIDVITLHANDTSQSASHTRDISVDLEELSDRLEQLLNQFTLSEQQRAN

K

>tr|Q87R35|Q87R35_VIBPA Methyl-accepting chemotaxis protein OS=Vibrio parahaemolyticus serotype O3:K6 (strain RIMD 2210633) OX=223926 GN=VP0963 PE=4 SV=1

MRGSVIKRMYAGFALIIILFAVTIAIMMGGMHDIHGKFETVSKSSLPLVSLSNQTSVELL

SADKSFKDFLTTENKQRMEEMRQEFARSQQRFESTLAQLESASQIYPSLAEPFSQLKTLE

QSYFTEALEAMDNYEAMFSAQEEVQKSSRRFQKLNTELSVGLKEYVADQSSISVKVMAKS

YFIKLKDAEVITSDALASSNPEFVTQAVTKNRKAVTHLNYAFRGLVTQLPELEKAFGESV

EQFTRDVGMRGGVLDQHNNYLNARAALYDNIANLANKVDSTMAILEQFTTTATDKLNESL

DDAGDIYSAGVTKAIVIGVVVVLFAAAIGYHIAQSVREPLTRILNALESLTKGDMTQRID

IRFNNEFSRVSGHINTLADSLHDILVKLNEASENLASTATTNERTSSQAQSKLNAQREQT

ANVATAMTEMSHSVQEVAQSAQGSLEMVQRVESASEEGRNVMSSNISTINQLETRLHESV

SAVSELQKMSGQIGSILDVIRNIAEQTNLLALNAAIEAARAGEQGRGFAVVADEVRVLAS

KTTQSTTEIESMISNLQSSSQSANQVIQSCMSDMEMSVEQASKANSSMEEIQALIIEISQ

MSTHISQAAAEQSETSADIARNIEDINNIADESYHAMSSITHTSESLTQLAHQQNELVHR

FKL

>tr|Q87LH7|Q87LH7_VIBPA PTS system, cellobiose-specific IIA component OS=Vibrio parahaemolyticus serotype O3:K6 (strain RIMD 2210633) OX=223926 GN=VP2635 PE=4 SV=1

MEQELVVMEIICNAGEARSLSYEALRLAREQKFEAAEEKLLQARECINKAHLIQTQLIEE

DQGEGKVPMTLVMVHAQDHLMTTILAQEMAVEIVALNKQLANK

>tr|Q87NB6|Q87NB6_VIBPA Uncharacterized protein OS=Vibrio parahaemolyticus serotype O3:K6 (strain RIMD 2210633) OX=223926 GN=VP1952 PE=4 SV=1

MSKTNLDLNWIFGTSGDDDLTGTKGSETTDIFFGFGGNDKFVGYGGNDYIFGGWGDDTLL

GGRGNDFISGGFGNDFISGGLGNDSLFGGCGDDVIFDYSGNNFLSGGWGNDTLVVGDGES

VLSGGCGDDTFVFTNRLAIGVPDQPVSSNDIEIEADVLDFNIFHDKLVFDLGKDTDGDGI

RDTFLDSADDLTLSYNECGWAVFSSEEYNVEVTLNGVNSAYINYAEHHGIDIFEFA

>tr|Q87J22|Q87J22_VIBPA Putative chemotaxis protein CheV OS=Vibrio parahaemolyticus serotype O3:K6 (strain RIMD 2210633) OX=223926 GN=VPA0431 PE=4 SV=1

MAKVVSKANQSQGMLMFKLTLQQNFAIGTLKVREIVPYMPTTKIPYSHHHVIGTVTIRDL

TVPVIDMSAAVGFRPITQEEYKNCYLIVTDCLRTVVAFMVRSIEKIIECDWKAIETPPAS

AGKNIFVTGITRYGDQTVQMLDVELLLSKIYPQYENANIPMLTDVERERLKALNILLVDD

SSIARKQLSDALDSINISYQICKNGSDALQLMKNEAEANRPIDILVSDIEMPGLDGYELA

FEVQNDSALNHSYRILHTSLSSEICVDRAHQVGAHEALEKFNAGELIEAMLRGAKELEAN

AMAS

>tr|Q87LN8|Q87LN8_VIBPA Signal peptidase I OS=Vibrio parahaemolyticus serotype O3:K6 (strain RIMD 2210633) OX=223926 GN=VP2573 PE=3 SV=1

MANTFSLMLVIVTLVTGVVWLLEKLVFAKKRQAKVAEIQAQTTNGLDAVTLQKVERQPWW

IENSVSIFPVIAFVLVLRSFIYEPFQIPSGSMMPTLLVGDFILVEKYAYGLKDPVWRTQL

VETGKPERGDIVVFKYPPQPSVDYIKRVVGLPGDIVRYSGDKQLCIQSQGESSCKPVKLS

NVEESQFKSNGIPMIQLDEKLGNVEHNILVNPLVRNRVEQYFPRSGTTEWVVPQGQYFVM

GDNRDNSADSRYWGFVPEANLVGKAVAIWISFEFDRGADSVLPSWIPTGVRFNRIGGIH

>tr|Q87TF2|Q87TF2_VIBPA GGDEF family protein OS=Vibrio parahaemolyticus serotype O3:K6 (strain RIMD 2210633) OX=223926 GN=VP0117 PE=4 SV=1

MSTAKTQITLRTAVLIPFVMIFLLAIGVIVYVQKQSYEEVVTDISDKQLSVLTESVYDHL

NSYLRKPFTAVMTLAHNVSYHNLYHPHDTRDVQAYLLSAFQNLYRSIPQLDVIAFGSETG

DFAGFRREISDDYTLMVQDKYTDGKLIIYGTDHVSNDIRTVITPYDPRTRPWYQPVAKDE

KPMWSKIYTNADERQDITLSAMTPVYEHQEFAGVLVTDIRINTFNEFLRVIKKNTKASVF

IMDQEHRLVAHSGPGSVVSWGTKFSDKGQRLFASESADPIIKISASRVRGFDLYHTDQPY

TFEFDHSGQRIFSRITPYYDPNGLTWFIGTSISESDLLGLLPKSQEKSWIVGIVVSLFGI

GFSWVIFERVTRPINATADAAQHLANGDWDSSMPQPGRVYETTVLAKAFNEMASNLKASF

KALRDQLVYDSLTRLYSRQGLIEICENKPNLCHGSLFLLGINKFRDINDSVGHHNGDQLL

VSIAERLKQQFPDNTMLARIGGDEFALFMPDMDKDEDIRLTERRLQQLFAAPFMVEGESV

VMKISLGIVQTKHGENMPLWLRNASIALSYAKQDPLTGICYYSPELASASKFRTQLLTKM

QTGIDKREFVPHYQPIIDLASGNVCGAEALARWNSESGMISPLDFIPIAEESGMIKAIGQ

QILLQACRDTYKAIENKQWPSDFQLHVNISVNQLSCPAFVDTVTRVLDVTKLPAKNLTLE

ITESRIIDSAPTTLENMMKLRDMGIGIAIDDFGTGYSSLGYLHSLPFTCLKIDRTFINQL

TKENLDSSVVAAVINITAGLKTNVVAEGVEDSTQAQLLRSLGCHQVQGFYYSRPIPLDEW

PTHLVNMK

>tr|Q87NT1|Q87NT1_VIBPA Putative transposase OS=Vibrio parahaemolyticus serotype O3:K6 (strain RIMD 2210633) OX=223926 GN=VP1787 PE=4 SV=1

MLEHLLCFNVVRMAKVFGVSRSGFYYWIKHRHKAIQREANRQELDIKVKEAFDSSKGRDG

ARRIQKELAENGNSHNVKTIAASMKRQDLTPKAARKFKCTTDSKHKMPVAPNLLAQDFNA

AAPNQKWAGDITYVATSEGWLYLAVIIDLYSRQVVGWSMDTKMTATLVCDALSMALFRRG

FPEQVIVHSDRGSQYCSKDYRDLMTAYNLKQSMSRKGNCWDNACVESFFHSMKVEAIQYE

PIMTRDKTRQAIFEYIEVDYNRARRHSALGYLSPVNFEQQNVA

>tr|Q87G25|Q87G25_VIBPA Methyl-accepting chemotaxis protein OS=Vibrio parahaemolyticus serotype O3:K6 (strain RIMD 2210633) OX=223926 GN=VPA1492 PE=4 SV=1

MQLSLKNLSVRTQILVPVLFTAIVLFIALWITKNNLQAEQDVIASNQESLVFHKDTLARI

DDQIYPLRISAVYAIYDASRRETFLNELKAGLKQVEADLSAIEARNLFREDAIEVRKAIE

AYVQYSQRSVAFFNQYDQGLKSDNEYRAFISEYRRVGNEMVQGINTLSKHVNDQAVESTA

KSNAQNERVQTNAMLTVLAVFVFSLIGAWFLSGMIVTPIQKLQRVMRELAAGNLSVRADV

EGDNEIAQLSKDVNQTASQLYSIVDQLTRISEEVASASTELAAVMTQAEANAQQELAEIE

QVASAVNELASTANNVSDNATSADATAREADGLAQSGLAIFQESADASAQMSQALNDAAQ

VVLRLKEQSVQINDVIEVIRGVSEQTNLLALNAAIEAARAGESGRGFAVVADEVRMLAAR

TQDSTQEISTIIEELQAQSGLANDSMQVSLEMLTRNNELTQQANDALIGITESVANINDS

NTQVATAAEEQSQVTQDINRNVVNMSELVNQNVAGISQSASASSELSLLAEKQKEQLSFF

KL

>tr|Q87QZ1|Q87QZ1_VIBPA ATP-dependent DNA helicase DinG OS=Vibrio parahaemolyticus serotype O3:K6 (strain RIMD 2210633) OX=223926 GN=dinG PE=3 SV=1

MLTSNIQKSIRNSYQNLQTQLDNFVPRRAQNYLVAEIAKTLCGQYHKSNRILVAEAGTGI

GKSLSYLMAAIPVAVHNNRKVVISTATVALQEQLVNKDLPLFRRITDREFSFILAKGRQR

YCCAEKLATASGVDGGQLAMFETKPKKKDIELLETMYRSLAQGKWDGDRDAWPKPIDDRI

WQLIVSDKHSCNNSLPGHRGCPFQKARSELDKNDVIIANHSLVMADADLGGGVILPEPEN

TIYVFDEAHHLPHVARDHASASASLKGAAAWLEKLNQSISKFSSLADEKRVARFRNDLQD

SVQNLIPALTQLTKQFDPAQFEEGIYRFEHGELPTWLENQSKELKQFSKKANQSVAKIAD

LIAERVKDGELAARLAEPALAELGFYIQRLENLAQVWHLMAEPTREKGAPLARWLETHPD

REGDFIVSVSPLEIGWQLDQQIWSRCIGAVLVSATMRALNSFHYFCHQVGIDGKPESGTQ

FLALASPFDYQNQAELLIPAMKYEPSAPQFTEYLIEILPKYLEDKKANLVLFSSYWQMNQ

VAEALSSEFIKRGWALQVQGESSRQEILKKHKKLVETNKTSVLFGTGSFSEGLDLPGELL

ENLVITKIPFGVPTSPVEQAHSEYIEKKGGNPFMQITVPDASKKLIQSVGRLLRKERDSG

RVTILDRRLVTKRYGQALIDSLPPFKRKIEY

>tr|Q87PN0|Q87PN0_VIBPA Phosphorelay protein OS=Vibrio parahaemolyticus serotype O3:K6 (strain RIMD 2210633) OX=223926 GN=VP1472 PE=4 SV=1

MVDELILQQMIKDTCAEIIPTLIEHYIEESRERMQKIERALGEHDLEKLEFEVHTLGSSA

LALGNRPLSRQARSIEKHCVEGKTQQALELCAALPLLAERSFEALNLRKAQGFE

>tr|Q87FT3|Q87FT3_VIBPA Putative prolyl aminopeptidase OS=Vibrio parahaemolyticus serotype O3:K6 (strain RIMD 2210633) OX=223926 GN=VPA1595 PE=4 SV=1

MNAQSFIDAGLHYTPHSFTVPLDYQDASKGTIEVFARAVCLVGDEESTKPWLVYFQGGPG

FPSPRQNGNSGWVKRALQEYRVLLLDQRGTGNSSVINHQTLAHLTPEQQVDYLSHFRADN

IVRDAEYIREQFGVEKWAILGQSFGGFCSLTYLSLFPDRLLQSYITGGVPSVSRHADDVY

EATFKRTMEKNQAFFQQFPSAQQLCQNIANHLLENEEYLPNGQRFTVEQFQQIGINFGVS

DTFLPTYYWLESALIEVNGKQQLRYEFLNDMLTQQNFQTNPIYAILHESIYCQDFASNWS

AHRVRQTRPEFNYEPDQPFYFTGEMVFPWMFDQYVNLKPLKDAAEILAAKEDWAALYDQT

QLAQNKVPVSCAVYADDMFVEMDISRETLALIPNSKAWITNEYEHNGLRADGERILDRLI

AMGKQTAATLK

>tr|Q87N65|Q87N65_VIBPA Putative tetrathionate reductase complex: sensory transduction histidine kinase OS=Vibrio parahaemolyticus serotype O3:K6 (strain RIMD 2210633) OX=223926 GN=VP2010 PE=4 SV=1

MQQQKTVQPEAKQKVEVGVLAIRGHLYAEQRWQPTIDWLNQQISDVHFELHPLNLDEMGE

AVKFQTMDFILTNPGQAVRLGRQYALSWMATLTGRAPQNSNYSIGSALVVRANSPYQTLK

DVSGFPVAAVSEKAFGGYLTLRYQIVEMGLDPNDFFADVRYLGFPIDANLYQLRDGNVEA

AVVPACLLEQMQNEGLLQHGDFRVLNQQPNEHSSCAVSTPLYPNWSFAKTERGSSLLAKK

IAQVLLAMPPEHPAIIAAGASGWTSPVSLLRIDKLYQALDLHPLQQPWWSEALRWLRSHQ

EWAWALFMFVIVLNAYHFGLEYRFSKSKQALELTSLRLKEKSEQLEHSQRVAIVGEIGSS

LAHELNQPLAAIRNYSEGGLLRLAKKRPHEDIVPVLEKIQGQVERADAIIQRLRTLIRKR

SVDKTPCDIQALIADTIELLHFRMQKQNVAIVTSVEGEIRPLLADSVGVQQVLVNVINNA

IDACALFQEKYHSSGYQGKIALHCDYQANQLSIRILDNGTGLQQENPTQAFVSSKAEGLG

LGLAICRDVMEMHGGEFLIASTTPHGCLVELVFPYQN

>tr|Q87JF7|Q87JF7_VIBPA Putative oxidoreductase protein OS=Vibrio parahaemolyticus serotype O3:K6 (strain RIMD 2210633) OX=223926 GN=VPA0296 PE=4 SV=1

MKNNAFNALLGTELPVIQAPMAGVQDSQMAIAVSNVGGLGSLPCGMLNKDAIVKELQLIQ

QATNRPFNLNFFCHEMPKYDANKHEEWQKTLQPYFAEVGATVQAQPNAPTRMPFNHDIAD

AIEPFAPPVISFHFGFPADDLLARIRGWGGKILSTATTVDEALWLEAKGVDGIIAQGLEA

GGHRGMFLSDDLSTQLGLLALLPQIVKRVNLPVIAAGGIANKSGVEAALKLGASAVQVGS

AYLLCDEAKTSSLHRYAIASERAQHTAVTNVFSGRPARGIVNRAMSELGYICESAPKFPY

ASIEMTQLRALMEKQNRDEFTPLWCGQNSSGCKEVSAAEMTLSLVEGIDLN

>tr|Q87NS7|Q87NS7_VIBPA Uncharacterized protein OS=Vibrio parahaemolyticus serotype O3:K6 (strain RIMD 2210633) OX=223926 GN=VP1791 PE=4 SV=1

MMFGIFGKKFKKKDTEEYLWHSPSADFTIFDPKKIGSFSLRYGWGIYFSFEKDRARMHQR

KVEHIFKLPIAALEGKTFIYLHEGIKSQKDHKIQDLAISIYGRVEYCNPDFDVNGHQFYK

DIVSRYPTEEDAVKYILQSGITGAVVKELSETIVLLYDLSLWKLVDKVSFS

>tr|Q87IP2|Q87IP2_VIBPA Uncharacterized protein OS=Vibrio parahaemolyticus serotype O3:K6 (strain RIMD 2210633) OX=223926 GN=VPA0564 PE=4 SV=1

MIPFPLELERIKAVIFDLDNTLVSSDMNFQDLRQQLGCPQTEDLLDFVERIDHPHHKEHA

HNVIFDHEISDAEQSSPMIGCHELLNYLEKKTIKTAIVTRNCLIATQRKLEHNQITVEQV

ITRECYPPKPDPLSLQVLAKEWRLMPNEVLYVGDFLYDLQAAYNAQMPSCLVHHGITEQF

QNSASLAVRELSDLLAYFESAH

>tr|Q87G57|Q87G57_VIBPA Phosphate transport system permease protein OS=Vibrio parahaemolyticus serotype O3:K6 (strain RIMD 2210633) OX=223926 GN=VPA1460 PE=3 SV=1

MTIATNSEKLMNTDAKAISKPGLREKRRVDWKERIFHGLFLTSAVIGIVSLAVIAYFIVR

ESIPAFQEAGVSGIVLGQNWLPPALYGVATMIVASVVSTAGAVMVGVPVGVLTAIFIAEI

APKRLADVIRPAVELLAGIPSVVYGFFGLVIIVPLIQDIFNVPAGNTILAGIIVLGVMIL

PTVITVSETSIRAVPRAYKEGSLALGASKIYTIFKLLVPAARSGIMTGVILGIGRALGET

MAIIMVMGNAPAMPEGILDSARTLTANIAIEMSYASGVHANALYATGVVLLVFIMSLNAV

LLYLNREKAK

>tr|Q87GY3|Q87GY3_VIBPA Methyl-accepting chemotaxis protein OS=Vibrio parahaemolyticus serotype O3:K6 (strain RIMD 2210633) OX=223926 GN=VPA1182 PE=4 SV=1

MLANFSQKAQETLVGELEELVSTTNLKGVITYCNDAFCRVAEYTHEELVGQNHNIVRHSD

MPKAAFGDMWARLKEGKAWRGMVKNSTKSGGYYWVDAYVTPIYEKNQVVGYQSVRVKPKR

EWVDIAAKAYKGMLAAEKAGRTWSLKINETVRYAILLGALTAPAVAYALSVEGPLAWLAS

ALPASVLALLFRQELIDTPQQLKKLQKQYDSVSRLIYSGNSAFSIADFHIKMLSARIRTV

LGRMTDSALPLQNCAEELSQTTSEVSAALNQQNSDIRRVRDATQEVESSANSVSSSTNDA

HMLIDDTLKSCMMAKETIDQTHTNLAQLSLQAEKATETTYQLSDQAQKVNHLMVEIGGIA

EQTNLLALNAAIEAARAGEQGRGFAVVADEVRALSGRTSNATEQIQASISAMLSTIEGWQ

KDILANKEQTDACSQVAEQSALRLSEVEQMMQSMSGLMVDVAEAANNQLKLSSDVNQHIH

SIASTAEQNLAATHSVEQNSRQLKEQVQDFYQLAIRFEDKQS

>tr|Q87QC7|Q87QC7_VIBPA Cytochrome c-type protein OS=Vibrio parahaemolyticus serotype O3:K6 (strain RIMD 2210633) OX=223926 GN=VP1222 PE=4 SV=1

MSLWRKPNRKWMLGIPIGGLLAFILGGAALSVFHFGMDYTNRNEFCYSCHIGMDTIVEEY

QASPHFKNAQGVIAATCSDCHVPREFFAKIALKIGATADIYHKLKGDITLDNFEAEHRPR

LAAEVTQQFIENKSKQCRYCHSVDKMDFEAQGRTVARRHQMMESRDQSCIDCHAGIAHHL

PISLDSPETVPPEE

>tr|Q87JH8|Q87JH8_VIBPA Putative flagellin OS=Vibrio parahaemolyticus serotype O3:K6 (strain RIMD 2210633) OX=223926 GN=VPA0275 PE=4 SV=1

MVMSTVEVRAHSIRLGGQEQASIMPTRSADSSSTIKPRPIQVQADTASAYSVSGIQLTQG

QQQATAVQIASKSLQTIGKELTHIKRGLTQAVTQGTQNVPGLQDTLVRSKANIQRVVEQA

RFDGQKVIDNELHLKLDKADIRRFSIPGLNVHRLSDRAEQIRLDFPQGQAVMIQFDGQSD

GARTVKMLDRSLIAMGMRASLAEDGTILFEARDNAYQQMQQKVLVTGEGHRFPAGQPNVL

NLKSEPDGIAELSFDLGSRDGIKQTISKVNQHLRQVQTSLEEAKAFHGELNTQMRSIQSK

SHQLSVDEVNAKLDGFIETSGQFTSTFQALNAQANVRRHTVVALLK

>tr|Q87TH8|Q87TH8_VIBPA Putative permease protein OS=Vibrio parahaemolyticus serotype O3:K6 (strain RIMD 2210633) OX=223926 GN=VP0091 PE=4 SV=1

MNGKLNLPINTVIGVSLLVLLLVPALYFAKPVLLPMVISTFVALLFSPLINYFEDKGLPR

TITVVVTLTLLVSVSILGLAAISEPAKQWWAELPSIVQNVSQEVNEVTKASSEHMNAPLE

LASDMNIDEMGNNTVFSLLKALATTTPTLLTQAMIVLFMVYFMLNHGRSLFRKSVSRLRG

FSKQRQAVELVQALQKDLSRYIGTITLVNAGLGLCVGLVFFVLGLEDPFLWGAFAGVMNF

APYLGPVISMASFGFVAYLQLDSVSFMLTVVSIYLLLNLIESQFVTPTLLGRRFNLNPLV

IFMWLVFWGWLWGGMGLLIGVPLLVCINILADRLALCGSTNI

>tr|Q87TM8|Q87TM8_VIBPA ATP-dependent DNA helicase Rep OS=Vibrio parahaemolyticus serotype O3:K6 (strain RIMD 2210633) OX=223926 GN=rep PE=3 SV=1

MKLNPRQDEAVKYVSGPCLVLAGAGSGKTRVITNKIAYLVQQCGYKARNIAAVTFTNKAA

REMKERVGQTLGKAESKGLMVSTFHTLGLNIIKREYKQLGLKAGFSLFDDQDQMALLKEL

TEKQLDGDKDLLKQLLSTISNWKNDMLTPEQAKAMAKGEQQQLFAFCFEMYQKQMKAYNA

LDFDDLILLPVLLLRNNEDVRQRWQNRIRYLLVDEYQDTNTSQYELVKLIVGERGRLTVV

GDDDQSIYSWRGAKPQNLVLLGQDYPNLRLIKLEQNYRSTSRILRAANILIANNPHVYEK

SLFSEIPDGEKLKVLLAKNEEHEAERVTGELIAHKFLNRTEYRDYAILYRGNHQSRLIEK

SLMQNRVPYKLSGGTSFFARAEIKDIMAYLRVLVNPDDDNAFLRIVNTPRREIGPVTLEK

LGSYANMRGKSLFEASFEMGLEQHLSGRGLENLRRFTQWLVAIADQAERGNTVEAVRSLV

RDIHYEDWLYETSASPKAAEMRMKNVSDLYSWIVADLEGDNYDQEEKTLKEVVQRLTLRD

MMERGEEDEDSDAVQLMTLHASKGLEFPYVYLIGSEEGILPHQTSIDEDNVEEERRLMYV

GITRAQRELTFTMCKERRQFGELIKPTQSRFLDELPFDDVEWEVNKKPVSQEERMAKGQA

HIANLRSMFKK

>tr|Q87JI9|Q87JI9_VIBPA Uncharacterized protein OS=Vibrio parahaemolyticus serotype O3:K6 (strain RIMD 2210633) OX=223926 GN=VPA0260 PE=4 SV=1

MKINRFKRWSSCLQIQPPSRLDTVGLFISACLLFSTSFLCQAKPNTTDIEQQYQVLAPYK

LQIDQRLTSVNPLLDHIFKQLKSKSLPKSLVLVPMLESSYNAKAVSHANAAGLWQLIPAT

AMRFGLTVDSNVDERFDTQASTKAALDYLAFLYNKFDQNLALTLAAYNAGEGRIARAIKK

AGTDDFQQLTLPKETHQYVSRFFALEKLIDIGQLQHSSFQPLLLFAAESAAPSQPLIDFS

RLPPLVNL

>tr|Q87T37|Q87T37_VIBPA Uncharacterized protein OS=Vibrio parahaemolyticus serotype O3:K6 (strain RIMD 2210633) OX=223926 GN=VP0233 PE=4 SV=1

MEISKSAVWVFDLDDTLYSEKDYQRSGYLHIAHHLKNLYQQDILDIIDKADAQDKDVLHE

ICSALSLPDSVKQSLLWMYRLHIPDIELAPDVRHTLDMIKSCCSAMAVITDGRSVSQRNK

LFSLGLERLDSLISEEWGESKPGDIRFKEIERRYPDKCQYIYVGDNVKKDFITPKKMNWL

TIGIVDSGVNIHSQDLSNFSDEYLPHFWLNSISELQDYIC

>tr|Q87PB1|Q87PB1_VIBPA Tail-specific protease OS=Vibrio parahaemolyticus serotype O3:K6 (strain RIMD 2210633) OX=223926 GN=VP1606 PE=4 SV=1

MNCRSKVTLIAASLWLAASAQALEAKLHKDDLPVLAPEVQHETASKRVTSRFTRSHYKHF

NLNDDFSKAIFERYVEMLDYNRNIFTQADIDSFKDWSIELDDQLKAGNNKIAFDVYNLSM

QKRFERFAYAMTLLDKEIKFDNADEIELDRSEAAWPKDEAELNELWRKRVKYDALNLKLT

GKEWPEIKEVLEKRYNNAMKRITQTNNEDAFQLYMNAFARQVDPHTSYLSPRNAEQFQSE

MNLSLEGIGAVLQLTDDYTVIRSLVAGGPASKSKQLGEGDRIIGVGQDGEEIVDIIGWRL

DDVVQLIKGPKGTKVNLQILPEGAGAKSYVVTIVRDKVRLEDRAVKSEIIEKDGKKIGVL

EVPSFYVGLSQDTDKLLNDLKAKNVDGIIVDLRNNGGGALTEATALTGLFIKEGPVVQVR

DSYGRIKVNADTDGLVSYDGPLTVLINRYSASASEIFAAAMQDYNRAVILGENSFGKGTV

QQHRSLNHIYDLFDKELGYVQYTIQKFYRIDGGSTQNRGVAPDIAYPTPIDPSETGESVE

DNALPWDSIDKATYQTFPDNDKLIANLTELHNKRIADEMEFRFINEDIEKYRKEKDDNML

SLNEKVRKDESDKAEALRLKRINERQTALGKKTFKSLDDVPKDYEAPDVYLDESVAIMVD

MLKAPKQS

>tr|Q87H65|Q87H65_VIBPA Putative sensor histidine kinase OS=Vibrio parahaemolyticus serotype O3:K6 (strain RIMD 2210633) OX=223926 GN=VPA1100 PE=4 SV=1

MNKFRLVNSCLFIAMLVACTFAYMAHINSKSTQQLHSALSEVGHQLIEERDVIVNQYAIK

ERKNFELTKSLVDIEVEAEKLADTFDNAVWFPISPNRQKIQQTLAKFEQRVIQTTSQLDM

LIGVQVENQYALLMLLDIYEEEFSTHIGETQLDKHYVEFFSRDLVNQSGEQSESGANFLG

RLHESDKKIELLTNELLDHNYFVFVEEAEHSLLDLAQNEARFTWLFVFVAVMLLVGSFLY

QLQYRMHNLKQLNSELEAETDKAERAAKAKSSFLAAMSHELRTPMNGVLGISQLIAEETK

EPVTKEHIKVILDSGQHLMTILNDILDFSKVEENKLELEKAPFHLEQVLTPVCSAIQPLI

DEKSIDLIVENDVPNNTEFTGDCARLRQILFNLAGNAVKFTNEGHVLIRTELNSEDKHLL

IIVSDTGIGIAPDKQGRVFNSFEQADSSTTRRFGGTGLGLAIVKKLTELMGGSITLKSVE

GVGTQFIVTLPIPWNESEKPSPQHTPVQTRSTQNLRILLAEDNRVNALVAKGFCEKLGHA

VDVAENGLVAVEKARDNDYDLILMDNHMPEMNGVEATRFIREKLGVKTLLFAYTADVFRE

AHDHFIAAGADHVLTKPLQRESFADALKQFSARLKVKQTEEVSPVSNVLQLQRKPIENLR

LTEEELSNSEMLASLKEHPNELLDLLNSIITDFELAVDDLIENFMQSDFDALKLTMHTTK

GMALNLGLKILASQALELETQLKMNQVPAIEQLQMLINRLQVNIHQGHRLRDELVKAQQN

SEQVF

>tr|Q79YX4|Q79YX4_VIBPA Chemotaxis protein CheW OS=Vibrio parahaemolyticus serotype O3:K6 (strain RIMD 2210633) OX=223926 GN=VP2225 PE=4 SV=1

MSQAFEVEVKKDTSNDEVLQWVTFQLEEETYGINVMQVREVLRYTEIAPVPGAPDYVLGI

INLRGNVVTVIDTRSRFGLMEGEVTDNTRIIVIESERQVIGILVDSVAEVVYLRSSEIDT

TPSVGTDESAKFIQGVSNRDGKLLILVDLNKLLTDDEWDEMAHL

>tr|Q87P85|Q87P85_VIBPA Putative RTX toxin OS=Vibrio parahaemolyticus serotype O3:K6 (strain RIMD 2210633) OX=223926 GN=VP1633 PE=4 SV=1

MKFYMDRTALVSLGNQVVVIGLDGKLRVLTEGQQPRPGEVVVTSTDVGPLDLNVQLTQEQ

GSKDVTDDVLQIISAIEQGEDPSAVDEEFAPAAGENGGSSLQTSATIVRDGTELLASTSF

ETIGIESLGLSQTQVLALNDLFRSNLQTSADSTDKPLATTPVTLDAIEEDGGSIIITTEE

LLSNVDDEDKDTLSVENLIIDKGNGTLVDNGDGTWTFTPQIDDDTEVSFTFDIIDDEDLV

VSGSANLDILPINDAPNAENDVITTEEDTAVTIDVLVNDSDVEGDVLSIQSASVPSEQGS

VDIVNGKLVFTPAENFNGDATITYIVTDGDLTDEAKVTVTVTPVNDSPVAVDDTTSIQED

TAVTIDVLTNDTDVDGDKLSIESASVPKEQGTVEVVDGKLVFTPVENFNGHAEIIYTVTD

GELTDEAKVTVTVNPVNDAPTIKVDAVESITEDAVNTDTVVATLTVRDTDTPEDQLTVSL

ENNSNGYFVLVGNEVKLTQAGVDAVNNDELNLKDLTISASVSDGVNPTASDSDSLVVNRV

NDAPTVENAIADQVLSEDFDAYTIDLNEVFKDSDSSLEFSVSGNNSIQISIVSGVATITP

TADWNGKETITFTAKDPSGESVSQTVNFTVAPVVDIEADSADVVEDTPTIINVLGNDTFE

STDKVVSLDADNGPKNGTVIVNNDGTVTYTPDDNYVGEDTFTYIVTSGGMSESTTVEVNV

TPVNDAPVAKDDIATTQEDTAVTIDVLPNDTDVDGDKLSIQSATVPEAQGKVEIVDGKLV

FTPAENFNGDAEITYTVTDGSLTDQATVKVTVNAVNDTPEVESNIADQTLAEDFTPYSID

LNNAFSDVDGELTFSVSGNSNIQVAIVNGIATFTPTADWSGSEALTFTATDPSGESVSQT

VNFTVASVADIVADKATVVEDTPTIIKVLGNDTFEGTDKVVSLDTNNGPANGTVSVNPDG

SVTYTPNDNYHGTDSFTYIVTSGGVSESAIVEVNVTPANDAPVAKDDIATTQEDTAVTID

VLPNDTDVDGDKLSIESVSVPKEQGTVEVVDGKLVFTPAENFNGDAEITYTVTDGALTDQ

ATVKVTVNAVNDTPVVESNIADQTLAEDFTPYTIDLNTAFSDVDNVDGELTFSVSGNSNI

QVAIVNGIATITPTADWNGSETLTFTATDPSGESVSQPVNFTVAPVADIVADKATVVEDT

STVIKVLGNDTFEGDGKVVSLDTNNGPANGTVSVNPDGSVTYTPNDNYQGTDSFTYIVTS

GGVSESTTVSVDVTPVNDAPVAKDDTAITDEDTPVTIDVLPNDNGIDGDKLSIQSASVPE

AQGKVEIVDGKLVFTPAENFNGDAEITYTVTDGELTDAAKVTVTVNPVNDAPTIKVDAVE

SITEDAVSTDTVVATLTVRDTDTPEDQLTVSLENNSNGYFVLVGNEVKLTQAGVDSVNND

ELNLKNLTISASVSDGVNPTASDSDSLVVNRVNDAPTIKVDAVESITEDAVNTDTVVATL

TVRDTDTPEDQLTVSLENNSNGYFVLVGNEVKLTQAGVDAVNNDELNLKDLTISASVSDG

VNPTANDSDSLIVNRVNDAPTIKVDAVESITEDAVNTDTVVATLTVRDTDTSEDQLTVSL

ENNSNGYFVLVGDEVKLTQAGVDAVNNDELNLKDLTISASVSDGVNPTANDSDSLIVNRV

NDAPTVENAIADQELSEDFATYTIDLNDAFKDSDSALNFSVSGNSNVLVSIENNGIATIS

PTADWNGSETLTFTATDPNGESVSQTVDFTVAPVVDIEADSTNVVEDTSTIINVLGNDTF

EGKDKVVSLDAENGPKNGTVIVNNDGTVTYTPDDNYVGKDTFTYVVTSGGVSESTTVTVN

VTPVNDKPESEDFTHVADDQLTQVVFDTDTKPLGSGDSKDHIADVEDDLKGNDLHVRITE

LPTSGTLFFKDSDGELHEIKEVSDTLYDKDSLYYEADNVGFLLGIKDRPNTPNGSESTSD

FNNWGLSEDGGPSHSRTEHLANGASITISSDSGELAQYNRQVSHIGNGIADNDGQGIEKG

ETITIDLSNNPVGSVNLGLDGLGGLFDYGDDNAALITVTYLDSNNVQQTQTFEFLKPEGN

FMLFQETSVGYGKDLALPEGSVITQLDFSTKNEGNWELRYVEGVPAEDSFGYVAVDSESG

VSDPSTVNIVNEMLDGNVAENGPAVQSITGVTVVEGDDVTFSVSLNNATDQTVKYQVDFS

AEGSSTSSQDIDLSNATFTNGVKYLGGYLIVPAGVNGFDITIPSIDDQLVESTESLVIKV

GDVSGVGYIDDNDVPPTVQSNEISGLEDEAIVITLEHLGVVDSEVSVEFDTSALNGSLQV

KGSDGWEVVSGSVLASDIESGLVRYVPAEHESGADVFDKDGVGNGLDTYEQIHFTVSDGN

SSASGTLSIDIEPVADPIKIDLTFGPVTSVPVTLGGSWSTDEELKDLLEASNLPGLDFEH

VVTQAFNNGHAGNDLMLSGDTESPLSFVGDQQAGANVDAQGSDIFVTGGGNDSIYGGIGS

LDDQAETDTVVYSGKLSDYDFSYYPASDHSEVPYWIVEDSRFIDTKDVVSSTHKESGDHL

YEIEQLIFQDAIIKLDNNTGEYVVLTEQQVSFELNLDLIDVDGSESFINDSVTLTGIPSG

LTLEVNGKTVTENSDGSYTVDLSSEPNQTSHQLSGTITVPADYNGSLDFDVTATAGSVEV

NNNTQMGADTASVSVRDYEFVSGTHGDNNIVGSDDNDVIVGDVQGLQIVEGQDYNIAFML

DTSGSMGYDVGRAVTELKTVLNTLIESASGPHSGKVNVLLTTFSTESKQVLELDLSSDNA

KSQVESILDAIVKLGDGNTNYEAGFQSALNWFENADSGATNLSYFISDGRPNQATDNNVN

WYSSKESVVLGVSEQQLVTLADVLPSDYRFGDTVTYNNKTVIDFRGTVYSLSTGEKMGRM

LNSYEYDDYGNNVLEQANNAYSALAEFSEVRSIGIGGHLNEDSLKHFDSDGVVRTNIDVN

QLAEVILGKEVSLMQGKDEISSLDGNDIIFGDAIRFDINGEQGVSALQNYVASQLGKDVA

LVTKEEVHHYITENQAEFEQSRYYDQADTIYGGAGNDILFGQGGNDKLFGGADNDILIGG

LGSDILTGGDGEDIFKWIDVANERDTVTDFSSSEDSLDFSDLFDDLSKDEVGDLLSDLQS

GSHTGDAGGYHVEVSQDGSTDTNLSITKGSSTLDIHFNSASVDDITQHLIASLDSQYKDM

>tr|Q87IN7|Q87IN7_VIBPA Putative permease OS=Vibrio parahaemolyticus serotype O3:K6 (strain RIMD 2210633) OX=223926 GN=VPA0569 PE=4 SV=1

MKSVQFRFTLYGCIAILSWSCLLGIARLVTESLGPVGGAAMLYSLSSIFLLIVVGIPKLS

YFSPKYLIVGGAMFVCYEIFLALALGYSNSRAQAIEVSIVNYLWPALTVLFAVLGSNKKP

NWLLYPAVTLAFIGVAWTVSGDNGLSPTQIISNVSSNPLVYFMAFTGAVIWAVYCNLTQR

QQSKHNAITLFFIATAVSLWVKYAFADEPTMTFSWSALGYLFASAALMAGGYGLWNIAIV

GGNMVFLATLSYFTPIFSALFSSLILGVTLSNSFWQGVVMVTVGSLLCWLVTKEKREAAG

>tr|Q87GB1|Q87GB1_VIBPA Putative exopolysaccharide biosynthesis protein OS=Vibrio parahaemolyticus serotype O3:K6 (strain RIMD 2210633) OX=223926 GN=VPA1406 PE=4 SV=1

MTGNQMNGTHPNNFQMNKVELIRFGHHFKTLKKNWLSIAAFTLIFSLTCTWYIYSKTSIY

QATATLLIQEEQKSALSIEEVYGVDTTKKEYFQTQIAILKSNHIADKVINELNLTQHPEF

TSSGGLKQKIDDIKAVPLVQDLLNVSPSPKETSQYSESYYQALQAFKRKLEIEPVRNTQL

VRISFRSADPKLATRVANAVGQAYIDANFEAKLVVTQNAATWLTNNSQKLEERLKKSEHA

LQEFLLKEGLIDINGIDDIYANELEELNRKLNTAVNKRIEAQTLIQLLKRKSSQNLDSLL

SIDEFANQAQIRDLKLSEAQAAKNVSELAQRYGPKHDRMVQAKAQLASIQERTQQLIREI

SFSKQQDLLAAKAQEDMLREELDRKKSDFQSLGSQKARYEQLKREVESNKDLYEAFLNRE

KETSATSDYKNVTARFTDKAIIPLFPVAPQRMKLVLIATFFGFAIACALVIILETLREVI

RTSNDVQDKLGVTCLGVIPMVKKRTLRKNGVSYTAYLDKDEKLFSEACRSVRTSLLLRLT

NTKQKILRFYFSDSGGRQNLHQHQYGGVLLDYGESVDYRLRLASSIFSQTIQYS

>tr|Q87RL0|Q87RL0_VIBPA Uncharacterized protein OS=Vibrio parahaemolyticus serotype O3:K6 (strain RIMD 2210633) OX=223926 GN=VP0768 PE=4 SV=1

MKKWLVAASMMTLLTSCAYSPIYNGKEPYSGSQFMLMDSPRHTMDFFIESMTEDLMVSNV

AVSARTPIAITSFVDLQNMDATNWLGNSVSEGFIHQFQRRGFKVVDFKTTGSIQVTQQGD

FALSRDWQDLAQEQQIQYVLTGTMLRQEGGVLVNARVVGMQSRVVVATAQGFLPADRIGR

DLDTLNSIRTQDGVLIRSDPTIRQPYTVILRP

>tr|Q87K90|Q87K90_VIBPA Putative amino acid ABC transporter, periplasmic amino acid-binding portion OS=Vibrio parahaemolyticus serotype O3:K6 (strain RIMD 2210633) OX=223926 GN=VPA0008 PE=4 SV=1

MKGLLRVLAFSCMAVSAATAAEEVNYYVIADQARPFQIEQQGQQHAGIVTDIVNAIFANS

DYKVNFHTYPFKRMISVLEAGGEENWLTYGSPDWGRVQSENLSKEPIYTVKHVLVSSSDA

PFKFNTMQDIKGRSIVLLLGFDYPNLMPFFEDKTVNEMRVKDYDAAYRVIQRTPGDTAFV

EMESRVMYNLKRLELPLEQFEIQSFSAVIPDYSIYLAFSPEMKPEVQNFINQRLSALKAS

GEIDNIVKHYL

>tr|Q87GP5|Q87GP5_VIBPA Uncharacterized protein OS=Vibrio parahaemolyticus serotype O3:K6 (strain RIMD 2210633) OX=223926 GN=VPA1270 PE=4 SV=1

MSSVKVRATIIEDNTGIKSQLPILLTEQGELSTVTDYLLKLEADGVSNSVMNGFIQAVSL

LLDYMEANRGLFDDPKLLFQTFSKRLYTGTIGEDGLDPSGLYWVPSSTSNVNKHIHRLTA

FTNWLADKQGTAPMNPLRDATPHEQRLNYAAWYRKNQNDFLGHIEDKTVNKTIRKARTIK

GRTPLTKTEDDAIAFPEKHWEDFYMNGIGGASDPRVALRDKLILLLMHGGGLRESEALTL

WVTDVFEDPYEPDSAIVRIYNEVDGKAPNDPEVRSKNQNRETYLKEKYARIPRQRMKGTA

HLGWKNRVVDHKDNYIQVQWFPTDYGKVFMSLWKNYQKYRASIDCPHPYAFISFHHSALG

NPYTINAFHDNYANGLKRIGLEPSKAEGKDPHGHRHNYGRRLERSGLNPLVIRRCMHHKS

LDSQTPYTGKGQQEISDELTQATLQLANPESKVKALDWKALAEHGFDDIDPQGYFTGKHP

KLRGK

>tr|Q87R18|Q87R18_VIBPA Uncharacterized protein OS=Vibrio parahaemolyticus serotype O3:K6 (strain RIMD 2210633) OX=223926 GN=VP0980 PE=4 SV=1

MPRKLIKRFMPDHEVIKRQKALKVFGNVLYNPNLWCLNRRSASGAFAVGLFMAFVPLPSQ

MIMSAGLAILMGVNLPLSVALVWVSNPITMPVLFYFAYKLGAWVMHVPPQPFYFELSWDF

IMQQMSTIGPPFLLGCAICGVGSAIIGYFGIRGLWRYSVVRSWQKRKVR

>tr|Q87J19|Q87J19_VIBPA Putative site-specific recombinase OS=Vibrio parahaemolyticus serotype O3:K6 (strain RIMD 2210633) OX=223926 GN=VPA0434 PE=4 SV=1

MTKIAYSYTRLSTEKQIKGHGFTRQREAIEKVCCQHGWQLSDQTFNDLGVSAWKGANATT

GALSQFIDLAKKGAIRPNSVLIVESVDRLSRQQVDKSLRLMLELLEVGVSIFTLSDSKLY

TTNSDSVMLDLMMWLMTAQRAHEESEIKSLRVRAAKKKNKELIRKGIIVTRKCPEWLTVS

DDKSCFLVNNERAEIVRKIFTWYSQGFSTKPIALKLNELGIPYWGKSSTWSYRHIRSLLK

HRGVIGEIQLLKRIDGRDVADGDPVEDYYPAIIDRGLWATCQQIKSSKTTAKGRVVERGM

ANLFRGLLKCECGSGLAINSSTVKGRTYRFLRCSQLKTTGCQAKNWHYEKTERILLIALR

HSLSSFSEEDHAVSLRECQHYLARISEELKEKERQAENAINYLLDKPSKLLEARLQTLED

DIARLKAIQEQAEHRLEAVHYEKSTALDKVEELQRVLEDIANDDDSRHKANGLLRRLIDT

ITLGSISNPRSKEFMEVSGNDYHGLIIIKRRDGLELRIEVLKDYGLSISWEGDDFTVIEC

SPTQQLNSQHEHQKFINVGEQTVDLWDDEGWVASS

>tr|Q87J91|Q87J91_VIBPA Putative outer membrane protein OS=Vibrio parahaemolyticus serotype O3:K6 (strain RIMD 2210633) OX=223926 GN=VPA0362 PE=3 SV=1

MKKFILTPVVLALSLAGCAVGPDYQAPTTSMAETYLNAENAGLSTDSQHVKFWWTKFHDP

VLNQMVQDMQSQNIPLKVAAERVKMANNYKTMVESFKVPTINLGAGYMNYQFSKNDSSLG

PILNPLSDSVSGLPPQIGNVTLMDNQHDGVFAGASIAWEVDLFGRIDRQTNAAQIRLEQA

QIYQSGLNTVITADLIHNYLQYQGASERLELAKSNLEDQRRTLDLVGKVVRSGYGSDLDL

AQAKATLAAMESLVPQLEIAQQAHKHRLAVLLGEPLTQVEIRLSKQHSVPVMQNMVPVGL

PSDLLKRRTDIRLAEREMAALNEELAASVADQYPKFFLTGAPGLSASSFDDLFSSDSFGW

MGAAGVSWNLFDGGRGEAIEAINQARFDSATLSYQHTVEAALAETESTLFTYGRSQENQR

RIDEALKATNNAVNKAKSLYRAGLIDHLSVLDAQRQQRMMQDQQIAAKLQTAQATIALYK

SLGGDWTLQPEAQPETNAKG

>tr|Q87JR4|Q87JR4_VIBPA Uncharacterized protein OS=Vibrio parahaemolyticus serotype O3:K6 (strain RIMD 2210633) OX=223926 GN=VPA0184 PE=4 SV=1

MTGKGVARVMVRCGAHMESHYLDIRTLNFIIILFSCIYAISLLCYQYTQSKIKGLKTFAI

SLLFIGLGPFLLGFRDSAPDWLTIILSNTIIIIGFLLTLYGVSIFRKFPLKCAHVLTFLV

PVFSGLFYYFTFYSPSIRIRIIFLSIYLSLVTFCSGVAMIKGKRDDLKLPVQVMAYAFFG

FSAFMAGRTVWSIWAPEVTSFMNAGIIHQLTFLFSICLIVALSFSMLWLINARLVKSIND

LSHLDALTGLYNRRAMEVIVPNLVNQAREKNTPISIVMTDVDDFKTINDQYGHTTGDSVM

ATIATIFKQTLPESACTVRFGGDEFMIVLLANTENAKAYTELVRRSIERETSLLAFKNQV

TMSFGISELMPNDSLQDALTRADEALYCSKHTGRNQVTTFQDESEVGGKFIQTPTKSA

>tr|Q87Q18|Q87Q18_VIBPA Probable binding protein component of ABC transporter OS=Vibrio parahaemolyticus serotype O3:K6 (strain RIMD 2210633) OX=223926 GN=VP1332 PE=4 SV=1

MSRMTKTPLVMLISGMLLGTSAYAEDKLTVVSWGGAFTKSQVEAYHKPFIQKTGVEIVSE

DFSGGLAEIKAQVEANNVRWDLVSLDKPDIVRGCAEGLLEPVNPSILPPGADGTPAKEDF

IDGAIHECAINTIVVSTVLAVNEDAFKGKTAPTKLTDLFDLTNFPGRRALQKQPQGNLEW

ALLADGVKPDEVYRLLETEEGRARAFAKLDTIKPQVLWWTTGAQPPQMLADKEVVIASAF

NGRIHNARKDEGQPFRIIWDHQMGYMNGWAIPKGSANTKLALDFIAFSSGTKPLADQAKY

VAYGPTRKSSSAEVSPEILANLPTAPQNFKTAFLINDEWWSDYADELNEEFNTWLLN

>tr|Q87GE5|Q87GE5_VIBPA Uncharacterized protein OS=Vibrio parahaemolyticus serotype O3:K6 (strain RIMD 2210633) OX=223926 GN=VPA1370 PE=1 SV=1

MLKIKLPQQTSLAPSSETTQRLPVKISIKSICNKSICKTLHSLADKCHRFSKEIKQRSAN

HTPSSSHENLTTINLKSQNSAATFESTVELSIKHGSSIPPAPPLPGAIPPAPPLNLSKGA

PKSSSNSLDSVNEDHSKLMEQIRQGVKLKSATKSLSADKSSADAHSKLMEELLTGGRKLK

KVATSDIPAPPPLPSASTSKSPDSRNALLSEIAGFSKDRLRKTGSLETLNSSQSKDKESF

EPTTHERLLSEDLFKQSPKLSEQELDELANNLADYLFQAADIDWHQVISEKTRGLTTEEM

AKSEHRYVQAFCREILKYPDCYKSADVASPESPKSGGGSVIDVALKRLQTGRERLFTTTD

EKGNRELKKGDAILESAINAARMAISTEEKNTILSNNVKSATFEVFCELPCMDGFAEQNG

KTAFYALRAGFYSAFKNTDTAKQDITKFMKDNLQAGFSGYSYQGLTNRVAQLEAQLAALS

AKLS

>tr|Q87GV1|Q87GV1_VIBPA Putative transcriptional regulator OS=Vibrio parahaemolyticus serotype O3:K6 (strain RIMD 2210633) OX=223926 GN=VPA1214 PE=4 SV=1

MDKIDRQLLHLIQKDATLTTAELADQVGLSASPCARRLKRLEQEGVIRGYRAMISRGAVG

IAMTVFVEVSLNNHQASSIDEFETAIVDMDEVISCHVVSGAYDYLLEVVSKDLPGYESFT

RKLQRLENVKDIHTHLAIRQVKGNGCLPI

>tr|Q87IG6|Q87IG6_VIBPA Putative protocatechuate 3,4-dioxygenase beta chain protein OS=Vibrio parahaemolyticus serotype O3:K6 (strain RIMD 2210633) OX=223926 GN=VPA0640 PE=4 SV=1

MERRHFLALWTLMFAPFLKAESSMRPTPSQTEGPFYPVVKIPLRQNLILKPDALIGKAIT

LQGKVLDVFGRPISGVKIEIWQCDGQGIYDHPNQPSQNKFDPSFSGFGATVTEEDGRYFF

QTLFPVPYSSRPPHIHVKLWRGDQELLTTQLYLKGQTGNEWWGGKARDHLQFELINIDGR

IVGTFDFVIV

>tr|Q87FD9|Q87FD9_VIBPA Uncharacterized protein OS=Vibrio parahaemolyticus serotype O3:K6 (strain RIMD 2210633) OX=223926 GN=VPA1740 PE=4 SV=1

MSLIPRTERATFLIRPTTYGHSVLGAPLLYFPAQVESASRGLILAGIHGDETASIAGLSC

ALRSLPAKNLRHDVILSLNPDGNQLGTRANANQVDLNRAFPTQNWTEHGTVYRWSSHTPI

RDVKVKTGSKNQLEPEVGALINLIELRRPKFVVSFHEPLAFVDDPKHSNLAKWLGEQFQL

PIVDDVDYETPGSFGTWCSERDLPCITLELPAISADLTIEKHLSAFIALLMHDPDL

>tr|Q87FH8|Q87FH8_VIBPA Putative transcriptional regulatory protein OS=Vibrio parahaemolyticus serotype O3:K6 (strain RIMD 2210633) OX=223926 GN=VPA1701 PE=4 SV=1

MEYSDNIIMMPFEPKRPYQEIGLVLRQELINGRYVVGDRLPPERDIAERLDVSRTVVREA

IIMLELENLVEVKKGSGVYVINIPSQPNSRENVISDDAGPFEMLQARQLLESNIAEFAAM

QVTPGDIVKMRAALELEREELASGTADCNGDEKFHMCIAEATQNSVLVDMLKQSWERRES

SPMWKKLHSRIAGQDYREEWLDDHAKILAALQRKDPIAAKNAMWQHLENVKQRLLELSDI

DDPNFDGYLFSSNPVVLQGAENG

>tr|Q87H60|Q87H60_VIBPA Putative membrane protein OS=Vibrio parahaemolyticus serotype O3:K6 (strain RIMD 2210633) OX=223926 GN=VPA1105 PE=4 SV=1

MKRTWIWLLPLLLFAGQTLANTVWIVPVKGAIGPANSDYLTREIEQAQINGVSLVILKMD

TPGGLDSAMRDIIHAITTSTTPIATWVGPSGSRAASAGTYILLASHVAAMAEATNLGAAT

PVAIGGAPQAPSSDDGKDKEASDENKPNAEKSGDQVPAKTAMEKKVINDAKAYIKGLAKL

HDRNAQWAEKAVSEAASLDAEEALELNVIDVIANSPEELVTAINGKSVKVNNLPVTLSLD

NPIWVERTPDWRAEMLAVITNPNVAYILMLIGIYGLLLEFYNPGVGLPGVLGGICLLLAM

YALQMMPVNYAGLGLLLLGIALMIAEAFSPSFGILGLGGVVAFVLGSIFLMDSELPGFQI

ALPLIFGIAIFSIALILLTIGFLLRIRGKGVTTGLETYPGKHAVVSDDFIDGQGRVQFEG

AFWQAKTEQDQELKQGESVTIVHVKGLTLTVKPNDRPKE

>tr|Q87QH6|Q87QH6_VIBPA Phage shock protein A OS=Vibrio parahaemolyticus serotype O3:K6 (strain RIMD 2210633) OX=223926 GN=VP1173 PE=4 SV=1

MGIFSRFADIVNSNISALLDKAEDPEKMIRLIIQEMEDTLVEVRTNSAKAIADKKELARK

VEMLEEQIGEWGQKASLALVKEREDLARAALIEKQKLEQVVKGLHTEQTLVEETINKLTG

EIGKLENKIAETRAKQQALAIRNQTASNRRDVQKHLHTSRTNEAMAKFDQYSRKIDEMEA

EADLYAQTGNAKSLEQEFAELQAQDEIEKELAKLKEQMSSQDK

>tr|Q87S54|Q87S54_VIBPA Histidine protein kinase PhoR OS=Vibrio parahaemolyticus serotype O3:K6 (strain RIMD 2210633) OX=223926 GN=VP0570 PE=4 SV=1

MVERLTWKKLAWELAFFYTPWVIVGWIFGYMPWLLLAATALQLVWHLHNQVRLSSWLWDE

KRLTPPSGSGNWESLFNGLYRLQQRQRRKRKELTNLIRRFRNGAESLPDAVVVFRAEGNI

VWCNRLAQHLLGFHWPEDSGQPISNLIRTPDFIKYLNKKDFSEPLEMRSPLNVERMLELR

IVPYTEGEHLMVVRDVSQLKQLEGMRRNFFANVSHELRTPMTVLQGYLEMTEDPDMIVGP

MWTKAHGVMTEQLNRMNSLVNQLLTLSKIEAAPMHELEDVVNVPAMLEVLEKEAISLSGD

DQHKLKFDVDTSLRVLGDDDQLRSAISNLVYNAVKYTPPGANIHVRWYQTAQGACLEVED

SGDGIEPQHLHRLTERFYRVDKARSRDTGGSGLGLAIVKHALSHHDSHLEIQSEVGVGSK

FSFVLPSRLVVK

>tr|Q87MN0|Q87MN0_VIBPA Uncharacterized protein OS=Vibrio parahaemolyticus serotype O3:K6 (strain RIMD 2210633) OX=223926 GN=VP2201 PE=4 SV=1

MLLSVLYIIGITAEAMTGALSAGRRKMDWFGVMLVASATAIGGGTVRDILLGHYPLGWVK

NPEFLAITCVAGVLTTGLAKWVIKLKGLFIRLDALGLIVFSIIGTKIAMGMGLHPGICMV

SALVTGVFGGLLRDLICRQTPLVLHEELYASIALIASGLYLTLLEFSVPDVTATIVTLVV

GYVLRMAAVRFKWRLPSFQLETESSLH

>tr|Q87SR1|Q87SR1_VIBPA Putative two component response regulator transcription regulator protein OS=Vibrio parahaemolyticus serotype O3:K6 (strain RIMD 2210633) OX=223926 GN=VP0361 PE=4 SV=1

MKILIVEDEHKAGEYLQKGLIESGYVVDLVHDGVDGLYHATSEEYDLILLDIMLPKLDGW

QVLNTLRSSGIHTPVIMLTAKEQVEDRVRGFELGANDYVVKPYAFAELLARVQNVFRHHI

AAQVVASPQTLRVADLELDMIKRVATRAGQSMSLTAKEYALLELLMRKTGQVLSRTTIAS

LVWDMNFDSDTNVIDVAVKRLRSKVDKPFDRPLIHTVRGMGYKLEESRDA

>tr|Q87IF5|Q87IF5_VIBPA Uncharacterized protein OS=Vibrio parahaemolyticus serotype O3:K6 (strain RIMD 2210633) OX=223926 GN=VPA0652 PE=4 SV=1

MLKQMKIATLSLIALIASPMSLAHDYEAGKIHIDHPWSREAPPNAPVIGGFFQLTNHGDT

EDALIAAESPIAGRVEIHTHTNEDGVMKMIKIDEVRVAAHETVVFKPGSFHLMIFNPTQT

LQEGDRFPMTLTFKNAGKIDVEMAVEKKGHMEKHMHH

>tr|Q87MG1|Q87MG1_VIBPA Putative SAM-dependent methyltransferase OS=Vibrio parahaemolyticus serotype O3:K6 (strain RIMD 2210633) OX=223926 GN=VP2294 PE=4 SV=1

MKPALSNKTLPHPSTWSAMNNGPWVLESIQTRLDEWCPKLFGYHMLKLGGLSCELTSCNC

NIQHQVNVDIQNPLHNVIADGYELPFLEKSFDVVILAHQLDYASDPHRLLREVDRVMMDD

GCLIITGFNPISFTGLASLFPWRKNNLPWSGRMFTSSRINDWLGLLNYQVIHCDRYALFP

MTRYRTMWTWLENSLGDWASPAGSLYYIVARKRTYPLKPIKPHWRLKKKLTPLGVMNREG

FGVKRVSSQRY

>tr|Q87JE1|Q87JE1_VIBPA Putative exported protein OS=Vibrio parahaemolyticus serotype O3:K6 (strain RIMD 2210633) OX=223926 GN=VPA0312 PE=4 SV=1

MNMKSIAIATAVVAVLAGCKDKTPPAPTQANITLVEAVTETVKNPHQVCKDVVVTQQVEP

TDDNKIIGTVGGAAAGAALGNQIGGGSGKVIATAAGTIAGALTGRKIQENVQKGDVITTT

KKQCHTEYTTSEKVVGYDVTYEVAGAPTTVRMASKPATKTFPIQDGKVVLPQ

>tr|Q87H24|Q87H24_VIBPA Gamma-carboxymuconolactone decarboxylase OS=Vibrio parahaemolyticus serotype O3:K6 (strain RIMD 2210633) OX=223926 GN=VPA1141 PE=4 SV=1

MENTRFQTGLEQLSKIDGEAGQKVIESLNDICPDLAKFTIEYPFGDIYTREGLDLKSREI

ATVSALTAMGNCTPQLKVHLHAALNVGCTEEEIKEVIIQMSVYAGFPAALNGMFAFKEVL

SERQV

>tr|Q87NF2|Q87NF2_VIBPA Putative amidase OS=Vibrio parahaemolyticus serotype O3:K6 (strain RIMD 2210633) OX=223926 GN=VP1916 PE=4 SV=1

MASSARIVSVFARVLIMTALMVPFCSFSTVVSLDASAQTEQEGAVEQLSVEKMIHYPTVI

DQLYQSTNYRLNWENESDVEQFIFQMKLVALADVSKEFDNQLHRINQVRWKGDNLDFDLV

MTDSLLMYLSYLEQVPQEGVNWLFANKAHVSFPAPSVDTLSVLSNEITVGKLDQFLASLR

SPLQMDASFNTVFASLSEFSQYQYPFYEQKGLSRVGDPIENKSTLVERMAIVGVDVSYID

SEIPQYDENLELAVKEFQRIHGLNQDGVIGPNTIRWINFSPQQRLHLLALNAERSRIWAK

ERDNVVFVNVPGYEVTYWHDGQPLFESKVVVGRASRKTPIMTGTLDSVILNPTWNVPWKI

MVKDIIPKVKRNPMYLMEHNIQIIRSWTSREIIDPTTINWATVNPRTFPYRMRQASGSHN

ALGLYKFNMPNPQAIYLHDTPSKNLFQQDRRAFSSGCVRVENADQLAELLFKTQGLEERL

EKKRESARSSTTSVPLGERIPVHIIYQTAWLEEGTLYYRDDIYKYDHQG

>tr|Q87HC6|Q87HC6_VIBPA Uncharacterized protein OS=Vibrio parahaemolyticus serotype O3:K6 (strain RIMD 2210633) OX=223926 GN=VPA1039 PE=4 SV=1

MSIKEIGRVFTQRWFLGLVGVAACSIFIWVVGPLITVAGYEPLKSDFQRLVTILVIVFAW

ALINLTKQHKQKVREDESIQTLLEVDSQSDKEAASEIDVMRDRIEQAIKVVTKTHKGKRS

LYDLPWYVLIGPPGTGKTTVLKQSGLEFPLTESLGADSIAGVGGTRHCDWWFANKAVLID

TAGRYTTQDSQEKVDSKAWHGFLGLLKKYRTQRPINGAIVTVSLASVMSQTRTERSLHAR

SIKSRLQELKNQLGMQFPIYVLLTKMDLVAGFNEFFADLSKEEREELFGFMFPREVDDER

GVISLFNKEFHGMLERLDAHMLRILETEDDLEKRTLIFEFPKQLRVLQANLDEFLSEIFA

QNTFEEPALIRGVFLLSSVQEGIPVDRLMSESTNGLGLGRLPLATNVNSSHSYFVKNLFE

RVIFKEQLLGTVNRHYQKQSGWMRTGIYVGCVGVLVGASALWFLSYQWNSKLIVDTNSQV

NHIEAMIGAESLDFESDVISAVDTLDKIMMLPLGKNSKYGHSDAVKKFGLYQGDKVSQAA

NNAYSDALSQHFATLLSESLVSEMEANKQHREYLYETLKTYLMLFNPEKYQQEEVIAWFN

FYFERQYPGELNKELRERLLVHTKNLLENDEKGFSMDATAISAAREVLTQMSLPERAYQR

MKMQFAKSHVPSFRLTDVLGPKGLEQFERASGKPLSQGISGFYTYNGFHSIFQIQINRTV

KGLMEENWVYGDDLKAHEIDHDSAIQGVQARYYQDYVNEWKTLIEDIQLKQAPSLALATE

QSRVLSGVERPIESLLRAIQKEVGLSKVTLSENQKAATEVAGKVAKVKFSNTADKLDMYL

PEENGFNVALPGKEVESHFTEILRLSEQDFDDIQQAMVNLRSYLSDLSSSGNNQKIAYKS

ILDGTVTQDVAASFARAKDLLPKPFNQWLGELSQESVKFAESGSKDHLNQLWMTNVVRPY

QRTIAGRYPFEPNATKEVRLKDFQRFFGYGGTLDSFFQEYLEPFVDTSKSRWRLEKEIGV

RPETLAVFQRAKRIRQSFFESDNSLRVEFGMKPVYLDQHITRFVLELGDQDLVYKHGPAR

SKELRWPSGQDQTRIVFTPPESKREIAHTYEGEWGIFKLLDQSLKARPESRNDNIVMIDL

KGNKVQLELIPSSAINPFWSNEMERFRCPQTL

>tr|Q87HG6|Q87HG6_VIBPA Alpha-amylase OS=Vibrio parahaemolyticus serotype O3:K6 (strain RIMD 2210633) OX=223926 GN=VPA0999 PE=4 SV=1

MKTFKLKRTFLPLTLLLSAPAFAGQNGTMMQYFHWYVPNDGALWTQVESNAPALAENGFT

ALWLPPAYKGAGGSNDVGYGVYDMYDLGEFDQKGSVRTKYGTKAQYISAINAAHNNNIQI

YGDVVFNHRGGADGKSWVDTKRVDWDNRNIELGDKWIEAWVEFNFPGRNDKYSNFHWTWY

HFDGVDWDDAGKEKAIFKFKGEGKAWDWEVSSEKGNYDYLMYADLDMDHPEVKQELKDWG

EWYINMTGVDGFRMDAVKHIKYQYLQEWIDHLRWKTGKELFTVGEYWNYDVNQLHNFITK

TSGSMSLFDAPLHMNFYNASKSGGNYDMRQIMNGTLMKDNPVKAVTLVENHDTQPLQALE

STVDWWFKPLAYAFILLREEGYPSVFYADYYGAQYSDKGYNINMAKVPYIEELVTLRKEY

AYGKQNSYLDHWDVIGWTREGDAEHPNSMAVIMSDGPGGTKWMYTGKPSTRYVDKLGIRT

EEVWTDANGWAEFPVNGGSVSVWVGVK

>tr|Q87IE3|Q87IE3_VIBPA Putative transcriptional regulator, AraC/XylS family OS=Vibrio parahaemolyticus serotype O3:K6 (strain RIMD 2210633) OX=223926 GN=VPA0663 PE=4 SV=1

MIIILRYNKQVFYKYKGDSVMRNRTSVMRKRQMQEVTAKRTKLGKIAMTQKLEATEKQFI

VTQGQPCQTQLAEGKFLSYQYNDQIFVHGGRCIELVDSNIVSTAHASLLITILLEGKLSF

GYDDLQFDLDASDTPQGVVVNLIHPANFRRSLIENNQINKINILVKPQWLEYRSEESCCS

TAFFETHQAFYHLELNQALTELATELTNRDTPHRFQDKLEIESLIYQILFHATQQMPQDF

CPQNTNSYRDKITERHSDSRVENIVSYIETHLEQELSLEHIAEVFSMSVSNLQRRFKQSL

NMTVNGYIRYRRLEIARVHLERGLVSITEAAYEAGYHHPSNFTNAFKKVFGVPPHEIVKE

>tr|Q87HA8|Q87HA8_VIBPA Putative nitrate transporter OS=Vibrio parahaemolyticus serotype O3:K6 (strain RIMD 2210633) OX=223926 GN=VPA1057 PE=3 SV=1

MNRSRLVLLPVIGLLIFLAMWHLAAREVQTSLGTLPGPVQTFQQFSNLVDDHWQEREKEQ

AFIERQEKRNAAKLAKNPDTEVKIRPYTGKPTFFDQIVTSLVTVTAGFLLATVIAIPLGI

VLGLNQGLYQAFNPIIQLLKPVSPLAWLPIVTMVVSATYVSDDPMFAKSFINSLITVALC

SLWPTLINTAVGVTSVDKDLINVSKVLQLSWWQHIRTIVLPSAIPMIFTGLRLSLGIAWM

VLIAAEMLAQNPGLGKFVWDEFQNGSSASLGRIMVAVITIGFIGLMLDRGMLQLQKWLSW

NKQQALR

>tr|Q87RB7|Q87RB7_VIBPA Uncharacterized protein OS=Vibrio parahaemolyticus serotype O3:K6 (strain RIMD 2210633) OX=223926 GN=VP0880 PE=4 SV=1

MPATSRFYEQHADCLAEQYTSLKFEDVHASWRAFWPQARDRVLDVGAGSGRDAKWMAEHG

CEVIAIEPAQALLRFGKSYCGDSVTWLEDGLPKLEKTINLGFRFDLILVSAVWMHLAPTH

RERAFRKLANLLAPNGRLVISLRHGDFSDGRKAYPVSVEELEKFAKNRALMVRNVSLDDD

QLQRTGVSWQTIVMSLPDDGSGDLNKVRHIIVNDSKSATYKLALLRTLLRIADAHSGAVV

DRSDGKVAIPLGLVGLYWIRQFKRLIDKENIQQNSNSSKGLGFIKPEGWGRLSHLSPDDL

AIGATFFGEDAAALDKAIKDSLKTIKDGPVTFTYQGDKSNPYFEMIRSAKKPKSSIVVDS

EFLKSYGLFVLDESLWDCFRLYNSWIEPLVVNQWIMEMQRFRSNQERGIPLQTYHDCLVW

IDKDHDTRVVRKRIEQLKLSHCDIVSVWSGSKLRNEYHVDHCLPFAYWPNNDKWNLLPTS

KAENLNKRDRIPASYRLNASKPRILDWWQLAWGETDSFSRTFFTEASLSLPNVPASCQDF

EHVFEAMGLQIRGVKSRLCIAEW

>tr|Q87FS2|Q87FS2_VIBPA Uncharacterized protein OS=Vibrio parahaemolyticus serotype O3:K6 (strain RIMD 2210633) OX=223926 GN=VPA1606 PE=4 SV=1

MAKLLQVDFEFHGPFGEEMSNTLVDLAKSINQEPGMIWKVWTESEKDKLGGGIYLFEDEA

TAQAYLEMHAARLRKMGVVEVRGQIFDINVPLTTINQGPIGG

>tr|Q87J81|Q87J81_VIBPA Phosphoenolpyruvate synthase OS=Vibrio parahaemolyticus serotype O3:K6 (strain RIMD 2210633) OX=223926 GN=VPA0372 PE=3 SV=1

MFLEKDMQKNTLWFNGLSMDDVDKVGGKNASLGEMVSNLANVGVSVPNGFATTSYAFNQF

LDHEGLDERIHQLLDELDVDDVEALRKTGATIRQWVLQAPFPADLEQEIRNNYEELIEGN

TELSVAVRSSATAEDLPDASFAGQQETFLNVKGIDAVLEATKHVYASLFNDRAISYRVHQ

GFDHRGISLSAGIQRMVRSDKASSGVMFTLDTESGFDQVVFITSSWGLGEMVVQGAVNPD

EFYVHKPMLEAGEHPIVKKTFGSKLIKMIYSNNQEIGKQVDIIDTSEEERNTFSLNEEEI

KELAKQAMIIEKHYQRPMDIEWAKDGIDGKLYIVQARPETVCSQTEQNVIERYELNNKAD

VLVEGRAIGQRIGKGPVRLVDSLDQMSLVQEGDVLVTDMTDPDWEPVMKKASAIVTNRGG

RTCHAAIIARELGIPAIVGCGDATSKLTDGATVTVSCSEGETGYVYQGDLDFEVKRSSVD

ELPLLPTKVMMNVGNPDRAFDFAQIPNEGVGLARLEFIINKMIGIHPKALLNFDAQSDEL

KAEIKQRIRGYKDPIDFYVSKLTEGIATIASAFWPKRVIVRMSDFKSNEYSNLVGGKAYE

PHEENPMLGFRGASRYISPVFEDCFELETQAIKRVRNEMGLKNVEIMIPFVRTPSEAASV

IDLLAKFDLRRGDQGLKVIMMCELPSNAVLADEFLKYFDGFSIGSNDMTQLTLGLDRDSG

DVAHLFDERNAAVKIMLKMAIDAATKAGKYVGICGQGPSDHEDLAEWLMEQGISSVSLNP

DTVIDTWLQLGKVSK

>tr|Q87G61|Q87G61_VIBPA Uncharacterized protein OS=Vibrio parahaemolyticus serotype O3:K6 (strain RIMD 2210633) OX=223926 GN=VPA1456 PE=4 SV=1

MKKGVHLVFILVASLLSSFSVMAQSLQASVNKTEVAKNEVINLRIMADSEVASDAIDFSV

LEKDFFLGQPRYGRSSNNINGRKYQRTEWSISIAPMKEGIITIPSFSADGMKTEPIQLKV

TANKSAPDLDDLFSFNMSVDNHTLYPQQSANLRMQLIIKADTRRLDNPQVVPPRIEGMKL

EPVGEMQQGQRVIAGLEVTVVEQSFRLTAEQPGTFTLLGPQLTGSYIYGDSLTGSTKIMP

ISTKVEQMPITVKAIPSEFKGSWLPGSALQMTQSWQDDQGNTLSANTVNNVKQGSSITRT

IQIKARGTQAEYLPRITMDYPNSLRVYPEQPQFDTARDGTVIMTVKQVLIPTEAGEFTLP

GYTLNWWDSKSDEAKQANLSELKLNVEQSDAGLITLPETALPIPTVSNAAPAQQNGVDKL

WQTLTFVFAGLWILTSAIAFVIWKKRPVASTEPLLVSERPLCVEALKCIINEGDEAKIER

SVNEYLSTHRDRLNPEDVQAVKLELDAMNRARFSSNQQPWSHKALLEKVQKLAKAKRDKS

SHQLEKL

>tr|Q87M85|Q87M85_VIBPA RecBCD enzyme subunit RecD OS=Vibrio parahaemolyticus serotype O3:K6 (strain RIMD 2210633) OX=223926 GN=recD PE=3 SV=1

MTTNHNLHSGQDSLLVVLERLAHKGAIRQLDYQFACFIDSQTHDEQSDSQALAFIAGVVS

SELGKGHICLSLFDAQGQSTDLASKLGLFGESALALNTQLQGIDWIQVLKNSTVVGAQGE

ALPLMFDGERLYLHRYWHYEVTLAEKLNQLGAAVNLQAQEFSRLSELLNHLFARQYHFLF

NALGKAVEAGSSNQVLRQQLVCDHLDVVASESLDWLAIDSVLSNARKVQDLQTLNELVPL

SACVNWQKVAAAVALTRRFAVISGGPGTGKTTTVTKLLAALIEQATHEKNLTIKLVAPTG

KAAARLTESIGKAVQELPVSPELKVKIPTESSTLHRLLGAIPNSAEFRHNKQNPLHLDIL

VIDEASMVDLPMMYKVVDALPKHARLILLGDKDQLASVEAGAVLGDICSFHALGYGKEQA

SAIAKLTGFDTLAHTGNSASSIADSLCMLQKSYRFDARSGIGQLAKAVNSGSAASVDNVW

ARDFSDIEHFALSSQHYNQMMQTLVQEYGRYLKRIEQQETDPKTGEPESLTHKAKAVLDT

FNQCRLLCAIREGDFGVAGLNQRIEKALAARKFIQVQDEIWYHGRPVMVTRNDHGLGLYN

GDIGICMRDDSEEEPRLKVFFELPDGSVKSVLPSRVPEHETAYAMTIHKSQGSEFDYTLM

ILPPDFSPILTRELIYTGITRAKKRLALYAELNVLKRGIKVKTTRASGLVQRLTN

>tr|Q87IB2|Q87IB2_VIBPA Uncharacterized protein OS=Vibrio parahaemolyticus serotype O3:K6 (strain RIMD 2210633) OX=223926 GN=VPA0694 PE=4 SV=1

MKKMILASMFALLSSSAFAAFNGPEVSVINTVKDAQNATEDSAVMLTGHIIQSLGNETYL

FKDSTGEIEVEIDNEEWMGLDVTPNDKVAIRGEIDSEWTTMQIDVDTIQKL

>tr|Q87FD0|Q87FD0_VIBPA Uncharacterized protein OS=Vibrio parahaemolyticus serotype O3:K6 (strain RIMD 2210633) OX=223926 GN=VPA1749 PE=4 SV=1

MWKLCLELSEDKTKVIARLPGEVDLGGELNQNELNEQLVAMNAERMFVVEGEVVRFINCA

KERKAEAYEGIVIAEKRNAQVEVELSNNDMLASMNVVGAYGGRGLRGPEIVQALAQARVS

KGINKLALKKVLVVSTQLKPGETFSQPVAVGKQPIKGKDALFTPLVKDITKRILKPKDGQ

SASDKIDLRNLGETIAVDVGDEVMRRTPATKGTPGFTVQGRVIPPQAGKDSPMKPGKGTE

ICKHDPNLLIASVSGTPLIKDKTIEVDNALCLNSVGVNTGHVKFKGNVIIGGNIESGMIV

KATGSITVGGFIESADVQAQDDIQVGKGIIGHTVSDGQPKSCHVKAKGSIRASYAQYCDL

QAGEDIELAVHSMNNDIVCGRNLTVLDANGRNGTLSGGDAKVGDKVVCLQLGVEGDTATK

VEAFARYSAFKERLAELKDQYKRAQEGTMDVIRKELEFKKRPKAERTDEEQAQIDVMKTQ

NNEQLEAIKQKLEQTEHDFEVALEENIVEAKERVFTRVTVQFGDEQVTTKRTHGGCSFRF

NQYEIKVSANLEEEDVAAV

>tr|Q87I43|Q87I43_VIBPA Putative sensor kinase CitA OS=Vibrio parahaemolyticus serotype O3:K6 (strain RIMD 2210633) OX=223926 GN=VPA0763 PE=4 SV=1

MPFASFSFTKQLAVLLSGVMLLGMLSWWSYSAHQLDDILTHQISLRAQVQSQQLSQLSSL

IAAVGSGSASKTSEIINAVQAVSDADFITVSDRAGIRLAHPVAERVGLPVLGGDIERALE

NGESYLSYGVGSLGPSVRYISPIFSNEGDVIGMIKVGYLIDTLDLWTSERLLPLISFGII

AVAICIWLSWKFSRYVRTQMQELEPWQLKQALKTHQGVLQATYEGLVAINSEGSLYLIND

SARAMLNYHQELGNVFTDGIDNPESFSLKGDDYINGLIRVNGKNLVMNRVTLRTSTGEPY

GAVFSLRDQNEMHVLSEKISQVTQYMENMRVARHEYQNKLSTISGLLQMGAYDKALSVCL

SQAKASQSQLDSLHALNSRPALSALILAKASKANELGVALSIDCQSDLSALSRRLSEEQL

CGLIGNLAQNALEAVKGQENGHVHIGISESACEYTIQVSNNGPLLESEFDVLCELGFTTK

QNKADHGVGMYLVRSIVEQGNGHMELDSDEQETAFTIYFPKELG

>tr|Q87HU0|Q87HU0_VIBPA Putative oxidoreductase iron/ascorbate family OS=Vibrio parahaemolyticus serotype O3:K6 (strain RIMD 2210633) OX=223926 GN=VPA0866 PE=3 SV=1

MKLETVDYLADDAAQQFVASLRETGFGVLKNHPIPQELVESIYKNWHEFFCSEEKNDFQF

NVETQDGYFPPSVSEVAKGHKVKDIKEYFHVYPWGQIPEQLKAEILEYYERANAFAQELL

GWVEEYAPKDVQEKFSIALSEMINNSDKTLLRVLHYPPMTGEEEPGAIRAAAHEDINLLT

VLPAANEPGLQVMSKDGEWIDVPCDFGNLIINIGDMLQEASGGYFPSTTHRVINPTGARQ

EKSRISLPLFLHPKPDTVLSERYTAHSYLMERLRELGVI

>tr|Q87PE5|Q87PE5_VIBPA Uncharacterized protein OS=Vibrio parahaemolyticus serotype O3:K6 (strain RIMD 2210633) OX=223926 GN=VP1565 PE=4 SV=1

MESALVNALSGLGFSSESLVLFALMGMNLKYQIAINKQLTQGLQEVRESVLVLTATRNND

N

>tr|Q87ML3|Q87ML3_VIBPA Cytochrome c-type biogenesis protein CcmF OS=Vibrio parahaemolyticus serotype O3:K6 (strain RIMD 2210633) OX=223926 GN=VP2218 PE=4 SV=1

MIAEIGHFALILSLAMAVLLSVLPLWGASNNNTMLMNTARPLSWSMFIMLFFSFVILCWG

FYTNDFTLQYVASNSNSQLPWYYRLTAVWGAHEGSLLLWVLIQAGWTVAVATFSRGMPQE

SVARVLAVMGMISVGFLLFIILTSNPFLRTLPFFPVDGRDLNPLLQDPGLIVHPPMLYMG

YVGFSVAFSFAIASLMTGRLDTAWARWSRPWTTAAWVFLTLGIALGSWWAYYELGWGGWW

FWDPVENASFMPWLAGTALMHSLAVTEKRGTFKAWTVLLAISAFSLSLLGTFLVRSGILV

SVHAFASDPSRGMFILGFLVFVIGGSLLLFAVKGAAVRVRGNFDLVSRENALLANNVLLI

AALVVVLVGTLLPLVHKQLGLGSVSIGAPFFDMLFAWLMMPFAFLLGIGPLIRWKRDQLS

SIVKPMLVSGTMSLALAAVCVFLFADFFSVMAYIGWVMSIWIVAMHAFELHERATHRHSF

AEGVRKLQRSHWAMMFGHIGLAVTIIGIAMVQNYSIERDVRLAPGEHFQIEGYDFYFSGL

RDKDGPNYDGYIADFEITHDGKYINTLHAEKRFYRTAKSMMTEAAIDRGITRDLYIAMGE

RLDDNRSWAVRIYYKPFVRWIWAGSLIMALGGALAISDKRYRFRKSSKKNAKSNEQVA

>tr|Q87JH9|Q87JH9_VIBPA Putative flagellar hook-associated protein OS=Vibrio parahaemolyticus serotype O3:K6 (strain RIMD 2210633) OX=223926 GN=VPA0274 PE=1 SV=1

MRISDNQFSQMMLQSLQSNSAGLGKVLQQMSTRERLTKLSDDPMASIKLLNLERENSAIA

QYQSNIANLKTTLSSQETHLDSVNESLKSMRDIVLWGANGSLTDQDRSGMITELKSYRDS

IESSFNAQDEEGHFLFSGTKTDTAALNKSSGAYVVEGNSDVRVVTVAKGVTMDSNMTAQE

ILDIGGGKNVLNQIDALIAEFEKPSPNFQAEVDASLNAIDDTMANVLGAMTEIGGRHNNL

DLMDGAHSENKLFVDKVSGDLSALDYGEASVRLSNYMAALQATQASYVKINDLSLFDRI

>tr|Q87R97|Q87R97_VIBPA Putative oxidoreductase protein OS=Vibrio parahaemolyticus serotype O3:K6 (strain RIMD 2210633) OX=223926 GN=VP0900 PE=4 SV=1

MKTEHKQVVVVGAGPSGSTVSALLKSRGIDVVVIEKATFPRFSIGESLLPACMEVVELAG

MTEAVKQHGFQFKDGAAFRRNGVYTHFDFTDKFTAGPGTTFQVQRASFDKVLADSAAEQG

VDIRYQHELVSLAFEGKKSRLEVVGPDKQAYQIEADFVLDASGFGRVLPRLLDLEEPSCL

PPRKAIFTHIEDHISPQEPEYDRNKILISVHPENHDVWYWLIPFSNGTCSFGVVGEPEFF

EQYPEDKLAALQQLANEEPGLASLLRNAKYPNPVGELGGYSANVKRLATEHYALLGNAGE

FLDPVFSSGVTIAMKSAQFAADCVVRQLNGEVVDWQEEYSERLMVGVNTFRTYVEGWYNG

TLQDVIFYQAPNPRIKQMISAILAGYAWDTENPYVKQSEQRLSTLAELVRGEGF

>tr|Q87PA2|Q87PA2_VIBPA Uncharacterized protein OS=Vibrio parahaemolyticus serotype O3:K6 (strain RIMD 2210633) OX=223926 GN=VP1615 PE=4 SV=1

MLSAVRRANLYLNLFGFSKVPLIWLCHPKIIAIDSKHVEVRIPLRRRTKNHLNSMYFGAL

AVGADVAGGFLAMSKAQEKGEAISLAFKGVKAEFLKRPEADVHFVCNDGHVIDEMLDHTI

ATGERVNRDVKIIALCPSLHGQEPMAEFALTLSIKKAAQHTKKAA

>tr|Q87M34|Q87M34_VIBPA Uncharacterized protein OS=Vibrio parahaemolyticus serotype O3:K6 (strain RIMD 2210633) OX=223926 GN=VP2424 PE=4 SV=1

MFRLKLKGCHMERVTKAYWIGEPLHTLPEEFKAEFEQHFDLLDCSNDISVLSDKEFCYFF

YYITHDPTDHAKSIATLCENLDKKLIVVTKENTECCLPPSHITAECYELNENTLPVWLSQ

AKLKYFLHAKVDDAQISADNIFSRNGKSNFSDVVNYIANNVHKDLREEEAAALCHYSPTY

FSKVFHRKVGMCFRDYVTAKRISLAKKMLIENESMKIAYIAYQCGYRDVSYFSRIFKKKT

GLSPANYRQQF

>tr|Q87S49|Q87S49_VIBPA Phosphate transport system permease protein PstA OS=Vibrio parahaemolyticus serotype O3:K6 (strain RIMD 2210633) OX=223926 GN=VP0575 PE=3 SV=1

MLNWIRSGAPWIWLTGGAVSISLLSVLGLLLLIGWKGLTYFWPAPLYQWNVTSLTPVQGE

VLHENTILIGQIYERSFVPRSYLPVDAVKKLGEDEDFATRLNIKIANRELYPADFISVLQ

MQLDEPTTPKEWAVIERSSGGYFFGKLVAFQDGDKLYQTDIQTVLNKKLDDAETLRHEID

SLVVDQLKDLGWKLEQLRLDKRKHELNNTVTDDFLAQNLQQKEQVEQELAKLDLQLDGLR

LQLSGYALIVEDMTGSHVSIPLEDILDYWYPNQMSLPDKVMHWGKQVWKFLSEDPRESNS

EGGVFPAIFGTVFLVLIMSIIVMPLGVVAAIYLHEYAKNNALTRIIRIAVINLAGVPSIV

YGVFGLGFFVYTIGASIDNVFYAERLPAPTFGTPGLLWSALTLAVLTLPVVIVTTEEGLT

RIPSSVRHGSLALGATQFETLWRVVLPMATPAIITGLILAIARAAGEVAPLMLVGVVKLA

SSLPVDGQFPYVHLDRKFMHLGFHIYDVGFQTSNIEAARPLVYATSFLLVTVIVGLNLTA

ISIRNNLREKYRTLGQD

>tr|Q87QC6|Q87QC6_VIBPA Uncharacterized protein OS=Vibrio parahaemolyticus serotype O3:K6 (strain RIMD 2210633) OX=223926 GN=VP1223 PE=4 SV=1

MKKVLVLGASGYIGSQLLPQLLDKGYSVTAASRHIDYLESRTSPHPNLTLTYLDLADAES

TLSLVDDFDLVFFLVHGMAQGDDFVEYELNLALNFKYALEQSRVKHVIYLSAIQPQTGNS

RHLAARKATGKIIRQSGVPVTELRAGVIIGPGSAAFEIMRDFVYNLPILIAPKWVDSKAN

PIALPNLNHYLLQLAEASPPEESQIYEVGGPDTLSYREQFKIICRITNKPYRLWSTPLLT

PQMASYWLGLVTSVPANIGKALLAGLEHDYIADSKAIEARFPQTLISYEQAVSDTIQDEG

TFVRSNVWGFEPAALRRWQPGYGYYPKQAGASIKTQASAAALWKVAQKIGSREKGYYFAN

ILWRTREWLDIFFGGGKPIRVSPPGPELKVGDYIDSWKVIRCEEDQFLSLFFGMKGPGLG

RLEITIKDHGDERELDITAWWHPQGFLGLLYWFAMMPAHLFIFKGMVKAIAKEAESISIT

PQEEQSLENLEK

>tr|Q87IF0|Q87IF0_VIBPA Putative cyclic nucleotide binding protein OS=Vibrio parahaemolyticus serotype O3:K6 (strain RIMD 2210633) OX=223926 GN=VPA0656 PE=4 SV=1

MKISPYPTGRFKNYLEQKSAAFRDLIYQCQVNTRYFDAGEAILRQGEQLQYLYIVPVGRV

SMSILAANGRRFQLGEANCDYHIYGEMEYFTQTPCQWNVVADEHMQVDVICIQKLTEALQ

QHPEMMVFFASALAEDYQDSMDIYTNRLLHPITYNIAYDLLVRNQTDTLLGGFDKVNQEA

ERFGTSGRVYRRAVKDLIDKGLIAKTDNGLVILDEMALKAFIDSHE

>tr|Q87HF4|Q87HF4_VIBPA L-allo-threonine aldolase OS=Vibrio parahaemolyticus serotype O3:K6 (strain RIMD 2210633) OX=223926 GN=VPA1011 PE=4 SV=1

MDFRSDTVTQPTQAMREVMFSAPVGDDVYGDDPTVNELEQFAAELAGFEAALFTTSGTQA

NLLGLMAHCERGDEYLCGQQAHNYKYEAGGAAVLGSIQPQPIENNPDGTLPFDKLEAAIK

PDDAHFARTRLLSLENTINGKVIPLSYLAEAREFVNKHNLKLHLDGARVFNAAVALDVPV

KDIGQYFDSMTICLSKGLAAPVGSLLLGSKEYIAKARRLRKMVGGGMRQAGILAAAGKLA

ITEQVAQLKQDHINAKALAEGLAELPGFSVNPDFVQTNIVFAKLDAKVDIQSIAEQLKQQ

NIIISPGNPIRFVTHKDISAEDIQTFLSSLKSLL

>tr|Q87N80|Q87N80_VIBPA Macrolide export ATP-binding/permease protein MacB OS=Vibrio parahaemolyticus serotype O3:K6 (strain RIMD 2210633) OX=223926 GN=macB PE=3 SV=1

MSNKSLVELTNICKYYSSGEAEVRALDGVDLTIHQGEFLSILGPSGSGKSTLMNMLGCLD

KPTDGEYQLGGQNVASLSANELAGIRNQKIGFVFQSFNLLEYATALDNVALPLVYSGIKA

KERRQRAAKLLEQVGLGDRLDHKPNQLSGGQKQRVAIARALVNDPQIILADEPTGALDSK

SGAEIEALFNQLHAEGRTLIIVTHDNALAERTKRIITIKDGKVISDRAPIGKENAISHC

>tr|Q87QD2|Q87QD2_VIBPA Uncharacterized protein OS=Vibrio parahaemolyticus serotype O3:K6 (strain RIMD 2210633) OX=223926 GN=VP1217 PE=4 SV=1

MAYDHRYVCLFCAFSTVLLDFRKKGVMSNYTKEIQNLVNSALNELEQEHRSGKLANAPVA

NNHYLVRWVTKALKSQRFGRTVGDDLTRWQKAGRSKGNDAGLLFIFKRISAFYAQFFPEG

EDSKVIKDSDVEAFLDVMEQAGWEVSTSEQLVGCGKIQIFTEGQNSLALCADQCESCFDG

ELLVKPMSWFVRGNHAEFVEKAAQAGFMVHKVTDYKSMVKYHGEYLIFPANEGNQLAEIP

LSFQA

>tr|Q87S69|Q87S69_VIBPA Chorismate mutase/prephenate dehydratase OS=Vibrio parahaemolyticus serotype O3:K6 (strain RIMD 2210633) OX=223926 GN=VP0555 PE=4 SV=1

MTDQPISLEEIRLRLNELDDQLLSLLSERRKLSIEVAKSKVQTSKPVRDAVREQQLLVKL

ISNGRDKYELDAQYITKLFHTIIEDSVLLQQGYLQNLVNPQQSRKPLARVAFLGAKGSYS

HLASREYFSRKNTELIELNCEHFKEVTRTVESGHADYGVLPIENTSSGSINEVYDLLQHT

TLYIVGELTQPIEHCLVATKDIRLEDIKTLYSHPQPHQQCSEFLSRMKGVKLESCASTAD

AMQKVQEMNRDDVAAIGNASSGKLYGLQAIQGNIANQTENHTRFIVVARKPVEVSTQIPA

KTTLIMSTSQEAGSLVETLLVLQRYGINMTKLESRPIMGNPWEEMFYVDLEAHLGSTEMQ

QALQELTKITKHLKVLGCYPSENIKPTQVKLS

>tr|Q87JF3|Q87JF3_VIBPA Hypothetical signal peptide protein OS=Vibrio parahaemolyticus serotype O3:K6 (strain RIMD 2210633) OX=223926 GN=VPA0300 PE=4 SV=1

MNLKAVPKALLVTLLASSLPISSVAYAAPNANESSLVQQQWEGNWPTQNEAEKLVQELYY

QRAISAYYQMQPAMNVIGMRDGSESKFGKGYNVLPIWKDRMDSRTLVPTPNADVVYSMNY

LDLKETGPLVVAAPPKVIGMFTDFFQRTITDVGLIGPDRARGGLYLLLPPNYDGPVPKGY

FTFTSSTNNVFLFFRTVMKKGENGADPSDAVKIAEQTRIYPLWDEEKNIQPMKFPNASGQ

RINMMYPTDFSYWEKMKQFVDEEPISAISPELRGVLASIGIVKGKPFNPTVEEKQLLEKA

VKTAPKMITAQRLLGRKDERNLYYKDRQYERAWAGATSEYMQESYLDIDQRAAFFQYAYS

SAPAMVMRTMNAGSKYPFTVRDSKGDILNGSHQYKLHLPANPPAKLFWAVTLYNVVDGTM

PETSQLLPSKNGFDNIEKNTDGSIDLYFGPQLPNGAPESNYIKTIPGRDFLTAIRLYGTG

IEFFDQTWKPDDVLKIK

>tr|Q79YX8|Q79YX8_VIBPA Bacteriophage f237 ORF6 OS=Vibrio parahaemolyticus serotype O3:K6 (strain RIMD 2210633) OX=223926 GN=VP1557 PE=4 SV=1

MEYIYSALEFIANIGQTFLDFFDVAIEWIKNAFEYGAMWLISVWLDIKIASIQIALKIAQ

LLLEEYGVYTLVEDRFNALPSDVRYILTEYGVTSGLRVIFDAFATSLVMRFFNW

>tr|Q87FY5|Q87FY5_VIBPA Putative flagellar motor switch protein OS=Vibrio parahaemolyticus serotype O3:K6 (strain RIMD 2210633) OX=223926 GN=VPA1535 PE=4 SV=1

MNTAQLTSKQTLSYVEQTALVLLGMGEDAAAKVLQHFTRDETQRVTRAMAKLNGIKSDSA

RGVIQNFFEDFREHSGIRGASKEYLSNTLRKALGNDLAKGLLNNLYGDEIRNNMQRLQWV

EAETLARFIVNEHPQMQAIFLAYLPADSSSAVLKHLPQDYHDEIIFRIAQLQDIDHQVAT

DLHELVERCIEKVSASQSVPLSGVKQAADIINRFEGDRGSLMEMLKLHDEEVVNAIEENM

FDFMVLGRQREETMDMLVQQIPLELWATALKGSDITLQQAIKRSMPQRMVKALEDDMEAR

GAVALSRVQKARQDIMQMVRELDESGEVQLLLYEEPTVE

>tr|Q87S75|Q87S75_VIBPA Cyclic diguanosine monophosphate-binding protein OS=Vibrio parahaemolyticus serotype O3:K6 (strain RIMD 2210633) OX=223926 GN=VP0549 PE=4 SV=1

MTEKRRFSRIIYQAPAQLEQRDLRLNATIQDLSLHGLLLQAEGLDTLNPALLVDVGFSFH

QSEQEITLTAQIVDVTQDQIRLKITNIGIESISQLKRFIELNVGNNELLNRELDHLSDLG

EK

>tr|Q87K01|Q87K01_VIBPA Putative secretion protein OS=Vibrio parahaemolyticus serotype O3:K6 (strain RIMD 2210633) OX=223926 GN=VPA0097 PE=4 SV=1

MSGDIAKHSSSRSKLVKTLFFTLCVIAFGAMYWSWQYSDSHPSTEDAYVRAKILSVAPQV

KGQVVSVDVKDFQSVNKGDLLLKIDSRPYLLAVKQAKAAYQLAVQQHDVADKQVTEAVAG

LDAARSNLTEAQVEYKRTDSLVKRKLASDQDLDTAKNKLANAQASLEQARATVEKAIANR

GEEGAEAAVVQQAAAQLAQAELNLSYTDITSPVDGIAGEINTHVGSVVSVGQTLFPVILK

DSYWVRANFKETDLTHIKPGMHADVVIDMYPDVVWKATVEQLSPASGTSFSLMPPENATG

NWVKIKQRFPVRLALEVPADAPQLRVGASSEVTIDLQSSVQ

>tr|Q87FT9|Q87FT9_VIBPA Transcriptional regulator, LysR family OS=Vibrio parahaemolyticus serotype O3:K6 (strain RIMD 2210633) OX=223926 GN=VPA1589 PE=4 SV=1

MKALNDLNIFVETARQGSFSKAANSMDMTPAAISASIKRLEGQIGFPLFVRSTRSLRLTS

EGELFLDKTTQALATLQEGLDQISSARGELSGQLHITAPSDFGRNMLLDWVDEFIDLYPN

VSIKLELSDSLTDMYTKPVDIAIRYGEPADSNLVVLALCGSNERILCASPEYVATHPELT

SPADLTQHNCLCYMVADSVYNKWTLTRDGEKEQVVVSGNLMSNDSDLPHRLAIKGKGIAN

KSLMDISRDLIEGRLVRVLPEWDSGPVPLYMVCADRRLLTPTIRTFRDFIQQKCCQQRAN

VLATFCH

>tr|Q87FR6|Q87FR6_VIBPA Uncharacterized protein OS=Vibrio parahaemolyticus serotype O3:K6 (strain RIMD 2210633) OX=223926 GN=VPA1612 PE=4 SV=1

MSDVKVSKPMVTDIFVEGAKKGWVIAITSTVPNVLMAFVIIKALQITGALDLMGSVFSPV

MAVFGLPGEAAAVLIGAWMSMGGAVGVVITLFDQGILNGTHIAILAPAIYLMGSQVQYMG

RIMGPIGTEGRYIPIMIAISVLNAFGAMLVMNLFV

>tr|Q87NI7|Q87NI7_VIBPA Uncharacterized protein OS=Vibrio parahaemolyticus serotype O3:K6 (strain RIMD 2210633) OX=223926 GN=VP1881 PE=4 SV=1

MIRFELGNQICVCLSENDISLEINNTLHHAIPVSSNEYAILSTIATYGSLNAPISQRVIE

RKITQHYKMALPENGFKNAVAALRKKFRKLTEDHVSPTRNIIENIHRTGYFIPFTMLHTH

QSGIYQQKRINQHTKNSVRKALRICLRNKRIYTDIAWVLLVTTAIFFSVCYYAINSIVKH

NYLDSALDIADSLSQMSCYADEDQLKGLFDNVKLVESSMMLDRFNIRCLVTPEAVVPVSQ

KAFNEWSDNSNYTTQSFDINNATILVRVKNINLQNNVESHISRFFLSGMKLYTNTGTSFE

IGNTNGRYFHYQIKDTGYKEVYYISGPLKSIILLSLFFLVILRHRSLQAFITYLFAIREF

HIKLEPIYNTSTQQNIHYEALSRFKVKNTQRFIETLISNGLLLIHTILVIRAIYAKQPTL

LVPISINVCPSLLRGRNFSTLYQELASRDCRLLTIEITENASMYYTSEIYDNVAKLKLLN

CKISIDDFGTGNNNVSLISKINPDYLKIDREFVIGLKSDDKKVETLRQLIAMGNTYRCTV

IVEGVETADSAHLLTTLGAYIHQGYFYPLHF

>tr|Q87KV6|Q87KV6_VIBPA Sodium/solute symporter OS=Vibrio parahaemolyticus serotype O3:K6 (strain RIMD 2210633) OX=223926 GN=VP2869 PE=3 SV=1

MAGLIAFMGYGGSVFLMGWTGGYVLLALLLAPYLRKFGKFTVPEFVGERFYSKTARIVAV

VCLIIASVTYVIGQMKGVGVAFGRFLEVDYSTGLLIGMCIVFMYAVLGGMKGITYTQIAQ

YCVLILAYTIPAIFISLQLTGNPIPQIGLGSTMAGTDVYLLDRLDQVVTELGFSEYTTQV

RGDTLNMFVYTMSLMIGTAGLPHVIIRFFTVPKVRDARTSAGWALVFIAILYTTAPAVSA

MARLNLMDTVNPAPGQHLAYDERPTWFKNWEKTGLLGFDDKNGDGNINYTSDAATNELKV

DNDIMVLANPEIAKLPNWVIALVAAGGLAAALSTAAGLLLAISSAISHDLIKGVINPNIS

EKKELLASRISMAVAIAVAGYLGLHPPGFAAGTVALAFGLAASSIFPALMMGIFSKNINK

EGAIAGMIAGISITLFYVFQHKGILFIADWTYLESWGSNWFLGIEPNAFGAIGALFNFIV

AFAVSKVTAETPQEVKDLVEHVRVPVGAGSAVDH

>tr|Q87TH5|Q87TH5_VIBPA Uncharacterized protein OS=Vibrio parahaemolyticus serotype O3:K6 (strain RIMD 2210633) OX=223926 GN=VP0094 PE=4 SV=1

MQWILEHQSLILSSLFAATASGVAVGWWVKQRFITQTQLLEHQLVSEKQLHQQQLEQVKQ

SLADAQQELDELDNERDKAAFEVRQSHGKLMAAMEKLRYFEAVKQERQQYFDELGQMREQ

KSRLETQLREQQARHEQMNQANAEKLQILEQAEVRLKQQFEHLANQLFEEKTAKVDLQNR

QSLEGLLSPLKEQLEGFKKQVNDSFSQEAKERHTLVHELKNLQRLNEQMTREAVNLTQAL

KGDNKQQGNWGEVVLARVLAESGLREGHEYETQVNLQSEAGKRYQPDVIVHLPQNKQVVV

DSKMALVAYERYFNAETDAERDRALNAHLTALRAHIKGLSMKDYHKLKGIQSLDYVLMFI

PVEPAFQVAIQADPSLIKDAMEQNIILVSPTTLLVALRTIDNLWRNERQNENAKLIAQRA

TKLYDKLRLFIDDMEGLGGALDRANQTYQGAMNKLATGRGNVIRQAESFRQLGVEIKRPI

SSDLAQLAQSDAFSENESPNESLVERHPAEDKVN

>tr|Q87IH9|Q87IH9_VIBPA Ubiquinol oxidase subunit 2 OS=Vibrio parahaemolyticus serotype O3:K6 (strain RIMD 2210633) OX=223926 GN=VPA0627 PE=3 SV=1

MEASRYKRILSRGSLACVILLLSGCNSALLDPKGAIGVQEKELIITALLLMLIVVIPVIL

MTIYFAYRYRASNTDEEYAPEWSHSTKIEVVVWTIPIIIIAILATITWRSTHELEPSKPL

VSDVKPMTIEVVSLDWKWLFIYPEENIATVNYVAFPKDVPVTFKLTSDNIMNAFFIPRLG

TQIYAMPGMVTKLNLIANHEGDYKGFASNYSGEGFSQMKFTASAMPDRAAFLNWVQKVKA

SPDRIEDWEQYSSLAEPSVAAPVTLFSSVPPFLFSNVVTQHPGSMNCLPENQG

>tr|Q87P05|Q87P05_VIBPA Uncharacterized protein OS=Vibrio parahaemolyticus serotype O3:K6 (strain RIMD 2210633) OX=223926 GN=VP1713 PE=4 SV=1

MKQTGKFWVLGLISSSISLACHSAPISFDTAWQLLLENNYSLKAQRANVENYQYQESATS

NLNLPQVSIGANYTRLDTDVTISGKQILDSTGTHINVPPAFQPIFGALANTTSTITERDI

FSSSIRAIWPIFTGGRISAAQSAAAGKTDEAKSQLAMEQQARFEDLSKYYFSVVLAKEVL

ATRQAVEQGLKKHRDFAIKLEEQGQIARVERLQAEASLDKAKVETRKAASDLSIAQAALG

KLLAQEDSVEPAETLFVNDNLPPLSAFIDQTLLTYPGLDILDAKHKQASSLIKAEKGKYY

PEVYLYGDYSLYEDDSLASQMKPDWLVGVGVNIPLIESTGRSEKVKAAQSVVSQVDALKS

QAKQDLSLLVQKTYLEAQQAIDEVQGLESSIALANENLLLREKAFTQGLSSSLDVVDAQL

YVASIETQRSAARFRYLISLTKLLALSSEMNSFEQYQNTAYAPAKISKEVK

>tr|Q87QN0|Q87QN0_VIBPA Putative transcriptional regulator OS=Vibrio parahaemolyticus serotype O3:K6 (strain RIMD 2210633) OX=223926 GN=VP1119 PE=4 SV=1

MDIQSVGDIYQKLNKANLHLLKDVYHQDVVFEDSAHRLEGLPALSDYFQSLYANVIRCDF

IIHDHQQIGDTGFLTWTMDLQHPKLHKGAPIAVSGVSHMKFSDGQVIYHRDYFDLGEMLY

EHLPLLGSVIKTIKQRLGR

>tr|Q87GU9|Q87GU9_VIBPA Uncharacterized protein OS=Vibrio parahaemolyticus serotype O3:K6 (strain RIMD 2210633) OX=223926 GN=VPA1216 PE=4 SV=1

MPATHTKDVLHKFLKPGMKLSINFEFGPKDKFSYSTVYLGIKENSYLILDIPMRLLEDQV

MRKLHNADVIVRGVSDTELGHVLAFKSSVLMTAVRPSPLLFIRIPGTFATKPVREHERYK

LQMDCTIDHSGNIYDGSLVDFSLAGVGVQTFIEPEFKVGDRVSVVSHLSIHIGEENPCLI

ANIKKQPKGWVIGIRFEQPITMTEDLKTKLLEQSFLTSNV

>tr|Q87NW1|Q87NW1_VIBPA Uncharacterized protein OS=Vibrio parahaemolyticus serotype O3:K6 (strain RIMD 2210633) OX=223926 GN=VP1757 PE=4 SV=1

MLMFFMSSVRRNEMKRSLLAVLISSVAFPSLATVELTDNLSLSGFGSIAWAQSDNETSLL

VNRFIDDDSCFDCDTTFGLQLDYFYQAFRASVQVVKRPQDHWSEPKVEWAYLAYTYNNVE

IRGGRLRLPVFLISEYNYVGQAFTTARPPNEVYDSILGITAYDGLSFRWNYDLNENVMIT

AMPFVGYGYSSDVTISEDTDISIDTNYSAGLNLTLSGDNYRWNFAYLNAEYDQTTVLSNA

MAPIPGQGSAVGTIRLKAEDQHIQLFSLGAKYEVDRITVTAEGQTSDISTSWYAATSYNL

NKFTPYVVYGQQFDDNEKKTGDSVLTGLRFDVDYNVSVNAEWQHFKAFNNDSGAFSLPPA

DTNANLYTVMLNFVF

>tr|Q87GN1|Q87GN1_VIBPA Uncharacterized protein OS=Vibrio parahaemolyticus serotype O3:K6 (strain RIMD 2210633) OX=223926 GN=VPA1284 PE=4 SV=1

MRTLGNIIWFLFGGVFMGLLWWLFGILAFISIIGIPWGRACFVMGNFSFFPFGKEAISRD

ELTNEMDIGTSPLGVIGNVLWFVFAGLWLAIGHVLSAAACFVTIIGIPFALQHLKLAVIS

LAPIGKTVVTSEEAAIARYNINR

>tr|Q87P01|Q87P01_VIBPA Uncharacterized protein OS=Vibrio parahaemolyticus serotype O3:K6 (strain RIMD 2210633) OX=223926 GN=VP1717 PE=4 SV=1

MTLWQLIKAEIRSVLTNPVVTLTVFGGVVFYSFLYPLPYAQQTPREQPIAVVNLDGSQTS

LKLERMVDATPQVKIVSRLHTVEDAKQAFLNREITGFLVIPEHFYKDVMLGKSPTLAYAA

DASYFLVYGTVVEGLAQAGGTLGAQVKVSKMVIEGVPLSLASHNYSAIKLNMKPTFNPTM

GYIEYVVPAVFVLILQQTLIMAVGLQTGSQRHGRGYWSQVSTGSLLLVRTLVFIAIYYLL

SAYYFGFSFERLSVNHIAKAGELLTMLFPFLLGCCGLGFWLGYLLPRRELVTLVVLVSSM

PLIFLAGFIWPVESIPAPLLWIADLSPSTWSIKGFLALNQMGATWQQVAKHWTALWLLVA

LWGGVAYWIAKRNNKPVVTESLS

>tr|Q87H54|Q87H54_VIBPA Putative glycine betaine-binding ABC transporter OS=Vibrio parahaemolyticus serotype O3:K6 (strain RIMD 2210633) OX=223926 GN=VPA1111 PE=4 SV=1

MSKMTKILSTIALSSLATGAYANQCKTVRFADVGWTDITATTAVTTELLKGLGYKTKTDL

LSVPVTYSSMANGDIDIFLGNWMPTMEGDIAKYRDAGTVETVKANLVGAKYTLAVPKYVY

DAGVRSFADLEKHADKFKDRIYGIEPGNDGNRLIQSMIDSNAFGLKDFSLVESSEAGMVS

QVSRAVRRNQWIVYLGWAPHPMNNNVEMEYLAGGDDFFGPNYGGANVYTNVRKNYLAECP

NAGQLLKNLEFSLEMENELMEAILNQKQKPSKAAQAWLNANADKIEAWLKDVKTVDGQDA

KAAISAYLKTNA

>tr|Q87GM7|Q87GM7_VIBPA Uncharacterized protein OS=Vibrio parahaemolyticus serotype O3:K6 (strain RIMD 2210633) OX=223926 GN=VPA1288 PE=4 SV=1

MEHVGSRALIVEGGAMRGVFSCGILDHFLAADFSPFDSFWGVSAGASNLAAYLAKMPGRN

LKIYLDYSLRNEFITPSQLIRGGDVMDLDWMWQVTLEELGIDKEVLAADPRPFFLVVTRQ

DTGQAEYLTPDVDMLAETMKASSALPVLYRNGVLLNDTRYVDGGVADALPIAEAIKRGAT

KIMVLRSQIASYRKPRSKFSAITKRMLKETPALIEPMLTRDVRYNQTLELINNPPPGVEI

IQVCPPETFKLKRLSRLPEPLREAYELGIEAGKQAIERWNSM

>tr|Q87RK9|Q87RK9_VIBPA Uncharacterized protein OS=Vibrio parahaemolyticus serotype O3:K6 (strain RIMD 2210633) OX=223926 GN=VP0769 PE=4 SV=1

MKKLLFIIATLVLVGCQPLQQMRQDEILVAVGYASVSEQTGRTVEEKRVRAMRASKIDAY

RELAEQVYGMRVSGRAELEDQRLGIESTSGAVDGVIRGAEVVRSYLVEDSYVTELRLDIR

KMDKLRDYGEVQQVPEKRQQTLF

>tr|Q87QK2|Q87QK2_VIBPA Uncharacterized protein OS=Vibrio parahaemolyticus serotype O3:K6 (strain RIMD 2210633) OX=223926 GN=VP1147 PE=4 SV=1

MKYSYVARQPILDREKRTIGYELLFRDGPKNTFPEVEPELATSRLLSDHFLSTHYNTLGN

KLGFVNFPHQSLVNLVPTLFPKDSLVIEVLEDCEPTQELLDAIKHLHECGYRIALDDFVP

TKAWKRFLPYVSMIKFDIRLVPIEKAAIFIQALSQFNIDFLAEKVETYEEFEQALDAGFN

YFQGYFFSKPEMIQKKRLNPAFLTVIQLCKEIADKPIDFNEVERLFSIDVTLSYKLLTYV

NSGYTLTTKIKSFRQALIYLGEERLRRFISLVAIASVQEDKPDSLYSLAIQRARMCEILL

SQMKTKYDPGQAFLTGMFSLLGSLLDQPLSDVIEDIPVDEDIKLALTSRKGVLGYLLSMC

IAYEQADWELAERYCSVLKLTETQLADAFNESTEWAQELLSQTP

>tr|Q87H74|Q87H74_VIBPA Uncharacterized protein OS=Vibrio parahaemolyticus serotype O3:K6 (strain RIMD 2210633) OX=223926 GN=VPA1091 PE=4 SV=1

MKKLFLLLIWTCLSVSPFAMAQHPNSKAENRVSQQVIEKTYKEAKLNGVIDFKLFRDAFI

AYQKTPDRKKSILTIIDYSKPSTEKRFYVVDVNKKKLIYNTYVAHGVNSGKKTATQFSNV

VNSRKTSLGTFLTDTTYYGSNGYSLRLDGLSSGLNDKARERYIVVHGADYANPSFIKKNG

YLGRSWGCPALPQKLSREIIDTIKGGSVIYASA

>tr|Q87JG5|Q87JG5_VIBPA Uncharacterized protein OS=Vibrio parahaemolyticus serotype O3:K6 (strain RIMD 2210633) OX=223926 GN=VPA0288 PE=4 SV=1

MNKKILLPLMLGISATAAASDNKVVESVLIDNIALEGYVKKSYEVVMQSEPELFPYEFRG

SRVQMKLLVELFHNSKNDKKAARVITLGNGYVPGKPGVKHTFDAAIVDRFTQNYPVAFDY

SIYQAGGTPVFSEDYSPQNEANDVSVSTTTVAPSIGLTATATPGSAPSFSGSLTTDLGVA

YTSRYSSKDYVTAAKPKASMERGMGVKWTTSLSQIYTNDYQYYGKWAGVYHYTGCSNENL

LPEENLPRLIYGFKPKFQYVFTPEFERGEEPKTEMIARAGMKEVDEGFSRNACNWYDQGT

TSHYTEAQIRFTIDWDKLTVITQ

>tr|Q87I26|Q87I26_VIBPA Uncharacterized protein OS=Vibrio parahaemolyticus serotype O3:K6 (strain RIMD 2210633) OX=223926 GN=VPA0780 PE=4 SV=1

MSIMSFKYLTLLLATTSMNSFADVDLPSGDDWLNHVTNGLAPYWVMESAHGQPVGNFPTF

RCDNGVILDPQKPCEELNKGWIRPHFDREYTRMKSRQTYAYGVLFHLTGDKQALELAKKG

AYYLIDHLQDEKNGGFVSFTRSGKPGMEWQQRTSQDQAYALVGLAMYYYLTQDPKVEQAL

IDQQAFIFKQYRRSDDRGLAWVLKDGDGESEKQQELVAQLDQINGYLLLTAPLLPEPAQA

KWKQDLQWLTQVMLDNYHSEKEQRFYGAIHHKAVMMPDAKHNDFGHTIKAYWMTYLTGQL

LENSNWTEFGFKGMKHTLKQAQYQQNFEQVKALFSPEWQSKWTQESIPAWQSRPHKHYSS

SWEWAELDQAAMTVSLVDGSMKETLQYTLPTFMDVWVDHQYGGVGLNPKSTKAFHWGNGY

HQFEHALVGYLYAQQMAKQSAKLYYARPAESKMPLNPYYFNADIKALSSQSDGVTTATFT

NIRP

>tr|Q87PT8|Q87PT8_VIBPA Uncharacterized protein OS=Vibrio parahaemolyticus serotype O3:K6 (strain RIMD 2210633) OX=223926 GN=VP1413 PE=4 SV=1

MSLYNPVVWQDGMFMKPQHFQQLDRSQSKLSSMLSANASPLHWGIKRLEINSQLLALGKI

GITRAEGILQDRTPFELPLLAELPEVKDVDPSIADKVVYLCCPLPSERSELFGTKKDGAR

YTLESQEAVDACYDSEDMANIVVGKLNFCLMYDHEDKSAYTSIPILKISEVKPDGSVILD

ESFIPTCIDIHASTVLNKFATEFASMLKHRAESIVQRLGVVDQQGVSSVSDFMLLQALNR

YEPLFWHFASAEGVHPESFYRILLQAEGELSTLCSASRRPIEFTKYNHGDLTSCLSRTLD

SAKMTLSVMSEQRAIPLTLKDQNYGIRTAAIPDSKIIDTTTFILAVKADVTLDVLHTQFV

SQTKIGSIDNIRDLINLQLPGIEIKPMPVVPRALPYHAGYTYFELDKTGEEWAALKNTAA

IAVHVAGDFANLSLQLWAVRL

>tr|Q87R70|Q87R70_VIBPA Isochorismate synthase MenF OS=Vibrio parahaemolyticus serotype O3:K6 (strain RIMD 2210633) OX=223926 GN=menF PE=3 SV=1

MSEFHQAVKRIIERVQHTEGNQVRLVEPLSEKPNFAFIDWLDAQPLFPKFYWQSRDTREE

VVALGQLHSFVEPGPAYTILGEGQRVWGGRSFDGQHEKNRRCMPSFFFLPQIELIRFDQQ

WSLAVNLTEDKARTLAGLRKLICDVAALPPISSHIRSIVHTPVKSEWDALVHKVLTGIEN

DEFKKVVLARRSTVQLDNRLSAAQLLKASYLNNHHSFHFLLSLDSKHSFMGSTPERLYSR

VGHELHTEALAGTIGRGDNATHDMELANWLSQDSKNLNENQYVVDDIVERLSPHSEIVEV

ETEPRLVRLRKVQHLKRNIHASLKTGTNGVQLLSALQPTAAVAGLPRKESMQFILDNEPF

ARGWYAGSMGYISHERAEFCVAIRSALVLGDQVQLFAGAGIVPGSVAEHEWAELDKKMST

LLTLISDHPPLGVAS

>tr|Q87MS6|Q87MS6_VIBPA Uncharacterized protein OS=Vibrio parahaemolyticus serotype O3:K6 (strain RIMD 2210633) OX=223926 GN=VP2155 PE=4 SV=1

MKKTYWLLASLTTLTLTGCFEGNVTTEELCQKTPELRCENLNMDDGQCRIPRTKLIWHRF

ELLKNPSEANQITEYSIVADYRKCLELASQITPIDQSELKRQRFNALVHSIDELKRIVEE

LKGSQQPETLYFLWSQTGDTQARRQFLQMEGTPQLNTAEMQYALATFYTNRDAKKTLVLL

NNALALSNKDNLNPVILKSLASINQGLGEKKQAYLWAMVAKRFDVPLADEKQLQRMYHFE

DPNQHEKLDELADTIAKAIRKGSYSPDMVPKDM

>tr|Q87LA5|Q87LA5_VIBPA MSHA biogenesis protein MshI OS=Vibrio parahaemolyticus serotype O3:K6 (strain RIMD 2210633) OX=223926 GN=VP2707 PE=4 SV=1

MEFKAVFDKLMRSSGGGRSCFLVIQPDAVYLSSPAKSGRPSQFEISESNWERAVEQALSV

AASAYDSLTVVLSHNYYQMYQIDKPAMPRQEWSAALPFLLKELISERAIDIIADAVELPN

SSKVQAYVLSRKMLDKIVLLASRAQLPLEAIIPEDDVWGDTAGELGNFLLLQRSARGHFR

ISAFVEHTIAFHRSIRSVTPPLTGVPSSELQMDSLSLELQRSIDYLSAQLRQVQLHQLKV

CCDEEDENELVQSLNYRLSTKVSSLLPEEHELSGHVLADAAAASTNRRVNLYPDHLKPKK

DLLTLKNVAISWGAAAAVILLSYGYVSWQSEGLEDEIAIVKGKGDILKSDLDYYQVKLRK

HQPDPNKLAAKARLEREVKAKRDSLKAVGKYDDSQRTGYSGVMQSLAKLGNQEISLSEIR

INNNTLDLKGLAKAPSSVPSWVSQFKNEISLVGRTFDDVKIGRNEKDIVTFELKTREDKE

K

>tr|Q87I36|Q87I36_VIBPA Uncharacterized protein OS=Vibrio parahaemolyticus serotype O3:K6 (strain RIMD 2210633) OX=223926 GN=VPA0770 PE=4 SV=1

MKLYRLTQKKFADTPFSPIGAKLFGGRWNSKGTEALYFSESESLCSLEVFVHVNNDPAIT

KLYDLYRIEMPEYLIATLDEEDLPVTWRAIPASESTQYIGDQFLNDPHPEFAALQVPSTI

SPRDKNYVVNPNHPKMKEIIKKAEKLDFAFDPRIFK

>tr|Q87I80|Q87I80_VIBPA Putative TadC OS=Vibrio parahaemolyticus serotype O3:K6 (strain RIMD 2210633) OX=223926 GN=VPA0726 PE=4 SV=1

MVDIKLIFLALGLIISLSAAYYSLKVFDKTKANISTKKIIDSTQEKTPFVDKLSLHLSRL

SFNKEETKNNLVRAGIHSEFIAQAYYLLKIIPLVFCLAFIGYSYIQGEMEFSSAFISFSL

SMLVCLIAPDMYISSRGNANVRHVSSRLPFLLDLMNVCVHTGMTLEASLDYLSNELKTVD

ENLAYVVKKTSERAKIVGLDRALTEFYDLVPTTEAQSFVMTLTQSLKYGSSVGAVLASLA

SDIREINMLELEEKIGKMGAKMSIPMIAFIMIPIVVLIAAPGIMRMLG

>tr|Q87N84|Q87N84_VIBPA 5-methyltetrahydropteroyltriglutamate-homocysteine methyltransferase OS=Vibrio parahaemolyticus serotype O3:K6 (strain RIMD 2210633) OX=223926 GN=VP1991 PE=3 SV=1

MKTLLPTSTAGSLPKPSWLAEPEKLWSPWKLQGEELIDGKRDALRVALQEQELAGVDIVS

DGEQTRQHFVTTFIEHLSGVDFENRKTVTIRNRYEASVPTVVGPVSRTKPVFVEDAKFLR

QQTKQPIKWALPGPMTMIDTLYDDHYGSREKLAWEFAKILNQEAKELEAAGVDIIQFDEP

AFNVFFDDVNEWGIACLERAIEGLKCETAVHICYGYGIKANTDWKQTLGTEWRQYEEVFP

KLQQSNIDIISLECHNSRVPIELLELIRGKKVMVGAIDVATNEIETPEEVANTLREALKY

VDADKLYPCTNCGMAPLSREVSTAKLNALSAGAEIVRRELSA

>tr|Q87PX2|Q87PX2_VIBPA Putative HD-GYP domain containing protein OS=Vibrio parahaemolyticus serotype O3:K6 (strain RIMD 2210633) OX=223926 GN=VP1378 PE=4 SV=1

MQYNPEDSIKIAIADVAVGMFVTAIEHNKRVNLANAGRVSSAEGIKKLRASGVKFVWVDQ

SLSAQQCVFKPVEEPIDIEVGDKTLSVSSKVQRAYRSREVQHKRAKKLIAEAKGLAQKLL

NQTFEGKVIQVDEIESWADDMIESVFIDSDALQCVSALRQKDSYLLEHSVNVACLLVSFG

KHLGLDKDTLKQLAIGGIIHDVGKIKVDDKILHKPARLTPEEFEHMKLHQVFAGEIIVEV

KGLSDVSRDVCLMHHEKLDGTGYPRGLSGDEIPIHGRMSCIVDIYDALTADRCYKKGMSS

AEAFKILLSLTPFHLDPDLVYKFINCIGVYPVGSIVELSDGRVGIVWSSNPSQALKPEVK

CFYSRKYKRYIDVAMVDLKTSTHKIERAIAPSSLEIDPKPFYD

>tr|Q87SM8|Q87SM8_VIBPA Haemagglutinin associated protein OS=Vibrio parahaemolyticus serotype O3:K6 (strain RIMD 2210633) OX=223926 GN=VP0394 PE=4 SV=1

MMNLFQDDAVTWLSTLDAASVDLLITDPPYESLEKHRKIGTTTRLKVSKASSNQWFDIFP

NDRFEALLSEVYRVLKNHSHFYLFCDQETMFVIKPIAEKIGFKFWKPIVWDKVSIGMGYH

YRARHEYILFFEKGKRKLNDLSIPDILTHKRVYRGYPTEKPVSLLEVLVAQSSRDGELVV

DPFFGSGSTLVAAKNLNRRFKGNDISQSAHEHIHQRMDFGA

>tr|Q87KX4|Q87KX4_VIBPA Uncharacterized protein OS=Vibrio parahaemolyticus serotype O3:K6 (strain RIMD 2210633) OX=223926 GN=VP2850 PE=4 SV=1

MEIKMKKLMTSLAVAATIASPVALANSTPVMFSTIDHTNAPSNSAVGGVRLAVLHGQVNE

VKGVDFSVLGMSETDRTTGVNFGLFFGAAKVNQEMKGASLGLVNWNQGQTTGVNFGAVNI

TNNVKGLNWSAVNYSEGYTMADVGLASISKKSNFQLGFFNMTDQIDGVQIGLLNCADNGF

FKCFPIINFAK

>tr|Q87TN0|Q87TN0_VIBPA Uncharacterized protein OS=Vibrio parahaemolyticus serotype O3:K6 (strain RIMD 2210633) OX=223926 GN=VP0039 PE=3 SV=1

MKKWTFFMLLIAVLLFGSVIGFNLFKQQKIAEYMANRPEPEFPVTVTEVKAVDWVPVIEA

IGFIEPNQGVTVANETSGVIDKIAFESGTQVEAGQPLVLLDSEVEKANLKSSQAKLPAAE

AKYKRYQGLFKKGSISKEAYDEAEANYYSLKADIESLKATIDRREIKAPFAGVVGIRNVY

LGQYIQAGSDIVRLEDSSVMRLRFTVPQTDISRIKLDQEVDIFVDAYPDQPFKGSISAIE

PAVNVQSGLIQVQADIPNSDGKLRSGMFARANIIMPKLANQVTLPQTAITFTLYGDNVYI

VTEEEGEKRVKQHVVKVGERTKDIAHILAGVKPGDVVVTSGQVRLSNHAKVSIVESNAIT

PPAETPML

>tr|Q87S14|Q87S14_VIBPA Uncharacterized protein OS=Vibrio parahaemolyticus serotype O3:K6 (strain RIMD 2210633) OX=223926 GN=VP0610 PE=4 SV=1

MELYDTEEQQVEAIKDWWKENGKAVIIGAVVGLGGLFGWRYYQDTVIQASETASQSYTTA

MNTLQEKGVDAQSDVQAFIESNEVKEYSVLAALQLAKAQVEAKDFAAALEQLKWAQSNTK

DAALSPLISYRIARIETEMGNFDAANTELGKVTDTAWAGRIAELRGDIALRQGDKDAAYA

AYTEAQQAADASPTLQMKLDDLAK

>tr|Q87JG0|Q87JG0_VIBPA Transcriptional regulator OS=Vibrio parahaemolyticus serotype O3:K6 (strain RIMD 2210633) OX=223926 GN=VPA0293 PE=4 SV=1

MTKRQQTRDKILKAAWASFAQNGYDMTTTRQIAREAGVADGTVFSHFPTKLSILREGMLT

QLQQISQETLVSVEGKTAIEIGLALTEKYYRYYFANVELSRALLKEVIWDLDYYQSFNQA

LFQSASVSSVLEDKIPLIFDCYFMTLIAHLSRAEPDVEAALTDLHAKFRQIIPIESLG

>tr|Q87RS7|Q87RS7_VIBPA Uncharacterized protein OS=Vibrio parahaemolyticus serotype O3:K6 (strain RIMD 2210633) OX=223926 GN=VP0700 PE=4 SV=1

MFWDSSRLIKLIGFFCLLLAQPLHATILSSALLNEAQQLAEIEPAQAKQAAKNYLSQREL

TERKESGSPSAMSREETDRSIRTPASTIEAHKIIAQADYTMGNVRSAIDNLNEAEKLAKE

YQLPYMTLDVQLMRNQMIWMYDHNYSAAEDALNQIEKQLDEADSVLQRTDSVRYRLVMQR

ALLAAHRGDNVGAERLYAQAKSMLEDSRSDLALIDYYTSVGEFYLNSKKYNLALSELLYG

YWQSIESDSGTRLAKVNRLLARLFQERRVYDKAIEYLSQAADFYDSYPSSPILADVLEQM

GDIYFYQGKFNLALVHYFNVLDHESTSRNINRTIKIRLSLAATYLQLYNYALAEQYLERA

TELLEYADIPQLEAKAALLKSGLAYHQNDSKKVIENAKKALELVEKAPENKNFIKQQSYR

LLGLGYEQAGEYQLSLQAYKKYTSLVRLEQKQLNQISEDAFRQQKEFAEQTIHYVGQSER

LEQVEMEHRKFQKISFALFITVLVMFLFIMRRGVIMQRQAGEIEKLRNDLFTHSRSRLRN

LRMLNVKLSRSLKKSRDTFEQWQMGELIHEPLNDRLRFVMIDLPFLRSMYVQNGYKAGLE

LERAFGEYLAERIEKPARLYHFSDANLLYIEPNADRDASAEVMFEKFQAWVDEFATQHNV

NRIIRMGISDYPFLPRAYTAINDEELLDLLLLATHIAREVSLKDKQSHWVFLKAIDNAPA

ASFATGNIRTACQHAINQGLIKIHSSYKNEDDIKKILKNG

>tr|Q87IZ8|Q87IZ8_VIBPA Uncharacterized protein OS=Vibrio parahaemolyticus serotype O3:K6 (strain RIMD 2210633) OX=223926 GN=VPA0457 PE=4 SV=1

MPFQRVVMLEGRLYERYLPGYLESNSEEEKQRLWQQADPNTIYQLNSELQKLHGEGNDLI

SFSDPHRSHLPIAAWPDMLSADIAFWRKHQHWLLQSNDPVTPTNEDDLSVNYKIPLNYIN

QYNGEHLRLFYGSRLVYARLFSALHYILNHVKSVIEDWSHDRYPYQLPIELYIPTTSEPQ

SAVRSTYHHFNSANYQSLKNYFQQHYSSWIEEQYLNWMFELNTQLNHQTAATYIIEYEDE

FGIPRVDFICKNENTLRMIRPAHFVEDMRQMVSSTDYLDHQVQQVALRIERQCRAKGW

>tr|Q87SX0|Q87SX0_VIBPA Uncharacterized protein OS=Vibrio parahaemolyticus serotype O3:K6 (strain RIMD 2210633) OX=223926 GN=VP0302 PE=4 SV=1

MPSKQVLAIVIGLSLSTVATAEEYRQHSAHVHGHVEFNIAQDGSDLLLEITAPGADVVGF

EHAPENAEQEKTLQHAVATLEDSNALFAINPQAQCEIEEVHVEHSLGGQHEEHEHHDHEG

HDHDEHAHHDHDKHEHDGHEGHDHSEHSDHGEFTVQYRFHCAQVGELSHIQTDWFNQFPS

TESVNVNLFTDTTQSATSLTKSNTQIAIK

>tr|Q87G31|Q87G31_VIBPA Ferredoxin-type protein OS=Vibrio parahaemolyticus serotype O3:K6 (strain RIMD 2210633) OX=223926 GN=VPA1486 PE=4 SV=1

MKSSQSKTSQSRRRFLRDTVRTAAGVGAAACVLGLQSLQSQARETKGVPIRPPGALPEGD

FESACIRCGLCVQACPYDTLKLATLLSPVATGSPYFTARDIPCEMCEDIPCVVACPSGAL

DPTLTDIDDARMGTAVLIDHETCLNWQGLRCDVCYRVCPLIDEAITLEMVHNDRTGYHAK

LIPTVNSEVCTGCGKCEQACVLDVAAIKVVPTDLAKGKVGSHYNFGWKEIDKPLENILPS

ETPVPEGALESLKGGK

>tr|Q87G84|Q87G84_VIBPA Putative two-component sensor kinase OS=Vibrio parahaemolyticus serotype O3:K6 (strain RIMD 2210633) OX=223926 GN=VPA1433 PE=4 SV=1

MMYFMFAHWRDNTFLFSAIVIVGAVVASWLLDQLFDSTAAVLLILQLAVVVVAFQCNSRF

AYAAAVIEALSFNFFFTTPRYSLQMFRPEDIFNLVVFMVVAFITSTFADLYRRQQGELKQ

TKLQNSILLSVSHDLRTPLATIIGTLTTLNEYMPKLNDLERKELLDSATSESHRLHQYIE

NLLQATKLQHGTLKITKKDEPIANIVRDAVSRLPNYTEKVSMNMDDSVGYLSVSRSLIEQ

AIFNVLDNAMRFSPENESVEVSLSKQGLSCVIDVRDMGIGITAEDAEKIFSLFYSGANNK

SADSGTGMGLAVAKGIITAHQGEIQSMPVSEGTLIRIRLPLNQGAEQA

>tr|Q87QT3|Q87QT3_VIBPA Uncharacterized protein OS=Vibrio parahaemolyticus serotype O3:K6 (strain RIMD 2210633) OX=223926 GN=VP1066 PE=4 SV=1

MKRSSNVKRSSMDKSLKLGKIIPFAAFGGIFFLATQESKTEGYIFSDADECKSNSPEFSE

QCDIAYQEALARAERNAPRYNNEFECENDFYEDDCYYSSSSRAYVPHFGGFFYSRSVNDL

KGYNKSYYSEPMYRYKSKFYNGAGQFFGSYRNQSTKVATSNLNKRGGGTIGRAMSRGGFG

KAVSVSRGG

>tr|Q79YV9|Q79YV9_VIBPA Flagellar hook-basal body complex protein FliE OS=Vibrio parahaemolyticus serotype O3:K6 (strain RIMD 2210633) OX=223926 GN=fliE PE=3 SV=1

MKVDGIQAEMRAMMVEATNTTPTGTGAKVGADFNDLLTKAINNVNSLQKSSGDLQTRFDR

GDADVSLSDVMIARNKSSVAFEATVQIRNKLVEAYKDLMNMPV

>tr|Q79YY2|Q79YY2_VIBPA Bacteriophage f237 ORF1 OS=Vibrio parahaemolyticus serotype O3:K6 (strain RIMD 2210633) OX=223926 GN=VP1551 PE=4 SV=1

MSWCPSPTRPLNNVSTWGCLMTTATNILKSFDEQSVHIDYLCFTFAVKDLRHCHDAVRRL

HKHEEYKGFAKSGLLQRHCRAPKFPAPPVFNPTVAQTSDEIDAYNKAFDICYRNYLEDCL

RIFTNQVLGLSLSAPRGLGFQFYTESMKLTSPDGEDFCGFVGIGGNNDTVHFQINGTGCK

HVFARRPTWSLHDWLTNVLGVQTLARVDLAYDDYDGIFDCEYAYKAWRDDCFRTAERGRG

PVLHEDMTIASIGKDGKPIYTKEQYSIGSRTSRIYWRIYNKALEQKLANTGLVWYRSEVE

LKKWNVDVLLNPAGAYAALNDFAASISTAKKFNTKPVPTKRAALDLLASAHWMRRQYGKI

LNSLIEFHEGDIETVVGSLVRDGTKFTFPDTYGKLVTHILET

>tr|Q87NX7|Q87NX7_VIBPA Sodium/alanine symporter OS=Vibrio parahaemolyticus serotype O3:K6 (strain RIMD 2210633) OX=223926 GN=VP1741 PE=3 SV=1

MNDLQSLLQTIDNFVWGPPLLILLVGTGVYFTFSLGLIQFKHLPTALAMVFSKDKSSDKQ

GDVSSFAALCTALSATIGTGNIVGVATAIKLGGPGALFWMWLVALFGMATKYAECLLAVK

YRRVDDNGQMIGGPMYYLQYGVGSKALAIMFAVFSLGVACFGIGTFPQVNAILDASEISL

GVNRELAAFILTLLVAFVTLGGIKSIASVAGKVVPAMALFYVLACLSVIIMNADQLLNAV

ELVLVSAFTSTAATGGFLGASIMLAIQSGIARGVFSNESGLGSAPMAAAAAKTDSCVKQG

LISMTGTFFDTIIICTMTGLALILTGAWQSDLSGAAMTTHAFAVGLNAETFGPMLVSIGL

MFFAFTTILGWNYYGERCVVFLMGTKAVLPYKIIFLALVASGAFLHLDMIWILADIVNGL

MAIPNLIGLIALRHVVLAETKLFFNPSVQSDDLDAVKA

>tr|Q87TH9|Q87TH9_VIBPA Uncharacterized protein OS=Vibrio parahaemolyticus serotype O3:K6 (strain RIMD 2210633) OX=223926 GN=VP0090 PE=4 SV=1

MRSLTKLDRAMQIKRIEGKAALEQCLLDKQTLKFTSNRFVQENPWTALGLSAFAAIAVAK

AGRLSRVTSSFSSISLLLNGAMDLSGQLAKSSSSSAASSKEAGEEAAQPSDFPPHQ

>tr|Q87PA6|Q87PA6_VIBPA Uncharacterized protein OS=Vibrio parahaemolyticus serotype O3:K6 (strain RIMD 2210633) OX=223926 GN=VP1611 PE=4 SV=1

MSDQNNSQTSYVPDVKRSKGISPLWLLPILTMVLAGWLVVKSIHDAGQRVQIYFSDAAGL

VAGRTTIRYQGLEVGMVRDINLSEDLGSIYVDADIYPEATKLLNDKTRFWLVKPTASLTG

VSGLDALVSGNYISIQPGDGQEFETTFHALDSAPTDLRVSQGLNIKLKSRDLGGVSIGSQ

IVYKKIPIGAVYSYQLDEDAKSITIQANIQEQYRHIINDRSRFWNVSGIGASIGFEGVDV

RLESMSALLGGAIAVDSPDDGEPVEENTEFRLYKDLKTAGRGIAIKIALPDDNKVSSEGA

PIMYRGIEIGQVTDLSLSEGREVILASAAIQPAFSDMLTTGTRFVLEEAKVSLSGVENIA

NLVRGNFLTIVPGDGERSRRFTAIRKNVFNQQQEKSIAIRLISDNSFGLDSGANVLYKGI

VVGSIINVGLVDEKKQTKHEVFMDVLIDHEYKHLIKSNNRFYVTGSASAELTESGLSVTV

PPAKQLLTGSISFVSEGSESIQKEYQLFQNESLAELAQYNKTGSKTLMLFASELPPISKG

SPLLYRNLPVGNVSDFHLVDGGVLIKATIENRFAYLVTPQTVFWNRSGIEIDASLSGVSV

KAHPLKSLIEGGIAFDSVPGVENKVGERWKLYADQQKARKFGRVISLETDGTQEVLKGMP

IEYQGVKVGEVTLVVPNFRRNLVEVTARILPEYVENIAVEGTHFWLTEPEIGLGGVKNLG

ALVSKSISVEPGNGKAKFDFQLEKGFDRVEGVMFTLQSEQRGSVQVGTPVLYRQMEVGQV

TDVRLGEFADRVVSTIKIKPEYAYLVRQNSVFWNVSGVDVSIGITGANIKAGTIDSLVRG

GIAFSTPEQSQIPPAAKRGHSFYLYPRADESWVQWRTPIPKP

>tr|Q87NX1|Q87NX1_VIBPA Putative amino acid transporter OS=Vibrio parahaemolyticus serotype O3:K6 (strain RIMD 2210633) OX=223926 GN=VP1747 PE=4 SV=1

MSNIAKSAVKLSVFSVIMITVTSVDSIRNIPGAALFGSHAISFFLLAGLCFFVPTALVCA

ELSTTYPQQGGVYLWGKETIGPNFGFATVWYQYAENIVYYPPLISFIVATGAYPFFPELA

QNNIFMLIMINVIFWALTLVNIFGLRLSSMITNVFGTLGLIFPILLIIGLGGYWAYTNPG

ESHISLSHVSDWLPDFSQDGIGAGFTAVVLSLTGLEITTSYASEVENPQKAYPKALLAST

ALILVSLTACSLSISSVVSSDHASLSEGVILAFKTFFDDLNLSFMLPVIALAIVFGTLAS

LNNWIIAPTKSLHVAAKDQFMPLALSKENQNQAPVALLLLQGAIVSVLSLVFILVPNVNQ

GMWLLNILMTQLYMVMYVCIFISFLVSRRKHANIERPFRVPGGKVGMSVVAGLGLISCMI

TIVVSFDVPAGISAETGAYALVLGFIAFSLPAIAAVMYRNRKVRSQAQLIEALAS

>tr|Q87LD7|Q87LD7_VIBPA Magnesium transporter MgtE OS=Vibrio parahaemolyticus serotype O3:K6 (strain RIMD 2210633) OX=223926 GN=VP2675 PE=3 SV=1

MAEQIEFDQAHQALQEVTEALENGRFVHVRRQLQDMEPEDIAHLLEASPRRSREVLWQLT

DPEDYGEILDELNEDVKDSLVSKMAPEDLAEATEGMDTDDVAYVLRSLPDDLSREVLSQM

DSADRARVETALSYPEDTAGGLMNTDVITIRGDVDVDVVLRYLRMKGELPEATDALYVID

DESKLIGELPITTLITTQPDVKIIDVMEDADDAITVDTSDSDVASLFERRNWVSAPVVDE

NQHLVGRITIDDVVDVIREDAEHSMMSMAGMDDDEDTFAPVFKSARKRSVWLGANVLAAL

AAASVSNMFEATLDQMAAIAVLMTIVPSMGGVAGNQTVALVIRGLALGHIGDSNKRELLM

KEAAIGLLNGIMWALIIGGIVVVWKGNWMLGGIISAAMLTNLFVAGVAGVTIPVLLKKMN

IDPALAGGMALTTVTDVIGLSVFLGLATLLI

>tr|Q87R17|Q87R17_VIBPA Rec2-related protein OS=Vibrio parahaemolyticus serotype O3:K6 (strain RIMD 2210633) OX=223926 GN=VP0981 PE=4 SV=1

MTLLEKSWTLALFVASVISSAWWPTMPDWRWLLLGIITTGSIIKLRRGLISIGVIVGFMV

VIVHGNIMEYQRQALFQAGENSTIIGRVDSSFTQISHGYEGVVAIKQVNSHTLLPFLKPK

VRLITPFPLAVNSEFTTNVLIKPIIGLRNEAGFDAEKQSMGSGVVARAVVTKDSYWVIRT

SSSWREAIIQTVERDISRLEHFALIKALAFADRTGLTKEDWQSLRDSGLLHLVSISGLHI

GMALTFGLALGGLIRLAMPRYWFLPSVSGLAFAIVYAWLADFSLPTTRAVSVCIIYIALK

YWLVHWSPWRVLLLAVALQLFFQPFASFSLSFWLSYLSVGAVLFAVNTVQDSKEGRLGKL

RILLLTQLILSLLIVPISGYFFSGFSWSSLVYNLVFIPWFGFVVVPIMFAALIASLLFPM

LATVLWYLLDIFLVPLSWSVRYAIGTWQPISAEWTFVIAVVSAVLVSRHVMPRYVWMFVC

VIVVMTGLFPKQYNQTWRIDVLDVGHGLAVLVEKEGRVLLYDTGKAWQNGSIAEQVITPV

LHRRGYSSVDTMILSHADNDHAGGRKVIEQYFSPKHKLSSQSFLHYQPCIAAEKWKWQGL

NMEVLWPPKPVVRAYNPHSCVISLEDPSTGFKMLFTGDIEAISEWILLREPEKLRSDVML

VPHHGSKSSSNPKFINVVEPSLAIASTAKLNQWGMPAPEVVQAYTDSGVSWLDTGSDGQI

TILLDGNNWRFESKRRETIEPWYRQMLRNRVE

>tr|Q87IV5|Q87IV5_VIBPA Mannitol-specific PTS system enzyme II component OS=Vibrio parahaemolyticus serotype O3:K6 (strain RIMD 2210633) OX=223926 GN=VPA0501 PE=4 SV=1

MKANMRANVQAFGGHLTAMVLPNIGAFIAWGFITALFIPTGWMPNEAFGELVGPMITYLL

PLLIGYTGGQIVGEKRGAVAGAIGTMGVIVGADIPMFVGAMIMGPLSAWVVVQVDKRIQH

RIPSGFEMVVNNFSLGIVGMLLCLFAYEIVGPSVTAANLFVKSGIEALVATGFLPLLAII

NEPAKVLFLNNAIDQGIYYPLGLQAAAETGKSIFFMVASNPGPGLGMLLAYAKFGQGLSK

RSAPSAIIIHFFGGIHELYFPYVLMKPIMIVAMIAGAATGIATFNLFDAGLVAGPSPGSI

FSYLALTPKGSFIATIAGVTSATIVSFLVASAILKVSKSEEKESEFEKSVSDMKEMKAEG

AVKPVAAAATTQEPSKPISFVAFACDAGMGSSAMGASTFKRKLVQAGIDIEVKNFAIEKV

PSEADIVVTHESLESRAVNATGLPVVTIKNFLNDPALDELMDKIKQQTVATEQPA

>tr|Q87T79|Q87T79_VIBPA Putative lipopolysaccharide A protein OS=Vibrio parahaemolyticus serotype O3:K6 (strain RIMD 2210633) OX=223926 GN=VP0191 PE=4 SV=1

MKDSKFPYYFSNALLMLVPRFVYRAQAKRLLSTLTEAEKQYCLERVNYYNKCGQPFTVDS

REQSYAKIGEFKKTKGWTYFFDTRQVIRYFPSEFTFNYINGDVTHIPNVPSFIKSRPIHG

DNQNSVVLKLNQIRHFKFVDDEMSYHDKKDMVAWRGVGFQPHRCKVIQAFYDHPRCNIGQ

TRPQEGQPWEKGFMSIEEQLQYKFLLCIEGNDVATNLKWAMSSNSLVIMSKPKYETWFME

GKLEAGIHYVEVQDDYSDLPEKMDYYLANEQEALAIIENAHQWVEQFKDKRKERLISLMV

ADKYFTLSHQQ

>tr|Q87JB0|Q87JB0_VIBPA Putative membrane fusion protein OS=Vibrio parahaemolyticus serotype O3:K6 (strain RIMD 2210633) OX=223926 GN=VPA0343 PE=3 SV=1

MLAKPIVRWLLPFVVVGGSYAGYAAIAATAPEKESTKETITEPTVRASSLFPTDHKVVIT

SHGELVPFEKTHLSAQVSGEVISWHPNFVTGGIVKRGEVLFTIESDNYEAAVLQAEAGLA

SARASLIEEQAKAEVAKRQAKKLSDKQVTDLYLRKPQVLSAQAQVKSAQAALKRANRDLE

NCRVVAPYDALVVERDVGVGQFVTSGSRVATLNNIEVAEVHVPIAGFDSAFLPESIKELT

ATVTQQGILSTTREGKVVRDLGMIDSATRMINMVIQVEDPYGIDNQQPPIKFGSYVEVSF

TGKELKHIYRLPQELVKNRTVWVVNDENQLQPRTVTVLRAEGEFMLVGEGLEQSDQLVLT

LPEYPQKGMAVQIAKKNDDSETVVQ

>tr|Q87GZ3|Q87GZ3_VIBPA Uncharacterized protein OS=Vibrio parahaemolyticus serotype O3:K6 (strain RIMD 2210633) OX=223926 GN=VPA1172 PE=4 SV=1

MKRTSLILGSLFVSVYANAAPFDTCPSKAYLFQSTPVQVYGVNLVTGSTTLLADDVGLAS

GINAVGFDFTDRYIYGYDTTNLKIVRLGKDFQAETLNVAGLPSHTFFVGDVYNHVYYLYR

KGKGLFSIDLSPLDTDPNATLTLTQITATANVNLTDFAFHPGDGSLYGIDNNSGILYKFN

TANGQATMIGDTGETGTFGAGYFDVNGNYYVSRNQDGKIYRINLSADNASNINAGIVPAV

EFVSNGPSSSQNDGARCANAPVIDEDDPIDFGDAPDSYHTLLSSNGPRHEIDGVTWLGVI

APDGDPDGQIAPSGDNKVGTADEDGVGFVAALDPGLSSLVNVMASTSGYLSAWFDWNRDG

DFADEGEQVFTDQLLAAGNNTLPFIVSELATAGPSWSRFRFSQQTGLKYDGGSTSGEVED

HPITITENGVSVQHFPSAEGYATVAFEDNWPYTADYDMNDVVIQFRITETRMEGVVENIQ

ITGDLAAYGAGYKNGFAIRLPGIAKASIENGLTTLSFNGVQQASNGLETISEDAIFIISD

DLSQYTQSNCTYFRTQSGCDDNVAFSFTLTVYFKEGSDRSGLQAMPYDPFIFATPNTYHG

DGITFQPGRKWEVHLPDQAPTEQFDDTNLYGLGVDASDPVQGTYFKTSNNLPWALLIVED

WEWPLENVDLVDAYPQFATYAESAGSQNANWHASPKANKCYIP

>tr|Q87PN9|Q87PN9_VIBPA Putative polysaccharide export protein OS=Vibrio parahaemolyticus serotype O3:K6 (strain RIMD 2210633) OX=223926 GN=VP1462 PE=4 SV=1

MSDLRENISVLLHGAWRRRYMIVIPMLVLPILGFGVSKLVPTTYVAHTSMLIQETAKMNP

FLQDIAVSTMLKDRLSALSTLLKSRHVLYSVAKEQGLIDDDMGAKEQEFIIKDLASRLSV

QQLGKDFIQIQLRSGKAQGMESMLTSVSNRFVEQLLAPERSSIKDSSHFLTIHINKRREE

LDKAEHAFAEYKNTYSYATPEMQAQSLTRLASLKQTLAEKEAELAGVTRSLGSLDQQLSK

TNPVIGKIEEQIIEIRSELTLLRAKYTEAHSLVQGKLRELKRLEQERTVLLSSKQPELNS

NQLWDIASNATVSSLGEAQPLLVSQLHQLQIMRSRFESLTEETVSLQKMIQELESNAHRF

GSTATEINRLARDVAVKREMYDDLVERYEMAQLTGSLGVFEENKRVKIIDAPFTPTIPSN

LPSIVFIILGFIGGAGLGVGLAAILELADNSVRSRRALEKHLGVPVITTLPKVTFAS

>tr|Q87K32|Q87K32_VIBPA Uncharacterized protein OS=Vibrio parahaemolyticus serotype O3:K6 (strain RIMD 2210633) OX=223926 GN=VPA0066 PE=4 SV=1

MSQVRIKFIASDMDGTLLDQYGRLDPEFFDLFLQLEEQGILFSAASGRQYYSLRDTFAPI

KDRVLYVAENGTLVMYQDKELYSCTIPKAEVAEIVKAAREIDGANIVLCGKRSAYIETHD

QQSLEEFQKYYHRCETVTDLLEVEDEFIKVAICHFDGSEELLFPTMNAKFGATHKVVVSA

KIWLDVMNAEASKGAAIKHLQETMNFTPAETMTFGDYLNDLEMLQVSEHSYAVANAHEEI

KKIARYSAPSNQEAGVLKVIKEKVLAK

>tr|Q87L47|Q87L47_VIBPA Bacterioferritin OS=Vibrio parahaemolyticus serotype O3:K6 (strain RIMD 2210633) OX=223926 GN=VP2768 PE=3 SV=1

MKGDPIIIQHLNKVLGNELIAINQYFLHARMYKDWGLKHLADKEYHESIDEMKHADHLIE

RILFLEGLPNLQDLGKLMIGEDTKEMLECDLKLEMAAIPDLKDAIAYAEDIRDYVSRDLF

QDILEDEEEHVDWLETQLGLIEMSGIENYLQAQYVDEE

>tr|Q87QH9|Q87QH9_VIBPA Peptide ABC transporter, permease protein OS=Vibrio parahaemolyticus serotype O3:K6 (strain RIMD 2210633) OX=223926 GN=VP1170 PE=3 SV=1

MFWYTVRRFNLFFITLMILTLVGFSLLRLDPLSHWANVEFWSGWQSYLINLSQLDFGLSK

NGNAIYDELAVVFPATLELCFFAFLVSLLIGIPLGTIAGMKQGKWLDTGISFLSMSGYSA

PIFWVALLLIMVFSLEYHIFPVAGRYDLLYEIDHVTGFAIIDAFMAKGEYRAHALQSVIE

HMVLPCLVLALAPTTQVVGLMRASVAEVMSQNYIRAARIKGLSNREIVTQHVMRNAIPPI

IPKVGVQLSSMITLAIITESIFNWPGIGRWLLDALANQDYASIQAGVMVLATLVLTANIL

SDLIGAMINPLVRKEWYANK

>tr|Q87KF1|Q87KF1_VIBPA Thiamin-phosphate pyrophosphorylase OS=Vibrio parahaemolyticus serotype O3:K6 (strain RIMD 2210633) OX=223926 GN=VP3026 PE=4 SV=1

MAKILIPSSLIPLTGAVLQCLLLAKEQGFSIDEIELGVSPTQFIQLVLGQNTFRVGTDLI

DVCEEAETADFVLYYQSGLSVSECRQQPSSAIFIGIQDVESKLDDSVKTTSADVLDIWRH

PVNDEIRALSVASTSRTTTLQTDQHLAWTVTLLALDFPIEDALTLARPMTNVSRETLING

ETMVKQEWASQFADFPTPVLEDCRLGIKVGWSSHGQSVNFPHLSKQSLGLYPVVDDVSWI

ERLLPLGINTIQLRIKDPYQPDLEQQIARAIELGRQYDAQVFINDYWQLAIKHGAFGVHL

GQEDIEDSNLSQLSTAGICLGLSTHGYYELLRIVQINPSYIALGHIFSTTTKQMPSKPQG

LVRLALYQKLNDSIPYGESVGYPTVAIGGIDQSNAEQVWQCGVSSLAVVRAITLSESPKQ

VIEFFDQLMNTTSTSLVMEDYRAY

>tr|Q87H61|Q87H61_VIBPA Uncharacterized protein OS=Vibrio parahaemolyticus serotype O3:K6 (strain RIMD 2210633) OX=223926 GN=VPA1104 PE=4 SV=1

MQTPRLFNLNLRYKTALYFSLGLLGMIISFLFISRYFFLYSLDELENMEIDHSSQQAIAV

IDMMVTQQEGSSYDWAYWDETYDLFAHRDIAGYSERNLYVETLDALNLDLMSFITLDGQS

LVSLSRENTPESSSSLNNKVVSVPLLQQHILAMNSKLDVHRESLAGIFKINDNIWGLSLA

PVRNSEGDRPSNGWMLWGRNLSERFPGDFKAMMSASNTLVTKSFNPVDHAKTKSIDKTSE

SIIKWTPISDLAGEPIAWLKTQTKREHYSKGNTLFIYLFATVGVVASAIATSTFFIFKRK

VATRFSHFAEGINEIASQYQLEGLQSVKFDELELATKLVQKLSENTSMTQLQLKDSMEKF

GALYHSSSLGMLIVIEREIVDANHRALELLSYKKSDLLEQSLDTLCPTTADECRVDAMYR

ELQHGRNQFEAQMLDSHGETIDCLIEATLIQHNGHTALMLLLQDLSENKQQAEMIQKLTD

FDPVSGFCNRPVILGALEELVKHQPNHFSFIYITSKSLKQIAEVYGHLIFDEAIQYISTL

LRDHLGTHQIGRISEHEFIVILPNASGHSDALEAANRLLNQLSYKIELSGIMLSLDSKAV

MVDPKITHQSLEHLLLVARYSAQSMSGRHMHEVLVADGELSEQAETSMIIHRDLEVAIRQ

DNIIPYFQPIVNAKSGEIIGFEALARWPHPTLGIISPLIFIPLAEQGKLIVELGESILNQ

SCDFIGKLNTKRHSQGLPPLTVHVNLSAQHFYHSRLISFLQSMIEEHNISAGHLVLEITE

SMLMGGETESIHCINEIKQLGVQLALDDFGTGYASFSSVCNFPLDIVKLDKSYVDEIETN

DRAKTLVRNIANMSQELGLTIVAEGVETASQVRKLKVWNIDELQGFYFYKPMTREDAFAQ

FSGLPHC

>tr|Q87IE7|Q87IE7_VIBPA Iron(III) ABC transporter, permease protein OS=Vibrio parahaemolyticus serotype O3:K6 (strain RIMD 2210633) OX=223926 GN=VPA0659 PE=3 SV=1

MRDSTKIVLLGVIALVFAFLFIGIGLNADNYQYFLSRRVPKVLAIVLAGVAIAQSSMVFQ

TITHNRILTPSIMGFDALYVLTQVLIVLLFGGLSTLVLNIYVNFTIAACIMVAFSLLLFG

FYFSKGSRNLITLLLVGLIFGQLFSNVASFFSLLMDPNTFAFVQSKLYASFNNVKVNLVY

FSAPMLILACWLLFRMHRTLDVFWLDQDNAKSLGVDVPKVTRNVFILSAVLIAISTALVG

PIMFFGLLVTNLSREMFHSYQHKTLLIGCSLLAISSLLSGQWIIENVFNFETTLSVVINF

LGGIYFLYLLLKNKVV

>tr|Q87PQ0|Q87PQ0_VIBPA Putative ferredoxin-type protein NapF OS=Vibrio parahaemolyticus serotype O3:K6 (strain RIMD 2210633) OX=223926 GN=VP1451 PE=4 SV=1

MRQSESVDLSKRRLFSFRRAAVEQAQDPRVKARPPYAVEESMFTRLCDGCGKCASACPSQ

IIEMVDGVAALDISYSVCDLCGECKSACPTLALSNQTESTGLIATISNSCENLYGYCGSC

EDSCPYDALQWQDDAKPKIDAAKCKGCGQCAQSCYTSMISFDLKR

>tr|Q87QR1|Q87QR1_VIBPA Putative chemotaxis transducer OS=Vibrio parahaemolyticus serotype O3:K6 (strain RIMD 2210633) OX=223926 GN=VP1088 PE=4 SV=1

MFQNFTIKQKIVIPLSLIIGLFTVSSVLNVMTTSKQSELSDTLNEQIVPNLFTIEDAYRD

LYQATSAVQGIALAETQADIDHHIHEYKDNAYKALPRMEKVIELSRAGVMPASHGADVQK

LVSLGQKWLQSYEVMLSKPQSQWLSYYNEHKNTFEEQFVDVRAQLNVVKSAIEDKQGELK

SDISAATARAESILEMGIIVVILAALGMVFLLLRTVLKPLNDIKDAMAQIASGDGDLSQR

IQINTQDEIGQLAKAFNEFVSKIQATVSQVIDSSNTLRQEMANLSSLTETIADSTVSQQR

DSEAVAAAVHEMQVTSRNVSESANEAAVASQTANDELSNTNVILEQTVGSIRDLAGEIES

ASHVINTLDNDVSDIASVLDVIRGIAEQTNLLALNAAIEAARAGEQGRGFAVVADEVRSL

ASRTQQSTGEIQAMIEKLQSGAGQAVEVMRGSQNSSEETIQSAGRASESLAEILNAISRM

NEMNTHIATAASQQSTVSDEVNTNVQGIADSSTSIVDIVTQAQQSLAMLSQQTKRLDQQV

SQFRV

>tr|Q87KZ7|Q87KZ7_VIBPA Methyl-accepting chemotaxis protein OS=Vibrio parahaemolyticus serotype O3:K6 (strain RIMD 2210633) OX=223926 GN=VP2827 PE=4 SV=1

MRNTIKLKIQIAIAVIIAIVSGVQAWVSVNQLHEETTSTLNREIQNISESTNRYISDWLS

IRSDMMLANEQIIAGSDDADRELLLTKRAGKFLSVYAGFSDGAIAYGDKSESWPSDYDPR

TRPWYQDAMAQSGLIITEPYQDFDGSIVVSFAKAFNQNKQGVLAADLAVTDIINEVLNIQ

LDNNGFAFLVDGNNNLVAYKDEKLSQKPLTTLNPELTRDKMANLAQHAKLETITWPKQGD

QLIYVAQVPNTDWSLGVVQDKQMAFASVSEQVTFTAIASIVMYLIIAAISTYVITRLLRP

LQTLSDALSELSQGEGDLTQRIEIERMDEIGELATHVNQFLAQMQSMLKNIVENSQQLSE

QAQQANELSAMAAGRVEHQQNDVNQIATAIHEMSATAAEVASHAELTASASQNSASACVE

GQSVIQKNREAIVSLAEQVSDAANVISELEANTQSINQILSTIQGIAEQTNLLALNAAIE

AARAGEQGRGFAVVADEVRVLSQRTHGSTEEIRTMIETLQSNTKLAVNSMQASTSLADTS

VDYAQQAHDSLTSITNSITEINDMAMQIASAAEEQRAVSEDISRNTQGIKDDADVIAEQS

LKSSEGARRMFNTANTMRENISRFKV

>tr|Q87FI6|Q87FI6_VIBPA Uncharacterized protein OS=Vibrio parahaemolyticus serotype O3:K6 (strain RIMD 2210633) OX=223926 GN=VPA1693 PE=4 SV=1

MFGSKKLKMENEALKQELASLKDKYQTDVETLERQLKEAKQLLNTAQQRYESSDELMSSS

LKGGDMLQTIRTAMVESAQSMAHENEELKLLDDMFKQTHQALARLDDRAVKISSQATQSI

ESVQILDNTATSISHLVSTIQEISDQTNLLALNAAIEAARAGEAGRGFAVVADEVRNLAG

KASEASEQIDSLVNQVLTQVSSIKSAIDENQICAEEVSASSAQIGSIVNEVVVKSEHMKR

VIHFASTRSFLDTVKLDHAIWKNNIYRLLQSGSFGETVNSHSECRLGQWYYRGDGKAYSQ

LRSYAQLEAPHKGVHDSGRDAMNHAKSGNMAGMVTSINSMEDTSEQVVIHIDRLMDEIIA

N

>tr|Q87N38|Q87N38_VIBPA Chemotaxis protein CheV OS=Vibrio parahaemolyticus serotype O3:K6 (strain RIMD 2210633) OX=223926 GN=VP2037 PE=4 SV=1

MSGVLNTVDQRTNLVGENRLELLLFTLNSRQLFAINVFKVKEVIKLPPLTKLPGSHYNIR

GVASLRGEAVPVIDLRCSIGFPPLRGEAEEENLIVTEYNRSVQGFLVGPVRNIINTAWTE

IQPPPSTSGRSNYLTAITQVKDGDTSQIVEIIDVEKVLAEIVHYDVTISEDILDHDLSQS

MVGRNVLIVDDSSTARNQVRDTLSQLGMNIIECRDGLEALTVLKRWCDEGRDVEKELLMM

ITDAEMPEMDGYKLTHEVRSDPRMSKLFITLNTSLSGSFNEAMVQKVGCDRFISKFQPDL

LVEVAQDRLRQVLSANA

>tr|Q87IZ7|Q87IZ7_VIBPA Uncharacterized protein OS=Vibrio parahaemolyticus serotype O3:K6 (strain RIMD 2210633) OX=223926 GN=VPA0458 PE=4 SV=1

MSLWGFLGAGLAYLLMTFAFVFGGIFWLCAEGNTLRETKRQSSIMSGIIVCTMGTWVIAF

SIYIYGYFWDNSSHYYSYLLAPWPLAIVGITLRNHWVSQYSSVKQEKNEKWQRHWREILG

EDTEDLPPYRYDYGLYSGIWQANETLREQCFAALTHGNSVYERVKAFQKMTTHEHNTDDQ

ILLSKLAQLENEIIQALEQHSQKNVSIETGSGTLCKESKHNVYRHENGPTEEQLYNSINL

QHDLDRELRNIIYDRLGDDGLDEYFFLRAPLEELTENETAINWMLWGLVSDHFDVDPYQT

ALELNLMNAEPRWGQDERFVVVTTAA

>tr|Q87R95|Q87R95_VIBPA Putative hemolysin OS=Vibrio parahaemolyticus serotype O3:K6 (strain RIMD 2210633) OX=223926 GN=VP0902 PE=4 SV=1

MLLTCALLEGAMDILLLVGLIALNGIFAMSEIALVAAKSGRLKMMAEDNAPAALALELKN

NPTQFLSTIQIGITAIGLLSGIFGEATLSIPFEHWLVAQGLEREVATILATTSVVILITY

FAIIIGELVPKRFAQNNAESIAIVVAYPIHWLAKLARPFVFLLTVSTDALLKLLRQNENQ

GEIVTEEDIFAVVNEGSESGAIEPQEQLMIRKLLHLNDRLALSLMTPRCDIHFLDTNLPL

DAILKHLRQTQHSVWPVCKGGLDNIIGTISSKVLLDEYDHLSVSRLGKLLKHPRFVPESM

KGLPLLNYMQQTSAEMVFIVDEYGDVQGLVTLYDLLKSIAGELGMAPEQIWAKQQKDGSW

LMDALIPLNELKYKLGLTTIEGEESEGFQTLNGFLTWWLGRLPHAGELVDYEGWQFEVLN

VKHHRIVQVKVSRETESETTTDTNDGDANP

>tr|Q87JP9|Q87JP9_VIBPA Putative hemolysin secretion protein HylB OS=Vibrio parahaemolyticus serotype O3:K6 (strain RIMD 2210633) OX=223926 GN=VPA0199 PE=4 SV=1

MFAFVRQFPLYIMVSVVAGVPLLLAIFLAVQHTINLNTQSHQALKDQQLVTLITHYDNLA

HNLAVERGLTAGVLGSQGKTEIVQKLTQQRKKVDDAVNSLVQLNTPLISSSDSYDLLQDV

QVQLNRLNQVRQGVDRLSPQIAPFGYYSNLNQLIIDNIDILIAQTQSRELGTLGDALISV

IVMKERAGQARGALNGVFAKGSATSVLFSNIEGYIQSGDYASRKAQIAFPEQYRQSLQSH

QSNPAWKKVEQVQQSFLNQSANLDNIQGPQATEWFPMATERIGLMNQLRNQMVDQMLSAA

EHSAQQATLNRNLLMAATTVISLLMIMMVWGLVASLRSRVGRLKQTLRSMSEHHDMTVEL

DSRGKDEIASISNSINALISNFRKLLFDVTKTNNESSNRLQNIVESAQDLDSSSRSTIAK

CDNIATAMTELAQSSVEIAQSAERAMGDTTTMNNKVVDCQTQSELSYRSVQSLLEQINAT

EQCMAELANDTQSIGQIVETINSVSEQTNLLALNAAIEAARAGEHGRGFAVVSSEVRDLA

QRSQEATENISKLLDQIGEKTRFSVESMAKSKQASDDTFESVQQVNESVSLLESSIEHVN

NHISTITHSTIEQSKACEAIDQDIDVLASIAHKTGQHADDLNQIVSGYQAEAKELKHQLS

AFKLA

>tr|Q87SV7|Q87SV7_VIBPA Flavin prenyltransferase UbiX OS=Vibrio parahaemolyticus serotype O3:K6 (strain RIMD 2210633) OX=223926 GN=ubiX PE=3 SV=1

MHNKIQPSQKKAITLALTGASGAPYGLRLLECLVAADYHVYVLISSAARVVMATEHNLKL

PSGPEAAQQALVEHLNCNPDNITVCGKDDWFSPVASGSAAPKQMVVCPCSAGSVAAIAHG

MSDNLIERAADVVMKERGQLLLVVRETPFSTLHLENMHKLSQMGVTIMPAAPGFYHQPKS

IEDLVDFMVARILDHLGIEQGLVPRWGYDQRS

>tr|Q87G79|Q87G79_VIBPA Iron(III) ABC transporter, permease protein OS=Vibrio parahaemolyticus serotype O3:K6 (strain RIMD 2210633) OX=223926 GN=VPA1438 PE=3 SV=1

MRTKPYIGYVALIALLSLLSLQIDTSLSLSEQWQLFTQPESASEFRDVFFMQSQLPRLSI

TLLVGAMLGLTGSLMQQLTQNNLTSPLTLGTSSGAWLALVIVNIWFVDWVADYSAFAAMA

GALVAFGLIISIAGVRNMTGLPLVVSGMVINILLGSIATALIILNAQFAQNIFMWGAGDL

SQYSWDWFEWLLPRSTIAIVILIVAPRILTLMKLGQEGAAARGLAVLPAFGALMVMGIWL

VSASITAVGIISFIGLLTPNIARAMGARTPRDELISSMLLGAALLLITDSAAIYLSLLLE

ETIPSGVAAAAIGAPALIWFTRKKLTATDQLNLSMSQGKMALSNAAVWGIAVMGIIGILT

YSFVTHGISGIEFATPGEFQWQLRWPRMISAISVGVALSVAGIILQRIVYNPLASPDILG

VSSGATFAIIITGVMVGSVLAAFNWGVAFLGSLTVLMLLLIIGKRSHFNPSNFVLSGIAL

SALLQALVQFALAQGSGESYKILLWLTGSTYRVTSTSALMLLIAVLVLLAIVFALSRWLT

LISIGRVFSNARGLNPSSANTILLVIVALLCAFSTATVGPVSFVGLVAPHMAMMLGARKV

KEQLFVGSLIGATLMVWADWLGQIAIYPSQIAAGTLVAIIGSTYFLFLMLKSKFR

>tr|Q87IE8|Q87IE8_VIBPA Iron(III) ABC transporter, permease protein OS=Vibrio parahaemolyticus serotype O3:K6 (strain RIMD 2210633) OX=223926 GN=VPA0658 PE=3 SV=1

MKKLLLTLVVLSTVSLFVGVADMTPQQLFSGDAKALELFFTSRIPRLFAILLAGAGLSIA

GLVMQQISQNRFAAPSTTGTIECAMLGYVMSVVFFGDGDHLWLVFGISVLGTLTFVHFIQ

RIQFKSVVFVPLVGIIFGNVIESMTTFIAYKYDALQSLSAWSVANFANILRGDFELLYIA

VPMVILSYLFAARISAVGIGKDFAVNLGLNYQQVVTIGVLLVSIMSASVVMIVGQLPFLG

LIVPNLVSHFYGDNLKKNIPLTAMYGAILVLGCDLVSRLIIFPHEMPISIVISILGGVVF

IAMLLRGKQHA

>tr|Q87NH6|Q87NH6_VIBPA Methyl-accepting chemotaxis protein OS=Vibrio parahaemolyticus serotype O3:K6 (strain RIMD 2210633) OX=223926 GN=VP1892 PE=4 SV=1

MRSTITFKLLLALIVVFSCVLAASTAYQHYQQKTLINDVLSEQLHDKASNYFDSLNMMML

TGTMSQKETLRQKALAQEGIEQVRVLRADAVTKLYGAGQSNQQPIDEIDQRALAGELVIE

PITADWGKGIVVALPMKSSQNYRGTNCVSCHVAPEGEVLGAIRLEYNMNHVSSMINKQAM

YAMGIMSAIALVGFLITMGLIRKIIVRPIQKTSHFMSNVSASKDLSQRLVHKQNDEVGQL

SQSINSFMDTVSESLERVQDTSHSLAGSAGRLTDVAQSTDEAANNQQLETNEVQNNIIDM

LQQQVVVEEATINATTLVNHTVDVATNSASQAHNVSEDIKSLVSDIEQVREKITSLNQRT

EEVSSILGVIKGIAEQTNLLALNAAIEAARAGEQGRGFAVVADEVRNLASRTAEATSNIE

SIISQFQQGSEESLSSVDHVCQFAHQRSLDVEALSETMHNVVDEMHQVLKHAENIQLQTQ

TTSDVSKHIQSKIDVITLHANDTSQSASHTRDISVDLEELSDRLEQLLNQFTLSEQQRAN

K

>tr|Q87R35|Q87R35_VIBPA Methyl-accepting chemotaxis protein OS=Vibrio parahaemolyticus serotype O3:K6 (strain RIMD 2210633) OX=223926 GN=VP0963 PE=4 SV=1

MRGSVIKRMYAGFALIIILFAVTIAIMMGGMHDIHGKFETVSKSSLPLVSLSNQTSVELL

SADKSFKDFLTTENKQRMEEMRQEFARSQQRFESTLAQLESASQIYPSLAEPFSQLKTLE

QSYFTEALEAMDNYEAMFSAQEEVQKSSRRFQKLNTELSVGLKEYVADQSSISVKVMAKS

YFIKLKDAEVITSDALASSNPEFVTQAVTKNRKAVTHLNYAFRGLVTQLPELEKAFGESV

EQFTRDVGMRGGVLDQHNNYLNARAALYDNIANLANKVDSTMAILEQFTTTATDKLNESL

DDAGDIYSAGVTKAIVIGVVVVLFAAAIGYHIAQSVREPLTRILNALESLTKGDMTQRID

IRFNNEFSRVSGHINTLADSLHDILVKLNEASENLASTATTNERTSSQAQSKLNAQREQT

ANVATAMTEMSHSVQEVAQSAQGSLEMVQRVESASEEGRNVMSSNISTINQLETRLHESV

SAVSELQKMSGQIGSILDVIRNIAEQTNLLALNAAIEAARAGEQGRGFAVVADEVRVLAS

KTTQSTTEIESMISNLQSSSQSANQVIQSCMSDMEMSVEQASKANSSMEEIQALIIEISQ

MSTHISQAAAEQSETSADIARNIEDINNIADESYHAMSSITHTSESLTQLAHQQNELVHR

FKL

>tr|Q87LH7|Q87LH7_VIBPA PTS system, cellobiose-specific IIA component OS=Vibrio parahaemolyticus serotype O3:K6 (strain RIMD 2210633) OX=223926 GN=VP2635 PE=4 SV=1

MEQELVVMEIICNAGEARSLSYEALRLAREQKFEAAEEKLLQARECINKAHLIQTQLIEE

DQGEGKVPMTLVMVHAQDHLMTTILAQEMAVEIVALNKQLANK
[truncated: 1,155,666 more chars]
