# Supplementary material for: Comprehensive genome based analysis of Vibrio parahaemolyticus for identifying novel drug and vaccine molecules: Subtractive proteomics and vaccinomics approach
Source: PLoS One. 2020 Aug 19;15(8):e0237181. doi: 10.1371/journal.pone.0237181 (PMC7444560; doi:10.1371/journal.pone.0237181)
Supplement: S5 File — (DOCX) [file pone.0237181.s018.docx]

**S5 File.** Essential proteins in DEG 15.2 server (E value ≤10^-100^, Bit score >100).

>tr|Q87KJ0|Q87KJ0_VIBPA Adenylate cyclase OS=Vibrio parahaemolyticus serotype O3:K6 (strain RIMD 2210633) OX=223926 GN=VP2987 PE=4 SV=1

MQAYTYTIIQRLDNLNQQRIDRALALMDSQSQQVFHLIPALLNYNHPVIPGYYDADVPFG

VHGLELNPIQQQFIDDIQLAIGQPLKTAEKPAILGLYTMGSTSSIGQSTSSDLDIWVCIS

PEMDCDERELLTNKCLLITDWAQSQGVEANFFLMDEERFRSNHSEEMTGDNCGSSQHLLL

LDEFYRSAVRIAGQRLLWQIVPPEMEECYDEYVSQLCSDGYIDCSEWIDFGKLNRIPAEE

YFGSNLWQLYKSIDSPYKSVLKAILLEAYSWEYPHTQLLSIDTKRRFFAHEPDLYGMDAY

YLMLEKVTRYLERIQDDTRLDLVRRCFYLKTHEKLSREPDVGSVAWRREALSDMIAKWNW

DDSVVAELDDRRNWKVEQVKVVHHALLDALMQSYRNLIQFARRNDITSAISPQDISILAR

KLYAAFEVLPGKVTLLNPQISPDLHEADLSFIEVKEGGVNKSGWYLYKQPLIAHRILGQP

CLEHHEYLSKLVSWAFFNGLITESTRLHAVVREAQLDIDKFYQMVSDLRNTFALRKRRPT

MQALASPCEISQLAMFINFENDPTSELSGRSLKVDVKNTDIFSFGPEHKNLVGSVDLVYR

NSWHEVRTLHFKGETAMLDALKTILGKMHQDALPPESVDVFCYAKNMRGVMRNMVYQLLA

ECIDLRLKPVEQEKRRRFKAMRLGNQTYGLFFERRGVSVQKLENSIDFYRSISTNKLKGS

PLLMLDREQEYQLPEAVDGFASEGLVQFFFEDNEDGFNIYVLDESNQVEVYHQFSGSKDE

MIASVNSFYTSVKDDSRVASKFINFNLPQYYQIIHPEEGNAYIIPYRNDGCSPHRPTKAV

NA

>tr|Q87FY4|Q87FY4_VIBPA Flagellar M-ring protein OS=Vibrio parahaemolyticus serotype O3:K6 (strain RIMD 2210633) OX=223926 GN=VPA1536 PE=3 SV=1

MSELTPQVAGNTAMTTSTTQAFSPAGNMDDVTNKLKQLWSSSQRNLVLSAVLAAIVAAII

VVALWSSSQSFRPLYSQQERFDIGEIVSVLESEGVSYRMQEQNGQVLVPEGEVARIRMLL

ASKGVKAKLPTGLDSLKEDSSLGTSQFMETARYRHGLEGELVRTIMSLNSVANARVHLAI

PRQTLFVRQNGENPSASVMLELKPGEDLKPEQVEAIINLIVGSVTAMKPEFVSVIDQYGR

LLSADVASAEAGKVNAKYLEYQKNVEKQIIQRAADMLTPIVGPSNFRVQVAADMDFSQVE

ETREILDNAPVVRNEHTIQNNSIDQIALGVPGSLSNQPPVTGEAATNDSQNTNARSEVNR

QYAVGSSVRRTQYQQGQIEKLSVSVLLNSKASPDGVAWSDADKAQISTMITDAVGISAAR

GDSLSLMSFNFTPIDIDAPTALPWWQDPTVQQPLRYVIGGMLGLAMIFFVLRPLIMHLTG

ADKPVPELNFAEPPQEEPDYDNLQTREEREHEEVLNRRLSEKGISASTGLDVNSDMLPPA

GSPLEIQLKHLQLIANEEPERVAEILKQWVNINEHSSVDVKTNA

>tr|Q87T73|Q87T73_VIBPA Putative capsular polysaccharide biosynthesis protein D OS=Vibrio parahaemolyticus serotype O3:K6 (strain RIMD 2210633) OX=223926 GN=VP0197 PE=4 SV=1

MNTILSLIGRTDALFTQDIAHHEAELFRIVSESRFLVLGGAGSIGQAVTKEIFKRNPKKL

HVVDISENNMVELVRDIRSSFGYIDGDFQTFALDIGSLEYDAFIKADGKFDYVLNLSALK

HVRSEKDPYTLMRMIDVNVFNTDKTIQQSIDAGAKKYFCVSTDKAANPVNMMGASKRIME

MFLMRKSEQIAISTARFANVAFSDGSLLHGFNQRIQKRQPIVAPNDIKRYFVTPQESGEL

CLMSCIFGENRDIFFPKLSEALHLISFADIAVKYLEQLGYEPHLCDSEEEARQLAHMLPE

QGKWPCLFTASDTTGEKDFEEFFTDKEELDMSRFENLGIIKNEPLYEQELLTLFENSISE

MKGEQAWSKEEIVKLFFTMIPDFGHKETGKYLDSKM

>tr|Q87SY5|Q87SY5_VIBPA ElaA protein OS=Vibrio parahaemolyticus serotype O3:K6 (strain RIMD 2210633) OX=223926 GN=VP0287 PE=4 SV=1

MTTWITLPFAQLTTLQLYEMLRLRVDVFVVEQTCPYPDLDGKDIIDGVHHLLGYHNDELV

ACARLLPAGTTYDNVSIGRVVTKQTARGGGLGHRLITQAIQACSTLWPEKTIDIGAQEHL

IDFYAHHGFEVMSDSYLEDGIPHVDMRRKHSTQ

>tr|Q87FJ2|Q87FJ2_VIBPA Putative transcriptional regulator OS=Vibrio parahaemolyticus serotype O3:K6 (strain RIMD 2210633) OX=223926 GN=VPA1687 PE=4 SV=1

MDKIRSLRFFIATLEGGSFAAAAKAYGTDPSTVSKAIHRLESDLGIQLFQRSTRQIRLTE

AVRRYANTARFVLDELAACEDSLKSHNDALSGLLKINVPVSYGRLYIRPLLKEFCRRYPS

ITIDIHYDDAYVDIIEQGIDVSIRSGVVQDSQLIVRQLSPIDFIICGSQDYLTRHGVPSG

PDAFNDHSWVRFRFKQTGKLLPIRMPEPDGTSEYNPERNFIVNDGESMAELCAEGLGLTQ

IPHFIARDWLKVGRLVPIFPSMRQAGNGVYLLYANREYVPARVRVFIDFITQAIQDMDET

PFHTWAETLPIYQPNQGD

>tr|Q87P44|Q87P44_VIBPA Translocation protein in type III secretion OS=Vibrio parahaemolyticus serotype O3:K6 (strain RIMD 2210633) OX=223926 GN=VP1674 PE=3 SV=1

MSYDDLHQALFLYSLTLPRLMACFIFLPILSKQMLGGAMIRNGVLCSLALFIFPVVNEQA

LPAETDGLWLIVILGKEVLLGMLIGFVAAIPFWAIEATGFLVDNQRGAAMASMFNPTLGS

QSTPTAVLLTQTLITLFFSGGGFVAFIYALFKSYTTWPILGFFPMVTDAWVSFFYDQFQQ

LMWLGVLMSAPLVLAMFLAEFGLALISRFAPQLNVFFLAMPIKSAIASVLLIVYLGLMMD

HFEALFYGITRFGDQLNTIWK

>tr|Q87JU3|Q87JU3_VIBPA Protein TonB OS=Vibrio parahaemolyticus serotype O3:K6 (strain RIMD 2210633) OX=223926 GN=VPA0155 PE=3 SV=1

MGRLLIALPASLLIAVSLFSFMAWMVDNGNQRAPKPSEAVRFDMVMVENDADVQRRQRSV

PEQPEPPQAPEPMELSQANTQVEPMSQVTPISALGLNTALDGIAINAPNLKGTMGNQQAL

PLYKVEPRYPSKALKRKVEGYVIMRFTIDTTGRPKDIEVIDAEPKRMFEKEAISALKKWK

YQPKVENGVSIEQFGQTAKVEFKLGK

>tr|Q87I65|Q87I65_VIBPA Putative fimbrial protein Z, transcriptional regulator (LuxR/UhpA family) OS=Vibrio parahaemolyticus serotype O3:K6 (strain RIMD 2210633) OX=223926 GN=VPA0741 PE=4 SV=1

MRFTLNNVLIIDDQPLYSEALASLVENAINTAEVIQTTDSAEVMELVRSQRIDLIILDVV

LGDRDGMRLAKNILATGYRGRLLFVSSRDYSSLSKAAYEMGANGFLNKNEARETIADAIV

SVSRGYSMFKSTHTPSSGDVTLSNREAMVFHYLAQGYSNKKISEQLSLSAKTISTYKTRI

LKKYHADSLIELLHTIPQSENIQFCR

>tr|Q87QE5|Q87QE5_VIBPA Uncharacterized protein OS=Vibrio parahaemolyticus serotype O3:K6 (strain RIMD 2210633) OX=223926 GN=VP1204 PE=4 SV=1

MEQLEFFTVPSPCVGVCTSDEKGYCKGCMRKREERFNWLNLTPAQQLHVIKLCRQRYRRK

MLARKTKPEQLQDNSSPQQDLF

>tr|Q87J66|Q87J66_VIBPA Transcriptional regulator, LysR family OS=Vibrio parahaemolyticus serotype O3:K6 (strain RIMD 2210633) OX=223926 GN=VPA0387 PE=4 SV=1

MDKFSDMTLFVSIVKHQGLAAAGRELGLSPATVTARLQAIEERYGVKLLNRSTRHVSLTD

AGAMYHQACLNIIDSVKETENLLQTGISEVRGTLKISAPRDIGKQIISPMVSAFSEQYPD

VTPYLYLNDNLSNLAESGLDLVIRYGELADSNLISRRLASSQRVLCASPDYLSKQGVPNC

PQDLAAHRCLAMVRSNEELKTWHFKDEESHQSITVTPKRFSDDGEVIRQWALDGAGIALK

SILDIQQDLKQQRLVTVLDGYMKNFSAFSQGAEADLHVIYQSRQYQPKRVRLFLDFLVEQ

FSALSDTSNQI

>tr|Q87NZ4|Q87NZ4_VIBPA Putative DNA polymerase III, epsilon subunit OS=Vibrio parahaemolyticus serotype O3:K6 (strain RIMD 2210633) OX=223926 GN=VP1724 PE=4 SV=1

MMWLEKSKASTPLLDTSQTPEWSVLFEQLAEQAQDQRLKRYYSTPMVNGDTPLKEVPFVS

VDFETTGLNAEDDAILTIGLVPFTIDRVQCSGSAHWIVNPNRELNEESVVIHGITDSEVK

NAPQLTQILGEILDALAGKVVLVHYKNIERQFFYNALLNTIGEGIQFPVVDTLDIEYALQ

RRECSGIWNKLKGKKPGSVRLGHARERYGLPAYQPHHALTDALATAELFQAQLQYHFNRD

MPISAIWQ

>tr|Q87KM6|Q87KM6_VIBPA GlpG protein OS=Vibrio parahaemolyticus serotype O3:K6 (strain RIMD 2210633) OX=223926 GN=VP2951 PE=4 SV=1

MKRLVTLNNPRMAQAFIDYMASRQIDIEMMPEGEGQFALWLTDSQHEVEAEAELKQFLAN

PSASKYSAASWDVADTRKSQFHYASPSIIGMIKAKAGPVTLLIMTVCAVIYGLQMLGFGN

GVFALLHFPAFEGQQWQLWRWVSHALLHFSVTHIIFNLLWWWQLGGDIERRLGSGKLLQI

FVVSAALSGAGQFYVEGANFGGLSGVVYALLGYLWVLGYRLPHLGLTLPKSIIGFMLVWL

VLGFVQPFMAIANTAHLAGLLAGMAIALFDSGKQKYQQQA

>tr|Q87LZ6|Q87LZ6_VIBPA Dihydropteroate synthase OS=Vibrio parahaemolyticus serotype O3:K6 (strain RIMD 2210633) OX=223926 GN=VP2462 PE=3 SV=1

MIITANNKSLDLSRPHVMAILNVTPDSFSDGGKFNSLELALAQVEKMITAGVSIIDVGGE

STRPGAPDVSLEEELQRVVPVVKAIREKYDVWISVDTSKAEVMRQAIAVGADLINDIRAL

QEPGALQVAAASKLPVCLMHMKGQPRTMQESPQYENLMDDVAQFLEERIAACEAVGINKS

QLILDPGFGFGKTIEHNYHMLAHLEKFHEFGLPILAGMSRKSMIFKLLDKPAADCTNASV

VCATIAAMKGAQIIRVHDFEETIEAMKIVEMTQNNI

>tr|Q87HR8|Q87HR8_VIBPA Sui1 family protein OS=Vibrio parahaemolyticus serotype O3:K6 (strain RIMD 2210633) OX=223926 GN=VPA0888 PE=4 SV=1

MTLVYSTETGRIKPEEEKVARPKGDGIVRIQRQTKGRKGKGVCIVSGLDLDDAPLKLLAA

ELKKVCGCGGSVKDGTIEIQGDARDKIKTHLEKKGMTVKLAGG

>tr|Q87QL5|Q87QL5_VIBPA Uncharacterized protein OS=Vibrio parahaemolyticus serotype O3:K6 (strain RIMD 2210633) OX=223926 GN=VP1134 PE=4 SV=1

MNLIDFSHSPVSLLPPIVALTLAILTRRVLVSLGVGIALGAVLLNSWSIGGTASYVGTQV

SSVFIEDGGINTWNMSIVGFLILLGMTTALLTLSGGTRAFAEWAQSRVKSKRGSKLLAAF

LGVFIFVDDYFNSLAVGAISRPVTDRFYVSRAKLAYILDSTAAPMCVIMPASSWGAYIIT

IIGGILVSHGITEYSALGAYVRLIPMNFYAVFALLMVFAVAWFGLDIGKMREHEIAASQG

RGFDKDKENDTQEAHELNEELDIRESEKGKVSDLILPIVTLIIATIASMMYTGGQALAAD

GKEFALLGAFENTDVGTSLIYGSLLGLAVALFTVLKQGLPLTEITRTLWIGAKSMFGAIL

ILVFAWTIGSVIGDMKTGSYLSTMAQGNINPHWLPVILFLLSGLMAFSTGTSWGTFGIML

PIAGDMAGATDIALMLPMLSAVLAGAVFGDHCSPISDTTILSSTGARCNHIDHVSTQLPY

ALSVALVSCVGFIALGMTTSIAFSFIAASITFVIVCAVLSWLSKSKIESCQSA

>tr|Q87IU7|Q87IU7_VIBPA Putative tyrosine-specific transport protein OS=Vibrio parahaemolyticus serotype O3:K6 (strain RIMD 2210633) OX=223926 GN=VPA0509 PE=3 SV=1

MNLKLVGSSLIVAGTALGAGMLAIPMVLAQFGLLWGTLLMLFIWAGTTYAALLLLEASCK

VGGGVSMNAIARETLGKGGQLVTNGLLYALLVCLLMAYIIGAGDLVQKITASVGLSVSTV

SSQVGFTILVGLIVSAGTGVVDKLNRGLFIGMIVALVLTLFALAPSVSFEGLNEVVSSDK

MALIKTSSVLFTSFGFMVVIPSLVTYNKEASKTQLRNMIVVGSTIPLVCYLLWLFAVVGN

LPPHELVQYSNVTELISVLGQQYNGLEFILSMFTGLALLTSFLGVAMALYDQNADLLKTS

KPVVFVTTFILPLLGAVFAPEHFLAILSYAGIILVFLAVFVPLSMTMKVRRVPVEDNSVY

EAGGGVMGMSMIFLFGCFLLFAQAV

>tr|Q87HV4|Q87HV4_VIBPA Transcriptional regulator, LysR family OS=Vibrio parahaemolyticus serotype O3:K6 (strain RIMD 2210633) OX=223926 GN=VPA0852 PE=4 SV=1

MRYSLKQLAVFDAVADTGSVSQAADKLALTQSATSMSLAQLEKMLGRPLFERQGKQMALT

HWGMWLRPKAKRLLQDALQIEMGFYEQHLLSGEIRLGASQTPAEHLVPDLISIIDNDFPE

MRISLGVQSTKGVIDGVLDYKYDLGIIEGRCDDNRLHQEIWCRDHLTVVAASHHPFARNP

SVSLAQLEQAKWVLREHGSGTRKTFDSSIHHLIEDLDVWREYEHVPVLRSLVANGQYLTC

LPYLDVERYIEAGRLVALNVPDLKMERTLSFIWRADMAENPLVDCIKREGLRMMKGKPSV

L

>tr|Q87T69|Q87T69_VIBPA Putative acetyltransferase OS=Vibrio parahaemolyticus serotype O3:K6 (strain RIMD 2210633) OX=223926 GN=VP0201 PE=4 SV=1

MLVDLLRNQNRTILAVMSPDDISTRQAFDGIMQLSNDKDISRYSPDEVRLVNGIGMMPKS

LLRRKVNQYFLDLGYQFETVISDQALVSKFAHLQDGAQILKGAIVQCGAVIGEHSIINTG

AVIEHDTVVGEHNHIAPRAVLCGGIVTQSDVYVGANATVIQNLKLAQNVVVGAGAIVTCH

LDAHQVCYSGRATIKNSK

>tr|Q87K05|Q87K05_VIBPA Uncharacterized protein OS=Vibrio parahaemolyticus serotype O3:K6 (strain RIMD 2210633) OX=223926 GN=VPA0093 PE=4 SV=1

MLDTTKPSLALFDFDGTITREDMFSLFLHYSAYGLRKRVGKLAIMPFYALYKLGVLPARV

MRPLSSFIAFSGKETQHIEAIGATFAHEVIPLYLRPEAMERLAWHQHRGDTIVVVSASLN

AYLKPWCEANGYHLLCSELISEQPKLSGFYQQGDCSLERKVSRVKAAFSLDEFASVYAYG

DTHEDIPMLKLADYAMLNWSEWRASE

>tr|Q87KB1|Q87KB1_VIBPA Putative amino acid ABC transporter, permease protein OS=Vibrio parahaemolyticus serotype O3:K6 (strain RIMD 2210633) OX=223926 GN=VP3066 PE=3 SV=1

MTFRTLWLISLLLLTGCSDYQWGWYVLDPSTEQGITNLKFLVAGFNDTIQVSLLSMCFAM

TLGLLIALPALSRSPTLKWMNRIYVEVIRSIPVLVLLLWVYYGMPTLLDVSLNHFWAGVI

ALTIAESAFMAEVFRGGIQAINRGQHEAAESLGLNYWQKMRLVILPQAFRQILPPLGNQF

VYVLKMSSLVSVIGLSDLTRRANELVVNEYLPLEIYTFLVLEYLLLILFVSQAVRWLEKR

IAIPSY

>tr|Q79YY3|Q79YY3_VIBPA BfdA OS=Vibrio parahaemolyticus serotype O3:K6 (strain RIMD 2210633) OX=223926 GN=VP1393 PE=4 SV=1

MPTPAYMSINGETQGHITKDTYSADSVGNTWQEAHVDEFLVQELDHVLTVPRDPQSGQPT

GQRVHRPLVVTKVQDRSSPLLFNALVSGEKLPECLIRFYRTSVQGKQEHYYSIKLIDALL

VDIQTRMNHCQDAATADRVTEEVLKFTYRAIEVTHENCGTAGNDDWRAPREA

>tr|Q87SV8|Q87SV8_VIBPA UDP-N-acetylmuramate--L-alanyl-gamma-D-glutamyl-meso-2,6-diaminoheptandioate ligase OS=Vibrio parahaemolyticus serotype O3:K6 (strain RIMD 2210633) OX=223926 GN=mpl PE=3 SV=1

MHIHILGICGTFMGGAAILARQLGHKVTGSDANVYPPMSTLLESQGIEIIEGFDPSQLDP

QPDLVVIGNAMSRGNPCVEHVLNSNMRYTSGPQWLNEFLLHDRWVLAVSGTHGKTTTSSM

LAWILEDCGYQPGFLVGGVLGNFGVSARLGESMFFVVEADEYDSAFFDKRSKFVHYHPRT

LIMNNLEFDHADIFDDLEAIKRQFHHLVRTVPGNGLILAPKQDQALTDVLERGCWTEKQF

SGEDGDWQAHKLVLDGSKFEVALQGEKVGTVEWDLVGDHNVDNALMAIAAARHVGVTPEL

ACQALGRFINTKRRLELKGEEQGITVYDDFAHHPTAIELTLGGLRNKVGEKRILAVLEPR

SATMKRGVHKNTLADSLHSADEVFLFQPDNIEWSVQDIADQCKQPAFVDADMDNFVAKIV

ERAQPGDQILVMSNGGFGGIHGKLLEQLKLKA

>tr|Q87I85|Q87I85_VIBPA Rough colony protein RcpA OS=Vibrio parahaemolyticus serotype O3:K6 (strain RIMD 2210633) OX=223926 GN=VPA0721 PE=3 SV=1

MFNKNILLVTLGVALLLPASVSAGELLSLDKGAAKTINVKRNIDTVFVADTQIADYKVIA

NGKLVIYGIGRGATSIIAYDRAGNEIYNAEVVVNKSLRLLKQTIIARYPDEDIKLTNIGE

QIVIDGVVSSEEIKDKVYRHVGEMLKKSKQRNTFELSGANGESVDPLDYTATYVFEDIIN

NLKVLTTDQINVKLTVAEVSSSFLTELGVSYAETNGKSIGGAGQFVNKILDFTAEDIVAV

ISASGNDSIGQVLAEPNLSVISGESASFLVGGEIPITVRDNDGISVTYKEYGVKLSMVAK

VTDSENIRLSLLPEVSSIDKTNGVNSGLVSVPSLRTRKAQTTVQLKDGQSFVLAGLLTSE

EQESLAKIPYLGDIPILGALFSKTNTERRKTELIIVATVNLVDPVKETDIKLPKFERTSD

LERLLKLDLSKVDDEELENTIKAGGFN

>tr|Q87KG7|Q87KG7_VIBPA Uncharacterized protein OS=Vibrio parahaemolyticus serotype O3:K6 (strain RIMD 2210633) OX=223926 GN=VP3010 PE=4 SV=1

MNESTILLTLASIHFIALMSPGPDFALVVQNATRHGRQTGLYIALGLSVGILLHSLFSLT

GVSYLVHQHPLLYSVLQLLGGSYLLYLGVGALRGVIATIKNPQTDQQNKTNSFVISNKRQ

AFAKGFATNILNPKALVFFISLMSSLVPAGMSVSGKSIALVILFSLSLVWFSSLAWMLST

QRLQRRLQQAGIYIDGICGVVFTLVGGSILVQTISTLI

>tr|Q79YX1|Q79YX1_VIBPA Chemotaxis protein CheY OS=Vibrio parahaemolyticus serotype O3:K6 (strain RIMD 2210633) OX=223926 GN=VP2231 PE=4 SV=1

MNKNMKILIVDDFSTMRRIVKNLLRDLGFNNTQEADDGLTALPMLKKGDFDFVVTDWNMP

GMQGIDLLKHIRADAELKHLPVLMITAEAKREQIIEAAQAGVNGYIVKPFTAATLKEKLE

KIFERL

>tr|Q87HS2|Q87HS2_VIBPA Putative acetyltransferase OS=Vibrio parahaemolyticus serotype O3:K6 (strain RIMD 2210633) OX=223926 GN=VPA0884 PE=4 SV=1

MNKLHKLFKPSSVAVIGASQKDLRAGQVVMRNLLQSGFDGAIMPVTPRYKAVSGVIAYRD

VASLPYTPDIAILCTNASRNEQLLKELDERGTPFAIVISDDAQTLDLSSLNIRVLGPNSL

GIILPWHNFNCTFSPVAAKPGKIAFISQSAAVCTTVLDWANDKNIGFSSFISIGRGQDID

FADLLDYLSMDGNTEAILLYVDSIQDARRFMSAARAASRNRRILVLKAGRSKEMNTFEQQ

DGDTLDVIYDSAIRRTGMLRVSNTHELFAAVETLTHSVPLRGERLAIITNGGGPAVMAVD

TLVERGGNLATLDEVTTDQLRAILPSNWRGVNPIDLSGDATKKRYVDAINAVMNNDCADA

ILIMHSPSAVSDSYETALAVIEAIKNHPRHKHFNVLTNWSGEQTSRDARLAFTQAGIPTY

RTPESAVVAYMHLVEYRRNQKQLMETPTTAEPLHSGSVNSAKEWVDERLLDKNTVTLDTH

QTSPLFKLFGFNVLPTWIASDEIEAVHMAENIGYPVAVKLRSPDIPHKSDVHGVALNLRN

SREVSNAAQSILDTVKFSYPSANVHGLLVQGMAKLGSAEELRISIAVDKVFGPVILLGQG

GSEWNIAQDAVAALPPLNMTLARYLVVVALKSGKIRLQKHKDALDITELSKFLVRISQMA

VELPEIQRLDIHPVLVSGDDLTILDADVTLCKYEGDAQKRLAIRPFPAEFVETVTLRDGQ

PILLRPILPEDEPLHAQFINSVSKEDLYKRFFSEVGEFNHEALANFTQIDYDREMAFVAV

AFDKSGPSIIGVARALITPDNSDAEFAILVRSDLKGKGLGKILMEKIISYCKIKGTKQMS

GMTMPTNRGMLMLAQRLGFEVDVQFADGTADMVLPLN

>tr|Q87SQ2|Q87SQ2_VIBPA PTS system, mannitol-specific IIABC component OS=Vibrio parahaemolyticus serotype O3:K6 (strain RIMD 2210633) OX=223926 GN=VP0370 PE=4 SV=1

MISPDAKIKIQNFGRFLSNMVMPNIGAFIAWGFITALFIPTGWLPNETLASMVGPMITYL

LPLLIGYTGGKLVGGDRGAVVGAITTMGVIVGTDIPMFMGAMMVGPMGGWAIKKFDNYID

GKVKSGFEMLVNNFSAGIIGMLCAILAFFLIGPFVKVLSGALAAGVNFLVSAHLLPLTSI

FVEPAKILFLNNAINHGIFSPLGIQQASETGQSIFFLIEANPGPGLGILLAYMVFGKGTA

RQTAGGASIIHFFGGIHEIYFPYILMNPRLILAAIAGGMTGVFVLTMFNAGIVSPASPGS

IFAILLMTQKGSIVGVLASIAAATGVSFAVASLLMKTQTSTEEDGDEAALEKATSQMKDM

KSSSKNGAVVNNESKGDVDLATVQSIIVACDAGMGSSAMGASMLRKKVQDAGLNVHVTNL

AINSLPESADIVITHKDLTGRARKHAPNAHHISLTNFLDSEMYNQLVTKLLAAQKQSAAN

DDQMVKVSVLAANDDSFEPQQPSVFQIQRENIHLGLKAANKEEAIRFAGNKLVELGYAEP

EYVDAMFEREALVPTYLGESIAVPHGTVEAKDRVKKTGIVICQYPSGIQFTEDDDDVAKL

VIGIAAKNDEHIQVITTITNALDEPEAIEKLTSTNDVEEILNILGGQQAA

>tr|Q87L70|Q87L70_VIBPA Site-specific DNA-methyltransferase (adenine-specific) OS=Vibrio parahaemolyticus serotype O3:K6 (strain RIMD 2210633) OX=223926 GN=VP2742 PE=3 SV=1

MKKQRAFLKWAGGKYGLVEDIQRHLPPARKLVEPFVGAGSVFLNTDYDHYLLADINPDLI

NLYNLLKERPEEYISEAKRWFVAENNRKEAYLSIRAEFNKTDDVMYRSLAFLYMNRFGFN

GLCRYNKKGGFNVPFGSYKKPYFPEAELEFFAEKAKKATFVCEGYPETFRRARKGSVVYC

DPPYAPLSNTANFTSYAGNGFTLDDQAALADMAERTATERGIPVLISNHDTTLTRRLYHG

ADLSVVKVKRTISRNGSGRNKVDELLALFKAPESDSAAS

>tr|Q87QH1|Q87QH1_VIBPA Transporter, AcrB/D/F family OS=Vibrio parahaemolyticus serotype O3:K6 (strain RIMD 2210633) OX=223926 GN=VP1178 PE=3 SV=1

MSEQNKEPQSDDDVTGIAAYFIRNRVISWMVSLIFLIGGIAAFFGLGRLEDPAFTIKDAM

VVTSYPGATPQQVEEEVTYPLEKAIQQLTYVDEVNSISNRGLSQITVTMKNNYGPDDLPQ

IWDELRRKVNDLKVTLPPGVNEPQVIDDFGDVYGILLAVTGDGYSYKELLDYVDYLRREL

ELVDGVSKVSVSGQQQEQVFIEVSMKKLSSIGLSPNTVFNLLSTQNIVSDAGAIRIGDEY

IRIQPTGEFQSVDELGDLLITESGAQGLIFLKDVAEIKRGYVEVPSNIINFNGSLALNVG

VSFAQGVNVVEVGKAFDRRLAELKYQQPVGVEISEIYSQPKEVDKSVSGFVISLAQAVGI

VIIVLLFFMGLRSGLLIGLILLLTVLGTFIFMKYLAIDLQRISLGALVIALGMLVDNAIV

VVEGILIGTQKGRTRLQAATDIVTQTKWPLLGATVIAVTAFAPIGLSEDSTGEYCGTLFT

VLLISLMLSWFTAISLTPFFADIFFKGQKIKQGEGEENDPYNGIIFVAYKKFLEFCMRRA

WLTVVVLIVGLGASVYGFTLVKQSFFPSSTTPIFQLDVWLPEGTDIRATNDKLKELESWL

AEQEHVDHITTTAGKGLQRFMLTYAPEKSYAAYGEITTRVDNYEALAPLMARFRDHLKAN

YPEINYKLKQIELGPGGGAKIEARIIGSDPTVLRTIAAQVMDIMYADPSATNIRHDWRER

TQVLEPQFNESQARRYGITKSDVDDFLSMSFSGMTIGLYRDGTTLMPIVARLPEDERIDI

RNIEGMKIWSPAQSEFIPLQQVTMGYDMRWEDPIIVRKNRKRMLTVMADPDILGEETAST

LQKRLQPQIEAIQMPPGYSLEWGGEYESSGDAQESLFTTMPMGYLFMFLITVFLFNSIKE

PLIVWLTVPLALIGVTTGLLALNTPFGFMALLGFLSLSGMVLKNGIVLLDQIEIEMKSGK

EAYDAVVDAAVSRVRPVCMAAITTILGMIPLLPDIFFKPMAVTIMFGLGFATILTLIVVP

VLYRLFHKVSVPK

>tr|Q87IF8|Q87IF8_VIBPA Putative DOPA-dioxygenase-related protein OS=Vibrio parahaemolyticus serotype O3:K6 (strain RIMD 2210633) OX=223926 GN=VPA0648 PE=4 SV=1

MYHVHVYFPLQQLEKAQALNEIIRQERQDVLRVYPLVDRLVGPHKMPMFEMHLESISEEF

LAWLDTIRGDFSVLIHPVSERELRDHTESAIWLGRELGVFEEKLEN

>tr|Q87LU9|Q87LU9_VIBPA Lipid A biosynthesis lauroyltransferase OS=Vibrio parahaemolyticus serotype O3:K6 (strain RIMD 2210633) OX=223926 GN=lpxL PE=3 SV=1

MNIVPPPLFSYQLLKPKYWSVWLAFGALAIIVNVLPYVVLRILGRSIGCVAMQLMKRRYK

IALRNLQLCFPDYTDWQCKDVVRKNFQYTGMALIETGIAWFWPDWRINRITSIVGKDRLL

TEEKNGRGVLVVCSHHLNLEITARIFSQFAKGYGVYRPNSNPAYEFIQHRGRTRFGHQMI

DRKDVKSMLKVLKNGHRLWYLPDHDYGASHSVFAPFFAVEQAASTVGSSVLIDATKCAVI

SGVTVSRNHHYTLYIGKDLSEYFEKRNAMKAASILNQELEKMIRRDIPAWMWLHKRFKTR

PEGFDCVYT

>tr|Q87GG8|Q87GG8_VIBPA Putative transcriptional activator ToxR OS=Vibrio parahaemolyticus serotype O3:K6 (strain RIMD 2210633) OX=223926 GN=VPA1348 PE=4 SV=1

MLRNYLLGNQVIFDTLKREVLTTDKIISLGGREAAILKLLCENANTVIAKEEINDKVWGK

VFVSETSLTKAISNLRKSLQLIEGVMCEIKTIPKEGYMLILEGENLGLMVAEDEPPLEVK

RIESKDLALLKAPVGNNRFLSTLAKSDNKMNEGHIKPSWMLLAVLSSAFLSSVTSTAMIL

LLK

>tr|Q87IK0|Q87IK0_VIBPA Putative AraC-type regulatory protein OS=Vibrio parahaemolyticus serotype O3:K6 (strain RIMD 2210633) OX=223926 GN=VPA0606 PE=4 SV=1

MPNIEIIRFNHFTARKSERYTATHNGLYVVEEGALVVHQPNGEQFELQAGDFTLYNSSDL

RSAEAIPGENGFKAVALVFDISLFCEFKKAHPGLHSEAEHRRFYPFSPESNSEITQLKNT

LLALASRNAPDYTQSHIAMALLSLMVEVQPDILSIIDDASSLTASQKAIKYIEKNIEKDI

TLEGLAEHMSMSIATLKRRLAAENLSFSQILKVKRINYAATQLRVSQKSITEIAFESGFK

SAAHFSTAFKSIYNITPKDFRNQVVRG

>tr|Q87TR0|Q87TR0_VIBPA Amino acid ABC transporter, periplasmic amino acid-binding portion OS=Vibrio parahaemolyticus serotype O3:K6 (strain RIMD 2210633) OX=223926 GN=VP0008 PE=3 SV=1

MKNWVKVAVAAIALSAATVQAATEVKVGMSGRYFPFTFVKQDKLQGFEVDMWDEIGKRND

YKIEYVTSNFSGLFGLLETGRIDTISNQITMTDERKAKYLFADPYVIDGAQITVRKGNDS

IKGVDDLAGKTVAVNLGSNFEQLLRQYDKDGKINIKTYDTGIEHDVALGRADAFVMDRLS

ALELIKKTGLPLELAGEPFETIQNAWPFVNNEKGQKLQAEVNKALAEMRADGTVEKISVT

WFGADITK

>tr|Q87G97|Q87G97_VIBPA PTS system, fructose-specific IIABC component OS=Vibrio parahaemolyticus serotype O3:K6 (strain RIMD 2210633) OX=223926 GN=VPA1420 PE=4 SV=1

MDITNLIELETICLDLKAQTKDEALKELVEMLEAAGKLNSQSQFLADIWKREEIGNTGFD

DGIAIPHAKSDAVAKPAVAVGISRNGIDYGAEDGELSDVFFMLASPDGDDHHHIEVLAQI

STKIIEDGFVEKLKQAQSREEALEMLTDIQTQSNEFLPTSLEFASEPLSPWAQKLGRIKE

HLLFGTSHMIPFIVAGGVLLSLSVMISGHGGVPQEGILADIAQMGIAGLTLFTAVLGGYI

AYSIADKPGLAPGMIGSWIAVSHYNTGFLGAIVVGFFAGLVVWLLKKIQLPDSMSSLGSI

FIYPLVGTFVTCGAVMWVIGAPIASAMTTMNEVLTGMAGSGKVMLGTVLGAMTAFDMGGP

INKVATLFAQTQVNTQPWLMGGVGIAICTPPLGMALATFLAPSKFKRDEREAGKAAGIMG

MIGISEGAIPFAAGDPARVLPAIVAGGIVGNVIGFMFHVMNHAPWGGWIVLPVVDGKIGY

IIGTIAGSVTTALIVIALKKTVTEDESYTGHSQVYGSVQGEGEADVLAVTSCPSGVAHTF

LAAKSLEKAACALGIKIKVETQGANGVINRITEKDIEKAKFVIFAHDVAIKEPERFRKIK

VLDVTTKDAMLNATALLQARRVS

>tr|Q87R92|Q87R92_VIBPA KtrB OS=Vibrio parahaemolyticus serotype O3:K6 (strain RIMD 2210633) OX=223926 GN=VP0905 PE=4 SV=1

MTQFHQRGVFYVPDGKRDKAKGGEPRIILLSFLGVLLPSAVLLTLPVFSVSGLSITDALF

TATSAISVTGLGVVDTGQHFTLAGKILLMCLMQIGGLGQMTLSAVLLYMFGVRLSLRQQA

LAKEALGQERQVNLRRLVKKIVTFALVAEAIGFVFLSYRWVPEMGWQTGMFYALFHSISA

FNNAGFALFSDSMMSFVNDPLVSFTLAGLFIFGGLGFTVIGDVWRHWRKGFHFLHIHTKI

MLIATPLLLLVGTVLFWLLERHNPNTMGALTTGGQWLAAFFQSASARTAGFNSVDLTQFT

QPALLIMIVLMLIGAGSTSTGGGIKVSTFAVAFMATWTFLRQKKHVVMFKRTVNWPTVTK

SLAIIVVSGAILTTAMFLLMLTEKASFDKVMFETISAFATVGLTAGLTAELSEPGKYIMI

VVMIIGRIGPLTLAYMLARPEPTLIKYPEDTVLTG

>tr|Q87JL0|Q87JL0_VIBPA ABC transporter, permease protein OS=Vibrio parahaemolyticus serotype O3:K6 (strain RIMD 2210633) OX=223926 GN=VPA0239 PE=3 SV=1

MDAKTMTMNSLTTQDKAKSMMGSISRDNIVLFGLLAGLSTMMILFILMPLWAMLAKSVQN

SDGEFVGLANFATYFSSSSLWVSVGNTFSLGLVVTTVVGILAFGYAYALTRSCMPFKGLF

HILGTAPILAPSLLPAISLIFLFGNQGVAKELLGGHSVYGVIGISMGLIFWTFPHALMIL

TTSLRTSDARLYEAARALKTSPMKTFFMVTLPAAKYGLISTLIVVFTLVITDFGVPKVIG

GSYNVLATDIFKQVVGQQNFAMGAVTSIMLLFPAVMAFGADRWVQKKQKSLFDTRSVPYQ

PEPNKTRDGLCFVYCSLISVAVLAVLGMAVYGSLVTFWPWNKALTLNNYNFAEMSTYGWS

PFFNSLTLAGWTALIGTAVIFVGAYCIEKGRAFGPVRQAMQMLSVVPMAVPGMVLGLGYI

FYFNDVNNPLNVLYGTMAFLVINTVVHYYTVGHMTALTALKQLPSEIEATAASVRLPQYK

LFFKVTLPVCMPAVLDIATYLFVNALTTTSAVVFLYSTDTIPASVSILNMDDAGQTGAAA

AMAVMIMVAAAIAKIVQMTLGKWLESRTQAWRKR

>tr|Q87PK2|Q87PK2_VIBPA Uncharacterized protein OS=Vibrio parahaemolyticus serotype O3:K6 (strain RIMD 2210633) OX=223926 GN=VP1501 PE=1 SV=1

MKKIALTLAATSITLVSYSAFSAQDAEHVRLATTTSTYHSGLLDYLLPQFEKDTGYKVDV

IAAGTGKALKMGENGDVDLVMTHAPKAEGTFVEKGYGVLPRKLMYNDFVIVGPKADPAKI

KDDESVLDVFKEIANKNATFISRGDDSGTHKKEMGFWAQTKIEPNFGGYRSVGQGMGPTL

NMASEMQGYTMSDRGTWLAYQNKLDLEILFQGDEKLFNPYQVILVNPERYPTINYQGAKA

FSDWLVNPRGQELINGFRLNGKQLFVANAESK

>tr|Q87MD3|Q87MD3_VIBPA Uncharacterized protein OS=Vibrio parahaemolyticus serotype O3:K6 (strain RIMD 2210633) OX=223926 GN=VP2323 PE=4 SV=1

MTTSTKLVELLYQLEAQLQKHELWQQTMPSPEALQSVEPFAIDTLDPHEWLQWIFIARMH

ALVESSQPLPRGFSIEPYFAEVWKQEPQYAELLNTIRTIDELCK

>tr|Q87PD0|Q87PD0_VIBPA Uncharacterized protein OS=Vibrio parahaemolyticus serotype O3:K6 (strain RIMD 2210633) OX=223926 GN=VP1587 PE=4 SV=1

MTHKKLQSVHLSKMDLRMRYVVTLFLLLLPTASTLADDSETNPVAKKIKSTLQKKVDKQF

DQYDGYCDLMIEMEHKGKVAIVKRVTGSGDTKVCRFARSNLKTGKRYRYKYPEKYIRIHI

TTGS

>tr|Q87N29|Q87N29_VIBPA PTS system, glucose-specific IIBC component OS=Vibrio parahaemolyticus serotype O3:K6 (strain RIMD 2210633) OX=223926 GN=VP2046 PE=4 SV=1

MFKNLFANLQKVGKALMLPVSVLPVAGILLGVGAAHLSFIPEIVSNLMEQAGGSVFGQMA

LLFAVGVALGFTNNDGVAGLAAIVGYGIMTATLGVMAGVMGVEKIDTGVLGGILVGGVAA

WAFNRFFKIQLPEYLGFFAGKRAVPIITGFAAIILGVILSVIWPPIGGAISAFSDWAAHQ

NPQLAFGIYGVVERSLIPFGLHHVWNVPFFFEAGTCVNAAGETQHGVLTCYLVADEASRA

AGNGFGQLAGGYMFKMFGLPAAAIAIAHCAKPENRAKVMGIMASAALTSFLTGITEPIEF

SFLFVAPVLYGIHALLAGSAYIVANTLGFVHGTSFSHGLIDFLVLSGNAQKMGLMIAVGL

VYAVIYYVVFRAVITALDLKTPGREDETEEAAATSSSDMAGELVAAFGGKANITGLDACI

TRLRVAVADTAAVDQDKLKQLGAAGVVVVAGGVQAIFGTKSDNLKTEMDEWIRNHG

>tr|Q87SH3|Q87SH3_VIBPA Ribosomal RNA small subunit methyltransferase I OS=Vibrio parahaemolyticus serotype O3:K6 (strain RIMD 2210633) OX=223926 GN=rsmI PE=3 SV=1

MTDKNKLPNEGPTLYIVPTPIGNLADITQRAIEVLSNVDIIAAEDTRHTGKLLSHFNIQT

KTFALHDHNEQQKAQVLVEKLLSGQSIALVSDAGTPLISDPGYHLVTKCRQAGVRVVPLP

GACAVITALSASGLPSDRFSFEGFLPPKSKGRKDKFLEIASVERTCIFYESPHRILDSLQ

DMLDVLGPEREVVLARELTKTFETIQGMPLGELIEWVKSDDNQQRGEMVLLLHGHRETSD

EALPDEALRTLGILTKELPLKKAAALVAEIHNLKKNALYKWGLENLD

>tr|Q87SJ6|Q87SJ6_VIBPA Uncharacterized protein OS=Vibrio parahaemolyticus serotype O3:K6 (strain RIMD 2210633) OX=223926 GN=VP0427 PE=4 SV=1

MAQVAEKQRYHVDLAGLMRTYETNYAKLNALLPVSAEVGDVRCYQAANMVYQLTVNEITK

YTTVVEICQSDETPVFPLPTMSVRLYHDARVAEVCSSGEFSRIKAKYDYPNDQLMQRDEK

HQLNTFLGEWLTFCLRSGISRTPLAFN

>tr|Q87LW8|Q87LW8_VIBPA Iron(III) ABC transporter, permease protein OS=Vibrio parahaemolyticus serotype O3:K6 (strain RIMD 2210633) OX=223926 GN=VP2490 PE=3 SV=1

MKDRHSLWKTSSGAITLLLVLPILAIFYTAIGETDNLFTHLMSTVMPTYIYNTVVLTIGV

MGLSLIFGIPSAWLMAMCKLPTEKWLQWALVLPLAMPGYIIGYIFTDWFDFAGPIQIFLR

DVTGWGPGEYWFPDIRTLPGATFVLSLVLYPYVYLLCRAAFMEQNVSLLQSARLLKCSPW

ESFWRISMPLVRPSIAVGLSLVAMETIGDFGTVSYFAVNTLTTAVYDTWLGYSNLNAAAK

ISAIMLLIVVLLLSTERYSRRKQKLFQSQFNSHEDFRYELSGWKKWAALVWCWGLVAVAF

ILPLLQLIDYSITYFEQSWTPEFREYAWNSLVVSVIAAIIGVAVALIVNFTHRVNGKRES

LAFMRLSSMGYAVPGTVLAIGVMVAVLFMDYRVNDIAKAMEWGRPGLIFSGSMFALIFAM

VVRFSAVAIGSIESNLNKISPSLDMASRTMGCTPNTMLWRVHFPLVKRGALIAALLVFIE

SMKELNASLLLRPFNFETLATYVYNFASDEHLELAALPAVLLVLVGLIPLVVVNRSLEQN

H

>tr|Q87HL9|Q87HL9_VIBPA Putative cation efflux system (AcrB/AcrD/AcrF family) OS=Vibrio parahaemolyticus serotype O3:K6 (strain RIMD 2210633) OX=223926 GN=VPA0944 PE=3 SV=1

MPDKLKRSFVQSVMNSFFPPIMILLALVVGAAALWLTPKEEDPQIVVPMADVLVSAPGLS

ASQVENQITEPLEKLVSQIDGVEYVYSSSMEGAAQVIVRFYVGENREDALVKLYNKLYSN

QDKVPPSVTNWLVKPVEIDDVPIVVAAIYSTDPDILDRHQLRRIADQATLGIKSLDATNK

VEVIGGEPRKIQIELDSVAMANFKVTIDDLEQAIQLSNSKTQGKNVRVNGQNFTLESGRF

LTNAEEVGDLVIAVLNGKPVYLKDVARIYDGEGEATSDTWYRDKNHDEAYPAVFISVAKQ

KGSNAVNVAQSVRDKLAALQSEQFPPQVQVAVIRDYGETANAKVNNLVSSLGISILTVVV

FVGLFLNWRSALVVGIAIPISYGAALGMDLAFGYSINRVTLFALILALGLIVDDPIASID

NIERYLKRKNLTRTNAIVLAMAEIRSALLMSTVAIVIVFTPMFFITGMMGPYMAPLAFNV

PISVIFSTVVAFMITPWLAKKLLKGAEENGHYDIQSSPMYRLYRGVLIPLLESRKKAWLF

LGLVALLFVLAALLPALRLVPLKLLPYDNKNEFQLVLNMPESSSFVDTSNALSSFTDYLM

SVPEVTSVSGFAGTASPMDFNGMVRHYFMRSEPYQGELRVVLAEKNRRAMQSHELVTRLR

ADLEQIADKFDADVQLVEVPPGPPVIATITAEVYGDEATSYEDLMIQAEKVADRLRKEQL

VSEVDTSIQGDLETWQFIVDQEKAALSGVSVADINNTLITAANAKVLGYIADPREVDPLP

IEVQLRREDRDNLNQLEQLYVRGRPGIAKVESNGAVVDAPQPIVQLSEVGHFVKRAADKP

IFHKNLKPVVYVYAEVVGRVPGEVIADVMADQDTTHAEDVHRHWQDRTYLSNGAGVTWSV

PDNIEVVWSGEGEWKITVDVFRDLGIAYGAALLGVFVVMLIQTGLPAVSGIIMLAIPLTV

IGIMPGFWILNVLSSDIGNYPNPALFTATAMIGMIALAGIVVRNSLVLIEFVQQSLAEGR

SLHDALIESGVVRMRPILLTAGTTLLGNVVITLDPIFNGLAWAIIFGITASTIFTLLVVP

VVYNLAYQNTKGHGLPQMEEEQ

>tr|Q87T58|Q87T58_VIBPA ADP-heptose-LPS heptosyltransferase II OS=Vibrio parahaemolyticus serotype O3:K6 (strain RIMD 2210633) OX=223926 GN=VP0212 PE=4 SV=1

MKKILIIGPAWVGDMVMSQSLYITLKQLHPESQIDVIAPGWCKPILERMPEIHQAIEMPI

GHGEFNLLGRREIGKSLREKQYDHAYILPKSAKSALIPWFANIPLRTGWKGEMRYGLLND

LRPNMKSFQYMVERYVALAYSKSEMVDSSSLGGLDTLPRPSLSLNKEEQQTTINKFNLDQ

KRPAVGLCPGAEFGPAKKWPETHYAEVAAQMCKTGHQVWLFGSQKDLETCNNIRALIPTQ

FHEHIHVLAGQTSLIEAVDLLAACKTVVANDSGLMHVAAAVGCNVVAVYGSTSPKYTPPL

AEKVEMVHTDIDCRPCFKRECQYQHLKCLTELSPKQVLDSIQKLEAIATSSC

>tr|Q87SD5|Q87SD5_VIBPA Aerobic respiration control protein FexA OS=Vibrio parahaemolyticus serotype O3:K6 (strain RIMD 2210633) OX=223926 GN=VP0489 PE=4 SV=1

MQTPQILIVEDEQVTRNTLKSIFEAEGYAVFEASDGEEMHQVLSDNSINLVIMDINLPGK

NGLLLARELREQANIALMFLTGRDNEVDKILGLEIGADDYITKPFNPRELTIRARNLLSR

SMSTNAVQEEKRSVEKYEFNGWVLDINSRSLVSPAGDSYKLPRSEFRALLHFCENPGKIQ

TRADLLKKMTGGELKPHDRTVDVTIRRIRKHFESVSGTPEIIATIHGEGYRFCGDLED

>tr|Q87T39|Q87T39_VIBPA Putative UDP-galactose phosphate transferase OS=Vibrio parahaemolyticus serotype O3:K6 (strain RIMD 2210633) OX=223926 GN=VP0231 PE=4 SV=1

MMKRLFDFLVSLIALILLSPIIVLVAWKIRKKLGSPVLFRQTRPGLNGKPFEMVKFRTMK

DAVDEQGNLLPDSDRMTPFGEKLRNSSLDELPGLWSVLKGDMSLVGPRPLLMRYLPLYNE

EQARRHDARPGVTGWAQINGRNAISWEEKFALDVWYVDNQTFWLDIKILLLTVKKVFVKE

GISADDHVTMPEFEGSKDDK

>tr|Q87PK0|Q87PK0_VIBPA Sigma-54 dependent response regulator OS=Vibrio parahaemolyticus serotype O3:K6 (strain RIMD 2210633) OX=223926 GN=VP1502 PE=4 SV=1

MSTQSHISTNQQYNAFSVLVVDDELGMQAILKKALGKLFSHVDTAGSIEEAEQLRNSRHY

DLILLDINLPGRSGIEWEEAFEDDEKRADVIFMTGYADLEIAIRALQLGASDFILKPFNL

EQMLKAVSRCMDRRLNERMQYAMKRDYQRHNTSEIIGGSEKTRQLKQLITQFAPSRASVL

VEGESGTGKELVARGIHQASGRTGPFVPINCGAIAPELLESELFGHTSGAFTGAKKSREG

LFRVANGGTLFLDEIGEMPLSMQASLLRVLEQRTIRPVGSEREISIDVRIVAATNRNLQE

EVNQGNFRSDLYYRLNVLKIEVCPLRERKTDLFELVPFFSNMLTRELGMPAPKWAHEDME

AMSEYDWPGNIRELKNLIERCILLGKPPAHYWRELNGGHSLPNVSITVSHSAELPTFREG

KEFTGEGYPNNWTLKEVEKAHIKQVVNLHEGNKSAAARDLGVARKTLERKYKEWDSEDEG

YAD

>tr|Q87TK7|Q87TK7_VIBPA Phosphogluconate dehydratase OS=Vibrio parahaemolyticus serotype O3:K6 (strain RIMD 2210633) OX=223926 GN=VP0062 PE=3 SV=1

MTHSVVLEVTQRLTERSREARAAFLARTEVQAEAGKGRVGLSCGNLAHAVAASCSSEKKN

ILDFTHANVALISAYNDMLSAHQPYQDYPAQIKQVLADYGHTAQVAGCVPAMCDGVTQGQ

AGMDMSLFSRDLIAQSTALSLSHNVFDATLLLGICDKIAPGQLMGALSYAHLPTAFVPAG

LMATGISNEEKVDVRQKYAAGEVGKDALLDMECRAYHSAGTCTFYGTANTNQLVFEAMGL

MLPGSAFIHPHTQLRKALTDHAALKIASMTAGSAHFRPLAEVVTEKSLVNGIIALLASGG

STNHTIHMIAVARAAGILLTWQDISDLSDVVPLLARVYPNGPADMNAFQDAGGVPALLHR

LNESELLHRDVKPVFGKFEDQMTLPSLVDGQLTWTPCQGSQDGDVIAKPDATFQNTGGTR

VLTGNLGKAVVKVSAVKEEQRVIVAPAIVFQCQHEVEAAYKRGELNKDCIVVVTHNGPAA

NGMPELHKLMPILGNVQKAGFKVALVTDGRLSGASGKIPSAIHVSPEAIRGGAIGLVRNG

DLIRLDCQTGELNNLSDTTGRELIHFDTESTQQTWGRGLFSVIRQNVSSAEEGASFIV

>tr|Q87M84|Q87M84_VIBPA RecBCD enzyme subunit RecB OS=Vibrio parahaemolyticus serotype O3:K6 (strain RIMD 2210633) OX=223926 GN=recB PE=3 SV=1

MDQMSGAECPLHTLDRTEQPQPTPLEPMTFPLHGARLIEASAGTGKTFTIAGLYLRLLLG

HGSAETRHRVPLTVDQILVVTFTEAATAELRDRIRARIHDARIAFARGQSSDPVIQPLLN

EFDDHKQAAEILLQAERQMDEAAVYTIHGFCQRMLTQNAFESGSRFNNEFVTDESHLKAQ

VVADYWRRNFYPLPFTLAGEIRQLWSSPSALLSDISNYLTGAPLSLSVPAMKGSLADLHT

ENLKKIDELKAQWRESQDDFFTLISDSDINKRSYTKKSLPTWLEAVNAWAATETTGYDYP

DKLEKFAQNVLLEKTPKGSAPQHAVFEAIETFLANPISLKAPLLAHAIEHCRVMLANAKN

QKQWLSFDDLLTQLSASIDTDESELLAARIRTLYPVAMIDEFQDTDPLQYSIFSRIYLND

PECGLFMIGDPKQAIYGFRGADIFTYIKARNQVSAHYTLGTNWRSSADMVQAVNQVFALP

DSPFIYDSDIPFLPVKYSPNAEKRIWTMGGQKQPALTYWLQEADDKPLPKGEYLTRMAEA

TASQIQTILTQAQQGQACLVNGEKQKAVQAGDIAVLVRTGSEGRMVKQALADQGIASVYL

SNRDSVFTSSVAQDLQRLLQAVLTPENDRALRASLASELFALDAASLDALNNDEVVWENA

VNEFKEYRKLWVQRGVLPMLRAVISKRHIAERLLEEGASSQGENGERVLTDLMHIGELLQ

QASNELDSDHGLLRWLAQSISDAENGLGGSDDQIQRLESERNLVQIVTIHKSKGLEYDLV

FLPFVFSYREASEAKYYDAANDRTVLDITGNDASMKQADKERLAEDLRLIYVALTRAVYA

CFIGASPLRNGRSTKEPTGVHRSAIGYLIQNGQEGGINDLHQGLTKQQDELDCVVVADPP

QQLEDKYVAPQEEIHDLSAKELQNPIDRNWRITSYSGLVKQGSHHAEHDATIEITGFDID

SSEEQDEADLVEPERSIFTFPRGARPGTFLHSLFEEIEFTQPATTEENTQIILGLMESEQ

LDEEWLPILQQLIDTVLVTPLDGKSLLLNQKAPSQRLVEMEFLLPIEVLSAPALNRVIQR

HDPLSAKAGDLGFQTVQGMLKGFIDLVFEHQGKYYVLDWKSNHLGDDVTHYHGEALKSAM

ADHRYDLQYQIYALALHRFLRSRLANYQYEQHFGGVYYLFLRGMDGQSDHGIFAAKPTLD

FLREMDRLIDGQVLETRSTQAGQMELL

>tr|Q87H15|Q87H15_VIBPA Putative high-affinity branched-chain amino acid transport permease protein OS=Vibrio parahaemolyticus serotype O3:K6 (strain RIMD 2210633) OX=223926 GN=VPA1150 PE=3 SV=1

MAQLSMRPCGDFRTTYKSDTPIFETKTIRSLAIAGVIAMLAAPLVLDIYFLNLFIQIAYL

GIAALGLNILVGFTGQISLGHGAFFGFGAFASAWLNNQFNIPVVFAIPLAGYLTMIVGML

FGLPAARIKGLYLAIATLAAQFILEDFFARAEWFSGGSYGASASPINLFGFEFSTDESFF

YVALFALIFMYLWASNLIRSRDGRAFVSVRDHYLSAEIMGINLTKYRLLSFGVCAFYAGI

GGALYGHYLGFVSAEGFTIMMSIQFLAMIIIGGLGSVKGTLMGTIFIVLLPEVLEFGVTG

LAAFSDNTSFIDGLAYFKEMAIGLVIMLFLIFEPQGLSHRWQQIRAYWKHYPFSY

>tr|Q87TQ6|Q87TQ6_VIBPA Beta sliding clamp OS=Vibrio parahaemolyticus serotype O3:K6 (strain RIMD 2210633) OX=223926 GN=VP0012 PE=3 SV=1

MKFTIERSHLIKPLQQVSGALGGRPTLPILGNLLIKVEENVLSMTATDLEVELVSKVTLE

GDFEAGSITVPSRKFLDICRGLPDDAIITFVLEGDRVQVRSGRSRFSLATLPANDFPNIE

DWQSEVEVSLSQADLRTLIDKTQFSMANQDVRYYLNGMLFEIDGTTLRSVATDGHRMAVS

QTQLGADFAQKQIIVPRKGVQELVKLMDAPEQPVVLQIGSSNVRAEVNNFIFTSKLVDGR

FPDYRRVLPQHTNKTLIASCDELRQAFSRAAILSNEKFRGVRVNLAGSEMRITANNPEQE

EAEEMLDVTFEGDPIEIGFNVSYVLDVLNTLRCEKVQVSMSDANASALIENADDDSAMYV

VMPIRL

>tr|Q87SL7|Q87SL7_VIBPA DNA primase OS=Vibrio parahaemolyticus serotype O3:K6 (strain RIMD 2210633) OX=223926 GN=dnaG PE=3 SV=1

MAGHIPRSFIDDLLARLDIVDIIDARVKLKKKGKNYGACCPFHNEKTPSFSVSQEKQFYH

CFGCGAHGNAIDFMMEFERLEFVEAIEELASYLGLDVPREQRSGGSGQFKSGPQASSSEK

RSLYDLMGSIAQFYRNQLKQPSSKVAIEYLKDRGLSGEIVQKFGIGYVADEWDLVRKNFG

QNKDNQDMLVTGGMLIENDKGNRYDRFRGRIMFPIRDRRGRVIGFGGRVLGEGTPKYLNS

PETPIFHKGKELYGLYEVLQAHREPAQILVVEGYMDVVALAQYGVDYSVASLGTSTTGDH

IQMLFRQTNTVVCCYDGDRAGKEAAWRALENALQFLKTGNTLKFLFLPDGEDPDSYVRKY

GKAAFEQQIEQATPLSSYLFDNLIELHQINLGNNEGKSALRAYASALIDKIPDPYFQELL

EKLLDERTGFDNRLRQPRKKISETRPQPHKEIKRTPMREVIALLIQNPSYAQMVPDLSSV

RDLSIPGLSLFADVLDKCQAHPHINTGQLLEHWRNSQNEALLSRLASWDIPLDEDNQEEI

>tr|Q87JV9|Q87JV9_VIBPA Putative PmbA-related protein OS=Vibrio parahaemolyticus serotype O3:K6 (strain RIMD 2210633) OX=223926 GN=VPA0139 PE=4 SV=1

MSQEQQLLNAVDYVLSEAKRQGAEADVIVNRNSSFSLKANQGKLDEYKVSSSQVLGVRVI

KDARVATSYSESLEQPSLDLMLTNALQSARFSKQDEHQTISCVNSKITTDIAEIAQDDTT

SVDEKIELSLALEQGVVALPHASSSPYNGYSDGETQLIIANTQGTLCQHFERSFTCYAYT

LFEKDGKQSMAGRMSLGRRFDELNPTYCIEGGYNLARDLLDGVPVATGNYPAIFHINALA

SLFGAFGSAFSGVSAMKGISPLGDKLGQSVASELLTFTDAAYMPNGMAIAGFDSEGFATQ

DNVLIANGQLNTLLHNSQTASYLGAVSTASASRSAKSSLDVSANHKVIATGNSSASEVKA

GEYLELVELQGVHSGADAVSGDFSFGASGFLCRDGQRVQPVRGITVAGNFYKMLQEVEAV

GDTQLINDSRTFFAPDVRFARLSIGGK

FLDSLDKIIAQCVEKQIENLQAKARSVGLSAEEKRELLALMLDLKA

>tr|Q87K37|Q87K37_VIBPA Hypothetical membrane protein OS=Vibrio parahaemolyticus serotype O3:K6 (strain RIMD 2210633) OX=223926 GN=VPA0061 PE=4 SV=1

MPKRQNINQRMSIVALAWPILVEILLRTALGTSDVFMLSGYSDKAVSAVGVITQITFFLI

IVSTFVSSGTGILIAQYNGAGREQESVNVGVASIALSVIIGVLLSVIAVFGAIFLLPYYG

LEAQVEQYAREYLLISGAMTFNVTIGIVFTTILRSHGYSRSPMVVNLISGVFNIIGNYIA

LYQPFGLPVYGVQGVAIATVVSQVIGTLMLWFILARSSIELPMSTMKQVPAEIYKKILKI

GGMNAGEVLSYNVAQICIVYFVVQMGTASLAAFTYAQNIARFSFAFALAIGQAAQIQTGY

YIGKGWVSSILKRVQIYFLVGFVASTAATTLIYLFREEILRVFTDQPEILALAGSLVMGS

ILLEAGRVFNLIFIAGLKGAGDIKFPVQMGILSMWGLGVLFSYIFGIHLGYGVLGAWMAI

ALDEWVRGIIMARRWRSQVWTKFKVS

>tr|Q87GT3|Q87GT3_VIBPA Uncharacterized protein OS=Vibrio parahaemolyticus serotype O3:K6 (strain RIMD 2210633) OX=223926 GN=VPA1232 PE=4 SV=1

MTLTVWLSLFTVCLLGAMSPGPSLAIVAKHALAGGRMNGLATAWAHAFGIGIYAFITLIG

LAVVLQQSPMLFKTISLAGAAYLAYLGFNALRSKGGVAAKLESGEETTVLQSAREGFLIS

ILSPKIALFFIALFSQFVALGNDLSNQMIIVATPFVVDGLWYTFITLVLSSSRVVDKIRS

KAVLIDRLSGVVLMLLALRVVVTV

>tr|Q87K15|Q87K15_VIBPA 4-hydroxy-2-oxoglutarate aldolase/2-deydro-3-deoxyphosphogluconate aldolase OS=Vibrio parahaemolyticus serotype O3:K6 (strain RIMD 2210633) OX=223926 GN=VPA0083 PE=4 SV=1

MKDLNQQLSEIKVVPVIAIKDAGKAVKLAQVLIENGLPCAEVTFRTEDAALAIKNMREAY

PEMLIGAGTVLTSAQVDEAIDAGVDFIVSPGFNPTTVKYCQQRNVTIVPGVNNPSLVEQA

MEMGLRTLKFFPAEPSGGVAMLKALSAVYPVKFMPTGGVSPSNVKDYLSISSVLACGGTW

MVPGDLIDNEQWDELAKLVREVAGIIE

>tr|Q87G85|Q87G85_VIBPA Putative two-component response regulator OS=Vibrio parahaemolyticus serotype O3:K6 (strain RIMD 2210633) OX=223926 GN=VPA1432 PE=4 SV=1

MSYKVLVVDDEPRIHTFIRISLSAEGFDYIGASTIAEAKACFEAYSPHVILLDLGLPDGD

GTEFLTTLRQTYKTPVLVLTARDQEEEKIRLLEAGANDYLSKPFGVKELIARIKVLVRDL

VDEQSIADELVAGRVKIIKSTHQFWLDQREIPLTKKEFSFIEQLILKPGKLIEQTHLLAV

IWGKSHVEDTHYLRVLVSQLRKKLNDSADEQRLLKTEPGLGYRLVLETSKHH

>tr|Q87MA0|Q87MA0_VIBPA Putative transcriptional activator ChrR OS=Vibrio parahaemolyticus serotype O3:K6 (strain RIMD 2210633) OX=223926 GN=VP2357 PE=4 SV=1

MNKHPDNNLLEAYASGSIDAVSGLVVATHLETCSKCRAYVNQVEASQANTVSESPSEYSP

EFDDMLNDIINAEPVNDNVVIQDTAFVNVAGKSFELPKTLVRFSDLVGSWRSYGGKVFSA

QIDLGEDARVSLMYIGENVQIPQHTHRGLESTLVLHGGFSDEDGQYEEGDLMVRDASVKH

SPFTQEGEDCLCLTVLTEPMIFTQGVARIFNLFGKGLYP

>tr|Q87LW3|Q87LW3_VIBPA Aconitate hydratase B OS=Vibrio parahaemolyticus serotype O3:K6 (strain RIMD 2210633) OX=223926 GN=VP2495 PE=3 SV=1

MLEAYRKHVEERAAEGVVPKPLDAEQVAGLVELLKNPPQGEEEFILDLLENRIPPGVDEA

AYVKAGFLTAVAKGEVSSPLVSREKAAELLGTMQGGYNIAPLVELLDDEALAEIAVKALS

HTLLMFDAFYDVEEKAKAGNAHAQKVLQSWADAEWFLSKPKLEEKITLTVFKVTGETNTD

DLSPAPDAWSRPDIPVHALAMLKNEREGINPDQPGTIGPIKQIEELKSKGHQLVYVGDVV

GTGSSRKSATNSVLWFMGDDIPYVPNKRAGGYVLGGKIAPIFFNTMEDAGALPIEVDVTK

LNMGDVIDVYPYEGKVCNHETGEVLAEFSLKTDVLIDEVRAGGRIPLIIGRGLTDKARQA

LGLESSDVFRKPGEVADSGKGYTLAQKMVGKACGVAGVRPGTYCEPKMTTVGSQDTTGPM

TRDELKDLACLGFSADLVMQSFCHTSAYPKPVDVNTHHTLPDFIMNRGGVSLRPGDGIIH

SWLNRMLLPDTVGTGGDSHTRFPLGISFPAGSGLVAFAAATGVMPLDMPESILVRFKGKM

QPGITLRDLVHAIPYYAIQQGLLTVEKAGKVNEFSGRILEIEGLETLTVEQAFELSDASA

ERSAAGCTVKLSQESIEEYLNSNIVMLKWMISEGYGDRRTIERRITAMEEWLAKPELMSA

DSDAEYAHVIEIDMAEIHEPVLCAPNDPDDARLLSEVQGTAIDEVFIGSCMTNIGHFRAA

GKLLDKFNGQLATRLWVAPPTKMDKDQLTEEGYYGIFGRAGVRIETPGCSLCMGNQARVA

DKSTVMSTSTRNFPNRLGTGANVYLSSAELAAVGAILGKIPTKEEYLEYAKQIDATATDT

YRYLNFHKMEQYTKKADEVIFQEPA

>tr|Q87NR1|Q87NR1_VIBPA Uncharacterized protein OS=Vibrio parahaemolyticus serotype O3:K6 (strain RIMD 2210633) OX=223926 GN=VP1807 PE=4 SV=1

MSINIKWDGDCRFKVSTEGGFTFNVDATSETAPCPTEVLLSALGSCSATDVVLLLQDQGF

EVKGLKNKVTFALTESEPRLYKSANLHFTVNGSGFKESDILRAAQEAVEKHCHVCLMLSP

TIDITCSAEVGKNCT

>tr|Q87QA8|Q87QA8_VIBPA Uncharacterized protein OS=Vibrio parahaemolyticus serotype O3:K6 (strain RIMD 2210633) OX=223926 GN=VP1241 PE=4 SV=1

MDMTDQAFFKYVRNFNEYQKRSMFGGIGLFSDDAMFALVSNDCCYLRGGNGLDEEFTLLN

CEKYKHVKKQTTATVNYYDVTDLFESGFTGLDDLLRKSIDCSIKERKYQKSSASKRLRDL

PNMQLTLERMVKKAGIDDVETFLELGPVEVFNKVRVAYGNDVDVKLLWKFAGAIDGVHWK

LIQEPRKKQLLALCE

>tr|Q87SG0|Q87SG0_VIBPA Cell division protein FtsZ OS=Vibrio parahaemolyticus serotype O3:K6 (strain RIMD 2210633) OX=223926 GN=ftsZ PE=3 SV=1

MFEPMMEMSDDAVIKVVGVGGGGGNAVEHMVRESIEGVEFISVNTDAQALRKTSVGNVIQ

IGGDITKGLGAGANPQVGREAALEDRDRIKDSLTGADMVFIAAGMGGGTGTGAAPVIAEV

AKELGILTVAVVTKPFSFEGKKRLAFAEQGIDELSKHVDSLITIPNEKLLKVLGRGVTLL

EAFASANDVLKNAVQGIAELITRPGMINVDFADVRTVMSEMGHAMMGSGIAKGEDRAEEA

AEMAISSPLLEDIDLAGARGVLVNITAGLDMRLDEFETVGNTVKAFASDNATVVIGTSLD

PDMTDEIRVTVVATGIGNERKPDITLVAGGKAKVASAPQAQPQQVAATQAEEKPAQTLQN

QVQEKPQVTPQPTNTVSSSPAAGQSSAAPKQEKESGYLDIPAFLRRQAD

>tr|Q87S85|Q87S85_VIBPA Uncharacterized protein OS=Vibrio parahaemolyticus serotype O3:K6 (strain RIMD 2210633) OX=223926 GN=VP0539 PE=4 SV=1

MELVISLLQQMCVYLVLAYMLSKTPIILPLLSISSRLSHRLICYVLFSGFCILGTYFGLH

INDAIANTRAIGAVMGGLFGGPVVGFAVGLTGGIHRYSLGGFTDLACAISTTAEGVIGGL

LHVYLIKRNKGALLFNPSVVFSVTFVAEVVQMILLLAVAKPFDQAYELVSAIAAPMIIAN

SFGAALFMSILQDRKTIFEKYSATFSRRALTIADRSVGILSNGFNTENAEKIARIIYEET

KVGAVAITDQEKILAFVGIGDDHHRPNTPISSQSTLDSMEKNDIIYLDGTERPYQCSLAK

DCKLGSALIIPLRAGKAVIGTIKLYEPKRKLFSTANMSMAEGIAQLLSSQILYGDYQQQQ

ALLAQAEIKLLHAQVNPHFLFNALNTISAITRRDPDKARELIQNLSHFFRSNLKQNINTV

TLKEELAHVNSYLSIEKARFTDRLEVEIDIDPELLDIKLPSFTLQPLVENAIKHGISNML

EGGKVKIYSEMHPQGHLITVEDNAGSFQPPKDNHSGLGLEIVDKRLTNQFGRDSALKIAC

VTHQFTKMSFIIPPKS

>tr|Q87PA9|Q87PA9_VIBPA Uncharacterized protein OS=Vibrio parahaemolyticus serotype O3:K6 (strain RIMD 2210633) OX=223926 GN=VP1608 PE=4 SV=1

MNIEQYQRLTKQAVALIESEPDFIANLANLSSLLFMELEDLNWAGFYLTKGDELVLGPFQ

GKPACVRIPMGRGVCGTAAKTNTTQRVYDVHEFEGHIACDAASNSEIVIPFSIKGKVAGV

LDIDSPSIGRFNETDEEGLTHFMSEVEKLLNSHANDA

>tr|Q87GY9|Q87GY9_VIBPA Uncharacterized protein OS=Vibrio parahaemolyticus serotype O3:K6 (strain RIMD 2210633) OX=223926 GN=VPA1176 PE=4 SV=1

MVGLKKNIWTLYMMLLTVSIVTFSLFGYYHYQATLDKYKDKQLLQLELFASSVESLLKGQ

ESLLEVVGHQLVEQNNFTRTAAIQTRPMLDKLLNIHPAIIGFGLTNPNGDYISVSSNLIL

EKLHNLKQDPLTRETFLEALNSDRMVIGRTYFMEAQDSLVIPIRKAIPDKNGVVQAVMTA

GFNMNTSSVFRNDIHANEHNRVSLIRNDGYLTFSSSEDTTIKDYQKPADDLNKQALLDQI

QQDYGWDTDQVKQLTRAINVVVDTQRLNELITLKYLPDYGLWAASSTDLGFIKRGFYSQF

AFYCIVFLIVQAAFYALFRSIANNEHETKERLLYQACHDHLTRLPNREYLRSNIQRWMCG

SSNPFTLMFIDIDNFKSVNDTHGHEFGDEVLKQISTRLNHFSGEGRLIVREASDEFIFIV

NRTDEETIKDLASELIQTLSKPYNVNDNQFLLSCSIGIAFYPMHGDNLDALLLSADIAMY

QAKKQRNAYSLFNQEMQASHLHKMKVEQRLRLAIEKQTLFMAYQPQLNINGKIYGVEALV

RWEDEELGKVPPNEFVPVAESSGLMVRLGELIIEKSLEDMGLLTTHLATPIQMSINISVK

QFLHAKFIERLMAAMDKYHLDCNRITLEITENLFIEDLEKFSPTCERLHALGFKISLDDF

GTGYSSLSMLRTLPIDEVKIDKSFVDNIEHDKKALNMVKNIIAIGKNFEMKVLAEGVETQ

RQRDQLEACGCDLIQGYFYSKPLSFDQLVSFVKDNKEEKAIID

>tr|Q87MT1|Q87MT1_VIBPA Putative nitroreductase OS=Vibrio parahaemolyticus serotype O3:K6 (strain RIMD 2210633) OX=223926 GN=VP2150 PE=4 SV=1

MEALDLLLNRRSIAKLSAPAPEGKALENIIRAGLRAPDHAGLTPWRFVIAQGDGLKKLSD

ILVKAAIADHSDEAVIEKVKNAPFRAPIVITVIAKVTEHEKVPALEQYLSAGCAVQAMQM

AAVAQGFQGFWRSGKWMFHPEVHQAFGLEGEDEIVGFLYLGTPGCTPMKVPERDLSKFVE

FQ

>tr|Q87GL9|Q87GL9_VIBPA D-alanyl-D-alanine carboxypeptidase OS=Vibrio parahaemolyticus serotype O3:K6 (strain RIMD 2210633) OX=223926 GN=VPA1296 PE=3 SV=1

MRKITLFNTTFLTGLVTFSHFAFAAPTVVPNAPELSSRGYVLMDYHTGKVLVERDADKRL

NPASLTKLMTAYVAGQEVNAGNISLDDQVVISRNAWAKNFPDSSKMFIEVNTSVPLSDLY

RGLVVQSGNDASVAIAEHVAGSEAGFVSLMNSWASQLGLTNSSFTNPHGLDSDGLYSTPH

DIAKLGQAIIRDLPDIYPMYSETSFTYNGITQYNRNGLLRDRSMNVDGMKTGYTSGAGYS

LATSATNGDMRLIAVVMGAKSQSVRESESKQLLSYGFRFYDTLMPTAAGTDIANARVWMG

QKDELKVGVNRDVYLTLPKGDVNKLKAEVEYNGDLLAPIAQDQVVGTLLYKVDGKVVKET

ELVALEPVEEGGIFKRIMDWFKRLVASWF

>tr|Q87KK2|Q87KK2_VIBPA Uncharacterized protein OS=Vibrio parahaemolyticus serotype O3:K6 (strain RIMD 2210633) OX=223926 GN=VP2975 PE=4 SV=1

MSTSLPCKEITKIVASDLDGTLLAPNHQLSAYSKETLKALHEKGYTFVFATGRHHVDVAS

IRRQVGIPAYMITSNGARVHDQNDQLMYSENVPADLVQGVIDTIKHDHEILIHMYQNDSW

LMNKDDETLRDFHDEFTYVLFDEDQAPTDGIAKIFFTHPAQDHERLVVFENKLREQFGDK

LNIAFSTPWCLEVMSAGVSKGHALQAVAETLGLTLENCIAFGDGMNDVEMLSMAGKGLVM

GTSHEKVMKALPNNEVIGSNADDAVAHYLQDHLL

>tr|Q87TF0|Q87TF0_VIBPA Nitrogen regulation protein OS=Vibrio parahaemolyticus serotype O3:K6 (strain RIMD 2210633) OX=223926 GN=VP0119 PE=1 SV=1

MDTSLPSAILNNMVTATLILDDGLAIRYANPAAELLFSQSAKRIVEQSLSQLIQHASLDL

ALLTQPLQSGQSITDSDVTFVVDGRPLMLEVTVSPITWQRQLMLLVEMRKIDQQRRLTQE

LNQHAQQQAAKLLVRGLAHEIKNPLGGLRGAAQLLEKMLPDPSLTEYTHIIIEQADRLRA

LVDRLLGPQKPGKKTQENLHQILEKVRQLVELESQNSIIIERDYDPSLPEILMDADQIEQ

AMLNIVSNAAQILAHQEHGNITIRTRTVHQANIHGKRCKLAARIEITDNGPGIPPELQDT

LFYPMVSGREGGTGLGLSISQNLIDQHNGKIDVESWPGHTTFTIYLPI

>tr|Q87GP9|Q87GP9_VIBPA Putative ATP-dependent exoDNAse (Exonuclease V), alpha subunit OS=Vibrio parahaemolyticus serotype O3:K6 (strain RIMD 2210633) OX=223926 GN=VPA1266 PE=4 SV=1

MNTALHEDQMRVTSIPYRSTKMVIFSGVPLAKDSYKTNSGKYYVTIKADPDSIPVLPTLG

QHWSVKGARQIESVEMGDYVMQQHTYESPKHIECTLPETGEQLIRFIARESDFKGIGESK

ARALWQLLGKDFHTTLRNDTLESRKRLTSILSEDSVEALFKGYAKYKNLAHCNWMSEHNI

PASVQQRLLKHHGEASIEVIKDNPYALMGFGLSFSAIEDIIKVTDFKSDVAKDDPRRLSA

ALEMAIRKEIEKGHTYTTHANVRHYLSKLLKDKTLVTQAFQSGHDKAQYILNPDTGAYHP

TAQLLMESVVAKRLNTLIKRNDLFDENANAAYCAAVTELPYELTLKQIEAVTTCLDNSVS

CITGGAGTGKTTVLRTTLRAYHQLGFEIHAVALSGRAAMRLHESIGFVTSTIAKLLREDP

IEPSVEKTNHLLVIDEASMIDLPTMYRLVNHIHPSVRLIFTGDPDQLPPIGCGKVLADIV

EAKTVANTMLDIVKRQEGSTGIPEYSKLINQGVMPDQLSTGAIHFHETNKADIAKVCCEL

YQQCPENSRVMAPTKAIVTEINKLTQQAVNPNSDRLEFEINGDKFFLPLRMNDAVLFTQN

HYDKGIQNGSLGMLTNAKTSGDSYGEVTLDTGEKVEITQSVLDCMELGYAITLHKAQGSQ

FPRIIIALQKGRIVD

>tr|Q87NX5|Q87NX5_VIBPA Uncharacterized protein OS=Vibrio parahaemolyticus serotype O3:K6 (strain RIMD 2210633) OX=223926 GN=VP1743 PE=4 SV=1

MDILNFEAFLIAITILTLTPGLDTALVLRNTSRSGLKDGCTTSLGICFGLFVHAFFSAVG

ISAILAQSAELFQIVKMIGAAYLIWLGISSLKALMASGGGITVAEQIQQVYSGKRSFREG

FLSNVLNPKTAVFYLAFLPQFVNPEGSPLLQSMTMAAIHFVIAMVWQCGLAGALNSAKNL

LKNASFMKWMEGVTGAVLVALGIKLLIEEPL

>tr|Q87RU1|Q87RU1_VIBPA Phosphatidylglycerophosphatase A OS=Vibrio parahaemolyticus serotype O3:K6 (strain RIMD 2210633) OX=223926 GN=VP0685 PE=4 SV=1

MTNPLSLISLKNPWHLLATGFGSGLSPVVPGTMGTLAAVPFFLLLAQLPFPAYVVVVLLS

CVIGIKICQVTSDDMKVHDHGSIVWDEFAGFWITMSIVPALNIPITEWKWLLTGFILFRF

FDMVKPWPIGWLDKRIHGGLGIMLDDIVAGIMAGIALFLVAKYAGWMS

>tr|Q87SB0|Q87SB0_VIBPA Sigma-54 dependent transcriptional regulator OS=Vibrio parahaemolyticus serotype O3:K6 (strain RIMD 2210633) OX=223926 GN=VP0514 PE=4 SV=1

MAGQFKMDSIPGSLVVVGGTYEPWLSVLEQVGWKCHQVGDLRKANTLLEDIGPCIGIVDL

SHDEFSLNGLANLVSSHKHVRWLAFIRESQLGTDTICQFIVNFCIDFFTAPIPDAQLLST

IGHQLGMLKLEKKVWPSFGNSLDMGLIGESIPMKRLRDQVKRIGPTDVSILISGESGTGK

EAVARAIHKVSSRSHKPFMSINCRALNEQRFQAEVFGIAADAEMGPSLLEQADGGTVLFN

DILTISKDQQMNLLRFLQEGTIETREGVKNVNVRILAANSSDVEKALIDGDFNEELYHYI

NVLRINVPSLKERASDIALLARFYLQEFSKEYNSQAKSFSEDALKALTRYFWPGNVRELM

NQVKRAVLMSDSVMIEEHHLDLPQRNDSKRSLKSIREKSERDALLVVLESHSGQVSNAAK

ELGVSRATMYRLLNKHNLISDQAM

>tr|Q87TJ7|Q87TJ7_VIBPA Sensory box/GGDEF family protein OS=Vibrio parahaemolyticus serotype O3:K6 (strain RIMD 2210633) OX=223926 GN=VP0071 PE=4 SV=1

MQRFLILVAFFWSACAWSELTLAQEPKVLVVHSYHQGFFWTDSIQRGIDQQLDDREMDMR

VLYLDSKRNQSEQFFTQLESLYRTKLSDERFDAILVTDNNALELMQHLAPLIKDTPVIFC

GINNYRPSFHQSLNATGVIENVDLEANLALIERLHPDNKQIYIISDHSVTGAILRDEIDD

FIQQHDRYRNKITQLVPDNVEALKKKIAQLNKENVVLFWTYYRDKDGVVGSERDWIQINK

ASNAPLFMVHDVGLGHGAVGGVIQSGYRQGVEAARLLEQVLDHPQEPLPPVVNGDSEIKL

DYQAVVRWGLGAEQESSAVFFNKPMEFSERFAKEIRLFGSLFVLMLVAILLLSYYLQRIR

RSETAARESQAILESIFDQSMQYMGILDKHGLLKSGNDRLQSLLYHQELRLDKPLWLHQN

WSEQARHAIADYFDAPQQQVSTFEAEIWSKEHGSMVLEISLKPFSQQVGKQKQYLFEARD

ITSRKLMEDKLYQRESSLRNYYEQQPVMMVTLDSHNRIQEVNRFAQLLLGYEPIEMLGHR

LREFYLDDKALFPRQVLLQPSHQVHDVWRREIEYRHADGHSVWVRENIRPLNDSDQLLIV

GEDISETKLLAEKLEYQARYDLLTGTYNRNHFELELKTALREVDSHRRVHAMLYLDLDQL

KVLNDTVGHDAGDAAIQFCASMLEEVLPYNSVLARMGGDEFAVLLKDCDEYGAINIAKVI

ISTLGEHLFIWEDTRLFISCSVGIRIIDHTAETPQMVHAQADSACHAAKEQGRNRYNLFS

LDNEELQRRQMEMQSVNLVHEALSNQRLELFAQRVMNLQSPESLMYFEILVRIRDAQGNY

VSPAIFIPASERYNIAHWIDKQVINQALEWFEQRPDVVEKLGRCSINLSGQSMGNQEFID

FLFERLKNSTMPCEKICLEITETAAMGNLDQAIDFFTRAKSLGCMIALDDFGSGLSSFGY

LKKLPVDIVKIDGLFVRDIDVNEMDRVMVRSINDLAKQLGKVTVAEFVENEQVMQHLIDL

GVDYGQGYGIGHPKPLAELVENLSSL

>tr|Q87GU7|Q87GU7_VIBPA Putative secretion protein OS=Vibrio parahaemolyticus serotype O3:K6 (strain RIMD 2210633) OX=223926 GN=VPA1218 PE=4 SV=1

MTPDQKFARWIKYSCVAFVLVFAYFLVADLAMPLTPQAMATRVVTKVAPRVNGQITHLYV

ANNQEIQKGDLLFQIDPQPYKLAVEKAQLNLQQVIQNNEQLDASITAAKADVEASKIVAE

QKIREANRLNTLFSRNGTSQQQLDDAQSSATAAKANLLAAKARLKELEVSRGELGEANVN

VRVAQNQLKQAELNLSYTQVSAEHDSVITNLQLETGAYAAAGTPLIALVSEQVDIIADFR

EKSLRHFNRDSRALVAFDSLPGEVFEARITSLDAGVSSGQFDADGRLATPTDSNRWVRDA

QRLRLHLSINEQPQAFPAGARATVQLLPDSTISGWLARLQIRFLSTLHYIY

>tr|Q87SD0|Q87SD0_VIBPA Bifunctional aspartokinase/homoserine dehydrogenase OS=Vibrio parahaemolyticus serotype O3:K6 (strain RIMD 2210633) OX=223926 GN=VP0494 PE=3 SV=1

MRVLKFGGSSLADADRFLRAADIIANNAQQEEVAVVLSAPGKTTNKLVAVIEGALRNGEA

ELQINELEESFKTLFADIQAVLPNLEGAAFDNQVKTSLSQLRQFVHGINLLGMCPNNVNA

RIISKGERVSIQLMKAVLEAKGQKANLIDPVEYLYAKGDHLEAMVDVDVSTQNFRQNPLP

KDHVNIMPGFTAGNEKGELVTLGRNGSDYSAAVLAACLRADCCEIWTDVDGVYNCDPRLV

EDARLLKSLSYQEAMELSYFGASVLHPKTIAPIAQFHIPCLIKNSFNPQGAGTLIGQDTG

EDNLAIKGITTLNDLTMVNVSGPGMKGMVGMASRVFGAMSSAGVSIVLITQSSSEYSISF

CIEADDKLKAQQVLADAFELELKDGLLEPVDFIDDVSIVTLVGDGMRTSRGVASRFFSSL

AEVNVNIVAIAQGSSERAISAVIPEDKISEAIKACHENLFNSKYFLDVFVVGVGGVGGEL

VDQIQRQQSKLAEKGIVIRVCGLANSKGLLLDSEGLPLEHWRDRMSAATEEFSLARLIAL

VQRNHIINPVLVDCTSSEDIANQYADFLAAGFHVVTPNKKANTASMAYYHQLRDVARSSR

RKLMYETTVGAGLPVIENLQNLIAAGDELERFSGILSGSLSYIFGKLDEGMSLSEATNIA

KENGFTEPDPRDDLSGMDVARKLLILAREAGMSLELEDVVVDQALPPGFDDSGSVDEFMA

RLPEADAYFKELSAKAAEEGKVLRYVGEINDGKCTVSMAAVDENDPMFKIKDGENALAFY

SRYYQPIPLVLRGYGAGTEVTAAGVFSDVMRTLGWKLGV

>tr|Q87HJ0|Q87HJ0_VIBPA Putative MFS family transport protein OS=Vibrio parahaemolyticus serotype O3:K6 (strain RIMD 2210633) OX=223926 GN=VPA0973 PE=4 SV=1

MENTVVSAASPRVTVPVVALSLYAVASGYLMSLIPLMLGEYNISADYASWLASVFYGGLL

IGAMFIERIVRNVGHRKAFIGCLAAFSLTIVALPAFPNGLVWLVARFIAGVAVAGVFVIV

ESWLMSGDEASRAKRLSLYMLSLYGGSALGQFGIGVLGVSGGVPFVAITTLILMAMLVLM

FIDCEQPNSHESTSLSFKQIAKLNHAAIIGCVVSGLTLGAIYGLMPVELANRKITHQDIG

TLMALVILGGMLVQPMVTTLNKYMSRTVLMAFFCILGIFSIGLTFISTSTAVLAASLFLL

GMATFALYPVAINLGCEGLDERFIVSATQVMLFSYSVGSVAGPVVADKFMGQVHGLLGYL

FAALVATCVYMLLAASKTKQQMAAGL

>tr|Q87RZ6|Q87RZ6_VIBPA Uncharacterized protein OS=Vibrio parahaemolyticus serotype O3:K6 (strain RIMD 2210633) OX=223926 GN=VP0628 PE=4 SV=1

MNKNWIPIALSGLALTGCFSEEAKEVEAVAEPLKVTAADKIYYGGDILTMAGDKPEYAEA

VATLGEKIIYVGSKDGAMEHKFGKTQLVDLKGKTMLPGFVDPHSHVYGVGLQAMVANVLP

SPDGEADTVAKIIETLKNAENNNTQRLFVEKTGWILGFGYDDAQLDYYPTKADLDKVSTD

KPVLIIHTSGHLSVANSKALELAGITSESEDPKGGIIRRMENSQEPNGVLEENAHFAMLF

NLNKLIDSELQDRMLEASQGLYAKYGYTTAQEGRATSEGYEAMKRASKNDKLMIDLVAYA

DMVSSSDFMDSEYNTPEYTNHFRIGGVKLNFDGSPQGKTAWLSQPYFHPPHGQDKDYAGY

PTFEDQQAYDYVETAFKNEWQVLTHANGDAAIEQFINAVTKANEKLGKQDRRPVLIHGQT

MRQDQVDRLAAQGIFPSLFPMHTFYWGDWHVDSVLGHPRADFISPTQAVRKAGLKFSTHH

DAPVALPSSFRVLDATVNRTTRTDKVLGADQRVDTYTALQAMTIWPAYQHFEESYKGSIE

VGKNADLIILDNNPMKIEPKALKDLNVEETISRGQSVYQRQ

>tr|Q87SG1|Q87SG1_VIBPA Cell division protein FtsA OS=Vibrio parahaemolyticus serotype O3:K6 (strain RIMD 2210633) OX=223926 GN=ftsA PE=3 SV=1

MTKAADDNIIVGLDIGTATVSALVGEILPDGQINIIGAGSSPSRGMDKGGVNDLESVVKS

VQRAIDQAELMAECQISRVFISLSGKHIASRIEKGMGTISDEEVSQEDMDRAIHTAKSIK

IGDEQRILHVIPQEFTIDYQEGIKNPLGLSGVRMEVSVHLISCHNDMARNIIKAVERCGL

KVEQLVFSGLAASNAVITEDERELGVCVVDIGAGTMDVAIWTGGALRHTEVFSYAGNAVT

SDIAFAFGTPVSDAEEIKVKYGCALSELVSKDDTVNVPSVGGRPSRSLQRQTLSEVIEPR

YSELMGLVNQTIDSVQAKLREEGIKHHLAAGVVLTGGAAQIDGVVECAERVFRNQVRVGK

PLEVSGLTDYVKEPYHSTAVGLLHYARDMQSSDDSDYNEPKRSSVTGFFDKLRNWIQKEF

>tr|Q87PH7|Q87PH7_VIBPA Putrescine-binding periplasmic protein OS=Vibrio parahaemolyticus serotype O3:K6 (strain RIMD 2210633) OX=223926 GN=VP1525 PE=3 SV=1

MKSKFYASALCAATLIATPAMAADQELYFYNWSEYIPNEVLEDFTKETGIKVIYSTYESN

ESMYAKLKTQGSGYDLVVPSTYFVSKMRKEGMLQEIDKKKLSHFSDLDTNFLDKPFDPNN

NYSIPYIWGATGIGINADMLDKSSVSKWDDFWDSKWEGQLMLMDDSREVFHIALTKLGYS

PNTTNPDEIKAAYEELKKLMPNVLVFNSDFPANPYLAGEVSLGMLWNGSAYMARQEGANI

DIIWPEKGTIFWMDSLAIPAGAKNVDAAHKMIDFLLRPENAAKIALEIGYPTPVKTAHEL

LPKEFANDPSIFPPQEVMDSGTWQDEVGEASVIYDEYFQKLKVNN

>tr|Q87KI8|Q87KI8_VIBPA Uroporphyrinogen-III synthase OS=Vibrio parahaemolyticus serotype O3:K6 (strain RIMD 2210633) OX=223926 GN=VP2989 PE=4 SV=1

MAVLVTRPGEQGSALCSLLERHGISAHHHPLIDIVADLTDTHLTTHLHQAQIVIAVSRHA

VQCAQQILTSNGASWPKQAVYLAVGQKTAHYLSKCTQQKVHYPEVSDSEHLLRLPALQNV

EQQQVLILRGNGGRELIKDALVRRGAKVHYSETYKREFILFDPVSCVSLWKTLQINQIVV

TSGEQLDYLCSQLTSEQLAWLNQQELYIPSQRIADIAIQRGFTRVRCTGSASNQELLAAL

QP

>tr|Q87G05|Q87G05_VIBPA Histidine kinase OS=Vibrio parahaemolyticus serotype O3:K6 (strain RIMD 2210633) OX=223926 GN=VPA1515 PE=4 SV=1

MSFKSRLVVFTTVWFCLAMAAIALTYNWQKETIELRTKQSLHQDLASHMRDDNPLMIGTD

YNPKALKSIFHTLMLIGPDFEIYFLDSQGNITTHAAPEGTELMGAVNLAPIRQFLSGEPF

PILGDDPRNRDEHKVFSVAAIEELGSTIGYLYVVIGSSRHTAIANAQVDSPYLALAGLVL

ISILGFAFGSYFLVKRSLLNPIERVTDQLQKQAEHDFRLQPDFAHQVPELVPIARSYQLM

AKHIQQQFLQLEYQSSHRRQSLLQLSHDLKTPLSSVLGYLETWRLQHPDPDPLIEVAFRN

SEKLSQQLHALLDVAKQEAPLPSYEYLPIDISQLMAECAETMQSQFQRKGVTLNITVDEP

IQVIGDKGLLERLILNLLENALRHSPSDATVSCDVKRGDNTSQVRFTFSNHIELNAQAGA

LGIGTKIVQSILMLHHSHLETDATSHQFTQRFTLPAA

>tr|Q87P96|Q87P96_VIBPA Amino acid ABC transporter, permease protein OS=Vibrio parahaemolyticus serotype O3:K6 (strain RIMD 2210633) OX=223926 GN=VP1622 PE=3 SV=1

MSTHQFQPDLPPPSNTVGVVGWLRKNLFNGPVNSIVTLILAYIVFNALWHIVDWAFINAD

WIGSTRDDCSREGACWVFISVRWEQFMYGFYPEAELWRPRLFYITLAIFTVLLAYEKTPK

RLWIWLFFVNIYPFIVAALLYGGVFGLEVVETHKWGGLLVTLIIALVGIVVSLPIGVALA

LGRRSDMPIIRSICTIYIEVWRGVPLITVLFMASVMLPLFLAEGSETDKLIRALRALIGV

VMFSAAYMAEVVRGGLQAIPKGQYEAADALGLSYWKKTGLIILPQALKITIPSIVNTFIG

LFKDTSLVLIIGMFDVLGIGQAANTDPEWLGFATESYVFVALVFWVFCFGMSRYSIWLEN

KLHTGHKR

>tr|Q87QH7|Q87QH7_VIBPA Psp operon transcriptional activator OS=Vibrio parahaemolyticus serotype O3:K6 (strain RIMD 2210633) OX=223926 GN=VP1172 PE=4 SV=1

MKQNLIGESPAFLAVLDKVSQLAPIERPVLIIGERGTGKELIAQRLHYLSKRWDKPLLSL

NCATLSEGLIDSELFGHESGSFTGSKGKHKGRFERAEGGTLFLDELATAPLLVQEKLLRV

IEYGEYERVGGHTALNADVRLVCATNADLPRLAEQGDFRADLLDRLAFDVIMLPPLRERK

EDILSLAEHYAMKMCRELQLEYFVGFTHQAQQALLDYSWPGNVRELKNVIERAIYQHGLN

AEPIDELIFNPFATGWNNALGHTAANEEPQEEASSQTTSIHFPLDYKQWQEEQDINLLNR

ALEEAKFNQRQAAELLGLSYHQLRGMVRKYGLVGQS

>tr|Q87NJ5|Q87NJ5_VIBPA Fumarate hydratase class I OS=Vibrio parahaemolyticus serotype O3:K6 (strain RIMD 2210633) OX=223926 GN=VP1873 PE=3 SV=1

MTVIRKQDVISSVADALQYISYYHPLDFVQALEKAYHREESQAAKDAIAQILINSRMSAE

GHRPICQDTGIVTCFVNIGMGVQWDSTDMTVQQMVDEGVRQAYTNPDNPLRASVLMDPAG

KRINTKDNTPAVVHINMVPGDKVEIQIAAKGGGSENKTKMVMLNPSDDIAEWVEKTLPTM

GAGWCPPGMLGIGIGGTAEKAAVLAKESLMEHIDIQELIDRGPQNAEEELRLDIFNRVNK

LGIGAQGLGGLTTVVDVKIKTAPTHAASKPVCLIPNCAATRHVHFTLDGSGPAELTPPKL

EDWPDITWEAGENTRRVNLDTITKEEVQEWKTGETVLLSGKILTGRDAAHKRIQGMLDNG

EGLPEGVDLNGKFIYYVGPVDAVGDEVVGPAGPTTSTRMDKFTDMMLEKTGIMGMIGKAE

RGPATVESIKNHKAVYLMAVGGAAYLVAKAIKKARVVAFEDLGMEAIYEFEVEDMPVTVA

VDSNGVNAHQIGPDTWKVKIQEMEA

>tr|Q87PV7|Q87PV7_VIBPA Putative VgrG protein OS=Vibrio parahaemolyticus serotype O3:K6 (strain RIMD 2210633) OX=223926 GN=VP1394 PE=4 SV=1

MVNDVEFKFEVPGCGHEFRVESFQVNEELSKPFHISLSLLSLDPDISFDSLIRKAGTLTL

YGQGLSAARIFNGVVNEVRYLGTGRRFSRYQLILVPQAWFLSQRQDCRIFQQKSAKDIIT

EVLDDGSVTDYRFELSGIYPPKEYALQYRESDLHFVQRMMAEHGMWYYFDHTDSNHTMII

VDSNDAIAPLVSSPLNASYIGPIVYHADSGGVADREHISDLELVNRVRTGQVTYTDYNYE

QPKIPQEMTHAGDLDQDLKQFDYPGRYVDPVMGQVRTTEWMFEHIVDNQQVEASSDVMRL

ASGYSFNISDHPRSEINRDYIMLSVMHTGQDPQVHEDEASGMPTTYYNQFTCIPRDVVFK

APKLAAPVVDGPQTAVVVGPAGEEIYTDKLGRIKVQFHWDRYGNNDEHASCWIRVSQSMA

APTWGAVYLPRIGHEVVVTFLEGDPDRPLVTGAVYNGLHFPPYSLPENKTRTTFRTQTHK

GTGYNELSFEDEANQEEVYIHAQKDMSTKVLNNRYRDIGQDEFLKVARHQTNEVHGDHKE

TIDGHKTTQVNSTFTETVEQDVTVTYNANETQYVKNNSDLEIGDNRTTKIGKNDDLDVGE

NSNLTVGASKSSDIGADDNQTVGGNLTVSVKGNTSYKADGATQIISGDKIVLKTGGSSLV

MNSDGSIKLSGSSITIEGSDKVVVKGGNVAIN

>tr|Q87FM3|Q87FM3_VIBPA Ferric vibrioferrin receptor OS=Vibrio parahaemolyticus serotype O3:K6 (strain RIMD 2210633) OX=223926 GN=VPA1656 PE=3 SV=1

MSYQNLDGKVRVRAGLSTTYSVLAIAIASATSMSAVAAPAAKNETTVMETVVVTGSVIGN

SDIEDVKEYPGARTVITRDQIEKTAAGSIDNALQRVPGIKVQDESGTGVLPNISVRGLKA

SRSGHAQFLMDGVPLTLAPYGHTGQSIFPATLSMLDRIDIVRGGAAVQYGPNNVGGVINL

VTKPIPHTWQTEISNRLTVFDGGDAPLNDFYLRTGGWLSDTFALQLEGNFLKGESFREHS

DTDVKNFQAKAQWLLSDTQEIQAFLQRYDAETQMPGALSPQDYEQDRHQSKRPYDDYEGK

STRWSVKYIHDLPFADSAELEVLTFGHKSERLFKWGFNSAGGHWADPALPATDVRTSPRE

FTVYGIEPKMAMYFGEGKSVTQNWIVGTRYVNEDIDYKLTQTPIVGGATKVPRDWHLDTD

AFAGYVSNEIGLFNDTLKVTPGLRVESVRMTFTDRGKKQTADNKVTEWLPGLTVAYNVTD

QWVTYANAQKSLRAPQIAYIRGLGEEGSELAWNYEVGARYTQDATSFNAALYRIDFEDQL

QWQSSTQTFDNIGKTLHQGLELSARYVPEVLPALSLGASYNYLDATLEEEGANKGNQLPY

TSKHQLGWDATYAFYGMDTTLSGFYFSDSYTDNANTSAEDATGATGKVPSYMVWNFNLGT

DLYKDDKGKLRMNVAVNNLFDEEYYFRGIDTSPVGRYPAPGRSYTLDLNYQF

>tr|Q87FX4|Q87FX4_VIBPA Flagellar biosynthesis protein FlhA OS=Vibrio parahaemolyticus serotype O3:K6 (strain RIMD 2210633) OX=223926 GN=flhA PE=3 SV=1

MLTRLKQLQTSTKGYIGIPIVLLMILAMVILPLPPLLLDALFTFNIVLAILVLLVSTTAK

RPLDFSVFPTILLVATLLRLTLNVASTRIVLLEGHNGGDAAGKVIQAFGEVVIGGNYVVG

MVVFIILMIINFVVITKGGERISEVSARFTLDALPGKQMAIDADLNAGLIDQETARLRRK

EVANEADFHGSMDGASKFVRGDAVAGLLILFINIIGGISIGVFEHGLPASEAFKTYALLT

IGDGLVAQIPSLLLATAAAIIVTRINDSDNGMSETMQKQLLATPATLFTVAGIMAVIGMV

PGMPHLAFFAFAGALGFAGWRQSKKPVQDTQIEQVEALSQAMQEEDTPLTWDDIPHVHTL

SLALGYRLVHLVNKDQGAPLSQRIRGVRRNLSEQVGFLLPEVRIRDNLSLKPNQYTISLN

GEVIEQGFIEPERLMAIAVGDTYGEIDGILGSDPAYQLPAVWIEHQDKAKALNMGYQVVD

DGTVIATHISKIMKTNLAELFTHDDVEAMTQRLTQQAPKLAEALAAALNPAQQLKVYRQL

LLDQVPLKDIRTIANTMLESSENTKDPILLAADVRCALKRTLVNLIAGQKPELNVYALSD

ELEQMLLTSLQQAQASGTVVLDSFPIEPNILGQFQQNLPLIRQQLKQQGLPPILLVMPQL

RPLLARYARTFTQGLAVLSYNEIPENKQINVVGNLG

>tr|Q87NV6|Q87NV6_VIBPA Uncharacterized protein OS=Vibrio parahaemolyticus serotype O3:K6 (strain RIMD 2210633) OX=223926 GN=VP1762 PE=4 SV=1

MSMNIKELESYLEAFMCAESDFPFGPDALVFKVKGKMFAIIAERGGREYVSVKVKPEDGE

VLTSQFTDITPGYHLNKRHWVTVYFNGDVEDGLIQDLCERSYDLVVAKLPKAQRTLLEH

>tr|Q87JG4|Q87JG4_VIBPA Uncharacterized protein OS=Vibrio parahaemolyticus serotype O3:K6 (strain RIMD 2210633) OX=223926 GN=VPA0289 PE=4 SV=1

MEFVTLALLGVLIVISPGADFVLVLRNSLNQGREAGVYSAIGISMAISIHIAYSMLGISY

LISQNEWLFNLVRYLGAAYLVYLGIKGIFSSQPASNSETIQQSEYSMWRFFMQGFLCNLL

NPKTMLFFLSIFSQVISPDSSQQHIALFYGIYMIALHGIWFSIVAVLFTSLQLQAFLLKI

KHRLNQACGAGLVIFGAMLGLKA

>tr|Q87HD2|Q87HD2_VIBPA Uncharacterized protein OS=Vibrio parahaemolyticus serotype O3:K6 (strain RIMD 2210633) OX=223926 GN=VPA1033 PE=4 SV=1

MVVMTKIDALNFLDPEELTHEFTPMGLAEFDSHWLTQFVECSDSILAARIWLEGTQSKDF

ASFRHSIVSTINTIDTLLQKQLNEIIHHPDFQKLECSWVGVRYLCEQVDKPSSDSVKIKV

LSATWNEVSKDALKAIEFDQSALFKLLYQNEYGMAGGEPFGVVVGDYQLRYDPRTNYFDR

DISVLSKISQSAAAAFSPFVMSASPEIFGVNTFAELSSTRDVAAQFEQIDYVKWRQLRDN

DDTKFIGLTAPNVLFRQPYKSDGSRNDQFEFQENIDDSNNDLLWGSGAFCFAAVSIRAFQ

EHGWFTHMRGIKQGDYSQGIIVAPTRNKTRINGKNDRDRCPLNLKVSERKEQELSDCGFI

PISPVPETDMVGMTSNVSLYKPKLYEEKHVATNAKLTSMLQYTMCVSRIAHYVKVMGRDK

IGGYQDAASLEREFQTWLHQYTTASDEASDELRAKYPLNEAKIKVREKRDTPGHFYSVIH

LRPHFQLDQMVSSIKLITELSPEHLV

>tr|Q87N63|Q87N63_VIBPA Tetrathionate reductase, subunit B OS=Vibrio parahaemolyticus serotype O3:K6 (strain RIMD 2210633) OX=223926 GN=VP2011 PE=4 SV=1

MDSTKRRFLSAITAGAALVPIAGIGTATAGNVIRNNQSADRKGQVGKRYAMVIDLRKCVG

CQACTVGCSIENQAPIGQFRTTVKQYEVTLDDGSTTTQEAKAFMLPRLCNHCDNPPCVAV

CPVQATFQREDGIVMVDNSRCVACAYCVQACPYDARFINEDTLTADKCTFCAHRLEQGLL

PACVETCVGGARVIGDLNDPSSEVRRLITKHQDNIKVLKPDEKTKPHVFYIGMDERFTSH

IDGQPAIYAPQGDRA

>tr|Q87KN6|Q87KN6_VIBPA Transcriptional regulator, TetR family OS=Vibrio parahaemolyticus serotype O3:K6 (strain RIMD 2210633) OX=223926 GN=VP2941 PE=4 SV=1

MKSMGIRAQQKEKTRRSLIDAAFSQLSADRSFSSLSLREVAREAGIAPTSFYRHFKDMDE

LGLTMVDEGGLLLRQLMRQARQRIVKEGSVIRTSVETFMEFIESSPNVFRLLLRERSGTS

SEFRTAVAREIQHFAAELTEYLMSTGMTREEAYTQAEASVTLVFSSGAEALDLDRRERDE

LAERLIMQLRMIAKGAYWYRKERERNRLKGGIE

>tr|Q87LG7|Q87LG7_VIBPA DNA polymerase III, chi subunit OS=Vibrio parahaemolyticus serotype O3:K6 (strain RIMD 2210633) OX=223926 GN=VP2645 PE=4 SV=1

MQTATFYIINEESPQATTAGFEEYIVFLVQHFARQGAKVYLNCQDKPHAEQLAEAFWQID

ADQFMAHNLVGEGPKYATNIEIGHDGVKPSWNRQLVINLAENETTFANKFAQVVDFVPCE

EKAKQLARERYKIYRQAGYQLQTIEIQYP

>tr|Q87G74|Q87G74_VIBPA Uncharacterized protein OS=Vibrio parahaemolyticus serotype O3:K6 (strain RIMD 2210633) OX=223926 GN=VPA1443 PE=4 SV=1

MSNQQPVVNELPAEKVYRFSSDANLSFKKPILLGVATLSFFLGGLGYWAATAKLESAAIA

YGDLSVLTKRQEIQHLEGGIIEKLYVQEGDLVKKGQPLVQLSQRQPMAKLDAVSGQYIHT

LAKENRLSAELDELADISWSGDLEAIPRVNIVKEAQLVQNKIFTARKRFFESKLSIIEQS

ISGASLELENLKQTKVIERERLNFIEEEIASNQALVQKGFSGKSTLLQLKRLAAEVRSTL

SQLDRQSLTVGKRLDENQAKIEELKLERLNEIVEELRNTKKEVVAIREEYRSAQDVVART

SINAPISGRVVNMQVFTEKGVIGSGQTLLELVPQDDKLLVEARVNPQDIDLINPGQQAHV

RLTALNARTLAPLDGTVLTVSADKLSQENQDDYYLARISISQDDVAKYRLTSGMNAEVLI

LSEPRTPLSYLIKPLTESMNRAFREE

>tr|Q87FI5|Q87FI5_VIBPA Putative D-serine dehydratase (Deaminase) transcriptional activator OS=Vibrio parahaemolyticus serotype O3:K6 (strain RIMD 2210633) OX=223926 GN=VPA1694 PE=4 SV=1

MDRKQQMLSNMYTFAVAGKCLSFTKAAEELFITQGAVSQRIKSLEEQLGFSLFVRMTRRL

ELTKEGERLLHALNQSFEVIFSELEDIKFNELRGELYIGVAPTFAQSWLLPRMVEFQRLY

PSLNIKLRVKASRLDFLHEPVDIAIYYSDSEHPGFHHQRLFDEELVPVCSPEYYQTHFSD

GVSSASQFEAVTFIHCTESLEANEPNHEWQSWLASQSNSELKSLNVMEKTYLFNHADMAM

IAAKNSMGIAMARASLVQTSLEKRELVAPFERVNAGRGYDLICLNGQQHRPKNAAFIEWL

ETQLPPLVSQ

>tr|Q87G08|Q87G08_VIBPA ScrC (Sensory box/GGDEF family protein) (Involved in swarmer cell regulation) OS=Vibrio parahaemolyticus serotype O3:K6 (strain RIMD 2210633) OX=223926 GN=VPA1511 PE=4 SV=1

MKKAVKKISSKKIITISAIIVSIYLAILIVVTSLGQNKLKDSQYRELDLKVKSYASTLDN

LFTIASEDVDNLSSDKTVQTFFANLASGMSMEYGLGASLINLKRRFDEKIDGKLINSSNI

YNKLTLVGSDGTTIVTTGPKDLKPMDIHDLMTQHGHPEEVCATRDGDNIRIQVIKQVKFS

GKPVAFLIADINKDIVIEQLTNQEHEDSYSRMVLKIKDVEMVVWDSITKNSTINADENHF

LSKILDGKIYFEVPVEQHHFKLMAWFEPLNERDIFTSRLFIVGLSILAVMIVIALCYAFV

TNNNKLELKIKLEEEKKRKDILSQQNHKLTKEIERRKASEKELAFQAKYDTLTELPNRSY

GSERLALELIRASRTGSKVLVMFIDLDHFKQINDSMGHFVGDEILKLSAQRLQNVARKTD

LLARIGGDEFLLVIPDLPDNDTAKRVASSVLSAFSEPFVWNNHEFFLTGSVGMSVFPDDG

DNAEQLLACADMAMYRVKQDGRDAFCFYNHNMNQDLQRYLDLESRLRNAISNQLLEMYYQ

PIIELKSGKIVGAEALMRWNDEKFGFVNPEEFISIAEKNGLIHQLGEFAIQQACHQASQW

QSISPLFVSVNFSSVQFRYCDRLLAFIRQSLEESGLPAEQFDVEVTESLLFNHDDELVDM

LDNLRALGTKLTIDDFGTGYSALSYLQKFPFDRLKIDRSFMQNVFENDSDRELVNVIIAM

AKALRLKIVAEGIEEQRHVDYLNELNCEFGQGFHYSRPVPAKEFEQLLNQPTWS

>tr|Q87IX5|Q87IX5_VIBPA Putative cation efflux system transmembrane protein OS=Vibrio parahaemolyticus serotype O3:K6 (strain RIMD 2210633) OX=223926 GN=VPA0481 PE=3 SV=1

MNSNSIKIATIALLVGGALGFGVNQYLSGANHDMSGTGGSAASSSNEPLYWVAPMDPNYK

RDKPGKSPMGMDLIPVYAEDLAGEQDKPGTVKIDPSVENNLGVKTAQVSLEQLSPRIETV

GYIAFDESHLWQTNVRVAGWVEKLYINAVGEKVNKGDVLFTLYSPELVKAQEELLNAYRT

GRSGLVKGATERLVTLGVDRSQIRAITQRGKASQTIEIKAPEDGVIASLNVREGGYLSPA

QAVISAGPLENVWVDAEVFERQAHWMKAGSDATMTLDAIPGKEWQGSVDYVYPILDPKTR

TLRVRLKFSNPNGELKPNMFANIALQPVSDNAVLTIPKSSVIRSGGMTRVVLSEGDGKYR

STRIEVGREADDKIEVLQGLTQDDRIVTSAHFMLDSESSQTADLSRISSPTEATAQTAWT

KGEITDVMKGHRMLTINHQPVPEWDWPGMVMNFTLADGVDMGSLQKGQAIEFEIQKAASG

QYEIVDYKADDTVLATDVWVTGDISMLMADFGMITLNHLPVSEWNWDAGEMNFSVGDEVD

LSGFKEGQKVRFLVEKQGSDYVLKQIEAIEG

>tr|Q87FK6|Q87FK6_VIBPA L-arabinose-binding periplasmic protein OS=Vibrio parahaemolyticus serotype O3:K6 (strain RIMD 2210633) OX=223926 GN=VPA1673 PE=3 SV=1

MKLKKLLTIAALSGMTMLSASANAFFGSNDDNVRLGYLVKQPEEPWFQTEWSFAEKAAKQ

YDFELVKMAVPDGEKTLNAIDTLAASGAKGFVICTPDPKLGPAIMAKAKSYDLKVITVDD

QFLNAKGEPMKEVPLVMMAASEIGYRQGSELFKEMTNRGWDAATTGVMAITADELDTARR

RVDGSVKALEDAGFPKSQIYRVPTKTNDIPGALDAANSLLVQYPKVKQWLIVGMNDNTVL

GGVRATEGQGFAAENVIGIGINGVDAVNELAKSNATGFFGSLLPSPDVHGFKSIESLYKW

VKEDVQPEKFVEVTDVVLITRDNFREELQKKGL

>tr|Q87LT5|Q87LT5_VIBPA Type 4 prepilin-like proteins leader peptide-processing enzyme OS=Vibrio parahaemolyticus serotype O3:K6 (strain RIMD 2210633) OX=223926 GN=VP2526 PE=3 SV=1

MEVFQYYPWLFVVFASIFGLIVGSFLNVVIYRLPKIMELEWRRECAESFPEYKIKPPQEV

LTLSVPRSSCQNCATPIRIRDNIPVISWLLLKGKCHHCHTAISPRYPLIELLTAACAGFV

AYHFGFSYFTVALIFFTFFLIAATFIDLDTMLLPDQLTLPLTWAGIALALTEISPVSLQD

AVIGAIAGYLCLWSVYWGFKLLTGKEGMGYGDFKLLAALGAWLGWQSLPMIILLSSVVGV

IFGLVQLRLQKQGIERAFPFGPYLAIAGWVSLIWGDQILSWYFTSILGV

>tr|Q87S33|Q87S33_VIBPA Protein-export membrane protein SecF OS=Vibrio parahaemolyticus serotype O3:K6 (strain RIMD 2210633) OX=223926 GN=secF PE=3 SV=1

MFQILKAEKSIGFMRWSKVAFVFSIFMIAASIFTLSTKWLNWGLDFTGGTLIEVGFEKPA

NLEEIRTALDAKGFGDATVQNFGSARDVMVRLRPRDDVSGETLGNQIIGAIKEGTGESVE

MRRIEFVGPNVGDELTEAGGLAILVSLICILIYVSMRFEWRLAAGAVMALAHDIIITLGV

FSFLQIEVDLTIVAALLTVVGYSLNDTIVVFDRIRENFRKMRKGDPADIMDASITQTLSR

TLITSGTTLFVVIALFVQGGAMIHGFATALLLGITVGTYSSIYVASALALKLGIQKEHLM

PPQVEKEGAEFDEMP

>tr|Q87SU1|Q87SU1_VIBPA Uncharacterized protein OS=Vibrio parahaemolyticus serotype O3:K6 (strain RIMD 2210633) OX=223926 GN=VP0331 PE=4 SV=1

MNLETIPSGELTVSNQRSISRLVAQAGQMLLAHGAESTLVCDIMRRIGLACGVHEVAVAL

SANALVVTTVMDGHCITTTRSCADRGINMRVITQIQRICIMMERGLLDAAMAQKKLNTIS

PERYNRWLVVLMIGLSCAAFSHLAGGDWGVFIMTFLASAGGMIVRQEIGHRHFNPLLNFA

ATAFVTTLISAQAEIYQIGNLPTVAMASSVLMLVPGFPLINSVADMLKGHINMGIARFVM

ASLLTLATCLGIVAAMSVTGIWGWAI

>tr|Q87PW5|Q87PW5_VIBPA Uncharacterized protein OS=Vibrio parahaemolyticus serotype O3:K6 (strain RIMD 2210633) OX=223926 GN=VP1385 PE=4 SV=1

MTSLILSSRNHNLALPLITIALTIIAGVSFSHYLLTPEEQSEVISHSYQGVLFDKEANNS

IEKTYDSHFQRTKITTGVINYAFVTSLINAGLSQQEIKSLIKLIESEFDIIGSVRKGDKF

SLKTRTNSYNEKYISSFYYSGSKKDFFIINDGKNNAYDEYGDRLTRKPYYSFPLAKEYKI

SSGFSLKRKHPITGLNTPHLGTDYAVPVGTPIYSIADGVIVKSRYNRFAGNYINIRHTNG

SISRYLHLSRSSVRKGDNVVKGQEIGRSGNTGRTTGPHLHLELFVDGAPVDYARYIKSNQ

APSLNIQMMLAAKTERAELIKELL

>tr|Q87LB2|Q87LB2_VIBPA MSHA biogenesis protein MshG OS=Vibrio parahaemolyticus serotype O3:K6 (strain RIMD 2210633) OX=223926 GN=VP2700 PE=4 SV=1

MPTFRYQGRTLDGSSTSGKVDAVNSEAAAEALMNKGIIPLNLRLEKEGVKNHVSLSKLLV

PAIPLEVIILFSRQLFSLTKAGVPLLRSMRGLLQNCENKQLKEALEDVVSELSNGRGLSS

AMQPHNKVFSPLFVSMINVGENTGRLDEALLQLANYYEQELETRKRIKAAMRYPTFVIVF

ITIAMFILNILVIPEFASMFTRFGVELPLPTRILIATSNFFVHYWGLLIAAMVGAFFVFK

AWVATAGGREKFDKFRLRLPIVGDIVNRAQLSRFARTFSLMLKSGVPLNQSLALAGEALG

NRFLENRILEMKAAIEAGSTISVTAINSNIFTPLVIQMIAVGEETGRIDELLLEVSDFYD

REVDYDLKTLTARIEPLLLVIVAGMVMVLALGIFLPMWGMLDIIKGG

>tr|Q87IK2|Q87IK2_VIBPA ABC transporter, periplasmic substrate-binding protein OS=Vibrio parahaemolyticus serotype O3:K6 (strain RIMD 2210633) OX=223926 GN=VPA0604 PE=4 SV=1

MLRFTVATLLAMAVSLPSAMAKEVNISGSTSVARVMDVLAEEYNKTHPDNYIAVQGIGSS

AGITMVNKGVVKLGMSSRYLTESEKGEDLNVFPIAYDGLAVVVNRTNSVTNLSAQQLFDI

YKGEIKNWKEVGGADQPIAVVTREASSGSRYSFESLLGLTKIINDRLVSDISPNNLVVNS

NSMVKTIVNHNTRAIGFISVGSVDLSVKAIQFNGIEPTAANIANHKYKLARPFLVLYKVD

SLDQAGKDFVAFLKSEAGQKTIADYGYIPVKNFNQ

>tr|Q87R61|Q87R61_VIBPA Uncharacterized protein OS=Vibrio parahaemolyticus serotype O3:K6 (strain RIMD 2210633) OX=223926 GN=VP0937 PE=4 SV=1

MSSDRQPSLLTQRKFLPYFVTQFFGAFNDNIFKNVLLLFVAFAGSSALPISSNLFINLAA

GLFILPFFLFSASAGVLADKYEKSWFIRKVKLFEIAIMLLGAVGFITESYGVLLLLLFLM

GTQSAFFGPVKYALLPQELNAKELVPGNALVEAGTFIAILIGTLGAGIIASADNAKYLAA

FCVVLFALLGYLSSRSIPFAAASAPELKFRWRPYQQTKHTLAIAKSDRIVFQCIMAISWF

WFLGAAYLTQFPNFTKVYLNGTESAVSFLLALFSVGIALGSLACNWISNHRIEVGIVPIG

ALGITIFGYLMATSVPTELPEFANFIEFVRFEPFWPLFFYLLMIGASGGLFIVPLYALMQ

HRAKETERAQVIAGLNIFNSLFMVGSAILGIICLSVVEMSIPQLFALLAVLNFLVAAYIF

LQIPIFVVRFAMWMLTHTIYRVKHKNLHNLPEHGGALIVCNHVSYMDALLLSAVCPRLIR

FVMEEDYANLPPLRRFLRRAGVIPISASNRSSIRRAFNDVEEALNEGHIVCIFPEGRLTS

DGEMNDFMRGIDIILRRSPVPVIPMALKGLWGSYFSRAKGRACRGFPTRFWSKLEIEAGE

PVDPSSATAQTMFEKVHALRGDWR

>tr|Q87L61|Q87L61_VIBPA Penicillin-binding protein 1A OS=Vibrio parahaemolyticus serotype O3:K6 (strain RIMD 2210633) OX=223926 GN=VP2751 PE=4 SV=1

MKFIKRLLFLTLICIILGVTTIFGFYQYVKPELPDVATLKDVELQTPMQVFSQDGKLIAQ

FGEKRRIPVTYDEIPQDLIHALIATEDSRFYEHPGIDPIGITRAAVVVALSGSAKQGAST

ITQQLARNFFLSNEKKLMRKIKEIFIAIHIEQLLSKQEIMELYVNKIFLGYRSYGFGAAA

QVYFGKSLNELSLSEIATLAGMPKAPSTMNPIYSLERATKRRNVVLMRMLDEKYITQEQF

DEARNEPIIARYHSAEIEVSAPYVAELARAWAVKEYGEEKAYTSGLNIYMTVDSKLQDAA

NKAAVNNLMAYDERHGYRGAEKVLWKEGEAAWDAEQIEKHLKGQPTYGDLYPAVVTKVEG

KTAQVVVKNNDAQTIPWTGINWARKFLTDNRQGSTPKSAQEVLAAGEQIWVRELTTTDED

GNTQSSWKLSQVPSANTAFVAMNPENGGILSLVGGFNFVHSKFNRATMSVRQVGSSIKPF

IYSAAIDKGLTLATLINDAPINKWDAGSGSAWRPKNSPPTYGGPTRLRIGLAQSKNVMAV

RTLREVGLDETRQYLTRFGFDINEVPRSETIALGAGSLTPMKVAQGYSVFANGGYYVEPF

YVERVEDAFGEVLFKANPKSVCHQDCPQMSPQPEMDRFASEFGEQDVAVDGQAPENALEN

DEPKYAPQVISEQNAFLMREMMYSNIWGGGNWREGTGWNGTGWRAQKLERRDIGGKTGTT

NDSKDAWYNGYGPGVVAIAWVGFDDHSRALGRTTVNSNLGQGQVSGAESGAKTAEPAWID

FMQVALEGKPEQGKNIPDDIVRVRIDRNSGLLTHKVDSTSMFEYFEKGTEPTEYVGNSLE

DSIYSSGSGGTTEELF

>tr|Q87SZ3|Q87SZ3_VIBPA Protein translocase subunit SecY OS=Vibrio parahaemolyticus serotype O3:K6 (strain RIMD 2210633) OX=223926 GN=secY PE=3 SV=1

MAKKPGQDFRSAQSGLSELKSRLLFVIGALLVFRAGSFVPIPGIDAAVLADLFEQQKGTI

VEMFNMFSGGALERASILALGIMPYISASIVVQLLTVVHPALAELKKEGEAGRRKISQYT

RYGTLVLATFQAIGIATGLPNMVDNLVVINQTMFTLIATVSLVTGTMFLMWLGEQITERG

IGNGISLLIFAGIVAGLPSAIGQTIEQARQGELHVLLLLLIAVLAFAVIYFVVFMERGQR

RIVVNYAKRQQGRKVFAAQSSHLPLKINMAGVIPAIFASSIILFPGTLAQWFGQNGESSA

FGWLTDVSLALSPGQPLYVMLYAAAIIFFCFFYTALVFNPRETADNLKKSGAFVPGIRPG

EQTAKYIDKVMTRLTLAGALYITFICLIPEFMMVAWNVRFYFGGTSLLIVVVVIMDFMAQ

VQTHLMSQQYDSVLKKANLKGYGR

>tr|Q87JM6|Q87JM6_VIBPA Putative long-chain fatty acid transport protein OS=Vibrio parahaemolyticus serotype O3:K6 (strain RIMD 2210633) OX=223926 GN=VPA0222 PE=4 SV=1

MRKNMQRQFTLSPIYLLIGLASSTAYAGGFQINEHSATGLGRAFAGDAVIGDNASVVSRN

AAAMTLFKQNALSFGVTYVKPDVTVKNAQYHRANINADVDVSMGGGLIPNLQPPSVSVTP

SETVADIDDVDGVGQPAVVPNFYFIHPINDDWYLGLSTYSNFGTDMEFKPNYGAPVFGGV

TSVASVNLGASLAYKVNDRFSIGGGIDVIYGSGELYRDMDVGVCVGGNILGNELEQRCGA

VKGNALDVEAGGIGLGANIGMMYEFNERHRLGLSYKHSPNIDAKGDIHFAGESYDSLAMP

LPDIAEFSGYHRVLPKFALHYSVQWIGWSAFDSLKADDQLLKDFQWQDSAHYSIGATWYA

NERWTLRTGYMFDKTPVDELTSLSIPDSNRHWLSAGASYQWSSDTTVDIGMTYLIGEDVN

VEEYAYEGVPAPMVTGVTHSNAFLIGAQLSHRF

>tr|Q87TM0|Q87TM0_VIBPA Peptide ABC transporter, permease protein OS=Vibrio parahaemolyticus serotype O3:K6 (strain RIMD 2210633) OX=223926 GN=VP0049 PE=3 SV=1

MNFLSKFRDLWIVKGQGMFSFLVKRLFQALIVMFVISLVAFAIQDNLGDPLRELVGQSVS

ESERQALRDELGLNDPFITKYTRFVGNALQGDLGTSYFFKRPAVEVILDKLVATLELVFG

ATLIIIVLSIPLGVYSAIHPKSIFTKFVMAMSSVGISIPVFLTAIMLMYVFSIELGWLPS

YGRGETVNVLGWESGFFTIDGIKHLILPCIALASIMLPLFIRLVRSEMLEVLSSEYIKFA

KAKGLNLQKIYYQHALKNTMLPVLTVGGVQIGTMVAYTILTETVFQWPGTGFLFLEAINR

VDTPLITAYVIFVGLIFVVTNTIVDLLYGIINPTVNLTGKGA

>tr|Q87M80|Q87M80_VIBPA Transcriptional regulator, LysR family OS=Vibrio parahaemolyticus serotype O3:K6 (strain RIMD 2210633) OX=223926 GN=VP2378 PE=4 SV=1

MASHISLKQLKVFTTITQHKTLTAASDSLFLSKAAVSMALSELEKQIGHHLFDRVNNRLI

LNQEGQKLLPLADELLNRAKDIESIFDGDQALSGQLRIGASDTIGNQVAPYLLSEFRQQT

NHRSQSLFISNSAQICDMLTDYELDIALIEGKTLHPELQSTQFSEDEMCVICAPDYPAAH

EGPVNLTELENSEWILREAGSGSREFFMRVVAPRLEHWHEAFQLNTTEALINSVSAGLGL

GCLSRLSAEPAIRDGRVKLLDMPLDMKRRFWLLVHKEKYQSPLLKTFIEFCQDWERPTNL

PLGNKSAK

>tr|Q87HI0|Q87HI0_VIBPA Putative formate dehydrogenase OS=Vibrio parahaemolyticus serotype O3:K6 (strain RIMD 2210633) OX=223926 GN=VPA0985 PE=4 SV=1

MSTDFKPAEFVQTMINVGEAKTNTSTRDLLLRGTMAGIILSLAVVVAITAMVQTGIGLVG

ALVFPVGFVILSVMGYDLVTGVFGLAPLAKFANRPGITWGRIFRCWGLVGLGNLIGSLIV

AYLVAISLTGNFSLELNAVAKKFVAVSTARSLGFENMGMDGWITCFVRGIFCNLMVCLGV

IGNMTARSVSGRVAMMWFPIFIFFALVFEHTVVNMFLFPLGMILGADFGIATWLNFNLIP

TILGNIIGGLVMTCLPLYLTHAKTAPSLSVEQDVIAEPAIAK

>tr|Q87G52|Q87G52_VIBPA Uncharacterized protein OS=Vibrio parahaemolyticus serotype O3:K6 (strain RIMD 2210633) OX=223926 GN=VPA1465 PE=4 SV=1

MKLKLLALSLALAGVQVTNAAQWDYPSAAITVTNQAEALRYLQANYASAGELKFRYQTRS

KLGDHYNFDVWVDGQYQPQRTIVLSTNQDRQIERIFKSLEDTVIRNGEAMPAAELELPVR

LEVTEPPALSSGELVTVPVSVFDPDLRTMQQQAAPDSAWNSLEDYPQPLQYVEKQIQVLQ

SDGKFYLANERVKQVDALALLPSPAVGAEPVRDTSSILPPEGVQSFADVKAMQSTQLGDN

AFLQLMAFYHLDNSLQYLSSLSYDLFEEPLRFDGRGLALDNSSYYTGSRALMLGVGGVSP

DAADADVILHELGHGIHYQIVPDWAYGHTGAIGEGVGDYWAGSASYRTQYLDAARRGQEF

EIDTVFNWDGMFGVRRATRSLWNQRARYFEGAEYRAHESVGGELGDELWSTPLFQALKAS

VARYGDGSDRVFRDFDSIVLEGMYGVGRGVKMHDLAESTVFAATMLFPEKEYAQILKDSF

AQHNLLKVPFNARYQSRYINAGETVELTLSHTGRSASVKGNWSLNNDTSKAVDVQLANSA

SFSANLPTGLTCGAPFESKVSLDYQFAEGLKAQSWQNVTALVNGIPLLLNKPKTIEDALT

DASINSRGQEVQGNKIFVQTLSDKTQKIDDSFAVYLDIEHANLADLHITLTSPQGQSVVL

FNHQPSQSGGFTGYFTVQHDPQLQALVGEPSWGTWRLEVSDRVVGNSGALKAWGVSHFNQ

YQCGADTTSKSNGNGSGGSGSPWALFGLLVLSMLRVVRSRNNDSSKRR

>tr|Q87KA1|Q87KA1_VIBPA ParB family protein OS=Vibrio parahaemolyticus serotype O3:K6 (strain RIMD 2210633) OX=223926 GN=VP3077 PE=3 SV=1

MSKRGLGKGLDALLSTSSFAREKQQIASQSQALSADGELTELAIGQLQPGIYQPRKDMAP

EALEELAASIQSQGIIQPIVVRQVVGGQFEIIAGERRWRAAKQAGLKRVPCLVKKVEDRA

AIAMALIENIQREDLNVIEEAQALERLQDEFSLTHQQVADVIGKSRTTVSNLLRLNQLDA

DVKRLVAEKQLEMGHARALLALEGEQQVEVAQMVAKKQMTVRQTEQLVKKCLAPQNEQKA

QQEDTEAEQMSHKLSQLLDAKVSLSRSANGKAKLTISIDEPHKLDQLIAKLEA

>tr|Q87TA2|Q87TA2_VIBPA Uncharacterized protein OS=Vibrio parahaemolyticus serotype O3:K6 (strain RIMD 2210633) OX=223926 GN=VP0168 PE=3 SV=1

MYKNTTALSVAISLALGTAAAVAPLTAQAEEQQVEKLQKMKVTGSRLTRASMEGSTPVAV

IGRAEIERAGDVSIADVLRKSSFNSFGSHNERSGSSAQSQATISLRGLGSERTLVLINGK

RLPGSASMGGGAANINVIPSAIVERVEVMADGGSAVYGSDAVAGVVNIILKEEFDGINVT

LGSGIPSREGADEENVSIVLGTSGEKGNIMFSFEHDSKDEIYQRDRDYLSSTNTGSANYF

DMSGVSIYGRNVYHDGQLKALKGYDTDASCDPSKGFVGLTNYPGLGDICGYDYTSEAAQT

ASLERNTVFMNGNIFLSDDTTFNAQVLLNRNESFGRFAPAAGYFEVDPTTTGGAAFFAEN

GLDATKGPAAVYYRFNNVGTRDNSVTDFQADLKAGLDGTLYTDSFGEVIWETGYHLNFSN

SNETGTGYVFRSPAEQLANSGQFVNGEFSADATSQLSAGTGRETQMEMHQVYGGLQFDLA

EVGNIVIPLYVGAEYTTYDYFDQYDPQSEAGNIIGSSGNSAGGDRETYAIYAESLIAFTD

ELEMNIAARYDHYSDFGDEISPKVSFRYQPLDNLMFRTGAGLGFRAPTLSDLYGADSFSS

DYAKDYVFCSQNGISASECPETQYDVTRTSNEDLDAEKSVSFNFGISYSPIDDLDLTLDY

YNISIDDVITLNTLQSMIDQERSSGVSNPNIVRENGRIKEATAGLQNLGTLDTSGLDLKI

GYRYDFNVATIRYDFTGSYVLDYSSPEYVGGPVNNQVGRNGLPEYRFNTGVGVNFLEEHD

IYLSADHIADQAQDVDDNYNKTGHISSQTTWNIAYNYLAPWDAKFTAGVRNLTDEDPAFE

SDGVTYDKDLYSIQGRVYFLKYSQNF

>tr|Q87KG0|Q87KG0_VIBPA Putative transmembrane protein OS=Vibrio parahaemolyticus serotype O3:K6 (strain RIMD 2210633) OX=223926 GN=VP3017 PE=4 SV=1

MTDSVKSLISRYDEYTNSLQTHAMPLLLLFCRLWVAWVFFNSGLIKIASWDSTLYLFEFE

YQVPLLPWEFAAYSGTFAELVLPVFLALGLFTRPMAAMLFVFNIIAVLSYPLLWEKGFHD

HQLWGLMILIVTLWGAGPFSLDRLLKDKFVR

>tr|Q87LC2|Q87LC2_VIBPA Cell shape-determining protein MreC OS=Vibrio parahaemolyticus serotype O3:K6 (strain RIMD 2210633) OX=223926 GN=VP2690 PE=3 SV=1

MKPIFGRGPSLQLRLFFAVIVSASLMLADSRLDTFSNVRYLLNSMVAPIQYAANMPRSMF

DGVFERFNTRQALVEGNRNLKREVLRLKSELILLDQYKEENQRLRKLLGSSFVRDEKKVV

TEVMAVDTSPYRHQVVIDKGQIDGVYVGQPVINEKGIVGQVTFVAAHNARVLLLTDAKNA

IPVQVIRNDIRVIASGNGEMDEIQLEHIPTSTDIQVGDLLVTSGLGGIYPEGYPVATVTN

VDHDTRQEFASIKAEPVVEFDRLRYLLLIWPNEDRQHKVLQSNANDGLEQEQEVTNGQ

>tr|Q87MQ0|Q87MQ0_VIBPA Inner membrane protein OS=Vibrio parahaemolyticus serotype O3:K6 (strain RIMD 2210633) OX=223926 GN=VP2181 PE=4 SV=1

MEFTIRIKRYFFNIVGGLCIALGIAGIALPLLPTTPFILLASACFMRGSPAFHHWLHNHK

TFGPILDNWHRHRAVSPKVKQRGAVFITLSFTVSIIVAPIIWVKIALLVMLIVLLSWFMR

LPVIELVADREENH

>tr|Q87RD0|Q87RD0_VIBPA Uncharacterized protein OS=Vibrio parahaemolyticus serotype O3:K6 (strain RIMD 2210633) OX=223926 GN=VP0867 PE=4 SV=1

MASINGLPPSLIPGTNRTNKVGKKGQVKKAQNKQSVGQPSKVANAVAHSIKQVDESQIHR

AQVQYDLPEGNARKAMEQYMDVMNRAKKEELAQLLGVDIYV

>tr|Q87FM4|Q87FM4_VIBPA Putative FecB OS=Vibrio parahaemolyticus serotype O3:K6 (strain RIMD 2210633) OX=223926 GN=VPA1655 PE=4 SV=1

MKNKKQFTIKHFSTVLLMLLSSALMSFSAFSQARSVQDEQGTFELEAIPQRIVVLEFSFV

DALAAVDVSPVGVADDNDAIRVIPAVRAKIEPWQSVGMRSQPSLEAIAVLKPDLIIADAE

RHCAIYQDLQRIAPTLLLKSRGETYQENLESAQKIGVAIGKQAQMTQRIELHKQTMAEFK

QHFATQETIQFGVVSDKGMWLHSPVSYAGGVLSTLGIQSPLAPSERNAYIPTSFELLLKT

NPDWLLVGLYSQPNIVDEWRRNPLFKLLTASKKQQLVEVSPELWSLNRGMLAAEEIARNL

EALLGRS

>tr|Q87PN3|Q87PN3_VIBPA Putative hexosyltransferase OS=Vibrio parahaemolyticus serotype O3:K6 (strain RIMD 2210633) OX=223926 GN=VP1468 PE=4 SV=1

MKLISNNPDLPIVGEVWIFVDSRIFGGIESHILELAKGILSFGHAVRIVLPTEYIPQAQL

VEKAQAANIPTSYLPQISGVPLNTIAIKHLTSAVAHHQPSVLHTHGYKASILARTAKLLT

RTFPRLVSTYHAGESSSGKLWLYDGLDRCSGYLSDHCFAVSKSIQDKVLCPSQLLNNFVA

LPSMNNSYQEISFVGRLSHEKGADQFIELAKACPDYRFSIYGDGPERQNLETTAPANVIF

HGHQTDMNAVWENISVLIISSRFEGLPMAALEAMARGIVVISLKVGRLPDIIQSGNNGFI

ADDVPALALNLQYWMTMEKSQQERIRKNARQTIVEQFSTESVIPVLLTQYQIEMDN

>tr|Q87MQ9|Q87MQ9_VIBPA Uncharacterized protein OS=Vibrio parahaemolyticus serotype O3:K6 (strain RIMD 2210633) OX=223926 GN=VP2172 PE=4 SV=1

MLAQWFVSVCRILTNLLSILCRPNLQFLLREVTLLLKKLTKSDLDILNSMKNVVDGIARM

YGEHTEVVLHSLDAEAPEIIKIANGHVTERSEGAPITNLARMKLREGKDVSDSYLTKTSN

GKTLHSITTIVRNPKNKPIGLLCINVDMDAPMQAFLKAMLPQQHTECCVGGARSPETFAR

NIDETIISTIETVQTEVWENEAIAPSKRNRELVTRLHGLGIFKYKDAVLMVANHLGISRD

TIYLYLRELGND

>tr|Q87PM6|Q87PM6_VIBPA Uncharacterized protein OS=Vibrio parahaemolyticus serotype O3:K6 (strain RIMD 2210633) OX=223926 GN=VP1475 PE=3 SV=1

MVNLIRRSVVSGLVVGMFGCSSFDYPDHGQGGLAESYQDISIENYQFSPVMPDEPLGPEH

GLRFDWQLTKLHLDALIQEGARWCFPAAVVQALEKQNRIARELEGGLLLDAANDLVIQRR

RLNQLEQQLDYVLTQTTCTPPDDIDALRNDLNIVADIYALLNVDNQFAIDSAEINPKYMG

HLAEAAYILRDHPALSLVVTGHADVTGTPEHNKKLAQERAEQVKRYLSVFGVTSDRVQTK

SVGDTLPLYEGQTPAVRLTNRRVSIEVLSANSDSNAPSNHNLPNKGNMPTSASGSIGGAL

>tr|Q87QM6|Q87QM6_VIBPA Cyclopropane-fatty-acyl-phospholipid synthase OS=Vibrio parahaemolyticus serotype O3:K6 (strain RIMD 2210633) OX=223926 GN=VP1123 PE=4 SV=1

MLNTTSITLPRKLTTTQKAARGVIFQCLQKMEMGCLTIIESFHTETKERSERFTAPHGEY

SGQPVVATIEVKHPGFYSRILQGGSIAAGEAYMDGWWDSPDLTALMKLMALNIRALDKLE

EQGSWLTKLLYKFSHWTNRNSQENSRKNIHAHYDLGNDLYEAFLDTNMLYSSALYHTSDD

SLEQAQINKMERLCQQLDLQSTDHVIEIGTGWGAMAIYMAEQYGCRVTTTTISEEQHEYA

RQQIVQRGLADRITLLKEDYRNLTGTYDKLVSIEMIEAVGKQYLASYIKKCESLLKPSGL

MAIQAITIADQRYDYYSNNVDFIQKYIFPGGFLPSVTSLTQATTKHSDLVVRDLFDIGLD

YAKTLNEWHRRFNRAEDAVRGFGYDERFVRMWRYYLSYCEGGFLARTISAVHMTFQRP

>tr|Q87PJ4|Q87PJ4_VIBPA Uncharacterized protein OS=Vibrio parahaemolyticus serotype O3:K6 (strain RIMD 2210633) OX=223926 GN=VP1508 PE=4 SV=1

MSDKKDFENNYEKTEDAWPIGVELVEHEISTGIWVTTQWQLTGFALNPTEETPDVCLLQL

HKDERTDYRFNLSSQQPKLFLVMDNVESGEKPAIQLLTASQSVAAQYMDGDNLVLSNDMP

LAVQAWMEAFIGRHGELLEVRRKKRKGAGRANGN

>tr|Q87HN3|Q87HN3_VIBPA Molybdenum ABC transporter, permease protein OS=Vibrio parahaemolyticus serotype O3:K6 (strain RIMD 2210633) OX=223926 GN=VPA0930 PE=3 SV=1

MTELEYQALMLSLKVGAYAVLWLIPLGVFLAWLLSRKEFFGKSILDSLIHLPLVLPPVVI

GYLLLVSLGRQGFLGRLLYEHLGLVFSFNWKGAVVACIVVALPLMVRSVRLSLESVDPKL

EHAASTLGASPLKVFLTITLPLTIPGIITGTMLSFARSLGEFGATISFVSNIPGETQTIP

LAMYNFIETPGAEMEAARLCIISIALALSTLMISEWLNRKAASRLGAKR

>tr|Q87QJ5|Q87QJ5_VIBPA Putative transcriptional regulator OS=Vibrio parahaemolyticus serotype O3:K6 (strain RIMD 2210633) OX=223926 GN=VP1154 PE=4 SV=1

MFTIEQLQAFVATFESGSFSAAARKLGRAQSVISQHIMNMELDCGVDLFDRSGRYPKLTE

NGHALMPYAQAAISQLDRLNNKATQLFSAQASELVLAIDEGIPLTRLPDVLKNLEQQFPQ

LQVECLTASSPDIIELVKSERATTGIILSDLQMPRHIDFTNLGNIAFDVYVSSDHPLAAQ

RITHIDQLKQYRQLVIRSKSAEPGSLNQALSPDIWYADNYYILLELANKGFGWCFLPEHL

VADSPNTLKKVGDDFTKLAWQVNVDLIQHQKWHSDPLHQQAKAELITLFEQTVK

>tr|Q87SI0|Q87SI0_VIBPA Ubiquinol-cytochrome c reductase, cytochrome c1 OS=Vibrio parahaemolyticus serotype O3:K6 (strain RIMD 2210633) OX=223926 GN=VP0443 PE=4 SV=1

MKKWIVILFAMLPSLAMAAGANVPLDKANVDLTDKASLQNGAKLFMNYCFACHSTQYQRY

ERVATDLDIPADLMKENLIFNPEAKIGDLMVNAMPPKQAANWFGAPPPDLTLVARVRGAD

WLYTYLRSFYVDPSRPFGVNNIVFPSVGMPHVLEELQGIPTPVYGTKMVDGEEVKVVVGT

ETNGTGELSEGEYDKAVGDLVNFLVYAGDPVQLERHALGWWVMGFLVLLTIVVVMLKKEY

WRDVH

>tr|Q87HW0|Q87HW0_VIBPA Uncharacterized protein OS=Vibrio parahaemolyticus serotype O3:K6 (strain RIMD 2210633) OX=223926 GN=VPA0846 PE=4 SV=1

MMSISQLLGCISKQVDGQYIAQYEELTLRSVFQPIYKKDLSIIGLEALVRISTADGSMIR

PDLFFQSPSISEHVQLNVERLSRLIHIKNFGQSRYRNKKLFLNVLPRAAEMLAKDLSYSH

LLKQVICEADLCREQIVMELVELSAGDESFLYKATKELSDGGFKLAIDDYGINASTIERV

KCVRPDIIKMDRSLLLKYEDGDFSALIEALALAKELSSKTVIEGIETEHQLNLMKKLGFD

MYQGYLLAMPQTLEMYEEAKTA

>tr|Q87P78|Q87P78_VIBPA Uncharacterized protein OS=Vibrio parahaemolyticus serotype O3:K6 (strain RIMD 2210633) OX=223926 GN=VP1640 PE=4 SV=1

MYKLIALDMDGTLLNSDKAISEENKHAIAKAREAGVTVVLASGRPLEGMQAKLDELNIHS

EKDFVLFYNGSMVKNVGTNEIIHQQIIDGKAAKLVARKAKELGAYVHAFSQVHGLITNEN

NPYTDIEANINGLDVTEMNFESLEDDHPIIKAMMVAEPSKLTEVIAALPSEMREEFTVVQ

SAPFFLEFLNPASNKGIGVAAIAEYLGIQPEEVICMGDAENDHHMLKYAGLGIAMANAME

ETKKIADYITESNDDHGVAKAIEKFVLNA

>tr|Q87I44|Q87I44_VIBPA Putative 2-oxoglutarate/malate translocator OS=Vibrio parahaemolyticus serotype O3:K6 (strain RIMD 2210633) OX=223926 GN=VPA0762 PE=4 SV=1

MNSSQMKMILLVAIGCILWFIPTPEGLTDQAWQMMAIFVTTVLSLILAPLPLGAMALMGL

TLATLLGVLPIKTALTGFAHPTIWMIAAAFFISRGFITTGFGRRVGYWFISKLGHNSLGL

AYGLVLTDLLFAPATPSTTARCGGIISPLFRSVASAYDSDPEKGTENRIGAFLVQCIFQC

NAITCAMFLTSMAGNPLAANFAKEQGVEITWAGWATAAIVPGLLCLFLIPLVMYMVFPPE

LKKTPEMREIARQKLAEMGKMTRDELMVCITFIGMVSLWVLGPTLGIHATVTALLGLVFL

LLTHTITWDAVLGEKEAWHTITWFAVLVMMAAQLNKLGFISWFSESMAESLSGFGWVTTI

VVLLLVYYYSHYLMASAMAHISAMYSAFLAIAISAGAPPMLAAIVLGIFSNLYMSTTHYS

SGPAPILFGAGFHTLQNWWKIGFIFSLIVIPIFVFVGGAWWKLLGLW

>tr|Q87KH3|Q87KH3_VIBPA Probable queuosine precursor transporter OS=Vibrio parahaemolyticus serotype O3:K6 (strain RIMD 2210633) OX=223926 GN=VP3004 PE=3 SV=1

MSNFTPAQQRNALIYLVLFHLVIIASSNYLVQLPFTIFGLHTTWGAFTFPFIFLATDLTV

RIFGAQLARKIIFLVMLPALAVSYFLSVVFFEGQFQGFGHLGEFNLFVARIAIASFMAYL

LGQIMDVHVFNRLRQMKQWWVAPTCSTLFGNLDTIAFFAIAFYQSPDPFMAEHWTEIAL

VDYGFKLVISLGLFVPMYGVLLNYLIKKLTAVNPDFKVSTAA

>tr|Q87HP4|Q87HP4_VIBPA Transcriptional regulator OS=Vibrio parahaemolyticus serotype O3:K6 (strain RIMD 2210633) OX=223926 GN=VPA0919 PE=4 SV=1

MKQTLLLVEDDKNLADGLLVSLEQAGYECLHVERIADVEPQWKKADLVILDRQLPDGDSV

QHLPEWKKIKDVPVILLTALVTVKDKVAGLDSGANDYLTKPFAEAELFARIRAQLRAPDS

ADQANADKVMTKDLEIDRATREVIFKGDLITLTRTEFDLLLFLASNLGRVFTRDELLDHV

WGYNHFPTTRTVDTHVLQLRQKLPGLEIETLRGVGYKMKA

>tr|Q87IE5|Q87IE5_VIBPA Transcriptional regulator, MerR family OS=Vibrio parahaemolyticus serotype O3:K6 (strain RIMD 2210633) OX=223926 GN=VPA0662 PE=4 SV=1

MNIGAVAKLTGLSSKSIRLYEDKGIISPPARSDSGYREYSDNHIQELNLVSRAKNAGFSL

QECKEFVQLAHNPNRKSSEVKERTMDKLREVEEKIAHLMEIKKQLEGWVSACPGDAKSRC

PIIDELTK

>tr|Q87IH6|Q87IH6_VIBPA Uncharacterized protein OS=Vibrio parahaemolyticus serotype O3:K6 (strain RIMD 2210633) OX=223926 GN=VPA0630 PE=4 SV=1

MGQHVETSSSDYVKGFVASLILTVIPFYFVWAQTLPASATYVVMFTCALVQIFVHFKYFL

HMEAKTSDGRWNLVSLMFTAIVVLILIAGSIWIIYNMNVNMKL

>tr|Q87R47|Q87R47_VIBPA Methylated-DNA-protein-cysteine methyltransferase-related protein OS=Vibrio parahaemolyticus serotype O3:K6 (strain RIMD 2210633) OX=223926 GN=VP0951 PE=4 SV=1

MDQFLVQIFAVIHQIPKGKVSTYGEIAKMAGYPGYARHVGKALGNLPEGSKLPWFRVINS

QGKISLKGRDLDRQKQKLEAEGVEVSEIGKIALRKYKWQP

>tr|Q87FE6|Q87FE6_VIBPA Secretion protein, HlyD family OS=Vibrio parahaemolyticus serotype O3:K6 (strain RIMD 2210633) OX=223926 GN=VPA1733 PE=4 SV=1

MSRDNYNKLSNDELDFVDDKTAALLLNTPNSARLMLWVMVLFFVAAIGWASWAQIDQVTV

GQGKVIPSSQIQVVQNLEGGLVKEILVKEGQLVKKGQQLLLIDDTRFRSDYREREQQVAN

LTASVLQLSASINSVAVNRDFNIQDWEKSVVLDYGKLTFPPVLEETQPQLTQRQKAEYRE

DLDNLRNQLSVIDQQVEQKQQDLVEIEARVRNLRQSYQYAKKELDITQPLADEGVVPRIE

LLKLQRQVNDTRREMTSSELKIPVIKSAIKESMLNRIDVALKFRSEQQEKLNNAQDQLSA

LVESAVGLEDRVNRTVVVSPVTGKIKTLNVNTVGGVIQPGMDIVEIVPTEDTLLVEAKIA

PKDIAFLRPNLNAIVKFTAYDFTKYGGLVGELEHISADTTQDEEGNSFYIVRVRTEKTSF

GQDADLPIIPGMTASVDIITGKRTVLEYLLKPILSAKTNALKE

>tr|Q87JD8|Q87JD8_VIBPA Putative transcriptional regulator, LysR family OS=Vibrio parahaemolyticus serotype O3:K6 (strain RIMD 2210633) OX=223926 GN=VPA0315 PE=4 SV=1

MKLPPLRAVHCFEVVARNLSFSLAAEELNVTQSAVSHQIRLLEDYLGESLFIRQGRKLSL

SDSGARYLEDISPAISSIAMASQKVREGDKGSIRLAIYSSLAVKWLIPRLADFKRLYPEI

DLTLNMVAGDPEQTDSVGDCFITVQRPKRNYMAVKLYAETLYPVCSHKIWKEIQDQSIPD

ALWQYPILSTDSIYRERGKDWAEWCKAGGYTLPNDVDVQHFSHMLLAIEAARYDQGIAFA

NDFMLNERDKAQDLVYIPSHGLETGDSFYFVHKKNRAKQAEIVKLTNWLKQQCL

>tr|Q87FM8|Q87FM8_VIBPA Putative methyl-accepting chemotaxis protein OS=Vibrio parahaemolyticus serotype O3:K6 (strain RIMD 2210633) OX=223926 GN=VPA1651 PE=4 SV=1

MFKNLSLKNKLAISASAAIILGGVLVEGLSFRDSLQRLDAEVAQRLESTSASYNQYVSDW

LLSKERALTSLSAESEKRAIVTHLKQVRDSGAFDNVFLAYPDGSQDNANGVILPPGNNDP

RKWGWYTNAIANPSKVFMDNPTVAAATGANVVSLGKALQLHGQTTVLGADVEIGDILNSL

NQVILPGEGYMFIANDQGNIFTHNDSKLLNQPVSKLGLNNNDITNAARSGTERRVSISGT

DYVIYARPIEGTKLTTVTVLDHNSLVAPLYDAVWDQIIATAIVVIICVALFNLLCNILFR

PLYNVSNALSQIANGSGDLTQRIKVENRDEVGELAENFNQFVESLQQLIGHIRHQAEELS

QQSELSTTRANQSVSDLNHQQQEITMVATAVTEMASATQEIAAHAEQTAKAAQDSSASTQ

NGHELVINSKSSINNLSSEVNQASVVIGELNQHAQDISTVLSTIRDIAEQTNLLALNAAI

EAARAGEQGRGFAVVADEVRVLSQRTHTSTEEIRSTIETLQQTTQRAVTIMDKSSQLAQG

SVEDADRAALALDEINAAVALISDMATQIATAAEEQTHVTNEITQNVTSIKDVTDQLVVG

AEESMNQSAELKSQAEDLNSKVATFKLA

>tr|Q87PL7|Q87PL7_VIBPA Lipoprotein NlpC OS=Vibrio parahaemolyticus serotype O3:K6 (strain RIMD 2210633) OX=223926 GN=VP1485 PE=4 SV=1

MRRIYFPLIISALLSACSSGPEPSEQVEVTVPTNQLLSNNPDLFRFYNEWHGTPYRLGGT

QKSGIDCSAFVQRAFVEAYQLALPRTTKQQSTQGVELSWTDAKQGDLVFFKTRRSTYHVG

IYLGNKQFMHASTSKGVIISRIDNPYWASKFWQVRRVTL

>tr|Q87M99|Q87M99_VIBPA RNA polymerase sigma-70 factor, ECF subfamily OS=Vibrio parahaemolyticus serotype O3:K6 (strain RIMD 2210633) OX=223926 GN=VP2358 PE=3 SV=1

MMSDSPQKLGRNEWNAYMDKVKAKDREAFAFVFRFYAPKLKQFAYKHVGNEQVAMEMVQE

TMATVWQKAHLYDGKKSALSTWIYTIIRNLCFDLLRKQKGKELHIHSDDIWPSEYYPPDM

VDHYSPEQDMLKEQVVKFLDILPKNQRDVLQAVYLEELPHQQVAELFDIPLGTVKSRLRL

AVEKLRHSMHTEQL

>tr|Q87FM6|Q87FM6_VIBPA Putative ferrichrome ABC transporter (Permease) OS=Vibrio parahaemolyticus serotype O3:K6 (strain RIMD 2210633) OX=223926 GN=VPA1653 PE=3 SV=1

MMNQTKLMALLGLLLIASSLTLFVGAANLSAQQVFALLFSFSDSDFVIHQYRLPRMLLAI

GVGAGLGLSGVLVQGVIRNPLASPDLMGISAGAGLAATACLVLYPNAPVAMLPMVAMAGG

LLAAGFIAVLAYWSKPTPARLALIGVAVSAFLASGIDFLLIVHPIEINTAMVWLTGSLWG

RNWQQVPFIWSALMLLLPLAFWLAWRLDVMGLGEESATTLGMKPKQIQILALIAAVLLAS

ISVSVAGTISFVGLLAPHLARLLFGHNHKLLIPASATLGALLVICADGLARGLQPPIELP

AGVLTSVIGAPYFIFLLYRYRGW

>tr|Q87G68|Q87G68_VIBPA Methyl-accepting chemotaxis protein OS=Vibrio parahaemolyticus serotype O3:K6 (strain RIMD 2210633) OX=223926 GN=VPA1449 PE=4 SV=1

MLKLHSLSIKQKVVLGITFAVLASTIIVGVMAQRHARDVLSHRLIDIELPAMLQQINTEI

DREVVQMQQAAKQLATNEFVVEALKNTDHPQFSETQLVQQLNNIKSQYGLNDASVANRKT

AYYWNQNGFLRQLNHSQDAWFFGFTSSGRETSVSVFQEANGEVKMFTNYQDLNGISMSGL

SKSMDDMVSLLNSFQIEDTGYVFLTNEKGDIQIHRQQGKNKTSIAQLFGSNANQLLNKNS

FNLINVEFEGKDNFIASLYVPSMNWFVIGVVPVDEVFADLNATGQKMMITTIVVALVFIL

MGVLLANSITKPIRLIADRFTDLGQGEGDLSQRIEIRGNDEIAQLSKGFNGFIEKIHATM

KEVSLTSGALSQAADSVSSKATSTYDNSQEQRDQTIQVVAAINQMGATISEIASNAATAA

DTANQASDNTQTGREVVMKAKEVISRLADDVETTNIVVTQLASTTKDIGSILGVIRDISE

QTNLLALNAAIEAARAGEQGRGFAVVADEVRNLASRTADSTEEIQRMINQLQSDAQDAVS

AMEAGKAVTFEGVASTDEAVEVLVNISERITDISDRNTQVATATEEQSTVVHTINQNIEE

INAINEMTTATAEELAGASRDLQELSSRLDKMVGSFKL

>tr|Q87MJ1|Q87MJ1_VIBPA Uncharacterized protein OS=Vibrio parahaemolyticus serotype O3:K6 (strain RIMD 2210633) OX=223926 GN=VP2264 PE=4 SV=1

MKKRTPATPVPSLETQTEAMKIAKATQKPGQTKEQTKLIAQGIEKGIAQYKKQQKERNRQ

ADKAKKKQQREKLRQHEEGVIESDNITDNTPTHTPSKLPWALLALSWIGFAAYIMLSK

>tr|Q87RR9|Q87RR9_VIBPA D,D-heptose 1,7-bisphosphate phosphatase OS=Vibrio parahaemolyticus serotype O3:K6 (strain RIMD 2210633) OX=223926 GN=VP0708 PE=3 SV=1

MAKPAVFIDRDGVINVDHGYVHDEHDFEYIDGVFEATKALKDKGYLLVLVTNQSGIARGK

FSEDRFLSLTQWMDWNFVDNGVEFDGFYYCPHHPEHGIGDYKQDCDCRKPKPGMFISARD

FLKIDMEKSVMIGDKAEDMMAAEAAGVGTKILVRTGKPITEQGEALATVVLDSIADVPAY

LQK

>tr|Q87QC5|Q87QC5_VIBPA Adhesin OS=Vibrio parahaemolyticus serotype O3:K6 (strain RIMD 2210633) OX=223926 GN=VP1224 PE=4 SV=1

MTFLSQLSTHADKAFLRRLFAIALPITLQSIMFSSRSLVDVLMLGQLGEAEIAAVGVAAR

ATFVTTIMLVGVTTGGALLTAQYWGAGDKIGVRQSTSLTWMIAMVFAALAVCLFVFFPQP

IMGLTTDSQEVIELGSSYLVISSASMFAVACVASMAVGLRAMHQPGLSTFFSGIGILSNV

FLNWVLIFGHLGFPALGITGAAIATVISGAIEVGCLFGYLWLKKHIIAFGWGDIRASLVL

DKITRFLSLSLPTTFNFLAWAGGLFAYHAIMGQAGVQGLAALSVMTPVESIALAMMIGLS

NAAAVLVGNQLGAKNFEPVYYQAWATVILNVLIAFGVAVLLFFTNQLILDAFSALSAETR

HLAEQFMVILALGVILRSVPMMVIVGVLRAGGDVKFCLYQDVFAQWIIGIPLAAFAAIYL

GWEPQWVYLLFLTEEVVKWCICLPRMKSKKWMKNLIEK

>tr|Q87FL8|Q87FL8_VIBPA Putative AcsD OS=Vibrio parahaemolyticus serotype O3:K6 (strain RIMD 2210633) OX=223926 GN=VPA1661 PE=4 SV=1

MLQTQSSQTSELRAHDVQLNREKAQLNTIMGVLNCYLREFALPNQQVEWHYQSTSLPQTL

KRNYSAKQRVAVHLSQQNGVLVLPIHYASKLGKIKLAELPWAKMPGSGWAKLDATQTLTL

LLNYLKQVLAIPFNHELIEQLENSVLVTEQFLNVAPKSHHNEFIASEQSLIWGHTFHPTP

KSRSGVTMDDLLAFSPEVGAQVPLYWFEVDTSLLDVLSSDGEANPAQRMLQQLAPQHTTS

NGKTLYPCHPWESYTILSNPSVRRAIEQGKITPLGLGGEKMQPTSSVRTLYHPDMDWFAK

FSINVRLTNCVRKNAWYELDSAVQLTSILKPIRENEQLHNPVFKVMTEPYATTLNLESVA

ERNWDDNTKARESFGILYRENFSLSEVDILKPTLAGALFAYDKNGNSCIAQRLKNKARTT

QNRYSDIATLWFERYLHCLIPGVFNYYFKHGVAFEPHLQNTLIGFEQEMPCCVWIRDLEG

TKLLPEFWPAETLTDLSERARQSVYYSREQGWNRIGYCTFINNISEAIFFIAEGDEPLEQ

TLWNAVQSAIVRWQSVNGKQPELEALLNGGHFPSKNNFTTRLMQKADKESGYTQVAAPWQ

QHEKGAQHA

>tr|Q87NJ3|Q87NJ3_VIBPA Para-aminobenzoate synthase, component I OS=Vibrio parahaemolyticus serotype O3:K6 (strain RIMD 2210633) OX=223926 GN=VP1875 PE=4 SV=1

MDNQFIDFKALEYAPEFALHLFSRIQHQPWTMLLRSASKTHIDSRFDVLVANPIATLETI

ADSTQVETPSNAYSIQDDPFTLLHQLQEQWLPHVELNKELDLPFVGGALGYFSYDLGRRV

ETMPEQAEKDLNTPDMAVGLYEWAVVVDHKLKKACLVGQNIEQAWQWLDKQEAEQSVDFA

LSGDWQSNMTKESYATRFDKVQEYLLSGDCYQINLAQRFNAPYLGSEWQAYLKLESANQA

PFSAFIRMPESSILSISPERFLELKDRVIETKPIKGTRPRSEDPKQDNANAHDLQTAEKD

QAENLMIVDLLRNDIGRVASPGSVHVPKLFDIESFPAVHHLVSTIRANLDEQYSPADLLR

ACFPGGSITGAPKVRAMQIIEELEPHRRSAYCGSIGYISRHGRMDTSITIRTLVAENHKL

YAWAGGGVVADSDCASEYQETLDKLSKILPALQS

>tr|Q87H89|Q87H89_VIBPA Putative two-component response regulator OS=Vibrio parahaemolyticus serotype O3:K6 (strain RIMD 2210633) OX=223926 GN=VPA1076 PE=4 SV=1

MKILIVDDNHNVSETIADYLELEGMTIDCAYHGEAALALLEDNHYDVIIMDIMMPKLDGI

STVQKLRQEQFCGTPILFLTAKDTLDDKIAAFKAGGDDYLMKPFAMQELSLRIHALASRG

PRQDIGTLTFADICLDARTGKVTRDGKEIKLSRIQTKILKLLLKYAPASVSRTEVIESVW

GDEPPSSDALRSHIYGLRTALDKGFEESRLETIHGQGYRLKA

>tr|Q87HK6|Q87HK6_VIBPA Putative transporter binding protein OS=Vibrio parahaemolyticus serotype O3:K6 (strain RIMD 2210633) OX=223926 GN=VPA0957 PE=4 SV=1

MTETCLRRLSQLLKHYKHSQTYQVNLDDLELVFSTSRRNISNILRILDSYNWIHWEPGRG

RGKASTLKVTVTIHQALYFTIRNEINNGSFDVISRLLEHYRSTAVSALSQALAEVSEENK

DSNTLIVSQYPWVDELTPSLTYRFSELQVIRSLYDTLFTVDHYGQLKNHLACEYKNEGSC

IYIWLRPDIYCHDGLALQAEDVVHSLNKLITTDGPVQKLLQQVTHISFDNAKQAIRIDLK

QPNNLFIYCLATANASITTRRQKSFKGRSITIGTGPFVLRHWDTNKIVLKKHHNYFAKKA

LLEQITLSHQGTELDQYISYNQETDDTECYMIQAFSFLAHNRRAECSLSEQTWQRLFSFI

ESRRFEFAKANGLETMHILDDNSHNENVPQLEGTLVITHPKWTIDYLSKANQWIVDLIRQ

TGLHVEFVELTDASNPQLVKEQADLLFVEDVIEPPLTYGIYEWLLTGTGIRFALHSDEFE

QHVNHVHDAVSDLTPEKRLEGILSELRADTTILPLFWGQEKITRAKGVSGVQLRKSGYSD

FYKLRVRAGQD

>tr|Q87QU0|Q87QU0_VIBPA TolA protein OS=Vibrio parahaemolyticus serotype O3:K6 (strain RIMD 2210633) OX=223926 GN=VP1059 PE=4 SV=1

MKDKKKQSKEYTKPIVISVGLHALLVAALLWGTDFAMTKPEPTGQMVQAVVIDPKLVQQQ

AKEIRQQREKAAKKEQDRLDKLRREAEQLEKNRKAEEEQIRKLKEQQAKDAKAAREAEAA

RKQKEQERKAEEERVRQEKERTAKLEKERKAKEEAVRKAEQERLAKEAAIAKAEQERVAR

EKAAKEAEEKAKREREAAQKAEQERIAKEKAAKEAAEKARKEKERLKRLERERKEQEAAL

DDIFAGLESEASANQQAQGKFVADEVSRYSSIYIQLIQSRLLKDDYLLGKECRVNIKLIP

TGTDMIVSSVNVLSGDSRVCAAAKSAIAQVPSFPMSTDSTVNQRLKDINLTVALQQ

>tr|Q87NB4|Q87NB4_VIBPA Putative metal-dependent phosphoesterase YciV OS=Vibrio parahaemolyticus serotype O3:K6 (strain RIMD 2210633) OX=223926 GN=VP1954 PE=4 SV=1

MRIDLHSHTTASDGRLEPKDLVDRALGFDIEVLAITDHDTVDGLARAKQYVEENDLPIKI

INGIEISTVWQNKDIHIVGLNIDPENPALAALIEQQKQHRVARSELIASRLQKATREGVL

EEVQQLAGDAPITRAHFAKWLVDNGYAKTMQMVFKKYLTRNNPGYVPPNWCSMKEAVDAI

HAAGGKAVLAHPGRYQLTAKWIKRLLAAFVEANGDAMEVAQPQQAQQERRNLADYAIQYK

LLASQGSDFHYPSPWMELGRNLWLPAGVEPVWKDWGIDPSLDVSK

>tr|Q87L76|Q87L76_VIBPA Uncharacterized protein OS=Vibrio parahaemolyticus serotype O3:K6 (strain RIMD 2210633) OX=223926 GN=VP2736 PE=4 SV=1

MSRYWLLGLSLLCTTASYAEESVQPEQNIDVPNPLQTEVEFGYQAHTGNTDSRSLNARLS

AEYTSGRHRSNGEWKYYNLYKDGEEDKRSSTYSVQSDYKLGPKTYLYGSFKGVDSRYSAY

FKDYTLSGGLGYQFSYTENFILEAEIGPGFRYQEPNLDEIDDDDIVFPNIVREGIFRGNL

NTTWHALDNLSFAADITLVTGKSNTRVDSELSVTNDITEDIALKLAHSRQYHDKVPEGLS

KADSVFSVNLLFAF

>tr|Q87PH1|Q87PH1_VIBPA Putative Bax protein OS=Vibrio parahaemolyticus serotype O3:K6 (strain RIMD 2210633) OX=223926 GN=VP1531 PE=4 SV=1

MPKSLKLMTLRVAAACIAATFISVGPYLHYDHQFKSANDPNVVQGPVTHPDLSHLPYIGD

IPDFNSIKDAKEKKAAFFDFLRPKIALENHRIEKERAFLSSLEIGEVSPDEEAYAERLAQ

LYGFPLHDGHVDEAWLTEMLKRVNVLPEALVLTQAANESAWGTSRFATQANNLFGQWCYK

QGCGIVPAQRAAGKTHEVQKFDSVQQSIHGYFMNVNRNPAYADLRDIRAMLADKHKNLFS

VATASELTHGLLSYSERGIAYVNDLRSMIRHNNAYWTQ

>tr|Q87FT6|Q87FT6_VIBPA Uncharacterized protein OS=Vibrio parahaemolyticus serotype O3:K6 (strain RIMD 2210633) OX=223926 GN=VPA1592 PE=4 SV=1

MLPQDNVITSFLCKKLGHNHLRIAGEYWHIEKLVALQCMMLKEAPNLQEGLLWWSKCVSL

FDRRLYVTLQHGDAQIHLQVECRTADKPSWAESVFSLLKMQLEMLELSESIQVQVHANNP

LAADIFLEETRDSKTSSSVFELVKHVYLLLSHQPIEQPELLSVLNALFIKNSNYALKLEQ

AARQLGVSKRTLQRRLQEKQMSYSQCVDFAKRKQALTLLADTQLTTQQIAYQLGYEEPSN

FHRTFRRWYPFSPMQYRQQCLDNRTQLNQQPIRLYYAKANTFDDNHIDQPVGKIWMEVDN

IAFEKVVSVECRDRDGAWRRYPAFFERFLSHGTELWATTELPVAHPLTFRLCYEVDGERY

IENNHQRDYVVSKGLLIGATEYIVPTRQLINIGAQYTLFVELACRLENVARIDGYVDDDP

TPHAMRLTQHSLHYACWVLQLSLTQTAKQCRFRLYDESGNELAKEHYPIQYPIVQPLN

>tr|Q87R59|Q87R59_VIBPA Uncharacterized protein OS=Vibrio parahaemolyticus serotype O3:K6 (strain RIMD 2210633) OX=223926 GN=VP0939 PE=4 SV=1

MKRLFSIVALLMFTVAVTPIAEAKKFGGGKSFGKSYKTAPAPKQQQQNTNTIGKEQTTKS

SSKKGLMGGLLGGLLAGGLLAAFFGGAFEGIQFMDILIIGLIAFIIFKLMRGMLGAKQGS

MNQQRQQPAFGGNASKFEQPNMQNFEQQPNTNAGGFTGFGAQTDVPHNYPPGFDQAAFIN

GSREHYRILQGAWNHNQLDTIEEYVSPSLFEDLKAERAKLEGEQHTDVMYVDAEIVRAEY

DANKAQLSLQFSGRYRDAAEGVEEDIEDIWHLERDLTVPNAPWLIVGIQG

>tr|Q87LU0|Q87LU0_VIBPA AmpD protein OS=Vibrio parahaemolyticus serotype O3:K6 (strain RIMD 2210633) OX=223926 GN=VP2521 PE=4 SV=1

MPPIIDNGWLTHAKHVPSPFFDARNSEHDISLLVVHNISLPPGQFGGSYIEDFFSGNLDP

NAHPFFEVIHKMGVSAHCLIKRDGEIVQFVSFLDRAWHAGQSSFAGRDRCNDYSIGIELE

GTEFVAYTEEQYQSLARLTQAIMHQYPQITLPRITGHQYIAPLRKSDPGLSFDWVKYRQL

VQR

>tr|Q87JR3|Q87JR3_VIBPA Branched-chain amino acid transport system carrier protein OS=Vibrio parahaemolyticus serotype O3:K6 (strain RIMD 2210633) OX=223926 GN=VPA0185 PE=3 SV=1

MLSARNIAALGFMTFAMYLGAGNLIFPPFLGYQAGENFLSGMSGFLLTGVGLPALALVMV

AIVNGSDKLTAALPKPLATSFWVMVFIVIGPAFVIPRAITVAYQFSFAPIFGEAALVPFT

IVFCVATIWFALYPGKLVDNLGKILTPALMAILIIMSVTALIYPAGELTQASGPYVSGAF

AEGLTQGYMTMDALGSIGFGWIIFRAIRSMGVDCPKATAKYTLIAALMYAVAMAFVYISL

SYIGSTSSYLGSEFSNGGDILTAFTFNHFGAFGSVLLGAVMVLACLTTAIGVTTAGSEFY

DNTFSKVNYKSCVVITMVLSGFIANIGLEQLLAITLPAVVALHPVAIALMMMAPVRNKMS

QFMLVLTAFTALAFGCVDALHILGYMPEAVDQWMSHNMPLYNEFASWIVPSVIMATIGLL

FTKKAEEIKEFELVNE

>tr|Q87J60|Q87J60_VIBPA Uncharacterized protein OS=Vibrio parahaemolyticus serotype O3:K6 (strain RIMD 2210633) OX=223926 GN=VPA0393 PE=4 SV=1

MRSMLQDSLVLLDYLRGMLLHNEELWLLLFPVMIIIELPLYLLVLTGIFRWSYMREEPEL

KRFPSVSFVITCYGEGEAIGITIDTLVEQIYPGHIEILAVVDGAVQNQDTYKAALNGERR

HTGVRNRKVRVLPKWQRGGRVSTLNAGLSMASGEIVINVDGDTSFDNDMVFTMMKQFADK

NVIASGGALRVRNHNANLLTKMQSLEYMLSMQAGKTGMATWGVLNNISGAFGAFRKNLLK

QVGGWDTHTAEDLDLTMRLKQYKCRYPDNKLAFSTHSIGHTDVPDTLKGLVLQRLRWDGD

LLFLFLRKHNEGLSPRLLGWGNFVFTLAYGVIQNVLLPLLVVIFSVYMVIVYPLKFVLAL

MLMLYFVYLFLSALIFVVYIGLVSERKKEDLKSVKWLFLYPVYQFFMRLITAFSMVNEVV

RRSHEESSMAPWWVLKRGKKF

>tr|Q87RE6|Q87RE6_VIBPA Zinc ABC transporter, permease protein OS=Vibrio parahaemolyticus serotype O3:K6 (strain RIMD 2210633) OX=223926 GN=VP0851 PE=3 SV=1

MIEFLLPSILAGIGIAIIAGPLGSFVVWRKMAYFGDTLAHASLMGLALGFLLNVNLYLAL

LVCCLALAVLLVTLQRQQLVATDTLLGILAHSSLSIGLVSVSFLDNVRVDLMSYLFGDLL

AVSPEDLMFIYAGVIAVSACLYIFWRPLLSSTVSEELAAVEGVNTDLIRLVLMLMVGVVI

AVGMKFVGALIMTSLLIIPAATARKLSSTPEQMAFFASIIGAISVLMGLSLSWHFDTPAG

PSVVISATSLFMLSQLIQRKA

>tr|Q87LX8|Q87LX8_VIBPA Peptide ABC transporter, permease protein OS=Vibrio parahaemolyticus serotype O3:K6 (strain RIMD 2210633) OX=223926 GN=VP2480 PE=3 SV=1

MGYFLRRLSFYLVALLVAATLNFIIPRAMPGDPVTMMFANASVQVTPERIAAMKELLGFV

DGPIYIQYLSYIKNILSWELGTSIQFYPLSVNSLLGSAFGWSLFLAGTAVVLSFSIASVL

GIFAAWKRGSRYDAFVTPGTLIIQAIPQMVIAMLALFTFSIGLKWFPSGYAYTPGTVPDW

SSWAFIKDVGYHAVLPLFCATIVQIGGFLVNMRNNMINLLAEDYITMAKGKGLSENRVVF

NYAARNALLPSVTALSMSLGMAIGGQLIIEMIFNYPGLGTVLLNAIHARDYQVLQGQLII

MTMFMLCFNLMADMLYMILDPRLRKGGK

>tr|Q87P97|Q87P97_VIBPA Amino acid ABC transporter, permease protein OS=Vibrio parahaemolyticus serotype O3:K6 (strain RIMD 2210633) OX=223926 GN=VP1621 PE=3 SV=1

MKPTKDTSPSTMSKPSGSKSLIYNPAFRSAIFQIIAIAALVFFFYTIINNALNNLDARGI

ATGFGFLNQEAGFGIGLTLIEYNETYSYGRTFIVGLLNTALVSVLGIILATAIGFTMGVA

RLSTNWLVSRLAAVYIETFRNIPLLLQIFFWYFAVLQALPSARQSLSLGEAIFLNVRGLY

FPAPVFNEGSGVVIAAFAIGLIATISISIWARNKQRLTGQQTPMGRIGLGLLVGLPLLVY

FVSGMPISLEYPELKGFNFKGGISIIPELAALLLALSVYTAAFIAEIVRSGINAVSHGQT

EAAMSLGLPRAKTLKLVVIPQALRIIIPPLTSQYLNLTKNSSLAMAIGYPDLVSVFAGTT

LNQTGQAIEIIAMTMGVYLTLSLLTSALMNLYNRKVALVER

>tr|Q87IT8|Q87IT8_VIBPA GGDEF family protein OS=Vibrio parahaemolyticus serotype O3:K6 (strain RIMD 2210633) OX=223926 GN=VPA0518 PE=4 SV=1

MDHVQISVQKLDEILKLNGSALLNRVTLDIHQQLKSHCTCVVEVAHLQHAAHTISFASGG

EISDNLSYHLSGTPCEKVAKDIGEHIFYQDQVYKRFPEDQMFQDDGVQAYLGLPLKTQSG

EVLGILLSTFTRSIHAKEAQDVLELHRFYANVIIHSLREKWVSERSDKLLNQLSYEVSHD

NLTGLLNRSCLADTLETLTQQATRPFNLAYLDIDNFKSINDINGNYIGDQIIKFTANAIQ

QSLSSPNNAFRVAGDEFAFITYDEDPVAVCQEVLDKIEAGYSDKSNRISFTVSIGIARAP

VHMLNSDELILNASLALKDCKKHRDTRIQHYDTHLSALYHRQTQLIEAMRIQLSTSITES

HELYVVLQPIVDVNNDRWDYFEILARWNSSVYGNISPAEFIEAAEQSGLIIELGERIIEL

ACIAKQELEAHLGYTVKLSINCSAHELVDSNRYINHLTTLLERYGHDASDFVIELTETVL

LSQSGREQMVLNSLRYLGFKVALDDFGTGYSSLNYIHNYPIDSIKIDATFVRNMLSNKTS

EQVVYLIAQLAQLLDVDLIAEGVEDDRALNKLIDMGCHYIQGYFYSRPHNVDELVHMIND

RQVKRA

>tr|Q87MI1|Q87MI1_VIBPA Putative glycine cleavage system transcriptional repressor OS=Vibrio parahaemolyticus serotype O3:K6 (strain RIMD 2210633) OX=223926 GN=VP2274 PE=4 SV=1

MKQHLVLTAVGTDRPGICNQVVKLVTQAGCNIVDSRIAIFGNEFTLIMLLTGNASHITRV

ETQLPLLGQEHDLITIMKRTSAHELLDNSYTMEVFIESEDRPGLTEKFTQFFADQQIGLD

SLSAQTISKSKLQLDADQFHIAITASVSADCNLMQLQEDFDELCKSLNVQGSLNFIKNTL

>tr|Q87QU3|Q87QU3_VIBPA Uncharacterized protein OS=Vibrio parahaemolyticus serotype O3:K6 (strain RIMD 2210633) OX=223926 GN=VP1056 PE=4 SV=1

MQGTSNPFRWPITVYYEDTDAGGVVYHSNYLKFFERARTEMLRAKGISQHVLLEQNIGFV

VRHMDIDFKQGARLDEQLTVLTRVSEIKRASLQFCQELVNDQGKILCKAFVKVACIDNKK

MKPIAIPTFINSELTNSDC

>tr|Q87FE1|Q87FE1_VIBPA Putative tryptophan repressor binding protein OS=Vibrio parahaemolyticus serotype O3:K6 (strain RIMD 2210633) OX=223926 GN=VPA1738 PE=4 SV=1

MSKIAIIYFSKTDVTGQLARAIAAGVEQQGIKQQGECEILSHRIEGSEIIEGRFVNPHLM

DELAECDAIIFGSPTYMGGVAAQFKAFADASSESWYHQKWANKVAAGFTSGGALNGDQSC

TLQYLQTFAYQHGMMWVGLDKISNSGEQNLNRYGVQGGIVAQGGEDGQLHSSDVATAEYL

GKRVAALVSKLSATSAT

>tr|Q87LU4|Q87LU4_VIBPA Carbonic anhydrase OS=Vibrio parahaemolyticus serotype O3:K6 (strain RIMD 2210633) OX=223926 GN=VP2514 PE=3 SV=1

MPEIKQLFENNSKWSEEIKSDRPEYFAKLAEGQKPDFLWIGCSDSRVPAERLTGLYSGEL

FVHRNVANQVIHTDLNCLSVVQYAVDVLKVKHIIVCGHYGCGGVNAAIDNPQLGLINNWL

LHIRDLYFKHRSYLDQMPVEDRADKLGEINVAEQVYNLGNSTIMQNAWERGQDVEIHGVV

YGIEDGRLEYLGIRSNSKETVEASYQKALSTILNPDNKLLCR

>tr|Q87JY2|Q87JY2_VIBPA Permease OS=Vibrio parahaemolyticus serotype O3:K6 (strain RIMD 2210633) OX=223926 GN=VPA0116 PE=4 SV=1

MNLILILLGVIAFIVLTTTKFKLHPFLALIIAAFLAAFAYGLPADSIAKTIASGFGGILG

YIGLVIVLGTIIGVILEKSGAAITMADTVIKVLGERFPTLTMSIIGYIVSVPVFCDSGFV

ILNSLKESLAKRLKTSSVAMSVALATGLYATHTFVPPTPGPIAAAGNLGLESNLGLVIGV

GVFVAAVAALAGMLWANRFQHVEPDGIEAADEIQHDWQALKASYGKLPTASQAFAPIFVP

ILLICFGSIAKFPSLPLGEGFVFDVLTFLGQPLTALVIGLFLSVRLLKSDNKIEEFGERI

SQGITAAAPILLITGAGGAFGAVLKATPLGEYLGTTLSALGVGIFMPFIVAAALKSAQGS

STVALVTTSALVAPMLTQLGLDSEMGRVLTVMAIGAGAMTVSHANDSFFWVVSQFSRMSV

GLAYRAQTMATLVQGVTAMALVYILSLVLL

>tr|Q87HC5|Q87HC5_VIBPA Uncharacterized protein OS=Vibrio parahaemolyticus serotype O3:K6 (strain RIMD 2210633) OX=223926 GN=VPA1040 PE=4 SV=1

MVSMEQTIVKPTPGGRAAVSKAQPQRSADSTVVISKNPELVNNDSVVAYGDNPLLAEANG

LLSIIGQIRATATHSDPLFLKETLAQKLRDYENRLRQHDVDLETIDTARYCLCCSLDEAV

LNTNWGSQSFWTHDSLLSSFYASSQGGEAFFKHLDSCLAHPESHLDLLELMYVCLSLGFI

GQYRLEKNGLEAHRRLRKQVVSVLKSHGRGVQQELSNKVEQHILAGAQVSERAPLWVVCS

VTSALLVCIFMYFSYELNKASNQTFAQLVNLIQPTPAVSNPMVESKSAPIAERISMYLAT

EIGKDLVTVEALQDRVRISLKAQDLFESGSASVVAYIQPVISKVARTLEATQGKIIITGH

TDDRPIFTSKYPSNWHLSLARATSLSEQLISNSALKGRVIPEGLGDARPLVENDSEKNRA

MNRRIEIDLIVGN

>tr|Q87GN4|Q87GN4_VIBPA Oxidoreductase, Gfo/Idh/MocA family OS=Vibrio parahaemolyticus serotype O3:K6 (strain RIMD 2210633) OX=223926 GN=VPA1281 PE=4 SV=1

MKIGIIGLGDIAQKVYLPVITQLPNVELVFCTRDAETLSSLAQQYRIMENCQDYRQLTAF

GVDAVMIHAATHVHFQIAEYFLKQSIPTFVDKPLADSAQQVEQLYEIAAIANQPLYVGFN

RRHIPLYNDYLPNVQKGDVANLKSLRWEKNRHNLPGELRTFIFDDFIHPLDSVNVVAKAD

LQDVYITHQFDGQQLARLDVQWQHGDTLLHASMNRHFGITNERVQACYANQAFEFDSFVE

GKLWQDNQERKLNLKDWTPMLTSKGFHAMLFDWFKVVESGKLATSTVQRNIASHQLAEQI

YQRIEQAVHCN

>tr|Q87M10|Q87M10_VIBPA Putative protease OS=Vibrio parahaemolyticus serotype O3:K6 (strain RIMD 2210633) OX=223926 GN=VP2448 PE=4 SV=1

MENSMKYALGPLLYFWPKQDIEAFYLQAKESSADIIYLGETVCSKRREMKPAHWFDIAKD

LSASGKQVVLSTMALLEAPSEVNIMKKYIDNGDFAIEANDVSAVQLASEHKVPFVVGPAI

NTYNAHTLNLFLKQGMTRWCMPVELSREWLSDTLTQCEDLGIRNKFEVEVFSHGYLPLAY

SARCFTARAENKAKDDCETCCIKYPTGIQVSSQEGQEVFNLNGIQTQSGYCYNLINDLPS

MQGLVDVVRLSPLGVSTFSELDRFRSNEQGSNPDKLSSRQCNGYWHQLAGLEVKNI

>tr|Q87LK9|Q87LK9_VIBPA Iron-regulated outer membrane virulence protein homolog OS=Vibrio parahaemolyticus serotype O3:K6 (strain RIMD 2210633) OX=223926 GN=VP2602 PE=3 SV=1

MSTLRLPVLTATGVLAFVSVPHVFANDSVSKMETVVVTASSYEQSQADAPASISVISREE

LDSRYYRDVTDALKSVPGVVVTGGGDTTDISIRGMGSKYTLILVDGKRQSTRETRPNSDG

PGIEQGWLPPLQAIERIEVIRGPMSTLYGSDAIGGVINVITRKDALEWTGNVQLGTVIQE

NSRSGGEQSANFFVNGPLAENLLLQVYGQYTAREEDDIDYGYEDKDMQSISSKLIYQIND

RHSVQLEGGTSAQSRRGNVGLSVPTTGCRRGCEDSLNEYRRNYVTLSHTGEWELLGNSDT

YLQREESENKSREMTIVNTTFKSSLVKGLGAHTLTTGVDATHAELEDFTSNKASSKTKAS

NTQWAVFIEDEWKIAEPFSLTLGGRLDHDENYGAHFSPRVYGVWRVAPAWTVKGGVATGF

RSPQLREITPGWAQVSGGGNIYGNPDLDPETSLNKEISVLYQGDSGLDVTLTAFHNEFKD

KITRVVCPDTICTDGPNQWGADPTYRINVDEAVTQGVEATLAKPLTETIYLSSSYTFTDS

EQKTGEYKGMPLQQLPKHLFNVDVTWQTTDNLESWTKVTYRGKEMDPVTGPSRNSIVEPA

YTFVDAGVTYQLTDNTKIKGAIYNLFDEDINYKEYGYVEDGRRYWLGLDVAF

>tr|Q87GT6|Q87GT6_VIBPA Uncharacterized protein OS=Vibrio parahaemolyticus serotype O3:K6 (strain RIMD 2210633) OX=223926 GN=VPA1229 PE=4 SV=1

MERWMLRLSIKTRLALLALLPAVVIVAFAIHQFYANSVRVDHLNQTVSNIQGFQLISQAS

HFIYSMEKDRRQYGQETPLSIEVDVQNNVVLSMHHKFSTNPHTSEYADDLKEAMLGMVSG

DLTNTDEVGDWAFQLLQEMSLSLLQNYQLYGNSDGHAMQNFIAYLAQLSYWTQKEAWLTY

RLVMDPDAKLNQSVFFQTIDRQQQNLDAFLHLGASYAHVDKLLGLFSSPRYQRNLESRAR

LMNGDMPRSDYAAYLDELDFRVQRLQMMIEGFTRQAEQSLLTQVNEQKRNVLLITCGVAV

VLLLLGWLGFATWYRVNSKIGAIIHALNALVNEEEKEKKVAVDGSDEFTLFAQQVNRVVE

EKQRQTHEILHAKESAVAANRAKSVFLANMSHEIRTPLNGIMGMTEILSQSELSPHQQEV

VDDIDTSSHTLLALLNDILDLSKIESGRLELSLVEADIREVVYQSVILFQSKATSKQLEL

DISLDENIPARVMVDDHRIKQIITNLVSNAVKFTEQGYILVDVSYEEALEQGRGSLTFLI

KDSGIGIERDKLATIFEPFTQEDEGVSRQFGGTGLGLAICRQLVSMMGGKLVATSTKGVG

TCFGFSIEVEALPLFGWHSDVVKRGLFICDNYAYAEQIVQECRLAQIELVGVNSLSEAKA

LDEDFDVIFLCNDGQMDIDSCLSELAEVYDVRRVVVCQHHLTSSYTNAENVHAVLTQPFL

GNRFKHAIEELAKLEKNTLRDNVTNIASRAESKISRTHRRILIAEDNLMNQKIASFFLDK

AGYDYLITSNGQEALDAITKGEQFDAILMDCMMPVMDGLTATKEIRRWEKKVGCKKTTII

ALTASVLEEDIHNCFAAGMDAYLPKPYKSNQLFELFNELKLA

>tr|Q87H41|Q87H41_VIBPA Putative transcriptional regulator OS=Vibrio parahaemolyticus serotype O3:K6 (strain RIMD 2210633) OX=223926 GN=VPA1124 PE=4 SV=1

MNTFKISELAKEFDITTRSIRFYEDLGLLTPERKGNTRIYNGRDRIRLKLILRGKRLGFS

LADIKELFELYDTDQSTEQLNYMIRLIEEKKAALQQQANDIQAVMMELNAAQLRCQNTLR

SMKGEKVT

>tr|Q87LX7|Q87LX7_VIBPA Peptide ABC transporter, permease protein OS=Vibrio parahaemolyticus serotype O3:K6 (strain RIMD 2210633) OX=223926 GN=VP2481 PE=3 SV=1

MKNLFKLILGNSFARIGLAIITIFIFVAVAAPLITKHAPDKRTGNPHEYPSFVVKQAQSN

PDGWVAKNLADDRRTLIMSKKADHVLGTSRMGRDIWSQVAYGARVSLGVGFGAGIIVCFL

ATVIGISAGYFGGKVDDVLSAAMNIMLVIPQYPLLFVLAAFIGEAGPLTIALIIAGTSWA

WGARVVRSQTMALREKEFVKAAEVLGESPFRIIFVEILPNLIPIVGASFIGSVMLAINTE

AVISFLGLGDANTISWGIMLYNVQTSSAMLIGAWWEVLAPCIALTLLVTGLALLNFAVDE

IANPQLRSHKGMKRWKKLAAKDKKEREPELAPQNALWSGDK

>tr|Q87FF6|Q87FF6_VIBPA Putative ribosomal protein N-acetyltransferase OS=Vibrio parahaemolyticus serotype O3:K6 (strain RIMD 2210633) OX=223926 GN=VPA1723 PE=4 SV=1

MIFRSFFTHRIASMETKRLKLIPACLERAEEAHQAVVRSQKTLEVYLPWVPHVLTLEAMI

EGTEKAIANFENFEEELRYYIIEKESDRLLGAVGLMIRDPDVPSFEIGYWLDDAAVGNGY

VAEAVLEVERYAFDDLGAKRIAIHADSTNQKSRAVAERCGYEFEGELRNERLTTSGELSN

TVIYSKIRE

>tr|Q87TN5|Q87TN5_VIBPA Transcriptional activator IlvY OS=Vibrio parahaemolyticus serotype O3:K6 (strain RIMD 2210633) OX=223926 GN=VP0034 PE=4 SV=1

MNIKSLQLFIHLCESKSFAKTAAAMHISPSALSRQIQKLEEETNQQLFVRDNRSVELTTQ

GKKLMPVALKILSEWQQYQNHIKGTEGELKGEIRLFCSVTASYSHLPELISDFRLQHPFI

EFKLSTGDPAQAIDKILADEADIAISAKSEQMPNKIAFETISEIPLSVIAPVGVSSFAEE

LQKEQPDWSIIPFILPEAGTARDRANTWLKKMKIKPNIYAQTSGHEAIVSMVALGCGVGI

APDVVINNSPVREKINRLKVLPIKPFELGVCCTKNQLENPLVKAFWKVAESKYIAP

>tr|Q87LE4|Q87LE4_VIBPA Lipopolysaccharide export system protein LptA OS=Vibrio parahaemolyticus serotype O3:K6 (strain RIMD 2210633) OX=223926 GN=lptA PE=3 SV=1

MKPLHLSLLALVLAAPQAFALKSDTQQPVYINSDTQQVDMKSNQVIFKGDVSLKQGSINI

DADRIVVTRDPKTEAIKQIQAFGKPATFSQLMDDGKTLSGQATELDYRISTDELTMKGQA

QLAQDGNTIKGSSIRYQIGQQKLVADSSKNERVTTILQPNQIEN

>tr|Q87QT0|Q87QT0_VIBPA Sensor histidine kinase OS=Vibrio parahaemolyticus serotype O3:K6 (strain RIMD 2210633) OX=223926 GN=VP1069 PE=4 SV=1

MEIRSSLRKKSILALTLYLCFFIATIGSVVYLVVEPPVRDKLERNLDLRTQLLASQIKEP

LITSTGVLNSLVGLAQSSNQSDSLKSTIPQILRLSDEIIVSGGLWPKPELKEERWRFTSL

FFNKNSEGNIDQIHSYNNPESGGYDNEPWYRAAAEQSSGTVSWSAVYIDTFTQVQMITAS

APYYRNGEFAGVATVDLSLEALFQFIREHTNQYSLGVVIRDANSNVIIEHNFQITKQMYI

SKLDFGEFHWKLEVVNAKAKVADQVFEQVMSVEGGIIPFLLLCVLVGYYLLNRYIVEPIV

RIATKIDDSKTGGIIDIDYGSEDEIGHLITKFNEKTIYLEQERVKAQASTNAKTAFLATL

SHEIRTPMNGVLGTAQILLKTPLTDEQRKHLSTLYDSGDHMMTLLNEILDYSKIEQGHVE

FSNSPFPIESIIGSIKSVYHTLCAEKGLQFKVTSLVPAGRWYDNDKARLRQVLFNLLNNA

VKFTDRGIVEVTLSEQTHYDKTVLVIAIKDTGIGISKEAQKRIFRPFEQAESSTTRRFGG

TGLGLAIVKEIAEHMGGHVTVQSQENIGTTFTVEVEISPCEPGKVESGHRHKLNCNGLKA

LIVEDNRTNAIIMETFLRAKGFECSSVENGQLAVNKIAVEPFDLILMDNHMPVLDGVGAI

SAIRSMSSAAKSVLIFGCTADVFKETQERMLGVGADHIIAKPIVESELDDALYRHADLLY

QYQTKQNQQALEVLGTDSLLISFYVALDNGNLGDALDALLAIMDSLQPNTDEVLSEVITR

IKRDLLRQSPPDQEDIDTLTMLLATP

>tr|Q87K89|Q87K89_VIBPA Putative two-component sensor OS=Vibrio parahaemolyticus serotype O3:K6 (strain RIMD 2210633) OX=223926 GN=VPA0009 PE=4 SV=1

MDVKERTQRRFSIGNQLMLAVLTLSLIFTLVISAISLYRDFQEELSHLDTDLKQVESSYL

SSFSASLWVEDRELLLTQALGAMRLPSVDYLRIATKDEVIIELGTEITQDVVERRWPMQF

SVGEKTFELAELTVQSDLSAVYQDLWQQFFFLLTTEAIKILLLMVGVLWVAFRLLVNPLQ

LLSGAVSDFSGGNAPSTVTLPKRWCFDEVSLLAQKYNRSVQKVREHQAELEAERDKAEVA

NRKKSEFLATMSHEIRTPMNGIIGVASLLSDTKLDPQQKEFVEIIDNSSQSLMTIIDDIL

DFSKVEAGKVELASETYHFRQLLDDVISLHTVKAQQKNLQLLSDIDPKLPAEVQGDEGRL

KQVLNNLLSNAVKFTERGHVKLLVSLHEQNNDIAQVRFRVVDSGIGIAKEHQQAVFERFQ

QADGSTTRKYGGTGLGLAICAQLVHIMGGDIKLTSELGLGSCFDFTIPLTVVSGLPTYTD

PLNVLDFPRTEANEATNKNPDKPWVLIVEDTEVNQRVVRIMLEQLGLKVSVASHGEEAFQ

LCREHAFDLIFMDCQMPVMDGFIATEQIRDMNEWGAHVPIIALTANVVKEDQQRCFEAGM

NEFVAKPVTKARLQQIFEQYLPKALKNIATPK

>tr|Q87L39|Q87L39_VIBPA Uncharacterized protein OS=Vibrio parahaemolyticus serotype O3:K6 (strain RIMD 2210633) OX=223926 GN=VP2777 PE=4 SV=1

MTTTETINAEMLLEMESVNVMPFTEHDKIILRSYEAVVDGIASLIGPFCEIVLHSLEDLN

TSAIKIANGENTGRQVGSPITDLALKMLKDIEGSERNFSRSYFTRAKGGVLMKSITVAIR

NGENRVIGLLCINVNLDAPFSQVLQSFMPTQEAKEAASSVNFASDVEELVDQTVERTIEE

INADKSVSNNTKNRQIVMELYDKGIFDIKDAINRVADRLNISKHTVYLYIRQRKTEDE

>tr|Q87FF9|Q87FF9_VIBPA Putative acetyltransferase OS=Vibrio parahaemolyticus serotype O3:K6 (strain RIMD 2210633) OX=223926 GN=VPA1720 PE=4 SV=1

MTIQPTLTTERLVLRPFNIGDCQQVALLAGDKRIADMTANIPHPYELPMAQAWIDRHYPM

YQQHQGVAYAITLRNTGELLGAVSLPRIEEGYGTLGYWVGVPHWGKGYAFEASKVLLEFA

RMHFELNGITVMHLVDNQRSKSVIQKLKIPYVGDKTLRMQGKERKVCVYQLTFVASE

>tr|Q87HZ5|Q87HZ5_VIBPA PTS system, fructose-specific IIBC component OS=Vibrio parahaemolyticus serotype O3:K6 (strain RIMD 2210633) OX=223926 GN=VPA0811 PE=4 SV=1

MNIAIITACPSGVANSILAAGLLEQAVAKLGWNAKIECQSSVIAPTPLTDADIEQADAIV

IAANTTVDTSRFVGKKVYQAEISAVAKDATAFLTTAVESAATLEQATTVEAPVESASATK

KIVAITACPTGVAHTFMAAEALEEEGKRRGHQIKVETRGSVGAKNQLTDQEIADADLVII

AADIEVPLDRFNGKKMYRTKTGPALKKTAEEMDKAFEQATIYQHSGAASSASATDEKKGA

YKHLMTGVSHMLPVVVAGGLIIALSFVFGIEAFKEEGTLAAALMTIGGGSAFALMIPVLA

GYIAFSIADRPGLAPGLVGGMLASSTGAGFLGGIAAGFIAGYAAKLLADKVKLPQSMEAL

KPILIIPFVATLFTGLVMIYIVGGPVSGIMNGLTDFLNNMGSDSAVLLGIILGAMMCFDL

GGPVNKAAYAFGVGLLASQTYAPMAAIMAAGMVPALGMGLATFLAKNKFEPNEREAGKAS

FVLGLCFISEGAIPFAAKDPMRVIPSCMAGGALTGALSMLFGAKLMAPHGGLFVLLIPNA

ISPVLMYLVAIAAGTAVTGFTYAFLKNKADAKQEVAA

>tr|Q87LQ8|Q87LQ8_VIBPA RNA polymerase sigma factor RpoS OS=Vibrio parahaemolyticus serotype O3:K6 (strain RIMD 2210633) OX=223926 GN=rpoS PE=3 SV=1

MSISNTVSKVEEFEYDNASETTIDNELEKSSSTTEGKTAVREEFDASSKSLDATQLYLGE

IGFSPLLTAEEEVLYARRALRGDEAARKRMIESNLRLVVKISRRYSNRGLALLDLIEEGN

LGLIRAVEKFDPERGFRFSTYATWWIRQTIERALMNQTRTIRLPIHVVKELNIYLRTARE

LSQKLDHEPTAEEIAAQLDIPVEDVSKMLRLNERISSVDTPIGGDGEKALLDIIPDANNS

DPEVSTQDDDIKSSLIHWLEELNPKQKEVLARRFGLLGYEPSTLEEVGLRLKKWDERLVL

PVSVYVKFKWRVYVDFVKS

>tr|Q87HS8|Q87HS8_VIBPA Uncharacterized protein OS=Vibrio parahaemolyticus serotype O3:K6 (strain RIMD 2210633) OX=223926 GN=VPA0878 PE=4 SV=1

MSLRAKLIWPILVFITAIFVASKGYTSYTAYQSSKHELVEQTRKLINNVSYSIKDALTTK

NKRKAQTILADLIEQPNVSRVKLYDRANELFVLLEGSGESAPVPNQNERSKLDALGYALS

AKFLYVLEPIIHEGHVIGSIRVTLSYIPIINAQHSFLKDAGVLLLILAAGGIIFYITIDR

IILRPLLDLNGAIQDVTFGNASHVQIRHHSKDELGEVIHAFNRMMTKLRKREKQRQHSLA

TLEQKRAFSEEVIESIQYALVITDNLGTIIHSNAATQHIFQKTPEALENANIRDLIKTKM

PNELSQILSRCLECDDIHLQNIDNEQQLSLTTRRLSNHGYLLFAIQDITEIEEAMNRQRV

AGRVFEASQDGLIVLNHKGVITMVNPAVTKLVGLEIDQLVGQSFIQTIRWRKLQEMMPSI

IESIENYGVWQGEVIEQNHLGLLVPMFARVNRIVKCENNGFYDLVIILTDLSNAKEMERL

EYLAHHDALTGLANRSKFHLELEDLVQRSGYQRDEFAVLYLDLDGFKEINDTYGHDAGDE

VLKRVADRLTSATRHSDLIARLSGDEFVMLVNPANQKVVTRIAEQLLESICAPIEYKGNE

LKVGVSIGVKLVGVNERDATRILKSADTAMYQAKKAGKGQAILMGCELQETV

>tr|Q79YZ2|Q79YZ2_VIBPA Chemotaxis CheV OS=Vibrio parahaemolyticus serotype O3:K6 (strain RIMD 2210633) OX=223926 GN=VP0773 PE=4 SV=1

MTGILDSVNQRTQLVGQNRLELLTFRLMGRQRYGINVFKVKEVLQCPKLTSMPNLHPLVK

GIAHIRGHTVSVIDLSLAIGGRPTTDIDKCFVVIAEFNRTIQAFLVSSVERIINMHWEAI

LPPPDGAGKAHYLTAVTNIDNELVEILDVEKILAEIAPVDETMDSTIGEEIAQAEQEKPI

VRRILIADDSTVARKQVERAITSIGFEVVSVKDGKEAYNKLLEMAQEGSIYDQISLVISD

IEMPEMDGYTLTAEIRRNADLKDLYVILHSSLSGVFNQAMVERVGANTFIAKFNPDELGN

AVKSALTQ

>tr|Q87Q12|Q87Q12_VIBPA Probable permease of ABC transporter OS=Vibrio parahaemolyticus serotype O3:K6 (strain RIMD 2210633) OX=223926 GN=VP1338 PE=3 SV=1

MALPNYASKSERMAYAGYLVFCGLVLFFLIAPILTIIPLSFNATPYFTFTEGMLNLDADA

YSVRWYQEMFTNEQWLLALKNSTFIALMATLIATGLGTLAALGLANSNLPFRNAIMALLI

SPMIVPVIISAAAMYFFYTRLGLSQTYFGIILAHAALGTPFVVITVSATLSGFDQSLVKA

AASLGANPVYTFRHVTFPLIRPGMISGGLFAFGTSFDEVVVALFLTGAEQKTVPRQMWSG

IREQISPTILAVATLLIFMSVCLLVTLEILRRRNVRIRGIQE

>tr|Q87SQ0|Q87SQ0_VIBPA Putative hemolysin OS=Vibrio parahaemolyticus serotype O3:K6 (strain RIMD 2210633) OX=223926 GN=VP0372 PE=4 SV=1

MDSSTPFRLPRKTPFGIGENVAEWATGLSQLDKFYAQRPVNADTKTFLRFTLDILGIDYR

IAHGSLGSVPKQGATVIVANHPLGCVEGVILAELLLMVRDDIQILANQYLKTVPELDQLF

IGVDVFEGKDAVKSNMKALRAANKHLANGGLLLVFPAGEVSQLVDAKQQRLEDKEWSRSV

SALIRKNKAVTVPVFIRGQNSKRFYMAGKIHPLLRTLMLGRELLNKSAKTIELSFGQAIK

FKELNNLNDDQIVNYLRLNTYLLNRDVSATQQTVSDNDLLPIAAGLPIGQLLEELHSLPA

ETQLLQNGEFDVYCASAQQIPSLLHEIGRLREHNFRQVGEGTGQAIDIDHFDHDYLHLFV

WDRENQCMVGAYRLGLVDQLLAKYGVEGLYSRTLFNYDQRFLDQMGKSIEMGRSVIAEQY

QKSMSALLLLWKGIATFVHQHPEYTHLFGPVSISNDYSHTARQLLAQSMTLHHYDNDCAE

YVTPSNPLPETNLNWNTSMLTALGDLQLLSRVIARIDEGKGVPVLLRQYLSLNGKLVCFN

VDPAFNNALDGLIMVDLRDVPEKTLARYMGSENAREYLAMNN

>tr|Q87TF1|Q87TF1_VIBPA DNA-binding transcriptional regulator NtrC OS=Vibrio parahaemolyticus serotype O3:K6 (strain RIMD 2210633) OX=223926 GN=ntrC PE=4 SV=1

MSKGYVWVVDDDSSIRWVMEKTLSSANIKCETYADGESVLMALEREVPDVLVSDIRMPGI

DGLELLKQVQRDYPDLPVIIMTAHSDLDAAVNAYQKGAFEYLPKPFDIDETLTLVERAIA

HSHENKREQLSSEDAPADTPEIIGEAPAMQEVFRAIGRLSRSSISVLINGESGTGKELVA

HALHRHSPRANKPFIALNMAAIPKDLIESELFGHEKGAFTGANSVRQGRFEQANGGTLFL

DEIGDMPLDIQTRLLRVLADGQFYRVGGHSPIRVDVRIVAATHQNLEKLVHQGDFREDLF

HRLNVIRVQIPALRERKQDIEKLTQHFLVRAADELGVETKTLHPSTVEILNRLNWPGNVR

QLENICRWLTVMASGSEVLPNDLPSELLEEKKTVSDSTKGSWQEQLADWARQSLAAGDKE

LLSFALPEFERILLEAALEHTKGHKQDAAKVLGWGRNTLTRKLKELY

>tr|Q87PF5|Q87PF5_VIBPA Sensor histidine kinase/response regulator OS=Vibrio parahaemolyticus serotype O3:K6 (strain RIMD 2210633) OX=223926 GN=VP1547 PE=4 SV=1

MKDKYLDTYQQEALQEALVELKQTKQREKLLADENKAILSAISAMSEAKNRNEIFSGLNS

VLKKYISFEDFIVITRDDNRYPFKTLISTNSVFDKVEWLHGNTMDRALNGECILLFEPAK

LLEFENLNSFVKTHVNSVILTGIRSEVTQSIILLIGAQKGHFSIENKETLRRFRPLIERA

VIDIETKEKLQRIVEVRTTQLARAREEAELANQSKSEFLAMMSHEIRTPLNSVLGMLDIL

RQSTLSDEQFDALNQMECSAELLLAIISDILDLSKIESGSFQLNEQWIHLNDTVTFVISQ

QKQVAITKNLSFNFDCQISSDKQYWIDSTRLSQILFNLIGNAIKFTDSGSVSVSVAEEND

EVVVSISDTGIGISRAKQAHLFTAFHQGDRSITRRFGGTGLGLAITKHLVEMMRGEISVK

SRENEGSDFTIRIPVLTRYNQSRPVKIEHNRPSKALNLLIVEDTQSNQLVIKLILNKLGH

NVHIASHGAEALTFLEENDTRIDMILMDVSMPVMDGITATRLIRKKGITIPIVALTAHAL

ESDKDKCLDAGMDSFVSKPVRRQDIYEAIQSLIETA

>tr|Q87SG2|Q87SG2_VIBPA Cell division protein FtsQ OS=Vibrio parahaemolyticus serotype O3:K6 (strain RIMD 2210633) OX=223926 GN=ftsQ PE=3 SV=1

MLNIALNEERLNTDNNRGRQDKILGALFFVVVVTLISSVLYSAISWMWDDQRLPLSKIVL

QGKLEYVKADDVQAAFSRIDHIGTFMSQDIDVLQQSVEALPWVAHAAIRKQWPDTVKVFL

TEHQPEAIWNGNELLDKNGLVFDGDVALLKDEKVKLYGPKDTGPEVLQTYRELSPKFQQL

GLAISSLVLNERRAWQIILDNGIRLELGKESLLERIERFFSLYNKLGSDTQRISYIDLRY

DTGAAVGWFPEEELEESTDD

>tr|Q87NE5|Q87NE5_VIBPA Formate-dependent nitrite reductase complex subunit OS=Vibrio parahaemolyticus serotype O3:K6 (strain RIMD 2210633) OX=223926 GN=VP1923 PE=3 SV=1

MALFKYIFITVFALALYSPASNGSEIKQNVDLFEFQSVEVQQRATSLAKTLRCPQCQNQN

LIESNAPAAKDLRLKVYTMVNEGSSDQQVKDYLVERYGNIVLYQPPFNYSTALLWIFPII

FLIFFALFSIRLIKRN

>tr|Q87PL6|Q87PL6_VIBPA Methyl-accepting chemotaxis protein OS=Vibrio parahaemolyticus serotype O3:K6 (strain RIMD 2210633) OX=223926 GN=VP1486 PE=4 SV=1

MQGRIDDSVGGFRYGLSSIGPEMNRISSFLSVDDPESSDAANRFIASASSMESTFLVMMM

HTDLLKAEKEYREMRNRIAGINLAYEDFKALHPEVSDYASLTAPYDMVKSGFDQEGILQL

ILAKLAQSEQQQREFHQASLLADETMRLLDSISQSASNLIDEREAMVNNTIGNVSMMVLV

AACVISLVILVSWFGLKTWTNRGLKNVLVRLSALTDHDFRAKADEIGPFELKEVARKLNQ

VIDSTHDSIQTVTRNCETLYQTAEISHDAAEQTNEGLTTQNEALASMVTTITQLEASIRE

IATVTNASSEDSLLATRHTEKGVQVVEQNRKRLESLESSLDVNEQSMLELDQRVKQIREM

VDMISGIAENTNLLALNAAIEAARAGEQGRGFAVVADEVRKLAKDTSQQTTNIREMMSEL

ITAAERSRQAVNDSREEMTHALHSSNEVKSTFSDINLAVKHIQERVEQISVATEEQERAT

ADVSQSINNISELGERTKLQLESMVESSEQVAEIAGHQQAMLHKYELHQSA

>tr|Q87LN3|Q87LN3_VIBPA RNA polymerase sigma factor OS=Vibrio parahaemolyticus serotype O3:K6 (strain RIMD 2210633) OX=223926 GN=VP2578 PE=3 SV=1

MNEQLTDQVLIERVQNGDKQAFNLLVTKYQNKVCNLISRYVSNPGDVPDVAQEAFIKAYR

AIPSFRGESAFYTWLYRIAVNTAKNHIVAQGRRPPATDVDAEEAEFYETGSALKEISNPE

NLTLSKELQRVVFSAIEALPEDLKTAMTLRELDGLSYEEIAEVMDCPVGTVRSRIFRARE

AVEKKIKPLLQR

>tr|Q87HG5|Q87HG5_VIBPA Methyl-accepting chemotaxis protein OS=Vibrio parahaemolyticus serotype O3:K6 (strain RIMD 2210633) OX=223926 GN=VPA1000 PE=4 SV=1

MSIRNLSIAKKISLSFLLIALINIVFGVFLSKELKEIKSELLNYTDDTLPAMERVDAIRD

DLSHWRRSQFATYTYKDADKIRNKIASNIREREKISKELEAYGSTIWPGEEQQTFQRLMR

QWKQYLVTMDQYNESMLAGNKTEALAVLSNSLNDFEAVDSDLNELIRLLKVAMDSNKNHI

LSSVNGLSSSSIASNVTILVIMIVMTLVLTRLICGPLQLVVEQANSIAKGDLSKDIDRKL

IGNDELGELADATTKMQNDLRQVIDNVIAAVTQLSSAVEEMNQISELSASGMKDQQLQIT

HVATAMTEMKAAVADVARNTEESASQANDANHRTQLGVRETQSMVDAIGEVANVIGAAGD

TVSELEQQSNQINVVVDVIRDIADQTNLLALNAAIEAARAGESGRGFAVVADEVRTLAGR

TQDSTSEITAIIEQLQSLAKDAKSATELSRTSIAECADQGIQSKQLMNDIEHAISDISDM

GSQIATACNQQDSVAEELSRSIENIHLASQEVAQGSEQTAQACRELSQLSVSLQDVMSRF

KLN

>tr|Q87LE2|Q87LE2_VIBPA RNA polymerase sigma-54 factor OS=Vibrio parahaemolyticus serotype O3:K6 (strain RIMD 2210633) OX=223926 GN=VP2670 PE=3 SV=1

MKPSLQLKLGQQLAMTPQLQQAIRLLQLSTLDLQQEIQEALDSNPLLEVEEGHEEPQANG

EDKSALETADNSANEPTEIEVPDSSDVIEKSEISSELEIDTTWDDVYSANTGSTGLALDD

DMPVYQGETTESLHDYLMWQLDLTPFSETDRTIAIAIIDAIDDYGYLTLSPEEIHESFDN

EDIELDEVEAVRKRIQQFDPLGVASRNLQECLLLQLATFPEDTPWLAEAKMILADHIDHL

GNRDYKLVIKETKLKEADLREVLKLIQQLDPRPGSRITPDDTEYVIPDVSVFKDHGKWTV

SINPDSIPKLKVNQQYAQLSKGNSADSQYIRSNLQEAKWLIKSLESRNETLLKVARCIVE

HQQDFFEYGEEAMKPMVLNDVALAVDMHESTISRVTTQKFMHTPRGIFELKYFFSSHVST

DNGGECSSTAIRALIKKLVAAENTAKPLSDSKIAALLADQGIQVARRTIAKYRESLGIAP

SSQRKRLL

>tr|Q87HX7|Q87HX7_VIBPA Iron-containing alcohol dehydrogenase OS=Vibrio parahaemolyticus serotype O3:K6 (strain RIMD 2210633) OX=223926 GN=VPA0829 PE=4 SV=1

MNFSYVNPTKIFFGQQQIAAIKDAIPADQKVLVIYGGGSIKKNGVYDQVAEALTGHEWSE

FSGVEPNPTKETLDKAVAIVKDQNIDFILAVGGGSVIDGSKYVAAASKYDGDGWDIMIGK

HQVTEATPLAAILTLPATGSESNMGAVITKAETQDKLPFMSPAVQPKFAVLDPDVMKTLP

ERQLINGIVDAWVHVCEQYITLPTDAMVQDGYAETLLKTLKMLGEQFAERDDDKWRANLM

WSANQALNGLIGSGVPHDWATHMIGHELTALWGVDHARSLAIIQPSLLRNQMQFKRAKLE

QMGRNVFGLESGDDLAERTIEAIEAFYHQLGVATQLDNYGESREQAIDAIIDQLNKHGMT

VLGENQAITLERSREILELAVS

>tr|Q87RQ7|Q87RQ7_VIBPA Endolytic peptidoglycan transglycosylase RlpA OS=Vibrio parahaemolyticus serotype O3:K6 (strain RIMD 2210633) OX=223926 GN=rlpA PE=3 SV=1

MQKRALYSLVFSALILAGCSSTSQKTQEGRYELESDVAPDTPLSVEHIEDAHPKYEPYSL

GGNTDYHLRGNDYKIVRDAKGFTEKGRASWYGKKFQGHLTSNGEIYDMYSMTAAHKTLPL

PSYVKVTNTDNGKTTVVRVNDRGPFHDGRIIDLSYAAAHKLDVIKTGTANVEIEVISVEK

PTDQKSLESHPKYVIQVASSKNEDRARTLGAELGQKLDTETFLENAKESYRLLLGPFTDY

SLTQATLDKVKLLGYSSAFIKKHNTAK

>tr|Q87FD8|Q87FD8_VIBPA Uncharacterized protein OS=Vibrio parahaemolyticus serotype O3:K6 (strain RIMD 2210633) OX=223926 GN=VPA1741 PE=4 SV=1

MSVINKIVARRTQLSQSGRLVGDWIVENAEKAAQLTSQELAAQVKVSQSSIVKFTQRIGF

KGYSEFKLALNEEIGRKHAMQSTPLHSDILADDPIAVISQKLVKAKTDAMFQTTNALSYE

ACHQAVKWLSEARRVQVVGIGGSALTAKDLSFKLLKLGITALSEQDSHVQIAVARTLSSE

DVQIAISYSGERKEILVAAEAAKEQGAKVIALSAPGRSRLRGIADITFDTIANETEHRSS

SIASRTAQNVITDLLFIILVQQRDESARQLISDISTDIKQILT

>tr|Q87M13|Q87M13_VIBPA Putative lipid carrier protein OS=Vibrio parahaemolyticus serotype O3:K6 (strain RIMD 2210633) OX=223926 GN=VP2445 PE=4 SV=1

MNVLNKIRSQLVKNAANILRSPVQLLPQTVQQKALLEGLKMVFKEALEDGDFEFLEDKWL

KVAIKDLNLAWYISYQDEKLVVAEKPVQEDVSFSGNLNDLVLIAGRKEDPDTLFFQRRLS

IEGDTELGLEVKNLMDSVDLEQLPKAMQVALNQLADFVQKGVQEPAQQPGVANAYSN

>tr|Q87QU6|Q87QU6_VIBPA Cytochrome d ubiquinol oxidase, subunit I OS=Vibrio parahaemolyticus serotype O3:K6 (strain RIMD 2210633) OX=223926 GN=VP1053 PE=4 SV=1

MSFSRHIGVTMIDVVDLSRLQFALTAMYHFLFVPLTLGMAFLLAIMESMYVMTNKQIYKD

MTKFWGKLFGINFALGVATGLTMEFQFGTNWSYYSHYVGDIFGAPLAIEALVAFFLESTF

VGLFFFGWDRLSKRQHLAVTWLVALGSNFSALWILIANGWMQNPVGADFNFETMRMEMVS

FSEVVLNPVAQVKFVHTVASGYTCGAMFILGISSYYLLKGRDIAFARRSFAIAASFGIAA

ILSTIVLGDESGYELGEVQKVKLAAIEAEWHTEPAPAAFTLFGVPNQETMHTDYAIKIPY

VMGIIATRSFDEQVTGLRDLRDEHVERIRGGMYAYELLEKLRAGDKSEENMTAFDEVKGD

LGYGLLLKRYTDNVVDATEEQIQAAADDSIPTVWPLFWSFRIMVACGFIMLFVFGAAFIQ

TCRQKIEQKQWILKAALLSIPLPWIAIEAGWFVAEYGRQPWAVGEILPVHVAASALTAGE

IWTSLFAILALYTVFLIAEVYLMLKFARKGPSSLKTGRYHFEQNADSVEDKVSRQVEA

>tr|Q87G93|Q87G93_VIBPA PTS system, fructose-specific IIABC component OS=Vibrio parahaemolyticus serotype O3:K6 (strain RIMD 2210633) OX=223926 GN=VPA1424 PE=4 SV=1

MITKLINEDLIKLDLKASSKEDVFKELVAVLHAQGRISDQTQFLADIKAREELGNTGFED

GVAIPHAKSAAVIEPAVVIGVSKSGIEYGAEDGLPSKLFFMIASPDGGDNHHIEVLAELS

SKLIEDGFIDAFLDAANSQDALALLLAKEEPQPVTDAPANQGFIIGVTGCPAGVAHTYLA

AEALEKGAAAMGYEIKVETNGSIGVKNSPTAEEIERADAIIVACDKQVDMNRFAGKRVVK

TNVKAPIRDAQGLINEALNAPTYQAESNGNTQASVADKASQARSDLYRYLMNGVSHMIPF

VVTGGLLIALALAVGGQPSEAGMAIPEGSMWNQILNVGVVAFTLMIPILAGYIAYAIADR

PALAPGLIGGWIANNGSFYGADAGTGFIGAIIAGLLVGYFVKWITSINYHKFIQPLVPIM

IAPITGSLFIAGLFIFVIGAPIASLMDGLTALLTSMSTGNVVLLGIVLGGMAGFDMGGPF

NKVAFLFSVGMIASGQTQFMGAMACAIPVAPLGMALATALGRKFDLFEESETEAGKAAGA

MGLVGISEGAIPFAAQDPMSVIPANVLGSMVAAVMAFSFGITNSVAHGGPVVALLGAMNH

PVLALICMTAGATVTAVTCVTLKKVRKAKMMQAAA

>tr|Q87TE3|Q87TE3_VIBPA Uncharacterized protein OS=Vibrio parahaemolyticus serotype O3:K6 (strain RIMD 2210633) OX=223926 GN=VP0127 PE=4 SV=1

MFKLITPTTENQLNKYYQFRWQMLREPWRMPVGSERDEYDVVSHHRMIVDSRGRPMAIGR

LYITPDNDGQIRYMAVKGNRRSKGMGSLVLVALESLARQEGAKRLVCNAREDAIAFYEKN

GFERRGELTDERGPVRHQQMVKPLDPMANVLRRPEWCTELQQRWEAQIPIADKMGIKINQ

YTGYQFECSAQLNPNLNPHNTMFAGSAFTLATLTGWGMTWLLLKERGLHGDIVLADSSIR

YRHPVEQNPVASTSLDGISGDLDRLASGRKARIVIHVVIYSGDTPAVDFVGTYMLLPNYS

QLLSC

>tr|Q87SL2|Q87SL2_VIBPA 7,8-dihydroneopterin aldolase OS=Vibrio parahaemolyticus serotype O3:K6 (strain RIMD 2210633) OX=223926 GN=VP0411 PE=3 SV=1

MALDKVFIEQLEVITTIGVYDWEQQIKQKLVLDIEMAHDNKPAGKSDDVQDALDYSQVSE

AVLNHIENGRFLLVERVAEEVAELIMQRFSVPWVKIRLAKPGAVPQARSVGVVIERGQA

>tr|Q87FC7|Q87FC7_VIBPA ParA family protein OS=Vibrio parahaemolyticus serotype O3:K6 (strain RIMD 2210633) OX=223926 GN=VPA1752 PE=4 SV=1

MKREKTIENLQELAELTQQVQADRIEIVLEERSDNYFPPMSKAMMETRSGLTRRKLDEAI

GKMEAAGHQFTKNNANHYSITLEEAHMLMDAAEVPKFYERKKNNGNKPWIINVQNQKGGT

GKSMTAVHLAACLALNLDKRYRICLIDLDPQGSLRLFLNPQISVAEHDNIYSAVDIMLGN

VPDGVEIDREFLHKNVLLPTQYPNLKSISAFPEDAMFNAEAWQTLSEDPSLDIVRLLKEQ

LIDKIANDFDIIMIDTGPHVDPLVWNAMYASNALLIPCAAKRLDWASTVNFFQHLPTVYE

MFPEDWNGLEFVRLMPTMFEDDNKKQVSVLTEMNYLLGDQVMMATIPRSRAFETCADTYS

TVFDLTVSDFEGGKKTLATAQDAVHKSALELERVLHSNWPSLNQG

>tr|Q87LH5|Q87LH5_VIBPA PTS system, cellobiose-specific IIB component OS=Vibrio parahaemolyticus serotype O3:K6 (strain RIMD 2210633) OX=223926 GN=VP2637 PE=4 SV=1

MKKILLCCSAGMSTSMLVKKMEQAAEIKGIECKIDAMSVNAFEEAIKEYDVCLLGPQVRF

QLEELRKTAQEYGKNIDAISPQAYGMMKGDEVLEQALELIN

>tr|Q87P07|Q87P07_VIBPA Transcriptional regulatory protein OS=Vibrio parahaemolyticus serotype O3:K6 (strain RIMD 2210633) OX=223926 GN=VP1711 PE=4 SV=1

MNMATRVMIIEDDIAIAELHHKYLSQLAGLDVVGIATTRLEAEMQLEVLKPDLLLMDVYL

PDGTGLEILNTLRSNNQTCDVILITAARDVDTLQQAMRGGVVDYLLKPVMFPRLETALKK

YITQRQQLDVAKSLDQGLVDRMLQSNTGTDSCPKRLPKGIDSVTLDKIRDLFVGEAALTA

DEAGEKIGASRTTARRYLEYLISSGELEADLNYGTVGRPERCYKKVIR

>tr|Q87HJ8|Q87HJ8_VIBPA Putative sensor histidine protein kinase UhpB OS=Vibrio parahaemolyticus serotype O3:K6 (strain RIMD 2210633) OX=223926 GN=VPA0965 PE=4 SV=1

MRAYSVTTICGLFVMACAWFCLWVIAYYFVNDPELAILLFPFALRLGIALHTRTAYWPTI

YVSEWALTIALATLLEQPQWLTVLIASVASIPVTLIAKKYYYGDQNRHLAVMGIVIIITA

FINVMAVGFHVPSVYMVWLASISGGLMLVPMCYLLWNYLFQSRWSPLTSHLLNNTVVFSI

RHIVFYAVLLIGSILVQTSLPEELKRFAPFCMAIPIIVLALRYGWQGALLATMLNSIALI

AARSGVSNLEITDLLLSLSAQTITGIMLGLAVQKQKDLNHKLRGELSRNQNLSRQLIEAE

ESVRRDIARELHDEIGQNITAIRTQANIIKRIDNAEMSAHCADTIEGLSLNVYDTTKRLL

SKLRPKMLDDLDLKESVEQLTREMEFANHGTTVQLNWQGDYTSLSDTLKVTLFRLCQESL

NNAAKYAEAQLINIELTIGEAAVSLMIHDDGVGFKVQDSMKGMGVRGMQERVHALGGKMV

IYSTSDQVIGTQISITLPKV

>tr|Q87FV9|Q87FV9_VIBPA Transaldolase OS=Vibrio parahaemolyticus serotype O3:K6 (strain RIMD 2210633) OX=223926 GN=VPA1569 PE=4 SV=1

MIELYLDTADVAEVKRFNQCLPLKGVTTNPSILAKSKQGLNQTLAGMQEALGGTPRFHAQ

VVSTTVEGMVEEARQIHELPYDMVIKVPATETGLSAIKLMKKEGIQVLATAIYSAQQGFL

AALCGADYLAPYVNRIDAMNGNGVEVVADLQLLLDQNQLPAKILAASFKNTQQAMEVMKL

GIEAITLPVDVAAQMFSHPAVQPAVDQFDKDWKSTFGNKLSFES

>tr|Q87Q99|Q87Q99_VIBPA Thioredoxin reductase OS=Vibrio parahaemolyticus serotype O3:K6 (strain RIMD 2210633) OX=223926 GN=VP1251 PE=3 SV=1

MSDVKHCKLLILGSGPAGYTAAVYAARANLNPVLVTGMQQGGQLTTTTEVENWPGDAEGL

TGPALMERMKEHAERFETEIVFDHINEVELSQRPFRLKGDSGEYTCDALIISTGASAKYL

GLESEETFKGRGVSACATCDGFFYRNQKVAVVGGGNTAVEEALYLSNIASEVHLIHRRDS

FRAEKILINRLMDKVENGNIILHTDRTLDEVLGDDMGVTGVRIKDVNTGTTEDLEVMGAF

IAIGHQPNTQIFEGQLEMKDGYIVVKSGLEGNATQTSIEGVFAAGDVMDHNYRQAITSAG

TGCMAALDAERFLDALSDK

>tr|Q87R21|Q87R21_VIBPA Uncharacterized protein OS=Vibrio parahaemolyticus serotype O3:K6 (strain RIMD 2210633) OX=223926 GN=VP0977 PE=4 SV=1

MSCMYHPISLFIGLRYLRGRSGDRFSRFVSYMSTAGITIGVMALVTVLSVMNGFEAQLKE

RILGVLPHAVISQHDGRTPLTESAPQFIQAMSDVAEPEPVVRGEAVIQSSAQLTAGYLIG

IEPKKGDPIANHLIAGRLSSLQAGEYKVFLGHSLARSLKVSIGDKVRLMVTNATQFTPLG

RIPSQRNFTVAGIFNTGSDVDGQLMIVNMADAAKLMRLPKDTVSGWRVFFSDPFMVTDFA

DKPMPEGWQWSDWRAQRGELFQAVKMEKNMMGLMLGLIVGVAAFNIISALIMVVMEKQSE

VAILKTQGMTQSQVMTIFMVQGASSGVIGAIVGGAVGVALSLNLNVILESAGVALFSFGG

HLPIVIDSFQILLVVVLAIALSLAATVYPSYRASSVKPAEALRYE

>tr|Q87GB2|Q87GB2_VIBPA Putative polysaccharide export-related protein OS=Vibrio parahaemolyticus serotype O3:K6 (strain RIMD 2210633) OX=223926 GN=VPA1405 PE=4 SV=1

MNPLFKLIGLALLLFSTFVSANSNEQDYLLDTGDTISVQVYGEEDLSIKNILITSDGYFD

YPYLGRIKAINKTPKQLKYEIETGLKGDYLINPKVMVTINYFRLFYVNGEVRKPGGFEYR

PGLTIEKAIALAGGLTDRASRKSINLTKHKTGKTVEGVSMQRTVEPGDIVFIDQSFF

>tr|Q87K13|Q87K13_VIBPA Putative exported protein OS=Vibrio parahaemolyticus serotype O3:K6 (strain RIMD 2210633) OX=223926 GN=VPA0085 PE=4 SV=1

MKVRYLTSALLLAMTSGLAAAASVDIRHEYVPDRDGDEHRDRIYVSHRFDNQIGFSVEAK

WNYKDGHMGNAGHETGVSYRWKATDNFSLTPGINIDASPSGASVFKYNLTGAYKINDEWD

VAARIRHGYKNTDDSRYNQLNLYANRKFEWGKLGVEAEYKDIHGGEGGWKDKGHDQLIDF

KGEYTKLESGVIPFFAIAAITHKGDGSEFKDEYVPRFRVGLKYNF

>tr|Q87RG5|Q87RG5_VIBPA PTS system, N-acetylglucosamine-specific IIABC component OS=Vibrio parahaemolyticus serotype O3:K6 (strain RIMD 2210633) OX=223926 GN=VP0831 PE=4 SV=1

MNILGYAQKLGKALMLPIATLPIAGLLLRLGQPDVFDIAFMAQAGNSIFSNLPLLFGLGI

AIGLSKDGQGAAGLAGAVAYFVLTATASTIDASVNMSFFGGIIAGIIAGHSYNAFHATRL

PEWLAFFSGKRLVPIMAGLFALVAGAVSGIVWPTIQGGLDALAHGISTSGAIGQFVYGTL

NRALIPVGLHHVLNSFFWFGMGSCQEVLVSGATAAGQALPALQQLCVDPALAKTLVAGQT

HTFEFANSVTPEITATVKEVTETVKSGDLHRFFGGDKSAGVFMNGFFPVMMFGLPGAALA

MYLAAPVEKRSQVGGALFSVAFCSFLTGITEPLEFMFVFLAPALYAIHAVFTGLSLVVAN

MLGTLHGFTFSAGLIDYLLNYGLATKPLLLGAVGLGFGALYFFTFSFAIRAFNLKSPGRE

DDDSQAAAPAGEAKSGDLARQYLKALGGHDNLTSIDACITRLRLTLKDRSVADEEVLKKL

GAKGVVKLGENNLQVILGPLAEIVAGEMKAIGANEDLSNVKLP

>tr|Q87R27|Q87R27_VIBPA NADH dehydrogenase OS=Vibrio parahaemolyticus serotype O3:K6 (strain RIMD 2210633) OX=223926 GN=VP0971 PE=4 SV=1

MTRIIVVGGGAGGLELATKLGRTLGRKNRAQITLVDRKASHLWKPLLHEVATGSLDEGVD

ALSYRAHAKNHSFDFQMGSLQDIDRERKVIILSELKDEHGELLMPSRELEYDLLVMAIGS

TSNDFNTPGVRENCIFLDSPEQAHRFRTEMNNEFLKLHAKNGNGTVDIAIVGAGATGVEL

SAELHNAVKELRTYGFGDLDSSKLNVNLIEAGERILPALPPRISSAAHQELVKLGVNVRT

ATMVTKAEKDGLTTKDGEKIPAQIMVWAAGIKAPDFIKDIAGLETNRINQLVVKGTLQTT

RDDDIFVIGDLAQCTQPDGSFVPPRAQAAHQMASQAFSNIVAKLNGRELKNYVYKDHGSL

VSLSRFSTVGSLMGNLTKGSMMVEGRIARVVYISLYRMHLMALHGVFKTGLMMLVGRINR

VLRPNLKLH

>tr|Q87QA5|Q87QA5_VIBPA Response regulator OS=Vibrio parahaemolyticus serotype O3:K6 (strain RIMD 2210633) OX=223926 GN=VP1245 PE=4 SV=1

MNRYAVLCLDNNPISAEQFRLELSAFSSKFDIFSVESIEEAQSALEYLEEREQTVALVIA

SHHAHFNGVDFLIGLDKTPHTERARKILISCSSDIDAILTAVNEGRLDHCLTKPLPDNVL

FNTAQKELTQFILRNCKEDLLSYSQILDQHKLLRAHIENQMSNYQAGFLHDYHSMSDNEL

AEQVISALQQFFKENDETKACRTYSPEHLLTVEGEPNSFLWFITSGEVALYKRDEQGMQR

EVVRHTKGNIVGGMSFVTGECSFSTALTLTETEVIKLDRNVFRKVMQSDSNLLPLFTNLL

LRHFNRRLQRSINTKLQLQKTLESLESAHQQLIEREKMAMLGQLVAGVAHELNNPIAAIL

RGVENLTHTLEGLLTQLPTAEVQIKGVQLLTRAQTAKPASTAELRSRVKELNSTLPDRTI

AKKVVNLGLESDSELVSQLAKKKDNASSTLETLEQYHQAGASLRSINVCAARIADMVKSL

KGYARSDDERMHYADIHEGIEDTLVIFENKLKVHQVSTDYAPLPRMLCQPIALQQVWTNL

ISNAIDAFPDKGSLKIQTREVEKNQQRYAVISFEDNGCGIPDSQKKAIFELNFTTKKEGN

FGLGIGLSICQQIVAAHKGWIDVQSELNAFTCMTVWLPVIEEGDET

>tr|Q87K42|Q87K42_VIBPA Uncharacterized protein OS=Vibrio parahaemolyticus serotype O3:K6 (strain RIMD 2210633) OX=223926 GN=VPA0056 PE=4 SV=1

MAIPARRKRKQDKEPQLSFWARHQLKMVKSAFVIFVCSLVAFFAYQLHVSYQDYIDPEHV

YGEWIEIGAPPYQTERLIFTSDGVYRNHRLITTEFAFDGKVITLNTGIGETAYQLSGSHL

SPQIRRIEPRIPDQRFIRKGFEHTVQGSEVGAASKRRAALSEHFSRD

>tr|Q87H50|Q87H50_VIBPA Uncharacterized protein OS=Vibrio parahaemolyticus serotype O3:K6 (strain RIMD 2210633) OX=223926 GN=VPA1115 PE=4 SV=1

MSQFYKLRALSLVFWFVFLTTKVYAQSPEPKDKFLQSLEGIESQIYALPQSSLAQIESLE

EDSLLQNQPKDLLIRYWLAKSTVLELLGRDKESLAVVDKGLSLTPEQSQEHLLFKLIQIR

AMMGNRDIDTALSSLDALLETSREKGDKKLESEVLLLKGRYYDEQGDYKKSYAALMSSME

AAESSGAQGLVERAALELGDVLVKIQGYDRSEVVLKQAYRYFKDRRMSFNELLSVLTIAK

LHKAQFQYDEAIKSYQAALKLAQIIGDGRFRFRVNLELAALYRETNNEKNMLRHLKLAEN

LQYRETSNAYLATFKLLQAEYMLERKQYQALLTMITPLLPEIIESRYIKQQQMELLKVAA

MAYAGDQNFELAYQTYGQYHEKFIQFSNQREVENLERQQTLFELERLEYENENLNWNNVL

QRLELENNRRTFYLLGEVLLIMIGILLLMALVFLYVNRSRLRMRRLAKTDMLTGLFNRRF

LEEWFAKPAEQKPKLIEKPIPETKKGKLVHKLNKQVMRVQYGYLALNHWVERKLDKQKMV

AKKPETGPITLVMMDVDHFKQVNDTYGHVFGDVVLTGVAKVLDSSVRESDIVARLGGEEF

VIVLPNTDLEEATALAERLRIALSQRGFVTENNQAVNVTCSFGVITSDDVDVAFEALCSQ

ADKLLYEAKSSGRNCVKALSFS

>tr|Q87TD5|Q87TD5_VIBPA General secretion pathway protein F OS=Vibrio parahaemolyticus serotype O3:K6 (strain RIMD 2210633) OX=223926 GN=VP0135 PE=3 SV=1

MAAFEYKALDAKGKQKKGTIEGDNARQVRQRLKEQGMIPVEVVEAKAKAAKSSGSVGFKR

GIKTAELALITRQLSTLVQSGMPLEECLRAVSEQAEKPRIRTMIAAVRSKVTEGYPLADS

LGDYPHVFDELFRSMVAAGEKSGHLDTVLERLAEYVENRQKMRSKLLQAMIYPVVLVVFA

VAIVSFLLATVVPKIIEPIIQMGQELPQSTQFLLAASEFVQEWGLIIFAVLVVCFYGLKL

ALQKPDFRLSWDRKIISLPLVGKISKGLNTARFARTLSICTSSAIPILEGMRVAVDVMSN

RYVKQQVLIAADNVREGASLRKALDQTRLFPPMMLHMIASGEQSGELESMLTRAADNQDQ

NFESTVNIALGVFTPALIALMAGLVLFIVMATLMPMLEMNNLMSG

>tr|Q87L21|Q87L21_VIBPA Arginine N-succinyltransferase OS=Vibrio parahaemolyticus serotype O3:K6 (strain RIMD 2210633) OX=223926 GN=VP2796 PE=4 SV=1

MLVVRPIAMSDYDALHTCAVESGHGFTSLPVNEELLTNRIKHSEYSFGKPDVTEPGDEGY

LMVGFDSESGEVAGTTGIEASIGWDVPFYSYHISKVVHSSPKLGVNNVVKLLTFGNNYTG

NSEICTLFLREKYRQGLNGRLMSKCRFLMMAEHPERFSKTIFAEMRGVSDANGNSPFWQW

LQEHFFSIDFTLADYLTGIGKKGFIADLMPKLPIYINLLSPEAQAVIGQVHDNTRPALKL

LEREGFTNRGYVDIFDGGPTVECDLRNIESVRHSFRAQVKISAHTSTQDFLMCNSSFENF

RAAAAKAAYDAETKSVVLAPELADALLVKEGDFVRLLPQ

>tr|Q87SQ6|Q87SQ6_VIBPA Phosphoenolpyruvate-protein phosphotransferase OS=Vibrio parahaemolyticus serotype O3:K6 (strain RIMD 2210633) OX=223926 GN=VP0366 PE=3 SV=1

MASNQIEGAVVGIRVNDGIAAAPVVLFTHEMPAVPERDFQSEQGEIERVKRAIGVVVQHL

QEQAKQPKGEIFSAHSMMLSDPELWASVESRIQTGMIAEQAWIESLQTLADEFRQAESQY

MREREADVHDIARQVMVEMTGVTPNAIDIQEPSILLARDLMPSDVAGLDKSKVLGICLSE

GGKTSHSAILARAMGIPAMVKAQGCLDAVRAGQVVTIDGFRGHLWFSPSDAIQQELEAQQ

IEWQSTRQSALASAQQAAATCDGVHIPVFANIGGPKDIDDALTSGAEGVGLFRTEFLFQN

SDELPTEEAQYQVYRDIAAALGDKPLTIRSLDVGGDKPLAAYPMPAEDNPFLGLRGVRLC

LQHESLFTAQLRAILRAFHEQPNIQLMIPMVAQVEEVRKVKALLAHQANQLGLDATHLPV

GIMIEVPAAVLNADALAQEVDFFSIGTNDLTQYVMAADRGNAAVAELVNYFEPSVLKAIE

LTCAAGDRAGIPVSMCGEMAGDPNATETLLRVGLQKFSASPSLLPGLKAQIRQLSVDV

>tr|Q87PM2|Q87PM2_VIBPA Riboflavin synthase, alpha chain OS=Vibrio parahaemolyticus serotype O3:K6 (strain RIMD 2210633) OX=223926 GN=VP1480 PE=4 SV=1

MFTGIVQGTAKVVQIDKKERFQTHVIELDGALIEGLEIGASVAHNGCCLTVTNIDGNRVS

FDLMQATLALTNLGLIEEGSAVNVERAAKFGDEIGGHSMSGHISLMANIVDVIDTPNNRT

IWFELPQESMKYVLAKGYIGIDGCSLTIGEVEANRFSVHLIPETLQRTLFGSRQVGDKVN

IEFDPQTQAIVDTVERVLAAKQL

>tr|Q87J01|Q87J01_VIBPA Putative DNA-binding stress protein OS=Vibrio parahaemolyticus serotype O3:K6 (strain RIMD 2210633) OX=223926 GN=VPA0454 PE=3 SV=1

MTHIDIGIDREHRLSTAEGLKQLLADSYTLYLQTHNFHWNVEGPNFRELHLMFEEHYTEL

ATAVDEIAERIRTLDVPAPGTFKEFAKLSVIEEVEGVPSATEMVDILTHNHEQVVKTARK

VLKLAQQADDESSVALVSDRMRIHEKTAWMLRSLQK

>tr|Q87T91|Q87T91_VIBPA Lipid A biosynthesis lauroyltransferase OS=Vibrio parahaemolyticus serotype O3:K6 (strain RIMD 2210633) OX=223926 GN=lpxL PE=3 SV=1

MSKDKYTQPEFSLSLLHPRNWGVWLGFGLLAIIVNILPYRLLLSLGRSLGKLGMRYGKKR

VHIAKRNLELAFPEKTPEEVQHIVEENFKNTGMALIETGITWFWPTWRFKTLIVEKDIHA

LKEKGAEGKGVLLCCVHALNLEITARAFAVLGVAGYGAFRPHNNPAYNFIQYWGRTHNGN

KLIDRKDVKKMIRVLRSGERLFYLPDHDYGRNKSVFVPFFAIDDACTTTGTSILAYTSKC

TIIPGSGFRNDEGKYEIIADKCIEADYPQKDEVAAAAYMNKYVEEVILRAPEQWMWLHKR

YKTMQDENEPKGIRYK

>tr|Q87JF0|Q87JF0_VIBPA Putative regulatory protein OS=Vibrio parahaemolyticus serotype O3:K6 (strain RIMD 2210633) OX=223926 GN=VPA0303 PE=4 SV=1

MNPRQNEILQLVNDRKRVQVTELSDIIGVSGVTIRQDLNFLEQQGYLKRVHGAATALQSD

DIDTRLEVRFDIKQTLANKAADLVAPNETVLIEGGSANALLARTLAERGDVTIITPSAYI

AHLIRNTSANIILLGGVYQHQGESLVGPLTKLCIENIHFSTAFLGIDGFHQDTGFTSRDM

MRADIAEAILAKKRRNIVLTDSSKFGQIYPSSIGKTNEISVLLTDKAAPKSDLEQLKKLG

VEVVLG

>tr|Q87FY2|Q87FY2_VIBPA Putative two-component response regulator OS=Vibrio parahaemolyticus serotype O3:K6 (strain RIMD 2210633) OX=223926 GN=VPA1538 PE=4 SV=1

MMTKTNILLVEPNEHLAQPVLDVLKNAGYTAKHTRTGRSALLEERASITLVSSTLPDMCV

REFVACHQKQRNAGVVIAIVDQEQGILAAETMKSGATDYLLRPFEANQLINLLKRVEALG

KPMANIVAESWRSKQVLQLAHRAACTNASVLITGESGTGKEVLARYVHEHSPRINGPFVA

VNCAAIPESMLEAVLFGHVKGAFTGATNSQSGKFEEANGGTILLDEIGEMSPAVQAKLLR

VLQEREVERVGSHKAIKLDIRVIAATNKDLREEVQKGTFREDLYYRLDVLPLHWPPLRER

KEDILPISQFFIEKYQDSSRCHLSQDAISALSQYHWPGNIRELENVIQRALVMRHGDYIT

AHDLMLPIELIAPVPSMEPKSSFGHVEAKKQAEYQFILDKLRQFGGNRTKTANALGVSTR

ALRYKLAAMREHGIDLQSALGSAA

>tr|Q87KX6|Q87KX6_VIBPA Alanine racemase OS=Vibrio parahaemolyticus serotype O3:K6 (strain RIMD 2210633) OX=223926 GN=VP2848 PE=3 SV=1

MGQTAAHAAPLLVDFDDSEREERVQASNAWLEIDTQAFSNNIQLLQKDLKGNTQICAIMK

ADAYGNGIAGLMPSIIANNVACVGITSNEEARVVREHGFEGKIMRVRAASRNEIENGVQY

EIEELIGTKMQADQIIEIAYNYNTVIPVHLALNTSGMGRNGLDLTTYEGQVEGVEIASDP

NLKIVGMMTHFPNEGLDEIRRKVDRFKVETKWLMDSAGLKRKDITLHVANSYITLNLPEA

HLDMVRPGGMLYGDYPATLPYERIVSFKTRIASLHFFPAGSTIGYGSTKVLERDSILANL

PIGYSDGFARSLSNKADVLINGQRARVMGKASMNTTMVDVTDIIGVQANDEVVVFGRQGF

EEITAEETEEKSGRILPEHYTIWGATNPRVYR

>tr|Q87PH6|Q87PH6_VIBPA Putrescine-binding periplasmic protein OS=Vibrio parahaemolyticus serotype O3:K6 (strain RIMD 2210633) OX=223926 GN=VP1526 PE=3 SV=1

MIWTTFGLELTSMKKWATLLAGSACALSLFSGSAAADDKELVFMNWGPYINSNILEQFTK

ETGIKVIYSTYESNETLYAKLKTHNQGYDLVVPSTYFVAKMRDEGMLQKIDKTKLKNFGN

LDKNYLDKPYDPNNDYSIPHVVAITGLAVNADMYDPNDFQSWADLWKPELEGQVMLMDDT

REVFHIALRKLGYSGNSTDPKQIDEAYAELQKLMPNVLVFNSDNPGAPYMSGEVGVGMLW

NGSAAAAQNEGLNLKLVFPKEGGIGWVDNFAISSGAKNVEAAHKMIDFLLRPEIAEQISR

DTGYLTAVTESNSKFKDVAPLFPSQEDLDRVEWQDSVGDMTVKYEDYFLKLKAGQ

>tr|Q87JL8|Q87JL8_VIBPA Phosphotransferase enzyme II, A component OS=Vibrio parahaemolyticus serotype O3:K6 (strain RIMD 2210633) OX=223926 GN=VPA0231 PE=4 SV=1

MLRELITSDVIRIHSDATDWKDAISKSCEALIENGAIEPSYVEAIYRSHEELGPYYVVGP

GMAMPHARPEDGVNRLSLAITVIQNGVNFNSEENDPVKMLVTLAATDSNSHVDAISKLAE

LFMNEEHVEAICNAQSKEDVLAIIDKY

>tr|Q87QZ2|Q87QZ2_VIBPA Probable membrane transporter protein OS=Vibrio parahaemolyticus serotype O3:K6 (strain RIMD 2210633) OX=223926 GN=VP1006 PE=3 SV=1

MEMIEPTMLLVLALVAFVAGFIDAVAGGGGMLTVPALLSLGLPPHIALGTNKLAATFASS

TAAFTYYKKRLFKPQCWGRAFAATLVGATLGTLFVDAISTDWLEKVLPLIILAAALYTVF

HKTLHSPHQSPIPEPCPKLHKKQYLQGLSIGFYDGLAGPGTGAFWTVSSMALYRLNILLA

SGLAKAMNFTSNFTSLITFAILGHINWVLGLTMGVCLMAGAFVGAHSAIRFGSKFIRPVF

VTVVSVLAIKLAYDAWFVGLS

>tr|Q87QZ7|Q87QZ7_VIBPA Amino acid ABC transporter, permease protein OS=Vibrio parahaemolyticus serotype O3:K6 (strain RIMD 2210633) OX=223926 GN=VP1001 PE=3 SV=1

MDFSLIIESFPVYFQGLWTTVWLVGISLVIGLCVSVPLAIARNSTNYALSLPSWGFIYFF

RGTPLLVQLYLIYYGMDQFFPVKDTLWEHAWFCALVAFVLNTSAYTAEIIRGAINGLPKG

EVEAAKAYGMSKFMTYKRIILPSALRRALPAYSNEVIFMLHGSAVAGIVTIVDLTGAARL

VNSRYYAPFESFLTAGLFYMSLTFIILWCFKRAEKRFLAYLRPLS

>tr|Q87SH8|Q87SH8_VIBPA Stringent starvation protein B OS=Vibrio parahaemolyticus serotype O3:K6 (strain RIMD 2210633) OX=223926 GN=VP0445 PE=4 SV=1

MDIAKMTARRPYMLRAFYDWLVDNDLTPHLVVDATMPGVRVPVEFVQDGQIILNIAPRAV

GNLELGNDAITFHARFSGRPHSVIVPVYAVQAIYARENGAGTMFEPEEAYTHIEEETIEE

EDLSPSFKAVTEETSEEIGAESQEEEAPRPKGRPSLRVIK

>tr|Q87R19|Q87R19_VIBPA Uncharacterized protein OS=Vibrio parahaemolyticus serotype O3:K6 (strain RIMD 2210633) OX=223926 GN=VP0979 PE=4 SV=1

MFSSLALMIGGRFSRAKKRNKMVSFISLSSTIGIAVGVAVIIIGLSAMNGFERELQSRVL

SVIPHGELEGVNGPLQNYTKTMNQALQHEHVVAAAPYVRFTGLAEKGSKLKAIEVRGVDP

AYEQAVSSMSDFIDPEAWQNFYSGQQQVILGRGVANELKVQVGDYVTLMIPQTGGTNKVQ

APKRVRVKVAGFLTLNGQIDHSLALVPLADAQQYVRLGDGVTGISLKTDDVLDAPSIVRE

VGNLVNVYVYLKSWQQQFGFLYRDIQLVRTIMYLVMVLVIGVACFNIVSTLMMAVKDRAA

EIAILRTMGAKDGLIKRIFVWQGVFSGVFGSLVGSLVGVLVALNLTPIIKGLEGLIGHQF

LSGDIYFVDFLPSQLHWPDVALVSTTAIVLSLLATWYPASRAAKLNPAAVLSAK

>tr|Q87TN8|Q87TN8_VIBPA Uncharacterized protein OS=Vibrio parahaemolyticus serotype O3:K6 (strain RIMD 2210633) OX=223926 GN=VP0031 PE=4 SV=1

MNDQPYLIPSAPALFEEEIKKSVFITYLAHTPSVDAAKAFVDQIKAKHSDARHNCWGFVA

GRPEDSMKWGFSDDGEPSGTAGKPILAQLSGSRVGEITAVVTRYSGGIKLGTGGLVKAYG

GGVQQALKLLQTIEKKITTKLRLTLDYGFMPIAQSIMPQFGAVEVAAEYSDQVILVVEIE

LREVSAFTQAIINKSGAKAIVTPLDGQ

>tr|Q87H83|Q87H83_VIBPA Ribose operon repressor OS=Vibrio parahaemolyticus serotype O3:K6 (strain RIMD 2210633) OX=223926 GN=VPA1082 PE=4 SV=1

MATMKDIAKLAGVSTSTVSHVINKTRFVSEEISERVNNAAKELNYYAPSALARSLKVNRT

KTIGMLVTTSTNPFFGEVVKGVERSCYHKGYSLILCNTEGDNERMRQSINTLLQKRVDGL

ILMCSSLEGERIDVFERYPDIPVVVMDWGPMLFTSDKIQDNSLRGGYLAAKYLIDCGHTE

IGCITGPLIKHQAQMRYEGYKRAMNEAGLEFNANWIIESDFECEGGYQAFKKMAERGTLP

SSIFVSNDMMAMGVINAANELGIKVPDDLSIIGYDDIHIAKFMSPSLTTIHQPKYRLGQA

AVETLVRRLDDKSNEAQVVQLEPTLVVRNSVTNFS

>tr|Q87RY8|Q87RY8_VIBPA Putative outer membrane protein A OS=Vibrio parahaemolyticus serotype O3:K6 (strain RIMD 2210633) OX=223926 GN=VP0636 PE=4 SV=1

MKSNVVTLVGLFSLTTFSTLSYAADSKDHGVYVGANYGYLKVDGQDDFDDDSDAMQALVG

YRFNRYLALEGGYIDFGSYGNNLANAETDGYTAALKVTAPITDRVDVYAKGGQMWYSTDY

NVAGFHGNKDDEGVFAGAGVGFKVTDNFLVNAEYTWYDVELNAENVFDGANTNTDFKQAS

LGVEYRF

>tr|Q87PH5|Q87PH5_VIBPA Spermidine/putrescine ABC transporter, permease protein OS=Vibrio parahaemolyticus serotype O3:K6 (strain RIMD 2210633) OX=223926 GN=VP1527 PE=3 SV=1

MGRTVRFSFMALVYAFLYLPIIVLIVNSFNANKFGMKWGGFTTKWYETLVNNDSLMQAAW

HSLNVAVFSATAATIIGSLTAVALFRYSFKGKGAVNGMLFVVMMSPDIVMAISLLALFLV

LGAQLGFFTLLIAHITFCLPFVVVTVYSRLNGFDVKMLEAAKDLGASEWVILKQIILPLA

KPAVAAGWLLSFTLSLDDVIISSFVTGPTYEILPLKIYSMVKVGISPEVNALATVMLIVS

LVLVVISQLLAREKVK

>tr|Q87T99|Q87T99_VIBPA Putative peptide ABC transporter, permease protein OS=Vibrio parahaemolyticus serotype O3:K6 (strain RIMD 2210633) OX=223926 GN=VP0171 PE=3 SV=1

MIKVSPLTQKKIRHFKEIKRGYWSFVILSIMLILSLFAELLINSKALIVKYDGSYYFPVV

SDVRLGSEFGQDSSSEADYRVLQQVFEEEGGDNFVILPIVPWNPYEQDFSGDFPPTAPSA

ENKHYLGTDVIGRDILARLVYGFRTAMGFALLTMAVSYAIGTAVGCAMGFWGGKFDLFVQ

RLIEVWSMVPFLYVIMILVSIVQPTFTLFVAINVLFGWMGMTWYMRTMTYKESAREYVMA

ARALGASTGRILFNHILPNTMVMIVTLAPFTIAANITALTALDYLGLGLMPPTPSWGELL

QQGKSNLDSPWIVASVVTSIVLVLVMVTFIGEAIRAAFDPKKFTRYV

>tr|Q87NU8|Q87NU8_VIBPA Putative exopolyphosphatase Ppx1 OS=Vibrio parahaemolyticus serotype O3:K6 (strain RIMD 2210633) OX=223926 GN=VP1770 PE=4 SV=1

MDLTTLSNNNTDNLVWVGHLSPDTDSAVSVILASHIYGGEAALTGEANPESKFVFEFCGM

DAPKVKADFSSHHIGLVDFNQSTQLAKSVDPTSIVAIIDHHAMGSSPISMPQIVTMDIRA

WGSAATILTANAEKLNVKLPKNIACAGLGAILSDTVVFQSSTTTEYDKQYAQKLADIAGI

KDIKGFGEQMLLAKSDLSHFSAETILTMDYKNFEFAGKKVGIGVAETLNAQQLIDRKQDF

NEAIQAYKKAQNLDYLFFSITDTKHKRANMLWADDADKKVLSKAFDVKIDNDMLVLDGVT

SRKRQIGPAIQQAIESL

>tr|Q87RD2|Q87RD2_VIBPA ATP-dependent helicase, DinG family OS=Vibrio parahaemolyticus serotype O3:K6 (strain RIMD 2210633) OX=223926 GN=VP0865 PE=4 SV=1

MIAKTFSSDGALGKAIPGFQARQPQIDMAEAVSSAIKEQSQLVVEAGTGTGKTFAYLVPA

LLSGKKVIISTGSKNLQEQLYHRDLPLMVNALGFYGQVALLKGRSNYLCLDRLSRQMVES

HTNESDPTLLTQLVKVRSWSSETKTGDLGDCEDLPEDSMIIPTITSTNDNCLGKECPSYT

DCFVLKARKRAMDSDIVVVNHHLFLADLAIKETGFGELIPEADVFIFDEAHQLPDIASEY

FGQSVSSRQIHDLAKDIEIAYRTEAKDMRQLQKVGDKLLQSAMDMRIVLGEPGFRGNWRE

AMQSESIKRELVRLTDSLDLAIDVLKLALGRSQLLDTAFERANLIKGRIERVCDVDITGY

SYWYDTSPRHFTLHITPLSVADKFHEQIEIKQGAWIFTSATLAVSGDFKHFTDRLGLKPK

QQFSLPSPFDYEKQARLCVPRYLPEPNSPGLADKLVRMLAPVIEENDGRCFFLCTSHSMM

RELGEKFREVLDLPVLMQGEMSKQKTLAEFMELGNALLVATGAFWEGIDVRGDALSCVII

DKLPFTAPDDPLLKARIEDCRLRGGEPFAEVQIPDAVITLKQGVGRLIRDQKDHGALIIC

DNRLVTRDYGGTFLGSLPPIPRTRDLERIKAFLKAE

>tr|Q87QX3|Q87QX3_VIBPA Uncharacterized protein OS=Vibrio parahaemolyticus serotype O3:K6 (strain RIMD 2210633) OX=223926 GN=VP1026 PE=4 SV=1

MKIFIAKNPAEAHIVCELLKTEDICCEVRGEGLFGLKGELPFGDDTDPYVWLLDPEQQLK

AHSIIEAFRQQSQSNIYEDWQCPHCLEHNEGQFGACWQCGYQIGEP

>tr|Q87RU5|Q87RU5_VIBPA 3,4-dihydroxy-2-butanone 4-phosphate synthase OS=Vibrio parahaemolyticus serotype O3:K6 (strain RIMD 2210633) OX=223926 GN=ribB PE=3 SV=1

MPISTPQEIIDDIRAGKMVILMDDEDRENEGDLIMAAEHITPEAINFMATYGRGLICLTM

TKARCENLGLPPMVQDNNAQYTTNFTVSIEAAEGVTTGISAADRARTVQAAVAPNAKAAD

LVQPGHIFPLAAQDGGVLTRAGHTEAGCDLARLAGLEPASVIVEILNDDGTMARRPDLEI

FAEKHGLKLGTIADLIEYRNNTETTIERVAECKLPTEHGEFTLVTYKDTIDSQVHYAMCK

GDLAGEAPLVRVHLQDVFTDVLRSDRNAERSWTLDKAMKRIGEEGGVLVVLGNEESTELL

IHRVKMFEAQDKGEAPTLAKKQGTSRRVGVGSQILADLGVHDMRLLSSTNKKYHALGGFG

LNVVEYVCE

>tr|Q87IQ8|Q87IQ8_VIBPA Uncharacterized protein OS=Vibrio parahaemolyticus serotype O3:K6 (strain RIMD 2210633) OX=223926 GN=VPA0548 PE=3 SV=1

MKHWFFIVCGILSGCSSLNPDMVPGGNMLDTAPKTNTELRHPEWGYASASHVSTAPRVNQ

TARAAEKKSNNVTSLEMFLDRHNIPHETIGGGHLMIRLKEQVHFQTGSAQLSSNSQDWLR

KLGHYLAGRTDVDVVIDGHADSTGAASFNDTLSEKRAREVEKQMLASSIPRQRVFSRGFG

EYVPQCSNATSSGKACNRRVELMLIVDE

>tr|Q87M12|Q87M12_VIBPA C-di-GMP phosphodiesterase A-related protein OS=Vibrio parahaemolyticus serotype O3:K6 (strain RIMD 2210633) OX=223926 GN=VP2446 PE=4 SV=1

MPSQQLQHWFATLTSNSPFFFAILDKKHNYRMVSDRYCDIAGLNHEEIIGLNDCQVLGEQ

FYKKLAPYYQRAFKGVHVEAEITLDETDLETSLHFSLSPVYEGNEVRFVVFHAVDTSEKQ

ILVRSLEEAENKFAKLTQLLPDGLLLIEDDTIISANPASARLLGLNSPHELLGEELSRLF

IDENTKKVFSHRLSTLISDKPFVCLTSARCGFERKVQLHADSTAILGSESQIILIQDADD

TPKHLSSASSEDSHIDSLTKLYNRFGFTKRLEQLIKSQTPLLVFYLDIDNFKNINDSLGH

HIGDKVIQEVSARLKRLLPNQAIIGHLGGDEFGIILPEPENSRMAEVLSDRIISLINQPF

DLHHFSKRLACSIGSVRYPEDGQDARILLQNADTAMYEAKDRGRNRLIKFNDQMNKEARM

RLWLEIELQKALQQNGLEVWYQPKVNARDFSINGAEALVRWKHPVEGYISPGSFIPVAER

AGLIEHLGRVVMRDVFNTVKRWKQQGILPGRVAINLSPEQFGNPQLIDFMEKLLRTTELD

PSCITFELTESAVMSDSEHTLQMLNAIKKLGFALSIDDFGTGYSSLSYLARFPIDELKID

RAFINDIDALPKQVTVIENIINLGKSLELTVVAEGVETHQQATLLSNLQCNSIQGFHFYR

PQPKHEIEELFVQNRRHKN

>tr|Q87GS3|Q87GS3_VIBPA Cytosine permease/transport OS=Vibrio parahaemolyticus serotype O3:K6 (strain RIMD 2210633) OX=223926 GN=VPA1242 PE=4 SV=1

MAGDNNYSLGPVPNTARKGVASLTMVMLGLTFFSASMWTGGSLGTGLSFNDFFLAVLIGN

LILGIYTSFLGYIGASTGLSTHLLARFSFGSKGSWLPSALLGGTQVGWFGVGVAMFAIPV

HKATGIDTNTLILVSGLLMTATVYFGISALMVLSAIAVPAIALLGGYSVVEAVNSVGGIR

ELQQVQPTEPLDFSMALAMVVGSFVSAGTLTADFVRFGKKPRSAVMITMVAFFIGNSLMF

IFGAAGASVTGQSDISEVMIAQGLLIPAIIVLGLNIWTTNDNALYASGLGFSNITGLPSK

YISMANGLVGTLCALWLYNNFVGWLTFLSLAIPPIGGVIIADFFTNRKRYANFEAAQFQS

VNWAGIIAVAIGVGAGHFLPGVVPINAVLGGAISFLILNPILNKKVLATQPA

>tr|Q87TL1|Q87TL1_VIBPA Gluconate utilization system Gnt-I transcriptional repressor OS=Vibrio parahaemolyticus serotype O3:K6 (strain RIMD 2210633) OX=223926 GN=VP0058 PE=4 SV=1

MILLWILCLAFGGLSASCVFVIMLHVTLLINHIFMAQQNKKTRTTLQDVADQVGVTKMTV

SRYMRNPESVAEKTRVKIAAAIEEMGYIENRAPAMLSKSSSKAIGILLPSLSNQIFASFV

QGIETVTKANGYETLLAHFSYDELEEERKIASLLSYQVDGLILTESHHTPRTLQMIKNAG

VPVVETMELPPQPIDMAVGLDHEDASYNAVKRMLDAGKRTIVYFGARLDTRTKLRMQGYD

RAMNEAGLEPKHVLTGVHSSFSLAHDLLERALKTYPTLDGVFCTNDDIAIGTMLSAQQRG

IQVPQQLAVVGYNALDIGQTISPKLTSVDTPRFQIGVKSAELLIARLKGEAQEEKVFDMG

YQITSGESV

>tr|Q87GL4|Q87GL4_VIBPA Uncharacterized protein OS=Vibrio parahaemolyticus serotype O3:K6 (strain RIMD 2210633) OX=223926 GN=VPA1301 PE=4 SV=1

MKSIASYQIKFKVLFTLTCSCIFATACNSDNTSTEIQSKLLVEKDFANNSRLRANPEQGT

VILFLEPPSATVAADDFNGESGSDVIPYRYSRSLYHTFCYEDDNSNSKHSTVLNDSSGAE

VLRISANEECVSAVISEGEYHLVMTHGQHVDSTDITFLVTTPDNGSQTEINSINYSITSR

VLRSIGSLPINSAYADAADNNVTTLISTNACKDCDLSGADLSSATLTFADLSGADLSDAI

LTNVDLFESTLTGTNFSGADLSNGDFRSSEMAYTDLSNANLSGAYFSNAHLSPSNLNHAT

VIDTNFDYANLVGATWIDGGICDITSVGFCNSTDGGDATPCDSLQQGTSDDGNIVYKCLL

PTVDKEVCTTELGGPDGGTLITSCEAKDASELVTSVDLVDIFDQASSSFSVTLDNDTPMA

ILAWGGEGGIGSSGGLWTSGGDGGRGGFASTVTTLTDFLDNYGQTYFIFYIGENGTLSNE

YGDGGSSTLVMTVENSPTSLEDDVVLIAGGGGGGESSSFFVDGTDGSMGGIAASSIMGQG

TIGVGQSFTDGASGGSSNEWGDGGNGADSGKDGIGGQGGQGFLGRNSEWVNGDPVVGSDG

RGGNADDSFSAGGGGGGGGGYGGGGAGDGGAGAGGGSWSIIPTITCNSAPTQDTMPSSPG

SSGDDYGSKNGAVEVWIFPNGC

>tr|Q87J68|Q87J68_VIBPA N-ethylmaleimide reductase OS=Vibrio parahaemolyticus serotype O3:K6 (strain RIMD 2210633) OX=223926 GN=VPA0385 PE=4 SV=1

MKQRPNKLFEPAQLKALSLQNRIVMAPMTRARTTQPGNIPNEMMATYYQQRASAGLIISE

ATQISDDSQGYSFTPGVYTDAQVAGWQGVTQAVKLQGAAMFCQLWHVGRVSHPTFQNGEQ

PIAPSALAPVETQVWIADEQGNGNMVDCVEPRAMTQADINRVVGDFANAAKRAIESGFDG

VEIHGGNGYLIDQFLRTNSNHRTDNYGGSRENRIRFLIEVVDAVIAAVGAHRVGVRLAPF

ITFKDMDCPDIVPTILEASKQLQERDIAYLHLSEADWADAPTIPETFRIELRKRFRNAII

VAGRYDPQRANEVLEKGYADLVAFGRPFVANPDLVSRLQHHHPLAELDGSTLFGGNERGY

TDYPALQQECAEQA

>tr|Q87HC6|Q87HC6_VIBPA Uncharacterized protein OS=Vibrio parahaemolyticus serotype O3:K6 (strain RIMD 2210633) OX=223926 GN=VPA1039 PE=4 SV=1

MSIKEIGRVFTQRWFLGLVGVAACSIFIWVVGPLITVAGYEPLKSDFQRLVTILVIVFAW

ALINLTKQHKQKVREDESIQTLLEVDSQSDKEAASEIDVMRDRIEQAIKVVTKTHKGKRS

LYDLPWYVLIGPPGTGKTTVLKQSGLEFPLTESLGADSIAGVGGTRHCDWWFANKAVLID

TAGRYTTQDSQEKVDSKAWHGFLGLLKKYRTQRPINGAIVTVSLASVMSQTRTERSLHAR

SIKSRLQELKNQLGMQFPIYVLLTKMDLVAGFNEFFADLSKEEREELFGFMFPREVDDER

GVISLFNKEFHGMLERLDAHMLRILETEDDLEKRTLIFEFPKQLRVLQANLDEFLSEIFA

QNTFEEPALIRGVFLLSSVQEGIPVDRLMSESTNGLGLGRLPLATNVNSSHSYFVKNLFE

RVIFKEQLLGTVNRHYQKQSGWMRTGIYVGCVGVLVGASALWFLSYQWNSKLIVDTNSQV

NHIEAMIGAESLDFESDVISAVDTLDKIMMLPLGKNSKYGHSDAVKKFGLYQGDKVSQAA

NNAYSDALSQHFATLLSESLVSEMEANKQHREYLYETLKTYLMLFNPEKYQQEEVIAWFN

FYFERQYPGELNKELRERLLVHTKNLLENDEKGFSMDATAISAAREVLTQMSLPERAYQR

MKMQFAKSHVPSFRLTDVLGPKGLEQFERASGKPLSQGISGFYTYNGFHSIFQIQINRTV

KGLMEENWVYGDDLKAHEIDHDSAIQGVQARYYQDYVNEWKTLIEDIQLKQAPSLALATE

QSRVLSGVERPIESLLRAIQKEVGLSKVTLSENQKAATEVAGKVAKVKFSNTADKLDMYL

PEENGFNVALPGKEVESHFTEILRLSEQDFDDIQQAMVNLRSYLSDLSSSGNNQKIAYKS

ILDGTVTQDVAASFARAKDLLPKPFNQWLGELSQESVKFAESGSKDHLNQLWMTNVVRPY

QRTIAGRYPFEPNATKEVRLKDFQRFFGYGGTLDSFFQEYLEPFVDTSKSRWRLEKEIGV

RPETLAVFQRAKRIRQSFFESDNSLRVEFGMKPVYLDQHITRFVLELGDQDLVYKHGPAR

SKELRWPSGQDQTRIVFTPPESKREIAHTYEGEWGIFKLLDQSLKARPESRNDNIVMIDL

KGNKVQLELIPSSAINPFWSNEMERFRCPQTL

>tr|Q87FS2|Q87FS2_VIBPA Uncharacterized protein OS=Vibrio parahaemolyticus serotype O3:K6 (strain RIMD 2210633) OX=223926 GN=VPA1606 PE=4 SV=1

MAKLLQVDFEFHGPFGEEMSNTLVDLAKSINQEPGMIWKVWTESEKDKLGGGIYLFEDEA

TAQAYLEMHAARLRKMGVVEVRGQIFDINVPLTTINQGPIGG

>tr|Q87J81|Q87J81_VIBPA Phosphoenolpyruvate synthase OS=Vibrio parahaemolyticus serotype O3:K6 (strain RIMD 2210633) OX=223926 GN=VPA0372 PE=3 SV=1

MFLEKDMQKNTLWFNGLSMDDVDKVGGKNASLGEMVSNLANVGVSVPNGFATTSYAFNQF

LDHEGLDERIHQLLDELDVDDVEALRKTGATIRQWVLQAPFPADLEQEIRNNYEELIEGN

TELSVAVRSSATAEDLPDASFAGQQETFLNVKGIDAVLEATKHVYASLFNDRAISYRVHQ

GFDHRGISLSAGIQRMVRSDKASSGVMFTLDTESGFDQVVFITSSWGLGEMVVQGAVNPD

EFYVHKPMLEAGEHPIVKKTFGSKLIKMIYSNNQEIGKQVDIIDTSEEERNTFSLNEEEI

KELAKQAMIIEKHYQRPMDIEWAKDGIDGKLYIVQARPETVCSQTEQNVIERYELNNKAD

VLVEGRAIGQRIGKGPVRLVDSLDQMSLVQEGDVLVTDMTDPDWEPVMKKASAIVTNRGG

RTCHAAIIARELGIPAIVGCGDATSKLTDGATVTVSCSEGETGYVYQGDLDFEVKRSSVD

ELPLLPTKVMMNVGNPDRAFDFAQIPNEGVGLARLEFIINKMIGIHPKALLNFDAQSDEL

KAEIKQRIRGYKDPIDFYVSKLTEGIATIASAFWPKRVIVRMSDFKSNEYSNLVGGKAYE

PHEENPMLGFRGASRYISPVFEDCFELETQAIKRVRNEMGLKNVEIMIPFVRTPSEAASV

IDLLAKFDLRRGDQGLKVIMMCELPSNAVLADEFLKYFDGFSIGSNDMTQLTLGLDRDSG

DVAHLFDERNAAVKIMLKMAIDAATKAGKYVGICGQGPSDHEDLAEWLMEQGISSVSLNP

DTVIDTWLQLGKVSK

>tr|Q87M85|Q87M85_VIBPA RecBCD enzyme subunit RecD OS=Vibrio parahaemolyticus serotype O3:K6 (strain RIMD 2210633) OX=223926 GN=recD PE=3 SV=1

MTTNHNLHSGQDSLLVVLERLAHKGAIRQLDYQFACFIDSQTHDEQSDSQALAFIAGVVS

SELGKGHICLSLFDAQGQSTDLASKLGLFGESALALNTQLQGIDWIQVLKNSTVVGAQGE

ALPLMFDGERLYLHRYWHYEVTLAEKLNQLGAAVNLQAQEFSRLSELLNHLFARQYHFLF

NALGKAVEAGSSNQVLRQQLVCDHLDVVASESLDWLAIDSVLSNARKVQDLQTLNELVPL

SACVNWQKVAAAVALTRRFAVISGGPGTGKTTTVTKLLAALIEQATHEKNLTIKLVAPTG

KAAARLTESIGKAVQELPVSPELKVKIPTESSTLHRLLGAIPNSAEFRHNKQNPLHLDIL

VIDEASMVDLPMMYKVVDALPKHARLILLGDKDQLASVEAGAVLGDICSFHALGYGKEQA

SAIAKLTGFDTLAHTGNSASSIADSLCMLQKSYRFDARSGIGQLAKAVNSGSAASVDNVW

ARDFSDIEHFALSSQHYNQMMQTLVQEYGRYLKRIEQQETDPKTGEPESLTHKAKAVLDT

FNQCRLLCAIREGDFGVAGLNQRIEKALAARKFIQVQDEIWYHGRPVMVTRNDHGLGLYN

GDIGICMRDDSEEEPRLKVFFELPDGSVKSVLPSRVPEHETAYAMTIHKSQGSEFDYTLM

ILPPDFSPILTRELIYTGITRAKKRLALYAELNVLKRGIKVKTTRASGLVQRLTN

>tr|Q87ML3|Q87ML3_VIBPA Cytochrome c-type biogenesis protein CcmF OS=Vibrio parahaemolyticus serotype O3:K6 (strain RIMD 2210633) OX=223926 GN=VP2218 PE=4 SV=1

MIAEIGHFALILSLAMAVLLSVLPLWGASNNNTMLMNTARPLSWSMFIMLFFSFVILCWG

FYTNDFTLQYVASNSNSQLPWYYRLTAVWGAHEGSLLLWVLIQAGWTVAVATFSRGMPQE

SVARVLAVMGMISVGFLLFIILTSNPFLRTLPFFPVDGRDLNPLLQDPGLIVHPPMLYMG

YVGFSVAFSFAIASLMTGRLDTAWARWSRPWTTAAWVFLTLGIALGSWWAYYELGWGGWW

FWDPVENASFMPWLAGTALMHSLAVTEKRGTFKAWTVLLAISAFSLSLLGTFLVRSGILV

SVHAFASDPSRGMFILGFLVFVIGGSLLLFAVKGAAVRVRGNFDLVSRENALLANNVLLI

AALVVVLVGTLLPLVHKQLGLGSVSIGAPFFDMLFAWLMMPFAFLLGIGPLIRWKRDQLS

SIVKPMLVSGTMSLALAAVCVFLFADFFSVMAYIGWVMSIWIVAMHAFELHERATHRHSF

AEGVRKLQRSHWAMMFGHIGLAVTIIGIAMVQNYSIERDVRLAPGEHFQIEGYDFYFSGL

RDKDGPNYDGYIADFEITHDGKYINTLHAEKRFYRTAKSMMTEAAIDRGITRDLYIAMGE

RLDDNRSWAVRIYYKPFVRWIWAGSLIMALGGALAISDKRYRFRKSSKKNAKSNEQVA

>tr|Q87JH9|Q87JH9_VIBPA Putative flagellar hook-associated protein OS=Vibrio parahaemolyticus serotype O3:K6 (strain RIMD 2210633) OX=223926 GN=VPA0274 PE=1 SV=1

MRISDNQFSQMMLQSLQSNSAGLGKVLQQMSTRERLTKLSDDPMASIKLLNLERENSAIA

QYQSNIANLKTTLSSQETHLDSVNESLKSMRDIVLWGANGSLTDQDRSGMITELKSYRDS

IESSFNAQDEEGHFLFSGTKTDTAALNKSSGAYVVEGNSDVRVVTVAKGVTMDSNMTAQE

ILDIGGGKNVLNQIDALIAEFEKPSPNFQAEVDASLNAIDDTMANVLGAMTEIGGRHNNL

DLMDGAHSENKLFVDKVSGDLSALDYGEASVRLSNYMAALQATQASYVKINDLSLFDRI

>tr|Q87R97|Q87R97_VIBPA Putative oxidoreductase protein OS=Vibrio parahaemolyticus serotype O3:K6 (strain RIMD 2210633) OX=223926 GN=VP0900 PE=4 SV=1

MKTEHKQVVVVGAGPSGSTVSALLKSRGIDVVVIEKATFPRFSIGESLLPACMEVVELAG

MTEAVKQHGFQFKDGAAFRRNGVYTHFDFTDKFTAGPGTTFQVQRASFDKVLADSAAEQG

VDIRYQHELVSLAFEGKKSRLEVVGPDKQAYQIEADFVLDASGFGRVLPRLLDLEEPSCL

PPRKAIFTHIEDHISPQEPEYDRNKILISVHPENHDVWYWLIPFSNGTCSFGVVGEPEFF

EQYPEDKLAALQQLANEEPGLASLLRNAKYPNPVGELGGYSANVKRLATEHYALLGNAGE

FLDPVFSSGVTIAMKSAQFAADCVVRQLNGEVVDWQEEYSERLMVGVNTFRTYVEGWYNG

TLQDVIFYQAPNPRIKQMISAILAGYAWDTENPYVKQSEQRLSTLAELVRGEGF

>tr|Q87PA2|Q87PA2_VIBPA Uncharacterized protein OS=Vibrio parahaemolyticus serotype O3:K6 (strain RIMD 2210633) OX=223926 GN=VP1615 PE=4 SV=1

MLSAVRRANLYLNLFGFSKVPLIWLCHPKIIAIDSKHVEVRIPLRRRTKNHLNSMYFGAL

AVGADVAGGFLAMSKAQEKGEAISLAFKGVKAEFLKRPEADVHFVCNDGHVIDEMLDHTI

ATGERVNRDVKIIALCPSLHGQEPMAEFALTLSIKKAAQHTKKAA

>tr|Q87M34|Q87M34_VIBPA Uncharacterized protein OS=Vibrio parahaemolyticus serotype O3:K6 (strain RIMD 2210633) OX=223926 GN=VP2424 PE=4 SV=1

MFRLKLKGCHMERVTKAYWIGEPLHTLPEEFKAEFEQHFDLLDCSNDISVLSDKEFCYFF

YYITHDPTDHAKSIATLCENLDKKLIVVTKENTECCLPPSHITAECYELNENTLPVWLSQ

AKLKYFLHAKVDDAQISADNIFSRNGKSNFSDVVNYIANNVHKDLREEEAAALCHYSPTY

FSKVFHRKVGMCFRDYVTAKRISLAKKMLIENESMKIAYIAYQCGYRDVSYFSRIFKKKT

GLSPANYRQQF

>tr|Q87S49|Q87S49_VIBPA Phosphate transport system permease protein PstA OS=Vibrio parahaemolyticus serotype O3:K6 (strain RIMD 2210633) OX=223926 GN=VP0575 PE=3 SV=1

MLNWIRSGAPWIWLTGGAVSISLLSVLGLLLLIGWKGLTYFWPAPLYQWNVTSLTPVQGE

VLHENTILIGQIYERSFVPRSYLPVDAVKKLGEDEDFATRLNIKIANRELYPADFISVLQ

MQLDEPTTPKEWAVIERSSGGYFFGKLVAFQDGDKLYQTDIQTVLNKKLDDAETLRHEID

SLVVDQLKDLGWKLEQLRLDKRKHELNNTVTDDFLAQNLQQKEQVEQELAKLDLQLDGLR

LQLSGYALIVEDMTGSHVSIPLEDILDYWYPNQMSLPDKVMHWGKQVWKFLSEDPRESNS

EGGVFPAIFGTVFLVLIMSIIVMPLGVVAAIYLHEYAKNNALTRIIRIAVINLAGVPSIV

YGVFGLGFFVYTIGASIDNVFYAERLPAPTFGTPGLLWSALTLAVLTLPVVIVTTEEGLT

RIPSSVRHGSLALGATQFETLWRVVLPMATPAIITGLILAIARAAGEVAPLMLVGVVKLA

SSLPVDGQFPYVHLDRKFMHLGFHIYDVGFQTSNIEAARPLVYATSFLLVTVIVGLNLTA

ISIRNNLREKYRTLGQD

>tr|Q87N80|Q87N80_VIBPA Macrolide export ATP-binding/permease protein MacB OS=Vibrio parahaemolyticus serotype O3:K6 (strain RIMD 2210633) OX=223926 GN=macB PE=3 SV=1

MSNKSLVELTNICKYYSSGEAEVRALDGVDLTIHQGEFLSILGPSGSGKSTLMNMLGCLD

KPTDGEYQLGGQNVASLSANELAGIRNQKIGFVFQSFNLLEYATALDNVALPLVYSGIKA

KERRQRAAKLLEQVGLGDRLDHKPNQLSGGQKQRVAIARALVNDPQIILADEPTGALDSK

SGAEIEALFNQLHAEGRTLIIVTHDNALAERTKRIITIKDGKVISDRAPIGKENAISHC

>tr|Q87S69|Q87S69_VIBPA Chorismate mutase/prephenate dehydratase OS=Vibrio parahaemolyticus serotype O3:K6 (strain RIMD 2210633) OX=223926 GN=VP0555 PE=4 SV=1

MTDQPISLEEIRLRLNELDDQLLSLLSERRKLSIEVAKSKVQTSKPVRDAVREQQLLVKL

ISNGRDKYELDAQYITKLFHTIIEDSVLLQQGYLQNLVNPQQSRKPLARVAFLGAKGSYS

HLASREYFSRKNTELIELNCEHFKEVTRTVESGHADYGVLPIENTSSGSINEVYDLLQHT

TLYIVGELTQPIEHCLVATKDIRLEDIKTLYSHPQPHQQCSEFLSRMKGVKLESCASTAD

AMQKVQEMNRDDVAAIGNASSGKLYGLQAIQGNIANQTENHTRFIVVARKPVEVSTQIPA

KTTLIMSTSQEAGSLVETLLVLQRYGINMTKLESRPIMGNPWEEMFYVDLEAHLGSTEMQ

QALQELTKITKHLKVLGCYPSENIKPTQVKLS

>tr|Q87FY5|Q87FY5_VIBPA Putative flagellar motor switch protein OS=Vibrio parahaemolyticus serotype O3:K6 (strain RIMD 2210633) OX=223926 GN=VPA1535 PE=4 SV=1

MNTAQLTSKQTLSYVEQTALVLLGMGEDAAAKVLQHFTRDETQRVTRAMAKLNGIKSDSA

RGVIQNFFEDFREHSGIRGASKEYLSNTLRKALGNDLAKGLLNNLYGDEIRNNMQRLQWV

EAETLARFIVNEHPQMQAIFLAYLPADSSSAVLKHLPQDYHDEIIFRIAQLQDIDHQVAT

DLHELVERCIEKVSASQSVPLSGVKQAADIINRFEGDRGSLMEMLKLHDEEVVNAIEENM

FDFMVLGRQREETMDMLVQQIPLELWATALKGSDITLQQAIKRSMPQRMVKALEDDMEAR

GAVALSRVQKARQDIMQMVRELDESGEVQLLLYEEPTVE

>tr|Q87K01|Q87K01_VIBPA Putative secretion protein OS=Vibrio parahaemolyticus serotype O3:K6 (strain RIMD 2210633) OX=223926 GN=VPA0097 PE=4 SV=1

MSGDIAKHSSSRSKLVKTLFFTLCVIAFGAMYWSWQYSDSHPSTEDAYVRAKILSVAPQV

KGQVVSVDVKDFQSVNKGDLLLKIDSRPYLLAVKQAKAAYQLAVQQHDVADKQVTEAVAG

LDAARSNLTEAQVEYKRTDSLVKRKLASDQDLDTAKNKLANAQASLEQARATVEKAIANR

GEEGAEAAVVQQAAAQLAQAELNLSYTDITSPVDGIAGEINTHVGSVVSVGQTLFPVILK

DSYWVRANFKETDLTHIKPGMHADVVIDMYPDVVWKATVEQLSPASGTSFSLMPPENATG

NWVKIKQRFPVRLALEVPADAPQLRVGASSEVTIDLQSSVQ

>tr|Q87FT9|Q87FT9_VIBPA Transcriptional regulator, LysR family OS=Vibrio parahaemolyticus serotype O3:K6 (strain RIMD 2210633) OX=223926 GN=VPA1589 PE=4 SV=1

MKALNDLNIFVETARQGSFSKAANSMDMTPAAISASIKRLEGQIGFPLFVRSTRSLRLTS

EGELFLDKTTQALATLQEGLDQISSARGELSGQLHITAPSDFGRNMLLDWVDEFIDLYPN

VSIKLELSDSLTDMYTKPVDIAIRYGEPADSNLVVLALCGSNERILCASPEYVATHPELT

SPADLTQHNCLCYMVADSVYNKWTLTRDGEKEQVVVSGNLMSNDSDLPHRLAIKGKGIAN

KSLMDISRDLIEGRLVRVLPEWDSGPVPLYMVCADRRLLTPTIRTFRDFIQQKCCQQRAN

VLATFCH

>tr|Q87NI7|Q87NI7_VIBPA Uncharacterized protein OS=Vibrio parahaemolyticus serotype O3:K6 (strain RIMD 2210633) OX=223926 GN=VP1881 PE=4 SV=1

MIRFELGNQICVCLSENDISLEINNTLHHAIPVSSNEYAILSTIATYGSLNAPISQRVIE

RKITQHYKMALPENGFKNAVAALRKKFRKLTEDHVSPTRNIIENIHRTGYFIPFTMLHTH

QSGIYQQKRINQHTKNSVRKALRICLRNKRIYTDIAWVLLVTTAIFFSVCYYAINSIVKH

NYLDSALDIADSLSQMSCYADEDQLKGLFDNVKLVESSMMLDRFNIRCLVTPEAVVPVSQ

KAFNEWSDNSNYTTQSFDINNATILVRVKNINLQNNVESHISRFFLSGMKLYTNTGTSFE

IGNTNGRYFHYQIKDTGYKEVYYISGPLKSIILLSLFFLVILRHRSLQAFITYLFAIREF

HIKLEPIYNTSTQQNIHYEALSRFKVKNTQRFIETLISNGLLLIHTILVIRAIYAKQPTL

LVPISINVCPSLLRGRNFSTLYQELASRDCRLLTIEITENASMYYTSEIYDNVAKLKLLN

CKISIDDFGTGNNNVSLISKINPDYLKIDREFVIGLKSDDKKVETLRQLIAMGNTYRCTV

IVEGVETADSAHLLTTLGAYIHQGYFYPLHF

>tr|Q87TH5|Q87TH5_VIBPA Uncharacterized protein OS=Vibrio parahaemolyticus serotype O3:K6 (strain RIMD 2210633) OX=223926 GN=VP0094 PE=4 SV=1

MQWILEHQSLILSSLFAATASGVAVGWWVKQRFITQTQLLEHQLVSEKQLHQQQLEQVKQ

SLADAQQELDELDNERDKAAFEVRQSHGKLMAAMEKLRYFEAVKQERQQYFDELGQMREQ

KSRLETQLREQQARHEQMNQANAEKLQILEQAEVRLKQQFEHLANQLFEEKTAKVDLQNR

QSLEGLLSPLKEQLEGFKKQVNDSFSQEAKERHTLVHELKNLQRLNEQMTREAVNLTQAL

KGDNKQQGNWGEVVLARVLAESGLREGHEYETQVNLQSEAGKRYQPDVIVHLPQNKQVVV

DSKMALVAYERYFNAETDAERDRALNAHLTALRAHIKGLSMKDYHKLKGIQSLDYVLMFI

PVEPAFQVAIQADPSLIKDAMEQNIILVSPTTLLVALRTIDNLWRNERQNENAKLIAQRA

TKLYDKLRLFIDDMEGLGGALDRANQTYQGAMNKLATGRGNVIRQAESFRQLGVEIKRPI

SSDLAQLAQSDAFSENESPNESLVERHPAEDKVN

>tr|Q87IH9|Q87IH9_VIBPA Ubiquinol oxidase subunit 2 OS=Vibrio parahaemolyticus serotype O3:K6 (strain RIMD 2210633) OX=223926 GN=VPA0627 PE=3 SV=1

MEASRYKRILSRGSLACVILLLSGCNSALLDPKGAIGVQEKELIITALLLMLIVVIPVIL

MTIYFAYRYRASNTDEEYAPEWSHSTKIEVVVWTIPIIIIAILATITWRSTHELEPSKPL

VSDVKPMTIEVVSLDWKWLFIYPEENIATVNYVAFPKDVPVTFKLTSDNIMNAFFIPRLG

TQIYAMPGMVTKLNLIANHEGDYKGFASNYSGEGFSQMKFTASAMPDRAAFLNWVQKVKA

SPDRIEDWEQYSSLAEPSVAAPVTLFSSVPPFLFSNVVTQHPGSMNCLPENQG

>tr|Q87GN1|Q87GN1_VIBPA Uncharacterized protein OS=Vibrio parahaemolyticus serotype O3:K6 (strain RIMD 2210633) OX=223926 GN=VPA1284 PE=4 SV=1

MRTLGNIIWFLFGGVFMGLLWWLFGILAFISIIGIPWGRACFVMGNFSFFPFGKEAISRD

ELTNEMDIGTSPLGVIGNVLWFVFAGLWLAIGHVLSAAACFVTIIGIPFALQHLKLAVIS

LAPIGKTVVTSEEAAIARYNINR

>tr|Q87GM7|Q87GM7_VIBPA Uncharacterized protein OS=Vibrio parahaemolyticus serotype O3:K6 (strain RIMD 2210633) OX=223926 GN=VPA1288 PE=4 SV=1

MEHVGSRALIVEGGAMRGVFSCGILDHFLAADFSPFDSFWGVSAGASNLAAYLAKMPGRN

LKIYLDYSLRNEFITPSQLIRGGDVMDLDWMWQVTLEELGIDKEVLAADPRPFFLVVTRQ

DTGQAEYLTPDVDMLAETMKASSALPVLYRNGVLLNDTRYVDGGVADALPIAEAIKRGAT

KIMVLRSQIASYRKPRSKFSAITKRMLKETPALIEPMLTRDVRYNQTLELINNPPPGVEI

IQVCPPETFKLKRLSRLPEPLREAYELGIEAGKQAIERWNSM

>tr|Q87QK2|Q87QK2_VIBPA Uncharacterized protein OS=Vibrio parahaemolyticus serotype O3:K6 (strain RIMD 2210633) OX=223926 GN=VP1147 PE=4 SV=1

MKYSYVARQPILDREKRTIGYELLFRDGPKNTFPEVEPELATSRLLSDHFLSTHYNTLGN

KLGFVNFPHQSLVNLVPTLFPKDSLVIEVLEDCEPTQELLDAIKHLHECGYRIALDDFVP

TKAWKRFLPYVSMIKFDIRLVPIEKAAIFIQALSQFNIDFLAEKVETYEEFEQALDAGFN

YFQGYFFSKPEMIQKKRLNPAFLTVIQLCKEIADKPIDFNEVERLFSIDVTLSYKLLTYV

NSGYTLTTKIKSFRQALIYLGEERLRRFISLVAIASVQEDKPDSLYSLAIQRARMCEILL

SQMKTKYDPGQAFLTGMFSLLGSLLDQPLSDVIEDIPVDEDIKLALTSRKGVLGYLLSMC

IAYEQADWELAERYCSVLKLTETQLADAFNESTEWAQELLSQTP

>tr|Q87R70|Q87R70_VIBPA Isochorismate synthase MenF OS=Vibrio parahaemolyticus serotype O3:K6 (strain RIMD 2210633) OX=223926 GN=menF PE=3 SV=1

MSEFHQAVKRIIERVQHTEGNQVRLVEPLSEKPNFAFIDWLDAQPLFPKFYWQSRDTREE

VVALGQLHSFVEPGPAYTILGEGQRVWGGRSFDGQHEKNRRCMPSFFFLPQIELIRFDQQ

WSLAVNLTEDKARTLAGLRKLICDVAALPPISSHIRSIVHTPVKSEWDALVHKVLTGIEN

DEFKKVVLARRSTVQLDNRLSAAQLLKASYLNNHHSFHFLLSLDSKHSFMGSTPERLYSR

VGHELHTEALAGTIGRGDNATHDMELANWLSQDSKNLNENQYVVDDIVERLSPHSEIVEV

ETEPRLVRLRKVQHLKRNIHASLKTGTNGVQLLSALQPTAAVAGLPRKESMQFILDNEPF

ARGWYAGSMGYISHERAEFCVAIRSALVLGDQVQLFAGAGIVPGSVAEHEWAELDKKMST

LLTLISDHPPLGVAS

>tr|Q87I80|Q87I80_VIBPA Putative TadC OS=Vibrio parahaemolyticus serotype O3:K6 (strain RIMD 2210633) OX=223926 GN=VPA0726 PE=4 SV=1

MVDIKLIFLALGLIISLSAAYYSLKVFDKTKANISTKKIIDSTQEKTPFVDKLSLHLSRL

SFNKEETKNNLVRAGIHSEFIAQAYYLLKIIPLVFCLAFIGYSYIQGEMEFSSAFISFSL

SMLVCLIAPDMYISSRGNANVRHVSSRLPFLLDLMNVCVHTGMTLEASLDYLSNELKTVD

ENLAYVVKKTSERAKIVGLDRALTEFYDLVPTTEAQSFVMTLTQSLKYGSSVGAVLASLA

SDIREINMLELEEKIGKMGAKMSIPMIAFIMIPIVVLIAAPGIMRMLG

>tr|Q87N84|Q87N84_VIBPA 5-methyltetrahydropteroyltriglutamate-homocysteine methyltransferase OS=Vibrio parahaemolyticus serotype O3:K6 (strain RIMD 2210633) OX=223926 GN=VP1991 PE=3 SV=1

MKTLLPTSTAGSLPKPSWLAEPEKLWSPWKLQGEELIDGKRDALRVALQEQELAGVDIVS

DGEQTRQHFVTTFIEHLSGVDFENRKTVTIRNRYEASVPTVVGPVSRTKPVFVEDAKFLR

QQTKQPIKWALPGPMTMIDTLYDDHYGSREKLAWEFAKILNQEAKELEAAGVDIIQFDEP

AFNVFFDDVNEWGIACLERAIEGLKCETAVHICYGYGIKANTDWKQTLGTEWRQYEEVFP

KLQQSNIDIISLECHNSRVPIELLELIRGKKVMVGAIDVATNEIETPEEVANTLREALKY

VDADKLYPCTNCGMAPLSREVSTAKLNALSAGAEIVRRELSA

>tr|Q87SM8|Q87SM8_VIBPA Haemagglutinin associated protein OS=Vibrio parahaemolyticus serotype O3:K6 (strain RIMD 2210633) OX=223926 GN=VP0394 PE=4 SV=1

MMNLFQDDAVTWLSTLDAASVDLLITDPPYESLEKHRKIGTTTRLKVSKASSNQWFDIFP

NDRFEALLSEVYRVLKNHSHFYLFCDQETMFVIKPIAEKIGFKFWKPIVWDKVSIGMGYH

YRARHEYILFFEKGKRKLNDLSIPDILTHKRVYRGYPTEKPVSLLEVLVAQSSRDGELVV

DPFFGSGSTLVAAKNLNRRFKGNDISQSAHEHIHQRMDFGA

>tr|Q87TN0|Q87TN0_VIBPA Uncharacterized protein OS=Vibrio parahaemolyticus serotype O3:K6 (strain RIMD 2210633) OX=223926 GN=VP0039 PE=3 SV=1

MKKWTFFMLLIAVLLFGSVIGFNLFKQQKIAEYMANRPEPEFPVTVTEVKAVDWVPVIEA

IGFIEPNQGVTVANETSGVIDKIAFESGTQVEAGQPLVLLDSEVEKANLKSSQAKLPAAE

AKYKRYQGLFKKGSISKEAYDEAEANYYSLKADIESLKATIDRREIKAPFAGVVGIRNVY

LGQYIQAGSDIVRLEDSSVMRLRFTVPQTDISRIKLDQEVDIFVDAYPDQPFKGSISAIE

PAVNVQSGLIQVQADIPNSDGKLRSGMFARANIIMPKLANQVTLPQTAITFTLYGDNVYI

VTEEEGEKRVKQHVVKVGERTKDIAHILAGVKPGDVVVTSGQVRLSNHAKVSIVESNAIT

PPAETPML

>tr|Q87S14|Q87S14_VIBPA Uncharacterized protein OS=Vibrio parahaemolyticus serotype O3:K6 (strain RIMD 2210633) OX=223926 GN=VP0610 PE=4 SV=1

MELYDTEEQQVEAIKDWWKENGKAVIIGAVVGLGGLFGWRYYQDTVIQASETASQSYTTA

MNTLQEKGVDAQSDVQAFIESNEVKEYSVLAALQLAKAQVEAKDFAAALEQLKWAQSNTK

DAALSPLISYRIARIETEMGNFDAANTELGKVTDTAWAGRIAELRGDIALRQGDKDAAYA

AYTEAQQAADASPTLQMKLDDLAK

>tr|Q87G31|Q87G31_VIBPA Ferredoxin-type protein OS=Vibrio parahaemolyticus serotype O3:K6 (strain RIMD 2210633) OX=223926 GN=VPA1486 PE=4 SV=1

MKSSQSKTSQSRRRFLRDTVRTAAGVGAAACVLGLQSLQSQARETKGVPIRPPGALPEGD

FESACIRCGLCVQACPYDTLKLATLLSPVATGSPYFTARDIPCEMCEDIPCVVACPSGAL

DPTLTDIDDARMGTAVLIDHETCLNWQGLRCDVCYRVCPLIDEAITLEMVHNDRTGYHAK

LIPTVNSEVCTGCGKCEQACVLDVAAIKVVPTDLAKGKVGSHYNFGWKEIDKPLENILPS

ETPVPEGALESLKGGK

>tr|Q87G84|Q87G84_VIBPA Putative two-component sensor kinase OS=Vibrio parahaemolyticus serotype O3:K6 (strain RIMD 2210633) OX=223926 GN=VPA1433 PE=4 SV=1

MMYFMFAHWRDNTFLFSAIVIVGAVVASWLLDQLFDSTAAVLLILQLAVVVVAFQCNSRF

AYAAAVIEALSFNFFFTTPRYSLQMFRPEDIFNLVVFMVVAFITSTFADLYRRQQGELKQ

TKLQNSILLSVSHDLRTPLATIIGTLTTLNEYMPKLNDLERKELLDSATSESHRLHQYIE

NLLQATKLQHGTLKITKKDEPIANIVRDAVSRLPNYTEKVSMNMDDSVGYLSVSRSLIEQ

AIFNVLDNAMRFSPENESVEVSLSKQGLSCVIDVRDMGIGITAEDAEKIFSLFYSGANNK

SADSGTGMGLAVAKGIITAHQGEIQSMPVSEGTLIRIRLPLNQGAEQA

>tr|Q79YY2|Q79YY2_VIBPA Bacteriophage f237 ORF1 OS=Vibrio parahaemolyticus serotype O3:K6 (strain RIMD 2210633) OX=223926 GN=VP1551 PE=4 SV=1

MSWCPSPTRPLNNVSTWGCLMTTATNILKSFDEQSVHIDYLCFTFAVKDLRHCHDAVRRL

HKHEEYKGFAKSGLLQRHCRAPKFPAPPVFNPTVAQTSDEIDAYNKAFDICYRNYLEDCL

RIFTNQVLGLSLSAPRGLGFQFYTESMKLTSPDGEDFCGFVGIGGNNDTVHFQINGTGCK

HVFARRPTWSLHDWLTNVLGVQTLARVDLAYDDYDGIFDCEYAYKAWRDDCFRTAERGRG

PVLHEDMTIASIGKDGKPIYTKEQYSIGSRTSRIYWRIYNKALEQKLANTGLVWYRSEVE

LKKWNVDVLLNPAGAYAALNDFAASISTAKKFNTKPVPTKRAALDLLASAHWMRRQYGKI

LNSLIEFHEGDIETVVGSLVRDGTKFTFPDTYGKLVTHILET

>tr|Q87NX7|Q87NX7_VIBPA Sodium/alanine symporter OS=Vibrio parahaemolyticus serotype O3:K6 (strain RIMD 2210633) OX=223926 GN=VP1741 PE=3 SV=1

MNDLQSLLQTIDNFVWGPPLLILLVGTGVYFTFSLGLIQFKHLPTALAMVFSKDKSSDKQ

GDVSSFAALCTALSATIGTGNIVGVATAIKLGGPGALFWMWLVALFGMATKYAECLLAVK

YRRVDDNGQMIGGPMYYLQYGVGSKALAIMFAVFSLGVACFGIGTFPQVNAILDASEISL

GVNRELAAFILTLLVAFVTLGGIKSIASVAGKVVPAMALFYVLACLSVIIMNADQLLNAV

ELVLVSAFTSTAATGGFLGASIMLAIQSGIARGVFSNESGLGSAPMAAAAAKTDSCVKQG

LISMTGTFFDTIIICTMTGLALILTGAWQSDLSGAAMTTHAFAVGLNAETFGPMLVSIGL

MFFAFTTILGWNYYGERCVVFLMGTKAVLPYKIIFLALVASGAFLHLDMIWILADIVNGL

MAIPNLIGLIALRHVVLAETKLFFNPSVQSDDLDAVKA

>tr|Q87NX1|Q87NX1_VIBPA Putative amino acid transporter OS=Vibrio parahaemolyticus serotype O3:K6 (strain RIMD 2210633) OX=223926 GN=VP1747 PE=4 SV=1

MSNIAKSAVKLSVFSVIMITVTSVDSIRNIPGAALFGSHAISFFLLAGLCFFVPTALVCA

ELSTTYPQQGGVYLWGKETIGPNFGFATVWYQYAENIVYYPPLISFIVATGAYPFFPELA

QNNIFMLIMINVIFWALTLVNIFGLRLSSMITNVFGTLGLIFPILLIIGLGGYWAYTNPG

ESHISLSHVSDWLPDFSQDGIGAGFTAVVLSLTGLEITTSYASEVENPQKAYPKALLAST

ALILVSLTACSLSISSVVSSDHASLSEGVILAFKTFFDDLNLSFMLPVIALAIVFGTLAS

LNNWIIAPTKSLHVAAKDQFMPLALSKENQNQAPVALLLLQGAIVSVLSLVFILVPNVNQ

GMWLLNILMTQLYMVMYVCIFISFLVSRRKHANIERPFRVPGGKVGMSVVAGLGLISCMI

TIVVSFDVPAGISAETGAYALVLGFIAFSLPAIAAVMYRNRKVRSQAQLIEALAS

>tr|Q87LD7|Q87LD7_VIBPA Magnesium transporter MgtE OS=Vibrio parahaemolyticus serotype O3:K6 (strain RIMD 2210633) OX=223926 GN=VP2675 PE=3 SV=1

MAEQIEFDQAHQALQEVTEALENGRFVHVRRQLQDMEPEDIAHLLEASPRRSREVLWQLT

DPEDYGEILDELNEDVKDSLVSKMAPEDLAEATEGMDTDDVAYVLRSLPDDLSREVLSQM

DSADRARVETALSYPEDTAGGLMNTDVITIRGDVDVDVVLRYLRMKGELPEATDALYVID

DESKLIGELPITTLITTQPDVKIIDVMEDADDAITVDTSDSDVASLFERRNWVSAPVVDE

NQHLVGRITIDDVVDVIREDAEHSMMSMAGMDDDEDTFAPVFKSARKRSVWLGANVLAAL

AAASVSNMFEATLDQMAAIAVLMTIVPSMGGVAGNQTVALVIRGLALGHIGDSNKRELLM

KEAAIGLLNGIMWALIIGGIVVVWKGNWMLGGIISAAMLTNLFVAGVAGVTIPVLLKKMN

IDPALAGGMALTTVTDVIGLSVFLGLATLLI

>tr|Q87R17|Q87R17_VIBPA Rec2-related protein OS=Vibrio parahaemolyticus serotype O3:K6 (strain RIMD 2210633) OX=223926 GN=VP0981 PE=4 SV=1

MTLLEKSWTLALFVASVISSAWWPTMPDWRWLLLGIITTGSIIKLRRGLISIGVIVGFMV

VIVHGNIMEYQRQALFQAGENSTIIGRVDSSFTQISHGYEGVVAIKQVNSHTLLPFLKPK

VRLITPFPLAVNSEFTTNVLIKPIIGLRNEAGFDAEKQSMGSGVVARAVVTKDSYWVIRT

SSSWREAIIQTVERDISRLEHFALIKALAFADRTGLTKEDWQSLRDSGLLHLVSISGLHI

GMALTFGLALGGLIRLAMPRYWFLPSVSGLAFAIVYAWLADFSLPTTRAVSVCIIYIALK

YWLVHWSPWRVLLLAVALQLFFQPFASFSLSFWLSYLSVGAVLFAVNTVQDSKEGRLGKL

RILLLTQLILSLLIVPISGYFFSGFSWSSLVYNLVFIPWFGFVVVPIMFAALIASLLFPM

LATVLWYLLDIFLVPLSWSVRYAIGTWQPISAEWTFVIAVVSAVLVSRHVMPRYVWMFVC

VIVVMTGLFPKQYNQTWRIDVLDVGHGLAVLVEKEGRVLLYDTGKAWQNGSIAEQVITPV

LHRRGYSSVDTMILSHADNDHAGGRKVIEQYFSPKHKLSSQSFLHYQPCIAAEKWKWQGL

NMEVLWPPKPVVRAYNPHSCVISLEDPSTGFKMLFTGDIEAISEWILLREPEKLRSDVML

VPHHGSKSSSNPKFINVVEPSLAIASTAKLNQWGMPAPEVVQAYTDSGVSWLDTGSDGQI

TILLDGNNWRFESKRRETIEPWYRQMLRNRVE

>tr|Q87IV5|Q87IV5_VIBPA Mannitol-specific PTS system enzyme II component OS=Vibrio parahaemolyticus serotype O3:K6 (strain RIMD 2210633) OX=223926 GN=VPA0501 PE=4 SV=1

MKANMRANVQAFGGHLTAMVLPNIGAFIAWGFITALFIPTGWMPNEAFGELVGPMITYLL

PLLIGYTGGQIVGEKRGAVAGAIGTMGVIVGADIPMFVGAMIMGPLSAWVVVQVDKRIQH

RIPSGFEMVVNNFSLGIVGMLLCLFAYEIVGPSVTAANLFVKSGIEALVATGFLPLLAII

NEPAKVLFLNNAIDQGIYYPLGLQAAAETGKSIFFMVASNPGPGLGMLLAYAKFGQGLSK

RSAPSAIIIHFFGGIHELYFPYVLMKPIMIVAMIAGAATGIATFNLFDAGLVAGPSPGSI

FSYLALTPKGSFIATIAGVTSATIVSFLVASAILKVSKSEEKESEFEKSVSDMKEMKAEG

AVKPVAAAATTQEPSKPISFVAFACDAGMGSSAMGASTFKRKLVQAGIDIEVKNFAIEKV

PSEADIVVTHESLESRAVNATGLPVVTIKNFLNDPALDELMDKIKQQTVATEQPA

>tr|Q87K32|Q87K32_VIBPA Uncharacterized protein OS=Vibrio parahaemolyticus serotype O3:K6 (strain RIMD 2210633) OX=223926 GN=VPA0066 PE=4 SV=1

MSQVRIKFIASDMDGTLLDQYGRLDPEFFDLFLQLEEQGILFSAASGRQYYSLRDTFAPI

KDRVLYVAENGTLVMYQDKELYSCTIPKAEVAEIVKAAREIDGANIVLCGKRSAYIETHD

QQSLEEFQKYYHRCETVTDLLEVEDEFIKVAICHFDGSEELLFPTMNAKFGATHKVVVSA

KIWLDVMNAEASKGAAIKHLQETMNFTPAETMTFGDYLNDLEMLQVSEHSYAVANAHEEI

KKIARYSAPSNQEAGVLKVIKEKVLAK

>tr|Q87QH9|Q87QH9_VIBPA Peptide ABC transporter, permease protein OS=Vibrio parahaemolyticus serotype O3:K6 (strain RIMD 2210633) OX=223926 GN=VP1170 PE=3 SV=1

MFWYTVRRFNLFFITLMILTLVGFSLLRLDPLSHWANVEFWSGWQSYLINLSQLDFGLSK

NGNAIYDELAVVFPATLELCFFAFLVSLLIGIPLGTIAGMKQGKWLDTGISFLSMSGYSA

PIFWVALLLIMVFSLEYHIFPVAGRYDLLYEIDHVTGFAIIDAFMAKGEYRAHALQSVIE

HMVLPCLVLALAPTTQVVGLMRASVAEVMSQNYIRAARIKGLSNREIVTQHVMRNAIPPI

IPKVGVQLSSMITLAIITESIFNWPGIGRWLLDALANQDYASIQAGVMVLATLVLTANIL

SDLIGAMINPLVRKEWYANK

>tr|Q87KF1|Q87KF1_VIBPA Thiamin-phosphate pyrophosphorylase OS=Vibrio parahaemolyticus serotype O3:K6 (strain RIMD 2210633) OX=223926 GN=VP3026 PE=4 SV=1

MAKILIPSSLIPLTGAVLQCLLLAKEQGFSIDEIELGVSPTQFIQLVLGQNTFRVGTDLI

DVCEEAETADFVLYYQSGLSVSECRQQPSSAIFIGIQDVESKLDDSVKTTSADVLDIWRH

PVNDEIRALSVASTSRTTTLQTDQHLAWTVTLLALDFPIEDALTLARPMTNVSRETLING

ETMVKQEWASQFADFPTPVLEDCRLGIKVGWSSHGQSVNFPHLSKQSLGLYPVVDDVSWI

ERLLPLGINTIQLRIKDPYQPDLEQQIARAIELGRQYDAQVFINDYWQLAIKHGAFGVHL

GQEDIEDSNLSQLSTAGICLGLSTHGYYELLRIVQINPSYIALGHIFSTTTKQMPSKPQG

LVRLALYQKLNDSIPYGESVGYPTVAIGGIDQSNAEQVWQCGVSSLAVVRAITLSESPKQ

VIEFFDQLMNTTSTSLVMEDYRAY

>tr|Q87H61|Q87H61_VIBPA Uncharacterized protein OS=Vibrio parahaemolyticus serotype O3:K6 (strain RIMD 2210633) OX=223926 GN=VPA1104 PE=4 SV=1

MQTPRLFNLNLRYKTALYFSLGLLGMIISFLFISRYFFLYSLDELENMEIDHSSQQAIAV

IDMMVTQQEGSSYDWAYWDETYDLFAHRDIAGYSERNLYVETLDALNLDLMSFITLDGQS

LVSLSRENTPESSSSLNNKVVSVPLLQQHILAMNSKLDVHRESLAGIFKINDNIWGLSLA

PVRNSEGDRPSNGWMLWGRNLSERFPGDFKAMMSASNTLVTKSFNPVDHAKTKSIDKTSE

SIIKWTPISDLAGEPIAWLKTQTKREHYSKGNTLFIYLFATVGVVASAIATSTFFIFKRK

VATRFSHFAEGINEIASQYQLEGLQSVKFDELELATKLVQKLSENTSMTQLQLKDSMEKF

GALYHSSSLGMLIVIEREIVDANHRALELLSYKKSDLLEQSLDTLCPTTADECRVDAMYR

ELQHGRNQFEAQMLDSHGETIDCLIEATLIQHNGHTALMLLLQDLSENKQQAEMIQKLTD

FDPVSGFCNRPVILGALEELVKHQPNHFSFIYITSKSLKQIAEVYGHLIFDEAIQYISTL

LRDHLGTHQIGRISEHEFIVILPNASGHSDALEAANRLLNQLSYKIELSGIMLSLDSKAV

MVDPKITHQSLEHLLLVARYSAQSMSGRHMHEVLVADGELSEQAETSMIIHRDLEVAIRQ

DNIIPYFQPIVNAKSGEIIGFEALARWPHPTLGIISPLIFIPLAEQGKLIVELGESILNQ

SCDFIGKLNTKRHSQGLPPLTVHVNLSAQHFYHSRLISFLQSMIEEHNISAGHLVLEITE

SMLMGGETESIHCINEIKQLGVQLALDDFGTGYASFSSVCNFPLDIVKLDKSYVDEIETN

DRAKTLVRNIANMSQELGLTIVAEGVETASQVRKLKVWNIDELQGFYFYKPMTREDAFAQ

FSGLPHC

>tr|Q87IE7|Q87IE7_VIBPA Iron(III) ABC transporter, permease protein OS=Vibrio parahaemolyticus serotype O3:K6 (strain RIMD 2210633) OX=223926 GN=VPA0659 PE=3 SV=1

MRDSTKIVLLGVIALVFAFLFIGIGLNADNYQYFLSRRVPKVLAIVLAGVAIAQSSMVFQ

TITHNRILTPSIMGFDALYVLTQVLIVLLFGGLSTLVLNIYVNFTIAACIMVAFSLLLFG

FYFSKGSRNLITLLLVGLIFGQLFSNVASFFSLLMDPNTFAFVQSKLYASFNNVKVNLVY

FSAPMLILACWLLFRMHRTLDVFWLDQDNAKSLGVDVPKVTRNVFILSAVLIAISTALVG

PIMFFGLLVTNLSREMFHSYQHKTLLIGCSLLAISSLLSGQWIIENVFNFETTLSVVINF

LGGIYFLYLLLKNKVV

>tr|Q87PQ0|Q87PQ0_VIBPA Putative ferredoxin-type protein NapF OS=Vibrio parahaemolyticus serotype O3:K6 (strain RIMD 2210633) OX=223926 GN=VP1451 PE=4 SV=1

MRQSESVDLSKRRLFSFRRAAVEQAQDPRVKARPPYAVEESMFTRLCDGCGKCASACPSQ

IIEMVDGVAALDISYSVCDLCGECKSACPTLALSNQTESTGLIATISNSCENLYGYCGSC

EDSCPYDALQWQDDAKPKIDAAKCKGCGQCAQSCYTSMISFDLKR

>tr|Q87QR1|Q87QR1_VIBPA Putative chemotaxis transducer OS=Vibrio parahaemolyticus serotype O3:K6 (strain RIMD 2210633) OX=223926 GN=VP1088 PE=4 SV=1

MFQNFTIKQKIVIPLSLIIGLFTVSSVLNVMTTSKQSELSDTLNEQIVPNLFTIEDAYRD

LYQATSAVQGIALAETQADIDHHIHEYKDNAYKALPRMEKVIELSRAGVMPASHGADVQK

LVSLGQKWLQSYEVMLSKPQSQWLSYYNEHKNTFEEQFVDVRAQLNVVKSAIEDKQGELK

SDISAATARAESILEMGIIVVILAALGMVFLLLRTVLKPLNDIKDAMAQIASGDGDLSQR

IQINTQDEIGQLAKAFNEFVSKIQATVSQVIDSSNTLRQEMANLSSLTETIADSTVSQQR

DSEAVAAAVHEMQVTSRNVSESANEAAVASQTANDELSNTNVILEQTVGSIRDLAGEIES

ASHVINTLDNDVSDIASVLDVIRGIAEQTNLLALNAAIEAARAGEQGRGFAVVADEVRSL

ASRTQQSTGEIQAMIEKLQSGAGQAVEVMRGSQNSSEETIQSAGRASESLAEILNAISRM

NEMNTHIATAASQQSTVSDEVNTNVQGIADSSTSIVDIVTQAQQSLAMLSQQTKRLDQQV

SQFRV

>tr|Q87KZ7|Q87KZ7_VIBPA Methyl-accepting chemotaxis protein OS=Vibrio parahaemolyticus serotype O3:K6 (strain RIMD 2210633) OX=223926 GN=VP2827 PE=4 SV=1

MRNTIKLKIQIAIAVIIAIVSGVQAWVSVNQLHEETTSTLNREIQNISESTNRYISDWLS

IRSDMMLANEQIIAGSDDADRELLLTKRAGKFLSVYAGFSDGAIAYGDKSESWPSDYDPR

TRPWYQDAMAQSGLIITEPYQDFDGSIVVSFAKAFNQNKQGVLAADLAVTDIINEVLNIQ

LDNNGFAFLVDGNNNLVAYKDEKLSQKPLTTLNPELTRDKMANLAQHAKLETITWPKQGD

QLIYVAQVPNTDWSLGVVQDKQMAFASVSEQVTFTAIASIVMYLIIAAISTYVITRLLRP

LQTLSDALSELSQGEGDLTQRIEIERMDEIGELATHVNQFLAQMQSMLKNIVENSQQLSE

QAQQANELSAMAAGRVEHQQNDVNQIATAIHEMSATAAEVASHAELTASASQNSASACVE

GQSVIQKNREAIVSLAEQVSDAANVISELEANTQSINQILSTIQGIAEQTNLLALNAAIE

AARAGEQGRGFAVVADEVRVLSQRTHGSTEEIRTMIETLQSNTKLAVNSMQASTSLADTS

VDYAQQAHDSLTSITNSITEINDMAMQIASAAEEQRAVSEDISRNTQGIKDDADVIAEQS

LKSSEGARRMFNTANTMRENISRFKV

>tr|Q87FI6|Q87FI6_VIBPA Uncharacterized protein OS=Vibrio parahaemolyticus serotype O3:K6 (strain RIMD 2210633) OX=223926 GN=VPA1693 PE=4 SV=1

MFGSKKLKMENEALKQELASLKDKYQTDVETLERQLKEAKQLLNTAQQRYESSDELMSSS

LKGGDMLQTIRTAMVESAQSMAHENEELKLLDDMFKQTHQALARLDDRAVKISSQATQSI

ESVQILDNTATSISHLVSTIQEISDQTNLLALNAAIEAARAGEAGRGFAVVADEVRNLAG

KASEASEQIDSLVNQVLTQVSSIKSAIDENQICAEEVSASSAQIGSIVNEVVVKSEHMKR

VIHFASTRSFLDTVKLDHAIWKNNIYRLLQSGSFGETVNSHSECRLGQWYYRGDGKAYSQ

LRSYAQLEAPHKGVHDSGRDAMNHAKSGNMAGMVTSINSMEDTSEQVVIHIDRLMDEIIA

N

>tr|Q87N38|Q87N38_VIBPA Chemotaxis protein CheV OS=Vibrio parahaemolyticus serotype O3:K6 (strain RIMD 2210633) OX=223926 GN=VP2037 PE=4 SV=1

MSGVLNTVDQRTNLVGENRLELLLFTLNSRQLFAINVFKVKEVIKLPPLTKLPGSHYNIR

GVASLRGEAVPVIDLRCSIGFPPLRGEAEEENLIVTEYNRSVQGFLVGPVRNIINTAWTE

IQPPPSTSGRSNYLTAITQVKDGDTSQIVEIIDVEKVLAEIVHYDVTISEDILDHDLSQS

MVGRNVLIVDDSSTARNQVRDTLSQLGMNIIECRDGLEALTVLKRWCDEGRDVEKELLMM

ITDAEMPEMDGYKLTHEVRSDPRMSKLFITLNTSLSGSFNEAMVQKVGCDRFISKFQPDL

LVEVAQDRLRQVLSANA

>tr|Q87R95|Q87R95_VIBPA Putative hemolysin OS=Vibrio parahaemolyticus serotype O3:K6 (strain RIMD 2210633) OX=223926 GN=VP0902 PE=4 SV=1

MLLTCALLEGAMDILLLVGLIALNGIFAMSEIALVAAKSGRLKMMAEDNAPAALALELKN

NPTQFLSTIQIGITAIGLLSGIFGEATLSIPFEHWLVAQGLEREVATILATTSVVILITY

FAIIIGELVPKRFAQNNAESIAIVVAYPIHWLAKLARPFVFLLTVSTDALLKLLRQNENQ

GEIVTEEDIFAVVNEGSESGAIEPQEQLMIRKLLHLNDRLALSLMTPRCDIHFLDTNLPL

DAILKHLRQTQHSVWPVCKGGLDNIIGTISSKVLLDEYDHLSVSRLGKLLKHPRFVPESM

KGLPLLNYMQQTSAEMVFIVDEYGDVQGLVTLYDLLKSIAGELGMAPEQIWAKQQKDGSW

LMDALIPLNELKYKLGLTTIEGEESEGFQTLNGFLTWWLGRLPHAGELVDYEGWQFEVLN

VKHHRIVQVKVSRETESETTTDTNDGDANP

>tr|Q87JP9|Q87JP9_VIBPA Putative hemolysin secretion protein HylB OS=Vibrio parahaemolyticus serotype O3:K6 (strain RIMD 2210633) OX=223926 GN=VPA0199 PE=4 SV=1

MFAFVRQFPLYIMVSVVAGVPLLLAIFLAVQHTINLNTQSHQALKDQQLVTLITHYDNLA

HNLAVERGLTAGVLGSQGKTEIVQKLTQQRKKVDDAVNSLVQLNTPLISSSDSYDLLQDV

QVQLNRLNQVRQGVDRLSPQIAPFGYYSNLNQLIIDNIDILIAQTQSRELGTLGDALISV

IVMKERAGQARGALNGVFAKGSATSVLFSNIEGYIQSGDYASRKAQIAFPEQYRQSLQSH

QSNPAWKKVEQVQQSFLNQSANLDNIQGPQATEWFPMATERIGLMNQLRNQMVDQMLSAA

EHSAQQATLNRNLLMAATTVISLLMIMMVWGLVASLRSRVGRLKQTLRSMSEHHDMTVEL

DSRGKDEIASISNSINALISNFRKLLFDVTKTNNESSNRLQNIVESAQDLDSSSRSTIAK

CDNIATAMTELAQSSVEIAQSAERAMGDTTTMNNKVVDCQTQSELSYRSVQSLLEQINAT

EQCMAELANDTQSIGQIVETINSVSEQTNLLALNAAIEAARAGEHGRGFAVVSSEVRDLA

QRSQEATENISKLLDQIGEKTRFSVESMAKSKQASDDTFESVQQVNESVSLLESSIEHVN

NHISTITHSTIEQSKACEAIDQDIDVLASIAHKTGQHADDLNQIVSGYQAEAKELKHQLS

AFKLA

>tr|Q87SV7|Q87SV7_VIBPA Flavin prenyltransferase UbiX OS=Vibrio parahaemolyticus serotype O3:K6 (strain RIMD 2210633) OX=223926 GN=ubiX PE=3 SV=1

MHNKIQPSQKKAITLALTGASGAPYGLRLLECLVAADYHVYVLISSAARVVMATEHNLKL

PSGPEAAQQALVEHLNCNPDNITVCGKDDWFSPVASGSAAPKQMVVCPCSAGSVAAIAHG

MSDNLIERAADVVMKERGQLLLVVRETPFSTLHLENMHKLSQMGVTIMPAAPGFYHQPKS

IEDLVDFMVARILDHLGIEQGLVPRWGYDQRS

>tr|Q87G79|Q87G79_VIBPA Iron(III) ABC transporter, permease protein OS=Vibrio parahaemolyticus serotype O3:K6 (strain RIMD 2210633) OX=223926 GN=VPA1438 PE=3 SV=1

MRTKPYIGYVALIALLSLLSLQIDTSLSLSEQWQLFTQPESASEFRDVFFMQSQLPRLSI

TLLVGAMLGLTGSLMQQLTQNNLTSPLTLGTSSGAWLALVIVNIWFVDWVADYSAFAAMA

GALVAFGLIISIAGVRNMTGLPLVVSGMVINILLGSIATALIILNAQFAQNIFMWGAGDL

SQYSWDWFEWLLPRSTIAIVILIVAPRILTLMKLGQEGAAARGLAVLPAFGALMVMGIWL

VSASITAVGIISFIGLLTPNIARAMGARTPRDELISSMLLGAALLLITDSAAIYLSLLLE

ETIPSGVAAAAIGAPALIWFTRKKLTATDQLNLSMSQGKMALSNAAVWGIAVMGIIGILT

YSFVTHGISGIEFATPGEFQWQLRWPRMISAISVGVALSVAGIILQRIVYNPLASPDILG

VSSGATFAIIITGVMVGSVLAAFNWGVAFLGSLTVLMLLLIIGKRSHFNPSNFVLSGIAL

SALLQALVQFALAQGSGESYKILLWLTGSTYRVTSTSALMLLIAVLVLLAIVFALSRWLT

LISIGRVFSNARGLNPSSANTILLVIVALLCAFSTATVGPVSFVGLVAPHMAMMLGARKV

KEQLFVGSLIGATLMVWADWLGQIAIYPSQIAAGTLVAIIGSTYFLFLMLKSKFR

>tr|Q87IE8|Q87IE8_VIBPA Iron(III) ABC transporter, permease protein OS=Vibrio parahaemolyticus serotype O3:K6 (strain RIMD 2210633) OX=223926 GN=VPA0658 PE=3 SV=1

MKKLLLTLVVLSTVSLFVGVADMTPQQLFSGDAKALELFFTSRIPRLFAILLAGAGLSIA

GLVMQQISQNRFAAPSTTGTIECAMLGYVMSVVFFGDGDHLWLVFGISVLGTLTFVHFIQ

RIQFKSVVFVPLVGIIFGNVIESMTTFIAYKYDALQSLSAWSVANFANILRGDFELLYIA

VPMVILSYLFAARISAVGIGKDFAVNLGLNYQQVVTIGVLLVSIMSASVVMIVGQLPFLG

LIVPNLVSHFYGDNLKKNIPLTAMYGAILVLGCDLVSRLIIFPHEMPISIVISILGGVVF

IAMLLRGKQHA

>tr|Q87NH6|Q87NH6_VIBPA Methyl-accepting chemotaxis protein OS=Vibrio parahaemolyticus serotype O3:K6 (strain RIMD 2210633) OX=223926 GN=VP1892 PE=4 SV=1

MRSTITFKLLLALIVVFSCVLAASTAYQHYQQKTLINDVLSEQLHDKASNYFDSLNMMML

TGTMSQKETLRQKALAQEGIEQVRVLRADAVTKLYGAGQSNQQPIDEIDQRALAGELVIE

PITADWGKGIVVALPMKSSQNYRGTNCVSCHVAPEGEVLGAIRLEYNMNHVSSMINKQAM

YAMGIMSAIALVGFLITMGLIRKIIVRPIQKTSHFMSNVSASKDLSQRLVHKQNDEVGQL

SQSINSFMDTVSESLERVQDTSHSLAGSAGRLTDVAQSTDEAANNQQLETNEVQNNIIDM

LQQQVVVEEATINATTLVNHTVDVATNSASQAHNVSEDIKSLVSDIEQVREKITSLNQRT

EEVSSILGVIKGIAEQTNLLALNAAIEAARAGEQGRGFAVVADEVRNLASRTAEATSNIE

SIISQFQQGSEESLSSVDHVCQFAHQRSLDVEALSETMHNVVDEMHQVLKHAENIQLQTQ

TTSDVSKHIQSKIDVITLHANDTSQSASHTRDISVDLEELSDRLEQLLNQFTLSEQQRAN

K

>tr|Q87R35|Q87R35_VIBPA Methyl-accepting chemotaxis protein OS=Vibrio parahaemolyticus serotype O3:K6 (strain RIMD 2210633) OX=223926 GN=VP0963 PE=4 SV=1

MRGSVIKRMYAGFALIIILFAVTIAIMMGGMHDIHGKFETVSKSSLPLVSLSNQTSVELL

SADKSFKDFLTTENKQRMEEMRQEFARSQQRFESTLAQLESASQIYPSLAEPFSQLKTLE

QSYFTEALEAMDNYEAMFSAQEEVQKSSRRFQKLNTELSVGLKEYVADQSSISVKVMAKS

YFIKLKDAEVITSDALASSNPEFVTQAVTKNRKAVTHLNYAFRGLVTQLPELEKAFGESV

EQFTRDVGMRGGVLDQHNNYLNARAALYDNIANLANKVDSTMAILEQFTTTATDKLNESL

DDAGDIYSAGVTKAIVIGVVVVLFAAAIGYHIAQSVREPLTRILNALESLTKGDMTQRID

IRFNNEFSRVSGHINTLADSLHDILVKLNEASENLASTATTNERTSSQAQSKLNAQREQT

ANVATAMTEMSHSVQEVAQSAQGSLEMVQRVESASEEGRNVMSSNISTINQLETRLHESV

SAVSELQKMSGQIGSILDVIRNIAEQTNLLALNAAIEAARAGEQGRGFAVVADEVRVLAS

KTTQSTTEIESMISNLQSSSQSANQVIQSCMSDMEMSVEQASKANSSMEEIQALIIEISQ

MSTHISQAAAEQSETSADIARNIEDINNIADESYHAMSSITHTSESLTQLAHQQNELVHR

FKL

>tr|Q87LH7|Q87LH7_VIBPA PTS system, cellobiose-specific IIA component OS=Vibrio parahaemolyticus serotype O3:K6 (strain RIMD 2210633) OX=223926 GN=VP2635 PE=4 SV=1

MEQELVVMEIICNAGEARSLSYEALRLAREQKFEAAEEKLLQARECINKAHLIQTQLIEE

DQGEGKVPMTLVMVHAQDHLMTTILAQEMAVEIVALNKQLANK

>tr|Q87LN8|Q87LN8_VIBPA Signal peptidase I OS=Vibrio parahaemolyticus serotype O3:K6 (strain RIMD 2210633) OX=223926 GN=VP2573 PE=3 SV=1

MANTFSLMLVIVTLVTGVVWLLEKLVFAKKRQAKVAEIQAQTTNGLDAVTLQKVERQPWW

IENSVSIFPVIAFVLVLRSFIYEPFQIPSGSMMPTLLVGDFILVEKYAYGLKDPVWRTQL

VETGKPERGDIVVFKYPPQPSVDYIKRVVGLPGDIVRYSGDKQLCIQSQGESSCKPVKLS

NVEESQFKSNGIPMIQLDEKLGNVEHNILVNPLVRNRVEQYFPRSGTTEWVVPQGQYFVM

GDNRDNSADSRYWGFVPEANLVGKAVAIWISFEFDRGADSVLPSWIPTGVRFNRIGGIH

>tr|Q87TF2|Q87TF2_VIBPA GGDEF family protein OS=Vibrio parahaemolyticus serotype O3:K6 (strain RIMD 2210633) OX=223926 GN=VP0117 PE=4 SV=1

MSTAKTQITLRTAVLIPFVMIFLLAIGVIVYVQKQSYEEVVTDISDKQLSVLTESVYDHL

NSYLRKPFTAVMTLAHNVSYHNLYHPHDTRDVQAYLLSAFQNLYRSIPQLDVIAFGSETG

DFAGFRREISDDYTLMVQDKYTDGKLIIYGTDHVSNDIRTVITPYDPRTRPWYQPVAKDE

KPMWSKIYTNADERQDITLSAMTPVYEHQEFAGVLVTDIRINTFNEFLRVIKKNTKASVF

IMDQEHRLVAHSGPGSVVSWGTKFSDKGQRLFASESADPIIKISASRVRGFDLYHTDQPY

TFEFDHSGQRIFSRITPYYDPNGLTWFIGTSISESDLLGLLPKSQEKSWIVGIVVSLFGI

GFSWVIFERVTRPINATADAAQHLANGDWDSSMPQPGRVYETTVLAKAFNEMASNLKASF

KALRDQLVYDSLTRLYSRQGLIEICENKPNLCHGSLFLLGINKFRDINDSVGHHNGDQLL

VSIAERLKQQFPDNTMLARIGGDEFALFMPDMDKDEDIRLTERRLQQLFAAPFMVEGESV

VMKISLGIVQTKHGENMPLWLRNASIALSYAKQDPLTGICYYSPELASASKFRTQLLTKM

QTGIDKREFVPHYQPIIDLASGNVCGAEALARWNSESGMISPLDFIPIAEESGMIKAIGQ

QILLQACRDTYKAIENKQWPSDFQLHVNISVNQLSCPAFVDTVTRVLDVTKLPAKNLTLE

ITESRIIDSAPTTLENMMKLRDMGIGIAIDDFGTGYSSLGYLHSLPFTCLKIDRTFINQL

TKENLDSSVVAAVINITAGLKTNVVAEGVEDSTQAQLLRSLGCHQVQGFYYSRPIPLDEW

PTHLVNMK

>tr|Q87NT1|Q87NT1_VIBPA Putative transposase OS=Vibrio parahaemolyticus serotype O3:K6 (strain RIMD 2210633) OX=223926 GN=VP1787 PE=4 SV=1

MLEHLLCFNVVRMAKVFGVSRSGFYYWIKHRHKAIQREANRQELDIKVKEAFDSSKGRDG

ARRIQKELAENGNSHNVKTIAASMKRQDLTPKAARKFKCTTDSKHKMPVAPNLLAQDFNA

AAPNQKWAGDITYVATSEGWLYLAVIIDLYSRQVVGWSMDTKMTATLVCDALSMALFRRG

FPEQVIVHSDRGSQYCSKDYRDLMTAYNLKQSMSRKGNCWDNACVESFFHSMKVEAIQYE

PIMTRDKTRQAIFEYIEVDYNRARRHSALGYLSPVNFEQQNVA

>tr|Q87G25|Q87G25_VIBPA Methyl-accepting chemotaxis protein OS=Vibrio parahaemolyticus serotype O3:K6 (strain RIMD 2210633) OX=223926 GN=VPA1492 PE=4 SV=1

MQLSLKNLSVRTQILVPVLFTAIVLFIALWITKNNLQAEQDVIASNQESLVFHKDTLARI

DDQIYPLRISAVYAIYDASRRETFLNELKAGLKQVEADLSAIEARNLFREDAIEVRKAIE

AYVQYSQRSVAFFNQYDQGLKSDNEYRAFISEYRRVGNEMVQGINTLSKHVNDQAVESTA

KSNAQNERVQTNAMLTVLAVFVFSLIGAWFLSGMIVTPIQKLQRVMRELAAGNLSVRADV

EGDNEIAQLSKDVNQTASQLYSIVDQLTRISEEVASASTELAAVMTQAEANAQQELAEIE

QVASAVNELASTANNVSDNATSADATAREADGLAQSGLAIFQESADASAQMSQALNDAAQ

VVLRLKEQSVQINDVIEVIRGVSEQTNLLALNAAIEAARAGESGRGFAVVADEVRMLAAR

TQDSTQEISTIIEELQAQSGLANDSMQVSLEMLTRNNELTQQANDALIGITESVANINDS

NTQVATAAEEQSQVTQDINRNVVNMSELVNQNVAGISQSASASSELSLLAEKQKEQLSFF

KL

>tr|Q87QZ1|Q87QZ1_VIBPA ATP-dependent DNA helicase DinG OS=Vibrio parahaemolyticus serotype O3:K6 (strain RIMD 2210633) OX=223926 GN=dinG PE=3 SV=1

MLTSNIQKSIRNSYQNLQTQLDNFVPRRAQNYLVAEIAKTLCGQYHKSNRILVAEAGTGI

GKSLSYLMAAIPVAVHNNRKVVISTATVALQEQLVNKDLPLFRRITDREFSFILAKGRQR

YCCAEKLATASGVDGGQLAMFETKPKKKDIELLETMYRSLAQGKWDGDRDAWPKPIDDRI

WQLIVSDKHSCNNSLPGHRGCPFQKARSELDKNDVIIANHSLVMADADLGGGVILPEPEN

TIYVFDEAHHLPHVARDHASASASLKGAAAWLEKLNQSISKFSSLADEKRVARFRNDLQD

SVQNLIPALTQLTKQFDPAQFEEGIYRFEHGELPTWLENQSKELKQFSKKANQSVAKIAD

LIAERVKDGELAARLAEPALAELGFYIQRLENLAQVWHLMAEPTREKGAPLARWLETHPD

REGDFIVSVSPLEIGWQLDQQIWSRCIGAVLVSATMRALNSFHYFCHQVGIDGKPESGTQ

FLALASPFDYQNQAELLIPAMKYEPSAPQFTEYLIEILPKYLEDKKANLVLFSSYWQMNQ

VAEALSSEFIKRGWALQVQGESSRQEILKKHKKLVETNKTSVLFGTGSFSEGLDLPGELL

ENLVITKIPFGVPTSPVEQAHSEYIEKKGGNPFMQITVPDASKKLIQSVGRLLRKERDSG

RVTILDRRLVTKRYGQALIDSLPPFKRKIEY

>tr|Q87N65|Q87N65_VIBPA Putative tetrathionate reductase complex: sensory transduction histidine kinase OS=Vibrio parahaemolyticus serotype O3:K6 (strain RIMD 2210633) OX=223926 GN=VP2010 PE=4 SV=1

MQQQKTVQPEAKQKVEVGVLAIRGHLYAEQRWQPTIDWLNQQISDVHFELHPLNLDEMGE

AVKFQTMDFILTNPGQAVRLGRQYALSWMATLTGRAPQNSNYSIGSALVVRANSPYQTLK

DVSGFPVAAVSEKAFGGYLTLRYQIVEMGLDPNDFFADVRYLGFPIDANLYQLRDGNVEA

AVVPACLLEQMQNEGLLQHGDFRVLNQQPNEHSSCAVSTPLYPNWSFAKTERGSSLLAKK

IAQVLLAMPPEHPAIIAAGASGWTSPVSLLRIDKLYQALDLHPLQQPWWSEALRWLRSHQ

EWAWALFMFVIVLNAYHFGLEYRFSKSKQALELTSLRLKEKSEQLEHSQRVAIVGEIGSS

LAHELNQPLAAIRNYSEGGLLRLAKKRPHEDIVPVLEKIQGQVERADAIIQRLRTLIRKR

SVDKTPCDIQALIADTIELLHFRMQKQNVAIVTSVEGEIRPLLADSVGVQQVLVNVINNA

IDACALFQEKYHSSGYQGKIALHCDYQANQLSIRILDNGTGLQQENPTQAFVSSKAEGLG

LGLAICRDVMEMHGGEFLIASTTPHGCLVELVFPYQN

>tr|Q87JF7|Q87JF7_VIBPA Putative oxidoreductase protein OS=Vibrio parahaemolyticus serotype O3:K6 (strain RIMD 2210633) OX=223926 GN=VPA0296 PE=4 SV=1

MKNNAFNALLGTELPVIQAPMAGVQDSQMAIAVSNVGGLGSLPCGMLNKDAIVKELQLIQ

QATNRPFNLNFFCHEMPKYDANKHEEWQKTLQPYFAEVGATVQAQPNAPTRMPFNHDIAD

AIEPFAPPVISFHFGFPADDLLARIRGWGGKILSTATTVDEALWLEAKGVDGIIAQGLEA

GGHRGMFLSDDLSTQLGLLALLPQIVKRVNLPVIAAGGIANKSGVEAALKLGASAVQVGS

AYLLCDEAKTSSLHRYAIASERAQHTAVTNVFSGRPARGIVNRAMSELGYICESAPKFPY

ASIEMTQLRALMEKQNRDEFTPLWCGQNSSGCKEVSAAEMTLSLVEGIDLN

>tr|Q87G57|Q87G57_VIBPA Phosphate transport system permease protein OS=Vibrio parahaemolyticus serotype O3:K6 (strain RIMD 2210633) OX=223926 GN=VPA1460 PE=3 SV=1

MTIATNSEKLMNTDAKAISKPGLREKRRVDWKERIFHGLFLTSAVIGIVSLAVIAYFIVR

ESIPAFQEAGVSGIVLGQNWLPPALYGVATMIVASVVSTAGAVMVGVPVGVLTAIFIAEI

APKRLADVIRPAVELLAGIPSVVYGFFGLVIIVPLIQDIFNVPAGNTILAGIIVLGVMIL

PTVITVSETSIRAVPRAYKEGSLALGASKIYTIFKLLVPAARSGIMTGVILGIGRALGET

MAIIMVMGNAPAMPEGILDSARTLTANIAIEMSYASGVHANALYATGVVLLVFIMSLNAV

LLYLNREKAK

>tr|Q87GY3|Q87GY3_VIBPA Methyl-accepting chemotaxis protein OS=Vibrio parahaemolyticus serotype O3:K6 (strain RIMD 2210633) OX=223926 GN=VPA1182 PE=4 SV=1

MLANFSQKAQETLVGELEELVSTTNLKGVITYCNDAFCRVAEYTHEELVGQNHNIVRHSD

MPKAAFGDMWARLKEGKAWRGMVKNSTKSGGYYWVDAYVTPIYEKNQVVGYQSVRVKPKR

EWVDIAAKAYKGMLAAEKAGRTWSLKINETVRYAILLGALTAPAVAYALSVEGPLAWLAS

ALPASVLALLFRQELIDTPQQLKKLQKQYDSVSRLIYSGNSAFSIADFHIKMLSARIRTV

LGRMTDSALPLQNCAEELSQTTSEVSAALNQQNSDIRRVRDATQEVESSANSVSSSTNDA

HMLIDDTLKSCMMAKETIDQTHTNLAQLSLQAEKATETTYQLSDQAQKVNHLMVEIGGIA

EQTNLLALNAAIEAARAGEQGRGFAVVADEVRALSGRTSNATEQIQASISAMLSTIEGWQ

KDILANKEQTDACSQVAEQSALRLSEVEQMMQSMSGLMVDVAEAANNQLKLSSDVNQHIH

SIASTAEQNLAATHSVEQNSRQLKEQVQDFYQLAIRFEDKQS

>tr|Q87TH8|Q87TH8_VIBPA Putative permease protein OS=Vibrio parahaemolyticus serotype O3:K6 (strain RIMD 2210633) OX=223926 GN=VP0091 PE=4 SV=1

MNGKLNLPINTVIGVSLLVLLLVPALYFAKPVLLPMVISTFVALLFSPLINYFEDKGLPR

TITVVVTLTLLVSVSILGLAAISEPAKQWWAELPSIVQNVSQEVNEVTKASSEHMNAPLE

LASDMNIDEMGNNTVFSLLKALATTTPTLLTQAMIVLFMVYFMLNHGRSLFRKSVSRLRG

FSKQRQAVELVQALQKDLSRYIGTITLVNAGLGLCVGLVFFVLGLEDPFLWGAFAGVMNF

APYLGPVISMASFGFVAYLQLDSVSFMLTVVSIYLLLNLIESQFVTPTLLGRRFNLNPLV

IFMWLVFWGWLWGGMGLLIGVPLLVCINILADRLALCGSTNI

>tr|Q87TM8|Q87TM8_VIBPA ATP-dependent DNA helicase Rep OS=Vibrio parahaemolyticus serotype O3:K6 (strain RIMD 2210633) OX=223926 GN=rep PE=3 SV=1

MKLNPRQDEAVKYVSGPCLVLAGAGSGKTRVITNKIAYLVQQCGYKARNIAAVTFTNKAA

REMKERVGQTLGKAESKGLMVSTFHTLGLNIIKREYKQLGLKAGFSLFDDQDQMALLKEL

TEKQLDGDKDLLKQLLSTISNWKNDMLTPEQAKAMAKGEQQQLFAFCFEMYQKQMKAYNA

LDFDDLILLPVLLLRNNEDVRQRWQNRIRYLLVDEYQDTNTSQYELVKLIVGERGRLTVV

GDDDQSIYSWRGAKPQNLVLLGQDYPNLRLIKLEQNYRSTSRILRAANILIANNPHVYEK

SLFSEIPDGEKLKVLLAKNEEHEAERVTGELIAHKFLNRTEYRDYAILYRGNHQSRLIEK

SLMQNRVPYKLSGGTSFFARAEIKDIMAYLRVLVNPDDDNAFLRIVNTPRREIGPVTLEK

LGSYANMRGKSLFEASFEMGLEQHLSGRGLENLRRFTQWLVAIADQAERGNTVEAVRSLV

RDIHYEDWLYETSASPKAAEMRMKNVSDLYSWIVADLEGDNYDQEEKTLKEVVQRLTLRD

MMERGEEDEDSDAVQLMTLHASKGLEFPYVYLIGSEEGILPHQTSIDEDNVEEERRLMYV

GITRAQRELTFTMCKERRQFGELIKPTQSRFLDELPFDDVEWEVNKKPVSQEERMAKGQA

HIANLRSMFKK

>tr|Q87JI9|Q87JI9_VIBPA Uncharacterized protein OS=Vibrio parahaemolyticus serotype O3:K6 (strain RIMD 2210633) OX=223926 GN=VPA0260 PE=4 SV=1

MKINRFKRWSSCLQIQPPSRLDTVGLFISACLLFSTSFLCQAKPNTTDIEQQYQVLAPYK

LQIDQRLTSVNPLLDHIFKQLKSKSLPKSLVLVPMLESSYNAKAVSHANAAGLWQLIPAT

AMRFGLTVDSNVDERFDTQASTKAALDYLAFLYNKFDQNLALTLAAYNAGEGRIARAIKK

AGTDDFQQLTLPKETHQYVSRFFALEKLIDIGQLQHSSFQPLLLFAAESAAPSQPLIDFS

RLPPLVNL

>tr|Q87PB1|Q87PB1_VIBPA Tail-specific protease OS=Vibrio parahaemolyticus serotype O3:K6 (strain RIMD 2210633) OX=223926 GN=VP1606 PE=4 SV=1

MNCRSKVTLIAASLWLAASAQALEAKLHKDDLPVLAPEVQHETASKRVTSRFTRSHYKHF

NLNDDFSKAIFERYVEMLDYNRNIFTQADIDSFKDWSIELDDQLKAGNNKIAFDVYNLSM

QKRFERFAYAMTLLDKEIKFDNADEIELDRSEAAWPKDEAELNELWRKRVKYDALNLKLT

GKEWPEIKEVLEKRYNNAMKRITQTNNEDAFQLYMNAFARQVDPHTSYLSPRNAEQFQSE

MNLSLEGIGAVLQLTDDYTVIRSLVAGGPASKSKQLGEGDRIIGVGQDGEEIVDIIGWRL

DDVVQLIKGPKGTKVNLQILPEGAGAKSYVVTIVRDKVRLEDRAVKSEIIEKDGKKIGVL

EVPSFYVGLSQDTDKLLNDLKAKNVDGIIVDLRNNGGGALTEATALTGLFIKEGPVVQVR

DSYGRIKVNADTDGLVSYDGPLTVLINRYSASASEIFAAAMQDYNRAVILGENSFGKGTV

QQHRSLNHIYDLFDKELGYVQYTIQKFYRIDGGSTQNRGVAPDIAYPTPIDPSETGESVE

DNALPWDSIDKATYQTFPDNDKLIANLTELHNKRIADEMEFRFINEDIEKYRKEKDDNML

SLNEKVRKDESDKAEALRLKRINERQTALGKKTFKSLDDVPKDYEAPDVYLDESVAIMVD

MLKAPKQS

>tr|Q87H65|Q87H65_VIBPA Putative sensor histidine kinase OS=Vibrio parahaemolyticus serotype O3:K6 (strain RIMD 2210633) OX=223926 GN=VPA1100 PE=4 SV=1

MNKFRLVNSCLFIAMLVACTFAYMAHINSKSTQQLHSALSEVGHQLIEERDVIVNQYAIK

ERKNFELTKSLVDIEVEAEKLADTFDNAVWFPISPNRQKIQQTLAKFEQRVIQTTSQLDM

LIGVQVENQYALLMLLDIYEEEFSTHIGETQLDKHYVEFFSRDLVNQSGEQSESGANFLG

RLHESDKKIELLTNELLDHNYFVFVEEAEHSLLDLAQNEARFTWLFVFVAVMLLVGSFLY

QLQYRMHNLKQLNSELEAETDKAERAAKAKSSFLAAMSHELRTPMNGVLGISQLIAEETK

EPVTKEHIKVILDSGQHLMTILNDILDFSKVEENKLELEKAPFHLEQVLTPVCSAIQPLI

DEKSIDLIVENDVPNNTEFTGDCARLRQILFNLAGNAVKFTNEGHVLIRTELNSEDKHLL

IIVSDTGIGIAPDKQGRVFNSFEQADSSTTRRFGGTGLGLAIVKKLTELMGGSITLKSVE

GVGTQFIVTLPIPWNESEKPSPQHTPVQTRSTQNLRILLAEDNRVNALVAKGFCEKLGHA

VDVAENGLVAVEKARDNDYDLILMDNHMPEMNGVEATRFIREKLGVKTLLFAYTADVFRE

AHDHFIAAGADHVLTKPLQRESFADALKQFSARLKVKQTEEVSPVSNVLQLQRKPIENLR

LTEEELSNSEMLASLKEHPNELLDLLNSIITDFELAVDDLIENFMQSDFDALKLTMHTTK

GMALNLGLKILASQALELETQLKMNQVPAIEQLQMLINRLQVNIHQGHRLRDELVKAQQN

SEQVF

>tr|Q79YX4|Q79YX4_VIBPA Chemotaxis protein CheW OS=Vibrio parahaemolyticus serotype O3:K6 (strain RIMD 2210633) OX=223926 GN=VP2225 PE=4 SV=1

MSQAFEVEVKKDTSNDEVLQWVTFQLEEETYGINVMQVREVLRYTEIAPVPGAPDYVLGI

INLRGNVVTVIDTRSRFGLMEGEVTDNTRIIVIESERQVIGILVDSVAEVVYLRSSEIDT

TPSVGTDESAKFIQGVSNRDGKLLILVDLNKLLTDDEWDEMAHL

>tr|Q87GB1|Q87GB1_VIBPA Putative exopolysaccharide biosynthesis protein OS=Vibrio parahaemolyticus serotype O3:K6 (strain RIMD 2210633) OX=223926 GN=VPA1406 PE=4 SV=1

MTGNQMNGTHPNNFQMNKVELIRFGHHFKTLKKNWLSIAAFTLIFSLTCTWYIYSKTSIY

QATATLLIQEEQKSALSIEEVYGVDTTKKEYFQTQIAILKSNHIADKVINELNLTQHPEF

TSSGGLKQKIDDIKAVPLVQDLLNVSPSPKETSQYSESYYQALQAFKRKLEIEPVRNTQL

VRISFRSADPKLATRVANAVGQAYIDANFEAKLVVTQNAATWLTNNSQKLEERLKKSEHA

LQEFLLKEGLIDINGIDDIYANELEELNRKLNTAVNKRIEAQTLIQLLKRKSSQNLDSLL

SIDEFANQAQIRDLKLSEAQAAKNVSELAQRYGPKHDRMVQAKAQLASIQERTQQLIREI

SFSKQQDLLAAKAQEDMLREELDRKKSDFQSLGSQKARYEQLKREVESNKDLYEAFLNRE

KETSATSDYKNVTARFTDKAIIPLFPVAPQRMKLVLIATFFGFAIACALVIILETLREVI

RTSNDVQDKLGVTCLGVIPMVKKRTLRKNGVSYTAYLDKDEKLFSEACRSVRTSLLLRLT

NTKQKILRFYFSDSGGRQNLHQHQYGGVLLDYGESVDYRLRLASSIFSQTIQYS

>tr|Q87J91|Q87J91_VIBPA Putative outer membrane protein OS=Vibrio parahaemolyticus serotype O3:K6 (strain RIMD 2210633) OX=223926 GN=VPA0362 PE=3 SV=1

MKKFILTPVVLALSLAGCAVGPDYQAPTTSMAETYLNAENAGLSTDSQHVKFWWTKFHDP

VLNQMVQDMQSQNIPLKVAAERVKMANNYKTMVESFKVPTINLGAGYMNYQFSKNDSSLG

PILNPLSDSVSGLPPQIGNVTLMDNQHDGVFAGASIAWEVDLFGRIDRQTNAAQIRLEQA

QIYQSGLNTVITADLIHNYLQYQGASERLELAKSNLEDQRRTLDLVGKVVRSGYGSDLDL

AQAKATLAAMESLVPQLEIAQQAHKHRLAVLLGEPLTQVEIRLSKQHSVPVMQNMVPVGL

PSDLLKRRTDIRLAEREMAALNEELAASVADQYPKFFLTGAPGLSASSFDDLFSSDSFGW

MGAAGVSWNLFDGGRGEAIEAINQARFDSATLSYQHTVEAALAETESTLFTYGRSQENQR

RIDEALKATNNAVNKAKSLYRAGLIDHLSVLDAQRQQRMMQDQQIAAKLQTAQATIALYK

SLGGDWTLQPEAQPETNAKG

>tr|Q87JR4|Q87JR4_VIBPA Uncharacterized protein OS=Vibrio parahaemolyticus serotype O3:K6 (strain RIMD 2210633) OX=223926 GN=VPA0184 PE=4 SV=1

MTGKGVARVMVRCGAHMESHYLDIRTLNFIIILFSCIYAISLLCYQYTQSKIKGLKTFAI

SLLFIGLGPFLLGFRDSAPDWLTIILSNTIIIIGFLLTLYGVSIFRKFPLKCAHVLTFLV

PVFSGLFYYFTFYSPSIRIRIIFLSIYLSLVTFCSGVAMIKGKRDDLKLPVQVMAYAFFG

FSAFMAGRTVWSIWAPEVTSFMNAGIIHQLTFLFSICLIVALSFSMLWLINARLVKSIND

LSHLDALTGLYNRRAMEVIVPNLVNQAREKNTPISIVMTDVDDFKTINDQYGHTTGDSVM

ATIATIFKQTLPESACTVRFGGDEFMIVLLANTENAKAYTELVRRSIERETSLLAFKNQV

TMSFGISELMPNDSLQDALTRADEALYCSKHTGRNQVTTFQDESEVGGKFIQTPTKSA

>tr|Q87Q18|Q87Q18_VIBPA Probable binding protein component of ABC transporter OS=Vibrio parahaemolyticus serotype O3:K6 (strain RIMD 2210633) OX=223926 GN=VP1332 PE=4 SV=1

MSRMTKTPLVMLISGMLLGTSAYAEDKLTVVSWGGAFTKSQVEAYHKPFIQKTGVEIVSE

DFSGGLAEIKAQVEANNVRWDLVSLDKPDIVRGCAEGLLEPVNPSILPPGADGTPAKEDF

IDGAIHECAINTIVVSTVLAVNEDAFKGKTAPTKLTDLFDLTNFPGRRALQKQPQGNLEW

ALLADGVKPDEVYRLLETEEGRARAFAKLDTIKPQVLWWTTGAQPPQMLADKEVVIASAF

NGRIHNARKDEGQPFRIIWDHQMGYMNGWAIPKGSANTKLALDFIAFSSGTKPLADQAKY

VAYGPTRKSSSAEVSPEILANLPTAPQNFKTAFLINDEWWSDYADELNEEFNTWLLN

>tr|Q87GV1|Q87GV1_VIBPA Putative transcriptional regulator OS=Vibrio parahaemolyticus serotype O3:K6 (strain RIMD 2210633) OX=223926 GN=VPA1214 PE=4 SV=1

MDKIDRQLLHLIQKDATLTTAELADQVGLSASPCARRLKRLEQEGVIRGYRAMISRGAVG

IAMTVFVEVSLNNHQASSIDEFETAIVDMDEVISCHVVSGAYDYLLEVVSKDLPGYESFT

RKLQRLENVKDIHTHLAIRQVKGNGCLPI

>tr|Q87FD9|Q87FD9_VIBPA Uncharacterized protein OS=Vibrio parahaemolyticus serotype O3:K6 (strain RIMD 2210633) OX=223926 GN=VPA1740 PE=4 SV=1

MSLIPRTERATFLIRPTTYGHSVLGAPLLYFPAQVESASRGLILAGIHGDETASIAGLSC

ALRSLPAKNLRHDVILSLNPDGNQLGTRANANQVDLNRAFPTQNWTEHGTVYRWSSHTPI

RDVKVKTGSKNQLEPEVGALINLIELRRPKFVVSFHEPLAFVDDPKHSNLAKWLGEQFQL

PIVDDVDYETPGSFGTWCSERDLPCITLELPAISADLTIEKHLSAFIALLMHDPDL

>tr|Q87FH8|Q87FH8_VIBPA Putative transcriptional regulatory protein OS=Vibrio parahaemolyticus serotype O3:K6 (strain RIMD 2210633) OX=223926 GN=VPA1701 PE=4 SV=1

MEYSDNIIMMPFEPKRPYQEIGLVLRQELINGRYVVGDRLPPERDIAERLDVSRTVVREA

IIMLELENLVEVKKGSGVYVINIPSQPNSRENVISDDAGPFEMLQARQLLESNIAEFAAM

QVTPGDIVKMRAALELEREELASGTADCNGDEKFHMCIAEATQNSVLVDMLKQSWERRES

SPMWKKLHSRIAGQDYREEWLDDHAKILAALQRKDPIAAKNAMWQHLENVKQRLLELSDI

DDPNFDGYLFSSNPVVLQGAENG

>tr|Q87QH6|Q87QH6_VIBPA Phage shock protein A OS=Vibrio parahaemolyticus serotype O3:K6 (strain RIMD 2210633) OX=223926 GN=VP1173 PE=4 SV=1

MGIFSRFADIVNSNISALLDKAEDPEKMIRLIIQEMEDTLVEVRTNSAKAIADKKELARK

VEMLEEQIGEWGQKASLALVKEREDLARAALIEKQKLEQVVKGLHTEQTLVEETINKLTG

EIGKLENKIAETRAKQQALAIRNQTASNRRDVQKHLHTSRTNEAMAKFDQYSRKIDEMEA

EADLYAQTGNAKSLEQEFAELQAQDEIEKELAKLKEQMSSQDK

>tr|Q87S54|Q87S54_VIBPA Histidine protein kinase PhoR OS=Vibrio parahaemolyticus serotype O3:K6 (strain RIMD 2210633) OX=223926 GN=VP0570 PE=4 SV=1

MVERLTWKKLAWELAFFYTPWVIVGWIFGYMPWLLLAATALQLVWHLHNQVRLSSWLWDE

KRLTPPSGSGNWESLFNGLYRLQQRQRRKRKELTNLIRRFRNGAESLPDAVVVFRAEGNI

VWCNRLAQHLLGFHWPEDSGQPISNLIRTPDFIKYLNKKDFSEPLEMRSPLNVERMLELR

IVPYTEGEHLMVVRDVSQLKQLEGMRRNFFANVSHELRTPMTVLQGYLEMTEDPDMIVGP

MWTKAHGVMTEQLNRMNSLVNQLLTLSKIEAAPMHELEDVVNVPAMLEVLEKEAISLSGD

DQHKLKFDVDTSLRVLGDDDQLRSAISNLVYNAVKYTPPGANIHVRWYQTAQGACLEVED

SGDGIEPQHLHRLTERFYRVDKARSRDTGGSGLGLAIVKHALSHHDSHLEIQSEVGVGSK

FSFVLPSRLVVK

>tr|Q87MN0|Q87MN0_VIBPA Uncharacterized protein OS=Vibrio parahaemolyticus serotype O3:K6 (strain RIMD 2210633) OX=223926 GN=VP2201 PE=4 SV=1

MLLSVLYIIGITAEAMTGALSAGRRKMDWFGVMLVASATAIGGGTVRDILLGHYPLGWVK

NPEFLAITCVAGVLTTGLAKWVIKLKGLFIRLDALGLIVFSIIGTKIAMGMGLHPGICMV

SALVTGVFGGLLRDLICRQTPLVLHEELYASIALIASGLYLTLLEFSVPDVTATIVTLVV

GYVLRMAAVRFKWRLPSFQLETESSLH

>tr|Q87SR1|Q87SR1_VIBPA Putative two component response regulator transcription regulator protein OS=Vibrio parahaemolyticus serotype O3:K6 (strain RIMD 2210633) OX=223926 GN=VP0361 PE=4 SV=1

MKILIVEDEHKAGEYLQKGLIESGYVVDLVHDGVDGLYHATSEEYDLILLDIMLPKLDGW

QVLNTLRSSGIHTPVIMLTAKEQVEDRVRGFELGANDYVVKPYAFAELLARVQNVFRHHI

AAQVVASPQTLRVADLELDMIKRVATRAGQSMSLTAKEYALLELLMRKTGQVLSRTTIAS

LVWDMNFDSDTNVIDVAVKRLRSKVDKPFDRPLIHTVRGMGYKLEESRDA

>tr|Q87MG1|Q87MG1_VIBPA Putative SAM-dependent methyltransferase OS=Vibrio parahaemolyticus serotype O3:K6 (strain RIMD 2210633) OX=223926 GN=VP2294 PE=4 SV=1

MKPALSNKTLPHPSTWSAMNNGPWVLESIQTRLDEWCPKLFGYHMLKLGGLSCELTSCNC

NIQHQVNVDIQNPLHNVIADGYELPFLEKSFDVVILAHQLDYASDPHRLLREVDRVMMDD

GCLIITGFNPISFTGLASLFPWRKNNLPWSGRMFTSSRINDWLGLLNYQVIHCDRYALFP

MTRYRTMWTWLENSLGDWASPAGSLYYIVARKRTYPLKPIKPHWRLKKKLTPLGVMNREG

FGVKRVSSQRY

>tr|Q87LB1|Q87LB1_VIBPA MSHA biogenesis protein MshE OS=Vibrio parahaemolyticus serotype O3:K6 (strain RIMD 2210633) OX=223926 GN=VP2701 PE=4 SV=1

MKIQLRKRLGDLLVEEGIVSEDQIQQALSAQRSTGQKLGDALIDLGFITEKQMLEFLSQQ

LGLPLIDLGRAPVDADAVQILPEVHARRLRAMVVARNGDTLRVAMSDPADLFTQESLMNL

LGEYNLEFIIASERQLISSFDRYYRRTKEIASFAEQLQAEHQDVQSFDYGIDEADSEEVT

VVKLVNSMFEDAVQVGASDIHIEPDDKVLRLRQRVDGVLHETILNEVNIASALVLRLKLM

AHLDISEKRLPQDGRFNIKVRGQSIDIRMSTLPTQYGESVVMRLLNQSSGLRPLEESGLP

PELLARLRRQLSRPHGMILVTGPTGSGKTTTLYGALSELNEPGKKIITAEDPVEYRLPRI

TQVQINSKIDLTFSRVLRTFLRQDPDIILIGEMRDQETVEIGLRAALTGHLVLSTLHTND

AVDSALRMIDMGAPGYLVASAVRAVVAQRLVRRVCPDCKTQDHLDESRQQWLAGRFPNQV

GVTFHKGAGCQNCNLTGYRGRIGVFEMLELEHEMMDKLRANDAVGFAQAARRSENYKPLL

ASAMELALQGAVSLDEVMTLGEGDASGKTDPIFM

>tr|Q87PB8|Q87PB8_VIBPA Cell division protein ZapC OS=Vibrio parahaemolyticus serotype O3:K6 (strain RIMD 2210633) OX=223926 GN=zapC PE=3 SV=1

MLKPNDKWNWYFDEHKACLMLDLGEEMIFQTNLSRKLLVNCAFSKNEFTVDDASAFQTFN

ERIRCLDINEYRQAELTLYCVAAKRFHKPVQPKSWFFDAQSTGHEPEEGDMVFLSNRYSE

GVFIVLEPGEASSLCAYVGQEEFILDGHKTLQFGQPIKVMHDRMVCANHLFVMAPMAMVG

>tr|Q87L60|Q87L60_VIBPA Transcriptional regulator, LysR family OS=Vibrio parahaemolyticus serotype O3:K6 (strain RIMD 2210633) OX=223926 GN=VP2752 PE=4 SV=1

MNIRDFEYLVALAEHKHFRKAAEACFVSQPTLSGQIRKLEDELGTALLERSSRRVLFTDS

GLQLVDQAKRILSEVKTFKDMASGQSGAMTGPMHIGFIPTVGPYLLPKILPQLKEEFPEL

ELFLHEAQTHQLVRQLEEGKLDCLVLASVAETAPFKEIEVYNEPLSVAVPCGHEWAQLDQ

VDMLELNGKTVLALGDGHCLRDQALGFCFAAGAKDDERFKATSLETLRNMVAAGAGITLL

PQLSIPAEKQKDGVCYIPAVNPTPSRSIVLAYRPGSPLRARFEALAAKIKAILESQPSSM

AA

>tr|Q87J77|Q87J77_VIBPA Putative transcriptional regulator, LysR family OS=Vibrio parahaemolyticus serotype O3:K6 (strain RIMD 2210633) OX=223926 GN=VPA0376 PE=4 SV=1

MLDLNWLRTFVTLAEVKHFGKTATELHMTQPNVSLHIKQLEQATRTKLIERNPVQLTQAG

KRLLATAQRMLNELQICQSDLNAINDLTQGTLSIAASDIVSRLLLIKPFQQFKSEYPGID

LALFNTTSSQAVDLVKSARADIGFVIAQKESQPLHFTPLTQIHWCAFGDNIFEWQDNRKE

SQTLILLGHDTRTRELIDASLPELNLPKHRVMEVGSVEAQIDWAEAGFGTAIVPDFSLDP

RLKLSREVTPLPSFPNTDFGYIVRQNQVLSKAAKQLLAWVGENVSVVEG

>tr|Q87JG3|Q87JG3_VIBPA Putative transcriptional regulator OS=Vibrio parahaemolyticus serotype O3:K6 (strain RIMD 2210633) OX=223926 GN=VPA0290 PE=4 SV=1

MRHLKSFYVFHVAASSSSYTEAADKLNITHGAVSKQIKQLEQYLSQPLFLKEGRKSVLTA

EGELLKTYTKTAFQTLETGVNTLIQEKHQHLEVSCEPTLTMRWLMPRLAAFNDEHRADVR

LSTAGGPVTLGATGLSMAIRRDDFAQQHDYNKTILVEEWVGPVFSPEYWQSIQDDLDAVR

LLNSQTRPHAWVDWQQSTRCETFNGNTQQSFAHFYFCIQAAVDGLGAAMGSYPFVMDDIK

RGNLVAPFGFVLSGCNYVLLSQDKKRTELEHIFITWLQTQMVECVPLPSQQNTQGE

>tr|Q87NE0|Q87NE0_VIBPA Cytochrome c-type protein NrfB OS=Vibrio parahaemolyticus serotype O3:K6 (strain RIMD 2210633) OX=223926 GN=VP1928 PE=4 SV=1

MTMLKFLFAFAIYGFSISAHAISSEPVEGAQTTRHQVELIRDRDYKCLQCHKDAKETLNL

SHEPEALLSQGKTLNCTNCHSNIGPDHREGAPDVIKFTAAQSKAVHDKVFLDPSTILKAN

SQCVDCHAPTQLRESNWTHDVHAKNLTCSNCHDVHAAKTKALSYDRKQLIKQCVDCHSQF

AAEPELAKEEER

>tr|Q87HT7|Q87HT7_VIBPA GGDEF family protein OS=Vibrio parahaemolyticus serotype O3:K6 (strain RIMD 2210633) OX=223926 GN=VPA0869 PE=4 SV=1

MGRFQTLDYEIPESMQRSWQDIVNLLAQIADVPTTLIMRVHQNHIEVNTSSDTQGNPYKA

GDKEDLGHGLYCEHVIETKSQLVVPDALADEKWSNNPDIKLGMLSYCGLPLFWPNGETFG

TICMLDSKANEYSETYRQLLNTFKDSIEAQLAVVYQQYKLLRLNSELKNRVENRTTDLAQ

LSFSLTQEIDRRKAAERQVNYQKTHDQGTGFLNRAALEAVVEDTLQSLEHGHQSLIVINV

GFSNARSIQTRYGFEAFDEILKEFRKRLGMNDSNYFITGRPSSNDLVLLLWGEDADSKLH

NLLDKITNISVGNFYINDTEVHLHSYVGVVQAHRSSSAKQLLQNACYAMLLCKESGEAYS

YFCESHTHELNNHNQLESYLLQAVRNDDLMLYFQPKVNPHTHKWIGAEALLRWRHPVLGD

VSNEALIHMAEQNGLIFEVGSFVLRTAIDKAKQWSELFDNFKVAVNVSPIQLQNVNFAEQ

VEHLLEAFHLPARFLELEVTESALIADEVVARTTLKKLNKLGVTLSLDDFGTGYASFSYL

KKYPFDAIKIDKSFVQQMEKSDDDRAIICSIIHIAKKLELQVVIEGIESPQQEQFLIGEG

CDIGQGFLYGKPMPCNEFEQSLFNQRQIGGQYAYSS

>tr|Q87IP8|Q87IP8_VIBPA Uncharacterized protein OS=Vibrio parahaemolyticus serotype O3:K6 (strain RIMD 2210633) OX=223926 GN=VPA0558 PE=4 SV=1

MLIKLAWRNLWRQKRRTLLTATALALVLFLSLLTRAFQEGSYSSNIKNAAKFYTGLIQLQ

NPEFGDSSSIDDALPQTDTFISASRANSNIDVILPRVESFALAAKGERSKGVMVLGVDPE

SENAYSHISDKLVQGEFIASGQNAVLVGGGLAQYLRLKIGDELVLYGAGYRGQTAAGLYR

VAGILHFPLQQLDSQLVYMPLDTAQTLYSLDKQVTAWVLNTSNLRVLPSVIEQLKRDYGN

SVAVKGWQELSPELSQQIALDKAGGIFLIYILYGIVGFGLFATILMTTLERQREFAVMLA

TGMLRSKLIGLISIESLFIAIIGIVLGLIVSAPVLGYFYFNPIEITGEAAQVMLESGFEP

IVPVSLDPHLLLNQIIVVLIILSLCLVYPMIRLLRLPIASGLKGGAHVD

>tr|Q87JC4|Q87JC4_VIBPA Putative muconate cycloisomerase I OS=Vibrio parahaemolyticus serotype O3:K6 (strain RIMD 2210633) OX=223926 GN=VPA0329 PE=3 SV=1

MKIHVEPVTIAMATPFRISRGSRTECHVVRVTIEHRGMIAQGECTPYPRYGESVESVIKA

ISHAAHELQSLYAKGDADVMALKSQLQSLLPAGAARNALDCALWHLHALLSQKQQVHDLF

TLPEAIVTAMTVSIDTPQAMAKQAQAYLSQGAKLLKVKLDGENVIERVAAVRDAAPHAQI

VLDANEAWQSLDLATVFAQLEPFNITMIEQPLPQDCDDVLASIPHPIPLCADESCHTREQ

LPQLVGKYEMVNIKLDKTGGLSEALLLADDAKELGFSLMSGCMLGTSLAMRAALPIAAQA

SVVDLDGPVLLGQDVTPSLVYRDGEIVLSK

>tr|Q87I13|Q87I13_VIBPA Uncharacterized protein OS=Vibrio parahaemolyticus serotype O3:K6 (strain RIMD 2210633) OX=223926 GN=VPA0793 PE=4 SV=1

MRNITQLENVMPQANSVVVLDFETTGLSPTLGDRAIEIGAVKLVDGEVVDSFQQLMNPGF

RVSSFIENYTGITNNMLRTAPSCEEVMASFSEFIDGENLIAHNASFDKRFLDAELERINC

GYSGEFACSLLVARRLIQDAPSHKLGELVRYKNIDNDGVFHRALADAQMTAKLWLLMVEG

LENSGIVKPSFQLMQTVSKTAKGKVDLLLAKSRA

>tr|Q87HH1|Q87HH1_VIBPA Putative membrane protein OS=Vibrio parahaemolyticus serotype O3:K6 (strain RIMD 2210633) OX=223926 GN=VPA0994 PE=4 SV=1

MFIQTVRIVIPLVLLQFLTGCQSDDNDKLKSEIESLKQEISQLTKEVGKIENEVKEMKNL

AFNPPKKEPPTLPNQPNFTEDGTLPLLGSKDAKIAIIEFSDFQCPYCKRFTDSAFKQIKE

NYIDTGKVQYIARDFPLSFHAKAMGAAIAATCSLHQNSYWPMRDMLFSNVKDLGEELYQK

AATDLSLNLEEFNKCMKDKSIANKVEQDLTLGKSLGIRGTPSFLIGRVENDQLIEPQIVV

GAQRYAVFESLLEQLSNSDKAK

>tr|Q87SG8|Q87SG8_VIBPA UDP-N-acetylmuramoyl-tripeptide--D-alanyl-D-alanine ligase OS=Vibrio parahaemolyticus serotype O3:K6 (strain RIMD 2210633) OX=223926 GN=murF PE=3 SV=1

MIKVTLSQIAEITGGALFGNDLTIDAVSTDTRTIDNGALFVALVGERFDAHNFCQQAFEA

GAGALLVEKRLDVNAPQVVVSDTKIALGELASWVHHSCQTPTVAITGSCGKTTVKEMVAS

ILQQKGNVLFTAGNFNNDIGVPLTLLRSQQDDDYAVIELGANHIGEIAYTTQLVKPVIAL

VNNVAAAHLEGFGSIEGVKQAKGEIYQGLQAGGIAIVNLDSNGDALWQSVLADKKVITFS

HNNSQADFYASNVTMNEEGKASFTLHISSDSREIELSLGIIGQHNVANAIAASIIALHMG

ATVEEIQFGLLNLNKVKGRVDVEQLSSTIKLIDDSYNASVPAMKAAVDLLGAFHGQRWLI

LGNMAELGEESLALHRQVGEHAAPQKFEHVLTYGADAKIISELCHGIHFETHQEMIDYIK

QHLDQTVSDQHTILVKGANGARMFEVAAALKEYF

>tr|Q87IW1|Q87IW1_VIBPA Putative transcriptional regulator, AraC/XylS family OS=Vibrio parahaemolyticus serotype O3:K6 (strain RIMD 2210633) OX=223926 GN=VPA0495 PE=4 SV=1

MNKKGEKMSHQYQVTLFRAEQQQKLRNVRIHSPSIIQIIKGSKRLFWKDEAFDILHPNIM

LCEANASLNFENLPQQGRFLSRMFSFHCVPSDDMLELSMSNALGENVPAVETDKALQATL

NALFSFEQESLSEATQRYWVLGLYQQLAERGLLHRLFTSSNTSFSEKLSRYLSRSPGDDH

PLERVAERFAMSRATLIRKLKQEGTQYREVLAEVRLNHALYLMQNGSYNVALLAQLCGYQ

SEGRFSQRFKGKFGLTPSEYIKTVAS

>tr|Q87Q42|Q87Q42_VIBPA Putative oligopeptidase OS=Vibrio parahaemolyticus serotype O3:K6 (strain RIMD 2210633) OX=223926 GN=VP1308 PE=3 SV=1

MTTPSWDLSIVYNDLADPRIQDDIALVEQCIDLLNKQSQDCENVEVMQNAILTNEAASRL

AGTVYNFANCYSSVNATNAEAKALSGRMMRIFSELSQAFSAFELTLTHADDEFIARVLDH

ENPDISGQAFSIMESRKLADTRLSMEEEKLLAAMSVDGKSAWGNLYDNLTGSLKVTLDHA

DGTTEELGFSQAASILYGSEFDRQEAAWRGVQKAMETHQESFAAILNALAGWRLTENKKR

STKREVHFLDPSLYGSRIQSETLAAMMKVAKDSRDIGQKAGKLMAQVHGLDEMKPWNHLA

AMRALSGDAKVYDFNEAIDVICEAFETVNPEMSEFVRLMVQNGWIDAAPNANKRLGAYCT

KLPATRTPLVFMTWSGSRSDLMTLAHELGHAFHNWVIRDLPLCQTYYPMTLAETASIFAE

NIVRDHLISKAESVDDKLEMLWEELSSALALMINIPVRYEFEKAFYERRQEGELTAQDFC

NLMSETWEDWYGDVMSEADPYFWASKLHFSIADVSFYNYPYLFGFLFSKGIYAQREAKGE

NFYIDYVNLLRDTGNMMAEEVVEKHLSMDLTKPDFWQQSVELVRDKVDEFERLLAQRSQ

>tr|Q87T36|Q87T36_VIBPA Pilin glycosylation protein OS=Vibrio parahaemolyticus serotype O3:K6 (strain RIMD 2210633) OX=223926 GN=VP0234 PE=3 SV=1

MLNTQFSPWPSFTKEEADAVSRVLLSNKVNYWTGNECREFEKEFATWVGCEYAIALGNGT

LALDVALKALGVGEGDEVITTPRTFLASASSIVTSGAAPVFADVDLNSQAITAESIHAVL

TPKTKAVIVVHLAGMPAEMDAIMALSEQHGFYVIEDCAQAHGAKYKGRSVGTIGHIGAWS

FCQDKIMTTGGEGGMVTTNDKTLWSTMWSYKDHGKSFDAIYNREHPPGFRWLHDSFGTNW

RMTELQAVIGRIQLTRMPVWTAQRRANSTQIDEAVADLSVVRRVEVPEYCEHAAYKHYLF

VESQNLAEGWTRDRIVEAIVERGVPAYQGSCSEVYLEKAFDGTRWRPTERLKNAVTLGET

SLMFLVHPTLTKPEVVKTCQVLREVLLLAQK

>tr|Q87PG6|Q87PG6_VIBPA Fumarate and nitrate reduction regulatory protein OS=Vibrio parahaemolyticus serotype O3:K6 (strain RIMD 2210633) OX=223926 GN=VP1536 PE=4 SV=1

MISEKPATKRIQSGGCAIHCQDCSISQLCIPFTLNESELDQLDQIIERKKPIQKGQELFK

AGDELKSLYAIRSGTIKSYTITEQGDEQITAFHLAGDLVGFDAITGDCHPSFAQALETSM

VCEIPYEILDDLSGKMPKLRQQIMRLMSNEIKGDQEMILLLSKKNAEERLAAFLYNLSTR

FSQRGFSPREFRLTMTRGDIGNYLGLTVETISRLLGRFQKSEILSVKGKYITILDHDALM

ELAGVTKE

>tr|Q87FX8|Q87FX8_VIBPA Flagellar biosynthetic protein FliP OS=Vibrio parahaemolyticus serotype O3:K6 (strain RIMD 2210633) OX=223926 GN=fliP PE=3 SV=1

MNNLSAWHRWLPLLVLVSLLFAFPTMAADNGLTILSVTDGDAQQEYSVKLQILLLMTALS

FLPAFILMATSFTRIIVVLAILRQALGLQQSPPNRVLVGIALTLTLLIMRPVWTDIYENA

FQPYDNGEITLVQAFSVAEKPVRNFMLAQTHQSSLEQMLRIANEPLDQKVEDISFAVVLP

AFVISELKTAFQIGFMLFIPFLIIDLVVASVLMAMGMMMLSPLIVSLPFKLVVFVLVDGW

AMTVGTLSASFG

>tr|Q87K64|Q87K64_VIBPA Putative transcriptional regulator, GntR family OS=Vibrio parahaemolyticus serotype O3:K6 (strain RIMD 2210633) OX=223926 GN=VPA0034 PE=4 SV=1

MFIMTHWHDRQPIFRQLADQITQQILQGVWKEGEALPSVRSISADMKINHLTVMKGYQLL

VDEGLVEKKRGQGMFVAQGAIQQLRSAEKARFLEQQIPQIADTLQRLDMSVDELVQQLNP

HMKGDQ

>tr|Q87M78|Q87M78_VIBPA 2,3,4,5-tetrahydropyridine-2,6-dicarboxylate N-succinyltransferase OS=Vibrio parahaemolyticus serotype O3:K6 (strain RIMD 2210633) OX=223926 GN=dapD PE=3 SV=1

MAFFSLAFGTATKNRDGKIIEAFFPNPVLNPSDTLVNALAAVAGYEQGNQAIEISAAQSA

ELAAAFEANGDAANASFATKAAESAQPLVLVILATDEQPQSVAEGFLKLQLISNRLVKPH

GTVLDGIFGLLHNIAWTNEGPIDLPELAERQIEARLAGRTLSVDCVDKFPKMVDYVVPTG

VRIADTSRVRLGAHVGEGTTVMHEGFINFNAGTTGVSMVEGRISAGVVVGNGSDIGGGAS

IMGTLSGGGKVVVSIGENSLLGANAGLGFPMGDRCTIESGLYVTAGSKVRMLDSAGQEVE

VVKARDLAGVSDLLFRRNSVTGQIECLANKSAVELNSELHKNN

>tr|Q87KT8|Q87KT8_VIBPA Putative sensory box/GGDEF family protein OS=Vibrio parahaemolyticus serotype O3:K6 (strain RIMD 2210633) OX=223926 GN=VP2888 PE=4 SV=1

MNFLSQRYTGKSLHPDETKCYKNFTQTAQKLTHRLAESINHYVNHIDCVINTNLYNRLLA

EKKESNHMIYKERETWLEAILNTLPDHVFILDESGRYIESFGGTHHSKTFNAERYIGLQL

SDVLSPTKADELMGFIFDVMQDNETKVVKYNLSLHDHLLLPIEELEALENPEEMWFEAII

KPVNAPENANKLVIWSVRDITKTHLLEQRLKQLSETDALTGLLNRRAFISNLDNAIIQHT

KRSQTLSCLMIDIDHFKDINDSVGHFSGDHVITRIAHVCQGVIRGSDFIGRLGGEEFAVI

LTDTNAIQAYEVAERIRQAIQATICHVDDVEITTTVSIGVAELNSQQSNAKELLIEADKA

MYYSKHSGRNQVTLAYENLPDLKLYQAGHLKIQRVS

>tr|Q87N10|Q87N10_VIBPA AsmA protein OS=Vibrio parahaemolyticus serotype O3:K6 (strain RIMD 2210633) OX=223926 GN=VP2066 PE=4 SV=1

MKKLLLVLAIPVVVFVAAILALTIFVNPNQFKPLIVEQAQKQTGLELVIEGDISWQFFPS

IGFELGRTELRNPQGFSQPNLFKVDTVGVDVSVTPLFSKQLEIGNITLDGAEFYVETKKD

GSKNIDALTKAQTQQAEQPNDTAPDQSGESQSAGSDWSINLAGVTVSNGSLEIQDKQAGN

YTKLYDVSLNLSEFAFDSWTTADFGMKGEMNDQKFTAEGKADFKLAKGLASYALKNINVD

ATYTDPNNTIESAKIGLDTFEFDKVNNLSYALQGSAAGMKLDMQGGGQLTVDQAISKVLL

NKLTLKSSFEGDALPQSPMKVDMVSDLSFDLNKSHLSFILEKLTANAIALDGKADVTLGD

IPKIRFALHSPNIDLDEFLGLDKQAPASEKPASGGKSASAGPEVEPDLSALKALDVKGDI

TIDKFKAANAKMEAVKASFTVNRGVFDLTSFSSKLYQGTISATARLDARKTPATYSVKKS

IKGVKVQPLLIDVANNDKLEGTGNIDVNVQGSSLTPTGIKQNLAGTVVINFADGAVNGIN

VAQLIRENYARFKGQKVESTNEVKKTDFSAMTATLKLNKGVVSTDNLHAQSPLLRVRGKG

SANYINETVDFTISTSIVGTLEGQGGKNIDELKDITIPINVSGKWADPKFKLVFDDVLKQ

KAQKEIDRGVEKLTDKIKDEKTKEAVDGLLKNLFN

>tr|Q87R04|Q87R04_VIBPA Formate acetyltransferase OS=Vibrio parahaemolyticus serotype O3:K6 (strain RIMD 2210633) OX=223926 GN=VP0994 PE=4 SV=1

MAEQFAKAWEGFVAGDWQNEVNVRDFIQKNYTPYEGDESFLVSEGTEATNKLWAKVMEGI

KQENSTHAPVDFDTSIISTITAHDAGYIEKDLETIVGLQTDAPLKRAIIPNGGIRMVEGS

CKAYDRELDPQVKKIFTEYRKTHNAGVFDIYTPEILACRKSGVLTGLPDAYGRGRIIGDY

RRVALYGIDFLMKDKLAQFKSLQERFENGEDLQMTMQLREEIAEQHRALGQMKVMAAKYG

CDISRPAETAQEAIQWTYFGYLAAVKSQNGAAMSLGRTSTFLDVFIERDIAAGKLTEEQA

QEMIDHFVMKLRMVRFLRTPEYDDLFSGDPIWATESMGGMGLDGRTLVTRTNFRFLNSLY

TMGPSPEPNITVLWSEQLPEGFKKFCAKVSIDTSSIQYENDDLMRPDMNSDDYAIACCVS

PMVVGKQMQFFGARANLAKTMLYTINGGVDEKLKIQVGPKMPKIEGDVLSYDELWEKMDH

FMDWLAKQYVTALNAIHFMHDKYSYEAALMALHDRDVKRTMACGIAGLSVAADSLSAIKY

ATVKPIRDEDGLAIDFEIEGDYPKFGNNDPRVDDIACELVSVFMGKIRKLKTYRDAIPTQ

SILTITSNVVYGKKTGNTPDGRRAGAPFAPGANPMHGRDEKGAVASLTSVAKLPFADAQD

GISYTFSIVPNALGKDEGSQRANLAGLMDGYFHHEAGIEGGQHLNVNVLNRETLEDAVKH

PEKYPQLTIRVSGYAVRFNSLTAEQQADVIARTFTESL

>tr|Q87H96|Q87H96_VIBPA Putative virulence-mediating protein VirC OS=Vibrio parahaemolyticus serotype O3:K6 (strain RIMD 2210633) OX=223926 GN=VPA1069 PE=4 SV=1

MHFPIKTLSCALLAVIGLFFQTGYVFANTDKHIYLEEHISPESQPMLSTYFRSMGKPRSI

KENVLTVPQDASPETLALYYFARIYLERYEGVPLPDDMPDLIEFGRKHNMPWVVAEAKLN

KAIRMIELDDDWHAELLLHDVIGESRDIGYLALQGRAYRWMGNLEIARNQIHNGLKHYRT

AYELLENTVFEIQVAMTLNNIGTVYLDSSDWNRASNYLKQALDVYESSEYEYDNSFFIGV

IYANLSIVHLGLGDSEKAEYYFHEAIRRSMQTGSDVIKHHSLSNFSQMLSSIGKTDDALL

LAQRCVELPNPDGIEIIKMPCHEAFAEAYLADKQYDKAIRTALLVLEQTKSTNELELKQR

IDMLSVLVNANQVLQNYEAAFRYLSQLRALEEEFSEHIHGEEMINIKFDLEAKLAQKELN

LLETKNALQASELRSQRYREMFYFIAIAAIGVVGFRYVLRVKKINKALTQESTTDLLTGL

HNRRYLEVWLEKMVRRTPDRTFALAVLDVDHFKAFNDTYGHDIGDQMLMHIASIFNESTR

SGDLLIRWGGEEFVLLVEVNDPNDCAKSLERLRHVIENTPLIIDSKPINATISLGAVDRL

SAQTIKQEWDQWFFLADQALYDAKQAGRNQFKIHSTS

>tr|Q87HB2|Q87HB2_VIBPA Uncharacterized protein OS=Vibrio parahaemolyticus serotype O3:K6 (strain RIMD 2210633) OX=223926 GN=VPA1053 PE=4 SV=1

MSIEVIAVRLTKGMDLKLSLAKLVAEHDIKAGSIASCVGCISDLNLRLAGAESTLTKREP

FEIVSVMGTLTPEHQHVHISVSDREGRVWGGHLLEGTVIDTTAELIIHSYSELEFTRAMD

DSTGYTELQVNPSK

>tr|Q87Q95|Q87Q95_VIBPA GGDEF family protein OS=Vibrio parahaemolyticus serotype O3:K6 (strain RIMD 2210633) OX=223926 GN=VP1255 PE=4 SV=1

MKWIHKLIFALSICTISLVALYSVLGDTRKLVITPEQFNIYATKDASEGGLSTADITYDA

QSLVLNCELKKSSYAWPYCGISVYTDVAKPTHGIDLSNYHTIRLKLHYEEAGDGQNPSHD

LRLYLRNYNPEYSKPDDEYTIKYNGMQFSPSSFSETIEIPIKNLQVMTWWLADNKVDIEH

SAPEFSNITRIDIATGSGAVLGQHKIVIDKIEFEGAYLAQETLLFALLFSWMALGLAFSL

HELRKNRAAYEKAKRRHRHLEKVNGTLRAQNYEFAELAHRDALTGAMNRHAVQTWLEQQA

RQVRWGHSTLSILYMDLDNFKKINDKFGHQMGDDILREFVMVVASSIAPDDRLVRWGGEE

FVVFCPDTNIEQAVKKAEMIRKNVANHLWVHGEALTCSIGVAQMQNERVTETMARADEVL

YLAKRNGRNRVEVNYGLLSCQKNEA

>tr|Q87N78|Q87N78_VIBPA Uncharacterized protein OS=Vibrio parahaemolyticus serotype O3:K6 (strain RIMD 2210633) OX=223926 GN=VP1997 PE=4 SV=1

MSSLLQQTWQTLMAHRMKSVLAIIAIAWGVISVVVLIALGEGFYRHQTQSFSFMVNNVQV

VFPSSTSKPWQGLASRRQIDIPQEKVDMIKQSGFVQQASSVYAKWDANITNLKGQNLSST

VSGIDTSYFPLIQRKLQSGSRNLSPSDMKNHTRVAVLGDQIAQMGGIQIGDRLKVNGIPF

LVIGITVGEDTGISFGDSRTVFIPQTTYRDLWDAKPWMVLMKPRDGMDAPSFRQNIVNFF

AKQLHFDPSDKDAVELPDFSEGAAVISGILRGIQIFLGASGAMTMAVGALGVANIMFLSV

TERTREIGVRLAIGATQKSILSQFILEGFILVAVGTALGLMFSYAVVSVLSSMALPEWIG

LPVITPDSIAWSLLVTLILALMASYFPARRASRLTPVIALSARA

>tr|Q87IA2|Q87IA2_VIBPA Uncharacterized protein OS=Vibrio parahaemolyticus serotype O3:K6 (strain RIMD 2210633) OX=223926 GN=VPA0704 PE=4 SV=1

MDFFHHQDTARQRTGLLVMLFTLAVLAITGLVSVISIGIYFYFTGEPFTTQSIISYCLLS

FVGVLLVVSISSFIRLSELNANGGRGVAESIGGKLISTDTSNAKHRQLLNVVEEMSIASG

IPVPPVYVMAEEHGINAFAAGMSIDDAVIGVTQGALDAFSRDELQGVIAHEFSHILNGDM

RLNTRLIGALFGITCIAHFGHLILDNSNSTRHVSRSSSDSNKGFAVIILIAIVCLVLGWL

GTLFGNMIKAAISRQREFLADASAVQFTRNDQGIAGALKKIGSNVQGSTLNTKASDEMSH

MMFGQSKLSGFSGLFATHPPLDERIRRIEPNWDGSYAQHSHAQNTAFDNEQVSGFAVGGG

SPASQSASPSEQLSETGQQLISQLPPELVDIAREPYSARFIAFALIFDGSDIQREMIKSY

VPLASQSTLLPWLDYDLPLHLRFPLLELALPALKSLSEAQKISLCKVLRELSETDNQYSL

AEWCVINLLEKQLLASFGFIKQHKSLKQLEESVFWLLRELAWVSHSQADKAQRAYHCALA

HLGFPEVKLEPANSNWHLSRAALELLLQLKPNDRRMFVKACRLAIESDGEITVAEGEIYR

VIACFLEVPEPPLTISG

>tr|Q87TN1|Q87TN1_VIBPA Putative multidrug resistance protein OS=Vibrio parahaemolyticus serotype O3:K6 (strain RIMD 2210633) OX=223926 GN=VP0038 PE=3 SV=1

MRFTDVFIKRPVLAVSISFLIALLGLQAVFKMQVREYPEMTNTVVTVTTSYYGASANLIQ

GFITQPLEQAVAQADNIDYMTSQSVLGKSTITVNMKLNTDPNAALSDILAKTNSVRSQLP

KEAEDPTVTMSTGSTTAVLYIGFTSDELSSSQITDYLERVINPQLFTVNGVSKVDLYGGM

KYALRVWLDPAKMGALKLTATDVMSVLNANNYQSATGQATGEFVLYNGSADTQVSNVAEL

EALVVKTGEGDVIRLGDIAKVTLEKSHDVYRASANGQEAVVAAINAAPSANPINIAADVL

DLLPQLERNLPSNIKMNVMYDSTIAINESIHEVVKTILEAAVIVLVVITLFLGSFRAVII

PIVTIPLSLIGVAMVMQAMGFSWNLMTLLAMVLAIGLVVDDAIVVLENVDRHIKEGESPF

RAAIIGTREIAVPVIAMTLTLGAVYAPIALMGGITGSLFKEFALTLAGSVFVSGIIALTL

SPMMCSKMLKANEKPSKFEQKVHHILDGMTTRYEGMLKAVMAHRPVVIAFAIIVFASLPV

LFKFIPSELAPSEDKGVVVLMGTAPSNANLDYIQNTMNDVNKILSDQPEVEYAQVFNGVP

NSNQAFGLATLKPWSEREASQSEITKRVGGLVASVPGMSITAFQMPELPGAGSGLPIQFV

ITTPNSFESLFTIASDVLTDVASSPMFVYSDLDLNYDSATMKIKIDKDKAGAYGVTMQDI

GITLSTMMADGYVNRIDLNGRSYEVIPQVERKWRLNPESMKNYYVRSVDGKAVPLGSLIT

IDVVAEPRSLPHFNQLNSATVGAVPSPGVAMGDAINWFEDVAQNKLPAGYNHDYMGEARQ

FVTEGSALYATFGLALAIIFLVLAIQFESLRDPIVIMVSVPLAVCGALIALAWGTASMNI

YSQVGLITLVGLITKHGILICEVAKEEQLHNKLSRIDAVMEAAKVRLRPILMTTAAMIAG

LIPLMYATGAGAAQRFSIGIVIVAGLAIGTLFTLFVLPVIYSYLAEKHKPLPVFVEDKDL

EKLARVDEAKAAHRELAENK

>tr|Q87FH6|Q87FH6_VIBPA Putative small integral C4-dicarboxylate membrane transport protein OS=Vibrio parahaemolyticus serotype O3:K6 (strain RIMD 2210633) OX=223926 GN=VPA1703 PE=4 SV=1

MKMNKLVTYINRGLAAFTVSLSTFLVFCVVWQVLSRYVLGKPSTVTDELARYLFMWVALI

GAAYTTGLKRHLAIDLLTMKLKGKKKLVNEIVIQVAIALFSYVVLVHGGTQLALKTLATG

QLTPALGLEMGYIYFCLPISGALMIFYSVIFAYERVKQLMSGETLVTDSQLDS

>tr|Q87J40|Q87J40_VIBPA Uncharacterized protein OS=Vibrio parahaemolyticus serotype O3:K6 (strain RIMD 2210633) OX=223926 GN=VPA0413 PE=1 SV=1

MKKIAIFGSAFNPPSLGHKSVIESLSHFDLVLLEPSIAHAWGKNMLDYPIRCKLVDAFIK

DMGLSNVQRSDLEQALYQPGQSVTTYALLEKIQEIYPTADITFVIGPDNFFKFAKFYKAE

EITERWTVMACPEKVKIRSTDIRNALIEGKDISTYTTPTVSELLLNEGLYRETLSGK

>tr|Q87H00|Q87H00_VIBPA Uncharacterized protein OS=Vibrio parahaemolyticus serotype O3:K6 (strain RIMD 2210633) OX=223926 GN=VPA1165 PE=4 SV=1

MRFASRTLLFFCCVAASFSSLASQRWWQEVLSSTPYQKLSEQIQHDGNWYPCDVQIGETE

NLSRSFCLDEFSYYQQTLYGEAVFEEKKAQFSFLTEYQRQTLSELILNLRKDGLVMRSLA

IGNEQYDVAASLKNKSAGEVDKEVVLLINRYPPEIERSMVWVKADEFTEPSPHVVITLRS

DGEMIELRLTRL

>tr|Q87KW1|Q87KW1_VIBPA Anaerobic C4-dicarboxylate transporter OS=Vibrio parahaemolyticus serotype O3:K6 (strain RIMD 2210633) OX=223926 GN=VP2864 PE=3 SV=1

MVMVELFVVLLFIYLGARIGGIGIGFAGGAGVIALSLILGVPTSQAYIPVDVILIIMSVI

TAIAAMQVAGGLDWMVQVAEDFLRKHPERITFYAPIVTFLMTLMAGTGHTAFSTLPVIAE

VAKGQGVRPSRPLSIAVVASQIAITASPISAAVVAFAAMLHPFGVDYLTLLAICIPTTFI

ACMIGAFVANFMGCELKDDPEYQERLEKGLIKLNTEAKREILPTAKKATFIFLGAIAFVV

CYAAAISGSVGLIENPALGRNEAIMTVMLAAAAAIVMSCKIDAGKIPAAATFRSGMTACV

CVLGVAWLGSTFVNAHVDGIKEVAGALLADYPWMLALVLFFASMLLYSQGATTVALMPAA

LAIGVAPLTAVASFAAVSALFVLPTYPTLLAAVEMDDTGSTRIGKYVFNHPFFVPGVVTI

>tr|Q87SP5|Q87SP5_VIBPA Putative CFA/I fimbrial subunit D OS=Vibrio parahaemolyticus serotype O3:K6 (strain RIMD 2210633) OX=223926 GN=VP0377 PE=4 SV=1

MIRERSINLGHQVEGAIFTRHNHIVSVDGDGILSFQNNITKVKPNEAIFIPSKVMVRFSF

NSNENKSIKVSLYHINGINLNCKQDKVTSLINAYIEVEKLNLTNRETIIELIGSRLEKIK

NNRVLYNYDPLEKKIYRLVSKDLKKKWSLEEVCKLTYTSKSTLNRKLKDNQTSMGQIISN

ARLDYATILITNTSLSVDEISMLSGFNSTSYFCKKFKESHGFSPKQYRKLLAYNQVPLNR

I

>tr|Q87KW4|Q87KW4_VIBPA tRNA (cytidine(34)-2'-O)-methyltransferase OS=Vibrio parahaemolyticus serotype O3:K6 (strain RIMD 2210633) OX=223926 GN=trmL PE=3 SV=1

MFDIALYEPEIAPNTGNIIRLCANCGANLHLIEPLGFDLEEKKVRRAGLDYHDLARVTRH

KNYQAFLEYLDKEKQGNYRLFACTTKTTGHHVDAQYKAGDVLMFGPETRGLPAEIIEGLP

MEQRIRIPMMPDARSLNLSNAVAIIAFEAWRQMGFEGAL

>tr|Q87JT0|Q87JT0_VIBPA Multidrug resistance protein D OS=Vibrio parahaemolyticus serotype O3:K6 (strain RIMD 2210633) OX=223926 GN=VPA0168 PE=4 SV=1

MSASFPLAKLTFLIAILTAVGQMTQTMYVPSIGHMAGEFLVSASSLQAVMACYLIPYGLS

QFAYGTLSDRLGRKPIIIVGLLIYILGTLVALFAHQFEWFLAGSFIQGLGIGCGGAMSRT

LTRDCFEGAELHRANSLISMCVIFSPLMAPVLGGYLTEAFGWRSSYLFLALFGIAVVITM

MTSMMETLPKEKRKFEPVAKSYKHVLSDRRFQGFLICLVATFAGVGVFEAAAGVLLGGVL

ALPATTVSLLFILPIPGYLVGAGLSSYIAQRRSERRALNVGLVSIFIGSAVVLIPGLFGV

TTALTLIGGATIYFLGAGILFPAATTGAIAPFPYHAGTAGATLGGMQNLGAGIATLLASL

FPAHNQMPLGVLMMAMSIVAMLGLLWVHRAHDHSNEMPQVI

>tr|Q87MP5|Q87MP5_VIBPA Bacteriocin production protein OS=Vibrio parahaemolyticus serotype O3:K6 (strain RIMD 2210633) OX=223926 GN=VP2186 PE=4 SV=1

MNWIDFIILGVIGFSALISLVRGFAKEALSLVIWFGAFFISSNYYAKLAVYFTNIKDDMF

RNGAAIAALFVATLVVGAVVNYVIGQLVQKTGLSGTDRILGMVFGGLRGVLIVSAVLFFM

DAFTAFPSSDWWKESQLVPEFKLVIEPFFEHLQATSSFLSGTI

>tr|Q87HW4|Q87HW4_VIBPA Methyl-accepting chemotaxis protein OS=Vibrio parahaemolyticus serotype O3:K6 (strain RIMD 2210633) OX=223926 GN=VPA0842 PE=4 SV=1

MREVEFRTIDRLFIKMSINDKMWVIFLLFLVALTSVAGSRYLNDLHQFEQQSIANVQAKL

DGIIEANPTDIYQITGISKANHQQKSLFADGVTTVYGTTSAGELVRLTEHAGNQYNALRS

DALTSFLLSFLWVLPFAVFCYWVATFIGGALWVLYTTTEKIGDGDLTSRLGFHPGRDEFG

TIGCALDKSMDTLSELVNSVKESANTLSETSSAFEQDMKLSETQITHQYQTLDSVATAME

EMTASAKEVSSISQQATMQSDQDAQKIETSRSRVQHVIAEIETLSSYIEQASSSVTNLNE

NTTQINDVITTINAISEQTNLLALNAAIEAARAGEQGRGFAVVADEVRTLASRTQQATVE

IQAMIEKLQSESQNIASITGRTVKQAQTSSQLIDEIGQDVTAIADSARSLMDMSIQISTS

AEEQSAVANDIASELADIRTQSSTIREVAEQSTVGVANLTKASVSLGEVLERYRTA

>tr|Q87IW5|Q87IW5_VIBPA Methyl-accepting chemotaxis protein OS=Vibrio parahaemolyticus serotype O3:K6 (strain RIMD 2210633) OX=223926 GN=VPA0491 PE=4 SV=1

MKLSNLSIKYKISALILIISLSVIALSVFFTSEIKMIEGKLTVFSETTVPSVLLVKNTEI

ELGILRKDEFSLLTNVNHPQFMEWVAGLEKSEQKIDKYLDQYEKGLWDQRDRDAFNKVKS

AWVKYSAFNNEYAKLLLNNKIDEANKTLLNGFSTFTQLSDAIRDLVELNQTYVQEDIASA

HEAVRSAITYSIIAIVALLALSFTLGLFLTKQICTPLNYVVNMASKIASGDLTYQLPRNK

IGHDELGTLADACVDMQAKLLTLVDSISSTTAQVGTAIEEVSAISEQTSTGMDEQQVQLN

LIATAMNEMQATVNEVASNTEAASETANSASHDAKEGRGVVQECINQIHEASLAIQSVGN

MVTELEQDASNISVVVDVIQDIAEQTNLLALNAAIEAARAGEQGRGFAVVADEVRTLASR

TQASTEEIITIISKLQNCSKSAVSATNNSSDLIQECVEQAQKAGATIDQIEKGADNIAEM

SIQIASACSEQSSVTEELHRNVEHINQFSSEVATGSRQTAIACRDLSELAVGLQEIVGQF

KTA

>tr|Q87IQ2|Q87IQ2_VIBPA Methyl-accepting chemotaxis protein OS=Vibrio parahaemolyticus serotype O3:K6 (strain RIMD 2210633) OX=223926 GN=VPA0554 PE=4 SV=1

MFGLRTRNQEAESEYRRFIKGLSDSMAMIEFDTRGIILNANDLFLSCVGYTREAIVGKHH

SIFCDRGYVQSPRYQQFWDDLKMGKHKRGTFERLTSQRERLVLEASYFPIESEHGQIEKV

VKIASDVTQQRLESEQKEAILNALDLSLATIEFDREGYILTANQNFLKTLGYELSDVQGK

HHKLFCFDDFYQENPGFWKDLAAGQFKSGQFLRRSASGDKVYIEATYNPIFDPAGNVIKV

VKFASDITDKVQRDLNISQAIADSSFIARNASEEATSNVCGAEHSLNEFRTMIEEVLRAV

TACDDKVQELFKTSQQVTEIVKVIDSIASQTNLLALNAAIEAARAGEHGRGFAVVADEVR

TLATRTSTSIDEINHIVLSNQSLTTETRSFIEVINQGFLSSMSKLLEVDEFMKSIDEGSH

LTVESISALRAIVNQDAHLHLNSASNG

>tr|Q87QG4|Q87QG4_VIBPA Putative chemotaxis transducer OS=Vibrio parahaemolyticus serotype O3:K6 (strain RIMD 2210633) OX=223926 GN=VP1185 PE=4 SV=1

MSVKMSVRKKLYASFGAILATMLVMIAIITFEVINSHKVAQEVRTDDVPETIGYMALIDE

AGDIYRDAVGMIINTSGAQREYQSNKKEFASALADVKRLETAGGEDYRNIEKIEQLMANF

TSSFESQIQPNIGHRSLEENVEQVRQLYEVNLAPIEDMLNAATAEGRQSTHDALLQLTDS

FNDIENTIYALGTLAAIATVFIAYLLSNSITGRLTALDTLAQRVAEGDLTASPIKDESGD

ELSNLATSLNKMQASLTGLIGSISVVSEEVKSVTSELSVVSQDIVSGASSQADKANLIAT

AAEELSLTISQVAEQGASTFEEARKSEATAEQGRTVIVEMVESIQQVSKQMADMSVQMNH

LGAHGEKIGAVIKVIEDIAEQTNLLALNAAIEAARAGEFGRGFAVVADEVRALAERTTKA

TQEVGEIIQAIQVGTQEAVTYTEDGCRLVEIGVSQSSGAVESLEEIVAGAGHVQSMVNSI

ATAAEEQTAVTKEIAADITSISDISVRSLQLANDSSQSVEGLNRKVQELETLVGKFKLA

>tr|Q87KM4|Q87KM4_VIBPA RNA polymerase sigma factor RpoH OS=Vibrio parahaemolyticus serotype O3:K6 (strain RIMD 2210633) OX=223926 GN=rpoH PE=3 SV=1

MTKEAYPMALVTQDSLDSYIRSVNSYPMLTADEERELAERLHYKGEIEAAKGLILSHLRF

VVHVARGYSGYGLPMADLVQEGNIGLMKAVKRFNPEVGVRLVSFAVHWIKAEIHEYVLRN

WRIVKIATTKAQRKLFFNLRKSKKRLGWFNNGEVETVARELGVEPSEVREMESRLAAVDS

TFDMPTDDDDSATSSYTAPMLYLEDKSSDVADNIEAANWEMHTNNRLGHALASLDERSQR

IVRSRWLDDEKATLQDLADEFGVSAERIRQLEKNAMKKLKLAVGEF

>tr|Q87SK1|Q87SK1_VIBPA Methyl-accepting chemotaxis protein OS=Vibrio parahaemolyticus serotype O3:K6 (strain RIMD 2210633) OX=223926 GN=VP0421 PE=4 SV=1

MIEPKRKHNYKRCKNYIRLSGELTMEKLAFKPWERVISDIRLVPKMVMLMVFSTVLIVAK

QLWDANTFYDSLLAATQNAQVAQQHYEAYLTQVVWQTALLIVVFVALLLAAARVMLRQTQ

YLNEAIKLMASKNLSVPFGMDCKDEYGDVARELEKTRRQLHDVIQMQINASDELATLTEV

MTLSMSETKESAQEEFNEIDQLATAMSEMSSTVQTVADHAQTASSLTEQASTQAVTGQQF

LQSTVAKMSELSSDIASSAQAVNQVEERVESIGSVVGTIQGISEQTNLLALNAAIEAARA

GEAGRGFAVVADEVRNLAQRTQQATVEIQEMITQLQASATSAVDLMEKSVVEAAEGVELV

SNAGSELDGIVAQVTQINDMNFQIATASGQQSSVAEEMSQNLTNVRELVEASVVVVTELL

ETSEMMQSNAEELDKKIKSFSV

>tr|Q87LS5|Q87LS5_VIBPA Putative hemolysin OS=Vibrio parahaemolyticus serotype O3:K6 (strain RIMD 2210633) OX=223926 GN=VP2536 PE=4 SV=1

MDDISTGILFALLACLIVISGYFSGSETGMMSLNRYRLKHLAKSGHKGAKRVEKLLDRPD

RLIGLILIGNNLVNILASAIATIIGMRLYGDLGVAIATGALTMVVLVFAEVTPKTVAALY

PERVSYASSILLTILMKVLSPLVMLVNFITNGFIRLLGVKADHTVEDHLSSEELRTVVNE

AGGLIPRRHQDMLVSILDLEHVTVNDIMVPRNEITGIDINDDWKSIVRQLTHSPHGRIVF

YRDQIDEVVGMLRLREAYRLMLEKNEFNKETLLRAADEVYYIPEGTPLNVQMLKFQRNKQ

RIGLIVDEYGDIIGLITLEDILEEIIGEFTTSISPSLSDEISPQGDGSFLIEGSTNIRDI

NKGLKWDLPTDGPRTLNGLILEHLEDIPESHLSVRVAGHPMEIVEIEENRIKLVRVYPKK

KKSTSK

>tr|Q87K74|Q87K74_VIBPA Methyl-accepting chemotaxis protein OS=Vibrio parahaemolyticus serotype O3:K6 (strain RIMD 2210633) OX=223926 GN=VPA0024 PE=4 SV=1

MNLTIRKRLYILSIIPVLTIALGMMWFTYLQTNAYNQQQIDQTHTTMMAMKKAELKNYVQ

MARSAIEPLLKRNATLEEALPVLRELEFGETGYIFGYNSKGVRVVVGKNDKGIGENFYNL

QDKKGNYLIQDLLKNAKTGEFTTYYFPKLGQTEALPKLSYSMFIPEWDLMIGTGFYTDDI

DAVIAEMEASAHDALNTTLVAIALFCVSIAAVVAIFAVFVNRSIMRPIEQFDASIQSFAQ

GDADLTARMHESNVPEFKQLAHNFNIFVESLQGIIKSVTQVGEEVVAETNNMSQRASQVD

ELAGGQREETEQVAAAMTEMTATAHEISNNANQAAESARHADENAQQAKHIVDSAANSVE

ELASEVSEASTVIARVESDVQNISSSLEVIQDIAEQTNLLALNAAIEAARAGDQGRGFAV

VADEVRKLASRTQDSTGDIHKMIEQLKSGSDAAVRAMESSQARGEATVEEARAASVALQD

IQAAIANIMDMNTLIATATEEQSQVGQEISQRVEVISQQSSQSASLANQNRSGSQNLNHK

ANELYDLVGRFTV

>tr|Q87LI3|Q87LI3_VIBPA Methyl-accepting chemotaxis protein OS=Vibrio parahaemolyticus serotype O3:K6 (strain RIMD 2210633) OX=223926 GN=VP2629 PE=4 SV=1

MKLKTQAYLLSAIILLALLALTATGLWTLRVASNLDNKARVTELFKSAYSILTEVEKMAV

EGKMPEEEAKALATRLLRNNIYKDNEYVYVADENMMFIAAPLDPQLHGTSFHDFKDGSGN

SVGQLILDVLGRKTGQIVEYTWSQKQADGSIEEKHSIAEKTPHWGWVVGTGIGFNEVNAR

FWSTAQWQLMLCLVIAGSILGGLIISIRKMLNLLGGEPNDVREAVQAVAQGNIQTSFDTV

APKGSIYHAVQNMSQSLATMVTNLEQSMHALRDELSAVESRSNSIADLTVSQQQSTAMIA

TAMTEMASSANHVASSASDTARNTDEADKQSQHTQSLIHSTVDNIQGLATQLNTASKAVA

DLDQDVNNIVKVLDVIGDIAEQTNLLALNAAIEAARAGEQGRGFAVVADEVRNLAGRTQD

STKEIQHMISNLQEGSRNAIHTMEICAETSESTVTESMNASEALQQIVTALESITAMSQQ

IATAAAEQTQVSDDIAHRINMIEESGSKLNSVVTESQSSTQSLASLANELEGWVNKFSVK

H

>tr|Q87QU1|Q87QU1_VIBPA Tol-Pal system protein TolR OS=Vibrio parahaemolyticus serotype O3:K6 (strain RIMD 2210633) OX=223926 GN=tolR PE=3 SV=1

MAGYQPKKRKMTAEINVVPYIDVMLVLLIIFMATAPFVTQGVDVELPKTSTAESMEKIAG

NTDSSFIIIEIDRDGNLGLSVNDEEVQRGLSLQDVIVRVKAERSIKPDSPVAVGGDAATP

YADVVMLLDELSRAGIPKVGLMTDIRE

>tr|Q87H77|Q87H77_VIBPA C-di-GMP phosphodiesterase A-related protein OS=Vibrio parahaemolyticus serotype O3:K6 (strain RIMD 2210633) OX=223926 GN=VPA1088 PE=4 SV=1

MKLSNRVILLVAPVIILSALVSSYIIYSIQKVTLIKREDSYIQLQMEKLAGQFRQANIFL

NSYAYTLSKSDVLQDYFINHDNPYRERVLFSRLDETADVLSHGYKGDASMAILDGKQNLL

YYTENQYYESSGVVDPKVLEYIATSFAARGEYSHTGFIYNSSGQSILLRYELIDKRTGAP

PVSFDPSNVFFVVVSVSLEVFNDIKHDVEFDTHSVITFADKPIYLDIPLAQTIELLPHFY

AVLSPAEYLMWNKVDKVWLELALSFGIAAFCTITLIVLVLYRYVLHPVSRLDKQLSELES

NQRDNIEKLGTNDEIGRLSSRFFDMYEELNVIYKKTKRLAETDHLTQLANRHRFHQLATR

ELASPPSHLWVIYVDLDNFKYVNDKYGHELGDNLLKVFSTHIKNACQKFSQQYDSPCFGA

RLSGDEFAILLSSNKDVGSIPDLLAKELLKPISNLSQTWANAFPVTASVGIAQYPQDGQD

IAKLLSNADAAMYQAKRAGKNQYAYYSAELNLESQRRSQIERALRKSNVEDEFRLVFQPY

MNNKDNEIEGLEVLLRWDAEGLGPVPPTEFIPIAEQAGLFDRIDRWVFANATEEYHHLRS

IFGKDIVLSINLSSAELNSLEMAQFIHKHVTRNGIKPECIELEITETFAAEQQGTALLDE

LSKLGYRLAIDDFGSGYTSLTQLVQYPVQKIKFDREFLLTLMNTNNGHVIKPIIELCHAQ

NKTVTAEGIEHDVMHKWLSAYRCDFMQGYLFGKPMDKEQLSLWWKSANDDNRFSQPLTTK

LAAEGGI

>tr|Q87KX5|Q87KX5_VIBPA Uncharacterized protein OS=Vibrio parahaemolyticus serotype O3:K6 (strain RIMD 2210633) OX=223926 GN=VP2849 PE=4 SV=1

MHTTFEQIFDLGPFSWPALFCCAINGLMIGIERQTRGKPVGIRTAILIISGTYLFMSMAV

SLSPNTLDQARVLGQIITGVGFLGAGVMMTLDGKIHGVTSAAVIWVLAGLGLMIGLGYLM

QSVVITLLALSVLLGVDKAENRIKALRRGVHQKIQQRKTSSRLIK

>tr|Q87NG0|Q87NG0_VIBPA Sensor histidine kinase OS=Vibrio parahaemolyticus serotype O3:K6 (strain RIMD 2210633) OX=223926 GN=VP1908 PE=4 SV=1

MPLKAKLILLTLIPVVLVSASISWISIYQAKTLGQREVEIFHQNLIQSKEAALKDTVDVA

FDAISHIYNDSTIEERVAKARVKAILNRLTYGSDGYFFAYDKHGTNLVHPVLPELVGENL

LHLEDENGDRLIEALLYQAQSGGGFHQYLWQKPSTGDIVPKLSYAAWLDKWEWMIGTGLY

IEDVSQEVANMRAAVNKNIETTFFSVVVILVVTVAVIIVLTLAINLHEHRLADKNLKELA

HKTVMFQEDEKKHLARELHDGINQLLVSSKCHLDLMSHRLQDEKLKSHLDKSQRSLVTAI

NEVRHISHQLRPSALDDIGLEAALTTLLQDFHSHSGIDIDSHFDTQQHKLTSEVATTLYR

VAQESLNNIEKHAKAKKVTVILQKMGNMLQLLIRDDGVGFVVNQAVHRQGIGLRNMQERV

EFIGGEFELMSELGLGTEITVLLNLDELVYGQTD

>tr|Q87SK8|Q87SK8_VIBPA Putative general secretion pathway protein A OS=Vibrio parahaemolyticus serotype O3:K6 (strain RIMD 2210633) OX=223926 GN=VP0415 PE=4 SV=1

MYKDFFGFVEQPFSIVPNSRYLYLSQRHKEAITHLNAGLGDGGGFAMLTGEVGTGKTTVA

KAMLANLDESTKAGLILNPTFSSRDLLEAICDEFKISYPQDATLKQLNQVIHHYLLRNHK

VGWQTLLVIDEAQHLAADVLEQLRLLTNLETDTRKLLKVLLVGQPELQRLLQTTQLRQLA

QRITGRYHLLPLDEKETADYIAFRLHTAGGDQQLFHRSSSKLIAKYSHGIPRLINLICDK

ALNMSYHQGSVVVDKQTVQQACEEVMQFQADIYQQDKPRQSFTWPAWGSAAIGVMAAVGV

GWAAINYMPMKPKAPMSEVPVAASSSTPAPLMATEQLTDAQRDMLLAQKQSNLAVNDLYR

LWGYRASVRDNLCLSEPQSTMRCERKMATWPLLMQQNRPVILELNYQGDVGYVILYAVGN

DQVEVLNGKQRLRLPVSWLKPMWQGNIIELWQAPLKETLRLDMEGPAIEVLDQLLAKAVS

ESPLETSIFDGALKERVELFQRWQGIGVDGIAGHRTLERLQQSVQPNAPTLASINKEEA

>tr|Q87LJ8|Q87LJ8_VIBPA Twitching motility protein PilT OS=Vibrio parahaemolyticus serotype O3:K6 (strain RIMD 2210633) OX=223926 GN=VP2614 PE=4 SV=1

MDLNKFLEGMLTLKASDLYITVGAPILFRVDGELRPQGEKLTEHDVAKLLDSAMDPDRRQ

EFRKSRESNFAIVRDCGRFRVSAFFQRELPGAVIRRIETIIPTFEQLKLPLVLQDLAIAK

RGLVLVVGATGSGKSTTMAAMTGYRNSNKTGHILTVEDPIEFVHEHKRCIVTQREVGLDT

ESYEVALKNSLRQAPDMILIGEIRSRETMEYAMTFAETGHLCMATLHANNANQALERILH

LVPKDQKEQFLFDLSMNLKGVIGQQLIRDKNGQGRHGVFEILLNSPRVSDLIRRGDLHEL

KSTMARSNEFGMLTFDQSLYKLVMQGKISEEDALHSADSANDLRLMLKTQRGEPFSTGSL

ANVKIDMD

>tr|Q87IS5|Q87IS5_VIBPA Transcriptional regulator, AraC/XylS family OS=Vibrio parahaemolyticus serotype O3:K6 (strain RIMD 2210633) OX=223926 GN=VPA0531 PE=4 SV=1

METMKEKNKEIATFKIAQELGGLELLDAKYEKQNFSRHSHEGYTIGVIEKGAQQFFRTGG

NHIAPQDSIILVNADEVHNGHSATEGGWEYKAMYPVPEQFQTLGQELGSPNISLPYFPQP

VVYDPELASQLRLVFETLEKSDNRLLRETLVYGTLIKLASKHSTHRAPLKESTKAQRQLQ

LVKEFLEDFPQTDVSLEELAKLAGLSPFHLLREFQKQFGFPPHAYQIQQRLRMAKKLLKQ

GQRISDVAQECGFHDQSHLHRHFKKAIGVTPGQYLRFN

>tr|Q87FH5|Q87FH5_VIBPA Putative integral membrane protein, possible transporter OS=Vibrio parahaemolyticus serotype O3:K6 (strain RIMD 2210633) OX=223926 GN=VPA1704 PE=4 SV=1

MEWQLILTLFGSFAVLLAIGVPVSFAIGLSSLATILMGLPLEPAIAVVAQRMAAGLDNFA

LLAIPFFILAGNIMNQGGIALRLINFAKVLGGRLPGSLAHVNVMANMMFGSISGSAVASA

AAVGGTMSPLQKKDGYDENFSAAVNITSCPSGLLIPPSNTLIVFSLVSGGTSIAALFLAG

YIPGILMGLSIMVVAGIIAKRRGYPIAARPTLAMVWDTFLKAAPSLALIVVIMGGIIGGI

FTATEASAIAVVYTFVLAVLVYREVKWRDLPKIILESAVTTSIVLLLVGASMGMSWAMAN

ADIPYMIADALLAISDNPMMILLIINIILLIVGIFMDMTPAVLIFTPIFLPIALDMGIDP

VHFGIMMTFNLAIGICTPPVGSALFIGCSVANVAIDKVIKPLLPFYVALIAALMAVTFIP

ELSLFLPKLVLGY

>tr|Q87HY8|Q87HY8_VIBPA Uncharacterized protein OS=Vibrio parahaemolyticus serotype O3:K6 (strain RIMD 2210633) OX=223926 GN=VPA0818 PE=4 SV=1

MELKKQSDYEQYIRQDGLGEYYVVINQFTFHSVYQPIFDKHQRVIGMEALLRIHGADGVQ

IRPDIFLSNASIDPYFRLCVEFLSRAMHIRNFARHFAGTPIKLFLNVMPQTLLTLTKDMG

FKDNGLLYKRLADLGMTPSDVVFEVVEESCGDTDLLIKAVQLMRANGFIFAIDDFGAQHS

DIARVQQLCPDIIKIDRSYLLDYCAGDTFSIRSAVDLAATMGAKVIIEGVEENNQLQAMR

QLELDYYQGFYLGKPSAIHHWTEYATCQCTVH

>tr|Q87I35|Q87I35_VIBPA Putative lipopolysaccharide modification acyltransferase OS=Vibrio parahaemolyticus serotype O3:K6 (strain RIMD 2210633) OX=223926 GN=VPA0771 PE=4 SV=1

MTYRAEVDGLRAIAVTLVILFHSGVEQFAGGFIGVDVFFVISGYLITTIVINDLENQKFS

LGDFYERRIRRILPLLLVVIAVSYLMSWWLFLPQAHKDVGQLAVSSILSSSNILLYLKGH

NYFGLEDQANPLFHTWSLGVEEQYYIVIPLLLMLLARGKNAFYLTFFIAVFALSLMAIVY

TSNDPDFAFYMIFSRAWELATGSLLALVMRKAQVQSNDTLATIGIVLILASALLFEKSQD

GAGITLLVPVIGSALVILFASKDNVCGKLLSLKWVVFVGLISYSLYLWHIPLFVFYRYML

DATQDVNISLYIAALFVLSYFTWRFVEKPFRSRKAMSMRTVSAVVVLLTLPLLTFGVIGH

QNGGFPERSAFFEAMRINNGYGLDCNGNTDVNDVCSSAPQPTIAVLGNSHSMVYVKRLSE

VTPTGVVQLTQDSCAVGYVDILEAAGSISCRQFFKQAVDTILRTPSIRRVVISSNFNKEL

SQADYEASLTGLLNELEGKEVVVFGPTPSAPFAVGECLWKARLFGEQEESVCDFSPKRHH

PQNVAKLVNYFDRFEHVEFVDLTDAICRNGVCRMKVGANNAMYTDDSHLSYKGAELVLGH

YHNSTNETQQFTYDRQ

>tr|Q87G70|Q87G70_VIBPA Putative transcriptional regulator, LuxR family OS=Vibrio parahaemolyticus serotype O3:K6 (strain RIMD 2210633) OX=223926 GN=VPA1447 PE=4 SV=1

MEHQTTRNVILITEGSLQSSLLKDVLETKLGINVLLITPENLASPFVRNQSISAIVLDYS

VITDEVFARYMEFKTPNLTGTLEILINCDKSISTDELFVWGALAGIFYTSDDIQTLQTGI

DKVLQGDMWFSRKFAQQYITHLRRHSRPINKNVPAILTKREQQIITFLSMGASNQQIAEQ

LFVSENTVKTHLHNIFKKIDVKNRVQALIWAKENISDHSIEMV

>tr|Q87MI7|Q87MI7_VIBPA Putative arsenate reductase OS=Vibrio parahaemolyticus serotype O3:K6 (strain RIMD 2210633) OX=223926 GN=VP2268 PE=3 SV=1

MTITMFGIPNCDTIKKAKKWLEAENIAFDFHDYRKQGIDAQMVTEFCQSLGWEQVLNKRG

TTFRQLTQEQKDTLNEENAIALLVDNPAMIKRPILNVDGQLHIGFKADQYATIFNS

>tr|Q87SP3|Q87SP3_VIBPA Lipoprotein OS=Vibrio parahaemolyticus serotype O3:K6 (strain RIMD 2210633) OX=223926 GN=VP0379 PE=3 SV=1

MTSLGHIKKLSWVLAAVLSLSACGEKDNSVIKVGATVGPHAQVVEAVAKEAAKQGLNVEV

IEFSDYVTPNAALSDGSIDINSYQHQPFLDNFNSSHNSQLVSIGQSILMRMGIYSNKYRS

LEELPNRARIAIPNDPTNGGRGLLLLADAGLIELKPGVGHKAALTDIKSNQKELEFVEVD

AAQLPRTLDDVDAAAITMNYVMSSGLDPKKQSIFLESKDAPLAVMVIATREADRNNEAYK

KFVSIYQSQEIRDFLDSTFKGTIEPAF

>tr|Q87HI7|Q87HI7_VIBPA Uncharacterized protein OS=Vibrio parahaemolyticus serotype O3:K6 (strain RIMD 2210633) OX=223926 GN=VPA0976 PE=4 SV=1

MSDILLGKLATEFKTVKAMVEVYCHDHHGTKDALCSECRELLEYAEVRLDRCPYGEEKPT

CNKCPIHCYKPEPKEQMRLVMRYSGPRMLLKHPILAVRHLLHEKREVPEKPAANASNRHK

RINAERDTKKS

>tr|Q87H23|Q87H23_VIBPA Putative transcriptional regulator, MerR family OS=Vibrio parahaemolyticus serotype O3:K6 (strain RIMD 2210633) OX=223926 GN=VPA1142 PE=4 SV=1

MNMKEFSSAVGLSSYTLRYYEKIGLLKHVHRNSSGHRVYTHRDIDWVNFIKRLKETGMPL

EEIQEYASLRELGSQTTADRQKLLEAHRDNLIEHIRQQNEHLKRLEEKINLYKSGKVR

>tr|Q87J90|Q87J90_VIBPA Putative efflux protein OS=Vibrio parahaemolyticus serotype O3:K6 (strain RIMD 2210633) OX=223926 GN=VPA0363 PE=3 SV=1

MINLAEFAIRQRKFVLFFIVLSVIAGIYSYFDLGKLEDPSFTVKTAVVVTLYPGASAEEV

EHQVTDTVETKLQEMAELDRLRSLSRPGLSMVFVDLKESLNSKALPQEWDLLRRKVDDVK

LQLPSSAQISVVQDEFSEVYGMLFSIHSTDAAPEELRRYAEELQRQIKAVDGIKKIELHG

VQPRVVHIDMPDERLAQYGLSIAQVWNQLSTQNSTFEAGKFDAGTERIRIAQTSEFQSLE

DIRNLIINGGTGEFGSGLIRLGDIADITMGYQTPALAENRYNGEPAVTLAVSPVQGINVV

SLGDTIQDIIANYQASLPLGVDISTVAYQPEEVQKSIDDFVGNLLESVAIVFVVLLVFMG

FKSATIVGASLLLTILLTLVYMNIASIDLHRVSLGTFILALGMLVDNAIVITDMMIAKLN

KGIERTRAAIDSVKETAVPLLGATVIAIMGASPVLFSKTDSAEFASSVFYIVASSLLLSW

IVAMTFTVLMAWMFIKPKANNEETKPSRYKQLVFWTVDNPRKALAALVPLILVTAVAIPY

VAVNFIPQSDRSIVFLDYWLPNGAKIEQTSADMRKVEEWLVAQPEVESISSYVGTSAPRF

SVTVEPEPLDPAYGQILINTKDYESISHLVTRGDDWLKEAFPDAEPRFRALKLATKDKFA

VEVRFSGPDETVLHQLAAEAKTIFASNPDAKYIRDDWRQESKVLKPILNQDKMRQAGINR

ADVAFALKRASDGMPLGQMNLNDELIPIQLRGTSQNMASLETLPVRSLLGFNSVPLGQVV

DGFELVTEESMIWRRDRVKTITVQAGVSRDSTPANVRNAIKDQVEAIQLPAGYSMEWGGE

YYDEDKAVTDIMKQQPKAMLIMVIILVAMFNGFKQPIIILATLPLAASGAVFALLGFDKP

FGFMALIGAITLTGMIIKNGIVLMDQIELERTNGKSLSDAIKEATVNRTMAISMGALTTA

LGMIPLLSDLLFDQMAATIIGGLAAATILSLFVMPALYRLVYKEKAPSTQTNAELEEASS

>tr|Q87IB5|Q87IB5_VIBPA Putative riboflavin deaminase OS=Vibrio parahaemolyticus serotype O3:K6 (strain RIMD 2210633) OX=223926 GN=VPA0691 PE=4 SV=1

MSQEFMRRALEVSKNALPECQPNPPVGCVLVKDNQIVSEGHTQAIGGNHAEVEALNAYQG

SLESVTAYVTLEPCSFVGRTPACAVTLVKSGIGKVVVAMLDPDPRNSGRGIEILKQAGIE

VEIGLCSEEVSEFLTPYLGKS

>tr|Q87P19|Q87P19_VIBPA Transcriptional regulator ExsA OS=Vibrio parahaemolyticus serotype O3:K6 (strain RIMD 2210633) OX=223926 GN=VP1699 PE=4 SV=1

MDVSGQLNTETVGSSLRKIRSFSHYEKHDEVFHSDQSHIVVVHNGQLRVQTGDCTIDVVA

GSGVFLSQGDYLLEYSPQGGNYHSSIIEFDNELVSQLLQKHSDLLMTLPKVDKLNSGLFS

FGLNILIEQVLSGMKTLEEQSYPDAIMRLKYEEMLILLLHSQGGEVLYALLSQQTNRTSD

RLRRFMEQHYLKEWKLTDYAQEFGASLTTFKELFNEHYGISPRAWISERRLLHAHKLLLT

SKMSIVDVAMEAGFSSQSYFTQSYRRRFGTTPSKVRSGDEQVAIAN

>tr|Q87HU1|Q87HU1_VIBPA Uncharacterized protein OS=Vibrio parahaemolyticus serotype O3:K6 (strain RIMD 2210633) OX=223926 GN=VPA0865 PE=4 SV=1

MKSTSTLLGLTIFYAIVFLFSALEPSSRAVWFAEIIPAIGILIAIWAISIRYQFSNTAYL

LMFIWLCLHTIGAKYTFAEVPFDWFNNLIGSERNNFDRVAHFAIGLYAYPIAEYLIRNKK

FNPTFSCWFALFAIMSLAAGYEIIEWWYAELAGGDEGIAFLGSQGDIWDAQKDMLCDTTG

AILSLFLMSAQRRFAKPF

>tr|Q87QJ2|Q87QJ2_VIBPA Putative formate transporter 1 OS=Vibrio parahaemolyticus serotype O3:K6 (strain RIMD 2210633) OX=223926 GN=VP1157 PE=4 SV=1

MNFNQFDSLLPPQAAERAAEVGVGKATKAPIKSFLLAISAGLHIGIAFIFYTTVTTGAGD

LPWGITRLIGGLAFSLGLILVVVTGGELFTSSVLTLVARASGKISWKTLVKNWFVVYFGN

MVGAILLVACMLVTKQYMFDHGQVGLNAMAISQHKLHHGFFAAVALGIMCNVLVCIAVWM

TFSGRTLTDKIAVMILPVAMFVSAGFEHCIANMFQVPMAIGIKYFAPEAFWQMTGADIAN

YADLNMMGFIVNNLIPVTIGNIIGGGVFVGMWYWMIYLRDEDKHLR

>tr|Q87K22|Q87K22_VIBPA Putative regulator OS=Vibrio parahaemolyticus serotype O3:K6 (strain RIMD 2210633) OX=223926 GN=VPA0076 PE=4 SV=1

MKGATMEKSTQPEAVSSVLKVFHILQALGEQKAIGVSELSQRLMMSKATTYRFLQTMKSL

GYVSQEGEADKYSLTLKLFELGAKSLEYVDLIELADKEMRHISEQTNEALHLGALDENAI

IYIHKIDSGYNLRMQSRIGRRNPLYSTAIGKVLLSERDESFVRDVLSDVEFIKHTEKTLE

NTDQVLEELAKVRDFHYAEDNEEQEPGLRCIAAPIYDRFGQIIAGLSISFPTIRFDEERM

EYYVGLLHQAGKNISEQLGYHDYPA

>tr|Q87M56|Q87M56_VIBPA Regulator of ebg operon OS=Vibrio parahaemolyticus serotype O3:K6 (strain RIMD 2210633) OX=223926 GN=VP2402 PE=4 SV=1

MATLKDIATEAGVSLATVSRVLNDDPTLSVKEETKRRILEIAEKLEYRTSSSKKATKEAK

QKHHFLALYNYKQEAEVNDPYYLSIRHGIETQCDKLGITLTNCYNSEIDVETQKITGVLL

VGKVDQKVVNKLPKRLADSICYIDFSDPTSPYDCVDIDLVRISKQVVDFFVQQGYERIGF

IGGQDEPNTADIRENAFVDYGNLKGVVSEDDIYRGDFSSLSGYDLAKEMLAKGDFPKAMF

IASDSIAIGVLRAVHEFGLNIPDDIALISVNDIPTARFTFPPLSTVRIHSEMMGIQGVNL

LVEKYRDGRALPLQVYVPSKLKLRGTTR

>tr|Q87HF9|Q87HF9_VIBPA Transcriptional regulator, LysR family OS=Vibrio parahaemolyticus serotype O3:K6 (strain RIMD 2210633) OX=223926 GN=VPA1006 PE=4 SV=1

MRNTDDYIIFYHLVEQGSFSSAARHMELTKSVVSKRIAKLEQELGVQLLYRTTRTLTLTE

AGQSFFVHAKAVYKAVATAEESIVGLGKNLSGNIKITVPTISGELILPGVISEFNDKYPD

INIDMELDNRFVDIVNERFDLAIRTGMLPDSSLIARKLVDANWVVCASPKYLAKHGIPKQ

PIELTKHNCLVYSYQETGAREWAFKAGDKMYQVTVDGNLCTNNSSALRNVALLGQGVIYV

PRVLVYEDLKQGSLIQLFKEETAKCLGIYAVYPYTRQQPEKIKIFIDHLYNSFQSQNHRF

>tr|Q87GX0|Q87GX0_VIBPA Nitrate/nitrite response regulator protein OS=Vibrio parahaemolyticus serotype O3:K6 (strain RIMD 2210633) OX=223926 GN=VPA1195 PE=4 SV=1

MCKVMLVDDHPLMRRGIHQLLSFEPEFEVVAEASNGADAVAKAHELELDLVLLDLNMKGM

SGVDTLKALRADGCEARIVILTVSDSPADIEAIVRSGADGYLLKDTEPDELVELLKQAHQ

GDKAYSQEVAKYLSERSDHEDVFDSLTDRETQILREVARGFRNKQIADRLFISESTVKVH

MKSLLKKLQVPSRTAATVLYLERFGDIK

>tr|Q87RU7|Q87RU7_VIBPA Riboflavin biosynthesis protein RibD OS=Vibrio parahaemolyticus serotype O3:K6 (strain RIMD 2210633) OX=223926 GN=VP0679 PE=3 SV=1

MPQHTSASPFSPQDFSMMSRALKLARRGIYTTAPNPNVGCVIVRDGVIIGEGYHHRAGEP

HAEVYAMRMAGDKAEGATAYVTLEPCSHYGRTPPCAEGLIKAKVARVVCAMEDPNPKVAG

RGIQMLREAGVEVQVGLLENDAIELNRGFIKFMQTGMPFIQLKMAASLDGQSALSNGKSQ

WITSPQARQDVQRYRAQSGGILSTSKTVLDDNASLNVRWDDLPQSVQAHYELTEVRQPAR

VILDRQQKLSDDLKLFSTEGERIIVSSEGDVCPQLDQSGKIDLTATLKAVVTQHNINHLW

VEAGATLASSLIKANLVDELIVYLAPKLMGSDGRGLIGALGLTEMAQVIDLNITDVRMVG

RDIRITATLVRKEI

>tr|Q87K25|Q87K25_VIBPA Putative transcriptional regulator OS=Vibrio parahaemolyticus serotype O3:K6 (strain RIMD 2210633) OX=223926 GN=VPA0073 PE=4 SV=1

MANWEGVSEFVAVAETNSFTGAAAKLKTSVAQISRRVSALEERLAVKLLHRTTRKVSLSE

AGQLYYQQCKHLVEGLELAELAVTQMQTEPRGLLRVTAPVTYGEMNLAPLLHQFLEKYPR

VDLDLVLTNQKLDLIEQGVDVAIRLGRLQDSSMIAKRLSSRQTYVCASPMYLERFGEPHT

LSELGRHQCLVGSVDYWHFKENKRDKSLRVSGRIKCNSGFALVDAAKRGLGLVQLPDYYV

QEALDSGELVEVLTDYRDDREGIWALYPQSRNLSPKVRLLIDFLAQELA

>tr|Q87MK0|Q87MK0_VIBPA Flagellar biosynthesis protein FlhA OS=Vibrio parahaemolyticus serotype O3:K6 (strain RIMD 2210633) OX=223926 GN=flhA PE=3 SV=1

MKLSLPFADKLPRIRQREMPAIGAPVMVLATLAMVVLPIPAFLLDMFFTFNIALAMVVLL

VTVYTRRPLDFAAFPTVLLIATLLRLALNVASTRVVLLHGHEGGNAAGNVIEAFGNVVIG

GNYAVGLVVFLILMIINFMVVTKGAGRISEVSARFTLDALPGKQMAIDADLNAGLIDQEQ

ARVRRFEVTKEADFYGSMDGASKFVKGDAIAGILILFINIIGGLSIGMAQYGLGFGEAIQ

IYTLLTIGDGLVAQIPSLLLSIAAAMMVTRQNTDEDMGEQLVFQMFDNPKALMITAAILG

VMGIVPGMPHFAFLTLAVAAGTGAYLIDKRQKQKAKEPALPAKAEDGSEPISQRELSWDD

VQPVDIIGLEVGYRLIPLVDRDQGGELLERVKGVRKKLSQDFGFLIPAVHIRDNLELTPN

SYRITLMGVAVGEAEIRPDQELAINPGQVYGMIDGEPTMDPAFGLEAVWIREEQREHAQA

LGYTVVDSSTVLATHLSQLLTNNASQLIGHEEVQNLLEMLGRSAPKLVENFVPDQLQLGV

VVKVLQNLLNEAIPIRDIRTIVQTLAEYSSKSQEPDILTAAVRISLKRLIVQEINGIEPE

LPVITLIPELEQILHQTMQASGGESAGIEPGLAERLQSSLSHATQEQELKGEPAVLLTSG

VLRSTLAKFVKNTIPNLRVLSYQEIPDEKQIRIVQAVGN

>tr|Q87MC2|Q87MC2_VIBPA Uncharacterized protein OS=Vibrio parahaemolyticus serotype O3:K6 (strain RIMD 2210633) OX=223926 GN=VP2334 PE=4 SV=1

MDLKSLLNQALNSDLLKQGAGALGKQTQNIKSSSSSSQFKTLGAGAIGGGLIGMLMGSKK

SKKMAKKMGSGALKVGGAAALGALAYKVYNDWQAKQSDQGVHEPFDPQDSKHSVLILKAM

IGAAKADGHVDDVEMARIEQALTEMGADEHVRQLVQQELHKPLDPVEIASQASTPQQASE

IYLASLIVADEQNFMEKAYLQELAKQLQLSPEVTYQLEAQMQ

>tr|Q79YW3|Q79YW3_VIBPA Polar flagellar assembly protein FliJ OS=Vibrio parahaemolyticus serotype O3:K6 (strain RIMD 2210633) OX=223926 GN=VP2245 PE=4 SV=1

MNNAMEFLLEQTKEREDQAVLALNKARSELEDYYRQVEQIEKYRLDYCQQLVDRGQAGLT

ASQYGHLNRFLCQLDETLSKQKQAEHHFKEQVENCKDYWLKMRQERMSYEWMIEKKAKEK

QIAEAKREQKQMDEFSTLLFSRKAKPF

>tr|Q87PU6|Q87PU6_VIBPA Uncharacterized protein OS=Vibrio parahaemolyticus serotype O3:K6 (strain RIMD 2210633) OX=223926 GN=VP1405 PE=4 SV=1

MSNSKYFQDELTYLRESGSEFAKYHPKLTHFLSEGTFDPDVERLLEGFAFLTGRIREKID

DELPELTQSLMTLLWPHYMRSIPSLCISELKPHTGSVTEKTVVKRGAEMASEQVEGTQCL

FRTCYDVNLYPITITNIEQTNSRTSSAIDVTLSTEHGLELSRIGLDTLRLHLHGEIHITR

TVFLWLFRYLDYVELDVGGGYKHRLGPEYVKPVGHEEDEALLPYSKNSFAGYRLLQELFS

LPDKFMFFDITGLEWLKGIPQRSTVKIKFHFKRALPSEVVLKDKHLRLHCTPAVNLFEKD

GDPIRLEHRRNEYKVRPQSNTQEHYEVYSIEQVESWSKDERRRKPLIEFESFEHQINQRD

KREFYKSKVGERVSGRGLERYISFHTHNGDIADLGTETVLMKLQCSNADLAERLSVGDIT

YATHKSPTYATFKNITKPTQSVSPQVNGELQWQLIANMSLNYLSLANIDVLKVLLSTYDF

HSRVDRQAHRASIHRLDGIVSSEMKPIDRVFRGVSVRGNQFKLVTNSKFFVNEGDMFLMA

TVLNEFIRLYSSVNSFTELEVFDEATGEVYNWKSLIGQQTIL

>tr|Q87NG1|Q87NG1_VIBPA Transcriptional regulator, LuxR family OS=Vibrio parahaemolyticus serotype O3:K6 (strain RIMD 2210633) OX=223926 GN=VP1907 PE=4 SV=1

MDKPISVVIVDDHQVVLDGFIARLELEPDIDVIGTASNGLEAIEVVKHLRPDVVLMDISM

PIMNGIEATGVIKEELPESKILMLTMHDNREYIMKVMQAGAVGYMLKEISAEKMVQAIKT

VNQGSTYFCESVTQTLFTQEIKPAHHKPNPLSRREEAVLRLVAQGNSSKKIATLLNISYR

TVETHRHNIKHKLDLHSTAELAKYAFETGLTE

>tr|Q87IG5|Q87IG5_VIBPA Putative transcriptional regulator, LysR family OS=Vibrio parahaemolyticus serotype O3:K6 (strain RIMD 2210633) OX=223926 GN=VPA0641 PE=4 SV=1

MDIDALRSFLAFVETGSFTRAAKQINRTQSAFSAQMRKLEDELHVSLFEKEGRNLVLSEA

GMSLRAHAEQLVSLHNQAVKQVKRYENKRPLRLGCPEDFNDSLLPKVIRVLQQTEPTCSI

QVYSEPSVTLREWLDEGKLDAAIVTRAPDSEEGYWLASDRGVWISHPDFAIDESQPVPLA

LFQTDCKYHAAAINALTKRGTPYQLLACCNTSSAQRAIVRSGMAIGAMGRISVTDDLKIL

EDLPPLPSVDIVLVTGVEPHPILDKECLSQLAELMPC

>tr|Q87KX1|Q87KX1_VIBPA Uncharacterized protein OS=Vibrio parahaemolyticus serotype O3:K6 (strain RIMD 2210633) OX=223926 GN=VP2853 PE=4 SV=1

MLEKNKPENHATDKHGLLSAPIPETLRKMTVPMTMGMIAILMFNLVDTFFISLLGTHALA

AISYTFPVTFAVNCITMGIGMGLSTNIGRLLGQGHAPQAARFTTHGLLLAVLLVAIASSI

GFATIAPLFRFLGATDDLIPLIEQYMQVWYLTIPLLVIPMAGNSAIRATGDTKTPAKIMM

LAGLINGMLDPLLIFGYGPFPELGIQGAAIASAFSWLGALCGSFYVLVKREKLLAQPQWA

SLKQDWQQTLKIGTPAALSNAMNPLSGAILMMLLSSHGTAAVAAYGAAQRIESILILVLM

SLTSALTPFMAQNLGAKNPQRAFSGLFLSMRFAVVFQGFIFLMMVPLSIPLAALFSQEET

VKNLLWHYLLVVPFSYGFQGIVMMLVSGLNAMHKPLRAFQWSFMRLFVFTLPAAWVGSQV

YSIEGLFIGIALGNTLGGLLGYMFALRERKLTLIEQNSAS

>tr|Q87MZ7|Q87MZ7_VIBPA Putative ABC transporter, permease protein OS=Vibrio parahaemolyticus serotype O3:K6 (strain RIMD 2210633) OX=223926 GN=VP2079 PE=3 SV=1

MSAFAFFGALEIGLIYGLVALGVYLTFRVLDFPDLSVDGSFPMGAAVAATAIVAGINPWI

ATGMAIIAGGMTGWVTAFLAVRCGILHLLASILTMIAAFSINIRIMGKPNMALLGEDTIL

TPFESMGDAMFIRPLVVGVLVLISAFLVVRLLNSDFGLGLRATGVNGRMVSAQGASTGFY

TYFCLALSNGFVGFAGALFAQTNSFADVTSGVGTIVVGLAAVILGQTLIPGRKIWVAVCA

VILGSVLYRLAVAFALSSGMFGLQASDLNLVTAVLVAVALIAPKLKQSMKAKQRVTTAKQ

AASKNGSGEAL

>tr|Q87GV9|Q87GV9_VIBPA Uncharacterized protein OS=Vibrio parahaemolyticus serotype O3:K6 (strain RIMD 2210633) OX=223926 GN=VPA1206 PE=4 SV=1

MNLALLSMFIPTFFFVSITPGMCMTLALTLGMSVGYRRTLWMMIGELAGVALVSVSAVLG

IAAVMLNYPWLFTVLKFAGGAYLLYLGIEMWRSRGKLAINLENSTSPPKGNWNLVLQGFV

TAIANPKGWAFMISLLPPFIDQSKALASQLMVLVSIILLFEFICMSLYATGGKGLKRVLG

QSKNVRLMNRFAGTLMMGVGVWLFVS

>tr|Q87LP7|Q87LP7_VIBPA GTP pyrophosphokinase OS=Vibrio parahaemolyticus serotype O3:K6 (strain RIMD 2210633) OX=223926 GN=VP2564 PE=3 SV=1

MVAVRSAHLNQNEQFELENWVASLGQEKSTASRLIEVYRDCQTILAGHEQAELLLWRGRE

MIEILITLSMDKATLVAAQLFPLVSSGAFNRELLEEKYSKEIIKLIDGVEEMAAIGQLNV

TMEGSAASSQVDNVRRMLLAMVDDFRCVVIKLAERICNLIEVKKAPDEVRRAAAKECANI

YAPLANRLGIGQLKWEIEDYAFRYQQPDTYKQIAKQLAERRIVREQYIKDFVEDLSQEMK

ACGINAEVSGRPKHIYSIWRKMQKKSLAFDELFDVRAVRIIADKLQDCYAALGVVHTKYK

HLPSEFDDYVANPKPNGYQSIHTVILGPEGKTIEIQIRTKQMHEESELGVAAHWKYKEGA

SSRSGYDEKITWLRKLLDWQEEMSDSGEMLDELRSQVFDDRVYAFTPRGDVVDLPMGATP

LDFAYHIHSEVGHRCIGAKVGGRIVPFTHKLHMGDQVEIITAKEPNPSRDWLNPSLGFVH

SGRARAKINAWFRKQSREKNLEAGREILEHELVKIGATLKDAEHYALKRFNVNTPDELYV

GVGSGDLRINQIINHINALVNKPTAEEEDQQALEKLQEAEHKAPAQSRPKKDAVVVEGVD

NLMTHLARCCQPIPGDDICGYITQGRGISVHRSDCEQLEELRHHAPERIIDTVWGSGFVG

SYILTVRVEAMERGGLLKDITTLFANEKIKVTSMKSRVDYRRQLAIMDFDLEVTNIEVLS

RVSKRVEQIKDVMSVKRLG

>tr|Q87S30|Q87S30_VIBPA tRNA (cytidine/uridine-2'-O-)-methyltransferase TrmJ OS=Vibrio parahaemolyticus serotype O3:K6 (strain RIMD 2210633) OX=223926 GN=trmJ PE=4 SV=1

MLDQVKVVLVGTSHSGNIGSAARAMKVMGLSQLVLVDPQCEVDEQTLALAAGAADIAQNA

QVVSTLEEAIEDCGLVVGSSARSRTLEWPMLEPRECGEKFAVEGQKHPVALVFGRERTGL

TNEELQKCHYHVCIPANPEYSSLNLAMAVQTLSYEVRVAHLNLEQKQFSPVEVEEYPRHK

ELEMFFDHLEKVIVETQFINKEQPGQVMNKLRRLFSRARPETQELNILRGILTSIEKKL

>tr|Q79YV0|Q79YV0_VIBPA Flagellar basal-body rod protein FlgC OS=Vibrio parahaemolyticus serotype O3:K6 (strain RIMD 2210633) OX=223926 GN=VPA0265 PE=3 SV=1

MSFTDIYSITGSAMTAQTVRLNTVASNLANADAVSANPDDAYKALKPVFATVYQKTQMTT

ENGIYPNAEVRIVDVVQNKGQAEKRFEPNNPLANGEGYVYYPDIDVVAEMADMMSATRSF

ETNVEVLANVKSMQQGLLRLGQGS

>tr|Q87P98|Q87P98_VIBPA Amino acid ABC transporter, periplasmic amino acid-binding protein OS=Vibrio parahaemolyticus serotype O3:K6 (strain RIMD 2210633) OX=223926 GN=VP1620 PE=4 SV=1

MANKLTILASVVAASTAMMATSAQAADTTLDKVTKQGFLTCGVSTGLPGFSNPNSKGEWE

GIDVEYCQALAAAVLGDKTKVKYVPLTAKERFTALQSGEIDVLSRNTTWTLHRDTALGLN

FVGVNYYDGQGFMVKKDLGISSAKELDGASVCVQSGTTTELNLADYFRNSGMSYKPVVFD

TAAQTSKGFDAGRCDVLTTDQSGLYALRLNLKDPSSAAVLPEIISKEPLGPVVRQGDDQW

FNIAKWTLAAMVNAEEYGITSKNADEMLKSQDPNIKRILGVDGPKGKGLGIRDDWGYQVV

KQVGNYGESFERTVGKGSPLEIARGVNALWNAGGFMYAPPIR

>tr|Q87M68|Q87M68_VIBPA Uncharacterized protein OS=Vibrio parahaemolyticus serotype O3:K6 (strain RIMD 2210633) OX=223926 GN=VP2390 PE=4 SV=1

MLNITDKKVEEKIPAWLRLGFRPFFLFGSIYAIVAIALWVWMFQTGQPNALAVPALWWHV

HEMLFGFSMAIVVGFVLTAVQNWTGINGTKHYTLLVLFGLWLAPRILLWTPVPLWLTSSI

EAVFLLFVAYEVGIRVYRAKGWRNLFFVPLFLLAIFANFASYAAIKGMPPFTSSAVWQAM

LWWFTLLLSVMGGRVIPFFTARRFNFEKAQPLRWLDWFANLPLVMLFVLSFFPVTFAELG

NPLMLFAGVAQLVRFVRWKPWLTLSEPLVWSLHAAYLCLPLSLILRGAWGDAFASHNLIH

LFAIGALGGLILAMIARVTMGHTGRMIYKGPNMSLAFAAITAAALVRSFAVIFDPANMML

WIDISGGLWIVAFGLFVWRFGMMLVTPRVDGHPG

>tr|Q87N99|Q87N99_VIBPA Transcriptional activator MetR OS=Vibrio parahaemolyticus serotype O3:K6 (strain RIMD 2210633) OX=223926 GN=VP1976 PE=4 SV=1

MIELKHLRTLTSLRDTGSLTATATALHLTQSALSHQIKDLESRIGGQLFLRKTRPVKFTS

EGEILLRLAEDILPKLARAENELASLKEDVNGRLHMAIECHSCFQWLMPALREYQLAWPS

VTLDFSSGFGFEPLPALLAGELDLVITSDIQPRSEVHYEPLFDFEMRLITATNHPLADKE

IIEPQDLADQTMLSYPVQKQRLDVVKHFLQPAGVEPARWKQADNTLMLVQMVSAGLGVAA

LPNWAISEFSRQGLITSKPFGKGLWRRLFAATRNSEKDKRYLQAFFATARQQCKSHLDGI

KMA

>tr|Q87FD1|Q87FD1_VIBPA Chloramphenicol acetyltransferase OS=Vibrio parahaemolyticus serotype O3:K6 (strain RIMD 2210633) OX=223926 GN=VPA1748 PE=4 SV=1

MSVFDTWLKGQVIKDHITNPNIIVGDYSYYSGFYHQKSFEEQPVRYLLGDPVTLQEWQSR

EPDSIDKLIIGKFCSIASGATFMMAGNQGHRIDWISTFPFSPEEFGEGVQDGFERAGDTV

VGNDVWIGSEAMIMPGVNIGDGAVIGARTVVTKDVQPYSVVVGNNQVVRQRFSDKEIETL

LNIQWWNWPIEHIKQAMKVMCSSQVGALADYYQEHIVDL

>tr|Q87MH9|Q87MH9_VIBPA Permease PerM OS=Vibrio parahaemolyticus serotype O3:K6 (strain RIMD 2210633) OX=223926 GN=VP2276 PE=4 SV=1

MFEMVSRWYQRRFSDPHAVSLVAILLFGFITIYFFGHLIAPLLVAIVLAYLLEWPVVKLS

RMGVPRTLSVIIVLLIFISIMLIALFGLVPTIWTQVGNLINDIPNMYNGLQKFIMTLPER

HPELANLQIVETVVTNAKNQALGLGESVVKGSLASLVSIATLAVYLILVPLLIFFLLKDK

EEMLRMASGILPKNRKLANKVWHEMNEQISNYIRGKVLEILIVGGVSYVTFAILDLRYSA

LLAVAVGLSVLIPYIGAAAVTVPVAIVGLFQWGMTPQFYWLLLAYGIIQALDGNVLVPVL

FSEAVNLHPVAIIVAVLVFGGLWGFWGVFFAIPLATLVKAVWNALPSTEESEPVQE

>tr|Q87RX9|Q87RX9_VIBPA Outer membrane protein assembly factor BamE OS=Vibrio parahaemolyticus serotype O3:K6 (strain RIMD 2210633) OX=223926 GN=bamE PE=3 SV=1

MQLKKWLVAVPLAMTLLTGCSVLEKLVYRIDINQGNYVEQSAVDKLKFGMTKAQVRFVLG

SPMLIENGYPNTWYYIYHHTPGHGDPVQKDLVVNFNDQGTLADISGDFPKSDAFYEQIQ

>tr|Q87K30|Q87K30_VIBPA GGDEF family protein OS=Vibrio parahaemolyticus serotype O3:K6 (strain RIMD 2210633) OX=223926 GN=VPA0068 PE=4 SV=1

MKLSEAYPIKQKNYSTTSKMLLLVFATSLLLANVILLQQTRVLAQSFTDEQKQATWFLFQ

LSKELSELVSEARRLDENVLKIEGAELQYELAWSRFDLLINSKDVYTFFSRNHIQQYFLQ

LFNEFKELEPLLVEAKTGDSQAAAQFYRATQTLYLNLVEFVNQNFRLTSQVYETQKDESQ

RLMKAQYMLFATFVLSILSLTYFFYRESHFHRKLALSDPLTGLGNRNALFVTLRNNTRNR

TAFSLCLLDLNGFKNVNDTYGHQAGDTVLCEIAKRLKDMNIDSFSTYRMGGDEFAVVIES

SDVDAEEAKKHIHSVFDTPVLNANNVSSLSTSIGVAHYPSDSDNVDFLISIADKRMYKMK

FQR

>tr|Q87HS3|Q87HS3_VIBPA Transcriptional regulator, LysR family OS=Vibrio parahaemolyticus serotype O3:K6 (strain RIMD 2210633) OX=223926 GN=VPA0883 PE=4 SV=1

MQSPITLEALHILDAIDRRGSFAAAANELDRAPSSLSYQIQKLEQDLDINIFDRSGHKAL

FTEAGKLILERGRAILQASEKLVNDATLLANGWELDITVAFDGIIPISNFFQMVDALAEV

SSTRIRLQEEILAGSWESLNTGRADLLVCPSLDTLPQEVKAEKIGKMSMIWVAATEHYVH

KRSGEFDDSAREKYRIIAIADTAREQPALSINIIQKQPRLTVTNFTAKVEALTAGLGIGT

LPRQIALPLIEKGVLKQIEGTEEQPMDIILAWRRNTMGEAKSWCIQYLKKNWKLK

>tr|Q87HN5|Q87HN5_VIBPA Uncharacterized protein OS=Vibrio parahaemolyticus serotype O3:K6 (strain RIMD 2210633) OX=223926 GN=VPA0928 PE=4 SV=1

MSEATNNESKKIDLETISPELRKVIEFDEVPEQMFEMVTSIHEVSEEAVRASWDEMPASA

QNILDNFEQFHALVSVGQAFAGINVMEEFPTLNLPENMTDEDKEEYRSQLLDNVLHNCVK

DMVKQLKKARRDPILKREFKEVFAK

>tr|Q87NK6|Q87NK6_VIBPA Putative threonine efflux protein OS=Vibrio parahaemolyticus serotype O3:K6 (strain RIMD 2210633) OX=223926 GN=VP1862 PE=4 SV=1

MLEQYLFLLPIATLLLIGAMTPGPSFILVAQTAISKSRSEAMCISIGLGLGASKFAFIAS

MGLITLFDAVPEFYVAFKILGGLYLCYLGVKMWRASKSKVVSADAELTSKPAHFKAIVLG

LATQLSNPKTAIVFSSVFAAMLPVKVPVHTTSILVVGVFVLNFSWYFLVSILLSSPKAQA

SYLRFKSYINKGSGVLMGSMGSKFVAESFEP

>tr|Q87FM2|Q87FM2_VIBPA Ferric siderophore receptor homolog OS=Vibrio parahaemolyticus serotype O3:K6 (strain RIMD 2210633) OX=223926 GN=VPA1657 PE=3 SV=1

MNKLLTLTPLAVAIGSSLVVPSAVASEETNSTPSATETIQVYGHQYEGYAEHMPQSGTKT

DVEWLDVPQAVSVVTKTEMQDRGAVRLVDALDGVAGVNNTLGEGSRDQFMIRGFDSLNDM

YRDGMRDDGTLQSYRSLANVERVEIVKGPAGALYGRGSAGGIINLVTKRANGDNFTHVKG

SVGSNSQYVGQVDSSMAFSDKVNGRINLEYRQADSYVDHVDSNDFFIAPTIRVLPADGHT

IDIDVEYAHQELVPYRGVPSKNGKPVDLPVSTYFGGTNDYQESDSLRVAVDYEWRLNDQW

VWNNRAAFNHIELEQKGTRQGKVTGNEVSQTVNNFGYDPRTTTTLQSELIWETNDNQLML

GADFNQIDIDLTLASDKTLPPQNIYNPVVGPTPDPGFKPFRDNTTTTTGVYVQDVYTWGD

LSVIGNVRYDSMELEQQKAGSGKEKLDDDKVSYRAGLVYRINYDTSVYASLARSWQLPYA

GIYINPKLAEFFHTDLKEVGAKAYLLDNALMLNAALFQIDQEQPQTNVDGDVIDKIEVRH

QGIELEARGQITKQWDISVGYSYLDAEDKATGKKPNDVSDHLFSLWSTYQLDDNWRLGGG

VKYVGDRYAGNDEAVALGDYTTVDLMAAYTTGRHKIQANAYNILDEKYILGATNGTSGLN

QIGYGAPAEFMLSYGYQF

>tr|Q87G55|Q87G55_VIBPA Methyl-accepting chemotaxis protein OS=Vibrio parahaemolyticus serotype O3:K6 (strain RIMD 2210633) OX=223926 GN=VPA1462 PE=4 SV=1

MRQFLSTLSIKLQVFLPVLFTVVLLVIGLSVGIGKLDQAFNKVSTSTNKLIIHKEELSAI

VDNTYAMRIKAIYSLFRAKDVQSLNQELSDRQNKNRDFLNSIDSLPGVQTEVNAMRKAMD

HYVDFTRVTMTPLLTTKHSSGYTTSDFNQKYESAMAEYRLAGEAMINAIDNLSKQLNQIV

TDEVEANGEQHSTTLTYSAVSLAVILSAASLISWILASYIVAPIRNLQGTMQEVAKGNLL

VKAEAIGKNEVSQLAQDVNQTIDKLRETVSALVRISEDVASASTELATVMTQSSVNSDQE

KQEVEQVASAINQLESTAAEVSTNAQEADSASNQARMLTSQSLSMFEESTRASEKMAEQL

NEAAAVVTSLKDQSEHIGRVIEVIEGISEQTNLLALNAAIEAARAGESGRGFAVVADEVR

MLAARTQESTKEIQTIIEELQQQSGHANESMHSSLAMLSDNQALAQEVSQSLANISNAIA

ELNSINTQVATASEEQKQVTADINNNLTNIYELVSQNVTGITQSAAAAQELSGLAENQQQ

QLRQFQV

>tr|Q87RV4|Q87RV4_VIBPA Uncharacterized protein OS=Vibrio parahaemolyticus serotype O3:K6 (strain RIMD 2210633) OX=223926 GN=VP0672 PE=4 SV=1

MLERLFKLSENGTNVRTEIIAGITTFLTMAYIIFVNPAILSDTGMDRGAIFVATCLAAAI

GCFIMGLVANYPIAQAPGMGLNAFFTYSVVLGMGYTWQVALAAVFVSGVLFILLSIFKIR

EWIINSIPHSLRTGISAGIGLFLAFIALKNAGIVVDNPATLVSMGDITSLPSVLAAIGFF

LTIALVHRGVKGAVMIAILGVTALGLLFGDVQWNGVMSTPPSIAPTFLQLDFSGLFEVGM

ISVVFAFLFVDLFDTAGTLVGVSQKAGLTDENGNIPRLNKALLADSTATSVGALLGTSNT

TSYIESVSGVAAGGRTGLTAVVVGVLFLLALLFSPLAGMIPAYATSGALFYVAILMLSGL

VSIDWRDLTEASPVVVTCLVMPLTFSIAEGITLGFIAYAAIKLFSGKGRDVSMSVWVMSA

IFIVKYLAG

>tr|Q87IG7|Q87IG7_VIBPA Arginine ABC transporter, permease protein OS=Vibrio parahaemolyticus serotype O3:K6 (strain RIMD 2210633) OX=223926 GN=VPA0639 PE=3 SV=1

MNEQHVWQMLDGLGTSLQLTAASLAVGCILALLMTLTLILRTPGLHWVSRGIITLFTGTP

LLVQIFLVYYGPGQFDAVRESVLWTWLSQPWFCAMLALALNTAAYSTQLFKGAFNAIPSG

QWQACRALGMNKSATLRVLLPYAIRRAVPAYSNEVILVFKGTSLASTITIMDLMGYAQRI

NAQTYDTLMVFGVAGAFYLSVNGILTLIFRQVEKKALAFETV

>tr|Q87HB6|Q87HB6_VIBPA Putative two-component response regulator OS=Vibrio parahaemolyticus serotype O3:K6 (strain RIMD 2210633) OX=223926 GN=VPA1049 PE=4 SV=1

MKPILLVDDSKTVLLYMTSALQKQGYEVIAVEDGEAALEVLTHRKDIQFVLSDLMMPGLS

GIDLCRLLKSAAFERYIFFVLLSSRNDQGSIVKGIDAGADDFVDKKTSVEELQARIRAGF

RTLDLHNMLLTRNQELDVAYQTIRQDLESAGDLMRQLLPVEQQIGATKLSYSYLPCSQLG

GDMLGYAALDEQHVAFYVFDVSGHGISSALMSFSIQKTLSRSCGPESVTTDLVEGEYVVS

EPNVVVERLNLRYQQTPDSQLYFTIVYAVLNTQTGKLRYCTAGHPKFLWRHQSEATIDMV

GDENFIVGALAPMQYKGGEITLEPNDSIWCFSDGVVEARYENEFFSVNRLIANVQSVSGL

PFEAQVSSVVESIKRWQVKEELDDDLTLLQLQWFGPNTSTCEGEDD

>tr|Q87Q10|Q87Q10_VIBPA Collagenase OS=Vibrio parahaemolyticus serotype O3:K6 (strain RIMD 2210633) OX=223926 GN=VP1340 PE=4 SV=1

MELKTLSVAIAATLSSTAAFAMSEPVAQVTEKVEHHQHEHGVETAQPEYAPTELLPQLPK

QTLRTRATQSVEASSVVCDVESFTTTNSNDLISAIKTQGANCINELFSAQSRVQEAAFDS

DHMYNVAKHTVTLAKAYTGGGSDELEALYLYLRAGYYAEFYNNNISFVSWVTPAVQEAVD

AFVNNANFYENSDPHGKVLSEVIITMDSAGLQHAYLPQVTEWLTRWNDQYAQNWYMRNAV

NGVFTILFGGQWNDQYLQIIGNQAELAKALGDFALRESSIGASDEFMVANAGRELGRLTK

YSGSAATTVSSKLKDIFARYEMYGKGDAVWLAAADTVSYYAECSEYGICDFETKLKGLVL

SQTYTCSPTIRILSQNMTQEQHVAACSKMGYEEGYFHQSLETGEQPVADDHNTQLQVNIF

DSSDDYGKYAGPIFDISTNNGGMYLEGDPSKPGNIPNFVAYEASYANPDHFVWNLEHEYV

HYLDGRFDLYGGFGHPTEKVVWWSEGIAEYIANEKDNQAALDTIRDGSTYTLSEVFETTY

DGFDVDRIYRWGYLAVRFMFERHKDDVNQMLVETRQGNWSNYKATINQWANLYQSEFEQW

QQSLVSGGAPNAVITANNEGKVGESITFSSENSTDTDGQIVSVLWDFGDGTTSTQTQPTH

QYGSEGQYTVSLTVTDNDGLTAIATHNVTISATGGSSTLPQNCAVQSKVSGGRLNAGEPV

CLSNQQTIWLSVPAVNEHANIAISTGNGTGDLKIEYSNLGWPDGSNLHGWSDNAGNKECI

TVSSQANYWGYIKVSGSFENAAIVVDFDAEACRE

>tr|Q87J37|Q87J37_VIBPA Uncharacterized protein OS=Vibrio parahaemolyticus serotype O3:K6 (strain RIMD 2210633) OX=223926 GN=VPA0416 PE=4 SV=1

MTANTDPYIEIRPYNDEEIPAALDRLIQDDEFITAILDHRFENKAKWFKTLMSPFLRIYL

KAKWSKLDSVEAIQIEVKKYLQDTLNKTTDGVTFSGLDKLDKNTSYLFVSNHRDIAMDPA

LVNYGLYQSGHRTVRIAIGDNLLKKPCATELMRLNKSFIVKRSAKAPREMMKALGQLSGY

IKHSLDTGNSIWIAQKEGRAKDGNDFTDPAILKMFHVEGRKQKVAFGEYMRSLKIVPVSI

AYENDPCDIAKAKELFEKAENGRYEKGEFEDIESIIQGIVGYKGRVHVAFGDVIEQEFET

PESLANEIDRQIHENYKLYPINLLAAGREDSSIITEAVKRQLADKLEQLPEGARPYLVAS

YANPVNNQD

>tr|Q87MM6|Q87MM6_VIBPA Phosphohistidine phosphatase OS=Vibrio parahaemolyticus serotype O3:K6 (strain RIMD 2210633) OX=223926 GN=VP2205 PE=4 SV=1

MKIFIMRHGEAEHFADSDAARELTQRGRTESEAVARACAEQGFAQFDKVLVSPYIRAQQT

WQEISTIFSTKSIETSDDITPYGQSDCVFDFANALIEVEKLESLLFVSHLPLVGYLTSEF

VKEMTPPMFPTSGLVCIEYEPQTQRGEVLWHITP

QQVYLFAATSVSSSGFWSLVFMTFHYPILMLLFIFMARRLTHQNQYQEELKLSE

>tr|Q87K27|Q87K27_VIBPA S-(hydroxymethyl)glutathione dehydrogenase OS=Vibrio parahaemolyticus serotype O3:K6 (strain RIMD 2210633) OX=223926 GN=VPA0071 PE=3 SV=1

MTLEIKPGQTHIKSKAMVAWAAGEPLKMEEVDVQLPKAGEVLVRIVATGVCHTDAFTLSG

DDPEGIFPSILGHEGGGIVEMVGEGVTSVEVGDHVIPLYTAECGECKFCKSGKTNLCQAV

RETQGKGLMPDGTSRFSINGETIFHYMGCSTFSEYTVLPEISLAKVNKQAPLEEVCLLGC

GVTTGMGAVLNTAKVEKGDTVAIFGLGGIGLSAIIGARMAGASRIIGIDINESKFDLAKQ

LGATDVINPQKFDKPIQEVIVEMTDGGVDYSFECIGNVNVMRQALECCHKGWGESVIIGV

AGAGQEISTRPFQLVTGRVWRGSAFGGVKGRSELPEIVNRYMAGEFGLQEFITHTMGLED

VNKAFDLMHEGKSIRTVIHMDK

>tr|Q87K35|Q87K35_VIBPA Uncharacterized protein OS=Vibrio parahaemolyticus serotype O3:K6 (strain RIMD 2210633) OX=223926 GN=VPA0063 PE=4 SV=1

MVTVHYFFDPMCGWCYGASPLIEALMDTSQFKVELHPGGMIEKRAIESEFRQHIINSDAR

IATETGATFGEAYLQRVKSEDAFVLDSYLPTQAILSAEKMGLNPWHMLKAIQSAHYQLGL

KVNEASTLKAIAESLGLANDVWEQNMTLSEADLDEKIRSSRQLMRQLHVGGYPTLIAEVN

GEYLTLPHSTYYDKPAQWKLALQKLQ

>tr|Q87JK6|Q87JK6_VIBPA Putative virK protein OS=Vibrio parahaemolyticus serotype O3:K6 (strain RIMD 2210633) OX=223926 GN=VPA0243 PE=4 SV=1

MKMTANKSIFDHTKVIYADEYANTYNPIWVYRMKFIMRALYYKRAFKHLANNIESSLLDM

LCTRTHRFLEKPFRPYIIKNNSAFDRSNLVVDHYKTISELLSKETIAEIYTDSKGLTLTS

FEIDDIEYSVRLVYEARYQKEGDMSLVLHSAEDGNFYTLSFTVGHVDTGRCIMIGGLQGP

RSSEENNAKIKKLTRKLYGQRPKSLMVSLLTLIAQVWEVNTILAVKTQSHTYAARRYSKG

RIKTDYDALWQELGGIEYNRHFYALKVNDTRRDTEGMSRSKRSMYRRRYEWLDNTKAEFA

TRLR

>tr|Q87FQ4|Q87FQ4_VIBPA Uncharacterized protein OS=Vibrio parahaemolyticus serotype O3:K6 (strain RIMD 2210633) OX=223926 GN=VPA1624 PE=4 SV=1

MNYLITFFKGIAMGAADVVPGVSGGTIAFITGIYDTLLESIRRINPSLFSIWRKDGFKAA

FNHINGFFLIALFAGILSSIATLAKLITWLLDTHPIPIWSFFFGLILVSVYHILRQVEKR

DAIRFVTLLLGVGFAYSITVLKPLHLEPTAINTLIAGAIAICAMILPGISGSFILLLIGM

YTPVLAAVKGFQVDTLALFLSGCVIGLLSFSHLLSWLLRKFRDFTLMFLTGLMIGTLPKI

WPWKETLTWRTNSKGEQVPLLQHNLSPFEFEHITSQPSQLVIAVVMMLAAIALVLGLEKF

ADSDK

>tr|Q87JH6|Q87JH6_VIBPA Uncharacterized protein OS=Vibrio parahaemolyticus serotype O3:K6 (strain RIMD 2210633) OX=223926 GN=VPA0277 PE=3 SV=1

MQVGNEALVLSAVGVIGLGCQWLAWRLRLPAILFLLLAGLIVGPFMQWLKPDEILGNLLF

PLVSLAVAVILFEGSLTLNFKEIRGVSGSVWSIVSIGAIISWAATSVATHYFLGFTWELA

MLFGSLTVVTGPTVIVPLLRTVRPNSTLANILRWEGILIDPLGALFVVMVYEFIVSHSAV

NSMEVFGTIIAVGVILGVTSGAAVATVLRRAWLPEYLQPFAVLMVVLGVFSVSNHIESEA

GLLTVTVMGMWLANAKDINIQQILHFKEHLTILLITGLFIFLAARISLDDFAALGSGALL

LFVFMQLVSRPLSIFLSTMRSNLNLKDKLFLSWVAPRGIVAASISSLFAIKLTEYGVDGA

SLLVPMTFMVIIGTVVLQSATARPMAIALGVAEPAPRGFLLIGANRVAREIGQALARYDR

RVLMTDSNWEYISQVRMMGLDYYYGNPISSHADDNLNMIGIGQVVALTPDQHFNIMACMQ

FVDEFGEDKVHCLQKVKTNGNGSEKHSVAQEYHGKLLMGGNVSYTQLASLLSRGAEIKHT

KLSENFTYQDYLEHHKTNLVIPLFNVEDKGKIQFCDDPDQFAPSTTSTIVSLILPESAQ

>tr|Q87G56|Q87G56_VIBPA Phosphate ABC transporter, periplasmic phosphate-binding protein OS=Vibrio parahaemolyticus serotype O3:K6 (strain RIMD 2210633) OX=223926 GN=VPA1461 PE=4 SV=1

MKKTVIGAIALLGAMAVTPVSAKETISAVGSSSVTPLMEVFSETYMKTNPNVFIEVQGPG

SSAGVKAAKNGSADLGMSSRNLKESEKEPTLVEEVVARDGIAVVVNPQNKLAGLTAEQVT

AIYKGEVSNWKEVGGEDKPIVAITRDTASGTRGAFEDIMALKMKISGKKVSAISQRAQVA

NGNGALKTMVASNPYAIGYISLGTVDNTVNALAIDGVDATVANVKNGSYKVARPFLVLYK

EGKPSAETQKFLDWMLTEDAQKLVDQNGYISVH

>tr|Q87HT8|Q87HT8_VIBPA Uncharacterized protein OS=Vibrio parahaemolyticus serotype O3:K6 (strain RIMD 2210633) OX=223926 GN=VPA0868 PE=4 SV=1

MDQLTATLKKIEKQNYRAYQQIKGQYDFTDFTLFIDHVQGDPYASASRFRATRAWSLTGL

EWLKDESPAFQRAARDFIARSFEQFAKQENTVSIALNGQTVLDSTAVLFTEEGIELRFRV

NLPAEGRSVLGKKANNILTFHLPKFIRRATLERELDKEAMVKHCQVVEDQSALREQLEAH

NLVAFVANGSVLPRIAGNCDLPMKEAVEFTAPESLQVTLHAPNKGYVTGLGIPKGITLIV

GGGFHGKSTLLNAIERSIYDHIPGDGREYIVTDQKAMKIRAEDGRCVHHLNLSNYINHLP

MGKDTADFSTQDASGSTSQAAWLQESIEAGATSLLIDEDTSATNFMIRDERMQALVAKGD

EPITPLVDRIGQLRDELDISTIIVMGGSGDYLDVANTVIQMHDYQAVDVTEKAKQVIAQH

PTQRHNESEESLQTFRPRALNRVALMNILTDGKFRVSAKGKDSLRFGKEFTDLSALEQIE

SADEVNAIGWLWFQLAQLPGWCNNPAKEIEEMLSGEWHASLPKQGDLAKPRTLDVMAALN

RMRKSQFKPSH

>tr|Q87S72|Q87S72_VIBPA Soluble lytic murein transglycosylase OS=Vibrio parahaemolyticus serotype O3:K6 (strain RIMD 2210633) OX=223926 GN=VP0552 PE=4 SV=1

MTQMRFNVSELAKNVCYAAALCAVSMTANASLSLEKQREVYEQAQDLLDKNDIDGYLSIR

PKIADYPLTPYVDYRTFIRQLSMKSPQQVDAFINEHEAFPFSRRIRAPYLDNLYKQKDWK

TITEFQKVIPSGERYQCIFYVAQLKQGKQVAAFKGAEDMWLSGSSIASECDPLFNAWDKA

GGRTDDLVLQRMLLAFDARNGSLMAYLQKLPSSAKAKQQAQEMKALFDKPATVVEFAKKK

PANDFYRAQSEYALEKLARMDVNQAQKAYAQVVKGQKFSAEKAQALADYIAIRLIRTESD

SLAKWRDDKTKTSKNVALIENRIRLAIQNADWKGVQQWIAVLNKDEQASLRWQYWLGRSE

IALGDDIAGKQRLATLVGQRNFYSVAAANAIGQSIKYPSHRIKLDTKVIHPYQNSLTRIE

ELIATDKIAAAKSEWAHLLSRVGKDEKAMLAAYASSKNWHHLTVTASIQAQMWDNIELRF

PIAHRWWFNFYAKKHDIDPITMMSLARQESALDSEARSPVGARGIMQIMPATAKYTARKY

KLTYQGSDDLYNVGKNIEIGSHYLQGLLEDYDNNRIFALAAYNAGPNRVKTWRERTQGKV

DAYAFIEAIPFNETRGYVQNILMFETYYRDLLGIDGAFLNQHEINTKY

>tr|Q87PJ0|Q87PJ0_VIBPA Uncharacterized protein OS=Vibrio parahaemolyticus serotype O3:K6 (strain RIMD 2210633) OX=223926 GN=VP1512 PE=4 SV=1

MMKDNKEINTSRRDLLKGFTTAAVAGAVVAGTTKVAVASETVEPSEKDVKKKGYRETQHI

RDYYDTL

>tr|Q87IA1|Q87IA1_VIBPA Putative cytoplasmic membrane protein OS=Vibrio parahaemolyticus serotype O3:K6 (strain RIMD 2210633) OX=223926 GN=VPA0705 PE=4 SV=1

MELTMTALIVFIVIVLLVVVYAISIYNKLVTLRNRFKNSFAQIEVQLKRRYDLIPNLVNT

AKGYMDHEKETFERVIQARNQAIAGLKAASDAPDSNAAISQLSQAEGMLQNALGKLNVVV

EAYPELKANETMSQLQEELTSTENKVAFARQAFNDAVTSYNTYKQTFPPVIFANTFGFQD

GKLLEFADTEAIQEAPKVAF

>tr|Q87Q61|Q87Q61_VIBPA GGDEF family protein OS=Vibrio parahaemolyticus serotype O3:K6 (strain RIMD 2210633) OX=223926 GN=VP1289 PE=4 SV=1

MNSFRWDIYFETGIEDVDDQHQYLVEFINKYGKLLTRNTISIADIQSALFELSRYAEFYF

KEEENLMREVGIHHEHLQKHIQVHRTFMGDIVSMQSFINEDNRQSAEQLLDFLIHWLAYH

ILGIDQNMAKQIKAIERGVSPLEAYREQEQQANASTEPLLEALNALFSQVSERNRDLLKL

NLELEEKVEARTNQLLTANKQLEALSLTDSLTQLPNRRSAIKTLKKLWDDTEHKTLPLVC

IMIDADYFKQVNDTCGHEAGDLVLIELAQALKYHFRSDDIVCRLGGDEFFVICPDTGLDG

GLHVAEMVRKQVSQMLVPTCYEPWRGSISVGVAERTENMSTYTDLIRAADESVYLAKQEG

KNRVRAVQKYVTLEPVSVRQN

>tr|Q87IY4|Q87IY4_VIBPA Putative long-chain fatty acid transport protein OS=Vibrio parahaemolyticus serotype O3:K6 (strain RIMD 2210633) OX=223926 GN=VPA0472 PE=4 SV=1

MKNLSKIVVASSLLTCAAAQAGGLYLYETTASDIGLASAGMAARAQDASVMAANPAGLAN

VSGKSFSGNLIGLYGDAQLDTMTGDAGNVIGFVPMASAFYSQQVNDKWTLGIGLYGNYGL

GLEYEGLLNNHLDIPTATTQALTIQPSASYRINDHWSVGAALGIQYGMYEVETKGALNFN

DEDQDTQLNGRVGVLYEATPGTRLGLSYSSETEFEFDNSNSIAPQQLIFSAYHEVNDDLA

VMWNVNWQDWSEYTTTLKAADLVDVETQDTYQIALGTQYKLNEKMMWNAGFAFDTSMYES

QSNGDITVPTGKAYRIGTGIDYKLDSENSIGFAFEAVLMESSETPRLQAGFDEPALYFMS

MSYNWKN

>tr|Q87M17|Q87M17_VIBPA Ribosomal-protein-alanine acetyltransferase OS=Vibrio parahaemolyticus serotype O3:K6 (strain RIMD 2210633) OX=223926 GN=VP2441 PE=3 SV=1

MTIEILPICSEHVEQVWQIEQQAHSHPWAESLVRDLSSRGACHHVMVEDGSVVGYFYAQN

IVGEVTLLNIAVAPALQGKGYGQKLLDAFLNHCEQAKADSAWLEVRESNHPAIHIYEQAG

FNEVDRRYDYYPAKTGNGKEDAIIMSYLFFN

>tr|Q87SL8|Q87SL8_VIBPA RNA polymerase sigma factor RpoD OS=Vibrio parahaemolyticus serotype O3:K6 (strain RIMD 2210633) OX=223926 GN=rpoD PE=3 SV=1

MDQNPQSQLKLLVIKGKEQGYLTYAEVNDHLPAEIVDSEQVEDIIQMINDMGIKVVETAP

DADDLALNDDTNITDEDAAEAAAAALSSVESEIGRTTDPVRMYMREMGTVELLTREGEID

IAKRIEEGINQVQSSVAEYPGTIPYILEQFDKVQAEELRLTDLISGFVDPDADDTAAPTA

THIGSELSETQLEEEDEEDLEDDEESDDDSDDSEEDVGIDPELALEKFNQLRSTYQNLQL

AINEYGYDSPKATVANEMMLDVFKEFRLTPKQFDHLVNELRTAMDRVRTQERLIMKSVVE

YGKMPKKSFIALFTGNESSDAWLDEILASDKPYAEKIKRNEEEIRRSIAKLKMIEEETSL

NVQNIKDISRRMSIGEAKARRAKKEMVEANLRLVISIAKKYTNRGLQFLDLIQEGNIGLM

KAVDKFEYRRGYKFSTYATWWIRQAITRSIADQARTIRIPVHMIETINKLNRISRQMLQE

MGREPLPEELAERMQMPEDKIRKVLKIAKEPISMETPIGDDEDSHLGDFIEDTTLELPLD

SATATSLKMATKDVLAGLTPREAKVLRMRFGIDMNTDHTLEEVGKQFDVTRERIRQIEAK

ALRKLRHPSRSETLRSFLDE

>tr|Q87S40|Q87S40_VIBPA Isocitrate lyase OS=Vibrio parahaemolyticus serotype O3:K6 (strain RIMD 2210633) OX=223926 GN=VP0584 PE=4 SV=1

MTLTRRQQIEALEKDWATNPRWKNVKRTYTAEEVVELRGSMVPANTIAQRGADKLWSLVN

GSAKKGYVNCLGALTGGQAVQQAKAGIEAIYLSGWQVAADNNTASTMYPDQSLYPVDSVP

SVVKRINNSFRRADQIQWANGKSPEDEGGIDYFLPIVADAEAGFGGVLNAYELMKSMIEA

GAAGVHFEDQLASVKKCGHMGGKVLVPTQEAVQKLVAARLAADVSGTTTLVIARTDANAA

DLLTSDCDPYDKDFIEGERTQEGFYRVRAGIDQAISRGLAYAPYADLIWCETATPCLEEA

RKFAEAIHAEYPDQLLAYNCSPSFNWEKNLDAETIAKFQQELSDMGYKYQFITLAGIHNM

WFNMFELAHAYAQGEGMRHYVEKVQRPEFQAAEKGYTFVAHQQEVGTGYFDRMTNTIQGG

NSSVTALTGSTEEDQF

>tr|Q87QT8|Q87QT8_VIBPA Peptidoglycan-associated protein OS=Vibrio parahaemolyticus serotype O3:K6 (strain RIMD 2210633) OX=223926 GN=pal PE=3 SV=1

MQLNKVLKGLLIALPVMAMTACSSSDDAASNTGAATNNNAAAETTVATPIDQSGQLTEQE

LKEQALRETQTIYFAFDNSTIAGDYEEMLAAHASYLSKNPALKVTIEGHADERGTPEYNI

ALGERRAQAVANYLQALGVQADQISIVSYGEEKPLLLGQSDEVYAKNRRAVLVY

>tr|Q87MS2|Q87MS2_VIBPA Putative methyl-accepting chemotaxis transmembrane protein OS=Vibrio parahaemolyticus serotype O3:K6 (strain RIMD 2210633) OX=223926 GN=VP2159 PE=4 SV=1

MLEKYRNQSVGFQLKLVITLCLVIAFSSIAALVYRNASGVLLESTLREHQSKVESMAKTI

AGQFDAYLHTAQVLESTFRNGYLAGVYVENYTVDFMGHEVPNITQYSESLINDTKLVDSF

TRDTGAIATLFAPLGDDFIRVSTSLKDPQGKRAVGTTLGRNHPGYQLLKSGKPYYAQIKL

YGERYITYYAPIKDANGNVSGLSFIGLPVDQATQTLFDALEEIKWGDTGYTIIVDNDENN

LGKYLLHPTKSATDPSIVEVRDYDGNKPFHKIFEQKSGLIRYPFQYGSTVGEKYLVFTEV

PGWNWKLLGGTFIKEVTKGSDTLLKLIAIISSVAASITFVVLTLFLNRSLQPLTVLNSYM

TRLANGEVSLQIPSSRKRSKNEIVNLNNGVASMANQLNELVGQIRSTSDLVESNSTSVAS

DAHSNLTQADRQQEEVEQVVTAIEEMASSAQSVAQQVESIAENVRSANLDSQSGLTIVEG

VCVDVAQLNDQLDQSAEAIEQVNRDSESIQTVTRMIDEIAEQTNLLALNAAIEAARAGEQ

GRGFAVVADEVRTLAHRTQSSVQDVVGIIEKLKGSTQNAVTMMTDSQRSANQVLDKAQDA

GTALEAIAVQVQSIASQADAIAATSEEQAQVSQEIAANAHSISELNRESRNTSAKTSQSA

IELQQQARNLKEQVDFFH

>tr|Q87N94|Q87N94_VIBPA Putative methyl-accepting chemotaxis protein OS=Vibrio parahaemolyticus serotype O3:K6 (strain RIMD 2210633) OX=223926 GN=VP1981 PE=4 SV=1

MFNSIRTRIAVSAGGAMAFTLLIAMGMTTNAFTQVNEQITTKVKSQLNEATTTDLSNTAI

QQGLNISNQLEPVLANLKQARSIIELSAETAATPDIIVKQFIASLEAQNKAVFAGYMVWE

KPSWVEPTIQDKSLGFNSEGYLAPFFSPNSHNSFDVVAMDSFSNTELNNNGERKDDWHLM

PYETGKTFVMEPYMYPVRGKQELITTISQPIKLRGEIIGSLGFDLSLNELQSQSEDYASD

LFDGQGRILISSWKGITLANSSQADQVGKKVSHELASQWARIQSIAKQKGAGLVTFGGDE

YAITAIETSDAPWIVMVSVPSSFLKKNVDDYERWSEEQSAEALEKGVWAGIIAALIGIVA

MTMIATSLGKVLTNLVERFKDAAQGEGDLTYRIEVKGKDETAQLAHWFNTFLARIQEMLL

TVMATADQVDKNASEGQARAAASRDQLNVQVNEVNSLATAINEMSATAQEVANSAVQAAA

AASQVQSNSANGMSRMDNAASAVDNLASQVNDAQHQTQNLVASSTAIQGILSEIGGIADQ

TNLLALNAAIEAARAGEAGRGFAVVADEVRNLATRTQGSTEEIRAMLARLEQETQSIVVL

MEQSQKQAVDTKEETQAAQLALAEINQAIEVINDMNNQIASAAEEQSSVSEEINRNVVVI

NDTAMEVMDTMSSSVAISNELTVKASDLHGELSKFKLS

>tr|Q87QX1|Q87QX1_VIBPA Uncharacterized protein OS=Vibrio parahaemolyticus serotype O3:K6 (strain RIMD 2210633) OX=223926 GN=VP1028 PE=1 SV=1

MDKPILKDSMKLFEALGTIKSRSMFGGFGLFADETMFALVVNNQLHIRADQQTSSDFETQ

GLKPYVYKKRGFPVVTKYYAISSELWESSDRLIEVAKKSLENAKLEKEQQASTKPNRLKD

LPNLRLATERMLKKAGIDSVAQLEEEGALSAYKAIRDTHSTTVSLELLWALEGAINGTHW

SVVPQSRREELMNGLS

>tr|Q87HV5|Q87HV5_VIBPA Putative formate transporter 1 OS=Vibrio parahaemolyticus serotype O3:K6 (strain RIMD 2210633) OX=223926 GN=VPA0851 PE=4 SV=1

MAVTSSQTHQDFSPKEMMAEAEKFALSKANKTSSMTLGLAIMAGAFIGLAFLFYITVTTG

SANTGWGLSRLAGGIAFSMGLILIVICGGELFTSSVLSSISWANKQISFGKMLSIWGKVY

IGNFIGAMFLLALVTAAGLYQMDAGQWGLNALNIAQHKLHHTLLQAFALGVLCNLLVCLA

IWLTFSSANAMTKALMTILPVAMFVSSGFEHCVANMFMVPLGIVIANFAPESFWASVGVP

ASQYADLNVAHFISANLIPVTFGNIVGGAVLVGLANWCIYRRPELKAANVSSITNTTQLS

SVKEITMKNASFVKDIMNPKPVTLSVEMPVAAALDTLLDNNLTSAPVVDLHNRLVGFFSA

HDVMVELWCQDYIPVKDQKVVDLMSRDVVAIDASDRLVDVVEFLCIDKEQLYPTSSMGIA

TRLTSLSLEERAKSMKVSKPQVLPVLENGQMVGVVTRQEVLKALRPVFGERLNLVEDKAL

ETA

>tr|Q87IJ4|Q87IJ4_VIBPA Putative chemotaxis transducer OS=Vibrio parahaemolyticus serotype O3:K6 (strain RIMD 2210633) OX=223926 GN=VPA0612 PE=4 SV=1

MFFNKSLQQENQRLKQELYSLQQIQQSLDEEMLRLTLDAKGNVTSANQKFLQQLSLSDKD

ILAKHISDLVPSNVRNTDHFYRMKQSVQAKAFWNGAFQITKGDGEEAWLRVVIQPVRNAS

DQVESFFVFASELTRTITTSREYEDMLNALSRSMAVIEFTLDGTVLKANDNFLSTMGYKH

EQIVGKHHRIFCLPEEANSSAYHDFWKRLASGQFVTDRFKRVDQHGSVVWLEASYNPIHN

DRGELYKVVKFATNITQQVLQEQSMAQAAQLANEVSQETGTQTVQGRQVIGSTLEKMQTL

SEQMQQANAAIEDLKAHSSKISELVNSISGIADQTNLLALNAAIEAARAGDHGRGFAVVA

DEVRQLAMRTNTTTDEIVSMVTENLERTNNAVALISQCQKEALDTLELSEKAGNVMNDIQ

DGASRVVEAVAQFNRTL

>tr|Q87RS8|Q87RS8_VIBPA GGDEF family protein OS=Vibrio parahaemolyticus serotype O3:K6 (strain RIMD 2210633) OX=223926 GN=VP0699 PE=4 SV=1

MGILESDIQSQLHQLSCQLDQLRLTHRDTSLKFKREQQVLKRVVASLSSACQGSNTQVAN

YLFEIQQELQHQKDISTLIPRLAVLERMLKQQSKTMDKQNGYLDEQIKHSGETLQRVTGL

PAQLKRELRNLMSFSEVHAGKKVDQATRLLSIYERAIKIITSNSRLQLSEFENNPDKSQL

ECLAVDLQHTITELDFEGECGERLLDIRAKLLLGVNAESLIELTLSTLKLVVEATKFERK

ASSQFLDQLNSALASNLKTVHQNVDQHHTYFEHRQELNTEMNGLVELSQQSLDQAQDLVE

LKQEVAPLLAKMASLTERLKMTEEREQALQERLSYSKNQLEAVFETTQDYRRRLDDQTQR

MLLDPLTKVYNRTAFNDRLELEYRRWIRSQQNLRVVLFDVDNFKAVNDSYGYTAGDKALK

IIARAINKRVSDTETVARFGGEEFILLIPEQSEEYTLELMKNIQRDICQLPFKFREQNII

ITLTAVSTSFKESDAPEYVLDRLGQMLTDAKKRGTNQLNWN

>tr|Q87H32|Q87H32_VIBPA Putative integral membrane protein OS=Vibrio parahaemolyticus serotype O3:K6 (strain RIMD 2210633) OX=223926 GN=VPA1133 PE=4 SV=1

MFERVISFLETTLFTFNEQPITLWAILMVPTWVIVALWLSKILIKTVTSRMSSSGKDPNV

IHLVKRILFVIALAIIFMTTLSMINVPITAFAFLSGAIAIGFGFGAQNIINNFISGWILI

GEKPIRIGDFLEVEGAKGIVEQINTRSTRIRRTDGVHMLIPNSQLIENVVINWTLCDRLV

RGSVVVGVAYGSDVKQVAALMAEVTSQQAQVLSSPAPDVFFQDFGDNALIFESYFWINST

VEGGIRSVASAIRFEIYEAFEKHGIEIAFPQRDVHIDGEIKLSRERNG

>tr|Q87FE7|Q87FE7_VIBPA Putative two-component response regulator OS=Vibrio parahaemolyticus serotype O3:K6 (strain RIMD 2210633) OX=223926 GN=VPA1732 PE=4 SV=1

MHNAVIFVDDEPHIRDAVSQALLIEGIEVTCFPNAVEALRVIDANSPAIIITDIHMPVMD

GLEFMRTLLSKNHHFQFIVLTGHGDVKTAVEAMKSGAYDFLEKPFSTDQLMKSLNNASDK

LNLLQENIWLKKELDMQTHVGPKMIGHSKVMVSIRRALISAPTNQPLIFVGQDGTGRRLA

AQFAHDIHATPDSELIAASGEDLYELSLDALDKAIDHLTEDSNRCSLYLHSAEHISLAQW

QCLFNHPKLERVFGATTKVDADVKAFATLIHLPTLDERKEDIGPLYKHFVRHAASRYQLQ

PPHISLDEVRRITELSWPENVKQLRQFAELRALKPETFRMEDWDSEKSIEIQSMTQRTEH

FEYCLLFDALQRHRGRLKEVQLELQVSRKTLYDKLKKHQLDKSKFKAQ

>tr|Q87IT9|Q87IT9_VIBPA Uncharacterized protein OS=Vibrio parahaemolyticus serotype O3:K6 (strain RIMD 2210633) OX=223926 GN=VPA0517 PE=4 SV=1

MSTNEITKRLPNLTSRRALLLYSLPVLVAIGVSHSLKESSLTKTIALNLPESQVVERILD

ATTAEVVTPPNFEYQIQAGDNLSTIFSQLGFGYSSLMKVMETDLNYLALDTLKPGNTLRF

WRNDATGELEKMELQFSIADKVVYQLNSDGGYDFTDISIPGVWRQEPLVGVIQGSFSSSA

NRLGLSSAEISQVVSLLKEQVNFGRDLRAGDRFEVVRRSQTINGVSTGKNEIEAIKIYNR

GREITAYLHTDGQFYNAKGESLQRAFQRYPVSRSWRISSGFNPNRLHPVTGRVAPHNGTD

FAVPTGTPVVSTGDGTVIMTRKHPYAGNYVVVEHGNKYKTRYLHLSKILVKKGQKVSRGQ

RIGLSGKTGRVTGPHLHYELIEYGRPVNAMRANIPMASSVPKKEMATFIANRDEMDKLLK

NKEKAVL

>tr|Q87JM3|Q87JM3_VIBPA Putative efflux pump channel protein OS=Vibrio parahaemolyticus serotype O3:K6 (strain RIMD 2210633) OX=223926 GN=VPA0225 PE=3 SV=1

MSNTMKFKTRLVAAALSAVWLSGCGSLMRSDYQAPAVQVPANWQQMQVAGDVSMDPWWLA

FNDPTLNQYITQVLAQNNDLTLATLTLKKARLQLGLTQDDLFPTLSSSTSGQVQKSLDGG

ESADSYSTNLSVNYELDLWGKISADVDQAKWTAVASQQDREATAQSLVATTASLYWQIGY

LKQRLNLSQRNVEDAAQTLQLIEKQYQLGAVDQLDVLEAKRTLASLQAQQSEFEQSLLEA

NNAFAILFNQPPKSISANIQMLPEGDMPSLSVGTPSDLLIRRPDIKAAIYEVKAALANKD

AADLDYLPKLTLTGALGGSSKALKDLLSNPIGSLGADMTLPFLQWNEMKNSQAIADLDYQ

SAIVSYRQVLYAAFQDVENALSAREKLKYQAARLQEQYDAASAAESIYAARYQYGSTSIM

DYLNAQEETRNAQASLLENRYNQFLTQVTLYQSLGGTDVAPL

>tr|Q87PQ1|Q87PQ1_VIBPA Putative component of anaerobic dehydrogenases OS=Vibrio parahaemolyticus serotype O3:K6 (strain RIMD 2210633) OX=223926 GN=VP1450 PE=4 SV=1

MQISQLEPQDTSIVLKLFGALFYYQPKDYPAANLDTLLSNTDTPIEALNDMLRSFQNESE

EALQMEHDRMFAGIGEMPAPPWGSAYLDKEAVLFGESTIEYRYFLQRCGFALESDQREPE

DQIGLMLMVLGMLIETDQQKLAAEMLREHLMTWFGFFNKRFKKAVTLTPYSKLSNLTEEL

LQPLSEQYQVVVPAKRDYSDAPV

>tr|Q87IV8|Q87IV8_VIBPA Putative transport protein OS=Vibrio parahaemolyticus serotype O3:K6 (strain RIMD 2210633) OX=223926 GN=VPA0498 PE=4 SV=1

MSDLNLFRYYQRLLSFGVGNEAKTTLQEIADLLFTSPRHARSLLAQMQEIAWLSWRPKPG

RNQRSTLLLNVELSALKESLALERIKLGKYEKALSILDEDEAAFGRLLKTTSGASVKEGR

LNIQLTYKRMFERVVPHQLQRSSERFLLRQIYCCLVSSDYYGRVKPELAHHWRYDEQKFE

WTFYLRPGLTFHNGNPIDADTVVSLFAKLSSLEYYEKELAHVANVTAPNPLKVVFTLNRP

DLGFAGLISGVKYGIQPVSQVNVANNKLVVGSGPFSVVEHSENRLKLQAFDGYYACRVLT

DLVTIWIVNDEKMENPSLAANTLQPQPVLVDDMCGHYMSTPNSETSPASKRSRTEDGCLF

ALFNHHAKHALSQAQRRYISDLIRPEQLAEVMKSEKINFGSVAAYNLLPNWKPVLRPFGD

VVKLPKLITIAGYNYTALRRCARAISSRLERAGSRVEIVMYSYRELSEKAKNGTLDETLV

VTNINLDDNRHASAFSNFFSNAVLYHTLGAQNAAWLDAQMENVRAFSPLEDYLSALEPIA

STLISEYWIAPMFHHTQTLRFQGVLEDVALTNWGWPDIRSVWSAD

>tr|Q87QI4|Q87QI4_VIBPA Uncharacterized protein OS=Vibrio parahaemolyticus serotype O3:K6 (strain RIMD 2210633) OX=223926 GN=VP1165 PE=4 SV=1

MILVVGHKNPDSDSICSALVATELLKARGVEAMPIRQGEINRETQHILEVAGAEVPELRT

SVAGETVWLVDYSDLAQAPDDIAEAEIAGIVDHHRLGDVMTVNPMEAWIWPVGCTNTVLF

NMFKIEGHEIKPQIAKLMMSAILSDTVGFASPTCTQKDKDAVAELAAIADVQDVDAFTKD

LLIAKTNIEGLSAAQLVEKDLKGYPFNGRDVVVGQVELATLEQVDGMIEALEADLESRCE

KDNLAFAAVMLTDITTAQTRLLYKGEWAEKLVKHEKDGMLMMENTLSRKKQGWPWLQTEL

A

>tr|Q79YZ0|Q79YZ0_VIBPA Flagellar basal-body rod protein FlgC OS=Vibrio parahaemolyticus serotype O3:K6 (strain RIMD 2210633) OX=223926 GN=VP0776 PE=3 SV=1

MSLFNVFNVTGSAMSAESVRLNTTSSNLANADSVSSSAKDTYKARHAVFGAELSNAMRGG

DTVPVKVLGIVESDKPLSAEYNPDHPLANDEGYIYKPNVNVMEEMANMISASRAYQTNVQ

VADSSKQMLLRTLQMGQ

>tr|Q87LI0|Q87LI0_VIBPA Transcriptional regulator, LacI family OS=Vibrio parahaemolyticus serotype O3:K6 (strain RIMD 2210633) OX=223926 GN=VP2632 PE=4 SV=1

MARIKDVAELAGVNRSTVSRIINGEGKFREETRKKVEQAMAQLNYRPSAIARSLATSSSN

MVGLLVTYYTGGFFGEMMEQVQTELDIHKKFLITAQGHHSAEGEKEAIQRFNDLRCDGYV

LHSRYLSDDDLRELAKLPTPFVLLDRYVEGIEERCITFNHHHASRIAVEHLIAGGHKNIA

CITGPSLRHNSLLRKLGYVDAMKTAGIDIDESWCEEGNYGRQSGYDAMASILKRHPEVTA

VFSCSEEMTVGAMQYLHEHKISVPEQISLTSFDSVDLCESLYPTVSAVHFPISDMARAAV

QTLMGLVKKQEHIEKPVFEAKLKLRHADRVLKQDSD

>tr|Q87H81|Q87H81_VIBPA Ribose ABC transporter, periplasmic D-ribose-binding protein OS=Vibrio parahaemolyticus serotype O3:K6 (strain RIMD 2210633) OX=223926 GN=VPA1084 PE=4 SV=1

MKKLATLISAALLSSTVSVAAQAQDTMAIVVSTLNNPFFVTMKDGAEAKAKELGYDLIVL

DSQNDPSKELSNIEDLTIRGVKAILINPTDSDAVSNAIRMANRSNIPVLTLDRGASRGEV

VSHIASDNVVGGEMAGHYIMEKVGEKAKVIQLEGIAGTSAARERGEGFMTAVKGSDMELL

ASQPADFDRTKGLNVMENLLAANPDVQAVFAQNDEMALGALRAVQASGKNVMIVGFDGTE

DGIAAVNRGKLAATIAQQPDLIGALGVETADKVLKGEKVDEYIPVPLKVVTK

>tr|Q87MS9|Q87MS9_VIBPA Protease 4 OS=Vibrio parahaemolyticus serotype O3:K6 (strain RIMD 2210633) OX=223926 GN=VP2152 PE=3 SV=1

MKKLFKFIGLIFKGIWKSITFIRLALANLIFLLMIAVFYFAFTYTGEGRPVIEKESALVM

NLSGPIVEQRRYVNPMDSVAGSLLGNEMPKENVLFDIVDTIRYAKDDAKVSGLVLALRDL

PETNLTKLRYIAKALNEFKASGKPVYAVGDFYNQSQYYLASYADKVYMAPDGGVLIKGYS

AYSMYYKTLLEKLDVSTHVFRVGTYKSAIEPFIRDDMSDAAKESATRWVTQLWSAFVDDV

TTNRNINAKVLNPTMEELLAEMKSVDGDLAQLAVKMGLVDELATRQDIRTLFAKEFGSDG

KDSYNAISYYDYLATIRPDYTLANHDIAVVVASGAIMDGQQPRGTVGGDTVASLLRQARN

DEKVKAVVLRVDSPGGSAFASEVIRNEVEALKKAGKPVVVSMSSLAASGGYWISMSADKI

VAQPTTLTGSIGIFSVITTFEKGFSKLGINTDGVGTSPFSGDGITTGLSEGASQAFQLGI

EHGYKRFISLVGSNRDMTVEEVDKVAQGRVWTGQDALSFGLVDQMGDFDDAVELAAKLAN

VTDYGIYWVEEPLSPTELFLQEFMNQVKVSLGVDATSLLPKSLQPVAQQFEQDASLLQSF

NDPKGQYAFCLNCQVQ

>sp|Q87TN7|TRKH_VIBPA Trk system potassium uptake protein TrkH OS=Vibrio parahaemolyticus serotype O3:K6 (strain RIMD 2210633) OX=223926 GN=trkH PE=1 SV=1

MQFRSIIRIVGLLLALFSVTMLAPALVALLYRDGAGVPFVTTFFVLLFCGAMCWFPNRRH

KHELKSRDGFLIVVLFWTVLGSAGSLPFLIADNPNISVTDAFFESFSALTTTGATVIVGL

DELPKAILFYRQFLQWFGGMGIIVLAVAILPVLGIGGMQLYRAEIPGPVKDTKMTPRIAE

TAKALWYIYLSLTIACAVAFWLAGMTPFDAISHSFSTIAIGGFSTHDASMGYFDSYAINL

ITVVFLLISACNFTLHFAAFASGGVHPKYYWKDPEFRAFIFIQVLLFLVCFLLLLKHHSY

TSPYDAFDQALFQTVSISTTAGFTTTGFADWPLFLPVLLLFSSFIGGCAGSTGGGMKVIR

ILLLTLQGARELKRLVHPRAVYTIKVGGSALPQRVVDAVWGFFSAYALVFVVCMLGLIAT

GMDELSAFSAVAATLNNLGPGLGEVALHFGDVNDKAKWVLIVSMLFGRLEIFTLLILLTP

TFWRS

>sp|Q87KB0|GLMU_VIBPA Bifunctional protein GlmU OS=Vibrio parahaemolyticus serotype O3:K6 (strain RIMD 2210633) OX=223926 GN=glmU PE=3 SV=1

MKFSAVILAAGKGTRMHSNMPKVLHTLAGKPMVKHVIDTCTGLGAQNIHLVFGHGGDQMQ

TTLADETVNWILQADQLGTGHAVDQASPRFEDDEKILVLYGDVPLISPETIENLLDAQPT

GGIALLTVMLDNPTGYGRIIRKNGPVVAIVEQKDASEEQKQIKEINTGVMVATGGDLKRW

LSGLNNNNAQGEYYLTDVIAAAHDEGRAVEAVHPVNAIEVEGVNDRAQLARLERAFQSMQ

AQKLLEQGVMLRDPARFDLRGELQCGMDCEIDANVIIEGNVSLGDNVIIGTGCVLKDCEI

DDNTIVRPYSVIEGATVGEECTVGPFTRLRPGAELRNDAHVGNFVEVKNARIGEGSKANH

LTYLGDAEIGQRTNIGAGTITCNYDGANKFKTIIGNDVFVGSDSQLVAPVTIADGATIGA

GTTLTKDVEEGELVITRVKERKITGWQRPVKQK

>sp|Q87KN2|LEXA_VIBPA LexA repressor OS=Vibrio parahaemolyticus serotype O3:K6 (strain RIMD 2210633) OX=223926 GN=lexA PE=3 SV=1

MKPLTPRQQQVFDLIKSKIDDTGMPPTRAEIARELGFRSANAAEEHLKALARKQAIEIIP

GASRGIRILLEDAANDEQGLPLIGQVAAGEPILAQEHVEAHYQVDPAMFKPQADFLLRVN

GESMKDIGIMDGDLLAVHKTQDVRDGQVVVARVDDDVTVKRLERKGSTVLLHAENEEFAP

IQVDLTSQHLTIEGLAVGIIRNTDWM

>sp|Q87SG9|MURE_VIBPA UDP-N-acetylmuramoyl-L-alanyl-D-glutamate--2,6-diaminopimelate ligase OS=Vibrio parahaemolyticus serotype O3:K6 (strain RIMD 2210633) OX=223926 GN=murE PE=3 SV=1

MTKAISMDALLSPWVDCPSLASVLVSELELDSRKVQPGTTFVALVGHVVDGRKFIASAIE

KGANAVIAQACDVKAHGTIDIIDDIPVVYLDALDKCLSEIAGQLYTYPDMKLIGVTGTNG

KTTITQLIAQWIGLVGSKAAVMGTTGNGFLDDLKEAANTTGNAVEIQHTLASLAEQQAQY

TALEVSSHGLIQGRVKSLSFAAGVFTNLSRDHLDYHGTMEEYANAKLTLFTQHQCAQAII

NVDDEVGAAWAKQLTNAIAVSLAPTTEFEHALWASQVAYAESGITIRFDGQFGEGTLHAP

LIGEFNAANLMLAFATLLSLGFDKSDLLATAAQLQPVLGRMELFQAEHRAKVVVDYAHTP

DALEKALQALRVHCDGQLWAIFGCGGDRDAGKRPMMAEIAERLGDKVVLTDDNPRSEDPV

LIVKDMLAGLSKPAEAIVQHDRFKALFYALENAAPQDIILLAGKGHEDYQIRNGETIHYS

DRESAMQLLGLSS

>tr|Q87MV7|Q87MV7_VIBPA Aspartate-semialdehyde dehydrogenase OS=Vibrio parahaemolyticus serotype O3:K6 (strain RIMD 2210633) OX=223926 GN=asd PE=3 SV=1

MRVGLVGWRGMVGSVLMQRMVEEKDFDLIEPVFYSTSQVGIPAPNLGKDAGMLQDAFDIE

SLKQLDAIITCQGGSYTEKVYPALRQAGWKGYWIDAASTLRMAEDSIITLDPVNLKQIQH

GIHGGTNTFVGGNCTVSLMLMGLGGLFEKGLVEWTSAMTYQAASGAGAQNMRELISQMGV

INDAVSSELANPASSILDIDKKVADTMRSASFPTDKFGVPLAGSLIPWIDVKRDNGQSKE

EWKAGVEANKILGSQGAPVPIDGTCVRIGAMRCHSQALTIKLKQNVPMDEIEEIIATHND

WVKVIPNDRDITAQELTPAKVTGTMSVPVGRLRKMAMGDDFLNAFTVGDQLLWGAAEPLR

RTLRIILSEKA

>sp|Q87S37|QUEA_VIBPA S-adenosylmethionine:tRNA ribosyltransferase-isomerase OS=Vibrio parahaemolyticus serotype O3:K6 (strain RIMD 2210633) OX=223926 GN=queA PE=3 SV=1

MQVSDFHFDLPDELIARYPQPERTASRLLQMDGNTGELIDGTFTDVLNQVQAGDLVVFNN

TRVIPARMFGRKESGGKLEVLVERMLDEKSILAHVRCSKSPKPGTTIIVGENDEYSAEMV

ARHDALFELKFNSDKTVLDILEEIGHMPLPPYIDRPDEDADKERYQTVYNQKPGAVAAPT

AGLHFDDVLLDKIKAKGAEFAYVTLHVGAGTFQPVKVDNINDHHMHAEYVEVPQEVVDAI

NATKARGGRIIAVGTTSVRSLESAAQDALKKGTELVPFFGDTEIFIYPGYEYQLVDCLIT

NFHLPESTLIMLVSAFAGYENTMNAYKHAVENKYRFFSYGDSMFIKKKTI

>sp|Q87ME0|PYRH_VIBPA Uridylate kinase OS=Vibrio parahaemolyticus serotype O3:K6 (strain RIMD 2210633) OX=223926 GN=pyrH PE=3 SV=1

MTTNPKPAYQRILLKLSGEALQGEDGFGIDPAILDRMAQEVKELVELGVQVGVVIGGGNL

FRGAGLAEAGMNRVVGDHMGMLATVMNGLAMRDALHRAYVNARVMSAIPLKGVCDDYNWA

DAIRELRQGRVVIFSAGTGNPFFTTDSAACLRGIEIEADVVLKATKVDGVFTADPVANPD

AELYDKLSYAEVLDKELKVMDLAAFTLARDHKMPIRVFNMNKPGALRRVVMGEAEGTLIN

SDA

>sp|Q87QW9|PURR_VIBPA HTH-type transcriptional repressor PurR OS=Vibrio parahaemolyticus serotype O3:K6 (strain RIMD 2210633) OX=223926 GN=purR PE=3 SV=1

MATIKDVARLAGVSTTTVSHVINKTRFVAEATQEKVMKAVDELNYAPSAVARSLKCNSTR

TIGMLVTQSTNLFFSEVIDGVESYCYRQGYTLILCNTGGIYEKQRDYIRMLAEKRVDGIL

VMCSDLTEELKEMLDRHSDIPKVVMDWGPESSRADKIIDNSEEGGYLATKYLIDNGHTDI

ACLSGHFEKLACQERIAGFRRAMAEAKLPINEDWILEGNFECDTAVLVADKITAMEKRPT

AVFCFNDTMALGLMSRLQQNGIKVPDDVSVIGYDNIELAEYFSPPLTTIHQPKRRVGKNA

FEILLERIKDKEHEKRVFEMQPEIVIRNTVKKLN

>sp|Q87J46|PYRC_VIBPA Dihydroorotase OS=Vibrio parahaemolyticus serotype O3:K6 (strain RIMD 2210633) OX=223926 GN=pyrC PE=3 SV=1

MTTLTITRPDDWHVHLRDGEVLKDTVRDISRYNGRALIMPNTVPPVTNTEMALAYRDRIL

KEQHGEQFEPLMALYLTDNTTPEEIRAAKATGKIVAAKLYPAGATTNSDSGVTDAKNIYH

VLEAMEEVGMLLLVHGEVTHHHVDIFDREKEFLDTVLAPIVNDFPNLKIVLEHITTADAA

QFVNNASDNVAATITAHHLLFNRNHMLVGGIKPHFYCLPILKRNTHQQALIEAATSGSKK

FFLGTDSAPHAKGAKESACGCAGSYTAHAALELYAEVFEKEGKLENLEAFASFNGPDFYG

IARNADTVTLEKSAWDVPESMPFGNDIVVPIRANEQIEWKVK

>sp|Q87TP8|SYGB_VIBPA Glycine--tRNA ligase beta subunit OS=Vibrio parahaemolyticus serotype O3:K6 (strain RIMD 2210633) OX=223926 GN=glyS PE=3 SV=1

MAKEFLIELGTEELPPTQLRTLAEAFAANFEAELKGAELAHEGVKWFAAPRRLALKVAAL

AESQSDKVVEKRGPAVSAAFDAEGNPTKAAQGWARGCGITVDQADRMVTDKGEWLLFKQE

VKGQPTSEIVVELAAKALANLPIAKPMRWGNKTTQFIRPVKTLTMLMGSDLIEGEILGVA

SSRTIRGHRFMGEKEFTIDSAEQYPAILEERGKVMADYEARKAIILADAQKAAAAVGGIA

DLEDDLVEEVTSLVEWPVVLTAKFEEEFLKVPSEALVYTMKGDQKYFPVYDENKKLLPNF

IFVSNIESKEPRYVIEGNEKVVRPRLADAEFFFNTDRKRPLIDRLPELEQAIFQQQLGTI

KDKTDRITELAGYIAEQIGADVEKSKRAGLLAKCDLMTSMVFEFTDTQGVMGMHYARHDG

EAEEVAVALNEQYMPRFAGDELPSNGVSTAVAMADKLDTIVGIFGIGQAPKGSDPFALRR

ASLGVLRIIVEYGYNLDLVDLVAKAKSLFGDRLTNDNVEQDVIEFMLGRFRAWYQDEGFS

VDIIQAVLARRPTKPADFDQRVKAVSHFRELEAAESLAAANKRVGNILAKFDGELAEEID

LALLQEDAEKALAESVEVMTEALEPAFATGNYQEALSKLADLREPVDAFFDNVMVMADDE

ALKKNRLTLLNNLRNLFLQIADISLLQK

>sp|Q87LQ5|SURE_VIBPA 5'-nucleotidase SurE OS=Vibrio parahaemolyticus serotype O3:K6 (strain RIMD 2210633) OX=223926 GN=surE PE=3 SV=1

MELDSLNTKPLRILISNDDGVHAQGIHALADELRSIAEVIIVAPDRNRSGASNSLTLEQP

LRVSEIAPNTYSVQGTPTDCVHFALNELMKDDLPDLVLSGINHGANLGDDVLYSGTVAAA

MEGHFLGVQAIAFSLVGKRHFESAAKIARQLVEQHLAAPIPTNRLLNVNVPDLPLESLGE

IEVTRLGARHHAENMIKQKDPRGHDIYWLGPPGKEQDAGEGTDFYAIEHGRVSITPLQVD

LTAHESLRAMDSWLKEEK

>sp|Q87QU8|RUVA_VIBPA Holliday junction ATP-dependent DNA helicase RuvA OS=Vibrio parahaemolyticus serotype O3:K6 (strain RIMD 2210633) OX=223926 GN=ruvA PE=3 SV=1

MIGRLRGILLEKQPPEVLIEVNGIGYEVQMPMSCFYELPNIGEEAIIYTHFVVREDAQLL

YGFNTVKERALFREVIKANGVGPKLGLGILSGMTASQFVSCVEREDVSTLVKLPGVGKKT

AERLVVEMKDRLKGWGAGDLFTPFTDAAPTDSAAASSNSAEEEAVSALLALGYKPTQASK

VVSQIAKPDMSSEQLIREALKSMV

>sp|Q87TP7|SYGA_VIBPA Glycine--tRNA ligase alpha subunit OS=Vibrio parahaemolyticus serotype O3:K6 (strain RIMD 2210633) OX=223926 GN=glyQ PE=3 SV=1

MQKYDIKTFQGMILALQDYWAQNGCTIVQPLDMEVGAGTSHPMTCLRALGPEPMSTAYVQ

PSRRPTDGRYGENPNRLQHYYQFQVALKPSPDNIQELYLGSLEVLGIDPLVHDIRFVEDN

WENPTLGAWGLGWEVWLNGMEVTQFTYFQQVGGLECKPVTGEITYGIERLAMYIQEVDSV

YDLTWNIAPDGSKVTYGDIFHQNEVEQSTYNFEHADVDFLFSFFDQCEKESKELLELEKP

LPLPAYERILKAAHAFNLLDARKAISVTERQRYILRIRNLTKAVAEAYYASREALGFPMC

KKEQA

>sp|P46232|RNT_VIBPA Ribonuclease T OS=Vibrio parahaemolyticus serotype O3:K6 (strain RIMD 2210633) OX=223926 GN=rnt PE=3 SV=2

MTIENEALTLKKRFRGYFPVVVDVETAGFNAQTDALLEICAVTLRMDEEGVLHPASTIHF

HIEPFEGANLEKEALEFNGIRDPFSPLRGAVSEQEALKEIYKLIRKEQKASDCSRAIMVA

HNAAFDLSFVNAANERCKLKRVPFHPFATFDTATLSGLAYGQTVLAKACKTAGMEFDNRE

AHSALYDTQKTAELFCGIVNKWKALGGWPLVNEE

>sp|Q87SZ0|RPOA_VIBPA DNA-directed RNA polymerase subunit alpha OS=Vibrio parahaemolyticus serotype O3:K6 (strain RIMD 2210633) OX=223926 GN=rpoA PE=3 SV=1

MQGSVTEFLKPRLVDIEQISSTHAKVTLEPLERGFGHTLGNALRRILLSSMPGCAVTEVE

IEGVLHEYSTKEGVQEDILEILLNLKGLAVRVAEGKDEVFITLNKSGSGPVVAGDITHDG

DVEIANPEHVICHLTDDNAEIAMRIKVERGRGYVPASARIHNEEDERPIGRLLVDATYSP

VDKIAYAVEAARVEQRTDLDKLVIDMETNGTLEPEEAIRRAATILAEQLDAFVDLRDVRV

PEEKEEKPEFDPILLRPVDDLELTVRSANCLKAEAIHYIGDLVQRTEVELLKTPNLGKKS

LTEIKDVLASRGLSLGMRLENWPPASIAED

>sp|Q87LS9|RIMM_VIBPA Ribosome maturation factor RimM OS=Vibrio parahaemolyticus serotype O3:K6 (strain RIMD 2210633) OX=223926 GN=rimM PE=3 SV=1

MSMKGKETMSNEKIVVGKFGATYGIRGWLKVFSYTDNAESIFDYSPWYINQKGKWVEYKV

ESWKRHNKGMVAKLEGMDVREDAHLMTNFEIAIDPAVLPELSEDEFYWRELFGMHVVTTK

GYDLGVVTDMLETGSNDVLVVKANLKDAFGQKERLIPFLEEQVIIKVDREAQRIEVDWDP

GF

>sp|Q87GR5|RIBB_VIBPA 3,4-dihydroxy-2-butanone 4-phosphate synthase OS=Vibrio parahaemolyticus serotype O3:K6 (strain RIMD 2210633) OX=223926 GN=ribB PE=3 SV=1

MNQSSLLAEFGDPITRVENALIALKEGRGVLLLDDEDRENEGDIIYSVEHLTNEQMALMI

RECSGIVCLCLTDAQADKLELPPMVVNNNSANQTAFTVSIEAKVGVTTGVSAADRVTTIK

TAANPHAKPEDLARPGHVFPLRARPGGVMTRRGHTEGTIDLMQMAGLQPAGVLCEVTNPD

GTMAKAPEIVAFGHLHNMPVLTIEDMVAYRNQFDLKLA

>sp|Q87KQ2|RL10_VIBPA 50S ribosomal protein L10 OS=Vibrio parahaemolyticus serotype O3:K6 (strain RIMD 2210633) OX=223926 GN=rplJ PE=3 SV=1

MALNLQDKKAIVAEVNEAASGALSAVVADSRGVEVGAMTSLRKQAREAGVYMKVVRNTLA

RRAVQGTDYECLTDTFTGPTLIAFSNEHPGAAARLFKDFAKENKDFEIKAAAFEGALTDA

EVLATLPTYDEAIARLMMCMKEASAGKLVRTIAAIRDQKEAA

>sp|Q87KQ1|RL1_VIBPA 50S ribosomal protein L1 OS=Vibrio parahaemolyticus serotype O3:K6 (strain RIMD 2210633) OX=223926 GN=rplA PE=3 SV=1

MAKLTKRMRVIREKVDVTKEYEINEAVALLQELATAKFVESVDVAVNLGIDARKSDQNVR

GATVLPHGTGRDIRVAVFTQGANAEAAKEAGADIVGMEDLAEQVKKGEMNFDVVVASPDA

MRVVGQLGTILGPRGLMPNPKVGTVTPNVAEAVKNAKAGQVRYRNDKNGIIHTTIGKANF

SAEQIKENLEALLVALKKAKPSSAKGTFLKKVSISTTMGAGVAVDQASLNTQA

>sp|Q87NE7|RIBA_VIBPA GTP cyclohydrolase-2 OS=Vibrio parahaemolyticus serotype O3:K6 (strain RIMD 2210633) OX=223926 GN=ribA PE=3 SV=1

MAEVRARVDFKVGAKSNIDAEILSFRGLKTDKEHVAVIFKQADQTQDTPLVRMHSECLTG

DVFHSSRCDCGEQLEETIQRMGESGGVILYLRQEGRGIGLYNKIDAYRLQSQGMNTYEAN

NHLGFDDDLRDFTEAAQMLEALGIKKIRLVTNNPKKIRELAEYGIEIVEVVNTSAHIKDG

NENYLRAKVSHGKHNLKV

>sp|Q87SZ8|RL6_VIBPA 50S ribosomal protein L6 OS=Vibrio parahaemolyticus serotype O3:K6 (strain RIMD 2210633) OX=223926 GN=rplF PE=3 SV=1

MSRVAKAPVAIPAGVEVKLNGQEITVKGAKGELTRVLNDAVVIAQEENNLTFGPKEGVAN

AWAQAGTARALVNNMVVGVTEGFTKKLTLKGVGYRAAIKGNAVGLTLGFSHPVEHELPAG

IKAECPSQTEIVITGCDKQLVGQVAADIRSYRQPEPYKGKGVRYADENVRTKEAKKK

>sp|Q87MW9|RNFE_VIBPA Ion-translocating oxidoreductase complex subunit E OS=Vibrio parahaemolyticus serotype O3:K6 (strain RIMD 2210633) OX=223926 GN=rnfE PE=3 SV=1

MSENKQLMKNGMWSNNPALVQLLGLCPLLAVSSTITNALGLGIATLLVLVGSNVTVSLIR

NYVPKEIRIPVFVMIIASLVTCVQLLMNAYAYGLYLSLGIFIPLIVTNCIIIGRAEAYAS

KNDVLPAALDGLWMGLGMTSVLVVLGSMRELIGNGTLFDGADLLLGDWAAALRIQVFQFD

SSFLLALLPPGAFIGVGLLIALKNVIDSSIQARQPKEEKPAIERARVTNA

>sp|Q87MF1|RNH2_VIBPA Ribonuclease HII OS=Vibrio parahaemolyticus serotype O3:K6 (strain RIMD 2210633) OX=223926 GN=rnhB PE=3 SV=1

MVAKAKTTKAKVELPPFEYPQGYQLIAGVDEVGRGPLVGDVVTAAVILDPNNPIEGLNDS

KKLSEKKRLALLPEIKEKALAWAVGRCSPEEIDELNILQATMVAMQRAITGLKVQPDLAL

IDGNRCPELPMDSQAVVKGDLRVAEISAASIIAKVVRDQEMEELDKQYPQFGFAKHKGYP

TKAHFEAIEQHGVISEHRKSFKPVKKALGLD

>sp|Q87NB7|RLUB_VIBPA Ribosomal large subunit pseudouridine synthase B OS=Vibrio parahaemolyticus serotype O3:K6 (strain RIMD 2210633) OX=223926 GN=rluB PE=3 SV=1

MSEKLQKVLARAGHGSRREIESLIKSGRVSVNGVVAKLGERLEDESSVVRIDGHIVSAKV

QEEVICRVLAYYKPEGELCTRHDPEGRRTVFDRLPKIRGSRWISVGRLDANTSGLLLFTT

DGELANRLMHPSRQVEREYLVRVFGEVTEQKVRNLVKGVELEDGLARFEDVVYAGGEGMN

HTFYVVINEGRNREVRRLWESQECTVSRLKRVRYGDIFLDKKLPRGGWMELDLKEVNYLR

ELVELRPEKETMLDLSKDNTSRKRERARSQKIRRAVKRHEERVSTSKGRSNNPARRKPKK

NAGEQGARNKHR

>sp|Q87MX1|RNFD_VIBPA Ion-translocating oxidoreductase complex subunit D OS=Vibrio parahaemolyticus serotype O3:K6 (strain RIMD 2210633) OX=223926 GN=rnfD PE=3 SV=1

MSFFIASSPHAHSRRSTPDLMKWVALCAIPGLAAQTYYFGWGTLIQLIFAIAVAVSLEAL

VMICRKRSPMRALRDNSAIVTAWLLAVAIPPWSPWWIIVIGLIFAIVIAKHLYGGIGQNL

FNPAMVAYVVLLISFPVQMTSWSAPTLLIPDHVNFADTLSLIFTGFDYDGLSLQQVRAGV

DGVTMATPLDAFKTGIHTGATPSEVLSQPIFGGLAGIGWQWVNIAYLIGGLVMIKKRIIQ

WYIPAGFLASLTLFSLIFSVITPGETASPIFHLLSGATMLGAFFIATDPVSASTTVKGRL

IFGALIGALVFIIRSWGGFPDGVAFAVLLANMCVPLIDYYTKPRTYGH

>sp|Q87SZ1|RS4_VIBPA 30S ribosomal protein S4 OS=Vibrio parahaemolyticus serotype O3:K6 (strain RIMD 2210633) OX=223926 GN=rpsD PE=3 SV=1

MARYLGPKLKLSRREGTDLFLKSGVRAIDTKCKIDNAPGVHGARRGRLSEYGVQLREKQK

VRRMYGVLEKQFRNYYKEAARLKGNTGENLLQLLEGRLDNVVYRMGFGATRAEARQLVSH

KAILVNGKVVNVPSFKVAANDVVSIREKAKQQARIKAALEVAEQREKPTWIEVDGGKMEG

TFKRMPERSDLSADINEQLIVELYSK

>sp|Q87SF7|SECA_VIBPA Protein translocase subunit SecA OS=Vibrio parahaemolyticus serotype O3:K6 (strain RIMD 2210633) OX=223926 GN=secA PE=3 SV=1

MITKLLTKVIGSRNDRTLRRLRKIVKEINNYEPTFEALSDEELKAKTVEFRERLEQGETL

DKLLPEAFATVREASKRVYGMRHFDVQLIGGMVLNAGQIAEMRTGEGKTLTATLPAYLNA

LPGKGVHVVTVNDYLAKRDAETNRPLFEFLGMTVGVNVPNMPPQAKKEAYQADILYGTNN

EFGFDYLRDNMAFRNEDRVQRERFFAVVDEVDSILIDEARTPLIISGPAEDSSELYTRIN

LLIPHLQKQDKEDSEEYRGDGHYTVDEKSKQVHLTETGQEYVEELLVKNGLMEEGDTLYS

PANISLLHHVNAALRAHVLFERNVDYIVNDDGEVVIVDEHTGRTMPGRRWSEGLHQAVEA

KEGVKIQNENQTLASITFQNYFRLYEKLSGMTGTADTEAFEFQSIYGLETVVIPTNKPMI

RNDMPDVVYRTEAEKFAAIIEDIKERVAKGQPTLVGTVSIEKSELLSNALKKAKIKHNVL

NAKFHEKEAEIVAEAGMPGAVTIATNMAGRGTDIVLGGSWQAKVESLQDPTKEQIDAIKA

EWKKVHDQVLDAGGLHIIGTERHESRRIDNQLRGRSGRQGDAGSSRFYLSMEDSLLRIFT

SDRMASLIQSGMEEGEAIESKMLSRSIEKAQRKVEGRNFDIRKQLLEYDDVANDQRKVVY

ELRDELMNVDDISDMIEQNREDVLTAIIDEYIPPQSLEDMWDVEGLQERLKADFDLDAPI

KQWLEEDDKLYEEALREKIISLAVEVYKAKEEVVGAQVLRNFEKSVMLQTLDTLWKEHLA

AMDHLRQGIHLRGYAQKNPKQEYKRESFELFEGLLEALKTDVITVLSRVRVQQQEEVERM

EEQRRAQAEEAARRAQAQHAAAENQLADGEESEGSNQPVVRDERKVGRNEPCPCGSGKKY

KQCHGQIN

>sp|Q87JS8|SPEA_VIBPA Biosynthetic arginine decarboxylase OS=Vibrio parahaemolyticus serotype O3:K6 (strain RIMD 2210633) OX=223926 GN=speA PE=3 SV=1

MRIELEKATKLDRIRADYNVHYWSQGFYGIDDQGEVYVSPRSDRAHQIPFSAIVNELEAQ

QLNLPVLVRFPQIVHQRVHGICHAFNQAIEEYQYPNKYLLVYPIKVNQQREVVDEILASQ

AQLETKQLGLEAGSKPELLAVLALAQQGSSVIVCNGYKDREYVRLALIGEKLGHKVFIVL

EKLSELDLVLEEAKSLGVKPRLGLRIRLASQGAGKWQASGGEKSKFGLSASQVLSVIERL

KREGSLDAMQLVHFHLGSQMANIRDVRNGVNESARFYCELRALGANIEYFDVGGGLAVDY

DGTRSQSSNSMNYGLAEYARNIVNTVGDVCQQYEQPMPVIISESGRSLTAHHAVLISNVI

GTETYQPEEVHELGVDAPLLLQNMWRNWENLQDGTDARALIEIYNDTQSDLAEVHSQFAT

GVLNLEQRAWAEQLSLRIYFELSRKMSTKNRFHRPILDELSERLADKFFVNFSLFQSLPD

AWGIDQVFPVLPLSGLGDAEERRAVMLDITCDSDGAIDHYVDGQGIESTLPVPAWSKDKP

YLMGFFLVGAYQEILGDMHNLFGDTHSAVVNVDEHGQFEISYINEGDSVEDMMRYVHIDV

DAIRDNYKQLVSQRVEANEQAQILAELEQGLAGYTYLEDF

>sp|Q87KF4|THIG_VIBPA Thiazole synthase OS=Vibrio parahaemolyticus serotype O3:K6 (strain RIMD 2210633) OX=223926 GN=thiG PE=3 SV=1

MLKIGDKEFKSRLFTGTGKFSNSHLMAEAIQVSGSQLATMALKRVDVHDQQDDILQPLIH

AGVNLLPNTSGAKNAKDAVFAAQLAREALGTNWVKLEIHPDPKYLMPDPIETLAAAEQLV

RDGFIVLPYCHADPVLCKRLEEVGCAAVMPLGAPIGSNKGIASHDFLEIIIDQANVPVVV

DAGIGAPSHAARAMEMGADAVLVNTAIAAASNPVAMAKAFKMAVESGRMAYKAGLAGKVS

HAVASSPLTAFLDEL

>sp|Q87R81|TIG_VIBPA Trigger factor OS=Vibrio parahaemolyticus serotype O3:K6 (strain RIMD 2210633) OX=223926 GN=tig PE=3 SV=1

MQVTVETLEGLERRLNITVPAANIEDAVTAELRNIAKNRRFDGFRKGKVPLKMVAKMYGK

AVRQDVLGEVMQRHFIEAIVKEKINPAGAPTFAPVENKEGADLVFTATFEVYPEVELKGL

ENITVEKPLTEVKEADVEEMIETLRKQQATWVEVEEAAEAGKRVSIDFVGSIDGEEFEGG

KAENFPLEMGAGRMIPGFEDGIAGKTAGMEFDIDVTFPEDYHAENLKGKAAKFAIKVNKV

EARELPELNDEFVAKFGVAEGGVDALKAEVRKNMERELKQAVKTRIKEQAIEGLVKENEI

DVPAALIEQEIHVLRQQAAQRFGGNPEAAAQLPRELFEEQAKRRVVVGLLLGEVIKSEEL

KADDEKVKALIEEMATAYEDPSEVIAYYEQNEQMMNNMRNVALEEQAIDAIIAKAQVTEK

EVGFNELLNQQPAA

>sp|Q87MF4|TILS_VIBPA tRNA(Ile)-lysidine synthase OS=Vibrio parahaemolyticus serotype O3:K6 (strain RIMD 2210633) OX=223926 GN=tilS PE=3 SV=1

MESLYQQFSHVLNTYYQPQTKVVVAFSGGVDSRLLLELLRRYREENSLSCHAVYVHHGLS

ENADIWADKCQVWAKQAGISCSVERVNLDTNSGESIELLAREARYEALARHINRGDLLLT

GQHADDQVETFLLALKRGSGPKGLSSMAESMPFAGGTLVRPLLNTKREQIEATAKNIGLE

WVEDESNQDTRYDRNFLRHRIVPELSERWPSIHQAVQRSASLCAQQEALLDELLGSVFAR

ALQADLSLSIDELAIHSELAQARLIRMWLSKLNANMPSQTQLRLIWQEVALAQQDANPKL

KLKQGEIRRFQNKLYWVTHRADVTSWQGHIQIDEPLILPESLGTLTLSSGSHQPNISLPS

HPELLRVTFNPEGLSAHPTTRSRSRKLKKLFQEYNVPSWLRRQIPILMYKDQVVAVADLF

VDQTFSGQDCELIWRKPL

>sp|Q87QT9|TOLB_VIBPA Tol-Pal system protein TolB OS=Vibrio parahaemolyticus serotype O3:K6 (strain RIMD 2210633) OX=223926 GN=tolB PE=3 SV=1

MIKRLLLGMFVLLGSLTNVAHAALELVITDGIDSARPIAVVPFKWEGSQPLPTDISAVIA

SDLQRSGKFSPVPTNKMPQTPFNESEVNFDSWTSLGVDALLTGSIKQNEQGDYVVNYQLV

DVVRGQLTGGQSKALGSDGELVLSKDHVLFNKVATVKGPRMREYAHRISDLVYEQLTGER

GAFMTRIAYVVVNDKDRFPYQLRVADYDGYNERLVLRSKQPLMSPAWSPDGQKLAYVSFQ

NGQAEIFIMNIYTGEREKITSYPRHNGAPRFSPDGNKLALVLSKTGTLQVYTFDLKTRKL

TQITRSRSNNTEPFWHPDGKSLIFTSDRGGKPQIYQVNLGSGSIDRLTWQGSQNLGGQIT

PDGRFLVMVNRSDSGFNLAKQDLETGALQVLTKTLLDESPSIAPNGGMVVYSSIYNKKNV

LSMVSIDGRFKARLPATNGRVRAPAWSPFL

>sp|Q87L00|RSGA1_VIBPA Small ribosomal subunit biogenesis GTPase RsgA 1 OS=Vibrio parahaemolyticus serotype O3:K6 (strain RIMD 2210633) OX=223926 GN=rsgA1 PE=3 SV=1

MAKKKKLTKGQVRRVRSNQQKRLKKQEESIQWDENMLGASKQGLVITRFGQHADIEDLET

GEVQRCNLRRGIESLVSGDRVLWREGLESMAGISGVVEAVEPRTSMLTRPDYYDGLKPVA

ANIDQMVIVSSVLPELSLNIIDRYLVAAETLNIAPLLVLNKVDLLEVDDRAMYEEWLKEY

ERIGYKVLFVSKNSGEGISDLEVQLRDRINIFVGQSGVGKSSLVNALMPELEQEVEEGAI

SENSGLGQHTTTAARLYHIPTGGDLIDSPGVREFGLWHLEAEEVTKAFVEFRPYLGGCKF

RDCKHNDDPGCILREAVEKGEVSEVRFENYHRILESMMENKANRQYSRNKKADL

>sp|Q87N44|KCY_VIBPA Cytidylate kinase OS=Vibrio parahaemolyticus serotype O3:K6 (strain RIMD 2210633) OX=223926 GN=cmk PE=3 SV=1

MSSQTPVVTVDGPSGAGKGTLCMLLAKKLGFQLLDSGAIYRVLALAAIHHGVDTESEDAL

VPLATHLDVQFIAEGDLVKVILEGEDVSGELRKEETGMAASKVAALPRVREALLRRQRAF

EAAPGLVADGRDMGTVVFPSAQAKIFLDASAEERANRRLKQLQDKGLDVRFADLLSEIQE

RDDRDRNRPVAPLRPAEDALVLDSTSMTIDEVVEKALQYIESKLAE

>sp|Q87T74|KDKA_VIBPA 3-deoxy-D-manno-octulosonic acid kinase OS=Vibrio parahaemolyticus serotype O3:K6 (strain RIMD 2210633) OX=223926 GN=kdkA PE=3 SV=1

MIQQYRDSNQVIWFDEELIEDPSQPIFDAEYWQSTNKVTGSASGRGTTWFVQLDTMQAAL

RHYRRGGLFGKLVKDNYLFSGWEQTRCAQEFQLLLTLINAGVHVPRPIAARAVKSGLTYQ

ADLLSERIPNARDLVSILQEKPLPEGMYQKIGQEIAKMHNAGVNHTDLNIHNILIDDKDK

VWIIDFDKCRKQEHGDWKKQNLERLLRSFKKELLKRQIHWKERDFAVLTEALSCLDIK

>sp|Q87RN0|KDSA_VIBPA 2-dehydro-3-deoxyphosphooctonate aldolase OS=Vibrio parahaemolyticus serotype O3:K6 (strain RIMD 2210633) OX=223926 GN=kdsA PE=3 SV=1

MEQKIVNIGDIQVANDKPFTLFAGMNVLESRDLAMQICEHYVKVTDKLGIPYVFKASFDK

ANRSSVHSYRGPGLEEGMKIFQELKDTFGVKIITDVHTEAQAQPVADVVDVIQLPAFLAR

QTDLVEAMAKTGAVINVKKPQFMSPGQVGNIVEKFAECGNDKIILCERGSCHGYDNLVVD

MLGFGVMKNASKGSPIIFDVTHSLQMRDPSGAASGGRREQTVELAKAGLATGIAGLFIEA

HPNPDQARCDGPSALPLDKLEPFLAQMKSLDDLIKSFENIDIK

>sp|Q87N26|KTHY_VIBPA Thymidylate kinase OS=Vibrio parahaemolyticus serotype O3:K6 (strain RIMD 2210633) OX=223926 GN=tmk PE=3 SV=1

MMKANFIVVEGLEGAGKSTAIKTVLDTLKQAGIENIVNTREPGGTPLAEKMRALVKEEHE

GEELKDMTELLLLYAARVQLVENVIKPALANGQWVVGDRHDLSSQAYQGGGRQIDASLMK

NLRDTTLGDFKPAFTLYMDIDPRIGLERARGRGELDRIEKMDISFFERTRERYLEIANAD

PSIVVINAEQSIEEVSRDIQDALNEWLSRQ

>sp|Q87S16|ISPG_VIBPA 4-hydroxy-3-methylbut-2-en-1-yl diphosphate synthase (flavodoxin) OS=Vibrio parahaemolyticus serotype O3:K6 (strain RIMD 2210633) OX=223926 GN=ispG PE=3 SV=1

MQHESPIIRRKSTRIYVGDVPIGDGAPIAVQSMTNTRTTDVEATVAQIRALEKVGADIVR

VSVPTMEAAEAFKLIKQQVSVPLVADIHFDYRIALKVAEYGVDCLRINPGNIGNEERIRS

VVDCARDKNIPIRIGVNGGSLEKDLQMKYGEPTPEALVESAMRHVDHLDRLNFDQFKVSV

KASDVFLAVDSYRLLAKKIDQPLHLGITEAGGARAGAVKSAVGLGMLLSEGIGDTLRISL

AADPVEEIKVGFDILKSLRIRSRGINFIACPSCSRQEFDVIGTVNALEQRLEDIITPMDV

SIIGCVVNGPGEAEVSHLGLAGSNKKSAFYEDGKRQKERFDNNDLVNQLEAKIRAKASMM

DSENRIEIKVQD

>sp|Q87PC5|FABA_VIBPA 3-hydroxydecanoyl-[acyl-carrier-protein] dehydratase OS=Vibrio parahaemolyticus serotype O3:K6 (strain RIMD 2210633) OX=223926 GN=fabA PE=3 SV=1

MQNKRDSYNRDDLLASSQGELFGPGYPQLPAPNMLMMDRVTKMSETEGDFGKGLILAELD

ITPDLWFFDCHFPGDPVMPGCLGLDAMWQLVGFFLGWVGGKGKGRALGVGEVKFTGQILP

TAKKVTYEIHMKRVVNRKLVMGLADGRVCVDGKEIYVAKDLKVGLFQDTSSF

>sp|Q87N20|FABH1_VIBPA 3-oxoacyl-[acyl-carrier-protein] synthase 3 protein 1 OS=Vibrio parahaemolyticus serotype O3:K6 (strain RIMD 2210633) OX=223926 GN=fabH1 PE=3 SV=1

MYSKILGTGSYLPSQVRTNADLEKMVDTSDEWIVARTGIKERRIAAEDETVADMAFYAAE

NAIDMAGIDKNDIDLIIVATTSSSHTFPSSACQVQAKLGIKGCPAFDLAAACSGFVYALS

VADQHIKSGMCKNVLVIGADALSKTCDPTDRSTIILFGDGAGAVVVGASQEPGIISTHIY

ADGQFGDLLSLPVPERGKDVDKWLHMAGNEVFKVAVTQLSKLVKDTLEANDMHKSELDWL

VPHQANYRIISATAKKLSMSLDQVVVTLDRHGNTSAATVPTALDEAVRDGRIKRGQTLLL

EAFGGGFTWGSALVKF

>sp|Q87QB9|FABV1_VIBPA Enoyl-[acyl-carrier-protein] reductase [NADH] 1 OS=Vibrio parahaemolyticus serotype O3:K6 (strain RIMD 2210633) OX=223926 GN=fabV1 PE=3 SV=1

MIIKPRIRGFICTTTHPVGCEANVKEQIAYTKAQGPIKNAPKRVLVVGASSGYGLSSRIA

AAFGGGASTIGVFFEKEGTEKKPGTAGFYNAAAFEKLAREEGLYAKSLNGDAFSNEAKQK

TIDLIKEDLGQVDMVVYSLASPVRKMPETGELIRSALKPIGETYTSTAVDTNKDVIIEAS

VEPATEEEIKDTVTVMGGEDWELWINALSDAGVLAEGCKTVAYSYIGTELTWPIYWDGAL

GKAKMDLDRAAKALNEKLGATGGSANVAVLKSVVTQASSAIPVMPLYIAMVFKKMREEGV

HEGCMEQIYRMFSQRLYKEDGSAAEVDDMNRLRLDDWELREDIQQHCRELWPQITTENLK

ELTDYVEYKEEFLKLFGFGVEGVDYEADVNPAVETDFIQI

>sp|Q87HT6|FABV2_VIBPA Enoyl-[acyl-carrier-protein] reductase [NADH] 2 OS=Vibrio parahaemolyticus serotype O3:K6 (strain RIMD 2210633) OX=223926 GN=fabV2 PE=3 SV=1

MRIEPLIQGVVARSAHPYGCHASIKEQIEYVKKAPKIKSGPKRVLIIGASSGFGLAARIA

LTFGGAEADTIGVSFERGPSEKGVGSAGWYNNIFFKQEATHAGRTAINIVGDAFSDSVRN

EVIEAIETYFEGEVDLVIYSLAAGVRPKPHSDTFWRSVIKPIGESVTGASILLENDQWVE

TTLEPATEEEAEATIKVMGGEDWESWIDTLINTESVAQGCKTIAFSYMGPEVTHPIYLDG

TLGRAKIDLHQTSHALNLKLANFDGGAYATVCKALVTKASVFIPALSPYLLALYRVMKEK

GTHERCIEQMQRLFTTKLYDQPKVPVDGERLIRIDDLELDPQTQAEVSHLLEQMNTENFK

ECGDYQGFKDEFMKLNGFNFDDVDYSQDISLETLASLKP

>sp|Q87S09|EX7L_VIBPA Exodeoxyribonuclease 7 large subunit OS=Vibrio parahaemolyticus serotype O3:K6 (strain RIMD 2210633) OX=223926 GN=xseA PE=3 SV=1

MLSKTNQNIFTVSRLNAEVRLLLENEMGIVWLVGEISNFSAPVSGHWYLTLKDSRAQVKC

AMFRGNNRRVTFKPANGNQVLVKARLSLYEPRGDYQLIIESMQPEGDGRLQQEFEELKMK

LAAEGLFAQTNKLPLPEHPKRVGIITSKTGAALYDILDVLKRRDPSLPVVIYPTMVQGDD

AAIQIAQAIGRANSRNECDVLIVGRGGGSLEDLWCFNNEILARTIAASQIPIISAVGHEV

DMTIADFVADVRAPTPSAAAELVSRDNSHKDQSLVAKQHKLASAMRYYLSQQKQQSAQLL

HRLERQHPSYQLQRQSQQLDELDMRLRRAMQRFIDTRQQAVERKHHRLQLNSPVKHLAQQ

KSRLERVEHKLLDTMDRKLLTMRHQLAIAAEKLDTVSPLATLKRGYSITQTEQGKVVTSA

DDVKTGDLLVTRLANGEIHSTVS

>sp|Q87QX5|GLGA_VIBPA Glycogen synthase OS=Vibrio parahaemolyticus serotype O3:K6 (strain RIMD 2210633) OX=223926 GN=glgA PE=3 SV=1

MATNNLSILFVASEVEGLIKSGGLADVAKALPEALQNLQQDVRITIPAYTSIERLADAEV

VLETNLTSWPHTKYRVLLLTLGNNPVYLIDCDPYFNRPSMYAENNQAYTDNGERFAFFSA

ACLDMLPKLAFQPDIIHANDWHTGLVPFLLKHRYGNDPFFAHTKSVISIHNAVFKGVFSY

DDVQCLPEFHCRNVPDAAVSATHITMLKAGVMNADKINAVSPTYAEELKTELGSHGMAWE

FQQRAGDLVGILNGCDYSAWNPETDIYLPMNYSADKQSMVLGKNTCKRALQQRLNLAEKD

VAMFGMVCRLTQQKGVHYLLPALADFLKHDVQVVVVGTGDPVLAAQLEEVAAQFSDKFVF

VEAYDNELAHLVEAGSDFFLMPSEFEPCGLNQIYSMAYGTLPIVRGVGGLKDSVNDYDVD

PCDATGFVFYEPTSQALLLTMLRALLLYAQNLTEVQRVQLHAMQKDFCWRKAAESYLQLY

RSALN

>sp|Q87KB6|ILVD_VIBPA Dihydroxy-acid dehydratase OS=Vibrio parahaemolyticus serotype O3:K6 (strain RIMD 2210633) OX=223926 GN=ilvD PE=3 SV=1

MPKYRSATTTHGRNMAGARALWRATGVKDEDFGKPIIAVVNSFTQFVPGHVHLKDLGQLV

AQEIEAAGGIAKEFNTIAVDDGIAMGHGGMLYSLPSRELIADSVEYMVNAHCADAMVCIS

NCDKITPGMLMASMRLNIPVIFVSGGPMEAGKTKLSDQIIKLDLVDAMIQGADPKVSDEQ

SEQIERSACPTCGSCSGMFTANSMNCLTEALGLSQPGNGSLLATHADRKELFINAGKRIV

ELTKRYYEQDDETALPRNIATKAAFENAMALDIAMGGSTNTVLHLLAAAQEGEVDFDMTD

IDRMSRQVPHLCKVAPSTQKYHMEDVHRAGGVVGILGELNRAGLLHNQSKTVLGLTWEEQ

LAKYDIMLTDSEEVKSFYRAGPAGIRTTQAFSQDCRWDTLDDDRAEGCIRTKENAFSQDG

GLAVLKGNIALDGCIVKTAGVDESILKFTGPAVVFESQEDAVDGILGGKVKAGDVVVIRY

EGPKGGPGMQEMLYPTTYLKSMGLGKECALLTDGRFSGGTSGLSIGHASPEAANGGAIGL

VQDGDLIAIDIPNRSISLEISEQELAERRVKQDELGWKPANRQREVSFALKAYASMATSA

DKGAVRDKSKLEG

>sp|Q87S87|ISPH_VIBPA 4-hydroxy-3-methylbut-2-enyl diphosphate reductase OS=Vibrio parahaemolyticus serotype O3:K6 (strain RIMD 2210633) OX=223926 GN=ispH PE=3 SV=2

MKILLANPRGFCAGVDRAISIVERALELYQPPIYVRHEVVHNRFVVEGLKQRGAIFVEEL

HEVPDNNIVIFSAHGVSQAVRQEAKQRDLTVFDATCPLVTKVHMEVARASRRNMEVVLIG

HAGHPEVEGTMGQYSSETGGMYLVETPADVEKLKAIVKDPSDLHYVSQTTLSVDETADVI

EELRRVFPDIQGPRKDDICYATQNRQDAVRELAGDVDVMVVVGSKNSSNSTRLKELAEKL

GTPGYLTDCPEDIKPEWFEGKTKVGVTAGASAPEELVNQILERIKELVGARSVDEVLGRE

ENMFFEVPKELQIKQVD

>sp|Q87RN7|ISPE_VIBPA 4-diphosphocytidyl-2-C-methyl-D-erythritol kinase OS=Vibrio parahaemolyticus serotype O3:K6 (strain RIMD 2210633) OX=223926 GN=ispE PE=3 SV=1

MEHHPMIETSTRWPSPAKLNLFLYINGRTENGYHELQTLFQFVDHGDELTIQANHSGDVT

ISPEIEGVPLQDNLIWKAATALQNYAHCSFGAHIELHKVLPMGGGIGGGSSNAATTLVAL

NYLWQLNLTDDELAEIGLKLGADVPVFVRGFSAFAEGVGEKLSPANPEEKWYLVVRPNVS

IATADIFRHPDLTRNTPKRDLETLLNAPSVNDCEKIVRMLYPEVDKQLSWLLQYAPSRLT

GTGSCVFAEFSSKSEAETILAQLSDKVSAFVAQGRNISPLKETLAEYQSASHRPI

>sp|Q87SW1|IPYR_VIBPA Inorganic pyrophosphatase OS=Vibrio parahaemolyticus serotype O3:K6 (strain RIMD 2210633) OX=223926 GN=ppa PE=3 SV=1

MSLNHVPAGKSLPEDIYVVIEIPANADPIKYEVDKDSGAVFVDRFMSAPMFYPCNYGYVN

NTLSLDGDPVDVLVPTPYPLMPGSVIRCRPVGVLKMTDESGEDAKVVAVPHSKISKEYEH

IQDVGDIPELLKAQITHFFERYKELESGKWVKVDGWADVEAAKAEILQSYERAQNK

>sp|Q87TE0|HSLO_VIBPA 33 kDa chaperonin OS=Vibrio parahaemolyticus serotype O3:K6 (strain RIMD 2210633) OX=223926 GN=hslO PE=3 SV=1

MANNVLNRYLFEDLSVRGELVQLDEAYQRIISSKEYPAAVQKLLGELLVSTTLLTATLKF

EGSITIQLQGDGPVSLAVINGDHNQQVRGVARWEGDIADDASLHEMMGKGYLVITIEPKK

GERYQGVVGLEGENLTEVLEGYFANSEQLKTRLWIRTGEFEGKPHAAGMLIQVIPDGTGS

PDDFEHLEQLTNTVKDEELFGLEANDLLYRLYNQDKVRVYEPQPVAFHCGCSRERSGAAI

ITVEKAEIYDILAEVGSVSLHCDYCGTTYTFDETEVTELYTQASGGNKTLH

>sp|Q87KH4|GPPA_VIBPA Guanosine-5'-triphosphate,3'-diphosphate pyrophosphatase OS=Vibrio parahaemolyticus serotype O3:K6 (strain RIMD 2210633) OX=223926 GN=gppA PE=3 SV=1

MSQAGSSPLYAAIDLGSNSFHMLVVRHIDGSVQTMAKIKRKVRLAAGLDEHNSLSMEAMQ

RGWDCLSLFAERLQDIPTQNIRIVGTATLRTATNVDVFLEKANQILGQPIEVISGEEEAA

TIYKGVAHTSGGSGRRLVVDIGGASTELIIGEGFEAKALTSLKMGCVTWLENFFKDRQLN

ARNFEAAIEGAKQTIKPILEQYTDLGWDVCVGASGTVQALQEIMLAQGMDEVITHSKLKR

LQKQAMLADHLEELDIEGLTLERALVFPSGLSILIAIFELLEIDAMTLAGGALREGLVYE

MVDELRQNDIRARTICSVQSRYQLDCQYGEQVATLAGKLLEQAGGDEWIAEPQGKVLLET

TAKLHEIGLTIDFKKGGEHSAYLLQNLDLPGYTRAQKFFIGEIARRYREQLSSLPEQHAI

SGTSAKRVLRLLRLAVLLTHRRNPSLEPQVELLAEGDKLTLSIDAKWLEANPLTAAELEI

ESNRQTDIGWPLTITAC

>sp|Q87KZ5|GPMI_VIBPA 2,3-bisphosphoglycerate-independent phosphoglycerate mutase OS=Vibrio parahaemolyticus serotype O3:K6 (strain RIMD 2210633) OX=223926 GN=gpmI PE=3 SV=1

MSAKKPLALVILDGYGYREDTASNAIANAKTPVMDALIANNPHTLISASGMDVGLPDGQM

GNSEVGHTNIGAGRVVYQDLTRITKSIADGEFEQTPALVEAIDAAVKAEKAVHIMGLMSP

GGVHSHEDHIYAAVEMAAARGAEKIYLHCFLDGRDTPPRSAENSLQRFQDLFAKLGKGRV

ASLVGRYYAMDRDNNWERVQVAYDLLTQAKAEFTAETAVAGLEAAYARDENDEFVKATAI

KAEGQEDAIMQDGDAVIFMNYRADRARQITRAFVPGFDGFERAVFPAINFVMLTQYAADI

PLATAFPPASLENTYGEWLSKQGQTQLRISETEKYAHVTFFFNGGVENEFEGEERQLVAS

PKVATYDLQPEMSSPELTEKLVAAIKSGKYDTIICNYPNADMVGHTGVYEAAEKAIEALD

ESVGKVVEAIKEVGGQLLITADHGNAEMMIDPETGGVHTAHTNLPVPLIYVGDKAVEFKE

GGKLSDLAPTMLSLAGLEIPAEMSGDVLVK

>sp|Q87LK1|GSHB_VIBPA Glutathione synthetase OS=Vibrio parahaemolyticus serotype O3:K6 (strain RIMD 2210633) OX=223926 GN=gshB PE=3 SV=1

MIKLGIVMDPISSINIKKDSSFAMMLEAQRRGYEIHYMEMNDLHLDQGKAIADTKVVELK

EDPNGWYEFKSEQMIELSELDAVLMRKDPPFDTEYIYATYILERAEEQGALIVNKPQSLR

DCNEKLFTAWFPELTPTTIVTRKAEKIKAFREEHGDVILKPLDGMGGASIFRVKENDPNV

SVIIETLTNHGQNYAMAQTFVPDISNGDKRILVVDGEPMPYCLARIPAKGETRGNLAAGG

TGEARPLSETDMKIAQAVAPTLKEKGLIFVGLDVIGDKLTEINVTSPTCIREIEAAFDIS

ITGKLMDAIERRVKGE

>sp|Q87RN5|HEM1_VIBPA Glutamyl-tRNA reductase OS=Vibrio parahaemolyticus serotype O3:K6 (strain RIMD 2210633) OX=223926 GN=hemA PE=3 SV=1

MSLLAIGINHNTASVDLREKVAFGPDKLGPALEQLREHEAVNGSVIVSTCNRTELYCDVK

QGARNKLIDWLAQFHQVSREDLMPSLYVHEEQAAIKHLMRVSCGLDSLVLGEPQILGQVK

QAFSDSRDHQAVDSSIDKLFQKTFSVAKRVRTETDIGGNAVSVAYAACTLAKHIFESLSD

STVLLVGAGETIELVAKHLASNGCTKMIVANRTKERAQGLAEQFGAEVISLNEIPDYLAR

ADIVISSTASPLPIIGKGMVETALKQRRHQPILLVDIAVPRDVEAQVGELNDAYLYSVDD

LQSIIDSNIEQRKVEAIQAEAIVSEESASFMTWLRSLQAVDSIRDYRKSANEIREELLSK

SLQSLAAGADPEKVLRELSNKLTNKLIHAPTRALQSAAEQGEPAKLTIIRQTLGLDDL

>sp|Q87MG7|GMHA_VIBPA Phosphoheptose isomerase OS=Vibrio parahaemolyticus serotype O3:K6 (strain RIMD 2210633) OX=223926 GN=gmhA PE=3 SV=1

MYQDLIRSELNEAAEVLNKFLSDDHNIAQIEAAAKMIADSFKQDGKVLSCGNGGSHCDAM

HFAEELTGRYRDNRPGYAGIAISDPSHLSCVSNDFGYDFVFSRYVEAVGRKGDVLFGLST

SGNSGNILKAIEAAKAKGMKTVALTGKDGGKMAGLADVEIRVPHFGYADRIQEVHIKIIH

IIIQLIEKEME

>sp|Q87RP8|LNT_VIBPA Apolipoprotein N-acyltransferase OS=Vibrio parahaemolyticus serotype O3:K6 (strain RIMD 2210633) OX=223926 GN=lnt PE=3 SV=1

MMNLLFHRLKRPLAAAFVGASTTLAFAPYQLWPIAILSPAILLILLANQTPKRALWIGYA

WGLGQFATGVSWVYVSISGFGGMPLIANLFLMGMLIAYLAVYSGLFAWLNNKFFPQFSLS

KALLAAPALWLITDWLRGWVMTGFPWLWLGYSQIDAPLASFAPIGGVELLTLFVLISAGA

LAYAWIHKQWLMIIIPVVLMSAGFGIRQYDWVTPRPEDTTKVALIQGNVDQNLKWLPSQR

WPTIMKYADLTRENWDADIIVWPEAAIPAFEVEVPSFLSNIDSAAKMNNSAIITGIVNQS

EDRQFYNSILSLGVTPYGDYSFDMSERYHKHHLLPFGEFVPFEDILRPLAPFFNLPMSSF

SRGAFVQPNIVANGMHMAPALCYEIIFNEQVRQNVTDETDFILTLSNDAWFGHSIGPLQH

MEIARMRALELGKPLIRSTNNGLTAVTDYKGKIVEQVPQFETAVLRAELTPTDGTTPYRT

FGTWPLYFWVALSLMLAWWLPRKKD

>sp|Q87SF9|LPXC_VIBPA UDP-3-O-acyl-N-acetylglucosamine deacetylase OS=Vibrio parahaemolyticus serotype O3:K6 (strain RIMD 2210633) OX=223926 GN=lpxC PE=3 SV=1

MIRQRTLKEIVKTTGVGLHSGRKVTLTLRPAAANTGIIYRRTDVNPPVDFPADPASVRDT

MLCTALVNDEGVRISTVEHLNAALAGMGIDNIIVEVDAPEIPIMDGSASPFVYLLQQAGI

EMQNVPKRFIRIKKPVRFEDGDKWAEFVPFNGFRMDFEIDFNHPAIESDEQRLLFDFSSQ

GFVREISRARTFGFMRDIEYLQSQNLVLGGSFDNAIVLDDYRILNEEGLRFENEFVTHKV

LDAIGDLYMCGHPIIGEFRAYKSGHGLNNQLLRAVLADQEAWEWTTFEEEVGSPVAFAEP

NMVLA

>sp|Q87NW7|METAS_VIBPA Homoserine O-succinyltransferase OS=Vibrio parahaemolyticus serotype O3:K6 (strain RIMD 2210633) OX=223926 GN=metAS PE=3 SV=1

MPIRIPDQLPASDVLRTENIFVMSETRAASQEIRPLRVLILNLMPKKIETETQFLRLLSN

SPLQVNVELLRIDNRPSKNTPTEHLDTFYRQFEMVKGKNFDGLIITGAPLGLVQFEDVIY

WDHLKTIMEWAKDHVTSTLYVCWAAQAGLKLLYDLPKKTRKEKLSGVYHHQIHNPFHPIL

RGFDDTFLAPHSRYADFSPHFLEEHTDLDILATSDVAGVYLATTKDKRNVFVTGHPEYDS

HTLHNEYIRDLGEGMEPAIPVNYYPNNNPDNPPIASWRSHGHLLFLNWLNYCVYQQTPYD

LDHFSEDAFTKDD

>sp|Q87ME9|LPXA_VIBPA Acyl-[acyl-carrier-protein]--UDP-N-acetylglucosamine O-acyltransferase OS=Vibrio parahaemolyticus serotype O3:K6 (strain RIMD 2210633) OX=223926 GN=lpxA PE=3 SV=1

MIHETAKIHPAAVVEEGAKIGANVTVGPFTYITSTVEIGEGTEVMSHVVIKGHTKIGKDN

RIFPHAVIGEENQDKKYGGEDTTVVIGDRNVIREAVQVHRGTVQDKATTVIGDDNLLCVN

AHIAHDVVVGNHTHIGNNAILGGHVTVEDHAGVMALSAIHPFCTVGAYAYVGGCSAVVQD

VPAYVLAQGNHATPFGLNLVGLKRNGFEKPEIRALQKAYKEIYRSGKTLEEVKPILAEMA

QEWPAVKRFSDILETTERGIIR

>sp|Q87MF0|LPXB_VIBPA Lipid-A-disaccharide synthase OS=Vibrio parahaemolyticus serotype O3:K6 (strain RIMD 2210633) OX=223926 GN=lpxB PE=3 SV=1

MEKPLRIGIIAGELSGDTLGEGFIKAVKERYPNAEFVGIGGPKMIAQGCESLFDMEELAV

MGLVEVLGRLPRLLKVKAELVKYFTQNPPDVFVGIDAPDFNLRLELDLKQAGIKTVHYVS

PSVWAWRQKRIFKIEAATNLVLAFLPFEKAFYDKFNVPCEFIGHTLADAIPLQSEQAPAR

DLLGLEQDKKWLAVLPGSRGSELKMLSQPFIETCKLLHQKYPGLGFVVALVNQKRREQFE

QAWKEHAPELDFKLVDDTARNVITASDAVMLASGTVALECMLLKRPMVVGYRVNTFTAFL

AKRLLKTKYVSLPNILADDELVKEYLQDDCTPDNLFNEVSRLLESDNKPMLDKFTEMHHW

IRKDADQQAANAVLKLIEK

>sp|Q87QK1|LPXH_VIBPA UDP-2,3-diacylglucosamine hydrolase OS=Vibrio parahaemolyticus serotype O3:K6 (strain RIMD 2210633) OX=223926 GN=lpxH PE=3 SV=1

MTTLFISDLHLTPSRPDITECFITFMRTEAKNAEALYVLGDLFEFWVGDDDKTPFANQIR

TEFKALTDQGVPVFFIQGNRDFLLGERFCKETGITLLDDVCTIDLYGTKAVILHGDTLCI

DDVEYQKFRKTVHQPWLQWIFKRIPWYLKKKIVSKVQSDIRDDKQMKSLDIMDVNQSEVE

KVMSQNCVNLMIHGHTHRPNTHFFDANGAKNTRIVLGDWYTQGSVLQVNSDGFELQNRPF

NT

>sp|Q87R15|LPXK_VIBPA Tetraacyldisaccharide 4'-kinase OS=Vibrio parahaemolyticus serotype O3:K6 (strain RIMD 2210633) OX=223926 GN=lpxK PE=3 SV=1

MVEKIWFENHPLKYLLWPLLWPLSVLFGAISRSKRQQFQTGRKQAYQAPVPVVVVGNITA

GGNGKTPVVVWLVEQLQHLGYKPGVVSRGYGAKAPQYPLVLNDDTPTQHCGDEPKLIHRR

TGAPVAVDPVRANAVKALVELDVDIIITDDGLQHYALERDVELVIVDGNRRFGNECLIPL

GPLREGVERLQEVDFIITNGGLAHQGEISMSLAPSKAINLKTKQQVDVSELKALVAFAGI

GHPPRFFNTLESMHADVKVTKGFADHQDFDQKELEALALQGANVIMTEKDAVKCSDYAQD

NWWYLPVSAQLEPKDAERILNRIKEVKATYGSPSA

>sp|Q87ME7|LPXD_VIBPA UDP-3-O-acylglucosamine N-acyltransferase OS=Vibrio parahaemolyticus serotype O3:K6 (strain RIMD 2210633) OX=223926 GN=lpxD PE=3 SV=1

MKKLTLAELATITGGELFGDESLVVGRVAPMDKAQEGDVTFLSNPKYAKHLSECKATVVM

VKAEHKDQCAGNALVVADPYVAFARVVQAMDTTPKPAEDIAPSAVIASDVKMGENVAIGA

NAVIETGVELGDNVVIGAGCFIGKNAKLGNNTKLWANVTIYHEVSLGDDCLVQSGTVIGS

DGFGYANDRGEWIKIPQLGSVRIGNRVEIGACTTIDRGALEDTIIEDNVILDNQLQIAHN

VQIGYGTVMPGGTIVAGSTKIGKYCQIGGASVLNGHITIADGVAITGMGMVMRSIEEKGL

YSSGIPLQTNREWRKTATRVHRIDEMNKRLKAVEKQLEQKEES

>sp|Q87QJ8|KITH_VIBPA Thymidine kinase OS=Vibrio parahaemolyticus serotype O3:K6 (strain RIMD 2210633) OX=223926 GN=tdk PE=3 SV=1

MAQMYFYYSAMNAGKSTTLLQSSFNYQERGMTPVIFTAALDDRYGVGKVSSRIGLQSDAH

LFRPDTNLYQEIAALHEVEKRHCILIDECQFLSKEQVYQLTEVVDKLHIPVLCYGLRTDF

LGELFEGSKYLLSWADKLVELKTICHCGRKANMVIRTDEHGVAIKEGDQVAIGGNDRYVS

VCRQHYKEALGK

>sp|Q87SA1|LGT_VIBPA Prolipoprotein diacylglyceryl transferase OS=Vibrio parahaemolyticus serotype O3:K6 (strain RIMD 2210633) OX=223926 GN=lgt PE=3 SV=1

MSQGYLQFPNIDPVLVSIGPVSIRWYGLMYLVGFMFALWLANRRADKPGSGWTREQVSDL

LFAGFLGVVIGGRVGYVIFYNFELFLDDPLYLFKVWTGGMSFHGGLLGVITAMFWYAHKN

GRTFFGVADFVAPLVPFGLGMGRMGNFMNSELWGRVTDVPWAIVFPNGGPLPRHPSQLYE

MLLEGVVLFFILNWFIKKPRPLGSVSGLFLAGYGTFRFLVEFVREPDAQLGLFGGYISMG

QILSMPMIVLGILMMVWAYKRGLYQDKAQVKTK

>sp|Q87ST0|LEUD_VIBPA 3-isopropylmalate dehydratase small subunit OS=Vibrio parahaemolyticus serotype O3:K6 (strain RIMD 2210633) OX=223926 GN=leuD PE=3 SV=1

MSGFKQHTGLVVPLDAANVDTDAIIPKQFLQKVSRLGFGKHLFHDWRFLDDAGERPNPEF

VMNAPRYQGASILLARENFGCGSSREHAPWALADYGIKAMIAPSFADIFYGNSINNQMVP

VRLTEQEVDELFQFVEANEGAQIEVDLEALKVRANGKEYDFEIDEFRRHCLLNGLDNIGL

TLQHEDKIAEYEANIPSFLR

>sp|Q87ST3|LPTD_VIBPA LPS-assembly protein LptD OS=Vibrio parahaemolyticus serotype O3:K6 (strain RIMD 2210633) OX=223926 GN=lptD PE=3 SV=1

MQHFSRTFIAASISTALFVPTTQAEANINDSVQEMPATDQCLVETSGEEDALNTPVVVEA

DNLQAINGDKAQYSGNVQVTQGPKKITADSVTLHQQDNVVVAEGNVTFNDGQVKARSDRV

TNDINQDTFSLENTEYQFLCQQGRGTAAYIARTGQSVYELEDGSITSCPEGDNSWRLVAS

GIDVDQDEETATLHHPRFEIMDVPVFYVPYMTMPIGNTRKTGFLFPSLSYGSSDGMEVEV

PFYWNIAPQYDMTLTALYMQQRGTKLDTDFRYLTDGWGEGKLKGEYLNSDKKYQDDSRWG

YQVKHDGIINKQWIVKVDYSQVSDIDYFLDLDSDIGNREDGQLVQEGHVQYRSDFWDASL

TVRDFQILLKEENRPYRLLPQLDLNYYTPLWGDHLNFDVKSQVSRFDTSDTARPNATRVH

IEPGLTMPLSNSWATWTTEARVLSTYYSQDLTGLTDVDLKNQLDEQVSRVIPEFRTHAQI

YLERDTSWVKGYTQTLEPQLQYLYVPEEDQTNIYNYDTTLLQTDYYGLFRSRKYSGIDKI

ASANQLSYGASTRFFDDDYKERLNVSFGQIYYFDKKTKISNSPNIPDETTNYSSWAVEAD

FNYNDYLFYHGGVQYDIDLSSMQLANSTLEYQFNGGFIQGNYRYVTREYIEDTIILENLD

TITRKGISQAGIVAAYEFNRNWSASGQYYYDLNENTDMEWLASLRYQSDCWYIGLTYSNQ

LLGWENQAIGSTGSSPEYENNFSVNFGIQGFATNQSTGTAVKELDGSDNAIKYGRPFYLN

N

>sp|Q87QK7|HIS4_VIBPA 1-(5-phosphoribosyl)-5-[(5-phosphoribosylamino)methylideneamino] imidazole-4-carboxamide isomerase OS=Vibrio parahaemolyticus serotype O3:K6 (strain RIMD 2210633) OX=223926 GN=hisA PE=3 SV=1

MIIPALDLIEGQVVRLYQGDYGQVTEYKVDPAEQFNLYHQAGADWLHLVDLTGAKDTSAR

QLDLIAKLLASTPANIQIGGGVRTEQDVVDLLEAGAQRVVVGSTAVKQPELVKGWMEKYG

AEKIVLALDINIDQDGTRKVAISGWQEDSGVTIEALINDYLTVGLQHVLCTDISRDGTLE

GSNVELYVDLCKQYPQVQFQSSGGIGSLADIEALKGSGVAGVIVGRALLDGKFTAEEAFA

CWQSE

>sp|Q87QL2|HIS1_VIBPA ATP phosphoribosyltransferase OS=Vibrio parahaemolyticus serotype O3:K6 (strain RIMD 2210633) OX=223926 GN=hisG PE=3 SV=1

MQTQRLRIAIQKKGRLSKESQALLKQCGVKFNVMGERLVVHSENMPIDLLLVRDDDIPGL

IMDGVVDLGFIGENELEEVRLDRKALGEPCEFVQLRRLDFGGCRLSIAIDKDEEYNGPQD

LAGKRIATTYPQLLKAYMDEAGVPFSTCMLTGSVEVAPRAGLADAIADLVSTGATLEANG

LKEAEVIFRSKATLIQRIGEFDADKAELINKLLTRMQGVQQAKESKYIMLHAPAGKLEQI

KALLPGAEDPTVLPLSADKQKVAVHLVSTENLFWETMEQLKELGASSILVLPIEKMME

>sp|Q87T56|HLDD_VIBPA ADP-L-glycero-D-manno-heptose-6-epimerase OS=Vibrio parahaemolyticus serotype O3:K6 (strain RIMD 2210633) OX=223926 GN=hldD PE=3 SV=1

MIIVTGGAGMIGSNIVKALNEAGINDILVVDNLKNGKKFKNLVDLDITDYMDRDDFLTQI

MAGDDFGPIEAIFHEGACSATTEWDGKYMMLNNYEYSKELLHYCLDREIPFLYASSAATY

GETETFVEEREYEGALNVYGYSKQQFDNYVRRLWKDAEEHGEQLSQITGFRYFNVYGPRE

DHKGSMASVAFHLNNQINAGENPKLFEGSGHFKRDFVYVGDVCKVNLWFLENGVSGIFNC

GTGRAESFEEVAKAVVKHHNKGEIQTIPFPDHLKGAYQEFTQADLTKLRAAGCDVEFKTV

AEGVAEYLTIQNS

>sp|Q87QL1|HISX_VIBPA Histidinol dehydrogenase OS=Vibrio parahaemolyticus serotype O3:K6 (strain RIMD 2210633) OX=223926 GN=hisD PE=3 SV=1

MRTVVWQSLSEEQQDAILERPAIAEGANITAAVADVIAKVRTQGDAALLELTEKFDRVKP

ESIRVPSKEINAASERLSAEMKQALEQAYSNIAKFHKAQKPQPIKVETQPGVMCEQVTRP

IQKVGLYIPGGSAPLPSTVLMLGVPAKIAGCRKVVLCSPPPIADEILYVAKLCGIDEVYN

VGGGQAVAAMAYGTKSVSKVDKIFGPGNAYVTEAKRQVSNDFRGAAIDMPAGPSEVLVIA

DETADPDFIAADLLSQAEHGPDSQVVLVTPSPIVADQVTDAVQRQLKALSRADIAQKALA

SSLIIISESITQAVSISNYYGPEHLIVQTKNPRELLPLLDNAGSIFLGDWSPESAGDYAS

GTNHVLPTYGYTRTYSSLGLADFSKRMTVQELSAEGLQNLAPTVVTMAEAEGLDAHKRAV

TIRVEKLTQNR

>sp|Q87SG6|MURD_VIBPA UDP-N-acetylmuramoylalanine--D-glutamate ligase OS=Vibrio parahaemolyticus serotype O3:K6 (strain RIMD 2210633) OX=223926 GN=murD PE=3 SV=1

MERWQNIHNVVVVGLGITGLSVVKHLRKTQPQLTVKVIDTRDNPPGAERLPEQVELHRGG

WNTQWLAEADLVVTNPGIALATPEIQTVLAKGTPVVGDIELFAWAVNKPVVAITGSNGKS

TVTDLTGVMAKAAGLTVGVGGNIGVPALELLEQDADLYVLELSSFQLETTSSLKLKAAAF

LNLSEDHMDRYEGMADYRAAKLRIFDHAELAVVNRDDQETYPEVEMPVVTFGSDEQAYGL

EVDGSRTWLLDHGQRVIASDELKLVGKHNLANALVVLALLKAAGVDYHNALNALKNYTGL

THRCQVVADNRGVKWVNDSKATNIASTMAALSGLESTGKLYLLVGGVGKGADFTPLKPIF

ATLNLQLCCFGLDGDDFMPLHESAIRFNTMEDVIQQISSQLKSGDMVMLSPACASFDQFD

NFMARGDAFAVLAQKYA

>sp|Q87QT6|NADA_VIBPA Quinolinate synthase A OS=Vibrio parahaemolyticus serotype O3:K6 (strain RIMD 2210633) OX=223926 GN=nadA PE=3 SV=1

MSHILDKIDTVYPFPPKPIPLSEEEKSSYIASIKELLKQKDAVLIAHYYTDPEIQALAEE

TGGFVGDSLEMAKFGNRHPASTLIIAGVRFMGESAKILTPEKRILMPTLEAECSLDLGCP

ADKFSEFCDAHPDHTVVVYANTSAAVKARADWVVTSSIALEIVEHLDAEDKPIIWGPDRH

LGSYIANKTGADMLLWQGECVVHDEFSADALRKMKSVYPDAAILVHPESPASVVELADAV

GSTSQLIKAAKELPYQQMIVATDKGIFFKMQQLVPEKELIEAPTAGAGATCRSCAHCPWM

AMNGLKAIEKALSEGGEEHEIFVDEALRVKSLIPLNRMLDFAEQLNMQVKGNA

>sp|Q87KU2|PRMA_VIBPA Ribosomal protein L11 methyltransferase OS=Vibrio parahaemolyticus serotype O3:K6 (strain RIMD 2210633) OX=223926 GN=prmA PE=3 SV=1

MPWIQIKLNATNENAEQIGDMLMEETGALSVTFLDAQDTPVFEPLPGETRLWGDTDILAL

YDAEADTNFIIDQIKASNMLAENFAYKVEQLEDKDWEREWMENFHPMKFGERLWICPSWR

EVPEPDAVNVMLDPGLAFGTGTHPTTALCLEWLESMDLSGKTVIDFGCGSGILAIAAIKL

GAEKVIGIDIDPQALLASKDNAERNGVADKLEVYLPQNQPEGLIADVVVANILAGPLREL

APIIKGLVKPNGALAMSGVLDTQAEDVASYYRDELHIDPIVEQSEWCRISGRKQG

>sp|Q87S51|PPK1_VIBPA Polyphosphate kinase OS=Vibrio parahaemolyticus serotype O3:K6 (strain RIMD 2210633) OX=223926 GN=ppk PE=3 SV=1

MSAEKLYIEKELSWLSFNERVLQEAADKTVPLIERIRFLGIFSNNLDEFYKVRFADVKRR

ILINQERGGSDNSKRLLSKMQAKALKLNEQFDELYSELIREMARRRIFLVNEHQLDEAQE

KWITKYFRKEVMPHITPLLMKDEIDVLQFLKDEYAYIAVELRKEDHSQYALIEIPTDHLP

RFVMVPEQKGKRRKTIILLDNIIRYCLDELFKGFFDYDELAGYAMKMTRDAEYDLRNEIE

YSLLEQMSAGVNQRLTAMPVRFVYEREMPQEMLDFLCSKLRISNYDNLIPGGRYHNFKDF

IAFPNVGREYLENKPMPPMKCADFEGYANSFEAIKAKDILLYYPYHTFDHIGELVRQASF

DPKVLSIKINIYRVAKDSRLMNSLIDAVHNGKNVTVVVELQARFDEEANIEWSKVLTEAG

VHVIFGAPGLKIHSKLLMISRREGDDIIRYAHIGTGNFHEKTARIYTDFSLLTADQEITN

EVRNVFGYIENPYRPVKFNHLMVSPRNSRTQIYRLIDNEIANAKAGKKAGLTIKVNNLVD

KGIVTRLYAASNAGVKINMIIRGMCALVPGIEGVSENIRIISIVDRFLEHPRVVITHNDG

DPQVYISSADWMTRNIDHRIEVAAPVRDPRLKQRIIDITNIHFTDTVKARLIDKEMSNSY

VPRGNRKKVRSQVAIYDYLKNIEKQTRRQKSDVSDT

>sp|Q87KP5|MURB_VIBPA UDP-N-acetylenolpyruvoylglucosamine reductase OS=Vibrio parahaemolyticus serotype O3:K6 (strain RIMD 2210633) OX=223926 GN=murB PE=3 SV=1

MQIKEHASLKAFHTFGIEQTCSYLAIVDSIDDVISLYQNPAFQSLPKLFLGKGSNVLFTE

HFDGLVIVNRLLGKSVSETHEDYLLHVQGGEDWPSLVAWCVAQGMGGIENLALIPGCAGS

APIQNIGAYGVELKDLCSYVDVLDLTTLKTRRMSAEDCEFGYRDSVFKHDLYEKCFVTAI

GLKLPKRWTPKNQYGPLQNIPENELSPNAIFERVCQVRMEKLPDPAKVGNAGSFFKNPVI

SQDHYDQLVRKHSDMVAYPANEGMKVAAGWLIDQCGLKGISVNGAQVNPLQALVLTNVDN

CSADDVVALASLVKRAVWDKYQIELEHEVRFMNRQGETNLAKIEAAQ

>sp|Q87MA9|NQRD_VIBPA Na(+)-translocating NADH-quinone reductase subunit D OS=Vibrio parahaemolyticus serotype O3:K6 (strain RIMD 2210633) OX=223926 GN=nqrD PE=3 SV=1

MSSAQNIKKSIMAPVLDNNPIALQVLGVCSALAVTTKLETAFVMTLAVTFVTALSNFSVS

LIRNHIPNSVRIIVQMAIIASLVIVVDQVLKAYLYDISKQLSVFVGLIITNCIVMGRAEA

FAMKSAPVPSLIDGIGNGLGYGFVLITVGFFRELFGSGKLFGMEVLPLVSNGGWYQPNGL

MLLAPSAFFLIGFLIWVIRVFKPEQVEAKE

>sp|Q87RS6|QUEF_VIBPA NADPH-dependent 7-cyano-7-deazaguanine reductase OS=Vibrio parahaemolyticus serotype O3:K6 (strain RIMD 2210633) OX=223926 GN=queF PE=3 SV=1

MSKYSDAKELAGLTLGKKTEYANQYDASLLQPVPRSLNRDDLELGDTLPFLGHDIWTLYE

LSWLNSKGLPQVAVGEVYIPATSANLIESKSFKLYLNSYNQTRFASWEEVAERLTQDLSA

CAGEKVLVEVNPVGHYTNQPIVTMEGECIDDQDIEINSYDFDADLLAGAAGEDQVEEVLH

SHLLKSNCLITNQPDWGSVEIRYQGAKIDREKLLRYLVSFREHNEFHEQCVERIFTDLMK

YCQPNKLTVFARYTRRGGLDINPYRSTEQDKPAHNHRMARQ

>sp|Q87TQ5|RECF_VIBPA DNA replication and repair protein RecF OS=Vibrio parahaemolyticus serotype O3:K6 (strain RIMD 2210633) OX=223926 GN=recF PE=3 SV=1

MPLSRLIIQQFRNIKACDIQLSAGFNFLIGPNGSGKTSVLEAIYLLGHGRSFKSSLTGRV

IQNECDELFVHGRFLNSDQFELPIGINKQRDGSTEVKIGGQSGQKLAQLAQVLPLQLIHP

EGFDLLTDGPKHRRAFIDWGVFHTEPAFYDAWGRFKRLNKQRNALLKTASSYRELSYWDQ

EMARLAENISQWRSLYIEQMKTVAETICQTFLPEFEIQLKYYRGWDKDTPYQEILEKNFE

RDQSLGYTFSGPNKADLRIKVNGTPVEDVLSRGQLKLMVCALRVAQGQHLTAMTGKQCIY

LIDDFASELDSQRRKRLADCLKETGAQVFVSSITENQIADMLDDNGKLFHVEHGRIESN

>sp|Q87ST5|PDXA_VIBPA 4-hydroxythreonine-4-phosphate dehydrogenase OS=Vibrio parahaemolyticus serotype O3:K6 (strain RIMD 2210633) OX=223926 GN=pdxA PE=3 SV=1

MTTNSIRRIVVTAGEPAGIGPDLVLALSKEDWAHQIVVCADKNMLLERAKMLGIDVQLFD

YNPEEAPKAQKAGTLIVDHVEIAENAIAGQLNEANGHYVLKTLERAALGCMNDEFDAIVT

GPVHKGVINRAGVAFSGHTEFFAEKSNTPLVVMMLATEGLRVALVTTHIPLAYVSKAVTE

ERLEKIIDILHKDLVEKFAIAEPNIYVCGLNPHAGEDGCLGREEIETITPTLEKIQKEKG

IKLIGPLPADTIFNEKYLNDADAVLGMYHDQVLPVLKYKGFGRSVNITLGLPFIRTSVDH

GTALELAGTGQADTGSFRTALTHAIELVEKKQ

>sp|Q87LP2|PDXJ_VIBPA Pyridoxine 5'-phosphate synthase OS=Vibrio parahaemolyticus serotype O3:K6 (strain RIMD 2210633) OX=223926 GN=pdxJ PE=3 SV=1

MSSIYLGVNIDHIATLRNARGTKYPDPVHAAEIAERAGADGITIHLREDRRHILDRDVRI

LRETIQTRMNLEMAVTEEMVEIALKTKPEFVCLVPEKREELTTEGGLDVVGQLDKVKAAT

QKLTEAGIKVSLFIDADRQQIEAAKQCGAPFIELHTGHYADAETEEEQQAELKKIAAGAS

YADDLGIIVNAGHGLTYHNVAPIAALPEIYELNIGHSIIGRAVFDGLEKSVAEMKALMIA

ARK

>sp|Q87GX2|PEPT_VIBPA Peptidase T OS=Vibrio parahaemolyticus serotype O3:K6 (strain RIMD 2210633) OX=223926 GN=pepT PE=3 SV=1

MKHLVERFLRYVTFDTQSNPHVAQCPSSPGQLVFAELLKQEMLDFGLSDVTLDEHGYLMA

KLPSNVDYDVPPIGFIAHMDTAPDASGKNVNPQFVEDYQGGDIALGLGDEVLSPVQYPDL

HNLHGHNLITTDGTTLLGADNKAGIAEILSAIAMLIENPDIPHGDICIGFTPDEEIGRGA

DLFDVEKFGAKWAYTIDGGPQGELEYENFNAASADVIFHGVSVHPGTAKGKMVNAMNLAA

QFQVKMPADQTPETTEGYEGFFHLKSGELGIARSELGYIIRDFDREGLEERKALMQKLVD

EMNAGLKHGSVELNITDSYYNMREMVEPYPHIIELAKQAMEACDVEPLIKPIRGGTDGAR

LSFMGLPCPNIFTGGFNFHGIHEFISVEMMEKSVLVIVKIAELTAKKHG

>sp|Q87SG3|MURC_VIBPA UDP-N-acetylmuramate--L-alanine ligase OS=Vibrio parahaemolyticus serotype O3:K6 (strain RIMD 2210633) OX=223926 GN=murC PE=3 SV=1

MTIQHTQDLAQIRAMVPEMRRVKCIHFIGIGGAGMSGIAEVLLNEGYEITGSDLSENPVT

ERLVSKGATVFIGHQASNVEKASVVVVSTAINEENPEVMAARELRIPIVRRAEMLAELMR

FRHGIAVAGTHGKTTTTALVTQIYSEAGLDPTFVNGGLVKSAGTNARLGSSRILIAEADE

SDASFLHLQPMVSIVTNIEADHMDTYGGDFETLKQTFIDFLHNLPFYGQAIVCIDDPVIR

ELIPRISRQVITYGFSDDADVRIENYHQEGQQGKFTVVRKGRANLDITLNIPGRHNALNA

SAAIAVATEDDIEDDAILKAMAGTQGTGRRFDHLGEFDTGNGHAMLVDDYGHHPTEVDVT

IKAARSGWQDKRLVMIFQPHRYSRTRDLYDDFANVLEQVDVLIMLDVYAAGEKPIAGADG

RSLCRTIRSRGKVDPIFVPEIEQLPSVLANVIQDGDLILTQGAGDVGKVAKQLANLELNI

NKMLG

>sp|Q87KP1|MURI_VIBPA Glutamate racemase OS=Vibrio parahaemolyticus serotype O3:K6 (strain RIMD 2210633) OX=223926 GN=murI PE=3 SV=1

MRASSKKKVLVFDSGVGGLSVFQEIHQLLPHLDYFYLFDNEAYPYGELDQNVLISRVNQL

VSALVAEHHIDIVVIACNTASTIVLPSLRDNLSVPVVGVVPAIKPASLLATQGVGLIATP

ATVTRQYTHELIRDFAQGKPVELLGSTRLVDMAEEKLRGESVPLDELKSILSPLCNKVDV

AVLGCTHFPLIKNEIQQVLGSNVVLIDSGEAIARRVKALLSCGELEEKEEGIKRIFASAP

PWQEDALNICLAKLGFNPVQIYRHLGVSDR

>sp|Q87SG7|MRAY_VIBPA Phospho-N-acetylmuramoyl-pentapeptide-transferase OS=Vibrio parahaemolyticus serotype O3:K6 (strain RIMD 2210633) OX=223926 GN=mraY PE=3 SV=1

MIIWLAELLQPYLSFFRLFEYLSFRAILSVLTALGLSLWMGPIMIKRLQMLQIGQVVRNE

GPESHFSKRGTPTMGGIMILAAISITILLWTDLSNPYVWAVLTVLLGYGAVGFVDDYRKV

VRKNTDGLIARWKYFWQSLIAFVVAFALYAYGKDTAATQLVVPFFKDVMPQLGLMYIILT

YFVIVGTSNAVNLTDGLDGLAIMPTVLVAAGFAVIAWATGNVNFSEYLHIPYLPHASELV

VVCTAIVGAGLGFLWFNTYPAQVFMGDVGSLALGGALGTIAVLVRQELVLVIMGGVFVME

TLSVILQVGSYKLRGQRIFRMAPIHHHYELKGWPEPRVIVRFWIISMVLVLIGLATLKVR

>sp|Q87LF4|MURA_VIBPA UDP-N-acetylglucosamine 1-carboxyvinyltransferase OS=Vibrio parahaemolyticus serotype O3:K6 (strain RIMD 2210633) OX=223926 GN=murA PE=3 SV=1

MEKFRVIGSDKPLVGEVTISGAKNAALPILFASILAEEPVEVANVPHLRDIDTTMELLKR

LGAKVSRNGSVHVDPSSINEYCAPYDLVKTMRASIWALGPLVARFGQGQVSLPGGCAIGA

RPVDLHITGLEQLGATITLEDGYVKAEVDGRLKGAHIVMDKVSVGATITIMCAAALAEGT

TTLDNAAREPEIVDTADFLNKLGAKISGAGTDTITIEGVERLGGGKHSVVADRIETGTFL

VAAAVSGGKVVCRNTNGHLLEAVLAKLEEAGALVETGEDWISVDMTDRELKAVSIRTAPH

PGFPTDMQAQFTLLNMMAKGGGVITETIFENRFMHVPELMRMGAKAEIEGNTVICGDVES

LSGAQVMATDLRASASLVIAGCIAKGETIVDRIYHIDRGYDKIENKLAALGANIERVS

>sp|Q87QW3|MUKE_VIBPA Chromosome partition protein MukE OS=Vibrio parahaemolyticus serotype O3:K6 (strain RIMD 2210633) OX=223926 GN=mukE PE=3 SV=3

MSDNLAKAISNPLFPALDSMLRAGRHISTEDLDNHALLSDFELELSSFYQRYNTELVKAP

EGFFYLRPRSTSLIGRSVLSELDMLVGKVLCFLYLSPERLAHEGIFTNQELYDELLALAD

EKKLMKLVTNRATGSDLDKEKLFEKVRTSLRRLRRLGMIINIGETGKFSISEAVFRFGAD

VRVGDDIREAQLRLIRDGEAVVHTKEPSQGSLLSEEDQEEQAQEEMTEEGEA

>sp|Q87R69|MEND_VIBPA 2-succinyl-5-enolpyruvyl-6-hydroxy-3-cyclohexene-1-carboxylate synthase OS=Vibrio parahaemolyticus serotype O3:K6 (strain RIMD 2210633) OX=223926 GN=menD PE=3 SV=1

MSYDQAVLNRIWSETILTELHRFGVKHVCIAPGSRSTPLTLEAAEQPNFSIHTHFDERGL

GFMALGLAKASQEPVAVIVTSGTAVANLLPAVAEAKLTGEKLVLLTADRPVELVGCGANQ

AINQLGIFSQHVSANLNLPSPSLNTPLNWLLTSVDEVMFNQQLHGSAVHINCAFPEPLYS

DGEKSAYQSYLSSVEAWRKGGQTYTQRFVSPSFRDIPFCADRKGVVVIGSLSAEHAQEAK

AFAQQMGWPVLADPQSGVSSDWSHYDLWLQQPKLASQLDECDLVLQFGSRIISKRLNQWI

NKQVSQSQQGRDVQYWFISPSLSRDNQTHLPQLHWVASPKSWVERVDVKSSSTQGWADGL

LTDIAHVRAHISDEFLFSSASTLNEIALAADIEERTQSVDVFLGNSLFVRLVDMFGRLNT

EVFTNRGASGIDGLFATASGVQRSRGKPLLMYIGDTSALYDLNSLALFSRNDLPSVLVVT

NNDGGAIFDMLPVPQEHRTAYYQMPHGYQFEHAAKQFGLKYEKPTTLQMYQAMVADHLSS

GQGTMLVEVQTPPSQAAELIKAFNKSLHASL

>sp|Q87N19|PLSX_VIBPA Phosphate acyltransferase OS=Vibrio parahaemolyticus serotype O3:K6 (strain RIMD 2210633) OX=223926 GN=plsX PE=3 SV=1

MQSITVALDAMGGDFGPRVTVPAAVQALSHFPELKVILIGDQSLITSQLSQLGTSTSSRL

TILHSEKVISNSEKPSLALRNSQNSSMRMAIDLVSDQEADACVSGGNTGALMALSRFILK

LLPGIERPALVSALPTISGKRTWMLDLGANVSCDADSLFQFAVMGSALAEEHLCRPPRVA

VLNIGAEEIKGNDLVKRCAEMLSQTDAINFVGYIEGNQILHDVADVIVCDGFVGNVCLKA

SEGTAQLFIEKLKTSMMASTIKGWIARKLFSRLFNELKTLNPDQYNGASLLGLRGIVIKS

HGSADVSAIVNALGEAVHEVKRQVPSRISDRLEAVLLERHY

>sp|Q56725|NHAA_VIBPA Na(+)/H(+) antiporter NhaA OS=Vibrio parahaemolyticus serotype O3:K6 (strain RIMD 2210633) OX=223926 GN=nhaA PE=1 SV=1

MNDVIRDFFKMESAGGILLVIAAAIAMTIANSPLGETYQSLLHTYVFGMSVSHWINDGLM

AVFFLLIGLEVKRELLEGALKSKETAIFPAIAAVGGMLAPALIYVAFNANDPEAISGWAI

PAATDIAFALGIMALLGKRVPVSLKVFLLALAIIDDLGVVVIIALFYTGDLSSMALLVGF

VMTGVLFMLNAKEVTKLTPYMIVGAILWFAVLKSGVHATLAGVVIGFAIPLKGKQGEHSP

LKHMEHALHPYVAFGILPLFAFANAGISLEGVSMSGLTSMLPLGIALGLLIGKPLGIFSF

SWAAVKLGVAKLPEGINFKHIFAVSVLCGIGFTMSIFISSLAFGNVSPEFDTYARLGILM

GSTTAAVLGYALLHFSLPKKAQD

>sp|Q87KX9|EFP_VIBPA Elongation factor P OS=Vibrio parahaemolyticus serotype O3:K6 (strain RIMD 2210633) OX=223926 GN=efp PE=3 SV=1

MATVSTNEFKGGLKLMLDNEPCVILENEYVKPGKGQAFNRVKIRKLLSGKVLEKTFKSGD

TCEVADVMDIDLDYLYSDGEFYHFMNNETFEQIAADAKAVGENAKWLVENNTCMITLWNG

NPITVTPPNFVELEVTDTDPGLKGDTQGTGGKPATLATGAVVRVPLFIAIGEVIKVDTRT

GEYVGRVK

>sp|Q87QP4|FTSK_VIBPA DNA translocase FtsK OS=Vibrio parahaemolyticus serotype O3:K6 (strain RIMD 2210633) OX=223926 GN=ftsK PE=3 SV=1

MFKENAKKVETIIKTSEEPQSSRLNGFQRLKECCFIVGVLSSVLLAVALFTFSPADPSWS

QTAWGGEIDNAGGLFGAWLADTLFFTFGSLAYPIPFLLAAAAWVICRKRGEDEPIDFMLW

GTRLLGLTVLIMTSCGLADINFDDIWYFSSGGVVGDVLSSLALPTLNVLGTTLVLLFLWG

AGFTLFTGISWLNIVEWLGDRSLAVLAAIANKFRGSEQETLEPQLDEFVEDKVSTKHVED

DQQDDETLPHLTAYEVEEPKEKAAVHEYPIYMPQAKSETSAVKPTPEPQPQRVAAVNATP

TYVEPEPQLKAVSTDNVDPMVERTKQLNVTIEELEAAAQQADDWASEEQTSQSYADTNAV

YQEQVQAKHEEVVEHDTPQLESSYAEYAQFAAQQEQQLHVEPTPHEEPVIDTRALDDITD

HAEPSEHIEPTISDFDVVDEEETYVAPQPQSRSPEPQPMVQPQSVSQIQPEQAPEPSVAF

EPAPQEVEVEEVQDGDQDVAAFQSMVSSAQAKVAATQNPFLMKQEQNLPVPEEPLPTLEL

LYHPEKRENFIDREALEQVARLVESKLADYKIKADVVGIYPGPVITRFELDLAPGVKVSR

ISGLSMDLARALSAMAVRVVEVIPGKPYVGLELPNMSRQTVYLSDVISSPQFEQAKSPTT

VVLGQDIAGEAVIADIAKMPHVLVAGTTGSGKSVGVNVMILSMLYKASPEDLRFIMIDPK

MLELSIYEGIPHLLAEVVTDMKDASNALRWCVGEMERRYKLMSALGVRNVKGFNEKLKMA

AEAGHPIHDPFWQEGDSMDTEPPLLEKLPYIVVVVDEFADLMMVVGKKVEELIARLAQKA

RAAGIHLILATQRPSVDVITGLIKANIPTRVAFTVSTKTDSRTILDQGGAESLLGMGDML

YLPPGSSHTIRVHGAFASDDDVHAVVNNWKARGKPNYIDEIISGDQGPESLLPGEQMESD

EEMDPLFDQVVEHVVQSRRGSVSGVQRRFKIGYNRAARIVEQLEAQGIVSAPGHNGNREV

LAPAPPKD

>sp|Q87NC0|EFPL_VIBPA Elongation factor P-like protein OS=Vibrio parahaemolyticus serotype O3:K6 (strain RIMD 2210633) OX=223926 GN=VP1948 PE=3 SV=1

MPKASEIKKGFAIESNGKTLLVKDIEVTTPGGRGGAKIYKMRCTDLTTGARVDERYKSDD

VVETVEMNKRAVVYSYADGDEHIFMDNEDYSQYTFKHNEVEDDMLFINEDTQGIHIILVD

GSAVGLELPSSVELVIEETDPSIKGASASARTKPARFASGLVVQVPEYIATGDRVVINTA

ERKYMSRA

>sp|Q87ME3|DXR_VIBPA 1-deoxy-D-xylulose 5-phosphate reductoisomerase OS=Vibrio parahaemolyticus serotype O3:K6 (strain RIMD 2210633) OX=223926 GN=dxr PE=3 SV=1

MQKLTILGATGSIGASTLKVVEQNPELFSVVALAAGTNVEKMVALCRQWQPKFAVMADKA

AAVALQSEIHTISPNTEVLGGVDALCHVASLEEVDSVMAAIVGAAGLLPTMAAVKAGKRV

LLANKEALVMSGQLFIDAVEQYGAELLPVDSEHNAIFQCLPQQVQTNLGRCNLDEHGISS

ILLTGSGGPFRYADIADLDSVTPAQAIAHPNWSMGPKISVDSATMMNKGLEYIEAKWLFN

AARDQLKVIIHPQSVIHSMVQYRDGSVLAQMGEPDMATPIALTMSYPSRVDAGVKPLDFT

QVGELTFLQPDFARYPCLKLAIDACYEGQHATTALNAANEVAVDAFLNNRLGFTDIARIN

ELVLHKITASCKPENANSLESLLELDRMSRTIALEIIRERS

>sp|Q87LV1|PANC_VIBPA Pantothenate synthetase OS=Vibrio parahaemolyticus serotype O3:K6 (strain RIMD 2210633) OX=223926 GN=panC PE=3 SV=1

MQTFAEISAVRGHLKTFKREGRKIAFVPTMGNLHEGHLTLVRKAREYADIVVVSIFVNPM

QFDRADDLNNYPRTLEEDLSKLTAEGVDVVFTPTPEIIYPEGLDKQTFVDVPGLSTILEG

ASRPGHFRGVTTIVNKLFNIVQPDVACFGEKDFQQLAVIRKMVDDLAMDIEIIGVPTVRE

MDGLAMSSRNGLLTLDERQRAPVLARTMRWISSAIRGGRDDYASIIEDANDQLRAAGLHP

DEIFIRDARTLQVITPETTQAVILMSAFLGQARLIDNQTVDMVVESKDEAESNDGTAANA

E

>sp|Q87TE1|PCKA_VIBPA Phosphoenolpyruvate carboxykinase (ATP) OS=Vibrio parahaemolyticus serotype O3:K6 (strain RIMD 2210633) OX=223926 GN=pckA PE=3 SV=1

MTVMEHTKAATLDLTKHGLHNVKEVVRNPSYELLFEEETRADLTGYERGVVTELGAVAVD

TGIFTGRSPKDKYIVKDATTEEHMWWTSDTVKNDNKPITQEVWNDLKELVTNQLSGKRLF

VVDGYCGANPDTRLSIRVITEVAWQAHFVKNMFIRPTEEELATFEPDFVVMNGAKCTNPK

WEEQGLNSENFTVFNLTERTQLIGGTWYGGEMKKGMFAMMNYFLPLKDIASMHCSANMGK

DGDVAVFFGLSGTGKTTLSTDPKRALIGDDEHGWDDDGVFNFEGGCYAKTIKLSKEAEPD

IYNAIRRDALLENVTVRSDGSIDFDDGSKTENTRVSYPIYHIENIVKPVSKGGHANKVIF

LSADAFGVLPPVSKLTPEQTKYHFLSGFTAKLAGTERGITEPTPTFSACFGAAFLTLHPT

KYAEVLVKRMEAAGAEAYLVNTGWNGTGKRISIQDTRGIIDAILDGSIEDAPTKHIPIFN

LEVPTSLPGVDPSILDPRDTYVDPLQWESKAKDLAERFINNFDKYTDNAEGKALVAAGPQ

LD

>sp|Q87KP9|NUSG_VIBPA Transcription termination/antitermination protein NusG OS=Vibrio parahaemolyticus serotype O3:K6 (strain RIMD 2210633) OX=223926 GN=nusG PE=3 SV=2

MSEAPKKRWYVVQAFSGFEGRVAQSLREHIKMHGMEELFGEVLVPTEEVVEMRAGQRRKS

ERKFFPGYVLVQMIMNDESWHLVRSVPRVMGFIGGTSDRPAPITDKEADAILNRLEKASE

APRPRTMYEAGEVVRVNEGPFADFNGTVEEVDYEKSRLKVSVSIFGRATPVELEFGQVEK

LD

>sp|Q87RD1|TSAB_VIBPA tRNA threonylcarbamoyladenosine biosynthesis protein TsaB OS=Vibrio parahaemolyticus serotype O3:K6 (strain RIMD 2210633) OX=223926 GN=tsaB PE=1 SV=1

MSAKILAIDTATENCSVALLVNDQVISRSEVAPRDHTKKVLPMVDEVLKEAGLTLQDLDA

LAFGRGPGSFTGVRIGIGIAQGLAFGAELPMIGVSTLAAMAQASYRLHGATDVAVAIDAR

MSEVYWARYSRQENGEWIGVDEECVIPPARLAEEAQADSKTWTTAGTGWSAYQEELAGLP

FNTADSEVLYPDSQDIVILAKQELEKGNTVPVEESSPVYLRDNVTWKKLPGRE

>sp|Q87LI7|TRMB_VIBPA tRNA (guanine-N(7)-)-methyltransferase OS=Vibrio parahaemolyticus serotype O3:K6 (strain RIMD 2210633) OX=223926 GN=trmB PE=3 SV=1

MSEVTTNEYNEDGKLIRKIRSFVRREGRLTKGQENAMNECWPTMGIDYKAELLDWKEVFG

NDNPVVLEIGFGMGASLVEMAKNAPEKNFFGIEVHSPGVGACLSDAREAGITNLRVMCHD

AVEVFEHMIPNDSLATLQLFFPDPWHKKRHHKRRIVQLEFAEMVRQKLIPNEGIFHMATD

WENYAEHMIEIMNQAPGFENIAQDGDFVPRPEDRPLTKFEARGHRLGHGVWDIKYKRIA

>sp|Q87LT0|TRMD_VIBPA tRNA (guanine-N(1)-)-methyltransferase OS=Vibrio parahaemolyticus serotype O3:K6 (strain RIMD 2210633) OX=223926 GN=trmD PE=3 SV=1

MWVGVISLFPEMFRSVTDFGVTGQAVKKGLLSIETWNPRDFTHDKHRTVDDRPYGGGPGM

LMMVQPLRDAIHTAKKASPGKTKVIYLSPQGRKLDQKGVEELATNENLLLICGRYEGVDE

RIIQSEVDEEWSIGDFVMTGGEIPAMTLIDSVSRFIPGVLGDFASAEEDSFANGLLDCPH

YTRPEVLDDKEVPSVLMSGNHKDIRQWRLKQSLGRTWLRRPELLENLALTDEQEQLLAEF

ISEHNAK

>sp|P22095|TRPA_VIBPA Tryptophan synthase alpha chain OS=Vibrio parahaemolyticus serotype O3:K6 (strain RIMD 2210633) OX=223926 GN=trpA PE=3 SV=2

MSRYEKMFARLNEKNQGAFVPFVTVCDPNAEQSYKIMETLVESGADALELGIPFSDPLAD

GPTIQGANIRALDSGATPDICFEQIGKIRAKYPDLPIGLLMYANLVYSRGIESFYERCAK

AGIDSVLIADVPTNESAEFVAAAEKFGIHPIFIAPPTASDETLKQVSELGGGYTYLLSRA

GVTGAETKANMPVDHMLEKLNQFNAPPALLGFGISEPAQVKQAIEAGAAGAISGSAVVKI

IEAHVEQPQIMLDKLGEFVSAMKAATQK

>sp|Q87IM1|TRPB2_VIBPA Tryptophan synthase beta chain 2 OS=Vibrio parahaemolyticus serotype O3:K6 (strain RIMD 2210633) OX=223926 GN=trpB2 PE=3 SV=1

MKNSFDHNMPNNEGYFGEYGGSFVPPELEQIMRDINAAYEECCQDPEFKDELARLYKHFV

GRPSPIFHAANLSKKYGADIYLKREDLNHTGAHKINHCLGEAILAKKMGKKKLIAETGAG

QHGVALATAAALVGLECDIYMGEVDIAKEHPNVVRMRILGANVIPATHGRKTLKEAVDAA

FEAYLKDPETQLYAIGSVVGPHPFPKMVRDFQSIIGNEARVQFKEMTGKLPNNLVACVGG

GSNAMGLFSAFLEDENVAIHGVEPAGRSLDKVGEHAATLTLGEPGIMHGFKSYMLKDEQG

EPQEVHSVASGLDYPSVGPQHSYLKDIGRVNYGSINDDEAIDAFFELSREEGIIPAIESS

HAVAYAIKLAQQGESGSILVNLSGRGDKDIDFVVENYGAKYGIESLI

>sp|Q87NC4|UVRC_VIBPA UvrABC system protein C OS=Vibrio parahaemolyticus serotype O3:K6 (strain RIMD 2210633) OX=223926 GN=uvrC PE=3 SV=1

MNPPFDSASFLKTVTHQPGVYRMYNADAVVIYVGKAKDLQKRLSSYFRKKVDSEKTRALV

SNIAKIDVTVTHTETEALILEHNYIKQYLPKYNVLLRDDKSYPYILISGHKHPRLSMHRG

AKKRKGEYFGPYPDSGAVRETLHLLQKIFPVRQCEDTVYSNRTRPCLMYQIGRCAGPCVS

SIISDEEYAELVGFVRLFLQGKDQQVLKQLIEKMEVASQQLRFEDAAKFRDQIQAIRRVQ

EQQYVSEDSMDDMDVLGFAQENGIACIHILMIRQGKVLGSRSHFPKIPQNTSQQEVFDSF

LTQYYLSHNEARTIPSRIILNQELADDLEPIQKALSEVAGRKVHFHTSPTGARGRYLKLS

NTNALTAITTKINHKMTINQRFKALRETLGMESIMRMECFDISHTMGESTIASCVVFNNE

GPVKQEYRRYNITGITGGDDYAAMGQALERRYSKQLDVEKIPDIIFIDGGKGQLNRAHEI

IAQYWGDWPKRPIMIGIAKGVTRKPGLETLITVDGEEFNLPSDAPALHLIQHIRDESHNH

AIAGHRAKRGKTRRTSALEGIEGVGPKRRQALLKYMGGLQELKRASVEEIAKVPGISHSL

AEIIFQALKQ

>sp|P22096|TRPD_VIBPA Anthranilate phosphoribosyltransferase OS=Vibrio parahaemolyticus serotype O3:K6 (strain RIMD 2210633) OX=223926 GN=trpD PE=3 SV=1

MEAIINKLYEQQSLTQEESQQLFDIIIRGELDPILMASALTALKIKGETPDEIAGAAKAL

LANANPFPRPDYDFADIVGTGGDGHNTINISTTAAFVAAACGLKVAKHGNRSVSSKSGSS

DLLDSFGINLAMSAEDTRKAVDDIGVAFLFAPQYHGGVRHAMPVRQTMKTRTIFNILGPL

INPARPNIELMGVYSEELVRPIAETMLQMGMKRAAVVHGSGLDEVAIHGTTTVAEIKDGK

ITEYTLTPEDFGLESHPLEAIKGGDPEENKAIITNILTGKGTDAQLGAVAVNVALLMRLF

GHEDLKANTQQAIEAMNSGKAYQLVQQLAAHA

>sp|Q87KJ6|XERC_VIBPA Tyrosine recombinase XerC OS=Vibrio parahaemolyticus serotype O3:K6 (strain RIMD 2210633) OX=223926 GN=xerC PE=3 SV=1

MTTTPNTPLPNSLQKPLERFYEFLRSEKGLSLHTQRNYKQQLETMAQHLAEMGLKDWSQV

DAGWVRQLAGKGMREGMKASSLATRLSSLRSFFDFLILRGEMSANPAKGVSAPRKKRPLP

KNLDVDEVNQLLEVNEDDPLAIRDRAMMELMYGAGLRLAELVSVDVRDVQLRSGELRVIG

KGDKERKVPFSGMATEWVGKWLRVRGDLAAPGEPALFVSKLGTRISHRSVQKRMAEWGQK

QSVASHISPHKLRHSFATHMLESSNNLRAVQELLGHENISTTQIYTHLDFQHLAQAYDQA

HPRARKKNGE

>sp|P46231|Y2115_VIBPA Uncharacterized membrane protein VP2115 OS=Vibrio parahaemolyticus serotype O3:K6 (strain RIMD 2210633) OX=223926 GN=VP2115 PE=4 SV=2

MNPVVISVCVMLVLALMRVNVVVALTFSAIVGGLVAGMSLGDTVAAFESGLGGGATIALS

YAMLGTFAVAISKSGITDLLAKSVIKRLNGKESAASTTGLKYAVLVALVLVTMSSQNVIP

VHIAFIPILIPPLLGVFAKLKLDRRLIACVLTFGLITPYMVLPVGFGGIFLNNILLKNLH

DNGLENVVASQVPTAMLLPGAGMIFGLLLAIFVSYRKPREYKETELTVVHETDHSINKQH

ILVAALGIIAALGVQLYTGSMIIGALAGFMVFTFGGVIAWKETHDVFTKGVHMMAMIGFI

MIAAAGFAAVMKQTGGVETLVQSLSTSIGDNKPLAALLMLVVGLLVTMGIGSSFSTIPIL

ATIYVPLSLAFGFSPMATIALVGTAAALGDAGSPASDSTLGPTSGLNADGQHEHIWETVV

PTFIHYNIPLIIFGWIAAMVL

>sp|Q87HV6|Y4850_VIBPA UPF0312 protein VPA0850 OS=Vibrio parahaemolyticus serotype O3:K6 (strain RIMD 2210633) OX=223926 GN=VPA0850 PE=3 SV=1

MKKSLFATGLAIAMALPLGAQAADYVIDTKGAHASINFKVSHLGYSFIKGRFNTFSGDFS

FDEKNIADSKVNVVVDTTSLDSNHAERDKHIRSGDFIDAGKYSEATFKSTKVVDKGNGKL

DVTGDLTLHGVTKPITIEAEFVGAGNDPWGGERAGFVGTTRLELADFDIPVMGSSSYVDM

ELHIEGVKK

>sp|Q87SC0|Y504_VIBPA UPF0246 protein VP0504 OS=Vibrio parahaemolyticus serotype O3:K6 (strain RIMD 2210633) OX=223926 GN=VP0504 PE=3 SV=1

MLIVVSPAKTLDYESPLATEKFTQPELIEYSKELIDVCRKLTPADVASLMKVSDKIADLN

VGRFQEWSETFTTENSRQAILAFKGDVYTGLEAETLSDADFEYAQKHLRMLSGLYGLLKP

LDLMQPYRLEMGTKLANDKGSNLYQFWGNVITDKLNEAIAEQGDNVLINLASNEYFKAVK

PKNLDAQVITPIFKDCKNGQYKVISFYAKKARGMMARYIIENRIESVADLTKFDTAGYYF

VEEESTPTDLVFKREEQN

>sp|Q87RJ5|ZIPA_VIBPA Cell division protein ZipA OS=Vibrio parahaemolyticus serotype O3:K6 (strain RIMD 2210633) OX=223926 GN=zipA PE=3 SV=1

MQELRFVLIVVGALAIAALLFHGLWSSKKEGKAKFGNKPLGKLDVDQGDKDSVEQERSFA

PATEDDFEIIRKDRKEPDFGMENTFDSKFEADPLLGGVAEEKHSVKEEAEEIPSFVAMKN

DVEDVAIQPSEVEEPMQEVVEEEIMPSAFDAPKQEMEMVEEVAPAVVEQPEEPKPEPEMQ

VIVLNVHCAGEEPFIGTELFDSMQQNGLIYGEMHIFHRHVDLSGNGKVLFSVANMMHPGT

LEHGDPAEFSTKGISFFMTLPCYGEAEQNFNLMLRTAQQIADDMGGNVLDDKRNLMTPDR

LAAYRRQIVEFNAANA

>sp|Q87LJ9|YQGF_VIBPA Putative pre-16S rRNA nuclease OS=Vibrio parahaemolyticus serotype O3:K6 (strain RIMD 2210633) OX=223926 GN=VP2613 PE=3 SV=1

MSRTIMAFDFGTKSIGSAIGQEITGTASPLKAFKANDGIPNWDDIEKQIKEWQPDLLVVG

LPTDLHGKDLETITPRAKKFAKRLQGRYGLPVELHDERLSTSEARAELFSMGGYKALSKG

NIDCQSAVVILESWFEALWGE

>sp|P22097|TRPB1_VIBPA Tryptophan synthase beta chain 1 OS=Vibrio parahaemolyticus serotype O3:K6 (strain RIMD 2210633) OX=223926 GN=trpB1 PE=3 SV=2

MAKLNAYFGEYGGQYVPQILVPALKQLEQAFIDAQEDPEFRSEFMTLLQEYAGRPTALTL

TRNLTKGTKTKLYLKREDLLHGGAHKTNQVLGQALLAKRMGKHEIIAETGAGQHGVATAL

ACALLGLKCRVYMGAKDVERQSPNVFRMKLMGAEVIPVHSGSATLKDACNEALRDWSGSY

EDAHYLLGTAAGPHPFPTIVREFQRMIGEETKNQILAREGRLPDAVIACVGGGSNAIGMF

ADFIEEESVRLIGVEPAGKGIDTDQHGAPLKHGKTGIFFGMKAPLMQDENGQVEESYSVS

AGLDFPSVGPQHAHLNAIGRAEYDNVTDDEALEAFQELARSEGIIPALESSHALAHALRM

ARENPEKEQLLVVNLSGRGDKDIFTVHAILEEKGVI

>sp|Q87LD9|Y2673_VIBPA Nucleotide-binding protein VP2673 OS=Vibrio parahaemolyticus serotype O3:K6 (strain RIMD 2210633) OX=223926 GN=VP2673 PE=3 SV=1

MRLIVVSGHSGAGKSIALRVLEDLGYYCVDNLPVNLLDAFVHSIADSKQNVAVSIDIRNI

PKKLKELTGTLEQLKTELDVTVLFLDANKETLLKRYSETRRIHPLSLGGQSLSLDQAIER

EKEILTPLKAHADLILNSSGQSLHELSETVRMRVEGRDRKGLVMVFESFGFKYGLPSDAD

YVFDVRFLPNPHWEPALRPLTGLDAPIAAFLEQHQSVLSLKYQIESFIETWLPLLEKNNR

SYLTVAIGCTGGKHRSVYLTQQIGEYFADKGHQVQIRHTSLEKNAKE

>sp|Q87KI0|UBID_VIBPA 3-octaprenyl-4-hydroxybenzoate carboxy-lyase OS=Vibrio parahaemolyticus serotype O3:K6 (strain RIMD 2210633) OX=223926 GN=ubiD PE=3 SV=1

MSFKDLREFIDHLEQKGRLKRITHPVDPAYEMTEISDRTLRAGGPALLFENPIGYNVPVL

TNLFGTPERVAIGMGREDVKELREVGKLLAYLKEPEPPKGFKDALEKLPVFKQVLNMPAK

RLRKAPCQDIVWQGDEVDLDKIPVMSCWAEDVAPLLTWGLTVTKGPNKKRQNLGIYRQQK

IAKNKIIMRWLAHRGGALDLRDWMETNPGKPFPVSVAFGADPATILGAVTPVPDTLSEYA

FAGLLRGSKTEVVKSISNDLEVPASAEIVMEGYIDPNEFADEGPYGDHTGYYNEKEKHHV

FTITHITMRKDPIYHSTYTGRPPDEPAVLGVALNEVFVPILQKQFPEIEDFYLPPEGCSY

RMAVVTMKKQYPGHAKRVMMGVWSFLRQFMYTKFVIVCDESVNARDWNDVVKAMTEHMDP

VRDTLMIDNTPIDSLDFASPVVGLGSKMGLDATIKWDAELATRPQISKQDSKVITEADLE

SLKQQRPEIIDIYLPPTTNNRFAVVTMKKDQAGQSQALMEYLWDFFAQYTDNKFVILCDE

DVNARDWNDIIWAVTTRMDPDRDTTRVSGKAESSSKLGLDATNKFESEVTREWGTPIKKD

PKLVAKVDEIWDQLGIL

>sp|Q87HS0|DDL_VIBPA D-alanine--D-alanine ligase OS=Vibrio parahaemolyticus serotype O3:K6 (strain RIMD 2210633) OX=223926 GN=ddl PE=3 SV=1

MIKNILLLCGGGSSEHEISLLSANFVEQQLNLIQNVKVTRVEIKNEGWVTDQGELVYLDL

NTKQLCSNESNQTIDFIVPCIHGFPGETGDIQSLFEIAGIPYLGCGPEASSNSFNKITSK

LWYDALDIPNTPYLFLTRNDEHAHRQAEQAFEKWGKVFVKAARQGSSVGCYSVAEKQAIA

KAVNDAFGYSDQVLVEKAVKPRELEVAAYEMNGELHITKPGEVIAPDGAFYSYDEKYSSS

SHSLTEVEAKNLTQEQIDKIRHASETVFKQMNLRHLSRIDFFLTEDNEIYLNEVNTFPGM

TPISMFPKMLQNNGHKFHEFLEDCINSAK

>sp|Q87L91|CYSI_VIBPA Sulfite reductase [NADPH] hemoprotein beta-component OS=Vibrio parahaemolyticus serotype O3:K6 (strain RIMD 2210633) OX=223926 GN=cysI PE=3 SV=1

MTFSTENNKQIVLGEELGPLSDNERLKKQSNLLRGTIAEDLQDRITGGFTADNFQLIRFH

GMYQQDDRDIRNERTKQKLEPLHNVMLRARMPGGIITPTQWLAIDKFATEHSLYGSIRLT

TRQTFQFHGVLKPNIKLMHQTLNNIGIDSIATAGDVNRNVLCTTNPVESELHQEAYEWAK

KISEHLLPKTRAYAEIWLDGEKVESTEEDEPILGKTYLPRKFKTTVVIPPQNDVDVHAND

LNFVAIADNGKLVGFNVLVGGGLAMTHGDTSTYPRRADDFGFIPLEKTLDVAAAVVTTQR

DWGNRSNRKNAKTKYTLDRVGTDVFKAEVEKRAGIQFEASRPYEFTERGDRIGWVEGIDG

KFHLALFIENGRLLDYPGKPLKTGVAEIAKIHKGDFRMTANQNLIVAGVPKSEKAKIEKI

AREHGLMDDNVSEQRKNSMACVAFPTCPLAMAEAERFLPQFVTDVEGILEKHGLPENDNI

ILRVTGCPNGCGRAMLAEIGLVGKAPGRYNLHLGGNRAGTRVPKMYKENITDKQILEEID

LLVARWSKEREEGEAFGDFTIRAGIIQEVFVSKRDFYA

>sp|Q87SY0|CYSD_VIBPA Sulfate adenylyltransferase subunit 2 OS=Vibrio parahaemolyticus serotype O3:K6 (strain RIMD 2210633) OX=223926 GN=cysD PE=3 SV=1

MDQQRLTHLKQLEAESIHIIREVAAEFDNPVMMYSIGKDSSVMLHLARKAFYPGKIPFPL

LHVDTDWKFREMIEFRDRTAEKYGFELLVHKNPEGIAMGCSPFVHGSSKHTDIMKTQGLK

QALNKYGFDAAFGGARRDEEKSRAKERVYSFRDKNHTWDPKNQRPELWKTYNGQVNKGES

IRVFPLSNWTELDIWQYIYLENIEIVPLYLADKRPVVERDGMLIMVDDDRMELQPGEVIE

EKSVRFRTLGCYPLTGAIESEANTLTGIIEEMLVATSSERQGRAIDHDQSGSMELKKRQG

YF

>sp|Q87SF5|DAPB_VIBPA 4-hydroxy-tetrahydrodipicolinate reductase OS=Vibrio parahaemolyticus serotype O3:K6 (strain RIMD 2210633) OX=223926 GN=dapB PE=3 SV=1

MVRIAIAGAAGRMGRNLVKASHINPDASVTAGSERPESSLVGVDIGELCGEGKFDVFLTD

DLEKEVDNFDVVIDFTVPVSTLANLELCKQHGKSIVIGTTGFSEEERALIDAVAKHVPVV

MAPNYSVGVNLVFKLLEKAAKVMGDYCDVEIVEAHHRHKVDAPSGTAIGMGEAIAGAMGN

KLSDVAVYAREGITGERTKDEIGFATIRAGDIVGEHTAMFADIGERVEITHKATDRMTFA

NGAVKAAVWLHSKPAGFYTMTDVLGLNEL

>sp|Q87KJ4|DAPF_VIBPA Diaminopimelate epimerase OS=Vibrio parahaemolyticus serotype O3:K6 (strain RIMD 2210633) OX=223926 GN=dapF PE=3 SV=1

MHFHFSKMHGLGNDFMVVDCITQNVFFSQDLIRRLADRHTGVGFDQLLVVEAPYDPETDF

HYRIFNADGSEVEQCGNGARCFARFVRLKGLTNKYSISVSTKKGKMILDVEDDGEVTVNM

GVPEFEPNKIPFKAKQKEKTYIMRAGDKTLFCGAVSMGNPHVVTVVDDVDTADVDTLGPL

LESHERFPERVNAGFMQVVSRDHIRLRVYERGAGETQACGSGACGAVAVGILQGLLDESV

KVSLPGGELHISWQGPGKPLFMTGPATHVFDGQLSC

>sp|Q87L55|ARGC_VIBPA N-acetyl-gamma-glutamyl-phosphate reductase OS=Vibrio parahaemolyticus serotype O3:K6 (strain RIMD 2210633) OX=223926 GN=argC PE=3 SV=1

MLKTTIIGASGYTGAELAFMVNKHPQLTLSGLYVSANSVDAGKTIAQLHGKLANVVDMVV

NALTDPKQVAQDSDVVFLATAHEVSHDLAPIFLEAGCQVFDLSGAFRVKSDGFYDTFYGF

EHQFNNWLDKAAYGLAEWNQEEIKNAPLVAVAGCYPTASQLAIKPLLVDGLLDTQQWPVI

NATSGVSGAGRKASMTNSFCEVSLQPYGVFNHRHQPEIAQHLGCDVIFTPHLGNFKRGIL

ATVTMKLAQGVTEQQVAQAFEQAYQGKPAVRLKGDGIPRIQDVENTPFCDIGWKVQGEHI

IVISAIDNLLKGASSQAMQCLNIHYGYPELTALL

>sp|Q87S80|ANMK_VIBPA Anhydro-N-acetylmuramic acid kinase OS=Vibrio parahaemolyticus serotype O3:K6 (strain RIMD 2210633) OX=223926 GN=anmK PE=3 SV=1

MKFNELYIGVMSGTSMDGVDTALVEITDNHVRLIAHGDYPMPAAMKEMLLSVCTGQATNL

KAIGELDHQLGHLFADAVLQLLNKSGYVAEQIRAIGNHGQTVFHQPTGDLPFTTQLGDAN

IIAVKTGIDTVADFRRKDMALGGQGAPLVPAFHKSIFAMQDSTTVVLNIGGIANISVLHP

QQPVHGYDTGPGNMLMDAWCERHTGHGFDKDAQLALRGSVNEALLAHLLKEPYLAMSAPK

STGRELFNMDWLHHQLANYDVSVEDVQRTLCEYTAITIAHDVTKFTYGETPQLLVCGGGA

RNPLLMQRLAELLPQWHVTTTTDKGVDGDYMEAMAFAWLAQRHIHDLPSNLPEVTGASRL

ASLGVLYSKN

>sp|Q87L78|ALR1_VIBPA Alanine racemase 1 OS=Vibrio parahaemolyticus serotype O3:K6 (strain RIMD 2210633) OX=223926 GN=alr1 PE=3 SV=1

MKAAKACIDLSALQHNLQRVKAQAPESKVMAVVKANGYGHGLRHVAKHANHADAFGVARI

EEALQLRACGVVKPILLLEGFYSPGDLPVLVTNNIQTVVHCEEQLIALEQADLETPVVVW

LKIDSGMHRLGVRPEQYDEFISRLKTCPNVAKPLRYMSHFGCADELDSSITPQQIELFMS

LTSGCQGERSLAASAGLLAWPQSQLEWVRPGIIMYGVSPFSDKTAQDLGYQPVMTLKSHL

IAVREVKQGESVGYGGIWTSERDTKVGVIAVGYGDGYPRSAPNGTPVWVNGRTVPIAGRV

SMDMLTVDLGPDATDKVSDEAILWGKELPVEEVANHIGTIAYELVTKLTPRVEMEYTK

>sp|Q87L54|CAPP_VIBPA Phosphoenolpyruvate carboxylase OS=Vibrio parahaemolyticus serotype O3:K6 (strain RIMD 2210633) OX=223926 GN=ppc PE=3 SV=2

MNEKYAALKSNVRMLGHLLGNTIRDAHGEEIFEKVETIRKLSKSAQAGNQADRESLIEEI

KHLPDEQLTPVTRAFNQFLNLTNIAEQYHTISRHCEEHICEPDAINSLFSKLVQNDVSKL

DTAQAVRDLNIELVLTAHPTEITRRTMINKLVKINECLSKLELSDLSSKERKKTERRLEQ

LIAQSWHSDVIRQQRPTPLDEAKWGFAVVENSLWEAVPDFLREMNDRLKSYLGEGLPIDA

RPVHFSSWMGGDRDGNPFVTHSVTREVLLLSRWKAADLYLNDINELISELSMTVSNDQVR

ELAGEDQHEPYRAILKQLRALLNETKDILDAKIHGQKLAVKAPLQKVEQLWDPLYACYQS

LHECGMGVIADGSLLDTLRRVKAFGVHLVRLDIRQESTRHADVLSELTRYLGIGDYEQWS

EQDKIAFLTNELASKRPLLPRDWEPSEPVKEVLDTCKIIALQPREAFGAYVISMARTASD

VLAVHLLLQEAGCPYRMDVCPLFETLDDLNNAESVIKQLMSIDLYRGFIQNHQMVMIGYS

DSAKDAGVMSAGWAQYHAMESLVKVAEDEGVELTLFHGRGGTVGRGGAPAHAALLSQPPK

SLKGGLRVTEQGEMIRFKLGLPDVAVNSFNLYASAILEANLLPPPEPKQEWRDLMEVLSE

VSCEAYRGVVRGEPDFVPYFRQATPELELGKLPLGSRPAKRNPNGGVESLRAIPWIFSWS

QNRLVLPAWLGAGEAIQYSVDKGHQALLEEMCREWPFFSTRLGMLEMVYTKCNMEISRYY

DQRLVEPQLQPLGDRLREQLQRDIKSVLNVENNENLMQSDPWGQESIRLRNIYVEPLNML

QAELLYRTRQTEEASANLEEALMVTIAGIAAGMRNTG

>sp|Q87L68|AROB_VIBPA 3-dehydroquinate synthase OS=Vibrio parahaemolyticus serotype O3:K6 (strain RIMD 2210633) OX=223926 GN=aroB PE=3 SV=1

MERITVNLAERSYPISIGAGLFEDPAYLSQVLSNKNTNQKVVVISNVTVAPLYADKILHQ

LKQLGCDASLLELPDGEQYKNLDVFNQVMNFLLEGSYARDVVIIALGGGVIGDLVGFASA

CYQRGVDFIQIPTTLLSQVDSSVGGKTAVNHPLGKNMIGAFYQPKAVIIDTNCLSTLPER

EFAAGIAEVIKYGIIYDGAFFDWLEENLDRLYTLDEDALTYAIARCCQIKAEVVAQDEKE

SGIRALLNLGHTFGHAIEAELGYGNWLHGEAVSSGTVMAAKTSLLRGLISEEQFERIVAL

LRRAKLPVHTPDSMSFDDFIKHMMRDKKVLSGQLRLVLPTGIGSAEVIADTSQEVIQQAI

DFGRNI

>sp|Q87QN6|BIOB_VIBPA Biotin synthase OS=Vibrio parahaemolyticus serotype O3:K6 (strain RIMD 2210633) OX=223926 GN=bioB PE=3 SV=1

MEVRHNWTHAEVRDLMEKPFMDLLFEAQLVHRQYQQTNHVQVSTLLSIKTGACPEDCKYC

PQSARYTTDIEKERLMEVERVLDAAQKAKNAGSTRFCMGAAWKNPKERDMPHLTDMIKGV

KDMGLETCMTLGMLTPEQAKQLANAGLDYYNHNLDTSPEFYGNIITTRTYQDRLDTLSHV

RDAGMKICSGGIIGMGESANDRAGLLVELANLPTHPESVPINMLVKVKGTPLETVDDVDP

FDFIRLIAIARIMMPQSAVRLSAGRENMNEQMQALCFMAGANSVFYGCKLLTTPNPSEDK

DMMLFKKLGINSQEVSQKPDEIEENELLDRVVERVAARPTKDDLFYDASV

>sp|Q87HN1|COBQ_VIBPA Cobyric acid synthase OS=Vibrio parahaemolyticus serotype O3:K6 (strain RIMD 2210633) OX=223926 GN=cobQ PE=3 SV=1

MKSAIPSLMVQGTTSDAGKSVLVAGLCRVLARKGINVAPFKPQNMALNSAVTKDGGEIGR

AQAVQAQACNIEPTVHMNPVLIKPNSDTGAQIILQGKALSNMDAASFHDYKKVAMNTVLD

SFSKLTKEFDSIMIEGAGSPAEINLREGDIANMGFAEAADVPVIIVADIDRGGVFAHLYG

TLALLSESEQTRVKGFVINRFRGDIRLLQSGLDWLEEKTGKPVLGVLPYLHGLNLEAEDA

ITAQQELNSEVKLNVVVPVLTRISNHTDFDVLRLNPDINLSYVGKGEKIDKADLIILPGT

KSVRDDLAYLKSQGWDKDILRHIRLGGKVMGICGGYQMLGKTIDDPDGVEGEPGSSEGLG

LLNVHTVLTGSKQLTKTEAVLNLNNQKAKVKGYEIHVGRSQVLDEQPLELDNGECDGAIS

ECGQIMGTYLHGFFDEAEALNLITEWVNGTQVKQQDFEVLKEQGINRIADAIEQHMNLDF

LFK

>sp|Q87QV5|CMOB_VIBPA tRNA U34 carboxymethyltransferase OS=Vibrio parahaemolyticus serotype O3:K6 (strain RIMD 2210633) OX=223926 GN=cmoB PE=3 SV=1

MFNFANFYQLIAQDTRLQPWLNVLPQQLTDWQNAEHGDFGRWLKALNKIPEGSPDQVDIK

NSVTISNDTPFHEGELKKLENLLRTFHPWRKGPYTVHGIHIDTEWRSDWKWDRVLPHISP

LKNRSVLDVGCGNGYHMWRMLGEGARLCVGIDPSHLFLIQFEAIRKLMGGDQRAHLLPLG

IEQLPKLEAFDTVFSMGVLYHRRSPLDHLIQLKDQLVSGGELVLETLVIEGDENAVLVPT

SRYAQMRNVYFFPSAKALKVWLELVGFEDVHIVDENVTSVDEQRTTDWMTHNSLPDYLDP

NDPSKTVEGYPAPRRAVLVARKP

>sp|Q87MP2|ACCD1_VIBPA Acetyl-coenzyme A carboxylase carboxyl transferase subunit beta 1 OS=Vibrio parahaemolyticus serotype O3:K6 (strain RIMD 2210633) OX=223926 GN=accD1 PE=3 SV=1

MSWLEKILEKSNLVSSRKASIPEGVWTKCTSCEQVLYHAELERNLEVCPKCNHHMRMKAR

RRLETFLDEGNRVELGTELEPQDKLKFKDSKRYKERISAAQKSSGEKDALIVMQGELLGM

PLVACAFEFSFMGGSMGSVVGARFVKAVEAAIENNCALVCFSASGGARMQEALMSLMQMA

KTSAALERLSEKGLPFISVLTDPTMGGVSASLAMLGDINIGEPKALIGFAGRRVIEQTVR

EDLPEGFQRSEFLLEHGAIDMIVDRREMRQRVGGLIAKMTNHKSPLVVSVNESPNEEPYS

VPEVDEKG

>sp|Q87IJ5|ACKA2_VIBPA Acetate kinase 2 OS=Vibrio parahaemolyticus serotype O3:K6 (strain RIMD 2210633) OX=223926 GN=ackA2 PE=3 SV=1

MSNSFVLVINSGSSSLKFAVIDSVSGDAVLSGLGECFGLSDARMSWKFNGEKKEISIEGD

DSHHKIAIGKLVGLTEELGLAQDIVAVGHRIVHGGEKFTKTVRITEEVTQEIEKLADLAP

LHNPAGAIGIRAAVEAFPSLPQFAVFDTAFHQTMPQRAFTGAIAKELYTDFGIRRYGFHG

TSHYFVSREAAKMINKPIEESSFISVHLGNGASVCAINNGESVDTSMGFTPLSGLMMGTR

CGDLDPGIIEYLLKKGWSQEKVFNSLNKASGFLGVSGLTSDARGILEAMEEGHEGAALAF

QVFTYRVSKYIASYLAALDSFDGIIFTGGIGENSMPIRREILKNLKLLGFVEDVKGNEDA

RFGNAGVIATSELLGAKALVIPTNEEWVIAQQSVELL

>sp|Q87MZ4|ACKA1_VIBPA Acetate kinase 1 OS=Vibrio parahaemolyticus serotype O3:K6 (strain RIMD 2210633) OX=223926 GN=ackA1 PE=3 SV=1

MSKLVLVLNCGSSSLKFAVVDAENGEEHLSGLAECLHLPEARIKWKLDGKHEAQLGNGAA

HEEALAFMVETILASKPELSENLAAIGHRVVHGGEQFTQSALITDDVLKGIEDCATLAPL

HNPAHIIGIKAAQKSFPALKNVAVFDTAFHQTMPEESYLYALPYNLYKEHGIRRYGMHGT

SHLFITREVAGLLNKPVEEVNIINCHLGNGASVCAVKNGQSVDTSMGLTPLEGLVMGTRC

GDIDPAIIFHLHDTLGYSVEKINTMLTKESGLQGLTEVTSDCRFVEDNYGEKEEATRAMD

VFCHRLAKYVAGYTATLDGRLDAITFTGGIGENSAPIREMVLNRLGIFGIEVDSEANLKA

RFGGEGVITTENSRIPAMVISTNEELVIAEDTARLAGL

>sp|Q87TQ7|DNAA_VIBPA Chromosomal replication initiator protein DnaA OS=Vibrio parahaemolyticus serotype O3:K6 (strain RIMD 2210633) OX=223926 GN=dnaA PE=3 SV=1

MSSSLWLQCLQQLQEELPATEFSMWVRPLQAELNDNTLTLFAPNRFVLDWVRDKYLNSIT

RLLQEYCGNDIPNLRFEVGSRPVSAPKPAPTRTPADVAAESSAPAQLQARKPVHKTWDDD

PQAIAAINHRSNMNPKHKFDNFVEGKSNQLGLAAARQVSDNPGAAYNPLFLYGGTGLGKT

HLLHAVGNAIVDNNPNAKVVYMHSERFVQDMVKALQNNAIEEFKRYYRSVDALLIDDIQF

FANKERSQEEFFHTFNALLEGNQQIILTSDRYPKEISGVEDRLKSRFGWGLTVAIEPPEL

ETRVAILMKKAEDHQIHLADEVAFFIAKRLRSNVRELEGALNRVIANANFTGRPITIDFV

REALRDLLALQEKLVTIDNIQKTVAEYYKIKVADLLSKRRSRSVARPRQLAMALAKELTN

HSLPEIGDAFGGRDHTTVLHACRKIEQLREESHDIKEDYSNLIRTLSS

>sp|Q87N39|DNAE2_VIBPA Error-prone DNA polymerase OS=Vibrio parahaemolyticus serotype O3:K6 (strain RIMD 2210633) OX=223926 GN=dnaE2 PE=3 SV=1

MSYAELFCQSNFSFLTGASHAEELVLQAAFYRYHAIAITDECSVAGVVKAHATIEQHKLD

IKQIVGSMFWLNEECQIVLLCPCRKAYAEMCRIITNARRRSEKGSYQLSEWDLMSIRHCL

VLWLPTHQASDHYWGRWLNQHHNNRLWVAIQRHLGGDDDAYTNHCEKLAHELQQPITACG

GVLMHTAERLPLQHILTAIKHGCSVDQLGFERLSNAERALRPLNKLVRIYKPEWLEESKY

IADLCEFKLSDLKYEYPTELIPNGYTPNSYLRMLVEQGKERRFPEGVPEDINQTIENELR

LIEDLKYHYYFLTIHDIVMFAKQQGILYQGRGSAANSVVCYCLEITAVDPRQISVLFERF

ISKERKEPPDIDVDFEHERREEVIQYIYKKYGRERAALAATVISYRFKSAVREVGKALGI

EETQLDFFIKNVNRRDRSQGWQAQIIELGLQPESLKGQQFIQLVNEIIGFPRHLSQHVGG

FVISSGPLYELVPVENAAMEDRTIIQWDKDDLESLELLKVDVLALGMLNAIRKCFQLIEK

HHQRSLSIAEITRRQDDPHVYRMLQKADTVGVFQIESRAQMSMLPRLKPACYYDLVIQIA

IVRPGPIQGDMVHPFLKRRNGEEPVSYPSEAVKSVLERTMGVPIFQEQVIKLAMVAAGFS

GGEADQLRRAMASWKKNGDLAKFKPKLLNGMQERGYDLAFAERIFEQICGFGEYGFPESH

SASFAVLAYCSAWLKYYYPAEFYTALLNSQPMGFYSPSQLVQDARRHGVEVLPICVNHSY

YQHHLIQRPNGRLGVQLGFRLVKGFNEEGATRLVERRPKTGYHSIQEVKQILRSRRDIEL

LASANAFQILSGNRYNARWAAMDSLSDLPLFHHIEEPSVGYQVQPSEYESLIEDYASTGL

SLNRHPITLLEEAGILPRFTRMKQLVDKEHKSLVTVAGVVTGRQSPGTAAGVTFFTLEDD

TGNINVVVWSATARAQKQAYLTSKILMVKGILEREGEVIHVIAGKLIDCTHYLSNLQSKS

RDFH

>sp|Q87KA4|ATPF_VIBPA ATP synthase subunit b OS=Vibrio parahaemolyticus serotype O3:K6 (strain RIMD 2210633) OX=223926 GN=atpF PE=3 SV=1

MNINATLLGQAISFALFVWFCMKYVWPPLMQAIEERQKKIADGLQAAERAAKDLDLAQAN

ASDQLKEAKRTATEIIEQANKRKSQIIDEAREEAQAERQKILAQAEAELEAERNRARDEL

RKQVATLAVAGAEKILERTIDKDAQKDILDNITAKL

>sp|Q87KA3|ATP6_VIBPA ATP synthase subunit a OS=Vibrio parahaemolyticus serotype O3:K6 (strain RIMD 2210633) OX=223926 GN=atpB PE=3 SV=1

MAAPGEALTSSGYIAHHLSNLSLYKLGLVGSETSFWNVHIDSLFFSWFTGLIFLGIFYKV

AKRTTAGVPGKLQCAVEMIVEFVADNVKDTFHGRNPLIAPLALTIFCWVFLMNVMDLVPI

DFLPYPAEHWLGIPYLKVVPSADVNITMAMALGVFALMIYYSIKVKGLGGFAKELALHPF

NHPLMIPFNLLIEVVSLLAKPLSLGMRLFGNMFAGEVVFILCAAMLPWYLQWMGSLPWAI

FHILVITIQAFVFMMLTIVYLSMAHEDSDH

>sp|Q87LG0|ARCA_VIBPA Arginine deiminase OS=Vibrio parahaemolyticus serotype O3:K6 (strain RIMD 2210633) OX=223926 GN=arcA PE=3 SV=1

MSKLYVGSEVGQLRRVLLNRPERALTHLTPSNCHELLFDDVLAVEAAGEEHDAFARTLRE

QDVEVLLLHDLLVETLAVPEAKQWLLNTQISDFRYGPTFARDLRQYLLEMDDEHLATILL

GGLAYSELPIQSSSMLPKMKRPLDFVIEPLPNHLFTRDTSCWVYGGVSLNPMMMPARQRE

TNHLRAIYRWHPIFAGQDFIKYFGDDDLHYDNANVEGGDVLVIGKGAVLIGMSERTTPQG

VENLAASLFKAGQASEVIAIDLPKHRSCMHLDTVMTHMDVDTFSVYPEIMRKDLDTWRLT

PKGTDGEMHVEASHNYLHAIESALGLDQLKIITTGGDSYEAEREQWNDANNVLTVKPGVV

IGYERNVYTNEKYDKAGIQVLTVPGNELGRGRGGARCMSCPIERDDI

>sp|Q87MM9|AROC_VIBPA Chorismate synthase OS=Vibrio parahaemolyticus serotype O3:K6 (strain RIMD 2210633) OX=223926 GN=aroC PE=3 SV=1

MAGNSIGQHFRVTTFGESHGIALGCIVDGCPPGLEITEADLQTDLDRRRPGTSRYTTQRR

EPDEVKILSGVFEGKTTGTSIGLLIENTDQRSKDYSDIKDKFRPGHADYTYHQKYGIRDY

RGGGRSSARETAMRVAAGAIAKKYLKDEFGVEIRAYLSQMGDVSIDKVDWDEIENNAFFC

PDADKVEAFDQLIRDLKKEGDSIGAKIQVVATNVPVGLGEPVFDRLDADIAHALMSINAV

KGVEIGDGFDVVNQKGSQHRDPLSPQGFGSNHAGGILGGISTGQDIVANIALKPTSSITV

PGDTITKEGEPTQLITKGRHDPCVGIRAVPIAEAMLAIVVMDHLLRHRGQNHGVTTETPK

I

>tr|Q87MG3|Q87MG3_VIBPA DNA polymerase III subunit epsilon OS=Vibrio parahaemolyticus serotype O3:K6 (strain RIMD 2210633) OX=223926 GN=dnaQ PE=4 SV=1

MNTSSNSEHNRIVVLDTETTGMNQEGGPHYLGHRIIEIGAVEIINRKLTGRHFHVYIKPD

REIQPEAIQVHGITDEFLVDKPEYASIHQEFLDFIKGAELVAHNAPFDTGFMDYEFEKLD

PTIGKTDDYCKVTDTLAMAKKIFPGKRNNLDVLCERYGIDNSHRTLHGALLDAEILADVY

LLMTGGQTSLEFNANKQEGGVETIRRIEGRKALKVLRATADELEAHQKRLELVNDCIWHQ

>tr|Q87NX3|Q87NX3_VIBPA Lipid A biosynthesis lauroyltransferase OS=Vibrio parahaemolyticus serotype O3:K6 (strain RIMD 2210633) OX=223926 GN=lpxL PE=3 SV=1

MNKYKKPEFKAKFLLPRYWGTLILIGVMYSLSLLPFNIQLALGRGIGRLAMRIMKKRQVT

IRRNLELCFPHMGESQREAILKDNIDNSGIALFETAMAWLWSDKRVSKHVTIKGMEHLEA

LEKSGKGALMLAVHSMNLELGARAFGIQKSGTGVYRPNNNPCFDYFQYKGRSRSNRTLID

RKNVKAMLEALNSGERVWYAPDHDYGNRRSTFAPLFAVKNACTTTGTSLLVDNTNCAIVP

FTMVRGEDGHYTLTIDAPVDGFPKDDPDSAAAFVNKIVETSIMASPSQYMWLHRRFKTRP

EGEDCLYKPRLVPAMDISI

>tr|Q87QQ8|Q87QQ8_VIBPA Putative transmembrane protein affecting septum formation and cell membrane permeability OS=Vibrio parahaemolyticus serotype O3:K6 (strain RIMD 2210633) OX=223926 GN=VP1091 PE=3 SV=1

MRRRTTLSLLIASSLFMAGCQPSQEGTEQGGGGAPATEVNVLTVEPVRQALTVELPGRSR

AFKEAEVRPQVTGIISERNFVEGGVVEKGQSLYQIDDSSFQADLLSAEAELIRAQASEES

TFATVKRYRALITKKSISQQDLDEAEAAYKEAKAQVLVAKAKINTAKINLTYTRVKAPIS

GVISKSNVTAGALVTANQADKLTTIQQLDPINVDIVQSSAQLLRLKAALSQGHMQEDQSA

QVALTLEDGSTYEHKGTMKFTEVNVDESTGSVTLRAEFPNPDGLLLPGMFVRATVITGVD

PSAILIPQNTVTRDATGKASVMTVSADNTVAITPVITAEVIDNQWRIIDGLKAGDQVITA

GLQKVRPGSPVTIQTGEQG

>tr|Q87MB1|Q87MB1_VIBPA FAD:protein FMN transferase OS=Vibrio parahaemolyticus serotype O3:K6 (strain RIMD 2210633) OX=223926 GN=VP2345 PE=3 SV=1

MKKWLVAFTSLLILAGCEQPADQIHLSGPTMGTSYNIKYIEQDGIPTPKALQTEIDRLLE

EVNDQMSTYREDSELSRFNQHQTSEPFEVSAQTATVVKEAVRLNGLTLGALDVTVGPLVN

LWGFGPEARPDVVPSDEELAARKANTGIHHLTVDGNKLSKDIPNLYVDLSTIAKGWGVDV

VADYLQSVGIKNYMVEVGGEMRLKGINREGVPWRIAIEKPTVDERSIQEIIEPGDMAIAT

SGDYRNYFESNGVRYSHIINPQTGKPIHHKVVSVTVLDKSSMTADGLATGLMVLGEEKGM

EIANENNIPVFMIVKTEDGFKELASEAYKPFMNK

>tr|Q87R57|Q87R57_VIBPA Putative multidrug resistance protein OS=Vibrio parahaemolyticus serotype O3:K6 (strain RIMD 2210633) OX=223926 GN=VP0941 PE=3 SV=1

MLLSDVSVKRPVAALVLSMLLCVFGFVSFTKLAVREMPDIESPVVSISTRYEGASATIIE

SQITSVLEDQLSGISGIDEISSTTRNSMSRITITFELGYDLNTGVSDVRDAVARAQRSLP

DEADDPIVYKNNGSGEASLYINLSSSEMDRTQLTDYAERVLMDRFSLITGVSSIDLSGGL

YKVMYVKLKPELMAGRAVTASDITSALRSENLESPGGEVRNDSTVMSVRTARTYNTPEDF

QYLVIKRASDNTPIYLKDVADVFIGAENENSTFKSDGVVNISLGVVPQSDANPLEVAKLV

RSEVDNIQKFLPEGTRLAIDYDATVFIERSIEEVYSTLFITGGLVILVLYIFIGQARATL

IPAVTVPVSLISSFIAAYYFGFSINLITLMALILSIGLVVDDAIVVVENIFHHIERGESP

LLAAYKGTREVGFAVIATTLVLVMVFLPISFMDGMVGLLFTEFSVLLAMSVIFSSLIALT

LTPVLGSQILKANVKPNRFNEVVDRLFSKLENGYRSLLKGALKARLAAPLVILACMGGSY

FLMNQVPAQLTPQEDRGVIFAFVRGADATSYNRMSANMDIVEDRLMPLLGQGYLKSFSIQ

TPAFGGQAGDQTGFVIMILEDWNERDVTAQEALNKVRGALAGIPDVRVFPFMPGFQGGSS

EPVQFVLGGSDYDELLVWAELLKNKAEESPMMEGAEIDYSEKTPELLVTVDKQRAAELGV

SVKDISDTLEIMLGGKSETTYVERGEEYDVYLRGDENSFNNAADLSQIYLRTNSGELVTL

DTVTKIEEVAASIRLSHYNKQKSITITANLSEGYTLGEALNYLDQQAIDNLPGDISVSYS

GESKDFKENQASVAVVFALALLVAYLVLAAQFESFVNPLVVMFTVPMGVFGGFLGLVVMG

QGMNIYSQIGMIMLIGMVTKNGILIVEFANQLRDRGVEFEKAIIDAAARRLRPILMTAFT

TLAGAIPLIVSTGAGYESRIAVGTVIFFGMGFATLVTLFVIPAMYRLISAKTQAPGHVEA

ELNKALSHDSKGRTVHP

>tr|Q87RT5|Q87RT5_VIBPA Transcriptional regulator, LysR family OS=Vibrio parahaemolyticus serotype O3:K6 (strain RIMD 2210633) OX=223926 GN=VP0692 PE=4 SV=1

MSRRLPPLNSLRVFEAAARHLSFTRAAEELFVTQAAVSHQIKALEEFLSLKLFRRRNRSL

LLTEEGQSYFLDIKDIFTSIAEATDKVLERSEKGALTISLPPSFAIQWLVPRLADFNAQE

PDIDVRIKAVDMDEGSLTDDVDVAIYYGRGNWPGLRADKLYQEFLIPLCSPSLLLGNKPL

ESLSDLKLHTLLHDTSRKDWKQFARHYNIEGINVNHGPIFSHSTMVLQAAAHGQGVALGN

NVLAKPEMEAGRLIAPFDEVLISKNAFYVVCHEQQADMGRIATFRDWMLATARKEQEEVL

DEPVDS

>tr|Q87JK9|Q87JK9_VIBPA Putative repressor protein PhnR OS=Vibrio parahaemolyticus serotype O3:K6 (strain RIMD 2210633) OX=223926 GN=VPA0240 PE=4 SV=1

MQYVKIKDSIVEQIEAGMLSPRQKLPAERKLAESFDTTRVTLREALSLLEAEGRIYREDR

RGWFISPEPLRYDPTQTLNFPNMAKAQNRVPKTELIAAKGTLANKQSARLLQLQPFSDVF

RVDRVRYLEDRPVVYVTNYIRPEIFPNLLSFDLTNSLTDIYREHFGVVYQKTRYRISTST

LLGEVAQALRATSGTPAMVVERTNYNQHGELIDCDIEYWRHDAISIESVAELNQ

>tr|Q87L04|Q87L04_VIBPA N-acetylmuramoyl-L-alanine amidase OS=Vibrio parahaemolyticus serotype O3:K6 (strain RIMD 2210633) OX=223926 GN=VP2820 PE=4 SV=1

MLSLSNFRAVATFVATFLLIIPNVAFANVVKSFRVWPSPDETRVVIDLGSEADYSYFSLS

GPDRLVVDMKDTTMQAKLPVTVSDSPVLKLVRKSSPPEKGTYRLVFELKKNVQAELFKLS

PTPGGQYGHRLVIDLPHGKKTATTAAKPSKPATTSKDMSTVQRAQEVLIVIDPGHGGEDP

GSIGPSRRKYEKDAVLSISRKLAAQLDATPGIKTRMTRTGDYFVNLNRRVAFARENDAHL

LISVHADAFTSPQPRGGSVFVLNTRRANTEIARWVENHEKQSELLGGSGNAFVTNTKDRN

VNQTLLDLQFSHSQKEGYKLATEILGEMGRVAHLHNTKPINTSLAVLRSPQIPSVLVETG

FISNPTEEKLLFQRAHQDKLAQAISKAVVKYLKDNPPEGTVFSPSSKPMVHIVKRGESLS

VIANKYGTNSKALMAENKLKSTSLAVGQKLRIPSAGKIQVPSKPITIETETITHIVKSGE

FLGKIASHYKVKLSDIRRENNLKSDTLWVGQKLKITVAVKDKPIRKHKVARGEYLGKIAS

KYGVSVASIRQANNLRSDELAVGQVLIIPNK

>tr|Q87PC3|Q87PC3_VIBPA Uncharacterized protein OS=Vibrio parahaemolyticus serotype O3:K6 (strain RIMD 2210633) OX=223926 GN=VP1594 PE=4 SV=1

MNCSNKFKLTAVALMVGTAMNANAALYQVLEVDPQINGNSVDTETARTSYGVAIQQGDVP

YALGCFDEGAKSGCKPVNSPESEHFKLALETRVTPISTGQEVDGVSYREEVPFALDSGFY

YVQEYDDFERYCFNERRYSTCESWASVHWTPWNNEAFNRDFTSNALAYVEGNTTNTIDSA

AYTNKYNNVINSLTAEGKPVGNQSVISTTDPTVLETRNTVVAPGTSPNFDTSDAVPKLIE

SRAWKTNGTFTVGSVSRFANNDNGDHSTSKAAIWDSTGSAFELNWPSGQAQEDERLAQGS

MRDLVVKGSTVYGVGYNTYDSDNNYMNATVFVGTLAKEGSVVDSTWVNKEVDGAQQRISG

DTVHTNSRLTDVNANFVAIGEAKRSGAYLMPTGSAPNRLFVVDDVTKSSVSAFYPTSGIF

FSGAGGQMGGINSYNEIVGQLDAEKTREEEGKPRRKRGFIYPYAQGGTVSERAKEIFDGK

AWFLDDLTNGANDQSASARDISDSNNEFRIINASDINDAGVISATAMKCAGGYDTTAHNS

FCSSGEEKIVAVKLKPIPGATKANIQPRSVEEAAAERQGAGLGWLALTMLGLFGFRRK

>tr|Q87KG4|Q87KG4_VIBPA DNA helicase OS=Vibrio parahaemolyticus serotype O3:K6 (strain RIMD 2210633) OX=223926 GN=VP3013 PE=3 SV=1

MMDPSLLLDDLNDKQREAVAAPLENLLVLAGAGSGKTRVLVHRIAWLMSVEQASPFSIMS

VTFTNKAAAEMRGRIEELMMGSASGMWNGTFHGICHRILRAHYLDAKLPEDFQIIDSDDQ

QRLLKRLIKAQNLDEKQWPARQVAWWINGKKDEGLRPAHIDAYHDPVTKTYLQLYTAYQE

ACDRAGLVDFAEILLRAHELLRDNKFVREHYQARFKHILVDEFQDTNNIQYAWLRMMTGP

ECHVMIVGDDDQSIYGWRGAKVENIEKFTREFPSVTTIRLEQNYRSTKTILEASNTLIAN

NTERMGKELWTDGVVGEPISVYSAYNELDEARFAVNKIKEWQDKGGALNDAAMLYRNNAQ

SRVLEEALIQAGLPYRIYGGMRFFERQEIKDALAYMRLMANRNDDAAFERVVNTPTRGLG

DKTLETIRRAARDRGCTMWEASVAMLDEQVLAGRAAGALGRFIELITALEDDTLEMPLHE

QTDHVIKYSGLFAMYEQEKGEKSKARIENLEELVTATRQFEKPEEAEEMSLLTAFLTHAA

LEAGEGQADEFEDAVQLMTLHSAKGLEFPLVFMVGVEEGMFPSQMSAEEAGRLEEERRLC

YVGMTRAMQKLYITYAEMRRLYGQDKYHKPSRFIRELPETCLDEVRMKAQVSRPASSGRF

SQTAVKENFNETGFSLGSRVMHPKFGEGTIINFEGSGPQSRVQIAFNGEGIKWLVTAYAR

LEKL

>tr|Q87LK2|Q87LK2_VIBPA Ribosomal RNA small subunit methyltransferase E OS=Vibrio parahaemolyticus serotype O3:K6 (strain RIMD 2210633) OX=223926 GN=VP2610 PE=3 SV=1

MRIPRIYHPETIHQLGTIALSEDAAGHIGRVLRMKEGQEVLLFDGSGAEFPAVISEVSKK

NVLVDVTERVESNIESPLDLHLGQVISRGDKMEFTIQKSVELGVNTITPLISERCGVKLD

QKRFEKKLAQWQKIAISACEQCGRNVVPEIRPIMSLEQWCQEEYDGLKLNLHPRAKYSIN

TLPTPVEKVRLLIGPEGGLSSEEIDMTREYQFEETLLGPRVLRTETAALTAITALQVRFG

DLG

>tr|Q87LJ4|Q87LJ4_VIBPA FkuB OS=Vibrio parahaemolyticus serotype O3:K6 (strain RIMD 2210633) OX=223926 GN=VP2618 PE=4 SV=1

MNSMSFLISTLFDLYIMVVILRIWLQAARADFYNPFSQFIVKATQPVIGPLRRVIPSIGN

IDLATVLFAYVLCVLKFVALILIASNGSVSFSVDFLFLGLLSLLKAAGGLLFWVLLIRAI

LSWVSQGRSPIEYVFHQLTEPMCAPIRRVLPAFGGLDLSVLVLFIGLQFANFLMGDIIGP

IWFQL

>tr|Q87LE9|Q87LE9_VIBPA Putative ABC superfamily transport protein OS=Vibrio parahaemolyticus serotype O3:K6 (strain RIMD 2210633) OX=223926 GN=VP2663 PE=3 SV=1

MVASFIQFVASLGRRTLAICEAFGRATLMLVGAIFSKPQPSKSFPLLIKQLYSVGVQSLA

IIVVSGLFIGMVLSLQGYVVLVDYGAEGSLGQMVALSLLRELGPVVTALLFAGRAGSALT

AEIGLMKATEQLSSLEMMAVDPLKRVIAPRFWAGAISMPLLAMIFMAVGIWGAQLVGVDW

KGVDHGSFWAAMQSSVELGQDIGNSMIKCFVFAITVTWIALFNGYDAIPTSEGISRATTR

TVVHSSLAVLGLDFVLTALMFGN

>tr|Q87ME5|Q87ME5_VIBPA Outer membrane protein assembly factor BamA OS=Vibrio parahaemolyticus serotype O3:K6 (strain RIMD 2210633) OX=223926 GN=bamA PE=3 SV=1

MAIKRILFASLLATSVSANGAENFVVQDIEIDGLQRVALGAALLKMPVRVGDTIDQGDVA

EIIRALYASGNFEDVKVLRDGGVLMVQVKERPTIASISFSGNKAIKDEQLQENLNASGVR

EGEALDRTTLSNIEKGLEDFYYSVGKYNATVKAVVTPLPRNRSDLKFVFTEGVSAKIQQI

NFIGNEVFSDEELLSRFNLNVDVPWWNFLADEKYQKQVLAGDIEALKSFYLDRGYLKFNV

DSTQVAISPDKKGVYITLGLEEGEVYTVKDVKFRGDLIGEEATFERLVPFEDNETYNGSL

VTSMEEGIKRVLGESGYAYPQVNTIPEFDDENKQVSLVVNVDPGNRIYVRDIRFTGNNST

KDEVLRREMRQMEGSWLNSKSIETGKTRLNRLGYFENVEVQTVRVPGSDDQVDLVYSVKE

ANSGSVNFGVGYGTESGVSFQVGLQQDNFLGSGNRVGINAMMNDYQKNVSLDYRDPYWNL

DGVSLGGKIFYDEFEASEAGIVDYTNQSYGASLTWGFPFDELNRFEFGVGYTHNKIGNLS

PYLQVEQFLQAQADNIDSSGALNTNDFDFNISWTRNNLNRGYFPTAGNHQRAFYKMTVPG

SDVQYFKMQYDVRQYVPLTKKHEFTLLFRGRLGYGNGYGQTDGNDNLFPFYENYYAGGFT

TLRGFGSNSVGPKAVYRDYSGSNNGADTATDDSVGGNAVALASIELIVPTPFASDEVRNQ

IRTSIFFDMASIWDTEFDYRDSGAEYGDRYYYDYSDPTNYRSSYGAALQWMSPMGPLVFS

LAKPIKKFDGDDEEFFTFTIGRTF

>tr|Q87SW5|Q87SW5_VIBPA Uncharacterized protein OS=Vibrio parahaemolyticus serotype O3:K6 (strain RIMD 2210633) OX=223926 GN=VP0307 PE=4 SV=1

MIKKALPIIVGLLVVSQSAYADVSLKLKGIDGALEDNVKAYLSSIPEKDYSTSLRFQARL

DQSITEALNALGYYHAKISYSISEGNDELIVNIHKGLPVKIKVMDVVISGEAKEDEEFTN

LIAKSPLKVGRILNQGEYDSLKSGIRNLALQRGYFNGDFKLNKLEVIPELNEANVRLHYD

SGIRYHFGPVEITGSQIWENRVESMRPFEIGEPYLVSDVGEYNQNLSNTDWFSSVFVEPD

LSKLEDGRELPIKVSLAPAAKNQIETGIGYSTDTGVRGTLKWKKPWVSARGHSFNTALSL

SKPEQTITAGYKIPLDDVLREYYQLQFGLKHLDNRDTESLESNLAVERHWLTDGGWHKTV

YVRHLYENFSQGLQDDGVQFVLPGATFSRTRVRGGSMPMWGDKQSVTVEYGDPALLSETR

VLRLLGRSSWIRGIGENHRGLFRLEGGANITEEFEKLSPSLRFFAGGDNNIRGYGYESIS

PVDESGALTGAKYILSSTLEYQYRVYGNWWAATFYDIGDAFNDTPEWKSGAGVGIRWASP

VGPVSFDFAWGLDEKPNNEFRIHFSLGPEL

>tr|Q87S66|Q87S66_VIBPA Outer membrane protein assembly factor BamD OS=Vibrio parahaemolyticus serotype O3:K6 (strain RIMD 2210633) OX=223926 GN=bamD PE=3 SV=1

MKRQTLTGLLAVSLLFGCASKEEIVPDVPPSELYADAQVSLQSGNWLSAIEKLEALDSRY

PFGAYSEQVQLDLIYAYYKNDDLALGLATISRFMRLNPTHEKMDWVLYMRGLSHMAQDRN

FMHDLFSIDRSDRDPEPVKKAFDDFKKLLQRYPNSPYAEDAQKRMVALKNRLANYDLATA

DFYLRREAWIAAINRSQELQKSFPDTEAARKSLEIQLEAYKQLQLEDAVARTEALIKLNP

VK

>tr|Q87R06|Q87R06_VIBPA Pyruvate formate-lyase-activating enzyme OS=Vibrio parahaemolyticus serotype O3:K6 (strain RIMD 2210633) OX=223926 GN=VP0992 PE=3 SV=1

MSTTGRIHSFESCGTVDGPGIRFIVFLQGCLMRCKYCHNRDTWDTHDGKEVTVEEIIAEA

KSYRHFMNASGGGITCSGGEAMLQPEFVRDFFRAAHAEGIHTCLDTNGYIRKHTEVIDEV

LDATDLVMLDIKHMKDEIHQDFIGVSNRRTLDFARYLHKIGQKTWIRYVVVPGYTDDPEA

AHMLGEFIKDMDNIEKVELLPYHKLGAHKWEALGLEYPLEGVNPPSKETMDEIQSILSQY

HSNVKY

>tr|Q87IL2|Q87IL2_VIBPA Uncharacterized protein OS=Vibrio parahaemolyticus serotype O3:K6 (strain RIMD 2210633) OX=223926 GN=VPA0594 PE=4 SV=1

MLNKMLLKKAAIFFLPLPLVFAEVLYLAHESVQSNLDHLLEKNIQIADEILFQIETENRT

ALIHPERCEQLQQNLMFERDIDEMLIVKGDEIICSSKLGHLSKPLSEYLTFRPNHALTFG

QINGLDEPLLLVVTQGQQTYKAITIIDRDYFGATIGFNNDLRLKRSALFIHDDVVPAGAS

RKGTNPIAFNESKVFEYQALAEASDLFVEQKLISYIIYSVPIIAMIYLCLYALNKFVDPQ

RNLLAELKKALKKNELTLYYQPQIDVESGRVFGYEALIRWEHQQKGFIAPDEFVPAAEQN

GLVSLLTDYVLEKAADDFSKLTLTHPVHLGVNVPPGYLVGSHVIRKLQLIHRKLSENNVS

LGIEITERQLINGEARKHIAALRVHGIDVLIDDFGTGQTSLAFLQHMKIDYLKIDKCFVE

TIGIQSVSASVLNAIVHFADELQVKLIAEGVETPAQAEHLKNMGVQYHQGYLYSKPLPYS

LLAQQPIS

>tr|Q87QP8|Q87QP8_VIBPA Cys regulon transcriptional activator OS=Vibrio parahaemolyticus serotype O3:K6 (strain RIMD 2210633) OX=223926 GN=VP1101 PE=4 SV=1

MKLQQLKYIVEVVNHNLNVSATAESLYTSQPGISKQVRLLEDELGIQIFERSGKHLTQVT

PAGEEIVRISQEILARVESIKAVAGEHTHPEMGTLNISTTHTQARYALPDVIKGFTARYP

KVSLHMHQGTPSQMSEAIAKGTANFAIATEALHLYQDAIMLPCYHWNRSIVVPKDHPLAK

KDLVTIHDLAAYPLVTYVFGFTGRSELDTAFNREGLTPRVVFTATDADVIKTYVRMGIGV

GVIASMAVDEEQDRDLVAIDASHLFGASTTSIGFRRGTFLRSYMFDFMERFAPHLTRPVV

EQAISLKSNAEIEEMFKDIELPVR

>tr|Q87RY9|Q87RY9_VIBPA Putative transcriptional regulator, LysR family OS=Vibrio parahaemolyticus serotype O3:K6 (strain RIMD 2210633) OX=223926 GN=VP0635 PE=1 SV=1

MIKGSEYPSIRALRTFVAVANYLSFSKAADDLCVTQGAVSKQMASLEQLVGLPLIHRGLN

GVELTEEGKRYLPQITEALELIQHATASLIQTNTDQELLVVDVTPSFASLWLVPNINDFH

QRHPNIRVKILTGDGAVKNIHGESDLHVRCLPLSTHYEYSQLLCEETLLLIGNTNLPKLS

DNQAISHYPFIPQTTRPQLWEQFKQENDLECPITYHSVGFEHFYLACEAVRMEKGLALLP

DFMAQFSILRGDIQHIGNLKLHSGYGYYVVIPNFRLTSRKVALFHDWLKDKLTHHT

>tr|Q87ME4|Q87ME4_VIBPA Zinc metalloprotease OS=Vibrio parahaemolyticus serotype O3:K6 (strain RIMD 2210633) OX=223926 GN=VP2311 PE=3 SV=1

MTGILWNLVSFIVALGILVAVHEFGHFWVARRCGVKVERFSIGFGKSIWRKVGQDGTEYT

ISMIPLGGYVKMVDSRVDDVPESEKHLAFDQKPLWKRTSIVAAGPIFNFLFAIFAYWLVF

LIGVPAVKPVIGEVTPNSIVAEAGIESGMELKAVSGIKTPDWESVNMGLISHIGDDAMTL

TLTSDSEVGAEVTKTLDLRDWKFDPETESAMHSLGFAPYTPEIYRVIKQVSEDGAAAKAG

VLPEDEIIAIGGEPINDWKQVVDAVRSNPNTPIELTVLRRGIEQSLTLTPDSRELANKQV

VGFAGIAPEVAEWPESYRFELQFGVFESIGKAVDKTGQVIGLTVSMLKKLIVGDVGLNNL

SGPISIAKGAGATADYGLVYFLGFLALISVNLGIINLVPLPMLDGGHLLFFAIEAVIRRP

VPEKVQEMGFRIGGAIIFSLMALALFNDFTRL

>tr|Q87SN4|Q87SN4_VIBPA Type I restriction enzyme M protein OS=Vibrio parahaemolyticus serotype O3:K6 (strain RIMD 2210633) OX=223926 GN=VP0388 PE=4 SV=1

MSISSVIKSIQDIMRKDAGVDGDAQRLGQMSWLLFLKVFDAQEEELEFELDDYREPIPAK

YLWRNWAADNQGITGDELLEFINDDLFPTLKNLTAPKDTNPRGFVVKEAFSDAFNYMKNG

TLLRQVINKLNEIDFTDSKERHLFGDIYEQILRDLQSAGNAGEFYTPRAVTRFIVNRLDP

KLGEQIMDPACGTGGFLACSFDHVKENYVTSAADHQTLQKQIHGVEKKQLPHLLCITNMM

LHGIEVPVQIKHGNTLNKPLSNWDSNINVIATNPPFGGTEEDGIEKNFPAEMQTRETADL

FLQLIVEVLDKDGRAGVVLPDGTLFGEGVKTKIKKMLTEECNLHTIVRLPNGVFNPYTGI

KTNILFFTKGQPTKEVWFYEHPYPEGVKNYSKTKPMKFEEFQQEIDWWGNEADGFASRTE

TKQAWKVSIEDIIERNFNLDIKNPYQDEVVSHDPEELLANYQQQQQDIKALRDQLKSILG

DALSSTIQGKKQGGEQ

>tr|Q87SR4|Q87SR4_VIBPA Transcriptional regulator, DeoR family OS=Vibrio parahaemolyticus serotype O3:K6 (strain RIMD 2210633) OX=223926 GN=VP0358 PE=4 SV=1

MSKRNTQLRRHAISNMVNELGEVSVDELAQKFETSEVTIRKDLASLEKNGQLLRRYGGAI

AIPTEVIHEELSSNVSTRKLSLAKAAASLIRDHNRIVIDSGSTTAALIQQLNDKRGLVVM

TNSLHVANALNELESEPTLLMTGGTWDTHSESFQGKVAESVLRAYDFDQLFIGADGIDLE

RGTTTFNELVGLSKVMAEVSREVIVMIESEKIGRKIPNLELAWHQIDVLVTDADIQPEHK

VQIEQHGVKVICA

>tr|Q87Q93|Q87Q93_VIBPA SanA protein OS=Vibrio parahaemolyticus serotype O3:K6 (strain RIMD 2210633) OX=223926 GN=VP1257 PE=4 SV=1

MLFSRIRSKWKLKCSWKRIALILSISLFGLLLSVVAIDRWVALQAKDNIFIDYDQIPQHE

VAVVLGTSKYIGKTLNTYYTHRINAAIELYKQGKVKQFLLSGDNAHRSYNEPWTMKRDLL

KAGVPEEVIHLDYAGFRTLDSIVRAKKIFASERFLIVTQRFHCERALFIADAYNIDAQCL

AVAGPTANKQKTSMRIRELLARVKAFLDLYVMNTQPRFLGPQEPILAVEDPTEASNAERN

IPIE

>tr|Q87LZ9|Q87LZ9_VIBPA Preprotein translocase, SecG subunit OS=Vibrio parahaemolyticus serotype O3:K6 (strain RIMD 2210633) OX=223926 GN=VP2460 PE=4 SV=1

MFSVLLVIYLLAALGVIGLVLIQQGKGADMGASFGAGASNTVFGASGSGNFLTRTTAILA

TVFFVVSLLLGRMSTHKTESQWVDPTLGQPVVEQVKDAASEVPAPTGDEIPQ

>tr|Q87QM4|Q87QM4_VIBPA Uncharacterized protein OS=Vibrio parahaemolyticus serotype O3:K6 (strain RIMD 2210633) OX=223926 GN=VP1125 PE=4 SV=1

MGSGILFVFAIGAIPSQAQDIGSTDRLNVENWQEWETVGEAQLTWFVFDIYRSRLKAPNG

QYLVSTDVSPHPFALEINYQRDISKQQLLEVTDEQWQKLGFPKSNRQQWITQLSFIFPSI

KNGDELTYVTDGDKGQIIYRQAGTKTQKMVGEITDERMNDAFLSIWLSPKTEFPKLRKQL

IGQVRP

>tr|Q87S52|Q87S52_VIBPA Exopolyphosphatase OS=Vibrio parahaemolyticus serotype O3:K6 (strain RIMD 2210633) OX=223926 GN=VP0572 PE=3 SV=1

MSQILEQDERYIAAIDLGSNSFHMVVAKVVGSDLQLISRHKQRVRLASGLDSELNLSHAS

MERALECLAMFAERLQGFEESNVRIAATHTLRRAKNAHLFIQRAKAVMPFPIEIIPGEEE

ARLIYLGVAHTQVESNSKLVVDIGGGSTEMIIGQEFEPELLNSKQMGCVSFTQQYFKNGK

LSSKNFSKAMLAAEQKLESIATKYRKKGWDIALGSSGTIKAIQEVLIGLGFEDGLITAKR

LSKLIDTLNEFASIDDIQLAGLTDERKPVFAAGVAILAAIFQALKIDQMFFSDGALREGL

LYEMEERFARSDIRMRTTENLAQKHRVDLEHAARVKGHAREMLEQVHSELGIKKKSELFD

LLEWAALLHEVGLSINLRSFHRHSFYILLHSTLQGFNREQQLVLATLARFQRKALRLNEL

PEFNLYKQNDVLSLIKILRLSIVLNGQRNDDPLPDITLLIKGDEWMLTCTDEAWLDNNKL

LHADLLEEQDRWASAKWTLTF

>tr|Q87J86|Q87J86_VIBPA Putrescine-binding periplasmic protein OS=Vibrio parahaemolyticus serotype O3:K6 (strain RIMD 2210633) OX=223926 GN=VPA0367 PE=3 SV=1

MKLIIKGLGLSALVLSMFAHADSKTLNVYAWGGYLPEASLKAFEKQEGVTINYSTFENNE

SMYTKLKLLKGSGYDVVFASAYFIEKMGREGLLSKIDHAQVPNMQDTMDGLLGQAHDPKN

DYSLPYIWGITGISYNESMVEQPVTKWADLWESQYEQQVMLIDDVRDVFGMALKKNGHSV

NTKDEAEIKQAFDSLVALKNNVLLYNSDAPQVPYVSGETSVGMQWNGNAFQGQVEMPELK

FVMPEEGAVLWMDNFTIPSGSKNKTLAHKFINFMYQSENQAEIVTSLGYASATNAGRDKL

PEELKNNRTIFPSSEDMKKGEFINDVGAETLAIYEKYWQRLRTQ

>tr|Q87RF9|Q87RF9_VIBPA Negative modulator of initiation of replication OS=Vibrio parahaemolyticus serotype O3:K6 (strain RIMD 2210633) OX=223926 GN=seqA PE=3 SV=1

MKTIEVDEDLYRYIASQTKHIGESASDILRRLLNLDGQLQVAESAPAVEKPQGIVVSKDA

GKAESIDVVKEMRSLLISDEFAGLKKAIDRFMLVLSTLHKLNPEGFAQATNVKGRKRVYF

ADNEETLLANGNTTKPKAIPGTPFWVITNNNTSRKRQMVEQVMTHMEFQPDLIEKVTGSI

>tr|Q87I81|Q87I81_VIBPA Putative TadB OS=Vibrio parahaemolyticus serotype O3:K6 (strain RIMD 2210633) OX=223926 GN=VPA0725 PE=4 SV=1

MFIGHVKRQKELTSIIEQSNTFEGVTGSRAVIDSESFDVSYKKKVKDKFKQLKKLLSPNA

GLKLVGFFAASSIAVYCVNDWFFMFEYWKVLLALEPILFVMFILKLSGIQAQRFKDNFPD

ALNILSGALSSGQSIVHAFEYVGTQLDNEVGEEFKKMSERLLIGEDPDDVLARSAASFPY

VEYFFFAATIRINLSRGGQLKDVINRINRIMFEARAIEKKKNALTSEARASAKIIAALPV

IFIMILKVTSPENYNFVMFEEGGKPIFYYVLVSEAIGFFFIYMILRGVR

>tr|Q87S06|Q87S06_VIBPA Uncharacterized protein OS=Vibrio parahaemolyticus serotype O3:K6 (strain RIMD 2210633) OX=223926 GN=VP0618 PE=4 SV=1

MEQADVTSLIPILITLFLALTTRNVVVGLFAGVVSGVAMLEGTFVEKGPLDSFSALMKSY

LLPQLTDSYNAGVILLLVFIGGFVALMEKSGGGVAFAKKVTQWVASKCQAQLSAWFGGVV

IFFSDLGTPLIVGPVFRPLFDKLKVSRQKLAFIIDSTSSPVAILIPFIGWGVYIMGLIQK

EFTALNVSMSDWDAFIGAIPYQFYAFLAIAIVPIVSFFKLDFGPMAKAEQLAEQGSDFGK

EQESMNVFTHKNAKSSFVWAPLLVMLVVLCTILVPHGFPFQKVAGSTFRAALSSAYFFAA

FTLIALMAFYGVRKLSDGIQVYLKGMSNMMSVAVILVLAWALSSVGKELGAAAYIAEQAQ

AGFPYWLLPAVAFLLAGIISFATGSSWGTFAIMMPLVIPTAVAIDAPLLVCIGAVLSGGL

FGDHCSPISETTILSSTGAGCEQYEHFRTQLPYAVLNGVIALVCFLLAGVAASPVIVLAA

IVAQFAIYYVLSKQKPTEQLESVSAE

>tr|Q87KB3|Q87KB3_VIBPA Multidrug resistance protein OS=Vibrio parahaemolyticus serotype O3:K6 (strain RIMD 2210633) OX=223926 GN=VP3064 PE=4 SV=1

MKISKLQLVYLAALSMLGFVATDMYLPAFKAMEIDFATGPEQIALSLTVFLVGMAFGQLM

WGLASDKFGHRNTLAAGLVLFTIASFGLAFCDEVWQLLTLRFVQAIGVCAPAVIWQAMVI

KRYSSSSQQIFATIMPLVALSPALAPQLGVVLADSFGWHSIFIALTLVGVVLVAATMAQK

NEQAEIKQTSMSADIKALLGSKTYLGNVFMFATASAAFFAYLTGMPEIMAQLGYEAKDIG

LSFIPQTIAFMAGGYLGKVGVRKFGDEKVLRQLIGLFSVAALLIFVASQWELTSIWPILA

PFCLIAVANGALYPIVVNRALASAHQSPATAAGLQNSLQITVSSLSSALVAAMASQAQMV

TGVAIVICMGGLWMGYILSNRELSKHFTTPDNARVVSEDEL

>tr|Q87PH4|Q87PH4_VIBPA Spermidine/putrescine ABC transporter, permease protein OS=Vibrio parahaemolyticus serotype O3:K6 (strain RIMD 2210633) OX=223926 GN=VP1528 PE=3 SV=1

MISKKINLQNAIITLIVGWLTLFVLVPNLMIIGTSFLTRDEANLIELTFTFDNYLRLLDP

LYAKVLMHSFYMAIIATLLCLIIGYPFAYIVAKMPEKWRPFMLFLVIVPFWTNSLIRTYG

LKIVLGTQGILNKSLMAMDIIDKPLRLMYTETAVMIGLVYILLPFMILPLYSAIEKLDNT

YIEAAKDLGASKLQTITKVILPLTMPGIIGGCLLVLLPALGMFYISDLLGGAKNLLIGNV

IKSQVLNARDWPFGAATSIALTIAMAIMLYAYYRAGKLLNKKVELD

>tr|Q87H04|Q87H04_VIBPA Uncharacterized protein OS=Vibrio parahaemolyticus serotype O3:K6 (strain RIMD 2210633) OX=223926 GN=VPA1161 PE=4 SV=1

MLNMPAEFEQFHWMVDMVQNVDMGLVVIDRDYNVQVWNGFMTHHSGLQSHEAIGRSIFDI

FPEIPPEWFKLKTKPVYDLGCRSFITWRQRPYLFRCRNVRPVTQQAEFMYQNVTLNPMRT

PTGKINSLFLSIQDATAEALMSQHK

>tr|Q87JM0|Q87JM0_VIBPA Putative membrane protein OS=Vibrio parahaemolyticus serotype O3:K6 (strain RIMD 2210633) OX=223926 GN=VPA0229 PE=4 SV=1

MEFFSFLMNDVLSEPAVLVGLIALIGLIAQKKPVTECIKGTVKTILGFIVLGAGAGLVVS

SLGDFATIFQHAFGITGVVPNNEAIVSIAQEAFGKEMAMIMFFGMLVNILIARFTPWKFI

FLTGHHTLFMSMMVAAILASSGMKGVPLIALGSVVVGSVMVFFPAIAHKYMKQVTGSDDV

AIGHFSTLSYVLAGFIGSKFGNKEHSTEEMNVPKSLLFLRDTPVAISFTMGIIFMVTCLF

AGGDFVREVSGGKHWSMFALMQSITFAGGVYVILQGVRMVIAEIVPAFKGISDKLVPNAK

PALDCPVVFPYAPNAVLVGFLSSFAAGLVGMFLLYVMGLTVIIPGVVPHFFVGAAAGVFG

NATGGRRGAILGAFAQGLLITFLPVFLMPVLGDLGFANTTFSDADFGAVGILLGLIVR

>tr|Q87I69|Q87I69_VIBPA Putative two-component response regulator OS=Vibrio parahaemolyticus serotype O3:K6 (strain RIMD 2210633) OX=223926 GN=VPA0737 PE=4 SV=1

MNTQLNVMVIDDHPLQTTILTQILNRYCAQVTSFNGVDDAIQCAQRQHFDVIFCDIQMPG

KDGIDMMEMLDQIQYQGQVVLVSAMELTIISAVRAMCEGFSFEVLGKLPKPYDENQVVEL

LSLMKGEKTKKATFIQPIEVQDQEFLFALGEGRVKNYYQPLVDAQSGEVLGYEALARWSH

PIYGVLSPYHFLPIVERCHLSAELFQAVLNNAIYDIKHRGLTQKVSINVDHENLEDPEFS

HRFLQQCLENEIEPSQITIEITERDTFQTSASLYKNLLKLRMNGVTVSIDDFGTGSSTFE

KLAQLPFNELKIDRSFVQGVECDMKKRNIVVAICALAKSLNIRLVAEGIEDEVTLQVMRE

YGIDLCQGFYIDKPMPLEAITILNERYE

>tr|Q87HG8|Q87HG8_VIBPA Lipase-related protein OS=Vibrio parahaemolyticus serotype O3:K6 (strain RIMD 2210633) OX=223926 GN=VPA0997 PE=4 SV=1

MKPLKRYQYERYAVLCNLAYPRVFKQTRYGFDPNGQRVIRNEHGKIMIRVLWSKNRDEVV

VVIKGSHSITDWLLNFAMWTRSCRRLGLNYRIHAGFYHLLFQESLPSRNEDRLGLSVIER

LEATVVPLILQGKRISVTGHSSGGAIGSVFADYIDKKYPKSIKRVVTFGQPAIGDWTFKR

RYRLAHKTYRICCDIDIVTFMPPVPFLYWHVGKMLWLYNGRIYENTPTLIRLGRSLFSWL

IRPFSYHLMSKYIRNKDFFDER

>tr|Q87Q73|Q87Q73_VIBPA Histidine utilization repressor OS=Vibrio parahaemolyticus serotype O3:K6 (strain RIMD 2210633) OX=223926 GN=VP1277 PE=4 SV=1

MSAPLYMQIKQFILDKIDSGDWMVGQRIATEIELTEQFGVSRMTVNKAIRDLVNEGKLQR

RPRLGTFVCDPSEKSESPLLDIRNIAEEVSDRGRQYSSKVVQQVAIKADDSIAIKLGVML

GTTVFYSEIIHYEDSTPIQLELRWVNSQYAPSYLSQDFTQITPNQYLSNNCPLSAIEHTV

EAIVPDNRIKLDLKMQANEPCLLLNRRTWSQDKLVSTALLYHPGNKYKLSSKVLL

>tr|Q87SB5|Q87SB5_VIBPA Tyrosine recombinase XerD OS=Vibrio parahaemolyticus serotype O3:K6 (strain RIMD 2210633) OX=223926 GN=xerD PE=3 SV=1

MTAQQPVNQQDFALVEQFLDAMWMERGLSENTLASYRNDLMKLLTWMDHHRYRLDFISLS

GLQEYQTYLVDLDYKQTSRARMLSAIRRLFQYLHREKVRADDPSALLVSPKLPQRLPKDI

SEEQVDALLDAPDPNDPVELRDKAMLELLYATGLRVTELVSLTMENISLRQGVVRVTGKG

GKERLVPMGENAVDWIETFIQQGRPALLGETSSDVVFPSKRARQMTRQTFWHRIKYYAVI

AGIDTDQLSPHVLRHAFATHLLNYGADLRVVQMLLGHSDLSTTQIYTHVATERLKQIHSQ

HHPRA

>tr|Q87SH4|Q87SH4_VIBPA Penicillin-binding protein activator LpoA OS=Vibrio parahaemolyticus serotype O3:K6 (strain RIMD 2210633) OX=223926 GN=lpoA PE=3 SV=1

MINHKRLSVPRILTPVALAITLAACSSGPRQPDGVDVTLEPTQSVQNYMIQADSTEGSLQ

NDWLIMATKAAIQANQLDQADLLIKRLARQQLTEVQQAEWQLARATIQQKQGDYSQLLQL

LNFKPWWKLPNEQWKDYYSLRADAYQSLNQPFEANRQLVAFSQYASSAEQREISSRIWMN

FGSYSEYELTSLQTEPSEDVLDGWLQLAVYAKTLSGNLSQLKNTLERWLNENPSHPAAIY

TPEEIQNILSLDIVKPNNTALLLPLTGKFAPQAQLIRDGFVFAMMNDRNRDPSATLTVID

TNAYSADEVKQRLINENIDFVVGPLQKENVELLQTTMDGSANSPAIPALALNIPEDVQPG

TDICYLALSPEQEVAQAAKYLFSEGYSFPLILAPKGSYGERVVQAFNEEWSKYSSNKVAT

SYFGDKRQLQKDINDVFGLQESKQRIAQMQSLMRIKLETQPRSRRDVDAVYIVARSSELT

LIKPFIEVAINPDAKPPKLFSNSRSNSGGATYEDLTGIIYSDIPLLVNPDPIVTAEMNEL

WADQSNMEKRLKALGMDAYKLIGELPQMKVVPGYSVSGQTGTLSIDNNCVVQRELSWAER

GAL

>tr|Q87PV9|Q87PV9_VIBPA Putative transcriptional regulator OS=Vibrio parahaemolyticus serotype O3:K6 (strain RIMD 2210633) OX=223926 GN=VP1391 PE=4 SV=1

MRSANHSELSNALLAISQSLADRSQLTQTLDAVLTAARQMTLAKHGIIYVLDQTGQALIP

STAHHNDKTIVSHPWEALQIDSASENDPFNFAIRNGEVVLINELYKYKGYDCESIYQTEQ

TLGLKSENLLAWPLIDSESKTIGLLVLLDLNVIDNEAALTEFCRMAASNIRQAVWLEQYG

QVIKSLSADNQALVRENTQLKKRTQKAYQGPIAESEEMLNVLNRLDKVLSLPVDVLLRGE

TGAGKEVIAKYIHENSNRSEQPLIVQNCAAIPEQLLESELFGHKKGSFTGADKDKVGLFE

AANGGTLFLDEIGDMPMLLQAKLLRVLQERKVRPIGTSKEIEVDVRVIAATHCNLMQQIK

DGGFRADLFYRLNVFPITLPPLRARKSDIIPLAEHFVQHTTNTLGLPQAPGLSANVRKQL

LAYQYPGNVRELKNIIERSVLLSDFETITHIEFGEQIPEDVPNIDMKAASPTPDQPAYER

QAQADLEDASKSLKDVVSQYERTVIIDCLNACNWHTKKAAEQLALPMSTLNHKMKKYDIS

AAG

>tr|Q87QD8|Q87QD8_VIBPA Sensor histidine kinase OS=Vibrio parahaemolyticus serotype O3:K6 (strain RIMD 2210633) OX=223926 GN=VP1211 PE=4 SV=1

MPNKNKLPLHALKSLSIKSRLVLAAVVWLTAMILAAGVTIPTQVYNYMVDDTRSQLSIFM

DEIAAQLEVDHTGHLSLAAQLSDPRFSRPYSGLYWSASTDSSLERSRSLWDKRIEYKGLD

KDAYGARDEKLITLEKALYLPDYDGPIHIIVGIDEEPIKSTLQTLIGQLWLILGLLFAGV

LTVILLQIVWSLSPLTKLQKELAELKAGNKKSLEETYPKEISPLISDLNALLFHYQELLE

RARNHAGNLSHALKTPLSVLKNEVQTLEPKTQARLNAPLNQIQDHIDYHLGRARMAGSMN

ILSVKANPAERVDAISMAFDKVYAERDITLVNELDSELNVAVEKTDLDEMIGNLLENGYK

WANSMIRVHSTLDKDSIHLIIEDDGPGIPQAQLGQVIKRGVRLDETTPGSGLGLNIVSEM

AHSYRGLLALEKSKMGGLKATLTLHRSRT

>tr|Q87RU2|Q87RU2_VIBPA Thiamine-monophosphate kinase OS=Vibrio parahaemolyticus serotype O3:K6 (strain RIMD 2210633) OX=223926 GN=thiL PE=3 SV=1

MSGEFNLIDKYFVGRQNQRKDVHLAAGDDCALVKAPANVQIAISTDTLVAGTHFLPHANP

AWVAHKALASNISDLAAMGATPAWVSFALTMPEVDEEWLAPFCNAFFELADYFGIQLIGG

DTTKGPLSLTLTVQGFVPEGKALTRSGAKVGDWVYVTGNLGDAKAGLDVILDETLRSRIG

ADELEKAHYLSTPRVLAGQALVGLASSAIDISDGLISDIKHILKRSQVGVSIDVSQLPIS

SELVQFLDDKVSAQQYALSSGEEYELCFTVSEQNRGSLQSALSYSGCKVTCIGQIRPNGT

FELHDNNQPLDWDLSGFDHFK

>tr|Q87H86|Q87H86_VIBPA Uridine phosphorylase OS=Vibrio parahaemolyticus serotype O3:K6 (strain RIMD 2210633) OX=223926 GN=VPA1079 PE=4 SV=1

MAKQPHIGVDETQVAPLVIVCGEPDRANRIAALFDDAEMVSENREYRVFTGNYKGQTVSV

CSTGIGAPSMIIAVEELKQCGVTHVVRVGSAGAMQSQIQLGELIVAEGAVRDEGGSKSYV

DSAYPAYASFTLLKEVERYLSTQTTPYHFGVVRSHDSFYTDDEEAICQYWNKKGILGADM

ETSALFTVGRLRGLHVASILNNVVLYQQDVKEGVGQYVDEAKVMMEGEKLASFTALEALI

AQA

>tr|Q87LE0|Q87LE0_VIBPA Nitrogen regulatory IIA protein PtsN OS=Vibrio parahaemolyticus serotype O3:K6 (strain RIMD 2210633) OX=223926 GN=VP2672 PE=4 SV=1

MQLSEILSLDCTKSAVHCTSKKRALEMISQIVAEHTGQDSTELFECMLNREKMGSTGIGN

GIAIPHARMQSSDKAIAVLLQCDEAIEFDAIDNRPVDLLFALLVPEEQCKEHLKTLSSMA

ERLSDKQVLKLLRNAQSDEELYDIMIHQ

>tr|Q87K47|Q87K47_VIBPA NhaD OS=Vibrio parahaemolyticus serotype O3:K6 (strain RIMD 2210633) OX=223926 GN=VPA0051 PE=4 SV=1

MRKSKPVLLAAGLIWILIGYTFAQHHQQDVAKAALEHNLLEYAELLLFLLVAMTYINAME

ERKLFDALQAWMVGKGFGFKKLFWLTGFLAFVISPIADNLTTALLMCAVVMKVSGDNPRF

VNLACINIVIAANAGGAFSPFGDITTLMVWQAGHVRFSEFMPLFVPSLINYVVPAFLMSL

FVPNTKPNTIHEHVELKRGARRIVLLFVLTIATAVSFHAVLHFPPVVGMMMGLAYLQFFG

YFLRKTLKHSLAKKAAMAIANGDDHALKRLGSVVPFDVFHRVSRAEWDTLLFFYGVVMCV

GGLSLLGYLELVSNVMYTQWNPVWANVMVGVLSAIVDNIPVMFAVLTMDPSMSTGNWLLV

TLTAGVGGSLLSIGSAAGVALMGAARGQYTFFGHLKWTPVIALGYAASIAAHLWMNGGLF

T

>tr|Q87PQ4|Q87PQ4_VIBPA Putaive anaerobic dimethyl sulfoxide reductase, subunit A OS=Vibrio parahaemolyticus serotype O3:K6 (strain RIMD 2210633) OX=223926 GN=VP1447 PE=3 SV=1

MSQNGKFNITRRDALKGGSALGALALAGNALSLPFATKAVAKETPAKPNEKIVWSACTVN

CGSRCPLRMHVVDGEIKWVETDNTGDDVYNHNHQVRACLRGRSMRRRVYSPDRLKYPLLR

VGKRGEGKFKRITWDEAFDLIGDNLKRIIKDYGNDAVYLNYGTGTLGGTVTKSWDPSATL

VARMMNLCGGYLNHYGDYSAANIECMSEYFYGTWVDNNSIDDVQNADLCLMFGNNPAETR

MSGGGQIHNYVDAKNISHTRTICIDPRYTDTAAGREDQWIPIKHGTDAALVAAIAHVLIS

EDKVDQDFLDRYCVGYDRKTLPASAPENGSYKDYIMGTGPDGIEKTPEWAQPITGIPADV

ILKLAREIGDAKRIYITQGWGLQRSANGEQACKAIMMLSLLRGQVGLQGGGTGAREGNHS

YPFQRFPKVPNPISASIPMFLWTDAIFRGTEMTDLTDGIKGVEKLQNNIKFIWNYAGNCL

INQHSDINRTHDILQDEKACEMIVVIDNHMTSSAKYADIVLPDCTTSEQSDFCMDGAAAS

MPYFIFASQAIQPRFECKPIYDIMSGVAKRMGVFDEFTEGRTQEEWLQWMYAQTVKQNND

PNLPSYDEMRKQGIYKKQFDRPHVAFEDFRRDPEANPLPSPSGKIEIYSETLAKINEEWE

LDEDESITPLPEYVSTFNGWDSPDRKEFPLQLTGFHYKSRAHSTYGNVDILKAAAPQELW

INPIDAASRNIDNGDLVSVRSKFGEMNVRVKVTPRIMPHVVGLGEGAWYAPNAKGVDQAG

SINVLTTQRPSPLAKSNPSHTNLVEVTLIKKMGAK

>tr|Q87N77|Q87N77_VIBPA Putative outer membrane protein TolC OS=Vibrio parahaemolyticus serotype O3:K6 (strain RIMD 2210633) OX=223926 GN=VP1998 PE=4 SV=1

MVKLQRSTIAFALLAATLSLPTYAISIEQAWQQAKQNDPNYEKAKIGVQLGEVGVQSSRS

ALLPGLSASASTDWNESSSHSNSYGATLSQTIWDSSLWSELDQANANYLKVQLELSQAHN

ELAQKLLTAYLDVASAQGDLKLAQSKLEEGKELLNIIEKRYRAGKVKSVDVEEMRANQVS

EQASILNAKADLEVKKAELAALINQMPESVDQIRTDSLIQPPMLVDSLEQWLKLAKDSSP

ELLVAAQMVKAGEFAKDSAKGGYYPTVKGSVGYSDGDNRSNGEFNAGISLSLPIDLNGAV

RAKVDEASLNILNAKQDMRRVEIDIQKRVIQQFTQVDINWNQVLIANELVESRARVLQSK

EKLYDAGLLEVSEVISAHNSLFEAKNSLQTNLYSYWRKRIGLLQTAGKLDDDTMALISRA

FHS

>tr|Q87FT4|Q87FT4_VIBPA D-alanyl-D-alanine carboxypeptidase OS=Vibrio parahaemolyticus serotype O3:K6 (strain RIMD 2210633) OX=223926 GN=VPA1594 PE=3 SV=1

MKVSLARWTIVTCLISVPTFAVVTPNPPQLQAKGYVLMDFQSGAIIAEHNGRAGLAPASL

TKLMTAYVVGQEIKAGRLKWDDVVTVSENAWSAKFPGSSKMFIKPKDEISVANLMRGVII

QSGNDACVALAEHVAGSERGFVALMNGWAEKLGLQDTYFVNSHGLDSDGIQTSPTDMAKL

MQSIISDVPDVYALYSEKVFKWNDITQYNRNKLLWDNSIDVDGGKTGYTSNAGYSLVSSA

KEGRMRLISVVMGTPSKQARISQSKNLLSYGFRFYDTKQVAKQGQVESKVRVWKGNANQV

EAIFAKDAYLTLPRSMTAGLDKSVVLNEPLIAPIAQGDEIGKVVWKSDDSTVASYPLVSS

QSVEEGSWFAQLWDSLVLWVKSFFN

>tr|Q87NY2|Q87NY2_VIBPA Uncharacterized protein OS=Vibrio parahaemolyticus serotype O3:K6 (strain RIMD 2210633) OX=223926 GN=VP1736 PE=1 SV=1

MKSSSKRPMFGPLQPLAVFSLFSLAFLSISRILLAFWQFDRIESFNDFLYILGQGVRVDI

ATLCWLFILPALLSSFMPLKGKVGECWKWVLRLWMVAGLWILVYMELATAPFIQEYDLRP

NRLFVEYLIYPKEVMSMLWTGYKLELFIGAIGTALTLVLGWKWSKKLTDSAQQINWKWRP

LLAIFVVLLCVAGARSSLGHRPLNPAMVAFSNDPLLNDLALNSSYSLLFAVNNMKSEKSA

EQFYGKMDNQKMLDLVRASSTKIDFDPTLLPTMNSNPATYQGKRKNLVILLQESLGAQFV

GSLGGLPLTPNLDELMQEGWQFTQMYATGTRSVRGIEAVTTGFPPSPSRAVVKLSKSQTG

FFTIADLLKEQGYHTQFIYGGEANFDNMKTFFFGNGFDQIVEEKNYTNPGFVGSWGVSDE

DLYNKADEEFERLSKGDKPFFSLVFTSSNHSPYEYPEGKIEQYDSEHMTRNNAVKYSDYA

LGTFFDKAKKSSYWDDTIFIVIADHDARVFGANLVPVKHFHIPALIIGKDIQPRKDDRIA

NNIDMPPTLLSLIGVDAKTPMIGRDLTKPLAREDERAMMQYDKNFGYLTRDNLVVLSPGE

KVSTMEYDFESQTMKPLEVDESVIDRAKANALFASKAYQNNWYSSKRTN

>tr|Q87PQ2|Q87PQ2_VIBPA Putative anaerobic dimethyl sulfoxide reductase, chain C OS=Vibrio parahaemolyticus serotype O3:K6 (strain RIMD 2210633) OX=223926 GN=VP1449 PE=4 SV=1

MLYEAPLVAFTVLAQTAVGAHLTVNAFEKFGKPPRVTEPRMNIARFAILVVMGLGFLFST

THLGSPLRAFNALNRVGSAALSNEILTGASFLSLAGLYWLLTILKIGSEGVRKIVNWLSI

AVGVIFMFAMANVYQIETVPAWYSPMTTVAFWFTVVTSGLMFGYTLINVLDVTAEKTNRR

LMWAGVSLIALNLAFTVMQTIHFAGISTAIHSGLDQITMLSGYVAGHVLLLVVAAGLWVF

AELFTAEGRQKNLLVIVAFVALFVAEILGRNVFYGMHFTSGLY

>tr|Q87PJ9|Q87PJ9_VIBPA Sensor histidine kinase OS=Vibrio parahaemolyticus serotype O3:K6 (strain RIMD 2210633) OX=223926 GN=VP1503 PE=4 SV=1

MPTNWQQHKGLSKWTHRFKTMVRYRLLILTSAPIFLTLIALIGITIYWSIHYTWQNALLD

VSERLGVANNSVTLLQQKQANYVRAFADSYDFRTRINQGTPQDELQKWVTEQKKRYSLDF

LSFQRVNSMENKFRFMDLTKRESFFDVLNREELEQLDPELAKRAEVPILADGGMEARGLV

SRTVIPVYSQANDLIGFLDGGLLLNNSTVLVDQIRDLIYLSDNDRLRPVGTLTVFLDDLR

VSTNVPLDSDHRLGRAIGTRVSAEVYNQVLSKGQQWVDRAYVYDAWYITAYQPIKDQYDN

VIGMLYTGYLMWPFVKAYMTNIAEISLITLMLLLVSGVMVYRGSRDLFRPIERIHKVVKL

VQLGKEKRIGPLGLDDHHELAQLARQFDNMLDALEDRKIELKNAAAQLECKVQERTASLR

EKTEELELHIQLLNQTRDKLVVHEKLAALGELTAGIAHEINNPTAVILGNVELIHFELGE

DASRVQEEIDAIHAQIDRIRNITRSLLQYSRQGGVQDEITWQHVNPIIDESITLVKTGTK

KRDVEFVTDLQAHTPVEINRHHLLQILVNLQMNAIHAMNGKGKLIVMSEDWIEEGEIKGA

AIHVIDDGCGIKPENLNRIFSPFYTTKRDGTGLGLSVSQSILSQTGGELKAESEWGKGST

FSIYLPKKAELLLEVTNIA

>tr|Q87MY5|Q87MY5_VIBPA Oligopeptide ABC transporter, periplasmic oligopeptide-binding protein OS=Vibrio parahaemolyticus serotype O3:K6 (strain RIMD 2210633) OX=223926 GN=VP2091 PE=4 SV=1

MYKNKITRALFIGAGLSLALAGCGDNKPEEKQAAQPAPAPEAKTDSSEPKLAAVQELVRG

NGTEVATIDPHKSQGVPESHVIRDLLEGLVNQDADGNTIPGVAESWETEDNKTYTFHLRK

DAKWSNGDPVTAGDFVYSFQRAVDPATASPYAWYMEYTKMKNAKDIVAGKKDKSELGVKA

VDDHTLVVELDTAVPYFVMMMGHTTVKPVHQATVEKFGDQWTKPENFVGNGAYVVDNWVV

NERLVLKRNEQYWDNENTTLEKVTFLPIENQVAEMNRFLAGEIDFTNELPTEHFKRLKKE

HPQEVSVTGNLCTYYYIFNTKKAPFDDVRVRQAISYAIDRNIVTDAILAQGQKPAYFLTP

EITAGFNPEIPAYGKMTQEERNAEAARLLEEAGYGADNPLKFNLLYNTSENHKKIAVALG

SMWKKTLGLDVTLENQEWKTYLSTKDSGNFEVARAGWCGDYNEASSFLTLMMSNNTTGGI

HYDSKEYDAIMNKAFSSTSNEERQALYLEAEKLMAKDMPIAPIYQYVKSRLLSTKVGGFP

SNNAEEKIYSKDLYITE

>tr|Q87N67|Q87N67_VIBPA Transcriptional regulator, LysR family OS=Vibrio parahaemolyticus serotype O3:K6 (strain RIMD 2210633) OX=223926 GN=VP2008 PE=4 SV=1

MLNKVNLADIRSFVLIAQLGNFTKAAEALSVSRSHVSRQISGLEAQMGVTLLNRTTRTLR

LTHAGERFYHDCEKALRDIDQALIAAVDDTQEIRGVIRVNCVGGYIGEDIIAKYVCEFMQ

QYPNVSIDLDFSSPRIDLIEDQFDVAFRMGELDDAGFVARKLMTVDMVTLASPSYVQQYG

MPIHPKELAEHRTLTGSVARWSYRKADNPNDHTDVVVKGNLRCKNGRALVMGALHGNGII

RVPLSYCAEEVELGQLIKVMPNWEIPSVPFSAIFHRDRYQPKRLRTFIDFIKQKFEDS

>tr|Q87SB4|Q87SB4_VIBPA Thiol:disulfide interchange protein OS=Vibrio parahaemolyticus serotype O3:K6 (strain RIMD 2210633) OX=223926 GN=VP0510 PE=3 SV=1

MSVLRRLTLLTLPFFITVCGAEESQAKTETPAQQAAPTAQQHFDEAALKAKFSKLGLSII

DIQPSDVAGLLEIQTNGGILFASNNGDHFIAGTLYAIDDNGGYKDVLAERQAPLNAEKIA

EFADSMIEYKADNEKYVVTVFTDITCGYCVRLHNQMQGYNDLGITVRYMAYPRQGATGPV

AEQMATIWCAEDPKAAMHNAKVSRTFDNPAKDLEQCKETIQAHYNVGRQLGISGTPAIFL

PNGEMVGGYLPPAELLKRLEQL

>tr|Q87G86|Q87G86_VIBPA Putative potassium uptake protein OS=Vibrio parahaemolyticus serotype O3:K6 (strain RIMD 2210633) OX=223926 GN=VPA1431 PE=4 SV=1

MAHFTVIGLGRFGVAASLELIHLGHTVTGVDSDPKIVEKYVEELTQAVICDATDEGALRE

LALANSDVVLVAIGEDMQASLLCTLALKNLGVKSIWVKASTKAHHTIVSKLGVQRIIHPE

EEMGVRVAQALNYPMVNNYLEMGHGLYVVEIHVKSNLHDTPLSKLISRGSYNIQPILVKR

EEAVYTNINDEFVLNEFDTLLLCGSRAELKYVAPRLV

>tr|Q87R87|Q87R87_VIBPA C4-dicarboxylate-binding periplasmic protein OS=Vibrio parahaemolyticus serotype O3:K6 (strain RIMD 2210633) OX=223926 GN=VP0910 PE=4 SV=1

MLKPLTLLSVSALAVTSFNAAANCDPGETVIKFSHVTNADKHPKGIAASLLEKRVNEEMN

GKVCMQVFPNSTLYDDDKVLEALLNGDVQLAAPSLSKFEKFTKKYRLFDLPFLFDDVAAV

DRFQNSEAGEKLKNSMKRRGLQGIAFWHNGMKQMSANKPLMVPSDAKGLKFRVQASDVLV

AQFEQLGANPQKMSFKEVYGGLQTKVIDGQENTWSNIYGKKFFEVQDGITETNHGILDYL

VVTSNDFWQKLPEDQREQLNTIIQEVTVERNAESTKVNLANKNNIIEAGGVVRTLTPEQR

QEWVTALQPVWKKFEKDIGSDLIEAALASNQQ

>tr|Q87NX4|Q87NX4_VIBPA Metal-dependent carboxypeptidase OS=Vibrio parahaemolyticus serotype O3:K6 (strain RIMD 2210633) OX=223926 GN=VP1744 PE=3 SV=1

MKDPFRVFRERTKIRVVTMNAFTKLVEHSKKVANFGHLLEIVGWDQAAVMPSGGAEARSN

AMAELEVHIHSLMTQPHLEDLFAQAEEETLATQERAMLREMKREWQLANLLPESLVQASS

LAGSKCEHAWRTQRANNDWAGFEKNWAEVVKLSQEEAQIRADANGTTPYDAMLNVYEPGT

TSASLDTLFSDVKTWLPSLIDEAIEKQKSNNILLPNGHYPAEKQKALGLQVMKLLQFDFE

HGRLDESVHPFCGGVPTDVRITTRYDESEFMQSLMGIVHETGHARYEQGLPKELAGTPAG

EARSMGIHESQSLFFEMQIGRNNAFIDHLARLASNHFSGNEFAKDNLAKIYTRVEKGFIR

VDADELTYPAHVILRYEIERDLMNSVIKHTDVPELWNEKMKAYLGLSTEGNFKNGCMQDI

HWTDGSFGYFPSYTLGAMYAAQFMAAMKKTIDVDGVIQSGDLSPIFTWLSDNIWSKGSLL

TTDELVKQATGETLNAQHFQAYLKSRYL

>tr|Q87RC9|Q87RC9_VIBPA Putative outer membrane lipoprotein Slp OS=Vibrio parahaemolyticus serotype O3:K6 (strain RIMD 2210633) OX=223926 GN=VP0868 PE=4 SV=1

MKVFGRLFLVLVAALGLSACSSLPEELNASTEQVVTDYKAFAESQGELTNDVRLGGIIAK

VDNFKDKTRLEIVNLPINKSGKPDIDQEPTGRFAVYFDGYLEPVAFSQGRLVTIVGKGAG

EEEGKIGEHEYVFPLVKGQGYRLWKIEERVRMYDSPTYFYPCYSINCRMLRNDFPQDGKV

IKQVK

>tr|Q87JW9|Q87JW9_VIBPA Putative phosphatidylglycerophosphatase B OS=Vibrio parahaemolyticus serotype O3:K6 (strain RIMD 2210633) OX=223926 GN=VPA0129 PE=4 SV=1

MKTKKYGLMMLALFVLGIIPMSLFGSYHNLTSQVPESLGITFTLLTDSAGSKGFLITLTL

LLLSLFRFKPSRTEWMQKLSMLGLLLVIGFASKTGLKLMTESPRPYTELLAAEQLIETPE

TFYQLDTKQQANVINQISEHVSDWRTRHWQGEKDYSFPSGHTIFVSICLAFFGGLFLQNK

CYISALSLWMWGMSVAYSRLWLGMHRPEDLIGSVLFVAIVFTLLPTFQIKKQMPFVSLAS

R

>tr|Q87PY8|Q87PY8_VIBPA Putative ABC transport system permease protein OS=Vibrio parahaemolyticus serotype O3:K6 (strain RIMD 2210633) OX=223926 GN=VP1361 PE=3 SV=1

MDESNLEIELAESITSAMVGDLWEKANDILESYPDSPIVVNASNLTFVDISGVAFLSDLQ

TRFRPPGAEISIIGLSASLAELVPPSNIENAPQIPRGEDGFFERVGNATREMLVYVGSVV

RFIKECVLVFKLGINRRKNVNWTTVSNIATRAGADAVPIILLIGFLMGVIIAFEIGLVAQ

QFGAVLFVADGIGISMFRELGPLMTAIVFAGRTGAAFAAEIGTQKVNEEINALHTFGICP

VEFLVIPRIYASVLVLPLLTVLADIIGVLGGALVLLKFDISFVQYYHQLLNALSVWDLFF

GLIKATTFGFIIAVIGCERGLATGQGSTSVGLAATSAVVSSIIWIVVIDGFFTVLLT

>tr|Q87TL9|Q87TL9_VIBPA Peptide ABC transporter, permease protein OS=Vibrio parahaemolyticus serotype O3:K6 (strain RIMD 2210633) OX=223926 GN=VP0050 PE=3 SV=1

MSQTNTPAAPSAVPSAWERFKNSDFLYYFKRDKVAMASFTVFLMFLVLALAAPILAPTDP

YDLTSIDIMDSELPPSWMDGGEERFVLGTDEQGRDILSTILYGSRLSLTIGFLAVGLQLV

LGIIIGLSAGYFGGRIDSFLMRFADVQLSFSTMMVAIIVSAIFKASFGSDFYSQYAVVML

VVIIGVAEWPQYARTIRASVLAEKKKEYVEAARVMGFKAPRIMFRHILPNCLSPILVIST

VQVANAIMSEAALSFLGLGLPVDQPSLGALISIGFNYIFSGAWWITAFPGIVLVTLVLVI

NLLGDWLRDVFNPKLYKG

>tr|Q87TR1|Q87TR1_VIBPA Amino acid ABC transproter, permease protein OS=Vibrio parahaemolyticus serotype O3:K6 (strain RIMD 2210633) OX=223926 GN=VP0007 PE=3 SV=1

MGFDFNYMLELLPILLKYLGTTMEMATWGLVFSLILAVILANIRVFKLPVLDQLSQLYIS

FFRGTPLLVQLFLLYYGLPQIFPVMVGIDAFSAAVIGLTLHFAAYMAESIRAAIIGIDRN

QMEASLSVGMTTPQAMRRVILPQATRVALPSLMNYFIDMIKSTSLAFTLGVAEIMAKAQM

EASSSFRFFEAFLAVALIYWGVVVILTRVQIWAEAKLNKAYVR

>tr|Q87MY6|Q87MY6_VIBPA Oligopeptide ABC transporter, permease protein OS=Vibrio parahaemolyticus serotype O3:K6 (strain RIMD 2210633) OX=223926 GN=VP2090 PE=3 SV=1

MLKFIAKRIFEAIPTMLVLITVSFFLMRFAPGNPFSTERPLPPEVMANIEAKYGLDKPVF

EQYTTYLTNVLQGDFGPSFKYLDYTVNELIAVALPVSAKVGFIAFIFTLIMGVTVGTIAA

LKQNTWVDYTIMSTAMLGVVMPSFVLAPALIYLFSLHWHIFPAGGWQDGSWQFLVLPVIG

MSLLYVATFARITRGSMIETLNSNFIRTARAKGLSYRYIIIKHALKPALLPVVSYMGPAF

VGIITGSVVIETIFGLPGIGKLFVNAAFNRDYSLVMGVTILIGFLFILFNAIVDILLAMI

DPKIRY

>tr|Q87RS3|Q87RS3_VIBPA Lipoprotein OS=Vibrio parahaemolyticus serotype O3:K6 (strain RIMD 2210633) OX=223926 GN=VP0704 PE=3 SV=1

MKFSLKGLLTVATAASALVLAGCGDKEVDVNKVKVGVIAGAEAQVAEVAAKVAKEKYNLD

VELVTFTDYVTPNAALDDGSVDANAFQHKPYLDQQVKDRGYKLAIAGNSFVYPIAGYSKQ

VKSVDEIQDGARIAVPNDPTNLGRSLLLLEQQGLITLRDGVGLLATVRDIVGNPKNIEII

ELEAPQLPRSLDDVTLSIINTTYASSIDLSPERDGVFVEDKESPYVNLIVAREENVNAQN

VQNFVKAYQTEEVYEAAKKLFKGGVVKGW

>tr|Q87GC8|Q87GC8_VIBPA Uncharacterized protein OS=Vibrio parahaemolyticus serotype O3:K6 (strain RIMD 2210633) OX=223926 GN=VPA1389 PE=4 SV=1

MKLPNGLSYMKSIEASDVIFLVNWPDGRKTPLPYTSRVALGMKEGSKSAYKYDGQIDADV

TAYSLAQGNPHEIDFCCVPYGAESIECEFSVSFASSLRKPFKCSDPEVKRTLVQLIKLYE

EKVGWEELANRFLENICNGRWLWRNNECTYSTSIGIKPWPWEDEKAISPFHDIRKNYAGT

NHFRDHKDWDNLIKLITDAFSQPNGLCIFEVSATFRLGTNAPIYPSQVFKDSVKGEKNRI

YQSTDVDGESSPILGCYKTGAAIATIDDWYPDADKPIRISHYGAHREDVYCYRHPNTGKD

LFTLLEKADQYLEQLQATDVLPDEMINDLHFIVANLIKGGLLQQKGT

>tr|Q87LQ7|Q87LQ7_VIBPA Lipoprotein NlpD OS=Vibrio parahaemolyticus serotype O3:K6 (strain RIMD 2210633) OX=223926 GN=VP2554 PE=4 SV=1

MSKLLRHIGISFVLAAGLVGCAAHSPAPVSSLKKDYSSVERGSYRGSYYEVKKGDTLYFI

AYVTDKDVNDLVRYNELSQPYTIYPGQKLKLWAPKYVAPKYGQKVEPVVVPVVTTTPPPV

VKPTTTTKPVTSSKNSSQKPTTTPTKVAQKEPPKKVEQTKAKEYVGSKDNQPTKPKPPTT

TVQNDKVSKWLWPTKGRVIKNFSAGEQGNKGIDIAGQRGQPIVSTAAGTVVYSGNALRGY

GNLIIVKHNDNYLSAYAHNDKLLVTEGQSVKSGQKIATMGSSGAKSVKLHFEIRYQGKSV

NPKRYLP

>tr|Q87MH5|Q87MH5_VIBPA Trp repressor-binding protein OS=Vibrio parahaemolyticus serotype O3:K6 (strain RIMD 2210633) OX=223926 GN=VP2280 PE=4 SV=1

MDCQILILYFSRHGSTKQLARQIARGVESTPQCQAILRTVEELSPHSHTPSDPIVTLEEL

KKCDGLAFGSPVWFGNMAAPLKHFWDQTTPLWVSGDLIDKPACVFTSSSSMHGGQETTLQ

SMMTPLLHHGMMVLGIPYAEPELHSTQSGGTPYGASSVGHEPSLTAEETRLAQQLGKRLA

TAALKLKACH

>tr|Q87MQ6|Q87MQ6_VIBPA Putative HAAAP family transport protein OS=Vibrio parahaemolyticus serotype O3:K6 (strain RIMD 2210633) OX=223926 GN=VP2175 PE=4 SV=1

MSVTAPTQTLPIGKEPPHVKAGMALDEWKAATKFDSTDWGWVIMSIGMAIGAGIVFLPVK

VGVLGLWVFLASAVIGYPAMYLFQRLFINTLSSSPKCQDYAGVISGYLGNKWGALLGVLY

FIMLVIWVFVYSTAINNDSASFLHSFGITEGLLSENPLYGLALVCALVAIASRGEKILFK

VSTGLVLIKLCTVALLGVMMIEKWDVANVGSIPETGAGLKDAIELLPFTLTSILFIQSLS

PMVISYRSKEKSIEVARFKAMRAMKIAFGILFVTVFFYAISFTLAMSHEQAVKAAEENIS

ALAMVAQGMDGTTLKLMSLMLNIFSVMTAFFGVYLGFRDSCQGLAMLALKKVMPEEKINK

DLVTKGIILFAILMAWGAIVLNLPVLSFTSVCSPIFGLIGCLIPAYLVYQVPSLHKYKGA

SLYLIIATGILLCVSPFVAFS

>tr|Q87RD9|Q87RD9_VIBPA Ferrous iron transport protein B OS=Vibrio parahaemolyticus serotype O3:K6 (strain RIMD 2210633) OX=223926 GN=VP0858 PE=3 SV=1

MQYQVLTVGNPNSGKTTLFNGLTGAKQQVGNWAGVTVEKKTGQFKHAGDEFLLTDLPGIY

SLDSGNDSNSIDESIASRAVLTHPADLIINVIDATSLERSLYMTLQLRELGRPMVVVLNK

MDALKRERQMINIAELEKTLGCPVISLSATNKAQVAEFKEKLHKAIVQGVALKDRSLNYG

DKMETAIEHISSLFNDQTVSHRALAIRALEKDTMVLNSLSDLDRSKVLEAIATLDLDIDL

HVADTKYTHLHEQCKKVRRSEGKLSRSVTEKLDQFILNKWVGIPFFFVVMYLMFMFSINI

GSAFIDFFDIGVGALLVDGGHHLLDGHLPVWLVTVIADGIGGGIQTVATFIPVIACLYLF

LAVLESSGYMARAAFVLDKVMQKIGLPGKAFVPLVLGFGCNVPSIMATRTLDQERERKLA

ASMAPFMSCGARLPVYALFAAAFFPGSGQNIVFALYLLGIVAAIFTGLFLKHTLYPGSSD

SLVMEMPDYELPTLQNVVIKTWQKLKRFVLGAGKTIVVVVAILSFLNSLGTDGSFGNEDS

ENSVLSKAAQVVTPVFAPIGIQEDNWPATVGIITGIFAKEAVVGTLNSLYTSPSDGEEAE

FDLMGSLKDAVMSIPENLAGLSYSDPLGIEVGDLTDSNAVAEEQEVDESIFGNIKSQFVS

GHAAFAYLIFILLYTPCVAAMGAYVREFGHNFARFIAVWTMGLAYLCATFYNQLTHFSES

PITSGVWMAIIVAIFIGTYHVFKRIGKKQHGALEAQVA

>tr|Q87FH7|Q87FH7_VIBPA Putative dicarboxylate-binding periplasmic protein OS=Vibrio parahaemolyticus serotype O3:K6 (strain RIMD 2210633) OX=223926 GN=VPA1702 PE=4 SV=1

MMTRKTLLSAVVGAAMTFGATASAYAATTLKLSHNHPRDHAVHKAMDFMAKEVREMTDGE

VRIRIYPDAQLGTQRESMELMQNGALDMVKSNAAELEAFSPAYAAFNMPYLFRDKDHYYK

VTDGEVGREILNSSADSGFIGVTYYDAGARSFYTNKPINTPEDLKGLKVRVQPSPSAIAM

VKALGGNPTPLAYGELYTALQQGVVDAAENNIPSFSLSRHSEVSKYFSLDEHTMVPDVLV

ISTKTYDKLTPEQQKALMKAAADSSEYMKKLWAESEAKERTKAEKMGVTFVEPNKAAFVE

AVQPMYADLEKTNPELNELVEKIKAVK

>tr|Q87IZ0|Q87IZ0_VIBPA Universal stress protein OS=Vibrio parahaemolyticus serotype O3:K6 (strain RIMD 2210633) OX=223926 GN=VPA0466 PE=3 SV=1

MKYKHILVALELSDESTVLIDRAVSMANYLDSDISFIHIDGTHGEIYRELVDIKENPDQR

PLNEHSMECLRTFSDYMDYPLKHFFVGTGDLADKLEVTIKEQEVDLLICGHHQDFWSKII

SYSRHLINKSPVDILVVPIHE

>tr|Q87SJ8|Q87SJ8_VIBPA Outer membrane protein TolC OS=Vibrio parahaemolyticus serotype O3:K6 (strain RIMD 2210633) OX=223926 GN=VP0425 PE=4 SV=1

MKKLLPLFISAALGGISSSAWADSLAEIYDLAKQNDPQLLSVAAQRDRAFEAITSSRSAL

LPQINLTAGYNLTRGDTEYDSNLISDVSNDSNALTAGVNFSQELYNRASWITLDTAEKSA

RQADATYAAAQQGLILRVSQAYFEVLRAQDNLVFVRAEKAAVGRQLEQTKQRFEVGLSAI

TDVHDAQAQYDAVLADEVLAENDLINSYESLREITGQEHKNLNVLDTNRFSATRTNSPAE

TLIDEAKTKNLSLLSARISQDIARDNISLASSGHLPTLSLDGGYNYGDTSNSARDNTTDN

FNIGVNLAVPLYTGGNVTSQTKQAEFAYVAASEDLEAQYRSVVKDVRAQNNNINASIGAL

KAYEQSVVSARSALEATEAGFDVGTRTIVDVLDATRRLYDANKNLSDARYNYILSVLQLR

QAVGTLSEQDILDVDAGLKPAK

>tr|Q87PC7|Q87PC7_VIBPA Endopeptidase La OS=Vibrio parahaemolyticus serotype O3:K6 (strain RIMD 2210633) OX=223926 GN=VP1590 PE=3 SV=1

MNQLNWKDVTPSFEQYEDILKSASSLPKKKFVELQPRLLATVERFKKIKGLTRVLVINCA

DNTVYRKFICDVVTDGLEPTIMTESLDAKLLFDRYSVDLKGDVVVEAGLLSKANGGYLIL

PANLILANPGYWPSIKSAIQGKPVNPLNVSPTRLPILTADEKEFDVKIIVTGDRNQLADL

EYVDEDFSTGLTMYTEVEEDIHLSASNLELYVGLVNWICSEYGFPSLDDGAFQRLLLAGM

RLTEDQHYLPLGVMWHCQLLSLAAQFSDQNIIDYVAIDKAIDDKYYRESYLPQRAVYDIL

DGQVIIETTGEQIGQINGLTVIDMAGHPVSYGEPARISCVIHFGDGDISDVERKAELGGN

LHAKGMMIMQAFLSSALNLDEPLPYSASIVFEQSYSEVDGDSASLAELCCLVSALSESPV

NQQIAVTGAVDQFGRVQAVGGLNEKIEGFYQVCKHQGFTGHQGVIMPKSNLKHLALHKDV

IESIKNGEFHIWSVSTVDEAIPILMGKSFRGEDDSVIGKIAERIENFERHEHPEGIVQRI

KNWFV

>tr|Q87SE2|Q87SE2_VIBPA Glutamate synthase, large subunit OS=Vibrio parahaemolyticus serotype O3:K6 (strain RIMD 2210633) OX=223926 GN=VP0482 PE=4 SV=1

MALYDPSLEKDNCGFGLIAHMEGEQSHKLVRTAISALDRMTHRGGIAADGKTGDGCGLLL

QKPDSYLRLIAEENGFNLGKQYAVGMLFFSQDPVKAQLSKDIVNKELAQETLTVAGWREV

PTNSAVLGPIAANSLPNIQQVFISAPAGWRERDIERRLYIARRRIEKQITEDPDFYICSL

STQVLVYKGLCMPADLPRFYLDLADLRMESAICLFHQRFSTNTQPRWPLAQPFRYLAHNG

EINTIEGNRQWARARAYKFSSPLLPDLQTAAPFVNETGSDSSSLDNMLDLFLAGGMDIFR

AMRMLVPPAWQNHPDMDPDLRAFYDFNSKHMEPWDGPAGIVLSDGRYAACNLDRNGLRPA

RYVITKDKLITLASEIGIWDYAPDEVAKKGRVGPGELLVVDTRKGKLWQSSEIDNDLKSR

HPYREWMENNVRKLTPFSQLPEDQVGERSFDEDLLKTYQKQFAMTNEEVDQVLRVLGDMG

QEAVGSMGDDTPMAVLSSKERLVTDYFRQKFAQVTNPPIDPLREKHVMSLATSIGQEMNV

FCETDGHAHRVTFDSPVLLYSDMQQLLELGDDHYRNTILDINYDPQEKDLKQAIDDLCDQ

AEQVVREGTVLVVLSDRALVKGKLPIPAALAVGAVQTRLVTANLRCDANIVAETATARDP

HQFAVLLGFGATAVYPYLAYEALGKLIDDGALDKDYRDVMQNYQYGINKGLYKIMSKMGI

STIASYRCSQLFEAVGLSREVVDLCFKGVTTRIEGANFDDFQQDLTNLSRKAWAKRKSIE

HGGLLKYVHGGEYHAYNPDVVGTLQKAVKSGESNDYQSFAKQVNERPVAMLRDLMALKKT

DSPLPLERIEPKTELFKRFDSAAMSIGALSPEAHEALATAMNRLGGYSNSGEGGEDPRRF

GTERNSRIKQIASGRFGVTPHYLTNADVLQIKVAQGAKPGEGGQLPGHKVTAEIAKLRYS

VQGVTLISPPPHHDIYSIEDLAQLIFDLKQVNPNALVSVKLVSEPGVGTIATGVAKAYAD

LITISGYDGGTAASPLTSVKYAGSPWELGLAETQQALVANGLRHKIRLQVDGGLKTGLDV

VKAAILGAESFGFGTAPMVAMGCKFLRICHLNNCATGVATQDETLRKEYFKGLPEMVMNY

FTGLADEVRELLAALGVEKLTDLIGRTDLLEAVEGFTAKQTKLDLSNILEAPISAEGHPL

FWTEPNKPFDKAELNQKIVDDALNAVENSQSASFFYNVINTDRSVGARLSGEIAKRYGNQ

GLAASPIKIYLDGTAGQSFGVWNAGGVELYLTGDANDYVGKGMAGGKIAIKPHLGTAFKC

NEATIIGNTCLYGATGGKLFAAGKAGERFGVRNSGTIAVIEGAGDNACEYMTGGIVAILG

ATGVNFGAGMTGGFAYVLDENQDFQGRVNNESVEAISLSDLYIHQEHLRGLIAEHLEETG

SSHAEHILANFDEWIPKFYLVKPQAADLNTLLGHQSRSAAELRVQAQ

>tr|Q87LK3|Q87LK3_VIBPA Extracellular deoxyribonuclease OS=Vibrio parahaemolyticus serotype O3:K6 (strain RIMD 2210633) OX=223926 GN=VP2609 PE=4 SV=1

MKYLFTLFLFVLSTSAFSAPPSSFSAAKREAVKIYQDHPSSFYCGCDIQWQGKKGVPDLA

SCGYQVRKQEKRAARIEWEHVVPAWQFGHQLQCWQSGGRKNCSRNDTAFKLMEADLHNLT

PAVGEVNGDRSNFNFSQWNGMDGVSYGRCDMQVNFKQRKVMPPDRARGSIARTYLYMSKE

YGFKLSKQQTQLMSAWNKTYPVDKWECERDKRIAKVQGNHNPFVQEACRAL

>tr|Q87IY0|Q87IY0_VIBPA Uncharacterized protein OS=Vibrio parahaemolyticus serotype O3:K6 (strain RIMD 2210633) OX=223926 GN=VPA0476 PE=4 SV=1

MRLERTFDPNDLSTQNMESPICLPIGFVHFLAQSQTLQQVLDTVAEWINRIFESDRTSIT

LYENSDYLKVYSFSGNKAIPADFLVPIDQAFVGRVFKNQQLIICDDVSQSDELDCVMLTS

SGMGTCMDAPLMHGQMCLGTLNVAHHQTHFYTKEQAAQLQCIANWIALNIALHIQIMKME

HLATTDDLTGIPNRREFMRQIEHRLSEFRTQGIKFHVAILDLDNFKKLNDKFGHDAGDKS

LIHAANTIKGHLRECDLLARVGGEEFAIVLRSRSSQEAMDTLETIRNALETTTIRYSGRD

MTFTASIGVTQVCGLDDSFEPLVKRADLALYKAKETGRNRVEINHSEHQGVSNEQ

>tr|Q87PZ1|Q87PZ1_VIBPA Uncharacterized protein OS=Vibrio parahaemolyticus serotype O3:K6 (strain RIMD 2210633) OX=223926 GN=VP1359 PE=3 SV=1

MIKRYLITLILLCTAGTVLYTHYQTYTNNPWTRDGQVSAYVISITPRVTGQVTQVYVADN

SKVKKGDLLFEIDPSTYAASYHKALANQKQATALLAKAQNEEQRARKLEKRTHGAVPALT

LSNLHNAVQTAQANVELAKANIEEAQLNLDFTKVYAPTDGYITNLNVRVGSQVVANSPVV

ALIDENSFWIEGYFKETDLAGIAPNDRATVTLLMHDDVALEGKIKSIGFGIAKKDGTTGN

DLLPTVNPNFQWIRLAQRIPVKVTLNEMPKDLQLRVGMTASIKIIKH

>tr|Q87JE5|Q87JE5_VIBPA Uncharacterized protein OS=Vibrio parahaemolyticus serotype O3:K6 (strain RIMD 2210633) OX=223926 GN=VPA0308 PE=4 SV=1

MIVTVDIIPFRLSGCADKGLEVLLIKRSNPNRPYHGVWALPGGFVFDKDLTSEGGRPADE

NFEAARRRICREKIHTYPRHFSEAFIDGDPKRDPEDWSLNITHYALVDRNNVEQINNAGV

PECQLKWFPLQAILNGEETLAFDHQKTIEKAWQKLRASIEYTSVLLFALDKEFLVADIIS

AYQEFGIDISRMTIKRRLIDSGVLKPTNKVASTNKGKGGKPAMVYTLTSDEVTFFQNCLR

G

>tr|Q87JN9|Q87JN9_VIBPA Hexapeptide-repeat containing-acetyltransferase OS=Vibrio parahaemolyticus serotype O3:K6 (strain RIMD 2210633) OX=223926 GN=VPA0209 PE=4 SV=1

MTELEKMMSGQVFDGMDKEIDAIRTQATLALRAFNNNTDESKRDELQKQLFGNAGLCIVQ

APFHCEFGKTIEIGEETFINMNVVMLDGAKITIGNHVLIGPSVQLYTASHSVDYRSRRRW

ETFCKPITIEDDVWIGGNSVINQGVTIGARSVIAANSVVNHDVPPDCLYGGTPAKLIRRL

NEEE

>tr|Q87JW4|Q87JW4_VIBPA ABC transporter permease protein OS=Vibrio parahaemolyticus serotype O3:K6 (strain RIMD 2210633) OX=223926 GN=VPA0134 PE=3 SV=1

MSDMTQYNSVSARISKKERVTHPVLRLLISAAVILGLWQLIVVVFDMPNFILPSPIEVLD

RLITRYDVLLKHTWVTAQEILLGLALGLSMGLLFALQMLMFEPLKRWLLPILIASQAIPV

FAIAPVLMLWLGYGIASKVVMAAIIIFFPVTTCCYDGLRNTPTGYLDLAKTMGASKWQLL

RHIQLPAALPTLASGIRVAVVIAPIGAVVGEWVGSSEGLGYLMLQANARMIIDEMFAALF

ILAALSIALYFTTDKLLKKAIPWENK

>tr|Q87PN2|Q87PN2_VIBPA Putative LuxO repressor protein OS=Vibrio parahaemolyticus serotype O3:K6 (strain RIMD 2210633) OX=223926 GN=VP1469 PE=1 SV=1

MRPRVLLVEDSTSLAILYKQYVKDEPYDIFHVETGRDAIQFIERSKPQLIILDLKLPDMS

GEDVLDWINQNDIPTSVIIATAHGSVDLAVNLIQKGAEDFLEKPINADRLKTSVALHLKR

AKLEDLVENIQSTFDRHNYHGFIGSSLPMQAVYKTIDAVAPTSASVFIVGESGTGKEVCA

EAVHRQSDRRDKPFIAINCGAIPRDLMESEIFGHVKGAFTGATTDRKGAATLAHGGTLFL

DELCEMELEMQKTLLRFLQTGTYTPLGGTKEMKVDVRIICATNRDPLTEVEEGRFREDLY

YRVHVVPIDMPPLRERGSDIVTLAKHFLTTYAKEDKKKFSNIDTEAQHVIKHYEWPGNVR

QLQNIIRNIVVLNNDEKVTVAHLPAQLTQKKTQARTVTPVHVESSSPSNNLNGHNAPAMQ

TPPIEPVQPVQEAPAQQTQPTVGVETPSHSLSPYFNADGSIRPMWQVEREAIQNAIAYCD

GNVLNAAVMLELSPSTVYRKKQAWEADEVNLEQS

>tr|Q87KV1|Q87KV1_VIBPA Sensor histidine kinase OS=Vibrio parahaemolyticus serotype O3:K6 (strain RIMD 2210633) OX=223926 GN=VP2874 PE=4 SV=1

MQGWLVVPVSLAYLGALFIIAWYGDIQKRWLARWRPWIYSLSIAVYCTSWTFYGTVGQAS

SNPWSFLPIYIAPILVFTIGWRVLARLILIAKREHITSIADFIGARYGKSQGLAVVVTLI

AVAGILPYIALQLRGITMGLEIIAPELATDFGYQNDSVSWFVVVALAIFTMLFGTRHIDN

TEHHRGMMMAIAFESIIKLLAFLVVGIFIVWLAMSTENLSLIEVASETYQSPNIPTLLIH

TVLTMLAIVCLPRQFHTMVVENERAQDLHVARWLFPLYLILMGVFVLPIAWVGQGLLSGT

SADTYVISVPMAVGASEIALLAFLGGTSAASGMVIVSTIALAIMVSNDLVMPLILRRMRL

AQRNHHHFSELLLRIRRALILILLIGAWGFYQALDSIHSLSAIGFLSFAAITQFAPALIG

GMYWRQGNKKGVYVGLAVGFTIWLITLMSQTDMLAGNASNNFLIWLITPPEVLSSFDIAI

SDWGMIMSVVANAVCFVAVSLATRPSLSERLQSASFVGTPLPESENISLYQSRVTVAELE

MLASRFVGRTRVKVAFQAYWSQQREELLPNQQAPSSLIRHTERVLAGVFGASSAKLVLTS

ALQGRNMQLEEVATIVDEASELYDFSRGLLQGAIEHIGQGIAVVDKQLRLVAWNQRYLEL

FVFPPGLIQVGRPIADVIRHNAEQGLCGPGDPEDHVRRRVYHLEQGTRHTSSRVRPDGRV

IEVQGNPMPGGGFVMSFTDITVFRDAEQALKDANETLEERVRARTRELEQLNKQLVAATQ

RSDLESKSKSRFLAAVSHDLMQPLNAARLFASSLSEVAKDSETRKLSAHIESALGAAEDL

IGDLLDISRLESGKLEVNVHGFAVNDVLANLNAEFSALAKQQGIEFTMIPSSLMVKSDPK

LLRRVVQNFLTNAFRYSPNGKVALGVRRVQGRVRIDVWDNGMGIEEEKQQEIFEEFNRGT

QVRSDQGLGLGLAISKGIAQVLGHEISMRSWFGKGSVFSITLDKAHHVQPHPAKVETSPQ

SELCHLRILCVDNEPDILVGMENLLSRWGCDIRVATDLIESLQALEGDWVPDVIFSDYRL

DNGRTGLEVLQQCRLRLGNHFEGVIISADRTDDMMEGIKANGFSFIAKPVKPLKLRSVLN

RVA

>tr|Q87NY9|Q87NY9_VIBPA Thermostable hemolysin delta-VPH OS=Vibrio parahaemolyticus serotype O3:K6 (strain RIMD 2210633) OX=223926 GN=VP1729 PE=4 SV=1

MKHHAQTSADGFTLDIVYPMHPLWSQVIEHVSQRYQEAFFAELKQFMPAYLTLIEGGQIV

SVCGFRIAEDEPLFLEQYLEDDAQKLVSNVFNCDVKRSNLVEFGHLASFAKGMSSLHFYL

IAEMLVNLGFEWCIFTATDPLHAMMARLGLEPHIIAQADQNKVPDAESTWGSYYEHQPRV

LAGNLQKGLERLRLVQERKRKQA

>tr|Q87QD3|Q87QD3_VIBPA Multidrug resistance protein OS=Vibrio parahaemolyticus serotype O3:K6 (strain RIMD 2210633) OX=223926 GN=VP1216 PE=4 SV=1

MADGKDTTRGKRMTEKAQSAPQSQISFLLFLVLGAIGALTPLAIDMYLPAMPTIAKDLGV

APGAVQITLTAYTAGFAIGQLIHGPLADSFGRRPVLILGVLFFGLAAVVSATTNGIDALT

YVRTAQGFAGAAAAVIIQAVVRDMFDREDFARAMSFVTLVITIAPLVAPMIGGHLAIWFG

WRSIFWVLAIFAVVVILLVFWKIPETLKPENRQPLRFRTTLKNYARLCSSSEALGLMLSG

AFSFSGMFAFLTAGSFVYIDLYGVRPDQFGYLFGLNIVAMIIMTSINGRLVKKVGSHAML

RFGLFVQLLAGIGLFVSWLMDFGLWGIVPFVVLFIGTLSTIGSNAMALLLSGYPSMAGTA

SSLAGTLRFGIGSLVGALVASLPGNVAWPMIIVMFACSVLSALFYWTLGRKA

>tr|Q87PM0|Q87PM0_VIBPA Putative response regulator OS=Vibrio parahaemolyticus serotype O3:K6 (strain RIMD 2210633) OX=223926 GN=VP1482 PE=4 SV=1

MKILICDDSAVARKLIARTIVQDTSLHLIEAQDGNEALKILAEQNIDVLFLDLTMPIMDG

FEVLESLPVSHYPTQIVIVSGDVQQEAKQRCLELGAMDFIAKPLSEEQVIPLYERLGLHY

AQRTSMELNSAQSQEELSSLAKFREIANIALGKGAAIMADHLHEFIQLPVPHVGPLSYGE

LHMTLVDVIGRDSSVAVSQRFVGGGIHGEALVCLRGKDINQIGERLGYLLEFTPHNEIIL

NVSNLLVSSFLTSLGNQLDKQFSLRQPGIVDTITPYNEEQGESGELFTIEYTYMAEALDF

ECEVLFLIDKPSVEIIYEIMELI

>tr|Q87N00|Q87N00_VIBPA Putative GGDEF family protein OS=Vibrio parahaemolyticus serotype O3:K6 (strain RIMD 2210633) OX=223926 GN=VP2076 PE=4 SV=1

MVNQRRFSLSFVVFFPLVLFIVVMLVTTKNYYDAVNRYIDTEYSRIDRALTRGIKVLTAL

DYSFTNYSNFANPLSEEHNSIVKGDICYIWPIDVLLLENVYSDGLPAVDVDYMIVGEKSL

CQAGTKINKLAEQKAGFAPSLSFLHDIESHIVGIHYIDKRGYVISSPDKYAKNFTKELWS

TLKARPFWQKTAQDKVNITLAGPGPMLDSMEGQIISLTVPIYEKGVHQGVLSIDFDVDAL

LTTSENLVGKLHLVSNFQTIPEDAVRIKPIEKERLSANHSIYYEYNLWSEIKNLTFFEKY

SIMVAVFIYVLSTVVLFYVNMHTERRYFKDLAARDPMTGLLNRRGMQAVWRNKMTKKNVA

LAVFDIDNFKTINDTYGHDVGDSAIQLVAKCIRDNIRHSDVASRFGGEEFILAIYDEDIE

GMKHILERVKKAIDERSRRVTKNGFTVSGGVVFSSASKAESFDELFKAADEKLYEAKSTG

KNKICY

>tr|Q87KC5|Q87KC5_VIBPA FixG-related protein OS=Vibrio parahaemolyticus serotype O3:K6 (strain RIMD 2210633) OX=223926 GN=VP3052 PE=1 SV=1

MPPYIIKKPKKDECMSQDKIDIKDVTPKTFNPKTHKGNGDRFNPSNRIYVRESKGTYQKL

RRYGGWFLLLLFALVPWIPYGERQAILLDIGNQQFNFFGTTLYPQDLTLLALLFVIAAFG

LFFITTFLGRVWCGYLCPQTVWTFMYIWFEEKLEGSANKRRKQDANKLTANLVMRKTLKH

IAWFAIALATGFTFVGYFVPMKQLVIDFFTFNANFWPVFWVMFFAICTYGNAGWMRSIMC

IHMCPYARFQSAMFDKDTFIVGYDTTRGEKRGPRSRKADPKQLGLGDCIDCDLCVQVCPT

GIDIRDGLQYECINCGACIDACDNTMDRMGYEKGLISYTTEHRLSGKHTKVMRPKLLGYG

AVLLVMIGLFFAQIAAVDPAGMSVIRDRNQLFRVNSAGEVENTYTLKVINKTQQVQEYNL

DVKGLNDVSWYGKQTIQVEPGEVLNLPMSLGADPDKLNSAITTIQFILTDKSNEFTIEVE

SRFIKKL

>tr|Q87FN2|Q87FN2_VIBPA Hypothetical multidrug resistance protein D OS=Vibrio parahaemolyticus serotype O3:K6 (strain RIMD 2210633) OX=223926 GN=VPA1647 PE=4 SV=1

MNRPINFRTILLACLIISVGQLSMGLVMPSLPWIAKDFSVSLDQAQLLVSIYLLGFGPSQ

FIYGPMSDALGRKKVLLAGLLIAMSGLLMIIFWSHTFTGMVLGRFLQGLGTGCCAVLARA

STRDQFSGNELPVAMSYIAMAASITPLIAPVIGGFINFHFGWSMVFISLLGYVSLAWIII

AFRFKETITRRSAIPSPAKMLVQYRDLLSSRYFMSFASISWLNFSLMITTVSVMPFIMQD

QIGMTSDEYAMWALIPALGMLGGTTICNRIRPVIGNKKMLLCTPVLHISAALWLFFCPVE

PLYLMIGQLLMILGNGIALPCAQALVMQPYKEQAGAAAAMSGGGQMVVSSLVSMTLVQLG

LSEAWHLSLVVVLFTAITLTNILRGFNTEEAQQHREVAEKAS

>tr|Q87NT6|Q87NT6_VIBPA Uncharacterized protein OS=Vibrio parahaemolyticus serotype O3:K6 (strain RIMD 2210633) OX=223926 GN=VP1782 PE=4 SV=1

MHFMTTQTKALDAPLKINTQHIIVSLISVATFIFFTTIGLNHEEGEAYGWMSLLPTALVL

VFALTTHRTVEALFSGAIAGVLLLNPTEAVEQIVDISMSVMMDETIAWIILVCGLMGGLI

AVLEKGGSILSFSDMLVTKVKSRKQSMLMTFALGILIFIDDYLNAIAISSSMKKVTDGYK

ISREKLSYLVDSTAAPICILVPISTWAIFFSSLLEANGVAEAGQGIQTYMQAIPYMAYGW

VTLVIVLLVAMEKIPDLGAMKKAEERAKNGQVRPDGATDVDFGSEISAHTNPTMGLINFL

LPMIVLVAASWYFGIDLLAGVFVALIFTISFYGAQKLISMNDMFEAVYDGIKVMLLPLAT

VIGGFMLKSVNDSLGVTQYVIETVSPYLSASYFPAIIFMITGALVFATASSWGTFAVAMP

IVLPLGEQLGVPLHLTVAALLSASAAGSHSCFFSDSTVLSAQGSGCTSMQHATTQFPYAL

IGIVATAIFFFIIG

>tr|Q87HX9|Q87HX9_VIBPA Phosphoglycerate transport system transcriptional regulatory protein PgtA OS=Vibrio parahaemolyticus serotype O3:K6 (strain RIMD 2210633) OX=223926 GN=VPA0827 PE=4 SV=1

MSNNPYSVLLVDDDQDVLDSYSYLMNISSIKSKAINDPTQALQYVSPEWAGVVILDMYMP

QMHGLELLRMIKNVDERIPVIVITGHGDIPMAVDAVKQGACEFLEKPINPAELLTLVKQQ

LETRSSQVELQLQAEKSISRSLIGKSAHMEQIRKLVAQYALLDTHVVVYGESGTGRHSVA

GLIKDMMSKSEETEYGSFALSANTTMDSIDDAIDKQQSCLLVLENLPELPEEGQRHLAQL

LLARERCGKKNLRVVTIFDSEPEEYITKNQLLPELYYLLNQGVVHVQPLRQRPDDIVTIF

HYFLKLSCKKLGKTLPNVDSSYLALLRGYPWPGNIRELRNIAELYAIGIVKLTGKERIYS

QNEIQLPLDDLVDDFEKQLIEDALFLHSGRITDAANHLQIPRKKLYLRMKKHGIEKGNYK

SR

>tr|Q87RP5|Q87RP5_VIBPA PhoH family protein OS=Vibrio parahaemolyticus serotype O3:K6 (strain RIMD 2210633) OX=223926 GN=VP0732 PE=4 SV=1

MSNKIVTLEINLEPSDNRRLASLCGPFDDNIKHLERRLGVEINHRSDHFTIVGKPHTSAA

ALDILKTLYVDTAPVRGEIPDIEPEQIHLAIKESGVLEQNTESSIAHGKEVFVKTKKGVI

KPRTPNQAQYLVNMVTHDISFGIGPAGTGKTYLAVAAAVDALERQEIRRILLTRPAVEAG

EKLGFLPGDLSQKVDPYLRPLYDALFEMLGFERVEKLIERNVIEVAPLAYMRGRTLNDAF

IILDESQNTTVEQMKMFLTRIGFNSRAVITGDVTQIDLPRGAKSGLRHAIEVLSEVDDIS

FNFFQADDVVRHPVVARIVNAYEKWEAQDQKERKEFEKRRREERDAKLLEAAKAELSAQV

AATKE

>tr|Q87IN3|Q87IN3_VIBPA Transcriptional regulator, LysR family OS=Vibrio parahaemolyticus serotype O3:K6 (strain RIMD 2210633) OX=223926 GN=VPA0573 PE=4 SV=1

MNLTQVEAFCTIAECGSVSEAARQLDCNRTKLSMSIKALEKELDVELFTRTGNQLTLSEA

GKAIYKDCEYLLVTAQRIRKTCAQISEGFNAEVWVARDDSLPDDLWQELSHRLNNRFPST

TFNLILASSGDLANLVSTHQVDFAFGVDYERIDDPHIVYKPLGKIRMMSVCSVDHRLSKM

RRVSDEDLRSQMQALMVYLNEKDNPGLQPFSTRYIGFSSFDYMLNTILEEDAWGVLPEPL

IRHYLRQQKLAVIKHTYGLTQEDYCMFAPNGQIEHPAMTWLADKLSEYLFDF

>tr|Q87KF5|Q87KF5_VIBPA ThiH protein OS=Vibrio parahaemolyticus serotype O3:K6 (strain RIMD 2210633) OX=223926 GN=VP3022 PE=4 SV=1

MSFYDRFQQLDWDDISMSIYAKTAQDVERALANPKRDLEDFKALISPAAEPYLEQMAKLS

YSLTRKRFGNTMSLYIPLYLSNLCANACTYCGFSMENRIKRRTLNRDEVEAEIDAIKRMK

FDSVLLVTGEHETKVGMKYFREMVPIIKQRFNYLAMEVQPLDQDEYAELKTLGLDAVMVY

QETYHPKTYAQHHLRGNKMDFRYRLETPDRLAKAGIDKIGIGALIGLEEWRTDCFFAAAH

LDYLERTYWQSRYSISFPRLRPCAGNAPASGLQPKSVMTDKQLVQLICAYRLFNPEVELS

LSTRESPQFRDNVLPLGITSMSAASKTQPGGYATEEVELEQFEISDERSAASVEDMIRAK

GFDPVWRDWHSAYSG

>tr|Q87Q44|Q87Q44_VIBPA Bifunctional adenosylcobalamin biosynthesis protein OS=Vibrio parahaemolyticus serotype O3:K6 (strain RIMD 2210633) OX=223926 GN=VP1306 PE=3 SV=1

MKQLILGGARSGKSKLAEQTARQLSEKQNKSLHYVATALPFDDEMRERIKHHQAQRGEGW

HEHECHLRLPDLLAHFDANDVVLVDCLTLWLNNWIFELGEACSNELLEQEIEKLTQAVEN

SRATLIFVSNEVGMGIVPLGAVSRYFVDNAGRMNQQLAQVCSRVTFVSAGLPLVLKE

>tr|Q87JM8|Q87JM8_VIBPA Uncharacterized protein OS=Vibrio parahaemolyticus serotype O3:K6 (strain RIMD 2210633) OX=223926 GN=VPA0220 PE=4 SV=1

MKTPSVLSLLLALAPTFAFSHNLSVGQTTPSVSIDTYGEMTLQGDDVAYQPWATQQMLGK

VRVIQAIAGRSKAKEMNAPLMSAITAAHFPEDQYQTTTVINQEDAIWGTGTFVKSSAEDS

KKTFSWSSMVLDKNGVLATTWDLQQESSAIIVQNKQGEILFVKEGALTQDEVTHVIELIK

QNI

>tr|Q87P86|Q87P86_VIBPA Putative outer membrane protein OS=Vibrio parahaemolyticus serotype O3:K6 (strain RIMD 2210633) OX=223926 GN=VP1632 PE=3 SV=1

MARKSGVLIMKNITIIALSLVVAACSSSSDRGDEYDYIDTPIADQWADHQDDDSDGVINQ

RDLCPGTPLGAEIDNDGCGSYANSSQQMQIRVLFANDSDEINPVFAKQIRELSDFLKEYP

STSIELQGYASRTGGSEHNLDLSKRRAENVRKALLQNGITPDRVTIVGYGDTVLATTGTD

EVSHALNRRVTATVVGHKGEVKKEWTIFTTLPKS

>tr|Q87S50|Q87S50_VIBPA Phosphate ABC transporter, permease protein OS=Vibrio parahaemolyticus serotype O3:K6 (strain RIMD 2210633) OX=223926 GN=VP0574 PE=3 SV=1

MAQAEFSLQEKDKKRLIKDRLVRLAVTSGGVGVLAALILIFVYLAMVIIPLFSDAEIKPN

HATRTTQVGMPLAISVDDYSQLAFVLTQSGEIQYLSMDAPDKPAIYTQQLATNPVSFSQS

APGLGWYGLVDDQGLAHIFKPEFNATLRENTRPPEVVALSTDMNLTLTDAQDPVTQFVFS

ASTQTPTIVWQTRSGKVKARWQEKSALGVAPTTIDFSFSAGFDAPQQMLLTPDGETLYLR

DGSELIVLTKAAQKFTVREVIDLTQGDKKHSVRTIDLLAGAYSLLVTHNDGRVSQWFDTL

QNDKRTLTHIRDFKLASELKYLLPDSHRKGFYSFYTNGTLQSHYTTSEKLVLFKRAYKQA

PAMAAMSNNERYLITWNDDSLKVAEVDNSYPEVSLSSLWQKVWYEGYPEPEFVWQSTSAS

DNFEAKFSLVPIAFGTIKAAMFAMLFSVPLAVLGAIYTAYFMSPRMRRVVKPSIELMEAL

PTVIIGFLAGLWFAPIVEDHLITVVVMLFVLPLSTMVMGGLWALIPQSLRNRLPNGWHAL

VLMPVILLLIGIGVWISPSIEQTFFGGDMRLFLTNHGIGFDQRNSLVVGLAMGFAVIPTI

FTIAEDAIFSVPKHLSDGSLALGATPWQTLIYVVLLTASPGIFSAIMMGLGRAVGETMIV

LMATGNTPLMDWNILEGLRSLSATIAVELPESEVGSSHYRLLFLAALILFVFTFAVNALA

ELVRQRLRDKYRAL

>tr|Q87QD6|Q87QD6_VIBPA Uncharacterized protein OS=Vibrio parahaemolyticus serotype O3:K6 (strain RIMD 2210633) OX=223926 GN=VP1213 PE=4 SV=1

MYKPVTSQNLALFFSLAPMLISAPSFAQQNDIYTTQNGHAIVQDVEKPGTRIEFDEDQDE

VYRAVQKGYIRPFSELYAAVENDLYGRIIKVELEEDDNEWVYELKILFNNSVLKVEYDAA

TLEMLEVKGRNFNKALKPPQN

>tr|Q79YY5|Q79YY5_VIBPA Hook-associated protein type 3 FlgL OS=Vibrio parahaemolyticus serotype O3:K6 (strain RIMD 2210633) OX=223926 GN=VP0786 PE=4 SV=1

MLTRISSFHNYQSVQNDLRRQENKIHHNQEQLASGKKLLKPSDDPLAAHYIQNIGQQQEQ

LKQYLSSIVLVRNRLENHEVNIANAESFADESKRLTMEMINGAFSAEDRQAKKRELEEIA

NNFLNLVNAQDESGNYVFAGTKPKSQPFYRDKDGSVQYAGDDYQRKMKVSSMLDMPMNDP

GSKLFMEIPNPFGDYQPSYDLQSGSDLLLSKATNVDAKDTASYRVTFVDMNNGKFGYQLE

RNGKVVDADEFSPEKGIEYKGLKVHVKGQITPGDSIGIEKRESFSIFDTFKEAMSWSDKS

VSDTSATAKLHQMTEEFQAAFIHLNKARTDVGARLSTLDIQEQNHEDFNLSLAKAKSNFE

DLDYSKAVIEFSENSRALQASQQAFGKTKDLTLFNYI

>tr|Q87TK2|Q87TK2_VIBPA Putative LysR-family regulatory protein OS=Vibrio parahaemolyticus serotype O3:K6 (strain RIMD 2210633) OX=223926 GN=VP0067 PE=4 SV=1

MMKFARTQCMKSEPFERVTLKMLRYFYEVAQTKHFTKAAENLNITNSPLSNRIKELEEVL

NVKLFERDSRNVMLTEAGRLLESKCQLIFQTLDVSLNQVQQVGLNQRNILRVGVVSSAFW

GGFDRLLQRFHAEHGTYHADIFDLNPETQKKHLSEKKIDVGIVRFADALNIYPYQSVSLG

KDVFVVAVSEQHELASKSLLQLQDLRACEFAFMSLANSASANFIINACLQEGFSPHIGKQ

VVEPSTLMAYVANSNTVTLVPSTFSQHHWHGIRFIKLKESLRADLCLIHDGKQESAIKAE

FIRTLLENAS

>tr|Q87S00|Q87S00_VIBPA Putative transcriptional regulator, LysR family OS=Vibrio parahaemolyticus serotype O3:K6 (strain RIMD 2210633) OX=223926 GN=VP0624 PE=4 SV=1

MMRSNVISGLWLFNQVAEHASFTGAAKHLHLTTGAVSQQIIHLEESLGFTLFERHSRGIL

LTEKGIQLQQATQLHFSELGKLLDDLRVEKHINEVRLKLTPSFAFKWLVPKLESFQLENP

EIQVQIFAEGALVNSDLKDYDVAIDYGLHPYRDHDAELILDEKLLPVMSPKYLEDHRWLK

TPFGDQTSSMSANQWQAATLLHDAMPWEKATRDYEWLYWASAMGFDFKTDVGHFFNRTDM

AMSAAEAGVGIAMARMALIEDELTTKRLVSPFAPIPANAGYYLIMNTRSQSTERFREWLL

KQIESRR

>tr|Q87KJ8|Q87KJ8_VIBPA GGDEF family protein OS=Vibrio parahaemolyticus serotype O3:K6 (strain RIMD 2210633) OX=223926 GN=VP2979 PE=4 SV=1

MRAFSKRVHDVSQLNDFLVTSQLHPDKQYLVQILSTQSRECAAALAKAVLSMLPNAHVIG

HSTRNVILDGDIFTSGCLINLIEFGQTTLTSAVQAYSFSPDIDGGELKRSLQLQSNSQVI

ISFAVQIERRDYPLYQTFATENVRVSGGLAQTIEDGCWVLYQNQTYDNAVVAVALHSDTL

KTWTDAYSEWNPIGMPHKVTHAKGTRLYCLGERKALDVYKHYLADGHDVTINQLLSFPLY

RETLGRKEVCTVTELHVDGSMSFDRPWCLGDEVQFCYNHPSLTLEQVRHGVVELAMHQPE

VVMIYNCASRLDFIDSSDEVQAFMDIAATFGSYCMGEIHGDIGQPEILHHSLTYLAMREG

DEVQSLNLPKPQVSASISPLFSLIRNAIGDLNHMNAHMGYQLDRQTRKLQESYRRDSRTG

LQNRVALQERLSHIDCDEHLLTLKLLNFSHINEKYGYRVGDQLLKDLSMHFTNRLRKRLG

MPAALQLYSIGVGEWAFVFKAEQSSETIKQHFTRFADAVEQTNFEPLGLREIDYLSISLC

GGLVSRRDFPSASPDELLLKGIEARRSGVRSNTHICNAKDIEVSEDVRKEQLGWLSCVSR

AILKQNIITYSQPIVHAYSHLPASQECLVRILEEDGTVIAPGRFLPIISDTHLYTRLSRH

MIRSTLHYMRDKKGDFSINLSPQDLLSDKTLMLLEHEISQLNDPTRIGLEVLESEQIKDY

GRMIEVCSHFRKLGARIIVDDFGSGYSNIDEILKLEPEVIKLDGSLIRDIDKDLKQRKIA

SQLVRLCQVFNAETVAEFVHNREVCKIAQDMGVDYLQGYYLGEPTRLF

>tr|Q87RK1|Q87RK1_VIBPA PTS system, glucose-specific IIA component OS=Vibrio parahaemolyticus serotype O3:K6 (strain RIMD 2210633) OX=223926 GN=VP0793 PE=4 SV=1

MGLFDKLKKLVSDDSADAGAIEIIAPLSGEIVNIEDVPDVVFAEKIVGDGIAIKPAGNKM

VAPVNGTIGKIFETNHAFSIESDDGVELFVHFGIDTVELKGEGFTRIAEEGQTVKAGDTV

IEFDLALLEEKAKSTLTPVVISNMDEIKELNKLSGSVTVGETPVLRVTK

>tr|Q87J98|Q87J98_VIBPA Putative transcriptional regulator OS=Vibrio parahaemolyticus serotype O3:K6 (strain RIMD 2210633) OX=223926 GN=VPA0355 PE=4 SV=1

MKTTGAGQLGTQLGKIKTSLRQQIHTGVVSQGQKLPSERELSELFSTTRITIKDALVSLE

TEGLIYREERRGWYVSPERICYNPLSRSHFHQMIREQHRIAATQLISVRSEMAAGDYAKA

LDIEQMTPIHIIERLRCIDGRAVLFVENVLKASLFDGILAENLTMSLTGIYREKYGYETM

RSRFDVIPTSAPAHVAKALNLAEGQPVLKICRVNYKQDGELMDCELEYWRPDAVMIRIDS

VG

>tr|Q87GG1|Q87GG1_VIBPA Putative type III secretion system EscV protein OS=Vibrio parahaemolyticus serotype O3:K6 (strain RIMD 2210633) OX=223926 GN=VPA1355 PE=4 SV=1

MMAKLRKSSLLFNPSSVMIAFLLPTIILVTLPGIVIDFFLMISFISSALLLVIMLENDEP

LKVTFLPTMVLLLTTFRLLLSIATTRNIIANEDVGQVIETVGQFVMGGNLLSGLLIFIII

TIVQFLVVTKGGERVAEVGARFSLDALPGKQMTIDGDLKSGLITGEQAQKLRADLGTENR

LFGSLDGAMKFVKGDAIAGILISLVNLFGGIYVGINQFDLSLGDSVSRFSVLTVGDGLVS

QIPSLLLSMACGVYLTRIKGSDDESSSFMSQLMLQIRTFWKSLFVIGGIIIVLGIANPNL

LYVCLPLAGVCAALAIWLQKKDGSKNPTLSLEPQGHGLFQSLKFVFREEQYQHLIQRHVE

EKEKSLWGQALGLPAVVVDPTLDCDIKVYISDIEMYSYKNTEPGVVIEILNDELLMSSHD

VVSNINHIISYFIHNRFVEKYNLQYSSNIVTGLEQQSEIMKSEIDSAVGLNRVHDVLKQM

IRSPEFYLDRVSFFEALIYWSRVESDPRNWLTRMRSQARYDITSKLLNSEGKFNVILLTP

ELTEEISGFVAGEFDDAERLISVQTTLRSEIKRLLFQYGSKPVVVVNENELDYIKSFFQQ

VMSRIFVCSHADIVNSNCIENTDTIHI

>tr|Q87S55|Q87S55_VIBPA DNA-binding response regulator PhoB OS=Vibrio parahaemolyticus serotype O3:K6 (strain RIMD 2210633) OX=223926 GN=VP0569 PE=4 SV=1

MSRRILVVEDEAPIREMLCFVLEQKGYQAVEAEDYDTAVTKLAEPFPDLVLLDWMLPGGS

GINFIKHMKREELTRNIPVVMLTARGEEEDKVRGLEVGADDYITKPFSPKELVARLKAVI

RRVTPTALEDVIDVQGLKLDPVSHRVTANDEAVDMGPTEFKMLHFFMTHQERVYSREQLL

NNVWGTNVYVEDRTVDVHIRRLRKALEAAGHDKLIQTVRGAGYRFSTKA

>tr|Q87RG2|Q87RG2_VIBPA Flavodoxin OS=Vibrio parahaemolyticus serotype O3:K6 (strain RIMD 2210633) OX=223926 GN=VP0835 PE=3 SV=1

MASVGIFFGSDTGNTEAVAKMIQKQLGKHLVHVQDIAKSSKEDIDNFDLLLLGIPTWYYG

EAQCDWDDFFPELEQIDFSTKLVAIFGCGDQEDYAEYFCDAMGTVRDIVESKGGTILGHT

STEGYEFEASKALVEGDDSQFVGLCIDEDRQPELTDQRVENWVKQIYEEMCLAELEG

>tr|Q87TP1|Q87TP1_VIBPA Zinc-binding alcohol dehydrogenase OS=Vibrio parahaemolyticus serotype O3:K6 (strain RIMD 2210633) OX=223926 GN=VP0028 PE=4 SV=1

MFNALVLNQEDKRTIANIEQIDETQLPEGDVLIDVDYSSLNYKDGLAITGKGKIIRNFPM

VPGIDLAGTVVSSDDARYQAGDKVVLTGWGVGENHWGGMAQRARLKADWLVPLPKGLDSK

KAMMVGTAGFTAMLCVQALLDADIKPEAGEILVTGASGGVGSVAVTLLAQLGYKVAAVTG

RVEQNGPLLEKLGASRIIDRVEFEEPARPLEKQIWAGAVDTVGSKVLAKVLAQMDYNSAV

AACGLAGGFDLPTTVMPFILRNVRLQGVDSVMCPTEKRIAAWEKLVELLPDSYFEQACTE

VELAEAPKYAEDITNGQVTGRIVIKL

>tr|Q87QC3|Q87QC3_VIBPA Uncharacterized protein OS=Vibrio parahaemolyticus serotype O3:K6 (strain RIMD 2210633) OX=223926 GN=VP1226 PE=4 SV=1

MRKLRHTWKGKFVFKEGNCMLWPVVKALLGHYRRYPLQIILVWLGLTLGVSLLVGVTSIN

QHAKQSYETGEKLFSNPLPYRIRTKHSANKIPQGFYIQLRRAGFNQCAPFDVLHLDAKTD

MNLMLVGIDPVAMIPLQHGKSLNEMPVLSLMKPPYPILISQDLASYMNWQDGDFIELNDG

SRLGPVQVDQNKLLSGTRLVTDISLLRMLKRSSGLSAISCGEMPEEKLIALKNILPNGMT

LVRNTRTELESLTKAFHMNLTAMGMLSFLVGLFIFYQAMSLSFIQRQRLVGILRQTGVNG

TQLAQALLLELSILVLVAWACGNFFGLMLANELIPAVSSSLSDLYDANVGLTIGWSWQSS

LYSLIMSAMGALISCLWPLIRLLKSQPIRLSSRLSLMRFAGREFSWQALAACAFCVAAVA

VYQAPKTQETGFAIIALMLVSVALFMPFLMWHMFQSFSYTLRWVRVRWFFADAAASMSYR

GVATMAFMLALAANIGVETMVGSFRDTTDKWLSQRLAADIYIYPTNNSAGRMSAWLQDQP

EVESVWWRWEKDVPTEHGALQVVSTGPSEGELDSLTVKLGVPNYWYQLHQTKSLMVSESM

ALKLDIRPGDYIDLQAPLGEGWQVVGVYYDYGNPYNQVMMSHRNWLYAFAGTGNVGLGVL

LKDSVNGEGLKNRLENVFRLPQDRVFDNSNIYNQAMRVFDHTFAIAGTLGNITLIIAVFG

LFFATLAGELSRQRHFSLLRCMGVSGKELVALGGLQLFAFGAISAIIAVPLGLALAHLIV

DIIIKQSFGWSLELQTIPWEYAQTIAWAMLAIMIAGALPVIRMIKSTPMKSLRDAL

>tr|Q87P69|Q87P69_VIBPA Transcriptional regulator, GntR family OS=Vibrio parahaemolyticus serotype O3:K6 (strain RIMD 2210633) OX=223926 GN=VP1649 PE=4 SV=1

MNSELKARYKVLGSEKEHTKSETLTETLIEVIVNGDVEPGSKISEPELARKYQVSRGPLR

EALMRLEGLGLIERIPHVGARVTTFSPAKLIELYSVREALEGMAARLAARHITQEELLSL

EMLLSTHSKHIDQVEGASYFHQHGDFDFHYRIIQASRNSKLISLLCDELYHLLRMYRYQS

PRAQSRPNEALTEHKFILEAIRNRDEELAEMLMRRHISGSRKLIQSQIIHTESLDKD

>tr|Q87JY1|Q87JY1_VIBPA Uncharacterized protein OS=Vibrio parahaemolyticus serotype O3:K6 (strain RIMD 2210633) OX=223926 GN=VPA0117 PE=3 SV=1

MKVVIAPDSFKESLTAKQVSEAIKTGLARVWQDAEFVTVPVADGGEGTVQSLIDATQGEQ

VFTTVTAPLGNDVEAFYGILGDGETAVIEMAEASGLHLVPSDARDPKLTSSIGTGQLIKH

ALDRGIQRLIIGLGGSATNDGGVGMLTALGVTFLDESGHAITPNGGGLAALASIDISGLD

PRLAACEVLVACDVDNPLCGDKGASAIFGPQKGATASDVTLLDNALRKFGELTEQATGKH

VLTREGAGAAGGMGAALLGYTPARLQPGIEIVLETVKLAQHVADADIVFTGEGRIDHQTA

HGKTPMGVAKVAKQFDLPVIALAGCVGDNYQAVYECGIDAVFACVPRAMSLSEAMKEADV

NVANLAENVARMWQIKSH

>tr|Q87NC7|Q87NC7_VIBPA Putative carboxynorspermidine dehydrogenase OS=Vibrio parahaemolyticus serotype O3:K6 (strain RIMD 2210633) OX=223926 GN=VP1941 PE=4 SV=1

MAILQIGAGGVGWVVAHKAAQNNDVLGDITIASRTVGKCEKIIESIQKKNNLKDSTKKLE

ARAVNADDVDSLVALIEEVKPDLVINAGPPWVNMPIMEACYRAKVSYLDTSVAVDLCSEG

QQVPEAYDWQWGYREKFEEAGITGILGAGFDPGVVSVFAAYAVKHLFDEIDTIDVMDVNA

GDHGKKFATNFDPETNMLEIQGDSFYWENGEWKQVPCHSRMLEFEFPNCGSHKVYSMAHD

EVRSMKEFIPAKRIEFWMGFGDRYLNYFNVMRDIGLLSPDPLTLHDGTVVQPLHVLKALL

PDPTSLAPGYTGLTCIGTWVQGKKDGKERSVFIYNNADHEVAYEDVEHQAISYTTGVPAI

TAALQFFRGKWADKGVFNMEQLDPDPFLETMPEIGLDWHVQELEPAQGLPVIHELKK

>tr|Q87HI1|Q87HI1_VIBPA Uroporphyrin-III C-methyltransferase OS=Vibrio parahaemolyticus serotype O3:K6 (strain RIMD 2210633) OX=223926 GN=VPA0984 PE=3 SV=1

MTTANSSLNSGFVSLVGAGPGDPDLLTVKGLKAIQAAEVVVYDRLVSKEILELAQASAEM

IYVGKKLDFHCVPQDQINQILVEKAQEGKRVVRLKGGDSFIFGRGGEELEELAEYGIQYE

VVPGITAAAGATAYAGIPLTHRDHAQSVQFITGHVQKDGREIEWHSLAQSNNTLVFYMGL

KQSGHIMDKLITHGLDPEMSCAIIENGTRPEQRVFQGKLNELSSMAEQAVSPALIVVGSV

TQLHNKLAWFGK

>tr|Q87IT6|Q87IT6_VIBPA Multidrug resistance protein D OS=Vibrio parahaemolyticus serotype O3:K6 (strain RIMD 2210633) OX=223926 GN=VPA0520 PE=4 SV=1

MRKTPLLLAMMIIATGQVGVSIYLPSLPLIGHDLNLPQPSIQSLVTLFLVGFGVSQLFYG

PLSDAIGRRPVFILGQSICLIGTVICIVFGDNISALEAGRLLQGLGAGSASVLGRSVLRD

SYDGMQLTKALSYISVTASVMPIVAPLFGGWIAYHFSWQAVFVFVLLYLLAIFILGYLVL

PETLPYPKRKFEVRQVMVNYFYLLTNKQVIGSASYNWLSYLSGLVTLSLYPFLMQQELGL

TAADYGSLMIIPSAGLLMGSLVLNLINNRYKAQQILLMSFAIVALSGCLLLVTPFTVASL

LVAFTCLSFAQGMSFPVSISLLLSPHKQQAGAVSALSGSIQMCLAGLFGGYLVEKWVTDQ

HQLGIFYLFIALISCIVLVVSQYRRAPRQTAQPEASS

>tr|Q87M89|Q87M89_VIBPA Membrane-bound lytic murein transglycosylase A OS=Vibrio parahaemolyticus serotype O3:K6 (strain RIMD 2210633) OX=223926 GN=VP2369 PE=4 SV=1

MLKKLLPIISLSLLFGCAQQNDRAQQYLDGEFPQILNKVDVVESNKPRDFTEFNKQAEQV

VMKSPSMAKIYQPLYQRLSEWAQQSGDTSALSAFGIQAAQLGGGDKKGNVLFTGYFSPVM

ELRHTPNDIFKYPVYGKPNCTSDCPTRAEIYNGALDGQGLVLGYAPNRIDPFMMEVQGSG

YVHFEDDDTLEYFAYAGKNNKAYVSIGRILIERGEVPREKMSLKAIKEWVMDNDDATVRE

LLEQNPSYVFFAPKAEAPVTGSAGIPLLPMAAVAGDRSILPMGTPILAEVPLLNADGTWS

GAHQLRMLIVLDTGGAVKQNHLDLYHGIGARAGTEAGHYKHFGRVWKLGLENSPTQAPWA

LPPEKLQ

>tr|Q87KB4|Q87KB4_VIBPA Transcriptional regulator, LysR family OS=Vibrio parahaemolyticus serotype O3:K6 (strain RIMD 2210633) OX=223926 GN=VP3063 PE=4 SV=1

MFSKASLEMLDTVARLGSFTAAAEVLHKVPSAISYGVRQVEQDLDVVLFRRLPRKVELTP

AGELFMAEARSLLRQMEEVKAQTRRAAHGWKTTLKLTLDNVVKLEKLKPLVEDFYREFEF

AELQINMEVFNGSWEAIAQGRADIVVGATSAVPVGGDFEVKDMGVLDWAFVMSPAHPCVR

QQVLTEEFVSQFPAICLDDTSNVLPKRHTGHYAKQRRLLLPNWYSAIECLKNGVGVGYMP

RHIAMPLIHEGVLVEKLLQDDKPLSRCCLVWRKDDDHKLIQWMVDYLGSPNQLHQDWLQC

>tr|Q87S78|Q87S78_VIBPA Phospho-2-dehydro-3-deoxyheptonate aldolase OS=Vibrio parahaemolyticus serotype O3:K6 (strain RIMD 2210633) OX=223926 GN=VP0546 PE=3 SV=1

MQKSELSNINISDEQVLITPDELKVKLPLSEKARRFIQESRQTIANIIHKKDHRLLVVCG

PCSIHDIEAAKDYAKRLKALSEQLSDQLYIVMRVYFEKPRTTVGWKGLINDPHLDGSFDI

EHGLHVGRELLVELAEMEIPLATEALDPISPQYLADTFSWAAIGARTTESQTHREMASGL

SMPIGFKNGTDGSLATAINAMQAASSSHRFMGINREGQVALLTTQGNPNGHVILRGGKQT

NYDSVSVTECEQEMAKSGLEASLMVDCSHANSRKDYRRQPLVAEDVIHQIREGNKSIIGL

MIESHINEGNQSSELALDEMKYGVSITDACINWESTEALLRHAHEELVPFLENRLKG

>tr|Q87KD2|Q87KD2_VIBPA Potassium uptake protein TrkA OS=Vibrio parahaemolyticus serotype O3:K6 (strain RIMD 2210633) OX=223926 GN=VP3045 PE=1 SV=1

MKIIILGAGQVGGTLAENLVGENNDITIVDNNADRLRELQDKYDLRVVNGHASHPDVLHE

AGAQDADMLVAVTNTDETNMAACQVAFTLFNTPNRVARIRSPEYLAEKEALFKSGAIPVD

HLIAPEELVTSYIERLIQYPGALQVVSFAEQKVSLVAVKAYYGGPLVGNALSALREHMPH

IDTRVAAIFRQGRPIRPQGTTIIEADDEVFFVAASNHIRSVMSELQRLEKPYRRIMIVGG

GNIGASLAKRLEQTYSVKLIERDYQRAEKLSEQLENTIVFCGDAADQELLTEENIDQVDV

FIALTNEDETNIMSAMLAKRMGAKKVMVLIQRGAYVDLVQGGVIDVAISPQQATISALLT

HVRRADIVNVSSLRRGAAEAIEAVAHGDETTSKVVGRAIGDIKLPPGTTIGAVVRGEEVL

IAHDRTVIEQDDHVVMFLVDKKYVPDVEALFQPSPFFL

>tr|Q87GW3|Q87GW3_VIBPA Polyhydroxyalkanoic acid synthase OS=Vibrio parahaemolyticus serotype O3:K6 (strain RIMD 2210633) OX=223926 GN=VPA1202 PE=4 SV=1

MFQHFFSDYLVKLQETNHQWWHDFEVNKAVVNSPLNKAMQEVNFEDTAKLFEQAANQPAA

ILKLQAQWWEQQLQIWQNVALAGNQAQIIEAEKGDKRFSNEAWQNEAMYSFIKQSYLLFS

KTYLDTIESLEGLDEKTKERIIFFSRQAINALSPSNFIATNPELLKLTLEQNGQNLLAGL

EQLKEDVESSADILKVRMTNNNAFRVGDDVATTAGDVVFQNELFELIQYRPLTEKVNATP

LLIVPPFINKYYILDLTAKNSMVRWLLEQGHSVFMISWRNPGKAQAHVEFGDYVTEGVVK

AVSAIEEITGQEQINAAGYCIGGTVLASTVAYYAAKRMKKRIKSATFFTTLLDFSQPGEV

GAYINDTIISAIETQNNAKGYMDGRSLSVTFSLLRENSLYWNYYVDNYLKGNSPVDFDLL

YWNSDSTNVSAASHNFLLRELYLENKLVQDKGVKIGGVWIDLNKIKIPSYFVSTKEDHIA

LWQGTYRGALHTGGNKTFVLGESGHIAGIVNHPAKNKYGYWLNDTLDDSADEWLSNAEHK

EGSWWTHWNEWLLQYNPQEQVEPFAVGSENYPVIDEAPGQYVKQVLPVKES

>tr|Q87K33|Q87K33_VIBPA Putative LysR-family transcriptional regulatory protein OS=Vibrio parahaemolyticus serotype O3:K6 (strain RIMD 2210633) OX=223926 GN=VPA0065 PE=4 SV=1

MDRVTAAKVFVDVVYSGSFTATAERLDMSRPMVTRYVEAMENWLQTRLLHRTTRKVSLTT

AGERSLPQIERWLKQADTLTSNIAVDGELSGKIRLASSTSFGFSQLIPAVNSFMQKHPKV

HIDIDLQDSVSDLTEQRIDLAIRIASDPDPSLIGKPIAVCESVLVASPQYLQTHHAIAHP

RDLSSHDCLGYKNFERHVWHLSQGDQFESVGIECRLTSNEATALLHGALQGMGISMLPTY

LSNIYLQSGQLESVLPDWKPNDLNIYALYSSRKHLSPAVRAFIDHSEDYFKLHPW

>tr|Q87N66|Q87N66_VIBPA Putative tetrathionate reductase complex: response regulator OS=Vibrio parahaemolyticus serotype O3:K6 (strain RIMD 2210633) OX=223926 GN=VP2009 PE=4 SV=1

MISSEKQLPVYVVDDDESVRDSLAFMLEEHDFNVTTFADGQSFLDEVNIHQAGCVILDSR

MPNLRGQQVHQFLNEAHSPLAVIYLTGHGDVPMAVDALQAGAVNFFQKPVKGDELAQAIL

HGQMQSAASLDMVAARAAYESLTPREKDILRLIIAGKRNIRIADELCIAMRTVEVHRASL

LKKFSAKTVAELAYIYGKLENPI

>tr|Q87NW0|Q87NW0_VIBPA Galactoside O-acetyltransferase OS=Vibrio parahaemolyticus serotype O3:K6 (strain RIMD 2210633) OX=223926 GN=VP1758 PE=4 SV=1

MYANFNLTLVDDTYIYIGNSVMIGPNVTIATAGHPIEPGLRREVAQFNIPVHIEDNVWIG

ANSVVLPGVTIGENSVIGAGSVVTKDIPSNVVAVGNPCRVLREIGEHDREFYFKDRKVEG

NVYSDQEEA

>tr|Q87NE3|Q87NE3_VIBPA NrfE OS=Vibrio parahaemolyticus serotype O3:K6 (strain RIMD 2210633) OX=223926 GN=VP1925 PE=4 SV=1

MEAELGFFLLIFVAMGASCSAVLHWYRRAAAKPIMLEHQLATSHLISLASVVCLLLLIVC

FIQDNFALEYVVTHSNSQLPTAYKVAAAWGGHQGSMLFWVVTLSLWASYIALSSPISQCY

TADCLGIMNVLIAVFAWFTLTTSNPFEFAKTLAVEGRDLNPMLQDIGLIIHPPLLYLGYV

GYSAILAFALAALLSKRPVNEWYRCTRKAAYFAWGTLTVGILVGSWWAYNELGWGGWWFW

DPVENASLLPWLTGTALLHSGVLAKKNQGALWSTYALAFATFSLSILGTFIVRSGVLTSV

HAFAVDPTKGLALLAVLSALVLMTFGVLIVRGDAIKPFRLRSLISRAYMVYVAIGLLVIS

TAIVFLGTFYPMIYQLLGLGNISVGAPYFNSLIFPLSIFALLALAMTPILRWTKGVRANW

KGQFAFASTIALGVVAVFILSVDTVRIGTITSVFLAAWVIATHTMLWLNAENKEPLIKMV

LAHVGFSVAVVGAVFNSEYSYQYNLRVEPGVSHQRANIGIDYQGMDWIIGPNYTAEQARI

ILRLDDQTRYVLQPEKRHYPVRVMNMTEPAILSLWHGDYYLTLGDKVGTNAYAIKVQYRA

GIWWIWSGGLIAVFGALTTVYRKRKRAVNVTQNYA

>tr|Q87IJ7|Q87IJ7_VIBPA Uncharacterized protein OS=Vibrio parahaemolyticus serotype O3:K6 (strain RIMD 2210633) OX=223926 GN=VPA0609 PE=4 SV=1

MPTIAFLLVILGLILLCWGWQMTKNICSKTEVPGWTWLLMLISCFIFAYAAFLHAIMKKP

VASLLETGVSLLLFGGSIFVVLVIKWSNDSILELHSVAEREKRNAVHDSLTGLPNRKYCF

ELIDDRIQSGDPFSVILFDVVNFKQVNDAMGHFCGDQLLIQIGQRLQDKLKGSDKIFRVG

GDEFVILTSSACEKTGTELIESLDCALSARFHLDEFQVSSRVVFGISTYPYDAISNDLLI

KHADIAMYHAKRNGNLFAFYHDDMNVGAKYQLEISSRIQCALEKEEFQLYYQPLIDANTN

LAVGFEAVIRWHDENGKTISPNDFIPIAERSNQVHNITMWVLGQVEKDLKTFIEKGIKLP

VHVNLSAKDLSSNLLFHRLEKLLENNPQFADLISLEITETMAIDRVVELNPLIHQIKGLG

IKISLDDFGTGYSSLSLLRDLPVDQIKIDRSFLCTANRTEGSRSIVENTIALAHGLGYSV

VAEGVQDMDTLYFLRSRGCDIIQGYLFCPALPLNEVINWVNEHDDKQSHTRRA

>tr|Q87FJ7|Q87FJ7_VIBPA Transcriptional regulator, MarR family OS=Vibrio parahaemolyticus serotype O3:K6 (strain RIMD 2210633) OX=223926 GN=VPA1682 PE=4 SV=1

MSQCYSPSTLNADEKLLLENQVCFPLYSAANAVIRAYRPLLDALDLTYSQYLVMMVLWEK

DGTSVKQLGSQLHLDSGTLTPLLKRLEAKGFVSRGRSETDERVRVLNLTEAGRALKDQAK

SVPDAIACKFTLDLEELVTLKTLCEKVLDKLS

>tr|Q87QW7|Q87QW7_VIBPA DNA-binding response regulator TorR OS=Vibrio parahaemolyticus serotype O3:K6 (strain RIMD 2210633) OX=223926 GN=VP1032 PE=4 SV=1

MSYHVLVVEDDVVTRSKLAGYFQNEGYKVSEAESGAEMREVLQGGDVDLIMLDINLPGED

GLMLTRELRSQSDIGIILVTGRTDSIDKIVGLEMGADDYVTKPFELRELLVRVKNLLWRI

SAARSGASKAASETNDEHIVRFGEWTFDIQRRALSRNGEPVKLTKAEYELLVALSSYPNQ

VLSRERILNMISHRVDAPNDRTIDVLIRRMRAKMEFDPKNPQIFVTVHGEGYMFAGD

>tr|Q87FQ9|Q87FQ9_VIBPA 4-alpha-glucanotransferase OS=Vibrio parahaemolyticus serotype O3:K6 (strain RIMD 2210633) OX=223926 GN=VPA1619 PE=3 SV=1

MKEQTALKKVAEMARLADSYVSAWGDEAKVSDETLRRLLTSLGYDTSSDEKLLASAEKKH

KKDVLAPVLVLRDGEPVEVELNLGTSARESEFSWRLETEQGEVLEGYLQSQIVRDERAEG

GPLVFALPKNLAWGYHKLIISRKRRKTPYEMSLIITPKACFKQEDLNQHKKLWGPSIQLY

TLRTQHNWGIGDFGDLKQLVSDIASRGGDFIGLNPIHSLFPANPEGASPYSPSSRRWLNI

MYIDVSSVPEFALSAEAQQRVGSAEFQQRLQKARDSHWVNYTEVSQLKMSVLPLLFSEFK

ARHLDKNTDRARAFLDFVEKGGESLLHQAAFDALHADLHAEDSGVWGWPVFPEKYRTFDA

AGTQKYIKDNQDRVHLYMYLQWIADDQIKEAQALAEEKGMAVGLYRDLAVGVADSGSETW

ADEGNLVLDASIGAPPDILGPLGQNWGLPPLNPQVLEATGYDAYIKLLRANMKHCGALRI

DHVLGLLRLWWIPKGEKATEGAYLYYPVEDMLAILALESHRHQCSVIGEDLGTVPDEIVD

ILRDAGVHSYKVFFFETSKEDGGFISPKHYAEQSMAALCTHDMPTLRGFWHCDDLKMGRE

IGLYPDEKQLEGLFADRLKCKQGILDSVRWHGYLPEGIGHDAQFVPMDSYLSEALQLHVA

AGDSALLSVQLEDWLEMDQPVNIPGTVDEYPNWRRKLSMNLDEIFSREDVNRISKRLTEV

RAQASK

>tr|Q87LC3|Q87LC3_VIBPA Rod shape-determining protein MreD OS=Vibrio parahaemolyticus serotype O3:K6 (strain RIMD 2210633) OX=223926 GN=VP2689 PE=3 SV=1

MASNVLRSRMVIGVSFFVALVLQTIPWPGVLDLLRPSWLFLVTCYWVLALPHRVNVGTAL

VLGLLWDILIGSTLGIRGMMMSIVIYLVALNFLLIRNMALWQQSILIAFFTALLEVLIFC

GEYLNQDVVFNPLSLWTAAINCILWPWMFLLMRRVRRAWHVR

>tr|Q87SF0|Q87SF0_VIBPA Probable membrane transporter protein OS=Vibrio parahaemolyticus serotype O3:K6 (strain RIMD 2210633) OX=223926 GN=VP0474 PE=3 SV=1

MELTLEVLTALFLVASVAGFIDAMAGGGGLLTLPALLAAGLSPTQALATNKLQSSFGSFS

ASWYFVRNGIVSLKEMRFAILCTFIGAAIGAEAVQFIDASVLTSLIPVLLIAISLYFLLA

PSSRKIDGEPKLAEAMFALCIGGGVGFYDGFFGPGTGSIFTVCFVALGHFSLVDATARTK

ILNFTSNIAALLFFVLAGLPVWEIGLTMAVGGFIGAQLGAKVVVTKGQKWIRPLVITMSM

LMATKLLWQQHQQWLLTLF

>tr|Q87N25|Q87N25_VIBPA Endolytic murein transglycosylase OS=Vibrio parahaemolyticus serotype O3:K6 (strain RIMD 2210633) OX=223926 GN=mltG PE=3 SV=1

MIKKLLAFVVLIAVISAAGFFYVVSQTKQYVNSPILIEQPQLFTVENGTSFHRVMRDLVK

ENIVKASDYTRLMPHLYPELLQVRAGTYQLEPNVSLYEALELLNTGKEHQFAITFVEGSR

FSEWLEQLQSAPYVQHDLSGLSEKEMAQKLGIERDKLEGLFLAETYHYTAGASESQLLKR

AHSKLNKILDANWDARQEKLPLKDKYEALILASIIEKETAIDSERERVASVFVNRLNKRM

RLQTDPTVIYGMGDAYDGNIRKKDLRTPTPYNTYVINGLPPTPIAMAGEASIEAALNPES

SDYLYFVASGTGGHVFSKSLAEHNRAVRAYLRELRKNK

>tr|Q87G32|Q87G32_VIBPA Ferredoxin-type protein NapH OS=Vibrio parahaemolyticus serotype O3:K6 (strain RIMD 2210633) OX=223926 GN=VPA1485 PE=4 SV=1

MAKNLAQNAGKEAIEKLGWWRAHRFLVLRRLCQLTIIALFMAGPTLGVLTGNLSSSMLLD

TVPLSDPLIVLQALATGHIPEFNALLGVVIVVVFYAILAPRAFCAWVCPLNIVTDLAAWL

RRKFNIKASYRWSPAIRYWLIPVLMLGSALSGAILWTWLDPVAALHRGLVFGMGAGWVLI

ALVFVLDLLLVEHGWCGHLCPLGAAYGVIGRKSLLRVTAVRREDCTKCMDCFYVCPEPEV

LRQPLKEGDRRVMVQNCISCGRCLDVCPEQVFEFKNRLNVKNID

>tr|Q87NI9|Q87NI9_VIBPA Serine transporter OS=Vibrio parahaemolyticus serotype O3:K6 (strain RIMD 2210633) OX=223926 GN=VP1879 PE=4 SV=1

MNTTTSAVSTAQSSSKFTYKDFTWCLSLFGTAVGAGVLFLPIKAGAGGFWPLVILALIAA

PMTWFAHKSLARFVLSAKNPEADITDTVEEHFGKTGANLITFAYFFAIYPIVLIYGVGIT

NTVDSFLVNQMGMESIPRWLLSGALIAAMTAGVVFGKELMLKATSAMVYPLVFILLALSF

YLIPEWNTSMIEVAPDWAAMPTIVWLAIPIIVFSFNHSPIISQFSKEQRMQYGDEAYKKT

DMITGGAAMMLMGFVMFFVFSVVLSLSPEQLASAKEQNISVLSYLANIHESPLISYMGPL

VAFAAITSSYFGHFLGAHEGLVGLIKSRSQSPVSKIEKGSLLFIVITTWIVAIVNPSILG

MIETMGAPMIAAILFLMPVFAMQKVPAMAKYKTSAPVQIFTAICGLAAITSVIYGAL

>tr|Q87PI9|Q87PI9_VIBPA Putative formate dehydrogenase large subunit OS=Vibrio parahaemolyticus serotype O3:K6 (strain RIMD 2210633) OX=223926 GN=VP1513 PE=3 SV=1

MKLVKRSDSVSKETNQLGVSRRAFMKNTSLAAGGAVVGASLFAPGMMKKAQAKSVDPEAK

TEVKRTICSHCSVGCGIYAEVQNGVWTGQEPAFDHPFNAGGHCAKGAALREHGHGERRLK

YPMKLENGKWKKLSWDQAIEEIGNKVMDIRKESGPDSVYFLGSAKHSNEQAYLFRKMASL

WGTNNVDHQARICHSTTVAGVANTWGYGAMTNSFNDMHNCKSMLFIGSNPAEAHPVAMQH

ILIAKEKNNCKIVVADPRRTRTAAKSDHYVSLRPGSDVAFIWGVLWHIFENQWEDKEFIR

QRVFGMDEIRAEVAKWTPAEVERVTGVSEEDVYQTAKLLSENRPGCIVWCMGGTQHTTGN

NNTRAYCVLELALGNIGKSGGGANIFRGHDNVQGATDLGVLSDTLPGYYGLSEGAWRHWS

KVWEIDYEWIKGRFDDNAYGGQKPMNSAGIPVSRWVDGVLENKDNIRQRENIRAMFYWGH

AVNSQTRGVEMKKAMQKLDMMVIVDPYPTVAAVMNDRTDGVYLLPATTQFETYGSVTASN

RSLQWRDQVIEPLFESKPDHEIMYLLSKKLGYSDLLFKNIRVENNQPVIEDLTREFNKGM

WTIGYTGQSPERLKAHQQNWHTFHKTTLEAEGGPVNGETYGLPWPCWGTPEMKHPGTHIL

YDTSKTVAEGGGNFRTRFGVEFEGKSLLAEDSYSKGCEIKDGYPEFTDKLLKQLGWWDDL

TAEEKAAAEGKNWKTDLSGGIQRVAIKHGCIPFGNAKARAIVWTFPDRVPLHREPLYTPR

RDLVTDYPTWDDKDAIFRVPTLYKSIQEQDKSGEYPIVLTSGRLVEYEGGGEETRSNPWL

AELQQEMFVEVNPKDANDLGFRDGDDVWVEGAEKGRIKVKAMVTRRVKPGLAFIPFHFGG

KFEGEDLRPKYPEGTQPYVVGEAANTATTYGYDPVTLMQETKVTLCNIRKA

>tr|Q79YW4|Q79YW4_VIBPA Flagellar protein FliL OS=Vibrio parahaemolyticus serotype O3:K6 (strain RIMD 2210633) OX=223926 GN=VP2243 PE=3 SV=1

MAEEQLQGADAPKGKSKLLIIIIAVVVLLGGGGAAAFFLMGSDDSAQAAEAESQQTQAAA

ANPIAYVNLPQPFIFNVTGDRRDRLVQIKAQLMVRGSENEQLARYHSPLVESSLLSTFAS

ATVEQLRSPTGRVELRDRASEDIKAALNAAVGKPVIEKVLFTDFVIQ

>tr|Q87M09|Q87M09_VIBPA Na+-coupled multidrug efflux pump OS=Vibrio parahaemolyticus serotype O3:K6 (strain RIMD 2210633) OX=223926 GN=VP2449 PE=4 SV=1

MQNSIYKQFWKYTIPTVAAMLVNGLYQVVDGIFIGRYVGADGLAGINVAWPVIGSILGIG

MMVGVGTGALTSIKQGEQDQQGAKRVLTTGLLFLLALMPIVATILFFFADDFIRWQGAQE

RVYELGLQYLQILIVACIFSLGSIAVPFLLRNDNSPNLATILMVIGAIINIVLDYVFIAW

MNWELTGAAIATALAQMVVTVLGVGYFFSSKAKLRLTFRELKVQLDAIPKIVLIGTSSFF

MYAYGSTMVALHNALFAQYGSPLLIGAYAILGYIVTVYYLVAEGIANGMQPLVSYNHGAR

NQDNIRKLLQIAMGTSVLGGVVFVVLMNLFPYQLVSVFNDADQQLIESAVMGIRLHMFAL

FMDGFLVVAGAYYQSVNKGSKAMFVTVGNMLIQLPFLFIMPKLLGVTGIWIAFPLSNIAL

SLVVMSMLWRDIKKLMVDSPVEPSIQAS

>tr|Q87MG8|Q87MG8_VIBPA Putative amidotransferase OS=Vibrio parahaemolyticus serotype O3:K6 (strain RIMD 2210633) OX=223926 GN=VP2287 PE=4 SV=1

MCELLGMSANVPTDICFSFTGLMQRGGRTGPHRDGWGITFYEGKGFRTFKDPKPSCESQI

AELVQNYPIKSRAVVSHIRQANRGGVNLENTHPFTRELWGKYWTFAHNGQLSDYQDLYTG

RHRPVGQTDSELAFCWLLKQMEDRYPEPPQDMESVFLYIAKCCDQLKEKGVFNMLLSDGE

FVMTYCTNHLYWITRRAPFGKAALLDEDVEINFQEETTPNDVVSVIATQPLTGNETWHRM

KPGEYGLFHLGELIQNNASDLKDVPFAAPKPGNQAPTEPLE

>tr|Q87G20|Q87G20_VIBPA Transcriptional regulator, LysR family OS=Vibrio parahaemolyticus serotype O3:K6 (strain RIMD 2210633) OX=223926 GN=VPA1497 PE=4 SV=1

MRADDLILFSQVIELGSFSKVAEANNLTNSVVSKRMARLEEEIGAQLLYRTTRKLTLTEA

GKVLLHSAKNVKQATQEAMDAVAGFGENVSGHIKMSVPTISGDLILADAVAEFCNLHPGL

TVDMSLDNRFVDLVADGYDLVIRTGYLEDSSLIARHILDSQWVVCASPSYIAKNGKPIEP

IDLTSHNCLQYAYQTTGASEWEFKSDEGNYIVRVSGCFSTDNATALRKAALGGHGIAYVP

RCLVYHDIRNGQLVDIFPELVGKKLGIYAVYPFTRQPPNKVKLLIEHIRERYLTISHYF

>tr|Q87HX1|Q87HX1_VIBPA Inosine-guanosine kinase OS=Vibrio parahaemolyticus serotype O3:K6 (strain RIMD 2210633) OX=223926 GN=VPA0835 PE=4 SV=1

MKFPGQRKSKHYFPTHARDPLVNQIRQAPKLYRPTIVGVGQTIVDIEARVDDAFLEKYNL

SKGHSLVLEESKADALYEELVEQGLITHQYPGDTIGNTLHNYSVLADSKSVLLGVMSKNI

QVGSFAYRYLCRTSSRMNLNHLQTVDGPIGRCYTLISQDGERTFAINEGHMNQLLPESIP

EEVFEKASALVVSSYLMRGKEEDPMPKAVQRAIDFAKKHDVPVVLTLGTKYVIEGNAEWW

QEYIRENVSVVAMNEEEGAALTGEHDPLAAADKALEWVDLVLCTAGPNGLYMAGYTDEKV

KRETTHDILESSIEEFNKFEFSRAMRKEDCVNPMKVYSHIGPYLGGPLEIKNTNGAGDAA

LSALLHDMAANSFHQKEVPTSEKHEVRCLTYSSLSQICKYANRVSYEVLTQHSPRLSRAL

PEREDSLEETYWDR

>tr|Q87I12|Q87I12_VIBPA Putative transcriptional regulator, LysR family OS=Vibrio parahaemolyticus serotype O3:K6 (strain RIMD 2210633) OX=223926 GN=VPA0794 PE=4 SV=1

MGLMEMLEKIDQQWLKSFHCVYENNSFKRAAEFLSLPTSNVSRHIALLEEQLDVRLFDRT

TRRICATDAGEQLYLRTQPLLDKLNDALEEVTRHSREVMGQLNVVMPDSPELAKAVVSFC

AQYLAISLNCETNLSPKEDLLDGFDVIVSFHRGKLEDSNWIAKEIKRWPSVVVASPKLLH

TRQRPFKITDLKHVPCISSFTALKGTPWVFRNSEGELITQRVQSAFKVNSGKLAKSGALA
[truncated: 201,682 more chars]
