# Supplementary material for: Comprehensive genome based analysis of Vibrio parahaemolyticus for identifying novel drug and vaccine molecules: Subtractive proteomics and vaccinomics approach
Source: PLoS One. 2020 Aug 19;15(8):e0237181. doi: 10.1371/journal.pone.0237181 (PMC7444560; doi:10.1371/journal.pone.0237181)
Supplement: S6 File — (DOCX) [file pone.0237181.s019.docx]

**S6 File.** Metabolic pathways for *V. parahaemolyticus* in KEGG server.

| **Entry** | **Name** | **Description** | **Object** | **Legend** |
| --- | --- | --- | --- | --- |
| [vpa00010](https://www.genome.jp/dbget-bin/www_bget?pathway:vpa00010) | Glycolysis / Gluconeogenesis - Vibrio parahaemolyticus RIMD 2210633 | Glycolysis is the process of converting glucose into pyruvate and generating small amounts of ATP (e... | C00033 (Acetate) C00031 (D-Glucose) C00103 (D-Glucose 1-phosphate) C00631 (2-Phospho-D-glycerate) C0... | 4.1.2.13 1.2.1.3 6.2.1.13 1.2.1.5 Acetate Pentose phosphate pathway Starch and sucrose metabolism... |
|  |  |  |  |  |
| [vpa00020](https://www.genome.jp/dbget-bin/www_bget?pathway:vpa00020) | Citrate cycle (TCA cycle) - Vibrio parahaemolyticus RIMD 2210633 | The citrate cycle (TCA cycle, Krebs cycle) is an important aerobic pathway for the final steps of th... | C00022 (Pyruvate) C00122 (Fumarate) C00036 (Oxaloacetate) C05379 (Oxalosuccinate) C00024 (Acetyl-CoA... | 1.8.1.4 1.2.4.2 1.2.4.2 2.3.1.61 6.2.1.5 6.2.1.4 1.1.1.42 1.1.1.41 1.1.1.42 2.3.3.8 4.2.1.2 4.2.1.3 ... |
| [vpa00030](https://www.genome.jp/dbget-bin/www_bget?pathway:vpa00030) | Pentose phosphate pathway - Vibrio parahaemolyticus RIMD 2210633 | The pentose phosphate pathway is a process of glucose turnover that produces NADPH as reducing equiv... | C01151 (D-Ribose 1,5-bisphosphate) C00668 (alpha-D-Glucose 6-phosphate) C00118 (D-Glyceraldehyde 3-p... | 2.7.4.23 5.3.1.9 4.1.2.14 Glycolysis 2.7.1.15 5.4.2.7 4.1.2.4 2.7.6.1 5.4.2.7 2.7.1.15 2.2.1.1 5.3.1... |
| [vpa00040](https://www.genome.jp/dbget-bin/www_bget?pathway:vpa00040) | Pentose and glucuronate interconversions - Vibrio parahaemolyticus RIMD 2210633 |  | C14899 (3-Dehydro-L-gulonate 6-phosphate) C03033 (beta-D-Glucuronoside) C00817 (D-Altronate) C00103 ... | 3.2.1.15 3-Dehydro-L-gulonate-6P 4.1.1.85 2.7.1.53 β-D-Glucuronoside 3.2.1.31 5.3.1.4 1.1.1.13 2.7.... |
| [vpa00051](https://www.genome.jp/dbget-bin/www_bget?pathway:vpa00051) | Fructose and mannose metabolism - Vibrio parahaemolyticus RIMD 2210633 |  | C00247 (L-Sorbose) C00267 (alpha-D-Glucose) C01094 (D-Fructose 1-phosphate) C00159 (D-Mannose) C0027... | 2.7.1.202 Galactose metabolism 1.1.99.21 1.1.1.21 1.1.1.14 5.3.1.5 2.7.1.3 5.3.1.7 1.1.1.11 1.1.1.6... |
| [vpa00052](https://www.genome.jp/dbget-bin/www_bget?pathway:vpa00052) | Galactose metabolism - Vibrio parahaemolyticus RIMD 2210633 |  | C06311 (Galactitol 1-phosphate) C01216 (2-Dehydro-3-deoxy-D-galactonate) C00137 (myo-Inositol) C0009... | 1.1.1.251 2.7.1.200 3.2.1.22 2.7.1.144 2.7.1.11 4.1.2.40 4.1.2.21 3.2.1.85 3.2.1.22 3.2.1.26 2.4.1.6... |
| [vpa00053](https://www.genome.jp/dbget-bin/www_bget?pathway:vpa00053) | Ascorbate and aldarate metabolism - Vibrio parahaemolyticus RIMD 2210633 |  | C00137 (myo-Inositol) C05385 (D-Glucuronate 1-phosphate) C00167 (UDP-glucuronate) C00029 (UDP-glucos... | 3.1.1.- 1.1.1.365 1.1.1.122 3.1.3.93 2.7.7.69 5.1.3.18 5.1.3.18 5.1.3.18 1.13.99.1 2.7.1.43 2.7.7.44... |
| [vpa00061](https://www.genome.jp/dbget-bin/www_bget?pathway:vpa00061) | Fatty acid biosynthesis - Vibrio parahaemolyticus RIMD 2210633 |  | C04088 (Octadecanoyl-[acyl-carrier protein]) C16221 ((2E)-Octadecenoyl-[acp]) C16220 ((R)-3-Hydroxyo... | FabK FabK FabK FabK FabK FabK FabL Octadecanoyl-[acp] trans-Octadec-2-enoyl-[acp] (R)-3-Hydroxy-octa... |
| [vpa00071](https://www.genome.jp/dbget-bin/www_bget?pathway:vpa00071) | Fatty acid degradation - Vibrio parahaemolyticus RIMD 2210633 |  | C02990 (L-Palmitoylcarnitine) C00638 (Long-chain fatty acid) C05280 (cis,cis-3,6-Dodecadienoyl-CoA) ... | 1.3.99.- 1.3.99.- 1.3.99.- 1.3.99.- 1.3.99.- 1.3.99.- 1.3.99.- 1.3.8.1 1.1.1.35 4.2.1.17 Synthesis a... |
| [vpa00072](https://www.genome.jp/dbget-bin/www_bget?pathway:vpa00072) | Synthesis and degradation of ketone bodies - Vibrio parahaemolyticus RIMD 2210633 |  | C00332 (Acetoacetyl-CoA) C00356 ((S)-3-Hydroxy-3-methylglutaryl-CoA) C00207 (Acetone) C00164 (Acetoa... | SYNTHESIS AND DEGRADATION OF KETONE BODIES Fatty acid degradation 2.3.3.10 4.1.3.4 4.1.1.4 2.3... |
| [vpa00130](https://www.genome.jp/dbget-bin/www_bget?pathway:vpa00130) | Ubiquinone and other terpenoid-quinone biosynthesis - Vibrio parahaemolyticus RIMD 2210633 | Ubiquinone (UQ), also called coenzyme Q, and plastoquinone (PQ) are electron carriers in oxidative p... | C16519 (2-Succinyl-5-enolpyruvyl-6-hydroxy-3-cyclohexene-1-carboxylate) C03657 (1,4-Dihydroxy-2-naph... | UBIQUINONE AND OTHER TERPENOID-QUINONE BIOSYNTHESIS Phenylpropanoid biosynthesis 2-Succinyl-5-e... |
| [vpa00190](https://www.genome.jp/dbget-bin/www_bget?pathway:vpa00190) | Oxidative phosphorylation - Vibrio parahaemolyticus RIMD 2210633 |  | C00061 (FMN) C00390 (Ubiquinol) C00399 (Ubiquinone) C00524 (Cytochrome c) C00080 (H+) C00080 (H+) C0... | F-type ATPase (Eukaryotes) V/A-type ATPase (Bacteria, Archaeas) V-type ATPase (Eukaryotes) F-type AT... |
| [vpa00220](https://www.genome.jp/dbget-bin/www_bget?pathway:vpa00220) | Arginine biosynthesis - Vibrio parahaemolyticus RIMD 2210633 |  | C03406 (N-(L-Arginino)succinate) C00327 (L-Citrulline) C00062 (L-Arginine) C00077 (L-Ornithine) C001... | L-Arginosuccinate Citruline Arginine Ornithine Carbamoyl-P Aspartate Fumarate NH3 N-Acetyl-glutamate... |
| [vpa00230](https://www.genome.jp/dbget-bin/www_bget?pathway:vpa00230) | Purine metabolism - Vibrio parahaemolyticus RIMD 2210633 |  | C12248 (5-Hydroxy-2-oxo-4-ureido-2,5-dihydro-1H-imidazole-5-carboxylate) C11821 (5-Hydroxyisourate) ... | 1.17.4.1 5-Hydroxy-2-oxo-4-ureido-2,5-dihydro-1H-imidazole-5-carboxylate 5-Hydroxyisourate 1.17.3.2 ... |
| [vpa00240](https://www.genome.jp/dbget-bin/www_bget?pathway:vpa00240) | Pyrimidine metabolism - Vibrio parahaemolyticus RIMD 2210633 |  | C00063 (CTP) C15607 (3-Oxo-3-ureidopropanoate) C00064 (L-Glutamine) C00438 (N-Carbamoyl-L-aspartate)... | (extracellular) CTP 1.17.4.1 3-Oxo-3-ureido-propanoate 3.5.1.95 2.7.4.22 3.5.4.30 2.1.1.148 L-Glutam... |
| [vpa00250](https://www.genome.jp/dbget-bin/www_bget?pathway:vpa00250) | Alanine, aspartate and glutamate metabolism - Vibrio parahaemolyticus RIMD 2210633 |  | C00122 (Fumarate) C00042 (Succinate) C00036 (Oxaloacetate) C00026 (2-Oxoglutarate) C00041 (L-Alanine... | Fumarate Succinate Oxaloacetate 2-Oxo-glutarate Citrate cycle L-Alanine L-Aspartate L-Asparagine D-A... |
| [vpa00260](https://www.genome.jp/dbget-bin/www_bget?pathway:vpa00260) | Glycine, serine and threonine metabolism - Vibrio parahaemolyticus RIMD 2210633 | Serine is derived from 3-phospho-D-glycerate, an intermediate of glycolysis [MD:M00020], and glycine... | C16432 (5-Hydroxyectoine) C06231 (Ectoine) C06442 (N(gamma)-Acetyldiaminobutyrate) C03283 (L-2,4-Dia... | 1.1.3.17 5.1.1.18 5-Hydroxyectoine 1.14.11.55 1.14.15.7 L-Ectoine Nγ-Acetyl-L-2,4-diaminobutyrate L... |
| [vpa00261](https://www.genome.jp/dbget-bin/www_bget?pathway:vpa00261) | Monobactam biosynthesis - Vibrio parahaemolyticus RIMD 2210633 | Monobactams are beta-lactam antibiotics containing a monocyclic beta-lactam nucleus, which is struct... | C01179 (3-(4-Hydroxyphenyl)pyruvate) C03198 ((S)-4-Hydroxymandelate) C03590 (4-Hydroxyphenylglyoxyla... | MONOBACTAM BIOSYNTHESIS Phenylalanine, tyrosine and tryptophan biosynthesis 4-Hydroxy-phenylpyruva... |

| [vpa00270](https://www.genome.jp/dbget-bin/www_bget?pathway:vpa00270) | Cysteine and methionine metabolism - Vibrio parahaemolyticus RIMD 2210633 | Cysteine and methionine are sulfur-containing amino acids. Cysteine is synthesized from serine throu... | C06547 (Ethylene) C01234 (1-Aminocyclopropane-1-carboxylate) C08276 (3-(Methylthio)propanoate) C1560... | 2.5.1.- Ethylene 1-Aminocyclopropane-1-carboxylate 1.14.17.4 4.4.1.14 MtnE 3-Methylthio-propionate 1... |
| --- | --- | --- | --- | --- |
| [vpa00280](https://www.genome.jp/dbget-bin/www_bget?pathway:vpa00280) | Valine, leucine and isoleucine degradation - Vibrio parahaemolyticus RIMD 2210633 |  | C15978 (2-Methyl-1-hydroxybutyl-ThPP) C15973 (Enzyme N6-(dihydrolipoyl)lysine) C15973 (Enzyme N6-(di... | 2-Methyl-1-hydroxybutyl-ThPP 1.2.4.4 1.8.1.4 Dihydro-lipoamide-E Dihydro-lipoamide-E Lipoamide-E Lip... |

| **Entry** | **Name** | **Description** | **Object** | **Legend** |
| --- | --- | --- | --- | --- |
| [vpa00281](https://www.genome.jp/dbget-bin/www_bget?pathway:vpa00281) | Geraniol degradation - Vibrio parahaemolyticus RIMD 2210633 |  | C03069 (3-Methylcrotonyl-CoA) C16471 (5-Methyl-3-oxo-4-hexenoyl-CoA) C16469 (3-Hydroxy-5-methylhex-4... | 3-Methylcrotonyl-CoA 5-Methyl-3-oxo-4-hexenoyl-CoA 2.3.1.16 1.1.1.35 6.4.1.5 Valine, leucine and is... |
| [vpa00290](https://www.genome.jp/dbget-bin/www_bget?pathway:vpa00290) | Valine, leucine and isoleucine biosynthesis - Vibrio parahaemolyticus RIMD 2210633 |  | C00188 (L-Threonine) C06032 (D-erythro-3-Methylmalate) C02226 (2-Methylmaleate) C02612 ((R)-2-Methyl... | 2.3.1.182 4.2.1.35 1.1.1.85 4.3.1.19 Threonine D-erythro-3-Methylmalate 2-Methyl-maleate (R)-2-Methy... |
| [vpa00300](https://www.genome.jp/dbget-bin/www_bget?pathway:vpa00300) | Lysine biosynthesis - Vibrio parahaemolyticus RIMD 2210633 |  | C19889 (LysW-gamma-L-lysine) C19888 (LysW-gamma-L-alpha-aminoadipate 6-semialdehyde) C19887 (LysW-ga... | 2.6.1.83 Tropane, piperidine and pyridine alkaloid biosynthesis LysW-γ-L-lysine LysW-γ-L-α-aminoa... |
| [vpa00310](https://www.genome.jp/dbget-bin/www_bget?pathway:vpa00310) | Lysine degradation - Vibrio parahaemolyticus RIMD 2210633 |  | C06181 (Piperideine) C05825 (2-Amino-5-oxohexanoate) C05161 ((2R,5S)-2,5-Diaminohexanoate) C00877 (C... | 1-Piperideine 2.6.1.21 2-Amino-5-oxohexanoate 2,5-Diaminohexanoate 1.4.1.12 5.4.3.3 5.1.1.9 1.5.1.1 ... |
| [vpa00330](https://www.genome.jp/dbget-bin/www_bget?pathway:vpa00330) | Arginine and proline metabolism - Vibrio parahaemolyticus RIMD 2210633 |  | C00062 (L-Arginine) C00077 (L-Ornithine) C00134 (Putrescine) C00555 (4-Aminobutyraldehyde) C00334 (4... | ARGININE AND PROLINE METABOLISM Arginine Ornithine Putrescine 4-Amino-butanal 4-Amino-butanoate �... |
| [vpa00332](https://www.genome.jp/dbget-bin/www_bget?pathway:vpa00332) | Carbapenem biosynthesis - Vibrio parahaemolyticus RIMD 2210633 | Carbapenems are broad-spectrum beta-lactam antibiotics, which are often considered as the antibiotic... | C00025 (L-Glutamate) C03287 (L-Glutamyl 5-phosphate) C01165 (L-Glutamate 5-semialdehyde) C03912 ((S)... | CARBAPENEM BIOSYNTHESIS Arginine and proline metabolism L-Glutamate 2.7.2.11 L-Glutamyl-P 1.2.1.41 ... |
| [vpa00340](https://www.genome.jp/dbget-bin/www_bget?pathway:vpa00340) | Histidine metabolism - Vibrio parahaemolyticus RIMD 2210633 |  | C00119 (5-Phospho-alpha-D-ribose 1-diphosphate) C02739 (1-(5-Phospho-D-ribosyl)-ATP) C02741 (Phospho... | 1.14.13.- 3.5.3.13 3.5.1.68 3.5.3.8 2.1.2.5 3.5.2.7 4.2.1.49 4.3.1.3 3.4.13.5 2.1.1.22 3.4.13.18 6.3... |
| [vpa00350](https://www.genome.jp/dbget-bin/www_bget?pathway:vpa00350) | Tyrosine metabolism - Vibrio parahaemolyticus RIMD 2210633 |  | C06044 (4-Hydroxyphenylethanol) C06046 (Salidroside) C04186 (5-Carboxymethyl-2-hydroxymuconate) C046... | Isoquinoline alkaloid biosynthesis 4-Hydroxy-phenylethanol Salidroside Dioxin degradation TYROSINE... |
| [vpa00360](https://www.genome.jp/dbget-bin/www_bget?pathway:vpa00360) | Phenylalanine metabolism - Vibrio parahaemolyticus RIMD 2210633 |  | C12621 (trans-3-Hydroxycinnamate) C00084 (Acetaldehyde) C00022 (Pyruvate) C03589 (4-Hydroxy-2-oxopen... | 4.1.3.39 4.3.1.25 Tropane, piperidine and pyridine alkaloid biosynthesis 1.2.1.5 6.2.1.30 2.3.1.14 ... |
| [vpa00361](https://www.genome.jp/dbget-bin/www_bget?pathway:vpa00361) | Chlorocyclohexane and chlorobenzene degradation - Vibrio parahaemolyticus RIMD 2210633 |  | C16266 (3-Chloro-2-hydroxymuconic semialdehyde) C12832 (3,4,6-Trichloro-cis-1,2-dihydroxycyclohexa-3... | 1.13.11.39 3-Chloro-2-hydroxy-muconic semialdehyde 3,4,6-Trichloro-cis-1,2-dihydroxy-cyclohexa-3,5-d... |
| [vpa00362](https://www.genome.jp/dbget-bin/www_bget?pathway:vpa00362) | Benzoate degradation - Vibrio parahaemolyticus RIMD 2210633 |  | C06714 (3-Hydroxypimeloyl-CoA) C04553 (3-Carboxy-2,5-dihydro-5-oxofuran-2-acetate) C04434 ((1E)-4-Ox... | 3-Hydroxy-pimeloyl-CoA BENZOATE DEGRADATION 3-Carboxy-2,5-dihydro-5-oxofuran-2-acetate 1.14.13.33... |
| [vpa00364](https://www.genome.jp/dbget-bin/www_bget?pathway:vpa00364) | Fluorobenzoate degradation - Vibrio parahaemolyticus RIMD 2210633 |  | C02364 (3-Fluorobenzoate) C16474 (3-Fluoro-cis,cis-muconate) C00090 (Catechol) C16484 (1-Fluorocyclo... | 3-Fluorobenzoate 1.14.12.- 1.14.12.- 3.1.1.45 3.1.1.45 3-Fluoro-cis,cis-muconate Catechol 1-Fluorocy... |
| [vpa00380](https://www.genome.jp/dbget-bin/www_bget?pathway:vpa00380) | Tryptophan metabolism - Vibrio parahaemolyticus RIMD 2210633 |  | C05663 (6-Hydroxykynurenate) C05639 (4,6-Dihydroxyquinoline) C05652 (4-(2-Amino-5-hydroxyphenyl)-2,4... | 1.11.1.6 1.1.1.35 1.13.11.52 4.1.1.74 Nicotinamide metabolism 2.6.1.27 1.4.3.2 1.13.11.52 1.14.14.1 ... |
| [vpa00400](https://www.genome.jp/dbget-bin/www_bget?pathway:vpa00400) | Phenylalanine, tyrosine and tryptophan biosynthesis - Vibrio parahaemolyticus RIMD 2210633 |  | C00279 (D-Erythrose 4-phosphate) C00074 (Phosphoenolpyruvate) C04691 (2-Dehydro-3-deoxy-D-arabino-he... | 2.5.1.54 Phenylpropanoid biosynthesis Phenylpropanoid biosynthesis Biosynthesis of siderophore grou... |
| [vpa00401](https://www.genome.jp/dbget-bin/www_bget?pathway:vpa00401) | Novobiocin biosynthesis - Vibrio parahaemolyticus RIMD 2210633 |  | C12477 (3-Methylpyrrole-2,4-dicarboxylic acid) C12483 (Pyrrole-2-carbonyl-[pcp]) C12479 (Coumermic a... | Polyketide sugar unit biosynthesis CouN7 CouP CouM CouL CouL CloN6 6.2.1.53 1.3.8.14 CloN7 CloP CloM... |
| [vpa00410](https://www.genome.jp/dbget-bin/www_bget?pathway:vpa00410) | beta-Alanine metabolism - Vibrio parahaemolyticus RIMD 2210633 |  | C00429 (5,6-Dihydrouracil) C02642 (3-Ureidopropionate) C00099 (beta-Alanine) C00083 (Malonyl-CoA) C0... | β-ALANINE METABOLISM Propanoate metabolism Fatty acid biosynthesis Pantothenate and CoA biosynthes... |
| [vpa00430](https://www.genome.jp/dbget-bin/www_bget?pathway:vpa00430) | Taurine and hypotaurine metabolism - Vibrio parahaemolyticus RIMD 2210633 |  | C00094 (Sulfite) C06735 (Aminoacetaldehyde) C00041 (L-Alanine) C00022 (Pyruvate) C14179 (Sulfoacetat... | 1.14.11.17 Sulfur metabolism 1.1.2.- 1.2.1.73 1.4.1.1 Sulfite Aminoacetaldehyde L-Alanine Pyruvate S... |
| [vpa00440](https://www.genome.jp/dbget-bin/www_bget?pathway:vpa00440) | Phosphonate and phosphinate metabolism - Vibrio parahaemolyticus RIMD 2210633 | Natural products containing carbon-phosphorous bonds, so-called C-P compounds, are derivatives of ph... | C00074 (Phosphoenolpyruvate) C02798 (3-Phosphonopyruvate) C05672 (2-Amino-3-phosphonopropanoate) C17... | PHOSPHONATE AND PHOSPHINATE METABOLISM Glycolysis Phosphoenol-pyruvate 3-Phosphono-pyruvate Phosp... |
| [vpa00450](https://www.genome.jp/dbget-bin/www_bget?pathway:vpa00450) | Selenocompound metabolism - Vibrio parahaemolyticus RIMD 2210633 |  | C01528 (Hydrogen selenide) C05697 (Selenate) C05684 (Selenite) C05172 (Selenophosphoric acid) C06481... | SELENOCOMPOUND METABOLISM 1.8.1.9 2.7.9.3 2.1.1.12 4.4.1.11 6.1.1.10 Hydrogen selenide Selenate Se... |
| [vpa00460](https://www.genome.jp/dbget-bin/www_bget?pathway:vpa00460) | Cyanoamino acid metabolism - Vibrio parahaemolyticus RIMD 2210633 |  | C05670 (3-Aminopropiononitrile) C01401 (Alanine) C00302 (Glutamate) C05714 (alpha-Aminopropiononitri... | 3-Aminopropiono-nitrile D-Alanine metabolism D-Gln & D-Glu metabolism D-Arg & D-Orn metabolism 3.5.... |

| **Entry** | **Name** | **Description** | **Object** | **Legend** |
| --- | --- | --- | --- | --- |
| [vpa00471](https://www.genome.jp/dbget-bin/www_bget?pathway:vpa00471) | D-Glutamine and D-glutamate metabolism - Vibrio parahaemolyticus RIMD 2210633 |  | C00064 (L-Glutamine) C00819 (D-Glutamine) C03933 (5-D-Glutamyl-D-glutamyl-peptide) C05723 (Poly-gamm... | D-GLUTAMINE AND D-GLUTAMATE METABOLISM Peptidoglycan biosynthesis Amino sugar and nucleotide suga... |
| [vpa00473](https://www.genome.jp/dbget-bin/www_bget?pathway:vpa00473) | D-Alanine metabolism - Vibrio parahaemolyticus RIMD 2210633 |  | C00041 (L-Alanine) C00133 (D-Alanine) C00993 (D-Alanyl-D-alanine) C00022 (Pyruvate) C04260 (O-D-Alan... | D-ALANINE METABOLISM Peptideglycan metabolism D-Arginine and D-ornithine metabolism Cyanoamino ac... |
| [vpa00480](https://www.genome.jp/dbget-bin/www_bget?pathway:vpa00480) | Glutathione metabolism - Vibrio parahaemolyticus RIMD 2210633 |  | C00024 (Acetyl-CoA) C00151 (L-Amino acid) C01879 (5-Oxoproline) C00097 (L-Cysteine) C00025 (L-Glutam... | Acetyl-CoA GLUTATHIONE METABOLISM Glutamate metabolism Taurine and hypotaurine metabolism Cyano... |
| [vpa00500](https://www.genome.jp/dbget-bin/www_bget?pathway:vpa00500) | Starch and sucrose metabolism - Vibrio parahaemolyticus RIMD 2210633 |  | C00092 (D-Glucose 6-phosphate) C00092 (D-Glucose 6-phosphate) C00095 (D-Fructose) C05731 (3-Ketosucr... | 3.6.1.- D-Glucose-6P 3.2.1.21 3.2.1.21 2.7.1.1 3.2.1.122 STARCH AND SUCROSE METABOLISM 3.2.1.26 2... |
| [vpa00511](https://www.genome.jp/dbget-bin/www_bget?pathway:vpa00511) | Other glycan degradation - Vibrio parahaemolyticus RIMD 2210633 |  | VP2403 (ebgA), VP2404 (ebgC) VP0755, VP2486 VP2403 (ebgA), VP2404 (ebgC) VP0755, VP2486 VP0755, VP24... | OTHER GLYCAN DEGRADATION GlcNAc GlcNAc Fuc Neu5Ac Gal Asn α2 β1 β1 Man α1 GlcNAc Neu5Ac Gal α... |
| [vpa00520](https://www.genome.jp/dbget-bin/www_bget?pathway:vpa00520) | Amino sugar and nucleotide sugar metabolism - Vibrio parahaemolyticus RIMD 2210633 |  | C00357 (N-Acetyl-D-glucosamine 6-phosphate) C00140 (N-Acetyl-D-glucosamine) C04501 (N-Acetyl-alpha-D... | 2.7.1.59 5.4.2.3 2.7.7.23 3.2.1.183 2.7.1.60 2.5.1.57 3.1.3.29 3.5.99.6 3.5.1.25 5.4.2.10 2.3.1.157 ... |
| [vpa00521](https://www.genome.jp/dbget-bin/www_bget?pathway:vpa00521) | Streptomycin biosynthesis - Vibrio parahaemolyticus RIMD 2210633 | Streptomycin is an aminocyclitol-aminoglycoside antibiotic produced by Streptomyces griseus. Strepto... | C01214 (1-Amino-1-deoxy-scyllo-inositol) C03319 (dTDP-L-rhamnose) C06592 (NDP-N-methyl-L-glucosamine... | Aminoglycosides 2.7.1.2 2.7.1.1 Polyketide sugar unit biosynthesis scyllo-Inosamine 2.7.1.65 3.1.3.2... |
| [vpa00523](https://www.genome.jp/dbget-bin/www_bget?pathway:vpa00523) | Polyketide sugar unit biosynthesis - Vibrio parahaemolyticus RIMD 2210633 |  | C07277 (dTDP-D-fucose) C00103 (D-Glucose 1-phosphate) C11911 (dTDP-D-desosamine) C00688 (dTDP-4-dehy... | dTDP-D-fucose Biosynthesis of 12-, 14- and 16-membered macrolides D-Glc-1P dTDP-D-desosamine dTDP-4-... |
| [vpa00525](https://www.genome.jp/dbget-bin/www_bget?pathway:vpa00525) | Acarbose and validamycin biosynthesis - Vibrio parahaemolyticus RIMD 2210633 |  | C20956 (alpha-D-Sedoheptulopyranose 7-phosphate) C17692 (2-epi-5-epi-Valiolone 7-phosphate) C17693 (... | ACARBOSE AND VALIDAMYCIN BIOSYNTHESIS 4.2.3.152 2.7.1.188 5.1.3.35 AcbL AcbN SalP 2.7.7.91 SalE 2... |
| [vpa00540](https://www.genome.jp/dbget-bin/www_bget?pathway:vpa00540) | Lipopolysaccharide biosynthesis - Vibrio parahaemolyticus RIMD 2210633 |  | C19877 (4-O-Phospho-alpha-Kdo-(2-&gt;6)-lipid IVA) C05382 (Sedoheptulose 7-phosphate) C07836 (D-glyc... | 4-Phospho-KDO-lipid IV [ ] Sedoheptulose-7P D-glycero-β-D-manno-Heptose-7P D-glyce... |
| [vpa00550](https://www.genome.jp/dbget-bin/www_bget?pathway:vpa00550) | Peptidoglycan biosynthesis - Vibrio parahaemolyticus RIMD 2210633 | Peptidoglycan is a macromolecule made of long aminosugar strands cross-linked by short peptides. It ... | C01050 (UDP-N-acetylmuramate) C04574 (di-trans,poly-cis-Undecaprenyl diphosphate) C17556 (di-trans,p... | 6.3.2.10 PEPTIDOGLYCAN BIOSYNTHESIS Aminosugar metabolism 3.6.1.27 2.4.1.227 2.7.8.13 6.3.2.10 6.3.... |
| [vpa00561](https://www.genome.jp/dbget-bin/www_bget?pathway:vpa00561) | Glycerolipid metabolism - Vibrio parahaemolyticus RIMD 2210633 |  | C00422 (Triacylglycerol) C00641 (1,2-Diacyl-sn-glycerol) C00111 (Glycerone phosphate) C00093 (sn-Gly... | 2.3.1.158 SQD1 SQD2 2.4.1.241 2.4.99.5 Glycerophospholipid metabolism 3.1.1.3 2.3.1.20 2.4.1.46 2.4.... |
| [vpa00562](https://www.genome.jp/dbget-bin/www_bget?pathway:vpa00562) | Inositol phosphate metabolism - Vibrio parahaemolyticus RIMD 2210633 |  | C11557 (1-Phosphatidyl-1D-myo-inositol 5-phosphate) C11555 (1D-myo-Inositol 1,4,5,6-tetrakisphosphat... | 1-Phosphatidyl-1D-myo-inositol-5P 2.7.1.149 1D-myo-Inositol- 1,4,5,6P 3.1.3.36 2.7.1.151 2.7.1.151 ... |
| [vpa00564](https://www.genome.jp/dbget-bin/www_bget?pathway:vpa00564) | Glycerophospholipid metabolism - Vibrio parahaemolyticus RIMD 2210633 |  | C06771 (Triethanolamine) C06772 (Diethanolamine) C00084 (Acetaldehyde) C00641 (1,2-Diacyl-sn-glycero... | Ether lipid metaboilsm 2.7.8.29 Ptdss1 Glycolysis 1.1.3.21 GPI-anchor biosynthesis Triethanolamine D... |
| [vpa00565](https://www.genome.jp/dbget-bin/www_bget?pathway:vpa00565) | Ether lipid metabolism - Vibrio parahaemolyticus RIMD 2210633 |  | C15647 (2-Acyl-1-(1-alkenyl)-sn-glycero-3-phosphate) C15646 (1-(1-Alkenyl)-sn-glycero-3-phosphate) C... | 2-Acyl-1-(1-alkenyl)-sn-glycero-3-phosphate (Plasmenic acid) 1-(1-Alkenyl)-sn-glycero-3-phosphate 1-... |
| [vpa00590](https://www.genome.jp/dbget-bin/www_bget?pathway:vpa00590) | Arachidonic acid metabolism - Vibrio parahaemolyticus RIMD 2210633 |  | C03577 (20-Hydroxyleukotriene E4) C14811 (Trioxilin B3) C14809 (Trioxilin A3) C14810 (Hepoxilin B3) ... | Eicosanoids 3.3.2.10 3.3.2.10 3.3.2.10 3.3.2.10 1.14.13.34 20-OH-LTE Trioxilin B Trioxilin A Hepoxil... |
| [vpa00592](https://www.genome.jp/dbget-bin/www_bget?pathway:vpa00592) | alpha-Linolenic acid metabolism - Vibrio parahaemolyticus RIMD 2210633 |  | C11512 (Methyl jasmonate) C16318 ((+)-7-Isomethyljasmonate) C08491 ((-)-Jasmonic acid) C16317 ((+)-7... | MFP2 MFP2 MFP2 HPL1 ACX ACX 2.3.1.16 2.3.1.16 4.2.1.92 1.13.11.12 (-)-Methyl-jasmonate (+)-7-Isometh... |
| [vpa00600](https://www.genome.jp/dbget-bin/www_bget?pathway:vpa00600) | Sphingolipid metabolism - Vibrio parahaemolyticus RIMD 2210633 |  | C00836 (Sphinganine) C12144 (Phytosphingosine) C12145 (Phytoceramide) C12126 (Dihydroceramide) C0129... | YDC1 YPC1 2.7.8.3 3.1.3.- 3.1.3.- 3.1.3.- 3.5.1.23 3.5.1.23 2.3.1.24 1.14.19.17 LAG1 1.14.18.5 1.14.... |
| [vpa00620](https://www.genome.jp/dbget-bin/www_bget?pathway:vpa00620) | Pyruvate metabolism - Vibrio parahaemolyticus RIMD 2210633 |  | C15973 (Enzyme N6-(dihydrolipoyl)lysine) C15972 (Enzyme N6-(lipoyl)lysine) C16255 ([Dihydrolipoyllys... | PYRUVATE METABOLISM Nicotinate and nicotinamide metabolism 1.2.4.1 Citrate cycle Glycine, serine a... |
| [vpa00623](https://www.genome.jp/dbget-bin/www_bget?pathway:vpa00623) | Toluene degradation - Vibrio parahaemolyticus RIMD 2210633 |  | C00587 (3-Hydroxybenzoate) C03067 (3-Hydroxybenzaldehyde) C03351 (3-Hydroxybenzyl alcohol) C01467 (3... | TOLUENE DEGRADATION 3-Hydroxy-benzoate 3-Hydroxy-benzaldehyde 3-Hydroxybenzyl-alcohol 1.14.13.- 1.1... |

| Entry | Name | Description | Object | Legend |
| --- | --- | --- | --- | --- |
| [vpa00625](https://www.genome.jp/dbget-bin/www_bget?pathway:vpa00625) | Chloroalkane and chloroalkene degradation - Vibrio parahaemolyticus RIMD 2210633 |  | C01380 (Ethylene glycol) C06548 (Ethylene oxide) C06547 (Ethylene) C06793 (Vinyl chloride) C06791 (t... | 1.97.1.- 1.97.1.- 1.97.1.- 1.21.99.5 1.21.99.5 1.21.99.5 1.18.6.1 Ethylene glycol 1.1.1.- 3.3.2.10 1... |
| [vpa00626](https://www.genome.jp/dbget-bin/www_bget?pathway:vpa00626) | Naphthalene degradation - Vibrio parahaemolyticus RIMD 2210633 |  | C00091 (Succinyl-CoA) C03203 (1-Hydroxy-2-naphthoate) C14101 (2-Naphthoic acid) C14099 (2-Naphthalde... | NmoAB 2.8.3.- Succinyl-CoA 1-Hydroxy-2-naphthoate 2-Naphthoate 2-Naphthaldehyde 2-Hydroxymethyl-naph... |
| [vpa00627](https://www.genome.jp/dbget-bin/www_bget?pathway:vpa00627) | Aminobenzoate degradation - Vibrio parahaemolyticus RIMD 2210633 |  | C00568 (4-Aminobenzoate) C00230 (3,4-Dihydroxybenzoate) C07103 (2-Hydroxy-1,4-benzoquinone) C03360 (... | AMINOBENZOATE DEGRADATION 4-Aminobenzoate 1.14.12.- 1.7.1.- 3,4-Dihydroxybenzoate 3.1.8.1 2-Hydrox... |
| [vpa00630](https://www.genome.jp/dbget-bin/www_bget?pathway:vpa00630) | Glyoxylate and dicarboxylate metabolism - Vibrio parahaemolyticus RIMD 2210633 |  | C00898 ((R,R)-Tartaric acid) C00036 (Oxaloacetate) C00158 (Citrate) C00149 ((S)-Malate) C04348 (L-Ma... | 5.3.1.22 4.2.1.3 2.2.1.5 2.3.3.12 2.3.3.7 4.1.3.13 4.1.3.16 4.1.3.1 3.5.1.68 3.5.1.56 3.5.1.49 3.5.1... |
| [vpa00633](https://www.genome.jp/dbget-bin/www_bget?pathway:vpa00633) | Nitrotoluene degradation - Vibrio parahaemolyticus RIMD 2210633 |  | C01468 (4-Cresol) C16401 (2,4,6-Trihydroxytoluene) C16400 (2,4,6-Triaminotoluene) C16395 (2-Amino-4,... | 4-Hydroxytoluene 2,4,6-Trihydroxytoluene 2,4,6-Triamino-toluene 2-Amino-4,6-dinitrotoluene 2-Amino-4... |
| [vpa00640](https://www.genome.jp/dbget-bin/www_bget?pathway:vpa00640) | Propanoate metabolism - Vibrio parahaemolyticus RIMD 2210633 |  | C00222 (3-Oxopropanoate) C00804 (Propynoate) C01013 (3-Hydroxypropanoate) C00099 (beta-Alanine) C000... | PROPANOATE METABOLISM 1.2.1.18 2.1.3.1 6.4.1.2 4.1.1.9 1.2.1.75 2.6.1.18 2.6.1.19 1.1.1.59 4.2.1.27... |
| [vpa00643](https://www.genome.jp/dbget-bin/www_bget?pathway:vpa00643) | Styrene degradation - Vibrio parahaemolyticus RIMD 2210633 |  | C05593 (3-Hydroxyphenylacetate) C02505 (2-Phenylacetamide) C16074 (Phenylacetonitrile) C16075 ((Z)-P... | 1.14.13.63 3-Hydroxyphenylacetate 3.5.5.1 3.5.1.4 4.2.1.84 4.99.1.7 Phenylacetamide Phenylacetonitri... |
| [vpa00650](https://www.genome.jp/dbget-bin/www_bget?pathway:vpa00650) | Butanoate metabolism - Vibrio parahaemolyticus RIMD 2210633 |  | C03046 ((S,S)-Butane-2,3-diol) C01769 ((S)-Acetoin) C00810 ((R)-Acetoin) C03044 ((R,R)-Butane-2,3-di... | Biosynthesis of type II polyketide backbone 4.2.1.27 1.1.1.30 3.1.1.22 2.8.3.5 6.2.1.16 4.1.3.4 2.3... |
| [vpa00660](https://www.genome.jp/dbget-bin/www_bget?pathway:vpa00660) | C5-Branched dibasic acid metabolism - Vibrio parahaemolyticus RIMD 2210633 |  | C00810 ((R)-Acetoin) C06010 ((S)-2-Acetolactate) C01011 ((3S)-Citramalyl-CoA) C00531 (Itaconyl-CoA) ... | Nicotinate and nicotinamide metabolism Valine, leucine and isoleucine biosynthesis 2.3.3.11 Alani... |
| [vpa00670](https://www.genome.jp/dbget-bin/www_bget?pathway:vpa00670) | One carbon pool by folate - Vibrio parahaemolyticus RIMD 2210633 |  | C00504 (Folate) C00415 (Dihydrofolate) C03479 (Folinic acid) C00143 (5,10-Methylenetetrahydrofolate)... | ONE CARBON POOL BY FOLATE Folate biosynthesis 2.1.2.5 2.1.2.4 2.1.2.2 4.3.1.4 1.5.1.15 1.5.1.3 1... |
| [vpa00680](https://www.genome.jp/dbget-bin/www_bget?pathway:vpa00680) | Methane metabolism - Vibrio parahaemolyticus RIMD 2210633 | Methane is metabolized principally by methanotrophs and methanogens in the global carbon cycle. Meth... | C01438 (Methane) C00132 (Methanol) C00067 (Formaldehyde) C00058 (Formate) C00237 (CO) C04330 (5,10-M... | METHANE METABOLISM Methane 1.14.13.25 1.14.18.3 Methanol Formaldehyde 1.1.3.13 1.1.2.7 1.1.1.244 1.... |
| [vpa00730](https://www.genome.jp/dbget-bin/www_bget?pathway:vpa00730) | Thiamine metabolism - Vibrio parahaemolyticus RIMD 2210633 |  | C00082 (L-Tyrosine) C15809 (Iminoglycine) C11437 (1-Deoxy-D-xylulose 5-phosphate) C15814 (Thiocarbox... | L-Tyrosine Iminoglycine 1-Deoxy-D-xylulose 5-phosphate [ThiS]-COSH [ThiS]-CO-AMP [IscS]-SSH [IscS]-... |
| [vpa00740](https://www.genome.jp/dbget-bin/www_bget?pathway:vpa00740) | Riboflavin metabolism - Vibrio parahaemolyticus RIMD 2210633 |  | C15556 (L-3,4-Dihydroxybutan-2-one 4-phosphate) C00199 (D-Ribulose 5-phosphate) C00044 (GTP) C01304 ... | 3,4-Dihydroxy-2-butanone 4-phosphate Ribulose 5-phosphate Pentose phosphate pathway RIBOFLAVIN META... |
| [vpa00750](https://www.genome.jp/dbget-bin/www_bget?pathway:vpa00750) | Vitamin B6 metabolism - Vibrio parahaemolyticus RIMD 2210633 |  | C00118 (D-Glyceraldehyde 3-phosphate) C00199 (D-Ribulose 5-phosphate) C07335 (2-Amino-3-oxo-4-phosph... | Glyceraldehyde 3-phosphate Ribulose 5-phosphate 4.3.3.6 Glycolysis Pentose phosphate pathway 2-Amino... |
| [vpa00760](https://www.genome.jp/dbget-bin/www_bget?pathway:vpa00760) | Nicotinate and nicotinamide metabolism - Vibrio parahaemolyticus RIMD 2210633 |  | C06178 (1-Methylpyrrolinium) C15523 (2,6-Dihydroxynicotinate) C00022 (Pyruvate) C00163 (Propanoate) ... | 1.5.99.4 Pyruvate metabolism C5-Branched dibasic acid metabolism 4.1.3.32 4.2.1.85 5.3.3.6 5.4.99.4... |
| [vpa00770](https://www.genome.jp/dbget-bin/www_bget?pathway:vpa00770) | Pantothenate and CoA biosynthesis - Vibrio parahaemolyticus RIMD 2210633 |  | C03688 (Apo-[acyl-carrier-protein]) C00022 (Pyruvate) C00900 (2-Acetolactate) C04039 (2,3-Dihydroxy-... | Apo-[acp] PANTOTHENATE AND CoA BIOSYNTHESIS Alanine, aspartate and glutamate metabolism Propanoate ... |
| [vpa00780](https://www.genome.jp/dbget-bin/www_bget?pathway:vpa00780) | Biotin metabolism - Vibrio parahaemolyticus RIMD 2210633 | Biotin (vitamin H or vitamin B7) is the essential cofactor of biotin-dependent carboxylases, such as... | C02656 (Pimelate) C01063 (6-Carboxyhexanoyl-CoA) C01092 (8-Amino-7-oxononanoate) C01909 (Dethiobioti... | Tropane, piperidine and pyridine alkaloid biosynthesis BIOTIN METABOLISM Lysine degradation Pimel... |
| [vpa00785](https://www.genome.jp/dbget-bin/www_bget?pathway:vpa00785) | Lipoic acid metabolism - Vibrio parahaemolyticus RIMD 2210633 |  | C16239 (Lipoyl-[acp]) C05752 (Octanoyl-[acp]) C16241 ((R)-Lipoate) C16238 (Lipoyl-AMP) C16236 (Prote... | 2.8.1.8 2.3.1.181 Lipoyl-[acp] LIPOIC ACID METABOLISM Fatty acid biosynthesis 2.8.1.8 2.3.1.181 O... |
| [vpa00790](https://www.genome.jp/dbget-bin/www_bget?pathway:vpa00790) | Folate biosynthesis - Vibrio parahaemolyticus RIMD 2210633 |  | C11355 (4-Amino-4-deoxychorismate) C00251 (Chorismate) C00044 (GTP) C04895 (7,8-Dihydroneopterin 3'-... | 4-Amino-4-deoxychorismate Chorismate FOLATE BIOSYNTHESIS One carbon pool by folate Purine metabolis... |
| [vpa00860](https://www.genome.jp/dbget-bin/www_bget?pathway:vpa00860) | Porphyrin and chlorophyll metabolism - Vibrio parahaemolyticus RIMD 2210633 |  | C05774 (Cobinamide) C16243 (Cobalt-precorrin 5B) C16244 (Cobalt-precorrin 7) C15670 (Heme A) C15672 ... | Alanine, aspartate and glutamate metabolism 3.1.1.82 Cobinamide Co-precorrin 5B Co-precorrin 7 Heme... |

| Entry | Name | Description | Object | Legend |
| --- | --- | --- | --- | --- |
| [vpa00900](https://www.genome.jp/dbget-bin/www_bget?pathway:vpa00900) | Terpenoid backbone biosynthesis - Vibrio parahaemolyticus RIMD 2210633 | Terpenoids, also known as isoprenoids, are a large class of natural products consisting of isoprene ... | C05859 (Dehydrodolichol diphosphate) C00621 (Dolichyl diphosphate) C00022 (Pyruvate) C00024 (Acetyl-... | MEP/DOXP pathway Mevalonate pathway Dehydro dolichol-PP Dolichol-PP Glycolysis TERPENOID BACKBONE ... |
| [vpa00903](https://www.genome.jp/dbget-bin/www_bget?pathway:vpa00903) | Limonene and pinene degradation - Vibrio parahaemolyticus RIMD 2210633 |  | C11950 (3-Isopropyl-3-butenoic acid) C11949 (3-Isopropylbut-3-enoyl-CoA) C11948 (2,6-Dimethyl-5-meth... | 3-Isopropylbut-3-enoic acid 3-Isopropylbut-3-enoyl-CoA 2,6-Dimethyl-5-methylene-3-oxo-heptanoyl-CoA ... |
| [vpa00910](https://www.genome.jp/dbget-bin/www_bget?pathway:vpa00910) | Nitrogen metabolism - Vibrio parahaemolyticus RIMD 2210633 | The biological process of the nitrogen cycle is a complex interplay among many microorganisms cataly... | C00058 (Formate) C00488 (Formamide) C00014 (Ammonia) C00192 (Hydroxylamine) C06058 (Nitroalkane) C00... | Formate Formamide 3.5.1.49 Ammonia Hydroxylamine Nitroalkane 1.7.2.6 1.9.6.1 1.7.99.- 1.7.7.2 1.13.1... |
| [vpa00920](https://www.genome.jp/dbget-bin/www_bget?pathway:vpa00920) | Sulfur metabolism - Vibrio parahaemolyticus RIMD 2210633 | Sulfur is an essential element for life and the metabolism of organic sulfur compounds plays an impo... | C00059 (Sulfate) C00094 (Sulfite) C00283 (Hydrogen sulfide) C00224 (Adenylyl sulfate) C00054 (Adenos... | SULFUR METABOLISM Sulfate Sulfite 1.8.2.1 1.8.3.1 1.8.7.1 1.8.99.5 1.8.1.2 Asr Sulfide 3.1.3.7 2.7.... |
| [vpa00930](https://www.genome.jp/dbget-bin/www_bget?pathway:vpa00930) | Caprolactam degradation - Vibrio parahaemolyticus RIMD 2210633 |  | C02232 (3-Oxoadipyl-CoA) C14145 ((3S)-3-Hydroxyadipyl-CoA) C11519 (N-Cyclohexylformamide) C14144 (5-... | 3-Oxoadipyl-CoA (3S)-3-Hydroxyadipyl-CoA N-Cyclohexylformamide 5-Carboxy-2-pentenoyl-CoA Adipyl-CoA ... |
| [vpa00970](https://www.genome.jp/dbget-bin/www_bget?pathway:vpa00970) | Aminoacyl-tRNA biosynthesis - Vibrio parahaemolyticus RIMD 2210633 |  | C06113 (L-Aspartyl-tRNA(Asn)) C02992 (L-Threonyl-tRNA(Thr)) C02839 (L-Tyrosyl-tRNA(Tyr)) C00078 (L-T... | L-Aspartyl-tRNA(Asn) 6.1.1.5 AMINOACYL-tRNA BIOSYNTHESIS 6.1.1.22 6.1.1.21 6.1.1.20 6.1.1.19 6.1.1.... |
| [vpa01040](https://www.genome.jp/dbget-bin/www_bget?pathway:vpa01040) | Biosynthesis of unsaturated fatty acids - Vibrio parahaemolyticus RIMD 2210633 |  | C16533 (13,16-Docosadienoic acid) C16645 ((13Z,16Z)-Docosadi-13,16-enoyl-CoA) C16645 ((13Z,16Z)-Doco... | Docosadienoic acid Δ13,16 Δ13,16 3.1.2.- YciA TesB TesB TesB TesA TesA TesA 3.1.2.- 3.1.2.- 3.1.2.... |
| [vpa01100](https://www.genome.jp/dbget-bin/www_bget?pathway:vpa01100) | Metabolic pathways - Vibrio parahaemolyticus RIMD 2210633 |  | C20876 (Very-long-chain acyl-CoA) C20879 (Very-long-chain trans-2,3-dehydroacyl-CoA) C20878 (Very-lo... | Lipoarabinomannan (LAM) biosynthesis Neomycin, kanamycin and gentamicin biosynthesis Glycosaminogly... |
| [vpa01110](https://www.genome.jp/dbget-bin/www_bget?pathway:vpa01110) | Biosynthesis of secondary metabolites - Vibrio parahaemolyticus RIMD 2210633 |  | C21833 (Kaempferol-3-O-rutinoside) C21835 (12,18-Didecarboxysiroheme) C05797 (Pheophytin a) C05306 (... | Carotenoid biosynthesis Porphyrin and chlorophyll metabolism Diterpenoid biosynthesis Flavone and ... |
| [vpa01120](https://www.genome.jp/dbget-bin/www_bget?pathway:vpa01120) | Microbial metabolism in diverse environments - Vibrio parahaemolyticus RIMD 2210633 |  | C03618 (L-threo-3-Methylaspartate) C01732 (Mesaconate) C21104 (2,5-Dichloro-p-benzoquinone) C21859 (... | Xylene degradation Glyoxylate and dicarboxylate metabolism Aminobenzoate degradation Lysine bios... |
| [vpa01130](https://www.genome.jp/dbget-bin/www_bget?pathway:vpa01130) | Biosynthesis of antibiotics - Vibrio parahaemolyticus RIMD 2210633 |  | C21875 (Aurachin A) C21874 (Aurachin B epoxide) C21140 (Aurachin B) C21141 (4-Hydroxy-2-methyl-3-oxo... | Macrolides and ketolides Pentose phosphate pathway MEP / DOXP pathway Mevalonate pathway Lysine b... |
| [vpa01200](https://www.genome.jp/dbget-bin/www_bget?pathway:vpa01200) | Carbon metabolism - Vibrio parahaemolyticus RIMD 2210633 | Carbon metabolism is the most basic aspect of life. This map presents an overall view of central car... | C00085 (D-Fructose 6-phosphate), C05345 (beta-D-Fructose 6-phosphate) C01182 (D-Ribulose 1,5-bisphos... | HCO HCHO HCHO HCO HCO AcCoA CARBON METABOLISM Fructose-6P Ribulose-1,5P Glycerate-3P THF Glucose Gl... |
| [vpa01210](https://www.genome.jp/dbget-bin/www_bget?pathway:vpa01210) | 2-Oxocarboxylic acid metabolism - Vibrio parahaemolyticus RIMD 2210633 | 2-Oxocarboxylic acids, also called 2-oxo acids and alpha-keto acids, are the most elementary set of ... | C17254 (8-Methylthiooctyl glucosinolate) C17232 (2-Oxo-10-methylthiodecanoic acid) C17252 (7-Methylt... | 8-Methylthiooctyl glucosinolate 2-Oxo-10-methylthio-decanoic acid 7-Methylthioheptyl glucosinolate... |
| [vpa01212](https://www.genome.jp/dbget-bin/www_bget?pathway:vpa01212) | Fatty acid metabolism - Vibrio parahaemolyticus RIMD 2210633 |  | C00229 (Acyl-carrier protein) C00024 (Acetyl-CoA) C03939 (Acetyl-[acyl-carrier protein]) C05744 (Ace... | FATTY ACID METABOLISM ACP Acetyl-CoA Acetyl-[acp] Acetoacetyl-[acp] (R)-3-Hydroxybutanoyl-[acp] Ma... |
| [vpa01220](https://www.genome.jp/dbget-bin/www_bget?pathway:vpa01220) | Degradation of aromatic compounds - Vibrio parahaemolyticus RIMD 2210633 | Microorganisms are known to be capable of degrading diverse chemical substances including man-made c... | C01455 (Toluene) C00261 (Benzaldehyde) C00556 (Benzyl alcohol) C00180 (Benzoate) C06321 ((1R,6S)-1,6... | DEGRADATION OF AROMATIC COMPOUNDS Toluene Benzaldehyde Benzyl alcohol Benzoate Catechol 3-Fluorob... |
| [vpa01230](https://www.genome.jp/dbget-bin/www_bget?pathway:vpa01230) | Biosynthesis of amino acids - Vibrio parahaemolyticus RIMD 2210633 | This map presents a modular architecture of the biosynthesis pathways of twenty amino acids, which m... | C04390 (N6-Acetyl-LL-2,6-diaminoheptanedioate) C04002 ((Z)-But-1-ene-1,2,4-tricarboxylate) C00118 (D... | N-Acetyl-LL-2,6-diaminopimelate M00763 Homo-cis-aconitate BIOSYNTHESIS OF AMINO ACIDS Glyceraldeh... |
| [vpa01501](https://www.genome.jp/dbget-bin/www_bget?pathway:vpa01501) | beta-Lactam resistance - Vibrio parahaemolyticus RIMD 2210633 | The beta-lactam antibiotics are the most widely used group of antibiotics, which exert their effect ... | C04702 (UDPMurNAc(oyl-L-Ala-D-gamma-Glu-L-Lys-D-Ala-D-Ala)) C00039 (DNA) D07746 (Colistin (INN)), D0... | DNA DNA MecA MecI MecR1 BlaZ BlaI BlaR1 beta-LACTAM RESISTANCE DNA AmpC AmpR AmpG GlcNAc-anhMurNAc ... |
| [vpa01502](https://www.genome.jp/dbget-bin/www_bget?pathway:vpa01502) | Vancomycin resistance - Vibrio parahaemolyticus RIMD 2210633 | Vancomycin (VCM) is a glycopeptide antibiotic agent that inhibits the synthesis of peptidolgycan in ... | C00022 (Pyruvate) C00256 ((R)-Lactate) C19694 (D-Alanyl-(R)-lactate) C00041 (L-Alanine) C00133 (D-Al... | VANCOMYCIN RESISTANCE Cell wall Inner membrane VanS VanR Pyruvate D-Lac VanH VanA/B/D D-Ala-D-Lac L... |
| [vpa01503](https://www.genome.jp/dbget-bin/www_bget?pathway:vpa01503) | Cationic antimicrobial peptide (CAMP) resistance - Vibrio parahaemolyticus RIMD 2210633 | Cationic antimicrobial peptides (CAMPs) play an important role in host defense against microbial inf... | C20931 (4-Amino-4-deoxy-L-arabinose) C00346 (Ethanolamine phosphate) D07746 (Colistin (INN)), D00128... | CATIONIC ANTIMICROBIAL PEPTIDE (CAMP) RESISTANCE Inner membrane Outer membrane PmrB PmrA ArnT Ep... |
| [vpa02010](https://www.genome.jp/dbget-bin/www_bget?pathway:vpa02010) | ABC transporters - Vibrio parahaemolyticus RIMD 2210633 | The ATP-binding cassette (ABC) transporters form one of the largest known protein families, and are ... | C16421 (AI-2) C01684 (D-Rhamnose) C00095 (D-Fructose) C01487 (D-Allose) C00181 (D-Xylose) C03619 (Me... | GguA GguB ChvE LsrA LsrD LsrC LsrB Autoinducer 2 RhaT RhaQ RhaP RhaS Rhamnose FrcA FrcC FrcB Fructos... |

| Entry | Name | Description | Object | Legend |
| --- | --- | --- | --- | --- |
| [vpa02020](https://www.genome.jp/dbget-bin/www_bget?pathway:vpa02020) | Two-component system - Vibrio parahaemolyticus RIMD 2210633 | Two-component signal transduction systems enable bacteria to sense, respond, and adapt to changes in... | C00164 (Acetoacetate) C00038 (Zinc cation), C06696 (Lead) C00244 (Nitrate), C00088 (Nitrite) C00031 ... | NarI NarJ NarH NarG Bacterial chemotaxis MCP PilA RpoN PilS PilI CheW SdiA Acetoacetate Zn / Pb Nitr... |
| [vpa02024](https://www.genome.jp/dbget-bin/www_bget?pathway:vpa02024) | Quorum sensing - Vibrio parahaemolyticus RIMD 2210633 | Quorum sensing (QS) is a regulatory system that allows bacteria to share information about cell dens... | C16640 (CAI-1) C16421 (AI-2) C21195 (N-(3-Hydroxybutanoyl)-L-homoserine lactone) C18049 (N-Acyl-L-ho... | QUORUM SENSING Sensing protein Qrr LuxQ LuxO LuxU CqsS CAI-1 LitR/HapR LuxP AI-2 LuxN/AinR AI-1 (sR... |
| [vpa02030](https://www.genome.jp/dbget-bin/www_bget?pathway:vpa02030) | Bacterial chemotaxis - Vibrio parahaemolyticus RIMD 2210633 | Chemotaxis is the process by which cells sense chemical gradients in their environment and then move... | C00716 (Serine) C00049 (L-Aspartate) C00208 (Maltose) C00121 (D-Ribose) C00124 (D-Galactose) C00107 ... | Escherichia coli BACTERIAL CHEMOTAXIS Flagellar assembly MotB MotA FliN FliM FliG CheZ CheY CheR Ch... |
| [vpa02040](https://www.genome.jp/dbget-bin/www_bget?pathway:vpa02040) | Flagellar assembly - Vibrio parahaemolyticus RIMD 2210633 |  | VP0771, VPA0262 VP0772 (flgA), VPA0263 VP0770, VPA0261 VP2245 (fliJ) VP2254 (fliS), VPA1551 VP2237 (... | Type III secretion system Bacterial chemotaxis FlhD FlhC FlgM FlgA FlgN FliT FliJ FliS FliR FliP Fli... |
| [vpa02060](https://www.genome.jp/dbget-bin/www_bget?pathway:vpa02060) | Phosphotransferase system (PTS) - Vibrio parahaemolyticus RIMD 2210633 | The phosphoenolpyruvate (PEP)-dependent phosphotransferase system (PTS) is a major mechanism used by... | C06377 (D-Galactosamine 6-phosphate) C11544 (2(alpha-D-Mannosyl)-D-glycerate) C01083 (alpha,alpha-Tr... | Galactosamine 6-phosphate 2-O-α-Mannosyl-D-glycerate Trehalose β-Glucoside Trehalose 6-phosphate P... |
| [vpa03010](https://www.genome.jp/dbget-bin/www_bget?pathway:vpa03010) | Ribosome - Vibrio parahaemolyticus RIMD 2210633 |  | VPAr03, VPr003, VPr006, VPr007, VPr008, VPr011, VPr014, VPr017, VPr020, VPr023, VPr026, VPr029 VPAr0... | L7A S30e L13e L28e S14e S23e S18e S29e S13e S11e S15e S6e S8e S17e S19e S24e S27e S27Ae S7e S10e S12... |
| [vpa03018](https://www.genome.jp/dbget-bin/www_bget?pathway:vpa03018) | RNA degradation - Vibrio parahaemolyticus RIMD 2210633 | The correct processing, quality control and turnover of cellular RNA molecules are critical to many ... | VP2062 VP2062 VP2062 VP3002 VP2561 (eno) VP2452 VPA0390, VPA0845 VP1890, VP2807 VP2340, VP3007, VPA0... | RNA helicase RNase E Catalytic domain C-terminal domain Degradosome component interaction RNA d... |
| [vpa03020](https://www.genome.jp/dbget-bin/www_bget?pathway:vpa03020) | RNA polymerase - Vibrio parahaemolyticus RIMD 2210633 |  | VP2922 (rpoB) VP2921 VP0282 VP0160 (rpoZ) vpa03020: RNA polymerase | RNA POLYMERASE β' ABC1 ABC2 ABC3 ABC5 B11 ABC4 AC1 C11 AC2 C25 C31 C34 A12 A14 A34 A43 A49 RNA pol... |
| [vpa03030](https://www.genome.jp/dbget-bin/www_bget?pathway:vpa03030) | DNA replication - Vibrio parahaemolyticus RIMD 2210633 | A complex network of interacting proteins and enzymes is required for DNA replication. Generally, DN... | VP1477 VP2709 VP0405 (dnaG) VP2303 (dnaE) VP1724, VP1903, VP2292, VP2877, VPA0074, VPA0793 VP2179 VP... | Lig1 Fen1 Dna2 RNaseH2A RNaseH2B RFC2/4 RNaseH2C RFC3/5 PCNA RPA3 Mcm5 δ4 δ3 ε4 ε3 SSB DnaG DNA ... |
| [vpa03060](https://www.genome.jp/dbget-bin/www_bget?pathway:vpa03060) | Protein export - Vibrio parahaemolyticus RIMD 2210633 | The protein export is the active transport of proteins from the cytoplasm to the exterior of the cel... | VP0003 VP0099 VP0100 VP0098 (tatA) VP0467 (secA) VP0277 (secY) VP2928 (secE) VP2460 (secG) VP0590 (s... | TatE Ffs YidC TatB TatC TatA SecA SecY SecE SecG SecD/F YajC SPase I SPase II SRP14 SRP72 SRP68 SRP5... |
| [vpa03070](https://www.genome.jp/dbget-bin/www_bget?pathway:vpa03070) | Bacterial secretion system - Vibrio parahaemolyticus RIMD 2210633 | Gram-negative bacteria secrete a wide range of proteins whose functions include biogenesis of organe... | C00002 (ATP) C00002 (ATP) C00002 (ATP) C00002 (ATP) C00002 (ATP) C00002 (ATP) C00002 (ATP) C00002 (A... | Type I Type III Type II Type Va Type IV Type VI TolC HlyD HlyB YscC GspGHIJK GspD TatABCE SecDEFGY Y... |
| [vpa03410](https://www.genome.jp/dbget-bin/www_bget?pathway:vpa03410) | Base excision repair - Vibrio parahaemolyticus RIMD 2210633 | Base excision repair (BER) is the predominant DNA damage repair pathway for the processing of small ... | VP1477 VP0800 (ligA), VP1477 VP0107 VP0107 VP0107 VP0498 VP1003 VP2626 VP0500 VP2108 VP0189 VP0189 V... | APEX Fen1 PARP Lig1 Lig3 Lig Polε Polδ Polβ Polβ Polβ Polβ PCNA PCNA PCNA DpoI DpoI DpoI APE2 ... |
| [vpa03420](https://www.genome.jp/dbget-bin/www_bget?pathway:vpa03420) | Nucleotide excision repair - Vibrio parahaemolyticus RIMD 2210633 | Nucleotide excision repair (NER) is a mechanism to recognize and repair bulky DNA damage caused by c... | VP2100 VP2712 (uvrA) VP0800 (ligA) VP0107 VP2712 (uvrA) VP1477 VP3013 (uvrD) VP2100 VP0975 VP1944 (u... | 5 ' 3 ' 3 ' 5 ' 5 ' 3 ' 3 ' 5 ' TFIIH4 TFIIH3 TFIIH2 TFIIH1 XPD TTDA XPB CCNH MNAT1 CDK7 DDB1 Cul4 R... |
| [vpa03430](https://www.genome.jp/dbget-bin/www_bget?pathway:vpa03430) | Mismatch repair - Vibrio parahaemolyticus RIMD 2210633 | DNA mismatch repair (MMR) is a highly conserved biological pathway that plays a key role in maintain... | VP2742 VP2552 VP0518 VP2819 (mutL) VP0800 (ligA) VP3013 (uvrD) VP1477 VP0012, VP0725 (holA), VP1724,... | Dam MutS MSH2 MLH3 MLH1 Colorectal cancer MutH MutL Lig UVRD MLH1 LigI PCNA DpoIII ExoI Polδ RPA Ex... |
| [vpa03440](https://www.genome.jp/dbget-bin/www_bget?pathway:vpa03440) | Homologous recombination - Vibrio parahaemolyticus RIMD 2210633 | Homologous recombination (HR) is essential for the accurate repair of DNA double-strand breaks (DSBs... | VP2739 VP1005 VP2373, VPA1266 VP0157 VP0157 VP1048 (ruvC) VP1048 (ruvC) VP1052 (ruvB) VP1052 (ruvB) ... | TOP3 BLM DSS1 Rad52 Mre11 Rad50 XRCC3 Rad51C XRCC2 XRCC2 Rad51D Rad51D Rad51C Rad51B Rad51C DnaT Pri... |
| [vpa04122](https://www.genome.jp/dbget-bin/www_bget?pathway:vpa04122) | Sulfur relay system - Vibrio parahaemolyticus RIMD 2210633 | Ubiquitin and ubiquitin-like proteins (Ubls) are signalling messengers that control many cellular fu... | C00097 (L-Cysteine) C00041 (L-Alanine) C00868 (tRNA uridine) C17322 (tRNA containing 2-thiouridine) ... | SULFUR RELAY SYSTEM E1-like urm1 -COOH ATP PPi -COAMP Protein modification Prokaryote Ahp1 -NHCO-... |
| [vpa05110](https://www.genome.jp/dbget-bin/www_bget?pathway:vpa05110) | Vibrio cholerae infection - Vibrio parahaemolyticus RIMD 2210633 | Cholera toxin (CTX) is one of the main virulence factors of Vibrio cholerae. Once secreted, CTX B-ch... | C00014 (Ammonia) C00080 (H+) C00238 (Potassium cation) C00238 (Potassium cation) C01330 (Sodium cati... | TcpF TcpE TcpD TcpC TcpB TcpA Hap ACE NanH GM1 Tight junction Zot RtxA CtxA CtxA CtxB VCC Vibrio cho... |
| [vpa05111](https://www.genome.jp/dbget-bin/www_bget?pathway:vpa05111) | Biofilm formation - Vibrio cholerae - Vibrio parahaemolyticus RIMD 2210633 | Surface colonization and subsequent biofilm formation and development provide numerous advantages to... | C00575 (3',5'-Cyclic AMP) C16463 (3',5'-Cyclic diGMP) C00044 (GTP) C18076 (5'-Phosphoguanylyl(3'-&gt... | CyaA cAMP CRP BIOFILM FORMATION - VIBRIO CHOLERAE VarS VarA CsrB/C/D (ncRNA) CsrA CdgL CdgM VCA0... |
