# Supplementary material for: Comprehensive genome based analysis of Vibrio parahaemolyticus for identifying novel drug and vaccine molecules: Subtractive proteomics and vaccinomics approach
Source: PLoS One. 2020 Aug 19;15(8):e0237181. doi: 10.1371/journal.pone.0237181 (PMC7444560; doi:10.1371/journal.pone.0237181)
Supplement: S7 File — (DOCX) [file pone.0237181.s020.docx]

**S7 File.** Metabolic pathways for humans in KEGG server.

| **Entry** | **Name** | **Description** | **Object** | **Legend** |
| --- | --- | --- | --- | --- |
| [hsa00010](https://www.genome.jp/dbget-bin/www_bget?pathway:hsa00010) | Glycolysis / Gluconeogenesis - Homo sapiens (human) | Glycolysis is the process of converting glucose into pyruvate and generating small amounts of ATP | C00033 (Acetate) C00031 (D-Glucose) C00103 (D-Glucose 1-phosphate) C00631 (2-Phospho-D-glycerate) C0... | 4.1.2.13 1.2.1.3 6.2.1.13 1.2.1.5 Acetate Pentose phosphate pathway Starch and sucrose metabolism... |
| [hsa00020](https://www.genome.jp/dbget-bin/www_bget?pathway:hsa00020) | Citrate cycle (TCA cycle) - Homo sapiens (human) | The citrate cycle (TCA cycle, Krebs cycle) is an important aerobic pathway for the final steps of th... | C00022 (Pyruvate) C00122 (Fumarate) C00036 (Oxaloacetate) C05379 (Oxalosuccinate) C00024 (Acetyl-CoA... | 1.8.1.4 1.2.4.2 1.2.4.2 2.3.1.61 6.2.1.5 6.2.1.4 1.1.1.42 1.1.1.41 1.1.1.42 2.3.3.8 4.2.1.2 4.2.1.3 ... |
| [hsa00030](https://www.genome.jp/dbget-bin/www_bget?pathway:hsa00030) | Pentose phosphate pathway - Homo sapiens (human) | The pentose phosphate pathway is a process of glucose turnover that produces NADPH as reducing equiv... | C01151 (D-Ribose 1,5-bisphosphate) C00668 (alpha-D-Glucose 6-phosphate) C00118 (D-Glyceraldehyde 3-p... | 2.7.4.23 5.3.1.9 4.1.2.14 Glycolysis 2.7.1.15 5.4.2.7 4.1.2.4 2.7.6.1 5.4.2.7 2.7.1.15 2.2.1.1 5.3.1... |
| [hsa00040](https://www.genome.jp/dbget-bin/www_bget?pathway:hsa00040) | Pentose and glucuronate interconversions - Homo sapiens (human) |  | C14899 (3-Dehydro-L-gulonate 6-phosphate) C03033 (beta-D-Glucuronoside) C00817 (D-Altronate) C00103 ... | 3.2.1.15 3-Dehydro-L-gulonate-6P 4.1.1.85 2.7.1.53 β-D-Glucuronoside 3.2.1.31 5.3.1.4 1.1.1.13 2.7.... |
| [hsa00051](https://www.genome.jp/dbget-bin/www_bget?pathway:hsa00051) | Fructose and mannose metabolism - Homo sapiens (human) |  | C00247 (L-Sorbose) C00267 (alpha-D-Glucose) C01094 (D-Fructose 1-phosphate) C00159 (D-Mannose) C0027... | 2.7.1.202 Galactose metabolism 1.1.99.21 1.1.1.21 1.1.1.14 5.3.1.5 2.7.1.3 5.3.1.7 1.1.1.11 1.1.1.6... |
| [hsa00052](https://www.genome.jp/dbget-bin/www_bget?pathway:hsa00052) | Galactose metabolism - Homo sapiens (human) |  | C06311 (Galactitol 1-phosphate) C01216 (2-Dehydro-3-deoxy-D-galactonate) C00137 (myo-Inositol) C0009... | 1.1.1.251 2.7.1.200 3.2.1.22 2.7.1.144 2.7.1.11 4.1.2.40 4.1.2.21 3.2.1.85 3.2.1.22 3.2.1.26 2.4.1.6... |
| [hsa00053](https://www.genome.jp/dbget-bin/www_bget?pathway:hsa00053) | Ascorbate and aldarate metabolism - Homo sapiens (human) |  | C00137 (myo-Inositol) C05385 (D-Glucuronate 1-phosphate) C00167 (UDP-glucuronate) C00029 (UDP-glucos... | 3.1.1.- 1.1.1.365 1.1.1.122 3.1.3.93 2.7.7.69 5.1.3.18 5.1.3.18 5.1.3.18 1.13.99.1 2.7.1.43 2.7.7.44... |
| [hsa00061](https://www.genome.jp/dbget-bin/www_bget?pathway:hsa00061) | Fatty acid biosynthesis - Homo sapiens (human) |  | C04088 (Octadecanoyl-[acyl-carrier protein]) C16221 ((2E)-Octadecenoyl-[acp]) C16220 ((R)-3-Hydroxyo... | FabK FabK FabK FabK FabK FabK FabL Octadecanoyl-[acp] trans-Octadec-2-enoyl-[acp] (R)-3-Hydroxy-octa... |
| [hsa00062](https://www.genome.jp/dbget-bin/www_bget?pathway:hsa00062) | Fatty acid elongation - Homo sapiens (human) |  | C00024 (Acetyl-CoA) C00136 (Butanoyl-CoA) C05270 (Hexanoyl-CoA) C05271 (trans-Hex-2-enoyl-CoA) C0526... | 4.2.1.74 4.2.1.74 Fatty acid degradation FATTY ACID ELONGATION 3.1.2.22 1.3.1.38 4.2.1.17 1.1.1.21... |
| [hsa00071](https://www.genome.jp/dbget-bin/www_bget?pathway:hsa00071) | Fatty acid degradation - Homo sapiens (human) |  | C02990 (L-Palmitoylcarnitine) C00638 (Long-chain fatty acid) C05280 (cis,cis-3,6-Dodecadienoyl-CoA) ... | 1.3.99.- 1.3.99.- 1.3.99.- 1.3.99.- 1.3.99.- 1.3.99.- 1.3.99.- 1.3.8.1 1.1.1.35 4.2.1.17 Synthesis a... |
| [hsa00072](https://www.genome.jp/dbget-bin/www_bget?pathway:hsa00072) | Synthesis and degradation of ketone bodies - Homo sapiens (human) |  | C00332 (Acetoacetyl-CoA) C00356 ((S)-3-Hydroxy-3-methylglutaryl-CoA) C00207 (Acetone) C00164 (Acetoa... | SYNTHESIS AND DEGRADATION OF KETONE BODIES Fatty acid degradation 2.3.3.10 4.1.3.4 4.1.1.4 2.3... |
| [hsa00100](https://www.genome.jp/dbget-bin/www_bget?pathway:hsa00100) | Steroid biosynthesis - Homo sapiens (human) |  | C01673 (Calcitriol) C01561 (Calcidiol) C01902 (Cycloartenol) C05442 (Stigmasterol) C15776 (4alpha-Me... | STE1 5.3.3.5 1.3.1.21 1.14.19.20 5.3.3.5 ERG4 ERG5 ERG3 ERG2 Brassinosteroid biosynthesis Calcitriol... |
| [hsa00120](https://www.genome.jp/dbget-bin/www_bget?pathway:hsa00120) | Primary bile acid biosynthesis - Homo sapiens (human) | Bile acids are steroid carboxylic acids derived from cholesterol in vertebrates. The primary bile ac... | C00187 (Cholesterol) C03594 (7alpha-Hydroxycholesterol) C15610 (Cholest-5-ene-3beta,26-diol) C15519 ... | 1.14.14.25 1.14.14.29 1.14.14.23 1.14.14.29 1.3.1.3 1.1.1.50 1.14.15.15 5.1.99.4 1.17.99.3 1.1.1.50 ... |
| [hsa00130](https://www.genome.jp/dbget-bin/www_bget?pathway:hsa00130) | Ubiquinone and other terpenoid-quinone biosynthesis - Homo sapiens (human) | Ubiquinone (UQ), also called coenzyme Q, and plastoquinone (PQ) are electron carriers in oxidative p... | C16519 (2-Succinyl-5-enolpyruvyl-6-hydroxy-3-cyclohexene-1-carboxylate) C03657 (1,4-Dihydroxy-2-naph... | UBIQUINONE AND OTHER TERPENOID-QUINONE BIOSYNTHESIS Phenylpropanoid biosynthesis 2-Succinyl-5-e... |
| [hsa00140](https://www.genome.jp/dbget-bin/www_bget?pathway:hsa00140) | Steroid hormone biosynthesis - Homo sapiens (human) | Steroid hormones derived from cholesterol are a class of biologically active compounds in vertebrate... | C05138 (17alpha-Hydroxypregnenolone) C05485 (21-Hydroxypregnenolone) C05489 (11beta,17alpha,21-Trihy... | 1.1.1.62 STEROID HORMONE BIOSYNTHESIS 17α-Hydroxy-pregnenolone 21-Hydroxy-pregnenolone 11β,17α,2... |
| [hsa00190](https://www.genome.jp/dbget-bin/www_bget?pathway:hsa00190) | Oxidative phosphorylation - Homo sapiens (human) |  | C00061 (FMN) C00390 (Ubiquinol) C00399 (Ubiquinone) C00524 (Cytochrome c) C00080 (H+) C00080 (H+) C0... | F-type ATPase (Eukaryotes) V/A-type ATPase (Bacteria, Archaeas) V-type ATPase (Eukaryotes) F-type AT... |
| [hsa00220](https://www.genome.jp/dbget-bin/www_bget?pathway:hsa00220) | Arginine biosynthesis - Homo sapiens (human) |  | C03406 (N-(L-Arginino)succinate) C00327 (L-Citrulline) C00062 (L-Arginine) C00077 (L-Ornithine) C001... | L-Arginosuccinate Citruline Arginine Ornithine Carbamoyl-P Aspartate Fumarate NH3 N-Acetyl-glutamate... |
| [hsa00230](https://www.genome.jp/dbget-bin/www_bget?pathway:hsa00230) | Purine metabolism - Homo sapiens (human) |  | C12248 (5-Hydroxy-2-oxo-4-ureido-2,5-dihydro-1H-imidazole-5-carboxylate) C11821 (5-Hydroxyisourate) ... | 1.17.4.1 5-Hydroxy-2-oxo-4-ureido-2,5-dihydro-1H-imidazole-5-carboxylate 5-Hydroxyisourate 1.17.3.2 ... |
| [hsa00232](https://www.genome.jp/dbget-bin/www_bget?pathway:hsa00232) | Caffeine metabolism - Homo sapiens (human) |  | C00385 (Xanthine) C13747 (1,7-Dimethylxanthine) C16362 (3,6,8-Trimethylallantoin) C16363 (N-Methylur... | Xanthine Paraxanthine 3,6,8-Trimethyl-allantoin N-Methylurea 1,3,7-Trimethyl-uric acid Glyoxylate N,... |
| [hsa00240](https://www.genome.jp/dbget-bin/www_bget?pathway:hsa00240) | Pyrimidine metabolism - Homo sapiens (human) |  | C00063 (CTP) C15607 (3-Oxo-3-ureidopropanoate) C00064 (L-Glutamine) C00438 (N-Carbamoyl-L-aspartate)... | (extracellular) CTP 1.17.4.1 3-Oxo-3-ureido-propanoate 3.5.1.95 2.7.4.22 3.5.4.30 2.1.1.148 L-Glutam... |
| [hsa00250](https://www.genome.jp/dbget-bin/www_bget?pathway:hsa00250) | Alanine, aspartate and glutamate metabolism - Homo sapiens (human) |  | C00122 (Fumarate) C00042 (Succinate) C00036 (Oxaloacetate) C00026 (2-Oxoglutarate) C00041 (L-Alanine... | Fumarate Succinate Oxaloacetate 2-Oxo-glutarate Citrate cycle L-Alanine L-Aspartate L-Asparagine D-A... |
| [hsa00260](https://www.genome.jp/dbget-bin/www_bget?pathway:hsa00260) | Glycine, serine and threonine metabolism - Homo sapiens (human) | Serine is derived from 3-phospho-D-glycerate, an intermediate of glycolysis [MD:M00020], and glycine... | C16432 (5-Hydroxyectoine) C06231 (Ectoine) C06442 (N(gamma)-Acetyldiaminobutyrate) C03283 (L-2,4-Dia... | 1.1.3.17 5.1.1.18 5-Hydroxyectoine 1.14.11.55 1.14.15.7 L-Ectoine Nγ-Acetyl-L-2,4-diaminobutyrate L... |
| [hsa00270](https://www.genome.jp/dbget-bin/www_bget?pathway:hsa00270) | Cysteine and methionine metabolism - Homo sapiens (human) | Cysteine and methionine are sulfur-containing amino acids. Cysteine is synthesized from serine throu... | C06547 (Ethylene) C01234 (1-Aminocyclopropane-1-carboxylate) C08276 (3-(Methylthio)propanoate) C1560... | 2.5.1.- Ethylene 1-Aminocyclopropane-1-carboxylate 1.14.17.4 4.4.1.14 MtnE 3-Methylthio-propionate 1... |
| [hsa00280](https://www.genome.jp/dbget-bin/www_bget?pathway:hsa00280) | Valine, leucine and isoleucine degradation - Homo sapiens (human) |  | C15978 (2-Methyl-1-hydroxybutyl-ThPP) C15973 (Enzyme N6-(dihydrolipoyl)lysine) C15973 (Enzyme N6-(di... | 2-Methyl-1-hydroxybutyl-ThPP 1.2.4.4 1.8.1.4 Dihydro-lipoamide-E Dihydro-lipoamide-E Lipoamide-E Lip... |
| [hsa00290](https://www.genome.jp/dbget-bin/www_bget?pathway:hsa00290) | Valine, leucine and isoleucine biosynthesis - Homo sapiens (human) |  | C00188 (L-Threonine) C06032 (D-erythro-3-Methylmalate) C02226 (2-Methylmaleate) C02612 ((R)-2-Methyl... | 2.3.1.182 4.2.1.35 1.1.1.85 4.3.1.19 Threonine D-erythro-3-Methylmalate 2-Methyl-maleate (R)-2-Methy... |
| [hsa00310](https://www.genome.jp/dbget-bin/www_bget?pathway:hsa00310) | Lysine degradation - Homo sapiens (human) |  | C06181 (Piperideine) C05825 (2-Amino-5-oxohexanoate) C05161 ((2R,5S)-2,5-Diaminohexanoate) C00877 (C... | 1-Piperideine 2.6.1.21 2-Amino-5-oxohexanoate 2,5-Diaminohexanoate 1.4.1.12 5.4.3.3 5.1.1.9 1.5.1.1 ... |
| [hsa00330](https://www.genome.jp/dbget-bin/www_bget?pathway:hsa00330) | Arginine and proline metabolism - Homo sapiens (human) |  | C00062 (L-Arginine) C00077 (L-Ornithine) C00134 (Putrescine) C00555 (4-Aminobutyraldehyde) C00334 (4... | ARGININE AND PROLINE METABOLISM Arginine Ornithine Putrescine 4-Amino-butanal 4-Amino-butanoate �... |
| [hsa00340](https://www.genome.jp/dbget-bin/www_bget?pathway:hsa00340) | Histidine metabolism - Homo sapiens (human) |  | C00119 (5-Phospho-alpha-D-ribose 1-diphosphate) C02739 (1-(5-Phospho-D-ribosyl)-ATP) C02741 (Phospho... | 1.14.13.- 3.5.3.13 3.5.1.68 3.5.3.8 2.1.2.5 3.5.2.7 4.2.1.49 4.3.1.3 3.4.13.5 2.1.1.22 3.4.13.18 6.3... |
| [hsa00350](https://www.genome.jp/dbget-bin/www_bget?pathway:hsa00350) | Tyrosine metabolism - Homo sapiens (human) |  | C06044 (4-Hydroxyphenylethanol) C06046 (Salidroside) C04186 (5-Carboxymethyl-2-hydroxymuconate) C046... | Isoquinoline alkaloid biosynthesis 4-Hydroxy-phenylethanol Salidroside Dioxin degradation TYROSINE... |
| [hsa00360](https://www.genome.jp/dbget-bin/www_bget?pathway:hsa00360) | Phenylalanine metabolism - Homo sapiens (human) |  | C12621 (trans-3-Hydroxycinnamate) C00084 (Acetaldehyde) C00022 (Pyruvate) C03589 (4-Hydroxy-2-oxopen... | 4.1.3.39 4.3.1.25 Tropane, piperidine and pyridine alkaloid biosynthesis 1.2.1.5 6.2.1.30 2.3.1.14 ... |
| [hsa00380](https://www.genome.jp/dbget-bin/www_bget?pathway:hsa00380) | Tryptophan metabolism - Homo sapiens (human) |  | C05663 (6-Hydroxykynurenate) C05639 (4,6-Dihydroxyquinoline) C05652 (4-(2-Amino-5-hydroxyphenyl)-2,4... | 1.11.1.6 1.1.1.35 1.13.11.52 4.1.1.74 Nicotinamide metabolism 2.6.1.27 1.4.3.2 1.13.11.52 1.14.14.1 ... |
| [hsa00400](https://www.genome.jp/dbget-bin/www_bget?pathway:hsa00400) | Phenylalanine, tyrosine and tryptophan biosynthesis - Homo sapiens (human) |  | C00279 (D-Erythrose 4-phosphate) C00074 (Phosphoenolpyruvate) C04691 (2-Dehydro-3-deoxy-D-arabino-he... | 2.5.1.54 Phenylpropanoid biosynthesis Phenylpropanoid biosynthesis Biosynthesis of siderophore grou... |
| [hsa00410](https://www.genome.jp/dbget-bin/www_bget?pathway:hsa00410) | beta-Alanine metabolism - Homo sapiens (human) |  | C00429 (5,6-Dihydrouracil) C02642 (3-Ureidopropionate) C00099 (beta-Alanine) C00083 (Malonyl-CoA) C0... | β-ALANINE METABOLISM Propanoate metabolism Fatty acid biosynthesis Pantothenate and CoA biosynthes... |
| [hsa00430](https://www.genome.jp/dbget-bin/www_bget?pathway:hsa00430) | Taurine and hypotaurine metabolism - Homo sapiens (human) |  | C00094 (Sulfite) C06735 (Aminoacetaldehyde) C00041 (L-Alanine) C00022 (Pyruvate) C14179 (Sulfoacetat... | 1.14.11.17 Sulfur metabolism 1.1.2.- 1.2.1.73 1.4.1.1 Sulfite Aminoacetaldehyde L-Alanine Pyruvate S... |
| [hsa00440](https://www.genome.jp/dbget-bin/www_bget?pathway:hsa00440) | Phosphonate and phosphinate metabolism - Homo sapiens (human) | Natural products containing carbon-phosphorous bonds, so-called C-P compounds, are derivatives of ph... | C00074 (Phosphoenolpyruvate) C02798 (3-Phosphonopyruvate) C05672 (2-Amino-3-phosphonopropanoate) C17... | PHOSPHONATE AND PHOSPHINATE METABOLISM Glycolysis Phosphoenol-pyruvate 3-Phosphono-pyruvate Phosp... |
| [hsa00450](https://www.genome.jp/dbget-bin/www_bget?pathway:hsa00450) | Selenocompound metabolism - Homo sapiens (human) |  | C01528 (Hydrogen selenide) C05697 (Selenate) C05684 (Selenite) C05172 (Selenophosphoric acid) C06481... | SELENOCOMPOUND METABOLISM 1.8.1.9 2.7.9.3 2.1.1.12 4.4.1.11 6.1.1.10 Hydrogen selenide Selenate Se... |
| [hsa00471](https://www.genome.jp/dbget-bin/www_bget?pathway:hsa00471) | D-Glutamine and D-glutamate metabolism - Homo sapiens (human) |  | C00064 (L-Glutamine) C00819 (D-Glutamine) C03933 (5-D-Glutamyl-D-glutamyl-peptide) C05723 (Poly-gamm... | D-GLUTAMINE AND D-GLUTAMATE METABOLISM Peptidoglycan biosynthesis Amino sugar and nucleotide suga... |
| [hsa00472](https://www.genome.jp/dbget-bin/www_bget?pathway:hsa00472) | D-Arginine and D-ornithine metabolism - Homo sapiens (human) |  | C03564 (1-Pyrroline-2-carboxylate) C01110 (5-Amino-2-oxopentanoic acid) C03341 (2-Amino-4-oxopentano... | D-ARGININE AND D-ORNITHINE METABOLISM 1.4.1.12 5.4.3.5 1.4.3.3 2.6.1.21 5.1.1.10 5.1.1.9 5.1.1.12 ... |
| [hsa00480](https://www.genome.jp/dbget-bin/www_bget?pathway:hsa00480) | Glutathione metabolism - Homo sapiens (human) |  | C00024 (Acetyl-CoA) C00151 (L-Amino acid) C01879 (5-Oxoproline) C00097 (L-Cysteine) C00025 (L-Glutam... | Acetyl-CoA GLUTATHIONE METABOLISM Glutamate metabolism Taurine and hypotaurine metabolism Cyano... |
| [hsa00500](https://www.genome.jp/dbget-bin/www_bget?pathway:hsa00500) | Starch and sucrose metabolism - Homo sapiens (human) |  | C00092 (D-Glucose 6-phosphate) C00092 (D-Glucose 6-phosphate) C00095 (D-Fructose) C05731 (3-Ketosucr... | 3.6.1.- D-Glucose-6P 3.2.1.21 3.2.1.21 2.7.1.1 3.2.1.122 STARCH AND SUCROSE METABOLISM 3.2.1.26 2... |
| [hsa00510](https://www.genome.jp/dbget-bin/www_bget?pathway:hsa00510) | N-Glycan biosynthesis - Homo sapiens (human) | N-glycans or asparagine-linked glycans are major constituents of glycoproteins in eukaryotes. N-glyc... | G10596 G00022 G00021 G00020 G00019 G00018 (DS 3) G00017 G00016 G00015 G00014 G00013 G00012 G00011 G0... | ALG14 DPM3 DPM2 Various types of N-glycan biosynthesis ALG6 ALG12 ALG9 ALG3 N-glycan degradation MG... |
| [hsa00511](https://www.genome.jp/dbget-bin/www_bget?pathway:hsa00511) | Other glycan degradation - Homo sapiens (human) |  | 10825 (NEU3), 129807 (NEU4), 4758 (NEU1), 4759 (NEU2) 2720 (GLB1) 23324 (MAN2B2), 4123 (MAN2C1), 412... | OTHER GLYCAN DEGRADATION GlcNAc GlcNAc Fuc Neu5Ac Gal Asn α2 β1 β1 Man α1 GlcNAc Neu5Ac Gal α... |
| [hsa00512](https://www.genome.jp/dbget-bin/www_bget?pathway:hsa00512) | Mucin type O-glycan biosynthesis - Homo sapiens (human) | O-glycans are a class of glycans that modify serine or threonine residues of proteins. Biosynthesis ... | G00034 G00033 G00032 G00031 G00030 G00029 G00028 G00027 G00026 G00025 G00023 (Tn antigen) G00035 (Si... | Keratan sulfate biosynthesis Core 8 Core 7 (F1α) Core 6 Core 5 Core 4 Core 3 (disialyl-T antigen) (... |
| [hsa00514](https://www.genome.jp/dbget-bin/www_bget?pathway:hsa00514) | Other types of O-glycan biosynthesis - Homo sapiens (human) | O-linked glycosylation is the attachment of monosaccharides to the hydroxyl groups of amino acids, m... | 23127 (COLGALT2), 79709 (COLGALT1), 8985 (PLOD3) 145173 (B3GLCT) 8985 (PLOD3) 6480 (ST6GAL1), 6487 (... | OTHER TYPES OF O-GLYCAN BIOSYNTHESIS Glc Hydroxylysine α1 Glc β1 Gal β1 O-linked Gal type O-l... |
| [hsa00515](https://www.genome.jp/dbget-bin/www_bget?pathway:hsa00515) | Mannose type O-glycan biosynthesis - Homo sapiens (human) | Biosynthesis of mammalian O-mannosyl glycans is initiated by the transfer of mannose from mannose-P-... | C02189 ([Protein]-L-serine) C03862 (Dolichyl phosphate D-mannose) G13082 G13027 G13026 G13085 G13088... | 2.4.1.- -Ser/Thr-peptide Manβ-P-Dol 2.4.1.109 POMT1/POMT2 2.4.1.- GalT POMGNT1 2.4.99.6 2.4.1.135 S... |
| [hsa00520](https://www.genome.jp/dbget-bin/www_bget?pathway:hsa00520) | Amino sugar and nucleotide sugar metabolism - Homo sapiens (human) |  | C00357 (N-Acetyl-D-glucosamine 6-phosphate) C00140 (N-Acetyl-D-glucosamine) C04501 (N-Acetyl-alpha-D... | 2.7.1.59 5.4.2.3 2.7.7.23 3.2.1.183 2.7.1.60 2.5.1.57 3.1.3.29 3.5.99.6 3.5.1.25 5.4.2.10 2.3.1.157 ... |
| [hsa00524](https://www.genome.jp/dbget-bin/www_bget?pathway:hsa00524) | Neomycin, kanamycin and gentamicin biosynthesis - Homo sapiens (human) |  | C00031 (D-Glucose) C00092 (D-Glucose 6-phosphate) C17209 (2-Deoxy-scyllo-inosose) C17580 (2-Deoxy-sc... | NEOMYCIN, KANAMYCIN AND GENTAMICIN BIOSYNTHESIS D-Glucose D-Glucose-6P 2-Deoxy-scyllo-inosose 2-... |
| [hsa00531](https://www.genome.jp/dbget-bin/www_bget?pathway:hsa00531) | Glycosaminoglycan degradation - Homo sapiens (human) |  | G01391 G13074 G13073 G01977 G01945 G02632 G09660 G13040 G13039 G13038 G13037 G13036 G13035 G13034 G0... | 3.10.1.1 HPSE2 HPSE Heparan sulfate biosynthesis Keratan sulfate biosynthesis Chondroitin sulfate bi... |
| [hsa00532](https://www.genome.jp/dbget-bin/www_bget?pathway:hsa00532) | Glycosaminoglycan biosynthesis - chondroitin sulfate / dermatan sulfate - Homo sapiens (human) | Glycosaminoglycans (GAGs) are linear polysaccharide chains consisting of repeating disaccharide unit... | C00054 (Adenosine 3',5'-bisphosphate) C00053 (3'-Phosphoadenylyl sulfate) G00160 G00159 G00158 G0015... | PAP PAPS Heparan sulfate biosynthesis 2.4.1.135 2.4.2.26 2.4.1.133 GLYCOSAMINOGLYCAN BIOSYNTHESIS -... |
| [hsa00533](https://www.genome.jp/dbget-bin/www_bget?pathway:hsa00533) | Glycosaminoglycan biosynthesis - keratan sulfate - Homo sapiens (human) | Keratan sulfate (KS) is a glycosaminoglycan with the basic disaccharide unit of N-acetyllactosamine,... | 10678 (B3GNT2) 2683 (B4GALT1), 8703 (B4GALT3), 8704 (B4GALT2) 6487 (ST3GAL3) 2683 (B4GALT1), 8703 (B... | Keratan sulfate II (KSII) O-Glycan biosynthesis GLYCOSAMINOGLYCAN BIOSYNTHESIS - KERATAN SULFATE K... |
| [hsa00534](https://www.genome.jp/dbget-bin/www_bget?pathway:hsa00534) | Glycosaminoglycan biosynthesis - heparan sulfate / heparin - Homo sapiens (human) | Heparan sulfate (HS) and heparin (Hep) are glycosaminoglycans with repeating disaccharide units that... | G00164 G00163 G00162 G00157 C00054 (Adenosine 3',5'-bisphosphate) C00053 (3'-Phosphoadenylyl sulfate... | Chondroitin sulfate biosynthesis 2.4.1.223 EXTL2,3 EXTL1,3 EXT1,2 EXT1,2 GlcNAc GlcNAc GlcA GlcA Ga... |
| [hsa00561](https://www.genome.jp/dbget-bin/www_bget?pathway:hsa00561) | Glycerolipid metabolism - Homo sapiens (human) |  | C00422 (Triacylglycerol) C00641 (1,2-Diacyl-sn-glycerol) C00111 (Glycerone phosphate) C00093 (sn-Gly... | 2.3.1.158 SQD1 SQD2 2.4.1.241 2.4.99.5 Glycerophospholipid metabolism 3.1.1.3 2.3.1.20 2.4.1.46 2.4.... |
| [hsa00562](https://www.genome.jp/dbget-bin/www_bget?pathway:hsa00562) | Inositol phosphate metabolism - Homo sapiens (human) |  | C11557 (1-Phosphatidyl-1D-myo-inositol 5-phosphate) C11555 (1D-myo-Inositol 1,4,5,6-tetrakisphosphat... | 1-Phosphatidyl-1D-myo-inositol-5P 2.7.1.149 1D-myo-Inositol- 1,4,5,6P 3.1.3.36 2.7.1.151 2.7.1.151 ... |
| [hsa00563](https://www.genome.jp/dbget-bin/www_bget?pathway:hsa00563) | Glycosylphosphatidylinositol (GPI)-anchor biosynthesis - Homo sapiens (human) | Cell surface proteins can be attached to the cell membrane via the glycolipid structure called glyco... | G13045 G12396 (6-(alpha-D-glucosaminyl)-1D-myo-inositol) G00145 G00143 G00144 G00146 G13046 G00147 C... | PIG-Y DPM2 GPI1 PIG-P PIG-H PIG-C PIG-X PIG-U PIG-T PIG-S 6-(α-D-Glucosaminyl)-1D-myo-inositol PIG-... |
| [hsa00564](https://www.genome.jp/dbget-bin/www_bget?pathway:hsa00564) | Glycerophospholipid metabolism - Homo sapiens (human) |  | C06771 (Triethanolamine) C06772 (Diethanolamine) C00084 (Acetaldehyde) C00641 (1,2-Diacyl-sn-glycero... | Ether lipid metaboilsm 2.7.8.29 Ptdss1 Glycolysis 1.1.3.21 GPI-anchor biosynthesis Triethanolamine D... |
| [hsa00565](https://www.genome.jp/dbget-bin/www_bget?pathway:hsa00565) | Ether lipid metabolism - Homo sapiens (human) |  | C15647 (2-Acyl-1-(1-alkenyl)-sn-glycero-3-phosphate) C15646 (1-(1-Alkenyl)-sn-glycero-3-phosphate) C... | 2-Acyl-1-(1-alkenyl)-sn-glycero-3-phosphate (Plasmenic acid) 1-(1-Alkenyl)-sn-glycero-3-phosphate 1-... |
| [hsa00590](https://www.genome.jp/dbget-bin/www_bget?pathway:hsa00590) | Arachidonic acid metabolism - Homo sapiens (human) |  | C03577 (20-Hydroxyleukotriene E4) C14811 (Trioxilin B3) C14809 (Trioxilin A3) C14810 (Hepoxilin B3) ... | Eicosanoids 3.3.2.10 3.3.2.10 3.3.2.10 3.3.2.10 1.14.13.34 20-OH-LTE Trioxilin B Trioxilin A Hepoxil... |
| [hsa00591](https://www.genome.jp/dbget-bin/www_bget?pathway:hsa00591) | Linoleic acid metabolism - Homo sapiens (human) |  | C04717 ((9Z,11E)-(13S)-13-Hydroperoxyoctadeca-9,11-dienoic acid) C14833 (9,12,13-TriHOME) C14832 (12... | 3.3.2.- 3.3.2.- CYP1A2 13(S)-HPODE 9,12,13-TriHOME 12,13-Epoxy-9-hydroxy-10-octadecenoate 9-OxoODE 1... |
| [hsa00592](https://www.genome.jp/dbget-bin/www_bget?pathway:hsa00592) | alpha-Linolenic acid metabolism - Homo sapiens (human) |  | C11512 (Methyl jasmonate) C16318 ((+)-7-Isomethyljasmonate) C08491 ((-)-Jasmonic acid) C16317 ((+)-7... | MFP2 MFP2 MFP2 HPL1 ACX ACX 2.3.1.16 2.3.1.16 4.2.1.92 1.13.11.12 (-)-Methyl-jasmonate (+)-7-Isometh... |
| [hsa00600](https://www.genome.jp/dbget-bin/www_bget?pathway:hsa00600) | Sphingolipid metabolism - Homo sapiens (human) |  | C00836 (Sphinganine) C12144 (Phytosphingosine) C12145 (Phytoceramide) C12126 (Dihydroceramide) C0129... | YDC1 YPC1 2.7.8.3 3.1.3.- 3.1.3.- 3.1.3.- 3.5.1.23 3.5.1.23 2.3.1.24 1.14.19.17 LAG1 1.14.18.5 1.14.... |
| [hsa00601](https://www.genome.jp/dbget-bin/www_bget?pathway:hsa00601) | Glycosphingolipid biosynthesis - lacto and neolacto series - Homo sapiens (human) |  | G00078 (iso-nLc8Cer) G00057 G00090 (V3Fuc,III3Fuc-nLc6Cer) G00091 G00089 (V3Fuc-nLc6Cer) G00088 (VI3... | 2.4.1.152 2.4.1.152 2.4.1.152 2.4.1.152 2.4.1.- 2.4.1.65 2.4.1.65 2.4.1.65 2.4.1.65 2.4.1.65 B4GALT4... |
| [hsa00603](https://www.genome.jp/dbget-bin/www_bget?pathway:hsa00603) | Glycosphingolipid biosynthesis - globo and isoglobo series - Homo sapiens (human) |  | G00181 (Disialosylglobopentaosylceramide) G00096 (IV3GalNAcb-Gb4Cer) G00099 (Globo-H) G00100 (V3(Neu... | 2.4.99.4 (type IV H) (SSEA-4) (SSEA-3) B3GALT5 2.4.1.- 3.2.1.49 2.4.1.228 Globoside (P antigen) 3.2.... |
| [hsa00604](https://www.genome.jp/dbget-bin/www_bget?pathway:hsa00604) | Glycosphingolipid biosynthesis - ganglio series - Homo sapiens (human) |  | G00129 (GQ1balpha) G00128 (GT1aalpha) G00127 (GD1a) G00132 (GD1beta) G00131 (GM1alpha) G00130 (GM2al... | 2.4.99.4 3.2.1.23 3.2.1.52 Siat8a GQ1bα GT1aα GD1α GD1β GM1α GM2α O-Acetylated GT3 GP1c GQ1c G... |
| [hsa00620](https://www.genome.jp/dbget-bin/www_bget?pathway:hsa00620) | Pyruvate metabolism - Homo sapiens (human) |  | C15973 (Enzyme N6-(dihydrolipoyl)lysine) C15972 (Enzyme N6-(lipoyl)lysine) C16255 ([Dihydrolipoyllys... | PYRUVATE METABOLISM Nicotinate and nicotinamide metabolism 1.2.4.1 Citrate cycle Glycine, serine a... |
| [hsa00630](https://www.genome.jp/dbget-bin/www_bget?pathway:hsa00630) | Glyoxylate and dicarboxylate metabolism - Homo sapiens (human) |  | C00898 ((R,R)-Tartaric acid) C00036 (Oxaloacetate) C00158 (Citrate) C00149 ((S)-Malate) C04348 (L-Ma... | 5.3.1.22 4.2.1.3 2.2.1.5 2.3.3.12 2.3.3.7 4.1.3.13 4.1.3.16 4.1.3.1 3.5.1.68 3.5.1.56 3.5.1.49 3.5.1... |
| [hsa00640](https://www.genome.jp/dbget-bin/www_bget?pathway:hsa00640) | Propanoate metabolism - Homo sapiens (human) |  | C00222 (3-Oxopropanoate) C00804 (Propynoate) C01013 (3-Hydroxypropanoate) C00099 (beta-Alanine) C000... | PROPANOATE METABOLISM 1.2.1.18 2.1.3.1 6.4.1.2 4.1.1.9 1.2.1.75 2.6.1.18 2.6.1.19 1.1.1.59 4.2.1.27... |
| [hsa00650](https://www.genome.jp/dbget-bin/www_bget?pathway:hsa00650) | Butanoate metabolism - Homo sapiens (human) |  | C03046 ((S,S)-Butane-2,3-diol) C01769 ((S)-Acetoin) C00810 ((R)-Acetoin) C03044 ((R,R)-Butane-2,3-di... | Biosynthesis of type II polyketide backbone 4.2.1.27 1.1.1.30 3.1.1.22 2.8.3.5 6.2.1.16 4.1.3.4 2.3... |
| [hsa00670](https://www.genome.jp/dbget-bin/www_bget?pathway:hsa00670) | One carbon pool by folate - Homo sapiens (human) |  | C00504 (Folate) C00415 (Dihydrofolate) C03479 (Folinic acid) C00143 (5,10-Methylenetetrahydrofolate)... | ONE CARBON POOL BY FOLATE Folate biosynthesis 2.1.2.5 2.1.2.4 2.1.2.2 4.3.1.4 1.5.1.15 1.5.1.3 1... |
| [hsa00730](https://www.genome.jp/dbget-bin/www_bget?pathway:hsa00730) | Thiamine metabolism - Homo sapiens (human) |  | C00082 (L-Tyrosine) C15809 (Iminoglycine) C11437 (1-Deoxy-D-xylulose 5-phosphate) C15814 (Thiocarbox... | L-Tyrosine Iminoglycine 1-Deoxy-D-xylulose 5-phosphate [ThiS]-COSH [ThiS]-CO-AMP [IscS]-SSH [IscS]-... |
| [hsa00740](https://www.genome.jp/dbget-bin/www_bget?pathway:hsa00740) | Riboflavin metabolism - Homo sapiens (human) |  | C15556 (L-3,4-Dihydroxybutan-2-one 4-phosphate) C00199 (D-Ribulose 5-phosphate) C00044 (GTP) C01304 ... | 3,4-Dihydroxy-2-butanone 4-phosphate Ribulose 5-phosphate Pentose phosphate pathway RIBOFLAVIN META... |
| [hsa00750](https://www.genome.jp/dbget-bin/www_bget?pathway:hsa00750) | Vitamin B6 metabolism - Homo sapiens (human) |  | C00118 (D-Glyceraldehyde 3-phosphate) C00199 (D-Ribulose 5-phosphate) C07335 (2-Amino-3-oxo-4-phosph... | Glyceraldehyde 3-phosphate Ribulose 5-phosphate 4.3.3.6 Glycolysis Pentose phosphate pathway 2-Amino... |
| [hsa00760](https://www.genome.jp/dbget-bin/www_bget?pathway:hsa00760) | Nicotinate and nicotinamide metabolism - Homo sapiens (human) |  | C06178 (1-Methylpyrrolinium) C15523 (2,6-Dihydroxynicotinate) C00022 (Pyruvate) C00163 (Propanoate) ... | 1.5.99.4 Pyruvate metabolism C5-Branched dibasic acid metabolism 4.1.3.32 4.2.1.85 5.3.3.6 5.4.99.4... |
| [hsa00770](https://www.genome.jp/dbget-bin/www_bget?pathway:hsa00770) | Pantothenate and CoA biosynthesis - Homo sapiens (human) |  | C03688 (Apo-[acyl-carrier-protein]) C00022 (Pyruvate) C00900 (2-Acetolactate) C04039 (2,3-Dihydroxy-... | Apo-[acp] PANTOTHENATE AND CoA BIOSYNTHESIS Alanine, aspartate and glutamate metabolism Propanoate ... |
| [hsa00780](https://www.genome.jp/dbget-bin/www_bget?pathway:hsa00780) | Biotin metabolism - Homo sapiens (human) | Biotin (vitamin H or vitamin B7) is the essential cofactor of biotin-dependent carboxylases, such as... | C02656 (Pimelate) C01063 (6-Carboxyhexanoyl-CoA) C01092 (8-Amino-7-oxononanoate) C01909 (Dethiobioti... | Tropane, piperidine and pyridine alkaloid biosynthesis BIOTIN METABOLISM Lysine degradation Pimel... |
| [hsa00785](https://www.genome.jp/dbget-bin/www_bget?pathway:hsa00785) | Lipoic acid metabolism - Homo sapiens (human) |  | C16239 (Lipoyl-[acp]) C05752 (Octanoyl-[acp]) C16241 ((R)-Lipoate) C16238 (Lipoyl-AMP) C16236 (Prote... | 2.8.1.8 2.3.1.181 Lipoyl-[acp] LIPOIC ACID METABOLISM Fatty acid biosynthesis 2.8.1.8 2.3.1.181 O... |
| [hsa00790](https://www.genome.jp/dbget-bin/www_bget?pathway:hsa00790) | Folate biosynthesis - Homo sapiens (human) |  | C11355 (4-Amino-4-deoxychorismate) C00251 (Chorismate) C00044 (GTP) C04895 (7,8-Dihydroneopterin 3'-... | 4-Amino-4-deoxychorismate Chorismate FOLATE BIOSYNTHESIS One carbon pool by folate Purine metabolis... |
| [hsa00830](https://www.genome.jp/dbget-bin/www_bget?pathway:hsa00830) | Retinol metabolism - Homo sapiens (human) |  | C05917 (Iodopsin) C00473 (Retinol) C00376 (Retinal) C02094 (beta-Carotene) C00777 (Retinoate) C02075... | Iodopsin RETINOL METABOLISM IN ANIMALS all-trans-Retinol (Vitamine A) ADH all-trans-Retinal 1... |
| [hsa00860](https://www.genome.jp/dbget-bin/www_bget?pathway:hsa00860) | Porphyrin and chlorophyll metabolism - Homo sapiens (human) |  | C05774 (Cobinamide) C16243 (Cobalt-precorrin 5B) C16244 (Cobalt-precorrin 7) C15670 (Heme A) C15672 ... | Alanine, aspartate and glutamate metabolism 3.1.1.82 Cobinamide Co-precorrin 5B Co-precorrin 7 Heme... |
| [hsa00900](https://www.genome.jp/dbget-bin/www_bget?pathway:hsa00900) | Terpenoid backbone biosynthesis - Homo sapiens (human) | Terpenoids, also known as isoprenoids, are a large class of natural products consisting of isoprene ... | C05859 (Dehydrodolichol diphosphate) C00621 (Dolichyl diphosphate) C00022 (Pyruvate) C00024 (Acetyl-... | MEP/DOXP pathway Mevalonate pathway Dehydro dolichol-PP Dolichol-PP Glycolysis TERPENOID BACKBONE ... |
| [hsa00910](https://www.genome.jp/dbget-bin/www_bget?pathway:hsa00910) | Nitrogen metabolism - Homo sapiens (human) | The biological process of the nitrogen cycle is a complex interplay among many microorganisms cataly... | C00058 (Formate) C00488 (Formamide) C00014 (Ammonia) C00192 (Hydroxylamine) C06058 (Nitroalkane) C00... | Formate Formamide 3.5.1.49 Ammonia Hydroxylamine Nitroalkane 1.7.2.6 1.9.6.1 1.7.99.- 1.7.7.2 1.13.1... |
| [hsa00920](https://www.genome.jp/dbget-bin/www_bget?pathway:hsa00920) | Sulfur metabolism - Homo sapiens (human) | Sulfur is an essential element for life and the metabolism of organic sulfur compounds plays an impo... | C00059 (Sulfate) C00094 (Sulfite) C00283 (Hydrogen sulfide) C00224 (Adenylyl sulfate) C00054 (Adenos... | SULFUR METABOLISM Sulfate Sulfite 1.8.2.1 1.8.3.1 1.8.7.1 1.8.99.5 1.8.1.2 Asr Sulfide 3.1.3.7 2.7.... |
| [hsa00970](https://www.genome.jp/dbget-bin/www_bget?pathway:hsa00970) | Aminoacyl-tRNA biosynthesis - Homo sapiens (human) |  | C06113 (L-Aspartyl-tRNA(Asn)) C02992 (L-Threonyl-tRNA(Thr)) C02839 (L-Tyrosyl-tRNA(Tyr)) C00078 (L-T... | L-Aspartyl-tRNA(Asn) 6.1.1.5 AMINOACYL-tRNA BIOSYNTHESIS 6.1.1.22 6.1.1.21 6.1.1.20 6.1.1.19 6.1.1.... |
| [hsa00980](https://www.genome.jp/dbget-bin/www_bget?pathway:hsa00980) | Metabolism of xenobiotics by cytochrome P450 - Homo sapiens (human) |  | C14039 (1,1-Dichloroethylene) C14876 (S-(2-Hydroxyethyl)-N-acetyl-L-cysteine) C14875 (S-(2-Hydroxyet... | 1,1-Dichloro-ethylene CYP2E1 CYP2E1 CYP2E1 CYP2E1 CYP2E1 S-(2-Hydroxyethyl)-N-acetyl-L-cysteine S-(2... |
| [hsa00982](https://www.genome.jp/dbget-bin/www_bget?pathway:hsa00982) | Drug metabolism - cytochrome P450 - Homo sapiens (human) |  | C16544 (alpha-Hydroxytamoxifen) D00343 (Ifosfamide (JAN/USP/INN)), C07047 (Ifosfamide) C16612 (Cital... | α-Hydroxytamoxifen Ifosfamide (prodrug) Citalopram-aldehyde 4-Ene-VPA 4-OH-VPA 5-OH-VPA Oxcarbazepi... |
| [hsa00983](https://www.genome.jp/dbget-bin/www_bget?pathway:hsa00983) | Drug metabolism - other enzymes - Homo sapiens (human) |  | D00346 (Isoniazid (JP17/USP/INN)), C07054 (Isoniazid) C04646 (6-Thioinosine-5'-monophosphate) D01223... | Isoniazid 6-Thioinosine-5'-monophosphate Capecitabine Isonicotinic acid DRUG METABOLISM - OTHER ENZ... |
| [hsa01040](https://www.genome.jp/dbget-bin/www_bget?pathway:hsa01040) | Biosynthesis of unsaturated fatty acids - Homo sapiens (human) |  | C16533 (13,16-Docosadienoic acid) C16645 ((13Z,16Z)-Docosadi-13,16-enoyl-CoA) C16645 ((13Z,16Z)-Doco... | Docosadienoic acid Δ13,16 Δ13,16 3.1.2.- YciA TesB TesB TesB TesA TesA TesA 3.1.2.- 3.1.2.- 3.1.2.... |
| [hsa01100](https://www.genome.jp/dbget-bin/www_bget?pathway:hsa01100) | Metabolic pathways - Homo sapiens (human) |  | C16157 (Undecaprenyl phosphate alpha-L-Ara4N) C16156 (Undecaprenyl phosphate alpha-L-Ara4FN) C16154 ... | Lipoarabinomannan (LAM) biosynthesis Neomycin, kanamycin and gentamicin biosynthesis Glycosaminogly... |
| [hsa01200](https://www.genome.jp/dbget-bin/www_bget?pathway:hsa01200) | Carbon metabolism - Homo sapiens (human) | Carbon metabolism is the most basic aspect of life. This map presents an overall view of central car... | C00085 (D-Fructose 6-phosphate), C05345 (beta-D-Fructose 6-phosphate) C01182 (D-Ribulose 1,5-bisphos... | HCO HCHO HCHO HCO HCO AcCoA CARBON METABOLISM Fructose-6P Ribulose-1,5P Glycerate-3P THF Glucose Gl... |
| [hsa01210](https://www.genome.jp/dbget-bin/www_bget?pathway:hsa01210) | 2-Oxocarboxylic acid metabolism - Homo sapiens (human) | 2-Oxocarboxylic acids, also called 2-oxo acids and alpha-keto acids, are the most elementary set of ... | C17254 (8-Methylthiooctyl glucosinolate) C17232 (2-Oxo-10-methylthiodecanoic acid) C17252 (7-Methylt... | 8-Methylthiooctyl glucosinolate 2-Oxo-10-methylthio-decanoic acid 7-Methylthioheptyl glucosinolate... |
| [hsa01212](https://www.genome.jp/dbget-bin/www_bget?pathway:hsa01212) | Fatty acid metabolism - Homo sapiens (human) |  | C00229 (Acyl-carrier protein) C00024 (Acetyl-CoA) C03939 (Acetyl-[acyl-carrier protein]) C05744 (Ace... | FATTY ACID METABOLISM ACP Acetyl-CoA Acetyl-[acp] Acetoacetyl-[acp] (R)-3-Hydroxybutanoyl-[acp] Ma... |
| [hsa01230](https://www.genome.jp/dbget-bin/www_bget?pathway:hsa01230) | Biosynthesis of amino acids - Homo sapiens (human) | This map presents a modular architecture of the biosynthesis pathways of twenty amino acids, which m... | C04390 (N6-Acetyl-LL-2,6-diaminoheptanedioate) C04002 ((Z)-But-1-ene-1,2,4-tricarboxylate) C00118 (D... | N-Acetyl-LL-2,6-diaminopimelate M00763 Homo-cis-aconitate BIOSYNTHESIS OF AMINO ACIDS Glyceraldeh... |
| [hsa01521](https://www.genome.jp/dbget-bin/www_bget?pathway:hsa01521) | EGFR tyrosine kinase inhibitor resistance - Homo sapiens (human) | EGFR is a tyrosine kinase that participates in the regulation of cellular homeostasis. EGFR also ser... | C00165 (Diacylglycerol) C05981 (Phosphatidylinositol-3,4,5-trisphosphate) D07907 (Erlotinib (INN)) D... | Non-small cell lung cancer Her3 Her2 EGFR Pancreatic cancer mTOR signaling pathway GSK-3 eI... |
| [hsa01522](https://www.genome.jp/dbget-bin/www_bget?pathway:hsa01522) | Endocrine resistance - Homo sapiens (human) | Endocrine therapy is a key treatment strategy to control or eradicate hormone-responsive breast canc... | C00951 (Estradiol-17beta) C00951 (Estradiol-17beta) C00575 (3',5'-Cyclic AMP) C00951 (Estradiol-17be... | ENDOCRINE RESISTANCE GPR30 Src MMP HB-EGF EGFR HB-EGF CoA DNA DNA cAMP PKA Ras Raf MEK ERK1/2 GPR30... |
| [hsa01523](https://www.genome.jp/dbget-bin/www_bget?pathway:hsa01523) | Antifolate resistance - Homo sapiens (human) | Since the 1940s, antifolates have played a pivotal role in drug treatment of malignant, microbial, p... | D00142 (Methotrexate (JP17/USP/INN)), D05589 (Pralatrexate (JAN/USAN/INN)), D04766 (Lometrexol sodiu... | ANTIFOLATE RESISTANCE RFC ABCC1 ABCC2 ABCC3 ABCC4 ABCC5 GARTF Antifolate Methotrexate (MTX) Pemetre... |
| [hsa01524](https://www.genome.jp/dbget-bin/www_bget?pathway:hsa01524) | Platinum drug resistance - Homo sapiens (human) | Platinum-based drugs cisplatin, carboplatin and oxaliplatin are widely used in the therapy of solid ... | D00275 (Cisplatin (JP17/USP/INN)) D01790 (Oxaliplatin (JAN/USAN/INN)) D01363 (Carboplatin (JP17/USP/... | Mitochondrion p53 signaling pathway ATM p53 Bcl-2 CASP9 CASP3 Bad IAP/XIAP Apaf-1 CytC Bid CASP8 FAD... |
| [hsa02010](https://www.genome.jp/dbget-bin/www_bget?pathway:hsa02010) | ABC transporters - Homo sapiens (human) | The ATP-binding cassette (ABC) transporters form one of the largest known protein families, and are ... | C16421 (AI-2) C01684 (D-Rhamnose) C00095 (D-Fructose) C01487 (D-Allose) C00181 (D-Xylose) C03619 (Me... | GguA GguB ChvE LsrA LsrD LsrC LsrB Autoinducer 2 RhaT RhaQ RhaP RhaS Rhamnose FrcA FrcC FrcB Fructos... |
| [hsa03008](https://www.genome.jp/dbget-bin/www_bget?pathway:hsa03008) | Ribosome biogenesis in eukaryotes - Homo sapiens (human) | Ribosomes are the cellular factories responsible for making proteins. In eukaryotes, ribosome biogen... | 5901 (RAN) 7514 (XPO1) 51068 (NMD3) 26851 (SNORD3B-1), 780851 (SNORD3A), 780852 (SNORD3B-2), 780853 ... | Cleavages Box C/D snoRNPs RIBOSOME BIOGENESIS IN EUKARYOTES pre-rRNA Ribosome Ran CRM1 NMD3 Pol ... |
| [hsa03010](https://www.genome.jp/dbget-bin/www_bget?pathway:hsa03010) | Ribosome - Homo sapiens (human) |  | 2197 (FAU) 6137 (RPL13) 6158 (RPL28) 6208 (RPS14) 6228 (RPS23) 6222 (RPS18) 6235 (RPS29) 6207 (RPS13... | L7A S30e L13e L28e S14e S23e S18e S29e S13e S11e S15e S6e S8e S17e S19e S24e S27e S27Ae S7e S10e S12... |
| [hsa03013](https://www.genome.jp/dbget-bin/www_bget?pathway:hsa03013) | RNA transport - Homo sapiens (human) | RNA transport from the nucleus to the cytoplasm is fundamental for gene expression. The different RN... | C01647 (tRNA(Met)) C00044 (GTP) C00035 (GDP) C01647 (tRNA(Met)) C00044 (GTP) 11260 (XPOT) 5901 (RAN)... | NPC RNA TRANSPORT Nucleus Cytoplasm pre-tRNA U snRNA pre-mRNA Nuclear Pore complex (N... |
| [hsa03015](https://www.genome.jp/dbget-bin/www_bget?pathway:hsa03015) | mRNA surveillance pathway - Homo sapiens (human) | The mRNA surveillance pathway is a quality control mechanism that detects and degrades abnormal mRNA... | 9939 (RBM8A) 4116 (MAGOH), 55110 (MAGOHB) 9775 (EIF4A3) 22794 (CASC3) 22985 (ACIN1) 10921 (RNPS1) 10... | Spliceosome m7G m7G AAAAAAA EJC complex Y14 MAGOH EIF4A3 MLN51 ACIN1 RNPS1 SAP18 SRm160 Tap Pinin Re... |
| [hsa03018](https://www.genome.jp/dbget-bin/www_bget?pathway:hsa03018) | RNA degradation - Homo sapiens (human) | The correct processing, quality control and turnover of cellular RNA molecules are critical to many ... | 2023 (ENO1), 2026 (ENO2), 2027 (ENO3), 387712 (ENO4) 87178 (PNPT1) 54512 (EXOSC4) 23016 (EXOSC7) 510... | RNA helicase RNase E Catalytic domain C-terminal domain Degradosome component interaction RNA d... |
| [hsa03020](https://www.genome.jp/dbget-bin/www_bget?pathway:hsa03020) | RNA polymerase - Homo sapiens (human) |  | 5431 (POLR2B) 5430 (POLR2A) 5432 (POLR2C) 5433 (POLR2D) 5434 (POLR2E) 5435 (POLR2F) 5436 (POLR2G) 54... | RNA POLYMERASE β' ABC1 ABC2 ABC3 ABC5 B11 ABC4 AC1 C11 AC2 C25 C31 C34 A12 A14 A34 A43 A49 RNA pol... |
| [hsa03022](https://www.genome.jp/dbget-bin/www_bget?pathway:hsa03022) | Basal transcription factors - Homo sapiens (human) |  | 387332 (TBPL2), 6908 (TBP), 9519 (TBPL1) 138474 (TAF1L), 6872 (TAF1) 6873 (TAF2) 83860 (TAF3) 6874 (... | General transcription factors for RNA polymerase II BASAL TRANSCRIPTION FACTORS (EUKARYOTES) TBP T... |
| [hsa03030](https://www.genome.jp/dbget-bin/www_bget?pathway:hsa03030) | DNA replication - Homo sapiens (human) | A complex network of interacting proteins and enzymes is required for DNA replication. Generally, DN... | 3978 (LIG1) 2237 (FEN1) 1763 (DNA2) 10535 (RNASEH2A) 79621 (RNASEH2B) 5982 (RFC2), 5984 (RFC4) 84153... | Lig1 Fen1 Dna2 RNaseH2A RNaseH2B RFC2/4 RNaseH2C RFC3/5 PCNA RPA3 Mcm5 δ4 δ3 ε4 ε3 SSB DnaG DNA ... |
| [hsa03040](https://www.genome.jp/dbget-bin/www_bget?pathway:hsa03040) | Spliceosome - Homo sapiens (human) | After transcription, eukaryotic mRNA precursors contain protein-coding exons and noncoding introns. ... | 9879 (DDX46) 7919 (DDX39B) 6625 (SNRNP70) 6626 (SNRPA) 6631 (SNRPC) 8449 (DHX16) 9785 (DHX38) 51362 ... | complex pre-mRNA Exon Exon 5' splice site 3' splice site Branch point complex complex complex B* Pos... |
| [hsa03050](https://www.genome.jp/dbget-bin/www_bget?pathway:hsa03050) | Proteasome - Homo sapiens (human) | The proteasome is a protein-destroying apparatus involved in many essential cellular functions, such... | 3458 (IFNG) 5709 (PSMD3) 5718 (PSMD12) 5717 (PSMD11) 9861 (PSMD6) 5713 (PSMD7) 5719 (PSMD13) 10213 (... | IFNγ Rpt2 Rpt1 β2 β3 β4 β5 β6 β7 α1 α2 α3 α4 α5 α6 α7 β1 Rpt6 Rpt4 Rpt5 Rpt3 Rpn1 Rpn... |
| [hsa03060](https://www.genome.jp/dbget-bin/www_bget?pathway:hsa03060) | Protein export - Homo sapiens (human) | The protein export is the active transport of proteins from the cytoplasm to the exterior of the cel... | 5018 (OXA1L) 6727 (SRP14) 6731 (SRP72) 6730 (SRP68) 6729 (SRP54) 6728 (SRP19) 6734 (SRPRA) 6726 (SRP... | TatE Ffs YidC TatB TatC TatA SecA SecY SecE SecG SecD/F YajC SPase I SPase II SRP14 SRP72 SRP68 SRP5... |
| [hsa03320](https://www.genome.jp/dbget-bin/www_bget?pathway:hsa03320) | PPAR signaling pathway - Homo sapiens (human) | Peroxisome proliferator-activated receptors (PPARs) are nuclear hormone receptors that are activated... | C02165 (Leukotriene B4), C14776 (8(S)-HETE) C15493 (9-cis-Retinoic acid) C15493 (9-cis-Retinoic acid... | Unsaturated fatty acid Unsaturated fatty acid Saturated fatty acid Eicosanoid Fibrate drug NSAID 9-c... |
| [hsa03410](https://www.genome.jp/dbget-bin/www_bget?pathway:hsa03410) | Base excision repair - Homo sapiens (human) | Base excision repair (BER) is the predominant DNA damage repair pathway for the processing of small ... | 27301 (APEX2), 328 (APEX1) 2237 (FEN1) 10038 (PARP2), 10039 (PARP3), 142 (PARP1), 143 (PARP4) 3978 (... | APEX Fen1 PARP Lig1 Lig3 Lig Polε Polδ Polβ Polβ Polβ Polβ PCNA PCNA PCNA DpoI DpoI DpoI APE2 ... |
| [hsa03420](https://www.genome.jp/dbget-bin/www_bget?pathway:hsa03420) | Nucleotide excision repair - Homo sapiens (human) | Nucleotide excision repair (NER) is a mechanism to recognize and repair bulky DNA damage caused by c... | 2968 (GTF2H4) 2967 (GTF2H3) 2966 (GTF2H2), 728340 (GTF2H2C), 730394 (GTF2H2C_2) 2965 (GTF2H1) 2068 (... | 5 ' 3 ' 3 ' 5 ' 5 ' 3 ' 3 ' 5 ' TFIIH4 TFIIH3 TFIIH2 TFIIH1 XPD TTDA XPB CCNH MNAT1 CDK7 DDB1 Cul4 R... |
| [hsa03430](https://www.genome.jp/dbget-bin/www_bget?pathway:hsa03430) | Mismatch repair - Homo sapiens (human) | DNA mismatch repair (MMR) is a highly conserved biological pathway that plays a key role in maintain... | 4436 (MSH2) 27030 (MLH3) 4292 (MLH1) 4292 (MLH1) 3978 (LIG1) 5111 (PCNA) 10714 (POLD3), 5424 (POLD1)... | Dam MutS MSH2 MLH3 MLH1 Colorectal cancer MutH MutL Lig UVRD MLH1 LigI PCNA DpoIII ExoI Polδ RPA Ex... |
| [hsa03440](https://www.genome.jp/dbget-bin/www_bget?pathway:hsa03440) | Homologous recombination - Homo sapiens (human) | Homologous recombination (HR) is essential for the accurate repair of DNA double-strand breaks (DSBs... | 7156 (TOP3A), 8940 (TOP3B) 641 (BLM) 7979 (SEM1) 5893 (RAD52) 4361 (MRE11) 10111 (RAD50) 7517 (XRCC3... | TOP3 BLM DSS1 Rad52 Mre11 Rad50 XRCC3 Rad51C XRCC2 XRCC2 Rad51D Rad51D Rad51C Rad51B Rad51C DnaT Pri... |
| [hsa03450](https://www.genome.jp/dbget-bin/www_bget?pathway:hsa03450) | Non-homologous end-joining - Homo sapiens (human) | Nonhomologous end joining (NHEJ) eliminates DNA double-strand breaks (DSBs) by direct ligation. NHEJ... | 3981 (LIG4) 27343 (POLL) 3981 (LIG4) 1791 (DNTT) 27434 (POLM) 64421 (DCLRE1C) 4361 (MRE11A) 10111 (R... | Lig4 Nej1 Polλ Dnl4 TdT Polμ Artemis Pol4 Lig NON-HOMOLOGOUS END-JOINING Mre11 Rad50 DNAPKcs XRS2... |
| [hsa03460](https://www.genome.jp/dbget-bin/www_bget?pathway:hsa03460) | Fanconi anemia pathway - Homo sapiens (human) | The Fanconi anemia pathway is required for the efficient repair of damaged DNA, especially interstra... | 57697 (FANCM) 2175 (FANCA) 2187 (FANCB) 2176 (FANCC) 2177 (FANCD2) 55215 (FANCI) 2178 (FANCE) 2188 (... | FANCM FANCA FANCB FANCC FANCONI ANEMIA PATHWAY FANCD2 FANCI FANCE FANCF FANCG FANCL FANCN FAAP100 ... |
| [hsa04010](https://www.genome.jp/dbget-bin/www_bget?pathway:hsa04010) | MAPK signaling pathway - Homo sapiens (human) | The mitogen-activated protein kinase (MAPK) cascade is a highly conserved module that is involved in... | C00338 (Lipopolysaccharide) C00165 (Diacylglycerol) C00575 (3',5'-Cyclic AMP) C01245 (D-myo-Inositol... | LPS RasGRF MKP PTP PP2CB FASL DAG cAMP HGK cPLA2 STMN1 Tau NFκB IKK NIK ERK MP1 MEK2 MEK1 Raf1 RafB... |
| [hsa04012](https://www.genome.jp/dbget-bin/www_bget?pathway:hsa04012) | ErbB signaling pathway - Homo sapiens (human) | The ErbB family of receptor tyrosine kinases (RTKs) couples binding of extracellular growth factor l... | C00076 (Calcium cation) C00165 (Diacylglycerol) C01245 (D-myo-Inositol 1,4,5-trisphosphate) C05981 (... | Non-small cell lung cancer Grb2 Elk ErbB-4 ErbB-4 ErbB-3 ErbB-3 STAT5 ErbB-2 ErbB-2 ErbB-2 ErbB-2... |
| [hsa04014](https://www.genome.jp/dbget-bin/www_bget?pathway:hsa04014) | Ras signaling pathway - Homo sapiens (human) | The Ras proteins are GTPases that function as molecular switches for signaling pathways regulating c... | C05981 (Phosphatidylinositol-3,4,5-trisphosphate) C00165 (Diacylglycerol) C01245 (D-myo-Inositol 1,4... | MAPK signaling pathway RAS SIGNALING PATHWAY ERK MEK Raf-1 Ras PI3K-Akt signaling pathway Grb2 SOS... |
| [hsa04015](https://www.genome.jp/dbget-bin/www_bget?pathway:hsa04015) | Rap1 signaling pathway - Homo sapiens (human) | Rap1 is a small GTPase that controls diverse processes, such as cell adhesion, cell-cell junction fo... | C00044 (GTP) C00035 (GDP) C00575 (3',5'-Cyclic AMP) C00165 (Diacylglycerol) C00076 (Calcium cation) ... | MAPK signaling pathway RAP1 SIGNALING PATHWAY ERK MEK Raf-1 Rap1 RIAM RTK RalGDS Rac RapL MEK3,6 p... |
| [hsa04020](https://www.genome.jp/dbget-bin/www_bget?pathway:hsa04020) | Calcium signaling pathway - Homo sapiens (human) | Ca2+ that enters the cell from the outside is a principal source of signal Ca2+. Entry of Ca2+ is dr... | C01330 (Sodium cation) C00076 (Calcium cation) C13050 (Cyclic ADP-ribose) C13051 (Nicotinic acid ade... | Long term depression Long term potentiation Phosphatidylinositol signaling pathway Apoptosis ... |
| [hsa04022](https://www.genome.jp/dbget-bin/www_bget?pathway:hsa04022) | cGMP-PKG signaling pathway - Homo sapiens (human) | Cyclic GMP (cGMP) is the intracellular second messenger that mediates the action of nitric oxide (NO... | C00533 (Nitric oxide) C00942 (3',5'-Cyclic GMP) C00076 (Calcium cation) C00238 (Potassium cation) C1... | cGMP-PKG SIGNALING PATHWAY MLCP MLC NPR-A cGMP PKG s-GC Sarcoplasmic reticulum (SR) Kca ANP BNP CN... |
| [hsa04024](https://www.genome.jp/dbget-bin/www_bget?pathway:hsa04024) | cAMP signaling pathway - Homo sapiens (human) | cAMP is one of the most common and universal second messengers, and its formation is promoted by ade... | C00076 (Calcium cation) C00076 (Calcium cation) C00238 (Potassium cation) C01330 (Sodium cation) C00... | cAMP SIGNALING PATHWAY Sarcoplasmic Reticulum (SR) VDCC RyR2 SERCA2a TnI Calcium signaling pathway... |
| [hsa04060](https://www.genome.jp/dbget-bin/www_bget?pathway:hsa04060) | Cytokine-cytokine receptor interaction - Homo sapiens (human) | Cytokines are soluble extracellular proteins or glycoproteins that are crucial intercellular regulat... | 53833 (IL20RB) 53833 (IL20RB) 3565 (IL4) 659 (BMPR2) 93 (ACVR2B) 91 (ACVR1B) 92 (ACVR2A) 3588 (IL10R... | IL20RB IL20RB IL4 BMPR2 ACVR2B ACVR1B ACVR2A IL10RB IL22RA1 IL2RG IL2RG IL2RG IL2RG IL2RG IL2RG IL2R... |
| [hsa04062](https://www.genome.jp/dbget-bin/www_bget?pathway:hsa04062) | Chemokine signaling pathway - Homo sapiens (human) | Inflammatory immune response requires the recruitment of leukocytes to the site of inflammation upon... | C00165 (Diacylglycerol) C01245 (D-myo-Inositol 1,4,5-trisphosphate) C00076 (Calcium cation) C00575 (... | Migration Apoptosis Degranulation Cellular shape changes Cell survival chemokineR chemokine JAK2/3 ... |
| [hsa04064](https://www.genome.jp/dbget-bin/www_bget?pathway:hsa04064) | NF-kappa B signaling pathway - Homo sapiens (human) | Nuclear factor-kappa B (NF-kappa B) is the generic name of a family of transcription factors that fu... | C00165 (Diacylglycerol) C01245 (D-myo-Inositol 1,4,5-trisphosphate) C00076 (Calcium cation) C00165 (... | Cytokine-cytokine receptor interaction Bcl-XL Bcl-2 c-IAP1/2 IκBα IRAK1/4 MyD88 TRAF2/5 RIP1 TRADD... |
| [hsa04066](https://www.genome.jp/dbget-bin/www_bget?pathway:hsa04066) | HIF-1 signaling pathway - Homo sapiens (human) | Hypoxia-inducible factor 1 (HIF-1) is a transcription factor that functions as a master regulator of... | C00165 (Diacylglycerol) C01245 (D-myo-Inositol 1,4,5-trisphosphate) C00704 (O2.-) C00031 (D-Glucose)... | CUL2 Rbx1 ElonginB ElonginC VEGF PHD VHL HIF-1β p300/CBP HIF-1α Glut1 HIF-1 SIGNALING PATHWAY mT... |
| [hsa04068](https://www.genome.jp/dbget-bin/www_bget?pathway:hsa04068) | FoxO signaling pathway - Homo sapiens (human) | The forkhead box O (FOXO) family of transcription factors regulates the expression of genes in cellu... | C00031 (D-Glucose) C05981 (Phosphatidylinositol-3,4,5-trisphosphate) C00008 (ADP) C00020 (AMP) C0002... | IKKα/β JNK ERK1/2 AMPK INS Glucose FOXO SOS Ras MEK1/2 INSR Raf PEPCK G6PC Akt P13K PDK1/2 IRS PIP... |
| [hsa04070](https://www.genome.jp/dbget-bin/www_bget?pathway:hsa04070) | Phosphatidylinositol signaling system - Homo sapiens (human) |  | C11554 (1-Phosphatidyl-1D-myo-inositol 3,4-bisphosphate) C11556 (1-Phosphatidyl-1D-myo-inositol 3,5-... | PTEN 2.7.1.158 PI(3,4)P PI(3,5)P PI(5)P PLC PLC 5PP-IP Focal adhesion I(1,3,4)P I(1,3,4,5)P I(4)P I(... |
| [hsa04071](https://www.genome.jp/dbget-bin/www_bget?pathway:hsa04071) | Sphingolipid signaling pathway - Homo sapiens (human) | Sphingomyelin (SM) and its metabolic products are now known to have second messenger functions in a ... | C05981 (Phosphatidylinositol-3,4,5-trisphosphate) C00195 (N-Acylsphingosine) C12126 (Dihydroceramide... | ERK1/2 MEK1/2 Raf AKT P13K PDK1 PIP SPHINGOLIPID SIGNALING PATHWAY MAPK signaling pathway P... |
| [hsa04072](https://www.genome.jp/dbget-bin/www_bget?pathway:hsa04072) | Phospholipase D signaling pathway - Homo sapiens (human) | Phospholipase D (PLD) is an essential enzyme responsible for the production of the lipid second mess... | C05981 (Phosphatidylinositol-3,4,5-trisphosphate) C00165 (Diacylglycerol) C01245 (D-myo-Inositol 1,4... | MAPK signaling pathway PHOSPHOLIPASE D SIGNALING PATHWAY ERK MEK Raf-1 Ras PI3K-Akt signaling pat... |
| [hsa04080](https://www.genome.jp/dbget-bin/www_bget?pathway:hsa04080) | Neuroactive ligand-receptor interaction - Homo sapiens (human) |  | C15906 (Glucose-dependent insulinotropic peptide) C01501 (Glucagon) C16048 (Glucagon-like peptide 1)... | PARRS Gastric inhibitory peptide Glucagon Glucagon-like peptide Parathyroid hormone Growth hormone-r... |
| [hsa04110](https://www.genome.jp/dbget-bin/www_bget?pathway:hsa04110) | Cell cycle - Homo sapiens (human) | Mitotic cell cycle progression is accomplished through a reproducible sequence of events, DNA replic... | 1029 (CDKN2A) 51343 (FZR1) 4171 (MCM2), 4172 (MCM3), 4173 (MCM4), 4174 (MCM5), 4175 (MCM6), 4176 (MC... | ARF Cdh1 MCM ORC APC/C APC/C SCF SCF Orc6 Orc3 Orc5 Orc4 Orc2 Orc1 Mcm7 Mcm6 Mcm5 Mcm4 Mcm3 Mcm2 CDK... |
| [hsa04114](https://www.genome.jp/dbget-bin/www_bget?pathway:hsa04114) | Oocyte meiosis - Homo sapiens (human) | During meiosis, a single round of DNA replication is followed by two rounds of chromosome segregatio... | C00410 (Progesterone) C01245 (D-myo-Inositol 1,4,5-trisphosphate) C00076 (Calcium cation) C00575 (3'... | Mos Cdc2 Mad1/2 CycB2/B5 Myt1 Rsk1/2 p42MAPK MEK1 Progesterone OOCYTE MEIOSIS Early translation of ... |
| [hsa04115](https://www.genome.jp/dbget-bin/www_bget?pathway:hsa04115) | p53 signaling pathway - Homo sapiens (human) | p53 activation is induced by a number of stress signals, including DNA damage, oxidative stress and ... | 6477 (SIAH1) 3486 (IGFBP3) 10912 (GADD45G), 1647 (GADD45A), 4616 (GADD45B) 4194 (MDM4) 1111 (CHEK1) ... | Siah-1 IGF-BP3 Gadd45 MDM-X CHK1 ATR CHK2 ATM Reprimo 14-3-3-σ CDK2 Cyclin E Cell cycle Apoptosis T... |
| [hsa04120](https://www.genome.jp/dbget-bin/www_bget?pathway:hsa04120) | Ubiquitin mediated proteolysis - Homo sapiens (human) | Protein ubiquitination plays an important role in eukaryotic cellular processes. It mainly functions... | 10054 (UBA2) 10055 (SAE1) 6921 (ELOC) 6923 (ELOB) 9978 (RBX1) 9978 (RBX1) 9978 (RBX1) 9978 (RBX1) 23... | UBLE1B UBLE1A EloC EloB RBX1 RBX1 RBX1 RBX1 F-box BTB DCAF SOCSbox VHLbox HERC4 HERC3 UBE2W UBE2F UB... |
| [hsa04122](https://www.genome.jp/dbget-bin/www_bget?pathway:hsa04122) | Sulfur relay system - Homo sapiens (human) | Ubiquitin and ubiquitin-like proteins (Ubls) are signalling messengers that control many cellular fu... | C00097 (L-Cysteine) C00041 (L-Alanine) C00868 (tRNA uridine) C17322 (tRNA containing 2-thiouridine) ... | SULFUR RELAY SYSTEM E1-like urm1 -COOH ATP PPi -COAMP Protein modification Prokaryote Ahp1 -NHCO-... |
| [hsa04130](https://www.genome.jp/dbget-bin/www_bget?pathway:hsa04130) | SNARE interactions in vesicular transport - Homo sapiens (human) |  | 662 (BNIP1) 55850 (USE1) 53407 (STX18) 55014 (STX17) 10282 (BET1), 51272 (BET1L) 10652 (YKT6) 9527 (... | Sec20 Use1 Stx18 Stx17 Bet1 Ykt6 Gos1 Stx5 Sec22 Bos1 Stx5 SNAP29 STX6 Stx16 Stx11 Ykt6 Vti1 STX8 St... |
| [hsa04136](https://www.genome.jp/dbget-bin/www_bget?pathway:hsa04136) | Autophagy - other - Homo sapiens (human) | Autophagy is a degradative pathway for the removal of cytoplasmic materials in eukaryotic cells, and... | C00350 (Phosphatidylethanolamine) C00350 (Phosphatidylethanolamine) C04549 (1-Phosphatidyl-1D-myo-in... | AUTOPHAGY - OTHER Nutrient starvation TORC1 complex Raptor TOR LST8 ATG1 complex ATG1 ATG13 ATG11 ... |
| [hsa04137](https://www.genome.jp/dbget-bin/www_bget?pathway:hsa04137) | Mitophagy - animal - Homo sapiens (human) | Mitochondria act as the energy powerhouse of the cell, and are essential for eukaryotic cells to gro... | 65018 (PINK1) 55669 (MFN1), 9927 (MFN2) 84749 (USP30) 9958 (USP15) 55626 (AMBRA1) 9101 (USP8) 5071 (... | MITOPHAGY - ANIMAL PINK1 Mfn1/2 Depolarization of mitochondria USP30 USP15 AMBRA1 USP8 Parkin Aut... |
| [hsa04140](https://www.genome.jp/dbget-bin/www_bget?pathway:hsa04140) | Autophagy - animal - Homo sapiens (human) | Autophagy (or macroautophagy) is a cellular catabolic pathway involving in protein degradation, orga... | C04549 (1-Phosphatidyl-1D-myo-inositol 3-phosphate) C01194 (1-Phosphatidyl-D-myo-inositol) C05981 (P... | PI3P AUTOPHAGY - ANIMAL Nutrient starvation (Amino acids, glucose) mTORC1 complex ER stress Fusio... |
| [hsa04141](https://www.genome.jp/dbget-bin/www_bget?pathway:hsa04141) | Protein processing in endoplasmic reticulum - Homo sapiens (human) | The endoplasmic reticulum (ER) is a subcellular organelle where proteins are folded with the help of... | G00010 C00076 (Calcium cation) G00010 G00009 G00011 G00011 G10694 G00012 468 (ATF4) 1649 (DDIT3) 559... | PROTEIN PROCESSING IN ENDOPLASMIC RETICULUM Protein export Apoptosis Proteasome ATF4 CHOP JNK TR... |
| [hsa04142](https://www.genome.jp/dbget-bin/www_bget?pathway:hsa04142) | Lysosome - Homo sapiens (human) | Lysosomes are membrane-delimited organelles in animal cells serving as the cell's main digestive com... | C00002 (ATP) C00008 (ADP) C00159 (D-Mannose) C00159 (D-Mannose) C00275 (D-Mannose 6-phosphate) C0027... | mitochondria pH~ 5.0 pH~ 7.2 acid hydrolase lysosome Regulation of autophagy ATPeV ATP ADP cytos... |
| [hsa04144](https://www.genome.jp/dbget-bin/www_bget?pathway:hsa04144) | Endocytosis - Homo sapiens (human) | Endocytosis is a mechanism for cells to remove ligands, nutrients, and plasma membrane (PM) proteins... | C04549 (1-Phosphatidyl-1D-myo-inositol 3-phosphate) C04549 (1-Phosphatidyl-1D-myo-inositol 3-phospha... | ENDOCYTOSIS STAM Hrs PI(3)P PI(3)P ESCRT-I ESCRT-0 ESCRT-II ESCRT-III Late endosome and multivesicu... |
| [hsa04145](https://www.genome.jp/dbget-bin/www_bget?pathway:hsa04145) | Phagosome - Homo sapiens (human) | Phagocytosis is the process of taking in relatively large particles by a cell, and is a central mech... | C04549 (1-Phosphatidyl-1D-myo-inositol 3-phosphate) C04549 (1-Phosphatidyl-1D-myo-inositol 3-phospha... | PHAGOSOME Fc gamma R-mediated phagocytosis Endocytosis Phagocytic cup pathogen Conventional ... |
| [hsa04146](https://www.genome.jp/dbget-bin/www_bget?pathway:hsa04146) | Peroxisome - Homo sapiens (human) | Peroxisomes are essential organelles that play a key role in redox signalling and lipid homeostasis.... | 5195 (PEX14) 5828 (PEX2) 5189 (PEX1) 5190 (PEX6) 55670 (PEX26) 5194 (PEX13) 5192 (PEX10) 5193 (PEX12... | PEROXISOME PEX14 PEX2 PEX1 PEX6 PEX26 PEX13 PTS1 PTS2 Matrix protein import PEX10 PEX12 PEX5 PEX7 PE... |
| [hsa04150](https://www.genome.jp/dbget-bin/www_bget?pathway:hsa04150) | mTOR signaling pathway - Homo sapiens (human) | The mammalian (mechanistic) target of rapamycin (mTOR) is a highly conserved serine/threonine protei... | C05981 (Phosphatidylinositol-3,4,5-trisphosphate) C00020 (AMP) C00123 (L-Leucine) 3480 (IGF1R), 3643... | mTOR SIGNALING PATHWAY IRS1 PI3K Raf MAPK signaling pathway ERK1/2 Ras MEK PTEN PDK1 Akt TSC1/2 R... |
| [hsa04151](https://www.genome.jp/dbget-bin/www_bget?pathway:hsa04151) | PI3K-Akt signaling pathway - Homo sapiens (human) | The phosphatidylinositol 3' -kinase(PI3K)-Akt signaling pathway is activated by many types of cellul... | C05981 (Phosphatidylinositol-3,4,5-trisphosphate) C00533 (Nitric oxide) C04637 (1-Phosphatidyl-D-myo... | PIP eIF4E 4EBPs eIF4B S6K1/2 Raptor mTOR GβL Rheb TSC2 TSC1 AKT PDK1 PI3K p53 signaling pathway mTO... |
| [hsa04152](https://www.genome.jp/dbget-bin/www_bget?pathway:hsa04152) | AMPK signaling pathway - Homo sapiens (human) | AMP-activated protein kinase (AMPK) is a serine threonine kinase that is highly conserved through ev... | C00031 (D-Glucose) C05981 (Phosphatidylinositol-3,4,5-trisphosphate) C00008 (ADP) C00020 (AMP) C0000... | AMPK INS Glucose INSR Akt P13K PDK1/2 IRS PIP AMPK SIGNALING PATHWAY Glycolysis / Gluconeogenesis ... |
| [hsa04210](https://www.genome.jp/dbget-bin/www_bget?pathway:hsa04210) | Apoptosis - Homo sapiens (human) | Apoptosis is a genetically programmed process for the elimination of damaged or redundant cells by a... | C00076 (Calcium cation) C05981 (Phosphatidylinositol-3,4,5-trisphosphate) C00319 (Sphingosine) C0002... | Mitochondrion p53 signaling pathway TNF signaling pathway Bcl-XL ICAD ATM p53 ENDO-G AIF Bcl-2 IAP/X... |
| [hsa04211](https://www.genome.jp/dbget-bin/www_bget?pathway:hsa04211) | Longevity regulating pathway - Homo sapiens (human) | Regulation of longevity depends on genetic and environmental factors. Caloric restriction (CR), that... | C05981 (Phosphatidylinositol-3,4,5-trisphosphate) C00575 (3',5'-Cyclic AMP) C03582 (Resveratrol) C07... | FOXO S6K mTOR Akt P13K PIP LONGEVITY REGULATING PATHWAY DNA IGF-I IGF-IR IRS1-4 Ras cAMP PKA AMPK ... |
| [hsa04213](https://www.genome.jp/dbget-bin/www_bget?pathway:hsa04213) | Longevity regulating pathway - multiple species - Homo sapiens (human) | Aging is a complex process of accumulation of molecular, cellular, and organ damage, leading to loss... | C05981 (Phosphatidylinositol-3,4,5-trisphosphate) C05981 (Phosphatidylinositol-3,4,5-trisphosphate) ... | FOXO S6K mTOR Akt P13K PIP LONGEVITY REGULATING PATHWAY - MULTIPLE SPECIES DNA IGF-I IGF-IR IRS1... |
| [hsa04215](https://www.genome.jp/dbget-bin/www_bget?pathway:hsa04215) | Apoptosis - multiple species - Homo sapiens (human) | Apoptosis is an evolutionarily conserved process used by multicellular organisms to developmentally ... | 112401 (BIRC8), 79444 (BIRC7) 840 (CASP7) 317 (APAF1) 666 (BOK) 54205 (CYCS) 842 (CASP9) 840 (CASP7)... | APOPTOSIS - MULTIPLE SPECIES Mitochondrion DIAP1 DRONC DRICE GRIM RPR HID dArk Dcp-1 DEBCL RHG fa... |
| [hsa04216](https://www.genome.jp/dbget-bin/www_bget?pathway:hsa04216) | Ferroptosis - Homo sapiens (human) | Ferroptosis is a regulated form of cell death and characterized by a production of reactive oxygen s... | C00025 (L-Glutamate) C00491 (L-Cystine) C00051 (Glutathione) C21478 (Erastin) C00097 (L-Cysteine) C0... | FERROPTOSIS SLC7A11 Glutamate Cystine p53 GPX4 GSH Mitochondria VDAC2/3 Erastin Cysteine GCL γGC SL... |
| [hsa04217](https://www.genome.jp/dbget-bin/www_bget?pathway:hsa04217) | Necroptosis - Homo sapiens (human) | Necroptosis is a programmed form of necrosis. It can be initiated by different stimuli, such as tumo... | C00076 (Calcium cation) C00305 (Magnesium cation) C00002 (ATP) C00027 (Hydrogen peroxide), C16844 (H... | NECROPTOSIS TNF TNFR1 TRADD TRAF2/5 MLKL TLR4 IFNR LPS IFN JAKs TRAILR TRAIL Fas FasL IAPs Casp8 cFL... |
| [hsa04218](https://www.genome.jp/dbget-bin/www_bget?pathway:hsa04218) | Cellular senescence - Homo sapiens (human) | Cellular senescence is a state of irreversible cellular arrest and can be triggered by a number of f... | C16844 (Hydroxyl radical), C00027 (Hydrogen peroxide) C07909 (Sirolimus) C00076 (Calcium cation) C16... | CELLULAR SENESCENCE NORE1A PP1A C-Myc HIPK2 ARF MDM2 p53 P53 signaling pathway PI3K AKT TSC1/2 RHEB... |
| [hsa04260](https://www.genome.jp/dbget-bin/www_bget?pathway:hsa04260) | Cardiac muscle contraction - Homo sapiens (human) | Contraction of the heart is a complex process initiated by the electrical excitation of cardiac myoc... | C00076 (Calcium cation) C00076 (Calcium cation) C00076 (Calcium cation) C00076 (Calcium cation) C013... | CARDIAC MUSCLE CONTRACTION T-tubule Sarcoplasmic Reticulum (SR) DHPR RyR2 NCX NCX Systole Diastole... |
| [hsa04261](https://www.genome.jp/dbget-bin/www_bget?pathway:hsa04261) | Adrenergic signaling in cardiomyocytes - Homo sapiens (human) | Cardiac myocytes express at least six subtypes of adrenergic receptor (AR) which include three subty... | C00076 (Calcium cation) C00076 (Calcium cation) C00076 (Calcium cation) C01330 (Sodium cation) C0133... | ADRENERGIC SIGNALING IN CARDIOMYOCYTES T-tubule Sarcoplasmic Reticulum (SR) DHPR RyR2 NCX NCX SER... |
| [hsa04270](https://www.genome.jp/dbget-bin/www_bget?pathway:hsa04270) | Vascular smooth muscle contraction - Homo sapiens (human) | The vascular smooth muscle cell (VSMC) is a highly specialized cell whose principal function is cont... | C01245 (D-myo-Inositol 1,4,5-trisphosphate) C00165 (Diacylglycerol) C00076 (Calcium cation) C00219 (... | Calcium signaling pathway MLCK MLC MLCP ROCK PKC RhoA RhoGEF VASCULAR SMOOTH MUSCLE CONTRACTION A... |
| [hsa04310](https://www.genome.jp/dbget-bin/www_bget?pathway:hsa04310) | Wnt signaling pathway - Homo sapiens (human) | Wnt proteins are secreted morphogens that are required for basic developmental processes, such as ce... | C00076 (Calcium cation) 4088 (SMAD3) 4772 (NFATC1), 4773 (NFATC2), 4775 (NFATC3), 4776 (NFATC4) 5578... | p53 signaling pathway Adherens junction SMAD3 TGF-β signaling pathway Cell cycle WNT SIGNALING PA... |
| [hsa04330](https://www.genome.jp/dbget-bin/www_bget?pathway:hsa04330) | Notch signaling pathway - Homo sapiens (human) | The Notch signaling pathway is an evolutionarily conserved, intercellular signaling mechanism essent... | 23385 (NCSTN) 51107 (APH1A), 83464 (APH1B) 5663 (PSEN1), 5664 (PSEN2) 3065 (HDAC1), 3066 (HDAC2) 954... | NCSTN APH-1 PSEN MAPK signaling pathway HDAC CIR Groucho CtBP Hairless SMRT PreTα Hes1/5 CSL SKIP H... |
| [hsa04340](https://www.genome.jp/dbget-bin/www_bget?pathway:hsa04340) | Hedgehog signaling pathway - Homo sapiens (human) | The Hedgehog (Hh) signaling pathway has numerous roles in the control of cell proliferation, tissue ... | C00575 (3',5'-Cyclic AMP) 6608 (SMO) 23432 (GPR161) 102800317, 122011 (CSNK1A1L), 1452 (CSNK1A1), 14... | Smo Gpr161 cAMP CK1 GSK3β PKA Gli Kif7 GliR Cilium Cytoplasm Without Hh Vesicle Cul1 β-TrCP Nucleu... |
| [hsa04350](https://www.genome.jp/dbget-bin/www_bget?pathway:hsa04350) | TGF-beta signaling pathway - Homo sapiens (human) | The transforming growth factor-beta (TGF-beta) family members, which include TGF-betas, activins and... | 4052 (LTBP1) 7057 (THBS1) 8454 (CUL1) 9978 (RBX1) 4089 (SMAD4) 4089 (SMAD4) 92 (ACVR2A), 93 (ACVR2B)... | LTBP1 THBS1 Cul1 Rbx1 TGF-BETA SIGNALING PATHWAY Apoptosis Cell Cycle Ubiquitin mediated proteolys... |
| [hsa04360](https://www.genome.jp/dbget-bin/www_bget?pathway:hsa04360) | Axon guidance - Homo sapiens (human) | Axon guidance represents a key stage in the formation of neuronal network. Axons are guided by a var... | C00076 (Calcium cation) C00076 (Calcium cation) C00076 (Calcium cation) C00076 (Calcium cation) 5085... | Par6 Par3 CXCR4 ERK Rac Rac Rac DCC NFAT CaN Netrin-G1 Netrin-4 Slit3 Slit2 Slit1 FAK Cdc42 ROCK PAK... |
| [hsa04370](https://www.genome.jp/dbget-bin/www_bget?pathway:hsa04370) | VEGF signaling pathway - Homo sapiens (human) | There is now much evidence that VEGFR-2 is the major mediator of VEGF-driven responses in endothelia... | C00533 (Nitric oxide) C00076 (Calcium cation) C01245 (D-myo-Inositol 1,4,5-trisphosphate) C00165 (Di... | Cdc42 DAG Calcium signaling pathway Focal adhesion MAPK signaling pathway VEGFR2 PLCγ SPK PIP PGI C... |
| [hsa04371](https://www.genome.jp/dbget-bin/www_bget?pathway:hsa04371) | Apelin signaling pathway - Homo sapiens (human) | Apelin is an endogenous peptide capable of binding the apelin receptor (APJ), which was originally d... | C01245 (D-myo-Inositol 1,4,5-trisphosphate) C00076 (Calcium cation) C01330 (Sodium cation) C00080 (H... | APELIN SIGNALING PATHWAY APJ AMPK Sphk1 PAI-1 Gα βγ PI3K Akt MEK1/2 ERK1/2 P70S6K PLCβ IP3 IP3... |
| [hsa04380](https://www.genome.jp/dbget-bin/www_bget?pathway:hsa04380) | Osteoclast differentiation - Homo sapiens (human) | The osteoclasts, multinucleared cells originating from the hematopoietic monocyte-macrophage lineage... | C01245 (D-myo-Inositol 1,4,5-trisphosphate) C00076 (Calcium cation) C00027 (Hydrogen peroxide) 1436 ... | OSTEOCLAST DIFFERENTIATION c-Fms TRAF2/6 RANK RANKL M-CSF OPG CYLD Gab2 NIK IKKα IKKγ IKKα IKKβ... |
| [hsa04390](https://www.genome.jp/dbget-bin/www_bget?pathway:hsa04390) | Hippo signaling pathway - Homo sapiens (human) | Hippo signaling is an evolutionarily conserved signaling pathway that controls organ size from flies... | 23286 (WWC1) 4771 (NF2) 122786 (FRMD6), 79981 (FRMD1) 60485 (SAV1) 6788 (STK3) 10413 (YAP1), 25937 (... | HIPPO SIGNALING PATHWAY KIBRA Mer FRMD SAV1 Mst1/2 YAP/TAZ Lats1/2 Crb α-Catenin RASSF1A CTGF Bi... |
| [hsa04392](https://www.genome.jp/dbget-bin/www_bget?pathway:hsa04392) | Hippo signaling pathway - multiple species - Homo sapiens (human) | Hippo signaling pathways control diverse aspects of cell proliferation, survival, and morphogenesis ... | 23286 (WWC1) 4771 (NF2) 122786 (FRMD6), 79981 (FRMD1) 60485 (SAV1) 6788 (STK3) 10413 (YAP1), 25937 (... | HIPPO SIGNALING PATHWAY - MULTIPLE SPECIES Kibra NF2 FRMD SAV1 Mst1/2 YAP/TAZ Lats1/2 DNA Mob1... |
| [hsa04510](https://www.genome.jp/dbget-bin/www_bget?pathway:hsa04510) | Focal adhesion - Homo sapiens (human) | Cell-matrix adhesions play essential roles in important biological processes including cell motility... | C05981 (Phosphatidylinositol-3,4,5-trisphosphate) C04637 (1-Phosphatidyl-D-myo-inositol 4,5-bisphosp... | ECM-receptor interaction Cytokine-cytokine receptor interaction MAPK signaling pathway ITGB ITGA Fil... |
| [hsa04512](https://www.genome.jp/dbget-bin/www_bget?pathway:hsa04512) | ECM-receptor interaction - Homo sapiens (human) | The extracellular matrix (ECM) consists of a complex mixture of structural and functional macromolec... | G10505 (Hyaluronic acid) G10505 (Hyaluronic acid) 10319 (LAMC3), 22798 (LAMB4), 284217 (LAMA1), 3908... | Laminin Laminin Laminin Laminin Laminin Laminin Laminin Laminin Laminin Laminin Collagen Collagen Co... |
| [hsa04514](https://www.genome.jp/dbget-bin/www_bget?pathway:hsa04514) | Cell adhesion molecules (CAMs) - Homo sapiens (human) | Cell adhesion molecules are (glyco)proteins expressed on the cell surface and play a critical role i... | 214 (ALCAM) 923 (CD6) 6693 (SPN) 6614 (SIGLEC1) 5788 (PTPRC) 933 (CD22) 925 (CD8A), 926 (CD8B) 3688 ... | TCR TCR TCR TCR Leukocyte transendothelial migration Leukocyte transendothelial migration T cell rec... |
| [hsa04520](https://www.genome.jp/dbget-bin/www_bget?pathway:hsa04520) | Adherens junction - Homo sapiens (human) | Cell-cell adherens junctions (AJs), the most common type of intercellular adhesions, are important f... | 4089 (SMAD4) 999 (CDH1) 1495 (CTNNA1), 1496 (CTNNA2), 29119 (CTNNA3) 1500 (CTNND1) 1500 (CTNND1) 999... | Smad4 Cadherin α-Catenin p120ctn p120ctn Cadherin Cadherin Cadherin TCF/LEF NLK TAK1 CBP Smad3 Yes ... |
| [hsa04530](https://www.genome.jp/dbget-bin/www_bget?pathway:hsa04530) | Tight junction - Homo sapiens (human) | Tight junctions (TJs) are essential for establishing a selectively permeable barrier to diffusion th... | C00076 (Calcium cation) 50855 (PARD6A), 84552 (PARD6G), 84612 (PARD6B) 10207 (PATJ) 998 (CDC42) 6794... | PAR6 PATJ Cdc42 TIGHT JUNCTION Inside Paracellular space LKB1 Cingulin Tiam1 Decreased paracellul... |
| [hsa04540](https://www.genome.jp/dbget-bin/www_bget?pathway:hsa04540) | Gap junction - Homo sapiens (human) | Gap junctions contain intercellular channels that allow direct communication between the cytosolic c... | C00681 (1-Acyl-sn-glycerol 3-phosphate) C00076 (Calcium cation) C01245 (D-myo-Inositol 1,4,5-trispho... | LPA v-Src cGMP DAG cAMP CK1 PKC Serotonin Glutamate Connexin Connexin Connexin Connexin Connexin Con... |
| [hsa04550](https://www.genome.jp/dbget-bin/www_bget?pathway:hsa04550) | Signaling pathways regulating pluripotency of stem cells - Homo sapiens (human) | Pluripotent stem cells (PSCs) are basic cells with an indefinite self-renewal capacity and the poten... | 51384 (WNT16), 54361 (WNT4), 7471 (WNT1), 7472 (WNT2), 7473 (WNT3), 7474 (WNT5A), 7475 (WNT6), 7476 ... | Wnt APC Axin Dvl Frizzled SIGNALING PATHWAYS REGULATING PLURIPOTENCY OF STEM CELLS β-catenin ... |
| [hsa04610](https://www.genome.jp/dbget-bin/www_bget?pathway:hsa04610) | Complement and coagulation cascades - Homo sapiens (human) | The complement system is a proteolytic cascade in blood plasma and a mediator of innate immunity, a ... | C00290 (Fibrin) C00306 (Bradykinin) 2161 (F12) 2160 (F11) 2158 (F9) 2159 (F10) 2155 (F7) 2243 (FGA),... | COMPLEMENT AND COAGULATION CASCADES Coagulation cascade F12 F11 F10 Fibrin monomer Fibrinogen F1... |
| [hsa04611](https://www.genome.jp/dbget-bin/www_bget?pathway:hsa04611) | Platelet activation - Homo sapiens (human) | Platelets play a key and beneficial role for primary hemostasis on the disruption of the integrity o... | C00575 (3',5'-Cyclic AMP) C00076 (Calcium cation) C01245 (D-myo-Inositol 1,4,5-trisphosphate) C00165... | PLATELET ACTIVATION cAMP DAG Calcium signaling pathway Platelet PAR1/4 P2Y1 Thrombin ADP TXA2 PLC�... |
| [hsa04612](https://www.genome.jp/dbget-bin/www_bget?pathway:hsa04612) | Antigen processing and presentation - Homo sapiens (human) |  | 972 (CD74) 972 (CD74) 3108 (HLA-DMA), 3109 (HLA-DMB) 4800 (NFYA), 4801 (NFYB), 4802 (NFYC) 5993 (RFX... | CLIP SLIP HLA-DM NFY RFX CREB CIITA CTSB/L/S AEP CTSB AEP GILT TCR TCR CD4 KIR CD8 TAPBP CALR ERp57 ... |
| [hsa04614](https://www.genome.jp/dbget-bin/www_bget?pathway:hsa04614) | Renin-angiotensin system - Homo sapiens (human) | The renin-angiotensin system (RAS) is a peptidergic system with endocrine characteristics regarding ... | C02135 (Angiotensin II) C15849 (Angiotensin IV) C15848 (Angiotensin III) C15850 (Angiotensin (1-7)) ... | ACE2 ACE2 ACE ACE ACE Angiotensin II Angiotensin IV Angiotensin III Angiotensin (1-7) Angiotensin (1... |
| [hsa04620](https://www.genome.jp/dbget-bin/www_bget?pathway:hsa04620) | Toll-like receptor signaling pathway - Homo sapiens (human) | Specific families of pattern recognition receptors are responsible for detecting microbial pathogens... | 3456 (IFNB1) 1147 (CHUK) 7189 (TRAF6) 3654 (IRAK1) 51135 (IRAK4) 4615 (MYD88) 4615 (MYD88) 4615 (MYD... | IFN-β IKKα TRAF6 IRAK1 IRAK4 MyD88 MyD88 MyD88 TRAF6 ERK Tpl2 p105 OPN TRAF3 RIP1 IRF7 IRF5 I-TAC... |
| [hsa04621](https://www.genome.jp/dbget-bin/www_bget?pathway:hsa04621) | NOD-like receptor signaling pathway - Homo sapiens (human) | Specific families of pattern recognition receptors are responsible for detecting various pathogens a... | C00238 (Potassium cation) C01245 (D-myo-Inositol 1,4,5-trisphosphate) C00076 (Calcium cation) C00002... | ASC pro-CASP1 ASC pro-CASP1 ASC pro-CASP1 ASC pro-CASP1 NOD1 NOD2 iE-DAP RIP2 IKKα/β TAK1 TAB NLRP... |
| [hsa04622](https://www.genome.jp/dbget-bin/www_bget?pathway:hsa04622) | RIG-I-like receptor signaling pathway - Homo sapiens (human) | Specific families of pattern recognition receptors are responsible for detecting viral pathogens and... | 23586 (DDX58) 64135 (IFIH1) 7706 (TRIM25) 54941 (RNF125) 57506 (MAVS) 4790 (NFKB1), 5970 (RELA) 5559... | 5'-ppp ssRNA long dsRNA RIG-I MDA5 TRIM25 RNF125 IPS-1 NFκB DUBA TRAF3 IRF-3 IKKε IRF-7 TANK NAP1 ... |
| [hsa04623](https://www.genome.jp/dbget-bin/www_bget?pathway:hsa04623) | Cytosolic DNA-sensing pathway - Homo sapiens (human) | Specific families of pattern recognition receptors are responsible for detecting foreign DNA from in... | C20640 (Cyclic GMP-AMP) 834 (CASP1) 29108 (PYCARD) 9447 (AIM2) 3553 (IL1B) 3606 (IL18) 81030 (ZBP1) ... | CASP1 ASC Inflammasome AIM2 IL-1β IL-18 p202 dsDNA Proinflammatory cytokine processing Cell death D... |
| [hsa04625](https://www.genome.jp/dbget-bin/www_bget?pathway:hsa04625) | C-type lectin receptor signaling pathway - Homo sapiens (human) | C-type lectin receptors (CLRs) are a large superfamily of proteins characterized by the presence of ... | C00965 (1,3-beta-D-Glucan) C00159 (D-Mannose) C01019 (6-Deoxy-L-galactose) C00159 (D-Mannose) C01245... | C-TYPE LECTIN RECEPTOR SIGNALING PATHWAY Dectin-1 Dectin-2 Mincle MCL FcRγ DC-SIGN LSP-1 KSR1 C... |
| [hsa04630](https://www.genome.jp/dbget-bin/www_bget?pathway:hsa04630) | JAK-STAT signaling pathway - Homo sapiens (human) | The Janus kinase/signal transducers and activators of transcription (JAK/STAT) pathway is one of a h... | 4609 (MYC) 10000 (AKT3), 207 (AKT1), 208 (AKT2) 5290 (PIK3CA), 5291 (PIK3CB), 5293 (PIK3CD), 5295 (P... | c-Myc AKT PI3K SOS GRB CIS Bcl-2 SOCS CBP/P300 IRF9 STAT PIAS SHP1 STAM JAK Ubiquitin mediated prot... |
| [hsa04640](https://www.genome.jp/dbget-bin/www_bget?pathway:hsa04640) | Hematopoietic cell lineage - Homo sapiens (human) | Blood-cell development progresses from a hematopoietic stem cell (HSC), which can undergo either sel... | 102723407 (IGH) 3655 (ITGA6), 3672 (ITGA1), 3673 (ITGA2), 3675 (ITGA3), 3676 (ITGA4), 3678 (ITGA5) 2... | IgM CD49 CD42 CD41 CD14 CD9 CD61 IL-11R CD59 CD55 CD35 CD44 CD235a CD36 CD36 CD117 EPOR CD125 CD114 ... |
| [hsa04650](https://www.genome.jp/dbget-bin/www_bget?pathway:hsa04650) | Natural killer cell mediated cytotoxicity - Homo sapiens (human) | Natural killer (NK) cells are lymphocytes of the innate immune system that are involved in early def... | C05981 (Phosphatidylinositol-3,4,5-trisphosphate) C00165 (Diacylglycerol) C00076 (Calcium cation) C0... | IgG NFAT CaN PI(3,4,5)P Fyn TRAIL IFNγR PLCγ LAT Perforin HLA-E IFN-γ GM-CSF TNF-α PKC 3BP2 PLC�... |
| [hsa04657](https://www.genome.jp/dbget-bin/www_bget?pathway:hsa04657) | IL-17 signaling pathway - Homo sapiens (human) | The interleukin 17 (IL-17) family, a subset of cytokines consisting of IL-17A-F, plays crucial roles... | 3551 (IKBKB) 6885 (MAP3K7) 1147 (CHUK) 7189 (TRAF6) 3569 (IL6) 257397 (TAB3) 23118 (TAB2) 4792 (NFKB... | IKKβ TAK1 IKKα TRAF6 IL-6 TAB3 TAB2 IκBα IKKγ IL-17 SIGNALING PATHWAY MAPKsignaling pathway M... |
| [hsa04658](https://www.genome.jp/dbget-bin/www_bget?pathway:hsa04658) | Th1 and Th2 cell differentiation - Homo sapiens (human) | Immunity to different classes of microorganisms is orchestrated by separate lineages of effector T h... | C00165 (Diacylglycerol) C00076 (Calcium cation) C01245 (D-myo-Inositol 1,4,5-trisphosphate) 3458 (IF... | DAG IFN-γ IL-5 IκB NFκB IKK PKCθ NFAT CaN ZAP70 CD3ε CD3δ TCRβ TCRα CD3ζ CD3ζ CD3γ CD3ε ... |
| [hsa04659](https://www.genome.jp/dbget-bin/www_bget?pathway:hsa04659) | Th17 cell differentiation - Homo sapiens (human) | Interleukin (IL)-17-producing helper T (Th17) cells serve as a subset of CD4+ T cells involved in ep... | C00165 (Diacylglycerol) C00076 (Calcium cation) C01245 (D-myo-Inositol 1,4,5-trisphosphate) C00777 (... | DAG IκB NFκB IKK PKCθ NFAT CaN ZAP70 TCRβ TCRα Cell adhesion molecules Th17 CELL DIFFERENT... |
| [hsa04660](https://www.genome.jp/dbget-bin/www_bget?pathway:hsa04660) | T cell receptor signaling pathway - Homo sapiens (human) | Activation of T lymphocytes is a key event for an efficient response of the immune system. It requir... | C05981 (Phosphatidylinositol-3,4,5-trisphosphate) C00165 (Diacylglycerol) C00076 (Calcium cation) C0... | PIP DAG CDK4 TNFα GM-CSF IFN-γ IL-10 IL-5 IL-4 IL-2 IκB NFκB IKKβ IKKγ IKKα NIK COT AKT MALT1... |
| [hsa04662](https://www.genome.jp/dbget-bin/www_bget?pathway:hsa04662) | B cell receptor signaling pathway - Homo sapiens (human) | B cells are an important component of adaptive immunity. They produce and secrete millions of differ... | C05981 (Phosphatidylinositol-3,4,5-trisphosphate) C00165 (Diacylglycerol) C00076 (Calcium cation) C0... | PIP DAG IκB NFκB IKKβ IKKγ IKKα GSK3β MALT1 BCL-10 CALMA1 AKT PKCβ VAV PI3K CD21 CD19 CD81 LE... |
| [hsa04664](https://www.genome.jp/dbget-bin/www_bget?pathway:hsa04664) | Fc epsilon RI signaling pathway - Homo sapiens (human) | Fc epsilon RI-mediated signaling pathways in mast cells are initiated by the interaction of antigen ... | C00696 (Prostaglandin D2) C05952 (Leukotriene E4) C05951 (Leukotriene D4) C02166 (Leukotriene C4) C0... | IgE IgE FcεRIγ FcεRIβ FcεRIα PGD LTE LTD LTC Arachidonic acid Heparin Histamine DAG FcεRIβ F... |
| [hsa04666](https://www.genome.jp/dbget-bin/www_bget?pathway:hsa04666) | Fc gamma R-mediated phagocytosis - Homo sapiens (human) | Phagocytosis plays an essential role in host-defense mechanisms through the uptake and destruction o... | C05981 (Phosphatidylinositol-3,4,5-trisphosphate) C01245 (D-myo-Inositol 1,4,5-trisphosphate) C00165... | Macrophage Monocyte Neutrophil FcγRIIIA FcγRIIB FcγRIIA FcγRI Src Syk CD45 IgG PI3K SHIP LAT PIP... |
| [hsa04668](https://www.genome.jp/dbget-bin/www_bget?pathway:hsa04668) | TNF signaling pathway - Homo sapiens (human) | Tumor necrosis factor (TNF), as a critical cytokine, can induce a wide range of intracellular signal... | 7124 (TNF) 7132 (TNFRSF1A) 8717 (TRADD) 7186 (TRAF2), 7188 (TRAF5) 8737 (RIPK1) 329 (BIRC2), 330 (BI... | TNF SIGNALING PATHWAY TNF TNFR1 TRADD TRAF2/5 RIP1 cIAP1/2 TAK1 MKK4/7 MKK3/6 p38 JNK1/2 IKKγ IK... |
| [hsa04670](https://www.genome.jp/dbget-bin/www_bget?pathway:hsa04670) | Leukocyte transendothelial migration - Homo sapiens (human) | Leukocyte migaration from the blood into tissues is vital for immune surveillance and inflammation. ... | C00027 (Hydrogen peroxide) C00076 (Calcium cation) C00076 (Calcium cation) C00575 (3',5'-Cyclic AMP)... | CAMs MMPs CAMs SHP-2 CDH5 CD99 PECAM1 ITGB2 p120ctn p130 AF-6 SPA-1 PECAM1 JAM1 ITGAL CDH5 SHP-2 CD9... |
| [hsa04672](https://www.genome.jp/dbget-bin/www_bget?pathway:hsa04672) | Intestinal immune network for IgA production - Homo sapiens (human) | The intestine is the largest lymphoid tissue in the body. One striking feature of intestinal immunit... | C00777 (Retinoate), C15493 (9-cis-Retinoic acid) 7040 (TGFB1) 3567 (IL5) 3569 (IL6) 8741 (TNFSF13) 1... | TGFβ Gut lumen M cell Antigen processing and presentation T cell receptor signaling pathway ... |
| [hsa04710](https://www.genome.jp/dbget-bin/www_bget?pathway:hsa04710) | Circadian rhythm - Homo sapiens (human) | Circadian rhythm is an internal biological clock, which enables to sustain an approximately 24-hour ... | C00076 (Calcium cation) C00575 (3',5'-Cyclic AMP) 406 (ARNTL) 4862 (NPAS2), 9575 (CLOCK) 6095 (RORA)... | CIRCADIAN RHYTHM Degradation Ubiquitin mediated proteolysis Bmal1 Clock Ror Rev-erba Per Cry DNA P... |
| [hsa04713](https://www.genome.jp/dbget-bin/www_bget?pathway:hsa04713) | Circadian entrainment - Homo sapiens (human) | Circadian entrainment is a fundamental property by which the period of the internal biological clock... | C00076 (Calcium cation) C00533 (Nitric oxide) C00942 (3',5'-Cyclic GMP) C00165 (Diacylglycerol) C015... | CIRCADIAN ENTRAINMENT NMDAR CaM CaMKII nNOS cGMP PKG PLC DAG PKC Melatonin T-VDCC RyR Postsynaptic ... |
| [hsa04714](https://www.genome.jp/dbget-bin/www_bget?pathway:hsa04714) | Thermogenesis - Homo sapiens (human) | Thermogenesis is essential for warm-blooded animals, ensuring normal cellular and physiological func... | C00547 (L-Noradrenaline) C00575 (3',5'-Cyclic AMP) C00040 (Acyl-CoA) C16003 (Atrial natriuretic pept... | THERMOGENESIS CREB PKA p38 β3-AR ATF2 cAMP Acyl-CoA FGF21 UCP1 PGC-1α Mitochondrion Nucleus UCP1 S... |
| [hsa04720](https://www.genome.jp/dbget-bin/www_bget?pathway:hsa04720) | Long-term potentiation - Homo sapiens (human) | Hippocampal long-term potentiation (LTP), a long-lasting increase in synaptic efficacy, is the molec... | C00305 (Magnesium cation) C00076 (Calcium cation) C01245 (D-myo-Inositol 1,4,5-trisphosphate) C01330... | PKC CaMKIV CBP DAG cAMP CaMKII Glutamate CaN CaM Rsk CREB NMDAR PKA VDCC PP1 AC1/8 AMPAR EPAC1 ERK1/... |
| [hsa04721](https://www.genome.jp/dbget-bin/www_bget?pathway:hsa04721) | Synaptic vesicle cycle - Homo sapiens (human) | Communication between neurons is mediated by the release of neurotransmitter from synaptic vesicles ... | C00076 (Calcium cation) C00080 (H+) C00080 (H+) C03758 (Dopamine), C01996 (Acetylcholine), C00334 (4... | SYNAPTIC VESICLE CYCLE Neuron (Presynaptic terminal) Neuron (Postsynaptic cell) VGCC Glutamatergic... |
| [hsa04722](https://www.genome.jp/dbget-bin/www_bget?pathway:hsa04722) | Neurotrophin signaling pathway - Homo sapiens (human) | Neurotrophins are a family of trophic factors involved in differentiation and survival of neural cel... | C00165 (Diacylglycerol) C01245 (D-myo-Inositol 1,4,5-trisphosphate) C00076 (Calcium cation) C00195 (... | Plasticity Cell survival TrkA TrkB TrkC Apoptosis BDNF NT4 NT3 Cellular differentiation PI3K PLCγ ... |
| [hsa04723](https://www.genome.jp/dbget-bin/www_bget?pathway:hsa04723) | Retrograde endocannabinoid signaling - Homo sapiens (human) | Endogenous cannabinoids (endocannabinoids) serve as retrograde messengers at synapses in various reg... | C01245 (D-myo-Inositol 1,4,5-trisphosphate) C00165 (Diacylglycerol) C00076 (Calcium cation) C00076 (... | RETROGRADE ENDOCANNABINOID SIGNALING Neuron (Presynaptic terminal) Neuron (Postsynaptic cell) vGLU... |
| [hsa04724](https://www.genome.jp/dbget-bin/www_bget?pathway:hsa04724) | Glutamatergic synapse - Homo sapiens (human) | Glutamate is the major excitatory neurotransmitter in the mammalian central nervous system(CNS). Glu... | C00025 (L-Glutamate) C00064 (L-Glutamine) C00064 (L-Glutamine) C00025 (L-Glutamate) C00025 (L-Glutam... | GLUTAMATERGIC SYNAPSE Glial cell Neuron (Presynaptic terminal) Neuron (Postsynaptic cell) mGluR7/8 ... |
| [hsa04725](https://www.genome.jp/dbget-bin/www_bget?pathway:hsa04725) | Cholinergic synapse - Homo sapiens (human) | Acetylcholine (ACh) is a neurotransmitter widely distributed in the central (and also peripheral, au... | C00024 (Acetyl-CoA) C01996 (Acetylcholine) C01996 (Acetylcholine) C00575 (3',5'-Cyclic AMP) C01245 (... | CHOLINERGIC SYNAPSE Neuron (Presynaptic terminal) Neuron (Postsynaptic cell) nAChR AChE vAChT AcCoA... |
| [hsa04726](https://www.genome.jp/dbget-bin/www_bget?pathway:hsa04726) | Serotonergic synapse - Homo sapiens (human) | Serotonin (5-Hydroxytryptamine, 5-HT) is a monoamine neurotransmitter that plays important roles in ... | C00643 (5-Hydroxy-L-tryptophan) C00780 (Serotonin) C00780 (Serotonin) C00575 (3',5'-Cyclic AMP) C012... | SEROTONERGIC SYNAPSE Glial cell Neuron (Presynaptic terminal) Neuron (Postsynaptic cell) 5-HT 4, 6... |
| [hsa04727](https://www.genome.jp/dbget-bin/www_bget?pathway:hsa04727) | GABAergic synapse - Homo sapiens (human) | Gamma aminobutyric acid (GABA) is the most abundant inhibitory neurotransmitter in the mammalian cen... | C00025 (L-Glutamate) C00334 (4-Aminobutanoate) C00334 (4-Aminobutanoate) C00334 (4-Aminobutanoate) C... | GABAERGIC SYNAPSE Glial cell Neuron (Presynaptic terminal) Neuron (Postsynaptic cell) GABA GAT2/3 V... |
| [hsa04728](https://www.genome.jp/dbget-bin/www_bget?pathway:hsa04728) | Dopaminergic synapse - Homo sapiens (human) | Dopamine (DA) is an important and prototypical slow neurotransmitter in the mammalian brain, where i... | C03758 (Dopamine) C00355 (3,4-Dihydroxy-L-phenylalanine) C03758 (Dopamine) C03758 (Dopamine) C00575 ... | DOPAMINERGIC SYNAPSE Glial cell Neuron (Presynaptic terminal) Neuron (Postsynaptic cell) DAT VMAT ... |
| [hsa04730](https://www.genome.jp/dbget-bin/www_bget?pathway:hsa04730) | Long-term depression - Homo sapiens (human) | Cerebellar long-term depression (LTD), thought to be a molecular and cellular basis for cerebellar l... | C00219 (Arachidonate) C00533 (Nitric oxide) C00641 (1,2-Diacyl-sn-glycerol) C01245 (D-myo-Inositol 1... | Parallel fiber PLC AMPAR RyR PLA2 PLA2 NOS DAG PLC mGluR1 Glutamate PKC cGMP IGF1R CRFR1 DAG Glutama... |
| [hsa04740](https://www.genome.jp/dbget-bin/www_bget?pathway:hsa04740) | Olfactory transduction - Homo sapiens (human) | Within the compact cilia of the olfactory receptor neurons (ORNs) a cascade of enzymatic activity tr... | C00575 (3',5'-Cyclic AMP) C00076 (Calcium cation) C00698 (Cl-) C01330 (Sodium cation) C00942 (3',5'-... | OLFACTORY TRANSDUCTION cAMP Golf ACⅢ CNG CaCC Olfactory receptor neuron (ORN) Depolarization ODO... |
| [hsa04742](https://www.genome.jp/dbget-bin/www_bget?pathway:hsa04742) | Taste transduction - Homo sapiens (human) | Five basic tastes are recognized by humans and most other animals - bitter, sweet, sour, salty and u... | C00076 (Calcium cation) C01330 (Sodium cation) C01330 (Sodium cation) C01451 (Salicin) C00076 (Calci... | TASTE TRANSDUCTION TRPM5 + Type Ⅱ receptor cell Bitter Depolarization T1R2 Salicin PLCβ2 2+ ... |
| [hsa04744](https://www.genome.jp/dbget-bin/www_bget?pathway:hsa04744) | Phototransduction - Homo sapiens (human) | Phototransduction is a biochemical process by which the photoreceptor cells generate electrical sign... | C00076 (Calcium cation) C01330 (Sodium cation) C00942 (3',5'-Cyclic GMP) C00144 (GMP) C00942 (3',5'-... | PHOTOTRANSDUCTION DARK LIGHT Disc membrane Disc membrane CNG CNG Hyperpolarization (Light response)... |
| [hsa04750](https://www.genome.jp/dbget-bin/www_bget?pathway:hsa04750) | Inflammatory mediator regulation of TRP channels - Homo sapiens (human) | The TRP channels that exhibit a unique response to temperature have been given the name thermo-TRPs.... | C00002 (ATP) C00306 (Bradykinin) C00780 (Serotonin) C00388 (Histamine) C00584 (Prostaglandin E2) C00... | Mast cell INFLAMMATORY MEDIATOR REGULATION OF TRP CHANNELS P2YR B1/B2 5HTR TRPV1 ASIC IL1R TrkA I... |
| [hsa04810](https://www.genome.jp/dbget-bin/www_bget?pathway:hsa04810) | Regulation of actin cytoskeleton - Homo sapiens (human) |  | C04637 (1-Phosphatidyl-D-myo-inositol 4,5-bisphosphate), C11554 (1-Phosphatidyl-1D-myo-inositol 3,4-... | PIP PIP PIP Bradykinin Acetylcholine TMSB4 Arp2/3 PFN WAVE2 WAVE1 HSPC300 APC NWASP Asef Abi2 Nap125... |
| [hsa04910](https://www.genome.jp/dbget-bin/www_bget?pathway:hsa04910) | Insulin signaling pathway - Homo sapiens (human) | Insulin binding to its receptor results in the tyrosine phosphorylation of insulin receptor substrat... | C00369 (Starch) C00575 (3',5'-Cyclic AMP) C00031 (D-Glucose) C05981 (Phosphatidylinositol-3,4,5-tris... | TSC1 SKIP SHIP2 IKK JNK LAR PTP1B SOCS Elk1 MNK ERK1/2 FAS AMPK Glycogen cAMP INS Glucose HSL PP1 PY... |
| [hsa04911](https://www.genome.jp/dbget-bin/www_bget?pathway:hsa04911) | Insulin secretion - Homo sapiens (human) | Pancreatic beta cells are specialised endocrine cells that continuously sense the levels of blood su... | C00031 (D-Glucose) C00575 (3',5'-Cyclic AMP) C00076 (Calcium cation) C00002 (ATP) C00092 (D-Glucose ... | INSULIN SECRETION GLUT1/2 Kir6.2 VDCC M3R GLP-1R PLC Glucose Citrate cycle (TCA cycle) PKA Gq/G11 G... |
| [hsa04912](https://www.genome.jp/dbget-bin/www_bget?pathway:hsa04912) | GnRH signaling pathway - Homo sapiens (human) | Gonadotropin-releasing hormone (GnRH) secretion from the hypothalamus acts upon its receptor in the ... | C00076 (Calcium cation) C00416 (Phosphatidate) C00219 (Arachidonate) C00165 (Diacylglycerol) C01245 ... | PKC αGSU LHβ PA, PE DAG PLCβ CDC42 cAMP CREB CaMK MKK3/6 p38MAPK MEKK BMK PLA2 IP3R CaM q/11 MT1 ... |
| [hsa04913](https://www.genome.jp/dbget-bin/www_bget?pathway:hsa04913) | Ovarian steroidogenesis - Homo sapiens (human) | The ovarian steroids, 17-beta estradiol (E2) and progesterone (P4), are critical for normal uterine ... | C00187 (Cholesterol) C01953 (Pregnenolone) C05138 (17alpha-Hydroxypregnenolone) C01227 (Dehydroepian... | OVARIAN STEROIDOGENESIS Steroid hormone biosynthesis Ovary (theca interstitial cell) Ovary (granul... |
| [hsa04914](https://www.genome.jp/dbget-bin/www_bget?pathway:hsa04914) | Progesterone-mediated oocyte maturation - Homo sapiens (human) | Xenopus oocytes are naturally arrested at G2 of meiosis I. Exposure to either insulin/IGF-1 or the s... | C05981 (Phosphatidylinositol-3,4,5-trisphosphate), C11554 (1-Phosphatidyl-1D-myo-inositol 3,4-bispho... | IGF1R IGF-1 Insulin Mos CPEB CycB1/B4 Cdc2 Ras PI3K Cdc2 PIP , PIP Akt/PKB PDE3 APC/C Fizzy Mad1/2/3... |
| [hsa04915](https://www.genome.jp/dbget-bin/www_bget?pathway:hsa04915) | Estrogen signaling pathway - Homo sapiens (human) | Estrogens are steroid hormones that regulate a plethora of physiological processes in mammals, inclu... | C00951 (Estradiol-17beta) C00951 (Estradiol-17beta) C00951 (Estradiol-17beta) C00575 (3',5'-Cyclic A... | ESTROGEN SIGNALING PATHWAY GPER c-Src MMP HB-EGF EGFR HB-EGF DNA DNA cAMP PKA Ras Raf MEK ERK1/2 p... |
| [hsa04916](https://www.genome.jp/dbget-bin/www_bget?pathway:hsa04916) | Melanogenesis - Homo sapiens (human) | Cutaneous melanin pigment plays a critical role in camouflage, mimicry, social communication, and pr... | C05606 (Melanin) C00082 (L-Tyrosine) C00076 (Calcium cation) C01245 (D-myo-Inositol 1,4,5-trisphosph... | Dvl DCT TYRP1 TYR MITF DCT TYRP1 TYR PKA CAMK CAM Gi/o ETB-R c-Kit MITF Melanin Tyrosine SCF Frizzle... |
| [hsa04917](https://www.genome.jp/dbget-bin/www_bget?pathway:hsa04917) | Prolactin signaling pathway - Homo sapiens (human) | Prolactin (PRL) is a polypeptide hormone known to be involved in a wide range of biological function... | C05981 (Phosphatidylinositol-3,4,5-trisphosphate) C05981 (Phosphatidylinositol-3,4,5-trisphosphate) ... | Jak_STAT signaling pathway MAPK signaling pathway PIP Akt/PKB PI3K PROLACTIN SIGNALING PATHWAY ERK... |
| [hsa04918](https://www.genome.jp/dbget-bin/www_bget?pathway:hsa04918) | Thyroid hormone synthesis - Homo sapiens (human) | Thyroid hormones triiodothyronine (T3) and thyroxine (T4) are essential for normal development, grow... | C00165 (Diacylglycerol) C01245 (D-myo-Inositol 1,4,5-trisphosphate) C00575 (3',5'-Cyclic AMP) C01330... | THYROID HORMONE SYNTHESIS Blood Follicular lumen Tight junction Apical membrane Basal membrane Thy... |
| [hsa04919](https://www.genome.jp/dbget-bin/www_bget?pathway:hsa04919) | Thyroid hormone signaling pathway - Homo sapiens (human) | The thyroid hormones (THs) are important regulators of growth, development and metabolism. The actio... | C05981 (Phosphatidylinositol-3,4,5-trisphosphate) C02465 (Triiodothyronine) C01829 (Thyroxine) C0246... | MAPK signaling pathway ITGB ITGA PIP Akt/PKB PDK1 PI3K PKC THYROID HORMONE SIGNALING PATHWAY Regu... |
| [hsa04920](https://www.genome.jp/dbget-bin/www_bget?pathway:hsa04920) | Adipocytokine signaling pathway - Homo sapiens (human) | Increased adipocyte volume and number are positively correlated with leptin production, and negative... | C00195 (N-Acylsphingosine) C00638 (Long-chain fatty acid) C15493 (9-cis-Retinoic acid) C00165 (Diacy... | Ceramide Long-chain fatty acid Retinoic acid NF-κB IκB SHP-2 SOCS3 CPT-1 G6PC PEPCK FACS Diglyceri... |
| [hsa04921](https://www.genome.jp/dbget-bin/www_bget?pathway:hsa04921) | Oxytocin signaling pathway - Homo sapiens (human) | Oxytocin (OT) is a nonapeptide synthesized by the magno-cellular neurons located in the supraoptic (... | C00076 (Calcium cation) C00165 (Diacylglycerol) C01245 (D-myo-Inositol 1,4,5-trisphosphate) C00575 (... | MAPK signalng pathway DAG cAMP MLCK IP3R CALM ADCY PKA OXYTOCIN SIGNALING PATHWAY ER/SR OTR OXT PL... |
| [hsa04922](https://www.genome.jp/dbget-bin/www_bget?pathway:hsa04922) | Glucagon signaling pathway - Homo sapiens (human) | Glucagon is conventionally regarded as a counterregulatory hormone for insulin and plays a critical ... | C00031 (D-Glucose) C00668 (alpha-D-Glucose 6-phosphate), C01172 (beta-D-Glucose 6-phosphate) C00022 ... | GLUCAGON SIGNALING PATHWAY GLUT1/2 Glucose G6P Pyruvate Glucose Citrate cycle (TCA cycle) Citrate ... |
| [hsa04923](https://www.genome.jp/dbget-bin/www_bget?pathway:hsa04923) | Regulation of lipolysis in adipocytes - Homo sapiens (human) | Lipolysis in adipocytes, the hydrolysis of triacylglycerol (TAG) to release fatty acids (FAs) and gl... | C00575 (3',5'-Cyclic AMP) C00788 (L-Adrenaline) C00547 (L-Noradrenaline) C00942 (3',5'-Cyclic GMP) C... | REGULATION OF LIPOLYSIS IN ADIPOCYTES PKA cAMP β-AR Epinephrine HSL Lypolysis Norepinephrine NP... |
| [hsa04924](https://www.genome.jp/dbget-bin/www_bget?pathway:hsa04924) | Renin secretion - Homo sapiens (human) | The aspartyl-protease renin is the key regulator of the renin-angiotensin-aldosterone system, which ... | C00575 (3',5'-Cyclic AMP) C00788 (L-Adrenaline) C00547 (L-Noradrenaline) C00942 (3',5'-Cyclic GMP) C... | RENIN SECRETION AC5/6 PKA cAMP β-AR Epinephrine Norepinephrine NPR-A cAMP signaling pathway cGMP c... |
| [hsa04925](https://www.genome.jp/dbget-bin/www_bget?pathway:hsa04925) | Aldosterone synthesis and secretion - Homo sapiens (human) | Aldosterone is a steroid hormone synthesized in and secreted from the outer layer of the adrenal cor... | C00942 (3',5'-Cyclic GMP) C00076 (Calcium cation) C00238 (Potassium cation) C01245 (D-myo-Inositol 1... | ALDOSTERONE SYNTHESIS AND SECRETION cGMP TASK PLCβ L-type IP3 q/11 T-type Calcium signaling path... |
| [hsa04926](https://www.genome.jp/dbget-bin/www_bget?pathway:hsa04926) | Relaxin signaling pathway - Homo sapiens (human) | Human relaxin-2 ("relaxin"), originally identified as a peptidic hormone of pregnancy, is now known ... | C05981 (Phosphatidylinositol-3,4,5-trisphosphate) C00533 (Nitric oxide) C00575 (3',5'-Cyclic AMP) C0... | PIP AKT RELAXIN SIGNALING PATHWAY RXFP1 IκB NF κB NFκB signaling pathway eNOS Vasodilation VEGF... |
| [hsa04927](https://www.genome.jp/dbget-bin/www_bget?pathway:hsa04927) | Cortisol synthesis and secretion - Homo sapiens (human) | Cortisol is the main endogenous glucocorticoid, which affects a plethora of physiological functions,... | C00076 (Calcium cation) C00238 (Potassium cation) C01245 (D-myo-Inositol 1,4,5-trisphosphate) C00076... | CORTISOL SYNTHESIS AND SECRETION PLCβ L-type IP3 q/11 T-type Calcium signaling pathway Mitochond... |
| [hsa04928](https://www.genome.jp/dbget-bin/www_bget?pathway:hsa04928) | Parathyroid hormone synthesis, secretion and action - Homo sapiens (human) | Parathyroid hormone (PTH) is a key regulator of calcium and phosphorus homeostasis. The principal re... | C00076 (Calcium cation) C01245 (D-myo-Inositol 1,4,5-trisphosphate) C00575 (3',5'-Cyclic AMP) C00076... | PARATHYROID HORMONE SYNTHESIS, SECRETION AND ACTION PLCβ IP3 q/11 Calcium signaling pathway cA... |
| [hsa04930](https://www.genome.jp/dbget-bin/www_bget?pathway:hsa04930) | Type II diabetes mellitus - Homo sapiens (human) | Insulin resistance is strongly associated with type II diabetes. "Diabetogenic" factors including FF... | C00002 (ATP) C00022 (Pyruvate) C00238 (Potassium cation) C00031 (D-Glucose) C00162 (Fatty acid) C000... | PYK P13K GLUT4 IRS1 INSR INS ATP Pyruvate Glucose SOCS ERK FFA Glucose ADIPO IKK PKCε/δ PDX-1 IRS ... |
| [hsa04931](https://www.genome.jp/dbget-bin/www_bget?pathway:hsa04931) | Insulin resistance - Homo sapiens (human) | Insulin resistance is a condition where cells become resistant to the effects of insulin. It is ofte... | C00162 (Fatty acid) C00162 (Fatty acid) C02843 (Long-chain acyl-CoA) C00165 (Diacylglycerol) C00031 ... | JNK1 TNFα FFA ER stress TNFR1 FATPs FFA IKKβ mTOR S6K INSR IRS-1 PI3K AKT2 GSK-3 +py LCFA-CoA DAG ... |
| [hsa04932](https://www.genome.jp/dbget-bin/www_bget?pathway:hsa04932) | Non-alcoholic fatty liver disease (NAFLD) - Homo sapiens (human) | Non-alcoholic fatty liver disease (NAFLD) represents a spectrum ranging from simple steatosis to mor... | C00031 (D-Glucose) C00162 (Fatty acid) 3630 (INS) 3667 (IRS1), 8660 (IRS2) 10000 (AKT3), 207 (AKT1),... | NON-ALCOHOLIC FATTY LIVER DISEASE (NAFLD) Simple steatosis (without inflammation and fibrosis) ... |
| [hsa04933](https://www.genome.jp/dbget-bin/www_bget?pathway:hsa04933) | AGE-RAGE signaling pathway in diabetic complications - Homo sapiens (human) | Advanced glycation end products (AGEs) are a complex group of compounds produced through the non-enz... | C00031 (D-Glucose) C00195 (N-Acylsphingosine) C21011 ([Protein]-N(epsilon)-(carboxymethyl)lysine), C... | AGE-RAGE SIGNALING PATHWAY IN DIABETIC COMPLICATIONS High glucose Aging Inflammation Oxidative... |
| [hsa04934](https://www.genome.jp/dbget-bin/www_bget?pathway:hsa04934) | Cushing syndrome - Homo sapiens (human) | Cushing syndrome (CS) is a rare disorder resulting from prolonged exposure to excess glucocorticoids... | C00076 (Calcium cation) C00238 (Potassium cation) C01245 (D-myo-Inositol 1,4,5-trisphosphate) C00076... | CUSHING SYNDROME PLCβ L-type IP3 q/11 T-type Calcium signaling pathway Mitochondria CREB cAMP AMP ... |
| [hsa04940](https://www.genome.jp/dbget-bin/www_bget?pathway:hsa04940) | Type I diabetes mellitus - Homo sapiens (human) | Type I diabetes mellitus is a disease that results from autoimmune destruction of the insulin-produc... | C00704 (O2.-), C00027 (Hydrogen peroxide), C00533 (Nitric oxide) 3382 (ICA1) 940 (CD28) 3592 (IL12A)... | TCR TCR ICA Oxygen / nitrogen free radicals CD28 Apoptosis IL-12 CPE CD80/86 IA-2 GAD HSP IL-2 ... |
| [hsa04950](https://www.genome.jp/dbget-bin/www_bget?pathway:hsa04950) | Maturity onset diabetes of the young - Homo sapiens (human) | About 2-5% of type II diabetic patients suffer from a monogenic disease with autosomal dominant inhe... | 6928 (HNF1B) 3280 (HES1) 168620 (BHLHA15) 3175 (ONECUT1) 2645 (GCK) 3651 (PDX1) 3171 (FOXA3) 4760 (N... | HNF1β Hes1 Mist1 HNF6 PDX1 FoxA3 NeuroD1 Hes1 MafA HNF4γ Nkx2.2 Ins HNF1α IAPP Pax4 Pax6 Glut2 Ne... |
| [hsa04960](https://www.genome.jp/dbget-bin/www_bget?pathway:hsa04960) | Aldosterone-regulated sodium reabsorption - Homo sapiens (human) | Sodium transport across the tight epithelia of Na+ reabsorbing tissues such as the distal part of th... | C01330 (Sodium cation) C01780 (Aldosterone) C00238 (Potassium cation) C01330 (Sodium cation) C00735 ... | ALDOSTERONE-REGULATED SODIUM REABSORPTION Blood DNA ENaC ATPase SGK1 Aldosterone Urinary lumen Ubi... |
| [hsa04961](https://www.genome.jp/dbget-bin/www_bget?pathway:hsa04961) | Endocrine and other factor-regulated calcium reabsorption - Homo sapiens (human) | Calcium (Ca2+) is essential for numerous physiological functions including intracellular signalling ... | C01673 (Calcitriol) C16051 (Parathyroid hormone) C00951 (Estradiol-17beta) C01673 (Calcitriol) C0007... | Blood DNA Urinary lumen Nucleus Distal convoluted tubular (DCT) cell Tight junction Tight junction ... |
| [hsa04962](https://www.genome.jp/dbget-bin/www_bget?pathway:hsa04962) | Vasopressin-regulated water reabsorption - Homo sapiens (human) | In the kidney, the antidiuretic hormone vasopressin (AVP) is a critical regulator of water homeostas... | C00575 (3',5'-Cyclic AMP) C00001 (H2O) C00001 (H2O) C00001 (H2O) C00001 (H2O) 551 (AVP) 554 (AVPR2) ... | VASOPRESSIN-REGULATED WATER REABSORPTION Renal interstitium fluid (blood) DNA Urinary lumen Nucleu... |
| [hsa04964](https://www.genome.jp/dbget-bin/www_bget?pathway:hsa04964) | Proximal tubule bicarbonate reclamation - Homo sapiens (human) | One of the major tasks of the renal proximal tubule (PT) is to secrete acid into the tubule lumen, t... | C00001 (H2O) C00001 (H2O) C00080 (H+) C01330 (Sodium cation) C00288 (HCO3-) C00011 (CO2) C00011 (CO2... | PROXIMAL TUBULE BICARBONATE RECLAMATION Blood Urinary lumen Proximal tubular cell Tight junction ... |
| [hsa04966](https://www.genome.jp/dbget-bin/www_bget?pathway:hsa04966) | Collecting duct acid secretion - Homo sapiens (human) | One of the important roles of the collecting duct segment of the kidney nephron is acid secretion. A... | C00001 (H2O) C00080 (H+) C00011 (CO2) C00080 (H+) C00288 (HCO3-) C00698 (Cl-) C00288 (HCO3-) C00014 ... | COLLECTING DUCT ACID SECRETION Blood Urinary lumen intercalated cell type A Tight junction Tight ... |
| [hsa04970](https://www.genome.jp/dbget-bin/www_bget?pathway:hsa04970) | Salivary secretion - Homo sapiens (human) | Saliva has manifold functions in maintaining the integrity of the oral tissues, in protecting teeth ... | C00001 (H2O) C01330 (Sodium cation) C00698 (Cl-) C00698 (Cl-) C00238 (Potassium cation) C01330 (Sodi... | SALIVARY SECRETION Interstitium Acinar lumen Tight junction Apical membrane Basolateral membrane Sa... |
| [hsa04971](https://www.genome.jp/dbget-bin/www_bget?pathway:hsa04971) | Gastric acid secretion - Homo sapiens (human) | Gastric acid is a key factor in normal upper gastrointestinal functions, including protein digestion... | C00388 (Histamine) C00575 (3',5'-Cyclic AMP) C01245 (D-myo-Inositol 1,4,5-trisphosphate) C00076 (Cal... | GASTRIC ACID SECRETION Gastric gland lumen Histamine Apical membrane Basolateral membrane Gastric ... |
| [hsa04972](https://www.genome.jp/dbget-bin/www_bget?pathway:hsa04972) | Pancreatic secretion - Homo sapiens (human) | The pancreas performs both exocrine and endocrine functions. The exocrine pancreas consists of two p... | C01330 (Sodium cation) C00698 (Cl-) C00238 (Potassium cation) C01330 (Sodium cation) C01245 (D-myo-I... | PANCREATIC SECRETION Interstitium Lumen Tight junction Apical membrane Basolateral membrane Pancrea... |
| [hsa04973](https://www.genome.jp/dbget-bin/www_bget?pathway:hsa04973) | Carbohydrate digestion and absorption - Homo sapiens (human) | Dietary carbohydrate in humans and omnivorous animals is a major nutrient. The carbohydrates that we... | C00092 (D-Glucose 6-phosphate) C00092 (D-Glucose 6-phosphate) C00031 (D-Glucose) C00031 (D-Glucose) ... | CARBOHYDRATE DIGESTION AND ABSORPTION Blood Lumen Salivary secretion Small intestinal epithelial ... |
| [hsa04974](https://www.genome.jp/dbget-bin/www_bget?pathway:hsa04974) | Protein digestion and absorption - Homo sapiens (human) | Protein is a dietary component essential for nutritional homeostasis in humans. Normally, ingested p... | C01330 (Sodium cation) C01330 (Sodium cation) C00025 (L-Glutamate), C00049 (L-Aspartate) C00037 (Gly... | PROTEIN DIGESTION AND ABSORPTION Blood Lumen Small intestinal epithelial cell Tight junction Tigh... |
| [hsa04975](https://www.genome.jp/dbget-bin/www_bget?pathway:hsa04975) | Fat digestion and absorption - Homo sapiens (human) | Fat is an important energy source from food. More than 95% of dietary fat is long-chain triacylglyce... | C00187 (Cholesterol) C00187 (Cholesterol) C00162 (Fatty acid) C01558 (Bile salt) C00162 (Fatty acid)... | FAT DIGESTION AND ABSORPTION Lumen Small intestinal epithelial cell Tight junction Tight junction... |
| [hsa04976](https://www.genome.jp/dbget-bin/www_bget?pathway:hsa04976) | Bile secretion - Homo sapiens (human) | Bile is a vital secretion, essential for digestion and absorption of fats and fat-soluble vitamins i... | C01330 (Sodium cation) C00080 (H+) C00698 (Cl-) C00288 (HCO3-) C01353 (Carbonic acid) C00001 (H2O) C... | AQP9 BILE SECRETION Tight junction Hepatocyte NHE1 HCO Cholangiocyte AE2 ATP CFTR HCO Secretin cAMP... |
| [hsa04977](https://www.genome.jp/dbget-bin/www_bget?pathway:hsa04977) | Vitamin digestion and absorption - Homo sapiens (human) | Vitamins are a diverse and chemically unrelated group of organic substances that share a common feat... | C00422 (Triacylglycerol) C02530 (Cholesterol ester) C00422 (Triacylglycerol) C02530 (Cholesterol est... | VITAMIN DIGESTION AND ABSORPTION Lumen Small intestinal epithelial cell Tight junction Tight junc... |
| [hsa04978](https://www.genome.jp/dbget-bin/www_bget?pathway:hsa04978) | Mineral absorption - Homo sapiens (human) | Minerals are one of the five fundamental groups of nutrients needed to sustain life. Of the minerals... | C00076 (Calcium cation) C00076 (Calcium cation) C00076 (Calcium cation) C01330 (Sodium cation) C0007... | MINERAL ABSORPTION Blood Lumen Small intestinal epithelial cell Tight junction Tight junction Tight... |
| [hsa04979](https://www.genome.jp/dbget-bin/www_bget?pathway:hsa04979) | Cholesterol metabolism - Homo sapiens (human) | Cholesterol is an essential component of mammalian cell membranes as well as a precursor of bile aci... | C15610 (Cholest-5-ene-3beta,26-diol) C00422 (Triacylglycerol) C02530 (Cholesterol ester) C00422 (Tri... | Hepatocyte Nascent HDL HDL IDL VLDL LDL Lp(a) Lysosome/ Later endosome CM-remnant Endosome Bile LD... |
| [hsa05010](https://www.genome.jp/dbget-bin/www_bget?pathway:hsa05010) | Alzheimer disease - Homo sapiens (human) | Alzheimer disease (AD) is a chronic disorder that slowly destroys neurons and causes serious cogniti... | C01245 (D-myo-Inositol 1,4,5-trisphosphate) C00076 (Calcium cation) C00076 (Calcium cation) C00533 (... | PEN2 PSEN NCSTN APH-1 γ-Secretase BACE ADAM10/17 β-Secretase α-Secretase TNF Microglia AICD C99 s... |
| [hsa05012](https://www.genome.jp/dbget-bin/www_bget?pathway:hsa05012) | Parkinson disease - Homo sapiens (human) | Parkinson disease (PD) is a progressive neurodegenerative movement disorder that results primarily f... | C04599 (1-Methyl-4-phenyl-1,2,3,6-tetrahydropyridine) C07593 (Rotenone) C00002 (ATP) C00008 (ADP) C0... | LRRK2 Ubiquitin mediated proteolysis Proteasome Apoptosis UBA1 UbcH7/8 Parkin CDCrel1 synphilin1 Pae... |
| [hsa05014](https://www.genome.jp/dbget-bin/www_bget?pathway:hsa05014) | Amyotrophic lateral sclerosis (ALS) - Homo sapiens (human) | Amyotrophic lateral sclerosis (ALS) is a progressive, lethal, degenerative disorder of motor neurons... | C00025 (L-Glutamate) C00533 (Nitric oxide) C00076 (Calcium cation) C00533 (Nitric oxide) C00062 (L-A... | SOD1 EAAT2 AMYOTROPHIC LATERAL SCLEROSIS (ALS) Motor neuron Astrocyte Presynaptic neuron Microgli... |
| [hsa05016](https://www.genome.jp/dbget-bin/www_bget?pathway:hsa05016) | Huntington disease - Homo sapiens (human) | Huntington disease (HD) is an autosomal-dominant neurodegenerative disorder that primarily affects m... | C00025 (L-Glutamate) C01245 (D-myo-Inositol 1,4,5-trisphosphate) C00076 (Calcium cation) 100532726 (... | HUNTINGTON DISEASE Cx I Cx II Cx III Cx IV Cx V Htt Htt Htt Htt Htt REST REST coREST Sin3A HDACs DN... |
| [hsa05020](https://www.genome.jp/dbget-bin/www_bget?pathway:hsa05020) | Prion diseases - Homo sapiens (human) | Prion diseases, also termed transmissible spongiform encephalopathies (TSEs), are a group of fatal n... | C00575 (3',5'-Cyclic AMP) C00027 (Hydrogen peroxide) C02140 (Corticosterone) 5621 (PRNP) 5621 (PRNP)... | cyPrP PRION DISEASES DNA Endoplasmic reticulum (ER) Neuron Endocytosis Proteasome Golgi Endosome Pr... |
| Entry | Name | Description | Object | Legend |
| [hsa05030](https://www.genome.jp/dbget-bin/www_bget?pathway:hsa05030) | Cocaine addiction - Homo sapiens (human) | Drug addiction is a chronic, relapsing disorder in which compulsive drug-seeking and drug-taking beh... | C00082 (L-Tyrosine) C00355 (3,4-Dihydroxy-L-phenylalanine) C03758 (Dopamine) C03758 (Dopamine) C0116... | COCAINE ADDICTION Neuron (Presynaptic terminal) Neuron (Postsynaptic cell) VMAT Control Dopaminergi... |
| [hsa05031](https://www.genome.jp/dbget-bin/www_bget?pathway:hsa05031) | Amphetamine addiction - Homo sapiens (human) | Amphetamine is a psychostimulant drug that exerts persistent addictive effects. Most addictive drugs... | C00082 (L-Tyrosine) C00355 (3,4-Dihydroxy-L-phenylalanine) C03758 (Dopamine) C03758 (Dopamine) C0375... | AMPHETAMINE ADDICTION Neuron (Presynaptic terminal) Neuron (Postsynaptic cell) VMAT Control Dopamin... |
| [hsa05032](https://www.genome.jp/dbget-bin/www_bget?pathway:hsa05032) | Morphine addiction - Homo sapiens (human) | Morphine is an alkaloid from the plant extracts of opium poppy. Although morphine is highly effectiv... | C00575 (3',5'-Cyclic AMP) C00575 (3',5'-Cyclic AMP) C00334 (4-Aminobutanoate) C00334 (4-Aminobutanoa... | MORPHINE ADDICTION GABA neuron (Presynaptic terminal) MOR Control GABAergic synapse Synaptic cleft ... |
| [hsa05033](https://www.genome.jp/dbget-bin/www_bget?pathway:hsa05033) | Nicotine addiction - Homo sapiens (human) | Nicotine is one of the main psychoactive ingredients in tobacco that contributes to the harmful toba... | C00334 (4-Aminobutanoate) C01330 (Sodium cation), C00076 (Calcium cation) C00076 (Calcium cation) C0... | NICOTINE ADDICTION Control Smoking Nucleus accumbens (NAc) Nicotine (Nic) GABA GABA Ca / Na Ca / ... |
| [hsa05034](https://www.genome.jp/dbget-bin/www_bget?pathway:hsa05034) | Alcoholism - Homo sapiens (human) | Alcoholism, also called dependence on alcohol (ethanol), is a chronic relapsing disorder that is pro... | C00082 (L-Tyrosine) C00355 (3,4-Dihydroxy-L-phenylalanine) C03758 (Dopamine) C03758 (Dopamine) C0116... | ALCOHOLISM Neuron (Presynaptic terminal) Neuron (Postsynaptic cell) VMAT Control Dopaminergic synaps... |
| [hsa05100](https://www.genome.jp/dbget-bin/www_bget?pathway:hsa05100) | Bacterial invasion of epithelial cells - Homo sapiens (human) | Many pathogenic bacteria can invade phagocytic and non-phagocytic cells and colonize them intracellu... | 10801 (SEPT9), 124404 (SEPT12), 1731 (SEPT1), 23157 (SEPT6), 23176 (SEPT8), 4735 (SEPT2), 55752 (SEP... | BACTERIAL INVASION OF EPITHELIAL CELLS ECM-receptor interaction Regulation of actin cytosk... |
| [hsa05110](https://www.genome.jp/dbget-bin/www_bget?pathway:hsa05110) | Vibrio cholerae infection - Homo sapiens (human) | Cholera toxin (CTX) is one of the main virulence factors of Vibrio cholerae. Once secreted, CTX B-ch... | C00014 (Ammonia) C00080 (H+) C00238 (Potassium cation) C00238 (Potassium cation) C01330 (Sodium cati... | TcpF TcpE TcpD TcpC TcpB TcpA Hap ACE NanH GM1 Tight junction Zot RtxA CtxA CtxA CtxB VCC Vibrio cho... |
| [hsa05120](https://www.genome.jp/dbget-bin/www_bget?pathway:hsa05120) | Epithelial cell signaling in Helicobacter pylori infection - Homo sapiens (human) | Two major virulence factors of H. pylori are the vacuolating cytotoxin (VacA) and the cag type-IV se... | C01342 (NH4+) C00080 (H+) C00014 (Ammonia) G00263 (IV2Fuc,III4Fuc-Lc4-Cer) C00698 (Cl-) C00086 (Urea... | HopZ AlpB AlpA VacA p38 AP-1 JNK MKK4 IKKα IKKγ IKKβ IκBα ATPeV CASP3 PAK1 NF-κB RANTES GRO-α... |
| [hsa05130](https://www.genome.jp/dbget-bin/www_bget?pathway:hsa05130) | Pathogenic Escherichia coli infection - Homo sapiens (human) | Enteropathogenic E. coli (EPEC) and enterohemorrhagic E. coli (EHEC) are closely related pathogenic ... | C00350 (Phosphatidylethanolamine) 929 (CD14) 23643 (LY96) 7099 (TLR4) 7100 (TLR5) 10376 (TUBA1B), 11... | Regulation of actin cytoskeleton CD14 MD-2 Flagellar assembly Adherens junction Tight junction Toll-... |
| [hsa05131](https://www.genome.jp/dbget-bin/www_bget?pathway:hsa05131) | Shigellosis - Homo sapiens (human) | Shigellosis, or bacillary dysentery, is an intestinal infection caused by Shigella, a genus of enter... | C04637 (1-Phosphatidyl-D-myo-inositol 4,5-bisphosphate) C04737 (alpha-D-Galactosyl-(1-&gt;4)-beta-D-... | SHIGELLOSIS Rac1 Dock180 Crk Cortactin c-Src Abl ELMO RhoG Cdc42 WAVE Arp2/3 Vinculin IpgB1 VirA Ipa... |
| [hsa05132](https://www.genome.jp/dbget-bin/www_bget?pathway:hsa05132) | Salmonella infection - Homo sapiens (human) | Salmonella infection usually presents as a self-limiting gastroenteritis or the more severe typhoid ... | C04637 (1-Phosphatidyl-D-myo-inositol 4,5-bisphosphate) C00338 (Lipopolysaccharide) C00533 (Nitric o... | SALMONELLA INFECTION SopE/E2 SopB Rac1 Cdc42 NF-κB JNK ZO-1 Redistribution Disruption of tight jun... |
| [hsa05133](https://www.genome.jp/dbget-bin/www_bget?pathway:hsa05133) | Pertussis - Homo sapiens (human) | Pertussis, also known as whooping cough, is an acute respiratory infectious disease caused by a bact... | C00076 (Calcium cation) C00002 (ATP) C00575 (3',5'-Cyclic AMP) C00338 (Lipopolysaccharide) C19596 (L... | PERTUSSIS FhaC FHA SphB1 CD11b Sulfated carbohydrates CD29 FimD Fim2/3 Fimbrial proteins Adhesion Pr... |
| [hsa05134](https://www.genome.jp/dbget-bin/www_bget?pathway:hsa05134) | Legionellosis - Homo sapiens (human) | Legionellosis is a potentially fatal infectious disease caused by the bacterium Legionella pneumophi... | C00338 (Lipopolysaccharide) C01277 (1-Phosphatidyl-1D-myo-inositol 4-phosphate) 58484 (NLRC4) 29108 ... | LEGIONELLOSIS FlaA IPAF ASC CASP1 IL-1β IL-18 Naip5 CASP7 Host cell death NF-κB DNA Anti-apoptotic... |
| [hsa05140](https://www.genome.jp/dbget-bin/www_bget?pathway:hsa05140) | Leishmaniasis - Homo sapiens (human) | Leishmania is an intracellular protozoan parasite of macrophages that causes visceral, mucosal, and ... | C00076 (Calcium cation) C00533 (Nitric oxide) C02737 (Phosphatidylserine) C00195 (N-Acylsphingosine)... | Macrophage FNR FcγR C3b PKC p47phox Complement cascade Leishmania promastigote LPG Calcium sig... |
| [hsa05142](https://www.genome.jp/dbget-bin/www_bget?pathway:hsa05142) | Chagas disease (American trypanosomiasis) - Homo sapiens (human) | Trypanosoma cruzi is an intracellular protozoan parasite that causes Chagas disease. The parasite li... | C00062 (L-Arginine) C00533 (Nitric oxide) C01245 (D-myo-Inositol 1,4,5-trisphosphate) C00076 (Calciu... | TLR2 TLR4 IFNγ gp160 TcCRT C3b C1q Complement pathway Toll-like receptor signaling pathway TLR... |
| [hsa05143](https://www.genome.jp/dbget-bin/www_bget?pathway:hsa05143) | African trypanosomiasis - Homo sapiens (human) | Trypanosoma brucei, the parasite responsible for African trypanosomiasis (sleeping sickness), are sp... | C00584 (Prostaglandin E2) C00696 (Prostaglandin D2) C00306 (Bradykinin) C16003 (Atrial natriuretic p... | AFRICAN TRYPANOSOMIASIS Macrophage TLR9 MyD88 IL12 IL18 CD8+ T cell IFNγ TNFα VSG Trypanosoma bur... |
| [hsa05144](https://www.genome.jp/dbget-bin/www_bget?pathway:hsa05144) | Malaria - Homo sapiens (human) | Plasmodium protozoa are parasites that account for malaria infection. Sporozoite forms of the parasi... | C00533 (Nitric oxide) C00634 (Chondroitin 4-sulfate) G10505 (Hyaluronic acid) G00054 (Type II A anti... | ICAM1 Subcutaneous tissue Liver Circulation Kupffer cell Vascular endothelial cell PfEMP1 SPECT Mal... |
| [hsa05145](https://www.genome.jp/dbget-bin/www_bget?pathway:hsa05145) | Toxoplasmosis - Homo sapiens (human) | Toxoplasma gondii is an obligate intracellular parasite that is prevalent worldwide. The tachyzoite ... | C06314 (Lipoxin A4) C05981 (Phosphatidylinositol-3,4,5-trisphosphate) C00076 (Calcium cation) 4615 (... | MyD88 IRAK TRAF6 TAK TAB1/2 IKK IκB NFκB ERK JNK p38 MKK3/6 GRA3/5 Toxoplasma gondii tachyzoite Pa... |
| [hsa05146](https://www.genome.jp/dbget-bin/www_bget?pathway:hsa05146) | Amoebiasis - Homo sapiens (human) | Entamoeba histolytica, an extracellular protozoan parasite is a human pathogen that invades the inte... | C00076 (Calcium cation) C00027 (Hydrogen peroxide) C00219 (Arachidonate) C00584 (Prostaglandin E2) C... | Extracellular matrix Intestinal epithelial cell Entamoeba histolytica trophozoite IL1β MUC2 Lamini... |
| [hsa05150](https://www.genome.jp/dbget-bin/www_bget?pathway:hsa05150) | Staphylococcus aureus infection - Homo sapiens (human) | Staphylococcus aureus can cause multiple forms of infections ranging from superficial skin infection... | C11596 (FMLP) C20898 (Lipoteichoic acid) C16059 (Defensin beta-1), C16060 (Defensin beta-2), C16061 ... | C1S C1R C1Q MBL MASP1/2 STAPHYLOCOCCUS AUREUS INFECTION Alternative pathway Lectin pathway Classic... |
| Entry | Name | Description | Object | Legend |
| [hsa05152](https://www.genome.jp/dbget-bin/www_bget?pathway:hsa05152) | Tuberculosis - Homo sapiens (human) | Tuberculosis, or TB, is an infectious disease caused by Mycobacterium tuberculosis. One third of the... | C04549 (1-Phosphatidyl-1D-myo-inositol 3-phosphate) C01194 (1-Phosphatidyl-D-myo-inositol) C00338 (L... | SapM TUBERCULOSIS DC-SIGN Raf1 IL-10 Pro-inflammatory cytokines Phagomsome maturation arrest Rab5 VP... |
| [hsa05160](https://www.genome.jp/dbget-bin/www_bget?pathway:hsa05160) | Hepatitis C - Homo sapiens (human) | Hepatitis C virus (HCV) is a major cause of chronic liver disease. The HCV employ several strategies... | C00076 (Calcium cation) C00027 (Hydrogen peroxide) C05981 (Phosphatidylinositol-3,4,5-trisphosphate)... | HEPATITIS C Hepatitis C virus Hepatocyte RIG-I IPS-1 TRAF3 TBK1 IKKε IRF3/7 NFκB TLR3 TRIF IκB I... |
| [hsa05161](https://www.genome.jp/dbget-bin/www_bget?pathway:hsa05161) | Hepatitis B - Homo sapiens (human) | Hepatitis B virus (HBV) is an enveloped virus and contains a partially double-stranded relaxed circu... | 7419 (VDAC3) 54205 (CYCS) 10542 (LAMTOR5) 842 (CASP9) 317 (APAF1) 100506742 (CASP12) 836 (CASP3) 581... | HBx Mitochondria VDAC3 CytC HBXIP CASP9 Apaf-1 CASP12 CASP3 Apoptosis Bax Survivin Ca release PKC... |
| [hsa05162](https://www.genome.jp/dbget-bin/www_bget?pathway:hsa05162) | Measles - Homo sapiens (human) | Measles virus (MV) is highly contagious virus that leads infant death worldwide. Humans are the uniq... | C05981 (Phosphatidylinositol-3,4,5-trisphosphate) 51284 (TLR7), 54106 (TLR9) 4615 (MYD88) 3654 (IRAK... | MEASLES MV proteins TLR7/9 MyD88 IRAK IRF7 IFNα DNA TLR2/4 RIG-I MyD88 IRAK IκB NFκB LPS MDA5 IPS... |
| [hsa05163](https://www.genome.jp/dbget-bin/www_bget?pathway:hsa05163) | Human cytomegalovirus infection - Homo sapiens (human) | Human cytomegalovirus (HCMV) is an enveloped, double-stranded DNA virus that is a member of beta-her... | C05981 (Phosphatidylinositol-3,4,5-trisphosphate) C00584 (Prostaglandin E2) C05981 (Phosphatidylinos... | PIP 4EBPs S6K1/2 mTOR Rheb TSC2 TSC1 AKT PI3K HUMAN CYTOMEGALOVIRUS INFECTION EGFR FasL IKK NF κB... |
| [hsa05164](https://www.genome.jp/dbget-bin/www_bget?pathway:hsa05164) | Influenza A - Homo sapiens (human) | Influenza is a contagious respiratory disease caused by influenza virus infection. Influenza A virus... | C00080 (H+) C05981 (Phosphatidylinositol-3,4,5-trisphosphate) C00027 (Hydrogen peroxide) 5644 (PRSS1... | INFLUENZA A Alveolar and bronchial epithelial cells Alveolar macrophages Type II pneumocytes Endoso... |
| [hsa05165](https://www.genome.jp/dbget-bin/www_bget?pathway:hsa05165) | Human papillomavirus infection - Homo sapiens (human) | Human papillomavirus (HPV) is a non-enveloped, double-stranded DNA virus. HPV infect mucoal and cuta... | C05981 (Phosphatidylinositol-3,4,5-trisphosphate) C00575 (3',5'-Cyclic AMP) C00584 (Prostaglandin E2... | PIP 4EBPs S6K1/2 mTOR Rheb TSC2 TSC1 AKT PI3K p53 signaling pathway HUMAN PAPILLOMAVIRUS INFECTION... |
| [hsa05166](https://www.genome.jp/dbget-bin/www_bget?pathway:hsa05166) | Human T-cell leukemia virus 1 infection - Homo sapiens (human) | Human T-cell leukemia virus type 1 (HTLV-1) is a pathogenic retrovirus that is associated with adult... | C00076 (Calcium cation) C18996 (Accessory protein p30II) C18995 (P13 protein) C00238 (Potassium cati... | HUMAN T-CELL LEUKEMIA VIRUS 1 INFECTION Human T-lymphotropic virus-I gp21 gp46 NRP1 CD4+ T cell... |
| [hsa05167](https://www.genome.jp/dbget-bin/www_bget?pathway:hsa05167) | Kaposi sarcoma-associated herpesvirus infection - Homo sapiens (human) | Kaposi sarcoma-associated herpesvirus (KSHV), also known as human herpesvirus 8 (HHV-8), is the most... | C00350 (Phosphatidylethanolamine) C05981 (Phosphatidylinositol-3,4,5-trisphosphate) C01245 (D-myo-In... | IFN-α/β TRAF3 IRF7 IFN-β IFN-α IKK JNK TBK1 IKKε AKT STAT1 IκBα IRF3 TLR3 TRIF KAPOSI SARCO... |
| [hsa05168](https://www.genome.jp/dbget-bin/www_bget?pathway:hsa05168) | Herpes simplex infection - Homo sapiens (human) | Herpes simplex virus (HSV) infections are very common worldwide, with the prevalence of HSV-1 reachi... | 4049 (LTA) 8740 (TNFSF14) 8764 (TNFRSF14) 5818 (NECTIN1), 5819 (NECTIN2) 29992 (PILRA) 7185 (TRAF1),... | HERPES SIMPLEX INFECTION Herpes simplex virus-1 Herpes simplex virus-2 LTα LIGHT HVEM Nectin1/2 P... |
| [hsa05169](https://www.genome.jp/dbget-bin/www_bget?pathway:hsa05169) | Epstein-Barr virus infection - Homo sapiens (human) | Epstein-Barr virus (EBV) is a ubiquitous human herpesvirus that is associated with oncogenesis. EBV ... | C05981 (Phosphatidylinositol-3,4,5-trisphosphate) C01245 (D-myo-Inositol 1,4,5-trisphosphate) C00076... | Killing of target cells gp350/220 gH/gL gp42 CD21 HLA-DR HLA-DP HLA-DQ HLA-DR CD38 CD43 MHC-I EBNA2 ... |
| [hsa05170](https://www.genome.jp/dbget-bin/www_bget?pathway:hsa05170) | Human immunodeficiency virus 1 infection - Homo sapiens (human) | Human immunodeficiency virus type 1 (HIV-1) , the causative agent of AIDS (acquired immunodeficiency... | C05981 (Phosphatidylinositol-3,4,5-trisphosphate) C01245 (D-myo-Inositol 1,4,5-trisphosphate) C00165... | PIP S6K1/2 mTOR AKT PI3K HUMAN IMMUNODEFICIENCY VIRUS 1 INFECTION NF κB NFκB signaling pathway... |
| [hsa05200](https://www.genome.jp/dbget-bin/www_bget?pathway:hsa05200) | Pathways in cancer - Homo sapiens (human) |  | C00951 (Estradiol-17beta), C00410 (Progesterone), C07653 (Flutamide), C01227 (Dehydroepiandrosterone... | PATHWAYS IN CANCER DCC CASP3 CASP9 CASP3 ECAD β-catenin Wnt α-catenin β-catenin ITGB Dvl ECM ITGA... |
| [hsa05202](https://www.genome.jp/dbget-bin/www_bget?pathway:hsa05202) | Transcriptional misregulation in cancer - Homo sapiens (human) | In tumor cells, genes encoding transcription factors (TFs) are often amplified, deleted, rearranged ... | 2130 (EWSR1) 2313 (FLI1) 2130 (EWSR1) 2078 (ERG) 2130 (EWSR1) 2115 (ETV1) 2130 (EWSR1) 2118 (ETV4) 2... | TRANSCRIPTIONAL MISREGULATION IN CANCER Ewing's sarcoma Clear-cell sarcoma Desmoplastic small ro... |
| [hsa05203](https://www.genome.jp/dbget-bin/www_bget?pathway:hsa05203) | Viral carcinogenesis - Homo sapiens (human) | There is a strong association between viruses and the development of human malignancies. We now know... | 7157 (TP53) 7337 (UBE3A) 1739 (DLG1) 23513 (SCRIB) 5829 (PXN) 578 (BAK1) 3661 (IRF3) 5925 (RB1) 5933... | VIRAL CARCINOGENESIS Hepatitis C virus (HCV) Hepatocellular carcinoma Human papillomavirus (HPV) p... |
| [hsa05204](https://www.genome.jp/dbget-bin/www_bget?pathway:hsa05204) | Chemical carcinogenesis - Homo sapiens (human) | It has been estimated that exposure to environmental chemical carcinogens may contribute significant... | C14869 (Trichloroethanol glucuronide) C06899 (Chloral hydrate) C07490 (Trichloroethanol) C11150 (Tri... | CYP1/2 Trichloroethanol-glucuronide Chloral hydrate Trichloroethanol Trichloroacetic acid Dichloroa... |
| [hsa05205](https://www.genome.jp/dbget-bin/www_bget?pathway:hsa05205) | Proteoglycans in cancer - Homo sapiens (human) | Many proteoglycans (PGs) in the tumor microenvironment have been shown to be key macromolecules that... | G10505 (Hyaluronic acid) C00925 (Heparan sulfate) C00925 (Heparan sulfate) G13064 (Binding domain fo... | CD44v3 Tiam1 HER2 RhoA ROCK Ankyrin Cytoskeleton activation PI3K AKT CD44 Lipid Raft IP3R CAMKII Fil... |
| [hsa05206](https://www.genome.jp/dbget-bin/www_bget?pathway:hsa05206) | MicroRNAs in cancer - Homo sapiens (human) | MicroRNA (miRNA) is a cluster of small non-encoding RNA molecules of 21 - 23 nucleotides in length, ... | 406922 (MIR133A1), 406923 (MIR133A2), 442890 (MIR133B) 993 (CDC25A), 994 (CDC25B), 995 (CDC25C) 599 ... | MicroRNAs IN CANCER Lung cancer Lung epithelial cell Tumorigenesis Survival Invasion / metastasi... |
| [hsa05210](https://www.genome.jp/dbget-bin/www_bget?pathway:hsa05210) | Colorectal cancer - Homo sapiens (human) | Colorectal cancer (CRC) is the second largest cause of cancer-related deaths in Western countries. C... | C05981 (Phosphatidylinositol-3,4,5-trisphosphate) 1630 (DCC) 836 (CASP3) 7040 (TGFB1), 7042 (TGFB2),... | DCC CASP3 TGFβ PI3K CASP9 APPL CASP3 APC Smad2 TGFβRI Smad4 hMLH1 hMSH2 K-Ras Raf JNK Axin Axin Ra... |
| [hsa05211](https://www.genome.jp/dbget-bin/www_bget?pathway:hsa05211) | Renal cell carcinoma - Homo sapiens (human) | Renal cell cancer (RCC) accounts for ~3% of human malignancies and its incidence appears to be risin... | C00007 (Oxygen) C00149 ((S)-Malate) C00122 (Fumarate) C00007 (Oxygen) 5781 (PTPN11) 7040 (TGFB1), 70... | SHP2 TGF-β VEGF Glut1 HIF-β p300/CBP CUL2 Rbx1 ElonginB ElonginC VHL HPH HIF-α Malate Fumarate CU... |
| Entry | Name | Description | Object | Legend |
| [hsa05212](https://www.genome.jp/dbget-bin/www_bget?pathway:hsa05212) | Pancreatic cancer - Homo sapiens (human) | Infiltrating ductal adenocarcinoma is the most common malignancy of the pancreas. When most investig... | C05981 (Phosphatidylinositol-3,4,5-trisphosphate) C00416 (Phosphatidate) C05981 (Phosphatidylinosito... | HER2/neu K-Ras PI3K RacGEF PKB/Akt Rac IKK NFκB Bad Bcl-xl Raf CASP9 MEK ERK RalGDS Ral RalBP1 PLD1... |
| [hsa05213](https://www.genome.jp/dbget-bin/www_bget?pathway:hsa05213) | Endometrial cancer - Homo sapiens (human) | Endometrial cancer (EC) is the most common gynaecological malignancy and the fourth most common mali... | C05981 (Phosphatidylinositol-3,4,5-trisphosphate) 1499 (CTNNB1) 1499 (CTNNB1) 3265 (HRAS), 3845 (KRA... | β-Catenin β-Catenin Ras APC Axin GSK-3β α-Catenin β-Catenin Cell cycle Fkhrl1 BAD Casp9 ILK Erb... |
| [hsa05214](https://www.genome.jp/dbget-bin/www_bget?pathway:hsa05214) | Glioma - Homo sapiens (human) | Gliomas are the most common of the primary brain tumors and account for more than 40% of all central... | C01245 (D-myo-Inositol 1,4,5-trisphosphate) C00076 (Calcium cation) C00165 (Diacylglycerol) C05981 (... | EGF TGFα PDGF IGF-1 EGFR PDGFR IGFR EGF TGFα PDGF IGF-1 EGFR PDGFR IGFR CAM CAMK PKC Shc Grb2 Sos ... |
| [hsa05215](https://www.genome.jp/dbget-bin/www_bget?pathway:hsa05215) | Prostate cancer - Homo sapiens (human) | Prostate cancer constitutes a major health problem in Western countries. It is the most frequently d... | C00951 (Estradiol-17beta), C00410 (Progesterone), C07653 (Flutamide), C01227 (Dehydroepiandrosterone... | p53 signaling pathway Cytokine-cytokine receptor interaction Steroid hormone biosynthesis SRD5A2 NF�... |
| [hsa05216](https://www.genome.jp/dbget-bin/www_bget?pathway:hsa05216) | Thyroid cancer - Homo sapiens (human) | Thyroid cancer is the most common endocrine malignancy and accounts for the majority of endocrine ca... | 3265 (HRAS), 3845 (KRAS), 4893 (NRAS) 5468 (PPARG) 673 (BRAF) 673 (BRAF) 5594 (MAPK1), 5595 (MAPK3) ... | Ras PPARγ BRAF BRAF ERK MEK PPFP RXR β-catenin c-Myc ECAD THYROID CANCER ERK CyclinD1 MEK BRAF Ra... |
| [hsa05217](https://www.genome.jp/dbget-bin/www_bget?pathway:hsa05217) | Basal cell carcinoma - Homo sapiens (human) | Cancer of the skin is the most common cancer in Caucasians and basal cell carcinomas (BCC) account f... | C00187 (Cholesterol) 374654 (KIF7) 2735 (GLI1) 5727 (PTCH1), 8643 (PTCH2) 650 (BMP2), 652 (BMP4) 513... | KIF7 Cholesterol GLI1 PTCH1 BMP Wnt SHH APC PTCH1 SUFU Axin Dvl Frizzled BASAL CELL CARCINOMA GLI S... |
| [hsa05218](https://www.genome.jp/dbget-bin/www_bget?pathway:hsa05218) | Melanoma - Homo sapiens (human) | Melanoma is a form of skin cancer that has a poor prognosis and which is on the rise in Western popu... | C05981 (Phosphatidylinositol-3,4,5-trisphosphate) 673 (BRAF) 999 (CDH1) 4893 (NRAS) 1019 (CDK4) 595 ... | p53 signaling pathway Melanogenesis BRAF ECAD Adherens junction NRAS CDK4 CyclinD1 MITF PTEN RTK Bad... |
| [hsa05219](https://www.genome.jp/dbget-bin/www_bget?pathway:hsa05219) | Bladder cancer - Homo sapiens (human) | The urothelium covers the luminal surface of almost the entire urinary tract, extending from the ren... | 3265 (HRAS) 4312 (MMP1), 4313 (MMP2), 4318 (MMP9) 1890 (TYMP) 7422 (VEGFA) 3576 (CXCL8) 7057 (THBS1)... | Adherens junction H-Ras VEGF signaling pathway ErbB signaling pathway MMPs VEGF IL-8 TSP-1 EGFR E-ca... |
| [hsa05220](https://www.genome.jp/dbget-bin/www_bget?pathway:hsa05220) | Chronic myeloid leukemia - Homo sapiens (human) | Chronic myeloid leukemia (CML) is a clonal myeloproliferative disorder of a pluripotent stem cell. T... | C05981 (Phosphatidylinositol-3,4,5-trisphosphate) 2122 (MECOM), 861 (RUNX1) 598 (BCL2L1) 5781 (PTPN1... | p53 signaling pathway AML-EVI1 Bcl-xl Shp2 BCR-ABL p53 TGF-β signaling pathway Hematopoietic cell l... |
| [hsa05221](https://www.genome.jp/dbget-bin/www_bget?pathway:hsa05221) | Acute myeloid leukemia - Homo sapiens (human) | Acute myeloid leukemia (AML) is a disease that is characterized by uncontrolled proliferation of clo... | C05981 (Phosphatidylinositol-3,4,5-trisphosphate) 5914 (RARA), 7704 (ZBTB16) 3845 (KRAS) 4893 (NRAS)... | PLZF-RARα K-Ras N-Ras FLT3 PU.1 C/EBPα AML1-ETO Jak-STAT signaling pathway mTOR signaling pathway ... |
| [hsa05222](https://www.genome.jp/dbget-bin/www_bget?pathway:hsa05222) | Small cell lung cancer - Homo sapiens (human) | Lung cancer is a leading cause of cancer death among men and women in industrialized countries. Smal... | C05981 (Phosphatidylinositol-3,4,5-trisphosphate) C00777 (Retinoate), C15493 (9-cis-Retinoic acid) 8... | p53 signaling pathway CyclinE CDK2 CyclinD1 Apoptosis ECM-receptor interaction Focal adhesion ITGB I... |
| [hsa05223](https://www.genome.jp/dbget-bin/www_bget?pathway:hsa05223) | Non-small cell lung cancer - Homo sapiens (human) | Lung cancer is a leading cause of cancer death among men and women in industrialized countries. Non-... | C00777 (Retinoate), C15493 (9-cis-Retinoic acid) C00076 (Calcium cation) C00165 (Diacylglycerol) C01... | PKC Retinoic acid DAG PIP Forkhead CASP9 BAD PKB/Akt PDK1 PI3K CDK4/6 CyclinD1 E2F Ras PLCγ TGFα E... |
| [hsa05224](https://www.genome.jp/dbget-bin/www_bget?pathway:hsa05224) | Breast cancer - Homo sapiens (human) | Breast cancer is the leading cause of cancer death among women worldwide. The vast majority of breas... | C00951 (Estradiol-17beta) C00951 (Estradiol-17beta) C00951 (Estradiol-17beta) C00951 (Estradiol-17be... | BREAST CANCER EGFR CoR Ras Raf MEK ERK1/2 Shc Grb2 SOS Estrogen signaling pathway Jagged Notch mTOR... |
| [hsa05225](https://www.genome.jp/dbget-bin/www_bget?pathway:hsa05225) | Hepatocellular carcinoma - Homo sapiens (human) | Hepatocellular carcinoma (HCC) is a major type of primary liver cancer and one of the rare human neo... | C05981 (Phosphatidylinositol-3,4,5-trisphosphate) C05981 (Phosphatidylinositol-3,4,5-trisphosphate) ... | HEPATOCELLULAR CARCINOMA EGFR Ras Raf MEK ERK1/2 Shc Grb2 SOS mTOR S6K IGF-II IGF1R PI3K-Akt signal... |
| [hsa05226](https://www.genome.jp/dbget-bin/www_bget?pathway:hsa05226) | Gastric cancer - Homo sapiens (human) | Gastric cancer (GC) is one of the world's most common cancers. According to Lauren's histological cl... | C05981 (Phosphatidylinositol-3,4,5-trisphosphate) C05981 (Phosphatidylinositol-3,4,5-trisphosphate) ... | GASTRIC CANCER Ras Raf MEK ERK1/2 Shc Grb2 SOS mTOR S6K PI3K-Akt signaling pathway Ras Raf MEK ERK1... |
| [hsa05230](https://www.genome.jp/dbget-bin/www_bget?pathway:hsa05230) | Central carbon metabolism in cancer - Homo sapiens (human) | Malignant transformation of cells requires specific adaptations of cellular metabolism to support gr... | C00031 (D-Glucose) C00092 (D-Glucose 6-phosphate) C00022 (Pyruvate) C00031 (D-Glucose) C00064 (L-Glu... | CENTRAL CARBON METABOLISM IN CANCER GLUT1/2 Glucose G6P Pyruvate Glucose GCK Glutamine Glutamate... |
| [hsa05231](https://www.genome.jp/dbget-bin/www_bget?pathway:hsa05231) | Choline metabolism in cancer - Homo sapiens (human) | Abnormal choline metabolism is emerging as a metabolic hallmark that is associated with oncogenesis ... | C05981 (Phosphatidylinositol-3,4,5-trisphosphate) C00114 (Choline) C00114 (Choline) C00588 (Choline ... | JNK ERK1/2 SOS Ras MEK1/2 Raf Akt P13K PDK1 PIP CHOLINE METABOLISM IN CACNCER MAPK signaling ... |
| [hsa05310](https://www.genome.jp/dbget-bin/www_bget?pathway:hsa05310) | Asthma - Homo sapiens (human) | Asthma is a complex syndrome with many clinical phenotypes in both adults and children. Its major ch... | C00388 (Histamine) C02166 (Leukotriene C4) C00696 (Prostaglandin D2) C04598 (2-Acetyl-1-alkyl-sn-gly... | Cytokine-cytokine receptor interaction Jak-STAT signaling pathway FcεRI signaling pathway T cell re... |
| [hsa05320](https://www.genome.jp/dbget-bin/www_bget?pathway:hsa05320) | Autoimmune thyroid disease - Homo sapiens (human) | The classification of autoimmune throid disease (AITD) includes Hashimoto's thyroiditis (HT) or chro... | C01382 (Iodine) D00636 (Amiodarone hydrochloride (JP17/USAN)) C02465 (Triiodothyronine) C01829 (Thyr... | T cell receptor signaling pathway MHCII TCR CD40L CD40 TCR MHCII APC B cell Antigen processing and ... |
| [hsa05321](https://www.genome.jp/dbget-bin/www_bget?pathway:hsa05321) | Inflammatory bowel disease (IBD) - Homo sapiens (human) | Inflammatory bowel disease (IBD), which includes Crohn disease (CD) and ulcerative colitis (UC), is ... | 7097 (TLR2) 4790 (NFKB1), 5970 (RELA) 64127 (NOD2) 7100 (TLR5) 7097 (TLR2) 4790 (NFKB1), 5970 (RELA)... | INFLAMMATORY BOWEL DISEASE (IBD) APC TLR2 NF-κB DNA NOD2 MDP PGN TLR5 Flagellin TLR2 Peptidoglyc... |
| Entry | Name | Description | Object | Legend |
| [hsa05322](https://www.genome.jp/dbget-bin/www_bget?pathway:hsa05322) | Systemic lupus erythematosus - Homo sapiens (human) | Systemic lupus erythematosus (SLE) is a prototypic autoimmune disease characterised by the productio... | C00027 (Hydrogen peroxide) C00434 (Double-stranded DNA) C02737 (Phosphatidylserine) 102723407 (IGH) ... | BCR FcγR FcγR IgG IgG IgG IgG Elastase Cathepsin C1s C1q C1r MAC TNF-α IFN-γ IL-10 C1q C1q CD80/... |
| [hsa05323](https://www.genome.jp/dbget-bin/www_bget?pathway:hsa05323) | Rheumatoid arthritis - Homo sapiens (human) | Rheumatoid arthritis (RA) is a chronic autoimmune joint disease where persistent inflammation affect... | C05443 (Vitamin D3) C16051 (Parathyroid hormone) C00584 (Prostaglandin E2) C00080 (H+) 8600 (TNFSF11... | Th17 cell RHEUMATOID ARTHRITIS RANKL RANK Osteoclast differentiation RANKL IL17 IL1 TNFα IL6 IL... |
| [hsa05330](https://www.genome.jp/dbget-bin/www_bget?pathway:hsa05330) | Allograft rejection - Homo sapiens (human) | Allograft rejection is the consequence of the recipient's alloimmune response to nonself antigens ex... | C00533 (Nitric oxide) 3105 (HLA-A), 3106 (HLA-B), 3107 (HLA-C), 3108 (HLA-DMA), 3109 (HLA-DMB), 3111... | MHCI/II IL-2 T cell receptor signaling pathway MHCII TCR Recipient APC Antigen processing and presen... |
| [hsa05332](https://www.genome.jp/dbget-bin/www_bget?pathway:hsa05332) | Graft-versus-host disease - Homo sapiens (human) | Graft-versus-host disease (GVHD) is a lethal complication of allogeneic hematopoietic stem cell tran... | C00533 (Nitric oxide) C00338 (Lipopolysaccharide) 3108 (HLA-DMA), 3109 (HLA-DMB), 3111 (HLA-DOA), 31... | Cytokine-cytokine receptor interaction T cell receptor signaling pathway MHCII TCR Host APC Antigen ... |
| [hsa05340](https://www.genome.jp/dbget-bin/www_bget?pathway:hsa05340) | Primary immunodeficiency - Homo sapiens (human) | Primary immunodeficiencies (PIs) are a heterogeneous group of disorders, which affect cellular and h... | 6890 (TAP1) 958 (CD40) 959 (CD40LG) 102723407 102723407 920 (CD4) 925 (CD8A), 926 (CD8B) 925 (CD8A),... | T cell receptor signaling pathway Hematopoietic cell lineage B cell receptor signaling pathway PRIMA... |
| [hsa05410](https://www.genome.jp/dbget-bin/www_bget?pathway:hsa05410) | Hypertrophic cardiomyopathy (HCM) - Homo sapiens (human) | Hypertrophic cardiomyopathy (HCM) is a primary myocardial disorder with an autosomal dominant patter... | C00002 (ATP) C16010 (Endothelin-1) C02135 (Angiotensin II) C00076 (Calcium cation) C00076 (Calcium c... | SGCD HYPERTROPHIC CARDIOMYOPATHY (HCM) DHPR RyR SERCA2a NCX Sarcoplasmic reticulum (SR) T-tubule Ca... |
| [hsa05412](https://www.genome.jp/dbget-bin/www_bget?pathway:hsa05412) | Arrhythmogenic right ventricular cardiomyopathy (ARVC) - Homo sapiens (human) | Arrhythmogenic right ventricular cardiomyopathy (ARVC) is an inherited heart muscle disease that may... | C00076 (Calcium cation) C00076 (Calcium cation) C00076 (Calcium cation) C00076 (Calcium cation) C013... | SGCD ARRHYTHMOGENIC RIGHT VENTRICULAR CARDIOMYOPATHY (ARVC) DHPR RyR2 SERCA2a NCX Sarcoplasmic re... |
| [hsa05414](https://www.genome.jp/dbget-bin/www_bget?pathway:hsa05414) | Dilated cardiomyopathy (DCM) - Homo sapiens (human) | Dilated cardiomyopathy (DCM) is a heart muscle disease characterised by dilation and impaired contra... | C00076 (Calcium cation) C00076 (Calcium cation) C00076 (Calcium cation) C00076 (Calcium cation) C013... | SGCD DILATED CARDIOMYOPATHY (DCM) DHPR RyR SERCA2a NCX Sarcoplasmic reticulum (SR) T-tubule Cardiac... |
| [hsa05416](https://www.genome.jp/dbget-bin/www_bget?pathway:hsa05416) | Viral myocarditis - Homo sapiens (human) | Myocarditis is a cardiac disease associated with inflammation and injury of the myocardium. It resul... | 6444 (SGCD) 6445 (SGCG) 6443 (SGCB) 6442 (SGCA) 1605 (DAG1) 1605 (DAG1) 3908 (LAMA2) 1756 (DMD) 1981... | SGCD VIRAL MYOCARDITIS Cardiac muscle contraction SGCG SGCB SGCA α-DAG β-DAG Laminin ECM-receptor... |
| [hsa05418](https://www.genome.jp/dbget-bin/www_bget?pathway:hsa05418) | Fluid shear stress and atherosclerosis - Homo sapiens (human) | Shear stress represents the frictional force that the flow of blood exerts at the endothelial surfac... | C16845 (Peroxynitrite) C00533 (Nitric oxide) C00076 (Calcium cation) C00704 (O2.-) C00533 (Nitric ox... | FLUID SHEAR STRESS AND ATHEROSCLEROSIS ONOO MAPK signaling pathway p38 VCAM-1 ICAM-1 AP-1 Akt ... |
