# Supplementary material for: Comprehensive genome based analysis of Vibrio parahaemolyticus for identifying novel drug and vaccine molecules: Subtractive proteomics and vaccinomics approach
Source: PLoS One. 2020 Aug 19;15(8):e0237181. doi: 10.1371/journal.pone.0237181 (PMC7444560; doi:10.1371/journal.pone.0237181)
Supplement: S8 File — (DOC) [file pone.0237181.s021.doc]

**S8 File**. Pathogen-specific (40) metabolic pathways.

| **Entry** | **Name** | **Description** | **Object** | **Legend** |
| --- | --- | --- | --- | --- |
| [vpa00261](https://www.genome.jp/dbget-bin/www_bget?pathway:vpa00261) | Monobactam biosynthesis - Vibrio parahaemolyticus RIMD 2210633 | Monobactams are beta-lactam antibiotics containing a monocyclic beta-lactam nucleus, which is struct... | C01179 (3-(4-Hydroxyphenyl)pyruvate) C03198 ((S)-4-Hydroxymandelate) C03590 (4-Hydroxyphenylglyoxyla... | MONOBACTAM BIOSYNTHESIS Phenylalanine, tyrosine and tryptophan biosynthesis 4-Hydroxy-phenylpyruva... |
| [vpa00281](https://www.genome.jp/dbget-bin/www_bget?pathway:vpa00281) | Geraniol degradation - Vibrio parahaemolyticus RIMD 2210633 |  | C03069 (3-Methylcrotonyl-CoA) C16471 (5-Methyl-3-oxo-4-hexenoyl-CoA) C16469 (3-Hydroxy-5-methylhex-4... | 3-Methylcrotonyl-CoA 5-Methyl-3-oxo-4-hexenoyl-CoA 2.3.1.16 1.1.1.35 6.4.1.5 Valine, leucine and is... |
| [vpa00300](https://www.genome.jp/dbget-bin/www_bget?pathway:vpa00300) | Lysine biosynthesis - Vibrio parahaemolyticus RIMD 2210633 |  | C19889 (LysW-gamma-L-lysine) C19888 (LysW-gamma-L-alpha-aminoadipate 6-semialdehyde) C19887 (LysW-ga... | 2.6.1.83 Tropane, piperidine and pyridine alkaloid biosynthesis LysW-γ-L-lysine LysW-γ-L-α-aminoa... |
| [vpa00332](https://www.genome.jp/dbget-bin/www_bget?pathway:vpa00332) | Carbapenem biosynthesis - Vibrio parahaemolyticus RIMD 2210633 | Carbapenems are broad-spectrum beta-lactam antibiotics, which are often considered as the antibiotic... | C00025 (L-Glutamate) C03287 (L-Glutamyl 5-phosphate) C01165 (L-Glutamate 5-semialdehyde) C03912 ((S)... | CARBAPENEM BIOSYNTHESIS Arginine and proline metabolism L-Glutamate 2.7.2.11 L-Glutamyl-P 1.2.1.41 ... |
| [vpa00361](https://www.genome.jp/dbget-bin/www_bget?pathway:vpa00361) | Chlorocyclohexane and chlorobenzene degradation - Vibrio parahaemolyticus RIMD 2210633 |  | C16266 (3-Chloro-2-hydroxymuconic semialdehyde) C12832 (3,4,6-Trichloro-cis-1,2-dihydroxycyclohexa-3... | 1.13.11.39 3-Chloro-2-hydroxy-muconic semialdehyde 3,4,6-Trichloro-cis-1,2-dihydroxy-cyclohexa-3,5-d... |
| [vpa00362](https://www.genome.jp/dbget-bin/www_bget?pathway:vpa00362) | Benzoate degradation - Vibrio parahaemolyticus RIMD 2210633 |  | C06714 (3-Hydroxypimeloyl-CoA) C04553 (3-Carboxy-2,5-dihydro-5-oxofuran-2-acetate) C04434 ((1E)-4-Ox... | 3-Hydroxy-pimeloyl-CoA BENZOATE DEGRADATION 3-Carboxy-2,5-dihydro-5-oxofuran-2-acetate 1.14.13.33... |
| [vpa00364](https://www.genome.jp/dbget-bin/www_bget?pathway:vpa00364) | Fluorobenzoate degradation - Vibrio parahaemolyticus RIMD 2210633 |  | C02364 (3-Fluorobenzoate) C16474 (3-Fluoro-cis,cis-muconate) C00090 (Catechol) C16484 (1-Fluorocyclo... | 3-Fluorobenzoate 1.14.12.- 1.14.12.- 3.1.1.45 3.1.1.45 3-Fluoro-cis,cis-muconate Catechol 1-Fluorocy... |
| [vpa00401](https://www.genome.jp/dbget-bin/www_bget?pathway:vpa00401) | Novobiocin biosynthesis - Vibrio parahaemolyticus RIMD 2210633 |  | C12477 (3-Methylpyrrole-2,4-dicarboxylic acid) C12483 (Pyrrole-2-carbonyl-[pcp]) C12479 (Coumermic a... | Polyketide sugar unit biosynthesis CouN7 CouP CouM CouL CouL CloN6 6.2.1.53 1.3.8.14 CloN7 CloP CloM... |
| [vpa00460](https://www.genome.jp/dbget-bin/www_bget?pathway:vpa00460) | Cyanoamino acid metabolism - Vibrio parahaemolyticus RIMD 2210633 |  | C05670 (3-Aminopropiononitrile) C01401 (Alanine) C00302 (Glutamate) C05714 (alpha-Aminopropiononitri... | 3-Aminopropiono-nitrile D-Alanine metabolism D-Gln & D-Glu metabolism D-Arg & D-Orn metabolism 3.5.... |
| [vpa00473](https://www.genome.jp/dbget-bin/www_bget?pathway:vpa00473) | D-Alanine metabolism - Vibrio parahaemolyticus RIMD 2210633 |  | C00041 (L-Alanine) C00133 (D-Alanine) C00993 (D-Alanyl-D-alanine) C00022 (Pyruvate) C04260 (O-D-Alan... | D-ALANINE METABOLISM Peptideglycan metabolism D-Arginine and D-ornithine metabolism Cyanoamino ac... |
| [vpa00521](https://www.genome.jp/dbget-bin/www_bget?pathway:vpa00521) | Streptomycin biosynthesis - Vibrio parahaemolyticus RIMD 2210633 | Streptomycin is an aminocyclitol-aminoglycoside antibiotic produced by Streptomyces griseus. Strepto... | C01214 (1-Amino-1-deoxy-scyllo-inositol) C03319 (dTDP-L-rhamnose) C06592 (NDP-N-methyl-L-glucosamine... | Aminoglycosides 2.7.1.2 2.7.1.1 Polyketide sugar unit biosynthesis scyllo-Inosamine 2.7.1.65 3.1.3.2... |
| [vpa00523](https://www.genome.jp/dbget-bin/www_bget?pathway:vpa00523) | Polyketide sugar unit biosynthesis - Vibrio parahaemolyticus RIMD 2210633 |  | C07277 (dTDP-D-fucose) C00103 (D-Glucose 1-phosphate) C11911 (dTDP-D-desosamine) C00688 (dTDP-4-dehy... | dTDP-D-fucose Biosynthesis of 12-, 14- and 16-membered macrolides D-Glc-1P dTDP-D-desosamine dTDP-4-... |
| [vpa00525](https://www.genome.jp/dbget-bin/www_bget?pathway:vpa00525) | Acarbose and validamycin biosynthesis - Vibrio parahaemolyticus RIMD 2210633 |  | C20956 (alpha-D-Sedoheptulopyranose 7-phosphate) C17692 (2-epi-5-epi-Valiolone 7-phosphate) C17693 (... | ACARBOSE AND VALIDAMYCIN BIOSYNTHESIS 4.2.3.152 2.7.1.188 5.1.3.35 AcbL AcbN SalP 2.7.7.91 SalE 2... |
| [vpa00540](https://www.genome.jp/dbget-bin/www_bget?pathway:vpa00540) | Lipopolysaccharide biosynthesis - Vibrio parahaemolyticus RIMD 2210633 |  | C19877 (4-O-Phospho-alpha-Kdo-(2-&gt;6)-lipid IVA) C05382 (Sedoheptulose 7-phosphate) C07836 (D-glyc... | 4-Phospho-KDO-lipid IV [ ] Sedoheptulose-7P D-glycero-β-D-manno-Heptose-7P D-glyce... |
| [vpa00550](https://www.genome.jp/dbget-bin/www_bget?pathway:vpa00550) | Peptidoglycan biosynthesis - Vibrio parahaemolyticus RIMD 2210633 | Peptidoglycan is a macromolecule made of long aminosugar strands cross-linked by short peptides. It ... | C01050 (UDP-N-acetylmuramate) C04574 (di-trans,poly-cis-Undecaprenyl diphosphate) C17556 (di-trans,p... | 6.3.2.10 PEPTIDOGLYCAN BIOSYNTHESIS Aminosugar metabolism 3.6.1.27 2.4.1.227 2.7.8.13 6.3.2.10 6.3.... |
| [vpa00623](https://www.genome.jp/dbget-bin/www_bget?pathway:vpa00623) | Toluene degradation - Vibrio parahaemolyticus RIMD 2210633 |  | C00587 (3-Hydroxybenzoate) C03067 (3-Hydroxybenzaldehyde) C03351 (3-Hydroxybenzyl alcohol) C01467 (3... | TOLUENE DEGRADATION 3-Hydroxy-benzoate 3-Hydroxy-benzaldehyde 3-Hydroxybenzyl-alcohol 1.14.13.- 1.1... |
| [vpa00625](https://www.genome.jp/dbget-bin/www_bget?pathway:vpa00625) | Chloroalkane and chloroalkene degradation - Vibrio parahaemolyticus RIMD 2210633 |  | C01380 (Ethylene glycol) C06548 (Ethylene oxide) C06547 (Ethylene) C06793 (Vinyl chloride) C06791 (t... | 1.97.1.- 1.97.1.- 1.97.1.- 1.21.99.5 1.21.99.5 1.21.99.5 1.18.6.1 Ethylene glycol 1.1.1.- 3.3.2.10 1... |
| [vpa00626](https://www.genome.jp/dbget-bin/www_bget?pathway:vpa00626) | Naphthalene degradation - Vibrio parahaemolyticus RIMD 2210633 |  | C00091 (Succinyl-CoA) C03203 (1-Hydroxy-2-naphthoate) C14101 (2-Naphthoic acid) C14099 (2-Naphthalde... | NmoAB 2.8.3.- Succinyl-CoA 1-Hydroxy-2-naphthoate 2-Naphthoate 2-Naphthaldehyde 2-Hydroxymethyl-naph... |
| [vpa00627](https://www.genome.jp/dbget-bin/www_bget?pathway:vpa00627) | Aminobenzoate degradation - Vibrio parahaemolyticus RIMD 2210633 |  | C00568 (4-Aminobenzoate) C00230 (3,4-Dihydroxybenzoate) C07103 (2-Hydroxy-1,4-benzoquinone) C03360 (... | AMINOBENZOATE DEGRADATION 4-Aminobenzoate 1.14.12.- 1.7.1.- 3,4-Dihydroxybenzoate 3.1.8.1 2-Hydrox... |
| [vpa00633](https://www.genome.jp/dbget-bin/www_bget?pathway:vpa00633) | Nitrotoluene degradation - Vibrio parahaemolyticus RIMD 2210633 |  | C01468 (4-Cresol) C16401 (2,4,6-Trihydroxytoluene) C16400 (2,4,6-Triaminotoluene) C16395 (2-Amino-4,... | 4-Hydroxytoluene 2,4,6-Trihydroxytoluene 2,4,6-Triamino-toluene 2-Amino-4,6-dinitrotoluene 2-Amino-4... |
| [vpa00643](https://www.genome.jp/dbget-bin/www_bget?pathway:vpa00643) | Styrene degradation - Vibrio parahaemolyticus RIMD 2210633 |  | C05593 (3-Hydroxyphenylacetate) C02505 (2-Phenylacetamide) C16074 (Phenylacetonitrile) C16075 ((Z)-P... | 1.14.13.63 3-Hydroxyphenylacetate 3.5.5.1 3.5.1.4 4.2.1.84 4.99.1.7 Phenylacetamide Phenylacetonitri... |
| [vpa00660](https://www.genome.jp/dbget-bin/www_bget?pathway:vpa00660) | C5-Branched dibasic acid metabolism - Vibrio parahaemolyticus RIMD 2210633 |  | C00810 ((R)-Acetoin) C06010 ((S)-2-Acetolactate) C01011 ((3S)-Citramalyl-CoA) C00531 (Itaconyl-CoA) ... | Nicotinate and nicotinamide metabolism Valine, leucine and isoleucine biosynthesis 2.3.3.11 Alani... |
| [vpa00670](https://www.genome.jp/dbget-bin/www_bget?pathway:vpa00670) | One carbon pool by folate - Vibrio parahaemolyticus RIMD 2210633 |  | C00504 (Folate) C00415 (Dihydrofolate) C03479 (Folinic acid) C00143 (5,10-Methylenetetrahydrofolate)... | ONE CARBON POOL BY FOLATE Folate biosynthesis 2.1.2.5 2.1.2.4 2.1.2.2 4.3.1.4 1.5.1.15 1.5.1.3 1... |
| [vpa00680](https://www.genome.jp/dbget-bin/www_bget?pathway:vpa00680) | Methane metabolism - Vibrio parahaemolyticus RIMD 2210633 | Methane is metabolized principally by methanotrophs and methanogens in the global carbon cycle. Meth... | C01438 (Methane) C00132 (Methanol) C00067 (Formaldehyde) C00058 (Formate) C00237 (CO) C04330 (5,10-M... | METHANE METABOLISM Methane 1.14.13.25 1.14.18.3 Methanol Formaldehyde 1.1.3.13 1.1.2.7 1.1.1.244 1.... |
| [vpa00903](https://www.genome.jp/dbget-bin/www_bget?pathway:vpa00903) | Limonene and pinene degradation - Vibrio parahaemolyticus RIMD 2210633 |  | C11950 (3-Isopropyl-3-butenoic acid) C11949 (3-Isopropylbut-3-enoyl-CoA) C11948 (2,6-Dimethyl-5-meth... | 3-Isopropylbut-3-enoic acid 3-Isopropylbut-3-enoyl-CoA 2,6-Dimethyl-5-methylene-3-oxo-heptanoyl-CoA ... |
| [vpa00930](https://www.genome.jp/dbget-bin/www_bget?pathway:vpa00930) | Caprolactam degradation - Vibrio parahaemolyticus RIMD 2210633 |  | C02232 (3-Oxoadipyl-CoA) C14145 ((3S)-3-Hydroxyadipyl-CoA) C11519 (N-Cyclohexylformamide) C14144 (5-... | 3-Oxoadipyl-CoA (3S)-3-Hydroxyadipyl-CoA N-Cyclohexylformamide 5-Carboxy-2-pentenoyl-CoA Adipyl-CoA ... |
| [vpa01110](https://www.genome.jp/dbget-bin/www_bget?pathway:vpa01110) | Biosynthesis of secondary metabolites - Vibrio parahaemolyticus RIMD 2210633 |  | C21833 (Kaempferol-3-O-rutinoside) C21835 (12,18-Didecarboxysiroheme) C05797 (Pheophytin a) C05306 (... | Carotenoid biosynthesis Porphyrin and chlorophyll metabolism Diterpenoid biosynthesis Flavone and ... |
| [vpa01120](https://www.genome.jp/dbget-bin/www_bget?pathway:vpa01120) | Microbial metabolism in diverse environments - Vibrio parahaemolyticus RIMD 2210633 |  | C03618 (L-threo-3-Methylaspartate) C01732 (Mesaconate) C21104 (2,5-Dichloro-p-benzoquinone) C21859 (... | Xylene degradation Glyoxylate and dicarboxylate metabolism Aminobenzoate degradation Lysine bios... |
| [vpa01130](https://www.genome.jp/dbget-bin/www_bget?pathway:vpa01130) | Biosynthesis of antibiotics - Vibrio parahaemolyticus RIMD 2210633 |  | C21875 (Aurachin A) C21874 (Aurachin B epoxide) C21140 (Aurachin B) C21141 (4-Hydroxy-2-methyl-3-oxo... | Macrolides and ketolides Pentose phosphate pathway MEP / DOXP pathway Mevalonate pathway Lysine b... |
| [vpa01220](https://www.genome.jp/dbget-bin/www_bget?pathway:vpa01220) | Degradation of aromatic compounds - Vibrio parahaemolyticus RIMD 2210633 | Microorganisms are known to be capable of degrading diverse chemical substances including man-made c... | C01455 (Toluene) C00261 (Benzaldehyde) C00556 (Benzyl alcohol) C00180 (Benzoate) C06321 ((1R,6S)-1,6... | DEGRADATION OF AROMATIC COMPOUNDS Toluene Benzaldehyde Benzyl alcohol Benzoate Catechol 3-Fluorob... |
| [vpa01501](https://www.genome.jp/dbget-bin/www_bget?pathway:vpa01501) | beta-Lactam resistance - Vibrio parahaemolyticus RIMD 2210633 | The beta-lactam antibiotics are the most widely used group of antibiotics, which exert their effect ... | C04702 (UDPMurNAc(oyl-L-Ala-D-gamma-Glu-L-Lys-D-Ala-D-Ala)) C00039 (DNA) D07746 (Colistin (INN)), D0... | DNA DNA MecA MecI MecR1 BlaZ BlaI BlaR1 beta-LACTAM RESISTANCE DNA AmpC AmpR AmpG GlcNAc-anhMurNAc ... |
| [vpa01502](https://www.genome.jp/dbget-bin/www_bget?pathway:vpa01502) | Vancomycin resistance - Vibrio parahaemolyticus RIMD 2210633 | Vancomycin (VCM) is a glycopeptide antibiotic agent that inhibits the synthesis of peptidolgycan in ... | C00022 (Pyruvate) C00256 ((R)-Lactate) C19694 (D-Alanyl-(R)-lactate) C00041 (L-Alanine) C00133 (D-Al... | VANCOMYCIN RESISTANCE Cell wall Inner membrane VanS VanR Pyruvate D-Lac VanH VanA/B/D D-Ala-D-Lac L... |
| [vpa01503](https://www.genome.jp/dbget-bin/www_bget?pathway:vpa01503) | Cationic antimicrobial peptide (CAMP) resistance - Vibrio parahaemolyticus RIMD 2210633 | Cationic antimicrobial peptides (CAMPs) play an important role in host defense against microbial inf... | C20931 (4-Amino-4-deoxy-L-arabinose) C00346 (Ethanolamine phosphate) D07746 (Colistin (INN)), D00128... | CATIONIC ANTIMICROBIAL PEPTIDE (CAMP) RESISTANCE Inner membrane Outer membrane PmrB PmrA ArnT Ep... |
| [vpa02020](https://www.genome.jp/dbget-bin/www_bget?pathway:vpa02020) | Two-component system - Vibrio parahaemolyticus RIMD 2210633 | Two-component signal transduction systems enable bacteria to sense, respond, and adapt to changes in... | C00164 (Acetoacetate) C00038 (Zinc cation), C06696 (Lead) C00244 (Nitrate), C00088 (Nitrite) C00031 ... | NarI NarJ NarH NarG Bacterial chemotaxis MCP PilA RpoN PilS PilI CheW SdiA Acetoacetate Zn / Pb Nitr... |
| [vpa02024](https://www.genome.jp/dbget-bin/www_bget?pathway:vpa02024) | Quorum sensing - Vibrio parahaemolyticus RIMD 2210633 | Quorum sensing (QS) is a regulatory system that allows bacteria to share information about cell dens... | C16640 (CAI-1) C16421 (AI-2) C21195 (N-(3-Hydroxybutanoyl)-L-homoserine lactone) C18049 (N-Acyl-L-ho... | QUORUM SENSING Sensing protein Qrr LuxQ LuxO LuxU CqsS CAI-1 LitR/HapR LuxP AI-2 LuxN/AinR AI-1 (sR... |
| [vpa02030](https://www.genome.jp/dbget-bin/www_bget?pathway:vpa02030) | Bacterial chemotaxis - Vibrio parahaemolyticus RIMD 2210633 | Chemotaxis is the process by which cells sense chemical gradients in their environment and then move... | C00716 (Serine) C00049 (L-Aspartate) C00208 (Maltose) C00121 (D-Ribose) C00124 (D-Galactose) C00107 ... | Escherichia coli BACTERIAL CHEMOTAXIS Flagellar assembly MotB MotA FliN FliM FliG CheZ CheY CheR Ch... |
| [vpa02040](https://www.genome.jp/dbget-bin/www_bget?pathway:vpa02040) | Flagellar assembly - Vibrio parahaemolyticus RIMD 2210633 |  | VP0771, VPA0262 VP0772 (flgA), VPA0263 VP0770, VPA0261 VP2245 (fliJ) VP2254 (fliS), VPA1551 VP2237 (... | Type III secretion system Bacterial chemotaxis FlhD FlhC FlgM FlgA FlgN FliT FliJ FliS FliR FliP Fli... |
| [vpa02060](https://www.genome.jp/dbget-bin/www_bget?pathway:vpa02060) | Phosphotransferase system (PTS) - Vibrio parahaemolyticus RIMD 2210633 | The phosphoenolpyruvate (PEP)-dependent phosphotransferase system (PTS) is a major mechanism used by... | C06377 (D-Galactosamine 6-phosphate) C11544 (2(alpha-D-Mannosyl)-D-glycerate) C01083 (alpha,alpha-Tr... | Galactosamine 6-phosphate 2-O-α-Mannosyl-D-glycerate Trehalose β-Glucoside Trehalose 6-phosphate P... |
| [vpa03070](https://www.genome.jp/dbget-bin/www_bget?pathway:vpa03070) | Bacterial secretion system - Vibrio parahaemolyticus RIMD 2210633 | Gram-negative bacteria secrete a wide range of proteins whose functions include biogenesis of organe... | C00002 (ATP) C00002 (ATP) C00002 (ATP) C00002 (ATP) C00002 (ATP) C00002 (ATP) C00002 (ATP) C00002 (A... | Type I Type III Type II Type Va Type IV Type VI TolC HlyD HlyB YscC GspGHIJK GspD TatABCE SecDEFGY Y... |
| [vpa05111](https://www.genome.jp/dbget-bin/www_bget?pathway:vpa05111) | Biofilm formation - Vibrio cholerae - Vibrio parahaemolyticus RIMD 2210633 | Surface colonization and subsequent biofilm formation and development provide numerous advantages to... | C00575 (3',5'-Cyclic AMP) C16463 (3',5'-Cyclic diGMP) C00044 (GTP) C18076 (5'-Phosphoguanylyl(3'-&gt... | CyaA cAMP CRP BIOFILM FORMATION - VIBRIO CHOLERAE VarS VarA CsrB/C/D (ncRNA) CsrA CdgL CdgM VCA0... |
