# Supplementary material for: Comprehensive genome based analysis of Vibrio parahaemolyticus for identifying novel drug and vaccine molecules: Subtractive proteomics and vaccinomics approach
Source: PLoS One. 2020 Aug 19;15(8):e0237181. doi: 10.1371/journal.pone.0237181 (PMC7444560; doi:10.1371/journal.pone.0237181)
Supplement: S9 File — (DOCX) [file pone.0237181.s022.docx]

**S9 File.** Essential Proteins involved only in unique metabolic pathways (KAAS at KEGG)

>tr|Q87LW1|Q87LW1_VIBPA Penicillin-binding protein 1B OS=Vibrio parahaemolyticus serotype O3:K6 (strain RIMD 2210633) OX=223926 GN=VP2497 PE=3 SV=1

MTDSKKPSAKKAPAKKSTASKGTTKRPRRTPSKKPTNDKRSWLKVLWSFSWKAGVALAAV

LLFVGIYLDSVVKERFDGQLFELPTVVYARILNLNPGENITIQELRNELDVLNYRKVSQP

RYPGEYSSSSTRVELIRRPFEFADGPEPDRHVMLHFSDSGLQRIQSLESKGDLGYLRLEP

KMLGMLEKDRDEQRLFLRRDQFPEILVDALLATEDRDFYQHDGVSPLAIARALVANIKAG

RTVQGGSTLTQQLAKNLFLTRDKTLWRKVREAYIALILDYRYSKDRILEAYLNEVYLGQS

GGEAIHGFGLASRYYFGQPIQELRIDQLAMLVGMVKGPSYYNPVRYPERTKERRDLVLRL

LMQQNMLTSQQYEQAVSRPLDTQSKPRIASRQPAYFQQLNIELKEKVGDRFKAETGLRVF

TSLDPVSQSKMEQAIAKKIPDLAKRGGKELEAAAVAVDRHSGEIRAMVGGKRVGYEGFNR

ALNASRPIGSLVKPAIYLTALEQPDKYNLGTTLHDTPLSLKGSKGSVWTPRNYDRKYRGD

VPLYLALAKSLNVPTVRLGMELGIPEVSGTLERLGVNKDEIRPVPSMFLGSFSLTPFEVA

QMYQTLTNSGKRAKLTALRSVIDMDGNVLYQSLPRSSRAVDEQAAWLTTYAMKQGVAQGT

GRYLQSQFAWAALAGKTGTSNDTRDSWFVGIDGREVTTIWLGRDDNKPINLTGSSGALRV

YSEYLAQRIPERLDLPWPKEVTTLGFKPTSNGGLEMNCRSDYKLPVWDKTGQIKQQCEKK

SNWLNSLFDW

>tr|Q87T41|Q87T41_VIBPA dTDP-4-dehydrorhamnose 3,5-epimerase OS=Vibrio parahaemolyticus serotype O3:K6 (strain RIMD 2210633) OX=223926 GN=VP0229 PE=3 SV=1

MKVITTEIEGLLVVEPKVFGDSRGFFLESWNKLKFDEATGMEVSFVQDNHSKSESGTLRG

LHIQTKNAQGKLVRVVKGAVYDVAVDLRKDSKTYGKWFGLILSAENKKQLWIPKGFAHGF

LALEDDTEFLYKCDGYYDPLYEVSIDWNDKALAIDWGQFESFKTLKLSDKDSKGIGLSEF

STLEKEF

>tr|Q87LD8|Q87LD8_VIBPA Phosphocarrier protein NPr OS=Vibrio parahaemolyticus serotype O3:K6 (strain RIMD 2210633) OX=223926 GN=VP2674 PE=4 SV=1

MELSRKVLIQNRLGLHARAAVKLVELAQSFDAVVTIDNEEDKTATADSVMGLLMLESAQG

QYVTIHATGDQAELALDAVCHLIEDKFDEGE

>tr|Q87GN8|Q87GN8_VIBPA Sensor histidine kinase OS=Vibrio parahaemolyticus serotype O3:K6 (strain RIMD 2210633) OX=223926 GN=VPA1277 PE=4 SV=1

MRRIYLESFLGLIILFLASLKGYELIVYELNTDYDYLLQEHSSQAFYDLLSPIYEEKGLE

YTKSELEKFATASHRLLQPHTTDELPPEVKEVFDEDPTANIAFDDERDFWFRFDESTPFF

KISDNPNSPIIQAVNFDDNMVWIFFIAGFALYCVLLIWFLSRRIRELERVTVEFASGNFK

ARASTASAKSVGTLNKSFNNMADKVSRLITSNKMLTNAVAHELRTPIFRLQWQADLLADS

SLNEQQTKYVNSIVEDIDEMEEMVEELLYYAKMERPETELRTESLELNSLLLDLKDKWQQ

ETPLPITVKDTDCKEAQIKTDPKLLKRALDNLLRNAMRYADSQIMLEVTEDEEHCMISIH

DDGNGIDEKDWPHLFEAFYSADKSRNKSTSGFGLGLAIVRQIMELQRGDVSISHSPLGGA

CFTVSLPK

>tr|Q79YV7|Q79YV7_VIBPA FlaL OS=Vibrio parahaemolyticus serotype O3:K6 (strain RIMD 2210633) OX=223926 GN=VP2252 PE=4 SV=1

MDNNTNQSHLDSVENQVERYKQVLDVMPAGVILLDTYGVVREANPEAHRILEIPLVNEKW

FNIIQAAFDPREDDGHEVSLRNGRKVRLAISASATGQLILITDLTETRLLQARVSDLQRL

SSLGRMVASLAHQVRTPLSSAMLYASNLGAPNLPPATRERFQSKLMDRLHDLEKQVNDML

LFAKGGDNKVIKPFTIAQLVAEYQPMVETALKNNNIDYFLEVEEEQTELLGNANAIASAL

SNLVMNAIQMSGKESQIDIFFRPVNGELRISVQDSGPGVPQELQAKIMEPFFTTRSQGTG

LGLAVVQMVCRAHDGRLELISEQGDGACFTMCIPLERVQSEAQ

>tr|Q87R58|Q87R58_VIBPA Uncharacterized protein OS=Vibrio parahaemolyticus serotype O3:K6 (strain RIMD 2210633) OX=223926 GN=VP0940 PE=3 SV=1

MSMKATLSLSVLTLSILMASPSAFAAKRGPSAVTVVTEQVETHEINQSLSLIGKLKAAES

VVVASEVAGKVKQIAVKANQNVQQNQLLIQLDDDKAQAALVEAKAYLKDEERKLKEFQRL

VKRNAITQTEIDAQKASVEIAQARLDAAKANLADLHITAPFSGTVGFIDFSRGKMVSAGT

ELLTLDDLSVMELDLQIPERYLSMLSVGMEVAAKTSAWGEQRFSGKVTGIDTRISAETLN

LRVRIEFDNPENQLKPGMLMNASLAFPAIKAPIIPVQALEYSGTKRFVYVIDENNKATRQ

EVLLGARVDNEVVIESGVEIGDKIVVQGIVNMRDGVEVKEIVAPLKANTTDVTRNDSESV

EEEN

>tr|Q87P28|Q87P28_VIBPA Lipoprotein OS=Vibrio parahaemolyticus serotype O3:K6 (strain RIMD 2210633) OX=223926 GN=VP1690 PE=3 SV=1

MMKNKMRTLCLVLALFLVGCQTELYTNVSQKEGNEMLSILLSEGVVATKEPDKDNKVKLM

VDSSQIAFAVDALKRKGYPREQFSTLKEVFPKDDLISSPLAERARLVYAKSQELSSTLSQ

IDGVLVARVHVVLEDQDLRPGERPTPASASVFIKHAADVALDSYVPQIKLLVNNSIEGLN

YDRISVVMVPSSEVRVATQSNQFKSILSVQVTKETANHLIGILVFMVLLLIGSNVATFTW

CRRSAKRG

>tr|Q87HY1|Q87HY1_VIBPA Putative phosphoglycerate transport regulatory protein PgtC OS=Vibrio parahaemolyticus serotype O3:K6 (strain RIMD 2210633) OX=223926 GN=VPA0825 PE=4 SV=1

MVNLMADFKQFGRRLLLGAALYAPLTMAAEKELVILTTFSQAPITALVDDFTQHYPDAEV

RVVHRRTQSSLQLLSKSYMKDIDLVLSSSPFLMQELSNEHRLADMSSRVQVPKWLSPYLL

PNNDQVVAFGYSGAGIVWNKDYLAANHLPEPKRFQDLTNPLYFGHVTMSTPSRSGTTQLM

VESVLSQYGWQEGWRILLNVGANLATISSRSFGVADYIAKGKFGIGPTIDSYALIAQRKF

DYVGFAYDQDFTLMPTYIAQINRGKSDKLAESFIAHLLSKEVQEQMESSTFSKTALDDTA

RYGGENPVLDLEQVMPREALINLIFDTAITKRLPELQDAWLSLIKLNRLADGKAEKQRSL

QAIEKQLFELPLSKAQATEIAQKLLTMDKDSEVGMTHYQALLAEFSHELGRAMSEKLDNV

NQQLAQWRGKEK

>tr|Q87GX4|Q87GX4_VIBPA Putative multidrug efflux membrane fusion protein OS=Vibrio parahaemolyticus serotype O3:K6 (strain RIMD 2210633) OX=223926 GN=VPA1191 PE=3 SV=1

MQKKPLVALMAATAILAGCGEANNAQRESQAPLVVTQDVTVIDYQPSKSYIGRIEAVEDT

NITAQISGYLQARHFEEGQMVEKGQLLYSIEPSSFEAQVASAKAALAQAKASLKKAELDH

QRGKNLLPRGSISQSEFDALTATLLGARAELEAANAQLKLAEVNLSYTQIRAPFSGRISD

TKVSTGDLVSPSSGVLTTLVSLDPVHTSFSVSERERLAMGMDRIKGDGSAESNGVEVQLE

LENGRFFEHLGQLDFLGNRIDTKTGTIAMRALVPNPEHKLLPGQHIKVNLRDKNTRDVIV

VPRRAVQTDLEGDFVMVTTEGNVAERRNVELGPQVEQGIIIREGLEKGEAVITQGLQRVR

NGVEVRVQTPTEDKQ

>tr|Q87LZ0|Q87LZ0_VIBPA D-alanyl-D-alanine carboxypeptidase/D-alanyl-D-alanine-endopeptidase OS=Vibrio parahaemolyticus serotype O3:K6 (strain RIMD 2210633) OX=223926 GN=VP2468 PE=4 SV=1

MRLRWPLLISSLIFPLLSYAYPHQEVLPEGARISLVAEKLTESSTLDGIRPTDQLFPPAS

TLKIVTALAAKLELGDSFAFRTKLETSSSDAVIYFVGDPTLQTQDLKALLSLAKKNGLTR

INGDLWLDNSAFTGYDRAVGWPWDILGVCYSAPASSITLNNNCVQASIYTQKDGGTRVYV

PEHQPIRVKSSVETVSKTIQKSRHCDLDLLANPDNRYELKGCLVEREKPLPLKFAVQDPE

RYTSQNISTLLKQLGIELKGKIKIGSAPQKQRKLMALHQSKPLSDLLDDMLKHSDNLIAD

TLTKTLGAKFFVQPGSFTNGTEAIKQIIFANTGIDIRNARLEDGSGLSRNNRISANKMAE

ILRYIWKNEKTLKLIAIMPKSGESGTLQYRQSMRNAPIKGQLIAKSGSLYGTYNMAGYGL

DKNGQPNTIFVQFVSDYFPEKRDDNKPVIAPITHFEQLFYRDIVNFSQAIPKK

>tr|Q87TD7|Q87TD7_VIBPA General secretion pathway protein D OS=Vibrio parahaemolyticus serotype O3:K6 (strain RIMD 2210633) OX=223926 GN=VP0133 PE=3 SV=1

MKHWFSKSAWLLAGSLLCVPGAMANEFSASFKGTDIQEFINIVGRNLEKTIIVDPSVRGK

IDVRSYDVLNEEQYYSFFLNVLEVYGYAVVEMDNGVLKVIKAKDSKTSAIPVMGDGSAKG

DSVITRVVAVRNVSVRELSPLLRQLIDNAGAGNVVHYDPANIILITGRAAVVNRLAEIIK

RVDQAGDKEIELVELRNASAAEMVRIVEALNKTTNQKSTPEFLEPKIVADERTNSILISG

DPKVRARLKRLIRQLDVEMATKGNNRVVYLKYAKAEDLVDVLKGVSDNLQAEKQAGQKGA

SSAQRGDVVIAAHEATNSLVLTAPPDIMMALQDVISQLDIRRAQVLIEALIVEMSEGDGI

NLGVQWGSLETGAVIQYGNAGAPIGQVMVGLEEAKDTVEKKPIRDSDTGAIKYYEETTTK

GDYSTLASALKNVNGAAMSIVMGDWTALVSAVASDSNSNILSSPSITVMDNGEASFIVGE

EVPVITGSTAGSNNDNPFQTVDRKEVGIKLKVVPQINEGDSVQLNIEQEVSNVLGANGAV

DVRFAKRQLNTSVMIQDGQMLVLGGLVDERALESESKVPLLGDIPVLGHLFKSTSTQTQK

RNLMVFIKPTIIRDGMTADGITQRKYNFIRAEQLYKADQGLKLMSDDKIPVMPAFGQDRK

HPAEIQAFIDQMEKN

>tr|Q87RK0|Q87RK0_VIBPA Phosphoenolpyruvate-protein phosphotransferase OS=Vibrio parahaemolyticus serotype O3:K6 (strain RIMD 2210633) OX=223926 GN=VP0794 PE=3 SV=1

MISGILASPGIAIGKALLLQEDEIVLNTNTISDDQVEAEVARFFDARNKSAAQLETIKQK

ALETFGEEKEAIFEGHIMLLEDEELEEEILALIKNDKMTADHAIHSVIEEQACALESLDD

EYLKERATDIRDIGSRFVKNALGINIVSLSDINEEVILVAYDLTPSETAQINLDYVLGFA

CDIGGRTSHTSIMARSLELPAIVGTNDITKKVKNGDMLILDAMNNKIVVNPSEAEVEEAK

AVKAAFLAEKEELAKLKDLHAETTDGHRVEVCGNIGTVKDCDGIIRNGGEGVGLYRTEFL

FMDRTALPTEEEQYQAYKEVAEAMNGQAVIIRTMDIGGDKDLPYMDLPQEMNPFLGWRAV

RISLDRREILRDQLRGILRASAHGKLRIMFPMIISVEEIRELKNAIEEYKAELRAEGLAF

DENIEIGVMVETPAAAAIAHHLAKEVSFFSIGTNDLTQYTLAVDRGNEMISHLYNPLSPA

VLTVIKQVIDASHAEGKWTGMCGELAGDERATLLLLGMGLDEFSMSGISIPKVKKVIRNS

NFAEVKAMAEEALSLPTAAEIEAVVEKFIAEKTQ

>tr|Q87MK4|Q87MK4_VIBPA Chemotaxis protein CheA OS=Vibrio parahaemolyticus serotype O3:K6 (strain RIMD 2210633) OX=223926 GN=VP2229 PE=4 SV=1

MSYDLDEDILQDFLIEAGEILELLSEQLVELENNPEDKDLLNAIFRGFHTVKGGAGFLSL

AELVDTCHGAENVFDVLRNGQRSVTPSLMDTMLKALDTVNTQFQAVQDREALEAADPALL

DELHRLCKPESEDEVATVVEEPIVTPEPIIPEPANEVPAAPAADTTEAGSIGSVDEITQD

EFEKLLDELHGKGAAPGASTPAPAAAAPVQASAPVLDNGDITDDEFERLLDELHGVGQSP

SAATAAPVETPKAPEPVAPAVSAASGDSDLMTDEEFEKLLDELHGAGKGPTLEELDMATK

PASALAPEAPKPAPVAQPAPVSTPASAPKPAAKPVAVKEEPKASAPAVKKPQAEATVRVD

TSTLDTIMNMVGELVLVRNRLLSLGLNSDNEEMSKAVANLDVVTADLQGAVMKTRMQPIK

KVFGRFPRVVRDLARNLNKDIVLEMRGEETDLDKNLVEALADPLIHLVRNSVDHGIEMPE

DRVKAGKSRTGKVILSASQEGDHIELAIVDDGGGMDPNKLRGIAVKRGMMDEDAAARLSD

KECFNLIFAPGFSSKEKISDISGRGVGMDVVKTAINTLNGSIDIDSELGKGTKITIKVPL

TLAILPTLMVGVGGHPFALPLASVNEIFHLDLSRTNVVDGQLTIIVRDKSIPLFYLQNWL

ASKSPRVEQRIGHGHVVIVQIGSQRVGFVVDTLIGQEEVVIKPLDNLLQGTPGMAGATIT

SDGHIALILDVPDLLKQYAAASRI

>tr|Q87GB4|Q87GB4_VIBPA Putative capsular polysaccharide biosynthesis glycosyltransferase OS=Vibrio parahaemolyticus serotype O3:K6 (strain RIMD 2210633) OX=223926 GN=VPA1403 PE=4 SV=1

MKHKGLIRSYEMEFAFLYRLSDLAVIVTFMLLLVLKDTNTSMDKDYVILSFVGGISFLFM

AESGNLYRSWRTSSFREQMFIVCMSWLMTSALLFMVLYFSEVYPLFDRSILALWVTITPA

LLLAWRVTFRTVLAYLRKMGFNTRTAIIIGQTPHGITLANEIQNHTEHGVLFDGFYDERS

SDRLPSSEYPIKGAVNQALERAKRGEVDYVYIAMPMHAKERIASILNQFSDTTANTYLIP

DFFTYNLLHSRWDQIGQVQTLSVFDTPFAGVSSWIKRFEDILCSSIILVLISPILLAIAI

GIKLTSKGPVIFKQHRYGLDGRKIEVWKFRSMTTMDQGPNIKQATKNDPRITPFGSFLRR

TSLDELPQFINVLQGTMSIVGPRPHAVAHNEEYRQIVARYMLRHKVKPGITGWAQINGYR

GETDTLDKMEKRVEFDLDYIHHWSVWMDIKIIFLTVFKGFTGSNAY

>tr|Q87L83|Q87L83_VIBPA Ferric uptake regulation protein OS=Vibrio parahaemolyticus serotype O3:K6 (strain RIMD 2210633) OX=223926 GN=fur PE=3 SV=1

MVKGLNPTIIEQIESICAERGVRLTPQRRRVFELICSNRRASSAYELLEQLKESEPQAKP

PTVYRALDFLLEQGFIHRVESTNSFITCCSFNTQQHFFQLLICDKCGDVVELEDETLISL

LAENAEKHGFKLTNQVIETHGECQACSSETKEKV

>tr|Q87TB5|Q87TB5_VIBPA Osmolarity sensor protein EnvZ OS=Vibrio parahaemolyticus serotype O3:K6 (strain RIMD 2210633) OX=223926 GN=VP0155 PE=4 SV=1

MRIRSSLTQSILLFISLLLASQVFSYYAVFNYALMPSLQQFNKILAHELNLVLDEDGPLK

SDAPLRRQLLERLGVTVHSINSEKAVEFNHAMSIDLMSEEMTQELGSPTEVRLLLGSESY

ILWMHIEKLPNSIIRIPLSELQEEDFAPLFRNSLIMALLIIAGGWLFIRLQNRPLLALQR

AAEGVGRGEIPPPLPEKGASEIRSVTRAFNQMSKGIQELEEDRALLMAGISHDLRTPLTR

IRLATEMMSPEDSYLAEGIISDTEECNEIISQFMDYLKPVNKESFESVDVSTIASDVASS

EGGYEVEIETDLNMHLPPAKGSPIAIKRAVSNLVVNALRYGNGWVKISTGVTADKQLVWI

CVEDNGPGIEQSQIAKLFEPFTRGDTARGSEGTGLGLAIVKRIVIQHSGSVVMRNRSEGG

LIAQISFPTK

>tr|Q87P22|Q87P22_VIBPA Putative type III secretion protein YscC OS=Vibrio parahaemolyticus serotype O3:K6 (strain RIMD 2210633) OX=223926 GN=VP1696 PE=3 SV=1

MVTVMRTLMPKIGRIAAKMTLCALCVAPMFSVQATELNWPEQPFRYYADNDSLKDLLNNF

GANYRVSVSVSDKVNDRVSGRFTPEDPAEFLDYLAQVYNLMWYFDGAVLHVYKATETRSR

LLQLELLTARELRSTLISTGVWDARYGWRAAENKGLVYLAGPPRYVELVVQTAEALESRL

LQKSNSTDELFVELIPLKYASATDRSISYRDQSITVPGIASVLSRVVGGVQTQITDSASV

QTSSVNGLPAEAAKPRGKTASVHGGATVEAEPGLNAIIVRDTQARLPLYRKLVAQLDQPQ

SRIEVALSIVDISANDLRQLGVDWRAGVSVGNNRIVDIKTTGDVDNGDVTLGSGQSFKSL

LDSTNLNYLLAQIRLLESKGSAQVVSRPTLLTQENVEAVLNNSSTFYVKLVGKETAALEE

VTYGTLLRIVPRIVGDRFATRPEINLSLHLEDGAKIPDGGVDDLPSVRKTEISTLATVKQ

GQSLLIGGVYRDEVSHQLRKVPLLGDIPYLGALFRSNTNTTRRTVRMFIIEPRIVVDGIG

DSVLIGNEHDLRPSIGQLNNISNNSAEFKSVVEVFSCTSKTQAERYQQDLLSQQKSSLLT

QCQLPSGQVGWRVKVAECDLSQAECVRPSEEP

>tr|Q87R82|Q87R82_VIBPA C4-dicarboxylate transport sensor protein OS=Vibrio parahaemolyticus serotype O3:K6 (strain RIMD 2210633) OX=223926 GN=VP0915 PE=4 SV=1

MSQRNRILLIFFALYFLCAIVGGRWVWQHSYQSLLDKHQSQLEQFSSHIKNKLDKYAHIP

NLLAKDDPLFEALLNPSNSAQLEITNRYLESVNNVIEAADTYLIDQWGTTIAASNWRKKR

SFIGRNFAFRPYFKQAISGESSQYYALGSTSGQRGYYYSYPIIYAGGVLGIVVVKMDLSK

IEDNWQQPDSVFVASDPHNIVFMSSRQDWLFKSLQPLSDADKKQVWTSRQYLDTPIPTLG

FVGNLNAPNSELKQGYPYNKGTLVVSSLPLPELKLTIRVLSPKQSIVWVTMGYIVVLTLA

FTVLFLIGQLIYHRQQRHLQLERIQQEANQKLEFQVMARTAELQAEIAQRTETEQTLRLT

QDELIQAAKLAVLGQMSASISHELNNPLAAIRSFAENGKLFLQKEKYDRVEDNLTRISAL

TDRMANISQQLRSFAKKASGNELTQTRLLPVIASSKELMKPAFKSARVLLATELPTDDIE

VQINTIQLEQVLVNLLTNAIEAVKEQQDKQVWLLVETDTDENKVMIHVDDNGPGLGSHTL

SELCEPFLTTKKNGLGLGLSISQQILAGMNGSLSAHNRAQGGARFSLCLPICQRSEQSEG

>tr|Q79YV8|Q79YV8_VIBPA FlaM OS=Vibrio parahaemolyticus serotype O3:K6 (strain RIMD 2210633) OX=223926 GN=VP2251 PE=4 SV=1

MAQSKVLIVEDDEGLREALVDTLALAGYEWLEADSAEDALVKLKSNAVDIVVSDVQMAGM

GGLALLRNIKQNWPNLPVLLMTAYANIEDAVSAMKDGAIDYMAKPFAPEVLLNMVSRYAP

IKSDDNGDAVVADEKSLRLLALADKVARTDANVMILGPSGSGKEVMSRYIHKASNRKDGP

FVAINCAAIPDNMLEATLFGYEKGAFTGAVQACPGKFEQAQGGTILLDEISEMDLNLQAK

LLRVLQEREVERLGSRKSIKLDVRVLATSNRDLKQYVSEGNFREDLYYRLNVFPIAWPAL

NERKGDIAPLAKHLAERHCSKMGMPVPQFSPVAVEKLLQYPWPGNVRELDNVVQRALILS

ENGDIGAEHILLEGVDWQDANSLQYVVQSAETLVPDVKPVAQAESVNRVNTGGEGLGGEL

RDQEYAIILETLVECNGRRKEMAEKLGISPRTLRYKLAKMRDAGIDIPS

>tr|Q87I33|Q87I33_VIBPA N-ethylmaleimide reductase OS=Vibrio parahaemolyticus serotype O3:K6 (strain RIMD 2210633) OX=223926 GN=VPA0773 PE=4 SV=1

MTDALFQPIQLGSLSLKNRIVMPPMTRSRASQPGNVANDMMATYYAQRAEAGLIVAEGTQ

ISPMGQGYAWTPGIYSPEQIAGWKKVTDAVHEKGGVIFAQLWHVGRVTHPDNIGGEQPIS

SSALKAENVKVFIDNGTDEPGFVDVVEPREMTKQDIKNVIEEYRQAALNAIKAGFDGVEL

HAANGYLVNQFIDSEANNRTDEYGGSIENRLRFLGEVVEAMTQAIGAERVGVRLAPFTSL

NGTVDSTPVDTYTAAAALLDKLNVVYIHIAEVDWDDAPDTPHDFKTAVRKAYKGTLIYAG

RYNAEKAQHAIESGLADMIGFGRPFVANPDLPSRIKHGYPLAEHDPATLFGGGEKGLVDY

PAYHAG

>tr|Q87GW9|Q87GW9_VIBPA Histidine kinase OS=Vibrio parahaemolyticus serotype O3:K6 (strain RIMD 2210633) OX=223926 GN=VPA1196 PE=4 SV=1

MFKNVKKSVTRTIASAMLLILLLSVATTGFAIFTLASSLNDAEAVNVAGSMRMQSYRLAH

DIQIRSVDYSSHIDAFEHSIYSSSMKALQHWSVPEDITHDYYRLIMRWHELKSVLRGEDP

SQYQLLVAGFVQQIDDFVFKLQNFSEQKLINLAWIGGLGLGGILCASMFVVHFIRLEVVR

PLRALVFASERIKNRSFDINLAVSSDNEMGILTRTFNRMATDLGKLYRGLEQAVDEKTRK

LQHANQSLEVLYDSSKELTASRINQDNFQAILKHIASLEGIKAVKLEIEQLGEPNWILTE

GEECCHDCDDECHAEPLTLDGEHLGSLYWKAGLPCPNETLIDNFVQILSRAVYYNRAQRQ

AEQILLMEERATIARELHDSLAQALSYLKIQVALLKRSVKNLPDEKAIAQANQVIAELDT

GLSAAYTQLRELLTTFRLTIKEGSFGQALQEMVETLNEQTTAEITLKNRLSSTELDAHQQ

VHLLQLIREAALNAIKHAQADHIHIQCLDCDGKVTVTVSDDGVGFEHQDEKINHYGMTIM

QERAARLHADLQIEASINKGCTVKLEFQHSKEVNFDSV

>tr|Q79YW1|Q79YW1_VIBPA Flagellar motor switch protein FliG OS=Vibrio parahaemolyticus serotype O3:K6 (strain RIMD 2210633) OX=223926 GN=VP2248 PE=3 SV=1

MANDIVPQDENGAGMPVEFDASTITGEEKAAILLLSLNEQDAAGIIRHLEPKQVQRVGSA

MARAKDLSQEKVSAVHRTFLEDIQKYTNIGMGSEDFMRNALVAALGEDKANNLVDQILLG

TGSKGLDSLKWMDPRQVASIIVNEHPQIQTIVLSYLEADQSAEILSQFPERVRLDLMMRI

ANLEEVQPSALAELNEIMEKQFAGQAGAQAAKIGGLKAAAEIMNYLDNNVEGILMEQIRD

QDEDMATQIQDLMFVFENLVEVDDQGIQKLLRDVPQDVLQKALKGADDSLREKVFKNMSK

RAAEMMRDDIEAMPPVRVADVEAAQKEILAIARRMADAGEIMLSGGADEFL

>tr|Q87JA2|Q87JA2_VIBPA Putative permease of ABC transporter OS=Vibrio parahaemolyticus serotype O3:K6 (strain RIMD 2210633) OX=223926 GN=VPA0351 PE=3 SV=1

MQDVNSRFHKSVVYTIVGIMLIPILATFIYSISSRWGATILPDGFTFDWYIKLLTDPRFL

QAFGRSLFIGLSALALSVVLILPAIFVVFYYFPKLDKLMNILILLPFAVPPVVSSVGLLQ

LYADSKISLIGTPWILVGTYFTIALPFMYRAISNSFEAINLHDLMDAAHLLGSSTTKAFL

LIILPNLKKGLMASLFLSFSFLLGEFVFANILVGTRYETLQIYLYNMRQTSGHFTSALVM

TYFLFIFLLTWLASRFSRGVK

>tr|Q87GN9|Q87GN9_VIBPA Putative DNA-binding response regulator OS=Vibrio parahaemolyticus serotype O3:K6 (strain RIMD 2210633) OX=223926 GN=VPA1276 PE=4 SV=1

MNEQEIKPSLIIVEDDPKLQDMLGEYFVEQGFDVRCIDNGTDAPNAILDTQPDLVLLDLM

LPGQDGLSVCRQIRECYKGKVLMLTASDDDFDHVAALEIGADDFVTKPIKPRVLLARIRM

LLRRKESAAPAPENGLQIGVLCLNRLRKSCTMNQQPVALTDGEFDLLWVLATHAEKTLSR

EWLTKTLRGIEYDGTDRTIDNRIVTLRKKLGDASCSPQKIITVRGKGYLLMPDAWNA

>tr|Q87TM1|Q87TM1_VIBPA Peptide ABC transporter, periplasmic peptide-binding protein OS=Vibrio parahaemolyticus serotype O3:K6 (strain RIMD 2210633) OX=223926 GN=VP0048 PE=4 SV=1

MKTMKSKLAVALMAAGLSFNALAADIKVGYAADPVSLDPHEQLSGGTLQMSHMVFDPLVR

FTQTMDFEPRLAESWERVNDTTVRFKLREGVKFHSGNSLTADDVVWTFERLQSSPDFKAI

FDPYEKIVKVDDYTFDLVTKGPYPLVLQTATYIFPMDSKFYSGKTEDGKDKSELVKHGNS

FASTHVSGTGPFIVTSREQGVKVEFERFKDYWDKASKGNVDKLTLVPIKEDATRVAALLS

GGVDMIHPVAPNDHQRVKDAKGIDLVTLPGTRIITFQMNQNSNEALKDVRVRQAIVHAIN

NEGIVKKIMKGFATAAGQQSPAGYVGHNEKLVPRYDLKKAKELMKEAGYENGFALTMIAP

NNRYVNDAKVAQAAAAMLSKIGIKVDLKTMPKAQYWPEFDKCAGDMLMIGWHSDTEDSAN

FNEFLTMTRNEETGRGQYNCGYYSNPEMDKIVEAANVETDPAKRAEMLKGVEATLYNDAA

FVPLHWQSEAWGAKSNVKAADIVNPMVMPYFGDLVVE

>tr|Q87RQ5|Q87RQ5_VIBPA Peptidoglycan D,D-transpeptidase MrdA OS=Vibrio parahaemolyticus serotype O3:K6 (strain RIMD 2210633) OX=223926 GN=mrdA PE=3 SV=1

MIRRRRSQIRDYQAEARLFASRAIVAFFGIVVLMGLLVANMYNIQVNQFQDYQTRSNDNR

IKVVPIAPNRGLIYDRNGVLLAENRPVFNLELTPEKIKDIDATIQELQTILEITPEQIER

FHRERKRTRRFKSVPLLTQLNEKQVAVFSVNQYRFPGVEISATLKRYYPYGEVLTHVIGY

VSRINDRDMQRLIREEKDANYQATRDIGKLGIEKYYEDLLHGTAGYQEVEVNSRGRVIRT

LKYVPPVPGKDIVLNLDINLQLYVHQLLDGRRGSAIVLDPRDNGVLAMVSSPSYDPNAFV

HGISGKAYRDLLNDKNRPLVNRTTLGIYPPASTIKPFMAVAALQEGVITPNTTRNDPGYW

RIPNSDTRPFRDWLRWGHGRVDVIKSIEESVDTFYYQIAYDLGIDRISNWMMMFGFGDYT

GIDIYEESKANMPTRDWKMSRHKTPWYKGDTIPVGIGQGYWTATPMQIAKATSVLVNNGA

VSAPHLLKSTIDNGDNFEEQETSEYVTYPPIQNVPEKYWDIAKEGMRRVNHGTRGTARRS

FYNMSYQTAGKSGTAQVFGLGENEEYNADEIAEHLRDHALFTGFAPFDDPKVIVTLVLEN

AGGGSSNGGPMARKIFDRVVLGPEEIKPEDDKTNAQKEVTR

>tr|Q87Q13|Q87Q13_VIBPA Putative permease of ABC transporter OS=Vibrio parahaemolyticus serotype O3:K6 (strain RIMD 2210633) OX=223926 GN=VP1337 PE=3 SV=1

MSRELTSLDSVLEIERHVNYQQSELRAQLQKAERNKNLRSIMLTLPLVCFILLTFAFPIL

EMLYRSVDNRDIPQAMPKTIQALAHWDYQGLPDSEVVEAFSVELLALYETKALPKIANRM

NIEVSGMRSLMMKTGRKLSRLEVLPTSIKELSRLDKRWADAKHWVAFKNLSGAITVNHYL

AALDMQVNEMGEIEAQPEKRQIYVDLFFKTFWMSILITVICLLMAYPVAYLLANLPDKRA

NLLLIVVLLPFWTSLLVRTTSWIVLLQNQGVINDLLIWSGLTSERIQMIHNTFGTVVSMV

HILLPFMILPLYSVMKGISPTYFRAARSLGATPLVAFVKVYMPLTLPGIGAGALLTFILS

IGFYITPALVGGRSGQMISNMIAYHMQTSLNWGMAGALGGLLLFVVLALFYVFNRVVGIN

NIKVGG

>tr|Q87QE7|Q87QE7_VIBPA Putative two-component response regulator OS=Vibrio parahaemolyticus serotype O3:K6 (strain RIMD 2210633) OX=223926 GN=VP1202 PE=4 SV=1

MSRNLCPLYIVDDEVPVLESMAFMLESYGYSVDVYSNGQDFLQSVNLHQAGCVLLDSRMP

EMRGQELHLLMRNQCSPISVIYLTGHGDIPMAVEALKEGALDFFQKPVDGNALVAAIDNA

MECSLKNQEKQSAKLTLQSLTRREKEVLILVVKGMKNQDMANQLCVSLRTIEVHRSNVMK

KLEVESLAALIHKVGHVI

>tr|Q87SH0|Q87SH0_VIBPA Peptidoglycan D,D-transpeptidase FtsI OS=Vibrio parahaemolyticus serotype O3:K6 (strain RIMD 2210633) OX=223926 GN=ftsI PE=3 SV=1

MIRKKTNAKQGNKRKSSAKPKAAVNDTKTAPKAAKVEVESSFLIRWRFHLLLFFVFCAFA

LLVARVAYIQIIEPDNLIKQGDLRSIRVKSIPSARGIISDRNGEPLAVSVPVEAVWADPA

TIFKENALSQKQNWYALADVLGVDRQGLIDKIKRNEKRRFIYLQRQVSPAMANYIRELKL

PGIGLKSESRRYYPAGEVSAHLVGVTGIDGHGLEGVERSYDEWLTGEEGKKTIRKDRYGR

VVENIAWQDKQEGKSLQLTIDQRLQAIAYRAIKQAVADHRATSGSVVMLDVKTGAVLAMV

NAPSYNPNNRTDWQSYKMRNRVITDSMEPGSTIKPFVILAALENGVADKDTIVDTGNGVL

RLGGSRVRDVSWVGKASLSMILKKSSNIGVTKLAMQMPVEALLGLYSSVGFGELSGLNLV

GEVTGIFPTRTRWSPIERATIAFGYGLSITPIQLAHAYATLGNLGKYEPIHIIESNDRDM

SRQVVSKENARLVLDMLETVTQKGGSARRAAVPGYRVGAKTGTSRKASAGGYSDEYITYT

AGLAPVSDPRIALVVIVNEPQGDDYYGGSVASPVFSEIMKGALQILNVAPDENKFQQ

>tr|Q87HD9|Q87HD9_VIBPA Uncharacterized protein OS=Vibrio parahaemolyticus serotype O3:K6 (strain RIMD 2210633) OX=223926 GN=VPA1026 PE=4 SV=1

MKKASQDNNFIRISSPFGKDALILNSFEYREGISELFSLRAKAYFNDQKNELNEIVGKEV

TISVENSSRVSKSPRFFHGIVSAAKLEGQRVMNSHNGENYKNIEIIVEPKVKFAAYRNNC

KIFQKKNIKDIISEVLSEHGVAFKFELKNTYPQYSYKVQYEESDLAFVQRLLAEEGLSFC

FSHSKSSHVLDIFDDVSFYKPSPEFMVDFDTGSSESSHISAWNETQVLTTKSSQKSGFNM

LKPASQPKNVAAGDTALFTVPASEYFEYLGETESDDQYSLRNTHAIESLQQNVYLCSGEA

SCRTFSVGKCFKFKKHEDKSRVGKEYVLASVTIFASVFNQTGLGGTASQGVRVAFTCVDS

KTILRPAVTYPKPQIKGLQTAIVTGNKDGEVYVDKHGRIKVQFHWDRLGKYDVNSSCWIR

VAQSVAGNGWGAVFHPRVGQEVIVEFVNGDPDQPIVTGALYNGSQLPPYSLPEKSSQSGF

KSRSVQKGNANFNELRFEDKPGEEHIYLHAEKLFQMLVEDCVDIVVENNKVEKVTNDVTQ

DVGKNATLKVGENYTSDTGKVLSLNAGKSIEIKVGGASIQMSSSGEINIKGNKISINGSA

>tr|Q87IT0|Q87IT0_VIBPA Putative OmpU OS=Vibrio parahaemolyticus serotype O3:K6 (strain RIMD 2210633) OX=223926 GN=VPA0526 PE=4 SV=1

MKKAALTTAILTALVSAPSFAATVYKNDGTELKVGGRVEFRGDFIGSDGAEVEGSMEDQS

RARLNLKGKTDIGNGMSAFGVYEAEQKTGKSEFKNRYMYAGVNTDVGAFSVGRQDMAAVI

ISDMTDITEFSGVQQVIDSSSDKQDSVFAYRGEFDALQLQATYQANSGDSQDKYGISGMY

SLPMGLDLGLAYSGGDVDKSNSEDQILGGIAYSLDNLYLAGTYSQGSLTDSEDFTAYELV

ASYKVATKVTLAALYTAQENDPDNGSKYDSVEGIELVGYYKLNSNFRTYLSYYINQLDEV

KDATSGLVTEGEDTLRLGVRYDF

>tr|Q87R85|Q87R85_VIBPA C4-dicarboxylate transport protein OS=Vibrio parahaemolyticus serotype O3:K6 (strain RIMD 2210633) OX=223926 GN=VP0912 PE=4 SV=1

MDILLLFLMVIGFMLIGVPIAISLGLSSVLFLMLHSDASLASVAQTLFNAFAGHYTLLAI

PFFILASSFMSTGGVAKRIIRFAIAIVGWFRGGLAMASVVACMMFAALSGSSPATVVAIG

SIVIAGMIKNGYSKEFAAGVICNAGTLGILIPPSIVMVVYAAATDVSVGRMFLGGVIPGL

LAGVMLMIAIYIAARIKNLPKQPFVGWKETFDAAKDASWGLLLVVIILGGIYGGIFTPTE

AAAVAAVYSFLIANFIYKDMGPFADKQNTKPAIVKVIQTFVHEDTKHTLYEAGKLTIMLL

FIIANALILKHVLTEERIPQMITESMLSAGLGPITFLIVVNLLLLIGGQFMEPSGLLIIV

APLVFPIAIALGIDPIHLGIMMVVNMEIGMITPPVGLNLFVTAGVARMSMMNVVKAALPW

VGVMFLFLIIVTYVPWVSTWLPTTLMGPEIITK

>tr|Q87FT7|Q87FT7_VIBPA Oxygen-insensitive NAD(P)H nitroreductase OS=Vibrio parahaemolyticus serotype O3:K6 (strain RIMD 2210633) OX=223926 GN=VPA1591 PE=4 SV=1

MDIVQAAKSRYSTKVFDPTRKLPQEKIDAVKELIRFSPSSVNSQPWHFILASTEEGKQRI

AKAAQENYAFNAAKILNASHVLVFCAKTGIDNDYIEALMAQEEKDGRFPTEEAKAAVRGG

RSYFVNMHRFDLKDANHWMEKQVYLNVGTLLLGASTLEIDAVPIEGFDPKVLDEEFGLRE

KGYTSVVIVPLGYHAEDDFNAKTPKSRWDAETVFTEI

>tr|Q87TC9|Q87TC9_VIBPA Type II secretion system protein L OS=Vibrio parahaemolyticus serotype O3:K6 (strain RIMD 2210633) OX=223926 GN=VP0141 PE=1 SV=1

MEGSVSEFLTVRLSSEQQSTIPWVVWSTEQQEVIASGELAGWEHLDELVSYAGQRQVIAL

LASNDVVLTQVDIPPGATRQFDSMLPYLIEDEVAQDVDSLHFTVLGKQADKAQVCAVERA

WVQTVLQRFASQGLTIKRILPDVLALPVSDDNSSAALIGEQWLIRHSETEGAVVDSAWLD

LYLSSYLQNHEGWQLDCYSSVPESTVESVWVPKPEEMTMALLAKGVASSKTNLLTGEFKP

KSSWGKYWKVWQKAAIAAGVLLVVVVAQQLLVVHKYEAQAQAYREESERIFRQVFPNKNR

IPTVSYLKHQMTDEERRLSGGSTDVAMLSWLAALPATLGQVKDLEITSFKYDGQRGEVRI

HARSSDFQPFEQARVKLAEKFNVEQGQLNRSDNVVMGSFVLKRQ

>tr|Q79YZ4|Q79YZ4_VIBPA Sodium-driven polar flagellar protein MotA OS=Vibrio parahaemolyticus serotype O3:K6 (strain RIMD 2210633) OX=223926 GN=VP0689 PE=4 SV=1

MDLATLIGLIGGFAFVIMAMVLGGSIGMFVDVTSILIVVGGSAFVVLMKFTLGQFFGAAK

IAGKAFMFKADEPEDLIAKIVEMADAARKGGFLALEEMEINNSFMQKGIDLLVDGHDADV

VRAALQKDIALTDERHTQGTGVFRAFGDVAPAMGMIGTLVGLVAMLSNMDDPKAIGPAMA

VALLTTLYGAVLSNMLFFPIADKLSLRRDQETLNRRLIMDGVLAIQDGQNPRVIDSYLKN

YLNEGKRALEIDE

>tr|Q87NV3|Q87NV3_VIBPA Uncharacterized protein OS=Vibrio parahaemolyticus serotype O3:K6 (strain RIMD 2210633) OX=223926 GN=VP1765 PE=4 SV=1

MKALIEVLDGQVKHCPINQKFDISSTDFYIVLNHNCALELCDSKGIKQLIDPPCLVAIGA

DFKGQIAINSFVENANISGFRLAACFIEKLNQQLNFRDLCDGVFSGNVAVSFTLRPGIVD

IYSALKAMVKKGCGNRSNDELLDINSMLLYLLMHFDQSASQAETRSYSSLSSRIRALISK

DLTKPWTLKEIAKLVYMSESTVKRKLNKEGTTFTDVLQAARLDTAQKMVCNSDASVSAIA

ELCGFKHASYFGACFRKEYGVTPLAYRKQAQLRAN

>tr|Q87K78|Q87K78_VIBPA Uncharacterized protein OS=Vibrio parahaemolyticus serotype O3:K6 (strain RIMD 2210633) OX=223926 GN=VPA0020 PE=4 SV=1

MCSAKSMVASGWQRSVIVTTLFCLFISGMTLSVWGGPYYVHVLVSFGFGYSALFFSWLID

KLFPTIPRMLEIALSLTACLLFGVINAQFWLGEYFGISGMLPVLLMGLLFSGMCYFYFHS

REKEAIAQRELESIKRENAEQERALLLSQLKQMQSQIEPHFLFNTLANISALMSQDVDKA

KQMLDQLTALLRATLKNSREEHTTVENEITLIDAYLGIQKIRLGERLSYTIEVQEGLGNT

ELPPMMLQPLVENAIIHGIEPKREGGEVQLLIKQEKQLLQIEVKDTGVGLSHVSNHSGSG

IGLSNLKQRVDALFAGKGQVSISESSEGGVSVRLSWPMISKEQ

>tr|Q87P56|Q87P56_VIBPA Low calcium response protein OS=Vibrio parahaemolyticus serotype O3:K6 (strain RIMD 2210633) OX=223926 GN=VP1662 PE=4 SV=1

MNLMNKLIDILNKVGQRKDIMLAVMLLAIVFMMILPLPTALVDVLIGANMSIAVVLLMLA

IYITTPLEFSAFPAVLLITTLFRLSLSITTTRLILLQGDAGQIVYTFGNFVVGGNLVVGI

VVFLIITIVQFMVITKGSERVAEVSARFSLDAMPGKQMSIDGDMRAGVIDVHEARHRRSL

IEKESQMYGSMDGAMKFVKGDSIAGLVIIIVNILGGVTIGVTQKGMSASEALELFAILTV

GDGLVSQIPALFIAITAGIIVTRVSHEDSADLGSDIGGQVTAQPRALLIGGVLLVLFALI

PGFPKITFLVLALVVGGGGFYLFYQQKKQTESESSDLPSFVAQGAGSPAAKPNKPTPSRG

SKGKLGEQEEFAMTVPLLIDLDSSLQESLEAVALNDELARVRRALYLDLGVPFPGIHLRF

NDGMKNGEYLIQLQEVPVARGRIEKDKLLVTEGSDQIELLGVPFEQDDDFLPGVSSLWVA

QSYQEKLTASHVGFLTPDRILTFHLSHVLKEYAQDFIGIQETRYLLEQMEGSYSELVKEA

QRIVPLQKMTEILQRLVSEDISIRNLRVILEAMVEWGQKEKDVVQLTEYIRSSLKRYICY

KYASGQNMLPAYLLDQSLEDTIRSGIRQTSAGSYLALDPSVTQQFVSDVKQTVGDLSRMP

NKPVLVVSMDVRRYVRKLIESEYYDLPVLSFQELTQQINIQPLGRVGM

>tr|Q79YT9|Q79YT9_VIBPA Anaerobic C4-dicarboxylate transporter OS=Vibrio parahaemolyticus serotype O3:K6 (strain RIMD 2210633) OX=223926 GN=VPA0981 PE=3 SV=1

MLYLEFLFLLVMLYIGSRYGGIGLGVVSGIGLVIEVFIFKMPPTSPPVTVMLIILAVVTC

ASILEAAGGLKYMLQVAERVLRKNPKRVTLIAPFVTYFMTFLLGTGHAVYSIMPIIGDVA

LKNGIRPERPMAAASVASQIAITASPISAAVVYYLAQLSDIQHEITLLSILLVTVPATLF

GTLLMSLYSIKRGKELEDDEEYQERLKDPVWREKILNTTATSLDEVLPTSARNSVLLFIA

SILVIVVIAMWPDIRTIVDGAKPISMAVVIQMMMLCFGGIILLATKTDPRDVPNGVVFKS

GMVAAIAIFGIAWMSDTYFQYAMPQFKSGIVEMVTNYPWTFALALFIVSVVVNSQAATAR

MMLPVGLGLGLDPALLIGLMPAVYGYFFIPNYPSDIATVNFDTSGTTKIGKWYFNHSFMS

VGLIGVIGACCLGYVLGQIIIPS

>tr|Q87FY4|Q87FY4_VIBPA Flagellar M-ring protein OS=Vibrio parahaemolyticus serotype O3:K6 (strain RIMD 2210633) OX=223926 GN=VPA1536 PE=3 SV=1

MSELTPQVAGNTAMTTSTTQAFSPAGNMDDVTNKLKQLWSSSQRNLVLSAVLAAIVAAII

VVALWSSSQSFRPLYSQQERFDIGEIVSVLESEGVSYRMQEQNGQVLVPEGEVARIRMLL

ASKGVKAKLPTGLDSLKEDSSLGTSQFMETARYRHGLEGELVRTIMSLNSVANARVHLAI

PRQTLFVRQNGENPSASVMLELKPGEDLKPEQVEAIINLIVGSVTAMKPEFVSVIDQYGR

LLSADVASAEAGKVNAKYLEYQKNVEKQIIQRAADMLTPIVGPSNFRVQVAADMDFSQVE

ETREILDNAPVVRNEHTIQNNSIDQIALGVPGSLSNQPPVTGEAATNDSQNTNARSEVNR

QYAVGSSVRRTQYQQGQIEKLSVSVLLNSKASPDGVAWSDADKAQISTMITDAVGISAAR

GDSLSLMSFNFTPIDIDAPTALPWWQDPTVQQPLRYVIGGMLGLAMIFFVLRPLIMHLTG

ADKPVPELNFAEPPQEEPDYDNLQTREEREHEEVLNRRLSEKGISASTGLDVNSDMLPPA

GSPLEIQLKHLQLIANEEPERVAEILKQWVNINEHSSVDVKTNA

>tr|Q87P44|Q87P44_VIBPA Translocation protein in type III secretion OS=Vibrio parahaemolyticus serotype O3:K6 (strain RIMD 2210633) OX=223926 GN=VP1674 PE=3 SV=1

MSYDDLHQALFLYSLTLPRLMACFIFLPILSKQMLGGAMIRNGVLCSLALFIFPVVNEQA

LPAETDGLWLIVILGKEVLLGMLIGFVAAIPFWAIEATGFLVDNQRGAAMASMFNPTLGS

QSTPTAVLLTQTLITLFFSGGGFVAFIYALFKSYTTWPILGFFPMVTDAWVSFFYDQFQQ

LMWLGVLMSAPLVLAMFLAEFGLALISRFAPQLNVFFLAMPIKSAIASVLLIVYLGLMMD

HFEALFYGITRFGDQLNTIWK

>tr|Q87I65|Q87I65_VIBPA Putative fimbrial protein Z, transcriptional regulator (LuxR/UhpA family) OS=Vibrio parahaemolyticus serotype O3:K6 (strain RIMD 2210633) OX=223926 GN=VPA0741 PE=4 SV=1

MRFTLNNVLIIDDQPLYSEALASLVENAINTAEVIQTTDSAEVMELVRSQRIDLIILDVV

LGDRDGMRLAKNILATGYRGRLLFVSSRDYSSLSKAAYEMGANGFLNKNEARETIADAIV

SVSRGYSMFKSTHTPSSGDVTLSNREAMVFHYLAQGYSNKKISEQLSLSAKTISTYKTRI

LKKYHADSLIELLHTIPQSENIQFCR

>tr|Q79YY3|Q79YY3_VIBPA BfdA OS=Vibrio parahaemolyticus serotype O3:K6 (strain RIMD 2210633) OX=223926 GN=VP1393 PE=4 SV=1

MPTPAYMSINGETQGHITKDTYSADSVGNTWQEAHVDEFLVQELDHVLTVPRDPQSGQPT

GQRVHRPLVVTKVQDRSSPLLFNALVSGEKLPECLIRFYRTSVQGKQEHYYSIKLIDALL

VDIQTRMNHCQDAATADRVTEEVLKFTYRAIEVTHENCGTAGNDDWRAPREA

>tr|Q79YX1|Q79YX1_VIBPA Chemotaxis protein CheY OS=Vibrio parahaemolyticus serotype O3:K6 (strain RIMD 2210633) OX=223926 GN=VP2231 PE=4 SV=1

MNKNMKILIVDDFSTMRRIVKNLLRDLGFNNTQEADDGLTALPMLKKGDFDFVVTDWNMP

GMQGIDLLKHIRADAELKHLPVLMITAEAKREQIIEAAQAGVNGYIVKPFTAATLKEKLE

KIFERL

>tr|Q87SD5|Q87SD5_VIBPA Aerobic respiration control protein FexA OS=Vibrio parahaemolyticus serotype O3:K6 (strain RIMD 2210633) OX=223926 GN=VP0489 PE=4 SV=1

MQTPQILIVEDEQVTRNTLKSIFEAEGYAVFEASDGEEMHQVLSDNSINLVIMDINLPGK

NGLLLARELREQANIALMFLTGRDNEVDKILGLEIGADDYITKPFNPRELTIRARNLLSR

SMSTNAVQEEKRSVEKYEFNGWVLDINSRSLVSPAGDSYKLPRSEFRALLHFCENPGKIQ

TRADLLKKMTGGELKPHDRTVDVTIRRIRKHFESVSGTPEIIATIHGEGYRFCGDLED

>tr|Q87FM8|Q87FM8_VIBPA Putative methyl-accepting chemotaxis protein OS=Vibrio parahaemolyticus serotype O3:K6 (strain RIMD 2210633) OX=223926 GN=VPA1651 PE=4 SV=1

MFKNLSLKNKLAISASAAIILGGVLVEGLSFRDSLQRLDAEVAQRLESTSASYNQYVSDW

LLSKERALTSLSAESEKRAIVTHLKQVRDSGAFDNVFLAYPDGSQDNANGVILPPGNNDP

RKWGWYTNAIANPSKVFMDNPTVAAATGANVVSLGKALQLHGQTTVLGADVEIGDILNSL

NQVILPGEGYMFIANDQGNIFTHNDSKLLNQPVSKLGLNNNDITNAARSGTERRVSISGT

DYVIYARPIEGTKLTTVTVLDHNSLVAPLYDAVWDQIIATAIVVIICVALFNLLCNILFR

PLYNVSNALSQIANGSGDLTQRIKVENRDEVGELAENFNQFVESLQQLIGHIRHQAEELS

QQSELSTTRANQSVSDLNHQQQEITMVATAVTEMASATQEIAAHAEQTAKAAQDSSASTQ

NGHELVINSKSSINNLSSEVNQASVVIGELNQHAQDISTVLSTIRDIAEQTNLLALNAAI

EAARAGEQGRGFAVVADEVRVLSQRTHTSTEEIRSTIETLQQTTQRAVTIMDKSSQLAQG

SVEDADRAALALDEINAAVALISDMATQIATAAEEQTHVTNEITQNVTSIKDVTDQLVVG

AEESMNQSAELKSQAEDLNSKVATFKLA

>tr|Q87G68|Q87G68_VIBPA Methyl-accepting chemotaxis protein OS=Vibrio parahaemolyticus serotype O3:K6 (strain RIMD 2210633) OX=223926 GN=VPA1449 PE=4 SV=1

MLKLHSLSIKQKVVLGITFAVLASTIIVGVMAQRHARDVLSHRLIDIELPAMLQQINTEI

DREVVQMQQAAKQLATNEFVVEALKNTDHPQFSETQLVQQLNNIKSQYGLNDASVANRKT

AYYWNQNGFLRQLNHSQDAWFFGFTSSGRETSVSVFQEANGEVKMFTNYQDLNGISMSGL

SKSMDDMVSLLNSFQIEDTGYVFLTNEKGDIQIHRQQGKNKTSIAQLFGSNANQLLNKNS

FNLINVEFEGKDNFIASLYVPSMNWFVIGVVPVDEVFADLNATGQKMMITTIVVALVFIL

MGVLLANSITKPIRLIADRFTDLGQGEGDLSQRIEIRGNDEIAQLSKGFNGFIEKIHATM

KEVSLTSGALSQAADSVSSKATSTYDNSQEQRDQTIQVVAAINQMGATISEIASNAATAA

DTANQASDNTQTGREVVMKAKEVISRLADDVETTNIVVTQLASTTKDIGSILGVIRDISE

QTNLLALNAAIEAARAGEQGRGFAVVADEVRNLASRTADSTEEIQRMINQLQSDAQDAVS

AMEAGKAVTFEGVASTDEAVEVLVNISERITDISDRNTQVATATEEQSTVVHTINQNIEE

INAINEMTTATAEELAGASRDLQELSSRLDKMVGSFKL

>tr|Q87NJ3|Q87NJ3_VIBPA Para-aminobenzoate synthase, component I OS=Vibrio parahaemolyticus serotype O3:K6 (strain RIMD 2210633) OX=223926 GN=VP1875 PE=4 SV=1

MDNQFIDFKALEYAPEFALHLFSRIQHQPWTMLLRSASKTHIDSRFDVLVANPIATLETI

ADSTQVETPSNAYSIQDDPFTLLHQLQEQWLPHVELNKELDLPFVGGALGYFSYDLGRRV

ETMPEQAEKDLNTPDMAVGLYEWAVVVDHKLKKACLVGQNIEQAWQWLDKQEAEQSVDFA

LSGDWQSNMTKESYATRFDKVQEYLLSGDCYQINLAQRFNAPYLGSEWQAYLKLESANQA

PFSAFIRMPESSILSISPERFLELKDRVIETKPIKGTRPRSEDPKQDNANAHDLQTAEKD

QAENLMIVDLLRNDIGRVASPGSVHVPKLFDIESFPAVHHLVSTIRANLDEQYSPADLLR

ACFPGGSITGAPKVRAMQIIEELEPHRRSAYCGSIGYISRHGRMDTSITIRTLVAENHKL

YAWAGGGVVADSDCASEYQETLDKLSKILPALQS

>tr|Q87J60|Q87J60_VIBPA Uncharacterized protein OS=Vibrio parahaemolyticus serotype O3:K6 (strain RIMD 2210633) OX=223926 GN=VPA0393 PE=4 SV=1

MRSMLQDSLVLLDYLRGMLLHNEELWLLLFPVMIIIELPLYLLVLTGIFRWSYMREEPEL

KRFPSVSFVITCYGEGEAIGITIDTLVEQIYPGHIEILAVVDGAVQNQDTYKAALNGERR

HTGVRNRKVRVLPKWQRGGRVSTLNAGLSMASGEIVINVDGDTSFDNDMVFTMMKQFADK

NVIASGGALRVRNHNANLLTKMQSLEYMLSMQAGKTGMATWGVLNNISGAFGAFRKNLLK

QVGGWDTHTAEDLDLTMRLKQYKCRYPDNKLAFSTHSIGHTDVPDTLKGLVLQRLRWDGD

LLFLFLRKHNEGLSPRLLGWGNFVFTLAYGVIQNVLLPLLVVIFSVYMVIVYPLKFVLAL

MLMLYFVYLFLSALIFVVYIGLVSERKKEDLKSVKWLFLYPVYQFFMRLITAFSMVNEVV

RRSHEESSMAPWWVLKRGKKF

>tr|Q87H50|Q87H50_VIBPA Uncharacterized protein OS=Vibrio parahaemolyticus serotype O3:K6 (strain RIMD 2210633) OX=223926 GN=VPA1115 PE=4 SV=1

MSQFYKLRALSLVFWFVFLTTKVYAQSPEPKDKFLQSLEGIESQIYALPQSSLAQIESLE

EDSLLQNQPKDLLIRYWLAKSTVLELLGRDKESLAVVDKGLSLTPEQSQEHLLFKLIQIR

AMMGNRDIDTALSSLDALLETSREKGDKKLESEVLLLKGRYYDEQGDYKKSYAALMSSME

AAESSGAQGLVERAALELGDVLVKIQGYDRSEVVLKQAYRYFKDRRMSFNELLSVLTIAK

LHKAQFQYDEAIKSYQAALKLAQIIGDGRFRFRVNLELAALYRETNNEKNMLRHLKLAEN

LQYRETSNAYLATFKLLQAEYMLERKQYQALLTMITPLLPEIIESRYIKQQQMELLKVAA

MAYAGDQNFELAYQTYGQYHEKFIQFSNQREVENLERQQTLFELERLEYENENLNWNNVL

QRLELENNRRTFYLLGEVLLIMIGILLLMALVFLYVNRSRLRMRRLAKTDMLTGLFNRRF

LEEWFAKPAEQKPKLIEKPIPETKKGKLVHKLNKQVMRVQYGYLALNHWVERKLDKQKMV

AKKPETGPITLVMMDVDHFKQVNDTYGHVFGDVVLTGVAKVLDSSVRESDIVARLGGEEF

VIVLPNTDLEEATALAERLRIALSQRGFVTENNQAVNVTCSFGVITSDDVDVAFEALCSQ

ADKLLYEAKSSGRNCVKALSFS

>tr|Q87TD5|Q87TD5_VIBPA General secretion pathway protein F OS=Vibrio parahaemolyticus serotype O3:K6 (strain RIMD 2210633) OX=223926 GN=VP0135 PE=3 SV=1

MAAFEYKALDAKGKQKKGTIEGDNARQVRQRLKEQGMIPVEVVEAKAKAAKSSGSVGFKR

GIKTAELALITRQLSTLVQSGMPLEECLRAVSEQAEKPRIRTMIAAVRSKVTEGYPLADS

LGDYPHVFDELFRSMVAAGEKSGHLDTVLERLAEYVENRQKMRSKLLQAMIYPVVLVVFA

VAIVSFLLATVVPKIIEPIIQMGQELPQSTQFLLAASEFVQEWGLIIFAVLVVCFYGLKL

ALQKPDFRLSWDRKIISLPLVGKISKGLNTARFARTLSICTSSAIPILEGMRVAVDVMSN

RYVKQQVLIAADNVREGASLRKALDQTRLFPPMMLHMIASGEQSGELESMLTRAADNQDQ

NFESTVNIALGVFTPALIALMAGLVLFIVMATLMPMLEMNNLMSG

>tr|Q87SQ6|Q87SQ6_VIBPA Phosphoenolpyruvate-protein phosphotransferase OS=Vibrio parahaemolyticus serotype O3:K6 (strain RIMD 2210633) OX=223926 GN=VP0366 PE=3 SV=1

MASNQIEGAVVGIRVNDGIAAAPVVLFTHEMPAVPERDFQSEQGEIERVKRAIGVVVQHL

QEQAKQPKGEIFSAHSMMLSDPELWASVESRIQTGMIAEQAWIESLQTLADEFRQAESQY

MREREADVHDIARQVMVEMTGVTPNAIDIQEPSILLARDLMPSDVAGLDKSKVLGICLSE

GGKTSHSAILARAMGIPAMVKAQGCLDAVRAGQVVTIDGFRGHLWFSPSDAIQQELEAQQ

IEWQSTRQSALASAQQAAATCDGVHIPVFANIGGPKDIDDALTSGAEGVGLFRTEFLFQN

SDELPTEEAQYQVYRDIAAALGDKPLTIRSLDVGGDKPLAAYPMPAEDNPFLGLRGVRLC

LQHESLFTAQLRAILRAFHEQPNIQLMIPMVAQVEEVRKVKALLAHQANQLGLDATHLPV

GIMIEVPAAVLNADALAQEVDFFSIGTNDLTQYVMAADRGNAAVAELVNYFEPSVLKAIE

LTCAAGDRAGIPVSMCGEMAGDPNATETLLRVGLQKFSASPSLLPGLKAQIRQLSVDV

>tr|Q87FY2|Q87FY2_VIBPA Putative two-component response regulator OS=Vibrio parahaemolyticus serotype O3:K6 (strain RIMD 2210633) OX=223926 GN=VPA1538 PE=4 SV=1

MMTKTNILLVEPNEHLAQPVLDVLKNAGYTAKHTRTGRSALLEERASITLVSSTLPDMCV

REFVACHQKQRNAGVVIAIVDQEQGILAAETMKSGATDYLLRPFEANQLINLLKRVEALG

KPMANIVAESWRSKQVLQLAHRAACTNASVLITGESGTGKEVLARYVHEHSPRINGPFVA

VNCAAIPESMLEAVLFGHVKGAFTGATNSQSGKFEEANGGTILLDEIGEMSPAVQAKLLR

VLQEREVERVGSHKAIKLDIRVIAATNKDLREEVQKGTFREDLYYRLDVLPLHWPPLRER

KEDILPISQFFIEKYQDSSRCHLSQDAISALSQYHWPGNIRELENVIQRALVMRHGDYIT

AHDLMLPIELIAPVPSMEPKSSFGHVEAKKQAEYQFILDKLRQFGGNRTKTANALGVSTR

ALRYKLAAMREHGIDLQSALGSAA

>tr|Q87LX8|Q87LX8_VIBPA Peptide ABC transporter, permease protein OS=Vibrio parahaemolyticus serotype O3:K6 (strain RIMD 2210633) OX=223926 GN=VP2480 PE=3 SV=1

MGYFLRRLSFYLVALLVAATLNFIIPRAMPGDPVTMMFANASVQVTPERIAAMKELLGFV

DGPIYIQYLSYIKNILSWELGTSIQFYPLSVNSLLGSAFGWSLFLAGTAVVLSFSIASVL

GIFAAWKRGSRYDAFVTPGTLIIQAIPQMVIAMLALFTFSIGLKWFPSGYAYTPGTVPDW

SSWAFIKDVGYHAVLPLFCATIVQIGGFLVNMRNNMINLLAEDYITMAKGKGLSENRVVF

NYAARNALLPSVTALSMSLGMAIGGQLIIEMIFNYPGLGTVLLNAIHARDYQVLQGQLII

MTMFMLCFNLMADMLYMILDPRLRKGGK

>tr|Q87MI1|Q87MI1_VIBPA Putative glycine cleavage system transcriptional repressor OS=Vibrio parahaemolyticus serotype O3:K6 (strain RIMD 2210633) OX=223926 GN=VP2274 PE=4 SV=1

MKQHLVLTAVGTDRPGICNQVVKLVTQAGCNIVDSRIAIFGNEFTLIMLLTGNASHITRV

ETQLPLLGQEHDLITIMKRTSAHELLDNSYTMEVFIESEDRPGLTEKFTQFFADQQIGLD

SLSAQTISKSKLQLDADQFHIAITASVSADCNLMQLQEDFDELCKSLNVQGSLNFIKNTL

>tr|Q87HC5|Q87HC5_VIBPA Uncharacterized protein OS=Vibrio parahaemolyticus serotype O3:K6 (strain RIMD 2210633) OX=223926 GN=VPA1040 PE=4 SV=1

MVSMEQTIVKPTPGGRAAVSKAQPQRSADSTVVISKNPELVNNDSVVAYGDNPLLAEANG

LLSIIGQIRATATHSDPLFLKETLAQKLRDYENRLRQHDVDLETIDTARYCLCCSLDEAV

LNTNWGSQSFWTHDSLLSSFYASSQGGEAFFKHLDSCLAHPESHLDLLELMYVCLSLGFI

GQYRLEKNGLEAHRRLRKQVVSVLKSHGRGVQQELSNKVEQHILAGAQVSERAPLWVVCS

VTSALLVCIFMYFSYELNKASNQTFAQLVNLIQPTPAVSNPMVESKSAPIAERISMYLAT

EIGKDLVTVEALQDRVRISLKAQDLFESGSASVVAYIQPVISKVARTLEATQGKIIITGH

TDDRPIFTSKYPSNWHLSLARATSLSEQLISNSALKGRVIPEGLGDARPLVENDSEKNRA

MNRRIEIDLIVGN

>tr|Q87LX7|Q87LX7_VIBPA Peptide ABC transporter, permease protein OS=Vibrio parahaemolyticus serotype O3:K6 (strain RIMD 2210633) OX=223926 GN=VP2481 PE=3 SV=1

MKNLFKLILGNSFARIGLAIITIFIFVAVAAPLITKHAPDKRTGNPHEYPSFVVKQAQSN

PDGWVAKNLADDRRTLIMSKKADHVLGTSRMGRDIWSQVAYGARVSLGVGFGAGIIVCFL

ATVIGISAGYFGGKVDDVLSAAMNIMLVIPQYPLLFVLAAFIGEAGPLTIALIIAGTSWA

WGARVVRSQTMALREKEFVKAAEVLGESPFRIIFVEILPNLIPIVGASFIGSVMLAINTE

AVISFLGLGDANTISWGIMLYNVQTSSAMLIGAWWEVLAPCIALTLLVTGLALLNFAVDE

IANPQLRSHKGMKRWKKLAAKDKKEREPELAPQNALWSGDK

>tr|Q87QT0|Q87QT0_VIBPA Sensor histidine kinase OS=Vibrio parahaemolyticus serotype O3:K6 (strain RIMD 2210633) OX=223926 GN=VP1069 PE=4 SV=1

MEIRSSLRKKSILALTLYLCFFIATIGSVVYLVVEPPVRDKLERNLDLRTQLLASQIKEP

LITSTGVLNSLVGLAQSSNQSDSLKSTIPQILRLSDEIIVSGGLWPKPELKEERWRFTSL

FFNKNSEGNIDQIHSYNNPESGGYDNEPWYRAAAEQSSGTVSWSAVYIDTFTQVQMITAS

APYYRNGEFAGVATVDLSLEALFQFIREHTNQYSLGVVIRDANSNVIIEHNFQITKQMYI

SKLDFGEFHWKLEVVNAKAKVADQVFEQVMSVEGGIIPFLLLCVLVGYYLLNRYIVEPIV

RIATKIDDSKTGGIIDIDYGSEDEIGHLITKFNEKTIYLEQERVKAQASTNAKTAFLATL

SHEIRTPMNGVLGTAQILLKTPLTDEQRKHLSTLYDSGDHMMTLLNEILDYSKIEQGHVE

FSNSPFPIESIIGSIKSVYHTLCAEKGLQFKVTSLVPAGRWYDNDKARLRQVLFNLLNNA

VKFTDRGIVEVTLSEQTHYDKTVLVIAIKDTGIGISKEAQKRIFRPFEQAESSTTRRFGG

TGLGLAIVKEIAEHMGGHVTVQSQENIGTTFTVEVEISPCEPGKVESGHRHKLNCNGLKA

LIVEDNRTNAIIMETFLRAKGFECSSVENGQLAVNKIAVEPFDLILMDNHMPVLDGVGAI

SAIRSMSSAAKSVLIFGCTADVFKETQERMLGVGADHIIAKPIVESELDDALYRHADLLY

QYQTKQNQQALEVLGTDSLLISFYVALDNGNLGDALDALLAIMDSLQPNTDEVLSEVITR

IKRDLLRQSPPDQEDIDTLTMLLATP

>tr|Q87K89|Q87K89_VIBPA Putative two-component sensor OS=Vibrio parahaemolyticus serotype O3:K6 (strain RIMD 2210633) OX=223926 GN=VPA0009 PE=4 SV=1

MDVKERTQRRFSIGNQLMLAVLTLSLIFTLVISAISLYRDFQEELSHLDTDLKQVESSYL

SSFSASLWVEDRELLLTQALGAMRLPSVDYLRIATKDEVIIELGTEITQDVVERRWPMQF

SVGEKTFELAELTVQSDLSAVYQDLWQQFFFLLTTEAIKILLLMVGVLWVAFRLLVNPLQ

LLSGAVSDFSGGNAPSTVTLPKRWCFDEVSLLAQKYNRSVQKVREHQAELEAERDKAEVA

NRKKSEFLATMSHEIRTPMNGIIGVASLLSDTKLDPQQKEFVEIIDNSSQSLMTIIDDIL

DFSKVEAGKVELASETYHFRQLLDDVISLHTVKAQQKNLQLLSDIDPKLPAEVQGDEGRL

KQVLNNLLSNAVKFTERGHVKLLVSLHEQNNDIAQVRFRVVDSGIGIAKEHQQAVFERFQ

QADGSTTRKYGGTGLGLAICAQLVHIMGGDIKLTSELGLGSCFDFTIPLTVVSGLPTYTD

PLNVLDFPRTEANEATNKNPDKPWVLIVEDTEVNQRVVRIMLEQLGLKVSVASHGEEAFQ

LCREHAFDLIFMDCQMPVMDGFIATEQIRDMNEWGAHVPIIALTANVVKEDQQRCFEAGM

NEFVAKPVTKARLQQIFEQYLPKALKNIATPK

>tr|Q87LQ8|Q87LQ8_VIBPA RNA polymerase sigma factor RpoS OS=Vibrio parahaemolyticus serotype O3:K6 (strain RIMD 2210633) OX=223926 GN=rpoS PE=3 SV=1

MSISNTVSKVEEFEYDNASETTIDNELEKSSSTTEGKTAVREEFDASSKSLDATQLYLGE

IGFSPLLTAEEEVLYARRALRGDEAARKRMIESNLRLVVKISRRYSNRGLALLDLIEEGN

LGLIRAVEKFDPERGFRFSTYATWWIRQTIERALMNQTRTIRLPIHVVKELNIYLRTARE

LSQKLDHEPTAEEIAAQLDIPVEDVSKMLRLNERISSVDTPIGGDGEKALLDIIPDANNS

DPEVSTQDDDIKSSLIHWLEELNPKQKEVLARRFGLLGYEPSTLEEVGLRLKKWDERLVL

PVSVYVKFKWRVYVDFVKS

>tr|Q79YZ2|Q79YZ2_VIBPA Chemotaxis CheV OS=Vibrio parahaemolyticus serotype O3:K6 (strain RIMD 2210633) OX=223926 GN=VP0773 PE=4 SV=1

MTGILDSVNQRTQLVGQNRLELLTFRLMGRQRYGINVFKVKEVLQCPKLTSMPNLHPLVK

GIAHIRGHTVSVIDLSLAIGGRPTTDIDKCFVVIAEFNRTIQAFLVSSVERIINMHWEAI

LPPPDGAGKAHYLTAVTNIDNELVEILDVEKILAEIAPVDETMDSTIGEEIAQAEQEKPI

VRRILIADDSTVARKQVERAITSIGFEVVSVKDGKEAYNKLLEMAQEGSIYDQISLVISD

IEMPEMDGYTLTAEIRRNADLKDLYVILHSSLSGVFNQAMVERVGANTFIAKFNPDELGN

AVKSALTQ

>tr|Q87Q12|Q87Q12_VIBPA Probable permease of ABC transporter OS=Vibrio parahaemolyticus serotype O3:K6 (strain RIMD 2210633) OX=223926 GN=VP1338 PE=3 SV=1

MALPNYASKSERMAYAGYLVFCGLVLFFLIAPILTIIPLSFNATPYFTFTEGMLNLDADA

YSVRWYQEMFTNEQWLLALKNSTFIALMATLIATGLGTLAALGLANSNLPFRNAIMALLI

SPMIVPVIISAAAMYFFYTRLGLSQTYFGIILAHAALGTPFVVITVSATLSGFDQSLVKA

AASLGANPVYTFRHVTFPLIRPGMISGGLFAFGTSFDEVVVALFLTGAEQKTVPRQMWSG

IREQISPTILAVATLLIFMSVCLLVTLEILRRRNVRIRGIQE

>tr|Q87TF1|Q87TF1_VIBPA DNA-binding transcriptional regulator NtrC OS=Vibrio parahaemolyticus serotype O3:K6 (strain RIMD 2210633) OX=223926 GN=ntrC PE=4 SV=1

MSKGYVWVVDDDSSIRWVMEKTLSSANIKCETYADGESVLMALEREVPDVLVSDIRMPGI

DGLELLKQVQRDYPDLPVIIMTAHSDLDAAVNAYQKGAFEYLPKPFDIDETLTLVERAIA

HSHENKREQLSSEDAPADTPEIIGEAPAMQEVFRAIGRLSRSSISVLINGESGTGKELVA

HALHRHSPRANKPFIALNMAAIPKDLIESELFGHEKGAFTGANSVRQGRFEQANGGTLFL

DEIGDMPLDIQTRLLRVLADGQFYRVGGHSPIRVDVRIVAATHQNLEKLVHQGDFREDLF

HRLNVIRVQIPALRERKQDIEKLTQHFLVRAADELGVETKTLHPSTVEILNRLNWPGNVR

QLENICRWLTVMASGSEVLPNDLPSELLEEKKTVSDSTKGSWQEQLADWARQSLAAGDKE

LLSFALPEFERILLEAALEHTKGHKQDAAKVLGWGRNTLTRKLKELY

>tr|Q87PF5|Q87PF5_VIBPA Sensor histidine kinase/response regulator OS=Vibrio parahaemolyticus serotype O3:K6 (strain RIMD 2210633) OX=223926 GN=VP1547 PE=4 SV=1

MKDKYLDTYQQEALQEALVELKQTKQREKLLADENKAILSAISAMSEAKNRNEIFSGLNS

VLKKYISFEDFIVITRDDNRYPFKTLISTNSVFDKVEWLHGNTMDRALNGECILLFEPAK

LLEFENLNSFVKTHVNSVILTGIRSEVTQSIILLIGAQKGHFSIENKETLRRFRPLIERA

VIDIETKEKLQRIVEVRTTQLARAREEAELANQSKSEFLAMMSHEIRTPLNSVLGMLDIL

RQSTLSDEQFDALNQMECSAELLLAIISDILDLSKIESGSFQLNEQWIHLNDTVTFVISQ

QKQVAITKNLSFNFDCQISSDKQYWIDSTRLSQILFNLIGNAIKFTDSGSVSVSVAEEND

EVVVSISDTGIGISRAKQAHLFTAFHQGDRSITRRFGGTGLGLAITKHLVEMMRGEISVK

SRENEGSDFTIRIPVLTRYNQSRPVKIEHNRPSKALNLLIVEDTQSNQLVIKLILNKLGH

NVHIASHGAEALTFLEENDTRIDMILMDVSMPVMDGITATRLIRKKGITIPIVALTAHAL

ESDKDKCLDAGMDSFVSKPVRRQDIYEAIQSLIETA

>tr|Q87HG5|Q87HG5_VIBPA Methyl-accepting chemotaxis protein OS=Vibrio parahaemolyticus serotype O3:K6 (strain RIMD 2210633) OX=223926 GN=VPA1000 PE=4 SV=1

MSIRNLSIAKKISLSFLLIALINIVFGVFLSKELKEIKSELLNYTDDTLPAMERVDAIRD

DLSHWRRSQFATYTYKDADKIRNKIASNIREREKISKELEAYGSTIWPGEEQQTFQRLMR

QWKQYLVTMDQYNESMLAGNKTEALAVLSNSLNDFEAVDSDLNELIRLLKVAMDSNKNHI

LSSVNGLSSSSIASNVTILVIMIVMTLVLTRLICGPLQLVVEQANSIAKGDLSKDIDRKL

IGNDELGELADATTKMQNDLRQVIDNVIAAVTQLSSAVEEMNQISELSASGMKDQQLQIT

HVATAMTEMKAAVADVARNTEESASQANDANHRTQLGVRETQSMVDAIGEVANVIGAAGD

TVSELEQQSNQINVVVDVIRDIADQTNLLALNAAIEAARAGESGRGFAVVADEVRTLAGR

TQDSTSEITAIIEQLQSLAKDAKSATELSRTSIAECADQGIQSKQLMNDIEHAISDISDM

GSQIATACNQQDSVAEELSRSIENIHLASQEVAQGSEQTAQACRELSQLSVSLQDVMSRF

KLN

>tr|Q87LE2|Q87LE2_VIBPA RNA polymerase sigma-54 factor OS=Vibrio parahaemolyticus serotype O3:K6 (strain RIMD 2210633) OX=223926 GN=VP2670 PE=3 SV=1

MKPSLQLKLGQQLAMTPQLQQAIRLLQLSTLDLQQEIQEALDSNPLLEVEEGHEEPQANG

EDKSALETADNSANEPTEIEVPDSSDVIEKSEISSELEIDTTWDDVYSANTGSTGLALDD

DMPVYQGETTESLHDYLMWQLDLTPFSETDRTIAIAIIDAIDDYGYLTLSPEEIHESFDN

EDIELDEVEAVRKRIQQFDPLGVASRNLQECLLLQLATFPEDTPWLAEAKMILADHIDHL

GNRDYKLVIKETKLKEADLREVLKLIQQLDPRPGSRITPDDTEYVIPDVSVFKDHGKWTV

SINPDSIPKLKVNQQYAQLSKGNSADSQYIRSNLQEAKWLIKSLESRNETLLKVARCIVE

HQQDFFEYGEEAMKPMVLNDVALAVDMHESTISRVTTQKFMHTPRGIFELKYFFSSHVST

DNGGECSSTAIRALIKKLVAAENTAKPLSDSKIAALLADQGIQVARRTIAKYRESLGIAP

SSQRKRLL

>tr|Q87P07|Q87P07_VIBPA Transcriptional regulatory protein OS=Vibrio parahaemolyticus serotype O3:K6 (strain RIMD 2210633) OX=223926 GN=VP1711 PE=4 SV=1

MNMATRVMIIEDDIAIAELHHKYLSQLAGLDVVGIATTRLEAEMQLEVLKPDLLLMDVYL

PDGTGLEILNTLRSNNQTCDVILITAARDVDTLQQAMRGGVVDYLLKPVMFPRLETALKK

YITQRQQLDVAKSLDQGLVDRMLQSNTGTDSCPKRLPKGIDSVTLDKIRDLFVGEAALTA

DEAGEKIGASRTTARRYLEYLISSGELEADLNYGTVGRPERCYKKVIR

>tr|Q87HJ8|Q87HJ8_VIBPA Putative sensor histidine protein kinase UhpB OS=Vibrio parahaemolyticus serotype O3:K6 (strain RIMD 2210633) OX=223926 GN=VPA0965 PE=4 SV=1

MRAYSVTTICGLFVMACAWFCLWVIAYYFVNDPELAILLFPFALRLGIALHTRTAYWPTI

YVSEWALTIALATLLEQPQWLTVLIASVASIPVTLIAKKYYYGDQNRHLAVMGIVIIITA

FINVMAVGFHVPSVYMVWLASISGGLMLVPMCYLLWNYLFQSRWSPLTSHLLNNTVVFSI

RHIVFYAVLLIGSILVQTSLPEELKRFAPFCMAIPIIVLALRYGWQGALLATMLNSIALI

AARSGVSNLEITDLLLSLSAQTITGIMLGLAVQKQKDLNHKLRGELSRNQNLSRQLIEAE

ESVRRDIARELHDEIGQNITAIRTQANIIKRIDNAEMSAHCADTIEGLSLNVYDTTKRLL

SKLRPKMLDDLDLKESVEQLTREMEFANHGTTVQLNWQGDYTSLSDTLKVTLFRLCQESL

NNAAKYAEAQLINIELTIGEAAVSLMIHDDGVGFKVQDSMKGMGVRGMQERVHALGGKMV

IYSTSDQVIGTQISITLPKV

>tr|Q87GB2|Q87GB2_VIBPA Putative polysaccharide export-related protein OS=Vibrio parahaemolyticus serotype O3:K6 (strain RIMD 2210633) OX=223926 GN=VPA1405 PE=4 SV=1

MNPLFKLIGLALLLFSTFVSANSNEQDYLLDTGDTISVQVYGEEDLSIKNILITSDGYFD

YPYLGRIKAINKTPKQLKYEIETGLKGDYLINPKVMVTINYFRLFYVNGEVRKPGGFEYR

PGLTIEKAIALAGGLTDRASRKSINLTKHKTGKTVEGVSMQRTVEPGDIVFIDQSFF

>tr|Q87HC6|Q87HC6_VIBPA Uncharacterized protein OS=Vibrio parahaemolyticus serotype O3:K6 (strain RIMD 2210633) OX=223926 GN=VPA1039 PE=4 SV=1

MSIKEIGRVFTQRWFLGLVGVAACSIFIWVVGPLITVAGYEPLKSDFQRLVTILVIVFAW

ALINLTKQHKQKVREDESIQTLLEVDSQSDKEAASEIDVMRDRIEQAIKVVTKTHKGKRS

LYDLPWYVLIGPPGTGKTTVLKQSGLEFPLTESLGADSIAGVGGTRHCDWWFANKAVLID

TAGRYTTQDSQEKVDSKAWHGFLGLLKKYRTQRPINGAIVTVSLASVMSQTRTERSLHAR

SIKSRLQELKNQLGMQFPIYVLLTKMDLVAGFNEFFADLSKEEREELFGFMFPREVDDER

GVISLFNKEFHGMLERLDAHMLRILETEDDLEKRTLIFEFPKQLRVLQANLDEFLSEIFA

QNTFEEPALIRGVFLLSSVQEGIPVDRLMSESTNGLGLGRLPLATNVNSSHSYFVKNLFE

RVIFKEQLLGTVNRHYQKQSGWMRTGIYVGCVGVLVGASALWFLSYQWNSKLIVDTNSQV

NHIEAMIGAESLDFESDVISAVDTLDKIMMLPLGKNSKYGHSDAVKKFGLYQGDKVSQAA

NNAYSDALSQHFATLLSESLVSEMEANKQHREYLYETLKTYLMLFNPEKYQQEEVIAWFN

FYFERQYPGELNKELRERLLVHTKNLLENDEKGFSMDATAISAAREVLTQMSLPERAYQR

MKMQFAKSHVPSFRLTDVLGPKGLEQFERASGKPLSQGISGFYTYNGFHSIFQIQINRTV

KGLMEENWVYGDDLKAHEIDHDSAIQGVQARYYQDYVNEWKTLIEDIQLKQAPSLALATE

QSRVLSGVERPIESLLRAIQKEVGLSKVTLSENQKAATEVAGKVAKVKFSNTADKLDMYL

PEENGFNVALPGKEVESHFTEILRLSEQDFDDIQQAMVNLRSYLSDLSSSGNNQKIAYKS

ILDGTVTQDVAASFARAKDLLPKPFNQWLGELSQESVKFAESGSKDHLNQLWMTNVVRPY

QRTIAGRYPFEPNATKEVRLKDFQRFFGYGGTLDSFFQEYLEPFVDTSKSRWRLEKEIGV

RPETLAVFQRAKRIRQSFFESDNSLRVEFGMKPVYLDQHITRFVLELGDQDLVYKHGPAR

SKELRWPSGQDQTRIVFTPPESKREIAHTYEGEWGIFKLLDQSLKARPESRNDNIVMIDL

KGNKVQLELIPSSAINPFWSNEMERFRCPQTL

>tr|Q87JH9|Q87JH9_VIBPA Putative flagellar hook-associated protein OS=Vibrio parahaemolyticus serotype O3:K6 (strain RIMD 2210633) OX=223926 GN=VPA0274 PE=1 SV=1

MRISDNQFSQMMLQSLQSNSAGLGKVLQQMSTRERLTKLSDDPMASIKLLNLERENSAIA

QYQSNIANLKTTLSSQETHLDSVNESLKSMRDIVLWGANGSLTDQDRSGMITELKSYRDS

IESSFNAQDEEGHFLFSGTKTDTAALNKSSGAYVVEGNSDVRVVTVAKGVTMDSNMTAQE

ILDIGGGKNVLNQIDALIAEFEKPSPNFQAEVDASLNAIDDTMANVLGAMTEIGGRHNNL

DLMDGAHSENKLFVDKVSGDLSALDYGEASVRLSNYMAALQATQASYVKINDLSLFDRI

>tr|Q87TN0|Q87TN0_VIBPA Uncharacterized protein OS=Vibrio parahaemolyticus serotype O3:K6 (strain RIMD 2210633) OX=223926 GN=VP0039 PE=3 SV=1

MKKWTFFMLLIAVLLFGSVIGFNLFKQQKIAEYMANRPEPEFPVTVTEVKAVDWVPVIEA

IGFIEPNQGVTVANETSGVIDKIAFESGTQVEAGQPLVLLDSEVEKANLKSSQAKLPAAE

AKYKRYQGLFKKGSISKEAYDEAEANYYSLKADIESLKATIDRREIKAPFAGVVGIRNVY

LGQYIQAGSDIVRLEDSSVMRLRFTVPQTDISRIKLDQEVDIFVDAYPDQPFKGSISAIE

PAVNVQSGLIQVQADIPNSDGKLRSGMFARANIIMPKLANQVTLPQTAITFTLYGDNVYI

VTEEEGEKRVKQHVVKVGERTKDIAHILAGVKPGDVVVTSGQVRLSNHAKVSIVESNAIT

PPAETPML

>tr|Q87G84|Q87G84_VIBPA Putative two-component sensor kinase OS=Vibrio parahaemolyticus serotype O3:K6 (strain RIMD 2210633) OX=223926 GN=VPA1433 PE=4 SV=1

MMYFMFAHWRDNTFLFSAIVIVGAVVASWLLDQLFDSTAAVLLILQLAVVVVAFQCNSRF

AYAAAVIEALSFNFFFTTPRYSLQMFRPEDIFNLVVFMVVAFITSTFADLYRRQQGELKQ

TKLQNSILLSVSHDLRTPLATIIGTLTTLNEYMPKLNDLERKELLDSATSESHRLHQYIE

NLLQATKLQHGTLKITKKDEPIANIVRDAVSRLPNYTEKVSMNMDDSVGYLSVSRSLIEQ

AIFNVLDNAMRFSPENESVEVSLSKQGLSCVIDVRDMGIGITAEDAEKIFSLFYSGANNK

SADSGTGMGLAVAKGIITAHQGEIQSMPVSEGTLIRIRLPLNQGAEQA

>tr|Q87NX1|Q87NX1_VIBPA Putative amino acid transporter OS=Vibrio parahaemolyticus serotype O3:K6 (strain RIMD 2210633) OX=223926 GN=VP1747 PE=4 SV=1

MSNIAKSAVKLSVFSVIMITVTSVDSIRNIPGAALFGSHAISFFLLAGLCFFVPTALVCA

ELSTTYPQQGGVYLWGKETIGPNFGFATVWYQYAENIVYYPPLISFIVATGAYPFFPELA

QNNIFMLIMINVIFWALTLVNIFGLRLSSMITNVFGTLGLIFPILLIIGLGGYWAYTNPG

ESHISLSHVSDWLPDFSQDGIGAGFTAVVLSLTGLEITTSYASEVENPQKAYPKALLAST

ALILVSLTACSLSISSVVSSDHASLSEGVILAFKTFFDDLNLSFMLPVIALAIVFGTLAS

LNNWIIAPTKSLHVAAKDQFMPLALSKENQNQAPVALLLLQGAIVSVLSLVFILVPNVNQ

GMWLLNILMTQLYMVMYVCIFISFLVSRRKHANIERPFRVPGGKVGMSVVAGLGLISCMI

TIVVSFDVPAGISAETGAYALVLGFIAFSLPAIAAVMYRNRKVRSQAQLIEALAS

>tr|Q87QR1|Q87QR1_VIBPA Putative chemotaxis transducer OS=Vibrio parahaemolyticus serotype O3:K6 (strain RIMD 2210633) OX=223926 GN=VP1088 PE=4 SV=1

MFQNFTIKQKIVIPLSLIIGLFTVSSVLNVMTTSKQSELSDTLNEQIVPNLFTIEDAYRD

LYQATSAVQGIALAETQADIDHHIHEYKDNAYKALPRMEKVIELSRAGVMPASHGADVQK

LVSLGQKWLQSYEVMLSKPQSQWLSYYNEHKNTFEEQFVDVRAQLNVVKSAIEDKQGELK

SDISAATARAESILEMGIIVVILAALGMVFLLLRTVLKPLNDIKDAMAQIASGDGDLSQR

IQINTQDEIGQLAKAFNEFVSKIQATVSQVIDSSNTLRQEMANLSSLTETIADSTVSQQR

DSEAVAAAVHEMQVTSRNVSESANEAAVASQTANDELSNTNVILEQTVGSIRDLAGEIES

ASHVINTLDNDVSDIASVLDVIRGIAEQTNLLALNAAIEAARAGEQGRGFAVVADEVRSL

ASRTQQSTGEIQAMIEKLQSGAGQAVEVMRGSQNSSEETIQSAGRASESLAEILNAISRM

NEMNTHIATAASQQSTVSDEVNTNVQGIADSSTSIVDIVTQAQQSLAMLSQQTKRLDQQV

SQFRV

>tr|Q87KZ7|Q87KZ7_VIBPA Methyl-accepting chemotaxis protein OS=Vibrio parahaemolyticus serotype O3:K6 (strain RIMD 2210633) OX=223926 GN=VP2827 PE=4 SV=1

MRNTIKLKIQIAIAVIIAIVSGVQAWVSVNQLHEETTSTLNREIQNISESTNRYISDWLS

IRSDMMLANEQIIAGSDDADRELLLTKRAGKFLSVYAGFSDGAIAYGDKSESWPSDYDPR

TRPWYQDAMAQSGLIITEPYQDFDGSIVVSFAKAFNQNKQGVLAADLAVTDIINEVLNIQ

LDNNGFAFLVDGNNNLVAYKDEKLSQKPLTTLNPELTRDKMANLAQHAKLETITWPKQGD

QLIYVAQVPNTDWSLGVVQDKQMAFASVSEQVTFTAIASIVMYLIIAAISTYVITRLLRP

LQTLSDALSELSQGEGDLTQRIEIERMDEIGELATHVNQFLAQMQSMLKNIVENSQQLSE

QAQQANELSAMAAGRVEHQQNDVNQIATAIHEMSATAAEVASHAELTASASQNSASACVE

GQSVIQKNREAIVSLAEQVSDAANVISELEANTQSINQILSTIQGIAEQTNLLALNAAIE

AARAGEQGRGFAVVADEVRVLSQRTHGSTEEIRTMIETLQSNTKLAVNSMQASTSLADTS

VDYAQQAHDSLTSITNSITEINDMAMQIASAAEEQRAVSEDISRNTQGIKDDADVIAEQS

LKSSEGARRMFNTANTMRENISRFKV

>tr|Q87N38|Q87N38_VIBPA Chemotaxis protein CheV OS=Vibrio parahaemolyticus serotype O3:K6 (strain RIMD 2210633) OX=223926 GN=VP2037 PE=4 SV=1

MSGVLNTVDQRTNLVGENRLELLLFTLNSRQLFAINVFKVKEVIKLPPLTKLPGSHYNIR

GVASLRGEAVPVIDLRCSIGFPPLRGEAEEENLIVTEYNRSVQGFLVGPVRNIINTAWTE

IQPPPSTSGRSNYLTAITQVKDGDTSQIVEIIDVEKVLAEIVHYDVTISEDILDHDLSQS

MVGRNVLIVDDSSTARNQVRDTLSQLGMNIIECRDGLEALTVLKRWCDEGRDVEKELLMM

ITDAEMPEMDGYKLTHEVRSDPRMSKLFITLNTSLSGSFNEAMVQKVGCDRFISKFQPDL

LVEVAQDRLRQVLSANA

>tr|Q87G25|Q87G25_VIBPA Methyl-accepting chemotaxis protein OS=Vibrio parahaemolyticus serotype O3:K6 (strain RIMD 2210633) OX=223926 GN=VPA1492 PE=4 SV=1

MQLSLKNLSVRTQILVPVLFTAIVLFIALWITKNNLQAEQDVIASNQESLVFHKDTLARI

DDQIYPLRISAVYAIYDASRRETFLNELKAGLKQVEADLSAIEARNLFREDAIEVRKAIE

AYVQYSQRSVAFFNQYDQGLKSDNEYRAFISEYRRVGNEMVQGINTLSKHVNDQAVESTA

KSNAQNERVQTNAMLTVLAVFVFSLIGAWFLSGMIVTPIQKLQRVMRELAAGNLSVRADV

EGDNEIAQLSKDVNQTASQLYSIVDQLTRISEEVASASTELAAVMTQAEANAQQELAEIE

QVASAVNELASTANNVSDNATSADATAREADGLAQSGLAIFQESADASAQMSQALNDAAQ

VVLRLKEQSVQINDVIEVIRGVSEQTNLLALNAAIEAARAGESGRGFAVVADEVRMLAAR

TQDSTQEISTIIEELQAQSGLANDSMQVSLEMLTRNNELTQQANDALIGITESVANINDS

NTQVATAAEEQS

>tr|Q87GY3|Q87GY3_VIBPA Methyl-accepting chemotaxis protein OS=Vibrio parahaemolyticus serotype O3:K6 (strain RIMD 2210633) OX=223926 GN=VPA1182 PE=4 SV=1

MLANFSQKAQETLVGELEELVSTTNLKGVITYCNDAFCRVAEYTHEELVGQNHNIVRHSD

MPKAAFGDMWARLKEGKAWRGMVKNSTKSGGYYWVDAYVTPIYEKNQVVGYQSVRVKPKR

EWVDIAAKAYKGMLAAEKAGRTWSLKINETVRYAILLGALTAPAVAYALSVEGPLAWLAS

ALPASVLALLFRQELIDTPQQLKKLQKQYDSVSRLIYSGNSAFSIADFHIKMLSARIRTV

LGRMTDSALPLQNCAEELSQTTSEVSAALNQQNSDIRRVRDATQEVESSANSVSSSTNDA

HMLIDDTLKSCMMAKETIDQTHTNLAQLSLQAEKATETTYQLSDQAQKVNHLMVEIGGIA

EQTNLLALNAAIEAARAGEQGRGFAVVADEVRALSGRTSNATEQIQASISAMLSTIEGWQ

KDILANKEQTDACSQVAEQSALRLSEVEQMMQSMSGLMVDVAEAANNQLKLSSDVNQHIH

SIASTAEQNLAATHSVEQNSRQLKEQVQDFYQLAIRFEDKQS

>tr|Q87H65|Q87H65_VIBPA Putative sensor histidine kinase OS=Vibrio parahaemolyticus serotype O3:K6 (strain RIMD 2210633) OX=223926 GN=VPA1100 PE=4 SV=1

MNKFRLVNSCLFIAMLVACTFAYMAHINSKSTQQLHSALSEVGHQLIEERDVIVNQYAIK

ERKNFELTKSLVDIEVEAEKLADTFDNAVWFPISPNRQKIQQTLAKFEQRVIQTTSQLDM

LIGVQVENQYALLMLLDIYEEEFSTHIGETQLDKHYVEFFSRDLVNQSGEQSESGANFLG

RLHESDKKIELLTNELLDHNYFVFVEEAEHSLLDLAQNEARFTWLFVFVAVMLLVGSFLY

QLQYRMHNLKQLNSELEAETDKAERAAKAKSSFLAAMSHELRTPMNGVLGISQLIAEETK

EPVTKEHIKVILDSGQHLMTILNDILDFSKVEENKLELEKAPFHLEQVLTPVCSAIQPLI

DEKSIDLIVENDVPNNTEFTGDCARLRQILFNLAGNAVKFTNEGHVLIRTELNSEDKHLL

IIVSDTGIGIAPDKQGRVFNSFEQADSSTTRRFGGTGLGLAIVKKLTELMGGSITLKSVE

GVGTQFIVTLPIPWNESEKPSPQHTPVQTRSTQNLRILLAEDNRVNALVAKGFCEKLGHA

VDVAENGLVAVEKARDNDYDLILMDNHMPEMNGVEATRFIREKLGVKTLLFAYTADVFRE

AHDHFIAAGADHVLTKPLQRESFADALKQFSARLKVKQTEEVSPVSNVLQLQRKPIENLR

LTEEELSNSEMLASLKEHPNELLDLLNSIITDFELAVDDLIENFMQSDFDALKLTMHTTK

GMALNLGLKILASQALELETQLKMNQVPAIEQLQMLINRLQVNIHQGHRLRDELVKAQQN

SEQVF

>tr|Q79YX4|Q79YX4_VIBPA Chemotaxis protein CheW OS=Vibrio parahaemolyticus serotype O3:K6 (strain RIMD 2210633) OX=223926 GN=VP2225 PE=4 SV=1

MSQAFEVEVKKDTSNDEVLQWVTFQLEEETYGINVMQVREVLRYTEIAPVPGAPDYVLGI

INLRGNVVTVIDTRSRFGLMEGEVTDNTRIIVIESERQVIGILVDSVAEVVYLRSSEIDT

TPSVGTDESAKFIQGVSNRDGKLLILVDLNKLLTDDEWDEMAHL

>tr|Q87Q18|Q87Q18_VIBPA Probable binding protein component of ABC transporter OS=Vibrio parahaemolyticus serotype O3:K6 (strain RIMD 2210633) OX=223926 GN=VP1332 PE=4 SV=1

MSRMTKTPLVMLISGMLLGTSAYAEDKLTVVSWGGAFTKSQVEAYHKPFIQKTGVEIVSE

DFSGGLAEIKAQVEANNVRWDLVSLDKPDIVRGCAEGLLEPVNPSILPPGADGTPAKEDF

IDGAIHECAINTIVVSTVLAVNEDAFKGKTAPTKLTDLFDLTNFPGRRALQKQPQGNLEW

ALLADGVKPDEVYRLLETEEGRARAFAKLDTIKPQVLWWTTGAQPPQMLADKEVVIASAF

NGRIHNARKDEGQPFRIIWDHQMGYMNGWAIPKGSANTKLALDFIAFSSGTKPLADQAKY

VAYGPTRKSSSAEVSPEILANLPTAPQNFKTAFLINDEWWSDYADELNEEFNTWLLN

>tr|Q87L60|Q87L60_VIBPA Transcriptional regulator, LysR family OS=Vibrio parahaemolyticus serotype O3:K6 (strain RIMD 2210633) OX=223926 GN=VP2752 PE=4 SV=1

MNIRDFEYLVALAEHKHFRKAAEACFVSQPTLSGQIRKLEDELGTALLERSSRRVLFTDS

GLQLVDQAKRILSEVKTFKDMASGQSGAMTGPMHIGFIPTVGPYLLPKILPQLKEEFPEL

ELFLHEAQTHQLVRQLEEGKLDCLVLASVAETAPFKEIEVYNEPLSVAVPCGHEWAQLDQ

VDMLELNGKTVLALGDGHCLRDQALGFCFAAGAKDDERFKATSLETLRNMVAAGAGITLL

PQLSIPAEKQKDGVCYIPAVNPTPSRSIVLAYRPGSPLRARFEALAAKIKAILESQPSSM

AA

>tr|Q87FX8|Q87FX8_VIBPA Flagellar biosynthetic protein FliP OS=Vibrio parahaemolyticus serotype O3:K6 (strain RIMD 2210633) OX=223926 GN=fliP PE=3 SV=1

MNNLSAWHRWLPLLVLVSLLFAFPTMAADNGLTILSVTDGDAQQEYSVKLQILLLMTALS

FLPAFILMATSFTRIIVVLAILRQALGLQQSPPNRVLVGIALTLTLLIMRPVWTDIYENA

FQPYDNGEITLVQAFSVAEKPVRNFMLAQTHQSSLEQMLRIANEPLDQKVEDISFAVVLP

AFVISELKTAFQIGFMLFIPFLIIDLVVASVLMAMGMMMLSPLIVSLPFKLVVFVLVDGW

AMTVGTLSASFG

>tr|Q87KT8|Q87KT8_VIBPA Putative sensory box/GGDEF family protein OS=Vibrio parahaemolyticus serotype O3:K6 (strain RIMD 2210633) OX=223926 GN=VP2888 PE=4 SV=1

MNFLSQRYTGKSLHPDETKCYKNFTQTAQKLTHRLAESINHYVNHIDCVINTNLYNRLLA

EKKESNHMIYKERETWLEAILNTLPDHVFILDESGRYIESFGGTHHSKTFNAERYIGLQL

SDVLSPTKADELMGFIFDVMQDNETKVVKYNLSLHDHLLLPIEELEALENPEEMWFEAII

KPVNAPENANKLVIWSVRDITKTHLLEQRLKQLSETDALTGLLNRRAFISNLDNAIIQHT

KRSQTLSCLMIDIDHFKDINDSVGHFSGDHVITRIAHVCQGVIRGSDFIGRLGGEEFAVI

LTDTNAIQAYEVAERIRQAIQATICHVDDVEITTTVSIGVAELNSQQSNAKELLIEADKA

MYYSKHSGRNQVTLAYENLPDLKLYQAGHLKIQRVS

>tr|Q87H96|Q87H96_VIBPA Putative virulence-mediating protein VirC OS=Vibrio parahaemolyticus serotype O3:K6 (strain RIMD 2210633) OX=223926 GN=VPA1069 PE=4 SV=1

MHFPIKTLSCALLAVIGLFFQTGYVFANTDKHIYLEEHISPESQPMLSTYFRSMGKPRSI

KENVLTVPQDASPETLALYYFARIYLERYEGVPLPDDMPDLIEFGRKHNMPWVVAEAKLN

KAIRMIELDDDWHAELLLHDVIGESRDIGYLALQGRAYRWMGNLEIARNQIHNGLKHYRT

AYELLENTVFEIQVAMTLNNIGTVYLDSSDWNRASNYLKQALDVYESSEYEYDNSFFIGV

IYANLSIVHLGLGDSEKAEYYFHEAIRRSMQTGSDVIKHHSLSNFSQMLSSIGKTDDALL

LAQRCVELPNPDGIEIIKMPCHEAFAEAYLADKQYDKAIRTALLVLEQTKSTNELELKQR

IDMLSVLVNANQVLQNYEAAFRYLSQLRALEEEFSEHIHGEEMINIKFDLEAKLAQKELN

LLETKNALQASELRSQRYREMFYFIAIAAIGVVGFRYVLRVKKINKALTQESTTDLLTGL

HNRRYLEVWLEKMVRRTPDRTFALAVLDVDHFKAFNDTYGHDIGDQMLMHIASIFNESTR

SGDLLIRWGGEEFVLLVEVNDPNDCAKSLERLRHVIENTPLIIDSKPINATISLGAVDRL

>tr|Q87TN1|Q87TN1_VIBPA Putative multidrug resistance protein OS=Vibrio parahaemolyticus serotype O3:K6 (strain RIMD 2210633) OX=223926 GN=VP0038 PE=3 SV=1

MRFTDVFIKRPVLAVSISFLIALLGLQAVFKMQVREYPEMTNTVVTVTTSYYGASANLIQ

GFITQPLEQAVAQADNIDYMTSQSVLGKSTITVNMKLNTDPNAALSDILAKTNSVRSQLP

KEAEDPTVTMSTGSTTAVLYIGFTSDELSSSQITDYLERVINPQLFTVNGVSKVDLYGGM

KYALRVWLDPAKMGALKLTATDVMSVLNANNYQSATGQATGEFVLYNGSADTQVSNVAEL

EALVVKTGEGDVIRLGDIAKVTLEKSHDVYRASANGQEAVVAAINAAPSANPINIAADVL

DLLPQLERNLPSNIKMNVMYDSTIAINESIHEVVKTILEAAVIVLVVITLFLGSFRAVII

PIVTIPLSLIGVAMVMQAMGFSWNLMTLLAMVLAIGLVVDDAIVVLENVDRHIKEGESPF

RAAIIGTREIAVPVIAMTLTLGAVYAPIALMGGITGSLFKEFALTLAGSVFVSGIIALTL

SPMMCSKMLKANEKPSKFEQKVHHILDGMTTRYEGMLKAVMAHRPVVIAFAIIVFASLPV

LFKFIPSELAPSEDKGVVVLMGTAPSNANLDYIQNTMNDVNKILSDQPEVEYAQVFNGVP

NSNQAFGLATLKPWSEREASQSEITKRVGGLVASVPGMSITAFQMPELPGAGSGLPIQFV

ITTPNSFESLFTIASDVLTDVASSPMFVYSDLDLNYDSATMKIKIDKDKAGAYGVTMQDI

GITLSTMMADGYVNRIDLNGRSYEVIPQVERKWRLNPESMKNYYVRSVDGKAVPLGSLIT

IDVVAEPRSLPHFNQLNSATVGAVPSPGVAMGDAINWFEDVAQNKLPAGYNHDYMGEARQ

FVTEGSALYATFGLALAIIFLVLAIQFESLRDPIVIMVSVPLAVCGALIALAWGTASMNI

YSQVGLITLVGLITKHGILICEVAKEEQLHNKLSRIDAVMEAAKVRLRPILMTTAAMIAG

LIPLMYATGAGAAQRFSIGIVIVAGLAIGTLFTLFVLPVIYSYLAEKHKPLPVFVEDKDL

EKLARVDEAKAAHRELAENK

>tr|Q87IW5|Q87IW5_VIBPA Methyl-accepting chemotaxis protein OS=Vibrio parahaemolyticus serotype O3:K6 (strain RIMD 2210633) OX=223926 GN=VPA0491 PE=4 SV=1

MKLSNLSIKYKISALILIISLSVIALSVFFTSEIKMIEGKLTVFSETTVPSVLLVKNTEI

ELGILRKDEFSLLTNVNHPQFMEWVAGLEKSEQKIDKYLDQYEKGLWDQRDRDAFNKVKS

AWVKYSAFNNEYAKLLLNNKIDEANKTLLNGFSTFTQLSDAIRDLVELNQTYVQEDIASA

HEAVRSAITYSIIAIVALLALSFTLGLFLTKQICTPLNYVVNMASKIASGDLTYQLPRNK

IGHDELGTLADACVDMQAKLLTLVDSISSTTAQVGTAIEEVSAISEQTSTGMDEQQVQLN

LIATAMNEMQATVNEVASNTEAASETANSASHDAKEGRGVVQECINQIHEASLAIQSVGN

MVTELEQDASNISVVVDVIQDIAEQTNLLALNAAIEAARAGEQGRGFAVVADEVRTLASR

TQASTEEIITIISKLQNCSKSAVSATNNSSDLIQECVEQAQKAGATIDQIEKGADNIAEM

SIQIASACSEQSSVTEELHRNVEHINQFSSEVATGSRQTAIACRDLSELAVGLQEIVGQF

KTA

>tr|Q87IQ2|Q87IQ2_VIBPA Methyl-accepting chemotaxis protein OS=Vibrio parahaemolyticus serotype O3:K6 (strain RIMD 2210633) OX=223926 GN=VPA0554 PE=4 SV=1

MFGLRTRNQEAESEYRRFIKGLSDSMAMIEFDTRGIILNANDLFLSCVGYTREAIVGKHH

SIFCDRGYVQSPRYQQFWDDLKMGKHKRGTFERLTSQRERLVLEASYFPIESEHGQIEKV

VKIASDVTQQRLESEQKEAILNALDLSLATIEFDREGYILTANQNFLKTLGYELSDVQGK

HHKLFCFDDFYQENPGFWKDLAAGQFKSGQFLRRSASGDKVYIEATYNPIFDPAGNVIKV

VKFASDITDKVQRDLNISQAIADSSFIARNASEEATSNVCGAEHSLNEFRTMIEEVLRAV

TACDDKVQELFKTSQQVTEIVKVIDSIASQTNLLALNAAIEAARAGEHGRGFAVVADEVR

TLATRTSTSIDEINHIVLSNQSLTTETRSFIEVINQGFLSSMSKLLEVDEFMKSIDEGSH

LTVESISALRAIVNQDAHLHLNSASNG

>tr|Q87QG4|Q87QG4_VIBPA Putative chemotaxis transducer OS=Vibrio parahaemolyticus serotype O3:K6 (strain RIMD 2210633) OX=223926 GN=VP1185 PE=4 SV=1

MSVKMSVRKKLYASFGAILATMLVMIAIITFEVINSHKVAQEVRTDDVPETIGYMALIDE

AGDIYRDAVGMIINTSGAQREYQSNKKEFASALADVKRLETAGGEDYRNIEKIEQLMANF

TSSFESQIQPNIGHRSLEENVEQVRQLYEVNLAPIEDMLNAATAEGRQSTHDALLQLTDS

FNDIENTIYALGTLAAIATVFIAYLLSNSITGRLTALDTLAQRVAEGDLTASPIKDESGD

ELSNLATSLNKMQASLTGLIGSISVVSEEVKSVTSELSVVSQDIVSGASSQADKANLIAT

AAEELSLTISQVAEQGASTFEEARKSEATAEQGRTVIVEMVESIQQVSKQMADMSVQMNH

LGAHGEKIGAVIKVIEDIAEQTNLLALNAAIEAARAGEFGRGFAVVADEVRALAERTTKA

TQEVGEIIQAIQVGTQEAVTYTEDGCRLVEIGVSQSSGAVESLEEIVAGAGHVQSMVNSI

ATAAEEQTAVTKEIAADITSISDISVRSLQLANDSSQSVEGLNRKVQELETLVGKFKLA

>tr|Q87K74|Q87K74_VIBPA Methyl-accepting chemotaxis protein OS=Vibrio parahaemolyticus serotype O3:K6 (strain RIMD 2210633) OX=223926 GN=VPA0024 PE=4 SV=1

MNLTIRKRLYILSIIPVLTIALGMMWFTYLQTNAYNQQQIDQTHTTMMAMKKAELKNYVQ

MARSAIEPLLKRNATLEEALPVLRELEFGETGYIFGYNSKGVRVVVGKNDKGIGENFYNL

QDKKGNYLIQDLLKNAKTGEFTTYYFPKLGQTEALPKLSYSMFIPEWDLMIGTGFYTDDI

DAVIAEMEASAHDALNTTLVAIALFCVSIAAVVAIFAVFVNRSIMRPIEQFDASIQSFAQ

GDADLTARMHESNVPEFKQLAHNFNIFVESLQGIIKSVTQVGEEVVAETNNMSQRASQVD

ELAGGQREETEQVAAAMTEMTATAHEISNNANQAAESARHADENAQQAKHIVDSAANSVE

ELASEVSEASTVIARVESDVQNISSSLEVIQDIAEQTNLLALNAAIEAARAGDQGRGFAV

VADEVRKLASRTQDSTGDIHKMIEQLKSGSDAAVRAMESSQARGEATVEEARAASVALQD

IQAAIANIMDMNTLIATATEEQSQVGQEISQRVEVISQQSSQSASLANQNRSGSQNLNHK

ANELYDLVGRFTV

>tr|Q87NG0|Q87NG0_VIBPA Sensor histidine kinase OS=Vibrio parahaemolyticus serotype O3:K6 (strain RIMD 2210633) OX=223926 GN=VP1908 PE=4 SV=1

MPLKAKLILLTLIPVVLVSASISWISIYQAKTLGQREVEIFHQNLIQSKEAALKDTVDVA

FDAISHIYNDSTIEERVAKARVKAILNRLTYGSDGYFFAYDKHGTNLVHPVLPELVGENL

LHLEDENGDRLIEALLYQAQSGGGFHQYLWQKPSTGDIVPKLSYAAWLDKWEWMIGTGLY

IEDVSQEVANMRAAVNKNIETTFFSVVVILVVTVAVIIVLTLAINLHEHRLADKNLKELA

HKTVMFQEDEKKHLARELHDGINQLLVSSKCHLDLMSHRLQDEKLKSHLDKSQRSLVTAI

NEVRHISHQLRPSALDDIGLEAALTTLLQDFHSHSGIDIDSHFDTQQHKLTSEVATTLYR

VAQESLNNIEKHAKAKKVTVILQKMGNMLQLLIRDDGVGFVVNQAVHRQGIGLRNMQERV

EFIGGEFELMSELGLGTEITVLLNLDELVYGQTD

>tr|Q87G70|Q87G70_VIBPA Putative transcriptional regulator, LuxR family OS=Vibrio parahaemolyticus serotype O3:K6 (strain RIMD 2210633) OX=223926 GN=VPA1447 PE=4 SV=1

MEHQTTRNVILITEGSLQSSLLKDVLETKLGINVLLITPENLASPFVRNQSISAIVLDYS

VITDEVFARYMEFKTPNLTGTLEILINCDKSISTDELFVWGALAGIFYTSDDIQTLQTGI

DKVLQGDMWFSRKFAQQYITHLRRHSRPINKNVPAILTKREQQIITFLSMGASNQQIAEQ

LFVSENTVKTHLHNIFKKIDVKNRVQALIWAKENISDHSIEMV

>tr|Q87P19|Q87P19_VIBPA Transcriptional regulator ExsA OS=Vibrio parahaemolyticus serotype O3:K6 (strain RIMD 2210633) OX=223926 GN=VP1699 PE=4 SV=1

MDVSGQLNTETVGSSLRKIRSFSHYEKHDEVFHSDQSHIVVVHNGQLRVQTGDCTIDVVA

GSGVFLSQGDYLLEYSPQGGNYHSSIIEFDNELVSQLLQKHSDLLMTLPKVDKLNSGLFS

FGLNILIEQVLSGMKTLEEQSYPDAIMRLKYEEMLILLLHSQGGEVLYALLSQQTNRTSD

RLRRFMEQHYLKEWKLTDYAQEFGASLTTFKELFNEHYGISPRAWISERRLLHAHKLLLT

SKMSIVDVAMEAGFSSQSYFTQSYRRRFGTTPSKVRSGDEQVAIAN

>tr|Q87GX0|Q87GX0_VIBPA Nitrate/nitrite response regulator protein OS=Vibrio parahaemolyticus serotype O3:K6 (strain RIMD 2210633) OX=223926 GN=VPA1195 PE=4 SV=1

MCKVMLVDDHPLMRRGIHQLLSFEPEFEVVAEASNGADAVAKAHELELDLVLLDLNMKGM

SGVDTLKALRADGCEARIVILTVSDSPADIEAIVRSGADGYLLKDTEPDELVELLKQAHQ

GDKAYSQEVAKYLSERSDHEDVFDSLTDRETQILREVARGFRNKQIADRLFISESTVKVH

MKSLLKKLQVPSRTAATVLYLERFGDIK

>tr|Q87SA3|Q87SA3_VIBPA Phosphoenolpyruvate-protein phosphotransferase OS=Vibrio parahaemolyticus serotype O3:K6 (strain RIMD 2210633) OX=223926 GN=VP0521 PE=3 SV=1

MLSQLREIVEKVSRVDDVHLALDILVKETCAALRTECCTIYLANEEMQRLELMATQGLIF

EGDSIHINFNEGLVGLVKRSAEPLNLAEASKHPEFKFFPQLGEQVYHSFLATPIIHRKQV

LGVLVIQQKTPRLFSEMEESFLVTLSAQLAVIIAHAQSLGHWQLASKPTVLKGLPASTGV

AIGEFWFDNTQPSLSDVFPSSTLDKEREQELLLVAIERALNDFRRMRKKFDSEINKDALA

IFDLFTHLLNDPMLRGDLKKQIEKGDRADWALRQVVETYSNRFARMSDVYLRERAQDIRE

LGQRLLYFLHNSEQENAAIDRPIILVANELTATLLASVPKEHLLAVVSLEGGANSHAAIL

SRALGVPAVMGANINTDVVNGKLGIVDGYTGEIFLEPNRQLLREYRSLVSEESELFAMVN

KDLALPAVTLDNQYIEVMLNAGLSADSNIAINTGVDGVGLYRTEIAFLLQHHFPSEDEQY

HQYRAILNSYSNQRVVMRTLDIGGDKPLPYLPIEEDNPFLGWRGIRFTLDHPDIFLIQLR

AMLRASAESGNLSILLPMVSGIKELDDAMTLINQAYSEVVLLDERIQAPKVGVMIEVPSM

VYLLPAIAHRVDFVSVGTNDLTQYLLAVDRNNSRVADVYESMHPAVLLALKQIHDVCDKY

HVPVCICGELAGDPFGALLLLGLGYTSLSMNTSNVAKVKYLIRHSQQQELEKLAEQAMTK

SYGEEIHQMMHDFFIQQGFAGFVRAGKK
