# Supplementary material for: Comprehensive genome based analysis of Vibrio parahaemolyticus for identifying novel drug and vaccine molecules: Subtractive proteomics and vaccinomics approach
Source: PLoS One. 2020 Aug 19;15(8):e0237181. doi: 10.1371/journal.pone.0237181 (PMC7444560; doi:10.1371/journal.pone.0237181)
Supplement: S12 File — (DOCX) [file pone.0237181.s025.docx]

**S12 File.** Essential cytoplasmic proteins using PSORTb, CELLO, ngLOC, PSLpred

>tr|Q87L83|Q87L83_VIBPA Ferric uptake regulation protein OS=Vibrio parahaemolyticus serotype O3:K6 (strain RIMD 2210633) OX=223926 GN=fur PE=3 SV=1

MVKGLNPTIIEQIESICAERGVRLTPQRRRVFELICSNRRASSAYELLEQLKESEPQAKP

PTVYRALDFLLEQGFIHRVESTNSFITCCSFNTQQHFFQLLICDKCGDVVELEDETLISL

LAENAEKHGFKLTNQVIETHGECQACSSETKEKV

>tr|Q87GW9|Q87GW9_VIBPA Histidine kinase OS=Vibrio parahaemolyticus serotype O3:K6 (strain RIMD 2210633) OX=223926 GN=VPA1196 PE=4 SV=1

MFKNVKKSVTRTIASAMLLILLLSVATTGFAIFTLASSLNDAEAVNVAGSMRMQSYRLAH

DIQIRSVDYSSHIDAFEHSIYSSSMKALQHWSVPEDITHDYYRLIMRWHELKSVLRGEDP

SQYQLLVAGFVQQIDDFVFKLQNFSEQKLINLAWIGGLGLGGILCASMFVVHFIRLEVVR

PLRALVFASERIKNRSFDINLAVSSDNEMGILTRTFNRMATDLGKLYRGLEQAVDEKTRK

LQHANQSLEVLYDSSKELTASRINQDNFQAILKHIASLEGIKAVKLEIEQLGEPNWILTE

GEECCHDCDDECHAEPLTLDGEHLGSLYWKAGLPCPNETLIDNFVQILSRAVYYNRAQRQ

AEQILLMEERATIARELHDSLAQALSYLKIQVALLKRSVKNLPDEKAIAQANQVIAELDT

GLSAAYTQLRELLTTFRLTIKEGSFGQALQEMVETLNEQTTAEITLKNRLSSTELDAHQQ

VHLLQLIREAALNAIKHAQADHIHIQCLDCDGKVTVTVSDDGVGFEHQDEKINHYGMTIM

QERAARLHADLQIEASINKGCTVKLEFQHSKEVNFDSV

>tr|Q79YW1|Q79YW1_VIBPA Flagellar motor switch protein FliG OS=Vibrio parahaemolyticus serotype O3:K6 (strain RIMD 2210633) OX=223926 GN=VP2248 PE=3 SV=1

MANDIVPQDENGAGMPVEFDASTITGEEKAAILLLSLNEQDAAGIIRHLEPKQVQRVGSA

MARAKDLSQEKVSAVHRTFLEDIQKYTNIGMGSEDFMRNALVAALGEDKANNLVDQILLG

TGSKGLDSLKWMDPRQVASIIVNEHPQIQTIVLSYLEADQSAEILSQFPERVRLDLMMRI

ANLEEVQPSALAELNEIMEKQFAGQAGAQAAKIGGLKAAAEIMNYLDNNVEGILMEQIRD

QDEDMATQIQDLMFVFENLVEVDDQGIQKLLRDVPQDVLQKALKGADDSLREKVFKNMSK

RAAEMMRDDIEAMPPVRVADVEAAQKEILAIARRMADAGEIMLSGGADEFL

>tr|Q87HD9|Q87HD9_VIBPA Uncharacterized protein OS=Vibrio parahaemolyticus serotype O3:K6 (strain RIMD 2210633) OX=223926 GN=VPA1026 PE=4 SV=1

MKKASQDNNFIRISSPFGKDALILNSFEYREGISELFSLRAKAYFNDQKNELNEIVGKEV

TISVENSSRVSKSPRFFHGIVSAAKLEGQRVMNSHNGENYKNIEIIVEPKVKFAAYRNNC

KIFQKKNIKDIISEVLSEHGVAFKFELKNTYPQYSYKVQYEESDLAFVQRLLAEEGLSFC

FSHSKSSHVLDIFDDVSFYKPSPEFMVDFDTGSSESSHISAWNETQVLTTKSSQKSGFNM

LKPASQPKNVAAGDTALFTVPASEYFEYLGETESDDQYSLRNTHAIESLQQNVYLCSGEA

SCRTFSVGKCFKFKKHEDKSRVGKEYVLASVTIFASVFNQTGLGGTASQGVRVAFTCVDS

KTILRPAVTYPKPQIKGLQTAIVTGNKDGEVYVDKHGRIKVQFHWDRLGKYDVNSSCWIR

VAQSVAGNGWGAVFHPRVGQEVIVEFVNGDPDQPIVTGALYNGSQLPPYSLPEKSSQSGF

KSRSVQKGNANFNELRFEDKPGEEHIYLHAEKLFQMLVEDCVDIVVENNKVEKVTNDVTQ

DVGKNATLKVGENYTSDTGKVLSLNAGKSIEIKVGGASIQMSSSGEINIKGNKISINGSA

>tr|Q87TC9|Q87TC9_VIBPA Type II secretion system protein L OS=Vibrio parahaemolyticus serotype O3:K6 (strain RIMD 2210633) OX=223926 GN=VP0141 PE=1 SV=1

MEGSVSEFLTVRLSSEQQSTIPWVVWSTEQQEVIASGELAGWEHLDELVSYAGQRQVIAL

LASNDVVLTQVDIPPGATRQFDSMLPYLIEDEVAQDVDSLHFTVLGKQADKAQVCAVERA

WVQTVLQRFASQGLTIKRILPDVLALPVSDDNSSAALIGEQWLIRHSETEGAVVDSAWLD

LYLSSYLQNHEGWQLDCYSSVPESTVESVWVPKPEEMTMALLAKGVASSKTNLLTGEFKP

KSSWGKYWKVWQKAAIAAGVLLVVVVAQQLLVVHKYEAQAQAYREESERIFRQVFPNKNR

IPTVSYLKHQMTDEERRLSGGSTDVAMLSWLAALPATLGQVKDLEITSFKYDGQRGEVRI

HARSSDFQPFEQARVKLAEKFNVEQGQLNRSDNVVMGSFVLKRQ

>tr|Q87NV3|Q87NV3_VIBPA Uncharacterized protein OS=Vibrio parahaemolyticus serotype O3:K6 (strain RIMD 2210633) OX=223926 GN=VP1765 PE=4 SV=1

MKALIEVLDGQVKHCPINQKFDISSTDFYIVLNHNCALELCDSKGIKQLIDPPCLVAIGA

DFKGQIAINSFVENANISGFRLAACFIEKLNQQLNFRDLCDGVFSGNVAVSFTLRPGIVD

IYSALKAMVKKGCGNRSNDELLDINSMLLYLLMHFDQSASQAETRSYSSLSSRIRALISK

DLTKPWTLKEIAKLVYMSESTVKRKLNKEGTTFTDVLQAARLDTAQKMVCNSDASVSAIA

ELCGFKHASYFGACFRKEYGVTPLAYRKQAQLRAN

>tr|Q87K78|Q87K78_VIBPA Uncharacterized protein OS=Vibrio parahaemolyticus serotype O3:K6 (strain RIMD 2210633) OX=223926 GN=VPA0020 PE=4 SV=1

MCSAKSMVASGWQRSVIVTTLFCLFISGMTLSVWGGPYYVHVLVSFGFGYSALFFSWLID

KLFPTIPRMLEIALSLTACLLFGVINAQFWLGEYFGISGMLPVLLMGLLFSGMCYFYFHS

REKEAIAQRELESIKRENAEQERALLLSQLKQMQSQIEPHFLFNTLANISALMSQDVDKA

KQMLDQLTALLRATLKNSREEHTTVENEITLIDAYLGIQKIRLGERLSYTIEVQEGLGNT

ELPPMMLQPLVENAIIHGIEPKREGGEVQLLIKQEKQLLQIEVKDTGVGLSHVSNHSGSG

IGLSNLKQRVDALFAGKGQVSISESSEGGVSVRLSWPMISKEQ

>tr|Q87I65|Q87I65_VIBPA Putative fimbrial protein Z, transcriptional regulator (LuxR/UhpA family) OS=Vibrio parahaemolyticus serotype O3:K6 (strain RIMD 2210633) OX=223926 GN=VPA0741 PE=4 SV=1

MRFTLNNVLIIDDQPLYSEALASLVENAINTAEVIQTTDSAEVMELVRSQRIDLIILDVV

LGDRDGMRLAKNILATGYRGRLLFVSSRDYSSLSKAAYEMGANGFLNKNEARETIADAIV

SVSRGYSMFKSTHTPSSGDVTLSNREAMVFHYLAQGYSNKKISEQLSLSAKTISTYKTRI

LKKYHADSLIELLHTIPQSENIQFCR

>tr|Q87TD5|Q87TD5_VIBPA General secretion pathway protein F OS=Vibrio parahaemolyticus serotype O3:K6 (strain RIMD 2210633) OX=223926 GN=VP0135 PE=3 SV=1

MAAFEYKALDAKGKQKKGTIEGDNARQVRQRLKEQGMIPVEVVEAKAKAAKSSGSVGFKR

GIKTAELALITRQLSTLVQSGMPLEECLRAVSEQAEKPRIRTMIAAVRSKVTEGYPLADS

LGDYPHVFDELFRSMVAAGEKSGHLDTVLERLAEYVENRQKMRSKLLQAMIYPVVLVVFA

VAIVSFLLATVVPKIIEPIIQMGQELPQSTQFLLAASEFVQEWGLIIFAVLVVCFYGLKL

ALQKPDFRLSWDRKIISLPLVGKISKGLNTARFARTLSICTSSAIPILEGMRVAVDVMSN

RYVKQQVLIAADNVREGASLRKALDQTRLFPPMMLHMIASGEQSGELESMLTRAADNQDQ

NFESTVNIALGVFTPALIALMAGLVLFIVMATLMPMLEMNNLMSG

>tr|Q87MI1|Q87MI1_VIBPA Putative glycine cleavage system transcriptional repressor OS=Vibrio parahaemolyticus serotype O3:K6 (strain RIMD 2210633) OX=223926 GN=VP2274 PE=4 SV=1

MKQHLVLTAVGTDRPGICNQVVKLVTQAGCNIVDSRIAIFGNEFTLIMLLTGNASHITRV

ETQLPLLGQEHDLITIMKRTSAHELLDNSYTMEVFIESEDRPGLTEKFTQFFADQQIGLD

SLSAQTISKSKLQLDADQFHIAITASVSADCNLMQLQEDFDELCKSLNVQGSLNFIKNTL

>tr|Q87HC5|Q87HC5_VIBPA Uncharacterized protein OS=Vibrio parahaemolyticus serotype O3:K6 (strain RIMD 2210633) OX=223926 GN=VPA1040 PE=4 SV=1

MVSMEQTIVKPTPGGRAAVSKAQPQRSADSTVVISKNPELVNNDSVVAYGDNPLLAEANG

LLSIIGQIRATATHSDPLFLKETLAQKLRDYENRLRQHDVDLETIDTARYCLCCSLDEAV

LNTNWGSQSFWTHDSLLSSFYASSQGGEAFFKHLDSCLAHPESHLDLLELMYVCLSLGFI

GQYRLEKNGLEAHRRLRKQVVSVLKSHGRGVQQELSNKVEQHILAGAQVSERAPLWVVCS

VTSALLVCIFMYFSYELNKASNQTFAQLVNLIQPTPAVSNPMVESKSAPIAERISMYLAT

EIGKDLVTVEALQDRVRISLKAQDLFESGSASVVAYIQPVISKVARTLEATQGKIIITGH

TDDRPIFTSKYPSNWHLSLARATSLSEQLISNSALKGRVIPEGLGDARPLVENDSEKNRA

MNRRIEIDLIVGN

>tr|Q87Q12|Q87Q12_VIBPA Probable permease of ABC transporter OS=Vibrio parahaemolyticus serotype O3:K6 (strain RIMD 2210633) OX=223926 GN=VP1338 PE=3 SV=1

MALPNYASKSERMAYAGYLVFCGLVLFFLIAPILTIIPLSFNATPYFTFTEGMLNLDADA

YSVRWYQEMFTNEQWLLALKNSTFIALMATLIATGLGTLAALGLANSNLPFRNAIMALLI

SPMIVPVIISAAAMYFFYTRLGLSQTYFGIILAHAALGTPFVVITVSATLSGFDQSLVKA

AASLGANPVYTFRHVTFPLIRPGMISGGLFAFGTSFDEVVVALFLTGAEQKTVPRQMWSG

IREQISPTILAVATLLIFMSVCLLVTLEILRRRNVRIRGIQE

>tr|Q87LE2|Q87LE2_VIBPA RNA polymerase sigma-54 factor OS=Vibrio parahaemolyticus serotype O3:K6 (strain RIMD 2210633) OX=223926 GN=VP2670 PE=3 SV=1

MKPSLQLKLGQQLAMTPQLQQAIRLLQLSTLDLQQEIQEALDSNPLLEVEEGHEEPQANG

EDKSALETADNSANEPTEIEVPDSSDVIEKSEISSELEIDTTWDDVYSANTGSTGLALDD

DMPVYQGETTESLHDYLMWQLDLTPFSETDRTIAIAIIDAIDDYGYLTLSPEEIHESFDN

EDIELDEVEAVRKRIQQFDPLGVASRNLQECLLLQLATFPEDTPWLAEAKMILADHIDHL

GNRDYKLVIKETKLKEADLREVLKLIQQLDPRPGSRITPDDTEYVIPDVSVFKDHGKWTV

SINPDSIPKLKVNQQYAQLSKGNSADSQYIRSNLQEAKWLIKSLESRNETLLKVARCIVE

HQQDFFEYGEEAMKPMVLNDVALAVDMHESTISRVTTQKFMHTPRGIFELKYFFSSHVST

DNGGECSSTAIRALIKKLVAAENTAKPLSDSKIAALLADQGIQVARRTIAKYRESLGIAP

SSQRKRLL

>tr|Q87HC6|Q87HC6_VIBPA Uncharacterized protein OS=Vibrio parahaemolyticus serotype O3:K6 (strain RIMD 2210633) OX=223926 GN=VPA1039 PE=4 SV=1

MSIKEIGRVFTQRWFLGLVGVAACSIFIWVVGPLITVAGYEPLKSDFQRLVTILVIVFAW

ALINLTKQHKQKVREDESIQTLLEVDSQSDKEAASEIDVMRDRIEQAIKVVTKTHKGKRS

LYDLPWYVLIGPPGTGKTTVLKQSGLEFPLTESLGADSIAGVGGTRHCDWWFANKAVLID

TAGRYTTQDSQEKVDSKAWHGFLGLLKKYRTQRPINGAIVTVSLASVMSQTRTERSLHAR

SIKSRLQELKNQLGMQFPIYVLLTKMDLVAGFNEFFADLSKEEREELFGFMFPREVDDER

GVISLFNKEFHGMLERLDAHMLRILETEDDLEKRTLIFEFPKQLRVLQANLDEFLSEIFA

QNTFEEPALIRGVFLLSSVQEGIPVDRLMSESTNGLGLGRLPLATNVNSSHSYFVKNLFE

RVIFKEQLLGTVNRHYQKQSGWMRTGIYVGCVGVLVGASALWFLSYQWNSKLIVDTNSQV

NHIEAMIGAESLDFESDVISAVDTLDKIMMLPLGKNSKYGHSDAVKKFGLYQGDKVSQAA

NNAYSDALSQHFATLLSESLVSEMEANKQHREYLYETLKTYLMLFNPEKYQQEEVIAWFN

FYFERQYPGELNKELRERLLVHTKNLLENDEKGFSMDATAISAAREVLTQMSLPERAYQR

MKMQFAKSHVPSFRLTDVLGPKGLEQFERASGKPLSQGISGFYTYNGFHSIFQIQINRTV

KGLMEENWVYGDDLKAHEIDHDSAIQGVQARYYQDYVNEWKTLIEDIQLKQAPSLALATE

QSRVLSGVERPIESLLRAIQKEVGLSKVTLSENQKAATEVAGKVAKVKFSNTADKLDMYL

PEENGFNVALPGKEVESHFTEILRLSEQDFDDIQQAMVNLRSYLSDLSSSGNNQKIAYKS

ILDGTVTQDVAASFARAKDLLPKPFNQWLGELSQESVKFAESGSKDHLNQLWMTNVVRPY

QRTIAGRYPFEPNATKEVRLKDFQRFFGYGGTLDSFFQEYLEPFVDTSKSRWRLEKEIGV

RPETLAVFQRAKRIRQSFFESDNSLRVEFGMKPVYLDQHITRFVLELGDQDLVYKHGPAR

SKELRWPSGQDQTRIVFTPPESKREIAHTYEGEWGIFKLLDQSLKARPESRNDNIVMIDL

KGNKVQLELIPSSAINPFWSNEMERFRCPQTL

>tr|Q79YX4|Q79YX4_VIBPA Chemotaxis protein CheW OS=Vibrio parahaemolyticus serotype O3:K6 (strain RIMD 2210633) OX=223926 GN=VP2225 PE=4 SV=1

MSQAFEVEVKKDTSNDEVLQWVTFQLEEETYGINVMQVREVLRYTEIAPVPGAPDYVLGI

INLRGNVVTVIDTRSRFGLMEGEVTDNTRIIVIESERQVIGILVDSVAEVVYLRSSEIDT

TPSVGTDESAKFIQGVSNRDGKLLILVDLNKLLTDDEWDEMAHL

>tr|Q87NG0|Q87NG0_VIBPA Sensor histidine kinase OS=Vibrio parahaemolyticus serotype O3:K6 (strain RIMD 2210633) OX=223926 GN=VP1908 PE=4 SV=1

MPLKAKLILLTLIPVVLVSASISWISIYQAKTLGQREVEIFHQNLIQSKEAALKDTVDVA

FDAISHIYNDSTIEERVAKARVKAILNRLTYGSDGYFFAYDKHGTNLVHPVLPELVGENL

LHLEDENGDRLIEALLYQAQSGGGFHQYLWQKPSTGDIVPKLSYAAWLDKWEWMIGTGLY

IEDVSQEVANMRAAVNKNIETTFFSVVVILVVTVAVIIVLTLAINLHEHRLADKNLKELA

HKTVMFQEDEKKHLARELHDGINQLLVSSKCHLDLMSHRLQDEKLKSHLDKSQRSLVTAI

NEVRHISHQLRPSALDDIGLEAALTTLLQDFHSHSGIDIDSHFDTQQHKLTSEVATTLYR

VAQESLNNIEKHAKAKKVTVILQKMGNMLQLLIRDDGVGFVVNQAVHRQGIGLRNMQERV

EFIGGEFELMSELGLGTEITVLLNLDELVYGQTD
