# Supplementary material for: Comprehensive genome based analysis of Vibrio parahaemolyticus for identifying novel drug and vaccine molecules: Subtractive proteomics and vaccinomics approach
Source: PLoS One. 2020 Aug 19;15(8):e0237181. doi: 10.1371/journal.pone.0237181 (PMC7444560; doi:10.1371/journal.pone.0237181)
Supplement: S14 File — (DOCX) [file pone.0237181.s027.docx]

**S14 File.** Essential membrane proteins using PSORTb, CELLO, ngLOC, PSLpred

>tr|Q87P28|Q87P28_VIBPA Lipoprotein OS=Vibrio parahaemolyticus serotype O3:K6 (strain RIMD 2210633) OX=223926 GN=VP1690 PE=3 SV=1

MMKNKMRTLCLVLALFLVGCQTELYTNVSQKEGNEMLSILLSEGVVATKEPDKDNKVKLM

VDSSQIAFAVDALKRKGYPREQFSTLKEVFPKDDLISSPLAERARLVYAKSQELSSTLSQ

IDGVLVARVHVVLEDQDLRPGERPTPASASVFIKHAADVALDSYVPQIKLLVNNSIEGLN

YDRISVVMVPSSEVRVATQSNQFKSILSVQVTKETANHLIGILVFMVLLLIGSNVATFTW

CRRSAKRG

>tr|Q87HY1|Q87HY1_VIBPA Putative phosphoglycerate transport regulatory protein PgtC OS=Vibrio parahaemolyticus serotype O3:K6 (strain RIMD 2210633) OX=223926 GN=VPA0825 PE=4 SV=1

MVNLMADFKQFGRRLLLGAALYAPLTMAAEKELVILTTFSQAPITALVDDFTQHYPDAEV

RVVHRRTQSSLQLLSKSYMKDIDLVLSSSPFLMQELSNEHRLADMSSRVQVPKWLSPYLL

PNNDQVVAFGYSGAGIVWNKDYLAANHLPEPKRFQDLTNPLYFGHVTMSTPSRSGTTQLM

VESVLSQYGWQEGWRILLNVGANLATISSRSFGVADYIAKGKFGIGPTIDSYALIAQRKF

DYVGFAYDQDFTLMPTYIAQINRGKSDKLAESFIAHLLSKEVQEQMESSTFSKTALDDTA

RYGGENPVLDLEQVMPREALINLIFDTAITKRLPELQDAWLSLIKLNRLADGKAEKQRSL

QAIEKQLFELPLSKAQATEIAQKLLTMDKDSEVGMTHYQALLAEFSHELGRAMSEKLDNV

NQQLAQWRGKEK

>tr|Q87TD7|Q87TD7_VIBPA General secretion pathway protein D OS=Vibrio parahaemolyticus serotype O3:K6 (strain RIMD 2210633) OX=223926 GN=VP0133 PE=3 SV=1

MKHWFSKSAWLLAGSLLCVPGAMANEFSASFKGTDIQEFINIVGRNLEKTIIVDPSVRGK

IDVRSYDVLNEEQYYSFFLNVLEVYGYAVVEMDNGVLKVIKAKDSKTSAIPVMGDGSAKG

DSVITRVVAVRNVSVRELSPLLRQLIDNAGAGNVVHYDPANIILITGRAAVVNRLAEIIK

RVDQAGDKEIELVELRNASAAEMVRIVEALNKTTNQKSTPEFLEPKIVADERTNSILISG

DPKVRARLKRLIRQLDVEMATKGNNRVVYLKYAKAEDLVDVLKGVSDNLQAEKQAGQKGA

SSAQRGDVVIAAHEATNSLVLTAPPDIMMALQDVISQLDIRRAQVLIEALIVEMSEGDGI

NLGVQWGSLETGAVIQYGNAGAPIGQVMVGLEEAKDTVEKKPIRDSDTGAIKYYEETTTK

GDYSTLASALKNVNGAAMSIVMGDWTALVSAVASDSNSNILSSPSITVMDNGEASFIVGE

EVPVITGSTAGSNNDNPFQTVDRKEVGIKLKVVPQINEGDSVQLNIEQEVSNVLGANGAV

DVRFAKRQLNTSVMIQDGQMLVLGGLVDERALESESKVPLLGDIPVLGHLFKSTSTQTQK

RNLMVFIKPTIIRDGMTADGITQRKYNFIRAEQLYKADQGLKLMSDDKIPVMPAFGQDRK

HPAEIQAFIDQMEKN

>tr|Q87GB4|Q87GB4_VIBPA Putative capsular polysaccharide biosynthesis glycosyltransferase OS=Vibrio parahaemolyticus serotype O3:K6 (strain RIMD 2210633) OX=223926 GN=VPA1403 PE=4 SV=1

MKHKGLIRSYEMEFAFLYRLSDLAVIVTFMLLLVLKDTNTSMDKDYVILSFVGGISFLFM

AESGNLYRSWRTSSFREQMFIVCMSWLMTSALLFMVLYFSEVYPLFDRSILALWVTITPA

LLLAWRVTFRTVLAYLRKMGFNTRTAIIIGQTPHGITLANEIQNHTEHGVLFDGFYDERS

SDRLPSSEYPIKGAVNQALERAKRGEVDYVYIAMPMHAKERIASILNQFSDTTANTYLIP

DFFTYNLLHSRWDQIGQVQTLSVFDTPFAGVSSWIKRFEDILCSSIILVLISPILLAIAI

GIKLTSKGPVIFKQHRYGLDGRKIEVWKFRSMTTMDQGPNIKQATKNDPRITPFGSFLRR

TSLDELPQFINVLQGTMSIVGPRPHAVAHNEEYRQIVARYMLRHKVKPGITGWAQINGYR

GETDTLDKMEKRVEFDLDYIHHWSVWMDIKIIFLTVFKGFTGSNAY

>tr|Q87P22|Q87P22_VIBPA Putative type III secretion protein YscC OS=Vibrio parahaemolyticus serotype O3:K6 (strain RIMD 2210633) OX=223926 GN=VP1696 PE=3 SV=1

MVTVMRTLMPKIGRIAAKMTLCALCVAPMFSVQATELNWPEQPFRYYADNDSLKDLLNNF

GANYRVSVSVSDKVNDRVSGRFTPEDPAEFLDYLAQVYNLMWYFDGAVLHVYKATETRSR

LLQLELLTARELRSTLISTGVWDARYGWRAAENKGLVYLAGPPRYVELVVQTAEALESRL

LQKSNSTDELFVELIPLKYASATDRSISYRDQSITVPGIASVLSRVVGGVQTQITDSASV

QTSSVNGLPAEAAKPRGKTASVHGGATVEAEPGLNAIIVRDTQARLPLYRKLVAQLDQPQ

SRIEVALSIVDISANDLRQLGVDWRAGVSVGNNRIVDIKTTGDVDNGDVTLGSGQSFKSL

LDSTNLNYLLAQIRLLESKGSAQVVSRPTLLTQENVEAVLNNSSTFYVKLVGKETAALEE

VTYGTLLRIVPRIVGDRFATRPEINLSLHLEDGAKIPDGGVDDLPSVRKTEISTLATVKQ

GQSLLIGGVYRDEVSHQLRKVPLLGDIPYLGALFRSNTNTTRRTVRMFIIEPRIVVDGIG

DSVLIGNEHDLRPSIGQLNNISNNSAEFKSVVEVFSCTSKTQAERYQQDLLSQQKSSLLT

QCQLPSGQVGWRVKVAECDLSQAECVRPSEEP

>tr|Q87JA2|Q87JA2_VIBPA Putative permease of ABC transporter OS=Vibrio parahaemolyticus serotype O3:K6 (strain RIMD 2210633) OX=223926 GN=VPA0351 PE=3 SV=1

MQDVNSRFHKSVVYTIVGIMLIPILATFIYSISSRWGATILPDGFTFDWYIKLLTDPRFL

QAFGRSLFIGLSALALSVVLILPAIFVVFYYFPKLDKLMNILILLPFAVPPVVSSVGLLQ

LYADSKISLIGTPWILVGTYFTIALPFMYRAISNSFEAINLHDLMDAAHLLGSSTTKAFL

LIILPNLKKGLMASLFLSFSFLLGEFVFANILVGTRYETLQIYLYNMRQTSGHFTSALVM

TYFLFIFLLTWLASRFSRGVK

>tr|Q87Q13|Q87Q13_VIBPA Putative permease of ABC transporter OS=Vibrio parahaemolyticus serotype O3:K6 (strain RIMD 2210633) OX=223926 GN=VP1337 PE=3 SV=1

MSRELTSLDSVLEIERHVNYQQSELRAQLQKAERNKNLRSIMLTLPLVCFILLTFAFPIL

EMLYRSVDNRDIPQAMPKTIQALAHWDYQGLPDSEVVEAFSVELLALYETKALPKIANRM

NIEVSGMRSLMMKTGRKLSRLEVLPTSIKELSRLDKRWADAKHWVAFKNLSGAITVNHYL

AALDMQVNEMGEIEAQPEKRQIYVDLFFKTFWMSILITVICLLMAYPVAYLLANLPDKRA

NLLLIVVLLPFWTSLLVRTTSWIVLLQNQGVINDLLIWSGLTSERIQMIHNTFGTVVSMV

HILLPFMILPLYSVMKGISPTYFRAARSLGATPLVAFVKVYMPLTLPGIGAGALLTFILS

IGFYITPALVGGRSGQMISNMIAYHMQTSLNWGMAGALGGLLLFVVLALFYVFNRVVGIN

NIKVGG

>tr|Q87R85|Q87R85_VIBPA C4-dicarboxylate transport protein OS=Vibrio parahaemolyticus serotype O3:K6 (strain RIMD 2210633) OX=223926 GN=VP0912 PE=4 SV=1

MDILLLFLMVIGFMLIGVPIAISLGLSSVLFLMLHSDASLASVAQTLFNAFAGHYTLLAI

PFFILASSFMSTGGVAKRIIRFAIAIVGWFRGGLAMASVVACMMFAALSGSSPATVVAIG

SIVIAGMIKNGYSKEFAAGVICNAGTLGILIPPSIVMVVYAAATDVSVGRMFLGGVIPGL

LAGVMLMIAIYIAARIKNLPKQPFVGWKETFDAAKDASWGLLLVVIILGGIYGGIFTPTE

AAAVAAVYSFLIANFIYKDMGPFADKQNTKPAIVKVIQTFVHEDTKHTLYEAGKLTIMLL

FIIANALILKHVLTEERIPQMITESMLSAGLGPITFLIVVNLLLLIGGQFMEPSGLLIIV

APLVFPIAIALGIDPIHLGIMMVVNMEIGMITPPVGLNLFVTAGVARMSMMNVVKAALPW

VGVMFLFLIIVTYVPWVSTWLPTTLMGPEIITK

>tr|Q79YZ4|Q79YZ4_VIBPA Sodium-driven polar flagellar protein MotA OS=Vibrio parahaemolyticus serotype O3:K6 (strain RIMD 2210633) OX=223926 GN=VP0689 PE=4 SV=1

MDLATLIGLIGGFAFVIMAMVLGGSIGMFVDVTSILIVVGGSAFVVLMKFTLGQFFGAAK

IAGKAFMFKADEPEDLIAKIVEMADAARKGGFLALEEMEINNSFMQKGIDLLVDGHDADV

VRAALQKDIALTDERHTQGTGVFRAFGDVAPAMGMIGTLVGLVAMLSNMDDPKAIGPAMA

VALLTTLYGAVLSNMLFFPIADKLSLRRDQETLNRRLIMDGVLAIQDGQNPRVIDSYLKN

YLNEGKRALEIDE

>tr|Q87P56|Q87P56_VIBPA Low calcium response protein OS=Vibrio parahaemolyticus serotype O3:K6 (strain RIMD 2210633) OX=223926 GN=VP1662 PE=4 SV=1

MNLMNKLIDILNKVGQRKDIMLAVMLLAIVFMMILPLPTALVDVLIGANMSIAVVLLMLA

IYITTPLEFSAFPAVLLITTLFRLSLSITTTRLILLQGDAGQIVYTFGNFVVGGNLVVGI

VVFLIITIVQFMVITKGSERVAEVSARFSLDAMPGKQMSIDGDMRAGVIDVHEARHRRSL

IEKESQMYGSMDGAMKFVKGDSIAGLVIIIVNILGGVTIGVTQKGMSASEALELFAILTV

GDGLVSQIPALFIAITAGIIVTRVSHEDSADLGSDIGGQVTAQPRALLIGGVLLVLFALI

PGFPKITFLVLALVVGGGGFYLFYQQKKQTESESSDLPSFVAQGAGSPAAKPNKPTPSRG

SKGKLGEQEEFAMTVPLLIDLDSSLQESLEAVALNDELARVRRALYLDLGVPFPGIHLRF

NDGMKNGEYLIQLQEVPVARGRIEKDKLLVTEGSDQIELLGVPFEQDDDFLPGVSSLWVA

QSYQEKLTASHVGFLTPDRILTFHLSHVLKEYAQDFIGIQETRYLLEQMEGSYSELVKEA

QRIVPLQKMTEILQRLVSEDISIRNLRVILEAMVEWGQKEKDVVQLTEYIRSSLKRYICY

KYASGQNMLPAYLLDQSLEDTIRSGIRQTSAGSYLALDPSVTQQFVSDVKQTVGDLSRMP

NKPVLVVSMDVRRYVRKLIESEYYDLPVLSFQELTQQINIQPLGRVGM

>tr|Q79YT9|Q79YT9_VIBPA Anaerobic C4-dicarboxylate transporter OS=Vibrio parahaemolyticus serotype O3:K6 (strain RIMD 2210633) OX=223926 GN=VPA0981 PE=3 SV=1

MLYLEFLFLLVMLYIGSRYGGIGLGVVSGIGLVIEVFIFKMPPTSPPVTVMLIILAVVTC

ASILEAAGGLKYMLQVAERVLRKNPKRVTLIAPFVTYFMTFLLGTGHAVYSIMPIIGDVA

LKNGIRPERPMAAASVASQIAITASPISAAVVYYLAQLSDIQHEITLLSILLVTVPATLF

GTLLMSLYSIKRGKELEDDEEYQERLKDPVWREKILNTTATSLDEVLPTSARNSVLLFIA

SILVIVVIAMWPDIRTIVDGAKPISMAVVIQMMMLCFGGIILLATKTDPRDVPNGVVFKS

GMVAAIAIFGIAWMSDTYFQYAMPQFKSGIVEMVTNYPWTFALALFIVSVVVNSQAATAR

MMLPVGLGLGLDPALLIGLMPAVYGYFFIPNYPSDIATVNFDTSGTTKIGKWYFNHSFMS

VGLIGVIGACCLGYVLGQIIIPS

>tr|Q87FY4|Q87FY4_VIBPA Flagellar M-ring protein OS=Vibrio parahaemolyticus serotype O3:K6 (strain RIMD 2210633) OX=223926 GN=VPA1536 PE=3 SV=1

MSELTPQVAGNTAMTTSTTQAFSPAGNMDDVTNKLKQLWSSSQRNLVLSAVLAAIVAAII

VVALWSSSQSFRPLYSQQERFDIGEIVSVLESEGVSYRMQEQNGQVLVPEGEVARIRMLL

ASKGVKAKLPTGLDSLKEDSSLGTSQFMETARYRHGLEGELVRTIMSLNSVANARVHLAI

PRQTLFVRQNGENPSASVMLELKPGEDLKPEQVEAIINLIVGSVTAMKPEFVSVIDQYGR

LLSADVASAEAGKVNAKYLEYQKNVEKQIIQRAADMLTPIVGPSNFRVQVAADMDFSQVE

ETREILDNAPVVRNEHTIQNNSIDQIALGVPGSLSNQPPVTGEAATNDSQNTNARSEVNR

QYAVGSSVRRTQYQQGQIEKLSVSVLLNSKASPDGVAWSDADKAQISTMITDAVGISAAR

GDSLSLMSFNFTPIDIDAPTALPWWQDPTVQQPLRYVIGGMLGLAMIFFVLRPLIMHLTG

ADKPVPELNFAEPPQEEPDYDNLQTREEREHEEVLNRRLSEKGISASTGLDVNSDMLPPA

GSPLEIQLKHLQLIANEEPERVAEILKQWVNINEHSSVDVKTNA

>tr|Q87P44|Q87P44_VIBPA Translocation protein in type III secretion OS=Vibrio parahaemolyticus serotype O3:K6 (strain RIMD 2210633) OX=223926 GN=VP1674 PE=3 SV=1

MSYDDLHQALFLYSLTLPRLMACFIFLPILSKQMLGGAMIRNGVLCSLALFIFPVVNEQA

LPAETDGLWLIVILGKEVLLGMLIGFVAAIPFWAIEATGFLVDNQRGAAMASMFNPTLGS

QSTPTAVLLTQTLITLFFSGGGFVAFIYALFKSYTTWPILGFFPMVTDAWVSFFYDQFQQ

LMWLGVLMSAPLVLAMFLAEFGLALISRFAPQLNVFFLAMPIKSAIASVLLIVYLGLMMD

HFEALFYGITRFGDQLNTIWK

>tr|Q79YY3|Q79YY3_VIBPA BfdA OS=Vibrio parahaemolyticus serotype O3:K6 (strain RIMD 2210633) OX=223926 GN=VP1393 PE=4 SV=1

MPTPAYMSINGETQGHITKDTYSADSVGNTWQEAHVDEFLVQELDHVLTVPRDPQSGQPT

GQRVHRPLVVTKVQDRSSPLLFNALVSGEKLPECLIRFYRTSVQGKQEHYYSIKLIDALL

VDIQTRMNHCQDAATADRVTEEVLKFTYRAIEVTHENCGTAGNDDWRAPREA

>tr|Q87FM8|Q87FM8_VIBPA Putative methyl-accepting chemotaxis protein OS=Vibrio parahaemolyticus serotype O3:K6 (strain RIMD 2210633) OX=223926 GN=VPA1651 PE=4 SV=1

MFKNLSLKNKLAISASAAIILGGVLVEGLSFRDSLQRLDAEVAQRLESTSASYNQYVSDW

LLSKERALTSLSAESEKRAIVTHLKQVRDSGAFDNVFLAYPDGSQDNANGVILPPGNNDP

RKWGWYTNAIANPSKVFMDNPTVAAATGANVVSLGKALQLHGQTTVLGADVEIGDILNSL

NQVILPGEGYMFIANDQGNIFTHNDSKLLNQPVSKLGLNNNDITNAARSGTERRVSISGT

DYVIYARPIEGTKLTTVTVLDHNSLVAPLYDAVWDQIIATAIVVIICVALFNLLCNILFR

PLYNVSNALSQIANGSGDLTQRIKVENRDEVGELAENFNQFVESLQQLIGHIRHQAEELS

QQSELSTTRANQSVSDLNHQQQEITMVATAVTEMASATQEIAAHAEQTAKAAQDSSASTQ

NGHELVINSKSSINNLSSEVNQASVVIGELNQHAQDISTVLSTIRDIAEQTNLLALNAAI

EAARAGEQGRGFAVVADEVRVLSQRTHTSTEEIRSTIETLQQTTQRAVTIMDKSSQLAQG

SVEDADRAALALDEINAAVALISDMATQIATAAEEQTHVTNEITQNVTSIKDVTDQLVVG

AEESMNQSAELKSQAEDLNSKVATFKLA

>tr|Q87J60|Q87J60_VIBPA Uncharacterized protein OS=Vibrio parahaemolyticus serotype O3:K6 (strain RIMD 2210633) OX=223926 GN=VPA0393 PE=4 SV=1

MRSMLQDSLVLLDYLRGMLLHNEELWLLLFPVMIIIELPLYLLVLTGIFRWSYMREEPEL

KRFPSVSFVITCYGEGEAIGITIDTLVEQIYPGHIEILAVVDGAVQNQDTYKAALNGERR

HTGVRNRKVRVLPKWQRGGRVSTLNAGLSMASGEIVINVDGDTSFDNDMVFTMMKQFADK

NVIASGGALRVRNHNANLLTKMQSLEYMLSMQAGKTGMATWGVLNNISGAFGAFRKNLLK

QVGGWDTHTAEDLDLTMRLKQYKCRYPDNKLAFSTHSIGHTDVPDTLKGLVLQRLRWDGD

LLFLFLRKHNEGLSPRLLGWGNFVFTLAYGVIQNVLLPLLVVIFSVYMVIVYPLKFVLAL

MLMLYFVYLFLSALIFVVYIGLVSERKKEDLKSVKWLFLYPVYQFFMRLITAFSMVNEVV

RRSHEESSMAPWWVLKRGKKF

>tr|Q87LX8|Q87LX8_VIBPA Peptide ABC transporter, permease protein OS=Vibrio parahaemolyticus serotype O3:K6 (strain RIMD 2210633) OX=223926 GN=VP2480 PE=3 SV=1

MGYFLRRLSFYLVALLVAATLNFIIPRAMPGDPVTMMFANASVQVTPERIAAMKELLGFV

DGPIYIQYLSYIKNILSWELGTSIQFYPLSVNSLLGSAFGWSLFLAGTAVVLSFSIASVL

GIFAAWKRGSRYDAFVTPGTLIIQAIPQMVIAMLALFTFSIGLKWFPSGYAYTPGTVPDW

SSWAFIKDVGYHAVLPLFCATIVQIGGFLVNMRNNMINLLAEDYITMAKGKGLSENRVVF

NYAARNALLPSVTALSMSLGMAIGGQLIIEMIFNYPGLGTVLLNAIHARDYQVLQGQLII

MTMFMLCFNLMADMLYMILDPRLRKGGK

>tr|Q87LX7|Q87LX7_VIBPA Peptide ABC transporter, permease protein OS=Vibrio parahaemolyticus serotype O3:K6 (strain RIMD 2210633) OX=223926 GN=VP2481 PE=3 SV=1

MKNLFKLILGNSFARIGLAIITIFIFVAVAAPLITKHAPDKRTGNPHEYPSFVVKQAQSN

PDGWVAKNLADDRRTLIMSKKADHVLGTSRMGRDIWSQVAYGARVSLGVGFGAGIIVCFL

ATVIGISAGYFGGKVDDVLSAAMNIMLVIPQYPLLFVLAAFIGEAGPLTIALIIAGTSWA

WGARVVRSQTMALREKEFVKAAEVLGESPFRIIFVEILPNLIPIVGASFIGSVMLAINTE

AVISFLGLGDANTISWGIMLYNVQTSSAMLIGAWWEVLAPCIALTLLVTGLALLNFAVDE

IANPQLRSHKGMKRWKKLAAKDKKEREPELAPQNALWSGDK

>tr|Q87HJ8|Q87HJ8_VIBPA Putative sensor histidine protein kinase UhpB OS=Vibrio parahaemolyticus serotype O3:K6 (strain RIMD 2210633) OX=223926 GN=VPA0965 PE=4 SV=1

MRAYSVTTICGLFVMACAWFCLWVIAYYFVNDPELAILLFPFALRLGIALHTRTAYWPTI

YVSEWALTIALATLLEQPQWLTVLIASVASIPVTLIAKKYYYGDQNRHLAVMGIVIIITA

FINVMAVGFHVPSVYMVWLASISGGLMLVPMCYLLWNYLFQSRWSPLTSHLLNNTVVFSI

RHIVFYAVLLIGSILVQTSLPEELKRFAPFCMAIPIIVLALRYGWQGALLATMLNSIALI

AARSGVSNLEITDLLLSLSAQTITGIMLGLAVQKQKDLNHKLRGELSRNQNLSRQLIEAE

ESVRRDIARELHDEIGQNITAIRTQANIIKRIDNAEMSAHCADTIEGLSLNVYDTTKRLL

SKLRPKMLDDLDLKESVEQLTREMEFANHGTTVQLNWQGDYTSLSDTLKVTLFRLCQESL

NNAAKYAEAQLINIELTIGEAAVSLMIHDDGVGFKVQDSMKGMGVRGMQERVHALGGKMV

IYSTSDQVIGTQISITLPKV

>tr|Q87GB2|Q87GB2_VIBPA Putative polysaccharide export-related protein OS=Vibrio parahaemolyticus serotype O3:K6 (strain RIMD 2210633) OX=223926 GN=VPA1405 PE=4 SV=1

MNPLFKLIGLALLLFSTFVSANSNEQDYLLDTGDTISVQVYGEEDLSIKNILITSDGYFD

YPYLGRIKAINKTPKQLKYEIETGLKGDYLINPKVMVTINYFRLFYVNGEVRKPGGFEYR

PGLTIEKAIALAGGLTDRASRKSINLTKHKTGKTVEGVSMQRTVEPGDIVFIDQSFF

>tr|Q87JH9|Q87JH9_VIBPA Putative flagellar hook-associated protein OS=Vibrio parahaemolyticus serotype O3:K6 (strain RIMD 2210633) OX=223926 GN=VPA0274 PE=1 SV=1

MRISDNQFSQMMLQSLQSNSAGLGKVLQQMSTRERLTKLSDDPMASIKLLNLERENSAIA

QYQSNIANLKTTLSSQETHLDSVNESLKSMRDIVLWGANGSLTDQDRSGMITELKSYRDS

IESSFNAQDEEGHFLFSGTKTDTAALNKSSGAYVVEGNSDVRVVTVAKGVTMDSNMTAQE

ILDIGGGKNVLNQIDALIAEFEKPSPNFQAEVDASLNAIDDTMANVLGAMTEIGGRHNNL

DLMDGAHSENKLFVDKVSGDLSALDYGEASVRLSNYMAALQATQASYVKINDLSLFDRI

>tr|Q87GY3|Q87GY3_VIBPA Methyl-accepting chemotaxis protein OS=Vibrio parahaemolyticus serotype O3:K6 (strain RIMD 2210633) OX=223926 GN=VPA1182 PE=4 SV=1

MLANFSQKAQETLVGELEELVSTTNLKGVITYCNDAFCRVAEYTHEELVGQNHNIVRHSD

MPKAAFGDMWARLKEGKAWRGMVKNSTKSGGYYWVDAYVTPIYEKNQVVGYQSVRVKPKR

EWVDIAAKAYKGMLAAEKAGRTWSLKINETVRYAILLGALTAPAVAYALSVEGPLAWLAS

ALPASVLALLFRQELIDTPQQLKKLQKQYDSVSRLIYSGNSAFSIADFHIKMLSARIRTV

LGRMTDSALPLQNCAEELSQTTSEVSAALNQQNSDIRRVRDATQEVESSANSVSSSTNDA

HMLIDDTLKSCMMAKETIDQTHTNLAQLSLQAEKATETTYQLSDQAQKVNHLMVEIGGIA

EQTNLLALNAAIEAARAGEQGRGFAVVADEVRALSGRTSNATEQIQASISAMLSTIEGWQ

KDILANKEQTDACSQVAEQSALRLSEVEQMMQSMSGLMVDVAEAANNQLKLSSDVNQHIH

SIASTAEQNLAATHSVEQNSRQLKEQVQDFYQLAIRFEDKQS

>tr|Q87Q18|Q87Q18_VIBPA Probable binding protein component of ABC transporter OS=Vibrio parahaemolyticus serotype O3:K6 (strain RIMD 2210633) OX=223926 GN=VP1332 PE=4 SV=1

MSRMTKTPLVMLISGMLLGTSAYAEDKLTVVSWGGAFTKSQVEAYHKPFIQKTGVEIVSE

DFSGGLAEIKAQVEANNVRWDLVSLDKPDIVRGCAEGLLEPVNPSILPPGADGTPAKEDF

IDGAIHECAINTIVVSTVLAVNEDAFKGKTAPTKLTDLFDLTNFPGRRALQKQPQGNLEW

ALLADGVKPDEVYRLLETEEGRARAFAKLDTIKPQVLWWTTGAQPPQMLADKEVVIASAF

NGRIHNARKDEGQPFRIIWDHQMGYMNGWAIPKGSANTKLALDFIAFSSGTKPLADQAKY

VAYGPTRKSSSAEVSPEILANLPTAPQNFKTAFLINDEWWSDYADELNEEFNTWLLN

>tr|Q87FX8|Q87FX8_VIBPA Flagellar biosynthetic protein FliP OS=Vibrio parahaemolyticus serotype O3:K6 (strain RIMD 2210633) OX=223926 GN=fliP PE=3 SV=1

MNNLSAWHRWLPLLVLVSLLFAFPTMAADNGLTILSVTDGDAQQEYSVKLQILLLMTALS

FLPAFILMATSFTRIIVVLAILRQALGLQQSPPNRVLVGIALTLTLLIMRPVWTDIYENA

FQPYDNGEITLVQAFSVAEKPVRNFMLAQTHQSSLEQMLRIANEPLDQKVEDISFAVVLP

AFVISELKTAFQIGFMLFIPFLIIDLVVASVLMAMGMMMLSPLIVSLPFKLVVFVLVDGW

AMTVGTLSASFG

>tr|Q87IQ2|Q87IQ2_VIBPA Methyl-accepting chemotaxis protein OS=Vibrio parahaemolyticus serotype O3:K6 (strain RIMD 2210633) OX=223926 GN=VPA0554 PE=4 SV=1

MFGLRTRNQEAESEYRRFIKGLSDSMAMIEFDTRGIILNANDLFLSCVGYTREAIVGKHH

SIFCDRGYVQSPRYQQFWDDLKMGKHKRGTFERLTSQRERLVLEASYFPIESEHGQIEKV

VKIASDVTQQRLESEQKEAILNALDLSLATIEFDREGYILTANQNFLKTLGYELSDVQGK

HHKLFCFDDFYQENPGFWKDLAAGQFKSGQFLRRSASGDKVYIEATYNPIFDPAGNVIKV

VKFASDITDKVQRDLNISQAIADSSFIARNASEEATSNVCGAEHSLNEFRTMIEEVLRAV

TACDDKVQELFKTSQQVTEIVKVIDSIASQTNLLALNAAIEAARAGEHGRGFAVVADEVR

TLATRTSTSIDEINHIVLSNQSLTTETRSFIEVINQGFLSSMSKLLEVDEFMKSIDEGSH

LTVESISALRAIVNQDAHLHLNSASNG
