# Supplementary material for: Comprehensive genome based analysis of Vibrio parahaemolyticus for identifying novel drug and vaccine molecules: Subtractive proteomics and vaccinomics approach
Source: PLoS One. 2020 Aug 19;15(8):e0237181. doi: 10.1371/journal.pone.0237181 (PMC7444560; doi:10.1371/journal.pone.0237181)
Supplement: S15 File — (DOCX) [file pone.0237181.s028.docx]

**S15 File.** Predicted MHC-I epitopes for VIBPA Putative sensor histidine protein kinase UhpB

| Epitope | Topology | No. HLA cell | Vaxijen | Start | End | Length |
| --- | --- | --- | --- | --- | --- | --- |
| CYLLWNYLF | O | 81 | 2.8253 | 152 | 160 | 9 |
| VQLNWQGDY | O | 81 | 0.8385 | 393 | 401 | 9 |
| IVLALRYGW | O | 81 | 1.0261 | 217 | 225 | 9 |
| SEWALTIAL | O | 81 | 1.3134 | 63 | 71 | 9 |
| GEAAVSLMI | O | 81 | 0.8176 | 439 | 447 | 9 |
| ATLLEQPQW | O | 81 | 0.0490 | 72 | 80 | 9 |
| YWPTIYVSEW | O | 27 | 0.6126 | 56 | 65 | 10 |
| VPMCYLLWNY | O | 27 | 1.7417 | 149 | 158 | 10 |
| VLIASVASI | O | 81 | 0.0383 | 83 | 91 | 9 |
| NVMAVGFHV | O | 81 | 1.3035 | 123 | 131 | 9 |
| AEAQLINIEL | O | 27 | 1.4738 | 427 | 436 | 10 |
| APFCMAIPII | O | 27 | 1.1771 | 208 | 217 | 10 |
| FPFALRLGI | O | 81 | 1.7615 | 40 | 48 | 9 |
| WPTIYVSEW | O | 54 | 0.3033 | 57 | 65 | 9 |
| MAIPIIVLA | O | 81 | 1.5287 | 212 | 220 | 9 |
| HLLNNTVVF | O | 81 | -0.5514 | 170 | 178 | 9 |
| FHVPSVYMVW | O | 27 | 0.7891 | 129 | 138 | 10 |
| MLVPMCYLLW | O | 27 | 1.7874 | 147 | 156 | 10 |
| LEITDLLLSL | O | 27 | 0.6858 | 249 | 258 | 10 |
| LLATMLNSI | O | 81 | 0.1927 | 229 | 237 | 9 |
| ILLFPFALR | O | 81 | 2.9721 | 37 | 45 | 9 |
| YLFQSRWSPL | O | 27 | 0.7936 | 158 | 167 | 9 |
| RWSPLTSHLL | O | 27 | 0.3459 | 163 | 172 | 10 |
| IMLGLAVQK | O | 81 | 0.4849 | 266 | 274 | 9 |
| YYFVNDPEL | O | 81 | 1.1887 | 27 | 35 | 9 |
| GLMLVPMCYL | O | 27 | 0.8005 | 145 | 154 | 10 |
| TTICGLFVM | O | 81 | 0.4833 | 7 | 15 | 9 |
| YTSLSDTLK | O | 81 | 0.2997 | 401 | 409 | 9 |
| RFAPFCMAI | O | 81 | 1.1732 | 206 | 214 | 9 |
| SVASIPVTL | O | 81 | 0.8230 | 87 | 95 | 9 |
| LAVMGIVII | O | 81 | 0.5408 | 109 | 117 | 9 |
| LIASVASIPV | O | 27 | 0.2107 | 84 | 93 | 10 |
| FCLWVIAYYF | O | 27 | 2.8883 | 20 | 29 | 10 |
| SISGGLMLV | O | 81 | 0.5847 | 141 | 149 | 9 |
| RPKMLDDLDL | O | 27 | 0.9636 | 364 | 373 | 10 |
| MLVPMCYLL | O | 54 | 1.4830 | 147 | 155 | 9 |
| VLLIGSILV | O | 81 | -0.2848 | 188 | 196 | 9 |
| IVIIITAFI | O | 81 | 0.5971 | 114 | 122 | 9 |
| QPQWLTVLI | O | 81 | 0.4192 | 77 | 85 | 9 |
| VASIPVTLI | O | 54 | 0.9605 | 88 | 96 | 9 |
| APFCMAIPI | O | 54 | 1.2866 | 208 | 216 | 9 |
| EELKRFAPF | O | 81 | -0.4807 | 202 | 210 | 9 |
| LSKLRPKML | O | 81 | 0.9679 | 360 | 368 | 9 |
| LFVMACAWF | O | 54 | 1.6293 | 12 | 20 | 9 |
| QLIEAEESV | O | 81 | -0.0070 | 295 | 303 | 9 |
| YAVLLIGSI | O | 81 | -0.3968 | 186 | 194 | 9 |
| WLTVLIASV | O | 81 | 0.1077 | 80 | 88 | 9 |
| IPIIVLALRY | O | 27 | 1.6493 | 214 | 223 | 10 |
| WLASISGGL | O | 81 | 0.4494 | 138 | 146 | 9 |
| MAVGFHVPS | O | 81 | 0.9142 | 125 | 133 | 9 |
| ITAFINVMA | O | 54 | 0.1558 | 118 | 126 | 9 |
| IVFYAVLLI | O | 81 | 1.1485 | 183 | 191 | 9 |
| AVGFHVPSVY | O | 27 | 0.6616 | 126 | 135 | 10 |
| DTIEGLSLN | O | 81 | 0.9174 | 343 | 351 | 9 |
| SVTTICGLF | O | 81 | 0.0526 | 5 | 13 | 9 |
| ELAILLFPF | O | 81 | 2.7265 | 34 | 42 | 9 |
| VGFHVPSVY | O | 54 | 0.4827 | 127 | 135 | 9 |
| KMLDDLDLK | O | 81 | 1.7845 | 366 | 374 | 9 |
| SLMIHDDGV | O | 81 | 0.3508 | 444 | 452 | 9 |
| YLLWNYLFQS | O | 27 | 1.8146 | 153 | 162 | 10 |
| LSDTLKVTLF | O | 27 | 0.4139 | 404 | 413 | 10 |
| LLFPFALRL | O | 54 | 2.7071 | 38 | 46 | 9 |
| WFCLWVIAY | O | 54 | 2.5591 | 19 | 27 | 9 |
| FCMAIPIIV | O | 81 | 1.8760 | 210 | 218 | 9 |
| GLFVMACAW | O | 54 | 0.7724 | 11 | 19 | 9 |
| AVMGIVIII | O | 54 | 0.8100 | 110 | 118 | 9 |
| MLNSIALIAA | O | 27 | -0.1097 | 233 | 242 | 10 |
| EITDLLLSL | O | 54 | 0.2451 | 250 | 258 | 9 |
| AIPIIVLALR | O | 27 | 1.4030 | 213 | 222 | 10 |
| AFINVMAVGF | O | 27 | 0.6245 | 120 | 129 | 10 |
| LLWNYLFQS | O | 27 | 1.8429 | 154 | 162 | 9 |
| FALRLGIAL | O | 81 | 1.7690 | 42 | 50 | 9 |
| LFQSRWSPL | O | 54 | 0.7190 | 159 | 167 | 9 |
| MIHDDGVGFK | O | 27 | 1.3116 | 446 | 455 | 10 |
| IPIIVLALR | O | 27 | 1.5995 | 214 | 222 | 9 |
| MLNSIALIA | O | 54 | -0.1865 | 233 | 241 | 9 |
| LAILLFPFA | O | 54 | 2.6785 | 35 | 43 | 9 |
| VFSIRHIVF | O | 27 | 0.3844 | 177 | 185 | 9 |
| QTSLPEELK | O | 81 | -0.0512 | 197 | 205 | 9 |
| FVNDPELAI | O | 81 | 0.6265 | 29 | 37 | 9 |
| WQGALLATM | O | 81 | -0.3398 | 225 | 233 | 9 |
| QLINIELTI | O | 81 | 1.4019 | 430 | 438 | 9 |
| LMIHDDGVGF | O | 27 | 0.6405 | 445 | 454 | 9 |
| LVPMCYLLW | O | 54 | 1.7983 | 148 | 156 | 9 |
| YVSEWALTI | O | 54 | 1.3191 | 61 | 69 | 9 |
| HVPSVYMVW | O | 54 | 0.7725 | 130 | 138 | 9 |
| MIHDDGVGF | O | 27 | 0.4518 | 446 | 454 | 9 |
| VPSVYMVWL | OI | 54 | 1.1784 | 131 | 139 | 9 |
| FINVMAVGF | O | 54 | 0.7884 | 121 | 129 | 9 |
| TIEGLSLNVY | O | 27 | 1.7172 | 344 | 353 | 10 |
| PMCYLLWNY | O | 54 | 1.6402 | 150 | 158 | 9 |
| LEQPQWLTVL | O | 27 | 0.1305 | 75 | 84 | 10 |
| MGIVIIITAF | O | 27 | 0.4172 | 112 | 121 | 10 |
| IGSILVQTSL | O | 27 | -0.1941 | 191 | 200 | 10 |
| GLMLVPMCY | O | 54 | 0.5115 | 145 | 154 | 9 |
| LEQPQWLTV | O | 54 | 0.1735 | 75 | 83 | 9 |
| IASVASIPV | O | 27 | 0.3140 | 85 | 93 | 9 |
| TMLNSIALI | O | 27 | -0.0248 | 232 | 240 | 9 |
| RWSPLTSHL | O | 54 | 0.3909 | 163 | 171 | 9 |
| FQSRWSPLT | O | 54 | 0.6458 | 160 | 168 | 9 |
| IYVSEWALT | O | 54 | 1.3277 | 60 | 68 | 9 |
| IEGLSLNVY | O | 54 | 2.0168 | 345 | 353 | 9 |
| IELTIGEAAV | O | 27 | 1.0483 | 434 | 443 | 10 |
| LMLVPMCYL | O | 27 | 1.6507 | 146 | 154 | 9 |
| AILLFPFAL | O | 27 | 2.9930 | 36 | 44 | 9 |
| LTIALATLL | O | 81 | 0.6386 | 67 | 75 | 9 |
| LWVIAYYFV | O | 54 | 2.4774 | 22 | 30 | 9 |
| ITDLLLSLS | O | 54 | 0.0769 | 251 | 259 | 9 |
| VPMCYLLWN | O | 27 | 1.4956 | 149 | 157 | 9 |
| DPELAILLF | O | 81 | 1.4367 | 32 | 40 | 9 |
| HALGGKMVI | O | 54 | 1.0690 | 473 | 481 | 9 |
| AVGFHVPSV | O | 27 | 0.7566 | 126 | 134 | 9 |
| WALTIALATL | O | 27 | 1.2847 | 65 | 74 | 10 |
| ALTIALATL | O | 27 | 0.7795 | 66 | 74 | 9 |
| LLLSLSAQTI | O | 27 | 0.6246 | 254 | 263 | 9 |
| LTSHLLNNTV | O | 27 | -0.1394 | 167 | 176 | 10 |
| TLLEQPQWL | O | 54 | -0.1226 | 73 | 81 | 9 |
| DYTSLSDTL | O | 54 | 0.5366 | 400 | 408 | 9 |
| AYWPTIYVS | O | 54 | 0.6082 | 55 | 63 | 9 |
| TSHLLNNTV | O | 54 | -0.4706 | 168 | 176 | 9 |
| TITGIMLGL | O | 81 | 0.3746 | 262 | 270 | 9 |
| SLSDTLKVTL | O | 27 | 0.7380 | 403 | 412 | 10 |
| ITGIMLGLAV | O | 27 | 0.4605 | 263 | 272 | 10 |
| LMIHDDGVG | O | 27 | 1.3945 | 445 | 453 | 9 |
| LASISGGLM | O | 54 | 0.1433 | 139 | 147 | 9 |
| HLAVMGIVI | O | 54 | 0.8933 | 108 | 116 | 9 |
| LSDTLKVTL | O | 27 | 0.6072 | 404 | 412 | 9 |
| ASIPVTLIA | O | 27 | 0.6624 | 89 | 97 | 9 |
| TLFRLCQESL | O | 27 | -0.2939 | 411 | 420 | 10 |
| GIVIIITAF | O | 27 | 0.2373 | 113 | 121 | 9 |
| LLIGSILVQ | O | 54 | 0.1609 | 189 | 197 | 9 |
| GSILVQTSL | O | 54 | -0.2162 | 192 | 200 | 9 |
| PIIVLALRY | O | 54 | 1.3946 | 215 | 223 | 9 |
| LLSLSAQTI | O | 54 | 0.5025 | 255 | 263 | 9 |
| IELTIGEAA | O | 54 | 1.1396 | 434 | 442 | 9 |
| SVYMVWLAS | O | 54 | 0.6124 | 133 | 141 | 9 |
| DLNHKLRGEL | O | 27 | 0.8028 | 277 | 286 | 9 |
| PFCMAIPII | O | 27 | 1.4096 | 209 | 717 | 9 |
| EAQLINIEL | O | 54 | 1.5331 | 428 | 436 | 9 |
| VTTICGLFV | O | 27 | 0.4148 | 6 | 14 | 9 |
| CADTIEGLSL | O | 27 | 0.7634 | 341 | 350 | 10 |
| ASISGGLML | O | 27 | 0.5369 | 140 | 148 | 9 |
| MVWLASISG | O | 81 | 0.7765 | 136 | 144 | 9 |
| HIVFYAVLL | O | 27 | 1.1385 | 182 | 190 | 9 |
| CMAIPIIVL | O | 27 | 1.6532 | 211 | 219 | 9 |
| AIPIIVLAL | O | 27 | 1.7071 | 213 | 221 | 9 |
| ELTIGEAAV | O | 54 | 0.8775 | 435 | 443 | 9 |
| IALATLLEQ | O | 81 | -0.0544 | 69 | 77 | 9 |
| FHVPSVYMV | O | 54 | 0.5432 | 129 | 137 | 9 |
| SLPEELKRF | O | 54 | -1.0159 | 199 | 207 | 9 |
| YWPTIYVSE | O | 27 | 0.4125 | 56 | 64 | 9 |
| SDTLKVTLF | O | 27 | 0.9416 | 405 | 413 | 9 |
| WSPLTSHLL | O | 54 | 0.0097 | 164 | 172 | 9 |
| AVLLIGSIL | O | 27 | -0.5133 | 187 | 195 | 9 |
| MCYLLWNYL | O | 27 | 2.1120 | 151 | 159 | 9 |
| VQTSLPEEL | O | 54 | 0.2750 | 196 | 204 | 9 |
| NWQGDYTSL | O | 81 | 0.0365 | 396 | 404 | 9 |
| TSLSDTLKV | O | 54 | 0.5292 | 402 | 410 | 9 |
| NLEITDLLL | O | 81 | 1.3384 | 248 | 256 | 9 |
| ALRLGIALH | O | 54 | 1.4014 | 43 | 51 | 9 |
| SLSDTLKVT | O | 27 | 0.6293 | 403 | 411 | 9 |
| EWALTIALA | O | 27 | 1.4257 | 64 | 72 | 9 |
| VSNLEITDL | O | 81 | 1.1284 | 246 | 254 | 9 |
| LFRLCQESL | O | 54 | 0.1350 | 412 | 420 | 9 |
| VMAVGFHVP | O | 27 | 1.0122 | 124 | 132 | 9 |
| LTIGEAAVSL | O | 27 | 0.5333 | 436 | 445 | 10 |
| TIYVSEWAL | O | 54 | 0.7877 | 59 | 67 | 9 |
| LEITDLLLS | O | 27 | 0.6633 | 249 | 257 | 9 |
| LLNNTVVFS | O | 27 | -0.4603 | 171 | 179 | 9 |
| SPLTSHLLN | O | 54 | -0.3292 | 165 | 173 | 9 |
| TLKVTLFRL | O | 54 | 1.3728 | 407 | 415 | 9 |
| AWFCLWVIA | O | 27 | 2.0547 | 18 | 26 | 9 |
| VFYAVLLIG | O | 54 | 0.2749 | 184 | 192 | 9 |
| TIGEAAVSLM | O | 27 | 0.4502 | 437 | 446 | 10 |
| LSLNVYDTT | O | 54 | 1.1201 | 348 | 356 | 9 |
| EQPQWLTVL | O | 27 | 0.0185 | 76 | 84 | 9 |
| LLLSLSAQT | O | 54 | 0.7420 | 254 | 262 | 9 |
| ITGIMLGLA | O | 27 | 0.3249 | 263 | 271 | 9 |
| LLSKLRPKM | O | 27 | 0.6729 | 359 | 367 | 9 |
| YMVWLASIS | O | 27 | 0.8788 | 135 | 143 | 9 |
| PTIYVSEWA | O | 27 | 0.3669 | 58 | 66 | 9 |
| LDLKESVEQL | O | 27 | 0.3742 | 371 | 380 | 10 |
| LPEELKRFAP | O | 27 | -0.5540 | 200 | 209 | 10 |
| LPEELKRFA | O | 27 | -1.1408 | 200 | 208 | 9 |
| ASVASIPVT | O | 27 | 0.6383 | 86 | 94 | 9 |
| ELKRFAPFCM | O | 27 | 0.1343 | 203 | 212 | 10 |
| LTVLIASVA | O | 27 | 0.0546 | 81 | 89 | 9 |
| IAYYFVNDP | O | 81 | 0.9615 | 25 | 33 | 9 |
| EAAVSLMIH | O | 54 | 0.7132 | 440 | 448 | 9 |
| TGIMLGLAV | O | 54 | 0.3033 | 264 | 272 | 9 |
| YLFQSRWSP | O | 27 | 1.0073 | 158 | 166 | 9 |
| TICGLFVMA | O | 54 | 0.3672 | 8 | 16 | 9 |
| ADTIEGLSL | O | 27 | 0.5363 | 342 | 350 | 9 |
| YFVNDPELA | O | 27 | 0.7786 | 28 | 36 | 9 |
| LNHKLRGEL | O | 54 | 0.8137 | 278 | 286 | 9 |
| PELAILLFP | O | 27 | 1.6885 | 33 | 41 | 9 |
| PSVYMVWLA | O | 27 | 0.7982 | 132 | 140 | 9 |
| MLDDLDLKES | O | 27 | 1.4024 | 367 | 376 | 10 |
| ILVQTSLPE | O | 81 | -0.0886 | 194 | 202 | 9 |
| IGEAAVSLM | O | 27 | 0.1237 | 438 | 446 | 9 |
| MLDDLDLKE | O | 27 | 1.4822 | 367 | 375 | 9 |
| SGGLMLVPM | O | 81 | 0.5008 | 143 | 151 | 9 |
| YLLWNYLFQ | O | 27 | 2.3399 | 153 | 161 | 9 |
| FAPFCMAIP | O | 27 | 1.4985 | 207 | 215 | 9 |
| YGWQGALLA | O | 54 | -0.0920 | 223 | 231 | 9 |
| ELKRFAPFC | O | 27 | -0.2794 | 203 | 211 | 9 |
| GLSLNVYDT | O | 54 | 1.6271 | 347 | 355 | 9 |
| ALATLLEQP | O | 54 | -0.3181 | 70 | 78 | 9 |
| LATLLEQPQ | O | 27 | -0.6084 | 71 | 79 | 9 |
| WQGDYTSLS | O | 54 | -0.0380 | 397 | 405 | 9 |
| TIALATLLE | O | 27 | 0.0794 | 68 | 76 | 9 |
| TIEGLSLNV | O | 27 | 1.8107 | 344 | 352 | 9 |
| LTIGEAAVS | O | 27 | 0.6064 | 436 | 444 | 9 |
| TIGEAAVSL | O | 27 | 0.6235 | 437 | 445 | 9 |
| LKRFAPFCM | O | 27 | 0.0950 | 204 | 212 | 9 |
| SGVSNLEITD | O | 27 | 1.2180 | 244 | 253 | 10 |
| ISGGLMLVP | O | 27 | 0.2984 | 142 | 150 | 9 |
| LTSHLLNNT | O | 54 | -0.2528 | 167 | 175 | 9 |
| HDDGVGFKV | O | 81 | 2.3630 | 448 | 456 | 9 |
| LIASVASIP | O | 27 | -0.2060 | 84 | 92 | 9 |
| TDLLLSLSA | O | 54 | 0.0626 | 252 | 260 | 9 |
| DLKESVEQL | O | 54 | 0.0631 | 372 | 380 | 9 |
| ALLATMLNS | O | 54 | 0.1670 | 228 | 236 | 9 |
| FYAVLLIGS | O | 27 | 0.0261 | 185 | 193 | 9 |
| SNLEITDLL | O | 27 | 0.6924 | 247 | 255 | 9 |
| PEELKRFAP | O | 27 | -0.3292 | 201 | 209 | 9 |
| GFHVPSVYM | O | 27 | 0.6430 | 128 | 136 | 9 |
| ELHDEIGQN | O | 27 | 1.5237 | 310 | 318 | 9 |
| IHDDGVGFK | O | 27 | 1.6985 | 447 | 455 | 9 |
| LSRQLIEAE | O | 54 | -0.0326 | 292 | 300 | 9 |
| VHALGGKMV | O | 27 | 1.0232 | 472 | 480 | 9 |
| GIMLGLAVQ | O | 27 | 0.5942 | 265 | 273 | 9 |
| LFPFALRLG | O | 27 | 1.8643 | 39 | 47 | 9 |
| AYYFVNDPE | O | 27 | 0.4297 | 26 | 34 | 9 |
| AFINVMAVG | O | 27 | 0.3737 | 120 | 128 | 9 |
| LIGSILVQT | O | 54 | -0.0344 | 190 | 198 | 9 |
| SILVQTSLP | O | 27 | 0.0435 | 193 | 201 | 9 |
| VWLASISGG | O | 27 | 1.0125 | 137 | 145 | 9 |
| NIELTIGEA | O | 54 | 1.3163 | 433 | 441 | 9 |
| PQWLTVLIAS | O | 27 | 0.1039 | 78 | 87 | 10 |
| LKVTLFRLC | O | 54 | 1.0548 | 408 | 416 | 9 |
| LNSIALIAA | O | 27 | -0.0610 | 234 | 242 | 9 |
| DLLLSLSAQ | O | 27 | 0.5177 | 253 | 261 | 9 |
| LLEQPQWLT | O | 27 | -0.2186 | 74 | 82 | 9 |
| ICGLFVMACA | O | 27 | 0.6846 | 9 | 18 | 10 |
| LINIELTIGE | O | 27 | 1.2717 | 431 | 440 | 10 |
| GALLATMLN | O | 27 | 0.1225 | 227 | 235 | 9 |
| CGLFVMACA | O | 27 | 0.4226 | 10 | 18 | 9 |
| QWLTVLIAS | O | 27 | -0.2529 | 79 | 87 | 9 |
| LINIELTIG | O | 27 | 1.4709 | 431 | 439 | 9 |
| AVSLMIHDDG | O | 27 | 0.2154 | 442 | 451 | 10 |
| VSLMIHDDG | O | 27 | 0.0417 | 443 | 451 | 9 |
| LVQTSLPEE | O | 27 | -0.0637 | 195 | 203 | 9 |
| NDPELAILL | O | 54 | 0.5514 | 31 | 39 | 9 |
| QGALLATML | O | 27 | 0.1102 | 226 | 234 | 9 |
| RPKMLDDLD | O | 54 | 0.4559 | 364 | 372 | 9 |
| ICGLFVMAC | O | 27 | 0.7732 | 9 | 17 | 9 |
| LNWQGDYTS | O | 27 | 0.1446 | 395 | 403 | 9 |
| VNDPELAIL | O | 27 | 0.6485 | 30 | 38 | 9 |
| GWQGALLAT | O | 27 | -0.0996 | 224 | 232 | 9 |
| SRWSPLTSH | O | 27 | 0.2338 | 162 | 170 | 9 |
| DDLDLKESV | O | 27 | 1.1985 | 369 | 377 | 9 |
| IIVLALRYG | O | 27 | 0.7362 | 216 | 224 | 9 |
| GTQISITLP | O | 27 | 1.6324 | 490 | 498 | 9 |
| INIELTIGE | O | 27 | 1.6838 | 432 | 440 | 9 |
| TVQLNWQGD | O | 27 | 1.0641 | 392 | 400 | 9 |
| EGLSLNVYD | O | 27 | 1.6363 | 346 | 354 | 9 |
| PQWLTVLIA | O | 27 | 0.2297 | 78 | 86 | 9 |
| INVMAVGFH | O | 27 | 1.3246 | 122 | 130 | 9 |
| LRPKMLDDL | O | 27 | 1.1485 | 363 | 371 | 9 |
| AVSLMIHDD | O | 27 | 0.2161 | 442 | 450 | 9 |
| LRLGIALHT | O | 27 | 1.3761 | 44 | 52 | 9 |
| IGSILVQTS | O | 27 | -0.2531 | 191 | 199 | 9 |
| SHLLNNTVV | O | 27 | -0.1867 | 169 | 177 | 9 |
| PFALRLGIA | O | 27 | 1.4408 | 41 | 49 | 9 |
| LDLKESVEQ | O | 54 | 0.7715 | 371 | 379 | 9 |
| PLTSHLLNN | O | 27 | -0.3966 | 166 | 174 | 9 |
| PKMLDDLDL | O | 27 | 1.1031 | 365 | 373 | 9 |
| GDYTSLSDT | O | 54 | 0.7869 | 399 | 407 | 9 |
| GGLMLVPMC | O | 27 | 0.3617 | 144 | 152 | 9 |
| SGVSNLEIT | O | 27 | 1.3632 | 244 | 252 | 9 |
| DLDLKESVE | O | 27 | 1.4403 | 370 | 378 | 9 |
| QGDYTSLSD | O | 27 | 0.8248 | 398 | 406 | 9 |
| LGGKMVIYS | O | 27 | 0.1188 | 475 | 483 | 9 |
| GVGFKVQDS | O | 54 | 1.8551 | 451 | 459 | 9 |
| DGVGFKVQD | O | 54 | 2.1287 | 450 | 458 | 9 |
| LDDLDLKES | O | 27 | 1.7071 | 368 | 376 | 9 |
| DDGVGFKVQ | O | 27 | 2.3250 | 449 | 457 | 9 |

**Supplementary Table:** Predicted MHC-II Epitopes for VIBPA putative sensor histidine protein kinase UhpB

| Epitope | topology | Start | End | No of HLA cells | Vaxigen Score |
| --- | --- | --- | --- | --- | --- |
| VIAYYFVNDPELAIL | Outside | 24 | 38 | 26 | 0.9321 |
| IAYYFVNDPELAILL | Outside | 25 | 39 | 27 | 0.8769 |
| YYFVNDPELAILLFP | Outside | 27 | 41 | 27 | 1.1935 |
| YFVNDPELAILLFPF | Outside | 28 | 42 | 27 | 1.5726 |
| FVNDPELAILLFPFA | Outside | 29 | 43 | 28 | 1.3690 |
| AWFCLWVIAYYFVND | Outside | 18 | 32 | 27 | 1.7277 |
| KRFAPFCMAIPIIVL | Outside | 205 | 219 | 27 | 1.0644 |
| RFAPFCMAIPIIVLA | Outside | 206 | 220 | 27 | 1.3183 |
| RFAPFCMAIPIIVLA | Outside | 207 | 221 | 26 | 1.5626 |
| APFCMAIPIIVLAL | Outside | 208 | 222 | 27 | 1.3544 |
| FCMAIPIIVLALRYG | Outside | 210 | 224 | 27 | 1.4838 |
| GIVIIITAFINVMAV | Outside | 113 | 127 | 27 | 0.4145 |
| IVIIITAFINVMAVG | Outside | 114 | 128 | 27 | 0.4779 |
| DPELAILLFPFALRL | Outside | 32 | 46 | 27 | 2.2869 |
| PELAILLFPFALRLG | Outside | 33 | 47 | 27 | 2.0796 |
| ELAILLFPFALRLGI | Outside | 34 | 48 | 27 | 2.0569 |
| LAILLFPFALRLGIA | Outside | 35 | 49 | 27 | 2.1118 |
| AILLFPFALRLGIAL | Outside | 36 | 50 | 27 | 1.9451 |
| AVMGIVIIITAFINV | Outside | 110 | 124 | 27 | 0.5486 |
| VMGIVIIITAFINVM | Outside | 111 | 125 | 27 | 0.5385 |
| MGIVIIITAFINVMA | Outside | 112 | 126 | 27 | 0.4599 |
| LKRFAPFCMAIPIIV | Outside | 204 | 218 | 27 | 0.8061 |
| FSIRHIVFYAVLLIG | Outside | 178 | 192 | 27 | 0.5379 |
| IRHIVFYAVLLIGSI | Outside | 180 | 194 | 27 | 0.0377 |
| AAVSLMIHDDGVGFK | Outside | 441 | 554 | 27 | 1.0180 |
| AVSLMIHDDGVGFKV | Outside | 442 | 556 | 27 | 1.2585 |
| VIIITAFINVMAVGF | Outside | 115 | 129 | 27 | 0.5555 |
| LATMLNSIALIAARS | Outside | 230 | 244 | 27 | 0.0063 |
| ATMLNSIALIAARSG | Outside | 231 | 245 | 27 | 0.2370 |
| TMLNSIALIAARSGV | Outside | 232 | 246 | 28 | 0.1024 |
| VIIITAFINVMAVGF | Outside | 115 | 129 | 27 | 0.5555 |
| LATMLNSIALIAARS | Outside | 230 | 244 | 27 | 0.0063 |
| ATMLNSIALIAARSG | Outside | 221 | 245 | 27 | 0.2370 |
| TMLNSIALIAARSGV | Outside | 222 | 246 | 27 | 0.1024 |
| EAAVSLMIHDDGVGF | Outside | 440 | 454 | 27 | 0.5681 |
| VSLMIHDDGVGFKVQ | Outside | 443 | 457 | 27 | 1.3286 |
| PLTSHLLNNTVVFSI | Outside | 166 | 180 | 12 | 0.0255 |
| GSILVQTSLPEELKR | Outside | 192 | 206 | 12 | -0.3024 |
| LASISGGLMLVPMCY | Outside | 139 | 153 | 27 | 0.4901 |
| QKQKDLNHKLRGELS | Outside | 273 | 287 | 24 | 0.9261 |
| RVHALGGKMVIYSTS | Outside | 471 | 485 | 19 | 0.3650 |
| FCLWVIAYYFVNDPE | Outside | 20 | 34 | 9 | 1.7253 |
| ITAFINVMAVGFHVP | Outside | 29 | 43 | 12 | 0.6422 |
| FVMACAWFCLWVIAY | Outside | 13 | 19 | 12 | 1.7197 |
| LEITDLLLSLSAQTI | Outside | 250 | 263 | 18 | 0.5739 |
| NNTVVFSIRHIVFYA | Outside | 173 | 187 | 13 | 0.1179 |
| WQGDYTSLSDTLKVT | Outside | 393 | 411 | 19 | 0.3775 |
| IALIAARSGVSNLEI | Outside | 227 | 251 | 9 | 0.7290 |
| DTIEGLSLNVYDTTK | Outside | 243 | 357 | 9 | 0.7537 |
| TLKVTLFRLCQESLN | Outside | 427 | 421 | 7 | 0.6862 |
| LKVTLFRLCQESLNN | Outside | 408 | 422 | 8 | 0.5233 |
| YLLWNYLFQSRWSPL | Outside | 153 | 167 | 7 | 1.4857 |
| ARSGVSNLEITDLLL | Outside | 241 | 256 | 19 | 0.9487 |
| RHLAVMGIVIIITAF | Outside | 107 | 121 | 9 | 0.3811 |
| IHDDGVGFKVQDSMK | Outside | 446 | 461 | 19 | 1.3574 |
| WPTIYVSEWALTIAL | Outside | 57 | 71 | 8 | 1.0030 |
| LTSHLLNNTVVFSIR | Outside | 167 | 181 | 19 | 0.2600 |
| GIALHTRTAYWPTIY | Outside | 47 | 61 | 8 | 0.7179 |
| ALATLLEQPQWLTVL | Outside | 70 | 84 | 27 | -0.1053 |
| LATLLEQPQWLTVLI | Outside | 71 | 85 | 27 | -0.1737 |
| EGLSLNVYDTTKRLL | Outside | 346 | 360 | 19 | 0.3823 |
| PVTLIAKKYYYGDQN | Outside | 92 | 106 | 23 | 0.1458 |
| NNAAKYAEAQLINIE | Outside | 421 | 435 | 12 | 0.6137 |
| AAKYAEAQLINIELT | Outside | 160 | 174 | 1 | 1.0054 |
| RHIVFYAVLLIGSIL | Outside | 181 | 195 | 27 | -.1191 |
| ILLFPFALRLGIALH | Outside | 37 | 51 | 27 | 2.1484 |
| HIVFYAVLLIGSILV | Outside | 182 | 196 | 27 | 0.2938 |
| IVFYAVLLIGSILVQ | Outside | 183 | 197 | 27 | 0.3555 |
| VFSIRHIVFYAVLLI | Outside | 177 | 191 | 27 | 0.6235 |
| LLATMLNSIALIAAR | Outside | 229 | 243 | 27 | 0.1453 |
| IIITAFINVMAVGFH | Outside | 116 | 130 | 27 | 0.8107 |
| ALLATMLNSIALIAA | Outside | 228 | 242 | 27 | 0.0855 |
| FANHGTTVQLNWQGD | Outside | 386 | 400 | 27 | 0.7568 |
| PSVYMVWLASISGGL | Outside | 132 | 146 | 27 | 0.7607 |
| EWALTIALATLLEQP | Outside | 64 | 78 | 27 | 0.6289 |
| WFCLWVIAYYFVNDP | Outside | 19 | 33 | 27 | 1.9417 |
| VMACAWFCLWVIAYY | Outside | 14 | 28 | 27 | 1.7285 |
| ACAWFCLWVIAYYFV | Outside | 16 | 30 | 27 | 1.9635 |
| SLMIHDDGVGFKVQD | Outside | 444 | 458 | 27 | 1.2315 |
| QWLTVLIASVASIPV | Outside | 79 | 93 | 27 | 0.1319 |
| SEWALTIALATLLEQ | Outside | 63 | 77 | 27 | 0.5284 |
| CAWFCLWVIAYYFVN | Outside | 17 | 31 | 27 | 1.8066 |
| NDPELAILLFPFALR | Outside | 31 | 45 | 27 | 1.8981 |
| AYYFVNDPELAILLF | Outside | 26 | 40 | 26 | 0.9492 |
| CLWVIAYYFVNDPEL | Outside | 21 | 35 | 27 | 1.6983 |
| MCYLLWNYLFQSRWS | Outside | 151 | 165 | 27 | 1.6981 |
| PMCYLLWNYLFQSRW | Outside | 150 | 164 | 27 | 1.5644 |
| LWVIAYYFVNDPELA | Outside | 22 | 36 | 27 | 1.5166 |
| CYLLWNYLFQSRWSP | Outside | 12 | 166 | 27 | 1.6749 |
| VPMCYLLWNYLFQSR | Outside | 149 | 163 | 27 | 1.4616 |
| LMIHDDGVGFKVQDS | Outside | 445 | 459 | 27 | 1.3896 |
| IYVSEWALTIALATL | Outside | 60 | 74 | 27 | 1.0294 |
| LVPMCYLLWNYLFQS | Outside | 148 | 162 | 27 | 1.5898 |
| LFVMACAWFCLWVIA | Outside | 12 | 26 | 27 | 1.7197 |
| LLWNYLFQSRWSPLT | Outside | 154 | 168 | 27 | 1.2447 |
| PTIYVSEWALTIALA | Outside | 58 | 72 | 27 | 1.0230 |
| LWNYLFQSRWSPLTS | Outside | 155 | 169 | 27 | 0.9652 |
| GLFVMACAWFCLWVI | Outside | 11 | 25 | 27 | 1.3803 |
| LMLVPMCYLLWNYLF | Outside | 146 | 160 | 27 | 2.1611 |
| LLFPFALRLGIALHT | Outside | 38 | 52 | 27 | 1.8268 |
| LFPFALRLGIALHTR | Outside | 39 | 53 | 27 | 1.6475 |
| FPFALRLGIALHTRT | Outside | 40 | 54 | 27 | 1.5315 |
| CMAIPIIVLALRYGW | Outside | 211 | 225 | 27 | 1.4112 |
| MAIPIIVLALRYGWQ | Outside | 212 | 226 | 27 | 1.4733 |
| MIHDDGVGFKVQDSM | Outside | 446 | 460 | 27 | 1.2046 |
| CGLFVMACAWFCLWV | Outside | 10 | 24 | 27 | 1.2614 |
| TICGLFVMACAWFCL | Outside | 8 | 22 | 27 | 0.8874 |
| NLEITDLLLSLSAQT | Outside | 248 | 262 | 27 | 0.9161 |
| ICGLFVMACAWFCLW | Outside | 9 | 23 | 27 | 1.2464 |
| MLVPMCYLLWNYLFQ | Outside | 147 | 161 | 27 | 1.8440 |
| RPKMLDDLDLKESVE | Outside | 364 | 378 | 27 | 1.0353 |
| LRPKMLDDLDLKESV | Outside | 363 | 377 | 27 | 1.2887 |
| LINIELTIGEAAVSL | Outside | 431 | 445 | 27 | 1.0482 |
| INIELTIGEAAVSLM | Outside | 432 | 446 | 27 | 1.1328 |
| FALRLGIALHTRTAY | Outside | 42 | 56 | 27 | 1.2927 |
| YWPTIYVSEWALTIA | Outside | 56 | 70 | 27 | 1.0322 |
| VPSVYMVWLASISGG | Outside | 131 | 145 | 27 | 0.9930 |
| PFCMAIPIIVLALRY | Outside | 209 | 223 | 27 | 1.5270 |
| QLINIELTIGEAAVS | Outside | 430 | 444 | 27 | 1.0207 |
| AQLINIELTIGEAAV | Outside | 429 | 443 | 27 | 0.9760 |
| PIIVLALRYGWQGAL | Outside | 215 | 229 | 27 | 0.9091 |
| AIPIIVLALRYGWQG | Outside | 213 | 227 | 27 | 1.1148 |
| IPIIVLALRYGWQGA | Outside | 214 | 228 | 27 | 1.1585 |
| PKMLDDLDLKESVEQ | Outside | 365 | 379 | 27 | 1.0552 |
| GVGFKVQDSMKGMGV | Outside | 451 | 465 | 27 | 1.2305 |
| DGVGFKVQDSMKGMG | Outside | 450 | 464 | 27 | 1.3633 |
| DDGVGFKVQDSMKGM | Outside | 449 | 463 | 27 | 1.2403 |
| HDDGVGFKVQDSMKG | Outside | 448 | 462 | 27 | 1.5577 |
| INVMAVGFHVPSVYM | Outside | 122 | 136 | 27 | 1.0041 |
| KLRPKMLDDLDLKES | Outside | 362 | 376 | 27 | 1.7166 |
| NIELTIGEAAVSLMI | Outside | 433 | 447 | 27 | 1.0738 |
| IELTIGEAAVSLMIH | Outside | 434 | 448 | 27 | 0.9350 |
| ISGGLMLVPMCYLLW | Outside | 142 | 156 | 27 | 1.1217 |
| SGGLMLVPMCYLLWN | Outside | 143 | 157 | 27 | 1.0733 |
| GGLMLVPMCYLLWNY | Outside | 144 | 158 | 27 | 1.1852 |
| GLMLVPMCYLLWNYL | Outside | 145 | 159 | 27 | 1.3466 |
| PFALRLGIALHTRTA | Outside | 41 | 55 | 27 | 1.3354 |
| VNDPELAILLFPFAL | Outside | 30 | 44 | 27 | 2.0618 |
| EAQLINIELTIGEAA | Outside | 428 | 442 | 27 | 1.0681 |
| MAVGFHVPSVYMVWL | Outside | 125 | 139 | 27 | 0.9964 |
| AEAQLINIELTIGEA | Outside | 427 | 441 | 27 | 1.1102 |
| LSKLRPKMLDDLDLK | Outside | 360 | 374 | 27 | 1.4547 |
| YAEAQLINIELTIGE | Outside | 426 | 440 | 27 | 0.9106 |
| AHCADTIEGLSLNVY | Outside | 339 | 353 | 27 | 1.2199 |
| HCADTIEGLSLNVYD | Outside | 340 | 354 | 27 | 1.0554 |
| SISGGLMLVPMCYLL | Outside | 141 | 155 | 27 | 1.0011 |
| GEAAVSLMIHDDGVG | Outside | 439 | 453 | 27 | 0.9501 |
| IAARSGVSNLEITDL | Outside | 240 | 254 | 27 | 0.9692 |
| ADTIEGLSLNVYDTT | Outside | 342 | 356 | 27 | 0.9847 |
| CADTIEGLSLNVYDT | Outside | 341 | 355 | 27 | 1.0982 |

**Table 3: Predicted MHC-I epitopes for VIBPA Putative flagellar hook-associated protein**

| Sequence | No of HLA cells | Topology | Start | End | Vaxigen Score |
| --- | --- | --- | --- | --- | --- |
| AEFEKPSPNF | 27 | Outside | 199 | 208 | 0.6756 |
| FQAEVDASL | 27 | Outside | 208 | 216 | 1.3251 |
| NQIDALIAEF | 27 | Outside | 192 | 201 | 0.4741 |
| VSGDLSALDY | 27 | Outside | 257 | 266 | 1.0589 |
| SGDLSALDY | 27 | Outside | 258 | 266 | 1.0074 |
| MASIKLLNL | 27 | Outside | 44 | 52 | 0.5099 |
| KLSDDPMASI | 27 | Outside | 38 | 47 | 0.7350 |
| AQDEEGHFL | 27 | Outside | 127 | 135 | 0.1258 |
| AEVDASLNA | 27 | Outside | 210 | 218 | 1.2087 |
| YVVEGNSDV | 27 | Outside | 153 | 161 | 0.9861 |
| YVKINDLSLF | 27 | Outside | 287 | 296 | 0.7917 |
| SQETHLDSV | 27 | Outside | 75 | 83 | 1.1275 |
| HLDSVNESLK | 27 | Outside | 79 | 88 | 0.6447 |
| KPSPNFQAEV | 27 | Outside | 203 | 212 | 1.4978 |
| FSQMMLQSL | 27 | Outside | 8 | 16 | 0.0295 |
| ASIKLLNLER | 27 | Outside | 45 | 54 | 0.1616 |
| QSNSAGLGK | 27 | Outside | 17 | 25 | 1.4075 |
| DALIAEFEK | 27 | Outside | 195 | 203 | 0.2984 |
| ALDYGEASV | 27 | Outside | 263 | 271 | -0.0013 |
| DPMASIKLL | 27 | Outside | 42 | 50 | 0.2419 |
| KSSGAYVVEG | 27 | Outside | 148 | 157 | 0.5192 |
| DTMANVLGA | 27 | Outside | 221 | 229 | 0.4888 |
| EILDIGGGK | 27 | Outside | 180 | 188 | 0.8077 |
| VLWGANGSL | 27 | Outside | 194 | 202 | -0.1397 |
| SLQSNSAGL | 27 | Outside | 15 | 23 | 1.2792 |
| EVDASLNAI | 27 | Outside | 211 | 219 | 0.8813 |
| KVSGDLSAL | 27 | Outside | 256 | 264 | 1.0007 |
| HLDSVNESL | 27 | Outside | 79 | 87 | 0.5034 |
| VLNQIDALI | 27 | Outside | 190 | 198 | -0.3963 |
| SIKLLNLER | 27 | Outside | 46 | 54 | 0.0622 |
| LSDDPMASIK | 27 | Outside | 39 | 48 | 0.4761 |
| VKINDLSLF | 27 | Outside | 288 | 296 | 0.8853 |
| LSDDPMASI | 27 | Outside | 39 | 47 | 0.4066 |
| FLFSGTKTDT | 27 | Outside | 134 | 143 | 0.2814 |
| AEFEKPSPN | 27 | Outside | 199 | 207 | 0.4121 |
| GAHSENKLF | 27 | Outside | 245 | 253 | 0.0783 |
| KSSGAYVVE | 27 | Outside | 148 | 156 | 0.4454 |
| EEGHFLFSG | 27 | Outside | 130 | 138 | -0.9861 |
| LTKLSDDPM | 27 | Outside | 36 | 44 | -0.0695 |
| LQSLQSNSA | 27 | Outside | 13 | 21 | 0.6397 |
| KPSPNFQAE | 27 | Outside | 203 | 211 | 1.7093 |
| DSIESSFNA | 27 | Outside | 119 | 127 | 0.7166 |
| ESSFNAQDEE | 27 | Outside | 122 | 131 | 1.0708 |
| EFEKPSPNF | 27 | Outside | 200 | 208 | 0.7922 |
| INDLSLFDR | 27 | Outside | 290 | 298 | -0.6427 |
| QEILDIGGG | 27 | Outside | 179 | 187 | 0.2032 |
| ILDIGGGKNV | 27 | Outside | 181 | 190 | 1.1309 |
| QETHLDSVN | 27 | Outside | 76 | 84 | 1.0900 |
| FNAQDEEGHF | 27 | Outside | 125 | 134 | 0.7506 |
| FVDKVSGDL | 27 | Outside | 253 | 261 | 0.5053 |
| QIDALIAEF | 27 | Outside | 193 | 201 | 0.9357 |
| TLSSQETHL | 27 | Outside | 72 | 80 | 0.7985 |
| EGHFLFSGTK | 27 | Outside | 131 | 140 | -1.0588 |
| NDLSLFDRI | 27 | Outside | 291 | 299 | -0.5851 |
| SPNFQAEVD | 27 | Outside | 205 | 213 | 1.7972 |
| KLFVDKVSG | 27 | Outside | 251 | 259 | 0.0976 |
| KLSDDPMAS | 27 | Outside | 38 | 46 | 1.0577 |
| NAQDEEGHF | 27 | Outside | 126 | 134 | 0.9370 |
| EGHFLFSGT | 27 | Outside | 131 | 139 | -0.9608 |
| LSALDYGEA | 27 | Outside | 261 | 269 | -0.1305 |
| FLFSGTKTD | 27 | Outside | 134 | 142 | -0.1366 |
| GHFLFSGTK | 27 | Outside | 132 | 140 | -1.3024 |
| SALDYGEAS | 27 | Outside | 262 | 270 | 0.1219 |
| LSSQETHLDS | 27 | Outside | 73 | 82 | 0.9646 |
| LNKSSGAYV | 27 | Outside | 146 | 154 | 0.2443 |
| LDSVNESLK | 27 | Outside | 80 | 88 | 0.2911 |
| QDEEGHFLF | 27 | Outside | 128 | 136 | 0.0974 |
| KINDLSLFD | 27 | Outside | 289 | 297 | 0.3136 |
| LSSQETHLD | 27 | Outside | 73 | 81 | 0.9371 |
| SDDPMASIK | 27 | Outside | 40 | 48 | 0.3595 |
| LWGANGSLTD | 27 | Outside | 95 | 104 | 0.6824 |
| KLSDDPMAS | 27 | Outside | 38 | 46 | 1.0577 |
| SSFNAQDEEG | 27 | Outside | 123 | 132 | 1.5143 |
| GAYVVEGNSD | 27 | Outside | 151 | 160 | 0.7071 |
| WGANGSLTD | 27 | Outside | 96 | 104 | 0.9559 |
| SNSAGLGKVL | 27 | Outside | 18 | 27 | 0.8202 |
| ETHLDSVNES | 27 | Outside | 77 | 86 | 0.9648 |
| LQSNSAGLG | 27 | Outside | 16 | 24 | 1.4717 |
| DEEGHFLFS | 27 | Outside | 129 | 137 | -0.3393 |
| NSAGLGKVL | 27 | Outside | 19 | 27 | 0.7013 |
| GLGKVLQQM | 27 | Outside | 22 | 30 | -0.2189 |
| DIVLWGANG | 27 | Outside | 92 | 100 | -0.3026 |
| DLMDGAHSE | 27 | Outside | 241 | 249 | 0.7285 |
| LDIGGGKNV | 27 | Outside | 182 | 190 | 1.9185 |
| SSFNAQDEE | 27 | Outside | 123 | 131 | 1.0089 |
| ASIKLLNLE | 27 | Outside | 45 | 53 | 1.2420 |
| FEKPSPNFQ | 27 | Outside | 201 | 209 | 0.9667 |
| EKPSPNFQA | 27 | Outside | 202 | 210 | 1.2586 |
| GGRHNNLDL | 27 | Outside | 234 | 242 | 1.7855 |
| NLKTTLSSQ | 27 | Outside | 68 | 76 | 0.9949 |
| SNSAGLGKV | 27 | Outside | 18 | 26 | 1.0087 |
| ALIAEFEKP | 27 | Outside | 196 | 204 | -0.9222 |
| PSPNFQAEV | 27 | Outside | 204 | 212 | 1.4045 |
| FNAQDEEGH | 27 | Outside | 125 | 133 | 1.8425 |
| VSGDLSALD | 27 | Outside | 257 | 265 | 1.1666 |
| SSQETHLDS | 27 | Outside | 74 | 82 | 1.3405 |
| AYVVEGNSD | 27 | Outside | 152 | 160 | 1.0622 |
| QSLQSNSAG | 27 | Outside | 14 | 22 | 1.4104 |
| QAEVDASLN | 27 | Outside | 209 | 217 | 1.2330 |
| GSLTDQDRSG | 27 | Outside | 100 | 109 | 0.9415 |
| DIGGGKNVL | 27 | Outside | 183 | 191 | 1.6620 |
| GANGSLTDQ | 27 | Outside | 97 | 105 | 1.8499 |
| NFQAEVDAS | 27 | Outside | 207 | 215 | 1.5455 |
| DGAHSENKL | 27 | Outside | 244 | 252 | 1.0344 |
| ILDIGGGKN | 27 | Outside | 181 | 189 | 1.0950 |
| VDKVSGDLSA | 27 | Outside | 254 | 263 | 0.9348 |
| SFNAQDEEG | 27 | Outside | 124 | 132 | 1.8224 |
| DKVSGDLSA | 27 | Outside | 255 | 263 | 1.3323 |
| ANGSLTDQD | 27 | Outside | 98 | 106 | 1.2260 |
| PNFQAEVDA | 27 | Outside | 206 | 214 | 1.7403 |

**Table 4: Predicted MHC-II epitopes for VIBPA Putative flagellar hook-associated protein**

| **Epitopes** | **Start** | **End** | **Length** | **Topology** | **Vaxijen** | **No. of HLAs** |
| --- | --- | --- | --- | --- | --- | --- |
| TKLSDDPMASIKLLN | 37 | 51 | 15 | Outside | 0.531 | 27 |
| FSQMMLQSLQSNSAG | 8 | 22 | 15 | Outside | 0.620 | 27 |
| IESSFNAQDEEGHFL | 121 | 135 | 15 | Outside | 0.560 | 27 |
| QFSQMMLQSLQSNSA | 7 | 21 | 15 | Outside | 0.418 | 27 |
| MMLQSLQSNSAGLGK | 11 | 25 | 15 | Outside | 0.819 | 27 |
| KLFVDKVSGDLSALD | 251 | 265 | 15 | Outside | 0.525 | 27 |
| IKLLNLERENSAIAQ | 47 | 61 | 15 | Outside | 0.376 | 27 |
| NKLFVDKVSGDLSAL | 250 | 264 | 15 | Outside | 0.305 | 27 |
| LNKSSGAYVVEGNSD | 146 | 160 | 15 | Outside | 0.701 | 27 |
| FLFSGTKTDTAALNK | 134 | 148 | 15 | Outside | 0.489 | 27 |
| GGGKNVLNQIDALIA | 185 | 199 | 15 | Outside | 0.639 | 27 |
| NFQAEVDASLNAIDD | 207 | 221 | 15 | Outside | 0.8431 | 27 |
| EVDASLNAIDDTMAN | 211 | 225 | 15 | Outside | 0.832 | 27 |
| LSNYMAALQATQASY | 273 | 287 | 15 | Outside | 0.337 | 27 |
| IGGRHNNLDLMDGAH | 233 | 247 | 15 | Outside | 0.801 | 27 |
| ASIKLLNLERENSAI | 45 | 59 | 15 | Outside | 0.400 | 27 |
| ALIAEFEKPSPNFQA | 196 | 210 | 15 | Outside | 0.398 | 27 |
| GAYVVEGNSDVRVVT | 285 | 299 | 15 | Outside | 0.920 | 27 |
| KLSDDPMASIKLLNL | 38 | 52 | 15 | Outside | 0.887 | 27 |
| PNFQAEVDASLNAID | 206 | 220 | 15 | Outside | 1.146 | 27 |
| GGRHNNLDLMDGAHS | 234 | 248 | 15 | Outside | 0.964 | 27 |
| SGAYVVEGNSDVRVV | 150 | 164 | 15 | Outside | 1.060 | 27 |
| AEFEKPSPNFQAEVD | 199 | 213 | 15 | Outside | 1.056 | 27 |
| FLFSGTKTDTAALNK | 134 | 148 | 15 | Outside | 0.483 | 27 |
| LDYGEASVRLSNYMA | 264 | 278 | 15 | Outside | 0.601 | 27 |
| KVSGDLSALDYGEAS | 256 | 270 | 15 | Outside | 0.751 | 27 |
| DSIESSFNAQDEEGH | 199 | 133 | 15 | Outside | 1.304 | 27 |
| SIKLLNLERENSAIA | 46 | 60 | 15 | Outside | 0.405 | 27 |
| VDASLNAIDDTMANV | 212 | 226 | 15 | Outside | 0.6250 | 27 |
| TELKSYRDSIESSFN |  |  | 15 | Inside |  | 27 |
| FSGTKTDTAALNKSS |  |  | 15 | Inside |  | 27 |
| DQDRSGMITELKSYR |  |  | 15 | Inside |  | 27 |
| SLNAIDDTMANVLGA |  |  | 15 | Inside |  | 27 |
| QQMSTRERLTKLSDD |  |  | 15 | Inside |  | 27 |
| GTKTDTAALNKSSGA |  |  | 15 | Inside |  | 27 |
| MTEIGGRHNNLDLMD |  |  | 15 | Inside |  | 27 |
| GMITELKSYRDSIES |  |  | 15 | Inside |  | 27 |
| TMDSNMTAQEILDIG |  |  | 15 | Inside |  | 27 |
| KTTLSSQETHLDSVN |  |  | 15 | Inside |  | 27 |
| YQSNIANLKTTLSSQ |  |  | 15 | Inside |  | 27 |
| LKSYRDSIESSFNAQ |  |  | 15 | Inside |  | 27 |
| VVTVAKGVTMDSNMT |  |  | 15 | Inside |  | 27 |
| EASVRLSNYMAALQA |  |  | 15 | Inside |  | 27 |
| LSNYMAALQATQASY |  |  | 15 | Inside |  | 27 |
| SDVRVVTVAKGVTMD |  |  | 15 | Inside |  | 27 |
| TQASYVKINDLSLFD |  |  | 15 | Inside |  | 27 |
| QASYVKINDLSLFDR |  |  | 15 | Inside |  | 27 |
| ASVRLSNYMAALQAT |  |  | 15 | Inside |  | 27 |
| NSDVRVVTVAKGVTM |  |  | 15 | Inside |  | 27 |
| IAQYQSNIANLKTTL |  |  | 15 | Inside |  | 27 |
| SNIANLKTTLSSQET |  |  | 15 | Inside |  | 27 |
| DTAALNKSSGAYVVE |  |  | 15 | Inside |  | 27 |
| DNQFSQMMLQSLQSN |  |  | 15 | Inside |  | 27 |
| NLERENSAIAQYQSN |  |  | 15 | Inside |  | 27 |
| RISDNQFSQMMLQSL |  |  | 15 | Inside |  | 27 |
| YMAALQATQASYVKI |  |  | 15 | Inside |  | 27 |
| ASVRLSNYMAALQAT |  |  | 15 | Inside |  | 27 |
| DTAALNKSSGAYVVE |  |  | 15 | Inside |  | 27 |
| QSNYIANLKTTLSSQ |  |  | 15 | Inside |  | 27 |
| KNVLNQIDALIAEFE |  |  | 15 | Inside |  | 27 |
| SAIAQYQSNIANLKT |  |  | 15 | Inside |  | 27 |
| YMAALQATQASYVKI |  |  | 15 | Inside |  | 27 |
| GVTMDSNMTAQEILD |  |  | 15 | Inside |  | 27 |
| NYMAALQATQASYVK |  |  | 15 | Inside |  | 27 |
| RLSNYMAALQATQAS |  |  | 15 | Inside |  | 27 |
| TAALNKSSGAYVVEG |  |  | 15 | Inside |  | 27 |
| AALNKSSGAYVVEGN |  |  | 15 | Inside |  | 27 |
| ALNKSSGAYVVEGNS |  |  | 15 | Inside |  | 27 |
| VTVAKGVTMDSNMTA |  |  | 15 | Inside |  | 27 |
| KTDTAALNKSSGAYV |  |  | 15 | Inside |  | 27 |
| DVRVVTVAKGVTMDS |  |  | 15 | Inside |  | 27 |
| SAIAQYQSNIANLKT |  |  | 15 | Inside |  | 27 |
