# Supplementary material for: Comprehensive genome based analysis of Vibrio parahaemolyticus for identifying novel drug and vaccine molecules: Subtractive proteomics and vaccinomics approach
Source: PLoS One. 2020 Aug 19;15(8):e0237181. doi: 10.1371/journal.pone.0237181 (PMC7444560; doi:10.1371/journal.pone.0237181)
Supplement: S16 File — (DOCX) [file pone.0237181.s029.docx]

**S16 File.** Top MHC-I epitopes of VIBPA Putative sensor histidine protein kinase UhpB

| **Epitope** | **Topology** | **No. HLA cell** | **Vaxijen Score** | **Start** | **End** | **Length** | **Conservancy** |
| --- | --- | --- | --- | --- | --- | --- | --- |
| AILLFPFAL | Outside | 27 | 2.993 | 36 | 44 | 9 | 99.00% (99/100) |
| ILLFPFALR | Outside | 81 | 2.9721 | 37 | 45 | 9 | 99.00% (99/100) |
| FCLWVIAYYF | Outside | 27 | 2.8883 | 20 | 29 | 10 | 100.00% (100/100) |
| CYLLWNYLF | Outside | 81 | 2.8253 | 152 | 160 | 9 | 100.00% (100/100) |
| ELAILLFPF | Outside | 81 | 2.7265 | 34 | 42 | 9 | 100.00% (100/100) |
| LLFPFALRL | Outside | 54 | 2.7071 | 38 | 46 | 9 | 98.00% (98/100) |
| LAILLFPFA | Outside | 54 | 2.6785 | 35 | 43 | 9 | 99.00% (99/100) |
| WFCLWVIAY | Outside | 54 | 2.5591 | 19 | 27 | 9 | 100.00% (100/100) |
| LWVIAYYFV | Outside | 54 | 2.4774 | 22 | 30 | 9 | 99.00% (99/100) |
| HDDGVGFKV | Outside | 81 | 2.363 | 448 | 456 | 9 | 99.00% (99/100) |
| YLLWNYLFQ | Outside | 27 | 2.3399 | 153 | 161 | 9 | 100.00% (100/100) |
| DDGVGFKVQ | Outside | 27 | 2.325 | 449 | 457 | 9 | 100.00% (100/100) |
| DGVGFKVQD | Outside | 54 | 2.1287 | 450 | 458 | 9 | 100.00% (100/100) |

**Table 2: Top MHC-II epitopes of VIBPA Putative sensor histidine protein kinase UhpB**

| **Epitope** | **Topology** | **No of HLAs** | **Vaxigen Score** | **Start** | **End** | **Length** | **Conservancy** |
| --- | --- | --- | --- | --- | --- | --- | --- |
| DPELAILLFPFALRL | Outside | 27 | 2.2869 | 32 | 46 | 15 | 98.00% (98/100) |
| LMLVPMCYLLWNYLF | Outside | 27 | 2.1611 | 146 | 160 | 15 | 100.00% (100/100) |
| ILLFPFALRLGIALH | Outside | 27 | 2.1484 | 37 | 51 | 15 | 87.00% (87/100) |
| LAILLFPFALRLGIA | Outside | 27 | 2.1118 | 35 | 49 | 15 | 88.00% (88/100) |
| PELAILLFPFALRLG | Outside | 27 | 2.0796 | 33 | 47 | 15 | 98.00% (98/100) |
| VNDPELAILLFPFAL | Outside | 27 | 2.0618 | 30 | 44 | 15 | 98.00% (98/100) |
| ELAILLFPFALRLGI | Outside | 27 | 2.0569 | 34 | 48 | 15 | 98.00% (98/100) |
| ACAWFCLWVIAYYFV | Outside | 27 | 1.9635 | 16 | 30 | 15 | 99.00% (99/100) |
| AILLFPFALRLGIAL | Outside | 27 | 1.9451 | 36 | 50 | 15 | 87.00% (87/100) |
| WFCLWVIAYYFVNDP | Outside | 27 | 1.9417 | 19 | 33 | 15 | 99.00% (99/100) |
| NDPELAILLFPFALR | Outside | 27 | 1.8981 | 31 | 45 | 15 | 99.00% (99/100) |
| MLVPMCYLLWNYLFQ | Outside | 27 | 1.844 | 147 | 161 | 15 | 100.00% (100/100) |
| LLFPFALRLGIALHT | Outside | 27 | 1.8268 | 38 | 52 | 15 | 87.00% (87/100) |
| CAWFCLWVIAYYFVN | Outside | 27 | 1.8066 | 17 | 31 | 15 | 99.00% (99/100) |
| VMACAWFCLWVIAYY | Outside | 27 | 1.7285 | 14 | 28 | 15 | 99.00% (99/100) |

**Table 3: Top MHC-I epitopes of putative flagellar hook-associated protein**

| **Sequence** | **Topology** | **No of HLA cells** | **Vaxijen** | **Start** | **End** | **Length** | **Conservancy** |
| --- | --- | --- | --- | --- | --- | --- | --- |
| LDIGGGKNV | Outside | 27 | 1.9185 | 182 | 190 | 9 | 46.00% (46/100) |
| GANGSLTDQ | Outside | 27 | 1.8499 | 97 | 105 | 9 | 54.00% (54/100) |
| FNAQDEEGH | Outside | 27 | 1.8425 | 125 | 133 | 9 | 98.00% (98/100) |
| SFNAQDEEG | Outside | 27 | 1.8224 | 124 | 132 | 9 | 98.00% (98/100) |
| SPNFQAEVD | Outside | 27 | 1.7972 | 205 | 213 | 9 | 76.00% (76/100) |
| GGRHNNLDL | Outside | 27 | 1.7855 | 234 | 242 | 9 | 99.00% (99/100) |
| PNFQAEVDA | Outside | 27 | 1.7403 | 206 | 214 | 9 | 73.00% (73/100) |
| KPSPNFQAE | Outside | 27 | 1.7093 | 203 | 211 | 9 | 46.00% (46/100) |
| DIGGGKNVL | Outside | 27 | 1.662 | 183 | 191 | 9 | 46.00% (46/100) |
| NFQAEVDAS | Outside | 27 | 1.5455 | 207 | 215 | 9 | 74.00% (74/100) |
| SSFNAQDEEG | Outside | 27 | 1.5143 | 123 | 132 | 10 | 98.00% (98/100) |
| KPSPNFQAEV | Outside | 27 | 1.4978 | 203 | 212 | 10 | 45.00% (45/100) |
| LQSNSAGLG | Outside | 27 | 1.4717 | 16 | 24 | 9 | 50.00% (50/100) |

**Table 4: Top MHC-II epitopes of VIBPA putative flagellar hook-associated protein**

| **Epitopes** | **Start** | **End** | **Length** | **Topology** | **Vaxijen** | **No. of HLAs** | **Conservancy** |
| --- | --- | --- | --- | --- | --- | --- | --- |
| DSIESSFNAQDEEGH | 199 | 133 | 15 | Outside | 1.304 | 27 | 88.00% (88/100) |
| PNFQAEVDASLNAID | 206 | 220 | 15 | Outside | 1.146 | 27 | 45.00% (45/100) |
| SGAYVVEGNSDVRVV | 150 | 164 | 15 | Outside | 1.06 | 27 | 42.00% (42/100) |
| AEFEKPSPNFQAEVD | 199 | 213 | 15 | Outside | 1.056 | 27 | 43.00% (43/100) |
| GGRHNNLDLMDGAHS | 234 | 248 | 15 | Outside | 0.964 | 27 | 48.00% (48/100) |
| GAYVVEGNSDVRVVT | 285 | 299 | 15 | Outside | 0.92 | 27 | 42.00% (42/100) |
| KLSDDPMASIKLLNL | 38 | 52 | 15 | Outside | 0.887 | 27 | 88.00% (88/100) |
| NFQAEVDASLNAIDD | 207 | 221 | 15 | Outside | 0.8431 | 27 | 46.00% (46/100) |
| EVDASLNAIDDTMAN | 211 | 225 | 15 | Outside | 0.832 | 27 | 36.00% (36/100) |
| MMLQSLQSNSAGLGK | 11 | 25 | 15 | Outside | 0.819 | 27 | 47.00% (47/100) |
| IGGRHNNLDLMDGAH | 233 | 247 | 15 | Outside | 0.801 | 27 | 59.00% (59/100) |
| KVSGDLSALDYGEAS | 256 | 270 | 15 | Outside | 0.751 | 27 | 58.00% (58/100) |
| LNKSSGAYVVEGNSD | 146 | 160 | 15 | Outside | 0.701 | 27 | 42.00% (42/100) |
| GGGKNVLNQIDALIA | 185 | 199 | 15 | Outside | 0.639 | 27 | 40.00% (40/100) |
| VDASLNAIDDTMANV | 212 | 226 | 15 | Outside | 0.625 | 27 | 36.00% (36/100) |
